# MINTmap: fast and exhaustive profiling of nuclear and mitochondrial tRNA fragments from short RNA-seq data

**DOI:** 10.1038/srep41184

**Published:** 2017-02-21

**Authors:** Phillipe Loher, Aristeidis G. Telonis, Isidore Rigoutsos

**Affiliations:** 1 Computational Medicine Center, Sidney Kimmel Medical College, Thomas Jefferson University, 1020 Locust Street, Philadelphia, PA 19107, USA

## Abstract

Transfer RNA fragments (tRFs) are an established class of constitutive regulatory molecules that arise from precursor and mature tRNAs. RNA deep sequencing (RNA-seq) has greatly facilitated the study of tRFs. However, the repeat nature of the tRNA templates and the idiosyncrasies of tRNA sequences necessitate the development and use of methodologies that differ markedly from those used to analyze RNA-seq data when studying microRNAs (miRNAs) or messenger RNAs (mRNAs). Here we present MINTmap (for MItochondrial and Nuclear TRF mapping), a method and a software package that was developed specifically for the quick, deterministic and exhaustive identification of tRFs in short RNA-seq datasets. In addition to identifying them, MINTmap is able to unambiguously calculate and report both raw and normalized abundances for the discovered tRFs. Furthermore, to ensure specificity, MINTmap identifies the subset of discovered tRFs that could be originating outside of tRNA space and flags them as candidate false positives. Our comparative analysis shows that MINTmap exhibits superior sensitivity and specificity to other available methods while also being exceptionally fast. The MINTmap codes are available through https://github.com/TJU-CMC-Org/MINTmap/ under an open source GNU GPL v3.0 license.

In this paper, we build upon our previous work^1^,^2^ and present MINTmap, a portable software tool for identifying and quantitating tRFs in short RNA-seq datasets, where the molecules under study are typically less than 50 nucleotides (nt) in length. MINTmap can help researchers, who are interested in studying the new class of short non-coding RNA (ncRNA) molecules known as tRFs, leverage the information contained in deep-sequencing datasets.

In the last several years, deep-sequencing has been fueling new and unexpected discoveries in the field of ncRNAs. These discoveries have been leading us away from the linear view of the “Central Dogma of Biology” and towards a framework in which ncRNAs are as important as proteins. Not only have the advances of recent years made it possible to find *new categories* of such molecules, they have also helped improve our understanding of long-established classes of ncRNAs in unexpected ways. For example, the number of known locations in the human genome that harbor miRNA precursors was recently more than tripled^3^,^4^ while at the same time it was shown that miRNA precursors produce multiple isoforms in a manner that is constitutive and depends on a person’s sex, population origin, race, tissue, and disease type/subtype^5^,^6^.

## Background

### Transfer RNA fragments

tRNAs are ancient ncRNAs that are present in all three kingdoms of life (archaea, bacteria, eukaryotes) and whose activities have long been thought to revolve exclusively around the translation process of messenger RNA (mRNA) into an amino acid sequence. Conventionally, the mature tRNA was viewed as the sole product of the respective genomic locus that was used primarily in translation. Recent advances in deep-sequencing technologies have been reshaping this understanding revealing that tRNA loci produce fragments, which are known as tRNA fragments or “tRFs,” in parallel to producing mature tRNAs^7^,^8^,^9^,^10^.

**Figure 1:**
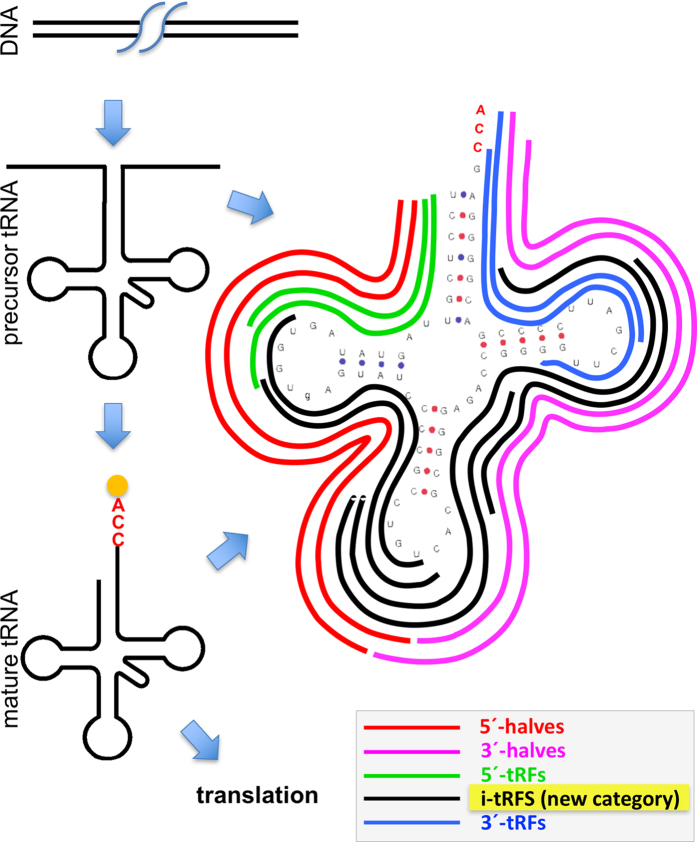
The five structural categories of tRFs. This is a schematic showing examples of tRFs aligned to the characteristic secondary structure of a tRNA molecule. Typically, each of the five categories of tRFs will comprise many more molecules than are shown here.

Work in this area analyzed tRNAs that are encoded by the nuclear genome and identified five *structural* categories of tRFs^8^ that are shown pictorially in Fig. 1 (see section ‘Nomenclature and Structural Categories of tRNA Fragments’ below for detailed definitions of the categories). The five structural categories comprise: (a) “5′-tRNA halves” (5′-tRHs; in red in Fig. 1) that are ~34 nt in length and arise from the mature tRNA through cleavage at the anticodon by Angiogenin (ANG)^11^,^12^,^13^; (b) “3′-tRNA halves” (3′-tRHs; in magenta) that are the remainder (i.e. second ‘half’) of the mature tRNA following cleavage at the anticodon; (c) “5′-tRFs” (in green) that are derived from mature tRNAs after cleavage at the D-loop or the anticodon stem; (d) the new category of i-tRFs (for *internal* tRFs; shown in black color) that are fully contained within the span of the mature tRNA^2^; and, (e) “3′-tRFs” (in blue) that are derived from mature tRNAs after cleavage at the T-loop or the anticodon stem.

### tRFs are constitutive regulators

Fast accumulating evidence is beginning to uncover significant roles for full-length tRNAs that go well beyond their conventional roles in translating mRNAs^14^,^15^. Similarly, tRNA-derived fragments, originally thought to be transcriptional noise, are now known to be *constitutive* in nature and to have expression profiles that depend on many variables (detailed below) in healthy individuals and in cancer patients^2^. We summarize the key findings so far in the following paragraphs.

tRFs have been reported in archaea (*Haloferax volcanii*^16^), bacteria (*Streptomyces coelicolor*^17^) and eukaryotes (human^2^,^18^,^19^,^20^,^21^,^22^; mouse^19^,^23^,^24^; yeast^19^,^25^; the protozoans *Giardia lamblia*^26^, *Tetrahymena thermophila*^27^,^28^, *Trypanosoma cruzi*^29^). These findings suggest that tRFs and the mechanisms underlying the production of tRFs are evolutionarily ancient.

**Figure 2:**
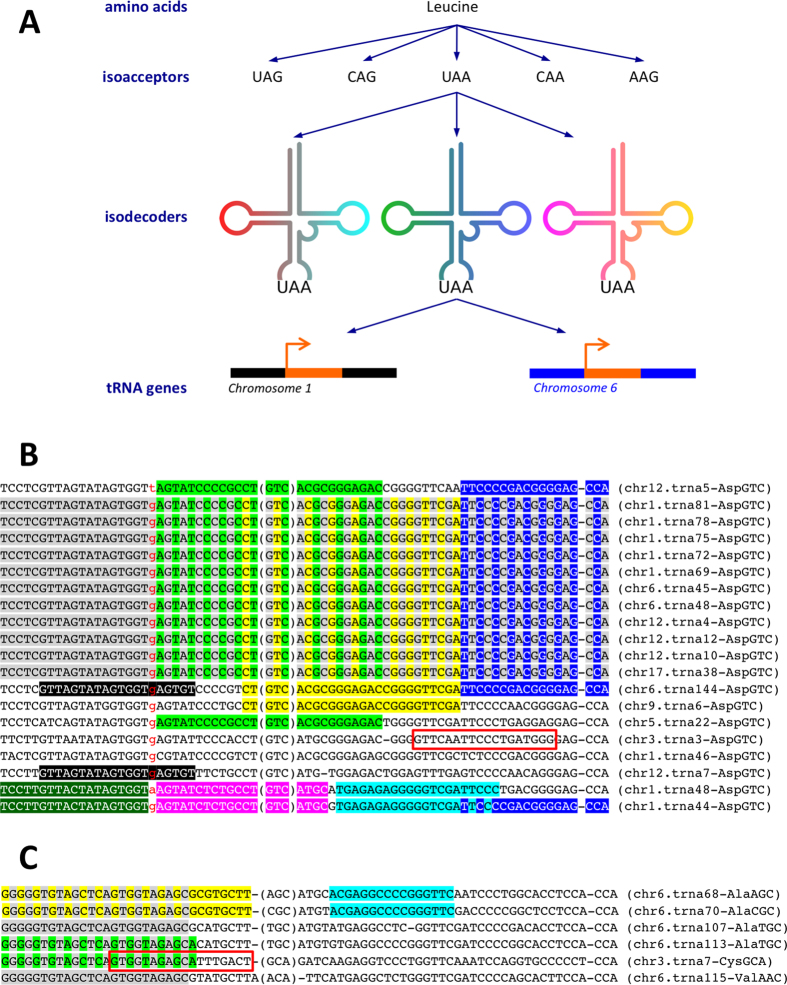
(**A**) A schematic of the tRNA hierarchy. The amino acids are at the top level. At the bottom level one finds individual tRNA genes (isodecoders for a given anticodon). (**B**) Alignment of several isodecoders for the same anticodon (tRNA^AspGTC^). Some of the segments that are shared by various subsets of the listed isodecoders are shown shaded in different colors. (**C**) Alignment of isodecoders from different anticodons (such as tRNA^AlaAGC^ and tRNA^CysGCA^). As in (**B**), sequence segments shared by the listed isodecoders are shown shaded in different colors.

We have shown that mitochondrially-encoded tRNAs produce a rich repertoire of tRFs^2^ just like the nuclearly-encoded tRNAs. We also showed that the locations of cleavage differ distinctly between mitochondrially-encoded and nuclearly-encoded isodecoders of the same isoacceptor, i.e. differ between counterpart sequences. There are multiple cleavage points that occur at specific sites and give rise to quantized tRF lengths: importantly, the resulting tRFs are *overlapping* and not consecutive on the mature tRNA. These findings suggest that the tRFs are not random products and do not result from post-transcriptional modifications of tRNA sites that might inhibit sequencing. Moreover, we have discovered the new category of *internal tRFs* or “i-tRFs” (Fig. 1 and also section ‘Nomenclature and Structural Categories of tRNA Fragments’ below). Our analyses of hundreds of human transcriptomes showed that i-tRFs are produced by both nuclearly-encoded and mitochondrially-encoded tRNAs, and are present in different tissues and in hundreds of individuals^2^. We also showed that i-tRF lengths can vary considerably from as short as 16 nt to as long as 36 nt, and possibly longer^2^. i-tRFs are fully contained within the span of the mature tRNA, can begin and end at a multitude of positions, and can precede, follow, or straddle the anticodon (e.g. see Fig. 2 of our previous publication^2^). These findings indicate that mature tRNAs produce a richer repertoire of fragments than originally thought.

Using hundreds of human transcriptomes, we showed that mature tRNAs will give rise to distinct tRF profiles in a manner that depends on the tissue at hand, the tissue’s state (health vs. cancer) and the disease subtype as well as on a individual’s attributes like sex, population origin, and race^2^. The profiles of tRFs also differ across cell types^2^,^19^. These findings indicate that the production of tRFs is affected by a number of important patient-centered variables. By comparing the tRF profiles of healthy individuals and cancer patients we found that choice of cleavage points and the relative abundances of tRFs remain *unchanged* across individuals who belong to the same sex, to the same race and to the same population^2^, in complete analogy to our reported findings on miRNA isoforms (isomiRs)^5^,^6^. These findings further support the *constitutive* nature of tRFs within human tissues. The cleavage points and relative abundances changed between health and disease, and also across disease subtypes.

From a functional standpoint, the longer tRNA halves have been shown to have roles that range from inhibition of protein synthesis in eukaryotes^8^,^12^ and archaea^16^, to enhancing cell proliferation^11^. On the other hand, the shorter tRNA fragments (i.e. 5′-tRFs, i-tRFs and 3′-tRFs) have been shown to have more diverse functions some of which include the regulation of expression of protein-coding genes^2^,^9^,^10^,^19^,^21^,^30^. These findings further support the *regulatory* nature of tRFs. 5′-tRFs, i-tRFs, and 3′-tRFs have been shown to enter the RNA interference (RNAi) pathway through Argonaute loading and, thus, to have regulatory roles through direct interaction with targets that are currently unknown^18^,^19^. On a related note, the production of 5′-tRFs was shown to be DICER-dependent in mammals and the fruit fly but DICER-independent in yeast^18^,^19^,^21^,^31^,^32^. Also, our analyses of the human breast cancer cell lines BT-474 (Her2+), MDA-MB-231 (ER−/PR−/Her2−) and MCF7 (ER+) showed that whether a given 5′-tRF, i-tRF or 3′-tRF will be loaded on Argonaute depends on the cell type^2^. These findings further support the participation of some tRF categories in regulation.

In addition to affecting targets through direct molecular contact, tRFs have also been shown to regulate other transcripts through molecular competition, also known as “decoying.” Specifically, it was recently shown that three i-tRFs that straddle the anticodon (i-tRF GTATCCCCGCCT**GTC**ACG from tRNA^AspGTC^, i-tRF GGATTCGGCGCT**CTC**ACC from tRNA^GluCTC^, and i-tRF TAGCATAGCTGCCT**TCC**A from tRNA^GlyTCC^) and are induced by hypoxic-stress can counteract post-transcriptionally the stabilization of oncogenic mRNAs by competing for binding to YBX1, an RNA binding protein^33^. These findings indicate that the newly discovered i-tRFs also have regulatory roles.

With regard to the longer tRNA halves (Fig. 1), their production has been shown to depend on cellular stress^12^,^13^,^34^,^35^. Their production is also modulated by the presence of the sex hormones estrogen and androgen^11^. These findings suggest that the production of the (longer) tRNA halves can be driven by external factors and by context.

In male mice that were subjected to a protein-restricted diet, the abundances of multiple (short) tRFs were found to be altered throughout their reproductive tract and in the mature sperm, compared to control mice^36^. In maturing mouse sperm, 5′-tRFs from tRNA^GlyGCC^ were shown to suppress protein-coding transcripts that are driven by the endogenous retroelement MERVL^36^. These findings suggest that the production of the (shorter) tRFs can be driven by external factors and that these tRFs affect protein expression.

tRNA halves as well as shorter tRNA fragments have been linked to viral and bacterial infections as well. For example, an 18 nt 3′-tRF from tRNA^LysTTT^ in HIV-infected human MT4 T-cells was shown to inhibit HIV-1 replication by ~80% and to be dependent on DICER and Argonaute 2 (AGO2)^37^. Also, RSV infections were found to increase the production of tRNA fragments by as much as 20x compared to mock infections^38^: among the fragments found to be up-regulated, a 5′-tRH from tRNA^GluCTC^ was shown to promote viral replication^39^. Similarly, production of 5′-tRHs, primarily from tRNA^ValGTG^ and tRNA^GlyGCC^, and of 5′-tRFs, i-tRFs and 3′-tRFs increased 2–4x in the lungs of mice following infection by Rickettsia^40^. These findings suggest that tRNA halves and the shorter tRFs play important roles in bacterial and viral infections.

The above multifaceted evidence that has accumulated in a few short years warrants viewing tRFs as a previously unrecognized, *bona fide* category of ncRNAs. It is reasonable to conjecture that the roles of tRNA fragments likely extend beyond what has been uncovered to date. Additionally, the current knowledge about the biogenesis of tRFs remains very limited, despite the multitude of settings in which these molecules have been encountered. We also note that fragments have also been found that are produced from the *precursor* tRNA molecule. These fragments are also “tRFs” and functions for a few of them have been reported already^7^,^9^. The emerging importance of tRFs as regulators and their complex dependencies on the source tissue, the tissue state, and an individual’s attributes are making the ability to analyze transcriptomic data (short RNA-seq) of paramount importance.

### Nomenclature and Structural Categories of tRNA Fragments

We distinguish among *three tRNA regions* (“−1/+1,” “internal,” and “CCA-ending”) and *five structural types of mature tRNA fragments* (5′-tRFs, i-tRFs, 3′-tRFs, 5′-tRHs and 3′-tRHs)–see Fig. 1. The “−1/+1” region gives rise to 5′-tRFs and 5′-tRHs–it is meant to capture molecules whose start coincides with the first position of the mature tRNA (+1) or have a nucleotide added post-transcriptionally to their 5′ end (−1) as is the case of tRNA^His^, which is discussed below. The “internal region” gives rise to i-tRFs. The “CCA-ending region” gives rise to 3′-tRFs and 3′-tRHs.

#### tRNA-halves

We use the Angiogenin cleavage sites that have been reported in the literature to define 5′-halves and 3′-halves. Specifically, if **A**_**1**_**A**_**2**_**A**_**3**_ is the anticodon triplet and n_1_n_2_**A**_**1**_**A**_**2**_**A**_**3**_ n_3_n_4_ denotes the anticodon loop and the sequence immediately surrounding the triplet, then 5′-tRHs begin at position +1 of a tRNA (or, −1 in the case of tRNA^His^) and terminate at any of the four underlined positions n_1_^∇^n_2_^∇^**A**_**1**_^∇^**A**_**2**_^∇^**A**_**3**_ n_3_n_4_ (each ∇ denotes a reported Angiogenin cleavage site). Analogously, 3′-tRHs can only begin at any of the four underlined positions n_1_^∇^n_2_^∇^**A**_**1**_^∇^**A**_**2**_^∇^**A**_**3**_ n_3_n_4_ and terminate in the first C, the second C, or the A of the non-templated CCA addition.

#### 5′-tRFs

5′-tRFs are fragments that begin at the 5′end of the tRNA (−1 for tRNA^His^–see also text) and end in a position other than n_1_, n_2_, **A**_**1**_, or **A**_**2**_. In other words, a 5′-tRF can end either *before* n_1_, or *after* **A**_**2**_ in which case it can potentially include positions to the right of n_4_.

#### i-tRFs

i-tRFs are fragments that begin at either position +2 or further to the right and end before the first C of the non-templated CCA addition.

#### 3′-tRFs

3′-tRFs are fragments that begin at a position other than n_2_, **A**_**1**_, **A**_**2**_ or **A**_**3**_ and terminate at the first C, the second C, or the A of the non-templated CCA addition. In other words, a 3′-tRF can begin either after **A**_**3**_ or before n_2_ including potentially positions to the left of n_1_.

We also distinguish between tRNAs whose isodecoders are harbored by the *nuclear* genome (“nuclearly-encoded”) and those harbored by the *mitochondrial* (“mitochondrially-encoded”) genome. Our use of “nuclear” or “mitochondrial” as an adjective will refer to the genomic source of a mature tRNA, of an isodecoder, or of a tRNA fragment, and not to the sub-cellular location of the corresponding transcript. This choice of nomenclature is necessitated by our recent discovery that the nuclear genomes of *H. sapiens*^41^ and of other organisms^42^ contain multiple sequences that best resemble the organism’s mitochondrial tRNAs. The nuclear genome of *H. sapiens* harbors 351 “tRNA-lookalikes” whose closest sequence match is one of the 22 human mitochondrial tRNAs. As these tRNA-lookalikes contain sub-sequences of various lengths that are identical to the organism’s *bona fide* mitochondrial tRNAs they represent a source of complication during the mapping of next generation sequencing data.

### The problem of tRF discovery in context

Seemingly simple, mapping short RNA-seq datasets to the human genome for the purpose of determining the profiles of the various classes of tRFs is a very involved undertaking. Several factors complicate this task and include: tRNAs are repeat elements themselves; tRNA isodecoders corresponding to the same amino acid can, and usually do, share extended regions of similarity confounding one’s attempts to determine a fragment’s source; for some short and long fragments it is not possible to unambiguously establish their tRNA nature; the human nuclear genome is riddled with hundreds of sequences that resemble mitochondrially-encoded tRNAs; the human nuclear genome is also riddled with hundreds of *partial* tRNA sequences whose lengths are ~1/2 of the 72 nt that are characteristic of a mature tRNA; mature tRNAs are post-transcriptionally modified through the addition of extra bases; etc. In our description of MINTmap’s implementation below, we describe each of these complicating factors in more detail and describe how each of them is tackled by MINTmap and by other methods.

## Results

So far, we have used MINTmap to analyze 843 public short RNA-seq datasets from a variety of cellular contexts^2^,^11^. We are currently hosting the results of these analyses in MINTbase^1^, which was developed in the context of a parallel project. MINTbase is an unrestricted, interactive web-based resource that allows users to interrogate the data by tRF structural category, isoacceptor, isodecoder, nucleic acid, sequence, genomic position, etc. User queries can be directed at tRFs produced by nuclearly-encoded tRNA, mitochondrially-encoded tRNAs, or both simultaneously. MINTbase is accessible at http://cm.jefferson.edu/MINTbase/. Next, we describe in detail the specific considerations that informed our design of MINTmap and the rationale behind our various design choices. As already mentioned, the sequence space of tRNAs is characterized by a number of idiosyncratic features. These features need to be taken into account explicitly when seeking to identify and quantitate tRFs in short RNA-seq datasets. Where relevant, we also describe the approaches taken by previous methods and discuss in detail the resulting complications. For clarity of presentation, we focus on *H. sapiens*. We also describe how we achieved exceptional runtime performance despite the computational demands of the task at hand.

### Design goals

We designed MINTmap with four goals in mind: (1) maximize sensitivity; (2) maximize specificity; (3) identify and report those fragments whose tRNA origin is inherently ambiguous (possible false positives); and (4) make the generated tRF expression profile adequately detailed and easy to incorporate into the users’ downstream analyses. MINTmap achieves this through an exhaustive, genome-wide search. MINTmap comprises twelve steps that are described in Methods. The provided detail should permit anyone to re-implement MINTmap at will. We note that the output is automatically saved both as a tab-separated plaintext file and as an html file (see also below). We stress that MINTmap was also designed to ensure data confidentiality. In fact, all of the data are generated locally on the user’s machine. The plaintext file allows the user to comprehensively study the mined tRFs without any need to connect to our MINTbase^1^ repository. Should the user decide to contribute his or her data to MINTbase, we make this possible through MINTsubmit an automated submission tool that is part of MINTbase and can be accessed from https://cm.jefferson.edu/MINTsubmit/.

The MINTmap codes are freely available under a GNU General Public License v3.0 from https://github.com/TJU-CMC-Org/MINTmap/.

### Establishing a tRNA reference set

Central to identifying tRFs among the transcripts of a tissue or cell of interest is the definition of the reference tRNA space, i.e. the union of genomic locations that harbor the DNA templates of tRNAs. In order to define the tRNA space, we need a comprehensive collection of tRNA genes for the genome of interest. Several groups currently maintain databases that are updated frequently and enumerate the tRNAs encoded in the nuclear or the mitochondrial genomes of various organisms; these databases include tRNAdb/mitotRNAdb^43^, tRNAdb-CE^44^, Mamit-tRNA^45^, and gtRNAdb^46^. For our work, we favor gtRNAdb because it is comprehensive and cross-links to other repositories such as NCBI Gene (http://www.ncbi.nlm.nih.gov/gene) and HGNC (http://www.genenames.org/). Specifically, we use release v1.0 of gtRNAdb^46^ and has been stable and vetted over the course of several years.

IMPORTANT: We highly recommend that the earlier release, v1.0, of gtRNAdb be used instead of the more recent release v2.0. Indeed, we have found release v2.0 to contain inconsistencies^47^ that would directly affect the quality of the reported results.

The current implementation of MINTmap is for the human genome. To allow interested users to extend the codes to other genomes as well, we have designed the approach in a very generic way and describe all its steps in detail in Methods.

We use a reference set of tRNAs that includes the following 640 tRNA sequences:

- The 508 true tRNAs and 102 pseudo-tRNAs from release v1.0 of gtRNAdb^46^. Selenocysteine tRNAs, tRNAs with undetermined anticodon identity, and tRNAs mapping to contigs that are not part of the human genome assembly are not included in the tRNA reference set.
- The 22 known human mitochondrial tRNA sequences. These are not listed among the tRNA sequences of gtRNAdb. Nonetheless, we strongly recommend the inclusion of mitochondrial tRNAs to the list of reference tRNAs. This recommendation is dictated by our recent discovery that mitochondrial tRNAs, just like their nuclearly-encoded counterparts, also produce tRNA fragments (5′-tRFs, i-tRFs, 3′-tRFs, 5′-tRHs and 3′-tRHs) with abundances that are modulated by an individual’s sex, population origin and race as well as by tissue type and disease subtype^2^. The mitochondrial tRNA sequences can be obtained from NCBI. For *H. sapiens* the relevant entry is NC_012920.1 and can be retrieved from http://www.ncbi.nlm.nih.gov/nuccore/251831106.
- The following eight genomic loci (the coordinates refer to the GRCh37 assembly of the human genome to permit references to recent publications^41^,^42^): chr1:+:566062–566129, chr1:+:568843–568912, chr1:−:564879–564950, chr1:−:566137–566205, chr14:+:32954252–32954320, chr1:−:566207–566279, chr1:−:567997–568065, and, chr5:−:93905172–93905240. We recently reported that the genomic sequences found at these eight loci are *exact* copies of seven full-length mitochondrial tRNAs: tRNA^TrpTCA^, tRNA^LysTTT^, tRNA^GlnTTG^, tRNA^AlaTGC^ (x2), tRNA^AsnGTT^, tRNA^SerTGA^, and tRNA^GluTTC 41^.

### Correct profiling of tRFs requires that all isodecoders of an isoacceptor be considered

The tRNA space is characterized by an inherent hierarchy at the top of which lay the amino acids (Fig. 2A). Many isoacceptors are grouped under a single amino acid (one distinct anticodon per isoacceptor). In turn, many isodecoders are grouped under a single anticodon whose respective DNA templates are spread across the nuclear chromosomes. For a given anticodon, the sequences of its isodecoders generally differ from one another. We highlight this last statement with an example. Figure 2B shows an alignment of the sequences of 20 isodecoders of the anticodon GTC of aspartic acid (Asp). First, we note that, even though these tRNA genes correspond to the same amino acid/anticodon combination, their sequences generally differ from one another. Second, we can recognize specific subsets of highly-similar sequences: e.g. rows 2 through 12 inclusive form one such subset; rows 13 and 14 form another subset; rows 19 and 20 form a third subset; etc. Third, we see that the same short segment can be present in various arrangements in multiple isodecoders of the same amino acid/anticodon combination: e.g. the segments highlighted in yellow, green, and dark blue in Fig. 2B are characteristic such examples.

The above examples show that it is not always possible to determine the source of a sequenced read at the tRNA gene level. For example, reads matching the dark blue subsequence, TTCCCCGACGGGGAG-*CCA* (all sequences that we use here are shown in 5′ → 3′ orientation), could be produced by any of 14 possible genomic loci. In such cases, one can only associate the respective fragment with the corresponding anticodon (GTC), but not with a specific isodecoder (tRNA gene).

Further complicating matters, a given sequence segment can be shared by tRNA genes from different anticodons of the same amino acid, or, by tRNA genes from different amino acids. Figure 2C shows several such examples. The gray segment (GGGGGTGTAGCTCAGTGGTAGAGC) is present in two isodecoders of tRNA^AlaTGC^ and in isodecoders for tRNA^AlaAGC^, tRNA^AlaCGC^, tRNA^CysGCA^, and tRNA^ValAAC^ respectively. The cyan (ACGAGGCCCCGGGTTC) and yellow (GGGGGTGTAGCTCAGTGGTAGAGCGCGTGCTT) segments are shared by two tRNA isoacceptors of Ala (tRNA^AlaAGC^ and tRNA^AlaCGC^). Finally, the green segment (GGGGGTGTAGCTCAGTGGTAGAGCA) is shared by an Ala tRNA gene (tRNA^AlaTGC^) and a Cys tRNA gene (tRNA^CysGCA^).

Another complication that arises from the potential provenance of a given sequence from multiple genomic locations within the tRNA reference set is that of “multiple counting.” These shared sequences and the corresponding genomic locations cannot be treated as independent; doing so would bias all downstream analyses as a result. Our strategy for overcoming this problem is to take a “sequence-centric” approach through extensive bookkeeping: we count each such molecule/sequence only once, no matter how many times the molecule’s sequence appears on the genome. In other words, MINTmap reports expression at the level of individual molecules/sequences. Let us look at the example of Fig. 2C: MINTmap calculates and reports expression values at the level of GGGGGTGTAGCTCAGTGGTAGAGCA (and not at the level of tRNA^AlaTGC^ and tRNA^CysGCA^) while still reporting in its output the fact that this segment can be originating from an isodecoder of either tRNA^AlaTGC^ or tRNA^CysGCA^.

This sequence-centric approach is necessary because such shared sequence segments are rather frequent in the human tRNA space, as evidenced by the examples of Fig. 2B,C. To facilitate this approach and also capture this ambiguity, we recently introduced a novel labeling scheme for tRFs that is *sequence*-centric^1^. The new scheme leads to compact tRF labels, requires no brokering service (see also: https://cm.jefferson.edu/MINTcodes/) and differs from the *genome*-centric labeling scheme we proposed originally^2^. In the next section, we describe these two labeling schemes. MINTbase^1^ uses both labeling schemes in order to provide the user with flexibility. As it is an intrinsic aim of mapping algorithms to identify the genomic origins of a sequenced read, MINTmap will generate and report *all* of the potential genomic tRNA origins of a tRF. In addition, MINTmap directly links each such fragment to its corresponding “tRF Summary Record” in MINTbase^1^ (http://cm.jefferson.edu/MINTbase/).

**Figure 3:**
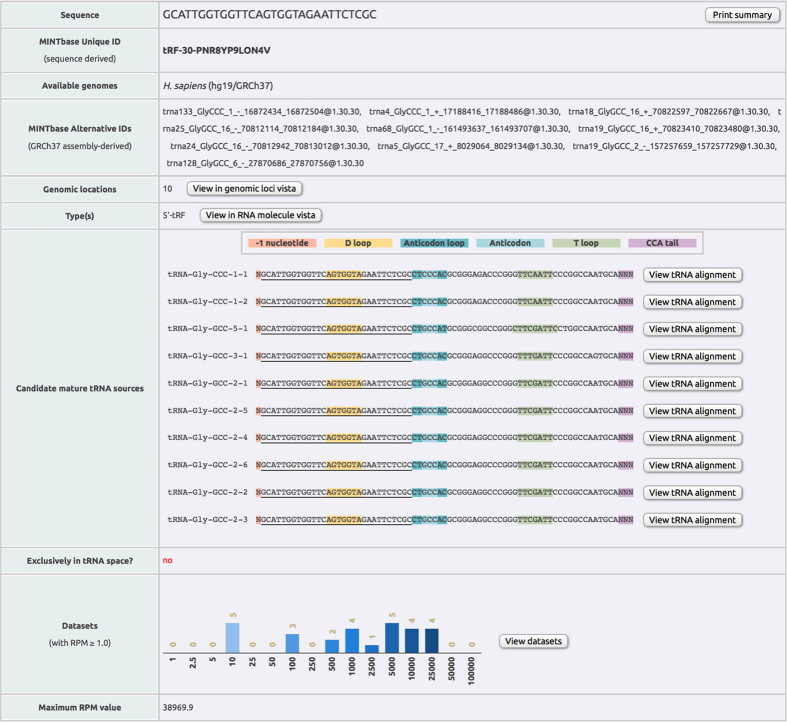
An example of a Summary Record from MINTbase (http://cm.jefferson.edu/MINTbase/). For each reported tRF, or candidate false positive tRF, the HTML files generated by MINTmap contain links to MINTbase “report cards” that summarize what is currently known for the corresponding tRF across public datasets.

Figure 3 shows an example tRF Summary record. The tRF is a 5′tRF with sequence GCATTGGTGGTTCAGTGGTAGAATTCTCGC and license plate tRF-30-PNR8YP9LON4V. This tRF is present in two isodecoders of tRNA^GlyCCC^ and eight isodecoders of tRNA^GlyGCC^ and has been found to be very abundant in more than a dozen public datasets. However, as the record indicates, it is potentially a *false positive* because this sequence also exists outside of the known tRNA space and thus could be transcribed from a locus that is unrelated to tRNAs (see the subsection named ‘The need to tag fragments that can originate inside and outside of tRNA space’ for a discussion). As mentioned above, the pattern of cleavage points of a given isodecoder, and thus the profile of tRFs it generates, changes as a function of tissue, sex, population origin, race, and disease subtype^2^. Thus, the information listed in the tRF Summary records has the potential to change as more short RNA-seq datasets are added to MINTbase.

### A “genome-centric” and a “sequence-centric” scheme for labeling tRFs

We use two complementary schemes to label tRFs. We introduced the first in our earlier work^2^ and based it on the genome assembly and the naming convention used by gtRNAdb^46^. Recently, we introduced the second scheme and based it on the actual sequence of a tRF^1^.

The genome-centric scheme augments the notation already in use by gtRNAdb. For example, to refer to a 19-nt-long i-tRF that spans positions 35 through 53 inclusive of the mature tRNA derived from the isodecoder trna5 of tRNA^AspGTC^ (“trna5-AspGTC” in gtRNAdb notation) we use trna5_AspGTC_12_+_98897281_98897352@35.53.19 as the label. The extended label also includes information about the chromosome (“12”), the strand (“+”), and the genomic location of the fragment (“98897281” through “98897352” inclusive).

The sequence-centric scheme decouples a tRF from its genomic origin and any given genome assembly while acknowledging the fact that tRFs can be found in distinct isodecoders of the same anticodon or of different anticodons (e.g. the tRFs highlighted in Fig. 2B,C). This sequence-centric scheme allows *anyone* to generate a unique label for their tRF of interest without any requirement for a brokering service such as the one that has been in use for miRNAs^48^. Importantly, since these labels are based on the tRF’s sequence, they are universally consistent and do not change with time (“persistent”). These properties give researchers at different institutions the ability to generate the same label for the same tRF without delay, to use the label as a shorthand in a manuscript, to easily compare data across publications, etc. We refer to this unique label as the tRF’s “license plate”. Any tRF can be mapped to a unique license-plate and any license plate can be mapped back unambiguously to the tRF sequence it represents. The license-plate uses a base-32 system that relies on the following alphanumeric symbols: B, D, 0, E, F, 1, H, I, 2, J, K, 3, L, M, 4, N, O, 5, P, Q, 6, R, S, 7, U, V, 8, W, X, 9, Y, Z (A, C, G, or T are not allowed symbols). As an example, tRF-16-E0PRRND is the label for ACGAGGCCCCGGGTTC, the cyan tRF of Fig. 2C. The label for the tRF GGGGGTGTAGCTCAGTGGTAGAGC would be tRF-24-RK9P4P9LH9. Detailed information on the method used to generate the license plates can be found in our previous publication^1^. The codes that a user can employ to generate license plates are freely available at https://cm.jefferson.edu/MINTcodes/. It is important to note that the algorithm for generating license plates is deterministic and entirely sequence-based. The immediate, practical implication of this is that any user can generate license plates for his/her favorite tRFs at will and without having to rely on “curation” by a centralized team. Also, because the license plate is derived from the tRF’s sequence the label will transcend changes to genome assemblies, possible changes to the nomenclature of tRNA genes, additions to the tRNA space, etc. Equally importantly, the scheme allows independent researchers to generate the exact same label for the same tRF, which will in turn ensure that labels remain consistent across publications and independently of the timing of manuscript submission.

### The need to tag fragments that can originate *inside* and *outside* of tRNA space

We now discuss in more detail the case of sequence segments that can exist both inside and outside of tRNA space (see Fig. 3 for such an example). As we demonstrate below, this phenomenon is pervasive and must be taken into account explicitly during tRF profiling.

The complexity of tRNA space and the presence of repeats in the human genome necessitates disambiguation of the potential origin of a sequenced read before it is allowed to contribute to the expression of a tRF^2^,^41^,^42^,^49^. As an example, the sequences GTTCAATTCCCTGATGGG and GTGGTAGAGCATTTGACT (in red rectangles in Fig. 2B,C respectively) are present in the shown tRNA^AspGTC^ and tRNA^CysGCA^ isodecoders but are not exclusive to tRNA space. Similarly, the sequence GCATTGGTGGTTCAGTGGTAGAATTCTCGCCT which was reported to be abundant in diseased liver^22^ exists both inside and outside of tRNA space^49^, and, thus, may not be of tRNA origin necessarily.

**Figure 4:**
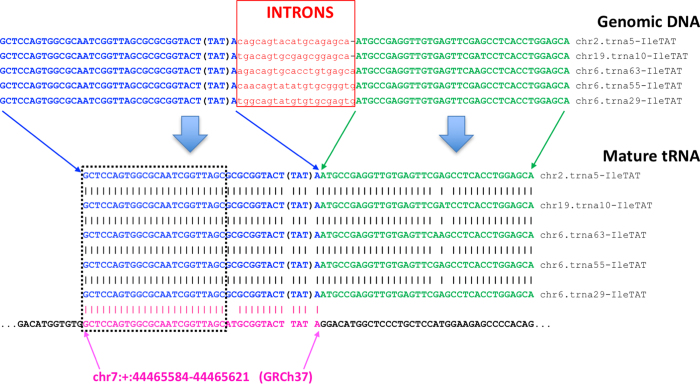
An example of an incomplete mature tRNA sequence that can be found in a genomic region outside of tRNA space. The sequence shown in magenta is present on chromosome 7 and matches the first exon of several distinct isodecoders of the intron-containing tRNA^IleTAT^. However, the second exon of tRNA^IleTAT^ is not present in the immediate vicinity of the shown sequence from chromosome 7. There are hundreds of such incomplete tRNA sequences in the human genome that need to be taken into account during tRF mapping and profiling.

The factors that complicate the analysis of tRFs in this regard and must be addressed computationally during tRF profiling include:

1. The human genome harbors numerous ***full-length*** tRNA-lookalike sequences. We recently reported that the human nuclear genome harbors numerous previously unreported sequences that resemble the known nuclear and mitochondrial tRNAs^41^. We found 497 such loci that we termed “tRNA-lookalikes.” Specifically, 351 of the 497 are lookalikes of the 22 mitochondrially-encoded tRNAs and the remaining 146 are lookalikes of the nuclearly-encoded tRNAs. The presence of lookalikes of mitochondrial tRNAs in the nuclear genome is not unique to humans: in fact, we found similarly high numbers of lookalikes in other primates and marsupials^42^, an observation that suggests the possibility of a process that is orchestrated and evolutionarily significant. Eight of the 497 lookalikes are full-length, exact copies of mitochondrial tRNAs and we have included them in the “true tRNA space” (see above). Neither the transcriptional characteristics nor the true nature of the lookalikes are known currently (e.g. they could be functioning as tRNAs, producing aberrant transcripts, or not be transcribed at all). Thus, MINTmap will tag any segments that are shared by the tRNA Reference set and the 489 (=497-8) tRNA-lookalikes as “not-exclusive to tRNA space” (Fig. 3) to alert users to the possibility that the corresponding sequence may be a false positive tRF.
2. The human genome harbors numerous ***incomplete*** tRNA sequences. The human nuclear genome also contains hundreds of incomplete tRNA sequences of variable lengths^2^,^41^,^49^. Indeed, the “tRNA” class of RepeatMasker (http://www.repeatmasker.org; Library 2014/01/31) includes 716 genomic entries each of which corresponds to sequence segments that are ≤50 nt long^49^. We illustrate this situation in Fig. 4 with the help of five isodecoders of tRNA^IleTAT^. The genomic DNA of each tRNA gene contains an intron that is excised following transcription. As can be seen from the alignment shown in this Figure, the two exons have virtually identical sequences. However, the first exon (shown in blue) contains a 24 nt segment (black rectangle) that also exists by itself in a different chromosome (chr7:+:44465584–44465621) and does not form part of a tRNA gene: indeed, the sequence after it (in 5′ → 3′ orientation) does not align with the second exon of tRNA^IleTAT^. Therefore, any sequenced reads that map exactly (i.e. without any mismatches or insertions/deletions) to this 24 nt segment have an ambiguous genomic origin, cannot be confidently declared *bona fide* tRFs, and ought to be treated as possible false positives. Reads mapping to segments that are shared by the tRNA Reference set and these *incomplete* tRNA sequences will be tagged by MINTmap as being “not-exclusive to tRNA space” (Fig. 3) to alert users to the possibility that the corresponding sequence may be a false positive tRF.

### The need to accommodate introns

The genomic DNA of 32 human tRNAs contains introns. Thus, special provisions are needed to ensure that reads can be mapped across exon-exon junctions as the resulting sequences do not exists on the genome. 31 of these introns range from 12 to 24 nt whereas one pseudo-tRNA has an intron that is 104 nt long. MINTmap incorporates additional bookkeeping to accommodate sequenced reads that correspond to tRFs spanning exon-exon junctions. Intron-spanning fragments that could exist on the genome at a location unrelated to tRNA space are flagged as candidate false positives^49^.

### The need to accommodate the non-templated CCA addition

Integral to the maturation of tRNAs is the addition of the trinucleotide “CCA” at the 3′ end of the pre-mature molecule^50^,^51^,^52^. The trinucleotide is added post-transcriptionally and is not present in the DNA sequence of the tRNA precursor. Were the sequenced reads to be mapped to an unmodified version of the human genome or to an unmodified version of the tRNA Reference set, the 3′-tRFs would not be captured accurately, especially if the mapping step did not allow mismatches (see below).

Previous studies tackled this matter by either allowing for mismatches at the 3′ end of the mapped reads, by masking these three nucleotides^53^, or by explicitly adding the trinucleotide sequence at the end of the tRNAs^54^,^55^,^56^. Of these three approaches, the first two carry the risk of introducing false positives or by making the classification of the fragment ambiguous (3′-tRF vs. i-tRF). MINTmap uses the third approach. CCA-ending fragments that could exist on the genome at a location unrelated to tRNA space are flagged as candidate false positives^49^.

### The need to accommodate the 5′ guanylation of tRNA^His^

In most eukaryotes, the tRNAs encoding the anticodon for histidine (His) require the post-transcriptional addition of a G nucleotide at their 5′ end before they can be recognized by their cognate tRNA synthetase^57^. This post-transcriptionally added nucleotide is described as the “−1 nucleotide” in the literature^58^,^59^. To properly enumerate and quantitate 5′-tRFs from tRNA^His^, MINTmap makes explicit provisions to permit sequenced reads to extend to the non-templated −1 nucleotide of the His isodecoders. Currently, tRNA^His^ is the only tRNA known to admit post-transcriptionally a 5′ addition of a nucleotide.

### Mapping exactly, deterministically, and exhaustively

Because of the highly repetitive nature of tRNA sequences and the shared sequence segments that we illustrated above (Fig. 2B,C), MINTmap maps reads exactly (no mismatches, and no insertions or deletions allowed), deterministically (no reliance on probabilistic schemes for identifying read matches), and exhaustively (looks at the full genome and enumerates all possible hits for a read).

MINTmap permits only exact matches thereby greatly improving one’s ability to disambiguate the genomic origin of a given read. A common practice encountered in mapping algorithms is to allow for a few mismatches between the sequenced read and the reference genome to accommodate the possibility of sequencing errors. However, as we demonstrated previously^2^, allowing mismatches introduces errors, can generate false positives, and complicates one’s efforts to determine a read’s genomic origin.

A characteristic example can be seen in Fig. 2C: the sequence GGGGGTGTAGCTCAGTGGTAGAGC is followed by GC in tRNA107-AlaTGC, by AC in tRNA113-AlaTGC, by AT in tRNA7-CysGCA, and by GT in tRNA115-ValAAC. If mismatches were allowed, a sequenced read that extends two nt past the end of GGGGGTGTAGCTCAGTGGTAGAGC would end up supporting a 5′-tRF from at least one wrong isodecoder.

The exhaustive character of MINTmap is complemented by its deterministic nature. Many alignment algorithms use metrics that evaluate the goodness of an alignment in its genomic context. However, such an approach is not appropriate for a class of transcripts such as the tRFs. Indeed, tRFs are very diverse in terms of length, spanning from as few as 16 nt to as many as 36 nt, or even more. Also, tRFs are very diverse in terms of their GC content, which ranges between ~4% and ~94%. Moreover, many read mappers employ seed-based matching, an approach that increases performance at the expense of sensitivity.

### How to achieve high performance

An important consideration for MINTmap is the need to analyze large datasets very speedily despite the competing requirement that the entire genome be examined in the process. MINTmap achieves its exceptional performance with the help of a lookup table that comprises all possible, distinct tRNA fragments with lengths between 16 and 50 nt. The choice of the upper limit of 50 nt is dictated by the currently available experimental evidence, and can be trivially changed to accommodate longer fragments. The lower limit of 16 nt is due to the fact that most of the 14 nt and 15 nt fragments also exist outside of tRNA space^49^ – see also http://biorxiv.org/content/early/2016/07/07/061572. The provided lookup table contains information for each tRNA fragment, whether it is found solely in tRNA space (exclusive) or also found in genomic regions outside of tRNA space (ambiguous). Given that the sequence space of tRNAs is known, we began by enumerating all possible sequence fragments that the space can generate. Then, for each such fragment, we searched the entire genome deterministically and exhaustively, recorded whether we could find an exact match outside of tRNA space, or not, and used the information to populate the table.

For the above-mentioned tRNA Reference set there can be no more than 594,972 distinct tRNA fragments with lengths between 16 and 50 nt inclusive. Once the tRNA Reference set has been defined and augmented as described in Methods (introns, CCA, −1 position in tRNA^His^), this lookup table comprising the distinct tRNA fragments can be built “*off-line*.” The lookup table need only be built once and re-used in all subsequent computations. Each of the 594,972 entries of the lookup table also contains metadata indicating whether the corresponding sequence is exclusive to the tRNA space being used.

### Methodological differences with previous methods

Several previous publications approached the profiling of tRNA fragments by mapping sequenced reads on only the space of tRNA sequences^22^,^60^,^61^,^62^. This is typically done by first creating a database that comprises tRNA sequences, downloaded from e.g. gtRNAdb or tRNAdb, and then mapping to it adapter-trimmed and quality-filtered sequenced reads. However, as we discussed above, such an approach has the potential to generate false positives, lead to a miscalculation of tRF abundances, and misrepresent the profile of the present fragments^2^,^49^ – see also http://biorxiv.org/content/early/2016/07/07/061572.

MINTmap looks across the complete genome instead, and not solely at tRNA space. This approach allows MINTmap to determine for each sequenced read whether it maps exclusively to tRNA space, or not, and to notify the user accordingly. More specifically, any tRFs that do not map exclusively to tRNA space are flagged as candidate false positives and are reported in their own separate table in the generated output. By taking this approach, MINTmap gives researchers the option to focus on fragments that originate exclusively from tRNA space, or to relax this constraint for improved sensitivity at concomitant increase of the level of noise.

### Runtime performance for the typical user

With an eye towards user-friendliness, we focused on helping the typical user, who simply wants to generate tRF expression profiles for their small RNA-seq datasets and does not generally have the time or skills to deal with a complex installation. To this end, we already executed steps 1–8 of Methods on behalf of the users (Methods), built the required lookup table, and include it in the MINTmap code bundle. What remains is for the user to apply their own preprocessing to the RNA-seq dataset at hand (step 9), and then execute steps 10–12 using the provided MINTmap codes.

With the provided MINTmap codes, a user can profile a 100-million-read short RNA-seq dataset (Illumina) and identify and report all present tRFs in under four minutes on a *single-*core 2.70 GHz Intel Xeon CPU E5-4650. If color-space reads are used, the time required to execute steps 10–12 will be a function of the third party aligner, e.g. Shrimp^63^ or BWA^64^, used to execute the mapping step (see Methods for details).

### The generated output

The output of MINTmap consists of two tables each listing the expression profiles of identified tRFs. The first table comprises sequences that are exclusive to tRNA space, and, thus, *bona fide* tRFs. The second table comprises sequences that, at the genome level, exist both inside and outside of tRNA space, and, thus, are *candidate false positives*.

In terms of format, each of the two tables is provided as (a) plaintext, and, (b) HTML (four output tables in total). The plaintext, tab-separated format allows for easy file manipulations and software import for downstream analyses (see also “Design goals” above for a discussion on data confidentiality). Table 1 shows the first few lines of the two HTML tables (*bona fide* tRFs and *candidate false positives*, respectively) that MINTmap generates for the dataset NA06986.6.MI. This dataset was described in an earlier report^65^ by the 1000 Genomes Project. Each HTML-based table contains all of the information found in the plaintext version plus an extra column with hyperlinks to the respective MINTbase^1^ record (http://cm.jefferson.edu/MINTbase) for each reported tRF (see Fig. 3). We designed and implemented MINTbase to provide users with the capability to interactively explore the tRF space while also serving as a knowledge repository for tRFs in the public domain.

### Comparisons between MINTmap and other tRF profiling methods

We compared MINTmap with tRFdb^54^, tDRmapper^55^, and tRFfinder^66^. Table 2 summarizes the attributes of each scheme. Our quantitative findings are summarized in Supp. Table 1. Methodological details about carrying out the comparisons are detailed in Methods. To compare the four schemes we used the human cell line short RNA-seq datasets under accession number GSE16579^67^ of the NCBI Gene Expression Omnibus (GEO) repository. The choice of this particular collection was dictated by the fact that both tRFdb (which contains only pre-computed data–no codes are available) and tRFfinder have reported results on these cell lines in their original analyses^54^,^66^. While there are 10 human cell line datasets listed under GSE16579, we used only nine of them: we excluded HEK293 because the primary data for it is not available on GEO.

In all comparisons involving tRFdb, we distinguish between the *tRFdb-AllAligned* collection of tRFs (see Methods) and the *tRFdb-withID* collection of tRFs. Only fragments that are supported by reads matching exactly are used for these comparisons. Also, only tRFs that arise from mature tRNA sequences are used for these comparisons. All of the results are listed in Supp. Table 1. Note, however, that the Venn diagrams that we discuss next do *not* include tRFfinder because tRFfinder reports only six tRFs using its default settings, i.e. it has a very low sensitivity compared to the other three schemes.

We distinguish between fragments that are ≤15 nt in length and fragments that are ≥16 nt. As we discussed above and demonstrated recently^49^, 92.4% of all 14-mers and 79.0% of all 15-mers that one can form out of mature tRNA sequences have exact genomic copies outside of tRNA space. As an example, we mention the 15-mer tRF from tRNA^LeuTAA^ with sequence ACCCCACTCCTGGTA: it has 275 exact genomic copies outside of tRNA space. Thus, the inclusion of 14-mer and 15-mer tRFs will increase the noise in the output (see also Supp. Table 1 of http://biorxiv.org/content/early/2016/07/07/061572). Allowing for indels/replacements during the mapping of reads further exacerbates the problem. We note that both tDRmapper and tRFdb-AllAligned report 14-mer and 15-mer tRFs, including ACCCCACTCCTGGTA. To not affect the tool comparisons by this prominent noise source, we used only tRFs that are ≥16 nt in length.

#### Sensitivity

**Figure 5:**
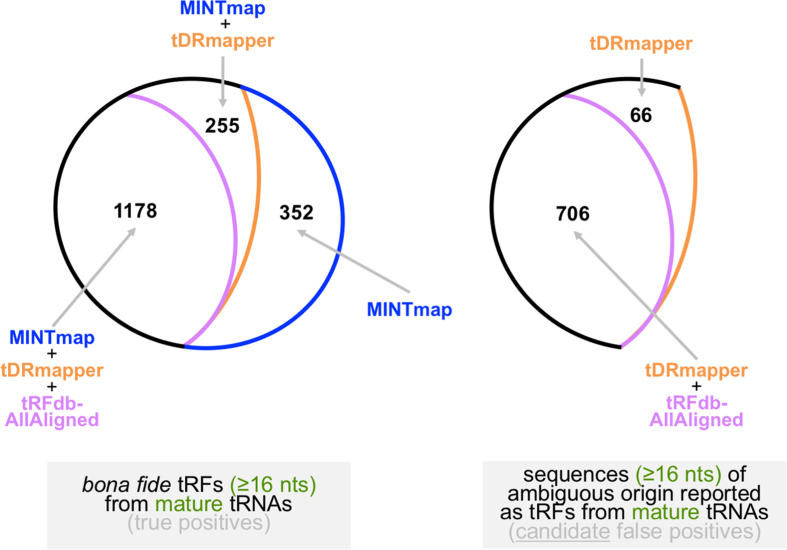
Comparison of the sensitivity and specificity attributes of MINTmap, tDRmapper, and tRFdb. The three methods were evaluated using nine public short RNA-seq datasets from human cell lines–see also text. Left: overlap of the output of each approach when focusing only on bona fide tRFs from mature tRNAs. Right: overlap of the output of tDRmapper and tRFdb when focusing only on reported sequences that exist identically inside as well as outside tRNA space.

As can be seen from the left Venn diagram of Fig. 5, there is a core group of 1,178 *bona fide* mature-tRNA-derived tRFs (≥16 nt) across the nine cell lines that are correctly identified by all three tools, i.e. MINTmap, tRFdb and tDRmapper. These are tRFs that have exact matches in tRNA space and no exact matches outside tRNA space. For tRFdb, we used the tRFdb-AllAligned collection in the Venn diagrams, since tRFdb-AllAligned maximizes tRFdb’s sensitivity and the overlap with the other two schemes. MINTmap and tDRmapper can jointly identify an *additional* 255 tRFs that are not predicted by tRFdb. Moreover, MINTmap can identify a further 352 tRFs that neither tDRmapper nor tRFdb report: 253 of these arise exclusively from mitochondrial tRNAs; 92 arise from either mitochondrial tRNAs or their eight *exact* copies that are present in the human nuclear genome (see discussion above); the remaining 7 are 5′-tRFs from the nuclearly-encoded tRNA^His^. Note: tDRmapper and tRFdb also report a combined total of 395 fragments that are either 5′-trailer or 3′-trailer tRFs and originate from the precursor tRNA whereas MINTmap restricts its analysis to only tRFs that overlap the mature tRNA sequence.

#### Specificity

To determine specificity, we focus on fragments (≥16 nt) whose exact genomic matches occur both in mature tRNA space and in loci that are either partial tRNA sequences (e.g. see Fig. 4), or non-tRNAs. Because of this provenance ambiguity we refer to such fragments as *candidate* false positives.

By design, MINTmap explicitly flags in its output those tRFs that are candidate false positives. The right Venn diagram of Fig. 5 shows the performance of tDRmapper and tRFdb with respect to *candidate* false positives. As can be seen, more than 30% of the sequences that would be reported as tRFs by tDRmapper and tRFdb, at a threshold of RPM ≥5 and using exact matching only, appear identically within mature tRNAs as well as at genomic loci that are *not* tRNAs. For comparison purposes, we mention that tDRmapper and tRFdb report 382 tRFs from mature tRNAs that are 14-mers or 15-mers, which is a high number in proportion to the 1178 *bona fide* tRFs found by all three schemes. As we explained above, 14-mers and 15-mers are nearly ubiquitous outside of tRNA space; thus, it follows that simple removal of 14-mers and 15-mers from the generated output will amount to a very considerable boost in the effective specificity of both tDRmapper and tRFdb.

#### Speed

The nine short RNA-seq datasets we analyzed also provided an opportunity to compare MINTmap’s runtime performance to that of tDRmapper, for which codes are available. For the abundance threshold set to RPM ≥5, MINTbase is ~10 times faster. Additional experiments using lower RPM thresholds showed an even higher speed advantage for MINTmap, a direct consequence of MINTmap’s use of a lookup table for processing. Of the other two tools, tRFfinder provides a web-based job-queuing system so a direct speed comparison is not feasible. Nonetheless, for the nine short RNA-seq datasets discussed here, results were returned within a few hours. tRFdb does not provide a way to profile individual datasets and, thus, runtime speed statistics cannot be obtained.

##### Optional Step. building the entire pipeline

A user would not need to perform Methods steps 1–8 (see Methods) from scratch *unless* he/she needed to replicate the pipeline for another organism, for a different genome assembly, or for a different tRNA Reference set. In such an event, the time requirements would be as follows:

- Steps 1–7 (preparatory stage): these depend on the skill of the bioinformatician and would require ~4 hours end-to-end to execute.

- Step 8 (actual building of the lookup table): this step would require an estimated ~9600 CPU-core-hours, or 2.5 days using 160 compute cores (2.70 GHz Intel Xeon CPU E5-4650).

Step 9 is not MINTmap-specific; rather it depends on the library preparation of the biological sample and would require ~45 minutes.

## Discussion

We presented MINTmap, a fast and exhaustive software resource for profiling tRFs that may be present in short RNA-seq datasets. We expect that practitioners who are interested in studying tRFs using deep-sequencing approaches will find MINTmap to be useful and very flexible. In fact, we sought to enable individual users who do not have the time or the skills needed for an involved software installation. Consequently, the software that we generated and make available can be run standalone on any modern laptop or workstation and is exceptionally fast: it can analyze 100 million adapter-cut-and-quality-trimmed Illumina reads and report the identified tRFs and their abundances in under four minutes. In addition, in this presentation, we provided a very detailed description of all of MINTmap’s steps to enable users who might be interested in a site-specific implementation of our approach. We have already used MINTmap to analyze and publish tRF profiles from 843 public short RNA-seq datasets. We made these tRF profiles publicly available through a companion web-based resource, MINTbase^1^. MINTbase allows users to interactively interrogate and study these tRF profiles across isodecoders, isoacceptors, tissues, diseases, etc. On a related note, MINTbase allows a user to directly submit over the web (https://cm.jefferson.edu/MINTsubmit/) the output of MINTmap on his/her short RNA-seq datasets, thereby contributing the user’s respective tRF profiles to this public resource.

## Methods

### Equipment

#### Personal computer with an Internet connection

A personal computer (either a desktop or a laptop) will be powerful enough to execute all steps of MINTmap and analyze datasets with one hundred million reads or more. We recommend a system with at least 2 Gb of main memory powered by at least 1 CPU Intel Core i5/i7, or equivalent.

#### Compute server

For step 8 of MINTmap, we recommend using a multi-core compute setup (compute time *decreases* in proportion to the number of processing cores). We recommend a compute server with a minimum of 16-cores Intel Xeon, Intel Core i5/i7, or equivalent, with at least 8 Gb of main memory. Nonetheless, it is possible to run step 8 on a personal computer such as the one recommended above: to do so, step 8 needs to be run separately on each chromosome (see below). For the human genome, this would amount to a total of 25 executions of step 8.

### Data and other downloads

An Internet connection is required to access the tRNA Reference set (if not using the one in Supp. Table 2). Access to tools such as SAMTools (http://samtools.sourceforge.net/) may also be desired for some of the steps.

### The generated output

MINTmap generates its output as both a plain text file and an HTML file. The textual output can be viewed using any standard text editor. The HTML output can be viewed using a web browser; all popular web browsers (Internet Explorer, Firefox, Chrome, and Safari) are currently supported.

### The 12 steps of MINTmap in detail

**Figure 6:**
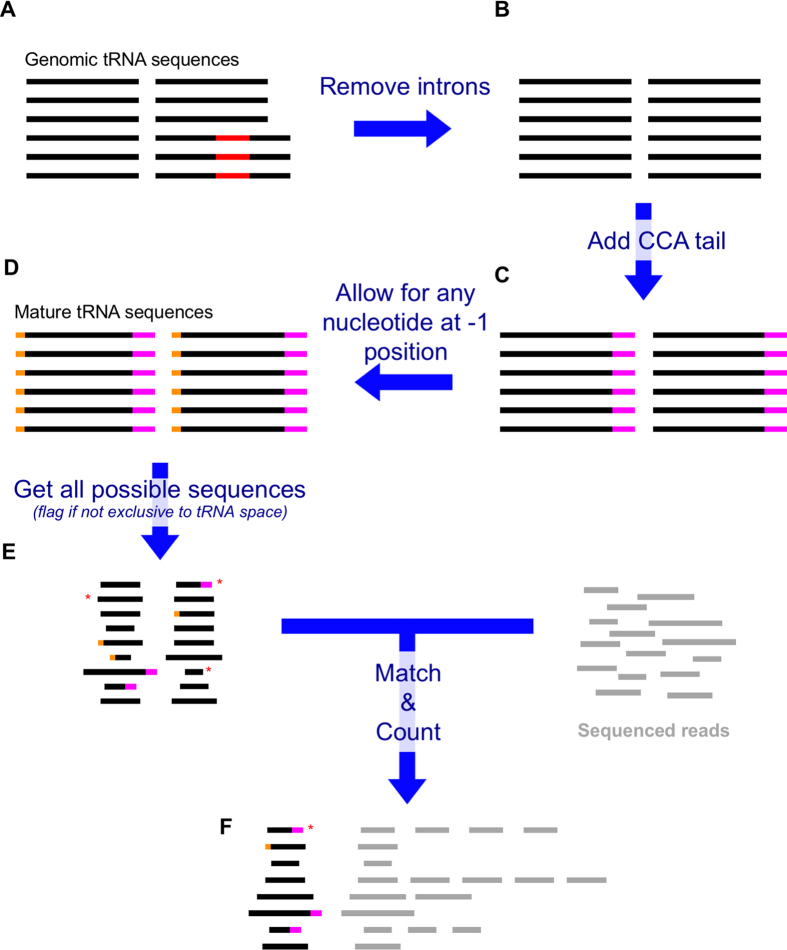
Flowchart of MINTmap. Genomic sequences of the tRNA reference set (**A**) are processed to simulate exon splicing (**B**), and then get modified to admit the non-templated CCA addition (**C**) and the “−1” nucleotide of tRNA^His^ (**D**). The resulting sequences are fragmented computationally into (overlapping) segments of variable lengths and entered into a lookup table: sequences that are not exclusive to tRNA space are flagged at this point using metadata added to the table. The lookup table is then used to process a (quality-filtered and adapter-trimmed) short RNA-seq dataset (**E**) to generate a tRF expression profile table (**F**). Red: introns. Magenta: CCA tail. Orange: nucleotide at -1 position. Asterisk: tRF not exclusive to tRNA space (possible false positive tRF).

To achieve its speed MINTmap makes use of a lookup table. To make it easier for users to install and run MINTmap with minimal requirements for setup, we provide a pre-generated lookup table for *H. sapiens* as part of the code distribution. It is conceivable that some users might desire to create their own instances of MINTmap; thus, we outline the steps required to build the table below (see also panels A through E of Fig. 6). We note that the table is generated after the tRNA Reference set is established.[Fig f7]

**Steps 1–8: create the lookup table**

**1. Initial tRNA sequence collection:** Create a starting collection of the unspliced genomic sequences that encode tRNA isodecoders, in 5′ → 3′ orientation. This collection is referred to as the ‘tRNA Reference set’ and for the human genome comprises 640 sequences: true tRNAs, pseudo-tRNAs, mitochondrial tRNAs, and exact tRNA-lookalikes (Supp. Table 2 and Fig. 6A).

**2. Accommodate exon-exon junctions:** Using the above sequence list, remove any intronic sequences so that only the tRNAs’ spliced sequences remain (Fig. 6B).

**3. Accommodate non-templated CCA addition:** Extend each tRNA sequence by appending the CCA trinucleotide (Fig. 6C).

**4. Accommodate post-transcriptional modifications at the −1 position:** Make 4 additional copies of each sequence, extending the 5′ end to the left by 1 nt with an A, T, C, and G respectively (Fig. 6D).

**5. Generate the list of all potential tRF sequences:** For each one of the above sequences, enumerate all possible substrings with lengths between 16 and 50 nt inclusive, independently of whether there is already available experimental evidence for the respective fragment or not. Add each one of these substrings to a new list called the ‘tRF sequence candidate pool’ and remove any duplicate entries before proceeding (Fig. 6E and Supp. Table 3, column C).

**6. Prepare the whole genome search space:** Put the sequences of the forward and reverse strands of each chromosome on consecutive lines of a new text file–no labels are necessary. For the human genome there will be a total of 50 lines: two lines for each of chromosomes 1–22, X, and Y and the mitochondrial (MT) genome.

CAUTION: All sequences, including those corresponding to the negative strand, must be listed in 5′ → 3′ orientation.

**Figure 7 f7:**
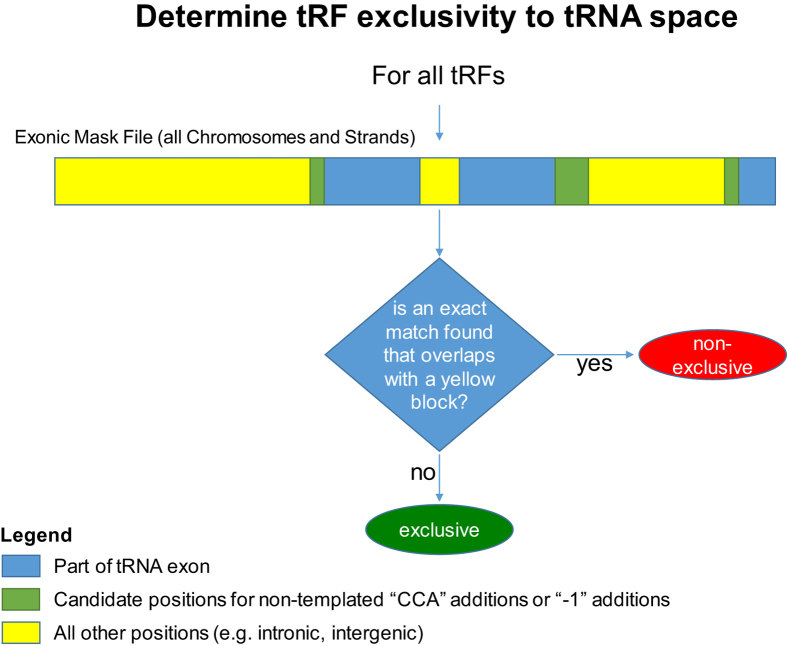
For each tRF, we determine whether it is exclusive to tRNA space. The exonic mask file (step 7 of the scheme) should contain a value of 1 (shown in blue) for positions representing tRNA exons, a value of 2 (shown in green) for positions representing tRNA −1/CCA post-transcriptional modifications, or a value of 0 (shown in yellow) for all other positions.

**7. Mark tRNA positions within the chromosome (Fig. 7)**: For each of the 50 sequences above, create an identically-sized corresponding file, internally referred to as “exonic mask file.” Begin by replacing each and every position of the exonic mask file by a ‘0.’ Next, enumerate all exons of the tRNA Reference, go to the matching chromosome, strand, and genomic coordinates in the exonic mask file and mark each of the corresponding positions with a ‘1.’ For every genomic positions that would correspond to a “−1” or “CCA” post-transcriptional addition mark the corresponding position(s) in the exonic mask file with a ‘2.’ Upon completion, in the exonic mask file, tRNA exons (blue regions in Fig. 7) will have been replaced by trails of 1’s, positions corresponding to post-transcriptional additions will have been replaced by one or more 2’s (green regions in Fig. 7), and all other positions (yellow regions in Fig. 7) will have been replaced by trails of 0’s.

CRITICAL STEP: When marking exonic positions, make sure that the correct chromosomal strand sequence gets marked with 1’s. Failure to do so will over-represent the tRNA exons.

**8. Determine exclusivity to tRNA space, or lack thereof:** Check each candidate tRF sequence from the list that was created during step 5 above and identify *all* of its exact-matches within the search space created in step 6. In turn for each exact-match that is found, examine the corresponding span of the match in the exonic mask file (from step 7). If any of the positions within the span for the exact-match being examined contains a ‘0,’ conclude that the candidate tRF is *non-exclusiv*e to mature tRNA space, terminate the search for this candidate tRF, and proceed to the next candidate tRF. If none of the positions within the span for the exact-match being examined contains a ‘0,’ continue with the next match for this candidate tRF. If upon investigation of all exact matches for the tRF being examined no positions are found that contain a ‘0,’ conclude that this sequence is exclusive to mature tRNA space and thus a *bona fide* tRF if transcribed (Fig. 6 and Supp. Table 3, column D).

CRITICAL STEP: A tool that performs an exhaustive and deterministic string search should be used for this stage.

OPTIONAL STEP: including information about tRF categories. The codes we provide currently include a second table that lists the structural category to which a tRF belongs and is used to populate the tables that are produced by MINTmap. Users who want to build their own version of MINTmap should also create this second table to reflect the structural categories for their tRF sequence collection. However, this second table is not necessary: if users do not provide it, MINTmap will still run correctly but will not report structural type information for the discovered tRFs.

CLARIFICATION: In the event that the available computational platform is a personal computer, steps 6 and 7 should be modified to create one file per chromosome (for a total of 25 files in the human genome); each file should contain two lines, one for the forward and one for the reverse strand of the corresponding chromosome. Then, during step 8, each of the candidate tRF sequences should be examined separately in each of the chromosome files before it can be determined whether it is indeed exclusive to tRNA space.

**Steps 9–10: generate raw and normalized expression values**

**9**. **Preprocess your reads.** For each short RNA-seq dataset of interest, perform the desired quality filtering and adapter trimming.

CAUTION: Any remaining adapter sequences must be removed from the reads prior to proceeding to the next step. The adapter sequences that will need to be removed depend on the library preparation for the corresponding biological sample.

**10. Generate table of tRF abundances.** This is the step where the actual tRF profiling takes place. This step should be performed using the option that is appropriate for the short RNA-seq dataset at hand.

If the short RNA-seq datase to be analyzed **does not** contain color space reads, execute:

- A1. Process the list of NGS read-sequences that resulted from step 9 and keep only those that exist in the lookup table that was generated above (Fig. 6E and column C of Supp. Table 3).
- B1. For each NGS read-sequence that was obtained in 10.A1, generate molecule-level expression information by counting the frequency of the sequence (Fig. 6F) in the RNA-seq dataset from step 9. The resulting table is referred to as the ‘count table’. Optionally, sort the count table in order of decreasing abundance. We suggest, and our provided codes already implement this, that normalization in reads-per-million (RPM) be computed in two different ways: (i) an RPM value computed using as a denominator the counts of reads that map either exclusively or non-exclusively to tRNA space; and, (ii) an RPM value computed using as a denominator the count of reads from the FASTQ file that is being processed)–see Table 1 for an example.

If the short RNA-seq dataset to be analyzed **does** contain color space reads, execute:

- A2. First, you must map the sequenced reads at hand using a mapping tool of your choosing that can handle color-space reads. Allow for non-unique mappings and map the reads to all modified tRNA sequences obtained in step 4 above (lookup-table creation). Only map in the 5′ → 3′ orientation relative to each tRNA.CAUTION: It is important that one be exhaustive during this step, so we recommend using mappers with the highest sensitivity possible.
- B2. From the collection of mapped reads from 10.A2 only keep those reads that could be mapped with no mismatches, no insertions, no deletions, and no crossovers, and create “(NGS read-label, NGS read-sequence)” pairs. Sort the formed list of pairs, and identify and remove any duplicates that may be present. From the resulting list keep only those of the (NGS read-label, NGS read-sequence) pairs for which the NGS read-sequence exists in the lookup table that was generated above (Fig. 6E and column C of Supp. Table 3). In this final list count the frequency of each distinct NGS read-sequence. The resulting table is referred as the ‘count table’ (Fig. 6F). Optionally, sort the count table in order of decreasing abundance.CRITICAL STEP: During mapping, a given sequenced read may be found in multiple tRNA genes: the read and its associated NGS read-label will be reported once for each of these hits. By removing duplicate (NGS read-label, NGS read-sequence) pairs we ensure that we do not misrepresent the abundance of the molecule represented by the NGS read-sequence.

**Steps 11–12: flag candidate false positives, link to metadata, and report**

**11. Account for exclusivity to tRNA space**: Use the lookup table’s exclusivity column (Supp. Table 3, column D) to split the count table, which was generated during either step 10.B1 or step 10.B2, into two distinct files. The first file will contain fragments whose sequences are present exclusively in the tRNA space, and nowhere else on the genome: these are *bona fide* tRFs. The second file will contain fragments whose sequences are present both inside and outside of tRNA space: these are possible false positive tRFs and we recommend that they be treated as such. Optionally, add a column in each of the two output files that normalizes the reported counts by, e.g., scaling them to an RPM value (see above).

**12. Associate each fragment in the output with relevant meta-data:** Add genomic information and other meta-data to each of the reported tRFs. The codes that we provide already accomplish this by providing the following information (see Table 1 for a detailed example): number of reads mapping to the respective tRF; RPM values computed in three different ways, one of which is user-defined; a hyperlink to the respective tRF’s Summary Record in MINTbase^1^ (Fig. 3 shows an example of such a record); information about the isodecoder(s) from which the tRF could be arising, starting and ending location within the isodecoder(s), and chromosome, strand, and genomic coordinates of the isodecoder(s).

### Troubleshooting MINTmap

In Table 3, we provide detailed information that will help users troubleshoot problems that they may encounter during execution of the codes. For some of the above-described steps, the table contains multiple troubleshooting entries.

### How the comparisons between the various approached were run

We generated results from comparing MINTmap, tRFdb^54^, tDRmapper^55^, and tRFfinder^66^. We note that only MINTmap and tDRmapper are available as downloadable tools that can be used to profile individual datasets. tRFfinder provides a web-interface for users to upload pre-processed data that can then be analyzed. tRFdb does not allow users to profile custom datasets. MINTmap, tRFFinder^66^, and tDRmapper^55^ were ran using their default settings.

The schemes were evaluated using the datasets listed under GSE16579^67^ in the NCBI Gene Expression Omnibus (GEO) repository. To facilitate comparisons, we enforced a normalized threshold of RPM ≥5 across all schemes. Because tRFdb contains only pre-computed data, we downloaded from their website the tRNA alignments for the datasets of GSE16579 and sub-selected only the tRFs that satisfied the RPM ≥5 threshold.

A tRF would enter our analysis (Supp. Table 1) if and only if

- it had a length ≥16 nt
- it was reported by *at least one* of the 4 schemes;
- it was detected in any of the nine datasets above at the enforced RPM threshold; and,
- it could be mapped to the genome exactly, i.e. without permitting any indels or replacements.

(Step d) is necessary because MINTbase, tRFdb, and tRFfinder (in its default settings) report only exactly-matching tRFs. Moreover, as we showed in our previous work^2^,^49^, mismatches produce erroneous results. Lastly, because tRFdb comprises tRFs that are shown in their “alignment view” but are not included in the tRFs to which tRFdb assigned unique identifiers, we reported statistics using two tRF collections: “tRFdb-AllAligned” is those tRFs from tRFdb that are listed in the reported alignments; “tRFdb-withID” is those tRFs from tRFdb that have been assigned a unique tRFdb identifier.

### Data availability

The available codes are written in Perl and allow a user to process a short RNA-seq dataset that has already been adapter-trimmed and quality-trimmed. MINTmap runs under Linux and OSX and requires Perl and Java. To facilitate use of the codes, we pre-generated and make available the lookup table for the human genome. Optionally, as noted in the help file that accompanies the codes, a custom user-generated lookup table can be employed instead. The output will comprise the identified tRFs and their respective abundances (both raw and normalized). The output distinguishes between tRFs that are exclusive to tRNA space, and tRFs that occur inside and outside of tRNA space. The MINTmap codes are freely available under a GNU General Public License v3.0 from https://github.com/TJU-CMC-Org/MINTmap/. We expect to be releasing updated lookup tables and code-updates for MINTmap at regular intervals. The current implementation of MINTmap is for the human genome. MINTmap has been designed very generically, which should allow interested users to easily adapt the codes to other genomes as well.

## Additional Information

**How to cite this article:** Loher, P. *et al*. MINTmap: fast and exhaustive profiling of nuclear and mitochondrial tRNA fragments from short RNA-seq data. *Sci. Rep.* **7**, 41184; doi: 10.1038/srep41184 (2017).

**Publisher's note:** Springer Nature remains neutral with regard to jurisdictional claims in published maps and institutional affiliations.

## Supplementary Material

Supplementary Information

## Figures and Tables

**Table 1:** The first few lines from the HTML output files following a run of MINTmap on the dataset NA06986.6.MI originally reported in an earlier study^65^ from the 1000 Genomes Project and which we analyzed recently^2^.

**A. Example output table (partial) of tRFs that are exclusive to tRNA space**							
GTTTCCGTAGTGTAGTGGTCATCACGTTCGCCT	5′-half	19032	87751.98	2381.56	na	Summary^a^	trna15_ValAAC_5_−_180615416_180615488@1.33.33
GCTTCTGTAGTGTAGTGGTTATCACGTTCGCCT	5′-half	11357	52364.40	1421.15	na	Summary^b^	trna152_ValCAC_6_−_27248049_27248121@1.33.33
TTGGTCGTGGTTGTAGTCCGTGCGAGAATACCA	3′-tRF	8214	37872.78	1027.86	na	Summary^c^	trnalookalike8_GluTTC_5_−_93905172_93905240@40.72.33, trnaMT_GluTTC_MT_−_14674_14742@40.72.33
GAGAAAGCTCACAAGAACTG	5′-half	8031	37029.01	1004.96	na	Summary^d^	trnaMT_SerGCT_MT_+_12207_12265@1.20.20
ATTGGTCGTGGTTGTAGTCCGTGCGAGAATACCA	3′-tRF	7129	32870.11	892.08	na	Summary^3^	trnalookalike8_GluTTC_5_−_93905172_93905240@39.72.34, trnaMT_GluTTC_MT_−_14674_14742@39.72.34
GAGAAAGCTCACAAGAACTGCTAACT	5′-tRF	5563	25649.66	696.12	na	Summary^f^	trnaMT_SerGCT_MT_+_12207_12265@1.26.26
GCATGGGTGGTTCAGTGGTAGAATTCTCGCCT	5′-half	4666	21513.80	583.88	na	Summary^g^	trna39_GlyGCC_1_+_161427898_161427968@1.32.32, trna37_GlyGCC_1_+_161420467_161420537@1.32.32, trna35_GlyGCC_1_+_161413094_161413164@1.32.32, trna41_GlyGCC_1_+_161435258_161435328@1.32.32, trna2_GlyGCC_21_−_18827107_18827177@1.32.32
**B. Example output table (partial) of tRFs that are not exclusive to tRNA space**							
GTTTCCGTAGTGTAGTGGTTATCACGTTCGCCT	5′-half	213191	440613.83	26677.56	na	Summary^h^	trna2_ValAAC_3_+_169490018_169490090@1.33.33, trna90_ValCAC_1_−_149684088_149684161@1.33.33, trna139_ValAAC_6_−_27618707_27618779@1.33.33, trna9_ValCAC_6_+_26538282_26538354@1.33.33, trna85_ValCAC_1_−_161369490_161369562@1.33.33, trna10_ValCAC_5_−_180649395_180649467@1.33.33, trna18_ValCAC_5_−_180529253_180529325@1.33.33, trna132_ValAAC_6_−_27721179_27721251@1.33.33, trna136_ValAAC_6_−_27648885_27648957@1.33.33, trna12_ValAAC_5_−_180645270_180645342@1.33.33, trna5_ValAAC_5_+_180596610_180596682@1.33.33, trna2_ValCAC_5_+_180524070_180524142@1.33.33, trna98_ValCAC_1_−_149298555_149298627@1.33.33, trna4_ValAAC_5_+_180591154_180591226@1.33.33, trna6_ValCAC_5_+_180600650_180600722@1.33.33
ATCCCGGACGAGCCCCCA	3′-tRF	78216	161653.40	9787.53	na	Summary^i^	trna12_ProAGG_6_+_26555498_26555569@58.75.18, trna4_ProAGG_16_+_3210386_3210480@65.82.18, trna3_ProTGG_14_+_21101165_21101236@58.75.18, trna9_ProAGG_16_+_3239634_3239705@58.75.18, trna23_ProAGG_14_−_21077495_21077566@58.75.18, trna14_ProTGG_5_−_180615854_180615925@58.75.18, trna30_ProCGG_6_+_27059521_27059592@58.75.18, trna65_ProAGG_1_−_167684725_167684796@58.75.18, trna8_ProTGG_16_+_3238094_3238165@58.75.18, trna2_ProAGG_7_+_128423504_128423575@58.75.18, trna37_ProCGG_17_−_8126151_8126222@58.75.18, trna12_ProTGG_11_−_75946869_75946940@58.75.18, trna6_ProCGG_16_+_3222049_3222120@58.75.18, trna22_ProAGG_14_−_21081560_21081631@58.75.18, trna52_ProCGG_1_+_167683962_167684033@58.75.18, trna28_ProTGG_16_−_3234133_3234204@58.75.18, trna9_ProAGG_11_+_75946557_75946628@58.75.18, trna29_ProAGG_16_−_3232635_3232706@58.75.18, trna3_ProTGG_16_+_3208923_3208994@58.75.18, trna6_ProTGG_14_+_21152175_21152246@58.75.18, trna11_ProAGG_16_+_3241989_3242060@58.75.18
GCATTGGTGGTTCAGTGGTAGAATTCTCGCCT	5′-half	35136	72617.55	4396.73	na	Summary^j^	trna19_GlyGCC_2_−_157257659_157257729@1.32.32, trna68_GlyGCC_1_−_161493637_161493707@1.32.32, trna5_GlyGCC_17_+_8029064_8029134@1.32.32, trna133_GlyCCC_1_−_16872434_16872504@1.32.32, trna24_GlyGCC_16_−_70812942_70813012@1.32.32, trna18_GlyGCC_16_+_70822597_70822667@1.32.32, trna4_GlyCCC_1_+_17188416_17188486@1.32.32, trna25_GlyGCC_16_−_70812114_70812184@1.32.32, trna128_GlyGCC_6_−_27870686_27870756@1.32.32, trna19_GlyGCC_16_+_70823410_70823480@1.32.32
GTTTCCGTAGTGTAGTGGTTATCACGTTCGCCTC	5′-half	17720	36622.92	2217.38	na	Summary^k^	trna90_ValCAC_1_−_149684088_149684161@1.34.34, trna9_ValCAC_6_+_26538282_26538354@1.34.34, trna85_ValCAC_1_−_161369490_161369562@1.34.34, trna10_ValCAC_5_−_180649395_180649467@1.34.34, trna18_ValCAC_5_−_180529253_180529325@1.34.34, trna2_ValCAC_5_+_180524070_180524142@1.34.34, trna98_ValCAC_1_−_149298555_149298627@1.34.34, trna6_ValCAC_5_+_180600650_180600722@1.34.34
AGTAAGGTCAGCTAAATAAGCTATCGGGCCC	5′-half	14173	29292.14	1773.53	na	Summary^l^	trnaMT_MetCAT_MT_+_4402_4469@1.31.31

(A): table for tRFs that are exclusive to tRNA space. (B): table for tRFs that exist both inside and outside of tRNA space. In both cases, the columns have as follows. Column 1: tRF sequence. Column 2: tRF type(s) possible for the tRF sequence. Column 3: count of reads mapping exactly (no insertions/deletions, no replacements) to shown fragment. Column 4: RPM read count computed using as a denominator the counts of reads that map either exclusively or non-exclusively to tRNA space. Column 5: RPM read count computed using as a denominator the count of reads from the corresponding FASTQ file. Column 6: RPM read count computed using as a denominator an optional user-provided count. Column 7: a link to the tRF’s “Summary Record” in MINTbase (http://cm.jefferson.edu/MINTbase) – because MINTbase already contains a record for each of the nearly 600,000 possible human tRFs of lengths 16 to 50 nt inclusive these links always point to a valid MINTbase record for human-genome derived RNA-seq datasets. Column 8: a comma-delimited list of the locations within known isodecoders from which the tRF could be arising. This column makes use of our genome-centric labeling scheme to make it convenient for the user to visually process the data (see the ‘scheme for labeling tRFs’ section for a summary of this labeling scheme). In the next release of the MINTmap codes, the generated output will include one more column that will be listing the license plate of the corresponding tRF. ^a^https://cm.jefferson.edu/MINTbase/InputController?g=GRCh37&v=s&fs=GTTTCCGTAGTGTAGTGGTCATCACGTTCGCCT. ^b^https://cm.jefferson.edu/MINTbase/InputController?g=GRCh37&v=s&fs=GCTTCTGTAGTGTAGTGGTTATCACGTTCGCCT. ^c^https://cm.jefferson.edu/MINTbase/InputController?g=GRCh37&v=s&fs=TTGGTCGTGGTTGTAGTCCGTGCGAGAATACCA. ^d^https://cm.jefferson.edu/MINTbase/InputController?g=GRCh37&v=s&fs=GAGAAAGCTCACAAGAACTG. ^e^https://cm.jefferson.edu/MINTbase/InputController?g=GRCh37&v=s&fs=ATTGGTCGTGGTTGTAGTCCGTGCGAGAATACCA. ^f^https://cm.jefferson.edu/MINTbase/InputController?g=GRCh37&v=s&fs=GAGAAAGCTCACAAGAACTGCTAACT. ^g^https://cm.jefferson.edu/MINTbase/InputController?g=GRCh37&v=s&fs=GCATGGGTGGTTCAGTGGTAGAATTCTCGCCT. ^h^https://cm.jefferson.edu/MINTbase/InputController?g=GRCh37&v=s&fs=GTTTCCGTAGTGTAGTGGTTATCACGTTCGCCT. ^i^https://cm.jefferson.edu/MINTbase/InputController?g=GRCh37&v=s&fs=ATCCCGGACGAGCCCCCA. ^j^https://cm.jefferson.edu/MINTbase/InputController?g=GRCh37&v=s&fs=GCATTGGTGGTTCAGTGGTAGAATTCTCGCCT. ^k^https://cm.jefferson.edu/MINTbase/InputController?g=GRCh37&v=s&fs=GTTTCCGTAGTGTAGTGGTTATCACGTTCGCCTC. ^l^https://cm.jefferson.edu/MINTbase/InputController?g=GRCh37&v=s&fs=AGTAAGGTCAGCTAAATAAGCTATCGGGCCC.

**Table 2:** Comparison of the attributes of MINTmap, tDRmapper, tRFdb, and tRFfinder.

	MINTmap	tDRmapper	tRFdb	tRFfinder
Standalone/downloadable tool available?	Yes	Yes	No	No
Generates or provides downloadable dataset profiles	Yes	Yes	No	Partial
Source code available	Yes	Yes	No	No
Speed	Fastest (<2 mins/sample using tRFs of all RPM values)	~18 mins/sample considering only tRFs with RPM ≥ 5*	NA**	NA**
Sensitivity	High	High	Moderate	Very low
Specificity	High	Moderate	Moderate	High
Full genome search	Yes (exhaustive)	No	Partial (via blast)	Partial (certain known transcripts via bowtie)
Reports tRFs from MT tRNA	Yes	No (nmt’s are included)	No	No
Accommodates 5′ Guanylation of tRNA^His^	Yes	No	No	No
Accommodates non-templated CCA addition	Yes	Yes	Yes	Yes
Accommodates tRNA introns	Yes	Yes	Yes	Yes
tRF structural category: 5′-tRFs	Yes	Yes	Yes	Yes
tRF structural category: 3′-tRFs	Yes	Yes	Yes	Yes
tRF structural category: i-tRFs	Yes	Yes	No (but alignment view shows i-tRF read overlap)	Yes
tRF structural category: tRF-1 (tRFs generated from trailer sequences)	No	Yes	Partial (3′ only)	Yes
Output visualization	HTML + links to the MINTbase framework	PDF	HTML	HTML

The shown comments on timings are based on the analysis of the nine short RNA-seq datasets discussed in the text.

^*^Processing did not complete in a reasonable time when considering all reads.

^**^Data not available (tools could not be downloaded).

**Table 3:** Troubleshooting Table.

Step	Problem	Possible reason	Solution
1	Starting tRNA reference sequences are shorter than those in Supplementary Table 1.	Sequences of mature tRNAs are used.	Use unspliced sequences.
1	Starting tRNA reference sequences don’t match Supplementary Table 1.	A different assembly or tRNA space definition is used.	The lookup table can be built with various species/assemblies. If GRCh37 is desired with the tRNA space as described in the text, then Supplementary Table 1 can be used.
5	The number of candidate sequences is less than those in Supp. Table 3.	Not all post-transcriptional modifications have been carried out.	Perform permutations only after accommodating exon splicing, and the “−1” and “CCA” modifications.
5	The number of candidate sequences is more than those in Supp. Table 3	Duplicate values have been included.	If a sequence appears multiple times in the reference tRNA space, remove duplicates and report it once.
8	Exclusivity values do not match Column D in Supp. Table 3.	An exhaustive and deterministic mapper was not used.	Use a search/mapper that performs exact string matching and does not rely on alignment scores.
10	No or few entries exist in the sample’s count table.	Adapters were not removed from the input dataset.	Remove any adapters from the sequenced reads in the input NGS file
10	If open source MINTmap script used: Error opening input file.	FASTQ file was not provided as input.	Provide the short RNA-seq dataset in FASTQ format (4 lines per read).
10	If open source MINTmap script used: Error opening input file.	Color-space data provided as input and cannot be directly provided to the tool.	Map the data as described in the color-space section of step 10.

For several of the steps in Methods, we list potential complications that the user may encounter together with an explanation of the behavior and the recommended solution.

## References

[b1] Pliatsika V., Loher P., Telonis A. G. & Rigoutsos I. MINTbase: a framework for the interactive exploration of mitochondrial and nuclear tRNA fragments. Bioinformatics 32, 2481–2489 (2016). 27153631 10.1093/bioinformatics/btw194 PMC4978933

[b2] Telonis A. G. . Dissecting tRNA-derived fragment complexities using personalized transcriptomes reveals novel fragment classes and unexpected dependencies. Oncotarget 6, 24797–24822 (2015). 26325506 10.18632/oncotarget.4695 PMC4694795

[b3] Friedlander M. R. . Evidence for the biogenesis of more than 1,000 novel human microRNAs. Genome Biol 15, R57 (2014). 24708865 10.1186/gb-2014-15-4-r57 PMC4054668

[b4] Londin E. . Analysis of 13 cell types reveals evidence for the expression of numerous novel primate- and tissue-specific microRNAs. Proc Natl Acad Sci USA 112, E1106–1115 (2015). 25713380 10.1073/pnas.1420955112 PMC4364231

[b5] Loher P., Londin E. R. & Rigoutsos I. IsomiR expression profiles in human lymphoblastoid cell lines exhibit population and gender dependencies. Oncotarget 5, 8790–8802 (2014). 25229428 10.18632/oncotarget.2405 PMC4226722

[b6] Telonis A. G., Loher P., Jing Y., Londin E. & Rigoutsos I. Beyond the one-locus-one-miRNA paradigm: microRNA isoforms enable deeper insights into breast cancer heterogeneity. Nucleic Acids Res 43, 9158–9175 (2015). 26400174 10.1093/nar/gkv922 PMC4627084

[b7] Gebetsberger J. & Polacek N. Slicing tRNAs to boost functional ncRNA diversity. RNA Biol 10, 1798–1806 (2013). 24351723 10.4161/rna.27177 PMC3917982

[b8] Keam S. P. & Hutvagner G. tRNA-Derived Fragments (tRFs): Emerging New Roles for an Ancient RNA in the Regulation of Gene Expression. Life (Basel) 5, 1638–1651 (2015). 26703738 10.3390/life5041638 PMC4695841

[b9] Shigematsu M., Honda S. & Kirino Y. Transfer RNA as a source of small functional RNA. Journal of Molecular Biology and Molecular Imaging 1 (2014). PMC4572697 26389128

[b10] Raina M. & Ibba M. tRNAs as regulators of biological processes. Front Genet 5, 171 (2014). 24966867 10.3389/fgene.2014.00171 PMC4052509

[b11] Honda S. . Sex hormone-dependent tRNA halves enhance cell proliferation in breast and prostate cancers. Proc Natl Acad Sci USA 112, E3816–3825 (2015). 26124144 10.1073/pnas.1510077112 PMC4517238

[b12] Ivanov P., Emara M. M., Villen J., Gygi S. P. & Anderson P. Angiogenin-induced tRNA fragments inhibit translation initiation. Mol Cell 43, 613–623 (2011). 21855800 10.1016/j.molcel.2011.06.022 PMC3160621

[b13] Yamasaki S., Ivanov P., Hu G. F. & Anderson P. Angiogenin cleaves tRNA and promotes stress-induced translational repression. J Cell Biol 185, 35–42 (2009). 19332886 10.1083/jcb.200811106 PMC2700517

[b14] Grewal S. S. Why should cancer biologists care about tRNAs? tRNA synthesis, mRNA translation and the control of growth. Biochim Biophys Acta 1849, 898–907 (2015). 25497380 10.1016/j.bbagrm.2014.12.005

[b15] Phizicky E. M. & Hopper A. K. tRNA biology charges to the front. Genes Dev 24, 1832–1860 (2010). 20810645 10.1101/gad.1956510 PMC2932967

[b16] Gebetsberger J., Zywicki M., Kunzi A. & Polacek N. tRNA-derived fragments target the ribosome and function as regulatory non-coding RNA in Haloferax volcanii. Archaea 2012, 260909 (2012). 23326205 10.1155/2012/260909 PMC3544259

[b17] Haiser H. J., Karginov F. V., Hannon G. J. & Elliot M. A. Developmentally regulated cleavage of tRNAs in the bacterium Streptomyces coelicolor. Nucleic Acids Res 36, 732–741 (2008). 18084030 10.1093/nar/gkm1096 PMC2241913

[b18] Haussecker D. . Human tRNA-derived small RNAs in the global regulation of RNA silencing. RNA 16, 673–695 (2010). 20181738 10.1261/rna.2000810 PMC2844617

[b19] Kumar P., Anaya J., Mudunuri S. B. & Dutta A. Meta-analysis of tRNA derived RNA fragments reveals that they are evolutionarily conserved and associate with AGO proteins to recognize specific RNA targets. BMC Biol 12, 78 (2014). 25270025 10.1186/s12915-014-0078-0 PMC4203973

[b20] Lee Y. S., Shibata Y., Malhotra A. & Dutta A. A novel class of small RNAs: tRNA-derived RNA fragments (tRFs). Genes Dev 23, 2639–2649 (2009). 19933153 10.1101/gad.1837609 PMC2779758

[b21] Maute R. L. . tRNA-derived microRNA modulates proliferation and the DNA damage response and is down-regulated in B cell lymphoma. Proc Natl Acad Sci USA 110, 1404–1409 (2013). 23297232 10.1073/pnas.1206761110 PMC3557069

[b22] Selitsky S. R. . Small tRNA-derived RNAs are increased and more abundant than microRNAs in chronic hepatitis B and C. Sci Rep 5, 7675 (2015). 25567797 10.1038/srep07675 PMC4286764

[b23] Babiarz J. E., Ruby J. G., Wang Y., Bartel D. P. & Blelloch R. Mouse ES cells express endogenous shRNAs, siRNAs, and other Microprocessor-independent, Dicer-dependent small RNAs. Genes Dev 22, 2773–2785 (2008). 18923076 10.1101/gad.1705308 PMC2569885

[b24] Hanada T. . CLP1 links tRNA metabolism to progressive motor-neuron loss. Nature 495, 474–480 (2013). 23474986 10.1038/nature11923 PMC3674495

[b25] Buhler M., Spies N., Bartel D. P. & Moazed D. TRAMP-mediated RNA surveillance prevents spurious entry of RNAs into the Schizosaccharomyces pombe siRNA pathway. Nat Struct Mol Biol 15, 1015–1023 (2008). 18776903 10.1038/nsmb.1481 PMC3240669

[b26] Liao J. Y. . Both endo-siRNAs and tRNA-derived small RNAs are involved in the differentiation of primitive eukaryote Giardia lamblia. Proc Natl Acad Sci USA 111, 14159–14164 (2014). 25225396 10.1073/pnas.1414394111 PMC4191773

[b27] Couvillion M. T., Bounova G., Purdom E., Speed T. P. & Collins K. A Tetrahymena Piwi bound to mature tRNA 3′ fragments activates the exonuclease Xrn2 for RNA processing in the nucleus. Mol Cell 48, 509–520 (2012). 23084833 10.1016/j.molcel.2012.09.010 PMC3513674

[b28] Couvillion M. T., Sachidanandam R. & Collins K. A growth-essential Tetrahymena Piwi protein carries tRNA fragment cargo. Genes Dev 24, 2742–2747 (2010). 21106669 10.1101/gad.1996210 PMC3003190

[b29] Reifur L. . Distinct subcellular localization of tRNA-derived fragments in the infective metacyclic forms of Trypanosoma cruzi. Mem Inst Oswaldo Cruz 107, 816–819 (2012). 22990974 10.1590/s0074-02762012000600018

[b30] Shigematsu M. & Kirino Y. tRNA-Derived Short Non-coding RNA as Interacting Partners of Argonaute Proteins. Gene Regul Syst Bio 9, 27–33 (2015). 10.4137/GRSB.S29411 PMC4567038 26401098

[b31] Cole C. . Filtering of deep sequencing data reveals the existence of abundant Dicer-dependent small RNAs derived from tRNAs. RNA 15, 2147–2160 (2009). 19850906 10.1261/rna.1738409 PMC2779667

[b32] Li Z. . Extensive terminal and asymmetric processing of small RNAs from rRNAs, snoRNAs, snRNAs, and tRNAs. Nucleic Acids Res 40, 6787–6799 (2012). 22492706 10.1093/nar/gks307 PMC3413118

[b33] Goodarzi H. . Endogenous tRNA-Derived Fragments Suppress Breast Cancer Progression via YBX1 Displacement. Cell 161, 790–802 (2015). 25957686 10.1016/j.cell.2015.02.053 PMC4457382

[b34] Dhahbi J. M., Spindler S. R., Atamna H., Boffelli D. & Martin D. I. Deep Sequencing of Serum Small RNAs Identifies Patterns of 5′ tRNA Half and YRNA Fragment Expression Associated with Breast Cancer. Biomark Cancer 6, 37–47 (2014). 25520563 10.4137/BIC.S20764 PMC4260766

[b35] Emara M. M. . Angiogenin-induced tRNA-derived stress-induced RNAs promote stress-induced stress granule assembly. J Biol Chem 285, 10959–10968 (2010). 20129916 10.1074/jbc.M109.077560 PMC2856301

[b36] Sharma U. . Biogenesis and function of tRNA fragments during sperm maturation and fertilization in mammals. Science 351, 391–396 (2016). 26721685 10.1126/science.aad6780 PMC4888079

[b37] Yeung M. L. . Pyrosequencing of small non-coding RNAs in HIV-1 infected cells: evidence for the processing of a viral-cellular double-stranded RNA hybrid. Nucleic Acids Res 37, 6575–6586 (2009). 19729508 10.1093/nar/gkp707 PMC2770672

[b38] Wang Q. . Identification and functional characterization of tRNA-derived RNA fragments (tRFs) in respiratory syncytial virus infection. Mol Ther 21, 368–379 (2013). 23183536 10.1038/mt.2012.237 PMC3594034

[b39] Deng J. . Respiratory Syncytial Virus Utilizes a tRNA Fragment to Suppress Antiviral Responses Through a Novel Targeting Mechanism. Mol Ther 23, 1622–1629 (2015). 26156244 10.1038/mt.2015.124 PMC4817927

[b40] Gong B. . Compartmentalized, functional role of angiogenin during spotted fever group rickettsia-induced endothelial barrier dysfunction: evidence of possible mediation by host tRNA-derived small noncoding RNAs. BMC Infect Dis 13, 285 (2013). 23800282 10.1186/1471-2334-13-285 PMC3699377

[b41] Telonis A. G., Loher P., Kirino Y. & Rigoutsos I. Nuclear and mitochondrial tRNA-lookalikes in the human genome. Front Genet 5, 344 (2014). 25339973 10.3389/fgene.2014.00344 PMC4189335

[b42] Telonis A. G., Kirino Y. & Rigoutsos I. Mitochondrial tRNA-lookalikes in nuclear chromosomes: could they be functional? RNA Biol 12, 375–380 (2015). 25849196 10.1080/15476286.2015.1017239 PMC4615777

[b43] Juhling F. . tRNAdb 2009: compilation of tRNA sequences and tRNA genes. Nucleic Acids Res 37, D159–162 (2009). 18957446 10.1093/nar/gkn772 PMC2686557

[b44] Abe T. . tRNADB-CE: tRNA gene database well-timed in the era of big sequence data. Front Genet 5, 114 (2014). 24822057 10.3389/fgene.2014.00114 PMC4013482

[b45] Putz J., Dupuis B., Sissler M. & Florentz C. Mamit-tRNA, a database of mammalian mitochondrial tRNA primary and secondary structures. RNA 13, 1184–1190 (2007). 17585048 10.1261/rna.588407 PMC1924894

[b46] Chan P. P. & Lowe T. M. GtRNAdb: a database of transfer RNA genes detected in genomic sequence. Nucleic Acids Res 37, D93–97 (2009). 18984615 10.1093/nar/gkn787 PMC2686519

[b47] Rigoutsos I. Comment on PMID 26673694:GtRNAdb 2.0: an expanded database of transfer RNA genes identified in complete and draft genomes. In: PubMed Commons [Internet]. Bethesda (MD): National Library of Medicine http://www.ncbi.nlm.nih.gov/pubmed/26673694-cm26673694_13813 (2016 Jan 21).

[b48] Kozomara A. & Griffiths-Jones S. miRBase: integrating microRNA annotation and deep-sequencing data. Nucleic Acids Res 39, D152–157 (2011). 21037258 10.1093/nar/gkq1027 PMC3013655

[b49] Telonis A. G., Loher P., Kirino Y. & Rigoutsos I. Consequential considerations when mapping tRNA fragments. BMC Bioinformatics 17, 123 (2016). 26961774 10.1186/s12859-016-0921-0 PMC4785646

[b50] Betat H., Rammelt C. & Morl M. tRNA nucleotidyltransferases: ancient catalysts with an unusual mechanism of polymerization. Cell Mol Life Sci 67, 1447–1463 (2010). 20155482 10.1007/s00018-010-0271-4 PMC11115931

[b51] Hou Y. M. CCA addition to tRNA: implications for tRNA quality control. IUBMB Life 62, 251–260 (2010). 20101632 10.1002/iub.301 PMC2848691

[b52] Tomita K. & Yamashita S. Molecular mechanisms of template-independent RNA polymerization by tRNA nucleotidyltransferases. Front Genet 5, 36 (2014). 24596576 10.3389/fgene.2014.00036 PMC3925840

[b53] Hirose Y. . Precise mapping and dynamics of tRNA-derived fragments (tRFs) in the development of Triops cancriformis (tadpole shrimp). BMC Genet 16, 83 (2015). 26168920 10.1186/s12863-015-0245-5 PMC4501094

[b54] Kumar P., Mudunuri S. B., Anaya J. & Dutta A. tRFdb: a database for transfer RNA fragments. Nucleic Acids Res 43, D141–145 (2015). 25392422 10.1093/nar/gku1138 PMC4383946

[b55] Selitsky S. R. & Sethupathy P. tDRmapper: challenges and solutions to mapping, naming, and quantifying tRNA-derived RNAs from human small RNA-sequencing data. BMC Bioinformatics 16, 354 (2015). 26530785 10.1186/s12859-015-0800-0 PMC4632369

[b56] Karaiskos S., Naqvi A. S., Swanson K. E. & Grigoriev A. Age-driven modulation of tRNA-derived fragments in Drosophila and their potential targets. Biol Direct 10, 51 (2015). 26374501 10.1186/s13062-015-0081-6 PMC4572633

[b57] Rao B. S. & Jackman J. E. Life without post-transcriptional addition of G-1: two alternatives for tRNAHis identity in Eukarya. RNA 21, 243–253 (2015). 25505023 10.1261/rna.048389.114 PMC4338351

[b58] Heinemann I. U., Nakamura A., O’Donoghue P., Eiler D. & Soll D. tRNAHis-guanylyltransferase establishes tRNAHis identity. Nucleic Acids Res 40, 333–344 (2012). 21890903 10.1093/nar/gkr696 PMC3245924

[b59] Hyde S. J. . tRNA(His) guanylyltransferase (THG1), a unique 3′-5′ nucleotidyl transferase, shares unexpected structural homology with canonical 5′-3′ DNA polymerases. Proc Natl Acad Sci USA 107, 20305–20310 (2010). 21059936 10.1073/pnas.1010436107 PMC2996709

[b60] Guo Y. . Transfer RNA detection by small RNA deep sequencing and disease association with myelodysplastic syndromes. BMC Genomics 16, 727 (2015). 26400237 10.1186/s12864-015-1929-y PMC4581457

[b61] Keam S. P., Sobala A., Humphreys D. T., Suter C. M. & Hutvagner G. Computational analysis, biochemical purification, and detection of tRNA-derived small RNA fragments. Methods Mol Biol 1173, 157–167 (2014). 24920368 10.1007/978-1-4939-0931-5_14

[b62] Olvedy M. . A comprehensive repertoire of tRNA-derived fragments in prostate cancer. Oncotarget (2016). 10.18632/oncotarget.8293 PMC5029740 27015120

[b63] David M., Dzamba M., Lister D., Ilie L. & Brudno M. SHRiMP2: sensitive yet practical SHort Read Mapping. Bioinformatics 27, 1011–1012 (2011). 21278192 10.1093/bioinformatics/btr046

[b64] Li H. & Durbin R. Fast and accurate short read alignment with Burrows-Wheeler transform. Bioinformatics 25, 1754–1760 (2009). 19451168 10.1093/bioinformatics/btp324 PMC2705234

[b65] Lappalainen T. . Transcriptome and genome sequencing uncovers functional variation in humans. Nature 501, 506–511 (2013). 24037378 10.1038/nature12531 PMC3918453

[b66] Zheng L. L. . tRF2Cancer: A web server to detect tRNA-derived small RNA fragments (tRFs) and their expression in multiple cancers. Nucleic Acids Res 44, W185–193 (2016). 27179031 10.1093/nar/gkw414 PMC4987945

[b67] Mayr C. & Bartel D. P. Widespread shortening of 3′UTRs by alternative cleavage and polyadenylation activates oncogenes in cancer cells. Cell 138, 673–684 (2009). 19703394 10.1016/j.cell.2009.06.016 PMC2819821

