# Supplementary material for: MINTmap: fast and exhaustive profiling of nuclear and mitochondrial tRNA fragments from short RNA-seq data
Source: Sci Rep. 2017 Feb 21;7:41184. doi: 10.1038/srep41184 (PMC5318995; doi:10.1038/srep41184)
Supplement: Supplementary Information [file srep41184-s1.pdf]

Supplemental Material

MINTmap: fast and exhaustive profiling of nuclear and mitochondrial tRNA fragments from RNA-seq data

Phillipe Loher[1], Aristeidis G. Telonis[1] and Isidore Rigoutsos[1,*]

[1] Computational Medicine Center, Sidney Kimmel Medical College, Thomas Jefferson University, 1020 Locust Street, Philadelphia, PA 19107, USA.

* Correspondence should be addressed to IR: isidore.rigoutsos@jefferson.edu / (215) 503-6152

Supplemental Table 1: Sequences and attributes for tRNA fragments reported by MINTmap, tRFdb, tDRmapper, and tRFfinder when looking at 9 of the 10 human cell lines listed under GEO entry # 16579.

Supplemental Table 2: Unspliced tRNA reference set.

Supplemental Table 3: All tRF candidate sequences and their exclusivity to tRNA space. This is MINTmap's lookup-table for the human genome.

Supplemental Table 1: Sequences and attributes for tRNA fragments reported by MINTmap, tRFdb, tDRmapper, and tRFfinder when looking at 9 of the 10 human cell lines listed under GEO entry # 16579 (see also text).

tRF Sequence	Reported by MINTmap-ExclusiveOnly?	Reported by MINTmap-All?	Reported by tRFdb-AllAligned?	Reported by tRFdb-withID?	Reported by tDRmapper?	Reported by tRFfinder (default settings)	< 16nt?	MT tRNA possible?	Exact-lookalike possible?	-1 location possible?	Within 5p Trailer sequence?	Within 3p Trailer sequence?	Pseudo tRNA possible?	Parent tRNA(s) aminoacid annotations (generated using MINTbase)
AAAAAAAAAAAAAAA	No	No	No	No	Yes	No	Yes	No	No	No	Yes	No	No	NA
AAAAAAAAAAAAAAAA	No	No	No	No	Yes	No	No	No	No	No	Yes	No	No	NA
AAAAAAAAAAAAAAAAA	No	No	No	No	Yes	No	No	No	No	No	Yes	No	No	NA
AAAAAAAAAAAAAAAAAA	No	No	No	No	Yes	No	No	No	No	No	Yes	No	No	NA
AAAACCTTTTTCCAAGGACACCA	Yes	Yes	No	No	No	No	No	Yes	No	No	No	No	No	ThrTGT
AAAAGTGGCTTTTTT	No	No	Yes	No	Yes	No	Yes	No	No	No	No	Yes	No	NA
AAAAGTGGCTTTTTTT	No	No	Yes	No	Yes	No	No	No	No	No	No	Yes	No	NA
AAAATGTTGGTTATACCCTTCCCGTACTACCA	Yes	Yes	No	No	No	No	No	Yes	No	No	No	No	No	MetCAT
AAAATGTTTAGACGGGC	Yes	Yes	No	No	No	No	No	Yes	No	No	No	No	No	PheGAA
AAAATGTTTAGACGGGCTCAC	Yes	Yes	No	No	No	No	No	Yes	No	No	No	No	No	PheGAA
AAAATGTTTAGACGGGCTCACATCACCCC	Yes	Yes	No	No	No	No	No	Yes	No	No	No	No	No	PheGAA
AAAATGTTTAGACGGGCTCACATCACCCCATAA	Yes	Yes	No	No	No	No	No	Yes	No	No	No	No	No	PheGAA
AAACCATCCTCTGCTACCA	Yes	Yes	Yes	No	Yes	No	No	No	No	No	No	No	No	MetCAT
AAACCATCCTCTGCTATCGGAGTTTTT	No	No	No	No	Yes	No	No	No	No	No	No	No	No	NA
AAACCGGGCGGAAACACCA	Yes	Yes	Yes	No	Yes	No	No	No	No	No	No	No	No	ValCAC,ValAAC
AAACCTTTTTCCAAGGACACCA	Yes	Yes	No	No	No	No	No	Yes	No	No	No	No	No	ThrTGT
AAAGGGTCTTTTTCACCCCGCTGTTGCTCTTT	No	No	Yes	No	Yes	No	No	No	No	No	No	Yes	No	NA
AAAGGGTCTTTTTCACCCCGCTGTTGCTCTTTA	No	No	Yes	No	Yes	No	No	No	No	No	No	Yes	No	NA
AAATGGTAGAGCGCTC	Yes	Yes	No	No	Yes	No	No	No	No	No	No	No	No	AlaAGC
AAATGTTGGTTATACCCTTCCCGTACTACCA	Yes	Yes	No	No	No	No	No	Yes	No	No	No	No	No	MetCAT
AACAAAACATTGTTT	No	No	No	No	Yes	No	Yes	No	No	No	No	Yes	No	NA
AACAAAACATTGTTTT	No	No	No	No	Yes	No	No	No	No	No	No	Yes	No	NA
AACACCTCTTTACAGTGACCA	Yes	Yes	No	No	No	No	No	Yes	Yes	No	No	No	No	LysTTT
AACAGGGTTTGTTAA	No	No	No	No	Yes	No	Yes	No	No	No	Yes	No	No	NA
AACAGGGTTTGTTAAGA	No	No	No	No	Yes	No	No	No	No	No	Yes	No	No	NA
AACCATCCTCTGCTACCA	Yes	Yes	Yes	No	Yes	No	No	No	No	No	No	No	No	MetCAT
AACCGAGCGGAAACACCA	Yes	Yes	No	No	Yes	No	No	No	No	No	No	No	No	ValTAC
AACCGAGCGTCCAAGC	No	No	Yes	No	Yes	No	No	No	No	No	No	Yes	No	NA
AACCGAGCGTCCAAGCT	No	No	Yes	No	Yes	No	No	No	No	No	No	Yes	No	NA
AACCGAGCGTCCAAGCTC	No	No	Yes	No	Yes	No	No	No	No	No	No	Yes	No	NA
AACCGAGCGTCCAAGCTCTTT	No	No	Yes	No	Yes	No	No	No	No	No	No	Yes	No	NA
AACCGAGCGTCCAAGCTCTTTCC	No	No	Yes	No	Yes	No	No	No	No	No	No	Yes	No	NA
AACCGAGCGTCCAAGCTCTTTCCA	No	No	Yes	No	Yes	No	No	No	No	No	No	Yes	No	NA
AACCGAGCGTCCAAGCTCTTTCCAT	No	No	Yes	No	Yes	No	No	No	No	No	No	Yes	No	NA
AACCGAGCGTCCAAGCTCTTTCCATT	No	No	Yes	No	Yes	No	No	No	No	No	No	Yes	No	NA
AACCGAGCGTCCAAGCTCTTTCCATTT	No	No	Yes	No	Yes	No	No	No	No	No	No	Yes	No	NA
AACCGAGCGTCCAAGCTCTTTCCATTTT	No	No	Yes	Yes	Yes	No	No	No	No	No	No	Yes	No	NA
AACCGAGCGTCCAAGCTCTTTCCATTTTT	No	No	Yes	No	Yes	No	No	No	No	No	No	Yes	No	NA
AACCGGGCAGAAGCACCA	Yes	Yes	Yes	No	Yes	No	No	No	No	No	No	No	No	ValCAC
AACCGGGCGGAAACA	No	No	Yes	No	Yes	No	Yes	No	No	No	No	No	No	NA
AACCGGGCGGAAACAC	Yes	Yes	Yes	No	Yes	No	No	No	No	No	No	No	No	ValCAC,ValAAC
AACCGGGCGGAAACACC	Yes	Yes	Yes	No	Yes	No	No	No	No	No	No	No	No	ValCAC,ValAAC
AACCGGGCGGAAACACCA	Yes	Yes	Yes	No	Yes	No	No	No	No	No	No	No	No	ValCAC,ValAAC
AACCGGGCGGAAACACCAA	No	No	Yes	No	No	No	No	No	No	No	No	No	No	NA
AACCGGGCGGAAACACCAAA	No	No	Yes	No	No	No	No	No	No	No	No	No	No	NA
AACCGGGCGGAAACACCAAG	No	No	Yes	No	No	No	No	No	No	No	No	No	No	NA
AACCGGGCGGAAACACCAT	No	No	Yes	No	No	No	No	No	No	No	No	No	No	NA
AACCTTTTTCCAAGGACACCA	Yes	Yes	No	No	No	No	No	Yes	No	No	No	No	No	ThrTGT
AACTTAACTTGACCGCTCTGACCA	Yes	Yes	No	No	No	No	No	Yes	No	No	No	No	No	ValTAC
AACTTCAGAAGTTTT	No	No	No	No	Yes	No	Yes	No	No	No	No	Yes	No	NA
AACTTGACCGCTCTGACC	Yes	Yes	No	No	No	No	No	Yes	No	No	No	No	No	ValTAC
AACTTGACCGCTCTGACCA	Yes	Yes	No	No	No	No	No	Yes	No	No	No	No	No	ValTAC
AAGAGAACCAACACCTCTTTACAGTGACCA	Yes	Yes	No	No	No	No	No	Yes	Yes	No	No	No	No	LysTTT
AAGAGAGTCGCTTTT	No	No	Yes	No	Yes	No	Yes	No	No	No	No	Yes	No	NA
AAGAGAGTCGCTTTTT	No	No	Yes	No	Yes	No	No	No	No	No	No	Yes	No	NA
AAGAGAGTCGCTTTTTT	No	No	Yes	No	Yes	No	No	No	No	No	No	Yes	No	NA
AAGAGGAGTTGTTTT	No	No	Yes	Yes	Yes	No	Yes	No	No	No	No	Yes	No	NA
AAGAGGAGTTGTTTTT	No	No	Yes	No	Yes	No	No	No	No	No	No	Yes	No	NA
AAGAGGAGTTGTTTTTCCTGGCAAAAACCTTT	No	No	Yes	No	Yes	No	No	No	No	No	No	Yes	No	NA
AAGAGGTCCCCGGTT	No	No	Yes	No	Yes	No	Yes	No	No	No	No	No	No	NA
AAGAGGTCCCTGGTT	No	No	Yes	No	Yes	No	Yes	No	No	No	No	No	No	NA
AAGCAACCTGTATTTT	No	No	No	No	Yes	No	No	No	No	No	No	Yes	No	NA
AAGCAGCTTCAAACCTGCCGGGGCTTCCA	Yes	Yes	No	No	No	No	No	Yes	No	No	No	No	No	CysGCA
AAGCCAGGGATTGTGGGT	Yes	Yes	Yes	No	Yes	No	No	No	No	No	No	No	No	ArgCCT
AAGCGGGTGCTCTTA	No	No	Yes	No	Yes	No	Yes	No	No	No	No	Yes	No	NA
AAGCGGGTGCTCTTAT	No	No	Yes	No	Yes	No	No	No	No	No	No	Yes	No	NA
AAGCGGGTGCTCTTATT	No	No	Yes	No	Yes	No	No	No	No	No	No	Yes	No	NA
AAGCGGGTGCTCTTATTT	No	No	Yes	No	Yes	No	No	No	No	No	No	Yes	No	NA
AAGCGGGTGCTCTTATTTT	No	No	Yes	No	Yes	No	No	No	No	No	No	Yes	No	NA
AAGCGGGTGCTCTTATTTTT	No	No	Yes	No	Yes	No	No	No	No	No	No	Yes	No	NA
AAGCTCACAAGAACTGCT	Yes	Yes	No	No	No	No	No	Yes	No	No	No	No	No	SerGCT
AAGGACGAAAGTTTT	No	No	No	No	Yes	No	Yes	No	No	No	No	Yes	No	NA
AAGGACTTCGTCTGTAATTTT	No	No	No	No	Yes	No	No	No	No	No	No	Yes	No	NA
AAGGAGTTCACAATTTT	No	No	No	No	Yes	No	No	No	No	No	No	Yes	No	NA
AAGGTATTAGAAAAACCATT	Yes	Yes	No	No	No	No	No	Yes	No	No	No	No	No	AspGTC
AAGGTATTAGAAAAACCATTTC	Yes	Yes	No	No	No	No	No	Yes	No	No	No	No	No	AspGTC
AAGGTATTAGAAAAACCATTTCA	Yes	Yes	No	No	No	No	No	Yes	No	No	No	No	No	AspGTC
AAGGTATTAGAAAAACCATTTCATA	Yes	Yes	No	No	No	No	No	Yes	No	No	No	No	No	AspGTC

AAGGTATTAGAAAAACCATTTCATAA	Yes	Yes	No	No	No	No	No	Yes	No	No	No	No	No	AspGTC
AAGGTATTAGAAAAACCATTTCATAAC	Yes	Yes	No	No	No	No	No	Yes	No	No	No	No	No	AspGTC
AAGGTCCCCGGTTCGAAACCGGGCGGAAAC	Yes	Yes	Yes	No	Yes	No	No	No	No	No	No	No	No	ValCAC,ValAAC
AAGGTCCTGGGTTCGAGCCCCAGTGGAACCA	Yes	Yes	Yes	No	Yes	No	No	No	No	No	No	No	No	ValTAC
AAGGTCCTGGGTTCGAGCCCCAGTGGAACCAC	Yes	Yes	Yes	No	Yes	No	No	No	No	No	No	No	No	ValTAC
AAGGTCGCGGGTTCGATCCCC	Yes	Yes	Yes	No	Yes	No	No	No	No	No	No	No	No	IleAAT
AAGTCCCATTGGTCTAGCCA	Yes	Yes	No	No	No	No	No	Yes	Yes	No	No	No	No	AsnGTT
AAGTGGTTCCCGTTT	No	No	Yes	No	Yes	No	Yes	No	No	No	No	Yes	No	NA
AAGTGGTTCCCGTTTT	No	No	Yes	No	Yes	No	No	No	No	No	No	Yes	No	NA
AATAGTTTAAATTAGAATCTTAGC	Yes	Yes	No	No	No	No	No	Yes	No	No	No	No	No	ProTGG
AATCCAGCAATCCGAGTTCGAAT	Yes	Yes	Yes	No	Yes	No	No	No	No	No	No	No	No	GlnTTG
AATCCAGCGATCCGAG	Yes	Yes	Yes	No	Yes	No	No	No	No	No	No	No	No	GlnCTG,GlnTTG
AATCCAGCGATCCGAGT	Yes	Yes	Yes	No	Yes	No	No	No	No	No	No	No	No	GlnCTG,GlnTTG
AATCCAGCGATCCGAGTT	Yes	Yes	Yes	No	Yes	No	No	No	No	No	No	No	No	GlnCTG,GlnTTG
AATCCAGCGATCCGAGTTCA	Yes	Yes	Yes	No	Yes	No	No	No	No	No	No	No	No	GlnCTG,GlnTTG
AATCCAGCGATCCGAGTTCAAA	Yes	Yes	Yes	No	Yes	No	No	No	No	No	No	No	No	GlnCTG,GlnTTG
AATCCAGCGATCCGAGTTCAAAT	Yes	Yes	Yes	No	Yes	No	No	No	No	No	No	No	No	GlnCTG,GlnTTG
AATCCCACCAGAGTCGCCA	Yes	Yes	No	No	Yes	No	No	No	No	No	No	No	No	ArgTCT
AATCCCACCGCTGCCACCA	Yes	Yes	Yes	No	Yes	No	No	No	No	No	No	No	No	LeuTAG,LeuAAG
AATCCCACCTTCGTCGCCA	Yes	Yes	No	No	Yes	No	No	No	No	No	No	No	No	SerGCT
AATCCCACTCCTGACACCA	No	Yes	Yes	No	Yes	No	No	No	No	No	No	No	No	LeuCAG
AATCCCACTTCTGACACCA	No	Yes	Yes	No	Yes	No	No	No	No	No	No	No	No	LeuCAA,LeuCAG
AATCCCAGCGGTGCCTCCA	Yes	Yes	Yes	No	Yes	No	No	No	No	No	No	No	No	ThrAGT
AATCCCAGTAGAGCCTCCA	Yes	Yes	No	No	Yes	No	No	No	No	No	No	No	No	ThrTGT
AATCCCATCCTCGTCGCCA	Yes	Yes	Yes	No	Yes	No	No	No	No	No	No	No	No	SerGCT
AATCCCGGACGAGCCCCCA	No	Yes	Yes	No	Yes	No	No	No	No	No	No	No	No	ProAGG,ProCGG,ProTGG
AATCCGAGTCACGGCACCA	No	Yes	Yes	No	Yes	No	No	No	No	No	No	No	No	HisGTG
AATCCGGCTCGAAGGACCA	Yes	Yes	Yes	No	Yes	No	No	No	No	No	No	No	No	TyrGTA
AATCCGGCTCGGAGGACCA	Yes	Yes	Yes	No	Yes	No	No	No	No	No	No	No	No	TyrGTA
AATCCGGGTGCCCCCTCCA	Yes	Yes	Yes	No	Yes	No	No	No	No	No	No	No	No	CysGCA
AATCCTGCCGACTACGCCA	Yes	Yes	Yes	No	Yes	No	No	No	No	No	No	No	No	SerTGA,SerAGA
AATCTCGGTGGAACCTCCA	Yes	Yes	Yes	No	Yes	No	No	No	No	No	No	No	No	GlnCTG
AATCTCGGTGGGACCTCCA	Yes	Yes	Yes	No	Yes	No	No	No	No	No	No	No	No	GlnTTG
AATGGAGGCGTGGGT	No	No	Yes	No	Yes	No	Yes	No	No	No	No	No	No	NA
AATGGAGGCGTGGGTT	Yes	Yes	Yes	No	Yes	No	No	No	No	No	No	No	No	LeuCAA
AATGGATAAGGCACTG	No	Yes	Yes	No	Yes	No	No	No	No	No	No	No	No	ArgCCT
AATGGATAAGGCATCA	No	Yes	Yes	No	Yes	No	No	No	No	No	No	No	No	ArgCCG
AATGGATAAGGCATT	No	No	No	No	Yes	No	Yes	No	No	No	No	No	No	NA
AATGGATAAGGCATTG	No	Yes	No	No	Yes	No	No	No	No	No	No	No	No	ArgCCT
AATGGATAGCGCATTG	No	Yes	No	No	Yes	No	No	No	No	No	No	No	No	ArgTCT
AATGGCTTGGTTTTT	No	No	Yes	No	Yes	No	Yes	No	No	No	No	Yes	No	NA
AATGGTGGAGTTAAAGACTTTTTCTCTGACCA	Yes	Yes	No	No	No	No	No	Yes	No	No	No	No	No	ProTGG
AATGGTTAGCACTCTGG	No	Yes	Yes	No	Yes	No	No	No	No	No	No	No	No	GlnCTG,GlnTTG
AATGGTTAGCACTCTGGA	No	Yes	Yes	No	Yes	No	No	No	No	No	No	No	No	GlnCTG,GlnTTG
AATGTTTAGACGGGCTCACATCACCCC	Yes	Yes	No	No	No	No	No	Yes	No	No	No	No	No	PheGAA
AATTCTCGCCTGCCAC	No	Yes	Yes	No	Yes	No	No	No	No	No	No	No	No	GlyGCC
AATTCTCGCCTGCCACG	Yes	Yes	Yes	No	Yes	No	No	No	No	No	No	No	No	GlyGCC
AATTCTCGCCTGCCACGC	Yes	Yes	Yes	No	Yes	No	No	No	No	No	No	No	No	GlyGCC
AATTCTCGCCTGCCACGCGGGAGGCCCGGGT	Yes	Yes	Yes	No	Yes	No	No	No	No	No	No	No	No	GlyGCC
AATTTAGGTTAAATACAGACCAAGAG	Yes	Yes	No	No	No	No	No	Yes	Yes	No	No	No	No	TrpTCA
ACAAAACATTGTTTT	No	No	No	No	Yes	No	Yes	No	No	No	No	Yes	No	NA
ACAAGTGCGGTTTTT	No	No	Yes	No	Yes	No	Yes	No	No	No	No	Yes	No	NA
ACAAGTGCGGTTTTTT	No	No	Yes	Yes	Yes	No	No	No	No	No	No	Yes	No	NA
ACAAGTGCGGTTTTTTT	No	No	Yes	No	Yes	No	No	No	No	No	No	Yes	No	NA
ACACGCGAAAGGTCCCCG	Yes	Yes	Yes	No	Yes	No	No	No	No	No	No	No	No	ValCAC,ValAAC
ACACGCGAAAGGTCCCCGG	Yes	Yes	Yes	No	Yes	No	No	No	No	No	No	No	No	ValCAC,ValAAC
ACACGCGAAAGGTCCCCGGT	Yes	Yes	Yes	No	Yes	No	No	No	No	No	No	No	No	ValCAC,ValAAC
ACACGGGGCACAATAATTT	No	No	No	No	Yes	No	No	No	No	No	No	Yes	No	NA
ACAGAGAATAGTTTAAATTAGA	Yes	Yes	No	No	No	No	No	Yes	No	Yes	No	No	No	ProTGG
ACAGGGGTTAGGCCTCTTTTTACC	Yes	Yes	No	No	No	No	No	Yes	No	No	No	No	No	TyrGTA
ACAGGGGTTAGGCCTCTTTTTACCACCA	Yes	Yes	No	No	No	No	No	Yes	No	No	No	No	No	TyrGTA
ACATGGTCTAGCGGTTAGGATTCCTG	Yes	Yes	Yes	No	Yes	No	No	No	No	No	No	No	No	GluTTC
ACCAGAATGGCCGAGTGG	Yes	Yes	Yes	No	Yes	No	No	No	No	No	No	No	No	LeuTAA
ACCAGGATGGCCGAG	No	No	Yes	No	Yes	No	Yes	No	No	No	No	No	No	NA
ACCAGGATGGCCGAGTGG	Yes	Yes	Yes	No	Yes	No	No	No	No	No	No	No	No	LeuTAA
ACCAGGATGGCCGAGTGGTTAAGG	Yes	Yes	Yes	No	Yes	No	No	No	No	No	No	No	No	LeuTAA
ACCAGGATGGCCGAGTGGTTAAGGC	Yes	Yes	Yes	No	Yes	No	No	No	No	No	No	No	No	LeuTAA
ACCATCCTCTGCTACCA	No	Yes	Yes	No	Yes	No	No	No	No	No	No	No	No	MetCAT
ACCCCACTCCTGGTA	No	No	Yes	No	Yes	No	Yes	No	No	No	No	No	No	NA
ACCCCACTCCTGGTAC	No	Yes	Yes	No	Yes	No	No	No	No	No	No	No	No	LeuTAA
ACCCCACTCCTGGTACC	No	Yes	Yes	No	Yes	No	No	No	No	No	No	No	No	LeuTAA
ACCCCACTCCTGGTACCA	No	Yes	Yes	Yes	Yes	No	No	No	No	No	No	No	No	LeuTAA
ACCCCACTCTCGGTACCA	No	Yes	No	No	Yes	No	No	No	No	No	No	No	No	LeuTAA
ACCCCACTTCTGGTACCA	No	Yes	Yes	No	Yes	No	No	No	No	No	No	No	No	LeuTAA
ACCCCCTTATTTCTACCA	Yes	Yes	No	No	No	No	No	Yes	No	No	No	No	No	IleGAT
ACCCCGTACGGGCCACCA	Yes	Yes	Yes	No	Yes	No	No	No	No	No	No	No	No	IleAAT
ACCCCGTCCGTGCCTCCA	Yes	Yes	Yes	No	Yes	No	No	No	No	No	No	No	No	ThrCGT
ACCCCTTATTTACCCC	Yes	Yes	No	No	No	No	No	Yes	No	No	No	No	No	HisGTG
ACCCCTTATTTACCCCA	Yes	Yes	No	No	No	No	No	Yes	No	No	No	No	No	HisGTG
ACCCTGCTCGCTGCGC	Yes	Yes	Yes	No	Yes	No	No	No	No	No	No	No	No	SerTGA
ACCCTGCTCGCTGCGCC	Yes	Yes	Yes	No	Yes	No	No	No	No	No	No	No	No	SerTGA
ACCCTGCTCGCTGCGCCA	Yes	Yes	Yes	No	Yes	No	No	No	No	No	No	No	No	SerTGA
ACCCTTCCCGTACTACCA	Yes	Yes	No	No	No	No	No	Yes	No	No	No	No	No	MetCAT
ACCGGAGATGAAAACCTTTTTCCAAGGACACCA	Yes	Yes	No	No	No	No	No	Yes	No	No	No	No	No	ThrTGT
ACCGGGCAGAAGCACCA	Yes	Yes	Yes	No	Yes	No	No	No	No	No	No	No	No	ValCAC
ACCGGGCGGAAACAC	No	No	Yes	No	Yes	No	Yes	No	No	No	No	Yes	No	NA
ACCGGGCGGAAACACC	Yes	Yes	Yes	No	Yes	No	No	No	No	No	No	No	No	ValCAC,ValAAC
ACCGGGCGGAAACACCA	Yes	Yes	Yes	Yes	Yes	No	No	No	No	No	No	No	No	ValCAC,ValAAC

ACCGGGCGGAAACACCAA	No	No	Yes	No	No	No	No	No	No	No	No	No	No	NA
ACCGGGCGGAAACACCAAA	No	No	Yes	No	No	No	No	No	No	No	No	No	No	NA
ACCGGGCGGAAACACCAAG	No	No	Yes	No	No	No	No	No	No	No	No	No	No	NA
ACCGGGCGGAAACACCAT	No	No	Yes	No	No	No	No	No	No	No	No	No	No	NA
ACCGGGGTTCGATTCCCCGAC	Yes	Yes	Yes	No	Yes	No	No	No	No	No	No	No	No	AspGTC
ACCGGGGTTCGATTCCCCGACG	Yes	Yes	Yes	No	Yes	No	No	No	No	No	No	No	No	AspGTC
ACCGGGGTTCGATTCCCCGACGGG	Yes	Yes	Yes	No	Yes	No	No	No	No	No	No	No	No	AspGTC
ACCGGGGTTCGATTCCCCGACGGGGAGCCA	Yes	Yes	Yes	No	Yes	No	No	No	No	No	No	No	No	AspGTC
ACCTCAGAAGGTCTCA	No	No	Yes	No	Yes	No	No	No	No	No	No	Yes	No	NA
ACCTCAGAAGGTCTCAC	No	No	Yes	No	Yes	No	No	No	No	No	No	Yes	No	NA
ACCTCAGAAGGTCTCACT	No	No	Yes	No	Yes	No	No	No	No	No	No	Yes	No	NA
ACCTCAGAAGGTCTCACTT	No	No	Yes	No	Yes	No	No	No	No	No	No	Yes	No	NA
ACCTCAGAAGGTCTCACTTT	No	No	Yes	Yes	Yes	No	No	No	No	No	No	Yes	No	NA
ACCTCAGAAGGTCTCACTTTT	No	No	Yes	No	Yes	No	No	No	No	No	No	Yes	No	NA
ACCTCTTTACAGTGACCA	Yes	Yes	No	No	No	No	No	Yes	Yes	No	No	No	No	LysTTT
ACGCCAAGGTCGCGGGT	Yes	Yes	Yes	No	Yes	No	No	No	No	No	No	No	No	IleAAT
ACGCGGGAGACCCGGGT	Yes	Yes	Yes	No	Yes	No	No	No	No	No	No	No	Yes	GlyCCC
ACGCGGGAGACCCGGGTT	Yes	Yes	Yes	No	Yes	No	No	No	No	No	No	No	Yes	GlyCCC
ACGCGGGAGGCCCGGGT	Yes	Yes	Yes	No	Yes	No	No	No	No	No	No	No	No	GlyGCC
ACGGGCTCACATCACCCCATAAACACCA	Yes	Yes	No	No	No	No	No	Yes	No	No	No	No	No	PheGAA
ACGGGTCCCCGGTAGC	No	No	No	No	Yes	No	No	No	No	No	Yes	No	No	NA
ACGGGTCCCCGGTAGCT	No	No	No	No	Yes	No	No	No	No	No	Yes	No	No	NA
ACGGGTCCCCGGTAGCTC	No	No	No	No	Yes	No	No	No	No	No	Yes	No	No	NA
ACGGGTTCGAACCCCGTCCGTGCCTCCA	Yes	Yes	Yes	No	Yes	No	No	No	No	No	No	No	No	ThrCGT
ACGTCGGGGTCACCA	No	No	No	No	Yes	No	Yes	No	No	No	No	No	No	NA
ACGTCTGCTTTACACG	No	Yes	Yes	No	Yes	No	No	No	No	No	No	No	No	ValTAC
ACTAAAAAATCCCTTT	No	No	Yes	No	Yes	No	No	No	No	No	No	Yes	No	NA
ACTAAGTGTTTGTGGGTTTAAGTCCCATT	Yes	Yes	No	No	No	No	No	Yes	Yes	No	No	No	No	AsnGTT
ACTCAAGTTCTGGTC	No	No	No	No	Yes	No	Yes	No	No	No	No	No	No	NA
ACTCAAGTTCTGGTCTC	No	Yes	No	No	Yes	No	No	No	No	No	No	No	No	LeuCAA
ACTCAAGTTCTGGTCTCC	Yes	Yes	No	No	Yes	No	No	No	No	No	No	No	No	LeuCAA
ACTCCAGATCAGAAGGTTGCGTG	Yes	Yes	No	No	Yes	No	No	No	No	No	No	No	No	TrpCCA
ACTCCCGGTATGGGAACCA	Yes	Yes	Yes	No	Yes	No	No	No	No	No	No	No	No	GluTTC
ACTCCCGGTGTGGGAA	Yes	Yes	Yes	No	Yes	No	No	No	No	No	No	No	No	GluTTC
ACTCCCGGTGTGGGAACCA	Yes	Yes	Yes	Yes	Yes	No	No	No	No	No	No	No	No	GluTTC
ACTCCTGGCTGGCTCGCCA	No	Yes	Yes	No	Yes	No	No	No	No	No	No	No	No	ArgACG
ACTCTTTTAGTATAAATAGTACCGT	Yes	Yes	No	No	No	No	No	Yes	No	No	No	No	No	GlyTCC
ACTCTTTTAGTATAAATAGTACCGTTAAC	Yes	Yes	No	No	No	No	No	Yes	No	No	No	No	No	GlyTCC
ACTGGGCGGAAACACCA	Yes	Yes	Yes	No	Yes	No	No	No	No	No	No	No	No	ValCAC
ACTGTAAAGCTAACTTAGCATTAAC	Yes	Yes	No	No	No	No	No	Yes	Yes	No	No	No	No	LysTTT
ACTTAACTTGACCGCTCTGACCA	Yes	Yes	No	No	No	No	No	Yes	No	No	No	No	No	ValTAC
ACTTCGGATCAGAAGATTGAGGGT	Yes	Yes	No	No	Yes	No	No	No	No	No	No	No	No	ArgTCG
ACTTGACCGCTCTGACCA	Yes	Yes	No	No	No	No	No	Yes	No	No	No	No	No	ValTAC
ACTTTGAATCCAGCAATCCGAG	Yes	Yes	Yes	No	Yes	No	No	No	No	No	No	No	No	GlnTTG
ACTTTGAATCCAGCGA	No	Yes	Yes	No	Yes	No	No	No	No	No	No	No	No	GlnTTG
ACTTTGAATCCAGCGATCCGAGT	Yes	Yes	Yes	No	Yes	No	No	No	No	No	No	No	No	GlnTTG
ACTTTGAATCCAGCGATCCGAGTT	Yes	Yes	Yes	No	Yes	No	No	No	No	No	No	No	No	GlnTTG
ACTTTTAAAGGATAACAGCT	Yes	Yes	No	No	No	No	No	Yes	No	No	No	No	No	LeuTAG
ACTTTTAAAGGATAACAGCTAT	Yes	Yes	No	No	No	No	No	Yes	No	No	No	No	No	LeuTAG
ACTTTTAAAGGATAACAGCTATCC	Yes	Yes	No	No	No	No	No	Yes	No	No	No	No	No	LeuTAG
ACTTTTAAAGGATAACAGCTATCCATTGG	Yes	Yes	No	No	No	No	No	Yes	No	No	No	No	No	LeuTAG
ACTTTTAAAGGATAACAGCTATCCATTGGT	Yes	Yes	No	No	No	No	No	Yes	No	No	No	No	No	LeuTAG
ACTTTTAAAGGATAACAGCTATCCATTGGTC	Yes	Yes	No	No	No	No	No	Yes	No	No	No	No	No	LeuTAG
ACTTTTAATCTGAGGGTCC	Yes	Yes	Yes	No	Yes	No	No	No	No	No	No	No	Yes	LysTTT
AGAAAGGCCGAATTT	No	No	No	No	Yes	No	Yes	No	No	No	No	Yes	No	NA
AGAAAGGCCGAATTTT	No	No	No	No	Yes	No	No	No	No	No	No	Yes	No	NA
AGAAAGGCCGAATTTTA	No	No	No	No	Yes	No	No	No	No	No	No	Yes	No	NA
AGAAATTTAGGTTAAATAC	Yes	Yes	No	No	No	No	No	Yes	Yes	No	No	No	No	TrpTCA
AGAAATTTAGGTTAAATACAGA	Yes	Yes	No	No	No	No	No	Yes	Yes	No	No	No	No	TrpTCA
AGAAATTTAGGTTAAATACAGACC	Yes	Yes	No	No	No	No	No	Yes	Yes	No	No	No	No	TrpTCA
AGAAATTTAGGTTAAATACAGACCAAGA	Yes	Yes	No	No	No	No	No	Yes	Yes	No	No	No	No	TrpTCA
AGAAATTTAGGTTAAATACAGACCAAGAGC	Yes	Yes	No	No	No	No	No	Yes	Yes	No	No	No	No	TrpTCA
AGAAATTTAGGTTAAATACAGACCAAGAGCC	Yes	Yes	No	No	No	No	No	Yes	Yes	No	No	No	No	TrpTCA
AGAAGATTCCAGGTT	No	No	Yes	No	Yes	No	Yes	No	No	No	No	No	No	NA
AGAAGATTCTAGGTT	No	No	Yes	No	Yes	No	Yes	No	No	No	No	No	No	NA
AGAAGATTGAGGGTT	No	No	No	No	Yes	No	Yes	No	No	No	No	No	No	NA
AGAAGCAGCTTCAAACCTGCCGGGGCTTCCA	Yes	Yes	No	No	No	No	No	Yes	No	No	No	No	No	CysGCA
AGAATAGTTTAAATTAGAATCTTAGC	Yes	Yes	No	No	No	No	No	Yes	No	No	No	No	No	ProTGG
AGAATTCTCGCCTGCC	No	Yes	Yes	No	Yes	No	No	No	No	No	No	No	No	GlyGCC
AGAATTCTCGCCTGCCAC	No	Yes	Yes	No	Yes	No	No	No	No	No	No	No	No	GlyGCC
AGAATTCTCGCCTGCCACG	Yes	Yes	Yes	No	Yes	No	No	No	No	No	No	No	No	GlyGCC
AGACCCGAGGTAGGGCTTT	No	No	No	No	Yes	No	No	No	No	No	No	Yes	No	NA
AGACCGGGGTTCGATTCCCCGACGGGGAGCCA	Yes	Yes	Yes	No	Yes	No	No	No	No	No	No	No	No	AspGTC
AGACGGGCTCACATCACCCCATAAACACCA	Yes	Yes	No	No	No	No	No	Yes	No	No	No	No	No	PheGAA
AGAGCCCGGTAATCGC	Yes	Yes	No	No	No	No	No	Yes	No	No	No	No	No	LeuTAA
AGAGCCCGGTAATCGCATAAA	Yes	Yes	No	No	No	No	No	Yes	No	No	No	No	No	LeuTAA
AGAGCCCGGTAATCGCATAAAA	Yes	Yes	No	No	No	No	No	Yes	No	No	No	No	No	LeuTAA
AGAGCCCGGTAATCGCATAAAACTTA	Yes	Yes	No	No	No	No	No	Yes	No	No	No	No	No	LeuTAA
AGAGCCCGGTAATCGCATAAAACTTAAAAC	Yes	Yes	No	No	No	No	No	Yes	No	No	No	No	No	LeuTAA
AGAGGAGTTGTTTTT	No	No	Yes	No	Yes	No	Yes	No	No	No	No	Yes	No	NA
AGAGGGTCTTTTTCACCCCGCTGTTGCTCTTT	No	No	Yes	No	Yes	No	No	No	No	No	No	Yes	No	NA
AGAGGGTCTTTTTCACCCCGCTGTTGCTCTTTA	No	No	Yes	No	Yes	No	No	No	No	No	No	Yes	No	NA
AGAGGTCCCGGGTTC	No	No	Yes	No	Yes	No	Yes	No	No	No	No	No	No	NA
AGAGGTCCCGGGTTCAAATCCCGGACGAGCCC	Yes	Yes	Yes	No	Yes	No	No	No	No	No	No	No	No	ProAGG,ProCGG,ProTGG
AGAGTAACATCTTCTTT	No	No	Yes	No	Yes	No	No	No	No	No	No	Yes	No	NA
AGAGTGGCGCAGCGGA	Yes	Yes	Yes	No	Yes	No	No	No	No	No	No	No	No	MetCAT
AGAGTGGGGTTTTGCAGTCCTTACCA	Yes	Yes	No	No	No	No	No	Yes	Yes	No	No	No	No	AlaTGC
AGATCAAAAGGTCCCTGGT	Yes	Yes	No	No	Yes	No	No	No	No	No	No	No	No	CysGCA

AGATCAAGAGGTCCCC	No	Yes	Yes	No	Yes	No	No	No	No	No	No	No	Yes	CysGCA
AGATCAAGAGGTCCCCAGT	Yes	Yes	Yes	No	Yes	No	No	No	No	No	No	No	No	CysGCA
AGATCAAGAGGTCCCCGG	Yes	Yes	Yes	No	Yes	No	No	No	No	No	No	No	Yes	CysGCA
AGATCAAGAGGTCCCCGGT	Yes	Yes	Yes	No	Yes	No	No	No	No	No	No	No	Yes	CysGCA
AGATCAAGAGGTCCCCGGTT	Yes	Yes	Yes	No	Yes	No	No	No	No	No	No	No	Yes	CysGCA
AGATCAAGAGGTCCCTGGT	Yes	Yes	Yes	No	Yes	No	No	No	No	No	No	No	No	CysGCA,SerAGA
AGATCAAGAGGTCCCTGGTT	Yes	Yes	Yes	No	Yes	No	No	No	No	No	No	No	No	CysGCA,SerAGA
AGATCACGGGTTCGAACCCCGTCCGTGCCT	Yes	Yes	Yes	No	Yes	No	No	No	No	No	No	No	No	ThrCGT
AGATCACGGGTTCGAACCCCGTCCGTGCCTCCA	Yes	Yes	No	No	Yes	No	No	No	No	No	No	No	No	ThrCGT
AGATCCTGGGTTCGAATCCCAGCGGTGCCTCCA	Yes	Yes	Yes	No	Yes	No	No	No	No	No	No	No	No	ThrAGT
AGATCTAAAGGTCCCTGGT	No	Yes	Yes	No	Yes	No	No	No	No	No	No	No	No	PheGAA
AGATGAAAACCTTTTTCCAAGGACA	Yes	Yes	No	No	No	No	No	Yes	No	No	No	No	No	ThrTGT
AGATGAAAACCTTTTTCCAAGGACACCA	Yes	Yes	No	No	No	No	No	Yes	No	No	No	No	No	ThrTGT
AGATTCCAGGTTCGACTCCTGGCTGGCTCG	Yes	Yes	Yes	No	Yes	No	No	No	No	No	No	No	No	ArgACG
AGATTCCAGGTTCGACTCCTGGCTGGCTCGCCA	Yes	Yes	Yes	No	Yes	No	No	No	No	No	No	No	No	ArgACG
AGATTCTAGGTTCGACTCCTGGCTGG	Yes	Yes	Yes	No	Yes	No	No	No	No	No	No	No	No	ArgACG
AGATTCTAGGTTCGACTCCTGGCTGGCTCG	Yes	Yes	Yes	No	Yes	No	No	No	No	No	No	No	No	ArgACG
AGATTCTAGGTTCGACTCCTGGCTGGCTCGCCA	Yes	Yes	No	No	Yes	No	No	No	No	No	No	No	No	ArgACG
AGATTGAGGGTTCGAATCCCTCCGTGGTTA	Yes	Yes	No	No	Yes	No	No	No	No	No	No	No	No	ArgTCG
AGATTGAGGGTTCGAATCCCTCCGTGGTTACCA	Yes	Yes	No	No	Yes	No	No	No	No	No	No	No	No	ArgTCG
AGATTGAGGGTTCGAATCCCTTCGTGG	Yes	Yes	No	No	Yes	No	No	No	No	No	No	No	No	ArgTCG
AGATTGAGGGTTCGAATCCCTTCGTGGTTACCA	Yes	Yes	No	No	Yes	No	No	No	No	No	No	No	No	ArgTCG
AGATTGAGGGTTCGAATCCCTTCGTGGTTGCCA	Yes	Yes	No	No	Yes	No	No	No	No	No	No	No	No	ArgTCG
AGATTGAGGGTTCGAGTCCCTTCGTGGTC	Yes	Yes	No	No	Yes	No	No	No	No	No	No	No	No	ArgTCG,ArgCCG
AGATTGAGGGTTCGAGTCCCTTCGTGGTCG	Yes	Yes	No	No	Yes	No	No	No	No	No	No	No	No	ArgTCG,ArgCCG
AGATTGAGGGTTCGAGTCCCTTCGTGGTCGC	Yes	Yes	No	No	Yes	No	No	No	No	No	No	No	No	ArgTCG,ArgCCG
AGATTGAGGGTTCGAGTCCCTTCGTGGTCGCC	Yes	Yes	No	No	Yes	No	No	No	No	No	No	No	No	ArgTCG,ArgCCG
AGATTGAGGGTTCGAGTCCCTTCGTGGTCGCCA	Yes	Yes	No	No	Yes	No	No	No	No	No	No	No	No	ArgTCG,ArgCCG
AGATTGCAGGTTCGAGTCCTGCCGCGGTC	Yes	Yes	Yes	No	Yes	No	No	No	No	No	No	No	No	ArgTCG
AGATTGCAGGTTCGAGTCCTGCCGCGGTCGCCA	Yes	Yes	Yes	No	Yes	No	No	No	No	No	No	No	No	ArgTCG
AGATTTCAACTTAACTTGACCGCTCTGACCA	Yes	Yes	No	No	No	No	No	Yes	No	No	No	No	No	ValTAC
AGCAACCTGTATTTT	No	No	No	No	Yes	No	Yes	No	No	No	No	Yes	No	NA
AGCAACCTGTATTTTT	No	No	No	No	Yes	No	No	No	No	No	No	Yes	No	NA
AGCAGAGTGGCGCAGCGGA	Yes	Yes	Yes	No	Yes	No	No	No	No	No	No	No	No	MetCAT
AGCAGAGTGGCGCAGCGGAAGCGTGCTG	Yes	Yes	Yes	No	Yes	No	No	No	No	No	No	No	No	MetCAT
AGCAGAGTGGCGCAGCGGAAGCGTGCTGG	Yes	Yes	Yes	No	Yes	No	No	No	No	No	No	No	No	MetCAT
AGCAGAGTGGCGCAGCGGAAGCGTGCTGGG	Yes	Yes	Yes	No	Yes	No	No	No	No	No	No	No	No	MetCAT
AGCAGAGTGGCGCAGCGGAAGCGTGCTGGGCC	Yes	Yes	Yes	No	Yes	No	No	No	No	No	No	No	No	MetCAT
AGCAGCTTCAAACCTGCCGGGGCTTCCA	Yes	Yes	No	No	No	No	No	Yes	No	No	No	No	No	CysGCA
AGCCAGGGATTGTGGGT	Yes	Yes	Yes	No	Yes	No	No	No	No	No	No	No	No	ArgCCT
AGCCCACCCAGGGAC	No	No	Yes	No	Yes	No	Yes	No	No	No	No	No	No	NA
AGCCCCACGTTGGGC	No	No	Yes	No	Yes	No	Yes	No	No	No	No	No	No	NA
AGCCCCACGTTGGGCGCCA	No	Yes	Yes	Yes	Yes	No	No	No	No	No	No	No	No	LysCTT
AGCCCCAGTGGAACCACCA	No	Yes	Yes	Yes	Yes	No	No	No	No	No	No	No	No	ValTAC
AGCCCGGTAATCGCATA	Yes	Yes	No	No	No	No	No	Yes	No	No	No	No	No	LeuTAA
AGCCCGGTAATCGCATAAA	Yes	Yes	No	No	No	No	No	Yes	No	No	No	No	No	LeuTAA
AGCCCGGTAATCGCATAAAA	Yes	Yes	No	No	No	No	No	Yes	No	No	No	No	No	LeuTAA
AGCCCGGTAATCGCATAAAAC	Yes	Yes	No	No	No	No	No	Yes	No	No	No	No	No	LeuTAA
AGCCCGGTAATCGCATAAAACTTA	Yes	Yes	No	No	No	No	No	Yes	No	No	No	No	No	LeuTAA
AGCCCGGTAATCGCATAAAACTTAAAA	Yes	Yes	No	No	No	No	No	Yes	No	No	No	No	No	LeuTAA
AGCCCGGTAATCGCATAAAACTTAAAAC	Yes	Yes	No	No	No	No	No	Yes	No	No	No	No	No	LeuTAA
AGCCGTAAGGCGGCTGTTT	No	No	Yes	No	Yes	No	No	No	No	No	No	Yes	No	NA
AGCCGTAAGGCGGCTGTTTT	No	No	Yes	No	Yes	No	No	No	No	No	No	Yes	No	NA
AGCCGTAAGGCGGCTGTTTTT	No	No	Yes	No	Yes	No	No	No	No	No	No	Yes	No	NA
AGCCGTAAGGCGGCTGTTTTTGCTTT	No	No	Yes	No	Yes	No	No	No	No	No	No	Yes	No	NA
AGCCGTGATCGTATAGTGGTTAGT	Yes	Yes	No	No	No	No	No	No	No	Yes	No	No	No	HisGTG
AGCCTCCGGAGCTGGGGA	Yes	Yes	Yes	No	Yes	No	No	No	No	No	No	No	No	ArgCCG
AGCCTCCGGAGCTGGGGATTGTGGGT	Yes	Yes	Yes	No	Yes	No	No	No	No	No	No	No	No	ArgCCG
AGCGGCCTCTGGTGGCGT	No	No	No	No	Yes	No	No	No	No	No	Yes	No	No	NA
AGCGGGTGCTCTTAT	No	No	Yes	No	Yes	No	Yes	No	No	No	No	Yes	No	NA
AGCGGGTGCTCTTATT	No	No	Yes	No	Yes	No	No	No	No	No	No	Yes	No	NA
AGCGGGTGCTCTTATTT	No	No	Yes	No	Yes	No	No	No	No	No	No	Yes	No	NA
AGCGGGTGCTCTTATTTT	No	No	Yes	No	Yes	No	No	No	No	No	No	Yes	No	NA
AGCGGGTGCTCTTATTTTT	No	No	Yes	No	Yes	No	No	No	No	No	No	Yes	No	NA
AGCGGGTGCTCTTATTTTTT	No	No	Yes	No	Yes	No	No	No	No	No	No	Yes	No	NA
AGCGTGCTGGGCCCA	No	No	Yes	No	Yes	No	Yes	No	No	No	No	No	No	NA
AGCGTGCTGGGCCCAT	No	Yes	Yes	No	Yes	No	No	No	No	No	No	No	No	MetCAT
AGCGTGCTGGGCCCATA	Yes	Yes	Yes	No	Yes	No	No	No	No	No	No	No	No	MetCAT
AGCGTTGGTGTGTACTTTGACGTTTGCGGTTT	No	No	No	No	Yes	No	No	No	No	No	No	Yes	No	NA
AGCTATAGGGTGGAG	No	No	Yes	No	Yes	No	Yes	No	No	No	No	Yes	No	NA
AGCTATAGGGTGGAGAC	No	No	Yes	No	Yes	No	No	No	No	No	No	Yes	No	NA
AGCTCACAAGAACTGC	Yes	Yes	No	No	No	No	No	Yes	No	No	No	No	No	SerGCT
AGCTCACAAGAACTGCT	Yes	Yes	No	No	No	No	No	Yes	No	No	No	No	No	SerGCT
AGCTCAGGGGTAGAG	No	No	Yes	No	Yes	No	Yes	No	No	No	No	No	No	NA
AGCTCAGTCGGTAGAG	No	Yes	Yes	No	Yes	No	No	No	No	No	No	No	Yes	LysCTT,LysTTT
AGCTCAGTCGGTAGAGC	No	Yes	Yes	No	Yes	No	No	No	No	No	No	No	Yes	LysCTT,LysTTT
AGCTCAGTCGGTAGAGCATCAGA	Yes	Yes	Yes	No	Yes	No	No	No	No	No	No	No	No	LysTTT
AGCTCCGAGGTGATTT	Yes	Yes	No	No	No	No	No	Yes	No	No	No	No	No	CysGCA
AGCTCCGAGGTGATTTTCAT	Yes	Yes	No	No	No	No	No	Yes	No	No	No	No	No	CysGCA
AGCTCCGAGGTGATTTTCATA	Yes	Yes	No	No	No	No	No	Yes	No	No	No	No	No	CysGCA
AGCTCCGAGGTGATTTTCATATTG	Yes	Yes	No	No	No	No	No	Yes	No	No	No	No	No	CysGCA
AGCTCCGAGGTGATTTTCATATTGA	Yes	Yes	No	No	No	No	No	Yes	No	No	No	No	No	CysGCA
AGCTCCGAGGTGATTTTCATATTGAA	Yes	Yes	No	No	No	No	No	Yes	No	No	No	No	No	CysGCA
AGCTCCGAGGTGATTTTCATATTGAATTGC	Yes	Yes	No	No	No	No	No	Yes	No	No	No	No	No	CysGCA
AGCTTAAACCCCCTTATTTCTACCA	Yes	Yes	No	No	No	No	No	Yes	No	No	No	No	No	IleGAT
AGGACTTCGTCTGTAAT	No	No	No	No	Yes	No	No	No	No	No	No	Yes	No	NA
AGGACTTCGTCTGTAATT	No	No	No	No	Yes	No	No	No	No	No	No	Yes	No	NA
AGGACTTCGTCTGTAATTT	No	No	No	No	Yes	No	No	No	No	No	No	Yes	No	NA

AGGACTTCGTCTGTAATTTT	No	No	No	No	Yes	No	No	No	No	No	No	Yes	No	NA
AGGACTTCGTCTGTAATTTTT	No	No	No	No	Yes	No	No	No	No	No	No	Yes	No	NA
AGGAGATCCTGGGTT	No	No	Yes	No	Yes	No	Yes	No	No	No	No	No	No	NA
AGGAGCTGCCGTATTCTTT	No	No	No	No	Yes	No	No	No	No	No	No	Yes	No	NA
AGGAGTTCACAATTT	No	No	No	No	Yes	No	Yes	No	No	No	No	Yes	No	NA
AGGAGTTCACAATTTT	No	No	No	No	Yes	No	No	No	No	No	No	Yes	No	NA
AGGAGTTCACAATTTTT	No	No	No	No	Yes	No	No	No	No	No	No	Yes	No	NA
AGGAGTTCACAATTTTTT	No	No	No	No	Yes	No	No	No	No	No	No	Yes	No	NA
AGGATGGCCGAGCGG	No	No	Yes	No	Yes	No	Yes	No	No	No	No	No	No	NA
AGGATGGCCGAGCGGTC	No	Yes	Yes	No	Yes	No	No	No	No	No	No	No	No	LeuCAG
AGGATGGCCGAGCGGTCTAA	No	Yes	Yes	No	Yes	No	No	No	No	No	No	No	No	LeuCAG
AGGATGGCCGAGCGGTCTAAG	No	Yes	Yes	No	Yes	No	No	No	No	No	No	No	No	LeuCAG
AGGATGGCCGAGCGGTCTAAGG	No	Yes	Yes	No	Yes	No	No	No	No	No	No	No	No	LeuCAG
AGGATGGCCGAGTGGTCTAAG	Yes	Yes	Yes	No	Yes	No	No	No	No	No	No	No	No	LeuCAA
AGGATTCGGCGCTCTCA	Yes	Yes	Yes	No	Yes	No	No	No	No	No	No	No	Yes	GluCTC
AGGATTCGGCGCTCTCACCGCC	Yes	Yes	Yes	No	Yes	No	No	No	No	No	No	No	Yes	GluCTC
AGGATTCGGCGCTCTCACCGCCGC	Yes	Yes	Yes	No	Yes	No	No	No	No	No	No	No	No	GluCTC
AGGATTCGGCGCTCTCACCGCCGCGGCCC	Yes	Yes	Yes	No	Yes	No	No	No	No	No	No	No	No	GluCTC
AGGATTCGGCGCTTTCACCGCCGC	Yes	Yes	Yes	No	Yes	No	No	No	No	No	No	No	No	GluTTC
AGGCACTGGTGGTTCAGTGGTAGA	No	No	No	No	Yes	No	No	No	No	No	No	No	No	NA
AGGCGATCACGTAGATTT	No	No	Yes	No	Yes	No	No	No	No	No	No	Yes	No	NA
AGGCGATCACGTAGATTTT	No	No	Yes	No	Yes	No	No	No	No	No	No	Yes	No	NA
AGGCTGCGTGTTCGAA	Yes	Yes	No	No	Yes	No	No	No	No	No	No	No	No	TrpCCA
AGGCTGCGTGTTCGAATCACGTCGGGGTCACCA	Yes	Yes	No	No	Yes	No	No	No	No	No	No	No	No	TrpCCA
AGGGAGGTTATGATTA	No	No	Yes	No	Yes	No	No	No	No	No	No	Yes	No	NA
AGGGAGGTTATGATTAACTTT	No	No	Yes	No	Yes	No	No	No	No	No	No	Yes	No	NA
AGGGAGGTTATGATTAACTTTT	No	No	Yes	Yes	Yes	No	No	No	No	No	No	Yes	No	NA
AGGGGTAGAGCATTTGA	No	Yes	Yes	No	Yes	No	No	No	No	No	No	No	No	CysGCA
AGGGGTAGAGCATTTGACT	No	Yes	Yes	No	Yes	No	No	No	No	No	No	No	No	CysGCA
AGGGGTATGATTCTC	No	No	Yes	No	Yes	No	Yes	No	No	No	No	No	No	NA
AGGGGTATGATTCTCG	No	Yes	Yes	No	Yes	No	No	No	No	No	No	No	No	ProAGG,ProCGG,ProTGG
AGGGGTATGATTCTCGC	No	Yes	Yes	No	Yes	No	No	No	No	No	No	No	No	ProAGG,ProCGG,ProTGG
AGGGGTATGATTCTCGCT	No	Yes	Yes	No	Yes	No	No	No	No	No	No	No	No	ProAGG,ProCGG,ProTGG
AGGGGTATGATTCTCGG	Yes	Yes	Yes	No	Yes	No	No	No	No	No	No	No	No	ProTGG
AGGGGTATGATTCTCGGT	Yes	Yes	Yes	No	Yes	No	No	No	No	No	No	No	No	ProTGG
AGGGGTTAGGCCTCTTTTTACCACCA	Yes	Yes	No	No	No	No	No	Yes	No	No	No	No	No	TyrGTA
AGGGTCGTGGGTTCGAGCCCC	No	Yes	Yes	No	Yes	No	No	No	No	No	No	No	No	LysCTT
AGGGTGTGCGTGTTT	No	No	Yes	No	Yes	No	Yes	No	No	No	No	Yes	No	NA
AGGGTGTGCGTGTTTT	No	No	Yes	No	Yes	No	No	No	No	No	No	Yes	No	NA
AGGGTGTGCGTGTTTTT	No	No	Yes	No	Yes	No	No	No	No	No	No	Yes	No	NA
AGGTCCCCGGTTCGAAACCGGGCGGAAAC	Yes	Yes	Yes	No	Yes	No	No	No	No	No	No	No	No	ValCAC,ValAAC
AGGTCCCCGGTTCGAAACCGGGCGGAAACACCA	Yes	Yes	Yes	No	Yes	No	No	No	No	No	No	No	No	ValCAC,ValAAC
AGGTCCTGAGTTCGAACCTCAGAGGGGGCA	Yes	Yes	No	No	Yes	No	No	No	No	No	No	No	No	MetCAT
AGGTCCTGAGTTCGAGCCTCAGAGAGGG	Yes	Yes	Yes	No	Yes	No	No	No	No	No	No	No	No	MetCAT
AGGTCCTGAGTTCGAGCCTCAGAGAGGGCA	Yes	Yes	Yes	No	Yes	No	No	No	No	No	No	No	No	MetCAT
AGGTCCTGGGTTCAAGCCCCAGTGGAACCACCA	Yes	Yes	Yes	No	Yes	No	No	No	No	No	No	No	No	ValTAC
AGGTCCTGGGTTCGAGCCCCAGTGGAACC	Yes	Yes	Yes	No	Yes	No	No	No	No	No	No	No	No	ValTAC
AGGTCCTGGGTTCGAGCCCCAGTGGAACCA	Yes	Yes	Yes	No	Yes	No	No	No	No	No	No	No	No	ValTAC
AGGTCCTGGGTTCGAGCCCCAGTGGAACCAC	Yes	Yes	Yes	No	Yes	No	No	No	No	No	No	No	No	ValTAC
AGGTCCTGGGTTCGAGCCCCAGTGGAACCACC	Yes	Yes	Yes	No	Yes	No	No	No	No	No	No	No	No	ValTAC
AGGTCCTGGGTTCGAGCCCCAGTGGAACCACCA	Yes	Yes	Yes	No	Yes	No	No	No	No	No	No	No	No	ValTAC
AGGTCGCAGTCTCCC	No	No	Yes	No	Yes	No	Yes	No	No	No	No	No	No	NA
AGGTCGTGAGTTCGAGCCTCACACGGGG	Yes	Yes	No	No	Yes	No	No	No	No	No	No	No	No	MetCAT
AGGTCGTGAGTTCGAGCCTCACACGGGGCA	Yes	Yes	No	No	Yes	No	No	No	No	No	No	No	No	MetCAT
AGGTCGTGAGTTCGAGCCTCACACGGGGCACCA	Yes	Yes	No	No	Yes	No	No	No	No	No	No	No	No	MetCAT
AGGTCGTGAGTTCGATCCTCACACGGGGCACCA	Yes	Yes	No	No	Yes	No	No	No	No	No	No	No	No	MetCAT
AGGTGAAAGTTCCTTT	No	No	Yes	Yes	Yes	No	No	No	No	No	No	Yes	No	NA
AGGTGAAAGTTCCTTTT	No	No	Yes	No	Yes	No	No	No	No	No	No	Yes	No	NA
AGGTGGTAGAGCATT	No	No	Yes	No	Yes	No	Yes	No	No	No	No	No	No	NA
AGGTTGCGTGTTCAAATCACGTCGGGGTCACCA	Yes	Yes	No	No	Yes	No	No	No	No	No	No	No	No	TrpCCA
AGGTTGCGTGTTCAAGTCACGTCGGGGTCACCA	Yes	Yes	No	No	Yes	No	No	No	No	No	No	No	No	TrpCCA
AGGTTGGGTTTTTTTT	No	No	No	No	Yes	No	Yes	No	No	No	No	Yes	No	NA
AGGTTGGGTTTTTTTTT	No	No	No	No	Yes	No	No	No	No	No	No	Yes	No	NA
AGTACTCTGCGTTGTGGCCGCAGCAACCTCGGT	Yes	Yes	Yes	No	Yes	No	No	No	No	No	No	No	No	HisGTG
AGTAGGTAGCGCATCAGTC	Yes	Yes	No	No	Yes	No	No	No	No	No	No	No	No	LeuCAA
AGTAGGTAGCGCGTCAGTC	Yes	Yes	No	No	Yes	No	No	No	No	No	No	No	No	MetCAT
AGTATAGTGGTGAGTATCC	No	Yes	Yes	No	Yes	No	No	No	No	No	No	No	No	AspGTC
AGTATAGTGGTGAGTATCCCCGC	No	Yes	Yes	No	Yes	No	No	No	No	No	No	No	No	AspGTC
AGTATTCTCTGTGGCTTT	No	No	Yes	No	Yes	No	No	No	No	No	No	Yes	No	NA
AGTATTCTCTGTGGCTTTT	No	No	Yes	No	Yes	No	No	No	No	No	No	Yes	No	NA
AGTCCCACCAGAGTCGCCA	Yes	Yes	No	No	Yes	No	No	No	No	No	No	No	No	ArgTCT
AGTCCCACCCGGGGTACCA	Yes	Yes	No	No	Yes	No	No	No	No	No	No	No	No	ArgCCT
AGTCCCACCTGGGGTACCA	No	Yes	Yes	No	Yes	No	No	No	No	No	No	No	No	ArgCCT
AGTCCCATCTGGGTCGCCA	No	Yes	Yes	No	Yes	No	No	No	No	No	No	No	No	ArgCCG
AGTCCCATTGGTCTAGCCA	Yes	Yes	No	No	No	No	No	Yes	Yes	No	No	No	No	AsnGTT
AGTCCCTGTTCAGGCGCCA	No	Yes	No	No	Yes	No	No	No	No	No	No	No	No	LysTTT
AGTCCCTGTTCGGGCGCCA	No	Yes	Yes	No	Yes	No	No	No	No	No	No	No	No	LysTTT
AGTCCCTTCGTGGTCGCCA	No	Yes	No	No	Yes	No	No	No	No	No	No	No	No	ArgTCG,ArgCCG
AGTCCGTGCGAGAATACCA	Yes	Yes	No	No	No	No	No	Yes	Yes	No	No	No	No	GluTTC
AGTCCTGTGCTCAGTTTT	No	No	Yes	No	Yes	No	No	No	No	No	No	Yes	No	NA
AGTCGGTAGAGCATC	No	No	Yes	No	Yes	No	Yes	No	No	No	No	No	No	NA
AGTCGGTAGAGCATCA	Yes	Yes	Yes	No	Yes	No	No	No	No	No	No	No	No	LysTTT
AGTCGGTAGAGCATCAG	Yes	Yes	Yes	No	Yes	No	No	No	No	No	No	No	No	LysTTT
AGTCGGTAGAGCATCAGA	Yes	Yes	Yes	No	Yes	No	No	No	No	No	No	No	No	LysTTT
AGTCGGTAGAGCATCAGAC	Yes	Yes	Yes	No	Yes	No	No	No	No	No	No	No	No	LysTTT
AGTCGGTAGAGCATCAGACTT	Yes	Yes	Yes	No	Yes	No	No	No	No	No	No	No	No	LysTTT
AGTCGGTAGAGCATG	No	No	Yes	No	Yes	No	Yes	No	No	No	No	No	No	NA
AGTCGGTAGAGCATGA	No	Yes	Yes	No	Yes	No	No	No	No	No	No	No	No	LysCTT

AGTCGGTAGAGCATGAG	No	Yes	Yes	No	Yes	No	No	No	No	No	No	No	No	LysCTT
AGTCGGTAGAGCATGAGA	No	Yes	Yes	No	Yes	No	No	No	No	No	No	No	No	LysCTT
AGTCGGTAGAGCATGAGAC	No	Yes	Yes	No	Yes	No	No	No	No	No	No	No	No	LysCTT
AGTCGGTAGAGCATGAGACT	No	Yes	Yes	No	Yes	No	No	No	No	No	No	No	No	LysCTT
AGTCGGTAGAGCATGAGACTC	No	Yes	Yes	No	Yes	No	No	No	No	No	No	No	No	LysCTT
AGTCGGTAGAGCATGG	Yes	Yes	Yes	No	Yes	No	No	No	No	No	No	No	No	LysCTT
AGTCGGTAGAGCATGGG	Yes	Yes	Yes	No	Yes	No	No	No	No	No	No	No	No	LysCTT
AGTCGGTAGAGCATGGGA	Yes	Yes	Yes	No	Yes	No	No	No	No	No	No	No	No	LysCTT
AGTCGGTAGAGCATGGGAC	Yes	Yes	Yes	No	Yes	No	No	No	No	No	No	No	No	LysCTT
AGTCGGTAGAGCATGGGACT	Yes	Yes	Yes	No	Yes	No	No	No	No	No	No	No	No	LysCTT
AGTCGGTAGAGCATGGGACTC	Yes	Yes	Yes	No	Yes	No	No	No	No	No	No	No	No	LysCTT
AGTCTCGGTGGAACCTCCA	Yes	Yes	Yes	No	Yes	No	No	No	No	No	No	No	No	GlnCTG
AGTGCCCGATGCTTT	No	No	Yes	No	Yes	No	Yes	No	No	No	No	Yes	No	NA
AGTGCCCGATGCTTTT	No	No	Yes	No	Yes	No	No	No	No	No	No	Yes	No	NA
AGTGCGTGATGCTTT	No	No	Yes	No	Yes	No	Yes	No	No	No	No	Yes	No	NA
AGTGCGTGATGCTTTT	No	No	Yes	No	Yes	No	No	No	No	No	No	Yes	No	NA
AGTGGCGCAGCGGAAGCGTGCTGGGCC	Yes	Yes	Yes	No	Yes	No	No	No	No	No	No	No	No	MetCAT
AGTGGCGCAGCGGAAGCGTGCTGGGCCA	Yes	Yes	Yes	No	Yes	No	No	No	No	No	No	No	No	MetCAT
AGTGGCTAGGATTCGGC	No	Yes	Yes	No	Yes	No	No	No	No	No	No	No	Yes	GluTTC
AGTGGCTAGGATTCGGCG	Yes	Yes	Yes	No	Yes	No	No	No	No	No	No	No	Yes	GluTTC
AGTGGGGTTTTGCAGTCCTTACCA	Yes	Yes	No	No	No	No	No	Yes	Yes	No	No	No	No	AlaTGC
AGTGGGTAGAGCATT	No	No	Yes	No	Yes	No	Yes	No	No	No	No	No	No	NA
AGTGGTAAGCATAGCT	No	Yes	Yes	No	Yes	No	No	No	No	No	No	No	No	GlyTCC
AGTGGTAAGCATAGCTG	No	Yes	Yes	No	Yes	No	No	No	No	No	No	No	No	GlyTCC
AGTGGTAGAATTCTC	No	No	Yes	No	Yes	No	Yes	No	No	No	No	No	No	NA
AGTGGTAGAATTCTCGC	No	Yes	Yes	No	Yes	No	No	No	No	No	No	No	Yes	GlyCCC,ValCAC,GlyGCC,GluTTC
AGTGGTAGAATTCTCGCC	No	Yes	Yes	No	Yes	No	No	No	No	No	No	No	Yes	GlyCCC,ValCAC,GlyGCC,GluTTC
AGTGGTAGAATTCTCGCCTC	No	Yes	Yes	No	Yes	No	No	No	No	No	No	No	Yes	GlyCCC,ValCAC
AGTGGTAGAATTCTCGCCTGCC	No	Yes	Yes	No	Yes	No	No	No	No	No	No	No	No	GlyGCC
AGTGGTAGAGCACAT	No	No	Yes	No	Yes	No	Yes	No	No	No	No	No	No	NA
AGTGGTAGAGCATTT	No	No	Yes	No	Yes	No	Yes	No	No	No	No	No	No	NA
AGTGGTAGAGCATTTG	No	Yes	Yes	No	Yes	No	No	No	No	No	No	No	Yes	CysGCA,CysACA
AGTGGTAGAGCATTTGA	No	Yes	Yes	No	Yes	No	No	No	No	No	No	No	Yes	CysGCA,CysACA
AGTGGTAGAGCATTTGACT	No	Yes	Yes	No	Yes	No	No	No	No	No	No	No	Yes	CysGCA,CysACA
AGTGGTAGAGCATTTGACTGC	No	Yes	Yes	No	Yes	No	No	No	No	No	No	No	No	CysGCA
AGTGGTAGAGCGCAT	No	No	Yes	No	Yes	No	Yes	No	No	No	No	No	No	NA
AGTGGTAGAGCGCATGC	Yes	Yes	Yes	No	Yes	No	No	No	No	No	No	No	No	AlaTGC,AlaCGC,AlaAGC
AGTGGTAGAGCGCGTGCT	Yes	Yes	Yes	No	Yes	No	No	No	No	No	No	No	No	AlaCGC,AlaAGC
AGTGGTATGATTCTCGC	Yes	Yes	Yes	No	Yes	No	No	No	No	No	No	No	No	ProTGG
AGTGGTCATCACGTTCGCC	Yes	Yes	Yes	No	Yes	No	No	No	No	No	No	No	No	ValAAC
AGTGGTGAGCATAGCT	No	Yes	Yes	No	Yes	No	No	No	No	No	No	No	Yes	GlyTCC
AGTGGTGAGCATAGCTG	No	Yes	Yes	No	Yes	No	No	No	No	No	No	No	Yes	GlyTCC
AGTGGTGAGCATAGCTGC	No	Yes	Yes	No	Yes	No	No	No	No	No	No	No	Yes	GlyTCC
AGTGGTGAGCATAGCTGCC	No	Yes	Yes	No	Yes	No	No	No	No	No	No	No	Yes	GlyTCC
AGTGGTGAGTATCCCC	No	Yes	Yes	No	Yes	No	No	No	No	No	No	No	No	AspGTC
AGTGGTGAGTATCCCCGC	No	Yes	Yes	No	Yes	No	No	No	No	No	No	No	No	AspGTC
AGTGGTGAGTATCCCCGCC	Yes	Yes	Yes	No	Yes	No	No	No	No	No	No	No	No	AspGTC
AGTGGTGAGTATCCCCGCCTGT	Yes	Yes	Yes	No	Yes	No	No	No	No	No	No	No	No	AspGTC
AGTGGTTAGCATAGCTG	No	Yes	Yes	No	Yes	No	No	No	No	No	No	No	No	GlyTCC
AGTGGTTAGGATTCGG	No	Yes	Yes	No	Yes	No	No	No	No	No	No	No	Yes	GluCTC
AGTGGTTAGGATTCGGC	No	Yes	Yes	No	Yes	No	No	No	No	No	No	No	Yes	GluCTC
AGTGGTTAGGATTCGGCG	Yes	Yes	Yes	No	Yes	No	No	No	No	No	No	No	Yes	GluCTC
AGTGGTTAGGATTCGGCGC	Yes	Yes	Yes	No	Yes	No	No	No	No	No	No	No	Yes	GluCTC
AGTGGTTAGGATTCGGCGCT	Yes	Yes	Yes	No	Yes	No	No	No	No	No	No	No	Yes	GluCTC
AGTGGTTAGGATTCGGCGCTC	Yes	Yes	Yes	No	Yes	No	No	No	No	No	No	No	Yes	GluCTC
AGTGGTTAGGATTCGGCGCTCT	Yes	Yes	Yes	No	Yes	No	No	No	No	No	No	No	Yes	GluCTC
AGTGGTTAGGATTCGGCGCTCTCA	Yes	Yes	Yes	No	Yes	No	No	No	No	No	No	No	Yes	GluCTC
AGTGGTTAGGATTCGGCGCTCTCAC	Yes	Yes	Yes	No	Yes	No	No	No	No	No	No	No	No	GluCTC
AGTGGTTAGGATTCGGCGCTCTCACCGCC	Yes	Yes	Yes	No	Yes	No	No	No	No	No	No	No	No	GluCTC
AGTGGTTAGGATTCGGCGCTCTCACCGCCGC	Yes	Yes	Yes	No	Yes	No	No	No	No	No	No	No	No	GluCTC
AGTGGTTAGTACTCTGC	No	Yes	Yes	No	Yes	No	No	No	No	No	No	No	No	HisGTG
AGTGGTTAGTACTCTGCG	Yes	Yes	Yes	No	Yes	No	No	No	No	No	No	No	No	HisGTG
AGTGGTTAGTACTCTGCGT	Yes	Yes	Yes	No	Yes	No	No	No	No	No	No	No	No	HisGTG
AGTGGTTAGTACTCTGCGTT	Yes	Yes	Yes	No	Yes	No	No	No	No	No	No	No	No	HisGTG
AGTGGTTAGTATCCCCGC	Yes	Yes	Yes	No	Yes	No	No	No	No	No	No	No	No	AspGTC
AGTGGTTATCACGTTCGC	No	Yes	Yes	No	Yes	No	No	No	No	No	No	No	No	ValCAC,ValAAC
AGTGGTTATCACGTTCGCC	No	Yes	Yes	No	Yes	No	No	No	No	No	No	No	No	ValCAC,ValAAC
AGTGTAGTGGTCATCACGTTC	Yes	Yes	Yes	No	Yes	No	No	No	No	No	No	No	No	ValAAC
AGTGTAGTGGTCATCACGTTCGC	Yes	Yes	Yes	No	Yes	No	No	No	No	No	No	No	No	ValAAC
AGTGTAGTGGTTATC	No	No	Yes	No	Yes	No	Yes	No	No	No	No	No	No	NA
AGTGTAGTGGTTATCACGTTCGC	No	Yes	Yes	No	Yes	No	No	No	No	No	No	No	No	ValCAC,ValAAC
AGTGTAGTGGTTATCACGTTCGCC	No	Yes	Yes	No	Yes	No	No	No	No	No	No	No	No	ValCAC,ValAAC
AGTGTAGTGGTTATCACGTTCGCCT	No	Yes	Yes	No	Yes	No	No	No	No	No	No	No	No	ValCAC,ValAAC
AGTTGACCCGGGTTCGATTCCCGGCCAACG	Yes	Yes	Yes	No	Yes	No	No	No	No	No	No	No	No	GlyTCC
AGTTGGTAGAGCATC	No	No	No	No	Yes	No	Yes	No	No	No	No	No	No	NA
AGTTGGTAGAGCATCAG	No	Yes	No	No	Yes	No	No	No	No	No	No	No	No	SupTTA,LysTTT
AGTTGGTAGAGCATCAGA	Yes	Yes	No	No	Yes	No	No	No	No	No	No	No	No	SupTTA,LysTTT
AGTTGGTAGAGCATCAGAC	Yes	Yes	No	No	Yes	No	No	No	No	No	No	No	No	SupTTA,LysTTT
AGTTGGTAGAGCGGAGGAC	No	Yes	Yes	No	Yes	No	No	No	No	No	No	No	No	TyrGTA
AGTTTCTGTGGTGTAGTGGTTATTAT	Yes	Yes	No	No	Yes	No	No	No	No	Yes	No	No	Yes	ValCAC
ATAAACTAATACACCAGT	Yes	Yes	No	No	No	No	No	Yes	No	No	No	No	No	ThrTGT
ATAACAGCTATCCATTGG	Yes	Yes	No	No	No	No	No	Yes	No	No	No	No	No	LeuTAG
ATAACCCAGAGGTCG	No	No	Yes	No	Yes	No	Yes	No	No	No	No	No	No	NA
ATAACCCAGAGGTCGA	No	Yes	Yes	No	Yes	No	No	No	No	No	No	No	No	MetCAT
ATAACCCAGAGGTCGATG	No	Yes	Yes	No	Yes	No	No	No	No	No	No	No	No	MetCAT
ATAACCCAGAGGTCGATGGA	No	Yes	Yes	No	Yes	No	No	No	No	No	No	No	No	MetCAT
ATAACGCCAAGGTCGCGGGT	Yes	Yes	Yes	No	Yes	No	No	No	No	No	No	No	No	IleAAT
ATAACGCTAAGGTCGCGGGT	Yes	Yes	Yes	No	Yes	No	No	No	No	No	No	No	No	IleAAT

ATAATCTGAAGGTCCTGAGT	No	Yes	Yes	No	Yes	No	No	No	No	No	No	No	No	MetCAT
ATAATCTGAAGGTCGTGAGT	Yes	Yes	No	No	Yes	No	No	No	No	No	No	No	No	MetCAT
ATAGCTCAGTCGGTAGAGCATCAGA	Yes	Yes	Yes	No	Yes	No	No	No	No	No	No	No	No	LysTTT
ATAGCTCAGTTGGTAGAGCGGAGGA	No	Yes	Yes	No	Yes	No	No	No	No	No	No	No	No	TyrGTA
ATAGGTATTAAGGTTT	No	No	Yes	No	Yes	No	No	No	No	No	No	Yes	No	NA
ATAGGTATTAAGGTTTT	No	No	Yes	Yes	Yes	No	No	No	No	No	No	Yes	No	NA
ATAGGTGGCACGGAGA	Yes	Yes	No	No	No	No	No	Yes	Yes	No	No	No	No	GlnTTG
ATAGTGGTGAGTATCCCC	No	Yes	Yes	No	Yes	No	No	No	No	No	No	No	No	AspGTC
ATAGTGGTGAGTATCCCCG	No	Yes	Yes	No	Yes	No	No	No	No	No	No	No	No	AspGTC
ATAGTGGTGAGTATCCCCGC	No	Yes	Yes	No	Yes	No	No	No	No	No	No	No	No	AspGTC
ATAGTGGTGAGTATCCCCGCC	Yes	Yes	Yes	No	Yes	No	No	No	No	No	No	No	No	AspGTC
ATAGTGGTGAGTATCCCCGCCTG	Yes	Yes	Yes	No	Yes	No	No	No	No	No	No	No	No	AspGTC
ATAGTGGTGAGTATCCCCGCCTGT	Yes	Yes	Yes	No	Yes	No	No	No	No	No	No	No	No	AspGTC
ATAGTGGTGAGTATCCCCGCCTGTC	Yes	Yes	Yes	No	Yes	No	No	No	No	No	No	No	No	AspGTC
ATAGTGTAGTGGTTATC	No	Yes	Yes	No	Yes	No	No	No	No	No	No	No	No	ValTAC
ATAGTTTAAACAAAACGA	Yes	Yes	No	No	No	No	No	Yes	No	No	No	No	No	ArgTCG
ATAGTTTAAACAAAACGAATGA	Yes	Yes	No	No	No	No	No	Yes	No	No	No	No	No	ArgTCG
ATAGTTTAAACAAAACGAATGATTTCGACTC	Yes	Yes	No	No	No	No	No	Yes	No	No	No	No	No	ArgTCG
ATATAGTTTAAACAAAACGAATGA	Yes	Yes	No	No	No	No	No	Yes	No	No	No	No	No	ArgTCG
ATATCCAACCTTCAGCTATAGGGTGGAGAC	No	No	Yes	No	Yes	No	No	No	No	No	No	Yes	No	NA
ATATCCAACCTTCAGCTATAGGGTGGAGACTTT	No	No	Yes	No	Yes	No	No	No	No	No	No	Yes	No	NA
ATATGCAGTTTCTGTGGTGTAGTGGTTATTAT	No	No	No	No	Yes	No	No	No	No	No	No	No	No	NA
ATATGGTCTAGCGGTTAGG	Yes	Yes	Yes	No	Yes	No	No	No	No	No	No	No	No	GluTTC
ATGAAAACCTTTTTCCAAGGACA	Yes	Yes	No	No	No	No	No	Yes	No	No	No	No	No	ThrTGT
ATGAAAACCTTTTTCCAAGGACACCA	Yes	Yes	No	No	No	No	No	Yes	No	No	No	No	No	ThrTGT
ATGATTCTCGCTTCGGGTGCGAG	Yes	Yes	Yes	No	Yes	No	No	No	No	No	No	No	No	ProCGG
ATGATTCTCGCTTTGGGTGCGAG	Yes	Yes	Yes	No	Yes	No	No	No	No	No	No	No	Yes	ProTGG
ATGCCCGCATCCTCCACCA	Yes	Yes	No	No	Yes	No	No	No	No	No	No	No	No	AlaAGC
ATGCCCGCATTCTCCACCA	Yes	Yes	No	No	Yes	No	No	No	No	No	No	No	No	AlaAGC
ATGGATAAGGCATCA	No	No	Yes	No	Yes	No	Yes	No	No	No	No	No	No	NA
ATGGATAAGGCATCAGC	No	Yes	Yes	No	Yes	No	No	No	No	No	No	No	No	ArgCCG
ATGGATAAGGCATCAGCCT	Yes	Yes	Yes	No	Yes	No	No	No	No	No	No	No	No	ArgCCG
ATGGATAAGGCATCAGCCTCC	Yes	Yes	Yes	No	Yes	No	No	No	No	No	No	No	No	ArgCCG
ATGGATAAGGCATCAGCCTCCGGAGCTGG	Yes	Yes	Yes	No	Yes	No	No	No	No	No	No	No	No	ArgCCG
ATGGATAAGGCATTG	No	No	No	No	Yes	No	Yes	No	No	No	No	No	No	NA
ATGGATAAGGCATTGGC	No	Yes	No	No	Yes	No	No	No	No	No	No	No	No	ArgCCT
ATGGATAAGGCGTCTGA	Yes	Yes	Yes	No	Yes	No	No	No	No	No	No	No	No	ArgTCG,ArgCCG
ATGGATAGCGCATTGG	No	Yes	No	No	Yes	No	No	No	No	No	No	No	No	ArgTCT
ATGGATAGCGCATTGGA	No	Yes	No	No	Yes	No	No	No	No	No	No	No	No	ArgTCT
ATGGCCGCATATATT	No	No	Yes	No	Yes	No	Yes	No	No	No	No	Yes	No	NA
ATGGCCGCATATATTT	No	No	Yes	Yes	Yes	No	No	No	No	No	No	Yes	No	NA
ATGGCCGCATATATTTT	No	No	Yes	No	Yes	No	No	No	No	No	No	Yes	No	NA
ATGGCGTTTGTTTTT	No	No	No	No	Yes	No	Yes	No	No	No	No	Yes	No	NA
ATGGGTGGTTCAGTGGTAGA	No	Yes	Yes	No	Yes	No	No	No	No	No	No	No	No	GlyGCC
ATGGGTGGTTCAGTGGTAGAA	No	Yes	Yes	No	Yes	No	No	No	No	No	No	No	No	GlyGCC
ATGGGTGGTTCAGTGGTAGAAT	No	Yes	Yes	No	Yes	No	No	No	No	No	No	No	No	GlyGCC
ATGGGTGGTTCAGTGGTAGAATTCTC	Yes	Yes	Yes	No	Yes	No	No	No	No	No	No	No	No	GlyGCC
ATGGGTGGTTCAGTGGTAGAATTCTCG	Yes	Yes	Yes	No	Yes	No	No	No	No	No	No	No	No	GlyGCC
ATGGGTGGTTCAGTGGTAGAATTCTCGC	Yes	Yes	Yes	No	Yes	No	No	No	No	No	No	No	No	GlyGCC
ATGGGTGGTTCAGTGGTAGAATTCTCGCC	Yes	Yes	Yes	No	Yes	No	No	No	No	No	No	No	No	GlyGCC
ATGGGTGGTTCAGTGGTAGAATTCTCGCCT	Yes	Yes	Yes	No	Yes	No	No	No	No	No	No	No	No	GlyGCC
ATGGTAGAGCGCTCGC	Yes	Yes	No	No	Yes	No	No	No	No	No	No	No	No	AlaAGC
ATGGTCTAGCGGTTAGG	Yes	Yes	Yes	No	Yes	No	No	No	No	No	No	No	No	GluTTC
ATGGTCTAGCGGTTAGGA	Yes	Yes	Yes	No	Yes	No	No	No	No	No	No	No	No	GluTTC
ATGGTCTAGCGGTTAGGATTCCTGG	Yes	Yes	Yes	No	Yes	No	No	No	No	No	No	No	No	GluTTC
ATGGTGGAGTTAAAGACTTTTTCTCTGACCA	Yes	Yes	No	No	No	No	No	Yes	No	No	No	No	No	ProTGG
ATGGTGTAATGGTTAGC	No	Yes	Yes	No	Yes	No	No	No	No	No	No	No	No	GlnCTG,GlnTTG
ATGGTTAGCACTCTGG	No	Yes	Yes	No	Yes	No	No	No	No	No	No	No	No	GlnCTG,GlnTTG
ATGGTTAGCACTCTGGA	No	Yes	Yes	No	Yes	No	No	No	No	No	No	No	No	GlnCTG,GlnTTG
ATGGTTAGCACTCTGGACT	No	Yes	Yes	No	Yes	No	No	No	No	No	No	No	No	GlnCTG,GlnTTG
ATGGTTAGCACTCTGGACTC	No	Yes	Yes	No	Yes	No	No	No	No	No	No	No	No	GlnCTG
ATGGTTAGCACTCTGGACTCTGAATCCA	Yes	Yes	Yes	No	Yes	No	No	No	No	No	No	No	No	GlnCTG
ATGGTTAGCACTCTGGACTTTGAATCCA	Yes	Yes	Yes	No	Yes	No	No	No	No	No	No	No	No	GlnTTG
ATGTGGTGGCTTACTT	No	No	Yes	No	Yes	No	No	No	No	No	No	Yes	No	NA
ATGTGGTGGCTTACTTT	No	No	Yes	Yes	Yes	No	No	No	No	No	No	Yes	No	NA
ATGTGGTGGCTTACTTTT	No	No	Yes	No	Yes	No	No	No	No	No	No	Yes	No	NA
ATGTTTAGACGGGCTCAC	Yes	Yes	No	No	No	No	No	Yes	No	No	No	No	No	PheGAA
ATGTTTAGACGGGCTCACATCACCCC	Yes	Yes	No	No	No	No	No	Yes	No	No	No	No	No	PheGAA
ATTAAGAGAACCAACACCTCTTTACAGTGACCA	Yes	Yes	No	No	No	No	No	Yes	Yes	No	No	No	No	LysTTT
ATTCAAAGGTTCCGGGT	Yes	Yes	No	No	Yes	No	No	No	No	No	No	No	No	ArgTCT
ATTCCAGGTTCGACTCCTGGCTGGCTCGCCA	Yes	Yes	Yes	No	Yes	No	No	No	No	No	No	No	No	ArgACG
ATTCCCCGACGGGGA	No	No	Yes	No	Yes	No	Yes	No	No	No	No	No	No	NA
ATTCCCCGACGGGGAG	No	Yes	Yes	No	Yes	No	No	No	No	No	No	No	Yes	AspGTC
ATTCCCCGACGGGGAGC	Yes	Yes	Yes	No	Yes	No	No	No	No	No	No	No	Yes	AspGTC
ATTCCCCGACGGGGAGCCA	Yes	Yes	Yes	No	Yes	No	No	No	No	No	No	No	Yes	AspGTC
ATTCCCGGCCAACGCACCA	Yes	Yes	Yes	No	Yes	No	No	No	No	No	No	No	No	GlyTCC
ATTCCCGGCCAATGCACCA	No	Yes	Yes	No	Yes	No	No	No	No	No	No	No	No	GlyCCC,GlyGCC
ATTCCCGGCCCATGCACCA	Yes	Yes	Yes	No	Yes	No	No	No	No	No	No	No	No	GlyGCC
ATTCCCGGTCAGGGA	No	No	Yes	No	Yes	No	Yes	No	No	No	No	No	No	NA
ATTCCCGGTCAGGGAA	Yes	Yes	Yes	No	Yes	No	No	No	No	No	No	No	Yes	GluCTC,PheGAA,GluTTC
ATTCCCGGTCAGGGAAC	Yes	Yes	Yes	No	Yes	No	No	No	No	No	No	No	Yes	GluCTC,PheGAA,GluTTC
ATTCCCGGTCAGGGAACC	Yes	Yes	Yes	No	Yes	No	No	No	No	No	No	No	Yes	GluCTC,PheGAA,GluTTC
ATTCCCGGTCAGGGAACCA	Yes	Yes	Yes	No	Yes	No	No	No	No	No	No	No	Yes	GluCTC,PheGAA,GluTTC
ATTCCGGCTCGAAGGACCA	Yes	Yes	Yes	No	Yes	No	No	No	No	No	No	No	No	TyrGTA
ATTCCTTCCTTTTTTGCCA	Yes	Yes	No	No	Yes	No	No	Yes	Yes	No	No	No	Yes	SerTGA
ATTCGGCGCTCTCACCGCC	Yes	Yes	Yes	No	Yes	No	No	No	No	No	No	No	Yes	GluCTC
ATTCGGCGCTCTCACCGCCGCGGCCC	Yes	Yes	Yes	No	Yes	No	No	No	No	No	No	No	No	GluCTC
ATTCTAGGTTCGACTCCTGGCTGGCTCGCCA	Yes	Yes	Yes	No	Yes	No	No	No	No	No	No	No	No	ArgACG

ATTCTCATAGTCCTAGCCA	Yes	Yes	No	No	No	No	No	Yes	Yes	No	No	No	No	GlnTTG
ATTCTCGCCTGCCAC	No	No	Yes	No	Yes	No	Yes	No	No	No	No	No	No	NA
ATTCTCGCCTGCCACG	Yes	Yes	Yes	No	Yes	No	No	No	No	No	No	No	No	GlyGCC
ATTCTCGCCTGCCACGCGGGAGGCCCGGGT	Yes	Yes	Yes	No	Yes	No	No	No	No	No	No	No	No	GlyGCC
ATTGAGGGTTCGAATCCCTCCGTGGTTACCA	Yes	Yes	No	No	Yes	No	No	No	No	No	No	No	No	ArgTCG
ATTGAGGGTTCGAATCCCTTCGTGGTTGCCA	Yes	Yes	No	No	Yes	No	No	No	No	No	No	No	No	ArgTCG
ATTGAGGGTTCGAGTCCCTTCGTGGTCGCCA	Yes	Yes	No	No	Yes	No	No	No	No	No	No	No	No	ArgTCG,ArgCCG
ATTGGTCGTGGTTGTAGTCCGTGCGAGA	Yes	Yes	No	No	No	No	No	Yes	Yes	No	No	No	No	GluTTC
ATTGGTGGTTCAGTGGTAGA	No	Yes	Yes	No	Yes	No	No	No	No	No	No	No	No	GlyCCC,GlyGCC
ATTGGTGGTTCAGTGGTAGAA	No	Yes	Yes	No	Yes	No	No	No	No	No	No	No	No	GlyCCC,GlyGCC
ATTGGTGGTTCAGTGGTAGAATTCTC	No	Yes	Yes	No	Yes	No	No	No	No	No	No	No	No	GlyCCC,GlyGCC
ATTGGTGGTTCAGTGGTAGAATTCTCG	No	Yes	Yes	No	Yes	No	No	No	No	No	No	No	No	GlyCCC,GlyGCC
ATTGGTGGTTCAGTGGTAGAATTCTCGCC	No	Yes	Yes	No	Yes	No	No	No	No	No	No	No	No	GlyCCC,GlyGCC
ATTGTACAGACATTTGCG	No	No	Yes	No	Yes	No	No	No	No	No	No	No	No	NA
ATTGTACAGACATTTGCGG	No	No	Yes	No	Yes	No	No	No	No	No	No	No	No	NA
ATTGTATAGACATTTGCGG	No	No	Yes	No	Yes	No	No	No	No	No	No	No	No	NA
ATTTCAACTTAACTTGACCGCTCTGACCA	Yes	Yes	No	No	No	No	No	Yes	No	No	No	No	No	ValTAC
ATTTCGATGGCGTGGGT	Yes	Yes	Yes	No	Yes	No	No	No	No	No	No	No	No	LeuTAG
CAAAAAAAAAAAAAA	No	No	No	No	Yes	No	Yes	No	No	No	Yes	No	No	NA
CAAAAAAAAAAAAAAA	No	No	No	No	Yes	No	No	No	No	No	Yes	No	No	NA
CAAAAAAAAAAAAAAAA	No	No	No	No	Yes	No	No	No	No	No	Yes	No	No	NA
CAAATCCCGGACGAGCCCCCA	Yes	Yes	Yes	No	Yes	No	No	No	No	No	No	No	No	ProAGG,ProCGG,ProTGG
CAAATCTCGGTGGAACCTCCA	Yes	Yes	Yes	No	Yes	No	No	No	No	No	No	No	No	GlnCTG
CAACTTAACTTGACCGCTCTGACCA	Yes	Yes	No	No	No	No	No	Yes	No	No	No	No	No	ValTAC
CAAGAGGTCCCCGGT	No	No	Yes	No	Yes	No	Yes	No	No	No	No	No	No	NA
CAAGAGGTCCCTGGT	No	No	Yes	No	Yes	No	Yes	No	No	No	No	No	No	NA
CAAGAGGTCCCTGGTT	No	Yes	Yes	No	Yes	No	No	No	No	No	No	No	No	TyrGTA,CysGCA,SerAGA
CAATCCCCGGCATCTCCACCA	Yes	Yes	Yes	No	Yes	No	No	No	No	No	No	No	No	AlaTGC,AlaCGC
CAATGGAGGCGTGGGT	Yes	Yes	Yes	No	Yes	No	No	No	No	No	No	No	No	LeuCAA
CAATGGCTTGGTTTT	No	No	Yes	No	Yes	No	Yes	No	No	No	No	Yes	No	NA
CAATGGCTTGGTTTTT	No	No	Yes	No	Yes	No	No	No	No	No	No	Yes	No	NA
CACCCAGGGACGCCA	No	No	Yes	No	Yes	No	Yes	No	No	No	No	No	No	NA
CACCTCAGAAGGTCTCAC	No	No	Yes	No	Yes	No	No	No	No	No	No	Yes	No	NA
CACCTCAGAAGGTCTCACT	No	No	Yes	No	Yes	No	No	No	No	No	No	Yes	No	NA
CACCTCAGAAGGTCTCACTTT	No	No	Yes	No	Yes	No	No	No	No	No	No	Yes	No	NA
CACCTCAGAAGGTCTCACTTTT	No	No	Yes	No	Yes	No	No	No	No	No	No	Yes	No	NA
CACGCGAAAGGTCCCCGGT	Yes	Yes	Yes	No	Yes	No	No	No	No	No	No	No	No	ValCAC,ValAAC
CACGCGGGAGACCCGG	No	Yes	Yes	No	Yes	No	No	No	No	No	No	No	Yes	GlyCCC
CACGCGGGAGACCCGGG	Yes	Yes	Yes	No	Yes	No	No	No	No	No	No	No	Yes	GlyCCC
CACGCGGGAGACCCGGGT	Yes	Yes	Yes	No	Yes	No	No	No	No	No	No	No	Yes	GlyCCC
CACGCGGGAGACCCGGGTT	Yes	Yes	Yes	No	Yes	No	No	No	No	No	No	No	Yes	GlyCCC
CACGCGGGAGACCCGGGTTT	Yes	Yes	No	No	Yes	No	No	No	No	No	No	No	Yes	GlyCCC
CACGCGGGAGACCGG	No	No	Yes	No	Yes	No	Yes	No	No	No	No	No	No	NA
CACGCGGGAGGCCCGGGT	Yes	Yes	Yes	No	Yes	No	No	No	No	No	No	No	No	GlyGCC
CACTCTGGACTCTGAATCCA	No	Yes	Yes	No	Yes	No	No	No	No	No	No	No	No	GlnCTG
CACTCTGGACTTTGAATC	No	Yes	Yes	No	Yes	No	No	No	No	No	No	No	No	GlnTTG
CACTGGTGGTTCAGTGGTAGAATTCT	Yes	Yes	No	No	Yes	No	No	No	No	No	No	No	Yes	GlyCCC,ValCAC
CACTGTAAAGCTAACTTAGC	Yes	Yes	No	No	No	No	No	Yes	Yes	No	No	No	No	LysTTT
CACTGTAAAGCTAACTTAGCA	Yes	Yes	No	No	No	No	No	Yes	Yes	No	No	No	No	LysTTT
CACTGTAAAGCTAACTTAGCATT	Yes	Yes	No	No	No	No	No	Yes	Yes	No	No	No	No	LysTTT
CACTGTAAAGCTAACTTAGCATTA	Yes	Yes	No	No	No	No	No	Yes	Yes	No	No	No	No	LysTTT
CACTGTAAAGCTAACTTAGCATTAAC	Yes	Yes	No	No	No	No	No	Yes	Yes	No	No	No	No	LysTTT
CACTGTAAAGCTAACTTAGCATTAACC	Yes	Yes	No	No	No	No	No	Yes	Yes	No	No	No	No	LysTTT
CACTGTAAAGCTAACTTAGCATTAACCT	Yes	Yes	No	No	No	No	No	Yes	Yes	No	No	No	No	LysTTT
CACTGTAAAGCTAACTTAGCATTAACCTT	Yes	Yes	No	No	No	No	No	Yes	Yes	No	No	No	No	LysTTT
CACTTCTGACACAGCTATT	No	No	No	No	Yes	No	No	No	No	No	No	Yes	No	NA
CAGAAGATTGAGGGT	No	No	No	No	Yes	No	Yes	No	No	No	No	No	No	NA
CAGACCAAGAGCCTTCA	Yes	Yes	No	No	No	No	No	Yes	Yes	No	No	No	No	TrpTCA
CAGAGAATAGTTTAAATTAGA	Yes	Yes	No	No	No	No	No	Yes	No	No	No	No	No	ProTGG
CAGAGAATAGTTTAAATTAGAA	Yes	Yes	No	No	No	No	No	Yes	No	No	No	No	No	ProTGG
CAGAGAATAGTTTAAATTAGAAT	Yes	Yes	No	No	No	No	No	Yes	No	No	No	No	No	ProTGG
CAGAGAATAGTTTAAATTAGAATC	Yes	Yes	No	No	No	No	No	Yes	No	No	No	No	No	ProTGG
CAGAGAATAGTTTAAATTAGAATCTTA	Yes	Yes	No	No	No	No	No	Yes	No	No	No	No	No	ProTGG
CAGAGAATAGTTTAAATTAGAATCTTAGC	Yes	Yes	No	No	No	No	No	Yes	No	No	No	No	No	ProTGG
CAGAGAATAGTTTAAATTAGAATCTTAGCT	Yes	Yes	No	No	No	No	No	Yes	No	No	No	No	No	ProTGG
CAGAGAATAGTTTAAATTAGAATCTTAGCTTT	Yes	Yes	No	No	No	No	No	Yes	No	No	No	No	No	ProTGG
CAGAGTGGCGCAGCGGAAGCGTGCTGGGC	Yes	Yes	Yes	No	Yes	No	No	No	No	No	No	No	No	MetCAT
CAGATCAAGAGGTCCCC	No	Yes	Yes	No	Yes	No	No	No	No	No	No	No	Yes	CysGCA
CAGATCAAGAGGTCCCCGGT	Yes	Yes	Yes	No	Yes	No	No	No	No	No	No	No	Yes	CysGCA
CAGATCAAGAGGTCCCTGGT	Yes	Yes	Yes	No	Yes	No	No	No	No	No	No	No	No	CysGCA
CAGATCAAGAGGTCCCTGGTT	Yes	Yes	Yes	No	Yes	No	No	No	No	No	No	No	No	CysGCA
CAGCAGTACATGCAGA	No	No	No	No	Yes	No	No	No	No	No	No	No	No	NA
CAGCAGTACATGCAGAGCA	No	No	No	No	Yes	No	No	No	No	No	No	No	No	NA
CAGCCTCCGGAGCTGGGGATTGTGGGT	Yes	Yes	Yes	No	Yes	No	No	No	No	No	No	No	No	ArgCCG
CAGCTTCAAACCTGCCGGGGCTTCCA	Yes	Yes	No	No	No	No	No	Yes	No	No	No	No	No	CysGCA
CAGGAGATCCTGGGT	No	No	Yes	No	Yes	No	Yes	No	No	No	No	No	No	NA
CAGGAGATCCTGGGTT	No	Yes	Yes	No	Yes	No	No	No	No	No	No	No	No	ThrAGT,ThrTGT,ThrCGT
CAGGGGTTAGGCCTCTTTTTACC	Yes	Yes	No	No	No	No	No	Yes	No	No	No	No	No	TyrGTA
CAGGGGTTAGGCCTCTTTTTACCACC	Yes	Yes	No	No	No	No	No	Yes	No	No	No	No	No	TyrGTA
CAGGGGTTAGGCCTCTTTTTACCACCA	Yes	Yes	No	No	No	No	No	Yes	No	No	No	No	No	TyrGTA
CAGGTCGCAGTCTCCC	Yes	Yes	Yes	No	Yes	No	No	No	No	No	No	No	No	LeuCAG
CAGGTGCCCCCTCCA	No	No	Yes	No	Yes	No	Yes	No	No	No	No	No	No	NA
CAGTCGGTAGAGCAT	No	No	Yes	No	Yes	No	Yes	No	No	No	No	No	No	NA
CAGTCGGTAGAGCATC	Yes	Yes	Yes	No	Yes	No	No	No	No	No	No	No	No	LysTTT
CAGTCGGTAGAGCATCA	Yes	Yes	Yes	No	Yes	No	No	No	No	No	No	No	No	LysTTT
CAGTCGGTAGAGCATCAG	Yes	Yes	Yes	No	Yes	No	No	No	No	No	No	No	No	LysTTT
CAGTCGGTAGAGCATCAGA	Yes	Yes	Yes	No	Yes	No	No	No	No	No	No	No	No	LysTTT
CAGTCGGTAGAGCATCAGAC	Yes	Yes	Yes	No	Yes	No	No	No	No	No	No	No	No	LysTTT

CAGTCGGTAGAGCATCAGACTT	Yes	Yes	Yes	No	Yes	No	No	No	No	No	No	No	No	LysTTT
CAGTCGGTAGAGCATG	No	Yes	Yes	No	Yes	No	No	No	No	No	No	No	No	LysCTT
CAGTCGGTAGAGCATGAG	No	Yes	Yes	No	Yes	No	No	No	No	No	No	No	No	LysCTT
CAGTCGGTAGAGCATGAGA	No	Yes	Yes	No	Yes	No	No	No	No	No	No	No	No	LysCTT
CAGTCGGTAGAGCATGAGAC	No	Yes	Yes	No	Yes	No	No	No	No	No	No	No	No	LysCTT
CAGTCGGTAGAGCATGAGACTC	No	Yes	Yes	No	Yes	No	No	No	No	No	No	No	No	LysCTT
CAGTCGGTAGAGCATGGG	Yes	Yes	Yes	No	Yes	No	No	No	No	No	No	No	No	LysCTT
CAGTCGGTAGAGCATGGGA	Yes	Yes	Yes	No	Yes	No	No	No	No	No	No	No	No	LysCTT
CAGTCGGTAGAGCATGGGAC	Yes	Yes	Yes	No	Yes	No	No	No	No	No	No	No	No	LysCTT
CAGTCGGTAGAGCATGGGACTCTTAATCCC	Yes	Yes	Yes	No	Yes	No	No	No	No	No	No	No	No	LysCTT
CAGTCTCATAATCTGAAGGTCCTGAGT	No	Yes	Yes	No	Yes	No	No	No	No	No	No	No	No	MetCAT
CAGTCTCATAATCTGAAGGTCGTGAGT	Yes	Yes	No	No	Yes	No	No	No	No	No	No	No	No	MetCAT
CAGTGGTAGAATTCTC	No	Yes	Yes	No	Yes	No	No	No	No	No	No	No	Yes	ArgCCT,GlyCCC,ValCAC,GlyGCC,GluTTC
CAGTGGTAGAATTCTCGC	No	Yes	Yes	No	Yes	No	No	No	No	No	No	No	Yes	GlyCCC,ValCAC,GlyGCC,GluTTC
CAGTGGTAGAATTCTCGCC	No	Yes	Yes	No	Yes	No	No	No	No	No	No	No	Yes	GlyCCC,ValCAC,GlyGCC,GluTTC
CAGTGGTAGAATTCTCGCCTC	No	Yes	Yes	No	Yes	No	No	No	No	No	No	No	Yes	GlyCCC,ValCAC
CAGTGGTAGAATTCTCGCCTCC	No	Yes	Yes	No	Yes	No	No	No	No	No	No	No	Yes	GlyCCC
CAGTGGTAGAATTCTCGCCTGCC	No	Yes	Yes	No	Yes	No	No	No	No	No	No	No	No	GlyGCC
CAGTGGTAGAGCACA	No	No	Yes	No	Yes	No	Yes	No	No	No	No	No	No	NA
CAGTGGTAGAGCACAT	Yes	Yes	Yes	No	Yes	No	No	No	No	No	No	No	No	AlaTGC
CAGTGGTAGAGCATT	No	No	Yes	No	Yes	No	Yes	No	No	No	No	No	No	NA
CAGTGGTAGAGCATTT	No	Yes	Yes	No	Yes	No	No	No	No	No	No	No	Yes	TyrGTA,CysGCA,CysACA
CAGTGGTAGAGCATTTGA	No	Yes	Yes	No	Yes	No	No	No	No	No	No	No	Yes	CysGCA,CysACA
CAGTGGTAGAGCATTTGACT	No	Yes	Yes	No	Yes	No	No	No	No	No	No	No	Yes	CysGCA,CysACA
CAGTGGTAGAGCATTTGACTGC	No	Yes	Yes	No	Yes	No	No	No	No	No	No	No	No	CysGCA
CAGTGGTAGAGCATTTGACTGCA	No	Yes	Yes	No	Yes	No	No	No	No	No	No	No	No	CysGCA
CAGTGGTAGAGCGCA	No	No	Yes	No	Yes	No	Yes	No	No	No	No	No	No	NA
CAGTGGTAGAGCGCAT	Yes	Yes	Yes	No	Yes	No	No	No	No	No	No	No	No	AlaTGC,AlaCGC,AlaAGC
CAGTGGTAGAGCGCATGC	Yes	Yes	Yes	No	Yes	No	No	No	No	No	No	No	No	AlaTGC,AlaCGC,AlaAGC
CAGTTGGTAGAGCATC	Yes	Yes	No	No	Yes	No	No	No	No	No	No	No	No	SupTTA,LysTTT
CAGTTGGTAGAGCATCAGA	Yes	Yes	No	No	Yes	No	No	No	No	No	No	No	No	SupTTA,LysTTT
CAGTTGGTAGAGCGGAGGAC	No	Yes	Yes	No	Yes	No	No	No	No	No	No	No	No	TyrGTA
CATAAAACTTAAAAC	No	No	Yes	No	Yes	No	Yes	No	No	No	No	No	No	NA
CATAATCTGAAGGTCC	No	Yes	Yes	No	Yes	No	No	No	No	No	No	No	No	MetCAT
CATAATCTGAAGGTCCTG	No	Yes	Yes	No	Yes	No	No	No	No	No	No	No	No	MetCAT
CATAATCTGAAGGTCCTGAGT	No	Yes	Yes	No	Yes	No	No	No	No	No	No	No	No	MetCAT
CATAATCTGAAGGTCGTGAGT	Yes	Yes	No	No	Yes	No	No	No	No	No	No	No	No	MetCAT
CATCACCCCATAAACACCA	Yes	Yes	No	No	No	No	No	Yes	No	No	No	No	No	PheGAA
CATCAGCCTCCGGAGCTGGGGA	Yes	Yes	Yes	No	Yes	No	No	No	No	No	No	No	No	ArgCCG
CATCCTCTGCTACCA	No	No	Yes	No	Yes	No	Yes	No	No	No	No	No	No	NA
CATCTCCAGACTTTT	No	No	No	No	Yes	No	Yes	No	No	No	No	Yes	No	NA
CATCTCCAGTGTAGTTTT	No	No	No	No	Yes	No	No	No	No	No	No	Yes	No	NA
CATGAGACTCTTAATCT	No	Yes	Yes	No	Yes	No	No	No	No	No	No	No	No	LysCTT
CATGGAGGCGTGGGT	No	No	Yes	No	Yes	No	Yes	No	No	No	No	No	No	NA
CATTTGACTGCAGATCAAGAGGTCCCCGGT	Yes	Yes	Yes	No	Yes	No	No	No	No	No	No	No	Yes	CysGCA
CATTTGACTGCAGATCAAGAGGTCCCTGGT	Yes	Yes	Yes	No	Yes	No	No	No	No	No	No	No	No	CysGCA
CATTTGACTGCAGATCAAGAGGTCCCTGGTT	Yes	Yes	Yes	No	Yes	No	No	No	No	No	No	No	No	CysGCA
CCAATGGAGGCGTGGGT	Yes	Yes	Yes	No	Yes	No	No	No	No	No	No	No	No	LeuCAA
CCACATGGTCTAGCGGTTAGG	Yes	Yes	Yes	No	Yes	No	No	No	No	No	No	No	No	GluTTC
CCACATGGTCTAGCGGTTAGGATTCCTG	Yes	Yes	Yes	No	Yes	No	No	No	No	No	No	No	No	GluTTC
CCACATGGTCTAGCGGTTAGGATTCCTGG	Yes	Yes	Yes	No	Yes	No	No	No	No	No	No	No	No	GluTTC
CCACCACTGCCACCA	No	No	Yes	No	Yes	No	Yes	No	No	No	No	No	No	NA
CCACCAGAGTCGCCA	No	No	No	No	Yes	No	Yes	No	No	No	No	No	No	NA
CCACCCAGGGACGCCA	No	Yes	Yes	No	Yes	No	No	No	No	No	No	No	No	AsnGTT,AsnATT
CCACCCTCGTCGCCA	No	No	No	No	Yes	No	Yes	No	No	No	No	No	No	NA
CCACCGCTGCCACCA	No	No	Yes	No	Yes	No	Yes	No	No	No	No	No	No	NA
CCACCGCTGCCACCAA	No	No	Yes	No	No	No	No	No	No	No	No	No	No	NA
CCACCTCTGCCACCA	No	No	No	No	Yes	No	Yes	No	No	No	No	No	No	NA
CCACCTTCGTCGCCA	No	No	No	No	Yes	No	Yes	No	No	No	No	No	No	NA
CCACGCGGGAGACCC	No	No	Yes	No	Yes	No	Yes	No	No	No	No	No	No	NA
CCACGCGGGAGGCCCGGGT	Yes	Yes	Yes	No	Yes	No	No	No	No	No	No	No	No	GlyGCC
CCACGTGGCCTAATGG	No	Yes	No	No	Yes	No	No	No	No	No	No	No	No	ArgTCG
CCACGTGGCCTAATGGA	Yes	Yes	No	No	Yes	No	No	No	No	No	No	No	No	ArgTCG
CCACGTTGGGCGCCA	No	No	Yes	No	Yes	No	Yes	No	No	No	No	No	No	NA
CCACTCCTGACACCA	No	No	Yes	No	Yes	No	Yes	No	No	No	No	No	No	NA
CCACTCCTGACACCAA	No	No	Yes	No	No	No	No	No	No	No	No	No	No	NA
CCACTTCTGACACCA	No	No	Yes	No	Yes	No	Yes	No	No	No	No	No	No	NA
CCAGAGGTCGATGGA	No	No	Yes	No	Yes	No	Yes	No	No	No	No	No	No	NA
CCAGCATCTCCACCA	No	No	No	No	Yes	No	Yes	No	No	No	No	No	No	NA
CCAGCGATCCGAGTT	No	No	Yes	No	Yes	No	Yes	No	No	No	No	No	No	NA
CCAGCGGGGCCTCCA	No	No	Yes	No	Yes	No	Yes	No	No	No	No	No	No	NA
CCAGCGGTGCCTCCA	No	No	Yes	No	Yes	No	Yes	No	No	No	No	No	No	NA
CCAGCTCGAAGGACCA	Yes	Yes	Yes	No	Yes	No	No	No	No	No	No	No	No	TyrGTA
CCAGGGATTGTGGGT	No	No	Yes	No	Yes	No	Yes	No	No	No	No	No	No	NA
CCAGTAGAGCCTCCA	No	No	No	No	Yes	No	Yes	No	No	No	No	No	No	NA
CCAGTGGAACCACCA	No	No	Yes	No	Yes	No	Yes	No	No	No	No	No	No	NA
CCAGTGGCGCAATGG	No	No	Yes	No	Yes	No	Yes	No	No	No	No	No	No	NA
CCATAACCCAGAGGTCGATG	Yes	Yes	Yes	No	Yes	No	No	No	No	No	No	No	No	MetCAT
CCATAACCCAGAGGTCGATGGA	Yes	Yes	Yes	No	Yes	No	No	No	No	No	No	No	No	MetCAT
CCATAGTGTAGTGGTTATC	No	Yes	Yes	No	Yes	No	No	No	No	No	No	No	No	ValTAC
CCATATGGTCTAGCGGTTAGGA	Yes	Yes	Yes	No	Yes	No	No	No	No	No	No	No	No	GluTTC
CCATATGGTCTAGCGGTTAGGATTCCTG	Yes	Yes	Yes	No	Yes	No	No	No	No	No	No	No	No	GluTTC
CCATCCTCGTCGCCA	No	No	Yes	No	Yes	No	Yes	No	No	No	No	No	No	NA
CCATCCTCTGCTACCA	No	Yes	Yes	No	Yes	No	No	No	No	No	No	No	No	MetCAT
CCATCTGGGGTGCCA	No	No	No	No	Yes	No	Yes	No	No	No	No	No	No	NA
CCCACCACTGCCACCA	No	Yes	Yes	No	Yes	No	No	No	No	No	No	No	No	LeuTAG
CCCACCAGAGTCGCCA	Yes	Yes	No	No	Yes	No	No	No	No	No	No	No	No	ArgTCT
CCCACCCAGGGACGC	No	No	Yes	No	Yes	No	Yes	No	No	No	No	Yes	No	NA

CCCACCCAGGGACGCCA	No	Yes	Yes	Yes	Yes	No	No	No	No	No	No	No	No	AsnGTT
CCCACCCTCGTCGCCA	Yes	Yes	No	No	Yes	No	No	No	No	No	No	No	No	SerGCT
CCCACCGCTGCCACCA	No	Yes	Yes	No	Yes	No	No	No	No	No	No	No	No	LeuTAG,LeuAAG
CCCACCTTCGTCGCCA	Yes	Yes	No	No	Yes	No	No	No	No	No	No	No	No	SerGCT
CCCACGTTGGGCGCCA	No	Yes	Yes	No	Yes	No	No	No	No	No	No	No	No	LysCTT
CCCACTCCTGACACCA	No	Yes	Yes	No	Yes	No	No	No	No	No	No	No	No	LeuCAG
CCCACTTCTGACACATTCGTTT	No	No	No	No	Yes	No	No	No	No	No	No	Yes	No	NA
CCCACTTCTGACACCA	No	Yes	Yes	No	Yes	No	No	No	No	No	No	No	No	LeuCAA,LeuCAG
CCCAGCACCTCCACCA	No	Yes	Yes	No	Yes	No	No	No	No	No	No	No	No	AlaTGC,AlaAGC
CCCAGCATCTCCACCA	No	Yes	No	No	Yes	No	No	No	No	No	No	No	No	AlaAGC
CCCAGCGGGGCCTCCA	No	Yes	Yes	No	Yes	No	No	No	No	No	No	No	No	ThrAGT,ThrCGT
CCCAGCGGTGCCTCCA	No	Yes	Yes	No	Yes	No	No	No	No	No	No	No	No	ThrAGT
CCCAGTACCTCCACCA	No	Yes	Yes	No	Yes	No	No	No	No	No	No	No	No	AlaAGC
CCCAGTAGAGCCTCCA	No	Yes	No	No	Yes	No	No	No	No	No	No	No	No	ThrTGT
CCCAGTGGAACCACCA	No	Yes	Yes	No	Yes	No	No	No	No	No	No	No	No	ValTAC
CCCAGTGGCCTAATGGA	No	Yes	Yes	No	Yes	No	No	No	No	No	No	No	No	ArgCCT,ArgCCG
CCCAGTGGCCTAATGGATAAG	Yes	Yes	Yes	No	Yes	No	No	No	No	No	No	No	No	ArgCCT,ArgCCG
CCCATAACCCAGAGGTCGATG	Yes	Yes	Yes	No	Yes	No	No	No	No	No	No	No	No	MetCAT
CCCATAACCCAGAGGTCGATGG	Yes	Yes	Yes	No	Yes	No	No	No	No	No	No	No	No	MetCAT
CCCATCCTCGTCGCCA	Yes	Yes	Yes	No	Yes	No	No	No	No	No	No	No	No	SerGCT
CCCCACGTTGGGCGCCA	No	Yes	Yes	No	Yes	No	No	No	No	No	No	No	No	LysCTT
CCCCACTCCTGGTACCA	No	Yes	Yes	No	Yes	No	No	No	No	No	No	No	No	LeuTAA
CCCCAGCATCTCCACCA	No	Yes	No	No	Yes	No	No	No	No	No	No	No	No	AlaAGC
CCCCAGTACCTCCACCA	Yes	Yes	Yes	No	Yes	No	No	No	No	No	No	No	No	AlaAGC
CCCCAGTGGAACCACCA	No	Yes	Yes	No	Yes	No	No	No	No	No	No	No	No	ValTAC
CCCCCGGCTCCTCCACCA	Yes	Yes	Yes	No	Yes	No	No	No	No	No	No	No	No	AlaCGC
CCCCCTTATTTCTACCA	Yes	Yes	No	No	No	No	No	Yes	No	No	No	No	No	IleGAT
CCCCGACACCTCCACCA	Yes	Yes	Yes	No	Yes	No	No	No	No	No	No	No	No	AlaTGC
CCCCGGCACCTCCAC	No	No	Yes	No	Yes	No	Yes	No	No	No	No	No	No	NA
CCCCGGCACCTCCACC	No	Yes	Yes	No	Yes	No	No	No	No	No	No	No	No	AlaTGC,AlaAGC
CCCCGGCACCTCCACCA	Yes	Yes	Yes	No	Yes	No	No	No	No	No	No	No	No	AlaTGC,AlaAGC
CCCCGGCATCTCCACCA	Yes	Yes	Yes	No	Yes	No	No	No	No	No	No	No	No	AlaTGC,AlaCGC
CCCCGGCTCCTCCACCA	No	Yes	Yes	No	Yes	No	No	No	No	No	No	No	No	AlaCGC
CCCCGGGTTCAATCCCCGGCATCTCCACCA	Yes	Yes	Yes	No	Yes	No	No	No	No	No	No	No	No	AlaTGC
CCCCGGGTTCGATCCCCGGCATCTCCACCA	Yes	Yes	Yes	No	Yes	No	No	No	No	No	No	No	No	AlaTGC,AlaCGC
CCCCGTACGGGCCACCA	No	Yes	Yes	No	Yes	No	No	No	No	No	No	No	No	IleAAT
CCCCGTACTGGCCACCA	No	Yes	Yes	No	Yes	No	No	No	No	No	No	No	No	IleAAT
CCCCGTCCGTGCCTCCA	Yes	Yes	Yes	No	Yes	No	No	No	No	No	No	No	No	ThrCGT
CCCCTGTGGTCTAGTGGTTAGG	Yes	Yes	No	No	No	No	No	No	No	Yes	No	No	No	GluTTC
CCCCTGTGGTCTAGTGGTTAGGA	Yes	Yes	No	No	No	No	No	No	No	Yes	No	No	No	GluTTC
CCCCTTATTTACCCCA	Yes	Yes	No	No	No	No	No	Yes	No	No	No	No	No	HisGTG
CCCCTTATTTCTACCA	Yes	Yes	No	No	No	No	No	Yes	No	No	No	No	No	IleGAT
CCCGACGGGGAGCCA	No	No	Yes	No	Yes	No	Yes	No	No	No	No	No	No	NA
CCCGCATCCTCCACCA	No	Yes	No	No	Yes	No	No	No	No	No	No	No	No	AlaAGC
CCCGCATTCTCCACCA	No	Yes	No	No	Yes	No	No	No	No	No	No	No	No	AlaAGC
CCCGGACGAGCCCCCA	No	Yes	Yes	No	Yes	No	No	No	No	No	No	No	No	ProAGG,ProCGG,ProTGG
CCCGGCACCTCCACC	No	No	Yes	No	Yes	No	Yes	No	No	No	No	Yes	No	NA
CCCGGCACCTCCACCA	No	Yes	Yes	No	Yes	No	No	No	No	No	No	Yes	No	AlaTGC,AlaAGC
CCCGGCATCTCCACCA	No	Yes	Yes	No	Yes	No	No	No	No	No	No	No	No	AlaTGC,AlaCGC
CCCGGCCAACGCACCA	Yes	Yes	Yes	No	Yes	No	No	No	No	No	No	No	No	GlyTCC
CCCGGCCAATGCACCA	No	Yes	Yes	No	Yes	No	No	No	No	No	No	No	No	GlyCCC,GlyGCC
CCCGGCCCATGCACCA	No	Yes	Yes	No	Yes	No	No	No	No	No	No	No	No	GlyGCC
CCCGGCTCCTCCACCA	No	Yes	Yes	No	Yes	No	No	No	No	No	No	No	No	AlaCGC
CCCGGGCGGAAACACCA	Yes	Yes	No	No	Yes	No	No	No	No	No	No	No	No	ValCAC
CCCGGGCGGCGCACCA	Yes	Yes	Yes	No	Yes	No	No	No	No	No	No	No	No	GlyCCC
CCCGGGTTCAATCCCCGG	Yes	Yes	Yes	No	Yes	No	No	No	No	No	No	No	No	AlaTGC,AlaCGC,AlaAGC
CCCGGGTTCAATTCCCGG	Yes	Yes	Yes	No	Yes	No	No	No	No	No	No	No	Yes	GluCTC,GlyCCC,ValCAC
CCCGGGTTCGACTCCCGGTATGGGAACCA	Yes	Yes	Yes	No	Yes	No	No	No	No	No	No	No	No	GluTTC
CCCGGGTTCGACTCCCGGTGTGGGAACCA	Yes	Yes	Yes	No	Yes	No	No	No	No	No	No	No	No	GluTTC
CCCGGGTTCGATCCCCGGCATCTCCACCA	Yes	Yes	Yes	No	Yes	No	No	No	No	No	No	No	No	AlaTGC,AlaCGC
CCCGGGTTCGATTCCCGG	Yes	Yes	Yes	No	Yes	No	No	No	No	No	No	No	Yes	PheGAA,GlyTCC,GlyCCC,GlyGCC,GluCTC,GluTTC
CCCGGGTTCGATTCCCGGC	Yes	Yes	Yes	No	Yes	No	No	No	No	No	No	No	No	GlyTCC,GlyGCC,GluTTC
CCCGGGTTCGATTCCCGGTCAGGGAACCA	Yes	Yes	Yes	No	Yes	No	No	No	No	No	No	No	Yes	GluCTC,PheGAA,GluTTC
CCCGGGTTTCGGCAC	No	No	Yes	No	Yes	No	Yes	No	No	No	No	Yes	No	NA
CCCGGGTTTCGGCACC	No	Yes	Yes	No	Yes	No	No	No	No	No	No	No	No	PheGAA
CCCGGGTTTCGGCACCA	No	Yes	Yes	Yes	Yes	No	No	No	No	No	No	No	No	PheGAA
CCCGGGTTTCGGCACCAT	No	No	Yes	No	No	No	No	No	No	No	No	No	No	NA
CCCGGTCAGGGAACCA	No	Yes	Yes	No	Yes	No	No	No	No	No	No	Yes	Yes	GluCTC,PheGAA,GluTTC
CCCGGTGTGGGAACCA	No	Yes	Yes	No	Yes	No	No	No	No	No	No	No	No	GluTTC
CCCGTACGGGCCACCA	No	Yes	Yes	No	Yes	No	No	No	No	No	No	No	No	IleAAT
CCCGTACTGGCCACCA	No	Yes	Yes	No	Yes	No	No	No	No	No	No	No	No	IleAAT
CCCTGCTCGCTGCGCCA	Yes	Yes	Yes	No	Yes	No	No	No	No	No	No	No	No	SerTGA
CCCTGTTCAGGCGCCA	No	Yes	No	No	Yes	No	No	No	No	No	No	No	No	LysTTT
CCCTGTTCGGGCGCCA	No	Yes	Yes	No	Yes	No	No	No	No	No	No	No	No	LysTTT
CCGAGCGGAAACACCA	Yes	Yes	No	No	Yes	No	No	No	No	No	No	No	No	ValTAC
CCGAGTCACGGCACCA	No	Yes	Yes	No	Yes	No	No	No	No	No	No	No	No	HisGTG
CCGAGTGGTTAAGGC	No	No	Yes	No	Yes	No	Yes	No	No	No	No	No	No	NA
CCGCAGCAACCTCGGT	No	Yes	Yes	No	Yes	No	No	No	No	No	No	No	No	HisGTG
CCGCGGCCCGGGTTCGATTCCCGGTCAGG	Yes	Yes	Yes	No	Yes	No	No	No	No	No	No	No	No	GluCTC,GluTTC
CCGCGTGGCCTAATGGA	Yes	Yes	Yes	No	Yes	No	No	No	No	No	No	No	No	ArgTCG,ArgCCG
CCGGACGAGCCCCCA	No	No	Yes	No	Yes	No	Yes	No	No	No	No	No	No	NA
CCGGAGATGAAAACCTTTTTCCAAGGACACCA	Yes	Yes	No	No	No	No	No	Yes	No	No	No	No	No	ThrTGT
CCGGAGCTGGGGATTGTGGG	Yes	Yes	Yes	No	Yes	No	No	No	No	No	No	No	No	ArgCCG
CCGGAGCTGGGGATTGTGGGT	Yes	Yes	Yes	No	Yes	No	No	No	No	No	No	No	No	ArgCCG
CCGGATAGCTCAGTC	No	No	Yes	No	Yes	No	Yes	No	No	No	No	No	No	NA
CCGGATAGCTCAGTCGGTAGAGCA	Yes	Yes	Yes	No	Yes	No	No	No	No	No	No	No	No	LysTTT
CCGGATAGCTCAGTCGGTAGAGCAT	Yes	Yes	Yes	No	Yes	No	No	No	No	No	No	No	No	LysTTT
CCGGATAGCTCAGTCGGTAGAGCATC	Yes	Yes	Yes	No	Yes	No	No	No	No	No	No	No	No	LysTTT

CCGGATAGCTCAGTCGGTAGAGCATCAG	Yes	Yes	Yes	No	Yes	No	No	No	No	No	No	No	No	LysTTT
CCGGATAGCTCAGTCGGTAGAGCATCAGA	Yes	Yes	Yes	No	Yes	No	No	No	No	No	No	No	No	LysTTT
CCGGATAGCTCAGTCGGTAGAGCATCAGAC	Yes	Yes	Yes	No	Yes	No	No	No	No	No	No	No	No	LysTTT
CCGGATCAGAAGATTGAGGGT	Yes	Yes	No	No	Yes	No	No	No	No	No	No	No	No	ArgCCG
CCGGATCAGAAGATTGAGGGTT	Yes	Yes	No	No	Yes	No	No	No	No	No	No	No	No	ArgCCG
CCGGCACCTCCACCA	No	No	Yes	No	Yes	No	Yes	No	No	No	No	Yes	No	NA
CCGGCATCTCCACCA	No	No	Yes	No	Yes	No	Yes	No	No	No	No	No	No	NA
CCGGCCAACGCACCA	No	No	Yes	No	Yes	No	Yes	No	No	No	No	No	No	NA
CCGGCCAATGCACCA	No	No	Yes	No	Yes	No	Yes	No	No	No	No	No	No	NA
CCGGCTAGCTCAGTC	No	No	Yes	No	Yes	No	Yes	No	No	No	No	No	No	NA
CCGGCTAGCTCAGTCG	Yes	Yes	Yes	No	Yes	No	No	No	No	No	No	No	No	LysCTT
CCGGCTAGCTCAGTCGG	Yes	Yes	Yes	No	Yes	No	No	No	No	No	No	No	No	LysCTT
CCGGCTAGCTCAGTCGGT	Yes	Yes	Yes	No	Yes	No	No	No	No	No	No	No	No	LysCTT
CCGGCTAGCTCAGTCGGTAG	Yes	Yes	Yes	No	Yes	No	No	No	No	No	No	No	No	LysCTT
CCGGCTAGCTCAGTCGGTAGA	Yes	Yes	Yes	No	Yes	No	No	No	No	No	No	No	No	LysCTT
CCGGCTAGCTCAGTCGGTAGAG	Yes	Yes	Yes	No	Yes	No	No	No	No	No	No	No	No	LysCTT
CCGGCTAGCTCAGTCGGTAGAGC	Yes	Yes	Yes	No	Yes	No	No	No	No	No	No	No	No	LysCTT
CCGGCTAGCTCAGTCGGTAGAGCA	Yes	Yes	Yes	No	Yes	No	No	No	No	No	No	No	No	LysCTT
CCGGCTAGCTCAGTCGGTAGAGCAT	Yes	Yes	Yes	No	Yes	No	No	No	No	No	No	No	No	LysCTT
CCGGCTAGCTCAGTCGGTAGAGCATGA	Yes	Yes	Yes	No	Yes	No	No	No	No	No	No	No	No	LysCTT
CCGGCTAGCTCAGTCGGTAGAGCATGAG	Yes	Yes	Yes	No	Yes	No	No	No	No	No	No	No	No	LysCTT
CCGGCTAGCTCAGTCGGTAGAGCATGAGA	Yes	Yes	Yes	No	Yes	No	No	No	No	No	No	No	No	LysCTT
CCGGCTAGCTCAGTCGGTAGAGCATGAGAC	Yes	Yes	Yes	No	Yes	No	No	No	No	No	No	No	No	LysCTT
CCGGCTAGCTCAGTCGGTAGAGCATGAGACTC	Yes	Yes	Yes	No	Yes	No	No	No	No	No	No	No	No	LysCTT
CCGGCTAGCTCAGTCGGTAGAGCATGGG	Yes	Yes	Yes	No	Yes	No	No	No	No	No	No	No	No	LysCTT
CCGGCTAGCTCAGTCGGTAGAGCATGGGA	Yes	Yes	Yes	No	Yes	No	No	No	No	No	No	No	No	LysCTT
CCGGCTAGCTCAGTCGGTAGAGCATGGGAC	Yes	Yes	Yes	No	Yes	No	No	No	No	No	No	No	No	LysCTT
CCGGCTAGCTCAGTCGGTAGAGCATGGGACT	Yes	Yes	Yes	No	Yes	No	No	No	No	No	No	No	No	LysCTT
CCGGCTAGCTCAGTCGGTAGAGCATGGGACTC	Yes	Yes	Yes	No	Yes	No	No	No	No	No	No	No	No	LysCTT
CCGGCTCGAAGGACCA	Yes	Yes	Yes	No	Yes	No	No	No	No	No	No	No	No	TyrGTA
CCGGCTCGGAGGACCA	Yes	Yes	Yes	No	Yes	No	No	No	No	No	No	No	No	TyrGTA
CCGGGCAGAAGCACCA	Yes	Yes	Yes	No	Yes	No	No	No	No	No	No	No	No	ValCAC
CCGGGCGGAAACACC	No	No	Yes	No	Yes	No	Yes	No	No	No	No	Yes	No	NA
CCGGGCGGAAACACCA	No	Yes	Yes	No	Yes	No	No	No	No	No	No	No	No	ValCAC,ValAAC
CCGGGGTTCAATTCCCCGACGGGGAGCCA	Yes	Yes	Yes	No	Yes	No	No	No	No	No	No	No	No	AspGTC
CCGGGGTTCGATTCCCCGA	Yes	Yes	Yes	No	Yes	No	No	No	No	No	No	No	No	AspGTC
CCGGGGTTCGATTCCCCGACGGGGA	Yes	Yes	Yes	No	Yes	No	No	No	No	No	No	No	No	AspGTC
CCGGGGTTCGATTCCCCGACGGGGAGC	Yes	Yes	Yes	No	Yes	No	No	No	No	No	No	No	No	AspGTC
CCGGGGTTCGATTCCCCGACGGGGAGCCA	Yes	Yes	Yes	No	Yes	No	No	No	No	No	No	No	No	AspGTC
CCGGGTGCCCCCCTCCA	Yes	Yes	Yes	No	Yes	No	No	No	No	No	No	No	No	CysGCA
CCGGGTTCGATTCCC	No	No	Yes	No	Yes	No	Yes	No	No	No	No	No	No	NA
CCGGGTTTCGGCACC	No	No	Yes	No	Yes	No	Yes	No	No	No	No	No	No	NA
CCGGGTTTCGGCACCA	No	Yes	Yes	No	Yes	No	No	No	No	No	No	No	No	PheGAA
CCGGTCAGGGAACCA	No	No	Yes	No	Yes	No	Yes	No	No	No	No	Yes	No	NA
CCGGTGTGGGAACCA	No	No	Yes	No	Yes	No	Yes	No	No	No	No	No	No	NA
CCGTACGGGCCACCA	No	No	Yes	No	Yes	No	Yes	No	No	No	No	No	No	NA
CCGTAGTGTAGTGGTTATC	No	Yes	Yes	No	Yes	No	No	No	No	No	No	No	No	ValCAC,ValAAC
CCGTAGTGTAGTGGTTATCACGTTCGC	No	Yes	Yes	No	Yes	No	No	No	No	No	No	No	No	ValCAC,ValAAC
CCGTAGTGTAGTGGTTATCACGTTCGCC	No	Yes	Yes	No	Yes	No	No	No	No	No	No	No	No	ValCAC,ValAAC
CCGTGATCGTATAGT	No	No	Yes	No	Yes	No	Yes	No	No	No	No	No	No	NA
CCGTGATCGTATAGTGG	No	Yes	Yes	No	Yes	No	No	No	No	No	No	No	No	HisGTG
CCGTGATCGTATAGTGGTTAGTACT	No	Yes	Yes	No	Yes	No	No	No	No	No	No	No	No	HisGTG
CCGTGATCGTATAGTGGTTAGTACTCTG	No	Yes	Yes	No	Yes	No	No	No	No	No	No	No	No	HisGTG
CCGTGATCGTATAGTGGTTAGTACTCTGC	Yes	Yes	Yes	No	Yes	No	No	No	No	No	No	No	No	HisGTG
CCGTGATCGTATAGTGGTTAGTACTCTGCG	Yes	Yes	Yes	No	Yes	No	No	No	No	No	No	No	No	HisGTG
CCGTGATCGTATAGTGGTTAGTACTCTGCGT	Yes	Yes	Yes	No	Yes	No	No	No	No	No	No	No	No	HisGTG
CCGTGATCGTATAGTGGTTAGTACTCTGCGTTG	Yes	Yes	No	No	Yes	No	No	No	No	No	No	No	No	HisGTG
CCGTGCGAGAATACCA	Yes	Yes	No	No	No	No	No	Yes	Yes	No	No	No	No	GluTTC
CCGTGTTTCCCCCACGCTTT	No	No	No	No	Yes	No	No	No	No	No	No	Yes	No	NA
CCTAAGCCAGGGATTGTGGGT	Yes	Yes	Yes	No	Yes	No	No	No	No	No	No	No	No	ArgCCT
CCTATATATCTTACCA	Yes	Yes	No	No	No	No	No	Yes	No	No	No	No	No	AspGTC
CCTCACACGGGGCACCA	No	Yes	No	No	Yes	No	No	No	No	No	No	No	No	MetCAT
CCTCACCTGGAGCACCA	No	Yes	No	No	Yes	No	No	No	No	No	No	No	No	IleTAT
CCTCACCTGGAGCATGTTTTCT	No	No	No	No	Yes	No	No	No	No	No	No	No	No	NA
CCTCCGTGGTTACCA	No	No	No	No	Yes	No	Yes	No	No	No	No	No	No	NA
CCTCCTTCTGCTTTT	No	No	Yes	No	Yes	No	Yes	No	No	No	No	Yes	No	NA
CCTCGCTGGGGCCTCCA	No	Yes	No	No	Yes	No	No	No	No	No	No	No	No	ThrTGT
CCTCGTGGCGCAACGG	Yes	Yes	No	No	Yes	No	No	No	No	No	No	No	No	TrpCCA
CCTCGTGGCGCAATGG	Yes	Yes	No	No	Yes	No	No	No	No	No	No	No	No	TrpCCA
CCTCTTTGAGCTTTT	No	No	Yes	No	Yes	No	Yes	No	No	No	No	Yes	No	NA
CCTGCCACGCGGGAGGCCCGGGT	Yes	Yes	Yes	No	Yes	No	No	No	No	No	No	No	No	GlyGCC
CCTGCCGACTACGCCA	Yes	Yes	Yes	No	Yes	No	No	No	No	No	No	No	No	SerTGA,SerAGA
CCTGCTCGCTGCGCCA	Yes	Yes	Yes	No	Yes	No	No	No	No	No	No	No	No	SerTGA
CCTGGCTGGCTCGCCA	No	Yes	Yes	No	Yes	No	No	No	No	No	No	No	No	ArgACG
CCTGTCACGCGGGAG	No	No	Yes	No	Yes	No	Yes	No	No	No	No	No	No	NA
CCTGTCACGCGGGAGA	No	Yes	Yes	No	Yes	No	No	No	No	No	No	No	No	AspGTC
CCTGTCACGCGGGAGAC	No	Yes	Yes	No	Yes	No	No	No	No	No	No	No	No	AspGTC
CCTGTCACGCGGGAGACCGG	Yes	Yes	Yes	No	Yes	No	No	No	No	No	No	No	No	AspGTC
CCTGTCACGCGGGAGACCGGGGT	Yes	Yes	Yes	No	Yes	No	No	No	No	No	No	No	No	AspGTC
CCTGTCACGCGGGAGACCGGGGTT	Yes	Yes	Yes	No	Yes	No	No	No	No	No	No	No	No	AspGTC
CCTGTTCGGGCGCCA	No	No	Yes	No	Yes	No	Yes	No	No	No	No	No	No	NA
CCTGTTCGTGACGCCA	No	Yes	No	No	Yes	No	No	No	No	No	No	No	No	SerCGA
CCTGTTGGCTTACTTT	No	No	No	No	Yes	No	No	No	No	No	No	Yes	No	NA
CCTGTTGGCTTACTTTT	No	No	No	No	Yes	No	No	No	No	No	No	Yes	No	NA
CCTTCCAAGCAGTTG	No	No	Yes	No	Yes	No	Yes	No	No	No	No	No	No	NA
CCTTCCAAGCAGTTGA	No	Yes	Yes	No	Yes	No	No	No	No	No	No	No	No	GlyTCC
CCTTCCAAGCAGTTGACCC	Yes	Yes	Yes	No	Yes	No	No	No	No	No	No	No	No	GlyTCC
CCTTCCAAGCAGTTGACCCGGG	Yes	Yes	Yes	No	Yes	No	No	No	No	No	No	No	No	GlyTCC

CCTTCCAAGCAGTTGACCCGGGT	Yes	Yes	Yes	No	Yes	Yes	No	No	No	No	No	No	No	GlyTCC
CCTTCCAAGCAGTTGACCCGGGTT	Yes	Yes	Yes	No	Yes	No	No	No	No	No	No	No	No	GlyTCC
CCTTCGATAGCTCAG	No	No	Yes	Yes	Yes	No	Yes	No	No	No	No	No	No	NA
CCTTCGATAGCTCAGC	No	Yes	Yes	No	Yes	No	No	No	No	No	No	No	No	TyrGTA
CCTTCGATAGCTCAGCTGGTAGA	No	Yes	Yes	No	Yes	No	No	No	No	No	No	No	No	TyrGTA
CCTTCGATAGCTCAGCTGGTAGAGC	No	Yes	Yes	Yes	Yes	No	No	No	No	No	No	No	No	TyrGTA
CCTTCGATAGCTCAGT	No	Yes	Yes	No	Yes	No	No	No	No	No	No	No	No	TyrGTA
CCTTCGATAGCTCAGTTGGTAGAGC	No	Yes	Yes	No	Yes	No	No	No	No	No	No	No	No	TyrGTA
CCTTCGTGGTCGCCA	No	No	No	No	Yes	No	Yes	No	No	No	No	No	No	NA
CCTTCGTGGTTGCCA	No	No	No	No	Yes	No	Yes	No	No	No	No	No	No	NA
CGAAACCATCCTCTGCTACCA	Yes	Yes	Yes	No	Yes	No	No	No	No	No	No	No	No	MetCAT
CGAAACCGGGCGGAAACACCA	Yes	Yes	Yes	No	Yes	No	No	No	No	No	No	No	No	ValCAC,ValAAC
CGAAAGGTCCCCGGT	No	No	Yes	No	Yes	No	Yes	No	No	No	No	No	No	NA
CGAATCCCACCGCTGCCACCA	Yes	Yes	Yes	No	Yes	No	No	No	No	No	No	No	No	LeuTAG,LeuAAG
CGAATCCCACTCCTGACACCA	Yes	Yes	Yes	No	Yes	No	No	No	No	No	No	No	No	LeuCAG
CGAATCCCACTTCTGACACATACTTT	No	No	No	No	Yes	No	No	No	No	No	No	No	No	NA
CGAATCCCACTTCTGACACCA	Yes	Yes	Yes	No	Yes	No	No	No	No	No	No	No	No	LeuCAA,LeuCAG
CGAATCCCATCCTCGTCGCCA	Yes	Yes	Yes	No	Yes	No	No	No	No	No	No	No	No	SerGCT
CGAATCCGGCTCGAAGGACCA	Yes	Yes	Yes	No	Yes	No	No	No	No	No	No	No	No	TyrGTA
CGAATCCTGCCGACTACGCCA	Yes	Yes	Yes	No	Yes	No	No	No	No	No	No	No	No	SerTGA,SerAGA
CGACCCCTTATTTACCCCA	Yes	Yes	No	No	No	No	No	Yes	No	No	No	No	No	HisGTG
CGACTCCCGGTGTGGGAACCA	Yes	Yes	Yes	No	Yes	No	No	No	No	No	No	No	No	GluTTC
CGAGCCCCAGTGGAACCACCA	Yes	Yes	Yes	No	Yes	No	No	No	No	No	No	No	No	ValTAC
CGAGCGGTCTAAGGC	No	No	Yes	No	Yes	No	Yes	No	No	No	No	No	No	NA
CGAGGGTTCTCACCTTCTCTCTCCGATTT	No	No	Yes	No	Yes	No	No	No	No	No	No	Yes	No	NA
CGAGTCACGGCACCA	No	No	Yes	No	Yes	No	Yes	No	No	No	No	No	No	NA
CGAGTCACGGCACCAT	No	No	Yes	No	No	No	No	No	No	No	No	No	No	NA
CGAGTGGTCTAAGGC	No	No	Yes	No	Yes	No	Yes	No	No	No	No	No	No	NA
CGATTCCCCGACGGGGAGCCA	Yes	Yes	Yes	No	Yes	No	No	No	No	No	No	No	Yes	AspGTC
CGATTCCCGGCCAACGCACCA	Yes	Yes	Yes	No	Yes	No	No	No	No	No	No	No	No	GlyTCC
CGATTCCCGGTCAGGGAACCA	Yes	Yes	Yes	No	Yes	No	No	No	No	No	No	No	Yes	GluCTC,PheGAA,GluTTC
CGATTCCGGCTCGAAGGACCA	Yes	Yes	Yes	No	Yes	No	No	No	No	No	No	No	No	TyrGTA
CGCCTGCCACGCGGGAGGCCCGGGT	Yes	Yes	Yes	No	Yes	No	No	No	No	No	No	No	No	GlyGCC
CGGAAGCGTGCTGGGC	Yes	Yes	Yes	No	Yes	No	No	No	No	No	No	No	No	MetCAT,ThrCGT
CGGAGATGAAAACCTTTTTCCAAGGACACCA	Yes	Yes	No	No	No	No	No	Yes	No	No	No	No	No	ThrTGT
CGGAGCTGGGGATTGT	Yes	Yes	Yes	No	Yes	No	No	No	No	No	No	No	No	ArgCCG
CGGAGCTGGGGATTGTGGG	Yes	Yes	Yes	No	Yes	No	No	No	No	No	No	No	No	ArgCCG
CGGAGCTGGGGATTGTGGGT	Yes	Yes	Yes	No	Yes	No	No	No	No	No	No	No	No	ArgCCG
CGGATAGCTCAGTCGGTAGAGCA	Yes	Yes	Yes	No	Yes	No	No	No	No	No	No	No	No	LysTTT
CGGATAGCTCAGTCGGTAGAGCAT	Yes	Yes	Yes	No	Yes	No	No	No	No	No	No	No	No	LysTTT
CGGATAGCTCAGTCGGTAGAGCATC	Yes	Yes	Yes	No	Yes	No	No	No	No	No	No	No	No	LysTTT
CGGATAGCTCAGTCGGTAGAGCATCAG	Yes	Yes	Yes	No	Yes	No	No	No	No	No	No	No	No	LysTTT
CGGATAGCTCAGTCGGTAGAGCATCAGA	Yes	Yes	Yes	No	Yes	No	No	No	No	No	No	No	No	LysTTT
CGGATAGCTCAGTCGGTAGAGCATCAGAC	Yes	Yes	Yes	No	Yes	No	No	No	No	No	No	No	No	LysTTT
CGGATCAGAAGATTCCAGGT	Yes	Yes	Yes	No	Yes	No	No	No	No	No	No	No	No	ArgACG
CGGATCAGAAGATTCCAGGTT	Yes	Yes	Yes	No	Yes	No	No	No	No	No	No	No	No	ArgACG
CGGATCAGAAGATTCT	No	Yes	Yes	No	Yes	No	No	No	No	No	No	No	No	ArgACG
CGGATCAGAAGATTCTAGGT	Yes	Yes	Yes	No	Yes	No	No	No	No	No	No	No	No	ArgACG
CGGATCAGAAGATTCTAGGTT	Yes	Yes	Yes	No	Yes	No	No	No	No	No	No	No	No	ArgACG
CGGATCAGAAGATTGAGGGT	Yes	Yes	No	No	Yes	No	No	No	No	No	No	No	No	ArgTCG,ArgCCG
CGGATCAGAAGATTGAGGGTT	Yes	Yes	No	No	Yes	No	No	No	No	No	No	No	No	ArgTCG,ArgCCG
CGGATCAGAAGATTGCAGGT	Yes	Yes	Yes	No	Yes	No	No	No	No	No	No	No	No	ArgTCG
CGGATGGAGGCGTGGGT	Yes	Yes	Yes	No	Yes	No	No	No	No	No	No	No	No	LeuCAA
CGGCCCGGGTTCGACTCCCGGTGTGGGAACCA	Yes	Yes	Yes	No	Yes	No	No	No	No	No	No	No	No	GluTTC
CGGCGCTCTCACCGCC	Yes	Yes	Yes	No	Yes	No	No	No	No	No	No	No	Yes	GluCTC
CGGCTAGCTCAGTCGG	Yes	Yes	Yes	No	Yes	No	No	No	No	No	No	No	No	LysCTT
CGGCTAGCTCAGTCGGTAG	Yes	Yes	Yes	No	Yes	No	No	No	No	No	No	No	No	LysCTT
CGGCTAGCTCAGTCGGTAGA	Yes	Yes	Yes	No	Yes	No	No	No	No	No	No	No	No	LysCTT
CGGCTAGCTCAGTCGGTAGAGCA	Yes	Yes	Yes	No	Yes	No	No	No	No	No	No	No	No	LysCTT
CGGCTAGCTCAGTCGGTAGAGCAT	Yes	Yes	Yes	No	Yes	No	No	No	No	No	No	No	No	LysCTT
CGGCTAGCTCAGTCGGTAGAGCATGAG	Yes	Yes	Yes	No	Yes	No	No	No	No	No	No	No	No	LysCTT
CGGCTAGCTCAGTCGGTAGAGCATGAGA	Yes	Yes	Yes	No	Yes	No	No	No	No	No	No	No	No	LysCTT
CGGCTAGCTCAGTCGGTAGAGCATGAGAC	Yes	Yes	Yes	No	Yes	No	No	No	No	No	No	No	No	LysCTT
CGGCTAGCTCAGTCGGTAGAGCATGGG	Yes	Yes	Yes	No	Yes	No	No	No	No	No	No	No	No	LysCTT
CGGCTAGCTCAGTCGGTAGAGCATGGGA	Yes	Yes	Yes	No	Yes	No	No	No	No	No	No	No	No	LysCTT
CGGCTAGCTCAGTCGGTAGAGCATGGGAC	Yes	Yes	Yes	No	Yes	No	No	No	No	No	No	No	No	LysCTT
CGGCTAGCTCAGTCGGTAGAGCATGGGACT	Yes	Yes	Yes	No	Yes	No	No	No	No	No	No	No	No	LysCTT
CGGCTAGCTCAGTCGGTAGAGCATGGGACTC	Yes	Yes	Yes	No	Yes	No	No	No	No	No	No	No	No	LysCTT
CGGCTCGAAGGACCA	No	No	Yes	No	Yes	No	Yes	No	No	No	No	No	No	NA
CGGCTCGAAGGACCAA	No	No	Yes	No	No	No	No	No	No	No	No	No	No	NA
CGGCTCGGAGGACCA	No	No	Yes	No	Yes	No	Yes	No	No	No	No	No	No	NA
CGGCTTAGCCTGTGG	No	No	No	No	Yes	No	Yes	No	No	No	Yes	No	No	NA
CGGCTTAGCCTGTGGC	No	No	No	No	Yes	No	No	No	No	No	Yes	No	No	NA
CGGCTTAGCCTGTGGCT	No	No	No	No	Yes	No	No	No	No	No	Yes	No	No	NA
CGGCTTAGCCTGTGGCTC	No	No	No	No	Yes	No	No	No	No	No	Yes	No	No	NA
CGGGAGGCCCGGGTT	No	No	Yes	No	Yes	No	Yes	No	No	No	No	No	No	NA
CGGGCGGAAACACCA	No	No	Yes	No	Yes	No	Yes	No	No	No	No	No	No	NA
CGGGCTCACATCACCCCATAAACACCA	Yes	Yes	No	No	No	No	No	Yes	No	No	No	No	No	PheGAA
CGGGTGCCCCCTCCA	No	No	Yes	No	Yes	No	Yes	No	No	No	No	No	No	NA
CGGGTGCGAGAGGTCCCGGGT	Yes	Yes	Yes	No	Yes	No	No	No	No	No	No	No	No	ProCGG
CGGGTGCTCTTATTT	No	No	Yes	No	Yes	No	Yes	No	No	No	No	Yes	No	NA
CGGGTGCTCTTATTTT	No	No	Yes	No	Yes	No	No	No	No	No	No	Yes	No	NA
CGGGTGCTCTTATTTTT	No	No	Yes	No	Yes	No	No	No	No	No	No	Yes	No	NA
CGGGTGCTCTTATTTTTT	No	No	Yes	No	Yes	No	No	No	No	No	No	Yes	No	NA
CGGGTTCGATTCCCGGTCAGGGAACCA	Yes	Yes	Yes	No	Yes	No	No	No	No	No	No	No	Yes	GluCTC,PheGAA,GluTTC
CGGGTTTCGGCACCA	No	No	Yes	No	Yes	No	Yes	No	No	No	No	No	No	NA
CGGTAGAGCATCAGA	No	No	Yes	No	Yes	No	Yes	No	No	No	No	No	No	NA
CGGTAGAGCATGAGA	No	No	Yes	No	Yes	No	Yes	No	No	No	No	No	No	NA

CGGTAGAGCATGAGAC	No	Yes	Yes	No	Yes	No	No	No	No	No	No	No	No	LysCTT
CGGTAGAGCATGGGA	No	No	Yes	No	Yes	No	Yes	No	No	No	No	No	No	NA
CGGTAGAGCATGGGAC	No	Yes	Yes	No	Yes	No	No	No	No	No	No	No	No	LysCTT
CGGTAGAGCGCGTGC	No	No	Yes	No	Yes	No	Yes	No	No	No	No	No	No	NA
CGGTAGAGCGCGTGCT	Yes	Yes	Yes	No	Yes	No	No	No	No	No	No	No	No	AlaAGC
CGGTAGCGCGTCTGA	No	No	No	No	Yes	No	Yes	No	No	No	No	No	No	NA
CGGTCTAAGGCGCTG	No	No	Yes	No	Yes	No	Yes	No	No	No	No	No	No	NA
CGGTCTAAGGCGCTGC	Yes	Yes	Yes	No	Yes	No	No	No	No	No	No	No	No	LeuCAG
CGGTCTAAGGCGCTGCG	Yes	Yes	Yes	No	Yes	No	No	No	No	No	No	No	No	LeuCAG
CGGTTAGCGCATTCGG	Yes	Yes	Yes	No	Yes	No	No	No	No	No	No	No	No	AsnGTT
CGGTTAGCGCATTCGGC	Yes	Yes	Yes	No	Yes	No	No	No	No	No	No	No	No	AsnGTT
CGGTTAGCGCCTTCGG	Yes	Yes	No	No	Yes	No	No	No	No	No	No	No	No	AsnGTT
CGGTTAGCGCCTTCGGC	Yes	Yes	No	No	Yes	No	No	No	No	No	No	No	No	AsnGTT
CGGTTAGGATTCCTGG	No	Yes	Yes	No	Yes	No	No	No	No	No	No	No	No	GluTTC
CGGTTAGGATTCCTGGT	No	Yes	Yes	No	Yes	No	No	No	No	No	No	No	No	GluTTC
CGGTTAGGATTCCTGGTT	No	Yes	Yes	No	Yes	No	No	No	No	No	No	No	No	GluTTC
CGGTTAGGATTCCTGGTTTTCACCCAGGC	Yes	Yes	Yes	No	Yes	No	No	No	No	No	No	No	No	GluTTC
CGGTTATCACGTCTGC	Yes	Yes	Yes	No	Yes	No	No	No	No	No	No	No	No	ValTAC
CGTAGTGTAGTGGTTATC	No	Yes	Yes	No	Yes	No	No	No	No	No	No	No	No	ValCAC,ValAAC
CGTAGTGTAGTGGTTATCACGTTCGCC	No	Yes	Yes	No	Yes	No	No	No	No	No	No	No	No	ValCAC,ValAAC
CGTATAGTGGTTAGTAC	No	Yes	Yes	No	Yes	No	No	No	No	No	No	No	No	HisGTG
CGTATAGTGGTTAGTACTCTGC	No	Yes	Yes	No	Yes	No	No	No	No	No	No	No	No	HisGTG
CGTATGAGGCCCCGGGT	Yes	Yes	Yes	No	Yes	No	No	No	No	No	No	No	No	AlaTGC
CGTCTTGTTCTCTTT	No	No	No	No	Yes	No	Yes	No	No	No	No	Yes	No	NA
CGTGATCGTATAGTGGTTAGTACTCTGCGTTG	Yes	Yes	No	No	Yes	No	No	No	No	No	No	No	No	HisGTG
CGTGATTCATACCTTT	No	No	Yes	No	Yes	No	No	No	No	No	No	Yes	No	NA
CGTGATTCATACCTTTT	No	No	Yes	No	Yes	No	No	No	No	No	No	Yes	No	NA
CGTTCAGGTCGCAGTC	No	Yes	Yes	No	Yes	No	No	No	No	No	No	No	No	LeuCAG
CGTTCAGGTCGCAGTCT	No	Yes	Yes	No	Yes	No	No	No	No	No	No	No	No	LeuCAG
CGTTCAGGTCGCAGTCTC	Yes	Yes	Yes	No	Yes	No	No	No	No	No	No	No	No	LeuCAG
CGTTCAGGTCGCAGTCTCC	Yes	Yes	Yes	No	Yes	No	No	No	No	No	No	No	No	LeuCAG
CGTTCAGGTCGCAGTCTCCC	Yes	Yes	Yes	No	Yes	No	No	No	No	No	No	No	No	LeuCAG
CGTTGGTGGTATAGTGGTAAGCATAGCTG	No	Yes	Yes	No	Yes	No	No	No	No	No	No	No	No	GlyTCC
CTAAAGGTCCCTGGT	No	No	Yes	No	Yes	No	Yes	No	No	No	No	No	No	NA
CTAAATCCTATATATCTTACCA	Yes	Yes	No	No	No	No	No	Yes	No	No	No	No	No	AspGTC
CTAACTTAGCATTAAC	Yes	Yes	No	No	No	No	No	Yes	Yes	No	No	No	No	LysTTT
CTAACTTAGCATTAACCT	Yes	Yes	No	No	No	No	No	Yes	Yes	No	No	No	No	LysTTT
CTAACTTAGCATTAACCTTTTAAGTTAAAGA	Yes	Yes	No	No	No	No	No	Yes	Yes	No	No	No	No	LysTTT
CTAAGCCAGGGATTGTG	Yes	Yes	Yes	No	Yes	No	No	No	No	No	No	No	No	ArgCCT
CTAAGCCAGGGATTGTGG	Yes	Yes	Yes	No	Yes	No	No	No	No	No	No	No	No	ArgCCT
CTAAGCCAGGGATTGTGGG	Yes	Yes	Yes	No	Yes	No	No	No	No	No	No	No	No	ArgCCT
CTAAGCCAGGGATTGTGGGT	Yes	Yes	Yes	No	Yes	No	No	No	No	No	No	No	No	ArgCCT
CTAAGCCAGGGATTGTGGGTT	Yes	Yes	Yes	No	Yes	No	No	No	No	No	No	No	No	ArgCCT
CTAATGGATAAGGCA	No	No	Yes	No	Yes	No	Yes	No	No	No	No	No	No	NA
CTAATGGATAAGGCACT	No	Yes	Yes	No	Yes	No	No	No	No	No	No	No	No	ArgCCT
CTAATGGATAAGGCACTG	No	Yes	Yes	No	Yes	No	No	No	No	No	No	No	No	ArgCCT
CTAATGGATAAGGCATTG	Yes	Yes	No	No	Yes	No	No	No	No	No	No	No	No	ArgCCT
CTAATTCAAAGGTTCCGGGT	Yes	Yes	No	No	Yes	No	No	No	No	No	No	No	No	ArgTCT
CTAATTCAAAGGTTCCGGGTT	Yes	Yes	No	No	Yes	No	No	No	No	No	No	No	No	ArgTCT
CTAGCTCAGTCGGTAGAG	No	Yes	Yes	No	Yes	No	No	No	No	No	No	No	No	LysCTT
CTAGCTCAGTCGGTAGAGC	No	Yes	Yes	No	Yes	No	No	No	No	No	No	No	No	LysCTT
CTAGCTCAGTCGGTAGAGCA	No	Yes	Yes	No	Yes	No	No	No	No	No	No	No	No	LysCTT
CTAGCTCAGTCGGTAGAGCATGAG	No	Yes	Yes	No	Yes	No	No	No	No	No	No	No	No	LysCTT
CTAGGGGTATGATTCTC	No	Yes	Yes	No	Yes	No	No	No	No	No	No	No	No	ProAGG,ProCGG,ProTGG
CTAGGGGTATGATTCTCGC	No	Yes	Yes	No	Yes	No	No	No	No	No	No	No	No	ProAGG,ProCGG,ProTGG
CTAGGGGTATGATTCTCGG	Yes	Yes	Yes	No	Yes	No	No	No	No	No	No	No	No	ProTGG
CTATAGGGTGGAGACTTT	No	No	Yes	No	Yes	No	No	No	No	No	No	Yes	No	NA
CTATAGGGTGGAGACTTTT	No	No	Yes	No	Yes	No	No	No	No	No	No	Yes	No	NA
CTCAAGTTCTGGTCTC	No	Yes	No	No	Yes	No	No	No	No	No	No	No	No	LeuCAA
CTCAAGTTCTGGTCTCC	Yes	Yes	No	No	Yes	No	No	No	No	No	No	No	No	LeuCAA
CTCACACGGGGCACCA	No	Yes	No	No	Yes	No	No	No	No	No	No	No	No	MetCAT
CTCACATCACCCCATAAACACCA	Yes	Yes	No	No	No	No	No	Yes	No	No	No	No	No	PheGAA
CTCACCTGGAGCACCA	No	Yes	No	No	Yes	No	No	No	No	No	No	No	No	IleTAT
CTCACCTGGAGCATGTTTT	No	No	No	No	Yes	No	No	No	No	No	No	Yes	No	NA
CTCACCTGGAGCATGTTTTC	No	No	No	No	Yes	No	No	No	No	No	No	Yes	No	NA
CTCACCTGGAGCATGTTTTCT	No	No	No	No	Yes	No	No	No	No	No	No	Yes	No	NA
CTCACCTGGAGCATGTTTTCTT	No	No	No	No	Yes	No	No	No	No	No	No	Yes	No	NA
CTCAGTGGTAGAGCAT	No	Yes	Yes	No	Yes	No	No	No	No	No	No	No	Yes	TyrGTA,CysGCA,CysACA
CTCAGTGGTAGAGCGCAT	Yes	Yes	Yes	No	Yes	No	No	No	No	No	No	No	No	AlaTGC,AlaCGC,AlaAGC
CTCCAGATCAGAAGGTTGCGTGT	Yes	Yes	No	No	Yes	No	No	No	No	No	No	No	No	TrpCCA
CTCCAGATCAGAAGGTTGCGTGTT	Yes	Yes	No	No	Yes	No	No	No	No	No	No	No	No	TrpCCA
CTCCCAGCGGGGCCTCCA	No	Yes	No	No	Yes	No	No	No	No	No	No	No	No	ThrAGT
CTCCCAGTGGGGCCTCCA	Yes	Yes	No	No	Yes	No	No	No	No	No	No	No	No	ThrCGT
CTCCCGGTATGGGAACCA	Yes	Yes	Yes	No	Yes	No	No	No	No	No	No	No	No	GluTTC
CTCCCGGTGTGGGAACCA	Yes	Yes	Yes	No	Yes	No	No	No	No	No	No	No	No	GluTTC
CTCCGGAGCTGGGGATTGTGGGT	Yes	Yes	Yes	No	Yes	No	No	No	No	No	No	No	No	ArgCCG
CTCCTGGCTGGCTCGCCA	No	Yes	Yes	No	Yes	No	No	No	No	No	No	No	No	ArgACG
CTCGAAGGAAGTGCCCGATGCTTT	No	No	No	No	Yes	No	No	No	No	No	No	Yes	No	NA
CTCGAAGGAAGTGCCCGATGCTTTT	No	No	No	No	Yes	No	No	No	No	No	No	Yes	No	NA
CTCGAAGGACGAAAGTTT	No	No	No	No	Yes	No	No	No	No	No	No	Yes	No	NA
CTCGAAGGACGAAAGTTTT	No	No	No	No	Yes	No	No	No	No	No	No	Yes	No	NA
CTCGAAGGACTTCGTCTGTAAT	No	No	No	No	Yes	No	No	No	No	No	No	Yes	No	NA
CTCGAAGGACTTCGTCTGTAATT	No	No	No	No	Yes	No	No	No	No	No	No	Yes	No	NA
CTCGAAGGACTTCGTCTGTAATTT	No	No	No	No	Yes	No	No	No	No	No	No	Yes	No	NA
CTCGAAGGACTTCGTCTGTAATTTT	No	No	No	No	Yes	No	No	No	No	No	No	Yes	No	NA
CTCGAAGGACTTCGTCTGTAATTTTT	No	No	No	No	Yes	No	No	No	No	No	No	Yes	No	NA
CTCGAAGGAGTTCACAATTTT	No	No	No	No	Yes	No	No	No	No	No	No	Yes	No	NA
CTCGAAGGAGTTCACAATTTTT	No	No	No	No	Yes	No	No	No	No	No	No	Yes	No	NA

CTCGCTGGGGCCTCCA	No	Yes	Yes	No	Yes	No	No	No	No	No	No	No	No	ThrTGT
CTCGGAGGAGTTCCATTTT	No	No	No	No	Yes	No	No	No	No	No	No	Yes	No	NA
CTCGGAGGAGTTCCATTTTT	No	No	No	No	Yes	No	No	No	No	No	No	Yes	No	NA
CTCGGCTTTCCCTGCTAACTGGGCTTTT	No	No	No	No	Yes	No	No	No	No	No	No	Yes	No	NA
CTCGGCTTTCCCTGCTAACTGGGCTTTTT	No	No	No	No	Yes	No	No	No	No	No	No	Yes	No	NA
CTCGGCTTTCCCTGCTAACTGGGCTTTTTT	No	No	No	No	Yes	No	No	No	No	No	No	Yes	No	NA
CTCGGTGGAACCTCCA	Yes	Yes	Yes	No	Yes	No	No	No	No	No	No	No	No	GlnCTG
CTCGGTGGGACCTCCA	No	Yes	Yes	No	Yes	No	No	No	No	No	No	No	No	GlnTTG
CTCGTTAGTATAGTGGTGAGT	Yes	Yes	Yes	No	Yes	No	No	No	No	No	No	No	No	AspGTC
CTCGTTGGTCTAGGG	No	No	Yes	No	Yes	No	Yes	No	No	No	No	No	No	NA
CTCGTTGGTCTAGGGGTA	No	Yes	Yes	No	Yes	No	No	No	No	No	No	No	No	ProAGG,ProCGG,ProTGG
CTCGTTGGTCTAGGGGTATGA	No	Yes	Yes	No	Yes	No	No	No	No	No	No	No	No	ProAGG,ProCGG,ProTGG
CTCGTTGGTCTAGGGGTATGATTCTC	No	Yes	Yes	No	Yes	No	No	No	No	No	No	No	No	ProAGG,ProCGG,ProTGG
CTCGTTGGTCTAGGGGTATGATTCTCGC	No	Yes	Yes	No	Yes	No	No	No	No	No	No	No	No	ProAGG,ProCGG,ProTGG
CTCGTTGGTCTAGGGGTATGATTCTCGCT	No	Yes	Yes	No	Yes	No	No	No	No	No	No	No	No	ProAGG,ProCGG,ProTGG
CTCGTTGGTCTAGGGGTATGATTCTCGG	Yes	Yes	Yes	No	Yes	No	No	No	No	No	No	No	No	ProTGG
CTCGTTGGTCTAGGGGTATGATTCTCGGT	Yes	Yes	Yes	No	Yes	No	No	No	No	No	No	No	No	ProTGG
CTCTGGACTCTGAATCCAGCGATCCGAGT	Yes	Yes	Yes	No	Yes	No	No	No	No	No	No	No	No	GlnCTG
CTCTGTGGCGCAATGG	No	Yes	No	No	Yes	No	No	No	No	No	No	No	No	ArgTCT,AsnGTT
CTCTTTACAGTGACCA	Yes	Yes	No	No	No	No	No	Yes	Yes	No	No	No	No	LysTTT
CTGAAGATCTAAAGGTCCC	No	Yes	Yes	No	Yes	No	No	No	No	No	No	No	No	PheGAA
CTGAAGATCTAAAGGTCCCTGGT	No	Yes	Yes	No	Yes	No	No	No	No	No	No	No	No	PheGAA
CTGAAGGTCCTGAGT	No	No	Yes	No	Yes	No	Yes	No	No	No	No	No	No	NA
CTGAAGGTCGTGAGT	No	No	No	No	Yes	No	Yes	No	No	No	No	No	No	NA
CTGAATCCAGCGATCC	Yes	Yes	Yes	No	Yes	No	No	No	No	No	No	No	No	GlnCTG
CTGAATCCAGCGATCCGA	Yes	Yes	Yes	No	Yes	No	No	No	No	No	No	No	No	GlnCTG
CTGAATCCAGCGATCCGAG	Yes	Yes	Yes	No	Yes	No	No	No	No	No	No	No	No	GlnCTG
CTGAATCCAGCGATCCGAGT	Yes	Yes	Yes	No	Yes	No	No	No	No	No	No	No	No	GlnCTG
CTGAATCCAGCGATCCGAGTT	Yes	Yes	Yes	No	Yes	No	No	No	No	No	No	No	No	GlnCTG
CTGAATCCAGCGATCCGAGTTCA	Yes	Yes	Yes	No	Yes	No	No	No	No	No	No	No	No	GlnCTG
CTGACTTCGGATCAGA	Yes	Yes	Yes	No	Yes	No	No	No	No	No	No	No	No	ArgTCG
CTGACTTCGGATCAGAA	Yes	Yes	Yes	No	Yes	No	No	No	No	No	No	No	No	ArgTCG
CTGACTTCGGATCAGAAGATTGAGGGT	Yes	Yes	No	No	Yes	No	No	No	No	No	No	No	No	ArgTCG
CTGACTTCGGATCAGAAGATTGAGGGTT	Yes	Yes	No	No	Yes	No	No	No	No	No	No	No	No	ArgTCG
CTGAGGGTCCAGGGT	No	No	Yes	No	Yes	No	Yes	No	No	No	No	No	No	NA
CTGATTCCGGATCAGAAGATTGAGGGT	Yes	Yes	No	No	Yes	No	No	No	No	No	No	No	No	ArgCCG
CTGATTGCAACTTTT	No	No	No	No	Yes	No	Yes	No	No	No	No	Yes	No	NA
CTGCAGATCAAGAGGTCCCCGGT	Yes	Yes	Yes	No	Yes	No	No	No	No	No	No	No	Yes	CysGCA
CTGCAGATCAAGAGGTCCCTGGT	Yes	Yes	Yes	No	Yes	No	No	No	No	No	No	No	No	CysGCA
CTGCCACGCGGGAGGCCC	Yes	Yes	Yes	No	Yes	No	No	No	No	No	No	No	No	GlyGCC
CTGCCACGCGGGAGGCCCGGGT	Yes	Yes	Yes	No	Yes	No	No	No	No	No	No	No	No	GlyGCC
CTGCCGACTACGCCA	No	No	Yes	No	Yes	No	Yes	No	No	No	No	No	No	NA
CTGCCGACTACGCCAT	No	No	Yes	No	No	No	No	No	No	No	No	No	No	NA
CTGCGCTGTGGCCGCAGCA	Yes	Yes	Yes	No	Yes	No	No	No	No	No	No	No	No	HisGTG
CTGCTATCGGAGTTTTT	No	No	No	No	Yes	No	No	No	No	No	No	Yes	No	NA
CTGCTCACAGCGCCA	No	No	No	No	Yes	No	Yes	No	No	No	No	No	No	NA
CTGCTTTACACGCAGA	Yes	Yes	Yes	No	Yes	No	No	No	No	No	No	No	No	ValTAC
CTGGGGATTGTGGGT	No	No	Yes	No	Yes	No	Yes	No	No	No	No	No	No	NA
CTGGGTTCGAATCCC	No	No	Yes	No	Yes	No	Yes	No	No	No	No	No	No	NA
CTGGGTTCGAATCCCAGC	No	Yes	Yes	No	Yes	No	No	No	No	No	No	No	No	ThrAGT,ThrCGT
CTGGGTTCGACTCCC	No	No	No	No	Yes	No	Yes	No	No	No	No	No	No	NA
CTGGTTCGAATCCGGCTCGGAGGACCA	Yes	Yes	Yes	No	Yes	No	No	No	No	No	No	No	No	TyrGTA
CTGGTTCGATTCCGGCTCGAAGGACCA	Yes	Yes	Yes	No	Yes	No	No	No	No	No	No	No	No	TyrGTA
CTGTAGATCCTTAGGTCGCTGGT	Yes	Yes	No	No	Yes	No	No	No	No	No	No	No	No	TyrGTA
CTGTCACGCGGGAGA	No	No	Yes	No	Yes	No	Yes	No	No	No	No	No	No	NA
CTGTCACGCGGGAGAC	No	Yes	Yes	No	Yes	No	No	No	No	No	No	No	No	AspGTC
CTGTCACGCGGGAGACC	No	Yes	Yes	No	Yes	No	No	No	No	No	No	No	No	AspGTC
CTGTCACGCGGGAGACCGG	Yes	Yes	Yes	No	Yes	No	No	No	No	No	No	No	No	AspGTC
CTGTCACGCGGGAGACCGGGGT	Yes	Yes	Yes	No	Yes	No	No	No	No	No	No	No	No	AspGTC
CTGTCACGCGGGAGACCGGGGTT	Yes	Yes	Yes	No	Yes	No	No	No	No	No	No	No	No	AspGTC
CTGTCGGCTACGCCA	No	No	Yes	No	Yes	No	Yes	No	No	No	No	No	No	NA
CTGTGCTCCGGAGTTACCTCGTTT	No	No	Yes	No	Yes	No	No	No	No	No	No	Yes	No	NA
CTGTGCTCCGGAGTTACCTCGTTTT	No	No	Yes	No	Yes	No	No	No	No	No	No	Yes	No	NA
CTGTTAACCGAAAGGTTGG	Yes	Yes	Yes	No	Yes	No	No	No	No	No	No	No	No	AsnGTT
CTGTTAACCGAAAGGTTGGTGGT	Yes	Yes	Yes	No	Yes	No	No	No	No	No	No	No	No	AsnGTT
CTGTTCGTGACGCCA	No	No	No	No	Yes	No	Yes	No	No	No	No	No	No	NA
CTTAACTTGACCGCTCTGACCA	Yes	Yes	No	No	No	No	No	Yes	No	No	No	No	No	ValTAC
CTTACCTCCTCAAAGCAATA	Yes	Yes	No	No	No	No	No	Yes	No	No	No	No	No	PheGAA
CTTACCTCCTCAAAGCAATACACT	Yes	Yes	No	No	No	No	No	Yes	No	No	No	No	No	PheGAA
CTTACGACCCCTTATTTACC	Yes	Yes	No	No	No	No	No	Yes	No	No	No	No	No	HisGTG
CTTACGACCCCTTATTTACCCC	Yes	Yes	No	No	No	No	No	Yes	No	No	No	No	No	HisGTG
CTTACGACCCCTTATTTACCCCA	Yes	Yes	No	No	No	No	No	Yes	No	No	No	No	No	HisGTG
CTTACTGCTTCCTGTGTTCGGG	No	No	Yes	No	Yes	No	No	No	No	No	No	No	No	NA
CTTAGCCTGTGGCTC	No	No	No	No	Yes	No	Yes	No	No	No	Yes	No	No	NA
CTTATAATGCCGAGGTTGTGAGT	Yes	Yes	No	No	Yes	No	No	No	No	No	No	No	No	IleTAT
CTTCCAAGCAGTTGA	No	No	Yes	No	Yes	No	Yes	No	No	No	No	No	No	NA
CTTCCAAGCAGTTGACCC	Yes	Yes	Yes	No	Yes	No	No	No	No	No	No	No	No	GlyTCC
CTTCCAAGCAGTTGACCCGGGT	Yes	Yes	Yes	No	Yes	No	No	No	No	No	No	No	No	GlyTCC
CTTCGGATCAGAAGATTGAGGGT	Yes	Yes	No	No	Yes	No	No	No	No	No	No	No	No	ArgTCG
CTTCGGATCAGAAGATTGAGGGTT	Yes	Yes	No	No	Yes	No	No	No	No	No	No	No	No	ArgTCG
CTTCGGATCAGAAGATTGCAGGT	Yes	Yes	Yes	No	Yes	No	No	No	No	No	No	No	No	ArgTCG
CTTCGGATCAGAAGATTGCAGGTT	Yes	Yes	Yes	No	Yes	No	No	No	No	No	No	No	No	ArgTCG
CTTGACCGCTCTGACCA	Yes	Yes	No	No	No	No	No	Yes	No	No	No	No	No	ValTAC
CTTGTGCTCTCACTTTT	No	No	Yes	No	Yes	No	No	No	No	No	No	Yes	No	NA
CTTTGAATCCAGCAATCCGAGT	Yes	Yes	Yes	No	Yes	No	No	No	No	No	No	No	No	GlnTTG
CTTTGAATCCAGCGA	No	No	Yes	No	Yes	No	Yes	No	No	No	No	No	No	NA
CTTTGAATCCAGCGAT	Yes	Yes	Yes	No	Yes	No	No	No	No	No	No	No	No	GlnTTG
CTTTGAATCCAGCGATCCGAG	Yes	Yes	Yes	No	Yes	No	No	No	No	No	No	No	No	GlnTTG

CTTTGAATCCAGCGATCCGAGT	Yes	Yes	Yes	No	Yes	No	No	No	No	No	No	No	No	GlnTTG
CTTTGAATCCAGCGATCCGAGTT	Yes	Yes	Yes	No	Yes	No	No	No	No	No	No	No	No	GlnTTG
CTTTGCACGCGTGGGT	Yes	Yes	No	No	Yes	No	No	No	No	No	No	No	No	SerGCT
CTTTTAATCTGAGGGTCC	Yes	Yes	Yes	No	Yes	No	No	No	No	No	No	No	Yes	LysTTT
CTTTTAATCTGAGGGTCCA	Yes	Yes	Yes	No	Yes	No	No	No	No	No	No	No	Yes	LysTTT
CTTTTAATCTGAGGGTCCAG	Yes	Yes	Yes	No	Yes	No	No	No	No	No	No	No	Yes	LysTTT
CTTTTAATCTGAGGGTCCAGGGT	Yes	Yes	Yes	No	Yes	No	No	No	No	No	No	No	Yes	LysTTT
GAAAAAGTCATGGAGGCC	Yes	Yes	No	No	No	No	No	Yes	Yes	No	No	No	No	SerTGA
GAAAAAGTCATGGAGGCCA	Yes	Yes	No	No	No	No	No	Yes	Yes	No	No	No	No	SerTGA
GAAAAAGTCATGGAGGCCATGG	Yes	Yes	No	No	No	No	No	Yes	Yes	No	No	No	No	SerTGA
GAAAAAGTCATGGAGGCCATGGG	Yes	Yes	No	No	No	No	No	Yes	Yes	No	No	No	No	SerTGA
GAAAAAGTCATGGAGGCCATGGGGTT	Yes	Yes	No	No	No	No	No	Yes	Yes	No	No	No	No	SerTGA
GAAAAAGTCATGGAGGCCATGGGGTTG	Yes	Yes	No	No	No	No	No	Yes	Yes	No	No	No	No	SerTGA
GAAAAAGTCATGGAGGCCATGGGGTTGG	Yes	Yes	No	No	No	No	No	Yes	Yes	No	No	No	No	SerTGA
GAAAACCTTTTTCCAAGGACACCA	Yes	Yes	No	No	No	No	No	Yes	No	No	No	No	No	ThrTGT
GAAAATGTTTAGACGGGC	Yes	Yes	No	No	No	No	No	Yes	No	No	No	No	No	PheGAA
GAAAATGTTTAGACGGGCTCAC	Yes	Yes	No	No	No	No	No	Yes	No	No	No	No	No	PheGAA
GAAAATGTTTAGACGGGCTCACATC	Yes	Yes	No	No	No	No	No	Yes	No	No	No	No	No	PheGAA
GAAAATGTTTAGACGGGCTCACATCACCCC	Yes	Yes	No	No	No	No	No	Yes	No	No	No	No	No	PheGAA
GAAACCATCCTCTGCTACCA	Yes	Yes	Yes	No	Yes	No	No	No	No	No	No	No	No	MetCAT
GAAACCGGGCGGAAACACCA	Yes	Yes	Yes	No	Yes	No	No	No	No	No	No	No	No	ValCAC,ValAAC
GAAAGCTCACAAGAACTGCTAACTC	Yes	Yes	No	No	No	No	No	Yes	No	No	No	No	No	SerGCT
GAAGATCGCGGGTTCG	Yes	Yes	No	No	Yes	No	No	No	No	No	No	No	No	ThrCGT
GAAGATCGCGGGTTCGA	Yes	Yes	No	No	Yes	No	No	No	No	No	No	No	No	ThrCGT
GAAGCGGGTGCTCTTA	No	No	Yes	No	Yes	No	No	No	No	No	No	Yes	No	NA
GAAGCGGGTGCTCTTAT	No	No	Yes	No	Yes	No	No	No	No	No	No	Yes	No	NA
GAAGCGGGTGCTCTTATT	No	No	Yes	No	Yes	No	No	No	No	No	No	Yes	No	NA
GAAGCGGGTGCTCTTATTT	No	No	Yes	No	Yes	No	No	No	No	No	No	Yes	No	NA
GAAGCGGGTGCTCTTATTTT	No	No	Yes	Yes	Yes	No	No	No	No	No	No	Yes	No	NA
GAAGCGGGTGCTCTTATTTTT	No	No	Yes	No	Yes	No	No	No	No	No	No	Yes	No	NA
GAAGCGTGCTGGGCCCA	No	Yes	Yes	No	Yes	No	No	No	No	No	No	No	No	MetCAT
GAAGGTCCTGGGTTCGAGCCCCAGTGGAACCA	Yes	Yes	Yes	No	Yes	No	No	No	No	No	No	No	No	ValTAC
GAAGGTCCTGGGTTCGAGCCCCAGTGGAACCAC	Yes	Yes	Yes	No	Yes	No	No	No	No	No	No	No	No	ValTAC
GAAGGTCTCACTTTT	No	No	Yes	No	Yes	No	Yes	No	No	No	No	Yes	No	NA
GAATAGTTTAAATTAGAATC	Yes	Yes	No	No	No	No	No	Yes	No	No	No	No	No	ProTGG
GAATAGTTTAAATTAGAATCTTAGC	Yes	Yes	No	No	No	No	No	Yes	No	No	No	No	No	ProTGG
GAATCCAGCGATCCGAGT	Yes	Yes	Yes	No	Yes	No	No	No	No	No	No	No	No	GlnCTG,GlnTTG
GAATCCAGCGATCCGAGTT	Yes	Yes	Yes	No	Yes	No	No	No	No	No	No	No	No	GlnCTG,GlnTTG
GAATCCAGCGATCCGAGTTC	Yes	Yes	Yes	No	Yes	No	No	No	No	No	No	No	No	GlnCTG,GlnTTG
GAATCCCACTCCTGACACCA	Yes	Yes	Yes	No	Yes	No	No	No	No	No	No	No	No	LeuCAG
GAATCCCACTTCTGACACCA	No	Yes	Yes	No	Yes	No	No	No	No	No	No	No	No	LeuCAA,LeuCAG
GAATCCGGCTCGAAGGACCA	Yes	Yes	Yes	No	Yes	No	No	No	No	No	No	No	No	TyrGTA
GAATCCTGCCGACTACGCCA	Yes	Yes	Yes	No	Yes	No	No	No	No	No	No	No	No	SerTGA,SerAGA
GAATTCTCGCCTGCC	No	No	Yes	No	Yes	No	Yes	No	No	No	No	No	No	NA
GAATTCTCGCCTGCCA	No	Yes	Yes	No	Yes	No	No	No	No	No	No	No	No	GlyGCC
GAATTCTCGCCTGCCAC	No	Yes	Yes	No	Yes	No	No	No	No	No	No	No	No	GlyGCC
GACAACATTCAAAAAAGA	Yes	Yes	No	No	No	No	No	Yes	No	No	No	No	No	GlyTCC
GACAGGGGTTAGGCCTC	Yes	Yes	No	No	No	No	No	Yes	No	No	No	No	No	TyrGTA
GACAGGGGTTAGGCCTCTTTTTACCACCA	Yes	Yes	No	No	No	No	No	Yes	No	No	No	No	No	TyrGTA
GACCACGTGGCCTAA	No	No	No	No	Yes	No	Yes	No	No	No	No	No	No	NA
GACCACGTGGCCTAATGG	Yes	Yes	No	No	Yes	No	No	No	No	No	No	No	No	ArgTCG
GACCACGTGGCCTAATGGA	Yes	Yes	No	No	Yes	No	No	No	No	No	No	No	No	ArgTCG
GACCCAGTGGCCTAA	No	No	Yes	No	Yes	No	Yes	No	No	No	No	No	No	NA
GACCCAGTGGCCTAATGG	No	Yes	Yes	No	Yes	No	No	No	No	No	No	No	No	ArgCCG
GACCCAGTGGCCTAATGGA	No	Yes	Yes	Yes	Yes	No	No	No	No	No	No	No	No	ArgCCG
GACCCAGTGGCCTAATGGATAAG	Yes	Yes	Yes	No	Yes	No	No	No	No	No	No	No	No	ArgCCG
GACCCAGTGGCCTAATGGATAAGG	Yes	Yes	Yes	No	Yes	No	No	No	No	No	No	No	No	ArgCCG
GACCCAGTGGCCTAATGGATAAGGC	Yes	Yes	Yes	No	Yes	No	No	No	No	No	No	No	No	ArgCCG
GACCCAGTGGCCTAATGGATAAGGCA	Yes	Yes	Yes	No	Yes	No	No	No	No	No	No	No	No	ArgCCG
GACCCAGTGGCCTAATGGATAAGGCATC	Yes	Yes	Yes	No	Yes	No	No	No	No	No	No	No	No	ArgCCG
GACCCAGTGGCCTAATGGATAAGGCATCAG	Yes	Yes	Yes	No	Yes	No	No	No	No	No	No	No	No	ArgCCG
GACCCAGTGGCCTAATGGATAAGGCATCAGC	Yes	Yes	Yes	Yes	Yes	No	No	No	No	No	No	No	No	ArgCCG
GACCCAGTGGCCTAATGGATAAGGCATCAGCCT	Yes	Yes	Yes	No	Yes	No	No	No	No	No	No	No	No	ArgCCG
GACCCCTTATTTACCCCA	Yes	Yes	No	No	No	No	No	Yes	No	No	No	No	No	HisGTG
GACCGCGTGGCCTAA	No	No	No	No	Yes	No	Yes	No	No	No	No	No	No	NA
GACCGCGTGGCCTAATGGA	Yes	Yes	No	No	Yes	No	No	No	No	No	No	No	No	ArgTCG
GACCGCGTGGCCTAATGGATAAGGC	Yes	Yes	No	No	Yes	No	No	No	No	No	No	No	No	ArgTCG
GACCTCGTGGCGCAA	No	No	No	No	Yes	No	Yes	No	No	No	No	No	No	NA
GACCTCGTGGCGCAAC	Yes	Yes	No	No	Yes	No	No	No	No	No	No	No	No	TrpCCA
GACCTCGTGGCGCAACGG	Yes	Yes	No	No	Yes	No	No	No	No	No	No	No	No	TrpCCA
GACCTCGTGGCGCAACGGTAGC	Yes	Yes	No	No	Yes	No	No	No	No	No	No	No	No	TrpCCA
GACCTCGTGGCGCAATGG	Yes	Yes	No	No	Yes	No	No	No	No	No	No	No	No	TrpCCA
GACCTCGTGGCGCAATGGTAGC	Yes	Yes	No	No	Yes	No	No	No	No	No	No	No	No	TrpCCA
GACGAGGTGGCCGAG	No	No	Yes	No	Yes	No	Yes	No	No	No	No	No	No	NA
GACGAGGTGGCCGAGTGGTTAA	No	Yes	Yes	No	Yes	No	No	No	No	No	No	No	No	SerGCT
GACGAGGTGGCCGAGTGGTTAAG	No	Yes	Yes	No	Yes	No	No	No	No	No	No	No	No	SerGCT
GACGAGGTGGCCGAGTGGTTAAGG	Yes	Yes	Yes	No	Yes	No	No	No	No	No	No	No	No	SerGCT
GACGAGGTGGCCGAGTGGTTAAGGC	Yes	Yes	Yes	No	Yes	No	No	No	No	No	No	No	No	SerGCT
GACGGGCTCACATCACCCCATAAACACCA	Yes	Yes	No	No	No	No	No	Yes	No	No	No	No	No	PheGAA
GACTCCCGGTGTGGGAACCA	Yes	Yes	Yes	No	Yes	No	No	No	No	No	No	No	No	GluTTC
GACTCTGAATCCAGCGATCCGAG	Yes	Yes	Yes	No	Yes	No	No	No	No	No	No	No	No	GlnCTG
GACTGGACCTTTCTTT	No	No	Yes	No	Yes	No	No	No	No	No	No	Yes	No	NA
GACTGGACCTTTCTTTT	No	No	Yes	No	Yes	No	No	No	No	No	No	Yes	No	NA
GACTTCGTCTGTAATTT	No	No	No	No	Yes	No	No	No	No	No	No	Yes	No	NA
GACTTCGTCTGTAATTTT	No	No	No	No	Yes	No	No	No	No	No	No	Yes	No	NA
GAGAAAGCTCACAAGAAC	Yes	Yes	No	No	No	No	No	Yes	No	No	No	No	No	SerGCT
GAGAAAGCTCACAAGAACT	Yes	Yes	No	No	No	No	No	Yes	No	No	No	No	No	SerGCT
GAGAAAGCTCACAAGAACTG	Yes	Yes	No	No	No	No	No	Yes	No	No	No	No	No	SerGCT

GAGAAAGCTCACAAGAACTGC	Yes	Yes	No	No	No	No	No	Yes	No	No	No	No	No	SerGCT
GAGAAAGCTCACAAGAACTGCT	Yes	Yes	No	No	No	No	No	Yes	No	No	No	No	No	SerGCT
GAGAAAGCTCACAAGAACTGCTA	Yes	Yes	No	No	No	No	No	Yes	No	No	No	No	No	SerGCT
GAGAAAGCTCACAAGAACTGCTAA	Yes	Yes	No	No	No	No	No	Yes	No	No	No	No	No	SerGCT
GAGAAAGCTCACAAGAACTGCTAAC	Yes	Yes	No	No	No	No	No	Yes	No	No	No	No	No	SerGCT
GAGAAAGCTCACAAGAACTGCTAACT	Yes	Yes	No	No	No	No	No	Yes	No	No	No	No	No	SerGCT
GAGAAAGCTCACAAGAACTGCTAACTC	Yes	Yes	No	No	No	No	No	Yes	No	No	No	No	No	SerGCT
GAGAAAGCTCACAAGAACTGCTAACTCA	Yes	Yes	No	No	No	No	No	Yes	No	No	No	No	No	SerGCT
GAGAAAGCTCACAAGAACTGCTAACTCATGCC	Yes	Yes	No	No	No	No	No	Yes	No	No	No	No	No	SerGCT
GAGACCCGAGGTAGGGCT	No	No	No	No	Yes	No	No	No	No	No	No	Yes	No	NA
GAGACCCGAGGTAGGGCTTT	No	No	No	No	Yes	No	No	No	No	No	No	Yes	No	NA
GAGAGCGCTCGGTTT	No	No	Yes	No	Yes	No	Yes	No	No	No	No	Yes	No	NA
GAGAGCGCTCGGTTTT	No	No	Yes	No	Yes	No	No	No	No	No	No	Yes	No	NA
GAGAGCGCTCGGTTTTT	No	No	Yes	Yes	Yes	No	No	No	No	No	No	Yes	No	NA
GAGAGCGCTCGGTTTTTT	No	No	Yes	No	Yes	No	No	No	No	No	No	Yes	No	NA
GAGAGGCACTGGTGGTTCAGTGGTAGA	No	No	No	No	Yes	No	No	No	No	No	No	No	No	NA
GAGAGGTCCCGGGTT	No	No	Yes	No	Yes	No	Yes	No	No	No	No	No	No	NA
GAGATGAAAACCTTTTTCCAAGGACA	Yes	Yes	No	No	No	No	No	Yes	No	No	No	No	No	ThrTGT
GAGATGAAAACCTTTTTCCAAGGACACCA	Yes	Yes	No	No	No	No	No	Yes	No	No	No	No	No	ThrTGT
GAGATGTTACCTAGCGTTT	No	No	Yes	No	Yes	No	No	No	No	No	No	Yes	No	NA
GAGATTTCAACTTAACTTGACCGCTCTGACCA	Yes	Yes	No	No	No	No	No	Yes	No	No	No	No	No	ValTAC
GAGCCCGGTAATCGCA	Yes	Yes	No	No	No	No	No	Yes	No	No	No	No	No	LeuTAA
GAGCCCGGTAATCGCAT	Yes	Yes	No	No	No	No	No	Yes	No	No	No	No	No	LeuTAA
GAGCCCGGTAATCGCATA	Yes	Yes	No	No	No	No	No	Yes	No	No	No	No	No	LeuTAA
GAGCCCGGTAATCGCATAA	Yes	Yes	No	No	No	No	No	Yes	No	No	No	No	No	LeuTAA
GAGCCCGGTAATCGCATAAA	Yes	Yes	No	No	No	No	No	Yes	No	No	No	No	No	LeuTAA
GAGCCCGGTAATCGCATAAAA	Yes	Yes	No	No	No	No	No	Yes	No	No	No	No	No	LeuTAA
GAGCCCGGTAATCGCATAAAAC	Yes	Yes	No	No	No	No	No	Yes	No	No	No	No	No	LeuTAA
GAGCCCGGTAATCGCATAAAACT	Yes	Yes	No	No	No	No	No	Yes	No	No	No	No	No	LeuTAA
GAGCCCGGTAATCGCATAAAACTTA	Yes	Yes	No	No	No	No	No	Yes	No	No	No	No	No	LeuTAA
GAGCCCGGTAATCGCATAAAACTTAA	Yes	Yes	No	No	No	No	No	Yes	No	No	No	No	No	LeuTAA
GAGCCCGGTAATCGCATAAAACTTAAA	Yes	Yes	No	No	No	No	No	Yes	No	No	No	No	No	LeuTAA
GAGCCCGGTAATCGCATAAAACTTAAAA	Yes	Yes	No	No	No	No	No	Yes	No	No	No	No	No	LeuTAA
GAGCCCGGTAATCGCATAAAACTTAAAAC	Yes	Yes	No	No	No	No	No	Yes	No	No	No	No	No	LeuTAA
GAGCCCGGTAATCGCATAAAACTTAAAACT	Yes	Yes	No	No	No	No	No	Yes	No	No	No	No	No	LeuTAA
GAGCCCGGTAATCGCATAAAACTTAAAACTT	Yes	Yes	No	No	No	No	No	Yes	No	No	No	No	No	LeuTAA
GAGCTCAATTACTTT	No	No	Yes	No	Yes	No	Yes	No	No	No	No	Yes	No	NA
GAGCTCAATTACTTTT	No	No	Yes	No	Yes	No	No	No	No	No	No	Yes	No	NA
GAGGCTTAAACTTTT	No	No	Yes	Yes	Yes	No	Yes	No	No	No	No	Yes	No	NA
GAGGCTTAAACTTTTT	No	No	Yes	No	Yes	No	No	No	No	No	No	Yes	No	NA
GAGGGTTCTCACCTTCTCTCTCCGAT	No	No	Yes	No	Yes	No	No	No	No	No	No	Yes	No	NA
GAGGGTTCTCACCTTCTCTCTCCGATT	No	No	Yes	No	Yes	No	No	No	No	No	No	Yes	No	NA
GAGGGTTCTCACCTTCTCTCTCCGATTT	No	No	Yes	Yes	Yes	No	No	No	No	No	No	Yes	No	NA
GAGGGTTCTCACCTTCTCTCTCCGATTTT	No	No	Yes	No	Yes	No	No	No	No	No	No	Yes	No	NA
GAGGTTTTTCTGTTT	No	No	Yes	No	Yes	No	Yes	No	No	No	No	Yes	No	NA
GAGTGGCGCAGCGGA	No	No	Yes	No	Yes	No	Yes	No	No	No	No	No	No	NA
GAGTGGCGCAGCGGAA	Yes	Yes	Yes	No	Yes	No	No	No	No	No	No	No	No	MetCAT
GAGTGGCGCAGCGGAAG	Yes	Yes	Yes	No	Yes	No	No	No	No	No	No	No	No	MetCAT
GAGTGGCGCAGCGGAAGCGTGCT	Yes	Yes	Yes	No	Yes	No	No	No	No	No	No	No	No	MetCAT
GAGTGGCGCAGCGGAAGCGTGCTG	Yes	Yes	Yes	No	Yes	No	No	No	No	No	No	No	No	MetCAT
GAGTGGCGCAGCGGAAGCGTGCTGG	Yes	Yes	Yes	No	Yes	No	No	No	No	No	No	No	No	MetCAT
GAGTGGCGCAGCGGAAGCGTGCTGGG	Yes	Yes	Yes	No	Yes	No	No	No	No	No	No	No	No	MetCAT
GAGTGGCGCAGCGGAAGCGTGCTGGGC	Yes	Yes	Yes	No	Yes	No	No	No	No	No	No	No	No	MetCAT
GAGTGGCGCAGCGGAAGCGTGCTGGGCC	Yes	Yes	Yes	No	Yes	No	No	No	No	No	No	No	No	MetCAT
GAGTGGCGCAGCGGAAGCGTGCTGGGCCC	Yes	Yes	Yes	No	Yes	No	No	No	No	No	No	No	No	MetCAT
GAGTGGCGCAGCGGAAGCGTGCTGGGCCCA	Yes	Yes	Yes	No	Yes	No	No	No	No	No	No	No	No	MetCAT
GAGTGGGGTTTTGCAGTC	Yes	Yes	No	No	No	No	No	Yes	Yes	No	No	No	No	AlaTGC
GAGTGGGGTTTTGCAGTCC	Yes	Yes	No	No	No	No	No	Yes	Yes	No	No	No	No	AlaTGC
GAGTGGGGTTTTGCAGTCCT	Yes	Yes	No	No	No	No	No	Yes	Yes	No	No	No	No	AlaTGC
GAGTGGGGTTTTGCAGTCCTT	Yes	Yes	No	No	No	No	No	Yes	Yes	No	No	No	No	AlaTGC
GAGTGGGGTTTTGCAGTCCTTACCA	Yes	Yes	No	No	No	No	No	Yes	Yes	No	No	No	No	AlaTGC
GAGTGGTGGCTTTTA	No	No	Yes	No	Yes	No	Yes	No	No	No	No	Yes	No	NA
GAGTTAGCGGGGAGTGATATATT	No	No	No	No	Yes	No	No	No	No	No	No	Yes	No	NA
GAGTTCGAGTCTCGGTGGAACCTCCA	Yes	Yes	Yes	No	Yes	No	No	No	No	No	No	No	No	GlnCTG
GATAGCTCAGCTGGTAGAGCGGAGGA	Yes	Yes	Yes	No	Yes	No	No	No	No	No	No	No	No	TyrGTA
GATAGCTCAGTCGGTAGAGC	No	Yes	Yes	No	Yes	No	No	No	No	No	No	No	No	LysTTT
GATAGCTCAGTCGGTAGAGCA	No	Yes	Yes	No	Yes	No	No	No	No	No	No	No	No	LysTTT
GATAGCTCAGTCGGTAGAGCAT	No	Yes	Yes	No	Yes	No	No	No	No	No	No	No	No	LysTTT
GATAGCTCAGTCGGTAGAGCATC	Yes	Yes	Yes	No	Yes	No	No	No	No	No	No	No	No	LysTTT
GATAGCTCAGTCGGTAGAGCATCAGA	Yes	Yes	Yes	No	Yes	No	No	No	No	No	No	No	No	LysTTT
GATAGCTCAGTCGGTAGAGCATCAGACTTT	Yes	Yes	Yes	No	Yes	No	No	No	No	No	No	No	No	LysTTT
GATAGCTCAGTCGGTAGAGCATCAGACTTTT	Yes	Yes	Yes	No	Yes	No	No	No	No	No	No	No	No	LysTTT
GATAGCTCAGTTGGTAGAGCGGAGGAC	No	Yes	Yes	No	Yes	No	No	No	No	No	No	No	No	TyrGTA
GATATCCAACCTTCAGCTATAGGGTGGAGAC	No	No	Yes	No	Yes	No	No	No	No	No	No	Yes	No	NA
GATATCCAACCTTCAGCTATAGGGTGGAGACTT	No	No	Yes	No	Yes	No	No	No	No	No	No	Yes	No	NA
GATGAAAACCTTTTTCCAAGGACA	Yes	Yes	No	No	No	No	No	Yes	No	No	No	No	No	ThrTGT
GATGAAAACCTTTTTCCAAGGACACC	Yes	Yes	No	No	No	No	No	Yes	No	No	No	No	No	ThrTGT
GATGAAAACCTTTTTCCAAGGACACCA	Yes	Yes	No	No	No	No	No	Yes	No	No	No	No	No	ThrTGT
GATGGCCGAGCGGTCTAAG	No	Yes	Yes	No	Yes	No	No	No	No	No	No	No	No	LeuCAG
GATGGCCGAGCGGTCTAAGG	No	Yes	Yes	No	Yes	No	No	No	No	No	No	No	No	LeuCAG
GATTCCAGGTTCGACTCCTGGCTGGCTCG	Yes	Yes	Yes	No	Yes	No	No	No	No	No	No	No	No	ArgACG
GATTCCAGGTTCGACTCCTGGCTGGCTCGCC	Yes	Yes	Yes	No	Yes	No	No	No	No	No	No	No	No	ArgACG
GATTCCAGGTTCGACTCCTGGCTGGCTCGCCA	Yes	Yes	Yes	No	Yes	No	No	No	No	No	No	No	No	ArgACG
GATTCCCCGACGGGGAGCCA	Yes	Yes	Yes	No	Yes	No	No	No	No	No	No	No	Yes	AspGTC
GATTCCCGGCCAACGCACCA	Yes	Yes	Yes	No	Yes	No	No	No	No	No	No	No	No	GlyTCC
GATTCCCGGTCAGGGAACCA	Yes	Yes	Yes	No	Yes	No	No	No	No	No	No	No	Yes	GluCTC,PheGAA,GluTTC
GATTCCGGCTCGAAGGACCA	Yes	Yes	Yes	No	Yes	No	No	No	No	No	No	No	No	TyrGTA
GATTCGGCGCTCTCACCGCC	Yes	Yes	Yes	No	Yes	No	No	No	No	No	No	No	Yes	GluCTC

GATTCTAGGTTCGACTCCTGGC	Yes	Yes	Yes	No	Yes	No	No	No	No	No	No	No	No	ArgACG
GATTCTAGGTTCGACTCCTGGCTGGCTCGCCA	Yes	Yes	Yes	No	Yes	No	No	No	No	No	No	No	No	ArgACG
GATTGAGGGTTCGAATCCCTCCGTGGTTA	Yes	Yes	No	No	Yes	No	No	No	No	No	No	No	No	ArgTCG
GATTGAGGGTTCGAATCCCTCCGTGGTTACCA	Yes	Yes	No	No	Yes	No	No	No	No	No	No	No	No	ArgTCG
GATTGAGGGTTCGAATCCCTTCGTGGTTGCCA	Yes	Yes	No	No	Yes	No	No	No	No	No	No	No	No	ArgTCG
GATTGAGGGTTCGAGTCCCTTCGTGGTCGCCA	Yes	Yes	No	No	Yes	No	No	No	No	No	No	No	No	ArgTCG,ArgCCG
GATTGCAGGTTCGAGTCCTGCCGCGGTCGCCA	Yes	Yes	Yes	No	Yes	No	No	No	No	No	No	No	No	ArgTCG
GATTTCAACTTAACTTGACCGCTCTGACCA	Yes	Yes	No	No	No	No	No	Yes	No	No	No	No	No	ValTAC
GATTTCTGTACCTTACACATTT	No	No	No	No	Yes	No	No	No	No	No	No	Yes	No	NA
GCAACCTCGGTTCGAATCCGAGTCACGG	Yes	Yes	Yes	No	Yes	No	No	No	No	No	No	No	No	HisGTG
GCAACTGGTCGTTTT	No	No	Yes	Yes	Yes	No	Yes	No	No	No	No	Yes	No	NA
GCAACTGGTCGTTTTA	No	No	Yes	No	Yes	No	No	No	No	No	No	Yes	No	NA
GCACAGTAATTGTTT	No	No	Yes	No	Yes	No	Yes	No	No	No	No	Yes	No	NA
GCACAGTAATTGTTTT	No	No	Yes	No	Yes	No	No	No	No	No	No	Yes	No	NA
GCACAGTAATTGTTTTT	No	No	Yes	No	Yes	No	No	No	No	No	No	Yes	No	NA
GCACGAAAATGTGTTT	No	No	Yes	No	Yes	No	No	No	No	No	No	Yes	No	NA
GCACGAAAATGTGTTTT	No	No	Yes	Yes	Yes	No	No	No	No	No	No	Yes	No	NA
GCACGCCCTCCCATTT	No	No	Yes	No	Yes	No	No	No	No	No	No	Yes	No	NA
GCACGCCCTCCCATTTT	No	No	Yes	No	Yes	No	No	No	No	No	No	Yes	No	NA
GCAGATCAAGAGGTCCCCGGT	Yes	Yes	Yes	No	Yes	No	No	No	No	No	No	No	Yes	CysGCA
GCAGATCAAGAGGTCCCTGGT	Yes	Yes	Yes	No	Yes	No	No	No	No	No	No	No	No	CysGCA
GCAGCCGTGCTTTTT	No	No	Yes	No	Yes	No	Yes	No	No	No	No	Yes	No	NA
GCAGCTGAAAGCTTTT	No	No	Yes	No	Yes	No	No	No	No	No	No	Yes	No	NA
GCAGCTGAAAGCTTTTT	No	No	Yes	No	Yes	No	No	No	No	No	No	Yes	No	NA
GCATGAGGTCCCGGGT	Yes	Yes	No	No	Yes	No	No	No	No	No	No	No	No	AlaAGC
GCATGCACGAGGTCCTGGGT	Yes	Yes	Yes	No	Yes	No	No	No	No	No	No	No	No	AlaAGC
GCATGGGTGGTTCAG	No	No	Yes	No	Yes	No	Yes	No	No	No	No	No	No	NA
GCATGGGTGGTTCAGT	No	Yes	Yes	No	Yes	No	No	No	No	No	No	No	No	GlyGCC
GCATGGGTGGTTCAGTG	No	Yes	Yes	No	Yes	No	No	No	No	No	No	No	No	GlyGCC
GCATGGGTGGTTCAGTGG	No	Yes	Yes	No	Yes	No	No	No	No	No	No	No	No	GlyGCC
GCATGGGTGGTTCAGTGGT	No	Yes	Yes	No	Yes	No	No	No	No	No	No	No	No	GlyGCC
GCATGGGTGGTTCAGTGGTA	No	Yes	Yes	No	Yes	No	No	No	No	No	No	No	No	GlyGCC
GCATGGGTGGTTCAGTGGTAG	No	Yes	Yes	No	Yes	No	No	No	No	No	No	No	No	GlyGCC
GCATGGGTGGTTCAGTGGTAGA	No	Yes	Yes	Yes	Yes	No	No	No	No	No	No	No	No	GlyGCC
GCATGGGTGGTTCAGTGGTAGAA	No	Yes	Yes	No	Yes	No	No	No	No	No	No	No	No	GlyGCC
GCATGGGTGGTTCAGTGGTAGAAT	No	Yes	Yes	No	Yes	No	No	No	No	No	No	No	No	GlyGCC
GCATGGGTGGTTCAGTGGTAGAATT	No	Yes	Yes	No	Yes	No	No	No	No	No	No	No	No	GlyGCC
GCATGGGTGGTTCAGTGGTAGAATTC	No	Yes	Yes	No	Yes	No	No	No	No	No	No	No	No	GlyGCC
GCATGGGTGGTTCAGTGGTAGAATTCT	No	Yes	Yes	No	Yes	No	No	No	No	No	No	No	No	GlyGCC
GCATGGGTGGTTCAGTGGTAGAATTCTC	Yes	Yes	Yes	No	Yes	No	No	No	No	No	No	No	No	GlyGCC
GCATGGGTGGTTCAGTGGTAGAATTCTCG	Yes	Yes	Yes	No	Yes	No	No	No	No	No	No	No	No	GlyGCC
GCATGGGTGGTTCAGTGGTAGAATTCTCGC	Yes	Yes	Yes	No	Yes	No	No	No	No	No	No	No	No	GlyGCC
GCATGGGTGGTTCAGTGGTAGAATTCTCGCC	Yes	Yes	Yes	Yes	Yes	No	No	No	No	No	No	No	No	GlyGCC
GCATGGGTGGTTCAGTGGTAGAATTCTCGCCT	Yes	Yes	No	No	Yes	No	No	No	No	No	No	No	No	GlyGCC
GCATGGGTGGTTCAGTGGTAGAATTCTCGCCTG	Yes	Yes	No	No	Yes	No	No	No	No	No	No	No	No	GlyGCC
GCATGTCTTTGCTTT	No	No	Yes	No	Yes	No	Yes	No	No	No	No	Yes	No	NA
GCATGTCTTTGCTTTT	No	No	Yes	No	Yes	No	No	No	No	No	No	Yes	No	NA
GCATTGGTGGTTCAATGGTAGA	Yes	Yes	No	No	Yes	No	No	No	No	No	No	No	No	GlyCCC
GCATTGGTGGTTCAG	No	No	Yes	No	Yes	No	Yes	No	No	No	No	No	No	NA
GCATTGGTGGTTCAGT	No	Yes	Yes	No	Yes	No	No	No	No	No	No	No	No	GlyCCC,GlyGCC
GCATTGGTGGTTCAGTG	No	Yes	Yes	No	Yes	No	No	No	No	No	No	No	No	GlyCCC,GlyGCC
GCATTGGTGGTTCAGTGG	No	Yes	Yes	No	Yes	No	No	No	No	No	No	No	No	GlyCCC,GlyGCC
GCATTGGTGGTTCAGTGGT	No	Yes	Yes	No	Yes	No	No	No	No	No	No	No	No	GlyCCC,GlyGCC
GCATTGGTGGTTCAGTGGTA	No	Yes	Yes	No	Yes	No	No	No	No	No	No	No	No	GlyCCC,GlyGCC
GCATTGGTGGTTCAGTGGTAG	No	Yes	Yes	No	Yes	No	No	No	No	No	No	No	No	GlyCCC,GlyGCC
GCATTGGTGGTTCAGTGGTAGA	No	Yes	Yes	Yes	Yes	No	No	No	No	No	No	No	No	GlyCCC,GlyGCC
GCATTGGTGGTTCAGTGGTAGAA	No	Yes	Yes	No	Yes	No	No	No	No	No	No	No	No	GlyCCC,GlyGCC
GCATTGGTGGTTCAGTGGTAGAAT	No	Yes	Yes	No	Yes	No	No	No	No	No	No	No	No	GlyCCC,GlyGCC
GCATTGGTGGTTCAGTGGTAGAATT	No	Yes	Yes	No	Yes	No	No	No	No	No	No	No	No	GlyCCC,GlyGCC
GCATTGGTGGTTCAGTGGTAGAATTC	No	Yes	Yes	No	Yes	No	No	No	No	No	No	No	No	GlyCCC,GlyGCC
GCATTGGTGGTTCAGTGGTAGAATTCT	No	Yes	Yes	No	Yes	No	No	No	No	No	No	No	No	GlyCCC,GlyGCC
GCATTGGTGGTTCAGTGGTAGAATTCTC	No	Yes	Yes	No	Yes	No	No	No	No	No	No	No	No	GlyCCC,GlyGCC
GCATTGGTGGTTCAGTGGTAGAATTCTCG	No	Yes	Yes	No	Yes	No	No	No	No	No	No	No	No	GlyCCC,GlyGCC
GCATTGGTGGTTCAGTGGTAGAATTCTCGC	No	Yes	Yes	No	Yes	No	No	No	No	No	No	No	No	GlyCCC,GlyGCC
GCATTGGTGGTTCAGTGGTAGAATTCTCGCC	No	Yes	Yes	Yes	Yes	No	No	No	No	No	No	No	No	GlyCCC,GlyGCC
GCATTGGTGGTTCAGTGGTAGAATTCTCGCCT	No	Yes	No	No	Yes	No	No	No	No	No	No	No	No	GlyCCC,GlyGCC
GCATTGGTGGTTCAGTGGTAGAATTCTCGCCTC	No	Yes	Yes	No	Yes	No	No	No	No	No	No	No	No	GlyCCC
GCATTGGTGGTTCAGTGGTAGAATTCTCGCCTG	No	Yes	No	No	Yes	No	No	No	No	No	No	No	No	GlyGCC
GCCAAGTACGTTTTT	No	No	Yes	No	Yes	No	Yes	No	No	No	No	Yes	No	NA
GCCACAGTGTTTTTTT	No	No	Yes	No	Yes	No	No	No	No	No	No	Yes	No	NA
GCCACGCGGGAGGCCC	Yes	Yes	Yes	No	Yes	No	No	No	No	No	No	No	No	GlyGCC
GCCCACCCAGGGACGCCA	No	Yes	Yes	No	Yes	No	No	No	No	No	No	No	No	AsnGTT
GCCCCACGTTGGGCGCCA	No	Yes	Yes	No	Yes	No	No	No	No	No	No	No	No	LysCTT
GCCCCAGTGGAACCACCA	No	Yes	Yes	No	Yes	No	No	No	No	No	No	No	No	ValTAC
GCCCCAGTGGCCTAATGG	Yes	Yes	Yes	No	Yes	No	No	No	No	No	No	No	No	ArgCCT
GCCCCAGTGGCCTAATGGA	Yes	Yes	Yes	No	Yes	No	No	No	No	No	No	No	No	ArgCCT
GCCCCAGTGGCCTAATGGATAAGGCACTG	Yes	Yes	Yes	No	Yes	No	No	No	No	No	No	No	No	ArgCCT
GCCCCGGTGGCCTAA	No	No	No	No	Yes	No	Yes	No	No	No	No	No	No	NA
GCCCCGGTGGCCTAATGGA	Yes	Yes	No	No	Yes	No	No	No	No	No	No	No	No	ArgCCT
GCCCGCATCCTCCACCA	No	Yes	No	No	Yes	No	No	No	No	No	No	No	No	AlaAGC
GCCCGCATTCTCCACCA	Yes	Yes	No	No	Yes	No	No	No	No	No	No	No	No	AlaAGC
GCCCGGATAGCTCAG	No	No	Yes	No	Yes	No	Yes	No	No	No	No	No	No	NA
GCCCGGATAGCTCAGT	No	Yes	Yes	No	Yes	No	No	No	No	No	No	No	No	LysTTT
GCCCGGATAGCTCAGTC	No	Yes	Yes	No	Yes	No	No	No	No	No	No	No	No	LysTTT
GCCCGGATAGCTCAGTCGG	Yes	Yes	Yes	Yes	Yes	No	No	No	No	No	No	No	No	LysTTT
GCCCGGATAGCTCAGTCGGT	Yes	Yes	Yes	No	Yes	No	No	No	No	No	No	No	No	LysTTT
GCCCGGATAGCTCAGTCGGTA	Yes	Yes	Yes	No	Yes	No	No	No	No	No	No	No	No	LysTTT
GCCCGGATAGCTCAGTCGGTAG	Yes	Yes	Yes	No	Yes	No	No	No	No	No	No	No	No	LysTTT

GCCCGGATAGCTCAGTCGGTAGA	Yes	Yes	Yes	No	Yes	No	No	No	No	No	No	No	No	LysTTT
GCCCGGATAGCTCAGTCGGTAGAG	Yes	Yes	Yes	No	Yes	No	No	No	No	No	No	No	No	LysTTT
GCCCGGATAGCTCAGTCGGTAGAGC	Yes	Yes	Yes	No	Yes	No	No	No	No	No	No	No	No	LysTTT
GCCCGGATAGCTCAGTCGGTAGAGCA	Yes	Yes	Yes	No	Yes	No	No	No	No	No	No	No	No	LysTTT
GCCCGGATAGCTCAGTCGGTAGAGCAT	Yes	Yes	Yes	No	Yes	No	No	No	No	No	No	No	No	LysTTT
GCCCGGATAGCTCAGTCGGTAGAGCATC	Yes	Yes	Yes	No	Yes	No	No	No	No	No	No	No	No	LysTTT
GCCCGGATAGCTCAGTCGGTAGAGCATCA	Yes	Yes	Yes	No	Yes	No	No	No	No	No	No	No	No	LysTTT
GCCCGGATAGCTCAGTCGGTAGAGCATCAG	Yes	Yes	Yes	No	Yes	No	No	No	No	No	No	No	No	LysTTT
GCCCGGATAGCTCAGTCGGTAGAGCATCAGA	Yes	Yes	Yes	Yes	Yes	No	No	No	No	No	No	No	No	LysTTT
GCCCGGATAGCTCAGTCGGTAGAGCATCAGAC	Yes	Yes	Yes	No	Yes	No	No	No	No	No	No	No	No	LysTTT
GCCCGGATAGCTCAGTCGGTAGAGCATCAGACT	Yes	Yes	Yes	No	Yes	No	No	No	No	No	No	No	No	LysTTT
GCCCGGATGATCCTCAGTGGTCTGGGGTG	No	No	No	No	Yes	No	No	No	No	No	No	No	No	NA
GCCCGGCTAGCTCAG	No	No	Yes	Yes	Yes	No	Yes	No	No	No	No	No	No	NA
GCCCGGCTAGCTCAGT	Yes	Yes	Yes	No	Yes	No	No	No	No	No	No	No	No	LysCTT
GCCCGGCTAGCTCAGTC	Yes	Yes	Yes	No	Yes	No	No	No	No	No	No	No	No	LysCTT
GCCCGGCTAGCTCAGTCG	Yes	Yes	Yes	No	Yes	No	No	No	No	No	No	No	No	LysCTT
GCCCGGCTAGCTCAGTCGG	Yes	Yes	Yes	No	Yes	No	No	No	No	No	No	No	No	LysCTT
GCCCGGCTAGCTCAGTCGGT	Yes	Yes	Yes	No	Yes	No	No	No	No	No	No	No	No	LysCTT
GCCCGGCTAGCTCAGTCGGTA	Yes	Yes	Yes	No	Yes	No	No	No	No	No	No	No	No	LysCTT
GCCCGGCTAGCTCAGTCGGTAG	Yes	Yes	Yes	No	Yes	No	No	No	No	No	No	No	No	LysCTT
GCCCGGCTAGCTCAGTCGGTAGA	Yes	Yes	Yes	No	Yes	No	No	No	No	No	No	No	No	LysCTT
GCCCGGCTAGCTCAGTCGGTAGAG	Yes	Yes	Yes	No	Yes	No	No	No	No	No	No	No	No	LysCTT
GCCCGGCTAGCTCAGTCGGTAGAGC	Yes	Yes	Yes	Yes	Yes	No	No	No	No	No	No	No	No	LysCTT
GCCCGGCTAGCTCAGTCGGTAGAGCA	Yes	Yes	Yes	No	Yes	No	No	No	No	No	No	No	No	LysCTT
GCCCGGCTAGCTCAGTCGGTAGAGCAT	Yes	Yes	Yes	No	Yes	No	No	No	No	No	No	No	No	LysCTT
GCCCGGCTAGCTCAGTCGGTAGAGCATG	Yes	Yes	Yes	No	Yes	No	No	No	No	No	No	No	No	LysCTT
GCCCGGCTAGCTCAGTCGGTAGAGCATGA	Yes	Yes	Yes	No	Yes	No	No	No	No	No	No	No	No	LysCTT
GCCCGGCTAGCTCAGTCGGTAGAGCATGAG	Yes	Yes	Yes	No	Yes	No	No	No	No	No	No	No	No	LysCTT
GCCCGGCTAGCTCAGTCGGTAGAGCATGAGA	Yes	Yes	Yes	Yes	Yes	No	No	No	No	No	No	No	No	LysCTT
GCCCGGCTAGCTCAGTCGGTAGAGCATGAGAC	Yes	Yes	Yes	No	Yes	No	No	No	No	No	No	No	No	LysCTT
GCCCGGCTAGCTCAGTCGGTAGAGCATGAGACT	Yes	Yes	Yes	No	Yes	No	No	No	No	No	No	No	No	LysCTT
GCCCGGCTAGCTCAGTCGGTAGAGCATGG	Yes	Yes	Yes	No	Yes	No	No	No	No	No	No	No	No	LysCTT
GCCCGGCTAGCTCAGTCGGTAGAGCATGGG	Yes	Yes	Yes	No	Yes	No	No	No	No	No	No	No	No	LysCTT
GCCCGGCTAGCTCAGTCGGTAGAGCATGGGA	Yes	Yes	Yes	No	Yes	No	No	No	No	No	No	No	No	LysCTT
GCCCGGCTAGCTCAGTCGGTAGAGCATGGGAC	Yes	Yes	Yes	Yes	Yes	No	No	No	No	No	No	No	No	LysCTT
GCCCGGCTAGCTCAGTCGGTAGAGCATGGGACT	Yes	Yes	Yes	No	Yes	No	No	No	No	No	No	No	No	LysCTT
GCCCGGGTTCGACTCCCGGTGTGGGA	Yes	Yes	Yes	No	Yes	No	No	No	No	No	No	No	No	GluTTC
GCCCGGGTTCGACTCCCGGTGTGGGAA	Yes	Yes	Yes	No	Yes	No	No	No	No	No	No	No	No	GluTTC
GCCCGGGTTCGACTCCCGGTGTGGGAAC	Yes	Yes	Yes	No	Yes	No	No	No	No	No	No	No	No	GluTTC
GCCCGGGTTCGACTCCCGGTGTGGGAACC	Yes	Yes	Yes	No	Yes	No	No	No	No	No	No	No	No	GluTTC
GCCCGGGTTCGACTCCCGGTGTGGGAACCA	Yes	Yes	Yes	No	Yes	No	No	No	No	No	No	No	No	GluTTC
GCCCGGGTTCGATTCCCGGTCAGG	Yes	Yes	Yes	No	Yes	No	No	No	No	No	No	No	Yes	GluCTC,PheGAA,GluTTC
GCCCTCTTAGCGCAG	No	No	Yes	No	Yes	No	Yes	No	No	No	No	No	No	NA
GCCCTCTTAGCGCAGC	No	Yes	Yes	No	Yes	No	No	No	No	No	No	No	No	MetCAT
GCCCTCTTAGCGCAGCGG	Yes	Yes	Yes	No	Yes	No	No	No	No	No	No	No	No	MetCAT
GCCGAAATAGCTCAGTTGG	No	Yes	Yes	No	Yes	No	No	No	No	No	No	No	No	PheGAA
GCCGAAATAGCTCAGTTGGGAGA	No	Yes	Yes	No	Yes	No	No	No	No	No	No	No	No	PheGAA
GCCGAAATAGCTCAGTTGGGAGAGC	No	Yes	Yes	No	Yes	No	No	No	No	No	No	No	No	PheGAA
GCCGAGCGGTCTAAGGC	No	Yes	Yes	No	Yes	No	No	No	No	No	No	No	Yes	LeuTAG,LeuAAG,LeuCAG
GCCGGGTACTTTCGTATT	No	No	Yes	No	Yes	No	No	No	No	No	No	Yes	No	NA
GCCGGGTACTTTCGTATTT	No	No	Yes	No	Yes	No	No	No	No	No	No	Yes	No	NA
GCCGGGTACTTTCGTATTTT	No	No	Yes	No	Yes	No	No	No	No	No	No	Yes	No	NA
GCCGGGTACTTTCGTATTTTT	No	No	Yes	No	Yes	No	No	No	No	No	No	Yes	No	NA
GCCGTGATCGTATAGTGG	No	Yes	Yes	No	Yes	No	No	No	No	No	No	No	No	HisGTG
GCCGTGATCGTATAGTGGTTAGTACTCTGCGT	Yes	Yes	Yes	No	Yes	No	No	No	No	No	No	No	No	HisGTG
GCCTCCGGAGCTGGGGATTGTGGGT	Yes	Yes	Yes	No	Yes	No	No	No	No	No	No	No	No	ArgCCG
GCCTCCTTAGCGCAG	No	No	No	No	Yes	No	Yes	No	No	No	No	No	No	NA
GCCTCGTTAGCGCAG	No	No	No	No	Yes	No	Yes	No	No	No	No	No	No	NA
GCCTCGTTAGCGCAGTAGG	No	Yes	No	No	Yes	Yes	No	No	No	No	No	No	No	MetCAT
GCCTCGTTAGCGCAGTAGGTA	No	Yes	No	No	Yes	No	No	No	No	No	No	No	No	MetCAT
GCCTCGTTAGCGCAGTAGGTAGCGC	No	Yes	No	No	Yes	No	No	No	No	No	No	No	No	MetCAT
GCCTGGATAGCTCAG	No	No	No	No	Yes	No	Yes	No	No	No	No	No	No	NA
GCCTGGATAGCTCAGTCGG	No	Yes	No	No	Yes	No	No	No	No	No	No	No	No	LysTTT
GCCTGGATAGCTCAGTCGGTAGAGCATC	Yes	Yes	No	No	Yes	No	No	No	No	No	No	No	No	LysTTT
GCCTGGATAGCTCAGTCGGTAGAGCATCAG	Yes	Yes	No	No	Yes	No	No	No	No	No	No	No	No	LysTTT
GCCTGGATAGCTCAGTCGGTAGAGCATCAGA	Yes	Yes	No	No	Yes	No	No	No	No	No	No	No	No	LysTTT
GCCTGGATAGCTCAGTCGGTAGAGCATCAGAC	Yes	Yes	No	No	Yes	No	No	No	No	No	No	No	No	LysTTT
GCCTGGATAGCTCAGTTGGTAGAGC	Yes	Yes	No	No	Yes	No	No	No	No	No	No	No	No	LysTTT
GCCTGGATAGCTCAGTTGGTAGAGCAT	Yes	Yes	No	No	Yes	No	No	No	No	No	No	No	No	LysTTT
GCCTGGATAGCTCAGTTGGTAGAGCATCAG	Yes	Yes	No	No	Yes	No	No	No	No	No	No	No	No	LysTTT
GCCTGGATAGCTCAGTTGGTAGAGCATCAGA	Yes	Yes	No	No	Yes	No	No	No	No	No	No	No	No	LysTTT
GCCTGGATAGCTCAGTTGGTAGAGCATCAGAC	Yes	Yes	No	No	Yes	No	No	No	No	No	No	No	No	LysTTT
GCCTGGGTAGCTCAG	No	No	No	No	Yes	No	Yes	No	No	No	No	No	No	NA
GCCTGGGTAGCTCAGTCGG	Yes	Yes	No	No	Yes	Yes	No	No	No	No	No	No	Yes	LysTTT
GCCTGGGTAGCTCAGTCGGTAGAGCATCAGA	Yes	Yes	No	No	Yes	No	No	No	No	No	No	No	No	LysTTT
GCCTGGGTAGCTCAGTCGGTAGAGCATCAGAC	Yes	Yes	No	No	Yes	No	No	No	No	No	No	No	No	LysTTT
GCGAGAGGTAGCGGGA	Yes	Yes	No	No	Yes	No	No	No	No	No	No	No	No	AlaAGC
GCGAGAGGTCCCGGGT	Yes	Yes	Yes	No	Yes	No	No	No	No	No	No	No	No	ProAGG,ProCGG,ProTGG
GCGATCACGTAGATTTT	No	No	Yes	No	Yes	No	No	No	No	No	No	Yes	No	NA
GCGCCGCTGGTGTAG	No	No	Yes	Yes	Yes	No	Yes	No	No	No	No	No	No	NA
GCGCCGCTGGTGTAGT	Yes	Yes	Yes	No	Yes	No	No	No	No	No	No	No	No	GlyCCC
GCGCCGCTGGTGTAGTG	Yes	Yes	Yes	No	Yes	No	No	No	No	No	No	No	No	GlyCCC
GCGCCGCTGGTGTAGTGG	Yes	Yes	Yes	No	Yes	No	No	No	No	No	No	No	No	GlyCCC
GCGCCGCTGGTGTAGTGGTA	Yes	Yes	Yes	No	Yes	No	No	No	No	No	No	No	No	GlyCCC
GCGCCGCTGGTGTAGTGGTAT	Yes	Yes	Yes	No	Yes	No	No	No	No	No	No	No	No	GlyCCC
GCGCCGCTGGTGTAGTGGTATC	Yes	Yes	Yes	No	Yes	No	No	No	No	No	No	No	No	GlyCCC
GCGCCGCTGGTGTAGTGGTATCA	Yes	Yes	Yes	No	Yes	No	No	No	No	No	No	No	No	GlyCCC
GCGCCGCTGGTGTAGTGGTATCATG	Yes	Yes	Yes	No	Yes	No	No	No	No	No	No	No	No	GlyCCC

GCGCCGCTGGTGTAGTGGTATCATGC	Yes	Yes	Yes	No	Yes	No	No	No	No	No	No	No	No	GlyCCC
GCGCCGCTGGTGTAGTGGTATCATGCA	Yes	Yes	Yes	No	Yes	No	No	No	No	No	No	No	No	GlyCCC
GCGCCGCTGGTGTAGTGGTATCATGCAA	Yes	Yes	Yes	No	Yes	No	No	No	No	No	No	No	No	GlyCCC
GCGCCGCTGGTGTAGTGGTATCATGCAAG	Yes	Yes	Yes	No	Yes	No	No	No	No	No	No	No	No	GlyCCC
GCGCCGCTGGTGTAGTGGTATCATGCAAGA	Yes	Yes	Yes	Yes	Yes	No	No	No	No	No	No	No	No	GlyCCC
GCGCCGCTGGTGTAGTGGTATCATGCAAGATT	Yes	Yes	Yes	No	Yes	No	No	No	No	No	No	No	No	GlyCCC
GCGCCGCTGGTGTAGTGGTATCATGCAAGATTC	Yes	Yes	Yes	No	Yes	No	No	No	No	No	No	No	No	GlyCCC
GCGGCCCGGGTTCGACTCCCGGTGTGGGAACCA	Yes	Yes	Yes	No	Yes	No	No	No	No	No	No	No	No	GluTTC
GCGGCTTAGCCTGTGGCTC	No	No	No	No	Yes	No	No	No	No	No	Yes	No	No	NA
GCGGGAGGCCCGGGT	No	No	Yes	No	Yes	No	Yes	No	No	No	No	No	No	NA
GCGGGCGGACCTTTT	No	No	Yes	Yes	Yes	No	Yes	No	No	No	No	Yes	No	NA
GCGGGTGCTCTTATT	No	No	Yes	No	Yes	No	Yes	No	No	No	No	Yes	No	NA
GCGGGTGCTCTTATTT	No	No	Yes	No	Yes	No	No	No	No	No	No	Yes	No	NA
GCGGGTGCTCTTATTTT	No	No	Yes	No	Yes	No	No	No	No	No	No	Yes	No	NA
GCGGGTGCTCTTATTTTT	No	No	Yes	No	Yes	No	No	No	No	No	No	Yes	No	NA
GCGGTTAGGATTCCT	No	No	Yes	No	Yes	No	Yes	No	No	No	No	No	No	NA
GCGGTTAGGATTCCTGG	No	Yes	Yes	No	Yes	No	No	No	No	No	No	No	No	GluTTC
GCGTTCAGGTCGCAGTCTCC	Yes	Yes	Yes	No	Yes	No	No	No	No	No	No	No	No	LeuCAG
GCGTTCAGGTCGCAGTCTCCC	Yes	Yes	Yes	No	Yes	No	No	No	No	No	No	No	No	LeuCAG
GCGTTGGTGGTATAG	No	No	Yes	No	Yes	No	Yes	No	No	No	No	No	No	NA
GCGTTGGTGGTATAGTGG	No	Yes	Yes	No	Yes	No	No	No	No	No	No	No	No	GlyTCC
GCGTTGGTGGTATAGTGGT	No	Yes	Yes	No	Yes	No	No	No	No	No	No	No	No	GlyTCC
GCGTTGGTGGTATAGTGGTAAGC	Yes	Yes	Yes	No	Yes	No	No	No	No	No	No	No	No	GlyTCC
GCGTTGGTGGTATAGTGGTAAGCA	Yes	Yes	Yes	No	Yes	No	No	No	No	No	No	No	No	GlyTCC
GCGTTGGTGGTATAGTGGTAAGCAT	Yes	Yes	Yes	No	Yes	No	No	No	No	No	No	No	No	GlyTCC
GCGTTGGTGGTATAGTGGTAAGCATA	Yes	Yes	Yes	No	Yes	No	No	No	No	No	No	No	No	GlyTCC
GCGTTGGTGGTATAGTGGTAAGCATAGCT	Yes	Yes	Yes	No	Yes	No	No	No	No	No	No	No	No	GlyTCC
GCGTTGGTGGTATAGTGGTAAGCATAGCTG	Yes	Yes	Yes	No	Yes	No	No	No	No	No	No	No	No	GlyTCC
GCGTTGGTGGTATAGTGGTAAGCATAGCTGC	Yes	Yes	Yes	No	Yes	No	No	No	No	No	No	No	No	GlyTCC
GCGTTGGTGGTATAGTGGTGA	No	Yes	Yes	No	Yes	No	No	No	No	No	No	No	No	GlyTCC
GCGTTGGTGGTATAGTGGTGAGC	No	Yes	Yes	Yes	Yes	No	No	No	No	No	No	No	No	GlyTCC
GCGTTGGTGGTATAGTGGTGAGCA	No	Yes	Yes	No	Yes	No	No	No	No	No	No	No	No	GlyTCC
GCGTTGGTGGTATAGTGGTGAGCAT	No	Yes	Yes	No	Yes	No	No	No	No	No	No	No	No	GlyTCC
GCGTTGGTGGTATAGTGGTGAGCATA	No	Yes	Yes	No	Yes	No	No	No	No	No	No	No	No	GlyTCC
GCGTTGGTGGTATAGTGGTGAGCATAG	No	Yes	Yes	No	Yes	No	No	No	No	No	No	No	No	GlyTCC
GCGTTGGTGGTATAGTGGTGAGCATAGC	No	Yes	Yes	No	Yes	No	No	No	No	No	No	No	No	GlyTCC
GCGTTGGTGGTATAGTGGTGAGCATAGCT	No	Yes	Yes	No	Yes	No	No	No	No	No	No	No	No	GlyTCC
GCGTTGGTGGTATAGTGGTGAGCATAGCTG	No	Yes	Yes	Yes	Yes	No	No	No	No	No	No	No	No	GlyTCC
GCGTTGGTGGTATAGTGGTTAGC	No	Yes	Yes	No	Yes	No	No	No	No	No	No	No	No	GlyTCC
GCGTTGGTGGTATAGTGGTTAGCATA	No	Yes	Yes	No	Yes	No	No	No	No	No	No	No	No	GlyTCC
GCGTTGGTGGTATAGTGGTTAGCATAGCT	No	Yes	Yes	No	Yes	No	No	No	No	No	No	No	No	GlyTCC
GCGTTGGTGGTATAGTGGTTAGCATAGCTG	No	Yes	Yes	No	Yes	No	No	No	No	No	No	No	No	GlyTCC
GCGTTGTGGCCGCAGCAACCTCGGT	Yes	Yes	Yes	No	Yes	No	No	No	No	No	No	No	No	HisGTG
GCTAAGGAAGTCCTGTGCTCAGTT	No	No	Yes	No	Yes	No	No	No	No	No	No	Yes	No	NA
GCTAAGGAAGTCCTGTGCTCAGTTT	No	No	Yes	No	Yes	No	No	No	No	No	No	Yes	No	NA
GCTAAGGAAGTCCTGTGCTCAGTTTT	No	No	Yes	Yes	Yes	No	No	No	No	No	No	Yes	No	NA
GCTAAGGTCGCGGGT	No	No	Yes	No	Yes	No	Yes	No	No	No	No	No	No	NA
GCTAGCTCAGTCGGTA	No	Yes	Yes	No	Yes	No	No	No	No	No	No	No	No	LysCTT
GCTAGCTCAGTCGGTAGAG	No	Yes	Yes	No	Yes	No	No	No	No	No	No	No	No	LysCTT
GCTAGCTCAGTCGGTAGAGC	No	Yes	Yes	No	Yes	No	No	No	No	No	No	No	No	LysCTT
GCTAGCTCAGTCGGTAGAGCA	No	Yes	Yes	No	Yes	No	No	No	No	No	No	No	No	LysCTT
GCTAGCTCAGTCGGTAGAGCATG	No	Yes	Yes	No	Yes	No	No	No	No	No	No	No	No	LysCTT
GCTAGCTCAGTCGGTAGAGCATGAG	No	Yes	Yes	No	Yes	No	No	No	No	No	No	No	No	LysCTT
GCTAGCTCAGTCGGTAGAGCATGAGAC	No	Yes	Yes	No	Yes	No	No	No	No	No	No	No	No	LysCTT
GCTAGCTCAGTCGGTAGAGCATGAGACTC	No	Yes	Yes	No	Yes	No	No	No	No	No	No	No	No	LysCTT
GCTAGCTCAGTCGGTAGAGCATGGG	Yes	Yes	Yes	No	Yes	No	No	No	No	No	No	No	No	LysCTT
GCTCACATCACCCCATAAACACCA	Yes	Yes	No	No	No	No	No	Yes	No	No	No	No	No	PheGAA
GCTCAGTCGGTAGAGCATGGGA	Yes	Yes	Yes	No	Yes	No	No	No	No	No	No	No	No	LysCTT
GCTCAGTGGTAGAGC	No	No	Yes	No	Yes	No	Yes	No	No	No	No	No	No	NA
GCTCCAGTGGCGCAA	No	No	No	No	Yes	No	Yes	No	No	No	No	No	No	NA
GCTCCGAGGTGATTTTCATATTGA	Yes	Yes	No	No	No	No	No	Yes	No	No	No	No	No	CysGCA
GCTCGAAGGACGAAAGTTT	No	No	No	No	Yes	No	No	No	No	No	No	Yes	No	NA
GCTCGAAGGACTTCGTCTGTAATTTT	No	No	No	No	Yes	No	No	No	No	No	No	Yes	No	NA
GCTCGTTGGTCTAGGG	No	Yes	Yes	No	Yes	No	No	No	No	No	No	No	No	ProAGG,ProCGG,ProTGG
GCTCGTTGGTCTAGGGGTATGATTCTCGCT	No	Yes	Yes	No	Yes	No	No	No	No	No	No	No	No	ProAGG,ProCGG,ProTGG
GCTCTCACCGCCGCGG	Yes	Yes	Yes	No	Yes	No	No	No	No	No	No	No	No	GluCTC
GCTGCATAGCAAGCCTTT	No	No	No	No	Yes	No	No	No	No	No	No	Yes	No	NA
GCTGCATAGCAAGCCTTTT	No	No	No	No	Yes	No	No	No	No	No	No	Yes	No	NA
GCTGGTGTAGTGGTA	No	No	Yes	No	Yes	No	Yes	No	No	No	No	No	No	NA
GCTGGTGTAGTGGTATC	Yes	Yes	Yes	No	Yes	No	No	No	No	No	No	No	No	GlyCCC
GCTGTGATGGCCGAG	No	No	No	No	Yes	No	Yes	No	No	No	No	No	No	NA
GCTGTGATGGCCGAGTGG	No	Yes	No	No	Yes	No	No	No	No	No	No	No	No	SerCGA
GCTGTGATGGCCGAGTGGTTAA	No	Yes	No	No	Yes	No	No	No	No	No	No	No	No	SerCGA
GCTGTGATGGCCGAGTGGTTAAGG	No	Yes	No	No	Yes	No	No	No	No	No	No	No	No	SerCGA
GCTTAGCCTGTGGCT	No	No	No	No	Yes	No	Yes	No	No	No	Yes	No	No	NA
GCTTAGCCTGTGGCTC	No	No	No	No	Yes	No	No	No	No	No	Yes	No	No	NA
GCTTCGCTGCGTGAGC	No	No	Yes	No	Yes	No	No	No	No	No	No	Yes	No	NA
GCTTCTGTAGTGTAG	No	No	Yes	Yes	Yes	No	Yes	No	No	No	No	No	No	NA
GCTTCTGTAGTGTAGTGGTTATC	Yes	Yes	Yes	Yes	Yes	No	No	No	No	No	No	No	No	ValCAC
GCTTCTGTAGTGTAGTGGTTATCAC	Yes	Yes	Yes	No	Yes	No	No	No	No	No	No	No	No	ValCAC
GCTTCTGTAGTGTAGTGGTTATCACG	Yes	Yes	Yes	No	Yes	No	No	No	No	No	No	No	No	ValCAC
GCTTCTGTAGTGTAGTGGTTATCACGTTC	Yes	Yes	Yes	No	Yes	No	No	No	No	No	No	No	No	ValCAC
GCTTCTGTAGTGTAGTGGTTATCACGTTCG	Yes	Yes	Yes	No	Yes	No	No	No	No	No	No	No	No	ValCAC
GCTTCTGTAGTGTAGTGGTTATCACGTTCGC	Yes	Yes	Yes	Yes	Yes	No	No	No	No	No	No	No	No	ValCAC
GCTTCTGTAGTGTAGTGGTTATCACGTTCGCC	Yes	Yes	Yes	No	Yes	No	No	No	No	No	No	No	No	ValCAC
GCTTCTGTAGTGTAGTGGTTATCACGTTCGCCT	Yes	Yes	No	No	Yes	No	No	No	No	No	No	No	No	ValCAC
GCTTGTTGTGATTCCTCCATTTT	No	No	Yes	No	Yes	No	No	No	No	No	No	Yes	No	NA
GCTTGTTGTGGTTTGCCCATTTT	No	No	Yes	No	Yes	No	No	No	No	No	No	Yes	No	NA

GCTTTGCACGCGTGGGT	Yes	Yes	No	No	Yes	No	No	No	No	No	No	No	No	SerGCT
GCTTTGCACGTATGAGGCCCCGGGT	Yes	Yes	Yes	No	Yes	No	No	No	No	No	No	No	No	AlaTGC
GCTTTGCACGTATGAGGCCCCGGGTT	Yes	Yes	Yes	No	Yes	No	No	No	No	No	No	No	No	AlaTGC
GCTTTGGGTGCTAATGGTGGAGTTAA	Yes	Yes	No	No	No	No	No	Yes	No	No	No	No	No	ProTGG
GGAAGCGTGCTGGGC	No	No	Yes	No	Yes	No	Yes	No	No	No	No	No	No	NA
GGAATGTCAGCTTTT	No	No	Yes	Yes	Yes	No	Yes	No	No	No	No	Yes	No	NA
GGAATGTCAGCTTTTT	No	No	Yes	No	Yes	No	No	No	No	No	No	Yes	No	NA
GGACTCTGAATCCAGCGATCCGAGT	Yes	Yes	Yes	No	Yes	No	No	No	No	No	No	No	No	GlnCTG
GGAGACCGGGGTTCG	No	No	Yes	No	Yes	No	Yes	No	No	No	No	No	No	NA
GGAGACCGGGGTTCGA	No	Yes	Yes	No	Yes	No	No	No	No	No	No	No	No	AspGTC
GGAGACCGGGGTTCGATTCCCCGACGGG	Yes	Yes	Yes	No	Yes	No	No	No	No	No	No	No	No	AspGTC
GGAGATGAAAACCTTTTTCCAAGGACACCA	Yes	Yes	No	No	No	No	No	Yes	No	No	No	No	No	ThrTGT
GGAGATTTCAACTTAACTTGACCGCTCTGACCA	Yes	Yes	No	No	No	No	No	Yes	No	No	No	No	No	ValTAC
GGAGCGTTTTGTGGATTTGTTTT	No	No	No	No	Yes	No	No	No	No	No	No	Yes	No	NA
GGAGCTGGGGATTGT	No	No	Yes	No	Yes	No	Yes	No	No	No	No	No	No	NA
GGAGCTGGGGATTGTGGG	Yes	Yes	Yes	No	Yes	No	No	No	No	No	No	No	No	ArgCCG
GGAGCTGGGGATTGTGGGT	Yes	Yes	Yes	No	Yes	No	No	No	No	No	No	No	No	ArgCCG
GGAGTTAAAGACTTTTTCTCTGACCA	Yes	Yes	No	No	No	No	No	Yes	No	No	No	No	No	ProTGG
GGATAAGGCACTGGC	No	No	Yes	No	Yes	No	Yes	No	No	No	No	No	No	NA
GGATAAGGCATTGGC	No	No	No	No	Yes	No	Yes	No	No	No	No	No	No	NA
GGATAAGGCGTCTGA	No	No	Yes	No	Yes	No	Yes	No	No	No	No	No	No	NA
GGATAGCGCATTGGA	No	No	No	No	Yes	No	Yes	No	No	No	No	No	No	NA
GGATAGCTCAGTCGGTAGAGCA	No	Yes	Yes	No	Yes	No	No	No	No	No	No	No	No	LysTTT
GGATAGCTCAGTCGGTAGAGCAT	No	Yes	Yes	No	Yes	No	No	No	No	No	No	No	No	LysTTT
GGATAGCTCAGTCGGTAGAGCATC	Yes	Yes	Yes	No	Yes	No	No	No	No	No	No	No	No	LysTTT
GGATAGCTCAGTCGGTAGAGCATCAGA	Yes	Yes	Yes	No	Yes	No	No	No	No	No	No	No	No	LysTTT
GGATCAGAAGATTGAGGGT	Yes	Yes	No	No	Yes	No	No	No	No	No	No	No	No	ArgTCG,ArgCCG
GGATCGAAACCATCCTCTGCTACCA	Yes	Yes	Yes	No	Yes	No	No	No	No	No	No	No	No	MetCAT
GGATGATCCTCAGTGGTC	No	No	No	No	Yes	No	No	No	No	No	No	No	No	NA
GGATGGCCGAGCGGTCTAA	No	Yes	Yes	No	Yes	No	No	No	No	No	No	No	No	LeuCAG
GGATGGCCGAGCGGTCTAAG	No	Yes	Yes	No	Yes	No	No	No	No	No	No	No	No	LeuCAG
GGATGGCCGAGCGGTCTAAGG	No	Yes	Yes	No	Yes	No	No	No	No	No	No	No	No	LeuCAG
GGATGGCCGAGTGGTCTAAGG	Yes	Yes	Yes	No	Yes	No	No	No	No	No	No	No	No	LeuCAA
GGATTCGGCGCTCTCA	Yes	Yes	Yes	No	Yes	No	No	No	No	No	No	No	Yes	GluCTC
GGATTCGGCGCTCTCACCGCC	Yes	Yes	Yes	No	Yes	No	No	No	No	No	No	No	Yes	GluCTC
GGATTCGGCGCTCTCACCGCCGC	Yes	Yes	Yes	No	Yes	No	No	No	No	No	No	No	No	GluCTC
GGCCCATAACCCAGAGGTCGATG	Yes	Yes	Yes	No	Yes	No	No	No	No	No	No	No	No	MetCAT
GGCCCCATGGTGTAATGGTTAGCACTCTG	Yes	Yes	Yes	No	Yes	No	No	No	No	No	No	No	No	GlnTTG
GGCCCCATGGTGTAATGGTTAGCACTCTGG	Yes	Yes	Yes	No	Yes	No	No	No	No	No	No	No	No	GlnTTG
GGCCCCATGGTGTAATGGTTAGCACTCTGGA	Yes	Yes	Yes	No	Yes	No	No	No	No	No	No	No	No	GlnTTG
GGCCCTATAGCTCAGGGGTTAGAGC	No	Yes	Yes	No	Yes	No	No	No	No	No	No	No	No	ThrTGT
GGCCCTATAGCTCAGGGGTTAGAGCACTGG	Yes	Yes	Yes	No	Yes	No	No	No	No	No	No	No	No	ThrTGT
GGCCCTGTGGCTTAGCTGG	Yes	Yes	No	No	Yes	No	No	No	No	No	No	No	No	ThrAGT
GGCCGAGCGGTCTAA	No	No	Yes	No	Yes	No	Yes	No	No	No	No	No	No	NA
GGCCGAGCGGTCTAAG	No	Yes	Yes	No	Yes	No	No	No	No	No	No	No	Yes	LeuTAG,LeuAAG,LeuCAG
GGCCGAGCGGTCTAAGG	No	Yes	Yes	No	Yes	No	No	No	No	No	No	No	Yes	LeuTAG,LeuAAG,LeuCAG
GGCCGCGTGGCCTAATGGA	Yes	Yes	Yes	No	Yes	No	No	No	No	No	No	No	No	ArgTCG,ArgCCG
GGCCGGTTAGCTCAG	No	No	Yes	Yes	Yes	No	Yes	No	No	No	No	No	No	NA
GGCCGGTTAGCTCAGT	No	Yes	Yes	No	Yes	No	No	No	No	No	No	No	Yes	IleAAT,SerACT,IleGAT
GGCCGGTTAGCTCAGTCGGC	Yes	Yes	Yes	No	Yes	No	No	No	No	No	No	No	No	IleAAT
GGCCGTGATCGTATAGTGGTTAGTACTCTGCG	Yes	Yes	No	No	No	No	No	No	No	Yes	No	No	No	HisGTG
GGCCTCGTGGCGCAA	No	No	No	No	Yes	No	Yes	No	No	No	No	No	No	NA
GGCCTCGTGGCGCAACGG	Yes	Yes	No	No	Yes	No	No	No	No	No	No	No	No	TrpCCA
GGCGATCACGTAGATTT	No	No	Yes	No	Yes	No	No	No	No	No	No	Yes	No	NA
GGCGATCACGTAGATTTT	No	No	Yes	Yes	Yes	No	No	No	No	No	No	Yes	No	NA
GGCGCCGTGGCTTAG	No	No	Yes	No	Yes	No	Yes	No	No	No	No	No	No	NA
GGCGCGGTGGCCAAG	No	No	Yes	No	Yes	No	Yes	No	No	No	No	No	No	NA
GGCGCGGTGGCCAAGTGG	Yes	Yes	Yes	No	Yes	No	No	No	No	No	No	No	No	ThrCGT
GGCGCGGTGGCCAAGTGGTAAGG	Yes	Yes	Yes	No	Yes	No	No	No	No	No	No	No	No	ThrCGT
GGCGTGATTCATACCTTT	No	No	Yes	No	Yes	No	No	No	No	No	No	Yes	No	NA
GGCGTGATTCATACCTTTT	No	No	Yes	No	Yes	No	No	No	No	No	No	Yes	No	NA
GGCTAGCTCAGTCGG	No	No	Yes	No	Yes	No	Yes	No	No	No	No	No	No	NA
GGCTAGCTCAGTCGGTAGAGCATGAGA	Yes	Yes	Yes	No	Yes	No	No	No	No	No	No	No	No	LysCTT
GGCTAGCTCAGTCGGTAGAGCATGAGAC	Yes	Yes	Yes	No	Yes	No	No	No	No	No	No	No	No	LysCTT
GGCTAGCTCAGTCGGTAGAGCATGGGAC	Yes	Yes	Yes	No	Yes	No	No	No	No	No	No	No	No	LysCTT
GGCTCACATCACCCCATAAACACCA	Yes	Yes	No	No	No	No	No	Yes	No	No	No	No	No	PheGAA
GGCTCCATAGCTCAG	No	No	Yes	No	Yes	No	Yes	No	No	No	No	No	No	NA
GGCTCCATAGCTCAGG	No	Yes	Yes	No	Yes	No	No	No	No	No	No	No	No	ThrTGT
GGCTCCATAGCTCAGGG	No	Yes	Yes	No	Yes	No	No	No	No	No	No	No	No	ThrTGT
GGCTCCATAGCTCAGGGGTTAGAGC	No	Yes	Yes	No	Yes	No	No	No	No	No	No	No	No	ThrTGT
GGCTCCATAGCTCAGGGGTTAGAGCACTGG	No	Yes	Yes	No	Yes	No	No	No	No	No	No	No	No	ThrTGT
GGCTCCGTGGCGCAA	No	No	No	No	Yes	No	Yes	No	No	No	No	No	No	NA
GGCTCCGTGGCGCAATGG	Yes	Yes	No	No	Yes	No	No	No	No	No	No	No	No	ArgTCT
GGCTCCGTGGCGCAATGGA	Yes	Yes	No	No	Yes	No	No	No	No	No	No	No	No	ArgTCT
GGCTCCGTGGCGCAATGGATAGCGCATTG	Yes	Yes	No	No	Yes	No	No	No	No	No	No	No	No	ArgTCT
GGCTCCGTGGCGCAATGGATAGCGCATTGGA	Yes	Yes	No	No	Yes	No	No	No	No	No	No	No	No	ArgTCT
GGCTCCGTGGCTTAG	No	No	No	No	Yes	No	Yes	No	No	No	No	No	No	NA
GGCTCCGTGGCTTAGC	Yes	Yes	No	No	Yes	No	No	No	No	No	No	No	No	ThrAGT
GGCTCCGTGGCTTAGCTGG	Yes	Yes	No	No	Yes	No	No	No	No	No	No	No	No	ThrAGT
GGCTCGAAGGACTTCGTCTGTAA	No	No	No	No	Yes	No	No	No	No	No	No	Yes	No	NA
GGCTCGAAGGACTTCGTCTGTAAT	No	No	No	No	Yes	No	No	No	No	No	No	Yes	No	NA
GGCTCGTTGGTCTAG	No	No	Yes	Yes	Yes	No	Yes	No	No	No	No	No	No	NA
GGCTCGTTGGTCTAGG	No	Yes	Yes	No	Yes	No	No	No	No	No	No	No	No	ProAGG,ProCGG,ProTGG
GGCTCGTTGGTCTAGGG	No	Yes	Yes	Yes	Yes	No	No	No	No	No	No	No	No	ProAGG,ProCGG,ProTGG
GGCTCGTTGGTCTAGGGG	No	Yes	Yes	No	Yes	No	No	No	No	No	No	No	No	ProAGG,ProCGG,ProTGG
GGCTCGTTGGTCTAGGGGT	No	Yes	Yes	No	Yes	No	No	No	No	No	No	No	No	ProAGG,ProCGG,ProTGG
GGCTCGTTGGTCTAGGGGTA	No	Yes	Yes	No	Yes	No	No	No	No	No	No	No	No	ProAGG,ProCGG,ProTGG
GGCTCGTTGGTCTAGGGGTAT	No	Yes	Yes	Yes	Yes	No	No	No	No	No	No	No	No	ProAGG,ProCGG,ProTGG

GGCTCGTTGGTCTAGGGGTATG	No	Yes	Yes	No	Yes	No	No	No	No	No	No	No	No	ProAGG,ProCGG,ProTGG
GGCTCGTTGGTCTAGGGGTATGA	No	Yes	Yes	Yes	Yes	No	No	No	No	No	No	No	No	ProAGG,ProCGG,ProTGG
GGCTCGTTGGTCTAGGGGTATGAT	No	Yes	Yes	No	Yes	No	No	No	No	No	No	No	No	ProAGG,ProCGG,ProTGG
GGCTCGTTGGTCTAGGGGTATGATT	No	Yes	Yes	No	Yes	No	No	No	No	No	No	No	No	ProAGG,ProCGG,ProTGG
GGCTCGTTGGTCTAGGGGTATGATTC	No	Yes	Yes	No	Yes	No	No	No	No	No	No	No	No	ProAGG,ProCGG,ProTGG
GGCTCGTTGGTCTAGGGGTATGATTCT	No	Yes	Yes	No	Yes	No	No	No	No	No	No	No	No	ProAGG,ProCGG,ProTGG
GGCTCGTTGGTCTAGGGGTATGATTCTC	No	Yes	Yes	No	Yes	No	No	No	No	No	No	No	No	ProAGG,ProCGG,ProTGG
GGCTCGTTGGTCTAGGGGTATGATTCTCG	No	Yes	Yes	No	Yes	No	No	No	No	No	No	No	No	ProAGG,ProCGG,ProTGG
GGCTCGTTGGTCTAGGGGTATGATTCTCGC	No	Yes	Yes	Yes	Yes	No	No	No	No	No	No	No	No	ProAGG,ProCGG,ProTGG
GGCTCGTTGGTCTAGGGGTATGATTCTCGCT	No	Yes	Yes	No	Yes	No	No	No	No	No	No	No	No	ProAGG,ProCGG,ProTGG
GGCTCGTTGGTCTAGGGGTATGATTCTCGCTT	No	Yes	Yes	No	Yes	No	No	No	No	No	No	No	No	ProAGG,ProCGG,ProTGG
GGCTCGTTGGTCTAGGGGTATGATTCTCGCTTC	Yes	Yes	Yes	No	Yes	No	No	No	No	No	No	No	No	ProCGG
GGCTCGTTGGTCTAGGGGTATGATTCTCGG	Yes	Yes	Yes	No	Yes	No	No	No	No	No	No	No	No	ProTGG
GGCTCGTTGGTCTAGGGGTATGATTCTCGGT	Yes	Yes	Yes	No	Yes	No	No	No	No	No	No	No	No	ProTGG
GGCTCGTTGGTCTAGGGGTATGATTCTCGGTT	Yes	Yes	Yes	No	Yes	No	No	No	No	No	No	No	No	ProTGG
GGCTCGTTGGTCTAGT	No	Yes	Yes	No	Yes	No	No	No	No	No	No	No	No	ProTGG
GGCTCGTTGGTCTAGTGGTATGA	Yes	Yes	Yes	No	Yes	No	No	No	No	No	No	No	No	ProTGG
GGCTCGTTGGTCTAGTGGTATGATTCTC	Yes	Yes	Yes	No	Yes	No	No	No	No	No	No	No	No	ProTGG
GGCTCGTTGGTCTAGTGGTATGATTCTCGC	Yes	Yes	Yes	No	Yes	No	No	No	No	No	No	No	No	ProTGG
GGCTCGTTGGTCTAGTGGTATGATTCTCGCT	Yes	Yes	Yes	No	Yes	No	No	No	No	No	No	No	No	ProTGG
GGCTCTGTGGCGCAATGG	No	Yes	No	No	Yes	No	No	No	No	No	No	No	No	ArgTCT
GGCTCTGTGGCGCAATGGA	No	Yes	No	No	Yes	No	No	No	No	No	No	No	No	ArgTCT
GGCTCTGTGGCGCAATGGATAGCGCATTG	Yes	Yes	No	No	Yes	No	No	No	No	No	No	No	No	ArgTCT
GGCTCTGTGGCGCAATGGATAGCGCATTGGA	Yes	Yes	No	No	Yes	No	No	No	No	No	No	No	No	ArgTCT
GGCTCTGTGGCTTAGTTGGC	Yes	Yes	Yes	No	Yes	No	No	No	No	No	No	No	No	ThrCGT
GGCTGCGTGTTCGAATCACGTCGGGGTCACCA	Yes	Yes	No	No	Yes	No	No	No	No	No	No	No	No	TrpCCA
GGCTTAGCCTGTGGCTC	No	No	No	No	Yes	No	No	No	No	No	Yes	No	No	NA
GGGAATTAGCTCAAA	No	No	No	No	Yes	No	Yes	No	No	No	No	No	No	NA
GGGATGTAGCTCAGTGGTAGAGCGCATGC	Yes	Yes	Yes	No	Yes	No	No	No	No	No	No	No	No	AlaTGC,AlaCGC,AlaAGC
GGGATTAGCTCAAAT	No	No	No	No	Yes	No	Yes	No	No	No	No	No	No	NA
GGGATTAGCTCAAATGGTAGAGCGCTCGC	Yes	Yes	No	No	Yes	No	No	No	No	No	No	No	No	AlaAGC
GGGCCAGTGGCGCAA	No	No	Yes	No	Yes	No	Yes	No	No	No	No	No	No	NA
GGGCCAGTGGCGCAATGG	No	Yes	Yes	Yes	Yes	No	No	No	No	No	No	No	No	ArgACG
GGGCCAGTGGCGCAATGGA	No	Yes	Yes	No	Yes	No	No	No	No	No	No	No	No	ArgACG
GGGCCAGTGGCGCAATGGATA	No	Yes	Yes	No	Yes	No	No	No	No	No	No	No	No	ArgACG
GGGCCAGTGGCGCAATGGATAAC	No	Yes	Yes	No	Yes	No	No	No	No	No	No	No	No	ArgACG
GGGCCAGTGGCGCAATGGATAACGC	No	Yes	Yes	No	Yes	No	No	No	No	No	No	No	No	ArgACG
GGGCTCACATCACCCCATAAACACCA	Yes	Yes	No	No	No	No	No	Yes	No	No	No	No	No	PheGAA
GGGGAATTAGCTCAA	No	No	No	No	Yes	No	Yes	No	No	No	No	No	No	NA
GGGGAATTAGCTCAAA	No	Yes	No	No	Yes	No	No	No	No	No	No	No	No	AlaAGC
GGGGAATTAGCTCAAATGG	Yes	Yes	No	No	Yes	No	No	No	No	No	No	No	No	AlaAGC
GGGGAATTAGCTCAAATGGTAGA	Yes	Yes	No	No	Yes	No	No	No	No	No	No	No	No	AlaAGC
GGGGAATTAGCTCAAATGGTAGAGC	Yes	Yes	No	No	Yes	No	No	No	No	No	No	No	No	AlaAGC
GGGGAATTAGCTCAAATGGTAGAGCGCTC	Yes	Yes	No	No	Yes	No	No	No	No	No	No	No	No	AlaAGC
GGGGAATTAGCTCAAATGGTAGAGCGCTCGC	Yes	Yes	No	No	Yes	No	No	No	No	No	No	No	No	AlaAGC
GGGGAATTAGCTCAAGC	Yes	Yes	No	No	Yes	No	No	No	No	No	No	No	No	AlaAGC
GGGGAATTAGCTCAAGCGGTAG	Yes	Yes	No	No	Yes	No	No	No	No	No	No	No	No	AlaAGC
GGGGAATTAGCTCAAGCGGTAGA	Yes	Yes	No	No	Yes	No	No	No	No	No	No	No	No	AlaAGC
GGGGAATTAGCTCAAGCGGTAGAG	Yes	Yes	No	No	Yes	No	No	No	No	No	No	No	No	AlaAGC
GGGGATGTAGCTCAG	No	No	Yes	No	Yes	No	Yes	No	No	No	No	No	No	NA
GGGGATGTAGCTCAGTGG	Yes	Yes	Yes	No	Yes	No	No	No	No	No	No	No	No	AlaTGC,AlaCGC,AlaAGC
GGGGATGTAGCTCAGTGGT	Yes	Yes	Yes	No	Yes	No	No	No	No	No	No	No	No	AlaTGC,AlaCGC,AlaAGC
GGGGATGTAGCTCAGTGGTA	Yes	Yes	Yes	No	Yes	No	No	No	No	No	No	No	No	AlaTGC,AlaCGC,AlaAGC
GGGGATGTAGCTCAGTGGTAG	Yes	Yes	Yes	No	Yes	No	No	No	No	No	No	No	No	AlaTGC,AlaCGC,AlaAGC
GGGGATGTAGCTCAGTGGTAGA	Yes	Yes	Yes	No	Yes	No	No	No	No	No	No	No	No	AlaTGC,AlaCGC,AlaAGC
GGGGATGTAGCTCAGTGGTAGAG	Yes	Yes	Yes	No	Yes	No	No	No	No	No	No	No	No	AlaTGC,AlaCGC,AlaAGC
GGGGATGTAGCTCAGTGGTAGAGC	Yes	Yes	Yes	No	Yes	No	No	No	No	No	No	No	No	AlaTGC,AlaCGC,AlaAGC
GGGGATGTAGCTCAGTGGTAGAGCGCA	Yes	Yes	Yes	No	Yes	No	No	No	No	No	No	No	No	AlaTGC,AlaCGC,AlaAGC
GGGGATGTAGCTCAGTGGTAGAGCGCAT	Yes	Yes	Yes	No	Yes	No	No	No	No	No	No	No	No	AlaTGC,AlaCGC,AlaAGC
GGGGATGTAGCTCAGTGGTAGAGCGCATG	Yes	Yes	Yes	No	Yes	No	No	No	No	No	No	No	No	AlaTGC,AlaCGC,AlaAGC
GGGGATGTAGCTCAGTGGTAGAGCGCATGC	Yes	Yes	Yes	No	Yes	No	No	No	No	No	No	No	No	AlaTGC,AlaCGC,AlaAGC
GGGGATGTAGCTCAGTGGTAGAGCGCATGCT	Yes	Yes	Yes	No	Yes	No	No	No	No	No	No	No	No	AlaTGC,AlaCGC,AlaAGC
GGGGATGTAGCTCAGTGGTAGAGCGCATGCTT	Yes	Yes	No	No	Yes	No	No	No	No	No	No	No	No	AlaTGC,AlaCGC,AlaAGC
GGGGATTAGCTCAAA	No	No	No	No	Yes	No	Yes	No	No	No	No	No	No	NA
GGGGGATTAGCTCAA	No	No	No	No	Yes	No	Yes	No	No	No	No	No	No	NA
GGGGGATTAGCTCAAA	No	Yes	No	No	Yes	No	No	No	No	No	No	No	No	AlaAGC
GGGGGATTAGCTCAAATGG	Yes	Yes	No	No	Yes	No	No	No	No	No	No	No	No	AlaAGC
GGGGGATTAGCTCAAATGGTAGA	Yes	Yes	No	No	Yes	No	No	No	No	No	No	No	No	AlaAGC
GGGGGATTAGCTCAAATGGTAGAG	Yes	Yes	No	No	Yes	No	No	No	No	No	No	No	No	AlaAGC
GGGGGATTAGCTCAAATGGTAGAGC	Yes	Yes	No	No	Yes	No	No	No	No	No	No	No	No	AlaAGC
GGGGGATTAGCTCAAATGGTAGAGCGCTC	Yes	Yes	No	No	Yes	No	No	No	No	No	No	No	No	AlaAGC
GGGGGATTAGCTCAAATGGTAGAGCGCTCGC	Yes	Yes	No	No	Yes	No	No	No	No	No	No	No	No	AlaAGC
GGGGGATTAGCTCAAATGGTAGAGCGCTCGCTT	Yes	Yes	No	No	Yes	No	No	No	No	No	No	No	No	AlaAGC
GGGGGCATAGCTCAGTGG	No	Yes	No	No	Yes	No	No	No	No	No	No	No	No	CysGCA
GGGGGCATAGCTCAGTGGTAGAGCATTTGA	Yes	Yes	No	No	Yes	No	No	No	No	No	No	No	No	CysGCA
GGGGGCATAGCTCAGTGGTAGAGCATTTGACT	Yes	Yes	No	No	Yes	No	No	No	No	No	No	No	No	CysGCA
GGGGGTATAGCTCAG	No	No	Yes	Yes	Yes	No	Yes	No	No	No	No	No	No	NA
GGGGGTATAGCTCAGC	No	Yes	Yes	No	Yes	No	No	No	No	No	No	No	No	AlaAGC
GGGGGTATAGCTCAGCGG	No	Yes	Yes	No	Yes	No	No	No	No	No	No	No	No	AlaAGC
GGGGGTATAGCTCAGCGGTAGA	No	Yes	Yes	No	Yes	No	No	No	No	No	No	No	No	AlaAGC
GGGGGTATAGCTCAGCGGTAGAGC	No	Yes	Yes	No	Yes	No	No	No	No	No	No	No	No	AlaAGC
GGGGGTATAGCTCAGCGGTAGAGCGCGTG	Yes	Yes	Yes	No	Yes	No	No	No	No	No	No	No	No	AlaAGC
GGGGGTATAGCTCAGCGGTAGAGCGCGTGC	Yes	Yes	Yes	No	Yes	No	No	No	No	No	No	No	No	AlaAGC
GGGGGTATAGCTCAGCGGTAGAGCGCGTGCT	Yes	Yes	Yes	No	Yes	No	No	No	No	No	No	No	No	AlaAGC
GGGGGTATAGCTCAGG	No	Yes	Yes	No	Yes	No	No	No	No	No	No	No	Yes	TyrGTA,CysGCA,AlaAGC
GGGGGTATAGCTCAGGG	No	Yes	Yes	No	Yes	No	No	No	No	No	No	No	Yes	TyrGTA,CysGCA,AlaAGC
GGGGGTATAGCTCAGGGG	No	Yes	Yes	No	Yes	No	No	No	No	No	No	No	No	CysGCA
GGGGGTATAGCTCAGGGGTAG	No	Yes	Yes	No	Yes	No	No	No	No	No	No	No	No	CysGCA

GGGGGTATAGCTCAGGGGTAGA	No	Yes	Yes	No	Yes	No	No	No	No	No	No	No	No	CysGCA
GGGGGTATAGCTCAGGGGTAGAG	No	Yes	Yes	No	Yes	No	No	No	No	No	No	No	No	CysGCA
GGGGGTATAGCTCAGGGGTAGAGC	No	Yes	Yes	No	Yes	No	No	No	No	No	No	No	No	CysGCA
GGGGGTATAGCTCAGGGGTAGAGCA	No	Yes	Yes	No	Yes	No	No	No	No	No	No	No	No	CysGCA
GGGGGTATAGCTCAGGGGTAGAGCATT	Yes	Yes	Yes	No	Yes	No	No	No	No	No	No	No	No	CysGCA
GGGGGTATAGCTCAGGGGTAGAGCATTT	Yes	Yes	Yes	No	Yes	No	No	No	No	No	No	No	No	CysGCA
GGGGGTATAGCTCAGGGGTAGAGCATTTG	Yes	Yes	Yes	No	Yes	No	No	No	No	No	No	No	No	CysGCA
GGGGGTATAGCTCAGGGGTAGAGCATTTGA	Yes	Yes	Yes	No	Yes	No	No	No	No	No	No	No	No	CysGCA
GGGGGTATAGCTCAGGGGTAGAGCATTTGAC	Yes	Yes	Yes	No	Yes	No	No	No	No	No	No	No	No	CysGCA
GGGGGTATAGCTCAGGGGTAGAGCATTTGACT	Yes	Yes	Yes	No	Yes	No	No	No	No	No	No	No	No	CysGCA
GGGGGTATAGCTCAGTGG	No	Yes	Yes	No	Yes	No	No	No	No	No	No	No	No	CysGCA,AlaAGC
GGGGGTATAGCTCAGTGGG	Yes	Yes	Yes	No	Yes	No	No	No	No	No	No	No	No	CysGCA
GGGGGTATAGCTCAGTGGGTAGAGCATTTG	Yes	Yes	Yes	No	Yes	No	No	No	No	No	No	No	No	CysGCA
GGGGGTATAGCTCAGTGGGTAGAGCATTTGA	Yes	Yes	Yes	No	Yes	No	No	No	No	No	No	No	No	CysGCA
GGGGGTATAGCTCAGTGGGTAGAGCATTTGACT	Yes	Yes	Yes	No	Yes	No	No	No	No	No	No	No	No	CysGCA
GGGGGTATAGCTCAGTGGTA	No	Yes	Yes	No	Yes	No	No	No	No	No	No	No	No	CysGCA,AlaAGC
GGGGGTATAGCTCAGTGGTAGA	No	Yes	Yes	No	Yes	No	No	No	No	No	No	No	No	CysGCA,AlaAGC
GGGGGTATAGCTCAGTGGTAGAG	No	Yes	Yes	No	Yes	No	No	No	No	No	No	No	No	CysGCA,AlaAGC
GGGGGTATAGCTCAGTGGTAGAGC	No	Yes	Yes	No	Yes	No	No	No	No	No	No	No	No	CysGCA,AlaAGC
GGGGGTATAGCTCAGTGGTAGAGCA	No	Yes	Yes	No	Yes	No	No	No	No	No	No	No	No	CysGCA
GGGGGTATAGCTCAGTGGTAGAGCATT	No	Yes	Yes	No	Yes	No	No	No	No	No	No	No	No	CysGCA
GGGGGTATAGCTCAGTGGTAGAGCATTT	No	Yes	Yes	No	Yes	No	No	No	No	No	No	No	No	CysGCA
GGGGGTATAGCTCAGTGGTAGAGCATTTG	No	Yes	Yes	No	Yes	No	No	No	No	No	No	No	No	CysGCA
GGGGGTATAGCTCAGTGGTAGAGCATTTGA	No	Yes	Yes	No	Yes	No	No	No	No	No	No	No	No	CysGCA
GGGGGTATAGCTCAGTGGTAGAGCATTTGAC	No	Yes	Yes	No	Yes	No	No	No	No	No	No	No	No	CysGCA
GGGGGTATAGCTCAGTGGTAGAGCATTTGACT	No	Yes	Yes	Yes	Yes	No	No	No	No	No	No	No	No	CysGCA
GGGGGTATAGCTCAGTGGTAGAGCATTTGACTG	No	Yes	Yes	No	Yes	No	No	No	No	No	No	No	No	CysGCA
GGGGGTATAGCTCAGTGGTAGAGCGCGTGC	Yes	Yes	Yes	No	Yes	No	No	No	No	No	No	No	No	AlaAGC
GGGGGTATAGCTCAGTGGTAGAGCGCGTGCT	Yes	Yes	Yes	No	Yes	No	No	No	No	No	No	No	No	AlaAGC
GGGGGTGTAGCTCAG	No	No	Yes	No	Yes	No	Yes	No	No	No	No	No	No	NA
GGGGGTGTAGCTCAGTGG	No	Yes	Yes	No	Yes	No	No	No	No	No	No	No	No	AlaTGC,ValAAC,AlaCGC,CysGCA,AlaAGC
GGGGGTGTAGCTCAGTGGT	Yes	Yes	Yes	No	Yes	No	No	No	No	No	No	No	No	AlaTGC,ValAAC,AlaCGC,CysGCA,AlaAGC
GGGGGTGTAGCTCAGTGGTA	Yes	Yes	Yes	No	Yes	No	No	No	No	No	No	No	No	AlaTGC,ValAAC,AlaCGC,CysGCA,AlaAGC
GGGGGTGTAGCTCAGTGGTAGA	Yes	Yes	Yes	No	Yes	No	No	No	No	No	No	No	No	AlaTGC,ValAAC,AlaCGC,CysGCA,AlaAGC
GGGGGTGTAGCTCAGTGGTAGAGC	Yes	Yes	Yes	Yes	Yes	No	No	No	No	No	No	No	No	AlaTGC,ValAAC,AlaCGC,CysGCA,AlaAGC
GGGGGTGTAGCTCAGTGGTAGAGCA	Yes	Yes	Yes	No	Yes	No	No	No	No	No	No	No	No	AlaTGC,CysGCA
GGGGGTGTAGCTCAGTGGTAGAGCACATGCT	Yes	Yes	Yes	No	Yes	No	No	No	No	No	No	No	No	AlaTGC
GGGGGTGTAGCTCAGTGGTAGAGCAT	Yes	Yes	Yes	No	Yes	No	No	No	No	No	No	No	No	CysGCA
GGGGGTGTAGCTCAGTGGTAGAGCATT	Yes	Yes	Yes	No	Yes	No	No	No	No	No	No	No	No	CysGCA
GGGGGTGTAGCTCAGTGGTAGAGCATTTGA	Yes	Yes	Yes	No	Yes	No	No	No	No	No	No	No	No	CysGCA
GGGGGTGTAGCTCAGTGGTAGAGCATTTGACT	Yes	Yes	Yes	No	Yes	No	No	No	No	No	No	No	No	CysGCA
GGGGGTGTAGCTCAGTGGTAGAGCGCAT	Yes	Yes	Yes	No	Yes	No	No	No	No	No	No	No	No	AlaTGC
GGGGGTGTAGCTCAGTGGTAGAGCGCATG	Yes	Yes	Yes	No	Yes	No	No	No	No	No	No	No	No	AlaTGC
GGGGGTGTAGCTCAGTGGTAGAGCGCATGC	Yes	Yes	Yes	No	Yes	No	No	No	No	No	No	No	No	AlaTGC
GGGGGTGTAGCTCAGTGGTAGAGCGCATGCT	Yes	Yes	Yes	No	Yes	No	No	No	No	No	No	No	No	AlaTGC
GGGGGTGTAGCTCAGTGGTAGAGCGCGTG	Yes	Yes	Yes	No	Yes	No	No	No	No	No	No	No	No	AlaCGC,AlaAGC
GGGGGTGTAGCTCAGTGGTAGAGCGCGTGC	Yes	Yes	Yes	Yes	Yes	No	No	No	No	No	No	No	No	AlaCGC,AlaAGC
GGGGGTGTAGCTCAGTGGTAGAGCGCGTGCT	Yes	Yes	Yes	No	Yes	No	No	No	No	No	No	No	No	AlaCGC,AlaAGC
GGGGGTGTAGCTCAGTGGTAGAGCGCGTGCTT	Yes	Yes	Yes	No	Yes	No	No	No	No	No	No	No	No	AlaCGC,AlaAGC
GGGGGTGTAGCTCAGTGGTAGAGCGT	Yes	Yes	Yes	No	Yes	No	No	No	No	No	No	No	No	ValAAC
GGGGTATAGCTCAGCGGTAGA	No	Yes	Yes	No	Yes	No	No	No	No	No	No	No	No	AlaAGC
GGGGTATAGCTCAGG	No	No	Yes	No	Yes	No	Yes	No	No	No	No	No	No	NA
GGGGTATAGCTCAGGG	No	Yes	Yes	No	Yes	No	No	No	No	No	No	No	Yes	TyrGTA,CysGCA,AlaAGC
GGGGTATAGCTCAGGGGTAG	No	Yes	Yes	No	Yes	No	No	No	No	No	No	No	No	CysGCA
GGGGTATAGCTCAGGGGTAGAG	No	Yes	Yes	No	Yes	No	No	No	No	No	No	No	No	CysGCA
GGGGTATAGCTCAGGGGTAGAGCATTTGA	Yes	Yes	Yes	No	Yes	No	No	No	No	No	No	No	No	CysGCA
GGGGTATAGCTCAGGGGTAGAGCATTTGACT	Yes	Yes	Yes	No	Yes	No	No	No	No	No	No	No	No	CysGCA
GGGGTATGATTCTCGC	No	Yes	Yes	No	Yes	No	No	No	No	No	No	No	No	ProAGG,ProCGG,ProTGG
GGGGTATGATTCTCGCT	No	Yes	Yes	No	Yes	No	No	No	No	No	No	No	No	ProAGG,ProCGG,ProTGG
GGGGTATGATTCTCGCTTC	Yes	Yes	Yes	No	Yes	No	No	No	No	No	No	No	No	ProCGG
GGGGTATGATTCTCGG	Yes	Yes	Yes	No	Yes	No	No	No	No	No	No	No	No	ProTGG
GGGGTATGATTCTCGGT	Yes	Yes	Yes	No	Yes	No	No	No	No	No	No	No	No	ProTGG
GGGGTGTAGCTCAGTGGTAGAGCGCGTGCT	Yes	Yes	Yes	No	Yes	No	No	No	No	No	No	No	No	AlaCGC,AlaAGC
GGGTATAGCTCAGCGGTAGA	No	Yes	Yes	No	Yes	No	No	No	No	No	No	No	No	AlaAGC
GGGTATAGCTCAGCGGTAGAGCGCGTGC	Yes	Yes	Yes	No	Yes	No	No	No	No	No	No	No	No	AlaAGC
GGGTATAGCTCAGCGGTAGAGCGCGTGCT	Yes	Yes	Yes	No	Yes	No	No	No	No	No	No	No	No	AlaAGC
GGGTATAGCTCAGGG	No	No	Yes	No	Yes	No	Yes	No	No	No	No	No	No	NA
GGGTATAGCTCAGGGG	No	Yes	Yes	No	Yes	No	No	No	No	No	No	No	No	CysGCA
GGGTATAGCTCAGGGGTAGAG	No	Yes	Yes	No	Yes	No	No	No	No	No	No	No	No	CysGCA
GGGTATAGCTCAGGGGTAGAGCATTTGACT	Yes	Yes	Yes	No	Yes	No	No	No	No	No	No	No	No	CysGCA
GGGTATAGCTCAGTGGTAGA	No	Yes	Yes	No	Yes	No	No	No	No	No	No	No	No	CysGCA,AlaAGC
GGGTATAGCTCAGTGGTAGAGCGCGTGC	Yes	Yes	Yes	No	Yes	No	No	No	No	No	No	No	No	AlaAGC
GGGTATAGCTCAGTGGTAGAGCGCGTGCT	Yes	Yes	Yes	No	Yes	No	No	No	No	No	No	No	No	AlaAGC
GGGTATGATTCTCGC	No	No	Yes	No	Yes	No	Yes	No	No	No	No	No	No	NA
GGGTATGATTCTCGCT	No	Yes	Yes	No	Yes	No	No	No	No	No	No	No	No	ProAGG,ProCGG,ProTGG
GGGTATGATTCTCGG	No	No	Yes	No	Yes	No	Yes	No	No	No	No	No	No	NA
GGGTATGATTCTCGGT	Yes	Yes	Yes	No	Yes	No	No	No	No	No	No	No	No	ProTGG
GGGTCCCCGGTAGCT	No	No	No	No	Yes	No	Yes	No	No	No	Yes	No	No	NA
GGGTCCCCGGTAGCTC	No	No	No	No	Yes	No	No	No	No	No	Yes	No	No	NA
GGGTGCGAGAGGTCCCGGGT	Yes	Yes	Yes	No	Yes	No	No	No	No	No	No	No	No	ProAGG,ProCGG,ProTGG
GGGTGCTCTTATTTT	No	No	Yes	No	Yes	No	Yes	No	No	No	No	Yes	No	NA
GGGTGGTTGCGTTTT	No	No	No	No	Yes	No	Yes	No	No	No	No	Yes	No	NA
GGGTGTAGCTCAGTGGTAGAGCGCGTGC	Yes	Yes	Yes	No	Yes	No	No	No	No	No	No	No	No	AlaCGC,AlaAGC
GGGTGTGATAGGTGGCACGGAGA	Yes	Yes	No	No	No	No	No	Yes	Yes	No	No	No	No	GlnTTG
GGGTTAGAGCACTGGT	No	Yes	Yes	No	Yes	No	No	No	No	No	No	No	No	ThrTGT
GGGTTCGATTCCCCGACGGGGAGCCA	Yes	Yes	Yes	No	Yes	No	No	No	No	No	No	No	No	AspGTC
GGGTTCGATTCCCGGTCAGGGAACCA	Yes	Yes	Yes	No	Yes	No	No	No	No	No	No	No	Yes	GluCTC,PheGAA,GluTTC
GGGTTCGATTCTCATAGTCCTAGCCA	Yes	Yes	No	No	No	No	No	Yes	Yes	No	No	No	No	GlnTTG

GGGTTGCTGTCTTTT	No	No	Yes	Yes	Yes	No	Yes	No	No	No	No	Yes	No	NA
GGGTTGCTGTCTTTTT	No	No	Yes	No	Yes	No	No	No	No	No	No	Yes	No	NA
GGGTTTCTTTAAGTTT	No	No	Yes	No	Yes	No	No	No	No	No	No	Yes	No	NA
GGGTTTCTTTAAGTTTT	No	No	Yes	No	Yes	No	No	No	No	No	No	Yes	No	NA
GGTAAATATAGTTTAAC	Yes	Yes	No	No	No	No	No	Yes	No	Yes	No	No	No	HisGTG
GGTAAATATAGTTTAACC	Yes	Yes	No	No	No	No	No	Yes	No	Yes	No	No	No	HisGTG
GGTAAATATAGTTTAACCAAAACATCAGA	Yes	Yes	No	No	No	No	No	Yes	No	Yes	No	No	No	HisGTG
GGTAAATATAGTTTAACCAAAACATCAGATTG	Yes	Yes	No	No	No	No	No	Yes	No	Yes	No	No	No	HisGTG
GGTAGAATTCTCGCC	No	No	Yes	No	Yes	No	Yes	No	No	No	No	No	No	NA
GGTAGAATTCTCGCCTCC	No	Yes	Yes	No	Yes	No	No	No	No	No	No	No	Yes	GlyCCC
GGTAGAATTCTCGCCTGCC	No	Yes	Yes	No	Yes	No	No	No	No	No	No	No	No	GlyGCC
GGTAGAATTCTCGCCTGCCACGCGGGAGGCCC	Yes	Yes	Yes	No	Yes	No	No	No	No	No	No	No	No	GlyGCC
GGTAGAGCATCAGAC	No	No	Yes	No	Yes	No	Yes	No	No	No	No	No	No	NA
GGTAGAGCATCAGACTTTT	Yes	Yes	Yes	No	Yes	No	No	No	No	No	No	No	Yes	LysTTT
GGTAGAGCATGAGAC	No	No	Yes	No	Yes	No	Yes	No	No	No	No	No	No	NA
GGTAGAGCATGAGACTC	No	Yes	Yes	No	Yes	No	No	No	No	No	No	No	No	LysCTT
GGTAGAGCATGAGACTCT	No	Yes	Yes	No	Yes	No	No	No	No	No	No	No	No	LysCTT
GGTAGAGCATGAGACTCTTAATCTC	Yes	Yes	Yes	No	Yes	No	No	No	No	No	No	No	No	LysCTT
GGTAGAGCATGGGAC	No	No	Yes	No	Yes	No	Yes	No	No	No	No	No	No	NA
GGTAGAGCATGGGACT	No	Yes	Yes	No	Yes	No	No	No	No	No	No	No	Yes	LysCTT
GGTAGAGCATGGGACTC	Yes	Yes	Yes	No	Yes	No	No	No	No	No	No	No	Yes	LysCTT
GGTAGAGCATTTGACT	No	Yes	Yes	No	Yes	No	No	No	No	No	No	No	Yes	CysGCA,CysACA
GGTAGAGCGGAGGAC	No	No	Yes	No	Yes	No	Yes	No	No	No	No	No	No	NA
GGTAGCGTGGCCGAG	No	No	Yes	No	Yes	No	Yes	No	No	No	No	No	No	NA
GGTAGCGTGGCCGAGC	Yes	Yes	Yes	Yes	Yes	No	No	No	No	No	No	No	No	LeuTAG,LeuAAG
GGTAGCGTGGCCGAGCG	Yes	Yes	Yes	No	Yes	No	No	No	No	No	No	No	No	LeuTAG,LeuAAG
GGTAGCGTGGCCGAGCGG	Yes	Yes	Yes	No	Yes	No	No	No	No	No	No	No	No	LeuTAG,LeuAAG
GGTAGCGTGGCCGAGCGGTC	Yes	Yes	Yes	No	Yes	No	No	No	No	No	No	No	No	LeuTAG,LeuAAG
GGTAGCGTGGCCGAGCGGTCTA	Yes	Yes	Yes	No	Yes	No	No	No	No	No	No	No	No	LeuTAG,LeuAAG
GGTAGCGTGGCCGAGCGGTCTAA	Yes	Yes	Yes	No	Yes	No	No	No	No	No	No	No	No	LeuTAG,LeuAAG
GGTAGCGTGGCCGAGCGGTCTAAG	Yes	Yes	Yes	Yes	Yes	No	No	No	No	No	No	No	No	LeuTAG,LeuAAG
GGTAGCGTGGCCGAGCGGTCTAAGG	Yes	Yes	Yes	No	Yes	No	No	No	No	No	No	No	No	LeuTAG,LeuAAG
GGTAGCGTGGCCGAGCGGTCTAAGGC	Yes	Yes	Yes	No	Yes	No	No	No	No	No	No	No	No	LeuTAG,LeuAAG
GGTAGCGTGGCCGAGCGGTCTAAGGCGCT	Yes	Yes	Yes	No	Yes	No	No	No	No	No	No	No	No	LeuTAG,LeuAAG
GGTAGCGTGGCCGAGCGGTCTAAGGCGCTGG	Yes	Yes	Yes	No	Yes	No	No	No	No	No	No	No	No	LeuTAG,LeuAAG
GGTAGCGTGGCCGAGCGGTCTAAGGCGCTGGA	Yes	Yes	Yes	No	Yes	No	No	No	No	No	No	No	No	LeuTAG,LeuAAG
GGTAGCGTGGCCGAGT	Yes	Yes	Yes	No	Yes	No	No	No	No	No	No	No	No	LeuTAG,LeuAAG
GGTAGCGTGGCCGAGTGGTCTAAGGCGCT	Yes	Yes	Yes	No	Yes	No	No	No	No	No	No	No	No	LeuTAG
GGTAGCGTGGCCGAGTGGTCTAAGGCGCTGG	Yes	Yes	Yes	No	Yes	No	No	No	No	No	No	No	No	LeuTAG
GGTAGCGTGGCCGAGTGGTCTAAGGCGCTGGA	Yes	Yes	Yes	No	Yes	No	No	No	No	No	No	No	No	LeuTAG
GGTAGTGTGGCCGAGC	No	Yes	Yes	No	Yes	No	No	No	No	No	No	No	No	LeuTAG
GGTAGTGTGGCCGAGCGG	Yes	Yes	Yes	No	Yes	No	No	No	No	No	No	No	No	LeuTAG
GGTAGTGTGGCCGAGCGGTCTAA	Yes	Yes	Yes	No	Yes	No	No	No	No	No	No	No	No	LeuTAG
GGTAGTGTGGCCGAGCGGTCTAAG	Yes	Yes	Yes	No	Yes	No	No	No	No	No	No	No	No	LeuTAG
GGTAGTGTGGCCGAGCGGTCTAAGG	Yes	Yes	Yes	No	Yes	No	No	No	No	No	No	No	No	LeuTAG
GGTATAGCTCAGCGGTAGA	No	Yes	Yes	No	Yes	No	No	No	No	No	No	No	No	AlaAGC
GGTATAGCTCAGCGGTAGAGCGCGTGC	Yes	Yes	Yes	No	Yes	No	No	No	No	No	No	No	No	AlaAGC
GGTATAGCTCAGGGG	No	No	Yes	No	Yes	No	Yes	No	No	No	No	No	No	NA
GGTATAGCTCAGGGGTAGAG	No	Yes	Yes	No	Yes	No	No	No	No	No	No	No	No	CysGCA
GGTATAGCTCAGTGGTAGA	No	Yes	Yes	No	Yes	No	No	No	No	No	No	No	No	CysGCA,AlaAGC
GGTATAGCTCAGTGGTAGAGCGCGTGC	Yes	Yes	Yes	No	Yes	No	No	No	No	No	No	No	No	AlaAGC
GGTATGATTCTCGCT	No	No	Yes	No	Yes	No	Yes	No	No	No	No	No	No	NA
GGTATGATTCTCGCTT	No	Yes	Yes	No	Yes	No	No	No	No	No	No	No	Yes	ProAGG,ProCGG,ProTGG
GGTATGATTCTCGGT	No	No	Yes	No	Yes	No	Yes	No	No	No	No	No	No	NA
GGTCCCATGGTGTAAT	No	Yes	Yes	No	Yes	No	No	No	No	No	No	No	No	GlnTTG
GGTCCCATGGTGTAATGG	Yes	Yes	Yes	No	Yes	No	No	No	No	No	No	No	No	GlnTTG
GGTCCCATGGTGTAATGGT	Yes	Yes	Yes	No	Yes	No	No	No	No	No	No	No	No	GlnTTG
GGTCCCATGGTGTAATGGTTAG	Yes	Yes	Yes	No	Yes	No	No	No	No	No	No	No	No	GlnTTG
GGTCCCATGGTGTAATGGTTAGC	Yes	Yes	Yes	No	Yes	No	No	No	No	No	No	No	No	GlnTTG
GGTCCCATGGTGTAATGGTTAGCACTCTG	Yes	Yes	Yes	No	Yes	No	No	No	No	No	No	No	No	GlnTTG
GGTCCCATGGTGTAATGGTTAGCACTCTGG	Yes	Yes	Yes	No	Yes	No	No	No	No	No	No	No	No	GlnTTG
GGTCCCATGGTGTAATGGTTAGCACTCTGGA	Yes	Yes	Yes	No	Yes	No	No	No	No	No	No	No	No	GlnTTG
GGTCCCATGGTGTAATGGTTAGCACTCTGGAC	Yes	Yes	Yes	No	Yes	No	No	No	No	No	No	No	No	GlnTTG
GGTCCCATGGTGTAATGGTTAGCACTCTGGACT	Yes	Yes	Yes	No	Yes	No	No	No	No	No	No	No	No	GlnTTG
GGTCCCCGGTAGCTC	No	No	No	No	Yes	No	Yes	No	No	No	Yes	No	No	NA
GGTCCCCGGTTCGAAACCGGGCGGAAAC	Yes	Yes	Yes	No	Yes	No	No	No	No	No	No	No	No	ValCAC,ValAAC
GGTCCCCGGTTCGAAACCGGGCGGAAACACCA	Yes	Yes	Yes	No	Yes	No	No	No	No	No	No	No	No	ValCAC,ValAAC
GGTCCCGGGTTCAAATCCCGGACGAGCCCCCA	Yes	Yes	Yes	No	Yes	No	No	No	No	No	No	No	No	ProAGG,ProCGG,ProTGG
GGTCCCTGGATCAAAACCAGGCGGAAACACCA	Yes	Yes	Yes	No	Yes	No	No	No	No	No	No	No	No	ValAAC
GGTCCTCGGGTCGAAACCGAGCGGAAACACCA	Yes	Yes	No	No	Yes	No	No	No	No	No	No	No	No	ValTAC
GGTCCTGGGTTCAAGCCCCAGTGGAACCACCA	Yes	Yes	Yes	No	Yes	No	No	No	No	No	No	No	No	ValTAC
GGTCCTGGGTTCGAGCCCCAGTGGAACCACCA	Yes	Yes	Yes	No	Yes	No	No	No	No	No	No	No	No	ValTAC
GGTCGCGGGTTCGATCCCCGTACGGGCCACCA	Yes	Yes	Yes	No	Yes	No	No	No	No	No	No	No	No	IleAAT
GGTCGTGAGTTCGAGCCTCACACGGGGCACCA	Yes	Yes	No	No	Yes	No	No	No	No	No	No	No	No	MetCAT
GGTCTAGCGGTTAGGA	No	Yes	Yes	No	Yes	No	No	No	No	No	No	No	No	GluTTC
GGTCTAGCGGTTAGGATTCCTGG	Yes	Yes	Yes	No	Yes	No	No	No	No	No	No	No	No	GluTTC
GGTCTAGGGGTATGA	No	No	Yes	No	Yes	No	Yes	No	No	No	No	No	No	NA
GGTCTAGGGGTATGAT	No	Yes	Yes	No	Yes	No	No	No	No	No	No	No	Yes	ProAGG,ProCGG,ProTGG
GGTCTAGGGGTATGATTC	No	Yes	Yes	No	Yes	No	No	No	No	No	No	No	No	ProAGG,ProCGG,ProTGG
GGTCTAGGGGTATGATTCT	No	Yes	Yes	No	Yes	No	No	No	No	No	No	No	No	ProAGG,ProCGG,ProTGG
GGTCTAGGGGTATGATTCTC	No	Yes	Yes	No	Yes	No	No	No	No	No	No	No	No	ProAGG,ProCGG,ProTGG
GGTCTAGGGGTATGATTCTCGC	No	Yes	Yes	No	Yes	No	No	No	No	No	No	No	No	ProAGG,ProCGG,ProTGG
GGTCTAGGGGTATGATTCTCGCT	No	Yes	Yes	No	Yes	No	No	No	No	No	No	No	No	ProAGG,ProCGG,ProTGG
GGTCTAGGGGTATGATTCTCGGT	Yes	Yes	Yes	No	Yes	No	No	No	No	No	No	No	No	ProTGG
GGTGAGTATCCCCGC	No	No	Yes	No	Yes	No	Yes	No	No	No	No	No	No	NA
GGTGAGTATCCCCGCC	Yes	Yes	Yes	No	Yes	No	No	No	No	No	No	No	No	AspGTC
GGTGCATGGGTGGTTCAGTGGTAGAATTC	No	No	No	No	Yes	No	No	No	No	No	No	No	No	NA
GGTGCGAGAGGTCCCGGGT	Yes	Yes	Yes	No	Yes	No	No	No	No	No	No	No	No	ProAGG,ProCGG,ProTGG

GGTGGAGTTAAAGACTTTTTCTCTGACCA	Yes	Yes	No	No	No	No	No	Yes	No	No	No	No	No	ProTGG
GGTGGTATAGTGGTAAGCATAGCTG	No	Yes	Yes	No	Yes	No	No	No	No	No	No	No	No	GlyTCC
GGTGGTATAGTGGTGAGCATAGCTG	No	Yes	Yes	No	Yes	No	No	No	No	No	No	No	No	GlyTCC
GGTGGTCTAGTGGTTAGG	No	Yes	Yes	No	Yes	No	No	No	No	No	No	No	No	GluCTC
GGTGGTCTAGTGGTTAGGA	No	Yes	Yes	No	Yes	No	No	No	No	No	No	No	No	GluCTC
GGTGGTCTAGTGGTTAGGATTC	No	Yes	Yes	No	Yes	No	No	No	No	No	No	No	No	GluCTC
GGTGGTCTAGTGGTTAGGATTCGGC	No	Yes	Yes	No	Yes	No	No	No	No	No	No	No	No	GluCTC
GGTGGTTCAGTGGTA	No	No	Yes	No	Yes	No	Yes	No	No	No	No	No	No	NA
GGTGGTTCAGTGGTAGA	No	Yes	Yes	No	Yes	No	No	No	No	No	No	No	Yes	GlyCCC,ValCAC,GlyGCC,GluTTC
GGTGGTTCAGTGGTAGAA	No	Yes	Yes	No	Yes	No	No	No	No	No	No	No	Yes	GlyCCC,ValCAC,GlyGCC,GluTTC
GGTGGTTCAGTGGTAGAAT	No	Yes	Yes	No	Yes	No	No	No	No	No	No	No	Yes	GlyCCC,ValCAC,GlyGCC,GluTTC
GGTGGTTCAGTGGTAGAATTCTCGCCTCC	No	Yes	Yes	No	Yes	No	No	No	No	No	No	No	Yes	GlyCCC
GGTGTAATGGTTAGC	No	No	Yes	No	Yes	No	Yes	No	No	No	No	No	No	NA
GGTGTAATGGTTAGCA	No	Yes	Yes	No	Yes	No	No	No	No	No	No	No	No	GlnCTG,GlnTTG
GGTGTAATGGTTAGCACTCTGG	No	Yes	Yes	No	Yes	No	No	No	No	No	No	No	No	GlnCTG,GlnTTG
GGTGTAATGGTTAGCACTCTGGA	No	Yes	Yes	No	Yes	No	No	No	No	No	No	No	No	GlnCTG,GlnTTG
GGTGTGAGAGGTCCC	No	No	Yes	No	Yes	No	Yes	No	No	No	No	No	No	NA
GGTGTGATAGGTGGCACGGAGA	Yes	Yes	No	No	No	No	No	Yes	Yes	No	No	No	No	GlnTTG
GGTGTGATAGGTGGCACGGAGAA	Yes	Yes	No	No	No	No	No	Yes	Yes	No	No	No	No	GlnTTG
GGTGTGGTCTGTTGTT	No	No	Yes	No	Yes	No	No	No	No	No	No	Yes	No	NA
GGTGTGGTCTGTTGTTT	No	No	Yes	Yes	Yes	No	No	No	No	No	No	Yes	No	NA
GGTGTGGTCTGTTGTTTT	No	No	Yes	No	Yes	No	No	No	No	No	No	Yes	No	NA
GGTGTTAATCTTGGCTTT	No	No	Yes	No	Yes	No	No	No	No	No	No	Yes	No	NA
GGTGTTAATCTTGGCTTTT	No	No	Yes	No	Yes	No	No	No	No	No	No	Yes	No	NA
GGTGTTAATCTTGGCTTTTT	No	No	Yes	No	Yes	No	No	No	No	No	No	Yes	No	NA
GGTTAGCACTCTGGA	No	No	Yes	No	Yes	No	Yes	No	No	No	No	No	No	NA
GGTTAGCACTCTGGACT	No	Yes	Yes	No	Yes	No	No	No	No	No	No	No	No	GlnCTG,GlnTTG
GGTTAGGATTCGGCG	No	No	Yes	No	Yes	No	Yes	No	No	No	No	No	No	NA
GGTTAGGATTCGGCGC	Yes	Yes	Yes	No	Yes	No	No	No	No	No	No	No	Yes	GluCTC
GGTTAGGATTCGGCGCT	Yes	Yes	Yes	No	Yes	No	No	No	No	No	No	No	Yes	GluCTC
GGTTAGGATTCGGCGCTC	Yes	Yes	Yes	No	Yes	No	No	No	No	No	No	No	Yes	GluCTC
GGTTAGGATTCGGCGCTCTCA	Yes	Yes	Yes	No	Yes	No	No	No	No	No	No	No	Yes	GluCTC
GGTTAGGATTCGGCGCTCTCACCGCC	Yes	Yes	Yes	No	Yes	No	No	No	No	No	No	No	Yes	GluCTC
GGTTAGTACTCTGCG	No	No	Yes	No	Yes	No	Yes	No	No	No	No	No	No	NA
GGTTAGTACTCTGCGT	Yes	Yes	Yes	No	Yes	No	No	No	No	No	No	No	No	HisGTG
GGTTAGTATCCCCGC	No	No	Yes	No	Yes	No	Yes	No	No	No	No	No	No	NA
GGTTAGTATCCCCGCC	Yes	Yes	Yes	No	Yes	No	No	No	No	No	No	No	No	AspGTC
GGTTATCACGTCTGC	No	No	Yes	No	Yes	No	Yes	No	No	No	No	No	No	NA
GGTTATCACGTTCGC	No	No	Yes	No	Yes	No	Yes	No	No	No	No	No	No	NA
GGTTATCACGTTCGCC	No	Yes	Yes	No	Yes	No	No	No	No	No	No	No	No	ValCAC,ValAAC
GGTTCAATTCCCCGACGGGGAGCCA	Yes	Yes	Yes	No	Yes	No	No	No	No	No	No	No	No	AspGTC
GGTTCAGTGGTAGAA	No	No	Yes	No	Yes	No	Yes	No	No	No	No	No	No	NA
GGTTCAGTGGTAGAAT	No	Yes	Yes	No	Yes	No	No	No	No	No	No	No	Yes	GlyCCC,ValCAC,GlyGCC,GluTTC
GGTTCAGTGGTAGAATTCTCGCC	No	Yes	Yes	No	Yes	No	No	No	No	No	No	No	Yes	GlyCCC,ValCAC,GlyGCC,GluTTC
GGTTCAGTGGTAGAATTCTCGCCTC	No	Yes	Yes	No	Yes	No	No	No	No	No	No	No	Yes	GlyCCC,ValCAC
GGTTCAGTGGTAGAATTCTCGCCTCC	No	Yes	Yes	No	Yes	No	No	No	No	No	No	No	Yes	GlyCCC
GGTTCAGTGGTAGAATTCTCGCCTCCC	No	Yes	Yes	No	Yes	No	No	No	No	No	No	No	Yes	GlyCCC
GGTTCCATAGTGTAG	No	No	Yes	No	Yes	No	Yes	No	No	No	No	No	No	NA
GGTTCCATAGTGTAGC	Yes	Yes	Yes	No	Yes	No	No	No	No	No	No	No	No	ValTAC
GGTTCCATAGTGTAGCGG	Yes	Yes	Yes	No	Yes	No	No	No	No	No	No	No	No	ValTAC
GGTTCCATAGTGTAGCGGT	Yes	Yes	Yes	No	Yes	No	No	No	No	No	No	No	No	ValTAC
GGTTCCATAGTGTAGCGGTTATC	Yes	Yes	Yes	No	Yes	No	No	No	No	No	No	No	No	ValTAC
GGTTCCATAGTGTAGCGGTTATCAC	Yes	Yes	Yes	No	Yes	No	No	No	No	No	No	No	No	ValTAC
GGTTCCATAGTGTAGCGGTTATCACGTCT	Yes	Yes	Yes	No	Yes	No	No	No	No	No	No	No	No	ValTAC
GGTTCCATAGTGTAGCGGTTATCACGTCTGC	Yes	Yes	Yes	No	Yes	No	No	No	No	No	No	No	No	ValTAC
GGTTCCATAGTGTAGTGG	No	Yes	Yes	No	Yes	No	No	No	No	No	No	No	No	ValTAC
GGTTCCATAGTGTAGTGGT	No	Yes	Yes	No	Yes	No	No	No	No	No	No	No	No	ValTAC
GGTTCCATAGTGTAGTGGTTATC	Yes	Yes	Yes	Yes	Yes	No	No	No	No	No	No	No	No	ValTAC
GGTTCCATAGTGTAGTGGTTATCAC	Yes	Yes	Yes	No	Yes	No	No	No	No	No	No	No	No	ValTAC
GGTTCCATAGTGTAGTGGTTATCACATCTGC	Yes	Yes	Yes	No	Yes	No	No	No	No	No	No	No	No	ValTAC
GGTTCCATAGTGTAGTGGTTATCACATCTGCT	Yes	Yes	No	No	Yes	No	No	No	No	No	No	No	No	ValTAC
GGTTCCATAGTGTAGTGGTTATCACG	Yes	Yes	Yes	No	Yes	No	No	No	No	No	No	No	No	ValTAC
GGTTCCATAGTGTAGTGGTTATCACGTC	Yes	Yes	Yes	No	Yes	No	No	No	No	No	No	No	No	ValTAC
GGTTCCATAGTGTAGTGGTTATCACGTCT	Yes	Yes	Yes	No	Yes	No	No	No	No	No	No	No	No	ValTAC
GGTTCCATAGTGTAGTGGTTATCACGTCTG	Yes	Yes	Yes	No	Yes	No	No	No	No	No	No	No	No	ValTAC
GGTTCCATAGTGTAGTGGTTATCACGTCTGC	Yes	Yes	Yes	Yes	Yes	No	No	No	No	No	No	No	No	ValTAC
GGTTCCATAGTGTAGTGGTTATCACGTCTGCT	Yes	Yes	No	No	Yes	No	No	No	No	No	No	No	No	ValTAC
GGTTCCATAGTGTAGTGGTTATCACGTCTGCTT	Yes	Yes	No	No	Yes	No	No	No	No	No	No	No	No	ValTAC
GGTTCCATGGTGTAA	No	No	Yes	No	Yes	No	Yes	No	No	No	No	No	No	NA
GGTTCCATGGTGTAAT	No	Yes	Yes	No	Yes	No	No	No	No	No	No	No	Yes	GlnCTG
GGTTCCATGGTGTAATGG	No	Yes	Yes	Yes	Yes	No	No	No	No	No	No	No	Yes	GlnCTG
GGTTCCATGGTGTAATGGT	No	Yes	Yes	No	Yes	No	No	No	No	No	No	No	Yes	GlnCTG
GGTTCCATGGTGTAATGGTTAGC	No	Yes	Yes	No	Yes	No	No	No	No	No	No	No	No	GlnCTG
GGTTCCATGGTGTAATGGTTAGCA	No	Yes	Yes	No	Yes	No	No	No	No	No	No	No	No	GlnCTG
GGTTCCATGGTGTAATGGTTAGCAC	No	Yes	Yes	No	Yes	No	No	No	No	No	No	No	No	GlnCTG
GGTTCCATGGTGTAATGGTTAGCACT	No	Yes	Yes	No	Yes	No	No	No	No	No	No	No	No	GlnCTG
GGTTCCATGGTGTAATGGTTAGCACTC	No	Yes	Yes	No	Yes	No	No	No	No	No	No	No	No	GlnCTG
GGTTCCATGGTGTAATGGTTAGCACTCTG	No	Yes	Yes	No	Yes	No	No	No	No	No	No	No	No	GlnCTG
GGTTCCATGGTGTAATGGTTAGCACTCTGG	Yes	Yes	Yes	No	Yes	No	No	No	No	No	No	No	No	GlnCTG
GGTTCCATGGTGTAATGGTTAGCACTCTGGA	Yes	Yes	Yes	No	Yes	No	No	No	No	No	No	No	No	GlnCTG
GGTTCCATGGTGTAATGGTTAGCACTCTGGAC	Yes	Yes	Yes	No	Yes	No	No	No	No	No	No	No	No	GlnCTG
GGTTCCATGGTGTAATGGTTAGCACTCTGGACT	Yes	Yes	Yes	No	Yes	No	No	No	No	No	No	No	No	GlnCTG
GGTTCCTCGCAGCTTCGCTGCGTGA	No	No	Yes	No	Yes	No	No	No	No	No	No	Yes	No	NA
GGTTCGAAACCGGGC	No	No	Yes	No	Yes	No	Yes	No	No	No	No	No	No	NA
GGTTCGAAACCGGGCGGAAACACCA	Yes	Yes	Yes	No	Yes	No	No	No	No	No	No	No	No	ValCAC,ValAAC
GGTTCGAATCCGAGT	No	No	Yes	No	Yes	No	Yes	No	No	No	No	No	No	NA
GGTTCGAATCCGAGTC	No	Yes	Yes	No	Yes	No	No	No	No	No	No	No	No	HisGTG
GGTTCGACTCCCGGTATGGGAACCA	Yes	Yes	Yes	No	Yes	No	No	No	No	No	No	No	No	GluTTC

GGTTCGACTCCCGGTGTGGGAAC	Yes	Yes	Yes	No	Yes	No	No	No	No	No	No	No	No	GluTTC
GGTTCGACTCCCGGTGTGGGAACC	Yes	Yes	Yes	No	Yes	No	No	No	No	No	No	No	No	GluTTC
GGTTCGACTCCCGGTGTGGGAACCA	Yes	Yes	Yes	No	Yes	No	No	No	No	No	No	No	No	GluTTC
GGTTCGACTCCTGGC	No	No	Yes	No	Yes	No	Yes	No	No	No	No	No	No	NA
GGTTCGATTCCCCGACGGG	Yes	Yes	Yes	No	Yes	No	No	No	No	No	No	No	No	AspGTC
GGTTCGATTCCCCGACGGGGA	Yes	Yes	Yes	No	Yes	No	No	No	No	No	No	No	No	AspGTC
GGTTCGATTCCCCGACGGGGAG	Yes	Yes	Yes	No	Yes	No	No	No	No	No	No	No	No	AspGTC
GGTTCGATTCCCCGACGGGGAGC	Yes	Yes	Yes	No	Yes	No	No	No	No	No	No	No	No	AspGTC
GGTTCGATTCCCCGACGGGGAGCCA	Yes	Yes	Yes	No	Yes	No	No	No	No	No	No	No	No	AspGTC
GGTTCGATTCCCGGC	No	No	Yes	No	Yes	No	Yes	No	No	No	No	No	No	NA
GGTTCGATTCCCGGCC	Yes	Yes	Yes	No	Yes	No	No	No	No	No	No	No	No	GlyTCC,GlyGCC,GluTTC
GGTTCGATTCCCGGT	No	No	Yes	No	Yes	No	Yes	No	No	No	No	No	No	NA
GGTTCGATTCCCGGTC	No	Yes	Yes	No	Yes	No	No	No	No	No	No	No	Yes	GluCTC,GlyCCC,PheGAA,GluTTC
GGTTCGATTCCCGGTCAGGGAAC	Yes	Yes	Yes	No	Yes	No	No	No	No	No	No	No	Yes	GluCTC,PheGAA,GluTTC
GGTTCGATTCCCGGTCAGGGAACC	Yes	Yes	Yes	No	Yes	No	No	No	No	No	No	No	Yes	GluCTC,PheGAA,GluTTC
GGTTCGATTCCCGGTCAGGGAACCA	Yes	Yes	Yes	No	Yes	No	No	No	No	No	No	No	Yes	GluCTC,PheGAA,GluTTC
GGTTCGATTCCCGGTCATGGAACCA	Yes	Yes	No	No	Yes	No	No	No	No	No	No	No	Yes	GlyCCC
GGTTCGATTCTCATAGTCCTAGCCA	Yes	Yes	No	No	No	No	No	Yes	Yes	No	No	No	No	GlnTTG
GGTTGCGTGTTCAAATCACGTCGGGGTCACCA	Yes	Yes	No	No	Yes	No	No	No	No	No	No	No	No	TrpCCA
GGTTGCGTGTTCAAGTCACGTCGGGGTCACCA	Yes	Yes	No	No	Yes	No	No	No	No	No	No	No	No	TrpCCA
GGTTGCTGTCTTTTT	No	No	Yes	No	Yes	No	Yes	No	No	No	No	Yes	No	NA
GGTTGGGTTTTTTTT	No	No	No	No	Yes	No	Yes	No	No	No	No	Yes	No	NA
GGTTTTGCAGTCCTTAC	Yes	Yes	No	No	No	No	No	Yes	Yes	No	No	No	No	AlaTGC
GTAAACAGGAGATCCTGGGT	Yes	Yes	Yes	No	Yes	No	No	No	No	No	No	No	No	ThrAGT,ThrTGT,ThrCGT
GTAAACCAGGGGTCG	No	No	Yes	No	Yes	No	Yes	No	No	No	No	No	No	NA
GTAAACCAGGGGTCGCG	Yes	Yes	Yes	No	Yes	No	No	No	No	No	No	No	No	ThrTGT
GTAAACCAGGGGTCGCGA	Yes	Yes	Yes	No	Yes	No	No	No	No	No	No	No	No	ThrTGT
GTAAACCAGGGGTCGCGAGT	Yes	Yes	Yes	No	Yes	No	No	No	No	No	No	No	No	ThrTGT
GTAAACCAGGGGTCGCGAGTT	Yes	Yes	Yes	No	Yes	No	No	No	No	No	No	No	No	ThrTGT
GTAAACCGAAGATCACGGGT	Yes	Yes	Yes	No	Yes	No	No	No	No	No	No	No	No	ThrCGT
GTAAACCGAAGATCGCGGGT	Yes	Yes	No	No	Yes	No	No	No	No	No	No	No	No	ThrCGT
GTAATCGCATAAAACTTA	Yes	Yes	No	No	No	No	No	Yes	No	No	No	No	No	LeuTAA
GTAATGGTGAGCACTCTGGACTC	No	Yes	Yes	No	Yes	No	No	No	No	No	No	No	No	GlnCTG
GTAATGGTTAGCACTC	No	Yes	Yes	No	Yes	No	No	No	No	No	No	No	No	GlnCTG,GlnTTG
GTAATGGTTAGCACTCTGG	No	Yes	Yes	No	Yes	No	No	No	No	No	No	No	No	GlnCTG,GlnTTG
GTAATGGTTAGCACTCTGGA	No	Yes	Yes	No	Yes	No	No	No	No	No	No	No	No	GlnCTG,GlnTTG
GTAATGGTTAGCACTCTGGACTC	No	Yes	Yes	No	Yes	No	No	No	No	No	No	No	No	GlnCTG
GTAGAGCATGAGACTC	No	Yes	Yes	No	Yes	No	No	No	No	No	No	No	No	LysCTT
GTAGGTAGCGCATCAGTC	Yes	Yes	No	No	Yes	No	No	No	No	No	No	No	No	LeuCAA
GTAGTCGTGGCCGAG	No	No	Yes	No	Yes	No	Yes	No	No	No	No	No	No	NA
GTAGTCGTGGCCGAGT	Yes	Yes	Yes	No	Yes	No	No	No	No	No	No	No	No	SerTGA,SerAGA
GTAGTCGTGGCCGAGTGG	Yes	Yes	Yes	No	Yes	No	No	No	No	No	No	No	No	SerTGA,SerAGA
GTAGTCGTGGCCGAGTGGTTAA	Yes	Yes	Yes	No	Yes	No	No	No	No	No	No	No	No	SerTGA,SerAGA
GTAGTCGTGGCCGAGTGGTTAAG	Yes	Yes	Yes	No	Yes	No	No	No	No	No	No	No	No	SerTGA,SerAGA
GTAGTCGTGGCCGAGTGGTTAAGG	Yes	Yes	Yes	No	Yes	No	No	No	No	No	No	No	No	SerTGA,SerAGA
GTAGTCGTGGCCGAGTGGTTAAGGC	Yes	Yes	Yes	Yes	Yes	No	No	No	No	No	No	No	No	SerTGA,SerAGA
GTAGTCGTGGCCGAGTGGTTAAGGCGATGG	Yes	Yes	Yes	No	Yes	No	No	No	No	No	No	No	No	SerTGA,SerAGA
GTAGTGTAGTGGTCATC	Yes	Yes	Yes	No	Yes	No	No	No	No	No	No	No	No	ValAAC
GTAGTGTAGTGGTTATC	No	Yes	Yes	No	Yes	No	No	No	No	No	No	No	No	ValCAC,ValAAC
GTATAGTGGTGAGTATCCCCGCC	Yes	Yes	Yes	No	Yes	No	No	No	No	No	No	No	No	AspGTC
GTATAGTGGTTAGTACTCTG	No	Yes	Yes	No	Yes	No	No	No	No	No	No	No	No	HisGTG
GTATAGTGGTTAGTACTCTGC	No	Yes	Yes	No	Yes	No	No	No	No	No	No	No	No	HisGTG
GTATGAGGCCCCGGGT	Yes	Yes	Yes	No	Yes	No	No	No	No	No	No	No	No	AlaTGC,AlaCGC
GTATGAGGTCCCGGGT	Yes	Yes	Yes	No	Yes	No	No	No	No	No	No	No	No	AlaTGC,AlaCGC
GTATTCTCTGTGGCTTT	No	No	Yes	No	Yes	No	No	No	No	No	No	Yes	No	NA
GTATTCTCTGTGGCTTTT	No	No	Yes	No	Yes	No	No	No	No	No	No	Yes	No	NA
GTCACCTGGCAGGTGCCTC	No	No	No	No	Yes	No	No	No	No	No	No	Yes	No	NA
GTCACCTGGCAGGTGCCTCTT	No	No	No	No	Yes	No	No	No	No	No	No	Yes	No	NA
GTCACCTGGCAGGTGCCTCTTT	No	No	No	No	Yes	No	No	No	No	No	No	Yes	No	NA
GTCACCTGGCAGGTGCCTCTTTT	No	No	No	No	Yes	No	No	No	No	No	No	Yes	No	NA
GTCACGGTGGCCGAG	No	No	No	No	Yes	No	Yes	No	No	No	No	No	No	NA
GTCACGGTGGCCGAGTGGTTAAGG	Yes	Yes	No	No	Yes	No	No	No	No	No	No	No	No	SerCGA
GTCAGGATGGCCGAG	No	No	Yes	No	Yes	No	Yes	No	No	No	No	No	No	NA
GTCAGGATGGCCGAGC	No	Yes	Yes	Yes	Yes	No	No	No	No	No	No	No	No	LeuCAA,LeuCAG
GTCAGGATGGCCGAGCG	No	Yes	Yes	No	Yes	No	No	No	No	No	No	No	No	LeuCAG
GTCAGGATGGCCGAGCGG	No	Yes	Yes	No	Yes	No	No	No	No	No	No	No	No	LeuCAG
GTCAGGATGGCCGAGCGGT	No	Yes	Yes	No	Yes	No	No	No	No	No	No	No	No	LeuCAG
GTCAGGATGGCCGAGCGGTC	No	Yes	Yes	No	Yes	No	No	No	No	No	No	No	No	LeuCAG
GTCAGGATGGCCGAGCGGTCTA	No	Yes	Yes	No	Yes	No	No	No	No	No	No	No	No	LeuCAG
GTCAGGATGGCCGAGCGGTCTAA	No	Yes	Yes	Yes	Yes	No	No	No	No	No	No	No	No	LeuCAG
GTCAGGATGGCCGAGCGGTCTAAG	No	Yes	Yes	No	Yes	No	No	No	No	No	No	No	No	LeuCAG
GTCAGGATGGCCGAGCGGTCTAAGG	No	Yes	Yes	No	Yes	No	No	No	No	No	No	No	No	LeuCAG
GTCAGGATGGCCGAGCGGTCTAAGGC	No	Yes	Yes	No	Yes	No	No	No	No	No	No	No	No	LeuCAG
GTCAGGATGGCCGAGCGGTCTAAGGCGCT	No	Yes	Yes	No	Yes	No	No	No	No	No	No	No	No	LeuCAG
GTCAGGATGGCCGAGCGGTCTAAGGCGCTG	No	Yes	Yes	No	Yes	No	No	No	No	No	No	No	No	LeuCAG
GTCAGGATGGCCGAGCGGTCTAAGGCGCTGC	Yes	Yes	Yes	No	Yes	No	No	No	No	No	No	No	No	LeuCAG
GTCAGGATGGCCGAGCGGTCTAAGGCGCTGCG	Yes	Yes	Yes	No	Yes	No	No	No	No	No	No	No	No	LeuCAG
GTCAGGATGGCCGAGCGGTCTAAGGCGCTGCGT	Yes	Yes	Yes	No	Yes	No	No	No	No	No	No	No	No	LeuCAG
GTCAGGATGGCCGAGT	No	Yes	Yes	No	Yes	No	No	No	No	No	No	No	No	LeuCAA
GTCAGGATGGCCGAGTGG	No	Yes	Yes	No	Yes	No	No	No	No	No	No	No	No	LeuCAA
GTCAGGATGGCCGAGTGGTCTA	No	Yes	Yes	No	Yes	No	No	No	No	No	No	No	No	LeuCAA
GTCAGGATGGCCGAGTGGTCTAAGG	Yes	Yes	Yes	No	Yes	No	No	No	No	No	No	No	No	LeuCAA
GTCAGGATGGCCGAGTGGTCTAAGGCGCCAGA	Yes	Yes	Yes	No	Yes	No	No	No	No	No	No	No	No	LeuCAA
GTCAGTGCTTCTTTT	No	No	Yes	No	Yes	No	Yes	No	No	No	No	Yes	No	NA
GTCCCACCAGAGTCGCCA	Yes	Yes	No	No	Yes	No	No	No	No	No	No	No	No	ArgTCT
GTCCCACCCGGGGTACCA	Yes	Yes	No	No	Yes	No	No	No	No	No	No	No	No	ArgCCT
GTCCCACCTGGGGTACC	No	Yes	Yes	No	Yes	No	No	No	No	No	No	No	No	ArgCCT
GTCCCACCTGGGGTACCA	No	Yes	Yes	No	Yes	No	No	No	No	No	No	No	No	ArgCCT

GTCCCACCTGGGGTGCCA	No	Yes	Yes	No	Yes	No	No	No	No	No	No	No	No	ArgCCT
GTCCCATCTGGGTCGCCA	No	Yes	Yes	No	Yes	No	No	No	No	No	No	No	No	ArgCCG
GTCCCGGCAGAGATGCCA	Yes	Yes	No	No	Yes	No	No	No	No	No	No	No	No	ArgTCT
GTCCCGGCGGAGTCGCCA	Yes	Yes	No	No	Yes	No	No	No	No	No	No	No	No	ArgTCT
GTCCCTGTCCAGGCGCCA	Yes	Yes	No	No	Yes	No	No	No	No	No	No	No	No	LysTTT
GTCCCTGTTCAGGCGCC	No	Yes	No	No	Yes	No	No	No	No	No	No	No	No	LysTTT
GTCCCTGTTCAGGCGCCA	No	Yes	No	No	Yes	No	No	No	No	No	No	No	No	LysTTT
GTCCCTGTTCGGGCGCC	No	Yes	Yes	No	Yes	No	No	No	No	No	No	No	No	LysTTT
GTCCCTGTTCGGGCGCCA	No	Yes	Yes	No	Yes	No	No	No	No	No	No	No	No	LysTTT
GTCCCTGTTCGGGCGCCAT	No	No	Yes	No	No	No	No	No	No	No	No	No	No	NA
GTCCCTGTTCGGGCGCCATT	No	No	Yes	No	No	No	No	No	No	No	No	No	No	NA
GTCCCTTCGTGGTCGCCA	No	Yes	No	No	Yes	No	No	No	No	No	No	No	No	ArgTCG,ArgCCG
GTCCTGCCGCGGTCGCCA	Yes	Yes	Yes	No	Yes	No	No	No	No	No	No	No	No	ArgTCG
GTCCTGGGTTCGAGCCCCAGTGGAACCACCA	Yes	Yes	Yes	No	Yes	No	No	No	No	No	No	No	No	ValTAC
GTCCTTGTAGTATAAAC	Yes	Yes	No	No	No	No	No	Yes	No	No	No	No	No	ThrTGT
GTCCTTGTAGTATAAACT	Yes	Yes	No	No	No	No	No	Yes	No	No	No	No	No	ThrTGT
GTCCTTGTAGTATAAACTA	Yes	Yes	No	No	No	No	No	Yes	No	No	No	No	No	ThrTGT
GTCCTTGTAGTATAAACTAATACACCAGT	Yes	Yes	No	No	No	No	No	Yes	No	No	No	No	No	ThrTGT
GTCGGTAGAGCATCA	No	No	Yes	No	Yes	No	Yes	No	No	No	No	No	No	NA
GTCGGTAGAGCATCAG	Yes	Yes	Yes	No	Yes	No	No	No	No	No	No	No	No	LysTTT
GTCGGTAGAGCATCAGA	Yes	Yes	Yes	No	Yes	No	No	No	No	No	No	No	No	LysTTT
GTCGGTAGAGCATCAGAC	Yes	Yes	Yes	No	Yes	No	No	No	No	No	No	No	No	LysTTT
GTCGGTAGAGCATGA	No	No	Yes	No	Yes	No	Yes	No	No	No	No	No	No	NA
GTCGGTAGAGCATGAG	No	Yes	Yes	No	Yes	No	No	No	No	No	No	No	No	LysCTT
GTCGGTAGAGCATGAGA	No	Yes	Yes	No	Yes	No	No	No	No	No	No	No	No	LysCTT
GTCGGTAGAGCATGAGAC	No	Yes	Yes	No	Yes	No	No	No	No	No	No	No	No	LysCTT
GTCGGTAGAGCATGGG	Yes	Yes	Yes	No	Yes	No	No	No	No	No	No	No	No	LysCTT
GTCGGTAGAGCATGGGA	Yes	Yes	Yes	No	Yes	No	No	No	No	No	No	No	No	LysCTT
GTCGGTAGAGCATGGGAC	Yes	Yes	Yes	No	Yes	No	No	No	No	No	No	No	No	LysCTT
GTCTAAGGCGCTGGA	No	No	Yes	No	Yes	No	Yes	No	No	No	No	No	No	NA
GTCTAGCGGTTAGGA	No	No	Yes	No	Yes	No	Yes	No	No	No	No	No	No	NA
GTCTAGCGGTTAGGATTCCTGG	Yes	Yes	Yes	No	Yes	No	No	No	No	No	No	No	No	GluTTC
GTCTAGGGGTATGATTCTCGCT	No	Yes	Yes	No	Yes	No	No	No	No	No	No	No	No	ProAGG,ProCGG,ProTGG
GTCTCATAATCTGAAGGTCCTGAGT	No	Yes	Yes	No	Yes	No	No	No	No	No	No	No	No	MetCAT
GTCTCGGTGGAACCTCCA	Yes	Yes	Yes	No	Yes	No	No	No	No	No	No	No	No	GlnCTG
GTCTCTGTGGCGCAA	No	No	Yes	No	Yes	No	Yes	No	No	No	No	No	No	NA
GTCTCTGTGGCGCAATCGG	Yes	Yes	Yes	No	Yes	Yes	No	No	No	No	No	No	No	AsnGTT,AsnATT
GTCTCTGTGGCGCAATCGGT	Yes	Yes	Yes	No	Yes	No	No	No	No	No	No	No	No	AsnGTT,AsnATT
GTCTCTGTGGCGCAATGGA	Yes	Yes	No	No	Yes	No	No	No	No	No	No	No	No	ArgTCT
GTCTCTGTGGCGCAATGGAC	Yes	Yes	No	No	Yes	Yes	No	No	No	No	No	No	No	ArgTCT
GTGAGTATCCCCGCC	No	No	Yes	No	Yes	No	Yes	No	No	No	No	No	No	NA
GTGAGTATCCCCGCCTGTC	Yes	Yes	Yes	No	Yes	No	No	No	No	No	No	No	No	AspGTC
GTGAGTATCCCCGCCTGTCACGCGGG	Yes	Yes	Yes	No	Yes	No	No	No	No	No	No	No	No	AspGTC
GTGAGTTCGAGCCTC	No	No	No	No	Yes	No	Yes	No	No	No	No	No	No	NA
GTGATCGTATAGTGGTTAGTACTCTGC	No	Yes	Yes	No	Yes	No	No	No	No	No	No	No	No	HisGTG
GTGCATTTGTGTTTT	No	No	Yes	No	Yes	No	Yes	No	No	No	No	Yes	No	NA
GTGCGAGAGGTCCCGGGT	Yes	Yes	Yes	No	Yes	No	No	No	No	No	No	No	No	ProAGG,ProCGG,ProTGG
GTGCGTGATGCTTTT	No	No	Yes	No	Yes	No	Yes	No	No	No	No	Yes	No	NA
GTGGAGTTAAAGACTTTTTCTCTGACC	Yes	Yes	No	No	No	No	No	Yes	No	No	No	No	No	ProTGG
GTGGAGTTAAAGACTTTTTCTCTGACCA	Yes	Yes	No	No	No	No	No	Yes	No	No	No	No	No	ProTGG
GTGGCCGCAGCAACC	No	No	Yes	No	Yes	No	Yes	No	No	No	No	No	No	NA
GTGGCCGCAGCAACCTCGGT	Yes	Yes	Yes	No	Yes	No	No	No	No	No	No	No	No	HisGTG
GTGGCGTCCCGGTAGC	No	No	No	No	Yes	No	No	No	No	No	Yes	No	No	NA
GTGGCGTCCCGGTAGCT	No	No	No	No	Yes	No	No	No	No	No	Yes	No	No	NA
GTGGCGTCCCGGTAGCTC	No	No	No	No	Yes	No	No	No	No	No	Yes	No	No	NA
GTGGCTAGGATTCGGC	No	Yes	Yes	No	Yes	No	No	No	No	No	No	No	Yes	GluTTC
GTGGGGTTCCTCGCAGCTTCGCTGCGTGA	No	No	Yes	No	Yes	No	No	No	No	No	No	Yes	No	NA
GTGGGGTTCCTCGCAGCTTCGCTGCGTGAGC	No	No	Yes	No	Yes	No	No	No	No	No	No	Yes	No	NA
GTGGGGTTCCTCGCAGCTTCGCTGCGTGAGCA	No	No	Yes	No	Yes	No	No	No	No	No	No	Yes	No	NA
GTGGGGTTCGTTTTCGGGCATGAAAATTT	No	No	No	No	Yes	No	No	No	No	No	No	Yes	No	NA
GTGGGGTTCGTTTTCGGGCATGAAAATTTT	No	No	No	No	Yes	No	No	No	No	No	No	Yes	No	NA
GTGGGGTTTTGCAGTCCTTACCA	Yes	Yes	No	No	No	No	No	Yes	Yes	No	No	No	No	AlaTGC
GTGGGTGGCTTTTTT	No	No	Yes	Yes	Yes	No	Yes	No	No	No	No	Yes	No	NA
GTGGGTGGCTTTTTTT	No	No	Yes	No	Yes	No	No	No	No	No	No	Yes	No	NA
GTGGGTGGCTTTTTTTT	No	No	Yes	No	Yes	No	No	No	No	No	No	Yes	No	NA
GTGGTAAGCATAGCTG	No	Yes	Yes	No	Yes	No	No	No	No	No	No	No	No	GlyTCC
GTGGTAGAATTCTCGCCTCC	No	Yes	Yes	No	Yes	No	No	No	No	No	No	No	Yes	GlyCCC
GTGGTAGAATTCTCGCCTGCC	No	Yes	Yes	No	Yes	No	No	No	No	No	No	No	No	GlyGCC
GTGGTAGAGCATTTGA	No	Yes	Yes	No	Yes	No	No	No	No	No	No	No	Yes	CysGCA,CysACA
GTGGTAGAGCATTTGACTGC	No	Yes	Yes	No	Yes	No	No	No	No	No	No	No	No	CysGCA
GTGGTAGAGCGCGTGC	Yes	Yes	Yes	No	Yes	No	No	No	No	No	No	No	No	AlaCGC,AlaAGC
GTGGTAGAGCGCGTGCT	Yes	Yes	Yes	No	Yes	No	No	No	No	No	No	No	No	AlaCGC,AlaAGC
GTGGTGAGCATAGCTG	No	Yes	Yes	No	Yes	No	No	No	No	No	No	No	Yes	GlyTCC
GTGGTGAGCATAGCTGC	No	Yes	Yes	No	Yes	No	No	No	No	No	No	No	Yes	GlyTCC
GTGGTGAGTATCCCC	No	No	Yes	No	Yes	No	Yes	No	No	No	No	No	No	NA
GTGGTGAGTATCCCCGC	No	Yes	Yes	No	Yes	No	No	No	No	No	No	No	No	AspGTC
GTGGTGAGTATCCCCGCC	Yes	Yes	Yes	No	Yes	No	No	No	No	No	No	No	No	AspGTC
GTGGTGAGTATCCCCGCCTGTC	Yes	Yes	Yes	No	Yes	No	No	No	No	No	No	No	No	AspGTC
GTGGTTAGCATAGCTG	No	Yes	Yes	No	Yes	No	No	No	No	No	No	No	No	GlyTCC
GTGGTTAGGATTCGG	No	No	Yes	No	Yes	No	Yes	No	No	No	No	No	No	NA
GTGGTTAGGATTCGGC	No	Yes	Yes	No	Yes	No	No	No	No	No	No	No	Yes	GluCTC
GTGGTTAGGATTCGGCG	Yes	Yes	Yes	No	Yes	No	No	No	No	No	No	No	Yes	GluCTC
GTGGTTAGGATTCGGCGC	Yes	Yes	Yes	No	Yes	No	No	No	No	No	No	No	Yes	GluCTC
GTGGTTAGGATTCGGCGCT	Yes	Yes	Yes	No	Yes	No	No	No	No	No	No	No	Yes	GluCTC
GTGGTTAGGATTCGGCGCTC	Yes	Yes	Yes	No	Yes	No	No	No	No	No	No	No	Yes	GluCTC
GTGGTTAGGATTCGGCGCTCTCA	Yes	Yes	Yes	No	Yes	No	No	No	No	No	No	No	Yes	GluCTC
GTGGTTAGGATTCGGCGCTCTCAC	Yes	Yes	Yes	No	Yes	No	No	No	No	No	No	No	Yes	GluCTC
GTGGTTAGGATTCGGCGCTCTCACCGCC	Yes	Yes	Yes	No	Yes	No	No	No	No	No	No	No	Yes	GluCTC

GTGGTTAGGATTCGGCGCTCTCACCGCCGC	Yes	Yes	Yes	No	Yes	No	No	No	No	No	No	No	No	GluCTC
GTGGTTAGTACTCTGC	No	Yes	Yes	No	Yes	No	No	No	No	No	No	No	No	HisGTG
GTGGTTATCACGTCTGC	Yes	Yes	Yes	No	Yes	No	No	No	No	No	No	No	No	ValTAC
GTGGTTATCACGTTCGC	No	Yes	Yes	No	Yes	No	No	No	No	No	No	No	No	ValCAC,ValAAC
GTGGTTATCACGTTCGCC	No	Yes	Yes	No	Yes	No	No	No	No	No	No	No	No	ValCAC,ValAAC
GTGGTTCAGTGGTAGA	No	Yes	Yes	No	Yes	No	No	No	No	No	No	No	Yes	GlyCCC,ValCAC,GlyGCC,GluTTC
GTGGTTCAGTGGTAGAA	No	Yes	Yes	No	Yes	No	No	No	No	No	No	No	Yes	GlyCCC,ValCAC,GlyGCC,GluTTC
GTGGTTCAGTGGTAGAATTCTT	No	Yes	No	No	Yes	No	No	No	No	No	No	No	No	GlyGCC
GTGGTTGTAGTCCGTGCGAGAATACCA	Yes	Yes	No	No	No	No	No	Yes	Yes	No	No	No	No	GluTTC
GTGTAAGCAGGGTCGTTT	No	No	Yes	No	Yes	No	No	No	No	No	No	Yes	No	NA
GTGTAAGCAGGGTCGTTTT	No	No	Yes	Yes	Yes	No	No	No	No	No	No	Yes	No	NA
GTGTAAGCAGGGTCGTTTTA	No	No	Yes	No	Yes	No	No	No	No	No	No	Yes	No	NA
GTGTAATGGTTAGCACTCTGGA	No	Yes	Yes	No	Yes	No	No	No	No	No	No	No	No	GlnCTG,GlnTTG
GTGTCCCTAGTAGCTC	No	No	No	No	Yes	No	No	No	No	No	Yes	No	No	NA
GTGTGAGGTATTTTT	No	No	Yes	No	Yes	No	Yes	No	No	No	No	Yes	No	NA
GTGTGAGGTATTTTTT	No	No	Yes	No	Yes	No	No	No	No	No	No	Yes	No	NA
GTGTGAGGTCCCGGGT	No	Yes	Yes	No	Yes	No	No	No	No	No	No	No	No	AlaCGC
GTGTGTAGCTGCACT	No	No	Yes	No	Yes	No	Yes	No	No	No	No	Yes	No	NA
GTGTGTAGCTGCACTTT	No	No	Yes	No	Yes	No	No	No	No	No	No	Yes	No	NA
GTGTGTAGCTGCACTTTT	No	No	Yes	Yes	Yes	No	No	No	No	No	No	Yes	No	NA
GTGTGTAGCTGCACTTTTT	No	No	Yes	No	Yes	No	No	No	No	No	No	Yes	No	NA
GTGTGTAGCTGCACTTTTTT	No	No	Yes	No	Yes	No	No	No	No	No	No	Yes	No	NA
GTTAAAGACTTTTTCTCTGAC	Yes	Yes	No	No	No	No	No	Yes	No	No	No	No	No	ProTGG
GTTAAGATGGCAGAGC	No	Yes	Yes	No	Yes	No	No	Yes	No	No	No	No	Yes	LeuTAA
GTTAAGATGGCAGAGCC	No	Yes	Yes	No	Yes	No	No	Yes	No	No	No	No	Yes	LeuTAA
GTTAAGATGGCAGAGCCC	No	Yes	Yes	No	Yes	No	No	Yes	No	No	No	No	Yes	LeuTAA
GTTAAGATGGCAGAGCCCG	No	Yes	Yes	Yes	Yes	No	No	Yes	No	No	No	No	No	LeuTAA
GTTAAGATGGCAGAGCCCGG	No	Yes	Yes	No	Yes	No	No	Yes	No	No	No	No	No	LeuTAA
GTTAAGATGGCAGAGCCCGGT	Yes	Yes	No	No	No	No	No	Yes	No	No	No	No	No	LeuTAA
GTTAAGATGGCAGAGCCCGGTA	Yes	Yes	No	No	No	No	No	Yes	No	No	No	No	No	LeuTAA
GTTAAGATGGCAGAGCCCGGTAA	Yes	Yes	No	No	No	No	No	Yes	No	No	No	No	No	LeuTAA
GTTAAGATGGCAGAGCCCGGTAAT	Yes	Yes	No	No	No	No	No	Yes	No	No	No	No	No	LeuTAA
GTTAAGATGGCAGAGCCCGGTAATC	Yes	Yes	No	No	No	No	No	Yes	No	No	No	No	No	LeuTAA
GTTAAGATGGCAGAGCCCGGTAATCG	Yes	Yes	No	No	No	No	No	Yes	No	No	No	No	No	LeuTAA
GTTAAGATGGCAGAGCCCGGTAATCGC	Yes	Yes	No	No	No	No	No	Yes	No	No	No	No	No	LeuTAA
GTTAAGATGGCAGAGCCCGGTAATCGCA	Yes	Yes	No	No	No	No	No	Yes	No	No	No	No	No	LeuTAA
GTTAAGATGGCAGAGCCCGGTAATCGCAT	Yes	Yes	No	No	No	No	No	Yes	No	No	No	No	No	LeuTAA
GTTAAGATGGCAGAGCCCGGTAATCGCATA	Yes	Yes	No	No	No	No	No	Yes	No	No	No	No	No	LeuTAA
GTTAAGATGGCAGAGCCCGGTAATCGCATAA	Yes	Yes	No	No	No	No	No	Yes	No	No	No	No	No	LeuTAA
GTTAAGATGGCAGAGCCCGGTAATCGCATAAA	Yes	Yes	No	No	No	No	No	Yes	No	No	No	No	No	LeuTAA
GTTAAGATGGCAGAGCCCGGTAATCGCATAAAA	Yes	Yes	No	No	No	No	No	Yes	No	No	No	No	No	LeuTAA
GTTAGGATTCGGCGC	No	No	Yes	No	Yes	No	Yes	No	No	No	No	No	No	NA
GTTAGGATTCGGCGCT	Yes	Yes	Yes	No	Yes	No	No	No	No	No	No	No	Yes	GluCTC
GTTAGGATTCGGCGCTC	Yes	Yes	Yes	No	Yes	No	No	No	No	No	No	No	Yes	GluCTC
GTTAGGATTCGGCGCTCTCACCGCC	Yes	Yes	Yes	No	Yes	No	No	No	No	No	No	No	Yes	GluCTC
GTTAGTATAGTGGTGAGT	No	Yes	Yes	No	Yes	No	No	No	No	No	No	No	No	AspGTC
GTTAGTATAGTGGTGAGTATC	Yes	Yes	Yes	No	Yes	No	No	No	No	No	No	No	No	AspGTC
GTTAGTATAGTGGTGAGTATCC	Yes	Yes	Yes	No	Yes	No	No	No	No	No	No	No	No	AspGTC
GTTAGTATAGTGGTGAGTATCCCC	Yes	Yes	Yes	No	Yes	No	No	No	No	No	No	No	No	AspGTC
GTTCAGGTCGCAGTC	No	No	Yes	No	Yes	No	Yes	No	No	No	No	No	No	NA
GTTCAGGTCGCAGTCTCC	Yes	Yes	Yes	No	Yes	No	No	No	No	No	No	No	No	LeuCAG
GTTCAGGTCGCAGTCTCCC	Yes	Yes	Yes	No	Yes	No	No	No	No	No	No	No	No	LeuCAG
GTTCAGTGGTAGAATTCTCGCCTGCC	No	Yes	Yes	No	Yes	No	No	No	No	No	No	No	No	GlyGCC
GTTCGAAACCGGGCGGAAACACCA	Yes	Yes	Yes	No	Yes	No	No	No	No	No	No	No	No	ValCAC,ValAAC
GTTCGACTCCCGGTATGGGAACCA	Yes	Yes	Yes	No	Yes	No	No	No	No	No	No	No	No	GluTTC
GTTCGACTCCCGGTGTGGGAACCA	Yes	Yes	Yes	No	Yes	No	No	No	No	No	No	No	No	GluTTC
GTTCGAGTCTCGGTGGAACCTCCA	Yes	Yes	Yes	No	Yes	No	No	No	No	No	No	No	No	GlnCTG
GTTCGATTCCCCGAC	No	No	Yes	No	Yes	No	Yes	No	No	No	No	No	No	NA
GTTCGATTCCCCGACGGGGAGCCA	Yes	Yes	Yes	No	Yes	No	No	No	No	No	No	No	No	AspGTC
GTTCGATTCCCGGTCAGGGAAC	Yes	Yes	Yes	No	Yes	No	No	No	No	No	No	No	Yes	GluCTC,PheGAA,GluTTC
GTTCGATTCCCGGTCAGGGAACC	Yes	Yes	Yes	No	Yes	No	No	No	No	No	No	No	Yes	GluCTC,PheGAA,GluTTC
GTTCGATTCCCGGTCAGGGAACCA	Yes	Yes	Yes	No	Yes	No	No	No	No	No	No	No	Yes	GluCTC,PheGAA,GluTTC
GTTCGATTCCGGCTCGAAGGACCA	Yes	Yes	Yes	No	Yes	No	No	No	No	No	No	No	No	TyrGTA
GTTCGATTCTCATAGTCCTAGCCA	Yes	Yes	No	No	No	No	No	Yes	Yes	No	No	No	No	GlnTTG
GTTGCAATACTTAATTTCTGCCA	Yes	Yes	No	No	No	No	No	Yes	Yes	No	No	No	No	TrpTCA
GTTGGTAGAGCATCAGA	Yes	Yes	No	No	Yes	No	No	No	No	No	No	No	No	SupTTA,LysTTT
GTTGGTCTAGGGGTATGATTCTCGCT	No	Yes	Yes	No	Yes	No	No	No	No	No	No	No	No	ProAGG,ProCGG,ProTGG
GTTTAAGTCCCATTGGTCTAGCCA	Yes	Yes	No	No	No	No	No	Yes	Yes	No	No	No	No	AsnGTT
GTTTATGTAGCTTACCT	Yes	Yes	No	No	No	No	No	Yes	No	No	No	No	No	PheGAA
GTTTATGTAGCTTACCTC	Yes	Yes	No	No	No	No	No	Yes	No	No	No	No	No	PheGAA
GTTTATGTAGCTTACCTCC	Yes	Yes	No	No	No	No	No	Yes	No	No	No	No	No	PheGAA
GTTTATGTAGCTTACCTCCTC	Yes	Yes	No	No	No	No	No	Yes	No	No	No	No	No	PheGAA
GTTTATGTAGCTTACCTCCTCAAAGC	Yes	Yes	No	No	No	No	No	Yes	No	No	No	No	No	PheGAA
GTTTATGTAGCTTACCTCCTCAAAGCA	Yes	Yes	No	No	No	No	No	Yes	No	No	No	No	No	PheGAA
GTTTATGTAGCTTACCTCCTCAAAGCAATA	Yes	Yes	No	No	No	No	No	Yes	No	No	No	No	No	PheGAA
GTTTATGTAGCTTACCTCCTCAAAGCAATAC	Yes	Yes	No	No	No	No	No	Yes	No	No	No	No	No	PheGAA
GTTTATGTAGCTTACCTCCTCAAAGCAATACA	Yes	Yes	No	No	No	No	No	Yes	No	No	No	No	No	PheGAA
GTTTCCGTAGTGGAGTGGTTATCACGTTCGC	Yes	Yes	No	No	Yes	No	No	No	No	No	No	No	No	ValCAC
GTTTCCGTAGTGTAG	No	No	Yes	No	Yes	No	Yes	No	No	No	No	No	No	NA
GTTTCCGTAGTGTAGC	Yes	Yes	No	No	Yes	No	No	No	No	No	No	No	No	ValCAC
GTTTCCGTAGTGTAGCGG	Yes	Yes	No	No	Yes	No	No	No	No	No	No	No	No	ValCAC
GTTTCCGTAGTGTAGCGGTTATCAC	Yes	Yes	No	No	Yes	No	No	No	No	No	No	No	No	ValCAC
GTTTCCGTAGTGTAGCGGTTATCACA	Yes	Yes	No	No	Yes	No	No	No	No	No	No	No	No	ValCAC
GTTTCCGTAGTGTAGCGGTTATCACATT	Yes	Yes	No	No	Yes	No	No	No	No	No	No	No	No	ValCAC
GTTTCCGTAGTGTAGCGGTTATCACATTC	Yes	Yes	No	No	Yes	No	No	No	No	No	No	No	No	ValCAC
GTTTCCGTAGTGTAGCGGTTATCACATTCGC	Yes	Yes	No	No	Yes	No	No	No	No	No	No	No	No	ValCAC
GTTTCCGTAGTGTAGCGGTTATCACATTCGCC	Yes	Yes	No	No	Yes	No	No	No	No	No	No	No	No	ValCAC
GTTTCCGTAGTGTAGTGG	No	Yes	Yes	Yes	Yes	No	No	No	No	No	No	No	No	ValCAC,ValAAC

GTTTCCGTAGTGTAGTGGT	No	Yes	Yes	No	Yes	No	No	No	No	No	No	No	No	ValCAC,ValAAC
GTTTCCGTAGTGTAGTGGTC	Yes	Yes	Yes	No	Yes	No	No	No	No	No	No	No	No	ValAAC
GTTTCCGTAGTGTAGTGGTCAT	Yes	Yes	Yes	No	Yes	No	No	No	No	No	No	No	No	ValAAC
GTTTCCGTAGTGTAGTGGTCATC	Yes	Yes	Yes	Yes	Yes	No	No	No	No	No	No	No	No	ValAAC
GTTTCCGTAGTGTAGTGGTCATCAC	Yes	Yes	Yes	No	Yes	No	No	No	No	No	No	No	No	ValAAC
GTTTCCGTAGTGTAGTGGTCATCACG	Yes	Yes	Yes	No	Yes	No	No	No	No	No	No	No	No	ValAAC
GTTTCCGTAGTGTAGTGGTCATCACGTT	Yes	Yes	Yes	No	Yes	No	No	No	No	No	No	No	No	ValAAC
GTTTCCGTAGTGTAGTGGTCATCACGTTC	Yes	Yes	Yes	No	Yes	No	No	No	No	No	No	No	No	ValAAC
GTTTCCGTAGTGTAGTGGTCATCACGTTCGC	Yes	Yes	Yes	Yes	Yes	No	No	No	No	No	No	No	No	ValAAC
GTTTCCGTAGTGTAGTGGTCATCACGTTCGCC	Yes	Yes	Yes	No	Yes	No	No	No	No	No	No	No	No	ValAAC
GTTTCCGTAGTGTAGTGGTCATCACGTTCGCCT	Yes	Yes	No	No	Yes	No	No	No	No	No	No	No	No	ValAAC
GTTTCCGTAGTGTAGTGGTTAT	No	Yes	Yes	No	Yes	No	No	No	No	No	No	No	No	ValCAC,ValAAC
GTTTCCGTAGTGTAGTGGTTATC	No	Yes	Yes	Yes	Yes	No	No	No	No	No	No	No	No	ValCAC,ValAAC
GTTTCCGTAGTGTAGTGGTTATCA	No	Yes	Yes	No	Yes	No	No	No	No	No	No	No	No	ValCAC,ValAAC
GTTTCCGTAGTGTAGTGGTTATCAC	No	Yes	Yes	No	Yes	No	No	No	No	No	No	No	No	ValCAC,ValAAC
GTTTCCGTAGTGTAGTGGTTATCACG	No	Yes	Yes	No	Yes	No	No	No	No	No	No	No	No	ValCAC,ValAAC
GTTTCCGTAGTGTAGTGGTTATCACGT	No	Yes	Yes	No	Yes	No	No	No	No	No	No	No	No	ValCAC,ValAAC
GTTTCCGTAGTGTAGTGGTTATCACGTT	No	Yes	Yes	No	Yes	No	No	No	No	No	No	No	No	ValCAC,ValAAC
GTTTCCGTAGTGTAGTGGTTATCACGTTC	No	Yes	Yes	No	Yes	No	No	No	No	No	No	No	No	ValCAC,ValAAC
GTTTCCGTAGTGTAGTGGTTATCACGTTCG	No	Yes	Yes	No	Yes	No	No	No	No	No	No	No	No	ValCAC,ValAAC
GTTTCCGTAGTGTAGTGGTTATCACGTTCGC	No	Yes	Yes	Yes	Yes	No	No	No	No	No	No	No	No	ValCAC,ValAAC
GTTTCCGTAGTGTAGTGGTTATCACGTTCGCC	No	Yes	Yes	No	Yes	No	No	No	No	No	No	No	No	ValCAC,ValAAC
GTTTCCGTAGTGTAGTGGTTATCACGTTCGCCT	No	Yes	No	No	Yes	No	No	No	No	No	No	No	No	ValCAC,ValAAC
GTTTCCGTAGTGTAGTGGTTATCACGTTT	Yes	Yes	Yes	No	Yes	No	No	No	No	No	No	No	No	ValAAC
GTTTCCGTAGTGTAGTGGTTATCACGTTTGC	Yes	Yes	Yes	No	Yes	No	No	No	No	No	No	No	No	ValAAC
GTTTCCGTAGTGTAGTGGTTATCACGTTTGCC	Yes	Yes	No	No	Yes	No	No	No	No	No	No	No	No	ValAAC
GTTTCCGTAGTGTAGTGGTTATT	Yes	Yes	Yes	No	Yes	No	No	No	No	No	No	No	No	ValCAC
GTTTCCGTGGTGTAGTGG	Yes	Yes	No	No	Yes	No	No	No	No	No	No	No	No	ValTAC
GTTTCCGTGGTGTAGTGGTTATC	Yes	Yes	No	No	Yes	No	No	No	No	No	No	No	No	ValTAC
GTTTCCGTGGTGTAGTGGTTATCAC	Yes	Yes	No	No	Yes	No	No	No	No	No	No	No	No	ValTAC
GTTTCCGTGGTGTAGTGGTTATCACA	Yes	Yes	No	No	Yes	No	No	No	No	No	No	No	No	ValTAC
GTTTCCGTGGTGTAGTGGTTATCACATTC	Yes	Yes	No	No	Yes	No	No	No	No	No	No	No	No	ValTAC
GTTTCCGTGGTGTAGTGGTTATCACATTCGC	Yes	Yes	No	No	Yes	No	No	No	No	No	No	No	No	ValTAC
GTTTCCGTGGTGTAGTGGTTATCACATTCGCC	Yes	Yes	No	No	Yes	No	No	No	No	No	No	No	No	ValTAC
GTTTCTGTGGTGTAGTGGTTATTA	Yes	Yes	No	No	Yes	No	No	No	No	No	No	No	Yes	ValCAC
GTTTCTGTGGTGTAGTGGTTATTAT	Yes	Yes	No	No	Yes	Yes	No	No	No	No	No	No	Yes	ValCAC
GTTTGTGGTAGTTTT	No	No	Yes	No	Yes	No	Yes	No	No	No	No	Yes	No	NA
TAAAAATGCAACTTTT	No	No	No	No	Yes	No	No	No	No	No	No	Yes	No	NA
TAAACAGGAGATCCTGGGT	Yes	Yes	Yes	No	Yes	No	No	No	No	No	No	No	No	ThrAGT,ThrTGT,ThrCGT
TAAAGACTTTTTCTCTGACCA	Yes	Yes	No	No	No	No	No	Yes	No	No	No	No	No	ProTGG
TAACTTGACCGCTCTGACCA	Yes	Yes	No	No	No	No	No	Yes	No	No	No	No	No	ValTAC
TAAGCCAGGGATTGTGGGT	Yes	Yes	Yes	No	Yes	No	No	No	No	No	No	No	No	ArgCCT
TAAGCCAGGGATTGTGGGTT	Yes	Yes	Yes	No	Yes	No	No	No	No	No	No	No	No	ArgCCT
TAAGGAAGTCCTGTGCTCAGTTT	No	No	Yes	No	Yes	No	No	No	No	No	No	Yes	No	NA
TAAGGAAGTCCTGTGCTCAGTTTT	No	No	Yes	No	Yes	No	No	No	No	No	No	Yes	No	NA
TAATCATATTTACCAACCA	Yes	Yes	No	No	No	No	No	Yes	No	No	No	No	No	ArgTCG
TAATCTGAAGGTCCTGAGT	No	Yes	Yes	No	Yes	No	No	No	No	No	No	No	No	MetCAT
TAATCTGAAGGTCGTGAGT	Yes	Yes	No	No	Yes	No	No	No	No	No	No	No	No	MetCAT
TAATGGATAAGGCAC	No	No	Yes	No	Yes	No	Yes	No	No	No	No	No	No	NA
TAATGGATAAGGCACT	No	Yes	Yes	No	Yes	No	No	No	No	No	No	No	No	ArgCCT
TAATGGATAAGGCACTG	No	Yes	Yes	No	Yes	No	No	No	No	No	No	No	No	ArgCCT
TAATGGATAAGGCATCAGCCT	Yes	Yes	Yes	No	Yes	No	No	No	No	No	No	No	No	ArgCCG
TAATGGATAAGGCATT	No	Yes	No	No	Yes	No	No	No	No	No	No	No	No	ArgCCT
TAATGGATAAGGCATTG	Yes	Yes	No	No	Yes	No	No	No	No	No	No	No	No	ArgCCT
TAATGGTGGAGTTAAAGACTTTTTCTCTGACC	Yes	Yes	No	No	No	No	No	Yes	No	No	No	No	No	ProTGG
TAATGGTGGAGTTAAAGACTTTTTCTCTGACCA	Yes	Yes	No	No	No	No	No	Yes	No	No	No	No	No	ProTGG
TACGACCCCTTATTTACCCCA	Yes	Yes	No	No	No	No	No	Yes	No	No	No	No	No	HisGTG
TAGAATTCTCGCCTCC	No	Yes	Yes	No	Yes	No	No	No	No	No	No	No	Yes	GlyCCC
TAGAATTCTCGCCTCCC	No	Yes	Yes	No	Yes	No	No	No	No	No	No	No	Yes	GlyCCC
TAGAATTCTCGCCTGC	No	Yes	Yes	No	Yes	No	No	No	No	No	No	No	No	GlyGCC
TAGAATTCTCGCCTGCC	No	Yes	Yes	No	Yes	No	No	No	No	No	No	No	No	GlyGCC
TAGAATTCTCGCCTGCCAC	No	Yes	Yes	No	Yes	No	No	No	No	No	No	No	No	GlyGCC
TAGAATTCTCGCCTGCCACG	Yes	Yes	Yes	No	Yes	No	No	No	No	No	No	No	No	GlyGCC
TAGAATTCTCGCCTGCCACGC	Yes	Yes	Yes	No	Yes	No	No	No	No	No	No	No	No	GlyGCC
TAGAATTCTCGCCTGCCACGCGGGAGGCCC	Yes	Yes	Yes	No	Yes	No	No	No	No	No	No	No	No	GlyGCC
TAGACGGGCTCACATCACCCCATAAACACCA	Yes	Yes	No	No	No	No	No	Yes	No	No	No	No	No	PheGAA
TAGAGCATGAGACTC	No	No	Yes	No	Yes	No	Yes	No	No	No	No	No	No	NA
TAGAGCATGGGACTC	No	No	Yes	No	Yes	No	Yes	No	No	No	No	No	No	NA
TAGAGCGGAGGACTGTA	No	Yes	Yes	No	Yes	No	No	No	No	No	No	No	No	TyrGTA
TAGATTGAAGCCAGTTGATTAGGGTGCTTAGC	Yes	Yes	No	No	No	No	No	Yes	Yes	No	No	No	No	AsnGTT
TAGCCGTAAGGCGGCTG	No	No	Yes	No	Yes	No	No	No	No	No	No	Yes	No	NA
TAGCCGTAAGGCGGCTGT	No	No	Yes	No	Yes	No	No	No	No	No	No	Yes	No	NA
TAGCCGTAAGGCGGCTGTT	No	No	Yes	No	Yes	No	No	No	No	No	No	Yes	No	NA
TAGCCGTAAGGCGGCTGTTT	No	No	Yes	No	Yes	No	No	No	No	No	No	Yes	No	NA
TAGCCGTAAGGCGGCTGTTTT	No	No	Yes	No	Yes	No	No	No	No	No	No	Yes	No	NA
TAGCCGTAAGGCGGCTGTTTTT	No	No	Yes	No	Yes	No	No	No	No	No	No	Yes	No	NA
TAGCCGTAAGGCGGCTGTTTTTGC	No	No	Yes	No	Yes	No	No	No	No	No	No	Yes	No	NA
TAGCCGTAAGGCGGCTGTTTTTGCTTT	No	No	Yes	No	Yes	No	No	No	No	No	No	Yes	No	NA
TAGCGGTTAGGATTCCT	No	Yes	Yes	No	Yes	No	No	No	No	No	No	No	No	GluTTC
TAGCTCAGGGGTAGAG	No	Yes	Yes	No	Yes	No	No	No	No	No	No	No	No	CysGCA
TAGCTCAGTCGGTAGAG	No	Yes	Yes	No	Yes	No	No	No	No	No	No	No	Yes	LysCTT,LysTTT
TAGCTCAGTCGGTAGAGC	No	Yes	Yes	No	Yes	No	No	No	No	No	No	No	Yes	LysCTT,LysTTT
TAGCTCAGTCGGTAGAGCA	No	Yes	Yes	No	Yes	No	No	No	No	No	No	No	No	LysCTT,LysTTT
TAGCTCAGTCGGTAGAGCATCAGA	Yes	Yes	Yes	No	Yes	No	No	No	No	No	No	No	No	LysTTT
TAGCTCAGTCGGTAGAGCATGAGAC	No	Yes	Yes	No	Yes	No	No	No	No	No	No	No	No	LysCTT
TAGCTCAGTGGTAGAGC	No	Yes	Yes	No	Yes	No	No	No	No	No	No	No	Yes	AlaTGC,ValAAC,TyrGTA,AlaCGC,CysACA,CysGCA,AlaAGC
TAGCTGCCTTCCAAGCAG	Yes	Yes	Yes	No	Yes	No	No	No	No	No	No	No	No	GlyTCC
TAGGATGGGGTGTGATAG	Yes	Yes	No	No	No	No	No	Yes	Yes	No	No	No	No	GlnTTG

TAGGATGGGGTGTGATAGG	Yes	Yes	No	No	No	No	No	Yes	Yes	No	No	No	No	GlnTTG
TAGGATGGGGTGTGATAGGT	Yes	Yes	No	No	No	No	No	Yes	Yes	No	No	No	No	GlnTTG
TAGGATGGGGTGTGATAGGTG	Yes	Yes	No	No	No	No	No	Yes	Yes	No	No	No	No	GlnTTG
TAGGATGGGGTGTGATAGGTGG	Yes	Yes	No	No	No	No	No	Yes	Yes	No	No	No	No	GlnTTG
TAGGATGGGGTGTGATAGGTGGC	Yes	Yes	No	No	No	No	No	Yes	Yes	No	No	No	No	GlnTTG
TAGGATGGGGTGTGATAGGTGGCA	Yes	Yes	No	No	No	No	No	Yes	Yes	No	No	No	No	GlnTTG
TAGGATGGGGTGTGATAGGTGGCAC	Yes	Yes	No	No	No	No	No	Yes	Yes	No	No	No	No	GlnTTG
TAGGATGGGGTGTGATAGGTGGCACG	Yes	Yes	No	No	No	No	No	Yes	Yes	No	No	No	No	GlnTTG
TAGGATGGGGTGTGATAGGTGGCACGG	Yes	Yes	No	No	No	No	No	Yes	Yes	No	No	No	No	GlnTTG
TAGGATGGGGTGTGATAGGTGGCACGGA	Yes	Yes	No	No	No	No	No	Yes	Yes	No	No	No	No	GlnTTG
TAGGATGGGGTGTGATAGGTGGCACGGAG	Yes	Yes	No	No	No	No	No	Yes	Yes	No	No	No	No	GlnTTG
TAGGATGGGGTGTGATAGGTGGCACGGAGA	Yes	Yes	No	No	No	No	No	Yes	Yes	No	No	No	No	GlnTTG
TAGGATGGGGTGTGATAGGTGGCACGGAGAA	Yes	Yes	No	No	No	No	No	Yes	Yes	No	No	No	No	GlnTTG
TAGGATGGGGTGTGATAGGTGGCACGGAGAAT	Yes	Yes	No	No	No	No	No	Yes	Yes	No	No	No	No	GlnTTG
TAGGGGTATGATTCTC	No	Yes	Yes	No	Yes	No	No	No	No	No	No	No	No	ProAGG,ProCGG,ProTGG
TAGGGGTATGATTCTCGC	No	Yes	Yes	No	Yes	No	No	No	No	No	No	No	No	ProAGG,ProCGG,ProTGG
TAGGGGTATGATTCTCGCT	No	Yes	Yes	No	Yes	No	No	No	No	No	No	No	No	ProAGG,ProCGG,ProTGG
TAGGGGTATGATTCTCGGT	Yes	Yes	Yes	No	Yes	No	No	No	No	No	No	No	No	ProTGG
TAGGGTGTGCGTGTTTT	No	No	Yes	No	Yes	No	No	No	No	No	No	Yes	No	NA
TAGGGTGTGCGTGTTTTT	No	No	Yes	Yes	Yes	No	No	No	No	No	No	Yes	No	NA
TAGTCCGTGCGAGAATACCA	Yes	Yes	No	No	No	No	No	Yes	Yes	No	No	No	No	GluTTC
TAGTGGTATGATTCTCGC	Yes	Yes	Yes	No	Yes	No	No	No	No	No	No	No	No	ProTGG
TAGTGGTATGATTCTCGCT	Yes	Yes	Yes	No	Yes	No	No	No	No	No	No	No	No	ProTGG
TAGTGGTGAGCATAGCT	No	Yes	Yes	No	Yes	No	No	No	No	No	No	No	Yes	GlyTCC
TAGTGGTGAGTATCC	No	No	Yes	No	Yes	No	Yes	No	No	No	No	No	No	NA
TAGTGGTGAGTATCCCC	No	Yes	Yes	No	Yes	No	No	No	No	No	No	No	No	AspGTC
TAGTGGTGAGTATCCCCG	No	Yes	Yes	No	Yes	No	No	No	No	No	No	No	No	AspGTC
TAGTGGTGAGTATCCCCGC	No	Yes	Yes	No	Yes	No	No	No	No	No	No	No	No	AspGTC
TAGTGGTGAGTATCCCCGCC	Yes	Yes	Yes	No	Yes	No	No	No	No	No	No	No	No	AspGTC
TAGTGGTGAGTATCCCCGCCTGTCACGCGGGAG	Yes	Yes	Yes	No	Yes	No	No	No	No	No	No	No	No	AspGTC
TAGTGGTTAGCATAGCT	No	Yes	Yes	No	Yes	No	No	No	No	No	No	No	No	GlyTCC
TAGTGGTTAGGATTC	No	No	Yes	No	Yes	No	Yes	No	No	No	No	No	No	NA
TAGTGGTTAGGATTCGG	No	Yes	Yes	No	Yes	No	No	No	No	No	No	No	Yes	GluCTC
TAGTGGTTAGGATTCGGC	No	Yes	Yes	No	Yes	No	No	No	No	No	No	No	Yes	GluCTC
TAGTGGTTAGGATTCGGCG	Yes	Yes	Yes	No	Yes	No	No	No	No	No	No	No	Yes	GluCTC
TAGTGGTTAGGATTCGGCGC	Yes	Yes	Yes	No	Yes	No	No	No	No	No	No	No	Yes	GluCTC
TAGTGGTTAGGATTCGGCGCT	Yes	Yes	Yes	No	Yes	No	No	No	No	No	No	No	Yes	GluCTC
TAGTGGTTAGGATTCGGCGCTC	Yes	Yes	Yes	No	Yes	No	No	No	No	No	No	No	Yes	GluCTC
TAGTGGTTAGGATTCGGCGCTCT	Yes	Yes	Yes	No	Yes	No	No	No	No	No	No	No	Yes	GluCTC
TAGTGGTTAGGATTCGGCGCTCTCA	Yes	Yes	Yes	No	Yes	No	No	No	No	No	No	No	Yes	GluCTC
TAGTGGTTAGGATTCGGCGCTCTCAC	Yes	Yes	Yes	No	Yes	No	No	No	No	No	No	No	No	GluCTC
TAGTGGTTAGGATTCGGCGCTCTCACC	Yes	Yes	Yes	No	Yes	No	No	No	No	No	No	No	No	GluCTC
TAGTGGTTAGGATTCGGCGCTCTCACCGCC	Yes	Yes	Yes	No	Yes	No	No	No	No	No	No	No	No	GluCTC
TAGTGGTTAGGATTCGGCGCTCTCACCGCCGC	Yes	Yes	Yes	No	Yes	No	No	No	No	No	No	No	No	GluCTC
TAGTGGTTAGTACTCTGC	No	Yes	Yes	No	Yes	No	No	No	No	No	No	No	No	HisGTG
TAGTGGTTAGTACTCTGCG	Yes	Yes	Yes	No	Yes	No	No	No	No	No	No	No	No	HisGTG
TAGTGGTTAGTACTCTGCGTT	Yes	Yes	Yes	No	Yes	No	No	No	No	No	No	No	No	HisGTG
TAGTGGTTAGTATCCCCGC	Yes	Yes	Yes	No	Yes	No	No	No	No	No	No	No	No	AspGTC
TAGTGGTTATCACGTTC	No	Yes	Yes	No	Yes	No	No	No	No	No	No	No	No	ValCAC,ValAAC
TAGTGTAGTGGTTAT	No	No	Yes	No	Yes	No	Yes	No	No	No	No	No	No	NA
TAGTGTAGTGGTTATC	No	Yes	Yes	No	Yes	No	No	No	No	No	No	No	No	ValCAC,ValAAC,ValTAC
TAGTGTAGTGGTTATCACGTCTGC	Yes	Yes	Yes	No	Yes	No	No	No	No	No	No	No	No	ValTAC
TAGTGTAGTGGTTATCACGTTCGC	No	Yes	Yes	No	Yes	No	No	No	No	No	No	No	No	ValCAC,ValAAC
TATACCCTTCCCGTACTACCA	Yes	Yes	No	No	No	No	No	Yes	No	No	No	No	No	MetCAT
TATAGTGGTGAGTATCCCCGC	No	Yes	Yes	No	Yes	No	No	No	No	No	No	No	No	AspGTC
TATAGTGGTGAGTATCCCCGCC	Yes	Yes	Yes	No	Yes	No	No	No	No	No	No	No	No	AspGTC
TATAGTTTAAACAAAACGAATGA	Yes	Yes	No	No	No	No	No	Yes	No	No	No	No	No	ArgTCG
TATATAGTTTAAACAAAACGA	Yes	Yes	No	No	No	No	No	Yes	No	No	No	No	No	ArgTCG
TATATAGTTTAAACAAAACGAATGA	Yes	Yes	No	No	No	No	No	Yes	No	No	No	No	No	ArgTCG
TATGGCCGCATATATTT	No	No	Yes	No	Yes	No	No	No	No	No	No	Yes	No	NA
TATGGCCGCATATATTTT	No	No	Yes	No	Yes	No	No	No	No	No	No	Yes	No	NA
TATGGTCTAGCGGTTAGG	Yes	Yes	Yes	No	Yes	No	No	No	No	No	No	No	No	GluTTC
TATGGTCTAGCGGTTAGGA	Yes	Yes	Yes	No	Yes	No	No	No	No	No	No	No	No	GluTTC
TATGGTCTAGCGGTTAGGATTCCTGG	Yes	Yes	Yes	No	Yes	No	No	No	No	No	No	No	No	GluTTC
TCAAAACCAGGCGGAAACACCA	Yes	Yes	Yes	No	Yes	No	No	No	No	No	No	No	No	ValAAC
TCAAATCACGTCGGGGTCACCA	Yes	Yes	No	No	Yes	No	No	No	No	No	No	No	No	TrpCCA
TCAAATCCCGGACGAGCCCCCA	Yes	Yes	Yes	Yes	Yes	No	No	No	No	No	No	No	No	ProAGG,ProCGG,ProTGG
TCAAATCCGGGTGCCCCCTCCA	Yes	Yes	Yes	Yes	Yes	No	No	No	No	No	No	No	No	CysGCA
TCAAATCTCGCTGGGGCCTCCA	Yes	Yes	Yes	No	Yes	No	No	No	No	No	No	No	No	ThrTGT
TCAAATCTCGGTGGAACCTCCA	Yes	Yes	Yes	No	Yes	No	No	No	No	No	No	No	No	GlnCTG
TCAAATCTCGGTGGGACCTCCA	Yes	Yes	Yes	Yes	Yes	No	No	No	No	No	No	No	No	GlnTTG
TCAACTTAACTTGACCGCTCTGACCA	Yes	Yes	No	No	No	No	No	Yes	No	No	No	No	No	ValTAC
TCAAGAGGTCCCCGGT	No	Yes	Yes	No	Yes	No	No	No	No	No	No	No	Yes	CysGCA
TCAAGAGGTCCCTGGT	No	Yes	Yes	No	Yes	No	No	No	No	No	No	No	No	CysGCA,SerAGA
TCAAGTCCCTGTTCGGGCGCCA	Yes	Yes	Yes	Yes	Yes	No	No	No	No	No	No	No	No	LysTTT
TCAAGTCTCGGTGGAACCTCCA	Yes	Yes	Yes	No	Yes	No	No	No	No	No	No	No	No	GlnCTG
TCAATCCCCAGCACCTCCACCA	Yes	Yes	Yes	No	Yes	No	No	No	No	No	No	No	No	AlaAGC
TCAATCCCCGGCACCTCCACCA	Yes	Yes	Yes	No	Yes	No	No	No	No	No	No	No	No	AlaAGC
TCAATCCCCGGCATCTCCACCA	Yes	Yes	Yes	No	Yes	No	No	No	No	No	No	No	No	AlaTGC,AlaCGC
TCAATCCCGGGTTTCGGCACCA	Yes	Yes	Yes	No	Yes	No	No	No	No	No	No	No	No	PheGAA
TCAATTCCCCGACGGGGAGCCA	Yes	Yes	Yes	No	Yes	No	No	No	No	No	No	No	No	AspGTC
TCAATTCCCGGCCAATGCACCA	Yes	Yes	Yes	No	Yes	No	No	No	No	No	No	No	No	GlyCCC
TCACAAGAACTGCTAACTC	Yes	Yes	No	No	No	No	No	Yes	No	No	No	No	No	SerGCT
TCACACGCGAAAGGTCCCCGGT	Yes	Yes	Yes	No	Yes	No	No	No	No	No	No	No	No	ValCAC
TCACCTGGAGCATGTTTTCT	No	No	No	No	Yes	No	No	No	No	No	No	Yes	No	NA
TCACCTGGAGCATGTTTTCTT	No	No	No	No	Yes	No	No	No	No	No	No	Yes	No	NA
TCACGCGGGAGACCGG	Yes	Yes	Yes	No	Yes	No	No	No	No	No	No	No	No	AspGTC
TCACGTCGGGGTCACCA	No	Yes	No	No	Yes	No	No	No	No	No	No	No	No	TrpCCA

TCAGCCTCCGGAGCTGGGGA	Yes	Yes	Yes	No	Yes	No	No	No	No	No	No	No	No	ArgCCG
TCAGCCTCCGGAGCTGGGGATTGTGGGT	Yes	Yes	Yes	No	Yes	No	No	No	No	No	No	No	No	ArgCCG
TCAGTCGGTAGAGCA	No	No	Yes	No	Yes	No	Yes	No	No	No	No	No	No	NA
TCAGTCGGTAGAGCAT	No	Yes	Yes	No	Yes	No	No	No	No	No	No	No	No	LysCTT,LysTTT
TCAGTCGGTAGAGCATC	Yes	Yes	Yes	No	Yes	No	No	No	No	No	No	No	No	LysTTT
TCAGTCGGTAGAGCATCA	Yes	Yes	Yes	No	Yes	No	No	No	No	No	No	No	No	LysTTT
TCAGTCGGTAGAGCATCAG	Yes	Yes	Yes	No	Yes	No	No	No	No	No	No	No	No	LysTTT
TCAGTCGGTAGAGCATCAGA	Yes	Yes	Yes	No	Yes	No	No	No	No	No	No	No	No	LysTTT
TCAGTCGGTAGAGCATCAGAC	Yes	Yes	Yes	No	Yes	No	No	No	No	No	No	No	No	LysTTT
TCAGTCGGTAGAGCATGGG	Yes	Yes	Yes	No	Yes	No	No	No	No	No	No	No	No	LysCTT
TCAGTCGGTAGAGCATGGGA	Yes	Yes	Yes	No	Yes	No	No	No	No	No	No	No	No	LysCTT
TCAGTGGTAGAATTCTCGCCTGCC	No	Yes	Yes	No	Yes	No	No	No	No	No	No	No	No	GlyGCC
TCAGTGGTAGAGCAT	No	No	Yes	No	Yes	No	Yes	No	No	No	No	No	No	NA
TCAGTGGTAGAGCGCAT	Yes	Yes	Yes	No	Yes	No	No	No	No	No	No	No	No	AlaTGC,AlaCGC,AlaAGC
TCATAATCTGAAGGTCGTGAGT	Yes	Yes	No	No	Yes	No	No	No	No	No	No	No	No	MetCAT
TCCAATGGAGGCGTGGGT	Yes	Yes	Yes	No	Yes	No	No	No	No	No	No	No	No	LeuCAA
TCCAGCGATCCGAGT	No	No	Yes	No	Yes	No	Yes	No	No	No	No	No	No	NA
TCCAGCGATCCGAGTT	Yes	Yes	Yes	No	Yes	No	No	No	No	No	No	No	No	GlnCTG,GlnTTG
TCCAGCGATCCGAGTTC	Yes	Yes	Yes	No	Yes	No	No	No	No	No	No	No	No	GlnCTG,GlnTTG
TCCAGCTCGAAGGACCA	Yes	Yes	Yes	No	Yes	No	No	No	No	No	No	No	No	TyrGTA
TCCAGGGTTCAAGTCCCTGTTCGGGCGCCA	Yes	Yes	Yes	No	Yes	No	No	No	No	No	No	No	No	LysTTT
TCCAGGTGCCCCCTCCA	No	Yes	Yes	No	Yes	No	No	No	No	No	No	No	No	CysGCA,SerAGA
TCCAGGTGCCCCTTCCA	Yes	Yes	No	No	Yes	No	No	No	No	No	No	No	No	CysGCA
TCCATTGGGGTTTCCCCGCGCAGGT	Yes	Yes	Yes	No	Yes	No	No	No	No	No	No	No	No	SerTGA,SerAGA
TCCCACATGGTCTAG	No	No	Yes	No	Yes	No	Yes	No	No	No	No	No	No	NA
TCCCACATGGTCTAGC	No	Yes	Yes	No	Yes	No	No	No	No	No	No	No	No	GluTTC
TCCCACATGGTCTAGCGG	Yes	Yes	Yes	Yes	Yes	No	No	No	No	No	No	No	No	GluTTC
TCCCACATGGTCTAGCGGT	Yes	Yes	Yes	No	Yes	No	No	No	No	No	No	No	No	GluTTC
TCCCACATGGTCTAGCGGTTAG	Yes	Yes	Yes	No	Yes	No	No	No	No	No	No	No	No	GluTTC
TCCCACATGGTCTAGCGGTTAGG	Yes	Yes	Yes	Yes	Yes	No	No	No	No	No	No	No	No	GluTTC
TCCCACATGGTCTAGCGGTTAGGA	Yes	Yes	Yes	No	Yes	No	No	No	No	No	No	No	No	GluTTC
TCCCACATGGTCTAGCGGTTAGGAT	Yes	Yes	Yes	No	Yes	No	No	No	No	No	No	No	No	GluTTC
TCCCACATGGTCTAGCGGTTAGGATT	Yes	Yes	Yes	No	Yes	No	No	No	No	No	No	No	No	GluTTC
TCCCACATGGTCTAGCGGTTAGGATTC	Yes	Yes	Yes	No	Yes	No	No	No	No	No	No	No	No	GluTTC
TCCCACATGGTCTAGCGGTTAGGATTCC	Yes	Yes	Yes	No	Yes	No	No	No	No	No	No	No	No	GluTTC
TCCCACATGGTCTAGCGGTTAGGATTCCT	Yes	Yes	Yes	No	Yes	No	No	No	No	No	No	No	No	GluTTC
TCCCACATGGTCTAGCGGTTAGGATTCCTG	Yes	Yes	Yes	No	Yes	No	No	No	No	No	No	No	No	GluTTC
TCCCACATGGTCTAGCGGTTAGGATTCCTGG	Yes	Yes	Yes	Yes	Yes	No	No	No	No	No	No	No	No	GluTTC
TCCCACATGGTCTAGCGGTTAGGATTCCTGGT	Yes	Yes	Yes	No	Yes	No	No	No	No	No	No	No	No	GluTTC
TCCCACATGGTCTAGCGGTTAGGATTCCTGGTT	Yes	Yes	Yes	No	Yes	No	No	No	No	No	No	No	No	GluTTC
TCCCACCACTGCCACC	No	Yes	Yes	No	Yes	No	No	No	No	No	No	No	No	LeuTAG
TCCCACCACTGCCACCA	No	Yes	Yes	No	Yes	No	No	No	No	No	No	No	No	LeuTAG
TCCCACCAGAGTCGCCA	Yes	Yes	No	No	Yes	No	No	No	No	No	No	No	No	ArgTCT
TCCCACCCAGGGACGCCA	Yes	Yes	Yes	No	Yes	No	No	No	No	No	No	No	No	AsnGTT
TCCCACCCTCGTCGCCA	Yes	Yes	No	No	Yes	No	No	No	No	No	No	No	No	SerGCT
TCCCACCGCTGCCACC	No	Yes	Yes	No	Yes	No	No	No	No	No	No	No	No	LeuTAG,LeuAAG
TCCCACCGCTGCCACCA	Yes	Yes	Yes	No	Yes	No	No	No	No	No	No	No	No	LeuTAG,LeuAAG
TCCCACCGCTGCCACCAA	No	No	Yes	No	No	No	No	No	No	No	No	No	No	NA
TCCCACTCCTGACACC	No	Yes	Yes	No	Yes	No	No	No	No	No	No	No	No	LeuCAG
TCCCACTCCTGACACCA	No	Yes	Yes	No	Yes	No	No	No	No	No	No	No	No	LeuCAG
TCCCACTTCTGACACCA	No	Yes	Yes	No	Yes	No	No	No	No	No	No	No	No	LeuCAA,LeuCAG
TCCCAGCGGGGCCTCCA	No	Yes	Yes	No	Yes	No	No	No	No	No	No	No	No	ThrAGT,ThrCGT
TCCCAGCGGTGCCTCCA	No	Yes	Yes	No	Yes	No	No	No	No	No	No	No	No	ThrAGT
TCCCATATGGTCTAGC	No	Yes	Yes	No	Yes	No	No	No	No	No	No	No	No	GluTTC
TCCCATATGGTCTAGCG	Yes	Yes	Yes	No	Yes	No	No	No	No	No	No	No	No	GluTTC
TCCCATATGGTCTAGCGG	Yes	Yes	Yes	Yes	Yes	No	No	No	No	No	No	No	No	GluTTC
TCCCATATGGTCTAGCGGTTAGG	Yes	Yes	Yes	Yes	Yes	No	No	No	No	No	No	No	No	GluTTC
TCCCATATGGTCTAGCGGTTAGGA	Yes	Yes	Yes	No	Yes	No	No	No	No	No	No	No	No	GluTTC
TCCCATATGGTCTAGCGGTTAGGAT	Yes	Yes	Yes	No	Yes	No	No	No	No	No	No	No	No	GluTTC
TCCCATATGGTCTAGCGGTTAGGATT	Yes	Yes	Yes	No	Yes	No	No	No	No	No	No	No	No	GluTTC
TCCCATATGGTCTAGCGGTTAGGATTCC	Yes	Yes	Yes	No	Yes	No	No	No	No	No	No	No	No	GluTTC
TCCCATATGGTCTAGCGGTTAGGATTCCT	Yes	Yes	Yes	No	Yes	No	No	No	No	No	No	No	No	GluTTC
TCCCATATGGTCTAGCGGTTAGGATTCCTG	Yes	Yes	Yes	No	Yes	No	No	No	No	No	No	No	No	GluTTC
TCCCATATGGTCTAGCGGTTAGGATTCCTGG	Yes	Yes	Yes	No	Yes	No	No	No	No	No	No	No	No	GluTTC
TCCCATATGGTCTAGCGGTTAGGATTCCTGGT	Yes	Yes	Yes	No	Yes	No	No	No	No	No	No	No	No	GluTTC
TCCCATATGGTCTAGCGGTTAGGATTCCTGGTT	Yes	Yes	Yes	No	Yes	No	No	No	No	No	No	No	No	GluTTC
TCCCATCCTCGTCGCCA	Yes	Yes	Yes	No	Yes	No	No	No	No	No	No	No	No	SerGCT
TCCCATTGGTCTAGCCA	Yes	Yes	No	No	No	No	No	Yes	Yes	No	No	No	No	AsnGTT
TCCCCAGCACCTCCACCA	No	Yes	Yes	No	Yes	No	No	No	No	No	No	No	No	AlaTGC,AlaAGC
TCCCCAGCATCTCCACCA	No	Yes	No	No	Yes	No	No	No	No	No	No	No	No	AlaAGC
TCCCCAGTACCTCCA	No	No	Yes	No	Yes	No	Yes	No	No	No	No	No	No	NA
TCCCCAGTACCTCCAC	No	Yes	Yes	No	Yes	No	No	No	No	No	No	No	No	AlaAGC
TCCCCAGTACCTCCACC	Yes	Yes	Yes	No	Yes	No	No	No	No	No	No	No	No	AlaAGC
TCCCCAGTACCTCCACCA	Yes	Yes	Yes	Yes	Yes	No	No	No	No	No	No	No	No	AlaAGC
TCCCCGACACCTCCACCA	Yes	Yes	Yes	No	Yes	No	No	No	No	No	No	No	No	AlaTGC
TCCCCGACGGGGAGCCAAA	No	No	Yes	No	No	No	No	No	No	No	No	No	No	NA
TCCCCGACGGGGAGCCAGA	No	No	Yes	No	No	No	No	No	No	No	No	No	No	NA
TCCCCGGCACCTCCA	No	No	Yes	No	Yes	No	Yes	No	No	No	No	No	No	NA
TCCCCGGCACCTCCAA	No	No	No	No	Yes	No	No	No	No	No	No	No	No	NA
TCCCCGGCACCTCCAAA	No	No	No	No	Yes	No	No	No	No	No	No	No	No	NA
TCCCCGGCACCTCCAC	Yes	Yes	Yes	No	Yes	No	No	No	No	No	No	No	No	AlaTGC,AlaAGC
TCCCCGGCACCTCCACC	Yes	Yes	Yes	No	Yes	No	No	No	No	No	No	No	No	AlaTGC,AlaAGC
TCCCCGGCACCTCCACCA	Yes	Yes	Yes	Yes	Yes	No	No	No	No	No	No	No	No	AlaTGC,AlaAGC
TCCCCGGCACCTCCACCAA	No	No	Yes	No	No	No	No	No	No	No	No	No	No	NA
TCCCCGGCACCTCCACCAAA	No	No	Yes	No	No	No	No	No	No	No	No	No	No	NA
TCCCCGGCACCTCCACCAT	No	No	Yes	No	No	No	No	No	No	No	No	No	No	NA
TCCCCGGCACCTCCACCATT	No	No	Yes	No	No	No	No	No	No	No	No	No	No	NA
TCCCCGGCATCTCCA	No	No	Yes	No	Yes	No	Yes	No	No	No	No	No	No	NA

TCCCCGGCATCTCCAC	No	Yes	Yes	No	Yes	No	No	No	No	No	No	No	No	AlaTGC,AlaCGC
TCCCCGGCATCTCCACC	Yes	Yes	Yes	No	Yes	No	No	No	No	No	No	No	No	AlaTGC,AlaCGC
TCCCCGGCATCTCCACCA	Yes	Yes	Yes	Yes	Yes	No	No	No	No	No	No	No	No	AlaTGC,AlaCGC
TCCCCGGCATCTCCACCAA	No	No	Yes	No	No	No	No	No	No	No	No	No	No	NA
TCCCCGGCATCTCCACCAT	No	No	Yes	No	No	No	No	No	No	No	No	No	No	NA
TCCCCGGTTCGAAACCGGGCGGAAACACCA	Yes	Yes	Yes	No	Yes	No	No	No	No	No	No	No	No	ValCAC,ValAAC
TCCCCGTACGGGCCACC	No	Yes	Yes	No	Yes	No	No	No	No	No	No	No	No	IleAAT
TCCCCGTACGGGCCACCA	No	Yes	Yes	Yes	Yes	No	No	No	No	No	No	No	No	IleAAT
TCCCCGTACTGGCCACCA	No	Yes	Yes	No	Yes	No	No	No	No	No	No	No	No	IleAAT
TCCCGGACGAGCCCCCA	No	Yes	Yes	No	Yes	No	No	No	No	No	No	No	No	ProAGG,ProCGG,ProTGG
TCCCGGCAGAGATGCCA	Yes	Yes	No	No	Yes	No	No	No	No	No	No	No	No	ArgTCT
TCCCGGCCAACGCACC	Yes	Yes	Yes	No	Yes	No	No	No	No	No	No	No	No	GlyTCC
TCCCGGCCAACGCACCA	Yes	Yes	Yes	No	Yes	No	No	No	No	No	No	No	No	GlyTCC
TCCCGGCCAATGCACCA	No	Yes	Yes	Yes	Yes	No	No	No	No	No	No	No	No	GlyCCC,GlyGCC
TCCCGGCCCATGCACCA	No	Yes	Yes	Yes	Yes	No	No	No	No	No	No	No	No	GlyGCC
TCCCGGCGGAGTCGCCA	Yes	Yes	No	No	Yes	No	No	No	No	No	No	No	No	ArgTCT
TCCCGGGCGGAAACACCA	Yes	Yes	No	No	Yes	No	No	No	No	No	No	No	No	ValCAC
TCCCGGGCGGCGCACCA	Yes	Yes	Yes	No	Yes	No	No	No	No	No	No	No	No	GlyCCC
TCCCGGGTTTCGGCA	No	No	Yes	No	Yes	No	Yes	No	No	No	No	No	No	NA
TCCCGGGTTTCGGCAC	No	Yes	Yes	No	Yes	No	No	No	No	No	No	No	No	PheGAA
TCCCGGGTTTCGGCACC	No	Yes	Yes	No	Yes	No	No	No	No	No	No	No	No	PheGAA
TCCCGGGTTTCGGCACCA	No	Yes	Yes	No	Yes	No	No	No	No	No	No	No	No	PheGAA
TCCCGGGTTTCGGCACCAT	No	No	Yes	No	No	No	No	No	No	No	No	No	No	NA
TCCCGGTCAGGGAACCA	No	Yes	Yes	No	Yes	No	No	No	No	No	No	Yes	Yes	GluCTC,PheGAA,GluTTC
TCCCGGTGTGGGAACCA	Yes	Yes	Yes	No	Yes	No	No	No	No	No	No	No	No	GluTTC
TCCCTCCGTGGTTACCA	No	Yes	No	No	Yes	No	No	No	No	No	No	No	No	ArgTCG
TCCCTGGTCTAGTGGTTAGG	Yes	Yes	No	No	Yes	No	No	No	No	No	No	No	Yes	GluTTC
TCCCTGGTCTAGTGGTTAGGA	Yes	Yes	No	No	Yes	No	No	No	No	No	No	No	Yes	GluTTC
TCCCTGGTGGTCTAG	No	No	Yes	No	Yes	No	Yes	No	No	No	No	No	No	NA
TCCCTGGTGGTCTAGT	No	Yes	Yes	No	Yes	No	No	No	No	No	No	No	No	GluCTC,GluTTC
TCCCTGGTGGTCTAGTG	No	Yes	Yes	No	Yes	No	No	No	No	No	No	No	No	GluCTC,GluTTC
TCCCTGGTGGTCTAGTGG	No	Yes	Yes	No	Yes	No	No	No	No	No	No	No	No	GluCTC,GluTTC
TCCCTGGTGGTCTAGTGGCTAGG	No	Yes	Yes	No	Yes	No	No	No	No	No	No	No	No	GluTTC
TCCCTGGTGGTCTAGTGGCTAGGA	No	Yes	Yes	No	Yes	No	No	No	No	No	No	No	No	GluTTC
TCCCTGGTGGTCTAGTGGCTAGGAT	No	Yes	Yes	No	Yes	No	No	No	No	No	No	No	No	GluTTC
TCCCTGGTGGTCTAGTGGCTAGGATTCG	No	Yes	Yes	No	Yes	No	No	No	No	No	No	No	No	GluTTC
TCCCTGGTGGTCTAGTGGCTAGGATTCGG	No	Yes	Yes	No	Yes	No	No	No	No	No	No	No	No	GluTTC
TCCCTGGTGGTCTAGTGGCTAGGATTCGGC	No	Yes	Yes	No	Yes	No	No	No	No	No	No	No	No	GluTTC
TCCCTGGTGGTCTAGTGGCTAGGATTCGGCG	Yes	Yes	Yes	No	Yes	No	No	No	No	No	No	No	No	GluTTC
TCCCTGGTGGTCTAGTGGCTAGGATTCGGCGC	Yes	Yes	Yes	No	Yes	No	No	No	No	No	No	No	No	GluTTC
TCCCTGGTGGTCTAGTGGT	No	Yes	Yes	No	Yes	No	No	No	No	No	No	No	No	GluCTC
TCCCTGGTGGTCTAGTGGTTAG	No	Yes	Yes	No	Yes	No	No	No	No	No	No	No	No	GluCTC
TCCCTGGTGGTCTAGTGGTTAGG	No	Yes	Yes	Yes	Yes	No	No	No	No	No	No	No	No	GluCTC
TCCCTGGTGGTCTAGTGGTTAGGA	No	Yes	Yes	No	Yes	No	No	No	No	No	No	No	No	GluCTC
TCCCTGGTGGTCTAGTGGTTAGGAT	No	Yes	Yes	No	Yes	No	No	No	No	No	No	No	No	GluCTC
TCCCTGGTGGTCTAGTGGTTAGGATT	No	Yes	Yes	No	Yes	No	No	No	No	No	No	No	No	GluCTC
TCCCTGGTGGTCTAGTGGTTAGGATTC	No	Yes	Yes	No	Yes	No	No	No	No	No	No	No	No	GluCTC
TCCCTGGTGGTCTAGTGGTTAGGATTCG	No	Yes	Yes	No	Yes	No	No	No	No	No	No	No	No	GluCTC
TCCCTGGTGGTCTAGTGGTTAGGATTCGG	No	Yes	Yes	No	Yes	No	No	No	No	No	No	No	No	GluCTC
TCCCTGGTGGTCTAGTGGTTAGGATTCGGC	No	Yes	Yes	No	Yes	No	No	No	No	No	No	No	No	GluCTC
TCCCTGGTGGTCTAGTGGTTAGGATTCGGCG	Yes	Yes	Yes	Yes	Yes	No	No	No	No	No	No	No	No	GluCTC
TCCCTGGTGGTCTAGTGGTTAGGATTCGGCGC	Yes	Yes	Yes	No	Yes	No	No	No	No	No	No	No	No	GluCTC
TCCCTGGTGGTCTAGTGGTTAGGATTCGGCGCT	Yes	Yes	Yes	No	Yes	No	No	No	No	No	No	No	No	GluCTC
TCCCTGGTTCGATCCCGGGTTTCGGCACCA	Yes	Yes	Yes	No	Yes	No	No	No	No	No	No	No	No	PheGAA
TCCCTGTCCAGGCGCC	No	Yes	No	No	Yes	No	No	No	No	No	No	No	No	LysTTT
TCCCTGTCCAGGCGCCA	Yes	Yes	No	No	Yes	No	No	No	No	No	No	No	No	LysTTT
TCCCTGTGGTCTAGTGG	Yes	Yes	No	No	No	No	No	No	No	Yes	No	No	No	GluTTC
TCCCTGTGGTCTAGTGGTTAGG	Yes	Yes	No	No	No	No	No	No	No	Yes	No	No	No	GluTTC
TCCCTGTGGTCTAGTGGTTAGGA	Yes	Yes	No	No	No	No	No	No	No	Yes	No	No	No	GluTTC
TCCCTGTTCAGGCACC	No	Yes	No	No	Yes	No	No	No	No	No	No	No	No	LysTTT
TCCCTGTTCAGGCACCA	No	Yes	No	No	Yes	No	No	No	No	No	No	No	No	LysTTT
TCCCTGTTCAGGCGC	No	No	No	No	Yes	No	Yes	No	No	No	No	No	No	NA
TCCCTGTTCAGGCGCC	No	Yes	No	No	Yes	No	No	No	No	No	No	No	No	LysTTT
TCCCTGTTCAGGCGCCA	No	Yes	No	No	Yes	No	No	No	No	No	No	No	No	LysTTT
TCCCTGTTCGGGCGC	No	No	Yes	No	Yes	No	Yes	No	No	No	No	No	No	NA
TCCCTGTTCGGGCGCC	No	Yes	Yes	No	Yes	No	No	No	No	No	No	No	No	LysTTT
TCCCTGTTCGGGCGCCA	No	Yes	Yes	Yes	Yes	No	No	No	No	No	No	No	No	LysTTT
TCCCTGTTCGGGCGCCAG	No	No	Yes	No	No	No	No	No	No	No	No	No	No	NA
TCCCTGTTCGGGCGCCAGA	No	No	Yes	No	No	No	No	No	No	No	No	No	No	NA
TCCCTGTTCGGGCGCCAGT	No	No	Yes	No	No	No	No	No	No	No	No	No	No	NA
TCCCTGTTCGGGCGCCAT	No	No	Yes	No	No	No	No	No	No	No	No	No	No	NA
TCCCTGTTCGGGCGCCATT	No	No	Yes	No	No	No	No	No	No	No	No	No	No	NA
TCCCTTCGTGGTCGCCA	No	Yes	No	No	Yes	No	No	No	No	No	No	No	No	ArgTCG,ArgCCG
TCCGAGTCACGGCAC	No	No	Yes	No	Yes	No	Yes	No	No	No	No	No	No	NA
TCCGAGTCACGGCACC	No	Yes	Yes	No	Yes	No	No	No	No	No	No	No	No	HisGTG
TCCGAGTCACGGCACCA	No	Yes	Yes	Yes	Yes	No	No	No	No	No	No	No	No	HisGTG
TCCGAGTCACGGCACCAA	No	No	Yes	No	No	No	No	No	No	No	No	No	No	NA
TCCGAGTCACGGCACCAAA	No	No	Yes	No	No	No	No	No	No	No	No	No	No	NA
TCCGAGTCACGGCACCAAT	No	No	Yes	No	No	No	No	No	No	No	No	No	No	NA
TCCGAGTCACGGCACCAT	No	No	Yes	No	No	No	No	No	No	No	No	No	No	NA
TCCGAGTCACGGCACCATT	No	No	Yes	No	No	No	No	No	No	No	No	No	No	NA
TCCGAGTTCAAATCTCGGT	Yes	Yes	Yes	No	Yes	No	No	No	No	No	No	No	Yes	GlnCTG,GlnTTG
TCCGGAGTTACCTCGTTT	No	No	Yes	No	Yes	No	No	No	No	No	No	Yes	No	NA
TCCGGCTCGAAGGAC	No	No	Yes	No	Yes	No	Yes	No	No	No	No	No	No	NA
TCCGGCTCGAAGGACC	Yes	Yes	Yes	No	Yes	No	No	No	No	No	No	No	No	TyrGTA
TCCGGCTCGAAGGACCA	Yes	Yes	Yes	No	Yes	No	No	No	No	No	No	No	No	TyrGTA
TCCGGCTCGAAGGACCAA	No	No	Yes	No	No	No	No	No	No	No	No	No	No	NA
TCCGGCTCGAAGGACCAAG	No	No	Yes	No	No	No	No	No	No	No	No	No	No	NA

TCCGGCTCGAAGGACCAC	No	No	Yes	No	No	No	No	No	No	No	No	No	No	NA
TCCGGCTCGAAGGACCAG	No	No	Yes	No	No	No	No	No	No	No	No	No	No	NA
TCCGGCTCGAAGGACCAGA	No	No	Yes	No	No	No	No	No	No	No	No	No	No	NA
TCCGGCTCGGAGGAC	No	No	Yes	No	Yes	No	Yes	No	No	No	No	No	No	NA
TCCGGCTCGGAGGACC	Yes	Yes	Yes	No	Yes	No	No	No	No	No	No	No	No	TyrGTA
TCCGGCTCGGAGGACCA	Yes	Yes	Yes	Yes	Yes	No	No	No	No	No	No	No	No	TyrGTA
TCCGGCTCGGAGGACCAG	No	No	Yes	No	No	No	No	No	No	No	No	No	No	NA
TCCGGGTGCCCCCTC	No	No	Yes	No	Yes	No	Yes	No	No	No	No	Yes	No	NA
TCCGGGTGCCCCCTCC	Yes	Yes	Yes	No	Yes	No	No	No	No	No	No	No	No	CysGCA
TCCGGGTGCCCCCTCCA	Yes	Yes	Yes	Yes	Yes	No	No	No	No	No	No	No	No	CysGCA
TCCGGGTGCCCCCTCCAT	No	No	Yes	No	No	No	No	No	No	No	No	No	No	NA
TCCGTAGTGTAGTGGTCATC	Yes	Yes	Yes	No	Yes	No	No	No	No	No	No	No	No	ValAAC
TCCGTAGTGTAGTGGTTATC	No	Yes	Yes	No	Yes	No	No	No	No	No	No	No	No	ValCAC,ValAAC
TCCGTAGTGTAGTGGTTATCAC	No	Yes	Yes	No	Yes	No	No	No	No	No	No	No	No	ValCAC,ValAAC
TCCGTGGTGTAGTGGTTATC	Yes	Yes	No	No	Yes	No	No	No	No	No	No	No	No	ValTAC
TCCTAAGCCAGGGATTGTGGGT	Yes	Yes	Yes	No	Yes	No	No	No	No	No	No	No	No	ArgCCT
TCCTCGTTAGTATAG	No	No	Yes	No	Yes	No	Yes	No	No	No	No	No	No	NA
TCCTCGTTAGTATAGT	No	Yes	Yes	No	Yes	No	No	No	No	No	No	No	No	AspGTC
TCCTCGTTAGTATAGTG	No	Yes	Yes	No	Yes	No	No	No	No	No	No	No	No	AspGTC
TCCTCGTTAGTATAGTGG	No	Yes	Yes	Yes	Yes	No	No	No	No	No	No	No	No	AspGTC
TCCTCGTTAGTATAGTGGTGA	No	Yes	Yes	No	Yes	No	No	No	No	No	No	No	No	AspGTC
TCCTCGTTAGTATAGTGGTGAG	Yes	Yes	Yes	No	Yes	No	No	No	No	No	No	No	No	AspGTC
TCCTCGTTAGTATAGTGGTGAGT	Yes	Yes	Yes	Yes	Yes	No	No	No	No	No	No	No	No	AspGTC
TCCTCGTTAGTATAGTGGTGAGTATCC	Yes	Yes	Yes	No	Yes	No	No	No	No	No	No	No	No	AspGTC
TCCTCGTTAGTATAGTGGTGAGTATCCC	Yes	Yes	Yes	No	Yes	No	No	No	No	No	No	No	No	AspGTC
TCCTCGTTAGTATAGTGGTGAGTATCCCC	Yes	Yes	Yes	No	Yes	No	No	No	No	No	No	No	No	AspGTC
TCCTCGTTAGTATAGTGGTGAGTATCCCCG	Yes	Yes	Yes	No	Yes	No	No	No	No	No	No	No	No	AspGTC
TCCTCGTTAGTATAGTGGTGAGTATCCCCGC	Yes	Yes	Yes	No	Yes	No	No	No	No	No	No	No	No	AspGTC
TCCTCGTTAGTATAGTGGTGAGTATCCCCGCC	Yes	Yes	Yes	Yes	Yes	No	No	No	No	No	No	No	No	AspGTC
TCCTCGTTAGTATAGTGGTGAGTATCCCCGCCT	Yes	Yes	No	No	Yes	No	No	No	No	No	No	No	No	AspGTC
TCCTCGTTAGTATAGTGGTTAGT	No	Yes	Yes	No	Yes	No	No	No	No	No	No	No	No	AspGTC
TCCTCGTTAGTATAGTGGTTAGTATCCCCGC	Yes	Yes	Yes	No	Yes	No	No	No	No	No	No	No	No	AspGTC
TCCTCGTTAGTATAGTGGTTAGTATCCCCGCC	Yes	Yes	No	No	Yes	No	No	No	No	No	No	No	No	AspGTC
TCCTGAGTTCGAGCCTCAGAGAGGGCACCA	Yes	Yes	Yes	No	Yes	No	No	No	No	No	No	No	No	MetCAT
TCCTGCCGACTACGCC	Yes	Yes	Yes	No	Yes	No	No	No	No	No	No	No	No	SerTGA,SerAGA
TCCTGCCGACTACGCCA	Yes	Yes	Yes	No	Yes	No	No	No	No	No	No	No	No	SerTGA,SerAGA
TCCTGCCGCGGTCGCCA	Yes	Yes	Yes	No	Yes	No	No	No	No	No	No	No	No	ArgTCG
TCCTGCTCACAGCGCC	No	Yes	No	No	Yes	No	No	No	No	No	No	No	No	SerCGA
TCCTGCTCACAGCGCCA	No	Yes	No	No	Yes	No	No	No	No	No	No	No	No	SerCGA
TCCTGGCTGGCTCGCCA	No	Yes	Yes	No	Yes	No	No	No	No	No	No	No	No	ArgACG
TCCTGGGTTCGAATCCC	No	Yes	Yes	No	Yes	No	No	No	No	No	No	No	No	ThrAGT,ThrTGT,ThrCGT
TCCTGGGTTCGAGCCCCAGTGGAACCACCA	Yes	Yes	Yes	No	Yes	No	No	No	No	No	No	No	No	ValTAC
TCCTGTCGGCTACGCCA	Yes	Yes	Yes	No	Yes	No	No	No	No	No	No	No	No	SerTGA
TCCTGTTCGTGACGCCA	No	Yes	No	No	Yes	No	No	No	No	No	No	No	No	SerCGA
TCGAAACCATCCTCTGCTACCA	Yes	Yes	Yes	No	Yes	No	No	No	No	No	No	No	No	MetCAT
TCGAAACCGAGCGGAAACACCA	Yes	Yes	No	No	Yes	No	No	No	No	No	No	No	No	ValTAC
TCGAAACCGGGCAGAA	Yes	Yes	Yes	No	Yes	No	No	No	No	No	No	No	No	ValCAC,ValAAC
TCGAAACCGGGCAGAAGCACCA	Yes	Yes	Yes	No	Yes	No	No	No	No	No	No	No	No	ValCAC
TCGAAACCGGGCGGAAACACCA	Yes	Yes	Yes	Yes	Yes	No	No	No	No	No	No	No	No	ValCAC,ValAAC
TCGAAACTGGGCGGAAACACCA	Yes	Yes	Yes	No	Yes	No	No	No	No	No	No	No	No	ValCAC
TCGAAATCCAATGGGGTTTCCCC	Yes	Yes	No	No	Yes	No	No	No	No	No	No	No	No	SerCGA
TCGAACCCCGTACGGGCCACCA	Yes	Yes	Yes	No	Yes	No	No	No	No	No	No	No	No	IleAAT
TCGAACCCTGCTCGCTGCGCCA	Yes	Yes	Yes	No	Yes	No	No	No	No	No	No	No	No	SerTGA
TCGAACCTCAGAGGGGGCA	Yes	Yes	No	No	Yes	No	No	No	No	No	No	No	No	MetCAT
TCGAACCTCAGAGGGGGCACCA	Yes	Yes	No	No	Yes	No	No	No	No	No	No	No	No	MetCAT
TCGAATCACGTCGGGGTCACCA	Yes	Yes	No	No	Yes	No	No	No	No	No	No	No	No	TrpCCA
TCGAATCCCACCACTGCCACCA	Yes	Yes	Yes	Yes	Yes	No	No	No	No	No	No	No	No	LeuTAG
TCGAATCCCACCAGAGTCGCCA	Yes	Yes	No	No	Yes	No	No	No	No	No	No	No	No	ArgTCT
TCGAATCCCACCCTCGTCGCCA	Yes	Yes	No	No	Yes	No	No	No	No	No	No	No	No	SerGCT
TCGAATCCCACCGCTGCCACCA	Yes	Yes	Yes	No	Yes	No	No	No	No	No	No	No	No	LeuTAG,LeuAAG
TCGAATCCCACCTTCGTCGCCA	Yes	Yes	No	No	Yes	No	No	No	No	No	No	No	No	SerGCT
TCGAATCCCACTCCTGACACCA	Yes	Yes	Yes	Yes	Yes	No	No	No	No	No	No	No	No	LeuCAG
TCGAATCCCACTTCTGACACCA	Yes	Yes	Yes	Yes	Yes	No	No	No	No	No	No	No	No	LeuCAA,LeuCAG
TCGAATCCCAGCGAGGCCTCCA	Yes	Yes	No	No	Yes	No	No	No	No	No	No	No	No	ThrAGT
TCGAATCCCAGCGGGGCCTCCA	Yes	Yes	Yes	No	Yes	No	No	No	No	No	No	No	No	ThrAGT,ThrCGT
TCGAATCCCAGCGGTGCCTCCA	Yes	Yes	Yes	No	Yes	No	No	No	No	No	No	No	No	ThrAGT
TCGAATCCCAGTAGAGCCTCCA	Yes	Yes	No	No	Yes	No	No	No	No	No	No	No	No	ThrTGT
TCGAATCCCATCCTCGTCGCCA	Yes	Yes	Yes	No	Yes	No	No	No	No	No	No	No	No	SerGCT
TCGAATCCGAGTCACGG	Yes	Yes	Yes	No	Yes	No	No	No	No	No	No	No	No	HisGTG
TCGAATCCGAGTCACGGCACC	Yes	Yes	Yes	No	Yes	No	No	No	No	No	No	No	No	HisGTG
TCGAATCCGAGTCACGGCACCA	Yes	Yes	Yes	Yes	Yes	No	No	No	No	No	No	No	No	HisGTG
TCGAATCCGAGTCACGGCACCAT	No	No	Yes	No	No	No	No	No	No	No	No	No	No	NA
TCGAATCCGGCTCGAAGGACCA	Yes	Yes	Yes	No	Yes	No	No	No	No	No	No	No	No	TyrGTA
TCGAATCCGGCTCGGAGGACCA	Yes	Yes	Yes	No	Yes	No	No	No	No	No	No	No	No	TyrGTA
TCGAATCCTGCCGACTACGCCA	Yes	Yes	Yes	Yes	Yes	No	No	No	No	No	No	No	No	SerTGA,SerAGA
TCGAATCCTGCTCACAGCGCCA	Yes	Yes	No	No	Yes	No	No	No	No	No	No	No	No	SerCGA
TCGAATCCTGTCGGCTACGCCA	Yes	Yes	Yes	No	Yes	No	No	No	No	No	No	No	No	SerTGA
TCGAATCCTGTTCGTGACGCCA	No	Yes	No	No	Yes	No	No	No	No	No	No	No	No	SerCGA
TCGACTCCCGGTATGGGAACCA	Yes	Yes	Yes	No	Yes	No	No	No	No	No	No	No	No	GluTTC
TCGACTCCCGGTGTGGGAACC	Yes	Yes	Yes	No	Yes	No	No	No	No	No	No	No	No	GluTTC
TCGACTCCCGGTGTGGGAACCA	Yes	Yes	Yes	Yes	Yes	No	No	No	No	No	No	No	No	GluTTC
TCGACTCCTGGCTGGCTCGCCA	Yes	Yes	Yes	No	Yes	No	No	No	No	No	No	No	No	ArgACG
TCGAGAGGGGCTGTGCTCGCAAGG	No	No	Yes	No	Yes	No	No	No	No	No	No	Yes	No	NA
TCGAGAGGGGCTGTGCTCGCAAGGTTTCTTT	No	No	Yes	Yes	Yes	No	No	No	No	No	No	Yes	No	NA
TCGAGAGGGGCTGTGCTCGCAAGGTTTCTTTT	No	No	No	No	Yes	No	No	No	No	No	No	Yes	No	NA
TCGAGCCCACCCAGGGACGCCA	Yes	Yes	Yes	No	Yes	No	No	No	No	No	No	No	No	AsnGTT
TCGAGCCCCACGTTGGGCGCCA	No	Yes	Yes	Yes	Yes	No	No	No	No	No	No	No	No	LysCTT
TCGAGCCCCAGTGGAACCACCA	Yes	Yes	Yes	Yes	Yes	No	No	No	No	No	No	No	No	ValTAC

TCGAGCCTCACCTGGAGCACCA	Yes	Yes	No	No	Yes	No	No	No	No	No	No	No	No	IleTAT
TCGAGCCTCAGAGAGGGCACCA	Yes	Yes	Yes	Yes	Yes	No	No	No	No	No	No	No	No	MetCAT
TCGAGTCCCACCAGAGTCGCCA	Yes	Yes	No	No	Yes	No	No	No	No	No	No	No	No	ArgTCT
TCGAGTCCCATCTGGGTCGCCA	Yes	Yes	Yes	No	Yes	No	No	No	No	No	No	No	No	ArgCCG
TCGAGTCCCGGCGGAGTCGCCA	Yes	Yes	No	No	Yes	No	No	No	No	No	No	No	No	ArgTCT
TCGAGTCCCTTCGTGGTCGCCA	Yes	Yes	No	No	Yes	No	No	No	No	No	No	No	No	ArgTCG,ArgCCG
TCGATCCCCAGTACCTCCACCA	Yes	Yes	Yes	Yes	Yes	No	No	No	No	No	No	No	No	AlaAGC
TCGATCCCCGGCATCTCCACCA	Yes	Yes	Yes	Yes	Yes	No	No	No	No	No	No	No	No	AlaTGC,AlaCGC
TCGATCCCCGTACGGGCCACCA	Yes	Yes	Yes	Yes	Yes	No	No	No	No	No	No	No	No	IleAAT
TCGATCCCCGTACTGGCCACCA	Yes	Yes	Yes	No	Yes	No	No	No	No	No	No	No	No	IleAAT
TCGATCCCGGGCGGAAACACCA	Yes	Yes	No	No	Yes	No	No	No	No	No	No	No	No	ValCAC
TCGATCCCGGGTTTCGGCACCA	Yes	Yes	Yes	Yes	Yes	No	No	No	No	No	No	No	No	PheGAA
TCGATGCCCGCATCCTCCACCA	Yes	Yes	No	No	Yes	No	No	No	No	No	No	No	No	AlaAGC
TCGATGGATCGAAACCATCCTCTGCTACCA	Yes	Yes	Yes	No	Yes	No	No	No	No	No	No	No	No	MetCAT
TCGATTCCAGCTCGAAGGACCA	Yes	Yes	Yes	No	Yes	No	No	No	No	No	No	No	No	TyrGTA
TCGATTCCCCGACGGG	Yes	Yes	Yes	No	Yes	No	No	No	No	No	No	No	Yes	AspGTC
TCGATTCCCCGACGGGGAGCCA	Yes	Yes	Yes	Yes	Yes	No	No	No	No	No	No	No	Yes	AspGTC
TCGATTCCCGGCCAACGCACCA	Yes	Yes	Yes	Yes	Yes	No	No	No	No	No	No	No	No	GlyTCC
TCGATTCCCGGCCAATGCACCA	Yes	Yes	Yes	Yes	Yes	No	No	No	No	No	No	No	No	GlyGCC
TCGATTCCCGGCCCATGCACCA	Yes	Yes	Yes	Yes	Yes	No	No	No	No	No	No	No	No	GlyGCC
TCGATTCCCGGGCGGCGCACCA	Yes	Yes	Yes	No	Yes	No	No	No	No	No	No	No	No	GlyCCC
TCGATTCCCGGTCAGG	No	Yes	Yes	No	Yes	No	No	No	No	No	No	No	Yes	GluCTC,PheGAA,GluTTC
TCGATTCCCGGTCAGGGAACCA	Yes	Yes	Yes	Yes	Yes	No	No	No	No	No	No	No	Yes	GluCTC,PheGAA,GluTTC
TCGATTCCGGCTCGAAGGACCA	Yes	Yes	Yes	Yes	Yes	No	No	No	No	No	No	No	No	TyrGTA
TCGCCTGCCACGCGGGAGGCCC	Yes	Yes	Yes	No	Yes	No	No	No	No	No	No	No	No	GlyGCC
TCGCCTGCCACGCGGGAGGCCCGGG	Yes	Yes	Yes	No	Yes	No	No	No	No	No	No	No	No	GlyGCC
TCGCCTGCCACGCGGGAGGCCCGGGT	Yes	Yes	Yes	No	Yes	No	No	No	No	No	No	No	No	GlyGCC
TCGCCTGCCACGCGGGAGGCCCGGGTT	Yes	Yes	Yes	No	Yes	No	No	No	No	No	No	No	No	GlyGCC
TCGCGGGTTCGATCCCCGTACGGGCCACCA	Yes	Yes	Yes	No	Yes	No	No	No	No	No	No	No	No	IleAAT
TCGCTGGGGCCTCCA	No	No	Yes	No	Yes	No	Yes	No	No	No	No	No	No	NA
TCGCTGGTTCGAATCCGGCTCGAAGGACCA	Yes	Yes	Yes	No	Yes	No	No	No	No	No	No	No	No	TyrGTA
TCGCTGGTTCGAATCCGGCTCGGAGGACCA	Yes	Yes	Yes	Yes	Yes	No	No	No	No	No	No	No	No	TyrGTA
TCGCTGGTTCGATTCCGGCTCGAAGGACCA	Yes	Yes	Yes	No	Yes	No	No	No	No	No	No	No	No	TyrGTA
TCGGATCAGAAGATTGAGGGT	Yes	Yes	No	No	Yes	No	No	No	No	No	No	No	No	ArgTCG
TCGGCGCTCTCACCGCC	Yes	Yes	Yes	No	Yes	No	No	No	No	No	No	No	Yes	GluCTC
TCGGCTTTCCCTGCTAACTGGGCTTTT	No	No	No	No	Yes	No	No	No	No	No	No	Yes	No	NA
TCGGCTTTCCCTGCTAACTGGGCTTTTT	No	No	No	No	Yes	No	No	No	No	No	No	Yes	No	NA
TCGGCTTTCCCTGCTAACTGGGCTTTTTT	No	No	No	No	Yes	No	No	No	No	No	No	Yes	No	NA
TCGGGCGGGAGTGGTGGCTTT	No	No	No	No	Yes	No	No	No	No	No	No	Yes	No	NA
TCGGGCGGGAGTGGTGGCTTTT	No	No	No	No	Yes	No	No	No	No	No	No	Yes	No	NA
TCGGGTGCGAGAGGTCCC	Yes	Yes	Yes	No	Yes	No	No	No	No	No	No	No	No	ProCGG
TCGGGTGCGAGAGGTCCCGGG	Yes	Yes	Yes	No	Yes	No	No	No	No	No	No	No	No	ProCGG
TCGGGTGCGAGAGGTCCCGGGT	Yes	Yes	Yes	No	Yes	No	No	No	No	No	No	No	No	ProCGG
TCGGGTGCGAGAGGTCCCGGGTT	Yes	Yes	Yes	No	Yes	No	No	No	No	No	No	No	No	ProCGG
TCGGGTGTGAGAGGTCCC	Yes	Yes	Yes	No	Yes	No	No	No	No	No	No	No	No	ProCGG
TCGGGTGTGAGAGGTCCCGGGT	Yes	Yes	Yes	No	Yes	No	No	No	No	No	No	No	No	ProCGG
TCGGGTGTGAGAGGTCCCGGGTT	Yes	Yes	Yes	No	Yes	No	No	No	No	No	No	No	No	ProCGG
TCGGTAGAGCATCAG	No	No	Yes	No	Yes	No	Yes	No	No	No	No	No	No	NA
TCGGTAGAGCATCAGA	Yes	Yes	Yes	No	Yes	No	No	No	No	No	No	No	No	LysTTT
TCGGTAGAGCATCAGAC	Yes	Yes	Yes	No	Yes	No	No	No	No	No	No	No	No	LysTTT
TCGGTAGAGCATCAGACTTTT	Yes	Yes	Yes	No	Yes	No	No	No	No	No	No	No	No	LysTTT
TCGGTAGAGCATGAG	No	No	Yes	No	Yes	No	Yes	No	No	No	No	No	No	NA
TCGGTAGAGCATGAGA	No	Yes	Yes	No	Yes	No	No	No	No	No	No	No	No	LysCTT
TCGGTAGAGCATGAGAC	No	Yes	Yes	No	Yes	No	No	No	No	No	No	No	No	LysCTT
TCGGTAGAGCATGAGACT	No	Yes	Yes	No	Yes	No	No	No	No	No	No	No	No	LysCTT
TCGGTAGAGCATGAGACTC	No	Yes	Yes	No	Yes	No	No	No	No	No	No	No	No	LysCTT
TCGGTAGAGCATGAGACTCTTAATCTC	Yes	Yes	Yes	No	Yes	No	No	No	No	No	No	No	No	LysCTT
TCGGTAGAGCATGGG	No	No	Yes	No	Yes	No	Yes	No	No	No	No	No	No	NA
TCGGTAGAGCATGGGA	Yes	Yes	Yes	No	Yes	No	No	No	No	No	No	No	No	LysCTT
TCGGTAGAGCATGGGAC	Yes	Yes	Yes	No	Yes	No	No	No	No	No	No	No	No	LysCTT
TCGGTAGAGCATGGGACTC	Yes	Yes	Yes	No	Yes	No	No	No	No	No	No	No	No	LysCTT
TCGGTAGAGCATGGGACTCTTAATCCC	Yes	Yes	Yes	No	Yes	No	No	No	No	No	No	No	No	LysCTT
TCGGTGGAACCTCCA	No	No	Yes	No	Yes	No	Yes	No	No	No	No	No	No	NA
TCGGTGGGACCTCCA	No	No	Yes	No	Yes	No	Yes	No	No	No	No	No	No	NA
TCGTAAACAGGAGATCCTGGGT	Yes	Yes	Yes	No	Yes	No	No	No	No	No	No	No	No	ThrCGT
TCGTAAACCGAAGATCACGGGT	Yes	Yes	Yes	No	Yes	No	No	No	No	No	No	No	No	ThrCGT
TCGTAAACCGAAGATCGCGGGT	Yes	Yes	No	No	Yes	No	No	No	No	No	No	No	No	ThrCGT
TCGTATAGTGGTTAGT	No	Yes	Yes	No	Yes	No	No	No	No	No	No	No	No	HisGTG
TCGTATAGTGGTTAGTAC	No	Yes	Yes	No	Yes	No	No	No	No	No	No	No	No	HisGTG
TCGTATAGTGGTTAGTACT	No	Yes	Yes	No	Yes	No	No	No	No	No	No	No	No	HisGTG
TCGTATAGTGGTTAGTACTCTGC	No	Yes	Yes	No	Yes	No	No	No	No	No	No	No	No	HisGTG
TCGTATAGTGGTTAGTACTCTGCGT	Yes	Yes	Yes	No	Yes	No	No	No	No	No	No	No	No	HisGTG
TCGTGAGTTCGATCCTCACACGGGGCACCA	Yes	Yes	No	No	Yes	No	No	No	No	No	No	No	No	MetCAT
TCGTGGCTACTGTTT	No	No	Yes	No	Yes	No	Yes	No	No	No	No	Yes	No	NA
TCGTGGCTACTGTTTT	No	No	Yes	No	Yes	No	Yes	No	No	No	No	Yes	No	NA
TCGTGGGTTCGAGCCCCACGTTGGGCGCCA	Yes	Yes	Yes	No	Yes	No	No	No	No	No	No	No	No	LysCTT
TCGTGGTTGTAGTCCGTGCGAGAATACCA	Yes	Yes	No	No	No	No	No	Yes	Yes	No	No	No	No	GluTTC
TCGTTAGTATAGTGGTGAGTATCCCCGC	Yes	Yes	Yes	No	Yes	No	No	No	No	No	No	No	No	AspGTC
TCGTTGGTCTAGGGG	No	No	Yes	No	Yes	No	Yes	No	No	No	No	No	No	NA
TCGTTGGTCTAGGGGT	No	Yes	Yes	No	Yes	No	No	No	No	No	No	No	No	ProAGG,ProCGG,ProTGG
TCGTTGGTCTAGGGGTA	No	Yes	Yes	No	Yes	No	No	No	No	No	No	No	No	ProAGG,ProCGG,ProTGG
TCGTTGGTCTAGGGGTATGATTC	No	Yes	Yes	No	Yes	No	No	No	No	No	No	No	No	ProAGG,ProCGG,ProTGG
TCGTTGGTCTAGGGGTATGATTCTCGC	No	Yes	Yes	No	Yes	No	No	No	No	No	No	No	No	ProAGG,ProCGG,ProTGG
TCGTTGGTCTAGGGGTATGATTCTCGCT	No	Yes	Yes	No	Yes	No	No	No	No	No	No	No	No	ProAGG,ProCGG,ProTGG
TCTAAAGGTCCCTGGT	No	Yes	Yes	No	Yes	No	No	No	No	No	No	No	No	PheGAA
TCTAGCGGTTAGGAT	No	No	Yes	No	Yes	No	Yes	No	No	No	No	No	No	NA
TCTAGCGGTTAGGATTCCT	Yes	Yes	Yes	No	Yes	No	No	No	No	No	No	No	No	GluTTC
TCTAGCGGTTAGGATTCCTG	Yes	Yes	Yes	No	Yes	No	No	No	No	No	No	No	No	GluTTC

TCTAGCGGTTAGGATTCCTGG	Yes	Yes	Yes	No	Yes	No	No	No	No	No	No	No	No	GluTTC
TCTAGCGGTTAGGATTCCTGGT	Yes	Yes	Yes	No	Yes	No	No	No	No	No	No	No	No	GluTTC
TCTAGCGGTTAGGATTCCTGGTT	Yes	Yes	Yes	No	Yes	No	No	No	No	No	No	No	No	GluTTC
TCTAGGGGTATGATTCTCGC	No	Yes	Yes	No	Yes	No	No	No	No	No	No	No	No	ProAGG,ProCGG,ProTGG
TCTAGGGGTATGATTCTCGCT	No	Yes	Yes	No	Yes	No	No	No	No	No	No	No	No	ProAGG,ProCGG,ProTGG
TCTAGGGGTATGATTCTCGG	Yes	Yes	Yes	No	Yes	No	No	No	No	No	No	No	No	ProTGG
TCTAGGGGTATGATTCTCGGT	Yes	Yes	Yes	No	Yes	No	No	No	No	No	No	No	No	ProTGG
TCTCATAATCTGAAGGTCCTGAGT	No	Yes	Yes	No	Yes	No	No	No	No	No	No	No	No	MetCAT
TCTCCAATGGAGGCGTGGGT	Yes	Yes	Yes	No	Yes	No	No	No	No	No	No	No	No	LeuCAA
TCTCCCACATGGTCTAGCGGTTAGGA	No	No	No	No	Yes	No	No	No	No	No	No	No	No	NA
TCTCCCTGGTGGTCTAGTGGTTAGG	No	No	No	No	Yes	No	No	No	No	No	No	No	No	NA
TCTCCCTGGTGGTCTAGTGGTTAGGA	No	No	No	No	Yes	No	No	No	No	No	No	No	No	NA
TCTCGCCTGCCACGC	No	No	Yes	No	Yes	No	Yes	No	No	No	No	No	No	NA
TCTCGCCTGCCACGCGGGAGGCCCGGGT	Yes	Yes	Yes	No	Yes	No	No	No	No	No	No	No	No	GlyGCC
TCTCGCTGGGGCCTCCA	No	Yes	Yes	Yes	Yes	No	No	No	No	No	No	No	No	ThrTGT
TCTCGGTGGAACCTCC	No	Yes	Yes	No	Yes	No	No	No	No	No	No	No	No	GlnCTG
TCTCGGTGGAACCTCCA	Yes	Yes	Yes	Yes	Yes	No	No	No	No	No	No	No	No	GlnCTG
TCTCGGTGGAACCTCCAT	No	No	Yes	No	No	No	No	No	No	No	No	No	No	NA
TCTCGGTGGAACCTCCATT	No	No	Yes	No	No	No	No	No	No	No	No	No	No	NA
TCTCGGTGGGACCTCC	No	Yes	Yes	No	Yes	No	No	No	No	No	No	No	No	GlnTTG
TCTCGGTGGGACCTCCA	No	Yes	Yes	Yes	Yes	No	No	No	No	No	No	No	No	GlnTTG
TCTGACTTCGGATCAGAAGATTGAGGGT	Yes	Yes	No	No	Yes	No	No	No	No	No	No	No	No	ArgTCG
TCTGATTCCGGATCAGAAGATTGAGGGT	Yes	Yes	No	No	Yes	No	No	No	No	No	No	No	No	ArgCCG
TCTGGGTGCCCCCTCCA	No	Yes	Yes	No	Yes	No	No	No	No	No	No	No	No	CysGCA
TCTTAATCTCAGGGTCGTGGGT	No	Yes	Yes	No	Yes	No	No	No	No	No	No	No	Yes	LysCTT
TCTTGCGACCCGGGT	No	No	Yes	No	Yes	No	Yes	No	No	No	No	No	No	NA
TCTTTGGTCTAGGGG	No	No	No	No	Yes	No	Yes	No	No	No	No	No	No	NA
TGAAAACCTTTTTCCAAGGACA	Yes	Yes	No	No	No	No	No	Yes	No	No	No	No	No	ThrTGT
TGAAAACCTTTTTCCAAGGACACC	Yes	Yes	No	No	No	No	No	Yes	No	No	No	No	No	ThrTGT
TGAAAACCTTTTTCCAAGGACACCA	Yes	Yes	No	No	No	No	No	Yes	No	No	No	No	No	ThrTGT
TGAATCCAGCGATCCG	Yes	Yes	Yes	No	Yes	No	No	No	No	No	No	No	No	GlnCTG,GlnTTG
TGAATCCAGCGATCCGAG	Yes	Yes	Yes	No	Yes	No	No	No	No	No	No	No	No	GlnCTG,GlnTTG
TGAATCCAGCGATCCGAGT	Yes	Yes	Yes	No	Yes	No	No	No	No	No	No	No	No	GlnCTG,GlnTTG
TGAATCCAGCGATCCGAGTT	Yes	Yes	Yes	No	Yes	No	No	No	No	No	No	No	No	GlnCTG,GlnTTG
TGAATCCAGCGATCCGAGTTC	Yes	Yes	Yes	No	Yes	No	No	No	No	No	No	No	No	GlnCTG,GlnTTG
TGAATCTGACAACAGAGGCTTA	Yes	Yes	No	No	No	No	No	Yes	No	No	No	No	No	HisGTG
TGAATCTGACAACAGAGGCTTACGACCCC	Yes	Yes	No	No	No	No	No	Yes	No	No	No	No	No	HisGTG
TGAATCTGACAACAGAGGCTTACGACCCCTT	Yes	Yes	No	No	No	No	No	Yes	No	No	No	No	No	HisGTG
TGAATCTGACAACAGAGGCTTACGACCCCTTA	Yes	Yes	No	No	No	No	No	Yes	No	No	No	No	No	HisGTG
TGACTCCAGATCAGA	No	No	No	No	Yes	No	Yes	No	No	No	No	No	No	NA
TGACTGCAGATCAAGAGGTCCCCGGT	Yes	Yes	Yes	No	Yes	No	No	No	No	No	No	No	Yes	CysGCA
TGACTGCAGATCAAGAGGTCCCTGGT	Yes	Yes	Yes	No	Yes	No	No	No	No	No	No	No	No	CysGCA
TGACTGGACCTTTCTTT	No	No	Yes	No	Yes	No	No	No	No	No	No	Yes	No	NA
TGACTGGACCTTTCTTTT	No	No	Yes	No	Yes	No	No	No	No	No	No	Yes	No	NA
TGACTTCGGATCAGA	No	No	Yes	No	Yes	No	Yes	No	No	No	No	No	No	NA
TGACTTCGGATCAGAAGATTGAGGGT	Yes	Yes	No	No	Yes	No	No	No	No	No	No	No	No	ArgTCG
TGACTTCGGATCAGAAGATTGAGGGTT	Yes	Yes	No	No	Yes	No	No	No	No	No	No	No	No	ArgTCG
TGACTTCGGATCAGAAGATTGCAGGT	Yes	Yes	Yes	No	Yes	No	No	No	No	No	No	No	No	ArgTCG
TGAGAGCGCTCGGTTTT	No	No	Yes	No	Yes	No	No	No	No	No	No	Yes	No	NA
TGAGAGCGCTCGGTTTTT	No	No	Yes	No	Yes	No	No	No	No	No	No	Yes	No	NA
TGAGAGCGCTCGGTTTTTT	No	No	Yes	No	Yes	No	No	No	No	No	No	Yes	No	NA
TGAGATGTTACCTAGCGTT	No	No	Yes	No	Yes	No	No	No	No	No	No	Yes	No	NA
TGAGATGTTACCTAGCGTTT	No	No	Yes	No	Yes	No	No	No	No	No	No	Yes	No	NA
TGAGGTTTTTCTGTTT	No	No	Yes	No	Yes	No	No	No	No	No	No	Yes	No	NA
TGAGTATCCCCGCCTGT	Yes	Yes	Yes	No	Yes	No	No	No	No	No	No	No	No	AspGTC
TGAGTATCCCCGCCTGTC	Yes	Yes	Yes	No	Yes	No	No	No	No	No	No	No	No	AspGTC
TGAGTATCCCCGCCTGTCACGCGGG	Yes	Yes	Yes	No	Yes	No	No	No	No	No	No	No	No	AspGTC
TGATTCCGGATCAGAAGATTGAGGGT	Yes	Yes	No	No	Yes	No	No	No	No	No	No	No	No	ArgCCG
TGATTCTCGCTTCGGGTGCGAG	Yes	Yes	Yes	No	Yes	No	No	No	No	No	No	No	No	ProCGG
TGATTCTCGCTTTGGGTGCGAG	Yes	Yes	Yes	No	Yes	No	No	No	No	No	No	No	Yes	ProTGG
TGATTCTCGGTTTGGGTCCGAG	Yes	Yes	Yes	No	Yes	No	No	No	No	No	No	No	No	ProTGG
TGCAGAGTGGGGTTTTGCAGTCCTTACCA	Yes	Yes	No	No	No	No	No	Yes	Yes	No	No	No	No	AlaTGC
TGCCACAGTGTTTTT	No	No	Yes	No	Yes	No	Yes	No	No	No	No	Yes	No	NA
TGCCACGCGGGAGGCCC	Yes	Yes	Yes	No	Yes	No	No	No	No	No	No	No	No	GlyGCC
TGCCACGCGGGAGGCCCGGGT	Yes	Yes	Yes	No	Yes	No	No	No	No	No	No	No	No	GlyGCC
TGCCATGTTAACGTTT	No	No	No	No	Yes	No	No	No	No	No	No	Yes	No	NA
TGCCCGCATCCTCCACCA	Yes	Yes	No	No	Yes	No	No	No	No	No	No	No	No	AlaAGC
TGCCCGCATTCTCCACCA	Yes	Yes	No	No	Yes	No	No	No	No	No	No	No	No	AlaAGC
TGCCGTGATCGTATA	No	No	No	No	Yes	No	Yes	No	No	No	No	No	No	NA
TGCCGTGATCGTATAGT	Yes	Yes	No	No	Yes	No	No	No	No	Yes	No	No	No	HisGTG
TGCCGTGATCGTATAGTGG	Yes	Yes	No	No	Yes	No	No	No	No	Yes	No	No	No	HisGTG
TGCCGTGATCGTATAGTGGTTAG	Yes	Yes	No	No	Yes	No	No	No	No	Yes	No	No	No	HisGTG
TGCCGTGATCGTATAGTGGTTAGT	Yes	Yes	No	No	Yes	No	No	No	No	Yes	No	No	No	HisGTG
TGCCGTGATCGTATAGTGGTTAGTA	Yes	Yes	No	No	Yes	No	No	No	No	Yes	No	No	No	HisGTG
TGCCGTGATCGTATAGTGGTTAGTACT	Yes	Yes	No	No	Yes	No	No	No	No	Yes	No	No	No	HisGTG
TGCGAAACTACTTTCTT	No	No	Yes	No	Yes	No	No	No	No	No	No	Yes	No	NA
TGCGGTACCACTTTT	No	No	Yes	Yes	Yes	No	Yes	No	No	No	No	Yes	No	NA
TGCGGTACCACTTTTG	No	No	Yes	No	Yes	No	No	No	No	No	No	Yes	No	NA
TGCTAATCCATTGTGCT	Yes	Yes	Yes	No	Yes	No	No	No	No	No	No	No	No	SerGCT
TGCTAATGGTGGAGTTA	Yes	Yes	No	No	No	No	No	Yes	No	No	No	No	No	ProTGG
TGCTACTAATGCCAGGGTCGAGGTTT	Yes	Yes	Yes	No	Yes	No	No	No	No	No	No	No	Yes	SerACT
TGCTATCGGAGTTTT	No	No	No	No	Yes	No	Yes	No	No	No	No	Yes	No	NA
TGCTATCGGAGTTTTT	No	No	No	No	Yes	No	No	No	No	No	No	Yes	No	NA
TGCTATCGGAGTTTTTT	No	No	No	No	Yes	No	No	No	No	No	No	Yes	No	NA
TGCTTTGCACGCGTGGG	Yes	Yes	No	No	Yes	No	No	No	No	No	No	No	No	SerGCT
TGCTTTGCACGCGTGGGT	Yes	Yes	No	No	Yes	No	No	No	No	No	No	No	No	SerGCT
TGGACATATGTCCGCGTGGG	Yes	Yes	Yes	No	Yes	No	No	No	No	No	No	No	No	LeuTAA
TGGACATATGTCCGCGTGGGT	Yes	Yes	Yes	No	Yes	No	No	No	No	No	No	No	No	LeuTAA

TGGACTCTGAATCCAGCGATCCGAGT	Yes	Yes	Yes	No	Yes	No	No	No	No	No	No	No	No	GlnCTG
TGGACTCTGAATCCAGCGATCCGAGTT	Yes	Yes	Yes	No	Yes	No	No	No	No	No	No	No	No	GlnCTG
TGGATAAGGCACTGGC	No	Yes	Yes	No	Yes	No	No	No	No	No	No	No	No	ArgCCT
TGGATAAGGCATCAGC	No	Yes	Yes	No	Yes	No	No	No	No	No	No	No	No	ArgCCG
TGGATAAGGCATCAGCCT	Yes	Yes	Yes	No	Yes	No	No	No	No	No	No	No	No	ArgCCG
TGGATAAGGCATTGG	No	No	No	No	Yes	No	Yes	No	No	No	No	No	No	NA
TGGATAAGGCATTGGC	No	Yes	No	No	Yes	No	No	No	No	No	No	No	No	ArgCCT
TGGATAAGGCGTCTG	No	No	Yes	No	Yes	No	Yes	No	No	No	No	No	No	NA
TGGATAAGGCGTCTGA	Yes	Yes	Yes	No	Yes	No	No	No	No	No	No	No	No	ArgTCG,ArgCCG
TGGATAAGGCGTCTGAC	Yes	Yes	Yes	No	Yes	No	No	No	No	No	No	No	No	ArgTCG
TGGATAAGGCGTCTGACTTCGGAT	Yes	Yes	Yes	No	Yes	No	No	No	No	No	No	No	No	ArgTCG
TGGATAAGGCGTCTGAT	Yes	Yes	No	No	Yes	No	No	No	No	No	No	No	No	ArgCCG
TGGATAAGGCGTCTGATT	Yes	Yes	No	No	Yes	No	No	No	No	No	No	No	No	ArgCCG
TGGATAAGGCGTCTGATTC	Yes	Yes	No	No	Yes	No	No	No	No	No	No	No	No	ArgCCG
TGGATAAGGCGTCTGATTCC	Yes	Yes	No	No	Yes	No	No	No	No	No	No	No	No	ArgCCG
TGGATAAGGCGTCTGATTCCGGAT	Yes	Yes	No	No	Yes	No	No	No	No	No	No	No	No	ArgCCG
TGGATAGCGCATTGG	No	No	No	No	Yes	No	Yes	No	No	No	No	No	No	NA
TGGATAGCGCATTGGA	No	Yes	No	No	Yes	No	No	No	No	No	No	No	No	ArgTCT
TGGATAGCGCATTGGAC	No	Yes	No	No	Yes	No	No	No	No	No	No	No	No	ArgTCT
TGGATAGGGCGTGGC	No	No	Yes	No	Yes	No	Yes	No	No	No	No	No	No	NA
TGGATAGGGCGTGGCA	No	No	Yes	No	Yes	No	No	No	No	No	No	No	No	NA
TGGATCGAAACCATCCTCTGCTACCA	Yes	Yes	Yes	No	Yes	No	No	No	No	No	No	No	No	MetCAT
TGGCAGGTGCCTCTTT	No	No	No	No	Yes	No	No	No	No	No	No	Yes	No	NA
TGGCCGCAGCAACCTCGGT	Yes	Yes	Yes	No	Yes	No	No	No	No	No	No	No	No	HisGTG
TGGCCGCAGCAACCTCGGTT	Yes	Yes	Yes	No	Yes	No	No	No	No	No	No	No	No	HisGTG
TGGCTAGGATTCGGC	No	No	Yes	No	Yes	No	Yes	No	No	No	No	No	No	NA
TGGCTAGGATTCGGCG	No	Yes	Yes	No	Yes	No	No	No	No	No	No	No	Yes	GluTTC
TGGCTAGGATTCGGCGCTTTCACCGCC	Yes	Yes	Yes	No	Yes	No	No	No	No	No	No	No	Yes	GluTTC
TGGGACTCTTAATCCCAGGGTCGTGGGT	No	Yes	Yes	No	Yes	No	No	No	No	No	No	No	No	LysCTT
TGGGGTGTGATAGGTGGCACGGAGA	Yes	Yes	No	No	No	No	No	Yes	Yes	No	No	No	No	GlnTTG
TGGGGTTTCCCCGCGCAGG	Yes	Yes	Yes	No	Yes	No	No	No	No	No	No	No	No	SerTGA,SerAGA
TGGGTGCGAGAGGTCCCGGGT	Yes	Yes	Yes	No	Yes	No	No	No	No	No	No	No	No	ProTGG
TGGGTGGTTCAGTGG	No	No	Yes	No	Yes	No	Yes	No	No	No	No	No	No	NA
TGGGTGGTTCAGTGGT	No	Yes	Yes	No	Yes	No	No	No	No	No	No	No	No	GlyGCC
TGGGTGGTTCAGTGGTA	No	Yes	Yes	No	Yes	No	No	No	No	No	No	No	No	GlyGCC
TGGGTGGTTCAGTGGTAG	No	Yes	Yes	No	Yes	No	No	No	No	No	No	No	No	GlyGCC
TGGGTGGTTCAGTGGTAGA	No	Yes	Yes	No	Yes	No	No	No	No	No	No	No	No	GlyGCC
TGGGTGGTTCAGTGGTAGAA	No	Yes	Yes	No	Yes	No	No	No	No	No	No	No	No	GlyGCC
TGGGTGGTTCAGTGGTAGAAT	No	Yes	Yes	No	Yes	No	No	No	No	No	No	No	No	GlyGCC
TGGGTGGTTCAGTGGTAGAATTC	No	Yes	Yes	No	Yes	No	No	No	No	No	No	No	No	GlyGCC
TGGGTGGTTCAGTGGTAGAATTCTC	Yes	Yes	Yes	No	Yes	No	No	No	No	No	No	No	No	GlyGCC
TGGGTGGTTCAGTGGTAGAATTCTCGC	Yes	Yes	Yes	No	Yes	No	No	No	No	No	No	No	No	GlyGCC
TGGGTGGTTCAGTGGTAGAATTCTCGCC	Yes	Yes	Yes	No	Yes	No	No	No	No	No	No	No	No	GlyGCC
TGGGTTCGATTCTCATAGTCCTAGCC	Yes	Yes	No	No	No	No	No	Yes	Yes	No	No	No	No	GlnTTG
TGGGTTCGATTCTCATAGTCCTAGCCA	Yes	Yes	No	No	No	No	No	Yes	Yes	No	No	No	No	GlnTTG
TGGTAAGCATAGCTGC	No	Yes	Yes	No	Yes	No	No	No	No	No	No	No	No	GlyTCC
TGGTAGAATTCTCGC	No	No	Yes	No	Yes	No	Yes	No	No	No	No	No	No	NA
TGGTAGAATTCTCGCC	No	Yes	Yes	No	Yes	No	No	No	No	No	No	No	Yes	GlyCCC,ValCAC,GlyGCC,GluTTC
TGGTAGAATTCTCGCCTC	No	Yes	Yes	No	Yes	No	No	No	No	No	No	No	Yes	GlyCCC,ValCAC
TGGTAGAATTCTCGCCTCC	No	Yes	Yes	No	Yes	No	No	No	No	No	No	No	Yes	GlyCCC
TGGTAGAATTCTCGCCTGCC	No	Yes	Yes	No	Yes	No	No	No	No	No	No	No	No	GlyGCC
TGGTAGAATTCTCGCCTGCCAC	No	Yes	Yes	No	Yes	No	No	No	No	No	No	No	No	GlyGCC
TGGTAGAATTCTCGCCTGCCACGC	Yes	Yes	Yes	No	Yes	No	No	No	No	No	No	No	No	GlyGCC
TGGTAGAATTCTCGCCTGCCACGCGGGAGGCCC	Yes	Yes	Yes	No	Yes	No	No	No	No	No	No	No	No	GlyGCC
TGGTAGAGCATTTGA	No	No	Yes	No	Yes	No	Yes	No	No	No	No	No	No	NA
TGGTAGAGCATTTGAC	No	Yes	Yes	No	Yes	No	No	No	No	No	No	No	Yes	CysGCA,CysACA
TGGTAGAGCATTTGACT	No	Yes	Yes	No	Yes	No	No	No	No	No	No	No	Yes	CysGCA,CysACA
TGGTAGAGCATTTGACTG	No	Yes	Yes	No	Yes	No	No	No	No	No	No	No	No	CysGCA
TGGTAGAGCATTTGACTGC	No	Yes	Yes	No	Yes	No	No	No	No	No	No	No	No	CysGCA
TGGTAGAGCATTTGACTGCA	No	Yes	Yes	No	Yes	No	No	No	No	No	No	No	No	CysGCA
TGGTAGAGCGCGTGC	No	No	Yes	No	Yes	No	Yes	No	No	No	No	No	No	NA
TGGTAGAGCGCGTGCT	Yes	Yes	Yes	No	Yes	No	No	No	No	No	No	No	No	AlaCGC,AlaAGC
TGGTAGAGCGCTCGC	No	No	No	No	Yes	No	Yes	No	No	No	No	No	No	NA
TGGTAGAGCGGAGGA	No	No	Yes	No	Yes	No	Yes	No	No	No	No	No	No	NA
TGGTAGAGCGGAGGAC	No	Yes	Yes	No	Yes	No	No	No	No	No	No	No	No	TyrGTA
TGGTAGAGCGGAGGACTGTA	No	Yes	Yes	No	Yes	No	No	No	No	No	No	No	No	TyrGTA
TGGTATAGTGGTAAGCATAGCTG	No	Yes	Yes	No	Yes	No	No	No	No	No	No	No	No	GlyTCC
TGGTATAGTGGTGAGCA	No	Yes	Yes	No	Yes	No	No	No	No	No	No	No	Yes	GlyTCC
TGGTATAGTGGTGAGCATAG	No	Yes	Yes	No	Yes	No	No	No	No	No	No	No	Yes	GlyTCC
TGGTATAGTGGTGAGCATAGCTG	No	Yes	Yes	No	Yes	No	No	No	No	No	No	No	Yes	GlyTCC
TGGTATATAGTTTAAACAAA	Yes	Yes	No	No	No	No	No	Yes	No	No	No	No	No	ArgTCG
TGGTATATAGTTTAAACAAAACG	Yes	Yes	No	No	No	No	No	Yes	No	No	No	No	No	ArgTCG
TGGTATATAGTTTAAACAAAACGA	Yes	Yes	No	No	No	No	No	Yes	No	No	No	No	No	ArgTCG
TGGTATATAGTTTAAACAAAACGAATG	Yes	Yes	No	No	No	No	No	Yes	No	No	No	No	No	ArgTCG
TGGTATATAGTTTAAACAAAACGAATGA	Yes	Yes	No	No	No	No	No	Yes	No	No	No	No	No	ArgTCG
TGGTATATAGTTTAAACAAAACGAATGAT	Yes	Yes	No	No	No	No	No	Yes	No	No	No	No	No	ArgTCG
TGGTATCATGCAAGA	No	No	Yes	No	Yes	No	Yes	No	No	No	No	No	No	NA
TGGTATGATTCTCGC	No	No	Yes	No	Yes	No	Yes	No	No	No	No	No	No	NA
TGGTATGATTCTCGCT	No	Yes	Yes	No	Yes	No	No	No	No	No	No	No	No	ProTGG
TGGTCATCACGTTCGC	Yes	Yes	Yes	No	Yes	No	No	No	No	No	No	No	No	ValAAC
TGGTCATCACGTTCGCC	Yes	Yes	Yes	No	Yes	No	No	No	No	No	No	No	No	ValAAC
TGGTCTAAGGCGCTGGA	No	Yes	Yes	No	Yes	No	No	No	No	No	No	No	No	LeuTAG
TGGTCTAGCGGTTAG	No	No	Yes	No	Yes	No	Yes	No	No	No	No	No	No	NA
TGGTCTAGCGGTTAGG	Yes	Yes	Yes	No	Yes	No	No	No	No	No	No	No	No	GluTTC
TGGTCTAGCGGTTAGGA	Yes	Yes	Yes	No	Yes	No	No	No	No	No	No	No	No	GluTTC
TGGTCTAGCGGTTAGGAT	Yes	Yes	Yes	No	Yes	No	No	No	No	No	No	No	No	GluTTC
TGGTCTAGCGGTTAGGATTC	Yes	Yes	Yes	No	Yes	No	No	No	No	No	No	No	No	GluTTC
TGGTCTAGCGGTTAGGATTCC	Yes	Yes	Yes	No	Yes	No	No	No	No	No	No	No	No	GluTTC

TGGTCTAGCGGTTAGGATTCCTG	Yes	Yes	Yes	No	Yes	No	No	No	No	No	No	No	No	GluTTC
TGGTCTAGCGGTTAGGATTCCTGG	Yes	Yes	Yes	No	Yes	No	No	No	No	No	No	No	No	GluTTC
TGGTCTAGGGGTATG	No	No	Yes	No	Yes	No	Yes	No	No	No	No	No	No	NA
TGGTCTAGGGGTATGA	No	Yes	Yes	No	Yes	No	No	No	No	No	No	No	Yes	ProAGG,ProCGG,ProTGG
TGGTCTAGGGGTATGAT	No	Yes	Yes	No	Yes	No	No	No	No	No	No	No	Yes	ProAGG,ProCGG,ProTGG
TGGTCTAGGGGTATGATT	No	Yes	Yes	No	Yes	No	No	No	No	No	No	No	No	ProAGG,ProCGG,ProTGG
TGGTCTAGGGGTATGATTC	No	Yes	Yes	No	Yes	No	No	No	No	No	No	No	No	ProAGG,ProCGG,ProTGG
TGGTCTAGGGGTATGATTCT	No	Yes	Yes	No	Yes	No	No	No	No	No	No	No	No	ProAGG,ProCGG,ProTGG
TGGTCTAGGGGTATGATTCTC	No	Yes	Yes	No	Yes	No	No	No	No	No	No	No	No	ProAGG,ProCGG,ProTGG
TGGTCTAGGGGTATGATTCTCGC	No	Yes	Yes	No	Yes	No	No	No	No	No	No	No	No	ProAGG,ProCGG,ProTGG
TGGTCTAGGGGTATGATTCTCGCT	No	Yes	Yes	No	Yes	No	No	No	No	No	No	No	No	ProAGG,ProCGG,ProTGG
TGGTCTAGGGGTATGATTCTCGG	Yes	Yes	Yes	No	Yes	No	No	No	No	No	No	No	No	ProTGG
TGGTCTAGGGGTATGATTCTCGGT	Yes	Yes	Yes	No	Yes	No	No	No	No	No	No	No	No	ProTGG
TGGTCTAGTGGTTAGGATTCGGCGCT	Yes	Yes	Yes	No	Yes	No	No	No	No	No	No	No	No	GluCTC
TGGTGAGCATAGCTG	No	No	Yes	No	Yes	No	Yes	No	No	No	No	No	No	NA
TGGTGAGCATAGCTGC	No	Yes	Yes	No	Yes	No	No	No	No	No	No	No	Yes	GlyTCC
TGGTGAGTATCCCCG	No	No	Yes	No	Yes	No	Yes	No	No	No	No	No	No	NA
TGGTGAGTATCCCCGC	No	Yes	Yes	No	Yes	No	No	No	No	No	No	No	No	AspGTC
TGGTGAGTATCCCCGCC	Yes	Yes	Yes	No	Yes	No	No	No	No	No	No	No	No	AspGTC
TGGTGAGTATCCCCGCCTGTC	Yes	Yes	Yes	No	Yes	No	No	No	No	No	No	No	No	AspGTC
TGGTGAGTATCCCCGCCTGTCACGCGGGAGA	Yes	Yes	Yes	No	Yes	No	No	No	No	No	No	No	No	AspGTC
TGGTGAGTATCCCCGCCTGTCACGCGGGAGACC	Yes	Yes	Yes	No	Yes	No	No	No	No	No	No	No	No	AspGTC
TGGTGGAGTTAAAGACTTTTTCTCTGACCA	Yes	Yes	No	No	No	No	No	Yes	No	No	No	No	No	ProTGG
TGGTGGTCTAGTGGCT	No	Yes	Yes	No	Yes	No	No	No	No	No	No	No	Yes	GluTTC
TGGTGGTCTAGTGGTTAGG	No	Yes	Yes	No	Yes	No	No	No	No	No	No	No	No	GluCTC
TGGTGGTCTAGTGGTTAGGATTCGGC	No	Yes	Yes	No	Yes	No	No	No	No	No	No	No	No	GluCTC
TGGTGGTTCAGTGGTA	No	Yes	Yes	No	Yes	No	No	No	No	No	No	No	Yes	GlyCCC,ValCAC,GlyGCC,GluTTC
TGGTGGTTCAGTGGTAGAA	No	Yes	Yes	No	Yes	No	No	No	No	No	No	No	Yes	GlyCCC,ValCAC,GlyGCC,GluTTC
TGGTGTAATGGTTAG	No	No	Yes	No	Yes	No	Yes	No	No	No	No	No	No	NA
TGGTGTAATGGTTAGC	No	Yes	Yes	No	Yes	No	No	No	No	No	No	No	No	GlnCTG,GlnTTG
TGGTGTAATGGTTAGCA	No	Yes	Yes	No	Yes	No	No	No	No	No	No	No	No	GlnCTG,GlnTTG
TGGTGTAATGGTTAGCACTCTGG	No	Yes	Yes	No	Yes	No	No	No	No	No	No	No	No	GlnCTG,GlnTTG
TGGTGTAATGGTTAGCACTCTGGA	No	Yes	Yes	No	Yes	No	No	No	No	No	No	No	No	GlnCTG,GlnTTG
TGGTGTAATGGTTAGCACTCTGGACT	No	Yes	Yes	No	Yes	No	No	No	No	No	No	No	No	GlnCTG,GlnTTG
TGGTGTAATGGTTAGCACTCTGGACTC	No	Yes	Yes	No	Yes	No	No	No	No	No	No	No	No	GlnCTG
TGGTGTAGTGGTATC	No	No	Yes	No	Yes	No	Yes	No	No	No	No	No	No	NA
TGGTGTAGTGGTATCA	Yes	Yes	Yes	No	Yes	No	No	No	No	No	No	No	No	GlyCCC
TGGTGTAGTGGTATCATGCA	Yes	Yes	Yes	No	Yes	No	No	No	No	No	No	No	No	GlyCCC
TGGTGTAGTGGTATCATGCAAGA	Yes	Yes	Yes	No	Yes	No	No	No	No	No	No	No	No	GlyCCC
TGGTGTAGTGGTTATCACATTCGCC	Yes	Yes	No	No	Yes	No	No	No	No	No	No	No	No	ValTAC
TGGTGTGGTCTGTTGTTT	No	No	Yes	No	Yes	No	No	No	No	No	No	Yes	No	NA
TGGTGTGGTCTGTTGTTTT	No	No	Yes	No	Yes	No	No	No	No	No	No	Yes	No	NA
TGGTTAGCACTCTGG	No	No	Yes	No	Yes	No	Yes	No	No	No	No	No	No	NA
TGGTTAGCACTCTGGA	No	Yes	Yes	No	Yes	No	No	No	No	No	No	No	No	GlnCTG,GlnTTG
TGGTTAGCATAGCTGC	No	Yes	Yes	No	Yes	No	No	No	No	No	No	No	No	GlyTCC
TGGTTAGGATTCGGC	No	No	Yes	No	Yes	No	Yes	No	No	No	No	No	No	NA
TGGTTAGGATTCGGCG	Yes	Yes	Yes	No	Yes	No	No	No	No	No	No	No	Yes	GluCTC
TGGTTAGGATTCGGCGC	Yes	Yes	Yes	No	Yes	No	No	No	No	No	No	No	Yes	GluCTC
TGGTTAGGATTCGGCGCT	Yes	Yes	Yes	No	Yes	No	No	No	No	No	No	No	Yes	GluCTC
TGGTTAGGATTCGGCGCTC	Yes	Yes	Yes	No	Yes	No	No	No	No	No	No	No	Yes	GluCTC
TGGTTAGGATTCGGCGCTCT	Yes	Yes	Yes	No	Yes	No	No	No	No	No	No	No	Yes	GluCTC
TGGTTAGGATTCGGCGCTCTC	Yes	Yes	Yes	No	Yes	No	No	No	No	No	No	No	Yes	GluCTC
TGGTTAGGATTCGGCGCTCTCA	Yes	Yes	Yes	No	Yes	No	No	No	No	No	No	No	Yes	GluCTC
TGGTTAGGATTCGGCGCTCTCAC	Yes	Yes	Yes	No	Yes	No	No	No	No	No	No	No	Yes	GluCTC
TGGTTAGGATTCGGCGCTCTCACC	Yes	Yes	Yes	No	Yes	No	No	No	No	No	No	No	Yes	GluCTC
TGGTTAGGATTCGGCGCTCTCACCG	Yes	Yes	Yes	No	Yes	No	No	No	No	No	No	No	Yes	GluCTC
TGGTTAGGATTCGGCGCTCTCACCGCC	Yes	Yes	Yes	No	Yes	No	No	No	No	No	No	No	Yes	GluCTC
TGGTTAGGATTCGGCGCTCTCACCGCCGC	Yes	Yes	Yes	No	Yes	No	No	No	No	No	No	No	No	GluCTC
TGGTTAGGATTCGGCGCTCTCACCGCCGCGGC	Yes	Yes	Yes	No	Yes	No	No	No	No	No	No	No	No	GluCTC
TGGTTAGTACTCTGC	No	No	Yes	No	Yes	No	Yes	No	No	No	No	No	No	NA
TGGTTAGTACTCTGCG	No	Yes	Yes	No	Yes	No	No	No	No	No	No	No	No	HisGTG
TGGTTAGTACTCTGCGT	Yes	Yes	Yes	No	Yes	No	No	No	No	No	No	No	No	HisGTG
TGGTTAGTACTCTGCGTT	Yes	Yes	Yes	No	Yes	No	No	No	No	No	No	No	No	HisGTG
TGGTTAGTATCCCCG	No	No	Yes	No	Yes	No	Yes	No	No	No	No	No	No	NA
TGGTTAGTATCCCCGC	Yes	Yes	Yes	No	Yes	No	No	No	No	No	No	No	No	AspGTC
TGGTTAGTATCCCCGCC	Yes	Yes	Yes	No	Yes	No	No	No	No	No	No	No	No	AspGTC
TGGTTATCACATTCGCC	No	Yes	No	No	Yes	No	No	No	No	No	No	No	Yes	ValCAC,ValTAC
TGGTTATCACGTCTGC	Yes	Yes	Yes	No	Yes	No	No	No	No	No	No	No	No	ValTAC
TGGTTATCACGTTCGC	No	Yes	Yes	No	Yes	No	No	No	No	No	No	No	No	ValCAC,ValAAC
TGGTTATCACGTTCGCC	No	Yes	Yes	No	Yes	No	No	No	No	No	No	No	No	ValCAC,ValAAC
TGGTTATCACGTTCGCCTCAC	No	Yes	Yes	No	Yes	No	No	No	No	No	No	No	No	ValCAC
TGGTTATCACGTTCGCCTCACAC	Yes	Yes	Yes	No	Yes	No	No	No	No	No	No	No	No	ValCAC
TGGTTATCACGTTCGCCTCACACGCGA	Yes	Yes	Yes	No	Yes	No	No	No	No	No	No	No	No	ValCAC
TGGTTATCACGTTCGCCTCACACGCGAA	Yes	Yes	Yes	No	Yes	No	No	No	No	No	No	No	No	ValCAC
TGGTTCAGTGGTAGA	No	No	Yes	No	Yes	No	Yes	No	No	No	No	No	No	NA
TGGTTCAGTGGTAGAA	No	Yes	Yes	No	Yes	No	No	No	No	No	No	No	Yes	GlyCCC,ValCAC,GlyGCC,GluTTC
TGGTTCAGTGGTAGAAT	No	Yes	Yes	No	Yes	No	No	No	No	No	No	No	Yes	GlyCCC,ValCAC,GlyGCC,GluTTC
TGGTTCAGTGGTAGAATTC	No	Yes	Yes	No	Yes	No	No	No	No	No	No	No	Yes	GlyCCC,ValCAC,GlyGCC,GluTTC
TGGTTCAGTGGTAGAATTCTCGC	No	Yes	Yes	No	Yes	No	No	No	No	No	No	No	Yes	GlyCCC,ValCAC,GlyGCC,GluTTC
TGGTTCAGTGGTAGAATTCTCGCC	No	Yes	Yes	No	Yes	No	No	No	No	No	No	No	Yes	GlyCCC,ValCAC,GlyGCC,GluTTC
TGGTTCAGTGGTAGAATTCTCGCCTC	No	Yes	Yes	No	Yes	No	No	No	No	No	No	No	Yes	GlyCCC,ValCAC
TGGTTCAGTGGTAGAATTCTCGCCTCC	No	Yes	Yes	No	Yes	No	No	No	No	No	No	No	Yes	GlyCCC
TGGTTCAGTGGTAGAATTCTCGCCTGCC	No	Yes	Yes	No	Yes	No	No	No	No	No	No	No	No	GlyGCC
TGGTTCAGTGGTAGAATTCTCGCCTGCCAC	No	Yes	Yes	No	Yes	No	No	No	No	No	No	No	No	GlyGCC
TGGTTCAGTGGTAGAATTCTCGCCTGCCACGC	Yes	Yes	Yes	No	Yes	No	No	No	No	No	No	No	No	GlyGCC
TGGTTGTAGTCCGTGCGAGAATAC	Yes	Yes	No	No	No	No	No	Yes	Yes	No	No	No	No	GluTTC
TGGTTGTAGTCCGTGCGAGAATACC	Yes	Yes	No	No	No	No	No	Yes	Yes	No	No	No	No	GluTTC
TGGTTGTAGTCCGTGCGAGAATACCA	Yes	Yes	No	No	No	No	No	Yes	Yes	No	No	No	No	GluTTC

TGTAAACCGGAGATGAAAACCT	Yes	Yes	No	No	No	No	No	Yes	No	No	No	No	No	ThrTGT
TGTAATGGTTAGCACTCTGGA	No	Yes	Yes	No	Yes	No	No	No	No	No	No	No	No	GlnCTG,GlnTTG
TGTAGTGGTTATTAT	No	No	Yes	No	Yes	No	Yes	No	No	No	No	No	No	NA
TGTATGAGGTCCCGGGT	Yes	Yes	Yes	No	Yes	No	No	No	No	No	No	No	No	AlaTGC,AlaCGC
TGTCACGCGGGAGAC	No	No	Yes	No	Yes	No	Yes	No	No	No	No	No	No	NA
TGTCACGCGGGAGACC	No	Yes	Yes	No	Yes	No	No	No	No	No	No	No	No	AspGTC
TGTCACGCGGGAGACCGG	Yes	Yes	Yes	No	Yes	No	No	No	No	No	No	No	No	AspGTC
TGTCACGCGGGAGACCGGGGT	Yes	Yes	Yes	No	Yes	No	No	No	No	No	No	No	No	AspGTC
TGTGATAGGTGGCACGGAGA	Yes	Yes	No	No	No	No	No	Yes	Yes	No	No	No	No	GlnTTG
TGTGCTCCGGAGTTACCTC	No	No	Yes	No	Yes	No	No	No	No	No	No	Yes	No	NA
TGTGCTCCGGAGTTACCTCGT	No	No	Yes	No	Yes	No	No	No	No	No	No	Yes	No	NA
TGTGCTCCGGAGTTACCTCGTT	No	No	Yes	No	Yes	No	No	No	No	No	No	Yes	No	NA
TGTGCTCCGGAGTTACCTCGTTT	No	No	Yes	Yes	Yes	No	No	No	No	No	No	Yes	No	NA
TGTGCTCCGGAGTTACCTCGTTTT	No	No	Yes	No	Yes	No	No	No	No	No	No	Yes	No	NA
TGTGCTCTGGAGTTTT	No	No	Yes	No	Yes	No	No	No	No	No	No	Yes	No	NA
TGTGCTTTGCACGCGTGGG	Yes	Yes	No	No	Yes	No	No	No	No	No	No	No	No	SerGCT
TGTGCTTTGCACGCGTGGGT	Yes	Yes	No	No	Yes	No	No	No	No	No	No	No	No	SerGCT
TGTGGCCGCAGCAACC	Yes	Yes	Yes	No	Yes	No	No	No	No	No	No	No	No	HisGTG
TGTGGCCGCAGCAACCTCGGT	Yes	Yes	Yes	No	Yes	No	No	No	No	No	No	No	No	HisGTG
TGTGGCCGCAGCAACCTCGGTT	Yes	Yes	Yes	No	Yes	No	No	No	No	No	No	No	No	HisGTG
TGTGGTGGCTTACTTT	No	No	Yes	No	Yes	No	No	No	No	No	No	Yes	No	NA
TGTTAACCGAAAGGTTGGTGGT	Yes	Yes	Yes	No	Yes	No	No	No	No	No	No	No	No	AsnGTT
TTAAACAAAACGAATGA	Yes	Yes	No	No	No	No	No	Yes	No	No	No	No	No	ArgTCG
TTAAACAAAACGAATGATTTCGACTC	Yes	Yes	No	No	No	No	No	Yes	No	No	No	No	No	ArgTCG
TTAAACAAAACGAATGATTTCGACTCA	Yes	Yes	No	No	No	No	No	Yes	No	No	No	No	No	ArgTCG
TTAAACAAAACGAATGATTTCGACTCAT	Yes	Yes	No	No	No	No	No	Yes	No	No	No	No	No	ArgTCG
TTAAAGACTTTTTCTCTGACCA	Yes	Yes	No	No	No	No	No	Yes	No	No	No	No	No	ProTGG
TTAAATACAGACCAAGAGC	Yes	Yes	No	No	No	No	No	Yes	Yes	No	No	No	No	TrpTCA
TTAACTTGACCGCTCTGACCA	Yes	Yes	No	No	No	No	No	Yes	No	No	No	No	No	ValTAC
TTAAGAGAACCAACACCTCTTTACAGTGACCA	Yes	Yes	No	No	No	No	No	Yes	Yes	No	No	No	No	LysTTT
TTAAGTCCCATTGGTCTAGCCA	Yes	Yes	No	No	No	No	No	Yes	Yes	No	No	No	No	AsnGTT
TTAATCCCAGGGTCGTGGGT	No	Yes	Yes	No	Yes	No	No	No	No	No	No	No	No	LysCTT
TTAATCTCAGGGTCG	No	No	Yes	No	Yes	No	Yes	No	No	No	No	No	No	NA
TTAATCTCAGGGTCGT	No	Yes	Yes	No	Yes	No	No	No	No	No	No	No	Yes	LysCTT
TTAATCTCAGGGTCGTGGGT	No	Yes	Yes	No	Yes	No	No	No	No	No	No	No	Yes	LysCTT
TTAATCTGAGGGTCC	No	No	Yes	No	Yes	No	Yes	No	No	No	No	No	No	NA
TTAATCTGAGGGTCCA	Yes	Yes	Yes	No	Yes	No	No	No	No	No	No	No	Yes	LysTTT
TTAATCTGAGGGTCCAGGGT	Yes	Yes	Yes	No	Yes	No	No	No	No	No	No	No	Yes	LysTTT
TTACGACCCCTTATTTACCCCA	Yes	Yes	No	No	No	No	No	Yes	No	No	No	No	No	HisGTG
TTAGACGGGCTCACATCACCCCATAAACACCA	Yes	Yes	No	No	No	No	No	Yes	No	No	No	No	No	PheGAA
TTATTCAATTGAAACAGCGTG	No	No	No	No	Yes	No	No	No	No	No	No	Yes	No	NA
TTATTCAATTGAAACAGCGTGA	No	No	No	No	Yes	No	No	No	No	No	No	Yes	No	NA
TTATTCAATTGAAACAGCGTGATT	No	No	No	No	Yes	No	No	No	No	No	No	Yes	No	NA
TTATTCAATTGAAACAGCGTGATTT	No	No	No	No	Yes	No	No	No	No	No	No	Yes	No	NA
TTATTCAATTGAAACAGCGTGATTTT	No	No	No	No	Yes	No	No	No	No	No	No	Yes	No	NA
TTCAAAGGTGAACGT	No	No	Yes	No	Yes	No	Yes	No	No	No	No	Yes	No	NA
TTCAAAGGTGAACGTT	No	No	Yes	No	Yes	No	No	No	No	No	No	Yes	No	NA
TTCAAAGGTGAACGTTT	No	No	Yes	Yes	Yes	No	No	No	No	No	No	Yes	No	NA
TTCAAAGGTGAACGTTTT	No	No	Yes	No	Yes	No	No	No	No	No	No	Yes	No	NA
TTCAAAGGTGAACGTTTTA	No	No	Yes	No	Yes	No	No	No	No	No	No	Yes	No	NA
TTCAACTTAACTTGACCGCTCTGACCA	Yes	Yes	No	No	No	No	No	Yes	No	No	No	No	No	ValTAC
TTCAGCTATAGGGTG	No	No	Yes	No	Yes	No	Yes	No	No	No	No	Yes	No	NA
TTCAGGTCGCAGTCT	No	No	Yes	No	Yes	No	Yes	No	No	No	No	No	No	NA
TTCAGGTCGCAGTCTC	Yes	Yes	Yes	No	Yes	No	No	No	No	No	No	No	No	LeuCAG
TTCAGGTCGCAGTCTCC	Yes	Yes	Yes	No	Yes	No	No	No	No	No	No	No	No	LeuCAG
TTCAGGTCGCAGTCTCCC	Yes	Yes	Yes	No	Yes	No	No	No	No	No	No	No	No	LeuCAG
TTCAGTGGTAGAATTCTCGCC	No	Yes	Yes	No	Yes	No	No	No	No	No	No	No	Yes	GlyCCC,ValCAC,GlyGCC,GluTTC
TTCCAGCTCGAAGGACCA	Yes	Yes	Yes	No	Yes	No	No	No	No	No	No	No	No	TyrGTA
TTCCCATTCTTGCGA	No	No	Yes	No	Yes	No	Yes	No	No	No	No	No	No	NA
TTCCCCGACGGGGAG	No	No	Yes	No	Yes	No	Yes	No	No	No	No	No	No	NA
TTCCCCGACGGGGAGC	Yes	Yes	Yes	No	Yes	No	No	No	No	No	No	No	Yes	AspGTC
TTCCCCGACGGGGAGCCA	Yes	Yes	Yes	Yes	Yes	No	No	No	No	No	No	No	Yes	AspGTC
TTCCCGGCCAACGCA	No	No	Yes	No	Yes	No	Yes	No	No	No	No	No	No	NA
TTCCCGGCCAACGCACC	Yes	Yes	Yes	No	Yes	No	No	No	No	No	No	No	No	GlyTCC
TTCCCGGCCAACGCACCA	Yes	Yes	Yes	Yes	Yes	No	No	No	No	No	No	No	No	GlyTCC
TTCCCGGCCAATGCACCA	No	Yes	Yes	No	Yes	No	No	No	No	No	No	No	No	GlyCCC,GlyGCC
TTCCCGGCCCATGCACC	No	Yes	Yes	No	Yes	No	No	No	No	No	No	No	No	GlyGCC
TTCCCGGCCCATGCACCA	No	Yes	Yes	No	Yes	No	No	No	No	No	No	No	No	GlyGCC
TTCCCGGGCGGCGCACCA	Yes	Yes	Yes	No	Yes	No	No	No	No	No	No	No	No	GlyCCC
TTCCCGGTCAGGGAA	No	No	Yes	No	Yes	No	Yes	No	No	No	No	No	No	NA
TTCCCGGTCAGGGAAC	No	Yes	Yes	No	Yes	No	No	No	No	No	No	No	Yes	GluCTC,PheGAA,GluTTC
TTCCCGGTCAGGGAACC	No	Yes	Yes	No	Yes	No	No	No	No	No	No	No	Yes	GluCTC,PheGAA,GluTTC
TTCCCGGTCAGGGAACCA	No	Yes	Yes	Yes	Yes	No	No	No	No	No	No	No	Yes	GluCTC,PheGAA,GluTTC
TTCCGGATCAGAAGATTGAGGGT	Yes	Yes	No	No	Yes	No	No	No	No	No	No	No	No	ArgCCG
TTCCGGATCAGAAGATTGAGGGTT	Yes	Yes	No	No	Yes	No	No	No	No	No	No	No	No	ArgCCG
TTCCGGCTCGAAGGA	No	No	Yes	No	Yes	No	Yes	No	No	No	No	No	No	NA
TTCCGGCTCGAAGGAC	Yes	Yes	Yes	No	Yes	No	No	No	No	No	No	No	No	TyrGTA
TTCCGGCTCGAAGGACC	Yes	Yes	Yes	No	Yes	No	No	No	No	No	No	No	No	TyrGTA
TTCCGGCTCGAAGGACCA	Yes	Yes	Yes	Yes	Yes	No	No	No	No	No	No	No	No	TyrGTA
TTCCGGCTCGAAGGACCAA	No	No	Yes	No	No	No	No	No	No	No	No	No	No	NA
TTCCGGGTTCGAGTCCCGGCGGAGTCGCCA	Yes	Yes	No	No	Yes	No	No	No	No	No	No	No	No	ArgTCT
TTCCGTAGTGTAGTGGTTATCACGTTCGC	No	Yes	Yes	No	Yes	No	No	No	No	No	No	No	No	ValCAC,ValAAC
TTCCGTGGGTTTGTT	No	No	Yes	No	Yes	No	Yes	No	No	No	No	Yes	No	NA
TTCCGTGGGTTTGTTT	No	No	Yes	No	Yes	No	No	No	No	No	No	Yes	No	NA
TTCCGTGGGTTTGTTTT	No	No	Yes	Yes	Yes	No	No	No	No	No	No	Yes	No	NA
TTCCGTGGGTTTGTTTTT	No	No	Yes	No	Yes	No	No	No	No	No	No	Yes	No	NA
TTCCGTGGGTTTGTTTTTT	No	No	Yes	No	Yes	No	No	No	No	No	No	Yes	No	NA
TTCCTTCCTTTTTTGCCA	No	Yes	No	No	Yes	No	No	Yes	Yes	No	No	No	Yes	SerTGA

TTCGATTCCCCGACGGGGAGCCA	Yes	Yes	Yes	No	Yes	No	No	No	No	No	No	No	No	AspGTC
TTCGGATCAGAAGATTGAGGGT	Yes	Yes	No	No	Yes	No	No	No	No	No	No	No	No	ArgTCG
TTCGGGTGCGAGAGGTCCCGGGT	Yes	Yes	Yes	No	Yes	No	No	No	No	No	No	No	No	ProCGG
TTCTCATAGTCCTAGCC	Yes	Yes	No	No	No	No	No	Yes	Yes	No	No	No	No	GlnTTG
TTCTCATAGTCCTAGCCA	Yes	Yes	No	No	No	No	No	Yes	Yes	No	No	No	No	GlnTTG
TTCTCGCCTCCCACGCGGGAG	Yes	Yes	Yes	No	Yes	No	No	No	No	No	No	No	Yes	GlyCCC
TTCTCGCCTGCCACG	No	No	Yes	No	Yes	No	Yes	No	No	No	No	No	No	NA
TTCTCGCCTGCCACGC	Yes	Yes	Yes	No	Yes	No	No	No	No	No	No	No	No	GlyGCC
TTCTCGCCTGCCACGCGGGA	Yes	Yes	Yes	No	Yes	No	No	No	No	No	No	No	No	GlyGCC
TTCTCGCCTGCCACGCGGGAGGCCCGGGT	Yes	Yes	Yes	No	Yes	No	No	No	No	No	No	No	No	GlyGCC
TTCTCGCTGGGGCCTCCA	No	Yes	Yes	No	Yes	No	No	No	No	No	No	No	No	ThrTGT
TTGAATCCAGCGATCCGAGT	Yes	Yes	Yes	No	Yes	No	No	No	No	No	No	No	No	GlnTTG
TTGAATCCAGCGATCCGAGTT	Yes	Yes	Yes	No	Yes	No	No	No	No	No	No	No	No	GlnTTG
TTGACCGCTCTGACCA	Yes	Yes	No	No	No	No	No	Yes	No	No	No	No	No	ValTAC
TTGACTGCAGATCAAGAGGTCCCCGGT	Yes	Yes	Yes	No	Yes	No	No	No	No	No	No	No	Yes	CysGCA
TTGACTGCAGATCAAGAGGTCCCTGGT	Yes	Yes	Yes	No	Yes	No	No	No	No	No	No	No	No	CysGCA
TTGCCATGTTAACGTTT	No	No	No	No	Yes	No	No	No	No	No	No	Yes	No	NA
TTGGGTCCGAGAGGTCCC	Yes	Yes	Yes	No	Yes	No	No	No	No	No	No	No	No	ProTGG
TTGGGTCCGAGAGGTCCCGGGT	Yes	Yes	Yes	No	Yes	No	No	No	No	No	No	No	No	ProTGG
TTGGGTCCGAGAGGTCCCGGGTT	Yes	Yes	Yes	No	Yes	No	No	No	No	No	No	No	No	ProTGG
TTGGGTGCGAGAGGTCCC	Yes	Yes	Yes	No	Yes	No	No	No	No	No	No	No	No	ProTGG
TTGGGTGCGAGAGGTCCCG	Yes	Yes	Yes	No	Yes	No	No	No	No	No	No	No	No	ProTGG
TTGGGTGCGAGAGGTCCCGG	Yes	Yes	Yes	No	Yes	No	No	No	No	No	No	No	No	ProTGG
TTGGGTGCGAGAGGTCCCGGG	Yes	Yes	Yes	No	Yes	No	No	No	No	No	No	No	No	ProTGG
TTGGGTGCGAGAGGTCCCGGGT	Yes	Yes	Yes	No	Yes	No	No	No	No	No	No	No	No	ProTGG
TTGGGTGCGAGAGGTCCCGGGTT	Yes	Yes	Yes	No	Yes	No	No	No	No	No	No	No	No	ProTGG
TTGGTAGAGCATCAGA	No	Yes	No	No	Yes	No	No	No	No	No	No	No	No	SupTTA,LysTTT
TTGGTAGAGCGGAGGAC	No	Yes	Yes	No	Yes	No	No	No	No	No	No	No	No	TyrGTA
TTGGTCGTGGTTGTAGTCCGTGCGAGAATACCA	Yes	Yes	No	No	No	No	No	Yes	Yes	No	No	No	No	GluTTC
TTGGTCTAGGGGTAT	No	No	Yes	No	Yes	No	Yes	No	No	No	No	No	No	NA
TTGGTCTAGGGGTATGA	No	Yes	Yes	No	Yes	No	No	No	No	No	No	No	Yes	ProAGG,ProCGG,ProTGG
TTGGTCTAGGGGTATGATTCTCGC	No	Yes	Yes	No	Yes	No	No	No	No	No	No	No	No	ProAGG,ProCGG,ProTGG
TTGGTCTAGGGGTATGATTCTCGCT	No	Yes	Yes	No	Yes	No	No	No	No	No	No	No	No	ProAGG,ProCGG,ProTGG
TTGGTGGTATAGTGGTAAGCATAGCTG	No	Yes	Yes	No	Yes	No	No	No	No	No	No	No	No	GlyTCC
TTGGTGGTTCAGTGG	No	No	Yes	No	Yes	No	Yes	No	No	No	No	No	No	NA
TTGGTGGTTCAGTGGT	No	Yes	Yes	No	Yes	No	No	No	No	No	No	No	No	GlyCCC,GlyGCC
TTGGTGGTTCAGTGGTA	No	Yes	Yes	No	Yes	No	No	No	No	No	No	No	No	GlyCCC,GlyGCC
TTGGTGGTTCAGTGGTAGA	No	Yes	Yes	No	Yes	No	No	No	No	No	No	No	No	GlyCCC,GlyGCC
TTGGTGGTTCAGTGGTAGAA	No	Yes	Yes	No	Yes	No	No	No	No	No	No	No	No	GlyCCC,GlyGCC
TTGGTGGTTCAGTGGTAGAAT	No	Yes	Yes	No	Yes	No	No	No	No	No	No	No	No	GlyCCC,GlyGCC
TTGGTGGTTCAGTGGTAGAATTCTC	No	Yes	Yes	No	Yes	No	No	No	No	No	No	No	No	GlyCCC,GlyGCC
TTGGTGGTTCAGTGGTAGAATTCTCGC	No	Yes	Yes	No	Yes	No	No	No	No	No	No	No	No	GlyCCC,GlyGCC
TTGGTGGTTCAGTGGTAGAATTCTCGCC	No	Yes	Yes	No	Yes	No	No	No	No	No	No	No	No	GlyCCC,GlyGCC
TTGGTGGTTCAGTGGTAGAATTCTCGCCT	No	Yes	Yes	No	Yes	No	No	No	No	No	No	No	No	GlyCCC,GlyGCC
TTGGTGGTTCAGTGGTAGAATTCTCGCCTCC	No	Yes	Yes	No	Yes	No	No	No	No	No	No	No	No	GlyCCC
TTGTAAACAGGAGATCCTGGGT	Yes	Yes	No	No	Yes	No	No	No	No	No	No	No	No	ThrTGT
TTGTAAACCAGGGGTCGCGAGT	Yes	Yes	Yes	No	Yes	No	No	No	No	No	No	No	No	ThrTGT
TTGTAAACCAGGGGTCGCGAGTT	Yes	Yes	Yes	No	Yes	No	No	No	No	No	No	No	No	ThrTGT
TTGTGGCCGCAGCAACCTCGGT	Yes	Yes	Yes	No	Yes	No	No	No	No	No	No	No	No	HisGTG
TTGTGGCCGCAGCAACCTCGGTT	Yes	Yes	Yes	No	Yes	No	No	No	No	No	No	No	No	HisGTG
TTTAAACAAAACGAATGA	Yes	Yes	No	No	No	No	No	Yes	No	No	No	No	No	ArgTCG
TTTAAACAAAACGAATGATTTCGAC	Yes	Yes	No	No	No	No	No	Yes	No	No	No	No	No	ArgTCG
TTTAAACAAAACGAATGATTTCGACTC	Yes	Yes	No	No	No	No	No	Yes	No	No	No	No	No	ArgTCG
TTTAAACAAAACGAATGATTTCGACTCA	Yes	Yes	No	No	No	No	No	Yes	No	No	No	No	No	ArgTCG
TTTAAACAAAACGAATGATTTCGACTCAT	Yes	Yes	No	No	No	No	No	Yes	No	No	No	No	No	ArgTCG
TTTAAACAAAACGAATGATTTCGACTCATTAA	Yes	Yes	No	No	No	No	No	Yes	No	No	No	No	No	ArgTCG
TTTAAGTCCCATTGGTCTAGCCA	Yes	Yes	No	No	No	No	No	Yes	Yes	No	No	No	No	AsnGTT
TTTACACGCAGAAGGTCC	Yes	Yes	Yes	No	Yes	No	No	No	No	No	No	No	No	ValTAC
TTTACACGCAGAAGGTCCTGGGT	Yes	Yes	Yes	No	Yes	No	No	No	No	No	No	No	No	ValTAC
TTTAGACGGGCTCACATCACCCCATAAACACCA	Yes	Yes	No	No	No	No	No	Yes	No	No	No	No	No	PheGAA
TTTCAACTTAACTTGACCGCTCTGACC	Yes	Yes	No	No	No	No	No	Yes	No	No	No	No	No	ValTAC
TTTCAACTTAACTTGACCGCTCTGACCA	Yes	Yes	No	No	No	No	No	Yes	No	No	No	No	No	ValTAC
TTTCCGTAGTGTAGTGGTTATCACGTTCGC	No	Yes	Yes	No	Yes	No	No	No	No	No	No	No	No	ValCAC,ValAAC
TTTCTGGAGGTTTTT	No	No	No	No	Yes	No	Yes	No	No	No	No	Yes	No	NA
TTTCTGGAGGTTTTTAT	No	No	No	No	Yes	No	No	No	No	No	No	Yes	No	NA
TTTGAATCCAGCGATCCGAGT	Yes	Yes	Yes	No	Yes	No	No	No	No	No	No	No	No	GlnTTG
TTTGCACGCGTGGGT	No	No	No	No	Yes	No	Yes	No	No	No	No	No	No	NA
TTTGCACGCGTGGGTT	Yes	Yes	No	No	Yes	No	No	No	No	No	No	No	No	SerGCT
TTTGCACGTATGAGGCCCCGGGT	Yes	Yes	Yes	No	Yes	No	No	No	No	No	No	No	No	AlaTGC
TTTGGCCTTTTCGAGATACTGTTT	No	No	Yes	No	Yes	No	No	No	No	No	No	Yes	No	NA
TTTGGGTCCGAGAGGTCCCGGGT	Yes	Yes	Yes	No	Yes	No	No	No	No	No	No	No	No	ProTGG
TTTGGGTGCGAGAGGTCCC	Yes	Yes	Yes	No	Yes	No	No	No	No	No	No	No	No	ProTGG
TTTGGGTGCGAGAGGTCCCGGGT	Yes	Yes	Yes	No	Yes	No	No	No	No	No	No	No	No	ProTGG
TTTGGGTGCGAGAGGTCCCGGGTT	Yes	Yes	Yes	No	Yes	No	No	No	No	No	No	No	No	ProTGG
TTTTAATCTGAGGGTCC	Yes	Yes	Yes	No	Yes	No	No	No	No	No	No	No	Yes	LysTTT
TTTTAATCTGAGGGTCCA	Yes	Yes	Yes	No	Yes	No	No	No	No	No	No	No	Yes	LysTTT
TTTTAATCTGAGGGTCCAGGGT	Yes	Yes	Yes	No	Yes	No	No	No	No	No	No	No	Yes	LysTTT

Supplementary Table S2: Unspliced tRNA reference set.

	Source	tRNA id and coordinates	tRNA type	Sequence
1	gtRNAdb v1.0	trna100_LeuCAA_6_-_28864000_28864105_intronat_28864045_28864067	TRUE	GTCAGGATGGCCGAGTGGTCTAAGGCGCCAGACTCAAGCTAAGCTTCCTCCGCGGTGGGGATTCTGGTCTCCAATGGAGGCGTGGGTTCGAATCCCACTTCTGACA
2	gtRNAdb v1.0	trna100_PheGAA_1_-_149287359_149287430	PSEUDO	TGCATGGTTGTCTAGTGGCTAGGATTCGGTGCTGAAAGAGCCACGGCCCGGGTTCGATTCCCGGTCAGGGAA
3	gtRNAdb v1.0	trna101_AlaAGC_6_-_28831462_28831533	TRUE	GGGGGTGTAGCTCAGTGGTAGAGCGCGTGCTTAGCATGCACGAGGCCCCGGGTTCAATCCCCGGCACCTCCA
4	gtRNAdb v1.0	trna101_AsnGTT_1_-_149284503_149284576	TRUE	ATCTCCGTGGAGCAATTGGTTAGCGCGTTCGGCTGTTAACCGGAAAGTTGGTGGTTCGAGCCTACCCAGGGACG
5	gtRNAdb v1.0	trna102_AlaAGC_6_-_28806221_28806292	TRUE	GGGGGTGTAGCTCAGTGGTAGAGCGCGTGCTTAGCATGCACGAGGCCCCGGGTTCAATCCCCGGCACCTCCA
6	gtRNAdb v1.0	trna102_GluTTC_1_-_149278785_149278857	PSEUDO	TCCGTGGTGGTCTAGTGGCTAGGATTCGGCGCTTTCACCGCCTGCAGCTCGAGTTCGATTCCTGGTCAGGGAA
7	gtRNAdb v1.0	trna103_AsnGTT_1_-_149230570_149230643	TRUE	GTCTCTGTGGCGCAATGGGTTAGCGCGTTCGGCTGTTAACCGAAAGGTTGGTGGTTCGAGCCCATCCAGGGACG
8	gtRNAdb v1.0	trna103_PheGAA_6_-_28791093_28791166	TRUE	GCCGAAATAGCTCAGTTGGGAGAGCGTTAGACCGAAGATCTTAAAGGTCCCTGGTTCAATCCCGGGTTTCGGCA
9	gtRNAdb v1.0	trna104_AlaTGC_6_-_28785012_28785083	TRUE	GGGGGTGTAGCTCAGTGGTAGAGCGCATGCTTTGCATGTATGAGGCCTCGGGTTCGATCCCCGACACCTCCA
10	gtRNAdb v1.0	trna104_AsnGTT_1_-_149211948_149212021	PSEUDO	GTCTCTGTGGCACAATCGGTTAGAGCGTTCGGCTGTTAATCTAAAGGTTGGTGGCTAGAACCCACTGAGGGACG
11	gtRNAdb v1.0	trna105_AlaAGC_6_-_28779849_28779920	TRUE	GGGGGTATAGCTCAGCGGTAGAGCGCGTGCTTAGCATGCACGAGGTCCTGGGTTCAATCCCCAATACCTCCA
12	gtRNAdb v1.0	trna105_GluTTC_1_-_149161628_149161699	PSEUDO	TGTCTGGTGGTCAAGTGGCTAGGATTTGGCGCTTTCACTGCCGCGGCCCGCGTTCGATTCCCGGTCAGGGAA
13	gtRNAdb v1.0	trna106_HisGTG_1_-_149155828_149155899	TRUE	GCCATGATCGTATAGTGGTTAGTACTCTGCGCTGTGGCCGCAGCAACCTCGGTTCGAATCCGAGTCACGGCA
14	gtRNAdb v1.0	trna106_PheGAA_6_-_28775610_28775682	TRUE	GCCGAGATAGCTCAGTTGGGAGAGCGTTAGACTGAAGATCTAAAGGTCCCTGGTTCAATCCCGGGTTTCGGCA
15	gtRNAdb v1.0	trna107_AlaTGC_6_-_28770577_28770647	TRUE	GGGGGTGTAGCTCAGTGGTAGAGCGCATGCTTTGCATGTATGAGGCCTCGGTTCGATCCCCGACACCTCCA
16	gtRNAdb v1.0	trna107_AsnGTT_1_-_148760356_148760429	TRUE	GTCTCTGTGGCGCAATCGGTTAGCGCATTCGGCTGTTAACCGAAAGGTTGGTGGTTCGAGCCCACCCAGGGACG
17	gtRNAdb v1.0	trna108_AlaAGC_6_-_28763741_28763812	TRUE	GGGGGTATAGCTCAGTGGTAGAGCGCGTGCTTAGCATGCACGAGGTCCTGGGTTCGATCCCCAGTACCTCCA
18	gtRNAdb v1.0	trna108_AsnGTT_1_-_148598314_148598387	TRUE	GTCTCTGTGGCGCAATCGGTTAGCGCATTCGGCTGTTAACCGAAAGGTTGGTGGTTCGAGCCCACCCAGGGACG
19	gtRNAdb v1.0	trna109_AsnGTT_1_-_147851060_147851133	PSEUDO	GTCTCTGTGGCACAATCGGTTAGCTTGTTCGGCTGTTAATCTAGAGGTTGGTGGTTAGAGCCCACTGAGGGATG
20	gtRNAdb v1.0	trna109_PheGAA_6_-_28758499_28758571	TRUE	GCCGAAATAGCTCAGTTGGGAGAGCGTTAGACTGAAGATCTAAAGGTCCCTGGTTCGATCCCGGGTTTCGGCA
21	gtRNAdb v1.0	trna10_AlaCGC_6_+_26553731_26553802	TRUE	GGGGATGTAGCTCAGTGGTAGAGCGCATGCTTCGCATGTATGAGGTCCCGGGTTCGATCCCCGGCATCTCCA
22	gtRNAdb v1.0	trna10_AsnGTT_1_+_143690028_143690101	TRUE	GTCTCTGTGGCGCAATCGGTTAGCGCGTTTGACTGTTAACTGAAAGGTTGGTGGTGCAAGCCCATCCAGGGATG
23	gtRNAdb v1.0	trna10_AspGTC_12_-_125424193_125424264	TRUE	TCCTCGTTAGTATAGTGGTGAGTATCCCCGCCTGTCACGCGGGAGACCGGGGTTCGATTCCCCGACGGGGAG
24	gtRNAdb v1.0	trna10_CysGCA_7_+_149281816_149281887	TRUE	GGGGGTATAGCTCAGGGGTAGAGCATTTGACTGCAGATCAAGAGGTCTCTGGTTCAAATCCAGGTGCCCCCT
25	gtRNAdb v1.0	trna10_GluCTC_3_-_103879524_103879595	PSEUDO	TTATTATTATACCTGTGGTTAGGATTCGGCGCTCTCACCGCCACGACCCGGGTTCAATTCCCGGTCAGGGAA
26	gtRNAdb v1.0	trna10_GlyTCC_17_+_8124866_8124937	TRUE	GCGTTGGTGGTATAGTGGTAAGCATAGCTGCCTTCCAAGCAGTTGACCCGGGTTCGATTCCCGGCCAACGCA
27	gtRNAdb v1.0	trna10_IleAAT_14_+_102783429_102783502	TRUE	GGCCGGTTAGCTCAGTTGGTTAGAGCGTGGTGCTAATAACGCCAAGGTCGCGGGTTCGATCCCCGTACGGGCCA
28	gtRNAdb v1.0	trna10_IleTAT_19_-_39902808_39902900_intronat_39902844_39902862	TRUE	GCTCCAGTGGCGCAATCGGTTAGCGCGCGGTACTTATATGACAGTGCGAGCGGAGCAATGCCGAGGTTGTGAGTTCGATCCTCACCTGGAGCA
29	gtRNAdb v1.0	trna10_LysCTT_16_+_3241501_3241573	TRUE	GCCCGGCTAGCTCAGTCGGTAGAGCATGGGACTCTTAATCTCAGGGTCGTGGGTTCGAGCCCCACGTTGGGCG
30	gtRNAdb v1.0	trna10_MetCAT_8_-_124169470_124169542	TRUE	GCCTCGTTAGCGCAGTAGGTAGCGCGTCAGTCTCATAATCTGAAGGTCGTGAGTTCGATCCTCACACGGGGCA
31	gtRNAdb v1.0	trna10_SerAGA_11_+_109036039_109036120	TRUE	TGAGTTGTAGCTGAGTGGTTAAGGCAACGAGCTAGAAATTCGTTGGTTTCTCTCTGTGCAGGTTTGAATCCTGCTAATTATG
32	gtRNAdb v1.0	trna10_SerGCT_15_-_40886023_40886104	TRUE	GACGAGGTGGCCGAGTGGTTAAGGCGATGGACTGCTAATCCATTGTGCTCTGCACGCGTGGGTTCGAATCCCATCCTCGTCG
33	gtRNAdb v1.0	trna10_SerTGA_2_+_132140142_132140211	PSEUDO	GAGAAGGTCATAGAGGTTATGGGATTGGCTTGAAACCAGTCTCTGGGGGGTTCGATTCCCTCCTTTTTCA
34	gtRNAdb v1.0	trna10_ValCAC_5_-_180649395_180649467	TRUE	GTTTCCGTAGTGTAGTGGTTATCACGTTCGCCTCACACGCGAAAGGTCCCCGGTTCGAAACCGGGCGGAAACA
35	gtRNAdb v1.0	trna110_AlaTGC_6_-_28757547_28757618	TRUE	GGGGGTGTAGCTCAGTGGTAGAGCGCATGCTTTGCATGTATGAGGTCCCGGGTTCGATCCCCGGCACCTCCA
36	gtRNAdb v1.0	trna110_GluTTC_1_-_147781030_147781101	PSEUDO	TCCTTGGTGGTCTAGTGGCTAGGATTCGGTGCTTTCACCTGTGCGGCCCGGGTTCAATTCCCGATGAAGGAA
37	gtRNAdb v1.0	trna111_AlaAGC_6_-_28746544_28746615	PSEUDO	GGGGGTATAGCTCAGGGACAGAGCACGTGGTTAGCATGCGTGAGGTCCTGCGTTCAACTTCCAGTATTTCCA
38	gtRNAdb v1.0	trna111_HisGTG_1_-_147774845_147774916	TRUE	GCCGTGATCGTATAGTGGTTAGTACTCTGCGTTGTGGCCGCAGCAACCTCGGTTCGAATCCGAGTCACGGCA
39	gtRNAdb v1.0	trna112_GlnCTG_1_-_147737382_147737453	TRUE	GGTTCCATGGTGTAATGGTAAGCACTCTGGACTCTGAATCCAGCGATCCGAGTTCGAGTCTCGGTGGAACCT
40	gtRNAdb v1.0	trna112_PheGAA_6_-_28731374_28731447	TRUE	GCTGAAATAGCTCAGTTGGGAGAGCGTTAGACTGAAGATCTTAAAGTTCCCTGGTTCAACCCTGGGTTTCAGCC
41	gtRNAdb v1.0	trna113_AlaTGC_6_-_28726141_28726212	TRUE	GGGGGTGTAGCTCAGTGGTAGAGCACATGCTTTGCATGTGTGAGGCCCCGGGTTCGATCCCCGGCACCTCCA
42	gtRNAdb v1.0	trna113_AsnGTT_1_-_147520767_147520840	TRUE	GTCTCTGTGGCGCAATCGGTTAGCGCGTTCGGCTGTTAACTGAAAGGTTAGTGGTTCGAGCCCACCCGGGGACG
43	gtRNAdb v1.0	trna114_ArgCCG_6_-_28710729_28710801	TRUE	GGCCGCGTGGCCTAATGGATAAGGCGTCTGATTCCGGATCAGAAGATTGAGGGTTCGAGTCCCTTCGTGGTCG
44	gtRNAdb v1.0	trna114_GlnCTG_1_-_146476760_146476831	TRUE	GGTTCCATGGTGTAATGGTGACCACTTTGGACTCTGAATACAGTGATCAGAGTTCAAGTCTCACTGGAACCT
45	gtRNAdb v1.0	trna115_AsnGTT_1_-_145979034_145979107	TRUE	GTCTCTGTGGCGCAATCGGTTAGCGCGTTCGGCTGTTAACTGAAAGGTTAGTGGTTCGAGCCCACCCGGGGACG
46	gtRNAdb v1.0	trna115_ValAAC_6_-_28703206_28703277	TRUE	GGGGGTGTAGCTCAGTGGTAGAGCGTATGCTTAACATTCATGAGGCTCTGGGTTCGATCCCCAGCACTTCCA
47	gtRNAdb v1.0	trna116_GluCTC_1_-_145399233_145399304	TRUE	TCCCTGGTGGTCTAGTGGTTAGGATTCGGCGCTCTCACCGCCGCGGCCCGGGTTCGATTCCCGGTCAGGGAA
48	gtRNAdb v1.0	trna116_PheGAA_6_-_28694855_28694927	PSEUDO	GCTGAGATAGCTCGGTTGGGAGGGCATCAGCCTGAAGATCTAAAGGAGACTGGTTCAATTCTGGGTTTTGGCA
49	gtRNAdb v1.0	trna117_AlaCGC_6_-_28663709_28663780	TRUE	GGGGGTGTAGATCAGTGGTAGAGCGCATGCTTCGCATGTACGAGGTCCCTGGTTCAATCCCTGGTACCTCCA
50	gtRNAdb v1.0	trna117_GlyTCC_1_-_145397864_145397935	TRUE	GCGTTGGTGGTATAGTGGTGAGCATAGCTGCCTTCCAAGCAGTTGACCCGGGTTCGATTCCCGGCCAACGCA
51	gtRNAdb v1.0	trna118_HisGTG_1_-_145396881_145396952	TRUE	GCCGTGATCGTATAGTGGTTAGTACTCTGCGTTGTGGCCGCAGCAACCTCGGTTCGAATCCGAGTCACGGCA
52	gtRNAdb v1.0	trna118_LysTTT_6_-_28660987_28661060	PSEUDO	GCCTGGGTAGCTCAGTCGGTAGAGCTATCAGACTTTTAGCCTGAGGATTCAGGGTTCAATCCCTTGCTGGGGCG
53	gtRNAdb v1.0	trna119_AlaCGC_6_-_28641613_28641684	TRUE	GGGGATGTAGCTCAGTGGTAGAGCGCATGCTTCGCATGTATGAGGCCCCGGGTTCGATCCCCGGCATCTCCA
54	gtRNAdb v1.0	trna119_LysCTT_1_-_145395522_145395594	TRUE	GCCCGGCTAGCTCAGTCGGTAGAGCATGAGACTCTTAATCTCAGGGTCGTGGGTTCGAGCCCCACGTTGGGCG
55	gtRNAdb v1.0	trna11_ArgACG_3_-_45730491_45730563	TRUE	GGGCCAGTGGCGCAATGGATAACGCGTCTGACTACGGATCAGAAGATTCTAGGTTCGACTCCTGGCTGGCTCG
56	gtRNAdb v1.0	trna11_AsnGTT_1_+_144301611_144301684	TRUE	GTCTCTGTGGTGCAATCGGTTAGCGCGTTCCGCTGTTAACCGAAAGCTTGGTGGTTCGAGCCCACCCAGGGATG
57	gtRNAdb v1.0	trna11_CysGCA_7_+_149295046_149295117	TRUE	GGGCGTATAGCTCAGGGGTAGAGCATTTGACTGCAGATCAAGAGGTCCCCAGTTCAAATCTGGGTGCCCCCT
58	gtRNAdb v1.0	trna11_GlnTTG_2_+_132143133_132143204	PSEUDO	TAGGACGTGGTGTGATAGGTAGCACGGAGAATTTTGGATTCTCAGGGATGGGTTCAATTCCTGTAGTTCTAG
59	gtRNAdb v1.0	trna11_GluTTC_15_-_26327381_26327452	TRUE	TCCCACATGGTCTAGCGGTTAGGATTCCTGGTTTTCACCCAGGCGGCCCGGGTTCGACTCCCGGTGTGGGAA
60	gtRNAdb v1.0	trna11_IleAAT_6_+_26554350_26554423	TRUE	GGCCGGTTAGCTCAGTTGGTTAGAGCGTGGTGCTAATAACGCCAAGGTCGCGGGTTCGATCCCCGTACGGGCCA
61	gtRNAdb v1.0	trna11_LysCTT_5_-_180648979_180649051	TRUE	GCCCGGCTAGCTCAGTCGGTAGAGCATGAGACTCTTAATCTCAGGGTCGTGGGTTCGAGCCCCACGTTGGGCG
62	gtRNAdb v1.0	trna11_LysTTT_11_+_122430655_122430727	TRUE	GCCTGGATAGCTCAGTTGGTAGAGCATCAGACTTTTAATCTGAGGGTCCAGGGTTCAAGTCCCTGTTCAGGCG
63	gtRNAdb v1.0	trna11_PheGAA_12_-_125412389_125412461	TRUE	GCCGAAATAGCTCAGTTGGGAGAGCGTTAGACTGAAGATCTAAAGGTCCCTGGTTCGATCCCGGGTTTCGGCA
64	gtRNAdb v1.0	trna11_ProAGG_16_+_3241989_3242060	TRUE	GGCTCGTTGGTCTAGGGGTATGATTCTCGCTTAGGATGCGAGAGGTCCCGGGTTCAAATCCCGGACGAGCCC
65	gtRNAdb v1.0	trna11_SerAGA_8_-_96281885_96281966	TRUE	GTAGTCGTGGCCGAGTGGTTAAGGCGATGGACTAGAAATCCATTGGGGTCTCCCCGCGCAGGTTCGAATCCTGCCGACTACG
66	gtRNAdb v1.0	trna11_SupCTA_14_-_78700838_78700910	PSEUDO	GGCGGGATAATGTAGTGGTTAAAGGCATGGGCTCTAGAGCCAGACTTCCTGGGTTCAAATCTCAGACCTGCTA
67	gtRNAdb v1.0	trna11_SupCTA_17_+_15408685_15408758	TRUE	GGCAGTGACGCCTACTGGTTTAGAGCACAGATTCTAGATCGAGACATTCCTGGGTTCAAATCCCAGCACTGTTG
68	gtRNAdb v1.0	trna120_AlaAGC_6_-_28626014_28626085	TRUE	GGGGATGTAGCTCAGTGGTAGAGCGCATGCTTAGCATGCATGAGGTCCCGGGTTCGATCCCCAGCATCTCCA
69	gtRNAdb v1.0	trna120_GlnCTG_1_-_145379320_145379391	TRUE	GGTTCCATGGTGTAATGGTGAGCACTTTGGACTCTGAATACAGTGATCAGAGTTCAAGTCTCACTGGGACCT
70	gtRNAdb v1.0	trna121_GlnCTG_1_-_144839436_144839507	TRUE	GGTTCCATGGTGTAATGGTGAGCACTTTGGACTCTGAATACAGTGATCAGAGTTCAAGTCTCACTGGGACCT
71	gtRNAdb v1.0	trna121_ThrCGT_6_-_28615984_28616057	TRUE	GGCTCTGTGGCTTAGTTGGCTAAAGCGCCTGTCTCGTAAACAGGAGATCCTGGGTTCGAATCCCAGCGGGGCCT
72	gtRNAdb v1.0	trna122_AlaTGC_6_-_28601859_28601930	PSEUDO	GTGGATGTAGTTTAGTGGTAGAACGCGCGCTTTGCATGTATGAGGTCCCGGTTTCGATCCCTGGCGTTTCCA
73	gtRNAdb v1.0	trna122_AsnGTT_1_-_144488843_144488916	TRUE	GTCTCTGTGGCGCAATCGGTTAGCGCGTTTGACTGTTAACTGAAAGGTTGGTGGTGCAAGCCCATCCAGGGATG
74	gtRNAdb v1.0	trna123_AsnGTT_1_-_144308614_144308687	TRUE	GTCTCTGTGGCGCAATCGGTTAGCGCGTTTGACTGTTAACTGAAAGGTTGGTGGTGCAAGCCCATCCAGGGATG
75	gtRNAdb v1.0	trna123_SerGCT_6_-_28565117_28565198	TRUE	GACGAGGTGGCCGAGTGGTTAAGGCGATGGACTGCTAATCCATTGTGCTCTGCACGCGTGGGTTCGAATCCCATCCTCGTCG
76	gtRNAdb v1.0	trna124_ArgTCG_6_-_28510891_28510963	TRUE	GACCACGTGGCCTAATGGATAAGGCGTCTGACTTCGGATCAGAAGATTGAGGGTTCGAATCCCTTCGTGGTTG
77	gtRNAdb v1.0	trna124_AsnGTT_1_-_143879832_143879905	TRUE	GTCTCTGTGGCGCAATCGGCTAGCGCGTTTGGCTGTTAACTAAAAGGTTGGCGGTTCGAACCCACCCAGAGGCG
78	gtRNAdb v1.0	trna125_AsnGTT_1_-_143663558_143663631	PSEUDO	GTCTCTGTGGCACAATCGGTTAGCGCGTTCGGCTGTTAATCTAGAGGTTGGTGGTTAGAGCCCACTGAGGGATG
79	gtRNAdb v1.0	trna125_ThrCGT_6_-_28456770_28456843	TRUE	GGCTCTATGGCTTAGTTGGTTAAAGCGCCTGTCTCGTAAACAGGAGATCCTGGGTTCGACTCCCAGTGGGGCCT

80	gtRNAdb v1.0	trna126_LeuAAG_6_-_28446400_28446481	TRUE	GGTAGCGTGGCCGAGTGGTCTAAGACGCTGGATTAAGGCTCCAGTCTCTTCGGGGGCGTGGGTTTGAATCCCACCGCTGCCA
81	gtRNAdb v1.0	trna127_CysGCA_1_-_93981834_93981906	TRUE	GGGGGTATAGCTCAGGTGGTAGAGCATTTGACTGCAGATCAAGAGGTCCCCGGTTCAAATCCGGGTGCCCCCT
82	gtRNAdb v1.0	trna127_ThrTGT_6_-_28442329_28442402	TRUE	GGCTCTATGGCTTAGTTGGTTAAAGCGCCTGTCTTGTAAACAGGAGATCCTGGGTTCGAATCCCAGTAGAGCCT
83	gtRNAdb v1.0	trna128_GlyGCC_6_-_27870686_27870756	TRUE	GCATTGGTGGTTCAGTGGTAGAATTCTCGCCTGCCACGCGGGAGGCCCGGGTTCGATTCCCGGCCAATGCA
84	gtRNAdb v1.0	trna128_LysCTT_1_-_55423542_55423614	TRUE	GCCCAGCTAGCTCAGTCGGTAGAGCATGAGACTCTTAATCTCAGGGTCATGGGTTTGAGCCCCACGTTTGGTG
85	gtRNAdb v1.0	trna129_GlnCTG_1_-_17180900_17180971	PSEUDO	GGTTCCATGATGTAATGGTGAGCGCTTTGGACTCTGAGTACGGTGATCAGCGTTCAAGTCTCAGTGGGACCT
86	gtRNAdb v1.0	trna129_MetCAT_6_-_27870271_27870342	TRUE	AGCAGAGTGGCGCAGCGGAAGCGTGCTGGGCCCATAACCCAGAGGTCGATGGATCGAAACCATCCTCTGCTA
87	gtRNAdb v1.0	trna12_ArgCCT_16_+_3243918_3243990	TRUE	GCCCCAGTGGCCTGATGGATAAGGTACTGGCCTCCTAAGCCAGGGATTGTGGGTTCGAGTTCCACCTGGGGTA
88	gtRNAdb v1.0	trna12_AsnGTT_1_+_144481840_144481913	TRUE	GTCTCTGTGGTGCAATCGGTTAGCGCGTTCCGCTGTTAACCGAAAGCTTGGTGGTTTGAGCCCACCCAGGGATG
89	gtRNAdb v1.0	trna12_AspGTC_12_-_125411891_125411962	TRUE	TCCTCGTTAGTATAGTGGTGAGTATCCCCGCCTGTCACGCGGGAGACCGGGGTTCGATTCCCCGACGGGGAG
90	gtRNAdb v1.0	trna12_CysGCA_3_-_17741392_17741464	PSEUDO	GGGGGTATATCTCAGGGGGCAGAGCATTTGACTGCAGATCAAGAGGTCCCCGGTTGAAATCCGGGTGCTGGAT
91	gtRNAdb v1.0	trna12_GlnTTG_19_-_9150356_9150428	PSEUDO	AGCAGTGTAGCCTAGTGGCTAGGTCCTCTGACTTTGAAACCACGTGGTCTGGGTTTAAGTCTCAGCTGTGCTA
92	gtRNAdb v1.0	trna12_GluCTC_2_+_151219277_151219349	PSEUDO	TTCCCCTTGGTCTAGTGGTTAGGATTCAACACTCTCACCGCCGCAGCCCGGGTTTGATTCCCAGGCAGGGAAG
93	gtRNAdb v1.0	trna12_LysTTT_14_-_74055529_74055601	PSEUDO	ACCCAGATAGCTCAGTTGATAGAGCATCAGACTTTTAATCTGAGGGTCCAGGGTTCATGTCCCTGTTCCTTAA
94	gtRNAdb v1.0	trna12_ProAGG_6_+_26555498_26555569	TRUE	GGCTCGTTGGTCTAGGGGTATGATTCTCGCTTAGGGTGCGAGAGGTCCCGGGTTCAAATCCCGGACGAGCCC
95	gtRNAdb v1.0	trna12_ProTGG_11_-_75946869_75946940	TRUE	GGCTCGTTGGTCTAGGGGTATGATTCTCGGTTTGGGTCCGAGAGGTCCCGGGTTCAAATCCCGGACGAGCCC
96	gtRNAdb v1.0	trna12_SerAGA_7_+_149305467_149305538	TRUE	GGGTGTATGGCTCAGGGGTAGAGAATTTGACTAGAGATCAAGAGGTCCCTGGTTCAAATCCAGGTGCCCCCT
97	gtRNAdb v1.0	trna12_TrpCCA_17_+_19411494_19411565	TRUE	GACCTCGTGGCGCAATGGTAGCGCGTCTGACTCCAGATCAGAAGGTTGCGTGTTCAAGTCACGTCGGGGTCA
98	gtRNAdb v1.0	trna12_TyrGTA_8_-_66609532_66609619_intronat_66609567_66609582	TRUE	TCTTCAATAGCTCAGCTGGTAGAGCGGAGGACTGTAGGTGCACGCCCGTGGCCATTCTTAGGTGCTGGTTTGATTCCGACTTGGAGAG
99	gtRNAdb v1.0	trna12_ValAAC_5_-_180645270_180645342	TRUE	GTTTCCGTAGTGTAGTGGTTATCACGTTCGCCTAACACGCGAAAGGTCCCCGGTTCGAAACCGGGCGGAAACA
100	gtRNAdb v1.0	trna130_GlnTTG_6_-_27763640_27763711	TRUE	GGCCCCATGGTGTAATGGTTAGCACTCTGGACTTTGAATCCAGCGATCCGAGTTCAAATCTCGGTGGGACCT
101	gtRNAdb v1.0	trna130_ValCAC_1_-_17006501_17006573	TRUE	GTTTCTGTGGTGTAGTGGTTATCATGTTCGCCTCACACGAGAAAAGTCCCTGATTCGAGACTGGGTGGGAACG
102	gtRNAdb v1.0	trna131_GlnCTG_6_-_27759135_27759206	TRUE	GGCCCCATGGTGTAATGGTCAGCACTCTGGACTCTGAATCCAGCGATCCGAGTTCAAATCTCGGTGGGACCC
103	gtRNAdb v1.0	trna131_GlyCCC_1_-_17004766_17004836	TRUE	GCGTTGGTGGTTTAGTGGTAGAATTCTCGCCTCCCATGCGGGAGACCCGGGTTCAATTCCCGGCCACTGCA
104	gtRNAdb v1.0	trna132_ValAAC_6_-_27721179_27721251	TRUE	GTTTCCGTAGTGTAGTGGTTATCACGTTCGCCTAACACGCGAAAGGTCCCCGGTTCGAAACCGGGCGGAAACA
105	gtRNAdb v1.0	trna132_ValCAC_1_-_16874160_16874232	PSEUDO	GTTTCTGTGGTGTAGTGGTTATTATGTTCGCTTCACATATGAAAGGTCTCTGGTTCGAGACTGCGTGGGAACA
106	gtRNAdb v1.0	trna133_GlyCCC_1_-_16872434_16872504	TRUE	GCATTGGTGGTTCAGTGGTAGAATTCTCGCCTCCCACGCGGGAGACCCGGGTTCAATTCCCGGCCAATGCA
107	gtRNAdb v1.0	trna133_ValCAC_6_-_27696327_27696399	TRUE	GTTTCCGTAGTGTAGTGGTTATTATGTTCGCCTCACACGCGAAAAGTCCCCGGTTCGAAATCAGGCGGGAACA
108	gtRNAdb v1.0	trna134_GluTTC_1_-_16861774_16861845	TRUE	TCCCTGGTGGTCTAGTGGCTAGGATTCGGCGCTTTCACCGCCGCGGCCCGGGTTCGATTCCCGGTCAGGGAA
109	gtRNAdb v1.0	trna134_LeuTAA_6_-_27688898_27688980	TRUE	ACCGGGATGGCCGAGTGGTTAAGGCGTTGGACTTAAGATCCAATGGGCTGGTGCCCGCGTGGGTTCGAACCCCACTCTCGGTA
110	gtRNAdb v1.0	trna135_AsnGTT_1_-_16858893_16858966	TRUE	GTCTCTGTGGTGCAATCGGTTAGCGCGTTCGGCTGTTAACCATAAGGTTGGTGGTTACAGACCACCCAGGGACG
111	gtRNAdb v1.0	trna135_ThrAGT_6_-_27652474_27652547	TRUE	GGCTCCGTGGCTTAGCTGGTTAAAGCGCCTGTCTAGTAAACAGGAGATCCTGGGTTCGAATCCCAGCGGGGCCT
112	gtRNAdb v1.0	trna136_AsnGTT_1_-_16847080_16847153	TRUE	GTCTCTGTGGCGCAATCGGTTAGCGCGTTCGGCTGTTAACTGAAAGGTTGGTGGTTCGAGCCCACCCAGGGACG
113	gtRNAdb v1.0	trna136_ValAAC_6_-_27648885_27648957	TRUE	GTTTCCGTAGTGTAGTGGTTATCACGTTCGCCTAACACGCGAAAGGTCCGCGGTTCGAAACCGGGCGGAAACA
114	gtRNAdb v1.0	trna137_SerCGA_6_-_27640229_27640310	TRUE	GCTGTGATGGCCGAGTGGTTAAGGTGTTGGACTCGAAATCCAATGGGGGTTCCCCGCGCAGGTTCAAATCCTGCTCACAGCG
115	gtRNAdb v1.0	trna138_ArgACG_6_-_27638344_27638416	TRUE	GGGCCAGTGGCGCAATGGATAACGCGTCTGACTACGGATCAGAAGATTCTAGGTTCGACTCCTGGCTGGCTCG
116	gtRNAdb v1.0	trna139_ValAAC_6_-_27618707_27618779	TRUE	GTTTCCGTAGTGTAGTGGTTATCACGTTCGCCTAACACGCGAAAGGTCCCTGGATCAAAACCAGGCGGAAACA
117	gtRNAdb v1.0	trna13_AlaCGC_2_+_157257281_157257352	TRUE	GGGGATGTAGCTCAGTGGTAGAGCGCGCGCTTCGCATGTGTGAGGTCCCGGGTTCAATCCCCGGCATCTCCA
118	gtRNAdb v1.0	trna13_AlaTGC_12_-_125406301_125406372	TRUE	GGGGATGTAGCTCAGTGGTAGAGCGCATGCTTTGCATGTATGAGGCCCCGGGTTCGATCCCCGGCATCTCCA
119	gtRNAdb v1.0	trna13_AsnGTT_1_+_145385729_145385804	TRUE	GTCTCTGTGGCGCCATCGGTTAGTGCCTTCGGCTGTTTGAACCGAAAGGCTGGTGGTTCAAGCCCACCCAGAGATG
120	gtRNAdb v1.0	trna13_CysGCA_7_+_149332778_149332849	TRUE	GGGGGTATAGCTCAGGGGTAGAGCATTTGACTGCAGATCAAGAGGTCCCTGGTTCAAATCCAGGTGCCCCCT
121	gtRNAdb v1.0	trna13_GlyCCC_17_+_19764175_19764245	TRUE	GCATTGGTGGTTCAATGGTAGAATTCTCGCCTCCCACGCAGGAGACCCAGGTTCGATTCCTGGCCAATGCA
122	gtRNAdb v1.0	trna13_LeuTAA_8_-_47740038_47740112	PSEUDO	GTTAAGATGGCATAGCCCAGCAATTGCATAAAACTTAAGACTTTATAATTAGAAGTTCAACACCTCTTCTTAACA
123	gtRNAdb v1.0	trna13_LysCTT_14_-_58706613_58706685	TRUE	GCCCGGCTAGCTCAGTCGGTAGAGCATGGGACTCTTAATCCCAGGGTCGTGGGTTCGAGCCCCACGTTGGGCG
124	gtRNAdb v1.0	trna13_LysCTT_16_+_3246153_3246225	PSEUDO	GCCCAGCTAGCTCAGCCGGTAGAGCACAAGACTCTTAATCTCAGGGTCGTGGGTTTGAGCCCTGTGTTGAGCA
125	gtRNAdb v1.0	trna13_LysCTT_6_+_26556774_26556846	TRUE	GCCCGGCTAGCTCAGTCGGTAGAGCATGAGACTCTTAATCTCAGGGTCGTGGGTTCGAGCCCCACGTTGGGCG
126	gtRNAdb v1.0	trna13_PheGAA_11_-_59333853_59333925	TRUE	GCCGAAATAGCTCAGTTGGGAGAGCGTTAGACTGAAGATCTAAAGGTCCCTGGTTCAATCCCGGGTTTCGGCA
127	gtRNAdb v1.0	trna13_ThrTGT_5_-_180618687_180618758	TRUE	GGCTCCATAGCTCAGGGGTTAGAGCACTGGTCTTGTAAACCAGGGTCGCGAGTTCAAATCTCGCTGGGGCCT
128	gtRNAdb v1.0	trna13_ValCAC_19_-_4724647_4724719	TRUE	GTTTCCGTAGTGTAGCGGTTATCACATTCGCCTCACACGCGAAAGGTCCCCGGTTCGATCCCGGGCGGAAACA
129	gtRNAdb v1.0	trna140_LeuCAA_6_-_27573417_27573524_intronat_27573463_27573486	TRUE	GTCAGGATGGCCGAGTGGTCTAAGGCGCCAGACTCAAGCTTACTGCTTCCTGTGTTCGGGTCTTCTGGTCTCCGTATGGAGGCGTGGGTTCGAATCCCACTTCTGACA
130	gtRNAdb v1.0	trna141_LeuCAA_6_-_27570348_27570454_intronat_27570394_27570416	TRUE	GTCAGGATGGCCGAGTGGTCTAAGGCGCCAGACTCAAGTTGCTACTTCCCAGGTTTGGGGCTTCTGGTCTCCGCATGGAGGCGTGGGTTCGAATCCCACTTCTGACA
131	gtRNAdb v1.0	trna142_MetCAT_6_-_27560600_27560671	TRUE	AGCAGAGTGGCGCAGCGGAAGCGTGCTGGGCCCATAACCCAGAGGTCGATGGATCGAAACCATCCTCTGCTA
132	gtRNAdb v1.0	trna143_LysTTT_6_-_27559593_27559665	TRUE	GCCTGGATAGCTCAGTCGGTAGAGCATCAGACTTTTAATCTGAGGGTCCAGGGTTCAAGTCCCTGTTCAGGCG
133	gtRNAdb v1.0	trna144_AspGTC_6_-_27551236_27551307	TRUE	TCCTCGTTAGTATAGTGGTGAGTGTCCCCGTCTGTCACGCGGGAGACCGGGGTTCGATTCCCCGACGGGGAG
134	gtRNAdb v1.0	trna145_SerAGA_6_-_27521192_27521273	TRUE	GTAGTCGTGGCCGAGTGGTTAAGGTGATGGACTAGAAACCCATTGGGGTCTCCCCGCGCAGGTTCGAATCCTGCCGACTACG
135	gtRNAdb v1.0	trna146_GlnCTG_6_-_27515531_27515602	TRUE	GGTTCCATGGTGTAATGGTTAGCACTCTGGACTCTGAATCCAGCGATCCGAGTTCAAGTCTCGGTGGAACCT
136	gtRNAdb v1.0	trna147_SerAGA_6_-_27509554_27509635	TRUE	GTAGTCGTGGCCGAGTGGTTAAGGCGATGGACTAGAAATCCATTGGGGTTTCCCCGCGCAGGTTCGAATCCTGCCGACTACG
137	gtRNAdb v1.0	trna148_SerTGA_6_-_27473607_27473688	TRUE	GTAGTCGTGGCCGAGTGGTTAAGGCGATGGACTTGAAATCCATTGGGGTTTCCCCGCGCAGGTTCGAATCCTGTCGGCTACG
138	gtRNAdb v1.0	trna149_LysTTT_6_-_27302769_27302841	TRUE	GCCTGGGTAGCTCAGTCGGTAGAGCATCAGACTTTTAATCTGAGGGTCCAGGGTTCAAGTCCCTGTCCAGGCG
139	gtRNAdb v1.0	trna14_ArgCCT_1_+_145941579_145941649	PSEUDO	GCATTGGTAGTTCAGCGGTGGCATTCTCCCCACCTACGCGGGAGACCTGGGTTCAACTCCCGGCCAAAGCA
140	gtRNAdb v1.0	trna14_CysGCA_7_+_149361915_149361986	TRUE	GGGGGTATAGCTCACAGGTAGAGCATTTGACTGCAGATCAAGAGGTCCCCGGTTCAAATCTGGGTGCCCCCT
141	gtRNAdb v1.0	trna14_GlnTTG_16_+_3419813_3419884	TRUE	TAGGACTTGGTGTAATGGGTAGCACAGAGAATTTTGGATTCTCAGGGGTGGGTTCAATTCCTTTCGTCCTAG
142	gtRNAdb v1.0	trna14_GlnTTG_8_-_32872646_32872717	PSEUDO	TAGTACATAGTGTAATAGGTAGCACAGATAATTTTGGATTCTCAGGGGTAGGTTCAATTCTTATAGTTCTAG
143	gtRNAdb v1.0	trna14_GluCTC_12_-_114386545_114386617	PSEUDO	CCGTGGATAGCCCAGCGGCTATGGGAGCCGGGCTCTCACTCTGACGTCCTGGGTTCAAGTCCCAGTGTGCACA
144	gtRNAdb v1.0	trna14_GluTTC_14_-_32236816_32236886	TRUE	ACCCTGTGGTCTAGTGGCTAAGACTTTGTGCTTTCATTGCTGCATCCTAGGTTCAATTCCCAGTCAGGGAA
145	gtRNAdb v1.0	trna14_LysTTT_11_-_59327808_59327880	TRUE	GCCCGGATAGCTCAGTCGGTAGAGCATCAGACTTTTAATCTGAGGGTCCAGGGTTCAAGTCCCTGTTCGGGCG
146	gtRNAdb v1.0	trna14_PheGAA_19_-_1383361_1383433	TRUE	GCCGAAATAGCTCAGTTGGGAGAGCGTTAGACTGAAGATCTAAAGGTCCCTGGTTCGATCCCGGGTTTCGGCA
147	gtRNAdb v1.0	trna14_ProTGG_5_-_180615854_180615925	TRUE	GGCTCGTTGGTCTAGGGGTATGATTCTCGCTTTGGGTGCGAGAGGTCCCGGGTTCAAATCCCGGACGAGCCC
148	gtRNAdb v1.0	trna14_ThrCGT_17_+_29877093_29877164	TRUE	GGCGCGGTGGCCAAGTGGTAAGGCGTCGGTCTCGTAAACCGAAGATCGCGGGTTCGAACCCCGTCCGTGCCT
149	gtRNAdb v1.0	trna14_TyrATA_2_+_219110549_219110641_intronat_219110586_219110605	TRUE	CCTTCAATAGTTCAGCTGGTAGAGCAGAGGACTATAGCTACTTCCTCAGTAGGAGACGTCCTTAGGTTGCTGGTTCGATTCCAGCTTGAAGGA
150	gtRNAdb v1.0	trna14_TyrGTA_6_+_26569086_26569176_intronat_26569123_26569140	TRUE	CCTTCGATAGCTCAGTTGGTAGAGCGGAGGACTGTAGTTGGCTGTGTCCTTAGACATCCTTAGGTCGCTGGTTCGAATCCGGCTCGAAGGA
151	gtRNAdb v1.0	trna150_MetCAT_6_-_27300764_27300835	TRUE	AGCAGAGTGGCGCAGCGGAAGCGTGCTGGGCCCATAACCCAGAGGTCGATGGATCGAAACCATCCTCTGCTA
152	gtRNAdb v1.0	trna151_ThrCGT_6_-_27271568_27271639	TRUE	GGCAGAGTGGTGCAGCGGAAGCGTGCTGGGCCCGTAACCCAGAGGTCAATGGATCGAAGCCATCCTTGGCTA
153	gtRNAdb v1.0	trna152_ValCAC_6_-_27248049_27248121	TRUE	GCTTCTGTAGTGTAGTGGTTATCACGTTCGCCTCACACGCGAAAGGTCCCCGGTTCGAAACCGGGCAGAAGCA
154	gtRNAdb v1.0	trna153_IleAAT_6_-_27242990_27243063	TRUE	GGCTGGTTAGCTCAGTTGGTTAGAGCGTGGTGCTAATAACGCCAAGGTCGCGGGTTCGATCCCCGTACTGGCCA
155	gtRNAdb v1.0	trna154_IleAAT_6_-_27205350_27205423	TRUE	GGCCGGTTAGCTCAGTTGGTTAGAGCGTGGTGCTAATAACGCCAAGGTCGCGGGTTCGATCCCCGTACGGGCCA
156	gtRNAdb v1.0	trna155_LeuTAA_6_-_27198334_27198416	TRUE	ACCGGGATGGCTGAGTGGTTAAGGCGTTGGACTTAAGATCCAATGGACAGGTGTCCGCGTGGGTTCGAGCCCCACTCCCGGTA
157	gtRNAdb v1.0	trna156_ArgACG_6_-_27181623_27181695	TRUE	GGGCCAGTGGCGCAATGGATAACGCGTCTGACTACGGATCAGAAGATTCTAGGTTCGACTCCTGGCTGGCTCG
158	gtRNAdb v1.0	trna157_ValCAC_6_-_27173867_27173939	TRUE	GTTTCCGTAGTGGAGTGGTTATCACGTTCGCCTCACACGCGAAAGGTCCCCGGTTTGAAACCAGGCGGAAACA
159	gtRNAdb v1.0	trna158_IleAAT_6_-_27144994_27145067	TRUE	GGCCGGTTAGCTCAGTTGGTTAGAGCGTGGTGCTAATAACGCCAAGGTCGCGGGTTCGATCCCCGTACGGGCCA
160	gtRNAdb v1.0	trna159_AlaAGC_6_-_26796006_26796078	TRUE	GGGGAATTAGCTCAAGTGGTAGAGCGCTTGCTTAGCATGCAAGAGGTAGTGGGATCGATGCCCACATTCTCCA
161	gtRNAdb v1.0	trna15_CysGCA_17_+_37023898_37023969	TRUE	GGGGGTATAGCTCAGTGGTAGAGCATTTGACTGCAGATCAAGAGGTCCCCGGTTCAAATCCGGGTGCCCCCT
162	gtRNAdb v1.0	trna15_CysGCA_7_+_149404760_149404831	TRUE	GGGGGTATAGCTCAGGGGTAGAGCATTTGACTGCAGATCAAGAGGTCCCTGGTTCAAATCCAGGTGCCCCCT
163	gtRNAdb v1.0	trna15_GlnCTG_1_+_145963304_145963375	TRUE	GGTTCCATGGTGTAATGGTGAGCACTCTGGACTCTGAATCCAGCGATCCGAGTTCGAGTCTCGGTGGAACCT
164	gtRNAdb v1.0	trna15_GlnCTG_2_+_219491132_219491207	PSEUDO	GTGAGACTGCACAGCCCAGTGGTGCAGGGCATGGCTCTGACACCTGGCGGCCTGGGTTCAAATCCCAGCTTCTACA
165	gtRNAdb v1.0	trna15_GlnTTG_12_-_97490691_97490764	PSEUDO	GGCAATGTAGCATTGTGGCTAAGTGCACAGGCTTTGGAAACTGGCAGGCCTGGGTTCAAATCCCAGCTTATTCA
166	gtRNAdb v1.0	trna15_LeuTAG_14_-_21145178_21145259	PSEUDO	GGTAGTGTGGTTGAATGGTCTAAGGCACTGAATTTAGGCTCCAGTCTCTTTGGGGACGTGGGTTTAAATCCCACTGCTGCAA
167	gtRNAdb v1.0	trna15_PheGAA_11_-_59324970_59325042	TRUE	GCCGAAATAGCTCAGTTGGGAGAGCGTTAGACTGAAGATCTAAAGGTCCCTGGTTCGATCCCGGGTTTCGGCA

168	gtRNAdb v1.0	trna15_ThrCGT_16_+_14379750_14379821	TRUE	GGCGCGGTGGCCAAGTGGTAAGGCGTCGGTCTCGTAAACCGAAGATCACGGGTTCGAACCCCGTCCGTGCCT
169	gtRNAdb v1.0	trna15_TyrGTA_6_+_26575798_26575887_intronat_26575835_26575851	TRUE	CTTTCGATAGCTCAGTTGGTAGAGCGGAGGACTGTAGGTTCATTAAACTAAGGCATCCTTAGGTCGCTGGTTCGAATCCGGCTCGAAGGA
170	gtRNAdb v1.0	trna15_ValAAC_5_-_180615416_180615488	TRUE	GTTTCCGTAGTGTAGTGGTCATCACGTTCGCCTAACACGCGAAAGGTCCCCGGTTCGAAACCGGGCGGAAACA
171	gtRNAdb v1.0	trna160_AlaAGC_6_-_26773771_26773843	TRUE	GGGGAATTGGCTCAAGCGGTAGAGCGCTTGCTTAGCATGCAAGAGGTAGCAGGATCGACGCCTGCACTCTCCA
172	gtRNAdb v1.0	trna161_AlaAGC_6_-_26771290_26771362	TRUE	GGGGAATTAGCTCAGGCGGTAGAGCGCTCGCTTAGCATGCGAGAGGTAGCGGGATCGACGCCCGCATTCTCCA
173	gtRNAdb v1.0	trna162_MetCAT_6_-_26758550_26758622	TRUE	GCCCTCTTAGCGCAGCGGGCAGCGCGTCAGTCTCATAATCTGAAGGTCCTGAGTTCGAGCCTCAGAGAGGGCA
174	gtRNAdb v1.0	trna163_IleAAT_6_-_26745255_26745328	TRUE	GGCCGGTTAGCTCAGTTGGTTAGAGCGTGGTGCTAATAACGCTAAGGTCGCGGGTTCGATCCCCGTACTGGCCA
175	gtRNAdb v1.0	trna164_MetCAT_6_-_26735574_26735646	TRUE	GCCCTCTTAGCGCAGCGGGCAGCGCGTCAGTCTCATAATCTGAAGGTCCTGAGTTCGAGCCTCAGAGAGGGCA
176	gtRNAdb v1.0	trna165_IleAAT_6_-_26721221_26721294	TRUE	GGCCGGTTAGCTCAGTTGGTCAGAGCGTGGTGCTAATAACGCCAAGGTCGCGGGTTCGATCCCCGTACGGGCCA
177	gtRNAdb v1.0	trna166_AlaAGC_6_-_26572092_26572164	TRUE	GGGGAATTAGCTCAAATGGTAGAGCGCTCGCTTAGCATGCGAGAGGTAGCGGGATCGATGCCCGCATTCTCCA
178	gtRNAdb v1.0	trna167_ThrAGT_6_-_26533145_26533218	TRUE	GGCTCCGTGGCTTAGCTGGTTAAAGCGCCTGTCTAGTAAACAGGAGATCCTGGGTTCGAATCCCAGCGGGGCCT
179	gtRNAdb v1.0	trna168_TrpCCA_6_-_26331672_26331743	TRUE	GACCTCGTGGCGCAACGGTAGCGCGTCTGACTCCAGATCAGAAGGTTGCGTGTTCAAATCACGTCGGGGTCA
180	gtRNAdb v1.0	trna169_MetCAT_6_-_26330529_26330600	TRUE	AGCAGAGTGGCGCAGCGGAAGCGTGCTGGGCCCATAACCCAGAGGTCGATGGATCGAAACCATCCTCTGCTA
181	gtRNAdb v1.0	trna16_CysGCA_7_-_149388272_149388343	TRUE	GGGGATATAGCTCAGGGGTAGAGCATTTGACTGCAGATCAAGAGGTCCCCGGTTCAAATCCGGGTGCCCCCC
182	gtRNAdb v1.0	trna16_GlnTTG_12_-_50211190_50211264	PSEUDO	TCTAGGATGTGGTGTGATAGGTAGCATGGAGAATTTTGGATTCTCAGGGGTAGGTTCAATTCCTATATTCTAGAA
183	gtRNAdb v1.0	trna16_GlnTTG_17_+_47269890_47269961	TRUE	GGTCCCATGGTGTAATGGTTAGCACTCTGGACTTTGAATCCAGCGATCCGAGTTCAAATCTCGGTGGGACCT
184	gtRNAdb v1.0	trna16_HisGTG_1_+_146544773_146544844	TRUE	GCCGTGATCGTATAGTGGTTAGTACTCTGCGTTGTGGCCGCAGCAACCTCGGTTCGAATCCGAGTCACGGCA
185	gtRNAdb v1.0	trna16_LeuAAG_16_+_22308461_22308542	TRUE	GGTAGCGTGGCCGAGCGGTCTAAGGCGCTGGATTAAGGCTCCAGTCTCTTCGGGGGCGTGGGTTCGAATCCCACCGCTGCCA
186	gtRNAdb v1.0	trna16_LeuAAG_5_-_180601044_180601125	TRUE	GGTAGCGTGGCCGAGCGGTCTAAGGCGCTGGATTAAGGCTCCAGTCTCTTCGGAGGCGTGGGTTCGAATCCCACCGCTGCCA
187	gtRNAdb v1.0	trna16_LysTTT_2_-_224186315_224186387	PSEUDO	GTTGGGGTAACTCAGTTGGTAGAGTAGCAGACTTTTCATCTGAGGGTCCAGGGTTTAAGTCCATGTCCAGGCA
188	gtRNAdb v1.0	trna16_TyrGTA_14_-_21131351_21131444_intronat_21131387_21131407	TRUE	CCTTCGATAGCTCAGCTGGTAGAGCGGAGGACTGTAGATTGTACAGACATTTGCGGACATCCTTAGGTCGCTGGTTCGATTCCGGCTCGAAGGA
189	gtRNAdb v1.0	trna16_TyrGTA_6_+_26577332_26577420_intronat_26577369_26577384	TRUE	CCTTCGATAGCTCAGTTGGTAGAGCGGAGGACTGTAGGCTCATTAAGCAAGGTATCCTTAGGTCGCTGGTTCGAATCCGGCTCGGAGGA
190	gtRNAdb v1.0	trna16_ValTAC_11_-_59318460_59318532	TRUE	GGTTCCATAGTGTAGCGGTTATCACGTCTGCTTTACACGCAGAAGGTCCTGGGTTCGAGCCCCAGTGGAACCA
191	gtRNAdb v1.0	trna170_TrpCCA_6_-_26319330_26319401	TRUE	GACCTCGTGGCGCAACGGTAGCGCGTCTGACTCCAGATCAGAAGGTTGCGTGTTCAAATCACGTCGGGGTCA
192	gtRNAdb v1.0	trna171_MetCAT_6_-_26313352_26313423	TRUE	AGCAGAGTGGCGCAGCGGAAGCGTGCTGGGCCCATAACCCAGAGGTCGATGGATCGAAACCATCCTCTGCTA
193	gtRNAdb v1.0	trna172_SerTGA_6_-_26312824_26312905	TRUE	GTAGTCGTGGCCGAGTGGTTAAGGCGATGGACTTGAAATCCATTGGGGTCTCCCCGCGCAGGTTCGAATCCTGCCGACTACG
194	gtRNAdb v1.0	trna173_GlnTTG_6_-_26311975_26312046	TRUE	GGCCCCATGGTGTAATGGTTAGCACTCTGGACTTTGAATCCAGCGATCCGAGTTCAAATCTCGGTGGGACCT
195	gtRNAdb v1.0	trna174_GlnTTG_6_-_26311424_26311495	TRUE	GGCCCCATGGTGTAATGGTTAGCACTCTGGACTTTGAATCCAGCGATCCGAGTTCAAATCTCGGTGGGACCT
196	gtRNAdb v1.0	trna175_SerGCT_6_-_26305718_26305801	TRUE	GGAGAGGCCTGGCCGAGTGGTTAAGGCGATGGACTGCTAATCCATTGTGCTCTGCACGCGTGGGTTCGAATCCCATCCTCGTCG
197	gtRNAdb v1.0	trna17_CysGCA_7_-_149344046_149344117	TRUE	GGGGGTATAGCTTAGGGGTAGAGCATTTGACTGCAGATCAAAAGGTCCCTGGTTCAAATCCAGGTGCCCCTT
198	gtRNAdb v1.0	trna17_GluTTC_1_+_146550178_146550249	PSEUDO	TCCCTGGTGGTCTGGTGGCTAGAATTTAGCGCTTTCACCGCCGCAGCTCGGGTTGGATTACCAGTCAGGGAA
199	gtRNAdb v1.0	trna17_GluTTC_2_-_204229131_204229201	PSEUDO	GCAATGGTGGTTCAGTGGTAGAATTCTCGCCTTTCACACAGGAGACCCGGGTTCAATTCCTGACCCATGTA
200	gtRNAdb v1.0	trna17_LeuAAG_5_-_180591564_180591645	PSEUDO	GATAGCAAGGCCGAGCGGTCTAAGGCTCCGGATTAAGGCGCCGGTGTCTTCGGAGGCATGGGTTCGAATTCCACCTCTGCCA
201	gtRNAdb v1.0	trna17_LeuCAG_16_+_57333863_57333945	TRUE	GTCAGGATGGCCGAGCGGTCTAAGGCGCTGCGTTCAGGTCGCAGTCTCCCCTGGAGGCGTGGGTTCGAATCCCACTTCTGACA
202	gtRNAdb v1.0	trna17_SupTTA_17_+_58863593_58863663	TRUE	GCCCGGATAGTTCAGTTGGTAGAGCATCAGACTTAATCAGAGGGTCCAGGGTTCAAGTCCCTGTTTGGGTG
203	gtRNAdb v1.0	trna17_TyrGTA_14_-_21128117_21128210_intronat_21128153_21128173	TRUE	CCTTCGATAGCTCAGCTGGTAGAGCGGAGGACTGTAGACTGCGGAAACGTTTGTGGACATCCTTAGGTCGCTGGTTCAATTCCGGCTCGAAGGA
204	gtRNAdb v1.0	trna17_TyrGTA_6_+_26595102_26595190_intronat_26595139_26595154	TRUE	CCTTCGATAGCTCAGCTGGTAGAGCGGAGGACTGTAGGGGTTTGAATGTGGTCATCCTTAGGTCGCTGGTTCGAATCCGGCTCGGAGGA
205	gtRNAdb v1.0	trna17_ValTAC_11_-_59318102_59318174	TRUE	GGTTCCATAGTGTAGTGGTTATCACGTCTGCTTTACACGCAGAAGGTCCTGGGTTCGAGCCCCAGTGGAACCA
206	gtRNAdb v1.0	trna18_AlaAGC_6_+_26673590_26673662	TRUE	GGGGAATTAGCTCAAGTGGTAGAGCGCTTGCTTAGCATGCAAGAGGTAGTGGGATCAATGCCCACATTCTCCA
207	gtRNAdb v1.0	trna18_AlaTGC_11_-_50233879_50233950	TRUE	GGGGGTGTAGCTCAGTGGTAGAGCGGATGCTTTGCATGTATGAGACTTTGGGTTGGATCCCCAGCACCTCCA
208	gtRNAdb v1.0	trna18_ArgCCT_1_+_147483291_147483361	PSEUDO	GCATTGGTAGTTCAGCGGTGGCATTCTCCCCACCTACGCGGGAGACCTGGGTTCAACTCCCGGCCAAAGCA
209	gtRNAdb v1.0	trna18_ArgCCT_17_+_73030001_73030073	TRUE	GCCCCAGTGGCCTAATGGATAAGGCACTGGCCTCCTAAGCCAGGGATTGTGGGTTCGAGTCCCACCTGGGGTA
210	gtRNAdb v1.0	trna18_CysGCA_7_-_149310156_149310227	TRUE	GGGGGTATAGCTCAGGGGTAGAGCATTTGACTGCAAATCAAGAGGTCCCTGATTCAAATCCAGGTGCCCCCT
211	gtRNAdb v1.0	trna18_GluCTC_2_-_159738171_159738242	PSEUDO	CCCCTGGTGGTCTAGTGCTTAGGATTTGGCACTCTCGCCACCGCAGCCTGCGTTCAATTCCCGGTCAGGGAA
212	gtRNAdb v1.0	trna18_GlyGCC_16_+_70822597_70822667	TRUE	GCATTGGTGGTTCAGTGGTAGAATTCTCGCCTGCCATGCGGGCGGCCGGGCTTCGATTCCTGGCCAATGCA
213	gtRNAdb v1.0	trna18_TyrGTA_14_-_21125623_21125716_intronat_21125659_21125679	TRUE	CCTTCGATAGCTCAGCTGGTAGAGCGGAGGACTGTAGATTGTATAGACATTTGCGGACATCCTTAGGTCGCTGGTTCGATTCCAGCTCGAAGGA
214	gtRNAdb v1.0	trna18_ValCAC_5_-_180529253_180529325	TRUE	GTTTCCGTAGTGTAGTGGTTATCACGTTCGCCTCACACGCGAAAGGTCCCCGGTTCGAAACCGGGCGGAAACA
215	gtRNAdb v1.0	trna19_AlaAGC_6_+_26682715_26682787	TRUE	GGGGAATTAGCTCAAGTGGTAGAGCGCTTGCTTAGCATGCAAGAGGTAGTGGGATCGATGCCCACATTCTCCA
216	gtRNAdb v1.0	trna19_ArgTCG_17_+_73031208_73031280	TRUE	GACCGCGTGGCCTAATGGATAAGGCGTCTGACTTCGGATCAGAAGATTGAGGGTTCGAGTCCCTTCGTGGTCG
217	gtRNAdb v1.0	trna19_CysGCA_7_-_149292305_149292376	TRUE	GGGGGTATAGCTCACAGGTAGAGCATTTGACTGCAGATCAAGAGGTCCCCGGTTCAAATCCGGTTACTCCCT
218	gtRNAdb v1.0	trna19_GlnCTG_1_+_147505038_147505109	TRUE	GGTTCCATGGTGTAATGGTGAGCACTCTGGACTCTGAATCCAGCGATCCGAGTTCGAGTCTCGGTGGAACCT
219	gtRNAdb v1.0	trna19_GlyGCC_16_+_70823410_70823480	TRUE	GCATTGGTGGTTCAGTGGTAGAATTCTCGCCTGCCACGCGGGAGGCCCGGGTTCGATTCCCGGCCAATGCA
220	gtRNAdb v1.0	trna19_GlyGCC_2_-_157257659_157257729	TRUE	GCATTGGTGGTTCAGTGGTAGAATTCTCGCCTGCCACGCGGGAGGCCCGGGTTCGATTCCCGGCCAATGCA
221	gtRNAdb v1.0	trna19_LeuAAG_5_-_180524474_180524555	TRUE	GGTAGCGTGGCCGAGCGGTCTAAGGCGCTGGATTAAGGCTCCAGTCTCTTCGGAGGCGTGGGTTCGAATCCCACCGCTGCCA
222	gtRNAdb v1.0	trna19_TrpCCA_11_-_45290200_45290273	TRUE	GGAAGGATGGGGCCAAGCTGGAAAGCCTGTGGGCTCCACAGTCATGTGCCTGGGTTCAATTCCCAGTTCTGCAT
223	gtRNAdb v1.0	trna19_TyrGTA_14_-_21121258_21121351_intronat_21121294_21121314	TRUE	CCTTCGATAGCTCAGCTGGTAGAGCGGAGGACTGTAGCCTGTAGAAACATTTGTGGACATCCTTAGGTCGCTGGTTCGATTCCGGCTCGAAGGA
224	gtRNAdb v1.0	trna1_ArgCCG_16_+_3200675_3200747	TRUE	GGCCGCGTGGCCTAATGGATAAGGCGTCTGATTCCGGATCAGAAGATTGAGGGTTCGAGTCCCTTCGTGGTCG
225	gtRNAdb v1.0	trna1_AsnGTT_19_+_1383562_1383635	TRUE	GTCTCTGTGGCGCAATCGGTTAGCGCGTTCGGCTGTTAACCGAAAGGTTGGTGGTTCGAGCCCACCCAGGGACG
226	gtRNAdb v1.0	trna1_CysACA_5_+_151988596_151988771_intronat_151988632_151988735	PSEUDO	GGCTGTATAGCTCAGTGGTAGAGCATTTGACTACAGAATCCTATACTCAGGGGAAGGAGAACTGGGGGTTTCTCAGTGGGTCAAAGGACTTGTAGTGGTAAATCAAAAGCAACTCTATAAGCTATGTAACAAACTTTAAAGTCATATGTAGCTGGGTTCAAATCCTGTTTCTGCCA
227	gtRNAdb v1.0	trna1_GlnCTG_20_+_17855142_17855219	TRUE	GGCAGTGTAGCCCAGAGGTTCAAGGGCATTCGCTCTGGTATCAGAAGGGTCTGGGTTCAAATCCCTTGTGCACTGCTT
228	gtRNAdb v1.0	trna1_GlnCTG_6_+_18836402_18836473	TRUE	GGTTCCATGGTGTAATGGTTAGCACTCTGGACTCTGAATCCAGCGATCCGAGTTCAAATCTCGGTGGAACCT
229	gtRNAdb v1.0	trna1_GlnTTG_4_+_156383888_156383959	PSEUDO	TAGGACGTGGTGTGATAGGTAGCACAGAGAATTTTGGATTCTCAGGGGTAGGTTAAATTCCTATAGTACTAG
230	gtRNAdb v1.0	trna1_GlnTTG_X_+_55206666_55206737	PSEUDO	TAGGACATGGTGTGATAGGTAGCATGGAGAATTTTGGATTCTCAGGGGTAGGTTCAATTCCTACAGTTCTAG
231	gtRNAdb v1.0	trna1_GlnTTG_Y_-_8240140_8240214	PSEUDO	TTTAGGACGTGGTGTAATAGGTAGCACAGAGAATTTTGGATTCTCAGGTGCAGGTTCAATTCCTATATTCTAGAG
232	gtRNAdb v1.0	trna1_GluCTC_18_+_43299751_43299822	TRUE	CCCCGGGTGGTGTAGTGGATGGGATTTGGCGCTCTCACCACCATGGCCCGGATTTGATTCCCGGTCAGGGAA
233	gtRNAdb v1.0	trna1_GluCTC_8_+_11793380_11793451	PSEUDO	CCCCTGGTAGTCTAGTGGTTAGGCTTTGCCGCTCTCAGTGCCGCTGCCTGGGTTGGATTCCCAGTCATGTGA
234	gtRNAdb v1.0	trna1_GlyCCC_2_+_11691827_11691898	PSEUDO	TACTCAGTGGTCTAGTGGTTAGGATTCAGCGCTCCCACCGCCGCAGCCCGGGTTCGATTCCCGGTCATGGAA
235	gtRNAdb v1.0	trna1_HisGTG_15_+_45493349_45493420	TRUE	GCCGTGATCGTATAGTGGTTAGTACTCTGCGTTGTGGCCGCAGCAACCTCGGTTCGAATCCGAGTCACGGCA
236	gtRNAdb v1.0	trna1_LeuAAG_14_+_21078291_21078372	TRUE	GGTAGCGTGGCCGAGCGGTCTAAGGCGCTGGATTAAGGCTCCAGTCTCTTCGGGGGCGTGGGTTCGAATCCCACCGCTGCCA
237	gtRNAdb v1.0	trna1_LeuCAA_11_+_9296790_9296863	TRUE	GCCTCCTTAGTGCAGTAGGTAGCGCATCAGTCTCAAAATCTGAATGGTCCTGAGTTCAAGCCTCAGAGGGGGCA
238	gtRNAdb v1.0	trna1_LysTTT_12_+_27843306_27843378	TRUE	ACCCAGATAGCTCAGTCAGTAGAGCATCAGACTTTTAATCTGAGGGTCCAAGGTTCATGTCCCTTTTTGGGTG
239	gtRNAdb v1.0	trna1_MetCAT_9_+_19403996_19404070	TRUE	AGCAGAGTGGTGCAGTGGAAGCATACCTATGGGCCCATAACCCAGAGGTTGATGGATGGAAACCATCCTCTGCTA
240	gtRNAdb v1.0	trna1_PheGAA_13_-_95201904_95201976	TRUE	GCCGAAATAGCTCAGTTGGGAGAGCGTTAGACTGAAGATCTAAAGGTCCCTGGTTCGATCCCGGGTTTCGGCA
241	gtRNAdb v1.0	trna1_ProGGG_10_+_22852579_22852651	PSEUDO	GGCAGTGTGGCATAGTGGTTAGAAATGTGCGCTCTGGGGCTGCTGATCCCAGGCTCAAACCCTGGCGCTGTCA
242	gtRNAdb v1.0	trna1_SupCTA_3_+_13833886_13833953	PSEUDO	GGCAGGGTAGGGTAGAGGTTAAAACCATGGATTCTAGAGCCAGATGGGTTCAAATCCCGGCTCTGCCG
243	gtRNAdb v1.0	trna1_SupTTA_21_+_15926516_15926586	TRUE	GGCAGTGACATGTAATGGTTATGAGGGTGGACTTTAACCACACTGCCTAGGTTCAAATCCTGACTCTGTCA
244	gtRNAdb v1.0	trna1_TrpCCA_7_+_99067307_99067378	TRUE	GACCTCGTGGCGCAACGGCAGCGCGTCTGACTCCAGATCAGAAGGTTGCGTGTTCAAATCACGTCGGGGTCA
245	gtRNAdb v1.0	trna1_ValCAC_1_+_17052061_17052133	PSEUDO	GTTTCTGTAGTATAGTGGTTATCATGTTTGCCTCACATGTGAAAGACCCTTGGCTCGAGACTGGAGGGAAACA
246	gtRNAdb v1.0	trna20_AlaAGC_6_+_26687485_26687557	TRUE	GGGGAATTAGCTCAAGTGGTAGAGCGCTTGCTTAGCACGCAAGAGGTAGTGGGATCGATGCCCACATTCTCCA
247	gtRNAdb v1.0	trna20_AsnATT_1_+_147719029_147719102	TRUE	GTCTCTGTGGCGCAATCGGTCAGAGCGTTCGGCTATTAACCGAACGGTGAGTAGTTCAAGACCACCCAGGGACG
248	gtRNAdb v1.0	trna20_CysGCA_7_-_149286164_149286235	TRUE	GGGGGTATAGCTCAGGGGTAGAGCACTTGACTGCAGATCAAGAGGTCCCTGGTTCAAATCCAGGTGCCCCCT
249	gtRNAdb v1.0	trna20_GluTTC_2_-_131094701_131094772	TRUE	TCCCATATGGTCTAGCGGTTAGGATTCCTGGTTTTCACCCAGGTGGCCCGGGTTCGACTCCCGGTATGGGAA
250	gtRNAdb v1.0	trna20_LeuCAG_5_-_159392041_159392118	TRUE	GGCAGTGGAGTTTAGTGGTTAAGGACCTGCTCAGACATCACAGGTAGGTAGATCTGGGTTCAAACCCTAGCCCTGGCA
251	gtRNAdb v1.0	trna20_MetCAT_16_+_71460396_71460468	TRUE	GCCCTCTTAGCGCAGTGGGCAGCGCGTCAGTCTCATAATCTGAAGGTCCTGAGTTCGAGCCTCAGAGAGGGCA
252	gtRNAdb v1.0	trna20_MetCAT_17_-_80452597_80452668	TRUE	AGCAGAGTGGCGCAGCGGAAGCGTGCTGGGCCCATAACCCAGAGGTCGATGGATCGAAACCATCCTCTGCTA
253	gtRNAdb v1.0	trna20_ThrTGT_14_-_21099319_21099391	TRUE	GGCTCCATAGCTCAGGGGTTAGAGCACTGGTCTTGTAAACCAGGGGTCGCGAGTTCAAATCTCGCTGGGGCCT
254	gtRNAdb v1.0	trna21_ArgCCT_17_-_73030526_73030598	TRUE	GCCCCAGTGGCCTAATGGATAAGGCACTGGCCTCCTAAGCCAGGGATTGTGGGTTCGAGTCCCACCTGGGGTG
255	gtRNAdb v1.0	trna21_CysGCA_7_-_149112229_149112300	TRUE	GGGGGTATAGCTTAGCGGTAGAGCATTTGACTGCAGATCAAGAGGTCCCCGGTTCAAATCCGGGTGCCCCCT

256	gtRNAdb v1.0	trna21_GlnCTG_16_+_71724890_71724963	TRUE	GGCAGTATGGTAGAGTGGTTAAGATCATGAACTCTGAAGTCAGAGATACTTGAATTTGAATGCTGGTTCTGTCA
257	gtRNAdb v1.0	trna21_GlnCTG_5_-_151248074_151248145	PSEUDO	GGTAGTGTAGTCTACTGGTTAAACGCTTGGGCTCTGACATTAACGTCCTGGGTTCAAATCCCAGCTTTGTCA
258	gtRNAdb v1.0	trna21_HisGTG_1_+_147753471_147753542	TRUE	GCCGTGATCGTATAGTGGTTAGTACTCTGCGTTGTGGCCGCAGCAACCTCGGTTCGAATCCGAGTCACGGCA
259	gtRNAdb v1.0	trna21_MetCAT_6_+_26701712_26701784	TRUE	GCCCTCTTAGCGCAGCTGGCAGCGCGTCAGTCTCATAATCTGAAGGTCCTGAGTTCAAGCCTCAGAGAGGGCA
260	gtRNAdb v1.0	trna21_SerTGA_2_-_131033024_131033093	TRUE	GAGAAGGTCACAGAGGTTATGGGATTGGCTTGAAACCAGTCTGTGGGGGGTTCGATTCCCTCCTTTTTCA
261	gtRNAdb v1.0	trna21_ThrTGT_14_-_21081949_21082021	TRUE	GGCTCCATAGCTCAGGGGTTAGAGCGCTGGTCTTGTAAACCAGGGGTCGCGAGTTCAATTCTCGCTGGGGCCT
262	gtRNAdb v1.0	trna22_AlaAGC_6_+_26705606_26705678	TRUE	GGGGAATTAGCTCAAGCGGTAGAGCGCTTGCTTAGCATGCAAGAGGTAGTGGGATCGATGCCCACATTCTCCA
263	gtRNAdb v1.0	trna22_AspGTC_5_-_141773988_141774059	TRUE	TCCTCATCAGTATAGTGGTGAGTATCCCCGCCTGTCACGCGGGAGACTGGGGTTCGATTCCCTGAGGAGGAG
264	gtRNAdb v1.0	trna22_CysGCA_7_-_149074601_149074672	TRUE	GGGGGTATAGCTCAGGGGTAGAGCATTTGACTGCAGATCAAGAGGTCCCTGGTTCAAATCCAGGTGCCCCCC
265	gtRNAdb v1.0	trna22_GlnCTG_1_+_147800937_147801008	TRUE	GGTTCCATGGTGTAATGGTAAGCACTCTGGACTCTGAATCCAGCCATCTGAGTTCGAGTCTCTGTGGAACCT
266	gtRNAdb v1.0	trna22_GlnTTG_2_-_131029911_131029982	PSEUDO	TAGGACGTGGTGTGATAGGTAGCATGGAGAATTTTGGATTCTCAGGGATGGGTTCAATTCCTATAGTCCTAG
267	gtRNAdb v1.0	trna22_MetCAT_16_-_87417628_87417700	TRUE	GCCTCGTTAGCGCAGTAGGCAGCGCGTCAGTCTCATAATCTGAAGGTCGTGAGTTCGAGCCTCACACGGGGCA
268	gtRNAdb v1.0	trna22_ProAGG_14_-_21081560_21081631	TRUE	GGCTCGTTGGTCTAGGGGTATGATTCTCGCTTAGGGTGCGAGAGGTCCCGGGTTCAAATCCCGGACGAGCCC
269	gtRNAdb v1.0	trna23_AlaAGC_6_+_26728256_26728328	TRUE	GGGGAATTGGCTCAAGCGGTAGAGCGCTTGCTTAGCATGCAAGAGGTAGCAGGATCGACGCCTGCACTCTCCA
270	gtRNAdb v1.0	trna23_ArgCCG_17_-_66016013_66016085	TRUE	GACCCAGTGGCCTAATGGATAAGGCATCAGCCTCCGGAGCTGGGGATTGTGGGTTCGAGTCCCATCTGGGTCG
271	gtRNAdb v1.0	trna23_CysGCA_7_-_149072850_149072921	TRUE	GGGGGTATAGTTCAGGGGTAGAGCATTTGACTGCAGATCAAGAGGTCCCTGGTTCAAATCCAGGTGCCCCCT
272	gtRNAdb v1.0	trna23_GlnCTG_1_+_147825689_147825760	PSEUDO	GGTTCCATGGGTTAATGGTGAGCACCCTGGACTCTGAATCAAGCGATCCGAGTTCAAATCTCGGTGGTACCT
273	gtRNAdb v1.0	trna23_GlnTTG_2_-_117782509_117782580	TRUE	TAGGACATGGTGTAATAGGTAGAATGGAGAATTTTGAATTCTCAGGGGTAGGTTCAATTCCTATAGTTCTAG
274	gtRNAdb v1.0	trna23_GlnTTG_5_-_77318776_77318848	PSEUDO	AGCTGTATATTATAGTGGAATAAATGTGGACTTTGAAGTTAGATACACCTGGGTTCAAATCCCAGTGCTGTCA
275	gtRNAdb v1.0	trna23_LysTTT_16_-_73512216_73512288	TRUE	GCCTGGATAGCTCAGTTGGTAGAGCATCAGACTTTTAATCTGAGGGTCCAGGGTTCAAGTCCCTGTTCAGGCA
276	gtRNAdb v1.0	trna23_ProAGG_14_-_21077495_21077566	TRUE	GGCTCGTTGGTCTAGGGGTATGATTCTCGCTTAGGGTGCGAGAGGTCCCGGGTTCAAATCCCGGACGAGCCC
277	gtRNAdb v1.0	trna24_AlaAGC_6_+_26730737_26730809	TRUE	GGGGAATTAGCTCAGGCGGTAGAGCGCTCGCTTAGCATGCGAGAGGTAGCGGGATCGACGCCCGCATTCTCCA
278	gtRNAdb v1.0	trna24_AsnGTT_1_+_147877537_147877610	TRUE	GTCTCTGTGGCGCAATCGGTTAGCGCGTTTGACTGTTAACTGAAAGGTTGGTGGTGCAAGCCCATCCAGGGATG
279	gtRNAdb v1.0	trna24_GluCTC_2_-_96217194_96217265	PSEUDO	CCCCTTGTAGTCTAGTGGTTAGAATTCTGCGGTCTCACAGCCGCGGCCCGGGTTCGATTCCCATTCCGGGAA
280	gtRNAdb v1.0	trna24_GlyGCC_16_-_70812942_70813012	TRUE	GCATTGGTGGTTCAGTGGTAGAATTCTCGCCTGCCACGCGGGAGGCCCGGGTTCGATTCCCGGCCAATGCA
281	gtRNAdb v1.0	trna24_LysCTT_5_-_26198539_26198611	TRUE	GCCCGACTACCTCAGTCGGTGGAGCATGGGACTCTTCATCCCAGGGTTGTGGGTTCGAGCCCCACATTGGGCA
282	gtRNAdb v1.0	trna24_ThrAGT_17_-_62526918_62526991	TRUE	AGCACCATGGCTTAGCTGGTTAAAGCACCTGTCTAGTAAACAGGAGATCCTGAGTTTCAATTCCAATGGTGCCT
283	gtRNAdb v1.0	trna24_TyrGTA_7_-_149053745_149053820	PSEUDO	TCAATTATAGCTCAGTGGTAGAGCATTTAACTGTAGATCAAGAGGTCCCTGGATCAACTCTGGGTGCCCCCTTTAA
284	gtRNAdb v1.0	trna25_AlaAGC_6_+_26751918_26751990	TRUE	GGGGGATTAGCTCAAGCGGTAGGGTGCCTGCTTAGCATGCAAGAGGTAGCAGGATCGACGCCTGCATTCTCCA
285	gtRNAdb v1.0	trna25_AsnGTT_1_+_148000805_148000878	TRUE	GTCTCTGTGGCGTAGTCGGTTAGCGCGTTCGGCTGTTAACCGAAAAGTTGGTGGTTCGAGCCCACCCAGGAACG
286	gtRNAdb v1.0	trna25_CysGCA_7_-_149052766_149052837	TRUE	GGGGGTATAGCTCAGGGGTAGAGCATTTGACTGCAGATCAAGAGGTCCCCAGTTCAAATCTGGGTGCCCCCT
287	gtRNAdb v1.0	trna25_GluCTC_2_-_71273488_71273560	PSEUDO	CCCCTGGCGGTCTAGTGGTTAGGATTCGGCGCTCTCATCCACCGCGGCCTGGGTTCGACTCGTGGTCAGAGTG
288	gtRNAdb v1.0	trna25_GlyGCC_16_-_70812114_70812184	TRUE	GCATTGGTGGTTCAGTGGTAGAATTCTCGCCTGCCACGCGGGAGGCCCGGGTTTGATTCCCGGCCAGTGCA
289	gtRNAdb v1.0	trna26_AsnGTT_1_+_148248115_148248188	TRUE	GTCTCTGTGGCGCAATCGGTTAGCGCGTTCGGCTGTTAACCGAAAGGTTGGTGGTTCGAGCCCACCCAGGGACG
290	gtRNAdb v1.0	trna26_CysGCA_17_-_37310744_37310815	TRUE	GGGGGTATAGCTCAGTGGTAGAGCATTTGACTGCAGATCAAGAGGTCCCCGGTTCAAATCCGGGTGCCCCCT
291	gtRNAdb v1.0	trna26_GlnTTG_7_-_57253980_57254051	PSEUDO	TAGGACGTGGTGTGATAGGTAGCATGGGGAATTTTGGATTCTCAGGGGTGGGTTCAATTCCTATAGTTCTAG
292	gtRNAdb v1.0	trna26_LeuCAG_16_-_57334392_57334474	TRUE	GTCAGGATGGCCGAGCGGTCTAAGGCGCTGCGTTCAGGTCGCAGTCTCCCCTGGAGGCGTGGGTTCGAATCCCACTTCTGACA
293	gtRNAdb v1.0	trna27_CysGCA_17_-_37309987_37310058	TRUE	GGGGGTATAGCTCAGTGGTAGAGCATTTGACTGCAGATCAAGAGGTCCCCGGTTCAAATCCGGGTGCCCCCT
294	gtRNAdb v1.0	trna27_GlnCTG_1_+_149079364_149079435	PSEUDO	GGTTCCATGGTGTAATGGTGAGGGCTTTGGACTCTGACTACAGTGATCAGAGTTCAAGTCTCAGTGGGACCT
295	gtRNAdb v1.0	trna27_GlyCCC_2_-_70476123_70476193	TRUE	GCGCCGCTGGTGTAGTGGTATCATGCAAGATTCCCATTCTTGCGACCCGGGTTCGATTCCCGGGCGGCGCA
296	gtRNAdb v1.0	trna27_LeuTAG_16_-_22207032_22207113	TRUE	GGTAGCGTGGCCGAGTGGTCTAAGGCGCTGGATTTAGGCTCCAGTCATTTCGATGGCGTGGGTTCGAATCCCACCGCTGCCA
297	gtRNAdb v1.0	trna27_MetCAT_6_+_26766444_26766516	TRUE	GCCCTCTTAGCGCAGCGGGCAGCGCGTCAGTCTCATAATCTGAAGGTCCTGAGTTCGAGCCTCAGAGAGGGCA
298	gtRNAdb v1.0	trna28_CysGCA_17_-_37025545_37025616	TRUE	GGGGGTATAGCTCAGTGGTAGAGCATTTGACTGCAGATCAAGAGGTCCCTGGTTCAAATCCGGGTGCCCCCT
299	gtRNAdb v1.0	trna28_GlnCTG_1_+_149186125_149186196	TRUE	GGTTCCATGGTGTAATGGTAAGCACTCTGGACTCTGAATCCAGCGATCCGAGTTCGAGTCTCGGTGGAACCT
300	gtRNAdb v1.0	trna28_GlnTTG_2_-_45937441_45937513	PSEUDO	GGCTGTGTACCTCAGTGGGCAAGGGTATGGACTTTGAAGCCAGACTATTTGGGTTCAAATCCCAGCTTGGCCT
301	gtRNAdb v1.0	trna28_IleAAT_6_+_26780832_26780905	TRUE	GGCCGGTTAGCTCAGTTGGTCAGAGCGTGGTGCTAATAACGCCAAGGTCGCGGGTTCGATCCCCGTACGGGCCA
302	gtRNAdb v1.0	trna28_ProTGG_16_-_3234133_3234204	TRUE	GGCTCGTTGGTCTAGGGGTATGATTCTCGCTTTGGGTGCGAGAGGTCCCGGGTTCAAATCCCGGACGAGCCC
303	gtRNAdb v1.0	trna29_CysGCA_17_-_37017937_37018008	TRUE	GGGGGTATAGCTCAGGGGTAGAGCATTTGACTGCAGATCAAGAAGTCCCCGGTTCAAATCCGGGTGCCCCCT
304	gtRNAdb v1.0	trna29_GluCTC_1_+_149334272_149334340	TRUE	TCCCTGGTAGTCTAGTGGCTAAAGTTTGGCGCTCTCACCGCCGGGACTGGTTGATTCCAGATCAGGGGA
305	gtRNAdb v1.0	trna29_IleTAT_6_+_26988125_26988218_intronat_26988163_26988182	TRUE	GCTCCAGTGGCGCAATCGGTTAGCGCGCGGTACTTATATGGCAGTATGTGTGCGAGTGATGCCGAGGTTGTGAGTTCGAGCCTCACCTGGAGCA
306	gtRNAdb v1.0	trna29_ProAGG_16_-_3232635_3232706	TRUE	GGCTCGTTGGTCTAGGGGTATGATTCTCGCTTAGGGTGCGAGAGGTCCCGGGTTCAAATCCCGGACGAGCCC
307	gtRNAdb v1.0	trna2_ArgCCT_16_+_3202901_3202973	TRUE	GCCCCGGTGGCCTAATGGATAAGGCATTGGCCTCCTAAGCCAGGGATTGTGGGTTCGAGTCCCACCCGGGGTA
308	gtRNAdb v1.0	trna2_ArgTCG_9_+_112960803_112960875	TRUE	GGCCGTGTGGCCTAATGGATAAGGCGTCTGACTTCGGATCAAAAGATTGCAGGTTTGAGTTCTGCCACGGTCG
309	gtRNAdb v1.0	trna2_GluCTC_13_-_58458550_58458621	PSEUDO	TCCCTGGTGGTCTAATGGTTAGGAGTCGGCACTCTCACCGCCGCGGCTGGGGTTTGATTCCCAGTCATGTAA
310	gtRNAdb v1.0	trna2_GlyCCC_1_+_17053780_17053850	TRUE	GCCTTGGTGGTGCAGTGGTAGAATTCTCGCCTCCCACGTGGGAGACCCGGGTTCAATTCCCGGCCAATGCA
311	gtRNAdb v1.0	trna2_GlyGCC_21_-_18827107_18827177	TRUE	GCATGGGTGGTTCAGTGGTAGAATTCTCGCCTGCCACGCGGGAGGCCCGGGTTCGATTCCCGGCCCATGCA
312	gtRNAdb v1.0	trna2_GlyTCC_18_+_55345854_55345925	PSEUDO	GTGTTGATGGTATAGTGGTGAGCATAGCTGCCTTCCAAGCAATTGACCCGACTTCGATTCCCAGCCAATGCA
313	gtRNAdb v1.0	trna2_GlyTCC_19_+_4724082_4724153	TRUE	GCGTTGGTGGTATAGTGGTTAGCATAGCTGCCTTCCAAGCAGTTGACCCGGGTTCGATTCCCGGCCAACGCA
314	gtRNAdb v1.0	trna2_LeuTAA_4_-_156384978_156385052	TRUE	GTTAAGATGGCAGAGCCTGGTAATTGCATAAAACTTAAAATTTTATAATCAGAGGTTCAACTCCTCTTCTTAACA
315	gtRNAdb v1.0	trna2_LeuTAA_X_-_55207755_55207829	TRUE	GTTAAGATGGCAGAGCCCGGCAATTGCATAAGACTTAAAACTTTATAATCAGAGGTTCAACTCCTCTCATTAACA
316	gtRNAdb v1.0	trna2_LeuTAG_14_+_21093529_21093610	TRUE	GGTAGTGTGGCCGAGCGGTCTAAGGCGCTGGATTTAGGCTCCAGTCTCTTCGGGGGCGTGGGTTCGAATCCCACCACTGCCA
317	gtRNAdb v1.0	trna2_LysCTT_11_+_51359900_51359972	TRUE	AACCGAATAGCTTAGTTGATGAAGCGTGAGACTCTTAATCTCAGGGTAGTGGGTTCAAGCCCCACATTGGACA
318	gtRNAdb v1.0	trna2_LysCTT_15_+_79152904_79152976	TRUE	GCCCGGCTAGCTCAGTCGGTAGAGCATGGGACTCTTAATCCCAGGGTCGTGGGTTCGAGCCCCACGTTGGGCG
319	gtRNAdb v1.0	trna2_LysTTT_17_+_8022473_8022545	TRUE	GCCCGGATAGCTCAGTCGGTAGAGCATCAGACTTTTAATCTGAGGGTCCAGGGTTCAAGTCCCTGTTCGGGCG
320	gtRNAdb v1.0	trna2_LysTTT_8_+_18120203_18120272	PSEUDO	TCCTATAGCCCAGTGATTAGGATTCTTTGCTTTTACTACCATGACCTGGGTTCAATACCCAGTCAGGGAA
321	gtRNAdb v1.0	trna2_MetCAT_6_+_26286754_26286825	TRUE	AGCAGAGTGGCGCAGCGGAAGCGTGCTGGGCCCATAACCCAGAGGTCGATGGATCGAAACCATCCTCTGCTA
322	gtRNAdb v1.0	trna2_ProAGG_7_+_128423504_128423575	TRUE	GGCTCGTTGGTCTAGGGGTATGATTCTCGCTTAGGGTGCGAGAGGTCCCGGGTTCAAATCCCGGACGAGCCC
323	gtRNAdb v1.0	trna2_SerAGA_20_+_30088703_30088774	PSEUDO	GGTAATGTAGCCTCGTGGTTAGGGGCTGCATTCTAGAGCTATGCTGCCCAGGTTCAAATTCTGGTGCCACTC
324	gtRNAdb v1.0	trna2_SerCGA_12_+_56584148_56584229	TRUE	GTCACGGTGGCCGAGTGGTTAAGGCGTTGGACTCGAAATCCAATGGGGTTTCCCCGCACAGGTTCGAATCCTGTTCGTGACG
325	gtRNAdb v1.0	trna2_SerTGA_10_+_69524261_69524342	TRUE	GCAGCGATGGCCGAGTGGTTAAGGCGTTGGACTTGAAATCCAATGGGGTCTCCCCGCGCAGGTTCGAACCCTGCTCGCTGCG
326	gtRNAdb v1.0	trna2_TyrGTA_2_+_27273650_27273738_intronat_27273687_27273702	TRUE	CCTTCGATAGCTCAGTTGGTAGAGCGGAGGACTGTAGTGGATAGGGCGTGGCAATCCTTAGGTCGCTGGTTCGATTCCGGCTCGAAGGA
327	gtRNAdb v1.0	trna2_ValAAC_3_+_169490018_169490090	TRUE	GTTTCCGTAGTGTAGTGGTTATCACGTTCGCCTAACACGCGAAAGGTCCCCGGTTCGAAACCGGGCGGAAACA
328	gtRNAdb v1.0	trna2_ValCAC_5_+_180524070_180524142	TRUE	GTTTCCGTAGTGTAGTGGTTATCACGTTCGCCTCACACGCGAAAGGTCCCCGGTTCGAAACCGGGCGGAAACA
329	gtRNAdb v1.0	trna30_AsnGTT_1_+_149608609_149608682	TRUE	GTCTCTGTGGCGCAATCGGTTAGCGCGTTTGACTGTTAACTGAAAGGTTGGTGGTGCAAGCCCATCCAGGGATG
330	gtRNAdb v1.0	trna30_CysGCA_17_-_36989975_36990046	TRUE	GGGGGTAGGGCTCAGGGATAGAGCATTTGACTGCAGATCAAGAGGTCCCCGGTTCGAATCTAGGTGCCCCCT
331	gtRNAdb v1.0	trna30_LysCTT_16_-_3230555_3230627	TRUE	GCCCGGCTAGCTCAGTCGATAGAGCATGAGACTCTTAATCTCAGGGTCGTGGGTTCGAGCCGCACGTTGGGCG
332	gtRNAdb v1.0	trna30_ProCGG_6_+_27059521_27059592	TRUE	GGCTCGTTGGTCTAGGGGTATGATTCTCGCTTCGGGTGTGAGAGGTCCCGGGTTCAAATCCCGGACGAGCCC
333	gtRNAdb v1.0	trna31_AsnGTT_17_-_36908034_36908107	TRUE	GTCTCTGTGGCGCAATCGGTTAGCGCGTTCGGCTGTTAACCGAAAGGTTGGTGGTTCGAGCCCACCCAGGGACG
334	gtRNAdb v1.0	trna31_GluTTC_1_+_149719802_149719870	TRUE	TCCCTGGTAGTCTAGTGGCTAAAGTTTGGCGCTTTCACCGCCGGGACTGGTTGATTCCAGATCAGGGGA
335	gtRNAdb v1.0	trna31_ProTGG_16_-_3220961_3221031	PSEUDO	GGCCTGTTGGTCTAGAGGTATGATTCTCGCTTTGGGTGCGAGAGGCCCCGGTGCGAGTCCCAGAGGAGCCC
336	gtRNAdb v1.0	trna31_SerGCT_6_+_27065085_27065166	TRUE	GACGAGGTGGCCGAGTGGTTAAGGCGATGGACTGCTAATCCATTGTGCTCTGCACGCGTGGGTTCGAATCCCACCCTCGTCG
337	gtRNAdb v1.0	trna32_LysCTT_16_-_3207406_3207478	TRUE	GCCCGGCTAGCTCAGTCGGTAGAGCATGAGACCCTTAATCTCAGGGTCGTGGGTTCGAGCCCCACGTTGGGCG
338	gtRNAdb v1.0	trna32_MetCAT_1_+_153643726_153643797	TRUE	AGCAGAGTGGCGCAGCGGAAGCGTGCTGGGCCCATAACCCAGAGGTCGATGGATCGAAACCATCCTCTGCTA
339	gtRNAdb v1.0	trna32_SerTGA_17_-_22028179_22028247	PSEUDO	GAAAAAGTCATAGGGGTTATGAGGCTGGCTTGAAACCAGCCTTAGGAGGTTCAATTCCTTCCTTTTTTG
340	gtRNAdb v1.0	trna32_ValCAC_6_+_27118022_27118094	TRUE	GTTTCTGTAGTATGGTGGTTATCACGTTAGTCTCACACGTGAAAGGTCCCTGGTTCGAAACCAGGTGGAAACA
341	gtRNAdb v1.0	trna33_GlnTTG_17_-_19506587_19506658	PSEUDO	TAGGATGTGGTGTAATAGGTGGCATGGAGAATTTTGGATTATCAGGGGTAGGTTCAATTCCTATAGTTCTAG
342	gtRNAdb v1.0	trna33_HisGTG_6_+_27125906_27125977	TRUE	GCCGTGATCGTATAGTGGTTAGTACTCTGCGTTGTGGCCGCAGCAACCTCGGTTCGAATCCGAGTCACGGCA
343	gtRNAdb v1.0	trna33_ProAGG_16_-_3202609_3202680	PSEUDO	CGCTCTTTGGTCTAGGGGTATGATCTTCGCTTAGGGTGCGAGAGGTGCCTGGATCAACTCCTTCACAAGCCG

344	gtRNAdb v1.0	trna34_GlyCCC_16_-_686736_686806	TRUE	GCGCCGCTGGTGTAGTGGTATCATGCAAGATTCCCATTCTTGCGACCCGGGTTCGATTCCCGGGCGGCGCA
345	gtRNAdb v1.0	trna34_IleAAT_17_-_8130309_8130382	TRUE	GGCCGGTTAGCTCAGTTGGTTAGAGCGTGGTGCTAATAACGCCAAGGTCGCGGGTTCGAACCCCGTACGGGCCA
346	gtRNAdb v1.0	trna34_LeuCAG_1_+_161411323_161411405	TRUE	GTCAGGATGGCCGAGCGGTCTAAGGCGCTGCGTTCAGGTCGCAGTCTCCCCTGGAGGCGTGGGTTCGAATCCCACTCCTGACA
347	gtRNAdb v1.0	trna34_ThrAGT_6_+_27130050_27130123	TRUE	GGCCCTGTGGCTTAGCTGGTCAAAGCGCCTGTCTAGTAAACAGGAGATCCTGGGTTCGAATCCCAGCGGGGCCT
348	gtRNAdb v1.0	trna35_GlyGCC_1_+_161413094_161413164	TRUE	GCATGGGTGGTTCAGTGGTAGAATTCTCGCCTGCCACGCGGGAGGCCCGGGTTCGATTCCCGGCCCATGCA
349	gtRNAdb v1.0	trna35_SerAGA_17_-_8129928_8130009	TRUE	GTAGTCGTGGCCGAGTGGTTAAGGCGATGGACTAGAAATCCATTGGGGTCTCCCCGCGCAGGTTCGAATCCTGCCGACTACG
350	gtRNAdb v1.0	trna35_SerCGA_6_+_27177628_27177709	TRUE	GCTGTGATGGCCGAGTGGTTAAGGCGTTGGACTCGAAATCCAATGGGGTCTCCCCGCGCAGGTTCAAATCCTGCTCACAGCG
351	gtRNAdb v1.0	trna36_ArgACG_6_+_27182952_27183024	TRUE	GGGCCAGTGGCGCAATGGATAACGCGTCTGACTACGGATCAGAAGATTCTAGGTTCGACTCCTGGCTGGCTCG
352	gtRNAdb v1.0	trna36_LeuCAG_1_+_161418741_161418823	TRUE	GTCAGGATGGCCGAGCGGTCTAAGGCGCTGCGTTCAGGTCGCAGTCTCCCCTGGAGGCGTGGGTTCGAATCCCACTCCTGACA
353	gtRNAdb v1.0	trna36_ThrAGT_17_-_8129553_8129626	TRUE	GGCGCCGTGGCTTAGTTGGTTAAAGCGCCTGTCTAGTAAACAGGAGATCCTGGGTTCGAATCCCAGCGGTGCCT
354	gtRNAdb v1.0	trna37_GlyGCC_1_+_161420467_161420537	TRUE	GCATGGGTGGTTCAGTGGTAGAATTCTCGCCTGCCACGCGGGAGGCCCGGGTTCGATTCCCGGCCCATGCA
355	gtRNAdb v1.0	trna37_ProCGG_17_-_8126151_8126222	TRUE	GGCTCGTTGGTCTAGGGGTATGATTCTCGCTTCGGGTGCGAGAGGTCCCGGGTTCAAATCCCGGACGAGCCC
356	gtRNAdb v1.0	trna37_ValAAC_6_+_27203288_27203360	TRUE	GTTTCCGTAGTGTAGTGGTTATCACGTTTGCCTAACACGCGAAAGGTCCCCGGTTCGAAACCGGGCAGAAACA
357	gtRNAdb v1.0	trna38_AspGTC_17_-_8125556_8125627	TRUE	TCCTCGTTAGTATAGTGGTGAGTATCCCCGCCTGTCACGCGGGAGACCGGGGTTCGATTCCCCGACGGGGAG
358	gtRNAdb v1.0	trna38_IleAAT_6_+_27241739_27241812	TRUE	GGCTGGTTAGTTCAGTTGGTTAGAGCGTGGTGCTAATAACGCCAAGGTCGTGGGTTCGATCCCCATATCGGCCA
359	gtRNAdb v1.0	trna38_LeuCAG_1_+_161426122_161426204	TRUE	GTCAGGATGGCCGAGCGGTCTAAGGCGCTGCGTTCAGGTCGCAGTCTCCCCTGGAGGCGTGGGTTCGAATCCCACTCCTGACA
360	gtRNAdb v1.0	trna39_GlyGCC_1_+_161427898_161427968	TRUE	GCATGGGTGGTTCAGTGGTAGAATTCTCGCCTGCCACGCGGGAGGCCCGGGTTCGATTCCCGGCCCATGCA
361	gtRNAdb v1.0	trna39_IleAAT_6_+_27251864_27251937	PSEUDO	GGCCGGTTAACTTAATTGGTTAGAGCGTGGTGCTAATAATGTCAAGGTTGCGGGTTGGATCCCCGAACGGGCCA
362	gtRNAdb v1.0	trna39_TrpCCA_17_-_8124187_8124258	TRUE	GGCCTCGTGGCGCAACGGTAGCGCGTCTGACTCCAGATCAGAAGGTTGCGTGTTCAAATCACGTCGGGGTCA
363	gtRNAdb v1.0	trna3_AlaAGC_2_+_27274082_27274154	TRUE	GGGGGATTAGCTCAAATGGTAGAGCGCTCGCTTAGCATGCGAGAGGTAGCGGGATCGATGCCCGCATCCTCCA
364	gtRNAdb v1.0	trna3_ArgCCT_7_+_139025446_139025518	TRUE	GCCCCAGTGGCCTAATGGATAAGGCATTGGCCTCCTAAGCCAGGGATTGTGGGTTCGAGTCCCATCTGGGGTG
365	gtRNAdb v1.0	trna3_ArgTCG_6_+_26299905_26299977	TRUE	GACCACGTGGCCTAATGGATAAGGCGTCTGACTTCGGATCAGAAGATTGAGGGTTCGAATCCCTTCGTGGTTA
366	gtRNAdb v1.0	trna3_ArgTCT_11_+_59318767_59318852_intronat_59318804_59318816	TRUE	GGCTCTGTGGCGCAATGGATAGCGCATTGGACTTCTAGATAGTTAGAGAAATTCAAAGGTTGTGGGTTCGAGTCCCACCAGAGTCG
367	gtRNAdb v1.0	trna3_AspGTC_3_-_184366095_184366165	TRUE	TTCTTGTTAATATAGTGGTGAGTATTCCCACCTGTCATGCGGGAGACGGGGTTCAATTCCCTGATGGGGAG
368	gtRNAdb v1.0	trna3_CysGCA_15_+_80036997_80037069	TRUE	GGGGGTATAGCTCAGTGGGTAGAGCATTTGACTGCAGATCAAGAGGTCCCCGGTTCAAATCCGGGTGCCCCCT
369	gtRNAdb v1.0	trna3_CysGCA_4_-_124430005_124430076	TRUE	GGGGGTATAGCTCAGTGGTAGAGCATTTGACTGCAGATCAAGAGGTCCCCGGTTCAAATCCGGGTGCCCCCT
370	gtRNAdb v1.0	trna3_GlnCTG_12_+_74851182_74851254	TRUE	GGTTCCATGGTGTAATGGTAAGCACCCTGGACTCTGAATCCAGCAACCAGAGTTCCAGTCTCAGCGTGGACCT
371	gtRNAdb v1.0	trna3_GlnCTG_17_+_8023070_8023141	TRUE	GGTTCCATGGTGTAATGGTTAGCACTCTGGACTCTGAATCCAGCGATCCGAGTTCAAATCTCGGTGGAACCT
372	gtRNAdb v1.0	trna3_GluCTC_8_+_59504798_59504868	TRUE	TCCTTGATGTCTAGTGGTTAGGATTTGGTGCTCTCACTGCAGCAGCCTGGGTTCATTTCTCAGTCAGGGAA
373	gtRNAdb v1.0	trna3_GluCTC_X_-_51306112_51306183	PSEUDO	TCCCTGGTGTTCCGGTGGTTAGGATTTGGCATTCTCACTGTTGTGGTGCGGATTCAATCCTGGCTTAGGGTA
374	gtRNAdb v1.0	trna3_GluTTC_13_-_45492062_45492133	TRUE	TCCCACATGGTCTAGCGGTTAGGATTCCTGGTTTTCACCCAGGCGGCCCGGGTTCGACTCCCGGTGTGGGAA
375	gtRNAdb v1.0	trna3_GlyTCC_18_+_55346177_55346248	PSEUDO	GTGTTGATGGTATAGTGGTGAGCATAGCTGCCTTCCAAGCAATTGACCCGACTTCAATTCCCAGCCAACGCA
376	gtRNAdb v1.0	trna3_LeuAAG_5_+_180528840_180528921	TRUE	GGTAGCGTGGCCGAGCGGTCTAAGGCGCTGGATTAAGGCTCCAGTCTCTTCGGAGGCGTGGGTTCGAATCCCACCGCTGCCA
377	gtRNAdb v1.0	trna3_LeuCAA_10_-_34591416_34591483	PSEUDO	CATAGGGTAGTGGCTAAGAACCTAAACTCTAAATTTAGATGTCCTGAGTTCAAATCCCAGCTGTATGC
378	gtRNAdb v1.0	trna3_LysTTT_19_+_19852207_19852277	TRUE	ACCCTGTGGTACAGGGGCTAATATGCTGGGCCTTTACCACTTCAGCCCAGGTTCGATTCCTGGTCAGGGAA
379	gtRNAdb v1.0	trna3_ProTGG_14_+_21101165_21101236	TRUE	GGCTCGTTGGTCTAGTGGTATGATTCTCGCTTTGGGTGCGAGAGGTCCCGGGTTCAAATCCCGGACGAGCCC
380	gtRNAdb v1.0	trna3_ProTGG_16_+_3208923_3208994	TRUE	GGCTCGTTGGTCTAGGGGTATGATTCTCGCTTTGGGTGCGAGAGGTCCCGGGTTCAAATCCCGGACGAGCCC
381	gtRNAdb v1.0	trna3_TrpCCA_9_+_115616989_115617087_intronat_115617028_115617051	TRUE	GGCAGAGGAGGGTGCAGTTGGCAGCCTGTCCAAGTCCAGCACGGTTGGAGCACAGGATTTAGAATGGGATGGTCCTGGGTTCAAACCCCAGCTGCGCCC
382	gtRNAdb v1.0	trna3_ValCAC_1_+_17186693_17186765	PSEUDO	GTTTCTGTGGTGTAGTGGTTATTATGTTCGCTTCACATATGAAAGGTCTCTGGTTCGAGACTGCGTGGGAACA
383	gtRNAdb v1.0	trna3_ValCAC_20_+_43951001_43951073	PSEUDO	GCTTCTGTAATGTAGTGGTTATCACATTCGCCTCACACATGAAAGGTCACCAGTTTGAGACCGGGCCAAAACA
384	gtRNAdb v1.0	trna40_LeuCAG_1_+_161433532_161433614	TRUE	GTCAGGATGGCCGAGCGGTCTAAGGCGCTGCGTTCAGGTCGCAGTCTCCCCTGGAGGCGTGGGTTCGAATCCCACTCCTGACA
385	gtRNAdb v1.0	trna40_ThrAGT_17_-_8042770_8042843	TRUE	GGCGCCGTGGCTTAGCTGGTTAAAGCGCCTGTCTAGTAAACAGGAGATCCTGGGTTCGAATCCCAGCGGTGCCT
386	gtRNAdb v1.0	trna40_ValTAC_6_+_27258405_27258477	TRUE	GTTTCCGTGGTGTAGTGGTTATCACATTCGCCTTACACGCGAAAGGTCCTCGGGTCGAAACCGAGCGGAAACA
387	gtRNAdb v1.0	trna41_GlyGCC_1_+_161435258_161435328	TRUE	GCATGGGTGGTTCAGTGGTAGAATTCTCGCCTGCCACGCGGGAGGCCCGGGTTCGATTCCCGGCCCATGCA
388	gtRNAdb v1.0	trna41_SerACT_6_+_27261671_27261744	PSEUDO	GGCCGGTTAGCTCAGTTGGTTAGAGCGTGCTGCTACTAATGCCAGGGTCGAGGTTTCGATCCCCGTACGGGCCT
389	gtRNAdb v1.0	trna41_SerCGA_17_-_8042199_8042280	TRUE	GCTGTGATGGCCGAGTGGTTAAGGCGTTGGACTCGAAATCCAATGGGGTCTCCCCGCGCAGGTTCGAATCCTGCTCACAGCG
390	gtRNAdb v1.0	trna42_GlnCTG_6_+_27263212_27263283	TRUE	GGTTCCATGGTGTAATGGTTAGCACTCTGGACTCTGAATCCGGTAATCCGAGTTCAAATCTCGGTGGAACCT
391	gtRNAdb v1.0	trna42_LeuCAG_1_+_161440913_161440995	TRUE	GTCAGGATGGCCGAGCGGTCTAAGGCGCTGCGTTCAGGTCGCAGTCTCCCCTGGAGGCGTGGGTTCGAATCCCACTCCTGACA
392	gtRNAdb v1.0	trna42_LeuTAG_17_-_8023632_8023713	TRUE	GGTAGCGTGGCCGAGCGGTCTAAGGCGCTGGATTTAGGCTCCAGTCTCTTCGGAGGCGTGGGTTCGAATCCCACCGCTGCCA
393	gtRNAdb v1.0	trna43_GlyGCC_1_+_161450356_161450426	TRUE	GCATAGGTGGTTCAGTGGTAGAATTCTTGCCTGCCACGCAGGAGGCCCAGGTTTGATTCCTGGCCCATGCA
394	gtRNAdb v1.0	trna43_SerGCT_6_+_27265775_27265856	TRUE	GACGAGGTGGCCGAGTGGTTAAGGCGATGGACTGCTAATCCATTGTGCTCTGCACGCGTGGGTTCGAATCCCACCTTCGTCG
395	gtRNAdb v1.0	trna44_AspGTC_1_+_161492935_161493006	PSEUDO	TCCTTGTTACTATAGTGGTGAGTATCTCTGCCTGTCATGCGTGAGAGAGGGGGTCGATTCCCCGACGGGGAG
396	gtRNAdb v1.0	trna44_SerAGA_6_+_27446591_27446672	TRUE	GTAGTCGTGGCCGAGTGGTTAAGGCGATGGACTAGAAATCCATTGGGGTCTCCCCGCGCAGGTTCGAATCCTGCCGACTACG
397	gtRNAdb v1.0	trna45_AspGTC_6_+_27447453_27447524	TRUE	TCCTCGTTAGTATAGTGGTGAGTATCCCCGCCTGTCACGCGGGAGACCGGGGTTCGATTCCCCGACGGGGAG
398	gtRNAdb v1.0	trna45_GlyTCC_1_+_161500903_161500974	TRUE	GCGTTGGTGGTATAGTGGTGAGCATAGCTGCCTTCCAAGCAGTTGACCCGGGTTCGATTCCCGGCCAACGCA
399	gtRNAdb v1.0	trna46_AspGTC_1_+_161501915_161501986	TRUE	TACTCGTTAGTATAGTGGTGCGTATCCCCGTCTGTCACGCGGGAGAGCGGGGTTCGCTCTCCCGACGGGGAG
400	gtRNAdb v1.0	trna46_SerAGA_6_+_27463593_27463674	TRUE	GTAGTCGTGGCCGAGTGGTTAAGGCGATGGACTAGAAATCCATTGGGGTCTCCCCGCGCAGGTTCGAATCCTGCCGACTACG
401	gtRNAdb v1.0	trna47_AsnGTT_1_+_161510031_161510104	TRUE	GTCTCTGTGGCGCAATCGGTTAGCGCGTTCGGCTGTTAACCGAAAGGTTGGTGGTTCGATCCCACCCAGGGACG
402	gtRNAdb v1.0	trna47_SerAGA_6_+_27470818_27470899	TRUE	GTAGTCGTGGCCGAGTGGTTAAGGCGATGGACTAGAAATCCATTGGGGTCTCCCCGCGCAGGTTCGAATCCTGCCGACTACG
403	gtRNAdb v1.0	trna48_AspGTC_1_+_161574588_161574659	TRUE	TCCTTGTTACTATAGTGGTAAGTATCTCTGCCTGTCATGCATGAGAGAGGGGGTCGATTCCCTGACGGGGAG
404	gtRNAdb v1.0	trna48_AspGTC_6_+_27471523_27471594	TRUE	TCCTCGTTAGTATAGTGGTGAGTATCCCCGCCTGTCACGCGGGAGACCGGGGTTCGATTCCCCGACGGGGAG
405	gtRNAdb v1.0	trna49_GlnCTG_6_+_27487308_27487379	TRUE	GGTTCCATGGTGTAATGGTTAGCACTCTGGACTCTGAATCCAGCGATCCGAGTTCAAATCTCGGTGGAACCT
406	gtRNAdb v1.0	trna49_GluTTC_1_+_161582508_161582579	TRUE	GCGTTGGTGGTGTAGTGGTGAGCACAGCTGCCTTTCAAGCAGTTAACGCGGGTTCGATTCCCGGGTAACGAA
407	gtRNAdb v1.0	trna4_ArgTCG_15_+_89878304_89878376	TRUE	GGCCGCGTGGCCTAATGGATAAGGCGTCTGACTTCGGATCAGAAGATTGCAGGTTCGAGTCCTGCCGCGGTCG
408	gtRNAdb v1.0	trna4_ArgTCG_6_+_26323046_26323118	TRUE	GACCACGTGGCCTAATGGATAAGGCGTCTGACTTCGGATCAGAAGATTGAGGGTTCGAATCCCTCCGTGGTTA
409	gtRNAdb v1.0	trna4_ArgTCT_17_+_8024243_8024330_intronat_8024280_8024294	TRUE	GGCTCTGTGGCGCAATGGATAGCGCATTGGACTTCTAGTGACGAATAGAGCAATTCAAAGGTTGTGGGTTCGAATCCCACCAGAGTCG
410	gtRNAdb v1.0	trna4_ArgTCT_9_-_131102355_131102445_intronat_131102391_131102408	TRUE	GGCTCTGTGGCGCAATGGATAGCGCATTGGACTTCTAGCTGAGCCTAGTGTGGTCATTCAAAGGTTGTGGGTTCGAGTCCCACCAGAGTCG
411	gtRNAdb v1.0	trna4_AsnGTT_10_-_22518438_22518511	TRUE	GTCTCTGTGGCGCAATCGGTTAGCGCGTTCGGCTGTTAACCGAAAGGTTGGTGGTTCGAGCCCACCCAGGGACG
412	gtRNAdb v1.0	trna4_AspGTC_12_+_96429799_96429870	TRUE	TCCTCGTTAGTATAGTGGTGAGTATCCCCGCCTGTCACGCGGGAGACCGGGGTTCGATTCCCCGACGGGGAG
413	gtRNAdb v1.0	trna4_GlnTTG_4_-_40908743_40908815	TRUE	GACCATGTGGCCTAAGGGAAAAGACATCTCACTTTGGGTCAGAAGATTGAGGGTTCAAGTCCTTTCATGGTCA
414	gtRNAdb v1.0	trna4_GlnTTG_7_+_141503357_141503428	PSEUDO	TAGGACGTGGTGTAGTAGGTAGCATGGAGAATGTTGAATTCTCAGGGGTAGGTTCAATTCCTATAGTTCTAG
415	gtRNAdb v1.0	trna4_GluCTC_13_-_42030061_42030132	TRUE	CCCCTGGTGGTCTAGTGCTTAGGATTCGGTGCTCTCACCGCTGCTGCCTGCGTTCGATTCCCGGTCAGGGAA
416	gtRNAdb v1.0	trna4_GlyCCC_1_+_17188416_17188486	TRUE	GCATTGGTGGTTCAGTGGTAGAATTCTCGCCTCCCACGCGGGAGACCCGGGTTCAATTCCCGGCCAATGCA
417	gtRNAdb v1.0	trna4_HisGTG_3_-_148316563_148316634	TRUE	GCAGTGACTGTATAGTGGTTAGCACTCTGTGTTGTGGCCACAGCAACCATGGTTCAAATCTGAGTCATGACA
418	gtRNAdb v1.0	trna4_LeuAAG_2_+_30277568_30277640	TRUE	GGGCCAGTGGCTCAATGGATAATGCGTCTGACTAAGAATCAGAAGATTCCAGCCTTGACTCCTGGCTGGCTCA
419	gtRNAdb v1.0	trna4_LeuTAA_11_+_59319228_59319310	TRUE	ACCAGAATGGCCGAGTGGTTAAGGCGTTGGACTTAAGATCCAATGGATTCATATCCGCGTGGGTTCGAACCCCACTTCTGGTA
420	gtRNAdb v1.0	trna4_LeuTAA_20_-_55933428_55933502	PSEUDO	GTTAAGATGGCAGAGCCCAGCGATTGCATAAAACTTAACACTTTATAATCAGAGGTTCAACTCCTCTTCTTAACA
421	gtRNAdb v1.0	trna4_LysCTT_18_-_43669271_43669343	TRUE	GACGAGCTAGCTCAGTCGGTAGAGCATGGGACTCTTAATCCCAGGGTCGTGGGTTTGAGCCCCATGTTGGGCA
422	gtRNAdb v1.0	trna4_ProAGG_16_+_3210386_3210480_intronat_3210422_3210437	TRUE	GGCTCGTTGGTCTAGGGGTGTGGTTCTCGCTTAGGGACCACAGGGACAAGCCCGGGAGACCCAAGAGGTCCCGGGTTCAAATCCCGGACGAGCCC
423	gtRNAdb v1.0	trna4_ThrAGT_19_+_33667963_33668036	TRUE	GGCGCCGTGGCTTAGTTGGTTAAAGCGCCTGTCTAGTAAACAGGAGATCCTGGGTTCGAATCCCAGCGGTGCCT
424	gtRNAdb v1.0	trna4_ThrTGT_14_+_21149849_21149921	TRUE	GGCCCTATAGCTCAGGGGTTAGAGCACTGGTCTTGTAAACCAGGGGTCGCGAGTTCAAATCTCGCTGGGGCCT
425	gtRNAdb v1.0	trna4_TyrGTA_8_+_67025602_67025694_intronat_67025639_67025658	TRUE	CCTTCGATAGCTCAGCTGGTAGAGCGGAGGACTGTAGCTACTTCCTCAGCAGGAGACATCCTTAGGTCGCTGGTTCGATTCCGGCTCGAAGGA
426	gtRNAdb v1.0	trna4_ValAAC_5_+_180591154_180591226	TRUE	GTTTCCGTAGTGTAGTGGTTATCACGTTCGCCTAACACGCGAAAGGTCCCCGGTTCGAAACCGGGCGGAAACA
427	gtRNAdb v1.0	trna4_ValTAC_X_-_18693029_18693101	TRUE	GGTTCCATAGTGTAGTGGTTATCACGTCTGCTTTACACGCAGAAGGTCCTGGGTTCGAGCCCCAGTGGAACCA
428	gtRNAdb v1.0	trna50_AsnGTT_1_+_161591465_161591538	TRUE	GTTTCTGTAGCGCGATCGGTTAGCGCCTTCGGCTGTTAAACGAAAGGTTGGTGGTTCGTTCCCACCCCGGGACA
429	gtRNAdb v1.0	trna50_SerAGA_6_+_27499987_27500068	TRUE	GTAGTCGTGGCCGAGTGGTTAAGGCGATGGACTAGAAATCCATTGGGGTTTCCCCACGCAGGTTCGAATCCTGCCGACTACG
430	gtRNAdb v1.0	trna51_LysCTT_1_+_165566150_165566222	PSEUDO	GTCTAGCTAGATCAGTTGGTAGAGCATAAGACTCTTAATCTCAGGGTCATGGGTTTGAGCCCTACGTTGGGCG
431	gtRNAdb v1.0	trna51_SerTGA_6_+_27513468_27513549	TRUE	GTAGTCGTGGCCGAGTGGTTAAGGCGATGGACTTGAAATCCATTGGGGTTTCCCCGCGCAGGTTCGAATCCTGCCGACTACG

432	gtRNAdb v1.0	trna52_ArgTCT_6_+_27529963_27530049_intronat_27530000_27530013	TRUE	GGCTCTGTGGCGCAATGGATAGCGCATTGGACTTCTAGCCTAAATCAAGAGATTCAAAGGTTGCGGGTTCGAGTCCCTCCAGAGTCG
433	gtRNAdb v1.0	trna52_ProCGG_1_+_167683962_167684033	TRUE	GGCTCGTTGGTCTAGGGGTATGATTCTCGCTTCGGGTGCGAGAGGTCCCGGGTTCAAATCCCGGACGAGCCC
434	gtRNAdb v1.0	trna53_LysTTT_6_+_27543846_27543918	TRUE	ACCTGGGTAGCTCAGTAGGTAGAACATCAGACTTTTAATCTGAGGGTCTAGGGTTCAAGTCCCTGTCCAGGCG
435	gtRNAdb v1.0	trna54_LysTTT_1_+_204475655_204475727	TRUE	GCCCGGATAGCTCAGTCGGTAGAGCATCAGACTTTTAATCTGAGGGTCCAGGGTTCAAGTCCCTGTTCGGGCG
436	gtRNAdb v1.0	trna54_ThrCGT_6_+_27586135_27586208	TRUE	GGCCCTGTAGCTCAGCGGTTGGAGCGCTGGTCTCGTAAACCTAGGGGTCGTGAGTTCAAATCTCACCAGGGCCT
437	gtRNAdb v1.0	trna55_IleTAT_6_+_27599200_27599293_intronat_27599238_27599257	TRUE	GCTCCAGTGGCGCAATCGGTTAGCGCGCGGTACTTATACAACAGTATATGTGCGGGTGATGCCGAGGTTGTGAGTTCGAGCCTCACCTGGAGCA
438	gtRNAdb v1.0	trna55_LysTTT_1_+_205443271_205443343	TRUE	GCCCGGAGAGCTCAGTGGGTAGAGCATCAGACTTTTAATCTGAGGGTCCAGGGTTCAAGTCCTCGTTCGGGCA
439	gtRNAdb v1.0	trna56_PheGAA_6_+_27632545_27632620	TRUE	GCCGAAATAGCTCAATTGGGAGAGTGTTAGACTGAAGATCTTCTGCAGGTCTCTGGTTCAATTCCGGGTTTCGACA
440	gtRNAdb v1.0	trna56_ThrTGT_1_+_222638347_222638419	TRUE	GGCTCCATAGCTCAGTGGTTAGAGCACTGGTCTTGTAAACCAGGGGTCGCGAGTTCGATCCTCGCTGGGGCCT
441	gtRNAdb v1.0	trna57_IleAAT_6_+_27636362_27636435	TRUE	GGCCGGTTAGCTCAGTCGGCTAGAGCGTGGTGCTAATAACGCCAAGGTCGCGGGTTCGATCCCCGTACGGGCCA
442	gtRNAdb v1.0	trna57_ProTGG_1_+_238105862_238105933	PSEUDO	TAGGACTTGGTGTAATAGGTAGCACGAAGAGATTTGGATTCTCAGGGGTAGGTTCAATTCCTATAGTTCTGG
443	gtRNAdb v1.0	trna58_LeuCAA_1_+_249168054_249168159_intronat_249168092_249168113	TRUE	GTCAGGATGGCCGAGTGGTCTAAGGCGCCAGACTCAAGGTAAGCACCTTGCCTGCGGGCTTTCTGGTCTCCGGATGGAGGCGTGGGTTCGAATCCCACTTCTGACA
444	gtRNAdb v1.0	trna58_ValCAC_6_+_27650488_27650561	TRUE	TTTTCTGTAGTGTAGTTGTTAACACGTTCGCCTCACACGCTTAAAGTTCTCTGGTTGGATACCAGATGGAAATG
445	gtRNAdb v1.0	trna59_GluCTC_1_+_249168447_249168518	TRUE	TCCCTGGTGGTCTAGTGGTTAGGATTCGGCGCTCTCACCGCCGCGGCCCGGGTTCGATTCCCGGTCAGGAAA
446	gtRNAdb v1.0	trna59_IleAAT_6_+_27655967_27656040	TRUE	GGCCGGTTAGCTCAGTTGGTTAGAGCGTGGTGCTAATAACGCCAAGGTCGCGGGTTCGATCCCCGTACTGGCCA
447	gtRNAdb v1.0	trna5_AspGTC_12_+_98897281_98897352	TRUE	TCCTCGTTAGTATAGTGGTTAGTATCCCCGCCTGTCACGCGGGAGACCGGGGTTCAATTCCCCGACGGGGAG
448	gtRNAdb v1.0	trna5_CysGCA_7_+_149007281_149007352	TRUE	GGGGGCATAGCTCAGTGGTAGAGCATTTGACTGCAGATCAAGAGGTCCCTGGTTCAAATCCAGGTGCCCCCT
449	gtRNAdb v1.0	trna5_GlnCTG_10_-_20036608_20036682	PSEUDO	CTAGGACGTGGTGTAATAGGTAGCACAGAGAATTCTGGATTCTCAGGGGTAGGTTCAATTCCTATAGAACCTAGG
450	gtRNAdb v1.0	trna5_GlnCTG_9_-_126655522_126655594	TRUE	GAGCTGTAGCATAGTGATTAGGGACATGGACTCTGGAGCCAAATCTGCCTGGGTTCTAGTCCCAGCTGTCTCA
451	gtRNAdb v1.0	trna5_GluTTC_1_+_17199078_17199149	TRUE	TCCCTGGTGGTCTAGTGGCTAGGATTCGGCGCTTTCACCGCCGCGGCCCGGGTTCGATTCCCGGCCAGGGAA
452	gtRNAdb v1.0	trna5_GluTTC_13_-_41634874_41634945	TRUE	TCCCATATGGTCTAGCGGTTAGGATTCCTGGTTTTCACCCAGGTGGCCCGGGTTCGACTCCCGGTATGGGAA
453	gtRNAdb v1.0	trna5_GlyGCC_17_+_8029064_8029134	TRUE	GCATTGGTGGTTCAGTGGTAGAATTCTCGCCTGCCACGCGGGAGGCCCGGGTTCGATTCCCGGCCAATGCA
454	gtRNAdb v1.0	trna5_IleAAT_20_-_51218338_51218415	PSEUDO	GACCAATTAGCAAGCACAGTTGGCTAGAACATGGTGCTAATAAGGCCACGGTCAGGGGTTCAATTCCCTTATGGGCTG
455	gtRNAdb v1.0	trna5_IleGAT_X_-_3833271_3833344	TRUE	GGCCGGTTAGCTCAGTTGGTAAGAGCGTGGTGCTGATAACACCAAGGTCGCGGGCTCGACTCCCGCACCGGCCA
456	gtRNAdb v1.0	trna5_IleTAT_2_+_43037676_43037768_intronat_43037714_43037732	TRUE	GCTCCAGTGGCGCAATCGGTTAGCGCGCGGTACTTATACAGCAGTACATGCAGAGCAATGCCGAGGTTGTGAGTTCGAGCCTCACCTGGAGCA
457	gtRNAdb v1.0	trna5_LeuAAG_3_-_148221228_148221309	TRUE	GGTAGCATGGCTGAGTGGTCTAAGATTCTGAATTAAGTCTCCAGTCTCTTTGGGGGCGTGGTTTTCAATCCCACCGCTGCTA
458	gtRNAdb v1.0	trna5_LysCTT_16_+_3214939_3215011	TRUE	GCCTGGCTAGCTCAGTCGGCAAAGCATGAGACTCTTAATCTCAGGGTCGTGGGCTCGAGCTCCATGTTGGGCG
459	gtRNAdb v1.0	trna5_LysCTT_19_+_36066750_36066822	TRUE	GCCCAGCTAGCTCAGTCGGTAGAGCATAAGACTCTTAATCTCAGGGTTGTGGATTCGTGCCCCATGCTGGGTG
460	gtRNAdb v1.0	trna5_LysTTT_11_+_59323902_59323974	TRUE	GCCCGGATAGCTCAGTCGGTAGAGCATCAGACTTTTAATCTGAGGGTCCGGGGTTCAAGTCCCTGTTCGGGCG
461	gtRNAdb v1.0	trna5_SerAGA_6_+_26327817_26327898	TRUE	GTAGTCGTGGCCGAGTGGTTAAGGCGATGGACTAGAAATCCATTGGGGTCTCCCCGCGCAGGTTCGAATCCTGCCGACTACG
462	gtRNAdb v1.0	trna5_SupTTA_4_-_7325838_7325909	PSEUDO	GGCAGCCTGGCTTAGTGGAAAGGGAATAGGCTTTAGAGCCAGACTGCCTGGGTTTGAATCCCAGCCCCGCCA
463	gtRNAdb v1.0	trna5_TyrGTA_14_+_21151432_21151520_intronat_21151469_21151484	TRUE	CCTTCGATAGCTCAGCTGGTAGAGCGGAGGACTGTAGTACTTAATGTGTGGTCATCCTTAGGTCGCTGGTTCGATTCCGGCTCGAAGGA
464	gtRNAdb v1.0	trna5_TyrGTA_15_-_92254302_92254374	PSEUDO	GTCAGTGTTGCACAACGGTTAAGTGAAGAGGCTGTAAACCCAGACTGGATGGGTTCAATTCCCATCTCTGCCG
465	gtRNAdb v1.0	trna5_TyrGTA_8_+_67026223_67026311_intronat_67026260_67026275	TRUE	CCTTCGATAGCTCAGCTGGTAGAGCGGAGGACTGTAGGCGCGCGCCCGTGGCCATCCTTAGGTCGCTGGTTCGATTCCGGCTCGAAGGA
466	gtRNAdb v1.0	trna5_ValAAC_5_+_180596610_180596682	TRUE	GTTTCCGTAGTGTAGTGGTTATCACGTTCGCCTAACACGCGAAAGGTCCCCGGTTCGAAACCGGGCGGAAACA
467	gtRNAdb v1.0	trna60_LeuTAA_1_-_238106956_238107030	PSEUDO	GTTAAGATGGCAGAGCCCAGCAATTGCATAAAACTTAAAACTTTACAATCAGAGGTTCAACTCCTCTTCTTAACA
468	gtRNAdb v1.0	trna60_ThrAGT_6_+_27694473_27694546	TRUE	GGCTTCGTGGCTTAGCTGGTTAAAGCGCCTGTCTAGTAAACAGGAGATCCTGGGTTCGAATCCCAGCGAGGCCT
469	gtRNAdb v1.0	trna61_MetCAT_6_+_27745664_27745735	TRUE	AGCAGAGTGGCGCAGCGGAAGCGTGCTGGGCCCATAACCCAGAGGTCGATGGATCTAAACCATCCTCTGCTA
470	gtRNAdb v1.0	trna61_ProTGG_1_-_207178154_207178230	PSEUDO	GCCAGGAGAGCTCAGTGGTGATGGGATGAGATCTGGACTCACACCTCTAGGCCTGGGTTCAAATCCCAGGTCTAGCG
471	gtRNAdb v1.0	trna62_LysTTT_1_-_204476158_204476230	TRUE	GCCCGGATAGCTCAGTCGGTAGAGCATCAGACTTTTAATCTGAGGGTCCAGGGTTCAAGTCCCTGTTCGGGCG
472	gtRNAdb v1.0	trna62_SerGCT_6_+_28180815_28180896	TRUE	GACGAGGTGGCCGAGTGGTTAAGGCGATGGACTGCTAATCCATTGTGCTCTGCACACGTGGGTTCGAATCCCATCCTCGTCG
473	gtRNAdb v1.0	trna63_IleTAT_6_+_28505367_28505460_intronat_28505405_28505424	TRUE	GCTCCAGTGGCGCAATCGGTTAGCGCGCGGTACTTATAAGACAGTGCACCTGTGAGCAATGCCGAGGTTGTGAGTTCAAGCCTCACCTGGAGCA
474	gtRNAdb v1.0	trna63_ValAAC_1_-_180184276_180184348	TRUE	GTTTCCATAGTGTACTGGTTATCACATTCACCTAACACGCGAAAGGTCCTTGGTTTGAAACCAGGCAGAAACA
475	gtRNAdb v1.0	trna64_GlnTTG_6_+_28557156_28557227	TRUE	GGTCCCATGGTGTAATGGTTAGCACTCTGGACTTTGAATCCAGCAATCCGAGTTCGAATCTCGGTGGGACCT
476	gtRNAdb v1.0	trna64_GluTTC_1_-_172157539_172157607	TRUE	CCCTGTGGTCTAGTGGTTAGGAGTTGGTGCTTTCGTCATGACAGCCCAGGTTCAATTCCTGGTTAGAGA
477	gtRNAdb v1.0	trna65_AlaAGC_6_+_28574933_28575004	TRUE	GGGGGTGTAGCTCAGTGGTAGAGCGCGTGCTTAGCATGTACGAGGTCCCGGGTTCAATCCCCGGCACCTCCA
478	gtRNAdb v1.0	trna65_ProAGG_1_-_167684725_167684796	TRUE	GGCTCGTTGGTCTAGGGGTATGATTCTCGCTTAGGGTGCGAGAGGTCCCGGGTTCAAATCCCGGACGAGCCC
479	gtRNAdb v1.0	trna66_AlaTGC_6_+_28611222_28611293	TRUE	GGGGATGTAGCTCAGTGGTAGAGCGCATGCTTTGCATGTATGAGGTCCCGGGTTCGATCCCCGGCATCTCCA
480	gtRNAdb v1.0	trna66_LeuCAA_1_-_161581736_161581819	TRUE	GTCAGGATGGCCGAGCAGTCTTAAGGCGCTGCGTTCAAATCGCACCCTCCGCTGGAGGCGTGGGTTCGAATCCCACTTTTGACA
481	gtRNAdb v1.0	trna67_AlaAGC_6_+_28678366_28678437	TRUE	GGGGGTGTAGCTCAGTGGTAGAGCGCGTGCTTAGCATGCACGAGGCCCTGGGTTCAATCCCCAGCACCTCCA
482	gtRNAdb v1.0	trna67_LeuCAG_1_-_161500132_161500214	TRUE	GTCAGGATGGCCGAGCGGTCTAAGGCGCTGCGTTCAGGTCGCAGTCTCCCCTGGAGGCGTGGGTTCGAATCCCACTCCTGACA
483	gtRNAdb v1.0	trna68_AlaAGC_6_+_28687481_28687552	TRUE	GGGGGTGTAGCTCAGTGGTAGAGCGCGTGCTTAGCATGCACGAGGCCCCGGGTTCAATCCCTGGCACCTCCA
484	gtRNAdb v1.0	trna68_GlyGCC_1_-_161493637_161493707	TRUE	GCATTGGTGGTTCAGTGGTAGAATTCTCGCCTGCCACGCGGGAGGCCCGGGTTCGATTCCCGGCCAATGCA
485	gtRNAdb v1.0	trna69_AspGTC_1_-_161440205_161440276	TRUE	TCCTCGTTAGTATAGTGGTGAGTATCCCCGCCTGTCACGCGGGAGACCGGGGTTCGATTCCCCGACGGGGAG
486	gtRNAdb v1.0	trna69_ThrAGT_6_+_28693795_28693868	TRUE	GGCTCCGTAGCTTAGTTGGTTAAAGCGCCTGTCTAGTAAACAGGAGATCCTGGGTTCGACTCCCAGCGGGGCCT
487	gtRNAdb v1.0	trna6_AlaAGC_8_+_67026424_67026496	TRUE	GGGGGATTAGCTCAAATGGTAGAGCGCTCGCTTAGCATGCGAGAGGTAGCGGGATCGATGCCCGCATCCTCCA
488	gtRNAdb v1.0	trna6_AlaTGC_11_+_60763508_60763577	PSEUDO	GGGGGAGTGGTGTGGTTACGAATGTGGCCTCTGCAAGCAGACAGCCTGGGTTCAATTCCCAGCTTGGCCA
489	gtRNAdb v1.0	trna6_ArgACG_6_+_26328368_26328440	TRUE	GGGCCAGTGGCGCAATGGATAACGCGTCTGACTACGGATCAGAAGATTCCAGGTTCGACTCCTGGCTGGCTCG
490	gtRNAdb v1.0	trna6_AsnGTT_1_+_17201958_17202031	TRUE	GTCTCTGTGGTGCAATCGGTTAGCGCGTTCGGCTGTTAACCATAAGGTTGGTGGTTAGAGACCACCCAGGGACG
491	gtRNAdb v1.0	trna6_AspGTC_9_-_77517990_77518061	TRUE	TCCTCGTTAGTATGGTGGTGAGTATCCCTGCCTGTCACGCGGGAGACCGGGGTTCGATTCCCCAACGGGGAG
492	gtRNAdb v1.0	trna6_CysGCA_3_-_131950642_131950713	TRUE	GGGGGTATAGCTCAGGGGTAGAGCATTTGACTGCAGATCAAGAGGTCCCTGGTTCAAATCCAGGTGCCCCCT
493	gtRNAdb v1.0	trna6_CysGCA_7_+_149028220_149028291	TRUE	GGGGGTATAGCTCAGGGGTAGAGCATTTGACTGCAGATCAAGAGGTCCCTGGTTCAAATCCAGGTGCCCCCT
494	gtRNAdb v1.0	trna6_GlnTTG_13_-_36639747_36639818	PSEUDO	TAGGATGTGGTGTGACAGGTAGCATGGAGAATTTTGGATTCTCAGGGTTAGGTTCAATTCCTATAGTTCTAG
495	gtRNAdb v1.0	trna6_GluTTC_2_+_75124046_75124114	TRUE	GCCTGTGGTCTAGTGGTTAGAATTCAGTGTTTTCAGTGCTCTAGTCCAGGTTCAATTCCTGGTCAGGGA
496	gtRNAdb v1.0	trna6_IleGAT_X_-_3794842_3794915	TRUE	GGCCGGTTAGCTCAGTTGGTAAGAGCGTGGTGCTGATAACACCAAGGTCGCGGGCTCGACTCCCGCACCGGCCA
497	gtRNAdb v1.0	trna6_LeuAAG_20_-_48952342_48952423	TRUE	GGTAGGGTGGCCGAGCGGTCTAAGGCACTGTATTAAGACTCCAGTCTCTTCAGAGGCATGGGTTTGAATCCCACTGCTGCCA
498	gtRNAdb v1.0	trna6_LysCTT_15_-_76674765_76674838	PSEUDO	GCCTGGCTACCTCAGTTGGTAGAGCATGGGACTCTTAATCCCAGAGTCAGTGGGTTCAAGCCTCACATTGAGTG
499	gtRNAdb v1.0	trna6_LysCTT_19_-_52425396_52425468	TRUE	CTGCAGCTAGCTCAGTCGGTAGAGCATGAGACTCTTAATCTCAGGGTCATGGGTTCGTGCCCCATGTTGGGTG
500	gtRNAdb v1.0	trna6_ProCGG_16_+_3222049_3222120	TRUE	GGCTCGTTGGTCTAGGGGTATGATTCTCGCTTCGGGTGCGAGAGGTCCCGGGTTCAAATCCCGGACGAGCCC
501	gtRNAdb v1.0	trna6_ProTGG_14_+_21152175_21152246	TRUE	GGCTCGTTGGTCTAGGGGTATGATTCTCGCTTTGGGTGCGAGAGGTCCCGGGTTCAAATCCCGGACGAGCCC
502	gtRNAdb v1.0	trna6_TrpCCA_12_+_98898030_98898101	TRUE	GACCTCGTGGCGCAACGGTAGCGCGTCTGACTCCAGATCAGAAGGCTGCGTGTTCGAATCACGTCGGGGTCA
503	gtRNAdb v1.0	trna6_TrpCCA_17_+_8089676_8089747	TRUE	GACCTCGTGGCGCAACGGTAGCGCGTCTGACTCCAGATCAGAAGGTTGCGTGTTCAAATCACGTCGGGGTCA
504	gtRNAdb v1.0	trna6_ValCAC_5_+_180600650_180600722	TRUE	GTTTCCGTAGTGTAGTGGTTATCACGTTCGCCTCACACGCGAAAGGTCCCCGGTTCGAAACCGGGCGGAAACA
505	gtRNAdb v1.0	trna6_ValTAC_10_-_5895674_5895746	TRUE	GGTTCCATAGTGTAGTGGTTATCACATCTGCTTTACACGCAGAAGGTCCTGGGTTCAAGCCCCAGTGGAACCA
506	gtRNAdb v1.0	trna70_AlaCGC_6_+_28697092_28697163	TRUE	GGGGGTGTAGCTCAGTGGTAGAGCGCGTGCTTCGCATGTACGAGGCCCCGGGTTCGACCCCCGGCTCCTCCA
507	gtRNAdb v1.0	trna70_GlyTCC_1_-_161439547_161439618	TRUE	GCGTTGGTGGTATAGTGGTGAGCATAGCTGCCTTCCAAGCAGTTGACCCGGGTTCGATTCCCGGCCAACGCA
508	gtRNAdb v1.0	trna71_GluCTC_1_-_161439189_161439260	TRUE	TCCCTGGTGGTCTAGTGGTTAGGATTCGGCGCTCTCACCGCCGCGGCCCGGGTTCGATTCCCGGTCAGGGAA
509	gtRNAdb v1.0	trna71_LysTTT_6_+_28715521_28715593	TRUE	GCCTGGATAGCTCAGTTGGTAGAACATCAGACTTTTAATCTGACGGTGCAGGGTTCAAGTCCCTGTTCAGGCG
510	gtRNAdb v1.0	trna72_AspGTC_1_-_161432824_161432895	TRUE	TCCTCGTTAGTATAGTGGTGAGTATCCCCGCCTGTCACGCGGGAGACCGGGGTTCGATTCCCCGACGGGGAG
511	gtRNAdb v1.0	trna72_PheGAA_6_+_28732164_28732236	TRUE	GCCAAAATTGCTCAGTTGGGAGAGCGTTAGACTGAAGATCTAAAGGTCCCTGGTTCGATCCCGGGTTTCACCA
512	gtRNAdb v1.0	trna73_ArgCCG_6_+_28849165_28849237	TRUE	GGCCGCGTGGCCTAATGGATAAGGCGTCTGATTCCGGATCAGAAGATTGAGGGTTCGAGTCCCTTCGTGGTCG
513	gtRNAdb v1.0	trna73_GlyTCC_1_-_161432166_161432237	TRUE	GCGTTGGTGGTATAGTGGTGAGCATAGCTGCCTTCCAAGCAGTTGACCCGGGTTCGATTCCCGGCCAACGCA
514	gtRNAdb v1.0	trna74_GluCTC_1_-_161431809_161431880	TRUE	TCCCTGGTGGTCTAGTGGTTAGGATTCGGCGCTCTCACCGCCGCGGCCCGGGTTCGATTCCCGGTCAGGGAA
515	gtRNAdb v1.0	trna74_LeuCAA_6_+_28908830_28908934_intronat_28908868_28908889	TRUE	GTCAGGATGGCCGAGTGGTCTAAGGCGCCAGACTCAAGCTTGGCTTCCTCGTGTTGAGGATTCTGGTCTCCAATGGAGGCGTGGGTTCGAATCCCACTTCTGACA
516	gtRNAdb v1.0	trna75_AspGTC_1_-_161425414_161425485	TRUE	TCCTCGTTAGTATAGTGGTGAGTATCCCCGCCTGTCACGCGGGAGACCGGGGTTCGATTCCCCGACGGGGAG
517	gtRNAdb v1.0	trna75_MetCAT_6_+_28912352_28912424	TRUE	GCCTCCTTAGCGCAGTAGGCAGCGCGTCAGTCTCATAATCTGAAGGTCCTGAGTTCGAACCTCAGAGGGGGCA
518	gtRNAdb v1.0	trna76_GlyTCC_1_-_161424756_161424827	TRUE	GCGTTGGTGGTATAGTGGTGAGCATAGCTGCCTTCCAAGCAGTTGACCCGGGTTCGATTCCCGGCCAACGCA
519	gtRNAdb v1.0	trna76_LysTTT_6_+_28918806_28918878	TRUE	GCCCGGATAGCTCAGTCGGTAGAGCATCAGACTTTTAATCTGAGGGTCCAGGGTTCAAGTCCCTGTTCGGGCG

520	gtRNAdb v1.0	trna77_GluCTC_1_-_161424398_161424469	TRUE	TCCCTGGTGGTCTAGTGGTTAGGATTCGGCGCTCTCACCGCCGCGGCCCGGGTTCGATTCCCGGTCAGGGAA
521	gtRNAdb v1.0	trna77_GluCTC_6_+_28949976_28950047	TRUE	TCCCTGGTGGTCTAGTGGTTAGGATTCGGCGCTCTCACCGCCGCGGCCCGGGTTCGATTCCCGGTCAGGGAA
522	gtRNAdb v1.0	trna78_AspGTC_1_-_161418033_161418104	TRUE	TCCTCGTTAGTATAGTGGTGAGTATCCCCGCCTGTCACGCGGGAGACCGGGGTTCGATTCCCCGACGGGGAG
523	gtRNAdb v1.0	trna78_LeuAAG_6_+_28956779_28956860	TRUE	GGTAGCGTGGCCGAGCGGTCTAAGGCGCTGGATTAAGGCTCCAGTCTCTTCGGGGGCGTGGGTTCAAATCCCACCGCTGCCA
524	gtRNAdb v1.0	trna79_GlnTTG_6_+_37287995_37288067	TRUE	GTGCAGAGTAGTACAGTGGTTAAACCATGGTCTTTGGAGCCAGACTGCCTGGGGTCGGATCCCAGCTCTCACA
525	gtRNAdb v1.0	trna79_GlyTCC_1_-_161417375_161417446	TRUE	GCGTTGGTGGTATAGTGGTGAGCATAGCTGCCTTCCAAGCAGTTGACCCGGGTTCGATTCCCGGCCAACGCA
526	gtRNAdb v1.0	trna7_ArgACG_14_+_23398910_23398982	TRUE	GGGCCAGTGGCGCAATGGATAACGCGTCTGACTACGGATCAGAAGATTCCAGGTTCGACTCCTGGCTGGCTCG
527	gtRNAdb v1.0	trna7_ArgCCT_11_+_63661767_63661835	PSEUDO	GCATTCGTAGTTCAGCGGCAGAAATTTCGTCTCCTACGCGGGAGACTCGGGTTCGACTTCGGCCATGCA
528	gtRNAdb v1.0	trna7_AsnGTT_1_+_17216172_17216245	TRUE	GTCTCTGTGGCGCAATCGGTTAGCGCGTTCGGCTGTTAACCGAAAGATTGGTGGTTCGAGCCCACCCAGGGACG
529	gtRNAdb v1.0	trna7_AsnGTT_13_-_31248101_31248174	TRUE	GTCTCTGTGGCGCAATCGGTTAGCGCGTTCGGCTGTTAACCGAAAGGTTGGTGGTTCGAGCCCACCCAGGGACG
530	gtRNAdb v1.0	trna7_AspGTC_12_+_122860924_122860994	TRUE	TCCTTGTTAGTATAGTGGTGAGTGTTTCTGCCTGTCATGTGGAGACTGGAGTTTGAGTCCCCAACAGGGAG
531	gtRNAdb v1.0	trna7_CysGCA_3_-_131947944_131948015	TRUE	GGGGGTGTAGCTCAGTGGTAGAGCATTTGACTGCAGATCAAGAGGTCCCTGGTTCAAATCCAGGTGCCCCCT
532	gtRNAdb v1.0	trna7_CysGCA_7_+_149243631_149243702	TRUE	GGGGGTATAGCTCAGGGGTAGAGCACTTGACTGCAGATCAAGAAGTCCTTGGTTCAAATCCAGGTGCCCCCT
533	gtRNAdb v1.0	trna7_GlnCTG_15_-_66161400_66161471	TRUE	GGTTCCATGGTGTAATGGTTAGCACTCTGGACTCTGAATCCAGCGATCCGAGTTCAAATCTCGGTGGAACCT
534	gtRNAdb v1.0	trna7_GluCTC_20_-_13970470_13970541	PSEUDO	TCCCTGCTTGTCTAGTGGTTAGAATTCAGCACTCTCACTGCCACAGCCCAGGTTCAATTCCCTGTCAGAGAA
535	gtRNAdb v1.0	trna7_GluCTC_8_+_70072340_70072414	PSEUDO	CTCTGGTGGTTTAGTGGCTAGGATTCACCTCTCTCACTGCTGCAGCCCAGGGTTCCATTCCCTGGGAGTCAGATG
536	gtRNAdb v1.0	trna7_HisGTG_9_-_14433938_14434009	TRUE	GCCGTGATCGTATAGTGGTTAGTACTCTGCGTTGTGGCCGCAGCAACCTCGGTTCGAATCCGAGTCACGGCA
537	gtRNAdb v1.0	trna7_IleGAT_X_-_3756418_3756491	TRUE	GGCCGGTTAGCTCAGTTGGTAAGAGCGTGGTGCTGATAACACCAAGGTCGCGGGCTCGACTCCCGCACCGGCCA
538	gtRNAdb v1.0	trna7_LeuAAG_5_+_180614701_180614782	TRUE	GGTAGCGTGGCCGAGCGGTCTAAGGCGCTGGATTAAGGCTCCAGTCTCTTCGGGGGCGTGGGTTCGAATCCCACCGCTGCCA
539	gtRNAdb v1.0	trna7_LeuCAG_6_+_26521436_26521518	TRUE	GTCAGGATGGCCGAGCGGTCTAAGGCGCTGCGTTCAGGTCGCAGTCTCCCCTGGAGGCGTGGGTTCGAATCCCACTCCTGACA
540	gtRNAdb v1.0	trna7_LysCTT_16_+_3225692_3225764	TRUE	GCCCGGCTAGCTCAGTCGGTAGAGCATGAGACTCTTAATCTCAGGGTCGTGGGTTCGAGCCCCACGTTGGGCG
541	gtRNAdb v1.0	trna7_LysTTT_19_-_50037933_50038005	TRUE	ACCTGGGTAGCTTAGTTGGTAGAGCATTGGACTTTTAATTTGAGGGCCCAGGTTTCAAGTCCCTGTTTGGGTG
542	gtRNAdb v1.0	trna7_ProAGG_2_+_77895593_77895663	PSEUDO	GGCTGGTTGGTCTAGGGCTATGATTCTCACTTAGGGTGCAAGAGGTCCTGGTTCAAATCCCAGAGGAGCCC
543	gtRNAdb v1.0	trna7_SerGCT_17_+_8090184_8090265	TRUE	GACGAGGTGGCCGAGTGGTTAAGGCGATGGACTGCTAATCCATTGTGCTCTGCACGCGTGGGTTCGAATCCCATCCTCGTCG
544	gtRNAdb v1.0	trna80_GluCTC_1_-_161417018_161417089	TRUE	TCCCTGGTGGTCTAGTGGTTAGGATTCGGCGCTCTCACCGCCGCGGCCCGGGTTCGATTCCCGGTCAGGGAA
545	gtRNAdb v1.0	trna80_IleAAT_6_+_58149254_58149327	TRUE	GGCCGGTTAGCTCAGTTGGTTAGAGCGTGGCGCTAATAACGCCAAGGTCGCGGGTTCGATCCCCGTACGGGCCA
546	gtRNAdb v1.0	trna81_AspGTC_1_-_161410615_161410686	TRUE	TCCTCGTTAGTATAGTGGTGAGTATCCCCGCCTGTCACGCGGGAGACCGGGGTTCGATTCCCCGACGGGGAG
547	gtRNAdb v1.0	trna81_LeuTAA_6_+_69914378_69914460	TRUE	ACTCATTTGGCTGAGTGGTTAAGGCATTGGACTTAAGATCCAATGGAGTAGTGGCTGTGTGGGTTTAAACCCCACTACTGGTA
548	gtRNAdb v1.0	trna82_GlyGCC_6_+_142578776_142578846	TRUE	GCATGGGTGATTCAGTGGTAGAATTTTCACCTGCCATGCAGGAGGTCCAGGTTCATTTCCTGGCCTATGCA
549	gtRNAdb v1.0	trna82_GlyTCC_1_-_161409961_161410032	TRUE	GCGTTGGTGGTATAGTGGTGAGCATAGTTGCCTTCCAAGCAGTTGACCCGGGCTCGATTCCCGCCCAACGCA
550	gtRNAdb v1.0	trna83_AsnGTT_1_-_161397867_161397940	TRUE	GTCTCTGTGGCGCAATCGGTTAGCGCGTTCGGCTGTTAACCGAAAGGTTGGTGGTTCGAGCCCACCCAGGGACG
551	gtRNAdb v1.0	trna83_LeuTAA_6_+_144537684_144537766	TRUE	ACCAGGATGGCCGAGTGGTTAAGGCGTTGGACTTAAGATCCAATGGACATATGTCCGCGTGGGTTCGAACCCCACTCCTGGTA
552	gtRNAdb v1.0	trna84_GlnTTG_6_+_145503859_145503930	TRUE	GGTCCCATGGTGTAATGGTTAGCACTCTGGGCTTTGAATCCAGCAATCCGAGTTCGAATCTTGGTGGGACCT
553	gtRNAdb v1.0	trna84_GluTTC_1_-_161391883_161391954	TRUE	TCCCTGGTGGTCTAGTGGCTAGGATTCGGCGCTTTCACCGCCGCGGCCCGGGTTCGATTCCCGGTCAGGGAA
554	gtRNAdb v1.0	trna85_GluTTC_6_-_163210014_163210082	PSEUDO	TCCCTGGTCTAGTGGTTAGGATTTATTATTTTCATGGCTGTGGCCTGAGTTCAATTTCCAATCAGGGAA
555	gtRNAdb v1.0	trna85_ValCAC_1_-_161369490_161369562	TRUE	GTTTCCGTAGTGTAGTGGTTATCACGTTCGCCTCACACGCGAAAGGTCCCCGGTTCGAAACCGGGCGGAAACA
556	gtRNAdb v1.0	trna86_ArgTCT_1_-_159111401_159111474	TRUE	GTCTCTGTGGCGCAATGGACGAGCGCGCTGGACTTCTAATCCAGAGGTTCCGGGTTCGAGTCCCGGCAGAGATG
557	gtRNAdb v1.0	trna86_ValTAC_6_-_156869046_156869120	PSEUDO	GTTAAGACGGCAGAGCCCGGCAATTGCGTAAAATTTACAACTTTATGGGCAGAGGTTCAATTCCTCTTCTTAACA
558	gtRNAdb v1.0	trna87_AlaAGC_1_-_150017382_150017452	TRUE	GGGGGTGTAGATCAGTGGTAGGGCGCACGCTTAGCATGCATGAGGCCCTGGGTCAATCCCCAGCACCTCCA
559	gtRNAdb v1.0	trna87_GluCTC_6_-_126101393_126101464	TRUE	TCCCTGGTGGTCTAGTGGTTAGGATTCGGCGCTCTCACCGCCGCGGCCCGGGTTCGATTCCCGGTCAGGGAA
560	gtRNAdb v1.0	trna88_ArgCCT_1_-_149728271_149728341	PSEUDO	GGATTTGTGGTCCAGTGGTAGAATTCTCACCGCCTGCATAGGAGACCCTGGTTTAATTCCTGGCCAATGCA
561	gtRNAdb v1.0	trna88_PheGAA_6_-_79668010_79668082	PSEUDO	GCCAAAATAGCTCAGCTGGGAGAGCATTAGACTGAAGATCTAAAGGTCTCTGGTTTGATCCTGGGTTTCAGAA
562	gtRNAdb v1.0	trna89_AlaAGC_6_-_58196623_58196695	TRUE	GGGGAATTAGCCCAAGTGGTAGAGCGCTTGCTTAGCATGCAAGAGGTAGTGGGATCGATGCCCACATTCTCCA
563	gtRNAdb v1.0	trna89_AsnGTT_1_-_149711798_149711871	TRUE	GTCTCTGTGGCGCAATCGGCTAGCGCGTTTGGCTGTTAACTAAAAGGTTGGTGGTTCGAACCCACCCAGAGGCG
564	gtRNAdb v1.0	trna8_AlaTGC_12_+_125424512_125424583	TRUE	GGGGATGTAGCTCAGTGGTAGAGCGCATGCTTTGCACGTATGAGGCCCCGGGTTCAATCCCCGGCATCTCCA
565	gtRNAdb v1.0	trna8_AlaTGC_5_+_180633868_180633939	TRUE	GGGGATGTAGCTCAGTGGTAGAGCGCATGCTTTGCATGTATGAGGCCCCGGGTTCGATCCCCGGCATCTCCA
566	gtRNAdb v1.0	trna8_ArgACG_6_+_26537726_26537798	TRUE	GGGCCAGTGGCGCAATGGATAACGCGTCTGACTACGGATCAGAAGATTCCAGGTTCGACTCCTGGCTGGCTCG
567	gtRNAdb v1.0	trna8_CysGCA_14_+_73429679_73429750	TRUE	GGGGGTATAGCTCAGGGGTAGAGCATTTGACTGCAGATCAAGAGGTCCCCGGTTCAAATCCGGGTGCCCCCT
568	gtRNAdb v1.0	trna8_CysGCA_7_+_149253802_149253873	TRUE	GGGCGTATAGCTCAGGGGTAGAGCATTTGACTGCAGATCAAGAGGTCCCCAGTTCAAATCTGGGTGCCCACT
569	gtRNAdb v1.0	trna8_GlnTTG_9_-_5095085_5095156	TRUE	TAGGATATGGTTTAATAGGTAGCATGGAGAATTTTGGAGTCTCAGGGATAGGTTCAATTCCTATAGTTCCAG
570	gtRNAdb v1.0	trna8_GluCTC_3_-_125413177_125413248	TRUE	CCCCTGGTGGTCTATCGGTTAGGATTCAGACCTCTCACCACTGCTACCCATGCTCGATTCCTGGTCAGGGAA
571	gtRNAdb v1.0	trna8_HisGTG_15_-_45492611_45492682	TRUE	GCCGTGATCGTATAGTGGTTAGTACTCTGCGTTGTGGCCGCAGCAACCTCGGTTCGAATCCGAGTCACGGCA
572	gtRNAdb v1.0	trna8_LysCTT_1_+_39970195_39970267	PSEUDO	ACCAGCATGTCTCAGTCGGTATAGTGTGAGACTCTTAATCTCAGGGTCGTGGGTTCAAGCCCCACATTGGGCG
573	gtRNAdb v1.0	trna8_ProAGG_2_+_87339573_87339644	PSEUDO	GGCTGGTTGGTCTAGGGCTATGATTCTCACTTAGGGTGCAAAAGGTCCTGGGTTCAAATCCCAGAGGAGCCC
574	gtRNAdb v1.0	trna8_ProTGG_16_+_3238094_3238165	TRUE	GGCTCGTTGGTCTAGGGGTATGATTCTCGCTTTGGGTGCGAGAGGTCCCGGGTTCAAATCCCGGACGAGCCC
575	gtRNAdb v1.0	trna8_SerGCT_11_+_66115591_66115672	TRUE	GACGAGGTGGCCGAGTGGTTAAGGCGATGGACTGCTAATCCATTGTGCTTTGCACGCGTGGGTTCGAATCCCATCCTCGTCG
576	gtRNAdb v1.0	trna8_ThrAGT_17_+_8090478_8090551	TRUE	GGCGCCGTGGCTTAGTTGGTTAAAGCGCCTGTCTAGTAAACAGGAGATCCTGGGTTCGAATCCCAGCGGTGCCT
577	gtRNAdb v1.0	trna90_AlaAGC_6_-_58187444_58187516	TRUE	GGGGAATTAGCGCAAGTGGTAGAGTGCTTGCTTAGCATGCAAGAGGTAGTGGGATCGATGCCCACATTCTCCA
578	gtRNAdb v1.0	trna90_ValCAC_1_-_149684088_149684161	TRUE	GTTTCCGTAGTGTAGTGGTTATCACGTTCGCCTCACACGCGTAAAGGTCCCCGGTTCGAAACCGGGCGGAAACA
579	gtRNAdb v1.0	trna91_AlaAGC_6_-_58182679_58182751	TRUE	GGGGAATTAGCTCAAGTGGTAGAGCGCTTGCTTAGCATGCAAGAGGTAGTGGGATCGATGCCCACATTCTCCA
580	gtRNAdb v1.0	trna91_GlyCCC_1_-_149680210_149680280	PSEUDO	GCACTGGTGGTTCAGTGGTAGAATTCTCGCCTCCCACGCGGGAGACCCGGGTTTAATTCCCGGTCAAGATA
581	gtRNAdb v1.0	trna92_MetCAT_6_-_58168492_58168564	TRUE	GCCCTCTTAGTGCAGCTGGCAGCGCGTCAGTTTCATAATCTGAAAGTCCTGAGTTCAAGCCTCAGAGAGGGCA
582	gtRNAdb v1.0	trna92_PheGAA_1_-_149672905_149672976	PSEUDO	TGCATGGTTGTCTAGTGGCTAGGATTCGGTGCTGAAAGCGTCACGGCCCGGGTTCGATTCCCGGTCAGGGAA
583	gtRNAdb v1.0	trna93_AlaAGC_6_-_58164628_58164700	TRUE	GGGGAATTAGCTCAAGCGGTAGAGCGCTTGCTTAGCATGCAAGAGGTAGTGGGATCGATGCCCACATTCTCCA
584	gtRNAdb v1.0	trna93_AsnGTT_1_-_149670057_149670130	TRUE	ATCTCCGTGGAGCAATTGGTTAGCGCGTTCGGCCGTTAACCGGAAAGTTGGTGGTTCGAGCCTACCCAGGGACG
585	gtRNAdb v1.0	trna94_AlaAGC_6_-_58142589_58142661	TRUE	GGGGGATTAGCTCAAGCGGTAGAGCGCCTGCTTAGCATGCAAGAGGTAGCAGGATCGATGCCTGCATTCTCCA
586	gtRNAdb v1.0	trna94_GluTTC_1_-_149664355_149664427	TRUE	TCCCTGGTGGTCTAGTGGCTAGGATTCGGCGCTTTCACCGCCTGCAGCTCGAGTTCGATTCCTGGTCAGGGAA
587	gtRNAdb v1.0	trna95_AlaAGC_6_-_58141877_58141949	TRUE	GGGGAATTAGCTCAAGCGGTAGAGCGCTCCCTTAGCATGCGAGAGGTAGCGGGATCGACGCCCCCATTCTCTA
588	gtRNAdb v1.0	trna95_AsnGTT_1_-_149615617_149615690	TRUE	GTCTCTGTGGTGCAATCGGTTAGCGCGTTCCGCTGTTAACCGAAAGCTTGGTGGTTCGAGCCCACCCAGGGATG
589	gtRNAdb v1.0	trna96_ArgCCT_1_-_149343081_149343151	PSEUDO	GGATTGGTGGTCCAGTGGTAGAATTCTCACCGCCTGCATAGGAGACCCTGGTTTAATTCCTGGCCAATGCA
590	gtRNAdb v1.0	trna96_PheGAA_6_-_28949449_28949521	TRUE	GCCGAAATAGCTCAGTTGGGAGAGCGTTAGACTGAAGATCTAAAGGTCCCTGGTTCGATCCCGGGTTTCGGCA
591	gtRNAdb v1.0	trna97_AsnGTT_1_-_149326272_149326345	TRUE	GTCTCTGTGGCGCAATCGGCTAGCGCGTTTGGCTGTTAACTAAAAAGTTGGTGGTTCGAACACACCCAGAGGCG
592	gtRNAdb v1.0	trna97_MetCAT_6_-_28921042_28921114	TRUE	GCCTCCTTAGCGCAGTAGGCAGCGCGTCAGTCTCATAATCTGAAGGTCCTGAGTTCGAACCTCAGAGGGGGCA
593	gtRNAdb v1.0	trna98_LeuAAG_6_-_28911399_28911480	TRUE	GGTAGCGTGGCCGAGCGGTCTAAGGCGCTGGATTAAGGCTCCAGTCTCTTCGGGGGCGTGGGTTCGAATCCCACCGCTGCCA
594	gtRNAdb v1.0	trna98_ValCAC_1_-_149298555_149298627	TRUE	GTTTCCGTAGTGTAGTGGTTATCACGTTCGCCTCACACGCGAAAGGTCCCCGGTTCGAAACTGGGCGGAAACA
595	gtRNAdb v1.0	trna99_GlnCTG_6_-_28909378_28909449	TRUE	GGTTCCATGGTGTAATGGTTAGCACTCTGGACTCTGAATCCAGCGATCCGAGTTCAAATCTCGGTGGAACCT
596	gtRNAdb v1.0	trna99_ValCAC_1_-_149294666_149294736	TRUE	GCACTGGTGGTTCAGTGGTAGAATTCTCGCCTCACACGCGGGACACCCGGGTTCAATTCCCGGTCAAGGCA
597	gtRNAdb v1.0	trna9_AlaAGC_14_+_89445442_89445514	TRUE	GGGGAATTAGCTCAAGTGGTAGAGCGCTCGCTTAGCATGCGAGAGGTAGTGGGATCGATGCCCGCATTCTCCA
598	gtRNAdb v1.0	trna9_ArgTCT_1_+_94313129_94313213_intronat_94313166_94313177	TRUE	GGCTCCGTGGCGCAATGGATAGCGCATTGGACTTCTAGAGGCTGAAGGCATTCAAAGGTTCCGGGTTCGAGTCCCGGCGGAGTCG
599	gtRNAdb v1.0	trna9_GlnTTG_3_-_106620878_106620949	PSEUDO	TAGGATGTGGTGTATTAGGTAGCACAGAGAATTTTGGATTCTCAGGGGTAGGTTCGATTCCTATAATTCTAC
600	gtRNAdb v1.0	trna9_HisGTG_15_-_45490804_45490875	TRUE	GCCGTGATCGTATAGTGGTTAGTACTCTGCGTTGTGGCCGCAGCAACCTCGGTTCGAATCCGAGTCACGGCA
601	gtRNAdb v1.0	trna9_IleAAT_12_+_129716198_129716272	PSEUDO	TGGCTGATGAGCTCAGCTGGTGGGAGCATGGTGTTAATGAGGCTGAGGTCGTGGGTTCAATCCCCACCGGGCTAT
602	gtRNAdb v1.0	trna9_IleAAT_17_+_8090911_8090984	TRUE	GGCCGGTTAGCTCAGTTGGTTAGAGCGTGGTGCTAATAACGCCAAGGTCGCGGGTTCGATCCCCGTACGGGCCA
603	gtRNAdb v1.0	trna9_LeuTAA_2_+_117781417_117781491	PSEUDO	GTTAAGATGGCAGAGCCCAGCAATTGCATAAATCTTAAAACTTTATAATCAGAGGTTCGACTCCTCTTCTTAACA
604	gtRNAdb v1.0	trna9_LysCTT_5_+_180634755_180634827	TRUE	GCCCGGCTAGCTCAGTCGGTAGAGCATGAGACTCTTAATCTCAGGGTCGTGGGTTCGAGCCCCACGTTGGGCG
605	gtRNAdb v1.0	trna9_LysTTT_19_-_41748142_41748214	PSEUDO	GCCAGGATAGTTCAGGTGGTAGAGCATCAGACTTTTAACCTGAGGGTTCAGGGTTCAAGTCTCTGTTTGGGCG
606	gtRNAdb v1.0	trna9_PheGAA_8_-_124270725_124270797	PSEUDO	GCCAAAATAGCTCAGCTGGGAGAGTATTAGGTTGAAGATACAAAGTTCCTTGGCTCAATCCAGAGTTTGGGGG
607	gtRNAdb v1.0	trna9_ProAGG_11_+_75946557_75946628	TRUE	GGCTCGTTGGTCTAGGGGTATGATTCTCGCTTAGGGTGCGAGAGGTCCCGGGTTCAAATCCCGGACGAGCCC

608	gtRNAdb v1.0	trna9_ProAGG_16_+_3239634_3239705	TRUE	GGCTCGTTGGTCTAGGGGTATGATTCTCGCTTAGGGTGCGAGAGGTCCCGGGTTCAAATCCCGGACGAGCCC
609	gtRNAdb v1.0	trna9_TyrGTA_7_+_149255133_149255205	TRUE	GGGGGTATAGCTCAGGGCTAGAGCTTTTTGACTGTAGAGCAAGAGGTCCCTGGTTCAAATCCAGGTTCTCCCT
610	gtRNAdb v1.0	trna9_ValCAC_6_+_26538282_26538354	TRUE	GTTTCCGTAGTGTAGTGGTTATCACGTTCGCCTCACACGCGAAAGGTCCCCGGTTCGAAACCGGGCGGAAACA
611	PMID: 25339973	trnalookalike1_XXXxxx_14_+_32954252_32954320	AlaTGC_MT_-_5587_5	AAGGGCTTAGCTTAATTAAAGTGGCTGATTTGCGTTCAGTTGATGCAGAGTGGGGTTTTGCAGTCCTTA
612	PMID: 25339973	trnalookalike2_XXXxxx_1_-_564879_564950	GlnTTG_MT_-_4329_4	TAGGATGGGGTGTGATAGGTGGCACGGAGAATTTTGGATTCTCAGGGATGGGTTCGATTCTCATAGTCCTAG
613	PMID: 25339973	trnalookalike3_XXXxxx_1_+_566062_566129	TrpTCA_MT_+_5512_5	AGAAATTTAGGTTAAATACAGACCAAGAGCCTTCAAAGCCCTCAGTAAGTTGCAATACTTAATTTCTG
614	PMID: 25339973	trnalookalike4_XXXxxx_1_-_566137_566205	AlaTGC_MT_-_5587_5	AAGGGCTTAGCTTAATTAAAGTGGCTGATTTGCGTTCAGTTGATGCAGAGTGGGGTTTTGCAGTCCTTA
615	PMID: 25339973	trnalookalike5_XXXxxx_1_-_566207_566279	AsnGTT_MT_-_5657_5	TAGATTGAAGCCAGTTGATTAGGGTGCTTAGCTGTTAACTAAGTGTTTGTGGGTTTAAGTCCCATTGGTCTAG
616	PMID: 25339973	trnalookalike6_XXXxxx_1_-_567997_568065	SerTGA_MT_-_7446_7	GAAAAAGTCATGGAGGCCATGGGGTTGGCTTGAAACCAGCTTTGGGGGGTTCGATTCCTTCCTTTTTTG
617	PMID: 25339973	trnalookalike7_XXXxxx_1_+_568844_568913	LysTTT_MT_+_8295_8	CACTGTAAAGCTAACTTAGCATTAACCTTTTAAGTTAAAGATTAAGAGAACCAACACCTCTTTACAGTGA
618	PMID: 25339973	trnalookalike8_XXXxxx_5_-_93905172_93905240	luTTC_MT_-_14674_1	GTTCTTGTAGTTGAAATACAACGATGGTTTTTCATATCATTGGTCGTGGTTGTAGTCCGTGCGAGAATA
619	NCBI entry NC_012920.1	trnaMT_AlaTGC_MT_-_5587_5655	TRUE	AAGGGCTTAGCTTAATTAAAGTGGCTGATTTGCGTTCAGTTGATGCAGAGTGGGGTTTTGCAGTCCTTA
620	NCBI entry NC_012920.1	trnaMT_ArgTCG_MT_+_10405_10469	TRUE	TGGTATATAGTTTAAACAAAACGAATGATTTCGACTCATTAAATTATGATAATCATATTTACCAA
621	NCBI entry NC_012920.1	trnaMT_AsnGTT_MT_-_5657_5729	TRUE	TAGATTGAAGCCAGTTGATTAGGGTGCTTAGCTGTTAACTAAGTGTTTGTGGGTTTAAGTCCCATTGGTCTAG
622	NCBI entry NC_012920.1	trnaMT_AspGTC_MT_+_7518_7585	TRUE	AAGGTATTAGAAAAACCATTTCATAACTTTGTCAAAGTTAAATTATAGGCTAAATCCTATATATCTTA
623	NCBI entry NC_012920.1	trnaMT_CysGCA_MT_-_5761_5826	TRUE	AGCTCCGAGGTGATTTTCATATTGAATTGCAAATTCGAAGAAGCAGCTTCAAACCTGCCGGGGCTT
624	NCBI entry NC_012920.1	trnaMT_GlnTTG_MT_-_4329_4400	TRUE	TAGGATGGGGTGTGATAGGTGGCACGGAGAATTTTGGATTCTCAGGGATGGGTTCGATTCTCATAGTCCTAG
625	NCBI entry NC_012920.1	trnaMT_GluTTC_MT_-_14674_14742	TRUE	GTTCTTGTAGTTGAAATACAACGATGGTTTTTCATATCATTGGTCGTGGTTGTAGTCCGTGCGAGAATA
626	NCBI entry NC_012920.1	trnaMT_GlyTCC_MT_+_9991_10058	TRUE	ACTCTTTTAGTATAAATAGTACCGTTAACTTCCAATTAACTAGTTTTGACAACATTCAAAAAAGAGTA
627	NCBI entry NC_012920.1	trnaMT_HisGTG_MT_+_12138_12206	TRUE	GTAAATATAGTTTAACCAAAACATCAGATTGTGAATCTGACAACAGAGGCTTACGACCCCTTATTTACC
628	NCBI entry NC_012920.1	trnaMT_IleGAT_MT_+_4263_4331	TRUE	AGAAATATGTCTGATAAAAGAGTTACTTTGATAGAGTAAATAATAGGAGCTTAAACCCCCTTATTTCTA
629	NCBI entry NC_012920.1	trnaMT_LeuTAA_MT_+_3230_3304	TRUE	GTTAAGATGGCAGAGCCCGGTAATCGCATAAAACTTAAAACTTTACAGTCAGAGGTTCAATTCCTCTTCTTAACA
630	NCBI entry NC_012920.1	trnaMT_LeuTAG_MT_+_12266_12336	TRUE	ACTTTTAAAGGATAACAGCTATCCATTGGTCTTAGGCCCCAAAAATTTTGGTGCAACTCCAAATAAAAGTA
631	NCBI entry NC_012920.1	trnaMT_LysTTT_MT_+_8295_8364	TRUE	CACTGTAAAGCTAACTTAGCATTAACCTTTTAAGTTAAAGATTAAGAGAACCAACACCTCTTTACAGTGA
632	NCBI entry NC_012920.1	trnaMT_MetCAT_MT_+_4402_4469	TRUE	AGTAAGGTCAGCTAAATAAGCTATCGGGCCCATACCCCGAAAATGTTGGTTATACCCTTCCCGTACTA
633	NCBI entry NC_012920.1	trnaMT_PheGAA_MT_+_577_647	TRUE	GTTTATGTAGCTTACCTCCTCAAAGCAATACACTGAAAATGTTTAGACGGGCTCACATCACCCCATAAACA
634	NCBI entry NC_012920.1	trnaMT_ProTGG_MT_-_15956_16023	TRUE	CAGAGAATAGTTTAAATTAGAATCTTAGCTTTGGGTGCTAATGGTGGAGTTAAAGACTTTTTCTCTGA
635	NCBI entry NC_012920.1	trnaMT_SerGCT_MT_+_12207_12265	TRUE	GAGAAAGCTCACAAGAACTGCTAACTCATGCCCCCATGTCTAACAACATGGCTTTCTCA
636	NCBI entry NC_012920.1	trnaMT_SerTGA_MT_-_7446_7514	TRUE	GAAAAAGTCATGGAGGCCATGGGGTTGGCTTGAAACCAGCTTTGGGGGGTTCGATTCCTTCCTTTTTTG
637	NCBI entry NC_012920.1	trnaMT_ThrTGT_MT_+_15888_15953	TRUE	GTCCTTGTAGTATAAACTAATACACCAGTCTTGTAAACCGGAGATGAAAACCTTTTTCCAAGGACA
638	NCBI entry NC_012920.1	trnaMT_TrpTCA_MT_+_5512_5579	TRUE	AGAAATTTAGGTTAAATACAGACCAAGAGCCTTCAAAGCCCTCAGTAAGTTGCAATACTTAATTTCTG
639	NCBI entry NC_012920.1	trnaMT_TyrGTA_MT_-_5826_5891	TRUE	GGTAAAATGGCTGAGTGAAGCATTGGACTGTAAATCTAAAGACAGGGGTTAGGCCTCTTTTTACCA
640	NCBI entry NC_012920.1	trnaMT_ValTAC_MT_+_1602_1670	TRUE	CAGAGTGTAGCTTAACACAAAGCACCCAACTTACACTTAGGAGATTTCAACTTAACTTGACCGCTCTGA

Supplementary Table S3: All tRF candidate sequences and their exclusivity to tRNA space. The table below constitutes MINTmap's lookup-table for the human genome (GRCh37).

tRF	sequence exclusive to tRNA	sequence
1	No	AAAAACCATTTCATAA
2	No	AAAAACCATTTCATAAC
3	No	AAAAACCATTTCATAACT
4	No	AAAAACCATTTCATAACTT
5	No	AAAAACCATTTCATAACTTT
6	No	AAAAACCATTTCATAACTTTG
7	No	AAAAACCATTTCATAACTTTGT
8	No	AAAAACCATTTCATAACTTTGTC
9	No	AAAAACCATTTCATAACTTTGTCA
10	No	AAAAACCATTTCATAACTTTGTCAA
11	No	AAAAACCATTTCATAACTTTGTCAAA
12	No	AAAAACCATTTCATAACTTTGTCAAAG
13	No	AAAAACCATTTCATAACTTTGTCAAAGT
14	No	AAAAACCATTTCATAACTTTGTCAAAGTT
15	No	AAAAACCATTTCATAACTTTGTCAAAGTTA
16	No	AAAAACCATTTCATAACTTTGTCAAAGTTAA
17	No	AAAAACCATTTCATAACTTTGTCAAAGTTAAA
18	No	AAAAACCATTTCATAACTTTGTCAAAGTTAAAT
19	No	AAAAACCATTTCATAACTTTGTCAAAGTTAAATT
20	No	AAAAACCATTTCATAACTTTGTCAAAGTTAAATTA
21	No	AAAAACCATTTCATAACTTTGTCAAAGTTAAATTAT
22	No	AAAAACCATTTCATAACTTTGTCAAAGTTAAATTATA
23	No	AAAAACCATTTCATAACTTTGTCAAAGTTAAATTATAG
24	No	AAAAACCATTTCATAACTTTGTCAAAGTTAAATTATAGG
25	No	AAAAACCATTTCATAACTTTGTCAAAGTTAAATTATAGGC
26	No	AAAAACCATTTCATAACTTTGTCAAAGTTAAATTATAGGCT
27	No	AAAAACCATTTCATAACTTTGTCAAAGTTAAATTATAGGCTA
28	No	AAAAACCATTTCATAACTTTGTCAAAGTTAAATTATAGGCTAA
29	No	AAAAACCATTTCATAACTTTGTCAAAGTTAAATTATAGGCTAAA
30	No	AAAAACCATTTCATAACTTTGTCAAAGTTAAATTATAGGCTAAAT
31	No	AAAAACCATTTCATAACTTTGTCAAAGTTAAATTATAGGCTAAATC
32	No	AAAAACCATTTCATAACTTTGTCAAAGTTAAATTATAGGCTAAATCC
33	No	AAAAACCATTTCATAACTTTGTCAAAGTTAAATTATAGGCTAAATCCT
34	No	AAAAACCATTTCATAACTTTGTCAAAGTTAAATTATAGGCTAAATCCTA
35	No	AAAAACCATTTCATAACTTTGTCAAAGTTAAATTATAGGCTAAATCCTAT

36	No	AAAAAGTCATAGGGGT
37	Yes	AAAAAGTCATAGGGGTT
38	Yes	AAAAAGTCATAGGGGTTA
39	Yes	AAAAAGTCATAGGGGTTAT
40	Yes	AAAAAGTCATAGGGGTTATG
41	Yes	AAAAAGTCATAGGGGTTATGA
42	Yes	AAAAAGTCATAGGGGTTATGAG
43	Yes	AAAAAGTCATAGGGGTTATGAGG
44	Yes	AAAAAGTCATAGGGGTTATGAGGC
45	Yes	AAAAAGTCATAGGGGTTATGAGGCT
46	Yes	AAAAAGTCATAGGGGTTATGAGGCTG
47	Yes	AAAAAGTCATAGGGGTTATGAGGCTGG
48	Yes	AAAAAGTCATAGGGGTTATGAGGCTGGC
49	Yes	AAAAAGTCATAGGGGTTATGAGGCTGGCT
50	Yes	AAAAAGTCATAGGGGTTATGAGGCTGGCTT
51	Yes	AAAAAGTCATAGGGGTTATGAGGCTGGCTTG
52	Yes	AAAAAGTCATAGGGGTTATGAGGCTGGCTTGA
53	Yes	AAAAAGTCATAGGGGTTATGAGGCTGGCTTGAA
54	Yes	AAAAAGTCATAGGGGTTATGAGGCTGGCTTGAAA
55	Yes	AAAAAGTCATAGGGGTTATGAGGCTGGCTTGAAAC
56	Yes	AAAAAGTCATAGGGGTTATGAGGCTGGCTTGAAACC
57	Yes	AAAAAGTCATAGGGGTTATGAGGCTGGCTTGAAACCA
58	Yes	AAAAAGTCATAGGGGTTATGAGGCTGGCTTGAAACCAG
59	Yes	AAAAAGTCATAGGGGTTATGAGGCTGGCTTGAAACCAGC
60	Yes	AAAAAGTCATAGGGGTTATGAGGCTGGCTTGAAACCAGCC
61	Yes	AAAAAGTCATAGGGGTTATGAGGCTGGCTTGAAACCAGCCT
62	Yes	AAAAAGTCATAGGGGTTATGAGGCTGGCTTGAAACCAGCCTT
63	Yes	AAAAAGTCATAGGGGTTATGAGGCTGGCTTGAAACCAGCCTTA
64	Yes	AAAAAGTCATAGGGGTTATGAGGCTGGCTTGAAACCAGCCTTAG
65	Yes	AAAAAGTCATAGGGGTTATGAGGCTGGCTTGAAACCAGCCTTAGG
66	Yes	AAAAAGTCATAGGGGTTATGAGGCTGGCTTGAAACCAGCCTTAGGA
67	Yes	AAAAAGTCATAGGGGTTATGAGGCTGGCTTGAAACCAGCCTTAGGAG
68	Yes	AAAAAGTCATAGGGGTTATGAGGCTGGCTTGAAACCAGCCTTAGGAGG
69	Yes	AAAAAGTCATAGGGGTTATGAGGCTGGCTTGAAACCAGCCTTAGGAGGT
70	Yes	AAAAAGTCATAGGGGTTATGAGGCTGGCTTGAAACCAGCCTTAGGAGGTT
71	No	AAAAAGTCATGGAGGC
72	Yes	AAAAAGTCATGGAGGCC
73	Yes	AAAAAGTCATGGAGGCCA
74	Yes	AAAAAGTCATGGAGGCCAT
75	Yes	AAAAAGTCATGGAGGCCATG
76	Yes	AAAAAGTCATGGAGGCCATGG
77	Yes	AAAAAGTCATGGAGGCCATGGG

78	Yes	AAAAAGTCATGGAGGCCATGGGG
79	Yes	AAAAAGTCATGGAGGCCATGGGGT
80	Yes	AAAAAGTCATGGAGGCCATGGGGTT
81	Yes	AAAAAGTCATGGAGGCCATGGGGTTG
82	Yes	AAAAAGTCATGGAGGCCATGGGGTTGG
83	Yes	AAAAAGTCATGGAGGCCATGGGGTTGGC
84	Yes	AAAAAGTCATGGAGGCCATGGGGTTGGCT
85	Yes	AAAAAGTCATGGAGGCCATGGGGTTGGCTT
86	Yes	AAAAAGTCATGGAGGCCATGGGGTTGGCTTG
87	Yes	AAAAAGTCATGGAGGCCATGGGGTTGGCTTGA
88	Yes	AAAAAGTCATGGAGGCCATGGGGTTGGCTTGAA
89	Yes	AAAAAGTCATGGAGGCCATGGGGTTGGCTTGAAA
90	Yes	AAAAAGTCATGGAGGCCATGGGGTTGGCTTGAAAC
91	Yes	AAAAAGTCATGGAGGCCATGGGGTTGGCTTGAAACC
92	Yes	AAAAAGTCATGGAGGCCATGGGGTTGGCTTGAAACCA
93	Yes	AAAAAGTCATGGAGGCCATGGGGTTGGCTTGAAACCAG
94	Yes	AAAAAGTCATGGAGGCCATGGGGTTGGCTTGAAACCAGC
95	Yes	AAAAAGTCATGGAGGCCATGGGGTTGGCTTGAAACCAGCT
96	Yes	AAAAAGTCATGGAGGCCATGGGGTTGGCTTGAAACCAGCTT
97	Yes	AAAAAGTCATGGAGGCCATGGGGTTGGCTTGAAACCAGCTTT
98	Yes	AAAAAGTCATGGAGGCCATGGGGTTGGCTTGAAACCAGCTTTG
99	Yes	AAAAAGTCATGGAGGCCATGGGGTTGGCTTGAAACCAGCTTTGG
100	Yes	AAAAAGTCATGGAGGCCATGGGGTTGGCTTGAAACCAGCTTTGGG
101	Yes	AAAAAGTCATGGAGGCCATGGGGTTGGCTTGAAACCAGCTTTGGGG
102	Yes	AAAAAGTCATGGAGGCCATGGGGTTGGCTTGAAACCAGCTTTGGGGG
103	Yes	AAAAAGTCATGGAGGCCATGGGGTTGGCTTGAAACCAGCTTTGGGGGG
104	Yes	AAAAAGTCATGGAGGCCATGGGGTTGGCTTGAAACCAGCTTTGGGGGGT
105	Yes	AAAAAGTCATGGAGGCCATGGGGTTGGCTTGAAACCAGCTTTGGGGGGTT
106	No	AAAAAGTTGGTGGTTC
107	Yes	AAAAAGTTGGTGGTTCG
108	Yes	AAAAAGTTGGTGGTTCGA
109	Yes	AAAAAGTTGGTGGTTCGAA
110	Yes	AAAAAGTTGGTGGTTCGAAC
111	Yes	AAAAAGTTGGTGGTTCGAACA
112	Yes	AAAAAGTTGGTGGTTCGAACAC
113	Yes	AAAAAGTTGGTGGTTCGAACACA
114	Yes	AAAAAGTTGGTGGTTCGAACACAC
115	Yes	AAAAAGTTGGTGGTTCGAACACACC
116	Yes	AAAAAGTTGGTGGTTCGAACACACCC
117	Yes	AAAAAGTTGGTGGTTCGAACACACCCA
118	Yes	AAAAAGTTGGTGGTTCGAACACACCCAG
119	Yes	AAAAAGTTGGTGGTTCGAACACACCCAGA

120	Yes	AAAAAGTTGGTGGTTCGAACACACCCAGAG
121	Yes	AAAAAGTTGGTGGTTCGAACACACCCAGAGG
122	Yes	AAAAAGTTGGTGGTTCGAACACACCCAGAGGC
123	Yes	AAAAAGTTGGTGGTTCGAACACACCCAGAGGCG
124	Yes	AAAAAGTTGGTGGTTCGAACACACCCAGAGGCGC
125	Yes	AAAAAGTTGGTGGTTCGAACACACCCAGAGGCGCC
126	Yes	AAAAAGTTGGTGGTTCGAACACACCCAGAGGCGCCA
127	No	AAAAATTTTGGTGCAA
128	No	AAAAATTTTGGTGCAAC
129	No	AAAAATTTTGGTGCAACT
130	No	AAAAATTTTGGTGCAACTC
131	No	AAAAATTTTGGTGCAACTCC
132	No	AAAAATTTTGGTGCAACTCCA
133	No	AAAAATTTTGGTGCAACTCCAA
134	No	AAAAATTTTGGTGCAACTCCAAA
135	No	AAAAATTTTGGTGCAACTCCAAAT
136	No	AAAAATTTTGGTGCAACTCCAAATA
137	No	AAAAATTTTGGTGCAACTCCAAATAA
138	No	AAAAATTTTGGTGCAACTCCAAATAAA
139	No	AAAAATTTTGGTGCAACTCCAAATAAAA
140	No	AAAAATTTTGGTGCAACTCCAAATAAAAG
141	No	AAAAATTTTGGTGCAACTCCAAATAAAAGT
142	No	AAAAATTTTGGTGCAACTCCAAATAAAAGTA
143	Yes	AAAAATTTTGGTGCAACTCCAAATAAAAGTAC
144	Yes	AAAAATTTTGGTGCAACTCCAAATAAAAGTACC
145	Yes	AAAAATTTTGGTGCAACTCCAAATAAAAGTACCA
146	No	AAAACATCAGATTGTG
147	No	AAAACATCAGATTGTGA
148	No	AAAACATCAGATTGTGAA
149	No	AAAACATCAGATTGTGAAT
150	No	AAAACATCAGATTGTGAATC
151	No	AAAACATCAGATTGTGAATCT
152	No	AAAACATCAGATTGTGAATCTG
153	No	AAAACATCAGATTGTGAATCTGA
154	No	AAAACATCAGATTGTGAATCTGAC
155	No	AAAACATCAGATTGTGAATCTGACA
156	No	AAAACATCAGATTGTGAATCTGACAA
157	No	AAAACATCAGATTGTGAATCTGACAAC
158	No	AAAACATCAGATTGTGAATCTGACAACA
159	No	AAAACATCAGATTGTGAATCTGACAACAG
160	No	AAAACATCAGATTGTGAATCTGACAACAGA
161	No	AAAACATCAGATTGTGAATCTGACAACAGAG

162	No	AAAACATCAGATTGTGAATCTGACAACAGAGG
163	No	AAAACATCAGATTGTGAATCTGACAACAGAGGC
164	No	AAAACATCAGATTGTGAATCTGACAACAGAGGCT
165	Yes	AAAACATCAGATTGTGAATCTGACAACAGAGGCTT
166	Yes	AAAACATCAGATTGTGAATCTGACAACAGAGGCTTA
167	Yes	AAAACATCAGATTGTGAATCTGACAACAGAGGCTTAC
168	Yes	AAAACATCAGATTGTGAATCTGACAACAGAGGCTTACG
169	Yes	AAAACATCAGATTGTGAATCTGACAACAGAGGCTTACGA
170	Yes	AAAACATCAGATTGTGAATCTGACAACAGAGGCTTACGAC
171	Yes	AAAACATCAGATTGTGAATCTGACAACAGAGGCTTACGACC
172	Yes	AAAACATCAGATTGTGAATCTGACAACAGAGGCTTACGACCC
173	Yes	AAAACATCAGATTGTGAATCTGACAACAGAGGCTTACGACCCC
174	Yes	AAAACATCAGATTGTGAATCTGACAACAGAGGCTTACGACCCCT
175	Yes	AAAACATCAGATTGTGAATCTGACAACAGAGGCTTACGACCCCTT
176	Yes	AAAACATCAGATTGTGAATCTGACAACAGAGGCTTACGACCCCTTA
177	Yes	AAAACATCAGATTGTGAATCTGACAACAGAGGCTTACGACCCCTTAT
178	Yes	AAAACATCAGATTGTGAATCTGACAACAGAGGCTTACGACCCCTTATT
179	Yes	AAAACATCAGATTGTGAATCTGACAACAGAGGCTTACGACCCCTTATTT
180	Yes	AAAACATCAGATTGTGAATCTGACAACAGAGGCTTACGACCCCTTATTTA
181	Yes	AAAACCAGGCGGAAAC
182	Yes	AAAACCAGGCGGAAACA
183	Yes	AAAACCAGGCGGAAACAC
184	Yes	AAAACCAGGCGGAAACACC
185	Yes	AAAACCAGGCGGAAACACCA
186	No	AAAACCATGGATTCTA
187	No	AAAACCATGGATTCTAG
188	No	AAAACCATGGATTCTAGA
189	No	AAAACCATGGATTCTAGAG
190	No	AAAACCATGGATTCTAGAGC
191	No	AAAACCATGGATTCTAGAGCC
192	Yes	AAAACCATGGATTCTAGAGCCA
193	Yes	AAAACCATGGATTCTAGAGCCAG
194	Yes	AAAACCATGGATTCTAGAGCCAGA
195	Yes	AAAACCATGGATTCTAGAGCCAGAT
196	Yes	AAAACCATGGATTCTAGAGCCAGATG
197	Yes	AAAACCATGGATTCTAGAGCCAGATGG
198	Yes	AAAACCATGGATTCTAGAGCCAGATGGG
199	Yes	AAAACCATGGATTCTAGAGCCAGATGGGT
200	Yes	AAAACCATGGATTCTAGAGCCAGATGGGTT
201	Yes	AAAACCATGGATTCTAGAGCCAGATGGGTTC
202	Yes	AAAACCATGGATTCTAGAGCCAGATGGGTTCA
203	Yes	AAAACCATGGATTCTAGAGCCAGATGGGTTCAA

204	Yes	AAAACCATGGATTCTAGAGCCAGATGGGTTCAAA
205	Yes	AAAACCATGGATTCTAGAGCCAGATGGGTTCAAAT
206	Yes	AAAACCATGGATTCTAGAGCCAGATGGGTTCAAATC
207	Yes	AAAACCATGGATTCTAGAGCCAGATGGGTTCAAATCC
208	Yes	AAAACCATGGATTCTAGAGCCAGATGGGTTCAAATCCC
209	Yes	AAAACCATGGATTCTAGAGCCAGATGGGTTCAAATCCCG
210	Yes	AAAACCATGGATTCTAGAGCCAGATGGGTTCAAATCCCGG
211	Yes	AAAACCATGGATTCTAGAGCCAGATGGGTTCAAATCCCGGC
212	Yes	AAAACCATGGATTCTAGAGCCAGATGGGTTCAAATCCCGGCT
213	Yes	AAAACCATGGATTCTAGAGCCAGATGGGTTCAAATCCCGGCTC
214	Yes	AAAACCATGGATTCTAGAGCCAGATGGGTTCAAATCCCGGCTCT
215	Yes	AAAACCATGGATTCTAGAGCCAGATGGGTTCAAATCCCGGCTCTG
216	Yes	AAAACCATGGATTCTAGAGCCAGATGGGTTCAAATCCCGGCTCTGC
217	Yes	AAAACCATGGATTCTAGAGCCAGATGGGTTCAAATCCCGGCTCTGCC
218	Yes	AAAACCATGGATTCTAGAGCCAGATGGGTTCAAATCCCGGCTCTGCCG
219	Yes	AAAACCATGGATTCTAGAGCCAGATGGGTTCAAATCCCGGCTCTGCCGC
220	Yes	AAAACCATGGATTCTAGAGCCAGATGGGTTCAAATCCCGGCTCTGCCGCC
221	No	AAAACCATTTCATAAC
222	No	AAAACCATTTCATAACT
223	No	AAAACCATTTCATAACTT
224	No	AAAACCATTTCATAACTTT
225	No	AAAACCATTTCATAACTTTG
226	No	AAAACCATTTCATAACTTTGT
227	No	AAAACCATTTCATAACTTTGTC
228	No	AAAACCATTTCATAACTTTGTCA
229	No	AAAACCATTTCATAACTTTGTCAA
230	No	AAAACCATTTCATAACTTTGTCAAA
231	No	AAAACCATTTCATAACTTTGTCAAAG
232	No	AAAACCATTTCATAACTTTGTCAAAGT
233	No	AAAACCATTTCATAACTTTGTCAAAGTT
234	No	AAAACCATTTCATAACTTTGTCAAAGTTA
235	No	AAAACCATTTCATAACTTTGTCAAAGTTAA
236	No	AAAACCATTTCATAACTTTGTCAAAGTTAAA
237	No	AAAACCATTTCATAACTTTGTCAAAGTTAAAT
238	No	AAAACCATTTCATAACTTTGTCAAAGTTAAATT
239	No	AAAACCATTTCATAACTTTGTCAAAGTTAAATTA
240	No	AAAACCATTTCATAACTTTGTCAAAGTTAAATTAT
241	No	AAAACCATTTCATAACTTTGTCAAAGTTAAATTATA
242	No	AAAACCATTTCATAACTTTGTCAAAGTTAAATTATAG
243	No	AAAACCATTTCATAACTTTGTCAAAGTTAAATTATAGG
244	No	AAAACCATTTCATAACTTTGTCAAAGTTAAATTATAGGC
245	No	AAAACCATTTCATAACTTTGTCAAAGTTAAATTATAGGCT

246	No	AAAACCATTTCATAACTTTGTCAAAGTTAAATTATAGGCTA
247	No	AAAACCATTTCATAACTTTGTCAAAGTTAAATTATAGGCTAA
248	No	AAAACCATTTCATAACTTTGTCAAAGTTAAATTATAGGCTAAA
249	No	AAAACCATTTCATAACTTTGTCAAAGTTAAATTATAGGCTAAAT
250	No	AAAACCATTTCATAACTTTGTCAAAGTTAAATTATAGGCTAAATC
251	No	AAAACCATTTCATAACTTTGTCAAAGTTAAATTATAGGCTAAATCC
252	No	AAAACCATTTCATAACTTTGTCAAAGTTAAATTATAGGCTAAATCCT
253	No	AAAACCATTTCATAACTTTGTCAAAGTTAAATTATAGGCTAAATCCTA
254	No	AAAACCATTTCATAACTTTGTCAAAGTTAAATTATAGGCTAAATCCTAT
255	No	AAAACCATTTCATAACTTTGTCAAAGTTAAATTATAGGCTAAATCCTATA
256	No	AAAACCTTTTTCCAAG
257	No	AAAACCTTTTTCCAAGG
258	Yes	AAAACCTTTTTCCAAGGA
259	Yes	AAAACCTTTTTCCAAGGAC
260	Yes	AAAACCTTTTTCCAAGGACA
261	Yes	AAAACCTTTTTCCAAGGACAC
262	Yes	AAAACCTTTTTCCAAGGACACC
263	Yes	AAAACCTTTTTCCAAGGACACCA
264	No	AAAACGAATGATTTCG
265	No	AAAACGAATGATTTCGA
266	No	AAAACGAATGATTTCGAC
267	No	AAAACGAATGATTTCGACT
268	No	AAAACGAATGATTTCGACTC
269	No	AAAACGAATGATTTCGACTCA
270	No	AAAACGAATGATTTCGACTCAT
271	No	AAAACGAATGATTTCGACTCATT
272	No	AAAACGAATGATTTCGACTCATTA
273	No	AAAACGAATGATTTCGACTCATTAA
274	No	AAAACGAATGATTTCGACTCATTAAA
275	No	AAAACGAATGATTTCGACTCATTAAAT
276	No	AAAACGAATGATTTCGACTCATTAAATT
277	No	AAAACGAATGATTTCGACTCATTAAATTA
278	No	AAAACGAATGATTTCGACTCATTAAATTAT
279	No	AAAACGAATGATTTCGACTCATTAAATTATG
280	No	AAAACGAATGATTTCGACTCATTAAATTATGA
281	No	AAAACGAATGATTTCGACTCATTAAATTATGAT
282	No	AAAACGAATGATTTCGACTCATTAAATTATGATA
283	No	AAAACGAATGATTTCGACTCATTAAATTATGATAA
284	No	AAAACGAATGATTTCGACTCATTAAATTATGATAAT
285	No	AAAACGAATGATTTCGACTCATTAAATTATGATAATC
286	No	AAAACGAATGATTTCGACTCATTAAATTATGATAATCA
287	No	AAAACGAATGATTTCGACTCATTAAATTATGATAATCAT

288	No	AAAACGAATGATTTCGACTCATTAAATTATGATAATCATA
289	No	AAAACGAATGATTTCGACTCATTAAATTATGATAATCATAT
290	No	AAAACGAATGATTTCGACTCATTAAATTATGATAATCATATT
291	No	AAAACGAATGATTTCGACTCATTAAATTATGATAATCATATTT
292	No	AAAACGAATGATTTCGACTCATTAAATTATGATAATCATATTTA
293	No	AAAACGAATGATTTCGACTCATTAAATTATGATAATCATATTTAC
294	No	AAAACGAATGATTTCGACTCATTAAATTATGATAATCATATTTACC
295	No	AAAACGAATGATTTCGACTCATTAAATTATGATAATCATATTTACCA
296	No	AAAACGAATGATTTCGACTCATTAAATTATGATAATCATATTTACCAA
297	Yes	AAAACGAATGATTTCGACTCATTAAATTATGATAATCATATTTACCAAC
298	Yes	AAAACGAATGATTTCGACTCATTAAATTATGATAATCATATTTACCAACC
299	No	AAAACTTAAAACTTTA
300	No	AAAACTTAAAACTTTAC
301	No	AAAACTTAAAACTTTACA
302	No	AAAACTTAAAACTTTACAA
303	No	AAAACTTAAAACTTTACAAT
304	No	AAAACTTAAAACTTTACAATC
305	No	AAAACTTAAAACTTTACAATCA
306	No	AAAACTTAAAACTTTACAATCAG
307	Yes	AAAACTTAAAACTTTACAATCAGA
308	Yes	AAAACTTAAAACTTTACAATCAGAG
309	Yes	AAAACTTAAAACTTTACAATCAGAGG
310	Yes	AAAACTTAAAACTTTACAATCAGAGGT
311	Yes	AAAACTTAAAACTTTACAATCAGAGGTT
312	Yes	AAAACTTAAAACTTTACAATCAGAGGTTC
313	Yes	AAAACTTAAAACTTTACAATCAGAGGTTCA
314	Yes	AAAACTTAAAACTTTACAATCAGAGGTTCAA
315	Yes	AAAACTTAAAACTTTACAATCAGAGGTTCAAC
316	Yes	AAAACTTAAAACTTTACAATCAGAGGTTCAACT
317	Yes	AAAACTTAAAACTTTACAATCAGAGGTTCAACTC
318	Yes	AAAACTTAAAACTTTACAATCAGAGGTTCAACTCC
319	Yes	AAAACTTAAAACTTTACAATCAGAGGTTCAACTCCT
320	Yes	AAAACTTAAAACTTTACAATCAGAGGTTCAACTCCTC
321	Yes	AAAACTTAAAACTTTACAATCAGAGGTTCAACTCCTCT
322	Yes	AAAACTTAAAACTTTACAATCAGAGGTTCAACTCCTCTT
323	Yes	AAAACTTAAAACTTTACAATCAGAGGTTCAACTCCTCTTC
324	Yes	AAAACTTAAAACTTTACAATCAGAGGTTCAACTCCTCTTCT
325	Yes	AAAACTTAAAACTTTACAATCAGAGGTTCAACTCCTCTTCTT
326	Yes	AAAACTTAAAACTTTACAATCAGAGGTTCAACTCCTCTTCTTA
327	Yes	AAAACTTAAAACTTTACAATCAGAGGTTCAACTCCTCTTCTTAA
328	Yes	AAAACTTAAAACTTTACAATCAGAGGTTCAACTCCTCTTCTTAAC
329	Yes	AAAACTTAAAACTTTACAATCAGAGGTTCAACTCCTCTTCTTAACA

330	Yes	AAAACTTAAAACTTTACAATCAGAGGTTCAACTCCTCTTCTTAACAC
331	Yes	AAAACTTAAAACTTTACAATCAGAGGTTCAACTCCTCTTCTTAACACC
332	Yes	AAAACTTAAAACTTTACAATCAGAGGTTCAACTCCTCTTCTTAACACCA
333	Yes	AAAACTTAAAACTTTACAG
334	Yes	AAAACTTAAAACTTTACAGT
335	Yes	AAAACTTAAAACTTTACAGTC
336	Yes	AAAACTTAAAACTTTACAGTCA
337	Yes	AAAACTTAAAACTTTACAGTCAG
338	Yes	AAAACTTAAAACTTTACAGTCAGA
339	Yes	AAAACTTAAAACTTTACAGTCAGAG
340	Yes	AAAACTTAAAACTTTACAGTCAGAGG
341	Yes	AAAACTTAAAACTTTACAGTCAGAGGT
342	Yes	AAAACTTAAAACTTTACAGTCAGAGGTT
343	Yes	AAAACTTAAAACTTTACAGTCAGAGGTTC
344	Yes	AAAACTTAAAACTTTACAGTCAGAGGTTCA
345	Yes	AAAACTTAAAACTTTACAGTCAGAGGTTCAA
346	Yes	AAAACTTAAAACTTTACAGTCAGAGGTTCAAT
347	Yes	AAAACTTAAAACTTTACAGTCAGAGGTTCAATT
348	Yes	AAAACTTAAAACTTTACAGTCAGAGGTTCAATTC
349	Yes	AAAACTTAAAACTTTACAGTCAGAGGTTCAATTCC
350	Yes	AAAACTTAAAACTTTACAGTCAGAGGTTCAATTCCT
351	Yes	AAAACTTAAAACTTTACAGTCAGAGGTTCAATTCCTC
352	Yes	AAAACTTAAAACTTTACAGTCAGAGGTTCAATTCCTCT
353	Yes	AAAACTTAAAACTTTACAGTCAGAGGTTCAATTCCTCTT
354	Yes	AAAACTTAAAACTTTACAGTCAGAGGTTCAATTCCTCTTC
355	Yes	AAAACTTAAAACTTTACAGTCAGAGGTTCAATTCCTCTTCT
356	Yes	AAAACTTAAAACTTTACAGTCAGAGGTTCAATTCCTCTTCTT
357	Yes	AAAACTTAAAACTTTACAGTCAGAGGTTCAATTCCTCTTCTTA
358	Yes	AAAACTTAAAACTTTACAGTCAGAGGTTCAATTCCTCTTCTTAA
359	Yes	AAAACTTAAAACTTTACAGTCAGAGGTTCAATTCCTCTTCTTAAC
360	Yes	AAAACTTAAAACTTTACAGTCAGAGGTTCAATTCCTCTTCTTAACA
361	Yes	AAAACTTAAAACTTTACAGTCAGAGGTTCAATTCCTCTTCTTAACAC
362	Yes	AAAACTTAAAACTTTACAGTCAGAGGTTCAATTCCTCTTCTTAACACC
363	Yes	AAAACTTAAAACTTTACAGTCAGAGGTTCAATTCCTCTTCTTAACACCA
364	No	AAAACTTAAAATTTTA
365	No	AAAACTTAAAATTTTAT
366	No	AAAACTTAAAATTTTATA
367	No	AAAACTTAAAATTTTATAA
368	No	AAAACTTAAAATTTTATAAT
369	Yes	AAAACTTAAAATTTTATAATC
370	Yes	AAAACTTAAAATTTTATAATCA
371	Yes	AAAACTTAAAATTTTATAATCAG

372	Yes	AAAACTTAAAATTTTATAATCAGA
373	Yes	AAAACTTAAAATTTTATAATCAGAG
374	Yes	AAAACTTAAAATTTTATAATCAGAGG
375	Yes	AAAACTTAAAATTTTATAATCAGAGGT
376	Yes	AAAACTTAAAATTTTATAATCAGAGGTT
377	Yes	AAAACTTAAAATTTTATAATCAGAGGTTC
378	Yes	AAAACTTAAAATTTTATAATCAGAGGTTCA
379	Yes	AAAACTTAAAATTTTATAATCAGAGGTTCAA
380	Yes	AAAACTTAAAATTTTATAATCAGAGGTTCAAC
381	Yes	AAAACTTAAAATTTTATAATCAGAGGTTCAACT
382	Yes	AAAACTTAAAATTTTATAATCAGAGGTTCAACTC
383	Yes	AAAACTTAAAATTTTATAATCAGAGGTTCAACTCC
384	Yes	AAAACTTAAAATTTTATAATCAGAGGTTCAACTCCT
385	Yes	AAAACTTAAAATTTTATAATCAGAGGTTCAACTCCTC
386	Yes	AAAACTTAAAATTTTATAATCAGAGGTTCAACTCCTCT
387	Yes	AAAACTTAAAATTTTATAATCAGAGGTTCAACTCCTCTT
388	Yes	AAAACTTAAAATTTTATAATCAGAGGTTCAACTCCTCTTC
389	Yes	AAAACTTAAAATTTTATAATCAGAGGTTCAACTCCTCTTCT
390	Yes	AAAACTTAAAATTTTATAATCAGAGGTTCAACTCCTCTTCTT
391	Yes	AAAACTTAAAATTTTATAATCAGAGGTTCAACTCCTCTTCTTA
392	Yes	AAAACTTAAAATTTTATAATCAGAGGTTCAACTCCTCTTCTTAA
393	Yes	AAAACTTAAAATTTTATAATCAGAGGTTCAACTCCTCTTCTTAAC
394	Yes	AAAACTTAAAATTTTATAATCAGAGGTTCAACTCCTCTTCTTAACA
395	Yes	AAAACTTAAAATTTTATAATCAGAGGTTCAACTCCTCTTCTTAACAC
396	Yes	AAAACTTAAAATTTTATAATCAGAGGTTCAACTCCTCTTCTTAACACC
397	Yes	AAAACTTAAAATTTTATAATCAGAGGTTCAACTCCTCTTCTTAACACCA
398	No	AAAACTTAACACTTTA
399	No	AAAACTTAACACTTTAT
400	Yes	AAAACTTAACACTTTATA
401	Yes	AAAACTTAACACTTTATAA
402	Yes	AAAACTTAACACTTTATAAT
403	Yes	AAAACTTAACACTTTATAATC
404	Yes	AAAACTTAACACTTTATAATCA
405	Yes	AAAACTTAACACTTTATAATCAG
406	Yes	AAAACTTAACACTTTATAATCAGA
407	Yes	AAAACTTAACACTTTATAATCAGAG
408	Yes	AAAACTTAACACTTTATAATCAGAGG
409	Yes	AAAACTTAACACTTTATAATCAGAGGT
410	Yes	AAAACTTAACACTTTATAATCAGAGGTT
411	Yes	AAAACTTAACACTTTATAATCAGAGGTTC
412	Yes	AAAACTTAACACTTTATAATCAGAGGTTCA
413	Yes	AAAACTTAACACTTTATAATCAGAGGTTCAA

414	Yes	AAAACTTAACACTTTATAATCAGAGGTTCAAC
415	Yes	AAAACTTAACACTTTATAATCAGAGGTTCAACT
416	Yes	AAAACTTAACACTTTATAATCAGAGGTTCAACTC
417	Yes	AAAACTTAACACTTTATAATCAGAGGTTCAACTCC
418	Yes	AAAACTTAACACTTTATAATCAGAGGTTCAACTCCT
419	Yes	AAAACTTAACACTTTATAATCAGAGGTTCAACTCCTC
420	Yes	AAAACTTAACACTTTATAATCAGAGGTTCAACTCCTCT
421	Yes	AAAACTTAACACTTTATAATCAGAGGTTCAACTCCTCTT
422	Yes	AAAACTTAACACTTTATAATCAGAGGTTCAACTCCTCTTC
423	Yes	AAAACTTAACACTTTATAATCAGAGGTTCAACTCCTCTTCT
424	Yes	AAAACTTAACACTTTATAATCAGAGGTTCAACTCCTCTTCTT
425	Yes	AAAACTTAACACTTTATAATCAGAGGTTCAACTCCTCTTCTTA
426	Yes	AAAACTTAACACTTTATAATCAGAGGTTCAACTCCTCTTCTTAA
427	Yes	AAAACTTAACACTTTATAATCAGAGGTTCAACTCCTCTTCTTAAC
428	Yes	AAAACTTAACACTTTATAATCAGAGGTTCAACTCCTCTTCTTAACA
429	Yes	AAAACTTAACACTTTATAATCAGAGGTTCAACTCCTCTTCTTAACAC
430	Yes	AAAACTTAACACTTTATAATCAGAGGTTCAACTCCTCTTCTTAACACC
431	Yes	AAAACTTAACACTTTATAATCAGAGGTTCAACTCCTCTTCTTAACACCA
432	No	AAAACTTAAGACTTTA
433	No	AAAACTTAAGACTTTAT
434	Yes	AAAACTTAAGACTTTATA
435	Yes	AAAACTTAAGACTTTATAA
436	Yes	AAAACTTAAGACTTTATAAT
437	Yes	AAAACTTAAGACTTTATAATT
438	Yes	AAAACTTAAGACTTTATAATTA
439	Yes	AAAACTTAAGACTTTATAATTAG
440	Yes	AAAACTTAAGACTTTATAATTAGA
441	Yes	AAAACTTAAGACTTTATAATTAGAA
442	Yes	AAAACTTAAGACTTTATAATTAGAAG
443	Yes	AAAACTTAAGACTTTATAATTAGAAGT
444	Yes	AAAACTTAAGACTTTATAATTAGAAGTT
445	Yes	AAAACTTAAGACTTTATAATTAGAAGTTC
446	Yes	AAAACTTAAGACTTTATAATTAGAAGTTCA
447	Yes	AAAACTTAAGACTTTATAATTAGAAGTTCAA
448	Yes	AAAACTTAAGACTTTATAATTAGAAGTTCAAC
449	Yes	AAAACTTAAGACTTTATAATTAGAAGTTCAACA
450	Yes	AAAACTTAAGACTTTATAATTAGAAGTTCAACAC
451	Yes	AAAACTTAAGACTTTATAATTAGAAGTTCAACACC
452	Yes	AAAACTTAAGACTTTATAATTAGAAGTTCAACACCT
453	Yes	AAAACTTAAGACTTTATAATTAGAAGTTCAACACCTC
454	Yes	AAAACTTAAGACTTTATAATTAGAAGTTCAACACCTCT
455	Yes	AAAACTTAAGACTTTATAATTAGAAGTTCAACACCTCTT

456	Yes	AAAACTTAAGACTTTATAATTAGAAGTTCAACACCTCTTC
457	Yes	AAAACTTAAGACTTTATAATTAGAAGTTCAACACCTCTTCT
458	Yes	AAAACTTAAGACTTTATAATTAGAAGTTCAACACCTCTTCTT
459	Yes	AAAACTTAAGACTTTATAATTAGAAGTTCAACACCTCTTCTTA
460	Yes	AAAACTTAAGACTTTATAATTAGAAGTTCAACACCTCTTCTTAA
461	Yes	AAAACTTAAGACTTTATAATTAGAAGTTCAACACCTCTTCTTAAC
462	Yes	AAAACTTAAGACTTTATAATTAGAAGTTCAACACCTCTTCTTAACA
463	Yes	AAAACTTAAGACTTTATAATTAGAAGTTCAACACCTCTTCTTAACAC
464	Yes	AAAACTTAAGACTTTATAATTAGAAGTTCAACACCTCTTCTTAACACC
465	Yes	AAAACTTAAGACTTTATAATTAGAAGTTCAACACCTCTTCTTAACACCA
466	No	AAAACTTTACAATCAG
467	Yes	AAAACTTTACAATCAGA
468	Yes	AAAACTTTACAATCAGAG
469	Yes	AAAACTTTACAATCAGAGG
470	Yes	AAAACTTTACAATCAGAGGT
471	Yes	AAAACTTTACAATCAGAGGTT
472	Yes	AAAACTTTACAATCAGAGGTTC
473	Yes	AAAACTTTACAATCAGAGGTTCA
474	Yes	AAAACTTTACAATCAGAGGTTCAA
475	Yes	AAAACTTTACAATCAGAGGTTCAAC
476	Yes	AAAACTTTACAATCAGAGGTTCAACT
477	Yes	AAAACTTTACAATCAGAGGTTCAACTC
478	Yes	AAAACTTTACAATCAGAGGTTCAACTCC
479	Yes	AAAACTTTACAATCAGAGGTTCAACTCCT
480	Yes	AAAACTTTACAATCAGAGGTTCAACTCCTC
481	Yes	AAAACTTTACAATCAGAGGTTCAACTCCTCT
482	Yes	AAAACTTTACAATCAGAGGTTCAACTCCTCTT
483	Yes	AAAACTTTACAATCAGAGGTTCAACTCCTCTTC
484	Yes	AAAACTTTACAATCAGAGGTTCAACTCCTCTTCT
485	Yes	AAAACTTTACAATCAGAGGTTCAACTCCTCTTCTT
486	Yes	AAAACTTTACAATCAGAGGTTCAACTCCTCTTCTTA
487	Yes	AAAACTTTACAATCAGAGGTTCAACTCCTCTTCTTAA
488	Yes	AAAACTTTACAATCAGAGGTTCAACTCCTCTTCTTAAC
489	Yes	AAAACTTTACAATCAGAGGTTCAACTCCTCTTCTTAACA
490	Yes	AAAACTTTACAATCAGAGGTTCAACTCCTCTTCTTAACAC
491	Yes	AAAACTTTACAATCAGAGGTTCAACTCCTCTTCTTAACACC
492	Yes	AAAACTTTACAATCAGAGGTTCAACTCCTCTTCTTAACACCA
493	No	AAAACTTTACAGTCAG
494	No	AAAACTTTACAGTCAGA
495	Yes	AAAACTTTACAGTCAGAG
496	Yes	AAAACTTTACAGTCAGAGG
497	Yes	AAAACTTTACAGTCAGAGGT

498	Yes	AAAACTTTACAGTCAGAGGTT
499	Yes	AAAACTTTACAGTCAGAGGTTC
500	Yes	AAAACTTTACAGTCAGAGGTTCA
501	Yes	AAAACTTTACAGTCAGAGGTTCAA
502	Yes	AAAACTTTACAGTCAGAGGTTCAAT
503	Yes	AAAACTTTACAGTCAGAGGTTCAATT
504	Yes	AAAACTTTACAGTCAGAGGTTCAATTC
505	Yes	AAAACTTTACAGTCAGAGGTTCAATTCC
506	Yes	AAAACTTTACAGTCAGAGGTTCAATTCCT
507	Yes	AAAACTTTACAGTCAGAGGTTCAATTCCTC
508	Yes	AAAACTTTACAGTCAGAGGTTCAATTCCTCT
509	Yes	AAAACTTTACAGTCAGAGGTTCAATTCCTCTT
510	Yes	AAAACTTTACAGTCAGAGGTTCAATTCCTCTTC
511	Yes	AAAACTTTACAGTCAGAGGTTCAATTCCTCTTCT
512	Yes	AAAACTTTACAGTCAGAGGTTCAATTCCTCTTCTT
513	Yes	AAAACTTTACAGTCAGAGGTTCAATTCCTCTTCTTA
514	Yes	AAAACTTTACAGTCAGAGGTTCAATTCCTCTTCTTAA
515	Yes	AAAACTTTACAGTCAGAGGTTCAATTCCTCTTCTTAAC
516	Yes	AAAACTTTACAGTCAGAGGTTCAATTCCTCTTCTTAACA
517	Yes	AAAACTTTACAGTCAGAGGTTCAATTCCTCTTCTTAACAC
518	Yes	AAAACTTTACAGTCAGAGGTTCAATTCCTCTTCTTAACACC
519	Yes	AAAACTTTACAGTCAGAGGTTCAATTCCTCTTCTTAACACCA
520	No	AAAACTTTATAATCAG
521	No	AAAACTTTATAATCAGA
522	No	AAAACTTTATAATCAGAG
523	No	AAAACTTTATAATCAGAGG
524	No	AAAACTTTATAATCAGAGGT
525	No	AAAACTTTATAATCAGAGGTT
526	No	AAAACTTTATAATCAGAGGTTC
527	No	AAAACTTTATAATCAGAGGTTCA
528	No	AAAACTTTATAATCAGAGGTTCAA
529	No	AAAACTTTATAATCAGAGGTTCAAC
530	No	AAAACTTTATAATCAGAGGTTCAACT
531	No	AAAACTTTATAATCAGAGGTTCAACTC
532	No	AAAACTTTATAATCAGAGGTTCAACTCC
533	No	AAAACTTTATAATCAGAGGTTCAACTCCT
534	No	AAAACTTTATAATCAGAGGTTCAACTCCTC
535	No	AAAACTTTATAATCAGAGGTTCAACTCCTCT
536	Yes	AAAACTTTATAATCAGAGGTTCAACTCCTCTC
537	Yes	AAAACTTTATAATCAGAGGTTCAACTCCTCTCA
538	Yes	AAAACTTTATAATCAGAGGTTCAACTCCTCTCAT
539	Yes	AAAACTTTATAATCAGAGGTTCAACTCCTCTCATT

540	Yes	AAAACTTTATAATCAGAGGTTCAACTCCTCTCATTA
541	Yes	AAAACTTTATAATCAGAGGTTCAACTCCTCTCATTAA
542	Yes	AAAACTTTATAATCAGAGGTTCAACTCCTCTCATTAAC
543	Yes	AAAACTTTATAATCAGAGGTTCAACTCCTCTCATTAACA
544	Yes	AAAACTTTATAATCAGAGGTTCAACTCCTCTCATTAACAC
545	Yes	AAAACTTTATAATCAGAGGTTCAACTCCTCTCATTAACACC
546	Yes	AAAACTTTATAATCAGAGGTTCAACTCCTCTCATTAACACCA
547	Yes	AAAACTTTATAATCAGAGGTTCG
548	Yes	AAAACTTTATAATCAGAGGTTCGA
549	Yes	AAAACTTTATAATCAGAGGTTCGAC
550	Yes	AAAACTTTATAATCAGAGGTTCGACT
551	Yes	AAAACTTTATAATCAGAGGTTCGACTC
552	Yes	AAAACTTTATAATCAGAGGTTCGACTCC
553	Yes	AAAACTTTATAATCAGAGGTTCGACTCCT
554	Yes	AAAACTTTATAATCAGAGGTTCGACTCCTC
555	Yes	AAAACTTTATAATCAGAGGTTCGACTCCTCT
556	Yes	AAAACTTTATAATCAGAGGTTCGACTCCTCTT
557	Yes	AAAACTTTATAATCAGAGGTTCGACTCCTCTTC
558	Yes	AAAACTTTATAATCAGAGGTTCGACTCCTCTTCT
559	Yes	AAAACTTTATAATCAGAGGTTCGACTCCTCTTCTT
560	Yes	AAAACTTTATAATCAGAGGTTCGACTCCTCTTCTTA
561	Yes	AAAACTTTATAATCAGAGGTTCGACTCCTCTTCTTAA
562	Yes	AAAACTTTATAATCAGAGGTTCGACTCCTCTTCTTAAC
563	Yes	AAAACTTTATAATCAGAGGTTCGACTCCTCTTCTTAACA
564	Yes	AAAACTTTATAATCAGAGGTTCGACTCCTCTTCTTAACAC
565	Yes	AAAACTTTATAATCAGAGGTTCGACTCCTCTTCTTAACACC
566	Yes	AAAACTTTATAATCAGAGGTTCGACTCCTCTTCTTAACACCA
567	No	AAAAGACATCTCACTT
568	No	AAAAGACATCTCACTTT
569	No	AAAAGACATCTCACTTTG
570	Yes	AAAAGACATCTCACTTTGG
571	Yes	AAAAGACATCTCACTTTGGG
572	Yes	AAAAGACATCTCACTTTGGGT
573	Yes	AAAAGACATCTCACTTTGGGTC
574	Yes	AAAAGACATCTCACTTTGGGTCA
575	Yes	AAAAGACATCTCACTTTGGGTCAG
576	Yes	AAAAGACATCTCACTTTGGGTCAGA
577	Yes	AAAAGACATCTCACTTTGGGTCAGAA
578	Yes	AAAAGACATCTCACTTTGGGTCAGAAG
579	Yes	AAAAGACATCTCACTTTGGGTCAGAAGA
580	Yes	AAAAGACATCTCACTTTGGGTCAGAAGAT
581	Yes	AAAAGACATCTCACTTTGGGTCAGAAGATT

582	Yes	AAAAGACATCTCACTTTGGGTCAGAAGATTG
583	Yes	AAAAGACATCTCACTTTGGGTCAGAAGATTGA
584	Yes	AAAAGACATCTCACTTTGGGTCAGAAGATTGAG
585	Yes	AAAAGACATCTCACTTTGGGTCAGAAGATTGAGG
586	Yes	AAAAGACATCTCACTTTGGGTCAGAAGATTGAGGG
587	Yes	AAAAGACATCTCACTTTGGGTCAGAAGATTGAGGGT
588	Yes	AAAAGACATCTCACTTTGGGTCAGAAGATTGAGGGTT
589	Yes	AAAAGACATCTCACTTTGGGTCAGAAGATTGAGGGTTC
590	Yes	AAAAGACATCTCACTTTGGGTCAGAAGATTGAGGGTTCA
591	Yes	AAAAGACATCTCACTTTGGGTCAGAAGATTGAGGGTTCAA
592	Yes	AAAAGACATCTCACTTTGGGTCAGAAGATTGAGGGTTCAAG
593	Yes	AAAAGACATCTCACTTTGGGTCAGAAGATTGAGGGTTCAAGT
594	Yes	AAAAGACATCTCACTTTGGGTCAGAAGATTGAGGGTTCAAGTC
595	Yes	AAAAGACATCTCACTTTGGGTCAGAAGATTGAGGGTTCAAGTCC
596	Yes	AAAAGACATCTCACTTTGGGTCAGAAGATTGAGGGTTCAAGTCCT
597	Yes	AAAAGACATCTCACTTTGGGTCAGAAGATTGAGGGTTCAAGTCCTT
598	Yes	AAAAGACATCTCACTTTGGGTCAGAAGATTGAGGGTTCAAGTCCTTT
599	Yes	AAAAGACATCTCACTTTGGGTCAGAAGATTGAGGGTTCAAGTCCTTTC
600	Yes	AAAAGACATCTCACTTTGGGTCAGAAGATTGAGGGTTCAAGTCCTTTCA
601	Yes	AAAAGACATCTCACTTTGGGTCAGAAGATTGAGGGTTCAAGTCCTTTCAT
602	No	AAAAGAGTTACTTTGA
603	No	AAAAGAGTTACTTTGAT
604	No	AAAAGAGTTACTTTGATA
605	No	AAAAGAGTTACTTTGATAG
606	No	AAAAGAGTTACTTTGATAGA
607	No	AAAAGAGTTACTTTGATAGAG
608	No	AAAAGAGTTACTTTGATAGAGT
609	No	AAAAGAGTTACTTTGATAGAGTA
610	No	AAAAGAGTTACTTTGATAGAGTAA
611	No	AAAAGAGTTACTTTGATAGAGTAAA
612	No	AAAAGAGTTACTTTGATAGAGTAAAT
613	No	AAAAGAGTTACTTTGATAGAGTAAATA
614	No	AAAAGAGTTACTTTGATAGAGTAAATAA
615	No	AAAAGAGTTACTTTGATAGAGTAAATAAT
616	No	AAAAGAGTTACTTTGATAGAGTAAATAATA
617	No	AAAAGAGTTACTTTGATAGAGTAAATAATAG
618	No	AAAAGAGTTACTTTGATAGAGTAAATAATAGG
619	No	AAAAGAGTTACTTTGATAGAGTAAATAATAGGA
620	No	AAAAGAGTTACTTTGATAGAGTAAATAATAGGAG
621	Yes	AAAAGAGTTACTTTGATAGAGTAAATAATAGGAGC
622	Yes	AAAAGAGTTACTTTGATAGAGTAAATAATAGGAGCT
623	Yes	AAAAGAGTTACTTTGATAGAGTAAATAATAGGAGCTT

624	Yes	AAAAGAGTTACTTTGATAGAGTAAATAATAGGAGCTTA
625	Yes	AAAAGAGTTACTTTGATAGAGTAAATAATAGGAGCTTAA
626	Yes	AAAAGAGTTACTTTGATAGAGTAAATAATAGGAGCTTAAA
627	Yes	AAAAGAGTTACTTTGATAGAGTAAATAATAGGAGCTTAAAC
628	Yes	AAAAGAGTTACTTTGATAGAGTAAATAATAGGAGCTTAAACC
629	Yes	AAAAGAGTTACTTTGATAGAGTAAATAATAGGAGCTTAAACCC
630	Yes	AAAAGAGTTACTTTGATAGAGTAAATAATAGGAGCTTAAACCCC
631	Yes	AAAAGAGTTACTTTGATAGAGTAAATAATAGGAGCTTAAACCCCC
632	Yes	AAAAGAGTTACTTTGATAGAGTAAATAATAGGAGCTTAAACCCCCT
633	Yes	AAAAGAGTTACTTTGATAGAGTAAATAATAGGAGCTTAAACCCCCTT
634	Yes	AAAAGAGTTACTTTGATAGAGTAAATAATAGGAGCTTAAACCCCCTTA
635	Yes	AAAAGAGTTACTTTGATAGAGTAAATAATAGGAGCTTAAACCCCCTTAT
636	Yes	AAAAGAGTTACTTTGATAGAGTAAATAATAGGAGCTTAAACCCCCTTATT
637	No	AAAAGATTGCAGGTTT
638	Yes	AAAAGATTGCAGGTTTG
639	Yes	AAAAGATTGCAGGTTTGA
640	Yes	AAAAGATTGCAGGTTTGAG
641	Yes	AAAAGATTGCAGGTTTGAGT
642	Yes	AAAAGATTGCAGGTTTGAGTT
643	Yes	AAAAGATTGCAGGTTTGAGTTC
644	Yes	AAAAGATTGCAGGTTTGAGTTCT
645	Yes	AAAAGATTGCAGGTTTGAGTTCTG
646	Yes	AAAAGATTGCAGGTTTGAGTTCTGC
647	Yes	AAAAGATTGCAGGTTTGAGTTCTGCC
648	Yes	AAAAGATTGCAGGTTTGAGTTCTGCCA
649	Yes	AAAAGATTGCAGGTTTGAGTTCTGCCAC
650	Yes	AAAAGATTGCAGGTTTGAGTTCTGCCACG
651	Yes	AAAAGATTGCAGGTTTGAGTTCTGCCACGG
652	Yes	AAAAGATTGCAGGTTTGAGTTCTGCCACGGT
653	Yes	AAAAGATTGCAGGTTTGAGTTCTGCCACGGTC
654	Yes	AAAAGATTGCAGGTTTGAGTTCTGCCACGGTCG
655	Yes	AAAAGATTGCAGGTTTGAGTTCTGCCACGGTCGC
656	Yes	AAAAGATTGCAGGTTTGAGTTCTGCCACGGTCGCC
657	Yes	AAAAGATTGCAGGTTTGAGTTCTGCCACGGTCGCCA
658	No	AAAAGGTCCCTGGTTC
659	Yes	AAAAGGTCCCTGGTTCA
660	Yes	AAAAGGTCCCTGGTTCAA
661	Yes	AAAAGGTCCCTGGTTCAAA
662	Yes	AAAAGGTCCCTGGTTCAAAT
663	Yes	AAAAGGTCCCTGGTTCAAATC
664	Yes	AAAAGGTCCCTGGTTCAAATCC
665	Yes	AAAAGGTCCCTGGTTCAAATCCA

666	Yes	AAAAGGTCCCTGGTTCAAATCCAG
667	Yes	AAAAGGTCCCTGGTTCAAATCCAGG
668	Yes	AAAAGGTCCCTGGTTCAAATCCAGGT
669	Yes	AAAAGGTCCCTGGTTCAAATCCAGGTG
670	Yes	AAAAGGTCCCTGGTTCAAATCCAGGTGC
671	Yes	AAAAGGTCCCTGGTTCAAATCCAGGTGCC
672	Yes	AAAAGGTCCCTGGTTCAAATCCAGGTGCCC
673	Yes	AAAAGGTCCCTGGTTCAAATCCAGGTGCCCC
674	Yes	AAAAGGTCCCTGGTTCAAATCCAGGTGCCCCT
675	Yes	AAAAGGTCCCTGGTTCAAATCCAGGTGCCCCTT
676	Yes	AAAAGGTCCCTGGTTCAAATCCAGGTGCCCCTTC
677	Yes	AAAAGGTCCCTGGTTCAAATCCAGGTGCCCCTTCC
678	Yes	AAAAGGTCCCTGGTTCAAATCCAGGTGCCCCTTCCA
679	No	AAAAGGTCCTGGGTTC
680	No	AAAAGGTCCTGGGTTCA
681	Yes	AAAAGGTCCTGGGTTCAA
682	Yes	AAAAGGTCCTGGGTTCAAA
683	Yes	AAAAGGTCCTGGGTTCAAAT
684	Yes	AAAAGGTCCTGGGTTCAAATC
685	Yes	AAAAGGTCCTGGGTTCAAATCC
686	Yes	AAAAGGTCCTGGGTTCAAATCCC
687	Yes	AAAAGGTCCTGGGTTCAAATCCCA
688	Yes	AAAAGGTCCTGGGTTCAAATCCCAG
689	Yes	AAAAGGTCCTGGGTTCAAATCCCAGA
690	Yes	AAAAGGTCCTGGGTTCAAATCCCAGAG
691	Yes	AAAAGGTCCTGGGTTCAAATCCCAGAGG
692	Yes	AAAAGGTCCTGGGTTCAAATCCCAGAGGA
693	Yes	AAAAGGTCCTGGGTTCAAATCCCAGAGGAG
694	Yes	AAAAGGTCCTGGGTTCAAATCCCAGAGGAGC
695	Yes	AAAAGGTCCTGGGTTCAAATCCCAGAGGAGCC
696	Yes	AAAAGGTCCTGGGTTCAAATCCCAGAGGAGCCC
697	Yes	AAAAGGTCCTGGGTTCAAATCCCAGAGGAGCCCC
698	Yes	AAAAGGTCCTGGGTTCAAATCCCAGAGGAGCCCCC
699	Yes	AAAAGGTCCTGGGTTCAAATCCCAGAGGAGCCCCCA
700	No	AAAAGGTTGGCGGTTC
701	Yes	AAAAGGTTGGCGGTTCG
702	Yes	AAAAGGTTGGCGGTTCGA
703	Yes	AAAAGGTTGGCGGTTCGAA
704	Yes	AAAAGGTTGGCGGTTCGAAC
705	Yes	AAAAGGTTGGCGGTTCGAACC
706	Yes	AAAAGGTTGGCGGTTCGAACCC
707	Yes	AAAAGGTTGGCGGTTCGAACCCA

708	Yes	AAAAGGTTGGCGGTTCGAACCCAC
709	Yes	AAAAGGTTGGCGGTTCGAACCCACC
710	Yes	AAAAGGTTGGCGGTTCGAACCCACCC
711	Yes	AAAAGGTTGGCGGTTCGAACCCACCCA
712	Yes	AAAAGGTTGGCGGTTCGAACCCACCCAG
713	Yes	AAAAGGTTGGCGGTTCGAACCCACCCAGA
714	Yes	AAAAGGTTGGCGGTTCGAACCCACCCAGAG
715	Yes	AAAAGGTTGGCGGTTCGAACCCACCCAGAGG
716	Yes	AAAAGGTTGGCGGTTCGAACCCACCCAGAGGC
717	Yes	AAAAGGTTGGCGGTTCGAACCCACCCAGAGGCG
718	Yes	AAAAGGTTGGCGGTTCGAACCCACCCAGAGGCGC
719	Yes	AAAAGGTTGGCGGTTCGAACCCACCCAGAGGCGCC
720	Yes	AAAAGGTTGGCGGTTCGAACCCACCCAGAGGCGCCA
721	No	AAAAGGTTGGTGGTTC
722	Yes	AAAAGGTTGGTGGTTCG
723	Yes	AAAAGGTTGGTGGTTCGA
724	Yes	AAAAGGTTGGTGGTTCGAA
725	Yes	AAAAGGTTGGTGGTTCGAAC
726	Yes	AAAAGGTTGGTGGTTCGAACC
727	Yes	AAAAGGTTGGTGGTTCGAACCC
728	Yes	AAAAGGTTGGTGGTTCGAACCCA
729	Yes	AAAAGGTTGGTGGTTCGAACCCAC
730	Yes	AAAAGGTTGGTGGTTCGAACCCACC
731	Yes	AAAAGGTTGGTGGTTCGAACCCACCC
732	Yes	AAAAGGTTGGTGGTTCGAACCCACCCA
733	Yes	AAAAGGTTGGTGGTTCGAACCCACCCAG
734	Yes	AAAAGGTTGGTGGTTCGAACCCACCCAGA
735	Yes	AAAAGGTTGGTGGTTCGAACCCACCCAGAG
736	Yes	AAAAGGTTGGTGGTTCGAACCCACCCAGAGG
737	Yes	AAAAGGTTGGTGGTTCGAACCCACCCAGAGGC
738	Yes	AAAAGGTTGGTGGTTCGAACCCACCCAGAGGCG
739	Yes	AAAAGGTTGGTGGTTCGAACCCACCCAGAGGCGC
740	Yes	AAAAGGTTGGTGGTTCGAACCCACCCAGAGGCGCC
741	Yes	AAAAGGTTGGTGGTTCGAACCCACCCAGAGGCGCCA
742	Yes	AAAAGTCATAGGGGTT
743	Yes	AAAAGTCATAGGGGTTA
744	Yes	AAAAGTCATAGGGGTTAT
745	Yes	AAAAGTCATAGGGGTTATG
746	Yes	AAAAGTCATAGGGGTTATGA
747	Yes	AAAAGTCATAGGGGTTATGAG
748	Yes	AAAAGTCATAGGGGTTATGAGG
749	Yes	AAAAGTCATAGGGGTTATGAGGC

750	Yes	AAAAGTCATAGGGGTTATGAGGCT
751	Yes	AAAAGTCATAGGGGTTATGAGGCTG
752	Yes	AAAAGTCATAGGGGTTATGAGGCTGG
753	Yes	AAAAGTCATAGGGGTTATGAGGCTGGC
754	Yes	AAAAGTCATAGGGGTTATGAGGCTGGCT
755	Yes	AAAAGTCATAGGGGTTATGAGGCTGGCTT
756	Yes	AAAAGTCATAGGGGTTATGAGGCTGGCTTG
757	Yes	AAAAGTCATAGGGGTTATGAGGCTGGCTTGA
758	Yes	AAAAGTCATAGGGGTTATGAGGCTGGCTTGAA
759	Yes	AAAAGTCATAGGGGTTATGAGGCTGGCTTGAAA
760	Yes	AAAAGTCATAGGGGTTATGAGGCTGGCTTGAAAC
761	Yes	AAAAGTCATAGGGGTTATGAGGCTGGCTTGAAACC
762	Yes	AAAAGTCATAGGGGTTATGAGGCTGGCTTGAAACCA
763	Yes	AAAAGTCATAGGGGTTATGAGGCTGGCTTGAAACCAG
764	Yes	AAAAGTCATAGGGGTTATGAGGCTGGCTTGAAACCAGC
765	Yes	AAAAGTCATAGGGGTTATGAGGCTGGCTTGAAACCAGCC
766	Yes	AAAAGTCATAGGGGTTATGAGGCTGGCTTGAAACCAGCCT
767	Yes	AAAAGTCATAGGGGTTATGAGGCTGGCTTGAAACCAGCCTT
768	Yes	AAAAGTCATAGGGGTTATGAGGCTGGCTTGAAACCAGCCTTA
769	Yes	AAAAGTCATAGGGGTTATGAGGCTGGCTTGAAACCAGCCTTAG
770	Yes	AAAAGTCATAGGGGTTATGAGGCTGGCTTGAAACCAGCCTTAGG
771	Yes	AAAAGTCATAGGGGTTATGAGGCTGGCTTGAAACCAGCCTTAGGA
772	Yes	AAAAGTCATAGGGGTTATGAGGCTGGCTTGAAACCAGCCTTAGGAG
773	Yes	AAAAGTCATAGGGGTTATGAGGCTGGCTTGAAACCAGCCTTAGGAGG
774	Yes	AAAAGTCATAGGGGTTATGAGGCTGGCTTGAAACCAGCCTTAGGAGGT
775	Yes	AAAAGTCATAGGGGTTATGAGGCTGGCTTGAAACCAGCCTTAGGAGGTT
776	Yes	AAAAGTCATAGGGGTTATGAGGCTGGCTTGAAACCAGCCTTAGGAGGTTC
777	No	AAAAGTCATGGAGGCC
778	No	AAAAGTCATGGAGGCCA
779	Yes	AAAAGTCATGGAGGCCAT
780	Yes	AAAAGTCATGGAGGCCATG
781	Yes	AAAAGTCATGGAGGCCATGG
782	Yes	AAAAGTCATGGAGGCCATGGG
783	Yes	AAAAGTCATGGAGGCCATGGGG
784	Yes	AAAAGTCATGGAGGCCATGGGGT
785	Yes	AAAAGTCATGGAGGCCATGGGGTT
786	Yes	AAAAGTCATGGAGGCCATGGGGTTG
787	Yes	AAAAGTCATGGAGGCCATGGGGTTGG
788	Yes	AAAAGTCATGGAGGCCATGGGGTTGGC
789	Yes	AAAAGTCATGGAGGCCATGGGGTTGGCT
790	Yes	AAAAGTCATGGAGGCCATGGGGTTGGCTT
791	Yes	AAAAGTCATGGAGGCCATGGGGTTGGCTTG

792	Yes	AAAAGTCATGGAGGCCATGGGGTTGGCTTGA
793	Yes	AAAAGTCATGGAGGCCATGGGGTTGGCTTGAA
794	Yes	AAAAGTCATGGAGGCCATGGGGTTGGCTTGAAA
795	Yes	AAAAGTCATGGAGGCCATGGGGTTGGCTTGAAAC
796	Yes	AAAAGTCATGGAGGCCATGGGGTTGGCTTGAAACC
797	Yes	AAAAGTCATGGAGGCCATGGGGTTGGCTTGAAACCA
798	Yes	AAAAGTCATGGAGGCCATGGGGTTGGCTTGAAACCAG
799	Yes	AAAAGTCATGGAGGCCATGGGGTTGGCTTGAAACCAGC
800	Yes	AAAAGTCATGGAGGCCATGGGGTTGGCTTGAAACCAGCT
801	Yes	AAAAGTCATGGAGGCCATGGGGTTGGCTTGAAACCAGCTT
802	Yes	AAAAGTCATGGAGGCCATGGGGTTGGCTTGAAACCAGCTTT
803	Yes	AAAAGTCATGGAGGCCATGGGGTTGGCTTGAAACCAGCTTTG
804	Yes	AAAAGTCATGGAGGCCATGGGGTTGGCTTGAAACCAGCTTTGG
805	Yes	AAAAGTCATGGAGGCCATGGGGTTGGCTTGAAACCAGCTTTGGG
806	Yes	AAAAGTCATGGAGGCCATGGGGTTGGCTTGAAACCAGCTTTGGGG
807	Yes	AAAAGTCATGGAGGCCATGGGGTTGGCTTGAAACCAGCTTTGGGGG
808	Yes	AAAAGTCATGGAGGCCATGGGGTTGGCTTGAAACCAGCTTTGGGGGG
809	Yes	AAAAGTCATGGAGGCCATGGGGTTGGCTTGAAACCAGCTTTGGGGGGT
810	Yes	AAAAGTCATGGAGGCCATGGGGTTGGCTTGAAACCAGCTTTGGGGGGTT
811	Yes	AAAAGTCATGGAGGCCATGGGGTTGGCTTGAAACCAGCTTTGGGGGGTTC
812	Yes	AAAAGTCCCCGGTTCG
813	Yes	AAAAGTCCCCGGTTCGA
814	Yes	AAAAGTCCCCGGTTCGAA
815	Yes	AAAAGTCCCCGGTTCGAAA
816	Yes	AAAAGTCCCCGGTTCGAAAT
817	Yes	AAAAGTCCCCGGTTCGAAATC
818	Yes	AAAAGTCCCCGGTTCGAAATCA
819	Yes	AAAAGTCCCCGGTTCGAAATCAG
820	Yes	AAAAGTCCCCGGTTCGAAATCAGG
821	Yes	AAAAGTCCCCGGTTCGAAATCAGGC
822	Yes	AAAAGTCCCCGGTTCGAAATCAGGCG
823	Yes	AAAAGTCCCCGGTTCGAAATCAGGCGG
824	Yes	AAAAGTCCCCGGTTCGAAATCAGGCGGG
825	Yes	AAAAGTCCCCGGTTCGAAATCAGGCGGGA
826	Yes	AAAAGTCCCCGGTTCGAAATCAGGCGGGAA
827	Yes	AAAAGTCCCCGGTTCGAAATCAGGCGGGAAC
828	Yes	AAAAGTCCCCGGTTCGAAATCAGGCGGGAACA
829	Yes	AAAAGTCCCCGGTTCGAAATCAGGCGGGAACAC
830	Yes	AAAAGTCCCCGGTTCGAAATCAGGCGGGAACACC
831	Yes	AAAAGTCCCCGGTTCGAAATCAGGCGGGAACACCA
832	No	AAAAGTCCCTGATTCG
833	Yes	AAAAGTCCCTGATTCGA

834	Yes	AAAAGTCCCTGATTCGAG
835	Yes	AAAAGTCCCTGATTCGAGA
836	Yes	AAAAGTCCCTGATTCGAGAC
837	Yes	AAAAGTCCCTGATTCGAGACT
838	Yes	AAAAGTCCCTGATTCGAGACTG
839	Yes	AAAAGTCCCTGATTCGAGACTGG
840	Yes	AAAAGTCCCTGATTCGAGACTGGG
841	Yes	AAAAGTCCCTGATTCGAGACTGGGT
842	Yes	AAAAGTCCCTGATTCGAGACTGGGTG
843	Yes	AAAAGTCCCTGATTCGAGACTGGGTGG
844	Yes	AAAAGTCCCTGATTCGAGACTGGGTGGG
845	Yes	AAAAGTCCCTGATTCGAGACTGGGTGGGA
846	Yes	AAAAGTCCCTGATTCGAGACTGGGTGGGAA
847	Yes	AAAAGTCCCTGATTCGAGACTGGGTGGGAAC
848	Yes	AAAAGTCCCTGATTCGAGACTGGGTGGGAACG
849	Yes	AAAAGTCCCTGATTCGAGACTGGGTGGGAACGC
850	Yes	AAAAGTCCCTGATTCGAGACTGGGTGGGAACGCC
851	Yes	AAAAGTCCCTGATTCGAGACTGGGTGGGAACGCCA
852	Yes	AAAAGTTGGTGGTTCG
853	Yes	AAAAGTTGGTGGTTCGA
854	Yes	AAAAGTTGGTGGTTCGAA
855	Yes	AAAAGTTGGTGGTTCGAAC
856	Yes	AAAAGTTGGTGGTTCGAACA
857	Yes	AAAAGTTGGTGGTTCGAACAC
858	Yes	AAAAGTTGGTGGTTCGAACACA
859	Yes	AAAAGTTGGTGGTTCGAACACAC
860	Yes	AAAAGTTGGTGGTTCGAACACACC
861	Yes	AAAAGTTGGTGGTTCGAACACACCC
862	Yes	AAAAGTTGGTGGTTCGAACACACCCA
863	Yes	AAAAGTTGGTGGTTCGAACACACCCAG
864	Yes	AAAAGTTGGTGGTTCGAACACACCCAGA
865	Yes	AAAAGTTGGTGGTTCGAACACACCCAGAG
866	Yes	AAAAGTTGGTGGTTCGAACACACCCAGAGG
867	Yes	AAAAGTTGGTGGTTCGAACACACCCAGAGGC
868	Yes	AAAAGTTGGTGGTTCGAACACACCCAGAGGCG
869	Yes	AAAAGTTGGTGGTTCGAACACACCCAGAGGCGC
870	Yes	AAAAGTTGGTGGTTCGAACACACCCAGAGGCGCC
871	Yes	AAAAGTTGGTGGTTCGAACACACCCAGAGGCGCCA
872	Yes	AAAAGTTGGTGGTTCGAG
873	Yes	AAAAGTTGGTGGTTCGAGC
874	Yes	AAAAGTTGGTGGTTCGAGCC
875	Yes	AAAAGTTGGTGGTTCGAGCCC

876	Yes	AAAAGTTGGTGGTTCGAGCCCA
877	Yes	AAAAGTTGGTGGTTCGAGCCCAC
878	Yes	AAAAGTTGGTGGTTCGAGCCCACC
879	Yes	AAAAGTTGGTGGTTCGAGCCCACCC
880	Yes	AAAAGTTGGTGGTTCGAGCCCACCCA
881	Yes	AAAAGTTGGTGGTTCGAGCCCACCCAG
882	Yes	AAAAGTTGGTGGTTCGAGCCCACCCAGG
883	Yes	AAAAGTTGGTGGTTCGAGCCCACCCAGGA
884	Yes	AAAAGTTGGTGGTTCGAGCCCACCCAGGAA
885	Yes	AAAAGTTGGTGGTTCGAGCCCACCCAGGAAC
886	Yes	AAAAGTTGGTGGTTCGAGCCCACCCAGGAACG
887	Yes	AAAAGTTGGTGGTTCGAGCCCACCCAGGAACGC
888	Yes	AAAAGTTGGTGGTTCGAGCCCACCCAGGAACGCC
889	Yes	AAAAGTTGGTGGTTCGAGCCCACCCAGGAACGCCA
890	No	AAAATAGCTCAGCTGG
891	No	AAAATAGCTCAGCTGGG
892	Yes	AAAATAGCTCAGCTGGGA
893	Yes	AAAATAGCTCAGCTGGGAG
894	Yes	AAAATAGCTCAGCTGGGAGA
895	Yes	AAAATAGCTCAGCTGGGAGAG
896	Yes	AAAATAGCTCAGCTGGGAGAGC
897	Yes	AAAATAGCTCAGCTGGGAGAGCA
898	Yes	AAAATAGCTCAGCTGGGAGAGCAT
899	Yes	AAAATAGCTCAGCTGGGAGAGCATT
900	Yes	AAAATAGCTCAGCTGGGAGAGCATTA
901	Yes	AAAATAGCTCAGCTGGGAGAGCATTAG
902	Yes	AAAATAGCTCAGCTGGGAGAGCATTAGA
903	Yes	AAAATAGCTCAGCTGGGAGAGCATTAGAC
904	Yes	AAAATAGCTCAGCTGGGAGAGCATTAGACT
905	Yes	AAAATAGCTCAGCTGGGAGAGCATTAGACTG
906	Yes	AAAATAGCTCAGCTGGGAGAGCATTAGACTGA
907	Yes	AAAATAGCTCAGCTGGGAGAGCATTAGACTGAA
908	Yes	AAAATAGCTCAGCTGGGAGAGCATTAGACTGAAG
909	Yes	AAAATAGCTCAGCTGGGAGAGCATTAGACTGAAGA
910	Yes	AAAATAGCTCAGCTGGGAGAGCATTAGACTGAAGAT
911	Yes	AAAATAGCTCAGCTGGGAGAGCATTAGACTGAAGATC
912	Yes	AAAATAGCTCAGCTGGGAGAGCATTAGACTGAAGATCT
913	Yes	AAAATAGCTCAGCTGGGAGAGCATTAGACTGAAGATCTA
914	Yes	AAAATAGCTCAGCTGGGAGAGCATTAGACTGAAGATCTAA
915	Yes	AAAATAGCTCAGCTGGGAGAGCATTAGACTGAAGATCTAAA
916	Yes	AAAATAGCTCAGCTGGGAGAGCATTAGACTGAAGATCTAAAG
917	Yes	AAAATAGCTCAGCTGGGAGAGCATTAGACTGAAGATCTAAAGG

918	Yes	AAAATAGCTCAGCTGGGAGAGCATTAGACTGAAGATCTAAAGGT
919	Yes	AAAATAGCTCAGCTGGGAGAGCATTAGACTGAAGATCTAAAGGTC
920	Yes	AAAATAGCTCAGCTGGGAGAGCATTAGACTGAAGATCTAAAGGTCT
921	Yes	AAAATAGCTCAGCTGGGAGAGCATTAGACTGAAGATCTAAAGGTCTC
922	Yes	AAAATAGCTCAGCTGGGAGAGCATTAGACTGAAGATCTAAAGGTCTCT
923	Yes	AAAATAGCTCAGCTGGGAGAGCATTAGACTGAAGATCTAAAGGTCTCTG
924	Yes	AAAATAGCTCAGCTGGGAGAGCATTAGACTGAAGATCTAAAGGTCTCTGG
925	Yes	AAAATAGCTCAGCTGGGAGAGT
926	Yes	AAAATAGCTCAGCTGGGAGAGTA
927	Yes	AAAATAGCTCAGCTGGGAGAGTAT
928	Yes	AAAATAGCTCAGCTGGGAGAGTATT
929	Yes	AAAATAGCTCAGCTGGGAGAGTATTA
930	Yes	AAAATAGCTCAGCTGGGAGAGTATTAG
931	Yes	AAAATAGCTCAGCTGGGAGAGTATTAGG
932	Yes	AAAATAGCTCAGCTGGGAGAGTATTAGGT
933	Yes	AAAATAGCTCAGCTGGGAGAGTATTAGGTT
934	Yes	AAAATAGCTCAGCTGGGAGAGTATTAGGTTG
935	Yes	AAAATAGCTCAGCTGGGAGAGTATTAGGTTGA
936	Yes	AAAATAGCTCAGCTGGGAGAGTATTAGGTTGAA
937	Yes	AAAATAGCTCAGCTGGGAGAGTATTAGGTTGAAG
938	Yes	AAAATAGCTCAGCTGGGAGAGTATTAGGTTGAAGA
939	Yes	AAAATAGCTCAGCTGGGAGAGTATTAGGTTGAAGAT
940	Yes	AAAATAGCTCAGCTGGGAGAGTATTAGGTTGAAGATA
941	Yes	AAAATAGCTCAGCTGGGAGAGTATTAGGTTGAAGATAC
942	Yes	AAAATAGCTCAGCTGGGAGAGTATTAGGTTGAAGATACA
943	Yes	AAAATAGCTCAGCTGGGAGAGTATTAGGTTGAAGATACAA
944	Yes	AAAATAGCTCAGCTGGGAGAGTATTAGGTTGAAGATACAAA
945	Yes	AAAATAGCTCAGCTGGGAGAGTATTAGGTTGAAGATACAAAG
946	Yes	AAAATAGCTCAGCTGGGAGAGTATTAGGTTGAAGATACAAAGT
947	Yes	AAAATAGCTCAGCTGGGAGAGTATTAGGTTGAAGATACAAAGTT
948	Yes	AAAATAGCTCAGCTGGGAGAGTATTAGGTTGAAGATACAAAGTTC
949	Yes	AAAATAGCTCAGCTGGGAGAGTATTAGGTTGAAGATACAAAGTTCC
950	Yes	AAAATAGCTCAGCTGGGAGAGTATTAGGTTGAAGATACAAAGTTCCT
951	Yes	AAAATAGCTCAGCTGGGAGAGTATTAGGTTGAAGATACAAAGTTCCTT
952	Yes	AAAATAGCTCAGCTGGGAGAGTATTAGGTTGAAGATACAAAGTTCCTTG
953	Yes	AAAATAGCTCAGCTGGGAGAGTATTAGGTTGAAGATACAAAGTTCCTTGG
954	No	AAAATCTGAATGGTCC
955	No	AAAATCTGAATGGTCCT
956	No	AAAATCTGAATGGTCCTG
957	Yes	AAAATCTGAATGGTCCTGA
958	Yes	AAAATCTGAATGGTCCTGAG
959	Yes	AAAATCTGAATGGTCCTGAGT

960	Yes	AAAATCTGAATGGTCCTGAGTT
961	Yes	AAAATCTGAATGGTCCTGAGTTC
962	Yes	AAAATCTGAATGGTCCTGAGTTCA
963	Yes	AAAATCTGAATGGTCCTGAGTTCAA
964	Yes	AAAATCTGAATGGTCCTGAGTTCAAG
965	Yes	AAAATCTGAATGGTCCTGAGTTCAAGC
966	Yes	AAAATCTGAATGGTCCTGAGTTCAAGCC
967	Yes	AAAATCTGAATGGTCCTGAGTTCAAGCCT
968	Yes	AAAATCTGAATGGTCCTGAGTTCAAGCCTC
969	Yes	AAAATCTGAATGGTCCTGAGTTCAAGCCTCA
970	Yes	AAAATCTGAATGGTCCTGAGTTCAAGCCTCAG
971	Yes	AAAATCTGAATGGTCCTGAGTTCAAGCCTCAGA
972	Yes	AAAATCTGAATGGTCCTGAGTTCAAGCCTCAGAG
973	Yes	AAAATCTGAATGGTCCTGAGTTCAAGCCTCAGAGG
974	Yes	AAAATCTGAATGGTCCTGAGTTCAAGCCTCAGAGGG
975	Yes	AAAATCTGAATGGTCCTGAGTTCAAGCCTCAGAGGGG
976	Yes	AAAATCTGAATGGTCCTGAGTTCAAGCCTCAGAGGGGG
977	Yes	AAAATCTGAATGGTCCTGAGTTCAAGCCTCAGAGGGGGC
978	Yes	AAAATCTGAATGGTCCTGAGTTCAAGCCTCAGAGGGGGCA
979	Yes	AAAATCTGAATGGTCCTGAGTTCAAGCCTCAGAGGGGGCAC
980	Yes	AAAATCTGAATGGTCCTGAGTTCAAGCCTCAGAGGGGGCACC
981	Yes	AAAATCTGAATGGTCCTGAGTTCAAGCCTCAGAGGGGGCACCA
982	No	AAAATGGCTGAGTGAA
983	No	AAAATGGCTGAGTGAAG
984	No	AAAATGGCTGAGTGAAGC
985	No	AAAATGGCTGAGTGAAGCA
986	No	AAAATGGCTGAGTGAAGCAT
987	No	AAAATGGCTGAGTGAAGCATT
988	No	AAAATGGCTGAGTGAAGCATTG
989	No	AAAATGGCTGAGTGAAGCATTGG
990	No	AAAATGGCTGAGTGAAGCATTGGA
991	No	AAAATGGCTGAGTGAAGCATTGGAC
992	No	AAAATGGCTGAGTGAAGCATTGGACT
993	No	AAAATGGCTGAGTGAAGCATTGGACTG
994	No	AAAATGGCTGAGTGAAGCATTGGACTGT
995	No	AAAATGGCTGAGTGAAGCATTGGACTGTA
996	No	AAAATGGCTGAGTGAAGCATTGGACTGTAA
997	No	AAAATGGCTGAGTGAAGCATTGGACTGTAAA
998	No	AAAATGGCTGAGTGAAGCATTGGACTGTAAAT
999	No	AAAATGGCTGAGTGAAGCATTGGACTGTAAATC
1000	No	AAAATGGCTGAGTGAAGCATTGGACTGTAAATCT
1001	No	AAAATGGCTGAGTGAAGCATTGGACTGTAAATCTA

1002	No	AAAATGGCTGAGTGAAGCATTGGACTGTAAATCTAA
1003	No	AAAATGGCTGAGTGAAGCATTGGACTGTAAATCTAAA
1004	No	AAAATGGCTGAGTGAAGCATTGGACTGTAAATCTAAAG
1005	No	AAAATGGCTGAGTGAAGCATTGGACTGTAAATCTAAAGA
1006	No	AAAATGGCTGAGTGAAGCATTGGACTGTAAATCTAAAGAC
1007	No	AAAATGGCTGAGTGAAGCATTGGACTGTAAATCTAAAGACA
1008	No	AAAATGGCTGAGTGAAGCATTGGACTGTAAATCTAAAGACAG
1009	No	AAAATGGCTGAGTGAAGCATTGGACTGTAAATCTAAAGACAGG
1010	No	AAAATGGCTGAGTGAAGCATTGGACTGTAAATCTAAAGACAGGG
1011	No	AAAATGGCTGAGTGAAGCATTGGACTGTAAATCTAAAGACAGGGG
1012	No	AAAATGGCTGAGTGAAGCATTGGACTGTAAATCTAAAGACAGGGGT
1013	No	AAAATGGCTGAGTGAAGCATTGGACTGTAAATCTAAAGACAGGGGTT
1014	No	AAAATGGCTGAGTGAAGCATTGGACTGTAAATCTAAAGACAGGGGTTA
1015	Yes	AAAATGGCTGAGTGAAGCATTGGACTGTAAATCTAAAGACAGGGGTTAG
1016	Yes	AAAATGGCTGAGTGAAGCATTGGACTGTAAATCTAAAGACAGGGGTTAGG
1017	No	AAAATGTTGGTTATAC
1018	No	AAAATGTTGGTTATACC
1019	No	AAAATGTTGGTTATACCC
1020	No	AAAATGTTGGTTATACCCT
1021	No	AAAATGTTGGTTATACCCTT
1022	No	AAAATGTTGGTTATACCCTTC
1023	No	AAAATGTTGGTTATACCCTTCC
1024	No	AAAATGTTGGTTATACCCTTCCC
1025	No	AAAATGTTGGTTATACCCTTCCCG
1026	No	AAAATGTTGGTTATACCCTTCCCGT
1027	Yes	AAAATGTTGGTTATACCCTTCCCGTA
1028	Yes	AAAATGTTGGTTATACCCTTCCCGTAC
1029	Yes	AAAATGTTGGTTATACCCTTCCCGTACT
1030	Yes	AAAATGTTGGTTATACCCTTCCCGTACTA
1031	Yes	AAAATGTTGGTTATACCCTTCCCGTACTAC
1032	Yes	AAAATGTTGGTTATACCCTTCCCGTACTACC
1033	Yes	AAAATGTTGGTTATACCCTTCCCGTACTACCA
1034	Yes	AAAATGTTTAGACGGG
1035	Yes	AAAATGTTTAGACGGGC
1036	Yes	AAAATGTTTAGACGGGCT
1037	Yes	AAAATGTTTAGACGGGCTC
1038	Yes	AAAATGTTTAGACGGGCTCA
1039	Yes	AAAATGTTTAGACGGGCTCAC
1040	Yes	AAAATGTTTAGACGGGCTCACA
1041	Yes	AAAATGTTTAGACGGGCTCACAT
1042	Yes	AAAATGTTTAGACGGGCTCACATC
1043	Yes	AAAATGTTTAGACGGGCTCACATCA

1044	Yes	AAAATGTTTAGACGGGCTCACATCAC
1045	Yes	AAAATGTTTAGACGGGCTCACATCACC
1046	Yes	AAAATGTTTAGACGGGCTCACATCACCC
1047	Yes	AAAATGTTTAGACGGGCTCACATCACCCC
1048	Yes	AAAATGTTTAGACGGGCTCACATCACCCCA
1049	Yes	AAAATGTTTAGACGGGCTCACATCACCCCAT
1050	Yes	AAAATGTTTAGACGGGCTCACATCACCCCATA
1051	Yes	AAAATGTTTAGACGGGCTCACATCACCCCATAA
1052	Yes	AAAATGTTTAGACGGGCTCACATCACCCCATAAA
1053	Yes	AAAATGTTTAGACGGGCTCACATCACCCCATAAAC
1054	Yes	AAAATGTTTAGACGGGCTCACATCACCCCATAAACA
1055	Yes	AAAATGTTTAGACGGGCTCACATCACCCCATAAACAC
1056	Yes	AAAATGTTTAGACGGGCTCACATCACCCCATAAACACC
1057	Yes	AAAATGTTTAGACGGGCTCACATCACCCCATAAACACCA
1058	No	AAAATTGCTCAGTTGG
1059	No	AAAATTGCTCAGTTGGG
1060	Yes	AAAATTGCTCAGTTGGGA
1061	Yes	AAAATTGCTCAGTTGGGAG
1062	Yes	AAAATTGCTCAGTTGGGAGA
1063	Yes	AAAATTGCTCAGTTGGGAGAG
1064	Yes	AAAATTGCTCAGTTGGGAGAGC
1065	Yes	AAAATTGCTCAGTTGGGAGAGCG
1066	Yes	AAAATTGCTCAGTTGGGAGAGCGT
1067	Yes	AAAATTGCTCAGTTGGGAGAGCGTT
1068	Yes	AAAATTGCTCAGTTGGGAGAGCGTTA
1069	Yes	AAAATTGCTCAGTTGGGAGAGCGTTAG
1070	Yes	AAAATTGCTCAGTTGGGAGAGCGTTAGA
1071	Yes	AAAATTGCTCAGTTGGGAGAGCGTTAGAC
1072	Yes	AAAATTGCTCAGTTGGGAGAGCGTTAGACT
1073	Yes	AAAATTGCTCAGTTGGGAGAGCGTTAGACTG
1074	Yes	AAAATTGCTCAGTTGGGAGAGCGTTAGACTGA
1075	Yes	AAAATTGCTCAGTTGGGAGAGCGTTAGACTGAA
1076	Yes	AAAATTGCTCAGTTGGGAGAGCGTTAGACTGAAG
1077	Yes	AAAATTGCTCAGTTGGGAGAGCGTTAGACTGAAGA
1078	Yes	AAAATTGCTCAGTTGGGAGAGCGTTAGACTGAAGAT
1079	Yes	AAAATTGCTCAGTTGGGAGAGCGTTAGACTGAAGATC
1080	Yes	AAAATTGCTCAGTTGGGAGAGCGTTAGACTGAAGATCT
1081	Yes	AAAATTGCTCAGTTGGGAGAGCGTTAGACTGAAGATCTA
1082	Yes	AAAATTGCTCAGTTGGGAGAGCGTTAGACTGAAGATCTAA
1083	Yes	AAAATTGCTCAGTTGGGAGAGCGTTAGACTGAAGATCTAAA
1084	Yes	AAAATTGCTCAGTTGGGAGAGCGTTAGACTGAAGATCTAAAG
1085	Yes	AAAATTGCTCAGTTGGGAGAGCGTTAGACTGAAGATCTAAAGG

1086	Yes	AAAATTGCTCAGTTGGGAGAGCGTTAGACTGAAGATCTAAAGGT
1087	Yes	AAAATTGCTCAGTTGGGAGAGCGTTAGACTGAAGATCTAAAGGTC
1088	Yes	AAAATTGCTCAGTTGGGAGAGCGTTAGACTGAAGATCTAAAGGTCC
1089	Yes	AAAATTGCTCAGTTGGGAGAGCGTTAGACTGAAGATCTAAAGGTCCC
1090	Yes	AAAATTGCTCAGTTGGGAGAGCGTTAGACTGAAGATCTAAAGGTCCCT
1091	Yes	AAAATTGCTCAGTTGGGAGAGCGTTAGACTGAAGATCTAAAGGTCCCTG
1092	Yes	AAAATTGCTCAGTTGGGAGAGCGTTAGACTGAAGATCTAAAGGTCCCTGG
1093	No	AAAATTTACAACTTTA
1094	No	AAAATTTACAACTTTAT
1095	No	AAAATTTACAACTTTATG
1096	Yes	AAAATTTACAACTTTATGG
1097	Yes	AAAATTTACAACTTTATGGG
1098	Yes	AAAATTTACAACTTTATGGGC
1099	Yes	AAAATTTACAACTTTATGGGCA
1100	Yes	AAAATTTACAACTTTATGGGCAG
1101	Yes	AAAATTTACAACTTTATGGGCAGA
1102	Yes	AAAATTTACAACTTTATGGGCAGAG
1103	Yes	AAAATTTACAACTTTATGGGCAGAGG
1104	Yes	AAAATTTACAACTTTATGGGCAGAGGT
1105	Yes	AAAATTTACAACTTTATGGGCAGAGGTT
1106	Yes	AAAATTTACAACTTTATGGGCAGAGGTTC
1107	Yes	AAAATTTACAACTTTATGGGCAGAGGTTCA
1108	Yes	AAAATTTACAACTTTATGGGCAGAGGTTCAA
1109	Yes	AAAATTTACAACTTTATGGGCAGAGGTTCAAT
1110	Yes	AAAATTTACAACTTTATGGGCAGAGGTTCAATT
1111	Yes	AAAATTTACAACTTTATGGGCAGAGGTTCAATTC
1112	Yes	AAAATTTACAACTTTATGGGCAGAGGTTCAATTCC
1113	Yes	AAAATTTACAACTTTATGGGCAGAGGTTCAATTCCT
1114	Yes	AAAATTTACAACTTTATGGGCAGAGGTTCAATTCCTC
1115	Yes	AAAATTTACAACTTTATGGGCAGAGGTTCAATTCCTCT
1116	Yes	AAAATTTACAACTTTATGGGCAGAGGTTCAATTCCTCTT
1117	Yes	AAAATTTACAACTTTATGGGCAGAGGTTCAATTCCTCTTC
1118	Yes	AAAATTTACAACTTTATGGGCAGAGGTTCAATTCCTCTTCT
1119	Yes	AAAATTTACAACTTTATGGGCAGAGGTTCAATTCCTCTTCTT
1120	Yes	AAAATTTACAACTTTATGGGCAGAGGTTCAATTCCTCTTCTTA
1121	Yes	AAAATTTACAACTTTATGGGCAGAGGTTCAATTCCTCTTCTTAA
1122	Yes	AAAATTTACAACTTTATGGGCAGAGGTTCAATTCCTCTTCTTAAC
1123	Yes	AAAATTTACAACTTTATGGGCAGAGGTTCAATTCCTCTTCTTAACA
1124	Yes	AAAATTTACAACTTTATGGGCAGAGGTTCAATTCCTCTTCTTAACAC
1125	Yes	AAAATTTACAACTTTATGGGCAGAGGTTCAATTCCTCTTCTTAACACC
1126	Yes	AAAATTTACAACTTTATGGGCAGAGGTTCAATTCCTCTTCTTAACACCA
1127	No	AAAATTTTATAATCAG

1128	No	AAAATTTTATAATCAGA
1129	No	AAAATTTTATAATCAGAG
1130	No	AAAATTTTATAATCAGAGG
1131	Yes	AAAATTTTATAATCAGAGGT
1132	Yes	AAAATTTTATAATCAGAGGTT
1133	Yes	AAAATTTTATAATCAGAGGTTC
1134	Yes	AAAATTTTATAATCAGAGGTTCA
1135	Yes	AAAATTTTATAATCAGAGGTTCAA
1136	Yes	AAAATTTTATAATCAGAGGTTCAAC
1137	Yes	AAAATTTTATAATCAGAGGTTCAACT
1138	Yes	AAAATTTTATAATCAGAGGTTCAACTC
1139	Yes	AAAATTTTATAATCAGAGGTTCAACTCC
1140	Yes	AAAATTTTATAATCAGAGGTTCAACTCCT
1141	Yes	AAAATTTTATAATCAGAGGTTCAACTCCTC
1142	Yes	AAAATTTTATAATCAGAGGTTCAACTCCTCT
1143	Yes	AAAATTTTATAATCAGAGGTTCAACTCCTCTT
1144	Yes	AAAATTTTATAATCAGAGGTTCAACTCCTCTTC
1145	Yes	AAAATTTTATAATCAGAGGTTCAACTCCTCTTCT
1146	Yes	AAAATTTTATAATCAGAGGTTCAACTCCTCTTCTT
1147	Yes	AAAATTTTATAATCAGAGGTTCAACTCCTCTTCTTA
1148	Yes	AAAATTTTATAATCAGAGGTTCAACTCCTCTTCTTAA
1149	Yes	AAAATTTTATAATCAGAGGTTCAACTCCTCTTCTTAAC
1150	Yes	AAAATTTTATAATCAGAGGTTCAACTCCTCTTCTTAACA
1151	Yes	AAAATTTTATAATCAGAGGTTCAACTCCTCTTCTTAACAC
1152	Yes	AAAATTTTATAATCAGAGGTTCAACTCCTCTTCTTAACACC
1153	Yes	AAAATTTTATAATCAGAGGTTCAACTCCTCTTCTTAACACCA
1154	No	AAAATTTTGGTGCAAC
1155	No	AAAATTTTGGTGCAACT
1156	No	AAAATTTTGGTGCAACTC
1157	No	AAAATTTTGGTGCAACTCC
1158	No	AAAATTTTGGTGCAACTCCA
1159	No	AAAATTTTGGTGCAACTCCAA
1160	No	AAAATTTTGGTGCAACTCCAAA
1161	No	AAAATTTTGGTGCAACTCCAAAT
1162	No	AAAATTTTGGTGCAACTCCAAATA
1163	No	AAAATTTTGGTGCAACTCCAAATAA
1164	No	AAAATTTTGGTGCAACTCCAAATAAA
1165	No	AAAATTTTGGTGCAACTCCAAATAAAA
1166	No	AAAATTTTGGTGCAACTCCAAATAAAAG
1167	No	AAAATTTTGGTGCAACTCCAAATAAAAGT
1168	No	AAAATTTTGGTGCAACTCCAAATAAAAGTA
1169	Yes	AAAATTTTGGTGCAACTCCAAATAAAAGTAC

1170	Yes	AAAATTTTGGTGCAACTCCAAATAAAAGTACC
1171	Yes	AAAATTTTGGTGCAACTCCAAATAAAAGTACCA
1172	No	AAACAAAACGAATGAT
1173	No	AAACAAAACGAATGATT
1174	No	AAACAAAACGAATGATTT
1175	No	AAACAAAACGAATGATTTC
1176	No	AAACAAAACGAATGATTTCG
1177	No	AAACAAAACGAATGATTTCGA
1178	No	AAACAAAACGAATGATTTCGAC
1179	No	AAACAAAACGAATGATTTCGACT
1180	No	AAACAAAACGAATGATTTCGACTC
1181	No	AAACAAAACGAATGATTTCGACTCA
1182	No	AAACAAAACGAATGATTTCGACTCAT
1183	No	AAACAAAACGAATGATTTCGACTCATT
1184	No	AAACAAAACGAATGATTTCGACTCATTA
1185	No	AAACAAAACGAATGATTTCGACTCATTAA
1186	No	AAACAAAACGAATGATTTCGACTCATTAAA
1187	No	AAACAAAACGAATGATTTCGACTCATTAAAT
1188	No	AAACAAAACGAATGATTTCGACTCATTAAATT
1189	No	AAACAAAACGAATGATTTCGACTCATTAAATTA
1190	No	AAACAAAACGAATGATTTCGACTCATTAAATTAT
1191	No	AAACAAAACGAATGATTTCGACTCATTAAATTATG
1192	No	AAACAAAACGAATGATTTCGACTCATTAAATTATGA
1193	No	AAACAAAACGAATGATTTCGACTCATTAAATTATGAT
1194	No	AAACAAAACGAATGATTTCGACTCATTAAATTATGATA
1195	No	AAACAAAACGAATGATTTCGACTCATTAAATTATGATAA
1196	No	AAACAAAACGAATGATTTCGACTCATTAAATTATGATAAT
1197	No	AAACAAAACGAATGATTTCGACTCATTAAATTATGATAATC
1198	No	AAACAAAACGAATGATTTCGACTCATTAAATTATGATAATCA
1199	No	AAACAAAACGAATGATTTCGACTCATTAAATTATGATAATCAT
1200	No	AAACAAAACGAATGATTTCGACTCATTAAATTATGATAATCATA
1201	No	AAACAAAACGAATGATTTCGACTCATTAAATTATGATAATCATAT
1202	No	AAACAAAACGAATGATTTCGACTCATTAAATTATGATAATCATATT
1203	No	AAACAAAACGAATGATTTCGACTCATTAAATTATGATAATCATATTT
1204	No	AAACAAAACGAATGATTTCGACTCATTAAATTATGATAATCATATTTA
1205	No	AAACAAAACGAATGATTTCGACTCATTAAATTATGATAATCATATTTAC
1206	No	AAACAAAACGAATGATTTCGACTCATTAAATTATGATAATCATATTTACC
1207	No	AAACAGGAGATCCTGA
1208	No	AAACAGGAGATCCTGAG
1209	No	AAACAGGAGATCCTGAGT
1210	No	AAACAGGAGATCCTGAGTT
1211	No	AAACAGGAGATCCTGAGTTT

1212	Yes	AAACAGGAGATCCTGAGTTTC
1213	Yes	AAACAGGAGATCCTGAGTTTCA
1214	Yes	AAACAGGAGATCCTGAGTTTCAA
1215	Yes	AAACAGGAGATCCTGAGTTTCAAT
1216	Yes	AAACAGGAGATCCTGAGTTTCAATT
1217	Yes	AAACAGGAGATCCTGAGTTTCAATTC
1218	Yes	AAACAGGAGATCCTGAGTTTCAATTCC
1219	Yes	AAACAGGAGATCCTGAGTTTCAATTCCA
1220	Yes	AAACAGGAGATCCTGAGTTTCAATTCCAA
1221	Yes	AAACAGGAGATCCTGAGTTTCAATTCCAAT
1222	Yes	AAACAGGAGATCCTGAGTTTCAATTCCAATG
1223	Yes	AAACAGGAGATCCTGAGTTTCAATTCCAATGG
1224	Yes	AAACAGGAGATCCTGAGTTTCAATTCCAATGGT
1225	Yes	AAACAGGAGATCCTGAGTTTCAATTCCAATGGTG
1226	Yes	AAACAGGAGATCCTGAGTTTCAATTCCAATGGTGC
1227	Yes	AAACAGGAGATCCTGAGTTTCAATTCCAATGGTGCC
1228	Yes	AAACAGGAGATCCTGAGTTTCAATTCCAATGGTGCCT
1229	Yes	AAACAGGAGATCCTGAGTTTCAATTCCAATGGTGCCTC
1230	Yes	AAACAGGAGATCCTGAGTTTCAATTCCAATGGTGCCTCC
1231	Yes	AAACAGGAGATCCTGAGTTTCAATTCCAATGGTGCCTCCA
1232	No	AAACAGGAGATCCTGG
1233	No	AAACAGGAGATCCTGGG
1234	No	AAACAGGAGATCCTGGGT
1235	No	AAACAGGAGATCCTGGGTT
1236	Yes	AAACAGGAGATCCTGGGTTC
1237	Yes	AAACAGGAGATCCTGGGTTCG
1238	Yes	AAACAGGAGATCCTGGGTTCGA
1239	Yes	AAACAGGAGATCCTGGGTTCGAA
1240	Yes	AAACAGGAGATCCTGGGTTCGAAT
1241	Yes	AAACAGGAGATCCTGGGTTCGAATC
1242	Yes	AAACAGGAGATCCTGGGTTCGAATCC
1243	Yes	AAACAGGAGATCCTGGGTTCGAATCCC
1244	Yes	AAACAGGAGATCCTGGGTTCGAATCCCA
1245	Yes	AAACAGGAGATCCTGGGTTCGAATCCCAG
1246	Yes	AAACAGGAGATCCTGGGTTCGAATCCCAGC
1247	Yes	AAACAGGAGATCCTGGGTTCGAATCCCAGCG
1248	Yes	AAACAGGAGATCCTGGGTTCGAATCCCAGCGA
1249	Yes	AAACAGGAGATCCTGGGTTCGAATCCCAGCGAG
1250	Yes	AAACAGGAGATCCTGGGTTCGAATCCCAGCGAGG
1251	Yes	AAACAGGAGATCCTGGGTTCGAATCCCAGCGAGGC
1252	Yes	AAACAGGAGATCCTGGGTTCGAATCCCAGCGAGGCC
1253	Yes	AAACAGGAGATCCTGGGTTCGAATCCCAGCGAGGCCT

1254	Yes	AAACAGGAGATCCTGGGTTCGAATCCCAGCGAGGCCTC
1255	Yes	AAACAGGAGATCCTGGGTTCGAATCCCAGCGAGGCCTCC
1256	Yes	AAACAGGAGATCCTGGGTTCGAATCCCAGCGAGGCCTCCA
1257	Yes	AAACAGGAGATCCTGGGTTCGAATCCCAGCGG
1258	Yes	AAACAGGAGATCCTGGGTTCGAATCCCAGCGGG
1259	Yes	AAACAGGAGATCCTGGGTTCGAATCCCAGCGGGG
1260	Yes	AAACAGGAGATCCTGGGTTCGAATCCCAGCGGGGC
1261	Yes	AAACAGGAGATCCTGGGTTCGAATCCCAGCGGGGCC
1262	Yes	AAACAGGAGATCCTGGGTTCGAATCCCAGCGGGGCCT
1263	Yes	AAACAGGAGATCCTGGGTTCGAATCCCAGCGGGGCCTC
1264	Yes	AAACAGGAGATCCTGGGTTCGAATCCCAGCGGGGCCTCC
1265	Yes	AAACAGGAGATCCTGGGTTCGAATCCCAGCGGGGCCTCCA
1266	Yes	AAACAGGAGATCCTGGGTTCGAATCCCAGCGGT
1267	Yes	AAACAGGAGATCCTGGGTTCGAATCCCAGCGGTG
1268	Yes	AAACAGGAGATCCTGGGTTCGAATCCCAGCGGTGC
1269	Yes	AAACAGGAGATCCTGGGTTCGAATCCCAGCGGTGCC
1270	Yes	AAACAGGAGATCCTGGGTTCGAATCCCAGCGGTGCCT
1271	Yes	AAACAGGAGATCCTGGGTTCGAATCCCAGCGGTGCCTC
1272	Yes	AAACAGGAGATCCTGGGTTCGAATCCCAGCGGTGCCTCC
1273	Yes	AAACAGGAGATCCTGGGTTCGAATCCCAGCGGTGCCTCCA
1274	Yes	AAACAGGAGATCCTGGGTTCGAATCCCAGT
1275	Yes	AAACAGGAGATCCTGGGTTCGAATCCCAGTA
1276	Yes	AAACAGGAGATCCTGGGTTCGAATCCCAGTAG
1277	Yes	AAACAGGAGATCCTGGGTTCGAATCCCAGTAGA
1278	Yes	AAACAGGAGATCCTGGGTTCGAATCCCAGTAGAG
1279	Yes	AAACAGGAGATCCTGGGTTCGAATCCCAGTAGAGC
1280	Yes	AAACAGGAGATCCTGGGTTCGAATCCCAGTAGAGCC
1281	Yes	AAACAGGAGATCCTGGGTTCGAATCCCAGTAGAGCCT
1282	Yes	AAACAGGAGATCCTGGGTTCGAATCCCAGTAGAGCCTC
1283	Yes	AAACAGGAGATCCTGGGTTCGAATCCCAGTAGAGCCTCC
1284	Yes	AAACAGGAGATCCTGGGTTCGAATCCCAGTAGAGCCTCCA
1285	Yes	AAACAGGAGATCCTGGGTTCGAC
1286	Yes	AAACAGGAGATCCTGGGTTCGACT
1287	Yes	AAACAGGAGATCCTGGGTTCGACTC
1288	Yes	AAACAGGAGATCCTGGGTTCGACTCC
1289	Yes	AAACAGGAGATCCTGGGTTCGACTCCC
1290	Yes	AAACAGGAGATCCTGGGTTCGACTCCCA
1291	Yes	AAACAGGAGATCCTGGGTTCGACTCCCAG
1292	Yes	AAACAGGAGATCCTGGGTTCGACTCCCAGC
1293	Yes	AAACAGGAGATCCTGGGTTCGACTCCCAGCG
1294	Yes	AAACAGGAGATCCTGGGTTCGACTCCCAGCGG
1295	Yes	AAACAGGAGATCCTGGGTTCGACTCCCAGCGGG

1296	Yes	AAACAGGAGATCCTGGGTTCGACTCCCAGCGGGG
1297	Yes	AAACAGGAGATCCTGGGTTCGACTCCCAGCGGGGC
1298	Yes	AAACAGGAGATCCTGGGTTCGACTCCCAGCGGGGCC
1299	Yes	AAACAGGAGATCCTGGGTTCGACTCCCAGCGGGGCCT
1300	Yes	AAACAGGAGATCCTGGGTTCGACTCCCAGCGGGGCCTC
1301	Yes	AAACAGGAGATCCTGGGTTCGACTCCCAGCGGGGCCTCC
1302	Yes	AAACAGGAGATCCTGGGTTCGACTCCCAGCGGGGCCTCCA
1303	Yes	AAACAGGAGATCCTGGGTTCGACTCCCAGT
1304	Yes	AAACAGGAGATCCTGGGTTCGACTCCCAGTG
1305	Yes	AAACAGGAGATCCTGGGTTCGACTCCCAGTGG
1306	Yes	AAACAGGAGATCCTGGGTTCGACTCCCAGTGGG
1307	Yes	AAACAGGAGATCCTGGGTTCGACTCCCAGTGGGG
1308	Yes	AAACAGGAGATCCTGGGTTCGACTCCCAGTGGGGC
1309	Yes	AAACAGGAGATCCTGGGTTCGACTCCCAGTGGGGCC
1310	Yes	AAACAGGAGATCCTGGGTTCGACTCCCAGTGGGGCCT
1311	Yes	AAACAGGAGATCCTGGGTTCGACTCCCAGTGGGGCCTC
1312	Yes	AAACAGGAGATCCTGGGTTCGACTCCCAGTGGGGCCTCC
1313	Yes	AAACAGGAGATCCTGGGTTCGACTCCCAGTGGGGCCTCCA
1314	No	AAACATCAGATTGTGA
1315	No	AAACATCAGATTGTGAA
1316	No	AAACATCAGATTGTGAAT
1317	No	AAACATCAGATTGTGAATC
1318	No	AAACATCAGATTGTGAATCT
1319	No	AAACATCAGATTGTGAATCTG
1320	No	AAACATCAGATTGTGAATCTGA
1321	No	AAACATCAGATTGTGAATCTGAC
1322	No	AAACATCAGATTGTGAATCTGACA
1323	No	AAACATCAGATTGTGAATCTGACAA
1324	No	AAACATCAGATTGTGAATCTGACAAC
1325	No	AAACATCAGATTGTGAATCTGACAACA
1326	No	AAACATCAGATTGTGAATCTGACAACAG
1327	No	AAACATCAGATTGTGAATCTGACAACAGA
1328	No	AAACATCAGATTGTGAATCTGACAACAGAG
1329	No	AAACATCAGATTGTGAATCTGACAACAGAGG
1330	No	AAACATCAGATTGTGAATCTGACAACAGAGGC
1331	No	AAACATCAGATTGTGAATCTGACAACAGAGGCT
1332	Yes	AAACATCAGATTGTGAATCTGACAACAGAGGCTT
1333	Yes	AAACATCAGATTGTGAATCTGACAACAGAGGCTTA
1334	Yes	AAACATCAGATTGTGAATCTGACAACAGAGGCTTAC
1335	Yes	AAACATCAGATTGTGAATCTGACAACAGAGGCTTACG
1336	Yes	AAACATCAGATTGTGAATCTGACAACAGAGGCTTACGA
1337	Yes	AAACATCAGATTGTGAATCTGACAACAGAGGCTTACGAC

1338	Yes	AAACATCAGATTGTGAATCTGACAACAGAGGCTTACGACC
1339	Yes	AAACATCAGATTGTGAATCTGACAACAGAGGCTTACGACCC
1340	Yes	AAACATCAGATTGTGAATCTGACAACAGAGGCTTACGACCCC
1341	Yes	AAACATCAGATTGTGAATCTGACAACAGAGGCTTACGACCCCT
1342	Yes	AAACATCAGATTGTGAATCTGACAACAGAGGCTTACGACCCCTT
1343	Yes	AAACATCAGATTGTGAATCTGACAACAGAGGCTTACGACCCCTTA
1344	Yes	AAACATCAGATTGTGAATCTGACAACAGAGGCTTACGACCCCTTAT
1345	Yes	AAACATCAGATTGTGAATCTGACAACAGAGGCTTACGACCCCTTATT
1346	Yes	AAACATCAGATTGTGAATCTGACAACAGAGGCTTACGACCCCTTATTT
1347	Yes	AAACATCAGATTGTGAATCTGACAACAGAGGCTTACGACCCCTTATTTA
1348	Yes	AAACATCAGATTGTGAATCTGACAACAGAGGCTTACGACCCCTTATTTAC
1349	Yes	AAACCACGTGGTCTGG
1350	Yes	AAACCACGTGGTCTGGG
1351	Yes	AAACCACGTGGTCTGGGT
1352	Yes	AAACCACGTGGTCTGGGTT
1353	Yes	AAACCACGTGGTCTGGGTTT
1354	Yes	AAACCACGTGGTCTGGGTTTA
1355	Yes	AAACCACGTGGTCTGGGTTTAA
1356	Yes	AAACCACGTGGTCTGGGTTTAAG
1357	Yes	AAACCACGTGGTCTGGGTTTAAGT
1358	Yes	AAACCACGTGGTCTGGGTTTAAGTC
1359	Yes	AAACCACGTGGTCTGGGTTTAAGTCT
1360	Yes	AAACCACGTGGTCTGGGTTTAAGTCTC
1361	Yes	AAACCACGTGGTCTGGGTTTAAGTCTCA
1362	Yes	AAACCACGTGGTCTGGGTTTAAGTCTCAG
1363	Yes	AAACCACGTGGTCTGGGTTTAAGTCTCAGC
1364	Yes	AAACCACGTGGTCTGGGTTTAAGTCTCAGCT
1365	Yes	AAACCACGTGGTCTGGGTTTAAGTCTCAGCTG
1366	Yes	AAACCACGTGGTCTGGGTTTAAGTCTCAGCTGT
1367	Yes	AAACCACGTGGTCTGGGTTTAAGTCTCAGCTGTG
1368	Yes	AAACCACGTGGTCTGGGTTTAAGTCTCAGCTGTGC
1369	Yes	AAACCACGTGGTCTGGGTTTAAGTCTCAGCTGTGCT
1370	Yes	AAACCACGTGGTCTGGGTTTAAGTCTCAGCTGTGCTA
1371	Yes	AAACCACGTGGTCTGGGTTTAAGTCTCAGCTGTGCTAC
1372	Yes	AAACCACGTGGTCTGGGTTTAAGTCTCAGCTGTGCTACC
1373	Yes	AAACCACGTGGTCTGGGTTTAAGTCTCAGCTGTGCTACCA
1374	No	AAACCAGCCTTAGGAG
1375	Yes	AAACCAGCCTTAGGAGG
1376	Yes	AAACCAGCCTTAGGAGGT
1377	Yes	AAACCAGCCTTAGGAGGTT
1378	Yes	AAACCAGCCTTAGGAGGTTC
1379	Yes	AAACCAGCCTTAGGAGGTTCA

1380	Yes	AAACCAGCCTTAGGAGGTTCAA
1381	Yes	AAACCAGCCTTAGGAGGTTCAAT
1382	Yes	AAACCAGCCTTAGGAGGTTCAATT
1383	Yes	AAACCAGCCTTAGGAGGTTCAATTC
1384	Yes	AAACCAGCCTTAGGAGGTTCAATTCC
1385	Yes	AAACCAGCCTTAGGAGGTTCAATTCCT
1386	Yes	AAACCAGCCTTAGGAGGTTCAATTCCTT
1387	Yes	AAACCAGCCTTAGGAGGTTCAATTCCTTC
1388	Yes	AAACCAGCCTTAGGAGGTTCAATTCCTTCC
1389	Yes	AAACCAGCCTTAGGAGGTTCAATTCCTTCCT
1390	Yes	AAACCAGCCTTAGGAGGTTCAATTCCTTCCTT
1391	Yes	AAACCAGCCTTAGGAGGTTCAATTCCTTCCTTT
1392	Yes	AAACCAGCCTTAGGAGGTTCAATTCCTTCCTTTT
1393	Yes	AAACCAGCCTTAGGAGGTTCAATTCCTTCCTTTTT
1394	Yes	AAACCAGCCTTAGGAGGTTCAATTCCTTCCTTTTTT
1395	Yes	AAACCAGCCTTAGGAGGTTCAATTCCTTCCTTTTTTG
1396	Yes	AAACCAGCCTTAGGAGGTTCAATTCCTTCCTTTTTTGC
1397	Yes	AAACCAGCCTTAGGAGGTTCAATTCCTTCCTTTTTTGCC
1398	Yes	AAACCAGCCTTAGGAGGTTCAATTCCTTCCTTTTTTGCCA
1399	No	AAACCAGCTTTGGGGG
1400	No	AAACCAGCTTTGGGGGG
1401	Yes	AAACCAGCTTTGGGGGGT
1402	Yes	AAACCAGCTTTGGGGGGTT
1403	Yes	AAACCAGCTTTGGGGGGTTC
1404	Yes	AAACCAGCTTTGGGGGGTTCG
1405	Yes	AAACCAGCTTTGGGGGGTTCGA
1406	Yes	AAACCAGCTTTGGGGGGTTCGAT
1407	Yes	AAACCAGCTTTGGGGGGTTCGATT
1408	Yes	AAACCAGCTTTGGGGGGTTCGATTC
1409	Yes	AAACCAGCTTTGGGGGGTTCGATTCC
1410	Yes	AAACCAGCTTTGGGGGGTTCGATTCCT
1411	Yes	AAACCAGCTTTGGGGGGTTCGATTCCTT
1412	Yes	AAACCAGCTTTGGGGGGTTCGATTCCTTC
1413	Yes	AAACCAGCTTTGGGGGGTTCGATTCCTTCC
1414	Yes	AAACCAGCTTTGGGGGGTTCGATTCCTTCCT
1415	Yes	AAACCAGCTTTGGGGGGTTCGATTCCTTCCTT
1416	Yes	AAACCAGCTTTGGGGGGTTCGATTCCTTCCTTT
1417	Yes	AAACCAGCTTTGGGGGGTTCGATTCCTTCCTTTT
1418	Yes	AAACCAGCTTTGGGGGGTTCGATTCCTTCCTTTTT
1419	Yes	AAACCAGCTTTGGGGGGTTCGATTCCTTCCTTTTTT
1420	Yes	AAACCAGCTTTGGGGGGTTCGATTCCTTCCTTTTTTG
1421	Yes	AAACCAGCTTTGGGGGGTTCGATTCCTTCCTTTTTTGC

1422	Yes	AAACCAGCTTTGGGGGGTTCGATTCCTTCCTTTTTTGCC
1423	Yes	AAACCAGCTTTGGGGGGTTCGATTCCTTCCTTTTTTGCCA
1424	No	AAACCAGGCAGAAACA
1425	No	AAACCAGGCAGAAACAC
1426	Yes	AAACCAGGCAGAAACACC
1427	Yes	AAACCAGGCAGAAACACCA
1428	No	AAACCAGGCGGAAACA
1429	No	AAACCAGGCGGAAACAC
1430	No	AAACCAGGCGGAAACACC
1431	No	AAACCAGGCGGAAACACCA
1432	Yes	AAACCAGGGGTCGCGA
1433	Yes	AAACCAGGGGTCGCGAG
1434	Yes	AAACCAGGGGTCGCGAGT
1435	Yes	AAACCAGGGGTCGCGAGTT
1436	Yes	AAACCAGGGGTCGCGAGTTC
1437	Yes	AAACCAGGGGTCGCGAGTTCA
1438	Yes	AAACCAGGGGTCGCGAGTTCAA
1439	Yes	AAACCAGGGGTCGCGAGTTCAAA
1440	Yes	AAACCAGGGGTCGCGAGTTCAAAT
1441	Yes	AAACCAGGGGTCGCGAGTTCAAATC
1442	Yes	AAACCAGGGGTCGCGAGTTCAAATCT
1443	Yes	AAACCAGGGGTCGCGAGTTCAAATCTC
1444	Yes	AAACCAGGGGTCGCGAGTTCAAATCTCG
1445	Yes	AAACCAGGGGTCGCGAGTTCAAATCTCGC
1446	Yes	AAACCAGGGGTCGCGAGTTCAAATCTCGCT
1447	Yes	AAACCAGGGGTCGCGAGTTCAAATCTCGCTG
1448	Yes	AAACCAGGGGTCGCGAGTTCAAATCTCGCTGG
1449	Yes	AAACCAGGGGTCGCGAGTTCAAATCTCGCTGGG
1450	Yes	AAACCAGGGGTCGCGAGTTCAAATCTCGCTGGGG
1451	Yes	AAACCAGGGGTCGCGAGTTCAAATCTCGCTGGGGC
1452	Yes	AAACCAGGGGTCGCGAGTTCAAATCTCGCTGGGGCC
1453	Yes	AAACCAGGGGTCGCGAGTTCAAATCTCGCTGGGGCCT
1454	Yes	AAACCAGGGGTCGCGAGTTCAAATCTCGCTGGGGCCTC
1455	Yes	AAACCAGGGGTCGCGAGTTCAAATCTCGCTGGGGCCTCC
1456	Yes	AAACCAGGGGTCGCGAGTTCAAATCTCGCTGGGGCCTCCA
1457	Yes	AAACCAGGGGTCGCGAGTTCAAT
1458	Yes	AAACCAGGGGTCGCGAGTTCAATT
1459	Yes	AAACCAGGGGTCGCGAGTTCAATTC
1460	Yes	AAACCAGGGGTCGCGAGTTCAATTCT
1461	Yes	AAACCAGGGGTCGCGAGTTCAATTCTC
1462	Yes	AAACCAGGGGTCGCGAGTTCAATTCTCG
1463	Yes	AAACCAGGGGTCGCGAGTTCAATTCTCGC

1464	Yes	AAACCAGGGGTCGCGAGTTCAATTCTCGCT
1465	Yes	AAACCAGGGGTCGCGAGTTCAATTCTCGCTG
1466	Yes	AAACCAGGGGTCGCGAGTTCAATTCTCGCTGG
1467	Yes	AAACCAGGGGTCGCGAGTTCAATTCTCGCTGGG
1468	Yes	AAACCAGGGGTCGCGAGTTCAATTCTCGCTGGGG
1469	Yes	AAACCAGGGGTCGCGAGTTCAATTCTCGCTGGGGC
1470	Yes	AAACCAGGGGTCGCGAGTTCAATTCTCGCTGGGGCC
1471	Yes	AAACCAGGGGTCGCGAGTTCAATTCTCGCTGGGGCCT
1472	Yes	AAACCAGGGGTCGCGAGTTCAATTCTCGCTGGGGCCTC
1473	Yes	AAACCAGGGGTCGCGAGTTCAATTCTCGCTGGGGCCTCC
1474	Yes	AAACCAGGGGTCGCGAGTTCAATTCTCGCTGGGGCCTCCA
1475	Yes	AAACCAGGGGTCGCGAGTTCG
1476	Yes	AAACCAGGGGTCGCGAGTTCGA
1477	Yes	AAACCAGGGGTCGCGAGTTCGAT
1478	Yes	AAACCAGGGGTCGCGAGTTCGATC
1479	Yes	AAACCAGGGGTCGCGAGTTCGATCC
1480	Yes	AAACCAGGGGTCGCGAGTTCGATCCT
1481	Yes	AAACCAGGGGTCGCGAGTTCGATCCTC
1482	Yes	AAACCAGGGGTCGCGAGTTCGATCCTCG
1483	Yes	AAACCAGGGGTCGCGAGTTCGATCCTCGC
1484	Yes	AAACCAGGGGTCGCGAGTTCGATCCTCGCT
1485	Yes	AAACCAGGGGTCGCGAGTTCGATCCTCGCTG
1486	Yes	AAACCAGGGGTCGCGAGTTCGATCCTCGCTGG
1487	Yes	AAACCAGGGGTCGCGAGTTCGATCCTCGCTGGG
1488	Yes	AAACCAGGGGTCGCGAGTTCGATCCTCGCTGGGG
1489	Yes	AAACCAGGGGTCGCGAGTTCGATCCTCGCTGGGGC
1490	Yes	AAACCAGGGGTCGCGAGTTCGATCCTCGCTGGGGCC
1491	Yes	AAACCAGGGGTCGCGAGTTCGATCCTCGCTGGGGCCT
1492	Yes	AAACCAGGGGTCGCGAGTTCGATCCTCGCTGGGGCCTC
1493	Yes	AAACCAGGGGTCGCGAGTTCGATCCTCGCTGGGGCCTCC
1494	Yes	AAACCAGGGGTCGCGAGTTCGATCCTCGCTGGGGCCTCCA
1495	Yes	AAACCAGGGTCGCGAG
1496	Yes	AAACCAGGGTCGCGAGT
1497	Yes	AAACCAGGGTCGCGAGTT
1498	Yes	AAACCAGGGTCGCGAGTTC
1499	Yes	AAACCAGGGTCGCGAGTTCA
1500	Yes	AAACCAGGGTCGCGAGTTCAA
1501	Yes	AAACCAGGGTCGCGAGTTCAAA
1502	Yes	AAACCAGGGTCGCGAGTTCAAAT
1503	Yes	AAACCAGGGTCGCGAGTTCAAATC
1504	Yes	AAACCAGGGTCGCGAGTTCAAATCT
1505	Yes	AAACCAGGGTCGCGAGTTCAAATCTC

1506	Yes	AAACCAGGGTCGCGAGTTCAAATCTCG
1507	Yes	AAACCAGGGTCGCGAGTTCAAATCTCGC
1508	Yes	AAACCAGGGTCGCGAGTTCAAATCTCGCT
1509	Yes	AAACCAGGGTCGCGAGTTCAAATCTCGCTG
1510	Yes	AAACCAGGGTCGCGAGTTCAAATCTCGCTGG
1511	Yes	AAACCAGGGTCGCGAGTTCAAATCTCGCTGGG
1512	Yes	AAACCAGGGTCGCGAGTTCAAATCTCGCTGGGG
1513	Yes	AAACCAGGGTCGCGAGTTCAAATCTCGCTGGGGC
1514	Yes	AAACCAGGGTCGCGAGTTCAAATCTCGCTGGGGCC
1515	Yes	AAACCAGGGTCGCGAGTTCAAATCTCGCTGGGGCCT
1516	Yes	AAACCAGGGTCGCGAGTTCAAATCTCGCTGGGGCCTC
1517	Yes	AAACCAGGGTCGCGAGTTCAAATCTCGCTGGGGCCTCC
1518	Yes	AAACCAGGGTCGCGAGTTCAAATCTCGCTGGGGCCTCCA
1519	No	AAACCAGGTGGAAACA
1520	No	AAACCAGGTGGAAACAC
1521	No	AAACCAGGTGGAAACACC
1522	Yes	AAACCAGGTGGAAACACCA
1523	No	AAACCAGTCTCTGGGG
1524	Yes	AAACCAGTCTCTGGGGG
1525	Yes	AAACCAGTCTCTGGGGGG
1526	Yes	AAACCAGTCTCTGGGGGGT
1527	Yes	AAACCAGTCTCTGGGGGGTT
1528	Yes	AAACCAGTCTCTGGGGGGTTC
1529	Yes	AAACCAGTCTCTGGGGGGTTCG
1530	Yes	AAACCAGTCTCTGGGGGGTTCGA
1531	Yes	AAACCAGTCTCTGGGGGGTTCGAT
1532	Yes	AAACCAGTCTCTGGGGGGTTCGATT
1533	Yes	AAACCAGTCTCTGGGGGGTTCGATTC
1534	Yes	AAACCAGTCTCTGGGGGGTTCGATTCC
1535	Yes	AAACCAGTCTCTGGGGGGTTCGATTCCC
1536	Yes	AAACCAGTCTCTGGGGGGTTCGATTCCCT
1537	Yes	AAACCAGTCTCTGGGGGGTTCGATTCCCTC
1538	Yes	AAACCAGTCTCTGGGGGGTTCGATTCCCTCC
1539	Yes	AAACCAGTCTCTGGGGGGTTCGATTCCCTCCT
1540	Yes	AAACCAGTCTCTGGGGGGTTCGATTCCCTCCTT
1541	Yes	AAACCAGTCTCTGGGGGGTTCGATTCCCTCCTTT
1542	Yes	AAACCAGTCTCTGGGGGGTTCGATTCCCTCCTTTT
1543	Yes	AAACCAGTCTCTGGGGGGTTCGATTCCCTCCTTTTT
1544	Yes	AAACCAGTCTCTGGGGGGTTCGATTCCCTCCTTTTTC
1545	Yes	AAACCAGTCTCTGGGGGGTTCGATTCCCTCCTTTTTCA
1546	Yes	AAACCAGTCTCTGGGGGGTTCGATTCCCTCCTTTTTCAC
1547	Yes	AAACCAGTCTCTGGGGGGTTCGATTCCCTCCTTTTTCACC

1548	Yes	AAACCAGTCTCTGGGGGGTTCGATTCCCTCCTTTTTCACCA
1549	Yes	AAACCAGTCTGTGGGG
1550	Yes	AAACCAGTCTGTGGGGG
1551	Yes	AAACCAGTCTGTGGGGGG
1552	Yes	AAACCAGTCTGTGGGGGGT
1553	Yes	AAACCAGTCTGTGGGGGGTT
1554	Yes	AAACCAGTCTGTGGGGGGTTC
1555	Yes	AAACCAGTCTGTGGGGGGTTCG
1556	Yes	AAACCAGTCTGTGGGGGGTTCGA
1557	Yes	AAACCAGTCTGTGGGGGGTTCGAT
1558	Yes	AAACCAGTCTGTGGGGGGTTCGATT
1559	Yes	AAACCAGTCTGTGGGGGGTTCGATTC
1560	Yes	AAACCAGTCTGTGGGGGGTTCGATTCC
1561	Yes	AAACCAGTCTGTGGGGGGTTCGATTCCC
1562	Yes	AAACCAGTCTGTGGGGGGTTCGATTCCCT
1563	Yes	AAACCAGTCTGTGGGGGGTTCGATTCCCTC
1564	Yes	AAACCAGTCTGTGGGGGGTTCGATTCCCTCC
1565	Yes	AAACCAGTCTGTGGGGGGTTCGATTCCCTCCT
1566	Yes	AAACCAGTCTGTGGGGGGTTCGATTCCCTCCTT
1567	Yes	AAACCAGTCTGTGGGGGGTTCGATTCCCTCCTTT
1568	Yes	AAACCAGTCTGTGGGGGGTTCGATTCCCTCCTTTT
1569	Yes	AAACCAGTCTGTGGGGGGTTCGATTCCCTCCTTTTT
1570	Yes	AAACCAGTCTGTGGGGGGTTCGATTCCCTCCTTTTTC
1571	Yes	AAACCAGTCTGTGGGGGGTTCGATTCCCTCCTTTTTCA
1572	Yes	AAACCAGTCTGTGGGGGGTTCGATTCCCTCCTTTTTCAC
1573	Yes	AAACCAGTCTGTGGGGGGTTCGATTCCCTCCTTTTTCACC
1574	Yes	AAACCAGTCTGTGGGGGGTTCGATTCCCTCCTTTTTCACCA
1575	No	AAACCATCCTCTGCTA
1576	No	AAACCATCCTCTGCTAC
1577	Yes	AAACCATCCTCTGCTACC
1578	Yes	AAACCATCCTCTGCTACCA
1579	No	AAACCATGGATTCTAG
1580	No	AAACCATGGATTCTAGA
1581	No	AAACCATGGATTCTAGAG
1582	No	AAACCATGGATTCTAGAGC
1583	No	AAACCATGGATTCTAGAGCC
1584	Yes	AAACCATGGATTCTAGAGCCA
1585	Yes	AAACCATGGATTCTAGAGCCAG
1586	Yes	AAACCATGGATTCTAGAGCCAGA
1587	Yes	AAACCATGGATTCTAGAGCCAGAT
1588	Yes	AAACCATGGATTCTAGAGCCAGATG
1589	Yes	AAACCATGGATTCTAGAGCCAGATGG

1590	Yes	AAACCATGGATTCTAGAGCCAGATGGG
1591	Yes	AAACCATGGATTCTAGAGCCAGATGGGT
1592	Yes	AAACCATGGATTCTAGAGCCAGATGGGTT
1593	Yes	AAACCATGGATTCTAGAGCCAGATGGGTTC
1594	Yes	AAACCATGGATTCTAGAGCCAGATGGGTTCA
1595	Yes	AAACCATGGATTCTAGAGCCAGATGGGTTCAA
1596	Yes	AAACCATGGATTCTAGAGCCAGATGGGTTCAAA
1597	Yes	AAACCATGGATTCTAGAGCCAGATGGGTTCAAAT
1598	Yes	AAACCATGGATTCTAGAGCCAGATGGGTTCAAATC
1599	Yes	AAACCATGGATTCTAGAGCCAGATGGGTTCAAATCC
1600	Yes	AAACCATGGATTCTAGAGCCAGATGGGTTCAAATCCC
1601	Yes	AAACCATGGATTCTAGAGCCAGATGGGTTCAAATCCCG
1602	Yes	AAACCATGGATTCTAGAGCCAGATGGGTTCAAATCCCGG
1603	Yes	AAACCATGGATTCTAGAGCCAGATGGGTTCAAATCCCGGC
1604	Yes	AAACCATGGATTCTAGAGCCAGATGGGTTCAAATCCCGGCT
1605	Yes	AAACCATGGATTCTAGAGCCAGATGGGTTCAAATCCCGGCTC
1606	Yes	AAACCATGGATTCTAGAGCCAGATGGGTTCAAATCCCGGCTCT
1607	Yes	AAACCATGGATTCTAGAGCCAGATGGGTTCAAATCCCGGCTCTG
1608	Yes	AAACCATGGATTCTAGAGCCAGATGGGTTCAAATCCCGGCTCTGC
1609	Yes	AAACCATGGATTCTAGAGCCAGATGGGTTCAAATCCCGGCTCTGCC
1610	Yes	AAACCATGGATTCTAGAGCCAGATGGGTTCAAATCCCGGCTCTGCCG
1611	Yes	AAACCATGGATTCTAGAGCCAGATGGGTTCAAATCCCGGCTCTGCCGC
1612	Yes	AAACCATGGATTCTAGAGCCAGATGGGTTCAAATCCCGGCTCTGCCGCC
1613	Yes	AAACCATGGATTCTAGAGCCAGATGGGTTCAAATCCCGGCTCTGCCGCCA
1614	No	AAACCATGGTCTTTGG
1615	No	AAACCATGGTCTTTGGA
1616	Yes	AAACCATGGTCTTTGGAG
1617	Yes	AAACCATGGTCTTTGGAGC
1618	Yes	AAACCATGGTCTTTGGAGCC
1619	Yes	AAACCATGGTCTTTGGAGCCA
1620	Yes	AAACCATGGTCTTTGGAGCCAG
1621	Yes	AAACCATGGTCTTTGGAGCCAGA
1622	Yes	AAACCATGGTCTTTGGAGCCAGAC
1623	Yes	AAACCATGGTCTTTGGAGCCAGACT
1624	Yes	AAACCATGGTCTTTGGAGCCAGACTG
1625	Yes	AAACCATGGTCTTTGGAGCCAGACTGC
1626	Yes	AAACCATGGTCTTTGGAGCCAGACTGCC
1627	Yes	AAACCATGGTCTTTGGAGCCAGACTGCCT
1628	Yes	AAACCATGGTCTTTGGAGCCAGACTGCCTG
1629	Yes	AAACCATGGTCTTTGGAGCCAGACTGCCTGG
1630	Yes	AAACCATGGTCTTTGGAGCCAGACTGCCTGGG
1631	Yes	AAACCATGGTCTTTGGAGCCAGACTGCCTGGGG

1632	Yes	AAACCATGGTCTTTGGAGCCAGACTGCCTGGGGT
1633	Yes	AAACCATGGTCTTTGGAGCCAGACTGCCTGGGGTC
1634	Yes	AAACCATGGTCTTTGGAGCCAGACTGCCTGGGGTCG
1635	Yes	AAACCATGGTCTTTGGAGCCAGACTGCCTGGGGTCGG
1636	Yes	AAACCATGGTCTTTGGAGCCAGACTGCCTGGGGTCGGA
1637	Yes	AAACCATGGTCTTTGGAGCCAGACTGCCTGGGGTCGGAT
1638	Yes	AAACCATGGTCTTTGGAGCCAGACTGCCTGGGGTCGGATC
1639	Yes	AAACCATGGTCTTTGGAGCCAGACTGCCTGGGGTCGGATCC
1640	Yes	AAACCATGGTCTTTGGAGCCAGACTGCCTGGGGTCGGATCCC
1641	Yes	AAACCATGGTCTTTGGAGCCAGACTGCCTGGGGTCGGATCCCA
1642	Yes	AAACCATGGTCTTTGGAGCCAGACTGCCTGGGGTCGGATCCCAG
1643	Yes	AAACCATGGTCTTTGGAGCCAGACTGCCTGGGGTCGGATCCCAGC
1644	Yes	AAACCATGGTCTTTGGAGCCAGACTGCCTGGGGTCGGATCCCAGCT
1645	Yes	AAACCATGGTCTTTGGAGCCAGACTGCCTGGGGTCGGATCCCAGCTC
1646	Yes	AAACCATGGTCTTTGGAGCCAGACTGCCTGGGGTCGGATCCCAGCTCT
1647	Yes	AAACCATGGTCTTTGGAGCCAGACTGCCTGGGGTCGGATCCCAGCTCTC
1648	Yes	AAACCATGGTCTTTGGAGCCAGACTGCCTGGGGTCGGATCCCAGCTCTCA
1649	No	AAACCATTTCATAACT
1650	No	AAACCATTTCATAACTT
1651	No	AAACCATTTCATAACTTT
1652	No	AAACCATTTCATAACTTTG
1653	No	AAACCATTTCATAACTTTGT
1654	No	AAACCATTTCATAACTTTGTC
1655	No	AAACCATTTCATAACTTTGTCA
1656	No	AAACCATTTCATAACTTTGTCAA
1657	No	AAACCATTTCATAACTTTGTCAAA
1658	No	AAACCATTTCATAACTTTGTCAAAG
1659	No	AAACCATTTCATAACTTTGTCAAAGT
1660	No	AAACCATTTCATAACTTTGTCAAAGTT
1661	No	AAACCATTTCATAACTTTGTCAAAGTTA
1662	No	AAACCATTTCATAACTTTGTCAAAGTTAA
1663	No	AAACCATTTCATAACTTTGTCAAAGTTAAA
1664	No	AAACCATTTCATAACTTTGTCAAAGTTAAAT
1665	No	AAACCATTTCATAACTTTGTCAAAGTTAAATT
1666	No	AAACCATTTCATAACTTTGTCAAAGTTAAATTA
1667	No	AAACCATTTCATAACTTTGTCAAAGTTAAATTAT
1668	No	AAACCATTTCATAACTTTGTCAAAGTTAAATTATA
1669	No	AAACCATTTCATAACTTTGTCAAAGTTAAATTATAG
1670	No	AAACCATTTCATAACTTTGTCAAAGTTAAATTATAGG
1671	No	AAACCATTTCATAACTTTGTCAAAGTTAAATTATAGGC
1672	No	AAACCATTTCATAACTTTGTCAAAGTTAAATTATAGGCT
1673	No	AAACCATTTCATAACTTTGTCAAAGTTAAATTATAGGCTA

1674	No	AAACCATTTCATAACTTTGTCAAAGTTAAATTATAGGCTAA
1675	No	AAACCATTTCATAACTTTGTCAAAGTTAAATTATAGGCTAAA
1676	No	AAACCATTTCATAACTTTGTCAAAGTTAAATTATAGGCTAAAT
1677	No	AAACCATTTCATAACTTTGTCAAAGTTAAATTATAGGCTAAATC
1678	No	AAACCATTTCATAACTTTGTCAAAGTTAAATTATAGGCTAAATCC
1679	No	AAACCATTTCATAACTTTGTCAAAGTTAAATTATAGGCTAAATCCT
1680	No	AAACCATTTCATAACTTTGTCAAAGTTAAATTATAGGCTAAATCCTA
1681	No	AAACCATTTCATAACTTTGTCAAAGTTAAATTATAGGCTAAATCCTAT
1682	No	AAACCATTTCATAACTTTGTCAAAGTTAAATTATAGGCTAAATCCTATA
1683	No	AAACCATTTCATAACTTTGTCAAAGTTAAATTATAGGCTAAATCCTATAT
1684	No	AAACCCAGACTGGATG
1685	Yes	AAACCCAGACTGGATGG
1686	Yes	AAACCCAGACTGGATGGG
1687	Yes	AAACCCAGACTGGATGGGT
1688	Yes	AAACCCAGACTGGATGGGTT
1689	Yes	AAACCCAGACTGGATGGGTTC
1690	Yes	AAACCCAGACTGGATGGGTTCA
1691	Yes	AAACCCAGACTGGATGGGTTCAA
1692	Yes	AAACCCAGACTGGATGGGTTCAAT
1693	Yes	AAACCCAGACTGGATGGGTTCAATT
1694	Yes	AAACCCAGACTGGATGGGTTCAATTC
1695	Yes	AAACCCAGACTGGATGGGTTCAATTCC
1696	Yes	AAACCCAGACTGGATGGGTTCAATTCCC
1697	Yes	AAACCCAGACTGGATGGGTTCAATTCCCA
1698	Yes	AAACCCAGACTGGATGGGTTCAATTCCCAT
1699	Yes	AAACCCAGACTGGATGGGTTCAATTCCCATC
1700	Yes	AAACCCAGACTGGATGGGTTCAATTCCCATCT
1701	Yes	AAACCCAGACTGGATGGGTTCAATTCCCATCTC
1702	Yes	AAACCCAGACTGGATGGGTTCAATTCCCATCTCT
1703	Yes	AAACCCAGACTGGATGGGTTCAATTCCCATCTCTG
1704	Yes	AAACCCAGACTGGATGGGTTCAATTCCCATCTCTGC
1705	Yes	AAACCCAGACTGGATGGGTTCAATTCCCATCTCTGCC
1706	Yes	AAACCCAGACTGGATGGGTTCAATTCCCATCTCTGCCG
1707	Yes	AAACCCAGACTGGATGGGTTCAATTCCCATCTCTGCCGC
1708	Yes	AAACCCAGACTGGATGGGTTCAATTCCCATCTCTGCCGCC
1709	Yes	AAACCCAGACTGGATGGGTTCAATTCCCATCTCTGCCGCCA
1710	Yes	AAACCCATTGGGGTCT
1711	Yes	AAACCCATTGGGGTCTC
1712	Yes	AAACCCATTGGGGTCTCC
1713	Yes	AAACCCATTGGGGTCTCCC
1714	Yes	AAACCCATTGGGGTCTCCCC
1715	Yes	AAACCCATTGGGGTCTCCCCG

1716	Yes	AAACCCATTGGGGTCTCCCCGC
1717	Yes	AAACCCATTGGGGTCTCCCCGCG
1718	Yes	AAACCCATTGGGGTCTCCCCGCGC
1719	Yes	AAACCCATTGGGGTCTCCCCGCGCA
1720	Yes	AAACCCATTGGGGTCTCCCCGCGCAG
1721	Yes	AAACCCATTGGGGTCTCCCCGCGCAGG
1722	Yes	AAACCCATTGGGGTCTCCCCGCGCAGGT
1723	Yes	AAACCCATTGGGGTCTCCCCGCGCAGGTT
1724	Yes	AAACCCATTGGGGTCTCCCCGCGCAGGTTC
1725	Yes	AAACCCATTGGGGTCTCCCCGCGCAGGTTCG
1726	Yes	AAACCCATTGGGGTCTCCCCGCGCAGGTTCGA
1727	Yes	AAACCCATTGGGGTCTCCCCGCGCAGGTTCGAA
1728	Yes	AAACCCATTGGGGTCTCCCCGCGCAGGTTCGAAT
1729	Yes	AAACCCATTGGGGTCTCCCCGCGCAGGTTCGAATC
1730	Yes	AAACCCATTGGGGTCTCCCCGCGCAGGTTCGAATCC
1731	Yes	AAACCCATTGGGGTCTCCCCGCGCAGGTTCGAATCCT
1732	Yes	AAACCCATTGGGGTCTCCCCGCGCAGGTTCGAATCCTG
1733	Yes	AAACCCATTGGGGTCTCCCCGCGCAGGTTCGAATCCTGC
1734	Yes	AAACCCATTGGGGTCTCCCCGCGCAGGTTCGAATCCTGCC
1735	Yes	AAACCCATTGGGGTCTCCCCGCGCAGGTTCGAATCCTGCCG
1736	Yes	AAACCCATTGGGGTCTCCCCGCGCAGGTTCGAATCCTGCCGA
1737	Yes	AAACCCATTGGGGTCTCCCCGCGCAGGTTCGAATCCTGCCGAC
1738	Yes	AAACCCATTGGGGTCTCCCCGCGCAGGTTCGAATCCTGCCGACT
1739	Yes	AAACCCATTGGGGTCTCCCCGCGCAGGTTCGAATCCTGCCGACTA
1740	Yes	AAACCCATTGGGGTCTCCCCGCGCAGGTTCGAATCCTGCCGACTAC
1741	Yes	AAACCCATTGGGGTCTCCCCGCGCAGGTTCGAATCCTGCCGACTACG
1742	Yes	AAACCCATTGGGGTCTCCCCGCGCAGGTTCGAATCCTGCCGACTACGC
1743	Yes	AAACCCATTGGGGTCTCCCCGCGCAGGTTCGAATCCTGCCGACTACGCC
1744	Yes	AAACCCATTGGGGTCTCCCCGCGCAGGTTCGAATCCTGCCGACTACGCCA
1745	Yes	AAACCCCACTACTGGT
1746	Yes	AAACCCCACTACTGGTA
1747	Yes	AAACCCCACTACTGGTAC
1748	Yes	AAACCCCACTACTGGTACC
1749	Yes	AAACCCCACTACTGGTACCA
1750	Yes	AAACCCCAGCTGCGCC
1751	Yes	AAACCCCAGCTGCGCCC
1752	Yes	AAACCCCAGCTGCGCCCC
1753	Yes	AAACCCCAGCTGCGCCCCC
1754	Yes	AAACCCCAGCTGCGCCCCCA
1755	No	AAACCCCCTTATTTCT
1756	No	AAACCCCCTTATTTCTA
1757	Yes	AAACCCCCTTATTTCTAC

1758	Yes	AAACCCCCTTATTTCTACC
1759	Yes	AAACCCCCTTATTTCTACCA
1760	Yes	AAACCCTAGCCCTGGC
1761	Yes	AAACCCTAGCCCTGGCA
1762	Yes	AAACCCTAGCCCTGGCAC
1763	Yes	AAACCCTAGCCCTGGCACC
1764	Yes	AAACCCTAGCCCTGGCACCA
1765	Yes	AAACCCTGGCGCTGTC
1766	Yes	AAACCCTGGCGCTGTCA
1767	Yes	AAACCCTGGCGCTGTCAC
1768	Yes	AAACCCTGGCGCTGTCACC
1769	Yes	AAACCCTGGCGCTGTCACCA
1770	Yes	AAACCGAAGATCACGG
1771	Yes	AAACCGAAGATCACGGG
1772	Yes	AAACCGAAGATCACGGGT
1773	Yes	AAACCGAAGATCACGGGTT
1774	Yes	AAACCGAAGATCACGGGTTC
1775	Yes	AAACCGAAGATCACGGGTTCG
1776	Yes	AAACCGAAGATCACGGGTTCGA
1777	Yes	AAACCGAAGATCACGGGTTCGAA
1778	Yes	AAACCGAAGATCACGGGTTCGAAC
1779	Yes	AAACCGAAGATCACGGGTTCGAACC
1780	Yes	AAACCGAAGATCACGGGTTCGAACCC
1781	Yes	AAACCGAAGATCACGGGTTCGAACCCC
1782	Yes	AAACCGAAGATCACGGGTTCGAACCCCG
1783	Yes	AAACCGAAGATCACGGGTTCGAACCCCGT
1784	Yes	AAACCGAAGATCACGGGTTCGAACCCCGTC
1785	Yes	AAACCGAAGATCACGGGTTCGAACCCCGTCC
1786	Yes	AAACCGAAGATCACGGGTTCGAACCCCGTCCG
1787	Yes	AAACCGAAGATCACGGGTTCGAACCCCGTCCGT
1788	Yes	AAACCGAAGATCACGGGTTCGAACCCCGTCCGTG
1789	Yes	AAACCGAAGATCACGGGTTCGAACCCCGTCCGTGC
1790	Yes	AAACCGAAGATCACGGGTTCGAACCCCGTCCGTGCC
1791	Yes	AAACCGAAGATCACGGGTTCGAACCCCGTCCGTGCCT
1792	Yes	AAACCGAAGATCACGGGTTCGAACCCCGTCCGTGCCTC
1793	Yes	AAACCGAAGATCACGGGTTCGAACCCCGTCCGTGCCTCC
1794	Yes	AAACCGAAGATCACGGGTTCGAACCCCGTCCGTGCCTCCA
1795	Yes	AAACCGAAGATCGCGG
1796	Yes	AAACCGAAGATCGCGGG
1797	Yes	AAACCGAAGATCGCGGGT
1798	Yes	AAACCGAAGATCGCGGGTT
1799	Yes	AAACCGAAGATCGCGGGTTC

1800	Yes	AAACCGAAGATCGCGGGTTCG
1801	Yes	AAACCGAAGATCGCGGGTTCGA
1802	Yes	AAACCGAAGATCGCGGGTTCGAA
1803	Yes	AAACCGAAGATCGCGGGTTCGAAC
1804	Yes	AAACCGAAGATCGCGGGTTCGAACC
1805	Yes	AAACCGAAGATCGCGGGTTCGAACCC
1806	Yes	AAACCGAAGATCGCGGGTTCGAACCCC
1807	Yes	AAACCGAAGATCGCGGGTTCGAACCCCG
1808	Yes	AAACCGAAGATCGCGGGTTCGAACCCCGT
1809	Yes	AAACCGAAGATCGCGGGTTCGAACCCCGTC
1810	Yes	AAACCGAAGATCGCGGGTTCGAACCCCGTCC
1811	Yes	AAACCGAAGATCGCGGGTTCGAACCCCGTCCG
1812	Yes	AAACCGAAGATCGCGGGTTCGAACCCCGTCCGT
1813	Yes	AAACCGAAGATCGCGGGTTCGAACCCCGTCCGTG
1814	Yes	AAACCGAAGATCGCGGGTTCGAACCCCGTCCGTGC
1815	Yes	AAACCGAAGATCGCGGGTTCGAACCCCGTCCGTGCC
1816	Yes	AAACCGAAGATCGCGGGTTCGAACCCCGTCCGTGCCT
1817	Yes	AAACCGAAGATCGCGGGTTCGAACCCCGTCCGTGCCTC
1818	Yes	AAACCGAAGATCGCGGGTTCGAACCCCGTCCGTGCCTCC
1819	Yes	AAACCGAAGATCGCGGGTTCGAACCCCGTCCGTGCCTCCA
1820	No	AAACCGAATAGCTTAG
1821	Yes	AAACCGAATAGCTTAGT
1822	Yes	AAACCGAATAGCTTAGTT
1823	Yes	AAACCGAATAGCTTAGTTG
1824	Yes	AAACCGAATAGCTTAGTTGA
1825	Yes	AAACCGAATAGCTTAGTTGAT
1826	Yes	AAACCGAATAGCTTAGTTGATG
1827	Yes	AAACCGAATAGCTTAGTTGATGA
1828	Yes	AAACCGAATAGCTTAGTTGATGAA
1829	Yes	AAACCGAATAGCTTAGTTGATGAAG
1830	Yes	AAACCGAATAGCTTAGTTGATGAAGC
1831	Yes	AAACCGAATAGCTTAGTTGATGAAGCG
1832	Yes	AAACCGAATAGCTTAGTTGATGAAGCGT
1833	Yes	AAACCGAATAGCTTAGTTGATGAAGCGTG
1834	Yes	AAACCGAATAGCTTAGTTGATGAAGCGTGA
1835	Yes	AAACCGAATAGCTTAGTTGATGAAGCGTGAG
1836	Yes	AAACCGAATAGCTTAGTTGATGAAGCGTGAGA
1837	Yes	AAACCGAATAGCTTAGTTGATGAAGCGTGAGAC
1838	Yes	AAACCGAATAGCTTAGTTGATGAAGCGTGAGACT
1839	Yes	AAACCGAATAGCTTAGTTGATGAAGCGTGAGACTC
1840	Yes	AAACCGAATAGCTTAGTTGATGAAGCGTGAGACTCT
1841	Yes	AAACCGAATAGCTTAGTTGATGAAGCGTGAGACTCTT

1842	Yes	AAACCGAATAGCTTAGTTGATGAAGCGTGAGACTCTTA
1843	Yes	AAACCGAATAGCTTAGTTGATGAAGCGTGAGACTCTTAA
1844	Yes	AAACCGAATAGCTTAGTTGATGAAGCGTGAGACTCTTAAT
1845	Yes	AAACCGAATAGCTTAGTTGATGAAGCGTGAGACTCTTAATC
1846	Yes	AAACCGAATAGCTTAGTTGATGAAGCGTGAGACTCTTAATCT
1847	Yes	AAACCGAATAGCTTAGTTGATGAAGCGTGAGACTCTTAATCTC
1848	Yes	AAACCGAATAGCTTAGTTGATGAAGCGTGAGACTCTTAATCTCA
1849	Yes	AAACCGAATAGCTTAGTTGATGAAGCGTGAGACTCTTAATCTCAG
1850	Yes	AAACCGAATAGCTTAGTTGATGAAGCGTGAGACTCTTAATCTCAGG
1851	Yes	AAACCGAATAGCTTAGTTGATGAAGCGTGAGACTCTTAATCTCAGGG
1852	Yes	AAACCGAATAGCTTAGTTGATGAAGCGTGAGACTCTTAATCTCAGGGT
1853	Yes	AAACCGAATAGCTTAGTTGATGAAGCGTGAGACTCTTAATCTCAGGGTA
1854	Yes	AAACCGAATAGCTTAGTTGATGAAGCGTGAGACTCTTAATCTCAGGGTAG
1855	Yes	AAACCGAGCGGAAACA
1856	Yes	AAACCGAGCGGAAACAC
1857	Yes	AAACCGAGCGGAAACACC
1858	Yes	AAACCGAGCGGAAACACCA
1859	No	AAACCGGAGATGAAAA
1860	Yes	AAACCGGAGATGAAAAC
1861	Yes	AAACCGGAGATGAAAACC
1862	Yes	AAACCGGAGATGAAAACCT
1863	Yes	AAACCGGAGATGAAAACCTT
1864	Yes	AAACCGGAGATGAAAACCTTT
1865	Yes	AAACCGGAGATGAAAACCTTTT
1866	Yes	AAACCGGAGATGAAAACCTTTTT
1867	Yes	AAACCGGAGATGAAAACCTTTTTC
1868	Yes	AAACCGGAGATGAAAACCTTTTTCC
1869	Yes	AAACCGGAGATGAAAACCTTTTTCCA
1870	Yes	AAACCGGAGATGAAAACCTTTTTCCAA
1871	Yes	AAACCGGAGATGAAAACCTTTTTCCAAG
1872	Yes	AAACCGGAGATGAAAACCTTTTTCCAAGG
1873	Yes	AAACCGGAGATGAAAACCTTTTTCCAAGGA
1874	Yes	AAACCGGAGATGAAAACCTTTTTCCAAGGAC
1875	Yes	AAACCGGAGATGAAAACCTTTTTCCAAGGACA
1876	Yes	AAACCGGAGATGAAAACCTTTTTCCAAGGACAC
1877	Yes	AAACCGGAGATGAAAACCTTTTTCCAAGGACACC
1878	Yes	AAACCGGAGATGAAAACCTTTTTCCAAGGACACCA
1879	No	AAACCGGGCAGAAACA
1880	Yes	AAACCGGGCAGAAACAC
1881	Yes	AAACCGGGCAGAAACACC
1882	Yes	AAACCGGGCAGAAACACCA
1883	No	AAACCGGGCAGAAGCA

1884	No	AAACCGGGCAGAAGCAC
1885	Yes	AAACCGGGCAGAAGCACC
1886	Yes	AAACCGGGCAGAAGCACCA
1887	No	AAACCGGGCGGAAACA
1888	Yes	AAACCGGGCGGAAACAC
1889	Yes	AAACCGGGCGGAAACACC
1890	Yes	AAACCGGGCGGAAACACCA
1891	Yes	AAACCTAGGGGTCGTG
1892	Yes	AAACCTAGGGGTCGTGA
1893	Yes	AAACCTAGGGGTCGTGAG
1894	Yes	AAACCTAGGGGTCGTGAGT
1895	Yes	AAACCTAGGGGTCGTGAGTT
1896	Yes	AAACCTAGGGGTCGTGAGTTC
1897	Yes	AAACCTAGGGGTCGTGAGTTCA
1898	Yes	AAACCTAGGGGTCGTGAGTTCAA
1899	Yes	AAACCTAGGGGTCGTGAGTTCAAA
1900	Yes	AAACCTAGGGGTCGTGAGTTCAAAT
1901	Yes	AAACCTAGGGGTCGTGAGTTCAAATC
1902	Yes	AAACCTAGGGGTCGTGAGTTCAAATCT
1903	Yes	AAACCTAGGGGTCGTGAGTTCAAATCTC
1904	Yes	AAACCTAGGGGTCGTGAGTTCAAATCTCA
1905	Yes	AAACCTAGGGGTCGTGAGTTCAAATCTCAC
1906	Yes	AAACCTAGGGGTCGTGAGTTCAAATCTCACC
1907	Yes	AAACCTAGGGGTCGTGAGTTCAAATCTCACCA
1908	Yes	AAACCTAGGGGTCGTGAGTTCAAATCTCACCAG
1909	Yes	AAACCTAGGGGTCGTGAGTTCAAATCTCACCAGG
1910	Yes	AAACCTAGGGGTCGTGAGTTCAAATCTCACCAGGG
1911	Yes	AAACCTAGGGGTCGTGAGTTCAAATCTCACCAGGGC
1912	Yes	AAACCTAGGGGTCGTGAGTTCAAATCTCACCAGGGCC
1913	Yes	AAACCTAGGGGTCGTGAGTTCAAATCTCACCAGGGCCT
1914	Yes	AAACCTAGGGGTCGTGAGTTCAAATCTCACCAGGGCCTC
1915	Yes	AAACCTAGGGGTCGTGAGTTCAAATCTCACCAGGGCCTCC
1916	Yes	AAACCTAGGGGTCGTGAGTTCAAATCTCACCAGGGCCTCCA
1917	No	AAACCTGCCGGGGCTT
1918	No	AAACCTGCCGGGGCTTC
1919	Yes	AAACCTGCCGGGGCTTCC
1920	Yes	AAACCTGCCGGGGCTTCCA
1921	No	AAACCTTTTTCCAAGG
1922	No	AAACCTTTTTCCAAGGA
1923	Yes	AAACCTTTTTCCAAGGAC
1924	Yes	AAACCTTTTTCCAAGGACA
1925	Yes	AAACCTTTTTCCAAGGACAC

1926	Yes	AAACCTTTTTCCAAGGACACC
1927	Yes	AAACCTTTTTCCAAGGACACCA
1928	Yes	AAACGAAAGGTTGGTG
1929	Yes	AAACGAAAGGTTGGTGG
1930	Yes	AAACGAAAGGTTGGTGGT
1931	Yes	AAACGAAAGGTTGGTGGTT
1932	Yes	AAACGAAAGGTTGGTGGTTC
1933	Yes	AAACGAAAGGTTGGTGGTTCG
1934	Yes	AAACGAAAGGTTGGTGGTTCGT
1935	Yes	AAACGAAAGGTTGGTGGTTCGTT
1936	Yes	AAACGAAAGGTTGGTGGTTCGTTC
1937	Yes	AAACGAAAGGTTGGTGGTTCGTTCC
1938	Yes	AAACGAAAGGTTGGTGGTTCGTTCCC
1939	Yes	AAACGAAAGGTTGGTGGTTCGTTCCCA
1940	Yes	AAACGAAAGGTTGGTGGTTCGTTCCCAC
1941	Yes	AAACGAAAGGTTGGTGGTTCGTTCCCACC
1942	Yes	AAACGAAAGGTTGGTGGTTCGTTCCCACCC
1943	Yes	AAACGAAAGGTTGGTGGTTCGTTCCCACCCC
1944	Yes	AAACGAAAGGTTGGTGGTTCGTTCCCACCCCG
1945	Yes	AAACGAAAGGTTGGTGGTTCGTTCCCACCCCGG
1946	Yes	AAACGAAAGGTTGGTGGTTCGTTCCCACCCCGGG
1947	Yes	AAACGAAAGGTTGGTGGTTCGTTCCCACCCCGGGA
1948	Yes	AAACGAAAGGTTGGTGGTTCGTTCCCACCCCGGGAC
1949	Yes	AAACGAAAGGTTGGTGGTTCGTTCCCACCCCGGGACA
1950	Yes	AAACGAAAGGTTGGTGGTTCGTTCCCACCCCGGGACAC
1951	Yes	AAACGAAAGGTTGGTGGTTCGTTCCCACCCCGGGACACC
1952	Yes	AAACGAAAGGTTGGTGGTTCGTTCCCACCCCGGGACACCA
1953	No	AAACGAATGATTTCGA
1954	No	AAACGAATGATTTCGAC
1955	No	AAACGAATGATTTCGACT
1956	No	AAACGAATGATTTCGACTC
1957	No	AAACGAATGATTTCGACTCA
1958	No	AAACGAATGATTTCGACTCAT
1959	No	AAACGAATGATTTCGACTCATT
1960	No	AAACGAATGATTTCGACTCATTA
1961	No	AAACGAATGATTTCGACTCATTAA
1962	No	AAACGAATGATTTCGACTCATTAAA
1963	No	AAACGAATGATTTCGACTCATTAAAT
1964	No	AAACGAATGATTTCGACTCATTAAATT
1965	No	AAACGAATGATTTCGACTCATTAAATTA
1966	No	AAACGAATGATTTCGACTCATTAAATTAT
1967	No	AAACGAATGATTTCGACTCATTAAATTATG

1968	No	AAACGAATGATTTCGACTCATTAAATTATGA
1969	No	AAACGAATGATTTCGACTCATTAAATTATGAT
1970	No	AAACGAATGATTTCGACTCATTAAATTATGATA
1971	No	AAACGAATGATTTCGACTCATTAAATTATGATAA
1972	No	AAACGAATGATTTCGACTCATTAAATTATGATAAT
1973	No	AAACGAATGATTTCGACTCATTAAATTATGATAATC
1974	No	AAACGAATGATTTCGACTCATTAAATTATGATAATCA
1975	No	AAACGAATGATTTCGACTCATTAAATTATGATAATCAT
1976	No	AAACGAATGATTTCGACTCATTAAATTATGATAATCATA
1977	No	AAACGAATGATTTCGACTCATTAAATTATGATAATCATAT
1978	No	AAACGAATGATTTCGACTCATTAAATTATGATAATCATATT
1979	No	AAACGAATGATTTCGACTCATTAAATTATGATAATCATATTT
1980	No	AAACGAATGATTTCGACTCATTAAATTATGATAATCATATTTA
1981	No	AAACGAATGATTTCGACTCATTAAATTATGATAATCATATTTAC
1982	No	AAACGAATGATTTCGACTCATTAAATTATGATAATCATATTTACC
1983	No	AAACGAATGATTTCGACTCATTAAATTATGATAATCATATTTACCA
1984	No	AAACGAATGATTTCGACTCATTAAATTATGATAATCATATTTACCAA
1985	Yes	AAACGAATGATTTCGACTCATTAAATTATGATAATCATATTTACCAAC
1986	Yes	AAACGAATGATTTCGACTCATTAAATTATGATAATCATATTTACCAACC
1987	Yes	AAACGAATGATTTCGACTCATTAAATTATGATAATCATATTTACCAACCA
1988	Yes	AAACGCTTGGGCTCTG
1989	Yes	AAACGCTTGGGCTCTGA
1990	Yes	AAACGCTTGGGCTCTGAC
1991	Yes	AAACGCTTGGGCTCTGACA
1992	Yes	AAACGCTTGGGCTCTGACAT
1993	Yes	AAACGCTTGGGCTCTGACATT
1994	Yes	AAACGCTTGGGCTCTGACATTA
1995	Yes	AAACGCTTGGGCTCTGACATTAA
1996	Yes	AAACGCTTGGGCTCTGACATTAAC
1997	Yes	AAACGCTTGGGCTCTGACATTAACG
1998	Yes	AAACGCTTGGGCTCTGACATTAACGT
1999	Yes	AAACGCTTGGGCTCTGACATTAACGTC
2000	Yes	AAACGCTTGGGCTCTGACATTAACGTCC
2001	Yes	AAACGCTTGGGCTCTGACATTAACGTCCT
2002	Yes	AAACGCTTGGGCTCTGACATTAACGTCCTG
2003	Yes	AAACGCTTGGGCTCTGACATTAACGTCCTGG
2004	Yes	AAACGCTTGGGCTCTGACATTAACGTCCTGGG
2005	Yes	AAACGCTTGGGCTCTGACATTAACGTCCTGGGT
2006	Yes	AAACGCTTGGGCTCTGACATTAACGTCCTGGGTT
2007	Yes	AAACGCTTGGGCTCTGACATTAACGTCCTGGGTTC
2008	Yes	AAACGCTTGGGCTCTGACATTAACGTCCTGGGTTCA
2009	Yes	AAACGCTTGGGCTCTGACATTAACGTCCTGGGTTCAA

2010	Yes	AAACGCTTGGGCTCTGACATTAACGTCCTGGGTTCAAA
2011	Yes	AAACGCTTGGGCTCTGACATTAACGTCCTGGGTTCAAAT
2012	Yes	AAACGCTTGGGCTCTGACATTAACGTCCTGGGTTCAAATC
2013	Yes	AAACGCTTGGGCTCTGACATTAACGTCCTGGGTTCAAATCC
2014	Yes	AAACGCTTGGGCTCTGACATTAACGTCCTGGGTTCAAATCCC
2015	Yes	AAACGCTTGGGCTCTGACATTAACGTCCTGGGTTCAAATCCCA
2016	Yes	AAACGCTTGGGCTCTGACATTAACGTCCTGGGTTCAAATCCCAG
2017	Yes	AAACGCTTGGGCTCTGACATTAACGTCCTGGGTTCAAATCCCAGC
2018	Yes	AAACGCTTGGGCTCTGACATTAACGTCCTGGGTTCAAATCCCAGCT
2019	Yes	AAACGCTTGGGCTCTGACATTAACGTCCTGGGTTCAAATCCCAGCTT
2020	Yes	AAACGCTTGGGCTCTGACATTAACGTCCTGGGTTCAAATCCCAGCTTT
2021	Yes	AAACGCTTGGGCTCTGACATTAACGTCCTGGGTTCAAATCCCAGCTTTG
2022	Yes	AAACGCTTGGGCTCTGACATTAACGTCCTGGGTTCAAATCCCAGCTTTGT
2023	No	AAACTAATACACCAGT
2024	No	AAACTAATACACCAGTC
2025	Yes	AAACTAATACACCAGTCT
2026	Yes	AAACTAATACACCAGTCTT
2027	Yes	AAACTAATACACCAGTCTTG
2028	Yes	AAACTAATACACCAGTCTTGT
2029	Yes	AAACTAATACACCAGTCTTGTA
2030	Yes	AAACTAATACACCAGTCTTGTAA
2031	Yes	AAACTAATACACCAGTCTTGTAAA
2032	Yes	AAACTAATACACCAGTCTTGTAAAC
2033	Yes	AAACTAATACACCAGTCTTGTAAACC
2034	Yes	AAACTAATACACCAGTCTTGTAAACCG
2035	Yes	AAACTAATACACCAGTCTTGTAAACCGG
2036	Yes	AAACTAATACACCAGTCTTGTAAACCGGA
2037	Yes	AAACTAATACACCAGTCTTGTAAACCGGAG
2038	Yes	AAACTAATACACCAGTCTTGTAAACCGGAGA
2039	Yes	AAACTAATACACCAGTCTTGTAAACCGGAGAT
2040	Yes	AAACTAATACACCAGTCTTGTAAACCGGAGATG
2041	Yes	AAACTAATACACCAGTCTTGTAAACCGGAGATGA
2042	Yes	AAACTAATACACCAGTCTTGTAAACCGGAGATGAA
2043	Yes	AAACTAATACACCAGTCTTGTAAACCGGAGATGAAA
2044	Yes	AAACTAATACACCAGTCTTGTAAACCGGAGATGAAAA
2045	Yes	AAACTAATACACCAGTCTTGTAAACCGGAGATGAAAAC
2046	Yes	AAACTAATACACCAGTCTTGTAAACCGGAGATGAAAACC
2047	Yes	AAACTAATACACCAGTCTTGTAAACCGGAGATGAAAACCT
2048	Yes	AAACTAATACACCAGTCTTGTAAACCGGAGATGAAAACCTT
2049	Yes	AAACTAATACACCAGTCTTGTAAACCGGAGATGAAAACCTTT
2050	Yes	AAACTAATACACCAGTCTTGTAAACCGGAGATGAAAACCTTTT
2051	Yes	AAACTAATACACCAGTCTTGTAAACCGGAGATGAAAACCTTTTT

2052	Yes	AAACTAATACACCAGTCTTGTAAACCGGAGATGAAAACCTTTTTC
2053	Yes	AAACTAATACACCAGTCTTGTAAACCGGAGATGAAAACCTTTTTCC
2054	Yes	AAACTAATACACCAGTCTTGTAAACCGGAGATGAAAACCTTTTTCCA
2055	Yes	AAACTAATACACCAGTCTTGTAAACCGGAGATGAAAACCTTTTTCCAA
2056	Yes	AAACTAATACACCAGTCTTGTAAACCGGAGATGAAAACCTTTTTCCAAG
2057	Yes	AAACTAATACACCAGTCTTGTAAACCGGAGATGAAAACCTTTTTCCAAGG
2058	No	AAACTCTAAATTTAGA
2059	No	AAACTCTAAATTTAGAT
2060	No	AAACTCTAAATTTAGATG
2061	Yes	AAACTCTAAATTTAGATGT
2062	Yes	AAACTCTAAATTTAGATGTC
2063	Yes	AAACTCTAAATTTAGATGTCC
2064	Yes	AAACTCTAAATTTAGATGTCCT
2065	Yes	AAACTCTAAATTTAGATGTCCTG
2066	Yes	AAACTCTAAATTTAGATGTCCTGA
2067	Yes	AAACTCTAAATTTAGATGTCCTGAG
2068	Yes	AAACTCTAAATTTAGATGTCCTGAGT
2069	Yes	AAACTCTAAATTTAGATGTCCTGAGTT
2070	Yes	AAACTCTAAATTTAGATGTCCTGAGTTC
2071	Yes	AAACTCTAAATTTAGATGTCCTGAGTTCA
2072	Yes	AAACTCTAAATTTAGATGTCCTGAGTTCAA
2073	Yes	AAACTCTAAATTTAGATGTCCTGAGTTCAAA
2074	Yes	AAACTCTAAATTTAGATGTCCTGAGTTCAAAT
2075	Yes	AAACTCTAAATTTAGATGTCCTGAGTTCAAATC
2076	Yes	AAACTCTAAATTTAGATGTCCTGAGTTCAAATCC
2077	Yes	AAACTCTAAATTTAGATGTCCTGAGTTCAAATCCC
2078	Yes	AAACTCTAAATTTAGATGTCCTGAGTTCAAATCCCA
2079	Yes	AAACTCTAAATTTAGATGTCCTGAGTTCAAATCCCAG
2080	Yes	AAACTCTAAATTTAGATGTCCTGAGTTCAAATCCCAGC
2081	Yes	AAACTCTAAATTTAGATGTCCTGAGTTCAAATCCCAGCT
2082	Yes	AAACTCTAAATTTAGATGTCCTGAGTTCAAATCCCAGCTG
2083	Yes	AAACTCTAAATTTAGATGTCCTGAGTTCAAATCCCAGCTGT
2084	Yes	AAACTCTAAATTTAGATGTCCTGAGTTCAAATCCCAGCTGTA
2085	Yes	AAACTCTAAATTTAGATGTCCTGAGTTCAAATCCCAGCTGTAT
2086	Yes	AAACTCTAAATTTAGATGTCCTGAGTTCAAATCCCAGCTGTATG
2087	Yes	AAACTCTAAATTTAGATGTCCTGAGTTCAAATCCCAGCTGTATGC
2088	Yes	AAACTCTAAATTTAGATGTCCTGAGTTCAAATCCCAGCTGTATGCC
2089	Yes	AAACTCTAAATTTAGATGTCCTGAGTTCAAATCCCAGCTGTATGCCC
2090	Yes	AAACTCTAAATTTAGATGTCCTGAGTTCAAATCCCAGCTGTATGCCCA
2091	No	AAACTGGCAGGCCTGG
2092	No	AAACTGGCAGGCCTGGG
2093	Yes	AAACTGGCAGGCCTGGGT

2094	Yes	AAACTGGCAGGCCTGGGTT
2095	Yes	AAACTGGCAGGCCTGGGTTC
2096	Yes	AAACTGGCAGGCCTGGGTTCA
2097	Yes	AAACTGGCAGGCCTGGGTTCAA
2098	Yes	AAACTGGCAGGCCTGGGTTCAAA
2099	Yes	AAACTGGCAGGCCTGGGTTCAAAT
2100	Yes	AAACTGGCAGGCCTGGGTTCAAATC
2101	Yes	AAACTGGCAGGCCTGGGTTCAAATCC
2102	Yes	AAACTGGCAGGCCTGGGTTCAAATCCC
2103	Yes	AAACTGGCAGGCCTGGGTTCAAATCCCA
2104	Yes	AAACTGGCAGGCCTGGGTTCAAATCCCAG
2105	Yes	AAACTGGCAGGCCTGGGTTCAAATCCCAGC
2106	Yes	AAACTGGCAGGCCTGGGTTCAAATCCCAGCT
2107	Yes	AAACTGGCAGGCCTGGGTTCAAATCCCAGCTT
2108	Yes	AAACTGGCAGGCCTGGGTTCAAATCCCAGCTTA
2109	Yes	AAACTGGCAGGCCTGGGTTCAAATCCCAGCTTAT
2110	Yes	AAACTGGCAGGCCTGGGTTCAAATCCCAGCTTATT
2111	Yes	AAACTGGCAGGCCTGGGTTCAAATCCCAGCTTATTC
2112	Yes	AAACTGGCAGGCCTGGGTTCAAATCCCAGCTTATTCA
2113	Yes	AAACTGGCAGGCCTGGGTTCAAATCCCAGCTTATTCAC
2114	Yes	AAACTGGCAGGCCTGGGTTCAAATCCCAGCTTATTCACC
2115	Yes	AAACTGGCAGGCCTGGGTTCAAATCCCAGCTTATTCACCA
2116	Yes	AAACTGGGCGGAAACA
2117	Yes	AAACTGGGCGGAAACAC
2118	Yes	AAACTGGGCGGAAACACC
2119	Yes	AAACTGGGCGGAAACACCA
2120	No	AAACTTAAAACTTTAC
2121	No	AAACTTAAAACTTTACA
2122	No	AAACTTAAAACTTTACAA
2123	No	AAACTTAAAACTTTACAAT
2124	No	AAACTTAAAACTTTACAATC
2125	No	AAACTTAAAACTTTACAATCA
2126	No	AAACTTAAAACTTTACAATCAG
2127	Yes	AAACTTAAAACTTTACAATCAGA
2128	Yes	AAACTTAAAACTTTACAATCAGAG
2129	Yes	AAACTTAAAACTTTACAATCAGAGG
2130	Yes	AAACTTAAAACTTTACAATCAGAGGT
2131	Yes	AAACTTAAAACTTTACAATCAGAGGTT
2132	Yes	AAACTTAAAACTTTACAATCAGAGGTTC
2133	Yes	AAACTTAAAACTTTACAATCAGAGGTTCA
2134	Yes	AAACTTAAAACTTTACAATCAGAGGTTCAA
2135	Yes	AAACTTAAAACTTTACAATCAGAGGTTCAAC

2136	Yes	AAACTTAAAACTTTACAATCAGAGGTTCAACT
2137	Yes	AAACTTAAAACTTTACAATCAGAGGTTCAACTC
2138	Yes	AAACTTAAAACTTTACAATCAGAGGTTCAACTCC
2139	Yes	AAACTTAAAACTTTACAATCAGAGGTTCAACTCCT
2140	Yes	AAACTTAAAACTTTACAATCAGAGGTTCAACTCCTC
2141	Yes	AAACTTAAAACTTTACAATCAGAGGTTCAACTCCTCT
2142	Yes	AAACTTAAAACTTTACAATCAGAGGTTCAACTCCTCTT
2143	Yes	AAACTTAAAACTTTACAATCAGAGGTTCAACTCCTCTTC
2144	Yes	AAACTTAAAACTTTACAATCAGAGGTTCAACTCCTCTTCT
2145	Yes	AAACTTAAAACTTTACAATCAGAGGTTCAACTCCTCTTCTT
2146	Yes	AAACTTAAAACTTTACAATCAGAGGTTCAACTCCTCTTCTTA
2147	Yes	AAACTTAAAACTTTACAATCAGAGGTTCAACTCCTCTTCTTAA
2148	Yes	AAACTTAAAACTTTACAATCAGAGGTTCAACTCCTCTTCTTAAC
2149	Yes	AAACTTAAAACTTTACAATCAGAGGTTCAACTCCTCTTCTTAACA
2150	Yes	AAACTTAAAACTTTACAATCAGAGGTTCAACTCCTCTTCTTAACAC
2151	Yes	AAACTTAAAACTTTACAATCAGAGGTTCAACTCCTCTTCTTAACACC
2152	Yes	AAACTTAAAACTTTACAATCAGAGGTTCAACTCCTCTTCTTAACACCA
2153	No	AAACTTAAAACTTTACAG
2154	No	AAACTTAAAACTTTACAGT
2155	No	AAACTTAAAACTTTACAGTC
2156	Yes	AAACTTAAAACTTTACAGTCA
2157	Yes	AAACTTAAAACTTTACAGTCAG
2158	Yes	AAACTTAAAACTTTACAGTCAGA
2159	Yes	AAACTTAAAACTTTACAGTCAGAG
2160	Yes	AAACTTAAAACTTTACAGTCAGAGG
2161	Yes	AAACTTAAAACTTTACAGTCAGAGGT
2162	Yes	AAACTTAAAACTTTACAGTCAGAGGTT
2163	Yes	AAACTTAAAACTTTACAGTCAGAGGTTC
2164	Yes	AAACTTAAAACTTTACAGTCAGAGGTTCA
2165	Yes	AAACTTAAAACTTTACAGTCAGAGGTTCAA
2166	Yes	AAACTTAAAACTTTACAGTCAGAGGTTCAAT
2167	Yes	AAACTTAAAACTTTACAGTCAGAGGTTCAATT
2168	Yes	AAACTTAAAACTTTACAGTCAGAGGTTCAATTC
2169	Yes	AAACTTAAAACTTTACAGTCAGAGGTTCAATTCC
2170	Yes	AAACTTAAAACTTTACAGTCAGAGGTTCAATTCCT
2171	Yes	AAACTTAAAACTTTACAGTCAGAGGTTCAATTCCTC
2172	Yes	AAACTTAAAACTTTACAGTCAGAGGTTCAATTCCTCT
2173	Yes	AAACTTAAAACTTTACAGTCAGAGGTTCAATTCCTCTT
2174	Yes	AAACTTAAAACTTTACAGTCAGAGGTTCAATTCCTCTTC
2175	Yes	AAACTTAAAACTTTACAGTCAGAGGTTCAATTCCTCTTCT
2176	Yes	AAACTTAAAACTTTACAGTCAGAGGTTCAATTCCTCTTCTT
2177	Yes	AAACTTAAAACTTTACAGTCAGAGGTTCAATTCCTCTTCTTA

2178	Yes	AAACTTAAAACTTTACAGTCAGAGGTTCAATTCCTCTTCTTAA
2179	Yes	AAACTTAAAACTTTACAGTCAGAGGTTCAATTCCTCTTCTTAAC
2180	Yes	AAACTTAAAACTTTACAGTCAGAGGTTCAATTCCTCTTCTTAACA
2181	Yes	AAACTTAAAACTTTACAGTCAGAGGTTCAATTCCTCTTCTTAACAC
2182	Yes	AAACTTAAAACTTTACAGTCAGAGGTTCAATTCCTCTTCTTAACACC
2183	Yes	AAACTTAAAACTTTACAGTCAGAGGTTCAATTCCTCTTCTTAACACCA
2184	No	AAACTTAAAATTTTAT
2185	No	AAACTTAAAATTTTATA
2186	No	AAACTTAAAATTTTATAA
2187	No	AAACTTAAAATTTTATAAT
2188	No	AAACTTAAAATTTTATAATC
2189	Yes	AAACTTAAAATTTTATAATCA
2190	Yes	AAACTTAAAATTTTATAATCAG
2191	Yes	AAACTTAAAATTTTATAATCAGA
2192	Yes	AAACTTAAAATTTTATAATCAGAG
2193	Yes	AAACTTAAAATTTTATAATCAGAGG
2194	Yes	AAACTTAAAATTTTATAATCAGAGGT
2195	Yes	AAACTTAAAATTTTATAATCAGAGGTT
2196	Yes	AAACTTAAAATTTTATAATCAGAGGTTC
2197	Yes	AAACTTAAAATTTTATAATCAGAGGTTCA
2198	Yes	AAACTTAAAATTTTATAATCAGAGGTTCAA
2199	Yes	AAACTTAAAATTTTATAATCAGAGGTTCAAC
2200	Yes	AAACTTAAAATTTTATAATCAGAGGTTCAACT
2201	Yes	AAACTTAAAATTTTATAATCAGAGGTTCAACTC
2202	Yes	AAACTTAAAATTTTATAATCAGAGGTTCAACTCC
2203	Yes	AAACTTAAAATTTTATAATCAGAGGTTCAACTCCT
2204	Yes	AAACTTAAAATTTTATAATCAGAGGTTCAACTCCTC
2205	Yes	AAACTTAAAATTTTATAATCAGAGGTTCAACTCCTCT
2206	Yes	AAACTTAAAATTTTATAATCAGAGGTTCAACTCCTCTT
2207	Yes	AAACTTAAAATTTTATAATCAGAGGTTCAACTCCTCTTC
2208	Yes	AAACTTAAAATTTTATAATCAGAGGTTCAACTCCTCTTCT
2209	Yes	AAACTTAAAATTTTATAATCAGAGGTTCAACTCCTCTTCTT
2210	Yes	AAACTTAAAATTTTATAATCAGAGGTTCAACTCCTCTTCTTA
2211	Yes	AAACTTAAAATTTTATAATCAGAGGTTCAACTCCTCTTCTTAA
2212	Yes	AAACTTAAAATTTTATAATCAGAGGTTCAACTCCTCTTCTTAAC
2213	Yes	AAACTTAAAATTTTATAATCAGAGGTTCAACTCCTCTTCTTAACA
2214	Yes	AAACTTAAAATTTTATAATCAGAGGTTCAACTCCTCTTCTTAACAC
2215	Yes	AAACTTAAAATTTTATAATCAGAGGTTCAACTCCTCTTCTTAACACC
2216	Yes	AAACTTAAAATTTTATAATCAGAGGTTCAACTCCTCTTCTTAACACCA
2217	No	AAACTTAACACTTTAT
2218	No	AAACTTAACACTTTATA
2219	No	AAACTTAACACTTTATAA

2220	Yes	AAACTTAACACTTTATAAT
2221	Yes	AAACTTAACACTTTATAATC
2222	Yes	AAACTTAACACTTTATAATCA
2223	Yes	AAACTTAACACTTTATAATCAG
2224	Yes	AAACTTAACACTTTATAATCAGA
2225	Yes	AAACTTAACACTTTATAATCAGAG
2226	Yes	AAACTTAACACTTTATAATCAGAGG
2227	Yes	AAACTTAACACTTTATAATCAGAGGT
2228	Yes	AAACTTAACACTTTATAATCAGAGGTT
2229	Yes	AAACTTAACACTTTATAATCAGAGGTTC
2230	Yes	AAACTTAACACTTTATAATCAGAGGTTCA
2231	Yes	AAACTTAACACTTTATAATCAGAGGTTCAA
2232	Yes	AAACTTAACACTTTATAATCAGAGGTTCAAC
2233	Yes	AAACTTAACACTTTATAATCAGAGGTTCAACT
2234	Yes	AAACTTAACACTTTATAATCAGAGGTTCAACTC
2235	Yes	AAACTTAACACTTTATAATCAGAGGTTCAACTCC
2236	Yes	AAACTTAACACTTTATAATCAGAGGTTCAACTCCT
2237	Yes	AAACTTAACACTTTATAATCAGAGGTTCAACTCCTC
2238	Yes	AAACTTAACACTTTATAATCAGAGGTTCAACTCCTCT
2239	Yes	AAACTTAACACTTTATAATCAGAGGTTCAACTCCTCTT
2240	Yes	AAACTTAACACTTTATAATCAGAGGTTCAACTCCTCTTC
2241	Yes	AAACTTAACACTTTATAATCAGAGGTTCAACTCCTCTTCT
2242	Yes	AAACTTAACACTTTATAATCAGAGGTTCAACTCCTCTTCTT
2243	Yes	AAACTTAACACTTTATAATCAGAGGTTCAACTCCTCTTCTTA
2244	Yes	AAACTTAACACTTTATAATCAGAGGTTCAACTCCTCTTCTTAA
2245	Yes	AAACTTAACACTTTATAATCAGAGGTTCAACTCCTCTTCTTAAC
2246	Yes	AAACTTAACACTTTATAATCAGAGGTTCAACTCCTCTTCTTAACA
2247	Yes	AAACTTAACACTTTATAATCAGAGGTTCAACTCCTCTTCTTAACAC
2248	Yes	AAACTTAACACTTTATAATCAGAGGTTCAACTCCTCTTCTTAACACC
2249	Yes	AAACTTAACACTTTATAATCAGAGGTTCAACTCCTCTTCTTAACACCA
2250	No	AAACTTAAGACTTTAT
2251	No	AAACTTAAGACTTTATA
2252	No	AAACTTAAGACTTTATAA
2253	No	AAACTTAAGACTTTATAAT
2254	Yes	AAACTTAAGACTTTATAATT
2255	Yes	AAACTTAAGACTTTATAATTA
2256	Yes	AAACTTAAGACTTTATAATTAG
2257	Yes	AAACTTAAGACTTTATAATTAGA
2258	Yes	AAACTTAAGACTTTATAATTAGAA
2259	Yes	AAACTTAAGACTTTATAATTAGAAG
2260	Yes	AAACTTAAGACTTTATAATTAGAAGT
2261	Yes	AAACTTAAGACTTTATAATTAGAAGTT

2262	Yes	AAACTTAAGACTTTATAATTAGAAGTTC
2263	Yes	AAACTTAAGACTTTATAATTAGAAGTTCA
2264	Yes	AAACTTAAGACTTTATAATTAGAAGTTCAA
2265	Yes	AAACTTAAGACTTTATAATTAGAAGTTCAAC
2266	Yes	AAACTTAAGACTTTATAATTAGAAGTTCAACA
2267	Yes	AAACTTAAGACTTTATAATTAGAAGTTCAACAC
2268	Yes	AAACTTAAGACTTTATAATTAGAAGTTCAACACC
2269	Yes	AAACTTAAGACTTTATAATTAGAAGTTCAACACCT
2270	Yes	AAACTTAAGACTTTATAATTAGAAGTTCAACACCTC
2271	Yes	AAACTTAAGACTTTATAATTAGAAGTTCAACACCTCT
2272	Yes	AAACTTAAGACTTTATAATTAGAAGTTCAACACCTCTT
2273	Yes	AAACTTAAGACTTTATAATTAGAAGTTCAACACCTCTTC
2274	Yes	AAACTTAAGACTTTATAATTAGAAGTTCAACACCTCTTCT
2275	Yes	AAACTTAAGACTTTATAATTAGAAGTTCAACACCTCTTCTT
2276	Yes	AAACTTAAGACTTTATAATTAGAAGTTCAACACCTCTTCTTA
2277	Yes	AAACTTAAGACTTTATAATTAGAAGTTCAACACCTCTTCTTAA
2278	Yes	AAACTTAAGACTTTATAATTAGAAGTTCAACACCTCTTCTTAAC
2279	Yes	AAACTTAAGACTTTATAATTAGAAGTTCAACACCTCTTCTTAACA
2280	Yes	AAACTTAAGACTTTATAATTAGAAGTTCAACACCTCTTCTTAACAC
2281	Yes	AAACTTAAGACTTTATAATTAGAAGTTCAACACCTCTTCTTAACACC
2282	Yes	AAACTTAAGACTTTATAATTAGAAGTTCAACACCTCTTCTTAACACCA
2283	No	AAACTTTACAATCAGA
2284	Yes	AAACTTTACAATCAGAG
2285	Yes	AAACTTTACAATCAGAGG
2286	Yes	AAACTTTACAATCAGAGGT
2287	Yes	AAACTTTACAATCAGAGGTT
2288	Yes	AAACTTTACAATCAGAGGTTC
2289	Yes	AAACTTTACAATCAGAGGTTCA
2290	Yes	AAACTTTACAATCAGAGGTTCAA
2291	Yes	AAACTTTACAATCAGAGGTTCAAC
2292	Yes	AAACTTTACAATCAGAGGTTCAACT
2293	Yes	AAACTTTACAATCAGAGGTTCAACTC
2294	Yes	AAACTTTACAATCAGAGGTTCAACTCC
2295	Yes	AAACTTTACAATCAGAGGTTCAACTCCT
2296	Yes	AAACTTTACAATCAGAGGTTCAACTCCTC
2297	Yes	AAACTTTACAATCAGAGGTTCAACTCCTCT
2298	Yes	AAACTTTACAATCAGAGGTTCAACTCCTCTT
2299	Yes	AAACTTTACAATCAGAGGTTCAACTCCTCTTC
2300	Yes	AAACTTTACAATCAGAGGTTCAACTCCTCTTCT
2301	Yes	AAACTTTACAATCAGAGGTTCAACTCCTCTTCTT
2302	Yes	AAACTTTACAATCAGAGGTTCAACTCCTCTTCTTA
2303	Yes	AAACTTTACAATCAGAGGTTCAACTCCTCTTCTTAA

2304	Yes	AAACTTTACAATCAGAGGTTCAACTCCTCTTCTTAAC
2305	Yes	AAACTTTACAATCAGAGGTTCAACTCCTCTTCTTAACA
2306	Yes	AAACTTTACAATCAGAGGTTCAACTCCTCTTCTTAACAC
2307	Yes	AAACTTTACAATCAGAGGTTCAACTCCTCTTCTTAACACC
2308	Yes	AAACTTTACAATCAGAGGTTCAACTCCTCTTCTTAACACCA
2309	No	AAACTTTACAGTCAGA
2310	Yes	AAACTTTACAGTCAGAG
2311	Yes	AAACTTTACAGTCAGAGG
2312	Yes	AAACTTTACAGTCAGAGGT
2313	Yes	AAACTTTACAGTCAGAGGTT
2314	Yes	AAACTTTACAGTCAGAGGTTC
2315	Yes	AAACTTTACAGTCAGAGGTTCA
2316	Yes	AAACTTTACAGTCAGAGGTTCAA
2317	Yes	AAACTTTACAGTCAGAGGTTCAAT
2318	Yes	AAACTTTACAGTCAGAGGTTCAATT
2319	Yes	AAACTTTACAGTCAGAGGTTCAATTC
2320	Yes	AAACTTTACAGTCAGAGGTTCAATTCC
2321	Yes	AAACTTTACAGTCAGAGGTTCAATTCCT
2322	Yes	AAACTTTACAGTCAGAGGTTCAATTCCTC
2323	Yes	AAACTTTACAGTCAGAGGTTCAATTCCTCT
2324	Yes	AAACTTTACAGTCAGAGGTTCAATTCCTCTT
2325	Yes	AAACTTTACAGTCAGAGGTTCAATTCCTCTTC
2326	Yes	AAACTTTACAGTCAGAGGTTCAATTCCTCTTCT
2327	Yes	AAACTTTACAGTCAGAGGTTCAATTCCTCTTCTT
2328	Yes	AAACTTTACAGTCAGAGGTTCAATTCCTCTTCTTA
2329	Yes	AAACTTTACAGTCAGAGGTTCAATTCCTCTTCTTAA
2330	Yes	AAACTTTACAGTCAGAGGTTCAATTCCTCTTCTTAAC
2331	Yes	AAACTTTACAGTCAGAGGTTCAATTCCTCTTCTTAACA
2332	Yes	AAACTTTACAGTCAGAGGTTCAATTCCTCTTCTTAACAC
2333	Yes	AAACTTTACAGTCAGAGGTTCAATTCCTCTTCTTAACACC
2334	Yes	AAACTTTACAGTCAGAGGTTCAATTCCTCTTCTTAACACCA
2335	No	AAACTTTATAATCAGA
2336	No	AAACTTTATAATCAGAG
2337	No	AAACTTTATAATCAGAGG
2338	No	AAACTTTATAATCAGAGGT
2339	No	AAACTTTATAATCAGAGGTT
2340	No	AAACTTTATAATCAGAGGTTC
2341	No	AAACTTTATAATCAGAGGTTCA
2342	No	AAACTTTATAATCAGAGGTTCAA
2343	No	AAACTTTATAATCAGAGGTTCAAC
2344	No	AAACTTTATAATCAGAGGTTCAACT
2345	No	AAACTTTATAATCAGAGGTTCAACTC

2346	No	AAACTTTATAATCAGAGGTTCAACTCC
2347	No	AAACTTTATAATCAGAGGTTCAACTCCT
2348	No	AAACTTTATAATCAGAGGTTCAACTCCTC
2349	No	AAACTTTATAATCAGAGGTTCAACTCCTCT
2350	Yes	AAACTTTATAATCAGAGGTTCAACTCCTCTC
2351	Yes	AAACTTTATAATCAGAGGTTCAACTCCTCTCA
2352	Yes	AAACTTTATAATCAGAGGTTCAACTCCTCTCAT
2353	Yes	AAACTTTATAATCAGAGGTTCAACTCCTCTCATT
2354	Yes	AAACTTTATAATCAGAGGTTCAACTCCTCTCATTA
2355	Yes	AAACTTTATAATCAGAGGTTCAACTCCTCTCATTAA
2356	Yes	AAACTTTATAATCAGAGGTTCAACTCCTCTCATTAAC
2357	Yes	AAACTTTATAATCAGAGGTTCAACTCCTCTCATTAACA
2358	Yes	AAACTTTATAATCAGAGGTTCAACTCCTCTCATTAACAC
2359	Yes	AAACTTTATAATCAGAGGTTCAACTCCTCTCATTAACACC
2360	Yes	AAACTTTATAATCAGAGGTTCAACTCCTCTCATTAACACCA
2361	Yes	AAACTTTATAATCAGAGGTTCG
2362	Yes	AAACTTTATAATCAGAGGTTCGA
2363	Yes	AAACTTTATAATCAGAGGTTCGAC
2364	Yes	AAACTTTATAATCAGAGGTTCGACT
2365	Yes	AAACTTTATAATCAGAGGTTCGACTC
2366	Yes	AAACTTTATAATCAGAGGTTCGACTCC
2367	Yes	AAACTTTATAATCAGAGGTTCGACTCCT
2368	Yes	AAACTTTATAATCAGAGGTTCGACTCCTC
2369	Yes	AAACTTTATAATCAGAGGTTCGACTCCTCT
2370	Yes	AAACTTTATAATCAGAGGTTCGACTCCTCTT
2371	Yes	AAACTTTATAATCAGAGGTTCGACTCCTCTTC
2372	Yes	AAACTTTATAATCAGAGGTTCGACTCCTCTTCT
2373	Yes	AAACTTTATAATCAGAGGTTCGACTCCTCTTCTT
2374	Yes	AAACTTTATAATCAGAGGTTCGACTCCTCTTCTTA
2375	Yes	AAACTTTATAATCAGAGGTTCGACTCCTCTTCTTAA
2376	Yes	AAACTTTATAATCAGAGGTTCGACTCCTCTTCTTAAC
2377	Yes	AAACTTTATAATCAGAGGTTCGACTCCTCTTCTTAACA
2378	Yes	AAACTTTATAATCAGAGGTTCGACTCCTCTTCTTAACAC
2379	Yes	AAACTTTATAATCAGAGGTTCGACTCCTCTTCTTAACACC
2380	Yes	AAACTTTATAATCAGAGGTTCGACTCCTCTTCTTAACACCA
2381	No	AAAGACAGGGGTTAGG
2382	No	AAAGACAGGGGTTAGGC
2383	No	AAAGACAGGGGTTAGGCC
2384	Yes	AAAGACAGGGGTTAGGCCT
2385	Yes	AAAGACAGGGGTTAGGCCTC
2386	Yes	AAAGACAGGGGTTAGGCCTCT
2387	Yes	AAAGACAGGGGTTAGGCCTCTT

2388	Yes	AAAGACAGGGGTTAGGCCTCTTT
2389	Yes	AAAGACAGGGGTTAGGCCTCTTTT
2390	Yes	AAAGACAGGGGTTAGGCCTCTTTTT
2391	Yes	AAAGACAGGGGTTAGGCCTCTTTTTA
2392	Yes	AAAGACAGGGGTTAGGCCTCTTTTTAC
2393	Yes	AAAGACAGGGGTTAGGCCTCTTTTTACC
2394	Yes	AAAGACAGGGGTTAGGCCTCTTTTTACCA
2395	Yes	AAAGACAGGGGTTAGGCCTCTTTTTACCAC
2396	Yes	AAAGACAGGGGTTAGGCCTCTTTTTACCACC
2397	Yes	AAAGACAGGGGTTAGGCCTCTTTTTACCACCA
2398	No	AAAGACATCTCACTTT
2399	No	AAAGACATCTCACTTTG
2400	Yes	AAAGACATCTCACTTTGG
2401	Yes	AAAGACATCTCACTTTGGG
2402	Yes	AAAGACATCTCACTTTGGGT
2403	Yes	AAAGACATCTCACTTTGGGTC
2404	Yes	AAAGACATCTCACTTTGGGTCA
2405	Yes	AAAGACATCTCACTTTGGGTCAG
2406	Yes	AAAGACATCTCACTTTGGGTCAGA
2407	Yes	AAAGACATCTCACTTTGGGTCAGAA
2408	Yes	AAAGACATCTCACTTTGGGTCAGAAG
2409	Yes	AAAGACATCTCACTTTGGGTCAGAAGA
2410	Yes	AAAGACATCTCACTTTGGGTCAGAAGAT
2411	Yes	AAAGACATCTCACTTTGGGTCAGAAGATT
2412	Yes	AAAGACATCTCACTTTGGGTCAGAAGATTG
2413	Yes	AAAGACATCTCACTTTGGGTCAGAAGATTGA
2414	Yes	AAAGACATCTCACTTTGGGTCAGAAGATTGAG
2415	Yes	AAAGACATCTCACTTTGGGTCAGAAGATTGAGG
2416	Yes	AAAGACATCTCACTTTGGGTCAGAAGATTGAGGG
2417	Yes	AAAGACATCTCACTTTGGGTCAGAAGATTGAGGGT
2418	Yes	AAAGACATCTCACTTTGGGTCAGAAGATTGAGGGTT
2419	Yes	AAAGACATCTCACTTTGGGTCAGAAGATTGAGGGTTC
2420	Yes	AAAGACATCTCACTTTGGGTCAGAAGATTGAGGGTTCA
2421	Yes	AAAGACATCTCACTTTGGGTCAGAAGATTGAGGGTTCAA
2422	Yes	AAAGACATCTCACTTTGGGTCAGAAGATTGAGGGTTCAAG
2423	Yes	AAAGACATCTCACTTTGGGTCAGAAGATTGAGGGTTCAAGT
2424	Yes	AAAGACATCTCACTTTGGGTCAGAAGATTGAGGGTTCAAGTC
2425	Yes	AAAGACATCTCACTTTGGGTCAGAAGATTGAGGGTTCAAGTCC
2426	Yes	AAAGACATCTCACTTTGGGTCAGAAGATTGAGGGTTCAAGTCCT
2427	Yes	AAAGACATCTCACTTTGGGTCAGAAGATTGAGGGTTCAAGTCCTT
2428	Yes	AAAGACATCTCACTTTGGGTCAGAAGATTGAGGGTTCAAGTCCTTT
2429	Yes	AAAGACATCTCACTTTGGGTCAGAAGATTGAGGGTTCAAGTCCTTTC

2430	Yes	AAAGACATCTCACTTTGGGTCAGAAGATTGAGGGTTCAAGTCCTTTCA
2431	Yes	AAAGACATCTCACTTTGGGTCAGAAGATTGAGGGTTCAAGTCCTTTCAT
2432	Yes	AAAGACATCTCACTTTGGGTCAGAAGATTGAGGGTTCAAGTCCTTTCATG
2433	Yes	AAAGACCCTTGGCTCG
2434	Yes	AAAGACCCTTGGCTCGA
2435	Yes	AAAGACCCTTGGCTCGAG
2436	Yes	AAAGACCCTTGGCTCGAGA
2437	Yes	AAAGACCCTTGGCTCGAGAC
2438	Yes	AAAGACCCTTGGCTCGAGACT
2439	Yes	AAAGACCCTTGGCTCGAGACTG
2440	Yes	AAAGACCCTTGGCTCGAGACTGG
2441	Yes	AAAGACCCTTGGCTCGAGACTGGA
2442	Yes	AAAGACCCTTGGCTCGAGACTGGAG
2443	Yes	AAAGACCCTTGGCTCGAGACTGGAGG
2444	Yes	AAAGACCCTTGGCTCGAGACTGGAGGG
2445	Yes	AAAGACCCTTGGCTCGAGACTGGAGGGA
2446	Yes	AAAGACCCTTGGCTCGAGACTGGAGGGAA
2447	Yes	AAAGACCCTTGGCTCGAGACTGGAGGGAAA
2448	Yes	AAAGACCCTTGGCTCGAGACTGGAGGGAAAC
2449	Yes	AAAGACCCTTGGCTCGAGACTGGAGGGAAACA
2450	Yes	AAAGACCCTTGGCTCGAGACTGGAGGGAAACAC
2451	Yes	AAAGACCCTTGGCTCGAGACTGGAGGGAAACACC
2452	Yes	AAAGACCCTTGGCTCGAGACTGGAGGGAAACACCA
2453	No	AAAGACTTTTTCTCTG
2454	No	AAAGACTTTTTCTCTGA
2455	No	AAAGACTTTTTCTCTGAC
2456	No	AAAGACTTTTTCTCTGACC
2457	No	AAAGACTTTTTCTCTGACCA
2458	Yes	AAAGAGCCACGGCCCG
2459	Yes	AAAGAGCCACGGCCCGG
2460	Yes	AAAGAGCCACGGCCCGGG
2461	Yes	AAAGAGCCACGGCCCGGGT
2462	Yes	AAAGAGCCACGGCCCGGGTT
2463	Yes	AAAGAGCCACGGCCCGGGTTC
2464	Yes	AAAGAGCCACGGCCCGGGTTCG
2465	Yes	AAAGAGCCACGGCCCGGGTTCGA
2466	Yes	AAAGAGCCACGGCCCGGGTTCGAT
2467	Yes	AAAGAGCCACGGCCCGGGTTCGATT
2468	Yes	AAAGAGCCACGGCCCGGGTTCGATTC
2469	Yes	AAAGAGCCACGGCCCGGGTTCGATTCC
2470	Yes	AAAGAGCCACGGCCCGGGTTCGATTCCC
2471	Yes	AAAGAGCCACGGCCCGGGTTCGATTCCCG

2472	Yes	AAAGAGCCACGGCCCGGGTTCGATTCCCGG
2473	Yes	AAAGAGCCACGGCCCGGGTTCGATTCCCGGT
2474	Yes	AAAGAGCCACGGCCCGGGTTCGATTCCCGGTC
2475	Yes	AAAGAGCCACGGCCCGGGTTCGATTCCCGGTCA
2476	Yes	AAAGAGCCACGGCCCGGGTTCGATTCCCGGTCAG
2477	Yes	AAAGAGCCACGGCCCGGGTTCGATTCCCGGTCAGG
2478	Yes	AAAGAGCCACGGCCCGGGTTCGATTCCCGGTCAGGG
2479	Yes	AAAGAGCCACGGCCCGGGTTCGATTCCCGGTCAGGGA
2480	Yes	AAAGAGCCACGGCCCGGGTTCGATTCCCGGTCAGGGAA
2481	Yes	AAAGAGCCACGGCCCGGGTTCGATTCCCGGTCAGGGAAC
2482	Yes	AAAGAGCCACGGCCCGGGTTCGATTCCCGGTCAGGGAACC
2483	Yes	AAAGAGCCACGGCCCGGGTTCGATTCCCGGTCAGGGAACCA
2484	No	AAAGAGTTACTTTGAT
2485	No	AAAGAGTTACTTTGATA
2486	No	AAAGAGTTACTTTGATAG
2487	No	AAAGAGTTACTTTGATAGA
2488	No	AAAGAGTTACTTTGATAGAG
2489	No	AAAGAGTTACTTTGATAGAGT
2490	No	AAAGAGTTACTTTGATAGAGTA
2491	No	AAAGAGTTACTTTGATAGAGTAA
2492	No	AAAGAGTTACTTTGATAGAGTAAA
2493	No	AAAGAGTTACTTTGATAGAGTAAAT
2494	No	AAAGAGTTACTTTGATAGAGTAAATA
2495	No	AAAGAGTTACTTTGATAGAGTAAATAA
2496	No	AAAGAGTTACTTTGATAGAGTAAATAAT
2497	No	AAAGAGTTACTTTGATAGAGTAAATAATA
2498	No	AAAGAGTTACTTTGATAGAGTAAATAATAG
2499	No	AAAGAGTTACTTTGATAGAGTAAATAATAGG
2500	No	AAAGAGTTACTTTGATAGAGTAAATAATAGGA
2501	No	AAAGAGTTACTTTGATAGAGTAAATAATAGGAG
2502	Yes	AAAGAGTTACTTTGATAGAGTAAATAATAGGAGC
2503	Yes	AAAGAGTTACTTTGATAGAGTAAATAATAGGAGCT
2504	Yes	AAAGAGTTACTTTGATAGAGTAAATAATAGGAGCTT
2505	Yes	AAAGAGTTACTTTGATAGAGTAAATAATAGGAGCTTA
2506	Yes	AAAGAGTTACTTTGATAGAGTAAATAATAGGAGCTTAA
2507	Yes	AAAGAGTTACTTTGATAGAGTAAATAATAGGAGCTTAAA
2508	Yes	AAAGAGTTACTTTGATAGAGTAAATAATAGGAGCTTAAAC
2509	Yes	AAAGAGTTACTTTGATAGAGTAAATAATAGGAGCTTAAACC
2510	Yes	AAAGAGTTACTTTGATAGAGTAAATAATAGGAGCTTAAACCC
2511	Yes	AAAGAGTTACTTTGATAGAGTAAATAATAGGAGCTTAAACCCC
2512	Yes	AAAGAGTTACTTTGATAGAGTAAATAATAGGAGCTTAAACCCCC
2513	Yes	AAAGAGTTACTTTGATAGAGTAAATAATAGGAGCTTAAACCCCCT

2514	Yes	AAAGAGTTACTTTGATAGAGTAAATAATAGGAGCTTAAACCCCCTT
2515	Yes	AAAGAGTTACTTTGATAGAGTAAATAATAGGAGCTTAAACCCCCTTA
2516	Yes	AAAGAGTTACTTTGATAGAGTAAATAATAGGAGCTTAAACCCCCTTAT
2517	Yes	AAAGAGTTACTTTGATAGAGTAAATAATAGGAGCTTAAACCCCCTTATT
2518	Yes	AAAGAGTTACTTTGATAGAGTAAATAATAGGAGCTTAAACCCCCTTATTT
2519	No	AAAGATTAAGAGAACC
2520	No	AAAGATTAAGAGAACCA
2521	No	AAAGATTAAGAGAACCAA
2522	No	AAAGATTAAGAGAACCAAC
2523	No	AAAGATTAAGAGAACCAACA
2524	No	AAAGATTAAGAGAACCAACAC
2525	No	AAAGATTAAGAGAACCAACACC
2526	No	AAAGATTAAGAGAACCAACACCT
2527	No	AAAGATTAAGAGAACCAACACCTC
2528	No	AAAGATTAAGAGAACCAACACCTCT
2529	No	AAAGATTAAGAGAACCAACACCTCTT
2530	No	AAAGATTAAGAGAACCAACACCTCTTT
2531	No	AAAGATTAAGAGAACCAACACCTCTTTA
2532	No	AAAGATTAAGAGAACCAACACCTCTTTAC
2533	No	AAAGATTAAGAGAACCAACACCTCTTTACA
2534	No	AAAGATTAAGAGAACCAACACCTCTTTACAG
2535	No	AAAGATTAAGAGAACCAACACCTCTTTACAGT
2536	No	AAAGATTAAGAGAACCAACACCTCTTTACAGTG
2537	No	AAAGATTAAGAGAACCAACACCTCTTTACAGTGA
2538	Yes	AAAGATTAAGAGAACCAACACCTCTTTACAGTGAC
2539	Yes	AAAGATTAAGAGAACCAACACCTCTTTACAGTGACC
2540	Yes	AAAGATTAAGAGAACCAACACCTCTTTACAGTGACCA
2541	No	AAAGATTGCAGGTTTG
2542	Yes	AAAGATTGCAGGTTTGA
2543	Yes	AAAGATTGCAGGTTTGAG
2544	Yes	AAAGATTGCAGGTTTGAGT
2545	Yes	AAAGATTGCAGGTTTGAGTT
2546	Yes	AAAGATTGCAGGTTTGAGTTC
2547	Yes	AAAGATTGCAGGTTTGAGTTCT
2548	Yes	AAAGATTGCAGGTTTGAGTTCTG
2549	Yes	AAAGATTGCAGGTTTGAGTTCTGC
2550	Yes	AAAGATTGCAGGTTTGAGTTCTGCC
2551	Yes	AAAGATTGCAGGTTTGAGTTCTGCCA
2552	Yes	AAAGATTGCAGGTTTGAGTTCTGCCAC
2553	Yes	AAAGATTGCAGGTTTGAGTTCTGCCACG
2554	Yes	AAAGATTGCAGGTTTGAGTTCTGCCACGG
2555	Yes	AAAGATTGCAGGTTTGAGTTCTGCCACGGT

2556	Yes	AAAGATTGCAGGTTTGAGTTCTGCCACGGTC
2557	Yes	AAAGATTGCAGGTTTGAGTTCTGCCACGGTCG
2558	Yes	AAAGATTGCAGGTTTGAGTTCTGCCACGGTCGC
2559	Yes	AAAGATTGCAGGTTTGAGTTCTGCCACGGTCGCC
2560	Yes	AAAGATTGCAGGTTTGAGTTCTGCCACGGTCGCCA
2561	Yes	AAAGATTGGTGGTTCG
2562	Yes	AAAGATTGGTGGTTCGA
2563	Yes	AAAGATTGGTGGTTCGAG
2564	Yes	AAAGATTGGTGGTTCGAGC
2565	Yes	AAAGATTGGTGGTTCGAGCC
2566	Yes	AAAGATTGGTGGTTCGAGCCC
2567	Yes	AAAGATTGGTGGTTCGAGCCCA
2568	Yes	AAAGATTGGTGGTTCGAGCCCAC
2569	Yes	AAAGATTGGTGGTTCGAGCCCACC
2570	Yes	AAAGATTGGTGGTTCGAGCCCACCC
2571	Yes	AAAGATTGGTGGTTCGAGCCCACCCA
2572	Yes	AAAGATTGGTGGTTCGAGCCCACCCAG
2573	Yes	AAAGATTGGTGGTTCGAGCCCACCCAGG
2574	Yes	AAAGATTGGTGGTTCGAGCCCACCCAGGG
2575	Yes	AAAGATTGGTGGTTCGAGCCCACCCAGGGA
2576	Yes	AAAGATTGGTGGTTCGAGCCCACCCAGGGAC
2577	Yes	AAAGATTGGTGGTTCGAGCCCACCCAGGGACG
2578	Yes	AAAGATTGGTGGTTCGAGCCCACCCAGGGACGC
2579	Yes	AAAGATTGGTGGTTCGAGCCCACCCAGGGACGCC
2580	Yes	AAAGATTGGTGGTTCGAGCCCACCCAGGGACGCCA
2581	No	AAAGCAATACACTGAA
2582	No	AAAGCAATACACTGAAA
2583	No	AAAGCAATACACTGAAAA
2584	No	AAAGCAATACACTGAAAAT
2585	No	AAAGCAATACACTGAAAATG
2586	No	AAAGCAATACACTGAAAATGT
2587	No	AAAGCAATACACTGAAAATGTT
2588	No	AAAGCAATACACTGAAAATGTTT
2589	Yes	AAAGCAATACACTGAAAATGTTTA
2590	Yes	AAAGCAATACACTGAAAATGTTTAG
2591	Yes	AAAGCAATACACTGAAAATGTTTAGA
2592	Yes	AAAGCAATACACTGAAAATGTTTAGAC
2593	Yes	AAAGCAATACACTGAAAATGTTTAGACG
2594	Yes	AAAGCAATACACTGAAAATGTTTAGACGG
2595	Yes	AAAGCAATACACTGAAAATGTTTAGACGGG
2596	Yes	AAAGCAATACACTGAAAATGTTTAGACGGGC
2597	Yes	AAAGCAATACACTGAAAATGTTTAGACGGGCT

2598	Yes	AAAGCAATACACTGAAAATGTTTAGACGGGCTC
2599	Yes	AAAGCAATACACTGAAAATGTTTAGACGGGCTCA
2600	Yes	AAAGCAATACACTGAAAATGTTTAGACGGGCTCAC
2601	Yes	AAAGCAATACACTGAAAATGTTTAGACGGGCTCACA
2602	Yes	AAAGCAATACACTGAAAATGTTTAGACGGGCTCACAT
2603	Yes	AAAGCAATACACTGAAAATGTTTAGACGGGCTCACATC
2604	Yes	AAAGCAATACACTGAAAATGTTTAGACGGGCTCACATCA
2605	Yes	AAAGCAATACACTGAAAATGTTTAGACGGGCTCACATCAC
2606	Yes	AAAGCAATACACTGAAAATGTTTAGACGGGCTCACATCACC
2607	Yes	AAAGCAATACACTGAAAATGTTTAGACGGGCTCACATCACCC
2608	Yes	AAAGCAATACACTGAAAATGTTTAGACGGGCTCACATCACCCC
2609	Yes	AAAGCAATACACTGAAAATGTTTAGACGGGCTCACATCACCCCA
2610	Yes	AAAGCAATACACTGAAAATGTTTAGACGGGCTCACATCACCCCAT
2611	Yes	AAAGCAATACACTGAAAATGTTTAGACGGGCTCACATCACCCCATA
2612	Yes	AAAGCAATACACTGAAAATGTTTAGACGGGCTCACATCACCCCATAA
2613	Yes	AAAGCAATACACTGAAAATGTTTAGACGGGCTCACATCACCCCATAAA
2614	Yes	AAAGCAATACACTGAAAATGTTTAGACGGGCTCACATCACCCCATAAAC
2615	Yes	AAAGCAATACACTGAAAATGTTTAGACGGGCTCACATCACCCCATAAACA
2616	No	AAAGCACCCAACTTAC
2617	No	AAAGCACCCAACTTACA
2618	No	AAAGCACCCAACTTACAC
2619	No	AAAGCACCCAACTTACACT
2620	No	AAAGCACCCAACTTACACTT
2621	No	AAAGCACCCAACTTACACTTA
2622	No	AAAGCACCCAACTTACACTTAG
2623	No	AAAGCACCCAACTTACACTTAGG
2624	No	AAAGCACCCAACTTACACTTAGGA
2625	No	AAAGCACCCAACTTACACTTAGGAG
2626	No	AAAGCACCCAACTTACACTTAGGAGA
2627	No	AAAGCACCCAACTTACACTTAGGAGAT
2628	No	AAAGCACCCAACTTACACTTAGGAGATT
2629	No	AAAGCACCCAACTTACACTTAGGAGATTT
2630	No	AAAGCACCCAACTTACACTTAGGAGATTTC
2631	No	AAAGCACCCAACTTACACTTAGGAGATTTCA
2632	No	AAAGCACCCAACTTACACTTAGGAGATTTCAA
2633	No	AAAGCACCCAACTTACACTTAGGAGATTTCAAC
2634	No	AAAGCACCCAACTTACACTTAGGAGATTTCAACT
2635	No	AAAGCACCCAACTTACACTTAGGAGATTTCAACTT
2636	No	AAAGCACCCAACTTACACTTAGGAGATTTCAACTTA
2637	No	AAAGCACCCAACTTACACTTAGGAGATTTCAACTTAA
2638	No	AAAGCACCCAACTTACACTTAGGAGATTTCAACTTAAC
2639	No	AAAGCACCCAACTTACACTTAGGAGATTTCAACTTAACT

2640	No	AAAGCACCCAACTTACACTTAGGAGATTTCAACTTAACTT
2641	No	AAAGCACCCAACTTACACTTAGGAGATTTCAACTTAACTTG
2642	No	AAAGCACCCAACTTACACTTAGGAGATTTCAACTTAACTTGA
2643	No	AAAGCACCCAACTTACACTTAGGAGATTTCAACTTAACTTGAC
2644	No	AAAGCACCCAACTTACACTTAGGAGATTTCAACTTAACTTGACC
2645	No	AAAGCACCCAACTTACACTTAGGAGATTTCAACTTAACTTGACCG
2646	No	AAAGCACCCAACTTACACTTAGGAGATTTCAACTTAACTTGACCGC
2647	No	AAAGCACCCAACTTACACTTAGGAGATTTCAACTTAACTTGACCGCT
2648	No	AAAGCACCCAACTTACACTTAGGAGATTTCAACTTAACTTGACCGCTC
2649	No	AAAGCACCCAACTTACACTTAGGAGATTTCAACTTAACTTGACCGCTCT
2650	No	AAAGCACCCAACTTACACTTAGGAGATTTCAACTTAACTTGACCGCTCTG
2651	No	AAAGCACCTGTCTAGT
2652	No	AAAGCACCTGTCTAGTA
2653	No	AAAGCACCTGTCTAGTAA
2654	No	AAAGCACCTGTCTAGTAAA
2655	Yes	AAAGCACCTGTCTAGTAAAC
2656	Yes	AAAGCACCTGTCTAGTAAACA
2657	Yes	AAAGCACCTGTCTAGTAAACAG
2658	Yes	AAAGCACCTGTCTAGTAAACAGG
2659	Yes	AAAGCACCTGTCTAGTAAACAGGA
2660	Yes	AAAGCACCTGTCTAGTAAACAGGAG
2661	Yes	AAAGCACCTGTCTAGTAAACAGGAGA
2662	Yes	AAAGCACCTGTCTAGTAAACAGGAGAT
2663	Yes	AAAGCACCTGTCTAGTAAACAGGAGATC
2664	Yes	AAAGCACCTGTCTAGTAAACAGGAGATCC
2665	Yes	AAAGCACCTGTCTAGTAAACAGGAGATCCT
2666	Yes	AAAGCACCTGTCTAGTAAACAGGAGATCCTG
2667	Yes	AAAGCACCTGTCTAGTAAACAGGAGATCCTGA
2668	Yes	AAAGCACCTGTCTAGTAAACAGGAGATCCTGAG
2669	Yes	AAAGCACCTGTCTAGTAAACAGGAGATCCTGAGT
2670	Yes	AAAGCACCTGTCTAGTAAACAGGAGATCCTGAGTT
2671	Yes	AAAGCACCTGTCTAGTAAACAGGAGATCCTGAGTTT
2672	Yes	AAAGCACCTGTCTAGTAAACAGGAGATCCTGAGTTTC
2673	Yes	AAAGCACCTGTCTAGTAAACAGGAGATCCTGAGTTTCA
2674	Yes	AAAGCACCTGTCTAGTAAACAGGAGATCCTGAGTTTCAA
2675	Yes	AAAGCACCTGTCTAGTAAACAGGAGATCCTGAGTTTCAAT
2676	Yes	AAAGCACCTGTCTAGTAAACAGGAGATCCTGAGTTTCAATT
2677	Yes	AAAGCACCTGTCTAGTAAACAGGAGATCCTGAGTTTCAATTC
2678	Yes	AAAGCACCTGTCTAGTAAACAGGAGATCCTGAGTTTCAATTCC
2679	Yes	AAAGCACCTGTCTAGTAAACAGGAGATCCTGAGTTTCAATTCCA
2680	Yes	AAAGCACCTGTCTAGTAAACAGGAGATCCTGAGTTTCAATTCCAA
2681	Yes	AAAGCACCTGTCTAGTAAACAGGAGATCCTGAGTTTCAATTCCAAT

2682	Yes	AAAGCACCTGTCTAGTAAACAGGAGATCCTGAGTTTCAATTCCAATG
2683	Yes	AAAGCACCTGTCTAGTAAACAGGAGATCCTGAGTTTCAATTCCAATGG
2684	Yes	AAAGCACCTGTCTAGTAAACAGGAGATCCTGAGTTTCAATTCCAATGGT
2685	Yes	AAAGCACCTGTCTAGTAAACAGGAGATCCTGAGTTTCAATTCCAATGGTG
2686	No	AAAGCATGAGACTCTT
2687	No	AAAGCATGAGACTCTTA
2688	Yes	AAAGCATGAGACTCTTAA
2689	Yes	AAAGCATGAGACTCTTAAT
2690	Yes	AAAGCATGAGACTCTTAATC
2691	Yes	AAAGCATGAGACTCTTAATCT
2692	Yes	AAAGCATGAGACTCTTAATCTC
2693	Yes	AAAGCATGAGACTCTTAATCTCA
2694	Yes	AAAGCATGAGACTCTTAATCTCAG
2695	Yes	AAAGCATGAGACTCTTAATCTCAGG
2696	Yes	AAAGCATGAGACTCTTAATCTCAGGG
2697	Yes	AAAGCATGAGACTCTTAATCTCAGGGT
2698	Yes	AAAGCATGAGACTCTTAATCTCAGGGTC
2699	Yes	AAAGCATGAGACTCTTAATCTCAGGGTCG
2700	Yes	AAAGCATGAGACTCTTAATCTCAGGGTCGT
2701	Yes	AAAGCATGAGACTCTTAATCTCAGGGTCGTG
2702	Yes	AAAGCATGAGACTCTTAATCTCAGGGTCGTGG
2703	Yes	AAAGCATGAGACTCTTAATCTCAGGGTCGTGGG
2704	Yes	AAAGCATGAGACTCTTAATCTCAGGGTCGTGGGC
2705	Yes	AAAGCATGAGACTCTTAATCTCAGGGTCGTGGGCT
2706	Yes	AAAGCATGAGACTCTTAATCTCAGGGTCGTGGGCTC
2707	Yes	AAAGCATGAGACTCTTAATCTCAGGGTCGTGGGCTCG
2708	Yes	AAAGCATGAGACTCTTAATCTCAGGGTCGTGGGCTCGA
2709	Yes	AAAGCATGAGACTCTTAATCTCAGGGTCGTGGGCTCGAG
2710	Yes	AAAGCATGAGACTCTTAATCTCAGGGTCGTGGGCTCGAGC
2711	Yes	AAAGCATGAGACTCTTAATCTCAGGGTCGTGGGCTCGAGCT
2712	Yes	AAAGCATGAGACTCTTAATCTCAGGGTCGTGGGCTCGAGCTC
2713	Yes	AAAGCATGAGACTCTTAATCTCAGGGTCGTGGGCTCGAGCTCC
2714	Yes	AAAGCATGAGACTCTTAATCTCAGGGTCGTGGGCTCGAGCTCCA
2715	Yes	AAAGCATGAGACTCTTAATCTCAGGGTCGTGGGCTCGAGCTCCAT
2716	Yes	AAAGCATGAGACTCTTAATCTCAGGGTCGTGGGCTCGAGCTCCATG
2717	Yes	AAAGCATGAGACTCTTAATCTCAGGGTCGTGGGCTCGAGCTCCATGT
2718	Yes	AAAGCATGAGACTCTTAATCTCAGGGTCGTGGGCTCGAGCTCCATGTT
2719	Yes	AAAGCATGAGACTCTTAATCTCAGGGTCGTGGGCTCGAGCTCCATGTTG
2720	Yes	AAAGCATGAGACTCTTAATCTCAGGGTCGTGGGCTCGAGCTCCATGTTGG
2721	No	AAAGCCCTCAGTAAGT
2722	No	AAAGCCCTCAGTAAGTT
2723	Yes	AAAGCCCTCAGTAAGTTG

2724	Yes	AAAGCCCTCAGTAAGTTGC
2725	Yes	AAAGCCCTCAGTAAGTTGCA
2726	Yes	AAAGCCCTCAGTAAGTTGCAA
2727	Yes	AAAGCCCTCAGTAAGTTGCAAT
2728	Yes	AAAGCCCTCAGTAAGTTGCAATA
2729	Yes	AAAGCCCTCAGTAAGTTGCAATAC
2730	Yes	AAAGCCCTCAGTAAGTTGCAATACT
2731	Yes	AAAGCCCTCAGTAAGTTGCAATACTT
2732	Yes	AAAGCCCTCAGTAAGTTGCAATACTTA
2733	Yes	AAAGCCCTCAGTAAGTTGCAATACTTAA
2734	Yes	AAAGCCCTCAGTAAGTTGCAATACTTAAT
2735	Yes	AAAGCCCTCAGTAAGTTGCAATACTTAATT
2736	Yes	AAAGCCCTCAGTAAGTTGCAATACTTAATTT
2737	Yes	AAAGCCCTCAGTAAGTTGCAATACTTAATTTC
2738	Yes	AAAGCCCTCAGTAAGTTGCAATACTTAATTTCT
2739	Yes	AAAGCCCTCAGTAAGTTGCAATACTTAATTTCTG
2740	Yes	AAAGCCCTCAGTAAGTTGCAATACTTAATTTCTGC
2741	Yes	AAAGCCCTCAGTAAGTTGCAATACTTAATTTCTGCC
2742	Yes	AAAGCCCTCAGTAAGTTGCAATACTTAATTTCTGCCA
2743	No	AAAGCCTGTGGGCTCC
2744	No	AAAGCCTGTGGGCTCCA
2745	No	AAAGCCTGTGGGCTCCAC
2746	No	AAAGCCTGTGGGCTCCACA
2747	Yes	AAAGCCTGTGGGCTCCACAG
2748	Yes	AAAGCCTGTGGGCTCCACAGT
2749	Yes	AAAGCCTGTGGGCTCCACAGTC
2750	Yes	AAAGCCTGTGGGCTCCACAGTCA
2751	Yes	AAAGCCTGTGGGCTCCACAGTCAT
2752	Yes	AAAGCCTGTGGGCTCCACAGTCATG
2753	Yes	AAAGCCTGTGGGCTCCACAGTCATGT
2754	Yes	AAAGCCTGTGGGCTCCACAGTCATGTG
2755	Yes	AAAGCCTGTGGGCTCCACAGTCATGTGC
2756	Yes	AAAGCCTGTGGGCTCCACAGTCATGTGCC
2757	Yes	AAAGCCTGTGGGCTCCACAGTCATGTGCCT
2758	Yes	AAAGCCTGTGGGCTCCACAGTCATGTGCCTG
2759	Yes	AAAGCCTGTGGGCTCCACAGTCATGTGCCTGG
2760	Yes	AAAGCCTGTGGGCTCCACAGTCATGTGCCTGGG
2761	Yes	AAAGCCTGTGGGCTCCACAGTCATGTGCCTGGGT
2762	Yes	AAAGCCTGTGGGCTCCACAGTCATGTGCCTGGGTT
2763	Yes	AAAGCCTGTGGGCTCCACAGTCATGTGCCTGGGTTC
2764	Yes	AAAGCCTGTGGGCTCCACAGTCATGTGCCTGGGTTCA
2765	Yes	AAAGCCTGTGGGCTCCACAGTCATGTGCCTGGGTTCAA

2766	Yes	AAAGCCTGTGGGCTCCACAGTCATGTGCCTGGGTTCAAT
2767	Yes	AAAGCCTGTGGGCTCCACAGTCATGTGCCTGGGTTCAATT
2768	Yes	AAAGCCTGTGGGCTCCACAGTCATGTGCCTGGGTTCAATTC
2769	Yes	AAAGCCTGTGGGCTCCACAGTCATGTGCCTGGGTTCAATTCC
2770	Yes	AAAGCCTGTGGGCTCCACAGTCATGTGCCTGGGTTCAATTCCC
2771	Yes	AAAGCCTGTGGGCTCCACAGTCATGTGCCTGGGTTCAATTCCCA
2772	Yes	AAAGCCTGTGGGCTCCACAGTCATGTGCCTGGGTTCAATTCCCAG
2773	Yes	AAAGCCTGTGGGCTCCACAGTCATGTGCCTGGGTTCAATTCCCAGT
2774	Yes	AAAGCCTGTGGGCTCCACAGTCATGTGCCTGGGTTCAATTCCCAGTT
2775	Yes	AAAGCCTGTGGGCTCCACAGTCATGTGCCTGGGTTCAATTCCCAGTTC
2776	Yes	AAAGCCTGTGGGCTCCACAGTCATGTGCCTGGGTTCAATTCCCAGTTCT
2777	Yes	AAAGCCTGTGGGCTCCACAGTCATGTGCCTGGGTTCAATTCCCAGTTCTG
2778	No	AAAGCGCCTGTCTAGT
2779	Yes	AAAGCGCCTGTCTAGTA
2780	Yes	AAAGCGCCTGTCTAGTAA
2781	Yes	AAAGCGCCTGTCTAGTAAA
2782	Yes	AAAGCGCCTGTCTAGTAAAC
2783	Yes	AAAGCGCCTGTCTAGTAAACA
2784	Yes	AAAGCGCCTGTCTAGTAAACAG
2785	Yes	AAAGCGCCTGTCTAGTAAACAGG
2786	Yes	AAAGCGCCTGTCTAGTAAACAGGA
2787	Yes	AAAGCGCCTGTCTAGTAAACAGGAG
2788	Yes	AAAGCGCCTGTCTAGTAAACAGGAGA
2789	Yes	AAAGCGCCTGTCTAGTAAACAGGAGAT
2790	Yes	AAAGCGCCTGTCTAGTAAACAGGAGATC
2791	Yes	AAAGCGCCTGTCTAGTAAACAGGAGATCC
2792	Yes	AAAGCGCCTGTCTAGTAAACAGGAGATCCT
2793	Yes	AAAGCGCCTGTCTAGTAAACAGGAGATCCTG
2794	Yes	AAAGCGCCTGTCTAGTAAACAGGAGATCCTGG
2795	Yes	AAAGCGCCTGTCTAGTAAACAGGAGATCCTGGG
2796	Yes	AAAGCGCCTGTCTAGTAAACAGGAGATCCTGGGT
2797	Yes	AAAGCGCCTGTCTAGTAAACAGGAGATCCTGGGTT
2798	Yes	AAAGCGCCTGTCTAGTAAACAGGAGATCCTGGGTTC
2799	Yes	AAAGCGCCTGTCTAGTAAACAGGAGATCCTGGGTTCG
2800	Yes	AAAGCGCCTGTCTAGTAAACAGGAGATCCTGGGTTCGA
2801	Yes	AAAGCGCCTGTCTAGTAAACAGGAGATCCTGGGTTCGAA
2802	Yes	AAAGCGCCTGTCTAGTAAACAGGAGATCCTGGGTTCGAAT
2803	Yes	AAAGCGCCTGTCTAGTAAACAGGAGATCCTGGGTTCGAATC
2804	Yes	AAAGCGCCTGTCTAGTAAACAGGAGATCCTGGGTTCGAATCC
2805	Yes	AAAGCGCCTGTCTAGTAAACAGGAGATCCTGGGTTCGAATCCC
2806	Yes	AAAGCGCCTGTCTAGTAAACAGGAGATCCTGGGTTCGAATCCCA
2807	Yes	AAAGCGCCTGTCTAGTAAACAGGAGATCCTGGGTTCGAATCCCAG

2808	Yes	AAAGCGCCTGTCTAGTAAACAGGAGATCCTGGGTTCGAATCCCAGC
2809	Yes	AAAGCGCCTGTCTAGTAAACAGGAGATCCTGGGTTCGAATCCCAGCG
2810	Yes	AAAGCGCCTGTCTAGTAAACAGGAGATCCTGGGTTCGAATCCCAGCGA
2811	Yes	AAAGCGCCTGTCTAGTAAACAGGAGATCCTGGGTTCGAATCCCAGCGAG
2812	Yes	AAAGCGCCTGTCTAGTAAACAGGAGATCCTGGGTTCGAATCCCAGCGAGG
2813	Yes	AAAGCGCCTGTCTAGTAAACAGGAGATCCTGGGTTCGAATCCCAGCGG
2814	Yes	AAAGCGCCTGTCTAGTAAACAGGAGATCCTGGGTTCGAATCCCAGCGGG
2815	Yes	AAAGCGCCTGTCTAGTAAACAGGAGATCCTGGGTTCGAATCCCAGCGGGG
2816	Yes	AAAGCGCCTGTCTAGTAAACAGGAGATCCTGGGTTCGAATCCCAGCGGT
2817	Yes	AAAGCGCCTGTCTAGTAAACAGGAGATCCTGGGTTCGAATCCCAGCGGTG
2818	Yes	AAAGCGCCTGTCTAGTAAACAGGAGATCCTGGGTTCGAC
2819	Yes	AAAGCGCCTGTCTAGTAAACAGGAGATCCTGGGTTCGACT
2820	Yes	AAAGCGCCTGTCTAGTAAACAGGAGATCCTGGGTTCGACTC
2821	Yes	AAAGCGCCTGTCTAGTAAACAGGAGATCCTGGGTTCGACTCC
2822	Yes	AAAGCGCCTGTCTAGTAAACAGGAGATCCTGGGTTCGACTCCC
2823	Yes	AAAGCGCCTGTCTAGTAAACAGGAGATCCTGGGTTCGACTCCCA
2824	Yes	AAAGCGCCTGTCTAGTAAACAGGAGATCCTGGGTTCGACTCCCAG
2825	Yes	AAAGCGCCTGTCTAGTAAACAGGAGATCCTGGGTTCGACTCCCAGC
2826	Yes	AAAGCGCCTGTCTAGTAAACAGGAGATCCTGGGTTCGACTCCCAGCG
2827	Yes	AAAGCGCCTGTCTAGTAAACAGGAGATCCTGGGTTCGACTCCCAGCGG
2828	Yes	AAAGCGCCTGTCTAGTAAACAGGAGATCCTGGGTTCGACTCCCAGCGGG
2829	Yes	AAAGCGCCTGTCTAGTAAACAGGAGATCCTGGGTTCGACTCCCAGCGGGG
2830	No	AAAGCGCCTGTCTCGT
2831	No	AAAGCGCCTGTCTCGTA
2832	No	AAAGCGCCTGTCTCGTAA
2833	No	AAAGCGCCTGTCTCGTAAA
2834	Yes	AAAGCGCCTGTCTCGTAAAC
2835	Yes	AAAGCGCCTGTCTCGTAAACA
2836	Yes	AAAGCGCCTGTCTCGTAAACAG
2837	Yes	AAAGCGCCTGTCTCGTAAACAGG
2838	Yes	AAAGCGCCTGTCTCGTAAACAGGA
2839	Yes	AAAGCGCCTGTCTCGTAAACAGGAG
2840	Yes	AAAGCGCCTGTCTCGTAAACAGGAGA
2841	Yes	AAAGCGCCTGTCTCGTAAACAGGAGAT
2842	Yes	AAAGCGCCTGTCTCGTAAACAGGAGATC
2843	Yes	AAAGCGCCTGTCTCGTAAACAGGAGATCC
2844	Yes	AAAGCGCCTGTCTCGTAAACAGGAGATCCT
2845	Yes	AAAGCGCCTGTCTCGTAAACAGGAGATCCTG
2846	Yes	AAAGCGCCTGTCTCGTAAACAGGAGATCCTGG
2847	Yes	AAAGCGCCTGTCTCGTAAACAGGAGATCCTGGG
2848	Yes	AAAGCGCCTGTCTCGTAAACAGGAGATCCTGGGT
2849	Yes	AAAGCGCCTGTCTCGTAAACAGGAGATCCTGGGTT

2850	Yes	AAAGCGCCTGTCTCGTAAACAGGAGATCCTGGGTTC
2851	Yes	AAAGCGCCTGTCTCGTAAACAGGAGATCCTGGGTTCG
2852	Yes	AAAGCGCCTGTCTCGTAAACAGGAGATCCTGGGTTCGA
2853	Yes	AAAGCGCCTGTCTCGTAAACAGGAGATCCTGGGTTCGAA
2854	Yes	AAAGCGCCTGTCTCGTAAACAGGAGATCCTGGGTTCGAAT
2855	Yes	AAAGCGCCTGTCTCGTAAACAGGAGATCCTGGGTTCGAATC
2856	Yes	AAAGCGCCTGTCTCGTAAACAGGAGATCCTGGGTTCGAATCC
2857	Yes	AAAGCGCCTGTCTCGTAAACAGGAGATCCTGGGTTCGAATCCC
2858	Yes	AAAGCGCCTGTCTCGTAAACAGGAGATCCTGGGTTCGAATCCCA
2859	Yes	AAAGCGCCTGTCTCGTAAACAGGAGATCCTGGGTTCGAATCCCAG
2860	Yes	AAAGCGCCTGTCTCGTAAACAGGAGATCCTGGGTTCGAATCCCAGC
2861	Yes	AAAGCGCCTGTCTCGTAAACAGGAGATCCTGGGTTCGAATCCCAGCG
2862	Yes	AAAGCGCCTGTCTCGTAAACAGGAGATCCTGGGTTCGAATCCCAGCGG
2863	Yes	AAAGCGCCTGTCTCGTAAACAGGAGATCCTGGGTTCGAATCCCAGCGGG
2864	Yes	AAAGCGCCTGTCTCGTAAACAGGAGATCCTGGGTTCGAATCCCAGCGGGG
2865	Yes	AAAGCGCCTGTCTCGTAAACAGGAGATCCTGGGTTCGAC
2866	Yes	AAAGCGCCTGTCTCGTAAACAGGAGATCCTGGGTTCGACT
2867	Yes	AAAGCGCCTGTCTCGTAAACAGGAGATCCTGGGTTCGACTC
2868	Yes	AAAGCGCCTGTCTCGTAAACAGGAGATCCTGGGTTCGACTCC
2869	Yes	AAAGCGCCTGTCTCGTAAACAGGAGATCCTGGGTTCGACTCCC
2870	Yes	AAAGCGCCTGTCTCGTAAACAGGAGATCCTGGGTTCGACTCCCA
2871	Yes	AAAGCGCCTGTCTCGTAAACAGGAGATCCTGGGTTCGACTCCCAG
2872	Yes	AAAGCGCCTGTCTCGTAAACAGGAGATCCTGGGTTCGACTCCCAGT
2873	Yes	AAAGCGCCTGTCTCGTAAACAGGAGATCCTGGGTTCGACTCCCAGTG
2874	Yes	AAAGCGCCTGTCTCGTAAACAGGAGATCCTGGGTTCGACTCCCAGTGG
2875	Yes	AAAGCGCCTGTCTCGTAAACAGGAGATCCTGGGTTCGACTCCCAGTGGG
2876	Yes	AAAGCGCCTGTCTCGTAAACAGGAGATCCTGGGTTCGACTCCCAGTGGGG
2877	Yes	AAAGCGCCTGTCTTGT
2878	Yes	AAAGCGCCTGTCTTGTA
2879	Yes	AAAGCGCCTGTCTTGTAA
2880	Yes	AAAGCGCCTGTCTTGTAAA
2881	Yes	AAAGCGCCTGTCTTGTAAAC
2882	Yes	AAAGCGCCTGTCTTGTAAACA
2883	Yes	AAAGCGCCTGTCTTGTAAACAG
2884	Yes	AAAGCGCCTGTCTTGTAAACAGG
2885	Yes	AAAGCGCCTGTCTTGTAAACAGGA
2886	Yes	AAAGCGCCTGTCTTGTAAACAGGAG
2887	Yes	AAAGCGCCTGTCTTGTAAACAGGAGA
2888	Yes	AAAGCGCCTGTCTTGTAAACAGGAGAT
2889	Yes	AAAGCGCCTGTCTTGTAAACAGGAGATC
2890	Yes	AAAGCGCCTGTCTTGTAAACAGGAGATCC
2891	Yes	AAAGCGCCTGTCTTGTAAACAGGAGATCCT

2892	Yes	AAAGCGCCTGTCTTGTAAACAGGAGATCCTG
2893	Yes	AAAGCGCCTGTCTTGTAAACAGGAGATCCTGG
2894	Yes	AAAGCGCCTGTCTTGTAAACAGGAGATCCTGGG
2895	Yes	AAAGCGCCTGTCTTGTAAACAGGAGATCCTGGGT
2896	Yes	AAAGCGCCTGTCTTGTAAACAGGAGATCCTGGGTT
2897	Yes	AAAGCGCCTGTCTTGTAAACAGGAGATCCTGGGTTC
2898	Yes	AAAGCGCCTGTCTTGTAAACAGGAGATCCTGGGTTCG
2899	Yes	AAAGCGCCTGTCTTGTAAACAGGAGATCCTGGGTTCGA
2900	Yes	AAAGCGCCTGTCTTGTAAACAGGAGATCCTGGGTTCGAA
2901	Yes	AAAGCGCCTGTCTTGTAAACAGGAGATCCTGGGTTCGAAT
2902	Yes	AAAGCGCCTGTCTTGTAAACAGGAGATCCTGGGTTCGAATC
2903	Yes	AAAGCGCCTGTCTTGTAAACAGGAGATCCTGGGTTCGAATCC
2904	Yes	AAAGCGCCTGTCTTGTAAACAGGAGATCCTGGGTTCGAATCCC
2905	Yes	AAAGCGCCTGTCTTGTAAACAGGAGATCCTGGGTTCGAATCCCA
2906	Yes	AAAGCGCCTGTCTTGTAAACAGGAGATCCTGGGTTCGAATCCCAG
2907	Yes	AAAGCGCCTGTCTTGTAAACAGGAGATCCTGGGTTCGAATCCCAGT
2908	Yes	AAAGCGCCTGTCTTGTAAACAGGAGATCCTGGGTTCGAATCCCAGTA
2909	Yes	AAAGCGCCTGTCTTGTAAACAGGAGATCCTGGGTTCGAATCCCAGTAG
2910	Yes	AAAGCGCCTGTCTTGTAAACAGGAGATCCTGGGTTCGAATCCCAGTAGA
2911	Yes	AAAGCGCCTGTCTTGTAAACAGGAGATCCTGGGTTCGAATCCCAGTAGAG
2912	Yes	AAAGCGTCACGGCCCG
2913	Yes	AAAGCGTCACGGCCCGG
2914	Yes	AAAGCGTCACGGCCCGGG
2915	Yes	AAAGCGTCACGGCCCGGGT
2916	Yes	AAAGCGTCACGGCCCGGGTT
2917	Yes	AAAGCGTCACGGCCCGGGTTC
2918	Yes	AAAGCGTCACGGCCCGGGTTCG
2919	Yes	AAAGCGTCACGGCCCGGGTTCGA
2920	Yes	AAAGCGTCACGGCCCGGGTTCGAT
2921	Yes	AAAGCGTCACGGCCCGGGTTCGATT
2922	Yes	AAAGCGTCACGGCCCGGGTTCGATTC
2923	Yes	AAAGCGTCACGGCCCGGGTTCGATTCC
2924	Yes	AAAGCGTCACGGCCCGGGTTCGATTCCC
2925	Yes	AAAGCGTCACGGCCCGGGTTCGATTCCCG
2926	Yes	AAAGCGTCACGGCCCGGGTTCGATTCCCGG
2927	Yes	AAAGCGTCACGGCCCGGGTTCGATTCCCGGT
2928	Yes	AAAGCGTCACGGCCCGGGTTCGATTCCCGGTC
2929	Yes	AAAGCGTCACGGCCCGGGTTCGATTCCCGGTCA
2930	Yes	AAAGCGTCACGGCCCGGGTTCGATTCCCGGTCAG
2931	Yes	AAAGCGTCACGGCCCGGGTTCGATTCCCGGTCAGG
2932	Yes	AAAGCGTCACGGCCCGGGTTCGATTCCCGGTCAGGG
2933	Yes	AAAGCGTCACGGCCCGGGTTCGATTCCCGGTCAGGGA

2934	Yes	AAAGCGTCACGGCCCGGGTTCGATTCCCGGTCAGGGAA
2935	Yes	AAAGCGTCACGGCCCGGGTTCGATTCCCGGTCAGGGAAC
2936	Yes	AAAGCGTCACGGCCCGGGTTCGATTCCCGGTCAGGGAACC
2937	Yes	AAAGCGTCACGGCCCGGGTTCGATTCCCGGTCAGGGAACCA
2938	Yes	AAAGCTAACTTAGCAT
2939	Yes	AAAGCTAACTTAGCATT
2940	Yes	AAAGCTAACTTAGCATTA
2941	Yes	AAAGCTAACTTAGCATTAA
2942	Yes	AAAGCTAACTTAGCATTAAC
2943	Yes	AAAGCTAACTTAGCATTAACC
2944	Yes	AAAGCTAACTTAGCATTAACCT
2945	Yes	AAAGCTAACTTAGCATTAACCTT
2946	Yes	AAAGCTAACTTAGCATTAACCTTT
2947	Yes	AAAGCTAACTTAGCATTAACCTTTT
2948	Yes	AAAGCTAACTTAGCATTAACCTTTTA
2949	Yes	AAAGCTAACTTAGCATTAACCTTTTAA
2950	Yes	AAAGCTAACTTAGCATTAACCTTTTAAG
2951	Yes	AAAGCTAACTTAGCATTAACCTTTTAAGT
2952	Yes	AAAGCTAACTTAGCATTAACCTTTTAAGTT
2953	Yes	AAAGCTAACTTAGCATTAACCTTTTAAGTTA
2954	Yes	AAAGCTAACTTAGCATTAACCTTTTAAGTTAA
2955	Yes	AAAGCTAACTTAGCATTAACCTTTTAAGTTAAA
2956	Yes	AAAGCTAACTTAGCATTAACCTTTTAAGTTAAAG
2957	Yes	AAAGCTAACTTAGCATTAACCTTTTAAGTTAAAGA
2958	Yes	AAAGCTAACTTAGCATTAACCTTTTAAGTTAAAGAT
2959	Yes	AAAGCTAACTTAGCATTAACCTTTTAAGTTAAAGATT
2960	Yes	AAAGCTAACTTAGCATTAACCTTTTAAGTTAAAGATTA
2961	Yes	AAAGCTAACTTAGCATTAACCTTTTAAGTTAAAGATTAA
2962	Yes	AAAGCTAACTTAGCATTAACCTTTTAAGTTAAAGATTAAG
2963	Yes	AAAGCTAACTTAGCATTAACCTTTTAAGTTAAAGATTAAGA
2964	Yes	AAAGCTAACTTAGCATTAACCTTTTAAGTTAAAGATTAAGAG
2965	Yes	AAAGCTAACTTAGCATTAACCTTTTAAGTTAAAGATTAAGAGA
2966	Yes	AAAGCTAACTTAGCATTAACCTTTTAAGTTAAAGATTAAGAGAA
2967	Yes	AAAGCTAACTTAGCATTAACCTTTTAAGTTAAAGATTAAGAGAAC
2968	Yes	AAAGCTAACTTAGCATTAACCTTTTAAGTTAAAGATTAAGAGAACC
2969	Yes	AAAGCTAACTTAGCATTAACCTTTTAAGTTAAAGATTAAGAGAACCA
2970	Yes	AAAGCTAACTTAGCATTAACCTTTTAAGTTAAAGATTAAGAGAACCAA
2971	Yes	AAAGCTAACTTAGCATTAACCTTTTAAGTTAAAGATTAAGAGAACCAAC
2972	Yes	AAAGCTAACTTAGCATTAACCTTTTAAGTTAAAGATTAAGAGAACCAACA
2973	No	AAAGCTCACAAGAACT
2974	No	AAAGCTCACAAGAACTG
2975	Yes	AAAGCTCACAAGAACTGC

2976	Yes	AAAGCTCACAAGAACTGCT
2977	Yes	AAAGCTCACAAGAACTGCTA
2978	Yes	AAAGCTCACAAGAACTGCTAA
2979	Yes	AAAGCTCACAAGAACTGCTAAC
2980	Yes	AAAGCTCACAAGAACTGCTAACT
2981	Yes	AAAGCTCACAAGAACTGCTAACTC
2982	Yes	AAAGCTCACAAGAACTGCTAACTCA
2983	Yes	AAAGCTCACAAGAACTGCTAACTCAT
2984	Yes	AAAGCTCACAAGAACTGCTAACTCATG
2985	Yes	AAAGCTCACAAGAACTGCTAACTCATGC
2986	Yes	AAAGCTCACAAGAACTGCTAACTCATGCC
2987	Yes	AAAGCTCACAAGAACTGCTAACTCATGCCC
2988	Yes	AAAGCTCACAAGAACTGCTAACTCATGCCCC
2989	Yes	AAAGCTCACAAGAACTGCTAACTCATGCCCCC
2990	Yes	AAAGCTCACAAGAACTGCTAACTCATGCCCCCA
2991	Yes	AAAGCTCACAAGAACTGCTAACTCATGCCCCCAT
2992	Yes	AAAGCTCACAAGAACTGCTAACTCATGCCCCCATG
2993	Yes	AAAGCTCACAAGAACTGCTAACTCATGCCCCCATGT
2994	Yes	AAAGCTCACAAGAACTGCTAACTCATGCCCCCATGTC
2995	Yes	AAAGCTCACAAGAACTGCTAACTCATGCCCCCATGTCT
2996	Yes	AAAGCTCACAAGAACTGCTAACTCATGCCCCCATGTCTA
2997	Yes	AAAGCTCACAAGAACTGCTAACTCATGCCCCCATGTCTAA
2998	Yes	AAAGCTCACAAGAACTGCTAACTCATGCCCCCATGTCTAAC
2999	Yes	AAAGCTCACAAGAACTGCTAACTCATGCCCCCATGTCTAACA
3000	Yes	AAAGCTCACAAGAACTGCTAACTCATGCCCCCATGTCTAACAA
3001	Yes	AAAGCTCACAAGAACTGCTAACTCATGCCCCCATGTCTAACAAC
3002	Yes	AAAGCTCACAAGAACTGCTAACTCATGCCCCCATGTCTAACAACA
3003	Yes	AAAGCTCACAAGAACTGCTAACTCATGCCCCCATGTCTAACAACAT
3004	Yes	AAAGCTCACAAGAACTGCTAACTCATGCCCCCATGTCTAACAACATG
3005	Yes	AAAGCTCACAAGAACTGCTAACTCATGCCCCCATGTCTAACAACATGG
3006	Yes	AAAGCTCACAAGAACTGCTAACTCATGCCCCCATGTCTAACAACATGGC
3007	Yes	AAAGCTCACAAGAACTGCTAACTCATGCCCCCATGTCTAACAACATGGCT
3008	Yes	AAAGCTTGGTGGTTCG
3009	Yes	AAAGCTTGGTGGTTCGA
3010	Yes	AAAGCTTGGTGGTTCGAG
3011	Yes	AAAGCTTGGTGGTTCGAGC
3012	Yes	AAAGCTTGGTGGTTCGAGCC
3013	Yes	AAAGCTTGGTGGTTCGAGCCC
3014	Yes	AAAGCTTGGTGGTTCGAGCCCA
3015	Yes	AAAGCTTGGTGGTTCGAGCCCAC
3016	Yes	AAAGCTTGGTGGTTCGAGCCCACC
3017	Yes	AAAGCTTGGTGGTTCGAGCCCACCC

3018	Yes	AAAGCTTGGTGGTTCGAGCCCACCCA
3019	Yes	AAAGCTTGGTGGTTCGAGCCCACCCAG
3020	Yes	AAAGCTTGGTGGTTCGAGCCCACCCAGG
3021	Yes	AAAGCTTGGTGGTTCGAGCCCACCCAGGG
3022	Yes	AAAGCTTGGTGGTTCGAGCCCACCCAGGGA
3023	Yes	AAAGCTTGGTGGTTCGAGCCCACCCAGGGAT
3024	Yes	AAAGCTTGGTGGTTCGAGCCCACCCAGGGATG
3025	Yes	AAAGCTTGGTGGTTCGAGCCCACCCAGGGATGC
3026	Yes	AAAGCTTGGTGGTTCGAGCCCACCCAGGGATGCC
3027	Yes	AAAGCTTGGTGGTTCGAGCCCACCCAGGGATGCCA
3028	No	AAAGCTTGGTGGTTTG
3029	No	AAAGCTTGGTGGTTTGA
3030	Yes	AAAGCTTGGTGGTTTGAG
3031	Yes	AAAGCTTGGTGGTTTGAGC
3032	Yes	AAAGCTTGGTGGTTTGAGCC
3033	Yes	AAAGCTTGGTGGTTTGAGCCC
3034	Yes	AAAGCTTGGTGGTTTGAGCCCA
3035	Yes	AAAGCTTGGTGGTTTGAGCCCAC
3036	Yes	AAAGCTTGGTGGTTTGAGCCCACC
3037	Yes	AAAGCTTGGTGGTTTGAGCCCACCC
3038	Yes	AAAGCTTGGTGGTTTGAGCCCACCCA
3039	Yes	AAAGCTTGGTGGTTTGAGCCCACCCAG
3040	Yes	AAAGCTTGGTGGTTTGAGCCCACCCAGG
3041	Yes	AAAGCTTGGTGGTTTGAGCCCACCCAGGG
3042	Yes	AAAGCTTGGTGGTTTGAGCCCACCCAGGGA
3043	Yes	AAAGCTTGGTGGTTTGAGCCCACCCAGGGAT
3044	Yes	AAAGCTTGGTGGTTTGAGCCCACCCAGGGATG
3045	Yes	AAAGCTTGGTGGTTTGAGCCCACCCAGGGATGC
3046	Yes	AAAGCTTGGTGGTTTGAGCCCACCCAGGGATGCC
3047	Yes	AAAGCTTGGTGGTTTGAGCCCACCCAGGGATGCCA
3048	No	AAAGGAGACTGGTTCA
3049	Yes	AAAGGAGACTGGTTCAA
3050	Yes	AAAGGAGACTGGTTCAAT
3051	Yes	AAAGGAGACTGGTTCAATT
3052	Yes	AAAGGAGACTGGTTCAATTC
3053	Yes	AAAGGAGACTGGTTCAATTCT
3054	Yes	AAAGGAGACTGGTTCAATTCTG
3055	Yes	AAAGGAGACTGGTTCAATTCTGG
3056	Yes	AAAGGAGACTGGTTCAATTCTGGG
3057	Yes	AAAGGAGACTGGTTCAATTCTGGGT
3058	Yes	AAAGGAGACTGGTTCAATTCTGGGTT
3059	Yes	AAAGGAGACTGGTTCAATTCTGGGTTT

3060	Yes	AAAGGAGACTGGTTCAATTCTGGGTTTT
3061	Yes	AAAGGAGACTGGTTCAATTCTGGGTTTTG
3062	Yes	AAAGGAGACTGGTTCAATTCTGGGTTTTGG
3063	Yes	AAAGGAGACTGGTTCAATTCTGGGTTTTGGC
3064	Yes	AAAGGAGACTGGTTCAATTCTGGGTTTTGGCA
3065	Yes	AAAGGAGACTGGTTCAATTCTGGGTTTTGGCAC
3066	Yes	AAAGGAGACTGGTTCAATTCTGGGTTTTGGCACC
3067	Yes	AAAGGAGACTGGTTCAATTCTGGGTTTTGGCACCA
3068	No	AAAGGATAACAGCTAT
3069	Yes	AAAGGATAACAGCTATC
3070	Yes	AAAGGATAACAGCTATCC
3071	Yes	AAAGGATAACAGCTATCCA
3072	Yes	AAAGGATAACAGCTATCCAT
3073	Yes	AAAGGATAACAGCTATCCATT
3074	Yes	AAAGGATAACAGCTATCCATTG
3075	Yes	AAAGGATAACAGCTATCCATTGG
3076	Yes	AAAGGATAACAGCTATCCATTGGT
3077	Yes	AAAGGATAACAGCTATCCATTGGTC
3078	Yes	AAAGGATAACAGCTATCCATTGGTCT
3079	Yes	AAAGGATAACAGCTATCCATTGGTCTT
3080	Yes	AAAGGATAACAGCTATCCATTGGTCTTA
3081	Yes	AAAGGATAACAGCTATCCATTGGTCTTAG
3082	Yes	AAAGGATAACAGCTATCCATTGGTCTTAGG
3083	Yes	AAAGGATAACAGCTATCCATTGGTCTTAGGC
3084	Yes	AAAGGATAACAGCTATCCATTGGTCTTAGGCC
3085	Yes	AAAGGATAACAGCTATCCATTGGTCTTAGGCCC
3086	Yes	AAAGGATAACAGCTATCCATTGGTCTTAGGCCCC
3087	Yes	AAAGGATAACAGCTATCCATTGGTCTTAGGCCCCA
3088	Yes	AAAGGATAACAGCTATCCATTGGTCTTAGGCCCCAA
3089	Yes	AAAGGATAACAGCTATCCATTGGTCTTAGGCCCCAAA
3090	Yes	AAAGGATAACAGCTATCCATTGGTCTTAGGCCCCAAAA
3091	Yes	AAAGGATAACAGCTATCCATTGGTCTTAGGCCCCAAAAA
3092	Yes	AAAGGATAACAGCTATCCATTGGTCTTAGGCCCCAAAAAT
3093	Yes	AAAGGATAACAGCTATCCATTGGTCTTAGGCCCCAAAAATT
3094	Yes	AAAGGATAACAGCTATCCATTGGTCTTAGGCCCCAAAAATTT
3095	Yes	AAAGGATAACAGCTATCCATTGGTCTTAGGCCCCAAAAATTTT
3096	Yes	AAAGGATAACAGCTATCCATTGGTCTTAGGCCCCAAAAATTTTG
3097	Yes	AAAGGATAACAGCTATCCATTGGTCTTAGGCCCCAAAAATTTTGG
3098	Yes	AAAGGATAACAGCTATCCATTGGTCTTAGGCCCCAAAAATTTTGGT
3099	Yes	AAAGGATAACAGCTATCCATTGGTCTTAGGCCCCAAAAATTTTGGTG
3100	Yes	AAAGGATAACAGCTATCCATTGGTCTTAGGCCCCAAAAATTTTGGTGC
3101	Yes	AAAGGATAACAGCTATCCATTGGTCTTAGGCCCCAAAAATTTTGGTGCA

3102	Yes	AAAGGATAACAGCTATCCATTGGTCTTAGGCCCCAAAAATTTTGGTGCAA
3103	No	AAAGGCATGGGCTCTA
3104	No	AAAGGCATGGGCTCTAG
3105	No	AAAGGCATGGGCTCTAGA
3106	No	AAAGGCATGGGCTCTAGAG
3107	Yes	AAAGGCATGGGCTCTAGAGC
3108	Yes	AAAGGCATGGGCTCTAGAGCC
3109	Yes	AAAGGCATGGGCTCTAGAGCCA
3110	Yes	AAAGGCATGGGCTCTAGAGCCAG
3111	Yes	AAAGGCATGGGCTCTAGAGCCAGA
3112	Yes	AAAGGCATGGGCTCTAGAGCCAGAC
3113	Yes	AAAGGCATGGGCTCTAGAGCCAGACT
3114	Yes	AAAGGCATGGGCTCTAGAGCCAGACTT
3115	Yes	AAAGGCATGGGCTCTAGAGCCAGACTTC
3116	Yes	AAAGGCATGGGCTCTAGAGCCAGACTTCC
3117	Yes	AAAGGCATGGGCTCTAGAGCCAGACTTCCT
3118	Yes	AAAGGCATGGGCTCTAGAGCCAGACTTCCTG
3119	Yes	AAAGGCATGGGCTCTAGAGCCAGACTTCCTGG
3120	Yes	AAAGGCATGGGCTCTAGAGCCAGACTTCCTGGG
3121	Yes	AAAGGCATGGGCTCTAGAGCCAGACTTCCTGGGT
3122	Yes	AAAGGCATGGGCTCTAGAGCCAGACTTCCTGGGTT
3123	Yes	AAAGGCATGGGCTCTAGAGCCAGACTTCCTGGGTTC
3124	Yes	AAAGGCATGGGCTCTAGAGCCAGACTTCCTGGGTTCA
3125	Yes	AAAGGCATGGGCTCTAGAGCCAGACTTCCTGGGTTCAA
3126	Yes	AAAGGCATGGGCTCTAGAGCCAGACTTCCTGGGTTCAAA
3127	Yes	AAAGGCATGGGCTCTAGAGCCAGACTTCCTGGGTTCAAAT
3128	Yes	AAAGGCATGGGCTCTAGAGCCAGACTTCCTGGGTTCAAATC
3129	Yes	AAAGGCATGGGCTCTAGAGCCAGACTTCCTGGGTTCAAATCT
3130	Yes	AAAGGCATGGGCTCTAGAGCCAGACTTCCTGGGTTCAAATCTC
3131	Yes	AAAGGCATGGGCTCTAGAGCCAGACTTCCTGGGTTCAAATCTCA
3132	Yes	AAAGGCATGGGCTCTAGAGCCAGACTTCCTGGGTTCAAATCTCAG
3133	Yes	AAAGGCATGGGCTCTAGAGCCAGACTTCCTGGGTTCAAATCTCAGA
3134	Yes	AAAGGCATGGGCTCTAGAGCCAGACTTCCTGGGTTCAAATCTCAGAC
3135	Yes	AAAGGCATGGGCTCTAGAGCCAGACTTCCTGGGTTCAAATCTCAGACC
3136	Yes	AAAGGCATGGGCTCTAGAGCCAGACTTCCTGGGTTCAAATCTCAGACCT
3137	Yes	AAAGGCATGGGCTCTAGAGCCAGACTTCCTGGGTTCAAATCTCAGACCTG
3138	Yes	AAAGGCTGGTGGTTCA
3139	Yes	AAAGGCTGGTGGTTCAA
3140	Yes	AAAGGCTGGTGGTTCAAG
3141	Yes	AAAGGCTGGTGGTTCAAGC
3142	Yes	AAAGGCTGGTGGTTCAAGCC
3143	Yes	AAAGGCTGGTGGTTCAAGCCC

3144	Yes	AAAGGCTGGTGGTTCAAGCCCA
3145	Yes	AAAGGCTGGTGGTTCAAGCCCAC
3146	Yes	AAAGGCTGGTGGTTCAAGCCCACC
3147	Yes	AAAGGCTGGTGGTTCAAGCCCACCC
3148	Yes	AAAGGCTGGTGGTTCAAGCCCACCCA
3149	Yes	AAAGGCTGGTGGTTCAAGCCCACCCAG
3150	Yes	AAAGGCTGGTGGTTCAAGCCCACCCAGA
3151	Yes	AAAGGCTGGTGGTTCAAGCCCACCCAGAG
3152	Yes	AAAGGCTGGTGGTTCAAGCCCACCCAGAGA
3153	Yes	AAAGGCTGGTGGTTCAAGCCCACCCAGAGAT
3154	Yes	AAAGGCTGGTGGTTCAAGCCCACCCAGAGATG
3155	Yes	AAAGGCTGGTGGTTCAAGCCCACCCAGAGATGC
3156	Yes	AAAGGCTGGTGGTTCAAGCCCACCCAGAGATGCC
3157	Yes	AAAGGCTGGTGGTTCAAGCCCACCCAGAGATGCCA
3158	No	AAAGGGAATAGGCTTT
3159	Yes	AAAGGGAATAGGCTTTA
3160	Yes	AAAGGGAATAGGCTTTAG
3161	Yes	AAAGGGAATAGGCTTTAGA
3162	Yes	AAAGGGAATAGGCTTTAGAG
3163	Yes	AAAGGGAATAGGCTTTAGAGC
3164	Yes	AAAGGGAATAGGCTTTAGAGCC
3165	Yes	AAAGGGAATAGGCTTTAGAGCCA
3166	Yes	AAAGGGAATAGGCTTTAGAGCCAG
3167	Yes	AAAGGGAATAGGCTTTAGAGCCAGA
3168	Yes	AAAGGGAATAGGCTTTAGAGCCAGAC
3169	Yes	AAAGGGAATAGGCTTTAGAGCCAGACT
3170	Yes	AAAGGGAATAGGCTTTAGAGCCAGACTG
3171	Yes	AAAGGGAATAGGCTTTAGAGCCAGACTGC
3172	Yes	AAAGGGAATAGGCTTTAGAGCCAGACTGCC
3173	Yes	AAAGGGAATAGGCTTTAGAGCCAGACTGCCT
3174	Yes	AAAGGGAATAGGCTTTAGAGCCAGACTGCCTG
3175	Yes	AAAGGGAATAGGCTTTAGAGCCAGACTGCCTGG
3176	Yes	AAAGGGAATAGGCTTTAGAGCCAGACTGCCTGGG
3177	Yes	AAAGGGAATAGGCTTTAGAGCCAGACTGCCTGGGT
3178	Yes	AAAGGGAATAGGCTTTAGAGCCAGACTGCCTGGGTT
3179	Yes	AAAGGGAATAGGCTTTAGAGCCAGACTGCCTGGGTTT
3180	Yes	AAAGGGAATAGGCTTTAGAGCCAGACTGCCTGGGTTTG
3181	Yes	AAAGGGAATAGGCTTTAGAGCCAGACTGCCTGGGTTTGA
3182	Yes	AAAGGGAATAGGCTTTAGAGCCAGACTGCCTGGGTTTGAA
3183	Yes	AAAGGGAATAGGCTTTAGAGCCAGACTGCCTGGGTTTGAAT
3184	Yes	AAAGGGAATAGGCTTTAGAGCCAGACTGCCTGGGTTTGAATC
3185	Yes	AAAGGGAATAGGCTTTAGAGCCAGACTGCCTGGGTTTGAATCC

3186	Yes	AAAGGGAATAGGCTTTAGAGCCAGACTGCCTGGGTTTGAATCCC
3187	Yes	AAAGGGAATAGGCTTTAGAGCCAGACTGCCTGGGTTTGAATCCCA
3188	Yes	AAAGGGAATAGGCTTTAGAGCCAGACTGCCTGGGTTTGAATCCCAG
3189	Yes	AAAGGGAATAGGCTTTAGAGCCAGACTGCCTGGGTTTGAATCCCAGC
3190	Yes	AAAGGGAATAGGCTTTAGAGCCAGACTGCCTGGGTTTGAATCCCAGCC
3191	Yes	AAAGGGAATAGGCTTTAGAGCCAGACTGCCTGGGTTTGAATCCCAGCCC
3192	Yes	AAAGGGAATAGGCTTTAGAGCCAGACTGCCTGGGTTTGAATCCCAGCCCC
3193	No	AAAGGGCTTAGCTTAA
3194	No	AAAGGGCTTAGCTTAAT
3195	No	AAAGGGCTTAGCTTAATT
3196	Yes	AAAGGGCTTAGCTTAATTA
3197	Yes	AAAGGGCTTAGCTTAATTAA
3198	Yes	AAAGGGCTTAGCTTAATTAAA
3199	Yes	AAAGGGCTTAGCTTAATTAAAG
3200	Yes	AAAGGGCTTAGCTTAATTAAAGT
3201	Yes	AAAGGGCTTAGCTTAATTAAAGTG
3202	Yes	AAAGGGCTTAGCTTAATTAAAGTGG
3203	Yes	AAAGGGCTTAGCTTAATTAAAGTGGC
3204	Yes	AAAGGGCTTAGCTTAATTAAAGTGGCT
3205	Yes	AAAGGGCTTAGCTTAATTAAAGTGGCTG
3206	Yes	AAAGGGCTTAGCTTAATTAAAGTGGCTGA
3207	Yes	AAAGGGCTTAGCTTAATTAAAGTGGCTGAT
3208	Yes	AAAGGGCTTAGCTTAATTAAAGTGGCTGATT
3209	Yes	AAAGGGCTTAGCTTAATTAAAGTGGCTGATTT
3210	Yes	AAAGGGCTTAGCTTAATTAAAGTGGCTGATTTG
3211	Yes	AAAGGGCTTAGCTTAATTAAAGTGGCTGATTTGC
3212	Yes	AAAGGGCTTAGCTTAATTAAAGTGGCTGATTTGCG
3213	Yes	AAAGGGCTTAGCTTAATTAAAGTGGCTGATTTGCGT
3214	Yes	AAAGGGCTTAGCTTAATTAAAGTGGCTGATTTGCGTT
3215	Yes	AAAGGGCTTAGCTTAATTAAAGTGGCTGATTTGCGTTC
3216	Yes	AAAGGGCTTAGCTTAATTAAAGTGGCTGATTTGCGTTCA
3217	Yes	AAAGGGCTTAGCTTAATTAAAGTGGCTGATTTGCGTTCAG
3218	Yes	AAAGGGCTTAGCTTAATTAAAGTGGCTGATTTGCGTTCAGT
3219	Yes	AAAGGGCTTAGCTTAATTAAAGTGGCTGATTTGCGTTCAGTT
3220	Yes	AAAGGGCTTAGCTTAATTAAAGTGGCTGATTTGCGTTCAGTTG
3221	Yes	AAAGGGCTTAGCTTAATTAAAGTGGCTGATTTGCGTTCAGTTGA
3222	Yes	AAAGGGCTTAGCTTAATTAAAGTGGCTGATTTGCGTTCAGTTGAT
3223	Yes	AAAGGGCTTAGCTTAATTAAAGTGGCTGATTTGCGTTCAGTTGATG
3224	Yes	AAAGGGCTTAGCTTAATTAAAGTGGCTGATTTGCGTTCAGTTGATGC
3225	Yes	AAAGGGCTTAGCTTAATTAAAGTGGCTGATTTGCGTTCAGTTGATGCA
3226	Yes	AAAGGGCTTAGCTTAATTAAAGTGGCTGATTTGCGTTCAGTTGATGCAG
3227	Yes	AAAGGGCTTAGCTTAATTAAAGTGGCTGATTTGCGTTCAGTTGATGCAGA

3228	No	AAAGGTATTAGAAAAA
3229	No	AAAGGTATTAGAAAAAC
3230	No	AAAGGTATTAGAAAAACC
3231	Yes	AAAGGTATTAGAAAAACCA
3232	Yes	AAAGGTATTAGAAAAACCAT
3233	Yes	AAAGGTATTAGAAAAACCATT
3234	Yes	AAAGGTATTAGAAAAACCATTT
3235	Yes	AAAGGTATTAGAAAAACCATTTC
3236	Yes	AAAGGTATTAGAAAAACCATTTCA
3237	Yes	AAAGGTATTAGAAAAACCATTTCAT
3238	Yes	AAAGGTATTAGAAAAACCATTTCATA
3239	Yes	AAAGGTATTAGAAAAACCATTTCATAA
3240	Yes	AAAGGTATTAGAAAAACCATTTCATAAC
3241	Yes	AAAGGTATTAGAAAAACCATTTCATAACT
3242	Yes	AAAGGTATTAGAAAAACCATTTCATAACTT
3243	Yes	AAAGGTATTAGAAAAACCATTTCATAACTTT
3244	Yes	AAAGGTATTAGAAAAACCATTTCATAACTTTG
3245	Yes	AAAGGTATTAGAAAAACCATTTCATAACTTTGT
3246	Yes	AAAGGTATTAGAAAAACCATTTCATAACTTTGTC
3247	Yes	AAAGGTATTAGAAAAACCATTTCATAACTTTGTCA
3248	Yes	AAAGGTATTAGAAAAACCATTTCATAACTTTGTCAA
3249	Yes	AAAGGTATTAGAAAAACCATTTCATAACTTTGTCAAA
3250	Yes	AAAGGTATTAGAAAAACCATTTCATAACTTTGTCAAAG
3251	Yes	AAAGGTATTAGAAAAACCATTTCATAACTTTGTCAAAGT
3252	Yes	AAAGGTATTAGAAAAACCATTTCATAACTTTGTCAAAGTT
3253	Yes	AAAGGTATTAGAAAAACCATTTCATAACTTTGTCAAAGTTA
3254	Yes	AAAGGTATTAGAAAAACCATTTCATAACTTTGTCAAAGTTAA
3255	Yes	AAAGGTATTAGAAAAACCATTTCATAACTTTGTCAAAGTTAAA
3256	Yes	AAAGGTATTAGAAAAACCATTTCATAACTTTGTCAAAGTTAAAT
3257	Yes	AAAGGTATTAGAAAAACCATTTCATAACTTTGTCAAAGTTAAATT
3258	Yes	AAAGGTATTAGAAAAACCATTTCATAACTTTGTCAAAGTTAAATTA
3259	Yes	AAAGGTATTAGAAAAACCATTTCATAACTTTGTCAAAGTTAAATTAT
3260	Yes	AAAGGTATTAGAAAAACCATTTCATAACTTTGTCAAAGTTAAATTATA
3261	Yes	AAAGGTATTAGAAAAACCATTTCATAACTTTGTCAAAGTTAAATTATAG
3262	Yes	AAAGGTATTAGAAAAACCATTTCATAACTTTGTCAAAGTTAAATTATAGG
3263	No	AAAGGTCACCAGTTTG
3264	Yes	AAAGGTCACCAGTTTGA
3265	Yes	AAAGGTCACCAGTTTGAG
3266	Yes	AAAGGTCACCAGTTTGAGA
3267	Yes	AAAGGTCACCAGTTTGAGAC
3268	Yes	AAAGGTCACCAGTTTGAGACC
3269	Yes	AAAGGTCACCAGTTTGAGACCG

3270	Yes	AAAGGTCACCAGTTTGAGACCGG
3271	Yes	AAAGGTCACCAGTTTGAGACCGGG
3272	Yes	AAAGGTCACCAGTTTGAGACCGGGC
3273	Yes	AAAGGTCACCAGTTTGAGACCGGGCC
3274	Yes	AAAGGTCACCAGTTTGAGACCGGGCCA
3275	Yes	AAAGGTCACCAGTTTGAGACCGGGCCAA
3276	Yes	AAAGGTCACCAGTTTGAGACCGGGCCAAA
3277	Yes	AAAGGTCACCAGTTTGAGACCGGGCCAAAA
3278	Yes	AAAGGTCACCAGTTTGAGACCGGGCCAAAAC
3279	Yes	AAAGGTCACCAGTTTGAGACCGGGCCAAAACA
3280	Yes	AAAGGTCACCAGTTTGAGACCGGGCCAAAACAC
3281	Yes	AAAGGTCACCAGTTTGAGACCGGGCCAAAACACC
3282	Yes	AAAGGTCACCAGTTTGAGACCGGGCCAAAACACCA
3283	Yes	AAAGGTCCCCGGTTCG
3284	Yes	AAAGGTCCCCGGTTCGA
3285	Yes	AAAGGTCCCCGGTTCGAA
3286	Yes	AAAGGTCCCCGGTTCGAAA
3287	Yes	AAAGGTCCCCGGTTCGAAAC
3288	Yes	AAAGGTCCCCGGTTCGAAACC
3289	Yes	AAAGGTCCCCGGTTCGAAACCG
3290	Yes	AAAGGTCCCCGGTTCGAAACCGG
3291	Yes	AAAGGTCCCCGGTTCGAAACCGGG
3292	Yes	AAAGGTCCCCGGTTCGAAACCGGGC
3293	Yes	AAAGGTCCCCGGTTCGAAACCGGGCA
3294	Yes	AAAGGTCCCCGGTTCGAAACCGGGCAG
3295	Yes	AAAGGTCCCCGGTTCGAAACCGGGCAGA
3296	Yes	AAAGGTCCCCGGTTCGAAACCGGGCAGAA
3297	Yes	AAAGGTCCCCGGTTCGAAACCGGGCAGAAA
3298	Yes	AAAGGTCCCCGGTTCGAAACCGGGCAGAAAC
3299	Yes	AAAGGTCCCCGGTTCGAAACCGGGCAGAAACA
3300	Yes	AAAGGTCCCCGGTTCGAAACCGGGCAGAAACAC
3301	Yes	AAAGGTCCCCGGTTCGAAACCGGGCAGAAACACC
3302	Yes	AAAGGTCCCCGGTTCGAAACCGGGCAGAAACACCA
3303	Yes	AAAGGTCCCCGGTTCGAAACCGGGCAGAAG
3304	Yes	AAAGGTCCCCGGTTCGAAACCGGGCAGAAGC
3305	Yes	AAAGGTCCCCGGTTCGAAACCGGGCAGAAGCA
3306	Yes	AAAGGTCCCCGGTTCGAAACCGGGCAGAAGCAC
3307	Yes	AAAGGTCCCCGGTTCGAAACCGGGCAGAAGCACC
3308	Yes	AAAGGTCCCCGGTTCGAAACCGGGCAGAAGCACCA
3309	Yes	AAAGGTCCCCGGTTCGAAACCGGGCG
3310	Yes	AAAGGTCCCCGGTTCGAAACCGGGCGG
3311	Yes	AAAGGTCCCCGGTTCGAAACCGGGCGGA

3312	Yes	AAAGGTCCCCGGTTCGAAACCGGGCGGAA
3313	Yes	AAAGGTCCCCGGTTCGAAACCGGGCGGAAA
3314	Yes	AAAGGTCCCCGGTTCGAAACCGGGCGGAAAC
3315	Yes	AAAGGTCCCCGGTTCGAAACCGGGCGGAAACA
3316	Yes	AAAGGTCCCCGGTTCGAAACCGGGCGGAAACAC
3317	Yes	AAAGGTCCCCGGTTCGAAACCGGGCGGAAACACC
3318	Yes	AAAGGTCCCCGGTTCGAAACCGGGCGGAAACACCA
3319	Yes	AAAGGTCCCCGGTTCGAAACT
3320	Yes	AAAGGTCCCCGGTTCGAAACTG
3321	Yes	AAAGGTCCCCGGTTCGAAACTGG
3322	Yes	AAAGGTCCCCGGTTCGAAACTGGG
3323	Yes	AAAGGTCCCCGGTTCGAAACTGGGC
3324	Yes	AAAGGTCCCCGGTTCGAAACTGGGCG
3325	Yes	AAAGGTCCCCGGTTCGAAACTGGGCGG
3326	Yes	AAAGGTCCCCGGTTCGAAACTGGGCGGA
3327	Yes	AAAGGTCCCCGGTTCGAAACTGGGCGGAA
3328	Yes	AAAGGTCCCCGGTTCGAAACTGGGCGGAAA
3329	Yes	AAAGGTCCCCGGTTCGAAACTGGGCGGAAAC
3330	Yes	AAAGGTCCCCGGTTCGAAACTGGGCGGAAACA
3331	Yes	AAAGGTCCCCGGTTCGAAACTGGGCGGAAACAC
3332	Yes	AAAGGTCCCCGGTTCGAAACTGGGCGGAAACACC
3333	Yes	AAAGGTCCCCGGTTCGAAACTGGGCGGAAACACCA
3334	Yes	AAAGGTCCCCGGTTCGAT
3335	Yes	AAAGGTCCCCGGTTCGATC
3336	Yes	AAAGGTCCCCGGTTCGATCC
3337	Yes	AAAGGTCCCCGGTTCGATCCC
3338	Yes	AAAGGTCCCCGGTTCGATCCCG
3339	Yes	AAAGGTCCCCGGTTCGATCCCGG
3340	Yes	AAAGGTCCCCGGTTCGATCCCGGG
3341	Yes	AAAGGTCCCCGGTTCGATCCCGGGC
3342	Yes	AAAGGTCCCCGGTTCGATCCCGGGCG
3343	Yes	AAAGGTCCCCGGTTCGATCCCGGGCGG
3344	Yes	AAAGGTCCCCGGTTCGATCCCGGGCGGA
3345	Yes	AAAGGTCCCCGGTTCGATCCCGGGCGGAA
3346	Yes	AAAGGTCCCCGGTTCGATCCCGGGCGGAAA
3347	Yes	AAAGGTCCCCGGTTCGATCCCGGGCGGAAAC
3348	Yes	AAAGGTCCCCGGTTCGATCCCGGGCGGAAACA
3349	Yes	AAAGGTCCCCGGTTCGATCCCGGGCGGAAACAC
3350	Yes	AAAGGTCCCCGGTTCGATCCCGGGCGGAAACACC
3351	Yes	AAAGGTCCCCGGTTCGATCCCGGGCGGAAACACCA
3352	Yes	AAAGGTCCCCGGTTTG
3353	Yes	AAAGGTCCCCGGTTTGA

3354	Yes	AAAGGTCCCCGGTTTGAA
3355	Yes	AAAGGTCCCCGGTTTGAAA
3356	Yes	AAAGGTCCCCGGTTTGAAAC
3357	Yes	AAAGGTCCCCGGTTTGAAACC
3358	Yes	AAAGGTCCCCGGTTTGAAACCA
3359	Yes	AAAGGTCCCCGGTTTGAAACCAG
3360	Yes	AAAGGTCCCCGGTTTGAAACCAGG
3361	Yes	AAAGGTCCCCGGTTTGAAACCAGGC
3362	Yes	AAAGGTCCCCGGTTTGAAACCAGGCG
3363	Yes	AAAGGTCCCCGGTTTGAAACCAGGCGG
3364	Yes	AAAGGTCCCCGGTTTGAAACCAGGCGGA
3365	Yes	AAAGGTCCCCGGTTTGAAACCAGGCGGAA
3366	Yes	AAAGGTCCCCGGTTTGAAACCAGGCGGAAA
3367	Yes	AAAGGTCCCCGGTTTGAAACCAGGCGGAAAC
3368	Yes	AAAGGTCCCCGGTTTGAAACCAGGCGGAAACA
3369	Yes	AAAGGTCCCCGGTTTGAAACCAGGCGGAAACAC
3370	Yes	AAAGGTCCCCGGTTTGAAACCAGGCGGAAACACC
3371	Yes	AAAGGTCCCCGGTTTGAAACCAGGCGGAAACACCA
3372	No	AAAGGTCCCTGGATCA
3373	No	AAAGGTCCCTGGATCAA
3374	No	AAAGGTCCCTGGATCAAA
3375	No	AAAGGTCCCTGGATCAAAA
3376	Yes	AAAGGTCCCTGGATCAAAAC
3377	Yes	AAAGGTCCCTGGATCAAAACC
3378	Yes	AAAGGTCCCTGGATCAAAACCA
3379	Yes	AAAGGTCCCTGGATCAAAACCAG
3380	Yes	AAAGGTCCCTGGATCAAAACCAGG
3381	Yes	AAAGGTCCCTGGATCAAAACCAGGC
3382	Yes	AAAGGTCCCTGGATCAAAACCAGGCG
3383	Yes	AAAGGTCCCTGGATCAAAACCAGGCGG
3384	Yes	AAAGGTCCCTGGATCAAAACCAGGCGGA
3385	Yes	AAAGGTCCCTGGATCAAAACCAGGCGGAA
3386	Yes	AAAGGTCCCTGGATCAAAACCAGGCGGAAA
3387	Yes	AAAGGTCCCTGGATCAAAACCAGGCGGAAAC
3388	Yes	AAAGGTCCCTGGATCAAAACCAGGCGGAAACA
3389	Yes	AAAGGTCCCTGGATCAAAACCAGGCGGAAACAC
3390	Yes	AAAGGTCCCTGGATCAAAACCAGGCGGAAACACC
3391	Yes	AAAGGTCCCTGGATCAAAACCAGGCGGAAACACCA
3392	No	AAAGGTCCCTGGTTCA
3393	Yes	AAAGGTCCCTGGTTCAA
3394	Yes	AAAGGTCCCTGGTTCAAA
3395	Yes	AAAGGTCCCTGGTTCAAAT

3396	Yes	AAAGGTCCCTGGTTCAAATC
3397	Yes	AAAGGTCCCTGGTTCAAATCC
3398	Yes	AAAGGTCCCTGGTTCAAATCCA
3399	Yes	AAAGGTCCCTGGTTCAAATCCAG
3400	Yes	AAAGGTCCCTGGTTCAAATCCAGG
3401	Yes	AAAGGTCCCTGGTTCAAATCCAGGT
3402	Yes	AAAGGTCCCTGGTTCAAATCCAGGTG
3403	Yes	AAAGGTCCCTGGTTCAAATCCAGGTGC
3404	Yes	AAAGGTCCCTGGTTCAAATCCAGGTGCC
3405	Yes	AAAGGTCCCTGGTTCAAATCCAGGTGCCC
3406	Yes	AAAGGTCCCTGGTTCAAATCCAGGTGCCCC
3407	Yes	AAAGGTCCCTGGTTCAAATCCAGGTGCCCCT
3408	Yes	AAAGGTCCCTGGTTCAAATCCAGGTGCCCCTT
3409	Yes	AAAGGTCCCTGGTTCAAATCCAGGTGCCCCTTC
3410	Yes	AAAGGTCCCTGGTTCAAATCCAGGTGCCCCTTCC
3411	Yes	AAAGGTCCCTGGTTCAAATCCAGGTGCCCCTTCCA
3412	Yes	AAAGGTCCCTGGTTCAAT
3413	Yes	AAAGGTCCCTGGTTCAATC
3414	Yes	AAAGGTCCCTGGTTCAATCC
3415	Yes	AAAGGTCCCTGGTTCAATCCC
3416	Yes	AAAGGTCCCTGGTTCAATCCCG
3417	Yes	AAAGGTCCCTGGTTCAATCCCGG
3418	Yes	AAAGGTCCCTGGTTCAATCCCGGG
3419	Yes	AAAGGTCCCTGGTTCAATCCCGGGT
3420	Yes	AAAGGTCCCTGGTTCAATCCCGGGTT
3421	Yes	AAAGGTCCCTGGTTCAATCCCGGGTTT
3422	Yes	AAAGGTCCCTGGTTCAATCCCGGGTTTC
3423	Yes	AAAGGTCCCTGGTTCAATCCCGGGTTTCG
3424	Yes	AAAGGTCCCTGGTTCAATCCCGGGTTTCGG
3425	Yes	AAAGGTCCCTGGTTCAATCCCGGGTTTCGGC
3426	Yes	AAAGGTCCCTGGTTCAATCCCGGGTTTCGGCA
3427	Yes	AAAGGTCCCTGGTTCAATCCCGGGTTTCGGCAC
3428	Yes	AAAGGTCCCTGGTTCAATCCCGGGTTTCGGCACC
3429	Yes	AAAGGTCCCTGGTTCAATCCCGGGTTTCGGCACCA
3430	Yes	AAAGGTCCCTGGTTCG
3431	Yes	AAAGGTCCCTGGTTCGA
3432	Yes	AAAGGTCCCTGGTTCGAA
3433	Yes	AAAGGTCCCTGGTTCGAAA
3434	Yes	AAAGGTCCCTGGTTCGAAAC
3435	Yes	AAAGGTCCCTGGTTCGAAACC
3436	Yes	AAAGGTCCCTGGTTCGAAACCA
3437	Yes	AAAGGTCCCTGGTTCGAAACCAG

3438	Yes	AAAGGTCCCTGGTTCGAAACCAGG
3439	Yes	AAAGGTCCCTGGTTCGAAACCAGGT
3440	Yes	AAAGGTCCCTGGTTCGAAACCAGGTG
3441	Yes	AAAGGTCCCTGGTTCGAAACCAGGTGG
3442	Yes	AAAGGTCCCTGGTTCGAAACCAGGTGGA
3443	Yes	AAAGGTCCCTGGTTCGAAACCAGGTGGAA
3444	Yes	AAAGGTCCCTGGTTCGAAACCAGGTGGAAA
3445	Yes	AAAGGTCCCTGGTTCGAAACCAGGTGGAAAC
3446	Yes	AAAGGTCCCTGGTTCGAAACCAGGTGGAAACA
3447	Yes	AAAGGTCCCTGGTTCGAAACCAGGTGGAAACAC
3448	Yes	AAAGGTCCCTGGTTCGAAACCAGGTGGAAACACC
3449	Yes	AAAGGTCCCTGGTTCGAAACCAGGTGGAAACACCA
3450	Yes	AAAGGTCCCTGGTTCGAT
3451	Yes	AAAGGTCCCTGGTTCGATC
3452	Yes	AAAGGTCCCTGGTTCGATCC
3453	Yes	AAAGGTCCCTGGTTCGATCCC
3454	Yes	AAAGGTCCCTGGTTCGATCCCG
3455	Yes	AAAGGTCCCTGGTTCGATCCCGG
3456	Yes	AAAGGTCCCTGGTTCGATCCCGGG
3457	Yes	AAAGGTCCCTGGTTCGATCCCGGGT
3458	Yes	AAAGGTCCCTGGTTCGATCCCGGGTT
3459	Yes	AAAGGTCCCTGGTTCGATCCCGGGTTT
3460	Yes	AAAGGTCCCTGGTTCGATCCCGGGTTTC
3461	Yes	AAAGGTCCCTGGTTCGATCCCGGGTTTCA
3462	Yes	AAAGGTCCCTGGTTCGATCCCGGGTTTCAC
3463	Yes	AAAGGTCCCTGGTTCGATCCCGGGTTTCACC
3464	Yes	AAAGGTCCCTGGTTCGATCCCGGGTTTCACCA
3465	Yes	AAAGGTCCCTGGTTCGATCCCGGGTTTCACCAC
3466	Yes	AAAGGTCCCTGGTTCGATCCCGGGTTTCACCACC
3467	Yes	AAAGGTCCCTGGTTCGATCCCGGGTTTCACCACCA
3468	Yes	AAAGGTCCCTGGTTCGATCCCGGGTTTCG
3469	Yes	AAAGGTCCCTGGTTCGATCCCGGGTTTCGG
3470	Yes	AAAGGTCCCTGGTTCGATCCCGGGTTTCGGC
3471	Yes	AAAGGTCCCTGGTTCGATCCCGGGTTTCGGCA
3472	Yes	AAAGGTCCCTGGTTCGATCCCGGGTTTCGGCAC
3473	Yes	AAAGGTCCCTGGTTCGATCCCGGGTTTCGGCACC
3474	Yes	AAAGGTCCCTGGTTCGATCCCGGGTTTCGGCACCA
3475	Yes	AAAGGTCCGCGGTTCG
3476	Yes	AAAGGTCCGCGGTTCGA
3477	Yes	AAAGGTCCGCGGTTCGAA
3478	Yes	AAAGGTCCGCGGTTCGAAA
3479	Yes	AAAGGTCCGCGGTTCGAAAC

3480	Yes	AAAGGTCCGCGGTTCGAAACC
3481	Yes	AAAGGTCCGCGGTTCGAAACCG
3482	Yes	AAAGGTCCGCGGTTCGAAACCGG
3483	Yes	AAAGGTCCGCGGTTCGAAACCGGG
3484	Yes	AAAGGTCCGCGGTTCGAAACCGGGC
3485	Yes	AAAGGTCCGCGGTTCGAAACCGGGCG
3486	Yes	AAAGGTCCGCGGTTCGAAACCGGGCGG
3487	Yes	AAAGGTCCGCGGTTCGAAACCGGGCGGA
3488	Yes	AAAGGTCCGCGGTTCGAAACCGGGCGGAA
3489	Yes	AAAGGTCCGCGGTTCGAAACCGGGCGGAAA
3490	Yes	AAAGGTCCGCGGTTCGAAACCGGGCGGAAAC
3491	Yes	AAAGGTCCGCGGTTCGAAACCGGGCGGAAACA
3492	Yes	AAAGGTCCGCGGTTCGAAACCGGGCGGAAACAC
3493	Yes	AAAGGTCCGCGGTTCGAAACCGGGCGGAAACACC
3494	Yes	AAAGGTCCGCGGTTCGAAACCGGGCGGAAACACCA
3495	Yes	AAAGGTCCTCGGGTCG
3496	Yes	AAAGGTCCTCGGGTCGA
3497	Yes	AAAGGTCCTCGGGTCGAA
3498	Yes	AAAGGTCCTCGGGTCGAAA
3499	Yes	AAAGGTCCTCGGGTCGAAAC
3500	Yes	AAAGGTCCTCGGGTCGAAACC
3501	Yes	AAAGGTCCTCGGGTCGAAACCG
3502	Yes	AAAGGTCCTCGGGTCGAAACCGA
3503	Yes	AAAGGTCCTCGGGTCGAAACCGAG
3504	Yes	AAAGGTCCTCGGGTCGAAACCGAGC
3505	Yes	AAAGGTCCTCGGGTCGAAACCGAGCG
3506	Yes	AAAGGTCCTCGGGTCGAAACCGAGCGG
3507	Yes	AAAGGTCCTCGGGTCGAAACCGAGCGGA
3508	Yes	AAAGGTCCTCGGGTCGAAACCGAGCGGAA
3509	Yes	AAAGGTCCTCGGGTCGAAACCGAGCGGAAA
3510	Yes	AAAGGTCCTCGGGTCGAAACCGAGCGGAAAC
3511	Yes	AAAGGTCCTCGGGTCGAAACCGAGCGGAAACA
3512	Yes	AAAGGTCCTCGGGTCGAAACCGAGCGGAAACAC
3513	Yes	AAAGGTCCTCGGGTCGAAACCGAGCGGAAACACC
3514	Yes	AAAGGTCCTCGGGTCGAAACCGAGCGGAAACACCA
3515	No	AAAGGTCCTGGGTTCA
3516	Yes	AAAGGTCCTGGGTTCAA
3517	Yes	AAAGGTCCTGGGTTCAAA
3518	Yes	AAAGGTCCTGGGTTCAAAT
3519	Yes	AAAGGTCCTGGGTTCAAATC
3520	Yes	AAAGGTCCTGGGTTCAAATCC
3521	Yes	AAAGGTCCTGGGTTCAAATCCC

3522	Yes	AAAGGTCCTGGGTTCAAATCCCA
3523	Yes	AAAGGTCCTGGGTTCAAATCCCAG
3524	Yes	AAAGGTCCTGGGTTCAAATCCCAGA
3525	Yes	AAAGGTCCTGGGTTCAAATCCCAGAG
3526	Yes	AAAGGTCCTGGGTTCAAATCCCAGAGG
3527	Yes	AAAGGTCCTGGGTTCAAATCCCAGAGGA
3528	Yes	AAAGGTCCTGGGTTCAAATCCCAGAGGAG
3529	Yes	AAAGGTCCTGGGTTCAAATCCCAGAGGAGC
3530	Yes	AAAGGTCCTGGGTTCAAATCCCAGAGGAGCC
3531	Yes	AAAGGTCCTGGGTTCAAATCCCAGAGGAGCCC
3532	Yes	AAAGGTCCTGGGTTCAAATCCCAGAGGAGCCCC
3533	Yes	AAAGGTCCTGGGTTCAAATCCCAGAGGAGCCCCC
3534	Yes	AAAGGTCCTGGGTTCAAATCCCAGAGGAGCCCCCA
3535	No	AAAGGTCCTTGGTTTG
3536	Yes	AAAGGTCCTTGGTTTGA
3537	Yes	AAAGGTCCTTGGTTTGAA
3538	Yes	AAAGGTCCTTGGTTTGAAA
3539	Yes	AAAGGTCCTTGGTTTGAAAC
3540	Yes	AAAGGTCCTTGGTTTGAAACC
3541	Yes	AAAGGTCCTTGGTTTGAAACCA
3542	Yes	AAAGGTCCTTGGTTTGAAACCAG
3543	Yes	AAAGGTCCTTGGTTTGAAACCAGG
3544	Yes	AAAGGTCCTTGGTTTGAAACCAGGC
3545	Yes	AAAGGTCCTTGGTTTGAAACCAGGCA
3546	Yes	AAAGGTCCTTGGTTTGAAACCAGGCAG
3547	Yes	AAAGGTCCTTGGTTTGAAACCAGGCAGA
3548	Yes	AAAGGTCCTTGGTTTGAAACCAGGCAGAA
3549	Yes	AAAGGTCCTTGGTTTGAAACCAGGCAGAAA
3550	Yes	AAAGGTCCTTGGTTTGAAACCAGGCAGAAAC
3551	Yes	AAAGGTCCTTGGTTTGAAACCAGGCAGAAACA
3552	Yes	AAAGGTCCTTGGTTTGAAACCAGGCAGAAACAC
3553	Yes	AAAGGTCCTTGGTTTGAAACCAGGCAGAAACACC
3554	Yes	AAAGGTCCTTGGTTTGAAACCAGGCAGAAACACCA
3555	Yes	AAAGGTCTCTGGTTCG
3556	Yes	AAAGGTCTCTGGTTCGA
3557	Yes	AAAGGTCTCTGGTTCGAG
3558	Yes	AAAGGTCTCTGGTTCGAGA
3559	Yes	AAAGGTCTCTGGTTCGAGAC
3560	Yes	AAAGGTCTCTGGTTCGAGACT
3561	Yes	AAAGGTCTCTGGTTCGAGACTG
3562	Yes	AAAGGTCTCTGGTTCGAGACTGC
3563	Yes	AAAGGTCTCTGGTTCGAGACTGCG

3564	Yes	AAAGGTCTCTGGTTCGAGACTGCGT
3565	Yes	AAAGGTCTCTGGTTCGAGACTGCGTG
3566	Yes	AAAGGTCTCTGGTTCGAGACTGCGTGG
3567	Yes	AAAGGTCTCTGGTTCGAGACTGCGTGGG
3568	Yes	AAAGGTCTCTGGTTCGAGACTGCGTGGGA
3569	Yes	AAAGGTCTCTGGTTCGAGACTGCGTGGGAA
3570	Yes	AAAGGTCTCTGGTTCGAGACTGCGTGGGAAC
3571	Yes	AAAGGTCTCTGGTTCGAGACTGCGTGGGAACA
3572	Yes	AAAGGTCTCTGGTTCGAGACTGCGTGGGAACAC
3573	Yes	AAAGGTCTCTGGTTCGAGACTGCGTGGGAACACC
3574	Yes	AAAGGTCTCTGGTTCGAGACTGCGTGGGAACACCA
3575	No	AAAGGTCTCTGGTTTG
3576	Yes	AAAGGTCTCTGGTTTGA
3577	Yes	AAAGGTCTCTGGTTTGAT
3578	Yes	AAAGGTCTCTGGTTTGATC
3579	Yes	AAAGGTCTCTGGTTTGATCC
3580	Yes	AAAGGTCTCTGGTTTGATCCT
3581	Yes	AAAGGTCTCTGGTTTGATCCTG
3582	Yes	AAAGGTCTCTGGTTTGATCCTGG
3583	Yes	AAAGGTCTCTGGTTTGATCCTGGG
3584	Yes	AAAGGTCTCTGGTTTGATCCTGGGT
3585	Yes	AAAGGTCTCTGGTTTGATCCTGGGTT
3586	Yes	AAAGGTCTCTGGTTTGATCCTGGGTTT
3587	Yes	AAAGGTCTCTGGTTTGATCCTGGGTTTC
3588	Yes	AAAGGTCTCTGGTTTGATCCTGGGTTTCA
3589	Yes	AAAGGTCTCTGGTTTGATCCTGGGTTTCAG
3590	Yes	AAAGGTCTCTGGTTTGATCCTGGGTTTCAGA
3591	Yes	AAAGGTCTCTGGTTTGATCCTGGGTTTCAGAA
3592	Yes	AAAGGTCTCTGGTTTGATCCTGGGTTTCAGAAC
3593	Yes	AAAGGTCTCTGGTTTGATCCTGGGTTTCAGAACC
3594	Yes	AAAGGTCTCTGGTTTGATCCTGGGTTTCAGAACCA
3595	Yes	AAAGGTTAGTGGTTCG
3596	Yes	AAAGGTTAGTGGTTCGA
3597	Yes	AAAGGTTAGTGGTTCGAG
3598	Yes	AAAGGTTAGTGGTTCGAGC
3599	Yes	AAAGGTTAGTGGTTCGAGCC
3600	Yes	AAAGGTTAGTGGTTCGAGCCC
3601	Yes	AAAGGTTAGTGGTTCGAGCCCA
3602	Yes	AAAGGTTAGTGGTTCGAGCCCAC
3603	Yes	AAAGGTTAGTGGTTCGAGCCCACC
3604	Yes	AAAGGTTAGTGGTTCGAGCCCACCC
3605	Yes	AAAGGTTAGTGGTTCGAGCCCACCCG

3606	Yes	AAAGGTTAGTGGTTCGAGCCCACCCGG
3607	Yes	AAAGGTTAGTGGTTCGAGCCCACCCGGG
3608	Yes	AAAGGTTAGTGGTTCGAGCCCACCCGGGG
3609	Yes	AAAGGTTAGTGGTTCGAGCCCACCCGGGGA
3610	Yes	AAAGGTTAGTGGTTCGAGCCCACCCGGGGAC
3611	Yes	AAAGGTTAGTGGTTCGAGCCCACCCGGGGACG
3612	Yes	AAAGGTTAGTGGTTCGAGCCCACCCGGGGACGC
3613	Yes	AAAGGTTAGTGGTTCGAGCCCACCCGGGGACGCC
3614	Yes	AAAGGTTAGTGGTTCGAGCCCACCCGGGGACGCCA
3615	Yes	AAAGGTTCCGGGTTCG
3616	Yes	AAAGGTTCCGGGTTCGA
3617	Yes	AAAGGTTCCGGGTTCGAG
3618	Yes	AAAGGTTCCGGGTTCGAGT
3619	Yes	AAAGGTTCCGGGTTCGAGTC
3620	Yes	AAAGGTTCCGGGTTCGAGTCC
3621	Yes	AAAGGTTCCGGGTTCGAGTCCC
3622	Yes	AAAGGTTCCGGGTTCGAGTCCCG
3623	Yes	AAAGGTTCCGGGTTCGAGTCCCGG
3624	Yes	AAAGGTTCCGGGTTCGAGTCCCGGC
3625	Yes	AAAGGTTCCGGGTTCGAGTCCCGGCG
3626	Yes	AAAGGTTCCGGGTTCGAGTCCCGGCGG
3627	Yes	AAAGGTTCCGGGTTCGAGTCCCGGCGGA
3628	Yes	AAAGGTTCCGGGTTCGAGTCCCGGCGGAG
3629	Yes	AAAGGTTCCGGGTTCGAGTCCCGGCGGAGT
3630	Yes	AAAGGTTCCGGGTTCGAGTCCCGGCGGAGTC
3631	Yes	AAAGGTTCCGGGTTCGAGTCCCGGCGGAGTCG
3632	Yes	AAAGGTTCCGGGTTCGAGTCCCGGCGGAGTCGC
3633	Yes	AAAGGTTCCGGGTTCGAGTCCCGGCGGAGTCGCC
3634	Yes	AAAGGTTCCGGGTTCGAGTCCCGGCGGAGTCGCCA
3635	Yes	AAAGGTTGCGGGTTCG
3636	Yes	AAAGGTTGCGGGTTCGA
3637	Yes	AAAGGTTGCGGGTTCGAG
3638	Yes	AAAGGTTGCGGGTTCGAGT
3639	Yes	AAAGGTTGCGGGTTCGAGTC
3640	Yes	AAAGGTTGCGGGTTCGAGTCC
3641	Yes	AAAGGTTGCGGGTTCGAGTCCC
3642	Yes	AAAGGTTGCGGGTTCGAGTCCCT
3643	Yes	AAAGGTTGCGGGTTCGAGTCCCTC
3644	Yes	AAAGGTTGCGGGTTCGAGTCCCTCC
3645	Yes	AAAGGTTGCGGGTTCGAGTCCCTCCA
3646	Yes	AAAGGTTGCGGGTTCGAGTCCCTCCAG
3647	Yes	AAAGGTTGCGGGTTCGAGTCCCTCCAGA

3648	Yes	AAAGGTTGCGGGTTCGAGTCCCTCCAGAG
3649	Yes	AAAGGTTGCGGGTTCGAGTCCCTCCAGAGT
3650	Yes	AAAGGTTGCGGGTTCGAGTCCCTCCAGAGTC
3651	Yes	AAAGGTTGCGGGTTCGAGTCCCTCCAGAGTCG
3652	Yes	AAAGGTTGCGGGTTCGAGTCCCTCCAGAGTCGC
3653	Yes	AAAGGTTGCGGGTTCGAGTCCCTCCAGAGTCGCC
3654	Yes	AAAGGTTGCGGGTTCGAGTCCCTCCAGAGTCGCCA
3655	Yes	AAAGGTTGGCGGTTCG
3656	Yes	AAAGGTTGGCGGTTCGA
3657	Yes	AAAGGTTGGCGGTTCGAA
3658	Yes	AAAGGTTGGCGGTTCGAAC
3659	Yes	AAAGGTTGGCGGTTCGAACC
3660	Yes	AAAGGTTGGCGGTTCGAACCC
3661	Yes	AAAGGTTGGCGGTTCGAACCCA
3662	Yes	AAAGGTTGGCGGTTCGAACCCAC
3663	Yes	AAAGGTTGGCGGTTCGAACCCACC
3664	Yes	AAAGGTTGGCGGTTCGAACCCACCC
3665	Yes	AAAGGTTGGCGGTTCGAACCCACCCA
3666	Yes	AAAGGTTGGCGGTTCGAACCCACCCAG
3667	Yes	AAAGGTTGGCGGTTCGAACCCACCCAGA
3668	Yes	AAAGGTTGGCGGTTCGAACCCACCCAGAG
3669	Yes	AAAGGTTGGCGGTTCGAACCCACCCAGAGG
3670	Yes	AAAGGTTGGCGGTTCGAACCCACCCAGAGGC
3671	Yes	AAAGGTTGGCGGTTCGAACCCACCCAGAGGCG
3672	Yes	AAAGGTTGGCGGTTCGAACCCACCCAGAGGCGC
3673	Yes	AAAGGTTGGCGGTTCGAACCCACCCAGAGGCGCC
3674	Yes	AAAGGTTGGCGGTTCGAACCCACCCAGAGGCGCCA
3675	No	AAAGGTTGGTGGCTAG
3676	Yes	AAAGGTTGGTGGCTAGA
3677	Yes	AAAGGTTGGTGGCTAGAA
3678	Yes	AAAGGTTGGTGGCTAGAAC
3679	Yes	AAAGGTTGGTGGCTAGAACC
3680	Yes	AAAGGTTGGTGGCTAGAACCC
3681	Yes	AAAGGTTGGTGGCTAGAACCCA
3682	Yes	AAAGGTTGGTGGCTAGAACCCAC
3683	Yes	AAAGGTTGGTGGCTAGAACCCACT
3684	Yes	AAAGGTTGGTGGCTAGAACCCACTG
3685	Yes	AAAGGTTGGTGGCTAGAACCCACTGA
3686	Yes	AAAGGTTGGTGGCTAGAACCCACTGAG
3687	Yes	AAAGGTTGGTGGCTAGAACCCACTGAGG
3688	Yes	AAAGGTTGGTGGCTAGAACCCACTGAGGG
3689	Yes	AAAGGTTGGTGGCTAGAACCCACTGAGGGA

3690	Yes	AAAGGTTGGTGGCTAGAACCCACTGAGGGAC
3691	Yes	AAAGGTTGGTGGCTAGAACCCACTGAGGGACG
3692	Yes	AAAGGTTGGTGGCTAGAACCCACTGAGGGACGC
3693	Yes	AAAGGTTGGTGGCTAGAACCCACTGAGGGACGCC
3694	Yes	AAAGGTTGGTGGCTAGAACCCACTGAGGGACGCCA
3695	Yes	AAAGGTTGGTGGTGCA
3696	Yes	AAAGGTTGGTGGTGCAA
3697	Yes	AAAGGTTGGTGGTGCAAG
3698	Yes	AAAGGTTGGTGGTGCAAGC
3699	Yes	AAAGGTTGGTGGTGCAAGCC
3700	Yes	AAAGGTTGGTGGTGCAAGCCC
3701	Yes	AAAGGTTGGTGGTGCAAGCCCA
3702	Yes	AAAGGTTGGTGGTGCAAGCCCAT
3703	Yes	AAAGGTTGGTGGTGCAAGCCCATC
3704	Yes	AAAGGTTGGTGGTGCAAGCCCATCC
3705	Yes	AAAGGTTGGTGGTGCAAGCCCATCCA
3706	Yes	AAAGGTTGGTGGTGCAAGCCCATCCAG
3707	Yes	AAAGGTTGGTGGTGCAAGCCCATCCAGG
3708	Yes	AAAGGTTGGTGGTGCAAGCCCATCCAGGG
3709	Yes	AAAGGTTGGTGGTGCAAGCCCATCCAGGGA
3710	Yes	AAAGGTTGGTGGTGCAAGCCCATCCAGGGAT
3711	Yes	AAAGGTTGGTGGTGCAAGCCCATCCAGGGATG
3712	Yes	AAAGGTTGGTGGTGCAAGCCCATCCAGGGATGC
3713	Yes	AAAGGTTGGTGGTGCAAGCCCATCCAGGGATGCC
3714	Yes	AAAGGTTGGTGGTGCAAGCCCATCCAGGGATGCCA
3715	Yes	AAAGGTTGGTGGTTCG
3716	Yes	AAAGGTTGGTGGTTCGA
3717	Yes	AAAGGTTGGTGGTTCGAA
3718	Yes	AAAGGTTGGTGGTTCGAAC
3719	Yes	AAAGGTTGGTGGTTCGAACC
3720	Yes	AAAGGTTGGTGGTTCGAACCC
3721	Yes	AAAGGTTGGTGGTTCGAACCCA
3722	Yes	AAAGGTTGGTGGTTCGAACCCAC
3723	Yes	AAAGGTTGGTGGTTCGAACCCACC
3724	Yes	AAAGGTTGGTGGTTCGAACCCACCC
3725	Yes	AAAGGTTGGTGGTTCGAACCCACCCA
3726	Yes	AAAGGTTGGTGGTTCGAACCCACCCAG
3727	Yes	AAAGGTTGGTGGTTCGAACCCACCCAGA
3728	Yes	AAAGGTTGGTGGTTCGAACCCACCCAGAG
3729	Yes	AAAGGTTGGTGGTTCGAACCCACCCAGAGG
3730	Yes	AAAGGTTGGTGGTTCGAACCCACCCAGAGGC
3731	Yes	AAAGGTTGGTGGTTCGAACCCACCCAGAGGCG

3732	Yes	AAAGGTTGGTGGTTCGAACCCACCCAGAGGCGC
3733	Yes	AAAGGTTGGTGGTTCGAACCCACCCAGAGGCGCC
3734	Yes	AAAGGTTGGTGGTTCGAACCCACCCAGAGGCGCCA
3735	Yes	AAAGGTTGGTGGTTCGAG
3736	Yes	AAAGGTTGGTGGTTCGAGC
3737	Yes	AAAGGTTGGTGGTTCGAGCC
3738	Yes	AAAGGTTGGTGGTTCGAGCCC
3739	Yes	AAAGGTTGGTGGTTCGAGCCCA
3740	Yes	AAAGGTTGGTGGTTCGAGCCCAC
3741	Yes	AAAGGTTGGTGGTTCGAGCCCACC
3742	Yes	AAAGGTTGGTGGTTCGAGCCCACCC
3743	Yes	AAAGGTTGGTGGTTCGAGCCCACCCA
3744	Yes	AAAGGTTGGTGGTTCGAGCCCACCCAG
3745	Yes	AAAGGTTGGTGGTTCGAGCCCACCCAGG
3746	Yes	AAAGGTTGGTGGTTCGAGCCCACCCAGGG
3747	Yes	AAAGGTTGGTGGTTCGAGCCCACCCAGGGA
3748	Yes	AAAGGTTGGTGGTTCGAGCCCACCCAGGGAC
3749	Yes	AAAGGTTGGTGGTTCGAGCCCACCCAGGGACG
3750	Yes	AAAGGTTGGTGGTTCGAGCCCACCCAGGGACGC
3751	Yes	AAAGGTTGGTGGTTCGAGCCCACCCAGGGACGCC
3752	Yes	AAAGGTTGGTGGTTCGAGCCCACCCAGGGACGCCA
3753	Yes	AAAGGTTGGTGGTTCGAGCCCAT
3754	Yes	AAAGGTTGGTGGTTCGAGCCCATC
3755	Yes	AAAGGTTGGTGGTTCGAGCCCATCC
3756	Yes	AAAGGTTGGTGGTTCGAGCCCATCCA
3757	Yes	AAAGGTTGGTGGTTCGAGCCCATCCAG
3758	Yes	AAAGGTTGGTGGTTCGAGCCCATCCAGG
3759	Yes	AAAGGTTGGTGGTTCGAGCCCATCCAGGG
3760	Yes	AAAGGTTGGTGGTTCGAGCCCATCCAGGGA
3761	Yes	AAAGGTTGGTGGTTCGAGCCCATCCAGGGAC
3762	Yes	AAAGGTTGGTGGTTCGAGCCCATCCAGGGACG
3763	Yes	AAAGGTTGGTGGTTCGAGCCCATCCAGGGACGC
3764	Yes	AAAGGTTGGTGGTTCGAGCCCATCCAGGGACGCC
3765	Yes	AAAGGTTGGTGGTTCGAGCCCATCCAGGGACGCCA
3766	Yes	AAAGGTTGGTGGTTCGAT
3767	Yes	AAAGGTTGGTGGTTCGATC
3768	Yes	AAAGGTTGGTGGTTCGATCC
3769	Yes	AAAGGTTGGTGGTTCGATCCC
3770	Yes	AAAGGTTGGTGGTTCGATCCCA
3771	Yes	AAAGGTTGGTGGTTCGATCCCAC
3772	Yes	AAAGGTTGGTGGTTCGATCCCACC
3773	Yes	AAAGGTTGGTGGTTCGATCCCACCC

3774	Yes	AAAGGTTGGTGGTTCGATCCCACCCA
3775	Yes	AAAGGTTGGTGGTTCGATCCCACCCAG
3776	Yes	AAAGGTTGGTGGTTCGATCCCACCCAGG
3777	Yes	AAAGGTTGGTGGTTCGATCCCACCCAGGG
3778	Yes	AAAGGTTGGTGGTTCGATCCCACCCAGGGA
3779	Yes	AAAGGTTGGTGGTTCGATCCCACCCAGGGAC
3780	Yes	AAAGGTTGGTGGTTCGATCCCACCCAGGGACG
3781	Yes	AAAGGTTGGTGGTTCGATCCCACCCAGGGACGC
3782	Yes	AAAGGTTGGTGGTTCGATCCCACCCAGGGACGCC
3783	Yes	AAAGGTTGGTGGTTCGATCCCACCCAGGGACGCCA
3784	Yes	AAAGGTTGGTGGTTCGT
3785	Yes	AAAGGTTGGTGGTTCGTT
3786	Yes	AAAGGTTGGTGGTTCGTTC
3787	Yes	AAAGGTTGGTGGTTCGTTCC
3788	Yes	AAAGGTTGGTGGTTCGTTCCC
3789	Yes	AAAGGTTGGTGGTTCGTTCCCA
3790	Yes	AAAGGTTGGTGGTTCGTTCCCAC
3791	Yes	AAAGGTTGGTGGTTCGTTCCCACC
3792	Yes	AAAGGTTGGTGGTTCGTTCCCACCC
3793	Yes	AAAGGTTGGTGGTTCGTTCCCACCCC
3794	Yes	AAAGGTTGGTGGTTCGTTCCCACCCCG
3795	Yes	AAAGGTTGGTGGTTCGTTCCCACCCCGG
3796	Yes	AAAGGTTGGTGGTTCGTTCCCACCCCGGG
3797	Yes	AAAGGTTGGTGGTTCGTTCCCACCCCGGGA
3798	Yes	AAAGGTTGGTGGTTCGTTCCCACCCCGGGAC
3799	Yes	AAAGGTTGGTGGTTCGTTCCCACCCCGGGACA
3800	Yes	AAAGGTTGGTGGTTCGTTCCCACCCCGGGACAC
3801	Yes	AAAGGTTGGTGGTTCGTTCCCACCCCGGGACACC
3802	Yes	AAAGGTTGGTGGTTCGTTCCCACCCCGGGACACCA
3803	Yes	AAAGGTTGTGGGTTCG
3804	Yes	AAAGGTTGTGGGTTCGA
3805	Yes	AAAGGTTGTGGGTTCGAA
3806	Yes	AAAGGTTGTGGGTTCGAAT
3807	Yes	AAAGGTTGTGGGTTCGAATC
3808	Yes	AAAGGTTGTGGGTTCGAATCC
3809	Yes	AAAGGTTGTGGGTTCGAATCCC
3810	Yes	AAAGGTTGTGGGTTCGAATCCCA
3811	Yes	AAAGGTTGTGGGTTCGAATCCCAC
3812	Yes	AAAGGTTGTGGGTTCGAATCCCACC
3813	Yes	AAAGGTTGTGGGTTCGAATCCCACCA
3814	Yes	AAAGGTTGTGGGTTCGAATCCCACCAG
3815	Yes	AAAGGTTGTGGGTTCGAATCCCACCAGA

3816	Yes	AAAGGTTGTGGGTTCGAATCCCACCAGAG
3817	Yes	AAAGGTTGTGGGTTCGAATCCCACCAGAGT
3818	Yes	AAAGGTTGTGGGTTCGAATCCCACCAGAGTC
3819	Yes	AAAGGTTGTGGGTTCGAATCCCACCAGAGTCG
3820	Yes	AAAGGTTGTGGGTTCGAATCCCACCAGAGTCGC
3821	Yes	AAAGGTTGTGGGTTCGAATCCCACCAGAGTCGCC
3822	Yes	AAAGGTTGTGGGTTCGAATCCCACCAGAGTCGCCA
3823	Yes	AAAGGTTGTGGGTTCGAG
3824	Yes	AAAGGTTGTGGGTTCGAGT
3825	Yes	AAAGGTTGTGGGTTCGAGTC
3826	Yes	AAAGGTTGTGGGTTCGAGTCC
3827	Yes	AAAGGTTGTGGGTTCGAGTCCC
3828	Yes	AAAGGTTGTGGGTTCGAGTCCCA
3829	Yes	AAAGGTTGTGGGTTCGAGTCCCAC
3830	Yes	AAAGGTTGTGGGTTCGAGTCCCACC
3831	Yes	AAAGGTTGTGGGTTCGAGTCCCACCA
3832	Yes	AAAGGTTGTGGGTTCGAGTCCCACCAG
3833	Yes	AAAGGTTGTGGGTTCGAGTCCCACCAGA
3834	Yes	AAAGGTTGTGGGTTCGAGTCCCACCAGAG
3835	Yes	AAAGGTTGTGGGTTCGAGTCCCACCAGAGT
3836	Yes	AAAGGTTGTGGGTTCGAGTCCCACCAGAGTC
3837	Yes	AAAGGTTGTGGGTTCGAGTCCCACCAGAGTCG
3838	Yes	AAAGGTTGTGGGTTCGAGTCCCACCAGAGTCGC
3839	Yes	AAAGGTTGTGGGTTCGAGTCCCACCAGAGTCGCC
3840	Yes	AAAGGTTGTGGGTTCGAGTCCCACCAGAGTCGCCA
3841	Yes	AAAGTCATAGGGGTTA
3842	Yes	AAAGTCATAGGGGTTAT
3843	Yes	AAAGTCATAGGGGTTATG
3844	Yes	AAAGTCATAGGGGTTATGA
3845	Yes	AAAGTCATAGGGGTTATGAG
3846	Yes	AAAGTCATAGGGGTTATGAGG
3847	Yes	AAAGTCATAGGGGTTATGAGGC
3848	Yes	AAAGTCATAGGGGTTATGAGGCT
3849	Yes	AAAGTCATAGGGGTTATGAGGCTG
3850	Yes	AAAGTCATAGGGGTTATGAGGCTGG
3851	Yes	AAAGTCATAGGGGTTATGAGGCTGGC
3852	Yes	AAAGTCATAGGGGTTATGAGGCTGGCT
3853	Yes	AAAGTCATAGGGGTTATGAGGCTGGCTT
3854	Yes	AAAGTCATAGGGGTTATGAGGCTGGCTTG
3855	Yes	AAAGTCATAGGGGTTATGAGGCTGGCTTGA
3856	Yes	AAAGTCATAGGGGTTATGAGGCTGGCTTGAA
3857	Yes	AAAGTCATAGGGGTTATGAGGCTGGCTTGAAA

3858	Yes	AAAGTCATAGGGGTTATGAGGCTGGCTTGAAAC
3859	Yes	AAAGTCATAGGGGTTATGAGGCTGGCTTGAAACC
3860	Yes	AAAGTCATAGGGGTTATGAGGCTGGCTTGAAACCA
3861	Yes	AAAGTCATAGGGGTTATGAGGCTGGCTTGAAACCAG
3862	Yes	AAAGTCATAGGGGTTATGAGGCTGGCTTGAAACCAGC
3863	Yes	AAAGTCATAGGGGTTATGAGGCTGGCTTGAAACCAGCC
3864	Yes	AAAGTCATAGGGGTTATGAGGCTGGCTTGAAACCAGCCT
3865	Yes	AAAGTCATAGGGGTTATGAGGCTGGCTTGAAACCAGCCTT
3866	Yes	AAAGTCATAGGGGTTATGAGGCTGGCTTGAAACCAGCCTTA
3867	Yes	AAAGTCATAGGGGTTATGAGGCTGGCTTGAAACCAGCCTTAG
3868	Yes	AAAGTCATAGGGGTTATGAGGCTGGCTTGAAACCAGCCTTAGG
3869	Yes	AAAGTCATAGGGGTTATGAGGCTGGCTTGAAACCAGCCTTAGGA
3870	Yes	AAAGTCATAGGGGTTATGAGGCTGGCTTGAAACCAGCCTTAGGAG
3871	Yes	AAAGTCATAGGGGTTATGAGGCTGGCTTGAAACCAGCCTTAGGAGG
3872	Yes	AAAGTCATAGGGGTTATGAGGCTGGCTTGAAACCAGCCTTAGGAGGT
3873	Yes	AAAGTCATAGGGGTTATGAGGCTGGCTTGAAACCAGCCTTAGGAGGTT
3874	Yes	AAAGTCATAGGGGTTATGAGGCTGGCTTGAAACCAGCCTTAGGAGGTTC
3875	Yes	AAAGTCATAGGGGTTATGAGGCTGGCTTGAAACCAGCCTTAGGAGGTTCA
3876	No	AAAGTCATGGAGGCCA
3877	No	AAAGTCATGGAGGCCAT
3878	Yes	AAAGTCATGGAGGCCATG
3879	Yes	AAAGTCATGGAGGCCATGG
3880	Yes	AAAGTCATGGAGGCCATGGG
3881	Yes	AAAGTCATGGAGGCCATGGGG
3882	Yes	AAAGTCATGGAGGCCATGGGGT
3883	Yes	AAAGTCATGGAGGCCATGGGGTT
3884	Yes	AAAGTCATGGAGGCCATGGGGTTG
3885	Yes	AAAGTCATGGAGGCCATGGGGTTGG
3886	Yes	AAAGTCATGGAGGCCATGGGGTTGGC
3887	Yes	AAAGTCATGGAGGCCATGGGGTTGGCT
3888	Yes	AAAGTCATGGAGGCCATGGGGTTGGCTT
3889	Yes	AAAGTCATGGAGGCCATGGGGTTGGCTTG
3890	Yes	AAAGTCATGGAGGCCATGGGGTTGGCTTGA
3891	Yes	AAAGTCATGGAGGCCATGGGGTTGGCTTGAA
3892	Yes	AAAGTCATGGAGGCCATGGGGTTGGCTTGAAA
3893	Yes	AAAGTCATGGAGGCCATGGGGTTGGCTTGAAAC
3894	Yes	AAAGTCATGGAGGCCATGGGGTTGGCTTGAAACC
3895	Yes	AAAGTCATGGAGGCCATGGGGTTGGCTTGAAACCA
3896	Yes	AAAGTCATGGAGGCCATGGGGTTGGCTTGAAACCAG
3897	Yes	AAAGTCATGGAGGCCATGGGGTTGGCTTGAAACCAGC
3898	Yes	AAAGTCATGGAGGCCATGGGGTTGGCTTGAAACCAGCT
3899	Yes	AAAGTCATGGAGGCCATGGGGTTGGCTTGAAACCAGCTT

3900	Yes	AAAGTCATGGAGGCCATGGGGTTGGCTTGAAACCAGCTTT
3901	Yes	AAAGTCATGGAGGCCATGGGGTTGGCTTGAAACCAGCTTTG
3902	Yes	AAAGTCATGGAGGCCATGGGGTTGGCTTGAAACCAGCTTTGG
3903	Yes	AAAGTCATGGAGGCCATGGGGTTGGCTTGAAACCAGCTTTGGG
3904	Yes	AAAGTCATGGAGGCCATGGGGTTGGCTTGAAACCAGCTTTGGGG
3905	Yes	AAAGTCATGGAGGCCATGGGGTTGGCTTGAAACCAGCTTTGGGGG
3906	Yes	AAAGTCATGGAGGCCATGGGGTTGGCTTGAAACCAGCTTTGGGGGG
3907	Yes	AAAGTCATGGAGGCCATGGGGTTGGCTTGAAACCAGCTTTGGGGGGT
3908	Yes	AAAGTCATGGAGGCCATGGGGTTGGCTTGAAACCAGCTTTGGGGGGTT
3909	Yes	AAAGTCATGGAGGCCATGGGGTTGGCTTGAAACCAGCTTTGGGGGGTTC
3910	Yes	AAAGTCATGGAGGCCATGGGGTTGGCTTGAAACCAGCTTTGGGGGGTTCG
3911	Yes	AAAGTCCCCGGTTCGA
3912	Yes	AAAGTCCCCGGTTCGAA
3913	Yes	AAAGTCCCCGGTTCGAAA
3914	Yes	AAAGTCCCCGGTTCGAAAT
3915	Yes	AAAGTCCCCGGTTCGAAATC
3916	Yes	AAAGTCCCCGGTTCGAAATCA
3917	Yes	AAAGTCCCCGGTTCGAAATCAG
3918	Yes	AAAGTCCCCGGTTCGAAATCAGG
3919	Yes	AAAGTCCCCGGTTCGAAATCAGGC
3920	Yes	AAAGTCCCCGGTTCGAAATCAGGCG
3921	Yes	AAAGTCCCCGGTTCGAAATCAGGCGG
3922	Yes	AAAGTCCCCGGTTCGAAATCAGGCGGG
3923	Yes	AAAGTCCCCGGTTCGAAATCAGGCGGGA
3924	Yes	AAAGTCCCCGGTTCGAAATCAGGCGGGAA
3925	Yes	AAAGTCCCCGGTTCGAAATCAGGCGGGAAC
3926	Yes	AAAGTCCCCGGTTCGAAATCAGGCGGGAACA
3927	Yes	AAAGTCCCCGGTTCGAAATCAGGCGGGAACAC
3928	Yes	AAAGTCCCCGGTTCGAAATCAGGCGGGAACACC
3929	Yes	AAAGTCCCCGGTTCGAAATCAGGCGGGAACACCA
3930	Yes	AAAGTCCCTGATTCGA
3931	Yes	AAAGTCCCTGATTCGAG
3932	Yes	AAAGTCCCTGATTCGAGA
3933	Yes	AAAGTCCCTGATTCGAGAC
3934	Yes	AAAGTCCCTGATTCGAGACT
3935	Yes	AAAGTCCCTGATTCGAGACTG
3936	Yes	AAAGTCCCTGATTCGAGACTGG
3937	Yes	AAAGTCCCTGATTCGAGACTGGG
3938	Yes	AAAGTCCCTGATTCGAGACTGGGT
3939	Yes	AAAGTCCCTGATTCGAGACTGGGTG
3940	Yes	AAAGTCCCTGATTCGAGACTGGGTGG
3941	Yes	AAAGTCCCTGATTCGAGACTGGGTGGG

3942	Yes	AAAGTCCCTGATTCGAGACTGGGTGGGA
3943	Yes	AAAGTCCCTGATTCGAGACTGGGTGGGAA
3944	Yes	AAAGTCCCTGATTCGAGACTGGGTGGGAAC
3945	Yes	AAAGTCCCTGATTCGAGACTGGGTGGGAACG
3946	Yes	AAAGTCCCTGATTCGAGACTGGGTGGGAACGC
3947	Yes	AAAGTCCCTGATTCGAGACTGGGTGGGAACGCC
3948	Yes	AAAGTCCCTGATTCGAGACTGGGTGGGAACGCCA
3949	No	AAAGTCCTGAGTTCAA
3950	No	AAAGTCCTGAGTTCAAG
3951	No	AAAGTCCTGAGTTCAAGC
3952	Yes	AAAGTCCTGAGTTCAAGCC
3953	Yes	AAAGTCCTGAGTTCAAGCCT
3954	Yes	AAAGTCCTGAGTTCAAGCCTC
3955	Yes	AAAGTCCTGAGTTCAAGCCTCA
3956	Yes	AAAGTCCTGAGTTCAAGCCTCAG
3957	Yes	AAAGTCCTGAGTTCAAGCCTCAGA
3958	Yes	AAAGTCCTGAGTTCAAGCCTCAGAG
3959	Yes	AAAGTCCTGAGTTCAAGCCTCAGAGA
3960	Yes	AAAGTCCTGAGTTCAAGCCTCAGAGAG
3961	Yes	AAAGTCCTGAGTTCAAGCCTCAGAGAGG
3962	Yes	AAAGTCCTGAGTTCAAGCCTCAGAGAGGG
3963	Yes	AAAGTCCTGAGTTCAAGCCTCAGAGAGGGC
3964	Yes	AAAGTCCTGAGTTCAAGCCTCAGAGAGGGCA
3965	Yes	AAAGTCCTGAGTTCAAGCCTCAGAGAGGGCAC
3966	Yes	AAAGTCCTGAGTTCAAGCCTCAGAGAGGGCACC
3967	Yes	AAAGTCCTGAGTTCAAGCCTCAGAGAGGGCACCA
3968	No	AAAGTGGCTGATTTGC
3969	No	AAAGTGGCTGATTTGCG
3970	No	AAAGTGGCTGATTTGCGT
3971	No	AAAGTGGCTGATTTGCGTT
3972	No	AAAGTGGCTGATTTGCGTTC
3973	No	AAAGTGGCTGATTTGCGTTCA
3974	No	AAAGTGGCTGATTTGCGTTCAG
3975	No	AAAGTGGCTGATTTGCGTTCAGT
3976	No	AAAGTGGCTGATTTGCGTTCAGTT
3977	No	AAAGTGGCTGATTTGCGTTCAGTTG
3978	No	AAAGTGGCTGATTTGCGTTCAGTTGA
3979	No	AAAGTGGCTGATTTGCGTTCAGTTGAT
3980	No	AAAGTGGCTGATTTGCGTTCAGTTGATG
3981	No	AAAGTGGCTGATTTGCGTTCAGTTGATGC
3982	No	AAAGTGGCTGATTTGCGTTCAGTTGATGCA
3983	No	AAAGTGGCTGATTTGCGTTCAGTTGATGCAG

3984	No	AAAGTGGCTGATTTGCGTTCAGTTGATGCAGA
3985	No	AAAGTGGCTGATTTGCGTTCAGTTGATGCAGAG
3986	No	AAAGTGGCTGATTTGCGTTCAGTTGATGCAGAGT
3987	Yes	AAAGTGGCTGATTTGCGTTCAGTTGATGCAGAGTG
3988	Yes	AAAGTGGCTGATTTGCGTTCAGTTGATGCAGAGTGG
3989	Yes	AAAGTGGCTGATTTGCGTTCAGTTGATGCAGAGTGGG
3990	Yes	AAAGTGGCTGATTTGCGTTCAGTTGATGCAGAGTGGGG
3991	Yes	AAAGTGGCTGATTTGCGTTCAGTTGATGCAGAGTGGGGT
3992	Yes	AAAGTGGCTGATTTGCGTTCAGTTGATGCAGAGTGGGGTT
3993	Yes	AAAGTGGCTGATTTGCGTTCAGTTGATGCAGAGTGGGGTTT
3994	Yes	AAAGTGGCTGATTTGCGTTCAGTTGATGCAGAGTGGGGTTTT
3995	Yes	AAAGTGGCTGATTTGCGTTCAGTTGATGCAGAGTGGGGTTTTG
3996	Yes	AAAGTGGCTGATTTGCGTTCAGTTGATGCAGAGTGGGGTTTTGC
3997	Yes	AAAGTGGCTGATTTGCGTTCAGTTGATGCAGAGTGGGGTTTTGCA
3998	Yes	AAAGTGGCTGATTTGCGTTCAGTTGATGCAGAGTGGGGTTTTGCAG
3999	Yes	AAAGTGGCTGATTTGCGTTCAGTTGATGCAGAGTGGGGTTTTGCAGT
4000	Yes	AAAGTGGCTGATTTGCGTTCAGTTGATGCAGAGTGGGGTTTTGCAGTC
4001	Yes	AAAGTGGCTGATTTGCGTTCAGTTGATGCAGAGTGGGGTTTTGCAGTCC
4002	Yes	AAAGTGGCTGATTTGCGTTCAGTTGATGCAGAGTGGGGTTTTGCAGTCCT
4003	No	AAAGTTAAATTATAGG
4004	No	AAAGTTAAATTATAGGC
4005	No	AAAGTTAAATTATAGGCT
4006	No	AAAGTTAAATTATAGGCTA
4007	No	AAAGTTAAATTATAGGCTAA
4008	No	AAAGTTAAATTATAGGCTAAA
4009	No	AAAGTTAAATTATAGGCTAAAT
4010	No	AAAGTTAAATTATAGGCTAAATC
4011	No	AAAGTTAAATTATAGGCTAAATCC
4012	No	AAAGTTAAATTATAGGCTAAATCCT
4013	No	AAAGTTAAATTATAGGCTAAATCCTA
4014	No	AAAGTTAAATTATAGGCTAAATCCTAT
4015	No	AAAGTTAAATTATAGGCTAAATCCTATA
4016	No	AAAGTTAAATTATAGGCTAAATCCTATAT
4017	No	AAAGTTAAATTATAGGCTAAATCCTATATA
4018	No	AAAGTTAAATTATAGGCTAAATCCTATATAT
4019	No	AAAGTTAAATTATAGGCTAAATCCTATATATC
4020	No	AAAGTTAAATTATAGGCTAAATCCTATATATCT
4021	No	AAAGTTAAATTATAGGCTAAATCCTATATATCTT
4022	No	AAAGTTAAATTATAGGCTAAATCCTATATATCTTA
4023	Yes	AAAGTTAAATTATAGGCTAAATCCTATATATCTTAC
4024	Yes	AAAGTTAAATTATAGGCTAAATCCTATATATCTTACC
4025	Yes	AAAGTTAAATTATAGGCTAAATCCTATATATCTTACCA

4026	No	AAAGTTCCCTGGTTCA
4027	Yes	AAAGTTCCCTGGTTCAA
4028	Yes	AAAGTTCCCTGGTTCAAC
4029	Yes	AAAGTTCCCTGGTTCAACC
4030	Yes	AAAGTTCCCTGGTTCAACCC
4031	Yes	AAAGTTCCCTGGTTCAACCCT
4032	Yes	AAAGTTCCCTGGTTCAACCCTG
4033	Yes	AAAGTTCCCTGGTTCAACCCTGG
4034	Yes	AAAGTTCCCTGGTTCAACCCTGGG
4035	Yes	AAAGTTCCCTGGTTCAACCCTGGGT
4036	Yes	AAAGTTCCCTGGTTCAACCCTGGGTT
4037	Yes	AAAGTTCCCTGGTTCAACCCTGGGTTT
4038	Yes	AAAGTTCCCTGGTTCAACCCTGGGTTTC
4039	Yes	AAAGTTCCCTGGTTCAACCCTGGGTTTCA
4040	Yes	AAAGTTCCCTGGTTCAACCCTGGGTTTCAG
4041	Yes	AAAGTTCCCTGGTTCAACCCTGGGTTTCAGC
4042	Yes	AAAGTTCCCTGGTTCAACCCTGGGTTTCAGCC
4043	Yes	AAAGTTCCCTGGTTCAACCCTGGGTTTCAGCCC
4044	Yes	AAAGTTCCCTGGTTCAACCCTGGGTTTCAGCCCC
4045	Yes	AAAGTTCCCTGGTTCAACCCTGGGTTTCAGCCCCA
4046	No	AAAGTTCCTTGGCTCA
4047	No	AAAGTTCCTTGGCTCAA
4048	No	AAAGTTCCTTGGCTCAAT
4049	Yes	AAAGTTCCTTGGCTCAATC
4050	Yes	AAAGTTCCTTGGCTCAATCC
4051	Yes	AAAGTTCCTTGGCTCAATCCA
4052	Yes	AAAGTTCCTTGGCTCAATCCAG
4053	Yes	AAAGTTCCTTGGCTCAATCCAGA
4054	Yes	AAAGTTCCTTGGCTCAATCCAGAG
4055	Yes	AAAGTTCCTTGGCTCAATCCAGAGT
4056	Yes	AAAGTTCCTTGGCTCAATCCAGAGTT
4057	Yes	AAAGTTCCTTGGCTCAATCCAGAGTTT
4058	Yes	AAAGTTCCTTGGCTCAATCCAGAGTTTG
4059	Yes	AAAGTTCCTTGGCTCAATCCAGAGTTTGG
4060	Yes	AAAGTTCCTTGGCTCAATCCAGAGTTTGGG
4061	Yes	AAAGTTCCTTGGCTCAATCCAGAGTTTGGGG
4062	Yes	AAAGTTCCTTGGCTCAATCCAGAGTTTGGGGG
4063	Yes	AAAGTTCCTTGGCTCAATCCAGAGTTTGGGGGC
4064	Yes	AAAGTTCCTTGGCTCAATCCAGAGTTTGGGGGCC
4065	Yes	AAAGTTCCTTGGCTCAATCCAGAGTTTGGGGGCCA
4066	No	AAAGTTCTCTGGTTGG
4067	Yes	AAAGTTCTCTGGTTGGA

4068	Yes	AAAGTTCTCTGGTTGGAT
4069	Yes	AAAGTTCTCTGGTTGGATA
4070	Yes	AAAGTTCTCTGGTTGGATAC
4071	Yes	AAAGTTCTCTGGTTGGATACC
4072	Yes	AAAGTTCTCTGGTTGGATACCA
4073	Yes	AAAGTTCTCTGGTTGGATACCAG
4074	Yes	AAAGTTCTCTGGTTGGATACCAGA
4075	Yes	AAAGTTCTCTGGTTGGATACCAGAT
4076	Yes	AAAGTTCTCTGGTTGGATACCAGATG
4077	Yes	AAAGTTCTCTGGTTGGATACCAGATGG
4078	Yes	AAAGTTCTCTGGTTGGATACCAGATGGA
4079	Yes	AAAGTTCTCTGGTTGGATACCAGATGGAA
4080	Yes	AAAGTTCTCTGGTTGGATACCAGATGGAAA
4081	Yes	AAAGTTCTCTGGTTGGATACCAGATGGAAAT
4082	Yes	AAAGTTCTCTGGTTGGATACCAGATGGAAATG
4083	Yes	AAAGTTCTCTGGTTGGATACCAGATGGAAATGC
4084	Yes	AAAGTTCTCTGGTTGGATACCAGATGGAAATGCC
4085	Yes	AAAGTTCTCTGGTTGGATACCAGATGGAAATGCCA
4086	Yes	AAAGTTGGTGGTTCGA
4087	Yes	AAAGTTGGTGGTTCGAA
4088	Yes	AAAGTTGGTGGTTCGAAC
4089	Yes	AAAGTTGGTGGTTCGAACA
4090	Yes	AAAGTTGGTGGTTCGAACAC
4091	Yes	AAAGTTGGTGGTTCGAACACA
4092	Yes	AAAGTTGGTGGTTCGAACACAC
4093	Yes	AAAGTTGGTGGTTCGAACACACC
4094	Yes	AAAGTTGGTGGTTCGAACACACCC
4095	Yes	AAAGTTGGTGGTTCGAACACACCCA
4096	Yes	AAAGTTGGTGGTTCGAACACACCCAG
4097	Yes	AAAGTTGGTGGTTCGAACACACCCAGA
4098	Yes	AAAGTTGGTGGTTCGAACACACCCAGAG
4099	Yes	AAAGTTGGTGGTTCGAACACACCCAGAGG
4100	Yes	AAAGTTGGTGGTTCGAACACACCCAGAGGC
4101	Yes	AAAGTTGGTGGTTCGAACACACCCAGAGGCG
4102	Yes	AAAGTTGGTGGTTCGAACACACCCAGAGGCGC
4103	Yes	AAAGTTGGTGGTTCGAACACACCCAGAGGCGCC
4104	Yes	AAAGTTGGTGGTTCGAACACACCCAGAGGCGCCA
4105	Yes	AAAGTTGGTGGTTCGAG
4106	Yes	AAAGTTGGTGGTTCGAGC
4107	Yes	AAAGTTGGTGGTTCGAGCC
4108	Yes	AAAGTTGGTGGTTCGAGCCC
4109	Yes	AAAGTTGGTGGTTCGAGCCCA

4110	Yes	AAAGTTGGTGGTTCGAGCCCAC
4111	Yes	AAAGTTGGTGGTTCGAGCCCACC
4112	Yes	AAAGTTGGTGGTTCGAGCCCACCC
4113	Yes	AAAGTTGGTGGTTCGAGCCCACCCA
4114	Yes	AAAGTTGGTGGTTCGAGCCCACCCAG
4115	Yes	AAAGTTGGTGGTTCGAGCCCACCCAGG
4116	Yes	AAAGTTGGTGGTTCGAGCCCACCCAGGA
4117	Yes	AAAGTTGGTGGTTCGAGCCCACCCAGGAA
4118	Yes	AAAGTTGGTGGTTCGAGCCCACCCAGGAAC
4119	Yes	AAAGTTGGTGGTTCGAGCCCACCCAGGAACG
4120	Yes	AAAGTTGGTGGTTCGAGCCCACCCAGGAACGC
4121	Yes	AAAGTTGGTGGTTCGAGCCCACCCAGGAACGCC
4122	Yes	AAAGTTGGTGGTTCGAGCCCACCCAGGAACGCCA
4123	Yes	AAAGTTGGTGGTTCGAGCCT
4124	Yes	AAAGTTGGTGGTTCGAGCCTA
4125	Yes	AAAGTTGGTGGTTCGAGCCTAC
4126	Yes	AAAGTTGGTGGTTCGAGCCTACC
4127	Yes	AAAGTTGGTGGTTCGAGCCTACCC
4128	Yes	AAAGTTGGTGGTTCGAGCCTACCCA
4129	Yes	AAAGTTGGTGGTTCGAGCCTACCCAG
4130	Yes	AAAGTTGGTGGTTCGAGCCTACCCAGG
4131	Yes	AAAGTTGGTGGTTCGAGCCTACCCAGGG
4132	Yes	AAAGTTGGTGGTTCGAGCCTACCCAGGGA
4133	Yes	AAAGTTGGTGGTTCGAGCCTACCCAGGGAC
4134	Yes	AAAGTTGGTGGTTCGAGCCTACCCAGGGACG
4135	Yes	AAAGTTGGTGGTTCGAGCCTACCCAGGGACGC
4136	Yes	AAAGTTGGTGGTTCGAGCCTACCCAGGGACGCC
4137	Yes	AAAGTTGGTGGTTCGAGCCTACCCAGGGACGCCA
4138	Yes	AAAGTTTGGCGCTCTC
4139	Yes	AAAGTTTGGCGCTCTCA
4140	Yes	AAAGTTTGGCGCTCTCAC
4141	Yes	AAAGTTTGGCGCTCTCACC
4142	Yes	AAAGTTTGGCGCTCTCACCG
4143	Yes	AAAGTTTGGCGCTCTCACCGC
4144	Yes	AAAGTTTGGCGCTCTCACCGCC
4145	Yes	AAAGTTTGGCGCTCTCACCGCCG
4146	Yes	AAAGTTTGGCGCTCTCACCGCCGG
4147	Yes	AAAGTTTGGCGCTCTCACCGCCGGG
4148	Yes	AAAGTTTGGCGCTCTCACCGCCGGGA
4149	Yes	AAAGTTTGGCGCTCTCACCGCCGGGAC
4150	Yes	AAAGTTTGGCGCTCTCACCGCCGGGACT
4151	Yes	AAAGTTTGGCGCTCTCACCGCCGGGACTG

4152	Yes	AAAGTTTGGCGCTCTCACCGCCGGGACTGG
4153	Yes	AAAGTTTGGCGCTCTCACCGCCGGGACTGGT
4154	Yes	AAAGTTTGGCGCTCTCACCGCCGGGACTGGTT
4155	Yes	AAAGTTTGGCGCTCTCACCGCCGGGACTGGTTG
4156	Yes	AAAGTTTGGCGCTCTCACCGCCGGGACTGGTTGA
4157	Yes	AAAGTTTGGCGCTCTCACCGCCGGGACTGGTTGAT
4158	Yes	AAAGTTTGGCGCTCTCACCGCCGGGACTGGTTGATT
4159	Yes	AAAGTTTGGCGCTCTCACCGCCGGGACTGGTTGATTC
4160	Yes	AAAGTTTGGCGCTCTCACCGCCGGGACTGGTTGATTCC
4161	Yes	AAAGTTTGGCGCTCTCACCGCCGGGACTGGTTGATTCCA
4162	Yes	AAAGTTTGGCGCTCTCACCGCCGGGACTGGTTGATTCCAG
4163	Yes	AAAGTTTGGCGCTCTCACCGCCGGGACTGGTTGATTCCAGA
4164	Yes	AAAGTTTGGCGCTCTCACCGCCGGGACTGGTTGATTCCAGAT
4165	Yes	AAAGTTTGGCGCTCTCACCGCCGGGACTGGTTGATTCCAGATC
4166	Yes	AAAGTTTGGCGCTCTCACCGCCGGGACTGGTTGATTCCAGATCA
4167	Yes	AAAGTTTGGCGCTCTCACCGCCGGGACTGGTTGATTCCAGATCAG
4168	Yes	AAAGTTTGGCGCTCTCACCGCCGGGACTGGTTGATTCCAGATCAGG
4169	Yes	AAAGTTTGGCGCTCTCACCGCCGGGACTGGTTGATTCCAGATCAGGG
4170	Yes	AAAGTTTGGCGCTCTCACCGCCGGGACTGGTTGATTCCAGATCAGGGG
4171	Yes	AAAGTTTGGCGCTCTCACCGCCGGGACTGGTTGATTCCAGATCAGGGGA
4172	Yes	AAAGTTTGGCGCTCTCACCGCCGGGACTGGTTGATTCCAGATCAGGGGAC
4173	Yes	AAAGTTTGGCGCTTTC
4174	Yes	AAAGTTTGGCGCTTTCA
4175	Yes	AAAGTTTGGCGCTTTCAC
4176	Yes	AAAGTTTGGCGCTTTCACC
4177	Yes	AAAGTTTGGCGCTTTCACCG
4178	Yes	AAAGTTTGGCGCTTTCACCGC
4179	Yes	AAAGTTTGGCGCTTTCACCGCC
4180	Yes	AAAGTTTGGCGCTTTCACCGCCG
4181	Yes	AAAGTTTGGCGCTTTCACCGCCGG
4182	Yes	AAAGTTTGGCGCTTTCACCGCCGGG
4183	Yes	AAAGTTTGGCGCTTTCACCGCCGGGA
4184	Yes	AAAGTTTGGCGCTTTCACCGCCGGGAC
4185	Yes	AAAGTTTGGCGCTTTCACCGCCGGGACT
4186	Yes	AAAGTTTGGCGCTTTCACCGCCGGGACTG
4187	Yes	AAAGTTTGGCGCTTTCACCGCCGGGACTGG
4188	Yes	AAAGTTTGGCGCTTTCACCGCCGGGACTGGT
4189	Yes	AAAGTTTGGCGCTTTCACCGCCGGGACTGGTT
4190	Yes	AAAGTTTGGCGCTTTCACCGCCGGGACTGGTTG
4191	Yes	AAAGTTTGGCGCTTTCACCGCCGGGACTGGTTGA
4192	Yes	AAAGTTTGGCGCTTTCACCGCCGGGACTGGTTGAT
4193	Yes	AAAGTTTGGCGCTTTCACCGCCGGGACTGGTTGATT

4194	Yes	AAAGTTTGGCGCTTTCACCGCCGGGACTGGTTGATTC
4195	Yes	AAAGTTTGGCGCTTTCACCGCCGGGACTGGTTGATTCC
4196	Yes	AAAGTTTGGCGCTTTCACCGCCGGGACTGGTTGATTCCA
4197	Yes	AAAGTTTGGCGCTTTCACCGCCGGGACTGGTTGATTCCAG
4198	Yes	AAAGTTTGGCGCTTTCACCGCCGGGACTGGTTGATTCCAGA
4199	Yes	AAAGTTTGGCGCTTTCACCGCCGGGACTGGTTGATTCCAGAT
4200	Yes	AAAGTTTGGCGCTTTCACCGCCGGGACTGGTTGATTCCAGATC
4201	Yes	AAAGTTTGGCGCTTTCACCGCCGGGACTGGTTGATTCCAGATCA
4202	Yes	AAAGTTTGGCGCTTTCACCGCCGGGACTGGTTGATTCCAGATCAG
4203	Yes	AAAGTTTGGCGCTTTCACCGCCGGGACTGGTTGATTCCAGATCAGG
4204	Yes	AAAGTTTGGCGCTTTCACCGCCGGGACTGGTTGATTCCAGATCAGGG
4205	Yes	AAAGTTTGGCGCTTTCACCGCCGGGACTGGTTGATTCCAGATCAGGGG
4206	Yes	AAAGTTTGGCGCTTTCACCGCCGGGACTGGTTGATTCCAGATCAGGGGA
4207	Yes	AAAGTTTGGCGCTTTCACCGCCGGGACTGGTTGATTCCAGATCAGGGGAC
4208	No	AAATAAGCTATCGGGC
4209	No	AAATAAGCTATCGGGCC
4210	No	AAATAAGCTATCGGGCCC
4211	No	AAATAAGCTATCGGGCCCA
4212	No	AAATAAGCTATCGGGCCCAT
4213	No	AAATAAGCTATCGGGCCCATA
4214	No	AAATAAGCTATCGGGCCCATAC
4215	No	AAATAAGCTATCGGGCCCATACC
4216	No	AAATAAGCTATCGGGCCCATACCC
4217	No	AAATAAGCTATCGGGCCCATACCCC
4218	No	AAATAAGCTATCGGGCCCATACCCCG
4219	No	AAATAAGCTATCGGGCCCATACCCCGA
4220	No	AAATAAGCTATCGGGCCCATACCCCGAA
4221	No	AAATAAGCTATCGGGCCCATACCCCGAAA
4222	No	AAATAAGCTATCGGGCCCATACCCCGAAAA
4223	No	AAATAAGCTATCGGGCCCATACCCCGAAAAT
4224	No	AAATAAGCTATCGGGCCCATACCCCGAAAATG
4225	No	AAATAAGCTATCGGGCCCATACCCCGAAAATGT
4226	No	AAATAAGCTATCGGGCCCATACCCCGAAAATGTT
4227	No	AAATAAGCTATCGGGCCCATACCCCGAAAATGTTG
4228	No	AAATAAGCTATCGGGCCCATACCCCGAAAATGTTGG
4229	No	AAATAAGCTATCGGGCCCATACCCCGAAAATGTTGGT
4230	No	AAATAAGCTATCGGGCCCATACCCCGAAAATGTTGGTT
4231	No	AAATAAGCTATCGGGCCCATACCCCGAAAATGTTGGTTA
4232	No	AAATAAGCTATCGGGCCCATACCCCGAAAATGTTGGTTAT
4233	No	AAATAAGCTATCGGGCCCATACCCCGAAAATGTTGGTTATA
4234	Yes	AAATAAGCTATCGGGCCCATACCCCGAAAATGTTGGTTATAC
4235	Yes	AAATAAGCTATCGGGCCCATACCCCGAAAATGTTGGTTATACC

4236	Yes	AAATAAGCTATCGGGCCCATACCCCGAAAATGTTGGTTATACCC
4237	Yes	AAATAAGCTATCGGGCCCATACCCCGAAAATGTTGGTTATACCCT
4238	Yes	AAATAAGCTATCGGGCCCATACCCCGAAAATGTTGGTTATACCCTT
4239	Yes	AAATAAGCTATCGGGCCCATACCCCGAAAATGTTGGTTATACCCTTC
4240	Yes	AAATAAGCTATCGGGCCCATACCCCGAAAATGTTGGTTATACCCTTCC
4241	Yes	AAATAAGCTATCGGGCCCATACCCCGAAAATGTTGGTTATACCCTTCCC
4242	Yes	AAATAAGCTATCGGGCCCATACCCCGAAAATGTTGGTTATACCCTTCCCG
4243	No	AAATAATAGGAGCTTA
4244	No	AAATAATAGGAGCTTAA
4245	No	AAATAATAGGAGCTTAAA
4246	No	AAATAATAGGAGCTTAAAC
4247	No	AAATAATAGGAGCTTAAACC
4248	Yes	AAATAATAGGAGCTTAAACCC
4249	Yes	AAATAATAGGAGCTTAAACCCC
4250	Yes	AAATAATAGGAGCTTAAACCCCC
4251	Yes	AAATAATAGGAGCTTAAACCCCCT
4252	Yes	AAATAATAGGAGCTTAAACCCCCTT
4253	Yes	AAATAATAGGAGCTTAAACCCCCTTA
4254	Yes	AAATAATAGGAGCTTAAACCCCCTTAT
4255	Yes	AAATAATAGGAGCTTAAACCCCCTTATT
4256	Yes	AAATAATAGGAGCTTAAACCCCCTTATTT
4257	Yes	AAATAATAGGAGCTTAAACCCCCTTATTTC
4258	Yes	AAATAATAGGAGCTTAAACCCCCTTATTTCT
4259	Yes	AAATAATAGGAGCTTAAACCCCCTTATTTCTA
4260	Yes	AAATAATAGGAGCTTAAACCCCCTTATTTCTAC
4261	Yes	AAATAATAGGAGCTTAAACCCCCTTATTTCTACC
4262	Yes	AAATAATAGGAGCTTAAACCCCCTTATTTCTACCA
4263	No	AAATACAACGATGGTT
4264	No	AAATACAACGATGGTTT
4265	No	AAATACAACGATGGTTTT
4266	No	AAATACAACGATGGTTTTT
4267	No	AAATACAACGATGGTTTTTC
4268	No	AAATACAACGATGGTTTTTCA
4269	No	AAATACAACGATGGTTTTTCAT
4270	No	AAATACAACGATGGTTTTTCATA
4271	No	AAATACAACGATGGTTTTTCATAT
4272	No	AAATACAACGATGGTTTTTCATATC
4273	No	AAATACAACGATGGTTTTTCATATCA
4274	No	AAATACAACGATGGTTTTTCATATCAT
4275	No	AAATACAACGATGGTTTTTCATATCATT
4276	No	AAATACAACGATGGTTTTTCATATCATTG
4277	No	AAATACAACGATGGTTTTTCATATCATTGG

4278	No	AAATACAACGATGGTTTTTCATATCATTGGT
4279	No	AAATACAACGATGGTTTTTCATATCATTGGTC
4280	No	AAATACAACGATGGTTTTTCATATCATTGGTCG
4281	No	AAATACAACGATGGTTTTTCATATCATTGGTCGT
4282	No	AAATACAACGATGGTTTTTCATATCATTGGTCGTG
4283	No	AAATACAACGATGGTTTTTCATATCATTGGTCGTGG
4284	No	AAATACAACGATGGTTTTTCATATCATTGGTCGTGGT
4285	No	AAATACAACGATGGTTTTTCATATCATTGGTCGTGGTT
4286	No	AAATACAACGATGGTTTTTCATATCATTGGTCGTGGTTG
4287	No	AAATACAACGATGGTTTTTCATATCATTGGTCGTGGTTGT
4288	No	AAATACAACGATGGTTTTTCATATCATTGGTCGTGGTTGTA
4289	No	AAATACAACGATGGTTTTTCATATCATTGGTCGTGGTTGTAG
4290	Yes	AAATACAACGATGGTTTTTCATATCATTGGTCGTGGTTGTAGT
4291	Yes	AAATACAACGATGGTTTTTCATATCATTGGTCGTGGTTGTAGTC
4292	Yes	AAATACAACGATGGTTTTTCATATCATTGGTCGTGGTTGTAGTCC
4293	Yes	AAATACAACGATGGTTTTTCATATCATTGGTCGTGGTTGTAGTCCG
4294	Yes	AAATACAACGATGGTTTTTCATATCATTGGTCGTGGTTGTAGTCCGT
4295	Yes	AAATACAACGATGGTTTTTCATATCATTGGTCGTGGTTGTAGTCCGTG
4296	Yes	AAATACAACGATGGTTTTTCATATCATTGGTCGTGGTTGTAGTCCGTGC
4297	Yes	AAATACAACGATGGTTTTTCATATCATTGGTCGTGGTTGTAGTCCGTGCG
4298	No	AAATACAGACCAAGAG
4299	Yes	AAATACAGACCAAGAGC
4300	Yes	AAATACAGACCAAGAGCC
4301	Yes	AAATACAGACCAAGAGCCT
4302	Yes	AAATACAGACCAAGAGCCTT
4303	Yes	AAATACAGACCAAGAGCCTTC
4304	Yes	AAATACAGACCAAGAGCCTTCA
4305	Yes	AAATACAGACCAAGAGCCTTCAA
4306	Yes	AAATACAGACCAAGAGCCTTCAAA
4307	Yes	AAATACAGACCAAGAGCCTTCAAAG
4308	Yes	AAATACAGACCAAGAGCCTTCAAAGC
4309	Yes	AAATACAGACCAAGAGCCTTCAAAGCC
4310	Yes	AAATACAGACCAAGAGCCTTCAAAGCCC
4311	Yes	AAATACAGACCAAGAGCCTTCAAAGCCCT
4312	Yes	AAATACAGACCAAGAGCCTTCAAAGCCCTC
4313	Yes	AAATACAGACCAAGAGCCTTCAAAGCCCTCA
4314	Yes	AAATACAGACCAAGAGCCTTCAAAGCCCTCAG
4315	Yes	AAATACAGACCAAGAGCCTTCAAAGCCCTCAGT
4316	Yes	AAATACAGACCAAGAGCCTTCAAAGCCCTCAGTA
4317	Yes	AAATACAGACCAAGAGCCTTCAAAGCCCTCAGTAA
4318	Yes	AAATACAGACCAAGAGCCTTCAAAGCCCTCAGTAAG
4319	Yes	AAATACAGACCAAGAGCCTTCAAAGCCCTCAGTAAGT

4320	Yes	AAATACAGACCAAGAGCCTTCAAAGCCCTCAGTAAGTT
4321	Yes	AAATACAGACCAAGAGCCTTCAAAGCCCTCAGTAAGTTG
4322	Yes	AAATACAGACCAAGAGCCTTCAAAGCCCTCAGTAAGTTGC
4323	Yes	AAATACAGACCAAGAGCCTTCAAAGCCCTCAGTAAGTTGCA
4324	Yes	AAATACAGACCAAGAGCCTTCAAAGCCCTCAGTAAGTTGCAA
4325	Yes	AAATACAGACCAAGAGCCTTCAAAGCCCTCAGTAAGTTGCAAT
4326	Yes	AAATACAGACCAAGAGCCTTCAAAGCCCTCAGTAAGTTGCAATA
4327	Yes	AAATACAGACCAAGAGCCTTCAAAGCCCTCAGTAAGTTGCAATAC
4328	Yes	AAATACAGACCAAGAGCCTTCAAAGCCCTCAGTAAGTTGCAATACT
4329	Yes	AAATACAGACCAAGAGCCTTCAAAGCCCTCAGTAAGTTGCAATACTT
4330	Yes	AAATACAGACCAAGAGCCTTCAAAGCCCTCAGTAAGTTGCAATACTTA
4331	Yes	AAATACAGACCAAGAGCCTTCAAAGCCCTCAGTAAGTTGCAATACTTAA
4332	Yes	AAATACAGACCAAGAGCCTTCAAAGCCCTCAGTAAGTTGCAATACTTAAT
4333	No	AAATAGCTCAATTGGG
4334	No	AAATAGCTCAATTGGGA
4335	No	AAATAGCTCAATTGGGAG
4336	No	AAATAGCTCAATTGGGAGA
4337	No	AAATAGCTCAATTGGGAGAG
4338	Yes	AAATAGCTCAATTGGGAGAGT
4339	Yes	AAATAGCTCAATTGGGAGAGTG
4340	Yes	AAATAGCTCAATTGGGAGAGTGT
4341	Yes	AAATAGCTCAATTGGGAGAGTGTT
4342	Yes	AAATAGCTCAATTGGGAGAGTGTTA
4343	Yes	AAATAGCTCAATTGGGAGAGTGTTAG
4344	Yes	AAATAGCTCAATTGGGAGAGTGTTAGA
4345	Yes	AAATAGCTCAATTGGGAGAGTGTTAGAC
4346	Yes	AAATAGCTCAATTGGGAGAGTGTTAGACT
4347	Yes	AAATAGCTCAATTGGGAGAGTGTTAGACTG
4348	Yes	AAATAGCTCAATTGGGAGAGTGTTAGACTGA
4349	Yes	AAATAGCTCAATTGGGAGAGTGTTAGACTGAA
4350	Yes	AAATAGCTCAATTGGGAGAGTGTTAGACTGAAG
4351	Yes	AAATAGCTCAATTGGGAGAGTGTTAGACTGAAGA
4352	Yes	AAATAGCTCAATTGGGAGAGTGTTAGACTGAAGAT
4353	Yes	AAATAGCTCAATTGGGAGAGTGTTAGACTGAAGATC
4354	Yes	AAATAGCTCAATTGGGAGAGTGTTAGACTGAAGATCT
4355	Yes	AAATAGCTCAATTGGGAGAGTGTTAGACTGAAGATCTT
4356	Yes	AAATAGCTCAATTGGGAGAGTGTTAGACTGAAGATCTTC
4357	Yes	AAATAGCTCAATTGGGAGAGTGTTAGACTGAAGATCTTCT
4358	Yes	AAATAGCTCAATTGGGAGAGTGTTAGACTGAAGATCTTCTG
4359	Yes	AAATAGCTCAATTGGGAGAGTGTTAGACTGAAGATCTTCTGC
4360	Yes	AAATAGCTCAATTGGGAGAGTGTTAGACTGAAGATCTTCTGCA
4361	Yes	AAATAGCTCAATTGGGAGAGTGTTAGACTGAAGATCTTCTGCAG

4362	Yes	AAATAGCTCAATTGGGAGAGTGTTAGACTGAAGATCTTCTGCAGG
4363	Yes	AAATAGCTCAATTGGGAGAGTGTTAGACTGAAGATCTTCTGCAGGT
4364	Yes	AAATAGCTCAATTGGGAGAGTGTTAGACTGAAGATCTTCTGCAGGTC
4365	Yes	AAATAGCTCAATTGGGAGAGTGTTAGACTGAAGATCTTCTGCAGGTCT
4366	Yes	AAATAGCTCAATTGGGAGAGTGTTAGACTGAAGATCTTCTGCAGGTCTC
4367	Yes	AAATAGCTCAATTGGGAGAGTGTTAGACTGAAGATCTTCTGCAGGTCTCT
4368	No	AAATAGCTCAGCTGGG
4369	Yes	AAATAGCTCAGCTGGGA
4370	Yes	AAATAGCTCAGCTGGGAG
4371	Yes	AAATAGCTCAGCTGGGAGA
4372	Yes	AAATAGCTCAGCTGGGAGAG
4373	Yes	AAATAGCTCAGCTGGGAGAGC
4374	Yes	AAATAGCTCAGCTGGGAGAGCA
4375	Yes	AAATAGCTCAGCTGGGAGAGCAT
4376	Yes	AAATAGCTCAGCTGGGAGAGCATT
4377	Yes	AAATAGCTCAGCTGGGAGAGCATTA
4378	Yes	AAATAGCTCAGCTGGGAGAGCATTAG
4379	Yes	AAATAGCTCAGCTGGGAGAGCATTAGA
4380	Yes	AAATAGCTCAGCTGGGAGAGCATTAGAC
4381	Yes	AAATAGCTCAGCTGGGAGAGCATTAGACT
4382	Yes	AAATAGCTCAGCTGGGAGAGCATTAGACTG
4383	Yes	AAATAGCTCAGCTGGGAGAGCATTAGACTGA
4384	Yes	AAATAGCTCAGCTGGGAGAGCATTAGACTGAA
4385	Yes	AAATAGCTCAGCTGGGAGAGCATTAGACTGAAG
4386	Yes	AAATAGCTCAGCTGGGAGAGCATTAGACTGAAGA
4387	Yes	AAATAGCTCAGCTGGGAGAGCATTAGACTGAAGAT
4388	Yes	AAATAGCTCAGCTGGGAGAGCATTAGACTGAAGATC
4389	Yes	AAATAGCTCAGCTGGGAGAGCATTAGACTGAAGATCT
4390	Yes	AAATAGCTCAGCTGGGAGAGCATTAGACTGAAGATCTA
4391	Yes	AAATAGCTCAGCTGGGAGAGCATTAGACTGAAGATCTAA
4392	Yes	AAATAGCTCAGCTGGGAGAGCATTAGACTGAAGATCTAAA
4393	Yes	AAATAGCTCAGCTGGGAGAGCATTAGACTGAAGATCTAAAG
4394	Yes	AAATAGCTCAGCTGGGAGAGCATTAGACTGAAGATCTAAAGG
4395	Yes	AAATAGCTCAGCTGGGAGAGCATTAGACTGAAGATCTAAAGGT
4396	Yes	AAATAGCTCAGCTGGGAGAGCATTAGACTGAAGATCTAAAGGTC
4397	Yes	AAATAGCTCAGCTGGGAGAGCATTAGACTGAAGATCTAAAGGTCT
4398	Yes	AAATAGCTCAGCTGGGAGAGCATTAGACTGAAGATCTAAAGGTCTC
4399	Yes	AAATAGCTCAGCTGGGAGAGCATTAGACTGAAGATCTAAAGGTCTCT
4400	Yes	AAATAGCTCAGCTGGGAGAGCATTAGACTGAAGATCTAAAGGTCTCTG
4401	Yes	AAATAGCTCAGCTGGGAGAGCATTAGACTGAAGATCTAAAGGTCTCTGG
4402	Yes	AAATAGCTCAGCTGGGAGAGCATTAGACTGAAGATCTAAAGGTCTCTGGT
4403	Yes	AAATAGCTCAGCTGGGAGAGT

4404	Yes	AAATAGCTCAGCTGGGAGAGTA
4405	Yes	AAATAGCTCAGCTGGGAGAGTAT
4406	Yes	AAATAGCTCAGCTGGGAGAGTATT
4407	Yes	AAATAGCTCAGCTGGGAGAGTATTA
4408	Yes	AAATAGCTCAGCTGGGAGAGTATTAG
4409	Yes	AAATAGCTCAGCTGGGAGAGTATTAGG
4410	Yes	AAATAGCTCAGCTGGGAGAGTATTAGGT
4411	Yes	AAATAGCTCAGCTGGGAGAGTATTAGGTT
4412	Yes	AAATAGCTCAGCTGGGAGAGTATTAGGTTG
4413	Yes	AAATAGCTCAGCTGGGAGAGTATTAGGTTGA
4414	Yes	AAATAGCTCAGCTGGGAGAGTATTAGGTTGAA
4415	Yes	AAATAGCTCAGCTGGGAGAGTATTAGGTTGAAG
4416	Yes	AAATAGCTCAGCTGGGAGAGTATTAGGTTGAAGA
4417	Yes	AAATAGCTCAGCTGGGAGAGTATTAGGTTGAAGAT
4418	Yes	AAATAGCTCAGCTGGGAGAGTATTAGGTTGAAGATA
4419	Yes	AAATAGCTCAGCTGGGAGAGTATTAGGTTGAAGATAC
4420	Yes	AAATAGCTCAGCTGGGAGAGTATTAGGTTGAAGATACA
4421	Yes	AAATAGCTCAGCTGGGAGAGTATTAGGTTGAAGATACAA
4422	Yes	AAATAGCTCAGCTGGGAGAGTATTAGGTTGAAGATACAAA
4423	Yes	AAATAGCTCAGCTGGGAGAGTATTAGGTTGAAGATACAAAG
4424	Yes	AAATAGCTCAGCTGGGAGAGTATTAGGTTGAAGATACAAAGT
4425	Yes	AAATAGCTCAGCTGGGAGAGTATTAGGTTGAAGATACAAAGTT
4426	Yes	AAATAGCTCAGCTGGGAGAGTATTAGGTTGAAGATACAAAGTTC
4427	Yes	AAATAGCTCAGCTGGGAGAGTATTAGGTTGAAGATACAAAGTTCC
4428	Yes	AAATAGCTCAGCTGGGAGAGTATTAGGTTGAAGATACAAAGTTCCT
4429	Yes	AAATAGCTCAGCTGGGAGAGTATTAGGTTGAAGATACAAAGTTCCTT
4430	Yes	AAATAGCTCAGCTGGGAGAGTATTAGGTTGAAGATACAAAGTTCCTTG
4431	Yes	AAATAGCTCAGCTGGGAGAGTATTAGGTTGAAGATACAAAGTTCCTTGG
4432	Yes	AAATAGCTCAGCTGGGAGAGTATTAGGTTGAAGATACAAAGTTCCTTGGC
4433	No	AAATAGCTCAGTTGGG
4434	No	AAATAGCTCAGTTGGGA
4435	No	AAATAGCTCAGTTGGGAG
4436	No	AAATAGCTCAGTTGGGAGA
4437	No	AAATAGCTCAGTTGGGAGAG
4438	No	AAATAGCTCAGTTGGGAGAGC
4439	No	AAATAGCTCAGTTGGGAGAGCG
4440	No	AAATAGCTCAGTTGGGAGAGCGT
4441	No	AAATAGCTCAGTTGGGAGAGCGTT
4442	No	AAATAGCTCAGTTGGGAGAGCGTTA
4443	No	AAATAGCTCAGTTGGGAGAGCGTTAG
4444	No	AAATAGCTCAGTTGGGAGAGCGTTAGA
4445	No	AAATAGCTCAGTTGGGAGAGCGTTAGAC

4446	Yes	AAATAGCTCAGTTGGGAGAGCGTTAGACC
4447	Yes	AAATAGCTCAGTTGGGAGAGCGTTAGACCG
4448	Yes	AAATAGCTCAGTTGGGAGAGCGTTAGACCGA
4449	Yes	AAATAGCTCAGTTGGGAGAGCGTTAGACCGAA
4450	Yes	AAATAGCTCAGTTGGGAGAGCGTTAGACCGAAG
4451	Yes	AAATAGCTCAGTTGGGAGAGCGTTAGACCGAAGA
4452	Yes	AAATAGCTCAGTTGGGAGAGCGTTAGACCGAAGAT
4453	Yes	AAATAGCTCAGTTGGGAGAGCGTTAGACCGAAGATC
4454	Yes	AAATAGCTCAGTTGGGAGAGCGTTAGACCGAAGATCT
4455	Yes	AAATAGCTCAGTTGGGAGAGCGTTAGACCGAAGATCTT
4456	Yes	AAATAGCTCAGTTGGGAGAGCGTTAGACCGAAGATCTTA
4457	Yes	AAATAGCTCAGTTGGGAGAGCGTTAGACCGAAGATCTTAA
4458	Yes	AAATAGCTCAGTTGGGAGAGCGTTAGACCGAAGATCTTAAA
4459	Yes	AAATAGCTCAGTTGGGAGAGCGTTAGACCGAAGATCTTAAAG
4460	Yes	AAATAGCTCAGTTGGGAGAGCGTTAGACCGAAGATCTTAAAGG
4461	Yes	AAATAGCTCAGTTGGGAGAGCGTTAGACCGAAGATCTTAAAGGT
4462	Yes	AAATAGCTCAGTTGGGAGAGCGTTAGACCGAAGATCTTAAAGGTC
4463	Yes	AAATAGCTCAGTTGGGAGAGCGTTAGACCGAAGATCTTAAAGGTCC
4464	Yes	AAATAGCTCAGTTGGGAGAGCGTTAGACCGAAGATCTTAAAGGTCCC
4465	Yes	AAATAGCTCAGTTGGGAGAGCGTTAGACCGAAGATCTTAAAGGTCCCT
4466	Yes	AAATAGCTCAGTTGGGAGAGCGTTAGACCGAAGATCTTAAAGGTCCCTG
4467	Yes	AAATAGCTCAGTTGGGAGAGCGTTAGACCGAAGATCTTAAAGGTCCCTGG
4468	No	AAATAGCTCAGTTGGGAGAGCGTTAGACT
4469	No	AAATAGCTCAGTTGGGAGAGCGTTAGACTG
4470	No	AAATAGCTCAGTTGGGAGAGCGTTAGACTGA
4471	No	AAATAGCTCAGTTGGGAGAGCGTTAGACTGAA
4472	No	AAATAGCTCAGTTGGGAGAGCGTTAGACTGAAG
4473	No	AAATAGCTCAGTTGGGAGAGCGTTAGACTGAAGA
4474	Yes	AAATAGCTCAGTTGGGAGAGCGTTAGACTGAAGAT
4475	Yes	AAATAGCTCAGTTGGGAGAGCGTTAGACTGAAGATC
4476	Yes	AAATAGCTCAGTTGGGAGAGCGTTAGACTGAAGATCT
4477	Yes	AAATAGCTCAGTTGGGAGAGCGTTAGACTGAAGATCTA
4478	Yes	AAATAGCTCAGTTGGGAGAGCGTTAGACTGAAGATCTAA
4479	Yes	AAATAGCTCAGTTGGGAGAGCGTTAGACTGAAGATCTAAA
4480	Yes	AAATAGCTCAGTTGGGAGAGCGTTAGACTGAAGATCTAAAG
4481	Yes	AAATAGCTCAGTTGGGAGAGCGTTAGACTGAAGATCTAAAGG
4482	Yes	AAATAGCTCAGTTGGGAGAGCGTTAGACTGAAGATCTAAAGGT
4483	Yes	AAATAGCTCAGTTGGGAGAGCGTTAGACTGAAGATCTAAAGGTC
4484	Yes	AAATAGCTCAGTTGGGAGAGCGTTAGACTGAAGATCTAAAGGTCC
4485	Yes	AAATAGCTCAGTTGGGAGAGCGTTAGACTGAAGATCTAAAGGTCCC
4486	Yes	AAATAGCTCAGTTGGGAGAGCGTTAGACTGAAGATCTAAAGGTCCCT
4487	Yes	AAATAGCTCAGTTGGGAGAGCGTTAGACTGAAGATCTAAAGGTCCCTG

4488	Yes	AAATAGCTCAGTTGGGAGAGCGTTAGACTGAAGATCTAAAGGTCCCTGG
4489	Yes	AAATAGCTCAGTTGGGAGAGCGTTAGACTGAAGATCTAAAGGTCCCTGGT
4490	Yes	AAATAGCTCAGTTGGGAGAGCGTTAGACTGAAGATCTT
4491	Yes	AAATAGCTCAGTTGGGAGAGCGTTAGACTGAAGATCTTA
4492	Yes	AAATAGCTCAGTTGGGAGAGCGTTAGACTGAAGATCTTAA
4493	Yes	AAATAGCTCAGTTGGGAGAGCGTTAGACTGAAGATCTTAAA
4494	Yes	AAATAGCTCAGTTGGGAGAGCGTTAGACTGAAGATCTTAAAG
4495	Yes	AAATAGCTCAGTTGGGAGAGCGTTAGACTGAAGATCTTAAAGT
4496	Yes	AAATAGCTCAGTTGGGAGAGCGTTAGACTGAAGATCTTAAAGTT
4497	Yes	AAATAGCTCAGTTGGGAGAGCGTTAGACTGAAGATCTTAAAGTTC
4498	Yes	AAATAGCTCAGTTGGGAGAGCGTTAGACTGAAGATCTTAAAGTTCC
4499	Yes	AAATAGCTCAGTTGGGAGAGCGTTAGACTGAAGATCTTAAAGTTCCC
4500	Yes	AAATAGCTCAGTTGGGAGAGCGTTAGACTGAAGATCTTAAAGTTCCCT
4501	Yes	AAATAGCTCAGTTGGGAGAGCGTTAGACTGAAGATCTTAAAGTTCCCTG
4502	Yes	AAATAGCTCAGTTGGGAGAGCGTTAGACTGAAGATCTTAAAGTTCCCTGG
4503	Yes	AAATAGTACCGTTAAC
4504	Yes	AAATAGTACCGTTAACT
4505	Yes	AAATAGTACCGTTAACTT
4506	Yes	AAATAGTACCGTTAACTTC
4507	Yes	AAATAGTACCGTTAACTTCC
4508	Yes	AAATAGTACCGTTAACTTCCA
4509	Yes	AAATAGTACCGTTAACTTCCAA
4510	Yes	AAATAGTACCGTTAACTTCCAAT
4511	Yes	AAATAGTACCGTTAACTTCCAATT
4512	Yes	AAATAGTACCGTTAACTTCCAATTA
4513	Yes	AAATAGTACCGTTAACTTCCAATTAA
4514	Yes	AAATAGTACCGTTAACTTCCAATTAAC
4515	Yes	AAATAGTACCGTTAACTTCCAATTAACT
4516	Yes	AAATAGTACCGTTAACTTCCAATTAACTA
4517	Yes	AAATAGTACCGTTAACTTCCAATTAACTAG
4518	Yes	AAATAGTACCGTTAACTTCCAATTAACTAGT
4519	Yes	AAATAGTACCGTTAACTTCCAATTAACTAGTT
4520	Yes	AAATAGTACCGTTAACTTCCAATTAACTAGTTT
4521	Yes	AAATAGTACCGTTAACTTCCAATTAACTAGTTTT
4522	Yes	AAATAGTACCGTTAACTTCCAATTAACTAGTTTTG
4523	Yes	AAATAGTACCGTTAACTTCCAATTAACTAGTTTTGA
4524	Yes	AAATAGTACCGTTAACTTCCAATTAACTAGTTTTGAC
4525	Yes	AAATAGTACCGTTAACTTCCAATTAACTAGTTTTGACA
4526	Yes	AAATAGTACCGTTAACTTCCAATTAACTAGTTTTGACAA
4527	Yes	AAATAGTACCGTTAACTTCCAATTAACTAGTTTTGACAAC
4528	Yes	AAATAGTACCGTTAACTTCCAATTAACTAGTTTTGACAACA
4529	Yes	AAATAGTACCGTTAACTTCCAATTAACTAGTTTTGACAACAT

4530	Yes	AAATAGTACCGTTAACTTCCAATTAACTAGTTTTGACAACATT
4531	Yes	AAATAGTACCGTTAACTTCCAATTAACTAGTTTTGACAACATTC
4532	Yes	AAATAGTACCGTTAACTTCCAATTAACTAGTTTTGACAACATTCA
4533	Yes	AAATAGTACCGTTAACTTCCAATTAACTAGTTTTGACAACATTCAA
4534	Yes	AAATAGTACCGTTAACTTCCAATTAACTAGTTTTGACAACATTCAAA
4535	Yes	AAATAGTACCGTTAACTTCCAATTAACTAGTTTTGACAACATTCAAAA
4536	Yes	AAATAGTACCGTTAACTTCCAATTAACTAGTTTTGACAACATTCAAAAA
4537	Yes	AAATAGTACCGTTAACTTCCAATTAACTAGTTTTGACAACATTCAAAAAA
4538	No	AAATATAGTTTAACCA
4539	No	AAATATAGTTTAACCAA
4540	No	AAATATAGTTTAACCAAA
4541	No	AAATATAGTTTAACCAAAA
4542	No	AAATATAGTTTAACCAAAAC
4543	No	AAATATAGTTTAACCAAAACA
4544	No	AAATATAGTTTAACCAAAACAT
4545	No	AAATATAGTTTAACCAAAACATC
4546	No	AAATATAGTTTAACCAAAACATCA
4547	No	AAATATAGTTTAACCAAAACATCAG
4548	No	AAATATAGTTTAACCAAAACATCAGA
4549	No	AAATATAGTTTAACCAAAACATCAGAT
4550	No	AAATATAGTTTAACCAAAACATCAGATT
4551	No	AAATATAGTTTAACCAAAACATCAGATTG
4552	No	AAATATAGTTTAACCAAAACATCAGATTGT
4553	No	AAATATAGTTTAACCAAAACATCAGATTGTG
4554	No	AAATATAGTTTAACCAAAACATCAGATTGTGA
4555	No	AAATATAGTTTAACCAAAACATCAGATTGTGAA
4556	No	AAATATAGTTTAACCAAAACATCAGATTGTGAAT
4557	No	AAATATAGTTTAACCAAAACATCAGATTGTGAATC
4558	No	AAATATAGTTTAACCAAAACATCAGATTGTGAATCT
4559	No	AAATATAGTTTAACCAAAACATCAGATTGTGAATCTG
4560	No	AAATATAGTTTAACCAAAACATCAGATTGTGAATCTGA
4561	No	AAATATAGTTTAACCAAAACATCAGATTGTGAATCTGAC
4562	No	AAATATAGTTTAACCAAAACATCAGATTGTGAATCTGACA
4563	No	AAATATAGTTTAACCAAAACATCAGATTGTGAATCTGACAA
4564	No	AAATATAGTTTAACCAAAACATCAGATTGTGAATCTGACAAC
4565	No	AAATATAGTTTAACCAAAACATCAGATTGTGAATCTGACAACA
4566	No	AAATATAGTTTAACCAAAACATCAGATTGTGAATCTGACAACAG
4567	No	AAATATAGTTTAACCAAAACATCAGATTGTGAATCTGACAACAGA
4568	No	AAATATAGTTTAACCAAAACATCAGATTGTGAATCTGACAACAGAG
4569	No	AAATATAGTTTAACCAAAACATCAGATTGTGAATCTGACAACAGAGG
4570	No	AAATATAGTTTAACCAAAACATCAGATTGTGAATCTGACAACAGAGGC
4571	No	AAATATAGTTTAACCAAAACATCAGATTGTGAATCTGACAACAGAGGCT

4572	Yes	AAATATAGTTTAACCAAAACATCAGATTGTGAATCTGACAACAGAGGCTT
4573	No	AAATATGTCTGATAAA
4574	No	AAATATGTCTGATAAAA
4575	No	AAATATGTCTGATAAAAG
4576	No	AAATATGTCTGATAAAAGA
4577	No	AAATATGTCTGATAAAAGAG
4578	No	AAATATGTCTGATAAAAGAGT
4579	No	AAATATGTCTGATAAAAGAGTT
4580	No	AAATATGTCTGATAAAAGAGTTA
4581	No	AAATATGTCTGATAAAAGAGTTAC
4582	No	AAATATGTCTGATAAAAGAGTTACT
4583	No	AAATATGTCTGATAAAAGAGTTACTT
4584	No	AAATATGTCTGATAAAAGAGTTACTTT
4585	No	AAATATGTCTGATAAAAGAGTTACTTTG
4586	No	AAATATGTCTGATAAAAGAGTTACTTTGA
4587	No	AAATATGTCTGATAAAAGAGTTACTTTGAT
4588	No	AAATATGTCTGATAAAAGAGTTACTTTGATA
4589	No	AAATATGTCTGATAAAAGAGTTACTTTGATAG
4590	No	AAATATGTCTGATAAAAGAGTTACTTTGATAGA
4591	No	AAATATGTCTGATAAAAGAGTTACTTTGATAGAG
4592	No	AAATATGTCTGATAAAAGAGTTACTTTGATAGAGT
4593	No	AAATATGTCTGATAAAAGAGTTACTTTGATAGAGTA
4594	No	AAATATGTCTGATAAAAGAGTTACTTTGATAGAGTAA
4595	No	AAATATGTCTGATAAAAGAGTTACTTTGATAGAGTAAA
4596	No	AAATATGTCTGATAAAAGAGTTACTTTGATAGAGTAAAT
4597	No	AAATATGTCTGATAAAAGAGTTACTTTGATAGAGTAAATA
4598	No	AAATATGTCTGATAAAAGAGTTACTTTGATAGAGTAAATAA
4599	No	AAATATGTCTGATAAAAGAGTTACTTTGATAGAGTAAATAAT
4600	No	AAATATGTCTGATAAAAGAGTTACTTTGATAGAGTAAATAATA
4601	No	AAATATGTCTGATAAAAGAGTTACTTTGATAGAGTAAATAATAG
4602	No	AAATATGTCTGATAAAAGAGTTACTTTGATAGAGTAAATAATAGG
4603	No	AAATATGTCTGATAAAAGAGTTACTTTGATAGAGTAAATAATAGGA
4604	No	AAATATGTCTGATAAAAGAGTTACTTTGATAGAGTAAATAATAGGAG
4605	Yes	AAATATGTCTGATAAAAGAGTTACTTTGATAGAGTAAATAATAGGAGC
4606	Yes	AAATATGTCTGATAAAAGAGTTACTTTGATAGAGTAAATAATAGGAGCT
4607	Yes	AAATATGTCTGATAAAAGAGTTACTTTGATAGAGTAAATAATAGGAGCTT
4608	No	AAATCAAGAGGTCCCT
4609	Yes	AAATCAAGAGGTCCCTG
4610	Yes	AAATCAAGAGGTCCCTGA
4611	Yes	AAATCAAGAGGTCCCTGAT
4612	Yes	AAATCAAGAGGTCCCTGATT
4613	Yes	AAATCAAGAGGTCCCTGATTC

4614	Yes	AAATCAAGAGGTCCCTGATTCA
4615	Yes	AAATCAAGAGGTCCCTGATTCAA
4616	Yes	AAATCAAGAGGTCCCTGATTCAAA
4617	Yes	AAATCAAGAGGTCCCTGATTCAAAT
4618	Yes	AAATCAAGAGGTCCCTGATTCAAATC
4619	Yes	AAATCAAGAGGTCCCTGATTCAAATCC
4620	Yes	AAATCAAGAGGTCCCTGATTCAAATCCA
4621	Yes	AAATCAAGAGGTCCCTGATTCAAATCCAG
4622	Yes	AAATCAAGAGGTCCCTGATTCAAATCCAGG
4623	Yes	AAATCAAGAGGTCCCTGATTCAAATCCAGGT
4624	Yes	AAATCAAGAGGTCCCTGATTCAAATCCAGGTG
4625	Yes	AAATCAAGAGGTCCCTGATTCAAATCCAGGTGC
4626	Yes	AAATCAAGAGGTCCCTGATTCAAATCCAGGTGCC
4627	Yes	AAATCAAGAGGTCCCTGATTCAAATCCAGGTGCCC
4628	Yes	AAATCAAGAGGTCCCTGATTCAAATCCAGGTGCCCC
4629	Yes	AAATCAAGAGGTCCCTGATTCAAATCCAGGTGCCCCC
4630	Yes	AAATCAAGAGGTCCCTGATTCAAATCCAGGTGCCCCCT
4631	Yes	AAATCAAGAGGTCCCTGATTCAAATCCAGGTGCCCCCTC
4632	Yes	AAATCAAGAGGTCCCTGATTCAAATCCAGGTGCCCCCTCC
4633	Yes	AAATCAAGAGGTCCCTGATTCAAATCCAGGTGCCCCCTCCA
4634	Yes	AAATCACGTCGGGGTC
4635	Yes	AAATCACGTCGGGGTCA
4636	Yes	AAATCACGTCGGGGTCAC
4637	Yes	AAATCACGTCGGGGTCACC
4638	Yes	AAATCACGTCGGGGTCACCA
4639	Yes	AAATCAGGCGGGAACA
4640	Yes	AAATCAGGCGGGAACAC
4641	Yes	AAATCAGGCGGGAACACC
4642	Yes	AAATCAGGCGGGAACACCA
4643	Yes	AAATCCAATGGGGGTT
4644	Yes	AAATCCAATGGGGGTTC
4645	Yes	AAATCCAATGGGGGTTCC
4646	Yes	AAATCCAATGGGGGTTCCC
4647	Yes	AAATCCAATGGGGGTTCCCC
4648	Yes	AAATCCAATGGGGGTTCCCCG
4649	Yes	AAATCCAATGGGGGTTCCCCGC
4650	Yes	AAATCCAATGGGGGTTCCCCGCG
4651	Yes	AAATCCAATGGGGGTTCCCCGCGC
4652	Yes	AAATCCAATGGGGGTTCCCCGCGCA
4653	Yes	AAATCCAATGGGGGTTCCCCGCGCAG
4654	Yes	AAATCCAATGGGGGTTCCCCGCGCAGG
4655	Yes	AAATCCAATGGGGGTTCCCCGCGCAGGT

4656	Yes	AAATCCAATGGGGGTTCCCCGCGCAGGTT
4657	Yes	AAATCCAATGGGGGTTCCCCGCGCAGGTTC
4658	Yes	AAATCCAATGGGGGTTCCCCGCGCAGGTTCA
4659	Yes	AAATCCAATGGGGGTTCCCCGCGCAGGTTCAA
4660	Yes	AAATCCAATGGGGGTTCCCCGCGCAGGTTCAAA
4661	Yes	AAATCCAATGGGGGTTCCCCGCGCAGGTTCAAAT
4662	Yes	AAATCCAATGGGGGTTCCCCGCGCAGGTTCAAATC
4663	Yes	AAATCCAATGGGGGTTCCCCGCGCAGGTTCAAATCC
4664	Yes	AAATCCAATGGGGGTTCCCCGCGCAGGTTCAAATCCT
4665	Yes	AAATCCAATGGGGGTTCCCCGCGCAGGTTCAAATCCTG
4666	Yes	AAATCCAATGGGGGTTCCCCGCGCAGGTTCAAATCCTGC
4667	Yes	AAATCCAATGGGGGTTCCCCGCGCAGGTTCAAATCCTGCT
4668	Yes	AAATCCAATGGGGGTTCCCCGCGCAGGTTCAAATCCTGCTC
4669	Yes	AAATCCAATGGGGGTTCCCCGCGCAGGTTCAAATCCTGCTCA
4670	Yes	AAATCCAATGGGGGTTCCCCGCGCAGGTTCAAATCCTGCTCAC
4671	Yes	AAATCCAATGGGGGTTCCCCGCGCAGGTTCAAATCCTGCTCACA
4672	Yes	AAATCCAATGGGGGTTCCCCGCGCAGGTTCAAATCCTGCTCACAG
4673	Yes	AAATCCAATGGGGGTTCCCCGCGCAGGTTCAAATCCTGCTCACAGC
4674	Yes	AAATCCAATGGGGGTTCCCCGCGCAGGTTCAAATCCTGCTCACAGCG
4675	Yes	AAATCCAATGGGGGTTCCCCGCGCAGGTTCAAATCCTGCTCACAGCGC
4676	Yes	AAATCCAATGGGGGTTCCCCGCGCAGGTTCAAATCCTGCTCACAGCGCC
4677	Yes	AAATCCAATGGGGGTTCCCCGCGCAGGTTCAAATCCTGCTCACAGCGCCA
4678	No	AAATCCAATGGGGTCT
4679	No	AAATCCAATGGGGTCTC
4680	Yes	AAATCCAATGGGGTCTCC
4681	Yes	AAATCCAATGGGGTCTCCC
4682	Yes	AAATCCAATGGGGTCTCCCC
4683	Yes	AAATCCAATGGGGTCTCCCCG
4684	Yes	AAATCCAATGGGGTCTCCCCGC
4685	Yes	AAATCCAATGGGGTCTCCCCGCG
4686	Yes	AAATCCAATGGGGTCTCCCCGCGC
4687	Yes	AAATCCAATGGGGTCTCCCCGCGCA
4688	Yes	AAATCCAATGGGGTCTCCCCGCGCAG
4689	Yes	AAATCCAATGGGGTCTCCCCGCGCAGG
4690	Yes	AAATCCAATGGGGTCTCCCCGCGCAGGT
4691	Yes	AAATCCAATGGGGTCTCCCCGCGCAGGTT
4692	Yes	AAATCCAATGGGGTCTCCCCGCGCAGGTTC
4693	Yes	AAATCCAATGGGGTCTCCCCGCGCAGGTTCA
4694	Yes	AAATCCAATGGGGTCTCCCCGCGCAGGTTCAA
4695	Yes	AAATCCAATGGGGTCTCCCCGCGCAGGTTCAAA
4696	Yes	AAATCCAATGGGGTCTCCCCGCGCAGGTTCAAAT
4697	Yes	AAATCCAATGGGGTCTCCCCGCGCAGGTTCAAATC

4698	Yes	AAATCCAATGGGGTCTCCCCGCGCAGGTTCAAATCC
4699	Yes	AAATCCAATGGGGTCTCCCCGCGCAGGTTCAAATCCT
4700	Yes	AAATCCAATGGGGTCTCCCCGCGCAGGTTCAAATCCTG
4701	Yes	AAATCCAATGGGGTCTCCCCGCGCAGGTTCAAATCCTGC
4702	Yes	AAATCCAATGGGGTCTCCCCGCGCAGGTTCAAATCCTGCT
4703	Yes	AAATCCAATGGGGTCTCCCCGCGCAGGTTCAAATCCTGCTC
4704	Yes	AAATCCAATGGGGTCTCCCCGCGCAGGTTCAAATCCTGCTCA
4705	Yes	AAATCCAATGGGGTCTCCCCGCGCAGGTTCAAATCCTGCTCAC
4706	Yes	AAATCCAATGGGGTCTCCCCGCGCAGGTTCAAATCCTGCTCACA
4707	Yes	AAATCCAATGGGGTCTCCCCGCGCAGGTTCAAATCCTGCTCACAG
4708	Yes	AAATCCAATGGGGTCTCCCCGCGCAGGTTCAAATCCTGCTCACAGC
4709	Yes	AAATCCAATGGGGTCTCCCCGCGCAGGTTCAAATCCTGCTCACAGCG
4710	Yes	AAATCCAATGGGGTCTCCCCGCGCAGGTTCAAATCCTGCTCACAGCGC
4711	Yes	AAATCCAATGGGGTCTCCCCGCGCAGGTTCAAATCCTGCTCACAGCGCC
4712	Yes	AAATCCAATGGGGTCTCCCCGCGCAGGTTCAAATCCTGCTCACAGCGCCA
4713	Yes	AAATCCAATGGGGTCTCCCCGCGCAGGTTCG
4714	Yes	AAATCCAATGGGGTCTCCCCGCGCAGGTTCGA
4715	Yes	AAATCCAATGGGGTCTCCCCGCGCAGGTTCGAA
4716	Yes	AAATCCAATGGGGTCTCCCCGCGCAGGTTCGAAC
4717	Yes	AAATCCAATGGGGTCTCCCCGCGCAGGTTCGAACC
4718	Yes	AAATCCAATGGGGTCTCCCCGCGCAGGTTCGAACCC
4719	Yes	AAATCCAATGGGGTCTCCCCGCGCAGGTTCGAACCCT
4720	Yes	AAATCCAATGGGGTCTCCCCGCGCAGGTTCGAACCCTG
4721	Yes	AAATCCAATGGGGTCTCCCCGCGCAGGTTCGAACCCTGC
4722	Yes	AAATCCAATGGGGTCTCCCCGCGCAGGTTCGAACCCTGCT
4723	Yes	AAATCCAATGGGGTCTCCCCGCGCAGGTTCGAACCCTGCTC
4724	Yes	AAATCCAATGGGGTCTCCCCGCGCAGGTTCGAACCCTGCTCG
4725	Yes	AAATCCAATGGGGTCTCCCCGCGCAGGTTCGAACCCTGCTCGC
4726	Yes	AAATCCAATGGGGTCTCCCCGCGCAGGTTCGAACCCTGCTCGCT
4727	Yes	AAATCCAATGGGGTCTCCCCGCGCAGGTTCGAACCCTGCTCGCTG
4728	Yes	AAATCCAATGGGGTCTCCCCGCGCAGGTTCGAACCCTGCTCGCTGC
4729	Yes	AAATCCAATGGGGTCTCCCCGCGCAGGTTCGAACCCTGCTCGCTGCG
4730	Yes	AAATCCAATGGGGTCTCCCCGCGCAGGTTCGAACCCTGCTCGCTGCGC
4731	Yes	AAATCCAATGGGGTCTCCCCGCGCAGGTTCGAACCCTGCTCGCTGCGCC
4732	Yes	AAATCCAATGGGGTCTCCCCGCGCAGGTTCGAACCCTGCTCGCTGCGCCA
4733	Yes	AAATCCAATGGGGTCTCCCCGCGCAGGTTCGAAT
4734	Yes	AAATCCAATGGGGTCTCCCCGCGCAGGTTCGAATC
4735	Yes	AAATCCAATGGGGTCTCCCCGCGCAGGTTCGAATCC
4736	Yes	AAATCCAATGGGGTCTCCCCGCGCAGGTTCGAATCCT
4737	Yes	AAATCCAATGGGGTCTCCCCGCGCAGGTTCGAATCCTG
4738	Yes	AAATCCAATGGGGTCTCCCCGCGCAGGTTCGAATCCTGC
4739	Yes	AAATCCAATGGGGTCTCCCCGCGCAGGTTCGAATCCTGCT

4740	Yes	AAATCCAATGGGGTCTCCCCGCGCAGGTTCGAATCCTGCTC
4741	Yes	AAATCCAATGGGGTCTCCCCGCGCAGGTTCGAATCCTGCTCA
4742	Yes	AAATCCAATGGGGTCTCCCCGCGCAGGTTCGAATCCTGCTCAC
4743	Yes	AAATCCAATGGGGTCTCCCCGCGCAGGTTCGAATCCTGCTCACA
4744	Yes	AAATCCAATGGGGTCTCCCCGCGCAGGTTCGAATCCTGCTCACAG
4745	Yes	AAATCCAATGGGGTCTCCCCGCGCAGGTTCGAATCCTGCTCACAGC
4746	Yes	AAATCCAATGGGGTCTCCCCGCGCAGGTTCGAATCCTGCTCACAGCG
4747	Yes	AAATCCAATGGGGTCTCCCCGCGCAGGTTCGAATCCTGCTCACAGCGC
4748	Yes	AAATCCAATGGGGTCTCCCCGCGCAGGTTCGAATCCTGCTCACAGCGCC
4749	Yes	AAATCCAATGGGGTCTCCCCGCGCAGGTTCGAATCCTGCTCACAGCGCCA
4750	No	AAATCCAATGGGGTTT
4751	No	AAATCCAATGGGGTTTC
4752	Yes	AAATCCAATGGGGTTTCC
4753	Yes	AAATCCAATGGGGTTTCCC
4754	Yes	AAATCCAATGGGGTTTCCCC
4755	Yes	AAATCCAATGGGGTTTCCCCG
4756	Yes	AAATCCAATGGGGTTTCCCCGC
4757	Yes	AAATCCAATGGGGTTTCCCCGCA
4758	Yes	AAATCCAATGGGGTTTCCCCGCAC
4759	Yes	AAATCCAATGGGGTTTCCCCGCACA
4760	Yes	AAATCCAATGGGGTTTCCCCGCACAG
4761	Yes	AAATCCAATGGGGTTTCCCCGCACAGG
4762	Yes	AAATCCAATGGGGTTTCCCCGCACAGGT
4763	Yes	AAATCCAATGGGGTTTCCCCGCACAGGTT
4764	Yes	AAATCCAATGGGGTTTCCCCGCACAGGTTC
4765	Yes	AAATCCAATGGGGTTTCCCCGCACAGGTTCG
4766	Yes	AAATCCAATGGGGTTTCCCCGCACAGGTTCGA
4767	Yes	AAATCCAATGGGGTTTCCCCGCACAGGTTCGAA
4768	Yes	AAATCCAATGGGGTTTCCCCGCACAGGTTCGAAT
4769	Yes	AAATCCAATGGGGTTTCCCCGCACAGGTTCGAATC
4770	Yes	AAATCCAATGGGGTTTCCCCGCACAGGTTCGAATCC
4771	Yes	AAATCCAATGGGGTTTCCCCGCACAGGTTCGAATCCT
4772	Yes	AAATCCAATGGGGTTTCCCCGCACAGGTTCGAATCCTG
4773	Yes	AAATCCAATGGGGTTTCCCCGCACAGGTTCGAATCCTGT
4774	Yes	AAATCCAATGGGGTTTCCCCGCACAGGTTCGAATCCTGTT
4775	Yes	AAATCCAATGGGGTTTCCCCGCACAGGTTCGAATCCTGTTC
4776	Yes	AAATCCAATGGGGTTTCCCCGCACAGGTTCGAATCCTGTTCG
4777	Yes	AAATCCAATGGGGTTTCCCCGCACAGGTTCGAATCCTGTTCGT
4778	Yes	AAATCCAATGGGGTTTCCCCGCACAGGTTCGAATCCTGTTCGTG
4779	Yes	AAATCCAATGGGGTTTCCCCGCACAGGTTCGAATCCTGTTCGTGA
4780	Yes	AAATCCAATGGGGTTTCCCCGCACAGGTTCGAATCCTGTTCGTGAC
4781	Yes	AAATCCAATGGGGTTTCCCCGCACAGGTTCGAATCCTGTTCGTGACG

4782	Yes	AAATCCAATGGGGTTTCCCCGCACAGGTTCGAATCCTGTTCGTGACGC
4783	Yes	AAATCCAATGGGGTTTCCCCGCACAGGTTCGAATCCTGTTCGTGACGCC
4784	Yes	AAATCCAATGGGGTTTCCCCGCACAGGTTCGAATCCTGTTCGTGACGCCA
4785	No	AAATCCAGGTGCCCCC
4786	Yes	AAATCCAGGTGCCCCCC
4787	Yes	AAATCCAGGTGCCCCCCC
4788	Yes	AAATCCAGGTGCCCCCCCC
4789	Yes	AAATCCAGGTGCCCCCCCCA
4790	No	AAATCCAGGTGCCCCCT
4791	Yes	AAATCCAGGTGCCCCCTC
4792	Yes	AAATCCAGGTGCCCCCTCC
4793	Yes	AAATCCAGGTGCCCCCTCCA
4794	Yes	AAATCCAGGTGCCCCT
4795	Yes	AAATCCAGGTGCCCCTT
4796	Yes	AAATCCAGGTGCCCCTTC
4797	Yes	AAATCCAGGTGCCCCTTCC
4798	Yes	AAATCCAGGTGCCCCTTCCA
4799	No	AAATCCAGGTTCTCCC
4800	No	AAATCCAGGTTCTCCCT
4801	No	AAATCCAGGTTCTCCCTC
4802	Yes	AAATCCAGGTTCTCCCTCC
4803	Yes	AAATCCAGGTTCTCCCTCCA
4804	Yes	AAATCCATTGGGGTCT
4805	Yes	AAATCCATTGGGGTCTC
4806	Yes	AAATCCATTGGGGTCTCC
4807	Yes	AAATCCATTGGGGTCTCCC
4808	Yes	AAATCCATTGGGGTCTCCCC
4809	Yes	AAATCCATTGGGGTCTCCCCG
4810	Yes	AAATCCATTGGGGTCTCCCCGC
4811	Yes	AAATCCATTGGGGTCTCCCCGCG
4812	Yes	AAATCCATTGGGGTCTCCCCGCGC
4813	Yes	AAATCCATTGGGGTCTCCCCGCGCA
4814	Yes	AAATCCATTGGGGTCTCCCCGCGCAG
4815	Yes	AAATCCATTGGGGTCTCCCCGCGCAGG
4816	Yes	AAATCCATTGGGGTCTCCCCGCGCAGGT
4817	Yes	AAATCCATTGGGGTCTCCCCGCGCAGGTT
4818	Yes	AAATCCATTGGGGTCTCCCCGCGCAGGTTC
4819	Yes	AAATCCATTGGGGTCTCCCCGCGCAGGTTCG
4820	Yes	AAATCCATTGGGGTCTCCCCGCGCAGGTTCGA
4821	Yes	AAATCCATTGGGGTCTCCCCGCGCAGGTTCGAA
4822	Yes	AAATCCATTGGGGTCTCCCCGCGCAGGTTCGAAT
4823	Yes	AAATCCATTGGGGTCTCCCCGCGCAGGTTCGAATC

4824	Yes	AAATCCATTGGGGTCTCCCCGCGCAGGTTCGAATCC
4825	Yes	AAATCCATTGGGGTCTCCCCGCGCAGGTTCGAATCCT
4826	Yes	AAATCCATTGGGGTCTCCCCGCGCAGGTTCGAATCCTG
4827	Yes	AAATCCATTGGGGTCTCCCCGCGCAGGTTCGAATCCTGC
4828	Yes	AAATCCATTGGGGTCTCCCCGCGCAGGTTCGAATCCTGCC
4829	Yes	AAATCCATTGGGGTCTCCCCGCGCAGGTTCGAATCCTGCCG
4830	Yes	AAATCCATTGGGGTCTCCCCGCGCAGGTTCGAATCCTGCCGA
4831	Yes	AAATCCATTGGGGTCTCCCCGCGCAGGTTCGAATCCTGCCGAC
4832	Yes	AAATCCATTGGGGTCTCCCCGCGCAGGTTCGAATCCTGCCGACT
4833	Yes	AAATCCATTGGGGTCTCCCCGCGCAGGTTCGAATCCTGCCGACTA
4834	Yes	AAATCCATTGGGGTCTCCCCGCGCAGGTTCGAATCCTGCCGACTAC
4835	Yes	AAATCCATTGGGGTCTCCCCGCGCAGGTTCGAATCCTGCCGACTACG
4836	Yes	AAATCCATTGGGGTCTCCCCGCGCAGGTTCGAATCCTGCCGACTACGC
4837	Yes	AAATCCATTGGGGTCTCCCCGCGCAGGTTCGAATCCTGCCGACTACGCC
4838	Yes	AAATCCATTGGGGTCTCCCCGCGCAGGTTCGAATCCTGCCGACTACGCCA
4839	No	AAATCCATTGGGGTTT
4840	No	AAATCCATTGGGGTTTC
4841	No	AAATCCATTGGGGTTTCC
4842	No	AAATCCATTGGGGTTTCCC
4843	Yes	AAATCCATTGGGGTTTCCCC
4844	Yes	AAATCCATTGGGGTTTCCCCA
4845	Yes	AAATCCATTGGGGTTTCCCCAC
4846	Yes	AAATCCATTGGGGTTTCCCCACG
4847	Yes	AAATCCATTGGGGTTTCCCCACGC
4848	Yes	AAATCCATTGGGGTTTCCCCACGCA
4849	Yes	AAATCCATTGGGGTTTCCCCACGCAG
4850	Yes	AAATCCATTGGGGTTTCCCCACGCAGG
4851	Yes	AAATCCATTGGGGTTTCCCCACGCAGGT
4852	Yes	AAATCCATTGGGGTTTCCCCACGCAGGTT
4853	Yes	AAATCCATTGGGGTTTCCCCACGCAGGTTC
4854	Yes	AAATCCATTGGGGTTTCCCCACGCAGGTTCG
4855	Yes	AAATCCATTGGGGTTTCCCCACGCAGGTTCGA
4856	Yes	AAATCCATTGGGGTTTCCCCACGCAGGTTCGAA
4857	Yes	AAATCCATTGGGGTTTCCCCACGCAGGTTCGAAT
4858	Yes	AAATCCATTGGGGTTTCCCCACGCAGGTTCGAATC
4859	Yes	AAATCCATTGGGGTTTCCCCACGCAGGTTCGAATCC
4860	Yes	AAATCCATTGGGGTTTCCCCACGCAGGTTCGAATCCT
4861	Yes	AAATCCATTGGGGTTTCCCCACGCAGGTTCGAATCCTG
4862	Yes	AAATCCATTGGGGTTTCCCCACGCAGGTTCGAATCCTGC
4863	Yes	AAATCCATTGGGGTTTCCCCACGCAGGTTCGAATCCTGCC
4864	Yes	AAATCCATTGGGGTTTCCCCACGCAGGTTCGAATCCTGCCG
4865	Yes	AAATCCATTGGGGTTTCCCCACGCAGGTTCGAATCCTGCCGA

4866	Yes	AAATCCATTGGGGTTTCCCCACGCAGGTTCGAATCCTGCCGAC
4867	Yes	AAATCCATTGGGGTTTCCCCACGCAGGTTCGAATCCTGCCGACT
4868	Yes	AAATCCATTGGGGTTTCCCCACGCAGGTTCGAATCCTGCCGACTA
4869	Yes	AAATCCATTGGGGTTTCCCCACGCAGGTTCGAATCCTGCCGACTAC
4870	Yes	AAATCCATTGGGGTTTCCCCACGCAGGTTCGAATCCTGCCGACTACG
4871	Yes	AAATCCATTGGGGTTTCCCCACGCAGGTTCGAATCCTGCCGACTACGC
4872	Yes	AAATCCATTGGGGTTTCCCCACGCAGGTTCGAATCCTGCCGACTACGCC
4873	Yes	AAATCCATTGGGGTTTCCCCACGCAGGTTCGAATCCTGCCGACTACGCCA
4874	Yes	AAATCCATTGGGGTTTCCCCG
4875	Yes	AAATCCATTGGGGTTTCCCCGC
4876	Yes	AAATCCATTGGGGTTTCCCCGCG
4877	Yes	AAATCCATTGGGGTTTCCCCGCGC
4878	Yes	AAATCCATTGGGGTTTCCCCGCGCA
4879	Yes	AAATCCATTGGGGTTTCCCCGCGCAG
4880	Yes	AAATCCATTGGGGTTTCCCCGCGCAGG
4881	Yes	AAATCCATTGGGGTTTCCCCGCGCAGGT
4882	Yes	AAATCCATTGGGGTTTCCCCGCGCAGGTT
4883	Yes	AAATCCATTGGGGTTTCCCCGCGCAGGTTC
4884	Yes	AAATCCATTGGGGTTTCCCCGCGCAGGTTCG
4885	Yes	AAATCCATTGGGGTTTCCCCGCGCAGGTTCGA
4886	Yes	AAATCCATTGGGGTTTCCCCGCGCAGGTTCGAA
4887	Yes	AAATCCATTGGGGTTTCCCCGCGCAGGTTCGAAT
4888	Yes	AAATCCATTGGGGTTTCCCCGCGCAGGTTCGAATC
4889	Yes	AAATCCATTGGGGTTTCCCCGCGCAGGTTCGAATCC
4890	Yes	AAATCCATTGGGGTTTCCCCGCGCAGGTTCGAATCCT
4891	Yes	AAATCCATTGGGGTTTCCCCGCGCAGGTTCGAATCCTG
4892	Yes	AAATCCATTGGGGTTTCCCCGCGCAGGTTCGAATCCTGC
4893	Yes	AAATCCATTGGGGTTTCCCCGCGCAGGTTCGAATCCTGCC
4894	Yes	AAATCCATTGGGGTTTCCCCGCGCAGGTTCGAATCCTGCCG
4895	Yes	AAATCCATTGGGGTTTCCCCGCGCAGGTTCGAATCCTGCCGA
4896	Yes	AAATCCATTGGGGTTTCCCCGCGCAGGTTCGAATCCTGCCGAC
4897	Yes	AAATCCATTGGGGTTTCCCCGCGCAGGTTCGAATCCTGCCGACT
4898	Yes	AAATCCATTGGGGTTTCCCCGCGCAGGTTCGAATCCTGCCGACTA
4899	Yes	AAATCCATTGGGGTTTCCCCGCGCAGGTTCGAATCCTGCCGACTAC
4900	Yes	AAATCCATTGGGGTTTCCCCGCGCAGGTTCGAATCCTGCCGACTACG
4901	Yes	AAATCCATTGGGGTTTCCCCGCGCAGGTTCGAATCCTGCCGACTACGC
4902	Yes	AAATCCATTGGGGTTTCCCCGCGCAGGTTCGAATCCTGCCGACTACGCC
4903	Yes	AAATCCATTGGGGTTTCCCCGCGCAGGTTCGAATCCTGCCGACTACGCCA
4904	Yes	AAATCCATTGGGGTTTCCCCGCGCAGGTTCGAATCCTGT
4905	Yes	AAATCCATTGGGGTTTCCCCGCGCAGGTTCGAATCCTGTC
4906	Yes	AAATCCATTGGGGTTTCCCCGCGCAGGTTCGAATCCTGTCG
4907	Yes	AAATCCATTGGGGTTTCCCCGCGCAGGTTCGAATCCTGTCGG

4908	Yes	AAATCCATTGGGGTTTCCCCGCGCAGGTTCGAATCCTGTCGGC
4909	Yes	AAATCCATTGGGGTTTCCCCGCGCAGGTTCGAATCCTGTCGGCT
4910	Yes	AAATCCATTGGGGTTTCCCCGCGCAGGTTCGAATCCTGTCGGCTA
4911	Yes	AAATCCATTGGGGTTTCCCCGCGCAGGTTCGAATCCTGTCGGCTAC
4912	Yes	AAATCCATTGGGGTTTCCCCGCGCAGGTTCGAATCCTGTCGGCTACG
4913	Yes	AAATCCATTGGGGTTTCCCCGCGCAGGTTCGAATCCTGTCGGCTACGC
4914	Yes	AAATCCATTGGGGTTTCCCCGCGCAGGTTCGAATCCTGTCGGCTACGCC
4915	Yes	AAATCCATTGGGGTTTCCCCGCGCAGGTTCGAATCCTGTCGGCTACGCCA
4916	No	AAATCCCACCGCTGCC
4917	Yes	AAATCCCACCGCTGCCA
4918	Yes	AAATCCCACCGCTGCCAC
4919	Yes	AAATCCCACCGCTGCCACC
4920	Yes	AAATCCCACCGCTGCCACCA
4921	No	AAATCCCACTGCTGCA
4922	No	AAATCCCACTGCTGCAA
4923	Yes	AAATCCCACTGCTGCAAC
4924	Yes	AAATCCCACTGCTGCAACC
4925	Yes	AAATCCCACTGCTGCAACCA
4926	No	AAATCCCAGAGGAGCC
4927	No	AAATCCCAGAGGAGCCC
4928	No	AAATCCCAGAGGAGCCCC
4929	No	AAATCCCAGAGGAGCCCCC
4930	Yes	AAATCCCAGAGGAGCCCCCA
4931	No	AAATCCCAGCACTGTT
4932	No	AAATCCCAGCACTGTTG
4933	Yes	AAATCCCAGCACTGTTGC
4934	Yes	AAATCCCAGCACTGTTGCC
4935	Yes	AAATCCCAGCACTGTTGCCA
4936	No	AAATCCCAGCTGTATG
4937	Yes	AAATCCCAGCTGTATGC
4938	Yes	AAATCCCAGCTGTATGCC
4939	Yes	AAATCCCAGCTGTATGCCC
4940	Yes	AAATCCCAGCTGTATGCCCA
4941	No	AAATCCCAGCTTATTC
4942	Yes	AAATCCCAGCTTATTCA
4943	Yes	AAATCCCAGCTTATTCAC
4944	Yes	AAATCCCAGCTTATTCACC
4945	Yes	AAATCCCAGCTTATTCACCA
4946	No	AAATCCCAGCTTCTAC
4947	Yes	AAATCCCAGCTTCTACA
4948	Yes	AAATCCCAGCTTCTACAC
4949	Yes	AAATCCCAGCTTCTACACC

4950	Yes	AAATCCCAGCTTCTACACCA
4951	No	AAATCCCAGCTTGGCC
4952	Yes	AAATCCCAGCTTGGCCT
4953	Yes	AAATCCCAGCTTGGCCTC
4954	Yes	AAATCCCAGCTTGGCCTCC
4955	Yes	AAATCCCAGCTTGGCCTCCA
4956	No	AAATCCCAGCTTTGTC
4957	No	AAATCCCAGCTTTGTCA
4958	No	AAATCCCAGCTTTGTCAC
4959	No	AAATCCCAGCTTTGTCACC
4960	Yes	AAATCCCAGCTTTGTCACCA
4961	No	AAATCCCAGGTCTAGC
4962	No	AAATCCCAGGTCTAGCG
4963	Yes	AAATCCCAGGTCTAGCGC
4964	Yes	AAATCCCAGGTCTAGCGCC
4965	Yes	AAATCCCAGGTCTAGCGCCA
4966	No	AAATCCCAGTGCTGTC
4967	No	AAATCCCAGTGCTGTCA
4968	No	AAATCCCAGTGCTGTCAC
4969	No	AAATCCCAGTGCTGTCACC
4970	Yes	AAATCCCAGTGCTGTCACCA
4971	Yes	AAATCCCGGACGAGCC
4972	Yes	AAATCCCGGACGAGCCC
4973	Yes	AAATCCCGGACGAGCCCC
4974	Yes	AAATCCCGGACGAGCCCCC
4975	Yes	AAATCCCGGACGAGCCCCCA
4976	No	AAATCCCGGCTCTGCC
4977	No	AAATCCCGGCTCTGCCG
4978	Yes	AAATCCCGGCTCTGCCGC
4979	Yes	AAATCCCGGCTCTGCCGCC
4980	Yes	AAATCCCGGCTCTGCCGCCA
4981	No	AAATCCCTTGTGCACT
4982	No	AAATCCCTTGTGCACTG
4983	Yes	AAATCCCTTGTGCACTGC
4984	Yes	AAATCCCTTGTGCACTGCT
4985	Yes	AAATCCCTTGTGCACTGCTT
4986	Yes	AAATCCCTTGTGCACTGCTTC
4987	Yes	AAATCCCTTGTGCACTGCTTCC
4988	Yes	AAATCCCTTGTGCACTGCTTCCA
4989	Yes	AAATCCGGGTGCCCCC
4990	Yes	AAATCCGGGTGCCCCCC
4991	Yes	AAATCCGGGTGCCCCCCC

4992	Yes	AAATCCGGGTGCCCCCCC
4993	Yes	AAATCCGGGTGCCCCCCCA
4994	Yes	AAATCCGGGTGCCCCCT
4995	Yes	AAATCCGGGTGCCCCCTC
4996	Yes	AAATCCGGGTGCCCCCTCC
4997	Yes	AAATCCGGGTGCCCCCTCCA
4998	Yes	AAATCCGGGTGCTGGA
4999	Yes	AAATCCGGGTGCTGGAT
5000	Yes	AAATCCGGGTGCTGGATC
5001	Yes	AAATCCGGGTGCTGGATCC
5002	Yes	AAATCCGGGTGCTGGATCCA
5003	Yes	AAATCCGGTTACTCCC
5004	Yes	AAATCCGGTTACTCCCT
5005	Yes	AAATCCGGTTACTCCCTC
5006	Yes	AAATCCGGTTACTCCCTCC
5007	Yes	AAATCCGGTTACTCCCTCCA
5008	No	AAATCCTATATATCTT
5009	No	AAATCCTATATATCTTA
5010	Yes	AAATCCTATATATCTTAC
5011	Yes	AAATCCTATATATCTTACC
5012	Yes	AAATCCTATATATCTTACCA
5013	No	AAATCCTGACTCTGTC
5014	No	AAATCCTGACTCTGTCA
5015	No	AAATCCTGACTCTGTCAC
5016	No	AAATCCTGACTCTGTCACC
5017	Yes	AAATCCTGACTCTGTCACCA
5018	No	AAATCCTGCTCACAGC
5019	Yes	AAATCCTGCTCACAGCG
5020	Yes	AAATCCTGCTCACAGCGC
5021	Yes	AAATCCTGCTCACAGCGCC
5022	Yes	AAATCCTGCTCACAGCGCCA
5023	No	AAATCCTGTTTCTGCC
5024	No	AAATCCTGTTTCTGCCA
5025	No	AAATCCTGTTTCTGCCAC
5026	No	AAATCCTGTTTCTGCCACC
5027	Yes	AAATCCTGTTTCTGCCACCA
5028	Yes	AAATCGCACCCTCCGC
5029	Yes	AAATCGCACCCTCCGCT
5030	Yes	AAATCGCACCCTCCGCTG
5031	Yes	AAATCGCACCCTCCGCTGG
5032	Yes	AAATCGCACCCTCCGCTGGA
5033	Yes	AAATCGCACCCTCCGCTGGAG

5034	Yes	AAATCGCACCCTCCGCTGGAGG
5035	Yes	AAATCGCACCCTCCGCTGGAGGC
5036	Yes	AAATCGCACCCTCCGCTGGAGGCG
5037	Yes	AAATCGCACCCTCCGCTGGAGGCGT
5038	Yes	AAATCGCACCCTCCGCTGGAGGCGTG
5039	Yes	AAATCGCACCCTCCGCTGGAGGCGTGG
5040	Yes	AAATCGCACCCTCCGCTGGAGGCGTGGG
5041	Yes	AAATCGCACCCTCCGCTGGAGGCGTGGGT
5042	Yes	AAATCGCACCCTCCGCTGGAGGCGTGGGTT
5043	Yes	AAATCGCACCCTCCGCTGGAGGCGTGGGTTC
5044	Yes	AAATCGCACCCTCCGCTGGAGGCGTGGGTTCG
5045	Yes	AAATCGCACCCTCCGCTGGAGGCGTGGGTTCGA
5046	Yes	AAATCGCACCCTCCGCTGGAGGCGTGGGTTCGAA
5047	Yes	AAATCGCACCCTCCGCTGGAGGCGTGGGTTCGAAT
5048	Yes	AAATCGCACCCTCCGCTGGAGGCGTGGGTTCGAATC
5049	Yes	AAATCGCACCCTCCGCTGGAGGCGTGGGTTCGAATCC
5050	Yes	AAATCGCACCCTCCGCTGGAGGCGTGGGTTCGAATCCC
5051	Yes	AAATCGCACCCTCCGCTGGAGGCGTGGGTTCGAATCCCA
5052	Yes	AAATCGCACCCTCCGCTGGAGGCGTGGGTTCGAATCCCAC
5053	Yes	AAATCGCACCCTCCGCTGGAGGCGTGGGTTCGAATCCCACT
5054	Yes	AAATCGCACCCTCCGCTGGAGGCGTGGGTTCGAATCCCACTT
5055	Yes	AAATCGCACCCTCCGCTGGAGGCGTGGGTTCGAATCCCACTTT
5056	Yes	AAATCGCACCCTCCGCTGGAGGCGTGGGTTCGAATCCCACTTTT
5057	Yes	AAATCGCACCCTCCGCTGGAGGCGTGGGTTCGAATCCCACTTTTG
5058	Yes	AAATCGCACCCTCCGCTGGAGGCGTGGGTTCGAATCCCACTTTTGA
5059	Yes	AAATCGCACCCTCCGCTGGAGGCGTGGGTTCGAATCCCACTTTTGAC
5060	Yes	AAATCGCACCCTCCGCTGGAGGCGTGGGTTCGAATCCCACTTTTGACA
5061	Yes	AAATCGCACCCTCCGCTGGAGGCGTGGGTTCGAATCCCACTTTTGACAC
5062	Yes	AAATCGCACCCTCCGCTGGAGGCGTGGGTTCGAATCCCACTTTTGACACC
5063	No	AAATCTAAAGACAGGG
5064	No	AAATCTAAAGACAGGGG
5065	No	AAATCTAAAGACAGGGGT
5066	No	AAATCTAAAGACAGGGGTT
5067	No	AAATCTAAAGACAGGGGTTA
5068	Yes	AAATCTAAAGACAGGGGTTAG
5069	Yes	AAATCTAAAGACAGGGGTTAGG
5070	Yes	AAATCTAAAGACAGGGGTTAGGC
5071	Yes	AAATCTAAAGACAGGGGTTAGGCC
5072	Yes	AAATCTAAAGACAGGGGTTAGGCCT
5073	Yes	AAATCTAAAGACAGGGGTTAGGCCTC
5074	Yes	AAATCTAAAGACAGGGGTTAGGCCTCT
5075	Yes	AAATCTAAAGACAGGGGTTAGGCCTCTT

5076	Yes	AAATCTAAAGACAGGGGTTAGGCCTCTTT
5077	Yes	AAATCTAAAGACAGGGGTTAGGCCTCTTTT
5078	Yes	AAATCTAAAGACAGGGGTTAGGCCTCTTTTT
5079	Yes	AAATCTAAAGACAGGGGTTAGGCCTCTTTTTA
5080	Yes	AAATCTAAAGACAGGGGTTAGGCCTCTTTTTAC
5081	Yes	AAATCTAAAGACAGGGGTTAGGCCTCTTTTTACC
5082	Yes	AAATCTAAAGACAGGGGTTAGGCCTCTTTTTACCA
5083	Yes	AAATCTAAAGACAGGGGTTAGGCCTCTTTTTACCAC
5084	Yes	AAATCTAAAGACAGGGGTTAGGCCTCTTTTTACCACC
5085	Yes	AAATCTAAAGACAGGGGTTAGGCCTCTTTTTACCACCA
5086	No	AAATCTCACCAGGGCC
5087	No	AAATCTCACCAGGGCCT
5088	Yes	AAATCTCACCAGGGCCTC
5089	Yes	AAATCTCACCAGGGCCTCC
5090	Yes	AAATCTCACCAGGGCCTCCA
5091	No	AAATCTCAGACCTGCT
5092	Yes	AAATCTCAGACCTGCTA
5093	Yes	AAATCTCAGACCTGCTAC
5094	Yes	AAATCTCAGACCTGCTACC
5095	Yes	AAATCTCAGACCTGCTACCA
5096	Yes	AAATCTCGCTGGGGCC
5097	Yes	AAATCTCGCTGGGGCCT
5098	Yes	AAATCTCGCTGGGGCCTC
5099	Yes	AAATCTCGCTGGGGCCTCC
5100	Yes	AAATCTCGCTGGGGCCTCCA
5101	Yes	AAATCTCGGTGGAACC
5102	Yes	AAATCTCGGTGGAACCT
5103	Yes	AAATCTCGGTGGAACCTC
5104	Yes	AAATCTCGGTGGAACCTCC
5105	Yes	AAATCTCGGTGGAACCTCCA
5106	Yes	AAATCTCGGTGGGACC
5107	Yes	AAATCTCGGTGGGACCC
5108	Yes	AAATCTCGGTGGGACCCC
5109	Yes	AAATCTCGGTGGGACCCCC
5110	Yes	AAATCTCGGTGGGACCCCCA
5111	Yes	AAATCTCGGTGGGACCT
5112	Yes	AAATCTCGGTGGGACCTC
5113	Yes	AAATCTCGGTGGGACCTCC
5114	Yes	AAATCTCGGTGGGACCTCCA
5115	Yes	AAATCTCGGTGGTACC
5116	Yes	AAATCTCGGTGGTACCT
5117	Yes	AAATCTCGGTGGTACCTC

5118	Yes	AAATCTCGGTGGTACCTCC
5119	Yes	AAATCTCGGTGGTACCTCCA
5120	No	AAATCTGAATGGTCCT
5121	No	AAATCTGAATGGTCCTG
5122	Yes	AAATCTGAATGGTCCTGA
5123	Yes	AAATCTGAATGGTCCTGAG
5124	Yes	AAATCTGAATGGTCCTGAGT
5125	Yes	AAATCTGAATGGTCCTGAGTT
5126	Yes	AAATCTGAATGGTCCTGAGTTC
5127	Yes	AAATCTGAATGGTCCTGAGTTCA
5128	Yes	AAATCTGAATGGTCCTGAGTTCAA
5129	Yes	AAATCTGAATGGTCCTGAGTTCAAG
5130	Yes	AAATCTGAATGGTCCTGAGTTCAAGC
5131	Yes	AAATCTGAATGGTCCTGAGTTCAAGCC
5132	Yes	AAATCTGAATGGTCCTGAGTTCAAGCCT
5133	Yes	AAATCTGAATGGTCCTGAGTTCAAGCCTC
5134	Yes	AAATCTGAATGGTCCTGAGTTCAAGCCTCA
5135	Yes	AAATCTGAATGGTCCTGAGTTCAAGCCTCAG
5136	Yes	AAATCTGAATGGTCCTGAGTTCAAGCCTCAGA
5137	Yes	AAATCTGAATGGTCCTGAGTTCAAGCCTCAGAG
5138	Yes	AAATCTGAATGGTCCTGAGTTCAAGCCTCAGAGG
5139	Yes	AAATCTGAATGGTCCTGAGTTCAAGCCTCAGAGGG
5140	Yes	AAATCTGAATGGTCCTGAGTTCAAGCCTCAGAGGGG
5141	Yes	AAATCTGAATGGTCCTGAGTTCAAGCCTCAGAGGGGG
5142	Yes	AAATCTGAATGGTCCTGAGTTCAAGCCTCAGAGGGGGC
5143	Yes	AAATCTGAATGGTCCTGAGTTCAAGCCTCAGAGGGGGCA
5144	Yes	AAATCTGAATGGTCCTGAGTTCAAGCCTCAGAGGGGGCAC
5145	Yes	AAATCTGAATGGTCCTGAGTTCAAGCCTCAGAGGGGGCACC
5146	Yes	AAATCTGAATGGTCCTGAGTTCAAGCCTCAGAGGGGGCACCA
5147	No	AAATCTGAGTCATGAC
5148	No	AAATCTGAGTCATGACA
5149	Yes	AAATCTGAGTCATGACAC
5150	Yes	AAATCTGAGTCATGACACC
5151	Yes	AAATCTGAGTCATGACACCA
5152	No	AAATCTGCCTGGGTTC
5153	No	AAATCTGCCTGGGTTCT
5154	Yes	AAATCTGCCTGGGTTCTA
5155	Yes	AAATCTGCCTGGGTTCTAG
5156	Yes	AAATCTGCCTGGGTTCTAGT
5157	Yes	AAATCTGCCTGGGTTCTAGTC
5158	Yes	AAATCTGCCTGGGTTCTAGTCC
5159	Yes	AAATCTGCCTGGGTTCTAGTCCC

5160	Yes	AAATCTGCCTGGGTTCTAGTCCCA
5161	Yes	AAATCTGCCTGGGTTCTAGTCCCAG
5162	Yes	AAATCTGCCTGGGTTCTAGTCCCAGC
5163	Yes	AAATCTGCCTGGGTTCTAGTCCCAGCT
5164	Yes	AAATCTGCCTGGGTTCTAGTCCCAGCTG
5165	Yes	AAATCTGCCTGGGTTCTAGTCCCAGCTGT
5166	Yes	AAATCTGCCTGGGTTCTAGTCCCAGCTGTC
5167	Yes	AAATCTGCCTGGGTTCTAGTCCCAGCTGTCT
5168	Yes	AAATCTGCCTGGGTTCTAGTCCCAGCTGTCTC
5169	Yes	AAATCTGCCTGGGTTCTAGTCCCAGCTGTCTCA
5170	Yes	AAATCTGCCTGGGTTCTAGTCCCAGCTGTCTCAC
5171	Yes	AAATCTGCCTGGGTTCTAGTCCCAGCTGTCTCACC
5172	Yes	AAATCTGCCTGGGTTCTAGTCCCAGCTGTCTCACCA
5173	No	AAATCTGGGTGCCCAC
5174	Yes	AAATCTGGGTGCCCACT
5175	Yes	AAATCTGGGTGCCCACTC
5176	Yes	AAATCTGGGTGCCCACTCC
5177	Yes	AAATCTGGGTGCCCACTCCA
5178	No	AAATCTGGGTGCCCCC
5179	No	AAATCTGGGTGCCCCCT
5180	No	AAATCTGGGTGCCCCCTC
5181	Yes	AAATCTGGGTGCCCCCTCC
5182	Yes	AAATCTGGGTGCCCCCTCCA
5183	No	AAATCTTAAAACTTTA
5184	No	AAATCTTAAAACTTTAT
5185	Yes	AAATCTTAAAACTTTATA
5186	Yes	AAATCTTAAAACTTTATAA
5187	Yes	AAATCTTAAAACTTTATAAT
5188	Yes	AAATCTTAAAACTTTATAATC
5189	Yes	AAATCTTAAAACTTTATAATCA
5190	Yes	AAATCTTAAAACTTTATAATCAG
5191	Yes	AAATCTTAAAACTTTATAATCAGA
5192	Yes	AAATCTTAAAACTTTATAATCAGAG
5193	Yes	AAATCTTAAAACTTTATAATCAGAGG
5194	Yes	AAATCTTAAAACTTTATAATCAGAGGT
5195	Yes	AAATCTTAAAACTTTATAATCAGAGGTT
5196	Yes	AAATCTTAAAACTTTATAATCAGAGGTTC
5197	Yes	AAATCTTAAAACTTTATAATCAGAGGTTCG
5198	Yes	AAATCTTAAAACTTTATAATCAGAGGTTCGA
5199	Yes	AAATCTTAAAACTTTATAATCAGAGGTTCGAC
5200	Yes	AAATCTTAAAACTTTATAATCAGAGGTTCGACT
5201	Yes	AAATCTTAAAACTTTATAATCAGAGGTTCGACTC

5202	Yes	AAATCTTAAAACTTTATAATCAGAGGTTCGACTCC
5203	Yes	AAATCTTAAAACTTTATAATCAGAGGTTCGACTCCT
5204	Yes	AAATCTTAAAACTTTATAATCAGAGGTTCGACTCCTC
5205	Yes	AAATCTTAAAACTTTATAATCAGAGGTTCGACTCCTCT
5206	Yes	AAATCTTAAAACTTTATAATCAGAGGTTCGACTCCTCTT
5207	Yes	AAATCTTAAAACTTTATAATCAGAGGTTCGACTCCTCTTC
5208	Yes	AAATCTTAAAACTTTATAATCAGAGGTTCGACTCCTCTTCT
5209	Yes	AAATCTTAAAACTTTATAATCAGAGGTTCGACTCCTCTTCTT
5210	Yes	AAATCTTAAAACTTTATAATCAGAGGTTCGACTCCTCTTCTTA
5211	Yes	AAATCTTAAAACTTTATAATCAGAGGTTCGACTCCTCTTCTTAA
5212	Yes	AAATCTTAAAACTTTATAATCAGAGGTTCGACTCCTCTTCTTAAC
5213	Yes	AAATCTTAAAACTTTATAATCAGAGGTTCGACTCCTCTTCTTAACA
5214	Yes	AAATCTTAAAACTTTATAATCAGAGGTTCGACTCCTCTTCTTAACAC
5215	Yes	AAATCTTAAAACTTTATAATCAGAGGTTCGACTCCTCTTCTTAACACC
5216	Yes	AAATCTTAAAACTTTATAATCAGAGGTTCGACTCCTCTTCTTAACACCA
5217	No	AAATGGCTGAGTGAAG
5218	No	AAATGGCTGAGTGAAGC
5219	No	AAATGGCTGAGTGAAGCA
5220	No	AAATGGCTGAGTGAAGCAT
5221	No	AAATGGCTGAGTGAAGCATT
5222	No	AAATGGCTGAGTGAAGCATTG
5223	No	AAATGGCTGAGTGAAGCATTGG
5224	No	AAATGGCTGAGTGAAGCATTGGA
5225	No	AAATGGCTGAGTGAAGCATTGGAC
5226	No	AAATGGCTGAGTGAAGCATTGGACT
5227	No	AAATGGCTGAGTGAAGCATTGGACTG
5228	No	AAATGGCTGAGTGAAGCATTGGACTGT
5229	No	AAATGGCTGAGTGAAGCATTGGACTGTA
5230	No	AAATGGCTGAGTGAAGCATTGGACTGTAA
5231	No	AAATGGCTGAGTGAAGCATTGGACTGTAAA
5232	No	AAATGGCTGAGTGAAGCATTGGACTGTAAAT
5233	No	AAATGGCTGAGTGAAGCATTGGACTGTAAATC
5234	No	AAATGGCTGAGTGAAGCATTGGACTGTAAATCT
5235	No	AAATGGCTGAGTGAAGCATTGGACTGTAAATCTA
5236	No	AAATGGCTGAGTGAAGCATTGGACTGTAAATCTAA
5237	No	AAATGGCTGAGTGAAGCATTGGACTGTAAATCTAAA
5238	No	AAATGGCTGAGTGAAGCATTGGACTGTAAATCTAAAG
5239	No	AAATGGCTGAGTGAAGCATTGGACTGTAAATCTAAAGA
5240	No	AAATGGCTGAGTGAAGCATTGGACTGTAAATCTAAAGAC
5241	No	AAATGGCTGAGTGAAGCATTGGACTGTAAATCTAAAGACA
5242	No	AAATGGCTGAGTGAAGCATTGGACTGTAAATCTAAAGACAG
5243	No	AAATGGCTGAGTGAAGCATTGGACTGTAAATCTAAAGACAGG

5244	No	AAATGGCTGAGTGAAGCATTGGACTGTAAATCTAAAGACAGGG
5245	No	AAATGGCTGAGTGAAGCATTGGACTGTAAATCTAAAGACAGGGG
5246	No	AAATGGCTGAGTGAAGCATTGGACTGTAAATCTAAAGACAGGGGT
5247	No	AAATGGCTGAGTGAAGCATTGGACTGTAAATCTAAAGACAGGGGTT
5248	No	AAATGGCTGAGTGAAGCATTGGACTGTAAATCTAAAGACAGGGGTTA
5249	Yes	AAATGGCTGAGTGAAGCATTGGACTGTAAATCTAAAGACAGGGGTTAG
5250	Yes	AAATGGCTGAGTGAAGCATTGGACTGTAAATCTAAAGACAGGGGTTAGG
5251	Yes	AAATGGCTGAGTGAAGCATTGGACTGTAAATCTAAAGACAGGGGTTAGGC
5252	Yes	AAATGGTAGAGCGCTC
5253	Yes	AAATGGTAGAGCGCTCG
5254	Yes	AAATGGTAGAGCGCTCGC
5255	Yes	AAATGGTAGAGCGCTCGCT
5256	Yes	AAATGGTAGAGCGCTCGCTT
5257	Yes	AAATGGTAGAGCGCTCGCTTA
5258	Yes	AAATGGTAGAGCGCTCGCTTAG
5259	Yes	AAATGGTAGAGCGCTCGCTTAGC
5260	Yes	AAATGGTAGAGCGCTCGCTTAGCA
5261	Yes	AAATGGTAGAGCGCTCGCTTAGCAT
5262	Yes	AAATGGTAGAGCGCTCGCTTAGCATG
5263	Yes	AAATGGTAGAGCGCTCGCTTAGCATGC
5264	Yes	AAATGGTAGAGCGCTCGCTTAGCATGCG
5265	Yes	AAATGGTAGAGCGCTCGCTTAGCATGCGA
5266	Yes	AAATGGTAGAGCGCTCGCTTAGCATGCGAG
5267	Yes	AAATGGTAGAGCGCTCGCTTAGCATGCGAGA
5268	Yes	AAATGGTAGAGCGCTCGCTTAGCATGCGAGAG
5269	Yes	AAATGGTAGAGCGCTCGCTTAGCATGCGAGAGG
5270	Yes	AAATGGTAGAGCGCTCGCTTAGCATGCGAGAGGT
5271	Yes	AAATGGTAGAGCGCTCGCTTAGCATGCGAGAGGTA
5272	Yes	AAATGGTAGAGCGCTCGCTTAGCATGCGAGAGGTAG
5273	Yes	AAATGGTAGAGCGCTCGCTTAGCATGCGAGAGGTAGC
5274	Yes	AAATGGTAGAGCGCTCGCTTAGCATGCGAGAGGTAGCG
5275	Yes	AAATGGTAGAGCGCTCGCTTAGCATGCGAGAGGTAGCGG
5276	Yes	AAATGGTAGAGCGCTCGCTTAGCATGCGAGAGGTAGCGGG
5277	Yes	AAATGGTAGAGCGCTCGCTTAGCATGCGAGAGGTAGCGGGA
5278	Yes	AAATGGTAGAGCGCTCGCTTAGCATGCGAGAGGTAGCGGGAT
5279	Yes	AAATGGTAGAGCGCTCGCTTAGCATGCGAGAGGTAGCGGGATC
5280	Yes	AAATGGTAGAGCGCTCGCTTAGCATGCGAGAGGTAGCGGGATCG
5281	Yes	AAATGGTAGAGCGCTCGCTTAGCATGCGAGAGGTAGCGGGATCGA
5282	Yes	AAATGGTAGAGCGCTCGCTTAGCATGCGAGAGGTAGCGGGATCGAT
5283	Yes	AAATGGTAGAGCGCTCGCTTAGCATGCGAGAGGTAGCGGGATCGATG
5284	Yes	AAATGGTAGAGCGCTCGCTTAGCATGCGAGAGGTAGCGGGATCGATGC
5285	Yes	AAATGGTAGAGCGCTCGCTTAGCATGCGAGAGGTAGCGGGATCGATGCC

5286	Yes	AAATGGTAGAGCGCTCGCTTAGCATGCGAGAGGTAGCGGGATCGATGCCC
5287	Yes	AAATGTGCGCTCTGGG
5288	Yes	AAATGTGCGCTCTGGGG
5289	Yes	AAATGTGCGCTCTGGGGC
5290	Yes	AAATGTGCGCTCTGGGGCT
5291	Yes	AAATGTGCGCTCTGGGGCTG
5292	Yes	AAATGTGCGCTCTGGGGCTGC
5293	Yes	AAATGTGCGCTCTGGGGCTGCT
5294	Yes	AAATGTGCGCTCTGGGGCTGCTG
5295	Yes	AAATGTGCGCTCTGGGGCTGCTGA
5296	Yes	AAATGTGCGCTCTGGGGCTGCTGAT
5297	Yes	AAATGTGCGCTCTGGGGCTGCTGATC
5298	Yes	AAATGTGCGCTCTGGGGCTGCTGATCC
5299	Yes	AAATGTGCGCTCTGGGGCTGCTGATCCC
5300	Yes	AAATGTGCGCTCTGGGGCTGCTGATCCCA
5301	Yes	AAATGTGCGCTCTGGGGCTGCTGATCCCAG
5302	Yes	AAATGTGCGCTCTGGGGCTGCTGATCCCAGG
5303	Yes	AAATGTGCGCTCTGGGGCTGCTGATCCCAGGC
5304	Yes	AAATGTGCGCTCTGGGGCTGCTGATCCCAGGCT
5305	Yes	AAATGTGCGCTCTGGGGCTGCTGATCCCAGGCTC
5306	Yes	AAATGTGCGCTCTGGGGCTGCTGATCCCAGGCTCA
5307	Yes	AAATGTGCGCTCTGGGGCTGCTGATCCCAGGCTCAA
5308	Yes	AAATGTGCGCTCTGGGGCTGCTGATCCCAGGCTCAAA
5309	Yes	AAATGTGCGCTCTGGGGCTGCTGATCCCAGGCTCAAAC
5310	Yes	AAATGTGCGCTCTGGGGCTGCTGATCCCAGGCTCAAACC
5311	Yes	AAATGTGCGCTCTGGGGCTGCTGATCCCAGGCTCAAACCC
5312	Yes	AAATGTGCGCTCTGGGGCTGCTGATCCCAGGCTCAAACCCT
5313	Yes	AAATGTGCGCTCTGGGGCTGCTGATCCCAGGCTCAAACCCTG
5314	Yes	AAATGTGCGCTCTGGGGCTGCTGATCCCAGGCTCAAACCCTGG
5315	Yes	AAATGTGCGCTCTGGGGCTGCTGATCCCAGGCTCAAACCCTGGC
5316	Yes	AAATGTGCGCTCTGGGGCTGCTGATCCCAGGCTCAAACCCTGGCG
5317	Yes	AAATGTGCGCTCTGGGGCTGCTGATCCCAGGCTCAAACCCTGGCGC
5318	Yes	AAATGTGCGCTCTGGGGCTGCTGATCCCAGGCTCAAACCCTGGCGCT
5319	Yes	AAATGTGCGCTCTGGGGCTGCTGATCCCAGGCTCAAACCCTGGCGCTG
5320	Yes	AAATGTGCGCTCTGGGGCTGCTGATCCCAGGCTCAAACCCTGGCGCTGT
5321	Yes	AAATGTGCGCTCTGGGGCTGCTGATCCCAGGCTCAAACCCTGGCGCTGTC
5322	No	AAATGTGGACTTTGAA
5323	No	AAATGTGGACTTTGAAG
5324	Yes	AAATGTGGACTTTGAAGT
5325	Yes	AAATGTGGACTTTGAAGTT
5326	Yes	AAATGTGGACTTTGAAGTTA
5327	Yes	AAATGTGGACTTTGAAGTTAG

5328	Yes	AAATGTGGACTTTGAAGTTAGA
5329	Yes	AAATGTGGACTTTGAAGTTAGAT
5330	Yes	AAATGTGGACTTTGAAGTTAGATA
5331	Yes	AAATGTGGACTTTGAAGTTAGATAC
5332	Yes	AAATGTGGACTTTGAAGTTAGATACA
5333	Yes	AAATGTGGACTTTGAAGTTAGATACAC
5334	Yes	AAATGTGGACTTTGAAGTTAGATACACC
5335	Yes	AAATGTGGACTTTGAAGTTAGATACACCT
5336	Yes	AAATGTGGACTTTGAAGTTAGATACACCTG
5337	Yes	AAATGTGGACTTTGAAGTTAGATACACCTGG
5338	Yes	AAATGTGGACTTTGAAGTTAGATACACCTGGG
5339	Yes	AAATGTGGACTTTGAAGTTAGATACACCTGGGT
5340	Yes	AAATGTGGACTTTGAAGTTAGATACACCTGGGTT
5341	Yes	AAATGTGGACTTTGAAGTTAGATACACCTGGGTTC
5342	Yes	AAATGTGGACTTTGAAGTTAGATACACCTGGGTTCA
5343	Yes	AAATGTGGACTTTGAAGTTAGATACACCTGGGTTCAA
5344	Yes	AAATGTGGACTTTGAAGTTAGATACACCTGGGTTCAAA
5345	Yes	AAATGTGGACTTTGAAGTTAGATACACCTGGGTTCAAAT
5346	Yes	AAATGTGGACTTTGAAGTTAGATACACCTGGGTTCAAATC
5347	Yes	AAATGTGGACTTTGAAGTTAGATACACCTGGGTTCAAATCC
5348	Yes	AAATGTGGACTTTGAAGTTAGATACACCTGGGTTCAAATCCC
5349	Yes	AAATGTGGACTTTGAAGTTAGATACACCTGGGTTCAAATCCCA
5350	Yes	AAATGTGGACTTTGAAGTTAGATACACCTGGGTTCAAATCCCAG
5351	Yes	AAATGTGGACTTTGAAGTTAGATACACCTGGGTTCAAATCCCAGT
5352	Yes	AAATGTGGACTTTGAAGTTAGATACACCTGGGTTCAAATCCCAGTG
5353	Yes	AAATGTGGACTTTGAAGTTAGATACACCTGGGTTCAAATCCCAGTGC
5354	Yes	AAATGTGGACTTTGAAGTTAGATACACCTGGGTTCAAATCCCAGTGCT
5355	Yes	AAATGTGGACTTTGAAGTTAGATACACCTGGGTTCAAATCCCAGTGCTG
5356	Yes	AAATGTGGACTTTGAAGTTAGATACACCTGGGTTCAAATCCCAGTGCTGT
5357	No	AAATGTTGGTTATACC
5358	No	AAATGTTGGTTATACCC
5359	No	AAATGTTGGTTATACCCT
5360	No	AAATGTTGGTTATACCCTT
5361	No	AAATGTTGGTTATACCCTTC
5362	No	AAATGTTGGTTATACCCTTCC
5363	No	AAATGTTGGTTATACCCTTCCC
5364	No	AAATGTTGGTTATACCCTTCCCG
5365	No	AAATGTTGGTTATACCCTTCCCGT
5366	Yes	AAATGTTGGTTATACCCTTCCCGTA
5367	Yes	AAATGTTGGTTATACCCTTCCCGTAC
5368	Yes	AAATGTTGGTTATACCCTTCCCGTACT
5369	Yes	AAATGTTGGTTATACCCTTCCCGTACTA

5370	Yes	AAATGTTGGTTATACCCTTCCCGTACTAC
5371	Yes	AAATGTTGGTTATACCCTTCCCGTACTACC
5372	Yes	AAATGTTGGTTATACCCTTCCCGTACTACCA
5373	Yes	AAATGTTTAGACGGGC
5374	Yes	AAATGTTTAGACGGGCT
5375	Yes	AAATGTTTAGACGGGCTC
5376	Yes	AAATGTTTAGACGGGCTCA
5377	Yes	AAATGTTTAGACGGGCTCAC
5378	Yes	AAATGTTTAGACGGGCTCACA
5379	Yes	AAATGTTTAGACGGGCTCACAT
5380	Yes	AAATGTTTAGACGGGCTCACATC
5381	Yes	AAATGTTTAGACGGGCTCACATCA
5382	Yes	AAATGTTTAGACGGGCTCACATCAC
5383	Yes	AAATGTTTAGACGGGCTCACATCACC
5384	Yes	AAATGTTTAGACGGGCTCACATCACCC
5385	Yes	AAATGTTTAGACGGGCTCACATCACCCC
5386	Yes	AAATGTTTAGACGGGCTCACATCACCCCA
5387	Yes	AAATGTTTAGACGGGCTCACATCACCCCAT
5388	Yes	AAATGTTTAGACGGGCTCACATCACCCCATA
5389	Yes	AAATGTTTAGACGGGCTCACATCACCCCATAA
5390	Yes	AAATGTTTAGACGGGCTCACATCACCCCATAAA
5391	Yes	AAATGTTTAGACGGGCTCACATCACCCCATAAAC
5392	Yes	AAATGTTTAGACGGGCTCACATCACCCCATAAACA
5393	Yes	AAATGTTTAGACGGGCTCACATCACCCCATAAACAC
5394	Yes	AAATGTTTAGACGGGCTCACATCACCCCATAAACACC
5395	Yes	AAATGTTTAGACGGGCTCACATCACCCCATAAACACCA
5396	No	AAATTAGAATCTTAGC
5397	No	AAATTAGAATCTTAGCT
5398	No	AAATTAGAATCTTAGCTT
5399	No	AAATTAGAATCTTAGCTTT
5400	No	AAATTAGAATCTTAGCTTTG
5401	No	AAATTAGAATCTTAGCTTTGG
5402	No	AAATTAGAATCTTAGCTTTGGG
5403	No	AAATTAGAATCTTAGCTTTGGGT
5404	No	AAATTAGAATCTTAGCTTTGGGTG
5405	No	AAATTAGAATCTTAGCTTTGGGTGC
5406	No	AAATTAGAATCTTAGCTTTGGGTGCT
5407	Yes	AAATTAGAATCTTAGCTTTGGGTGCTA
5408	Yes	AAATTAGAATCTTAGCTTTGGGTGCTAA
5409	Yes	AAATTAGAATCTTAGCTTTGGGTGCTAAT
5410	Yes	AAATTAGAATCTTAGCTTTGGGTGCTAATG
5411	Yes	AAATTAGAATCTTAGCTTTGGGTGCTAATGG

5412	Yes	AAATTAGAATCTTAGCTTTGGGTGCTAATGGT
5413	Yes	AAATTAGAATCTTAGCTTTGGGTGCTAATGGTG
5414	Yes	AAATTAGAATCTTAGCTTTGGGTGCTAATGGTGG
5415	Yes	AAATTAGAATCTTAGCTTTGGGTGCTAATGGTGGA
5416	Yes	AAATTAGAATCTTAGCTTTGGGTGCTAATGGTGGAG
5417	Yes	AAATTAGAATCTTAGCTTTGGGTGCTAATGGTGGAGT
5418	Yes	AAATTAGAATCTTAGCTTTGGGTGCTAATGGTGGAGTT
5419	Yes	AAATTAGAATCTTAGCTTTGGGTGCTAATGGTGGAGTTA
5420	Yes	AAATTAGAATCTTAGCTTTGGGTGCTAATGGTGGAGTTAA
5421	Yes	AAATTAGAATCTTAGCTTTGGGTGCTAATGGTGGAGTTAAA
5422	Yes	AAATTAGAATCTTAGCTTTGGGTGCTAATGGTGGAGTTAAAG
5423	Yes	AAATTAGAATCTTAGCTTTGGGTGCTAATGGTGGAGTTAAAGA
5424	Yes	AAATTAGAATCTTAGCTTTGGGTGCTAATGGTGGAGTTAAAGAC
5425	Yes	AAATTAGAATCTTAGCTTTGGGTGCTAATGGTGGAGTTAAAGACT
5426	Yes	AAATTAGAATCTTAGCTTTGGGTGCTAATGGTGGAGTTAAAGACTT
5427	Yes	AAATTAGAATCTTAGCTTTGGGTGCTAATGGTGGAGTTAAAGACTTT
5428	Yes	AAATTAGAATCTTAGCTTTGGGTGCTAATGGTGGAGTTAAAGACTTTT
5429	Yes	AAATTAGAATCTTAGCTTTGGGTGCTAATGGTGGAGTTAAAGACTTTTT
5430	Yes	AAATTAGAATCTTAGCTTTGGGTGCTAATGGTGGAGTTAAAGACTTTTTC
5431	No	AAATTATAGGCTAAAT
5432	No	AAATTATAGGCTAAATC
5433	No	AAATTATAGGCTAAATCC
5434	No	AAATTATAGGCTAAATCCT
5435	No	AAATTATAGGCTAAATCCTA
5436	No	AAATTATAGGCTAAATCCTAT
5437	No	AAATTATAGGCTAAATCCTATA
5438	No	AAATTATAGGCTAAATCCTATAT
5439	No	AAATTATAGGCTAAATCCTATATA
5440	No	AAATTATAGGCTAAATCCTATATAT
5441	No	AAATTATAGGCTAAATCCTATATATC
5442	No	AAATTATAGGCTAAATCCTATATATCT
5443	No	AAATTATAGGCTAAATCCTATATATCTT
5444	No	AAATTATAGGCTAAATCCTATATATCTTA
5445	Yes	AAATTATAGGCTAAATCCTATATATCTTAC
5446	Yes	AAATTATAGGCTAAATCCTATATATCTTACC
5447	Yes	AAATTATAGGCTAAATCCTATATATCTTACCA
5448	No	AAATTATGATAATCAT
5449	No	AAATTATGATAATCATA
5450	No	AAATTATGATAATCATAT
5451	No	AAATTATGATAATCATATT
5452	No	AAATTATGATAATCATATTT
5453	No	AAATTATGATAATCATATTTA

5454	No	AAATTATGATAATCATATTTAC
5455	No	AAATTATGATAATCATATTTACC
5456	No	AAATTATGATAATCATATTTACCA
5457	No	AAATTATGATAATCATATTTACCAA
5458	Yes	AAATTATGATAATCATATTTACCAAC
5459	Yes	AAATTATGATAATCATATTTACCAACC
5460	Yes	AAATTATGATAATCATATTTACCAACCA
5461	No	AAATTCCTATAGTACT
5462	Yes	AAATTCCTATAGTACTA
5463	Yes	AAATTCCTATAGTACTAG
5464	Yes	AAATTCCTATAGTACTAGC
5465	Yes	AAATTCCTATAGTACTAGCC
5466	Yes	AAATTCCTATAGTACTAGCCA
5467	No	AAATTCGAAGAAGCAG
5468	No	AAATTCGAAGAAGCAGC
5469	No	AAATTCGAAGAAGCAGCT
5470	No	AAATTCGAAGAAGCAGCTT
5471	No	AAATTCGAAGAAGCAGCTTC
5472	No	AAATTCGAAGAAGCAGCTTCA
5473	No	AAATTCGAAGAAGCAGCTTCAA
5474	No	AAATTCGAAGAAGCAGCTTCAAA
5475	No	AAATTCGAAGAAGCAGCTTCAAAC
5476	No	AAATTCGAAGAAGCAGCTTCAAACC
5477	No	AAATTCGAAGAAGCAGCTTCAAACCT
5478	No	AAATTCGAAGAAGCAGCTTCAAACCTG
5479	No	AAATTCGAAGAAGCAGCTTCAAACCTGC
5480	No	AAATTCGAAGAAGCAGCTTCAAACCTGCC
5481	No	AAATTCGAAGAAGCAGCTTCAAACCTGCCG
5482	No	AAATTCGAAGAAGCAGCTTCAAACCTGCCGG
5483	No	AAATTCGAAGAAGCAGCTTCAAACCTGCCGGG
5484	No	AAATTCGAAGAAGCAGCTTCAAACCTGCCGGGG
5485	No	AAATTCGAAGAAGCAGCTTCAAACCTGCCGGGGC
5486	No	AAATTCGAAGAAGCAGCTTCAAACCTGCCGGGGCT
5487	No	AAATTCGAAGAAGCAGCTTCAAACCTGCCGGGGCTT
5488	No	AAATTCGAAGAAGCAGCTTCAAACCTGCCGGGGCTTC
5489	Yes	AAATTCGAAGAAGCAGCTTCAAACCTGCCGGGGCTTCC
5490	Yes	AAATTCGAAGAAGCAGCTTCAAACCTGCCGGGGCTTCCA
5491	Yes	AAATTCGTTGGTTTCT
5492	Yes	AAATTCGTTGGTTTCTC
5493	Yes	AAATTCGTTGGTTTCTCT
5494	Yes	AAATTCGTTGGTTTCTCTC
5495	Yes	AAATTCGTTGGTTTCTCTCT

5496	Yes	AAATTCGTTGGTTTCTCTCTG
5497	Yes	AAATTCGTTGGTTTCTCTCTGT
5498	Yes	AAATTCGTTGGTTTCTCTCTGTG
5499	Yes	AAATTCGTTGGTTTCTCTCTGTGC
5500	Yes	AAATTCGTTGGTTTCTCTCTGTGCA
5501	Yes	AAATTCGTTGGTTTCTCTCTGTGCAG
5502	Yes	AAATTCGTTGGTTTCTCTCTGTGCAGG
5503	Yes	AAATTCGTTGGTTTCTCTCTGTGCAGGT
5504	Yes	AAATTCGTTGGTTTCTCTCTGTGCAGGTT
5505	Yes	AAATTCGTTGGTTTCTCTCTGTGCAGGTTT
5506	Yes	AAATTCGTTGGTTTCTCTCTGTGCAGGTTTG
5507	Yes	AAATTCGTTGGTTTCTCTCTGTGCAGGTTTGA
5508	Yes	AAATTCGTTGGTTTCTCTCTGTGCAGGTTTGAA
5509	Yes	AAATTCGTTGGTTTCTCTCTGTGCAGGTTTGAAT
5510	Yes	AAATTCGTTGGTTTCTCTCTGTGCAGGTTTGAATC
5511	Yes	AAATTCGTTGGTTTCTCTCTGTGCAGGTTTGAATCC
5512	Yes	AAATTCGTTGGTTTCTCTCTGTGCAGGTTTGAATCCT
5513	Yes	AAATTCGTTGGTTTCTCTCTGTGCAGGTTTGAATCCTG
5514	Yes	AAATTCGTTGGTTTCTCTCTGTGCAGGTTTGAATCCTGC
5515	Yes	AAATTCGTTGGTTTCTCTCTGTGCAGGTTTGAATCCTGCT
5516	Yes	AAATTCGTTGGTTTCTCTCTGTGCAGGTTTGAATCCTGCTA
5517	Yes	AAATTCGTTGGTTTCTCTCTGTGCAGGTTTGAATCCTGCTAA
5518	Yes	AAATTCGTTGGTTTCTCTCTGTGCAGGTTTGAATCCTGCTAAT
5519	Yes	AAATTCGTTGGTTTCTCTCTGTGCAGGTTTGAATCCTGCTAATT
5520	Yes	AAATTCGTTGGTTTCTCTCTGTGCAGGTTTGAATCCTGCTAATTA
5521	Yes	AAATTCGTTGGTTTCTCTCTGTGCAGGTTTGAATCCTGCTAATTAT
5522	Yes	AAATTCGTTGGTTTCTCTCTGTGCAGGTTTGAATCCTGCTAATTATG
5523	Yes	AAATTCGTTGGTTTCTCTCTGTGCAGGTTTGAATCCTGCTAATTATGC
5524	Yes	AAATTCGTTGGTTTCTCTCTGTGCAGGTTTGAATCCTGCTAATTATGCC
5525	Yes	AAATTCGTTGGTTTCTCTCTGTGCAGGTTTGAATCCTGCTAATTATGCCA
5526	No	AAATTCTGGTGCCACT
5527	No	AAATTCTGGTGCCACTC
5528	No	AAATTCTGGTGCCACTCC
5529	No	AAATTCTGGTGCCACTCCC
5530	Yes	AAATTCTGGTGCCACTCCCA
5531	No	AAATTGCTCAGTTGGG
5532	Yes	AAATTGCTCAGTTGGGA
5533	Yes	AAATTGCTCAGTTGGGAG
5534	Yes	AAATTGCTCAGTTGGGAGA
5535	Yes	AAATTGCTCAGTTGGGAGAG
5536	Yes	AAATTGCTCAGTTGGGAGAGC
5537	Yes	AAATTGCTCAGTTGGGAGAGCG

5538	Yes	AAATTGCTCAGTTGGGAGAGCGT
5539	Yes	AAATTGCTCAGTTGGGAGAGCGTT
5540	Yes	AAATTGCTCAGTTGGGAGAGCGTTA
5541	Yes	AAATTGCTCAGTTGGGAGAGCGTTAG
5542	Yes	AAATTGCTCAGTTGGGAGAGCGTTAGA
5543	Yes	AAATTGCTCAGTTGGGAGAGCGTTAGAC
5544	Yes	AAATTGCTCAGTTGGGAGAGCGTTAGACT
5545	Yes	AAATTGCTCAGTTGGGAGAGCGTTAGACTG
5546	Yes	AAATTGCTCAGTTGGGAGAGCGTTAGACTGA
5547	Yes	AAATTGCTCAGTTGGGAGAGCGTTAGACTGAA
5548	Yes	AAATTGCTCAGTTGGGAGAGCGTTAGACTGAAG
5549	Yes	AAATTGCTCAGTTGGGAGAGCGTTAGACTGAAGA
5550	Yes	AAATTGCTCAGTTGGGAGAGCGTTAGACTGAAGAT
5551	Yes	AAATTGCTCAGTTGGGAGAGCGTTAGACTGAAGATC
5552	Yes	AAATTGCTCAGTTGGGAGAGCGTTAGACTGAAGATCT
5553	Yes	AAATTGCTCAGTTGGGAGAGCGTTAGACTGAAGATCTA
5554	Yes	AAATTGCTCAGTTGGGAGAGCGTTAGACTGAAGATCTAA
5555	Yes	AAATTGCTCAGTTGGGAGAGCGTTAGACTGAAGATCTAAA
5556	Yes	AAATTGCTCAGTTGGGAGAGCGTTAGACTGAAGATCTAAAG
5557	Yes	AAATTGCTCAGTTGGGAGAGCGTTAGACTGAAGATCTAAAGG
5558	Yes	AAATTGCTCAGTTGGGAGAGCGTTAGACTGAAGATCTAAAGGT
5559	Yes	AAATTGCTCAGTTGGGAGAGCGTTAGACTGAAGATCTAAAGGTC
5560	Yes	AAATTGCTCAGTTGGGAGAGCGTTAGACTGAAGATCTAAAGGTCC
5561	Yes	AAATTGCTCAGTTGGGAGAGCGTTAGACTGAAGATCTAAAGGTCCC
5562	Yes	AAATTGCTCAGTTGGGAGAGCGTTAGACTGAAGATCTAAAGGTCCCT
5563	Yes	AAATTGCTCAGTTGGGAGAGCGTTAGACTGAAGATCTAAAGGTCCCTG
5564	Yes	AAATTGCTCAGTTGGGAGAGCGTTAGACTGAAGATCTAAAGGTCCCTGG
5565	Yes	AAATTGCTCAGTTGGGAGAGCGTTAGACTGAAGATCTAAAGGTCCCTGGT
5566	No	AAATTTACAACTTTAT
5567	No	AAATTTACAACTTTATG
5568	Yes	AAATTTACAACTTTATGG
5569	Yes	AAATTTACAACTTTATGGG
5570	Yes	AAATTTACAACTTTATGGGC
5571	Yes	AAATTTACAACTTTATGGGCA
5572	Yes	AAATTTACAACTTTATGGGCAG
5573	Yes	AAATTTACAACTTTATGGGCAGA
5574	Yes	AAATTTACAACTTTATGGGCAGAG
5575	Yes	AAATTTACAACTTTATGGGCAGAGG
5576	Yes	AAATTTACAACTTTATGGGCAGAGGT
5577	Yes	AAATTTACAACTTTATGGGCAGAGGTT
5578	Yes	AAATTTACAACTTTATGGGCAGAGGTTC
5579	Yes	AAATTTACAACTTTATGGGCAGAGGTTCA

5580	Yes	AAATTTACAACTTTATGGGCAGAGGTTCAA
5581	Yes	AAATTTACAACTTTATGGGCAGAGGTTCAAT
5582	Yes	AAATTTACAACTTTATGGGCAGAGGTTCAATT
5583	Yes	AAATTTACAACTTTATGGGCAGAGGTTCAATTC
5584	Yes	AAATTTACAACTTTATGGGCAGAGGTTCAATTCC
5585	Yes	AAATTTACAACTTTATGGGCAGAGGTTCAATTCCT
5586	Yes	AAATTTACAACTTTATGGGCAGAGGTTCAATTCCTC
5587	Yes	AAATTTACAACTTTATGGGCAGAGGTTCAATTCCTCT
5588	Yes	AAATTTACAACTTTATGGGCAGAGGTTCAATTCCTCTT
5589	Yes	AAATTTACAACTTTATGGGCAGAGGTTCAATTCCTCTTC
5590	Yes	AAATTTACAACTTTATGGGCAGAGGTTCAATTCCTCTTCT
5591	Yes	AAATTTACAACTTTATGGGCAGAGGTTCAATTCCTCTTCTT
5592	Yes	AAATTTACAACTTTATGGGCAGAGGTTCAATTCCTCTTCTTA
5593	Yes	AAATTTACAACTTTATGGGCAGAGGTTCAATTCCTCTTCTTAA
5594	Yes	AAATTTACAACTTTATGGGCAGAGGTTCAATTCCTCTTCTTAAC
5595	Yes	AAATTTACAACTTTATGGGCAGAGGTTCAATTCCTCTTCTTAACA
5596	Yes	AAATTTACAACTTTATGGGCAGAGGTTCAATTCCTCTTCTTAACAC
5597	Yes	AAATTTACAACTTTATGGGCAGAGGTTCAATTCCTCTTCTTAACACC
5598	Yes	AAATTTACAACTTTATGGGCAGAGGTTCAATTCCTCTTCTTAACACCA
5599	No	AAATTTAGATGTCCTG
5600	No	AAATTTAGATGTCCTGA
5601	Yes	AAATTTAGATGTCCTGAG
5602	Yes	AAATTTAGATGTCCTGAGT
5603	Yes	AAATTTAGATGTCCTGAGTT
5604	Yes	AAATTTAGATGTCCTGAGTTC
5605	Yes	AAATTTAGATGTCCTGAGTTCA
5606	Yes	AAATTTAGATGTCCTGAGTTCAA
5607	Yes	AAATTTAGATGTCCTGAGTTCAAA
5608	Yes	AAATTTAGATGTCCTGAGTTCAAAT
5609	Yes	AAATTTAGATGTCCTGAGTTCAAATC
5610	Yes	AAATTTAGATGTCCTGAGTTCAAATCC
5611	Yes	AAATTTAGATGTCCTGAGTTCAAATCCC
5612	Yes	AAATTTAGATGTCCTGAGTTCAAATCCCA
5613	Yes	AAATTTAGATGTCCTGAGTTCAAATCCCAG
5614	Yes	AAATTTAGATGTCCTGAGTTCAAATCCCAGC
5615	Yes	AAATTTAGATGTCCTGAGTTCAAATCCCAGCT
5616	Yes	AAATTTAGATGTCCTGAGTTCAAATCCCAGCTG
5617	Yes	AAATTTAGATGTCCTGAGTTCAAATCCCAGCTGT
5618	Yes	AAATTTAGATGTCCTGAGTTCAAATCCCAGCTGTA
5619	Yes	AAATTTAGATGTCCTGAGTTCAAATCCCAGCTGTAT
5620	Yes	AAATTTAGATGTCCTGAGTTCAAATCCCAGCTGTATG
5621	Yes	AAATTTAGATGTCCTGAGTTCAAATCCCAGCTGTATGC

5622	Yes	AAATTTAGATGTCCTGAGTTCAAATCCCAGCTGTATGCC
5623	Yes	AAATTTAGATGTCCTGAGTTCAAATCCCAGCTGTATGCCC
5624	Yes	AAATTTAGATGTCCTGAGTTCAAATCCCAGCTGTATGCCCA
5625	No	AAATTTAGGTTAAATA
5626	No	AAATTTAGGTTAAATAC
5627	Yes	AAATTTAGGTTAAATACA
5628	Yes	AAATTTAGGTTAAATACAG
5629	Yes	AAATTTAGGTTAAATACAGA
5630	Yes	AAATTTAGGTTAAATACAGAC
5631	Yes	AAATTTAGGTTAAATACAGACC
5632	Yes	AAATTTAGGTTAAATACAGACCA
5633	Yes	AAATTTAGGTTAAATACAGACCAA
5634	Yes	AAATTTAGGTTAAATACAGACCAAG
5635	Yes	AAATTTAGGTTAAATACAGACCAAGA
5636	Yes	AAATTTAGGTTAAATACAGACCAAGAG
5637	Yes	AAATTTAGGTTAAATACAGACCAAGAGC
5638	Yes	AAATTTAGGTTAAATACAGACCAAGAGCC
5639	Yes	AAATTTAGGTTAAATACAGACCAAGAGCCT
5640	Yes	AAATTTAGGTTAAATACAGACCAAGAGCCTT
5641	Yes	AAATTTAGGTTAAATACAGACCAAGAGCCTTC
5642	Yes	AAATTTAGGTTAAATACAGACCAAGAGCCTTCA
5643	Yes	AAATTTAGGTTAAATACAGACCAAGAGCCTTCAA
5644	Yes	AAATTTAGGTTAAATACAGACCAAGAGCCTTCAAA
5645	Yes	AAATTTAGGTTAAATACAGACCAAGAGCCTTCAAAG
5646	Yes	AAATTTAGGTTAAATACAGACCAAGAGCCTTCAAAGC
5647	Yes	AAATTTAGGTTAAATACAGACCAAGAGCCTTCAAAGCC
5648	Yes	AAATTTAGGTTAAATACAGACCAAGAGCCTTCAAAGCCC
5649	Yes	AAATTTAGGTTAAATACAGACCAAGAGCCTTCAAAGCCCT
5650	Yes	AAATTTAGGTTAAATACAGACCAAGAGCCTTCAAAGCCCTC
5651	Yes	AAATTTAGGTTAAATACAGACCAAGAGCCTTCAAAGCCCTCA
5652	Yes	AAATTTAGGTTAAATACAGACCAAGAGCCTTCAAAGCCCTCAG
5653	Yes	AAATTTAGGTTAAATACAGACCAAGAGCCTTCAAAGCCCTCAGT
5654	Yes	AAATTTAGGTTAAATACAGACCAAGAGCCTTCAAAGCCCTCAGTA
5655	Yes	AAATTTAGGTTAAATACAGACCAAGAGCCTTCAAAGCCCTCAGTAA
5656	Yes	AAATTTAGGTTAAATACAGACCAAGAGCCTTCAAAGCCCTCAGTAAG
5657	Yes	AAATTTAGGTTAAATACAGACCAAGAGCCTTCAAAGCCCTCAGTAAGT
5658	Yes	AAATTTAGGTTAAATACAGACCAAGAGCCTTCAAAGCCCTCAGTAAGTT
5659	Yes	AAATTTAGGTTAAATACAGACCAAGAGCCTTCAAAGCCCTCAGTAAGTTG
5660	Yes	AAATTTCGTCTCCTAC
5661	Yes	AAATTTCGTCTCCTACG
5662	Yes	AAATTTCGTCTCCTACGC
5663	Yes	AAATTTCGTCTCCTACGCG

5664	Yes	AAATTTCGTCTCCTACGCGG
5665	Yes	AAATTTCGTCTCCTACGCGGG
5666	Yes	AAATTTCGTCTCCTACGCGGGA
5667	Yes	AAATTTCGTCTCCTACGCGGGAG
5668	Yes	AAATTTCGTCTCCTACGCGGGAGA
5669	Yes	AAATTTCGTCTCCTACGCGGGAGAC
5670	Yes	AAATTTCGTCTCCTACGCGGGAGACT
5671	Yes	AAATTTCGTCTCCTACGCGGGAGACTC
5672	Yes	AAATTTCGTCTCCTACGCGGGAGACTCG
5673	Yes	AAATTTCGTCTCCTACGCGGGAGACTCGG
5674	Yes	AAATTTCGTCTCCTACGCGGGAGACTCGGG
5675	Yes	AAATTTCGTCTCCTACGCGGGAGACTCGGGT
5676	Yes	AAATTTCGTCTCCTACGCGGGAGACTCGGGTT
5677	Yes	AAATTTCGTCTCCTACGCGGGAGACTCGGGTTC
5678	Yes	AAATTTCGTCTCCTACGCGGGAGACTCGGGTTCG
5679	Yes	AAATTTCGTCTCCTACGCGGGAGACTCGGGTTCGA
5680	Yes	AAATTTCGTCTCCTACGCGGGAGACTCGGGTTCGAC
5681	Yes	AAATTTCGTCTCCTACGCGGGAGACTCGGGTTCGACT
5682	Yes	AAATTTCGTCTCCTACGCGGGAGACTCGGGTTCGACTT
5683	Yes	AAATTTCGTCTCCTACGCGGGAGACTCGGGTTCGACTTC
5684	Yes	AAATTTCGTCTCCTACGCGGGAGACTCGGGTTCGACTTCG
5685	Yes	AAATTTCGTCTCCTACGCGGGAGACTCGGGTTCGACTTCGG
5686	Yes	AAATTTCGTCTCCTACGCGGGAGACTCGGGTTCGACTTCGGC
5687	Yes	AAATTTCGTCTCCTACGCGGGAGACTCGGGTTCGACTTCGGCC
5688	Yes	AAATTTCGTCTCCTACGCGGGAGACTCGGGTTCGACTTCGGCCA
5689	Yes	AAATTTCGTCTCCTACGCGGGAGACTCGGGTTCGACTTCGGCCAT
5690	Yes	AAATTTCGTCTCCTACGCGGGAGACTCGGGTTCGACTTCGGCCATG
5691	Yes	AAATTTCGTCTCCTACGCGGGAGACTCGGGTTCGACTTCGGCCATGC
5692	Yes	AAATTTCGTCTCCTACGCGGGAGACTCGGGTTCGACTTCGGCCATGCA
5693	Yes	AAATTTCGTCTCCTACGCGGGAGACTCGGGTTCGACTTCGGCCATGCAC
5694	Yes	AAATTTCGTCTCCTACGCGGGAGACTCGGGTTCGACTTCGGCCATGCACC
5695	No	AAATTTTATAATCAGA
5696	No	AAATTTTATAATCAGAG
5697	No	AAATTTTATAATCAGAGG
5698	No	AAATTTTATAATCAGAGGT
5699	No	AAATTTTATAATCAGAGGTT
5700	No	AAATTTTATAATCAGAGGTTC
5701	No	AAATTTTATAATCAGAGGTTCA
5702	No	AAATTTTATAATCAGAGGTTCAA
5703	Yes	AAATTTTATAATCAGAGGTTCAAC
5704	Yes	AAATTTTATAATCAGAGGTTCAACT
5705	Yes	AAATTTTATAATCAGAGGTTCAACTC

5706	Yes	AAATTTTATAATCAGAGGTTCAACTCC
5707	Yes	AAATTTTATAATCAGAGGTTCAACTCCT
5708	Yes	AAATTTTATAATCAGAGGTTCAACTCCTC
5709	Yes	AAATTTTATAATCAGAGGTTCAACTCCTCT
5710	Yes	AAATTTTATAATCAGAGGTTCAACTCCTCTT
5711	Yes	AAATTTTATAATCAGAGGTTCAACTCCTCTTC
5712	Yes	AAATTTTATAATCAGAGGTTCAACTCCTCTTCT
5713	Yes	AAATTTTATAATCAGAGGTTCAACTCCTCTTCTT
5714	Yes	AAATTTTATAATCAGAGGTTCAACTCCTCTTCTTA
5715	Yes	AAATTTTATAATCAGAGGTTCAACTCCTCTTCTTAA
5716	Yes	AAATTTTATAATCAGAGGTTCAACTCCTCTTCTTAAC
5717	Yes	AAATTTTATAATCAGAGGTTCAACTCCTCTTCTTAACA
5718	Yes	AAATTTTATAATCAGAGGTTCAACTCCTCTTCTTAACAC
5719	Yes	AAATTTTATAATCAGAGGTTCAACTCCTCTTCTTAACACC
5720	Yes	AAATTTTATAATCAGAGGTTCAACTCCTCTTCTTAACACCA
5721	No	AAATTTTGGTGCAACT
5722	No	AAATTTTGGTGCAACTC
5723	No	AAATTTTGGTGCAACTCC
5724	No	AAATTTTGGTGCAACTCCA
5725	No	AAATTTTGGTGCAACTCCAA
5726	No	AAATTTTGGTGCAACTCCAAA
5727	No	AAATTTTGGTGCAACTCCAAAT
5728	No	AAATTTTGGTGCAACTCCAAATA
5729	No	AAATTTTGGTGCAACTCCAAATAA
5730	No	AAATTTTGGTGCAACTCCAAATAAA
5731	No	AAATTTTGGTGCAACTCCAAATAAAA
5732	No	AAATTTTGGTGCAACTCCAAATAAAAG
5733	No	AAATTTTGGTGCAACTCCAAATAAAAGT
5734	No	AAATTTTGGTGCAACTCCAAATAAAAGTA
5735	Yes	AAATTTTGGTGCAACTCCAAATAAAAGTAC
5736	Yes	AAATTTTGGTGCAACTCCAAATAAAAGTACC
5737	Yes	AAATTTTGGTGCAACTCCAAATAAAAGTACCA
5738	No	AACAAAACGAATGATT
5739	No	AACAAAACGAATGATTT
5740	No	AACAAAACGAATGATTTC
5741	No	AACAAAACGAATGATTTCG
5742	No	AACAAAACGAATGATTTCGA
5743	No	AACAAAACGAATGATTTCGAC
5744	No	AACAAAACGAATGATTTCGACT
5745	No	AACAAAACGAATGATTTCGACTC
5746	No	AACAAAACGAATGATTTCGACTCA
5747	No	AACAAAACGAATGATTTCGACTCAT

5748	No	AACAAAACGAATGATTTCGACTCATT
5749	No	AACAAAACGAATGATTTCGACTCATTA
5750	No	AACAAAACGAATGATTTCGACTCATTAA
5751	No	AACAAAACGAATGATTTCGACTCATTAAA
5752	No	AACAAAACGAATGATTTCGACTCATTAAAT
5753	No	AACAAAACGAATGATTTCGACTCATTAAATT
5754	No	AACAAAACGAATGATTTCGACTCATTAAATTA
5755	No	AACAAAACGAATGATTTCGACTCATTAAATTAT
5756	No	AACAAAACGAATGATTTCGACTCATTAAATTATG
5757	No	AACAAAACGAATGATTTCGACTCATTAAATTATGA
5758	No	AACAAAACGAATGATTTCGACTCATTAAATTATGAT
5759	No	AACAAAACGAATGATTTCGACTCATTAAATTATGATA
5760	No	AACAAAACGAATGATTTCGACTCATTAAATTATGATAA
5761	No	AACAAAACGAATGATTTCGACTCATTAAATTATGATAAT
5762	No	AACAAAACGAATGATTTCGACTCATTAAATTATGATAATC
5763	No	AACAAAACGAATGATTTCGACTCATTAAATTATGATAATCA
5764	No	AACAAAACGAATGATTTCGACTCATTAAATTATGATAATCAT
5765	No	AACAAAACGAATGATTTCGACTCATTAAATTATGATAATCATA
5766	No	AACAAAACGAATGATTTCGACTCATTAAATTATGATAATCATAT
5767	No	AACAAAACGAATGATTTCGACTCATTAAATTATGATAATCATATT
5768	No	AACAAAACGAATGATTTCGACTCATTAAATTATGATAATCATATTT
5769	No	AACAAAACGAATGATTTCGACTCATTAAATTATGATAATCATATTTA
5770	No	AACAAAACGAATGATTTCGACTCATTAAATTATGATAATCATATTTAC
5771	No	AACAAAACGAATGATTTCGACTCATTAAATTATGATAATCATATTTACC
5772	No	AACAAAACGAATGATTTCGACTCATTAAATTATGATAATCATATTTACCA
5773	No	AACAACATGGCTTTCT
5774	No	AACAACATGGCTTTCTC
5775	No	AACAACATGGCTTTCTCA
5776	Yes	AACAACATGGCTTTCTCAC
5777	Yes	AACAACATGGCTTTCTCACC
5778	Yes	AACAACATGGCTTTCTCACCA
5779	No	AACACAAAGCACCCAA
5780	No	AACACAAAGCACCCAAC
5781	No	AACACAAAGCACCCAACT
5782	No	AACACAAAGCACCCAACTT
5783	No	AACACAAAGCACCCAACTTA
5784	No	AACACAAAGCACCCAACTTAC
5785	No	AACACAAAGCACCCAACTTACA
5786	No	AACACAAAGCACCCAACTTACAC
5787	No	AACACAAAGCACCCAACTTACACT
5788	No	AACACAAAGCACCCAACTTACACTT
5789	No	AACACAAAGCACCCAACTTACACTTA

5790	No	AACACAAAGCACCCAACTTACACTTAG
5791	No	AACACAAAGCACCCAACTTACACTTAGG
5792	No	AACACAAAGCACCCAACTTACACTTAGGA
5793	No	AACACAAAGCACCCAACTTACACTTAGGAG
5794	No	AACACAAAGCACCCAACTTACACTTAGGAGA
5795	No	AACACAAAGCACCCAACTTACACTTAGGAGAT
5796	No	AACACAAAGCACCCAACTTACACTTAGGAGATT
5797	No	AACACAAAGCACCCAACTTACACTTAGGAGATTT
5798	No	AACACAAAGCACCCAACTTACACTTAGGAGATTTC
5799	No	AACACAAAGCACCCAACTTACACTTAGGAGATTTCA
5800	No	AACACAAAGCACCCAACTTACACTTAGGAGATTTCAA
5801	No	AACACAAAGCACCCAACTTACACTTAGGAGATTTCAAC
5802	No	AACACAAAGCACCCAACTTACACTTAGGAGATTTCAACT
5803	Yes	AACACAAAGCACCCAACTTACACTTAGGAGATTTCAACTT
5804	Yes	AACACAAAGCACCCAACTTACACTTAGGAGATTTCAACTTA
5805	Yes	AACACAAAGCACCCAACTTACACTTAGGAGATTTCAACTTAA
5806	Yes	AACACAAAGCACCCAACTTACACTTAGGAGATTTCAACTTAAC
5807	Yes	AACACAAAGCACCCAACTTACACTTAGGAGATTTCAACTTAACT
5808	Yes	AACACAAAGCACCCAACTTACACTTAGGAGATTTCAACTTAACTT
5809	Yes	AACACAAAGCACCCAACTTACACTTAGGAGATTTCAACTTAACTTG
5810	Yes	AACACAAAGCACCCAACTTACACTTAGGAGATTTCAACTTAACTTGA
5811	Yes	AACACAAAGCACCCAACTTACACTTAGGAGATTTCAACTTAACTTGAC
5812	Yes	AACACAAAGCACCCAACTTACACTTAGGAGATTTCAACTTAACTTGACC
5813	Yes	AACACAAAGCACCCAACTTACACTTAGGAGATTTCAACTTAACTTGACCG
5814	No	AACACACCCAGAGGCG
5815	Yes	AACACACCCAGAGGCGC
5816	Yes	AACACACCCAGAGGCGCC
5817	Yes	AACACACCCAGAGGCGCCA
5818	Yes	AACACCAAGGTCGCGG
5819	Yes	AACACCAAGGTCGCGGG
5820	Yes	AACACCAAGGTCGCGGGC
5821	Yes	AACACCAAGGTCGCGGGCT
5822	Yes	AACACCAAGGTCGCGGGCTC
5823	Yes	AACACCAAGGTCGCGGGCTCG
5824	Yes	AACACCAAGGTCGCGGGCTCGA
5825	Yes	AACACCAAGGTCGCGGGCTCGAC
5826	Yes	AACACCAAGGTCGCGGGCTCGACT
5827	Yes	AACACCAAGGTCGCGGGCTCGACTC
5828	Yes	AACACCAAGGTCGCGGGCTCGACTCC
5829	Yes	AACACCAAGGTCGCGGGCTCGACTCCC
5830	Yes	AACACCAAGGTCGCGGGCTCGACTCCCG
5831	Yes	AACACCAAGGTCGCGGGCTCGACTCCCGC

5832	Yes	AACACCAAGGTCGCGGGCTCGACTCCCGCA
5833	Yes	AACACCAAGGTCGCGGGCTCGACTCCCGCAC
5834	Yes	AACACCAAGGTCGCGGGCTCGACTCCCGCACC
5835	Yes	AACACCAAGGTCGCGGGCTCGACTCCCGCACCG
5836	Yes	AACACCAAGGTCGCGGGCTCGACTCCCGCACCGG
5837	Yes	AACACCAAGGTCGCGGGCTCGACTCCCGCACCGGC
5838	Yes	AACACCAAGGTCGCGGGCTCGACTCCCGCACCGGCC
5839	Yes	AACACCAAGGTCGCGGGCTCGACTCCCGCACCGGCCA
5840	Yes	AACACCAAGGTCGCGGGCTCGACTCCCGCACCGGCCAC
5841	Yes	AACACCAAGGTCGCGGGCTCGACTCCCGCACCGGCCACC
5842	Yes	AACACCAAGGTCGCGGGCTCGACTCCCGCACCGGCCACCA
5843	No	AACACCTCTTCTTAAC
5844	No	AACACCTCTTCTTAACA
5845	Yes	AACACCTCTTCTTAACAC
5846	Yes	AACACCTCTTCTTAACACC
5847	Yes	AACACCTCTTCTTAACACCA
5848	No	AACACCTCTTTACAGT
5849	No	AACACCTCTTTACAGTG
5850	No	AACACCTCTTTACAGTGA
5851	Yes	AACACCTCTTTACAGTGAC
5852	Yes	AACACCTCTTTACAGTGACC
5853	Yes	AACACCTCTTTACAGTGACCA
5854	Yes	AACACGCGAAAGGTCC
5855	Yes	AACACGCGAAAGGTCCC
5856	Yes	AACACGCGAAAGGTCCCC
5857	Yes	AACACGCGAAAGGTCCCCG
5858	Yes	AACACGCGAAAGGTCCCCGG
5859	Yes	AACACGCGAAAGGTCCCCGGT
5860	Yes	AACACGCGAAAGGTCCCCGGTT
5861	Yes	AACACGCGAAAGGTCCCCGGTTC
5862	Yes	AACACGCGAAAGGTCCCCGGTTCG
5863	Yes	AACACGCGAAAGGTCCCCGGTTCGA
5864	Yes	AACACGCGAAAGGTCCCCGGTTCGAA
5865	Yes	AACACGCGAAAGGTCCCCGGTTCGAAA
5866	Yes	AACACGCGAAAGGTCCCCGGTTCGAAAC
5867	Yes	AACACGCGAAAGGTCCCCGGTTCGAAACC
5868	Yes	AACACGCGAAAGGTCCCCGGTTCGAAACCG
5869	Yes	AACACGCGAAAGGTCCCCGGTTCGAAACCGG
5870	Yes	AACACGCGAAAGGTCCCCGGTTCGAAACCGGG
5871	Yes	AACACGCGAAAGGTCCCCGGTTCGAAACCGGGC
5872	Yes	AACACGCGAAAGGTCCCCGGTTCGAAACCGGGCA
5873	Yes	AACACGCGAAAGGTCCCCGGTTCGAAACCGGGCAG

5874	Yes	AACACGCGAAAGGTCCCCGGTTCGAAACCGGGCAGA
5875	Yes	AACACGCGAAAGGTCCCCGGTTCGAAACCGGGCAGAA
5876	Yes	AACACGCGAAAGGTCCCCGGTTCGAAACCGGGCAGAAA
5877	Yes	AACACGCGAAAGGTCCCCGGTTCGAAACCGGGCAGAAAC
5878	Yes	AACACGCGAAAGGTCCCCGGTTCGAAACCGGGCAGAAACA
5879	Yes	AACACGCGAAAGGTCCCCGGTTCGAAACCGGGCAGAAACAC
5880	Yes	AACACGCGAAAGGTCCCCGGTTCGAAACCGGGCAGAAACACC
5881	Yes	AACACGCGAAAGGTCCCCGGTTCGAAACCGGGCAGAAACACCA
5882	Yes	AACACGCGAAAGGTCCCCGGTTCGAAACCGGGCG
5883	Yes	AACACGCGAAAGGTCCCCGGTTCGAAACCGGGCGG
5884	Yes	AACACGCGAAAGGTCCCCGGTTCGAAACCGGGCGGA
5885	Yes	AACACGCGAAAGGTCCCCGGTTCGAAACCGGGCGGAA
5886	Yes	AACACGCGAAAGGTCCCCGGTTCGAAACCGGGCGGAAA
5887	Yes	AACACGCGAAAGGTCCCCGGTTCGAAACCGGGCGGAAAC
5888	Yes	AACACGCGAAAGGTCCCCGGTTCGAAACCGGGCGGAAACA
5889	Yes	AACACGCGAAAGGTCCCCGGTTCGAAACCGGGCGGAAACAC
5890	Yes	AACACGCGAAAGGTCCCCGGTTCGAAACCGGGCGGAAACACC
5891	Yes	AACACGCGAAAGGTCCCCGGTTCGAAACCGGGCGGAAACACCA
5892	Yes	AACACGCGAAAGGTCCCT
5893	Yes	AACACGCGAAAGGTCCCTG
5894	Yes	AACACGCGAAAGGTCCCTGG
5895	Yes	AACACGCGAAAGGTCCCTGGA
5896	Yes	AACACGCGAAAGGTCCCTGGAT
5897	Yes	AACACGCGAAAGGTCCCTGGATC
5898	Yes	AACACGCGAAAGGTCCCTGGATCA
5899	Yes	AACACGCGAAAGGTCCCTGGATCAA
5900	Yes	AACACGCGAAAGGTCCCTGGATCAAA
5901	Yes	AACACGCGAAAGGTCCCTGGATCAAAA
5902	Yes	AACACGCGAAAGGTCCCTGGATCAAAAC
5903	Yes	AACACGCGAAAGGTCCCTGGATCAAAACC
5904	Yes	AACACGCGAAAGGTCCCTGGATCAAAACCA
5905	Yes	AACACGCGAAAGGTCCCTGGATCAAAACCAG
5906	Yes	AACACGCGAAAGGTCCCTGGATCAAAACCAGG
5907	Yes	AACACGCGAAAGGTCCCTGGATCAAAACCAGGC
5908	Yes	AACACGCGAAAGGTCCCTGGATCAAAACCAGGCG
5909	Yes	AACACGCGAAAGGTCCCTGGATCAAAACCAGGCGG
5910	Yes	AACACGCGAAAGGTCCCTGGATCAAAACCAGGCGGA
5911	Yes	AACACGCGAAAGGTCCCTGGATCAAAACCAGGCGGAA
5912	Yes	AACACGCGAAAGGTCCCTGGATCAAAACCAGGCGGAAA
5913	Yes	AACACGCGAAAGGTCCCTGGATCAAAACCAGGCGGAAAC
5914	Yes	AACACGCGAAAGGTCCCTGGATCAAAACCAGGCGGAAACA
5915	Yes	AACACGCGAAAGGTCCCTGGATCAAAACCAGGCGGAAACAC

5916	Yes	AACACGCGAAAGGTCCCTGGATCAAAACCAGGCGGAAACACC
5917	Yes	AACACGCGAAAGGTCCCTGGATCAAAACCAGGCGGAAACACCA
5918	Yes	AACACGCGAAAGGTCCG
5919	Yes	AACACGCGAAAGGTCCGC
5920	Yes	AACACGCGAAAGGTCCGCG
5921	Yes	AACACGCGAAAGGTCCGCGG
5922	Yes	AACACGCGAAAGGTCCGCGGT
5923	Yes	AACACGCGAAAGGTCCGCGGTT
5924	Yes	AACACGCGAAAGGTCCGCGGTTC
5925	Yes	AACACGCGAAAGGTCCGCGGTTCG
5926	Yes	AACACGCGAAAGGTCCGCGGTTCGA
5927	Yes	AACACGCGAAAGGTCCGCGGTTCGAA
5928	Yes	AACACGCGAAAGGTCCGCGGTTCGAAA
5929	Yes	AACACGCGAAAGGTCCGCGGTTCGAAAC
5930	Yes	AACACGCGAAAGGTCCGCGGTTCGAAACC
5931	Yes	AACACGCGAAAGGTCCGCGGTTCGAAACCG
5932	Yes	AACACGCGAAAGGTCCGCGGTTCGAAACCGG
5933	Yes	AACACGCGAAAGGTCCGCGGTTCGAAACCGGG
5934	Yes	AACACGCGAAAGGTCCGCGGTTCGAAACCGGGC
5935	Yes	AACACGCGAAAGGTCCGCGGTTCGAAACCGGGCG
5936	Yes	AACACGCGAAAGGTCCGCGGTTCGAAACCGGGCGG
5937	Yes	AACACGCGAAAGGTCCGCGGTTCGAAACCGGGCGGA
5938	Yes	AACACGCGAAAGGTCCGCGGTTCGAAACCGGGCGGAA
5939	Yes	AACACGCGAAAGGTCCGCGGTTCGAAACCGGGCGGAAA
5940	Yes	AACACGCGAAAGGTCCGCGGTTCGAAACCGGGCGGAAAC
5941	Yes	AACACGCGAAAGGTCCGCGGTTCGAAACCGGGCGGAAACA
5942	Yes	AACACGCGAAAGGTCCGCGGTTCGAAACCGGGCGGAAACAC
5943	Yes	AACACGCGAAAGGTCCGCGGTTCGAAACCGGGCGGAAACACC
5944	Yes	AACACGCGAAAGGTCCGCGGTTCGAAACCGGGCGGAAACACCA
5945	Yes	AACACGCGAAAGGTCCT
5946	Yes	AACACGCGAAAGGTCCTT
5947	Yes	AACACGCGAAAGGTCCTTG
5948	Yes	AACACGCGAAAGGTCCTTGG
5949	Yes	AACACGCGAAAGGTCCTTGGT
5950	Yes	AACACGCGAAAGGTCCTTGGTT
5951	Yes	AACACGCGAAAGGTCCTTGGTTT
5952	Yes	AACACGCGAAAGGTCCTTGGTTTG
5953	Yes	AACACGCGAAAGGTCCTTGGTTTGA
5954	Yes	AACACGCGAAAGGTCCTTGGTTTGAA
5955	Yes	AACACGCGAAAGGTCCTTGGTTTGAAA
5956	Yes	AACACGCGAAAGGTCCTTGGTTTGAAAC
5957	Yes	AACACGCGAAAGGTCCTTGGTTTGAAACC

5958	Yes	AACACGCGAAAGGTCCTTGGTTTGAAACCA
5959	Yes	AACACGCGAAAGGTCCTTGGTTTGAAACCAG
5960	Yes	AACACGCGAAAGGTCCTTGGTTTGAAACCAGG
5961	Yes	AACACGCGAAAGGTCCTTGGTTTGAAACCAGGC
5962	Yes	AACACGCGAAAGGTCCTTGGTTTGAAACCAGGCA
5963	Yes	AACACGCGAAAGGTCCTTGGTTTGAAACCAGGCAG
5964	Yes	AACACGCGAAAGGTCCTTGGTTTGAAACCAGGCAGA
5965	Yes	AACACGCGAAAGGTCCTTGGTTTGAAACCAGGCAGAA
5966	Yes	AACACGCGAAAGGTCCTTGGTTTGAAACCAGGCAGAAA
5967	Yes	AACACGCGAAAGGTCCTTGGTTTGAAACCAGGCAGAAAC
5968	Yes	AACACGCGAAAGGTCCTTGGTTTGAAACCAGGCAGAAACA
5969	Yes	AACACGCGAAAGGTCCTTGGTTTGAAACCAGGCAGAAACAC
5970	Yes	AACACGCGAAAGGTCCTTGGTTTGAAACCAGGCAGAAACACC
5971	Yes	AACACGCGAAAGGTCCTTGGTTTGAAACCAGGCAGAAACACCA
5972	Yes	AACACGTTCGCCTCAC
5973	Yes	AACACGTTCGCCTCACA
5974	Yes	AACACGTTCGCCTCACAC
5975	Yes	AACACGTTCGCCTCACACG
5976	Yes	AACACGTTCGCCTCACACGC
5977	Yes	AACACGTTCGCCTCACACGCT
5978	Yes	AACACGTTCGCCTCACACGCTT
5979	Yes	AACACGTTCGCCTCACACGCTTA
5980	Yes	AACACGTTCGCCTCACACGCTTAA
5981	Yes	AACACGTTCGCCTCACACGCTTAAA
5982	Yes	AACACGTTCGCCTCACACGCTTAAAG
5983	Yes	AACACGTTCGCCTCACACGCTTAAAGT
5984	Yes	AACACGTTCGCCTCACACGCTTAAAGTT
5985	Yes	AACACGTTCGCCTCACACGCTTAAAGTTC
5986	Yes	AACACGTTCGCCTCACACGCTTAAAGTTCT
5987	Yes	AACACGTTCGCCTCACACGCTTAAAGTTCTC
5988	Yes	AACACGTTCGCCTCACACGCTTAAAGTTCTCT
5989	Yes	AACACGTTCGCCTCACACGCTTAAAGTTCTCTG
5990	Yes	AACACGTTCGCCTCACACGCTTAAAGTTCTCTGG
5991	Yes	AACACGTTCGCCTCACACGCTTAAAGTTCTCTGGT
5992	Yes	AACACGTTCGCCTCACACGCTTAAAGTTCTCTGGTT
5993	Yes	AACACGTTCGCCTCACACGCTTAAAGTTCTCTGGTTG
5994	Yes	AACACGTTCGCCTCACACGCTTAAAGTTCTCTGGTTGG
5995	Yes	AACACGTTCGCCTCACACGCTTAAAGTTCTCTGGTTGGA
5996	Yes	AACACGTTCGCCTCACACGCTTAAAGTTCTCTGGTTGGAT
5997	Yes	AACACGTTCGCCTCACACGCTTAAAGTTCTCTGGTTGGATA
5998	Yes	AACACGTTCGCCTCACACGCTTAAAGTTCTCTGGTTGGATAC
5999	Yes	AACACGTTCGCCTCACACGCTTAAAGTTCTCTGGTTGGATACC

6000	Yes	AACACGTTCGCCTCACACGCTTAAAGTTCTCTGGTTGGATACCA
6001	Yes	AACACGTTCGCCTCACACGCTTAAAGTTCTCTGGTTGGATACCAG
6002	Yes	AACACGTTCGCCTCACACGCTTAAAGTTCTCTGGTTGGATACCAGA
6003	Yes	AACACGTTCGCCTCACACGCTTAAAGTTCTCTGGTTGGATACCAGAT
6004	Yes	AACACGTTCGCCTCACACGCTTAAAGTTCTCTGGTTGGATACCAGATG
6005	Yes	AACACGTTCGCCTCACACGCTTAAAGTTCTCTGGTTGGATACCAGATGG
6006	Yes	AACACGTTCGCCTCACACGCTTAAAGTTCTCTGGTTGGATACCAGATGGA
6007	Yes	AACACTCTCACCGCCG
6008	Yes	AACACTCTCACCGCCGC
6009	Yes	AACACTCTCACCGCCGCA
6010	Yes	AACACTCTCACCGCCGCAG
6011	Yes	AACACTCTCACCGCCGCAGC
6012	Yes	AACACTCTCACCGCCGCAGCC
6013	Yes	AACACTCTCACCGCCGCAGCCC
6014	Yes	AACACTCTCACCGCCGCAGCCCG
6015	Yes	AACACTCTCACCGCCGCAGCCCGG
6016	Yes	AACACTCTCACCGCCGCAGCCCGGG
6017	Yes	AACACTCTCACCGCCGCAGCCCGGGT
6018	Yes	AACACTCTCACCGCCGCAGCCCGGGTT
6019	Yes	AACACTCTCACCGCCGCAGCCCGGGTTT
6020	Yes	AACACTCTCACCGCCGCAGCCCGGGTTTG
6021	Yes	AACACTCTCACCGCCGCAGCCCGGGTTTGA
6022	Yes	AACACTCTCACCGCCGCAGCCCGGGTTTGAT
6023	Yes	AACACTCTCACCGCCGCAGCCCGGGTTTGATT
6024	Yes	AACACTCTCACCGCCGCAGCCCGGGTTTGATTC
6025	Yes	AACACTCTCACCGCCGCAGCCCGGGTTTGATTCC
6026	Yes	AACACTCTCACCGCCGCAGCCCGGGTTTGATTCCC
6027	Yes	AACACTCTCACCGCCGCAGCCCGGGTTTGATTCCCA
6028	Yes	AACACTCTCACCGCCGCAGCCCGGGTTTGATTCCCAG
6029	Yes	AACACTCTCACCGCCGCAGCCCGGGTTTGATTCCCAGG
6030	Yes	AACACTCTCACCGCCGCAGCCCGGGTTTGATTCCCAGGC
6031	Yes	AACACTCTCACCGCCGCAGCCCGGGTTTGATTCCCAGGCA
6032	Yes	AACACTCTCACCGCCGCAGCCCGGGTTTGATTCCCAGGCAG
6033	Yes	AACACTCTCACCGCCGCAGCCCGGGTTTGATTCCCAGGCAGG
6034	Yes	AACACTCTCACCGCCGCAGCCCGGGTTTGATTCCCAGGCAGGG
6035	Yes	AACACTCTCACCGCCGCAGCCCGGGTTTGATTCCCAGGCAGGGA
6036	Yes	AACACTCTCACCGCCGCAGCCCGGGTTTGATTCCCAGGCAGGGAA
6037	Yes	AACACTCTCACCGCCGCAGCCCGGGTTTGATTCCCAGGCAGGGAAG
6038	Yes	AACACTCTCACCGCCGCAGCCCGGGTTTGATTCCCAGGCAGGGAAGC
6039	Yes	AACACTCTCACCGCCGCAGCCCGGGTTTGATTCCCAGGCAGGGAAGCC
6040	Yes	AACACTCTCACCGCCGCAGCCCGGGTTTGATTCCCAGGCAGGGAAGCCA
6041	No	AACACTTTATAATCAG

6042	Yes	AACACTTTATAATCAGA
6043	Yes	AACACTTTATAATCAGAG
6044	Yes	AACACTTTATAATCAGAGG
6045	Yes	AACACTTTATAATCAGAGGT
6046	Yes	AACACTTTATAATCAGAGGTT
6047	Yes	AACACTTTATAATCAGAGGTTC
6048	Yes	AACACTTTATAATCAGAGGTTCA
6049	Yes	AACACTTTATAATCAGAGGTTCAA
6050	Yes	AACACTTTATAATCAGAGGTTCAAC
6051	Yes	AACACTTTATAATCAGAGGTTCAACT
6052	Yes	AACACTTTATAATCAGAGGTTCAACTC
6053	Yes	AACACTTTATAATCAGAGGTTCAACTCC
6054	Yes	AACACTTTATAATCAGAGGTTCAACTCCT
6055	Yes	AACACTTTATAATCAGAGGTTCAACTCCTC
6056	Yes	AACACTTTATAATCAGAGGTTCAACTCCTCT
6057	Yes	AACACTTTATAATCAGAGGTTCAACTCCTCTT
6058	Yes	AACACTTTATAATCAGAGGTTCAACTCCTCTTC
6059	Yes	AACACTTTATAATCAGAGGTTCAACTCCTCTTCT
6060	Yes	AACACTTTATAATCAGAGGTTCAACTCCTCTTCTT
6061	Yes	AACACTTTATAATCAGAGGTTCAACTCCTCTTCTTA
6062	Yes	AACACTTTATAATCAGAGGTTCAACTCCTCTTCTTAA
6063	Yes	AACACTTTATAATCAGAGGTTCAACTCCTCTTCTTAAC
6064	Yes	AACACTTTATAATCAGAGGTTCAACTCCTCTTCTTAACA
6065	Yes	AACACTTTATAATCAGAGGTTCAACTCCTCTTCTTAACAC
6066	Yes	AACACTTTATAATCAGAGGTTCAACTCCTCTTCTTAACACC
6067	Yes	AACACTTTATAATCAGAGGTTCAACTCCTCTTCTTAACACCA
6068	Yes	AACAGAGGCTTACGAC
6069	Yes	AACAGAGGCTTACGACC
6070	Yes	AACAGAGGCTTACGACCC
6071	Yes	AACAGAGGCTTACGACCCC
6072	Yes	AACAGAGGCTTACGACCCCT
6073	Yes	AACAGAGGCTTACGACCCCTT
6074	Yes	AACAGAGGCTTACGACCCCTTA
6075	Yes	AACAGAGGCTTACGACCCCTTAT
6076	Yes	AACAGAGGCTTACGACCCCTTATT
6077	Yes	AACAGAGGCTTACGACCCCTTATTT
6078	Yes	AACAGAGGCTTACGACCCCTTATTTA
6079	Yes	AACAGAGGCTTACGACCCCTTATTTAC
6080	Yes	AACAGAGGCTTACGACCCCTTATTTACC
6081	Yes	AACAGAGGCTTACGACCCCTTATTTACCC
6082	Yes	AACAGAGGCTTACGACCCCTTATTTACCCC
6083	Yes	AACAGAGGCTTACGACCCCTTATTTACCCCA

6084	Yes	AACAGCTATCCATTGG
6085	Yes	AACAGCTATCCATTGGT
6086	Yes	AACAGCTATCCATTGGTC
6087	Yes	AACAGCTATCCATTGGTCT
6088	Yes	AACAGCTATCCATTGGTCTT
6089	Yes	AACAGCTATCCATTGGTCTTA
6090	Yes	AACAGCTATCCATTGGTCTTAG
6091	Yes	AACAGCTATCCATTGGTCTTAGG
6092	Yes	AACAGCTATCCATTGGTCTTAGGC
6093	Yes	AACAGCTATCCATTGGTCTTAGGCC
6094	Yes	AACAGCTATCCATTGGTCTTAGGCCC
6095	Yes	AACAGCTATCCATTGGTCTTAGGCCCC
6096	Yes	AACAGCTATCCATTGGTCTTAGGCCCCA
6097	Yes	AACAGCTATCCATTGGTCTTAGGCCCCAA
6098	Yes	AACAGCTATCCATTGGTCTTAGGCCCCAAA
6099	Yes	AACAGCTATCCATTGGTCTTAGGCCCCAAAA
6100	Yes	AACAGCTATCCATTGGTCTTAGGCCCCAAAAA
6101	Yes	AACAGCTATCCATTGGTCTTAGGCCCCAAAAAT
6102	Yes	AACAGCTATCCATTGGTCTTAGGCCCCAAAAATT
6103	Yes	AACAGCTATCCATTGGTCTTAGGCCCCAAAAATTT
6104	Yes	AACAGCTATCCATTGGTCTTAGGCCCCAAAAATTTT
6105	Yes	AACAGCTATCCATTGGTCTTAGGCCCCAAAAATTTTG
6106	Yes	AACAGCTATCCATTGGTCTTAGGCCCCAAAAATTTTGG
6107	Yes	AACAGCTATCCATTGGTCTTAGGCCCCAAAAATTTTGGT
6108	Yes	AACAGCTATCCATTGGTCTTAGGCCCCAAAAATTTTGGTG
6109	Yes	AACAGCTATCCATTGGTCTTAGGCCCCAAAAATTTTGGTGC
6110	Yes	AACAGCTATCCATTGGTCTTAGGCCCCAAAAATTTTGGTGCA
6111	Yes	AACAGCTATCCATTGGTCTTAGGCCCCAAAAATTTTGGTGCAA
6112	Yes	AACAGCTATCCATTGGTCTTAGGCCCCAAAAATTTTGGTGCAAC
6113	Yes	AACAGCTATCCATTGGTCTTAGGCCCCAAAAATTTTGGTGCAACT
6114	Yes	AACAGCTATCCATTGGTCTTAGGCCCCAAAAATTTTGGTGCAACTC
6115	Yes	AACAGCTATCCATTGGTCTTAGGCCCCAAAAATTTTGGTGCAACTCC
6116	Yes	AACAGCTATCCATTGGTCTTAGGCCCCAAAAATTTTGGTGCAACTCCA
6117	Yes	AACAGCTATCCATTGGTCTTAGGCCCCAAAAATTTTGGTGCAACTCCAA
6118	Yes	AACAGCTATCCATTGGTCTTAGGCCCCAAAAATTTTGGTGCAACTCCAAA
6119	No	AACAGGAGATCCTGAG
6120	No	AACAGGAGATCCTGAGT
6121	No	AACAGGAGATCCTGAGTT
6122	No	AACAGGAGATCCTGAGTTT
6123	Yes	AACAGGAGATCCTGAGTTTC
6124	Yes	AACAGGAGATCCTGAGTTTCA
6125	Yes	AACAGGAGATCCTGAGTTTCAA

6126	Yes	AACAGGAGATCCTGAGTTTCAAT
6127	Yes	AACAGGAGATCCTGAGTTTCAATT
6128	Yes	AACAGGAGATCCTGAGTTTCAATTC
6129	Yes	AACAGGAGATCCTGAGTTTCAATTCC
6130	Yes	AACAGGAGATCCTGAGTTTCAATTCCA
6131	Yes	AACAGGAGATCCTGAGTTTCAATTCCAA
6132	Yes	AACAGGAGATCCTGAGTTTCAATTCCAAT
6133	Yes	AACAGGAGATCCTGAGTTTCAATTCCAATG
6134	Yes	AACAGGAGATCCTGAGTTTCAATTCCAATGG
6135	Yes	AACAGGAGATCCTGAGTTTCAATTCCAATGGT
6136	Yes	AACAGGAGATCCTGAGTTTCAATTCCAATGGTG
6137	Yes	AACAGGAGATCCTGAGTTTCAATTCCAATGGTGC
6138	Yes	AACAGGAGATCCTGAGTTTCAATTCCAATGGTGCC
6139	Yes	AACAGGAGATCCTGAGTTTCAATTCCAATGGTGCCT
6140	Yes	AACAGGAGATCCTGAGTTTCAATTCCAATGGTGCCTC
6141	Yes	AACAGGAGATCCTGAGTTTCAATTCCAATGGTGCCTCC
6142	Yes	AACAGGAGATCCTGAGTTTCAATTCCAATGGTGCCTCCA
6143	No	AACAGGAGATCCTGGG
6144	No	AACAGGAGATCCTGGGT
6145	No	AACAGGAGATCCTGGGTT
6146	Yes	AACAGGAGATCCTGGGTTC
6147	Yes	AACAGGAGATCCTGGGTTCG
6148	Yes	AACAGGAGATCCTGGGTTCGA
6149	Yes	AACAGGAGATCCTGGGTTCGAA
6150	Yes	AACAGGAGATCCTGGGTTCGAAT
6151	Yes	AACAGGAGATCCTGGGTTCGAATC
6152	Yes	AACAGGAGATCCTGGGTTCGAATCC
6153	Yes	AACAGGAGATCCTGGGTTCGAATCCC
6154	Yes	AACAGGAGATCCTGGGTTCGAATCCCA
6155	Yes	AACAGGAGATCCTGGGTTCGAATCCCAG
6156	Yes	AACAGGAGATCCTGGGTTCGAATCCCAGC
6157	Yes	AACAGGAGATCCTGGGTTCGAATCCCAGCG
6158	Yes	AACAGGAGATCCTGGGTTCGAATCCCAGCGA
6159	Yes	AACAGGAGATCCTGGGTTCGAATCCCAGCGAG
6160	Yes	AACAGGAGATCCTGGGTTCGAATCCCAGCGAGG
6161	Yes	AACAGGAGATCCTGGGTTCGAATCCCAGCGAGGC
6162	Yes	AACAGGAGATCCTGGGTTCGAATCCCAGCGAGGCC
6163	Yes	AACAGGAGATCCTGGGTTCGAATCCCAGCGAGGCCT
6164	Yes	AACAGGAGATCCTGGGTTCGAATCCCAGCGAGGCCTC
6165	Yes	AACAGGAGATCCTGGGTTCGAATCCCAGCGAGGCCTCC
6166	Yes	AACAGGAGATCCTGGGTTCGAATCCCAGCGAGGCCTCCA
6167	Yes	AACAGGAGATCCTGGGTTCGAATCCCAGCGG

6168	Yes	AACAGGAGATCCTGGGTTCGAATCCCAGCGGG
6169	Yes	AACAGGAGATCCTGGGTTCGAATCCCAGCGGGG
6170	Yes	AACAGGAGATCCTGGGTTCGAATCCCAGCGGGGC
6171	Yes	AACAGGAGATCCTGGGTTCGAATCCCAGCGGGGCC
6172	Yes	AACAGGAGATCCTGGGTTCGAATCCCAGCGGGGCCT
6173	Yes	AACAGGAGATCCTGGGTTCGAATCCCAGCGGGGCCTC
6174	Yes	AACAGGAGATCCTGGGTTCGAATCCCAGCGGGGCCTCC
6175	Yes	AACAGGAGATCCTGGGTTCGAATCCCAGCGGGGCCTCCA
6176	Yes	AACAGGAGATCCTGGGTTCGAATCCCAGCGGT
6177	Yes	AACAGGAGATCCTGGGTTCGAATCCCAGCGGTG
6178	Yes	AACAGGAGATCCTGGGTTCGAATCCCAGCGGTGC
6179	Yes	AACAGGAGATCCTGGGTTCGAATCCCAGCGGTGCC
6180	Yes	AACAGGAGATCCTGGGTTCGAATCCCAGCGGTGCCT
6181	Yes	AACAGGAGATCCTGGGTTCGAATCCCAGCGGTGCCTC
6182	Yes	AACAGGAGATCCTGGGTTCGAATCCCAGCGGTGCCTCC
6183	Yes	AACAGGAGATCCTGGGTTCGAATCCCAGCGGTGCCTCCA
6184	Yes	AACAGGAGATCCTGGGTTCGAATCCCAGT
6185	Yes	AACAGGAGATCCTGGGTTCGAATCCCAGTA
6186	Yes	AACAGGAGATCCTGGGTTCGAATCCCAGTAG
6187	Yes	AACAGGAGATCCTGGGTTCGAATCCCAGTAGA
6188	Yes	AACAGGAGATCCTGGGTTCGAATCCCAGTAGAG
6189	Yes	AACAGGAGATCCTGGGTTCGAATCCCAGTAGAGC
6190	Yes	AACAGGAGATCCTGGGTTCGAATCCCAGTAGAGCC
6191	Yes	AACAGGAGATCCTGGGTTCGAATCCCAGTAGAGCCT
6192	Yes	AACAGGAGATCCTGGGTTCGAATCCCAGTAGAGCCTC
6193	Yes	AACAGGAGATCCTGGGTTCGAATCCCAGTAGAGCCTCC
6194	Yes	AACAGGAGATCCTGGGTTCGAATCCCAGTAGAGCCTCCA
6195	Yes	AACAGGAGATCCTGGGTTCGAC
6196	Yes	AACAGGAGATCCTGGGTTCGACT
6197	Yes	AACAGGAGATCCTGGGTTCGACTC
6198	Yes	AACAGGAGATCCTGGGTTCGACTCC
6199	Yes	AACAGGAGATCCTGGGTTCGACTCCC
6200	Yes	AACAGGAGATCCTGGGTTCGACTCCCA
6201	Yes	AACAGGAGATCCTGGGTTCGACTCCCAG
6202	Yes	AACAGGAGATCCTGGGTTCGACTCCCAGC
6203	Yes	AACAGGAGATCCTGGGTTCGACTCCCAGCG
6204	Yes	AACAGGAGATCCTGGGTTCGACTCCCAGCGG
6205	Yes	AACAGGAGATCCTGGGTTCGACTCCCAGCGGG
6206	Yes	AACAGGAGATCCTGGGTTCGACTCCCAGCGGGG
6207	Yes	AACAGGAGATCCTGGGTTCGACTCCCAGCGGGGC
6208	Yes	AACAGGAGATCCTGGGTTCGACTCCCAGCGGGGCC
6209	Yes	AACAGGAGATCCTGGGTTCGACTCCCAGCGGGGCCT

6210	Yes	AACAGGAGATCCTGGGTTCGACTCCCAGCGGGGCCTC
6211	Yes	AACAGGAGATCCTGGGTTCGACTCCCAGCGGGGCCTCC
6212	Yes	AACAGGAGATCCTGGGTTCGACTCCCAGCGGGGCCTCCA
6213	Yes	AACAGGAGATCCTGGGTTCGACTCCCAGT
6214	Yes	AACAGGAGATCCTGGGTTCGACTCCCAGTG
6215	Yes	AACAGGAGATCCTGGGTTCGACTCCCAGTGG
6216	Yes	AACAGGAGATCCTGGGTTCGACTCCCAGTGGG
6217	Yes	AACAGGAGATCCTGGGTTCGACTCCCAGTGGGG
6218	Yes	AACAGGAGATCCTGGGTTCGACTCCCAGTGGGGC
6219	Yes	AACAGGAGATCCTGGGTTCGACTCCCAGTGGGGCC
6220	Yes	AACAGGAGATCCTGGGTTCGACTCCCAGTGGGGCCT
6221	Yes	AACAGGAGATCCTGGGTTCGACTCCCAGTGGGGCCTC
6222	Yes	AACAGGAGATCCTGGGTTCGACTCCCAGTGGGGCCTCC
6223	Yes	AACAGGAGATCCTGGGTTCGACTCCCAGTGGGGCCTCCA
6224	No	AACATCAGACTTTTAA
6225	No	AACATCAGACTTTTAAT
6226	No	AACATCAGACTTTTAATC
6227	No	AACATCAGACTTTTAATCT
6228	No	AACATCAGACTTTTAATCTG
6229	No	AACATCAGACTTTTAATCTGA
6230	Yes	AACATCAGACTTTTAATCTGAC
6231	Yes	AACATCAGACTTTTAATCTGACG
6232	Yes	AACATCAGACTTTTAATCTGACGG
6233	Yes	AACATCAGACTTTTAATCTGACGGT
6234	Yes	AACATCAGACTTTTAATCTGACGGTG
6235	Yes	AACATCAGACTTTTAATCTGACGGTGC
6236	Yes	AACATCAGACTTTTAATCTGACGGTGCA
6237	Yes	AACATCAGACTTTTAATCTGACGGTGCAG
6238	Yes	AACATCAGACTTTTAATCTGACGGTGCAGG
6239	Yes	AACATCAGACTTTTAATCTGACGGTGCAGGG
6240	Yes	AACATCAGACTTTTAATCTGACGGTGCAGGGT
6241	Yes	AACATCAGACTTTTAATCTGACGGTGCAGGGTT
6242	Yes	AACATCAGACTTTTAATCTGACGGTGCAGGGTTC
6243	Yes	AACATCAGACTTTTAATCTGACGGTGCAGGGTTCA
6244	Yes	AACATCAGACTTTTAATCTGACGGTGCAGGGTTCAA
6245	Yes	AACATCAGACTTTTAATCTGACGGTGCAGGGTTCAAG
6246	Yes	AACATCAGACTTTTAATCTGACGGTGCAGGGTTCAAGT
6247	Yes	AACATCAGACTTTTAATCTGACGGTGCAGGGTTCAAGTC
6248	Yes	AACATCAGACTTTTAATCTGACGGTGCAGGGTTCAAGTCC
6249	Yes	AACATCAGACTTTTAATCTGACGGTGCAGGGTTCAAGTCCC
6250	Yes	AACATCAGACTTTTAATCTGACGGTGCAGGGTTCAAGTCCCT
6251	Yes	AACATCAGACTTTTAATCTGACGGTGCAGGGTTCAAGTCCCTG

6252	Yes	AACATCAGACTTTTAATCTGACGGTGCAGGGTTCAAGTCCCTGT
6253	Yes	AACATCAGACTTTTAATCTGACGGTGCAGGGTTCAAGTCCCTGTT
6254	Yes	AACATCAGACTTTTAATCTGACGGTGCAGGGTTCAAGTCCCTGTTC
6255	Yes	AACATCAGACTTTTAATCTGACGGTGCAGGGTTCAAGTCCCTGTTCA
6256	Yes	AACATCAGACTTTTAATCTGACGGTGCAGGGTTCAAGTCCCTGTTCAG
6257	Yes	AACATCAGACTTTTAATCTGACGGTGCAGGGTTCAAGTCCCTGTTCAGG
6258	Yes	AACATCAGACTTTTAATCTGACGGTGCAGGGTTCAAGTCCCTGTTCAGGC
6259	Yes	AACATCAGACTTTTAATCTGAG
6260	Yes	AACATCAGACTTTTAATCTGAGG
6261	Yes	AACATCAGACTTTTAATCTGAGGG
6262	Yes	AACATCAGACTTTTAATCTGAGGGT
6263	Yes	AACATCAGACTTTTAATCTGAGGGTC
6264	Yes	AACATCAGACTTTTAATCTGAGGGTCT
6265	Yes	AACATCAGACTTTTAATCTGAGGGTCTA
6266	Yes	AACATCAGACTTTTAATCTGAGGGTCTAG
6267	Yes	AACATCAGACTTTTAATCTGAGGGTCTAGG
6268	Yes	AACATCAGACTTTTAATCTGAGGGTCTAGGG
6269	Yes	AACATCAGACTTTTAATCTGAGGGTCTAGGGT
6270	Yes	AACATCAGACTTTTAATCTGAGGGTCTAGGGTT
6271	Yes	AACATCAGACTTTTAATCTGAGGGTCTAGGGTTC
6272	Yes	AACATCAGACTTTTAATCTGAGGGTCTAGGGTTCA
6273	Yes	AACATCAGACTTTTAATCTGAGGGTCTAGGGTTCAA
6274	Yes	AACATCAGACTTTTAATCTGAGGGTCTAGGGTTCAAG
6275	Yes	AACATCAGACTTTTAATCTGAGGGTCTAGGGTTCAAGT
6276	Yes	AACATCAGACTTTTAATCTGAGGGTCTAGGGTTCAAGTC
6277	Yes	AACATCAGACTTTTAATCTGAGGGTCTAGGGTTCAAGTCC
6278	Yes	AACATCAGACTTTTAATCTGAGGGTCTAGGGTTCAAGTCCC
6279	Yes	AACATCAGACTTTTAATCTGAGGGTCTAGGGTTCAAGTCCCT
6280	Yes	AACATCAGACTTTTAATCTGAGGGTCTAGGGTTCAAGTCCCTG
6281	Yes	AACATCAGACTTTTAATCTGAGGGTCTAGGGTTCAAGTCCCTGT
6282	Yes	AACATCAGACTTTTAATCTGAGGGTCTAGGGTTCAAGTCCCTGTC
6283	Yes	AACATCAGACTTTTAATCTGAGGGTCTAGGGTTCAAGTCCCTGTCC
6284	Yes	AACATCAGACTTTTAATCTGAGGGTCTAGGGTTCAAGTCCCTGTCCA
6285	Yes	AACATCAGACTTTTAATCTGAGGGTCTAGGGTTCAAGTCCCTGTCCAG
6286	Yes	AACATCAGACTTTTAATCTGAGGGTCTAGGGTTCAAGTCCCTGTCCAGG
6287	Yes	AACATCAGACTTTTAATCTGAGGGTCTAGGGTTCAAGTCCCTGTCCAGGC
6288	No	AACATCAGATTGTGAA
6289	No	AACATCAGATTGTGAAT
6290	No	AACATCAGATTGTGAATC
6291	No	AACATCAGATTGTGAATCT
6292	No	AACATCAGATTGTGAATCTG
6293	No	AACATCAGATTGTGAATCTGA

6294	No	AACATCAGATTGTGAATCTGAC
6295	No	AACATCAGATTGTGAATCTGACA
6296	No	AACATCAGATTGTGAATCTGACAA
6297	No	AACATCAGATTGTGAATCTGACAAC
6298	No	AACATCAGATTGTGAATCTGACAACA
6299	No	AACATCAGATTGTGAATCTGACAACAG
6300	No	AACATCAGATTGTGAATCTGACAACAGA
6301	No	AACATCAGATTGTGAATCTGACAACAGAG
6302	No	AACATCAGATTGTGAATCTGACAACAGAGG
6303	No	AACATCAGATTGTGAATCTGACAACAGAGGC
6304	No	AACATCAGATTGTGAATCTGACAACAGAGGCT
6305	Yes	AACATCAGATTGTGAATCTGACAACAGAGGCTT
6306	Yes	AACATCAGATTGTGAATCTGACAACAGAGGCTTA
6307	Yes	AACATCAGATTGTGAATCTGACAACAGAGGCTTAC
6308	Yes	AACATCAGATTGTGAATCTGACAACAGAGGCTTACG
6309	Yes	AACATCAGATTGTGAATCTGACAACAGAGGCTTACGA
6310	Yes	AACATCAGATTGTGAATCTGACAACAGAGGCTTACGAC
6311	Yes	AACATCAGATTGTGAATCTGACAACAGAGGCTTACGACC
6312	Yes	AACATCAGATTGTGAATCTGACAACAGAGGCTTACGACCC
6313	Yes	AACATCAGATTGTGAATCTGACAACAGAGGCTTACGACCCC
6314	Yes	AACATCAGATTGTGAATCTGACAACAGAGGCTTACGACCCCT
6315	Yes	AACATCAGATTGTGAATCTGACAACAGAGGCTTACGACCCCTT
6316	Yes	AACATCAGATTGTGAATCTGACAACAGAGGCTTACGACCCCTTA
6317	Yes	AACATCAGATTGTGAATCTGACAACAGAGGCTTACGACCCCTTAT
6318	Yes	AACATCAGATTGTGAATCTGACAACAGAGGCTTACGACCCCTTATT
6319	Yes	AACATCAGATTGTGAATCTGACAACAGAGGCTTACGACCCCTTATTT
6320	Yes	AACATCAGATTGTGAATCTGACAACAGAGGCTTACGACCCCTTATTTA
6321	Yes	AACATCAGATTGTGAATCTGACAACAGAGGCTTACGACCCCTTATTTAC
6322	Yes	AACATCAGATTGTGAATCTGACAACAGAGGCTTACGACCCCTTATTTACC
6323	No	AACATGGCTTTCTCAC
6324	Yes	AACATGGCTTTCTCACC
6325	Yes	AACATGGCTTTCTCACCA
6326	Yes	AACATGGTGCTAATAA
6327	Yes	AACATGGTGCTAATAAG
6328	Yes	AACATGGTGCTAATAAGG
6329	Yes	AACATGGTGCTAATAAGGC
6330	Yes	AACATGGTGCTAATAAGGCC
6331	Yes	AACATGGTGCTAATAAGGCCA
6332	Yes	AACATGGTGCTAATAAGGCCAC
6333	Yes	AACATGGTGCTAATAAGGCCACG
6334	Yes	AACATGGTGCTAATAAGGCCACGG
6335	Yes	AACATGGTGCTAATAAGGCCACGGT

6336	Yes	AACATGGTGCTAATAAGGCCACGGTC
6337	Yes	AACATGGTGCTAATAAGGCCACGGTCA
6338	Yes	AACATGGTGCTAATAAGGCCACGGTCAG
6339	Yes	AACATGGTGCTAATAAGGCCACGGTCAGG
6340	Yes	AACATGGTGCTAATAAGGCCACGGTCAGGG
6341	Yes	AACATGGTGCTAATAAGGCCACGGTCAGGGG
6342	Yes	AACATGGTGCTAATAAGGCCACGGTCAGGGGT
6343	Yes	AACATGGTGCTAATAAGGCCACGGTCAGGGGTT
6344	Yes	AACATGGTGCTAATAAGGCCACGGTCAGGGGTTC
6345	Yes	AACATGGTGCTAATAAGGCCACGGTCAGGGGTTCA
6346	Yes	AACATGGTGCTAATAAGGCCACGGTCAGGGGTTCAA
6347	Yes	AACATGGTGCTAATAAGGCCACGGTCAGGGGTTCAAT
6348	Yes	AACATGGTGCTAATAAGGCCACGGTCAGGGGTTCAATT
6349	Yes	AACATGGTGCTAATAAGGCCACGGTCAGGGGTTCAATTC
6350	Yes	AACATGGTGCTAATAAGGCCACGGTCAGGGGTTCAATTCC
6351	Yes	AACATGGTGCTAATAAGGCCACGGTCAGGGGTTCAATTCCC
6352	Yes	AACATGGTGCTAATAAGGCCACGGTCAGGGGTTCAATTCCCT
6353	Yes	AACATGGTGCTAATAAGGCCACGGTCAGGGGTTCAATTCCCTT
6354	Yes	AACATGGTGCTAATAAGGCCACGGTCAGGGGTTCAATTCCCTTA
6355	Yes	AACATGGTGCTAATAAGGCCACGGTCAGGGGTTCAATTCCCTTAT
6356	Yes	AACATGGTGCTAATAAGGCCACGGTCAGGGGTTCAATTCCCTTATG
6357	Yes	AACATGGTGCTAATAAGGCCACGGTCAGGGGTTCAATTCCCTTATGG
6358	Yes	AACATGGTGCTAATAAGGCCACGGTCAGGGGTTCAATTCCCTTATGGG
6359	Yes	AACATGGTGCTAATAAGGCCACGGTCAGGGGTTCAATTCCCTTATGGGC
6360	Yes	AACATGGTGCTAATAAGGCCACGGTCAGGGGTTCAATTCCCTTATGGGCT
6361	No	AACATTCAAAAAAGAG
6362	No	AACATTCAAAAAAGAGT
6363	Yes	AACATTCAAAAAAGAGTA
6364	Yes	AACATTCAAAAAAGAGTAC
6365	Yes	AACATTCAAAAAAGAGTACC
6366	Yes	AACATTCAAAAAAGAGTACCA
6367	Yes	AACATTCATGAGGCTC
6368	Yes	AACATTCATGAGGCTCT
6369	Yes	AACATTCATGAGGCTCTG
6370	Yes	AACATTCATGAGGCTCTGG
6371	Yes	AACATTCATGAGGCTCTGGG
6372	Yes	AACATTCATGAGGCTCTGGGT
6373	Yes	AACATTCATGAGGCTCTGGGTT
6374	Yes	AACATTCATGAGGCTCTGGGTTC
6375	Yes	AACATTCATGAGGCTCTGGGTTCG
6376	Yes	AACATTCATGAGGCTCTGGGTTCGA
6377	Yes	AACATTCATGAGGCTCTGGGTTCGAT

6378	Yes	AACATTCATGAGGCTCTGGGTTCGATC
6379	Yes	AACATTCATGAGGCTCTGGGTTCGATCC
6380	Yes	AACATTCATGAGGCTCTGGGTTCGATCCC
6381	Yes	AACATTCATGAGGCTCTGGGTTCGATCCCC
6382	Yes	AACATTCATGAGGCTCTGGGTTCGATCCCCA
6383	Yes	AACATTCATGAGGCTCTGGGTTCGATCCCCAG
6384	Yes	AACATTCATGAGGCTCTGGGTTCGATCCCCAGC
6385	Yes	AACATTCATGAGGCTCTGGGTTCGATCCCCAGCA
6386	Yes	AACATTCATGAGGCTCTGGGTTCGATCCCCAGCAC
6387	Yes	AACATTCATGAGGCTCTGGGTTCGATCCCCAGCACT
6388	Yes	AACATTCATGAGGCTCTGGGTTCGATCCCCAGCACTT
6389	Yes	AACATTCATGAGGCTCTGGGTTCGATCCCCAGCACTTC
6390	Yes	AACATTCATGAGGCTCTGGGTTCGATCCCCAGCACTTCC
6391	Yes	AACATTCATGAGGCTCTGGGTTCGATCCCCAGCACTTCCA
6392	Yes	AACATTCATGAGGCTCTGGGTTCGATCCCCAGCACTTCCAC
6393	Yes	AACATTCATGAGGCTCTGGGTTCGATCCCCAGCACTTCCACC
6394	Yes	AACATTCATGAGGCTCTGGGTTCGATCCCCAGCACTTCCACCA
6395	No	AACCAAAACATCAGAT
6396	No	AACCAAAACATCAGATT
6397	No	AACCAAAACATCAGATTG
6398	No	AACCAAAACATCAGATTGT
6399	No	AACCAAAACATCAGATTGTG
6400	No	AACCAAAACATCAGATTGTGA
6401	No	AACCAAAACATCAGATTGTGAA
6402	No	AACCAAAACATCAGATTGTGAAT
6403	No	AACCAAAACATCAGATTGTGAATC
6404	No	AACCAAAACATCAGATTGTGAATCT
6405	No	AACCAAAACATCAGATTGTGAATCTG
6406	No	AACCAAAACATCAGATTGTGAATCTGA
6407	No	AACCAAAACATCAGATTGTGAATCTGAC
6408	No	AACCAAAACATCAGATTGTGAATCTGACA
6409	No	AACCAAAACATCAGATTGTGAATCTGACAA
6410	No	AACCAAAACATCAGATTGTGAATCTGACAAC
6411	No	AACCAAAACATCAGATTGTGAATCTGACAACA
6412	No	AACCAAAACATCAGATTGTGAATCTGACAACAG
6413	No	AACCAAAACATCAGATTGTGAATCTGACAACAGA
6414	No	AACCAAAACATCAGATTGTGAATCTGACAACAGAG
6415	No	AACCAAAACATCAGATTGTGAATCTGACAACAGAGG
6416	No	AACCAAAACATCAGATTGTGAATCTGACAACAGAGGC
6417	No	AACCAAAACATCAGATTGTGAATCTGACAACAGAGGCT
6418	Yes	AACCAAAACATCAGATTGTGAATCTGACAACAGAGGCTT
6419	Yes	AACCAAAACATCAGATTGTGAATCTGACAACAGAGGCTTA

6420	Yes	AACCAAAACATCAGATTGTGAATCTGACAACAGAGGCTTAC
6421	Yes	AACCAAAACATCAGATTGTGAATCTGACAACAGAGGCTTACG
6422	Yes	AACCAAAACATCAGATTGTGAATCTGACAACAGAGGCTTACGA
6423	Yes	AACCAAAACATCAGATTGTGAATCTGACAACAGAGGCTTACGAC
6424	Yes	AACCAAAACATCAGATTGTGAATCTGACAACAGAGGCTTACGACC
6425	Yes	AACCAAAACATCAGATTGTGAATCTGACAACAGAGGCTTACGACCC
6426	Yes	AACCAAAACATCAGATTGTGAATCTGACAACAGAGGCTTACGACCCC
6427	Yes	AACCAAAACATCAGATTGTGAATCTGACAACAGAGGCTTACGACCCCT
6428	Yes	AACCAAAACATCAGATTGTGAATCTGACAACAGAGGCTTACGACCCCTT
6429	Yes	AACCAAAACATCAGATTGTGAATCTGACAACAGAGGCTTACGACCCCTTA
6430	No	AACCAACACCTCTTTA
6431	No	AACCAACACCTCTTTAC
6432	No	AACCAACACCTCTTTACA
6433	No	AACCAACACCTCTTTACAG
6434	No	AACCAACACCTCTTTACAGT
6435	No	AACCAACACCTCTTTACAGTG
6436	No	AACCAACACCTCTTTACAGTGA
6437	Yes	AACCAACACCTCTTTACAGTGAC
6438	Yes	AACCAACACCTCTTTACAGTGACC
6439	Yes	AACCAACACCTCTTTACAGTGACCA
6440	No	AACCACACTGCCTAGG
6441	Yes	AACCACACTGCCTAGGT
6442	Yes	AACCACACTGCCTAGGTT
6443	Yes	AACCACACTGCCTAGGTTC
6444	Yes	AACCACACTGCCTAGGTTCA
6445	Yes	AACCACACTGCCTAGGTTCAA
6446	Yes	AACCACACTGCCTAGGTTCAAA
6447	Yes	AACCACACTGCCTAGGTTCAAAT
6448	Yes	AACCACACTGCCTAGGTTCAAATC
6449	Yes	AACCACACTGCCTAGGTTCAAATCC
6450	Yes	AACCACACTGCCTAGGTTCAAATCCT
6451	Yes	AACCACACTGCCTAGGTTCAAATCCTG
6452	Yes	AACCACACTGCCTAGGTTCAAATCCTGA
6453	Yes	AACCACACTGCCTAGGTTCAAATCCTGAC
6454	Yes	AACCACACTGCCTAGGTTCAAATCCTGACT
6455	Yes	AACCACACTGCCTAGGTTCAAATCCTGACTC
6456	Yes	AACCACACTGCCTAGGTTCAAATCCTGACTCT
6457	Yes	AACCACACTGCCTAGGTTCAAATCCTGACTCTG
6458	Yes	AACCACACTGCCTAGGTTCAAATCCTGACTCTGT
6459	Yes	AACCACACTGCCTAGGTTCAAATCCTGACTCTGTC
6460	Yes	AACCACACTGCCTAGGTTCAAATCCTGACTCTGTCA
6461	Yes	AACCACACTGCCTAGGTTCAAATCCTGACTCTGTCAC

6462	Yes	AACCACACTGCCTAGGTTCAAATCCTGACTCTGTCACC
6463	Yes	AACCACACTGCCTAGGTTCAAATCCTGACTCTGTCACCA
6464	No	AACCACGTGGTCTGGG
6465	Yes	AACCACGTGGTCTGGGT
6466	Yes	AACCACGTGGTCTGGGTT
6467	Yes	AACCACGTGGTCTGGGTTT
6468	Yes	AACCACGTGGTCTGGGTTTA
6469	Yes	AACCACGTGGTCTGGGTTTAA
6470	Yes	AACCACGTGGTCTGGGTTTAAG
6471	Yes	AACCACGTGGTCTGGGTTTAAGT
6472	Yes	AACCACGTGGTCTGGGTTTAAGTC
6473	Yes	AACCACGTGGTCTGGGTTTAAGTCT
6474	Yes	AACCACGTGGTCTGGGTTTAAGTCTC
6475	Yes	AACCACGTGGTCTGGGTTTAAGTCTCA
6476	Yes	AACCACGTGGTCTGGGTTTAAGTCTCAG
6477	Yes	AACCACGTGGTCTGGGTTTAAGTCTCAGC
6478	Yes	AACCACGTGGTCTGGGTTTAAGTCTCAGCT
6479	Yes	AACCACGTGGTCTGGGTTTAAGTCTCAGCTG
6480	Yes	AACCACGTGGTCTGGGTTTAAGTCTCAGCTGT
6481	Yes	AACCACGTGGTCTGGGTTTAAGTCTCAGCTGTG
6482	Yes	AACCACGTGGTCTGGGTTTAAGTCTCAGCTGTGC
6483	Yes	AACCACGTGGTCTGGGTTTAAGTCTCAGCTGTGCT
6484	Yes	AACCACGTGGTCTGGGTTTAAGTCTCAGCTGTGCTA
6485	Yes	AACCACGTGGTCTGGGTTTAAGTCTCAGCTGTGCTAC
6486	Yes	AACCACGTGGTCTGGGTTTAAGTCTCAGCTGTGCTACC
6487	Yes	AACCACGTGGTCTGGGTTTAAGTCTCAGCTGTGCTACCA
6488	Yes	AACCAGAATGGCCGAG
6489	Yes	AACCAGAATGGCCGAGT
6490	Yes	AACCAGAATGGCCGAGTG
6491	Yes	AACCAGAATGGCCGAGTGG
6492	Yes	AACCAGAATGGCCGAGTGGT
6493	Yes	AACCAGAATGGCCGAGTGGTT
6494	Yes	AACCAGAATGGCCGAGTGGTTA
6495	Yes	AACCAGAATGGCCGAGTGGTTAA
6496	Yes	AACCAGAATGGCCGAGTGGTTAAG
6497	Yes	AACCAGAATGGCCGAGTGGTTAAGG
6498	Yes	AACCAGAATGGCCGAGTGGTTAAGGC
6499	Yes	AACCAGAATGGCCGAGTGGTTAAGGCG
6500	Yes	AACCAGAATGGCCGAGTGGTTAAGGCGT
6501	Yes	AACCAGAATGGCCGAGTGGTTAAGGCGTT
6502	Yes	AACCAGAATGGCCGAGTGGTTAAGGCGTTG
6503	Yes	AACCAGAATGGCCGAGTGGTTAAGGCGTTGG

6504	Yes	AACCAGAATGGCCGAGTGGTTAAGGCGTTGGA
6505	Yes	AACCAGAATGGCCGAGTGGTTAAGGCGTTGGAC
6506	Yes	AACCAGAATGGCCGAGTGGTTAAGGCGTTGGACT
6507	Yes	AACCAGAATGGCCGAGTGGTTAAGGCGTTGGACTT
6508	Yes	AACCAGAATGGCCGAGTGGTTAAGGCGTTGGACTTA
6509	Yes	AACCAGAATGGCCGAGTGGTTAAGGCGTTGGACTTAA
6510	Yes	AACCAGAATGGCCGAGTGGTTAAGGCGTTGGACTTAAG
6511	Yes	AACCAGAATGGCCGAGTGGTTAAGGCGTTGGACTTAAGA
6512	Yes	AACCAGAATGGCCGAGTGGTTAAGGCGTTGGACTTAAGAT
6513	Yes	AACCAGAATGGCCGAGTGGTTAAGGCGTTGGACTTAAGATC
6514	Yes	AACCAGAATGGCCGAGTGGTTAAGGCGTTGGACTTAAGATCC
6515	Yes	AACCAGAATGGCCGAGTGGTTAAGGCGTTGGACTTAAGATCCA
6516	Yes	AACCAGAATGGCCGAGTGGTTAAGGCGTTGGACTTAAGATCCAA
6517	Yes	AACCAGAATGGCCGAGTGGTTAAGGCGTTGGACTTAAGATCCAAT
6518	Yes	AACCAGAATGGCCGAGTGGTTAAGGCGTTGGACTTAAGATCCAATG
6519	Yes	AACCAGAATGGCCGAGTGGTTAAGGCGTTGGACTTAAGATCCAATGG
6520	Yes	AACCAGAATGGCCGAGTGGTTAAGGCGTTGGACTTAAGATCCAATGGA
6521	Yes	AACCAGAATGGCCGAGTGGTTAAGGCGTTGGACTTAAGATCCAATGGAT
6522	Yes	AACCAGAATGGCCGAGTGGTTAAGGCGTTGGACTTAAGATCCAATGGATT
6523	No	AACCAGAGTTCCAGTC
6524	No	AACCAGAGTTCCAGTCT
6525	Yes	AACCAGAGTTCCAGTCTC
6526	Yes	AACCAGAGTTCCAGTCTCA
6527	Yes	AACCAGAGTTCCAGTCTCAG
6528	Yes	AACCAGAGTTCCAGTCTCAGC
6529	Yes	AACCAGAGTTCCAGTCTCAGCG
6530	Yes	AACCAGAGTTCCAGTCTCAGCGT
6531	Yes	AACCAGAGTTCCAGTCTCAGCGTG
6532	Yes	AACCAGAGTTCCAGTCTCAGCGTGG
6533	Yes	AACCAGAGTTCCAGTCTCAGCGTGGA
6534	Yes	AACCAGAGTTCCAGTCTCAGCGTGGAC
6535	Yes	AACCAGAGTTCCAGTCTCAGCGTGGACC
6536	Yes	AACCAGAGTTCCAGTCTCAGCGTGGACCT
6537	Yes	AACCAGAGTTCCAGTCTCAGCGTGGACCTC
6538	Yes	AACCAGAGTTCCAGTCTCAGCGTGGACCTCC
6539	Yes	AACCAGAGTTCCAGTCTCAGCGTGGACCTCCA
6540	No	AACCAGCATGTCTCAG
6541	No	AACCAGCATGTCTCAGT
6542	Yes	AACCAGCATGTCTCAGTC
6543	Yes	AACCAGCATGTCTCAGTCG
6544	Yes	AACCAGCATGTCTCAGTCGG
6545	Yes	AACCAGCATGTCTCAGTCGGT

6546	Yes	AACCAGCATGTCTCAGTCGGTA
6547	Yes	AACCAGCATGTCTCAGTCGGTAT
6548	Yes	AACCAGCATGTCTCAGTCGGTATA
6549	Yes	AACCAGCATGTCTCAGTCGGTATAG
6550	Yes	AACCAGCATGTCTCAGTCGGTATAGT
6551	Yes	AACCAGCATGTCTCAGTCGGTATAGTG
6552	Yes	AACCAGCATGTCTCAGTCGGTATAGTGT
6553	Yes	AACCAGCATGTCTCAGTCGGTATAGTGTG
6554	Yes	AACCAGCATGTCTCAGTCGGTATAGTGTGA
6555	Yes	AACCAGCATGTCTCAGTCGGTATAGTGTGAG
6556	Yes	AACCAGCATGTCTCAGTCGGTATAGTGTGAGA
6557	Yes	AACCAGCATGTCTCAGTCGGTATAGTGTGAGAC
6558	Yes	AACCAGCATGTCTCAGTCGGTATAGTGTGAGACT
6559	Yes	AACCAGCATGTCTCAGTCGGTATAGTGTGAGACTC
6560	Yes	AACCAGCATGTCTCAGTCGGTATAGTGTGAGACTCT
6561	Yes	AACCAGCATGTCTCAGTCGGTATAGTGTGAGACTCTT
6562	Yes	AACCAGCATGTCTCAGTCGGTATAGTGTGAGACTCTTA
6563	Yes	AACCAGCATGTCTCAGTCGGTATAGTGTGAGACTCTTAA
6564	Yes	AACCAGCATGTCTCAGTCGGTATAGTGTGAGACTCTTAAT
6565	Yes	AACCAGCATGTCTCAGTCGGTATAGTGTGAGACTCTTAATC
6566	Yes	AACCAGCATGTCTCAGTCGGTATAGTGTGAGACTCTTAATCT
6567	Yes	AACCAGCATGTCTCAGTCGGTATAGTGTGAGACTCTTAATCTC
6568	Yes	AACCAGCATGTCTCAGTCGGTATAGTGTGAGACTCTTAATCTCA
6569	Yes	AACCAGCATGTCTCAGTCGGTATAGTGTGAGACTCTTAATCTCAG
6570	Yes	AACCAGCATGTCTCAGTCGGTATAGTGTGAGACTCTTAATCTCAGG
6571	Yes	AACCAGCATGTCTCAGTCGGTATAGTGTGAGACTCTTAATCTCAGGG
6572	Yes	AACCAGCATGTCTCAGTCGGTATAGTGTGAGACTCTTAATCTCAGGGT
6573	Yes	AACCAGCATGTCTCAGTCGGTATAGTGTGAGACTCTTAATCTCAGGGTC
6574	Yes	AACCAGCATGTCTCAGTCGGTATAGTGTGAGACTCTTAATCTCAGGGTCG
6575	No	AACCAGCCTTAGGAGG
6576	No	AACCAGCCTTAGGAGGT
6577	Yes	AACCAGCCTTAGGAGGTT
6578	Yes	AACCAGCCTTAGGAGGTTC
6579	Yes	AACCAGCCTTAGGAGGTTCA
6580	Yes	AACCAGCCTTAGGAGGTTCAA
6581	Yes	AACCAGCCTTAGGAGGTTCAAT
6582	Yes	AACCAGCCTTAGGAGGTTCAATT
6583	Yes	AACCAGCCTTAGGAGGTTCAATTC
6584	Yes	AACCAGCCTTAGGAGGTTCAATTCC
6585	Yes	AACCAGCCTTAGGAGGTTCAATTCCT
6586	Yes	AACCAGCCTTAGGAGGTTCAATTCCTT
6587	Yes	AACCAGCCTTAGGAGGTTCAATTCCTTC

6588	Yes	AACCAGCCTTAGGAGGTTCAATTCCTTCC
6589	Yes	AACCAGCCTTAGGAGGTTCAATTCCTTCCT
6590	Yes	AACCAGCCTTAGGAGGTTCAATTCCTTCCTT
6591	Yes	AACCAGCCTTAGGAGGTTCAATTCCTTCCTTT
6592	Yes	AACCAGCCTTAGGAGGTTCAATTCCTTCCTTTT
6593	Yes	AACCAGCCTTAGGAGGTTCAATTCCTTCCTTTTT
6594	Yes	AACCAGCCTTAGGAGGTTCAATTCCTTCCTTTTTT
6595	Yes	AACCAGCCTTAGGAGGTTCAATTCCTTCCTTTTTTG
6596	Yes	AACCAGCCTTAGGAGGTTCAATTCCTTCCTTTTTTGC
6597	Yes	AACCAGCCTTAGGAGGTTCAATTCCTTCCTTTTTTGCC
6598	Yes	AACCAGCCTTAGGAGGTTCAATTCCTTCCTTTTTTGCCA
6599	No	AACCAGCTTTGGGGGG
6600	Yes	AACCAGCTTTGGGGGGT
6601	Yes	AACCAGCTTTGGGGGGTT
6602	Yes	AACCAGCTTTGGGGGGTTC
6603	Yes	AACCAGCTTTGGGGGGTTCG
6604	Yes	AACCAGCTTTGGGGGGTTCGA
6605	Yes	AACCAGCTTTGGGGGGTTCGAT
6606	Yes	AACCAGCTTTGGGGGGTTCGATT
6607	Yes	AACCAGCTTTGGGGGGTTCGATTC
6608	Yes	AACCAGCTTTGGGGGGTTCGATTCC
6609	Yes	AACCAGCTTTGGGGGGTTCGATTCCT
6610	Yes	AACCAGCTTTGGGGGGTTCGATTCCTT
6611	Yes	AACCAGCTTTGGGGGGTTCGATTCCTTC
6612	Yes	AACCAGCTTTGGGGGGTTCGATTCCTTCC
6613	Yes	AACCAGCTTTGGGGGGTTCGATTCCTTCCT
6614	Yes	AACCAGCTTTGGGGGGTTCGATTCCTTCCTT
6615	Yes	AACCAGCTTTGGGGGGTTCGATTCCTTCCTTT
6616	Yes	AACCAGCTTTGGGGGGTTCGATTCCTTCCTTTT
6617	Yes	AACCAGCTTTGGGGGGTTCGATTCCTTCCTTTTT
6618	Yes	AACCAGCTTTGGGGGGTTCGATTCCTTCCTTTTTT
6619	Yes	AACCAGCTTTGGGGGGTTCGATTCCTTCCTTTTTTG
6620	Yes	AACCAGCTTTGGGGGGTTCGATTCCTTCCTTTTTTGC
6621	Yes	AACCAGCTTTGGGGGGTTCGATTCCTTCCTTTTTTGCC
6622	Yes	AACCAGCTTTGGGGGGTTCGATTCCTTCCTTTTTTGCCA
6623	No	AACCAGGATGGCCGAG
6624	Yes	AACCAGGATGGCCGAGT
6625	Yes	AACCAGGATGGCCGAGTG
6626	Yes	AACCAGGATGGCCGAGTGG
6627	Yes	AACCAGGATGGCCGAGTGGT
6628	Yes	AACCAGGATGGCCGAGTGGTT
6629	Yes	AACCAGGATGGCCGAGTGGTTA

6630	Yes	AACCAGGATGGCCGAGTGGTTAA
6631	Yes	AACCAGGATGGCCGAGTGGTTAAG
6632	Yes	AACCAGGATGGCCGAGTGGTTAAGG
6633	Yes	AACCAGGATGGCCGAGTGGTTAAGGC
6634	Yes	AACCAGGATGGCCGAGTGGTTAAGGCG
6635	Yes	AACCAGGATGGCCGAGTGGTTAAGGCGT
6636	Yes	AACCAGGATGGCCGAGTGGTTAAGGCGTT
6637	Yes	AACCAGGATGGCCGAGTGGTTAAGGCGTTG
6638	Yes	AACCAGGATGGCCGAGTGGTTAAGGCGTTGG
6639	Yes	AACCAGGATGGCCGAGTGGTTAAGGCGTTGGA
6640	Yes	AACCAGGATGGCCGAGTGGTTAAGGCGTTGGAC
6641	Yes	AACCAGGATGGCCGAGTGGTTAAGGCGTTGGACT
6642	Yes	AACCAGGATGGCCGAGTGGTTAAGGCGTTGGACTT
6643	Yes	AACCAGGATGGCCGAGTGGTTAAGGCGTTGGACTTA
6644	Yes	AACCAGGATGGCCGAGTGGTTAAGGCGTTGGACTTAA
6645	Yes	AACCAGGATGGCCGAGTGGTTAAGGCGTTGGACTTAAG
6646	Yes	AACCAGGATGGCCGAGTGGTTAAGGCGTTGGACTTAAGA
6647	Yes	AACCAGGATGGCCGAGTGGTTAAGGCGTTGGACTTAAGAT
6648	Yes	AACCAGGATGGCCGAGTGGTTAAGGCGTTGGACTTAAGATC
6649	Yes	AACCAGGATGGCCGAGTGGTTAAGGCGTTGGACTTAAGATCC
6650	Yes	AACCAGGATGGCCGAGTGGTTAAGGCGTTGGACTTAAGATCCA
6651	Yes	AACCAGGATGGCCGAGTGGTTAAGGCGTTGGACTTAAGATCCAA
6652	Yes	AACCAGGATGGCCGAGTGGTTAAGGCGTTGGACTTAAGATCCAAT
6653	Yes	AACCAGGATGGCCGAGTGGTTAAGGCGTTGGACTTAAGATCCAATG
6654	Yes	AACCAGGATGGCCGAGTGGTTAAGGCGTTGGACTTAAGATCCAATGG
6655	Yes	AACCAGGATGGCCGAGTGGTTAAGGCGTTGGACTTAAGATCCAATGGA
6656	Yes	AACCAGGATGGCCGAGTGGTTAAGGCGTTGGACTTAAGATCCAATGGAC
6657	Yes	AACCAGGATGGCCGAGTGGTTAAGGCGTTGGACTTAAGATCCAATGGACA
6658	No	AACCAGGCAGAAACAC
6659	No	AACCAGGCAGAAACACC
6660	Yes	AACCAGGCAGAAACACCA
6661	No	AACCAGGCGGAAACAC
6662	No	AACCAGGCGGAAACACC
6663	No	AACCAGGCGGAAACACCA
6664	Yes	AACCAGGGGTCGCGAG
6665	Yes	AACCAGGGGTCGCGAGT
6666	Yes	AACCAGGGGTCGCGAGTT
6667	Yes	AACCAGGGGTCGCGAGTTC
6668	Yes	AACCAGGGGTCGCGAGTTCA
6669	Yes	AACCAGGGGTCGCGAGTTCAA
6670	Yes	AACCAGGGGTCGCGAGTTCAAA
6671	Yes	AACCAGGGGTCGCGAGTTCAAAT

6672	Yes	AACCAGGGGTCGCGAGTTCAAATC
6673	Yes	AACCAGGGGTCGCGAGTTCAAATCT
6674	Yes	AACCAGGGGTCGCGAGTTCAAATCTC
6675	Yes	AACCAGGGGTCGCGAGTTCAAATCTCG
6676	Yes	AACCAGGGGTCGCGAGTTCAAATCTCGC
6677	Yes	AACCAGGGGTCGCGAGTTCAAATCTCGCT
6678	Yes	AACCAGGGGTCGCGAGTTCAAATCTCGCTG
6679	Yes	AACCAGGGGTCGCGAGTTCAAATCTCGCTGG
6680	Yes	AACCAGGGGTCGCGAGTTCAAATCTCGCTGGG
6681	Yes	AACCAGGGGTCGCGAGTTCAAATCTCGCTGGGG
6682	Yes	AACCAGGGGTCGCGAGTTCAAATCTCGCTGGGGC
6683	Yes	AACCAGGGGTCGCGAGTTCAAATCTCGCTGGGGCC
6684	Yes	AACCAGGGGTCGCGAGTTCAAATCTCGCTGGGGCCT
6685	Yes	AACCAGGGGTCGCGAGTTCAAATCTCGCTGGGGCCTC
6686	Yes	AACCAGGGGTCGCGAGTTCAAATCTCGCTGGGGCCTCC
6687	Yes	AACCAGGGGTCGCGAGTTCAAATCTCGCTGGGGCCTCCA
6688	Yes	AACCAGGGGTCGCGAGTTCAAT
6689	Yes	AACCAGGGGTCGCGAGTTCAATT
6690	Yes	AACCAGGGGTCGCGAGTTCAATTC
6691	Yes	AACCAGGGGTCGCGAGTTCAATTCT
6692	Yes	AACCAGGGGTCGCGAGTTCAATTCTC
6693	Yes	AACCAGGGGTCGCGAGTTCAATTCTCG
6694	Yes	AACCAGGGGTCGCGAGTTCAATTCTCGC
6695	Yes	AACCAGGGGTCGCGAGTTCAATTCTCGCT
6696	Yes	AACCAGGGGTCGCGAGTTCAATTCTCGCTG
6697	Yes	AACCAGGGGTCGCGAGTTCAATTCTCGCTGG
6698	Yes	AACCAGGGGTCGCGAGTTCAATTCTCGCTGGG
6699	Yes	AACCAGGGGTCGCGAGTTCAATTCTCGCTGGGG
6700	Yes	AACCAGGGGTCGCGAGTTCAATTCTCGCTGGGGC
6701	Yes	AACCAGGGGTCGCGAGTTCAATTCTCGCTGGGGCC
6702	Yes	AACCAGGGGTCGCGAGTTCAATTCTCGCTGGGGCCT
6703	Yes	AACCAGGGGTCGCGAGTTCAATTCTCGCTGGGGCCTC
6704	Yes	AACCAGGGGTCGCGAGTTCAATTCTCGCTGGGGCCTCC
6705	Yes	AACCAGGGGTCGCGAGTTCAATTCTCGCTGGGGCCTCCA
6706	Yes	AACCAGGGGTCGCGAGTTCG
6707	Yes	AACCAGGGGTCGCGAGTTCGA
6708	Yes	AACCAGGGGTCGCGAGTTCGAT
6709	Yes	AACCAGGGGTCGCGAGTTCGATC
6710	Yes	AACCAGGGGTCGCGAGTTCGATCC
6711	Yes	AACCAGGGGTCGCGAGTTCGATCCT
6712	Yes	AACCAGGGGTCGCGAGTTCGATCCTC
6713	Yes	AACCAGGGGTCGCGAGTTCGATCCTCG

6714	Yes	AACCAGGGGTCGCGAGTTCGATCCTCGC
6715	Yes	AACCAGGGGTCGCGAGTTCGATCCTCGCT
6716	Yes	AACCAGGGGTCGCGAGTTCGATCCTCGCTG
6717	Yes	AACCAGGGGTCGCGAGTTCGATCCTCGCTGG
6718	Yes	AACCAGGGGTCGCGAGTTCGATCCTCGCTGGG
6719	Yes	AACCAGGGGTCGCGAGTTCGATCCTCGCTGGGG
6720	Yes	AACCAGGGGTCGCGAGTTCGATCCTCGCTGGGGC
6721	Yes	AACCAGGGGTCGCGAGTTCGATCCTCGCTGGGGCC
6722	Yes	AACCAGGGGTCGCGAGTTCGATCCTCGCTGGGGCCT
6723	Yes	AACCAGGGGTCGCGAGTTCGATCCTCGCTGGGGCCTC
6724	Yes	AACCAGGGGTCGCGAGTTCGATCCTCGCTGGGGCCTCC
6725	Yes	AACCAGGGGTCGCGAGTTCGATCCTCGCTGGGGCCTCCA
6726	Yes	AACCAGGGTCGCGAGT
6727	Yes	AACCAGGGTCGCGAGTT
6728	Yes	AACCAGGGTCGCGAGTTC
6729	Yes	AACCAGGGTCGCGAGTTCA
6730	Yes	AACCAGGGTCGCGAGTTCAA
6731	Yes	AACCAGGGTCGCGAGTTCAAA
6732	Yes	AACCAGGGTCGCGAGTTCAAAT
6733	Yes	AACCAGGGTCGCGAGTTCAAATC
6734	Yes	AACCAGGGTCGCGAGTTCAAATCT
6735	Yes	AACCAGGGTCGCGAGTTCAAATCTC
6736	Yes	AACCAGGGTCGCGAGTTCAAATCTCG
6737	Yes	AACCAGGGTCGCGAGTTCAAATCTCGC
6738	Yes	AACCAGGGTCGCGAGTTCAAATCTCGCT
6739	Yes	AACCAGGGTCGCGAGTTCAAATCTCGCTG
6740	Yes	AACCAGGGTCGCGAGTTCAAATCTCGCTGG
6741	Yes	AACCAGGGTCGCGAGTTCAAATCTCGCTGGG
6742	Yes	AACCAGGGTCGCGAGTTCAAATCTCGCTGGGG
6743	Yes	AACCAGGGTCGCGAGTTCAAATCTCGCTGGGGC
6744	Yes	AACCAGGGTCGCGAGTTCAAATCTCGCTGGGGCC
6745	Yes	AACCAGGGTCGCGAGTTCAAATCTCGCTGGGGCCT
6746	Yes	AACCAGGGTCGCGAGTTCAAATCTCGCTGGGGCCTC
6747	Yes	AACCAGGGTCGCGAGTTCAAATCTCGCTGGGGCCTCC
6748	Yes	AACCAGGGTCGCGAGTTCAAATCTCGCTGGGGCCTCCA
6749	No	AACCAGGTGGAAACAC
6750	No	AACCAGGTGGAAACACC
6751	Yes	AACCAGGTGGAAACACCA
6752	No	AACCAGTCTCTGGGGG
6753	Yes	AACCAGTCTCTGGGGGG
6754	Yes	AACCAGTCTCTGGGGGGT
6755	Yes	AACCAGTCTCTGGGGGGTT

6756	Yes	AACCAGTCTCTGGGGGGTTC
6757	Yes	AACCAGTCTCTGGGGGGTTCG
6758	Yes	AACCAGTCTCTGGGGGGTTCGA
6759	Yes	AACCAGTCTCTGGGGGGTTCGAT
6760	Yes	AACCAGTCTCTGGGGGGTTCGATT
6761	Yes	AACCAGTCTCTGGGGGGTTCGATTC
6762	Yes	AACCAGTCTCTGGGGGGTTCGATTCC
6763	Yes	AACCAGTCTCTGGGGGGTTCGATTCCC
6764	Yes	AACCAGTCTCTGGGGGGTTCGATTCCCT
6765	Yes	AACCAGTCTCTGGGGGGTTCGATTCCCTC
6766	Yes	AACCAGTCTCTGGGGGGTTCGATTCCCTCC
6767	Yes	AACCAGTCTCTGGGGGGTTCGATTCCCTCCT
6768	Yes	AACCAGTCTCTGGGGGGTTCGATTCCCTCCTT
6769	Yes	AACCAGTCTCTGGGGGGTTCGATTCCCTCCTTT
6770	Yes	AACCAGTCTCTGGGGGGTTCGATTCCCTCCTTTT
6771	Yes	AACCAGTCTCTGGGGGGTTCGATTCCCTCCTTTTT
6772	Yes	AACCAGTCTCTGGGGGGTTCGATTCCCTCCTTTTTC
6773	Yes	AACCAGTCTCTGGGGGGTTCGATTCCCTCCTTTTTCA
6774	Yes	AACCAGTCTCTGGGGGGTTCGATTCCCTCCTTTTTCAC
6775	Yes	AACCAGTCTCTGGGGGGTTCGATTCCCTCCTTTTTCACC
6776	Yes	AACCAGTCTCTGGGGGGTTCGATTCCCTCCTTTTTCACCA
6777	No	AACCAGTCTGTGGGGG
6778	Yes	AACCAGTCTGTGGGGGG
6779	Yes	AACCAGTCTGTGGGGGGT
6780	Yes	AACCAGTCTGTGGGGGGTT
6781	Yes	AACCAGTCTGTGGGGGGTTC
6782	Yes	AACCAGTCTGTGGGGGGTTCG
6783	Yes	AACCAGTCTGTGGGGGGTTCGA
6784	Yes	AACCAGTCTGTGGGGGGTTCGAT
6785	Yes	AACCAGTCTGTGGGGGGTTCGATT
6786	Yes	AACCAGTCTGTGGGGGGTTCGATTC
6787	Yes	AACCAGTCTGTGGGGGGTTCGATTCC
6788	Yes	AACCAGTCTGTGGGGGGTTCGATTCCC
6789	Yes	AACCAGTCTGTGGGGGGTTCGATTCCCT
6790	Yes	AACCAGTCTGTGGGGGGTTCGATTCCCTC
6791	Yes	AACCAGTCTGTGGGGGGTTCGATTCCCTCC
6792	Yes	AACCAGTCTGTGGGGGGTTCGATTCCCTCCT
6793	Yes	AACCAGTCTGTGGGGGGTTCGATTCCCTCCTT
6794	Yes	AACCAGTCTGTGGGGGGTTCGATTCCCTCCTTT
6795	Yes	AACCAGTCTGTGGGGGGTTCGATTCCCTCCTTTT
6796	Yes	AACCAGTCTGTGGGGGGTTCGATTCCCTCCTTTTT
6797	Yes	AACCAGTCTGTGGGGGGTTCGATTCCCTCCTTTTTC

6798	Yes	AACCAGTCTGTGGGGGGTTCGATTCCCTCCTTTTTCA
6799	Yes	AACCAGTCTGTGGGGGGTTCGATTCCCTCCTTTTTCAC
6800	Yes	AACCAGTCTGTGGGGGGTTCGATTCCCTCCTTTTTCACC
6801	Yes	AACCAGTCTGTGGGGGGTTCGATTCCCTCCTTTTTCACCA
6802	No	AACCATAAGGTTGGTG
6803	Yes	AACCATAAGGTTGGTGG
6804	Yes	AACCATAAGGTTGGTGGT
6805	Yes	AACCATAAGGTTGGTGGTT
6806	Yes	AACCATAAGGTTGGTGGTTA
6807	Yes	AACCATAAGGTTGGTGGTTAC
6808	Yes	AACCATAAGGTTGGTGGTTACA
6809	Yes	AACCATAAGGTTGGTGGTTACAG
6810	Yes	AACCATAAGGTTGGTGGTTACAGA
6811	Yes	AACCATAAGGTTGGTGGTTACAGAC
6812	Yes	AACCATAAGGTTGGTGGTTACAGACC
6813	Yes	AACCATAAGGTTGGTGGTTACAGACCA
6814	Yes	AACCATAAGGTTGGTGGTTACAGACCAC
6815	Yes	AACCATAAGGTTGGTGGTTACAGACCACC
6816	Yes	AACCATAAGGTTGGTGGTTACAGACCACCC
6817	Yes	AACCATAAGGTTGGTGGTTACAGACCACCCA
6818	Yes	AACCATAAGGTTGGTGGTTACAGACCACCCAG
6819	Yes	AACCATAAGGTTGGTGGTTACAGACCACCCAGG
6820	Yes	AACCATAAGGTTGGTGGTTACAGACCACCCAGGG
6821	Yes	AACCATAAGGTTGGTGGTTACAGACCACCCAGGGA
6822	Yes	AACCATAAGGTTGGTGGTTACAGACCACCCAGGGAC
6823	Yes	AACCATAAGGTTGGTGGTTACAGACCACCCAGGGACG
6824	Yes	AACCATAAGGTTGGTGGTTACAGACCACCCAGGGACGC
6825	Yes	AACCATAAGGTTGGTGGTTACAGACCACCCAGGGACGCC
6826	Yes	AACCATAAGGTTGGTGGTTACAGACCACCCAGGGACGCCA
6827	Yes	AACCATAAGGTTGGTGGTTAG
6828	Yes	AACCATAAGGTTGGTGGTTAGA
6829	Yes	AACCATAAGGTTGGTGGTTAGAG
6830	Yes	AACCATAAGGTTGGTGGTTAGAGA
6831	Yes	AACCATAAGGTTGGTGGTTAGAGAC
6832	Yes	AACCATAAGGTTGGTGGTTAGAGACC
6833	Yes	AACCATAAGGTTGGTGGTTAGAGACCA
6834	Yes	AACCATAAGGTTGGTGGTTAGAGACCAC
6835	Yes	AACCATAAGGTTGGTGGTTAGAGACCACC
6836	Yes	AACCATAAGGTTGGTGGTTAGAGACCACCC
6837	Yes	AACCATAAGGTTGGTGGTTAGAGACCACCCA
6838	Yes	AACCATAAGGTTGGTGGTTAGAGACCACCCAG
6839	Yes	AACCATAAGGTTGGTGGTTAGAGACCACCCAGG

6840	Yes	AACCATAAGGTTGGTGGTTAGAGACCACCCAGGG
6841	Yes	AACCATAAGGTTGGTGGTTAGAGACCACCCAGGGA
6842	Yes	AACCATAAGGTTGGTGGTTAGAGACCACCCAGGGAC
6843	Yes	AACCATAAGGTTGGTGGTTAGAGACCACCCAGGGACG
6844	Yes	AACCATAAGGTTGGTGGTTAGAGACCACCCAGGGACGC
6845	Yes	AACCATAAGGTTGGTGGTTAGAGACCACCCAGGGACGCC
6846	Yes	AACCATAAGGTTGGTGGTTAGAGACCACCCAGGGACGCCA
6847	No	AACCATCCTCTGCTAC
6848	No	AACCATCCTCTGCTACC
6849	Yes	AACCATCCTCTGCTACCA
6850	No	AACCATGGATTCTAGA
6851	No	AACCATGGATTCTAGAG
6852	No	AACCATGGATTCTAGAGC
6853	No	AACCATGGATTCTAGAGCC
6854	Yes	AACCATGGATTCTAGAGCCA
6855	Yes	AACCATGGATTCTAGAGCCAG
6856	Yes	AACCATGGATTCTAGAGCCAGA
6857	Yes	AACCATGGATTCTAGAGCCAGAT
6858	Yes	AACCATGGATTCTAGAGCCAGATG
6859	Yes	AACCATGGATTCTAGAGCCAGATGG
6860	Yes	AACCATGGATTCTAGAGCCAGATGGG
6861	Yes	AACCATGGATTCTAGAGCCAGATGGGT
6862	Yes	AACCATGGATTCTAGAGCCAGATGGGTT
6863	Yes	AACCATGGATTCTAGAGCCAGATGGGTTC
6864	Yes	AACCATGGATTCTAGAGCCAGATGGGTTCA
6865	Yes	AACCATGGATTCTAGAGCCAGATGGGTTCAA
6866	Yes	AACCATGGATTCTAGAGCCAGATGGGTTCAAA
6867	Yes	AACCATGGATTCTAGAGCCAGATGGGTTCAAAT
6868	Yes	AACCATGGATTCTAGAGCCAGATGGGTTCAAATC
6869	Yes	AACCATGGATTCTAGAGCCAGATGGGTTCAAATCC
6870	Yes	AACCATGGATTCTAGAGCCAGATGGGTTCAAATCCC
6871	Yes	AACCATGGATTCTAGAGCCAGATGGGTTCAAATCCCG
6872	Yes	AACCATGGATTCTAGAGCCAGATGGGTTCAAATCCCGG
6873	Yes	AACCATGGATTCTAGAGCCAGATGGGTTCAAATCCCGGC
6874	Yes	AACCATGGATTCTAGAGCCAGATGGGTTCAAATCCCGGCT
6875	Yes	AACCATGGATTCTAGAGCCAGATGGGTTCAAATCCCGGCTC
6876	Yes	AACCATGGATTCTAGAGCCAGATGGGTTCAAATCCCGGCTCT
6877	Yes	AACCATGGATTCTAGAGCCAGATGGGTTCAAATCCCGGCTCTG
6878	Yes	AACCATGGATTCTAGAGCCAGATGGGTTCAAATCCCGGCTCTGC
6879	Yes	AACCATGGATTCTAGAGCCAGATGGGTTCAAATCCCGGCTCTGCC
6880	Yes	AACCATGGATTCTAGAGCCAGATGGGTTCAAATCCCGGCTCTGCCG
6881	Yes	AACCATGGATTCTAGAGCCAGATGGGTTCAAATCCCGGCTCTGCCGC

6882	Yes	AACCATGGATTCTAGAGCCAGATGGGTTCAAATCCCGGCTCTGCCGCC
6883	Yes	AACCATGGATTCTAGAGCCAGATGGGTTCAAATCCCGGCTCTGCCGCCA
6884	No	AACCATGGTCTTTGGA
6885	Yes	AACCATGGTCTTTGGAG
6886	Yes	AACCATGGTCTTTGGAGC
6887	Yes	AACCATGGTCTTTGGAGCC
6888	Yes	AACCATGGTCTTTGGAGCCA
6889	Yes	AACCATGGTCTTTGGAGCCAG
6890	Yes	AACCATGGTCTTTGGAGCCAGA
6891	Yes	AACCATGGTCTTTGGAGCCAGAC
6892	Yes	AACCATGGTCTTTGGAGCCAGACT
6893	Yes	AACCATGGTCTTTGGAGCCAGACTG
6894	Yes	AACCATGGTCTTTGGAGCCAGACTGC
6895	Yes	AACCATGGTCTTTGGAGCCAGACTGCC
6896	Yes	AACCATGGTCTTTGGAGCCAGACTGCCT
6897	Yes	AACCATGGTCTTTGGAGCCAGACTGCCTG
6898	Yes	AACCATGGTCTTTGGAGCCAGACTGCCTGG
6899	Yes	AACCATGGTCTTTGGAGCCAGACTGCCTGGG
6900	Yes	AACCATGGTCTTTGGAGCCAGACTGCCTGGGG
6901	Yes	AACCATGGTCTTTGGAGCCAGACTGCCTGGGGT
6902	Yes	AACCATGGTCTTTGGAGCCAGACTGCCTGGGGTC
6903	Yes	AACCATGGTCTTTGGAGCCAGACTGCCTGGGGTCG
6904	Yes	AACCATGGTCTTTGGAGCCAGACTGCCTGGGGTCGG
6905	Yes	AACCATGGTCTTTGGAGCCAGACTGCCTGGGGTCGGA
6906	Yes	AACCATGGTCTTTGGAGCCAGACTGCCTGGGGTCGGAT
6907	Yes	AACCATGGTCTTTGGAGCCAGACTGCCTGGGGTCGGATC
6908	Yes	AACCATGGTCTTTGGAGCCAGACTGCCTGGGGTCGGATCC
6909	Yes	AACCATGGTCTTTGGAGCCAGACTGCCTGGGGTCGGATCCC
6910	Yes	AACCATGGTCTTTGGAGCCAGACTGCCTGGGGTCGGATCCCA
6911	Yes	AACCATGGTCTTTGGAGCCAGACTGCCTGGGGTCGGATCCCAG
6912	Yes	AACCATGGTCTTTGGAGCCAGACTGCCTGGGGTCGGATCCCAGC
6913	Yes	AACCATGGTCTTTGGAGCCAGACTGCCTGGGGTCGGATCCCAGCT
6914	Yes	AACCATGGTCTTTGGAGCCAGACTGCCTGGGGTCGGATCCCAGCTC
6915	Yes	AACCATGGTCTTTGGAGCCAGACTGCCTGGGGTCGGATCCCAGCTCT
6916	Yes	AACCATGGTCTTTGGAGCCAGACTGCCTGGGGTCGGATCCCAGCTCTC
6917	Yes	AACCATGGTCTTTGGAGCCAGACTGCCTGGGGTCGGATCCCAGCTCTCA
6918	Yes	AACCATGGTCTTTGGAGCCAGACTGCCTGGGGTCGGATCCCAGCTCTCAC
6919	No	AACCATGGTTCAAATC
6920	Yes	AACCATGGTTCAAATCT
6921	Yes	AACCATGGTTCAAATCTG
6922	Yes	AACCATGGTTCAAATCTGA
6923	Yes	AACCATGGTTCAAATCTGAG

6924	Yes	AACCATGGTTCAAATCTGAGT
6925	Yes	AACCATGGTTCAAATCTGAGTC
6926	Yes	AACCATGGTTCAAATCTGAGTCA
6927	Yes	AACCATGGTTCAAATCTGAGTCAT
6928	Yes	AACCATGGTTCAAATCTGAGTCATG
6929	Yes	AACCATGGTTCAAATCTGAGTCATGA
6930	Yes	AACCATGGTTCAAATCTGAGTCATGAC
6931	Yes	AACCATGGTTCAAATCTGAGTCATGACA
6932	Yes	AACCATGGTTCAAATCTGAGTCATGACAC
6933	Yes	AACCATGGTTCAAATCTGAGTCATGACACC
6934	Yes	AACCATGGTTCAAATCTGAGTCATGACACCA
6935	No	AACCATTTCATAACTT
6936	No	AACCATTTCATAACTTT
6937	No	AACCATTTCATAACTTTG
6938	No	AACCATTTCATAACTTTGT
6939	No	AACCATTTCATAACTTTGTC
6940	No	AACCATTTCATAACTTTGTCA
6941	No	AACCATTTCATAACTTTGTCAA
6942	No	AACCATTTCATAACTTTGTCAAA
6943	No	AACCATTTCATAACTTTGTCAAAG
6944	No	AACCATTTCATAACTTTGTCAAAGT
6945	No	AACCATTTCATAACTTTGTCAAAGTT
6946	No	AACCATTTCATAACTTTGTCAAAGTTA
6947	No	AACCATTTCATAACTTTGTCAAAGTTAA
6948	No	AACCATTTCATAACTTTGTCAAAGTTAAA
6949	No	AACCATTTCATAACTTTGTCAAAGTTAAAT
6950	No	AACCATTTCATAACTTTGTCAAAGTTAAATT
6951	No	AACCATTTCATAACTTTGTCAAAGTTAAATTA
6952	No	AACCATTTCATAACTTTGTCAAAGTTAAATTAT
6953	No	AACCATTTCATAACTTTGTCAAAGTTAAATTATA
6954	No	AACCATTTCATAACTTTGTCAAAGTTAAATTATAG
6955	No	AACCATTTCATAACTTTGTCAAAGTTAAATTATAGG
6956	No	AACCATTTCATAACTTTGTCAAAGTTAAATTATAGGC
6957	No	AACCATTTCATAACTTTGTCAAAGTTAAATTATAGGCT
6958	No	AACCATTTCATAACTTTGTCAAAGTTAAATTATAGGCTA
6959	No	AACCATTTCATAACTTTGTCAAAGTTAAATTATAGGCTAA
6960	No	AACCATTTCATAACTTTGTCAAAGTTAAATTATAGGCTAAA
6961	No	AACCATTTCATAACTTTGTCAAAGTTAAATTATAGGCTAAAT
6962	No	AACCATTTCATAACTTTGTCAAAGTTAAATTATAGGCTAAATC
6963	No	AACCATTTCATAACTTTGTCAAAGTTAAATTATAGGCTAAATCC
6964	No	AACCATTTCATAACTTTGTCAAAGTTAAATTATAGGCTAAATCCT
6965	No	AACCATTTCATAACTTTGTCAAAGTTAAATTATAGGCTAAATCCTA

6966	No	AACCATTTCATAACTTTGTCAAAGTTAAATTATAGGCTAAATCCTAT
6967	No	AACCATTTCATAACTTTGTCAAAGTTAAATTATAGGCTAAATCCTATA
6968	No	AACCATTTCATAACTTTGTCAAAGTTAAATTATAGGCTAAATCCTATAT
6969	No	AACCATTTCATAACTTTGTCAAAGTTAAATTATAGGCTAAATCCTATATA
6970	Yes	AACCCACCCAGAGGCG
6971	Yes	AACCCACCCAGAGGCGC
6972	Yes	AACCCACCCAGAGGCGCC
6973	Yes	AACCCACCCAGAGGCGCCA
6974	Yes	AACCCACTGAGGGACG
6975	Yes	AACCCACTGAGGGACGC
6976	Yes	AACCCACTGAGGGACGCC
6977	Yes	AACCCACTGAGGGACGCCA
6978	No	AACCCAGACTGGATGG
6979	Yes	AACCCAGACTGGATGGG
6980	Yes	AACCCAGACTGGATGGGT
6981	Yes	AACCCAGACTGGATGGGTT
6982	Yes	AACCCAGACTGGATGGGTTC
6983	Yes	AACCCAGACTGGATGGGTTCA
6984	Yes	AACCCAGACTGGATGGGTTCAA
6985	Yes	AACCCAGACTGGATGGGTTCAAT
6986	Yes	AACCCAGACTGGATGGGTTCAATT
6987	Yes	AACCCAGACTGGATGGGTTCAATTC
6988	Yes	AACCCAGACTGGATGGGTTCAATTCC
6989	Yes	AACCCAGACTGGATGGGTTCAATTCCC
6990	Yes	AACCCAGACTGGATGGGTTCAATTCCCA
6991	Yes	AACCCAGACTGGATGGGTTCAATTCCCAT
6992	Yes	AACCCAGACTGGATGGGTTCAATTCCCATC
6993	Yes	AACCCAGACTGGATGGGTTCAATTCCCATCT
6994	Yes	AACCCAGACTGGATGGGTTCAATTCCCATCTC
6995	Yes	AACCCAGACTGGATGGGTTCAATTCCCATCTCT
6996	Yes	AACCCAGACTGGATGGGTTCAATTCCCATCTCTG
6997	Yes	AACCCAGACTGGATGGGTTCAATTCCCATCTCTGC
6998	Yes	AACCCAGACTGGATGGGTTCAATTCCCATCTCTGCC
6999	Yes	AACCCAGACTGGATGGGTTCAATTCCCATCTCTGCCG
7000	Yes	AACCCAGACTGGATGGGTTCAATTCCCATCTCTGCCGC
7001	Yes	AACCCAGACTGGATGGGTTCAATTCCCATCTCTGCCGCC
7002	Yes	AACCCAGACTGGATGGGTTCAATTCCCATCTCTGCCGCCA
7003	No	AACCCAGAGGTCAATG
7004	No	AACCCAGAGGTCAATGG
7005	Yes	AACCCAGAGGTCAATGGA
7006	Yes	AACCCAGAGGTCAATGGAT
7007	Yes	AACCCAGAGGTCAATGGATC

7008	Yes	AACCCAGAGGTCAATGGATCG
7009	Yes	AACCCAGAGGTCAATGGATCGA
7010	Yes	AACCCAGAGGTCAATGGATCGAA
7011	Yes	AACCCAGAGGTCAATGGATCGAAG
7012	Yes	AACCCAGAGGTCAATGGATCGAAGC
7013	Yes	AACCCAGAGGTCAATGGATCGAAGCC
7014	Yes	AACCCAGAGGTCAATGGATCGAAGCCA
7015	Yes	AACCCAGAGGTCAATGGATCGAAGCCAT
7016	Yes	AACCCAGAGGTCAATGGATCGAAGCCATC
7017	Yes	AACCCAGAGGTCAATGGATCGAAGCCATCC
7018	Yes	AACCCAGAGGTCAATGGATCGAAGCCATCCT
7019	Yes	AACCCAGAGGTCAATGGATCGAAGCCATCCTT
7020	Yes	AACCCAGAGGTCAATGGATCGAAGCCATCCTTG
7021	Yes	AACCCAGAGGTCAATGGATCGAAGCCATCCTTGG
7022	Yes	AACCCAGAGGTCAATGGATCGAAGCCATCCTTGGC
7023	Yes	AACCCAGAGGTCAATGGATCGAAGCCATCCTTGGCT
7024	Yes	AACCCAGAGGTCAATGGATCGAAGCCATCCTTGGCTA
7025	Yes	AACCCAGAGGTCAATGGATCGAAGCCATCCTTGGCTAC
7026	Yes	AACCCAGAGGTCAATGGATCGAAGCCATCCTTGGCTACC
7027	Yes	AACCCAGAGGTCAATGGATCGAAGCCATCCTTGGCTACCA
7028	No	AACCCAGAGGTCGATG
7029	No	AACCCAGAGGTCGATGG
7030	No	AACCCAGAGGTCGATGGA
7031	No	AACCCAGAGGTCGATGGAT
7032	No	AACCCAGAGGTCGATGGATC
7033	Yes	AACCCAGAGGTCGATGGATCG
7034	Yes	AACCCAGAGGTCGATGGATCGA
7035	Yes	AACCCAGAGGTCGATGGATCGAA
7036	Yes	AACCCAGAGGTCGATGGATCGAAA
7037	Yes	AACCCAGAGGTCGATGGATCGAAAC
7038	Yes	AACCCAGAGGTCGATGGATCGAAACC
7039	Yes	AACCCAGAGGTCGATGGATCGAAACCA
7040	Yes	AACCCAGAGGTCGATGGATCGAAACCAT
7041	Yes	AACCCAGAGGTCGATGGATCGAAACCATC
7042	Yes	AACCCAGAGGTCGATGGATCGAAACCATCC
7043	Yes	AACCCAGAGGTCGATGGATCGAAACCATCCT
7044	Yes	AACCCAGAGGTCGATGGATCGAAACCATCCTC
7045	Yes	AACCCAGAGGTCGATGGATCGAAACCATCCTCT
7046	Yes	AACCCAGAGGTCGATGGATCGAAACCATCCTCTG
7047	Yes	AACCCAGAGGTCGATGGATCGAAACCATCCTCTGC
7048	Yes	AACCCAGAGGTCGATGGATCGAAACCATCCTCTGCT
7049	Yes	AACCCAGAGGTCGATGGATCGAAACCATCCTCTGCTA

7050	Yes	AACCCAGAGGTCGATGGATCGAAACCATCCTCTGCTAC
7051	Yes	AACCCAGAGGTCGATGGATCGAAACCATCCTCTGCTACC
7052	Yes	AACCCAGAGGTCGATGGATCGAAACCATCCTCTGCTACCA
7053	Yes	AACCCAGAGGTCGATGGATCT
7054	Yes	AACCCAGAGGTCGATGGATCTA
7055	Yes	AACCCAGAGGTCGATGGATCTAA
7056	Yes	AACCCAGAGGTCGATGGATCTAAA
7057	Yes	AACCCAGAGGTCGATGGATCTAAAC
7058	Yes	AACCCAGAGGTCGATGGATCTAAACC
7059	Yes	AACCCAGAGGTCGATGGATCTAAACCA
7060	Yes	AACCCAGAGGTCGATGGATCTAAACCAT
7061	Yes	AACCCAGAGGTCGATGGATCTAAACCATC
7062	Yes	AACCCAGAGGTCGATGGATCTAAACCATCC
7063	Yes	AACCCAGAGGTCGATGGATCTAAACCATCCT
7064	Yes	AACCCAGAGGTCGATGGATCTAAACCATCCTC
7065	Yes	AACCCAGAGGTCGATGGATCTAAACCATCCTCT
7066	Yes	AACCCAGAGGTCGATGGATCTAAACCATCCTCTG
7067	Yes	AACCCAGAGGTCGATGGATCTAAACCATCCTCTGC
7068	Yes	AACCCAGAGGTCGATGGATCTAAACCATCCTCTGCT
7069	Yes	AACCCAGAGGTCGATGGATCTAAACCATCCTCTGCTA
7070	Yes	AACCCAGAGGTCGATGGATCTAAACCATCCTCTGCTAC
7071	Yes	AACCCAGAGGTCGATGGATCTAAACCATCCTCTGCTACC
7072	Yes	AACCCAGAGGTCGATGGATCTAAACCATCCTCTGCTACCA
7073	No	AACCCAGAGGTTGATG
7074	No	AACCCAGAGGTTGATGG
7075	Yes	AACCCAGAGGTTGATGGA
7076	Yes	AACCCAGAGGTTGATGGAT
7077	Yes	AACCCAGAGGTTGATGGATG
7078	Yes	AACCCAGAGGTTGATGGATGG
7079	Yes	AACCCAGAGGTTGATGGATGGA
7080	Yes	AACCCAGAGGTTGATGGATGGAA
7081	Yes	AACCCAGAGGTTGATGGATGGAAA
7082	Yes	AACCCAGAGGTTGATGGATGGAAAC
7083	Yes	AACCCAGAGGTTGATGGATGGAAACC
7084	Yes	AACCCAGAGGTTGATGGATGGAAACCA
7085	Yes	AACCCAGAGGTTGATGGATGGAAACCAT
7086	Yes	AACCCAGAGGTTGATGGATGGAAACCATC
7087	Yes	AACCCAGAGGTTGATGGATGGAAACCATCC
7088	Yes	AACCCAGAGGTTGATGGATGGAAACCATCCT
7089	Yes	AACCCAGAGGTTGATGGATGGAAACCATCCTC
7090	Yes	AACCCAGAGGTTGATGGATGGAAACCATCCTCT
7091	Yes	AACCCAGAGGTTGATGGATGGAAACCATCCTCTG

7092	Yes	AACCCAGAGGTTGATGGATGGAAACCATCCTCTGC
7093	Yes	AACCCAGAGGTTGATGGATGGAAACCATCCTCTGCT
7094	Yes	AACCCAGAGGTTGATGGATGGAAACCATCCTCTGCTA
7095	Yes	AACCCAGAGGTTGATGGATGGAAACCATCCTCTGCTAC
7096	Yes	AACCCAGAGGTTGATGGATGGAAACCATCCTCTGCTACC
7097	Yes	AACCCAGAGGTTGATGGATGGAAACCATCCTCTGCTACCA
7098	No	AACCCAGATAGCTCAG
7099	Yes	AACCCAGATAGCTCAGT
7100	Yes	AACCCAGATAGCTCAGTC
7101	Yes	AACCCAGATAGCTCAGTCA
7102	Yes	AACCCAGATAGCTCAGTCAG
7103	Yes	AACCCAGATAGCTCAGTCAGT
7104	Yes	AACCCAGATAGCTCAGTCAGTA
7105	Yes	AACCCAGATAGCTCAGTCAGTAG
7106	Yes	AACCCAGATAGCTCAGTCAGTAGA
7107	Yes	AACCCAGATAGCTCAGTCAGTAGAG
7108	Yes	AACCCAGATAGCTCAGTCAGTAGAGC
7109	Yes	AACCCAGATAGCTCAGTCAGTAGAGCA
7110	Yes	AACCCAGATAGCTCAGTCAGTAGAGCAT
7111	Yes	AACCCAGATAGCTCAGTCAGTAGAGCATC
7112	Yes	AACCCAGATAGCTCAGTCAGTAGAGCATCA
7113	Yes	AACCCAGATAGCTCAGTCAGTAGAGCATCAG
7114	Yes	AACCCAGATAGCTCAGTCAGTAGAGCATCAGA
7115	Yes	AACCCAGATAGCTCAGTCAGTAGAGCATCAGAC
7116	Yes	AACCCAGATAGCTCAGTCAGTAGAGCATCAGACT
7117	Yes	AACCCAGATAGCTCAGTCAGTAGAGCATCAGACTT
7118	Yes	AACCCAGATAGCTCAGTCAGTAGAGCATCAGACTTT
7119	Yes	AACCCAGATAGCTCAGTCAGTAGAGCATCAGACTTTT
7120	Yes	AACCCAGATAGCTCAGTCAGTAGAGCATCAGACTTTTA
7121	Yes	AACCCAGATAGCTCAGTCAGTAGAGCATCAGACTTTTAA
7122	Yes	AACCCAGATAGCTCAGTCAGTAGAGCATCAGACTTTTAAT
7123	Yes	AACCCAGATAGCTCAGTCAGTAGAGCATCAGACTTTTAATC
7124	Yes	AACCCAGATAGCTCAGTCAGTAGAGCATCAGACTTTTAATCT
7125	Yes	AACCCAGATAGCTCAGTCAGTAGAGCATCAGACTTTTAATCTG
7126	Yes	AACCCAGATAGCTCAGTCAGTAGAGCATCAGACTTTTAATCTGA
7127	Yes	AACCCAGATAGCTCAGTCAGTAGAGCATCAGACTTTTAATCTGAG
7128	Yes	AACCCAGATAGCTCAGTCAGTAGAGCATCAGACTTTTAATCTGAGG
7129	Yes	AACCCAGATAGCTCAGTCAGTAGAGCATCAGACTTTTAATCTGAGGG
7130	Yes	AACCCAGATAGCTCAGTCAGTAGAGCATCAGACTTTTAATCTGAGGGT
7131	Yes	AACCCAGATAGCTCAGTCAGTAGAGCATCAGACTTTTAATCTGAGGGTC
7132	Yes	AACCCAGATAGCTCAGTCAGTAGAGCATCAGACTTTTAATCTGAGGGTCC
7133	Yes	AACCCAGATAGCTCAGTT

7134	Yes	AACCCAGATAGCTCAGTTG
7135	Yes	AACCCAGATAGCTCAGTTGA
7136	Yes	AACCCAGATAGCTCAGTTGAT
7137	Yes	AACCCAGATAGCTCAGTTGATA
7138	Yes	AACCCAGATAGCTCAGTTGATAG
7139	Yes	AACCCAGATAGCTCAGTTGATAGA
7140	Yes	AACCCAGATAGCTCAGTTGATAGAG
7141	Yes	AACCCAGATAGCTCAGTTGATAGAGC
7142	Yes	AACCCAGATAGCTCAGTTGATAGAGCA
7143	Yes	AACCCAGATAGCTCAGTTGATAGAGCAT
7144	Yes	AACCCAGATAGCTCAGTTGATAGAGCATC
7145	Yes	AACCCAGATAGCTCAGTTGATAGAGCATCA
7146	Yes	AACCCAGATAGCTCAGTTGATAGAGCATCAG
7147	Yes	AACCCAGATAGCTCAGTTGATAGAGCATCAGA
7148	Yes	AACCCAGATAGCTCAGTTGATAGAGCATCAGAC
7149	Yes	AACCCAGATAGCTCAGTTGATAGAGCATCAGACT
7150	Yes	AACCCAGATAGCTCAGTTGATAGAGCATCAGACTT
7151	Yes	AACCCAGATAGCTCAGTTGATAGAGCATCAGACTTT
7152	Yes	AACCCAGATAGCTCAGTTGATAGAGCATCAGACTTTT
7153	Yes	AACCCAGATAGCTCAGTTGATAGAGCATCAGACTTTTA
7154	Yes	AACCCAGATAGCTCAGTTGATAGAGCATCAGACTTTTAA
7155	Yes	AACCCAGATAGCTCAGTTGATAGAGCATCAGACTTTTAAT
7156	Yes	AACCCAGATAGCTCAGTTGATAGAGCATCAGACTTTTAATC
7157	Yes	AACCCAGATAGCTCAGTTGATAGAGCATCAGACTTTTAATCT
7158	Yes	AACCCAGATAGCTCAGTTGATAGAGCATCAGACTTTTAATCTG
7159	Yes	AACCCAGATAGCTCAGTTGATAGAGCATCAGACTTTTAATCTGA
7160	Yes	AACCCAGATAGCTCAGTTGATAGAGCATCAGACTTTTAATCTGAG
7161	Yes	AACCCAGATAGCTCAGTTGATAGAGCATCAGACTTTTAATCTGAGG
7162	Yes	AACCCAGATAGCTCAGTTGATAGAGCATCAGACTTTTAATCTGAGGG
7163	Yes	AACCCAGATAGCTCAGTTGATAGAGCATCAGACTTTTAATCTGAGGGT
7164	Yes	AACCCAGATAGCTCAGTTGATAGAGCATCAGACTTTTAATCTGAGGGTC
7165	Yes	AACCCAGATAGCTCAGTTGATAGAGCATCAGACTTTTAATCTGAGGGTCC
7166	Yes	AACCCATTGGGGTCTC
7167	Yes	AACCCATTGGGGTCTCC
7168	Yes	AACCCATTGGGGTCTCCC
7169	Yes	AACCCATTGGGGTCTCCCC
7170	Yes	AACCCATTGGGGTCTCCCCG
7171	Yes	AACCCATTGGGGTCTCCCCGC
7172	Yes	AACCCATTGGGGTCTCCCCGCG
7173	Yes	AACCCATTGGGGTCTCCCCGCGC
7174	Yes	AACCCATTGGGGTCTCCCCGCGCA
7175	Yes	AACCCATTGGGGTCTCCCCGCGCAG

7176	Yes	AACCCATTGGGGTCTCCCGCGCAGG
7177	Yes	AACCCATTGGGGTCTCCCGCGCAGGT
7178	Yes	AACCCATTGGGGTCTCCCGCGCAGGTT
7179	Yes	AACCCATTGGGGTCTCCCGCGCAGGTTC
7180	Yes	AACCCATTGGGGTCTCCCGCGCAGGTTCG
7181	Yes	AACCCATTGGGGTCTCCCGCGCAGGTTCGA
7182	Yes	AACCCATTGGGGTCTCCCGCGCAGGTTCGAA
7183	Yes	AACCCATTGGGGTCTCCCGCGCAGGTTCGAAT
7184	Yes	AACCCATTGGGGTCTCCCGCGCAGGTTCGAATC
7185	Yes	AACCCATTGGGGTCTCCCGCGCAGGTTCGAATCC
7186	Yes	AACCCATTGGGGTCTCCCGCGCAGGTTCGAATCCT
7187	Yes	AACCCATTGGGGTCTCCCGCGCAGGTTCGAATCCTG
7188	Yes	AACCCATTGGGGTCTCCCGCGCAGGTTCGAATCCTGC
7189	Yes	AACCCATTGGGGTCTCCCGCGCAGGTTCGAATCCTGCC
7190	Yes	AACCCATTGGGGTCTCCCGCGCAGGTTCGAATCCTGCCG
7191	Yes	AACCCATTGGGGTCTCCCGCGCAGGTTCGAATCCTGCCGA
7192	Yes	AACCCATTGGGGTCTCCCGCGCAGGTTCGAATCCTGCCGAC
7193	Yes	AACCCATTGGGGTCTCCCGCGCAGGTTCGAATCCTGCCGACT
7194	Yes	AACCCATTGGGGTCTCCCGCGCAGGTTCGAATCCTGCCGACTA
7195	Yes	AACCCATTGGGGTCTCCCGCGCAGGTTCGAATCCTGCCGACTAC
7196	Yes	AACCCATTGGGGTCTCCCGCGCAGGTTCGAATCCTGCCGACTACG
7197	Yes	AACCCATTGGGGTCTCCCGCGCAGGTTCGAATCCTGCCGACTACGC
7198	Yes	AACCCATTGGGGTCTCCCGCGCAGGTTCGAATCCTGCCGACTACGCC
7199	Yes	AACCCATTGGGGTCTCCCGCGCAGGTTCGAATCCTGCCGACTACGCCA
7200	Yes	AACCCCACTACTGGTA
7201	Yes	AACCCCACTACTGGTAC
7202	Yes	AACCCCACTACTGGTACC
7203	Yes	AACCCCACTACTGGTACCA
7204	No	AACCCCACTCCTGGTA
7205	No	AACCCCACTCCTGGTAC
7206	No	AACCCCACTCCTGGTACC
7207	No	AACCCCACTCCTGGTACCA
7208	No	AACCCCACTCTCGGTA
7209	No	AACCCCACTCTCGGTAC
7210	No	AACCCCACTCTCGGTACC
7211	No	AACCCCACTCTCGGTACCA
7212	No	AACCCCACTTCTGGTA
7213	No	AACCCCACTTCTGGTAC
7214	No	AACCCCACTTCTGGTACC
7215	No	AACCCCACTTCTGGTACCA
7216	No	AACCCCAGCTGCGCCC
7217	No	AACCCCAGCTGCGCCCC

7218	Yes	AACCCCAGCTGCGCCCCC
7219	Yes	AACCCCAGCTGCGCCCCCA
7220	No	AACCCCCTTATTTCTA
7221	No	AACCCCCTTATTTCTAC
7222	Yes	AACCCCCTTATTTCTACC
7223	Yes	AACCCCCTTATTTCTACCA
7224	Yes	AACCCCGTACGGGCCA
7225	Yes	AACCCCGTACGGGCCAC
7226	Yes	AACCCCGTACGGGCCACC
7227	Yes	AACCCCGTACGGGCCACCA
7228	No	AACCCCGTCCGTGCCT
7229	Yes	AACCCCGTCCGTGCCTC
7230	Yes	AACCCCGTCCGTGCCTCC
7231	Yes	AACCCCGTCCGTGCCTCCA
7232	No	AACCCTAGCCCTGGCA
7233	Yes	AACCCTAGCCCTGGCAC
7234	Yes	AACCCTAGCCCTGGCACC
7235	Yes	AACCCTAGCCCTGGCACCA
7236	Yes	AACCCTGCTCGCTGCG
7237	Yes	AACCCTGCTCGCTGCGC
7238	Yes	AACCCTGCTCGCTGCGCC
7239	Yes	AACCCTGCTCGCTGCGCCA
7240	No	AACCCTGGCGCTGTCA
7241	No	AACCCTGGCGCTGTCAC
7242	Yes	AACCCTGGCGCTGTCACC
7243	Yes	AACCCTGGCGCTGTCACCA
7244	No	AACCCTGGGTTTCAGC
7245	No	AACCCTGGGTTTCAGCC
7246	Yes	AACCCTGGGTTTCAGCCC
7247	Yes	AACCCTGGGTTTCAGCCCC
7248	Yes	AACCCTGGGTTTCAGCCCCA
7249	No	AACCCTGTGGTACAGG
7250	Yes	AACCCTGTGGTACAGGG
7251	Yes	AACCCTGTGGTACAGGGG
7252	Yes	AACCCTGTGGTACAGGGGC
7253	Yes	AACCCTGTGGTACAGGGGCT
7254	Yes	AACCCTGTGGTACAGGGGCTA
7255	Yes	AACCCTGTGGTACAGGGGCTAA
7256	Yes	AACCCTGTGGTACAGGGGCTAAT
7257	Yes	AACCCTGTGGTACAGGGGCTAATA
7258	Yes	AACCCTGTGGTACAGGGGCTAATAT
7259	Yes	AACCCTGTGGTACAGGGGCTAATATG

7260	Yes	AACCCTGTGGTACAGGGGCTAATATGC
7261	Yes	AACCCTGTGGTACAGGGGCTAATATGCT
7262	Yes	AACCCTGTGGTACAGGGGCTAATATGCTG
7263	Yes	AACCCTGTGGTACAGGGGCTAATATGCTGG
7264	Yes	AACCCTGTGGTACAGGGGCTAATATGCTGGG
7265	Yes	AACCCTGTGGTACAGGGGCTAATATGCTGGGC
7266	Yes	AACCCTGTGGTACAGGGGCTAATATGCTGGGCC
7267	Yes	AACCCTGTGGTACAGGGGCTAATATGCTGGGCCT
7268	Yes	AACCCTGTGGTACAGGGGCTAATATGCTGGGCCTT
7269	Yes	AACCCTGTGGTACAGGGGCTAATATGCTGGGCCTTT
7270	Yes	AACCCTGTGGTACAGGGGCTAATATGCTGGGCCTTTA
7271	Yes	AACCCTGTGGTACAGGGGCTAATATGCTGGGCCTTTAC
7272	Yes	AACCCTGTGGTACAGGGGCTAATATGCTGGGCCTTTACC
7273	Yes	AACCCTGTGGTACAGGGGCTAATATGCTGGGCCTTTACCA
7274	Yes	AACCCTGTGGTACAGGGGCTAATATGCTGGGCCTTTACCAC
7275	Yes	AACCCTGTGGTACAGGGGCTAATATGCTGGGCCTTTACCACT
7276	Yes	AACCCTGTGGTACAGGGGCTAATATGCTGGGCCTTTACCACTT
7277	Yes	AACCCTGTGGTACAGGGGCTAATATGCTGGGCCTTTACCACTTC
7278	Yes	AACCCTGTGGTACAGGGGCTAATATGCTGGGCCTTTACCACTTCA
7279	Yes	AACCCTGTGGTACAGGGGCTAATATGCTGGGCCTTTACCACTTCAG
7280	Yes	AACCCTGTGGTACAGGGGCTAATATGCTGGGCCTTTACCACTTCAGC
7281	Yes	AACCCTGTGGTACAGGGGCTAATATGCTGGGCCTTTACCACTTCAGCC
7282	Yes	AACCCTGTGGTACAGGGGCTAATATGCTGGGCCTTTACCACTTCAGCCC
7283	Yes	AACCCTGTGGTACAGGGGCTAATATGCTGGGCCTTTACCACTTCAGCCCA
7284	Yes	AACCCTGTGGTCTAGT
7285	Yes	AACCCTGTGGTCTAGTG
7286	Yes	AACCCTGTGGTCTAGTGG
7287	Yes	AACCCTGTGGTCTAGTGGC
7288	Yes	AACCCTGTGGTCTAGTGGCT
7289	Yes	AACCCTGTGGTCTAGTGGCTA
7290	Yes	AACCCTGTGGTCTAGTGGCTAA
7291	Yes	AACCCTGTGGTCTAGTGGCTAAG
7292	Yes	AACCCTGTGGTCTAGTGGCTAAGA
7293	Yes	AACCCTGTGGTCTAGTGGCTAAGAC
7294	Yes	AACCCTGTGGTCTAGTGGCTAAGACT
7295	Yes	AACCCTGTGGTCTAGTGGCTAAGACTT
7296	Yes	AACCCTGTGGTCTAGTGGCTAAGACTTT
7297	Yes	AACCCTGTGGTCTAGTGGCTAAGACTTTG
7298	Yes	AACCCTGTGGTCTAGTGGCTAAGACTTTGT
7299	Yes	AACCCTGTGGTCTAGTGGCTAAGACTTTGTG
7300	Yes	AACCCTGTGGTCTAGTGGCTAAGACTTTGTGC
7301	Yes	AACCCTGTGGTCTAGTGGCTAAGACTTTGTGCT

7302	Yes	AACCCTGTGGTCTAGTGGCTAAGACTTTGTGCTT
7303	Yes	AACCCTGTGGTCTAGTGGCTAAGACTTTGTGCTTT
7304	Yes	AACCCTGTGGTCTAGTGGCTAAGACTTTGTGCTTTC
7305	Yes	AACCCTGTGGTCTAGTGGCTAAGACTTTGTGCTTTCA
7306	Yes	AACCCTGTGGTCTAGTGGCTAAGACTTTGTGCTTTCAT
7307	Yes	AACCCTGTGGTCTAGTGGCTAAGACTTTGTGCTTTCATT
7308	Yes	AACCCTGTGGTCTAGTGGCTAAGACTTTGTGCTTTCATTG
7309	Yes	AACCCTGTGGTCTAGTGGCTAAGACTTTGTGCTTTCATTGC
7310	Yes	AACCCTGTGGTCTAGTGGCTAAGACTTTGTGCTTTCATTGCT
7311	Yes	AACCCTGTGGTCTAGTGGCTAAGACTTTGTGCTTTCATTGCTG
7312	Yes	AACCCTGTGGTCTAGTGGCTAAGACTTTGTGCTTTCATTGCTGC
7313	Yes	AACCCTGTGGTCTAGTGGCTAAGACTTTGTGCTTTCATTGCTGCA
7314	Yes	AACCCTGTGGTCTAGTGGCTAAGACTTTGTGCTTTCATTGCTGCAT
7315	Yes	AACCCTGTGGTCTAGTGGCTAAGACTTTGTGCTTTCATTGCTGCATC
7316	Yes	AACCCTGTGGTCTAGTGGCTAAGACTTTGTGCTTTCATTGCTGCATCC
7317	Yes	AACCCTGTGGTCTAGTGGCTAAGACTTTGTGCTTTCATTGCTGCATCCT
7318	Yes	AACCCTGTGGTCTAGTGGCTAAGACTTTGTGCTTTCATTGCTGCATCCTA
7319	No	AACCGAAAAGTTGGTG
7320	Yes	AACCGAAAAGTTGGTGG
7321	Yes	AACCGAAAAGTTGGTGGT
7322	Yes	AACCGAAAAGTTGGTGGTT
7323	Yes	AACCGAAAAGTTGGTGGTTC
7324	Yes	AACCGAAAAGTTGGTGGTTCG
7325	Yes	AACCGAAAAGTTGGTGGTTCGA
7326	Yes	AACCGAAAAGTTGGTGGTTCGAG
7327	Yes	AACCGAAAAGTTGGTGGTTCGAGC
7328	Yes	AACCGAAAAGTTGGTGGTTCGAGCC
7329	Yes	AACCGAAAAGTTGGTGGTTCGAGCCC
7330	Yes	AACCGAAAAGTTGGTGGTTCGAGCCCA
7331	Yes	AACCGAAAAGTTGGTGGTTCGAGCCCAC
7332	Yes	AACCGAAAAGTTGGTGGTTCGAGCCCACC
7333	Yes	AACCGAAAAGTTGGTGGTTCGAGCCCACCC
7334	Yes	AACCGAAAAGTTGGTGGTTCGAGCCCACCCA
7335	Yes	AACCGAAAAGTTGGTGGTTCGAGCCCACCCAG
7336	Yes	AACCGAAAAGTTGGTGGTTCGAGCCCACCCAGG
7337	Yes	AACCGAAAAGTTGGTGGTTCGAGCCCACCCAGGA
7338	Yes	AACCGAAAAGTTGGTGGTTCGAGCCCACCCAGGAA
7339	Yes	AACCGAAAAGTTGGTGGTTCGAGCCCACCCAGGAAC
7340	Yes	AACCGAAAAGTTGGTGGTTCGAGCCCACCCAGGAACG
7341	Yes	AACCGAAAAGTTGGTGGTTCGAGCCCACCCAGGAACGC
7342	Yes	AACCGAAAAGTTGGTGGTTCGAGCCCACCCAGGAACGCC
7343	Yes	AACCGAAAAGTTGGTGGTTCGAGCCCACCCAGGAACGCCA

7344	Yes	AACCGAAAGATTGGTG
7345	Yes	AACCGAAAGATTGGTGG
7346	Yes	AACCGAAAGATTGGTGGT
7347	Yes	AACCGAAAGATTGGTGGTT
7348	Yes	AACCGAAAGATTGGTGGTTC
7349	Yes	AACCGAAAGATTGGTGGTTCG
7350	Yes	AACCGAAAGATTGGTGGTTCGA
7351	Yes	AACCGAAAGATTGGTGGTTCGAG
7352	Yes	AACCGAAAGATTGGTGGTTCGAGC
7353	Yes	AACCGAAAGATTGGTGGTTCGAGCC
7354	Yes	AACCGAAAGATTGGTGGTTCGAGCCC
7355	Yes	AACCGAAAGATTGGTGGTTCGAGCCCA
7356	Yes	AACCGAAAGATTGGTGGTTCGAGCCCAC
7357	Yes	AACCGAAAGATTGGTGGTTCGAGCCCACC
7358	Yes	AACCGAAAGATTGGTGGTTCGAGCCCACCC
7359	Yes	AACCGAAAGATTGGTGGTTCGAGCCCACCCA
7360	Yes	AACCGAAAGATTGGTGGTTCGAGCCCACCCAG
7361	Yes	AACCGAAAGATTGGTGGTTCGAGCCCACCCAGG
7362	Yes	AACCGAAAGATTGGTGGTTCGAGCCCACCCAGGG
7363	Yes	AACCGAAAGATTGGTGGTTCGAGCCCACCCAGGGA
7364	Yes	AACCGAAAGATTGGTGGTTCGAGCCCACCCAGGGAC
7365	Yes	AACCGAAAGATTGGTGGTTCGAGCCCACCCAGGGACG
7366	Yes	AACCGAAAGATTGGTGGTTCGAGCCCACCCAGGGACGC
7367	Yes	AACCGAAAGATTGGTGGTTCGAGCCCACCCAGGGACGCC
7368	Yes	AACCGAAAGATTGGTGGTTCGAGCCCACCCAGGGACGCCA
7369	Yes	AACCGAAAGCTTGGTG
7370	Yes	AACCGAAAGCTTGGTGG
7371	Yes	AACCGAAAGCTTGGTGGT
7372	Yes	AACCGAAAGCTTGGTGGTT
7373	Yes	AACCGAAAGCTTGGTGGTTC
7374	Yes	AACCGAAAGCTTGGTGGTTCG
7375	Yes	AACCGAAAGCTTGGTGGTTCGA
7376	Yes	AACCGAAAGCTTGGTGGTTCGAG
7377	Yes	AACCGAAAGCTTGGTGGTTCGAGC
7378	Yes	AACCGAAAGCTTGGTGGTTCGAGCC
7379	Yes	AACCGAAAGCTTGGTGGTTCGAGCCC
7380	Yes	AACCGAAAGCTTGGTGGTTCGAGCCCA
7381	Yes	AACCGAAAGCTTGGTGGTTCGAGCCCAC
7382	Yes	AACCGAAAGCTTGGTGGTTCGAGCCCACC
7383	Yes	AACCGAAAGCTTGGTGGTTCGAGCCCACCC
7384	Yes	AACCGAAAGCTTGGTGGTTCGAGCCCACCCA
7385	Yes	AACCGAAAGCTTGGTGGTTCGAGCCCACCCAG

7386	Yes	AACCGAAAGCTTGGTGGTTCGAGCCCACCCAGG
7387	Yes	AACCGAAAGCTTGGTGGTTCGAGCCCACCCAGGG
7388	Yes	AACCGAAAGCTTGGTGGTTCGAGCCCACCCAGGGA
7389	Yes	AACCGAAAGCTTGGTGGTTCGAGCCCACCCAGGGAT
7390	Yes	AACCGAAAGCTTGGTGGTTCGAGCCCACCCAGGGATG
7391	Yes	AACCGAAAGCTTGGTGGTTCGAGCCCACCCAGGGATGC
7392	Yes	AACCGAAAGCTTGGTGGTTCGAGCCCACCCAGGGATGCC
7393	Yes	AACCGAAAGCTTGGTGGTTCGAGCCCACCCAGGGATGCCA
7394	Yes	AACCGAAAGCTTGGTGGTTT
7395	Yes	AACCGAAAGCTTGGTGGTTTG
7396	Yes	AACCGAAAGCTTGGTGGTTTGA
7397	Yes	AACCGAAAGCTTGGTGGTTTGAG
7398	Yes	AACCGAAAGCTTGGTGGTTTGAGC
7399	Yes	AACCGAAAGCTTGGTGGTTTGAGCC
7400	Yes	AACCGAAAGCTTGGTGGTTTGAGCCC
7401	Yes	AACCGAAAGCTTGGTGGTTTGAGCCCA
7402	Yes	AACCGAAAGCTTGGTGGTTTGAGCCCAC
7403	Yes	AACCGAAAGCTTGGTGGTTTGAGCCCACC
7404	Yes	AACCGAAAGCTTGGTGGTTTGAGCCCACCC
7405	Yes	AACCGAAAGCTTGGTGGTTTGAGCCCACCCA
7406	Yes	AACCGAAAGCTTGGTGGTTTGAGCCCACCCAG
7407	Yes	AACCGAAAGCTTGGTGGTTTGAGCCCACCCAGG
7408	Yes	AACCGAAAGCTTGGTGGTTTGAGCCCACCCAGGG
7409	Yes	AACCGAAAGCTTGGTGGTTTGAGCCCACCCAGGGA
7410	Yes	AACCGAAAGCTTGGTGGTTTGAGCCCACCCAGGGAT
7411	Yes	AACCGAAAGCTTGGTGGTTTGAGCCCACCCAGGGATG
7412	Yes	AACCGAAAGCTTGGTGGTTTGAGCCCACCCAGGGATGC
7413	Yes	AACCGAAAGCTTGGTGGTTTGAGCCCACCCAGGGATGCC
7414	Yes	AACCGAAAGCTTGGTGGTTTGAGCCCACCCAGGGATGCCA
7415	No	AACCGAAAGGCTGGTG
7416	No	AACCGAAAGGCTGGTGG
7417	Yes	AACCGAAAGGCTGGTGGT
7418	Yes	AACCGAAAGGCTGGTGGTT
7419	Yes	AACCGAAAGGCTGGTGGTTC
7420	Yes	AACCGAAAGGCTGGTGGTTCA
7421	Yes	AACCGAAAGGCTGGTGGTTCAA
7422	Yes	AACCGAAAGGCTGGTGGTTCAAG
7423	Yes	AACCGAAAGGCTGGTGGTTCAAGC
7424	Yes	AACCGAAAGGCTGGTGGTTCAAGCC
7425	Yes	AACCGAAAGGCTGGTGGTTCAAGCCC
7426	Yes	AACCGAAAGGCTGGTGGTTCAAGCCCA
7427	Yes	AACCGAAAGGCTGGTGGTTCAAGCCCAC

7428	Yes	AACCGAAAGGCTGGTGGTTCAAGCCCACC
7429	Yes	AACCGAAAGGCTGGTGGTTCAAGCCCACCC
7430	Yes	AACCGAAAGGCTGGTGGTTCAAGCCCACCCA
7431	Yes	AACCGAAAGGCTGGTGGTTCAAGCCCACCCAG
7432	Yes	AACCGAAAGGCTGGTGGTTCAAGCCCACCCAGA
7433	Yes	AACCGAAAGGCTGGTGGTTCAAGCCCACCCAGAG
7434	Yes	AACCGAAAGGCTGGTGGTTCAAGCCCACCCAGAGA
7435	Yes	AACCGAAAGGCTGGTGGTTCAAGCCCACCCAGAGAT
7436	Yes	AACCGAAAGGCTGGTGGTTCAAGCCCACCCAGAGATG
7437	Yes	AACCGAAAGGCTGGTGGTTCAAGCCCACCCAGAGATGC
7438	Yes	AACCGAAAGGCTGGTGGTTCAAGCCCACCCAGAGATGCC
7439	Yes	AACCGAAAGGCTGGTGGTTCAAGCCCACCCAGAGATGCCA
7440	Yes	AACCGAAAGGTTGGTG
7441	Yes	AACCGAAAGGTTGGTGG
7442	Yes	AACCGAAAGGTTGGTGGT
7443	Yes	AACCGAAAGGTTGGTGGTT
7444	Yes	AACCGAAAGGTTGGTGGTTC
7445	Yes	AACCGAAAGGTTGGTGGTTCG
7446	Yes	AACCGAAAGGTTGGTGGTTCGA
7447	Yes	AACCGAAAGGTTGGTGGTTCGAG
7448	Yes	AACCGAAAGGTTGGTGGTTCGAGC
7449	Yes	AACCGAAAGGTTGGTGGTTCGAGCC
7450	Yes	AACCGAAAGGTTGGTGGTTCGAGCCC
7451	Yes	AACCGAAAGGTTGGTGGTTCGAGCCCA
7452	Yes	AACCGAAAGGTTGGTGGTTCGAGCCCAC
7453	Yes	AACCGAAAGGTTGGTGGTTCGAGCCCACC
7454	Yes	AACCGAAAGGTTGGTGGTTCGAGCCCACCC
7455	Yes	AACCGAAAGGTTGGTGGTTCGAGCCCACCCA
7456	Yes	AACCGAAAGGTTGGTGGTTCGAGCCCACCCAG
7457	Yes	AACCGAAAGGTTGGTGGTTCGAGCCCACCCAGG
7458	Yes	AACCGAAAGGTTGGTGGTTCGAGCCCACCCAGGG
7459	Yes	AACCGAAAGGTTGGTGGTTCGAGCCCACCCAGGGA
7460	Yes	AACCGAAAGGTTGGTGGTTCGAGCCCACCCAGGGAC
7461	Yes	AACCGAAAGGTTGGTGGTTCGAGCCCACCCAGGGACG
7462	Yes	AACCGAAAGGTTGGTGGTTCGAGCCCACCCAGGGACGC
7463	Yes	AACCGAAAGGTTGGTGGTTCGAGCCCACCCAGGGACGCC
7464	Yes	AACCGAAAGGTTGGTGGTTCGAGCCCACCCAGGGACGCCA
7465	Yes	AACCGAAAGGTTGGTGGTTCGAGCCCAT
7466	Yes	AACCGAAAGGTTGGTGGTTCGAGCCCATC
7467	Yes	AACCGAAAGGTTGGTGGTTCGAGCCCATCC
7468	Yes	AACCGAAAGGTTGGTGGTTCGAGCCCATCCA
7469	Yes	AACCGAAAGGTTGGTGGTTCGAGCCCATCCAG

7470	Yes	AACCGAAAGGTTGGTGGTTCGAGCCCATCCAGG
7471	Yes	AACCGAAAGGTTGGTGGTTCGAGCCCATCCAGGG
7472	Yes	AACCGAAAGGTTGGTGGTTCGAGCCCATCCAGGGA
7473	Yes	AACCGAAAGGTTGGTGGTTCGAGCCCATCCAGGGAC
7474	Yes	AACCGAAAGGTTGGTGGTTCGAGCCCATCCAGGGACG
7475	Yes	AACCGAAAGGTTGGTGGTTCGAGCCCATCCAGGGACGC
7476	Yes	AACCGAAAGGTTGGTGGTTCGAGCCCATCCAGGGACGCC
7477	Yes	AACCGAAAGGTTGGTGGTTCGAGCCCATCCAGGGACGCCA
7478	Yes	AACCGAAAGGTTGGTGGTTCGAT
7479	Yes	AACCGAAAGGTTGGTGGTTCGATC
7480	Yes	AACCGAAAGGTTGGTGGTTCGATCC
7481	Yes	AACCGAAAGGTTGGTGGTTCGATCCC
7482	Yes	AACCGAAAGGTTGGTGGTTCGATCCCA
7483	Yes	AACCGAAAGGTTGGTGGTTCGATCCCAC
7484	Yes	AACCGAAAGGTTGGTGGTTCGATCCCACC
7485	Yes	AACCGAAAGGTTGGTGGTTCGATCCCACCC
7486	Yes	AACCGAAAGGTTGGTGGTTCGATCCCACCCA
7487	Yes	AACCGAAAGGTTGGTGGTTCGATCCCACCCAG
7488	Yes	AACCGAAAGGTTGGTGGTTCGATCCCACCCAGG
7489	Yes	AACCGAAAGGTTGGTGGTTCGATCCCACCCAGGG
7490	Yes	AACCGAAAGGTTGGTGGTTCGATCCCACCCAGGGA
7491	Yes	AACCGAAAGGTTGGTGGTTCGATCCCACCCAGGGAC
7492	Yes	AACCGAAAGGTTGGTGGTTCGATCCCACCCAGGGACG
7493	Yes	AACCGAAAGGTTGGTGGTTCGATCCCACCCAGGGACGC
7494	Yes	AACCGAAAGGTTGGTGGTTCGATCCCACCCAGGGACGCC
7495	Yes	AACCGAAAGGTTGGTGGTTCGATCCCACCCAGGGACGCCA
7496	Yes	AACCGAACGGTGAGTA
7497	Yes	AACCGAACGGTGAGTAG
7498	Yes	AACCGAACGGTGAGTAGT
7499	Yes	AACCGAACGGTGAGTAGTT
7500	Yes	AACCGAACGGTGAGTAGTTC
7501	Yes	AACCGAACGGTGAGTAGTTCA
7502	Yes	AACCGAACGGTGAGTAGTTCAA
7503	Yes	AACCGAACGGTGAGTAGTTCAAG
7504	Yes	AACCGAACGGTGAGTAGTTCAAGA
7505	Yes	AACCGAACGGTGAGTAGTTCAAGAC
7506	Yes	AACCGAACGGTGAGTAGTTCAAGACC
7507	Yes	AACCGAACGGTGAGTAGTTCAAGACCA
7508	Yes	AACCGAACGGTGAGTAGTTCAAGACCAC
7509	Yes	AACCGAACGGTGAGTAGTTCAAGACCACC
7510	Yes	AACCGAACGGTGAGTAGTTCAAGACCACCC
7511	Yes	AACCGAACGGTGAGTAGTTCAAGACCACCCA

7512	Yes	AACCGAACGGTGAGTAGTTCAAGACCACCCAG
7513	Yes	AACCGAACGGTGAGTAGTTCAAGACCACCCAGG
7514	Yes	AACCGAACGGTGAGTAGTTCAAGACCACCCAGGG
7515	Yes	AACCGAACGGTGAGTAGTTCAAGACCACCCAGGGA
7516	Yes	AACCGAACGGTGAGTAGTTCAAGACCACCCAGGGAC
7517	Yes	AACCGAACGGTGAGTAGTTCAAGACCACCCAGGGACG
7518	Yes	AACCGAACGGTGAGTAGTTCAAGACCACCCAGGGACGC
7519	Yes	AACCGAACGGTGAGTAGTTCAAGACCACCCAGGGACGCC
7520	Yes	AACCGAACGGTGAGTAGTTCAAGACCACCCAGGGACGCCA
7521	Yes	AACCGAAGATCACGGG
7522	Yes	AACCGAAGATCACGGGT
7523	Yes	AACCGAAGATCACGGGTT
7524	Yes	AACCGAAGATCACGGGTTC
7525	Yes	AACCGAAGATCACGGGTTCG
7526	Yes	AACCGAAGATCACGGGTTCGA
7527	Yes	AACCGAAGATCACGGGTTCGAA
7528	Yes	AACCGAAGATCACGGGTTCGAAC
7529	Yes	AACCGAAGATCACGGGTTCGAACC
7530	Yes	AACCGAAGATCACGGGTTCGAACCC
7531	Yes	AACCGAAGATCACGGGTTCGAACCCC
7532	Yes	AACCGAAGATCACGGGTTCGAACCCCG
7533	Yes	AACCGAAGATCACGGGTTCGAACCCCGT
7534	Yes	AACCGAAGATCACGGGTTCGAACCCCGTC
7535	Yes	AACCGAAGATCACGGGTTCGAACCCCGTCC
7536	Yes	AACCGAAGATCACGGGTTCGAACCCCGTCCG
7537	Yes	AACCGAAGATCACGGGTTCGAACCCCGTCCGT
7538	Yes	AACCGAAGATCACGGGTTCGAACCCCGTCCGTG
7539	Yes	AACCGAAGATCACGGGTTCGAACCCCGTCCGTGC
7540	Yes	AACCGAAGATCACGGGTTCGAACCCCGTCCGTGCC
7541	Yes	AACCGAAGATCACGGGTTCGAACCCCGTCCGTGCCT
7542	Yes	AACCGAAGATCACGGGTTCGAACCCCGTCCGTGCCTC
7543	Yes	AACCGAAGATCACGGGTTCGAACCCCGTCCGTGCCTCC
7544	Yes	AACCGAAGATCACGGGTTCGAACCCCGTCCGTGCCTCCA
7545	Yes	AACCGAAGATCGCGGG
7546	Yes	AACCGAAGATCGCGGGT
7547	Yes	AACCGAAGATCGCGGGTT
7548	Yes	AACCGAAGATCGCGGGTTC
7549	Yes	AACCGAAGATCGCGGGTTCG
7550	Yes	AACCGAAGATCGCGGGTTCGA
7551	Yes	AACCGAAGATCGCGGGTTCGAA
7552	Yes	AACCGAAGATCGCGGGTTCGAAC
7553	Yes	AACCGAAGATCGCGGGTTCGAACC

7554	Yes	AACCGAAGATCGCGGGTTCGAACCC
7555	Yes	AACCGAAGATCGCGGGTTCGAACCCC
7556	Yes	AACCGAAGATCGCGGGTTCGAACCCCG
7557	Yes	AACCGAAGATCGCGGGTTCGAACCCCGT
7558	Yes	AACCGAAGATCGCGGGTTCGAACCCCGTC
7559	Yes	AACCGAAGATCGCGGGTTCGAACCCCGTCC
7560	Yes	AACCGAAGATCGCGGGTTCGAACCCCGTCCG
7561	Yes	AACCGAAGATCGCGGGTTCGAACCCCGTCCGT
7562	Yes	AACCGAAGATCGCGGGTTCGAACCCCGTCCGTG
7563	Yes	AACCGAAGATCGCGGGTTCGAACCCCGTCCGTGC
7564	Yes	AACCGAAGATCGCGGGTTCGAACCCCGTCCGTGCC
7565	Yes	AACCGAAGATCGCGGGTTCGAACCCCGTCCGTGCCT
7566	Yes	AACCGAAGATCGCGGGTTCGAACCCCGTCCGTGCCTC
7567	Yes	AACCGAAGATCGCGGGTTCGAACCCCGTCCGTGCCTCC
7568	Yes	AACCGAAGATCGCGGGTTCGAACCCCGTCCGTGCCTCCA
7569	Yes	AACCGAATAGCTTAGT
7570	Yes	AACCGAATAGCTTAGTT
7571	Yes	AACCGAATAGCTTAGTTG
7572	Yes	AACCGAATAGCTTAGTTGA
7573	Yes	AACCGAATAGCTTAGTTGAT
7574	Yes	AACCGAATAGCTTAGTTGATG
7575	Yes	AACCGAATAGCTTAGTTGATGA
7576	Yes	AACCGAATAGCTTAGTTGATGAA
7577	Yes	AACCGAATAGCTTAGTTGATGAAG
7578	Yes	AACCGAATAGCTTAGTTGATGAAGC
7579	Yes	AACCGAATAGCTTAGTTGATGAAGCG
7580	Yes	AACCGAATAGCTTAGTTGATGAAGCGT
7581	Yes	AACCGAATAGCTTAGTTGATGAAGCGTG
7582	Yes	AACCGAATAGCTTAGTTGATGAAGCGTGA
7583	Yes	AACCGAATAGCTTAGTTGATGAAGCGTGAG
7584	Yes	AACCGAATAGCTTAGTTGATGAAGCGTGAGA
7585	Yes	AACCGAATAGCTTAGTTGATGAAGCGTGAGAC
7586	Yes	AACCGAATAGCTTAGTTGATGAAGCGTGAGACT
7587	Yes	AACCGAATAGCTTAGTTGATGAAGCGTGAGACTC
7588	Yes	AACCGAATAGCTTAGTTGATGAAGCGTGAGACTCT
7589	Yes	AACCGAATAGCTTAGTTGATGAAGCGTGAGACTCTT
7590	Yes	AACCGAATAGCTTAGTTGATGAAGCGTGAGACTCTTA
7591	Yes	AACCGAATAGCTTAGTTGATGAAGCGTGAGACTCTTAA
7592	Yes	AACCGAATAGCTTAGTTGATGAAGCGTGAGACTCTTAAT
7593	Yes	AACCGAATAGCTTAGTTGATGAAGCGTGAGACTCTTAATC
7594	Yes	AACCGAATAGCTTAGTTGATGAAGCGTGAGACTCTTAATCT
7595	Yes	AACCGAATAGCTTAGTTGATGAAGCGTGAGACTCTTAATCTC

7596	Yes	AACCGAATAGCTTAGTTGATGAAGCGTGAGACTCTTAATCTCA
7597	Yes	AACCGAATAGCTTAGTTGATGAAGCGTGAGACTCTTAATCTCAG
7598	Yes	AACCGAATAGCTTAGTTGATGAAGCGTGAGACTCTTAATCTCAGG
7599	Yes	AACCGAATAGCTTAGTTGATGAAGCGTGAGACTCTTAATCTCAGGG
7600	Yes	AACCGAATAGCTTAGTTGATGAAGCGTGAGACTCTTAATCTCAGGGT
7601	Yes	AACCGAATAGCTTAGTTGATGAAGCGTGAGACTCTTAATCTCAGGGTA
7602	Yes	AACCGAATAGCTTAGTTGATGAAGCGTGAGACTCTTAATCTCAGGGTAG
7603	Yes	AACCGAATAGCTTAGTTGATGAAGCGTGAGACTCTTAATCTCAGGGTAGT
7604	Yes	AACCGAGCGGAAACAC
7605	Yes	AACCGAGCGGAAACACC
7606	Yes	AACCGAGCGGAAACACCA
7607	Yes	AACCGGAAAGTTGGTG
7608	Yes	AACCGGAAAGTTGGTGG
7609	Yes	AACCGGAAAGTTGGTGGT
7610	Yes	AACCGGAAAGTTGGTGGTT
7611	Yes	AACCGGAAAGTTGGTGGTTC
7612	Yes	AACCGGAAAGTTGGTGGTTCG
7613	Yes	AACCGGAAAGTTGGTGGTTCGA
7614	Yes	AACCGGAAAGTTGGTGGTTCGAG
7615	Yes	AACCGGAAAGTTGGTGGTTCGAGC
7616	Yes	AACCGGAAAGTTGGTGGTTCGAGCC
7617	Yes	AACCGGAAAGTTGGTGGTTCGAGCCT
7618	Yes	AACCGGAAAGTTGGTGGTTCGAGCCTA
7619	Yes	AACCGGAAAGTTGGTGGTTCGAGCCTAC
7620	Yes	AACCGGAAAGTTGGTGGTTCGAGCCTACC
7621	Yes	AACCGGAAAGTTGGTGGTTCGAGCCTACCC
7622	Yes	AACCGGAAAGTTGGTGGTTCGAGCCTACCCA
7623	Yes	AACCGGAAAGTTGGTGGTTCGAGCCTACCCAG
7624	Yes	AACCGGAAAGTTGGTGGTTCGAGCCTACCCAGG
7625	Yes	AACCGGAAAGTTGGTGGTTCGAGCCTACCCAGGG
7626	Yes	AACCGGAAAGTTGGTGGTTCGAGCCTACCCAGGGA
7627	Yes	AACCGGAAAGTTGGTGGTTCGAGCCTACCCAGGGAC
7628	Yes	AACCGGAAAGTTGGTGGTTCGAGCCTACCCAGGGACG
7629	Yes	AACCGGAAAGTTGGTGGTTCGAGCCTACCCAGGGACGC
7630	Yes	AACCGGAAAGTTGGTGGTTCGAGCCTACCCAGGGACGCC
7631	Yes	AACCGGAAAGTTGGTGGTTCGAGCCTACCCAGGGACGCCA
7632	Yes	AACCGGAGATGAAAAC
7633	Yes	AACCGGAGATGAAAACC
7634	Yes	AACCGGAGATGAAAACCT
7635	Yes	AACCGGAGATGAAAACCTT
7636	Yes	AACCGGAGATGAAAACCTTT
7637	Yes	AACCGGAGATGAAAACCTTTT

7638	Yes	AACCGGAGATGAAAACCTTTTT
7639	Yes	AACCGGAGATGAAAACCTTTTTC
7640	Yes	AACCGGAGATGAAAACCTTTTTCC
7641	Yes	AACCGGAGATGAAAACCTTTTTCCA
7642	Yes	AACCGGAGATGAAAACCTTTTTCCAA
7643	Yes	AACCGGAGATGAAAACCTTTTTCCAAG
7644	Yes	AACCGGAGATGAAAACCTTTTTCCAAGG
7645	Yes	AACCGGAGATGAAAACCTTTTTCCAAGGA
7646	Yes	AACCGGAGATGAAAACCTTTTTCCAAGGAC
7647	Yes	AACCGGAGATGAAAACCTTTTTCCAAGGACA
7648	Yes	AACCGGAGATGAAAACCTTTTTCCAAGGACAC
7649	Yes	AACCGGAGATGAAAACCTTTTTCCAAGGACACC
7650	Yes	AACCGGAGATGAAAACCTTTTTCCAAGGACACCA
7651	No	AACCGGGATGGCCGAG
7652	Yes	AACCGGGATGGCCGAGT
7653	Yes	AACCGGGATGGCCGAGTG
7654	Yes	AACCGGGATGGCCGAGTGG
7655	Yes	AACCGGGATGGCCGAGTGGT
7656	Yes	AACCGGGATGGCCGAGTGGTT
7657	Yes	AACCGGGATGGCCGAGTGGTTA
7658	Yes	AACCGGGATGGCCGAGTGGTTAA
7659	Yes	AACCGGGATGGCCGAGTGGTTAAG
7660	Yes	AACCGGGATGGCCGAGTGGTTAAGG
7661	Yes	AACCGGGATGGCCGAGTGGTTAAGGC
7662	Yes	AACCGGGATGGCCGAGTGGTTAAGGCG
7663	Yes	AACCGGGATGGCCGAGTGGTTAAGGCGT
7664	Yes	AACCGGGATGGCCGAGTGGTTAAGGCGTT
7665	Yes	AACCGGGATGGCCGAGTGGTTAAGGCGTTG
7666	Yes	AACCGGGATGGCCGAGTGGTTAAGGCGTTGG
7667	Yes	AACCGGGATGGCCGAGTGGTTAAGGCGTTGGA
7668	Yes	AACCGGGATGGCCGAGTGGTTAAGGCGTTGGAC
7669	Yes	AACCGGGATGGCCGAGTGGTTAAGGCGTTGGACT
7670	Yes	AACCGGGATGGCCGAGTGGTTAAGGCGTTGGACTT
7671	Yes	AACCGGGATGGCCGAGTGGTTAAGGCGTTGGACTTA
7672	Yes	AACCGGGATGGCCGAGTGGTTAAGGCGTTGGACTTAA
7673	Yes	AACCGGGATGGCCGAGTGGTTAAGGCGTTGGACTTAAG
7674	Yes	AACCGGGATGGCCGAGTGGTTAAGGCGTTGGACTTAAGA
7675	Yes	AACCGGGATGGCCGAGTGGTTAAGGCGTTGGACTTAAGAT
7676	Yes	AACCGGGATGGCCGAGTGGTTAAGGCGTTGGACTTAAGATC
7677	Yes	AACCGGGATGGCCGAGTGGTTAAGGCGTTGGACTTAAGATCC
7678	Yes	AACCGGGATGGCCGAGTGGTTAAGGCGTTGGACTTAAGATCCA
7679	Yes	AACCGGGATGGCCGAGTGGTTAAGGCGTTGGACTTAAGATCCAA

7680	Yes	AACCGGGATGGCCGAGTGGTTAAGGCGTTGGACTTAAGATCCAAT
7681	Yes	AACCGGGATGGCCGAGTGGTTAAGGCGTTGGACTTAAGATCCAATG
7682	Yes	AACCGGGATGGCCGAGTGGTTAAGGCGTTGGACTTAAGATCCAATGG
7683	Yes	AACCGGGATGGCCGAGTGGTTAAGGCGTTGGACTTAAGATCCAATGGG
7684	Yes	AACCGGGATGGCCGAGTGGTTAAGGCGTTGGACTTAAGATCCAATGGGC
7685	Yes	AACCGGGATGGCCGAGTGGTTAAGGCGTTGGACTTAAGATCCAATGGGCT
7686	No	AACCGGGATGGCTGAG
7687	No	AACCGGGATGGCTGAGT
7688	Yes	AACCGGGATGGCTGAGTG
7689	Yes	AACCGGGATGGCTGAGTGG
7690	Yes	AACCGGGATGGCTGAGTGGT
7691	Yes	AACCGGGATGGCTGAGTGGTT
7692	Yes	AACCGGGATGGCTGAGTGGTTA
7693	Yes	AACCGGGATGGCTGAGTGGTTAA
7694	Yes	AACCGGGATGGCTGAGTGGTTAAG
7695	Yes	AACCGGGATGGCTGAGTGGTTAAGG
7696	Yes	AACCGGGATGGCTGAGTGGTTAAGGC
7697	Yes	AACCGGGATGGCTGAGTGGTTAAGGCG
7698	Yes	AACCGGGATGGCTGAGTGGTTAAGGCGT
7699	Yes	AACCGGGATGGCTGAGTGGTTAAGGCGTT
7700	Yes	AACCGGGATGGCTGAGTGGTTAAGGCGTTG
7701	Yes	AACCGGGATGGCTGAGTGGTTAAGGCGTTGG
7702	Yes	AACCGGGATGGCTGAGTGGTTAAGGCGTTGGA
7703	Yes	AACCGGGATGGCTGAGTGGTTAAGGCGTTGGAC
7704	Yes	AACCGGGATGGCTGAGTGGTTAAGGCGTTGGACT
7705	Yes	AACCGGGATGGCTGAGTGGTTAAGGCGTTGGACTT
7706	Yes	AACCGGGATGGCTGAGTGGTTAAGGCGTTGGACTTA
7707	Yes	AACCGGGATGGCTGAGTGGTTAAGGCGTTGGACTTAA
7708	Yes	AACCGGGATGGCTGAGTGGTTAAGGCGTTGGACTTAAG
7709	Yes	AACCGGGATGGCTGAGTGGTTAAGGCGTTGGACTTAAGA
7710	Yes	AACCGGGATGGCTGAGTGGTTAAGGCGTTGGACTTAAGAT
7711	Yes	AACCGGGATGGCTGAGTGGTTAAGGCGTTGGACTTAAGATC
7712	Yes	AACCGGGATGGCTGAGTGGTTAAGGCGTTGGACTTAAGATCC
7713	Yes	AACCGGGATGGCTGAGTGGTTAAGGCGTTGGACTTAAGATCCA
7714	Yes	AACCGGGATGGCTGAGTGGTTAAGGCGTTGGACTTAAGATCCAA
7715	Yes	AACCGGGATGGCTGAGTGGTTAAGGCGTTGGACTTAAGATCCAAT
7716	Yes	AACCGGGATGGCTGAGTGGTTAAGGCGTTGGACTTAAGATCCAATG
7717	Yes	AACCGGGATGGCTGAGTGGTTAAGGCGTTGGACTTAAGATCCAATGG
7718	Yes	AACCGGGATGGCTGAGTGGTTAAGGCGTTGGACTTAAGATCCAATGGA
7719	Yes	AACCGGGATGGCTGAGTGGTTAAGGCGTTGGACTTAAGATCCAATGGAC
7720	Yes	AACCGGGATGGCTGAGTGGTTAAGGCGTTGGACTTAAGATCCAATGGACA
7721	Yes	AACCGGGCAGAAACAC

7722	Yes	AACCGGGCAGAAACACC
7723	Yes	AACCGGGCAGAAACACCA
7724	No	AACCGGGCAGAAGCAC
7725	Yes	AACCGGGCAGAAGCACC
7726	Yes	AACCGGGCAGAAGCACCA
7727	Yes	AACCGGGCGGAAACAC
7728	Yes	AACCGGGCGGAAACACC
7729	Yes	AACCGGGCGGAAACACCA
7730	No	AACCTAAACTCTAAAT
7731	No	AACCTAAACTCTAAATT
7732	No	AACCTAAACTCTAAATTT
7733	Yes	AACCTAAACTCTAAATTTA
7734	Yes	AACCTAAACTCTAAATTTAG
7735	Yes	AACCTAAACTCTAAATTTAGA
7736	Yes	AACCTAAACTCTAAATTTAGAT
7737	Yes	AACCTAAACTCTAAATTTAGATG
7738	Yes	AACCTAAACTCTAAATTTAGATGT
7739	Yes	AACCTAAACTCTAAATTTAGATGTC
7740	Yes	AACCTAAACTCTAAATTTAGATGTCC
7741	Yes	AACCTAAACTCTAAATTTAGATGTCCT
7742	Yes	AACCTAAACTCTAAATTTAGATGTCCTG
7743	Yes	AACCTAAACTCTAAATTTAGATGTCCTGA
7744	Yes	AACCTAAACTCTAAATTTAGATGTCCTGAG
7745	Yes	AACCTAAACTCTAAATTTAGATGTCCTGAGT
7746	Yes	AACCTAAACTCTAAATTTAGATGTCCTGAGTT
7747	Yes	AACCTAAACTCTAAATTTAGATGTCCTGAGTTC
7748	Yes	AACCTAAACTCTAAATTTAGATGTCCTGAGTTCA
7749	Yes	AACCTAAACTCTAAATTTAGATGTCCTGAGTTCAA
7750	Yes	AACCTAAACTCTAAATTTAGATGTCCTGAGTTCAAA
7751	Yes	AACCTAAACTCTAAATTTAGATGTCCTGAGTTCAAAT
7752	Yes	AACCTAAACTCTAAATTTAGATGTCCTGAGTTCAAATC
7753	Yes	AACCTAAACTCTAAATTTAGATGTCCTGAGTTCAAATCC
7754	Yes	AACCTAAACTCTAAATTTAGATGTCCTGAGTTCAAATCCC
7755	Yes	AACCTAAACTCTAAATTTAGATGTCCTGAGTTCAAATCCCA
7756	Yes	AACCTAAACTCTAAATTTAGATGTCCTGAGTTCAAATCCCAG
7757	Yes	AACCTAAACTCTAAATTTAGATGTCCTGAGTTCAAATCCCAGC
7758	Yes	AACCTAAACTCTAAATTTAGATGTCCTGAGTTCAAATCCCAGCT
7759	Yes	AACCTAAACTCTAAATTTAGATGTCCTGAGTTCAAATCCCAGCTG
7760	Yes	AACCTAAACTCTAAATTTAGATGTCCTGAGTTCAAATCCCAGCTGT
7761	Yes	AACCTAAACTCTAAATTTAGATGTCCTGAGTTCAAATCCCAGCTGTA
7762	Yes	AACCTAAACTCTAAATTTAGATGTCCTGAGTTCAAATCCCAGCTGTAT
7763	Yes	AACCTAAACTCTAAATTTAGATGTCCTGAGTTCAAATCCCAGCTGTATG

7764	Yes	AACCTAAACTCTAAATTTAGATGTCCTGAGTTCAAATCCCAGCTGTATGC
7765	Yes	AACCTAGGGGTCGTGA
7766	Yes	AACCTAGGGGTCGTGAG
7767	Yes	AACCTAGGGGTCGTGAGT
7768	Yes	AACCTAGGGGTCGTGAGTT
7769	Yes	AACCTAGGGGTCGTGAGTTC
7770	Yes	AACCTAGGGGTCGTGAGTTCA
7771	Yes	AACCTAGGGGTCGTGAGTTCAA
7772	Yes	AACCTAGGGGTCGTGAGTTCAAA
7773	Yes	AACCTAGGGGTCGTGAGTTCAAAT
7774	Yes	AACCTAGGGGTCGTGAGTTCAAATC
7775	Yes	AACCTAGGGGTCGTGAGTTCAAATCT
7776	Yes	AACCTAGGGGTCGTGAGTTCAAATCTC
7777	Yes	AACCTAGGGGTCGTGAGTTCAAATCTCA
7778	Yes	AACCTAGGGGTCGTGAGTTCAAATCTCAC
7779	Yes	AACCTAGGGGTCGTGAGTTCAAATCTCACC
7780	Yes	AACCTAGGGGTCGTGAGTTCAAATCTCACCA
7781	Yes	AACCTAGGGGTCGTGAGTTCAAATCTCACCAG
7782	Yes	AACCTAGGGGTCGTGAGTTCAAATCTCACCAGG
7783	Yes	AACCTAGGGGTCGTGAGTTCAAATCTCACCAGGG
7784	Yes	AACCTAGGGGTCGTGAGTTCAAATCTCACCAGGGC
7785	Yes	AACCTAGGGGTCGTGAGTTCAAATCTCACCAGGGCC
7786	Yes	AACCTAGGGGTCGTGAGTTCAAATCTCACCAGGGCCT
7787	Yes	AACCTAGGGGTCGTGAGTTCAAATCTCACCAGGGCCTC
7788	Yes	AACCTAGGGGTCGTGAGTTCAAATCTCACCAGGGCCTCC
7789	Yes	AACCTAGGGGTCGTGAGTTCAAATCTCACCAGGGCCTCCA
7790	No	AACCTCAGAGGGGGCA
7791	Yes	AACCTCAGAGGGGGCAC
7792	Yes	AACCTCAGAGGGGGCACC
7793	Yes	AACCTCAGAGGGGGCACCA
7794	Yes	AACCTCGGTTCGAATC
7795	Yes	AACCTCGGTTCGAATCC
7796	Yes	AACCTCGGTTCGAATCCG
7797	Yes	AACCTCGGTTCGAATCCGA
7798	Yes	AACCTCGGTTCGAATCCGAG
7799	Yes	AACCTCGGTTCGAATCCGAGT
7800	Yes	AACCTCGGTTCGAATCCGAGTC
7801	Yes	AACCTCGGTTCGAATCCGAGTCA
7802	Yes	AACCTCGGTTCGAATCCGAGTCAC
7803	Yes	AACCTCGGTTCGAATCCGAGTCACG
7804	Yes	AACCTCGGTTCGAATCCGAGTCACGG
7805	Yes	AACCTCGGTTCGAATCCGAGTCACGGC

7806	Yes	AACCTCGGTTCGAATCCGAGTCACGGCA
7807	Yes	AACCTCGGTTCGAATCCGAGTCACGGCAC
7808	Yes	AACCTCGGTTCGAATCCGAGTCACGGCACC
7809	Yes	AACCTCGGTTCGAATCCGAGTCACGGCACCA
7810	No	AACCTGAGGGTTCAGG
7811	No	AACCTGAGGGTTCAGGG
7812	Yes	AACCTGAGGGTTCAGGGT
7813	Yes	AACCTGAGGGTTCAGGGTT
7814	Yes	AACCTGAGGGTTCAGGGTTC
7815	Yes	AACCTGAGGGTTCAGGGTTCA
7816	Yes	AACCTGAGGGTTCAGGGTTCAA
7817	Yes	AACCTGAGGGTTCAGGGTTCAAG
7818	Yes	AACCTGAGGGTTCAGGGTTCAAGT
7819	Yes	AACCTGAGGGTTCAGGGTTCAAGTC
7820	Yes	AACCTGAGGGTTCAGGGTTCAAGTCT
7821	Yes	AACCTGAGGGTTCAGGGTTCAAGTCTC
7822	Yes	AACCTGAGGGTTCAGGGTTCAAGTCTCT
7823	Yes	AACCTGAGGGTTCAGGGTTCAAGTCTCTG
7824	Yes	AACCTGAGGGTTCAGGGTTCAAGTCTCTGT
7825	Yes	AACCTGAGGGTTCAGGGTTCAAGTCTCTGTT
7826	Yes	AACCTGAGGGTTCAGGGTTCAAGTCTCTGTTT
7827	Yes	AACCTGAGGGTTCAGGGTTCAAGTCTCTGTTTG
7828	Yes	AACCTGAGGGTTCAGGGTTCAAGTCTCTGTTTGG
7829	Yes	AACCTGAGGGTTCAGGGTTCAAGTCTCTGTTTGGG
7830	Yes	AACCTGAGGGTTCAGGGTTCAAGTCTCTGTTTGGGC
7831	Yes	AACCTGAGGGTTCAGGGTTCAAGTCTCTGTTTGGGCG
7832	Yes	AACCTGAGGGTTCAGGGTTCAAGTCTCTGTTTGGGCGC
7833	Yes	AACCTGAGGGTTCAGGGTTCAAGTCTCTGTTTGGGCGCC
7834	Yes	AACCTGAGGGTTCAGGGTTCAAGTCTCTGTTTGGGCGCCA
7835	No	AACCTGCCGGGGCTTC
7836	No	AACCTGCCGGGGCTTCC
7837	Yes	AACCTGCCGGGGCTTCCA
7838	No	AACCTGGGTAGCTCAG
7839	Yes	AACCTGGGTAGCTCAGT
7840	Yes	AACCTGGGTAGCTCAGTA
7841	Yes	AACCTGGGTAGCTCAGTAG
7842	Yes	AACCTGGGTAGCTCAGTAGG
7843	Yes	AACCTGGGTAGCTCAGTAGGT
7844	Yes	AACCTGGGTAGCTCAGTAGGTA
7845	Yes	AACCTGGGTAGCTCAGTAGGTAG
7846	Yes	AACCTGGGTAGCTCAGTAGGTAGA
7847	Yes	AACCTGGGTAGCTCAGTAGGTAGAA

7848	Yes	AACCTGGGTAGCTCAGTAGGTAGAAC
7849	Yes	AACCTGGGTAGCTCAGTAGGTAGAACA
7850	Yes	AACCTGGGTAGCTCAGTAGGTAGAACAT
7851	Yes	AACCTGGGTAGCTCAGTAGGTAGAACATC
7852	Yes	AACCTGGGTAGCTCAGTAGGTAGAACATCA
7853	Yes	AACCTGGGTAGCTCAGTAGGTAGAACATCAG
7854	Yes	AACCTGGGTAGCTCAGTAGGTAGAACATCAGA
7855	Yes	AACCTGGGTAGCTCAGTAGGTAGAACATCAGAC
7856	Yes	AACCTGGGTAGCTCAGTAGGTAGAACATCAGACT
7857	Yes	AACCTGGGTAGCTCAGTAGGTAGAACATCAGACTT
7858	Yes	AACCTGGGTAGCTCAGTAGGTAGAACATCAGACTTT
7859	Yes	AACCTGGGTAGCTCAGTAGGTAGAACATCAGACTTTT
7860	Yes	AACCTGGGTAGCTCAGTAGGTAGAACATCAGACTTTTA
7861	Yes	AACCTGGGTAGCTCAGTAGGTAGAACATCAGACTTTTAA
7862	Yes	AACCTGGGTAGCTCAGTAGGTAGAACATCAGACTTTTAAT
7863	Yes	AACCTGGGTAGCTCAGTAGGTAGAACATCAGACTTTTAATC
7864	Yes	AACCTGGGTAGCTCAGTAGGTAGAACATCAGACTTTTAATCT
7865	Yes	AACCTGGGTAGCTCAGTAGGTAGAACATCAGACTTTTAATCTG
7866	Yes	AACCTGGGTAGCTCAGTAGGTAGAACATCAGACTTTTAATCTGA
7867	Yes	AACCTGGGTAGCTCAGTAGGTAGAACATCAGACTTTTAATCTGAG
7868	Yes	AACCTGGGTAGCTCAGTAGGTAGAACATCAGACTTTTAATCTGAGG
7869	Yes	AACCTGGGTAGCTCAGTAGGTAGAACATCAGACTTTTAATCTGAGGG
7870	Yes	AACCTGGGTAGCTCAGTAGGTAGAACATCAGACTTTTAATCTGAGGGT
7871	Yes	AACCTGGGTAGCTCAGTAGGTAGAACATCAGACTTTTAATCTGAGGGTC
7872	Yes	AACCTGGGTAGCTCAGTAGGTAGAACATCAGACTTTTAATCTGAGGGTCT
7873	Yes	AACCTGGGTAGCTTAG
7874	Yes	AACCTGGGTAGCTTAGT
7875	Yes	AACCTGGGTAGCTTAGTT
7876	Yes	AACCTGGGTAGCTTAGTTG
7877	Yes	AACCTGGGTAGCTTAGTTGG
7878	Yes	AACCTGGGTAGCTTAGTTGGT
7879	Yes	AACCTGGGTAGCTTAGTTGGTA
7880	Yes	AACCTGGGTAGCTTAGTTGGTAG
7881	Yes	AACCTGGGTAGCTTAGTTGGTAGA
7882	Yes	AACCTGGGTAGCTTAGTTGGTAGAG
7883	Yes	AACCTGGGTAGCTTAGTTGGTAGAGC
7884	Yes	AACCTGGGTAGCTTAGTTGGTAGAGCA
7885	Yes	AACCTGGGTAGCTTAGTTGGTAGAGCAT
7886	Yes	AACCTGGGTAGCTTAGTTGGTAGAGCATT
7887	Yes	AACCTGGGTAGCTTAGTTGGTAGAGCATTG
7888	Yes	AACCTGGGTAGCTTAGTTGGTAGAGCATTGG
7889	Yes	AACCTGGGTAGCTTAGTTGGTAGAGCATTGGA

7890	Yes	AACCTGGGTAGCTTAGTTGGTAGAGCATTGGAC
7891	Yes	AACCTGGGTAGCTTAGTTGGTAGAGCATTGGACT
7892	Yes	AACCTGGGTAGCTTAGTTGGTAGAGCATTGGACTT
7893	Yes	AACCTGGGTAGCTTAGTTGGTAGAGCATTGGACTTT
7894	Yes	AACCTGGGTAGCTTAGTTGGTAGAGCATTGGACTTTT
7895	Yes	AACCTGGGTAGCTTAGTTGGTAGAGCATTGGACTTTTA
7896	Yes	AACCTGGGTAGCTTAGTTGGTAGAGCATTGGACTTTTAA
7897	Yes	AACCTGGGTAGCTTAGTTGGTAGAGCATTGGACTTTTAAT
7898	Yes	AACCTGGGTAGCTTAGTTGGTAGAGCATTGGACTTTTAATT
7899	Yes	AACCTGGGTAGCTTAGTTGGTAGAGCATTGGACTTTTAATTT
7900	Yes	AACCTGGGTAGCTTAGTTGGTAGAGCATTGGACTTTTAATTTG
7901	Yes	AACCTGGGTAGCTTAGTTGGTAGAGCATTGGACTTTTAATTTGA
7902	Yes	AACCTGGGTAGCTTAGTTGGTAGAGCATTGGACTTTTAATTTGAG
7903	Yes	AACCTGGGTAGCTTAGTTGGTAGAGCATTGGACTTTTAATTTGAGG
7904	Yes	AACCTGGGTAGCTTAGTTGGTAGAGCATTGGACTTTTAATTTGAGGG
7905	Yes	AACCTGGGTAGCTTAGTTGGTAGAGCATTGGACTTTTAATTTGAGGGC
7906	Yes	AACCTGGGTAGCTTAGTTGGTAGAGCATTGGACTTTTAATTTGAGGGCC
7907	Yes	AACCTGGGTAGCTTAGTTGGTAGAGCATTGGACTTTTAATTTGAGGGCCC
7908	No	AACCTTTTAAGTTAAA
7909	No	AACCTTTTAAGTTAAAG
7910	No	AACCTTTTAAGTTAAAGA
7911	No	AACCTTTTAAGTTAAAGAT
7912	No	AACCTTTTAAGTTAAAGATT
7913	No	AACCTTTTAAGTTAAAGATTA
7914	No	AACCTTTTAAGTTAAAGATTAA
7915	No	AACCTTTTAAGTTAAAGATTAAG
7916	No	AACCTTTTAAGTTAAAGATTAAGA
7917	No	AACCTTTTAAGTTAAAGATTAAGAG
7918	No	AACCTTTTAAGTTAAAGATTAAGAGA
7919	No	AACCTTTTAAGTTAAAGATTAAGAGAA
7920	No	AACCTTTTAAGTTAAAGATTAAGAGAAC
7921	No	AACCTTTTAAGTTAAAGATTAAGAGAACC
7922	No	AACCTTTTAAGTTAAAGATTAAGAGAACCA
7923	No	AACCTTTTAAGTTAAAGATTAAGAGAACCAA
7924	No	AACCTTTTAAGTTAAAGATTAAGAGAACCAAC
7925	No	AACCTTTTAAGTTAAAGATTAAGAGAACCAACA
7926	No	AACCTTTTAAGTTAAAGATTAAGAGAACCAACAC
7927	No	AACCTTTTAAGTTAAAGATTAAGAGAACCAACACC
7928	No	AACCTTTTAAGTTAAAGATTAAGAGAACCAACACCT
7929	No	AACCTTTTAAGTTAAAGATTAAGAGAACCAACACCTC
7930	No	AACCTTTTAAGTTAAAGATTAAGAGAACCAACACCTCT
7931	No	AACCTTTTAAGTTAAAGATTAAGAGAACCAACACCTCTT

7932	No	AACCTTTTAAGTTAAAGATTAAGAGAACCAACACCTCTTT
7933	No	AACCTTTTAAGTTAAAGATTAAGAGAACCAACACCTCTTTA
7934	No	AACCTTTTAAGTTAAAGATTAAGAGAACCAACACCTCTTTAC
7935	No	AACCTTTTAAGTTAAAGATTAAGAGAACCAACACCTCTTTACA
7936	No	AACCTTTTAAGTTAAAGATTAAGAGAACCAACACCTCTTTACAG
7937	No	AACCTTTTAAGTTAAAGATTAAGAGAACCAACACCTCTTTACAGT
7938	No	AACCTTTTAAGTTAAAGATTAAGAGAACCAACACCTCTTTACAGTG
7939	No	AACCTTTTAAGTTAAAGATTAAGAGAACCAACACCTCTTTACAGTGA
7940	Yes	AACCTTTTAAGTTAAAGATTAAGAGAACCAACACCTCTTTACAGTGAC
7941	Yes	AACCTTTTAAGTTAAAGATTAAGAGAACCAACACCTCTTTACAGTGACC
7942	Yes	AACCTTTTAAGTTAAAGATTAAGAGAACCAACACCTCTTTACAGTGACCA
7943	No	AACCTTTTTCCAAGGA
7944	Yes	AACCTTTTTCCAAGGAC
7945	Yes	AACCTTTTTCCAAGGACA
7946	Yes	AACCTTTTTCCAAGGACAC
7947	Yes	AACCTTTTTCCAAGGACACC
7948	Yes	AACCTTTTTCCAAGGACACCA
7949	Yes	AACGAAAGGTTGGTGG
7950	Yes	AACGAAAGGTTGGTGGT
7951	Yes	AACGAAAGGTTGGTGGTT
7952	Yes	AACGAAAGGTTGGTGGTTC
7953	Yes	AACGAAAGGTTGGTGGTTCG
7954	Yes	AACGAAAGGTTGGTGGTTCGT
7955	Yes	AACGAAAGGTTGGTGGTTCGTT
7956	Yes	AACGAAAGGTTGGTGGTTCGTTC
7957	Yes	AACGAAAGGTTGGTGGTTCGTTCC
7958	Yes	AACGAAAGGTTGGTGGTTCGTTCCC
7959	Yes	AACGAAAGGTTGGTGGTTCGTTCCCA
7960	Yes	AACGAAAGGTTGGTGGTTCGTTCCCAC
7961	Yes	AACGAAAGGTTGGTGGTTCGTTCCCACC
7962	Yes	AACGAAAGGTTGGTGGTTCGTTCCCACCC
7963	Yes	AACGAAAGGTTGGTGGTTCGTTCCCACCCC
7964	Yes	AACGAAAGGTTGGTGGTTCGTTCCCACCCCG
7965	Yes	AACGAAAGGTTGGTGGTTCGTTCCCACCCCGG
7966	Yes	AACGAAAGGTTGGTGGTTCGTTCCCACCCCGGG
7967	Yes	AACGAAAGGTTGGTGGTTCGTTCCCACCCCGGGA
7968	Yes	AACGAAAGGTTGGTGGTTCGTTCCCACCCCGGGAC
7969	Yes	AACGAAAGGTTGGTGGTTCGTTCCCACCCCGGGACA
7970	Yes	AACGAAAGGTTGGTGGTTCGTTCCCACCCCGGGACAC
7971	Yes	AACGAAAGGTTGGTGGTTCGTTCCCACCCCGGGACACC
7972	Yes	AACGAAAGGTTGGTGGTTCGTTCCCACCCCGGGACACCA
7973	No	AACGAATGATTTCGAC

7974	No	AACGAATGATTTCGACT
7975	No	AACGAATGATTTCGACTC
7976	No	AACGAATGATTTCGACTCA
7977	No	AACGAATGATTTCGACTCAT
7978	No	AACGAATGATTTCGACTCATT
7979	No	AACGAATGATTTCGACTCATTA
7980	No	AACGAATGATTTCGACTCATTAA
7981	No	AACGAATGATTTCGACTCATTAAA
7982	No	AACGAATGATTTCGACTCATTAAAT
7983	No	AACGAATGATTTCGACTCATTAAATT
7984	No	AACGAATGATTTCGACTCATTAAATTA
7985	No	AACGAATGATTTCGACTCATTAAATTAT
7986	No	AACGAATGATTTCGACTCATTAAATTATG
7987	No	AACGAATGATTTCGACTCATTAAATTATGA
7988	No	AACGAATGATTTCGACTCATTAAATTATGAT
7989	No	AACGAATGATTTCGACTCATTAAATTATGATA
7990	No	AACGAATGATTTCGACTCATTAAATTATGATAA
7991	No	AACGAATGATTTCGACTCATTAAATTATGATAAT
7992	No	AACGAATGATTTCGACTCATTAAATTATGATAATC
7993	No	AACGAATGATTTCGACTCATTAAATTATGATAATCA
7994	No	AACGAATGATTTCGACTCATTAAATTATGATAATCAT
7995	No	AACGAATGATTTCGACTCATTAAATTATGATAATCATA
7996	No	AACGAATGATTTCGACTCATTAAATTATGATAATCATAT
7997	No	AACGAATGATTTCGACTCATTAAATTATGATAATCATATT
7998	No	AACGAATGATTTCGACTCATTAAATTATGATAATCATATTT
7999	No	AACGAATGATTTCGACTCATTAAATTATGATAATCATATTTA
8000	No	AACGAATGATTTCGACTCATTAAATTATGATAATCATATTTAC
8001	No	AACGAATGATTTCGACTCATTAAATTATGATAATCATATTTACC
8002	No	AACGAATGATTTCGACTCATTAAATTATGATAATCATATTTACCA
8003	No	AACGAATGATTTCGACTCATTAAATTATGATAATCATATTTACCAA
8004	Yes	AACGAATGATTTCGACTCATTAAATTATGATAATCATATTTACCAAC
8005	Yes	AACGAATGATTTCGACTCATTAAATTATGATAATCATATTTACCAACC
8006	Yes	AACGAATGATTTCGACTCATTAAATTATGATAATCATATTTACCAACCA
8007	Yes	AACGAGCTAGAAATTC
8008	Yes	AACGAGCTAGAAATTCG
8009	Yes	AACGAGCTAGAAATTCGT
8010	Yes	AACGAGCTAGAAATTCGTT
8011	Yes	AACGAGCTAGAAATTCGTTG
8012	Yes	AACGAGCTAGAAATTCGTTGG
8013	Yes	AACGAGCTAGAAATTCGTTGGT
8014	Yes	AACGAGCTAGAAATTCGTTGGTT
8015	Yes	AACGAGCTAGAAATTCGTTGGTTT

8016	Yes	AACGAGCTAGAAATTCGTTGGTTTC
8017	Yes	AACGAGCTAGAAATTCGTTGGTTTCT
8018	Yes	AACGAGCTAGAAATTCGTTGGTTTCTC
8019	Yes	AACGAGCTAGAAATTCGTTGGTTTCTCT
8020	Yes	AACGAGCTAGAAATTCGTTGGTTTCTCTC
8021	Yes	AACGAGCTAGAAATTCGTTGGTTTCTCTCT
8022	Yes	AACGAGCTAGAAATTCGTTGGTTTCTCTCTG
8023	Yes	AACGAGCTAGAAATTCGTTGGTTTCTCTCTGT
8024	Yes	AACGAGCTAGAAATTCGTTGGTTTCTCTCTGTG
8025	Yes	AACGAGCTAGAAATTCGTTGGTTTCTCTCTGTGC
8026	Yes	AACGAGCTAGAAATTCGTTGGTTTCTCTCTGTGCA
8027	Yes	AACGAGCTAGAAATTCGTTGGTTTCTCTCTGTGCAG
8028	Yes	AACGAGCTAGAAATTCGTTGGTTTCTCTCTGTGCAGG
8029	Yes	AACGAGCTAGAAATTCGTTGGTTTCTCTCTGTGCAGGT
8030	Yes	AACGAGCTAGAAATTCGTTGGTTTCTCTCTGTGCAGGTT
8031	Yes	AACGAGCTAGAAATTCGTTGGTTTCTCTCTGTGCAGGTTT
8032	Yes	AACGAGCTAGAAATTCGTTGGTTTCTCTCTGTGCAGGTTTG
8033	Yes	AACGAGCTAGAAATTCGTTGGTTTCTCTCTGTGCAGGTTTGA
8034	Yes	AACGAGCTAGAAATTCGTTGGTTTCTCTCTGTGCAGGTTTGAA
8035	Yes	AACGAGCTAGAAATTCGTTGGTTTCTCTCTGTGCAGGTTTGAAT
8036	Yes	AACGAGCTAGAAATTCGTTGGTTTCTCTCTGTGCAGGTTTGAATC
8037	Yes	AACGAGCTAGAAATTCGTTGGTTTCTCTCTGTGCAGGTTTGAATCC
8038	Yes	AACGAGCTAGAAATTCGTTGGTTTCTCTCTGTGCAGGTTTGAATCCT
8039	Yes	AACGAGCTAGAAATTCGTTGGTTTCTCTCTGTGCAGGTTTGAATCCTG
8040	Yes	AACGAGCTAGAAATTCGTTGGTTTCTCTCTGTGCAGGTTTGAATCCTGC
8041	Yes	AACGAGCTAGAAATTCGTTGGTTTCTCTCTGTGCAGGTTTGAATCCTGCT
8042	No	AACGATGGTTTTTCAT
8043	No	AACGATGGTTTTTCATA
8044	No	AACGATGGTTTTTCATAT
8045	No	AACGATGGTTTTTCATATC
8046	No	AACGATGGTTTTTCATATCA
8047	No	AACGATGGTTTTTCATATCAT
8048	No	AACGATGGTTTTTCATATCATT
8049	No	AACGATGGTTTTTCATATCATTG
8050	No	AACGATGGTTTTTCATATCATTGG
8051	No	AACGATGGTTTTTCATATCATTGGT
8052	No	AACGATGGTTTTTCATATCATTGGTC
8053	No	AACGATGGTTTTTCATATCATTGGTCG
8054	No	AACGATGGTTTTTCATATCATTGGTCGT
8055	No	AACGATGGTTTTTCATATCATTGGTCGTG
8056	No	AACGATGGTTTTTCATATCATTGGTCGTGG
8057	No	AACGATGGTTTTTCATATCATTGGTCGTGGT

8058	No	AACGATGGTTTTTCATATCATTGGTCGTGGTT
8059	No	AACGATGGTTTTTCATATCATTGGTCGTGGTTG
8060	No	AACGATGGTTTTTCATATCATTGGTCGTGGTTGT
8061	No	AACGATGGTTTTTCATATCATTGGTCGTGGTTGTA
8062	No	AACGATGGTTTTTCATATCATTGGTCGTGGTTGTAG
8063	Yes	AACGATGGTTTTTCATATCATTGGTCGTGGTTGTAGT
8064	Yes	AACGATGGTTTTTCATATCATTGGTCGTGGTTGTAGTC
8065	Yes	AACGATGGTTTTTCATATCATTGGTCGTGGTTGTAGTCC
8066	Yes	AACGATGGTTTTTCATATCATTGGTCGTGGTTGTAGTCCG
8067	Yes	AACGATGGTTTTTCATATCATTGGTCGTGGTTGTAGTCCGT
8068	Yes	AACGATGGTTTTTCATATCATTGGTCGTGGTTGTAGTCCGTG
8069	Yes	AACGATGGTTTTTCATATCATTGGTCGTGGTTGTAGTCCGTGC
8070	Yes	AACGATGGTTTTTCATATCATTGGTCGTGGTTGTAGTCCGTGCG
8071	Yes	AACGATGGTTTTTCATATCATTGGTCGTGGTTGTAGTCCGTGCGA
8072	Yes	AACGATGGTTTTTCATATCATTGGTCGTGGTTGTAGTCCGTGCGAG
8073	Yes	AACGATGGTTTTTCATATCATTGGTCGTGGTTGTAGTCCGTGCGAGA
8074	Yes	AACGATGGTTTTTCATATCATTGGTCGTGGTTGTAGTCCGTGCGAGAA
8075	Yes	AACGATGGTTTTTCATATCATTGGTCGTGGTTGTAGTCCGTGCGAGAAT
8076	Yes	AACGATGGTTTTTCATATCATTGGTCGTGGTTGTAGTCCGTGCGAGAATA
8077	No	AACGCCAAGGTCGCGG
8078	Yes	AACGCCAAGGTCGCGGG
8079	Yes	AACGCCAAGGTCGCGGGT
8080	Yes	AACGCCAAGGTCGCGGGTT
8081	Yes	AACGCCAAGGTCGCGGGTTC
8082	Yes	AACGCCAAGGTCGCGGGTTCG
8083	Yes	AACGCCAAGGTCGCGGGTTCGA
8084	Yes	AACGCCAAGGTCGCGGGTTCGAA
8085	Yes	AACGCCAAGGTCGCGGGTTCGAAC
8086	Yes	AACGCCAAGGTCGCGGGTTCGAACC
8087	Yes	AACGCCAAGGTCGCGGGTTCGAACCC
8088	Yes	AACGCCAAGGTCGCGGGTTCGAACCCC
8089	Yes	AACGCCAAGGTCGCGGGTTCGAACCCCG
8090	Yes	AACGCCAAGGTCGCGGGTTCGAACCCCGT
8091	Yes	AACGCCAAGGTCGCGGGTTCGAACCCCGTA
8092	Yes	AACGCCAAGGTCGCGGGTTCGAACCCCGTAC
8093	Yes	AACGCCAAGGTCGCGGGTTCGAACCCCGTACG
8094	Yes	AACGCCAAGGTCGCGGGTTCGAACCCCGTACGG
8095	Yes	AACGCCAAGGTCGCGGGTTCGAACCCCGTACGGG
8096	Yes	AACGCCAAGGTCGCGGGTTCGAACCCCGTACGGGC
8097	Yes	AACGCCAAGGTCGCGGGTTCGAACCCCGTACGGGCC
8098	Yes	AACGCCAAGGTCGCGGGTTCGAACCCCGTACGGGCCA
8099	Yes	AACGCCAAGGTCGCGGGTTCGAACCCCGTACGGGCCAC

8100	Yes	AACGCCAAGGTCGCGGGTTCGAACCCCGTACGGGCCACC
8101	Yes	AACGCCAAGGTCGCGGGTTCGAACCCCGTACGGGCCACCA
8102	Yes	AACGCCAAGGTCGCGGGTTCGAT
8103	Yes	AACGCCAAGGTCGCGGGTTCGATC
8104	Yes	AACGCCAAGGTCGCGGGTTCGATCC
8105	Yes	AACGCCAAGGTCGCGGGTTCGATCCC
8106	Yes	AACGCCAAGGTCGCGGGTTCGATCCCC
8107	Yes	AACGCCAAGGTCGCGGGTTCGATCCCCG
8108	Yes	AACGCCAAGGTCGCGGGTTCGATCCCCGT
8109	Yes	AACGCCAAGGTCGCGGGTTCGATCCCCGTA
8110	Yes	AACGCCAAGGTCGCGGGTTCGATCCCCGTAC
8111	Yes	AACGCCAAGGTCGCGGGTTCGATCCCCGTACG
8112	Yes	AACGCCAAGGTCGCGGGTTCGATCCCCGTACGG
8113	Yes	AACGCCAAGGTCGCGGGTTCGATCCCCGTACGGG
8114	Yes	AACGCCAAGGTCGCGGGTTCGATCCCCGTACGGGC
8115	Yes	AACGCCAAGGTCGCGGGTTCGATCCCCGTACGGGCC
8116	Yes	AACGCCAAGGTCGCGGGTTCGATCCCCGTACGGGCCA
8117	Yes	AACGCCAAGGTCGCGGGTTCGATCCCCGTACGGGCCAC
8118	Yes	AACGCCAAGGTCGCGGGTTCGATCCCCGTACGGGCCACC
8119	Yes	AACGCCAAGGTCGCGGGTTCGATCCCCGTACGGGCCACCA
8120	Yes	AACGCCAAGGTCGCGGGTTCGATCCCCGTACT
8121	Yes	AACGCCAAGGTCGCGGGTTCGATCCCCGTACTG
8122	Yes	AACGCCAAGGTCGCGGGTTCGATCCCCGTACTGG
8123	Yes	AACGCCAAGGTCGCGGGTTCGATCCCCGTACTGGC
8124	Yes	AACGCCAAGGTCGCGGGTTCGATCCCCGTACTGGCC
8125	Yes	AACGCCAAGGTCGCGGGTTCGATCCCCGTACTGGCCA
8126	Yes	AACGCCAAGGTCGCGGGTTCGATCCCCGTACTGGCCAC
8127	Yes	AACGCCAAGGTCGCGGGTTCGATCCCCGTACTGGCCACC
8128	Yes	AACGCCAAGGTCGCGGGTTCGATCCCCGTACTGGCCACCA
8129	No	AACGCCAAGGTCGTGG
8130	No	AACGCCAAGGTCGTGGG
8131	Yes	AACGCCAAGGTCGTGGGT
8132	Yes	AACGCCAAGGTCGTGGGTT
8133	Yes	AACGCCAAGGTCGTGGGTTC
8134	Yes	AACGCCAAGGTCGTGGGTTCG
8135	Yes	AACGCCAAGGTCGTGGGTTCGA
8136	Yes	AACGCCAAGGTCGTGGGTTCGAT
8137	Yes	AACGCCAAGGTCGTGGGTTCGATC
8138	Yes	AACGCCAAGGTCGTGGGTTCGATCC
8139	Yes	AACGCCAAGGTCGTGGGTTCGATCCC
8140	Yes	AACGCCAAGGTCGTGGGTTCGATCCCC
8141	Yes	AACGCCAAGGTCGTGGGTTCGATCCCCA

8142	Yes	AACGCCAAGGTCGTGGGTTCGATCCCCAT
8143	Yes	AACGCCAAGGTCGTGGGTTCGATCCCCATA
8144	Yes	AACGCCAAGGTCGTGGGTTCGATCCCCATAT
8145	Yes	AACGCCAAGGTCGTGGGTTCGATCCCCATATC
8146	Yes	AACGCCAAGGTCGTGGGTTCGATCCCCATATCG
8147	Yes	AACGCCAAGGTCGTGGGTTCGATCCCCATATCGG
8148	Yes	AACGCCAAGGTCGTGGGTTCGATCCCCATATCGGC
8149	Yes	AACGCCAAGGTCGTGGGTTCGATCCCCATATCGGCC
8150	Yes	AACGCCAAGGTCGTGGGTTCGATCCCCATATCGGCCA
8151	Yes	AACGCCAAGGTCGTGGGTTCGATCCCCATATCGGCCAC
8152	Yes	AACGCCAAGGTCGTGGGTTCGATCCCCATATCGGCCACC
8153	Yes	AACGCCAAGGTCGTGGGTTCGATCCCCATATCGGCCACCA
8154	Yes	AACGCGCGCTTTGCAT
8155	Yes	AACGCGCGCTTTGCATG
8156	Yes	AACGCGCGCTTTGCATGT
8157	Yes	AACGCGCGCTTTGCATGTA
8158	Yes	AACGCGCGCTTTGCATGTAT
8159	Yes	AACGCGCGCTTTGCATGTATG
8160	Yes	AACGCGCGCTTTGCATGTATGA
8161	Yes	AACGCGCGCTTTGCATGTATGAG
8162	Yes	AACGCGCGCTTTGCATGTATGAGG
8163	Yes	AACGCGCGCTTTGCATGTATGAGGT
8164	Yes	AACGCGCGCTTTGCATGTATGAGGTC
8165	Yes	AACGCGCGCTTTGCATGTATGAGGTCC
8166	Yes	AACGCGCGCTTTGCATGTATGAGGTCCC
8167	Yes	AACGCGCGCTTTGCATGTATGAGGTCCCG
8168	Yes	AACGCGCGCTTTGCATGTATGAGGTCCCGG
8169	Yes	AACGCGCGCTTTGCATGTATGAGGTCCCGGT
8170	Yes	AACGCGCGCTTTGCATGTATGAGGTCCCGGTT
8171	Yes	AACGCGCGCTTTGCATGTATGAGGTCCCGGTTT
8172	Yes	AACGCGCGCTTTGCATGTATGAGGTCCCGGTTTC
8173	Yes	AACGCGCGCTTTGCATGTATGAGGTCCCGGTTTCG
8174	Yes	AACGCGCGCTTTGCATGTATGAGGTCCCGGTTTCGA
8175	Yes	AACGCGCGCTTTGCATGTATGAGGTCCCGGTTTCGAT
8176	Yes	AACGCGCGCTTTGCATGTATGAGGTCCCGGTTTCGATC
8177	Yes	AACGCGCGCTTTGCATGTATGAGGTCCCGGTTTCGATCC
8178	Yes	AACGCGCGCTTTGCATGTATGAGGTCCCGGTTTCGATCCC
8179	Yes	AACGCGCGCTTTGCATGTATGAGGTCCCGGTTTCGATCCCT
8180	Yes	AACGCGCGCTTTGCATGTATGAGGTCCCGGTTTCGATCCCTG
8181	Yes	AACGCGCGCTTTGCATGTATGAGGTCCCGGTTTCGATCCCTGG
8182	Yes	AACGCGCGCTTTGCATGTATGAGGTCCCGGTTTCGATCCCTGGC
8183	Yes	AACGCGCGCTTTGCATGTATGAGGTCCCGGTTTCGATCCCTGGCG

8184	Yes	AACGCGCGCTTTGCATGTATGAGGTCCCGGTTTCGATCCCTGGCGT
8185	Yes	AACGCGCGCTTTGCATGTATGAGGTCCCGGTTTCGATCCCTGGCGTT
8186	Yes	AACGCGCGCTTTGCATGTATGAGGTCCCGGTTTCGATCCCTGGCGTTT
8187	Yes	AACGCGCGCTTTGCATGTATGAGGTCCCGGTTTCGATCCCTGGCGTTTC
8188	Yes	AACGCGCGCTTTGCATGTATGAGGTCCCGGTTTCGATCCCTGGCGTTTCC
8189	Yes	AACGCGGGTTCGATTC
8190	Yes	AACGCGGGTTCGATTCC
8191	Yes	AACGCGGGTTCGATTCCC
8192	Yes	AACGCGGGTTCGATTCCCG
8193	Yes	AACGCGGGTTCGATTCCCGG
8194	Yes	AACGCGGGTTCGATTCCCGGG
8195	Yes	AACGCGGGTTCGATTCCCGGGT
8196	Yes	AACGCGGGTTCGATTCCCGGGTA
8197	Yes	AACGCGGGTTCGATTCCCGGGTAA
8198	Yes	AACGCGGGTTCGATTCCCGGGTAAC
8199	Yes	AACGCGGGTTCGATTCCCGGGTAACG
8200	Yes	AACGCGGGTTCGATTCCCGGGTAACGA
8201	Yes	AACGCGGGTTCGATTCCCGGGTAACGAA
8202	Yes	AACGCGGGTTCGATTCCCGGGTAACGAAC
8203	Yes	AACGCGGGTTCGATTCCCGGGTAACGAACC
8204	Yes	AACGCGGGTTCGATTCCCGGGTAACGAACCA
8205	No	AACGCGTCTGACTACG
8206	No	AACGCGTCTGACTACGG
8207	No	AACGCGTCTGACTACGGA
8208	No	AACGCGTCTGACTACGGAT
8209	No	AACGCGTCTGACTACGGATC
8210	No	AACGCGTCTGACTACGGATCA
8211	No	AACGCGTCTGACTACGGATCAG
8212	No	AACGCGTCTGACTACGGATCAGA
8213	No	AACGCGTCTGACTACGGATCAGAA
8214	No	AACGCGTCTGACTACGGATCAGAAG
8215	No	AACGCGTCTGACTACGGATCAGAAGA
8216	No	AACGCGTCTGACTACGGATCAGAAGAT
8217	No	AACGCGTCTGACTACGGATCAGAAGATT
8218	No	AACGCGTCTGACTACGGATCAGAAGATTC
8219	Yes	AACGCGTCTGACTACGGATCAGAAGATTCC
8220	Yes	AACGCGTCTGACTACGGATCAGAAGATTCCA
8221	Yes	AACGCGTCTGACTACGGATCAGAAGATTCCAG
8222	Yes	AACGCGTCTGACTACGGATCAGAAGATTCCAGG
8223	Yes	AACGCGTCTGACTACGGATCAGAAGATTCCAGGT
8224	Yes	AACGCGTCTGACTACGGATCAGAAGATTCCAGGTT
8225	Yes	AACGCGTCTGACTACGGATCAGAAGATTCCAGGTTC

8226	Yes	AACGCGTCTGACTACGGATCAGAAGATTCCAGGTTCG
8227	Yes	AACGCGTCTGACTACGGATCAGAAGATTCCAGGTTCGA
8228	Yes	AACGCGTCTGACTACGGATCAGAAGATTCCAGGTTCGAC
8229	Yes	AACGCGTCTGACTACGGATCAGAAGATTCCAGGTTCGACT
8230	Yes	AACGCGTCTGACTACGGATCAGAAGATTCCAGGTTCGACTC
8231	Yes	AACGCGTCTGACTACGGATCAGAAGATTCCAGGTTCGACTCC
8232	Yes	AACGCGTCTGACTACGGATCAGAAGATTCCAGGTTCGACTCCT
8233	Yes	AACGCGTCTGACTACGGATCAGAAGATTCCAGGTTCGACTCCTG
8234	Yes	AACGCGTCTGACTACGGATCAGAAGATTCCAGGTTCGACTCCTGG
8235	Yes	AACGCGTCTGACTACGGATCAGAAGATTCCAGGTTCGACTCCTGGC
8236	Yes	AACGCGTCTGACTACGGATCAGAAGATTCCAGGTTCGACTCCTGGCT
8237	Yes	AACGCGTCTGACTACGGATCAGAAGATTCCAGGTTCGACTCCTGGCTG
8238	Yes	AACGCGTCTGACTACGGATCAGAAGATTCCAGGTTCGACTCCTGGCTGG
8239	Yes	AACGCGTCTGACTACGGATCAGAAGATTCCAGGTTCGACTCCTGGCTGGC
8240	No	AACGCGTCTGACTACGGATCAGAAGATTCT
8241	No	AACGCGTCTGACTACGGATCAGAAGATTCTA
8242	No	AACGCGTCTGACTACGGATCAGAAGATTCTAG
8243	Yes	AACGCGTCTGACTACGGATCAGAAGATTCTAGG
8244	Yes	AACGCGTCTGACTACGGATCAGAAGATTCTAGGT
8245	Yes	AACGCGTCTGACTACGGATCAGAAGATTCTAGGTT
8246	Yes	AACGCGTCTGACTACGGATCAGAAGATTCTAGGTTC
8247	Yes	AACGCGTCTGACTACGGATCAGAAGATTCTAGGTTCG
8248	Yes	AACGCGTCTGACTACGGATCAGAAGATTCTAGGTTCGA
8249	Yes	AACGCGTCTGACTACGGATCAGAAGATTCTAGGTTCGAC
8250	Yes	AACGCGTCTGACTACGGATCAGAAGATTCTAGGTTCGACT
8251	Yes	AACGCGTCTGACTACGGATCAGAAGATTCTAGGTTCGACTC
8252	Yes	AACGCGTCTGACTACGGATCAGAAGATTCTAGGTTCGACTCC
8253	Yes	AACGCGTCTGACTACGGATCAGAAGATTCTAGGTTCGACTCCT
8254	Yes	AACGCGTCTGACTACGGATCAGAAGATTCTAGGTTCGACTCCTG
8255	Yes	AACGCGTCTGACTACGGATCAGAAGATTCTAGGTTCGACTCCTGG
8256	Yes	AACGCGTCTGACTACGGATCAGAAGATTCTAGGTTCGACTCCTGGC
8257	Yes	AACGCGTCTGACTACGGATCAGAAGATTCTAGGTTCGACTCCTGGCT
8258	Yes	AACGCGTCTGACTACGGATCAGAAGATTCTAGGTTCGACTCCTGGCTG
8259	Yes	AACGCGTCTGACTACGGATCAGAAGATTCTAGGTTCGACTCCTGGCTGG
8260	Yes	AACGCGTCTGACTACGGATCAGAAGATTCTAGGTTCGACTCCTGGCTGGC
8261	Yes	AACGCTAAGGTCGCGG
8262	Yes	AACGCTAAGGTCGCGGG
8263	Yes	AACGCTAAGGTCGCGGGT
8264	Yes	AACGCTAAGGTCGCGGGTT
8265	Yes	AACGCTAAGGTCGCGGGTTC
8266	Yes	AACGCTAAGGTCGCGGGTTCG
8267	Yes	AACGCTAAGGTCGCGGGTTCGA

8268	Yes	AACGCTAAGGTCGCGGGTTCGAT
8269	Yes	AACGCTAAGGTCGCGGGTTCGATC
8270	Yes	AACGCTAAGGTCGCGGGTTCGATCC
8271	Yes	AACGCTAAGGTCGCGGGTTCGATCCC
8272	Yes	AACGCTAAGGTCGCGGGTTCGATCCCC
8273	Yes	AACGCTAAGGTCGCGGGTTCGATCCCCG
8274	Yes	AACGCTAAGGTCGCGGGTTCGATCCCCGT
8275	Yes	AACGCTAAGGTCGCGGGTTCGATCCCCGTA
8276	Yes	AACGCTAAGGTCGCGGGTTCGATCCCCGTAC
8277	Yes	AACGCTAAGGTCGCGGGTTCGATCCCCGTACT
8278	Yes	AACGCTAAGGTCGCGGGTTCGATCCCCGTACTG
8279	Yes	AACGCTAAGGTCGCGGGTTCGATCCCCGTACTGG
8280	Yes	AACGCTAAGGTCGCGGGTTCGATCCCCGTACTGGC
8281	Yes	AACGCTAAGGTCGCGGGTTCGATCCCCGTACTGGCC
8282	Yes	AACGCTAAGGTCGCGGGTTCGATCCCCGTACTGGCCA
8283	Yes	AACGCTAAGGTCGCGGGTTCGATCCCCGTACTGGCCAC
8284	Yes	AACGCTAAGGTCGCGGGTTCGATCCCCGTACTGGCCACC
8285	Yes	AACGCTAAGGTCGCGGGTTCGATCCCCGTACTGGCCACCA
8286	No	AACGCTTGGGCTCTGA
8287	Yes	AACGCTTGGGCTCTGAC
8288	Yes	AACGCTTGGGCTCTGACA
8289	Yes	AACGCTTGGGCTCTGACAT
8290	Yes	AACGCTTGGGCTCTGACATT
8291	Yes	AACGCTTGGGCTCTGACATTA
8292	Yes	AACGCTTGGGCTCTGACATTAA
8293	Yes	AACGCTTGGGCTCTGACATTAAC
8294	Yes	AACGCTTGGGCTCTGACATTAACG
8295	Yes	AACGCTTGGGCTCTGACATTAACGT
8296	Yes	AACGCTTGGGCTCTGACATTAACGTC
8297	Yes	AACGCTTGGGCTCTGACATTAACGTCC
8298	Yes	AACGCTTGGGCTCTGACATTAACGTCCT
8299	Yes	AACGCTTGGGCTCTGACATTAACGTCCTG
8300	Yes	AACGCTTGGGCTCTGACATTAACGTCCTGG
8301	Yes	AACGCTTGGGCTCTGACATTAACGTCCTGGG
8302	Yes	AACGCTTGGGCTCTGACATTAACGTCCTGGGT
8303	Yes	AACGCTTGGGCTCTGACATTAACGTCCTGGGTT
8304	Yes	AACGCTTGGGCTCTGACATTAACGTCCTGGGTTC
8305	Yes	AACGCTTGGGCTCTGACATTAACGTCCTGGGTTCA
8306	Yes	AACGCTTGGGCTCTGACATTAACGTCCTGGGTTCAA
8307	Yes	AACGCTTGGGCTCTGACATTAACGTCCTGGGTTCAAA
8308	Yes	AACGCTTGGGCTCTGACATTAACGTCCTGGGTTCAAAT
8309	Yes	AACGCTTGGGCTCTGACATTAACGTCCTGGGTTCAAATC

8310	Yes	AACGCTTGGGCTCTGACATTAACGTCCTGGGTTCAAATCC
8311	Yes	AACGCTTGGGCTCTGACATTAACGTCCTGGGTTCAAATCCC
8312	Yes	AACGCTTGGGCTCTGACATTAACGTCCTGGGTTCAAATCCCA
8313	Yes	AACGCTTGGGCTCTGACATTAACGTCCTGGGTTCAAATCCCAG
8314	Yes	AACGCTTGGGCTCTGACATTAACGTCCTGGGTTCAAATCCCAGC
8315	Yes	AACGCTTGGGCTCTGACATTAACGTCCTGGGTTCAAATCCCAGCT
8316	Yes	AACGCTTGGGCTCTGACATTAACGTCCTGGGTTCAAATCCCAGCTT
8317	Yes	AACGCTTGGGCTCTGACATTAACGTCCTGGGTTCAAATCCCAGCTTT
8318	Yes	AACGCTTGGGCTCTGACATTAACGTCCTGGGTTCAAATCCCAGCTTTG
8319	Yes	AACGCTTGGGCTCTGACATTAACGTCCTGGGTTCAAATCCCAGCTTTGT
8320	Yes	AACGCTTGGGCTCTGACATTAACGTCCTGGGTTCAAATCCCAGCTTTGTC
8321	Yes	AACGGCAGCGCGTCTG
8322	Yes	AACGGCAGCGCGTCTGA
8323	Yes	AACGGCAGCGCGTCTGAC
8324	Yes	AACGGCAGCGCGTCTGACT
8325	Yes	AACGGCAGCGCGTCTGACTC
8326	Yes	AACGGCAGCGCGTCTGACTCC
8327	Yes	AACGGCAGCGCGTCTGACTCCA
8328	Yes	AACGGCAGCGCGTCTGACTCCAG
8329	Yes	AACGGCAGCGCGTCTGACTCCAGA
8330	Yes	AACGGCAGCGCGTCTGACTCCAGAT
8331	Yes	AACGGCAGCGCGTCTGACTCCAGATC
8332	Yes	AACGGCAGCGCGTCTGACTCCAGATCA
8333	Yes	AACGGCAGCGCGTCTGACTCCAGATCAG
8334	Yes	AACGGCAGCGCGTCTGACTCCAGATCAGA
8335	Yes	AACGGCAGCGCGTCTGACTCCAGATCAGAA
8336	Yes	AACGGCAGCGCGTCTGACTCCAGATCAGAAG
8337	Yes	AACGGCAGCGCGTCTGACTCCAGATCAGAAGG
8338	Yes	AACGGCAGCGCGTCTGACTCCAGATCAGAAGGT
8339	Yes	AACGGCAGCGCGTCTGACTCCAGATCAGAAGGTT
8340	Yes	AACGGCAGCGCGTCTGACTCCAGATCAGAAGGTTG
8341	Yes	AACGGCAGCGCGTCTGACTCCAGATCAGAAGGTTGC
8342	Yes	AACGGCAGCGCGTCTGACTCCAGATCAGAAGGTTGCG
8343	Yes	AACGGCAGCGCGTCTGACTCCAGATCAGAAGGTTGCGT
8344	Yes	AACGGCAGCGCGTCTGACTCCAGATCAGAAGGTTGCGTG
8345	Yes	AACGGCAGCGCGTCTGACTCCAGATCAGAAGGTTGCGTGT
8346	Yes	AACGGCAGCGCGTCTGACTCCAGATCAGAAGGTTGCGTGTT
8347	Yes	AACGGCAGCGCGTCTGACTCCAGATCAGAAGGTTGCGTGTTC
8348	Yes	AACGGCAGCGCGTCTGACTCCAGATCAGAAGGTTGCGTGTTCA
8349	Yes	AACGGCAGCGCGTCTGACTCCAGATCAGAAGGTTGCGTGTTCAA
8350	Yes	AACGGCAGCGCGTCTGACTCCAGATCAGAAGGTTGCGTGTTCAAA
8351	Yes	AACGGCAGCGCGTCTGACTCCAGATCAGAAGGTTGCGTGTTCAAAT

8352	Yes	AACGGCAGCGCGTCTGACTCCAGATCAGAAGGTTGCGTGTTCAAATC
8353	Yes	AACGGCAGCGCGTCTGACTCCAGATCAGAAGGTTGCGTGTTCAAATCA
8354	Yes	AACGGCAGCGCGTCTGACTCCAGATCAGAAGGTTGCGTGTTCAAATCAC
8355	Yes	AACGGCAGCGCGTCTGACTCCAGATCAGAAGGTTGCGTGTTCAAATCACG
8356	Yes	AACGGTAGCGCGTCTG
8357	Yes	AACGGTAGCGCGTCTGA
8358	Yes	AACGGTAGCGCGTCTGAC
8359	Yes	AACGGTAGCGCGTCTGACT
8360	Yes	AACGGTAGCGCGTCTGACTC
8361	Yes	AACGGTAGCGCGTCTGACTCC
8362	Yes	AACGGTAGCGCGTCTGACTCCA
8363	Yes	AACGGTAGCGCGTCTGACTCCAG
8364	Yes	AACGGTAGCGCGTCTGACTCCAGA
8365	Yes	AACGGTAGCGCGTCTGACTCCAGAT
8366	Yes	AACGGTAGCGCGTCTGACTCCAGATC
8367	Yes	AACGGTAGCGCGTCTGACTCCAGATCA
8368	Yes	AACGGTAGCGCGTCTGACTCCAGATCAG
8369	Yes	AACGGTAGCGCGTCTGACTCCAGATCAGA
8370	Yes	AACGGTAGCGCGTCTGACTCCAGATCAGAA
8371	Yes	AACGGTAGCGCGTCTGACTCCAGATCAGAAG
8372	Yes	AACGGTAGCGCGTCTGACTCCAGATCAGAAGG
8373	Yes	AACGGTAGCGCGTCTGACTCCAGATCAGAAGGC
8374	Yes	AACGGTAGCGCGTCTGACTCCAGATCAGAAGGCT
8375	Yes	AACGGTAGCGCGTCTGACTCCAGATCAGAAGGCTG
8376	Yes	AACGGTAGCGCGTCTGACTCCAGATCAGAAGGCTGC
8377	Yes	AACGGTAGCGCGTCTGACTCCAGATCAGAAGGCTGCG
8378	Yes	AACGGTAGCGCGTCTGACTCCAGATCAGAAGGCTGCGT
8379	Yes	AACGGTAGCGCGTCTGACTCCAGATCAGAAGGCTGCGTG
8380	Yes	AACGGTAGCGCGTCTGACTCCAGATCAGAAGGCTGCGTGT
8381	Yes	AACGGTAGCGCGTCTGACTCCAGATCAGAAGGCTGCGTGTT
8382	Yes	AACGGTAGCGCGTCTGACTCCAGATCAGAAGGCTGCGTGTTC
8383	Yes	AACGGTAGCGCGTCTGACTCCAGATCAGAAGGCTGCGTGTTCG
8384	Yes	AACGGTAGCGCGTCTGACTCCAGATCAGAAGGCTGCGTGTTCGA
8385	Yes	AACGGTAGCGCGTCTGACTCCAGATCAGAAGGCTGCGTGTTCGAA
8386	Yes	AACGGTAGCGCGTCTGACTCCAGATCAGAAGGCTGCGTGTTCGAAT
8387	Yes	AACGGTAGCGCGTCTGACTCCAGATCAGAAGGCTGCGTGTTCGAATC
8388	Yes	AACGGTAGCGCGTCTGACTCCAGATCAGAAGGCTGCGTGTTCGAATCA
8389	Yes	AACGGTAGCGCGTCTGACTCCAGATCAGAAGGCTGCGTGTTCGAATCAC
8390	Yes	AACGGTAGCGCGTCTGACTCCAGATCAGAAGGCTGCGTGTTCGAATCACG
8391	Yes	AACGGTAGCGCGTCTGACTCCAGATCAGAAGGT
8392	Yes	AACGGTAGCGCGTCTGACTCCAGATCAGAAGGTT
8393	Yes	AACGGTAGCGCGTCTGACTCCAGATCAGAAGGTTG

8394	Yes	AACGGTAGCGCGTCTGACTCCAGATCAGAAGGTTGC
8395	Yes	AACGGTAGCGCGTCTGACTCCAGATCAGAAGGTTGCG
8396	Yes	AACGGTAGCGCGTCTGACTCCAGATCAGAAGGTTGCGT
8397	Yes	AACGGTAGCGCGTCTGACTCCAGATCAGAAGGTTGCGTG
8398	Yes	AACGGTAGCGCGTCTGACTCCAGATCAGAAGGTTGCGTGT
8399	Yes	AACGGTAGCGCGTCTGACTCCAGATCAGAAGGTTGCGTGTT
8400	Yes	AACGGTAGCGCGTCTGACTCCAGATCAGAAGGTTGCGTGTTC
8401	Yes	AACGGTAGCGCGTCTGACTCCAGATCAGAAGGTTGCGTGTTCA
8402	Yes	AACGGTAGCGCGTCTGACTCCAGATCAGAAGGTTGCGTGTTCAA
8403	Yes	AACGGTAGCGCGTCTGACTCCAGATCAGAAGGTTGCGTGTTCAAA
8404	Yes	AACGGTAGCGCGTCTGACTCCAGATCAGAAGGTTGCGTGTTCAAAT
8405	Yes	AACGGTAGCGCGTCTGACTCCAGATCAGAAGGTTGCGTGTTCAAATC
8406	Yes	AACGGTAGCGCGTCTGACTCCAGATCAGAAGGTTGCGTGTTCAAATCA
8407	Yes	AACGGTAGCGCGTCTGACTCCAGATCAGAAGGTTGCGTGTTCAAATCAC
8408	Yes	AACGGTAGCGCGTCTGACTCCAGATCAGAAGGTTGCGTGTTCAAATCACG
8409	No	AACGGTGAGTAGTTCA
8410	No	AACGGTGAGTAGTTCAA
8411	Yes	AACGGTGAGTAGTTCAAG
8412	Yes	AACGGTGAGTAGTTCAAGA
8413	Yes	AACGGTGAGTAGTTCAAGAC
8414	Yes	AACGGTGAGTAGTTCAAGACC
8415	Yes	AACGGTGAGTAGTTCAAGACCA
8416	Yes	AACGGTGAGTAGTTCAAGACCAC
8417	Yes	AACGGTGAGTAGTTCAAGACCACC
8418	Yes	AACGGTGAGTAGTTCAAGACCACCC
8419	Yes	AACGGTGAGTAGTTCAAGACCACCCA
8420	Yes	AACGGTGAGTAGTTCAAGACCACCCAG
8421	Yes	AACGGTGAGTAGTTCAAGACCACCCAGG
8422	Yes	AACGGTGAGTAGTTCAAGACCACCCAGGG
8423	Yes	AACGGTGAGTAGTTCAAGACCACCCAGGGA
8424	Yes	AACGGTGAGTAGTTCAAGACCACCCAGGGAC
8425	Yes	AACGGTGAGTAGTTCAAGACCACCCAGGGACG
8426	Yes	AACGGTGAGTAGTTCAAGACCACCCAGGGACGC
8427	Yes	AACGGTGAGTAGTTCAAGACCACCCAGGGACGCC
8428	Yes	AACGGTGAGTAGTTCAAGACCACCCAGGGACGCCA
8429	Yes	AACGGTTAAGTGAAGA
8430	Yes	AACGGTTAAGTGAAGAG
8431	Yes	AACGGTTAAGTGAAGAGG
8432	Yes	AACGGTTAAGTGAAGAGGC
8433	Yes	AACGGTTAAGTGAAGAGGCT
8434	Yes	AACGGTTAAGTGAAGAGGCTG
8435	Yes	AACGGTTAAGTGAAGAGGCTGT

8436	Yes	AACGGTTAAGTGAAGAGGCTGTA
8437	Yes	AACGGTTAAGTGAAGAGGCTGTAA
8438	Yes	AACGGTTAAGTGAAGAGGCTGTAAA
8439	Yes	AACGGTTAAGTGAAGAGGCTGTAAAC
8440	Yes	AACGGTTAAGTGAAGAGGCTGTAAACC
8441	Yes	AACGGTTAAGTGAAGAGGCTGTAAACCC
8442	Yes	AACGGTTAAGTGAAGAGGCTGTAAACCCA
8443	Yes	AACGGTTAAGTGAAGAGGCTGTAAACCCAG
8444	Yes	AACGGTTAAGTGAAGAGGCTGTAAACCCAGA
8445	Yes	AACGGTTAAGTGAAGAGGCTGTAAACCCAGAC
8446	Yes	AACGGTTAAGTGAAGAGGCTGTAAACCCAGACT
8447	Yes	AACGGTTAAGTGAAGAGGCTGTAAACCCAGACTG
8448	Yes	AACGGTTAAGTGAAGAGGCTGTAAACCCAGACTGG
8449	Yes	AACGGTTAAGTGAAGAGGCTGTAAACCCAGACTGGA
8450	Yes	AACGGTTAAGTGAAGAGGCTGTAAACCCAGACTGGAT
8451	Yes	AACGGTTAAGTGAAGAGGCTGTAAACCCAGACTGGATG
8452	Yes	AACGGTTAAGTGAAGAGGCTGTAAACCCAGACTGGATGG
8453	Yes	AACGGTTAAGTGAAGAGGCTGTAAACCCAGACTGGATGGG
8454	Yes	AACGGTTAAGTGAAGAGGCTGTAAACCCAGACTGGATGGGT
8455	Yes	AACGGTTAAGTGAAGAGGCTGTAAACCCAGACTGGATGGGTT
8456	Yes	AACGGTTAAGTGAAGAGGCTGTAAACCCAGACTGGATGGGTTC
8457	Yes	AACGGTTAAGTGAAGAGGCTGTAAACCCAGACTGGATGGGTTCA
8458	Yes	AACGGTTAAGTGAAGAGGCTGTAAACCCAGACTGGATGGGTTCAA
8459	Yes	AACGGTTAAGTGAAGAGGCTGTAAACCCAGACTGGATGGGTTCAAT
8460	Yes	AACGGTTAAGTGAAGAGGCTGTAAACCCAGACTGGATGGGTTCAATT
8461	Yes	AACGGTTAAGTGAAGAGGCTGTAAACCCAGACTGGATGGGTTCAATTC
8462	Yes	AACGGTTAAGTGAAGAGGCTGTAAACCCAGACTGGATGGGTTCAATTCC
8463	Yes	AACGGTTAAGTGAAGAGGCTGTAAACCCAGACTGGATGGGTTCAATTCCC
8464	No	AACGTCCTGGGTTCAA
8465	Yes	AACGTCCTGGGTTCAAA
8466	Yes	AACGTCCTGGGTTCAAAT
8467	Yes	AACGTCCTGGGTTCAAATC
8468	Yes	AACGTCCTGGGTTCAAATCC
8469	Yes	AACGTCCTGGGTTCAAATCCC
8470	Yes	AACGTCCTGGGTTCAAATCCCA
8471	Yes	AACGTCCTGGGTTCAAATCCCAG
8472	Yes	AACGTCCTGGGTTCAAATCCCAGC
8473	Yes	AACGTCCTGGGTTCAAATCCCAGCT
8474	Yes	AACGTCCTGGGTTCAAATCCCAGCTT
8475	Yes	AACGTCCTGGGTTCAAATCCCAGCTTT
8476	Yes	AACGTCCTGGGTTCAAATCCCAGCTTTG
8477	Yes	AACGTCCTGGGTTCAAATCCCAGCTTTGT

8478	Yes	AACGTCCTGGGTTCAAATCCCAGCTTTGTC
8479	Yes	AACGTCCTGGGTTCAAATCCCAGCTTTGTCA
8480	Yes	AACGTCCTGGGTTCAAATCCCAGCTTTGTCAC
8481	Yes	AACGTCCTGGGTTCAAATCCCAGCTTTGTCACC
8482	Yes	AACGTCCTGGGTTCAAATCCCAGCTTTGTCACCA
8483	No	AACTAAAAAGTTGGTG
8484	No	AACTAAAAAGTTGGTGG
8485	Yes	AACTAAAAAGTTGGTGGT
8486	Yes	AACTAAAAAGTTGGTGGTT
8487	Yes	AACTAAAAAGTTGGTGGTTC
8488	Yes	AACTAAAAAGTTGGTGGTTCG
8489	Yes	AACTAAAAAGTTGGTGGTTCGA
8490	Yes	AACTAAAAAGTTGGTGGTTCGAA
8491	Yes	AACTAAAAAGTTGGTGGTTCGAAC
8492	Yes	AACTAAAAAGTTGGTGGTTCGAACA
8493	Yes	AACTAAAAAGTTGGTGGTTCGAACAC
8494	Yes	AACTAAAAAGTTGGTGGTTCGAACACA
8495	Yes	AACTAAAAAGTTGGTGGTTCGAACACAC
8496	Yes	AACTAAAAAGTTGGTGGTTCGAACACACC
8497	Yes	AACTAAAAAGTTGGTGGTTCGAACACACCC
8498	Yes	AACTAAAAAGTTGGTGGTTCGAACACACCCA
8499	Yes	AACTAAAAAGTTGGTGGTTCGAACACACCCAG
8500	Yes	AACTAAAAAGTTGGTGGTTCGAACACACCCAGA
8501	Yes	AACTAAAAAGTTGGTGGTTCGAACACACCCAGAG
8502	Yes	AACTAAAAAGTTGGTGGTTCGAACACACCCAGAGG
8503	Yes	AACTAAAAAGTTGGTGGTTCGAACACACCCAGAGGC
8504	Yes	AACTAAAAAGTTGGTGGTTCGAACACACCCAGAGGCG
8505	Yes	AACTAAAAAGTTGGTGGTTCGAACACACCCAGAGGCGC
8506	Yes	AACTAAAAAGTTGGTGGTTCGAACACACCCAGAGGCGCC
8507	Yes	AACTAAAAAGTTGGTGGTTCGAACACACCCAGAGGCGCCA
8508	Yes	AACTAAAAGGTTGGCG
8509	Yes	AACTAAAAGGTTGGCGG
8510	Yes	AACTAAAAGGTTGGCGGT
8511	Yes	AACTAAAAGGTTGGCGGTT
8512	Yes	AACTAAAAGGTTGGCGGTTC
8513	Yes	AACTAAAAGGTTGGCGGTTCG
8514	Yes	AACTAAAAGGTTGGCGGTTCGA
8515	Yes	AACTAAAAGGTTGGCGGTTCGAA
8516	Yes	AACTAAAAGGTTGGCGGTTCGAAC
8517	Yes	AACTAAAAGGTTGGCGGTTCGAACC
8518	Yes	AACTAAAAGGTTGGCGGTTCGAACCC
8519	Yes	AACTAAAAGGTTGGCGGTTCGAACCCA

8520	Yes	AACTAAAAGGTTGGCGGTTCGAACCCAC
8521	Yes	AACTAAAAGGTTGGCGGTTCGAACCCACC
8522	Yes	AACTAAAAGGTTGGCGGTTCGAACCCACCC
8523	Yes	AACTAAAAGGTTGGCGGTTCGAACCCACCCA
8524	Yes	AACTAAAAGGTTGGCGGTTCGAACCCACCCAG
8525	Yes	AACTAAAAGGTTGGCGGTTCGAACCCACCCAGA
8526	Yes	AACTAAAAGGTTGGCGGTTCGAACCCACCCAGAG
8527	Yes	AACTAAAAGGTTGGCGGTTCGAACCCACCCAGAGG
8528	Yes	AACTAAAAGGTTGGCGGTTCGAACCCACCCAGAGGC
8529	Yes	AACTAAAAGGTTGGCGGTTCGAACCCACCCAGAGGCG
8530	Yes	AACTAAAAGGTTGGCGGTTCGAACCCACCCAGAGGCGC
8531	Yes	AACTAAAAGGTTGGCGGTTCGAACCCACCCAGAGGCGCC
8532	Yes	AACTAAAAGGTTGGCGGTTCGAACCCACCCAGAGGCGCCA
8533	Yes	AACTAAAAGGTTGGTG
8534	Yes	AACTAAAAGGTTGGTGG
8535	Yes	AACTAAAAGGTTGGTGGT
8536	Yes	AACTAAAAGGTTGGTGGTT
8537	Yes	AACTAAAAGGTTGGTGGTTC
8538	Yes	AACTAAAAGGTTGGTGGTTCG
8539	Yes	AACTAAAAGGTTGGTGGTTCGA
8540	Yes	AACTAAAAGGTTGGTGGTTCGAA
8541	Yes	AACTAAAAGGTTGGTGGTTCGAAC
8542	Yes	AACTAAAAGGTTGGTGGTTCGAACC
8543	Yes	AACTAAAAGGTTGGTGGTTCGAACCC
8544	Yes	AACTAAAAGGTTGGTGGTTCGAACCCA
8545	Yes	AACTAAAAGGTTGGTGGTTCGAACCCAC
8546	Yes	AACTAAAAGGTTGGTGGTTCGAACCCACC
8547	Yes	AACTAAAAGGTTGGTGGTTCGAACCCACCC
8548	Yes	AACTAAAAGGTTGGTGGTTCGAACCCACCCA
8549	Yes	AACTAAAAGGTTGGTGGTTCGAACCCACCCAG
8550	Yes	AACTAAAAGGTTGGTGGTTCGAACCCACCCAGA
8551	Yes	AACTAAAAGGTTGGTGGTTCGAACCCACCCAGAG
8552	Yes	AACTAAAAGGTTGGTGGTTCGAACCCACCCAGAGG
8553	Yes	AACTAAAAGGTTGGTGGTTCGAACCCACCCAGAGGC
8554	Yes	AACTAAAAGGTTGGTGGTTCGAACCCACCCAGAGGCG
8555	Yes	AACTAAAAGGTTGGTGGTTCGAACCCACCCAGAGGCGC
8556	Yes	AACTAAAAGGTTGGTGGTTCGAACCCACCCAGAGGCGCC
8557	Yes	AACTAAAAGGTTGGTGGTTCGAACCCACCCAGAGGCGCCA
8558	Yes	AACTAAGTGTTTGTGG
8559	Yes	AACTAAGTGTTTGTGGG
8560	Yes	AACTAAGTGTTTGTGGGT
8561	Yes	AACTAAGTGTTTGTGGGTT

8562	Yes	AACTAAGTGTTTGTGGGTTT
8563	Yes	AACTAAGTGTTTGTGGGTTTA
8564	Yes	AACTAAGTGTTTGTGGGTTTAA
8565	Yes	AACTAAGTGTTTGTGGGTTTAAG
8566	Yes	AACTAAGTGTTTGTGGGTTTAAGT
8567	Yes	AACTAAGTGTTTGTGGGTTTAAGTC
8568	Yes	AACTAAGTGTTTGTGGGTTTAAGTCC
8569	Yes	AACTAAGTGTTTGTGGGTTTAAGTCCC
8570	Yes	AACTAAGTGTTTGTGGGTTTAAGTCCCA
8571	Yes	AACTAAGTGTTTGTGGGTTTAAGTCCCAT
8572	Yes	AACTAAGTGTTTGTGGGTTTAAGTCCCATT
8573	Yes	AACTAAGTGTTTGTGGGTTTAAGTCCCATTG
8574	Yes	AACTAAGTGTTTGTGGGTTTAAGTCCCATTGG
8575	Yes	AACTAAGTGTTTGTGGGTTTAAGTCCCATTGGT
8576	Yes	AACTAAGTGTTTGTGGGTTTAAGTCCCATTGGTC
8577	Yes	AACTAAGTGTTTGTGGGTTTAAGTCCCATTGGTCT
8578	Yes	AACTAAGTGTTTGTGGGTTTAAGTCCCATTGGTCTA
8579	Yes	AACTAAGTGTTTGTGGGTTTAAGTCCCATTGGTCTAG
8580	Yes	AACTAAGTGTTTGTGGGTTTAAGTCCCATTGGTCTAGC
8581	Yes	AACTAAGTGTTTGTGGGTTTAAGTCCCATTGGTCTAGCC
8582	Yes	AACTAAGTGTTTGTGGGTTTAAGTCCCATTGGTCTAGCCA
8583	No	AACTAATACACCAGTC
8584	Yes	AACTAATACACCAGTCT
8585	Yes	AACTAATACACCAGTCTT
8586	Yes	AACTAATACACCAGTCTTG
8587	Yes	AACTAATACACCAGTCTTGT
8588	Yes	AACTAATACACCAGTCTTGTA
8589	Yes	AACTAATACACCAGTCTTGTAA
8590	Yes	AACTAATACACCAGTCTTGTAAA
8591	Yes	AACTAATACACCAGTCTTGTAAAC
8592	Yes	AACTAATACACCAGTCTTGTAAACC
8593	Yes	AACTAATACACCAGTCTTGTAAACCG
8594	Yes	AACTAATACACCAGTCTTGTAAACCGG
8595	Yes	AACTAATACACCAGTCTTGTAAACCGGA
8596	Yes	AACTAATACACCAGTCTTGTAAACCGGAG
8597	Yes	AACTAATACACCAGTCTTGTAAACCGGAGA
8598	Yes	AACTAATACACCAGTCTTGTAAACCGGAGAT
8599	Yes	AACTAATACACCAGTCTTGTAAACCGGAGATG
8600	Yes	AACTAATACACCAGTCTTGTAAACCGGAGATGA
8601	Yes	AACTAATACACCAGTCTTGTAAACCGGAGATGAA
8602	Yes	AACTAATACACCAGTCTTGTAAACCGGAGATGAAA
8603	Yes	AACTAATACACCAGTCTTGTAAACCGGAGATGAAAA

8604	Yes	AACTAATACACCAGTCTTGTAAACCGGAGATGAAAAC
8605	Yes	AACTAATACACCAGTCTTGTAAACCGGAGATGAAAACC
8606	Yes	AACTAATACACCAGTCTTGTAAACCGGAGATGAAAACCT
8607	Yes	AACTAATACACCAGTCTTGTAAACCGGAGATGAAAACCTT
8608	Yes	AACTAATACACCAGTCTTGTAAACCGGAGATGAAAACCTTT
8609	Yes	AACTAATACACCAGTCTTGTAAACCGGAGATGAAAACCTTTT
8610	Yes	AACTAATACACCAGTCTTGTAAACCGGAGATGAAAACCTTTTT
8611	Yes	AACTAATACACCAGTCTTGTAAACCGGAGATGAAAACCTTTTTC
8612	Yes	AACTAATACACCAGTCTTGTAAACCGGAGATGAAAACCTTTTTCC
8613	Yes	AACTAATACACCAGTCTTGTAAACCGGAGATGAAAACCTTTTTCCA
8614	Yes	AACTAATACACCAGTCTTGTAAACCGGAGATGAAAACCTTTTTCCAA
8615	Yes	AACTAATACACCAGTCTTGTAAACCGGAGATGAAAACCTTTTTCCAAG
8616	Yes	AACTAATACACCAGTCTTGTAAACCGGAGATGAAAACCTTTTTCCAAGG
8617	Yes	AACTAATACACCAGTCTTGTAAACCGGAGATGAAAACCTTTTTCCAAGGA
8618	No	AACTAGTTTTGACAAC
8619	Yes	AACTAGTTTTGACAACA
8620	Yes	AACTAGTTTTGACAACAT
8621	Yes	AACTAGTTTTGACAACATT
8622	Yes	AACTAGTTTTGACAACATTC
8623	Yes	AACTAGTTTTGACAACATTCA
8624	Yes	AACTAGTTTTGACAACATTCAA
8625	Yes	AACTAGTTTTGACAACATTCAAA
8626	Yes	AACTAGTTTTGACAACATTCAAAA
8627	Yes	AACTAGTTTTGACAACATTCAAAAA
8628	Yes	AACTAGTTTTGACAACATTCAAAAAA
8629	Yes	AACTAGTTTTGACAACATTCAAAAAAG
8630	Yes	AACTAGTTTTGACAACATTCAAAAAAGA
8631	Yes	AACTAGTTTTGACAACATTCAAAAAAGAG
8632	Yes	AACTAGTTTTGACAACATTCAAAAAAGAGT
8633	Yes	AACTAGTTTTGACAACATTCAAAAAAGAGTA
8634	Yes	AACTAGTTTTGACAACATTCAAAAAAGAGTAC
8635	Yes	AACTAGTTTTGACAACATTCAAAAAAGAGTACC
8636	Yes	AACTAGTTTTGACAACATTCAAAAAAGAGTACCA
8637	No	AACTCAGTTGGTAGAG
8638	Yes	AACTCAGTTGGTAGAGT
8639	Yes	AACTCAGTTGGTAGAGTA
8640	Yes	AACTCAGTTGGTAGAGTAG
8641	Yes	AACTCAGTTGGTAGAGTAGC
8642	Yes	AACTCAGTTGGTAGAGTAGCA
8643	Yes	AACTCAGTTGGTAGAGTAGCAG
8644	Yes	AACTCAGTTGGTAGAGTAGCAGA
8645	Yes	AACTCAGTTGGTAGAGTAGCAGAC

8646	Yes	AACTCAGTTGGTAGAGTAGCAGACT
8647	Yes	AACTCAGTTGGTAGAGTAGCAGACTT
8648	Yes	AACTCAGTTGGTAGAGTAGCAGACTTT
8649	Yes	AACTCAGTTGGTAGAGTAGCAGACTTTT
8650	Yes	AACTCAGTTGGTAGAGTAGCAGACTTTTC
8651	Yes	AACTCAGTTGGTAGAGTAGCAGACTTTTCA
8652	Yes	AACTCAGTTGGTAGAGTAGCAGACTTTTCAT
8653	Yes	AACTCAGTTGGTAGAGTAGCAGACTTTTCATC
8654	Yes	AACTCAGTTGGTAGAGTAGCAGACTTTTCATCT
8655	Yes	AACTCAGTTGGTAGAGTAGCAGACTTTTCATCTG
8656	Yes	AACTCAGTTGGTAGAGTAGCAGACTTTTCATCTGA
8657	Yes	AACTCAGTTGGTAGAGTAGCAGACTTTTCATCTGAG
8658	Yes	AACTCAGTTGGTAGAGTAGCAGACTTTTCATCTGAGG
8659	Yes	AACTCAGTTGGTAGAGTAGCAGACTTTTCATCTGAGGG
8660	Yes	AACTCAGTTGGTAGAGTAGCAGACTTTTCATCTGAGGGT
8661	Yes	AACTCAGTTGGTAGAGTAGCAGACTTTTCATCTGAGGGTC
8662	Yes	AACTCAGTTGGTAGAGTAGCAGACTTTTCATCTGAGGGTCC
8663	Yes	AACTCAGTTGGTAGAGTAGCAGACTTTTCATCTGAGGGTCCA
8664	Yes	AACTCAGTTGGTAGAGTAGCAGACTTTTCATCTGAGGGTCCAG
8665	Yes	AACTCAGTTGGTAGAGTAGCAGACTTTTCATCTGAGGGTCCAGG
8666	Yes	AACTCAGTTGGTAGAGTAGCAGACTTTTCATCTGAGGGTCCAGGG
8667	Yes	AACTCAGTTGGTAGAGTAGCAGACTTTTCATCTGAGGGTCCAGGGT
8668	Yes	AACTCAGTTGGTAGAGTAGCAGACTTTTCATCTGAGGGTCCAGGGTT
8669	Yes	AACTCAGTTGGTAGAGTAGCAGACTTTTCATCTGAGGGTCCAGGGTTT
8670	Yes	AACTCAGTTGGTAGAGTAGCAGACTTTTCATCTGAGGGTCCAGGGTTTA
8671	Yes	AACTCAGTTGGTAGAGTAGCAGACTTTTCATCTGAGGGTCCAGGGTTTAA
8672	No	AACTCATGCCCCCATG
8673	No	AACTCATGCCCCCATGT
8674	No	AACTCATGCCCCCATGTC
8675	No	AACTCATGCCCCCATGTCT
8676	No	AACTCATGCCCCCATGTCTA
8677	No	AACTCATGCCCCCATGTCTAA
8678	No	AACTCATGCCCCCATGTCTAAC
8679	No	AACTCATGCCCCCATGTCTAACA
8680	No	AACTCATGCCCCCATGTCTAACAA
8681	No	AACTCATGCCCCCATGTCTAACAAC
8682	No	AACTCATGCCCCCATGTCTAACAACA
8683	No	AACTCATGCCCCCATGTCTAACAACAT
8684	No	AACTCATGCCCCCATGTCTAACAACATG
8685	No	AACTCATGCCCCCATGTCTAACAACATGG
8686	No	AACTCATGCCCCCATGTCTAACAACATGGC
8687	No	AACTCATGCCCCCATGTCTAACAACATGGCT

8688	No	AACTCATGCCCCCATGTCTAACAACATGGCTT
8689	No	AACTCATGCCCCCATGTCTAACAACATGGCTTT
8690	No	AACTCATGCCCCCATGTCTAACAACATGGCTTTC
8691	No	AACTCATGCCCCCATGTCTAACAACATGGCTTTCT
8692	No	AACTCATGCCCCCATGTCTAACAACATGGCTTTCTC
8693	No	AACTCATGCCCCCATGTCTAACAACATGGCTTTCTCA
8694	Yes	AACTCATGCCCCCATGTCTAACAACATGGCTTTCTCAC
8695	Yes	AACTCATGCCCCCATGTCTAACAACATGGCTTTCTCACC
8696	Yes	AACTCATGCCCCCATGTCTAACAACATGGCTTTCTCACCA
8697	No	AACTCATTTGGCTGAG
8698	Yes	AACTCATTTGGCTGAGT
8699	Yes	AACTCATTTGGCTGAGTG
8700	Yes	AACTCATTTGGCTGAGTGG
8701	Yes	AACTCATTTGGCTGAGTGGT
8702	Yes	AACTCATTTGGCTGAGTGGTT
8703	Yes	AACTCATTTGGCTGAGTGGTTA
8704	Yes	AACTCATTTGGCTGAGTGGTTAA
8705	Yes	AACTCATTTGGCTGAGTGGTTAAG
8706	Yes	AACTCATTTGGCTGAGTGGTTAAGG
8707	Yes	AACTCATTTGGCTGAGTGGTTAAGGC
8708	Yes	AACTCATTTGGCTGAGTGGTTAAGGCA
8709	Yes	AACTCATTTGGCTGAGTGGTTAAGGCAT
8710	Yes	AACTCATTTGGCTGAGTGGTTAAGGCATT
8711	Yes	AACTCATTTGGCTGAGTGGTTAAGGCATTG
8712	Yes	AACTCATTTGGCTGAGTGGTTAAGGCATTGG
8713	Yes	AACTCATTTGGCTGAGTGGTTAAGGCATTGGA
8714	Yes	AACTCATTTGGCTGAGTGGTTAAGGCATTGGAC
8715	Yes	AACTCATTTGGCTGAGTGGTTAAGGCATTGGACT
8716	Yes	AACTCATTTGGCTGAGTGGTTAAGGCATTGGACTT
8717	Yes	AACTCATTTGGCTGAGTGGTTAAGGCATTGGACTTA
8718	Yes	AACTCATTTGGCTGAGTGGTTAAGGCATTGGACTTAA
8719	Yes	AACTCATTTGGCTGAGTGGTTAAGGCATTGGACTTAAG
8720	Yes	AACTCATTTGGCTGAGTGGTTAAGGCATTGGACTTAAGA
8721	Yes	AACTCATTTGGCTGAGTGGTTAAGGCATTGGACTTAAGAT
8722	Yes	AACTCATTTGGCTGAGTGGTTAAGGCATTGGACTTAAGATC
8723	Yes	AACTCATTTGGCTGAGTGGTTAAGGCATTGGACTTAAGATCC
8724	Yes	AACTCATTTGGCTGAGTGGTTAAGGCATTGGACTTAAGATCCA
8725	Yes	AACTCATTTGGCTGAGTGGTTAAGGCATTGGACTTAAGATCCAA
8726	Yes	AACTCATTTGGCTGAGTGGTTAAGGCATTGGACTTAAGATCCAAT
8727	Yes	AACTCATTTGGCTGAGTGGTTAAGGCATTGGACTTAAGATCCAATG
8728	Yes	AACTCATTTGGCTGAGTGGTTAAGGCATTGGACTTAAGATCCAATGG
8729	Yes	AACTCATTTGGCTGAGTGGTTAAGGCATTGGACTTAAGATCCAATGGA

8730	Yes	AACTCATTTGGCTGAGTGGTTAAGGCATTGGACTTAAGATCCAATGGAG
8731	Yes	AACTCATTTGGCTGAGTGGTTAAGGCATTGGACTTAAGATCCAATGGAGT
8732	No	AACTCCAAATAAAAGT
8733	No	AACTCCAAATAAAAGTA
8734	Yes	AACTCCAAATAAAAGTAC
8735	Yes	AACTCCAAATAAAAGTACC
8736	Yes	AACTCCAAATAAAAGTACCA
8737	No	AACTCCCGGCCAAAGC
8738	No	AACTCCCGGCCAAAGCA
8739	Yes	AACTCCCGGCCAAAGCAC
8740	Yes	AACTCCCGGCCAAAGCACC
8741	Yes	AACTCCCGGCCAAAGCACCA
8742	No	AACTCCTCTCATTAAC
8743	No	AACTCCTCTCATTAACA
8744	Yes	AACTCCTCTCATTAACAC
8745	Yes	AACTCCTCTCATTAACACC
8746	Yes	AACTCCTCTCATTAACACCA
8747	No	AACTCCTCTTCTTAAC
8748	No	AACTCCTCTTCTTAACA
8749	Yes	AACTCCTCTTCTTAACAC
8750	Yes	AACTCCTCTTCTTAACACC
8751	Yes	AACTCCTCTTCTTAACACCA
8752	Yes	AACTCCTTCACAAGCC
8753	Yes	AACTCCTTCACAAGCCG
8754	Yes	AACTCCTTCACAAGCCGC
8755	Yes	AACTCCTTCACAAGCCGCC
8756	Yes	AACTCCTTCACAAGCCGCCA
8757	No	AACTCTAAATTTAGAT
8758	No	AACTCTAAATTTAGATG
8759	Yes	AACTCTAAATTTAGATGT
8760	Yes	AACTCTAAATTTAGATGTC
8761	Yes	AACTCTAAATTTAGATGTCC
8762	Yes	AACTCTAAATTTAGATGTCCT
8763	Yes	AACTCTAAATTTAGATGTCCTG
8764	Yes	AACTCTAAATTTAGATGTCCTGA
8765	Yes	AACTCTAAATTTAGATGTCCTGAG
8766	Yes	AACTCTAAATTTAGATGTCCTGAGT
8767	Yes	AACTCTAAATTTAGATGTCCTGAGTT
8768	Yes	AACTCTAAATTTAGATGTCCTGAGTTC
8769	Yes	AACTCTAAATTTAGATGTCCTGAGTTCA
8770	Yes	AACTCTAAATTTAGATGTCCTGAGTTCAA
8771	Yes	AACTCTAAATTTAGATGTCCTGAGTTCAAA

8772	Yes	AACTCTAAATTTAGATGTCCTGAGTTCAAAT
8773	Yes	AACTCTAAATTTAGATGTCCTGAGTTCAAATC
8774	Yes	AACTCTAAATTTAGATGTCCTGAGTTCAAATCC
8775	Yes	AACTCTAAATTTAGATGTCCTGAGTTCAAATCCC
8776	Yes	AACTCTAAATTTAGATGTCCTGAGTTCAAATCCCA
8777	Yes	AACTCTAAATTTAGATGTCCTGAGTTCAAATCCCAG
8778	Yes	AACTCTAAATTTAGATGTCCTGAGTTCAAATCCCAGC
8779	Yes	AACTCTAAATTTAGATGTCCTGAGTTCAAATCCCAGCT
8780	Yes	AACTCTAAATTTAGATGTCCTGAGTTCAAATCCCAGCTG
8781	Yes	AACTCTAAATTTAGATGTCCTGAGTTCAAATCCCAGCTGT
8782	Yes	AACTCTAAATTTAGATGTCCTGAGTTCAAATCCCAGCTGTA
8783	Yes	AACTCTAAATTTAGATGTCCTGAGTTCAAATCCCAGCTGTAT
8784	Yes	AACTCTAAATTTAGATGTCCTGAGTTCAAATCCCAGCTGTATG
8785	Yes	AACTCTAAATTTAGATGTCCTGAGTTCAAATCCCAGCTGTATGC
8786	Yes	AACTCTAAATTTAGATGTCCTGAGTTCAAATCCCAGCTGTATGCC
8787	Yes	AACTCTAAATTTAGATGTCCTGAGTTCAAATCCCAGCTGTATGCCC
8788	Yes	AACTCTAAATTTAGATGTCCTGAGTTCAAATCCCAGCTGTATGCCCA
8789	No	AACTCTGAAGTCAGAG
8790	No	AACTCTGAAGTCAGAGA
8791	No	AACTCTGAAGTCAGAGAT
8792	Yes	AACTCTGAAGTCAGAGATA
8793	Yes	AACTCTGAAGTCAGAGATAC
8794	Yes	AACTCTGAAGTCAGAGATACT
8795	Yes	AACTCTGAAGTCAGAGATACTT
8796	Yes	AACTCTGAAGTCAGAGATACTTG
8797	Yes	AACTCTGAAGTCAGAGATACTTGA
8798	Yes	AACTCTGAAGTCAGAGATACTTGAA
8799	Yes	AACTCTGAAGTCAGAGATACTTGAAT
8800	Yes	AACTCTGAAGTCAGAGATACTTGAATT
8801	Yes	AACTCTGAAGTCAGAGATACTTGAATTT
8802	Yes	AACTCTGAAGTCAGAGATACTTGAATTTG
8803	Yes	AACTCTGAAGTCAGAGATACTTGAATTTGA
8804	Yes	AACTCTGAAGTCAGAGATACTTGAATTTGAA
8805	Yes	AACTCTGAAGTCAGAGATACTTGAATTTGAAT
8806	Yes	AACTCTGAAGTCAGAGATACTTGAATTTGAATG
8807	Yes	AACTCTGAAGTCAGAGATACTTGAATTTGAATGC
8808	Yes	AACTCTGAAGTCAGAGATACTTGAATTTGAATGCT
8809	Yes	AACTCTGAAGTCAGAGATACTTGAATTTGAATGCTG
8810	Yes	AACTCTGAAGTCAGAGATACTTGAATTTGAATGCTGG
8811	Yes	AACTCTGAAGTCAGAGATACTTGAATTTGAATGCTGGT
8812	Yes	AACTCTGAAGTCAGAGATACTTGAATTTGAATGCTGGTT
8813	Yes	AACTCTGAAGTCAGAGATACTTGAATTTGAATGCTGGTTC

8814	Yes	AACTCTGAAGTCAGAGATACTTGAATTTGAATGCTGGTTCT
8815	Yes	AACTCTGAAGTCAGAGATACTTGAATTTGAATGCTGGTTCTG
8816	Yes	AACTCTGAAGTCAGAGATACTTGAATTTGAATGCTGGTTCTGT
8817	Yes	AACTCTGAAGTCAGAGATACTTGAATTTGAATGCTGGTTCTGTC
8818	Yes	AACTCTGAAGTCAGAGATACTTGAATTTGAATGCTGGTTCTGTCA
8819	Yes	AACTCTGAAGTCAGAGATACTTGAATTTGAATGCTGGTTCTGTCAC
8820	Yes	AACTCTGAAGTCAGAGATACTTGAATTTGAATGCTGGTTCTGTCACC
8821	Yes	AACTCTGAAGTCAGAGATACTTGAATTTGAATGCTGGTTCTGTCACCA
8822	Yes	AACTCTGGGTGCCCCC
8823	Yes	AACTCTGGGTGCCCCCT
8824	Yes	AACTCTGGGTGCCCCCTT
8825	Yes	AACTCTGGGTGCCCCCTTT
8826	Yes	AACTCTGGGTGCCCCCTTTA
8827	Yes	AACTCTGGGTGCCCCCTTTAA
8828	Yes	AACTCTGGGTGCCCCCTTTAAC
8829	Yes	AACTCTGGGTGCCCCCTTTAACC
8830	Yes	AACTCTGGGTGCCCCCTTTAACCA
8831	No	AACTCTTTTAGTATAA
8832	No	AACTCTTTTAGTATAAA
8833	Yes	AACTCTTTTAGTATAAAT
8834	Yes	AACTCTTTTAGTATAAATA
8835	Yes	AACTCTTTTAGTATAAATAG
8836	Yes	AACTCTTTTAGTATAAATAGT
8837	Yes	AACTCTTTTAGTATAAATAGTA
8838	Yes	AACTCTTTTAGTATAAATAGTAC
8839	Yes	AACTCTTTTAGTATAAATAGTACC
8840	Yes	AACTCTTTTAGTATAAATAGTACCG
8841	Yes	AACTCTTTTAGTATAAATAGTACCGT
8842	Yes	AACTCTTTTAGTATAAATAGTACCGTT
8843	Yes	AACTCTTTTAGTATAAATAGTACCGTTA
8844	Yes	AACTCTTTTAGTATAAATAGTACCGTTAA
8845	Yes	AACTCTTTTAGTATAAATAGTACCGTTAAC
8846	Yes	AACTCTTTTAGTATAAATAGTACCGTTAACT
8847	Yes	AACTCTTTTAGTATAAATAGTACCGTTAACTT
8848	Yes	AACTCTTTTAGTATAAATAGTACCGTTAACTTC
8849	Yes	AACTCTTTTAGTATAAATAGTACCGTTAACTTCC
8850	Yes	AACTCTTTTAGTATAAATAGTACCGTTAACTTCCA
8851	Yes	AACTCTTTTAGTATAAATAGTACCGTTAACTTCCAA
8852	Yes	AACTCTTTTAGTATAAATAGTACCGTTAACTTCCAAT
8853	Yes	AACTCTTTTAGTATAAATAGTACCGTTAACTTCCAATT
8854	Yes	AACTCTTTTAGTATAAATAGTACCGTTAACTTCCAATTA
8855	Yes	AACTCTTTTAGTATAAATAGTACCGTTAACTTCCAATTAA

8856	Yes	AACTCTTTTAGTATAAATAGTACCGTTAACTTCCAATTAAC
8857	Yes	AACTCTTTTAGTATAAATAGTACCGTTAACTTCCAATTAACT
8858	Yes	AACTCTTTTAGTATAAATAGTACCGTTAACTTCCAATTAACTA
8859	Yes	AACTCTTTTAGTATAAATAGTACCGTTAACTTCCAATTAACTAG
8860	Yes	AACTCTTTTAGTATAAATAGTACCGTTAACTTCCAATTAACTAGT
8861	Yes	AACTCTTTTAGTATAAATAGTACCGTTAACTTCCAATTAACTAGTT
8862	Yes	AACTCTTTTAGTATAAATAGTACCGTTAACTTCCAATTAACTAGTTT
8863	Yes	AACTCTTTTAGTATAAATAGTACCGTTAACTTCCAATTAACTAGTTTT
8864	Yes	AACTCTTTTAGTATAAATAGTACCGTTAACTTCCAATTAACTAGTTTTG
8865	Yes	AACTCTTTTAGTATAAATAGTACCGTTAACTTCCAATTAACTAGTTTTGA
8866	No	AACTGAAAGGTTAGTG
8867	Yes	AACTGAAAGGTTAGTGG
8868	Yes	AACTGAAAGGTTAGTGGT
8869	Yes	AACTGAAAGGTTAGTGGTT
8870	Yes	AACTGAAAGGTTAGTGGTTC
8871	Yes	AACTGAAAGGTTAGTGGTTCG
8872	Yes	AACTGAAAGGTTAGTGGTTCGA
8873	Yes	AACTGAAAGGTTAGTGGTTCGAG
8874	Yes	AACTGAAAGGTTAGTGGTTCGAGC
8875	Yes	AACTGAAAGGTTAGTGGTTCGAGCC
8876	Yes	AACTGAAAGGTTAGTGGTTCGAGCCC
8877	Yes	AACTGAAAGGTTAGTGGTTCGAGCCCA
8878	Yes	AACTGAAAGGTTAGTGGTTCGAGCCCAC
8879	Yes	AACTGAAAGGTTAGTGGTTCGAGCCCACC
8880	Yes	AACTGAAAGGTTAGTGGTTCGAGCCCACCC
8881	Yes	AACTGAAAGGTTAGTGGTTCGAGCCCACCCG
8882	Yes	AACTGAAAGGTTAGTGGTTCGAGCCCACCCGG
8883	Yes	AACTGAAAGGTTAGTGGTTCGAGCCCACCCGGG
8884	Yes	AACTGAAAGGTTAGTGGTTCGAGCCCACCCGGGG
8885	Yes	AACTGAAAGGTTAGTGGTTCGAGCCCACCCGGGGA
8886	Yes	AACTGAAAGGTTAGTGGTTCGAGCCCACCCGGGGAC
8887	Yes	AACTGAAAGGTTAGTGGTTCGAGCCCACCCGGGGACG
8888	Yes	AACTGAAAGGTTAGTGGTTCGAGCCCACCCGGGGACGC
8889	Yes	AACTGAAAGGTTAGTGGTTCGAGCCCACCCGGGGACGCC
8890	Yes	AACTGAAAGGTTAGTGGTTCGAGCCCACCCGGGGACGCCA
8891	No	AACTGAAAGGTTGGTG
8892	Yes	AACTGAAAGGTTGGTGG
8893	Yes	AACTGAAAGGTTGGTGGT
8894	Yes	AACTGAAAGGTTGGTGGTG
8895	Yes	AACTGAAAGGTTGGTGGTGC
8896	Yes	AACTGAAAGGTTGGTGGTGCA
8897	Yes	AACTGAAAGGTTGGTGGTGCAA

8898	Yes	AACTGAAAGGTTGGTGGTGCAAG
8899	Yes	AACTGAAAGGTTGGTGGTGCAAGC
8900	Yes	AACTGAAAGGTTGGTGGTGCAAGCC
8901	Yes	AACTGAAAGGTTGGTGGTGCAAGCCC
8902	Yes	AACTGAAAGGTTGGTGGTGCAAGCCCA
8903	Yes	AACTGAAAGGTTGGTGGTGCAAGCCCAT
8904	Yes	AACTGAAAGGTTGGTGGTGCAAGCCCATC
8905	Yes	AACTGAAAGGTTGGTGGTGCAAGCCCATCC
8906	Yes	AACTGAAAGGTTGGTGGTGCAAGCCCATCCA
8907	Yes	AACTGAAAGGTTGGTGGTGCAAGCCCATCCAG
8908	Yes	AACTGAAAGGTTGGTGGTGCAAGCCCATCCAGG
8909	Yes	AACTGAAAGGTTGGTGGTGCAAGCCCATCCAGGG
8910	Yes	AACTGAAAGGTTGGTGGTGCAAGCCCATCCAGGGA
8911	Yes	AACTGAAAGGTTGGTGGTGCAAGCCCATCCAGGGAT
8912	Yes	AACTGAAAGGTTGGTGGTGCAAGCCCATCCAGGGATG
8913	Yes	AACTGAAAGGTTGGTGGTGCAAGCCCATCCAGGGATGC
8914	Yes	AACTGAAAGGTTGGTGGTGCAAGCCCATCCAGGGATGCC
8915	Yes	AACTGAAAGGTTGGTGGTGCAAGCCCATCCAGGGATGCCA
8916	Yes	AACTGAAAGGTTGGTGGTT
8917	Yes	AACTGAAAGGTTGGTGGTTC
8918	Yes	AACTGAAAGGTTGGTGGTTCG
8919	Yes	AACTGAAAGGTTGGTGGTTCGA
8920	Yes	AACTGAAAGGTTGGTGGTTCGAG
8921	Yes	AACTGAAAGGTTGGTGGTTCGAGC
8922	Yes	AACTGAAAGGTTGGTGGTTCGAGCC
8923	Yes	AACTGAAAGGTTGGTGGTTCGAGCCC
8924	Yes	AACTGAAAGGTTGGTGGTTCGAGCCCA
8925	Yes	AACTGAAAGGTTGGTGGTTCGAGCCCAC
8926	Yes	AACTGAAAGGTTGGTGGTTCGAGCCCACC
8927	Yes	AACTGAAAGGTTGGTGGTTCGAGCCCACCC
8928	Yes	AACTGAAAGGTTGGTGGTTCGAGCCCACCCA
8929	Yes	AACTGAAAGGTTGGTGGTTCGAGCCCACCCAG
8930	Yes	AACTGAAAGGTTGGTGGTTCGAGCCCACCCAGG
8931	Yes	AACTGAAAGGTTGGTGGTTCGAGCCCACCCAGGG
8932	Yes	AACTGAAAGGTTGGTGGTTCGAGCCCACCCAGGGA
8933	Yes	AACTGAAAGGTTGGTGGTTCGAGCCCACCCAGGGAC
8934	Yes	AACTGAAAGGTTGGTGGTTCGAGCCCACCCAGGGACG
8935	Yes	AACTGAAAGGTTGGTGGTTCGAGCCCACCCAGGGACGC
8936	Yes	AACTGAAAGGTTGGTGGTTCGAGCCCACCCAGGGACGCC
8937	Yes	AACTGAAAGGTTGGTGGTTCGAGCCCACCCAGGGACGCCA
8938	No	AACTGCTAACTCATGC
8939	No	AACTGCTAACTCATGCC

8940	No	AACTGCTAACTCATGCCC
8941	No	AACTGCTAACTCATGCCCC
8942	No	AACTGCTAACTCATGCCCCC
8943	No	AACTGCTAACTCATGCCCCCA
8944	No	AACTGCTAACTCATGCCCCCAT
8945	No	AACTGCTAACTCATGCCCCCATG
8946	No	AACTGCTAACTCATGCCCCCATGT
8947	No	AACTGCTAACTCATGCCCCCATGTC
8948	No	AACTGCTAACTCATGCCCCCATGTCT
8949	No	AACTGCTAACTCATGCCCCCATGTCTA
8950	No	AACTGCTAACTCATGCCCCCATGTCTAA
8951	No	AACTGCTAACTCATGCCCCCATGTCTAAC
8952	No	AACTGCTAACTCATGCCCCCATGTCTAACA
8953	No	AACTGCTAACTCATGCCCCCATGTCTAACAA
8954	No	AACTGCTAACTCATGCCCCCATGTCTAACAAC
8955	No	AACTGCTAACTCATGCCCCCATGTCTAACAACA
8956	No	AACTGCTAACTCATGCCCCCATGTCTAACAACAT
8957	No	AACTGCTAACTCATGCCCCCATGTCTAACAACATG
8958	No	AACTGCTAACTCATGCCCCCATGTCTAACAACATGG
8959	No	AACTGCTAACTCATGCCCCCATGTCTAACAACATGGC
8960	No	AACTGCTAACTCATGCCCCCATGTCTAACAACATGGCT
8961	No	AACTGCTAACTCATGCCCCCATGTCTAACAACATGGCTT
8962	No	AACTGCTAACTCATGCCCCCATGTCTAACAACATGGCTTT
8963	No	AACTGCTAACTCATGCCCCCATGTCTAACAACATGGCTTTC
8964	No	AACTGCTAACTCATGCCCCCATGTCTAACAACATGGCTTTCT
8965	No	AACTGCTAACTCATGCCCCCATGTCTAACAACATGGCTTTCTC
8966	No	AACTGCTAACTCATGCCCCCATGTCTAACAACATGGCTTTCTCA
8967	Yes	AACTGCTAACTCATGCCCCCATGTCTAACAACATGGCTTTCTCAC
8968	Yes	AACTGCTAACTCATGCCCCCATGTCTAACAACATGGCTTTCTCACC
8969	Yes	AACTGCTAACTCATGCCCCCATGTCTAACAACATGGCTTTCTCACCA
8970	No	AACTGGCAGGCCTGGG
8971	Yes	AACTGGCAGGCCTGGGT
8972	Yes	AACTGGCAGGCCTGGGTT
8973	Yes	AACTGGCAGGCCTGGGTTC
8974	Yes	AACTGGCAGGCCTGGGTTCA
8975	Yes	AACTGGCAGGCCTGGGTTCAA
8976	Yes	AACTGGCAGGCCTGGGTTCAAA
8977	Yes	AACTGGCAGGCCTGGGTTCAAAT
8978	Yes	AACTGGCAGGCCTGGGTTCAAATC
8979	Yes	AACTGGCAGGCCTGGGTTCAAATCC
8980	Yes	AACTGGCAGGCCTGGGTTCAAATCCC
8981	Yes	AACTGGCAGGCCTGGGTTCAAATCCCA

8982	Yes	AACTGGCAGGCCTGGGTTCAAATCCCAG
8983	Yes	AACTGGCAGGCCTGGGTTCAAATCCCAGC
8984	Yes	AACTGGCAGGCCTGGGTTCAAATCCCAGCT
8985	Yes	AACTGGCAGGCCTGGGTTCAAATCCCAGCTT
8986	Yes	AACTGGCAGGCCTGGGTTCAAATCCCAGCTTA
8987	Yes	AACTGGCAGGCCTGGGTTCAAATCCCAGCTTAT
8988	Yes	AACTGGCAGGCCTGGGTTCAAATCCCAGCTTATT
8989	Yes	AACTGGCAGGCCTGGGTTCAAATCCCAGCTTATTC
8990	Yes	AACTGGCAGGCCTGGGTTCAAATCCCAGCTTATTCA
8991	Yes	AACTGGCAGGCCTGGGTTCAAATCCCAGCTTATTCAC
8992	Yes	AACTGGCAGGCCTGGGTTCAAATCCCAGCTTATTCACC
8993	Yes	AACTGGCAGGCCTGGGTTCAAATCCCAGCTTATTCACCA
8994	Yes	AACTGGGCGGAAACAC
8995	Yes	AACTGGGCGGAAACACC
8996	Yes	AACTGGGCGGAAACACCA
8997	Yes	AACTGTAGATCAAGAG
8998	Yes	AACTGTAGATCAAGAGG
8999	Yes	AACTGTAGATCAAGAGGT
9000	Yes	AACTGTAGATCAAGAGGTC
9001	Yes	AACTGTAGATCAAGAGGTCC
9002	Yes	AACTGTAGATCAAGAGGTCCC
9003	Yes	AACTGTAGATCAAGAGGTCCCT
9004	Yes	AACTGTAGATCAAGAGGTCCCTG
9005	Yes	AACTGTAGATCAAGAGGTCCCTGG
9006	Yes	AACTGTAGATCAAGAGGTCCCTGGA
9007	Yes	AACTGTAGATCAAGAGGTCCCTGGAT
9008	Yes	AACTGTAGATCAAGAGGTCCCTGGATC
9009	Yes	AACTGTAGATCAAGAGGTCCCTGGATCA
9010	Yes	AACTGTAGATCAAGAGGTCCCTGGATCAA
9011	Yes	AACTGTAGATCAAGAGGTCCCTGGATCAAC
9012	Yes	AACTGTAGATCAAGAGGTCCCTGGATCAACT
9013	Yes	AACTGTAGATCAAGAGGTCCCTGGATCAACTC
9014	Yes	AACTGTAGATCAAGAGGTCCCTGGATCAACTCT
9015	Yes	AACTGTAGATCAAGAGGTCCCTGGATCAACTCTG
9016	Yes	AACTGTAGATCAAGAGGTCCCTGGATCAACTCTGG
9017	Yes	AACTGTAGATCAAGAGGTCCCTGGATCAACTCTGGG
9018	Yes	AACTGTAGATCAAGAGGTCCCTGGATCAACTCTGGGT
9019	Yes	AACTGTAGATCAAGAGGTCCCTGGATCAACTCTGGGTG
9020	Yes	AACTGTAGATCAAGAGGTCCCTGGATCAACTCTGGGTGC
9021	Yes	AACTGTAGATCAAGAGGTCCCTGGATCAACTCTGGGTGCC
9022	Yes	AACTGTAGATCAAGAGGTCCCTGGATCAACTCTGGGTGCCC
9023	Yes	AACTGTAGATCAAGAGGTCCCTGGATCAACTCTGGGTGCCCC

9024	Yes	AACTGTAGATCAAGAGGTCCCTGGATCAACTCTGGGTGCCCCC
9025	Yes	AACTGTAGATCAAGAGGTCCCTGGATCAACTCTGGGTGCCCCCT
9026	Yes	AACTGTAGATCAAGAGGTCCCTGGATCAACTCTGGGTGCCCCCTT
9027	Yes	AACTGTAGATCAAGAGGTCCCTGGATCAACTCTGGGTGCCCCCTTT
9028	Yes	AACTGTAGATCAAGAGGTCCCTGGATCAACTCTGGGTGCCCCCTTTA
9029	Yes	AACTGTAGATCAAGAGGTCCCTGGATCAACTCTGGGTGCCCCCTTTAA
9030	Yes	AACTGTAGATCAAGAGGTCCCTGGATCAACTCTGGGTGCCCCCTTTAAC
9031	Yes	AACTGTAGATCAAGAGGTCCCTGGATCAACTCTGGGTGCCCCCTTTAACC
9032	No	AACTTAAAACTTTACA
9033	No	AACTTAAAACTTTACAA
9034	No	AACTTAAAACTTTACAAT
9035	No	AACTTAAAACTTTACAATC
9036	No	AACTTAAAACTTTACAATCA
9037	No	AACTTAAAACTTTACAATCAG
9038	Yes	AACTTAAAACTTTACAATCAGA
9039	Yes	AACTTAAAACTTTACAATCAGAG
9040	Yes	AACTTAAAACTTTACAATCAGAGG
9041	Yes	AACTTAAAACTTTACAATCAGAGGT
9042	Yes	AACTTAAAACTTTACAATCAGAGGTT
9043	Yes	AACTTAAAACTTTACAATCAGAGGTTC
9044	Yes	AACTTAAAACTTTACAATCAGAGGTTCA
9045	Yes	AACTTAAAACTTTACAATCAGAGGTTCAA
9046	Yes	AACTTAAAACTTTACAATCAGAGGTTCAAC
9047	Yes	AACTTAAAACTTTACAATCAGAGGTTCAACT
9048	Yes	AACTTAAAACTTTACAATCAGAGGTTCAACTC
9049	Yes	AACTTAAAACTTTACAATCAGAGGTTCAACTCC
9050	Yes	AACTTAAAACTTTACAATCAGAGGTTCAACTCCT
9051	Yes	AACTTAAAACTTTACAATCAGAGGTTCAACTCCTC
9052	Yes	AACTTAAAACTTTACAATCAGAGGTTCAACTCCTCT
9053	Yes	AACTTAAAACTTTACAATCAGAGGTTCAACTCCTCTT
9054	Yes	AACTTAAAACTTTACAATCAGAGGTTCAACTCCTCTTC
9055	Yes	AACTTAAAACTTTACAATCAGAGGTTCAACTCCTCTTCT
9056	Yes	AACTTAAAACTTTACAATCAGAGGTTCAACTCCTCTTCTT
9057	Yes	AACTTAAAACTTTACAATCAGAGGTTCAACTCCTCTTCTTA
9058	Yes	AACTTAAAACTTTACAATCAGAGGTTCAACTCCTCTTCTTAA
9059	Yes	AACTTAAAACTTTACAATCAGAGGTTCAACTCCTCTTCTTAAC
9060	Yes	AACTTAAAACTTTACAATCAGAGGTTCAACTCCTCTTCTTAACA
9061	Yes	AACTTAAAACTTTACAATCAGAGGTTCAACTCCTCTTCTTAACAC
9062	Yes	AACTTAAAACTTTACAATCAGAGGTTCAACTCCTCTTCTTAACACC
9063	Yes	AACTTAAAACTTTACAATCAGAGGTTCAACTCCTCTTCTTAACACCA
9064	No	AACTTAAAACTTTACAG
9065	No	AACTTAAAACTTTACAGT

9066	No	AACTTAAAACTTTACAGTC
9067	Yes	AACTTAAAACTTTACAGTCA
9068	Yes	AACTTAAAACTTTACAGTCAG
9069	Yes	AACTTAAAACTTTACAGTCAGA
9070	Yes	AACTTAAAACTTTACAGTCAGAG
9071	Yes	AACTTAAAACTTTACAGTCAGAGG
9072	Yes	AACTTAAAACTTTACAGTCAGAGGT
9073	Yes	AACTTAAAACTTTACAGTCAGAGGTT
9074	Yes	AACTTAAAACTTTACAGTCAGAGGTTC
9075	Yes	AACTTAAAACTTTACAGTCAGAGGTTCA
9076	Yes	AACTTAAAACTTTACAGTCAGAGGTTCAA
9077	Yes	AACTTAAAACTTTACAGTCAGAGGTTCAAT
9078	Yes	AACTTAAAACTTTACAGTCAGAGGTTCAATT
9079	Yes	AACTTAAAACTTTACAGTCAGAGGTTCAATTC
9080	Yes	AACTTAAAACTTTACAGTCAGAGGTTCAATTCC
9081	Yes	AACTTAAAACTTTACAGTCAGAGGTTCAATTCCT
9082	Yes	AACTTAAAACTTTACAGTCAGAGGTTCAATTCCTC
9083	Yes	AACTTAAAACTTTACAGTCAGAGGTTCAATTCCTCT
9084	Yes	AACTTAAAACTTTACAGTCAGAGGTTCAATTCCTCTT
9085	Yes	AACTTAAAACTTTACAGTCAGAGGTTCAATTCCTCTTC
9086	Yes	AACTTAAAACTTTACAGTCAGAGGTTCAATTCCTCTTCT
9087	Yes	AACTTAAAACTTTACAGTCAGAGGTTCAATTCCTCTTCTT
9088	Yes	AACTTAAAACTTTACAGTCAGAGGTTCAATTCCTCTTCTTA
9089	Yes	AACTTAAAACTTTACAGTCAGAGGTTCAATTCCTCTTCTTAA
9090	Yes	AACTTAAAACTTTACAGTCAGAGGTTCAATTCCTCTTCTTAAC
9091	Yes	AACTTAAAACTTTACAGTCAGAGGTTCAATTCCTCTTCTTAACA
9092	Yes	AACTTAAAACTTTACAGTCAGAGGTTCAATTCCTCTTCTTAACAC
9093	Yes	AACTTAAAACTTTACAGTCAGAGGTTCAATTCCTCTTCTTAACACC
9094	Yes	AACTTAAAACTTTACAGTCAGAGGTTCAATTCCTCTTCTTAACACCA
9095	No	AACTTAAAATTTTATA
9096	No	AACTTAAAATTTTATAA
9097	No	AACTTAAAATTTTATAAT
9098	No	AACTTAAAATTTTATAATC
9099	Yes	AACTTAAAATTTTATAATCA
9100	Yes	AACTTAAAATTTTATAATCAG
9101	Yes	AACTTAAAATTTTATAATCAGA
9102	Yes	AACTTAAAATTTTATAATCAGAG
9103	Yes	AACTTAAAATTTTATAATCAGAGG
9104	Yes	AACTTAAAATTTTATAATCAGAGGT
9105	Yes	AACTTAAAATTTTATAATCAGAGGTT
9106	Yes	AACTTAAAATTTTATAATCAGAGGTTC
9107	Yes	AACTTAAAATTTTATAATCAGAGGTTCA

9108	Yes	AACTTAAAATTTTATAATCAGAGGTTCAA
9109	Yes	AACTTAAAATTTTATAATCAGAGGTTCAAC
9110	Yes	AACTTAAAATTTTATAATCAGAGGTTCAACT
9111	Yes	AACTTAAAATTTTATAATCAGAGGTTCAACTC
9112	Yes	AACTTAAAATTTTATAATCAGAGGTTCAACTCC
9113	Yes	AACTTAAAATTTTATAATCAGAGGTTCAACTCCT
9114	Yes	AACTTAAAATTTTATAATCAGAGGTTCAACTCCTC
9115	Yes	AACTTAAAATTTTATAATCAGAGGTTCAACTCCTCT
9116	Yes	AACTTAAAATTTTATAATCAGAGGTTCAACTCCTCTT
9117	Yes	AACTTAAAATTTTATAATCAGAGGTTCAACTCCTCTTC
9118	Yes	AACTTAAAATTTTATAATCAGAGGTTCAACTCCTCTTCT
9119	Yes	AACTTAAAATTTTATAATCAGAGGTTCAACTCCTCTTCTT
9120	Yes	AACTTAAAATTTTATAATCAGAGGTTCAACTCCTCTTCTTA
9121	Yes	AACTTAAAATTTTATAATCAGAGGTTCAACTCCTCTTCTTAA
9122	Yes	AACTTAAAATTTTATAATCAGAGGTTCAACTCCTCTTCTTAAC
9123	Yes	AACTTAAAATTTTATAATCAGAGGTTCAACTCCTCTTCTTAACA
9124	Yes	AACTTAAAATTTTATAATCAGAGGTTCAACTCCTCTTCTTAACAC
9125	Yes	AACTTAAAATTTTATAATCAGAGGTTCAACTCCTCTTCTTAACACC
9126	Yes	AACTTAAAATTTTATAATCAGAGGTTCAACTCCTCTTCTTAACACCA
9127	No	AACTTAACACTTTATA
9128	No	AACTTAACACTTTATAA
9129	Yes	AACTTAACACTTTATAAT
9130	Yes	AACTTAACACTTTATAATC
9131	Yes	AACTTAACACTTTATAATCA
9132	Yes	AACTTAACACTTTATAATCAG
9133	Yes	AACTTAACACTTTATAATCAGA
9134	Yes	AACTTAACACTTTATAATCAGAG
9135	Yes	AACTTAACACTTTATAATCAGAGG
9136	Yes	AACTTAACACTTTATAATCAGAGGT
9137	Yes	AACTTAACACTTTATAATCAGAGGTT
9138	Yes	AACTTAACACTTTATAATCAGAGGTTC
9139	Yes	AACTTAACACTTTATAATCAGAGGTTCA
9140	Yes	AACTTAACACTTTATAATCAGAGGTTCAA
9141	Yes	AACTTAACACTTTATAATCAGAGGTTCAAC
9142	Yes	AACTTAACACTTTATAATCAGAGGTTCAACT
9143	Yes	AACTTAACACTTTATAATCAGAGGTTCAACTC
9144	Yes	AACTTAACACTTTATAATCAGAGGTTCAACTCC
9145	Yes	AACTTAACACTTTATAATCAGAGGTTCAACTCCT
9146	Yes	AACTTAACACTTTATAATCAGAGGTTCAACTCCTC
9147	Yes	AACTTAACACTTTATAATCAGAGGTTCAACTCCTCT
9148	Yes	AACTTAACACTTTATAATCAGAGGTTCAACTCCTCTT
9149	Yes	AACTTAACACTTTATAATCAGAGGTTCAACTCCTCTTC

9150	Yes	AACTTAACACTTTATAATCAGAGGTTCAACTCCTCTTCT
9151	Yes	AACTTAACACTTTATAATCAGAGGTTCAACTCCTCTTCTT
9152	Yes	AACTTAACACTTTATAATCAGAGGTTCAACTCCTCTTCTTA
9153	Yes	AACTTAACACTTTATAATCAGAGGTTCAACTCCTCTTCTTAA
9154	Yes	AACTTAACACTTTATAATCAGAGGTTCAACTCCTCTTCTTAAC
9155	Yes	AACTTAACACTTTATAATCAGAGGTTCAACTCCTCTTCTTAACA
9156	Yes	AACTTAACACTTTATAATCAGAGGTTCAACTCCTCTTCTTAACAC
9157	Yes	AACTTAACACTTTATAATCAGAGGTTCAACTCCTCTTCTTAACACC
9158	Yes	AACTTAACACTTTATAATCAGAGGTTCAACTCCTCTTCTTAACACCA
9159	No	AACTTAACTTGACCGC
9160	No	AACTTAACTTGACCGCT
9161	No	AACTTAACTTGACCGCTC
9162	No	AACTTAACTTGACCGCTCT
9163	No	AACTTAACTTGACCGCTCTG
9164	No	AACTTAACTTGACCGCTCTGA
9165	Yes	AACTTAACTTGACCGCTCTGAC
9166	Yes	AACTTAACTTGACCGCTCTGACC
9167	Yes	AACTTAACTTGACCGCTCTGACCA
9168	No	AACTTAAGACTTTATA
9169	No	AACTTAAGACTTTATAA
9170	No	AACTTAAGACTTTATAAT
9171	Yes	AACTTAAGACTTTATAATT
9172	Yes	AACTTAAGACTTTATAATTA
9173	Yes	AACTTAAGACTTTATAATTAG
9174	Yes	AACTTAAGACTTTATAATTAGA
9175	Yes	AACTTAAGACTTTATAATTAGAA
9176	Yes	AACTTAAGACTTTATAATTAGAAG
9177	Yes	AACTTAAGACTTTATAATTAGAAGT
9178	Yes	AACTTAAGACTTTATAATTAGAAGTT
9179	Yes	AACTTAAGACTTTATAATTAGAAGTTC
9180	Yes	AACTTAAGACTTTATAATTAGAAGTTCA
9181	Yes	AACTTAAGACTTTATAATTAGAAGTTCAA
9182	Yes	AACTTAAGACTTTATAATTAGAAGTTCAAC
9183	Yes	AACTTAAGACTTTATAATTAGAAGTTCAACA
9184	Yes	AACTTAAGACTTTATAATTAGAAGTTCAACAC
9185	Yes	AACTTAAGACTTTATAATTAGAAGTTCAACACC
9186	Yes	AACTTAAGACTTTATAATTAGAAGTTCAACACCT
9187	Yes	AACTTAAGACTTTATAATTAGAAGTTCAACACCTC
9188	Yes	AACTTAAGACTTTATAATTAGAAGTTCAACACCTCT
9189	Yes	AACTTAAGACTTTATAATTAGAAGTTCAACACCTCTT
9190	Yes	AACTTAAGACTTTATAATTAGAAGTTCAACACCTCTTC
9191	Yes	AACTTAAGACTTTATAATTAGAAGTTCAACACCTCTTCT

9192	Yes	AACTTAAGACTTTATAATTAGAAGTTCAACACCTCTTCTT
9193	Yes	AACTTAAGACTTTATAATTAGAAGTTCAACACCTCTTCTTA
9194	Yes	AACTTAAGACTTTATAATTAGAAGTTCAACACCTCTTCTTAA
9195	Yes	AACTTAAGACTTTATAATTAGAAGTTCAACACCTCTTCTTAAC
9196	Yes	AACTTAAGACTTTATAATTAGAAGTTCAACACCTCTTCTTAACA
9197	Yes	AACTTAAGACTTTATAATTAGAAGTTCAACACCTCTTCTTAACAC
9198	Yes	AACTTAAGACTTTATAATTAGAAGTTCAACACCTCTTCTTAACACC
9199	Yes	AACTTAAGACTTTATAATTAGAAGTTCAACACCTCTTCTTAACACCA
9200	No	AACTTAATTGGTTAGA
9201	No	AACTTAATTGGTTAGAG
9202	Yes	AACTTAATTGGTTAGAGC
9203	Yes	AACTTAATTGGTTAGAGCG
9204	Yes	AACTTAATTGGTTAGAGCGT
9205	Yes	AACTTAATTGGTTAGAGCGTG
9206	Yes	AACTTAATTGGTTAGAGCGTGG
9207	Yes	AACTTAATTGGTTAGAGCGTGGT
9208	Yes	AACTTAATTGGTTAGAGCGTGGTG
9209	Yes	AACTTAATTGGTTAGAGCGTGGTGC
9210	Yes	AACTTAATTGGTTAGAGCGTGGTGCT
9211	Yes	AACTTAATTGGTTAGAGCGTGGTGCTA
9212	Yes	AACTTAATTGGTTAGAGCGTGGTGCTAA
9213	Yes	AACTTAATTGGTTAGAGCGTGGTGCTAAT
9214	Yes	AACTTAATTGGTTAGAGCGTGGTGCTAATA
9215	Yes	AACTTAATTGGTTAGAGCGTGGTGCTAATAA
9216	Yes	AACTTAATTGGTTAGAGCGTGGTGCTAATAAT
9217	Yes	AACTTAATTGGTTAGAGCGTGGTGCTAATAATG
9218	Yes	AACTTAATTGGTTAGAGCGTGGTGCTAATAATGT
9219	Yes	AACTTAATTGGTTAGAGCGTGGTGCTAATAATGTC
9220	Yes	AACTTAATTGGTTAGAGCGTGGTGCTAATAATGTCA
9221	Yes	AACTTAATTGGTTAGAGCGTGGTGCTAATAATGTCAA
9222	Yes	AACTTAATTGGTTAGAGCGTGGTGCTAATAATGTCAAG
9223	Yes	AACTTAATTGGTTAGAGCGTGGTGCTAATAATGTCAAGG
9224	Yes	AACTTAATTGGTTAGAGCGTGGTGCTAATAATGTCAAGGT
9225	Yes	AACTTAATTGGTTAGAGCGTGGTGCTAATAATGTCAAGGTT
9226	Yes	AACTTAATTGGTTAGAGCGTGGTGCTAATAATGTCAAGGTTG
9227	Yes	AACTTAATTGGTTAGAGCGTGGTGCTAATAATGTCAAGGTTGC
9228	Yes	AACTTAATTGGTTAGAGCGTGGTGCTAATAATGTCAAGGTTGCG
9229	Yes	AACTTAATTGGTTAGAGCGTGGTGCTAATAATGTCAAGGTTGCGG
9230	Yes	AACTTAATTGGTTAGAGCGTGGTGCTAATAATGTCAAGGTTGCGGG
9231	Yes	AACTTAATTGGTTAGAGCGTGGTGCTAATAATGTCAAGGTTGCGGGT
9232	Yes	AACTTAATTGGTTAGAGCGTGGTGCTAATAATGTCAAGGTTGCGGGTT
9233	Yes	AACTTAATTGGTTAGAGCGTGGTGCTAATAATGTCAAGGTTGCGGGTTG

9234	Yes	AACTTAATTGGTTAGAGCGTGGTGCTAATAATGTCAAGGTTGCGGGTTGG
9235	No	AACTTACACTTAGGAG
9236	No	AACTTACACTTAGGAGA
9237	No	AACTTACACTTAGGAGAT
9238	No	AACTTACACTTAGGAGATT
9239	No	AACTTACACTTAGGAGATTT
9240	No	AACTTACACTTAGGAGATTTC
9241	No	AACTTACACTTAGGAGATTTCA
9242	No	AACTTACACTTAGGAGATTTCAA
9243	No	AACTTACACTTAGGAGATTTCAAC
9244	No	AACTTACACTTAGGAGATTTCAACT
9245	No	AACTTACACTTAGGAGATTTCAACTT
9246	No	AACTTACACTTAGGAGATTTCAACTTA
9247	No	AACTTACACTTAGGAGATTTCAACTTAA
9248	No	AACTTACACTTAGGAGATTTCAACTTAAC
9249	No	AACTTACACTTAGGAGATTTCAACTTAACT
9250	No	AACTTACACTTAGGAGATTTCAACTTAACTT
9251	No	AACTTACACTTAGGAGATTTCAACTTAACTTG
9252	No	AACTTACACTTAGGAGATTTCAACTTAACTTGA
9253	No	AACTTACACTTAGGAGATTTCAACTTAACTTGAC
9254	No	AACTTACACTTAGGAGATTTCAACTTAACTTGACC
9255	No	AACTTACACTTAGGAGATTTCAACTTAACTTGACCG
9256	No	AACTTACACTTAGGAGATTTCAACTTAACTTGACCGC
9257	No	AACTTACACTTAGGAGATTTCAACTTAACTTGACCGCT
9258	No	AACTTACACTTAGGAGATTTCAACTTAACTTGACCGCTC
9259	No	AACTTACACTTAGGAGATTTCAACTTAACTTGACCGCTCT
9260	No	AACTTACACTTAGGAGATTTCAACTTAACTTGACCGCTCTG
9261	No	AACTTACACTTAGGAGATTTCAACTTAACTTGACCGCTCTGA
9262	Yes	AACTTACACTTAGGAGATTTCAACTTAACTTGACCGCTCTGAC
9263	Yes	AACTTACACTTAGGAGATTTCAACTTAACTTGACCGCTCTGACC
9264	Yes	AACTTACACTTAGGAGATTTCAACTTAACTTGACCGCTCTGACCA
9265	No	AACTTAGCATTAACCT
9266	No	AACTTAGCATTAACCTT
9267	No	AACTTAGCATTAACCTTT
9268	Yes	AACTTAGCATTAACCTTTT
9269	Yes	AACTTAGCATTAACCTTTTA
9270	Yes	AACTTAGCATTAACCTTTTAA
9271	Yes	AACTTAGCATTAACCTTTTAAG
9272	Yes	AACTTAGCATTAACCTTTTAAGT
9273	Yes	AACTTAGCATTAACCTTTTAAGTT
9274	Yes	AACTTAGCATTAACCTTTTAAGTTA
9275	Yes	AACTTAGCATTAACCTTTTAAGTTAA

9276	Yes	AACTTAGCATTAACCTTTTAAGTTAAA
9277	Yes	AACTTAGCATTAACCTTTTAAGTTAAAG
9278	Yes	AACTTAGCATTAACCTTTTAAGTTAAAGA
9279	Yes	AACTTAGCATTAACCTTTTAAGTTAAAGAT
9280	Yes	AACTTAGCATTAACCTTTTAAGTTAAAGATT
9281	Yes	AACTTAGCATTAACCTTTTAAGTTAAAGATTA
9282	Yes	AACTTAGCATTAACCTTTTAAGTTAAAGATTAA
9283	Yes	AACTTAGCATTAACCTTTTAAGTTAAAGATTAAG
9284	Yes	AACTTAGCATTAACCTTTTAAGTTAAAGATTAAGA
9285	Yes	AACTTAGCATTAACCTTTTAAGTTAAAGATTAAGAG
9286	Yes	AACTTAGCATTAACCTTTTAAGTTAAAGATTAAGAGA
9287	Yes	AACTTAGCATTAACCTTTTAAGTTAAAGATTAAGAGAA
9288	Yes	AACTTAGCATTAACCTTTTAAGTTAAAGATTAAGAGAAC
9289	Yes	AACTTAGCATTAACCTTTTAAGTTAAAGATTAAGAGAACC
9290	Yes	AACTTAGCATTAACCTTTTAAGTTAAAGATTAAGAGAACCA
9291	Yes	AACTTAGCATTAACCTTTTAAGTTAAAGATTAAGAGAACCAA
9292	Yes	AACTTAGCATTAACCTTTTAAGTTAAAGATTAAGAGAACCAAC
9293	Yes	AACTTAGCATTAACCTTTTAAGTTAAAGATTAAGAGAACCAACA
9294	Yes	AACTTAGCATTAACCTTTTAAGTTAAAGATTAAGAGAACCAACAC
9295	Yes	AACTTAGCATTAACCTTTTAAGTTAAAGATTAAGAGAACCAACACC
9296	Yes	AACTTAGCATTAACCTTTTAAGTTAAAGATTAAGAGAACCAACACCT
9297	Yes	AACTTAGCATTAACCTTTTAAGTTAAAGATTAAGAGAACCAACACCTC
9298	Yes	AACTTAGCATTAACCTTTTAAGTTAAAGATTAAGAGAACCAACACCTCT
9299	Yes	AACTTAGCATTAACCTTTTAAGTTAAAGATTAAGAGAACCAACACCTCTT
9300	No	AACTTCCAATTAACTA
9301	No	AACTTCCAATTAACTAG
9302	No	AACTTCCAATTAACTAGT
9303	No	AACTTCCAATTAACTAGTT
9304	No	AACTTCCAATTAACTAGTTT
9305	No	AACTTCCAATTAACTAGTTTT
9306	No	AACTTCCAATTAACTAGTTTTG
9307	No	AACTTCCAATTAACTAGTTTTGA
9308	Yes	AACTTCCAATTAACTAGTTTTGAC
9309	Yes	AACTTCCAATTAACTAGTTTTGACA
9310	Yes	AACTTCCAATTAACTAGTTTTGACAA
9311	Yes	AACTTCCAATTAACTAGTTTTGACAAC
9312	Yes	AACTTCCAATTAACTAGTTTTGACAACA
9313	Yes	AACTTCCAATTAACTAGTTTTGACAACAT
9314	Yes	AACTTCCAATTAACTAGTTTTGACAACATT
9315	Yes	AACTTCCAATTAACTAGTTTTGACAACATTC
9316	Yes	AACTTCCAATTAACTAGTTTTGACAACATTCA
9317	Yes	AACTTCCAATTAACTAGTTTTGACAACATTCAA

9318	Yes	AACTTCCAATTAACTAGTTTTGACAACATTCAAA
9319	Yes	AACTTCCAATTAACTAGTTTTGACAACATTCAAAA
9320	Yes	AACTTCCAATTAACTAGTTTTGACAACATTCAAAAA
9321	Yes	AACTTCCAATTAACTAGTTTTGACAACATTCAAAAAA
9322	Yes	AACTTCCAATTAACTAGTTTTGACAACATTCAAAAAAG
9323	Yes	AACTTCCAATTAACTAGTTTTGACAACATTCAAAAAAGA
9324	Yes	AACTTCCAATTAACTAGTTTTGACAACATTCAAAAAAGAG
9325	Yes	AACTTCCAATTAACTAGTTTTGACAACATTCAAAAAAGAGT
9326	Yes	AACTTCCAATTAACTAGTTTTGACAACATTCAAAAAAGAGTA
9327	Yes	AACTTCCAATTAACTAGTTTTGACAACATTCAAAAAAGAGTAC
9328	Yes	AACTTCCAATTAACTAGTTTTGACAACATTCAAAAAAGAGTACC
9329	Yes	AACTTCCAATTAACTAGTTTTGACAACATTCAAAAAAGAGTACCA
9330	No	AACTTCCAGTATTTCC
9331	No	AACTTCCAGTATTTCCA
9332	Yes	AACTTCCAGTATTTCCAC
9333	Yes	AACTTCCAGTATTTCCACC
9334	Yes	AACTTCCAGTATTTCCACCA
9335	No	AACTTGACCGCTCTGA
9336	Yes	AACTTGACCGCTCTGAC
9337	Yes	AACTTGACCGCTCTGACC
9338	Yes	AACTTGACCGCTCTGACCA
9339	No	AACTTTACAATCAGAG
9340	No	AACTTTACAATCAGAGG
9341	No	AACTTTACAATCAGAGGT
9342	Yes	AACTTTACAATCAGAGGTT
9343	Yes	AACTTTACAATCAGAGGTTC
9344	Yes	AACTTTACAATCAGAGGTTCA
9345	Yes	AACTTTACAATCAGAGGTTCAA
9346	Yes	AACTTTACAATCAGAGGTTCAAC
9347	Yes	AACTTTACAATCAGAGGTTCAACT
9348	Yes	AACTTTACAATCAGAGGTTCAACTC
9349	Yes	AACTTTACAATCAGAGGTTCAACTCC
9350	Yes	AACTTTACAATCAGAGGTTCAACTCCT
9351	Yes	AACTTTACAATCAGAGGTTCAACTCCTC
9352	Yes	AACTTTACAATCAGAGGTTCAACTCCTCT
9353	Yes	AACTTTACAATCAGAGGTTCAACTCCTCTT
9354	Yes	AACTTTACAATCAGAGGTTCAACTCCTCTTC
9355	Yes	AACTTTACAATCAGAGGTTCAACTCCTCTTCT
9356	Yes	AACTTTACAATCAGAGGTTCAACTCCTCTTCTT
9357	Yes	AACTTTACAATCAGAGGTTCAACTCCTCTTCTTA
9358	Yes	AACTTTACAATCAGAGGTTCAACTCCTCTTCTTAA
9359	Yes	AACTTTACAATCAGAGGTTCAACTCCTCTTCTTAAC

9360	Yes	AACTTTACAATCAGAGGTTCAACTCCTCTTCTTAACA
9361	Yes	AACTTTACAATCAGAGGTTCAACTCCTCTTCTTAACAC
9362	Yes	AACTTTACAATCAGAGGTTCAACTCCTCTTCTTAACACC
9363	Yes	AACTTTACAATCAGAGGTTCAACTCCTCTTCTTAACACCA
9364	No	AACTTTACAGTCAGAG
9365	No	AACTTTACAGTCAGAGG
9366	Yes	AACTTTACAGTCAGAGGT
9367	Yes	AACTTTACAGTCAGAGGTT
9368	Yes	AACTTTACAGTCAGAGGTTC
9369	Yes	AACTTTACAGTCAGAGGTTCA
9370	Yes	AACTTTACAGTCAGAGGTTCAA
9371	Yes	AACTTTACAGTCAGAGGTTCAAT
9372	Yes	AACTTTACAGTCAGAGGTTCAATT
9373	Yes	AACTTTACAGTCAGAGGTTCAATTC
9374	Yes	AACTTTACAGTCAGAGGTTCAATTCC
9375	Yes	AACTTTACAGTCAGAGGTTCAATTCCT
9376	Yes	AACTTTACAGTCAGAGGTTCAATTCCTC
9377	Yes	AACTTTACAGTCAGAGGTTCAATTCCTCT
9378	Yes	AACTTTACAGTCAGAGGTTCAATTCCTCTT
9379	Yes	AACTTTACAGTCAGAGGTTCAATTCCTCTTC
9380	Yes	AACTTTACAGTCAGAGGTTCAATTCCTCTTCT
9381	Yes	AACTTTACAGTCAGAGGTTCAATTCCTCTTCTT
9382	Yes	AACTTTACAGTCAGAGGTTCAATTCCTCTTCTTA
9383	Yes	AACTTTACAGTCAGAGGTTCAATTCCTCTTCTTAA
9384	Yes	AACTTTACAGTCAGAGGTTCAATTCCTCTTCTTAAC
9385	Yes	AACTTTACAGTCAGAGGTTCAATTCCTCTTCTTAACA
9386	Yes	AACTTTACAGTCAGAGGTTCAATTCCTCTTCTTAACAC
9387	Yes	AACTTTACAGTCAGAGGTTCAATTCCTCTTCTTAACACC
9388	Yes	AACTTTACAGTCAGAGGTTCAATTCCTCTTCTTAACACCA
9389	No	AACTTTATAATCAGAG
9390	No	AACTTTATAATCAGAGG
9391	No	AACTTTATAATCAGAGGT
9392	No	AACTTTATAATCAGAGGTT
9393	No	AACTTTATAATCAGAGGTTC
9394	No	AACTTTATAATCAGAGGTTCA
9395	No	AACTTTATAATCAGAGGTTCAA
9396	No	AACTTTATAATCAGAGGTTCAAC
9397	No	AACTTTATAATCAGAGGTTCAACT
9398	No	AACTTTATAATCAGAGGTTCAACTC
9399	No	AACTTTATAATCAGAGGTTCAACTCC
9400	No	AACTTTATAATCAGAGGTTCAACTCCT
9401	No	AACTTTATAATCAGAGGTTCAACTCCTC

9402	No	AACTTTATAATCAGAGGTTCAACTCCTCT
9403	Yes	AACTTTATAATCAGAGGTTCAACTCCTCTC
9404	Yes	AACTTTATAATCAGAGGTTCAACTCCTCTCA
9405	Yes	AACTTTATAATCAGAGGTTCAACTCCTCTCAT
9406	Yes	AACTTTATAATCAGAGGTTCAACTCCTCTCATT
9407	Yes	AACTTTATAATCAGAGGTTCAACTCCTCTCATTA
9408	Yes	AACTTTATAATCAGAGGTTCAACTCCTCTCATTAA
9409	Yes	AACTTTATAATCAGAGGTTCAACTCCTCTCATTAAC
9410	Yes	AACTTTATAATCAGAGGTTCAACTCCTCTCATTAACA
9411	Yes	AACTTTATAATCAGAGGTTCAACTCCTCTCATTAACAC
9412	Yes	AACTTTATAATCAGAGGTTCAACTCCTCTCATTAACACC
9413	Yes	AACTTTATAATCAGAGGTTCAACTCCTCTCATTAACACCA
9414	Yes	AACTTTATAATCAGAGGTTCG
9415	Yes	AACTTTATAATCAGAGGTTCGA
9416	Yes	AACTTTATAATCAGAGGTTCGAC
9417	Yes	AACTTTATAATCAGAGGTTCGACT
9418	Yes	AACTTTATAATCAGAGGTTCGACTC
9419	Yes	AACTTTATAATCAGAGGTTCGACTCC
9420	Yes	AACTTTATAATCAGAGGTTCGACTCCT
9421	Yes	AACTTTATAATCAGAGGTTCGACTCCTC
9422	Yes	AACTTTATAATCAGAGGTTCGACTCCTCT
9423	Yes	AACTTTATAATCAGAGGTTCGACTCCTCTT
9424	Yes	AACTTTATAATCAGAGGTTCGACTCCTCTTC
9425	Yes	AACTTTATAATCAGAGGTTCGACTCCTCTTCT
9426	Yes	AACTTTATAATCAGAGGTTCGACTCCTCTTCTT
9427	Yes	AACTTTATAATCAGAGGTTCGACTCCTCTTCTTA
9428	Yes	AACTTTATAATCAGAGGTTCGACTCCTCTTCTTAA
9429	Yes	AACTTTATAATCAGAGGTTCGACTCCTCTTCTTAAC
9430	Yes	AACTTTATAATCAGAGGTTCGACTCCTCTTCTTAACA
9431	Yes	AACTTTATAATCAGAGGTTCGACTCCTCTTCTTAACAC
9432	Yes	AACTTTATAATCAGAGGTTCGACTCCTCTTCTTAACACC
9433	Yes	AACTTTATAATCAGAGGTTCGACTCCTCTTCTTAACACCA
9434	No	AACTTTATGGGCAGAG
9435	No	AACTTTATGGGCAGAGG
9436	Yes	AACTTTATGGGCAGAGGT
9437	Yes	AACTTTATGGGCAGAGGTT
9438	Yes	AACTTTATGGGCAGAGGTTC
9439	Yes	AACTTTATGGGCAGAGGTTCA
9440	Yes	AACTTTATGGGCAGAGGTTCAA
9441	Yes	AACTTTATGGGCAGAGGTTCAAT
9442	Yes	AACTTTATGGGCAGAGGTTCAATT
9443	Yes	AACTTTATGGGCAGAGGTTCAATTC

9444	Yes	AACTTTATGGGCAGAGGTTCAATTCC
9445	Yes	AACTTTATGGGCAGAGGTTCAATTCCT
9446	Yes	AACTTTATGGGCAGAGGTTCAATTCCTC
9447	Yes	AACTTTATGGGCAGAGGTTCAATTCCTCT
9448	Yes	AACTTTATGGGCAGAGGTTCAATTCCTCTT
9449	Yes	AACTTTATGGGCAGAGGTTCAATTCCTCTTC
9450	Yes	AACTTTATGGGCAGAGGTTCAATTCCTCTTCT
9451	Yes	AACTTTATGGGCAGAGGTTCAATTCCTCTTCTT
9452	Yes	AACTTTATGGGCAGAGGTTCAATTCCTCTTCTTA
9453	Yes	AACTTTATGGGCAGAGGTTCAATTCCTCTTCTTAA
9454	Yes	AACTTTATGGGCAGAGGTTCAATTCCTCTTCTTAAC
9455	Yes	AACTTTATGGGCAGAGGTTCAATTCCTCTTCTTAACA
9456	Yes	AACTTTATGGGCAGAGGTTCAATTCCTCTTCTTAACAC
9457	Yes	AACTTTATGGGCAGAGGTTCAATTCCTCTTCTTAACACC
9458	Yes	AACTTTATGGGCAGAGGTTCAATTCCTCTTCTTAACACCA
9459	No	AACTTTGTCAAAGTTA
9460	No	AACTTTGTCAAAGTTAA
9461	No	AACTTTGTCAAAGTTAAA
9462	No	AACTTTGTCAAAGTTAAAT
9463	No	AACTTTGTCAAAGTTAAATT
9464	No	AACTTTGTCAAAGTTAAATTA
9465	No	AACTTTGTCAAAGTTAAATTAT
9466	No	AACTTTGTCAAAGTTAAATTATA
9467	No	AACTTTGTCAAAGTTAAATTATAG
9468	No	AACTTTGTCAAAGTTAAATTATAGG
9469	No	AACTTTGTCAAAGTTAAATTATAGGC
9470	No	AACTTTGTCAAAGTTAAATTATAGGCT
9471	No	AACTTTGTCAAAGTTAAATTATAGGCTA
9472	No	AACTTTGTCAAAGTTAAATTATAGGCTAA
9473	No	AACTTTGTCAAAGTTAAATTATAGGCTAAA
9474	No	AACTTTGTCAAAGTTAAATTATAGGCTAAAT
9475	No	AACTTTGTCAAAGTTAAATTATAGGCTAAATC
9476	No	AACTTTGTCAAAGTTAAATTATAGGCTAAATCC
9477	No	AACTTTGTCAAAGTTAAATTATAGGCTAAATCCT
9478	No	AACTTTGTCAAAGTTAAATTATAGGCTAAATCCTA
9479	No	AACTTTGTCAAAGTTAAATTATAGGCTAAATCCTAT
9480	No	AACTTTGTCAAAGTTAAATTATAGGCTAAATCCTATA
9481	No	AACTTTGTCAAAGTTAAATTATAGGCTAAATCCTATAT
9482	No	AACTTTGTCAAAGTTAAATTATAGGCTAAATCCTATATA
9483	No	AACTTTGTCAAAGTTAAATTATAGGCTAAATCCTATATAT
9484	No	AACTTTGTCAAAGTTAAATTATAGGCTAAATCCTATATATC
9485	No	AACTTTGTCAAAGTTAAATTATAGGCTAAATCCTATATATCT

9486	No	AACTTTGTCAAAGTTAAATTATAGGCTAAATCCTATATATCTT
9487	No	AACTTTGTCAAAGTTAAATTATAGGCTAAATCCTATATATCTTA
9488	Yes	AACTTTGTCAAAGTTAAATTATAGGCTAAATCCTATATATCTTAC
9489	Yes	AACTTTGTCAAAGTTAAATTATAGGCTAAATCCTATATATCTTACC
9490	Yes	AACTTTGTCAAAGTTAAATTATAGGCTAAATCCTATATATCTTACCA
9491	No	AACTTTTAAAGGATAA
9492	No	AACTTTTAAAGGATAAC
9493	No	AACTTTTAAAGGATAACA
9494	No	AACTTTTAAAGGATAACAG
9495	No	AACTTTTAAAGGATAACAGC
9496	Yes	AACTTTTAAAGGATAACAGCT
9497	Yes	AACTTTTAAAGGATAACAGCTA
9498	Yes	AACTTTTAAAGGATAACAGCTAT
9499	Yes	AACTTTTAAAGGATAACAGCTATC
9500	Yes	AACTTTTAAAGGATAACAGCTATCC
9501	Yes	AACTTTTAAAGGATAACAGCTATCCA
9502	Yes	AACTTTTAAAGGATAACAGCTATCCAT
9503	Yes	AACTTTTAAAGGATAACAGCTATCCATT
9504	Yes	AACTTTTAAAGGATAACAGCTATCCATTG
9505	Yes	AACTTTTAAAGGATAACAGCTATCCATTGG
9506	Yes	AACTTTTAAAGGATAACAGCTATCCATTGGT
9507	Yes	AACTTTTAAAGGATAACAGCTATCCATTGGTC
9508	Yes	AACTTTTAAAGGATAACAGCTATCCATTGGTCT
9509	Yes	AACTTTTAAAGGATAACAGCTATCCATTGGTCTT
9510	Yes	AACTTTTAAAGGATAACAGCTATCCATTGGTCTTA
9511	Yes	AACTTTTAAAGGATAACAGCTATCCATTGGTCTTAG
9512	Yes	AACTTTTAAAGGATAACAGCTATCCATTGGTCTTAGG
9513	Yes	AACTTTTAAAGGATAACAGCTATCCATTGGTCTTAGGC
9514	Yes	AACTTTTAAAGGATAACAGCTATCCATTGGTCTTAGGCC
9515	Yes	AACTTTTAAAGGATAACAGCTATCCATTGGTCTTAGGCCC
9516	Yes	AACTTTTAAAGGATAACAGCTATCCATTGGTCTTAGGCCCC
9517	Yes	AACTTTTAAAGGATAACAGCTATCCATTGGTCTTAGGCCCCA
9518	Yes	AACTTTTAAAGGATAACAGCTATCCATTGGTCTTAGGCCCCAA
9519	Yes	AACTTTTAAAGGATAACAGCTATCCATTGGTCTTAGGCCCCAAA
9520	Yes	AACTTTTAAAGGATAACAGCTATCCATTGGTCTTAGGCCCCAAAA
9521	Yes	AACTTTTAAAGGATAACAGCTATCCATTGGTCTTAGGCCCCAAAAA
9522	Yes	AACTTTTAAAGGATAACAGCTATCCATTGGTCTTAGGCCCCAAAAAT
9523	Yes	AACTTTTAAAGGATAACAGCTATCCATTGGTCTTAGGCCCCAAAAATT
9524	Yes	AACTTTTAAAGGATAACAGCTATCCATTGGTCTTAGGCCCCAAAAATTT
9525	Yes	AACTTTTAAAGGATAACAGCTATCCATTGGTCTTAGGCCCCAAAAATTTT
9526	No	AAGAAATATGTCTGAT
9527	No	AAGAAATATGTCTGATA

9528	No	AAGAAATATGTCTGATAA
9529	No	AAGAAATATGTCTGATAAA
9530	No	AAGAAATATGTCTGATAAAA
9531	No	AAGAAATATGTCTGATAAAAG
9532	No	AAGAAATATGTCTGATAAAAGA
9533	No	AAGAAATATGTCTGATAAAAGAG
9534	No	AAGAAATATGTCTGATAAAAGAGT
9535	No	AAGAAATATGTCTGATAAAAGAGTT
9536	No	AAGAAATATGTCTGATAAAAGAGTTA
9537	No	AAGAAATATGTCTGATAAAAGAGTTAC
9538	No	AAGAAATATGTCTGATAAAAGAGTTACT
9539	No	AAGAAATATGTCTGATAAAAGAGTTACTT
9540	No	AAGAAATATGTCTGATAAAAGAGTTACTTT
9541	No	AAGAAATATGTCTGATAAAAGAGTTACTTTG
9542	No	AAGAAATATGTCTGATAAAAGAGTTACTTTGA
9543	No	AAGAAATATGTCTGATAAAAGAGTTACTTTGAT
9544	No	AAGAAATATGTCTGATAAAAGAGTTACTTTGATA
9545	No	AAGAAATATGTCTGATAAAAGAGTTACTTTGATAG
9546	No	AAGAAATATGTCTGATAAAAGAGTTACTTTGATAGA
9547	No	AAGAAATATGTCTGATAAAAGAGTTACTTTGATAGAG
9548	No	AAGAAATATGTCTGATAAAAGAGTTACTTTGATAGAGT
9549	No	AAGAAATATGTCTGATAAAAGAGTTACTTTGATAGAGTA
9550	No	AAGAAATATGTCTGATAAAAGAGTTACTTTGATAGAGTAA
9551	No	AAGAAATATGTCTGATAAAAGAGTTACTTTGATAGAGTAAA
9552	No	AAGAAATATGTCTGATAAAAGAGTTACTTTGATAGAGTAAAT
9553	No	AAGAAATATGTCTGATAAAAGAGTTACTTTGATAGAGTAAATA
9554	No	AAGAAATATGTCTGATAAAAGAGTTACTTTGATAGAGTAAATAA
9555	No	AAGAAATATGTCTGATAAAAGAGTTACTTTGATAGAGTAAATAAT
9556	No	AAGAAATATGTCTGATAAAAGAGTTACTTTGATAGAGTAAATAATA
9557	No	AAGAAATATGTCTGATAAAAGAGTTACTTTGATAGAGTAAATAATAG
9558	No	AAGAAATATGTCTGATAAAAGAGTTACTTTGATAGAGTAAATAATAGG
9559	No	AAGAAATATGTCTGATAAAAGAGTTACTTTGATAGAGTAAATAATAGGA
9560	No	AAGAAATATGTCTGATAAAAGAGTTACTTTGATAGAGTAAATAATAGGAG
9561	No	AAGAAATTTAGGTTAA
9562	No	AAGAAATTTAGGTTAAA
9563	No	AAGAAATTTAGGTTAAAT
9564	Yes	AAGAAATTTAGGTTAAATA
9565	Yes	AAGAAATTTAGGTTAAATAC
9566	Yes	AAGAAATTTAGGTTAAATACA
9567	Yes	AAGAAATTTAGGTTAAATACAG
9568	Yes	AAGAAATTTAGGTTAAATACAGA
9569	Yes	AAGAAATTTAGGTTAAATACAGAC

9570	Yes	AAGAAATTTAGGTTAAATACAGACC
9571	Yes	AAGAAATTTAGGTTAAATACAGACCA
9572	Yes	AAGAAATTTAGGTTAAATACAGACCAA
9573	Yes	AAGAAATTTAGGTTAAATACAGACCAAG
9574	Yes	AAGAAATTTAGGTTAAATACAGACCAAGA
9575	Yes	AAGAAATTTAGGTTAAATACAGACCAAGAG
9576	Yes	AAGAAATTTAGGTTAAATACAGACCAAGAGC
9577	Yes	AAGAAATTTAGGTTAAATACAGACCAAGAGCC
9578	Yes	AAGAAATTTAGGTTAAATACAGACCAAGAGCCT
9579	Yes	AAGAAATTTAGGTTAAATACAGACCAAGAGCCTT
9580	Yes	AAGAAATTTAGGTTAAATACAGACCAAGAGCCTTC
9581	Yes	AAGAAATTTAGGTTAAATACAGACCAAGAGCCTTCA
9582	Yes	AAGAAATTTAGGTTAAATACAGACCAAGAGCCTTCAA
9583	Yes	AAGAAATTTAGGTTAAATACAGACCAAGAGCCTTCAAA
9584	Yes	AAGAAATTTAGGTTAAATACAGACCAAGAGCCTTCAAAG
9585	Yes	AAGAAATTTAGGTTAAATACAGACCAAGAGCCTTCAAAGC
9586	Yes	AAGAAATTTAGGTTAAATACAGACCAAGAGCCTTCAAAGCC
9587	Yes	AAGAAATTTAGGTTAAATACAGACCAAGAGCCTTCAAAGCCC
9588	Yes	AAGAAATTTAGGTTAAATACAGACCAAGAGCCTTCAAAGCCCT
9589	Yes	AAGAAATTTAGGTTAAATACAGACCAAGAGCCTTCAAAGCCCTC
9590	Yes	AAGAAATTTAGGTTAAATACAGACCAAGAGCCTTCAAAGCCCTCA
9591	Yes	AAGAAATTTAGGTTAAATACAGACCAAGAGCCTTCAAAGCCCTCAG
9592	Yes	AAGAAATTTAGGTTAAATACAGACCAAGAGCCTTCAAAGCCCTCAGT
9593	Yes	AAGAAATTTAGGTTAAATACAGACCAAGAGCCTTCAAAGCCCTCAGTA
9594	Yes	AAGAAATTTAGGTTAAATACAGACCAAGAGCCTTCAAAGCCCTCAGTAA
9595	Yes	AAGAAATTTAGGTTAAATACAGACCAAGAGCCTTCAAAGCCCTCAGTAAG
9596	No	AAGAACCTAAACTCTA
9597	Yes	AAGAACCTAAACTCTAA
9598	Yes	AAGAACCTAAACTCTAAA
9599	Yes	AAGAACCTAAACTCTAAAT
9600	Yes	AAGAACCTAAACTCTAAATT
9601	Yes	AAGAACCTAAACTCTAAATTT
9602	Yes	AAGAACCTAAACTCTAAATTTA
9603	Yes	AAGAACCTAAACTCTAAATTTAG
9604	Yes	AAGAACCTAAACTCTAAATTTAGA
9605	Yes	AAGAACCTAAACTCTAAATTTAGAT
9606	Yes	AAGAACCTAAACTCTAAATTTAGATG
9607	Yes	AAGAACCTAAACTCTAAATTTAGATGT
9608	Yes	AAGAACCTAAACTCTAAATTTAGATGTC
9609	Yes	AAGAACCTAAACTCTAAATTTAGATGTCC
9610	Yes	AAGAACCTAAACTCTAAATTTAGATGTCCT
9611	Yes	AAGAACCTAAACTCTAAATTTAGATGTCCTG

9612	Yes	AAGAACCTAAACTCTAAATTTAGATGTCCTGA
9613	Yes	AAGAACCTAAACTCTAAATTTAGATGTCCTGAG
9614	Yes	AAGAACCTAAACTCTAAATTTAGATGTCCTGAGT
9615	Yes	AAGAACCTAAACTCTAAATTTAGATGTCCTGAGTT
9616	Yes	AAGAACCTAAACTCTAAATTTAGATGTCCTGAGTTC
9617	Yes	AAGAACCTAAACTCTAAATTTAGATGTCCTGAGTTCA
9618	Yes	AAGAACCTAAACTCTAAATTTAGATGTCCTGAGTTCAA
9619	Yes	AAGAACCTAAACTCTAAATTTAGATGTCCTGAGTTCAAA
9620	Yes	AAGAACCTAAACTCTAAATTTAGATGTCCTGAGTTCAAAT
9621	Yes	AAGAACCTAAACTCTAAATTTAGATGTCCTGAGTTCAAATC
9622	Yes	AAGAACCTAAACTCTAAATTTAGATGTCCTGAGTTCAAATCC
9623	Yes	AAGAACCTAAACTCTAAATTTAGATGTCCTGAGTTCAAATCCC
9624	Yes	AAGAACCTAAACTCTAAATTTAGATGTCCTGAGTTCAAATCCCA
9625	Yes	AAGAACCTAAACTCTAAATTTAGATGTCCTGAGTTCAAATCCCAG
9626	Yes	AAGAACCTAAACTCTAAATTTAGATGTCCTGAGTTCAAATCCCAGC
9627	Yes	AAGAACCTAAACTCTAAATTTAGATGTCCTGAGTTCAAATCCCAGCT
9628	Yes	AAGAACCTAAACTCTAAATTTAGATGTCCTGAGTTCAAATCCCAGCTG
9629	Yes	AAGAACCTAAACTCTAAATTTAGATGTCCTGAGTTCAAATCCCAGCTGT
9630	Yes	AAGAACCTAAACTCTAAATTTAGATGTCCTGAGTTCAAATCCCAGCTGTA
9631	No	AAGAACTGCTAACTCA
9632	No	AAGAACTGCTAACTCAT
9633	No	AAGAACTGCTAACTCATG
9634	No	AAGAACTGCTAACTCATGC
9635	No	AAGAACTGCTAACTCATGCC
9636	No	AAGAACTGCTAACTCATGCCC
9637	No	AAGAACTGCTAACTCATGCCCC
9638	No	AAGAACTGCTAACTCATGCCCCC
9639	No	AAGAACTGCTAACTCATGCCCCCA
9640	No	AAGAACTGCTAACTCATGCCCCCAT
9641	No	AAGAACTGCTAACTCATGCCCCCATG
9642	No	AAGAACTGCTAACTCATGCCCCCATGT
9643	Yes	AAGAACTGCTAACTCATGCCCCCATGTC
9644	Yes	AAGAACTGCTAACTCATGCCCCCATGTCT
9645	Yes	AAGAACTGCTAACTCATGCCCCCATGTCTA
9646	Yes	AAGAACTGCTAACTCATGCCCCCATGTCTAA
9647	Yes	AAGAACTGCTAACTCATGCCCCCATGTCTAAC
9648	Yes	AAGAACTGCTAACTCATGCCCCCATGTCTAACA
9649	Yes	AAGAACTGCTAACTCATGCCCCCATGTCTAACAA
9650	Yes	AAGAACTGCTAACTCATGCCCCCATGTCTAACAAC
9651	Yes	AAGAACTGCTAACTCATGCCCCCATGTCTAACAACA
9652	Yes	AAGAACTGCTAACTCATGCCCCCATGTCTAACAACAT
9653	Yes	AAGAACTGCTAACTCATGCCCCCATGTCTAACAACATG

9654	Yes	AAGAACTGCTAACTCATGCCCCCATGTCTAACAACATGG
9655	Yes	AAGAACTGCTAACTCATGCCCCCATGTCTAACAACATGGC
9656	Yes	AAGAACTGCTAACTCATGCCCCCATGTCTAACAACATGGCT
9657	Yes	AAGAACTGCTAACTCATGCCCCCATGTCTAACAACATGGCTT
9658	Yes	AAGAACTGCTAACTCATGCCCCCATGTCTAACAACATGGCTTT
9659	Yes	AAGAACTGCTAACTCATGCCCCCATGTCTAACAACATGGCTTTC
9660	Yes	AAGAACTGCTAACTCATGCCCCCATGTCTAACAACATGGCTTTCT
9661	Yes	AAGAACTGCTAACTCATGCCCCCATGTCTAACAACATGGCTTTCTC
9662	Yes	AAGAACTGCTAACTCATGCCCCCATGTCTAACAACATGGCTTTCTCA
9663	Yes	AAGAACTGCTAACTCATGCCCCCATGTCTAACAACATGGCTTTCTCAC
9664	Yes	AAGAACTGCTAACTCATGCCCCCATGTCTAACAACATGGCTTTCTCACC
9665	Yes	AAGAACTGCTAACTCATGCCCCCATGTCTAACAACATGGCTTTCTCACCA
9666	No	AAGAAGCAGCTTCAAA
9667	No	AAGAAGCAGCTTCAAAC
9668	No	AAGAAGCAGCTTCAAACC
9669	No	AAGAAGCAGCTTCAAACCT
9670	No	AAGAAGCAGCTTCAAACCTG
9671	No	AAGAAGCAGCTTCAAACCTGC
9672	No	AAGAAGCAGCTTCAAACCTGCC
9673	No	AAGAAGCAGCTTCAAACCTGCCG
9674	No	AAGAAGCAGCTTCAAACCTGCCGG
9675	No	AAGAAGCAGCTTCAAACCTGCCGGG
9676	No	AAGAAGCAGCTTCAAACCTGCCGGGG
9677	No	AAGAAGCAGCTTCAAACCTGCCGGGGC
9678	No	AAGAAGCAGCTTCAAACCTGCCGGGGCT
9679	No	AAGAAGCAGCTTCAAACCTGCCGGGGCTT
9680	No	AAGAAGCAGCTTCAAACCTGCCGGGGCTTC
9681	Yes	AAGAAGCAGCTTCAAACCTGCCGGGGCTTCC
9682	Yes	AAGAAGCAGCTTCAAACCTGCCGGGGCTTCCA
9683	Yes	AAGAAGTCCCCGGTTC
9684	Yes	AAGAAGTCCCCGGTTCA
9685	Yes	AAGAAGTCCCCGGTTCAA
9686	Yes	AAGAAGTCCCCGGTTCAAA
9687	Yes	AAGAAGTCCCCGGTTCAAAT
9688	Yes	AAGAAGTCCCCGGTTCAAATC
9689	Yes	AAGAAGTCCCCGGTTCAAATCC
9690	Yes	AAGAAGTCCCCGGTTCAAATCCG
9691	Yes	AAGAAGTCCCCGGTTCAAATCCGG
9692	Yes	AAGAAGTCCCCGGTTCAAATCCGGG
9693	Yes	AAGAAGTCCCCGGTTCAAATCCGGGT
9694	Yes	AAGAAGTCCCCGGTTCAAATCCGGGTG
9695	Yes	AAGAAGTCCCCGGTTCAAATCCGGGTGC

9696	Yes	AAGAAGTCCCCGGTTCAAATCCGGGTGCC
9697	Yes	AAGAAGTCCCCGGTTCAAATCCGGGTGCCC
9698	Yes	AAGAAGTCCCCGGTTCAAATCCGGGTGCCCC
9699	Yes	AAGAAGTCCCCGGTTCAAATCCGGGTGCCCCC
9700	Yes	AAGAAGTCCCCGGTTCAAATCCGGGTGCCCCCT
9701	Yes	AAGAAGTCCCCGGTTCAAATCCGGGTGCCCCCTC
9702	Yes	AAGAAGTCCCCGGTTCAAATCCGGGTGCCCCCTCC
9703	Yes	AAGAAGTCCCCGGTTCAAATCCGGGTGCCCCCTCCA
9704	No	AAGAAGTCCTTGGTTC
9705	Yes	AAGAAGTCCTTGGTTCA
9706	Yes	AAGAAGTCCTTGGTTCAA
9707	Yes	AAGAAGTCCTTGGTTCAAA
9708	Yes	AAGAAGTCCTTGGTTCAAAT
9709	Yes	AAGAAGTCCTTGGTTCAAATC
9710	Yes	AAGAAGTCCTTGGTTCAAATCC
9711	Yes	AAGAAGTCCTTGGTTCAAATCCA
9712	Yes	AAGAAGTCCTTGGTTCAAATCCAG
9713	Yes	AAGAAGTCCTTGGTTCAAATCCAGG
9714	Yes	AAGAAGTCCTTGGTTCAAATCCAGGT
9715	Yes	AAGAAGTCCTTGGTTCAAATCCAGGTG
9716	Yes	AAGAAGTCCTTGGTTCAAATCCAGGTGC
9717	Yes	AAGAAGTCCTTGGTTCAAATCCAGGTGCC
9718	Yes	AAGAAGTCCTTGGTTCAAATCCAGGTGCCC
9719	Yes	AAGAAGTCCTTGGTTCAAATCCAGGTGCCCC
9720	Yes	AAGAAGTCCTTGGTTCAAATCCAGGTGCCCCC
9721	Yes	AAGAAGTCCTTGGTTCAAATCCAGGTGCCCCCT
9722	Yes	AAGAAGTCCTTGGTTCAAATCCAGGTGCCCCCTC
9723	Yes	AAGAAGTCCTTGGTTCAAATCCAGGTGCCCCCTCC
9724	Yes	AAGAAGTCCTTGGTTCAAATCCAGGTGCCCCCTCCA
9725	No	AAGAATCAGAAGATTC
9726	No	AAGAATCAGAAGATTCC
9727	No	AAGAATCAGAAGATTCCA
9728	No	AAGAATCAGAAGATTCCAG
9729	Yes	AAGAATCAGAAGATTCCAGC
9730	Yes	AAGAATCAGAAGATTCCAGCC
9731	Yes	AAGAATCAGAAGATTCCAGCCT
9732	Yes	AAGAATCAGAAGATTCCAGCCTT
9733	Yes	AAGAATCAGAAGATTCCAGCCTTG
9734	Yes	AAGAATCAGAAGATTCCAGCCTTGA
9735	Yes	AAGAATCAGAAGATTCCAGCCTTGAC
9736	Yes	AAGAATCAGAAGATTCCAGCCTTGACT
9737	Yes	AAGAATCAGAAGATTCCAGCCTTGACTC

9738	Yes	AAGAATCAGAAGATTCCAGCCTTGACTCC
9739	Yes	AAGAATCAGAAGATTCCAGCCTTGACTCCT
9740	Yes	AAGAATCAGAAGATTCCAGCCTTGACTCCTG
9741	Yes	AAGAATCAGAAGATTCCAGCCTTGACTCCTGG
9742	Yes	AAGAATCAGAAGATTCCAGCCTTGACTCCTGGC
9743	Yes	AAGAATCAGAAGATTCCAGCCTTGACTCCTGGCT
9744	Yes	AAGAATCAGAAGATTCCAGCCTTGACTCCTGGCTG
9745	Yes	AAGAATCAGAAGATTCCAGCCTTGACTCCTGGCTGG
9746	Yes	AAGAATCAGAAGATTCCAGCCTTGACTCCTGGCTGGC
9747	Yes	AAGAATCAGAAGATTCCAGCCTTGACTCCTGGCTGGCT
9748	Yes	AAGAATCAGAAGATTCCAGCCTTGACTCCTGGCTGGCTC
9749	Yes	AAGAATCAGAAGATTCCAGCCTTGACTCCTGGCTGGCTCA
9750	Yes	AAGAATCAGAAGATTCCAGCCTTGACTCCTGGCTGGCTCAC
9751	Yes	AAGAATCAGAAGATTCCAGCCTTGACTCCTGGCTGGCTCACC
9752	Yes	AAGAATCAGAAGATTCCAGCCTTGACTCCTGGCTGGCTCACCA
9753	No	AAGACAGGGGTTAGGC
9754	No	AAGACAGGGGTTAGGCC
9755	Yes	AAGACAGGGGTTAGGCCT
9756	Yes	AAGACAGGGGTTAGGCCTC
9757	Yes	AAGACAGGGGTTAGGCCTCT
9758	Yes	AAGACAGGGGTTAGGCCTCTT
9759	Yes	AAGACAGGGGTTAGGCCTCTTT
9760	Yes	AAGACAGGGGTTAGGCCTCTTTT
9761	Yes	AAGACAGGGGTTAGGCCTCTTTTT
9762	Yes	AAGACAGGGGTTAGGCCTCTTTTTA
9763	Yes	AAGACAGGGGTTAGGCCTCTTTTTAC
9764	Yes	AAGACAGGGGTTAGGCCTCTTTTTACC
9765	Yes	AAGACAGGGGTTAGGCCTCTTTTTACCA
9766	Yes	AAGACAGGGGTTAGGCCTCTTTTTACCAC
9767	Yes	AAGACAGGGGTTAGGCCTCTTTTTACCACC
9768	Yes	AAGACAGGGGTTAGGCCTCTTTTTACCACCA
9769	No	AAGACATCTCACTTTG
9770	Yes	AAGACATCTCACTTTGG
9771	Yes	AAGACATCTCACTTTGGG
9772	Yes	AAGACATCTCACTTTGGGT
9773	Yes	AAGACATCTCACTTTGGGTC
9774	Yes	AAGACATCTCACTTTGGGTCA
9775	Yes	AAGACATCTCACTTTGGGTCAG
9776	Yes	AAGACATCTCACTTTGGGTCAGA
9777	Yes	AAGACATCTCACTTTGGGTCAGAA
9778	Yes	AAGACATCTCACTTTGGGTCAGAAG
9779	Yes	AAGACATCTCACTTTGGGTCAGAAGA

9780	Yes	AAGACATCTCACTTTGGGTCAGAAGAT
9781	Yes	AAGACATCTCACTTTGGGTCAGAAGATT
9782	Yes	AAGACATCTCACTTTGGGTCAGAAGATTG
9783	Yes	AAGACATCTCACTTTGGGTCAGAAGATTGA
9784	Yes	AAGACATCTCACTTTGGGTCAGAAGATTGAG
9785	Yes	AAGACATCTCACTTTGGGTCAGAAGATTGAGG
9786	Yes	AAGACATCTCACTTTGGGTCAGAAGATTGAGGG
9787	Yes	AAGACATCTCACTTTGGGTCAGAAGATTGAGGGT
9788	Yes	AAGACATCTCACTTTGGGTCAGAAGATTGAGGGTT
9789	Yes	AAGACATCTCACTTTGGGTCAGAAGATTGAGGGTTC
9790	Yes	AAGACATCTCACTTTGGGTCAGAAGATTGAGGGTTCA
9791	Yes	AAGACATCTCACTTTGGGTCAGAAGATTGAGGGTTCAA
9792	Yes	AAGACATCTCACTTTGGGTCAGAAGATTGAGGGTTCAAG
9793	Yes	AAGACATCTCACTTTGGGTCAGAAGATTGAGGGTTCAAGT
9794	Yes	AAGACATCTCACTTTGGGTCAGAAGATTGAGGGTTCAAGTC
9795	Yes	AAGACATCTCACTTTGGGTCAGAAGATTGAGGGTTCAAGTCC
9796	Yes	AAGACATCTCACTTTGGGTCAGAAGATTGAGGGTTCAAGTCCT
9797	Yes	AAGACATCTCACTTTGGGTCAGAAGATTGAGGGTTCAAGTCCTT
9798	Yes	AAGACATCTCACTTTGGGTCAGAAGATTGAGGGTTCAAGTCCTTT
9799	Yes	AAGACATCTCACTTTGGGTCAGAAGATTGAGGGTTCAAGTCCTTTC
9800	Yes	AAGACATCTCACTTTGGGTCAGAAGATTGAGGGTTCAAGTCCTTTCA
9801	Yes	AAGACATCTCACTTTGGGTCAGAAGATTGAGGGTTCAAGTCCTTTCAT
9802	Yes	AAGACATCTCACTTTGGGTCAGAAGATTGAGGGTTCAAGTCCTTTCATG
9803	Yes	AAGACATCTCACTTTGGGTCAGAAGATTGAGGGTTCAAGTCCTTTCATGG
9804	No	AAGACCACCCAGGGAC
9805	Yes	AAGACCACCCAGGGACG
9806	Yes	AAGACCACCCAGGGACGC
9807	Yes	AAGACCACCCAGGGACGCC
9808	Yes	AAGACCACCCAGGGACGCCA
9809	Yes	AAGACCCTTGGCTCGA
9810	Yes	AAGACCCTTGGCTCGAG
9811	Yes	AAGACCCTTGGCTCGAGA
9812	Yes	AAGACCCTTGGCTCGAGAC
9813	Yes	AAGACCCTTGGCTCGAGACT
9814	Yes	AAGACCCTTGGCTCGAGACTG
9815	Yes	AAGACCCTTGGCTCGAGACTGG
9816	Yes	AAGACCCTTGGCTCGAGACTGGA
9817	Yes	AAGACCCTTGGCTCGAGACTGGAG
9818	Yes	AAGACCCTTGGCTCGAGACTGGAGG
9819	Yes	AAGACCCTTGGCTCGAGACTGGAGGG
9820	Yes	AAGACCCTTGGCTCGAGACTGGAGGGA
9821	Yes	AAGACCCTTGGCTCGAGACTGGAGGGAA

9822	Yes	AAGACCCTTGGCTCGAGACTGGAGGGAAA
9823	Yes	AAGACCCTTGGCTCGAGACTGGAGGGAAAC
9824	Yes	AAGACCCTTGGCTCGAGACTGGAGGGAAACA
9825	Yes	AAGACCCTTGGCTCGAGACTGGAGGGAAACAC
9826	Yes	AAGACCCTTGGCTCGAGACTGGAGGGAAACACC
9827	Yes	AAGACCCTTGGCTCGAGACTGGAGGGAAACACCA
9828	Yes	AAGACGCTGGATTAAG
9829	Yes	AAGACGCTGGATTAAGG
9830	Yes	AAGACGCTGGATTAAGGC
9831	Yes	AAGACGCTGGATTAAGGCT
9832	Yes	AAGACGCTGGATTAAGGCTC
9833	Yes	AAGACGCTGGATTAAGGCTCC
9834	Yes	AAGACGCTGGATTAAGGCTCCA
9835	Yes	AAGACGCTGGATTAAGGCTCCAG
9836	Yes	AAGACGCTGGATTAAGGCTCCAGT
9837	Yes	AAGACGCTGGATTAAGGCTCCAGTC
9838	Yes	AAGACGCTGGATTAAGGCTCCAGTCT
9839	Yes	AAGACGCTGGATTAAGGCTCCAGTCTC
9840	Yes	AAGACGCTGGATTAAGGCTCCAGTCTCT
9841	Yes	AAGACGCTGGATTAAGGCTCCAGTCTCTT
9842	Yes	AAGACGCTGGATTAAGGCTCCAGTCTCTTC
9843	Yes	AAGACGCTGGATTAAGGCTCCAGTCTCTTCG
9844	Yes	AAGACGCTGGATTAAGGCTCCAGTCTCTTCGG
9845	Yes	AAGACGCTGGATTAAGGCTCCAGTCTCTTCGGG
9846	Yes	AAGACGCTGGATTAAGGCTCCAGTCTCTTCGGGG
9847	Yes	AAGACGCTGGATTAAGGCTCCAGTCTCTTCGGGGG
9848	Yes	AAGACGCTGGATTAAGGCTCCAGTCTCTTCGGGGGC
9849	Yes	AAGACGCTGGATTAAGGCTCCAGTCTCTTCGGGGGCG
9850	Yes	AAGACGCTGGATTAAGGCTCCAGTCTCTTCGGGGGCGT
9851	Yes	AAGACGCTGGATTAAGGCTCCAGTCTCTTCGGGGGCGTG
9852	Yes	AAGACGCTGGATTAAGGCTCCAGTCTCTTCGGGGGCGTGG
9853	Yes	AAGACGCTGGATTAAGGCTCCAGTCTCTTCGGGGGCGTGGG
9854	Yes	AAGACGCTGGATTAAGGCTCCAGTCTCTTCGGGGGCGTGGGT
9855	Yes	AAGACGCTGGATTAAGGCTCCAGTCTCTTCGGGGGCGTGGGTT
9856	Yes	AAGACGCTGGATTAAGGCTCCAGTCTCTTCGGGGGCGTGGGTTT
9857	Yes	AAGACGCTGGATTAAGGCTCCAGTCTCTTCGGGGGCGTGGGTTTG
9858	Yes	AAGACGCTGGATTAAGGCTCCAGTCTCTTCGGGGGCGTGGGTTTGA
9859	Yes	AAGACGCTGGATTAAGGCTCCAGTCTCTTCGGGGGCGTGGGTTTGAA
9860	Yes	AAGACGCTGGATTAAGGCTCCAGTCTCTTCGGGGGCGTGGGTTTGAAT
9861	Yes	AAGACGCTGGATTAAGGCTCCAGTCTCTTCGGGGGCGTGGGTTTGAATC
9862	Yes	AAGACGCTGGATTAAGGCTCCAGTCTCTTCGGGGGCGTGGGTTTGAATCC
9863	Yes	AAGACGGCAGAGCCCG

9864	Yes	AAGACGGCAGAGCCCGG
9865	Yes	AAGACGGCAGAGCCCGGC
9866	Yes	AAGACGGCAGAGCCCGGCA
9867	Yes	AAGACGGCAGAGCCCGGCAA
9868	Yes	AAGACGGCAGAGCCCGGCAAT
9869	Yes	AAGACGGCAGAGCCCGGCAATT
9870	Yes	AAGACGGCAGAGCCCGGCAATTG
9871	Yes	AAGACGGCAGAGCCCGGCAATTGC
9872	Yes	AAGACGGCAGAGCCCGGCAATTGCG
9873	Yes	AAGACGGCAGAGCCCGGCAATTGCGT
9874	Yes	AAGACGGCAGAGCCCGGCAATTGCGTA
9875	Yes	AAGACGGCAGAGCCCGGCAATTGCGTAA
9876	Yes	AAGACGGCAGAGCCCGGCAATTGCGTAAA
9877	Yes	AAGACGGCAGAGCCCGGCAATTGCGTAAAA
9878	Yes	AAGACGGCAGAGCCCGGCAATTGCGTAAAAT
9879	Yes	AAGACGGCAGAGCCCGGCAATTGCGTAAAATT
9880	Yes	AAGACGGCAGAGCCCGGCAATTGCGTAAAATTT
9881	Yes	AAGACGGCAGAGCCCGGCAATTGCGTAAAATTTA
9882	Yes	AAGACGGCAGAGCCCGGCAATTGCGTAAAATTTAC
9883	Yes	AAGACGGCAGAGCCCGGCAATTGCGTAAAATTTACA
9884	Yes	AAGACGGCAGAGCCCGGCAATTGCGTAAAATTTACAA
9885	Yes	AAGACGGCAGAGCCCGGCAATTGCGTAAAATTTACAAC
9886	Yes	AAGACGGCAGAGCCCGGCAATTGCGTAAAATTTACAACT
9887	Yes	AAGACGGCAGAGCCCGGCAATTGCGTAAAATTTACAACTT
9888	Yes	AAGACGGCAGAGCCCGGCAATTGCGTAAAATTTACAACTTT
9889	Yes	AAGACGGCAGAGCCCGGCAATTGCGTAAAATTTACAACTTTA
9890	Yes	AAGACGGCAGAGCCCGGCAATTGCGTAAAATTTACAACTTTAT
9891	Yes	AAGACGGCAGAGCCCGGCAATTGCGTAAAATTTACAACTTTATG
9892	Yes	AAGACGGCAGAGCCCGGCAATTGCGTAAAATTTACAACTTTATGG
9893	Yes	AAGACGGCAGAGCCCGGCAATTGCGTAAAATTTACAACTTTATGGG
9894	Yes	AAGACGGCAGAGCCCGGCAATTGCGTAAAATTTACAACTTTATGGGC
9895	Yes	AAGACGGCAGAGCCCGGCAATTGCGTAAAATTTACAACTTTATGGGCA
9896	Yes	AAGACGGCAGAGCCCGGCAATTGCGTAAAATTTACAACTTTATGGGCAG
9897	Yes	AAGACGGCAGAGCCCGGCAATTGCGTAAAATTTACAACTTTATGGGCAGA
9898	No	AAGACTCCAGTCTCTT
9899	No	AAGACTCCAGTCTCTTC
9900	Yes	AAGACTCCAGTCTCTTCA
9901	Yes	AAGACTCCAGTCTCTTCAG
9902	Yes	AAGACTCCAGTCTCTTCAGA
9903	Yes	AAGACTCCAGTCTCTTCAGAG
9904	Yes	AAGACTCCAGTCTCTTCAGAGG
9905	Yes	AAGACTCCAGTCTCTTCAGAGGC

9906	Yes	AAGACTCCAGTCTCTTCAGAGGCA
9907	Yes	AAGACTCCAGTCTCTTCAGAGGCAT
9908	Yes	AAGACTCCAGTCTCTTCAGAGGCATG
9909	Yes	AAGACTCCAGTCTCTTCAGAGGCATGG
9910	Yes	AAGACTCCAGTCTCTTCAGAGGCATGGG
9911	Yes	AAGACTCCAGTCTCTTCAGAGGCATGGGT
9912	Yes	AAGACTCCAGTCTCTTCAGAGGCATGGGTT
9913	Yes	AAGACTCCAGTCTCTTCAGAGGCATGGGTTT
9914	Yes	AAGACTCCAGTCTCTTCAGAGGCATGGGTTTG
9915	Yes	AAGACTCCAGTCTCTTCAGAGGCATGGGTTTGA
9916	Yes	AAGACTCCAGTCTCTTCAGAGGCATGGGTTTGAA
9917	Yes	AAGACTCCAGTCTCTTCAGAGGCATGGGTTTGAAT
9918	Yes	AAGACTCCAGTCTCTTCAGAGGCATGGGTTTGAATC
9919	Yes	AAGACTCCAGTCTCTTCAGAGGCATGGGTTTGAATCC
9920	Yes	AAGACTCCAGTCTCTTCAGAGGCATGGGTTTGAATCCC
9921	Yes	AAGACTCCAGTCTCTTCAGAGGCATGGGTTTGAATCCCA
9922	Yes	AAGACTCCAGTCTCTTCAGAGGCATGGGTTTGAATCCCAC
9923	Yes	AAGACTCCAGTCTCTTCAGAGGCATGGGTTTGAATCCCACT
9924	Yes	AAGACTCCAGTCTCTTCAGAGGCATGGGTTTGAATCCCACTG
9925	Yes	AAGACTCCAGTCTCTTCAGAGGCATGGGTTTGAATCCCACTGC
9926	Yes	AAGACTCCAGTCTCTTCAGAGGCATGGGTTTGAATCCCACTGCT
9927	Yes	AAGACTCCAGTCTCTTCAGAGGCATGGGTTTGAATCCCACTGCTG
9928	Yes	AAGACTCCAGTCTCTTCAGAGGCATGGGTTTGAATCCCACTGCTGC
9929	Yes	AAGACTCCAGTCTCTTCAGAGGCATGGGTTTGAATCCCACTGCTGCC
9930	Yes	AAGACTCCAGTCTCTTCAGAGGCATGGGTTTGAATCCCACTGCTGCCA
9931	Yes	AAGACTCCAGTCTCTTCAGAGGCATGGGTTTGAATCCCACTGCTGCCAC
9932	Yes	AAGACTCCAGTCTCTTCAGAGGCATGGGTTTGAATCCCACTGCTGCCACC
9933	No	AAGACTCTTAATCTCA
9934	Yes	AAGACTCTTAATCTCAG
9935	Yes	AAGACTCTTAATCTCAGG
9936	Yes	AAGACTCTTAATCTCAGGG
9937	Yes	AAGACTCTTAATCTCAGGGT
9938	Yes	AAGACTCTTAATCTCAGGGTC
9939	Yes	AAGACTCTTAATCTCAGGGTCA
9940	Yes	AAGACTCTTAATCTCAGGGTCAT
9941	Yes	AAGACTCTTAATCTCAGGGTCATG
9942	Yes	AAGACTCTTAATCTCAGGGTCATGG
9943	Yes	AAGACTCTTAATCTCAGGGTCATGGG
9944	Yes	AAGACTCTTAATCTCAGGGTCATGGGT
9945	Yes	AAGACTCTTAATCTCAGGGTCATGGGTT
9946	Yes	AAGACTCTTAATCTCAGGGTCATGGGTTT
9947	Yes	AAGACTCTTAATCTCAGGGTCATGGGTTTG

9948	Yes	AAGACTCTTAATCTCAGGGTCATGGGTTTGA
9949	Yes	AAGACTCTTAATCTCAGGGTCATGGGTTTGAG
9950	Yes	AAGACTCTTAATCTCAGGGTCATGGGTTTGAGC
9951	Yes	AAGACTCTTAATCTCAGGGTCATGGGTTTGAGCC
9952	Yes	AAGACTCTTAATCTCAGGGTCATGGGTTTGAGCCC
9953	Yes	AAGACTCTTAATCTCAGGGTCATGGGTTTGAGCCCT
9954	Yes	AAGACTCTTAATCTCAGGGTCATGGGTTTGAGCCCTA
9955	Yes	AAGACTCTTAATCTCAGGGTCATGGGTTTGAGCCCTAC
9956	Yes	AAGACTCTTAATCTCAGGGTCATGGGTTTGAGCCCTACG
9957	Yes	AAGACTCTTAATCTCAGGGTCATGGGTTTGAGCCCTACGT
9958	Yes	AAGACTCTTAATCTCAGGGTCATGGGTTTGAGCCCTACGTT
9959	Yes	AAGACTCTTAATCTCAGGGTCATGGGTTTGAGCCCTACGTTG
9960	Yes	AAGACTCTTAATCTCAGGGTCATGGGTTTGAGCCCTACGTTGG
9961	Yes	AAGACTCTTAATCTCAGGGTCATGGGTTTGAGCCCTACGTTGGG
9962	Yes	AAGACTCTTAATCTCAGGGTCATGGGTTTGAGCCCTACGTTGGGC
9963	Yes	AAGACTCTTAATCTCAGGGTCATGGGTTTGAGCCCTACGTTGGGCG
9964	Yes	AAGACTCTTAATCTCAGGGTCATGGGTTTGAGCCCTACGTTGGGCGC
9965	Yes	AAGACTCTTAATCTCAGGGTCATGGGTTTGAGCCCTACGTTGGGCGCC
9966	Yes	AAGACTCTTAATCTCAGGGTCATGGGTTTGAGCCCTACGTTGGGCGCCA
9967	Yes	AAGACTCTTAATCTCAGGGTCG
9968	Yes	AAGACTCTTAATCTCAGGGTCGT
9969	Yes	AAGACTCTTAATCTCAGGGTCGTG
9970	Yes	AAGACTCTTAATCTCAGGGTCGTGG
9971	Yes	AAGACTCTTAATCTCAGGGTCGTGGG
9972	Yes	AAGACTCTTAATCTCAGGGTCGTGGGT
9973	Yes	AAGACTCTTAATCTCAGGGTCGTGGGTT
9974	Yes	AAGACTCTTAATCTCAGGGTCGTGGGTTT
9975	Yes	AAGACTCTTAATCTCAGGGTCGTGGGTTTG
9976	Yes	AAGACTCTTAATCTCAGGGTCGTGGGTTTGA
9977	Yes	AAGACTCTTAATCTCAGGGTCGTGGGTTTGAG
9978	Yes	AAGACTCTTAATCTCAGGGTCGTGGGTTTGAGC
9979	Yes	AAGACTCTTAATCTCAGGGTCGTGGGTTTGAGCC
9980	Yes	AAGACTCTTAATCTCAGGGTCGTGGGTTTGAGCCC
9981	Yes	AAGACTCTTAATCTCAGGGTCGTGGGTTTGAGCCCT
9982	Yes	AAGACTCTTAATCTCAGGGTCGTGGGTTTGAGCCCTG
9983	Yes	AAGACTCTTAATCTCAGGGTCGTGGGTTTGAGCCCTGT
9984	Yes	AAGACTCTTAATCTCAGGGTCGTGGGTTTGAGCCCTGTG
9985	Yes	AAGACTCTTAATCTCAGGGTCGTGGGTTTGAGCCCTGTGT
9986	Yes	AAGACTCTTAATCTCAGGGTCGTGGGTTTGAGCCCTGTGTT
9987	Yes	AAGACTCTTAATCTCAGGGTCGTGGGTTTGAGCCCTGTGTTG
9988	Yes	AAGACTCTTAATCTCAGGGTCGTGGGTTTGAGCCCTGTGTTGA
9989	Yes	AAGACTCTTAATCTCAGGGTCGTGGGTTTGAGCCCTGTGTTGAG

9990	Yes	AAGACTCTTAATCTCAGGGTCGTGGGTTTGAGCCCTGTGTTGAGC
9991	Yes	AAGACTCTTAATCTCAGGGTCGTGGGTTTGAGCCCTGTGTTGAGCA
9992	Yes	AAGACTCTTAATCTCAGGGTCGTGGGTTTGAGCCCTGTGTTGAGCAC
9993	Yes	AAGACTCTTAATCTCAGGGTCGTGGGTTTGAGCCCTGTGTTGAGCACC
9994	Yes	AAGACTCTTAATCTCAGGGTCGTGGGTTTGAGCCCTGTGTTGAGCACCA
9995	Yes	AAGACTCTTAATCTCAGGGTT
9996	Yes	AAGACTCTTAATCTCAGGGTTG
9997	Yes	AAGACTCTTAATCTCAGGGTTGT
9998	Yes	AAGACTCTTAATCTCAGGGTTGTG
9999	Yes	AAGACTCTTAATCTCAGGGTTGTGG
10000	Yes	AAGACTCTTAATCTCAGGGTTGTGGA
10001	Yes	AAGACTCTTAATCTCAGGGTTGTGGAT
10002	Yes	AAGACTCTTAATCTCAGGGTTGTGGATT
10003	Yes	AAGACTCTTAATCTCAGGGTTGTGGATTC
10004	Yes	AAGACTCTTAATCTCAGGGTTGTGGATTCG
10005	Yes	AAGACTCTTAATCTCAGGGTTGTGGATTCGT
10006	Yes	AAGACTCTTAATCTCAGGGTTGTGGATTCGTG
10007	Yes	AAGACTCTTAATCTCAGGGTTGTGGATTCGTGC
10008	Yes	AAGACTCTTAATCTCAGGGTTGTGGATTCGTGCC
10009	Yes	AAGACTCTTAATCTCAGGGTTGTGGATTCGTGCCC
10010	Yes	AAGACTCTTAATCTCAGGGTTGTGGATTCGTGCCCC
10011	Yes	AAGACTCTTAATCTCAGGGTTGTGGATTCGTGCCCCA
10012	Yes	AAGACTCTTAATCTCAGGGTTGTGGATTCGTGCCCCAT
10013	Yes	AAGACTCTTAATCTCAGGGTTGTGGATTCGTGCCCCATG
10014	Yes	AAGACTCTTAATCTCAGGGTTGTGGATTCGTGCCCCATGC
10015	Yes	AAGACTCTTAATCTCAGGGTTGTGGATTCGTGCCCCATGCT
10016	Yes	AAGACTCTTAATCTCAGGGTTGTGGATTCGTGCCCCATGCTG
10017	Yes	AAGACTCTTAATCTCAGGGTTGTGGATTCGTGCCCCATGCTGG
10018	Yes	AAGACTCTTAATCTCAGGGTTGTGGATTCGTGCCCCATGCTGGG
10019	Yes	AAGACTCTTAATCTCAGGGTTGTGGATTCGTGCCCCATGCTGGGT
10020	Yes	AAGACTCTTAATCTCAGGGTTGTGGATTCGTGCCCCATGCTGGGTG
10021	Yes	AAGACTCTTAATCTCAGGGTTGTGGATTCGTGCCCCATGCTGGGTGC
10022	Yes	AAGACTCTTAATCTCAGGGTTGTGGATTCGTGCCCCATGCTGGGTGCC
10023	Yes	AAGACTCTTAATCTCAGGGTTGTGGATTCGTGCCCCATGCTGGGTGCCA
10024	No	AAGACTTAAAACTTTA
10025	No	AAGACTTAAAACTTTAT
10026	No	AAGACTTAAAACTTTATA
10027	No	AAGACTTAAAACTTTATAA
10028	Yes	AAGACTTAAAACTTTATAAT
10029	Yes	AAGACTTAAAACTTTATAATC
10030	Yes	AAGACTTAAAACTTTATAATCA
10031	Yes	AAGACTTAAAACTTTATAATCAG

10032	Yes	AAGACTTAAAACTTTATAATCAGA
10033	Yes	AAGACTTAAAACTTTATAATCAGAG
10034	Yes	AAGACTTAAAACTTTATAATCAGAGG
10035	Yes	AAGACTTAAAACTTTATAATCAGAGGT
10036	Yes	AAGACTTAAAACTTTATAATCAGAGGTT
10037	Yes	AAGACTTAAAACTTTATAATCAGAGGTTC
10038	Yes	AAGACTTAAAACTTTATAATCAGAGGTTCA
10039	Yes	AAGACTTAAAACTTTATAATCAGAGGTTCAA
10040	Yes	AAGACTTAAAACTTTATAATCAGAGGTTCAAC
10041	Yes	AAGACTTAAAACTTTATAATCAGAGGTTCAACT
10042	Yes	AAGACTTAAAACTTTATAATCAGAGGTTCAACTC
10043	Yes	AAGACTTAAAACTTTATAATCAGAGGTTCAACTCC
10044	Yes	AAGACTTAAAACTTTATAATCAGAGGTTCAACTCCT
10045	Yes	AAGACTTAAAACTTTATAATCAGAGGTTCAACTCCTC
10046	Yes	AAGACTTAAAACTTTATAATCAGAGGTTCAACTCCTCT
10047	Yes	AAGACTTAAAACTTTATAATCAGAGGTTCAACTCCTCTC
10048	Yes	AAGACTTAAAACTTTATAATCAGAGGTTCAACTCCTCTCA
10049	Yes	AAGACTTAAAACTTTATAATCAGAGGTTCAACTCCTCTCAT
10050	Yes	AAGACTTAAAACTTTATAATCAGAGGTTCAACTCCTCTCATT
10051	Yes	AAGACTTAAAACTTTATAATCAGAGGTTCAACTCCTCTCATTA
10052	Yes	AAGACTTAAAACTTTATAATCAGAGGTTCAACTCCTCTCATTAA
10053	Yes	AAGACTTAAAACTTTATAATCAGAGGTTCAACTCCTCTCATTAAC
10054	Yes	AAGACTTAAAACTTTATAATCAGAGGTTCAACTCCTCTCATTAACA
10055	Yes	AAGACTTAAAACTTTATAATCAGAGGTTCAACTCCTCTCATTAACAC
10056	Yes	AAGACTTAAAACTTTATAATCAGAGGTTCAACTCCTCTCATTAACACC
10057	Yes	AAGACTTAAAACTTTATAATCAGAGGTTCAACTCCTCTCATTAACACCA
10058	Yes	AAGACTTTATAATTAG
10059	Yes	AAGACTTTATAATTAGA
10060	Yes	AAGACTTTATAATTAGAA
10061	Yes	AAGACTTTATAATTAGAAG
10062	Yes	AAGACTTTATAATTAGAAGT
10063	Yes	AAGACTTTATAATTAGAAGTT
10064	Yes	AAGACTTTATAATTAGAAGTTC
10065	Yes	AAGACTTTATAATTAGAAGTTCA
10066	Yes	AAGACTTTATAATTAGAAGTTCAA
10067	Yes	AAGACTTTATAATTAGAAGTTCAAC
10068	Yes	AAGACTTTATAATTAGAAGTTCAACA
10069	Yes	AAGACTTTATAATTAGAAGTTCAACAC
10070	Yes	AAGACTTTATAATTAGAAGTTCAACACC
10071	Yes	AAGACTTTATAATTAGAAGTTCAACACCT
10072	Yes	AAGACTTTATAATTAGAAGTTCAACACCTC
10073	Yes	AAGACTTTATAATTAGAAGTTCAACACCTCT

10074	Yes	AAGACTTTATAATTAGAAGTTCAACACCTCTT
10075	Yes	AAGACTTTATAATTAGAAGTTCAACACCTCTTC
10076	Yes	AAGACTTTATAATTAGAAGTTCAACACCTCTTCT
10077	Yes	AAGACTTTATAATTAGAAGTTCAACACCTCTTCTT
10078	Yes	AAGACTTTATAATTAGAAGTTCAACACCTCTTCTTA
10079	Yes	AAGACTTTATAATTAGAAGTTCAACACCTCTTCTTAA
10080	Yes	AAGACTTTATAATTAGAAGTTCAACACCTCTTCTTAAC
10081	Yes	AAGACTTTATAATTAGAAGTTCAACACCTCTTCTTAACA
10082	Yes	AAGACTTTATAATTAGAAGTTCAACACCTCTTCTTAACAC
10083	Yes	AAGACTTTATAATTAGAAGTTCAACACCTCTTCTTAACACC
10084	Yes	AAGACTTTATAATTAGAAGTTCAACACCTCTTCTTAACACCA
10085	No	AAGACTTTGTGCTTTC
10086	Yes	AAGACTTTGTGCTTTCA
10087	Yes	AAGACTTTGTGCTTTCAT
10088	Yes	AAGACTTTGTGCTTTCATT
10089	Yes	AAGACTTTGTGCTTTCATTG
10090	Yes	AAGACTTTGTGCTTTCATTGC
10091	Yes	AAGACTTTGTGCTTTCATTGCT
10092	Yes	AAGACTTTGTGCTTTCATTGCTG
10093	Yes	AAGACTTTGTGCTTTCATTGCTGC
10094	Yes	AAGACTTTGTGCTTTCATTGCTGCA
10095	Yes	AAGACTTTGTGCTTTCATTGCTGCAT
10096	Yes	AAGACTTTGTGCTTTCATTGCTGCATC
10097	Yes	AAGACTTTGTGCTTTCATTGCTGCATCC
10098	Yes	AAGACTTTGTGCTTTCATTGCTGCATCCT
10099	Yes	AAGACTTTGTGCTTTCATTGCTGCATCCTA
10100	Yes	AAGACTTTGTGCTTTCATTGCTGCATCCTAG
10101	Yes	AAGACTTTGTGCTTTCATTGCTGCATCCTAGG
10102	Yes	AAGACTTTGTGCTTTCATTGCTGCATCCTAGGT
10103	Yes	AAGACTTTGTGCTTTCATTGCTGCATCCTAGGTT
10104	Yes	AAGACTTTGTGCTTTCATTGCTGCATCCTAGGTTC
10105	Yes	AAGACTTTGTGCTTTCATTGCTGCATCCTAGGTTCA
10106	Yes	AAGACTTTGTGCTTTCATTGCTGCATCCTAGGTTCAA
10107	Yes	AAGACTTTGTGCTTTCATTGCTGCATCCTAGGTTCAAT
10108	Yes	AAGACTTTGTGCTTTCATTGCTGCATCCTAGGTTCAATT
10109	Yes	AAGACTTTGTGCTTTCATTGCTGCATCCTAGGTTCAATTC
10110	Yes	AAGACTTTGTGCTTTCATTGCTGCATCCTAGGTTCAATTCC
10111	Yes	AAGACTTTGTGCTTTCATTGCTGCATCCTAGGTTCAATTCCC
10112	Yes	AAGACTTTGTGCTTTCATTGCTGCATCCTAGGTTCAATTCCCA
10113	Yes	AAGACTTTGTGCTTTCATTGCTGCATCCTAGGTTCAATTCCCAG
10114	Yes	AAGACTTTGTGCTTTCATTGCTGCATCCTAGGTTCAATTCCCAGT
10115	Yes	AAGACTTTGTGCTTTCATTGCTGCATCCTAGGTTCAATTCCCAGTC

10116	Yes	AAGACTTTGTGCTTTCATTGCTGCATCCTAGGTTCAATTCCCAGTCA
10117	Yes	AAGACTTTGTGCTTTCATTGCTGCATCCTAGGTTCAATTCCCAGTCAG
10118	Yes	AAGACTTTGTGCTTTCATTGCTGCATCCTAGGTTCAATTCCCAGTCAGG
10119	Yes	AAGACTTTGTGCTTTCATTGCTGCATCCTAGGTTCAATTCCCAGTCAGGG
10120	No	AAGACTTTTTCTCTGA
10121	No	AAGACTTTTTCTCTGAC
10122	No	AAGACTTTTTCTCTGACC
10123	No	AAGACTTTTTCTCTGACCA
10124	No	AAGAGAACCAACACCT
10125	No	AAGAGAACCAACACCTC
10126	No	AAGAGAACCAACACCTCT
10127	No	AAGAGAACCAACACCTCTT
10128	No	AAGAGAACCAACACCTCTTT
10129	No	AAGAGAACCAACACCTCTTTA
10130	No	AAGAGAACCAACACCTCTTTAC
10131	No	AAGAGAACCAACACCTCTTTACA
10132	No	AAGAGAACCAACACCTCTTTACAG
10133	No	AAGAGAACCAACACCTCTTTACAGT
10134	No	AAGAGAACCAACACCTCTTTACAGTG
10135	No	AAGAGAACCAACACCTCTTTACAGTGA
10136	Yes	AAGAGAACCAACACCTCTTTACAGTGAC
10137	Yes	AAGAGAACCAACACCTCTTTACAGTGACC
10138	Yes	AAGAGAACCAACACCTCTTTACAGTGACCA
10139	No	AAGAGATTTGGATTCT
10140	No	AAGAGATTTGGATTCTC
10141	Yes	AAGAGATTTGGATTCTCA
10142	Yes	AAGAGATTTGGATTCTCAG
10143	Yes	AAGAGATTTGGATTCTCAGG
10144	Yes	AAGAGATTTGGATTCTCAGGG
10145	Yes	AAGAGATTTGGATTCTCAGGGG
10146	Yes	AAGAGATTTGGATTCTCAGGGGT
10147	Yes	AAGAGATTTGGATTCTCAGGGGTA
10148	Yes	AAGAGATTTGGATTCTCAGGGGTAG
10149	Yes	AAGAGATTTGGATTCTCAGGGGTAGG
10150	Yes	AAGAGATTTGGATTCTCAGGGGTAGGT
10151	Yes	AAGAGATTTGGATTCTCAGGGGTAGGTT
10152	Yes	AAGAGATTTGGATTCTCAGGGGTAGGTTC
10153	Yes	AAGAGATTTGGATTCTCAGGGGTAGGTTCA
10154	Yes	AAGAGATTTGGATTCTCAGGGGTAGGTTCAA
10155	Yes	AAGAGATTTGGATTCTCAGGGGTAGGTTCAAT
10156	Yes	AAGAGATTTGGATTCTCAGGGGTAGGTTCAATT
10157	Yes	AAGAGATTTGGATTCTCAGGGGTAGGTTCAATTC

10158	Yes	AAGAGATTTGGATTCTCAGGGGTAGGTTCAATTCC
10159	Yes	AAGAGATTTGGATTCTCAGGGGTAGGTTCAATTCCT
10160	Yes	AAGAGATTTGGATTCTCAGGGGTAGGTTCAATTCCTA
10161	Yes	AAGAGATTTGGATTCTCAGGGGTAGGTTCAATTCCTAT
10162	Yes	AAGAGATTTGGATTCTCAGGGGTAGGTTCAATTCCTATA
10163	Yes	AAGAGATTTGGATTCTCAGGGGTAGGTTCAATTCCTATAG
10164	Yes	AAGAGATTTGGATTCTCAGGGGTAGGTTCAATTCCTATAGT
10165	Yes	AAGAGATTTGGATTCTCAGGGGTAGGTTCAATTCCTATAGTT
10166	Yes	AAGAGATTTGGATTCTCAGGGGTAGGTTCAATTCCTATAGTTC
10167	Yes	AAGAGATTTGGATTCTCAGGGGTAGGTTCAATTCCTATAGTTCT
10168	Yes	AAGAGATTTGGATTCTCAGGGGTAGGTTCAATTCCTATAGTTCTG
10169	Yes	AAGAGATTTGGATTCTCAGGGGTAGGTTCAATTCCTATAGTTCTGG
10170	Yes	AAGAGATTTGGATTCTCAGGGGTAGGTTCAATTCCTATAGTTCTGGC
10171	Yes	AAGAGATTTGGATTCTCAGGGGTAGGTTCAATTCCTATAGTTCTGGCC
10172	Yes	AAGAGATTTGGATTCTCAGGGGTAGGTTCAATTCCTATAGTTCTGGCCA
10173	Yes	AAGAGCCACGGCCCGG
10174	Yes	AAGAGCCACGGCCCGGG
10175	Yes	AAGAGCCACGGCCCGGGT
10176	Yes	AAGAGCCACGGCCCGGGTT
10177	Yes	AAGAGCCACGGCCCGGGTTC
10178	Yes	AAGAGCCACGGCCCGGGTTCG
10179	Yes	AAGAGCCACGGCCCGGGTTCGA
10180	Yes	AAGAGCCACGGCCCGGGTTCGAT
10181	Yes	AAGAGCCACGGCCCGGGTTCGATT
10182	Yes	AAGAGCCACGGCCCGGGTTCGATTC
10183	Yes	AAGAGCCACGGCCCGGGTTCGATTCC
10184	Yes	AAGAGCCACGGCCCGGGTTCGATTCCC
10185	Yes	AAGAGCCACGGCCCGGGTTCGATTCCCG
10186	Yes	AAGAGCCACGGCCCGGGTTCGATTCCCGG
10187	Yes	AAGAGCCACGGCCCGGGTTCGATTCCCGGT
10188	Yes	AAGAGCCACGGCCCGGGTTCGATTCCCGGTC
10189	Yes	AAGAGCCACGGCCCGGGTTCGATTCCCGGTCA
10190	Yes	AAGAGCCACGGCCCGGGTTCGATTCCCGGTCAG
10191	Yes	AAGAGCCACGGCCCGGGTTCGATTCCCGGTCAGG
10192	Yes	AAGAGCCACGGCCCGGGTTCGATTCCCGGTCAGGG
10193	Yes	AAGAGCCACGGCCCGGGTTCGATTCCCGGTCAGGGA
10194	Yes	AAGAGCCACGGCCCGGGTTCGATTCCCGGTCAGGGAA
10195	Yes	AAGAGCCACGGCCCGGGTTCGATTCCCGGTCAGGGAAC
10196	Yes	AAGAGCCACGGCCCGGGTTCGATTCCCGGTCAGGGAACC
10197	Yes	AAGAGCCACGGCCCGGGTTCGATTCCCGGTCAGGGAACCA
10198	No	AAGAGCCTTCAAAGCC
10199	No	AAGAGCCTTCAAAGCCC

10200	No	AAGAGCCTTCAAAGCCCT
10201	No	AAGAGCCTTCAAAGCCCTC
10202	No	AAGAGCCTTCAAAGCCCTCA
10203	No	AAGAGCCTTCAAAGCCCTCAG
10204	No	AAGAGCCTTCAAAGCCCTCAGT
10205	No	AAGAGCCTTCAAAGCCCTCAGTA
10206	No	AAGAGCCTTCAAAGCCCTCAGTAA
10207	No	AAGAGCCTTCAAAGCCCTCAGTAAG
10208	No	AAGAGCCTTCAAAGCCCTCAGTAAGT
10209	Yes	AAGAGCCTTCAAAGCCCTCAGTAAGTT
10210	Yes	AAGAGCCTTCAAAGCCCTCAGTAAGTTG
10211	Yes	AAGAGCCTTCAAAGCCCTCAGTAAGTTGC
10212	Yes	AAGAGCCTTCAAAGCCCTCAGTAAGTTGCA
10213	Yes	AAGAGCCTTCAAAGCCCTCAGTAAGTTGCAA
10214	Yes	AAGAGCCTTCAAAGCCCTCAGTAAGTTGCAAT
10215	Yes	AAGAGCCTTCAAAGCCCTCAGTAAGTTGCAATA
10216	Yes	AAGAGCCTTCAAAGCCCTCAGTAAGTTGCAATAC
10217	Yes	AAGAGCCTTCAAAGCCCTCAGTAAGTTGCAATACT
10218	Yes	AAGAGCCTTCAAAGCCCTCAGTAAGTTGCAATACTT
10219	Yes	AAGAGCCTTCAAAGCCCTCAGTAAGTTGCAATACTTA
10220	Yes	AAGAGCCTTCAAAGCCCTCAGTAAGTTGCAATACTTAA
10221	Yes	AAGAGCCTTCAAAGCCCTCAGTAAGTTGCAATACTTAAT
10222	Yes	AAGAGCCTTCAAAGCCCTCAGTAAGTTGCAATACTTAATT
10223	Yes	AAGAGCCTTCAAAGCCCTCAGTAAGTTGCAATACTTAATTT
10224	Yes	AAGAGCCTTCAAAGCCCTCAGTAAGTTGCAATACTTAATTTC
10225	Yes	AAGAGCCTTCAAAGCCCTCAGTAAGTTGCAATACTTAATTTCT
10226	Yes	AAGAGCCTTCAAAGCCCTCAGTAAGTTGCAATACTTAATTTCTG
10227	Yes	AAGAGCCTTCAAAGCCCTCAGTAAGTTGCAATACTTAATTTCTGC
10228	Yes	AAGAGCCTTCAAAGCCCTCAGTAAGTTGCAATACTTAATTTCTGCC
10229	Yes	AAGAGCCTTCAAAGCCCTCAGTAAGTTGCAATACTTAATTTCTGCCA
10230	No	AAGAGCGTGGTGCTGA
10231	Yes	AAGAGCGTGGTGCTGAT
10232	Yes	AAGAGCGTGGTGCTGATA
10233	Yes	AAGAGCGTGGTGCTGATAA
10234	Yes	AAGAGCGTGGTGCTGATAAC
10235	Yes	AAGAGCGTGGTGCTGATAACA
10236	Yes	AAGAGCGTGGTGCTGATAACAC
10237	Yes	AAGAGCGTGGTGCTGATAACACC
10238	Yes	AAGAGCGTGGTGCTGATAACACCA
10239	Yes	AAGAGCGTGGTGCTGATAACACCAA
10240	Yes	AAGAGCGTGGTGCTGATAACACCAAG
10241	Yes	AAGAGCGTGGTGCTGATAACACCAAGG

10242	Yes	AAGAGCGTGGTGCTGATAACACCAAGGT
10243	Yes	AAGAGCGTGGTGCTGATAACACCAAGGTC
10244	Yes	AAGAGCGTGGTGCTGATAACACCAAGGTCG
10245	Yes	AAGAGCGTGGTGCTGATAACACCAAGGTCGC
10246	Yes	AAGAGCGTGGTGCTGATAACACCAAGGTCGCG
10247	Yes	AAGAGCGTGGTGCTGATAACACCAAGGTCGCGG
10248	Yes	AAGAGCGTGGTGCTGATAACACCAAGGTCGCGGG
10249	Yes	AAGAGCGTGGTGCTGATAACACCAAGGTCGCGGGC
10250	Yes	AAGAGCGTGGTGCTGATAACACCAAGGTCGCGGGCT
10251	Yes	AAGAGCGTGGTGCTGATAACACCAAGGTCGCGGGCTC
10252	Yes	AAGAGCGTGGTGCTGATAACACCAAGGTCGCGGGCTCG
10253	Yes	AAGAGCGTGGTGCTGATAACACCAAGGTCGCGGGCTCGA
10254	Yes	AAGAGCGTGGTGCTGATAACACCAAGGTCGCGGGCTCGAC
10255	Yes	AAGAGCGTGGTGCTGATAACACCAAGGTCGCGGGCTCGACT
10256	Yes	AAGAGCGTGGTGCTGATAACACCAAGGTCGCGGGCTCGACTC
10257	Yes	AAGAGCGTGGTGCTGATAACACCAAGGTCGCGGGCTCGACTCC
10258	Yes	AAGAGCGTGGTGCTGATAACACCAAGGTCGCGGGCTCGACTCCC
10259	Yes	AAGAGCGTGGTGCTGATAACACCAAGGTCGCGGGCTCGACTCCCG
10260	Yes	AAGAGCGTGGTGCTGATAACACCAAGGTCGCGGGCTCGACTCCCGC
10261	Yes	AAGAGCGTGGTGCTGATAACACCAAGGTCGCGGGCTCGACTCCCGCA
10262	Yes	AAGAGCGTGGTGCTGATAACACCAAGGTCGCGGGCTCGACTCCCGCAC
10263	Yes	AAGAGCGTGGTGCTGATAACACCAAGGTCGCGGGCTCGACTCCCGCACC
10264	Yes	AAGAGCGTGGTGCTGATAACACCAAGGTCGCGGGCTCGACTCCCGCACCG
10265	No	AAGAGGCTGTAAACCC
10266	Yes	AAGAGGCTGTAAACCCA
10267	Yes	AAGAGGCTGTAAACCCAG
10268	Yes	AAGAGGCTGTAAACCCAGA
10269	Yes	AAGAGGCTGTAAACCCAGAC
10270	Yes	AAGAGGCTGTAAACCCAGACT
10271	Yes	AAGAGGCTGTAAACCCAGACTG
10272	Yes	AAGAGGCTGTAAACCCAGACTGG
10273	Yes	AAGAGGCTGTAAACCCAGACTGGA
10274	Yes	AAGAGGCTGTAAACCCAGACTGGAT
10275	Yes	AAGAGGCTGTAAACCCAGACTGGATG
10276	Yes	AAGAGGCTGTAAACCCAGACTGGATGG
10277	Yes	AAGAGGCTGTAAACCCAGACTGGATGGG
10278	Yes	AAGAGGCTGTAAACCCAGACTGGATGGGT
10279	Yes	AAGAGGCTGTAAACCCAGACTGGATGGGTT
10280	Yes	AAGAGGCTGTAAACCCAGACTGGATGGGTTC
10281	Yes	AAGAGGCTGTAAACCCAGACTGGATGGGTTCA
10282	Yes	AAGAGGCTGTAAACCCAGACTGGATGGGTTCAA
10283	Yes	AAGAGGCTGTAAACCCAGACTGGATGGGTTCAAT

10284	Yes	AAGAGGCTGTAAACCCAGACTGGATGGGTTCAATT
10285	Yes	AAGAGGCTGTAAACCCAGACTGGATGGGTTCAATTC
10286	Yes	AAGAGGCTGTAAACCCAGACTGGATGGGTTCAATTCC
10287	Yes	AAGAGGCTGTAAACCCAGACTGGATGGGTTCAATTCCC
10288	Yes	AAGAGGCTGTAAACCCAGACTGGATGGGTTCAATTCCCA
10289	Yes	AAGAGGCTGTAAACCCAGACTGGATGGGTTCAATTCCCAT
10290	Yes	AAGAGGCTGTAAACCCAGACTGGATGGGTTCAATTCCCATC
10291	Yes	AAGAGGCTGTAAACCCAGACTGGATGGGTTCAATTCCCATCT
10292	Yes	AAGAGGCTGTAAACCCAGACTGGATGGGTTCAATTCCCATCTC
10293	Yes	AAGAGGCTGTAAACCCAGACTGGATGGGTTCAATTCCCATCTCT
10294	Yes	AAGAGGCTGTAAACCCAGACTGGATGGGTTCAATTCCCATCTCTG
10295	Yes	AAGAGGCTGTAAACCCAGACTGGATGGGTTCAATTCCCATCTCTGC
10296	Yes	AAGAGGCTGTAAACCCAGACTGGATGGGTTCAATTCCCATCTCTGCC
10297	Yes	AAGAGGCTGTAAACCCAGACTGGATGGGTTCAATTCCCATCTCTGCCG
10298	Yes	AAGAGGCTGTAAACCCAGACTGGATGGGTTCAATTCCCATCTCTGCCGC
10299	Yes	AAGAGGCTGTAAACCCAGACTGGATGGGTTCAATTCCCATCTCTGCCGCC
10300	No	AAGAGGTAGCAGGATC
10301	Yes	AAGAGGTAGCAGGATCG
10302	Yes	AAGAGGTAGCAGGATCGA
10303	Yes	AAGAGGTAGCAGGATCGAC
10304	Yes	AAGAGGTAGCAGGATCGACG
10305	Yes	AAGAGGTAGCAGGATCGACGC
10306	Yes	AAGAGGTAGCAGGATCGACGCC
10307	Yes	AAGAGGTAGCAGGATCGACGCCT
10308	Yes	AAGAGGTAGCAGGATCGACGCCTG
10309	Yes	AAGAGGTAGCAGGATCGACGCCTGC
10310	Yes	AAGAGGTAGCAGGATCGACGCCTGCA
10311	Yes	AAGAGGTAGCAGGATCGACGCCTGCAC
10312	Yes	AAGAGGTAGCAGGATCGACGCCTGCACT
10313	Yes	AAGAGGTAGCAGGATCGACGCCTGCACTC
10314	Yes	AAGAGGTAGCAGGATCGACGCCTGCACTCT
10315	Yes	AAGAGGTAGCAGGATCGACGCCTGCACTCTC
10316	Yes	AAGAGGTAGCAGGATCGACGCCTGCACTCTCC
10317	Yes	AAGAGGTAGCAGGATCGACGCCTGCACTCTCCA
10318	Yes	AAGAGGTAGCAGGATCGACGCCTGCACTCTCCAC
10319	Yes	AAGAGGTAGCAGGATCGACGCCTGCACTCTCCACC
10320	Yes	AAGAGGTAGCAGGATCGACGCCTGCACTCTCCACCA
10321	Yes	AAGAGGTAGCAGGATCGACGCCTGCAT
10322	Yes	AAGAGGTAGCAGGATCGACGCCTGCATT
10323	Yes	AAGAGGTAGCAGGATCGACGCCTGCATTC
10324	Yes	AAGAGGTAGCAGGATCGACGCCTGCATTCT
10325	Yes	AAGAGGTAGCAGGATCGACGCCTGCATTCTC

10326	Yes	AAGAGGTAGCAGGATCGACGCCTGCATTCTCC
10327	Yes	AAGAGGTAGCAGGATCGACGCCTGCATTCTCCA
10328	Yes	AAGAGGTAGCAGGATCGACGCCTGCATTCTCCAC
10329	Yes	AAGAGGTAGCAGGATCGACGCCTGCATTCTCCACC
10330	Yes	AAGAGGTAGCAGGATCGACGCCTGCATTCTCCACCA
10331	Yes	AAGAGGTAGCAGGATCGAT
10332	Yes	AAGAGGTAGCAGGATCGATG
10333	Yes	AAGAGGTAGCAGGATCGATGC
10334	Yes	AAGAGGTAGCAGGATCGATGCC
10335	Yes	AAGAGGTAGCAGGATCGATGCCT
10336	Yes	AAGAGGTAGCAGGATCGATGCCTG
10337	Yes	AAGAGGTAGCAGGATCGATGCCTGC
10338	Yes	AAGAGGTAGCAGGATCGATGCCTGCA
10339	Yes	AAGAGGTAGCAGGATCGATGCCTGCAT
10340	Yes	AAGAGGTAGCAGGATCGATGCCTGCATT
10341	Yes	AAGAGGTAGCAGGATCGATGCCTGCATTC
10342	Yes	AAGAGGTAGCAGGATCGATGCCTGCATTCT
10343	Yes	AAGAGGTAGCAGGATCGATGCCTGCATTCTC
10344	Yes	AAGAGGTAGCAGGATCGATGCCTGCATTCTCC
10345	Yes	AAGAGGTAGCAGGATCGATGCCTGCATTCTCCA
10346	Yes	AAGAGGTAGCAGGATCGATGCCTGCATTCTCCAC
10347	Yes	AAGAGGTAGCAGGATCGATGCCTGCATTCTCCACC
10348	Yes	AAGAGGTAGCAGGATCGATGCCTGCATTCTCCACCA
10349	No	AAGAGGTAGTGGGATC
10350	Yes	AAGAGGTAGTGGGATCA
10351	Yes	AAGAGGTAGTGGGATCAA
10352	Yes	AAGAGGTAGTGGGATCAAT
10353	Yes	AAGAGGTAGTGGGATCAATG
10354	Yes	AAGAGGTAGTGGGATCAATGC
10355	Yes	AAGAGGTAGTGGGATCAATGCC
10356	Yes	AAGAGGTAGTGGGATCAATGCCC
10357	Yes	AAGAGGTAGTGGGATCAATGCCCA
10358	Yes	AAGAGGTAGTGGGATCAATGCCCAC
10359	Yes	AAGAGGTAGTGGGATCAATGCCCACA
10360	Yes	AAGAGGTAGTGGGATCAATGCCCACAT
10361	Yes	AAGAGGTAGTGGGATCAATGCCCACATT
10362	Yes	AAGAGGTAGTGGGATCAATGCCCACATTC
10363	Yes	AAGAGGTAGTGGGATCAATGCCCACATTCT
10364	Yes	AAGAGGTAGTGGGATCAATGCCCACATTCTC
10365	Yes	AAGAGGTAGTGGGATCAATGCCCACATTCTCC
10366	Yes	AAGAGGTAGTGGGATCAATGCCCACATTCTCCA
10367	Yes	AAGAGGTAGTGGGATCAATGCCCACATTCTCCAC

10368	Yes	AAGAGGTAGTGGGATCAATGCCCACATTCTCCACC
10369	Yes	AAGAGGTAGTGGGATCAATGCCCACATTCTCCACCA
10370	Yes	AAGAGGTAGTGGGATCG
10371	Yes	AAGAGGTAGTGGGATCGA
10372	Yes	AAGAGGTAGTGGGATCGAT
10373	Yes	AAGAGGTAGTGGGATCGATG
10374	Yes	AAGAGGTAGTGGGATCGATGC
10375	Yes	AAGAGGTAGTGGGATCGATGCC
10376	Yes	AAGAGGTAGTGGGATCGATGCCC
10377	Yes	AAGAGGTAGTGGGATCGATGCCCA
10378	Yes	AAGAGGTAGTGGGATCGATGCCCAC
10379	Yes	AAGAGGTAGTGGGATCGATGCCCACA
10380	Yes	AAGAGGTAGTGGGATCGATGCCCACAT
10381	Yes	AAGAGGTAGTGGGATCGATGCCCACATT
10382	Yes	AAGAGGTAGTGGGATCGATGCCCACATTC
10383	Yes	AAGAGGTAGTGGGATCGATGCCCACATTCT
10384	Yes	AAGAGGTAGTGGGATCGATGCCCACATTCTC
10385	Yes	AAGAGGTAGTGGGATCGATGCCCACATTCTCC
10386	Yes	AAGAGGTAGTGGGATCGATGCCCACATTCTCCA
10387	Yes	AAGAGGTAGTGGGATCGATGCCCACATTCTCCAC
10388	Yes	AAGAGGTAGTGGGATCGATGCCCACATTCTCCACC
10389	Yes	AAGAGGTAGTGGGATCGATGCCCACATTCTCCACCA
10390	No	AAGAGGTCCCCAGTTC
10391	Yes	AAGAGGTCCCCAGTTCA
10392	Yes	AAGAGGTCCCCAGTTCAA
10393	Yes	AAGAGGTCCCCAGTTCAAA
10394	Yes	AAGAGGTCCCCAGTTCAAAT
10395	Yes	AAGAGGTCCCCAGTTCAAATC
10396	Yes	AAGAGGTCCCCAGTTCAAATCT
10397	Yes	AAGAGGTCCCCAGTTCAAATCTG
10398	Yes	AAGAGGTCCCCAGTTCAAATCTGG
10399	Yes	AAGAGGTCCCCAGTTCAAATCTGGG
10400	Yes	AAGAGGTCCCCAGTTCAAATCTGGGT
10401	Yes	AAGAGGTCCCCAGTTCAAATCTGGGTG
10402	Yes	AAGAGGTCCCCAGTTCAAATCTGGGTGC
10403	Yes	AAGAGGTCCCCAGTTCAAATCTGGGTGCC
10404	Yes	AAGAGGTCCCCAGTTCAAATCTGGGTGCCC
10405	Yes	AAGAGGTCCCCAGTTCAAATCTGGGTGCCCA
10406	Yes	AAGAGGTCCCCAGTTCAAATCTGGGTGCCCAC
10407	Yes	AAGAGGTCCCCAGTTCAAATCTGGGTGCCCACT
10408	Yes	AAGAGGTCCCCAGTTCAAATCTGGGTGCCCACTC
10409	Yes	AAGAGGTCCCCAGTTCAAATCTGGGTGCCCACTCC

10410	Yes	AAGAGGTCCCCAGTTCAAATCTGGGTGCCCACTCCA
10411	Yes	AAGAGGTCCCCAGTTCAAATCTGGGTGCCCC
10412	Yes	AAGAGGTCCCCAGTTCAAATCTGGGTGCCCCC
10413	Yes	AAGAGGTCCCCAGTTCAAATCTGGGTGCCCCCT
10414	Yes	AAGAGGTCCCCAGTTCAAATCTGGGTGCCCCCTC
10415	Yes	AAGAGGTCCCCAGTTCAAATCTGGGTGCCCCCTCC
10416	Yes	AAGAGGTCCCCAGTTCAAATCTGGGTGCCCCCTCCA
10417	No	AAGAGGTCCCCGGTTC
10418	No	AAGAGGTCCCCGGTTCA
10419	Yes	AAGAGGTCCCCGGTTCAA
10420	Yes	AAGAGGTCCCCGGTTCAAA
10421	Yes	AAGAGGTCCCCGGTTCAAAT
10422	Yes	AAGAGGTCCCCGGTTCAAATC
10423	Yes	AAGAGGTCCCCGGTTCAAATCC
10424	Yes	AAGAGGTCCCCGGTTCAAATCCG
10425	Yes	AAGAGGTCCCCGGTTCAAATCCGG
10426	Yes	AAGAGGTCCCCGGTTCAAATCCGGG
10427	Yes	AAGAGGTCCCCGGTTCAAATCCGGGT
10428	Yes	AAGAGGTCCCCGGTTCAAATCCGGGTG
10429	Yes	AAGAGGTCCCCGGTTCAAATCCGGGTGC
10430	Yes	AAGAGGTCCCCGGTTCAAATCCGGGTGCC
10431	Yes	AAGAGGTCCCCGGTTCAAATCCGGGTGCCC
10432	Yes	AAGAGGTCCCCGGTTCAAATCCGGGTGCCCC
10433	Yes	AAGAGGTCCCCGGTTCAAATCCGGGTGCCCCC
10434	Yes	AAGAGGTCCCCGGTTCAAATCCGGGTGCCCCCC
10435	Yes	AAGAGGTCCCCGGTTCAAATCCGGGTGCCCCCCC
10436	Yes	AAGAGGTCCCCGGTTCAAATCCGGGTGCCCCCCCC
10437	Yes	AAGAGGTCCCCGGTTCAAATCCGGGTGCCCCCCCCA
10438	Yes	AAGAGGTCCCCGGTTCAAATCCGGGTGCCCCCT
10439	Yes	AAGAGGTCCCCGGTTCAAATCCGGGTGCCCCCTC
10440	Yes	AAGAGGTCCCCGGTTCAAATCCGGGTGCCCCCTCC
10441	Yes	AAGAGGTCCCCGGTTCAAATCCGGGTGCCCCCTCCA
10442	Yes	AAGAGGTCCCCGGTTCAAATCCGGT
10443	Yes	AAGAGGTCCCCGGTTCAAATCCGGTT
10444	Yes	AAGAGGTCCCCGGTTCAAATCCGGTTA
10445	Yes	AAGAGGTCCCCGGTTCAAATCCGGTTAC
10446	Yes	AAGAGGTCCCCGGTTCAAATCCGGTTACT
10447	Yes	AAGAGGTCCCCGGTTCAAATCCGGTTACTC
10448	Yes	AAGAGGTCCCCGGTTCAAATCCGGTTACTCC
10449	Yes	AAGAGGTCCCCGGTTCAAATCCGGTTACTCCC
10450	Yes	AAGAGGTCCCCGGTTCAAATCCGGTTACTCCCT
10451	Yes	AAGAGGTCCCCGGTTCAAATCCGGTTACTCCCTC

10452	Yes	AAGAGGTCCCCGGTTCAAATCCGGTTACTCCCTCC
10453	Yes	AAGAGGTCCCCGGTTCAAATCCGGTTACTCCCTCCA
10454	Yes	AAGAGGTCCCCGGTTCAAATCT
10455	Yes	AAGAGGTCCCCGGTTCAAATCTG
10456	Yes	AAGAGGTCCCCGGTTCAAATCTGG
10457	Yes	AAGAGGTCCCCGGTTCAAATCTGGG
10458	Yes	AAGAGGTCCCCGGTTCAAATCTGGGT
10459	Yes	AAGAGGTCCCCGGTTCAAATCTGGGTG
10460	Yes	AAGAGGTCCCCGGTTCAAATCTGGGTGC
10461	Yes	AAGAGGTCCCCGGTTCAAATCTGGGTGCC
10462	Yes	AAGAGGTCCCCGGTTCAAATCTGGGTGCCC
10463	Yes	AAGAGGTCCCCGGTTCAAATCTGGGTGCCCC
10464	Yes	AAGAGGTCCCCGGTTCAAATCTGGGTGCCCCC
10465	Yes	AAGAGGTCCCCGGTTCAAATCTGGGTGCCCCCT
10466	Yes	AAGAGGTCCCCGGTTCAAATCTGGGTGCCCCCTC
10467	Yes	AAGAGGTCCCCGGTTCAAATCTGGGTGCCCCCTCC
10468	Yes	AAGAGGTCCCCGGTTCAAATCTGGGTGCCCCCTCCA
10469	Yes	AAGAGGTCCCCGGTTCG
10470	Yes	AAGAGGTCCCCGGTTCGA
10471	Yes	AAGAGGTCCCCGGTTCGAA
10472	Yes	AAGAGGTCCCCGGTTCGAAT
10473	Yes	AAGAGGTCCCCGGTTCGAATC
10474	Yes	AAGAGGTCCCCGGTTCGAATCT
10475	Yes	AAGAGGTCCCCGGTTCGAATCTA
10476	Yes	AAGAGGTCCCCGGTTCGAATCTAG
10477	Yes	AAGAGGTCCCCGGTTCGAATCTAGG
10478	Yes	AAGAGGTCCCCGGTTCGAATCTAGGT
10479	Yes	AAGAGGTCCCCGGTTCGAATCTAGGTG
10480	Yes	AAGAGGTCCCCGGTTCGAATCTAGGTGC
10481	Yes	AAGAGGTCCCCGGTTCGAATCTAGGTGCC
10482	Yes	AAGAGGTCCCCGGTTCGAATCTAGGTGCCC
10483	Yes	AAGAGGTCCCCGGTTCGAATCTAGGTGCCCC
10484	Yes	AAGAGGTCCCCGGTTCGAATCTAGGTGCCCCC
10485	Yes	AAGAGGTCCCCGGTTCGAATCTAGGTGCCCCCT
10486	Yes	AAGAGGTCCCCGGTTCGAATCTAGGTGCCCCCTC
10487	Yes	AAGAGGTCCCCGGTTCGAATCTAGGTGCCCCCTCC
10488	Yes	AAGAGGTCCCCGGTTCGAATCTAGGTGCCCCCTCCA
10489	Yes	AAGAGGTCCCCGGTTG
10490	Yes	AAGAGGTCCCCGGTTGA
10491	Yes	AAGAGGTCCCCGGTTGAA
10492	Yes	AAGAGGTCCCCGGTTGAAA
10493	Yes	AAGAGGTCCCCGGTTGAAAT

10494	Yes	AAGAGGTCCCCGGTTGAAATC
10495	Yes	AAGAGGTCCCCGGTTGAAATCC
10496	Yes	AAGAGGTCCCCGGTTGAAATCCG
10497	Yes	AAGAGGTCCCCGGTTGAAATCCGG
10498	Yes	AAGAGGTCCCCGGTTGAAATCCGGG
10499	Yes	AAGAGGTCCCCGGTTGAAATCCGGGT
10500	Yes	AAGAGGTCCCCGGTTGAAATCCGGGTG
10501	Yes	AAGAGGTCCCCGGTTGAAATCCGGGTGC
10502	Yes	AAGAGGTCCCCGGTTGAAATCCGGGTGCT
10503	Yes	AAGAGGTCCCCGGTTGAAATCCGGGTGCTG
10504	Yes	AAGAGGTCCCCGGTTGAAATCCGGGTGCTGG
10505	Yes	AAGAGGTCCCCGGTTGAAATCCGGGTGCTGGA
10506	Yes	AAGAGGTCCCCGGTTGAAATCCGGGTGCTGGAT
10507	Yes	AAGAGGTCCCCGGTTGAAATCCGGGTGCTGGATC
10508	Yes	AAGAGGTCCCCGGTTGAAATCCGGGTGCTGGATCC
10509	Yes	AAGAGGTCCCCGGTTGAAATCCGGGTGCTGGATCCA
10510	Yes	AAGAGGTCCCGGGTTC
10511	Yes	AAGAGGTCCCGGGTTCA
10512	Yes	AAGAGGTCCCGGGTTCAA
10513	Yes	AAGAGGTCCCGGGTTCAAA
10514	Yes	AAGAGGTCCCGGGTTCAAAT
10515	Yes	AAGAGGTCCCGGGTTCAAATC
10516	Yes	AAGAGGTCCCGGGTTCAAATCC
10517	Yes	AAGAGGTCCCGGGTTCAAATCCC
10518	Yes	AAGAGGTCCCGGGTTCAAATCCCG
10519	Yes	AAGAGGTCCCGGGTTCAAATCCCGG
10520	Yes	AAGAGGTCCCGGGTTCAAATCCCGGA
10521	Yes	AAGAGGTCCCGGGTTCAAATCCCGGAC
10522	Yes	AAGAGGTCCCGGGTTCAAATCCCGGACG
10523	Yes	AAGAGGTCCCGGGTTCAAATCCCGGACGA
10524	Yes	AAGAGGTCCCGGGTTCAAATCCCGGACGAG
10525	Yes	AAGAGGTCCCGGGTTCAAATCCCGGACGAGC
10526	Yes	AAGAGGTCCCGGGTTCAAATCCCGGACGAGCC
10527	Yes	AAGAGGTCCCGGGTTCAAATCCCGGACGAGCCC
10528	Yes	AAGAGGTCCCGGGTTCAAATCCCGGACGAGCCCC
10529	Yes	AAGAGGTCCCGGGTTCAAATCCCGGACGAGCCCCC
10530	Yes	AAGAGGTCCCGGGTTCAAATCCCGGACGAGCCCCCA
10531	Yes	AAGAGGTCCCTGATTC
10532	Yes	AAGAGGTCCCTGATTCA
10533	Yes	AAGAGGTCCCTGATTCAA
10534	Yes	AAGAGGTCCCTGATTCAAA
10535	Yes	AAGAGGTCCCTGATTCAAAT

10536	Yes	AAGAGGTCCCTGATTCAAATC
10537	Yes	AAGAGGTCCCTGATTCAAATCC
10538	Yes	AAGAGGTCCCTGATTCAAATCCA
10539	Yes	AAGAGGTCCCTGATTCAAATCCAG
10540	Yes	AAGAGGTCCCTGATTCAAATCCAGG
10541	Yes	AAGAGGTCCCTGATTCAAATCCAGGT
10542	Yes	AAGAGGTCCCTGATTCAAATCCAGGTG
10543	Yes	AAGAGGTCCCTGATTCAAATCCAGGTGC
10544	Yes	AAGAGGTCCCTGATTCAAATCCAGGTGCC
10545	Yes	AAGAGGTCCCTGATTCAAATCCAGGTGCCC
10546	Yes	AAGAGGTCCCTGATTCAAATCCAGGTGCCCC
10547	Yes	AAGAGGTCCCTGATTCAAATCCAGGTGCCCCC
10548	Yes	AAGAGGTCCCTGATTCAAATCCAGGTGCCCCCT
10549	Yes	AAGAGGTCCCTGATTCAAATCCAGGTGCCCCCTC
10550	Yes	AAGAGGTCCCTGATTCAAATCCAGGTGCCCCCTCC
10551	Yes	AAGAGGTCCCTGATTCAAATCCAGGTGCCCCCTCCA
10552	No	AAGAGGTCCCTGGATC
10553	Yes	AAGAGGTCCCTGGATCA
10554	Yes	AAGAGGTCCCTGGATCAA
10555	Yes	AAGAGGTCCCTGGATCAAC
10556	Yes	AAGAGGTCCCTGGATCAACT
10557	Yes	AAGAGGTCCCTGGATCAACTC
10558	Yes	AAGAGGTCCCTGGATCAACTCT
10559	Yes	AAGAGGTCCCTGGATCAACTCTG
10560	Yes	AAGAGGTCCCTGGATCAACTCTGG
10561	Yes	AAGAGGTCCCTGGATCAACTCTGGG
10562	Yes	AAGAGGTCCCTGGATCAACTCTGGGT
10563	Yes	AAGAGGTCCCTGGATCAACTCTGGGTG
10564	Yes	AAGAGGTCCCTGGATCAACTCTGGGTGC
10565	Yes	AAGAGGTCCCTGGATCAACTCTGGGTGCC
10566	Yes	AAGAGGTCCCTGGATCAACTCTGGGTGCCC
10567	Yes	AAGAGGTCCCTGGATCAACTCTGGGTGCCCC
10568	Yes	AAGAGGTCCCTGGATCAACTCTGGGTGCCCCC
10569	Yes	AAGAGGTCCCTGGATCAACTCTGGGTGCCCCCT
10570	Yes	AAGAGGTCCCTGGATCAACTCTGGGTGCCCCCTT
10571	Yes	AAGAGGTCCCTGGATCAACTCTGGGTGCCCCCTTT
10572	Yes	AAGAGGTCCCTGGATCAACTCTGGGTGCCCCCTTTA
10573	Yes	AAGAGGTCCCTGGATCAACTCTGGGTGCCCCCTTTAA
10574	Yes	AAGAGGTCCCTGGATCAACTCTGGGTGCCCCCTTTAAC
10575	Yes	AAGAGGTCCCTGGATCAACTCTGGGTGCCCCCTTTAACC
10576	Yes	AAGAGGTCCCTGGATCAACTCTGGGTGCCCCCTTTAACCA
10577	No	AAGAGGTCCCTGGTTC

10578	Yes	AAGAGGTCCCTGGTTCA
10579	Yes	AAGAGGTCCCTGGTTCAA
10580	Yes	AAGAGGTCCCTGGTTCAAA
10581	Yes	AAGAGGTCCCTGGTTCAAAT
10582	Yes	AAGAGGTCCCTGGTTCAAATC
10583	Yes	AAGAGGTCCCTGGTTCAAATCC
10584	Yes	AAGAGGTCCCTGGTTCAAATCCA
10585	Yes	AAGAGGTCCCTGGTTCAAATCCAG
10586	Yes	AAGAGGTCCCTGGTTCAAATCCAGG
10587	Yes	AAGAGGTCCCTGGTTCAAATCCAGGT
10588	Yes	AAGAGGTCCCTGGTTCAAATCCAGGTG
10589	Yes	AAGAGGTCCCTGGTTCAAATCCAGGTGC
10590	Yes	AAGAGGTCCCTGGTTCAAATCCAGGTGCC
10591	Yes	AAGAGGTCCCTGGTTCAAATCCAGGTGCCC
10592	Yes	AAGAGGTCCCTGGTTCAAATCCAGGTGCCCC
10593	Yes	AAGAGGTCCCTGGTTCAAATCCAGGTGCCCCC
10594	Yes	AAGAGGTCCCTGGTTCAAATCCAGGTGCCCCCC
10595	Yes	AAGAGGTCCCTGGTTCAAATCCAGGTGCCCCCCC
10596	Yes	AAGAGGTCCCTGGTTCAAATCCAGGTGCCCCCCCC
10597	Yes	AAGAGGTCCCTGGTTCAAATCCAGGTGCCCCCCCCA
10598	Yes	AAGAGGTCCCTGGTTCAAATCCAGGTGCCCCCT
10599	Yes	AAGAGGTCCCTGGTTCAAATCCAGGTGCCCCCTC
10600	Yes	AAGAGGTCCCTGGTTCAAATCCAGGTGCCCCCTCC
10601	Yes	AAGAGGTCCCTGGTTCAAATCCAGGTGCCCCCTCCA
10602	Yes	AAGAGGTCCCTGGTTCAAATCCAGGTT
10603	Yes	AAGAGGTCCCTGGTTCAAATCCAGGTTC
10604	Yes	AAGAGGTCCCTGGTTCAAATCCAGGTTCT
10605	Yes	AAGAGGTCCCTGGTTCAAATCCAGGTTCTC
10606	Yes	AAGAGGTCCCTGGTTCAAATCCAGGTTCTCC
10607	Yes	AAGAGGTCCCTGGTTCAAATCCAGGTTCTCCC
10608	Yes	AAGAGGTCCCTGGTTCAAATCCAGGTTCTCCCT
10609	Yes	AAGAGGTCCCTGGTTCAAATCCAGGTTCTCCCTC
10610	Yes	AAGAGGTCCCTGGTTCAAATCCAGGTTCTCCCTCC
10611	Yes	AAGAGGTCCCTGGTTCAAATCCAGGTTCTCCCTCCA
10612	Yes	AAGAGGTCCCTGGTTCAAATCCG
10613	Yes	AAGAGGTCCCTGGTTCAAATCCGG
10614	Yes	AAGAGGTCCCTGGTTCAAATCCGGG
10615	Yes	AAGAGGTCCCTGGTTCAAATCCGGGT
10616	Yes	AAGAGGTCCCTGGTTCAAATCCGGGTG
10617	Yes	AAGAGGTCCCTGGTTCAAATCCGGGTGC
10618	Yes	AAGAGGTCCCTGGTTCAAATCCGGGTGCC
10619	Yes	AAGAGGTCCCTGGTTCAAATCCGGGTGCCC

10620	Yes	AAGAGGTCCCTGGTTCAAATCCGGGTGCCCC
10621	Yes	AAGAGGTCCCTGGTTCAAATCCGGGTGCCCCC
10622	Yes	AAGAGGTCCCTGGTTCAAATCCGGGTGCCCCCT
10623	Yes	AAGAGGTCCCTGGTTCAAATCCGGGTGCCCCCTC
10624	Yes	AAGAGGTCCCTGGTTCAAATCCGGGTGCCCCCTCC
10625	Yes	AAGAGGTCCCTGGTTCAAATCCGGGTGCCCCCTCCA
10626	No	AAGAGGTCCTGGTTCA
10627	Yes	AAGAGGTCCTGGTTCAA
10628	Yes	AAGAGGTCCTGGTTCAAA
10629	Yes	AAGAGGTCCTGGTTCAAAT
10630	Yes	AAGAGGTCCTGGTTCAAATC
10631	Yes	AAGAGGTCCTGGTTCAAATCC
10632	Yes	AAGAGGTCCTGGTTCAAATCCC
10633	Yes	AAGAGGTCCTGGTTCAAATCCCA
10634	Yes	AAGAGGTCCTGGTTCAAATCCCAG
10635	Yes	AAGAGGTCCTGGTTCAAATCCCAGA
10636	Yes	AAGAGGTCCTGGTTCAAATCCCAGAG
10637	Yes	AAGAGGTCCTGGTTCAAATCCCAGAGG
10638	Yes	AAGAGGTCCTGGTTCAAATCCCAGAGGA
10639	Yes	AAGAGGTCCTGGTTCAAATCCCAGAGGAG
10640	Yes	AAGAGGTCCTGGTTCAAATCCCAGAGGAGC
10641	Yes	AAGAGGTCCTGGTTCAAATCCCAGAGGAGCC
10642	Yes	AAGAGGTCCTGGTTCAAATCCCAGAGGAGCCC
10643	Yes	AAGAGGTCCTGGTTCAAATCCCAGAGGAGCCCC
10644	Yes	AAGAGGTCCTGGTTCAAATCCCAGAGGAGCCCCC
10645	Yes	AAGAGGTCCTGGTTCAAATCCCAGAGGAGCCCCCA
10646	No	AAGAGGTCTCTGGTTC
10647	Yes	AAGAGGTCTCTGGTTCA
10648	Yes	AAGAGGTCTCTGGTTCAA
10649	Yes	AAGAGGTCTCTGGTTCAAA
10650	Yes	AAGAGGTCTCTGGTTCAAAT
10651	Yes	AAGAGGTCTCTGGTTCAAATC
10652	Yes	AAGAGGTCTCTGGTTCAAATCC
10653	Yes	AAGAGGTCTCTGGTTCAAATCCA
10654	Yes	AAGAGGTCTCTGGTTCAAATCCAG
10655	Yes	AAGAGGTCTCTGGTTCAAATCCAGG
10656	Yes	AAGAGGTCTCTGGTTCAAATCCAGGT
10657	Yes	AAGAGGTCTCTGGTTCAAATCCAGGTG
10658	Yes	AAGAGGTCTCTGGTTCAAATCCAGGTGC
10659	Yes	AAGAGGTCTCTGGTTCAAATCCAGGTGCC
10660	Yes	AAGAGGTCTCTGGTTCAAATCCAGGTGCCC
10661	Yes	AAGAGGTCTCTGGTTCAAATCCAGGTGCCCC

10662	Yes	AAGAGGTCTCTGGTTCAAATCCAGGTGCCCCC
10663	Yes	AAGAGGTCTCTGGTTCAAATCCAGGTGCCCCCT
10664	Yes	AAGAGGTCTCTGGTTCAAATCCAGGTGCCCCCTC
10665	Yes	AAGAGGTCTCTGGTTCAAATCCAGGTGCCCCCTCC
10666	Yes	AAGAGGTCTCTGGTTCAAATCCAGGTGCCCCCTCCA
10667	No	AAGAGTTACTTTGATA
10668	No	AAGAGTTACTTTGATAG
10669	No	AAGAGTTACTTTGATAGA
10670	No	AAGAGTTACTTTGATAGAG
10671	No	AAGAGTTACTTTGATAGAGT
10672	No	AAGAGTTACTTTGATAGAGTA
10673	No	AAGAGTTACTTTGATAGAGTAA
10674	No	AAGAGTTACTTTGATAGAGTAAA
10675	No	AAGAGTTACTTTGATAGAGTAAAT
10676	No	AAGAGTTACTTTGATAGAGTAAATA
10677	No	AAGAGTTACTTTGATAGAGTAAATAA
10678	No	AAGAGTTACTTTGATAGAGTAAATAAT
10679	No	AAGAGTTACTTTGATAGAGTAAATAATA
10680	No	AAGAGTTACTTTGATAGAGTAAATAATAG
10681	No	AAGAGTTACTTTGATAGAGTAAATAATAGG
10682	No	AAGAGTTACTTTGATAGAGTAAATAATAGGA
10683	No	AAGAGTTACTTTGATAGAGTAAATAATAGGAG
10684	Yes	AAGAGTTACTTTGATAGAGTAAATAATAGGAGC
10685	Yes	AAGAGTTACTTTGATAGAGTAAATAATAGGAGCT
10686	Yes	AAGAGTTACTTTGATAGAGTAAATAATAGGAGCTT
10687	Yes	AAGAGTTACTTTGATAGAGTAAATAATAGGAGCTTA
10688	Yes	AAGAGTTACTTTGATAGAGTAAATAATAGGAGCTTAA
10689	Yes	AAGAGTTACTTTGATAGAGTAAATAATAGGAGCTTAAA
10690	Yes	AAGAGTTACTTTGATAGAGTAAATAATAGGAGCTTAAAC
10691	Yes	AAGAGTTACTTTGATAGAGTAAATAATAGGAGCTTAAACC
10692	Yes	AAGAGTTACTTTGATAGAGTAAATAATAGGAGCTTAAACCC
10693	Yes	AAGAGTTACTTTGATAGAGTAAATAATAGGAGCTTAAACCCC
10694	Yes	AAGAGTTACTTTGATAGAGTAAATAATAGGAGCTTAAACCCCC
10695	Yes	AAGAGTTACTTTGATAGAGTAAATAATAGGAGCTTAAACCCCCT
10696	Yes	AAGAGTTACTTTGATAGAGTAAATAATAGGAGCTTAAACCCCCTT
10697	Yes	AAGAGTTACTTTGATAGAGTAAATAATAGGAGCTTAAACCCCCTTA
10698	Yes	AAGAGTTACTTTGATAGAGTAAATAATAGGAGCTTAAACCCCCTTAT
10699	Yes	AAGAGTTACTTTGATAGAGTAAATAATAGGAGCTTAAACCCCCTTATT
10700	Yes	AAGAGTTACTTTGATAGAGTAAATAATAGGAGCTTAAACCCCCTTATTT
10701	Yes	AAGAGTTACTTTGATAGAGTAAATAATAGGAGCTTAAACCCCCTTATTTC
10702	No	AAGATACAAAGTTCCT
10703	No	AAGATACAAAGTTCCTT

10704	Yes	AAGATACAAAGTTCCTTG
10705	Yes	AAGATACAAAGTTCCTTGG
10706	Yes	AAGATACAAAGTTCCTTGGC
10707	Yes	AAGATACAAAGTTCCTTGGCT
10708	Yes	AAGATACAAAGTTCCTTGGCTC
10709	Yes	AAGATACAAAGTTCCTTGGCTCA
10710	Yes	AAGATACAAAGTTCCTTGGCTCAA
10711	Yes	AAGATACAAAGTTCCTTGGCTCAAT
10712	Yes	AAGATACAAAGTTCCTTGGCTCAATC
10713	Yes	AAGATACAAAGTTCCTTGGCTCAATCC
10714	Yes	AAGATACAAAGTTCCTTGGCTCAATCCA
10715	Yes	AAGATACAAAGTTCCTTGGCTCAATCCAG
10716	Yes	AAGATACAAAGTTCCTTGGCTCAATCCAGA
10717	Yes	AAGATACAAAGTTCCTTGGCTCAATCCAGAG
10718	Yes	AAGATACAAAGTTCCTTGGCTCAATCCAGAGT
10719	Yes	AAGATACAAAGTTCCTTGGCTCAATCCAGAGTT
10720	Yes	AAGATACAAAGTTCCTTGGCTCAATCCAGAGTTT
10721	Yes	AAGATACAAAGTTCCTTGGCTCAATCCAGAGTTTG
10722	Yes	AAGATACAAAGTTCCTTGGCTCAATCCAGAGTTTGG
10723	Yes	AAGATACAAAGTTCCTTGGCTCAATCCAGAGTTTGGG
10724	Yes	AAGATACAAAGTTCCTTGGCTCAATCCAGAGTTTGGGG
10725	Yes	AAGATACAAAGTTCCTTGGCTCAATCCAGAGTTTGGGGG
10726	Yes	AAGATACAAAGTTCCTTGGCTCAATCCAGAGTTTGGGGGC
10727	Yes	AAGATACAAAGTTCCTTGGCTCAATCCAGAGTTTGGGGGCC
10728	Yes	AAGATACAAAGTTCCTTGGCTCAATCCAGAGTTTGGGGGCCA
10729	Yes	AAGATCACGGGTTCGA
10730	Yes	AAGATCACGGGTTCGAA
10731	Yes	AAGATCACGGGTTCGAAC
10732	Yes	AAGATCACGGGTTCGAACC
10733	Yes	AAGATCACGGGTTCGAACCC
10734	Yes	AAGATCACGGGTTCGAACCCC
10735	Yes	AAGATCACGGGTTCGAACCCCG
10736	Yes	AAGATCACGGGTTCGAACCCCGT
10737	Yes	AAGATCACGGGTTCGAACCCCGTC
10738	Yes	AAGATCACGGGTTCGAACCCCGTCC
10739	Yes	AAGATCACGGGTTCGAACCCCGTCCG
10740	Yes	AAGATCACGGGTTCGAACCCCGTCCGT
10741	Yes	AAGATCACGGGTTCGAACCCCGTCCGTG
10742	Yes	AAGATCACGGGTTCGAACCCCGTCCGTGC
10743	Yes	AAGATCACGGGTTCGAACCCCGTCCGTGCC
10744	Yes	AAGATCACGGGTTCGAACCCCGTCCGTGCCT
10745	Yes	AAGATCACGGGTTCGAACCCCGTCCGTGCCTC

10746	Yes	AAGATCACGGGTTCGAACCCCGTCCGTGCCTCC
10747	Yes	AAGATCACGGGTTCGAACCCCGTCCGTGCCTCCA
10748	No	AAGATCATGAACTCTG
10749	No	AAGATCATGAACTCTGA
10750	No	AAGATCATGAACTCTGAA
10751	No	AAGATCATGAACTCTGAAG
10752	Yes	AAGATCATGAACTCTGAAGT
10753	Yes	AAGATCATGAACTCTGAAGTC
10754	Yes	AAGATCATGAACTCTGAAGTCA
10755	Yes	AAGATCATGAACTCTGAAGTCAG
10756	Yes	AAGATCATGAACTCTGAAGTCAGA
10757	Yes	AAGATCATGAACTCTGAAGTCAGAG
10758	Yes	AAGATCATGAACTCTGAAGTCAGAGA
10759	Yes	AAGATCATGAACTCTGAAGTCAGAGAT
10760	Yes	AAGATCATGAACTCTGAAGTCAGAGATA
10761	Yes	AAGATCATGAACTCTGAAGTCAGAGATAC
10762	Yes	AAGATCATGAACTCTGAAGTCAGAGATACT
10763	Yes	AAGATCATGAACTCTGAAGTCAGAGATACTT
10764	Yes	AAGATCATGAACTCTGAAGTCAGAGATACTTG
10765	Yes	AAGATCATGAACTCTGAAGTCAGAGATACTTGA
10766	Yes	AAGATCATGAACTCTGAAGTCAGAGATACTTGAA
10767	Yes	AAGATCATGAACTCTGAAGTCAGAGATACTTGAAT
10768	Yes	AAGATCATGAACTCTGAAGTCAGAGATACTTGAATT
10769	Yes	AAGATCATGAACTCTGAAGTCAGAGATACTTGAATTT
10770	Yes	AAGATCATGAACTCTGAAGTCAGAGATACTTGAATTTG
10771	Yes	AAGATCATGAACTCTGAAGTCAGAGATACTTGAATTTGA
10772	Yes	AAGATCATGAACTCTGAAGTCAGAGATACTTGAATTTGAA
10773	Yes	AAGATCATGAACTCTGAAGTCAGAGATACTTGAATTTGAAT
10774	Yes	AAGATCATGAACTCTGAAGTCAGAGATACTTGAATTTGAATG
10775	Yes	AAGATCATGAACTCTGAAGTCAGAGATACTTGAATTTGAATGC
10776	Yes	AAGATCATGAACTCTGAAGTCAGAGATACTTGAATTTGAATGCT
10777	Yes	AAGATCATGAACTCTGAAGTCAGAGATACTTGAATTTGAATGCTG
10778	Yes	AAGATCATGAACTCTGAAGTCAGAGATACTTGAATTTGAATGCTGG
10779	Yes	AAGATCATGAACTCTGAAGTCAGAGATACTTGAATTTGAATGCTGGT
10780	Yes	AAGATCATGAACTCTGAAGTCAGAGATACTTGAATTTGAATGCTGGTT
10781	Yes	AAGATCATGAACTCTGAAGTCAGAGATACTTGAATTTGAATGCTGGTTC
10782	Yes	AAGATCATGAACTCTGAAGTCAGAGATACTTGAATTTGAATGCTGGTTCT
10783	No	AAGATCCAATGGACAG
10784	Yes	AAGATCCAATGGACAGG
10785	Yes	AAGATCCAATGGACAGGT
10786	Yes	AAGATCCAATGGACAGGTG
10787	Yes	AAGATCCAATGGACAGGTGT

10788	Yes	AAGATCCAATGGACAGGTGTC
10789	Yes	AAGATCCAATGGACAGGTGTCC
10790	Yes	AAGATCCAATGGACAGGTGTCCG
10791	Yes	AAGATCCAATGGACAGGTGTCCGC
10792	Yes	AAGATCCAATGGACAGGTGTCCGCG
10793	Yes	AAGATCCAATGGACAGGTGTCCGCGT
10794	Yes	AAGATCCAATGGACAGGTGTCCGCGTG
10795	Yes	AAGATCCAATGGACAGGTGTCCGCGTGG
10796	Yes	AAGATCCAATGGACAGGTGTCCGCGTGGG
10797	Yes	AAGATCCAATGGACAGGTGTCCGCGTGGGT
10798	Yes	AAGATCCAATGGACAGGTGTCCGCGTGGGTT
10799	Yes	AAGATCCAATGGACAGGTGTCCGCGTGGGTTC
10800	Yes	AAGATCCAATGGACAGGTGTCCGCGTGGGTTCG
10801	Yes	AAGATCCAATGGACAGGTGTCCGCGTGGGTTCGA
10802	Yes	AAGATCCAATGGACAGGTGTCCGCGTGGGTTCGAG
10803	Yes	AAGATCCAATGGACAGGTGTCCGCGTGGGTTCGAGC
10804	Yes	AAGATCCAATGGACAGGTGTCCGCGTGGGTTCGAGCC
10805	Yes	AAGATCCAATGGACAGGTGTCCGCGTGGGTTCGAGCCC
10806	Yes	AAGATCCAATGGACAGGTGTCCGCGTGGGTTCGAGCCCC
10807	Yes	AAGATCCAATGGACAGGTGTCCGCGTGGGTTCGAGCCCCA
10808	Yes	AAGATCCAATGGACAGGTGTCCGCGTGGGTTCGAGCCCCAC
10809	Yes	AAGATCCAATGGACAGGTGTCCGCGTGGGTTCGAGCCCCACT
10810	Yes	AAGATCCAATGGACAGGTGTCCGCGTGGGTTCGAGCCCCACTC
10811	Yes	AAGATCCAATGGACAGGTGTCCGCGTGGGTTCGAGCCCCACTCC
10812	Yes	AAGATCCAATGGACAGGTGTCCGCGTGGGTTCGAGCCCCACTCCC
10813	Yes	AAGATCCAATGGACAGGTGTCCGCGTGGGTTCGAGCCCCACTCCCG
10814	Yes	AAGATCCAATGGACAGGTGTCCGCGTGGGTTCGAGCCCCACTCCCGG
10815	Yes	AAGATCCAATGGACAGGTGTCCGCGTGGGTTCGAGCCCCACTCCCGGT
10816	Yes	AAGATCCAATGGACAGGTGTCCGCGTGGGTTCGAGCCCCACTCCCGGTA
10817	Yes	AAGATCCAATGGACAGGTGTCCGCGTGGGTTCGAGCCCCACTCCCGGTAC
10818	No	AAGATCCAATGGACAT
10819	No	AAGATCCAATGGACATA
10820	No	AAGATCCAATGGACATAT
10821	No	AAGATCCAATGGACATATG
10822	No	AAGATCCAATGGACATATGT
10823	No	AAGATCCAATGGACATATGTC
10824	Yes	AAGATCCAATGGACATATGTCC
10825	Yes	AAGATCCAATGGACATATGTCCG
10826	Yes	AAGATCCAATGGACATATGTCCGC
10827	Yes	AAGATCCAATGGACATATGTCCGCG
10828	Yes	AAGATCCAATGGACATATGTCCGCGT
10829	Yes	AAGATCCAATGGACATATGTCCGCGTG

10830	Yes	AAGATCCAATGGACATATGTCCGCGTGG
10831	Yes	AAGATCCAATGGACATATGTCCGCGTGGG
10832	Yes	AAGATCCAATGGACATATGTCCGCGTGGGT
10833	Yes	AAGATCCAATGGACATATGTCCGCGTGGGTT
10834	Yes	AAGATCCAATGGACATATGTCCGCGTGGGTTC
10835	Yes	AAGATCCAATGGACATATGTCCGCGTGGGTTCG
10836	Yes	AAGATCCAATGGACATATGTCCGCGTGGGTTCGA
10837	Yes	AAGATCCAATGGACATATGTCCGCGTGGGTTCGAA
10838	Yes	AAGATCCAATGGACATATGTCCGCGTGGGTTCGAAC
10839	Yes	AAGATCCAATGGACATATGTCCGCGTGGGTTCGAACC
10840	Yes	AAGATCCAATGGACATATGTCCGCGTGGGTTCGAACCC
10841	Yes	AAGATCCAATGGACATATGTCCGCGTGGGTTCGAACCCC
10842	Yes	AAGATCCAATGGACATATGTCCGCGTGGGTTCGAACCCCA
10843	Yes	AAGATCCAATGGACATATGTCCGCGTGGGTTCGAACCCCAC
10844	Yes	AAGATCCAATGGACATATGTCCGCGTGGGTTCGAACCCCACT
10845	Yes	AAGATCCAATGGACATATGTCCGCGTGGGTTCGAACCCCACTC
10846	Yes	AAGATCCAATGGACATATGTCCGCGTGGGTTCGAACCCCACTCC
10847	Yes	AAGATCCAATGGACATATGTCCGCGTGGGTTCGAACCCCACTCCT
10848	Yes	AAGATCCAATGGACATATGTCCGCGTGGGTTCGAACCCCACTCCTG
10849	Yes	AAGATCCAATGGACATATGTCCGCGTGGGTTCGAACCCCACTCCTGG
10850	Yes	AAGATCCAATGGACATATGTCCGCGTGGGTTCGAACCCCACTCCTGGT
10851	Yes	AAGATCCAATGGACATATGTCCGCGTGGGTTCGAACCCCACTCCTGGTA
10852	Yes	AAGATCCAATGGACATATGTCCGCGTGGGTTCGAACCCCACTCCTGGTAC
10853	No	AAGATCCAATGGAGTA
10854	Yes	AAGATCCAATGGAGTAG
10855	Yes	AAGATCCAATGGAGTAGT
10856	Yes	AAGATCCAATGGAGTAGTG
10857	Yes	AAGATCCAATGGAGTAGTGG
10858	Yes	AAGATCCAATGGAGTAGTGGC
10859	Yes	AAGATCCAATGGAGTAGTGGCT
10860	Yes	AAGATCCAATGGAGTAGTGGCTG
10861	Yes	AAGATCCAATGGAGTAGTGGCTGT
10862	Yes	AAGATCCAATGGAGTAGTGGCTGTG
10863	Yes	AAGATCCAATGGAGTAGTGGCTGTGT
10864	Yes	AAGATCCAATGGAGTAGTGGCTGTGTG
10865	Yes	AAGATCCAATGGAGTAGTGGCTGTGTGG
10866	Yes	AAGATCCAATGGAGTAGTGGCTGTGTGGG
10867	Yes	AAGATCCAATGGAGTAGTGGCTGTGTGGGT
10868	Yes	AAGATCCAATGGAGTAGTGGCTGTGTGGGTT
10869	Yes	AAGATCCAATGGAGTAGTGGCTGTGTGGGTTT
10870	Yes	AAGATCCAATGGAGTAGTGGCTGTGTGGGTTTA
10871	Yes	AAGATCCAATGGAGTAGTGGCTGTGTGGGTTTAA

10872	Yes	AAGATCCAATGGAGTAGTGGCTGTGTGGGTTTAAA
10873	Yes	AAGATCCAATGGAGTAGTGGCTGTGTGGGTTTAAAC
10874	Yes	AAGATCCAATGGAGTAGTGGCTGTGTGGGTTTAAACC
10875	Yes	AAGATCCAATGGAGTAGTGGCTGTGTGGGTTTAAACCC
10876	Yes	AAGATCCAATGGAGTAGTGGCTGTGTGGGTTTAAACCCC
10877	Yes	AAGATCCAATGGAGTAGTGGCTGTGTGGGTTTAAACCCCA
10878	Yes	AAGATCCAATGGAGTAGTGGCTGTGTGGGTTTAAACCCCAC
10879	Yes	AAGATCCAATGGAGTAGTGGCTGTGTGGGTTTAAACCCCACT
10880	Yes	AAGATCCAATGGAGTAGTGGCTGTGTGGGTTTAAACCCCACTA
10881	Yes	AAGATCCAATGGAGTAGTGGCTGTGTGGGTTTAAACCCCACTAC
10882	Yes	AAGATCCAATGGAGTAGTGGCTGTGTGGGTTTAAACCCCACTACT
10883	Yes	AAGATCCAATGGAGTAGTGGCTGTGTGGGTTTAAACCCCACTACTG
10884	Yes	AAGATCCAATGGAGTAGTGGCTGTGTGGGTTTAAACCCCACTACTGG
10885	Yes	AAGATCCAATGGAGTAGTGGCTGTGTGGGTTTAAACCCCACTACTGGT
10886	Yes	AAGATCCAATGGAGTAGTGGCTGTGTGGGTTTAAACCCCACTACTGGTA
10887	Yes	AAGATCCAATGGAGTAGTGGCTGTGTGGGTTTAAACCCCACTACTGGTAC
10888	No	AAGATCCAATGGATTC
10889	No	AAGATCCAATGGATTCA
10890	No	AAGATCCAATGGATTCAT
10891	Yes	AAGATCCAATGGATTCATA
10892	Yes	AAGATCCAATGGATTCATAT
10893	Yes	AAGATCCAATGGATTCATATC
10894	Yes	AAGATCCAATGGATTCATATCC
10895	Yes	AAGATCCAATGGATTCATATCCG
10896	Yes	AAGATCCAATGGATTCATATCCGC
10897	Yes	AAGATCCAATGGATTCATATCCGCG
10898	Yes	AAGATCCAATGGATTCATATCCGCGT
10899	Yes	AAGATCCAATGGATTCATATCCGCGTG
10900	Yes	AAGATCCAATGGATTCATATCCGCGTGG
10901	Yes	AAGATCCAATGGATTCATATCCGCGTGGG
10902	Yes	AAGATCCAATGGATTCATATCCGCGTGGGT
10903	Yes	AAGATCCAATGGATTCATATCCGCGTGGGTT
10904	Yes	AAGATCCAATGGATTCATATCCGCGTGGGTTC
10905	Yes	AAGATCCAATGGATTCATATCCGCGTGGGTTCG
10906	Yes	AAGATCCAATGGATTCATATCCGCGTGGGTTCGA
10907	Yes	AAGATCCAATGGATTCATATCCGCGTGGGTTCGAA
10908	Yes	AAGATCCAATGGATTCATATCCGCGTGGGTTCGAAC
10909	Yes	AAGATCCAATGGATTCATATCCGCGTGGGTTCGAACC
10910	Yes	AAGATCCAATGGATTCATATCCGCGTGGGTTCGAACCC
10911	Yes	AAGATCCAATGGATTCATATCCGCGTGGGTTCGAACCCC
10912	Yes	AAGATCCAATGGATTCATATCCGCGTGGGTTCGAACCCCA
10913	Yes	AAGATCCAATGGATTCATATCCGCGTGGGTTCGAACCCCAC

10914	Yes	AAGATCCAATGGATTCATATCCGCGTGGGTTCGAACCCCACT
10915	Yes	AAGATCCAATGGATTCATATCCGCGTGGGTTCGAACCCCACTT
10916	Yes	AAGATCCAATGGATTCATATCCGCGTGGGTTCGAACCCCACTTC
10917	Yes	AAGATCCAATGGATTCATATCCGCGTGGGTTCGAACCCCACTTCT
10918	Yes	AAGATCCAATGGATTCATATCCGCGTGGGTTCGAACCCCACTTCTG
10919	Yes	AAGATCCAATGGATTCATATCCGCGTGGGTTCGAACCCCACTTCTGG
10920	Yes	AAGATCCAATGGATTCATATCCGCGTGGGTTCGAACCCCACTTCTGGT
10921	Yes	AAGATCCAATGGATTCATATCCGCGTGGGTTCGAACCCCACTTCTGGTA
10922	Yes	AAGATCCAATGGATTCATATCCGCGTGGGTTCGAACCCCACTTCTGGTAC
10923	Yes	AAGATCCAATGGGCTG
10924	Yes	AAGATCCAATGGGCTGG
10925	Yes	AAGATCCAATGGGCTGGT
10926	Yes	AAGATCCAATGGGCTGGTG
10927	Yes	AAGATCCAATGGGCTGGTGC
10928	Yes	AAGATCCAATGGGCTGGTGCC
10929	Yes	AAGATCCAATGGGCTGGTGCCC
10930	Yes	AAGATCCAATGGGCTGGTGCCCG
10931	Yes	AAGATCCAATGGGCTGGTGCCCGC
10932	Yes	AAGATCCAATGGGCTGGTGCCCGCG
10933	Yes	AAGATCCAATGGGCTGGTGCCCGCGT
10934	Yes	AAGATCCAATGGGCTGGTGCCCGCGTG
10935	Yes	AAGATCCAATGGGCTGGTGCCCGCGTGG
10936	Yes	AAGATCCAATGGGCTGGTGCCCGCGTGGG
10937	Yes	AAGATCCAATGGGCTGGTGCCCGCGTGGGT
10938	Yes	AAGATCCAATGGGCTGGTGCCCGCGTGGGTT
10939	Yes	AAGATCCAATGGGCTGGTGCCCGCGTGGGTTC
10940	Yes	AAGATCCAATGGGCTGGTGCCCGCGTGGGTTCG
10941	Yes	AAGATCCAATGGGCTGGTGCCCGCGTGGGTTCGA
10942	Yes	AAGATCCAATGGGCTGGTGCCCGCGTGGGTTCGAA
10943	Yes	AAGATCCAATGGGCTGGTGCCCGCGTGGGTTCGAAC
10944	Yes	AAGATCCAATGGGCTGGTGCCCGCGTGGGTTCGAACC
10945	Yes	AAGATCCAATGGGCTGGTGCCCGCGTGGGTTCGAACCC
10946	Yes	AAGATCCAATGGGCTGGTGCCCGCGTGGGTTCGAACCCC
10947	Yes	AAGATCCAATGGGCTGGTGCCCGCGTGGGTTCGAACCCCA
10948	Yes	AAGATCCAATGGGCTGGTGCCCGCGTGGGTTCGAACCCCAC
10949	Yes	AAGATCCAATGGGCTGGTGCCCGCGTGGGTTCGAACCCCACT
10950	Yes	AAGATCCAATGGGCTGGTGCCCGCGTGGGTTCGAACCCCACTC
10951	Yes	AAGATCCAATGGGCTGGTGCCCGCGTGGGTTCGAACCCCACTCT
10952	Yes	AAGATCCAATGGGCTGGTGCCCGCGTGGGTTCGAACCCCACTCTC
10953	Yes	AAGATCCAATGGGCTGGTGCCCGCGTGGGTTCGAACCCCACTCTCG
10954	Yes	AAGATCCAATGGGCTGGTGCCCGCGTGGGTTCGAACCCCACTCTCGG
10955	Yes	AAGATCCAATGGGCTGGTGCCCGCGTGGGTTCGAACCCCACTCTCGGT

10956	Yes	AAGATCCAATGGGCTGGTGCCCGCGTGGGTTCGAACCCCACTCTCGGTA
10957	Yes	AAGATCCAATGGGCTGGTGCCCGCGTGGGTTCGAACCCCACTCTCGGTAC
10958	Yes	AAGATCGCGGGTTCGA
10959	Yes	AAGATCGCGGGTTCGAA
10960	Yes	AAGATCGCGGGTTCGAAC
10961	Yes	AAGATCGCGGGTTCGAACC
10962	Yes	AAGATCGCGGGTTCGAACCC
10963	Yes	AAGATCGCGGGTTCGAACCCC
10964	Yes	AAGATCGCGGGTTCGAACCCCG
10965	Yes	AAGATCGCGGGTTCGAACCCCGT
10966	Yes	AAGATCGCGGGTTCGAACCCCGTC
10967	Yes	AAGATCGCGGGTTCGAACCCCGTCC
10968	Yes	AAGATCGCGGGTTCGAACCCCGTCCG
10969	Yes	AAGATCGCGGGTTCGAACCCCGTCCGT
10970	Yes	AAGATCGCGGGTTCGAACCCCGTCCGTG
10971	Yes	AAGATCGCGGGTTCGAACCCCGTCCGTGC
10972	Yes	AAGATCGCGGGTTCGAACCCCGTCCGTGCC
10973	Yes	AAGATCGCGGGTTCGAACCCCGTCCGTGCCT
10974	Yes	AAGATCGCGGGTTCGAACCCCGTCCGTGCCTC
10975	Yes	AAGATCGCGGGTTCGAACCCCGTCCGTGCCTCC
10976	Yes	AAGATCGCGGGTTCGAACCCCGTCCGTGCCTCCA
10977	No	AAGATCTAAAGGAGAC
10978	Yes	AAGATCTAAAGGAGACT
10979	Yes	AAGATCTAAAGGAGACTG
10980	Yes	AAGATCTAAAGGAGACTGG
10981	Yes	AAGATCTAAAGGAGACTGGT
10982	Yes	AAGATCTAAAGGAGACTGGTT
10983	Yes	AAGATCTAAAGGAGACTGGTTC
10984	Yes	AAGATCTAAAGGAGACTGGTTCA
10985	Yes	AAGATCTAAAGGAGACTGGTTCAA
10986	Yes	AAGATCTAAAGGAGACTGGTTCAAT
10987	Yes	AAGATCTAAAGGAGACTGGTTCAATT
10988	Yes	AAGATCTAAAGGAGACTGGTTCAATTC
10989	Yes	AAGATCTAAAGGAGACTGGTTCAATTCT
10990	Yes	AAGATCTAAAGGAGACTGGTTCAATTCTG
10991	Yes	AAGATCTAAAGGAGACTGGTTCAATTCTGG
10992	Yes	AAGATCTAAAGGAGACTGGTTCAATTCTGGG
10993	Yes	AAGATCTAAAGGAGACTGGTTCAATTCTGGGT
10994	Yes	AAGATCTAAAGGAGACTGGTTCAATTCTGGGTT
10995	Yes	AAGATCTAAAGGAGACTGGTTCAATTCTGGGTTT
10996	Yes	AAGATCTAAAGGAGACTGGTTCAATTCTGGGTTTT
10997	Yes	AAGATCTAAAGGAGACTGGTTCAATTCTGGGTTTTG

10998	Yes	AAGATCTAAAGGAGACTGGTTCAATTCTGGGTTTTGG
10999	Yes	AAGATCTAAAGGAGACTGGTTCAATTCTGGGTTTTGGC
11000	Yes	AAGATCTAAAGGAGACTGGTTCAATTCTGGGTTTTGGCA
11001	Yes	AAGATCTAAAGGAGACTGGTTCAATTCTGGGTTTTGGCAC
11002	Yes	AAGATCTAAAGGAGACTGGTTCAATTCTGGGTTTTGGCACC
11003	Yes	AAGATCTAAAGGAGACTGGTTCAATTCTGGGTTTTGGCACCA
11004	No	AAGATCTAAAGGTCCC
11005	No	AAGATCTAAAGGTCCCT
11006	No	AAGATCTAAAGGTCCCTG
11007	No	AAGATCTAAAGGTCCCTGG
11008	No	AAGATCTAAAGGTCCCTGGT
11009	No	AAGATCTAAAGGTCCCTGGTT
11010	Yes	AAGATCTAAAGGTCCCTGGTTC
11011	Yes	AAGATCTAAAGGTCCCTGGTTCA
11012	Yes	AAGATCTAAAGGTCCCTGGTTCAA
11013	Yes	AAGATCTAAAGGTCCCTGGTTCAAT
11014	Yes	AAGATCTAAAGGTCCCTGGTTCAATC
11015	Yes	AAGATCTAAAGGTCCCTGGTTCAATCC
11016	Yes	AAGATCTAAAGGTCCCTGGTTCAATCCC
11017	Yes	AAGATCTAAAGGTCCCTGGTTCAATCCCG
11018	Yes	AAGATCTAAAGGTCCCTGGTTCAATCCCGG
11019	Yes	AAGATCTAAAGGTCCCTGGTTCAATCCCGGG
11020	Yes	AAGATCTAAAGGTCCCTGGTTCAATCCCGGGT
11021	Yes	AAGATCTAAAGGTCCCTGGTTCAATCCCGGGTT
11022	Yes	AAGATCTAAAGGTCCCTGGTTCAATCCCGGGTTT
11023	Yes	AAGATCTAAAGGTCCCTGGTTCAATCCCGGGTTTC
11024	Yes	AAGATCTAAAGGTCCCTGGTTCAATCCCGGGTTTCG
11025	Yes	AAGATCTAAAGGTCCCTGGTTCAATCCCGGGTTTCGG
11026	Yes	AAGATCTAAAGGTCCCTGGTTCAATCCCGGGTTTCGGC
11027	Yes	AAGATCTAAAGGTCCCTGGTTCAATCCCGGGTTTCGGCA
11028	Yes	AAGATCTAAAGGTCCCTGGTTCAATCCCGGGTTTCGGCAC
11029	Yes	AAGATCTAAAGGTCCCTGGTTCAATCCCGGGTTTCGGCACC
11030	Yes	AAGATCTAAAGGTCCCTGGTTCAATCCCGGGTTTCGGCACCA
11031	Yes	AAGATCTAAAGGTCCCTGGTTCG
11032	Yes	AAGATCTAAAGGTCCCTGGTTCGA
11033	Yes	AAGATCTAAAGGTCCCTGGTTCGAT
11034	Yes	AAGATCTAAAGGTCCCTGGTTCGATC
11035	Yes	AAGATCTAAAGGTCCCTGGTTCGATCC
11036	Yes	AAGATCTAAAGGTCCCTGGTTCGATCCC
11037	Yes	AAGATCTAAAGGTCCCTGGTTCGATCCCG
11038	Yes	AAGATCTAAAGGTCCCTGGTTCGATCCCGG
11039	Yes	AAGATCTAAAGGTCCCTGGTTCGATCCCGGG

11040	Yes	AAGATCTAAAGGTCCCTGGTTCGATCCCGGGT
11041	Yes	AAGATCTAAAGGTCCCTGGTTCGATCCCGGGTT
11042	Yes	AAGATCTAAAGGTCCCTGGTTCGATCCCGGGTTT
11043	Yes	AAGATCTAAAGGTCCCTGGTTCGATCCCGGGTTTC
11044	Yes	AAGATCTAAAGGTCCCTGGTTCGATCCCGGGTTTCA
11045	Yes	AAGATCTAAAGGTCCCTGGTTCGATCCCGGGTTTCAC
11046	Yes	AAGATCTAAAGGTCCCTGGTTCGATCCCGGGTTTCACC
11047	Yes	AAGATCTAAAGGTCCCTGGTTCGATCCCGGGTTTCACCA
11048	Yes	AAGATCTAAAGGTCCCTGGTTCGATCCCGGGTTTCACCAC
11049	Yes	AAGATCTAAAGGTCCCTGGTTCGATCCCGGGTTTCACCACC
11050	Yes	AAGATCTAAAGGTCCCTGGTTCGATCCCGGGTTTCACCACCA
11051	Yes	AAGATCTAAAGGTCCCTGGTTCGATCCCGGGTTTCG
11052	Yes	AAGATCTAAAGGTCCCTGGTTCGATCCCGGGTTTCGG
11053	Yes	AAGATCTAAAGGTCCCTGGTTCGATCCCGGGTTTCGGC
11054	Yes	AAGATCTAAAGGTCCCTGGTTCGATCCCGGGTTTCGGCA
11055	Yes	AAGATCTAAAGGTCCCTGGTTCGATCCCGGGTTTCGGCAC
11056	Yes	AAGATCTAAAGGTCCCTGGTTCGATCCCGGGTTTCGGCACC
11057	Yes	AAGATCTAAAGGTCCCTGGTTCGATCCCGGGTTTCGGCACCA
11058	No	AAGATCTAAAGGTCTC
11059	No	AAGATCTAAAGGTCTCT
11060	No	AAGATCTAAAGGTCTCTG
11061	Yes	AAGATCTAAAGGTCTCTGG
11062	Yes	AAGATCTAAAGGTCTCTGGT
11063	Yes	AAGATCTAAAGGTCTCTGGTT
11064	Yes	AAGATCTAAAGGTCTCTGGTTT
11065	Yes	AAGATCTAAAGGTCTCTGGTTTG
11066	Yes	AAGATCTAAAGGTCTCTGGTTTGA
11067	Yes	AAGATCTAAAGGTCTCTGGTTTGAT
11068	Yes	AAGATCTAAAGGTCTCTGGTTTGATC
11069	Yes	AAGATCTAAAGGTCTCTGGTTTGATCC
11070	Yes	AAGATCTAAAGGTCTCTGGTTTGATCCT
11071	Yes	AAGATCTAAAGGTCTCTGGTTTGATCCTG
11072	Yes	AAGATCTAAAGGTCTCTGGTTTGATCCTGG
11073	Yes	AAGATCTAAAGGTCTCTGGTTTGATCCTGGG
11074	Yes	AAGATCTAAAGGTCTCTGGTTTGATCCTGGGT
11075	Yes	AAGATCTAAAGGTCTCTGGTTTGATCCTGGGTT
11076	Yes	AAGATCTAAAGGTCTCTGGTTTGATCCTGGGTTT
11077	Yes	AAGATCTAAAGGTCTCTGGTTTGATCCTGGGTTTC
11078	Yes	AAGATCTAAAGGTCTCTGGTTTGATCCTGGGTTTCA
11079	Yes	AAGATCTAAAGGTCTCTGGTTTGATCCTGGGTTTCAG
11080	Yes	AAGATCTAAAGGTCTCTGGTTTGATCCTGGGTTTCAGA
11081	Yes	AAGATCTAAAGGTCTCTGGTTTGATCCTGGGTTTCAGAA

11082	Yes	AAGATCTAAAGGTCTCTGGTTTGATCCTGGGTTTCAGAAC
11083	Yes	AAGATCTAAAGGTCTCTGGTTTGATCCTGGGTTTCAGAACC
11084	Yes	AAGATCTAAAGGTCTCTGGTTTGATCCTGGGTTTCAGAACCA
11085	No	AAGATCTTAAAGGTCC
11086	Yes	AAGATCTTAAAGGTCCC
11087	Yes	AAGATCTTAAAGGTCCCT
11088	Yes	AAGATCTTAAAGGTCCCTG
11089	Yes	AAGATCTTAAAGGTCCCTGG
11090	Yes	AAGATCTTAAAGGTCCCTGGT
11091	Yes	AAGATCTTAAAGGTCCCTGGTT
11092	Yes	AAGATCTTAAAGGTCCCTGGTTC
11093	Yes	AAGATCTTAAAGGTCCCTGGTTCA
11094	Yes	AAGATCTTAAAGGTCCCTGGTTCAA
11095	Yes	AAGATCTTAAAGGTCCCTGGTTCAAT
11096	Yes	AAGATCTTAAAGGTCCCTGGTTCAATC
11097	Yes	AAGATCTTAAAGGTCCCTGGTTCAATCC
11098	Yes	AAGATCTTAAAGGTCCCTGGTTCAATCCC
11099	Yes	AAGATCTTAAAGGTCCCTGGTTCAATCCCG
11100	Yes	AAGATCTTAAAGGTCCCTGGTTCAATCCCGG
11101	Yes	AAGATCTTAAAGGTCCCTGGTTCAATCCCGGG
11102	Yes	AAGATCTTAAAGGTCCCTGGTTCAATCCCGGGT
11103	Yes	AAGATCTTAAAGGTCCCTGGTTCAATCCCGGGTT
11104	Yes	AAGATCTTAAAGGTCCCTGGTTCAATCCCGGGTTT
11105	Yes	AAGATCTTAAAGGTCCCTGGTTCAATCCCGGGTTTC
11106	Yes	AAGATCTTAAAGGTCCCTGGTTCAATCCCGGGTTTCG
11107	Yes	AAGATCTTAAAGGTCCCTGGTTCAATCCCGGGTTTCGG
11108	Yes	AAGATCTTAAAGGTCCCTGGTTCAATCCCGGGTTTCGGC
11109	Yes	AAGATCTTAAAGGTCCCTGGTTCAATCCCGGGTTTCGGCA
11110	Yes	AAGATCTTAAAGGTCCCTGGTTCAATCCCGGGTTTCGGCAC
11111	Yes	AAGATCTTAAAGGTCCCTGGTTCAATCCCGGGTTTCGGCACC
11112	Yes	AAGATCTTAAAGGTCCCTGGTTCAATCCCGGGTTTCGGCACCA
11113	No	AAGATCTTAAAGTTCC
11114	Yes	AAGATCTTAAAGTTCCC
11115	Yes	AAGATCTTAAAGTTCCCT
11116	Yes	AAGATCTTAAAGTTCCCTG
11117	Yes	AAGATCTTAAAGTTCCCTGG
11118	Yes	AAGATCTTAAAGTTCCCTGGT
11119	Yes	AAGATCTTAAAGTTCCCTGGTT
11120	Yes	AAGATCTTAAAGTTCCCTGGTTC
11121	Yes	AAGATCTTAAAGTTCCCTGGTTCA
11122	Yes	AAGATCTTAAAGTTCCCTGGTTCAA
11123	Yes	AAGATCTTAAAGTTCCCTGGTTCAAC

11124	Yes	AAGATCTTAAAGTTCCCTGGTTCAACC
11125	Yes	AAGATCTTAAAGTTCCCTGGTTCAACCC
11126	Yes	AAGATCTTAAAGTTCCCTGGTTCAACCCT
11127	Yes	AAGATCTTAAAGTTCCCTGGTTCAACCCTG
11128	Yes	AAGATCTTAAAGTTCCCTGGTTCAACCCTGG
11129	Yes	AAGATCTTAAAGTTCCCTGGTTCAACCCTGGG
11130	Yes	AAGATCTTAAAGTTCCCTGGTTCAACCCTGGGT
11131	Yes	AAGATCTTAAAGTTCCCTGGTTCAACCCTGGGTT
11132	Yes	AAGATCTTAAAGTTCCCTGGTTCAACCCTGGGTTT
11133	Yes	AAGATCTTAAAGTTCCCTGGTTCAACCCTGGGTTTC
11134	Yes	AAGATCTTAAAGTTCCCTGGTTCAACCCTGGGTTTCA
11135	Yes	AAGATCTTAAAGTTCCCTGGTTCAACCCTGGGTTTCAG
11136	Yes	AAGATCTTAAAGTTCCCTGGTTCAACCCTGGGTTTCAGC
11137	Yes	AAGATCTTAAAGTTCCCTGGTTCAACCCTGGGTTTCAGCC
11138	Yes	AAGATCTTAAAGTTCCCTGGTTCAACCCTGGGTTTCAGCCC
11139	Yes	AAGATCTTAAAGTTCCCTGGTTCAACCCTGGGTTTCAGCCCC
11140	Yes	AAGATCTTAAAGTTCCCTGGTTCAACCCTGGGTTTCAGCCCCA
11141	No	AAGATCTTCTGCAGGT
11142	No	AAGATCTTCTGCAGGTC
11143	No	AAGATCTTCTGCAGGTCT
11144	Yes	AAGATCTTCTGCAGGTCTC
11145	Yes	AAGATCTTCTGCAGGTCTCT
11146	Yes	AAGATCTTCTGCAGGTCTCTG
11147	Yes	AAGATCTTCTGCAGGTCTCTGG
11148	Yes	AAGATCTTCTGCAGGTCTCTGGT
11149	Yes	AAGATCTTCTGCAGGTCTCTGGTT
11150	Yes	AAGATCTTCTGCAGGTCTCTGGTTC
11151	Yes	AAGATCTTCTGCAGGTCTCTGGTTCA
11152	Yes	AAGATCTTCTGCAGGTCTCTGGTTCAA
11153	Yes	AAGATCTTCTGCAGGTCTCTGGTTCAAT
11154	Yes	AAGATCTTCTGCAGGTCTCTGGTTCAATT
11155	Yes	AAGATCTTCTGCAGGTCTCTGGTTCAATTC
11156	Yes	AAGATCTTCTGCAGGTCTCTGGTTCAATTCC
11157	Yes	AAGATCTTCTGCAGGTCTCTGGTTCAATTCCG
11158	Yes	AAGATCTTCTGCAGGTCTCTGGTTCAATTCCGG
11159	Yes	AAGATCTTCTGCAGGTCTCTGGTTCAATTCCGGG
11160	Yes	AAGATCTTCTGCAGGTCTCTGGTTCAATTCCGGGT
11161	Yes	AAGATCTTCTGCAGGTCTCTGGTTCAATTCCGGGTT
11162	Yes	AAGATCTTCTGCAGGTCTCTGGTTCAATTCCGGGTTT
11163	Yes	AAGATCTTCTGCAGGTCTCTGGTTCAATTCCGGGTTTC
11164	Yes	AAGATCTTCTGCAGGTCTCTGGTTCAATTCCGGGTTTCG
11165	Yes	AAGATCTTCTGCAGGTCTCTGGTTCAATTCCGGGTTTCGA

11166	Yes	AAGATCTTCTGCAGGTCTCTGGTTCAATTCCGGGTTTCGAC
11167	Yes	AAGATCTTCTGCAGGTCTCTGGTTCAATTCCGGGTTTCGACA
11168	Yes	AAGATCTTCTGCAGGTCTCTGGTTCAATTCCGGGTTTCGACAC
11169	Yes	AAGATCTTCTGCAGGTCTCTGGTTCAATTCCGGGTTTCGACACC
11170	Yes	AAGATCTTCTGCAGGTCTCTGGTTCAATTCCGGGTTTCGACACCA
11171	No	AAGATGGCAGAGCCCA
11172	No	AAGATGGCAGAGCCCAG
11173	No	AAGATGGCAGAGCCCAGC
11174	No	AAGATGGCAGAGCCCAGCA
11175	No	AAGATGGCAGAGCCCAGCAA
11176	No	AAGATGGCAGAGCCCAGCAAT
11177	No	AAGATGGCAGAGCCCAGCAATT
11178	No	AAGATGGCAGAGCCCAGCAATTG
11179	No	AAGATGGCAGAGCCCAGCAATTGC
11180	No	AAGATGGCAGAGCCCAGCAATTGCA
11181	No	AAGATGGCAGAGCCCAGCAATTGCAT
11182	No	AAGATGGCAGAGCCCAGCAATTGCATA
11183	No	AAGATGGCAGAGCCCAGCAATTGCATAA
11184	No	AAGATGGCAGAGCCCAGCAATTGCATAAA
11185	No	AAGATGGCAGAGCCCAGCAATTGCATAAAA
11186	No	AAGATGGCAGAGCCCAGCAATTGCATAAAAC
11187	No	AAGATGGCAGAGCCCAGCAATTGCATAAAACT
11188	No	AAGATGGCAGAGCCCAGCAATTGCATAAAACTT
11189	No	AAGATGGCAGAGCCCAGCAATTGCATAAAACTTA
11190	No	AAGATGGCAGAGCCCAGCAATTGCATAAAACTTAA
11191	No	AAGATGGCAGAGCCCAGCAATTGCATAAAACTTAAA
11192	No	AAGATGGCAGAGCCCAGCAATTGCATAAAACTTAAAA
11193	No	AAGATGGCAGAGCCCAGCAATTGCATAAAACTTAAAAC
11194	No	AAGATGGCAGAGCCCAGCAATTGCATAAAACTTAAAACT
11195	No	AAGATGGCAGAGCCCAGCAATTGCATAAAACTTAAAACTT
11196	No	AAGATGGCAGAGCCCAGCAATTGCATAAAACTTAAAACTTT
11197	No	AAGATGGCAGAGCCCAGCAATTGCATAAAACTTAAAACTTTA
11198	Yes	AAGATGGCAGAGCCCAGCAATTGCATAAAACTTAAAACTTTAC
11199	Yes	AAGATGGCAGAGCCCAGCAATTGCATAAAACTTAAAACTTTACA
11200	Yes	AAGATGGCAGAGCCCAGCAATTGCATAAAACTTAAAACTTTACAA
11201	Yes	AAGATGGCAGAGCCCAGCAATTGCATAAAACTTAAAACTTTACAAT
11202	Yes	AAGATGGCAGAGCCCAGCAATTGCATAAAACTTAAAACTTTACAATC
11203	Yes	AAGATGGCAGAGCCCAGCAATTGCATAAAACTTAAAACTTTACAATCA
11204	Yes	AAGATGGCAGAGCCCAGCAATTGCATAAAACTTAAAACTTTACAATCAG
11205	Yes	AAGATGGCAGAGCCCAGCAATTGCATAAAACTTAAAACTTTACAATCAGA
11206	No	AAGATGGCAGAGCCCAGCAATTGCATAAAT
11207	No	AAGATGGCAGAGCCCAGCAATTGCATAAATC

11208	No	AAGATGGCAGAGCCCAGCAATTGCATAAATCT
11209	No	AAGATGGCAGAGCCCAGCAATTGCATAAATCTT
11210	No	AAGATGGCAGAGCCCAGCAATTGCATAAATCTTA
11211	No	AAGATGGCAGAGCCCAGCAATTGCATAAATCTTAA
11212	No	AAGATGGCAGAGCCCAGCAATTGCATAAATCTTAAA
11213	No	AAGATGGCAGAGCCCAGCAATTGCATAAATCTTAAAA
11214	No	AAGATGGCAGAGCCCAGCAATTGCATAAATCTTAAAAC
11215	No	AAGATGGCAGAGCCCAGCAATTGCATAAATCTTAAAACT
11216	No	AAGATGGCAGAGCCCAGCAATTGCATAAATCTTAAAACTT
11217	No	AAGATGGCAGAGCCCAGCAATTGCATAAATCTTAAAACTTT
11218	Yes	AAGATGGCAGAGCCCAGCAATTGCATAAATCTTAAAACTTTA
11219	Yes	AAGATGGCAGAGCCCAGCAATTGCATAAATCTTAAAACTTTAT
11220	Yes	AAGATGGCAGAGCCCAGCAATTGCATAAATCTTAAAACTTTATA
11221	Yes	AAGATGGCAGAGCCCAGCAATTGCATAAATCTTAAAACTTTATAA
11222	Yes	AAGATGGCAGAGCCCAGCAATTGCATAAATCTTAAAACTTTATAAT
11223	Yes	AAGATGGCAGAGCCCAGCAATTGCATAAATCTTAAAACTTTATAATC
11224	Yes	AAGATGGCAGAGCCCAGCAATTGCATAAATCTTAAAACTTTATAATCA
11225	Yes	AAGATGGCAGAGCCCAGCAATTGCATAAATCTTAAAACTTTATAATCAG
11226	Yes	AAGATGGCAGAGCCCAGCAATTGCATAAATCTTAAAACTTTATAATCAGA
11227	Yes	AAGATGGCAGAGCCCAGCG
11228	Yes	AAGATGGCAGAGCCCAGCGA
11229	Yes	AAGATGGCAGAGCCCAGCGAT
11230	Yes	AAGATGGCAGAGCCCAGCGATT
11231	Yes	AAGATGGCAGAGCCCAGCGATTG
11232	Yes	AAGATGGCAGAGCCCAGCGATTGC
11233	Yes	AAGATGGCAGAGCCCAGCGATTGCA
11234	Yes	AAGATGGCAGAGCCCAGCGATTGCAT
11235	Yes	AAGATGGCAGAGCCCAGCGATTGCATA
11236	Yes	AAGATGGCAGAGCCCAGCGATTGCATAA
11237	Yes	AAGATGGCAGAGCCCAGCGATTGCATAAA
11238	Yes	AAGATGGCAGAGCCCAGCGATTGCATAAAA
11239	Yes	AAGATGGCAGAGCCCAGCGATTGCATAAAAC
11240	Yes	AAGATGGCAGAGCCCAGCGATTGCATAAAACT
11241	Yes	AAGATGGCAGAGCCCAGCGATTGCATAAAACTT
11242	Yes	AAGATGGCAGAGCCCAGCGATTGCATAAAACTTA
11243	Yes	AAGATGGCAGAGCCCAGCGATTGCATAAAACTTAA
11244	Yes	AAGATGGCAGAGCCCAGCGATTGCATAAAACTTAAC
11245	Yes	AAGATGGCAGAGCCCAGCGATTGCATAAAACTTAACA
11246	Yes	AAGATGGCAGAGCCCAGCGATTGCATAAAACTTAACAC
11247	Yes	AAGATGGCAGAGCCCAGCGATTGCATAAAACTTAACACT
11248	Yes	AAGATGGCAGAGCCCAGCGATTGCATAAAACTTAACACTT
11249	Yes	AAGATGGCAGAGCCCAGCGATTGCATAAAACTTAACACTTT

11250	Yes	AAGATGGCAGAGCCCAGCGATTGCATAAAACTTAACACTTTA
11251	Yes	AAGATGGCAGAGCCCAGCGATTGCATAAAACTTAACACTTTAT
11252	Yes	AAGATGGCAGAGCCCAGCGATTGCATAAAACTTAACACTTTATA
11253	Yes	AAGATGGCAGAGCCCAGCGATTGCATAAAACTTAACACTTTATAA
11254	Yes	AAGATGGCAGAGCCCAGCGATTGCATAAAACTTAACACTTTATAAT
11255	Yes	AAGATGGCAGAGCCCAGCGATTGCATAAAACTTAACACTTTATAATC
11256	Yes	AAGATGGCAGAGCCCAGCGATTGCATAAAACTTAACACTTTATAATCA
11257	Yes	AAGATGGCAGAGCCCAGCGATTGCATAAAACTTAACACTTTATAATCAG
11258	Yes	AAGATGGCAGAGCCCAGCGATTGCATAAAACTTAACACTTTATAATCAGA
11259	No	AAGATGGCAGAGCCCG
11260	No	AAGATGGCAGAGCCCGG
11261	No	AAGATGGCAGAGCCCGGC
11262	No	AAGATGGCAGAGCCCGGCA
11263	No	AAGATGGCAGAGCCCGGCAA
11264	No	AAGATGGCAGAGCCCGGCAAT
11265	No	AAGATGGCAGAGCCCGGCAATT
11266	No	AAGATGGCAGAGCCCGGCAATTG
11267	No	AAGATGGCAGAGCCCGGCAATTGC
11268	No	AAGATGGCAGAGCCCGGCAATTGCA
11269	No	AAGATGGCAGAGCCCGGCAATTGCAT
11270	No	AAGATGGCAGAGCCCGGCAATTGCATA
11271	No	AAGATGGCAGAGCCCGGCAATTGCATAA
11272	Yes	AAGATGGCAGAGCCCGGCAATTGCATAAG
11273	Yes	AAGATGGCAGAGCCCGGCAATTGCATAAGA
11274	Yes	AAGATGGCAGAGCCCGGCAATTGCATAAGAC
11275	Yes	AAGATGGCAGAGCCCGGCAATTGCATAAGACT
11276	Yes	AAGATGGCAGAGCCCGGCAATTGCATAAGACTT
11277	Yes	AAGATGGCAGAGCCCGGCAATTGCATAAGACTTA
11278	Yes	AAGATGGCAGAGCCCGGCAATTGCATAAGACTTAA
11279	Yes	AAGATGGCAGAGCCCGGCAATTGCATAAGACTTAAA
11280	Yes	AAGATGGCAGAGCCCGGCAATTGCATAAGACTTAAAA
11281	Yes	AAGATGGCAGAGCCCGGCAATTGCATAAGACTTAAAAC
11282	Yes	AAGATGGCAGAGCCCGGCAATTGCATAAGACTTAAAACT
11283	Yes	AAGATGGCAGAGCCCGGCAATTGCATAAGACTTAAAACTT
11284	Yes	AAGATGGCAGAGCCCGGCAATTGCATAAGACTTAAAACTTT
11285	Yes	AAGATGGCAGAGCCCGGCAATTGCATAAGACTTAAAACTTTA
11286	Yes	AAGATGGCAGAGCCCGGCAATTGCATAAGACTTAAAACTTTAT
11287	Yes	AAGATGGCAGAGCCCGGCAATTGCATAAGACTTAAAACTTTATA
11288	Yes	AAGATGGCAGAGCCCGGCAATTGCATAAGACTTAAAACTTTATAA
11289	Yes	AAGATGGCAGAGCCCGGCAATTGCATAAGACTTAAAACTTTATAAT
11290	Yes	AAGATGGCAGAGCCCGGCAATTGCATAAGACTTAAAACTTTATAATC
11291	Yes	AAGATGGCAGAGCCCGGCAATTGCATAAGACTTAAAACTTTATAATCA

11292	Yes	AAGATGGCAGAGCCCGGCAATTGCATAAGACTTAAAACTTTATAATCAG
11293	Yes	AAGATGGCAGAGCCCGGCAATTGCATAAGACTTAAAACTTTATAATCAGA
11294	Yes	AAGATGGCAGAGCCCGGT
11295	Yes	AAGATGGCAGAGCCCGGTA
11296	Yes	AAGATGGCAGAGCCCGGTAA
11297	Yes	AAGATGGCAGAGCCCGGTAAT
11298	Yes	AAGATGGCAGAGCCCGGTAATC
11299	Yes	AAGATGGCAGAGCCCGGTAATCG
11300	Yes	AAGATGGCAGAGCCCGGTAATCGC
11301	Yes	AAGATGGCAGAGCCCGGTAATCGCA
11302	Yes	AAGATGGCAGAGCCCGGTAATCGCAT
11303	Yes	AAGATGGCAGAGCCCGGTAATCGCATA
11304	Yes	AAGATGGCAGAGCCCGGTAATCGCATAA
11305	Yes	AAGATGGCAGAGCCCGGTAATCGCATAAA
11306	Yes	AAGATGGCAGAGCCCGGTAATCGCATAAAA
11307	Yes	AAGATGGCAGAGCCCGGTAATCGCATAAAAC
11308	Yes	AAGATGGCAGAGCCCGGTAATCGCATAAAACT
11309	Yes	AAGATGGCAGAGCCCGGTAATCGCATAAAACTT
11310	Yes	AAGATGGCAGAGCCCGGTAATCGCATAAAACTTA
11311	Yes	AAGATGGCAGAGCCCGGTAATCGCATAAAACTTAA
11312	Yes	AAGATGGCAGAGCCCGGTAATCGCATAAAACTTAAA
11313	Yes	AAGATGGCAGAGCCCGGTAATCGCATAAAACTTAAAA
11314	Yes	AAGATGGCAGAGCCCGGTAATCGCATAAAACTTAAAAC
11315	Yes	AAGATGGCAGAGCCCGGTAATCGCATAAAACTTAAAACT
11316	Yes	AAGATGGCAGAGCCCGGTAATCGCATAAAACTTAAAACTT
11317	Yes	AAGATGGCAGAGCCCGGTAATCGCATAAAACTTAAAACTTT
11318	Yes	AAGATGGCAGAGCCCGGTAATCGCATAAAACTTAAAACTTTA
11319	Yes	AAGATGGCAGAGCCCGGTAATCGCATAAAACTTAAAACTTTAC
11320	Yes	AAGATGGCAGAGCCCGGTAATCGCATAAAACTTAAAACTTTACA
11321	Yes	AAGATGGCAGAGCCCGGTAATCGCATAAAACTTAAAACTTTACAG
11322	Yes	AAGATGGCAGAGCCCGGTAATCGCATAAAACTTAAAACTTTACAGT
11323	Yes	AAGATGGCAGAGCCCGGTAATCGCATAAAACTTAAAACTTTACAGTC
11324	Yes	AAGATGGCAGAGCCCGGTAATCGCATAAAACTTAAAACTTTACAGTCA
11325	Yes	AAGATGGCAGAGCCCGGTAATCGCATAAAACTTAAAACTTTACAGTCAG
11326	Yes	AAGATGGCAGAGCCCGGTAATCGCATAAAACTTAAAACTTTACAGTCAGA
11327	No	AAGATGGCAGAGCCTG
11328	No	AAGATGGCAGAGCCTGG
11329	No	AAGATGGCAGAGCCTGGT
11330	Yes	AAGATGGCAGAGCCTGGTA
11331	Yes	AAGATGGCAGAGCCTGGTAA
11332	Yes	AAGATGGCAGAGCCTGGTAAT
11333	Yes	AAGATGGCAGAGCCTGGTAATT

11334	Yes	AAGATGGCAGAGCCTGGTAATTG
11335	Yes	AAGATGGCAGAGCCTGGTAATTGC
11336	Yes	AAGATGGCAGAGCCTGGTAATTGCA
11337	Yes	AAGATGGCAGAGCCTGGTAATTGCAT
11338	Yes	AAGATGGCAGAGCCTGGTAATTGCATA
11339	Yes	AAGATGGCAGAGCCTGGTAATTGCATAA
11340	Yes	AAGATGGCAGAGCCTGGTAATTGCATAAA
11341	Yes	AAGATGGCAGAGCCTGGTAATTGCATAAAA
11342	Yes	AAGATGGCAGAGCCTGGTAATTGCATAAAAC
11343	Yes	AAGATGGCAGAGCCTGGTAATTGCATAAAACT
11344	Yes	AAGATGGCAGAGCCTGGTAATTGCATAAAACTT
11345	Yes	AAGATGGCAGAGCCTGGTAATTGCATAAAACTTA
11346	Yes	AAGATGGCAGAGCCTGGTAATTGCATAAAACTTAA
11347	Yes	AAGATGGCAGAGCCTGGTAATTGCATAAAACTTAAA
11348	Yes	AAGATGGCAGAGCCTGGTAATTGCATAAAACTTAAAA
11349	Yes	AAGATGGCAGAGCCTGGTAATTGCATAAAACTTAAAAT
11350	Yes	AAGATGGCAGAGCCTGGTAATTGCATAAAACTTAAAATT
11351	Yes	AAGATGGCAGAGCCTGGTAATTGCATAAAACTTAAAATTT
11352	Yes	AAGATGGCAGAGCCTGGTAATTGCATAAAACTTAAAATTTT
11353	Yes	AAGATGGCAGAGCCTGGTAATTGCATAAAACTTAAAATTTTA
11354	Yes	AAGATGGCAGAGCCTGGTAATTGCATAAAACTTAAAATTTTAT
11355	Yes	AAGATGGCAGAGCCTGGTAATTGCATAAAACTTAAAATTTTATA
11356	Yes	AAGATGGCAGAGCCTGGTAATTGCATAAAACTTAAAATTTTATAA
11357	Yes	AAGATGGCAGAGCCTGGTAATTGCATAAAACTTAAAATTTTATAAT
11358	Yes	AAGATGGCAGAGCCTGGTAATTGCATAAAACTTAAAATTTTATAATC
11359	Yes	AAGATGGCAGAGCCTGGTAATTGCATAAAACTTAAAATTTTATAATCA
11360	Yes	AAGATGGCAGAGCCTGGTAATTGCATAAAACTTAAAATTTTATAATCAG
11361	Yes	AAGATGGCAGAGCCTGGTAATTGCATAAAACTTAAAATTTTATAATCAGA
11362	Yes	AAGATGGCATAGCCCA
11363	Yes	AAGATGGCATAGCCCAG
11364	Yes	AAGATGGCATAGCCCAGC
11365	Yes	AAGATGGCATAGCCCAGCA
11366	Yes	AAGATGGCATAGCCCAGCAA
11367	Yes	AAGATGGCATAGCCCAGCAAT
11368	Yes	AAGATGGCATAGCCCAGCAATT
11369	Yes	AAGATGGCATAGCCCAGCAATTG
11370	Yes	AAGATGGCATAGCCCAGCAATTGC
11371	Yes	AAGATGGCATAGCCCAGCAATTGCA
11372	Yes	AAGATGGCATAGCCCAGCAATTGCAT
11373	Yes	AAGATGGCATAGCCCAGCAATTGCATA
11374	Yes	AAGATGGCATAGCCCAGCAATTGCATAA
11375	Yes	AAGATGGCATAGCCCAGCAATTGCATAAA

11376	Yes	AAGATGGCATAGCCCAGCAATTGCATAAAA
11377	Yes	AAGATGGCATAGCCCAGCAATTGCATAAAAC
11378	Yes	AAGATGGCATAGCCCAGCAATTGCATAAAACT
11379	Yes	AAGATGGCATAGCCCAGCAATTGCATAAAACTT
11380	Yes	AAGATGGCATAGCCCAGCAATTGCATAAAACTTA
11381	Yes	AAGATGGCATAGCCCAGCAATTGCATAAAACTTAA
11382	Yes	AAGATGGCATAGCCCAGCAATTGCATAAAACTTAAG
11383	Yes	AAGATGGCATAGCCCAGCAATTGCATAAAACTTAAGA
11384	Yes	AAGATGGCATAGCCCAGCAATTGCATAAAACTTAAGAC
11385	Yes	AAGATGGCATAGCCCAGCAATTGCATAAAACTTAAGACT
11386	Yes	AAGATGGCATAGCCCAGCAATTGCATAAAACTTAAGACTT
11387	Yes	AAGATGGCATAGCCCAGCAATTGCATAAAACTTAAGACTTT
11388	Yes	AAGATGGCATAGCCCAGCAATTGCATAAAACTTAAGACTTTA
11389	Yes	AAGATGGCATAGCCCAGCAATTGCATAAAACTTAAGACTTTAT
11390	Yes	AAGATGGCATAGCCCAGCAATTGCATAAAACTTAAGACTTTATA
11391	Yes	AAGATGGCATAGCCCAGCAATTGCATAAAACTTAAGACTTTATAA
11392	Yes	AAGATGGCATAGCCCAGCAATTGCATAAAACTTAAGACTTTATAAT
11393	Yes	AAGATGGCATAGCCCAGCAATTGCATAAAACTTAAGACTTTATAATT
11394	Yes	AAGATGGCATAGCCCAGCAATTGCATAAAACTTAAGACTTTATAATTA
11395	Yes	AAGATGGCATAGCCCAGCAATTGCATAAAACTTAAGACTTTATAATTAG
11396	Yes	AAGATGGCATAGCCCAGCAATTGCATAAAACTTAAGACTTTATAATTAGA
11397	No	AAGATTAAGAGAACCA
11398	No	AAGATTAAGAGAACCAA
11399	No	AAGATTAAGAGAACCAAC
11400	No	AAGATTAAGAGAACCAACA
11401	No	AAGATTAAGAGAACCAACAC
11402	No	AAGATTAAGAGAACCAACACC
11403	No	AAGATTAAGAGAACCAACACCT
11404	No	AAGATTAAGAGAACCAACACCTC
11405	No	AAGATTAAGAGAACCAACACCTCT
11406	No	AAGATTAAGAGAACCAACACCTCTT
11407	No	AAGATTAAGAGAACCAACACCTCTTT
11408	No	AAGATTAAGAGAACCAACACCTCTTTA
11409	No	AAGATTAAGAGAACCAACACCTCTTTAC
11410	No	AAGATTAAGAGAACCAACACCTCTTTACA
11411	No	AAGATTAAGAGAACCAACACCTCTTTACAG
11412	No	AAGATTAAGAGAACCAACACCTCTTTACAGT
11413	No	AAGATTAAGAGAACCAACACCTCTTTACAGTG
11414	No	AAGATTAAGAGAACCAACACCTCTTTACAGTGA
11415	Yes	AAGATTAAGAGAACCAACACCTCTTTACAGTGAC
11416	Yes	AAGATTAAGAGAACCAACACCTCTTTACAGTGACC
11417	Yes	AAGATTAAGAGAACCAACACCTCTTTACAGTGACCA

11418	No	AAGATTCCAGCCTTGA
11419	Yes	AAGATTCCAGCCTTGAC
11420	Yes	AAGATTCCAGCCTTGACT
11421	Yes	AAGATTCCAGCCTTGACTC
11422	Yes	AAGATTCCAGCCTTGACTCC
11423	Yes	AAGATTCCAGCCTTGACTCCT
11424	Yes	AAGATTCCAGCCTTGACTCCTG
11425	Yes	AAGATTCCAGCCTTGACTCCTGG
11426	Yes	AAGATTCCAGCCTTGACTCCTGGC
11427	Yes	AAGATTCCAGCCTTGACTCCTGGCT
11428	Yes	AAGATTCCAGCCTTGACTCCTGGCTG
11429	Yes	AAGATTCCAGCCTTGACTCCTGGCTGG
11430	Yes	AAGATTCCAGCCTTGACTCCTGGCTGGC
11431	Yes	AAGATTCCAGCCTTGACTCCTGGCTGGCT
11432	Yes	AAGATTCCAGCCTTGACTCCTGGCTGGCTC
11433	Yes	AAGATTCCAGCCTTGACTCCTGGCTGGCTCA
11434	Yes	AAGATTCCAGCCTTGACTCCTGGCTGGCTCAC
11435	Yes	AAGATTCCAGCCTTGACTCCTGGCTGGCTCACC
11436	Yes	AAGATTCCAGCCTTGACTCCTGGCTGGCTCACCA
11437	Yes	AAGATTCCAGGTTCGA
11438	Yes	AAGATTCCAGGTTCGAC
11439	Yes	AAGATTCCAGGTTCGACT
11440	Yes	AAGATTCCAGGTTCGACTC
11441	Yes	AAGATTCCAGGTTCGACTCC
11442	Yes	AAGATTCCAGGTTCGACTCCT
11443	Yes	AAGATTCCAGGTTCGACTCCTG
11444	Yes	AAGATTCCAGGTTCGACTCCTGG
11445	Yes	AAGATTCCAGGTTCGACTCCTGGC
11446	Yes	AAGATTCCAGGTTCGACTCCTGGCT
11447	Yes	AAGATTCCAGGTTCGACTCCTGGCTG
11448	Yes	AAGATTCCAGGTTCGACTCCTGGCTGG
11449	Yes	AAGATTCCAGGTTCGACTCCTGGCTGGC
11450	Yes	AAGATTCCAGGTTCGACTCCTGGCTGGCT
11451	Yes	AAGATTCCAGGTTCGACTCCTGGCTGGCTC
11452	Yes	AAGATTCCAGGTTCGACTCCTGGCTGGCTCG
11453	Yes	AAGATTCCAGGTTCGACTCCTGGCTGGCTCGC
11454	Yes	AAGATTCCAGGTTCGACTCCTGGCTGGCTCGCC
11455	Yes	AAGATTCCAGGTTCGACTCCTGGCTGGCTCGCCA
11456	No	AAGATTCCCATTCTTG
11457	Yes	AAGATTCCCATTCTTGC
11458	Yes	AAGATTCCCATTCTTGCG
11459	Yes	AAGATTCCCATTCTTGCGA

11460	Yes	AAGATTCCCATTCTTGCGAC
11461	Yes	AAGATTCCCATTCTTGCGACC
11462	Yes	AAGATTCCCATTCTTGCGACCC
11463	Yes	AAGATTCCCATTCTTGCGACCCG
11464	Yes	AAGATTCCCATTCTTGCGACCCGG
11465	Yes	AAGATTCCCATTCTTGCGACCCGGG
11466	Yes	AAGATTCCCATTCTTGCGACCCGGGT
11467	Yes	AAGATTCCCATTCTTGCGACCCGGGTT
11468	Yes	AAGATTCCCATTCTTGCGACCCGGGTTC
11469	Yes	AAGATTCCCATTCTTGCGACCCGGGTTCG
11470	Yes	AAGATTCCCATTCTTGCGACCCGGGTTCGA
11471	Yes	AAGATTCCCATTCTTGCGACCCGGGTTCGAT
11472	Yes	AAGATTCCCATTCTTGCGACCCGGGTTCGATT
11473	Yes	AAGATTCCCATTCTTGCGACCCGGGTTCGATTC
11474	Yes	AAGATTCCCATTCTTGCGACCCGGGTTCGATTCC
11475	Yes	AAGATTCCCATTCTTGCGACCCGGGTTCGATTCCC
11476	Yes	AAGATTCCCATTCTTGCGACCCGGGTTCGATTCCCG
11477	Yes	AAGATTCCCATTCTTGCGACCCGGGTTCGATTCCCGG
11478	Yes	AAGATTCCCATTCTTGCGACCCGGGTTCGATTCCCGGG
11479	Yes	AAGATTCCCATTCTTGCGACCCGGGTTCGATTCCCGGGC
11480	Yes	AAGATTCCCATTCTTGCGACCCGGGTTCGATTCCCGGGCG
11481	Yes	AAGATTCCCATTCTTGCGACCCGGGTTCGATTCCCGGGCGG
11482	Yes	AAGATTCCCATTCTTGCGACCCGGGTTCGATTCCCGGGCGGC
11483	Yes	AAGATTCCCATTCTTGCGACCCGGGTTCGATTCCCGGGCGGCG
11484	Yes	AAGATTCCCATTCTTGCGACCCGGGTTCGATTCCCGGGCGGCGC
11485	Yes	AAGATTCCCATTCTTGCGACCCGGGTTCGATTCCCGGGCGGCGCA
11486	Yes	AAGATTCCCATTCTTGCGACCCGGGTTCGATTCCCGGGCGGCGCAC
11487	Yes	AAGATTCCCATTCTTGCGACCCGGGTTCGATTCCCGGGCGGCGCACC
11488	Yes	AAGATTCCCATTCTTGCGACCCGGGTTCGATTCCCGGGCGGCGCACCA
11489	No	AAGATTCTAGGTTCGA
11490	Yes	AAGATTCTAGGTTCGAC
11491	Yes	AAGATTCTAGGTTCGACT
11492	Yes	AAGATTCTAGGTTCGACTC
11493	Yes	AAGATTCTAGGTTCGACTCC
11494	Yes	AAGATTCTAGGTTCGACTCCT
11495	Yes	AAGATTCTAGGTTCGACTCCTG
11496	Yes	AAGATTCTAGGTTCGACTCCTGG
11497	Yes	AAGATTCTAGGTTCGACTCCTGGC
11498	Yes	AAGATTCTAGGTTCGACTCCTGGCT
11499	Yes	AAGATTCTAGGTTCGACTCCTGGCTG
11500	Yes	AAGATTCTAGGTTCGACTCCTGGCTGG
11501	Yes	AAGATTCTAGGTTCGACTCCTGGCTGGC

11502	Yes	AAGATTCTAGGTTCGACTCCTGGCTGGCT
11503	Yes	AAGATTCTAGGTTCGACTCCTGGCTGGCTC
11504	Yes	AAGATTCTAGGTTCGACTCCTGGCTGGCTCG
11505	Yes	AAGATTCTAGGTTCGACTCCTGGCTGGCTCGC
11506	Yes	AAGATTCTAGGTTCGACTCCTGGCTGGCTCGCC
11507	Yes	AAGATTCTAGGTTCGACTCCTGGCTGGCTCGCCA
11508	No	AAGATTCTGAATTAAG
11509	Yes	AAGATTCTGAATTAAGT
11510	Yes	AAGATTCTGAATTAAGTC
11511	Yes	AAGATTCTGAATTAAGTCT
11512	Yes	AAGATTCTGAATTAAGTCTC
11513	Yes	AAGATTCTGAATTAAGTCTCC
11514	Yes	AAGATTCTGAATTAAGTCTCCA
11515	Yes	AAGATTCTGAATTAAGTCTCCAG
11516	Yes	AAGATTCTGAATTAAGTCTCCAGT
11517	Yes	AAGATTCTGAATTAAGTCTCCAGTC
11518	Yes	AAGATTCTGAATTAAGTCTCCAGTCT
11519	Yes	AAGATTCTGAATTAAGTCTCCAGTCTC
11520	Yes	AAGATTCTGAATTAAGTCTCCAGTCTCT
11521	Yes	AAGATTCTGAATTAAGTCTCCAGTCTCTT
11522	Yes	AAGATTCTGAATTAAGTCTCCAGTCTCTTT
11523	Yes	AAGATTCTGAATTAAGTCTCCAGTCTCTTTG
11524	Yes	AAGATTCTGAATTAAGTCTCCAGTCTCTTTGG
11525	Yes	AAGATTCTGAATTAAGTCTCCAGTCTCTTTGGG
11526	Yes	AAGATTCTGAATTAAGTCTCCAGTCTCTTTGGGG
11527	Yes	AAGATTCTGAATTAAGTCTCCAGTCTCTTTGGGGG
11528	Yes	AAGATTCTGAATTAAGTCTCCAGTCTCTTTGGGGGC
11529	Yes	AAGATTCTGAATTAAGTCTCCAGTCTCTTTGGGGGCG
11530	Yes	AAGATTCTGAATTAAGTCTCCAGTCTCTTTGGGGGCGT
11531	Yes	AAGATTCTGAATTAAGTCTCCAGTCTCTTTGGGGGCGTG
11532	Yes	AAGATTCTGAATTAAGTCTCCAGTCTCTTTGGGGGCGTGG
11533	Yes	AAGATTCTGAATTAAGTCTCCAGTCTCTTTGGGGGCGTGGT
11534	Yes	AAGATTCTGAATTAAGTCTCCAGTCTCTTTGGGGGCGTGGTT
11535	Yes	AAGATTCTGAATTAAGTCTCCAGTCTCTTTGGGGGCGTGGTTT
11536	Yes	AAGATTCTGAATTAAGTCTCCAGTCTCTTTGGGGGCGTGGTTTT
11537	Yes	AAGATTCTGAATTAAGTCTCCAGTCTCTTTGGGGGCGTGGTTTTC
11538	Yes	AAGATTCTGAATTAAGTCTCCAGTCTCTTTGGGGGCGTGGTTTTCA
11539	Yes	AAGATTCTGAATTAAGTCTCCAGTCTCTTTGGGGGCGTGGTTTTCAA
11540	Yes	AAGATTCTGAATTAAGTCTCCAGTCTCTTTGGGGGCGTGGTTTTCAAT
11541	Yes	AAGATTCTGAATTAAGTCTCCAGTCTCTTTGGGGGCGTGGTTTTCAATC
11542	Yes	AAGATTCTGAATTAAGTCTCCAGTCTCTTTGGGGGCGTGGTTTTCAATCC
11543	No	AAGATTGAGGGTTCAA

11544	Yes	AAGATTGAGGGTTCAAG
11545	Yes	AAGATTGAGGGTTCAAGT
11546	Yes	AAGATTGAGGGTTCAAGTC
11547	Yes	AAGATTGAGGGTTCAAGTCC
11548	Yes	AAGATTGAGGGTTCAAGTCCT
11549	Yes	AAGATTGAGGGTTCAAGTCCTT
11550	Yes	AAGATTGAGGGTTCAAGTCCTTT
11551	Yes	AAGATTGAGGGTTCAAGTCCTTTC
11552	Yes	AAGATTGAGGGTTCAAGTCCTTTCA
11553	Yes	AAGATTGAGGGTTCAAGTCCTTTCAT
11554	Yes	AAGATTGAGGGTTCAAGTCCTTTCATG
11555	Yes	AAGATTGAGGGTTCAAGTCCTTTCATGG
11556	Yes	AAGATTGAGGGTTCAAGTCCTTTCATGGT
11557	Yes	AAGATTGAGGGTTCAAGTCCTTTCATGGTC
11558	Yes	AAGATTGAGGGTTCAAGTCCTTTCATGGTCA
11559	Yes	AAGATTGAGGGTTCAAGTCCTTTCATGGTCAC
11560	Yes	AAGATTGAGGGTTCAAGTCCTTTCATGGTCACC
11561	Yes	AAGATTGAGGGTTCAAGTCCTTTCATGGTCACCA
11562	Yes	AAGATTGAGGGTTCGA
11563	Yes	AAGATTGAGGGTTCGAA
11564	Yes	AAGATTGAGGGTTCGAAT
11565	Yes	AAGATTGAGGGTTCGAATC
11566	Yes	AAGATTGAGGGTTCGAATCC
11567	Yes	AAGATTGAGGGTTCGAATCCC
11568	Yes	AAGATTGAGGGTTCGAATCCCT
11569	Yes	AAGATTGAGGGTTCGAATCCCTC
11570	Yes	AAGATTGAGGGTTCGAATCCCTCC
11571	Yes	AAGATTGAGGGTTCGAATCCCTCCG
11572	Yes	AAGATTGAGGGTTCGAATCCCTCCGT
11573	Yes	AAGATTGAGGGTTCGAATCCCTCCGTG
11574	Yes	AAGATTGAGGGTTCGAATCCCTCCGTGG
11575	Yes	AAGATTGAGGGTTCGAATCCCTCCGTGGT
11576	Yes	AAGATTGAGGGTTCGAATCCCTCCGTGGTT
11577	Yes	AAGATTGAGGGTTCGAATCCCTCCGTGGTTA
11578	Yes	AAGATTGAGGGTTCGAATCCCTCCGTGGTTAC
11579	Yes	AAGATTGAGGGTTCGAATCCCTCCGTGGTTACC
11580	Yes	AAGATTGAGGGTTCGAATCCCTCCGTGGTTACCA
11581	Yes	AAGATTGAGGGTTCGAATCCCTT
11582	Yes	AAGATTGAGGGTTCGAATCCCTTC
11583	Yes	AAGATTGAGGGTTCGAATCCCTTCG
11584	Yes	AAGATTGAGGGTTCGAATCCCTTCGT
11585	Yes	AAGATTGAGGGTTCGAATCCCTTCGTG

11586	Yes	AAGATTGAGGGTTCGAATCCCTTCGTGG
11587	Yes	AAGATTGAGGGTTCGAATCCCTTCGTGGT
11588	Yes	AAGATTGAGGGTTCGAATCCCTTCGTGGTT
11589	Yes	AAGATTGAGGGTTCGAATCCCTTCGTGGTTA
11590	Yes	AAGATTGAGGGTTCGAATCCCTTCGTGGTTAC
11591	Yes	AAGATTGAGGGTTCGAATCCCTTCGTGGTTACC
11592	Yes	AAGATTGAGGGTTCGAATCCCTTCGTGGTTACCA
11593	Yes	AAGATTGAGGGTTCGAATCCCTTCGTGGTTG
11594	Yes	AAGATTGAGGGTTCGAATCCCTTCGTGGTTGC
11595	Yes	AAGATTGAGGGTTCGAATCCCTTCGTGGTTGCC
11596	Yes	AAGATTGAGGGTTCGAATCCCTTCGTGGTTGCCA
11597	Yes	AAGATTGAGGGTTCGAG
11598	Yes	AAGATTGAGGGTTCGAGT
11599	Yes	AAGATTGAGGGTTCGAGTC
11600	Yes	AAGATTGAGGGTTCGAGTCC
11601	Yes	AAGATTGAGGGTTCGAGTCCC
11602	Yes	AAGATTGAGGGTTCGAGTCCCT
11603	Yes	AAGATTGAGGGTTCGAGTCCCTT
11604	Yes	AAGATTGAGGGTTCGAGTCCCTTC
11605	Yes	AAGATTGAGGGTTCGAGTCCCTTCG
11606	Yes	AAGATTGAGGGTTCGAGTCCCTTCGT
11607	Yes	AAGATTGAGGGTTCGAGTCCCTTCGTG
11608	Yes	AAGATTGAGGGTTCGAGTCCCTTCGTGG
11609	Yes	AAGATTGAGGGTTCGAGTCCCTTCGTGGT
11610	Yes	AAGATTGAGGGTTCGAGTCCCTTCGTGGTC
11611	Yes	AAGATTGAGGGTTCGAGTCCCTTCGTGGTCG
11612	Yes	AAGATTGAGGGTTCGAGTCCCTTCGTGGTCGC
11613	Yes	AAGATTGAGGGTTCGAGTCCCTTCGTGGTCGCC
11614	Yes	AAGATTGAGGGTTCGAGTCCCTTCGTGGTCGCCA
11615	Yes	AAGATTGCAGGTTCGA
11616	Yes	AAGATTGCAGGTTCGAG
11617	Yes	AAGATTGCAGGTTCGAGT
11618	Yes	AAGATTGCAGGTTCGAGTC
11619	Yes	AAGATTGCAGGTTCGAGTCC
11620	Yes	AAGATTGCAGGTTCGAGTCCT
11621	Yes	AAGATTGCAGGTTCGAGTCCTG
11622	Yes	AAGATTGCAGGTTCGAGTCCTGC
11623	Yes	AAGATTGCAGGTTCGAGTCCTGCC
11624	Yes	AAGATTGCAGGTTCGAGTCCTGCCG
11625	Yes	AAGATTGCAGGTTCGAGTCCTGCCGC
11626	Yes	AAGATTGCAGGTTCGAGTCCTGCCGCG
11627	Yes	AAGATTGCAGGTTCGAGTCCTGCCGCGG

11628	Yes	AAGATTGCAGGTTCGAGTCCTGCCGCGGT
11629	Yes	AAGATTGCAGGTTCGAGTCCTGCCGCGGTC
11630	Yes	AAGATTGCAGGTTCGAGTCCTGCCGCGGTCG
11631	Yes	AAGATTGCAGGTTCGAGTCCTGCCGCGGTCGC
11632	Yes	AAGATTGCAGGTTCGAGTCCTGCCGCGGTCGCC
11633	Yes	AAGATTGCAGGTTCGAGTCCTGCCGCGGTCGCCA
11634	No	AAGATTGCAGGTTTGA
11635	No	AAGATTGCAGGTTTGAG
11636	Yes	AAGATTGCAGGTTTGAGT
11637	Yes	AAGATTGCAGGTTTGAGTT
11638	Yes	AAGATTGCAGGTTTGAGTTC
11639	Yes	AAGATTGCAGGTTTGAGTTCT
11640	Yes	AAGATTGCAGGTTTGAGTTCTG
11641	Yes	AAGATTGCAGGTTTGAGTTCTGC
11642	Yes	AAGATTGCAGGTTTGAGTTCTGCC
11643	Yes	AAGATTGCAGGTTTGAGTTCTGCCA
11644	Yes	AAGATTGCAGGTTTGAGTTCTGCCAC
11645	Yes	AAGATTGCAGGTTTGAGTTCTGCCACG
11646	Yes	AAGATTGCAGGTTTGAGTTCTGCCACGG
11647	Yes	AAGATTGCAGGTTTGAGTTCTGCCACGGT
11648	Yes	AAGATTGCAGGTTTGAGTTCTGCCACGGTC
11649	Yes	AAGATTGCAGGTTTGAGTTCTGCCACGGTCG
11650	Yes	AAGATTGCAGGTTTGAGTTCTGCCACGGTCGC
11651	Yes	AAGATTGCAGGTTTGAGTTCTGCCACGGTCGCC
11652	Yes	AAGATTGCAGGTTTGAGTTCTGCCACGGTCGCCA
11653	Yes	AAGATTGGTGGTTCGA
11654	Yes	AAGATTGGTGGTTCGAG
11655	Yes	AAGATTGGTGGTTCGAGC
11656	Yes	AAGATTGGTGGTTCGAGCC
11657	Yes	AAGATTGGTGGTTCGAGCCC
11658	Yes	AAGATTGGTGGTTCGAGCCCA
11659	Yes	AAGATTGGTGGTTCGAGCCCAC
11660	Yes	AAGATTGGTGGTTCGAGCCCACC
11661	Yes	AAGATTGGTGGTTCGAGCCCACCC
11662	Yes	AAGATTGGTGGTTCGAGCCCACCCA
11663	Yes	AAGATTGGTGGTTCGAGCCCACCCAG
11664	Yes	AAGATTGGTGGTTCGAGCCCACCCAGG
11665	Yes	AAGATTGGTGGTTCGAGCCCACCCAGGG
11666	Yes	AAGATTGGTGGTTCGAGCCCACCCAGGGA
11667	Yes	AAGATTGGTGGTTCGAGCCCACCCAGGGAC
11668	Yes	AAGATTGGTGGTTCGAGCCCACCCAGGGACG
11669	Yes	AAGATTGGTGGTTCGAGCCCACCCAGGGACGC

11670	Yes	AAGATTGGTGGTTCGAGCCCACCCAGGGACGCC
11671	Yes	AAGATTGGTGGTTCGAGCCCACCCAGGGACGCCA
11672	No	AAGCAATACACTGAAA
11673	No	AAGCAATACACTGAAAA
11674	No	AAGCAATACACTGAAAAT
11675	No	AAGCAATACACTGAAAATG
11676	No	AAGCAATACACTGAAAATGT
11677	No	AAGCAATACACTGAAAATGTT
11678	No	AAGCAATACACTGAAAATGTTT
11679	Yes	AAGCAATACACTGAAAATGTTTA
11680	Yes	AAGCAATACACTGAAAATGTTTAG
11681	Yes	AAGCAATACACTGAAAATGTTTAGA
11682	Yes	AAGCAATACACTGAAAATGTTTAGAC
11683	Yes	AAGCAATACACTGAAAATGTTTAGACG
11684	Yes	AAGCAATACACTGAAAATGTTTAGACGG
11685	Yes	AAGCAATACACTGAAAATGTTTAGACGGG
11686	Yes	AAGCAATACACTGAAAATGTTTAGACGGGC
11687	Yes	AAGCAATACACTGAAAATGTTTAGACGGGCT
11688	Yes	AAGCAATACACTGAAAATGTTTAGACGGGCTC
11689	Yes	AAGCAATACACTGAAAATGTTTAGACGGGCTCA
11690	Yes	AAGCAATACACTGAAAATGTTTAGACGGGCTCAC
11691	Yes	AAGCAATACACTGAAAATGTTTAGACGGGCTCACA
11692	Yes	AAGCAATACACTGAAAATGTTTAGACGGGCTCACAT
11693	Yes	AAGCAATACACTGAAAATGTTTAGACGGGCTCACATC
11694	Yes	AAGCAATACACTGAAAATGTTTAGACGGGCTCACATCA
11695	Yes	AAGCAATACACTGAAAATGTTTAGACGGGCTCACATCAC
11696	Yes	AAGCAATACACTGAAAATGTTTAGACGGGCTCACATCACC
11697	Yes	AAGCAATACACTGAAAATGTTTAGACGGGCTCACATCACCC
11698	Yes	AAGCAATACACTGAAAATGTTTAGACGGGCTCACATCACCCC
11699	Yes	AAGCAATACACTGAAAATGTTTAGACGGGCTCACATCACCCCA
11700	Yes	AAGCAATACACTGAAAATGTTTAGACGGGCTCACATCACCCCAT
11701	Yes	AAGCAATACACTGAAAATGTTTAGACGGGCTCACATCACCCCATA
11702	Yes	AAGCAATACACTGAAAATGTTTAGACGGGCTCACATCACCCCATAA
11703	Yes	AAGCAATACACTGAAAATGTTTAGACGGGCTCACATCACCCCATAAA
11704	Yes	AAGCAATACACTGAAAATGTTTAGACGGGCTCACATCACCCCATAAAC
11705	Yes	AAGCAATACACTGAAAATGTTTAGACGGGCTCACATCACCCCATAAACA
11706	Yes	AAGCAATACACTGAAAATGTTTAGACGGGCTCACATCACCCCATAAACAC
11707	Yes	AAGCAATTGACCCGAC
11708	Yes	AAGCAATTGACCCGACT
11709	Yes	AAGCAATTGACCCGACTT
11710	Yes	AAGCAATTGACCCGACTTC
11711	Yes	AAGCAATTGACCCGACTTCA

11712	Yes	AAGCAATTGACCCGACTTCAA
11713	Yes	AAGCAATTGACCCGACTTCAAT
11714	Yes	AAGCAATTGACCCGACTTCAATT
11715	Yes	AAGCAATTGACCCGACTTCAATTC
11716	Yes	AAGCAATTGACCCGACTTCAATTCC
11717	Yes	AAGCAATTGACCCGACTTCAATTCCC
11718	Yes	AAGCAATTGACCCGACTTCAATTCCCA
11719	Yes	AAGCAATTGACCCGACTTCAATTCCCAG
11720	Yes	AAGCAATTGACCCGACTTCAATTCCCAGC
11721	Yes	AAGCAATTGACCCGACTTCAATTCCCAGCC
11722	Yes	AAGCAATTGACCCGACTTCAATTCCCAGCCA
11723	Yes	AAGCAATTGACCCGACTTCAATTCCCAGCCAA
11724	Yes	AAGCAATTGACCCGACTTCAATTCCCAGCCAAC
11725	Yes	AAGCAATTGACCCGACTTCAATTCCCAGCCAACG
11726	Yes	AAGCAATTGACCCGACTTCAATTCCCAGCCAACGC
11727	Yes	AAGCAATTGACCCGACTTCAATTCCCAGCCAACGCA
11728	Yes	AAGCAATTGACCCGACTTCAATTCCCAGCCAACGCAC
11729	Yes	AAGCAATTGACCCGACTTCAATTCCCAGCCAACGCACC
11730	Yes	AAGCAATTGACCCGACTTCAATTCCCAGCCAACGCACCA
11731	Yes	AAGCAATTGACCCGACTTCG
11732	Yes	AAGCAATTGACCCGACTTCGA
11733	Yes	AAGCAATTGACCCGACTTCGAT
11734	Yes	AAGCAATTGACCCGACTTCGATT
11735	Yes	AAGCAATTGACCCGACTTCGATTC
11736	Yes	AAGCAATTGACCCGACTTCGATTCC
11737	Yes	AAGCAATTGACCCGACTTCGATTCCC
11738	Yes	AAGCAATTGACCCGACTTCGATTCCCA
11739	Yes	AAGCAATTGACCCGACTTCGATTCCCAG
11740	Yes	AAGCAATTGACCCGACTTCGATTCCCAGC
11741	Yes	AAGCAATTGACCCGACTTCGATTCCCAGCC
11742	Yes	AAGCAATTGACCCGACTTCGATTCCCAGCCA
11743	Yes	AAGCAATTGACCCGACTTCGATTCCCAGCCAA
11744	Yes	AAGCAATTGACCCGACTTCGATTCCCAGCCAAT
11745	Yes	AAGCAATTGACCCGACTTCGATTCCCAGCCAATG
11746	Yes	AAGCAATTGACCCGACTTCGATTCCCAGCCAATGC
11747	Yes	AAGCAATTGACCCGACTTCGATTCCCAGCCAATGCA
11748	Yes	AAGCAATTGACCCGACTTCGATTCCCAGCCAATGCAC
11749	Yes	AAGCAATTGACCCGACTTCGATTCCCAGCCAATGCACC
11750	Yes	AAGCAATTGACCCGACTTCGATTCCCAGCCAATGCACCA
11751	No	AAGCACAGTTGGCTAG
11752	No	AAGCACAGTTGGCTAGA
11753	Yes	AAGCACAGTTGGCTAGAA

11754	Yes	AAGCACAGTTGGCTAGAAC
11755	Yes	AAGCACAGTTGGCTAGAACA
11756	Yes	AAGCACAGTTGGCTAGAACAT
11757	Yes	AAGCACAGTTGGCTAGAACATG
11758	Yes	AAGCACAGTTGGCTAGAACATGG
11759	Yes	AAGCACAGTTGGCTAGAACATGGT
11760	Yes	AAGCACAGTTGGCTAGAACATGGTG
11761	Yes	AAGCACAGTTGGCTAGAACATGGTGC
11762	Yes	AAGCACAGTTGGCTAGAACATGGTGCT
11763	Yes	AAGCACAGTTGGCTAGAACATGGTGCTA
11764	Yes	AAGCACAGTTGGCTAGAACATGGTGCTAA
11765	Yes	AAGCACAGTTGGCTAGAACATGGTGCTAAT
11766	Yes	AAGCACAGTTGGCTAGAACATGGTGCTAATA
11767	Yes	AAGCACAGTTGGCTAGAACATGGTGCTAATAA
11768	Yes	AAGCACAGTTGGCTAGAACATGGTGCTAATAAG
11769	Yes	AAGCACAGTTGGCTAGAACATGGTGCTAATAAGG
11770	Yes	AAGCACAGTTGGCTAGAACATGGTGCTAATAAGGC
11771	Yes	AAGCACAGTTGGCTAGAACATGGTGCTAATAAGGCC
11772	Yes	AAGCACAGTTGGCTAGAACATGGTGCTAATAAGGCCA
11773	Yes	AAGCACAGTTGGCTAGAACATGGTGCTAATAAGGCCAC
11774	Yes	AAGCACAGTTGGCTAGAACATGGTGCTAATAAGGCCACG
11775	Yes	AAGCACAGTTGGCTAGAACATGGTGCTAATAAGGCCACGG
11776	Yes	AAGCACAGTTGGCTAGAACATGGTGCTAATAAGGCCACGGT
11777	Yes	AAGCACAGTTGGCTAGAACATGGTGCTAATAAGGCCACGGTC
11778	Yes	AAGCACAGTTGGCTAGAACATGGTGCTAATAAGGCCACGGTCA
11779	Yes	AAGCACAGTTGGCTAGAACATGGTGCTAATAAGGCCACGGTCAG
11780	Yes	AAGCACAGTTGGCTAGAACATGGTGCTAATAAGGCCACGGTCAGG
11781	Yes	AAGCACAGTTGGCTAGAACATGGTGCTAATAAGGCCACGGTCAGGG
11782	Yes	AAGCACAGTTGGCTAGAACATGGTGCTAATAAGGCCACGGTCAGGGG
11783	Yes	AAGCACAGTTGGCTAGAACATGGTGCTAATAAGGCCACGGTCAGGGGT
11784	Yes	AAGCACAGTTGGCTAGAACATGGTGCTAATAAGGCCACGGTCAGGGGTT
11785	Yes	AAGCACAGTTGGCTAGAACATGGTGCTAATAAGGCCACGGTCAGGGGTTC
11786	No	AAGCACCATGGCTTAG
11787	Yes	AAGCACCATGGCTTAGC
11788	Yes	AAGCACCATGGCTTAGCT
11789	Yes	AAGCACCATGGCTTAGCTG
11790	Yes	AAGCACCATGGCTTAGCTGG
11791	Yes	AAGCACCATGGCTTAGCTGGT
11792	Yes	AAGCACCATGGCTTAGCTGGTT
11793	Yes	AAGCACCATGGCTTAGCTGGTTA
11794	Yes	AAGCACCATGGCTTAGCTGGTTAA
11795	Yes	AAGCACCATGGCTTAGCTGGTTAAA

11796	Yes	AAGCACCATGGCTTAGCTGGTTAAAG
11797	Yes	AAGCACCATGGCTTAGCTGGTTAAAGC
11798	Yes	AAGCACCATGGCTTAGCTGGTTAAAGCA
11799	Yes	AAGCACCATGGCTTAGCTGGTTAAAGCAC
11800	Yes	AAGCACCATGGCTTAGCTGGTTAAAGCACC
11801	Yes	AAGCACCATGGCTTAGCTGGTTAAAGCACCT
11802	Yes	AAGCACCATGGCTTAGCTGGTTAAAGCACCTG
11803	Yes	AAGCACCATGGCTTAGCTGGTTAAAGCACCTGT
11804	Yes	AAGCACCATGGCTTAGCTGGTTAAAGCACCTGTC
11805	Yes	AAGCACCATGGCTTAGCTGGTTAAAGCACCTGTCT
11806	Yes	AAGCACCATGGCTTAGCTGGTTAAAGCACCTGTCTA
11807	Yes	AAGCACCATGGCTTAGCTGGTTAAAGCACCTGTCTAG
11808	Yes	AAGCACCATGGCTTAGCTGGTTAAAGCACCTGTCTAGT
11809	Yes	AAGCACCATGGCTTAGCTGGTTAAAGCACCTGTCTAGTA
11810	Yes	AAGCACCATGGCTTAGCTGGTTAAAGCACCTGTCTAGTAA
11811	Yes	AAGCACCATGGCTTAGCTGGTTAAAGCACCTGTCTAGTAAA
11812	Yes	AAGCACCATGGCTTAGCTGGTTAAAGCACCTGTCTAGTAAAC
11813	Yes	AAGCACCATGGCTTAGCTGGTTAAAGCACCTGTCTAGTAAACA
11814	Yes	AAGCACCATGGCTTAGCTGGTTAAAGCACCTGTCTAGTAAACAG
11815	Yes	AAGCACCATGGCTTAGCTGGTTAAAGCACCTGTCTAGTAAACAGG
11816	Yes	AAGCACCATGGCTTAGCTGGTTAAAGCACCTGTCTAGTAAACAGGA
11817	Yes	AAGCACCATGGCTTAGCTGGTTAAAGCACCTGTCTAGTAAACAGGAG
11818	Yes	AAGCACCATGGCTTAGCTGGTTAAAGCACCTGTCTAGTAAACAGGAGA
11819	Yes	AAGCACCATGGCTTAGCTGGTTAAAGCACCTGTCTAGTAAACAGGAGAT
11820	Yes	AAGCACCATGGCTTAGCTGGTTAAAGCACCTGTCTAGTAAACAGGAGATC
11821	No	AAGCACCCAACTTACA
11822	No	AAGCACCCAACTTACAC
11823	No	AAGCACCCAACTTACACT
11824	No	AAGCACCCAACTTACACTT
11825	No	AAGCACCCAACTTACACTTA
11826	No	AAGCACCCAACTTACACTTAG
11827	No	AAGCACCCAACTTACACTTAGG
11828	No	AAGCACCCAACTTACACTTAGGA
11829	No	AAGCACCCAACTTACACTTAGGAG
11830	No	AAGCACCCAACTTACACTTAGGAGA
11831	No	AAGCACCCAACTTACACTTAGGAGAT
11832	No	AAGCACCCAACTTACACTTAGGAGATT
11833	No	AAGCACCCAACTTACACTTAGGAGATTT
11834	No	AAGCACCCAACTTACACTTAGGAGATTTC
11835	No	AAGCACCCAACTTACACTTAGGAGATTTCA
11836	No	AAGCACCCAACTTACACTTAGGAGATTTCAA
11837	No	AAGCACCCAACTTACACTTAGGAGATTTCAAC

11838	No	AAGCACCCAACTTACACTTAGGAGATTTCAACT
11839	No	AAGCACCCAACTTACACTTAGGAGATTTCAACTT
11840	No	AAGCACCCAACTTACACTTAGGAGATTTCAACTTA
11841	No	AAGCACCCAACTTACACTTAGGAGATTTCAACTTAA
11842	No	AAGCACCCAACTTACACTTAGGAGATTTCAACTTAAC
11843	No	AAGCACCCAACTTACACTTAGGAGATTTCAACTTAACT
11844	No	AAGCACCCAACTTACACTTAGGAGATTTCAACTTAACTT
11845	No	AAGCACCCAACTTACACTTAGGAGATTTCAACTTAACTTG
11846	No	AAGCACCCAACTTACACTTAGGAGATTTCAACTTAACTTGA
11847	No	AAGCACCCAACTTACACTTAGGAGATTTCAACTTAACTTGAC
11848	No	AAGCACCCAACTTACACTTAGGAGATTTCAACTTAACTTGACC
11849	No	AAGCACCCAACTTACACTTAGGAGATTTCAACTTAACTTGACCG
11850	No	AAGCACCCAACTTACACTTAGGAGATTTCAACTTAACTTGACCGC
11851	No	AAGCACCCAACTTACACTTAGGAGATTTCAACTTAACTTGACCGCT
11852	No	AAGCACCCAACTTACACTTAGGAGATTTCAACTTAACTTGACCGCTC
11853	No	AAGCACCCAACTTACACTTAGGAGATTTCAACTTAACTTGACCGCTCT
11854	No	AAGCACCCAACTTACACTTAGGAGATTTCAACTTAACTTGACCGCTCTG
11855	No	AAGCACCCAACTTACACTTAGGAGATTTCAACTTAACTTGACCGCTCTGA
11856	No	AAGCACCCTGGACTCT
11857	No	AAGCACCCTGGACTCTG
11858	Yes	AAGCACCCTGGACTCTGA
11859	Yes	AAGCACCCTGGACTCTGAA
11860	Yes	AAGCACCCTGGACTCTGAAT
11861	Yes	AAGCACCCTGGACTCTGAATC
11862	Yes	AAGCACCCTGGACTCTGAATCC
11863	Yes	AAGCACCCTGGACTCTGAATCCA
11864	Yes	AAGCACCCTGGACTCTGAATCCAG
11865	Yes	AAGCACCCTGGACTCTGAATCCAGC
11866	Yes	AAGCACCCTGGACTCTGAATCCAGCA
11867	Yes	AAGCACCCTGGACTCTGAATCCAGCAA
11868	Yes	AAGCACCCTGGACTCTGAATCCAGCAAC
11869	Yes	AAGCACCCTGGACTCTGAATCCAGCAACC
11870	Yes	AAGCACCCTGGACTCTGAATCCAGCAACCA
11871	Yes	AAGCACCCTGGACTCTGAATCCAGCAACCAG
11872	Yes	AAGCACCCTGGACTCTGAATCCAGCAACCAGA
11873	Yes	AAGCACCCTGGACTCTGAATCCAGCAACCAGAG
11874	Yes	AAGCACCCTGGACTCTGAATCCAGCAACCAGAGT
11875	Yes	AAGCACCCTGGACTCTGAATCCAGCAACCAGAGTT
11876	Yes	AAGCACCCTGGACTCTGAATCCAGCAACCAGAGTTC
11877	Yes	AAGCACCCTGGACTCTGAATCCAGCAACCAGAGTTCC
11878	Yes	AAGCACCCTGGACTCTGAATCCAGCAACCAGAGTTCCA
11879	Yes	AAGCACCCTGGACTCTGAATCCAGCAACCAGAGTTCCAG

11880	Yes	AAGCACCCTGGACTCTGAATCCAGCAACCAGAGTTCCAGT
11881	Yes	AAGCACCCTGGACTCTGAATCCAGCAACCAGAGTTCCAGTC
11882	Yes	AAGCACCCTGGACTCTGAATCCAGCAACCAGAGTTCCAGTCT
11883	Yes	AAGCACCCTGGACTCTGAATCCAGCAACCAGAGTTCCAGTCTC
11884	Yes	AAGCACCCTGGACTCTGAATCCAGCAACCAGAGTTCCAGTCTCA
11885	Yes	AAGCACCCTGGACTCTGAATCCAGCAACCAGAGTTCCAGTCTCAG
11886	Yes	AAGCACCCTGGACTCTGAATCCAGCAACCAGAGTTCCAGTCTCAGC
11887	Yes	AAGCACCCTGGACTCTGAATCCAGCAACCAGAGTTCCAGTCTCAGCG
11888	Yes	AAGCACCCTGGACTCTGAATCCAGCAACCAGAGTTCCAGTCTCAGCGT
11889	Yes	AAGCACCCTGGACTCTGAATCCAGCAACCAGAGTTCCAGTCTCAGCGTG
11890	Yes	AAGCACCCTGGACTCTGAATCCAGCAACCAGAGTTCCAGTCTCAGCGTGG
11891	No	AAGCACCTGTCTAGTA
11892	No	AAGCACCTGTCTAGTAA
11893	No	AAGCACCTGTCTAGTAAA
11894	Yes	AAGCACCTGTCTAGTAAAC
11895	Yes	AAGCACCTGTCTAGTAAACA
11896	Yes	AAGCACCTGTCTAGTAAACAG
11897	Yes	AAGCACCTGTCTAGTAAACAGG
11898	Yes	AAGCACCTGTCTAGTAAACAGGA
11899	Yes	AAGCACCTGTCTAGTAAACAGGAG
11900	Yes	AAGCACCTGTCTAGTAAACAGGAGA
11901	Yes	AAGCACCTGTCTAGTAAACAGGAGAT
11902	Yes	AAGCACCTGTCTAGTAAACAGGAGATC
11903	Yes	AAGCACCTGTCTAGTAAACAGGAGATCC
11904	Yes	AAGCACCTGTCTAGTAAACAGGAGATCCT
11905	Yes	AAGCACCTGTCTAGTAAACAGGAGATCCTG
11906	Yes	AAGCACCTGTCTAGTAAACAGGAGATCCTGA
11907	Yes	AAGCACCTGTCTAGTAAACAGGAGATCCTGAG
11908	Yes	AAGCACCTGTCTAGTAAACAGGAGATCCTGAGT
11909	Yes	AAGCACCTGTCTAGTAAACAGGAGATCCTGAGTT
11910	Yes	AAGCACCTGTCTAGTAAACAGGAGATCCTGAGTTT
11911	Yes	AAGCACCTGTCTAGTAAACAGGAGATCCTGAGTTTC
11912	Yes	AAGCACCTGTCTAGTAAACAGGAGATCCTGAGTTTCA
11913	Yes	AAGCACCTGTCTAGTAAACAGGAGATCCTGAGTTTCAA
11914	Yes	AAGCACCTGTCTAGTAAACAGGAGATCCTGAGTTTCAAT
11915	Yes	AAGCACCTGTCTAGTAAACAGGAGATCCTGAGTTTCAATT
11916	Yes	AAGCACCTGTCTAGTAAACAGGAGATCCTGAGTTTCAATTC
11917	Yes	AAGCACCTGTCTAGTAAACAGGAGATCCTGAGTTTCAATTCC
11918	Yes	AAGCACCTGTCTAGTAAACAGGAGATCCTGAGTTTCAATTCCA
11919	Yes	AAGCACCTGTCTAGTAAACAGGAGATCCTGAGTTTCAATTCCAA
11920	Yes	AAGCACCTGTCTAGTAAACAGGAGATCCTGAGTTTCAATTCCAAT
11921	Yes	AAGCACCTGTCTAGTAAACAGGAGATCCTGAGTTTCAATTCCAATG

11922	Yes	AAGCACCTGTCTAGTAAACAGGAGATCCTGAGTTTCAATTCCAATGG
11923	Yes	AAGCACCTGTCTAGTAAACAGGAGATCCTGAGTTTCAATTCCAATGGT
11924	Yes	AAGCACCTGTCTAGTAAACAGGAGATCCTGAGTTTCAATTCCAATGGTG
11925	Yes	AAGCACCTGTCTAGTAAACAGGAGATCCTGAGTTTCAATTCCAATGGTGC
11926	No	AAGCACTCTGGACTCT
11927	No	AAGCACTCTGGACTCTG
11928	No	AAGCACTCTGGACTCTGA
11929	No	AAGCACTCTGGACTCTGAA
11930	No	AAGCACTCTGGACTCTGAAT
11931	Yes	AAGCACTCTGGACTCTGAATC
11932	Yes	AAGCACTCTGGACTCTGAATCC
11933	Yes	AAGCACTCTGGACTCTGAATCCA
11934	Yes	AAGCACTCTGGACTCTGAATCCAG
11935	Yes	AAGCACTCTGGACTCTGAATCCAGC
11936	Yes	AAGCACTCTGGACTCTGAATCCAGCC
11937	Yes	AAGCACTCTGGACTCTGAATCCAGCCA
11938	Yes	AAGCACTCTGGACTCTGAATCCAGCCAT
11939	Yes	AAGCACTCTGGACTCTGAATCCAGCCATC
11940	Yes	AAGCACTCTGGACTCTGAATCCAGCCATCT
11941	Yes	AAGCACTCTGGACTCTGAATCCAGCCATCTG
11942	Yes	AAGCACTCTGGACTCTGAATCCAGCCATCTGA
11943	Yes	AAGCACTCTGGACTCTGAATCCAGCCATCTGAG
11944	Yes	AAGCACTCTGGACTCTGAATCCAGCCATCTGAGT
11945	Yes	AAGCACTCTGGACTCTGAATCCAGCCATCTGAGTT
11946	Yes	AAGCACTCTGGACTCTGAATCCAGCCATCTGAGTTC
11947	Yes	AAGCACTCTGGACTCTGAATCCAGCCATCTGAGTTCG
11948	Yes	AAGCACTCTGGACTCTGAATCCAGCCATCTGAGTTCGA
11949	Yes	AAGCACTCTGGACTCTGAATCCAGCCATCTGAGTTCGAG
11950	Yes	AAGCACTCTGGACTCTGAATCCAGCCATCTGAGTTCGAGT
11951	Yes	AAGCACTCTGGACTCTGAATCCAGCCATCTGAGTTCGAGTC
11952	Yes	AAGCACTCTGGACTCTGAATCCAGCCATCTGAGTTCGAGTCT
11953	Yes	AAGCACTCTGGACTCTGAATCCAGCCATCTGAGTTCGAGTCTC
11954	Yes	AAGCACTCTGGACTCTGAATCCAGCCATCTGAGTTCGAGTCTCT
11955	Yes	AAGCACTCTGGACTCTGAATCCAGCCATCTGAGTTCGAGTCTCTG
11956	Yes	AAGCACTCTGGACTCTGAATCCAGCCATCTGAGTTCGAGTCTCTGT
11957	Yes	AAGCACTCTGGACTCTGAATCCAGCCATCTGAGTTCGAGTCTCTGTG
11958	Yes	AAGCACTCTGGACTCTGAATCCAGCCATCTGAGTTCGAGTCTCTGTGG
11959	Yes	AAGCACTCTGGACTCTGAATCCAGCCATCTGAGTTCGAGTCTCTGTGGA
11960	Yes	AAGCACTCTGGACTCTGAATCCAGCCATCTGAGTTCGAGTCTCTGTGGAA
11961	Yes	AAGCACTCTGGACTCTGAATCCAGCG
11962	Yes	AAGCACTCTGGACTCTGAATCCAGCGA
11963	Yes	AAGCACTCTGGACTCTGAATCCAGCGAT

11964	Yes	AAGCACTCTGGACTCTGAATCCAGCGATC
11965	Yes	AAGCACTCTGGACTCTGAATCCAGCGATCC
11966	Yes	AAGCACTCTGGACTCTGAATCCAGCGATCCG
11967	Yes	AAGCACTCTGGACTCTGAATCCAGCGATCCGA
11968	Yes	AAGCACTCTGGACTCTGAATCCAGCGATCCGAG
11969	Yes	AAGCACTCTGGACTCTGAATCCAGCGATCCGAGT
11970	Yes	AAGCACTCTGGACTCTGAATCCAGCGATCCGAGTT
11971	Yes	AAGCACTCTGGACTCTGAATCCAGCGATCCGAGTTC
11972	Yes	AAGCACTCTGGACTCTGAATCCAGCGATCCGAGTTCG
11973	Yes	AAGCACTCTGGACTCTGAATCCAGCGATCCGAGTTCGA
11974	Yes	AAGCACTCTGGACTCTGAATCCAGCGATCCGAGTTCGAG
11975	Yes	AAGCACTCTGGACTCTGAATCCAGCGATCCGAGTTCGAGT
11976	Yes	AAGCACTCTGGACTCTGAATCCAGCGATCCGAGTTCGAGTC
11977	Yes	AAGCACTCTGGACTCTGAATCCAGCGATCCGAGTTCGAGTCT
11978	Yes	AAGCACTCTGGACTCTGAATCCAGCGATCCGAGTTCGAGTCTC
11979	Yes	AAGCACTCTGGACTCTGAATCCAGCGATCCGAGTTCGAGTCTCG
11980	Yes	AAGCACTCTGGACTCTGAATCCAGCGATCCGAGTTCGAGTCTCGG
11981	Yes	AAGCACTCTGGACTCTGAATCCAGCGATCCGAGTTCGAGTCTCGGT
11982	Yes	AAGCACTCTGGACTCTGAATCCAGCGATCCGAGTTCGAGTCTCGGTG
11983	Yes	AAGCACTCTGGACTCTGAATCCAGCGATCCGAGTTCGAGTCTCGGTGG
11984	Yes	AAGCACTCTGGACTCTGAATCCAGCGATCCGAGTTCGAGTCTCGGTGGA
11985	Yes	AAGCACTCTGGACTCTGAATCCAGCGATCCGAGTTCGAGTCTCGGTGGAA
11986	No	AAGCAGACAGCCTGGG
11987	No	AAGCAGACAGCCTGGGT
11988	No	AAGCAGACAGCCTGGGTT
11989	No	AAGCAGACAGCCTGGGTTC
11990	Yes	AAGCAGACAGCCTGGGTTCA
11991	Yes	AAGCAGACAGCCTGGGTTCAA
11992	Yes	AAGCAGACAGCCTGGGTTCAAT
11993	Yes	AAGCAGACAGCCTGGGTTCAATT
11994	Yes	AAGCAGACAGCCTGGGTTCAATTC
11995	Yes	AAGCAGACAGCCTGGGTTCAATTCC
11996	Yes	AAGCAGACAGCCTGGGTTCAATTCCC
11997	Yes	AAGCAGACAGCCTGGGTTCAATTCCCA
11998	Yes	AAGCAGACAGCCTGGGTTCAATTCCCAG
11999	Yes	AAGCAGACAGCCTGGGTTCAATTCCCAGC
12000	Yes	AAGCAGACAGCCTGGGTTCAATTCCCAGCT
12001	Yes	AAGCAGACAGCCTGGGTTCAATTCCCAGCTT
12002	Yes	AAGCAGACAGCCTGGGTTCAATTCCCAGCTTG
12003	Yes	AAGCAGACAGCCTGGGTTCAATTCCCAGCTTGG
12004	Yes	AAGCAGACAGCCTGGGTTCAATTCCCAGCTTGGC
12005	Yes	AAGCAGACAGCCTGGGTTCAATTCCCAGCTTGGCC

12006	Yes	AAGCAGACAGCCTGGGTTCAATTCCCAGCTTGGCCA
12007	Yes	AAGCAGACAGCCTGGGTTCAATTCCCAGCTTGGCCAC
12008	Yes	AAGCAGACAGCCTGGGTTCAATTCCCAGCTTGGCCACC
12009	Yes	AAGCAGACAGCCTGGGTTCAATTCCCAGCTTGGCCACCA
12010	Yes	AAGCAGAGTGGCGCAG
12011	Yes	AAGCAGAGTGGCGCAGC
12012	Yes	AAGCAGAGTGGCGCAGCG
12013	Yes	AAGCAGAGTGGCGCAGCGG
12014	Yes	AAGCAGAGTGGCGCAGCGGA
12015	Yes	AAGCAGAGTGGCGCAGCGGAA
12016	Yes	AAGCAGAGTGGCGCAGCGGAAG
12017	Yes	AAGCAGAGTGGCGCAGCGGAAGC
12018	Yes	AAGCAGAGTGGCGCAGCGGAAGCG
12019	Yes	AAGCAGAGTGGCGCAGCGGAAGCGT
12020	Yes	AAGCAGAGTGGCGCAGCGGAAGCGTG
12021	Yes	AAGCAGAGTGGCGCAGCGGAAGCGTGC
12022	Yes	AAGCAGAGTGGCGCAGCGGAAGCGTGCT
12023	Yes	AAGCAGAGTGGCGCAGCGGAAGCGTGCTG
12024	Yes	AAGCAGAGTGGCGCAGCGGAAGCGTGCTGG
12025	Yes	AAGCAGAGTGGCGCAGCGGAAGCGTGCTGGG
12026	Yes	AAGCAGAGTGGCGCAGCGGAAGCGTGCTGGGC
12027	Yes	AAGCAGAGTGGCGCAGCGGAAGCGTGCTGGGCC
12028	Yes	AAGCAGAGTGGCGCAGCGGAAGCGTGCTGGGCCC
12029	Yes	AAGCAGAGTGGCGCAGCGGAAGCGTGCTGGGCCCA
12030	Yes	AAGCAGAGTGGCGCAGCGGAAGCGTGCTGGGCCCAT
12031	Yes	AAGCAGAGTGGCGCAGCGGAAGCGTGCTGGGCCCATA
12032	Yes	AAGCAGAGTGGCGCAGCGGAAGCGTGCTGGGCCCATAA
12033	Yes	AAGCAGAGTGGCGCAGCGGAAGCGTGCTGGGCCCATAAC
12034	Yes	AAGCAGAGTGGCGCAGCGGAAGCGTGCTGGGCCCATAACC
12035	Yes	AAGCAGAGTGGCGCAGCGGAAGCGTGCTGGGCCCATAACCC
12036	Yes	AAGCAGAGTGGCGCAGCGGAAGCGTGCTGGGCCCATAACCCA
12037	Yes	AAGCAGAGTGGCGCAGCGGAAGCGTGCTGGGCCCATAACCCAG
12038	Yes	AAGCAGAGTGGCGCAGCGGAAGCGTGCTGGGCCCATAACCCAGA
12039	Yes	AAGCAGAGTGGCGCAGCGGAAGCGTGCTGGGCCCATAACCCAGAG
12040	Yes	AAGCAGAGTGGCGCAGCGGAAGCGTGCTGGGCCCATAACCCAGAGG
12041	Yes	AAGCAGAGTGGCGCAGCGGAAGCGTGCTGGGCCCATAACCCAGAGGT
12042	Yes	AAGCAGAGTGGCGCAGCGGAAGCGTGCTGGGCCCATAACCCAGAGGTC
12043	Yes	AAGCAGAGTGGCGCAGCGGAAGCGTGCTGGGCCCATAACCCAGAGGTCG
12044	Yes	AAGCAGAGTGGCGCAGCGGAAGCGTGCTGGGCCCATAACCCAGAGGTCGA
12045	No	AAGCAGAGTGGTGCAG
12046	Yes	AAGCAGAGTGGTGCAGT
12047	Yes	AAGCAGAGTGGTGCAGTG

12048	Yes	AAGCAGAGTGGTGCAGTGG
12049	Yes	AAGCAGAGTGGTGCAGTGGA
12050	Yes	AAGCAGAGTGGTGCAGTGGAA
12051	Yes	AAGCAGAGTGGTGCAGTGGAAG
12052	Yes	AAGCAGAGTGGTGCAGTGGAAGC
12053	Yes	AAGCAGAGTGGTGCAGTGGAAGCA
12054	Yes	AAGCAGAGTGGTGCAGTGGAAGCAT
12055	Yes	AAGCAGAGTGGTGCAGTGGAAGCATA
12056	Yes	AAGCAGAGTGGTGCAGTGGAAGCATAC
12057	Yes	AAGCAGAGTGGTGCAGTGGAAGCATACC
12058	Yes	AAGCAGAGTGGTGCAGTGGAAGCATACCT
12059	Yes	AAGCAGAGTGGTGCAGTGGAAGCATACCTA
12060	Yes	AAGCAGAGTGGTGCAGTGGAAGCATACCTAT
12061	Yes	AAGCAGAGTGGTGCAGTGGAAGCATACCTATG
12062	Yes	AAGCAGAGTGGTGCAGTGGAAGCATACCTATGG
12063	Yes	AAGCAGAGTGGTGCAGTGGAAGCATACCTATGGG
12064	Yes	AAGCAGAGTGGTGCAGTGGAAGCATACCTATGGGC
12065	Yes	AAGCAGAGTGGTGCAGTGGAAGCATACCTATGGGCC
12066	Yes	AAGCAGAGTGGTGCAGTGGAAGCATACCTATGGGCCC
12067	Yes	AAGCAGAGTGGTGCAGTGGAAGCATACCTATGGGCCCA
12068	Yes	AAGCAGAGTGGTGCAGTGGAAGCATACCTATGGGCCCAT
12069	Yes	AAGCAGAGTGGTGCAGTGGAAGCATACCTATGGGCCCATA
12070	Yes	AAGCAGAGTGGTGCAGTGGAAGCATACCTATGGGCCCATAA
12071	Yes	AAGCAGAGTGGTGCAGTGGAAGCATACCTATGGGCCCATAAC
12072	Yes	AAGCAGAGTGGTGCAGTGGAAGCATACCTATGGGCCCATAACC
12073	Yes	AAGCAGAGTGGTGCAGTGGAAGCATACCTATGGGCCCATAACCC
12074	Yes	AAGCAGAGTGGTGCAGTGGAAGCATACCTATGGGCCCATAACCCA
12075	Yes	AAGCAGAGTGGTGCAGTGGAAGCATACCTATGGGCCCATAACCCAG
12076	Yes	AAGCAGAGTGGTGCAGTGGAAGCATACCTATGGGCCCATAACCCAGA
12077	Yes	AAGCAGAGTGGTGCAGTGGAAGCATACCTATGGGCCCATAACCCAGAG
12078	Yes	AAGCAGAGTGGTGCAGTGGAAGCATACCTATGGGCCCATAACCCAGAGG
12079	Yes	AAGCAGAGTGGTGCAGTGGAAGCATACCTATGGGCCCATAACCCAGAGGT
12080	No	AAGCAGCTTCAAACCT
12081	No	AAGCAGCTTCAAACCTG
12082	No	AAGCAGCTTCAAACCTGC
12083	No	AAGCAGCTTCAAACCTGCC
12084	No	AAGCAGCTTCAAACCTGCCG
12085	No	AAGCAGCTTCAAACCTGCCGG
12086	No	AAGCAGCTTCAAACCTGCCGGG
12087	No	AAGCAGCTTCAAACCTGCCGGGG
12088	No	AAGCAGCTTCAAACCTGCCGGGGC
12089	No	AAGCAGCTTCAAACCTGCCGGGGCT

12090	No	AAGCAGCTTCAAACCTGCCGGGGCTT
12091	No	AAGCAGCTTCAAACCTGCCGGGGCTTC
12092	Yes	AAGCAGCTTCAAACCTGCCGGGGCTTCC
12093	Yes	AAGCAGCTTCAAACCTGCCGGGGCTTCCA
12094	No	AAGCAGTGTAGCCTAG
12095	No	AAGCAGTGTAGCCTAGT
12096	No	AAGCAGTGTAGCCTAGTG
12097	No	AAGCAGTGTAGCCTAGTGG
12098	Yes	AAGCAGTGTAGCCTAGTGGC
12099	Yes	AAGCAGTGTAGCCTAGTGGCT
12100	Yes	AAGCAGTGTAGCCTAGTGGCTA
12101	Yes	AAGCAGTGTAGCCTAGTGGCTAG
12102	Yes	AAGCAGTGTAGCCTAGTGGCTAGG
12103	Yes	AAGCAGTGTAGCCTAGTGGCTAGGT
12104	Yes	AAGCAGTGTAGCCTAGTGGCTAGGTC
12105	Yes	AAGCAGTGTAGCCTAGTGGCTAGGTCC
12106	Yes	AAGCAGTGTAGCCTAGTGGCTAGGTCCT
12107	Yes	AAGCAGTGTAGCCTAGTGGCTAGGTCCTC
12108	Yes	AAGCAGTGTAGCCTAGTGGCTAGGTCCTCT
12109	Yes	AAGCAGTGTAGCCTAGTGGCTAGGTCCTCTG
12110	Yes	AAGCAGTGTAGCCTAGTGGCTAGGTCCTCTGA
12111	Yes	AAGCAGTGTAGCCTAGTGGCTAGGTCCTCTGAC
12112	Yes	AAGCAGTGTAGCCTAGTGGCTAGGTCCTCTGACT
12113	Yes	AAGCAGTGTAGCCTAGTGGCTAGGTCCTCTGACTT
12114	Yes	AAGCAGTGTAGCCTAGTGGCTAGGTCCTCTGACTTT
12115	Yes	AAGCAGTGTAGCCTAGTGGCTAGGTCCTCTGACTTTG
12116	Yes	AAGCAGTGTAGCCTAGTGGCTAGGTCCTCTGACTTTGA
12117	Yes	AAGCAGTGTAGCCTAGTGGCTAGGTCCTCTGACTTTGAA
12118	Yes	AAGCAGTGTAGCCTAGTGGCTAGGTCCTCTGACTTTGAAA
12119	Yes	AAGCAGTGTAGCCTAGTGGCTAGGTCCTCTGACTTTGAAAC
12120	Yes	AAGCAGTGTAGCCTAGTGGCTAGGTCCTCTGACTTTGAAACC
12121	Yes	AAGCAGTGTAGCCTAGTGGCTAGGTCCTCTGACTTTGAAACCA
12122	Yes	AAGCAGTGTAGCCTAGTGGCTAGGTCCTCTGACTTTGAAACCAC
12123	Yes	AAGCAGTGTAGCCTAGTGGCTAGGTCCTCTGACTTTGAAACCACG
12124	Yes	AAGCAGTGTAGCCTAGTGGCTAGGTCCTCTGACTTTGAAACCACGT
12125	Yes	AAGCAGTGTAGCCTAGTGGCTAGGTCCTCTGACTTTGAAACCACGTG
12126	Yes	AAGCAGTGTAGCCTAGTGGCTAGGTCCTCTGACTTTGAAACCACGTGG
12127	Yes	AAGCAGTGTAGCCTAGTGGCTAGGTCCTCTGACTTTGAAACCACGTGGT
12128	Yes	AAGCAGTGTAGCCTAGTGGCTAGGTCCTCTGACTTTGAAACCACGTGGTC
12129	Yes	AAGCAGTTAACGCGGG
12130	Yes	AAGCAGTTAACGCGGGT
12131	Yes	AAGCAGTTAACGCGGGTT

12132	Yes	AAGCAGTTAACGCGGGTTC
12133	Yes	AAGCAGTTAACGCGGGTTCG
12134	Yes	AAGCAGTTAACGCGGGTTCGA
12135	Yes	AAGCAGTTAACGCGGGTTCGAT
12136	Yes	AAGCAGTTAACGCGGGTTCGATT
12137	Yes	AAGCAGTTAACGCGGGTTCGATTC
12138	Yes	AAGCAGTTAACGCGGGTTCGATTCC
12139	Yes	AAGCAGTTAACGCGGGTTCGATTCCC
12140	Yes	AAGCAGTTAACGCGGGTTCGATTCCCG
12141	Yes	AAGCAGTTAACGCGGGTTCGATTCCCGG
12142	Yes	AAGCAGTTAACGCGGGTTCGATTCCCGGG
12143	Yes	AAGCAGTTAACGCGGGTTCGATTCCCGGGT
12144	Yes	AAGCAGTTAACGCGGGTTCGATTCCCGGGTA
12145	Yes	AAGCAGTTAACGCGGGTTCGATTCCCGGGTAA
12146	Yes	AAGCAGTTAACGCGGGTTCGATTCCCGGGTAAC
12147	Yes	AAGCAGTTAACGCGGGTTCGATTCCCGGGTAACG
12148	Yes	AAGCAGTTAACGCGGGTTCGATTCCCGGGTAACGA
12149	Yes	AAGCAGTTAACGCGGGTTCGATTCCCGGGTAACGAA
12150	Yes	AAGCAGTTAACGCGGGTTCGATTCCCGGGTAACGAAC
12151	Yes	AAGCAGTTAACGCGGGTTCGATTCCCGGGTAACGAACC
12152	Yes	AAGCAGTTAACGCGGGTTCGATTCCCGGGTAACGAACCA
12153	Yes	AAGCAGTTGACCCGGG
12154	Yes	AAGCAGTTGACCCGGGC
12155	Yes	AAGCAGTTGACCCGGGCT
12156	Yes	AAGCAGTTGACCCGGGCTC
12157	Yes	AAGCAGTTGACCCGGGCTCG
12158	Yes	AAGCAGTTGACCCGGGCTCGA
12159	Yes	AAGCAGTTGACCCGGGCTCGAT
12160	Yes	AAGCAGTTGACCCGGGCTCGATT
12161	Yes	AAGCAGTTGACCCGGGCTCGATTC
12162	Yes	AAGCAGTTGACCCGGGCTCGATTCC
12163	Yes	AAGCAGTTGACCCGGGCTCGATTCCC
12164	Yes	AAGCAGTTGACCCGGGCTCGATTCCCG
12165	Yes	AAGCAGTTGACCCGGGCTCGATTCCCGC
12166	Yes	AAGCAGTTGACCCGGGCTCGATTCCCGCC
12167	Yes	AAGCAGTTGACCCGGGCTCGATTCCCGCCC
12168	Yes	AAGCAGTTGACCCGGGCTCGATTCCCGCCCA
12169	Yes	AAGCAGTTGACCCGGGCTCGATTCCCGCCCAA
12170	Yes	AAGCAGTTGACCCGGGCTCGATTCCCGCCCAAC
12171	Yes	AAGCAGTTGACCCGGGCTCGATTCCCGCCCAACG
12172	Yes	AAGCAGTTGACCCGGGCTCGATTCCCGCCCAACGC
12173	Yes	AAGCAGTTGACCCGGGCTCGATTCCCGCCCAACGCA

12174	Yes	AAGCAGTTGACCCGGGCTCGATTCCCGCCCAACGCAC
12175	Yes	AAGCAGTTGACCCGGGCTCGATTCCCGCCCAACGCACC
12176	Yes	AAGCAGTTGACCCGGGCTCGATTCCCGCCCAACGCACCA
12177	Yes	AAGCAGTTGACCCGGGT
12178	Yes	AAGCAGTTGACCCGGGTT
12179	Yes	AAGCAGTTGACCCGGGTTC
12180	Yes	AAGCAGTTGACCCGGGTTCG
12181	Yes	AAGCAGTTGACCCGGGTTCGA
12182	Yes	AAGCAGTTGACCCGGGTTCGAT
12183	Yes	AAGCAGTTGACCCGGGTTCGATT
12184	Yes	AAGCAGTTGACCCGGGTTCGATTC
12185	Yes	AAGCAGTTGACCCGGGTTCGATTCC
12186	Yes	AAGCAGTTGACCCGGGTTCGATTCCC
12187	Yes	AAGCAGTTGACCCGGGTTCGATTCCCG
12188	Yes	AAGCAGTTGACCCGGGTTCGATTCCCGG
12189	Yes	AAGCAGTTGACCCGGGTTCGATTCCCGGC
12190	Yes	AAGCAGTTGACCCGGGTTCGATTCCCGGCC
12191	Yes	AAGCAGTTGACCCGGGTTCGATTCCCGGCCA
12192	Yes	AAGCAGTTGACCCGGGTTCGATTCCCGGCCAA
12193	Yes	AAGCAGTTGACCCGGGTTCGATTCCCGGCCAAC
12194	Yes	AAGCAGTTGACCCGGGTTCGATTCCCGGCCAACG
12195	Yes	AAGCAGTTGACCCGGGTTCGATTCCCGGCCAACGC
12196	Yes	AAGCAGTTGACCCGGGTTCGATTCCCGGCCAACGCA
12197	Yes	AAGCAGTTGACCCGGGTTCGATTCCCGGCCAACGCAC
12198	Yes	AAGCAGTTGACCCGGGTTCGATTCCCGGCCAACGCACC
12199	Yes	AAGCAGTTGACCCGGGTTCGATTCCCGGCCAACGCACCA
12200	No	AAGCATACCTATGGGC
12201	No	AAGCATACCTATGGGCC
12202	Yes	AAGCATACCTATGGGCCC
12203	Yes	AAGCATACCTATGGGCCCA
12204	Yes	AAGCATACCTATGGGCCCAT
12205	Yes	AAGCATACCTATGGGCCCATA
12206	Yes	AAGCATACCTATGGGCCCATAA
12207	Yes	AAGCATACCTATGGGCCCATAAC
12208	Yes	AAGCATACCTATGGGCCCATAACC
12209	Yes	AAGCATACCTATGGGCCCATAACCC
12210	Yes	AAGCATACCTATGGGCCCATAACCCA
12211	Yes	AAGCATACCTATGGGCCCATAACCCAG
12212	Yes	AAGCATACCTATGGGCCCATAACCCAGA
12213	Yes	AAGCATACCTATGGGCCCATAACCCAGAG
12214	Yes	AAGCATACCTATGGGCCCATAACCCAGAGG
12215	Yes	AAGCATACCTATGGGCCCATAACCCAGAGGT

12216	Yes	AAGCATACCTATGGGCCCATAACCCAGAGGTT
12217	Yes	AAGCATACCTATGGGCCCATAACCCAGAGGTTG
12218	Yes	AAGCATACCTATGGGCCCATAACCCAGAGGTTGA
12219	Yes	AAGCATACCTATGGGCCCATAACCCAGAGGTTGAT
12220	Yes	AAGCATACCTATGGGCCCATAACCCAGAGGTTGATG
12221	Yes	AAGCATACCTATGGGCCCATAACCCAGAGGTTGATGG
12222	Yes	AAGCATACCTATGGGCCCATAACCCAGAGGTTGATGGA
12223	Yes	AAGCATACCTATGGGCCCATAACCCAGAGGTTGATGGAT
12224	Yes	AAGCATACCTATGGGCCCATAACCCAGAGGTTGATGGATG
12225	Yes	AAGCATACCTATGGGCCCATAACCCAGAGGTTGATGGATGG
12226	Yes	AAGCATACCTATGGGCCCATAACCCAGAGGTTGATGGATGGA
12227	Yes	AAGCATACCTATGGGCCCATAACCCAGAGGTTGATGGATGGAA
12228	Yes	AAGCATACCTATGGGCCCATAACCCAGAGGTTGATGGATGGAAA
12229	Yes	AAGCATACCTATGGGCCCATAACCCAGAGGTTGATGGATGGAAAC
12230	Yes	AAGCATACCTATGGGCCCATAACCCAGAGGTTGATGGATGGAAACC
12231	Yes	AAGCATACCTATGGGCCCATAACCCAGAGGTTGATGGATGGAAACCA
12232	Yes	AAGCATACCTATGGGCCCATAACCCAGAGGTTGATGGATGGAAACCAT
12233	Yes	AAGCATACCTATGGGCCCATAACCCAGAGGTTGATGGATGGAAACCATC
12234	Yes	AAGCATACCTATGGGCCCATAACCCAGAGGTTGATGGATGGAAACCATCC
12235	No	AAGCATAGCTGCCTTC
12236	No	AAGCATAGCTGCCTTCC
12237	No	AAGCATAGCTGCCTTCCA
12238	No	AAGCATAGCTGCCTTCCAA
12239	Yes	AAGCATAGCTGCCTTCCAAG
12240	Yes	AAGCATAGCTGCCTTCCAAGC
12241	Yes	AAGCATAGCTGCCTTCCAAGCA
12242	Yes	AAGCATAGCTGCCTTCCAAGCAG
12243	Yes	AAGCATAGCTGCCTTCCAAGCAGT
12244	Yes	AAGCATAGCTGCCTTCCAAGCAGTT
12245	Yes	AAGCATAGCTGCCTTCCAAGCAGTTG
12246	Yes	AAGCATAGCTGCCTTCCAAGCAGTTGA
12247	Yes	AAGCATAGCTGCCTTCCAAGCAGTTGAC
12248	Yes	AAGCATAGCTGCCTTCCAAGCAGTTGACC
12249	Yes	AAGCATAGCTGCCTTCCAAGCAGTTGACCC
12250	Yes	AAGCATAGCTGCCTTCCAAGCAGTTGACCCG
12251	Yes	AAGCATAGCTGCCTTCCAAGCAGTTGACCCGG
12252	Yes	AAGCATAGCTGCCTTCCAAGCAGTTGACCCGGG
12253	Yes	AAGCATAGCTGCCTTCCAAGCAGTTGACCCGGGT
12254	Yes	AAGCATAGCTGCCTTCCAAGCAGTTGACCCGGGTT
12255	Yes	AAGCATAGCTGCCTTCCAAGCAGTTGACCCGGGTTC
12256	Yes	AAGCATAGCTGCCTTCCAAGCAGTTGACCCGGGTTCG
12257	Yes	AAGCATAGCTGCCTTCCAAGCAGTTGACCCGGGTTCGA

12258	Yes	AAGCATAGCTGCCTTCCAAGCAGTTGACCCGGGTTCGAT
12259	Yes	AAGCATAGCTGCCTTCCAAGCAGTTGACCCGGGTTCGATT
12260	Yes	AAGCATAGCTGCCTTCCAAGCAGTTGACCCGGGTTCGATTC
12261	Yes	AAGCATAGCTGCCTTCCAAGCAGTTGACCCGGGTTCGATTCC
12262	Yes	AAGCATAGCTGCCTTCCAAGCAGTTGACCCGGGTTCGATTCCC
12263	Yes	AAGCATAGCTGCCTTCCAAGCAGTTGACCCGGGTTCGATTCCCG
12264	Yes	AAGCATAGCTGCCTTCCAAGCAGTTGACCCGGGTTCGATTCCCGG
12265	Yes	AAGCATAGCTGCCTTCCAAGCAGTTGACCCGGGTTCGATTCCCGGC
12266	Yes	AAGCATAGCTGCCTTCCAAGCAGTTGACCCGGGTTCGATTCCCGGCC
12267	Yes	AAGCATAGCTGCCTTCCAAGCAGTTGACCCGGGTTCGATTCCCGGCCA
12268	Yes	AAGCATAGCTGCCTTCCAAGCAGTTGACCCGGGTTCGATTCCCGGCCAA
12269	Yes	AAGCATAGCTGCCTTCCAAGCAGTTGACCCGGGTTCGATTCCCGGCCAAC
12270	No	AAGCATGAGACTCTTA
12271	No	AAGCATGAGACTCTTAA
12272	No	AAGCATGAGACTCTTAAT
12273	Yes	AAGCATGAGACTCTTAATC
12274	Yes	AAGCATGAGACTCTTAATCT
12275	Yes	AAGCATGAGACTCTTAATCTC
12276	Yes	AAGCATGAGACTCTTAATCTCA
12277	Yes	AAGCATGAGACTCTTAATCTCAG
12278	Yes	AAGCATGAGACTCTTAATCTCAGG
12279	Yes	AAGCATGAGACTCTTAATCTCAGGG
12280	Yes	AAGCATGAGACTCTTAATCTCAGGGT
12281	Yes	AAGCATGAGACTCTTAATCTCAGGGTC
12282	Yes	AAGCATGAGACTCTTAATCTCAGGGTCG
12283	Yes	AAGCATGAGACTCTTAATCTCAGGGTCGT
12284	Yes	AAGCATGAGACTCTTAATCTCAGGGTCGTG
12285	Yes	AAGCATGAGACTCTTAATCTCAGGGTCGTGG
12286	Yes	AAGCATGAGACTCTTAATCTCAGGGTCGTGGG
12287	Yes	AAGCATGAGACTCTTAATCTCAGGGTCGTGGGC
12288	Yes	AAGCATGAGACTCTTAATCTCAGGGTCGTGGGCT
12289	Yes	AAGCATGAGACTCTTAATCTCAGGGTCGTGGGCTC
12290	Yes	AAGCATGAGACTCTTAATCTCAGGGTCGTGGGCTCG
12291	Yes	AAGCATGAGACTCTTAATCTCAGGGTCGTGGGCTCGA
12292	Yes	AAGCATGAGACTCTTAATCTCAGGGTCGTGGGCTCGAG
12293	Yes	AAGCATGAGACTCTTAATCTCAGGGTCGTGGGCTCGAGC
12294	Yes	AAGCATGAGACTCTTAATCTCAGGGTCGTGGGCTCGAGCT
12295	Yes	AAGCATGAGACTCTTAATCTCAGGGTCGTGGGCTCGAGCTC
12296	Yes	AAGCATGAGACTCTTAATCTCAGGGTCGTGGGCTCGAGCTCC
12297	Yes	AAGCATGAGACTCTTAATCTCAGGGTCGTGGGCTCGAGCTCCA
12298	Yes	AAGCATGAGACTCTTAATCTCAGGGTCGTGGGCTCGAGCTCCAT
12299	Yes	AAGCATGAGACTCTTAATCTCAGGGTCGTGGGCTCGAGCTCCATG

12300	Yes	AAGCATGAGACTCTTAATCTCAGGGTCGTGGGCTCGAGCTCCATGT
12301	Yes	AAGCATGAGACTCTTAATCTCAGGGTCGTGGGCTCGAGCTCCATGTT
12302	Yes	AAGCATGAGACTCTTAATCTCAGGGTCGTGGGCTCGAGCTCCATGTTG
12303	Yes	AAGCATGAGACTCTTAATCTCAGGGTCGTGGGCTCGAGCTCCATGTTGG
12304	Yes	AAGCATGAGACTCTTAATCTCAGGGTCGTGGGCTCGAGCTCCATGTTGGG
12305	No	AAGCATTGGACTGTAA
12306	No	AAGCATTGGACTGTAAA
12307	No	AAGCATTGGACTGTAAAT
12308	No	AAGCATTGGACTGTAAATC
12309	No	AAGCATTGGACTGTAAATCT
12310	No	AAGCATTGGACTGTAAATCTA
12311	No	AAGCATTGGACTGTAAATCTAA
12312	No	AAGCATTGGACTGTAAATCTAAA
12313	No	AAGCATTGGACTGTAAATCTAAAG
12314	No	AAGCATTGGACTGTAAATCTAAAGA
12315	No	AAGCATTGGACTGTAAATCTAAAGAC
12316	No	AAGCATTGGACTGTAAATCTAAAGACA
12317	No	AAGCATTGGACTGTAAATCTAAAGACAG
12318	No	AAGCATTGGACTGTAAATCTAAAGACAGG
12319	No	AAGCATTGGACTGTAAATCTAAAGACAGGG
12320	No	AAGCATTGGACTGTAAATCTAAAGACAGGGG
12321	No	AAGCATTGGACTGTAAATCTAAAGACAGGGGT
12322	No	AAGCATTGGACTGTAAATCTAAAGACAGGGGTT
12323	No	AAGCATTGGACTGTAAATCTAAAGACAGGGGTTA
12324	Yes	AAGCATTGGACTGTAAATCTAAAGACAGGGGTTAG
12325	Yes	AAGCATTGGACTGTAAATCTAAAGACAGGGGTTAGG
12326	Yes	AAGCATTGGACTGTAAATCTAAAGACAGGGGTTAGGC
12327	Yes	AAGCATTGGACTGTAAATCTAAAGACAGGGGTTAGGCC
12328	Yes	AAGCATTGGACTGTAAATCTAAAGACAGGGGTTAGGCCT
12329	Yes	AAGCATTGGACTGTAAATCTAAAGACAGGGGTTAGGCCTC
12330	Yes	AAGCATTGGACTGTAAATCTAAAGACAGGGGTTAGGCCTCT
12331	Yes	AAGCATTGGACTGTAAATCTAAAGACAGGGGTTAGGCCTCTT
12332	Yes	AAGCATTGGACTGTAAATCTAAAGACAGGGGTTAGGCCTCTTT
12333	Yes	AAGCATTGGACTGTAAATCTAAAGACAGGGGTTAGGCCTCTTTT
12334	Yes	AAGCATTGGACTGTAAATCTAAAGACAGGGGTTAGGCCTCTTTTT
12335	Yes	AAGCATTGGACTGTAAATCTAAAGACAGGGGTTAGGCCTCTTTTTA
12336	Yes	AAGCATTGGACTGTAAATCTAAAGACAGGGGTTAGGCCTCTTTTTAC
12337	Yes	AAGCATTGGACTGTAAATCTAAAGACAGGGGTTAGGCCTCTTTTTACC
12338	Yes	AAGCATTGGACTGTAAATCTAAAGACAGGGGTTAGGCCTCTTTTTACCA
12339	Yes	AAGCATTGGACTGTAAATCTAAAGACAGGGGTTAGGCCTCTTTTTACCAC
12340	No	AAGCCAGACTATTTGG
12341	Yes	AAGCCAGACTATTTGGG

12342	Yes	AAGCCAGACTATTTGGGT
12343	Yes	AAGCCAGACTATTTGGGTT
12344	Yes	AAGCCAGACTATTTGGGTTC
12345	Yes	AAGCCAGACTATTTGGGTTCA
12346	Yes	AAGCCAGACTATTTGGGTTCAA
12347	Yes	AAGCCAGACTATTTGGGTTCAAA
12348	Yes	AAGCCAGACTATTTGGGTTCAAAT
12349	Yes	AAGCCAGACTATTTGGGTTCAAATC
12350	Yes	AAGCCAGACTATTTGGGTTCAAATCC
12351	Yes	AAGCCAGACTATTTGGGTTCAAATCCC
12352	Yes	AAGCCAGACTATTTGGGTTCAAATCCCA
12353	Yes	AAGCCAGACTATTTGGGTTCAAATCCCAG
12354	Yes	AAGCCAGACTATTTGGGTTCAAATCCCAGC
12355	Yes	AAGCCAGACTATTTGGGTTCAAATCCCAGCT
12356	Yes	AAGCCAGACTATTTGGGTTCAAATCCCAGCTT
12357	Yes	AAGCCAGACTATTTGGGTTCAAATCCCAGCTTG
12358	Yes	AAGCCAGACTATTTGGGTTCAAATCCCAGCTTGG
12359	Yes	AAGCCAGACTATTTGGGTTCAAATCCCAGCTTGGC
12360	Yes	AAGCCAGACTATTTGGGTTCAAATCCCAGCTTGGCC
12361	Yes	AAGCCAGACTATTTGGGTTCAAATCCCAGCTTGGCCT
12362	Yes	AAGCCAGACTATTTGGGTTCAAATCCCAGCTTGGCCTC
12363	Yes	AAGCCAGACTATTTGGGTTCAAATCCCAGCTTGGCCTCC
12364	Yes	AAGCCAGACTATTTGGGTTCAAATCCCAGCTTGGCCTCCA
12365	No	AAGCCAGGGATTGTGG
12366	No	AAGCCAGGGATTGTGGG
12367	Yes	AAGCCAGGGATTGTGGGT
12368	Yes	AAGCCAGGGATTGTGGGTT
12369	Yes	AAGCCAGGGATTGTGGGTTC
12370	Yes	AAGCCAGGGATTGTGGGTTCG
12371	Yes	AAGCCAGGGATTGTGGGTTCGA
12372	Yes	AAGCCAGGGATTGTGGGTTCGAG
12373	Yes	AAGCCAGGGATTGTGGGTTCGAGT
12374	Yes	AAGCCAGGGATTGTGGGTTCGAGTC
12375	Yes	AAGCCAGGGATTGTGGGTTCGAGTCC
12376	Yes	AAGCCAGGGATTGTGGGTTCGAGTCCC
12377	Yes	AAGCCAGGGATTGTGGGTTCGAGTCCCA
12378	Yes	AAGCCAGGGATTGTGGGTTCGAGTCCCAC
12379	Yes	AAGCCAGGGATTGTGGGTTCGAGTCCCACC
12380	Yes	AAGCCAGGGATTGTGGGTTCGAGTCCCACCC
12381	Yes	AAGCCAGGGATTGTGGGTTCGAGTCCCACCCG
12382	Yes	AAGCCAGGGATTGTGGGTTCGAGTCCCACCCGG
12383	Yes	AAGCCAGGGATTGTGGGTTCGAGTCCCACCCGGG

12384	Yes	AAGCCAGGGATTGTGGGTTCGAGTCCCACCCGGGG
12385	Yes	AAGCCAGGGATTGTGGGTTCGAGTCCCACCCGGGGT
12386	Yes	AAGCCAGGGATTGTGGGTTCGAGTCCCACCCGGGGTA
12387	Yes	AAGCCAGGGATTGTGGGTTCGAGTCCCACCCGGGGTAC
12388	Yes	AAGCCAGGGATTGTGGGTTCGAGTCCCACCCGGGGTACC
12389	Yes	AAGCCAGGGATTGTGGGTTCGAGTCCCACCCGGGGTACCA
12390	Yes	AAGCCAGGGATTGTGGGTTCGAGTCCCACCT
12391	Yes	AAGCCAGGGATTGTGGGTTCGAGTCCCACCTG
12392	Yes	AAGCCAGGGATTGTGGGTTCGAGTCCCACCTGG
12393	Yes	AAGCCAGGGATTGTGGGTTCGAGTCCCACCTGGG
12394	Yes	AAGCCAGGGATTGTGGGTTCGAGTCCCACCTGGGG
12395	Yes	AAGCCAGGGATTGTGGGTTCGAGTCCCACCTGGGGT
12396	Yes	AAGCCAGGGATTGTGGGTTCGAGTCCCACCTGGGGTA
12397	Yes	AAGCCAGGGATTGTGGGTTCGAGTCCCACCTGGGGTAC
12398	Yes	AAGCCAGGGATTGTGGGTTCGAGTCCCACCTGGGGTACC
12399	Yes	AAGCCAGGGATTGTGGGTTCGAGTCCCACCTGGGGTACCA
12400	Yes	AAGCCAGGGATTGTGGGTTCGAGTCCCACCTGGGGTG
12401	Yes	AAGCCAGGGATTGTGGGTTCGAGTCCCACCTGGGGTGC
12402	Yes	AAGCCAGGGATTGTGGGTTCGAGTCCCACCTGGGGTGCC
12403	Yes	AAGCCAGGGATTGTGGGTTCGAGTCCCACCTGGGGTGCCA
12404	Yes	AAGCCAGGGATTGTGGGTTCGAGTCCCAT
12405	Yes	AAGCCAGGGATTGTGGGTTCGAGTCCCATC
12406	Yes	AAGCCAGGGATTGTGGGTTCGAGTCCCATCT
12407	Yes	AAGCCAGGGATTGTGGGTTCGAGTCCCATCTG
12408	Yes	AAGCCAGGGATTGTGGGTTCGAGTCCCATCTGG
12409	Yes	AAGCCAGGGATTGTGGGTTCGAGTCCCATCTGGG
12410	Yes	AAGCCAGGGATTGTGGGTTCGAGTCCCATCTGGGG
12411	Yes	AAGCCAGGGATTGTGGGTTCGAGTCCCATCTGGGGT
12412	Yes	AAGCCAGGGATTGTGGGTTCGAGTCCCATCTGGGGTG
12413	Yes	AAGCCAGGGATTGTGGGTTCGAGTCCCATCTGGGGTGC
12414	Yes	AAGCCAGGGATTGTGGGTTCGAGTCCCATCTGGGGTGCC
12415	Yes	AAGCCAGGGATTGTGGGTTCGAGTCCCATCTGGGGTGCCA
12416	Yes	AAGCCAGGGATTGTGGGTTCGAGTT
12417	Yes	AAGCCAGGGATTGTGGGTTCGAGTTC
12418	Yes	AAGCCAGGGATTGTGGGTTCGAGTTCC
12419	Yes	AAGCCAGGGATTGTGGGTTCGAGTTCCA
12420	Yes	AAGCCAGGGATTGTGGGTTCGAGTTCCAC
12421	Yes	AAGCCAGGGATTGTGGGTTCGAGTTCCACC
12422	Yes	AAGCCAGGGATTGTGGGTTCGAGTTCCACCT
12423	Yes	AAGCCAGGGATTGTGGGTTCGAGTTCCACCTG
12424	Yes	AAGCCAGGGATTGTGGGTTCGAGTTCCACCTGG
12425	Yes	AAGCCAGGGATTGTGGGTTCGAGTTCCACCTGGG

12426	Yes	AAGCCAGGGATTGTGGGTTCGAGTTCCACCTGGGG
12427	Yes	AAGCCAGGGATTGTGGGTTCGAGTTCCACCTGGGGT
12428	Yes	AAGCCAGGGATTGTGGGTTCGAGTTCCACCTGGGGTA
12429	Yes	AAGCCAGGGATTGTGGGTTCGAGTTCCACCTGGGGTAC
12430	Yes	AAGCCAGGGATTGTGGGTTCGAGTTCCACCTGGGGTACC
12431	Yes	AAGCCAGGGATTGTGGGTTCGAGTTCCACCTGGGGTACCA
12432	No	AAGCCAGTTGATTAGG
12433	No	AAGCCAGTTGATTAGGG
12434	No	AAGCCAGTTGATTAGGGT
12435	No	AAGCCAGTTGATTAGGGTG
12436	Yes	AAGCCAGTTGATTAGGGTGC
12437	Yes	AAGCCAGTTGATTAGGGTGCT
12438	Yes	AAGCCAGTTGATTAGGGTGCTT
12439	Yes	AAGCCAGTTGATTAGGGTGCTTA
12440	Yes	AAGCCAGTTGATTAGGGTGCTTAG
12441	Yes	AAGCCAGTTGATTAGGGTGCTTAGC
12442	Yes	AAGCCAGTTGATTAGGGTGCTTAGCT
12443	Yes	AAGCCAGTTGATTAGGGTGCTTAGCTG
12444	Yes	AAGCCAGTTGATTAGGGTGCTTAGCTGT
12445	Yes	AAGCCAGTTGATTAGGGTGCTTAGCTGTT
12446	Yes	AAGCCAGTTGATTAGGGTGCTTAGCTGTTA
12447	Yes	AAGCCAGTTGATTAGGGTGCTTAGCTGTTAA
12448	Yes	AAGCCAGTTGATTAGGGTGCTTAGCTGTTAAC
12449	Yes	AAGCCAGTTGATTAGGGTGCTTAGCTGTTAACT
12450	Yes	AAGCCAGTTGATTAGGGTGCTTAGCTGTTAACTA
12451	Yes	AAGCCAGTTGATTAGGGTGCTTAGCTGTTAACTAA
12452	Yes	AAGCCAGTTGATTAGGGTGCTTAGCTGTTAACTAAG
12453	Yes	AAGCCAGTTGATTAGGGTGCTTAGCTGTTAACTAAGT
12454	Yes	AAGCCAGTTGATTAGGGTGCTTAGCTGTTAACTAAGTG
12455	Yes	AAGCCAGTTGATTAGGGTGCTTAGCTGTTAACTAAGTGT
12456	Yes	AAGCCAGTTGATTAGGGTGCTTAGCTGTTAACTAAGTGTT
12457	Yes	AAGCCAGTTGATTAGGGTGCTTAGCTGTTAACTAAGTGTTT
12458	Yes	AAGCCAGTTGATTAGGGTGCTTAGCTGTTAACTAAGTGTTTG
12459	Yes	AAGCCAGTTGATTAGGGTGCTTAGCTGTTAACTAAGTGTTTGT
12460	Yes	AAGCCAGTTGATTAGGGTGCTTAGCTGTTAACTAAGTGTTTGTG
12461	Yes	AAGCCAGTTGATTAGGGTGCTTAGCTGTTAACTAAGTGTTTGTGG
12462	Yes	AAGCCAGTTGATTAGGGTGCTTAGCTGTTAACTAAGTGTTTGTGGG
12463	Yes	AAGCCAGTTGATTAGGGTGCTTAGCTGTTAACTAAGTGTTTGTGGGT
12464	Yes	AAGCCAGTTGATTAGGGTGCTTAGCTGTTAACTAAGTGTTTGTGGGTT
12465	Yes	AAGCCAGTTGATTAGGGTGCTTAGCTGTTAACTAAGTGTTTGTGGGTTT
12466	Yes	AAGCCAGTTGATTAGGGTGCTTAGCTGTTAACTAAGTGTTTGTGGGTTTA
12467	No	AAGCCATCCTTGGCTA

12468	No	AAGCCATCCTTGGCTAC
12469	Yes	AAGCCATCCTTGGCTACC
12470	Yes	AAGCCATCCTTGGCTACCA
12471	No	AAGCCCACCCAGAGAT
12472	No	AAGCCCACCCAGAGATG
12473	No	AAGCCCACCCAGAGATGC
12474	No	AAGCCCACCCAGAGATGCC
12475	Yes	AAGCCCACCCAGAGATGCCA
12476	No	AAGCCCATCCAGGGAT
12477	Yes	AAGCCCATCCAGGGATG
12478	Yes	AAGCCCATCCAGGGATGC
12479	Yes	AAGCCCATCCAGGGATGCC
12480	Yes	AAGCCCATCCAGGGATGCCA
12481	No	AAGCCCCACATTGGAC
12482	Yes	AAGCCCCACATTGGACA
12483	Yes	AAGCCCCACATTGGACAC
12484	Yes	AAGCCCCACATTGGACACC
12485	Yes	AAGCCCCACATTGGACACCA
12486	No	AAGCCCCACATTGGGC
12487	Yes	AAGCCCCACATTGGGCG
12488	Yes	AAGCCCCACATTGGGCGC
12489	Yes	AAGCCCCACATTGGGCGCC
12490	Yes	AAGCCCCACATTGGGCGCCA
12491	Yes	AAGCCCCAGTGGAACC
12492	Yes	AAGCCCCAGTGGAACCA
12493	Yes	AAGCCCCAGTGGAACCAC
12494	Yes	AAGCCCCAGTGGAACCACC
12495	Yes	AAGCCCCAGTGGAACCACCA
12496	No	AAGCCCTCAGTAAGTT
12497	Yes	AAGCCCTCAGTAAGTTG
12498	Yes	AAGCCCTCAGTAAGTTGC
12499	Yes	AAGCCCTCAGTAAGTTGCA
12500	Yes	AAGCCCTCAGTAAGTTGCAA
12501	Yes	AAGCCCTCAGTAAGTTGCAAT
12502	Yes	AAGCCCTCAGTAAGTTGCAATA
12503	Yes	AAGCCCTCAGTAAGTTGCAATAC
12504	Yes	AAGCCCTCAGTAAGTTGCAATACT
12505	Yes	AAGCCCTCAGTAAGTTGCAATACTT
12506	Yes	AAGCCCTCAGTAAGTTGCAATACTTA
12507	Yes	AAGCCCTCAGTAAGTTGCAATACTTAA
12508	Yes	AAGCCCTCAGTAAGTTGCAATACTTAAT
12509	Yes	AAGCCCTCAGTAAGTTGCAATACTTAATT

12510	Yes	AAGCCCTCAGTAAGTTGCAATACTTAATTT
12511	Yes	AAGCCCTCAGTAAGTTGCAATACTTAATTTC
12512	Yes	AAGCCCTCAGTAAGTTGCAATACTTAATTTCT
12513	Yes	AAGCCCTCAGTAAGTTGCAATACTTAATTTCTG
12514	Yes	AAGCCCTCAGTAAGTTGCAATACTTAATTTCTGC
12515	Yes	AAGCCCTCAGTAAGTTGCAATACTTAATTTCTGCC
12516	Yes	AAGCCCTCAGTAAGTTGCAATACTTAATTTCTGCCA
12517	No	AAGCCTCACATTGAGT
12518	Yes	AAGCCTCACATTGAGTG
12519	Yes	AAGCCTCACATTGAGTGC
12520	Yes	AAGCCTCACATTGAGTGCC
12521	Yes	AAGCCTCACATTGAGTGCCA
12522	No	AAGCCTCACCTGGAGC
12523	No	AAGCCTCACCTGGAGCA
12524	No	AAGCCTCACCTGGAGCAC
12525	No	AAGCCTCACCTGGAGCACC
12526	No	AAGCCTCACCTGGAGCACCA
12527	No	AAGCCTCAGAGAGGGC
12528	No	AAGCCTCAGAGAGGGCA
12529	Yes	AAGCCTCAGAGAGGGCAC
12530	Yes	AAGCCTCAGAGAGGGCACC
12531	Yes	AAGCCTCAGAGAGGGCACCA
12532	No	AAGCCTCAGAGGGGGC
12533	No	AAGCCTCAGAGGGGGCA
12534	Yes	AAGCCTCAGAGGGGGCAC
12535	Yes	AAGCCTCAGAGGGGGCACC
12536	Yes	AAGCCTCAGAGGGGGCACCA
12537	No	AAGCCTGTGGGCTCCA
12538	No	AAGCCTGTGGGCTCCAC
12539	No	AAGCCTGTGGGCTCCACA
12540	Yes	AAGCCTGTGGGCTCCACAG
12541	Yes	AAGCCTGTGGGCTCCACAGT
12542	Yes	AAGCCTGTGGGCTCCACAGTC
12543	Yes	AAGCCTGTGGGCTCCACAGTCA
12544	Yes	AAGCCTGTGGGCTCCACAGTCAT
12545	Yes	AAGCCTGTGGGCTCCACAGTCATG
12546	Yes	AAGCCTGTGGGCTCCACAGTCATGT
12547	Yes	AAGCCTGTGGGCTCCACAGTCATGTG
12548	Yes	AAGCCTGTGGGCTCCACAGTCATGTGC
12549	Yes	AAGCCTGTGGGCTCCACAGTCATGTGCC
12550	Yes	AAGCCTGTGGGCTCCACAGTCATGTGCCT
12551	Yes	AAGCCTGTGGGCTCCACAGTCATGTGCCTG

12552	Yes	AAGCCTGTGGGCTCCACAGTCATGTGCCTGG
12553	Yes	AAGCCTGTGGGCTCCACAGTCATGTGCCTGGG
12554	Yes	AAGCCTGTGGGCTCCACAGTCATGTGCCTGGGT
12555	Yes	AAGCCTGTGGGCTCCACAGTCATGTGCCTGGGTT
12556	Yes	AAGCCTGTGGGCTCCACAGTCATGTGCCTGGGTTC
12557	Yes	AAGCCTGTGGGCTCCACAGTCATGTGCCTGGGTTCA
12558	Yes	AAGCCTGTGGGCTCCACAGTCATGTGCCTGGGTTCAA
12559	Yes	AAGCCTGTGGGCTCCACAGTCATGTGCCTGGGTTCAAT
12560	Yes	AAGCCTGTGGGCTCCACAGTCATGTGCCTGGGTTCAATT
12561	Yes	AAGCCTGTGGGCTCCACAGTCATGTGCCTGGGTTCAATTC
12562	Yes	AAGCCTGTGGGCTCCACAGTCATGTGCCTGGGTTCAATTCC
12563	Yes	AAGCCTGTGGGCTCCACAGTCATGTGCCTGGGTTCAATTCCC
12564	Yes	AAGCCTGTGGGCTCCACAGTCATGTGCCTGGGTTCAATTCCCA
12565	Yes	AAGCCTGTGGGCTCCACAGTCATGTGCCTGGGTTCAATTCCCAG
12566	Yes	AAGCCTGTGGGCTCCACAGTCATGTGCCTGGGTTCAATTCCCAGT
12567	Yes	AAGCCTGTGGGCTCCACAGTCATGTGCCTGGGTTCAATTCCCAGTT
12568	Yes	AAGCCTGTGGGCTCCACAGTCATGTGCCTGGGTTCAATTCCCAGTTC
12569	Yes	AAGCCTGTGGGCTCCACAGTCATGTGCCTGGGTTCAATTCCCAGTTCT
12570	Yes	AAGCCTGTGGGCTCCACAGTCATGTGCCTGGGTTCAATTCCCAGTTCTG
12571	Yes	AAGCCTGTGGGCTCCACAGTCATGTGCCTGGGTTCAATTCCCAGTTCTGC
12572	Yes	AAGCGATCCGAGTTCA
12573	Yes	AAGCGATCCGAGTTCAA
12574	Yes	AAGCGATCCGAGTTCAAA
12575	Yes	AAGCGATCCGAGTTCAAAT
12576	Yes	AAGCGATCCGAGTTCAAATC
12577	Yes	AAGCGATCCGAGTTCAAATCT
12578	Yes	AAGCGATCCGAGTTCAAATCTC
12579	Yes	AAGCGATCCGAGTTCAAATCTCG
12580	Yes	AAGCGATCCGAGTTCAAATCTCGG
12581	Yes	AAGCGATCCGAGTTCAAATCTCGGT
12582	Yes	AAGCGATCCGAGTTCAAATCTCGGTG
12583	Yes	AAGCGATCCGAGTTCAAATCTCGGTGG
12584	Yes	AAGCGATCCGAGTTCAAATCTCGGTGGT
12585	Yes	AAGCGATCCGAGTTCAAATCTCGGTGGTA
12586	Yes	AAGCGATCCGAGTTCAAATCTCGGTGGTAC
12587	Yes	AAGCGATCCGAGTTCAAATCTCGGTGGTACC
12588	Yes	AAGCGATCCGAGTTCAAATCTCGGTGGTACCT
12589	Yes	AAGCGATCCGAGTTCAAATCTCGGTGGTACCTC
12590	Yes	AAGCGATCCGAGTTCAAATCTCGGTGGTACCTCC
12591	Yes	AAGCGATCCGAGTTCAAATCTCGGTGGTACCTCCA
12592	Yes	AAGCGCCTGTCTAGTA
12593	Yes	AAGCGCCTGTCTAGTAA

12594	Yes	AAGCGCCTGTCTAGTAAA
12595	Yes	AAGCGCCTGTCTAGTAAAC
12596	Yes	AAGCGCCTGTCTAGTAAACA
12597	Yes	AAGCGCCTGTCTAGTAAACAG
12598	Yes	AAGCGCCTGTCTAGTAAACAGG
12599	Yes	AAGCGCCTGTCTAGTAAACAGGA
12600	Yes	AAGCGCCTGTCTAGTAAACAGGAG
12601	Yes	AAGCGCCTGTCTAGTAAACAGGAGA
12602	Yes	AAGCGCCTGTCTAGTAAACAGGAGAT
12603	Yes	AAGCGCCTGTCTAGTAAACAGGAGATC
12604	Yes	AAGCGCCTGTCTAGTAAACAGGAGATCC
12605	Yes	AAGCGCCTGTCTAGTAAACAGGAGATCCT
12606	Yes	AAGCGCCTGTCTAGTAAACAGGAGATCCTG
12607	Yes	AAGCGCCTGTCTAGTAAACAGGAGATCCTGG
12608	Yes	AAGCGCCTGTCTAGTAAACAGGAGATCCTGGG
12609	Yes	AAGCGCCTGTCTAGTAAACAGGAGATCCTGGGT
12610	Yes	AAGCGCCTGTCTAGTAAACAGGAGATCCTGGGTT
12611	Yes	AAGCGCCTGTCTAGTAAACAGGAGATCCTGGGTTC
12612	Yes	AAGCGCCTGTCTAGTAAACAGGAGATCCTGGGTTCG
12613	Yes	AAGCGCCTGTCTAGTAAACAGGAGATCCTGGGTTCGA
12614	Yes	AAGCGCCTGTCTAGTAAACAGGAGATCCTGGGTTCGAA
12615	Yes	AAGCGCCTGTCTAGTAAACAGGAGATCCTGGGTTCGAAT
12616	Yes	AAGCGCCTGTCTAGTAAACAGGAGATCCTGGGTTCGAATC
12617	Yes	AAGCGCCTGTCTAGTAAACAGGAGATCCTGGGTTCGAATCC
12618	Yes	AAGCGCCTGTCTAGTAAACAGGAGATCCTGGGTTCGAATCCC
12619	Yes	AAGCGCCTGTCTAGTAAACAGGAGATCCTGGGTTCGAATCCCA
12620	Yes	AAGCGCCTGTCTAGTAAACAGGAGATCCTGGGTTCGAATCCCAG
12621	Yes	AAGCGCCTGTCTAGTAAACAGGAGATCCTGGGTTCGAATCCCAGC
12622	Yes	AAGCGCCTGTCTAGTAAACAGGAGATCCTGGGTTCGAATCCCAGCG
12623	Yes	AAGCGCCTGTCTAGTAAACAGGAGATCCTGGGTTCGAATCCCAGCGA
12624	Yes	AAGCGCCTGTCTAGTAAACAGGAGATCCTGGGTTCGAATCCCAGCGAG
12625	Yes	AAGCGCCTGTCTAGTAAACAGGAGATCCTGGGTTCGAATCCCAGCGAGG
12626	Yes	AAGCGCCTGTCTAGTAAACAGGAGATCCTGGGTTCGAATCCCAGCGAGGC
12627	Yes	AAGCGCCTGTCTAGTAAACAGGAGATCCTGGGTTCGAATCCCAGCGG
12628	Yes	AAGCGCCTGTCTAGTAAACAGGAGATCCTGGGTTCGAATCCCAGCGGG
12629	Yes	AAGCGCCTGTCTAGTAAACAGGAGATCCTGGGTTCGAATCCCAGCGGGG
12630	Yes	AAGCGCCTGTCTAGTAAACAGGAGATCCTGGGTTCGAATCCCAGCGGGGC
12631	Yes	AAGCGCCTGTCTAGTAAACAGGAGATCCTGGGTTCGAATCCCAGCGGT
12632	Yes	AAGCGCCTGTCTAGTAAACAGGAGATCCTGGGTTCGAATCCCAGCGGTG
12633	Yes	AAGCGCCTGTCTAGTAAACAGGAGATCCTGGGTTCGAATCCCAGCGGTGC
12634	Yes	AAGCGCCTGTCTAGTAAACAGGAGATCCTGGGTTCGAC
12635	Yes	AAGCGCCTGTCTAGTAAACAGGAGATCCTGGGTTCGACT

12636	Yes	AAGCGCCTGTCTAGTAAACAGGAGATCCTGGGTTCGACTC
12637	Yes	AAGCGCCTGTCTAGTAAACAGGAGATCCTGGGTTCGACTCC
12638	Yes	AAGCGCCTGTCTAGTAAACAGGAGATCCTGGGTTCGACTCCC
12639	Yes	AAGCGCCTGTCTAGTAAACAGGAGATCCTGGGTTCGACTCCCA
12640	Yes	AAGCGCCTGTCTAGTAAACAGGAGATCCTGGGTTCGACTCCCAG
12641	Yes	AAGCGCCTGTCTAGTAAACAGGAGATCCTGGGTTCGACTCCCAGC
12642	Yes	AAGCGCCTGTCTAGTAAACAGGAGATCCTGGGTTCGACTCCCAGCG
12643	Yes	AAGCGCCTGTCTAGTAAACAGGAGATCCTGGGTTCGACTCCCAGCGG
12644	Yes	AAGCGCCTGTCTAGTAAACAGGAGATCCTGGGTTCGACTCCCAGCGGG
12645	Yes	AAGCGCCTGTCTAGTAAACAGGAGATCCTGGGTTCGACTCCCAGCGGGG
12646	Yes	AAGCGCCTGTCTAGTAAACAGGAGATCCTGGGTTCGACTCCCAGCGGGGC
12647	No	AAGCGCCTGTCTCGTA
12648	No	AAGCGCCTGTCTCGTAA
12649	No	AAGCGCCTGTCTCGTAAA
12650	Yes	AAGCGCCTGTCTCGTAAAC
12651	Yes	AAGCGCCTGTCTCGTAAACA
12652	Yes	AAGCGCCTGTCTCGTAAACAG
12653	Yes	AAGCGCCTGTCTCGTAAACAGG
12654	Yes	AAGCGCCTGTCTCGTAAACAGGA
12655	Yes	AAGCGCCTGTCTCGTAAACAGGAG
12656	Yes	AAGCGCCTGTCTCGTAAACAGGAGA
12657	Yes	AAGCGCCTGTCTCGTAAACAGGAGAT
12658	Yes	AAGCGCCTGTCTCGTAAACAGGAGATC
12659	Yes	AAGCGCCTGTCTCGTAAACAGGAGATCC
12660	Yes	AAGCGCCTGTCTCGTAAACAGGAGATCCT
12661	Yes	AAGCGCCTGTCTCGTAAACAGGAGATCCTG
12662	Yes	AAGCGCCTGTCTCGTAAACAGGAGATCCTGG
12663	Yes	AAGCGCCTGTCTCGTAAACAGGAGATCCTGGG
12664	Yes	AAGCGCCTGTCTCGTAAACAGGAGATCCTGGGT
12665	Yes	AAGCGCCTGTCTCGTAAACAGGAGATCCTGGGTT
12666	Yes	AAGCGCCTGTCTCGTAAACAGGAGATCCTGGGTTC
12667	Yes	AAGCGCCTGTCTCGTAAACAGGAGATCCTGGGTTCG
12668	Yes	AAGCGCCTGTCTCGTAAACAGGAGATCCTGGGTTCGA
12669	Yes	AAGCGCCTGTCTCGTAAACAGGAGATCCTGGGTTCGAA
12670	Yes	AAGCGCCTGTCTCGTAAACAGGAGATCCTGGGTTCGAAT
12671	Yes	AAGCGCCTGTCTCGTAAACAGGAGATCCTGGGTTCGAATC
12672	Yes	AAGCGCCTGTCTCGTAAACAGGAGATCCTGGGTTCGAATCC
12673	Yes	AAGCGCCTGTCTCGTAAACAGGAGATCCTGGGTTCGAATCCC
12674	Yes	AAGCGCCTGTCTCGTAAACAGGAGATCCTGGGTTCGAATCCCA
12675	Yes	AAGCGCCTGTCTCGTAAACAGGAGATCCTGGGTTCGAATCCCAG
12676	Yes	AAGCGCCTGTCTCGTAAACAGGAGATCCTGGGTTCGAATCCCAGC
12677	Yes	AAGCGCCTGTCTCGTAAACAGGAGATCCTGGGTTCGAATCCCAGCG

12678	Yes	AAGCGCCTGTCTCGTAAACAGGAGATCCTGGGTTCGAATCCCAGCGG
12679	Yes	AAGCGCCTGTCTCGTAAACAGGAGATCCTGGGTTCGAATCCCAGCGGG
12680	Yes	AAGCGCCTGTCTCGTAAACAGGAGATCCTGGGTTCGAATCCCAGCGGGG
12681	Yes	AAGCGCCTGTCTCGTAAACAGGAGATCCTGGGTTCGAATCCCAGCGGGGC
12682	Yes	AAGCGCCTGTCTCGTAAACAGGAGATCCTGGGTTCGAC
12683	Yes	AAGCGCCTGTCTCGTAAACAGGAGATCCTGGGTTCGACT
12684	Yes	AAGCGCCTGTCTCGTAAACAGGAGATCCTGGGTTCGACTC
12685	Yes	AAGCGCCTGTCTCGTAAACAGGAGATCCTGGGTTCGACTCC
12686	Yes	AAGCGCCTGTCTCGTAAACAGGAGATCCTGGGTTCGACTCCC
12687	Yes	AAGCGCCTGTCTCGTAAACAGGAGATCCTGGGTTCGACTCCCA
12688	Yes	AAGCGCCTGTCTCGTAAACAGGAGATCCTGGGTTCGACTCCCAG
12689	Yes	AAGCGCCTGTCTCGTAAACAGGAGATCCTGGGTTCGACTCCCAGT
12690	Yes	AAGCGCCTGTCTCGTAAACAGGAGATCCTGGGTTCGACTCCCAGTG
12691	Yes	AAGCGCCTGTCTCGTAAACAGGAGATCCTGGGTTCGACTCCCAGTGG
12692	Yes	AAGCGCCTGTCTCGTAAACAGGAGATCCTGGGTTCGACTCCCAGTGGG
12693	Yes	AAGCGCCTGTCTCGTAAACAGGAGATCCTGGGTTCGACTCCCAGTGGGG
12694	Yes	AAGCGCCTGTCTCGTAAACAGGAGATCCTGGGTTCGACTCCCAGTGGGGC
12695	Yes	AAGCGCCTGTCTTGTA
12696	Yes	AAGCGCCTGTCTTGTAA
12697	Yes	AAGCGCCTGTCTTGTAAA
12698	Yes	AAGCGCCTGTCTTGTAAAC
12699	Yes	AAGCGCCTGTCTTGTAAACA
12700	Yes	AAGCGCCTGTCTTGTAAACAG
12701	Yes	AAGCGCCTGTCTTGTAAACAGG
12702	Yes	AAGCGCCTGTCTTGTAAACAGGA
12703	Yes	AAGCGCCTGTCTTGTAAACAGGAG
12704	Yes	AAGCGCCTGTCTTGTAAACAGGAGA
12705	Yes	AAGCGCCTGTCTTGTAAACAGGAGAT
12706	Yes	AAGCGCCTGTCTTGTAAACAGGAGATC
12707	Yes	AAGCGCCTGTCTTGTAAACAGGAGATCC
12708	Yes	AAGCGCCTGTCTTGTAAACAGGAGATCCT
12709	Yes	AAGCGCCTGTCTTGTAAACAGGAGATCCTG
12710	Yes	AAGCGCCTGTCTTGTAAACAGGAGATCCTGG
12711	Yes	AAGCGCCTGTCTTGTAAACAGGAGATCCTGGG
12712	Yes	AAGCGCCTGTCTTGTAAACAGGAGATCCTGGGT
12713	Yes	AAGCGCCTGTCTTGTAAACAGGAGATCCTGGGTT
12714	Yes	AAGCGCCTGTCTTGTAAACAGGAGATCCTGGGTTC
12715	Yes	AAGCGCCTGTCTTGTAAACAGGAGATCCTGGGTTCG
12716	Yes	AAGCGCCTGTCTTGTAAACAGGAGATCCTGGGTTCGA
12717	Yes	AAGCGCCTGTCTTGTAAACAGGAGATCCTGGGTTCGAA
12718	Yes	AAGCGCCTGTCTTGTAAACAGGAGATCCTGGGTTCGAAT
12719	Yes	AAGCGCCTGTCTTGTAAACAGGAGATCCTGGGTTCGAATC

12720	Yes	AAGCGCCTGTCTTGTAAACAGGAGATCCTGGGTTCGAATCC
12721	Yes	AAGCGCCTGTCTTGTAAACAGGAGATCCTGGGTTCGAATCCC
12722	Yes	AAGCGCCTGTCTTGTAAACAGGAGATCCTGGGTTCGAATCCCA
12723	Yes	AAGCGCCTGTCTTGTAAACAGGAGATCCTGGGTTCGAATCCCAG
12724	Yes	AAGCGCCTGTCTTGTAAACAGGAGATCCTGGGTTCGAATCCCAGT
12725	Yes	AAGCGCCTGTCTTGTAAACAGGAGATCCTGGGTTCGAATCCCAGTA
12726	Yes	AAGCGCCTGTCTTGTAAACAGGAGATCCTGGGTTCGAATCCCAGTAG
12727	Yes	AAGCGCCTGTCTTGTAAACAGGAGATCCTGGGTTCGAATCCCAGTAGA
12728	Yes	AAGCGCCTGTCTTGTAAACAGGAGATCCTGGGTTCGAATCCCAGTAGAG
12729	Yes	AAGCGCCTGTCTTGTAAACAGGAGATCCTGGGTTCGAATCCCAGTAGAGC
12730	Yes	AAGCGGTAGAGCGCCT
12731	Yes	AAGCGGTAGAGCGCCTG
12732	Yes	AAGCGGTAGAGCGCCTGC
12733	Yes	AAGCGGTAGAGCGCCTGCT
12734	Yes	AAGCGGTAGAGCGCCTGCTT
12735	Yes	AAGCGGTAGAGCGCCTGCTTA
12736	Yes	AAGCGGTAGAGCGCCTGCTTAG
12737	Yes	AAGCGGTAGAGCGCCTGCTTAGC
12738	Yes	AAGCGGTAGAGCGCCTGCTTAGCA
12739	Yes	AAGCGGTAGAGCGCCTGCTTAGCAT
12740	Yes	AAGCGGTAGAGCGCCTGCTTAGCATG
12741	Yes	AAGCGGTAGAGCGCCTGCTTAGCATGC
12742	Yes	AAGCGGTAGAGCGCCTGCTTAGCATGCA
12743	Yes	AAGCGGTAGAGCGCCTGCTTAGCATGCAA
12744	Yes	AAGCGGTAGAGCGCCTGCTTAGCATGCAAG
12745	Yes	AAGCGGTAGAGCGCCTGCTTAGCATGCAAGA
12746	Yes	AAGCGGTAGAGCGCCTGCTTAGCATGCAAGAG
12747	Yes	AAGCGGTAGAGCGCCTGCTTAGCATGCAAGAGG
12748	Yes	AAGCGGTAGAGCGCCTGCTTAGCATGCAAGAGGT
12749	Yes	AAGCGGTAGAGCGCCTGCTTAGCATGCAAGAGGTA
12750	Yes	AAGCGGTAGAGCGCCTGCTTAGCATGCAAGAGGTAG
12751	Yes	AAGCGGTAGAGCGCCTGCTTAGCATGCAAGAGGTAGC
12752	Yes	AAGCGGTAGAGCGCCTGCTTAGCATGCAAGAGGTAGCA
12753	Yes	AAGCGGTAGAGCGCCTGCTTAGCATGCAAGAGGTAGCAG
12754	Yes	AAGCGGTAGAGCGCCTGCTTAGCATGCAAGAGGTAGCAGG
12755	Yes	AAGCGGTAGAGCGCCTGCTTAGCATGCAAGAGGTAGCAGGA
12756	Yes	AAGCGGTAGAGCGCCTGCTTAGCATGCAAGAGGTAGCAGGAT
12757	Yes	AAGCGGTAGAGCGCCTGCTTAGCATGCAAGAGGTAGCAGGATC
12758	Yes	AAGCGGTAGAGCGCCTGCTTAGCATGCAAGAGGTAGCAGGATCG
12759	Yes	AAGCGGTAGAGCGCCTGCTTAGCATGCAAGAGGTAGCAGGATCGA
12760	Yes	AAGCGGTAGAGCGCCTGCTTAGCATGCAAGAGGTAGCAGGATCGAT
12761	Yes	AAGCGGTAGAGCGCCTGCTTAGCATGCAAGAGGTAGCAGGATCGATG

12762	Yes	AAGCGGTAGAGCGCCTGCTTAGCATGCAAGAGGTAGCAGGATCGATGC
12763	Yes	AAGCGGTAGAGCGCCTGCTTAGCATGCAAGAGGTAGCAGGATCGATGCC
12764	Yes	AAGCGGTAGAGCGCCTGCTTAGCATGCAAGAGGTAGCAGGATCGATGCCT
12765	Yes	AAGCGGTAGAGCGCTC
12766	Yes	AAGCGGTAGAGCGCTCC
12767	Yes	AAGCGGTAGAGCGCTCCC
12768	Yes	AAGCGGTAGAGCGCTCCCT
12769	Yes	AAGCGGTAGAGCGCTCCCTT
12770	Yes	AAGCGGTAGAGCGCTCCCTTA
12771	Yes	AAGCGGTAGAGCGCTCCCTTAG
12772	Yes	AAGCGGTAGAGCGCTCCCTTAGC
12773	Yes	AAGCGGTAGAGCGCTCCCTTAGCA
12774	Yes	AAGCGGTAGAGCGCTCCCTTAGCAT
12775	Yes	AAGCGGTAGAGCGCTCCCTTAGCATG
12776	Yes	AAGCGGTAGAGCGCTCCCTTAGCATGC
12777	Yes	AAGCGGTAGAGCGCTCCCTTAGCATGCG
12778	Yes	AAGCGGTAGAGCGCTCCCTTAGCATGCGA
12779	Yes	AAGCGGTAGAGCGCTCCCTTAGCATGCGAG
12780	Yes	AAGCGGTAGAGCGCTCCCTTAGCATGCGAGA
12781	Yes	AAGCGGTAGAGCGCTCCCTTAGCATGCGAGAG
12782	Yes	AAGCGGTAGAGCGCTCCCTTAGCATGCGAGAGG
12783	Yes	AAGCGGTAGAGCGCTCCCTTAGCATGCGAGAGGT
12784	Yes	AAGCGGTAGAGCGCTCCCTTAGCATGCGAGAGGTA
12785	Yes	AAGCGGTAGAGCGCTCCCTTAGCATGCGAGAGGTAG
12786	Yes	AAGCGGTAGAGCGCTCCCTTAGCATGCGAGAGGTAGC
12787	Yes	AAGCGGTAGAGCGCTCCCTTAGCATGCGAGAGGTAGCG
12788	Yes	AAGCGGTAGAGCGCTCCCTTAGCATGCGAGAGGTAGCGG
12789	Yes	AAGCGGTAGAGCGCTCCCTTAGCATGCGAGAGGTAGCGGG
12790	Yes	AAGCGGTAGAGCGCTCCCTTAGCATGCGAGAGGTAGCGGGA
12791	Yes	AAGCGGTAGAGCGCTCCCTTAGCATGCGAGAGGTAGCGGGAT
12792	Yes	AAGCGGTAGAGCGCTCCCTTAGCATGCGAGAGGTAGCGGGATC
12793	Yes	AAGCGGTAGAGCGCTCCCTTAGCATGCGAGAGGTAGCGGGATCG
12794	Yes	AAGCGGTAGAGCGCTCCCTTAGCATGCGAGAGGTAGCGGGATCGA
12795	Yes	AAGCGGTAGAGCGCTCCCTTAGCATGCGAGAGGTAGCGGGATCGAC
12796	Yes	AAGCGGTAGAGCGCTCCCTTAGCATGCGAGAGGTAGCGGGATCGACG
12797	Yes	AAGCGGTAGAGCGCTCCCTTAGCATGCGAGAGGTAGCGGGATCGACGC
12798	Yes	AAGCGGTAGAGCGCTCCCTTAGCATGCGAGAGGTAGCGGGATCGACGCC
12799	Yes	AAGCGGTAGAGCGCTCCCTTAGCATGCGAGAGGTAGCGGGATCGACGCCC
12800	Yes	AAGCGGTAGAGCGCTT
12801	Yes	AAGCGGTAGAGCGCTTG
12802	Yes	AAGCGGTAGAGCGCTTGC
12803	Yes	AAGCGGTAGAGCGCTTGCT

12804	Yes	AAGCGGTAGAGCGCTTGCTT
12805	Yes	AAGCGGTAGAGCGCTTGCTTA
12806	Yes	AAGCGGTAGAGCGCTTGCTTAG
12807	Yes	AAGCGGTAGAGCGCTTGCTTAGC
12808	Yes	AAGCGGTAGAGCGCTTGCTTAGCA
12809	Yes	AAGCGGTAGAGCGCTTGCTTAGCAT
12810	Yes	AAGCGGTAGAGCGCTTGCTTAGCATG
12811	Yes	AAGCGGTAGAGCGCTTGCTTAGCATGC
12812	Yes	AAGCGGTAGAGCGCTTGCTTAGCATGCA
12813	Yes	AAGCGGTAGAGCGCTTGCTTAGCATGCAA
12814	Yes	AAGCGGTAGAGCGCTTGCTTAGCATGCAAG
12815	Yes	AAGCGGTAGAGCGCTTGCTTAGCATGCAAGA
12816	Yes	AAGCGGTAGAGCGCTTGCTTAGCATGCAAGAG
12817	Yes	AAGCGGTAGAGCGCTTGCTTAGCATGCAAGAGG
12818	Yes	AAGCGGTAGAGCGCTTGCTTAGCATGCAAGAGGT
12819	Yes	AAGCGGTAGAGCGCTTGCTTAGCATGCAAGAGGTA
12820	Yes	AAGCGGTAGAGCGCTTGCTTAGCATGCAAGAGGTAG
12821	Yes	AAGCGGTAGAGCGCTTGCTTAGCATGCAAGAGGTAGC
12822	Yes	AAGCGGTAGAGCGCTTGCTTAGCATGCAAGAGGTAGCA
12823	Yes	AAGCGGTAGAGCGCTTGCTTAGCATGCAAGAGGTAGCAG
12824	Yes	AAGCGGTAGAGCGCTTGCTTAGCATGCAAGAGGTAGCAGG
12825	Yes	AAGCGGTAGAGCGCTTGCTTAGCATGCAAGAGGTAGCAGGA
12826	Yes	AAGCGGTAGAGCGCTTGCTTAGCATGCAAGAGGTAGCAGGAT
12827	Yes	AAGCGGTAGAGCGCTTGCTTAGCATGCAAGAGGTAGCAGGATC
12828	Yes	AAGCGGTAGAGCGCTTGCTTAGCATGCAAGAGGTAGCAGGATCG
12829	Yes	AAGCGGTAGAGCGCTTGCTTAGCATGCAAGAGGTAGCAGGATCGA
12830	Yes	AAGCGGTAGAGCGCTTGCTTAGCATGCAAGAGGTAGCAGGATCGAC
12831	Yes	AAGCGGTAGAGCGCTTGCTTAGCATGCAAGAGGTAGCAGGATCGACG
12832	Yes	AAGCGGTAGAGCGCTTGCTTAGCATGCAAGAGGTAGCAGGATCGACGC
12833	Yes	AAGCGGTAGAGCGCTTGCTTAGCATGCAAGAGGTAGCAGGATCGACGCC
12834	Yes	AAGCGGTAGAGCGCTTGCTTAGCATGCAAGAGGTAGCAGGATCGACGCCT
12835	Yes	AAGCGGTAGAGCGCTTGCTTAGCATGCAAGAGGTAGT
12836	Yes	AAGCGGTAGAGCGCTTGCTTAGCATGCAAGAGGTAGTG
12837	Yes	AAGCGGTAGAGCGCTTGCTTAGCATGCAAGAGGTAGTGG
12838	Yes	AAGCGGTAGAGCGCTTGCTTAGCATGCAAGAGGTAGTGGG
12839	Yes	AAGCGGTAGAGCGCTTGCTTAGCATGCAAGAGGTAGTGGGA
12840	Yes	AAGCGGTAGAGCGCTTGCTTAGCATGCAAGAGGTAGTGGGAT
12841	Yes	AAGCGGTAGAGCGCTTGCTTAGCATGCAAGAGGTAGTGGGATC
12842	Yes	AAGCGGTAGAGCGCTTGCTTAGCATGCAAGAGGTAGTGGGATCG
12843	Yes	AAGCGGTAGAGCGCTTGCTTAGCATGCAAGAGGTAGTGGGATCGA
12844	Yes	AAGCGGTAGAGCGCTTGCTTAGCATGCAAGAGGTAGTGGGATCGAT
12845	Yes	AAGCGGTAGAGCGCTTGCTTAGCATGCAAGAGGTAGTGGGATCGATG

12846	Yes	AAGCGGTAGAGCGCTTGCTTAGCATGCAAGAGGTAGTGGGATCGATGC
12847	Yes	AAGCGGTAGAGCGCTTGCTTAGCATGCAAGAGGTAGTGGGATCGATGCC
12848	Yes	AAGCGGTAGAGCGCTTGCTTAGCATGCAAGAGGTAGTGGGATCGATGCCC
12849	Yes	AAGCGGTAGGGTGCCT
12850	Yes	AAGCGGTAGGGTGCCTG
12851	Yes	AAGCGGTAGGGTGCCTGC
12852	Yes	AAGCGGTAGGGTGCCTGCT
12853	Yes	AAGCGGTAGGGTGCCTGCTT
12854	Yes	AAGCGGTAGGGTGCCTGCTTA
12855	Yes	AAGCGGTAGGGTGCCTGCTTAG
12856	Yes	AAGCGGTAGGGTGCCTGCTTAGC
12857	Yes	AAGCGGTAGGGTGCCTGCTTAGCA
12858	Yes	AAGCGGTAGGGTGCCTGCTTAGCAT
12859	Yes	AAGCGGTAGGGTGCCTGCTTAGCATG
12860	Yes	AAGCGGTAGGGTGCCTGCTTAGCATGC
12861	Yes	AAGCGGTAGGGTGCCTGCTTAGCATGCA
12862	Yes	AAGCGGTAGGGTGCCTGCTTAGCATGCAA
12863	Yes	AAGCGGTAGGGTGCCTGCTTAGCATGCAAG
12864	Yes	AAGCGGTAGGGTGCCTGCTTAGCATGCAAGA
12865	Yes	AAGCGGTAGGGTGCCTGCTTAGCATGCAAGAG
12866	Yes	AAGCGGTAGGGTGCCTGCTTAGCATGCAAGAGG
12867	Yes	AAGCGGTAGGGTGCCTGCTTAGCATGCAAGAGGT
12868	Yes	AAGCGGTAGGGTGCCTGCTTAGCATGCAAGAGGTA
12869	Yes	AAGCGGTAGGGTGCCTGCTTAGCATGCAAGAGGTAG
12870	Yes	AAGCGGTAGGGTGCCTGCTTAGCATGCAAGAGGTAGC
12871	Yes	AAGCGGTAGGGTGCCTGCTTAGCATGCAAGAGGTAGCA
12872	Yes	AAGCGGTAGGGTGCCTGCTTAGCATGCAAGAGGTAGCAG
12873	Yes	AAGCGGTAGGGTGCCTGCTTAGCATGCAAGAGGTAGCAGG
12874	Yes	AAGCGGTAGGGTGCCTGCTTAGCATGCAAGAGGTAGCAGGA
12875	Yes	AAGCGGTAGGGTGCCTGCTTAGCATGCAAGAGGTAGCAGGAT
12876	Yes	AAGCGGTAGGGTGCCTGCTTAGCATGCAAGAGGTAGCAGGATC
12877	Yes	AAGCGGTAGGGTGCCTGCTTAGCATGCAAGAGGTAGCAGGATCG
12878	Yes	AAGCGGTAGGGTGCCTGCTTAGCATGCAAGAGGTAGCAGGATCGA
12879	Yes	AAGCGGTAGGGTGCCTGCTTAGCATGCAAGAGGTAGCAGGATCGAC
12880	Yes	AAGCGGTAGGGTGCCTGCTTAGCATGCAAGAGGTAGCAGGATCGACG
12881	Yes	AAGCGGTAGGGTGCCTGCTTAGCATGCAAGAGGTAGCAGGATCGACGC
12882	Yes	AAGCGGTAGGGTGCCTGCTTAGCATGCAAGAGGTAGCAGGATCGACGCC
12883	Yes	AAGCGGTAGGGTGCCTGCTTAGCATGCAAGAGGTAGCAGGATCGACGCCT
12884	Yes	AAGCGTCACGGCCCGG
12885	Yes	AAGCGTCACGGCCCGGG
12886	Yes	AAGCGTCACGGCCCGGGT
12887	Yes	AAGCGTCACGGCCCGGGTT

12888	Yes	AAGCGTCACGGCCCGGGTTC
12889	Yes	AAGCGTCACGGCCCGGGTTCG
12890	Yes	AAGCGTCACGGCCCGGGTTCGA
12891	Yes	AAGCGTCACGGCCCGGGTTCGAT
12892	Yes	AAGCGTCACGGCCCGGGTTCGATT
12893	Yes	AAGCGTCACGGCCCGGGTTCGATTC
12894	Yes	AAGCGTCACGGCCCGGGTTCGATTCC
12895	Yes	AAGCGTCACGGCCCGGGTTCGATTCCC
12896	Yes	AAGCGTCACGGCCCGGGTTCGATTCCCG
12897	Yes	AAGCGTCACGGCCCGGGTTCGATTCCCGG
12898	Yes	AAGCGTCACGGCCCGGGTTCGATTCCCGGT
12899	Yes	AAGCGTCACGGCCCGGGTTCGATTCCCGGTC
12900	Yes	AAGCGTCACGGCCCGGGTTCGATTCCCGGTCA
12901	Yes	AAGCGTCACGGCCCGGGTTCGATTCCCGGTCAG
12902	Yes	AAGCGTCACGGCCCGGGTTCGATTCCCGGTCAGG
12903	Yes	AAGCGTCACGGCCCGGGTTCGATTCCCGGTCAGGG
12904	Yes	AAGCGTCACGGCCCGGGTTCGATTCCCGGTCAGGGA
12905	Yes	AAGCGTCACGGCCCGGGTTCGATTCCCGGTCAGGGAA
12906	Yes	AAGCGTCACGGCCCGGGTTCGATTCCCGGTCAGGGAAC
12907	Yes	AAGCGTCACGGCCCGGGTTCGATTCCCGGTCAGGGAACC
12908	Yes	AAGCGTCACGGCCCGGGTTCGATTCCCGGTCAGGGAACCA
12909	No	AAGCGTGAGACTCTTA
12910	No	AAGCGTGAGACTCTTAA
12911	Yes	AAGCGTGAGACTCTTAAT
12912	Yes	AAGCGTGAGACTCTTAATC
12913	Yes	AAGCGTGAGACTCTTAATCT
12914	Yes	AAGCGTGAGACTCTTAATCTC
12915	Yes	AAGCGTGAGACTCTTAATCTCA
12916	Yes	AAGCGTGAGACTCTTAATCTCAG
12917	Yes	AAGCGTGAGACTCTTAATCTCAGG
12918	Yes	AAGCGTGAGACTCTTAATCTCAGGG
12919	Yes	AAGCGTGAGACTCTTAATCTCAGGGT
12920	Yes	AAGCGTGAGACTCTTAATCTCAGGGTA
12921	Yes	AAGCGTGAGACTCTTAATCTCAGGGTAG
12922	Yes	AAGCGTGAGACTCTTAATCTCAGGGTAGT
12923	Yes	AAGCGTGAGACTCTTAATCTCAGGGTAGTG
12924	Yes	AAGCGTGAGACTCTTAATCTCAGGGTAGTGG
12925	Yes	AAGCGTGAGACTCTTAATCTCAGGGTAGTGGG
12926	Yes	AAGCGTGAGACTCTTAATCTCAGGGTAGTGGGT
12927	Yes	AAGCGTGAGACTCTTAATCTCAGGGTAGTGGGTT
12928	Yes	AAGCGTGAGACTCTTAATCTCAGGGTAGTGGGTTC
12929	Yes	AAGCGTGAGACTCTTAATCTCAGGGTAGTGGGTTCA

12930	Yes	AAGCGTGAGACTCTTAATCTCAGGGTAGTGGGTTCAA
12931	Yes	AAGCGTGAGACTCTTAATCTCAGGGTAGTGGGTTCAAG
12932	Yes	AAGCGTGAGACTCTTAATCTCAGGGTAGTGGGTTCAAGC
12933	Yes	AAGCGTGAGACTCTTAATCTCAGGGTAGTGGGTTCAAGCC
12934	Yes	AAGCGTGAGACTCTTAATCTCAGGGTAGTGGGTTCAAGCCC
12935	Yes	AAGCGTGAGACTCTTAATCTCAGGGTAGTGGGTTCAAGCCCC
12936	Yes	AAGCGTGAGACTCTTAATCTCAGGGTAGTGGGTTCAAGCCCCA
12937	Yes	AAGCGTGAGACTCTTAATCTCAGGGTAGTGGGTTCAAGCCCCAC
12938	Yes	AAGCGTGAGACTCTTAATCTCAGGGTAGTGGGTTCAAGCCCCACA
12939	Yes	AAGCGTGAGACTCTTAATCTCAGGGTAGTGGGTTCAAGCCCCACAT
12940	Yes	AAGCGTGAGACTCTTAATCTCAGGGTAGTGGGTTCAAGCCCCACATT
12941	Yes	AAGCGTGAGACTCTTAATCTCAGGGTAGTGGGTTCAAGCCCCACATTG
12942	Yes	AAGCGTGAGACTCTTAATCTCAGGGTAGTGGGTTCAAGCCCCACATTGG
12943	Yes	AAGCGTGAGACTCTTAATCTCAGGGTAGTGGGTTCAAGCCCCACATTGGA
12944	No	AAGCGTGCTGGGCCCA
12945	Yes	AAGCGTGCTGGGCCCAT
12946	Yes	AAGCGTGCTGGGCCCATA
12947	Yes	AAGCGTGCTGGGCCCATAA
12948	Yes	AAGCGTGCTGGGCCCATAAC
12949	Yes	AAGCGTGCTGGGCCCATAACC
12950	Yes	AAGCGTGCTGGGCCCATAACCC
12951	Yes	AAGCGTGCTGGGCCCATAACCCA
12952	Yes	AAGCGTGCTGGGCCCATAACCCAG
12953	Yes	AAGCGTGCTGGGCCCATAACCCAGA
12954	Yes	AAGCGTGCTGGGCCCATAACCCAGAG
12955	Yes	AAGCGTGCTGGGCCCATAACCCAGAGG
12956	Yes	AAGCGTGCTGGGCCCATAACCCAGAGGT
12957	Yes	AAGCGTGCTGGGCCCATAACCCAGAGGTC
12958	Yes	AAGCGTGCTGGGCCCATAACCCAGAGGTCG
12959	Yes	AAGCGTGCTGGGCCCATAACCCAGAGGTCGA
12960	Yes	AAGCGTGCTGGGCCCATAACCCAGAGGTCGAT
12961	Yes	AAGCGTGCTGGGCCCATAACCCAGAGGTCGATG
12962	Yes	AAGCGTGCTGGGCCCATAACCCAGAGGTCGATGG
12963	Yes	AAGCGTGCTGGGCCCATAACCCAGAGGTCGATGGA
12964	Yes	AAGCGTGCTGGGCCCATAACCCAGAGGTCGATGGAT
12965	Yes	AAGCGTGCTGGGCCCATAACCCAGAGGTCGATGGATC
12966	Yes	AAGCGTGCTGGGCCCATAACCCAGAGGTCGATGGATCG
12967	Yes	AAGCGTGCTGGGCCCATAACCCAGAGGTCGATGGATCGA
12968	Yes	AAGCGTGCTGGGCCCATAACCCAGAGGTCGATGGATCGAA
12969	Yes	AAGCGTGCTGGGCCCATAACCCAGAGGTCGATGGATCGAAA
12970	Yes	AAGCGTGCTGGGCCCATAACCCAGAGGTCGATGGATCGAAAC
12971	Yes	AAGCGTGCTGGGCCCATAACCCAGAGGTCGATGGATCGAAACC

12972	Yes	AAGCGTGCTGGGCCCATAACCCAGAGGTCGATGGATCGAAACCA
12973	Yes	AAGCGTGCTGGGCCCATAACCCAGAGGTCGATGGATCGAAACCAT
12974	Yes	AAGCGTGCTGGGCCCATAACCCAGAGGTCGATGGATCGAAACCATC
12975	Yes	AAGCGTGCTGGGCCCATAACCCAGAGGTCGATGGATCGAAACCATCC
12976	Yes	AAGCGTGCTGGGCCCATAACCCAGAGGTCGATGGATCGAAACCATCCT
12977	Yes	AAGCGTGCTGGGCCCATAACCCAGAGGTCGATGGATCGAAACCATCCTC
12978	Yes	AAGCGTGCTGGGCCCATAACCCAGAGGTCGATGGATCGAAACCATCCTCT
12979	Yes	AAGCGTGCTGGGCCCATAACCCAGAGGTCGATGGATCT
12980	Yes	AAGCGTGCTGGGCCCATAACCCAGAGGTCGATGGATCTA
12981	Yes	AAGCGTGCTGGGCCCATAACCCAGAGGTCGATGGATCTAA
12982	Yes	AAGCGTGCTGGGCCCATAACCCAGAGGTCGATGGATCTAAA
12983	Yes	AAGCGTGCTGGGCCCATAACCCAGAGGTCGATGGATCTAAAC
12984	Yes	AAGCGTGCTGGGCCCATAACCCAGAGGTCGATGGATCTAAACC
12985	Yes	AAGCGTGCTGGGCCCATAACCCAGAGGTCGATGGATCTAAACCA
12986	Yes	AAGCGTGCTGGGCCCATAACCCAGAGGTCGATGGATCTAAACCAT
12987	Yes	AAGCGTGCTGGGCCCATAACCCAGAGGTCGATGGATCTAAACCATC
12988	Yes	AAGCGTGCTGGGCCCATAACCCAGAGGTCGATGGATCTAAACCATCC
12989	Yes	AAGCGTGCTGGGCCCATAACCCAGAGGTCGATGGATCTAAACCATCCT
12990	Yes	AAGCGTGCTGGGCCCATAACCCAGAGGTCGATGGATCTAAACCATCCTC
12991	Yes	AAGCGTGCTGGGCCCATAACCCAGAGGTCGATGGATCTAAACCATCCTCT
12992	Yes	AAGCGTGCTGGGCCCG
12993	Yes	AAGCGTGCTGGGCCCGT
12994	Yes	AAGCGTGCTGGGCCCGTA
12995	Yes	AAGCGTGCTGGGCCCGTAA
12996	Yes	AAGCGTGCTGGGCCCGTAAC
12997	Yes	AAGCGTGCTGGGCCCGTAACC
12998	Yes	AAGCGTGCTGGGCCCGTAACCC
12999	Yes	AAGCGTGCTGGGCCCGTAACCCA
13000	Yes	AAGCGTGCTGGGCCCGTAACCCAG
13001	Yes	AAGCGTGCTGGGCCCGTAACCCAGA
13002	Yes	AAGCGTGCTGGGCCCGTAACCCAGAG
13003	Yes	AAGCGTGCTGGGCCCGTAACCCAGAGG
13004	Yes	AAGCGTGCTGGGCCCGTAACCCAGAGGT
13005	Yes	AAGCGTGCTGGGCCCGTAACCCAGAGGTC
13006	Yes	AAGCGTGCTGGGCCCGTAACCCAGAGGTCA
13007	Yes	AAGCGTGCTGGGCCCGTAACCCAGAGGTCAA
13008	Yes	AAGCGTGCTGGGCCCGTAACCCAGAGGTCAAT
13009	Yes	AAGCGTGCTGGGCCCGTAACCCAGAGGTCAATG
13010	Yes	AAGCGTGCTGGGCCCGTAACCCAGAGGTCAATGG
13011	Yes	AAGCGTGCTGGGCCCGTAACCCAGAGGTCAATGGA
13012	Yes	AAGCGTGCTGGGCCCGTAACCCAGAGGTCAATGGAT
13013	Yes	AAGCGTGCTGGGCCCGTAACCCAGAGGTCAATGGATC

13014	Yes	AAGCGTGCTGGGCCCGTAACCCAGAGGTCAATGGATCG
13015	Yes	AAGCGTGCTGGGCCCGTAACCCAGAGGTCAATGGATCGA
13016	Yes	AAGCGTGCTGGGCCCGTAACCCAGAGGTCAATGGATCGAA
13017	Yes	AAGCGTGCTGGGCCCGTAACCCAGAGGTCAATGGATCGAAG
13018	Yes	AAGCGTGCTGGGCCCGTAACCCAGAGGTCAATGGATCGAAGC
13019	Yes	AAGCGTGCTGGGCCCGTAACCCAGAGGTCAATGGATCGAAGCC
13020	Yes	AAGCGTGCTGGGCCCGTAACCCAGAGGTCAATGGATCGAAGCCA
13021	Yes	AAGCGTGCTGGGCCCGTAACCCAGAGGTCAATGGATCGAAGCCAT
13022	Yes	AAGCGTGCTGGGCCCGTAACCCAGAGGTCAATGGATCGAAGCCATC
13023	Yes	AAGCGTGCTGGGCCCGTAACCCAGAGGTCAATGGATCGAAGCCATCC
13024	Yes	AAGCGTGCTGGGCCCGTAACCCAGAGGTCAATGGATCGAAGCCATCCT
13025	Yes	AAGCGTGCTGGGCCCGTAACCCAGAGGTCAATGGATCGAAGCCATCCTT
13026	Yes	AAGCGTGCTGGGCCCGTAACCCAGAGGTCAATGGATCGAAGCCATCCTTG
13027	No	AAGCTAACTTAGCATT
13028	Yes	AAGCTAACTTAGCATTA
13029	Yes	AAGCTAACTTAGCATTAA
13030	Yes	AAGCTAACTTAGCATTAAC
13031	Yes	AAGCTAACTTAGCATTAACC
13032	Yes	AAGCTAACTTAGCATTAACCT
13033	Yes	AAGCTAACTTAGCATTAACCTT
13034	Yes	AAGCTAACTTAGCATTAACCTTT
13035	Yes	AAGCTAACTTAGCATTAACCTTTT
13036	Yes	AAGCTAACTTAGCATTAACCTTTTA
13037	Yes	AAGCTAACTTAGCATTAACCTTTTAA
13038	Yes	AAGCTAACTTAGCATTAACCTTTTAAG
13039	Yes	AAGCTAACTTAGCATTAACCTTTTAAGT
13040	Yes	AAGCTAACTTAGCATTAACCTTTTAAGTT
13041	Yes	AAGCTAACTTAGCATTAACCTTTTAAGTTA
13042	Yes	AAGCTAACTTAGCATTAACCTTTTAAGTTAA
13043	Yes	AAGCTAACTTAGCATTAACCTTTTAAGTTAAA
13044	Yes	AAGCTAACTTAGCATTAACCTTTTAAGTTAAAG
13045	Yes	AAGCTAACTTAGCATTAACCTTTTAAGTTAAAGA
13046	Yes	AAGCTAACTTAGCATTAACCTTTTAAGTTAAAGAT
13047	Yes	AAGCTAACTTAGCATTAACCTTTTAAGTTAAAGATT
13048	Yes	AAGCTAACTTAGCATTAACCTTTTAAGTTAAAGATTA
13049	Yes	AAGCTAACTTAGCATTAACCTTTTAAGTTAAAGATTAA
13050	Yes	AAGCTAACTTAGCATTAACCTTTTAAGTTAAAGATTAAG
13051	Yes	AAGCTAACTTAGCATTAACCTTTTAAGTTAAAGATTAAGA
13052	Yes	AAGCTAACTTAGCATTAACCTTTTAAGTTAAAGATTAAGAG
13053	Yes	AAGCTAACTTAGCATTAACCTTTTAAGTTAAAGATTAAGAGA
13054	Yes	AAGCTAACTTAGCATTAACCTTTTAAGTTAAAGATTAAGAGAA
13055	Yes	AAGCTAACTTAGCATTAACCTTTTAAGTTAAAGATTAAGAGAAC

13056	Yes	AAGCTAACTTAGCATTAACCTTTTAAGTTAAAGATTAAGAGAACC
13057	Yes	AAGCTAACTTAGCATTAACCTTTTAAGTTAAAGATTAAGAGAACCA
13058	Yes	AAGCTAACTTAGCATTAACCTTTTAAGTTAAAGATTAAGAGAACCAA
13059	Yes	AAGCTAACTTAGCATTAACCTTTTAAGTTAAAGATTAAGAGAACCAAC
13060	Yes	AAGCTAACTTAGCATTAACCTTTTAAGTTAAAGATTAAGAGAACCAACA
13061	Yes	AAGCTAACTTAGCATTAACCTTTTAAGTTAAAGATTAAGAGAACCAACAC
13062	No	AAGCTATCGGGCCCAT
13063	No	AAGCTATCGGGCCCATA
13064	No	AAGCTATCGGGCCCATAC
13065	No	AAGCTATCGGGCCCATACC
13066	No	AAGCTATCGGGCCCATACCC
13067	No	AAGCTATCGGGCCCATACCCC
13068	No	AAGCTATCGGGCCCATACCCCG
13069	No	AAGCTATCGGGCCCATACCCCGA
13070	No	AAGCTATCGGGCCCATACCCCGAA
13071	No	AAGCTATCGGGCCCATACCCCGAAA
13072	No	AAGCTATCGGGCCCATACCCCGAAAA
13073	No	AAGCTATCGGGCCCATACCCCGAAAAT
13074	No	AAGCTATCGGGCCCATACCCCGAAAATG
13075	No	AAGCTATCGGGCCCATACCCCGAAAATGT
13076	No	AAGCTATCGGGCCCATACCCCGAAAATGTT
13077	No	AAGCTATCGGGCCCATACCCCGAAAATGTTG
13078	No	AAGCTATCGGGCCCATACCCCGAAAATGTTGG
13079	No	AAGCTATCGGGCCCATACCCCGAAAATGTTGGT
13080	No	AAGCTATCGGGCCCATACCCCGAAAATGTTGGTT
13081	No	AAGCTATCGGGCCCATACCCCGAAAATGTTGGTTA
13082	No	AAGCTATCGGGCCCATACCCCGAAAATGTTGGTTAT
13083	No	AAGCTATCGGGCCCATACCCCGAAAATGTTGGTTATA
13084	Yes	AAGCTATCGGGCCCATACCCCGAAAATGTTGGTTATAC
13085	Yes	AAGCTATCGGGCCCATACCCCGAAAATGTTGGTTATACC
13086	Yes	AAGCTATCGGGCCCATACCCCGAAAATGTTGGTTATACCC
13087	Yes	AAGCTATCGGGCCCATACCCCGAAAATGTTGGTTATACCCT
13088	Yes	AAGCTATCGGGCCCATACCCCGAAAATGTTGGTTATACCCTT
13089	Yes	AAGCTATCGGGCCCATACCCCGAAAATGTTGGTTATACCCTTC
13090	Yes	AAGCTATCGGGCCCATACCCCGAAAATGTTGGTTATACCCTTCC
13091	Yes	AAGCTATCGGGCCCATACCCCGAAAATGTTGGTTATACCCTTCCC
13092	Yes	AAGCTATCGGGCCCATACCCCGAAAATGTTGGTTATACCCTTCCCG
13093	Yes	AAGCTATCGGGCCCATACCCCGAAAATGTTGGTTATACCCTTCCCGT
13094	Yes	AAGCTATCGGGCCCATACCCCGAAAATGTTGGTTATACCCTTCCCGTA
13095	Yes	AAGCTATCGGGCCCATACCCCGAAAATGTTGGTTATACCCTTCCCGTAC
13096	Yes	AAGCTATCGGGCCCATACCCCGAAAATGTTGGTTATACCCTTCCCGTACT
13097	No	AAGCTCACAAGAACTG

13098	Yes	AAGCTCACAAGAACTGC
13099	Yes	AAGCTCACAAGAACTGCT
13100	Yes	AAGCTCACAAGAACTGCTA
13101	Yes	AAGCTCACAAGAACTGCTAA
13102	Yes	AAGCTCACAAGAACTGCTAAC
13103	Yes	AAGCTCACAAGAACTGCTAACT
13104	Yes	AAGCTCACAAGAACTGCTAACTC
13105	Yes	AAGCTCACAAGAACTGCTAACTCA
13106	Yes	AAGCTCACAAGAACTGCTAACTCAT
13107	Yes	AAGCTCACAAGAACTGCTAACTCATG
13108	Yes	AAGCTCACAAGAACTGCTAACTCATGC
13109	Yes	AAGCTCACAAGAACTGCTAACTCATGCC
13110	Yes	AAGCTCACAAGAACTGCTAACTCATGCCC
13111	Yes	AAGCTCACAAGAACTGCTAACTCATGCCCC
13112	Yes	AAGCTCACAAGAACTGCTAACTCATGCCCCC
13113	Yes	AAGCTCACAAGAACTGCTAACTCATGCCCCCA
13114	Yes	AAGCTCACAAGAACTGCTAACTCATGCCCCCAT
13115	Yes	AAGCTCACAAGAACTGCTAACTCATGCCCCCATG
13116	Yes	AAGCTCACAAGAACTGCTAACTCATGCCCCCATGT
13117	Yes	AAGCTCACAAGAACTGCTAACTCATGCCCCCATGTC
13118	Yes	AAGCTCACAAGAACTGCTAACTCATGCCCCCATGTCT
13119	Yes	AAGCTCACAAGAACTGCTAACTCATGCCCCCATGTCTA
13120	Yes	AAGCTCACAAGAACTGCTAACTCATGCCCCCATGTCTAA
13121	Yes	AAGCTCACAAGAACTGCTAACTCATGCCCCCATGTCTAAC
13122	Yes	AAGCTCACAAGAACTGCTAACTCATGCCCCCATGTCTAACA
13123	Yes	AAGCTCACAAGAACTGCTAACTCATGCCCCCATGTCTAACAA
13124	Yes	AAGCTCACAAGAACTGCTAACTCATGCCCCCATGTCTAACAAC
13125	Yes	AAGCTCACAAGAACTGCTAACTCATGCCCCCATGTCTAACAACA
13126	Yes	AAGCTCACAAGAACTGCTAACTCATGCCCCCATGTCTAACAACAT
13127	Yes	AAGCTCACAAGAACTGCTAACTCATGCCCCCATGTCTAACAACATG
13128	Yes	AAGCTCACAAGAACTGCTAACTCATGCCCCCATGTCTAACAACATGG
13129	Yes	AAGCTCACAAGAACTGCTAACTCATGCCCCCATGTCTAACAACATGGC
13130	Yes	AAGCTCACAAGAACTGCTAACTCATGCCCCCATGTCTAACAACATGGCT
13131	Yes	AAGCTCACAAGAACTGCTAACTCATGCCCCCATGTCTAACAACATGGCTT
13132	Yes	AAGCTCCGAGGTGATT
13133	Yes	AAGCTCCGAGGTGATTT
13134	Yes	AAGCTCCGAGGTGATTTT
13135	Yes	AAGCTCCGAGGTGATTTTC
13136	Yes	AAGCTCCGAGGTGATTTTCA
13137	Yes	AAGCTCCGAGGTGATTTTCAT
13138	Yes	AAGCTCCGAGGTGATTTTCATA
13139	Yes	AAGCTCCGAGGTGATTTTCATAT

13140	Yes	AAGCTCCGAGGTGATTTTCATATT
13141	Yes	AAGCTCCGAGGTGATTTTCATATTG
13142	Yes	AAGCTCCGAGGTGATTTTCATATTGA
13143	Yes	AAGCTCCGAGGTGATTTTCATATTGAA
13144	Yes	AAGCTCCGAGGTGATTTTCATATTGAAT
13145	Yes	AAGCTCCGAGGTGATTTTCATATTGAATT
13146	Yes	AAGCTCCGAGGTGATTTTCATATTGAATTG
13147	Yes	AAGCTCCGAGGTGATTTTCATATTGAATTGC
13148	Yes	AAGCTCCGAGGTGATTTTCATATTGAATTGCA
13149	Yes	AAGCTCCGAGGTGATTTTCATATTGAATTGCAA
13150	Yes	AAGCTCCGAGGTGATTTTCATATTGAATTGCAAA
13151	Yes	AAGCTCCGAGGTGATTTTCATATTGAATTGCAAAT
13152	Yes	AAGCTCCGAGGTGATTTTCATATTGAATTGCAAATT
13153	Yes	AAGCTCCGAGGTGATTTTCATATTGAATTGCAAATTC
13154	Yes	AAGCTCCGAGGTGATTTTCATATTGAATTGCAAATTCG
13155	Yes	AAGCTCCGAGGTGATTTTCATATTGAATTGCAAATTCGA
13156	Yes	AAGCTCCGAGGTGATTTTCATATTGAATTGCAAATTCGAA
13157	Yes	AAGCTCCGAGGTGATTTTCATATTGAATTGCAAATTCGAAG
13158	Yes	AAGCTCCGAGGTGATTTTCATATTGAATTGCAAATTCGAAGA
13159	Yes	AAGCTCCGAGGTGATTTTCATATTGAATTGCAAATTCGAAGAA
13160	Yes	AAGCTCCGAGGTGATTTTCATATTGAATTGCAAATTCGAAGAAG
13161	Yes	AAGCTCCGAGGTGATTTTCATATTGAATTGCAAATTCGAAGAAGC
13162	Yes	AAGCTCCGAGGTGATTTTCATATTGAATTGCAAATTCGAAGAAGCA
13163	Yes	AAGCTCCGAGGTGATTTTCATATTGAATTGCAAATTCGAAGAAGCAG
13164	Yes	AAGCTCCGAGGTGATTTTCATATTGAATTGCAAATTCGAAGAAGCAGC
13165	Yes	AAGCTCCGAGGTGATTTTCATATTGAATTGCAAATTCGAAGAAGCAGCT
13166	Yes	AAGCTCCGAGGTGATTTTCATATTGAATTGCAAATTCGAAGAAGCAGCTT
13167	No	AAGCTGGAAAGCCTGT
13168	No	AAGCTGGAAAGCCTGTG
13169	Yes	AAGCTGGAAAGCCTGTGG
13170	Yes	AAGCTGGAAAGCCTGTGGG
13171	Yes	AAGCTGGAAAGCCTGTGGGC
13172	Yes	AAGCTGGAAAGCCTGTGGGCT
13173	Yes	AAGCTGGAAAGCCTGTGGGCTC
13174	Yes	AAGCTGGAAAGCCTGTGGGCTCC
13175	Yes	AAGCTGGAAAGCCTGTGGGCTCCA
13176	Yes	AAGCTGGAAAGCCTGTGGGCTCCAC
13177	Yes	AAGCTGGAAAGCCTGTGGGCTCCACA
13178	Yes	AAGCTGGAAAGCCTGTGGGCTCCACAG
13179	Yes	AAGCTGGAAAGCCTGTGGGCTCCACAGT
13180	Yes	AAGCTGGAAAGCCTGTGGGCTCCACAGTC
13181	Yes	AAGCTGGAAAGCCTGTGGGCTCCACAGTCA

13182	Yes	AAGCTGGAAAGCCTGTGGGCTCCACAGTCAT
13183	Yes	AAGCTGGAAAGCCTGTGGGCTCCACAGTCATG
13184	Yes	AAGCTGGAAAGCCTGTGGGCTCCACAGTCATGT
13185	Yes	AAGCTGGAAAGCCTGTGGGCTCCACAGTCATGTG
13186	Yes	AAGCTGGAAAGCCTGTGGGCTCCACAGTCATGTGC
13187	Yes	AAGCTGGAAAGCCTGTGGGCTCCACAGTCATGTGCC
13188	Yes	AAGCTGGAAAGCCTGTGGGCTCCACAGTCATGTGCCT
13189	Yes	AAGCTGGAAAGCCTGTGGGCTCCACAGTCATGTGCCTG
13190	Yes	AAGCTGGAAAGCCTGTGGGCTCCACAGTCATGTGCCTGG
13191	Yes	AAGCTGGAAAGCCTGTGGGCTCCACAGTCATGTGCCTGGG
13192	Yes	AAGCTGGAAAGCCTGTGGGCTCCACAGTCATGTGCCTGGGT
13193	Yes	AAGCTGGAAAGCCTGTGGGCTCCACAGTCATGTGCCTGGGTT
13194	Yes	AAGCTGGAAAGCCTGTGGGCTCCACAGTCATGTGCCTGGGTTC
13195	Yes	AAGCTGGAAAGCCTGTGGGCTCCACAGTCATGTGCCTGGGTTCA
13196	Yes	AAGCTGGAAAGCCTGTGGGCTCCACAGTCATGTGCCTGGGTTCAA
13197	Yes	AAGCTGGAAAGCCTGTGGGCTCCACAGTCATGTGCCTGGGTTCAAT
13198	Yes	AAGCTGGAAAGCCTGTGGGCTCCACAGTCATGTGCCTGGGTTCAATT
13199	Yes	AAGCTGGAAAGCCTGTGGGCTCCACAGTCATGTGCCTGGGTTCAATTC
13200	Yes	AAGCTGGAAAGCCTGTGGGCTCCACAGTCATGTGCCTGGGTTCAATTCC
13201	Yes	AAGCTGGAAAGCCTGTGGGCTCCACAGTCATGTGCCTGGGTTCAATTCCC
13202	No	AAGCTGTATATTATAG
13203	No	AAGCTGTATATTATAGT
13204	No	AAGCTGTATATTATAGTG
13205	No	AAGCTGTATATTATAGTGG
13206	Yes	AAGCTGTATATTATAGTGGA
13207	Yes	AAGCTGTATATTATAGTGGAA
13208	Yes	AAGCTGTATATTATAGTGGAAT
13209	Yes	AAGCTGTATATTATAGTGGAATA
13210	Yes	AAGCTGTATATTATAGTGGAATAA
13211	Yes	AAGCTGTATATTATAGTGGAATAAA
13212	Yes	AAGCTGTATATTATAGTGGAATAAAT
13213	Yes	AAGCTGTATATTATAGTGGAATAAATG
13214	Yes	AAGCTGTATATTATAGTGGAATAAATGT
13215	Yes	AAGCTGTATATTATAGTGGAATAAATGTG
13216	Yes	AAGCTGTATATTATAGTGGAATAAATGTGG
13217	Yes	AAGCTGTATATTATAGTGGAATAAATGTGGA
13218	Yes	AAGCTGTATATTATAGTGGAATAAATGTGGAC
13219	Yes	AAGCTGTATATTATAGTGGAATAAATGTGGACT
13220	Yes	AAGCTGTATATTATAGTGGAATAAATGTGGACTT
13221	Yes	AAGCTGTATATTATAGTGGAATAAATGTGGACTTT
13222	Yes	AAGCTGTATATTATAGTGGAATAAATGTGGACTTTG
13223	Yes	AAGCTGTATATTATAGTGGAATAAATGTGGACTTTGA

13224	Yes	AAGCTGTATATTATAGTGGAATAAATGTGGACTTTGAA
13225	Yes	AAGCTGTATATTATAGTGGAATAAATGTGGACTTTGAAG
13226	Yes	AAGCTGTATATTATAGTGGAATAAATGTGGACTTTGAAGT
13227	Yes	AAGCTGTATATTATAGTGGAATAAATGTGGACTTTGAAGTT
13228	Yes	AAGCTGTATATTATAGTGGAATAAATGTGGACTTTGAAGTTA
13229	Yes	AAGCTGTATATTATAGTGGAATAAATGTGGACTTTGAAGTTAG
13230	Yes	AAGCTGTATATTATAGTGGAATAAATGTGGACTTTGAAGTTAGA
13231	Yes	AAGCTGTATATTATAGTGGAATAAATGTGGACTTTGAAGTTAGAT
13232	Yes	AAGCTGTATATTATAGTGGAATAAATGTGGACTTTGAAGTTAGATA
13233	Yes	AAGCTGTATATTATAGTGGAATAAATGTGGACTTTGAAGTTAGATAC
13234	Yes	AAGCTGTATATTATAGTGGAATAAATGTGGACTTTGAAGTTAGATACA
13235	Yes	AAGCTGTATATTATAGTGGAATAAATGTGGACTTTGAAGTTAGATACAC
13236	Yes	AAGCTGTATATTATAGTGGAATAAATGTGGACTTTGAAGTTAGATACACC
13237	No	AAGCTTGGTGGTTCGA
13238	Yes	AAGCTTGGTGGTTCGAG
13239	Yes	AAGCTTGGTGGTTCGAGC
13240	Yes	AAGCTTGGTGGTTCGAGCC
13241	Yes	AAGCTTGGTGGTTCGAGCCC
13242	Yes	AAGCTTGGTGGTTCGAGCCCA
13243	Yes	AAGCTTGGTGGTTCGAGCCCAC
13244	Yes	AAGCTTGGTGGTTCGAGCCCACC
13245	Yes	AAGCTTGGTGGTTCGAGCCCACCC
13246	Yes	AAGCTTGGTGGTTCGAGCCCACCCA
13247	Yes	AAGCTTGGTGGTTCGAGCCCACCCAG
13248	Yes	AAGCTTGGTGGTTCGAGCCCACCCAGG
13249	Yes	AAGCTTGGTGGTTCGAGCCCACCCAGGG
13250	Yes	AAGCTTGGTGGTTCGAGCCCACCCAGGGA
13251	Yes	AAGCTTGGTGGTTCGAGCCCACCCAGGGAT
13252	Yes	AAGCTTGGTGGTTCGAGCCCACCCAGGGATG
13253	Yes	AAGCTTGGTGGTTCGAGCCCACCCAGGGATGC
13254	Yes	AAGCTTGGTGGTTCGAGCCCACCCAGGGATGCC
13255	Yes	AAGCTTGGTGGTTCGAGCCCACCCAGGGATGCCA
13256	No	AAGCTTGGTGGTTTGA
13257	Yes	AAGCTTGGTGGTTTGAG
13258	Yes	AAGCTTGGTGGTTTGAGC
13259	Yes	AAGCTTGGTGGTTTGAGCC
13260	Yes	AAGCTTGGTGGTTTGAGCCC
13261	Yes	AAGCTTGGTGGTTTGAGCCCA
13262	Yes	AAGCTTGGTGGTTTGAGCCCAC
13263	Yes	AAGCTTGGTGGTTTGAGCCCACC
13264	Yes	AAGCTTGGTGGTTTGAGCCCACCC
13265	Yes	AAGCTTGGTGGTTTGAGCCCACCCA

13266	Yes	AAGCTTGGTGGTTTGAGCCCACCCAG
13267	Yes	AAGCTTGGTGGTTTGAGCCCACCCAGG
13268	Yes	AAGCTTGGTGGTTTGAGCCCACCCAGGG
13269	Yes	AAGCTTGGTGGTTTGAGCCCACCCAGGGA
13270	Yes	AAGCTTGGTGGTTTGAGCCCACCCAGGGAT
13271	Yes	AAGCTTGGTGGTTTGAGCCCACCCAGGGATG
13272	Yes	AAGCTTGGTGGTTTGAGCCCACCCAGGGATGC
13273	Yes	AAGCTTGGTGGTTTGAGCCCACCCAGGGATGCC
13274	Yes	AAGCTTGGTGGTTTGAGCCCACCCAGGGATGCCA
13275	No	AAGGACCTGCTCAGAC
13276	Yes	AAGGACCTGCTCAGACA
13277	Yes	AAGGACCTGCTCAGACAT
13278	Yes	AAGGACCTGCTCAGACATC
13279	Yes	AAGGACCTGCTCAGACATCA
13280	Yes	AAGGACCTGCTCAGACATCAC
13281	Yes	AAGGACCTGCTCAGACATCACA
13282	Yes	AAGGACCTGCTCAGACATCACAG
13283	Yes	AAGGACCTGCTCAGACATCACAGG
13284	Yes	AAGGACCTGCTCAGACATCACAGGT
13285	Yes	AAGGACCTGCTCAGACATCACAGGTA
13286	Yes	AAGGACCTGCTCAGACATCACAGGTAG
13287	Yes	AAGGACCTGCTCAGACATCACAGGTAGG
13288	Yes	AAGGACCTGCTCAGACATCACAGGTAGGT
13289	Yes	AAGGACCTGCTCAGACATCACAGGTAGGTA
13290	Yes	AAGGACCTGCTCAGACATCACAGGTAGGTAG
13291	Yes	AAGGACCTGCTCAGACATCACAGGTAGGTAGA
13292	Yes	AAGGACCTGCTCAGACATCACAGGTAGGTAGAT
13293	Yes	AAGGACCTGCTCAGACATCACAGGTAGGTAGATC
13294	Yes	AAGGACCTGCTCAGACATCACAGGTAGGTAGATCT
13295	Yes	AAGGACCTGCTCAGACATCACAGGTAGGTAGATCTG
13296	Yes	AAGGACCTGCTCAGACATCACAGGTAGGTAGATCTGG
13297	Yes	AAGGACCTGCTCAGACATCACAGGTAGGTAGATCTGGG
13298	Yes	AAGGACCTGCTCAGACATCACAGGTAGGTAGATCTGGGT
13299	Yes	AAGGACCTGCTCAGACATCACAGGTAGGTAGATCTGGGTT
13300	Yes	AAGGACCTGCTCAGACATCACAGGTAGGTAGATCTGGGTTC
13301	Yes	AAGGACCTGCTCAGACATCACAGGTAGGTAGATCTGGGTTCA
13302	Yes	AAGGACCTGCTCAGACATCACAGGTAGGTAGATCTGGGTTCAA
13303	Yes	AAGGACCTGCTCAGACATCACAGGTAGGTAGATCTGGGTTCAAA
13304	Yes	AAGGACCTGCTCAGACATCACAGGTAGGTAGATCTGGGTTCAAAC
13305	Yes	AAGGACCTGCTCAGACATCACAGGTAGGTAGATCTGGGTTCAAACC
13306	Yes	AAGGACCTGCTCAGACATCACAGGTAGGTAGATCTGGGTTCAAACCC
13307	Yes	AAGGACCTGCTCAGACATCACAGGTAGGTAGATCTGGGTTCAAACCCT

13308	Yes	AAGGACCTGCTCAGACATCACAGGTAGGTAGATCTGGGTTCAAACCCTA
13309	Yes	AAGGACCTGCTCAGACATCACAGGTAGGTAGATCTGGGTTCAAACCCTAG
13310	No	AAGGAGACTGGTTCAA
13311	No	AAGGAGACTGGTTCAAT
13312	Yes	AAGGAGACTGGTTCAATT
13313	Yes	AAGGAGACTGGTTCAATTC
13314	Yes	AAGGAGACTGGTTCAATTCT
13315	Yes	AAGGAGACTGGTTCAATTCTG
13316	Yes	AAGGAGACTGGTTCAATTCTGG
13317	Yes	AAGGAGACTGGTTCAATTCTGGG
13318	Yes	AAGGAGACTGGTTCAATTCTGGGT
13319	Yes	AAGGAGACTGGTTCAATTCTGGGTT
13320	Yes	AAGGAGACTGGTTCAATTCTGGGTTT
13321	Yes	AAGGAGACTGGTTCAATTCTGGGTTTT
13322	Yes	AAGGAGACTGGTTCAATTCTGGGTTTTG
13323	Yes	AAGGAGACTGGTTCAATTCTGGGTTTTGG
13324	Yes	AAGGAGACTGGTTCAATTCTGGGTTTTGGC
13325	Yes	AAGGAGACTGGTTCAATTCTGGGTTTTGGCA
13326	Yes	AAGGAGACTGGTTCAATTCTGGGTTTTGGCAC
13327	Yes	AAGGAGACTGGTTCAATTCTGGGTTTTGGCACC
13328	Yes	AAGGAGACTGGTTCAATTCTGGGTTTTGGCACCA
13329	No	AAGGATAACAGCTATC
13330	No	AAGGATAACAGCTATCC
13331	Yes	AAGGATAACAGCTATCCA
13332	Yes	AAGGATAACAGCTATCCAT
13333	Yes	AAGGATAACAGCTATCCATT
13334	Yes	AAGGATAACAGCTATCCATTG
13335	Yes	AAGGATAACAGCTATCCATTGG
13336	Yes	AAGGATAACAGCTATCCATTGGT
13337	Yes	AAGGATAACAGCTATCCATTGGTC
13338	Yes	AAGGATAACAGCTATCCATTGGTCT
13339	Yes	AAGGATAACAGCTATCCATTGGTCTT
13340	Yes	AAGGATAACAGCTATCCATTGGTCTTA
13341	Yes	AAGGATAACAGCTATCCATTGGTCTTAG
13342	Yes	AAGGATAACAGCTATCCATTGGTCTTAGG
13343	Yes	AAGGATAACAGCTATCCATTGGTCTTAGGC
13344	Yes	AAGGATAACAGCTATCCATTGGTCTTAGGCC
13345	Yes	AAGGATAACAGCTATCCATTGGTCTTAGGCCC
13346	Yes	AAGGATAACAGCTATCCATTGGTCTTAGGCCCC
13347	Yes	AAGGATAACAGCTATCCATTGGTCTTAGGCCCCA
13348	Yes	AAGGATAACAGCTATCCATTGGTCTTAGGCCCCAA
13349	Yes	AAGGATAACAGCTATCCATTGGTCTTAGGCCCCAAA

13350	Yes	AAGGATAACAGCTATCCATTGGTCTTAGGCCCCAAAA
13351	Yes	AAGGATAACAGCTATCCATTGGTCTTAGGCCCCAAAAA
13352	Yes	AAGGATAACAGCTATCCATTGGTCTTAGGCCCCAAAAAT
13353	Yes	AAGGATAACAGCTATCCATTGGTCTTAGGCCCCAAAAATT
13354	Yes	AAGGATAACAGCTATCCATTGGTCTTAGGCCCCAAAAATTT
13355	Yes	AAGGATAACAGCTATCCATTGGTCTTAGGCCCCAAAAATTTT
13356	Yes	AAGGATAACAGCTATCCATTGGTCTTAGGCCCCAAAAATTTTG
13357	Yes	AAGGATAACAGCTATCCATTGGTCTTAGGCCCCAAAAATTTTGG
13358	Yes	AAGGATAACAGCTATCCATTGGTCTTAGGCCCCAAAAATTTTGGT
13359	Yes	AAGGATAACAGCTATCCATTGGTCTTAGGCCCCAAAAATTTTGGTG
13360	Yes	AAGGATAACAGCTATCCATTGGTCTTAGGCCCCAAAAATTTTGGTGC
13361	Yes	AAGGATAACAGCTATCCATTGGTCTTAGGCCCCAAAAATTTTGGTGCA
13362	Yes	AAGGATAACAGCTATCCATTGGTCTTAGGCCCCAAAAATTTTGGTGCAA
13363	Yes	AAGGATAACAGCTATCCATTGGTCTTAGGCCCCAAAAATTTTGGTGCAAC
13364	No	AAGGATGGGGCCAAGC
13365	No	AAGGATGGGGCCAAGCT
13366	Yes	AAGGATGGGGCCAAGCTG
13367	Yes	AAGGATGGGGCCAAGCTGG
13368	Yes	AAGGATGGGGCCAAGCTGGA
13369	Yes	AAGGATGGGGCCAAGCTGGAA
13370	Yes	AAGGATGGGGCCAAGCTGGAAA
13371	Yes	AAGGATGGGGCCAAGCTGGAAAG
13372	Yes	AAGGATGGGGCCAAGCTGGAAAGC
13373	Yes	AAGGATGGGGCCAAGCTGGAAAGCC
13374	Yes	AAGGATGGGGCCAAGCTGGAAAGCCT
13375	Yes	AAGGATGGGGCCAAGCTGGAAAGCCTG
13376	Yes	AAGGATGGGGCCAAGCTGGAAAGCCTGT
13377	Yes	AAGGATGGGGCCAAGCTGGAAAGCCTGTG
13378	Yes	AAGGATGGGGCCAAGCTGGAAAGCCTGTGG
13379	Yes	AAGGATGGGGCCAAGCTGGAAAGCCTGTGGG
13380	Yes	AAGGATGGGGCCAAGCTGGAAAGCCTGTGGGC
13381	Yes	AAGGATGGGGCCAAGCTGGAAAGCCTGTGGGCT
13382	Yes	AAGGATGGGGCCAAGCTGGAAAGCCTGTGGGCTC
13383	Yes	AAGGATGGGGCCAAGCTGGAAAGCCTGTGGGCTCC
13384	Yes	AAGGATGGGGCCAAGCTGGAAAGCCTGTGGGCTCCA
13385	Yes	AAGGATGGGGCCAAGCTGGAAAGCCTGTGGGCTCCAC
13386	Yes	AAGGATGGGGCCAAGCTGGAAAGCCTGTGGGCTCCACA
13387	Yes	AAGGATGGGGCCAAGCTGGAAAGCCTGTGGGCTCCACAG
13388	Yes	AAGGATGGGGCCAAGCTGGAAAGCCTGTGGGCTCCACAGT
13389	Yes	AAGGATGGGGCCAAGCTGGAAAGCCTGTGGGCTCCACAGTC
13390	Yes	AAGGATGGGGCCAAGCTGGAAAGCCTGTGGGCTCCACAGTCA
13391	Yes	AAGGATGGGGCCAAGCTGGAAAGCCTGTGGGCTCCACAGTCAT

13392	Yes	AAGGATGGGGCCAAGCTGGAAAGCCTGTGGGCTCCACAGTCATG
13393	Yes	AAGGATGGGGCCAAGCTGGAAAGCCTGTGGGCTCCACAGTCATGT
13394	Yes	AAGGATGGGGCCAAGCTGGAAAGCCTGTGGGCTCCACAGTCATGTG
13395	Yes	AAGGATGGGGCCAAGCTGGAAAGCCTGTGGGCTCCACAGTCATGTGC
13396	Yes	AAGGATGGGGCCAAGCTGGAAAGCCTGTGGGCTCCACAGTCATGTGCC
13397	Yes	AAGGATGGGGCCAAGCTGGAAAGCCTGTGGGCTCCACAGTCATGTGCCT
13398	Yes	AAGGATGGGGCCAAGCTGGAAAGCCTGTGGGCTCCACAGTCATGTGCCTG
13399	No	AAGGCAACGAGCTAGA
13400	Yes	AAGGCAACGAGCTAGAA
13401	Yes	AAGGCAACGAGCTAGAAA
13402	Yes	AAGGCAACGAGCTAGAAAT
13403	Yes	AAGGCAACGAGCTAGAAATT
13404	Yes	AAGGCAACGAGCTAGAAATTC
13405	Yes	AAGGCAACGAGCTAGAAATTCG
13406	Yes	AAGGCAACGAGCTAGAAATTCGT
13407	Yes	AAGGCAACGAGCTAGAAATTCGTT
13408	Yes	AAGGCAACGAGCTAGAAATTCGTTG
13409	Yes	AAGGCAACGAGCTAGAAATTCGTTGG
13410	Yes	AAGGCAACGAGCTAGAAATTCGTTGGT
13411	Yes	AAGGCAACGAGCTAGAAATTCGTTGGTT
13412	Yes	AAGGCAACGAGCTAGAAATTCGTTGGTTT
13413	Yes	AAGGCAACGAGCTAGAAATTCGTTGGTTTC
13414	Yes	AAGGCAACGAGCTAGAAATTCGTTGGTTTCT
13415	Yes	AAGGCAACGAGCTAGAAATTCGTTGGTTTCTC
13416	Yes	AAGGCAACGAGCTAGAAATTCGTTGGTTTCTCT
13417	Yes	AAGGCAACGAGCTAGAAATTCGTTGGTTTCTCTC
13418	Yes	AAGGCAACGAGCTAGAAATTCGTTGGTTTCTCTCT
13419	Yes	AAGGCAACGAGCTAGAAATTCGTTGGTTTCTCTCTG
13420	Yes	AAGGCAACGAGCTAGAAATTCGTTGGTTTCTCTCTGT
13421	Yes	AAGGCAACGAGCTAGAAATTCGTTGGTTTCTCTCTGTG
13422	Yes	AAGGCAACGAGCTAGAAATTCGTTGGTTTCTCTCTGTGC
13423	Yes	AAGGCAACGAGCTAGAAATTCGTTGGTTTCTCTCTGTGCA
13424	Yes	AAGGCAACGAGCTAGAAATTCGTTGGTTTCTCTCTGTGCAG
13425	Yes	AAGGCAACGAGCTAGAAATTCGTTGGTTTCTCTCTGTGCAGG
13426	Yes	AAGGCAACGAGCTAGAAATTCGTTGGTTTCTCTCTGTGCAGGT
13427	Yes	AAGGCAACGAGCTAGAAATTCGTTGGTTTCTCTCTGTGCAGGTT
13428	Yes	AAGGCAACGAGCTAGAAATTCGTTGGTTTCTCTCTGTGCAGGTTT
13429	Yes	AAGGCAACGAGCTAGAAATTCGTTGGTTTCTCTCTGTGCAGGTTTG
13430	Yes	AAGGCAACGAGCTAGAAATTCGTTGGTTTCTCTCTGTGCAGGTTTGA
13431	Yes	AAGGCAACGAGCTAGAAATTCGTTGGTTTCTCTCTGTGCAGGTTTGAA
13432	Yes	AAGGCAACGAGCTAGAAATTCGTTGGTTTCTCTCTGTGCAGGTTTGAAT
13433	Yes	AAGGCAACGAGCTAGAAATTCGTTGGTTTCTCTCTGTGCAGGTTTGAATC

13434	No	AAGGCACTGAATTTAG
13435	Yes	AAGGCACTGAATTTAGG
13436	Yes	AAGGCACTGAATTTAGGC
13437	Yes	AAGGCACTGAATTTAGGCT
13438	Yes	AAGGCACTGAATTTAGGCTC
13439	Yes	AAGGCACTGAATTTAGGCTCC
13440	Yes	AAGGCACTGAATTTAGGCTCCA
13441	Yes	AAGGCACTGAATTTAGGCTCCAG
13442	Yes	AAGGCACTGAATTTAGGCTCCAGT
13443	Yes	AAGGCACTGAATTTAGGCTCCAGTC
13444	Yes	AAGGCACTGAATTTAGGCTCCAGTCT
13445	Yes	AAGGCACTGAATTTAGGCTCCAGTCTC
13446	Yes	AAGGCACTGAATTTAGGCTCCAGTCTCT
13447	Yes	AAGGCACTGAATTTAGGCTCCAGTCTCTT
13448	Yes	AAGGCACTGAATTTAGGCTCCAGTCTCTTT
13449	Yes	AAGGCACTGAATTTAGGCTCCAGTCTCTTTG
13450	Yes	AAGGCACTGAATTTAGGCTCCAGTCTCTTTGG
13451	Yes	AAGGCACTGAATTTAGGCTCCAGTCTCTTTGGG
13452	Yes	AAGGCACTGAATTTAGGCTCCAGTCTCTTTGGGG
13453	Yes	AAGGCACTGAATTTAGGCTCCAGTCTCTTTGGGGA
13454	Yes	AAGGCACTGAATTTAGGCTCCAGTCTCTTTGGGGAC
13455	Yes	AAGGCACTGAATTTAGGCTCCAGTCTCTTTGGGGACG
13456	Yes	AAGGCACTGAATTTAGGCTCCAGTCTCTTTGGGGACGT
13457	Yes	AAGGCACTGAATTTAGGCTCCAGTCTCTTTGGGGACGTG
13458	Yes	AAGGCACTGAATTTAGGCTCCAGTCTCTTTGGGGACGTGG
13459	Yes	AAGGCACTGAATTTAGGCTCCAGTCTCTTTGGGGACGTGGG
13460	Yes	AAGGCACTGAATTTAGGCTCCAGTCTCTTTGGGGACGTGGGT
13461	Yes	AAGGCACTGAATTTAGGCTCCAGTCTCTTTGGGGACGTGGGTT
13462	Yes	AAGGCACTGAATTTAGGCTCCAGTCTCTTTGGGGACGTGGGTTT
13463	Yes	AAGGCACTGAATTTAGGCTCCAGTCTCTTTGGGGACGTGGGTTTA
13464	Yes	AAGGCACTGAATTTAGGCTCCAGTCTCTTTGGGGACGTGGGTTTAA
13465	Yes	AAGGCACTGAATTTAGGCTCCAGTCTCTTTGGGGACGTGGGTTTAAA
13466	Yes	AAGGCACTGAATTTAGGCTCCAGTCTCTTTGGGGACGTGGGTTTAAAT
13467	Yes	AAGGCACTGAATTTAGGCTCCAGTCTCTTTGGGGACGTGGGTTTAAATC
13468	Yes	AAGGCACTGAATTTAGGCTCCAGTCTCTTTGGGGACGTGGGTTTAAATCC
13469	No	AAGGCACTGGCCTCCT
13470	Yes	AAGGCACTGGCCTCCTA
13471	Yes	AAGGCACTGGCCTCCTAA
13472	Yes	AAGGCACTGGCCTCCTAAG
13473	Yes	AAGGCACTGGCCTCCTAAGC
13474	Yes	AAGGCACTGGCCTCCTAAGCC
13475	Yes	AAGGCACTGGCCTCCTAAGCCA

13476	Yes	AAGGCACTGGCCTCCTAAGCCAG
13477	Yes	AAGGCACTGGCCTCCTAAGCCAGG
13478	Yes	AAGGCACTGGCCTCCTAAGCCAGGG
13479	Yes	AAGGCACTGGCCTCCTAAGCCAGGGA
13480	Yes	AAGGCACTGGCCTCCTAAGCCAGGGAT
13481	Yes	AAGGCACTGGCCTCCTAAGCCAGGGATT
13482	Yes	AAGGCACTGGCCTCCTAAGCCAGGGATTG
13483	Yes	AAGGCACTGGCCTCCTAAGCCAGGGATTGT
13484	Yes	AAGGCACTGGCCTCCTAAGCCAGGGATTGTG
13485	Yes	AAGGCACTGGCCTCCTAAGCCAGGGATTGTGG
13486	Yes	AAGGCACTGGCCTCCTAAGCCAGGGATTGTGGG
13487	Yes	AAGGCACTGGCCTCCTAAGCCAGGGATTGTGGGT
13488	Yes	AAGGCACTGGCCTCCTAAGCCAGGGATTGTGGGTT
13489	Yes	AAGGCACTGGCCTCCTAAGCCAGGGATTGTGGGTTC
13490	Yes	AAGGCACTGGCCTCCTAAGCCAGGGATTGTGGGTTCG
13491	Yes	AAGGCACTGGCCTCCTAAGCCAGGGATTGTGGGTTCGA
13492	Yes	AAGGCACTGGCCTCCTAAGCCAGGGATTGTGGGTTCGAG
13493	Yes	AAGGCACTGGCCTCCTAAGCCAGGGATTGTGGGTTCGAGT
13494	Yes	AAGGCACTGGCCTCCTAAGCCAGGGATTGTGGGTTCGAGTC
13495	Yes	AAGGCACTGGCCTCCTAAGCCAGGGATTGTGGGTTCGAGTCC
13496	Yes	AAGGCACTGGCCTCCTAAGCCAGGGATTGTGGGTTCGAGTCCC
13497	Yes	AAGGCACTGGCCTCCTAAGCCAGGGATTGTGGGTTCGAGTCCCA
13498	Yes	AAGGCACTGGCCTCCTAAGCCAGGGATTGTGGGTTCGAGTCCCAC
13499	Yes	AAGGCACTGGCCTCCTAAGCCAGGGATTGTGGGTTCGAGTCCCACC
13500	Yes	AAGGCACTGGCCTCCTAAGCCAGGGATTGTGGGTTCGAGTCCCACCT
13501	Yes	AAGGCACTGGCCTCCTAAGCCAGGGATTGTGGGTTCGAGTCCCACCTG
13502	Yes	AAGGCACTGGCCTCCTAAGCCAGGGATTGTGGGTTCGAGTCCCACCTGG
13503	Yes	AAGGCACTGGCCTCCTAAGCCAGGGATTGTGGGTTCGAGTCCCACCTGGG
13504	No	AAGGCACTGTATTAAG
13505	No	AAGGCACTGTATTAAGA
13506	No	AAGGCACTGTATTAAGAC
13507	Yes	AAGGCACTGTATTAAGACT
13508	Yes	AAGGCACTGTATTAAGACTC
13509	Yes	AAGGCACTGTATTAAGACTCC
13510	Yes	AAGGCACTGTATTAAGACTCCA
13511	Yes	AAGGCACTGTATTAAGACTCCAG
13512	Yes	AAGGCACTGTATTAAGACTCCAGT
13513	Yes	AAGGCACTGTATTAAGACTCCAGTC
13514	Yes	AAGGCACTGTATTAAGACTCCAGTCT
13515	Yes	AAGGCACTGTATTAAGACTCCAGTCTC
13516	Yes	AAGGCACTGTATTAAGACTCCAGTCTCT
13517	Yes	AAGGCACTGTATTAAGACTCCAGTCTCTT

13518	Yes	AAGGCACTGTATTAAGACTCCAGTCTCTTC
13519	Yes	AAGGCACTGTATTAAGACTCCAGTCTCTTCA
13520	Yes	AAGGCACTGTATTAAGACTCCAGTCTCTTCAG
13521	Yes	AAGGCACTGTATTAAGACTCCAGTCTCTTCAGA
13522	Yes	AAGGCACTGTATTAAGACTCCAGTCTCTTCAGAG
13523	Yes	AAGGCACTGTATTAAGACTCCAGTCTCTTCAGAGG
13524	Yes	AAGGCACTGTATTAAGACTCCAGTCTCTTCAGAGGC
13525	Yes	AAGGCACTGTATTAAGACTCCAGTCTCTTCAGAGGCA
13526	Yes	AAGGCACTGTATTAAGACTCCAGTCTCTTCAGAGGCAT
13527	Yes	AAGGCACTGTATTAAGACTCCAGTCTCTTCAGAGGCATG
13528	Yes	AAGGCACTGTATTAAGACTCCAGTCTCTTCAGAGGCATGG
13529	Yes	AAGGCACTGTATTAAGACTCCAGTCTCTTCAGAGGCATGGG
13530	Yes	AAGGCACTGTATTAAGACTCCAGTCTCTTCAGAGGCATGGGT
13531	Yes	AAGGCACTGTATTAAGACTCCAGTCTCTTCAGAGGCATGGGTT
13532	Yes	AAGGCACTGTATTAAGACTCCAGTCTCTTCAGAGGCATGGGTTT
13533	Yes	AAGGCACTGTATTAAGACTCCAGTCTCTTCAGAGGCATGGGTTTG
13534	Yes	AAGGCACTGTATTAAGACTCCAGTCTCTTCAGAGGCATGGGTTTGA
13535	Yes	AAGGCACTGTATTAAGACTCCAGTCTCTTCAGAGGCATGGGTTTGAA
13536	Yes	AAGGCACTGTATTAAGACTCCAGTCTCTTCAGAGGCATGGGTTTGAAT
13537	Yes	AAGGCACTGTATTAAGACTCCAGTCTCTTCAGAGGCATGGGTTTGAATC
13538	Yes	AAGGCACTGTATTAAGACTCCAGTCTCTTCAGAGGCATGGGTTTGAATCC
13539	No	AAGGCATCAGCCTCCG
13540	Yes	AAGGCATCAGCCTCCGG
13541	Yes	AAGGCATCAGCCTCCGGA
13542	Yes	AAGGCATCAGCCTCCGGAG
13543	Yes	AAGGCATCAGCCTCCGGAGC
13544	Yes	AAGGCATCAGCCTCCGGAGCT
13545	Yes	AAGGCATCAGCCTCCGGAGCTG
13546	Yes	AAGGCATCAGCCTCCGGAGCTGG
13547	Yes	AAGGCATCAGCCTCCGGAGCTGGG
13548	Yes	AAGGCATCAGCCTCCGGAGCTGGGG
13549	Yes	AAGGCATCAGCCTCCGGAGCTGGGGA
13550	Yes	AAGGCATCAGCCTCCGGAGCTGGGGAT
13551	Yes	AAGGCATCAGCCTCCGGAGCTGGGGATT
13552	Yes	AAGGCATCAGCCTCCGGAGCTGGGGATTG
13553	Yes	AAGGCATCAGCCTCCGGAGCTGGGGATTGT
13554	Yes	AAGGCATCAGCCTCCGGAGCTGGGGATTGTG
13555	Yes	AAGGCATCAGCCTCCGGAGCTGGGGATTGTGG
13556	Yes	AAGGCATCAGCCTCCGGAGCTGGGGATTGTGGG
13557	Yes	AAGGCATCAGCCTCCGGAGCTGGGGATTGTGGGT
13558	Yes	AAGGCATCAGCCTCCGGAGCTGGGGATTGTGGGTT
13559	Yes	AAGGCATCAGCCTCCGGAGCTGGGGATTGTGGGTTC

13560	Yes	AAGGCATCAGCCTCCGGAGCTGGGGATTGTGGGTTCG
13561	Yes	AAGGCATCAGCCTCCGGAGCTGGGGATTGTGGGTTCGA
13562	Yes	AAGGCATCAGCCTCCGGAGCTGGGGATTGTGGGTTCGAG
13563	Yes	AAGGCATCAGCCTCCGGAGCTGGGGATTGTGGGTTCGAGT
13564	Yes	AAGGCATCAGCCTCCGGAGCTGGGGATTGTGGGTTCGAGTC
13565	Yes	AAGGCATCAGCCTCCGGAGCTGGGGATTGTGGGTTCGAGTCC
13566	Yes	AAGGCATCAGCCTCCGGAGCTGGGGATTGTGGGTTCGAGTCCC
13567	Yes	AAGGCATCAGCCTCCGGAGCTGGGGATTGTGGGTTCGAGTCCCA
13568	Yes	AAGGCATCAGCCTCCGGAGCTGGGGATTGTGGGTTCGAGTCCCAT
13569	Yes	AAGGCATCAGCCTCCGGAGCTGGGGATTGTGGGTTCGAGTCCCATC
13570	Yes	AAGGCATCAGCCTCCGGAGCTGGGGATTGTGGGTTCGAGTCCCATCT
13571	Yes	AAGGCATCAGCCTCCGGAGCTGGGGATTGTGGGTTCGAGTCCCATCTG
13572	Yes	AAGGCATCAGCCTCCGGAGCTGGGGATTGTGGGTTCGAGTCCCATCTGG
13573	Yes	AAGGCATCAGCCTCCGGAGCTGGGGATTGTGGGTTCGAGTCCCATCTGGG
13574	No	AAGGCATGGGCTCTAG
13575	No	AAGGCATGGGCTCTAGA
13576	No	AAGGCATGGGCTCTAGAG
13577	Yes	AAGGCATGGGCTCTAGAGC
13578	Yes	AAGGCATGGGCTCTAGAGCC
13579	Yes	AAGGCATGGGCTCTAGAGCCA
13580	Yes	AAGGCATGGGCTCTAGAGCCAG
13581	Yes	AAGGCATGGGCTCTAGAGCCAGA
13582	Yes	AAGGCATGGGCTCTAGAGCCAGAC
13583	Yes	AAGGCATGGGCTCTAGAGCCAGACT
13584	Yes	AAGGCATGGGCTCTAGAGCCAGACTT
13585	Yes	AAGGCATGGGCTCTAGAGCCAGACTTC
13586	Yes	AAGGCATGGGCTCTAGAGCCAGACTTCC
13587	Yes	AAGGCATGGGCTCTAGAGCCAGACTTCCT
13588	Yes	AAGGCATGGGCTCTAGAGCCAGACTTCCTG
13589	Yes	AAGGCATGGGCTCTAGAGCCAGACTTCCTGG
13590	Yes	AAGGCATGGGCTCTAGAGCCAGACTTCCTGGG
13591	Yes	AAGGCATGGGCTCTAGAGCCAGACTTCCTGGGT
13592	Yes	AAGGCATGGGCTCTAGAGCCAGACTTCCTGGGTT
13593	Yes	AAGGCATGGGCTCTAGAGCCAGACTTCCTGGGTTC
13594	Yes	AAGGCATGGGCTCTAGAGCCAGACTTCCTGGGTTCA
13595	Yes	AAGGCATGGGCTCTAGAGCCAGACTTCCTGGGTTCAA
13596	Yes	AAGGCATGGGCTCTAGAGCCAGACTTCCTGGGTTCAAA
13597	Yes	AAGGCATGGGCTCTAGAGCCAGACTTCCTGGGTTCAAAT
13598	Yes	AAGGCATGGGCTCTAGAGCCAGACTTCCTGGGTTCAAATC
13599	Yes	AAGGCATGGGCTCTAGAGCCAGACTTCCTGGGTTCAAATCT
13600	Yes	AAGGCATGGGCTCTAGAGCCAGACTTCCTGGGTTCAAATCTC
13601	Yes	AAGGCATGGGCTCTAGAGCCAGACTTCCTGGGTTCAAATCTCA

13602	Yes	AAGGCATGGGCTCTAGAGCCAGACTTCCTGGGTTCAAATCTCAG
13603	Yes	AAGGCATGGGCTCTAGAGCCAGACTTCCTGGGTTCAAATCTCAGA
13604	Yes	AAGGCATGGGCTCTAGAGCCAGACTTCCTGGGTTCAAATCTCAGAC
13605	Yes	AAGGCATGGGCTCTAGAGCCAGACTTCCTGGGTTCAAATCTCAGACC
13606	Yes	AAGGCATGGGCTCTAGAGCCAGACTTCCTGGGTTCAAATCTCAGACCT
13607	Yes	AAGGCATGGGCTCTAGAGCCAGACTTCCTGGGTTCAAATCTCAGACCTG
13608	Yes	AAGGCATGGGCTCTAGAGCCAGACTTCCTGGGTTCAAATCTCAGACCTGC
13609	No	AAGGCATTGGACTTAA
13610	No	AAGGCATTGGACTTAAG
13611	Yes	AAGGCATTGGACTTAAGA
13612	Yes	AAGGCATTGGACTTAAGAT
13613	Yes	AAGGCATTGGACTTAAGATC
13614	Yes	AAGGCATTGGACTTAAGATCC
13615	Yes	AAGGCATTGGACTTAAGATCCA
13616	Yes	AAGGCATTGGACTTAAGATCCAA
13617	Yes	AAGGCATTGGACTTAAGATCCAAT
13618	Yes	AAGGCATTGGACTTAAGATCCAATG
13619	Yes	AAGGCATTGGACTTAAGATCCAATGG
13620	Yes	AAGGCATTGGACTTAAGATCCAATGGA
13621	Yes	AAGGCATTGGACTTAAGATCCAATGGAG
13622	Yes	AAGGCATTGGACTTAAGATCCAATGGAGT
13623	Yes	AAGGCATTGGACTTAAGATCCAATGGAGTA
13624	Yes	AAGGCATTGGACTTAAGATCCAATGGAGTAG
13625	Yes	AAGGCATTGGACTTAAGATCCAATGGAGTAGT
13626	Yes	AAGGCATTGGACTTAAGATCCAATGGAGTAGTG
13627	Yes	AAGGCATTGGACTTAAGATCCAATGGAGTAGTGG
13628	Yes	AAGGCATTGGACTTAAGATCCAATGGAGTAGTGGC
13629	Yes	AAGGCATTGGACTTAAGATCCAATGGAGTAGTGGCT
13630	Yes	AAGGCATTGGACTTAAGATCCAATGGAGTAGTGGCTG
13631	Yes	AAGGCATTGGACTTAAGATCCAATGGAGTAGTGGCTGT
13632	Yes	AAGGCATTGGACTTAAGATCCAATGGAGTAGTGGCTGTG
13633	Yes	AAGGCATTGGACTTAAGATCCAATGGAGTAGTGGCTGTGT
13634	Yes	AAGGCATTGGACTTAAGATCCAATGGAGTAGTGGCTGTGTG
13635	Yes	AAGGCATTGGACTTAAGATCCAATGGAGTAGTGGCTGTGTGG
13636	Yes	AAGGCATTGGACTTAAGATCCAATGGAGTAGTGGCTGTGTGGG
13637	Yes	AAGGCATTGGACTTAAGATCCAATGGAGTAGTGGCTGTGTGGGT
13638	Yes	AAGGCATTGGACTTAAGATCCAATGGAGTAGTGGCTGTGTGGGTT
13639	Yes	AAGGCATTGGACTTAAGATCCAATGGAGTAGTGGCTGTGTGGGTTT
13640	Yes	AAGGCATTGGACTTAAGATCCAATGGAGTAGTGGCTGTGTGGGTTTA
13641	Yes	AAGGCATTGGACTTAAGATCCAATGGAGTAGTGGCTGTGTGGGTTTAA
13642	Yes	AAGGCATTGGACTTAAGATCCAATGGAGTAGTGGCTGTGTGGGTTTAAA
13643	Yes	AAGGCATTGGACTTAAGATCCAATGGAGTAGTGGCTGTGTGGGTTTAAAC

13644	No	AAGGCATTGGCCTCCT
13645	No	AAGGCATTGGCCTCCTA
13646	No	AAGGCATTGGCCTCCTAA
13647	Yes	AAGGCATTGGCCTCCTAAG
13648	Yes	AAGGCATTGGCCTCCTAAGC
13649	Yes	AAGGCATTGGCCTCCTAAGCC
13650	Yes	AAGGCATTGGCCTCCTAAGCCA
13651	Yes	AAGGCATTGGCCTCCTAAGCCAG
13652	Yes	AAGGCATTGGCCTCCTAAGCCAGG
13653	Yes	AAGGCATTGGCCTCCTAAGCCAGGG
13654	Yes	AAGGCATTGGCCTCCTAAGCCAGGGA
13655	Yes	AAGGCATTGGCCTCCTAAGCCAGGGAT
13656	Yes	AAGGCATTGGCCTCCTAAGCCAGGGATT
13657	Yes	AAGGCATTGGCCTCCTAAGCCAGGGATTG
13658	Yes	AAGGCATTGGCCTCCTAAGCCAGGGATTGT
13659	Yes	AAGGCATTGGCCTCCTAAGCCAGGGATTGTG
13660	Yes	AAGGCATTGGCCTCCTAAGCCAGGGATTGTGG
13661	Yes	AAGGCATTGGCCTCCTAAGCCAGGGATTGTGGG
13662	Yes	AAGGCATTGGCCTCCTAAGCCAGGGATTGTGGGT
13663	Yes	AAGGCATTGGCCTCCTAAGCCAGGGATTGTGGGTT
13664	Yes	AAGGCATTGGCCTCCTAAGCCAGGGATTGTGGGTTC
13665	Yes	AAGGCATTGGCCTCCTAAGCCAGGGATTGTGGGTTCG
13666	Yes	AAGGCATTGGCCTCCTAAGCCAGGGATTGTGGGTTCGA
13667	Yes	AAGGCATTGGCCTCCTAAGCCAGGGATTGTGGGTTCGAG
13668	Yes	AAGGCATTGGCCTCCTAAGCCAGGGATTGTGGGTTCGAGT
13669	Yes	AAGGCATTGGCCTCCTAAGCCAGGGATTGTGGGTTCGAGTC
13670	Yes	AAGGCATTGGCCTCCTAAGCCAGGGATTGTGGGTTCGAGTCC
13671	Yes	AAGGCATTGGCCTCCTAAGCCAGGGATTGTGGGTTCGAGTCCC
13672	Yes	AAGGCATTGGCCTCCTAAGCCAGGGATTGTGGGTTCGAGTCCCA
13673	Yes	AAGGCATTGGCCTCCTAAGCCAGGGATTGTGGGTTCGAGTCCCAC
13674	Yes	AAGGCATTGGCCTCCTAAGCCAGGGATTGTGGGTTCGAGTCCCACC
13675	Yes	AAGGCATTGGCCTCCTAAGCCAGGGATTGTGGGTTCGAGTCCCACCC
13676	Yes	AAGGCATTGGCCTCCTAAGCCAGGGATTGTGGGTTCGAGTCCCACCCG
13677	Yes	AAGGCATTGGCCTCCTAAGCCAGGGATTGTGGGTTCGAGTCCCACCCGG
13678	Yes	AAGGCATTGGCCTCCTAAGCCAGGGATTGTGGGTTCGAGTCCCACCCGGG
13679	Yes	AAGGCATTGGCCTCCTAAGCCAGGGATTGTGGGTTCGAGTCCCAT
13680	Yes	AAGGCATTGGCCTCCTAAGCCAGGGATTGTGGGTTCGAGTCCCATC
13681	Yes	AAGGCATTGGCCTCCTAAGCCAGGGATTGTGGGTTCGAGTCCCATCT
13682	Yes	AAGGCATTGGCCTCCTAAGCCAGGGATTGTGGGTTCGAGTCCCATCTG
13683	Yes	AAGGCATTGGCCTCCTAAGCCAGGGATTGTGGGTTCGAGTCCCATCTGG
13684	Yes	AAGGCATTGGCCTCCTAAGCCAGGGATTGTGGGTTCGAGTCCCATCTGGG
13685	No	AAGGCCACGGTCAGGG

13686	Yes	AAGGCCACGGTCAGGGG
13687	Yes	AAGGCCACGGTCAGGGGT
13688	Yes	AAGGCCACGGTCAGGGGTT
13689	Yes	AAGGCCACGGTCAGGGGTTC
13690	Yes	AAGGCCACGGTCAGGGGTTCA
13691	Yes	AAGGCCACGGTCAGGGGTTCAA
13692	Yes	AAGGCCACGGTCAGGGGTTCAAT
13693	Yes	AAGGCCACGGTCAGGGGTTCAATT
13694	Yes	AAGGCCACGGTCAGGGGTTCAATTC
13695	Yes	AAGGCCACGGTCAGGGGTTCAATTCC
13696	Yes	AAGGCCACGGTCAGGGGTTCAATTCCC
13697	Yes	AAGGCCACGGTCAGGGGTTCAATTCCCT
13698	Yes	AAGGCCACGGTCAGGGGTTCAATTCCCTT
13699	Yes	AAGGCCACGGTCAGGGGTTCAATTCCCTTA
13700	Yes	AAGGCCACGGTCAGGGGTTCAATTCCCTTAT
13701	Yes	AAGGCCACGGTCAGGGGTTCAATTCCCTTATG
13702	Yes	AAGGCCACGGTCAGGGGTTCAATTCCCTTATGG
13703	Yes	AAGGCCACGGTCAGGGGTTCAATTCCCTTATGGG
13704	Yes	AAGGCCACGGTCAGGGGTTCAATTCCCTTATGGGC
13705	Yes	AAGGCCACGGTCAGGGGTTCAATTCCCTTATGGGCT
13706	Yes	AAGGCCACGGTCAGGGGTTCAATTCCCTTATGGGCTG
13707	Yes	AAGGCCACGGTCAGGGGTTCAATTCCCTTATGGGCTGC
13708	Yes	AAGGCCACGGTCAGGGGTTCAATTCCCTTATGGGCTGCC
13709	Yes	AAGGCCACGGTCAGGGGTTCAATTCCCTTATGGGCTGCCA
13710	Yes	AAGGCCGAGCGGTCTA
13711	Yes	AAGGCCGAGCGGTCTAA
13712	Yes	AAGGCCGAGCGGTCTAAG
13713	Yes	AAGGCCGAGCGGTCTAAGG
13714	Yes	AAGGCCGAGCGGTCTAAGGC
13715	Yes	AAGGCCGAGCGGTCTAAGGCT
13716	Yes	AAGGCCGAGCGGTCTAAGGCTC
13717	Yes	AAGGCCGAGCGGTCTAAGGCTCC
13718	Yes	AAGGCCGAGCGGTCTAAGGCTCCG
13719	Yes	AAGGCCGAGCGGTCTAAGGCTCCGG
13720	Yes	AAGGCCGAGCGGTCTAAGGCTCCGGA
13721	Yes	AAGGCCGAGCGGTCTAAGGCTCCGGAT
13722	Yes	AAGGCCGAGCGGTCTAAGGCTCCGGATT
13723	Yes	AAGGCCGAGCGGTCTAAGGCTCCGGATTA
13724	Yes	AAGGCCGAGCGGTCTAAGGCTCCGGATTAA
13725	Yes	AAGGCCGAGCGGTCTAAGGCTCCGGATTAAG
13726	Yes	AAGGCCGAGCGGTCTAAGGCTCCGGATTAAGG
13727	Yes	AAGGCCGAGCGGTCTAAGGCTCCGGATTAAGGC

13728	Yes	AAGGCCGAGCGGTCTAAGGCTCCGGATTAAGGCG
13729	Yes	AAGGCCGAGCGGTCTAAGGCTCCGGATTAAGGCGC
13730	Yes	AAGGCCGAGCGGTCTAAGGCTCCGGATTAAGGCGCC
13731	Yes	AAGGCCGAGCGGTCTAAGGCTCCGGATTAAGGCGCCG
13732	Yes	AAGGCCGAGCGGTCTAAGGCTCCGGATTAAGGCGCCGG
13733	Yes	AAGGCCGAGCGGTCTAAGGCTCCGGATTAAGGCGCCGGT
13734	Yes	AAGGCCGAGCGGTCTAAGGCTCCGGATTAAGGCGCCGGTG
13735	Yes	AAGGCCGAGCGGTCTAAGGCTCCGGATTAAGGCGCCGGTGT
13736	Yes	AAGGCCGAGCGGTCTAAGGCTCCGGATTAAGGCGCCGGTGTC
13737	Yes	AAGGCCGAGCGGTCTAAGGCTCCGGATTAAGGCGCCGGTGTCT
13738	Yes	AAGGCCGAGCGGTCTAAGGCTCCGGATTAAGGCGCCGGTGTCTT
13739	Yes	AAGGCCGAGCGGTCTAAGGCTCCGGATTAAGGCGCCGGTGTCTTC
13740	Yes	AAGGCCGAGCGGTCTAAGGCTCCGGATTAAGGCGCCGGTGTCTTCG
13741	Yes	AAGGCCGAGCGGTCTAAGGCTCCGGATTAAGGCGCCGGTGTCTTCGG
13742	Yes	AAGGCCGAGCGGTCTAAGGCTCCGGATTAAGGCGCCGGTGTCTTCGGA
13743	Yes	AAGGCCGAGCGGTCTAAGGCTCCGGATTAAGGCGCCGGTGTCTTCGGAG
13744	Yes	AAGGCCGAGCGGTCTAAGGCTCCGGATTAAGGCGCCGGTGTCTTCGGAGG
13745	Yes	AAGGCGATGGACTAGA
13746	Yes	AAGGCGATGGACTAGAA
13747	Yes	AAGGCGATGGACTAGAAA
13748	Yes	AAGGCGATGGACTAGAAAT
13749	Yes	AAGGCGATGGACTAGAAATC
13750	Yes	AAGGCGATGGACTAGAAATCC
13751	Yes	AAGGCGATGGACTAGAAATCCA
13752	Yes	AAGGCGATGGACTAGAAATCCAT
13753	Yes	AAGGCGATGGACTAGAAATCCATT
13754	Yes	AAGGCGATGGACTAGAAATCCATTG
13755	Yes	AAGGCGATGGACTAGAAATCCATTGG
13756	Yes	AAGGCGATGGACTAGAAATCCATTGGG
13757	Yes	AAGGCGATGGACTAGAAATCCATTGGGG
13758	Yes	AAGGCGATGGACTAGAAATCCATTGGGGT
13759	Yes	AAGGCGATGGACTAGAAATCCATTGGGGTC
13760	Yes	AAGGCGATGGACTAGAAATCCATTGGGGTCT
13761	Yes	AAGGCGATGGACTAGAAATCCATTGGGGTCTC
13762	Yes	AAGGCGATGGACTAGAAATCCATTGGGGTCTCC
13763	Yes	AAGGCGATGGACTAGAAATCCATTGGGGTCTCCC
13764	Yes	AAGGCGATGGACTAGAAATCCATTGGGGTCTCCCC
13765	Yes	AAGGCGATGGACTAGAAATCCATTGGGGTCTCCCCG
13766	Yes	AAGGCGATGGACTAGAAATCCATTGGGGTCTCCCCGC
13767	Yes	AAGGCGATGGACTAGAAATCCATTGGGGTCTCCCCGCG
13768	Yes	AAGGCGATGGACTAGAAATCCATTGGGGTCTCCCCGCGC
13769	Yes	AAGGCGATGGACTAGAAATCCATTGGGGTCTCCCCGCGCA

13770	Yes	AAGGCGATGGACTAGAAATCCATTGGGGTCTCCCCGCGCAG
13771	Yes	AAGGCGATGGACTAGAAATCCATTGGGGTCTCCCCGCGCAGG
13772	Yes	AAGGCGATGGACTAGAAATCCATTGGGGTCTCCCCGCGCAGGT
13773	Yes	AAGGCGATGGACTAGAAATCCATTGGGGTCTCCCCGCGCAGGTT
13774	Yes	AAGGCGATGGACTAGAAATCCATTGGGGTCTCCCCGCGCAGGTTC
13775	Yes	AAGGCGATGGACTAGAAATCCATTGGGGTCTCCCCGCGCAGGTTCG
13776	Yes	AAGGCGATGGACTAGAAATCCATTGGGGTCTCCCCGCGCAGGTTCGA
13777	Yes	AAGGCGATGGACTAGAAATCCATTGGGGTCTCCCCGCGCAGGTTCGAA
13778	Yes	AAGGCGATGGACTAGAAATCCATTGGGGTCTCCCCGCGCAGGTTCGAAT
13779	Yes	AAGGCGATGGACTAGAAATCCATTGGGGTCTCCCCGCGCAGGTTCGAATC
13780	Yes	AAGGCGATGGACTAGAAATCCATTGGGGTT
13781	Yes	AAGGCGATGGACTAGAAATCCATTGGGGTTT
13782	Yes	AAGGCGATGGACTAGAAATCCATTGGGGTTTC
13783	Yes	AAGGCGATGGACTAGAAATCCATTGGGGTTTCC
13784	Yes	AAGGCGATGGACTAGAAATCCATTGGGGTTTCCC
13785	Yes	AAGGCGATGGACTAGAAATCCATTGGGGTTTCCCC
13786	Yes	AAGGCGATGGACTAGAAATCCATTGGGGTTTCCCCA
13787	Yes	AAGGCGATGGACTAGAAATCCATTGGGGTTTCCCCAC
13788	Yes	AAGGCGATGGACTAGAAATCCATTGGGGTTTCCCCACG
13789	Yes	AAGGCGATGGACTAGAAATCCATTGGGGTTTCCCCACGC
13790	Yes	AAGGCGATGGACTAGAAATCCATTGGGGTTTCCCCACGCA
13791	Yes	AAGGCGATGGACTAGAAATCCATTGGGGTTTCCCCACGCAG
13792	Yes	AAGGCGATGGACTAGAAATCCATTGGGGTTTCCCCACGCAGG
13793	Yes	AAGGCGATGGACTAGAAATCCATTGGGGTTTCCCCACGCAGGT
13794	Yes	AAGGCGATGGACTAGAAATCCATTGGGGTTTCCCCACGCAGGTT
13795	Yes	AAGGCGATGGACTAGAAATCCATTGGGGTTTCCCCACGCAGGTTC
13796	Yes	AAGGCGATGGACTAGAAATCCATTGGGGTTTCCCCACGCAGGTTCG
13797	Yes	AAGGCGATGGACTAGAAATCCATTGGGGTTTCCCCACGCAGGTTCGA
13798	Yes	AAGGCGATGGACTAGAAATCCATTGGGGTTTCCCCACGCAGGTTCGAA
13799	Yes	AAGGCGATGGACTAGAAATCCATTGGGGTTTCCCCACGCAGGTTCGAAT
13800	Yes	AAGGCGATGGACTAGAAATCCATTGGGGTTTCCCCACGCAGGTTCGAATC
13801	Yes	AAGGCGATGGACTAGAAATCCATTGGGGTTTCCCCG
13802	Yes	AAGGCGATGGACTAGAAATCCATTGGGGTTTCCCCGC
13803	Yes	AAGGCGATGGACTAGAAATCCATTGGGGTTTCCCCGCG
13804	Yes	AAGGCGATGGACTAGAAATCCATTGGGGTTTCCCCGCGC
13805	Yes	AAGGCGATGGACTAGAAATCCATTGGGGTTTCCCCGCGCA
13806	Yes	AAGGCGATGGACTAGAAATCCATTGGGGTTTCCCCGCGCAG
13807	Yes	AAGGCGATGGACTAGAAATCCATTGGGGTTTCCCCGCGCAGG
13808	Yes	AAGGCGATGGACTAGAAATCCATTGGGGTTTCCCCGCGCAGGT
13809	Yes	AAGGCGATGGACTAGAAATCCATTGGGGTTTCCCCGCGCAGGTT
13810	Yes	AAGGCGATGGACTAGAAATCCATTGGGGTTTCCCCGCGCAGGTTC
13811	Yes	AAGGCGATGGACTAGAAATCCATTGGGGTTTCCCCGCGCAGGTTCG

13812	Yes	AAGGCGATGGACTAGAAATCCATTGGGGTTTCCCCGCGCAGGTTCGA
13813	Yes	AAGGCGATGGACTAGAAATCCATTGGGGTTTCCCCGCGCAGGTTCGAA
13814	Yes	AAGGCGATGGACTAGAAATCCATTGGGGTTTCCCCGCGCAGGTTCGAAT
13815	Yes	AAGGCGATGGACTAGAAATCCATTGGGGTTTCCCCGCGCAGGTTCGAATC
13816	Yes	AAGGCGATGGACTGCT
13817	Yes	AAGGCGATGGACTGCTA
13818	Yes	AAGGCGATGGACTGCTAA
13819	Yes	AAGGCGATGGACTGCTAAT
13820	Yes	AAGGCGATGGACTGCTAATC
13821	Yes	AAGGCGATGGACTGCTAATCC
13822	Yes	AAGGCGATGGACTGCTAATCCA
13823	Yes	AAGGCGATGGACTGCTAATCCAT
13824	Yes	AAGGCGATGGACTGCTAATCCATT
13825	Yes	AAGGCGATGGACTGCTAATCCATTG
13826	Yes	AAGGCGATGGACTGCTAATCCATTGT
13827	Yes	AAGGCGATGGACTGCTAATCCATTGTG
13828	Yes	AAGGCGATGGACTGCTAATCCATTGTGC
13829	Yes	AAGGCGATGGACTGCTAATCCATTGTGCT
13830	Yes	AAGGCGATGGACTGCTAATCCATTGTGCTC
13831	Yes	AAGGCGATGGACTGCTAATCCATTGTGCTCT
13832	Yes	AAGGCGATGGACTGCTAATCCATTGTGCTCTG
13833	Yes	AAGGCGATGGACTGCTAATCCATTGTGCTCTGC
13834	Yes	AAGGCGATGGACTGCTAATCCATTGTGCTCTGCA
13835	Yes	AAGGCGATGGACTGCTAATCCATTGTGCTCTGCAC
13836	Yes	AAGGCGATGGACTGCTAATCCATTGTGCTCTGCACA
13837	Yes	AAGGCGATGGACTGCTAATCCATTGTGCTCTGCACAC
13838	Yes	AAGGCGATGGACTGCTAATCCATTGTGCTCTGCACACG
13839	Yes	AAGGCGATGGACTGCTAATCCATTGTGCTCTGCACACGT
13840	Yes	AAGGCGATGGACTGCTAATCCATTGTGCTCTGCACACGTG
13841	Yes	AAGGCGATGGACTGCTAATCCATTGTGCTCTGCACACGTGG
13842	Yes	AAGGCGATGGACTGCTAATCCATTGTGCTCTGCACACGTGGG
13843	Yes	AAGGCGATGGACTGCTAATCCATTGTGCTCTGCACACGTGGGT
13844	Yes	AAGGCGATGGACTGCTAATCCATTGTGCTCTGCACACGTGGGTT
13845	Yes	AAGGCGATGGACTGCTAATCCATTGTGCTCTGCACACGTGGGTTC
13846	Yes	AAGGCGATGGACTGCTAATCCATTGTGCTCTGCACACGTGGGTTCG
13847	Yes	AAGGCGATGGACTGCTAATCCATTGTGCTCTGCACACGTGGGTTCGA
13848	Yes	AAGGCGATGGACTGCTAATCCATTGTGCTCTGCACACGTGGGTTCGAA
13849	Yes	AAGGCGATGGACTGCTAATCCATTGTGCTCTGCACACGTGGGTTCGAAT
13850	Yes	AAGGCGATGGACTGCTAATCCATTGTGCTCTGCACACGTGGGTTCGAATC
13851	Yes	AAGGCGATGGACTGCTAATCCATTGTGCTCTGCACG
13852	Yes	AAGGCGATGGACTGCTAATCCATTGTGCTCTGCACGC
13853	Yes	AAGGCGATGGACTGCTAATCCATTGTGCTCTGCACGCG

13854	Yes	AAGGCGATGGACTGCTAATCCATTGTGCTCTGCACGCGT
13855	Yes	AAGGCGATGGACTGCTAATCCATTGTGCTCTGCACGCGTG
13856	Yes	AAGGCGATGGACTGCTAATCCATTGTGCTCTGCACGCGTGG
13857	Yes	AAGGCGATGGACTGCTAATCCATTGTGCTCTGCACGCGTGGG
13858	Yes	AAGGCGATGGACTGCTAATCCATTGTGCTCTGCACGCGTGGGT
13859	Yes	AAGGCGATGGACTGCTAATCCATTGTGCTCTGCACGCGTGGGTT
13860	Yes	AAGGCGATGGACTGCTAATCCATTGTGCTCTGCACGCGTGGGTTC
13861	Yes	AAGGCGATGGACTGCTAATCCATTGTGCTCTGCACGCGTGGGTTCG
13862	Yes	AAGGCGATGGACTGCTAATCCATTGTGCTCTGCACGCGTGGGTTCGA
13863	Yes	AAGGCGATGGACTGCTAATCCATTGTGCTCTGCACGCGTGGGTTCGAA
13864	Yes	AAGGCGATGGACTGCTAATCCATTGTGCTCTGCACGCGTGGGTTCGAAT
13865	Yes	AAGGCGATGGACTGCTAATCCATTGTGCTCTGCACGCGTGGGTTCGAATC
13866	Yes	AAGGCGATGGACTGCTAATCCATTGTGCTT
13867	Yes	AAGGCGATGGACTGCTAATCCATTGTGCTTT
13868	Yes	AAGGCGATGGACTGCTAATCCATTGTGCTTTG
13869	Yes	AAGGCGATGGACTGCTAATCCATTGTGCTTTGC
13870	Yes	AAGGCGATGGACTGCTAATCCATTGTGCTTTGCA
13871	Yes	AAGGCGATGGACTGCTAATCCATTGTGCTTTGCAC
13872	Yes	AAGGCGATGGACTGCTAATCCATTGTGCTTTGCACG
13873	Yes	AAGGCGATGGACTGCTAATCCATTGTGCTTTGCACGC
13874	Yes	AAGGCGATGGACTGCTAATCCATTGTGCTTTGCACGCG
13875	Yes	AAGGCGATGGACTGCTAATCCATTGTGCTTTGCACGCGT
13876	Yes	AAGGCGATGGACTGCTAATCCATTGTGCTTTGCACGCGTG
13877	Yes	AAGGCGATGGACTGCTAATCCATTGTGCTTTGCACGCGTGG
13878	Yes	AAGGCGATGGACTGCTAATCCATTGTGCTTTGCACGCGTGGG
13879	Yes	AAGGCGATGGACTGCTAATCCATTGTGCTTTGCACGCGTGGGT
13880	Yes	AAGGCGATGGACTGCTAATCCATTGTGCTTTGCACGCGTGGGTT
13881	Yes	AAGGCGATGGACTGCTAATCCATTGTGCTTTGCACGCGTGGGTTC
13882	Yes	AAGGCGATGGACTGCTAATCCATTGTGCTTTGCACGCGTGGGTTCG
13883	Yes	AAGGCGATGGACTGCTAATCCATTGTGCTTTGCACGCGTGGGTTCGA
13884	Yes	AAGGCGATGGACTGCTAATCCATTGTGCTTTGCACGCGTGGGTTCGAA
13885	Yes	AAGGCGATGGACTGCTAATCCATTGTGCTTTGCACGCGTGGGTTCGAAT
13886	Yes	AAGGCGATGGACTGCTAATCCATTGTGCTTTGCACGCGTGGGTTCGAATC
13887	Yes	AAGGCGATGGACTTGA
13888	Yes	AAGGCGATGGACTTGAA
13889	Yes	AAGGCGATGGACTTGAAA
13890	Yes	AAGGCGATGGACTTGAAAT
13891	Yes	AAGGCGATGGACTTGAAATC
13892	Yes	AAGGCGATGGACTTGAAATCC
13893	Yes	AAGGCGATGGACTTGAAATCCA
13894	Yes	AAGGCGATGGACTTGAAATCCAT
13895	Yes	AAGGCGATGGACTTGAAATCCATT

13896	Yes	AAGGCGATGGACTTGAAATCCATTG
13897	Yes	AAGGCGATGGACTTGAAATCCATTGG
13898	Yes	AAGGCGATGGACTTGAAATCCATTGGG
13899	Yes	AAGGCGATGGACTTGAAATCCATTGGGG
13900	Yes	AAGGCGATGGACTTGAAATCCATTGGGGT
13901	Yes	AAGGCGATGGACTTGAAATCCATTGGGGTC
13902	Yes	AAGGCGATGGACTTGAAATCCATTGGGGTCT
13903	Yes	AAGGCGATGGACTTGAAATCCATTGGGGTCTC
13904	Yes	AAGGCGATGGACTTGAAATCCATTGGGGTCTCC
13905	Yes	AAGGCGATGGACTTGAAATCCATTGGGGTCTCCC
13906	Yes	AAGGCGATGGACTTGAAATCCATTGGGGTCTCCCC
13907	Yes	AAGGCGATGGACTTGAAATCCATTGGGGTCTCCCCG
13908	Yes	AAGGCGATGGACTTGAAATCCATTGGGGTCTCCCCGC
13909	Yes	AAGGCGATGGACTTGAAATCCATTGGGGTCTCCCCGCG
13910	Yes	AAGGCGATGGACTTGAAATCCATTGGGGTCTCCCCGCGC
13911	Yes	AAGGCGATGGACTTGAAATCCATTGGGGTCTCCCCGCGCA
13912	Yes	AAGGCGATGGACTTGAAATCCATTGGGGTCTCCCCGCGCAG
13913	Yes	AAGGCGATGGACTTGAAATCCATTGGGGTCTCCCCGCGCAGG
13914	Yes	AAGGCGATGGACTTGAAATCCATTGGGGTCTCCCCGCGCAGGT
13915	Yes	AAGGCGATGGACTTGAAATCCATTGGGGTCTCCCCGCGCAGGTT
13916	Yes	AAGGCGATGGACTTGAAATCCATTGGGGTCTCCCCGCGCAGGTTC
13917	Yes	AAGGCGATGGACTTGAAATCCATTGGGGTCTCCCCGCGCAGGTTCG
13918	Yes	AAGGCGATGGACTTGAAATCCATTGGGGTCTCCCCGCGCAGGTTCGA
13919	Yes	AAGGCGATGGACTTGAAATCCATTGGGGTCTCCCCGCGCAGGTTCGAA
13920	Yes	AAGGCGATGGACTTGAAATCCATTGGGGTCTCCCCGCGCAGGTTCGAAT
13921	Yes	AAGGCGATGGACTTGAAATCCATTGGGGTCTCCCCGCGCAGGTTCGAATC
13922	Yes	AAGGCGATGGACTTGAAATCCATTGGGGTT
13923	Yes	AAGGCGATGGACTTGAAATCCATTGGGGTTT
13924	Yes	AAGGCGATGGACTTGAAATCCATTGGGGTTTC
13925	Yes	AAGGCGATGGACTTGAAATCCATTGGGGTTTCC
13926	Yes	AAGGCGATGGACTTGAAATCCATTGGGGTTTCCC
13927	Yes	AAGGCGATGGACTTGAAATCCATTGGGGTTTCCCC
13928	Yes	AAGGCGATGGACTTGAAATCCATTGGGGTTTCCCCG
13929	Yes	AAGGCGATGGACTTGAAATCCATTGGGGTTTCCCCGC
13930	Yes	AAGGCGATGGACTTGAAATCCATTGGGGTTTCCCCGCG
13931	Yes	AAGGCGATGGACTTGAAATCCATTGGGGTTTCCCCGCGC
13932	Yes	AAGGCGATGGACTTGAAATCCATTGGGGTTTCCCCGCGCA
13933	Yes	AAGGCGATGGACTTGAAATCCATTGGGGTTTCCCCGCGCAG
13934	Yes	AAGGCGATGGACTTGAAATCCATTGGGGTTTCCCCGCGCAGG
13935	Yes	AAGGCGATGGACTTGAAATCCATTGGGGTTTCCCCGCGCAGGT
13936	Yes	AAGGCGATGGACTTGAAATCCATTGGGGTTTCCCCGCGCAGGTT
13937	Yes	AAGGCGATGGACTTGAAATCCATTGGGGTTTCCCCGCGCAGGTTC

13938	Yes	AAGGCGATGGACTTGAAATCCATTGGGGTTTCCCCGCGCAGGTTCG
13939	Yes	AAGGCGATGGACTTGAAATCCATTGGGGTTTCCCCGCGCAGGTTCGA
13940	Yes	AAGGCGATGGACTTGAAATCCATTGGGGTTTCCCCGCGCAGGTTCGAA
13941	Yes	AAGGCGATGGACTTGAAATCCATTGGGGTTTCCCCGCGCAGGTTCGAAT
13942	Yes	AAGGCGATGGACTTGAAATCCATTGGGGTTTCCCCGCGCAGGTTCGAATC
13943	Yes	AAGGCGCCAGACTCAA
13944	Yes	AAGGCGCCAGACTCAAG
13945	No	AAGGCGCCAGACTCAAGT
13946	No	AAGGCGCCAGACTCAAGTT
13947	Yes	AAGGCGCCAGACTCAAGTTC
13948	Yes	AAGGCGCCAGACTCAAGTTCT
13949	Yes	AAGGCGCCAGACTCAAGTTCTG
13950	Yes	AAGGCGCCAGACTCAAGTTCTGG
13951	Yes	AAGGCGCCAGACTCAAGTTCTGGT
13952	Yes	AAGGCGCCAGACTCAAGTTCTGGTC
13953	Yes	AAGGCGCCAGACTCAAGTTCTGGTCT
13954	Yes	AAGGCGCCAGACTCAAGTTCTGGTCTC
13955	Yes	AAGGCGCCAGACTCAAGTTCTGGTCTCC
13956	Yes	AAGGCGCCAGACTCAAGTTCTGGTCTCCA
13957	Yes	AAGGCGCCAGACTCAAGTTCTGGTCTCCAA
13958	Yes	AAGGCGCCAGACTCAAGTTCTGGTCTCCAAT
13959	Yes	AAGGCGCCAGACTCAAGTTCTGGTCTCCAATG
13960	Yes	AAGGCGCCAGACTCAAGTTCTGGTCTCCAATGG
13961	Yes	AAGGCGCCAGACTCAAGTTCTGGTCTCCAATGGA
13962	Yes	AAGGCGCCAGACTCAAGTTCTGGTCTCCAATGGAG
13963	Yes	AAGGCGCCAGACTCAAGTTCTGGTCTCCAATGGAGG
13964	Yes	AAGGCGCCAGACTCAAGTTCTGGTCTCCAATGGAGGC
13965	Yes	AAGGCGCCAGACTCAAGTTCTGGTCTCCAATGGAGGCG
13966	Yes	AAGGCGCCAGACTCAAGTTCTGGTCTCCAATGGAGGCGT
13967	Yes	AAGGCGCCAGACTCAAGTTCTGGTCTCCAATGGAGGCGTG
13968	Yes	AAGGCGCCAGACTCAAGTTCTGGTCTCCAATGGAGGCGTGG
13969	Yes	AAGGCGCCAGACTCAAGTTCTGGTCTCCAATGGAGGCGTGGG
13970	Yes	AAGGCGCCAGACTCAAGTTCTGGTCTCCAATGGAGGCGTGGGT
13971	Yes	AAGGCGCCAGACTCAAGTTCTGGTCTCCAATGGAGGCGTGGGTT
13972	Yes	AAGGCGCCAGACTCAAGTTCTGGTCTCCAATGGAGGCGTGGGTTC
13973	Yes	AAGGCGCCAGACTCAAGTTCTGGTCTCCAATGGAGGCGTGGGTTCG
13974	Yes	AAGGCGCCAGACTCAAGTTCTGGTCTCCAATGGAGGCGTGGGTTCGA
13975	Yes	AAGGCGCCAGACTCAAGTTCTGGTCTCCAATGGAGGCGTGGGTTCGAA
13976	Yes	AAGGCGCCAGACTCAAGTTCTGGTCTCCAATGGAGGCGTGGGTTCGAAT
13977	Yes	AAGGCGCCAGACTCAAGTTCTGGTCTCCAATGGAGGCGTGGGTTCGAATC
13978	Yes	AAGGCGCCAGACTCAAGTTCTGGTCTCCG
13979	Yes	AAGGCGCCAGACTCAAGTTCTGGTCTCCGC

13980	Yes	AAGGCGCCAGACTCAAGTTCTGGTCTCCGCA
13981	Yes	AAGGCGCCAGACTCAAGTTCTGGTCTCCGCAT
13982	Yes	AAGGCGCCAGACTCAAGTTCTGGTCTCCGCATG
13983	Yes	AAGGCGCCAGACTCAAGTTCTGGTCTCCGCATGG
13984	Yes	AAGGCGCCAGACTCAAGTTCTGGTCTCCGCATGGA
13985	Yes	AAGGCGCCAGACTCAAGTTCTGGTCTCCGCATGGAG
13986	Yes	AAGGCGCCAGACTCAAGTTCTGGTCTCCGCATGGAGG
13987	Yes	AAGGCGCCAGACTCAAGTTCTGGTCTCCGCATGGAGGC
13988	Yes	AAGGCGCCAGACTCAAGTTCTGGTCTCCGCATGGAGGCG
13989	Yes	AAGGCGCCAGACTCAAGTTCTGGTCTCCGCATGGAGGCGT
13990	Yes	AAGGCGCCAGACTCAAGTTCTGGTCTCCGCATGGAGGCGTG
13991	Yes	AAGGCGCCAGACTCAAGTTCTGGTCTCCGCATGGAGGCGTGG
13992	Yes	AAGGCGCCAGACTCAAGTTCTGGTCTCCGCATGGAGGCGTGGG
13993	Yes	AAGGCGCCAGACTCAAGTTCTGGTCTCCGCATGGAGGCGTGGGT
13994	Yes	AAGGCGCCAGACTCAAGTTCTGGTCTCCGCATGGAGGCGTGGGTT
13995	Yes	AAGGCGCCAGACTCAAGTTCTGGTCTCCGCATGGAGGCGTGGGTTC
13996	Yes	AAGGCGCCAGACTCAAGTTCTGGTCTCCGCATGGAGGCGTGGGTTCG
13997	Yes	AAGGCGCCAGACTCAAGTTCTGGTCTCCGCATGGAGGCGTGGGTTCGA
13998	Yes	AAGGCGCCAGACTCAAGTTCTGGTCTCCGCATGGAGGCGTGGGTTCGAA
13999	Yes	AAGGCGCCAGACTCAAGTTCTGGTCTCCGCATGGAGGCGTGGGTTCGAAT
14000	Yes	AAGGCGCCAGACTCAAGTTCTGGTCTCCGG
14001	Yes	AAGGCGCCAGACTCAAGTTCTGGTCTCCGGA
14002	Yes	AAGGCGCCAGACTCAAGTTCTGGTCTCCGGAT
14003	Yes	AAGGCGCCAGACTCAAGTTCTGGTCTCCGGATG
14004	Yes	AAGGCGCCAGACTCAAGTTCTGGTCTCCGGATGG
14005	Yes	AAGGCGCCAGACTCAAGTTCTGGTCTCCGGATGGA
14006	Yes	AAGGCGCCAGACTCAAGTTCTGGTCTCCGGATGGAG
14007	Yes	AAGGCGCCAGACTCAAGTTCTGGTCTCCGGATGGAGG
14008	Yes	AAGGCGCCAGACTCAAGTTCTGGTCTCCGGATGGAGGC
14009	Yes	AAGGCGCCAGACTCAAGTTCTGGTCTCCGGATGGAGGCG
14010	Yes	AAGGCGCCAGACTCAAGTTCTGGTCTCCGGATGGAGGCGT
14011	Yes	AAGGCGCCAGACTCAAGTTCTGGTCTCCGGATGGAGGCGTG
14012	Yes	AAGGCGCCAGACTCAAGTTCTGGTCTCCGGATGGAGGCGTGG
14013	Yes	AAGGCGCCAGACTCAAGTTCTGGTCTCCGGATGGAGGCGTGGG
14014	Yes	AAGGCGCCAGACTCAAGTTCTGGTCTCCGGATGGAGGCGTGGGT
14015	Yes	AAGGCGCCAGACTCAAGTTCTGGTCTCCGGATGGAGGCGTGGGTT
14016	Yes	AAGGCGCCAGACTCAAGTTCTGGTCTCCGGATGGAGGCGTGGGTTC
14017	Yes	AAGGCGCCAGACTCAAGTTCTGGTCTCCGGATGGAGGCGTGGGTTCG
14018	Yes	AAGGCGCCAGACTCAAGTTCTGGTCTCCGGATGGAGGCGTGGGTTCGA
14019	Yes	AAGGCGCCAGACTCAAGTTCTGGTCTCCGGATGGAGGCGTGGGTTCGAA
14020	Yes	AAGGCGCCAGACTCAAGTTCTGGTCTCCGGATGGAGGCGTGGGTTCGAAT
14021	Yes	AAGGCGCCAGACTCAAGTTCTGGTCTCCGT

14022	Yes	AAGGCGCCAGACTCAAGTTCTGGTCTCCGTA
14023	Yes	AAGGCGCCAGACTCAAGTTCTGGTCTCCGTAT
14024	Yes	AAGGCGCCAGACTCAAGTTCTGGTCTCCGTATG
14025	Yes	AAGGCGCCAGACTCAAGTTCTGGTCTCCGTATGG
14026	Yes	AAGGCGCCAGACTCAAGTTCTGGTCTCCGTATGGA
14027	Yes	AAGGCGCCAGACTCAAGTTCTGGTCTCCGTATGGAG
14028	Yes	AAGGCGCCAGACTCAAGTTCTGGTCTCCGTATGGAGG
14029	Yes	AAGGCGCCAGACTCAAGTTCTGGTCTCCGTATGGAGGC
14030	Yes	AAGGCGCCAGACTCAAGTTCTGGTCTCCGTATGGAGGCG
14031	Yes	AAGGCGCCAGACTCAAGTTCTGGTCTCCGTATGGAGGCGT
14032	Yes	AAGGCGCCAGACTCAAGTTCTGGTCTCCGTATGGAGGCGTG
14033	Yes	AAGGCGCCAGACTCAAGTTCTGGTCTCCGTATGGAGGCGTGG
14034	Yes	AAGGCGCCAGACTCAAGTTCTGGTCTCCGTATGGAGGCGTGGG
14035	Yes	AAGGCGCCAGACTCAAGTTCTGGTCTCCGTATGGAGGCGTGGGT
14036	Yes	AAGGCGCCAGACTCAAGTTCTGGTCTCCGTATGGAGGCGTGGGTT
14037	Yes	AAGGCGCCAGACTCAAGTTCTGGTCTCCGTATGGAGGCGTGGGTTC
14038	Yes	AAGGCGCCAGACTCAAGTTCTGGTCTCCGTATGGAGGCGTGGGTTCG
14039	Yes	AAGGCGCCAGACTCAAGTTCTGGTCTCCGTATGGAGGCGTGGGTTCGA
14040	Yes	AAGGCGCCAGACTCAAGTTCTGGTCTCCGTATGGAGGCGTGGGTTCGAA
14041	Yes	AAGGCGCCAGACTCAAGTTCTGGTCTCCGTATGGAGGCGTGGGTTCGAAT
14042	Yes	AAGGCGCCGGTGTCTT
14043	Yes	AAGGCGCCGGTGTCTTC
14044	Yes	AAGGCGCCGGTGTCTTCG
14045	Yes	AAGGCGCCGGTGTCTTCGG
14046	Yes	AAGGCGCCGGTGTCTTCGGA
14047	Yes	AAGGCGCCGGTGTCTTCGGAG
14048	Yes	AAGGCGCCGGTGTCTTCGGAGG
14049	Yes	AAGGCGCCGGTGTCTTCGGAGGC
14050	Yes	AAGGCGCCGGTGTCTTCGGAGGCA
14051	Yes	AAGGCGCCGGTGTCTTCGGAGGCAT
14052	Yes	AAGGCGCCGGTGTCTTCGGAGGCATG
14053	Yes	AAGGCGCCGGTGTCTTCGGAGGCATGG
14054	Yes	AAGGCGCCGGTGTCTTCGGAGGCATGGG
14055	Yes	AAGGCGCCGGTGTCTTCGGAGGCATGGGT
14056	Yes	AAGGCGCCGGTGTCTTCGGAGGCATGGGTT
14057	Yes	AAGGCGCCGGTGTCTTCGGAGGCATGGGTTC
14058	Yes	AAGGCGCCGGTGTCTTCGGAGGCATGGGTTCG
14059	Yes	AAGGCGCCGGTGTCTTCGGAGGCATGGGTTCGA
14060	Yes	AAGGCGCCGGTGTCTTCGGAGGCATGGGTTCGAA
14061	Yes	AAGGCGCCGGTGTCTTCGGAGGCATGGGTTCGAAT
14062	Yes	AAGGCGCCGGTGTCTTCGGAGGCATGGGTTCGAATT
14063	Yes	AAGGCGCCGGTGTCTTCGGAGGCATGGGTTCGAATTC

14064	Yes	AAGGCGCCGGTGTCTTCGGAGGCATGGGTTCGAATTCC
14065	Yes	AAGGCGCCGGTGTCTTCGGAGGCATGGGTTCGAATTCCA
14066	Yes	AAGGCGCCGGTGTCTTCGGAGGCATGGGTTCGAATTCCAC
14067	Yes	AAGGCGCCGGTGTCTTCGGAGGCATGGGTTCGAATTCCACC
14068	Yes	AAGGCGCCGGTGTCTTCGGAGGCATGGGTTCGAATTCCACCT
14069	Yes	AAGGCGCCGGTGTCTTCGGAGGCATGGGTTCGAATTCCACCTC
14070	Yes	AAGGCGCCGGTGTCTTCGGAGGCATGGGTTCGAATTCCACCTCT
14071	Yes	AAGGCGCCGGTGTCTTCGGAGGCATGGGTTCGAATTCCACCTCTG
14072	Yes	AAGGCGCCGGTGTCTTCGGAGGCATGGGTTCGAATTCCACCTCTGC
14073	Yes	AAGGCGCCGGTGTCTTCGGAGGCATGGGTTCGAATTCCACCTCTGCC
14074	Yes	AAGGCGCCGGTGTCTTCGGAGGCATGGGTTCGAATTCCACCTCTGCCA
14075	Yes	AAGGCGCCGGTGTCTTCGGAGGCATGGGTTCGAATTCCACCTCTGCCAC
14076	Yes	AAGGCGCCGGTGTCTTCGGAGGCATGGGTTCGAATTCCACCTCTGCCACC
14077	Yes	AAGGCGCTGCGTTCAA
14078	Yes	AAGGCGCTGCGTTCAAA
14079	Yes	AAGGCGCTGCGTTCAAAT
14080	Yes	AAGGCGCTGCGTTCAAATC
14081	Yes	AAGGCGCTGCGTTCAAATCG
14082	Yes	AAGGCGCTGCGTTCAAATCGC
14083	Yes	AAGGCGCTGCGTTCAAATCGCA
14084	Yes	AAGGCGCTGCGTTCAAATCGCAC
14085	Yes	AAGGCGCTGCGTTCAAATCGCACC
14086	Yes	AAGGCGCTGCGTTCAAATCGCACCC
14087	Yes	AAGGCGCTGCGTTCAAATCGCACCCT
14088	Yes	AAGGCGCTGCGTTCAAATCGCACCCTC
14089	Yes	AAGGCGCTGCGTTCAAATCGCACCCTCC
14090	Yes	AAGGCGCTGCGTTCAAATCGCACCCTCCG
14091	Yes	AAGGCGCTGCGTTCAAATCGCACCCTCCGC
14092	Yes	AAGGCGCTGCGTTCAAATCGCACCCTCCGCT
14093	Yes	AAGGCGCTGCGTTCAAATCGCACCCTCCGCTG
14094	Yes	AAGGCGCTGCGTTCAAATCGCACCCTCCGCTGG
14095	Yes	AAGGCGCTGCGTTCAAATCGCACCCTCCGCTGGA
14096	Yes	AAGGCGCTGCGTTCAAATCGCACCCTCCGCTGGAG
14097	Yes	AAGGCGCTGCGTTCAAATCGCACCCTCCGCTGGAGG
14098	Yes	AAGGCGCTGCGTTCAAATCGCACCCTCCGCTGGAGGC
14099	Yes	AAGGCGCTGCGTTCAAATCGCACCCTCCGCTGGAGGCG
14100	Yes	AAGGCGCTGCGTTCAAATCGCACCCTCCGCTGGAGGCGT
14101	Yes	AAGGCGCTGCGTTCAAATCGCACCCTCCGCTGGAGGCGTG
14102	Yes	AAGGCGCTGCGTTCAAATCGCACCCTCCGCTGGAGGCGTGG
14103	Yes	AAGGCGCTGCGTTCAAATCGCACCCTCCGCTGGAGGCGTGGG
14104	Yes	AAGGCGCTGCGTTCAAATCGCACCCTCCGCTGGAGGCGTGGGT
14105	Yes	AAGGCGCTGCGTTCAAATCGCACCCTCCGCTGGAGGCGTGGGTT

14106	Yes	AAGGCGCTGCGTTCAAATCGCACCCTCCGCTGGAGGCGTGGGTTC
14107	Yes	AAGGCGCTGCGTTCAAATCGCACCCTCCGCTGGAGGCGTGGGTTCG
14108	Yes	AAGGCGCTGCGTTCAAATCGCACCCTCCGCTGGAGGCGTGGGTTCGA
14109	Yes	AAGGCGCTGCGTTCAAATCGCACCCTCCGCTGGAGGCGTGGGTTCGAA
14110	Yes	AAGGCGCTGCGTTCAAATCGCACCCTCCGCTGGAGGCGTGGGTTCGAAT
14111	Yes	AAGGCGCTGCGTTCAAATCGCACCCTCCGCTGGAGGCGTGGGTTCGAATC
14112	Yes	AAGGCGCTGCGTTCAG
14113	Yes	AAGGCGCTGCGTTCAGG
14114	Yes	AAGGCGCTGCGTTCAGGT
14115	Yes	AAGGCGCTGCGTTCAGGTC
14116	Yes	AAGGCGCTGCGTTCAGGTCG
14117	Yes	AAGGCGCTGCGTTCAGGTCGC
14118	Yes	AAGGCGCTGCGTTCAGGTCGCA
14119	Yes	AAGGCGCTGCGTTCAGGTCGCAG
14120	Yes	AAGGCGCTGCGTTCAGGTCGCAGT
14121	Yes	AAGGCGCTGCGTTCAGGTCGCAGTC
14122	Yes	AAGGCGCTGCGTTCAGGTCGCAGTCT
14123	Yes	AAGGCGCTGCGTTCAGGTCGCAGTCTC
14124	Yes	AAGGCGCTGCGTTCAGGTCGCAGTCTCC
14125	Yes	AAGGCGCTGCGTTCAGGTCGCAGTCTCCC
14126	Yes	AAGGCGCTGCGTTCAGGTCGCAGTCTCCCC
14127	Yes	AAGGCGCTGCGTTCAGGTCGCAGTCTCCCCT
14128	Yes	AAGGCGCTGCGTTCAGGTCGCAGTCTCCCCTG
14129	Yes	AAGGCGCTGCGTTCAGGTCGCAGTCTCCCCTGG
14130	Yes	AAGGCGCTGCGTTCAGGTCGCAGTCTCCCCTGGA
14131	Yes	AAGGCGCTGCGTTCAGGTCGCAGTCTCCCCTGGAG
14132	Yes	AAGGCGCTGCGTTCAGGTCGCAGTCTCCCCTGGAGG
14133	Yes	AAGGCGCTGCGTTCAGGTCGCAGTCTCCCCTGGAGGC
14134	Yes	AAGGCGCTGCGTTCAGGTCGCAGTCTCCCCTGGAGGCG
14135	Yes	AAGGCGCTGCGTTCAGGTCGCAGTCTCCCCTGGAGGCGT
14136	Yes	AAGGCGCTGCGTTCAGGTCGCAGTCTCCCCTGGAGGCGTG
14137	Yes	AAGGCGCTGCGTTCAGGTCGCAGTCTCCCCTGGAGGCGTGG
14138	Yes	AAGGCGCTGCGTTCAGGTCGCAGTCTCCCCTGGAGGCGTGGG
14139	Yes	AAGGCGCTGCGTTCAGGTCGCAGTCTCCCCTGGAGGCGTGGGT
14140	Yes	AAGGCGCTGCGTTCAGGTCGCAGTCTCCCCTGGAGGCGTGGGTT
14141	Yes	AAGGCGCTGCGTTCAGGTCGCAGTCTCCCCTGGAGGCGTGGGTTC
14142	Yes	AAGGCGCTGCGTTCAGGTCGCAGTCTCCCCTGGAGGCGTGGGTTCG
14143	Yes	AAGGCGCTGCGTTCAGGTCGCAGTCTCCCCTGGAGGCGTGGGTTCGA
14144	Yes	AAGGCGCTGCGTTCAGGTCGCAGTCTCCCCTGGAGGCGTGGGTTCGAA
14145	Yes	AAGGCGCTGCGTTCAGGTCGCAGTCTCCCCTGGAGGCGTGGGTTCGAAT
14146	Yes	AAGGCGCTGCGTTCAGGTCGCAGTCTCCCCTGGAGGCGTGGGTTCGAATC
14147	No	AAGGCGCTGGATTAAG

14148	Yes	AAGGCGCTGGATTAAGG
14149	Yes	AAGGCGCTGGATTAAGGC
14150	Yes	AAGGCGCTGGATTAAGGCT
14151	Yes	AAGGCGCTGGATTAAGGCTC
14152	Yes	AAGGCGCTGGATTAAGGCTCC
14153	Yes	AAGGCGCTGGATTAAGGCTCCA
14154	Yes	AAGGCGCTGGATTAAGGCTCCAG
14155	Yes	AAGGCGCTGGATTAAGGCTCCAGT
14156	Yes	AAGGCGCTGGATTAAGGCTCCAGTC
14157	Yes	AAGGCGCTGGATTAAGGCTCCAGTCT
14158	Yes	AAGGCGCTGGATTAAGGCTCCAGTCTC
14159	Yes	AAGGCGCTGGATTAAGGCTCCAGTCTCT
14160	Yes	AAGGCGCTGGATTAAGGCTCCAGTCTCTT
14161	Yes	AAGGCGCTGGATTAAGGCTCCAGTCTCTTC
14162	Yes	AAGGCGCTGGATTAAGGCTCCAGTCTCTTCG
14163	Yes	AAGGCGCTGGATTAAGGCTCCAGTCTCTTCGG
14164	Yes	AAGGCGCTGGATTAAGGCTCCAGTCTCTTCGGA
14165	Yes	AAGGCGCTGGATTAAGGCTCCAGTCTCTTCGGAG
14166	Yes	AAGGCGCTGGATTAAGGCTCCAGTCTCTTCGGAGG
14167	Yes	AAGGCGCTGGATTAAGGCTCCAGTCTCTTCGGAGGC
14168	Yes	AAGGCGCTGGATTAAGGCTCCAGTCTCTTCGGAGGCG
14169	Yes	AAGGCGCTGGATTAAGGCTCCAGTCTCTTCGGAGGCGT
14170	Yes	AAGGCGCTGGATTAAGGCTCCAGTCTCTTCGGAGGCGTG
14171	Yes	AAGGCGCTGGATTAAGGCTCCAGTCTCTTCGGAGGCGTGG
14172	Yes	AAGGCGCTGGATTAAGGCTCCAGTCTCTTCGGAGGCGTGGG
14173	Yes	AAGGCGCTGGATTAAGGCTCCAGTCTCTTCGGAGGCGTGGGT
14174	Yes	AAGGCGCTGGATTAAGGCTCCAGTCTCTTCGGAGGCGTGGGTT
14175	Yes	AAGGCGCTGGATTAAGGCTCCAGTCTCTTCGGAGGCGTGGGTTC
14176	Yes	AAGGCGCTGGATTAAGGCTCCAGTCTCTTCGGAGGCGTGGGTTCG
14177	Yes	AAGGCGCTGGATTAAGGCTCCAGTCTCTTCGGAGGCGTGGGTTCGA
14178	Yes	AAGGCGCTGGATTAAGGCTCCAGTCTCTTCGGAGGCGTGGGTTCGAA
14179	Yes	AAGGCGCTGGATTAAGGCTCCAGTCTCTTCGGAGGCGTGGGTTCGAAT
14180	Yes	AAGGCGCTGGATTAAGGCTCCAGTCTCTTCGGAGGCGTGGGTTCGAATC
14181	Yes	AAGGCGCTGGATTAAGGCTCCAGTCTCTTCGGAGGCGTGGGTTCGAATCC
14182	Yes	AAGGCGCTGGATTAAGGCTCCAGTCTCTTCGGG
14183	Yes	AAGGCGCTGGATTAAGGCTCCAGTCTCTTCGGGG
14184	Yes	AAGGCGCTGGATTAAGGCTCCAGTCTCTTCGGGGG
14185	Yes	AAGGCGCTGGATTAAGGCTCCAGTCTCTTCGGGGGC
14186	Yes	AAGGCGCTGGATTAAGGCTCCAGTCTCTTCGGGGGCG
14187	Yes	AAGGCGCTGGATTAAGGCTCCAGTCTCTTCGGGGGCGT
14188	Yes	AAGGCGCTGGATTAAGGCTCCAGTCTCTTCGGGGGCGTG
14189	Yes	AAGGCGCTGGATTAAGGCTCCAGTCTCTTCGGGGGCGTGG

14190	Yes	AAGGCGCTGGATTAAGGCTCCAGTCTCTTCGGGGGCGTGGG
14191	Yes	AAGGCGCTGGATTAAGGCTCCAGTCTCTTCGGGGGCGTGGGT
14192	Yes	AAGGCGCTGGATTAAGGCTCCAGTCTCTTCGGGGGCGTGGGTT
14193	Yes	AAGGCGCTGGATTAAGGCTCCAGTCTCTTCGGGGGCGTGGGTTC
14194	Yes	AAGGCGCTGGATTAAGGCTCCAGTCTCTTCGGGGGCGTGGGTTCA
14195	Yes	AAGGCGCTGGATTAAGGCTCCAGTCTCTTCGGGGGCGTGGGTTCAA
14196	Yes	AAGGCGCTGGATTAAGGCTCCAGTCTCTTCGGGGGCGTGGGTTCAAA
14197	Yes	AAGGCGCTGGATTAAGGCTCCAGTCTCTTCGGGGGCGTGGGTTCAAAT
14198	Yes	AAGGCGCTGGATTAAGGCTCCAGTCTCTTCGGGGGCGTGGGTTCAAATC
14199	Yes	AAGGCGCTGGATTAAGGCTCCAGTCTCTTCGGGGGCGTGGGTTCAAATCC
14200	Yes	AAGGCGCTGGATTAAGGCTCCAGTCTCTTCGGGGGCGTGGGTTCG
14201	Yes	AAGGCGCTGGATTAAGGCTCCAGTCTCTTCGGGGGCGTGGGTTCGA
14202	Yes	AAGGCGCTGGATTAAGGCTCCAGTCTCTTCGGGGGCGTGGGTTCGAA
14203	Yes	AAGGCGCTGGATTAAGGCTCCAGTCTCTTCGGGGGCGTGGGTTCGAAT
14204	Yes	AAGGCGCTGGATTAAGGCTCCAGTCTCTTCGGGGGCGTGGGTTCGAATC
14205	Yes	AAGGCGCTGGATTAAGGCTCCAGTCTCTTCGGGGGCGTGGGTTCGAATCC
14206	Yes	AAGGCGCTGGATTTAG
14207	Yes	AAGGCGCTGGATTTAGG
14208	Yes	AAGGCGCTGGATTTAGGC
14209	Yes	AAGGCGCTGGATTTAGGCT
14210	Yes	AAGGCGCTGGATTTAGGCTC
14211	Yes	AAGGCGCTGGATTTAGGCTCC
14212	Yes	AAGGCGCTGGATTTAGGCTCCA
14213	Yes	AAGGCGCTGGATTTAGGCTCCAG
14214	Yes	AAGGCGCTGGATTTAGGCTCCAGT
14215	Yes	AAGGCGCTGGATTTAGGCTCCAGTC
14216	Yes	AAGGCGCTGGATTTAGGCTCCAGTCA
14217	Yes	AAGGCGCTGGATTTAGGCTCCAGTCAT
14218	Yes	AAGGCGCTGGATTTAGGCTCCAGTCATT
14219	Yes	AAGGCGCTGGATTTAGGCTCCAGTCATTT
14220	Yes	AAGGCGCTGGATTTAGGCTCCAGTCATTTC
14221	Yes	AAGGCGCTGGATTTAGGCTCCAGTCATTTCG
14222	Yes	AAGGCGCTGGATTTAGGCTCCAGTCATTTCGA
14223	Yes	AAGGCGCTGGATTTAGGCTCCAGTCATTTCGAT
14224	Yes	AAGGCGCTGGATTTAGGCTCCAGTCATTTCGATG
14225	Yes	AAGGCGCTGGATTTAGGCTCCAGTCATTTCGATGG
14226	Yes	AAGGCGCTGGATTTAGGCTCCAGTCATTTCGATGGC
14227	Yes	AAGGCGCTGGATTTAGGCTCCAGTCATTTCGATGGCG
14228	Yes	AAGGCGCTGGATTTAGGCTCCAGTCATTTCGATGGCGT
14229	Yes	AAGGCGCTGGATTTAGGCTCCAGTCATTTCGATGGCGTG
14230	Yes	AAGGCGCTGGATTTAGGCTCCAGTCATTTCGATGGCGTGG
14231	Yes	AAGGCGCTGGATTTAGGCTCCAGTCATTTCGATGGCGTGGG

14232	Yes	AAGGCGCTGGATTTAGGCTCCAGTCATTTCGATGGCGTGGGT
14233	Yes	AAGGCGCTGGATTTAGGCTCCAGTCATTTCGATGGCGTGGGTT
14234	Yes	AAGGCGCTGGATTTAGGCTCCAGTCATTTCGATGGCGTGGGTTC
14235	Yes	AAGGCGCTGGATTTAGGCTCCAGTCATTTCGATGGCGTGGGTTCG
14236	Yes	AAGGCGCTGGATTTAGGCTCCAGTCATTTCGATGGCGTGGGTTCGA
14237	Yes	AAGGCGCTGGATTTAGGCTCCAGTCATTTCGATGGCGTGGGTTCGAA
14238	Yes	AAGGCGCTGGATTTAGGCTCCAGTCATTTCGATGGCGTGGGTTCGAAT
14239	Yes	AAGGCGCTGGATTTAGGCTCCAGTCATTTCGATGGCGTGGGTTCGAATC
14240	Yes	AAGGCGCTGGATTTAGGCTCCAGTCATTTCGATGGCGTGGGTTCGAATCC
14241	Yes	AAGGCGCTGGATTTAGGCTCCAGTCT
14242	Yes	AAGGCGCTGGATTTAGGCTCCAGTCTC
14243	Yes	AAGGCGCTGGATTTAGGCTCCAGTCTCT
14244	Yes	AAGGCGCTGGATTTAGGCTCCAGTCTCTT
14245	Yes	AAGGCGCTGGATTTAGGCTCCAGTCTCTTC
14246	Yes	AAGGCGCTGGATTTAGGCTCCAGTCTCTTCG
14247	Yes	AAGGCGCTGGATTTAGGCTCCAGTCTCTTCGG
14248	Yes	AAGGCGCTGGATTTAGGCTCCAGTCTCTTCGGA
14249	Yes	AAGGCGCTGGATTTAGGCTCCAGTCTCTTCGGAG
14250	Yes	AAGGCGCTGGATTTAGGCTCCAGTCTCTTCGGAGG
14251	Yes	AAGGCGCTGGATTTAGGCTCCAGTCTCTTCGGAGGC
14252	Yes	AAGGCGCTGGATTTAGGCTCCAGTCTCTTCGGAGGCG
14253	Yes	AAGGCGCTGGATTTAGGCTCCAGTCTCTTCGGAGGCGT
14254	Yes	AAGGCGCTGGATTTAGGCTCCAGTCTCTTCGGAGGCGTG
14255	Yes	AAGGCGCTGGATTTAGGCTCCAGTCTCTTCGGAGGCGTGG
14256	Yes	AAGGCGCTGGATTTAGGCTCCAGTCTCTTCGGAGGCGTGGG
14257	Yes	AAGGCGCTGGATTTAGGCTCCAGTCTCTTCGGAGGCGTGGGT
14258	Yes	AAGGCGCTGGATTTAGGCTCCAGTCTCTTCGGAGGCGTGGGTT
14259	Yes	AAGGCGCTGGATTTAGGCTCCAGTCTCTTCGGAGGCGTGGGTTC
14260	Yes	AAGGCGCTGGATTTAGGCTCCAGTCTCTTCGGAGGCGTGGGTTCG
14261	Yes	AAGGCGCTGGATTTAGGCTCCAGTCTCTTCGGAGGCGTGGGTTCGA
14262	Yes	AAGGCGCTGGATTTAGGCTCCAGTCTCTTCGGAGGCGTGGGTTCGAA
14263	Yes	AAGGCGCTGGATTTAGGCTCCAGTCTCTTCGGAGGCGTGGGTTCGAAT
14264	Yes	AAGGCGCTGGATTTAGGCTCCAGTCTCTTCGGAGGCGTGGGTTCGAATC
14265	Yes	AAGGCGCTGGATTTAGGCTCCAGTCTCTTCGGAGGCGTGGGTTCGAATCC
14266	Yes	AAGGCGCTGGATTTAGGCTCCAGTCTCTTCGGG
14267	Yes	AAGGCGCTGGATTTAGGCTCCAGTCTCTTCGGGG
14268	Yes	AAGGCGCTGGATTTAGGCTCCAGTCTCTTCGGGGG
14269	Yes	AAGGCGCTGGATTTAGGCTCCAGTCTCTTCGGGGGC
14270	Yes	AAGGCGCTGGATTTAGGCTCCAGTCTCTTCGGGGGCG
14271	Yes	AAGGCGCTGGATTTAGGCTCCAGTCTCTTCGGGGGCGT
14272	Yes	AAGGCGCTGGATTTAGGCTCCAGTCTCTTCGGGGGCGTG
14273	Yes	AAGGCGCTGGATTTAGGCTCCAGTCTCTTCGGGGGCGTGG

14274	Yes	AAGGCGCTGGATTTAGGCTCCAGTCTCTTCGGGGGCGTGGG
14275	Yes	AAGGCGCTGGATTTAGGCTCCAGTCTCTTCGGGGGCGTGGGT
14276	Yes	AAGGCGCTGGATTTAGGCTCCAGTCTCTTCGGGGGCGTGGGTT
14277	Yes	AAGGCGCTGGATTTAGGCTCCAGTCTCTTCGGGGGCGTGGGTTC
14278	Yes	AAGGCGCTGGATTTAGGCTCCAGTCTCTTCGGGGGCGTGGGTTCG
14279	Yes	AAGGCGCTGGATTTAGGCTCCAGTCTCTTCGGGGGCGTGGGTTCGA
14280	Yes	AAGGCGCTGGATTTAGGCTCCAGTCTCTTCGGGGGCGTGGGTTCGAA
14281	Yes	AAGGCGCTGGATTTAGGCTCCAGTCTCTTCGGGGGCGTGGGTTCGAAT
14282	Yes	AAGGCGCTGGATTTAGGCTCCAGTCTCTTCGGGGGCGTGGGTTCGAATC
14283	Yes	AAGGCGCTGGATTTAGGCTCCAGTCTCTTCGGGGGCGTGGGTTCGAATCC
14284	Yes	AAGGCGTCGGTCTCGT
14285	Yes	AAGGCGTCGGTCTCGTA
14286	Yes	AAGGCGTCGGTCTCGTAA
14287	Yes	AAGGCGTCGGTCTCGTAAA
14288	Yes	AAGGCGTCGGTCTCGTAAAC
14289	Yes	AAGGCGTCGGTCTCGTAAACC
14290	Yes	AAGGCGTCGGTCTCGTAAACCG
14291	Yes	AAGGCGTCGGTCTCGTAAACCGA
14292	Yes	AAGGCGTCGGTCTCGTAAACCGAA
14293	Yes	AAGGCGTCGGTCTCGTAAACCGAAG
14294	Yes	AAGGCGTCGGTCTCGTAAACCGAAGA
14295	Yes	AAGGCGTCGGTCTCGTAAACCGAAGAT
14296	Yes	AAGGCGTCGGTCTCGTAAACCGAAGATC
14297	Yes	AAGGCGTCGGTCTCGTAAACCGAAGATCA
14298	Yes	AAGGCGTCGGTCTCGTAAACCGAAGATCAC
14299	Yes	AAGGCGTCGGTCTCGTAAACCGAAGATCACG
14300	Yes	AAGGCGTCGGTCTCGTAAACCGAAGATCACGG
14301	Yes	AAGGCGTCGGTCTCGTAAACCGAAGATCACGGG
14302	Yes	AAGGCGTCGGTCTCGTAAACCGAAGATCACGGGT
14303	Yes	AAGGCGTCGGTCTCGTAAACCGAAGATCACGGGTT
14304	Yes	AAGGCGTCGGTCTCGTAAACCGAAGATCACGGGTTC
14305	Yes	AAGGCGTCGGTCTCGTAAACCGAAGATCACGGGTTCG
14306	Yes	AAGGCGTCGGTCTCGTAAACCGAAGATCACGGGTTCGA
14307	Yes	AAGGCGTCGGTCTCGTAAACCGAAGATCACGGGTTCGAA
14308	Yes	AAGGCGTCGGTCTCGTAAACCGAAGATCACGGGTTCGAAC
14309	Yes	AAGGCGTCGGTCTCGTAAACCGAAGATCACGGGTTCGAACC
14310	Yes	AAGGCGTCGGTCTCGTAAACCGAAGATCACGGGTTCGAACCC
14311	Yes	AAGGCGTCGGTCTCGTAAACCGAAGATCACGGGTTCGAACCCC
14312	Yes	AAGGCGTCGGTCTCGTAAACCGAAGATCACGGGTTCGAACCCCG
14313	Yes	AAGGCGTCGGTCTCGTAAACCGAAGATCACGGGTTCGAACCCCGT
14314	Yes	AAGGCGTCGGTCTCGTAAACCGAAGATCACGGGTTCGAACCCCGTC
14315	Yes	AAGGCGTCGGTCTCGTAAACCGAAGATCACGGGTTCGAACCCCGTCC

14316	Yes	AAGGCGTCGGTCTCGTAAACCGAAGATCACGGGTTCGAACCCCGTCCG
14317	Yes	AAGGCGTCGGTCTCGTAAACCGAAGATCACGGGTTCGAACCCCGTCCGT
14318	Yes	AAGGCGTCGGTCTCGTAAACCGAAGATCACGGGTTCGAACCCCGTCCGTG
14319	Yes	AAGGCGTCGGTCTCGTAAACCGAAGATCG
14320	Yes	AAGGCGTCGGTCTCGTAAACCGAAGATCGC
14321	Yes	AAGGCGTCGGTCTCGTAAACCGAAGATCGCG
14322	Yes	AAGGCGTCGGTCTCGTAAACCGAAGATCGCGG
14323	Yes	AAGGCGTCGGTCTCGTAAACCGAAGATCGCGGG
14324	Yes	AAGGCGTCGGTCTCGTAAACCGAAGATCGCGGGT
14325	Yes	AAGGCGTCGGTCTCGTAAACCGAAGATCGCGGGTT
14326	Yes	AAGGCGTCGGTCTCGTAAACCGAAGATCGCGGGTTC
14327	Yes	AAGGCGTCGGTCTCGTAAACCGAAGATCGCGGGTTCG
14328	Yes	AAGGCGTCGGTCTCGTAAACCGAAGATCGCGGGTTCGA
14329	Yes	AAGGCGTCGGTCTCGTAAACCGAAGATCGCGGGTTCGAA
14330	Yes	AAGGCGTCGGTCTCGTAAACCGAAGATCGCGGGTTCGAAC
14331	Yes	AAGGCGTCGGTCTCGTAAACCGAAGATCGCGGGTTCGAACC
14332	Yes	AAGGCGTCGGTCTCGTAAACCGAAGATCGCGGGTTCGAACCC
14333	Yes	AAGGCGTCGGTCTCGTAAACCGAAGATCGCGGGTTCGAACCCC
14334	Yes	AAGGCGTCGGTCTCGTAAACCGAAGATCGCGGGTTCGAACCCCG
14335	Yes	AAGGCGTCGGTCTCGTAAACCGAAGATCGCGGGTTCGAACCCCGT
14336	Yes	AAGGCGTCGGTCTCGTAAACCGAAGATCGCGGGTTCGAACCCCGTC
14337	Yes	AAGGCGTCGGTCTCGTAAACCGAAGATCGCGGGTTCGAACCCCGTCC
14338	Yes	AAGGCGTCGGTCTCGTAAACCGAAGATCGCGGGTTCGAACCCCGTCCG
14339	Yes	AAGGCGTCGGTCTCGTAAACCGAAGATCGCGGGTTCGAACCCCGTCCGT
14340	Yes	AAGGCGTCGGTCTCGTAAACCGAAGATCGCGGGTTCGAACCCCGTCCGTG
14341	Yes	AAGGCGTCTGACTTCG
14342	Yes	AAGGCGTCTGACTTCGG
14343	Yes	AAGGCGTCTGACTTCGGA
14344	Yes	AAGGCGTCTGACTTCGGAT
14345	Yes	AAGGCGTCTGACTTCGGATC
14346	Yes	AAGGCGTCTGACTTCGGATCA
14347	Yes	AAGGCGTCTGACTTCGGATCAA
14348	Yes	AAGGCGTCTGACTTCGGATCAAA
14349	Yes	AAGGCGTCTGACTTCGGATCAAAA
14350	Yes	AAGGCGTCTGACTTCGGATCAAAAG
14351	Yes	AAGGCGTCTGACTTCGGATCAAAAGA
14352	Yes	AAGGCGTCTGACTTCGGATCAAAAGAT
14353	Yes	AAGGCGTCTGACTTCGGATCAAAAGATT
14354	Yes	AAGGCGTCTGACTTCGGATCAAAAGATTG
14355	Yes	AAGGCGTCTGACTTCGGATCAAAAGATTGC
14356	Yes	AAGGCGTCTGACTTCGGATCAAAAGATTGCA
14357	Yes	AAGGCGTCTGACTTCGGATCAAAAGATTGCAG

14358	Yes	AAGGCGTCTGACTTCGGATCAAAAGATTGCAGG
14359	Yes	AAGGCGTCTGACTTCGGATCAAAAGATTGCAGGT
14360	Yes	AAGGCGTCTGACTTCGGATCAAAAGATTGCAGGTT
14361	Yes	AAGGCGTCTGACTTCGGATCAAAAGATTGCAGGTTT
14362	Yes	AAGGCGTCTGACTTCGGATCAAAAGATTGCAGGTTTG
14363	Yes	AAGGCGTCTGACTTCGGATCAAAAGATTGCAGGTTTGA
14364	Yes	AAGGCGTCTGACTTCGGATCAAAAGATTGCAGGTTTGAG
14365	Yes	AAGGCGTCTGACTTCGGATCAAAAGATTGCAGGTTTGAGT
14366	Yes	AAGGCGTCTGACTTCGGATCAAAAGATTGCAGGTTTGAGTT
14367	Yes	AAGGCGTCTGACTTCGGATCAAAAGATTGCAGGTTTGAGTTC
14368	Yes	AAGGCGTCTGACTTCGGATCAAAAGATTGCAGGTTTGAGTTCT
14369	Yes	AAGGCGTCTGACTTCGGATCAAAAGATTGCAGGTTTGAGTTCTG
14370	Yes	AAGGCGTCTGACTTCGGATCAAAAGATTGCAGGTTTGAGTTCTGC
14371	Yes	AAGGCGTCTGACTTCGGATCAAAAGATTGCAGGTTTGAGTTCTGCC
14372	Yes	AAGGCGTCTGACTTCGGATCAAAAGATTGCAGGTTTGAGTTCTGCCA
14373	Yes	AAGGCGTCTGACTTCGGATCAAAAGATTGCAGGTTTGAGTTCTGCCAC
14374	Yes	AAGGCGTCTGACTTCGGATCAAAAGATTGCAGGTTTGAGTTCTGCCACG
14375	Yes	AAGGCGTCTGACTTCGGATCAAAAGATTGCAGGTTTGAGTTCTGCCACGG
14376	Yes	AAGGCGTCTGACTTCGGATCAG
14377	Yes	AAGGCGTCTGACTTCGGATCAGA
14378	Yes	AAGGCGTCTGACTTCGGATCAGAA
14379	Yes	AAGGCGTCTGACTTCGGATCAGAAG
14380	Yes	AAGGCGTCTGACTTCGGATCAGAAGA
14381	Yes	AAGGCGTCTGACTTCGGATCAGAAGAT
14382	Yes	AAGGCGTCTGACTTCGGATCAGAAGATT
14383	Yes	AAGGCGTCTGACTTCGGATCAGAAGATTG
14384	Yes	AAGGCGTCTGACTTCGGATCAGAAGATTGA
14385	Yes	AAGGCGTCTGACTTCGGATCAGAAGATTGAG
14386	Yes	AAGGCGTCTGACTTCGGATCAGAAGATTGAGG
14387	Yes	AAGGCGTCTGACTTCGGATCAGAAGATTGAGGG
14388	Yes	AAGGCGTCTGACTTCGGATCAGAAGATTGAGGGT
14389	Yes	AAGGCGTCTGACTTCGGATCAGAAGATTGAGGGTT
14390	Yes	AAGGCGTCTGACTTCGGATCAGAAGATTGAGGGTTC
14391	Yes	AAGGCGTCTGACTTCGGATCAGAAGATTGAGGGTTCG
14392	Yes	AAGGCGTCTGACTTCGGATCAGAAGATTGAGGGTTCGA
14393	Yes	AAGGCGTCTGACTTCGGATCAGAAGATTGAGGGTTCGAA
14394	Yes	AAGGCGTCTGACTTCGGATCAGAAGATTGAGGGTTCGAAT
14395	Yes	AAGGCGTCTGACTTCGGATCAGAAGATTGAGGGTTCGAATC
14396	Yes	AAGGCGTCTGACTTCGGATCAGAAGATTGAGGGTTCGAATCC
14397	Yes	AAGGCGTCTGACTTCGGATCAGAAGATTGAGGGTTCGAATCCC
14398	Yes	AAGGCGTCTGACTTCGGATCAGAAGATTGAGGGTTCGAATCCCT
14399	Yes	AAGGCGTCTGACTTCGGATCAGAAGATTGAGGGTTCGAATCCCTC

14400	Yes	AAGGCGTCTGACTTCGGATCAGAAGATTGAGGGTTCGAATCCCTCC
14401	Yes	AAGGCGTCTGACTTCGGATCAGAAGATTGAGGGTTCGAATCCCTCCG
14402	Yes	AAGGCGTCTGACTTCGGATCAGAAGATTGAGGGTTCGAATCCCTCCGT
14403	Yes	AAGGCGTCTGACTTCGGATCAGAAGATTGAGGGTTCGAATCCCTCCGTG
14404	Yes	AAGGCGTCTGACTTCGGATCAGAAGATTGAGGGTTCGAATCCCTCCGTGG
14405	Yes	AAGGCGTCTGACTTCGGATCAGAAGATTGAGGGTTCGAATCCCTT
14406	Yes	AAGGCGTCTGACTTCGGATCAGAAGATTGAGGGTTCGAATCCCTTC
14407	Yes	AAGGCGTCTGACTTCGGATCAGAAGATTGAGGGTTCGAATCCCTTCG
14408	Yes	AAGGCGTCTGACTTCGGATCAGAAGATTGAGGGTTCGAATCCCTTCGT
14409	Yes	AAGGCGTCTGACTTCGGATCAGAAGATTGAGGGTTCGAATCCCTTCGTG
14410	Yes	AAGGCGTCTGACTTCGGATCAGAAGATTGAGGGTTCGAATCCCTTCGTGG
14411	Yes	AAGGCGTCTGACTTCGGATCAGAAGATTGAGGGTTCGAG
14412	Yes	AAGGCGTCTGACTTCGGATCAGAAGATTGAGGGTTCGAGT
14413	Yes	AAGGCGTCTGACTTCGGATCAGAAGATTGAGGGTTCGAGTC
14414	Yes	AAGGCGTCTGACTTCGGATCAGAAGATTGAGGGTTCGAGTCC
14415	Yes	AAGGCGTCTGACTTCGGATCAGAAGATTGAGGGTTCGAGTCCC
14416	Yes	AAGGCGTCTGACTTCGGATCAGAAGATTGAGGGTTCGAGTCCCT
14417	Yes	AAGGCGTCTGACTTCGGATCAGAAGATTGAGGGTTCGAGTCCCTT
14418	Yes	AAGGCGTCTGACTTCGGATCAGAAGATTGAGGGTTCGAGTCCCTTC
14419	Yes	AAGGCGTCTGACTTCGGATCAGAAGATTGAGGGTTCGAGTCCCTTCG
14420	Yes	AAGGCGTCTGACTTCGGATCAGAAGATTGAGGGTTCGAGTCCCTTCGT
14421	Yes	AAGGCGTCTGACTTCGGATCAGAAGATTGAGGGTTCGAGTCCCTTCGTG
14422	Yes	AAGGCGTCTGACTTCGGATCAGAAGATTGAGGGTTCGAGTCCCTTCGTGG
14423	Yes	AAGGCGTCTGACTTCGGATCAGAAGATTGC
14424	Yes	AAGGCGTCTGACTTCGGATCAGAAGATTGCA
14425	Yes	AAGGCGTCTGACTTCGGATCAGAAGATTGCAG
14426	Yes	AAGGCGTCTGACTTCGGATCAGAAGATTGCAGG
14427	Yes	AAGGCGTCTGACTTCGGATCAGAAGATTGCAGGT
14428	Yes	AAGGCGTCTGACTTCGGATCAGAAGATTGCAGGTT
14429	Yes	AAGGCGTCTGACTTCGGATCAGAAGATTGCAGGTTC
14430	Yes	AAGGCGTCTGACTTCGGATCAGAAGATTGCAGGTTCG
14431	Yes	AAGGCGTCTGACTTCGGATCAGAAGATTGCAGGTTCGA
14432	Yes	AAGGCGTCTGACTTCGGATCAGAAGATTGCAGGTTCGAG
14433	Yes	AAGGCGTCTGACTTCGGATCAGAAGATTGCAGGTTCGAGT
14434	Yes	AAGGCGTCTGACTTCGGATCAGAAGATTGCAGGTTCGAGTC
14435	Yes	AAGGCGTCTGACTTCGGATCAGAAGATTGCAGGTTCGAGTCC
14436	Yes	AAGGCGTCTGACTTCGGATCAGAAGATTGCAGGTTCGAGTCCT
14437	Yes	AAGGCGTCTGACTTCGGATCAGAAGATTGCAGGTTCGAGTCCTG
14438	Yes	AAGGCGTCTGACTTCGGATCAGAAGATTGCAGGTTCGAGTCCTGC
14439	Yes	AAGGCGTCTGACTTCGGATCAGAAGATTGCAGGTTCGAGTCCTGCC
14440	Yes	AAGGCGTCTGACTTCGGATCAGAAGATTGCAGGTTCGAGTCCTGCCG
14441	Yes	AAGGCGTCTGACTTCGGATCAGAAGATTGCAGGTTCGAGTCCTGCCGC

14442	Yes	AAGGCGTCTGACTTCGGATCAGAAGATTGCAGGTTCGAGTCCTGCCGCG
14443	Yes	AAGGCGTCTGACTTCGGATCAGAAGATTGCAGGTTCGAGTCCTGCCGCGG
14444	Yes	AAGGCGTCTGATTCCG
14445	Yes	AAGGCGTCTGATTCCGG
14446	Yes	AAGGCGTCTGATTCCGGA
14447	Yes	AAGGCGTCTGATTCCGGAT
14448	Yes	AAGGCGTCTGATTCCGGATC
14449	Yes	AAGGCGTCTGATTCCGGATCA
14450	Yes	AAGGCGTCTGATTCCGGATCAG
14451	Yes	AAGGCGTCTGATTCCGGATCAGA
14452	Yes	AAGGCGTCTGATTCCGGATCAGAA
14453	Yes	AAGGCGTCTGATTCCGGATCAGAAG
14454	Yes	AAGGCGTCTGATTCCGGATCAGAAGA
14455	Yes	AAGGCGTCTGATTCCGGATCAGAAGAT
14456	Yes	AAGGCGTCTGATTCCGGATCAGAAGATT
14457	Yes	AAGGCGTCTGATTCCGGATCAGAAGATTG
14458	Yes	AAGGCGTCTGATTCCGGATCAGAAGATTGA
14459	Yes	AAGGCGTCTGATTCCGGATCAGAAGATTGAG
14460	Yes	AAGGCGTCTGATTCCGGATCAGAAGATTGAGG
14461	Yes	AAGGCGTCTGATTCCGGATCAGAAGATTGAGGG
14462	Yes	AAGGCGTCTGATTCCGGATCAGAAGATTGAGGGT
14463	Yes	AAGGCGTCTGATTCCGGATCAGAAGATTGAGGGTT
14464	Yes	AAGGCGTCTGATTCCGGATCAGAAGATTGAGGGTTC
14465	Yes	AAGGCGTCTGATTCCGGATCAGAAGATTGAGGGTTCG
14466	Yes	AAGGCGTCTGATTCCGGATCAGAAGATTGAGGGTTCGA
14467	Yes	AAGGCGTCTGATTCCGGATCAGAAGATTGAGGGTTCGAG
14468	Yes	AAGGCGTCTGATTCCGGATCAGAAGATTGAGGGTTCGAGT
14469	Yes	AAGGCGTCTGATTCCGGATCAGAAGATTGAGGGTTCGAGTC
14470	Yes	AAGGCGTCTGATTCCGGATCAGAAGATTGAGGGTTCGAGTCC
14471	Yes	AAGGCGTCTGATTCCGGATCAGAAGATTGAGGGTTCGAGTCCC
14472	Yes	AAGGCGTCTGATTCCGGATCAGAAGATTGAGGGTTCGAGTCCCT
14473	Yes	AAGGCGTCTGATTCCGGATCAGAAGATTGAGGGTTCGAGTCCCTT
14474	Yes	AAGGCGTCTGATTCCGGATCAGAAGATTGAGGGTTCGAGTCCCTTC
14475	Yes	AAGGCGTCTGATTCCGGATCAGAAGATTGAGGGTTCGAGTCCCTTCG
14476	Yes	AAGGCGTCTGATTCCGGATCAGAAGATTGAGGGTTCGAGTCCCTTCGT
14477	Yes	AAGGCGTCTGATTCCGGATCAGAAGATTGAGGGTTCGAGTCCCTTCGTG
14478	Yes	AAGGCGTCTGATTCCGGATCAGAAGATTGAGGGTTCGAGTCCCTTCGTGG
14479	No	AAGGCGTTGGACTCGA
14480	Yes	AAGGCGTTGGACTCGAA
14481	Yes	AAGGCGTTGGACTCGAAA
14482	Yes	AAGGCGTTGGACTCGAAAT
14483	Yes	AAGGCGTTGGACTCGAAATC

14484	Yes	AAGGCGTTGGACTCGAAATCC
14485	Yes	AAGGCGTTGGACTCGAAATCCA
14486	Yes	AAGGCGTTGGACTCGAAATCCAA
14487	Yes	AAGGCGTTGGACTCGAAATCCAAT
14488	Yes	AAGGCGTTGGACTCGAAATCCAATG
14489	Yes	AAGGCGTTGGACTCGAAATCCAATGG
14490	Yes	AAGGCGTTGGACTCGAAATCCAATGGG
14491	Yes	AAGGCGTTGGACTCGAAATCCAATGGGG
14492	Yes	AAGGCGTTGGACTCGAAATCCAATGGGGT
14493	Yes	AAGGCGTTGGACTCGAAATCCAATGGGGTC
14494	Yes	AAGGCGTTGGACTCGAAATCCAATGGGGTCT
14495	Yes	AAGGCGTTGGACTCGAAATCCAATGGGGTCTC
14496	Yes	AAGGCGTTGGACTCGAAATCCAATGGGGTCTCC
14497	Yes	AAGGCGTTGGACTCGAAATCCAATGGGGTCTCCC
14498	Yes	AAGGCGTTGGACTCGAAATCCAATGGGGTCTCCCC
14499	Yes	AAGGCGTTGGACTCGAAATCCAATGGGGTCTCCCCG
14500	Yes	AAGGCGTTGGACTCGAAATCCAATGGGGTCTCCCCGC
14501	Yes	AAGGCGTTGGACTCGAAATCCAATGGGGTCTCCCCGCG
14502	Yes	AAGGCGTTGGACTCGAAATCCAATGGGGTCTCCCCGCGC
14503	Yes	AAGGCGTTGGACTCGAAATCCAATGGGGTCTCCCCGCGCA
14504	Yes	AAGGCGTTGGACTCGAAATCCAATGGGGTCTCCCCGCGCAG
14505	Yes	AAGGCGTTGGACTCGAAATCCAATGGGGTCTCCCCGCGCAGG
14506	Yes	AAGGCGTTGGACTCGAAATCCAATGGGGTCTCCCCGCGCAGGT
14507	Yes	AAGGCGTTGGACTCGAAATCCAATGGGGTCTCCCCGCGCAGGTT
14508	Yes	AAGGCGTTGGACTCGAAATCCAATGGGGTCTCCCCGCGCAGGTTC
14509	Yes	AAGGCGTTGGACTCGAAATCCAATGGGGTCTCCCCGCGCAGGTTCA
14510	Yes	AAGGCGTTGGACTCGAAATCCAATGGGGTCTCCCCGCGCAGGTTCAA
14511	Yes	AAGGCGTTGGACTCGAAATCCAATGGGGTCTCCCCGCGCAGGTTCAAA
14512	Yes	AAGGCGTTGGACTCGAAATCCAATGGGGTCTCCCCGCGCAGGTTCAAAT
14513	Yes	AAGGCGTTGGACTCGAAATCCAATGGGGTCTCCCCGCGCAGGTTCAAATC
14514	Yes	AAGGCGTTGGACTCGAAATCCAATGGGGTCTCCCCGCGCAGGTTCG
14515	Yes	AAGGCGTTGGACTCGAAATCCAATGGGGTCTCCCCGCGCAGGTTCGA
14516	Yes	AAGGCGTTGGACTCGAAATCCAATGGGGTCTCCCCGCGCAGGTTCGAA
14517	Yes	AAGGCGTTGGACTCGAAATCCAATGGGGTCTCCCCGCGCAGGTTCGAAT
14518	Yes	AAGGCGTTGGACTCGAAATCCAATGGGGTCTCCCCGCGCAGGTTCGAATC
14519	Yes	AAGGCGTTGGACTCGAAATCCAATGGGGTT
14520	Yes	AAGGCGTTGGACTCGAAATCCAATGGGGTTT
14521	Yes	AAGGCGTTGGACTCGAAATCCAATGGGGTTTC
14522	Yes	AAGGCGTTGGACTCGAAATCCAATGGGGTTTCC
14523	Yes	AAGGCGTTGGACTCGAAATCCAATGGGGTTTCCC
14524	Yes	AAGGCGTTGGACTCGAAATCCAATGGGGTTTCCCC
14525	Yes	AAGGCGTTGGACTCGAAATCCAATGGGGTTTCCCCG

14526	Yes	AAGGCGTTGGACTCGAAATCCAATGGGGTTTCCCCGC
14527	Yes	AAGGCGTTGGACTCGAAATCCAATGGGGTTTCCCCGCA
14528	Yes	AAGGCGTTGGACTCGAAATCCAATGGGGTTTCCCCGCAC
14529	Yes	AAGGCGTTGGACTCGAAATCCAATGGGGTTTCCCCGCACA
14530	Yes	AAGGCGTTGGACTCGAAATCCAATGGGGTTTCCCCGCACAG
14531	Yes	AAGGCGTTGGACTCGAAATCCAATGGGGTTTCCCCGCACAGG
14532	Yes	AAGGCGTTGGACTCGAAATCCAATGGGGTTTCCCCGCACAGGT
14533	Yes	AAGGCGTTGGACTCGAAATCCAATGGGGTTTCCCCGCACAGGTT
14534	Yes	AAGGCGTTGGACTCGAAATCCAATGGGGTTTCCCCGCACAGGTTC
14535	Yes	AAGGCGTTGGACTCGAAATCCAATGGGGTTTCCCCGCACAGGTTCG
14536	Yes	AAGGCGTTGGACTCGAAATCCAATGGGGTTTCCCCGCACAGGTTCGA
14537	Yes	AAGGCGTTGGACTCGAAATCCAATGGGGTTTCCCCGCACAGGTTCGAA
14538	Yes	AAGGCGTTGGACTCGAAATCCAATGGGGTTTCCCCGCACAGGTTCGAAT
14539	Yes	AAGGCGTTGGACTCGAAATCCAATGGGGTTTCCCCGCACAGGTTCGAATC
14540	No	AAGGCGTTGGACTTAA
14541	Yes	AAGGCGTTGGACTTAAG
14542	Yes	AAGGCGTTGGACTTAAGA
14543	Yes	AAGGCGTTGGACTTAAGAT
14544	Yes	AAGGCGTTGGACTTAAGATC
14545	Yes	AAGGCGTTGGACTTAAGATCC
14546	Yes	AAGGCGTTGGACTTAAGATCCA
14547	Yes	AAGGCGTTGGACTTAAGATCCAA
14548	Yes	AAGGCGTTGGACTTAAGATCCAAT
14549	Yes	AAGGCGTTGGACTTAAGATCCAATG
14550	Yes	AAGGCGTTGGACTTAAGATCCAATGG
14551	Yes	AAGGCGTTGGACTTAAGATCCAATGGA
14552	Yes	AAGGCGTTGGACTTAAGATCCAATGGAC
14553	Yes	AAGGCGTTGGACTTAAGATCCAATGGACA
14554	Yes	AAGGCGTTGGACTTAAGATCCAATGGACAG
14555	Yes	AAGGCGTTGGACTTAAGATCCAATGGACAGG
14556	Yes	AAGGCGTTGGACTTAAGATCCAATGGACAGGT
14557	Yes	AAGGCGTTGGACTTAAGATCCAATGGACAGGTG
14558	Yes	AAGGCGTTGGACTTAAGATCCAATGGACAGGTGT
14559	Yes	AAGGCGTTGGACTTAAGATCCAATGGACAGGTGTC
14560	Yes	AAGGCGTTGGACTTAAGATCCAATGGACAGGTGTCC
14561	Yes	AAGGCGTTGGACTTAAGATCCAATGGACAGGTGTCCG
14562	Yes	AAGGCGTTGGACTTAAGATCCAATGGACAGGTGTCCGC
14563	Yes	AAGGCGTTGGACTTAAGATCCAATGGACAGGTGTCCGCG
14564	Yes	AAGGCGTTGGACTTAAGATCCAATGGACAGGTGTCCGCGT
14565	Yes	AAGGCGTTGGACTTAAGATCCAATGGACAGGTGTCCGCGTG
14566	Yes	AAGGCGTTGGACTTAAGATCCAATGGACAGGTGTCCGCGTGG
14567	Yes	AAGGCGTTGGACTTAAGATCCAATGGACAGGTGTCCGCGTGGG

14568	Yes	AAGGCGTTGGACTTAAGATCCAATGGACAGGTGTCCGCGTGGGT
14569	Yes	AAGGCGTTGGACTTAAGATCCAATGGACAGGTGTCCGCGTGGGTT
14570	Yes	AAGGCGTTGGACTTAAGATCCAATGGACAGGTGTCCGCGTGGGTTC
14571	Yes	AAGGCGTTGGACTTAAGATCCAATGGACAGGTGTCCGCGTGGGTTCG
14572	Yes	AAGGCGTTGGACTTAAGATCCAATGGACAGGTGTCCGCGTGGGTTCGA
14573	Yes	AAGGCGTTGGACTTAAGATCCAATGGACAGGTGTCCGCGTGGGTTCGAG
14574	Yes	AAGGCGTTGGACTTAAGATCCAATGGACAGGTGTCCGCGTGGGTTCGAGC
14575	Yes	AAGGCGTTGGACTTAAGATCCAATGGACAT
14576	Yes	AAGGCGTTGGACTTAAGATCCAATGGACATA
14577	Yes	AAGGCGTTGGACTTAAGATCCAATGGACATAT
14578	Yes	AAGGCGTTGGACTTAAGATCCAATGGACATATG
14579	Yes	AAGGCGTTGGACTTAAGATCCAATGGACATATGT
14580	Yes	AAGGCGTTGGACTTAAGATCCAATGGACATATGTC
14581	Yes	AAGGCGTTGGACTTAAGATCCAATGGACATATGTCC
14582	Yes	AAGGCGTTGGACTTAAGATCCAATGGACATATGTCCG
14583	Yes	AAGGCGTTGGACTTAAGATCCAATGGACATATGTCCGC
14584	Yes	AAGGCGTTGGACTTAAGATCCAATGGACATATGTCCGCG
14585	Yes	AAGGCGTTGGACTTAAGATCCAATGGACATATGTCCGCGT
14586	Yes	AAGGCGTTGGACTTAAGATCCAATGGACATATGTCCGCGTG
14587	Yes	AAGGCGTTGGACTTAAGATCCAATGGACATATGTCCGCGTGG
14588	Yes	AAGGCGTTGGACTTAAGATCCAATGGACATATGTCCGCGTGGG
14589	Yes	AAGGCGTTGGACTTAAGATCCAATGGACATATGTCCGCGTGGGT
14590	Yes	AAGGCGTTGGACTTAAGATCCAATGGACATATGTCCGCGTGGGTT
14591	Yes	AAGGCGTTGGACTTAAGATCCAATGGACATATGTCCGCGTGGGTTC
14592	Yes	AAGGCGTTGGACTTAAGATCCAATGGACATATGTCCGCGTGGGTTCG
14593	Yes	AAGGCGTTGGACTTAAGATCCAATGGACATATGTCCGCGTGGGTTCGA
14594	Yes	AAGGCGTTGGACTTAAGATCCAATGGACATATGTCCGCGTGGGTTCGAA
14595	Yes	AAGGCGTTGGACTTAAGATCCAATGGACATATGTCCGCGTGGGTTCGAAC
14596	Yes	AAGGCGTTGGACTTAAGATCCAATGGAT
14597	Yes	AAGGCGTTGGACTTAAGATCCAATGGATT
14598	Yes	AAGGCGTTGGACTTAAGATCCAATGGATTC
14599	Yes	AAGGCGTTGGACTTAAGATCCAATGGATTCA
14600	Yes	AAGGCGTTGGACTTAAGATCCAATGGATTCAT
14601	Yes	AAGGCGTTGGACTTAAGATCCAATGGATTCATA
14602	Yes	AAGGCGTTGGACTTAAGATCCAATGGATTCATAT
14603	Yes	AAGGCGTTGGACTTAAGATCCAATGGATTCATATC
14604	Yes	AAGGCGTTGGACTTAAGATCCAATGGATTCATATCC
14605	Yes	AAGGCGTTGGACTTAAGATCCAATGGATTCATATCCG
14606	Yes	AAGGCGTTGGACTTAAGATCCAATGGATTCATATCCGC
14607	Yes	AAGGCGTTGGACTTAAGATCCAATGGATTCATATCCGCG
14608	Yes	AAGGCGTTGGACTTAAGATCCAATGGATTCATATCCGCGT
14609	Yes	AAGGCGTTGGACTTAAGATCCAATGGATTCATATCCGCGTG

14610	Yes	AAGGCGTTGGACTTAAGATCCAATGGATTCATATCCGCGTGG
14611	Yes	AAGGCGTTGGACTTAAGATCCAATGGATTCATATCCGCGTGGG
14612	Yes	AAGGCGTTGGACTTAAGATCCAATGGATTCATATCCGCGTGGGT
14613	Yes	AAGGCGTTGGACTTAAGATCCAATGGATTCATATCCGCGTGGGTT
14614	Yes	AAGGCGTTGGACTTAAGATCCAATGGATTCATATCCGCGTGGGTTC
14615	Yes	AAGGCGTTGGACTTAAGATCCAATGGATTCATATCCGCGTGGGTTCG
14616	Yes	AAGGCGTTGGACTTAAGATCCAATGGATTCATATCCGCGTGGGTTCGA
14617	Yes	AAGGCGTTGGACTTAAGATCCAATGGATTCATATCCGCGTGGGTTCGAA
14618	Yes	AAGGCGTTGGACTTAAGATCCAATGGATTCATATCCGCGTGGGTTCGAAC
14619	Yes	AAGGCGTTGGACTTAAGATCCAATGGG
14620	Yes	AAGGCGTTGGACTTAAGATCCAATGGGC
14621	Yes	AAGGCGTTGGACTTAAGATCCAATGGGCT
14622	Yes	AAGGCGTTGGACTTAAGATCCAATGGGCTG
14623	Yes	AAGGCGTTGGACTTAAGATCCAATGGGCTGG
14624	Yes	AAGGCGTTGGACTTAAGATCCAATGGGCTGGT
14625	Yes	AAGGCGTTGGACTTAAGATCCAATGGGCTGGTG
14626	Yes	AAGGCGTTGGACTTAAGATCCAATGGGCTGGTGC
14627	Yes	AAGGCGTTGGACTTAAGATCCAATGGGCTGGTGCC
14628	Yes	AAGGCGTTGGACTTAAGATCCAATGGGCTGGTGCCC
14629	Yes	AAGGCGTTGGACTTAAGATCCAATGGGCTGGTGCCCG
14630	Yes	AAGGCGTTGGACTTAAGATCCAATGGGCTGGTGCCCGC
14631	Yes	AAGGCGTTGGACTTAAGATCCAATGGGCTGGTGCCCGCG
14632	Yes	AAGGCGTTGGACTTAAGATCCAATGGGCTGGTGCCCGCGT
14633	Yes	AAGGCGTTGGACTTAAGATCCAATGGGCTGGTGCCCGCGTG
14634	Yes	AAGGCGTTGGACTTAAGATCCAATGGGCTGGTGCCCGCGTGG
14635	Yes	AAGGCGTTGGACTTAAGATCCAATGGGCTGGTGCCCGCGTGGG
14636	Yes	AAGGCGTTGGACTTAAGATCCAATGGGCTGGTGCCCGCGTGGGT
14637	Yes	AAGGCGTTGGACTTAAGATCCAATGGGCTGGTGCCCGCGTGGGTT
14638	Yes	AAGGCGTTGGACTTAAGATCCAATGGGCTGGTGCCCGCGTGGGTTC
14639	Yes	AAGGCGTTGGACTTAAGATCCAATGGGCTGGTGCCCGCGTGGGTTCG
14640	Yes	AAGGCGTTGGACTTAAGATCCAATGGGCTGGTGCCCGCGTGGGTTCGA
14641	Yes	AAGGCGTTGGACTTAAGATCCAATGGGCTGGTGCCCGCGTGGGTTCGAA
14642	Yes	AAGGCGTTGGACTTAAGATCCAATGGGCTGGTGCCCGCGTGGGTTCGAAC
14643	Yes	AAGGCGTTGGACTTGA
14644	Yes	AAGGCGTTGGACTTGAA
14645	Yes	AAGGCGTTGGACTTGAAA
14646	Yes	AAGGCGTTGGACTTGAAAT
14647	Yes	AAGGCGTTGGACTTGAAATC
14648	Yes	AAGGCGTTGGACTTGAAATCC
14649	Yes	AAGGCGTTGGACTTGAAATCCA
14650	Yes	AAGGCGTTGGACTTGAAATCCAA
14651	Yes	AAGGCGTTGGACTTGAAATCCAAT

14652	Yes	AAGGCGTTGGACTTGAAATCCAATG
14653	Yes	AAGGCGTTGGACTTGAAATCCAATGG
14654	Yes	AAGGCGTTGGACTTGAAATCCAATGGG
14655	Yes	AAGGCGTTGGACTTGAAATCCAATGGGG
14656	Yes	AAGGCGTTGGACTTGAAATCCAATGGGGT
14657	Yes	AAGGCGTTGGACTTGAAATCCAATGGGGTC
14658	Yes	AAGGCGTTGGACTTGAAATCCAATGGGGTCT
14659	Yes	AAGGCGTTGGACTTGAAATCCAATGGGGTCTC
14660	Yes	AAGGCGTTGGACTTGAAATCCAATGGGGTCTCC
14661	Yes	AAGGCGTTGGACTTGAAATCCAATGGGGTCTCCC
14662	Yes	AAGGCGTTGGACTTGAAATCCAATGGGGTCTCCCC
14663	Yes	AAGGCGTTGGACTTGAAATCCAATGGGGTCTCCCCG
14664	Yes	AAGGCGTTGGACTTGAAATCCAATGGGGTCTCCCCGC
14665	Yes	AAGGCGTTGGACTTGAAATCCAATGGGGTCTCCCCGCG
14666	Yes	AAGGCGTTGGACTTGAAATCCAATGGGGTCTCCCCGCGC
14667	Yes	AAGGCGTTGGACTTGAAATCCAATGGGGTCTCCCCGCGCA
14668	Yes	AAGGCGTTGGACTTGAAATCCAATGGGGTCTCCCCGCGCAG
14669	Yes	AAGGCGTTGGACTTGAAATCCAATGGGGTCTCCCCGCGCAGG
14670	Yes	AAGGCGTTGGACTTGAAATCCAATGGGGTCTCCCCGCGCAGGT
14671	Yes	AAGGCGTTGGACTTGAAATCCAATGGGGTCTCCCCGCGCAGGTT
14672	Yes	AAGGCGTTGGACTTGAAATCCAATGGGGTCTCCCCGCGCAGGTTC
14673	Yes	AAGGCGTTGGACTTGAAATCCAATGGGGTCTCCCCGCGCAGGTTCG
14674	Yes	AAGGCGTTGGACTTGAAATCCAATGGGGTCTCCCCGCGCAGGTTCGA
14675	Yes	AAGGCGTTGGACTTGAAATCCAATGGGGTCTCCCCGCGCAGGTTCGAA
14676	Yes	AAGGCGTTGGACTTGAAATCCAATGGGGTCTCCCCGCGCAGGTTCGAAC
14677	Yes	AAGGCGTTGGACTTGAAATCCAATGGGGTCTCCCCGCGCAGGTTCGAACC
14678	No	AAGGCTCCAGTCTCTT
14679	No	AAGGCTCCAGTCTCTTC
14680	Yes	AAGGCTCCAGTCTCTTCG
14681	Yes	AAGGCTCCAGTCTCTTCGG
14682	Yes	AAGGCTCCAGTCTCTTCGGA
14683	Yes	AAGGCTCCAGTCTCTTCGGAG
14684	Yes	AAGGCTCCAGTCTCTTCGGAGG
14685	Yes	AAGGCTCCAGTCTCTTCGGAGGC
14686	Yes	AAGGCTCCAGTCTCTTCGGAGGCG
14687	Yes	AAGGCTCCAGTCTCTTCGGAGGCGT
14688	Yes	AAGGCTCCAGTCTCTTCGGAGGCGTG
14689	Yes	AAGGCTCCAGTCTCTTCGGAGGCGTGG
14690	Yes	AAGGCTCCAGTCTCTTCGGAGGCGTGGG
14691	Yes	AAGGCTCCAGTCTCTTCGGAGGCGTGGGT
14692	Yes	AAGGCTCCAGTCTCTTCGGAGGCGTGGGTT
14693	Yes	AAGGCTCCAGTCTCTTCGGAGGCGTGGGTTC

14694	Yes	AAGGCTCCAGTCTCTTCGGAGGCGTGGGTTCG
14695	Yes	AAGGCTCCAGTCTCTTCGGAGGCGTGGGTTCGA
14696	Yes	AAGGCTCCAGTCTCTTCGGAGGCGTGGGTTCGAA
14697	Yes	AAGGCTCCAGTCTCTTCGGAGGCGTGGGTTCGAAT
14698	Yes	AAGGCTCCAGTCTCTTCGGAGGCGTGGGTTCGAATC
14699	Yes	AAGGCTCCAGTCTCTTCGGAGGCGTGGGTTCGAATCC
14700	Yes	AAGGCTCCAGTCTCTTCGGAGGCGTGGGTTCGAATCCC
14701	Yes	AAGGCTCCAGTCTCTTCGGAGGCGTGGGTTCGAATCCCA
14702	Yes	AAGGCTCCAGTCTCTTCGGAGGCGTGGGTTCGAATCCCAC
14703	Yes	AAGGCTCCAGTCTCTTCGGAGGCGTGGGTTCGAATCCCACC
14704	Yes	AAGGCTCCAGTCTCTTCGGAGGCGTGGGTTCGAATCCCACCG
14705	Yes	AAGGCTCCAGTCTCTTCGGAGGCGTGGGTTCGAATCCCACCGC
14706	Yes	AAGGCTCCAGTCTCTTCGGAGGCGTGGGTTCGAATCCCACCGCT
14707	Yes	AAGGCTCCAGTCTCTTCGGAGGCGTGGGTTCGAATCCCACCGCTG
14708	Yes	AAGGCTCCAGTCTCTTCGGAGGCGTGGGTTCGAATCCCACCGCTGC
14709	Yes	AAGGCTCCAGTCTCTTCGGAGGCGTGGGTTCGAATCCCACCGCTGCC
14710	Yes	AAGGCTCCAGTCTCTTCGGAGGCGTGGGTTCGAATCCCACCGCTGCCA
14711	Yes	AAGGCTCCAGTCTCTTCGGAGGCGTGGGTTCGAATCCCACCGCTGCCAC
14712	Yes	AAGGCTCCAGTCTCTTCGGAGGCGTGGGTTCGAATCCCACCGCTGCCACC
14713	Yes	AAGGCTCCAGTCTCTTCGGG
14714	Yes	AAGGCTCCAGTCTCTTCGGGG
14715	Yes	AAGGCTCCAGTCTCTTCGGGGG
14716	Yes	AAGGCTCCAGTCTCTTCGGGGGC
14717	Yes	AAGGCTCCAGTCTCTTCGGGGGCG
14718	Yes	AAGGCTCCAGTCTCTTCGGGGGCGT
14719	Yes	AAGGCTCCAGTCTCTTCGGGGGCGTG
14720	Yes	AAGGCTCCAGTCTCTTCGGGGGCGTGG
14721	Yes	AAGGCTCCAGTCTCTTCGGGGGCGTGGG
14722	Yes	AAGGCTCCAGTCTCTTCGGGGGCGTGGGT
14723	Yes	AAGGCTCCAGTCTCTTCGGGGGCGTGGGTT
14724	Yes	AAGGCTCCAGTCTCTTCGGGGGCGTGGGTTC
14725	Yes	AAGGCTCCAGTCTCTTCGGGGGCGTGGGTTCA
14726	Yes	AAGGCTCCAGTCTCTTCGGGGGCGTGGGTTCAA
14727	Yes	AAGGCTCCAGTCTCTTCGGGGGCGTGGGTTCAAA
14728	Yes	AAGGCTCCAGTCTCTTCGGGGGCGTGGGTTCAAAT
14729	Yes	AAGGCTCCAGTCTCTTCGGGGGCGTGGGTTCAAATC
14730	Yes	AAGGCTCCAGTCTCTTCGGGGGCGTGGGTTCAAATCC
14731	Yes	AAGGCTCCAGTCTCTTCGGGGGCGTGGGTTCAAATCCC
14732	Yes	AAGGCTCCAGTCTCTTCGGGGGCGTGGGTTCAAATCCCA
14733	Yes	AAGGCTCCAGTCTCTTCGGGGGCGTGGGTTCAAATCCCAC
14734	Yes	AAGGCTCCAGTCTCTTCGGGGGCGTGGGTTCAAATCCCACC
14735	Yes	AAGGCTCCAGTCTCTTCGGGGGCGTGGGTTCAAATCCCACCG

14736	Yes	AAGGCTCCAGTCTCTTCGGGGGCGTGGGTTCAAATCCCACCGC
14737	Yes	AAGGCTCCAGTCTCTTCGGGGGCGTGGGTTCAAATCCCACCGCT
14738	Yes	AAGGCTCCAGTCTCTTCGGGGGCGTGGGTTCAAATCCCACCGCTG
14739	Yes	AAGGCTCCAGTCTCTTCGGGGGCGTGGGTTCAAATCCCACCGCTGC
14740	Yes	AAGGCTCCAGTCTCTTCGGGGGCGTGGGTTCAAATCCCACCGCTGCC
14741	Yes	AAGGCTCCAGTCTCTTCGGGGGCGTGGGTTCAAATCCCACCGCTGCCA
14742	Yes	AAGGCTCCAGTCTCTTCGGGGGCGTGGGTTCAAATCCCACCGCTGCCAC
14743	Yes	AAGGCTCCAGTCTCTTCGGGGGCGTGGGTTCAAATCCCACCGCTGCCACC
14744	Yes	AAGGCTCCAGTCTCTTCGGGGGCGTGGGTTCG
14745	Yes	AAGGCTCCAGTCTCTTCGGGGGCGTGGGTTCGA
14746	Yes	AAGGCTCCAGTCTCTTCGGGGGCGTGGGTTCGAA
14747	Yes	AAGGCTCCAGTCTCTTCGGGGGCGTGGGTTCGAAT
14748	Yes	AAGGCTCCAGTCTCTTCGGGGGCGTGGGTTCGAATC
14749	Yes	AAGGCTCCAGTCTCTTCGGGGGCGTGGGTTCGAATCC
14750	Yes	AAGGCTCCAGTCTCTTCGGGGGCGTGGGTTCGAATCCC
14751	Yes	AAGGCTCCAGTCTCTTCGGGGGCGTGGGTTCGAATCCCA
14752	Yes	AAGGCTCCAGTCTCTTCGGGGGCGTGGGTTCGAATCCCAC
14753	Yes	AAGGCTCCAGTCTCTTCGGGGGCGTGGGTTCGAATCCCACC
14754	Yes	AAGGCTCCAGTCTCTTCGGGGGCGTGGGTTCGAATCCCACCG
14755	Yes	AAGGCTCCAGTCTCTTCGGGGGCGTGGGTTCGAATCCCACCGC
14756	Yes	AAGGCTCCAGTCTCTTCGGGGGCGTGGGTTCGAATCCCACCGCT
14757	Yes	AAGGCTCCAGTCTCTTCGGGGGCGTGGGTTCGAATCCCACCGCTG
14758	Yes	AAGGCTCCAGTCTCTTCGGGGGCGTGGGTTCGAATCCCACCGCTGC
14759	Yes	AAGGCTCCAGTCTCTTCGGGGGCGTGGGTTCGAATCCCACCGCTGCC
14760	Yes	AAGGCTCCAGTCTCTTCGGGGGCGTGGGTTCGAATCCCACCGCTGCCA
14761	Yes	AAGGCTCCAGTCTCTTCGGGGGCGTGGGTTCGAATCCCACCGCTGCCAC
14762	Yes	AAGGCTCCAGTCTCTTCGGGGGCGTGGGTTCGAATCCCACCGCTGCCACC
14763	Yes	AAGGCTCCAGTCTCTTCGGGGGCGTGGGTTT
14764	Yes	AAGGCTCCAGTCTCTTCGGGGGCGTGGGTTTG
14765	Yes	AAGGCTCCAGTCTCTTCGGGGGCGTGGGTTTGA
14766	Yes	AAGGCTCCAGTCTCTTCGGGGGCGTGGGTTTGAA
14767	Yes	AAGGCTCCAGTCTCTTCGGGGGCGTGGGTTTGAAT
14768	Yes	AAGGCTCCAGTCTCTTCGGGGGCGTGGGTTTGAATC
14769	Yes	AAGGCTCCAGTCTCTTCGGGGGCGTGGGTTTGAATCC
14770	Yes	AAGGCTCCAGTCTCTTCGGGGGCGTGGGTTTGAATCCC
14771	Yes	AAGGCTCCAGTCTCTTCGGGGGCGTGGGTTTGAATCCCA
14772	Yes	AAGGCTCCAGTCTCTTCGGGGGCGTGGGTTTGAATCCCAC
14773	Yes	AAGGCTCCAGTCTCTTCGGGGGCGTGGGTTTGAATCCCACC
14774	Yes	AAGGCTCCAGTCTCTTCGGGGGCGTGGGTTTGAATCCCACCG
14775	Yes	AAGGCTCCAGTCTCTTCGGGGGCGTGGGTTTGAATCCCACCGC
14776	Yes	AAGGCTCCAGTCTCTTCGGGGGCGTGGGTTTGAATCCCACCGCT
14777	Yes	AAGGCTCCAGTCTCTTCGGGGGCGTGGGTTTGAATCCCACCGCTG

14778	Yes	AAGGCTCCAGTCTCTTCGGGGGCGTGGGTTTGAATCCCACCGCTGC
14779	Yes	AAGGCTCCAGTCTCTTCGGGGGCGTGGGTTTGAATCCCACCGCTGCC
14780	Yes	AAGGCTCCAGTCTCTTCGGGGGCGTGGGTTTGAATCCCACCGCTGCCA
14781	Yes	AAGGCTCCAGTCTCTTCGGGGGCGTGGGTTTGAATCCCACCGCTGCCAC
14782	Yes	AAGGCTCCAGTCTCTTCGGGGGCGTGGGTTTGAATCCCACCGCTGCCACC
14783	Yes	AAGGCTCCGGATTAAG
14784	Yes	AAGGCTCCGGATTAAGG
14785	Yes	AAGGCTCCGGATTAAGGC
14786	Yes	AAGGCTCCGGATTAAGGCG
14787	Yes	AAGGCTCCGGATTAAGGCGC
14788	Yes	AAGGCTCCGGATTAAGGCGCC
14789	Yes	AAGGCTCCGGATTAAGGCGCCG
14790	Yes	AAGGCTCCGGATTAAGGCGCCGG
14791	Yes	AAGGCTCCGGATTAAGGCGCCGGT
14792	Yes	AAGGCTCCGGATTAAGGCGCCGGTG
14793	Yes	AAGGCTCCGGATTAAGGCGCCGGTGT
14794	Yes	AAGGCTCCGGATTAAGGCGCCGGTGTC
14795	Yes	AAGGCTCCGGATTAAGGCGCCGGTGTCT
14796	Yes	AAGGCTCCGGATTAAGGCGCCGGTGTCTT
14797	Yes	AAGGCTCCGGATTAAGGCGCCGGTGTCTTC
14798	Yes	AAGGCTCCGGATTAAGGCGCCGGTGTCTTCG
14799	Yes	AAGGCTCCGGATTAAGGCGCCGGTGTCTTCGG
14800	Yes	AAGGCTCCGGATTAAGGCGCCGGTGTCTTCGGA
14801	Yes	AAGGCTCCGGATTAAGGCGCCGGTGTCTTCGGAG
14802	Yes	AAGGCTCCGGATTAAGGCGCCGGTGTCTTCGGAGG
14803	Yes	AAGGCTCCGGATTAAGGCGCCGGTGTCTTCGGAGGC
14804	Yes	AAGGCTCCGGATTAAGGCGCCGGTGTCTTCGGAGGCA
14805	Yes	AAGGCTCCGGATTAAGGCGCCGGTGTCTTCGGAGGCAT
14806	Yes	AAGGCTCCGGATTAAGGCGCCGGTGTCTTCGGAGGCATG
14807	Yes	AAGGCTCCGGATTAAGGCGCCGGTGTCTTCGGAGGCATGG
14808	Yes	AAGGCTCCGGATTAAGGCGCCGGTGTCTTCGGAGGCATGGG
14809	Yes	AAGGCTCCGGATTAAGGCGCCGGTGTCTTCGGAGGCATGGGT
14810	Yes	AAGGCTCCGGATTAAGGCGCCGGTGTCTTCGGAGGCATGGGTT
14811	Yes	AAGGCTCCGGATTAAGGCGCCGGTGTCTTCGGAGGCATGGGTTC
14812	Yes	AAGGCTCCGGATTAAGGCGCCGGTGTCTTCGGAGGCATGGGTTCG
14813	Yes	AAGGCTCCGGATTAAGGCGCCGGTGTCTTCGGAGGCATGGGTTCGA
14814	Yes	AAGGCTCCGGATTAAGGCGCCGGTGTCTTCGGAGGCATGGGTTCGAA
14815	Yes	AAGGCTCCGGATTAAGGCGCCGGTGTCTTCGGAGGCATGGGTTCGAAT
14816	Yes	AAGGCTCCGGATTAAGGCGCCGGTGTCTTCGGAGGCATGGGTTCGAATT
14817	Yes	AAGGCTCCGGATTAAGGCGCCGGTGTCTTCGGAGGCATGGGTTCGAATTC
14818	Yes	AAGGCTGCGTGTTCGA
14819	Yes	AAGGCTGCGTGTTCGAA

14820	Yes	AAGGCTGCGTGTTCGAAT
14821	Yes	AAGGCTGCGTGTTCGAATC
14822	Yes	AAGGCTGCGTGTTCGAATCA
14823	Yes	AAGGCTGCGTGTTCGAATCAC
14824	Yes	AAGGCTGCGTGTTCGAATCACG
14825	Yes	AAGGCTGCGTGTTCGAATCACGT
14826	Yes	AAGGCTGCGTGTTCGAATCACGTC
14827	Yes	AAGGCTGCGTGTTCGAATCACGTCG
14828	Yes	AAGGCTGCGTGTTCGAATCACGTCGG
14829	Yes	AAGGCTGCGTGTTCGAATCACGTCGGG
14830	Yes	AAGGCTGCGTGTTCGAATCACGTCGGGG
14831	Yes	AAGGCTGCGTGTTCGAATCACGTCGGGGT
14832	Yes	AAGGCTGCGTGTTCGAATCACGTCGGGGTC
14833	Yes	AAGGCTGCGTGTTCGAATCACGTCGGGGTCA
14834	Yes	AAGGCTGCGTGTTCGAATCACGTCGGGGTCAC
14835	Yes	AAGGCTGCGTGTTCGAATCACGTCGGGGTCACC
14836	Yes	AAGGCTGCGTGTTCGAATCACGTCGGGGTCACCA
14837	Yes	AAGGCTGGTGGTTCAA
14838	Yes	AAGGCTGGTGGTTCAAG
14839	Yes	AAGGCTGGTGGTTCAAGC
14840	Yes	AAGGCTGGTGGTTCAAGCC
14841	Yes	AAGGCTGGTGGTTCAAGCCC
14842	Yes	AAGGCTGGTGGTTCAAGCCCA
14843	Yes	AAGGCTGGTGGTTCAAGCCCAC
14844	Yes	AAGGCTGGTGGTTCAAGCCCACC
14845	Yes	AAGGCTGGTGGTTCAAGCCCACCC
14846	Yes	AAGGCTGGTGGTTCAAGCCCACCCA
14847	Yes	AAGGCTGGTGGTTCAAGCCCACCCAG
14848	Yes	AAGGCTGGTGGTTCAAGCCCACCCAGA
14849	Yes	AAGGCTGGTGGTTCAAGCCCACCCAGAG
14850	Yes	AAGGCTGGTGGTTCAAGCCCACCCAGAGA
14851	Yes	AAGGCTGGTGGTTCAAGCCCACCCAGAGAT
14852	Yes	AAGGCTGGTGGTTCAAGCCCACCCAGAGATG
14853	Yes	AAGGCTGGTGGTTCAAGCCCACCCAGAGATGC
14854	Yes	AAGGCTGGTGGTTCAAGCCCACCCAGAGATGCC
14855	Yes	AAGGCTGGTGGTTCAAGCCCACCCAGAGATGCCA
14856	No	AAGGGAAAAGACATCT
14857	No	AAGGGAAAAGACATCTC
14858	Yes	AAGGGAAAAGACATCTCA
14859	Yes	AAGGGAAAAGACATCTCAC
14860	Yes	AAGGGAAAAGACATCTCACT
14861	Yes	AAGGGAAAAGACATCTCACTT

14862	Yes	AAGGGAAAAGACATCTCACTTT
14863	Yes	AAGGGAAAAGACATCTCACTTTG
14864	Yes	AAGGGAAAAGACATCTCACTTTGG
14865	Yes	AAGGGAAAAGACATCTCACTTTGGG
14866	Yes	AAGGGAAAAGACATCTCACTTTGGGT
14867	Yes	AAGGGAAAAGACATCTCACTTTGGGTC
14868	Yes	AAGGGAAAAGACATCTCACTTTGGGTCA
14869	Yes	AAGGGAAAAGACATCTCACTTTGGGTCAG
14870	Yes	AAGGGAAAAGACATCTCACTTTGGGTCAGA
14871	Yes	AAGGGAAAAGACATCTCACTTTGGGTCAGAA
14872	Yes	AAGGGAAAAGACATCTCACTTTGGGTCAGAAG
14873	Yes	AAGGGAAAAGACATCTCACTTTGGGTCAGAAGA
14874	Yes	AAGGGAAAAGACATCTCACTTTGGGTCAGAAGAT
14875	Yes	AAGGGAAAAGACATCTCACTTTGGGTCAGAAGATT
14876	Yes	AAGGGAAAAGACATCTCACTTTGGGTCAGAAGATTG
14877	Yes	AAGGGAAAAGACATCTCACTTTGGGTCAGAAGATTGA
14878	Yes	AAGGGAAAAGACATCTCACTTTGGGTCAGAAGATTGAG
14879	Yes	AAGGGAAAAGACATCTCACTTTGGGTCAGAAGATTGAGG
14880	Yes	AAGGGAAAAGACATCTCACTTTGGGTCAGAAGATTGAGGG
14881	Yes	AAGGGAAAAGACATCTCACTTTGGGTCAGAAGATTGAGGGT
14882	Yes	AAGGGAAAAGACATCTCACTTTGGGTCAGAAGATTGAGGGTT
14883	Yes	AAGGGAAAAGACATCTCACTTTGGGTCAGAAGATTGAGGGTTC
14884	Yes	AAGGGAAAAGACATCTCACTTTGGGTCAGAAGATTGAGGGTTCA
14885	Yes	AAGGGAAAAGACATCTCACTTTGGGTCAGAAGATTGAGGGTTCAA
14886	Yes	AAGGGAAAAGACATCTCACTTTGGGTCAGAAGATTGAGGGTTCAAG
14887	Yes	AAGGGAAAAGACATCTCACTTTGGGTCAGAAGATTGAGGGTTCAAGT
14888	Yes	AAGGGAAAAGACATCTCACTTTGGGTCAGAAGATTGAGGGTTCAAGTC
14889	Yes	AAGGGAAAAGACATCTCACTTTGGGTCAGAAGATTGAGGGTTCAAGTCC
14890	Yes	AAGGGAAAAGACATCTCACTTTGGGTCAGAAGATTGAGGGTTCAAGTCCT
14891	Yes	AAGGGAATAGGCTTTA
14892	Yes	AAGGGAATAGGCTTTAG
14893	Yes	AAGGGAATAGGCTTTAGA
14894	Yes	AAGGGAATAGGCTTTAGAG
14895	Yes	AAGGGAATAGGCTTTAGAGC
14896	Yes	AAGGGAATAGGCTTTAGAGCC
14897	Yes	AAGGGAATAGGCTTTAGAGCCA
14898	Yes	AAGGGAATAGGCTTTAGAGCCAG
14899	Yes	AAGGGAATAGGCTTTAGAGCCAGA
14900	Yes	AAGGGAATAGGCTTTAGAGCCAGAC
14901	Yes	AAGGGAATAGGCTTTAGAGCCAGACT
14902	Yes	AAGGGAATAGGCTTTAGAGCCAGACTG
14903	Yes	AAGGGAATAGGCTTTAGAGCCAGACTGC

14904	Yes	AAGGGAATAGGCTTTAGAGCCAGACTGCC
14905	Yes	AAGGGAATAGGCTTTAGAGCCAGACTGCCT
14906	Yes	AAGGGAATAGGCTTTAGAGCCAGACTGCCTG
14907	Yes	AAGGGAATAGGCTTTAGAGCCAGACTGCCTGG
14908	Yes	AAGGGAATAGGCTTTAGAGCCAGACTGCCTGGG
14909	Yes	AAGGGAATAGGCTTTAGAGCCAGACTGCCTGGGT
14910	Yes	AAGGGAATAGGCTTTAGAGCCAGACTGCCTGGGTT
14911	Yes	AAGGGAATAGGCTTTAGAGCCAGACTGCCTGGGTTT
14912	Yes	AAGGGAATAGGCTTTAGAGCCAGACTGCCTGGGTTTG
14913	Yes	AAGGGAATAGGCTTTAGAGCCAGACTGCCTGGGTTTGA
14914	Yes	AAGGGAATAGGCTTTAGAGCCAGACTGCCTGGGTTTGAA
14915	Yes	AAGGGAATAGGCTTTAGAGCCAGACTGCCTGGGTTTGAAT
14916	Yes	AAGGGAATAGGCTTTAGAGCCAGACTGCCTGGGTTTGAATC
14917	Yes	AAGGGAATAGGCTTTAGAGCCAGACTGCCTGGGTTTGAATCC
14918	Yes	AAGGGAATAGGCTTTAGAGCCAGACTGCCTGGGTTTGAATCCC
14919	Yes	AAGGGAATAGGCTTTAGAGCCAGACTGCCTGGGTTTGAATCCCA
14920	Yes	AAGGGAATAGGCTTTAGAGCCAGACTGCCTGGGTTTGAATCCCAG
14921	Yes	AAGGGAATAGGCTTTAGAGCCAGACTGCCTGGGTTTGAATCCCAGC
14922	Yes	AAGGGAATAGGCTTTAGAGCCAGACTGCCTGGGTTTGAATCCCAGCC
14923	Yes	AAGGGAATAGGCTTTAGAGCCAGACTGCCTGGGTTTGAATCCCAGCCC
14924	Yes	AAGGGAATAGGCTTTAGAGCCAGACTGCCTGGGTTTGAATCCCAGCCCC
14925	Yes	AAGGGAATAGGCTTTAGAGCCAGACTGCCTGGGTTTGAATCCCAGCCCCG
14926	Yes	AAGGGCATTCGCTCTG
14927	Yes	AAGGGCATTCGCTCTGG
14928	Yes	AAGGGCATTCGCTCTGGT
14929	Yes	AAGGGCATTCGCTCTGGTA
14930	Yes	AAGGGCATTCGCTCTGGTAT
14931	Yes	AAGGGCATTCGCTCTGGTATC
14932	Yes	AAGGGCATTCGCTCTGGTATCA
14933	Yes	AAGGGCATTCGCTCTGGTATCAG
14934	Yes	AAGGGCATTCGCTCTGGTATCAGA
14935	Yes	AAGGGCATTCGCTCTGGTATCAGAA
14936	Yes	AAGGGCATTCGCTCTGGTATCAGAAG
14937	Yes	AAGGGCATTCGCTCTGGTATCAGAAGG
14938	Yes	AAGGGCATTCGCTCTGGTATCAGAAGGG
14939	Yes	AAGGGCATTCGCTCTGGTATCAGAAGGGT
14940	Yes	AAGGGCATTCGCTCTGGTATCAGAAGGGTC
14941	Yes	AAGGGCATTCGCTCTGGTATCAGAAGGGTCT
14942	Yes	AAGGGCATTCGCTCTGGTATCAGAAGGGTCTG
14943	Yes	AAGGGCATTCGCTCTGGTATCAGAAGGGTCTGG
14944	Yes	AAGGGCATTCGCTCTGGTATCAGAAGGGTCTGGG
14945	Yes	AAGGGCATTCGCTCTGGTATCAGAAGGGTCTGGGT

14946	Yes	AAGGGCATTCGCTCTGGTATCAGAAGGGTCTGGGTT
14947	Yes	AAGGGCATTCGCTCTGGTATCAGAAGGGTCTGGGTTC
14948	Yes	AAGGGCATTCGCTCTGGTATCAGAAGGGTCTGGGTTCA
14949	Yes	AAGGGCATTCGCTCTGGTATCAGAAGGGTCTGGGTTCAA
14950	Yes	AAGGGCATTCGCTCTGGTATCAGAAGGGTCTGGGTTCAAA
14951	Yes	AAGGGCATTCGCTCTGGTATCAGAAGGGTCTGGGTTCAAAT
14952	Yes	AAGGGCATTCGCTCTGGTATCAGAAGGGTCTGGGTTCAAATC
14953	Yes	AAGGGCATTCGCTCTGGTATCAGAAGGGTCTGGGTTCAAATCC
14954	Yes	AAGGGCATTCGCTCTGGTATCAGAAGGGTCTGGGTTCAAATCCC
14955	Yes	AAGGGCATTCGCTCTGGTATCAGAAGGGTCTGGGTTCAAATCCCT
14956	Yes	AAGGGCATTCGCTCTGGTATCAGAAGGGTCTGGGTTCAAATCCCTT
14957	Yes	AAGGGCATTCGCTCTGGTATCAGAAGGGTCTGGGTTCAAATCCCTTG
14958	Yes	AAGGGCATTCGCTCTGGTATCAGAAGGGTCTGGGTTCAAATCCCTTGT
14959	Yes	AAGGGCATTCGCTCTGGTATCAGAAGGGTCTGGGTTCAAATCCCTTGTG
14960	Yes	AAGGGCATTCGCTCTGGTATCAGAAGGGTCTGGGTTCAAATCCCTTGTGC
14961	No	AAGGGCTTAGCTTAAT
14962	No	AAGGGCTTAGCTTAATT
14963	No	AAGGGCTTAGCTTAATTA
14964	No	AAGGGCTTAGCTTAATTAA
14965	No	AAGGGCTTAGCTTAATTAAA
14966	No	AAGGGCTTAGCTTAATTAAAG
14967	No	AAGGGCTTAGCTTAATTAAAGT
14968	No	AAGGGCTTAGCTTAATTAAAGTG
14969	No	AAGGGCTTAGCTTAATTAAAGTGG
14970	No	AAGGGCTTAGCTTAATTAAAGTGGC
14971	No	AAGGGCTTAGCTTAATTAAAGTGGCT
14972	No	AAGGGCTTAGCTTAATTAAAGTGGCTG
14973	No	AAGGGCTTAGCTTAATTAAAGTGGCTGA
14974	No	AAGGGCTTAGCTTAATTAAAGTGGCTGAT
14975	No	AAGGGCTTAGCTTAATTAAAGTGGCTGATT
14976	No	AAGGGCTTAGCTTAATTAAAGTGGCTGATTT
14977	No	AAGGGCTTAGCTTAATTAAAGTGGCTGATTTG
14978	No	AAGGGCTTAGCTTAATTAAAGTGGCTGATTTGC
14979	No	AAGGGCTTAGCTTAATTAAAGTGGCTGATTTGCG
14980	No	AAGGGCTTAGCTTAATTAAAGTGGCTGATTTGCGT
14981	No	AAGGGCTTAGCTTAATTAAAGTGGCTGATTTGCGTT
14982	No	AAGGGCTTAGCTTAATTAAAGTGGCTGATTTGCGTTC
14983	No	AAGGGCTTAGCTTAATTAAAGTGGCTGATTTGCGTTCA
14984	No	AAGGGCTTAGCTTAATTAAAGTGGCTGATTTGCGTTCAG
14985	No	AAGGGCTTAGCTTAATTAAAGTGGCTGATTTGCGTTCAGT
14986	Yes	AAGGGCTTAGCTTAATTAAAGTGGCTGATTTGCGTTCAGTT
14987	Yes	AAGGGCTTAGCTTAATTAAAGTGGCTGATTTGCGTTCAGTTG

14988	Yes	AAGGGCTTAGCTTAATTAAAGTGGCTGATTTGCGTTCAGTTGA
14989	Yes	AAGGGCTTAGCTTAATTAAAGTGGCTGATTTGCGTTCAGTTGAT
14990	Yes	AAGGGCTTAGCTTAATTAAAGTGGCTGATTTGCGTTCAGTTGATG
14991	Yes	AAGGGCTTAGCTTAATTAAAGTGGCTGATTTGCGTTCAGTTGATGC
14992	Yes	AAGGGCTTAGCTTAATTAAAGTGGCTGATTTGCGTTCAGTTGATGCA
14993	Yes	AAGGGCTTAGCTTAATTAAAGTGGCTGATTTGCGTTCAGTTGATGCAG
14994	Yes	AAGGGCTTAGCTTAATTAAAGTGGCTGATTTGCGTTCAGTTGATGCAGA
14995	Yes	AAGGGCTTAGCTTAATTAAAGTGGCTGATTTGCGTTCAGTTGATGCAGAG
14996	No	AAGGGTATGGACTTTG
14997	No	AAGGGTATGGACTTTGA
14998	No	AAGGGTATGGACTTTGAA
14999	No	AAGGGTATGGACTTTGAAG
15000	Yes	AAGGGTATGGACTTTGAAGC
15001	Yes	AAGGGTATGGACTTTGAAGCC
15002	Yes	AAGGGTATGGACTTTGAAGCCA
15003	Yes	AAGGGTATGGACTTTGAAGCCAG
15004	Yes	AAGGGTATGGACTTTGAAGCCAGA
15005	Yes	AAGGGTATGGACTTTGAAGCCAGAC
15006	Yes	AAGGGTATGGACTTTGAAGCCAGACT
15007	Yes	AAGGGTATGGACTTTGAAGCCAGACTA
15008	Yes	AAGGGTATGGACTTTGAAGCCAGACTAT
15009	Yes	AAGGGTATGGACTTTGAAGCCAGACTATT
15010	Yes	AAGGGTATGGACTTTGAAGCCAGACTATTT
15011	Yes	AAGGGTATGGACTTTGAAGCCAGACTATTTG
15012	Yes	AAGGGTATGGACTTTGAAGCCAGACTATTTGG
15013	Yes	AAGGGTATGGACTTTGAAGCCAGACTATTTGGG
15014	Yes	AAGGGTATGGACTTTGAAGCCAGACTATTTGGGT
15015	Yes	AAGGGTATGGACTTTGAAGCCAGACTATTTGGGTT
15016	Yes	AAGGGTATGGACTTTGAAGCCAGACTATTTGGGTTC
15017	Yes	AAGGGTATGGACTTTGAAGCCAGACTATTTGGGTTCA
15018	Yes	AAGGGTATGGACTTTGAAGCCAGACTATTTGGGTTCAA
15019	Yes	AAGGGTATGGACTTTGAAGCCAGACTATTTGGGTTCAAA
15020	Yes	AAGGGTATGGACTTTGAAGCCAGACTATTTGGGTTCAAAT
15021	Yes	AAGGGTATGGACTTTGAAGCCAGACTATTTGGGTTCAAATC
15022	Yes	AAGGGTATGGACTTTGAAGCCAGACTATTTGGGTTCAAATCC
15023	Yes	AAGGGTATGGACTTTGAAGCCAGACTATTTGGGTTCAAATCCC
15024	Yes	AAGGGTATGGACTTTGAAGCCAGACTATTTGGGTTCAAATCCCA
15025	Yes	AAGGGTATGGACTTTGAAGCCAGACTATTTGGGTTCAAATCCCAG
15026	Yes	AAGGGTATGGACTTTGAAGCCAGACTATTTGGGTTCAAATCCCAGC
15027	Yes	AAGGGTATGGACTTTGAAGCCAGACTATTTGGGTTCAAATCCCAGCT
15028	Yes	AAGGGTATGGACTTTGAAGCCAGACTATTTGGGTTCAAATCCCAGCTT
15029	Yes	AAGGGTATGGACTTTGAAGCCAGACTATTTGGGTTCAAATCCCAGCTTG

15030	Yes	AAGGGTATGGACTTTGAAGCCAGACTATTTGGGTTCAAATCCCAGCTTGG
15031	No	AAGGGTCTGGGTTCAA
15032	No	AAGGGTCTGGGTTCAAA
15033	No	AAGGGTCTGGGTTCAAAT
15034	No	AAGGGTCTGGGTTCAAATC
15035	Yes	AAGGGTCTGGGTTCAAATCC
15036	Yes	AAGGGTCTGGGTTCAAATCCC
15037	Yes	AAGGGTCTGGGTTCAAATCCCT
15038	Yes	AAGGGTCTGGGTTCAAATCCCTT
15039	Yes	AAGGGTCTGGGTTCAAATCCCTTG
15040	Yes	AAGGGTCTGGGTTCAAATCCCTTGT
15041	Yes	AAGGGTCTGGGTTCAAATCCCTTGTG
15042	Yes	AAGGGTCTGGGTTCAAATCCCTTGTGC
15043	Yes	AAGGGTCTGGGTTCAAATCCCTTGTGCA
15044	Yes	AAGGGTCTGGGTTCAAATCCCTTGTGCAC
15045	Yes	AAGGGTCTGGGTTCAAATCCCTTGTGCACT
15046	Yes	AAGGGTCTGGGTTCAAATCCCTTGTGCACTG
15047	Yes	AAGGGTCTGGGTTCAAATCCCTTGTGCACTGC
15048	Yes	AAGGGTCTGGGTTCAAATCCCTTGTGCACTGCT
15049	Yes	AAGGGTCTGGGTTCAAATCCCTTGTGCACTGCTT
15050	Yes	AAGGGTCTGGGTTCAAATCCCTTGTGCACTGCTTC
15051	Yes	AAGGGTCTGGGTTCAAATCCCTTGTGCACTGCTTCC
15052	Yes	AAGGGTCTGGGTTCAAATCCCTTGTGCACTGCTTCCA
15053	No	AAGGTACTGGCCTCCT
15054	Yes	AAGGTACTGGCCTCCTA
15055	Yes	AAGGTACTGGCCTCCTAA
15056	Yes	AAGGTACTGGCCTCCTAAG
15057	Yes	AAGGTACTGGCCTCCTAAGC
15058	Yes	AAGGTACTGGCCTCCTAAGCC
15059	Yes	AAGGTACTGGCCTCCTAAGCCA
15060	Yes	AAGGTACTGGCCTCCTAAGCCAG
15061	Yes	AAGGTACTGGCCTCCTAAGCCAGG
15062	Yes	AAGGTACTGGCCTCCTAAGCCAGGG
15063	Yes	AAGGTACTGGCCTCCTAAGCCAGGGA
15064	Yes	AAGGTACTGGCCTCCTAAGCCAGGGAT
15065	Yes	AAGGTACTGGCCTCCTAAGCCAGGGATT
15066	Yes	AAGGTACTGGCCTCCTAAGCCAGGGATTG
15067	Yes	AAGGTACTGGCCTCCTAAGCCAGGGATTGT
15068	Yes	AAGGTACTGGCCTCCTAAGCCAGGGATTGTG
15069	Yes	AAGGTACTGGCCTCCTAAGCCAGGGATTGTGG
15070	Yes	AAGGTACTGGCCTCCTAAGCCAGGGATTGTGGG
15071	Yes	AAGGTACTGGCCTCCTAAGCCAGGGATTGTGGGT

15072	Yes	AAGGTACTGGCCTCCTAAGCCAGGGATTGTGGGTT
15073	Yes	AAGGTACTGGCCTCCTAAGCCAGGGATTGTGGGTTC
15074	Yes	AAGGTACTGGCCTCCTAAGCCAGGGATTGTGGGTTCG
15075	Yes	AAGGTACTGGCCTCCTAAGCCAGGGATTGTGGGTTCGA
15076	Yes	AAGGTACTGGCCTCCTAAGCCAGGGATTGTGGGTTCGAG
15077	Yes	AAGGTACTGGCCTCCTAAGCCAGGGATTGTGGGTTCGAGT
15078	Yes	AAGGTACTGGCCTCCTAAGCCAGGGATTGTGGGTTCGAGTT
15079	Yes	AAGGTACTGGCCTCCTAAGCCAGGGATTGTGGGTTCGAGTTC
15080	Yes	AAGGTACTGGCCTCCTAAGCCAGGGATTGTGGGTTCGAGTTCC
15081	Yes	AAGGTACTGGCCTCCTAAGCCAGGGATTGTGGGTTCGAGTTCCA
15082	Yes	AAGGTACTGGCCTCCTAAGCCAGGGATTGTGGGTTCGAGTTCCAC
15083	Yes	AAGGTACTGGCCTCCTAAGCCAGGGATTGTGGGTTCGAGTTCCACC
15084	Yes	AAGGTACTGGCCTCCTAAGCCAGGGATTGTGGGTTCGAGTTCCACCT
15085	Yes	AAGGTACTGGCCTCCTAAGCCAGGGATTGTGGGTTCGAGTTCCACCTG
15086	Yes	AAGGTACTGGCCTCCTAAGCCAGGGATTGTGGGTTCGAGTTCCACCTGG
15087	Yes	AAGGTACTGGCCTCCTAAGCCAGGGATTGTGGGTTCGAGTTCCACCTGGG
15088	No	AAGGTATTAGAAAAAC
15089	No	AAGGTATTAGAAAAACC
15090	Yes	AAGGTATTAGAAAAACCA
15091	Yes	AAGGTATTAGAAAAACCAT
15092	Yes	AAGGTATTAGAAAAACCATT
15093	Yes	AAGGTATTAGAAAAACCATTT
15094	Yes	AAGGTATTAGAAAAACCATTTC
15095	Yes	AAGGTATTAGAAAAACCATTTCA
15096	Yes	AAGGTATTAGAAAAACCATTTCAT
15097	Yes	AAGGTATTAGAAAAACCATTTCATA
15098	Yes	AAGGTATTAGAAAAACCATTTCATAA
15099	Yes	AAGGTATTAGAAAAACCATTTCATAAC
15100	Yes	AAGGTATTAGAAAAACCATTTCATAACT
15101	Yes	AAGGTATTAGAAAAACCATTTCATAACTT
15102	Yes	AAGGTATTAGAAAAACCATTTCATAACTTT
15103	Yes	AAGGTATTAGAAAAACCATTTCATAACTTTG
15104	Yes	AAGGTATTAGAAAAACCATTTCATAACTTTGT
15105	Yes	AAGGTATTAGAAAAACCATTTCATAACTTTGTC
15106	Yes	AAGGTATTAGAAAAACCATTTCATAACTTTGTCA
15107	Yes	AAGGTATTAGAAAAACCATTTCATAACTTTGTCAA
15108	Yes	AAGGTATTAGAAAAACCATTTCATAACTTTGTCAAA
15109	Yes	AAGGTATTAGAAAAACCATTTCATAACTTTGTCAAAG
15110	Yes	AAGGTATTAGAAAAACCATTTCATAACTTTGTCAAAGT
15111	Yes	AAGGTATTAGAAAAACCATTTCATAACTTTGTCAAAGTT
15112	Yes	AAGGTATTAGAAAAACCATTTCATAACTTTGTCAAAGTTA
15113	Yes	AAGGTATTAGAAAAACCATTTCATAACTTTGTCAAAGTTAA

15114	Yes	AAGGTATTAGAAAAACCATTTCATAACTTTGTCAAAGTTAAA
15115	Yes	AAGGTATTAGAAAAACCATTTCATAACTTTGTCAAAGTTAAAT
15116	Yes	AAGGTATTAGAAAAACCATTTCATAACTTTGTCAAAGTTAAATT
15117	Yes	AAGGTATTAGAAAAACCATTTCATAACTTTGTCAAAGTTAAATTA
15118	Yes	AAGGTATTAGAAAAACCATTTCATAACTTTGTCAAAGTTAAATTAT
15119	Yes	AAGGTATTAGAAAAACCATTTCATAACTTTGTCAAAGTTAAATTATA
15120	Yes	AAGGTATTAGAAAAACCATTTCATAACTTTGTCAAAGTTAAATTATAG
15121	Yes	AAGGTATTAGAAAAACCATTTCATAACTTTGTCAAAGTTAAATTATAGG
15122	Yes	AAGGTATTAGAAAAACCATTTCATAACTTTGTCAAAGTTAAATTATAGGC
15123	No	AAGGTCACAGAGGTTA
15124	Yes	AAGGTCACAGAGGTTAT
15125	Yes	AAGGTCACAGAGGTTATG
15126	Yes	AAGGTCACAGAGGTTATGG
15127	Yes	AAGGTCACAGAGGTTATGGG
15128	Yes	AAGGTCACAGAGGTTATGGGA
15129	Yes	AAGGTCACAGAGGTTATGGGAT
15130	Yes	AAGGTCACAGAGGTTATGGGATT
15131	Yes	AAGGTCACAGAGGTTATGGGATTG
15132	Yes	AAGGTCACAGAGGTTATGGGATTGG
15133	Yes	AAGGTCACAGAGGTTATGGGATTGGC
15134	Yes	AAGGTCACAGAGGTTATGGGATTGGCT
15135	Yes	AAGGTCACAGAGGTTATGGGATTGGCTT
15136	Yes	AAGGTCACAGAGGTTATGGGATTGGCTTG
15137	Yes	AAGGTCACAGAGGTTATGGGATTGGCTTGA
15138	Yes	AAGGTCACAGAGGTTATGGGATTGGCTTGAA
15139	Yes	AAGGTCACAGAGGTTATGGGATTGGCTTGAAA
15140	Yes	AAGGTCACAGAGGTTATGGGATTGGCTTGAAAC
15141	Yes	AAGGTCACAGAGGTTATGGGATTGGCTTGAAACC
15142	Yes	AAGGTCACAGAGGTTATGGGATTGGCTTGAAACCA
15143	Yes	AAGGTCACAGAGGTTATGGGATTGGCTTGAAACCAG
15144	Yes	AAGGTCACAGAGGTTATGGGATTGGCTTGAAACCAGT
15145	Yes	AAGGTCACAGAGGTTATGGGATTGGCTTGAAACCAGTC
15146	Yes	AAGGTCACAGAGGTTATGGGATTGGCTTGAAACCAGTCT
15147	Yes	AAGGTCACAGAGGTTATGGGATTGGCTTGAAACCAGTCTG
15148	Yes	AAGGTCACAGAGGTTATGGGATTGGCTTGAAACCAGTCTGT
15149	Yes	AAGGTCACAGAGGTTATGGGATTGGCTTGAAACCAGTCTGTG
15150	Yes	AAGGTCACAGAGGTTATGGGATTGGCTTGAAACCAGTCTGTGG
15151	Yes	AAGGTCACAGAGGTTATGGGATTGGCTTGAAACCAGTCTGTGGG
15152	Yes	AAGGTCACAGAGGTTATGGGATTGGCTTGAAACCAGTCTGTGGGG
15153	Yes	AAGGTCACAGAGGTTATGGGATTGGCTTGAAACCAGTCTGTGGGGG
15154	Yes	AAGGTCACAGAGGTTATGGGATTGGCTTGAAACCAGTCTGTGGGGGG
15155	Yes	AAGGTCACAGAGGTTATGGGATTGGCTTGAAACCAGTCTGTGGGGGGT

15156	Yes	AAGGTCACAGAGGTTATGGGATTGGCTTGAAACCAGTCTGTGGGGGGTT
15157	Yes	AAGGTCACAGAGGTTATGGGATTGGCTTGAAACCAGTCTGTGGGGGGTTC
15158	No	AAGGTCACCAGTTTGA
15159	No	AAGGTCACCAGTTTGAG
15160	No	AAGGTCACCAGTTTGAGA
15161	No	AAGGTCACCAGTTTGAGAC
15162	No	AAGGTCACCAGTTTGAGACC
15163	Yes	AAGGTCACCAGTTTGAGACCG
15164	Yes	AAGGTCACCAGTTTGAGACCGG
15165	Yes	AAGGTCACCAGTTTGAGACCGGG
15166	Yes	AAGGTCACCAGTTTGAGACCGGGC
15167	Yes	AAGGTCACCAGTTTGAGACCGGGCC
15168	Yes	AAGGTCACCAGTTTGAGACCGGGCCA
15169	Yes	AAGGTCACCAGTTTGAGACCGGGCCAA
15170	Yes	AAGGTCACCAGTTTGAGACCGGGCCAAA
15171	Yes	AAGGTCACCAGTTTGAGACCGGGCCAAAA
15172	Yes	AAGGTCACCAGTTTGAGACCGGGCCAAAAC
15173	Yes	AAGGTCACCAGTTTGAGACCGGGCCAAAACA
15174	Yes	AAGGTCACCAGTTTGAGACCGGGCCAAAACAC
15175	Yes	AAGGTCACCAGTTTGAGACCGGGCCAAAACACC
15176	Yes	AAGGTCACCAGTTTGAGACCGGGCCAAAACACCA
15177	No	AAGGTCAGCTAAATAA
15178	No	AAGGTCAGCTAAATAAG
15179	No	AAGGTCAGCTAAATAAGC
15180	No	AAGGTCAGCTAAATAAGCT
15181	No	AAGGTCAGCTAAATAAGCTA
15182	No	AAGGTCAGCTAAATAAGCTAT
15183	No	AAGGTCAGCTAAATAAGCTATC
15184	No	AAGGTCAGCTAAATAAGCTATCG
15185	No	AAGGTCAGCTAAATAAGCTATCGG
15186	No	AAGGTCAGCTAAATAAGCTATCGGG
15187	No	AAGGTCAGCTAAATAAGCTATCGGGC
15188	No	AAGGTCAGCTAAATAAGCTATCGGGCC
15189	No	AAGGTCAGCTAAATAAGCTATCGGGCCC
15190	No	AAGGTCAGCTAAATAAGCTATCGGGCCCA
15191	No	AAGGTCAGCTAAATAAGCTATCGGGCCCAT
15192	No	AAGGTCAGCTAAATAAGCTATCGGGCCCATA
15193	No	AAGGTCAGCTAAATAAGCTATCGGGCCCATAC
15194	No	AAGGTCAGCTAAATAAGCTATCGGGCCCATACC
15195	No	AAGGTCAGCTAAATAAGCTATCGGGCCCATACCC
15196	No	AAGGTCAGCTAAATAAGCTATCGGGCCCATACCCC
15197	No	AAGGTCAGCTAAATAAGCTATCGGGCCCATACCCCG

15198	No	AAGGTCAGCTAAATAAGCTATCGGGCCCATACCCCGA
15199	No	AAGGTCAGCTAAATAAGCTATCGGGCCCATACCCCGAA
15200	No	AAGGTCAGCTAAATAAGCTATCGGGCCCATACCCCGAAA
15201	No	AAGGTCAGCTAAATAAGCTATCGGGCCCATACCCCGAAAA
15202	No	AAGGTCAGCTAAATAAGCTATCGGGCCCATACCCCGAAAAT
15203	No	AAGGTCAGCTAAATAAGCTATCGGGCCCATACCCCGAAAATG
15204	No	AAGGTCAGCTAAATAAGCTATCGGGCCCATACCCCGAAAATGT
15205	No	AAGGTCAGCTAAATAAGCTATCGGGCCCATACCCCGAAAATGTT
15206	No	AAGGTCAGCTAAATAAGCTATCGGGCCCATACCCCGAAAATGTTG
15207	No	AAGGTCAGCTAAATAAGCTATCGGGCCCATACCCCGAAAATGTTGG
15208	No	AAGGTCAGCTAAATAAGCTATCGGGCCCATACCCCGAAAATGTTGGT
15209	No	AAGGTCAGCTAAATAAGCTATCGGGCCCATACCCCGAAAATGTTGGTT
15210	No	AAGGTCAGCTAAATAAGCTATCGGGCCCATACCCCGAAAATGTTGGTTA
15211	No	AAGGTCAGCTAAATAAGCTATCGGGCCCATACCCCGAAAATGTTGGTTAT
15212	No	AAGGTCATAGAGGTTA
15213	No	AAGGTCATAGAGGTTAT
15214	No	AAGGTCATAGAGGTTATG
15215	No	AAGGTCATAGAGGTTATGG
15216	No	AAGGTCATAGAGGTTATGGG
15217	No	AAGGTCATAGAGGTTATGGGA
15218	No	AAGGTCATAGAGGTTATGGGAT
15219	No	AAGGTCATAGAGGTTATGGGATT
15220	No	AAGGTCATAGAGGTTATGGGATTG
15221	No	AAGGTCATAGAGGTTATGGGATTGG
15222	No	AAGGTCATAGAGGTTATGGGATTGGC
15223	No	AAGGTCATAGAGGTTATGGGATTGGCT
15224	No	AAGGTCATAGAGGTTATGGGATTGGCTT
15225	No	AAGGTCATAGAGGTTATGGGATTGGCTTG
15226	No	AAGGTCATAGAGGTTATGGGATTGGCTTGA
15227	No	AAGGTCATAGAGGTTATGGGATTGGCTTGAA
15228	No	AAGGTCATAGAGGTTATGGGATTGGCTTGAAA
15229	No	AAGGTCATAGAGGTTATGGGATTGGCTTGAAAC
15230	No	AAGGTCATAGAGGTTATGGGATTGGCTTGAAACC
15231	No	AAGGTCATAGAGGTTATGGGATTGGCTTGAAACCA
15232	No	AAGGTCATAGAGGTTATGGGATTGGCTTGAAACCAG
15233	No	AAGGTCATAGAGGTTATGGGATTGGCTTGAAACCAGT
15234	Yes	AAGGTCATAGAGGTTATGGGATTGGCTTGAAACCAGTC
15235	Yes	AAGGTCATAGAGGTTATGGGATTGGCTTGAAACCAGTCT
15236	Yes	AAGGTCATAGAGGTTATGGGATTGGCTTGAAACCAGTCTC
15237	Yes	AAGGTCATAGAGGTTATGGGATTGGCTTGAAACCAGTCTCT
15238	Yes	AAGGTCATAGAGGTTATGGGATTGGCTTGAAACCAGTCTCTG
15239	Yes	AAGGTCATAGAGGTTATGGGATTGGCTTGAAACCAGTCTCTGG

15240	Yes	AAGGTCATAGAGGTTATGGGATTGGCTTGAAACCAGTCTCTGGG
15241	Yes	AAGGTCATAGAGGTTATGGGATTGGCTTGAAACCAGTCTCTGGGG
15242	Yes	AAGGTCATAGAGGTTATGGGATTGGCTTGAAACCAGTCTCTGGGGG
15243	Yes	AAGGTCATAGAGGTTATGGGATTGGCTTGAAACCAGTCTCTGGGGGG
15244	Yes	AAGGTCATAGAGGTTATGGGATTGGCTTGAAACCAGTCTCTGGGGGGT
15245	Yes	AAGGTCATAGAGGTTATGGGATTGGCTTGAAACCAGTCTCTGGGGGGTT
15246	Yes	AAGGTCATAGAGGTTATGGGATTGGCTTGAAACCAGTCTCTGGGGGGTTC
15247	Yes	AAGGTCCCCGGTTCGA
15248	Yes	AAGGTCCCCGGTTCGAA
15249	Yes	AAGGTCCCCGGTTCGAAA
15250	Yes	AAGGTCCCCGGTTCGAAAC
15251	Yes	AAGGTCCCCGGTTCGAAACC
15252	Yes	AAGGTCCCCGGTTCGAAACCG
15253	Yes	AAGGTCCCCGGTTCGAAACCGG
15254	Yes	AAGGTCCCCGGTTCGAAACCGGG
15255	Yes	AAGGTCCCCGGTTCGAAACCGGGC
15256	Yes	AAGGTCCCCGGTTCGAAACCGGGCA
15257	Yes	AAGGTCCCCGGTTCGAAACCGGGCAG
15258	Yes	AAGGTCCCCGGTTCGAAACCGGGCAGA
15259	Yes	AAGGTCCCCGGTTCGAAACCGGGCAGAA
15260	Yes	AAGGTCCCCGGTTCGAAACCGGGCAGAAA
15261	Yes	AAGGTCCCCGGTTCGAAACCGGGCAGAAAC
15262	Yes	AAGGTCCCCGGTTCGAAACCGGGCAGAAACA
15263	Yes	AAGGTCCCCGGTTCGAAACCGGGCAGAAACAC
15264	Yes	AAGGTCCCCGGTTCGAAACCGGGCAGAAACACC
15265	Yes	AAGGTCCCCGGTTCGAAACCGGGCAGAAACACCA
15266	Yes	AAGGTCCCCGGTTCGAAACCGGGCAGAAG
15267	Yes	AAGGTCCCCGGTTCGAAACCGGGCAGAAGC
15268	Yes	AAGGTCCCCGGTTCGAAACCGGGCAGAAGCA
15269	Yes	AAGGTCCCCGGTTCGAAACCGGGCAGAAGCAC
15270	Yes	AAGGTCCCCGGTTCGAAACCGGGCAGAAGCACC
15271	Yes	AAGGTCCCCGGTTCGAAACCGGGCAGAAGCACCA
15272	Yes	AAGGTCCCCGGTTCGAAACCGGGCG
15273	Yes	AAGGTCCCCGGTTCGAAACCGGGCGG
15274	Yes	AAGGTCCCCGGTTCGAAACCGGGCGGA
15275	Yes	AAGGTCCCCGGTTCGAAACCGGGCGGAA
15276	Yes	AAGGTCCCCGGTTCGAAACCGGGCGGAAA
15277	Yes	AAGGTCCCCGGTTCGAAACCGGGCGGAAAC
15278	Yes	AAGGTCCCCGGTTCGAAACCGGGCGGAAACA
15279	Yes	AAGGTCCCCGGTTCGAAACCGGGCGGAAACAC
15280	Yes	AAGGTCCCCGGTTCGAAACCGGGCGGAAACACC
15281	Yes	AAGGTCCCCGGTTCGAAACCGGGCGGAAACACCA

15282	Yes	AAGGTCCCCGGTTCGAAACT
15283	Yes	AAGGTCCCCGGTTCGAAACTG
15284	Yes	AAGGTCCCCGGTTCGAAACTGG
15285	Yes	AAGGTCCCCGGTTCGAAACTGGG
15286	Yes	AAGGTCCCCGGTTCGAAACTGGGC
15287	Yes	AAGGTCCCCGGTTCGAAACTGGGCG
15288	Yes	AAGGTCCCCGGTTCGAAACTGGGCGG
15289	Yes	AAGGTCCCCGGTTCGAAACTGGGCGGA
15290	Yes	AAGGTCCCCGGTTCGAAACTGGGCGGAA
15291	Yes	AAGGTCCCCGGTTCGAAACTGGGCGGAAA
15292	Yes	AAGGTCCCCGGTTCGAAACTGGGCGGAAAC
15293	Yes	AAGGTCCCCGGTTCGAAACTGGGCGGAAACA
15294	Yes	AAGGTCCCCGGTTCGAAACTGGGCGGAAACAC
15295	Yes	AAGGTCCCCGGTTCGAAACTGGGCGGAAACACC
15296	Yes	AAGGTCCCCGGTTCGAAACTGGGCGGAAACACCA
15297	Yes	AAGGTCCCCGGTTCGAT
15298	Yes	AAGGTCCCCGGTTCGATC
15299	Yes	AAGGTCCCCGGTTCGATCC
15300	Yes	AAGGTCCCCGGTTCGATCCC
15301	Yes	AAGGTCCCCGGTTCGATCCCG
15302	Yes	AAGGTCCCCGGTTCGATCCCGG
15303	Yes	AAGGTCCCCGGTTCGATCCCGGG
15304	Yes	AAGGTCCCCGGTTCGATCCCGGGC
15305	Yes	AAGGTCCCCGGTTCGATCCCGGGCG
15306	Yes	AAGGTCCCCGGTTCGATCCCGGGCGG
15307	Yes	AAGGTCCCCGGTTCGATCCCGGGCGGA
15308	Yes	AAGGTCCCCGGTTCGATCCCGGGCGGAA
15309	Yes	AAGGTCCCCGGTTCGATCCCGGGCGGAAA
15310	Yes	AAGGTCCCCGGTTCGATCCCGGGCGGAAAC
15311	Yes	AAGGTCCCCGGTTCGATCCCGGGCGGAAACA
15312	Yes	AAGGTCCCCGGTTCGATCCCGGGCGGAAACAC
15313	Yes	AAGGTCCCCGGTTCGATCCCGGGCGGAAACACC
15314	Yes	AAGGTCCCCGGTTCGATCCCGGGCGGAAACACCA
15315	Yes	AAGGTCCCCGGTTTGA
15316	Yes	AAGGTCCCCGGTTTGAA
15317	Yes	AAGGTCCCCGGTTTGAAA
15318	Yes	AAGGTCCCCGGTTTGAAAC
15319	Yes	AAGGTCCCCGGTTTGAAACC
15320	Yes	AAGGTCCCCGGTTTGAAACCA
15321	Yes	AAGGTCCCCGGTTTGAAACCAG
15322	Yes	AAGGTCCCCGGTTTGAAACCAGG
15323	Yes	AAGGTCCCCGGTTTGAAACCAGGC

15324	Yes	AAGGTCCCCGGTTTGAAACCAGGCG
15325	Yes	AAGGTCCCCGGTTTGAAACCAGGCGG
15326	Yes	AAGGTCCCCGGTTTGAAACCAGGCGGA
15327	Yes	AAGGTCCCCGGTTTGAAACCAGGCGGAA
15328	Yes	AAGGTCCCCGGTTTGAAACCAGGCGGAAA
15329	Yes	AAGGTCCCCGGTTTGAAACCAGGCGGAAAC
15330	Yes	AAGGTCCCCGGTTTGAAACCAGGCGGAAACA
15331	Yes	AAGGTCCCCGGTTTGAAACCAGGCGGAAACAC
15332	Yes	AAGGTCCCCGGTTTGAAACCAGGCGGAAACACC
15333	Yes	AAGGTCCCCGGTTTGAAACCAGGCGGAAACACCA
15334	No	AAGGTCCCTGGATCAA
15335	No	AAGGTCCCTGGATCAAA
15336	No	AAGGTCCCTGGATCAAAA
15337	Yes	AAGGTCCCTGGATCAAAAC
15338	Yes	AAGGTCCCTGGATCAAAACC
15339	Yes	AAGGTCCCTGGATCAAAACCA
15340	Yes	AAGGTCCCTGGATCAAAACCAG
15341	Yes	AAGGTCCCTGGATCAAAACCAGG
15342	Yes	AAGGTCCCTGGATCAAAACCAGGC
15343	Yes	AAGGTCCCTGGATCAAAACCAGGCG
15344	Yes	AAGGTCCCTGGATCAAAACCAGGCGG
15345	Yes	AAGGTCCCTGGATCAAAACCAGGCGGA
15346	Yes	AAGGTCCCTGGATCAAAACCAGGCGGAA
15347	Yes	AAGGTCCCTGGATCAAAACCAGGCGGAAA
15348	Yes	AAGGTCCCTGGATCAAAACCAGGCGGAAAC
15349	Yes	AAGGTCCCTGGATCAAAACCAGGCGGAAACA
15350	Yes	AAGGTCCCTGGATCAAAACCAGGCGGAAACAC
15351	Yes	AAGGTCCCTGGATCAAAACCAGGCGGAAACACC
15352	Yes	AAGGTCCCTGGATCAAAACCAGGCGGAAACACCA
15353	No	AAGGTCCCTGGTTCAA
15354	Yes	AAGGTCCCTGGTTCAAA
15355	Yes	AAGGTCCCTGGTTCAAAT
15356	Yes	AAGGTCCCTGGTTCAAATC
15357	Yes	AAGGTCCCTGGTTCAAATCC
15358	Yes	AAGGTCCCTGGTTCAAATCCA
15359	Yes	AAGGTCCCTGGTTCAAATCCAG
15360	Yes	AAGGTCCCTGGTTCAAATCCAGG
15361	Yes	AAGGTCCCTGGTTCAAATCCAGGT
15362	Yes	AAGGTCCCTGGTTCAAATCCAGGTG
15363	Yes	AAGGTCCCTGGTTCAAATCCAGGTGC
15364	Yes	AAGGTCCCTGGTTCAAATCCAGGTGCC
15365	Yes	AAGGTCCCTGGTTCAAATCCAGGTGCCC

15366	Yes	AAGGTCCCTGGTTCAAATCCAGGTGCCCC
15367	Yes	AAGGTCCCTGGTTCAAATCCAGGTGCCCCT
15368	Yes	AAGGTCCCTGGTTCAAATCCAGGTGCCCCTT
15369	Yes	AAGGTCCCTGGTTCAAATCCAGGTGCCCCTTC
15370	Yes	AAGGTCCCTGGTTCAAATCCAGGTGCCCCTTCC
15371	Yes	AAGGTCCCTGGTTCAAATCCAGGTGCCCCTTCCA
15372	Yes	AAGGTCCCTGGTTCAAT
15373	Yes	AAGGTCCCTGGTTCAATC
15374	Yes	AAGGTCCCTGGTTCAATCC
15375	Yes	AAGGTCCCTGGTTCAATCCC
15376	Yes	AAGGTCCCTGGTTCAATCCCG
15377	Yes	AAGGTCCCTGGTTCAATCCCGG
15378	Yes	AAGGTCCCTGGTTCAATCCCGGG
15379	Yes	AAGGTCCCTGGTTCAATCCCGGGT
15380	Yes	AAGGTCCCTGGTTCAATCCCGGGTT
15381	Yes	AAGGTCCCTGGTTCAATCCCGGGTTT
15382	Yes	AAGGTCCCTGGTTCAATCCCGGGTTTC
15383	Yes	AAGGTCCCTGGTTCAATCCCGGGTTTCG
15384	Yes	AAGGTCCCTGGTTCAATCCCGGGTTTCGG
15385	Yes	AAGGTCCCTGGTTCAATCCCGGGTTTCGGC
15386	Yes	AAGGTCCCTGGTTCAATCCCGGGTTTCGGCA
15387	Yes	AAGGTCCCTGGTTCAATCCCGGGTTTCGGCAC
15388	Yes	AAGGTCCCTGGTTCAATCCCGGGTTTCGGCACC
15389	Yes	AAGGTCCCTGGTTCAATCCCGGGTTTCGGCACCA
15390	Yes	AAGGTCCCTGGTTCGA
15391	Yes	AAGGTCCCTGGTTCGAA
15392	Yes	AAGGTCCCTGGTTCGAAA
15393	Yes	AAGGTCCCTGGTTCGAAAC
15394	Yes	AAGGTCCCTGGTTCGAAACC
15395	Yes	AAGGTCCCTGGTTCGAAACCA
15396	Yes	AAGGTCCCTGGTTCGAAACCAG
15397	Yes	AAGGTCCCTGGTTCGAAACCAGG
15398	Yes	AAGGTCCCTGGTTCGAAACCAGGT
15399	Yes	AAGGTCCCTGGTTCGAAACCAGGTG
15400	Yes	AAGGTCCCTGGTTCGAAACCAGGTGG
15401	Yes	AAGGTCCCTGGTTCGAAACCAGGTGGA
15402	Yes	AAGGTCCCTGGTTCGAAACCAGGTGGAA
15403	Yes	AAGGTCCCTGGTTCGAAACCAGGTGGAAA
15404	Yes	AAGGTCCCTGGTTCGAAACCAGGTGGAAAC
15405	Yes	AAGGTCCCTGGTTCGAAACCAGGTGGAAACA
15406	Yes	AAGGTCCCTGGTTCGAAACCAGGTGGAAACAC
15407	Yes	AAGGTCCCTGGTTCGAAACCAGGTGGAAACACC

15408	Yes	AAGGTCCCTGGTTCGAAACCAGGTGGAAACACCA
15409	Yes	AAGGTCCCTGGTTCGAT
15410	Yes	AAGGTCCCTGGTTCGATC
15411	Yes	AAGGTCCCTGGTTCGATCC
15412	Yes	AAGGTCCCTGGTTCGATCCC
15413	Yes	AAGGTCCCTGGTTCGATCCCG
15414	Yes	AAGGTCCCTGGTTCGATCCCGG
15415	Yes	AAGGTCCCTGGTTCGATCCCGGG
15416	Yes	AAGGTCCCTGGTTCGATCCCGGGT
15417	Yes	AAGGTCCCTGGTTCGATCCCGGGTT
15418	Yes	AAGGTCCCTGGTTCGATCCCGGGTTT
15419	Yes	AAGGTCCCTGGTTCGATCCCGGGTTTC
15420	Yes	AAGGTCCCTGGTTCGATCCCGGGTTTCA
15421	Yes	AAGGTCCCTGGTTCGATCCCGGGTTTCAC
15422	Yes	AAGGTCCCTGGTTCGATCCCGGGTTTCACC
15423	Yes	AAGGTCCCTGGTTCGATCCCGGGTTTCACCA
15424	Yes	AAGGTCCCTGGTTCGATCCCGGGTTTCACCAC
15425	Yes	AAGGTCCCTGGTTCGATCCCGGGTTTCACCACC
15426	Yes	AAGGTCCCTGGTTCGATCCCGGGTTTCACCACCA
15427	Yes	AAGGTCCCTGGTTCGATCCCGGGTTTCG
15428	Yes	AAGGTCCCTGGTTCGATCCCGGGTTTCGG
15429	Yes	AAGGTCCCTGGTTCGATCCCGGGTTTCGGC
15430	Yes	AAGGTCCCTGGTTCGATCCCGGGTTTCGGCA
15431	Yes	AAGGTCCCTGGTTCGATCCCGGGTTTCGGCAC
15432	Yes	AAGGTCCCTGGTTCGATCCCGGGTTTCGGCACC
15433	Yes	AAGGTCCCTGGTTCGATCCCGGGTTTCGGCACCA
15434	Yes	AAGGTCCGCGGTTCGA
15435	Yes	AAGGTCCGCGGTTCGAA
15436	Yes	AAGGTCCGCGGTTCGAAA
15437	Yes	AAGGTCCGCGGTTCGAAAC
15438	Yes	AAGGTCCGCGGTTCGAAACC
15439	Yes	AAGGTCCGCGGTTCGAAACCG
15440	Yes	AAGGTCCGCGGTTCGAAACCGG
15441	Yes	AAGGTCCGCGGTTCGAAACCGGG
15442	Yes	AAGGTCCGCGGTTCGAAACCGGGC
15443	Yes	AAGGTCCGCGGTTCGAAACCGGGCG
15444	Yes	AAGGTCCGCGGTTCGAAACCGGGCGG
15445	Yes	AAGGTCCGCGGTTCGAAACCGGGCGGA
15446	Yes	AAGGTCCGCGGTTCGAAACCGGGCGGAA
15447	Yes	AAGGTCCGCGGTTCGAAACCGGGCGGAAA
15448	Yes	AAGGTCCGCGGTTCGAAACCGGGCGGAAAC
15449	Yes	AAGGTCCGCGGTTCGAAACCGGGCGGAAACA

15450	Yes	AAGGTCCGCGGTTCGAAACCGGGCGGAAACAC
15451	Yes	AAGGTCCGCGGTTCGAAACCGGGCGGAAACACC
15452	Yes	AAGGTCCGCGGTTCGAAACCGGGCGGAAACACCA
15453	Yes	AAGGTCCTCGGGTCGA
15454	Yes	AAGGTCCTCGGGTCGAA
15455	Yes	AAGGTCCTCGGGTCGAAA
15456	Yes	AAGGTCCTCGGGTCGAAAC
15457	Yes	AAGGTCCTCGGGTCGAAACC
15458	Yes	AAGGTCCTCGGGTCGAAACCG
15459	Yes	AAGGTCCTCGGGTCGAAACCGA
15460	Yes	AAGGTCCTCGGGTCGAAACCGAG
15461	Yes	AAGGTCCTCGGGTCGAAACCGAGC
15462	Yes	AAGGTCCTCGGGTCGAAACCGAGCG
15463	Yes	AAGGTCCTCGGGTCGAAACCGAGCGG
15464	Yes	AAGGTCCTCGGGTCGAAACCGAGCGGA
15465	Yes	AAGGTCCTCGGGTCGAAACCGAGCGGAA
15466	Yes	AAGGTCCTCGGGTCGAAACCGAGCGGAAA
15467	Yes	AAGGTCCTCGGGTCGAAACCGAGCGGAAAC
15468	Yes	AAGGTCCTCGGGTCGAAACCGAGCGGAAACA
15469	Yes	AAGGTCCTCGGGTCGAAACCGAGCGGAAACAC
15470	Yes	AAGGTCCTCGGGTCGAAACCGAGCGGAAACACC
15471	Yes	AAGGTCCTCGGGTCGAAACCGAGCGGAAACACCA
15472	No	AAGGTCCTGAGTTCAA
15473	No	AAGGTCCTGAGTTCAAG
15474	Yes	AAGGTCCTGAGTTCAAGC
15475	Yes	AAGGTCCTGAGTTCAAGCC
15476	Yes	AAGGTCCTGAGTTCAAGCCT
15477	Yes	AAGGTCCTGAGTTCAAGCCTC
15478	Yes	AAGGTCCTGAGTTCAAGCCTCA
15479	Yes	AAGGTCCTGAGTTCAAGCCTCAG
15480	Yes	AAGGTCCTGAGTTCAAGCCTCAGA
15481	Yes	AAGGTCCTGAGTTCAAGCCTCAGAG
15482	Yes	AAGGTCCTGAGTTCAAGCCTCAGAGA
15483	Yes	AAGGTCCTGAGTTCAAGCCTCAGAGAG
15484	Yes	AAGGTCCTGAGTTCAAGCCTCAGAGAGG
15485	Yes	AAGGTCCTGAGTTCAAGCCTCAGAGAGGG
15486	Yes	AAGGTCCTGAGTTCAAGCCTCAGAGAGGGC
15487	Yes	AAGGTCCTGAGTTCAAGCCTCAGAGAGGGCA
15488	Yes	AAGGTCCTGAGTTCAAGCCTCAGAGAGGGCAC
15489	Yes	AAGGTCCTGAGTTCAAGCCTCAGAGAGGGCACC
15490	Yes	AAGGTCCTGAGTTCAAGCCTCAGAGAGGGCACCA
15491	Yes	AAGGTCCTGAGTTCGA

15492	Yes	AAGGTCCTGAGTTCGAA
15493	Yes	AAGGTCCTGAGTTCGAAC
15494	Yes	AAGGTCCTGAGTTCGAACC
15495	Yes	AAGGTCCTGAGTTCGAACCT
15496	Yes	AAGGTCCTGAGTTCGAACCTC
15497	Yes	AAGGTCCTGAGTTCGAACCTCA
15498	Yes	AAGGTCCTGAGTTCGAACCTCAG
15499	Yes	AAGGTCCTGAGTTCGAACCTCAGA
15500	Yes	AAGGTCCTGAGTTCGAACCTCAGAG
15501	Yes	AAGGTCCTGAGTTCGAACCTCAGAGG
15502	Yes	AAGGTCCTGAGTTCGAACCTCAGAGGG
15503	Yes	AAGGTCCTGAGTTCGAACCTCAGAGGGG
15504	Yes	AAGGTCCTGAGTTCGAACCTCAGAGGGGG
15505	Yes	AAGGTCCTGAGTTCGAACCTCAGAGGGGGC
15506	Yes	AAGGTCCTGAGTTCGAACCTCAGAGGGGGCA
15507	Yes	AAGGTCCTGAGTTCGAACCTCAGAGGGGGCAC
15508	Yes	AAGGTCCTGAGTTCGAACCTCAGAGGGGGCACC
15509	Yes	AAGGTCCTGAGTTCGAACCTCAGAGGGGGCACCA
15510	Yes	AAGGTCCTGAGTTCGAG
15511	Yes	AAGGTCCTGAGTTCGAGC
15512	Yes	AAGGTCCTGAGTTCGAGCC
15513	Yes	AAGGTCCTGAGTTCGAGCCT
15514	Yes	AAGGTCCTGAGTTCGAGCCTC
15515	Yes	AAGGTCCTGAGTTCGAGCCTCA
15516	Yes	AAGGTCCTGAGTTCGAGCCTCAG
15517	Yes	AAGGTCCTGAGTTCGAGCCTCAGA
15518	Yes	AAGGTCCTGAGTTCGAGCCTCAGAG
15519	Yes	AAGGTCCTGAGTTCGAGCCTCAGAGA
15520	Yes	AAGGTCCTGAGTTCGAGCCTCAGAGAG
15521	Yes	AAGGTCCTGAGTTCGAGCCTCAGAGAGG
15522	Yes	AAGGTCCTGAGTTCGAGCCTCAGAGAGGG
15523	Yes	AAGGTCCTGAGTTCGAGCCTCAGAGAGGGC
15524	Yes	AAGGTCCTGAGTTCGAGCCTCAGAGAGGGCA
15525	Yes	AAGGTCCTGAGTTCGAGCCTCAGAGAGGGCAC
15526	Yes	AAGGTCCTGAGTTCGAGCCTCAGAGAGGGCACC
15527	Yes	AAGGTCCTGAGTTCGAGCCTCAGAGAGGGCACCA
15528	No	AAGGTCCTGGGTTCAA
15529	No	AAGGTCCTGGGTTCAAA
15530	Yes	AAGGTCCTGGGTTCAAAT
15531	Yes	AAGGTCCTGGGTTCAAATC
15532	Yes	AAGGTCCTGGGTTCAAATCC
15533	Yes	AAGGTCCTGGGTTCAAATCCC

15534	Yes	AAGGTCCTGGGTTCAAATCCCA
15535	Yes	AAGGTCCTGGGTTCAAATCCCAG
15536	Yes	AAGGTCCTGGGTTCAAATCCCAGA
15537	Yes	AAGGTCCTGGGTTCAAATCCCAGAG
15538	Yes	AAGGTCCTGGGTTCAAATCCCAGAGG
15539	Yes	AAGGTCCTGGGTTCAAATCCCAGAGGA
15540	Yes	AAGGTCCTGGGTTCAAATCCCAGAGGAG
15541	Yes	AAGGTCCTGGGTTCAAATCCCAGAGGAGC
15542	Yes	AAGGTCCTGGGTTCAAATCCCAGAGGAGCC
15543	Yes	AAGGTCCTGGGTTCAAATCCCAGAGGAGCCC
15544	Yes	AAGGTCCTGGGTTCAAATCCCAGAGGAGCCCC
15545	Yes	AAGGTCCTGGGTTCAAATCCCAGAGGAGCCCCC
15546	Yes	AAGGTCCTGGGTTCAAATCCCAGAGGAGCCCCCA
15547	No	AAGGTCCTGGGTTCAAG
15548	Yes	AAGGTCCTGGGTTCAAGC
15549	Yes	AAGGTCCTGGGTTCAAGCC
15550	Yes	AAGGTCCTGGGTTCAAGCCC
15551	Yes	AAGGTCCTGGGTTCAAGCCCC
15552	Yes	AAGGTCCTGGGTTCAAGCCCCA
15553	Yes	AAGGTCCTGGGTTCAAGCCCCAG
15554	Yes	AAGGTCCTGGGTTCAAGCCCCAGT
15555	Yes	AAGGTCCTGGGTTCAAGCCCCAGTG
15556	Yes	AAGGTCCTGGGTTCAAGCCCCAGTGG
15557	Yes	AAGGTCCTGGGTTCAAGCCCCAGTGGA
15558	Yes	AAGGTCCTGGGTTCAAGCCCCAGTGGAA
15559	Yes	AAGGTCCTGGGTTCAAGCCCCAGTGGAAC
15560	Yes	AAGGTCCTGGGTTCAAGCCCCAGTGGAACC
15561	Yes	AAGGTCCTGGGTTCAAGCCCCAGTGGAACCA
15562	Yes	AAGGTCCTGGGTTCAAGCCCCAGTGGAACCAC
15563	Yes	AAGGTCCTGGGTTCAAGCCCCAGTGGAACCACC
15564	Yes	AAGGTCCTGGGTTCAAGCCCCAGTGGAACCACCA
15565	Yes	AAGGTCCTGGGTTCGA
15566	Yes	AAGGTCCTGGGTTCGAG
15567	Yes	AAGGTCCTGGGTTCGAGC
15568	Yes	AAGGTCCTGGGTTCGAGCC
15569	Yes	AAGGTCCTGGGTTCGAGCCC
15570	Yes	AAGGTCCTGGGTTCGAGCCCC
15571	Yes	AAGGTCCTGGGTTCGAGCCCCA
15572	Yes	AAGGTCCTGGGTTCGAGCCCCAG
15573	Yes	AAGGTCCTGGGTTCGAGCCCCAGT
15574	Yes	AAGGTCCTGGGTTCGAGCCCCAGTG
15575	Yes	AAGGTCCTGGGTTCGAGCCCCAGTGG

15576	Yes	AAGGTCCTGGGTTCGAGCCCCAGTGGA
15577	Yes	AAGGTCCTGGGTTCGAGCCCCAGTGGAA
15578	Yes	AAGGTCCTGGGTTCGAGCCCCAGTGGAAC
15579	Yes	AAGGTCCTGGGTTCGAGCCCCAGTGGAACC
15580	Yes	AAGGTCCTGGGTTCGAGCCCCAGTGGAACCA
15581	Yes	AAGGTCCTGGGTTCGAGCCCCAGTGGAACCAC
15582	Yes	AAGGTCCTGGGTTCGAGCCCCAGTGGAACCACC
15583	Yes	AAGGTCCTGGGTTCGAGCCCCAGTGGAACCACCA
15584	Yes	AAGGTCCTTGGTTTGA
15585	Yes	AAGGTCCTTGGTTTGAA
15586	Yes	AAGGTCCTTGGTTTGAAA
15587	Yes	AAGGTCCTTGGTTTGAAAC
15588	Yes	AAGGTCCTTGGTTTGAAACC
15589	Yes	AAGGTCCTTGGTTTGAAACCA
15590	Yes	AAGGTCCTTGGTTTGAAACCAG
15591	Yes	AAGGTCCTTGGTTTGAAACCAGG
15592	Yes	AAGGTCCTTGGTTTGAAACCAGGC
15593	Yes	AAGGTCCTTGGTTTGAAACCAGGCA
15594	Yes	AAGGTCCTTGGTTTGAAACCAGGCAG
15595	Yes	AAGGTCCTTGGTTTGAAACCAGGCAGA
15596	Yes	AAGGTCCTTGGTTTGAAACCAGGCAGAA
15597	Yes	AAGGTCCTTGGTTTGAAACCAGGCAGAAA
15598	Yes	AAGGTCCTTGGTTTGAAACCAGGCAGAAAC
15599	Yes	AAGGTCCTTGGTTTGAAACCAGGCAGAAACA
15600	Yes	AAGGTCCTTGGTTTGAAACCAGGCAGAAACAC
15601	Yes	AAGGTCCTTGGTTTGAAACCAGGCAGAAACACC
15602	Yes	AAGGTCCTTGGTTTGAAACCAGGCAGAAACACCA
15603	Yes	AAGGTCGCGGGCTCGA
15604	Yes	AAGGTCGCGGGCTCGAC
15605	Yes	AAGGTCGCGGGCTCGACT
15606	Yes	AAGGTCGCGGGCTCGACTC
15607	Yes	AAGGTCGCGGGCTCGACTCC
15608	Yes	AAGGTCGCGGGCTCGACTCCC
15609	Yes	AAGGTCGCGGGCTCGACTCCCG
15610	Yes	AAGGTCGCGGGCTCGACTCCCGC
15611	Yes	AAGGTCGCGGGCTCGACTCCCGCA
15612	Yes	AAGGTCGCGGGCTCGACTCCCGCAC
15613	Yes	AAGGTCGCGGGCTCGACTCCCGCACC
15614	Yes	AAGGTCGCGGGCTCGACTCCCGCACCG
15615	Yes	AAGGTCGCGGGCTCGACTCCCGCACCGG
15616	Yes	AAGGTCGCGGGCTCGACTCCCGCACCGGC
15617	Yes	AAGGTCGCGGGCTCGACTCCCGCACCGGCC

15618	Yes	AAGGTCGCGGGCTCGACTCCCGCACCGGCCA
15619	Yes	AAGGTCGCGGGCTCGACTCCCGCACCGGCCAC
15620	Yes	AAGGTCGCGGGCTCGACTCCCGCACCGGCCACC
15621	Yes	AAGGTCGCGGGCTCGACTCCCGCACCGGCCACCA
15622	Yes	AAGGTCGCGGGTTCGA
15623	Yes	AAGGTCGCGGGTTCGAA
15624	Yes	AAGGTCGCGGGTTCGAAC
15625	Yes	AAGGTCGCGGGTTCGAACC
15626	Yes	AAGGTCGCGGGTTCGAACCC
15627	Yes	AAGGTCGCGGGTTCGAACCCC
15628	Yes	AAGGTCGCGGGTTCGAACCCCG
15629	Yes	AAGGTCGCGGGTTCGAACCCCGT
15630	Yes	AAGGTCGCGGGTTCGAACCCCGTA
15631	Yes	AAGGTCGCGGGTTCGAACCCCGTAC
15632	Yes	AAGGTCGCGGGTTCGAACCCCGTACG
15633	Yes	AAGGTCGCGGGTTCGAACCCCGTACGG
15634	Yes	AAGGTCGCGGGTTCGAACCCCGTACGGG
15635	Yes	AAGGTCGCGGGTTCGAACCCCGTACGGGC
15636	Yes	AAGGTCGCGGGTTCGAACCCCGTACGGGCC
15637	Yes	AAGGTCGCGGGTTCGAACCCCGTACGGGCCA
15638	Yes	AAGGTCGCGGGTTCGAACCCCGTACGGGCCAC
15639	Yes	AAGGTCGCGGGTTCGAACCCCGTACGGGCCACC
15640	Yes	AAGGTCGCGGGTTCGAACCCCGTACGGGCCACCA
15641	Yes	AAGGTCGCGGGTTCGAT
15642	Yes	AAGGTCGCGGGTTCGATC
15643	Yes	AAGGTCGCGGGTTCGATCC
15644	Yes	AAGGTCGCGGGTTCGATCCC
15645	Yes	AAGGTCGCGGGTTCGATCCCC
15646	Yes	AAGGTCGCGGGTTCGATCCCCG
15647	Yes	AAGGTCGCGGGTTCGATCCCCGT
15648	Yes	AAGGTCGCGGGTTCGATCCCCGTA
15649	Yes	AAGGTCGCGGGTTCGATCCCCGTAC
15650	Yes	AAGGTCGCGGGTTCGATCCCCGTACG
15651	Yes	AAGGTCGCGGGTTCGATCCCCGTACGG
15652	Yes	AAGGTCGCGGGTTCGATCCCCGTACGGG
15653	Yes	AAGGTCGCGGGTTCGATCCCCGTACGGGC
15654	Yes	AAGGTCGCGGGTTCGATCCCCGTACGGGCC
15655	Yes	AAGGTCGCGGGTTCGATCCCCGTACGGGCCA
15656	Yes	AAGGTCGCGGGTTCGATCCCCGTACGGGCCAC
15657	Yes	AAGGTCGCGGGTTCGATCCCCGTACGGGCCACC
15658	Yes	AAGGTCGCGGGTTCGATCCCCGTACGGGCCACCA
15659	Yes	AAGGTCGCGGGTTCGATCCCCGTACT

15660	Yes	AAGGTCGCGGGTTCGATCCCCGTACTG
15661	Yes	AAGGTCGCGGGTTCGATCCCCGTACTGG
15662	Yes	AAGGTCGCGGGTTCGATCCCCGTACTGGC
15663	Yes	AAGGTCGCGGGTTCGATCCCCGTACTGGCC
15664	Yes	AAGGTCGCGGGTTCGATCCCCGTACTGGCCA
15665	Yes	AAGGTCGCGGGTTCGATCCCCGTACTGGCCAC
15666	Yes	AAGGTCGCGGGTTCGATCCCCGTACTGGCCACC
15667	Yes	AAGGTCGCGGGTTCGATCCCCGTACTGGCCACCA
15668	No	AAGGTCGTGAGTTCGA
15669	No	AAGGTCGTGAGTTCGAG
15670	Yes	AAGGTCGTGAGTTCGAGC
15671	Yes	AAGGTCGTGAGTTCGAGCC
15672	Yes	AAGGTCGTGAGTTCGAGCCT
15673	Yes	AAGGTCGTGAGTTCGAGCCTC
15674	Yes	AAGGTCGTGAGTTCGAGCCTCA
15675	Yes	AAGGTCGTGAGTTCGAGCCTCAC
15676	Yes	AAGGTCGTGAGTTCGAGCCTCACA
15677	Yes	AAGGTCGTGAGTTCGAGCCTCACAC
15678	Yes	AAGGTCGTGAGTTCGAGCCTCACACG
15679	Yes	AAGGTCGTGAGTTCGAGCCTCACACGG
15680	Yes	AAGGTCGTGAGTTCGAGCCTCACACGGG
15681	Yes	AAGGTCGTGAGTTCGAGCCTCACACGGGG
15682	Yes	AAGGTCGTGAGTTCGAGCCTCACACGGGGC
15683	Yes	AAGGTCGTGAGTTCGAGCCTCACACGGGGCA
15684	Yes	AAGGTCGTGAGTTCGAGCCTCACACGGGGCAC
15685	Yes	AAGGTCGTGAGTTCGAGCCTCACACGGGGCACC
15686	Yes	AAGGTCGTGAGTTCGAGCCTCACACGGGGCACCA
15687	Yes	AAGGTCGTGAGTTCGAT
15688	Yes	AAGGTCGTGAGTTCGATC
15689	Yes	AAGGTCGTGAGTTCGATCC
15690	Yes	AAGGTCGTGAGTTCGATCCT
15691	Yes	AAGGTCGTGAGTTCGATCCTC
15692	Yes	AAGGTCGTGAGTTCGATCCTCA
15693	Yes	AAGGTCGTGAGTTCGATCCTCAC
15694	Yes	AAGGTCGTGAGTTCGATCCTCACA
15695	Yes	AAGGTCGTGAGTTCGATCCTCACAC
15696	Yes	AAGGTCGTGAGTTCGATCCTCACACG
15697	Yes	AAGGTCGTGAGTTCGATCCTCACACGG
15698	Yes	AAGGTCGTGAGTTCGATCCTCACACGGG
15699	Yes	AAGGTCGTGAGTTCGATCCTCACACGGGG
15700	Yes	AAGGTCGTGAGTTCGATCCTCACACGGGGC
15701	Yes	AAGGTCGTGAGTTCGATCCTCACACGGGGCA

15702	Yes	AAGGTCGTGAGTTCGATCCTCACACGGGGCAC
15703	Yes	AAGGTCGTGAGTTCGATCCTCACACGGGGCACC
15704	Yes	AAGGTCGTGAGTTCGATCCTCACACGGGGCACCA
15705	No	AAGGTCGTGGGTTCGA
15706	Yes	AAGGTCGTGGGTTCGAT
15707	Yes	AAGGTCGTGGGTTCGATC
15708	Yes	AAGGTCGTGGGTTCGATCC
15709	Yes	AAGGTCGTGGGTTCGATCCC
15710	Yes	AAGGTCGTGGGTTCGATCCCC
15711	Yes	AAGGTCGTGGGTTCGATCCCCA
15712	Yes	AAGGTCGTGGGTTCGATCCCCAT
15713	Yes	AAGGTCGTGGGTTCGATCCCCATA
15714	Yes	AAGGTCGTGGGTTCGATCCCCATAT
15715	Yes	AAGGTCGTGGGTTCGATCCCCATATC
15716	Yes	AAGGTCGTGGGTTCGATCCCCATATCG
15717	Yes	AAGGTCGTGGGTTCGATCCCCATATCGG
15718	Yes	AAGGTCGTGGGTTCGATCCCCATATCGGC
15719	Yes	AAGGTCGTGGGTTCGATCCCCATATCGGCC
15720	Yes	AAGGTCGTGGGTTCGATCCCCATATCGGCCA
15721	Yes	AAGGTCGTGGGTTCGATCCCCATATCGGCCAC
15722	Yes	AAGGTCGTGGGTTCGATCCCCATATCGGCCACC
15723	Yes	AAGGTCGTGGGTTCGATCCCCATATCGGCCACCA
15724	Yes	AAGGTCTCTGGTTCGA
15725	Yes	AAGGTCTCTGGTTCGAG
15726	Yes	AAGGTCTCTGGTTCGAGA
15727	Yes	AAGGTCTCTGGTTCGAGAC
15728	Yes	AAGGTCTCTGGTTCGAGACT
15729	Yes	AAGGTCTCTGGTTCGAGACTG
15730	Yes	AAGGTCTCTGGTTCGAGACTGC
15731	Yes	AAGGTCTCTGGTTCGAGACTGCG
15732	Yes	AAGGTCTCTGGTTCGAGACTGCGT
15733	Yes	AAGGTCTCTGGTTCGAGACTGCGTG
15734	Yes	AAGGTCTCTGGTTCGAGACTGCGTGG
15735	Yes	AAGGTCTCTGGTTCGAGACTGCGTGGG
15736	Yes	AAGGTCTCTGGTTCGAGACTGCGTGGGA
15737	Yes	AAGGTCTCTGGTTCGAGACTGCGTGGGAA
15738	Yes	AAGGTCTCTGGTTCGAGACTGCGTGGGAAC
15739	Yes	AAGGTCTCTGGTTCGAGACTGCGTGGGAACA
15740	Yes	AAGGTCTCTGGTTCGAGACTGCGTGGGAACAC
15741	Yes	AAGGTCTCTGGTTCGAGACTGCGTGGGAACACC
15742	Yes	AAGGTCTCTGGTTCGAGACTGCGTGGGAACACCA
15743	Yes	AAGGTCTCTGGTTTGA

15744	Yes	AAGGTCTCTGGTTTGAT
15745	Yes	AAGGTCTCTGGTTTGATC
15746	Yes	AAGGTCTCTGGTTTGATCC
15747	Yes	AAGGTCTCTGGTTTGATCCT
15748	Yes	AAGGTCTCTGGTTTGATCCTG
15749	Yes	AAGGTCTCTGGTTTGATCCTGG
15750	Yes	AAGGTCTCTGGTTTGATCCTGGG
15751	Yes	AAGGTCTCTGGTTTGATCCTGGGT
15752	Yes	AAGGTCTCTGGTTTGATCCTGGGTT
15753	Yes	AAGGTCTCTGGTTTGATCCTGGGTTT
15754	Yes	AAGGTCTCTGGTTTGATCCTGGGTTTC
15755	Yes	AAGGTCTCTGGTTTGATCCTGGGTTTCA
15756	Yes	AAGGTCTCTGGTTTGATCCTGGGTTTCAG
15757	Yes	AAGGTCTCTGGTTTGATCCTGGGTTTCAGA
15758	Yes	AAGGTCTCTGGTTTGATCCTGGGTTTCAGAA
15759	Yes	AAGGTCTCTGGTTTGATCCTGGGTTTCAGAAC
15760	Yes	AAGGTCTCTGGTTTGATCCTGGGTTTCAGAACC
15761	Yes	AAGGTCTCTGGTTTGATCCTGGGTTTCAGAACCA
15762	No	AAGGTGATGGACTAGA
15763	No	AAGGTGATGGACTAGAA
15764	No	AAGGTGATGGACTAGAAA
15765	Yes	AAGGTGATGGACTAGAAAC
15766	Yes	AAGGTGATGGACTAGAAACC
15767	Yes	AAGGTGATGGACTAGAAACCC
15768	Yes	AAGGTGATGGACTAGAAACCCA
15769	Yes	AAGGTGATGGACTAGAAACCCAT
15770	Yes	AAGGTGATGGACTAGAAACCCATT
15771	Yes	AAGGTGATGGACTAGAAACCCATTG
15772	Yes	AAGGTGATGGACTAGAAACCCATTGG
15773	Yes	AAGGTGATGGACTAGAAACCCATTGGG
15774	Yes	AAGGTGATGGACTAGAAACCCATTGGGG
15775	Yes	AAGGTGATGGACTAGAAACCCATTGGGGT
15776	Yes	AAGGTGATGGACTAGAAACCCATTGGGGTC
15777	Yes	AAGGTGATGGACTAGAAACCCATTGGGGTCT
15778	Yes	AAGGTGATGGACTAGAAACCCATTGGGGTCTC
15779	Yes	AAGGTGATGGACTAGAAACCCATTGGGGTCTCC
15780	Yes	AAGGTGATGGACTAGAAACCCATTGGGGTCTCCC
15781	Yes	AAGGTGATGGACTAGAAACCCATTGGGGTCTCCCC
15782	Yes	AAGGTGATGGACTAGAAACCCATTGGGGTCTCCCCG
15783	Yes	AAGGTGATGGACTAGAAACCCATTGGGGTCTCCCCGC
15784	Yes	AAGGTGATGGACTAGAAACCCATTGGGGTCTCCCCGCG
15785	Yes	AAGGTGATGGACTAGAAACCCATTGGGGTCTCCCCGCGC

15786	Yes	AAGGTGATGGACTAGAAACCCATTGGGGTCTCCCCGCGCA
15787	Yes	AAGGTGATGGACTAGAAACCCATTGGGGTCTCCCCGCGCAG
15788	Yes	AAGGTGATGGACTAGAAACCCATTGGGGTCTCCCCGCGCAGG
15789	Yes	AAGGTGATGGACTAGAAACCCATTGGGGTCTCCCCGCGCAGGT
15790	Yes	AAGGTGATGGACTAGAAACCCATTGGGGTCTCCCCGCGCAGGTT
15791	Yes	AAGGTGATGGACTAGAAACCCATTGGGGTCTCCCCGCGCAGGTTC
15792	Yes	AAGGTGATGGACTAGAAACCCATTGGGGTCTCCCCGCGCAGGTTCG
15793	Yes	AAGGTGATGGACTAGAAACCCATTGGGGTCTCCCCGCGCAGGTTCGA
15794	Yes	AAGGTGATGGACTAGAAACCCATTGGGGTCTCCCCGCGCAGGTTCGAA
15795	Yes	AAGGTGATGGACTAGAAACCCATTGGGGTCTCCCCGCGCAGGTTCGAAT
15796	Yes	AAGGTGATGGACTAGAAACCCATTGGGGTCTCCCCGCGCAGGTTCGAATC
15797	Yes	AAGGTGTTGGACTCGA
15798	Yes	AAGGTGTTGGACTCGAA
15799	Yes	AAGGTGTTGGACTCGAAA
15800	Yes	AAGGTGTTGGACTCGAAAT
15801	Yes	AAGGTGTTGGACTCGAAATC
15802	Yes	AAGGTGTTGGACTCGAAATCC
15803	Yes	AAGGTGTTGGACTCGAAATCCA
15804	Yes	AAGGTGTTGGACTCGAAATCCAA
15805	Yes	AAGGTGTTGGACTCGAAATCCAAT
15806	Yes	AAGGTGTTGGACTCGAAATCCAATG
15807	Yes	AAGGTGTTGGACTCGAAATCCAATGG
15808	Yes	AAGGTGTTGGACTCGAAATCCAATGGG
15809	Yes	AAGGTGTTGGACTCGAAATCCAATGGGG
15810	Yes	AAGGTGTTGGACTCGAAATCCAATGGGGG
15811	Yes	AAGGTGTTGGACTCGAAATCCAATGGGGGT
15812	Yes	AAGGTGTTGGACTCGAAATCCAATGGGGGTT
15813	Yes	AAGGTGTTGGACTCGAAATCCAATGGGGGTTC
15814	Yes	AAGGTGTTGGACTCGAAATCCAATGGGGGTTCC
15815	Yes	AAGGTGTTGGACTCGAAATCCAATGGGGGTTCCC
15816	Yes	AAGGTGTTGGACTCGAAATCCAATGGGGGTTCCCC
15817	Yes	AAGGTGTTGGACTCGAAATCCAATGGGGGTTCCCCG
15818	Yes	AAGGTGTTGGACTCGAAATCCAATGGGGGTTCCCCGC
15819	Yes	AAGGTGTTGGACTCGAAATCCAATGGGGGTTCCCCGCG
15820	Yes	AAGGTGTTGGACTCGAAATCCAATGGGGGTTCCCCGCGC
15821	Yes	AAGGTGTTGGACTCGAAATCCAATGGGGGTTCCCCGCGCA
15822	Yes	AAGGTGTTGGACTCGAAATCCAATGGGGGTTCCCCGCGCAG
15823	Yes	AAGGTGTTGGACTCGAAATCCAATGGGGGTTCCCCGCGCAGG
15824	Yes	AAGGTGTTGGACTCGAAATCCAATGGGGGTTCCCCGCGCAGGT
15825	Yes	AAGGTGTTGGACTCGAAATCCAATGGGGGTTCCCCGCGCAGGTT
15826	Yes	AAGGTGTTGGACTCGAAATCCAATGGGGGTTCCCCGCGCAGGTTC
15827	Yes	AAGGTGTTGGACTCGAAATCCAATGGGGGTTCCCCGCGCAGGTTCA

15828	Yes	AAGGTGTTGGACTCGAAATCCAATGGGGGTTCCCCGCGCAGGTTCAA
15829	Yes	AAGGTGTTGGACTCGAAATCCAATGGGGGTTCCCCGCGCAGGTTCAAA
15830	Yes	AAGGTGTTGGACTCGAAATCCAATGGGGGTTCCCCGCGCAGGTTCAAAT
15831	Yes	AAGGTGTTGGACTCGAAATCCAATGGGGGTTCCCCGCGCAGGTTCAAATC
15832	No	AAGGTTAGTGGTTCGA
15833	Yes	AAGGTTAGTGGTTCGAG
15834	Yes	AAGGTTAGTGGTTCGAGC
15835	Yes	AAGGTTAGTGGTTCGAGCC
15836	Yes	AAGGTTAGTGGTTCGAGCCC
15837	Yes	AAGGTTAGTGGTTCGAGCCCA
15838	Yes	AAGGTTAGTGGTTCGAGCCCAC
15839	Yes	AAGGTTAGTGGTTCGAGCCCACC
15840	Yes	AAGGTTAGTGGTTCGAGCCCACCC
15841	Yes	AAGGTTAGTGGTTCGAGCCCACCCG
15842	Yes	AAGGTTAGTGGTTCGAGCCCACCCGG
15843	Yes	AAGGTTAGTGGTTCGAGCCCACCCGGG
15844	Yes	AAGGTTAGTGGTTCGAGCCCACCCGGGG
15845	Yes	AAGGTTAGTGGTTCGAGCCCACCCGGGGA
15846	Yes	AAGGTTAGTGGTTCGAGCCCACCCGGGGAC
15847	Yes	AAGGTTAGTGGTTCGAGCCCACCCGGGGACG
15848	Yes	AAGGTTAGTGGTTCGAGCCCACCCGGGGACGC
15849	Yes	AAGGTTAGTGGTTCGAGCCCACCCGGGGACGCC
15850	Yes	AAGGTTAGTGGTTCGAGCCCACCCGGGGACGCCA
15851	Yes	AAGGTTCATGTCCCTT
15852	Yes	AAGGTTCATGTCCCTTT
15853	Yes	AAGGTTCATGTCCCTTTT
15854	Yes	AAGGTTCATGTCCCTTTTT
15855	Yes	AAGGTTCATGTCCCTTTTTG
15856	Yes	AAGGTTCATGTCCCTTTTTGG
15857	Yes	AAGGTTCATGTCCCTTTTTGGG
15858	Yes	AAGGTTCATGTCCCTTTTTGGGT
15859	Yes	AAGGTTCATGTCCCTTTTTGGGTG
15860	Yes	AAGGTTCATGTCCCTTTTTGGGTGC
15861	Yes	AAGGTTCATGTCCCTTTTTGGGTGCC
15862	Yes	AAGGTTCATGTCCCTTTTTGGGTGCCA
15863	Yes	AAGGTTCCGGGTTCGA
15864	Yes	AAGGTTCCGGGTTCGAG
15865	Yes	AAGGTTCCGGGTTCGAGT
15866	Yes	AAGGTTCCGGGTTCGAGTC
15867	Yes	AAGGTTCCGGGTTCGAGTCC
15868	Yes	AAGGTTCCGGGTTCGAGTCCC
15869	Yes	AAGGTTCCGGGTTCGAGTCCCG

15870	Yes	AAGGTTCCGGGTTCGAGTCCCGG
15871	Yes	AAGGTTCCGGGTTCGAGTCCCGGC
15872	Yes	AAGGTTCCGGGTTCGAGTCCCGGCG
15873	Yes	AAGGTTCCGGGTTCGAGTCCCGGCGG
15874	Yes	AAGGTTCCGGGTTCGAGTCCCGGCGGA
15875	Yes	AAGGTTCCGGGTTCGAGTCCCGGCGGAG
15876	Yes	AAGGTTCCGGGTTCGAGTCCCGGCGGAGT
15877	Yes	AAGGTTCCGGGTTCGAGTCCCGGCGGAGTC
15878	Yes	AAGGTTCCGGGTTCGAGTCCCGGCGGAGTCG
15879	Yes	AAGGTTCCGGGTTCGAGTCCCGGCGGAGTCGC
15880	Yes	AAGGTTCCGGGTTCGAGTCCCGGCGGAGTCGCC
15881	Yes	AAGGTTCCGGGTTCGAGTCCCGGCGGAGTCGCCA
15882	Yes	AAGGTTGCGGGTTCGA
15883	Yes	AAGGTTGCGGGTTCGAG
15884	Yes	AAGGTTGCGGGTTCGAGT
15885	Yes	AAGGTTGCGGGTTCGAGTC
15886	Yes	AAGGTTGCGGGTTCGAGTCC
15887	Yes	AAGGTTGCGGGTTCGAGTCCC
15888	Yes	AAGGTTGCGGGTTCGAGTCCCT
15889	Yes	AAGGTTGCGGGTTCGAGTCCCTC
15890	Yes	AAGGTTGCGGGTTCGAGTCCCTCC
15891	Yes	AAGGTTGCGGGTTCGAGTCCCTCCA
15892	Yes	AAGGTTGCGGGTTCGAGTCCCTCCAG
15893	Yes	AAGGTTGCGGGTTCGAGTCCCTCCAGA
15894	Yes	AAGGTTGCGGGTTCGAGTCCCTCCAGAG
15895	Yes	AAGGTTGCGGGTTCGAGTCCCTCCAGAGT
15896	Yes	AAGGTTGCGGGTTCGAGTCCCTCCAGAGTC
15897	Yes	AAGGTTGCGGGTTCGAGTCCCTCCAGAGTCG
15898	Yes	AAGGTTGCGGGTTCGAGTCCCTCCAGAGTCGC
15899	Yes	AAGGTTGCGGGTTCGAGTCCCTCCAGAGTCGCC
15900	Yes	AAGGTTGCGGGTTCGAGTCCCTCCAGAGTCGCCA
15901	Yes	AAGGTTGCGGGTTGGA
15902	Yes	AAGGTTGCGGGTTGGAT
15903	Yes	AAGGTTGCGGGTTGGATC
15904	Yes	AAGGTTGCGGGTTGGATCC
15905	Yes	AAGGTTGCGGGTTGGATCCC
15906	Yes	AAGGTTGCGGGTTGGATCCCC
15907	Yes	AAGGTTGCGGGTTGGATCCCCG
15908	Yes	AAGGTTGCGGGTTGGATCCCCGA
15909	Yes	AAGGTTGCGGGTTGGATCCCCGAA
15910	Yes	AAGGTTGCGGGTTGGATCCCCGAAC
15911	Yes	AAGGTTGCGGGTTGGATCCCCGAACG

15912	Yes	AAGGTTGCGGGTTGGATCCCCGAACGG
15913	Yes	AAGGTTGCGGGTTGGATCCCCGAACGGG
15914	Yes	AAGGTTGCGGGTTGGATCCCCGAACGGGC
15915	Yes	AAGGTTGCGGGTTGGATCCCCGAACGGGCC
15916	Yes	AAGGTTGCGGGTTGGATCCCCGAACGGGCCA
15917	Yes	AAGGTTGCGGGTTGGATCCCCGAACGGGCCAC
15918	Yes	AAGGTTGCGGGTTGGATCCCCGAACGGGCCACC
15919	Yes	AAGGTTGCGGGTTGGATCCCCGAACGGGCCACCA
15920	Yes	AAGGTTGCGTGTTCAA
15921	Yes	AAGGTTGCGTGTTCAAA
15922	Yes	AAGGTTGCGTGTTCAAAT
15923	Yes	AAGGTTGCGTGTTCAAATC
15924	Yes	AAGGTTGCGTGTTCAAATCA
15925	Yes	AAGGTTGCGTGTTCAAATCAC
15926	Yes	AAGGTTGCGTGTTCAAATCACG
15927	Yes	AAGGTTGCGTGTTCAAATCACGT
15928	Yes	AAGGTTGCGTGTTCAAATCACGTC
15929	Yes	AAGGTTGCGTGTTCAAATCACGTCG
15930	Yes	AAGGTTGCGTGTTCAAATCACGTCGG
15931	Yes	AAGGTTGCGTGTTCAAATCACGTCGGG
15932	Yes	AAGGTTGCGTGTTCAAATCACGTCGGGG
15933	Yes	AAGGTTGCGTGTTCAAATCACGTCGGGGT
15934	Yes	AAGGTTGCGTGTTCAAATCACGTCGGGGTC
15935	Yes	AAGGTTGCGTGTTCAAATCACGTCGGGGTCA
15936	Yes	AAGGTTGCGTGTTCAAATCACGTCGGGGTCAC
15937	Yes	AAGGTTGCGTGTTCAAATCACGTCGGGGTCACC
15938	Yes	AAGGTTGCGTGTTCAAATCACGTCGGGGTCACCA
15939	Yes	AAGGTTGCGTGTTCAAG
15940	Yes	AAGGTTGCGTGTTCAAGT
15941	Yes	AAGGTTGCGTGTTCAAGTC
15942	Yes	AAGGTTGCGTGTTCAAGTCA
15943	Yes	AAGGTTGCGTGTTCAAGTCAC
15944	Yes	AAGGTTGCGTGTTCAAGTCACG
15945	Yes	AAGGTTGCGTGTTCAAGTCACGT
15946	Yes	AAGGTTGCGTGTTCAAGTCACGTC
15947	Yes	AAGGTTGCGTGTTCAAGTCACGTCG
15948	Yes	AAGGTTGCGTGTTCAAGTCACGTCGG
15949	Yes	AAGGTTGCGTGTTCAAGTCACGTCGGG
15950	Yes	AAGGTTGCGTGTTCAAGTCACGTCGGGG
15951	Yes	AAGGTTGCGTGTTCAAGTCACGTCGGGGT
15952	Yes	AAGGTTGCGTGTTCAAGTCACGTCGGGGTC
15953	Yes	AAGGTTGCGTGTTCAAGTCACGTCGGGGTCA

15954	Yes	AAGGTTGCGTGTTCAAGTCACGTCGGGGTCAC
15955	Yes	AAGGTTGCGTGTTCAAGTCACGTCGGGGTCACC
15956	Yes	AAGGTTGCGTGTTCAAGTCACGTCGGGGTCACCA
15957	Yes	AAGGTTGGCGGTTCGA
15958	Yes	AAGGTTGGCGGTTCGAA
15959	Yes	AAGGTTGGCGGTTCGAAC
15960	Yes	AAGGTTGGCGGTTCGAACC
15961	Yes	AAGGTTGGCGGTTCGAACCC
15962	Yes	AAGGTTGGCGGTTCGAACCCA
15963	Yes	AAGGTTGGCGGTTCGAACCCAC
15964	Yes	AAGGTTGGCGGTTCGAACCCACC
15965	Yes	AAGGTTGGCGGTTCGAACCCACCC
15966	Yes	AAGGTTGGCGGTTCGAACCCACCCA
15967	Yes	AAGGTTGGCGGTTCGAACCCACCCAG
15968	Yes	AAGGTTGGCGGTTCGAACCCACCCAGA
15969	Yes	AAGGTTGGCGGTTCGAACCCACCCAGAG
15970	Yes	AAGGTTGGCGGTTCGAACCCACCCAGAGG
15971	Yes	AAGGTTGGCGGTTCGAACCCACCCAGAGGC
15972	Yes	AAGGTTGGCGGTTCGAACCCACCCAGAGGCG
15973	Yes	AAGGTTGGCGGTTCGAACCCACCCAGAGGCGC
15974	Yes	AAGGTTGGCGGTTCGAACCCACCCAGAGGCGCC
15975	Yes	AAGGTTGGCGGTTCGAACCCACCCAGAGGCGCCA
15976	No	AAGGTTGGTGGCTAGA
15977	No	AAGGTTGGTGGCTAGAA
15978	Yes	AAGGTTGGTGGCTAGAAC
15979	Yes	AAGGTTGGTGGCTAGAACC
15980	Yes	AAGGTTGGTGGCTAGAACCC
15981	Yes	AAGGTTGGTGGCTAGAACCCA
15982	Yes	AAGGTTGGTGGCTAGAACCCAC
15983	Yes	AAGGTTGGTGGCTAGAACCCACT
15984	Yes	AAGGTTGGTGGCTAGAACCCACTG
15985	Yes	AAGGTTGGTGGCTAGAACCCACTGA
15986	Yes	AAGGTTGGTGGCTAGAACCCACTGAG
15987	Yes	AAGGTTGGTGGCTAGAACCCACTGAGG
15988	Yes	AAGGTTGGTGGCTAGAACCCACTGAGGG
15989	Yes	AAGGTTGGTGGCTAGAACCCACTGAGGGA
15990	Yes	AAGGTTGGTGGCTAGAACCCACTGAGGGAC
15991	Yes	AAGGTTGGTGGCTAGAACCCACTGAGGGACG
15992	Yes	AAGGTTGGTGGCTAGAACCCACTGAGGGACGC
15993	Yes	AAGGTTGGTGGCTAGAACCCACTGAGGGACGCC
15994	Yes	AAGGTTGGTGGCTAGAACCCACTGAGGGACGCCA
15995	No	AAGGTTGGTGGTGCAA

15996	Yes	AAGGTTGGTGGTGCAAG
15997	Yes	AAGGTTGGTGGTGCAAGC
15998	Yes	AAGGTTGGTGGTGCAAGCC
15999	Yes	AAGGTTGGTGGTGCAAGCCC
16000	Yes	AAGGTTGGTGGTGCAAGCCCA
16001	Yes	AAGGTTGGTGGTGCAAGCCCAT
16002	Yes	AAGGTTGGTGGTGCAAGCCCATC
16003	Yes	AAGGTTGGTGGTGCAAGCCCATCC
16004	Yes	AAGGTTGGTGGTGCAAGCCCATCCA
16005	Yes	AAGGTTGGTGGTGCAAGCCCATCCAG
16006	Yes	AAGGTTGGTGGTGCAAGCCCATCCAGG
16007	Yes	AAGGTTGGTGGTGCAAGCCCATCCAGGG
16008	Yes	AAGGTTGGTGGTGCAAGCCCATCCAGGGA
16009	Yes	AAGGTTGGTGGTGCAAGCCCATCCAGGGAT
16010	Yes	AAGGTTGGTGGTGCAAGCCCATCCAGGGATG
16011	Yes	AAGGTTGGTGGTGCAAGCCCATCCAGGGATGC
16012	Yes	AAGGTTGGTGGTGCAAGCCCATCCAGGGATGCC
16013	Yes	AAGGTTGGTGGTGCAAGCCCATCCAGGGATGCCA
16014	Yes	AAGGTTGGTGGTTACA
16015	Yes	AAGGTTGGTGGTTACAG
16016	Yes	AAGGTTGGTGGTTACAGA
16017	Yes	AAGGTTGGTGGTTACAGAC
16018	Yes	AAGGTTGGTGGTTACAGACC
16019	Yes	AAGGTTGGTGGTTACAGACCA
16020	Yes	AAGGTTGGTGGTTACAGACCAC
16021	Yes	AAGGTTGGTGGTTACAGACCACC
16022	Yes	AAGGTTGGTGGTTACAGACCACCC
16023	Yes	AAGGTTGGTGGTTACAGACCACCCA
16024	Yes	AAGGTTGGTGGTTACAGACCACCCAG
16025	Yes	AAGGTTGGTGGTTACAGACCACCCAGG
16026	Yes	AAGGTTGGTGGTTACAGACCACCCAGGG
16027	Yes	AAGGTTGGTGGTTACAGACCACCCAGGGA
16028	Yes	AAGGTTGGTGGTTACAGACCACCCAGGGAC
16029	Yes	AAGGTTGGTGGTTACAGACCACCCAGGGACG
16030	Yes	AAGGTTGGTGGTTACAGACCACCCAGGGACGC
16031	Yes	AAGGTTGGTGGTTACAGACCACCCAGGGACGCC
16032	Yes	AAGGTTGGTGGTTACAGACCACCCAGGGACGCCA
16033	No	AAGGTTGGTGGTTAGA
16034	No	AAGGTTGGTGGTTAGAG
16035	Yes	AAGGTTGGTGGTTAGAGA
16036	Yes	AAGGTTGGTGGTTAGAGAC
16037	Yes	AAGGTTGGTGGTTAGAGACC

16038	Yes	AAGGTTGGTGGTTAGAGACCA
16039	Yes	AAGGTTGGTGGTTAGAGACCAC
16040	Yes	AAGGTTGGTGGTTAGAGACCACC
16041	Yes	AAGGTTGGTGGTTAGAGACCACCC
16042	Yes	AAGGTTGGTGGTTAGAGACCACCCA
16043	Yes	AAGGTTGGTGGTTAGAGACCACCCAG
16044	Yes	AAGGTTGGTGGTTAGAGACCACCCAGG
16045	Yes	AAGGTTGGTGGTTAGAGACCACCCAGGG
16046	Yes	AAGGTTGGTGGTTAGAGACCACCCAGGGA
16047	Yes	AAGGTTGGTGGTTAGAGACCACCCAGGGAC
16048	Yes	AAGGTTGGTGGTTAGAGACCACCCAGGGACG
16049	Yes	AAGGTTGGTGGTTAGAGACCACCCAGGGACGC
16050	Yes	AAGGTTGGTGGTTAGAGACCACCCAGGGACGCC
16051	Yes	AAGGTTGGTGGTTAGAGACCACCCAGGGACGCCA
16052	Yes	AAGGTTGGTGGTTCGA
16053	Yes	AAGGTTGGTGGTTCGAA
16054	Yes	AAGGTTGGTGGTTCGAAC
16055	Yes	AAGGTTGGTGGTTCGAACC
16056	Yes	AAGGTTGGTGGTTCGAACCC
16057	Yes	AAGGTTGGTGGTTCGAACCCA
16058	Yes	AAGGTTGGTGGTTCGAACCCAC
16059	Yes	AAGGTTGGTGGTTCGAACCCACC
16060	Yes	AAGGTTGGTGGTTCGAACCCACCC
16061	Yes	AAGGTTGGTGGTTCGAACCCACCCA
16062	Yes	AAGGTTGGTGGTTCGAACCCACCCAG
16063	Yes	AAGGTTGGTGGTTCGAACCCACCCAGA
16064	Yes	AAGGTTGGTGGTTCGAACCCACCCAGAG
16065	Yes	AAGGTTGGTGGTTCGAACCCACCCAGAGG
16066	Yes	AAGGTTGGTGGTTCGAACCCACCCAGAGGC
16067	Yes	AAGGTTGGTGGTTCGAACCCACCCAGAGGCG
16068	Yes	AAGGTTGGTGGTTCGAACCCACCCAGAGGCGC
16069	Yes	AAGGTTGGTGGTTCGAACCCACCCAGAGGCGCC
16070	Yes	AAGGTTGGTGGTTCGAACCCACCCAGAGGCGCCA
16071	Yes	AAGGTTGGTGGTTCGAG
16072	Yes	AAGGTTGGTGGTTCGAGC
16073	Yes	AAGGTTGGTGGTTCGAGCC
16074	Yes	AAGGTTGGTGGTTCGAGCCC
16075	Yes	AAGGTTGGTGGTTCGAGCCCA
16076	Yes	AAGGTTGGTGGTTCGAGCCCAC
16077	Yes	AAGGTTGGTGGTTCGAGCCCACC
16078	Yes	AAGGTTGGTGGTTCGAGCCCACCC
16079	Yes	AAGGTTGGTGGTTCGAGCCCACCCA

16080	Yes	AAGGTTGGTGGTTCGAGCCCACCCAG
16081	Yes	AAGGTTGGTGGTTCGAGCCCACCCAGG
16082	Yes	AAGGTTGGTGGTTCGAGCCCACCCAGGG
16083	Yes	AAGGTTGGTGGTTCGAGCCCACCCAGGGA
16084	Yes	AAGGTTGGTGGTTCGAGCCCACCCAGGGAC
16085	Yes	AAGGTTGGTGGTTCGAGCCCACCCAGGGACG
16086	Yes	AAGGTTGGTGGTTCGAGCCCACCCAGGGACGC
16087	Yes	AAGGTTGGTGGTTCGAGCCCACCCAGGGACGCC
16088	Yes	AAGGTTGGTGGTTCGAGCCCACCCAGGGACGCCA
16089	Yes	AAGGTTGGTGGTTCGAGCCCAT
16090	Yes	AAGGTTGGTGGTTCGAGCCCATC
16091	Yes	AAGGTTGGTGGTTCGAGCCCATCC
16092	Yes	AAGGTTGGTGGTTCGAGCCCATCCA
16093	Yes	AAGGTTGGTGGTTCGAGCCCATCCAG
16094	Yes	AAGGTTGGTGGTTCGAGCCCATCCAGG
16095	Yes	AAGGTTGGTGGTTCGAGCCCATCCAGGG
16096	Yes	AAGGTTGGTGGTTCGAGCCCATCCAGGGA
16097	Yes	AAGGTTGGTGGTTCGAGCCCATCCAGGGAC
16098	Yes	AAGGTTGGTGGTTCGAGCCCATCCAGGGACG
16099	Yes	AAGGTTGGTGGTTCGAGCCCATCCAGGGACGC
16100	Yes	AAGGTTGGTGGTTCGAGCCCATCCAGGGACGCC
16101	Yes	AAGGTTGGTGGTTCGAGCCCATCCAGGGACGCCA
16102	Yes	AAGGTTGGTGGTTCGAT
16103	Yes	AAGGTTGGTGGTTCGATC
16104	Yes	AAGGTTGGTGGTTCGATCC
16105	Yes	AAGGTTGGTGGTTCGATCCC
16106	Yes	AAGGTTGGTGGTTCGATCCCA
16107	Yes	AAGGTTGGTGGTTCGATCCCAC
16108	Yes	AAGGTTGGTGGTTCGATCCCACC
16109	Yes	AAGGTTGGTGGTTCGATCCCACCC
16110	Yes	AAGGTTGGTGGTTCGATCCCACCCA
16111	Yes	AAGGTTGGTGGTTCGATCCCACCCAG
16112	Yes	AAGGTTGGTGGTTCGATCCCACCCAGG
16113	Yes	AAGGTTGGTGGTTCGATCCCACCCAGGG
16114	Yes	AAGGTTGGTGGTTCGATCCCACCCAGGGA
16115	Yes	AAGGTTGGTGGTTCGATCCCACCCAGGGAC
16116	Yes	AAGGTTGGTGGTTCGATCCCACCCAGGGACG
16117	Yes	AAGGTTGGTGGTTCGATCCCACCCAGGGACGC
16118	Yes	AAGGTTGGTGGTTCGATCCCACCCAGGGACGCC
16119	Yes	AAGGTTGGTGGTTCGATCCCACCCAGGGACGCCA
16120	Yes	AAGGTTGGTGGTTCGT
16121	Yes	AAGGTTGGTGGTTCGTT

16122	Yes	AAGGTTGGTGGTTCGTTC
16123	Yes	AAGGTTGGTGGTTCGTTCC
16124	Yes	AAGGTTGGTGGTTCGTTCCC
16125	Yes	AAGGTTGGTGGTTCGTTCCCA
16126	Yes	AAGGTTGGTGGTTCGTTCCCAC
16127	Yes	AAGGTTGGTGGTTCGTTCCCACC
16128	Yes	AAGGTTGGTGGTTCGTTCCCACCC
16129	Yes	AAGGTTGGTGGTTCGTTCCCACCCC
16130	Yes	AAGGTTGGTGGTTCGTTCCCACCCCG
16131	Yes	AAGGTTGGTGGTTCGTTCCCACCCCGG
16132	Yes	AAGGTTGGTGGTTCGTTCCCACCCCGGG
16133	Yes	AAGGTTGGTGGTTCGTTCCCACCCCGGGA
16134	Yes	AAGGTTGGTGGTTCGTTCCCACCCCGGGAC
16135	Yes	AAGGTTGGTGGTTCGTTCCCACCCCGGGACA
16136	Yes	AAGGTTGGTGGTTCGTTCCCACCCCGGGACAC
16137	Yes	AAGGTTGGTGGTTCGTTCCCACCCCGGGACACC
16138	Yes	AAGGTTGGTGGTTCGTTCCCACCCCGGGACACCA
16139	Yes	AAGGTTGTGGGTTCGA
16140	Yes	AAGGTTGTGGGTTCGAA
16141	Yes	AAGGTTGTGGGTTCGAAT
16142	Yes	AAGGTTGTGGGTTCGAATC
16143	Yes	AAGGTTGTGGGTTCGAATCC
16144	Yes	AAGGTTGTGGGTTCGAATCCC
16145	Yes	AAGGTTGTGGGTTCGAATCCCA
16146	Yes	AAGGTTGTGGGTTCGAATCCCAC
16147	Yes	AAGGTTGTGGGTTCGAATCCCACC
16148	Yes	AAGGTTGTGGGTTCGAATCCCACCA
16149	Yes	AAGGTTGTGGGTTCGAATCCCACCAG
16150	Yes	AAGGTTGTGGGTTCGAATCCCACCAGA
16151	Yes	AAGGTTGTGGGTTCGAATCCCACCAGAG
16152	Yes	AAGGTTGTGGGTTCGAATCCCACCAGAGT
16153	Yes	AAGGTTGTGGGTTCGAATCCCACCAGAGTC
16154	Yes	AAGGTTGTGGGTTCGAATCCCACCAGAGTCG
16155	Yes	AAGGTTGTGGGTTCGAATCCCACCAGAGTCGC
16156	Yes	AAGGTTGTGGGTTCGAATCCCACCAGAGTCGCC
16157	Yes	AAGGTTGTGGGTTCGAATCCCACCAGAGTCGCCA
16158	Yes	AAGGTTGTGGGTTCGAG
16159	Yes	AAGGTTGTGGGTTCGAGT
16160	Yes	AAGGTTGTGGGTTCGAGTC
16161	Yes	AAGGTTGTGGGTTCGAGTCC
16162	Yes	AAGGTTGTGGGTTCGAGTCCC
16163	Yes	AAGGTTGTGGGTTCGAGTCCCA

16164	Yes	AAGGTTGTGGGTTCGAGTCCCAC
16165	Yes	AAGGTTGTGGGTTCGAGTCCCACC
16166	Yes	AAGGTTGTGGGTTCGAGTCCCACCA
16167	Yes	AAGGTTGTGGGTTCGAGTCCCACCAG
16168	Yes	AAGGTTGTGGGTTCGAGTCCCACCAGA
16169	Yes	AAGGTTGTGGGTTCGAGTCCCACCAGAG
16170	Yes	AAGGTTGTGGGTTCGAGTCCCACCAGAGT
16171	Yes	AAGGTTGTGGGTTCGAGTCCCACCAGAGTC
16172	Yes	AAGGTTGTGGGTTCGAGTCCCACCAGAGTCG
16173	Yes	AAGGTTGTGGGTTCGAGTCCCACCAGAGTCGC
16174	Yes	AAGGTTGTGGGTTCGAGTCCCACCAGAGTCGCC
16175	Yes	AAGGTTGTGGGTTCGAGTCCCACCAGAGTCGCCA
16176	No	AAGTAAGGTCAGCTAA
16177	No	AAGTAAGGTCAGCTAAA
16178	No	AAGTAAGGTCAGCTAAAT
16179	No	AAGTAAGGTCAGCTAAATA
16180	No	AAGTAAGGTCAGCTAAATAA
16181	No	AAGTAAGGTCAGCTAAATAAG
16182	No	AAGTAAGGTCAGCTAAATAAGC
16183	No	AAGTAAGGTCAGCTAAATAAGCT
16184	No	AAGTAAGGTCAGCTAAATAAGCTA
16185	No	AAGTAAGGTCAGCTAAATAAGCTAT
16186	No	AAGTAAGGTCAGCTAAATAAGCTATC
16187	No	AAGTAAGGTCAGCTAAATAAGCTATCG
16188	No	AAGTAAGGTCAGCTAAATAAGCTATCGG
16189	No	AAGTAAGGTCAGCTAAATAAGCTATCGGG
16190	No	AAGTAAGGTCAGCTAAATAAGCTATCGGGC
16191	No	AAGTAAGGTCAGCTAAATAAGCTATCGGGCC
16192	No	AAGTAAGGTCAGCTAAATAAGCTATCGGGCCC
16193	No	AAGTAAGGTCAGCTAAATAAGCTATCGGGCCCA
16194	No	AAGTAAGGTCAGCTAAATAAGCTATCGGGCCCAT
16195	No	AAGTAAGGTCAGCTAAATAAGCTATCGGGCCCATA
16196	No	AAGTAAGGTCAGCTAAATAAGCTATCGGGCCCATAC
16197	No	AAGTAAGGTCAGCTAAATAAGCTATCGGGCCCATACC
16198	No	AAGTAAGGTCAGCTAAATAAGCTATCGGGCCCATACCC
16199	No	AAGTAAGGTCAGCTAAATAAGCTATCGGGCCCATACCCC
16200	No	AAGTAAGGTCAGCTAAATAAGCTATCGGGCCCATACCCCG
16201	No	AAGTAAGGTCAGCTAAATAAGCTATCGGGCCCATACCCCGA
16202	No	AAGTAAGGTCAGCTAAATAAGCTATCGGGCCCATACCCCGAA
16203	No	AAGTAAGGTCAGCTAAATAAGCTATCGGGCCCATACCCCGAAA
16204	No	AAGTAAGGTCAGCTAAATAAGCTATCGGGCCCATACCCCGAAAA
16205	No	AAGTAAGGTCAGCTAAATAAGCTATCGGGCCCATACCCCGAAAAT

16206	No	AAGTAAGGTCAGCTAAATAAGCTATCGGGCCCATACCCCGAAAATG
16207	No	AAGTAAGGTCAGCTAAATAAGCTATCGGGCCCATACCCCGAAAATGT
16208	No	AAGTAAGGTCAGCTAAATAAGCTATCGGGCCCATACCCCGAAAATGTT
16209	No	AAGTAAGGTCAGCTAAATAAGCTATCGGGCCCATACCCCGAAAATGTTG
16210	No	AAGTAAGGTCAGCTAAATAAGCTATCGGGCCCATACCCCGAAAATGTTGG
16211	No	AAGTATCTCTGCCTGT
16212	No	AAGTATCTCTGCCTGTC
16213	Yes	AAGTATCTCTGCCTGTCA
16214	Yes	AAGTATCTCTGCCTGTCAT
16215	Yes	AAGTATCTCTGCCTGTCATG
16216	Yes	AAGTATCTCTGCCTGTCATGC
16217	Yes	AAGTATCTCTGCCTGTCATGCA
16218	Yes	AAGTATCTCTGCCTGTCATGCAT
16219	Yes	AAGTATCTCTGCCTGTCATGCATG
16220	Yes	AAGTATCTCTGCCTGTCATGCATGA
16221	Yes	AAGTATCTCTGCCTGTCATGCATGAG
16222	Yes	AAGTATCTCTGCCTGTCATGCATGAGA
16223	Yes	AAGTATCTCTGCCTGTCATGCATGAGAG
16224	Yes	AAGTATCTCTGCCTGTCATGCATGAGAGA
16225	Yes	AAGTATCTCTGCCTGTCATGCATGAGAGAG
16226	Yes	AAGTATCTCTGCCTGTCATGCATGAGAGAGG
16227	Yes	AAGTATCTCTGCCTGTCATGCATGAGAGAGGG
16228	Yes	AAGTATCTCTGCCTGTCATGCATGAGAGAGGGG
16229	Yes	AAGTATCTCTGCCTGTCATGCATGAGAGAGGGGG
16230	Yes	AAGTATCTCTGCCTGTCATGCATGAGAGAGGGGGT
16231	Yes	AAGTATCTCTGCCTGTCATGCATGAGAGAGGGGGTC
16232	Yes	AAGTATCTCTGCCTGTCATGCATGAGAGAGGGGGTCG
16233	Yes	AAGTATCTCTGCCTGTCATGCATGAGAGAGGGGGTCGA
16234	Yes	AAGTATCTCTGCCTGTCATGCATGAGAGAGGGGGTCGAT
16235	Yes	AAGTATCTCTGCCTGTCATGCATGAGAGAGGGGGTCGATT
16236	Yes	AAGTATCTCTGCCTGTCATGCATGAGAGAGGGGGTCGATTC
16237	Yes	AAGTATCTCTGCCTGTCATGCATGAGAGAGGGGGTCGATTCC
16238	Yes	AAGTATCTCTGCCTGTCATGCATGAGAGAGGGGGTCGATTCCC
16239	Yes	AAGTATCTCTGCCTGTCATGCATGAGAGAGGGGGTCGATTCCCT
16240	Yes	AAGTATCTCTGCCTGTCATGCATGAGAGAGGGGGTCGATTCCCTG
16241	Yes	AAGTATCTCTGCCTGTCATGCATGAGAGAGGGGGTCGATTCCCTGA
16242	Yes	AAGTATCTCTGCCTGTCATGCATGAGAGAGGGGGTCGATTCCCTGAC
16243	Yes	AAGTATCTCTGCCTGTCATGCATGAGAGAGGGGGTCGATTCCCTGACG
16244	Yes	AAGTATCTCTGCCTGTCATGCATGAGAGAGGGGGTCGATTCCCTGACGG
16245	Yes	AAGTATCTCTGCCTGTCATGCATGAGAGAGGGGGTCGATTCCCTGACGGG
16246	Yes	AAGTCACGTCGGGGTC
16247	Yes	AAGTCACGTCGGGGTCA

16248	Yes	AAGTCACGTCGGGGTCAC
16249	Yes	AAGTCACGTCGGGGTCACC
16250	Yes	AAGTCACGTCGGGGTCACCA
16251	No	AAGTCAGAGATACTTG
16252	No	AAGTCAGAGATACTTGA
16253	No	AAGTCAGAGATACTTGAA
16254	Yes	AAGTCAGAGATACTTGAAT
16255	Yes	AAGTCAGAGATACTTGAATT
16256	Yes	AAGTCAGAGATACTTGAATTT
16257	Yes	AAGTCAGAGATACTTGAATTTG
16258	Yes	AAGTCAGAGATACTTGAATTTGA
16259	Yes	AAGTCAGAGATACTTGAATTTGAA
16260	Yes	AAGTCAGAGATACTTGAATTTGAAT
16261	Yes	AAGTCAGAGATACTTGAATTTGAATG
16262	Yes	AAGTCAGAGATACTTGAATTTGAATGC
16263	Yes	AAGTCAGAGATACTTGAATTTGAATGCT
16264	Yes	AAGTCAGAGATACTTGAATTTGAATGCTG
16265	Yes	AAGTCAGAGATACTTGAATTTGAATGCTGG
16266	Yes	AAGTCAGAGATACTTGAATTTGAATGCTGGT
16267	Yes	AAGTCAGAGATACTTGAATTTGAATGCTGGTT
16268	Yes	AAGTCAGAGATACTTGAATTTGAATGCTGGTTC
16269	Yes	AAGTCAGAGATACTTGAATTTGAATGCTGGTTCT
16270	Yes	AAGTCAGAGATACTTGAATTTGAATGCTGGTTCTG
16271	Yes	AAGTCAGAGATACTTGAATTTGAATGCTGGTTCTGT
16272	Yes	AAGTCAGAGATACTTGAATTTGAATGCTGGTTCTGTC
16273	Yes	AAGTCAGAGATACTTGAATTTGAATGCTGGTTCTGTCA
16274	Yes	AAGTCAGAGATACTTGAATTTGAATGCTGGTTCTGTCAC
16275	Yes	AAGTCAGAGATACTTGAATTTGAATGCTGGTTCTGTCACC
16276	Yes	AAGTCAGAGATACTTGAATTTGAATGCTGGTTCTGTCACCA
16277	No	AAGTCATAGGGGTTAT
16278	Yes	AAGTCATAGGGGTTATG
16279	Yes	AAGTCATAGGGGTTATGA
16280	Yes	AAGTCATAGGGGTTATGAG
16281	Yes	AAGTCATAGGGGTTATGAGG
16282	Yes	AAGTCATAGGGGTTATGAGGC
16283	Yes	AAGTCATAGGGGTTATGAGGCT
16284	Yes	AAGTCATAGGGGTTATGAGGCTG
16285	Yes	AAGTCATAGGGGTTATGAGGCTGG
16286	Yes	AAGTCATAGGGGTTATGAGGCTGGC
16287	Yes	AAGTCATAGGGGTTATGAGGCTGGCT
16288	Yes	AAGTCATAGGGGTTATGAGGCTGGCTT
16289	Yes	AAGTCATAGGGGTTATGAGGCTGGCTTG

16290	Yes	AAGTCATAGGGGTTATGAGGCTGGCTTGA
16291	Yes	AAGTCATAGGGGTTATGAGGCTGGCTTGAA
16292	Yes	AAGTCATAGGGGTTATGAGGCTGGCTTGAAA
16293	Yes	AAGTCATAGGGGTTATGAGGCTGGCTTGAAAC
16294	Yes	AAGTCATAGGGGTTATGAGGCTGGCTTGAAACC
16295	Yes	AAGTCATAGGGGTTATGAGGCTGGCTTGAAACCA
16296	Yes	AAGTCATAGGGGTTATGAGGCTGGCTTGAAACCAG
16297	Yes	AAGTCATAGGGGTTATGAGGCTGGCTTGAAACCAGC
16298	Yes	AAGTCATAGGGGTTATGAGGCTGGCTTGAAACCAGCC
16299	Yes	AAGTCATAGGGGTTATGAGGCTGGCTTGAAACCAGCCT
16300	Yes	AAGTCATAGGGGTTATGAGGCTGGCTTGAAACCAGCCTT
16301	Yes	AAGTCATAGGGGTTATGAGGCTGGCTTGAAACCAGCCTTA
16302	Yes	AAGTCATAGGGGTTATGAGGCTGGCTTGAAACCAGCCTTAG
16303	Yes	AAGTCATAGGGGTTATGAGGCTGGCTTGAAACCAGCCTTAGG
16304	Yes	AAGTCATAGGGGTTATGAGGCTGGCTTGAAACCAGCCTTAGGA
16305	Yes	AAGTCATAGGGGTTATGAGGCTGGCTTGAAACCAGCCTTAGGAG
16306	Yes	AAGTCATAGGGGTTATGAGGCTGGCTTGAAACCAGCCTTAGGAGG
16307	Yes	AAGTCATAGGGGTTATGAGGCTGGCTTGAAACCAGCCTTAGGAGGT
16308	Yes	AAGTCATAGGGGTTATGAGGCTGGCTTGAAACCAGCCTTAGGAGGTT
16309	Yes	AAGTCATAGGGGTTATGAGGCTGGCTTGAAACCAGCCTTAGGAGGTTC
16310	Yes	AAGTCATAGGGGTTATGAGGCTGGCTTGAAACCAGCCTTAGGAGGTTCA
16311	Yes	AAGTCATAGGGGTTATGAGGCTGGCTTGAAACCAGCCTTAGGAGGTTCAA
16312	No	AAGTCATGGAGGCCAT
16313	Yes	AAGTCATGGAGGCCATG
16314	Yes	AAGTCATGGAGGCCATGG
16315	Yes	AAGTCATGGAGGCCATGGG
16316	Yes	AAGTCATGGAGGCCATGGGG
16317	Yes	AAGTCATGGAGGCCATGGGGT
16318	Yes	AAGTCATGGAGGCCATGGGGTT
16319	Yes	AAGTCATGGAGGCCATGGGGTTG
16320	Yes	AAGTCATGGAGGCCATGGGGTTGG
16321	Yes	AAGTCATGGAGGCCATGGGGTTGGC
16322	Yes	AAGTCATGGAGGCCATGGGGTTGGCT
16323	Yes	AAGTCATGGAGGCCATGGGGTTGGCTT
16324	Yes	AAGTCATGGAGGCCATGGGGTTGGCTTG
16325	Yes	AAGTCATGGAGGCCATGGGGTTGGCTTGA
16326	Yes	AAGTCATGGAGGCCATGGGGTTGGCTTGAA
16327	Yes	AAGTCATGGAGGCCATGGGGTTGGCTTGAAA
16328	Yes	AAGTCATGGAGGCCATGGGGTTGGCTTGAAAC
16329	Yes	AAGTCATGGAGGCCATGGGGTTGGCTTGAAACC
16330	Yes	AAGTCATGGAGGCCATGGGGTTGGCTTGAAACCA
16331	Yes	AAGTCATGGAGGCCATGGGGTTGGCTTGAAACCAG

16332	Yes	AAGTCATGGAGGCCATGGGGTTGGCTTGAAACCAGC
16333	Yes	AAGTCATGGAGGCCATGGGGTTGGCTTGAAACCAGCT
16334	Yes	AAGTCATGGAGGCCATGGGGTTGGCTTGAAACCAGCTT
16335	Yes	AAGTCATGGAGGCCATGGGGTTGGCTTGAAACCAGCTTT
16336	Yes	AAGTCATGGAGGCCATGGGGTTGGCTTGAAACCAGCTTTG
16337	Yes	AAGTCATGGAGGCCATGGGGTTGGCTTGAAACCAGCTTTGG
16338	Yes	AAGTCATGGAGGCCATGGGGTTGGCTTGAAACCAGCTTTGGG
16339	Yes	AAGTCATGGAGGCCATGGGGTTGGCTTGAAACCAGCTTTGGGG
16340	Yes	AAGTCATGGAGGCCATGGGGTTGGCTTGAAACCAGCTTTGGGGG
16341	Yes	AAGTCATGGAGGCCATGGGGTTGGCTTGAAACCAGCTTTGGGGGG
16342	Yes	AAGTCATGGAGGCCATGGGGTTGGCTTGAAACCAGCTTTGGGGGGT
16343	Yes	AAGTCATGGAGGCCATGGGGTTGGCTTGAAACCAGCTTTGGGGGGTT
16344	Yes	AAGTCATGGAGGCCATGGGGTTGGCTTGAAACCAGCTTTGGGGGGTTC
16345	Yes	AAGTCATGGAGGCCATGGGGTTGGCTTGAAACCAGCTTTGGGGGGTTCG
16346	Yes	AAGTCATGGAGGCCATGGGGTTGGCTTGAAACCAGCTTTGGGGGGTTCGA
16347	No	AAGTCCAGATGGGATG
16348	No	AAGTCCAGATGGGATGG
16349	No	AAGTCCAGATGGGATGGT
16350	Yes	AAGTCCAGATGGGATGGTC
16351	Yes	AAGTCCAGATGGGATGGTCC
16352	Yes	AAGTCCAGATGGGATGGTCCT
16353	Yes	AAGTCCAGATGGGATGGTCCTG
16354	Yes	AAGTCCAGATGGGATGGTCCTGG
16355	Yes	AAGTCCAGATGGGATGGTCCTGGG
16356	Yes	AAGTCCAGATGGGATGGTCCTGGGT
16357	Yes	AAGTCCAGATGGGATGGTCCTGGGTT
16358	Yes	AAGTCCAGATGGGATGGTCCTGGGTTC
16359	Yes	AAGTCCAGATGGGATGGTCCTGGGTTCA
16360	Yes	AAGTCCAGATGGGATGGTCCTGGGTTCAA
16361	Yes	AAGTCCAGATGGGATGGTCCTGGGTTCAAA
16362	Yes	AAGTCCAGATGGGATGGTCCTGGGTTCAAAC
16363	Yes	AAGTCCAGATGGGATGGTCCTGGGTTCAAACC
16364	Yes	AAGTCCAGATGGGATGGTCCTGGGTTCAAACCC
16365	Yes	AAGTCCAGATGGGATGGTCCTGGGTTCAAACCCC
16366	Yes	AAGTCCAGATGGGATGGTCCTGGGTTCAAACCCCA
16367	Yes	AAGTCCAGATGGGATGGTCCTGGGTTCAAACCCCAG
16368	Yes	AAGTCCAGATGGGATGGTCCTGGGTTCAAACCCCAGC
16369	Yes	AAGTCCAGATGGGATGGTCCTGGGTTCAAACCCCAGCT
16370	Yes	AAGTCCAGATGGGATGGTCCTGGGTTCAAACCCCAGCTG
16371	Yes	AAGTCCAGATGGGATGGTCCTGGGTTCAAACCCCAGCTGC
16372	Yes	AAGTCCAGATGGGATGGTCCTGGGTTCAAACCCCAGCTGCG
16373	Yes	AAGTCCAGATGGGATGGTCCTGGGTTCAAACCCCAGCTGCGC

16374	Yes	AAGTCCAGATGGGATGGTCCTGGGTTCAAACCCCAGCTGCGCC
16375	Yes	AAGTCCAGATGGGATGGTCCTGGGTTCAAACCCCAGCTGCGCCC
16376	Yes	AAGTCCAGATGGGATGGTCCTGGGTTCAAACCCCAGCTGCGCCCC
16377	Yes	AAGTCCAGATGGGATGGTCCTGGGTTCAAACCCCAGCTGCGCCCCC
16378	Yes	AAGTCCAGATGGGATGGTCCTGGGTTCAAACCCCAGCTGCGCCCCCA
16379	No	AAGTCCATGTCCAGGC
16380	No	AAGTCCATGTCCAGGCA
16381	No	AAGTCCATGTCCAGGCAC
16382	Yes	AAGTCCATGTCCAGGCACC
16383	Yes	AAGTCCATGTCCAGGCACCA
16384	No	AAGTCCCAGTGTGCAC
16385	Yes	AAGTCCCAGTGTGCACA
16386	Yes	AAGTCCCAGTGTGCACAC
16387	Yes	AAGTCCCAGTGTGCACACC
16388	Yes	AAGTCCCAGTGTGCACACCA
16389	No	AAGTCCCATTGGTCTA
16390	No	AAGTCCCATTGGTCTAG
16391	Yes	AAGTCCCATTGGTCTAGC
16392	Yes	AAGTCCCATTGGTCTAGCC
16393	Yes	AAGTCCCATTGGTCTAGCCA
16394	Yes	AAGTCCCCGGTTCAAA
16395	Yes	AAGTCCCCGGTTCAAAT
16396	Yes	AAGTCCCCGGTTCAAATC
16397	Yes	AAGTCCCCGGTTCAAATCC
16398	Yes	AAGTCCCCGGTTCAAATCCG
16399	Yes	AAGTCCCCGGTTCAAATCCGG
16400	Yes	AAGTCCCCGGTTCAAATCCGGG
16401	Yes	AAGTCCCCGGTTCAAATCCGGGT
16402	Yes	AAGTCCCCGGTTCAAATCCGGGTG
16403	Yes	AAGTCCCCGGTTCAAATCCGGGTGC
16404	Yes	AAGTCCCCGGTTCAAATCCGGGTGCC
16405	Yes	AAGTCCCCGGTTCAAATCCGGGTGCCC
16406	Yes	AAGTCCCCGGTTCAAATCCGGGTGCCCC
16407	Yes	AAGTCCCCGGTTCAAATCCGGGTGCCCCC
16408	Yes	AAGTCCCCGGTTCAAATCCGGGTGCCCCCT
16409	Yes	AAGTCCCCGGTTCAAATCCGGGTGCCCCCTC
16410	Yes	AAGTCCCCGGTTCAAATCCGGGTGCCCCCTCC
16411	Yes	AAGTCCCCGGTTCAAATCCGGGTGCCCCCTCCA
16412	Yes	AAGTCCCCGGTTCGAA
16413	Yes	AAGTCCCCGGTTCGAAA
16414	Yes	AAGTCCCCGGTTCGAAAT
16415	Yes	AAGTCCCCGGTTCGAAATC

16416	Yes	AAGTCCCCGGTTCGAAATCA
16417	Yes	AAGTCCCCGGTTCGAAATCAG
16418	Yes	AAGTCCCCGGTTCGAAATCAGG
16419	Yes	AAGTCCCCGGTTCGAAATCAGGC
16420	Yes	AAGTCCCCGGTTCGAAATCAGGCG
16421	Yes	AAGTCCCCGGTTCGAAATCAGGCGG
16422	Yes	AAGTCCCCGGTTCGAAATCAGGCGGG
16423	Yes	AAGTCCCCGGTTCGAAATCAGGCGGGA
16424	Yes	AAGTCCCCGGTTCGAAATCAGGCGGGAA
16425	Yes	AAGTCCCCGGTTCGAAATCAGGCGGGAAC
16426	Yes	AAGTCCCCGGTTCGAAATCAGGCGGGAACA
16427	Yes	AAGTCCCCGGTTCGAAATCAGGCGGGAACAC
16428	Yes	AAGTCCCCGGTTCGAAATCAGGCGGGAACACC
16429	Yes	AAGTCCCCGGTTCGAAATCAGGCGGGAACACCA
16430	Yes	AAGTCCCTGATTCGAG
16431	Yes	AAGTCCCTGATTCGAGA
16432	Yes	AAGTCCCTGATTCGAGAC
16433	Yes	AAGTCCCTGATTCGAGACT
16434	Yes	AAGTCCCTGATTCGAGACTG
16435	Yes	AAGTCCCTGATTCGAGACTGG
16436	Yes	AAGTCCCTGATTCGAGACTGGG
16437	Yes	AAGTCCCTGATTCGAGACTGGGT
16438	Yes	AAGTCCCTGATTCGAGACTGGGTG
16439	Yes	AAGTCCCTGATTCGAGACTGGGTGG
16440	Yes	AAGTCCCTGATTCGAGACTGGGTGGG
16441	Yes	AAGTCCCTGATTCGAGACTGGGTGGGA
16442	Yes	AAGTCCCTGATTCGAGACTGGGTGGGAA
16443	Yes	AAGTCCCTGATTCGAGACTGGGTGGGAAC
16444	Yes	AAGTCCCTGATTCGAGACTGGGTGGGAACG
16445	Yes	AAGTCCCTGATTCGAGACTGGGTGGGAACGC
16446	Yes	AAGTCCCTGATTCGAGACTGGGTGGGAACGCC
16447	Yes	AAGTCCCTGATTCGAGACTGGGTGGGAACGCCA
16448	No	AAGTCCCTGTCCAGGC
16449	Yes	AAGTCCCTGTCCAGGCG
16450	Yes	AAGTCCCTGTCCAGGCGC
16451	Yes	AAGTCCCTGTCCAGGCGCC
16452	Yes	AAGTCCCTGTCCAGGCGCCA
16453	No	AAGTCCCTGTTCAGGC
16454	No	AAGTCCCTGTTCAGGCA
16455	No	AAGTCCCTGTTCAGGCAC
16456	No	AAGTCCCTGTTCAGGCACC
16457	No	AAGTCCCTGTTCAGGCACCA

16458	No	AAGTCCCTGTTCAGGCG
16459	No	AAGTCCCTGTTCAGGCGC
16460	No	AAGTCCCTGTTCAGGCGCC
16461	No	AAGTCCCTGTTCAGGCGCCA
16462	No	AAGTCCCTGTTCGGGC
16463	Yes	AAGTCCCTGTTCGGGCG
16464	Yes	AAGTCCCTGTTCGGGCGC
16465	Yes	AAGTCCCTGTTCGGGCGCC
16466	Yes	AAGTCCCTGTTCGGGCGCCA
16467	No	AAGTCCCTGTTTGGGT
16468	Yes	AAGTCCCTGTTTGGGTG
16469	Yes	AAGTCCCTGTTTGGGTGC
16470	Yes	AAGTCCCTGTTTGGGTGCC
16471	Yes	AAGTCCCTGTTTGGGTGCCA
16472	Yes	AAGTCCTCGTTCGGGC
16473	Yes	AAGTCCTCGTTCGGGCA
16474	Yes	AAGTCCTCGTTCGGGCAC
16475	Yes	AAGTCCTCGTTCGGGCACC
16476	Yes	AAGTCCTCGTTCGGGCACCA
16477	No	AAGTCCTGAGTTCAAG
16478	No	AAGTCCTGAGTTCAAGC
16479	Yes	AAGTCCTGAGTTCAAGCC
16480	Yes	AAGTCCTGAGTTCAAGCCT
16481	Yes	AAGTCCTGAGTTCAAGCCTC
16482	Yes	AAGTCCTGAGTTCAAGCCTCA
16483	Yes	AAGTCCTGAGTTCAAGCCTCAG
16484	Yes	AAGTCCTGAGTTCAAGCCTCAGA
16485	Yes	AAGTCCTGAGTTCAAGCCTCAGAG
16486	Yes	AAGTCCTGAGTTCAAGCCTCAGAGA
16487	Yes	AAGTCCTGAGTTCAAGCCTCAGAGAG
16488	Yes	AAGTCCTGAGTTCAAGCCTCAGAGAGG
16489	Yes	AAGTCCTGAGTTCAAGCCTCAGAGAGGG
16490	Yes	AAGTCCTGAGTTCAAGCCTCAGAGAGGGC
16491	Yes	AAGTCCTGAGTTCAAGCCTCAGAGAGGGCA
16492	Yes	AAGTCCTGAGTTCAAGCCTCAGAGAGGGCAC
16493	Yes	AAGTCCTGAGTTCAAGCCTCAGAGAGGGCACC
16494	Yes	AAGTCCTGAGTTCAAGCCTCAGAGAGGGCACCA
16495	No	AAGTCCTTGGTTCAAA
16496	No	AAGTCCTTGGTTCAAAT
16497	No	AAGTCCTTGGTTCAAATC
16498	No	AAGTCCTTGGTTCAAATCC
16499	Yes	AAGTCCTTGGTTCAAATCCA

16500	Yes	AAGTCCTTGGTTCAAATCCAG
16501	Yes	AAGTCCTTGGTTCAAATCCAGG
16502	Yes	AAGTCCTTGGTTCAAATCCAGGT
16503	Yes	AAGTCCTTGGTTCAAATCCAGGTG
16504	Yes	AAGTCCTTGGTTCAAATCCAGGTGC
16505	Yes	AAGTCCTTGGTTCAAATCCAGGTGCC
16506	Yes	AAGTCCTTGGTTCAAATCCAGGTGCCC
16507	Yes	AAGTCCTTGGTTCAAATCCAGGTGCCCC
16508	Yes	AAGTCCTTGGTTCAAATCCAGGTGCCCCC
16509	Yes	AAGTCCTTGGTTCAAATCCAGGTGCCCCCT
16510	Yes	AAGTCCTTGGTTCAAATCCAGGTGCCCCCTC
16511	Yes	AAGTCCTTGGTTCAAATCCAGGTGCCCCCTCC
16512	Yes	AAGTCCTTGGTTCAAATCCAGGTGCCCCCTCCA
16513	Yes	AAGTCCTTTCATGGTC
16514	Yes	AAGTCCTTTCATGGTCA
16515	Yes	AAGTCCTTTCATGGTCAC
16516	Yes	AAGTCCTTTCATGGTCACC
16517	Yes	AAGTCCTTTCATGGTCACCA
16518	Yes	AAGTCTCACTGGAACC
16519	Yes	AAGTCTCACTGGAACCT
16520	Yes	AAGTCTCACTGGAACCTC
16521	Yes	AAGTCTCACTGGAACCTCC
16522	Yes	AAGTCTCACTGGAACCTCCA
16523	Yes	AAGTCTCACTGGGACC
16524	Yes	AAGTCTCACTGGGACCT
16525	Yes	AAGTCTCACTGGGACCTC
16526	Yes	AAGTCTCACTGGGACCTCC
16527	Yes	AAGTCTCACTGGGACCTCCA
16528	No	AAGTCTCAGCTGTGCT
16529	No	AAGTCTCAGCTGTGCTA
16530	Yes	AAGTCTCAGCTGTGCTAC
16531	Yes	AAGTCTCAGCTGTGCTACC
16532	Yes	AAGTCTCAGCTGTGCTACCA
16533	No	AAGTCTCAGTGGGACC
16534	No	AAGTCTCAGTGGGACCT
16535	Yes	AAGTCTCAGTGGGACCTC
16536	Yes	AAGTCTCAGTGGGACCTCC
16537	Yes	AAGTCTCAGTGGGACCTCCA
16538	No	AAGTCTCCAGTCTCTT
16539	No	AAGTCTCCAGTCTCTTT
16540	No	AAGTCTCCAGTCTCTTTG
16541	Yes	AAGTCTCCAGTCTCTTTGG

16542	Yes	AAGTCTCCAGTCTCTTTGGG
16543	Yes	AAGTCTCCAGTCTCTTTGGGG
16544	Yes	AAGTCTCCAGTCTCTTTGGGGG
16545	Yes	AAGTCTCCAGTCTCTTTGGGGGC
16546	Yes	AAGTCTCCAGTCTCTTTGGGGGCG
16547	Yes	AAGTCTCCAGTCTCTTTGGGGGCGT
16548	Yes	AAGTCTCCAGTCTCTTTGGGGGCGTG
16549	Yes	AAGTCTCCAGTCTCTTTGGGGGCGTGG
16550	Yes	AAGTCTCCAGTCTCTTTGGGGGCGTGGT
16551	Yes	AAGTCTCCAGTCTCTTTGGGGGCGTGGTT
16552	Yes	AAGTCTCCAGTCTCTTTGGGGGCGTGGTTT
16553	Yes	AAGTCTCCAGTCTCTTTGGGGGCGTGGTTTT
16554	Yes	AAGTCTCCAGTCTCTTTGGGGGCGTGGTTTTC
16555	Yes	AAGTCTCCAGTCTCTTTGGGGGCGTGGTTTTCA
16556	Yes	AAGTCTCCAGTCTCTTTGGGGGCGTGGTTTTCAA
16557	Yes	AAGTCTCCAGTCTCTTTGGGGGCGTGGTTTTCAAT
16558	Yes	AAGTCTCCAGTCTCTTTGGGGGCGTGGTTTTCAATC
16559	Yes	AAGTCTCCAGTCTCTTTGGGGGCGTGGTTTTCAATCC
16560	Yes	AAGTCTCCAGTCTCTTTGGGGGCGTGGTTTTCAATCCC
16561	Yes	AAGTCTCCAGTCTCTTTGGGGGCGTGGTTTTCAATCCCA
16562	Yes	AAGTCTCCAGTCTCTTTGGGGGCGTGGTTTTCAATCCCAC
16563	Yes	AAGTCTCCAGTCTCTTTGGGGGCGTGGTTTTCAATCCCACC
16564	Yes	AAGTCTCCAGTCTCTTTGGGGGCGTGGTTTTCAATCCCACCG
16565	Yes	AAGTCTCCAGTCTCTTTGGGGGCGTGGTTTTCAATCCCACCGC
16566	Yes	AAGTCTCCAGTCTCTTTGGGGGCGTGGTTTTCAATCCCACCGCT
16567	Yes	AAGTCTCCAGTCTCTTTGGGGGCGTGGTTTTCAATCCCACCGCTG
16568	Yes	AAGTCTCCAGTCTCTTTGGGGGCGTGGTTTTCAATCCCACCGCTGC
16569	Yes	AAGTCTCCAGTCTCTTTGGGGGCGTGGTTTTCAATCCCACCGCTGCT
16570	Yes	AAGTCTCCAGTCTCTTTGGGGGCGTGGTTTTCAATCCCACCGCTGCTA
16571	Yes	AAGTCTCCAGTCTCTTTGGGGGCGTGGTTTTCAATCCCACCGCTGCTAC
16572	Yes	AAGTCTCCAGTCTCTTTGGGGGCGTGGTTTTCAATCCCACCGCTGCTACC
16573	Yes	AAGTCTCGGTGGAACC
16574	Yes	AAGTCTCGGTGGAACCT
16575	Yes	AAGTCTCGGTGGAACCTC
16576	Yes	AAGTCTCGGTGGAACCTCC
16577	Yes	AAGTCTCGGTGGAACCTCCA
16578	No	AAGTCTCTGTTTGGGC
16579	Yes	AAGTCTCTGTTTGGGCG
16580	Yes	AAGTCTCTGTTTGGGCGC
16581	Yes	AAGTCTCTGTTTGGGCGCC
16582	Yes	AAGTCTCTGTTTGGGCGCCA
16583	Yes	AAGTGAAGAGGCTGTA

16584	Yes	AAGTGAAGAGGCTGTAA
16585	Yes	AAGTGAAGAGGCTGTAAA
16586	Yes	AAGTGAAGAGGCTGTAAAC
16587	Yes	AAGTGAAGAGGCTGTAAACC
16588	Yes	AAGTGAAGAGGCTGTAAACCC
16589	Yes	AAGTGAAGAGGCTGTAAACCCA
16590	Yes	AAGTGAAGAGGCTGTAAACCCAG
16591	Yes	AAGTGAAGAGGCTGTAAACCCAGA
16592	Yes	AAGTGAAGAGGCTGTAAACCCAGAC
16593	Yes	AAGTGAAGAGGCTGTAAACCCAGACT
16594	Yes	AAGTGAAGAGGCTGTAAACCCAGACTG
16595	Yes	AAGTGAAGAGGCTGTAAACCCAGACTGG
16596	Yes	AAGTGAAGAGGCTGTAAACCCAGACTGGA
16597	Yes	AAGTGAAGAGGCTGTAAACCCAGACTGGAT
16598	Yes	AAGTGAAGAGGCTGTAAACCCAGACTGGATG
16599	Yes	AAGTGAAGAGGCTGTAAACCCAGACTGGATGG
16600	Yes	AAGTGAAGAGGCTGTAAACCCAGACTGGATGGG
16601	Yes	AAGTGAAGAGGCTGTAAACCCAGACTGGATGGGT
16602	Yes	AAGTGAAGAGGCTGTAAACCCAGACTGGATGGGTT
16603	Yes	AAGTGAAGAGGCTGTAAACCCAGACTGGATGGGTTC
16604	Yes	AAGTGAAGAGGCTGTAAACCCAGACTGGATGGGTTCA
16605	Yes	AAGTGAAGAGGCTGTAAACCCAGACTGGATGGGTTCAA
16606	Yes	AAGTGAAGAGGCTGTAAACCCAGACTGGATGGGTTCAAT
16607	Yes	AAGTGAAGAGGCTGTAAACCCAGACTGGATGGGTTCAATT
16608	Yes	AAGTGAAGAGGCTGTAAACCCAGACTGGATGGGTTCAATTC
16609	Yes	AAGTGAAGAGGCTGTAAACCCAGACTGGATGGGTTCAATTCC
16610	Yes	AAGTGAAGAGGCTGTAAACCCAGACTGGATGGGTTCAATTCCC
16611	Yes	AAGTGAAGAGGCTGTAAACCCAGACTGGATGGGTTCAATTCCCA
16612	Yes	AAGTGAAGAGGCTGTAAACCCAGACTGGATGGGTTCAATTCCCAT
16613	Yes	AAGTGAAGAGGCTGTAAACCCAGACTGGATGGGTTCAATTCCCATC
16614	Yes	AAGTGAAGAGGCTGTAAACCCAGACTGGATGGGTTCAATTCCCATCT
16615	Yes	AAGTGAAGAGGCTGTAAACCCAGACTGGATGGGTTCAATTCCCATCTC
16616	Yes	AAGTGAAGAGGCTGTAAACCCAGACTGGATGGGTTCAATTCCCATCTCT
16617	Yes	AAGTGAAGAGGCTGTAAACCCAGACTGGATGGGTTCAATTCCCATCTCTG
16618	No	AAGTGCACAGGCTTTG
16619	No	AAGTGCACAGGCTTTGG
16620	No	AAGTGCACAGGCTTTGGA
16621	No	AAGTGCACAGGCTTTGGAA
16622	No	AAGTGCACAGGCTTTGGAAA
16623	No	AAGTGCACAGGCTTTGGAAAC
16624	Yes	AAGTGCACAGGCTTTGGAAACT
16625	Yes	AAGTGCACAGGCTTTGGAAACTG

16626	Yes	AAGTGCACAGGCTTTGGAAACTGG
16627	Yes	AAGTGCACAGGCTTTGGAAACTGGC
16628	Yes	AAGTGCACAGGCTTTGGAAACTGGCA
16629	Yes	AAGTGCACAGGCTTTGGAAACTGGCAG
16630	Yes	AAGTGCACAGGCTTTGGAAACTGGCAGG
16631	Yes	AAGTGCACAGGCTTTGGAAACTGGCAGGC
16632	Yes	AAGTGCACAGGCTTTGGAAACTGGCAGGCC
16633	Yes	AAGTGCACAGGCTTTGGAAACTGGCAGGCCT
16634	Yes	AAGTGCACAGGCTTTGGAAACTGGCAGGCCTG
16635	Yes	AAGTGCACAGGCTTTGGAAACTGGCAGGCCTGG
16636	Yes	AAGTGCACAGGCTTTGGAAACTGGCAGGCCTGGG
16637	Yes	AAGTGCACAGGCTTTGGAAACTGGCAGGCCTGGGT
16638	Yes	AAGTGCACAGGCTTTGGAAACTGGCAGGCCTGGGTT
16639	Yes	AAGTGCACAGGCTTTGGAAACTGGCAGGCCTGGGTTC
16640	Yes	AAGTGCACAGGCTTTGGAAACTGGCAGGCCTGGGTTCA
16641	Yes	AAGTGCACAGGCTTTGGAAACTGGCAGGCCTGGGTTCAA
16642	Yes	AAGTGCACAGGCTTTGGAAACTGGCAGGCCTGGGTTCAAA
16643	Yes	AAGTGCACAGGCTTTGGAAACTGGCAGGCCTGGGTTCAAAT
16644	Yes	AAGTGCACAGGCTTTGGAAACTGGCAGGCCTGGGTTCAAATC
16645	Yes	AAGTGCACAGGCTTTGGAAACTGGCAGGCCTGGGTTCAAATCC
16646	Yes	AAGTGCACAGGCTTTGGAAACTGGCAGGCCTGGGTTCAAATCCC
16647	Yes	AAGTGCACAGGCTTTGGAAACTGGCAGGCCTGGGTTCAAATCCCA
16648	Yes	AAGTGCACAGGCTTTGGAAACTGGCAGGCCTGGGTTCAAATCCCAG
16649	Yes	AAGTGCACAGGCTTTGGAAACTGGCAGGCCTGGGTTCAAATCCCAGC
16650	Yes	AAGTGCACAGGCTTTGGAAACTGGCAGGCCTGGGTTCAAATCCCAGCT
16651	Yes	AAGTGCACAGGCTTTGGAAACTGGCAGGCCTGGGTTCAAATCCCAGCTT
16652	Yes	AAGTGCACAGGCTTTGGAAACTGGCAGGCCTGGGTTCAAATCCCAGCTTA
16653	No	AAGTGGCTAGGATTTG
16654	No	AAGTGGCTAGGATTTGG
16655	Yes	AAGTGGCTAGGATTTGGC
16656	Yes	AAGTGGCTAGGATTTGGCG
16657	Yes	AAGTGGCTAGGATTTGGCGC
16658	Yes	AAGTGGCTAGGATTTGGCGCT
16659	Yes	AAGTGGCTAGGATTTGGCGCTT
16660	Yes	AAGTGGCTAGGATTTGGCGCTTT
16661	Yes	AAGTGGCTAGGATTTGGCGCTTTC
16662	Yes	AAGTGGCTAGGATTTGGCGCTTTCA
16663	Yes	AAGTGGCTAGGATTTGGCGCTTTCAC
16664	Yes	AAGTGGCTAGGATTTGGCGCTTTCACT
16665	Yes	AAGTGGCTAGGATTTGGCGCTTTCACTG
16666	Yes	AAGTGGCTAGGATTTGGCGCTTTCACTGC
16667	Yes	AAGTGGCTAGGATTTGGCGCTTTCACTGCC

16668	Yes	AAGTGGCTAGGATTTGGCGCTTTCACTGCCG
16669	Yes	AAGTGGCTAGGATTTGGCGCTTTCACTGCCGC
16670	Yes	AAGTGGCTAGGATTTGGCGCTTTCACTGCCGCG
16671	Yes	AAGTGGCTAGGATTTGGCGCTTTCACTGCCGCGG
16672	Yes	AAGTGGCTAGGATTTGGCGCTTTCACTGCCGCGGC
16673	Yes	AAGTGGCTAGGATTTGGCGCTTTCACTGCCGCGGCC
16674	Yes	AAGTGGCTAGGATTTGGCGCTTTCACTGCCGCGGCCC
16675	Yes	AAGTGGCTAGGATTTGGCGCTTTCACTGCCGCGGCCCG
16676	Yes	AAGTGGCTAGGATTTGGCGCTTTCACTGCCGCGGCCCGC
16677	Yes	AAGTGGCTAGGATTTGGCGCTTTCACTGCCGCGGCCCGCG
16678	Yes	AAGTGGCTAGGATTTGGCGCTTTCACTGCCGCGGCCCGCGT
16679	Yes	AAGTGGCTAGGATTTGGCGCTTTCACTGCCGCGGCCCGCGTT
16680	Yes	AAGTGGCTAGGATTTGGCGCTTTCACTGCCGCGGCCCGCGTTC
16681	Yes	AAGTGGCTAGGATTTGGCGCTTTCACTGCCGCGGCCCGCGTTCG
16682	Yes	AAGTGGCTAGGATTTGGCGCTTTCACTGCCGCGGCCCGCGTTCGA
16683	Yes	AAGTGGCTAGGATTTGGCGCTTTCACTGCCGCGGCCCGCGTTCGAT
16684	Yes	AAGTGGCTAGGATTTGGCGCTTTCACTGCCGCGGCCCGCGTTCGATT
16685	Yes	AAGTGGCTAGGATTTGGCGCTTTCACTGCCGCGGCCCGCGTTCGATTC
16686	Yes	AAGTGGCTAGGATTTGGCGCTTTCACTGCCGCGGCCCGCGTTCGATTCC
16687	Yes	AAGTGGCTAGGATTTGGCGCTTTCACTGCCGCGGCCCGCGTTCGATTCCC
16688	No	AAGTGGCTGATTTGCG
16689	No	AAGTGGCTGATTTGCGT
16690	No	AAGTGGCTGATTTGCGTT
16691	No	AAGTGGCTGATTTGCGTTC
16692	No	AAGTGGCTGATTTGCGTTCA
16693	No	AAGTGGCTGATTTGCGTTCAG
16694	No	AAGTGGCTGATTTGCGTTCAGT
16695	No	AAGTGGCTGATTTGCGTTCAGTT
16696	No	AAGTGGCTGATTTGCGTTCAGTTG
16697	No	AAGTGGCTGATTTGCGTTCAGTTGA
16698	No	AAGTGGCTGATTTGCGTTCAGTTGAT
16699	No	AAGTGGCTGATTTGCGTTCAGTTGATG
16700	No	AAGTGGCTGATTTGCGTTCAGTTGATGC
16701	No	AAGTGGCTGATTTGCGTTCAGTTGATGCA
16702	No	AAGTGGCTGATTTGCGTTCAGTTGATGCAG
16703	No	AAGTGGCTGATTTGCGTTCAGTTGATGCAGA
16704	No	AAGTGGCTGATTTGCGTTCAGTTGATGCAGAG
16705	No	AAGTGGCTGATTTGCGTTCAGTTGATGCAGAGT
16706	Yes	AAGTGGCTGATTTGCGTTCAGTTGATGCAGAGTG
16707	Yes	AAGTGGCTGATTTGCGTTCAGTTGATGCAGAGTGG
16708	Yes	AAGTGGCTGATTTGCGTTCAGTTGATGCAGAGTGGG
16709	Yes	AAGTGGCTGATTTGCGTTCAGTTGATGCAGAGTGGGG

16710	Yes	AAGTGGCTGATTTGCGTTCAGTTGATGCAGAGTGGGGT
16711	Yes	AAGTGGCTGATTTGCGTTCAGTTGATGCAGAGTGGGGTT
16712	Yes	AAGTGGCTGATTTGCGTTCAGTTGATGCAGAGTGGGGTTT
16713	Yes	AAGTGGCTGATTTGCGTTCAGTTGATGCAGAGTGGGGTTTT
16714	Yes	AAGTGGCTGATTTGCGTTCAGTTGATGCAGAGTGGGGTTTTG
16715	Yes	AAGTGGCTGATTTGCGTTCAGTTGATGCAGAGTGGGGTTTTGC
16716	Yes	AAGTGGCTGATTTGCGTTCAGTTGATGCAGAGTGGGGTTTTGCA
16717	Yes	AAGTGGCTGATTTGCGTTCAGTTGATGCAGAGTGGGGTTTTGCAG
16718	Yes	AAGTGGCTGATTTGCGTTCAGTTGATGCAGAGTGGGGTTTTGCAGT
16719	Yes	AAGTGGCTGATTTGCGTTCAGTTGATGCAGAGTGGGGTTTTGCAGTC
16720	Yes	AAGTGGCTGATTTGCGTTCAGTTGATGCAGAGTGGGGTTTTGCAGTCC
16721	Yes	AAGTGGCTGATTTGCGTTCAGTTGATGCAGAGTGGGGTTTTGCAGTCCT
16722	Yes	AAGTGGCTGATTTGCGTTCAGTTGATGCAGAGTGGGGTTTTGCAGTCCTT
16723	Yes	AAGTGGTAAGGCGTCG
16724	Yes	AAGTGGTAAGGCGTCGG
16725	Yes	AAGTGGTAAGGCGTCGGT
16726	Yes	AAGTGGTAAGGCGTCGGTC
16727	Yes	AAGTGGTAAGGCGTCGGTCT
16728	Yes	AAGTGGTAAGGCGTCGGTCTC
16729	Yes	AAGTGGTAAGGCGTCGGTCTCG
16730	Yes	AAGTGGTAAGGCGTCGGTCTCGT
16731	Yes	AAGTGGTAAGGCGTCGGTCTCGTA
16732	Yes	AAGTGGTAAGGCGTCGGTCTCGTAA
16733	Yes	AAGTGGTAAGGCGTCGGTCTCGTAAA
16734	Yes	AAGTGGTAAGGCGTCGGTCTCGTAAAC
16735	Yes	AAGTGGTAAGGCGTCGGTCTCGTAAACC
16736	Yes	AAGTGGTAAGGCGTCGGTCTCGTAAACCG
16737	Yes	AAGTGGTAAGGCGTCGGTCTCGTAAACCGA
16738	Yes	AAGTGGTAAGGCGTCGGTCTCGTAAACCGAA
16739	Yes	AAGTGGTAAGGCGTCGGTCTCGTAAACCGAAG
16740	Yes	AAGTGGTAAGGCGTCGGTCTCGTAAACCGAAGA
16741	Yes	AAGTGGTAAGGCGTCGGTCTCGTAAACCGAAGAT
16742	Yes	AAGTGGTAAGGCGTCGGTCTCGTAAACCGAAGATC
16743	Yes	AAGTGGTAAGGCGTCGGTCTCGTAAACCGAAGATCA
16744	Yes	AAGTGGTAAGGCGTCGGTCTCGTAAACCGAAGATCAC
16745	Yes	AAGTGGTAAGGCGTCGGTCTCGTAAACCGAAGATCACG
16746	Yes	AAGTGGTAAGGCGTCGGTCTCGTAAACCGAAGATCACGG
16747	Yes	AAGTGGTAAGGCGTCGGTCTCGTAAACCGAAGATCACGGG
16748	Yes	AAGTGGTAAGGCGTCGGTCTCGTAAACCGAAGATCACGGGT
16749	Yes	AAGTGGTAAGGCGTCGGTCTCGTAAACCGAAGATCACGGGTT
16750	Yes	AAGTGGTAAGGCGTCGGTCTCGTAAACCGAAGATCACGGGTTC
16751	Yes	AAGTGGTAAGGCGTCGGTCTCGTAAACCGAAGATCACGGGTTCG

16752	Yes	AAGTGGTAAGGCGTCGGTCTCGTAAACCGAAGATCACGGGTTCGA
16753	Yes	AAGTGGTAAGGCGTCGGTCTCGTAAACCGAAGATCACGGGTTCGAA
16754	Yes	AAGTGGTAAGGCGTCGGTCTCGTAAACCGAAGATCACGGGTTCGAAC
16755	Yes	AAGTGGTAAGGCGTCGGTCTCGTAAACCGAAGATCACGGGTTCGAACC
16756	Yes	AAGTGGTAAGGCGTCGGTCTCGTAAACCGAAGATCACGGGTTCGAACCC
16757	Yes	AAGTGGTAAGGCGTCGGTCTCGTAAACCGAAGATCACGGGTTCGAACCCC
16758	Yes	AAGTGGTAAGGCGTCGGTCTCGTAAACCGAAGATCG
16759	Yes	AAGTGGTAAGGCGTCGGTCTCGTAAACCGAAGATCGC
16760	Yes	AAGTGGTAAGGCGTCGGTCTCGTAAACCGAAGATCGCG
16761	Yes	AAGTGGTAAGGCGTCGGTCTCGTAAACCGAAGATCGCGG
16762	Yes	AAGTGGTAAGGCGTCGGTCTCGTAAACCGAAGATCGCGGG
16763	Yes	AAGTGGTAAGGCGTCGGTCTCGTAAACCGAAGATCGCGGGT
16764	Yes	AAGTGGTAAGGCGTCGGTCTCGTAAACCGAAGATCGCGGGTT
16765	Yes	AAGTGGTAAGGCGTCGGTCTCGTAAACCGAAGATCGCGGGTTC
16766	Yes	AAGTGGTAAGGCGTCGGTCTCGTAAACCGAAGATCGCGGGTTCG
16767	Yes	AAGTGGTAAGGCGTCGGTCTCGTAAACCGAAGATCGCGGGTTCGA
16768	Yes	AAGTGGTAAGGCGTCGGTCTCGTAAACCGAAGATCGCGGGTTCGAA
16769	Yes	AAGTGGTAAGGCGTCGGTCTCGTAAACCGAAGATCGCGGGTTCGAAC
16770	Yes	AAGTGGTAAGGCGTCGGTCTCGTAAACCGAAGATCGCGGGTTCGAACC
16771	Yes	AAGTGGTAAGGCGTCGGTCTCGTAAACCGAAGATCGCGGGTTCGAACCC
16772	Yes	AAGTGGTAAGGCGTCGGTCTCGTAAACCGAAGATCGCGGGTTCGAACCCC
16773	Yes	AAGTGGTAGAGCGCTC
16774	Yes	AAGTGGTAGAGCGCTCG
16775	Yes	AAGTGGTAGAGCGCTCGC
16776	Yes	AAGTGGTAGAGCGCTCGCT
16777	Yes	AAGTGGTAGAGCGCTCGCTT
16778	Yes	AAGTGGTAGAGCGCTCGCTTA
16779	Yes	AAGTGGTAGAGCGCTCGCTTAG
16780	Yes	AAGTGGTAGAGCGCTCGCTTAGC
16781	Yes	AAGTGGTAGAGCGCTCGCTTAGCA
16782	Yes	AAGTGGTAGAGCGCTCGCTTAGCAT
16783	Yes	AAGTGGTAGAGCGCTCGCTTAGCATG
16784	Yes	AAGTGGTAGAGCGCTCGCTTAGCATGC
16785	Yes	AAGTGGTAGAGCGCTCGCTTAGCATGCG
16786	Yes	AAGTGGTAGAGCGCTCGCTTAGCATGCGA
16787	Yes	AAGTGGTAGAGCGCTCGCTTAGCATGCGAG
16788	Yes	AAGTGGTAGAGCGCTCGCTTAGCATGCGAGA
16789	Yes	AAGTGGTAGAGCGCTCGCTTAGCATGCGAGAG
16790	Yes	AAGTGGTAGAGCGCTCGCTTAGCATGCGAGAGG
16791	Yes	AAGTGGTAGAGCGCTCGCTTAGCATGCGAGAGGT
16792	Yes	AAGTGGTAGAGCGCTCGCTTAGCATGCGAGAGGTA
16793	Yes	AAGTGGTAGAGCGCTCGCTTAGCATGCGAGAGGTAG

16794	Yes	AAGTGGTAGAGCGCTCGCTTAGCATGCGAGAGGTAGT
16795	Yes	AAGTGGTAGAGCGCTCGCTTAGCATGCGAGAGGTAGTG
16796	Yes	AAGTGGTAGAGCGCTCGCTTAGCATGCGAGAGGTAGTGG
16797	Yes	AAGTGGTAGAGCGCTCGCTTAGCATGCGAGAGGTAGTGGG
16798	Yes	AAGTGGTAGAGCGCTCGCTTAGCATGCGAGAGGTAGTGGGA
16799	Yes	AAGTGGTAGAGCGCTCGCTTAGCATGCGAGAGGTAGTGGGAT
16800	Yes	AAGTGGTAGAGCGCTCGCTTAGCATGCGAGAGGTAGTGGGATC
16801	Yes	AAGTGGTAGAGCGCTCGCTTAGCATGCGAGAGGTAGTGGGATCG
16802	Yes	AAGTGGTAGAGCGCTCGCTTAGCATGCGAGAGGTAGTGGGATCGA
16803	Yes	AAGTGGTAGAGCGCTCGCTTAGCATGCGAGAGGTAGTGGGATCGAT
16804	Yes	AAGTGGTAGAGCGCTCGCTTAGCATGCGAGAGGTAGTGGGATCGATG
16805	Yes	AAGTGGTAGAGCGCTCGCTTAGCATGCGAGAGGTAGTGGGATCGATGC
16806	Yes	AAGTGGTAGAGCGCTCGCTTAGCATGCGAGAGGTAGTGGGATCGATGCC
16807	Yes	AAGTGGTAGAGCGCTCGCTTAGCATGCGAGAGGTAGTGGGATCGATGCCC
16808	Yes	AAGTGGTAGAGCGCTT
16809	Yes	AAGTGGTAGAGCGCTTG
16810	Yes	AAGTGGTAGAGCGCTTGC
16811	Yes	AAGTGGTAGAGCGCTTGCT
16812	Yes	AAGTGGTAGAGCGCTTGCTT
16813	Yes	AAGTGGTAGAGCGCTTGCTTA
16814	Yes	AAGTGGTAGAGCGCTTGCTTAG
16815	Yes	AAGTGGTAGAGCGCTTGCTTAGC
16816	Yes	AAGTGGTAGAGCGCTTGCTTAGCA
16817	Yes	AAGTGGTAGAGCGCTTGCTTAGCAC
16818	Yes	AAGTGGTAGAGCGCTTGCTTAGCACG
16819	Yes	AAGTGGTAGAGCGCTTGCTTAGCACGC
16820	Yes	AAGTGGTAGAGCGCTTGCTTAGCACGCA
16821	Yes	AAGTGGTAGAGCGCTTGCTTAGCACGCAA
16822	Yes	AAGTGGTAGAGCGCTTGCTTAGCACGCAAG
16823	Yes	AAGTGGTAGAGCGCTTGCTTAGCACGCAAGA
16824	Yes	AAGTGGTAGAGCGCTTGCTTAGCACGCAAGAG
16825	Yes	AAGTGGTAGAGCGCTTGCTTAGCACGCAAGAGG
16826	Yes	AAGTGGTAGAGCGCTTGCTTAGCACGCAAGAGGT
16827	Yes	AAGTGGTAGAGCGCTTGCTTAGCACGCAAGAGGTA
16828	Yes	AAGTGGTAGAGCGCTTGCTTAGCACGCAAGAGGTAG
16829	Yes	AAGTGGTAGAGCGCTTGCTTAGCACGCAAGAGGTAGT
16830	Yes	AAGTGGTAGAGCGCTTGCTTAGCACGCAAGAGGTAGTG
16831	Yes	AAGTGGTAGAGCGCTTGCTTAGCACGCAAGAGGTAGTGG
16832	Yes	AAGTGGTAGAGCGCTTGCTTAGCACGCAAGAGGTAGTGGG
16833	Yes	AAGTGGTAGAGCGCTTGCTTAGCACGCAAGAGGTAGTGGGA
16834	Yes	AAGTGGTAGAGCGCTTGCTTAGCACGCAAGAGGTAGTGGGAT
16835	Yes	AAGTGGTAGAGCGCTTGCTTAGCACGCAAGAGGTAGTGGGATC

16836	Yes	AAGTGGTAGAGCGCTTGCTTAGCACGCAAGAGGTAGTGGGATCG
16837	Yes	AAGTGGTAGAGCGCTTGCTTAGCACGCAAGAGGTAGTGGGATCGA
16838	Yes	AAGTGGTAGAGCGCTTGCTTAGCACGCAAGAGGTAGTGGGATCGAT
16839	Yes	AAGTGGTAGAGCGCTTGCTTAGCACGCAAGAGGTAGTGGGATCGATG
16840	Yes	AAGTGGTAGAGCGCTTGCTTAGCACGCAAGAGGTAGTGGGATCGATGC
16841	Yes	AAGTGGTAGAGCGCTTGCTTAGCACGCAAGAGGTAGTGGGATCGATGCC
16842	Yes	AAGTGGTAGAGCGCTTGCTTAGCACGCAAGAGGTAGTGGGATCGATGCCC
16843	Yes	AAGTGGTAGAGCGCTTGCTTAGCAT
16844	Yes	AAGTGGTAGAGCGCTTGCTTAGCATG
16845	Yes	AAGTGGTAGAGCGCTTGCTTAGCATGC
16846	Yes	AAGTGGTAGAGCGCTTGCTTAGCATGCA
16847	Yes	AAGTGGTAGAGCGCTTGCTTAGCATGCAA
16848	Yes	AAGTGGTAGAGCGCTTGCTTAGCATGCAAG
16849	Yes	AAGTGGTAGAGCGCTTGCTTAGCATGCAAGA
16850	Yes	AAGTGGTAGAGCGCTTGCTTAGCATGCAAGAG
16851	Yes	AAGTGGTAGAGCGCTTGCTTAGCATGCAAGAGG
16852	Yes	AAGTGGTAGAGCGCTTGCTTAGCATGCAAGAGGT
16853	Yes	AAGTGGTAGAGCGCTTGCTTAGCATGCAAGAGGTA
16854	Yes	AAGTGGTAGAGCGCTTGCTTAGCATGCAAGAGGTAG
16855	Yes	AAGTGGTAGAGCGCTTGCTTAGCATGCAAGAGGTAGT
16856	Yes	AAGTGGTAGAGCGCTTGCTTAGCATGCAAGAGGTAGTG
16857	Yes	AAGTGGTAGAGCGCTTGCTTAGCATGCAAGAGGTAGTGG
16858	Yes	AAGTGGTAGAGCGCTTGCTTAGCATGCAAGAGGTAGTGGG
16859	Yes	AAGTGGTAGAGCGCTTGCTTAGCATGCAAGAGGTAGTGGGA
16860	Yes	AAGTGGTAGAGCGCTTGCTTAGCATGCAAGAGGTAGTGGGAT
16861	Yes	AAGTGGTAGAGCGCTTGCTTAGCATGCAAGAGGTAGTGGGATC
16862	Yes	AAGTGGTAGAGCGCTTGCTTAGCATGCAAGAGGTAGTGGGATCA
16863	Yes	AAGTGGTAGAGCGCTTGCTTAGCATGCAAGAGGTAGTGGGATCAA
16864	Yes	AAGTGGTAGAGCGCTTGCTTAGCATGCAAGAGGTAGTGGGATCAAT
16865	Yes	AAGTGGTAGAGCGCTTGCTTAGCATGCAAGAGGTAGTGGGATCAATG
16866	Yes	AAGTGGTAGAGCGCTTGCTTAGCATGCAAGAGGTAGTGGGATCAATGC
16867	Yes	AAGTGGTAGAGCGCTTGCTTAGCATGCAAGAGGTAGTGGGATCAATGCC
16868	Yes	AAGTGGTAGAGCGCTTGCTTAGCATGCAAGAGGTAGTGGGATCAATGCCC
16869	Yes	AAGTGGTAGAGCGCTTGCTTAGCATGCAAGAGGTAGTGGGATCG
16870	Yes	AAGTGGTAGAGCGCTTGCTTAGCATGCAAGAGGTAGTGGGATCGA
16871	Yes	AAGTGGTAGAGCGCTTGCTTAGCATGCAAGAGGTAGTGGGATCGAT
16872	Yes	AAGTGGTAGAGCGCTTGCTTAGCATGCAAGAGGTAGTGGGATCGATG
16873	Yes	AAGTGGTAGAGCGCTTGCTTAGCATGCAAGAGGTAGTGGGATCGATGC
16874	Yes	AAGTGGTAGAGCGCTTGCTTAGCATGCAAGAGGTAGTGGGATCGATGCC
16875	Yes	AAGTGGTAGAGCGCTTGCTTAGCATGCAAGAGGTAGTGGGATCGATGCCC
16876	No	AAGTGGTAGAGTGCTT
16877	No	AAGTGGTAGAGTGCTTG

16878	No	AAGTGGTAGAGTGCTTGC
16879	No	AAGTGGTAGAGTGCTTGCT
16880	No	AAGTGGTAGAGTGCTTGCTT
16881	No	AAGTGGTAGAGTGCTTGCTTA
16882	Yes	AAGTGGTAGAGTGCTTGCTTAG
16883	Yes	AAGTGGTAGAGTGCTTGCTTAGC
16884	Yes	AAGTGGTAGAGTGCTTGCTTAGCA
16885	Yes	AAGTGGTAGAGTGCTTGCTTAGCAT
16886	Yes	AAGTGGTAGAGTGCTTGCTTAGCATG
16887	Yes	AAGTGGTAGAGTGCTTGCTTAGCATGC
16888	Yes	AAGTGGTAGAGTGCTTGCTTAGCATGCA
16889	Yes	AAGTGGTAGAGTGCTTGCTTAGCATGCAA
16890	Yes	AAGTGGTAGAGTGCTTGCTTAGCATGCAAG
16891	Yes	AAGTGGTAGAGTGCTTGCTTAGCATGCAAGA
16892	Yes	AAGTGGTAGAGTGCTTGCTTAGCATGCAAGAG
16893	Yes	AAGTGGTAGAGTGCTTGCTTAGCATGCAAGAGG
16894	Yes	AAGTGGTAGAGTGCTTGCTTAGCATGCAAGAGGT
16895	Yes	AAGTGGTAGAGTGCTTGCTTAGCATGCAAGAGGTA
16896	Yes	AAGTGGTAGAGTGCTTGCTTAGCATGCAAGAGGTAG
16897	Yes	AAGTGGTAGAGTGCTTGCTTAGCATGCAAGAGGTAGT
16898	Yes	AAGTGGTAGAGTGCTTGCTTAGCATGCAAGAGGTAGTG
16899	Yes	AAGTGGTAGAGTGCTTGCTTAGCATGCAAGAGGTAGTGG
16900	Yes	AAGTGGTAGAGTGCTTGCTTAGCATGCAAGAGGTAGTGGG
16901	Yes	AAGTGGTAGAGTGCTTGCTTAGCATGCAAGAGGTAGTGGGA
16902	Yes	AAGTGGTAGAGTGCTTGCTTAGCATGCAAGAGGTAGTGGGAT
16903	Yes	AAGTGGTAGAGTGCTTGCTTAGCATGCAAGAGGTAGTGGGATC
16904	Yes	AAGTGGTAGAGTGCTTGCTTAGCATGCAAGAGGTAGTGGGATCG
16905	Yes	AAGTGGTAGAGTGCTTGCTTAGCATGCAAGAGGTAGTGGGATCGA
16906	Yes	AAGTGGTAGAGTGCTTGCTTAGCATGCAAGAGGTAGTGGGATCGAT
16907	Yes	AAGTGGTAGAGTGCTTGCTTAGCATGCAAGAGGTAGTGGGATCGATG
16908	Yes	AAGTGGTAGAGTGCTTGCTTAGCATGCAAGAGGTAGTGGGATCGATGC
16909	Yes	AAGTGGTAGAGTGCTTGCTTAGCATGCAAGAGGTAGTGGGATCGATGCC
16910	Yes	AAGTGGTAGAGTGCTTGCTTAGCATGCAAGAGGTAGTGGGATCGATGCCC
16911	No	AAGTGTTTGTGGGTTT
16912	No	AAGTGTTTGTGGGTTTA
16913	No	AAGTGTTTGTGGGTTTAA
16914	Yes	AAGTGTTTGTGGGTTTAAG
16915	Yes	AAGTGTTTGTGGGTTTAAGT
16916	Yes	AAGTGTTTGTGGGTTTAAGTC
16917	Yes	AAGTGTTTGTGGGTTTAAGTCC
16918	Yes	AAGTGTTTGTGGGTTTAAGTCCC
16919	Yes	AAGTGTTTGTGGGTTTAAGTCCCA

16920	Yes	AAGTGTTTGTGGGTTTAAGTCCCAT
16921	Yes	AAGTGTTTGTGGGTTTAAGTCCCATT
16922	Yes	AAGTGTTTGTGGGTTTAAGTCCCATTG
16923	Yes	AAGTGTTTGTGGGTTTAAGTCCCATTGG
16924	Yes	AAGTGTTTGTGGGTTTAAGTCCCATTGGT
16925	Yes	AAGTGTTTGTGGGTTTAAGTCCCATTGGTC
16926	Yes	AAGTGTTTGTGGGTTTAAGTCCCATTGGTCT
16927	Yes	AAGTGTTTGTGGGTTTAAGTCCCATTGGTCTA
16928	Yes	AAGTGTTTGTGGGTTTAAGTCCCATTGGTCTAG
16929	Yes	AAGTGTTTGTGGGTTTAAGTCCCATTGGTCTAGC
16930	Yes	AAGTGTTTGTGGGTTTAAGTCCCATTGGTCTAGCC
16931	Yes	AAGTGTTTGTGGGTTTAAGTCCCATTGGTCTAGCCA
16932	No	AAGTTAAAGATTAAGA
16933	No	AAGTTAAAGATTAAGAG
16934	No	AAGTTAAAGATTAAGAGA
16935	No	AAGTTAAAGATTAAGAGAA
16936	No	AAGTTAAAGATTAAGAGAAC
16937	No	AAGTTAAAGATTAAGAGAACC
16938	No	AAGTTAAAGATTAAGAGAACCA
16939	No	AAGTTAAAGATTAAGAGAACCAA
16940	No	AAGTTAAAGATTAAGAGAACCAAC
16941	No	AAGTTAAAGATTAAGAGAACCAACA
16942	No	AAGTTAAAGATTAAGAGAACCAACAC
16943	No	AAGTTAAAGATTAAGAGAACCAACACC
16944	No	AAGTTAAAGATTAAGAGAACCAACACCT
16945	No	AAGTTAAAGATTAAGAGAACCAACACCTC
16946	No	AAGTTAAAGATTAAGAGAACCAACACCTCT
16947	No	AAGTTAAAGATTAAGAGAACCAACACCTCTT
16948	No	AAGTTAAAGATTAAGAGAACCAACACCTCTTT
16949	No	AAGTTAAAGATTAAGAGAACCAACACCTCTTTA
16950	No	AAGTTAAAGATTAAGAGAACCAACACCTCTTTAC
16951	No	AAGTTAAAGATTAAGAGAACCAACACCTCTTTACA
16952	No	AAGTTAAAGATTAAGAGAACCAACACCTCTTTACAG
16953	No	AAGTTAAAGATTAAGAGAACCAACACCTCTTTACAGT
16954	No	AAGTTAAAGATTAAGAGAACCAACACCTCTTTACAGTG
16955	No	AAGTTAAAGATTAAGAGAACCAACACCTCTTTACAGTGA
16956	Yes	AAGTTAAAGATTAAGAGAACCAACACCTCTTTACAGTGAC
16957	Yes	AAGTTAAAGATTAAGAGAACCAACACCTCTTTACAGTGACC
16958	Yes	AAGTTAAAGATTAAGAGAACCAACACCTCTTTACAGTGACCA
16959	No	AAGTTAAATTATAGGC
16960	No	AAGTTAAATTATAGGCT
16961	No	AAGTTAAATTATAGGCTA

16962	No	AAGTTAAATTATAGGCTAA
16963	No	AAGTTAAATTATAGGCTAAA
16964	No	AAGTTAAATTATAGGCTAAAT
16965	No	AAGTTAAATTATAGGCTAAATC
16966	No	AAGTTAAATTATAGGCTAAATCC
16967	No	AAGTTAAATTATAGGCTAAATCCT
16968	No	AAGTTAAATTATAGGCTAAATCCTA
16969	No	AAGTTAAATTATAGGCTAAATCCTAT
16970	No	AAGTTAAATTATAGGCTAAATCCTATA
16971	No	AAGTTAAATTATAGGCTAAATCCTATAT
16972	No	AAGTTAAATTATAGGCTAAATCCTATATA
16973	No	AAGTTAAATTATAGGCTAAATCCTATATAT
16974	No	AAGTTAAATTATAGGCTAAATCCTATATATC
16975	No	AAGTTAAATTATAGGCTAAATCCTATATATCT
16976	No	AAGTTAAATTATAGGCTAAATCCTATATATCTT
16977	No	AAGTTAAATTATAGGCTAAATCCTATATATCTTA
16978	Yes	AAGTTAAATTATAGGCTAAATCCTATATATCTTAC
16979	Yes	AAGTTAAATTATAGGCTAAATCCTATATATCTTACC
16980	Yes	AAGTTAAATTATAGGCTAAATCCTATATATCTTACCA
16981	No	AAGTTAGATACACCTG
16982	Yes	AAGTTAGATACACCTGG
16983	Yes	AAGTTAGATACACCTGGG
16984	Yes	AAGTTAGATACACCTGGGT
16985	Yes	AAGTTAGATACACCTGGGTT
16986	Yes	AAGTTAGATACACCTGGGTTC
16987	Yes	AAGTTAGATACACCTGGGTTCA
16988	Yes	AAGTTAGATACACCTGGGTTCAA
16989	Yes	AAGTTAGATACACCTGGGTTCAAA
16990	Yes	AAGTTAGATACACCTGGGTTCAAAT
16991	Yes	AAGTTAGATACACCTGGGTTCAAATC
16992	Yes	AAGTTAGATACACCTGGGTTCAAATCC
16993	Yes	AAGTTAGATACACCTGGGTTCAAATCCC
16994	Yes	AAGTTAGATACACCTGGGTTCAAATCCCA
16995	Yes	AAGTTAGATACACCTGGGTTCAAATCCCAG
16996	Yes	AAGTTAGATACACCTGGGTTCAAATCCCAGT
16997	Yes	AAGTTAGATACACCTGGGTTCAAATCCCAGTG
16998	Yes	AAGTTAGATACACCTGGGTTCAAATCCCAGTGC
16999	Yes	AAGTTAGATACACCTGGGTTCAAATCCCAGTGCT
17000	Yes	AAGTTAGATACACCTGGGTTCAAATCCCAGTGCTG
17001	Yes	AAGTTAGATACACCTGGGTTCAAATCCCAGTGCTGT
17002	Yes	AAGTTAGATACACCTGGGTTCAAATCCCAGTGCTGTC
17003	Yes	AAGTTAGATACACCTGGGTTCAAATCCCAGTGCTGTCA

17004	Yes	AAGTTAGATACACCTGGGTTCAAATCCCAGTGCTGTCAC
17005	Yes	AAGTTAGATACACCTGGGTTCAAATCCCAGTGCTGTCACC
17006	Yes	AAGTTAGATACACCTGGGTTCAAATCCCAGTGCTGTCACCA
17007	No	AAGTTCAACACCTCTT
17008	No	AAGTTCAACACCTCTTC
17009	Yes	AAGTTCAACACCTCTTCT
17010	Yes	AAGTTCAACACCTCTTCTT
17011	Yes	AAGTTCAACACCTCTTCTTA
17012	Yes	AAGTTCAACACCTCTTCTTAA
17013	Yes	AAGTTCAACACCTCTTCTTAAC
17014	Yes	AAGTTCAACACCTCTTCTTAACA
17015	Yes	AAGTTCAACACCTCTTCTTAACAC
17016	Yes	AAGTTCAACACCTCTTCTTAACACC
17017	Yes	AAGTTCAACACCTCTTCTTAACACCA
17018	No	AAGTTCCCTGGTTCAA
17019	Yes	AAGTTCCCTGGTTCAAC
17020	Yes	AAGTTCCCTGGTTCAACC
17021	Yes	AAGTTCCCTGGTTCAACCC
17022	Yes	AAGTTCCCTGGTTCAACCCT
17023	Yes	AAGTTCCCTGGTTCAACCCTG
17024	Yes	AAGTTCCCTGGTTCAACCCTGG
17025	Yes	AAGTTCCCTGGTTCAACCCTGGG
17026	Yes	AAGTTCCCTGGTTCAACCCTGGGT
17027	Yes	AAGTTCCCTGGTTCAACCCTGGGTT
17028	Yes	AAGTTCCCTGGTTCAACCCTGGGTTT
17029	Yes	AAGTTCCCTGGTTCAACCCTGGGTTTC
17030	Yes	AAGTTCCCTGGTTCAACCCTGGGTTTCA
17031	Yes	AAGTTCCCTGGTTCAACCCTGGGTTTCAG
17032	Yes	AAGTTCCCTGGTTCAACCCTGGGTTTCAGC
17033	Yes	AAGTTCCCTGGTTCAACCCTGGGTTTCAGCC
17034	Yes	AAGTTCCCTGGTTCAACCCTGGGTTTCAGCCC
17035	Yes	AAGTTCCCTGGTTCAACCCTGGGTTTCAGCCCC
17036	Yes	AAGTTCCCTGGTTCAACCCTGGGTTTCAGCCCCA
17037	No	AAGTTCCTTGGCTCAA
17038	No	AAGTTCCTTGGCTCAAT
17039	Yes	AAGTTCCTTGGCTCAATC
17040	Yes	AAGTTCCTTGGCTCAATCC
17041	Yes	AAGTTCCTTGGCTCAATCCA
17042	Yes	AAGTTCCTTGGCTCAATCCAG
17043	Yes	AAGTTCCTTGGCTCAATCCAGA
17044	Yes	AAGTTCCTTGGCTCAATCCAGAG
17045	Yes	AAGTTCCTTGGCTCAATCCAGAGT

17046	Yes	AAGTTCCTTGGCTCAATCCAGAGTT
17047	Yes	AAGTTCCTTGGCTCAATCCAGAGTTT
17048	Yes	AAGTTCCTTGGCTCAATCCAGAGTTTG
17049	Yes	AAGTTCCTTGGCTCAATCCAGAGTTTGG
17050	Yes	AAGTTCCTTGGCTCAATCCAGAGTTTGGG
17051	Yes	AAGTTCCTTGGCTCAATCCAGAGTTTGGGG
17052	Yes	AAGTTCCTTGGCTCAATCCAGAGTTTGGGGG
17053	Yes	AAGTTCCTTGGCTCAATCCAGAGTTTGGGGGC
17054	Yes	AAGTTCCTTGGCTCAATCCAGAGTTTGGGGGCC
17055	Yes	AAGTTCCTTGGCTCAATCCAGAGTTTGGGGGCCA
17056	No	AAGTTCTCTGGTTGGA
17057	Yes	AAGTTCTCTGGTTGGAT
17058	Yes	AAGTTCTCTGGTTGGATA
17059	Yes	AAGTTCTCTGGTTGGATAC
17060	Yes	AAGTTCTCTGGTTGGATACC
17061	Yes	AAGTTCTCTGGTTGGATACCA
17062	Yes	AAGTTCTCTGGTTGGATACCAG
17063	Yes	AAGTTCTCTGGTTGGATACCAGA
17064	Yes	AAGTTCTCTGGTTGGATACCAGAT
17065	Yes	AAGTTCTCTGGTTGGATACCAGATG
17066	Yes	AAGTTCTCTGGTTGGATACCAGATGG
17067	Yes	AAGTTCTCTGGTTGGATACCAGATGGA
17068	Yes	AAGTTCTCTGGTTGGATACCAGATGGAA
17069	Yes	AAGTTCTCTGGTTGGATACCAGATGGAAA
17070	Yes	AAGTTCTCTGGTTGGATACCAGATGGAAAT
17071	Yes	AAGTTCTCTGGTTGGATACCAGATGGAAATG
17072	Yes	AAGTTCTCTGGTTGGATACCAGATGGAAATGC
17073	Yes	AAGTTCTCTGGTTGGATACCAGATGGAAATGCC
17074	Yes	AAGTTCTCTGGTTGGATACCAGATGGAAATGCCA
17075	No	AAGTTCTGGTCTCCAA
17076	Yes	AAGTTCTGGTCTCCAAT
17077	Yes	AAGTTCTGGTCTCCAATG
17078	Yes	AAGTTCTGGTCTCCAATGG
17079	Yes	AAGTTCTGGTCTCCAATGGA
17080	Yes	AAGTTCTGGTCTCCAATGGAG
17081	Yes	AAGTTCTGGTCTCCAATGGAGG
17082	Yes	AAGTTCTGGTCTCCAATGGAGGC
17083	Yes	AAGTTCTGGTCTCCAATGGAGGCG
17084	Yes	AAGTTCTGGTCTCCAATGGAGGCGT
17085	Yes	AAGTTCTGGTCTCCAATGGAGGCGTG
17086	Yes	AAGTTCTGGTCTCCAATGGAGGCGTGG
17087	Yes	AAGTTCTGGTCTCCAATGGAGGCGTGGG

17088	Yes	AAGTTCTGGTCTCCAATGGAGGCGTGGGT
17089	Yes	AAGTTCTGGTCTCCAATGGAGGCGTGGGTT
17090	Yes	AAGTTCTGGTCTCCAATGGAGGCGTGGGTTC
17091	Yes	AAGTTCTGGTCTCCAATGGAGGCGTGGGTTCG
17092	Yes	AAGTTCTGGTCTCCAATGGAGGCGTGGGTTCGA
17093	Yes	AAGTTCTGGTCTCCAATGGAGGCGTGGGTTCGAA
17094	Yes	AAGTTCTGGTCTCCAATGGAGGCGTGGGTTCGAAT
17095	Yes	AAGTTCTGGTCTCCAATGGAGGCGTGGGTTCGAATC
17096	Yes	AAGTTCTGGTCTCCAATGGAGGCGTGGGTTCGAATCC
17097	Yes	AAGTTCTGGTCTCCAATGGAGGCGTGGGTTCGAATCCC
17098	Yes	AAGTTCTGGTCTCCAATGGAGGCGTGGGTTCGAATCCCA
17099	Yes	AAGTTCTGGTCTCCAATGGAGGCGTGGGTTCGAATCCCAC
17100	Yes	AAGTTCTGGTCTCCAATGGAGGCGTGGGTTCGAATCCCACT
17101	Yes	AAGTTCTGGTCTCCAATGGAGGCGTGGGTTCGAATCCCACTT
17102	Yes	AAGTTCTGGTCTCCAATGGAGGCGTGGGTTCGAATCCCACTTC
17103	Yes	AAGTTCTGGTCTCCAATGGAGGCGTGGGTTCGAATCCCACTTCT
17104	Yes	AAGTTCTGGTCTCCAATGGAGGCGTGGGTTCGAATCCCACTTCTG
17105	Yes	AAGTTCTGGTCTCCAATGGAGGCGTGGGTTCGAATCCCACTTCTGA
17106	Yes	AAGTTCTGGTCTCCAATGGAGGCGTGGGTTCGAATCCCACTTCTGAC
17107	Yes	AAGTTCTGGTCTCCAATGGAGGCGTGGGTTCGAATCCCACTTCTGACA
17108	Yes	AAGTTCTGGTCTCCAATGGAGGCGTGGGTTCGAATCCCACTTCTGACAC
17109	Yes	AAGTTCTGGTCTCCAATGGAGGCGTGGGTTCGAATCCCACTTCTGACACC
17110	Yes	AAGTTCTGGTCTCCGC
17111	Yes	AAGTTCTGGTCTCCGCA
17112	Yes	AAGTTCTGGTCTCCGCAT
17113	Yes	AAGTTCTGGTCTCCGCATG
17114	Yes	AAGTTCTGGTCTCCGCATGG
17115	Yes	AAGTTCTGGTCTCCGCATGGA
17116	Yes	AAGTTCTGGTCTCCGCATGGAG
17117	Yes	AAGTTCTGGTCTCCGCATGGAGG
17118	Yes	AAGTTCTGGTCTCCGCATGGAGGC
17119	Yes	AAGTTCTGGTCTCCGCATGGAGGCG
17120	Yes	AAGTTCTGGTCTCCGCATGGAGGCGT
17121	Yes	AAGTTCTGGTCTCCGCATGGAGGCGTG
17122	Yes	AAGTTCTGGTCTCCGCATGGAGGCGTGG
17123	Yes	AAGTTCTGGTCTCCGCATGGAGGCGTGGG
17124	Yes	AAGTTCTGGTCTCCGCATGGAGGCGTGGGT
17125	Yes	AAGTTCTGGTCTCCGCATGGAGGCGTGGGTT
17126	Yes	AAGTTCTGGTCTCCGCATGGAGGCGTGGGTTC
17127	Yes	AAGTTCTGGTCTCCGCATGGAGGCGTGGGTTCG
17128	Yes	AAGTTCTGGTCTCCGCATGGAGGCGTGGGTTCGA
17129	Yes	AAGTTCTGGTCTCCGCATGGAGGCGTGGGTTCGAA

17130	Yes	AAGTTCTGGTCTCCGCATGGAGGCGTGGGTTCGAAT
17131	Yes	AAGTTCTGGTCTCCGCATGGAGGCGTGGGTTCGAATC
17132	Yes	AAGTTCTGGTCTCCGCATGGAGGCGTGGGTTCGAATCC
17133	Yes	AAGTTCTGGTCTCCGCATGGAGGCGTGGGTTCGAATCCC
17134	Yes	AAGTTCTGGTCTCCGCATGGAGGCGTGGGTTCGAATCCCA
17135	Yes	AAGTTCTGGTCTCCGCATGGAGGCGTGGGTTCGAATCCCAC
17136	Yes	AAGTTCTGGTCTCCGCATGGAGGCGTGGGTTCGAATCCCACT
17137	Yes	AAGTTCTGGTCTCCGCATGGAGGCGTGGGTTCGAATCCCACTT
17138	Yes	AAGTTCTGGTCTCCGCATGGAGGCGTGGGTTCGAATCCCACTTC
17139	Yes	AAGTTCTGGTCTCCGCATGGAGGCGTGGGTTCGAATCCCACTTCT
17140	Yes	AAGTTCTGGTCTCCGCATGGAGGCGTGGGTTCGAATCCCACTTCTG
17141	Yes	AAGTTCTGGTCTCCGCATGGAGGCGTGGGTTCGAATCCCACTTCTGA
17142	Yes	AAGTTCTGGTCTCCGCATGGAGGCGTGGGTTCGAATCCCACTTCTGAC
17143	Yes	AAGTTCTGGTCTCCGCATGGAGGCGTGGGTTCGAATCCCACTTCTGACA
17144	Yes	AAGTTCTGGTCTCCGCATGGAGGCGTGGGTTCGAATCCCACTTCTGACAC
17145	No	AAGTTCTGGTCTCCGG
17146	Yes	AAGTTCTGGTCTCCGGA
17147	Yes	AAGTTCTGGTCTCCGGAT
17148	Yes	AAGTTCTGGTCTCCGGATG
17149	Yes	AAGTTCTGGTCTCCGGATGG
17150	Yes	AAGTTCTGGTCTCCGGATGGA
17151	Yes	AAGTTCTGGTCTCCGGATGGAG
17152	Yes	AAGTTCTGGTCTCCGGATGGAGG
17153	Yes	AAGTTCTGGTCTCCGGATGGAGGC
17154	Yes	AAGTTCTGGTCTCCGGATGGAGGCG
17155	Yes	AAGTTCTGGTCTCCGGATGGAGGCGT
17156	Yes	AAGTTCTGGTCTCCGGATGGAGGCGTG
17157	Yes	AAGTTCTGGTCTCCGGATGGAGGCGTGG
17158	Yes	AAGTTCTGGTCTCCGGATGGAGGCGTGGG
17159	Yes	AAGTTCTGGTCTCCGGATGGAGGCGTGGGT
17160	Yes	AAGTTCTGGTCTCCGGATGGAGGCGTGGGTT
17161	Yes	AAGTTCTGGTCTCCGGATGGAGGCGTGGGTTC
17162	Yes	AAGTTCTGGTCTCCGGATGGAGGCGTGGGTTCG
17163	Yes	AAGTTCTGGTCTCCGGATGGAGGCGTGGGTTCGA
17164	Yes	AAGTTCTGGTCTCCGGATGGAGGCGTGGGTTCGAA
17165	Yes	AAGTTCTGGTCTCCGGATGGAGGCGTGGGTTCGAAT
17166	Yes	AAGTTCTGGTCTCCGGATGGAGGCGTGGGTTCGAATC
17167	Yes	AAGTTCTGGTCTCCGGATGGAGGCGTGGGTTCGAATCC
17168	Yes	AAGTTCTGGTCTCCGGATGGAGGCGTGGGTTCGAATCCC
17169	Yes	AAGTTCTGGTCTCCGGATGGAGGCGTGGGTTCGAATCCCA
17170	Yes	AAGTTCTGGTCTCCGGATGGAGGCGTGGGTTCGAATCCCAC
17171	Yes	AAGTTCTGGTCTCCGGATGGAGGCGTGGGTTCGAATCCCACT

17172	Yes	AAGTTCTGGTCTCCGGATGGAGGCGTGGGTTCGAATCCCACTT
17173	Yes	AAGTTCTGGTCTCCGGATGGAGGCGTGGGTTCGAATCCCACTTC
17174	Yes	AAGTTCTGGTCTCCGGATGGAGGCGTGGGTTCGAATCCCACTTCT
17175	Yes	AAGTTCTGGTCTCCGGATGGAGGCGTGGGTTCGAATCCCACTTCTG
17176	Yes	AAGTTCTGGTCTCCGGATGGAGGCGTGGGTTCGAATCCCACTTCTGA
17177	Yes	AAGTTCTGGTCTCCGGATGGAGGCGTGGGTTCGAATCCCACTTCTGAC
17178	Yes	AAGTTCTGGTCTCCGGATGGAGGCGTGGGTTCGAATCCCACTTCTGACA
17179	Yes	AAGTTCTGGTCTCCGGATGGAGGCGTGGGTTCGAATCCCACTTCTGACAC
17180	Yes	AAGTTCTGGTCTCCGT
17181	Yes	AAGTTCTGGTCTCCGTA
17182	Yes	AAGTTCTGGTCTCCGTAT
17183	Yes	AAGTTCTGGTCTCCGTATG
17184	Yes	AAGTTCTGGTCTCCGTATGG
17185	Yes	AAGTTCTGGTCTCCGTATGGA
17186	Yes	AAGTTCTGGTCTCCGTATGGAG
17187	Yes	AAGTTCTGGTCTCCGTATGGAGG
17188	Yes	AAGTTCTGGTCTCCGTATGGAGGC
17189	Yes	AAGTTCTGGTCTCCGTATGGAGGCG
17190	Yes	AAGTTCTGGTCTCCGTATGGAGGCGT
17191	Yes	AAGTTCTGGTCTCCGTATGGAGGCGTG
17192	Yes	AAGTTCTGGTCTCCGTATGGAGGCGTGG
17193	Yes	AAGTTCTGGTCTCCGTATGGAGGCGTGGG
17194	Yes	AAGTTCTGGTCTCCGTATGGAGGCGTGGGT
17195	Yes	AAGTTCTGGTCTCCGTATGGAGGCGTGGGTT
17196	Yes	AAGTTCTGGTCTCCGTATGGAGGCGTGGGTTC
17197	Yes	AAGTTCTGGTCTCCGTATGGAGGCGTGGGTTCG
17198	Yes	AAGTTCTGGTCTCCGTATGGAGGCGTGGGTTCGA
17199	Yes	AAGTTCTGGTCTCCGTATGGAGGCGTGGGTTCGAA
17200	Yes	AAGTTCTGGTCTCCGTATGGAGGCGTGGGTTCGAAT
17201	Yes	AAGTTCTGGTCTCCGTATGGAGGCGTGGGTTCGAATC
17202	Yes	AAGTTCTGGTCTCCGTATGGAGGCGTGGGTTCGAATCC
17203	Yes	AAGTTCTGGTCTCCGTATGGAGGCGTGGGTTCGAATCCC
17204	Yes	AAGTTCTGGTCTCCGTATGGAGGCGTGGGTTCGAATCCCA
17205	Yes	AAGTTCTGGTCTCCGTATGGAGGCGTGGGTTCGAATCCCAC
17206	Yes	AAGTTCTGGTCTCCGTATGGAGGCGTGGGTTCGAATCCCACT
17207	Yes	AAGTTCTGGTCTCCGTATGGAGGCGTGGGTTCGAATCCCACTT
17208	Yes	AAGTTCTGGTCTCCGTATGGAGGCGTGGGTTCGAATCCCACTTC
17209	Yes	AAGTTCTGGTCTCCGTATGGAGGCGTGGGTTCGAATCCCACTTCT
17210	Yes	AAGTTCTGGTCTCCGTATGGAGGCGTGGGTTCGAATCCCACTTCTG
17211	Yes	AAGTTCTGGTCTCCGTATGGAGGCGTGGGTTCGAATCCCACTTCTGA
17212	Yes	AAGTTCTGGTCTCCGTATGGAGGCGTGGGTTCGAATCCCACTTCTGAC
17213	Yes	AAGTTCTGGTCTCCGTATGGAGGCGTGGGTTCGAATCCCACTTCTGACA

17214	Yes	AAGTTCTGGTCTCCGTATGGAGGCGTGGGTTCGAATCCCACTTCTGACAC
17215	No	AAGTTGCAATACTTAA
17216	Yes	AAGTTGCAATACTTAAT
17217	Yes	AAGTTGCAATACTTAATT
17218	Yes	AAGTTGCAATACTTAATTT
17219	Yes	AAGTTGCAATACTTAATTTC
17220	Yes	AAGTTGCAATACTTAATTTCT
17221	Yes	AAGTTGCAATACTTAATTTCTG
17222	Yes	AAGTTGCAATACTTAATTTCTGC
17223	Yes	AAGTTGCAATACTTAATTTCTGCC
17224	Yes	AAGTTGCAATACTTAATTTCTGCCA
17225	Yes	AAGTTGGTGGTTCGAA
17226	Yes	AAGTTGGTGGTTCGAAC
17227	Yes	AAGTTGGTGGTTCGAACA
17228	Yes	AAGTTGGTGGTTCGAACAC
17229	Yes	AAGTTGGTGGTTCGAACACA
17230	Yes	AAGTTGGTGGTTCGAACACAC
17231	Yes	AAGTTGGTGGTTCGAACACACC
17232	Yes	AAGTTGGTGGTTCGAACACACCC
17233	Yes	AAGTTGGTGGTTCGAACACACCCA
17234	Yes	AAGTTGGTGGTTCGAACACACCCAG
17235	Yes	AAGTTGGTGGTTCGAACACACCCAGA
17236	Yes	AAGTTGGTGGTTCGAACACACCCAGAG
17237	Yes	AAGTTGGTGGTTCGAACACACCCAGAGG
17238	Yes	AAGTTGGTGGTTCGAACACACCCAGAGGC
17239	Yes	AAGTTGGTGGTTCGAACACACCCAGAGGCG
17240	Yes	AAGTTGGTGGTTCGAACACACCCAGAGGCGC
17241	Yes	AAGTTGGTGGTTCGAACACACCCAGAGGCGCC
17242	Yes	AAGTTGGTGGTTCGAACACACCCAGAGGCGCCA
17243	No	AAGTTGGTGGTTCGAG
17244	Yes	AAGTTGGTGGTTCGAGC
17245	Yes	AAGTTGGTGGTTCGAGCC
17246	Yes	AAGTTGGTGGTTCGAGCCC
17247	Yes	AAGTTGGTGGTTCGAGCCCA
17248	Yes	AAGTTGGTGGTTCGAGCCCAC
17249	Yes	AAGTTGGTGGTTCGAGCCCACC
17250	Yes	AAGTTGGTGGTTCGAGCCCACCC
17251	Yes	AAGTTGGTGGTTCGAGCCCACCCA
17252	Yes	AAGTTGGTGGTTCGAGCCCACCCAG
17253	Yes	AAGTTGGTGGTTCGAGCCCACCCAGG
17254	Yes	AAGTTGGTGGTTCGAGCCCACCCAGGA
17255	Yes	AAGTTGGTGGTTCGAGCCCACCCAGGAA

17256	Yes	AAGTTGGTGGTTCGAGCCCACCCAGGAAC
17257	Yes	AAGTTGGTGGTTCGAGCCCACCCAGGAACG
17258	Yes	AAGTTGGTGGTTCGAGCCCACCCAGGAACGC
17259	Yes	AAGTTGGTGGTTCGAGCCCACCCAGGAACGCC
17260	Yes	AAGTTGGTGGTTCGAGCCCACCCAGGAACGCCA
17261	Yes	AAGTTGGTGGTTCGAGCCT
17262	Yes	AAGTTGGTGGTTCGAGCCTA
17263	Yes	AAGTTGGTGGTTCGAGCCTAC
17264	Yes	AAGTTGGTGGTTCGAGCCTACC
17265	Yes	AAGTTGGTGGTTCGAGCCTACCC
17266	Yes	AAGTTGGTGGTTCGAGCCTACCCA
17267	Yes	AAGTTGGTGGTTCGAGCCTACCCAG
17268	Yes	AAGTTGGTGGTTCGAGCCTACCCAGG
17269	Yes	AAGTTGGTGGTTCGAGCCTACCCAGGG
17270	Yes	AAGTTGGTGGTTCGAGCCTACCCAGGGA
17271	Yes	AAGTTGGTGGTTCGAGCCTACCCAGGGAC
17272	Yes	AAGTTGGTGGTTCGAGCCTACCCAGGGACG
17273	Yes	AAGTTGGTGGTTCGAGCCTACCCAGGGACGC
17274	Yes	AAGTTGGTGGTTCGAGCCTACCCAGGGACGCC
17275	Yes	AAGTTGGTGGTTCGAGCCTACCCAGGGACGCCA
17276	Yes	AAGTTTGGCGCTCTCA
17277	Yes	AAGTTTGGCGCTCTCAC
17278	Yes	AAGTTTGGCGCTCTCACC
17279	Yes	AAGTTTGGCGCTCTCACCG
17280	Yes	AAGTTTGGCGCTCTCACCGC
17281	Yes	AAGTTTGGCGCTCTCACCGCC
17282	Yes	AAGTTTGGCGCTCTCACCGCCG
17283	Yes	AAGTTTGGCGCTCTCACCGCCGG
17284	Yes	AAGTTTGGCGCTCTCACCGCCGGG
17285	Yes	AAGTTTGGCGCTCTCACCGCCGGGA
17286	Yes	AAGTTTGGCGCTCTCACCGCCGGGAC
17287	Yes	AAGTTTGGCGCTCTCACCGCCGGGACT
17288	Yes	AAGTTTGGCGCTCTCACCGCCGGGACTG
17289	Yes	AAGTTTGGCGCTCTCACCGCCGGGACTGG
17290	Yes	AAGTTTGGCGCTCTCACCGCCGGGACTGGT
17291	Yes	AAGTTTGGCGCTCTCACCGCCGGGACTGGTT
17292	Yes	AAGTTTGGCGCTCTCACCGCCGGGACTGGTTG
17293	Yes	AAGTTTGGCGCTCTCACCGCCGGGACTGGTTGA
17294	Yes	AAGTTTGGCGCTCTCACCGCCGGGACTGGTTGAT
17295	Yes	AAGTTTGGCGCTCTCACCGCCGGGACTGGTTGATT
17296	Yes	AAGTTTGGCGCTCTCACCGCCGGGACTGGTTGATTC
17297	Yes	AAGTTTGGCGCTCTCACCGCCGGGACTGGTTGATTCC

17298	Yes	AAGTTTGGCGCTCTCACCGCCGGGACTGGTTGATTCCA
17299	Yes	AAGTTTGGCGCTCTCACCGCCGGGACTGGTTGATTCCAG
17300	Yes	AAGTTTGGCGCTCTCACCGCCGGGACTGGTTGATTCCAGA
17301	Yes	AAGTTTGGCGCTCTCACCGCCGGGACTGGTTGATTCCAGAT
17302	Yes	AAGTTTGGCGCTCTCACCGCCGGGACTGGTTGATTCCAGATC
17303	Yes	AAGTTTGGCGCTCTCACCGCCGGGACTGGTTGATTCCAGATCA
17304	Yes	AAGTTTGGCGCTCTCACCGCCGGGACTGGTTGATTCCAGATCAG
17305	Yes	AAGTTTGGCGCTCTCACCGCCGGGACTGGTTGATTCCAGATCAGG
17306	Yes	AAGTTTGGCGCTCTCACCGCCGGGACTGGTTGATTCCAGATCAGGG
17307	Yes	AAGTTTGGCGCTCTCACCGCCGGGACTGGTTGATTCCAGATCAGGGG
17308	Yes	AAGTTTGGCGCTCTCACCGCCGGGACTGGTTGATTCCAGATCAGGGGA
17309	Yes	AAGTTTGGCGCTCTCACCGCCGGGACTGGTTGATTCCAGATCAGGGGAC
17310	Yes	AAGTTTGGCGCTCTCACCGCCGGGACTGGTTGATTCCAGATCAGGGGACC
17311	No	AAGTTTGGCGCTTTCA
17312	Yes	AAGTTTGGCGCTTTCAC
17313	Yes	AAGTTTGGCGCTTTCACC
17314	Yes	AAGTTTGGCGCTTTCACCG
17315	Yes	AAGTTTGGCGCTTTCACCGC
17316	Yes	AAGTTTGGCGCTTTCACCGCC
17317	Yes	AAGTTTGGCGCTTTCACCGCCG
17318	Yes	AAGTTTGGCGCTTTCACCGCCGG
17319	Yes	AAGTTTGGCGCTTTCACCGCCGGG
17320	Yes	AAGTTTGGCGCTTTCACCGCCGGGA
17321	Yes	AAGTTTGGCGCTTTCACCGCCGGGAC
17322	Yes	AAGTTTGGCGCTTTCACCGCCGGGACT
17323	Yes	AAGTTTGGCGCTTTCACCGCCGGGACTG
17324	Yes	AAGTTTGGCGCTTTCACCGCCGGGACTGG
17325	Yes	AAGTTTGGCGCTTTCACCGCCGGGACTGGT
17326	Yes	AAGTTTGGCGCTTTCACCGCCGGGACTGGTT
17327	Yes	AAGTTTGGCGCTTTCACCGCCGGGACTGGTTG
17328	Yes	AAGTTTGGCGCTTTCACCGCCGGGACTGGTTGA
17329	Yes	AAGTTTGGCGCTTTCACCGCCGGGACTGGTTGAT
17330	Yes	AAGTTTGGCGCTTTCACCGCCGGGACTGGTTGATT
17331	Yes	AAGTTTGGCGCTTTCACCGCCGGGACTGGTTGATTC
17332	Yes	AAGTTTGGCGCTTTCACCGCCGGGACTGGTTGATTCC
17333	Yes	AAGTTTGGCGCTTTCACCGCCGGGACTGGTTGATTCCA
17334	Yes	AAGTTTGGCGCTTTCACCGCCGGGACTGGTTGATTCCAG
17335	Yes	AAGTTTGGCGCTTTCACCGCCGGGACTGGTTGATTCCAGA
17336	Yes	AAGTTTGGCGCTTTCACCGCCGGGACTGGTTGATTCCAGAT
17337	Yes	AAGTTTGGCGCTTTCACCGCCGGGACTGGTTGATTCCAGATC
17338	Yes	AAGTTTGGCGCTTTCACCGCCGGGACTGGTTGATTCCAGATCA
17339	Yes	AAGTTTGGCGCTTTCACCGCCGGGACTGGTTGATTCCAGATCAG

17340	Yes	AAGTTTGGCGCTTTCACCGCCGGGACTGGTTGATTCCAGATCAGG
17341	Yes	AAGTTTGGCGCTTTCACCGCCGGGACTGGTTGATTCCAGATCAGGG
17342	Yes	AAGTTTGGCGCTTTCACCGCCGGGACTGGTTGATTCCAGATCAGGGG
17343	Yes	AAGTTTGGCGCTTTCACCGCCGGGACTGGTTGATTCCAGATCAGGGGA
17344	Yes	AAGTTTGGCGCTTTCACCGCCGGGACTGGTTGATTCCAGATCAGGGGAC
17345	Yes	AAGTTTGGCGCTTTCACCGCCGGGACTGGTTGATTCCAGATCAGGGGACC
17346	No	AATAAATGTGGACTTT
17347	No	AATAAATGTGGACTTTG
17348	Yes	AATAAATGTGGACTTTGA
17349	Yes	AATAAATGTGGACTTTGAA
17350	Yes	AATAAATGTGGACTTTGAAG
17351	Yes	AATAAATGTGGACTTTGAAGT
17352	Yes	AATAAATGTGGACTTTGAAGTT
17353	Yes	AATAAATGTGGACTTTGAAGTTA
17354	Yes	AATAAATGTGGACTTTGAAGTTAG
17355	Yes	AATAAATGTGGACTTTGAAGTTAGA
17356	Yes	AATAAATGTGGACTTTGAAGTTAGAT
17357	Yes	AATAAATGTGGACTTTGAAGTTAGATA
17358	Yes	AATAAATGTGGACTTTGAAGTTAGATAC
17359	Yes	AATAAATGTGGACTTTGAAGTTAGATACA
17360	Yes	AATAAATGTGGACTTTGAAGTTAGATACAC
17361	Yes	AATAAATGTGGACTTTGAAGTTAGATACACC
17362	Yes	AATAAATGTGGACTTTGAAGTTAGATACACCT
17363	Yes	AATAAATGTGGACTTTGAAGTTAGATACACCTG
17364	Yes	AATAAATGTGGACTTTGAAGTTAGATACACCTGG
17365	Yes	AATAAATGTGGACTTTGAAGTTAGATACACCTGGG
17366	Yes	AATAAATGTGGACTTTGAAGTTAGATACACCTGGGT
17367	Yes	AATAAATGTGGACTTTGAAGTTAGATACACCTGGGTT
17368	Yes	AATAAATGTGGACTTTGAAGTTAGATACACCTGGGTTC
17369	Yes	AATAAATGTGGACTTTGAAGTTAGATACACCTGGGTTCA
17370	Yes	AATAAATGTGGACTTTGAAGTTAGATACACCTGGGTTCAA
17371	Yes	AATAAATGTGGACTTTGAAGTTAGATACACCTGGGTTCAAA
17372	Yes	AATAAATGTGGACTTTGAAGTTAGATACACCTGGGTTCAAAT
17373	Yes	AATAAATGTGGACTTTGAAGTTAGATACACCTGGGTTCAAATC
17374	Yes	AATAAATGTGGACTTTGAAGTTAGATACACCTGGGTTCAAATCC
17375	Yes	AATAAATGTGGACTTTGAAGTTAGATACACCTGGGTTCAAATCCC
17376	Yes	AATAAATGTGGACTTTGAAGTTAGATACACCTGGGTTCAAATCCCA
17377	Yes	AATAAATGTGGACTTTGAAGTTAGATACACCTGGGTTCAAATCCCAG
17378	Yes	AATAAATGTGGACTTTGAAGTTAGATACACCTGGGTTCAAATCCCAGT
17379	Yes	AATAAATGTGGACTTTGAAGTTAGATACACCTGGGTTCAAATCCCAGTG
17380	Yes	AATAAATGTGGACTTTGAAGTTAGATACACCTGGGTTCAAATCCCAGTGC
17381	Yes	AATAACGCCAAGGTCG

17382	Yes	AATAACGCCAAGGTCGC
17383	Yes	AATAACGCCAAGGTCGCG
17384	Yes	AATAACGCCAAGGTCGCGG
17385	Yes	AATAACGCCAAGGTCGCGGG
17386	Yes	AATAACGCCAAGGTCGCGGGT
17387	Yes	AATAACGCCAAGGTCGCGGGTT
17388	Yes	AATAACGCCAAGGTCGCGGGTTC
17389	Yes	AATAACGCCAAGGTCGCGGGTTCG
17390	Yes	AATAACGCCAAGGTCGCGGGTTCGA
17391	Yes	AATAACGCCAAGGTCGCGGGTTCGAA
17392	Yes	AATAACGCCAAGGTCGCGGGTTCGAAC
17393	Yes	AATAACGCCAAGGTCGCGGGTTCGAACC
17394	Yes	AATAACGCCAAGGTCGCGGGTTCGAACCC
17395	Yes	AATAACGCCAAGGTCGCGGGTTCGAACCCC
17396	Yes	AATAACGCCAAGGTCGCGGGTTCGAACCCCG
17397	Yes	AATAACGCCAAGGTCGCGGGTTCGAACCCCGT
17398	Yes	AATAACGCCAAGGTCGCGGGTTCGAACCCCGTA
17399	Yes	AATAACGCCAAGGTCGCGGGTTCGAACCCCGTAC
17400	Yes	AATAACGCCAAGGTCGCGGGTTCGAACCCCGTACG
17401	Yes	AATAACGCCAAGGTCGCGGGTTCGAACCCCGTACGG
17402	Yes	AATAACGCCAAGGTCGCGGGTTCGAACCCCGTACGGG
17403	Yes	AATAACGCCAAGGTCGCGGGTTCGAACCCCGTACGGGC
17404	Yes	AATAACGCCAAGGTCGCGGGTTCGAACCCCGTACGGGCC
17405	Yes	AATAACGCCAAGGTCGCGGGTTCGAACCCCGTACGGGCCA
17406	Yes	AATAACGCCAAGGTCGCGGGTTCGAACCCCGTACGGGCCAC
17407	Yes	AATAACGCCAAGGTCGCGGGTTCGAACCCCGTACGGGCCACC
17408	Yes	AATAACGCCAAGGTCGCGGGTTCGAACCCCGTACGGGCCACCA
17409	Yes	AATAACGCCAAGGTCGCGGGTTCGAT
17410	Yes	AATAACGCCAAGGTCGCGGGTTCGATC
17411	Yes	AATAACGCCAAGGTCGCGGGTTCGATCC
17412	Yes	AATAACGCCAAGGTCGCGGGTTCGATCCC
17413	Yes	AATAACGCCAAGGTCGCGGGTTCGATCCCC
17414	Yes	AATAACGCCAAGGTCGCGGGTTCGATCCCCG
17415	Yes	AATAACGCCAAGGTCGCGGGTTCGATCCCCGT
17416	Yes	AATAACGCCAAGGTCGCGGGTTCGATCCCCGTA
17417	Yes	AATAACGCCAAGGTCGCGGGTTCGATCCCCGTAC
17418	Yes	AATAACGCCAAGGTCGCGGGTTCGATCCCCGTACG
17419	Yes	AATAACGCCAAGGTCGCGGGTTCGATCCCCGTACGG
17420	Yes	AATAACGCCAAGGTCGCGGGTTCGATCCCCGTACGGG
17421	Yes	AATAACGCCAAGGTCGCGGGTTCGATCCCCGTACGGGC
17422	Yes	AATAACGCCAAGGTCGCGGGTTCGATCCCCGTACGGGCC
17423	Yes	AATAACGCCAAGGTCGCGGGTTCGATCCCCGTACGGGCCA

17424	Yes	AATAACGCCAAGGTCGCGGGTTCGATCCCCGTACGGGCCAC
17425	Yes	AATAACGCCAAGGTCGCGGGTTCGATCCCCGTACGGGCCACC
17426	Yes	AATAACGCCAAGGTCGCGGGTTCGATCCCCGTACGGGCCACCA
17427	Yes	AATAACGCCAAGGTCGCGGGTTCGATCCCCGTACT
17428	Yes	AATAACGCCAAGGTCGCGGGTTCGATCCCCGTACTG
17429	Yes	AATAACGCCAAGGTCGCGGGTTCGATCCCCGTACTGG
17430	Yes	AATAACGCCAAGGTCGCGGGTTCGATCCCCGTACTGGC
17431	Yes	AATAACGCCAAGGTCGCGGGTTCGATCCCCGTACTGGCC
17432	Yes	AATAACGCCAAGGTCGCGGGTTCGATCCCCGTACTGGCCA
17433	Yes	AATAACGCCAAGGTCGCGGGTTCGATCCCCGTACTGGCCAC
17434	Yes	AATAACGCCAAGGTCGCGGGTTCGATCCCCGTACTGGCCACC
17435	Yes	AATAACGCCAAGGTCGCGGGTTCGATCCCCGTACTGGCCACCA
17436	Yes	AATAACGCCAAGGTCGT
17437	Yes	AATAACGCCAAGGTCGTG
17438	Yes	AATAACGCCAAGGTCGTGG
17439	Yes	AATAACGCCAAGGTCGTGGG
17440	Yes	AATAACGCCAAGGTCGTGGGT
17441	Yes	AATAACGCCAAGGTCGTGGGTT
17442	Yes	AATAACGCCAAGGTCGTGGGTTC
17443	Yes	AATAACGCCAAGGTCGTGGGTTCG
17444	Yes	AATAACGCCAAGGTCGTGGGTTCGA
17445	Yes	AATAACGCCAAGGTCGTGGGTTCGAT
17446	Yes	AATAACGCCAAGGTCGTGGGTTCGATC
17447	Yes	AATAACGCCAAGGTCGTGGGTTCGATCC
17448	Yes	AATAACGCCAAGGTCGTGGGTTCGATCCC
17449	Yes	AATAACGCCAAGGTCGTGGGTTCGATCCCC
17450	Yes	AATAACGCCAAGGTCGTGGGTTCGATCCCCA
17451	Yes	AATAACGCCAAGGTCGTGGGTTCGATCCCCAT
17452	Yes	AATAACGCCAAGGTCGTGGGTTCGATCCCCATA
17453	Yes	AATAACGCCAAGGTCGTGGGTTCGATCCCCATAT
17454	Yes	AATAACGCCAAGGTCGTGGGTTCGATCCCCATATC
17455	Yes	AATAACGCCAAGGTCGTGGGTTCGATCCCCATATCG
17456	Yes	AATAACGCCAAGGTCGTGGGTTCGATCCCCATATCGG
17457	Yes	AATAACGCCAAGGTCGTGGGTTCGATCCCCATATCGGC
17458	Yes	AATAACGCCAAGGTCGTGGGTTCGATCCCCATATCGGCC
17459	Yes	AATAACGCCAAGGTCGTGGGTTCGATCCCCATATCGGCCA
17460	Yes	AATAACGCCAAGGTCGTGGGTTCGATCCCCATATCGGCCAC
17461	Yes	AATAACGCCAAGGTCGTGGGTTCGATCCCCATATCGGCCACC
17462	Yes	AATAACGCCAAGGTCGTGGGTTCGATCCCCATATCGGCCACCA
17463	Yes	AATAACGCTAAGGTCG
17464	Yes	AATAACGCTAAGGTCGC
17465	Yes	AATAACGCTAAGGTCGCG

17466	Yes	AATAACGCTAAGGTCGCGG
17467	Yes	AATAACGCTAAGGTCGCGGG
17468	Yes	AATAACGCTAAGGTCGCGGGT
17469	Yes	AATAACGCTAAGGTCGCGGGTT
17470	Yes	AATAACGCTAAGGTCGCGGGTTC
17471	Yes	AATAACGCTAAGGTCGCGGGTTCG
17472	Yes	AATAACGCTAAGGTCGCGGGTTCGA
17473	Yes	AATAACGCTAAGGTCGCGGGTTCGAT
17474	Yes	AATAACGCTAAGGTCGCGGGTTCGATC
17475	Yes	AATAACGCTAAGGTCGCGGGTTCGATCC
17476	Yes	AATAACGCTAAGGTCGCGGGTTCGATCCC
17477	Yes	AATAACGCTAAGGTCGCGGGTTCGATCCCC
17478	Yes	AATAACGCTAAGGTCGCGGGTTCGATCCCCG
17479	Yes	AATAACGCTAAGGTCGCGGGTTCGATCCCCGT
17480	Yes	AATAACGCTAAGGTCGCGGGTTCGATCCCCGTA
17481	Yes	AATAACGCTAAGGTCGCGGGTTCGATCCCCGTAC
17482	Yes	AATAACGCTAAGGTCGCGGGTTCGATCCCCGTACT
17483	Yes	AATAACGCTAAGGTCGCGGGTTCGATCCCCGTACTG
17484	Yes	AATAACGCTAAGGTCGCGGGTTCGATCCCCGTACTGG
17485	Yes	AATAACGCTAAGGTCGCGGGTTCGATCCCCGTACTGGC
17486	Yes	AATAACGCTAAGGTCGCGGGTTCGATCCCCGTACTGGCC
17487	Yes	AATAACGCTAAGGTCGCGGGTTCGATCCCCGTACTGGCCA
17488	Yes	AATAACGCTAAGGTCGCGGGTTCGATCCCCGTACTGGCCAC
17489	Yes	AATAACGCTAAGGTCGCGGGTTCGATCCCCGTACTGGCCACC
17490	Yes	AATAACGCTAAGGTCGCGGGTTCGATCCCCGTACTGGCCACCA
17491	No	AATAAGCTATCGGGCC
17492	No	AATAAGCTATCGGGCCC
17493	No	AATAAGCTATCGGGCCCA
17494	No	AATAAGCTATCGGGCCCAT
17495	No	AATAAGCTATCGGGCCCATA
17496	No	AATAAGCTATCGGGCCCATAC
17497	No	AATAAGCTATCGGGCCCATACC
17498	No	AATAAGCTATCGGGCCCATACCC
17499	No	AATAAGCTATCGGGCCCATACCCC
17500	No	AATAAGCTATCGGGCCCATACCCCG
17501	No	AATAAGCTATCGGGCCCATACCCCGA
17502	No	AATAAGCTATCGGGCCCATACCCCGAA
17503	No	AATAAGCTATCGGGCCCATACCCCGAAA
17504	No	AATAAGCTATCGGGCCCATACCCCGAAAA
17505	No	AATAAGCTATCGGGCCCATACCCCGAAAAT
17506	No	AATAAGCTATCGGGCCCATACCCCGAAAATG
17507	No	AATAAGCTATCGGGCCCATACCCCGAAAATGT

17508	No	AATAAGCTATCGGGCCCATACCCCGAAAATGTT
17509	No	AATAAGCTATCGGGCCCATACCCCGAAAATGTTG
17510	No	AATAAGCTATCGGGCCCATACCCCGAAAATGTTGG
17511	No	AATAAGCTATCGGGCCCATACCCCGAAAATGTTGGT
17512	No	AATAAGCTATCGGGCCCATACCCCGAAAATGTTGGTT
17513	No	AATAAGCTATCGGGCCCATACCCCGAAAATGTTGGTTA
17514	No	AATAAGCTATCGGGCCCATACCCCGAAAATGTTGGTTAT
17515	No	AATAAGCTATCGGGCCCATACCCCGAAAATGTTGGTTATA
17516	Yes	AATAAGCTATCGGGCCCATACCCCGAAAATGTTGGTTATAC
17517	Yes	AATAAGCTATCGGGCCCATACCCCGAAAATGTTGGTTATACC
17518	Yes	AATAAGCTATCGGGCCCATACCCCGAAAATGTTGGTTATACCC
17519	Yes	AATAAGCTATCGGGCCCATACCCCGAAAATGTTGGTTATACCCT
17520	Yes	AATAAGCTATCGGGCCCATACCCCGAAAATGTTGGTTATACCCTT
17521	Yes	AATAAGCTATCGGGCCCATACCCCGAAAATGTTGGTTATACCCTTC
17522	Yes	AATAAGCTATCGGGCCCATACCCCGAAAATGTTGGTTATACCCTTCC
17523	Yes	AATAAGCTATCGGGCCCATACCCCGAAAATGTTGGTTATACCCTTCCC
17524	Yes	AATAAGCTATCGGGCCCATACCCCGAAAATGTTGGTTATACCCTTCCCG
17525	Yes	AATAAGCTATCGGGCCCATACCCCGAAAATGTTGGTTATACCCTTCCCGT
17526	Yes	AATAAGGCCACGGTCA
17527	Yes	AATAAGGCCACGGTCAG
17528	Yes	AATAAGGCCACGGTCAGG
17529	Yes	AATAAGGCCACGGTCAGGG
17530	Yes	AATAAGGCCACGGTCAGGGG
17531	Yes	AATAAGGCCACGGTCAGGGGT
17532	Yes	AATAAGGCCACGGTCAGGGGTT
17533	Yes	AATAAGGCCACGGTCAGGGGTTC
17534	Yes	AATAAGGCCACGGTCAGGGGTTCA
17535	Yes	AATAAGGCCACGGTCAGGGGTTCAA
17536	Yes	AATAAGGCCACGGTCAGGGGTTCAAT
17537	Yes	AATAAGGCCACGGTCAGGGGTTCAATT
17538	Yes	AATAAGGCCACGGTCAGGGGTTCAATTC
17539	Yes	AATAAGGCCACGGTCAGGGGTTCAATTCC
17540	Yes	AATAAGGCCACGGTCAGGGGTTCAATTCCC
17541	Yes	AATAAGGCCACGGTCAGGGGTTCAATTCCCT
17542	Yes	AATAAGGCCACGGTCAGGGGTTCAATTCCCTT
17543	Yes	AATAAGGCCACGGTCAGGGGTTCAATTCCCTTA
17544	Yes	AATAAGGCCACGGTCAGGGGTTCAATTCCCTTAT
17545	Yes	AATAAGGCCACGGTCAGGGGTTCAATTCCCTTATG
17546	Yes	AATAAGGCCACGGTCAGGGGTTCAATTCCCTTATGG
17547	Yes	AATAAGGCCACGGTCAGGGGTTCAATTCCCTTATGGG
17548	Yes	AATAAGGCCACGGTCAGGGGTTCAATTCCCTTATGGGC
17549	Yes	AATAAGGCCACGGTCAGGGGTTCAATTCCCTTATGGGCT

17550	Yes	AATAAGGCCACGGTCAGGGGTTCAATTCCCTTATGGGCTG
17551	Yes	AATAAGGCCACGGTCAGGGGTTCAATTCCCTTATGGGCTGC
17552	Yes	AATAAGGCCACGGTCAGGGGTTCAATTCCCTTATGGGCTGCC
17553	Yes	AATAAGGCCACGGTCAGGGGTTCAATTCCCTTATGGGCTGCCA
17554	No	AATAATAGGAGCTTAA
17555	No	AATAATAGGAGCTTAAA
17556	No	AATAATAGGAGCTTAAAC
17557	No	AATAATAGGAGCTTAAACC
17558	Yes	AATAATAGGAGCTTAAACCC
17559	Yes	AATAATAGGAGCTTAAACCCC
17560	Yes	AATAATAGGAGCTTAAACCCCC
17561	Yes	AATAATAGGAGCTTAAACCCCCT
17562	Yes	AATAATAGGAGCTTAAACCCCCTT
17563	Yes	AATAATAGGAGCTTAAACCCCCTTA
17564	Yes	AATAATAGGAGCTTAAACCCCCTTAT
17565	Yes	AATAATAGGAGCTTAAACCCCCTTATT
17566	Yes	AATAATAGGAGCTTAAACCCCCTTATTT
17567	Yes	AATAATAGGAGCTTAAACCCCCTTATTTC
17568	Yes	AATAATAGGAGCTTAAACCCCCTTATTTCT
17569	Yes	AATAATAGGAGCTTAAACCCCCTTATTTCTA
17570	Yes	AATAATAGGAGCTTAAACCCCCTTATTTCTAC
17571	Yes	AATAATAGGAGCTTAAACCCCCTTATTTCTACC
17572	Yes	AATAATAGGAGCTTAAACCCCCTTATTTCTACCA
17573	No	AATAATGTCAAGGTTG
17574	No	AATAATGTCAAGGTTGC
17575	Yes	AATAATGTCAAGGTTGCG
17576	Yes	AATAATGTCAAGGTTGCGG
17577	Yes	AATAATGTCAAGGTTGCGGG
17578	Yes	AATAATGTCAAGGTTGCGGGT
17579	Yes	AATAATGTCAAGGTTGCGGGTT
17580	Yes	AATAATGTCAAGGTTGCGGGTTG
17581	Yes	AATAATGTCAAGGTTGCGGGTTGG
17582	Yes	AATAATGTCAAGGTTGCGGGTTGGA
17583	Yes	AATAATGTCAAGGTTGCGGGTTGGAT
17584	Yes	AATAATGTCAAGGTTGCGGGTTGGATC
17585	Yes	AATAATGTCAAGGTTGCGGGTTGGATCC
17586	Yes	AATAATGTCAAGGTTGCGGGTTGGATCCC
17587	Yes	AATAATGTCAAGGTTGCGGGTTGGATCCCC
17588	Yes	AATAATGTCAAGGTTGCGGGTTGGATCCCCG
17589	Yes	AATAATGTCAAGGTTGCGGGTTGGATCCCCGA
17590	Yes	AATAATGTCAAGGTTGCGGGTTGGATCCCCGAA
17591	Yes	AATAATGTCAAGGTTGCGGGTTGGATCCCCGAAC

17592	Yes	AATAATGTCAAGGTTGCGGGTTGGATCCCCGAACG
17593	Yes	AATAATGTCAAGGTTGCGGGTTGGATCCCCGAACGG
17594	Yes	AATAATGTCAAGGTTGCGGGTTGGATCCCCGAACGGG
17595	Yes	AATAATGTCAAGGTTGCGGGTTGGATCCCCGAACGGGC
17596	Yes	AATAATGTCAAGGTTGCGGGTTGGATCCCCGAACGGGCC
17597	Yes	AATAATGTCAAGGTTGCGGGTTGGATCCCCGAACGGGCCA
17598	Yes	AATAATGTCAAGGTTGCGGGTTGGATCCCCGAACGGGCCAC
17599	Yes	AATAATGTCAAGGTTGCGGGTTGGATCCCCGAACGGGCCACC
17600	Yes	AATAATGTCAAGGTTGCGGGTTGGATCCCCGAACGGGCCACCA
17601	No	AATACAACGATGGTTT
17602	No	AATACAACGATGGTTTT
17603	No	AATACAACGATGGTTTTT
17604	No	AATACAACGATGGTTTTTC
17605	No	AATACAACGATGGTTTTTCA
17606	No	AATACAACGATGGTTTTTCAT
17607	No	AATACAACGATGGTTTTTCATA
17608	No	AATACAACGATGGTTTTTCATAT
17609	No	AATACAACGATGGTTTTTCATATC
17610	No	AATACAACGATGGTTTTTCATATCA
17611	No	AATACAACGATGGTTTTTCATATCAT
17612	No	AATACAACGATGGTTTTTCATATCATT
17613	No	AATACAACGATGGTTTTTCATATCATTG
17614	No	AATACAACGATGGTTTTTCATATCATTGG
17615	No	AATACAACGATGGTTTTTCATATCATTGGT
17616	No	AATACAACGATGGTTTTTCATATCATTGGTC
17617	No	AATACAACGATGGTTTTTCATATCATTGGTCG
17618	No	AATACAACGATGGTTTTTCATATCATTGGTCGT
17619	No	AATACAACGATGGTTTTTCATATCATTGGTCGTG
17620	No	AATACAACGATGGTTTTTCATATCATTGGTCGTGG
17621	No	AATACAACGATGGTTTTTCATATCATTGGTCGTGGT
17622	No	AATACAACGATGGTTTTTCATATCATTGGTCGTGGTT
17623	No	AATACAACGATGGTTTTTCATATCATTGGTCGTGGTTG
17624	No	AATACAACGATGGTTTTTCATATCATTGGTCGTGGTTGT
17625	No	AATACAACGATGGTTTTTCATATCATTGGTCGTGGTTGTA
17626	No	AATACAACGATGGTTTTTCATATCATTGGTCGTGGTTGTAG
17627	Yes	AATACAACGATGGTTTTTCATATCATTGGTCGTGGTTGTAGT
17628	Yes	AATACAACGATGGTTTTTCATATCATTGGTCGTGGTTGTAGTC
17629	Yes	AATACAACGATGGTTTTTCATATCATTGGTCGTGGTTGTAGTCC
17630	Yes	AATACAACGATGGTTTTTCATATCATTGGTCGTGGTTGTAGTCCG
17631	Yes	AATACAACGATGGTTTTTCATATCATTGGTCGTGGTTGTAGTCCGT
17632	Yes	AATACAACGATGGTTTTTCATATCATTGGTCGTGGTTGTAGTCCGTG
17633	Yes	AATACAACGATGGTTTTTCATATCATTGGTCGTGGTTGTAGTCCGTGC

17634	Yes	AATACAACGATGGTTTTTCATATCATTGGTCGTGGTTGTAGTCCGTGCG
17635	Yes	AATACAACGATGGTTTTTCATATCATTGGTCGTGGTTGTAGTCCGTGCGA
17636	No	AATACACCAGTCTTGT
17637	No	AATACACCAGTCTTGTA
17638	No	AATACACCAGTCTTGTAA
17639	No	AATACACCAGTCTTGTAAA
17640	Yes	AATACACCAGTCTTGTAAAC
17641	Yes	AATACACCAGTCTTGTAAACC
17642	Yes	AATACACCAGTCTTGTAAACCG
17643	Yes	AATACACCAGTCTTGTAAACCGG
17644	Yes	AATACACCAGTCTTGTAAACCGGA
17645	Yes	AATACACCAGTCTTGTAAACCGGAG
17646	Yes	AATACACCAGTCTTGTAAACCGGAGA
17647	Yes	AATACACCAGTCTTGTAAACCGGAGAT
17648	Yes	AATACACCAGTCTTGTAAACCGGAGATG
17649	Yes	AATACACCAGTCTTGTAAACCGGAGATGA
17650	Yes	AATACACCAGTCTTGTAAACCGGAGATGAA
17651	Yes	AATACACCAGTCTTGTAAACCGGAGATGAAA
17652	Yes	AATACACCAGTCTTGTAAACCGGAGATGAAAA
17653	Yes	AATACACCAGTCTTGTAAACCGGAGATGAAAAC
17654	Yes	AATACACCAGTCTTGTAAACCGGAGATGAAAACC
17655	Yes	AATACACCAGTCTTGTAAACCGGAGATGAAAACCT
17656	Yes	AATACACCAGTCTTGTAAACCGGAGATGAAAACCTT
17657	Yes	AATACACCAGTCTTGTAAACCGGAGATGAAAACCTTT
17658	Yes	AATACACCAGTCTTGTAAACCGGAGATGAAAACCTTTT
17659	Yes	AATACACCAGTCTTGTAAACCGGAGATGAAAACCTTTTT
17660	Yes	AATACACCAGTCTTGTAAACCGGAGATGAAAACCTTTTTC
17661	Yes	AATACACCAGTCTTGTAAACCGGAGATGAAAACCTTTTTCC
17662	Yes	AATACACCAGTCTTGTAAACCGGAGATGAAAACCTTTTTCCA
17663	Yes	AATACACCAGTCTTGTAAACCGGAGATGAAAACCTTTTTCCAA
17664	Yes	AATACACCAGTCTTGTAAACCGGAGATGAAAACCTTTTTCCAAG
17665	Yes	AATACACCAGTCTTGTAAACCGGAGATGAAAACCTTTTTCCAAGG
17666	Yes	AATACACCAGTCTTGTAAACCGGAGATGAAAACCTTTTTCCAAGGA
17667	Yes	AATACACCAGTCTTGTAAACCGGAGATGAAAACCTTTTTCCAAGGAC
17668	Yes	AATACACCAGTCTTGTAAACCGGAGATGAAAACCTTTTTCCAAGGACA
17669	Yes	AATACACCAGTCTTGTAAACCGGAGATGAAAACCTTTTTCCAAGGACAC
17670	Yes	AATACACCAGTCTTGTAAACCGGAGATGAAAACCTTTTTCCAAGGACACC
17671	No	AATACACTGAAAATGT
17672	No	AATACACTGAAAATGTT
17673	No	AATACACTGAAAATGTTT
17674	Yes	AATACACTGAAAATGTTTA
17675	Yes	AATACACTGAAAATGTTTAG

17676	Yes	AATACACTGAAAATGTTTAGA
17677	Yes	AATACACTGAAAATGTTTAGAC
17678	Yes	AATACACTGAAAATGTTTAGACG
17679	Yes	AATACACTGAAAATGTTTAGACGG
17680	Yes	AATACACTGAAAATGTTTAGACGGG
17681	Yes	AATACACTGAAAATGTTTAGACGGGC
17682	Yes	AATACACTGAAAATGTTTAGACGGGCT
17683	Yes	AATACACTGAAAATGTTTAGACGGGCTC
17684	Yes	AATACACTGAAAATGTTTAGACGGGCTCA
17685	Yes	AATACACTGAAAATGTTTAGACGGGCTCAC
17686	Yes	AATACACTGAAAATGTTTAGACGGGCTCACA
17687	Yes	AATACACTGAAAATGTTTAGACGGGCTCACAT
17688	Yes	AATACACTGAAAATGTTTAGACGGGCTCACATC
17689	Yes	AATACACTGAAAATGTTTAGACGGGCTCACATCA
17690	Yes	AATACACTGAAAATGTTTAGACGGGCTCACATCAC
17691	Yes	AATACACTGAAAATGTTTAGACGGGCTCACATCACC
17692	Yes	AATACACTGAAAATGTTTAGACGGGCTCACATCACCC
17693	Yes	AATACACTGAAAATGTTTAGACGGGCTCACATCACCCC
17694	Yes	AATACACTGAAAATGTTTAGACGGGCTCACATCACCCCA
17695	Yes	AATACACTGAAAATGTTTAGACGGGCTCACATCACCCCAT
17696	Yes	AATACACTGAAAATGTTTAGACGGGCTCACATCACCCCATA
17697	Yes	AATACACTGAAAATGTTTAGACGGGCTCACATCACCCCATAA
17698	Yes	AATACACTGAAAATGTTTAGACGGGCTCACATCACCCCATAAA
17699	Yes	AATACACTGAAAATGTTTAGACGGGCTCACATCACCCCATAAAC
17700	Yes	AATACACTGAAAATGTTTAGACGGGCTCACATCACCCCATAAACA
17701	Yes	AATACACTGAAAATGTTTAGACGGGCTCACATCACCCCATAAACAC
17702	Yes	AATACACTGAAAATGTTTAGACGGGCTCACATCACCCCATAAACACC
17703	Yes	AATACACTGAAAATGTTTAGACGGGCTCACATCACCCCATAAACACCA
17704	No	AATACAGACCAAGAGC
17705	Yes	AATACAGACCAAGAGCC
17706	Yes	AATACAGACCAAGAGCCT
17707	Yes	AATACAGACCAAGAGCCTT
17708	Yes	AATACAGACCAAGAGCCTTC
17709	Yes	AATACAGACCAAGAGCCTTCA
17710	Yes	AATACAGACCAAGAGCCTTCAA
17711	Yes	AATACAGACCAAGAGCCTTCAAA
17712	Yes	AATACAGACCAAGAGCCTTCAAAG
17713	Yes	AATACAGACCAAGAGCCTTCAAAGC
17714	Yes	AATACAGACCAAGAGCCTTCAAAGCC
17715	Yes	AATACAGACCAAGAGCCTTCAAAGCCC
17716	Yes	AATACAGACCAAGAGCCTTCAAAGCCCT
17717	Yes	AATACAGACCAAGAGCCTTCAAAGCCCTC

17718	Yes	AATACAGACCAAGAGCCTTCAAAGCCCTCA
17719	Yes	AATACAGACCAAGAGCCTTCAAAGCCCTCAG
17720	Yes	AATACAGACCAAGAGCCTTCAAAGCCCTCAGT
17721	Yes	AATACAGACCAAGAGCCTTCAAAGCCCTCAGTA
17722	Yes	AATACAGACCAAGAGCCTTCAAAGCCCTCAGTAA
17723	Yes	AATACAGACCAAGAGCCTTCAAAGCCCTCAGTAAG
17724	Yes	AATACAGACCAAGAGCCTTCAAAGCCCTCAGTAAGT
17725	Yes	AATACAGACCAAGAGCCTTCAAAGCCCTCAGTAAGTT
17726	Yes	AATACAGACCAAGAGCCTTCAAAGCCCTCAGTAAGTTG
17727	Yes	AATACAGACCAAGAGCCTTCAAAGCCCTCAGTAAGTTGC
17728	Yes	AATACAGACCAAGAGCCTTCAAAGCCCTCAGTAAGTTGCA
17729	Yes	AATACAGACCAAGAGCCTTCAAAGCCCTCAGTAAGTTGCAA
17730	Yes	AATACAGACCAAGAGCCTTCAAAGCCCTCAGTAAGTTGCAAT
17731	Yes	AATACAGACCAAGAGCCTTCAAAGCCCTCAGTAAGTTGCAATA
17732	Yes	AATACAGACCAAGAGCCTTCAAAGCCCTCAGTAAGTTGCAATAC
17733	Yes	AATACAGACCAAGAGCCTTCAAAGCCCTCAGTAAGTTGCAATACT
17734	Yes	AATACAGACCAAGAGCCTTCAAAGCCCTCAGTAAGTTGCAATACTT
17735	Yes	AATACAGACCAAGAGCCTTCAAAGCCCTCAGTAAGTTGCAATACTTA
17736	Yes	AATACAGACCAAGAGCCTTCAAAGCCCTCAGTAAGTTGCAATACTTAA
17737	Yes	AATACAGACCAAGAGCCTTCAAAGCCCTCAGTAAGTTGCAATACTTAAT
17738	Yes	AATACAGACCAAGAGCCTTCAAAGCCCTCAGTAAGTTGCAATACTTAATT
17739	No	AATACAGTGATCAGAG
17740	No	AATACAGTGATCAGAGT
17741	No	AATACAGTGATCAGAGTT
17742	Yes	AATACAGTGATCAGAGTTC
17743	Yes	AATACAGTGATCAGAGTTCA
17744	Yes	AATACAGTGATCAGAGTTCAA
17745	Yes	AATACAGTGATCAGAGTTCAAG
17746	Yes	AATACAGTGATCAGAGTTCAAGT
17747	Yes	AATACAGTGATCAGAGTTCAAGTC
17748	Yes	AATACAGTGATCAGAGTTCAAGTCT
17749	Yes	AATACAGTGATCAGAGTTCAAGTCTC
17750	Yes	AATACAGTGATCAGAGTTCAAGTCTCA
17751	Yes	AATACAGTGATCAGAGTTCAAGTCTCAC
17752	Yes	AATACAGTGATCAGAGTTCAAGTCTCACT
17753	Yes	AATACAGTGATCAGAGTTCAAGTCTCACTG
17754	Yes	AATACAGTGATCAGAGTTCAAGTCTCACTGG
17755	Yes	AATACAGTGATCAGAGTTCAAGTCTCACTGGA
17756	Yes	AATACAGTGATCAGAGTTCAAGTCTCACTGGAA
17757	Yes	AATACAGTGATCAGAGTTCAAGTCTCACTGGAAC
17758	Yes	AATACAGTGATCAGAGTTCAAGTCTCACTGGAACC
17759	Yes	AATACAGTGATCAGAGTTCAAGTCTCACTGGAACCT

17760	Yes	AATACAGTGATCAGAGTTCAAGTCTCACTGGAACCTC
17761	Yes	AATACAGTGATCAGAGTTCAAGTCTCACTGGAACCTCC
17762	Yes	AATACAGTGATCAGAGTTCAAGTCTCACTGGAACCTCCA
17763	Yes	AATACAGTGATCAGAGTTCAAGTCTCACTGGG
17764	Yes	AATACAGTGATCAGAGTTCAAGTCTCACTGGGA
17765	Yes	AATACAGTGATCAGAGTTCAAGTCTCACTGGGAC
17766	Yes	AATACAGTGATCAGAGTTCAAGTCTCACTGGGACC
17767	Yes	AATACAGTGATCAGAGTTCAAGTCTCACTGGGACCT
17768	Yes	AATACAGTGATCAGAGTTCAAGTCTCACTGGGACCTC
17769	Yes	AATACAGTGATCAGAGTTCAAGTCTCACTGGGACCTCC
17770	Yes	AATACAGTGATCAGAGTTCAAGTCTCACTGGGACCTCCA
17771	No	AATACCCAGTCAGGGA
17772	Yes	AATACCCAGTCAGGGAA
17773	Yes	AATACCCAGTCAGGGAAC
17774	Yes	AATACCCAGTCAGGGAACC
17775	Yes	AATACCCAGTCAGGGAACCA
17776	No	AATACTTAATTTCTGC
17777	No	AATACTTAATTTCTGCC
17778	No	AATACTTAATTTCTGCCA
17779	No	AATAGCTCAATTGGGA
17780	No	AATAGCTCAATTGGGAG
17781	No	AATAGCTCAATTGGGAGA
17782	No	AATAGCTCAATTGGGAGAG
17783	Yes	AATAGCTCAATTGGGAGAGT
17784	Yes	AATAGCTCAATTGGGAGAGTG
17785	Yes	AATAGCTCAATTGGGAGAGTGT
17786	Yes	AATAGCTCAATTGGGAGAGTGTT
17787	Yes	AATAGCTCAATTGGGAGAGTGTTA
17788	Yes	AATAGCTCAATTGGGAGAGTGTTAG
17789	Yes	AATAGCTCAATTGGGAGAGTGTTAGA
17790	Yes	AATAGCTCAATTGGGAGAGTGTTAGAC
17791	Yes	AATAGCTCAATTGGGAGAGTGTTAGACT
17792	Yes	AATAGCTCAATTGGGAGAGTGTTAGACTG
17793	Yes	AATAGCTCAATTGGGAGAGTGTTAGACTGA
17794	Yes	AATAGCTCAATTGGGAGAGTGTTAGACTGAA
17795	Yes	AATAGCTCAATTGGGAGAGTGTTAGACTGAAG
17796	Yes	AATAGCTCAATTGGGAGAGTGTTAGACTGAAGA
17797	Yes	AATAGCTCAATTGGGAGAGTGTTAGACTGAAGAT
17798	Yes	AATAGCTCAATTGGGAGAGTGTTAGACTGAAGATC
17799	Yes	AATAGCTCAATTGGGAGAGTGTTAGACTGAAGATCT
17800	Yes	AATAGCTCAATTGGGAGAGTGTTAGACTGAAGATCTT
17801	Yes	AATAGCTCAATTGGGAGAGTGTTAGACTGAAGATCTTC

17802	Yes	AATAGCTCAATTGGGAGAGTGTTAGACTGAAGATCTTCT
17803	Yes	AATAGCTCAATTGGGAGAGTGTTAGACTGAAGATCTTCTG
17804	Yes	AATAGCTCAATTGGGAGAGTGTTAGACTGAAGATCTTCTGC
17805	Yes	AATAGCTCAATTGGGAGAGTGTTAGACTGAAGATCTTCTGCA
17806	Yes	AATAGCTCAATTGGGAGAGTGTTAGACTGAAGATCTTCTGCAG
17807	Yes	AATAGCTCAATTGGGAGAGTGTTAGACTGAAGATCTTCTGCAGG
17808	Yes	AATAGCTCAATTGGGAGAGTGTTAGACTGAAGATCTTCTGCAGGT
17809	Yes	AATAGCTCAATTGGGAGAGTGTTAGACTGAAGATCTTCTGCAGGTC
17810	Yes	AATAGCTCAATTGGGAGAGTGTTAGACTGAAGATCTTCTGCAGGTCT
17811	Yes	AATAGCTCAATTGGGAGAGTGTTAGACTGAAGATCTTCTGCAGGTCTC
17812	Yes	AATAGCTCAATTGGGAGAGTGTTAGACTGAAGATCTTCTGCAGGTCTCT
17813	Yes	AATAGCTCAATTGGGAGAGTGTTAGACTGAAGATCTTCTGCAGGTCTCTG
17814	No	AATAGCTCAGCTGGGA
17815	No	AATAGCTCAGCTGGGAG
17816	Yes	AATAGCTCAGCTGGGAGA
17817	Yes	AATAGCTCAGCTGGGAGAG
17818	Yes	AATAGCTCAGCTGGGAGAGC
17819	Yes	AATAGCTCAGCTGGGAGAGCA
17820	Yes	AATAGCTCAGCTGGGAGAGCAT
17821	Yes	AATAGCTCAGCTGGGAGAGCATT
17822	Yes	AATAGCTCAGCTGGGAGAGCATTA
17823	Yes	AATAGCTCAGCTGGGAGAGCATTAG
17824	Yes	AATAGCTCAGCTGGGAGAGCATTAGA
17825	Yes	AATAGCTCAGCTGGGAGAGCATTAGAC
17826	Yes	AATAGCTCAGCTGGGAGAGCATTAGACT
17827	Yes	AATAGCTCAGCTGGGAGAGCATTAGACTG
17828	Yes	AATAGCTCAGCTGGGAGAGCATTAGACTGA
17829	Yes	AATAGCTCAGCTGGGAGAGCATTAGACTGAA
17830	Yes	AATAGCTCAGCTGGGAGAGCATTAGACTGAAG
17831	Yes	AATAGCTCAGCTGGGAGAGCATTAGACTGAAGA
17832	Yes	AATAGCTCAGCTGGGAGAGCATTAGACTGAAGAT
17833	Yes	AATAGCTCAGCTGGGAGAGCATTAGACTGAAGATC
17834	Yes	AATAGCTCAGCTGGGAGAGCATTAGACTGAAGATCT
17835	Yes	AATAGCTCAGCTGGGAGAGCATTAGACTGAAGATCTA
17836	Yes	AATAGCTCAGCTGGGAGAGCATTAGACTGAAGATCTAA
17837	Yes	AATAGCTCAGCTGGGAGAGCATTAGACTGAAGATCTAAA
17838	Yes	AATAGCTCAGCTGGGAGAGCATTAGACTGAAGATCTAAAG
17839	Yes	AATAGCTCAGCTGGGAGAGCATTAGACTGAAGATCTAAAGG
17840	Yes	AATAGCTCAGCTGGGAGAGCATTAGACTGAAGATCTAAAGGT
17841	Yes	AATAGCTCAGCTGGGAGAGCATTAGACTGAAGATCTAAAGGTC
17842	Yes	AATAGCTCAGCTGGGAGAGCATTAGACTGAAGATCTAAAGGTCT
17843	Yes	AATAGCTCAGCTGGGAGAGCATTAGACTGAAGATCTAAAGGTCTC

17844	Yes	AATAGCTCAGCTGGGAGAGCATTAGACTGAAGATCTAAAGGTCTCT
17845	Yes	AATAGCTCAGCTGGGAGAGCATTAGACTGAAGATCTAAAGGTCTCTG
17846	Yes	AATAGCTCAGCTGGGAGAGCATTAGACTGAAGATCTAAAGGTCTCTGG
17847	Yes	AATAGCTCAGCTGGGAGAGCATTAGACTGAAGATCTAAAGGTCTCTGGT
17848	Yes	AATAGCTCAGCTGGGAGAGCATTAGACTGAAGATCTAAAGGTCTCTGGTT
17849	Yes	AATAGCTCAGCTGGGAGAGT
17850	Yes	AATAGCTCAGCTGGGAGAGTA
17851	Yes	AATAGCTCAGCTGGGAGAGTAT
17852	Yes	AATAGCTCAGCTGGGAGAGTATT
17853	Yes	AATAGCTCAGCTGGGAGAGTATTA
17854	Yes	AATAGCTCAGCTGGGAGAGTATTAG
17855	Yes	AATAGCTCAGCTGGGAGAGTATTAGG
17856	Yes	AATAGCTCAGCTGGGAGAGTATTAGGT
17857	Yes	AATAGCTCAGCTGGGAGAGTATTAGGTT
17858	Yes	AATAGCTCAGCTGGGAGAGTATTAGGTTG
17859	Yes	AATAGCTCAGCTGGGAGAGTATTAGGTTGA
17860	Yes	AATAGCTCAGCTGGGAGAGTATTAGGTTGAA
17861	Yes	AATAGCTCAGCTGGGAGAGTATTAGGTTGAAG
17862	Yes	AATAGCTCAGCTGGGAGAGTATTAGGTTGAAGA
17863	Yes	AATAGCTCAGCTGGGAGAGTATTAGGTTGAAGAT
17864	Yes	AATAGCTCAGCTGGGAGAGTATTAGGTTGAAGATA
17865	Yes	AATAGCTCAGCTGGGAGAGTATTAGGTTGAAGATAC
17866	Yes	AATAGCTCAGCTGGGAGAGTATTAGGTTGAAGATACA
17867	Yes	AATAGCTCAGCTGGGAGAGTATTAGGTTGAAGATACAA
17868	Yes	AATAGCTCAGCTGGGAGAGTATTAGGTTGAAGATACAAA
17869	Yes	AATAGCTCAGCTGGGAGAGTATTAGGTTGAAGATACAAAG
17870	Yes	AATAGCTCAGCTGGGAGAGTATTAGGTTGAAGATACAAAGT
17871	Yes	AATAGCTCAGCTGGGAGAGTATTAGGTTGAAGATACAAAGTT
17872	Yes	AATAGCTCAGCTGGGAGAGTATTAGGTTGAAGATACAAAGTTC
17873	Yes	AATAGCTCAGCTGGGAGAGTATTAGGTTGAAGATACAAAGTTCC
17874	Yes	AATAGCTCAGCTGGGAGAGTATTAGGTTGAAGATACAAAGTTCCT
17875	Yes	AATAGCTCAGCTGGGAGAGTATTAGGTTGAAGATACAAAGTTCCTT
17876	Yes	AATAGCTCAGCTGGGAGAGTATTAGGTTGAAGATACAAAGTTCCTTG
17877	Yes	AATAGCTCAGCTGGGAGAGTATTAGGTTGAAGATACAAAGTTCCTTGG
17878	Yes	AATAGCTCAGCTGGGAGAGTATTAGGTTGAAGATACAAAGTTCCTTGGC
17879	Yes	AATAGCTCAGCTGGGAGAGTATTAGGTTGAAGATACAAAGTTCCTTGGCT
17880	No	AATAGCTCAGCTGGTA
17881	No	AATAGCTCAGCTGGTAG
17882	No	AATAGCTCAGCTGGTAGA
17883	No	AATAGCTCAGCTGGTAGAG
17884	No	AATAGCTCAGCTGGTAGAGC
17885	No	AATAGCTCAGCTGGTAGAGCG

17886	No	AATAGCTCAGCTGGTAGAGCGG
17887	No	AATAGCTCAGCTGGTAGAGCGGA
17888	No	AATAGCTCAGCTGGTAGAGCGGAG
17889	No	AATAGCTCAGCTGGTAGAGCGGAGG
17890	No	AATAGCTCAGCTGGTAGAGCGGAGGA
17891	No	AATAGCTCAGCTGGTAGAGCGGAGGAC
17892	No	AATAGCTCAGCTGGTAGAGCGGAGGACT
17893	No	AATAGCTCAGCTGGTAGAGCGGAGGACTG
17894	No	AATAGCTCAGCTGGTAGAGCGGAGGACTGT
17895	No	AATAGCTCAGCTGGTAGAGCGGAGGACTGTA
17896	No	AATAGCTCAGCTGGTAGAGCGGAGGACTGTAG
17897	Yes	AATAGCTCAGCTGGTAGAGCGGAGGACTGTAGA
17898	Yes	AATAGCTCAGCTGGTAGAGCGGAGGACTGTAGAT
17899	Yes	AATAGCTCAGCTGGTAGAGCGGAGGACTGTAGATT
17900	Yes	AATAGCTCAGCTGGTAGAGCGGAGGACTGTAGATTC
17901	Yes	AATAGCTCAGCTGGTAGAGCGGAGGACTGTAGATTCT
17902	Yes	AATAGCTCAGCTGGTAGAGCGGAGGACTGTAGATTCTT
17903	Yes	AATAGCTCAGCTGGTAGAGCGGAGGACTGTAGATTCTTA
17904	Yes	AATAGCTCAGCTGGTAGAGCGGAGGACTGTAGATTCTTAG
17905	Yes	AATAGCTCAGCTGGTAGAGCGGAGGACTGTAGATTCTTAGG
17906	Yes	AATAGCTCAGCTGGTAGAGCGGAGGACTGTAGATTCTTAGGT
17907	Yes	AATAGCTCAGCTGGTAGAGCGGAGGACTGTAGATTCTTAGGTG
17908	Yes	AATAGCTCAGCTGGTAGAGCGGAGGACTGTAGATTCTTAGGTGC
17909	Yes	AATAGCTCAGCTGGTAGAGCGGAGGACTGTAGATTCTTAGGTGCT
17910	Yes	AATAGCTCAGCTGGTAGAGCGGAGGACTGTAGATTCTTAGGTGCTG
17911	Yes	AATAGCTCAGCTGGTAGAGCGGAGGACTGTAGATTCTTAGGTGCTGG
17912	Yes	AATAGCTCAGCTGGTAGAGCGGAGGACTGTAGATTCTTAGGTGCTGGT
17913	Yes	AATAGCTCAGCTGGTAGAGCGGAGGACTGTAGATTCTTAGGTGCTGGTT
17914	Yes	AATAGCTCAGCTGGTAGAGCGGAGGACTGTAGATTCTTAGGTGCTGGTTT
17915	No	AATAGCTCAGTTGGGA
17916	No	AATAGCTCAGTTGGGAG
17917	No	AATAGCTCAGTTGGGAGA
17918	No	AATAGCTCAGTTGGGAGAG
17919	No	AATAGCTCAGTTGGGAGAGC
17920	No	AATAGCTCAGTTGGGAGAGCG
17921	No	AATAGCTCAGTTGGGAGAGCGT
17922	No	AATAGCTCAGTTGGGAGAGCGTT
17923	No	AATAGCTCAGTTGGGAGAGCGTTA
17924	No	AATAGCTCAGTTGGGAGAGCGTTAG
17925	No	AATAGCTCAGTTGGGAGAGCGTTAGA
17926	No	AATAGCTCAGTTGGGAGAGCGTTAGAC
17927	Yes	AATAGCTCAGTTGGGAGAGCGTTAGACC

17928	Yes	AATAGCTCAGTTGGGAGAGCGTTAGACCG
17929	Yes	AATAGCTCAGTTGGGAGAGCGTTAGACCGA
17930	Yes	AATAGCTCAGTTGGGAGAGCGTTAGACCGAA
17931	Yes	AATAGCTCAGTTGGGAGAGCGTTAGACCGAAG
17932	Yes	AATAGCTCAGTTGGGAGAGCGTTAGACCGAAGA
17933	Yes	AATAGCTCAGTTGGGAGAGCGTTAGACCGAAGAT
17934	Yes	AATAGCTCAGTTGGGAGAGCGTTAGACCGAAGATC
17935	Yes	AATAGCTCAGTTGGGAGAGCGTTAGACCGAAGATCT
17936	Yes	AATAGCTCAGTTGGGAGAGCGTTAGACCGAAGATCTT
17937	Yes	AATAGCTCAGTTGGGAGAGCGTTAGACCGAAGATCTTA
17938	Yes	AATAGCTCAGTTGGGAGAGCGTTAGACCGAAGATCTTAA
17939	Yes	AATAGCTCAGTTGGGAGAGCGTTAGACCGAAGATCTTAAA
17940	Yes	AATAGCTCAGTTGGGAGAGCGTTAGACCGAAGATCTTAAAG
17941	Yes	AATAGCTCAGTTGGGAGAGCGTTAGACCGAAGATCTTAAAGG
17942	Yes	AATAGCTCAGTTGGGAGAGCGTTAGACCGAAGATCTTAAAGGT
17943	Yes	AATAGCTCAGTTGGGAGAGCGTTAGACCGAAGATCTTAAAGGTC
17944	Yes	AATAGCTCAGTTGGGAGAGCGTTAGACCGAAGATCTTAAAGGTCC
17945	Yes	AATAGCTCAGTTGGGAGAGCGTTAGACCGAAGATCTTAAAGGTCCC
17946	Yes	AATAGCTCAGTTGGGAGAGCGTTAGACCGAAGATCTTAAAGGTCCCT
17947	Yes	AATAGCTCAGTTGGGAGAGCGTTAGACCGAAGATCTTAAAGGTCCCTG
17948	Yes	AATAGCTCAGTTGGGAGAGCGTTAGACCGAAGATCTTAAAGGTCCCTGG
17949	Yes	AATAGCTCAGTTGGGAGAGCGTTAGACCGAAGATCTTAAAGGTCCCTGGT
17950	No	AATAGCTCAGTTGGGAGAGCGTTAGACT
17951	No	AATAGCTCAGTTGGGAGAGCGTTAGACTG
17952	No	AATAGCTCAGTTGGGAGAGCGTTAGACTGA
17953	No	AATAGCTCAGTTGGGAGAGCGTTAGACTGAA
17954	No	AATAGCTCAGTTGGGAGAGCGTTAGACTGAAG
17955	No	AATAGCTCAGTTGGGAGAGCGTTAGACTGAAGA
17956	Yes	AATAGCTCAGTTGGGAGAGCGTTAGACTGAAGAT
17957	Yes	AATAGCTCAGTTGGGAGAGCGTTAGACTGAAGATC
17958	Yes	AATAGCTCAGTTGGGAGAGCGTTAGACTGAAGATCT
17959	Yes	AATAGCTCAGTTGGGAGAGCGTTAGACTGAAGATCTA
17960	Yes	AATAGCTCAGTTGGGAGAGCGTTAGACTGAAGATCTAA
17961	Yes	AATAGCTCAGTTGGGAGAGCGTTAGACTGAAGATCTAAA
17962	Yes	AATAGCTCAGTTGGGAGAGCGTTAGACTGAAGATCTAAAG
17963	Yes	AATAGCTCAGTTGGGAGAGCGTTAGACTGAAGATCTAAAGG
17964	Yes	AATAGCTCAGTTGGGAGAGCGTTAGACTGAAGATCTAAAGGT
17965	Yes	AATAGCTCAGTTGGGAGAGCGTTAGACTGAAGATCTAAAGGTC
17966	Yes	AATAGCTCAGTTGGGAGAGCGTTAGACTGAAGATCTAAAGGTCC
17967	Yes	AATAGCTCAGTTGGGAGAGCGTTAGACTGAAGATCTAAAGGTCCC
17968	Yes	AATAGCTCAGTTGGGAGAGCGTTAGACTGAAGATCTAAAGGTCCCT
17969	Yes	AATAGCTCAGTTGGGAGAGCGTTAGACTGAAGATCTAAAGGTCCCTG

17970	Yes	AATAGCTCAGTTGGGAGAGCGTTAGACTGAAGATCTAAAGGTCCCTGG
17971	Yes	AATAGCTCAGTTGGGAGAGCGTTAGACTGAAGATCTAAAGGTCCCTGGT
17972	Yes	AATAGCTCAGTTGGGAGAGCGTTAGACTGAAGATCTAAAGGTCCCTGGTT
17973	Yes	AATAGCTCAGTTGGGAGAGCGTTAGACTGAAGATCTT
17974	Yes	AATAGCTCAGTTGGGAGAGCGTTAGACTGAAGATCTTA
17975	Yes	AATAGCTCAGTTGGGAGAGCGTTAGACTGAAGATCTTAA
17976	Yes	AATAGCTCAGTTGGGAGAGCGTTAGACTGAAGATCTTAAA
17977	Yes	AATAGCTCAGTTGGGAGAGCGTTAGACTGAAGATCTTAAAG
17978	Yes	AATAGCTCAGTTGGGAGAGCGTTAGACTGAAGATCTTAAAGT
17979	Yes	AATAGCTCAGTTGGGAGAGCGTTAGACTGAAGATCTTAAAGTT
17980	Yes	AATAGCTCAGTTGGGAGAGCGTTAGACTGAAGATCTTAAAGTTC
17981	Yes	AATAGCTCAGTTGGGAGAGCGTTAGACTGAAGATCTTAAAGTTCC
17982	Yes	AATAGCTCAGTTGGGAGAGCGTTAGACTGAAGATCTTAAAGTTCCC
17983	Yes	AATAGCTCAGTTGGGAGAGCGTTAGACTGAAGATCTTAAAGTTCCCT
17984	Yes	AATAGCTCAGTTGGGAGAGCGTTAGACTGAAGATCTTAAAGTTCCCTG
17985	Yes	AATAGCTCAGTTGGGAGAGCGTTAGACTGAAGATCTTAAAGTTCCCTGG
17986	Yes	AATAGCTCAGTTGGGAGAGCGTTAGACTGAAGATCTTAAAGTTCCCTGGT
17987	Yes	AATAGCTTAGTTGATG
17988	Yes	AATAGCTTAGTTGATGA
17989	Yes	AATAGCTTAGTTGATGAA
17990	Yes	AATAGCTTAGTTGATGAAG
17991	Yes	AATAGCTTAGTTGATGAAGC
17992	Yes	AATAGCTTAGTTGATGAAGCG
17993	Yes	AATAGCTTAGTTGATGAAGCGT
17994	Yes	AATAGCTTAGTTGATGAAGCGTG
17995	Yes	AATAGCTTAGTTGATGAAGCGTGA
17996	Yes	AATAGCTTAGTTGATGAAGCGTGAG
17997	Yes	AATAGCTTAGTTGATGAAGCGTGAGA
17998	Yes	AATAGCTTAGTTGATGAAGCGTGAGAC
17999	Yes	AATAGCTTAGTTGATGAAGCGTGAGACT
18000	Yes	AATAGCTTAGTTGATGAAGCGTGAGACTC
18001	Yes	AATAGCTTAGTTGATGAAGCGTGAGACTCT
18002	Yes	AATAGCTTAGTTGATGAAGCGTGAGACTCTT
18003	Yes	AATAGCTTAGTTGATGAAGCGTGAGACTCTTA
18004	Yes	AATAGCTTAGTTGATGAAGCGTGAGACTCTTAA
18005	Yes	AATAGCTTAGTTGATGAAGCGTGAGACTCTTAAT
18006	Yes	AATAGCTTAGTTGATGAAGCGTGAGACTCTTAATC
18007	Yes	AATAGCTTAGTTGATGAAGCGTGAGACTCTTAATCT
18008	Yes	AATAGCTTAGTTGATGAAGCGTGAGACTCTTAATCTC
18009	Yes	AATAGCTTAGTTGATGAAGCGTGAGACTCTTAATCTCA
18010	Yes	AATAGCTTAGTTGATGAAGCGTGAGACTCTTAATCTCAG
18011	Yes	AATAGCTTAGTTGATGAAGCGTGAGACTCTTAATCTCAGG

18012	Yes	AATAGCTTAGTTGATGAAGCGTGAGACTCTTAATCTCAGGG
18013	Yes	AATAGCTTAGTTGATGAAGCGTGAGACTCTTAATCTCAGGGT
18014	Yes	AATAGCTTAGTTGATGAAGCGTGAGACTCTTAATCTCAGGGTA
18015	Yes	AATAGCTTAGTTGATGAAGCGTGAGACTCTTAATCTCAGGGTAG
18016	Yes	AATAGCTTAGTTGATGAAGCGTGAGACTCTTAATCTCAGGGTAGT
18017	Yes	AATAGCTTAGTTGATGAAGCGTGAGACTCTTAATCTCAGGGTAGTG
18018	Yes	AATAGCTTAGTTGATGAAGCGTGAGACTCTTAATCTCAGGGTAGTGG
18019	Yes	AATAGCTTAGTTGATGAAGCGTGAGACTCTTAATCTCAGGGTAGTGGG
18020	Yes	AATAGCTTAGTTGATGAAGCGTGAGACTCTTAATCTCAGGGTAGTGGGT
18021	Yes	AATAGCTTAGTTGATGAAGCGTGAGACTCTTAATCTCAGGGTAGTGGGTT
18022	No	AATAGGAGCTTAAACC
18023	Yes	AATAGGAGCTTAAACCC
18024	Yes	AATAGGAGCTTAAACCCC
18025	Yes	AATAGGAGCTTAAACCCCC
18026	Yes	AATAGGAGCTTAAACCCCCT
18027	Yes	AATAGGAGCTTAAACCCCCTT
18028	Yes	AATAGGAGCTTAAACCCCCTTA
18029	Yes	AATAGGAGCTTAAACCCCCTTAT
18030	Yes	AATAGGAGCTTAAACCCCCTTATT
18031	Yes	AATAGGAGCTTAAACCCCCTTATTT
18032	Yes	AATAGGAGCTTAAACCCCCTTATTTC
18033	Yes	AATAGGAGCTTAAACCCCCTTATTTCT
18034	Yes	AATAGGAGCTTAAACCCCCTTATTTCTA
18035	Yes	AATAGGAGCTTAAACCCCCTTATTTCTAC
18036	Yes	AATAGGAGCTTAAACCCCCTTATTTCTACC
18037	Yes	AATAGGAGCTTAAACCCCCTTATTTCTACCA
18038	No	AATAGGCTTTAGAGCC
18039	No	AATAGGCTTTAGAGCCA
18040	No	AATAGGCTTTAGAGCCAG
18041	Yes	AATAGGCTTTAGAGCCAGA
18042	Yes	AATAGGCTTTAGAGCCAGAC
18043	Yes	AATAGGCTTTAGAGCCAGACT
18044	Yes	AATAGGCTTTAGAGCCAGACTG
18045	Yes	AATAGGCTTTAGAGCCAGACTGC
18046	Yes	AATAGGCTTTAGAGCCAGACTGCC
18047	Yes	AATAGGCTTTAGAGCCAGACTGCCT
18048	Yes	AATAGGCTTTAGAGCCAGACTGCCTG
18049	Yes	AATAGGCTTTAGAGCCAGACTGCCTGG
18050	Yes	AATAGGCTTTAGAGCCAGACTGCCTGGG
18051	Yes	AATAGGCTTTAGAGCCAGACTGCCTGGGT
18052	Yes	AATAGGCTTTAGAGCCAGACTGCCTGGGTT
18053	Yes	AATAGGCTTTAGAGCCAGACTGCCTGGGTTT

18054	Yes	AATAGGCTTTAGAGCCAGACTGCCTGGGTTTG
18055	Yes	AATAGGCTTTAGAGCCAGACTGCCTGGGTTTGA
18056	Yes	AATAGGCTTTAGAGCCAGACTGCCTGGGTTTGAA
18057	Yes	AATAGGCTTTAGAGCCAGACTGCCTGGGTTTGAAT
18058	Yes	AATAGGCTTTAGAGCCAGACTGCCTGGGTTTGAATC
18059	Yes	AATAGGCTTTAGAGCCAGACTGCCTGGGTTTGAATCC
18060	Yes	AATAGGCTTTAGAGCCAGACTGCCTGGGTTTGAATCCC
18061	Yes	AATAGGCTTTAGAGCCAGACTGCCTGGGTTTGAATCCCA
18062	Yes	AATAGGCTTTAGAGCCAGACTGCCTGGGTTTGAATCCCAG
18063	Yes	AATAGGCTTTAGAGCCAGACTGCCTGGGTTTGAATCCCAGC
18064	Yes	AATAGGCTTTAGAGCCAGACTGCCTGGGTTTGAATCCCAGCC
18065	Yes	AATAGGCTTTAGAGCCAGACTGCCTGGGTTTGAATCCCAGCCC
18066	Yes	AATAGGCTTTAGAGCCAGACTGCCTGGGTTTGAATCCCAGCCCC
18067	Yes	AATAGGCTTTAGAGCCAGACTGCCTGGGTTTGAATCCCAGCCCCG
18068	Yes	AATAGGCTTTAGAGCCAGACTGCCTGGGTTTGAATCCCAGCCCCGC
18069	Yes	AATAGGCTTTAGAGCCAGACTGCCTGGGTTTGAATCCCAGCCCCGCC
18070	Yes	AATAGGCTTTAGAGCCAGACTGCCTGGGTTTGAATCCCAGCCCCGCCA
18071	Yes	AATAGGCTTTAGAGCCAGACTGCCTGGGTTTGAATCCCAGCCCCGCCAC
18072	Yes	AATAGGCTTTAGAGCCAGACTGCCTGGGTTTGAATCCCAGCCCCGCCACC
18073	Yes	AATAGGTAGAATGGAG
18074	Yes	AATAGGTAGAATGGAGA
18075	Yes	AATAGGTAGAATGGAGAA
18076	Yes	AATAGGTAGAATGGAGAAT
18077	Yes	AATAGGTAGAATGGAGAATT
18078	Yes	AATAGGTAGAATGGAGAATTT
18079	Yes	AATAGGTAGAATGGAGAATTTT
18080	Yes	AATAGGTAGAATGGAGAATTTTG
18081	Yes	AATAGGTAGAATGGAGAATTTTGA
18082	Yes	AATAGGTAGAATGGAGAATTTTGAA
18083	Yes	AATAGGTAGAATGGAGAATTTTGAAT
18084	Yes	AATAGGTAGAATGGAGAATTTTGAATT
18085	Yes	AATAGGTAGAATGGAGAATTTTGAATTC
18086	Yes	AATAGGTAGAATGGAGAATTTTGAATTCT
18087	Yes	AATAGGTAGAATGGAGAATTTTGAATTCTC
18088	Yes	AATAGGTAGAATGGAGAATTTTGAATTCTCA
18089	Yes	AATAGGTAGAATGGAGAATTTTGAATTCTCAG
18090	Yes	AATAGGTAGAATGGAGAATTTTGAATTCTCAGG
18091	Yes	AATAGGTAGAATGGAGAATTTTGAATTCTCAGGG
18092	Yes	AATAGGTAGAATGGAGAATTTTGAATTCTCAGGGG
18093	Yes	AATAGGTAGAATGGAGAATTTTGAATTCTCAGGGGT
18094	Yes	AATAGGTAGAATGGAGAATTTTGAATTCTCAGGGGTA
18095	Yes	AATAGGTAGAATGGAGAATTTTGAATTCTCAGGGGTAG

18096	Yes	AATAGGTAGAATGGAGAATTTTGAATTCTCAGGGGTAGG
18097	Yes	AATAGGTAGAATGGAGAATTTTGAATTCTCAGGGGTAGGT
18098	Yes	AATAGGTAGAATGGAGAATTTTGAATTCTCAGGGGTAGGTT
18099	Yes	AATAGGTAGAATGGAGAATTTTGAATTCTCAGGGGTAGGTTC
18100	Yes	AATAGGTAGAATGGAGAATTTTGAATTCTCAGGGGTAGGTTCA
18101	Yes	AATAGGTAGAATGGAGAATTTTGAATTCTCAGGGGTAGGTTCAA
18102	Yes	AATAGGTAGAATGGAGAATTTTGAATTCTCAGGGGTAGGTTCAAT
18103	Yes	AATAGGTAGAATGGAGAATTTTGAATTCTCAGGGGTAGGTTCAATT
18104	Yes	AATAGGTAGAATGGAGAATTTTGAATTCTCAGGGGTAGGTTCAATTC
18105	Yes	AATAGGTAGAATGGAGAATTTTGAATTCTCAGGGGTAGGTTCAATTCC
18106	Yes	AATAGGTAGAATGGAGAATTTTGAATTCTCAGGGGTAGGTTCAATTCCT
18107	Yes	AATAGGTAGAATGGAGAATTTTGAATTCTCAGGGGTAGGTTCAATTCCTA
18108	No	AATAGGTAGCACAGAG
18109	Yes	AATAGGTAGCACAGAGA
18110	Yes	AATAGGTAGCACAGAGAA
18111	Yes	AATAGGTAGCACAGAGAAT
18112	Yes	AATAGGTAGCACAGAGAATT
18113	Yes	AATAGGTAGCACAGAGAATTC
18114	Yes	AATAGGTAGCACAGAGAATTCT
18115	Yes	AATAGGTAGCACAGAGAATTCTG
18116	Yes	AATAGGTAGCACAGAGAATTCTGG
18117	Yes	AATAGGTAGCACAGAGAATTCTGGA
18118	Yes	AATAGGTAGCACAGAGAATTCTGGAT
18119	Yes	AATAGGTAGCACAGAGAATTCTGGATT
18120	Yes	AATAGGTAGCACAGAGAATTCTGGATTC
18121	Yes	AATAGGTAGCACAGAGAATTCTGGATTCT
18122	Yes	AATAGGTAGCACAGAGAATTCTGGATTCTC
18123	Yes	AATAGGTAGCACAGAGAATTCTGGATTCTCA
18124	Yes	AATAGGTAGCACAGAGAATTCTGGATTCTCAG
18125	Yes	AATAGGTAGCACAGAGAATTCTGGATTCTCAGG
18126	Yes	AATAGGTAGCACAGAGAATTCTGGATTCTCAGGG
18127	Yes	AATAGGTAGCACAGAGAATTCTGGATTCTCAGGGG
18128	Yes	AATAGGTAGCACAGAGAATTCTGGATTCTCAGGGGT
18129	Yes	AATAGGTAGCACAGAGAATTCTGGATTCTCAGGGGTA
18130	Yes	AATAGGTAGCACAGAGAATTCTGGATTCTCAGGGGTAG
18131	Yes	AATAGGTAGCACAGAGAATTCTGGATTCTCAGGGGTAGG
18132	Yes	AATAGGTAGCACAGAGAATTCTGGATTCTCAGGGGTAGGT
18133	Yes	AATAGGTAGCACAGAGAATTCTGGATTCTCAGGGGTAGGTT
18134	Yes	AATAGGTAGCACAGAGAATTCTGGATTCTCAGGGGTAGGTTC
18135	Yes	AATAGGTAGCACAGAGAATTCTGGATTCTCAGGGGTAGGTTCA
18136	Yes	AATAGGTAGCACAGAGAATTCTGGATTCTCAGGGGTAGGTTCAA
18137	Yes	AATAGGTAGCACAGAGAATTCTGGATTCTCAGGGGTAGGTTCAAT

18138	Yes	AATAGGTAGCACAGAGAATTCTGGATTCTCAGGGGTAGGTTCAATT
18139	Yes	AATAGGTAGCACAGAGAATTCTGGATTCTCAGGGGTAGGTTCAATTC
18140	Yes	AATAGGTAGCACAGAGAATTCTGGATTCTCAGGGGTAGGTTCAATTCC
18141	Yes	AATAGGTAGCACAGAGAATTCTGGATTCTCAGGGGTAGGTTCAATTCCT
18142	Yes	AATAGGTAGCACAGAGAATTCTGGATTCTCAGGGGTAGGTTCAATTCCTA
18143	Yes	AATAGGTAGCACAGAGAATTT
18144	Yes	AATAGGTAGCACAGAGAATTTT
18145	Yes	AATAGGTAGCACAGAGAATTTTG
18146	Yes	AATAGGTAGCACAGAGAATTTTGG
18147	Yes	AATAGGTAGCACAGAGAATTTTGGA
18148	Yes	AATAGGTAGCACAGAGAATTTTGGAT
18149	Yes	AATAGGTAGCACAGAGAATTTTGGATT
18150	Yes	AATAGGTAGCACAGAGAATTTTGGATTC
18151	Yes	AATAGGTAGCACAGAGAATTTTGGATTCT
18152	Yes	AATAGGTAGCACAGAGAATTTTGGATTCTC
18153	Yes	AATAGGTAGCACAGAGAATTTTGGATTCTCA
18154	Yes	AATAGGTAGCACAGAGAATTTTGGATTCTCAG
18155	Yes	AATAGGTAGCACAGAGAATTTTGGATTCTCAGG
18156	Yes	AATAGGTAGCACAGAGAATTTTGGATTCTCAGGT
18157	Yes	AATAGGTAGCACAGAGAATTTTGGATTCTCAGGTG
18158	Yes	AATAGGTAGCACAGAGAATTTTGGATTCTCAGGTGC
18159	Yes	AATAGGTAGCACAGAGAATTTTGGATTCTCAGGTGCA
18160	Yes	AATAGGTAGCACAGAGAATTTTGGATTCTCAGGTGCAG
18161	Yes	AATAGGTAGCACAGAGAATTTTGGATTCTCAGGTGCAGG
18162	Yes	AATAGGTAGCACAGAGAATTTTGGATTCTCAGGTGCAGGT
18163	Yes	AATAGGTAGCACAGAGAATTTTGGATTCTCAGGTGCAGGTT
18164	Yes	AATAGGTAGCACAGAGAATTTTGGATTCTCAGGTGCAGGTTC
18165	Yes	AATAGGTAGCACAGAGAATTTTGGATTCTCAGGTGCAGGTTCA
18166	Yes	AATAGGTAGCACAGAGAATTTTGGATTCTCAGGTGCAGGTTCAA
18167	Yes	AATAGGTAGCACAGAGAATTTTGGATTCTCAGGTGCAGGTTCAAT
18168	Yes	AATAGGTAGCACAGAGAATTTTGGATTCTCAGGTGCAGGTTCAATT
18169	Yes	AATAGGTAGCACAGAGAATTTTGGATTCTCAGGTGCAGGTTCAATTC
18170	Yes	AATAGGTAGCACAGAGAATTTTGGATTCTCAGGTGCAGGTTCAATTCC
18171	Yes	AATAGGTAGCACAGAGAATTTTGGATTCTCAGGTGCAGGTTCAATTCCT
18172	Yes	AATAGGTAGCACAGAGAATTTTGGATTCTCAGGTGCAGGTTCAATTCCTA
18173	No	AATAGGTAGCACAGAT
18174	No	AATAGGTAGCACAGATA
18175	Yes	AATAGGTAGCACAGATAA
18176	Yes	AATAGGTAGCACAGATAAT
18177	Yes	AATAGGTAGCACAGATAATT
18178	Yes	AATAGGTAGCACAGATAATTT
18179	Yes	AATAGGTAGCACAGATAATTTT

18180	Yes	AATAGGTAGCACAGATAATTTTG
18181	Yes	AATAGGTAGCACAGATAATTTTGG
18182	Yes	AATAGGTAGCACAGATAATTTTGGA
18183	Yes	AATAGGTAGCACAGATAATTTTGGAT
18184	Yes	AATAGGTAGCACAGATAATTTTGGATT
18185	Yes	AATAGGTAGCACAGATAATTTTGGATTC
18186	Yes	AATAGGTAGCACAGATAATTTTGGATTCT
18187	Yes	AATAGGTAGCACAGATAATTTTGGATTCTC
18188	Yes	AATAGGTAGCACAGATAATTTTGGATTCTCA
18189	Yes	AATAGGTAGCACAGATAATTTTGGATTCTCAG
18190	Yes	AATAGGTAGCACAGATAATTTTGGATTCTCAGG
18191	Yes	AATAGGTAGCACAGATAATTTTGGATTCTCAGGG
18192	Yes	AATAGGTAGCACAGATAATTTTGGATTCTCAGGGG
18193	Yes	AATAGGTAGCACAGATAATTTTGGATTCTCAGGGGT
18194	Yes	AATAGGTAGCACAGATAATTTTGGATTCTCAGGGGTA
18195	Yes	AATAGGTAGCACAGATAATTTTGGATTCTCAGGGGTAG
18196	Yes	AATAGGTAGCACAGATAATTTTGGATTCTCAGGGGTAGG
18197	Yes	AATAGGTAGCACAGATAATTTTGGATTCTCAGGGGTAGGT
18198	Yes	AATAGGTAGCACAGATAATTTTGGATTCTCAGGGGTAGGTT
18199	Yes	AATAGGTAGCACAGATAATTTTGGATTCTCAGGGGTAGGTTC
18200	Yes	AATAGGTAGCACAGATAATTTTGGATTCTCAGGGGTAGGTTCA
18201	Yes	AATAGGTAGCACAGATAATTTTGGATTCTCAGGGGTAGGTTCAA
18202	Yes	AATAGGTAGCACAGATAATTTTGGATTCTCAGGGGTAGGTTCAAT
18203	Yes	AATAGGTAGCACAGATAATTTTGGATTCTCAGGGGTAGGTTCAATT
18204	Yes	AATAGGTAGCACAGATAATTTTGGATTCTCAGGGGTAGGTTCAATTC
18205	Yes	AATAGGTAGCACAGATAATTTTGGATTCTCAGGGGTAGGTTCAATTCT
18206	Yes	AATAGGTAGCACAGATAATTTTGGATTCTCAGGGGTAGGTTCAATTCTT
18207	Yes	AATAGGTAGCACAGATAATTTTGGATTCTCAGGGGTAGGTTCAATTCTTA
18208	Yes	AATAGGTAGCACGAAG
18209	Yes	AATAGGTAGCACGAAGA
18210	Yes	AATAGGTAGCACGAAGAG
18211	Yes	AATAGGTAGCACGAAGAGA
18212	Yes	AATAGGTAGCACGAAGAGAT
18213	Yes	AATAGGTAGCACGAAGAGATT
18214	Yes	AATAGGTAGCACGAAGAGATTT
18215	Yes	AATAGGTAGCACGAAGAGATTTG
18216	Yes	AATAGGTAGCACGAAGAGATTTGG
18217	Yes	AATAGGTAGCACGAAGAGATTTGGA
18218	Yes	AATAGGTAGCACGAAGAGATTTGGAT
18219	Yes	AATAGGTAGCACGAAGAGATTTGGATT
18220	Yes	AATAGGTAGCACGAAGAGATTTGGATTC
18221	Yes	AATAGGTAGCACGAAGAGATTTGGATTCT

18222	Yes	AATAGGTAGCACGAAGAGATTTGGATTCTC
18223	Yes	AATAGGTAGCACGAAGAGATTTGGATTCTCA
18224	Yes	AATAGGTAGCACGAAGAGATTTGGATTCTCAG
18225	Yes	AATAGGTAGCACGAAGAGATTTGGATTCTCAGG
18226	Yes	AATAGGTAGCACGAAGAGATTTGGATTCTCAGGG
18227	Yes	AATAGGTAGCACGAAGAGATTTGGATTCTCAGGGG
18228	Yes	AATAGGTAGCACGAAGAGATTTGGATTCTCAGGGGT
18229	Yes	AATAGGTAGCACGAAGAGATTTGGATTCTCAGGGGTA
18230	Yes	AATAGGTAGCACGAAGAGATTTGGATTCTCAGGGGTAG
18231	Yes	AATAGGTAGCACGAAGAGATTTGGATTCTCAGGGGTAGG
18232	Yes	AATAGGTAGCACGAAGAGATTTGGATTCTCAGGGGTAGGT
18233	Yes	AATAGGTAGCACGAAGAGATTTGGATTCTCAGGGGTAGGTT
18234	Yes	AATAGGTAGCACGAAGAGATTTGGATTCTCAGGGGTAGGTTC
18235	Yes	AATAGGTAGCACGAAGAGATTTGGATTCTCAGGGGTAGGTTCA
18236	Yes	AATAGGTAGCACGAAGAGATTTGGATTCTCAGGGGTAGGTTCAA
18237	Yes	AATAGGTAGCACGAAGAGATTTGGATTCTCAGGGGTAGGTTCAAT
18238	Yes	AATAGGTAGCACGAAGAGATTTGGATTCTCAGGGGTAGGTTCAATT
18239	Yes	AATAGGTAGCACGAAGAGATTTGGATTCTCAGGGGTAGGTTCAATTC
18240	Yes	AATAGGTAGCACGAAGAGATTTGGATTCTCAGGGGTAGGTTCAATTCC
18241	Yes	AATAGGTAGCACGAAGAGATTTGGATTCTCAGGGGTAGGTTCAATTCCT
18242	Yes	AATAGGTAGCACGAAGAGATTTGGATTCTCAGGGGTAGGTTCAATTCCTA
18243	Yes	AATAGGTAGCATGGAG
18244	Yes	AATAGGTAGCATGGAGA
18245	Yes	AATAGGTAGCATGGAGAA
18246	Yes	AATAGGTAGCATGGAGAAT
18247	Yes	AATAGGTAGCATGGAGAATT
18248	Yes	AATAGGTAGCATGGAGAATTT
18249	Yes	AATAGGTAGCATGGAGAATTTT
18250	Yes	AATAGGTAGCATGGAGAATTTTG
18251	Yes	AATAGGTAGCATGGAGAATTTTGG
18252	Yes	AATAGGTAGCATGGAGAATTTTGGA
18253	Yes	AATAGGTAGCATGGAGAATTTTGGAG
18254	Yes	AATAGGTAGCATGGAGAATTTTGGAGT
18255	Yes	AATAGGTAGCATGGAGAATTTTGGAGTC
18256	Yes	AATAGGTAGCATGGAGAATTTTGGAGTCT
18257	Yes	AATAGGTAGCATGGAGAATTTTGGAGTCTC
18258	Yes	AATAGGTAGCATGGAGAATTTTGGAGTCTCA
18259	Yes	AATAGGTAGCATGGAGAATTTTGGAGTCTCAG
18260	Yes	AATAGGTAGCATGGAGAATTTTGGAGTCTCAGG
18261	Yes	AATAGGTAGCATGGAGAATTTTGGAGTCTCAGGG
18262	Yes	AATAGGTAGCATGGAGAATTTTGGAGTCTCAGGGA
18263	Yes	AATAGGTAGCATGGAGAATTTTGGAGTCTCAGGGAT

18264	Yes	AATAGGTAGCATGGAGAATTTTGGAGTCTCAGGGATA
18265	Yes	AATAGGTAGCATGGAGAATTTTGGAGTCTCAGGGATAG
18266	Yes	AATAGGTAGCATGGAGAATTTTGGAGTCTCAGGGATAGG
18267	Yes	AATAGGTAGCATGGAGAATTTTGGAGTCTCAGGGATAGGT
18268	Yes	AATAGGTAGCATGGAGAATTTTGGAGTCTCAGGGATAGGTT
18269	Yes	AATAGGTAGCATGGAGAATTTTGGAGTCTCAGGGATAGGTTC
18270	Yes	AATAGGTAGCATGGAGAATTTTGGAGTCTCAGGGATAGGTTCA
18271	Yes	AATAGGTAGCATGGAGAATTTTGGAGTCTCAGGGATAGGTTCAA
18272	Yes	AATAGGTAGCATGGAGAATTTTGGAGTCTCAGGGATAGGTTCAAT
18273	Yes	AATAGGTAGCATGGAGAATTTTGGAGTCTCAGGGATAGGTTCAATT
18274	Yes	AATAGGTAGCATGGAGAATTTTGGAGTCTCAGGGATAGGTTCAATTC
18275	Yes	AATAGGTAGCATGGAGAATTTTGGAGTCTCAGGGATAGGTTCAATTCC
18276	Yes	AATAGGTAGCATGGAGAATTTTGGAGTCTCAGGGATAGGTTCAATTCCT
18277	Yes	AATAGGTAGCATGGAGAATTTTGGAGTCTCAGGGATAGGTTCAATTCCTA
18278	No	AATAGGTGGCATGGAG
18279	No	AATAGGTGGCATGGAGA
18280	No	AATAGGTGGCATGGAGAA
18281	Yes	AATAGGTGGCATGGAGAAT
18282	Yes	AATAGGTGGCATGGAGAATT
18283	Yes	AATAGGTGGCATGGAGAATTT
18284	Yes	AATAGGTGGCATGGAGAATTTT
18285	Yes	AATAGGTGGCATGGAGAATTTTG
18286	Yes	AATAGGTGGCATGGAGAATTTTGG
18287	Yes	AATAGGTGGCATGGAGAATTTTGGA
18288	Yes	AATAGGTGGCATGGAGAATTTTGGAT
18289	Yes	AATAGGTGGCATGGAGAATTTTGGATT
18290	Yes	AATAGGTGGCATGGAGAATTTTGGATTA
18291	Yes	AATAGGTGGCATGGAGAATTTTGGATTAT
18292	Yes	AATAGGTGGCATGGAGAATTTTGGATTATC
18293	Yes	AATAGGTGGCATGGAGAATTTTGGATTATCA
18294	Yes	AATAGGTGGCATGGAGAATTTTGGATTATCAG
18295	Yes	AATAGGTGGCATGGAGAATTTTGGATTATCAGG
18296	Yes	AATAGGTGGCATGGAGAATTTTGGATTATCAGGG
18297	Yes	AATAGGTGGCATGGAGAATTTTGGATTATCAGGGG
18298	Yes	AATAGGTGGCATGGAGAATTTTGGATTATCAGGGGT
18299	Yes	AATAGGTGGCATGGAGAATTTTGGATTATCAGGGGTA
18300	Yes	AATAGGTGGCATGGAGAATTTTGGATTATCAGGGGTAG
18301	Yes	AATAGGTGGCATGGAGAATTTTGGATTATCAGGGGTAGG
18302	Yes	AATAGGTGGCATGGAGAATTTTGGATTATCAGGGGTAGGT
18303	Yes	AATAGGTGGCATGGAGAATTTTGGATTATCAGGGGTAGGTT
18304	Yes	AATAGGTGGCATGGAGAATTTTGGATTATCAGGGGTAGGTTC
18305	Yes	AATAGGTGGCATGGAGAATTTTGGATTATCAGGGGTAGGTTCA

18306	Yes	AATAGGTGGCATGGAGAATTTTGGATTATCAGGGGTAGGTTCAA
18307	Yes	AATAGGTGGCATGGAGAATTTTGGATTATCAGGGGTAGGTTCAAT
18308	Yes	AATAGGTGGCATGGAGAATTTTGGATTATCAGGGGTAGGTTCAATT
18309	Yes	AATAGGTGGCATGGAGAATTTTGGATTATCAGGGGTAGGTTCAATTC
18310	Yes	AATAGGTGGCATGGAGAATTTTGGATTATCAGGGGTAGGTTCAATTCC
18311	Yes	AATAGGTGGCATGGAGAATTTTGGATTATCAGGGGTAGGTTCAATTCCT
18312	Yes	AATAGGTGGCATGGAGAATTTTGGATTATCAGGGGTAGGTTCAATTCCTA
18313	Yes	AATAGTACCGTTAACT
18314	Yes	AATAGTACCGTTAACTT
18315	Yes	AATAGTACCGTTAACTTC
18316	Yes	AATAGTACCGTTAACTTCC
18317	Yes	AATAGTACCGTTAACTTCCA
18318	Yes	AATAGTACCGTTAACTTCCAA
18319	Yes	AATAGTACCGTTAACTTCCAAT
18320	Yes	AATAGTACCGTTAACTTCCAATT
18321	Yes	AATAGTACCGTTAACTTCCAATTA
18322	Yes	AATAGTACCGTTAACTTCCAATTAA
18323	Yes	AATAGTACCGTTAACTTCCAATTAAC
18324	Yes	AATAGTACCGTTAACTTCCAATTAACT
18325	Yes	AATAGTACCGTTAACTTCCAATTAACTA
18326	Yes	AATAGTACCGTTAACTTCCAATTAACTAG
18327	Yes	AATAGTACCGTTAACTTCCAATTAACTAGT
18328	Yes	AATAGTACCGTTAACTTCCAATTAACTAGTT
18329	Yes	AATAGTACCGTTAACTTCCAATTAACTAGTTT
18330	Yes	AATAGTACCGTTAACTTCCAATTAACTAGTTTT
18331	Yes	AATAGTACCGTTAACTTCCAATTAACTAGTTTTG
18332	Yes	AATAGTACCGTTAACTTCCAATTAACTAGTTTTGA
18333	Yes	AATAGTACCGTTAACTTCCAATTAACTAGTTTTGAC
18334	Yes	AATAGTACCGTTAACTTCCAATTAACTAGTTTTGACA
18335	Yes	AATAGTACCGTTAACTTCCAATTAACTAGTTTTGACAA
18336	Yes	AATAGTACCGTTAACTTCCAATTAACTAGTTTTGACAAC
18337	Yes	AATAGTACCGTTAACTTCCAATTAACTAGTTTTGACAACA
18338	Yes	AATAGTACCGTTAACTTCCAATTAACTAGTTTTGACAACAT
18339	Yes	AATAGTACCGTTAACTTCCAATTAACTAGTTTTGACAACATT
18340	Yes	AATAGTACCGTTAACTTCCAATTAACTAGTTTTGACAACATTC
18341	Yes	AATAGTACCGTTAACTTCCAATTAACTAGTTTTGACAACATTCA
18342	Yes	AATAGTACCGTTAACTTCCAATTAACTAGTTTTGACAACATTCAA
18343	Yes	AATAGTACCGTTAACTTCCAATTAACTAGTTTTGACAACATTCAAA
18344	Yes	AATAGTACCGTTAACTTCCAATTAACTAGTTTTGACAACATTCAAAA
18345	Yes	AATAGTACCGTTAACTTCCAATTAACTAGTTTTGACAACATTCAAAAA
18346	Yes	AATAGTACCGTTAACTTCCAATTAACTAGTTTTGACAACATTCAAAAAA
18347	Yes	AATAGTACCGTTAACTTCCAATTAACTAGTTTTGACAACATTCAAAAAAG

18348	No	AATAGTTCAGCTGGTA
18349	Yes	AATAGTTCAGCTGGTAG
18350	Yes	AATAGTTCAGCTGGTAGA
18351	Yes	AATAGTTCAGCTGGTAGAG
18352	Yes	AATAGTTCAGCTGGTAGAGC
18353	Yes	AATAGTTCAGCTGGTAGAGCA
18354	Yes	AATAGTTCAGCTGGTAGAGCAG
18355	Yes	AATAGTTCAGCTGGTAGAGCAGA
18356	Yes	AATAGTTCAGCTGGTAGAGCAGAG
18357	Yes	AATAGTTCAGCTGGTAGAGCAGAGG
18358	Yes	AATAGTTCAGCTGGTAGAGCAGAGGA
18359	Yes	AATAGTTCAGCTGGTAGAGCAGAGGAC
18360	Yes	AATAGTTCAGCTGGTAGAGCAGAGGACT
18361	Yes	AATAGTTCAGCTGGTAGAGCAGAGGACTA
18362	Yes	AATAGTTCAGCTGGTAGAGCAGAGGACTAT
18363	Yes	AATAGTTCAGCTGGTAGAGCAGAGGACTATA
18364	Yes	AATAGTTCAGCTGGTAGAGCAGAGGACTATAG
18365	Yes	AATAGTTCAGCTGGTAGAGCAGAGGACTATAGG
18366	Yes	AATAGTTCAGCTGGTAGAGCAGAGGACTATAGGT
18367	Yes	AATAGTTCAGCTGGTAGAGCAGAGGACTATAGGTC
18368	Yes	AATAGTTCAGCTGGTAGAGCAGAGGACTATAGGTCC
18369	Yes	AATAGTTCAGCTGGTAGAGCAGAGGACTATAGGTCCT
18370	Yes	AATAGTTCAGCTGGTAGAGCAGAGGACTATAGGTCCTT
18371	Yes	AATAGTTCAGCTGGTAGAGCAGAGGACTATAGGTCCTTA
18372	Yes	AATAGTTCAGCTGGTAGAGCAGAGGACTATAGGTCCTTAG
18373	Yes	AATAGTTCAGCTGGTAGAGCAGAGGACTATAGGTCCTTAGG
18374	Yes	AATAGTTCAGCTGGTAGAGCAGAGGACTATAGGTCCTTAGGT
18375	Yes	AATAGTTCAGCTGGTAGAGCAGAGGACTATAGGTCCTTAGGTT
18376	Yes	AATAGTTCAGCTGGTAGAGCAGAGGACTATAGGTCCTTAGGTTG
18377	Yes	AATAGTTCAGCTGGTAGAGCAGAGGACTATAGGTCCTTAGGTTGC
18378	Yes	AATAGTTCAGCTGGTAGAGCAGAGGACTATAGGTCCTTAGGTTGCT
18379	Yes	AATAGTTCAGCTGGTAGAGCAGAGGACTATAGGTCCTTAGGTTGCTG
18380	Yes	AATAGTTCAGCTGGTAGAGCAGAGGACTATAGGTCCTTAGGTTGCTGG
18381	Yes	AATAGTTCAGCTGGTAGAGCAGAGGACTATAGGTCCTTAGGTTGCTGGT
18382	Yes	AATAGTTCAGCTGGTAGAGCAGAGGACTATAGGTCCTTAGGTTGCTGGTT
18383	No	AATAGTTTAAATTAGA
18384	No	AATAGTTTAAATTAGAA
18385	No	AATAGTTTAAATTAGAAT
18386	Yes	AATAGTTTAAATTAGAATC
18387	Yes	AATAGTTTAAATTAGAATCT
18388	Yes	AATAGTTTAAATTAGAATCTT
18389	Yes	AATAGTTTAAATTAGAATCTTA

18390	Yes	AATAGTTTAAATTAGAATCTTAG
18391	Yes	AATAGTTTAAATTAGAATCTTAGC
18392	Yes	AATAGTTTAAATTAGAATCTTAGCT
18393	Yes	AATAGTTTAAATTAGAATCTTAGCTT
18394	Yes	AATAGTTTAAATTAGAATCTTAGCTTT
18395	Yes	AATAGTTTAAATTAGAATCTTAGCTTTG
18396	Yes	AATAGTTTAAATTAGAATCTTAGCTTTGG
18397	Yes	AATAGTTTAAATTAGAATCTTAGCTTTGGG
18398	Yes	AATAGTTTAAATTAGAATCTTAGCTTTGGGT
18399	Yes	AATAGTTTAAATTAGAATCTTAGCTTTGGGTG
18400	Yes	AATAGTTTAAATTAGAATCTTAGCTTTGGGTGC
18401	Yes	AATAGTTTAAATTAGAATCTTAGCTTTGGGTGCT
18402	Yes	AATAGTTTAAATTAGAATCTTAGCTTTGGGTGCTA
18403	Yes	AATAGTTTAAATTAGAATCTTAGCTTTGGGTGCTAA
18404	Yes	AATAGTTTAAATTAGAATCTTAGCTTTGGGTGCTAAT
18405	Yes	AATAGTTTAAATTAGAATCTTAGCTTTGGGTGCTAATG
18406	Yes	AATAGTTTAAATTAGAATCTTAGCTTTGGGTGCTAATGG
18407	Yes	AATAGTTTAAATTAGAATCTTAGCTTTGGGTGCTAATGGT
18408	Yes	AATAGTTTAAATTAGAATCTTAGCTTTGGGTGCTAATGGTG
18409	Yes	AATAGTTTAAATTAGAATCTTAGCTTTGGGTGCTAATGGTGG
18410	Yes	AATAGTTTAAATTAGAATCTTAGCTTTGGGTGCTAATGGTGGA
18411	Yes	AATAGTTTAAATTAGAATCTTAGCTTTGGGTGCTAATGGTGGAG
18412	Yes	AATAGTTTAAATTAGAATCTTAGCTTTGGGTGCTAATGGTGGAGT
18413	Yes	AATAGTTTAAATTAGAATCTTAGCTTTGGGTGCTAATGGTGGAGTT
18414	Yes	AATAGTTTAAATTAGAATCTTAGCTTTGGGTGCTAATGGTGGAGTTA
18415	Yes	AATAGTTTAAATTAGAATCTTAGCTTTGGGTGCTAATGGTGGAGTTAA
18416	Yes	AATAGTTTAAATTAGAATCTTAGCTTTGGGTGCTAATGGTGGAGTTAAA
18417	Yes	AATAGTTTAAATTAGAATCTTAGCTTTGGGTGCTAATGGTGGAGTTAAAG
18418	No	AATATAGTGGTGAGTA
18419	No	AATATAGTGGTGAGTAT
18420	No	AATATAGTGGTGAGTATT
18421	Yes	AATATAGTGGTGAGTATTC
18422	Yes	AATATAGTGGTGAGTATTCC
18423	Yes	AATATAGTGGTGAGTATTCCC
18424	Yes	AATATAGTGGTGAGTATTCCCA
18425	Yes	AATATAGTGGTGAGTATTCCCAC
18426	Yes	AATATAGTGGTGAGTATTCCCACC
18427	Yes	AATATAGTGGTGAGTATTCCCACCT
18428	Yes	AATATAGTGGTGAGTATTCCCACCTG
18429	Yes	AATATAGTGGTGAGTATTCCCACCTGT
18430	Yes	AATATAGTGGTGAGTATTCCCACCTGTC
18431	Yes	AATATAGTGGTGAGTATTCCCACCTGTCA

18432	Yes	AATATAGTGGTGAGTATTCCCACCTGTCAT
18433	Yes	AATATAGTGGTGAGTATTCCCACCTGTCATG
18434	Yes	AATATAGTGGTGAGTATTCCCACCTGTCATGC
18435	Yes	AATATAGTGGTGAGTATTCCCACCTGTCATGCG
18436	Yes	AATATAGTGGTGAGTATTCCCACCTGTCATGCGG
18437	Yes	AATATAGTGGTGAGTATTCCCACCTGTCATGCGGG
18438	Yes	AATATAGTGGTGAGTATTCCCACCTGTCATGCGGGA
18439	Yes	AATATAGTGGTGAGTATTCCCACCTGTCATGCGGGAG
18440	Yes	AATATAGTGGTGAGTATTCCCACCTGTCATGCGGGAGA
18441	Yes	AATATAGTGGTGAGTATTCCCACCTGTCATGCGGGAGAC
18442	Yes	AATATAGTGGTGAGTATTCCCACCTGTCATGCGGGAGACG
18443	Yes	AATATAGTGGTGAGTATTCCCACCTGTCATGCGGGAGACGG
18444	Yes	AATATAGTGGTGAGTATTCCCACCTGTCATGCGGGAGACGGG
18445	Yes	AATATAGTGGTGAGTATTCCCACCTGTCATGCGGGAGACGGGG
18446	Yes	AATATAGTGGTGAGTATTCCCACCTGTCATGCGGGAGACGGGGT
18447	Yes	AATATAGTGGTGAGTATTCCCACCTGTCATGCGGGAGACGGGGTT
18448	Yes	AATATAGTGGTGAGTATTCCCACCTGTCATGCGGGAGACGGGGTTC
18449	Yes	AATATAGTGGTGAGTATTCCCACCTGTCATGCGGGAGACGGGGTTCA
18450	Yes	AATATAGTGGTGAGTATTCCCACCTGTCATGCGGGAGACGGGGTTCAA
18451	Yes	AATATAGTGGTGAGTATTCCCACCTGTCATGCGGGAGACGGGGTTCAAT
18452	Yes	AATATAGTGGTGAGTATTCCCACCTGTCATGCGGGAGACGGGGTTCAATT
18453	No	AATATAGTTTAACCAA
18454	No	AATATAGTTTAACCAAA
18455	No	AATATAGTTTAACCAAAA
18456	No	AATATAGTTTAACCAAAAC
18457	No	AATATAGTTTAACCAAAACA
18458	No	AATATAGTTTAACCAAAACAT
18459	No	AATATAGTTTAACCAAAACATC
18460	No	AATATAGTTTAACCAAAACATCA
18461	No	AATATAGTTTAACCAAAACATCAG
18462	No	AATATAGTTTAACCAAAACATCAGA
18463	No	AATATAGTTTAACCAAAACATCAGAT
18464	No	AATATAGTTTAACCAAAACATCAGATT
18465	No	AATATAGTTTAACCAAAACATCAGATTG
18466	No	AATATAGTTTAACCAAAACATCAGATTGT
18467	No	AATATAGTTTAACCAAAACATCAGATTGTG
18468	No	AATATAGTTTAACCAAAACATCAGATTGTGA
18469	No	AATATAGTTTAACCAAAACATCAGATTGTGAA
18470	No	AATATAGTTTAACCAAAACATCAGATTGTGAAT
18471	No	AATATAGTTTAACCAAAACATCAGATTGTGAATC
18472	No	AATATAGTTTAACCAAAACATCAGATTGTGAATCT
18473	No	AATATAGTTTAACCAAAACATCAGATTGTGAATCTG

18474	No	AATATAGTTTAACCAAAACATCAGATTGTGAATCTGA
18475	No	AATATAGTTTAACCAAAACATCAGATTGTGAATCTGAC
18476	No	AATATAGTTTAACCAAAACATCAGATTGTGAATCTGACA
18477	No	AATATAGTTTAACCAAAACATCAGATTGTGAATCTGACAA
18478	No	AATATAGTTTAACCAAAACATCAGATTGTGAATCTGACAAC
18479	No	AATATAGTTTAACCAAAACATCAGATTGTGAATCTGACAACA
18480	No	AATATAGTTTAACCAAAACATCAGATTGTGAATCTGACAACAG
18481	No	AATATAGTTTAACCAAAACATCAGATTGTGAATCTGACAACAGA
18482	No	AATATAGTTTAACCAAAACATCAGATTGTGAATCTGACAACAGAG
18483	No	AATATAGTTTAACCAAAACATCAGATTGTGAATCTGACAACAGAGG
18484	No	AATATAGTTTAACCAAAACATCAGATTGTGAATCTGACAACAGAGGC
18485	No	AATATAGTTTAACCAAAACATCAGATTGTGAATCTGACAACAGAGGCT
18486	Yes	AATATAGTTTAACCAAAACATCAGATTGTGAATCTGACAACAGAGGCTT
18487	Yes	AATATAGTTTAACCAAAACATCAGATTGTGAATCTGACAACAGAGGCTTA
18488	No	AATATGCTGGGCCTTT
18489	Yes	AATATGCTGGGCCTTTA
18490	Yes	AATATGCTGGGCCTTTAC
18491	Yes	AATATGCTGGGCCTTTACC
18492	Yes	AATATGCTGGGCCTTTACCA
18493	Yes	AATATGCTGGGCCTTTACCAC
18494	Yes	AATATGCTGGGCCTTTACCACT
18495	Yes	AATATGCTGGGCCTTTACCACTT
18496	Yes	AATATGCTGGGCCTTTACCACTTC
18497	Yes	AATATGCTGGGCCTTTACCACTTCA
18498	Yes	AATATGCTGGGCCTTTACCACTTCAG
18499	Yes	AATATGCTGGGCCTTTACCACTTCAGC
18500	Yes	AATATGCTGGGCCTTTACCACTTCAGCC
18501	Yes	AATATGCTGGGCCTTTACCACTTCAGCCC
18502	Yes	AATATGCTGGGCCTTTACCACTTCAGCCCA
18503	Yes	AATATGCTGGGCCTTTACCACTTCAGCCCAG
18504	Yes	AATATGCTGGGCCTTTACCACTTCAGCCCAGG
18505	Yes	AATATGCTGGGCCTTTACCACTTCAGCCCAGGT
18506	Yes	AATATGCTGGGCCTTTACCACTTCAGCCCAGGTT
18507	Yes	AATATGCTGGGCCTTTACCACTTCAGCCCAGGTTC
18508	Yes	AATATGCTGGGCCTTTACCACTTCAGCCCAGGTTCG
18509	Yes	AATATGCTGGGCCTTTACCACTTCAGCCCAGGTTCGA
18510	Yes	AATATGCTGGGCCTTTACCACTTCAGCCCAGGTTCGAT
18511	Yes	AATATGCTGGGCCTTTACCACTTCAGCCCAGGTTCGATT
18512	Yes	AATATGCTGGGCCTTTACCACTTCAGCCCAGGTTCGATTC
18513	Yes	AATATGCTGGGCCTTTACCACTTCAGCCCAGGTTCGATTCC
18514	Yes	AATATGCTGGGCCTTTACCACTTCAGCCCAGGTTCGATTCCT
18515	Yes	AATATGCTGGGCCTTTACCACTTCAGCCCAGGTTCGATTCCTG

18516	Yes	AATATGCTGGGCCTTTACCACTTCAGCCCAGGTTCGATTCCTGG
18517	Yes	AATATGCTGGGCCTTTACCACTTCAGCCCAGGTTCGATTCCTGGT
18518	Yes	AATATGCTGGGCCTTTACCACTTCAGCCCAGGTTCGATTCCTGGTC
18519	Yes	AATATGCTGGGCCTTTACCACTTCAGCCCAGGTTCGATTCCTGGTCA
18520	Yes	AATATGCTGGGCCTTTACCACTTCAGCCCAGGTTCGATTCCTGGTCAG
18521	Yes	AATATGCTGGGCCTTTACCACTTCAGCCCAGGTTCGATTCCTGGTCAGG
18522	Yes	AATATGCTGGGCCTTTACCACTTCAGCCCAGGTTCGATTCCTGGTCAGGG
18523	No	AATATGTCTGATAAAA
18524	No	AATATGTCTGATAAAAG
18525	No	AATATGTCTGATAAAAGA
18526	No	AATATGTCTGATAAAAGAG
18527	No	AATATGTCTGATAAAAGAGT
18528	No	AATATGTCTGATAAAAGAGTT
18529	No	AATATGTCTGATAAAAGAGTTA
18530	No	AATATGTCTGATAAAAGAGTTAC
18531	No	AATATGTCTGATAAAAGAGTTACT
18532	No	AATATGTCTGATAAAAGAGTTACTT
18533	No	AATATGTCTGATAAAAGAGTTACTTT
18534	No	AATATGTCTGATAAAAGAGTTACTTTG
18535	No	AATATGTCTGATAAAAGAGTTACTTTGA
18536	No	AATATGTCTGATAAAAGAGTTACTTTGAT
18537	No	AATATGTCTGATAAAAGAGTTACTTTGATA
18538	No	AATATGTCTGATAAAAGAGTTACTTTGATAG
18539	No	AATATGTCTGATAAAAGAGTTACTTTGATAGA
18540	No	AATATGTCTGATAAAAGAGTTACTTTGATAGAG
18541	No	AATATGTCTGATAAAAGAGTTACTTTGATAGAGT
18542	No	AATATGTCTGATAAAAGAGTTACTTTGATAGAGTA
18543	No	AATATGTCTGATAAAAGAGTTACTTTGATAGAGTAA
18544	No	AATATGTCTGATAAAAGAGTTACTTTGATAGAGTAAA
18545	No	AATATGTCTGATAAAAGAGTTACTTTGATAGAGTAAAT
18546	No	AATATGTCTGATAAAAGAGTTACTTTGATAGAGTAAATA
18547	No	AATATGTCTGATAAAAGAGTTACTTTGATAGAGTAAATAA
18548	No	AATATGTCTGATAAAAGAGTTACTTTGATAGAGTAAATAAT
18549	No	AATATGTCTGATAAAAGAGTTACTTTGATAGAGTAAATAATA
18550	No	AATATGTCTGATAAAAGAGTTACTTTGATAGAGTAAATAATAG
18551	No	AATATGTCTGATAAAAGAGTTACTTTGATAGAGTAAATAATAGG
18552	No	AATATGTCTGATAAAAGAGTTACTTTGATAGAGTAAATAATAGGA
18553	No	AATATGTCTGATAAAAGAGTTACTTTGATAGAGTAAATAATAGGAG
18554	Yes	AATATGTCTGATAAAAGAGTTACTTTGATAGAGTAAATAATAGGAGC
18555	Yes	AATATGTCTGATAAAAGAGTTACTTTGATAGAGTAAATAATAGGAGCT
18556	Yes	AATATGTCTGATAAAAGAGTTACTTTGATAGAGTAAATAATAGGAGCTT
18557	Yes	AATATGTCTGATAAAAGAGTTACTTTGATAGAGTAAATAATAGGAGCTTA

18558	No	AATCAAGAGGTCCCTG
18559	No	AATCAAGAGGTCCCTGA
18560	Yes	AATCAAGAGGTCCCTGAT
18561	Yes	AATCAAGAGGTCCCTGATT
18562	Yes	AATCAAGAGGTCCCTGATTC
18563	Yes	AATCAAGAGGTCCCTGATTCA
18564	Yes	AATCAAGAGGTCCCTGATTCAA
18565	Yes	AATCAAGAGGTCCCTGATTCAAA
18566	Yes	AATCAAGAGGTCCCTGATTCAAAT
18567	Yes	AATCAAGAGGTCCCTGATTCAAATC
18568	Yes	AATCAAGAGGTCCCTGATTCAAATCC
18569	Yes	AATCAAGAGGTCCCTGATTCAAATCCA
18570	Yes	AATCAAGAGGTCCCTGATTCAAATCCAG
18571	Yes	AATCAAGAGGTCCCTGATTCAAATCCAGG
18572	Yes	AATCAAGAGGTCCCTGATTCAAATCCAGGT
18573	Yes	AATCAAGAGGTCCCTGATTCAAATCCAGGTG
18574	Yes	AATCAAGAGGTCCCTGATTCAAATCCAGGTGC
18575	Yes	AATCAAGAGGTCCCTGATTCAAATCCAGGTGCC
18576	Yes	AATCAAGAGGTCCCTGATTCAAATCCAGGTGCCC
18577	Yes	AATCAAGAGGTCCCTGATTCAAATCCAGGTGCCCC
18578	Yes	AATCAAGAGGTCCCTGATTCAAATCCAGGTGCCCCC
18579	Yes	AATCAAGAGGTCCCTGATTCAAATCCAGGTGCCCCCT
18580	Yes	AATCAAGAGGTCCCTGATTCAAATCCAGGTGCCCCCTC
18581	Yes	AATCAAGAGGTCCCTGATTCAAATCCAGGTGCCCCCTCC
18582	Yes	AATCAAGAGGTCCCTGATTCAAATCCAGGTGCCCCCTCCA
18583	Yes	AATCAAGCGATCCGAG
18584	Yes	AATCAAGCGATCCGAGT
18585	Yes	AATCAAGCGATCCGAGTT
18586	Yes	AATCAAGCGATCCGAGTTC
18587	Yes	AATCAAGCGATCCGAGTTCA
18588	Yes	AATCAAGCGATCCGAGTTCAA
18589	Yes	AATCAAGCGATCCGAGTTCAAA
18590	Yes	AATCAAGCGATCCGAGTTCAAAT
18591	Yes	AATCAAGCGATCCGAGTTCAAATC
18592	Yes	AATCAAGCGATCCGAGTTCAAATCT
18593	Yes	AATCAAGCGATCCGAGTTCAAATCTC
18594	Yes	AATCAAGCGATCCGAGTTCAAATCTCG
18595	Yes	AATCAAGCGATCCGAGTTCAAATCTCGG
18596	Yes	AATCAAGCGATCCGAGTTCAAATCTCGGT
18597	Yes	AATCAAGCGATCCGAGTTCAAATCTCGGTG
18598	Yes	AATCAAGCGATCCGAGTTCAAATCTCGGTGG
18599	Yes	AATCAAGCGATCCGAGTTCAAATCTCGGTGGT

18600	Yes	AATCAAGCGATCCGAGTTCAAATCTCGGTGGTA
18601	Yes	AATCAAGCGATCCGAGTTCAAATCTCGGTGGTAC
18602	Yes	AATCAAGCGATCCGAGTTCAAATCTCGGTGGTACC
18603	Yes	AATCAAGCGATCCGAGTTCAAATCTCGGTGGTACCT
18604	Yes	AATCAAGCGATCCGAGTTCAAATCTCGGTGGTACCTC
18605	Yes	AATCAAGCGATCCGAGTTCAAATCTCGGTGGTACCTCC
18606	Yes	AATCAAGCGATCCGAGTTCAAATCTCGGTGGTACCTCCA
18607	Yes	AATCACGTCGGGGTCA
18608	Yes	AATCACGTCGGGGTCAC
18609	Yes	AATCACGTCGGGGTCACC
18610	Yes	AATCACGTCGGGGTCACCA
18611	No	AATCAGAAGATTCCAG
18612	Yes	AATCAGAAGATTCCAGC
18613	Yes	AATCAGAAGATTCCAGCC
18614	Yes	AATCAGAAGATTCCAGCCT
18615	Yes	AATCAGAAGATTCCAGCCTT
18616	Yes	AATCAGAAGATTCCAGCCTTG
18617	Yes	AATCAGAAGATTCCAGCCTTGA
18618	Yes	AATCAGAAGATTCCAGCCTTGAC
18619	Yes	AATCAGAAGATTCCAGCCTTGACT
18620	Yes	AATCAGAAGATTCCAGCCTTGACTC
18621	Yes	AATCAGAAGATTCCAGCCTTGACTCC
18622	Yes	AATCAGAAGATTCCAGCCTTGACTCCT
18623	Yes	AATCAGAAGATTCCAGCCTTGACTCCTG
18624	Yes	AATCAGAAGATTCCAGCCTTGACTCCTGG
18625	Yes	AATCAGAAGATTCCAGCCTTGACTCCTGGC
18626	Yes	AATCAGAAGATTCCAGCCTTGACTCCTGGCT
18627	Yes	AATCAGAAGATTCCAGCCTTGACTCCTGGCTG
18628	Yes	AATCAGAAGATTCCAGCCTTGACTCCTGGCTGG
18629	Yes	AATCAGAAGATTCCAGCCTTGACTCCTGGCTGGC
18630	Yes	AATCAGAAGATTCCAGCCTTGACTCCTGGCTGGCT
18631	Yes	AATCAGAAGATTCCAGCCTTGACTCCTGGCTGGCTC
18632	Yes	AATCAGAAGATTCCAGCCTTGACTCCTGGCTGGCTCA
18633	Yes	AATCAGAAGATTCCAGCCTTGACTCCTGGCTGGCTCAC
18634	Yes	AATCAGAAGATTCCAGCCTTGACTCCTGGCTGGCTCACC
18635	Yes	AATCAGAAGATTCCAGCCTTGACTCCTGGCTGGCTCACCA
18636	No	AATCAGAGGGTCCAGG
18637	No	AATCAGAGGGTCCAGGG
18638	No	AATCAGAGGGTCCAGGGT
18639	No	AATCAGAGGGTCCAGGGTT
18640	No	AATCAGAGGGTCCAGGGTTC
18641	Yes	AATCAGAGGGTCCAGGGTTCA

18642	Yes	AATCAGAGGGTCCAGGGTTCAA
18643	Yes	AATCAGAGGGTCCAGGGTTCAAG
18644	Yes	AATCAGAGGGTCCAGGGTTCAAGT
18645	Yes	AATCAGAGGGTCCAGGGTTCAAGTC
18646	Yes	AATCAGAGGGTCCAGGGTTCAAGTCC
18647	Yes	AATCAGAGGGTCCAGGGTTCAAGTCCC
18648	Yes	AATCAGAGGGTCCAGGGTTCAAGTCCCT
18649	Yes	AATCAGAGGGTCCAGGGTTCAAGTCCCTG
18650	Yes	AATCAGAGGGTCCAGGGTTCAAGTCCCTGT
18651	Yes	AATCAGAGGGTCCAGGGTTCAAGTCCCTGTT
18652	Yes	AATCAGAGGGTCCAGGGTTCAAGTCCCTGTTT
18653	Yes	AATCAGAGGGTCCAGGGTTCAAGTCCCTGTTTG
18654	Yes	AATCAGAGGGTCCAGGGTTCAAGTCCCTGTTTGG
18655	Yes	AATCAGAGGGTCCAGGGTTCAAGTCCCTGTTTGGG
18656	Yes	AATCAGAGGGTCCAGGGTTCAAGTCCCTGTTTGGGT
18657	Yes	AATCAGAGGGTCCAGGGTTCAAGTCCCTGTTTGGGTG
18658	Yes	AATCAGAGGGTCCAGGGTTCAAGTCCCTGTTTGGGTGC
18659	Yes	AATCAGAGGGTCCAGGGTTCAAGTCCCTGTTTGGGTGCC
18660	Yes	AATCAGAGGGTCCAGGGTTCAAGTCCCTGTTTGGGTGCCA
18661	No	AATCAGAGGTTCAACT
18662	No	AATCAGAGGTTCAACTC
18663	No	AATCAGAGGTTCAACTCC
18664	No	AATCAGAGGTTCAACTCCT
18665	No	AATCAGAGGTTCAACTCCTC
18666	No	AATCAGAGGTTCAACTCCTCT
18667	Yes	AATCAGAGGTTCAACTCCTCTC
18668	Yes	AATCAGAGGTTCAACTCCTCTCA
18669	Yes	AATCAGAGGTTCAACTCCTCTCAT
18670	Yes	AATCAGAGGTTCAACTCCTCTCATT
18671	Yes	AATCAGAGGTTCAACTCCTCTCATTA
18672	Yes	AATCAGAGGTTCAACTCCTCTCATTAA
18673	Yes	AATCAGAGGTTCAACTCCTCTCATTAAC
18674	Yes	AATCAGAGGTTCAACTCCTCTCATTAACA
18675	Yes	AATCAGAGGTTCAACTCCTCTCATTAACAC
18676	Yes	AATCAGAGGTTCAACTCCTCTCATTAACACC
18677	Yes	AATCAGAGGTTCAACTCCTCTCATTAACACCA
18678	No	AATCAGAGGTTCAACTCCTCTT
18679	No	AATCAGAGGTTCAACTCCTCTTC
18680	No	AATCAGAGGTTCAACTCCTCTTCT
18681	No	AATCAGAGGTTCAACTCCTCTTCTT
18682	No	AATCAGAGGTTCAACTCCTCTTCTTA
18683	No	AATCAGAGGTTCAACTCCTCTTCTTAA

18684	No	AATCAGAGGTTCAACTCCTCTTCTTAAC
18685	No	AATCAGAGGTTCAACTCCTCTTCTTAACA
18686	Yes	AATCAGAGGTTCAACTCCTCTTCTTAACAC
18687	Yes	AATCAGAGGTTCAACTCCTCTTCTTAACACC
18688	Yes	AATCAGAGGTTCAACTCCTCTTCTTAACACCA
18689	Yes	AATCAGAGGTTCGACT
18690	Yes	AATCAGAGGTTCGACTC
18691	Yes	AATCAGAGGTTCGACTCC
18692	Yes	AATCAGAGGTTCGACTCCT
18693	Yes	AATCAGAGGTTCGACTCCTC
18694	Yes	AATCAGAGGTTCGACTCCTCT
18695	Yes	AATCAGAGGTTCGACTCCTCTT
18696	Yes	AATCAGAGGTTCGACTCCTCTTC
18697	Yes	AATCAGAGGTTCGACTCCTCTTCT
18698	Yes	AATCAGAGGTTCGACTCCTCTTCTT
18699	Yes	AATCAGAGGTTCGACTCCTCTTCTTA
18700	Yes	AATCAGAGGTTCGACTCCTCTTCTTAA
18701	Yes	AATCAGAGGTTCGACTCCTCTTCTTAAC
18702	Yes	AATCAGAGGTTCGACTCCTCTTCTTAACA
18703	Yes	AATCAGAGGTTCGACTCCTCTTCTTAACAC
18704	Yes	AATCAGAGGTTCGACTCCTCTTCTTAACACC
18705	Yes	AATCAGAGGTTCGACTCCTCTTCTTAACACCA
18706	Yes	AATCAGGCGGGAACAC
18707	Yes	AATCAGGCGGGAACACC
18708	Yes	AATCAGGCGGGAACACCA
18709	No	AATCATATTTACCAAC
18710	No	AATCATATTTACCAACC
18711	Yes	AATCATATTTACCAACCA
18712	Yes	AATCCAATGGGGGTTC
18713	Yes	AATCCAATGGGGGTTCC
18714	Yes	AATCCAATGGGGGTTCCC
18715	Yes	AATCCAATGGGGGTTCCCC
18716	Yes	AATCCAATGGGGGTTCCCCG
18717	Yes	AATCCAATGGGGGTTCCCCGC
18718	Yes	AATCCAATGGGGGTTCCCCGCG
18719	Yes	AATCCAATGGGGGTTCCCCGCGC
18720	Yes	AATCCAATGGGGGTTCCCCGCGCA
18721	Yes	AATCCAATGGGGGTTCCCCGCGCAG
18722	Yes	AATCCAATGGGGGTTCCCCGCGCAGG
18723	Yes	AATCCAATGGGGGTTCCCCGCGCAGGT
18724	Yes	AATCCAATGGGGGTTCCCCGCGCAGGTT
18725	Yes	AATCCAATGGGGGTTCCCCGCGCAGGTTC

18726	Yes	AATCCAATGGGGGTTCCCCGCGCAGGTTCA
18727	Yes	AATCCAATGGGGGTTCCCCGCGCAGGTTCAA
18728	Yes	AATCCAATGGGGGTTCCCCGCGCAGGTTCAAA
18729	Yes	AATCCAATGGGGGTTCCCCGCGCAGGTTCAAAT
18730	Yes	AATCCAATGGGGGTTCCCCGCGCAGGTTCAAATC
18731	Yes	AATCCAATGGGGGTTCCCCGCGCAGGTTCAAATCC
18732	Yes	AATCCAATGGGGGTTCCCCGCGCAGGTTCAAATCCT
18733	Yes	AATCCAATGGGGGTTCCCCGCGCAGGTTCAAATCCTG
18734	Yes	AATCCAATGGGGGTTCCCCGCGCAGGTTCAAATCCTGC
18735	Yes	AATCCAATGGGGGTTCCCCGCGCAGGTTCAAATCCTGCT
18736	Yes	AATCCAATGGGGGTTCCCCGCGCAGGTTCAAATCCTGCTC
18737	Yes	AATCCAATGGGGGTTCCCCGCGCAGGTTCAAATCCTGCTCA
18738	Yes	AATCCAATGGGGGTTCCCCGCGCAGGTTCAAATCCTGCTCAC
18739	Yes	AATCCAATGGGGGTTCCCCGCGCAGGTTCAAATCCTGCTCACA
18740	Yes	AATCCAATGGGGGTTCCCCGCGCAGGTTCAAATCCTGCTCACAG
18741	Yes	AATCCAATGGGGGTTCCCCGCGCAGGTTCAAATCCTGCTCACAGC
18742	Yes	AATCCAATGGGGGTTCCCCGCGCAGGTTCAAATCCTGCTCACAGCG
18743	Yes	AATCCAATGGGGGTTCCCCGCGCAGGTTCAAATCCTGCTCACAGCGC
18744	Yes	AATCCAATGGGGGTTCCCCGCGCAGGTTCAAATCCTGCTCACAGCGCC
18745	Yes	AATCCAATGGGGGTTCCCCGCGCAGGTTCAAATCCTGCTCACAGCGCCA
18746	No	AATCCAATGGGGTCTC
18747	Yes	AATCCAATGGGGTCTCC
18748	Yes	AATCCAATGGGGTCTCCC
18749	Yes	AATCCAATGGGGTCTCCCC
18750	Yes	AATCCAATGGGGTCTCCCCG
18751	Yes	AATCCAATGGGGTCTCCCCGC
18752	Yes	AATCCAATGGGGTCTCCCCGCG
18753	Yes	AATCCAATGGGGTCTCCCCGCGC
18754	Yes	AATCCAATGGGGTCTCCCCGCGCA
18755	Yes	AATCCAATGGGGTCTCCCCGCGCAG
18756	Yes	AATCCAATGGGGTCTCCCCGCGCAGG
18757	Yes	AATCCAATGGGGTCTCCCCGCGCAGGT
18758	Yes	AATCCAATGGGGTCTCCCCGCGCAGGTT
18759	Yes	AATCCAATGGGGTCTCCCCGCGCAGGTTC
18760	Yes	AATCCAATGGGGTCTCCCCGCGCAGGTTCA
18761	Yes	AATCCAATGGGGTCTCCCCGCGCAGGTTCAA
18762	Yes	AATCCAATGGGGTCTCCCCGCGCAGGTTCAAA
18763	Yes	AATCCAATGGGGTCTCCCCGCGCAGGTTCAAAT
18764	Yes	AATCCAATGGGGTCTCCCCGCGCAGGTTCAAATC
18765	Yes	AATCCAATGGGGTCTCCCCGCGCAGGTTCAAATCC
18766	Yes	AATCCAATGGGGTCTCCCCGCGCAGGTTCAAATCCT
18767	Yes	AATCCAATGGGGTCTCCCCGCGCAGGTTCAAATCCTG

18768	Yes	AATCCAATGGGGTCTCCCCGCGCAGGTTCAAATCCTGC
18769	Yes	AATCCAATGGGGTCTCCCCGCGCAGGTTCAAATCCTGCT
18770	Yes	AATCCAATGGGGTCTCCCCGCGCAGGTTCAAATCCTGCTC
18771	Yes	AATCCAATGGGGTCTCCCCGCGCAGGTTCAAATCCTGCTCA
18772	Yes	AATCCAATGGGGTCTCCCCGCGCAGGTTCAAATCCTGCTCAC
18773	Yes	AATCCAATGGGGTCTCCCCGCGCAGGTTCAAATCCTGCTCACA
18774	Yes	AATCCAATGGGGTCTCCCCGCGCAGGTTCAAATCCTGCTCACAG
18775	Yes	AATCCAATGGGGTCTCCCCGCGCAGGTTCAAATCCTGCTCACAGC
18776	Yes	AATCCAATGGGGTCTCCCCGCGCAGGTTCAAATCCTGCTCACAGCG
18777	Yes	AATCCAATGGGGTCTCCCCGCGCAGGTTCAAATCCTGCTCACAGCGC
18778	Yes	AATCCAATGGGGTCTCCCCGCGCAGGTTCAAATCCTGCTCACAGCGCC
18779	Yes	AATCCAATGGGGTCTCCCCGCGCAGGTTCAAATCCTGCTCACAGCGCCA
18780	Yes	AATCCAATGGGGTCTCCCCGCGCAGGTTCG
18781	Yes	AATCCAATGGGGTCTCCCCGCGCAGGTTCGA
18782	Yes	AATCCAATGGGGTCTCCCCGCGCAGGTTCGAA
18783	Yes	AATCCAATGGGGTCTCCCCGCGCAGGTTCGAAC
18784	Yes	AATCCAATGGGGTCTCCCCGCGCAGGTTCGAACC
18785	Yes	AATCCAATGGGGTCTCCCCGCGCAGGTTCGAACCC
18786	Yes	AATCCAATGGGGTCTCCCCGCGCAGGTTCGAACCCT
18787	Yes	AATCCAATGGGGTCTCCCCGCGCAGGTTCGAACCCTG
18788	Yes	AATCCAATGGGGTCTCCCCGCGCAGGTTCGAACCCTGC
18789	Yes	AATCCAATGGGGTCTCCCCGCGCAGGTTCGAACCCTGCT
18790	Yes	AATCCAATGGGGTCTCCCCGCGCAGGTTCGAACCCTGCTC
18791	Yes	AATCCAATGGGGTCTCCCCGCGCAGGTTCGAACCCTGCTCG
18792	Yes	AATCCAATGGGGTCTCCCCGCGCAGGTTCGAACCCTGCTCGC
18793	Yes	AATCCAATGGGGTCTCCCCGCGCAGGTTCGAACCCTGCTCGCT
18794	Yes	AATCCAATGGGGTCTCCCCGCGCAGGTTCGAACCCTGCTCGCTG
18795	Yes	AATCCAATGGGGTCTCCCCGCGCAGGTTCGAACCCTGCTCGCTGC
18796	Yes	AATCCAATGGGGTCTCCCCGCGCAGGTTCGAACCCTGCTCGCTGCG
18797	Yes	AATCCAATGGGGTCTCCCCGCGCAGGTTCGAACCCTGCTCGCTGCGC
18798	Yes	AATCCAATGGGGTCTCCCCGCGCAGGTTCGAACCCTGCTCGCTGCGCC
18799	Yes	AATCCAATGGGGTCTCCCCGCGCAGGTTCGAACCCTGCTCGCTGCGCCA
18800	Yes	AATCCAATGGGGTCTCCCCGCGCAGGTTCGAAT
18801	Yes	AATCCAATGGGGTCTCCCCGCGCAGGTTCGAATC
18802	Yes	AATCCAATGGGGTCTCCCCGCGCAGGTTCGAATCC
18803	Yes	AATCCAATGGGGTCTCCCCGCGCAGGTTCGAATCCT
18804	Yes	AATCCAATGGGGTCTCCCCGCGCAGGTTCGAATCCTG
18805	Yes	AATCCAATGGGGTCTCCCCGCGCAGGTTCGAATCCTGC
18806	Yes	AATCCAATGGGGTCTCCCCGCGCAGGTTCGAATCCTGCT
18807	Yes	AATCCAATGGGGTCTCCCCGCGCAGGTTCGAATCCTGCTC
18808	Yes	AATCCAATGGGGTCTCCCCGCGCAGGTTCGAATCCTGCTCA
18809	Yes	AATCCAATGGGGTCTCCCCGCGCAGGTTCGAATCCTGCTCAC

18810	Yes	AATCCAATGGGGTCTCCCCGCGCAGGTTCGAATCCTGCTCACA
18811	Yes	AATCCAATGGGGTCTCCCCGCGCAGGTTCGAATCCTGCTCACAG
18812	Yes	AATCCAATGGGGTCTCCCCGCGCAGGTTCGAATCCTGCTCACAGC
18813	Yes	AATCCAATGGGGTCTCCCCGCGCAGGTTCGAATCCTGCTCACAGCG
18814	Yes	AATCCAATGGGGTCTCCCCGCGCAGGTTCGAATCCTGCTCACAGCGC
18815	Yes	AATCCAATGGGGTCTCCCCGCGCAGGTTCGAATCCTGCTCACAGCGCC
18816	Yes	AATCCAATGGGGTCTCCCCGCGCAGGTTCGAATCCTGCTCACAGCGCCA
18817	No	AATCCAATGGGGTTTC
18818	Yes	AATCCAATGGGGTTTCC
18819	Yes	AATCCAATGGGGTTTCCC
18820	Yes	AATCCAATGGGGTTTCCCC
18821	Yes	AATCCAATGGGGTTTCCCCG
18822	Yes	AATCCAATGGGGTTTCCCCGC
18823	Yes	AATCCAATGGGGTTTCCCCGCA
18824	Yes	AATCCAATGGGGTTTCCCCGCAC
18825	Yes	AATCCAATGGGGTTTCCCCGCACA
18826	Yes	AATCCAATGGGGTTTCCCCGCACAG
18827	Yes	AATCCAATGGGGTTTCCCCGCACAGG
18828	Yes	AATCCAATGGGGTTTCCCCGCACAGGT
18829	Yes	AATCCAATGGGGTTTCCCCGCACAGGTT
18830	Yes	AATCCAATGGGGTTTCCCCGCACAGGTTC
18831	Yes	AATCCAATGGGGTTTCCCCGCACAGGTTCG
18832	Yes	AATCCAATGGGGTTTCCCCGCACAGGTTCGA
18833	Yes	AATCCAATGGGGTTTCCCCGCACAGGTTCGAA
18834	Yes	AATCCAATGGGGTTTCCCCGCACAGGTTCGAAT
18835	Yes	AATCCAATGGGGTTTCCCCGCACAGGTTCGAATC
18836	Yes	AATCCAATGGGGTTTCCCCGCACAGGTTCGAATCC
18837	Yes	AATCCAATGGGGTTTCCCCGCACAGGTTCGAATCCT
18838	Yes	AATCCAATGGGGTTTCCCCGCACAGGTTCGAATCCTG
18839	Yes	AATCCAATGGGGTTTCCCCGCACAGGTTCGAATCCTGT
18840	Yes	AATCCAATGGGGTTTCCCCGCACAGGTTCGAATCCTGTT
18841	Yes	AATCCAATGGGGTTTCCCCGCACAGGTTCGAATCCTGTTC
18842	Yes	AATCCAATGGGGTTTCCCCGCACAGGTTCGAATCCTGTTCG
18843	Yes	AATCCAATGGGGTTTCCCCGCACAGGTTCGAATCCTGTTCGT
18844	Yes	AATCCAATGGGGTTTCCCCGCACAGGTTCGAATCCTGTTCGTG
18845	Yes	AATCCAATGGGGTTTCCCCGCACAGGTTCGAATCCTGTTCGTGA
18846	Yes	AATCCAATGGGGTTTCCCCGCACAGGTTCGAATCCTGTTCGTGAC
18847	Yes	AATCCAATGGGGTTTCCCCGCACAGGTTCGAATCCTGTTCGTGACG
18848	Yes	AATCCAATGGGGTTTCCCCGCACAGGTTCGAATCCTGTTCGTGACGC
18849	Yes	AATCCAATGGGGTTTCCCCGCACAGGTTCGAATCCTGTTCGTGACGCC
18850	Yes	AATCCAATGGGGTTTCCCCGCACAGGTTCGAATCCTGTTCGTGACGCCA
18851	No	AATCCAGAGGTTCCGG

18852	No	AATCCAGAGGTTCCGGG
18853	Yes	AATCCAGAGGTTCCGGGT
18854	Yes	AATCCAGAGGTTCCGGGTT
18855	Yes	AATCCAGAGGTTCCGGGTTC
18856	Yes	AATCCAGAGGTTCCGGGTTCG
18857	Yes	AATCCAGAGGTTCCGGGTTCGA
18858	Yes	AATCCAGAGGTTCCGGGTTCGAG
18859	Yes	AATCCAGAGGTTCCGGGTTCGAGT
18860	Yes	AATCCAGAGGTTCCGGGTTCGAGTC
18861	Yes	AATCCAGAGGTTCCGGGTTCGAGTCC
18862	Yes	AATCCAGAGGTTCCGGGTTCGAGTCCC
18863	Yes	AATCCAGAGGTTCCGGGTTCGAGTCCCG
18864	Yes	AATCCAGAGGTTCCGGGTTCGAGTCCCGG
18865	Yes	AATCCAGAGGTTCCGGGTTCGAGTCCCGGC
18866	Yes	AATCCAGAGGTTCCGGGTTCGAGTCCCGGCA
18867	Yes	AATCCAGAGGTTCCGGGTTCGAGTCCCGGCAG
18868	Yes	AATCCAGAGGTTCCGGGTTCGAGTCCCGGCAGA
18869	Yes	AATCCAGAGGTTCCGGGTTCGAGTCCCGGCAGAG
18870	Yes	AATCCAGAGGTTCCGGGTTCGAGTCCCGGCAGAGA
18871	Yes	AATCCAGAGGTTCCGGGTTCGAGTCCCGGCAGAGAT
18872	Yes	AATCCAGAGGTTCCGGGTTCGAGTCCCGGCAGAGATG
18873	Yes	AATCCAGAGGTTCCGGGTTCGAGTCCCGGCAGAGATGC
18874	Yes	AATCCAGAGGTTCCGGGTTCGAGTCCCGGCAGAGATGCC
18875	Yes	AATCCAGAGGTTCCGGGTTCGAGTCCCGGCAGAGATGCCA
18876	No	AATCCAGAGTTTGGGG
18877	Yes	AATCCAGAGTTTGGGGG
18878	Yes	AATCCAGAGTTTGGGGGC
18879	Yes	AATCCAGAGTTTGGGGGCC
18880	Yes	AATCCAGAGTTTGGGGGCCA
18881	No	AATCCAGCAACCAGAG
18882	No	AATCCAGCAACCAGAGT
18883	Yes	AATCCAGCAACCAGAGTT
18884	Yes	AATCCAGCAACCAGAGTTC
18885	Yes	AATCCAGCAACCAGAGTTCC
18886	Yes	AATCCAGCAACCAGAGTTCCA
18887	Yes	AATCCAGCAACCAGAGTTCCAG
18888	Yes	AATCCAGCAACCAGAGTTCCAGT
18889	Yes	AATCCAGCAACCAGAGTTCCAGTC
18890	Yes	AATCCAGCAACCAGAGTTCCAGTCT
18891	Yes	AATCCAGCAACCAGAGTTCCAGTCTC
18892	Yes	AATCCAGCAACCAGAGTTCCAGTCTCA
18893	Yes	AATCCAGCAACCAGAGTTCCAGTCTCAG

18894	Yes	AATCCAGCAACCAGAGTTCCAGTCTCAGC
18895	Yes	AATCCAGCAACCAGAGTTCCAGTCTCAGCG
18896	Yes	AATCCAGCAACCAGAGTTCCAGTCTCAGCGT
18897	Yes	AATCCAGCAACCAGAGTTCCAGTCTCAGCGTG
18898	Yes	AATCCAGCAACCAGAGTTCCAGTCTCAGCGTGG
18899	Yes	AATCCAGCAACCAGAGTTCCAGTCTCAGCGTGGA
18900	Yes	AATCCAGCAACCAGAGTTCCAGTCTCAGCGTGGAC
18901	Yes	AATCCAGCAACCAGAGTTCCAGTCTCAGCGTGGACC
18902	Yes	AATCCAGCAACCAGAGTTCCAGTCTCAGCGTGGACCT
18903	Yes	AATCCAGCAACCAGAGTTCCAGTCTCAGCGTGGACCTC
18904	Yes	AATCCAGCAACCAGAGTTCCAGTCTCAGCGTGGACCTCC
18905	Yes	AATCCAGCAACCAGAGTTCCAGTCTCAGCGTGGACCTCCA
18906	Yes	AATCCAGCAATCCGAG
18907	Yes	AATCCAGCAATCCGAGT
18908	Yes	AATCCAGCAATCCGAGTT
18909	Yes	AATCCAGCAATCCGAGTTC
18910	Yes	AATCCAGCAATCCGAGTTCG
18911	Yes	AATCCAGCAATCCGAGTTCGA
18912	Yes	AATCCAGCAATCCGAGTTCGAA
18913	Yes	AATCCAGCAATCCGAGTTCGAAT
18914	Yes	AATCCAGCAATCCGAGTTCGAATC
18915	Yes	AATCCAGCAATCCGAGTTCGAATCT
18916	Yes	AATCCAGCAATCCGAGTTCGAATCTC
18917	Yes	AATCCAGCAATCCGAGTTCGAATCTCG
18918	Yes	AATCCAGCAATCCGAGTTCGAATCTCGG
18919	Yes	AATCCAGCAATCCGAGTTCGAATCTCGGT
18920	Yes	AATCCAGCAATCCGAGTTCGAATCTCGGTG
18921	Yes	AATCCAGCAATCCGAGTTCGAATCTCGGTGG
18922	Yes	AATCCAGCAATCCGAGTTCGAATCTCGGTGGG
18923	Yes	AATCCAGCAATCCGAGTTCGAATCTCGGTGGGA
18924	Yes	AATCCAGCAATCCGAGTTCGAATCTCGGTGGGAC
18925	Yes	AATCCAGCAATCCGAGTTCGAATCTCGGTGGGACC
18926	Yes	AATCCAGCAATCCGAGTTCGAATCTCGGTGGGACCT
18927	Yes	AATCCAGCAATCCGAGTTCGAATCTCGGTGGGACCTC
18928	Yes	AATCCAGCAATCCGAGTTCGAATCTCGGTGGGACCTCC
18929	Yes	AATCCAGCAATCCGAGTTCGAATCTCGGTGGGACCTCCA
18930	Yes	AATCCAGCAATCCGAGTTCGAATCTT
18931	Yes	AATCCAGCAATCCGAGTTCGAATCTTG
18932	Yes	AATCCAGCAATCCGAGTTCGAATCTTGG
18933	Yes	AATCCAGCAATCCGAGTTCGAATCTTGGT
18934	Yes	AATCCAGCAATCCGAGTTCGAATCTTGGTG
18935	Yes	AATCCAGCAATCCGAGTTCGAATCTTGGTGG

18936	Yes	AATCCAGCAATCCGAGTTCGAATCTTGGTGGG
18937	Yes	AATCCAGCAATCCGAGTTCGAATCTTGGTGGGA
18938	Yes	AATCCAGCAATCCGAGTTCGAATCTTGGTGGGAC
18939	Yes	AATCCAGCAATCCGAGTTCGAATCTTGGTGGGACC
18940	Yes	AATCCAGCAATCCGAGTTCGAATCTTGGTGGGACCT
18941	Yes	AATCCAGCAATCCGAGTTCGAATCTTGGTGGGACCTC
18942	Yes	AATCCAGCAATCCGAGTTCGAATCTTGGTGGGACCTCC
18943	Yes	AATCCAGCAATCCGAGTTCGAATCTTGGTGGGACCTCCA
18944	No	AATCCAGCCATCTGAG
18945	No	AATCCAGCCATCTGAGT
18946	Yes	AATCCAGCCATCTGAGTT
18947	Yes	AATCCAGCCATCTGAGTTC
18948	Yes	AATCCAGCCATCTGAGTTCG
18949	Yes	AATCCAGCCATCTGAGTTCGA
18950	Yes	AATCCAGCCATCTGAGTTCGAG
18951	Yes	AATCCAGCCATCTGAGTTCGAGT
18952	Yes	AATCCAGCCATCTGAGTTCGAGTC
18953	Yes	AATCCAGCCATCTGAGTTCGAGTCT
18954	Yes	AATCCAGCCATCTGAGTTCGAGTCTC
18955	Yes	AATCCAGCCATCTGAGTTCGAGTCTCT
18956	Yes	AATCCAGCCATCTGAGTTCGAGTCTCTG
18957	Yes	AATCCAGCCATCTGAGTTCGAGTCTCTGT
18958	Yes	AATCCAGCCATCTGAGTTCGAGTCTCTGTG
18959	Yes	AATCCAGCCATCTGAGTTCGAGTCTCTGTGG
18960	Yes	AATCCAGCCATCTGAGTTCGAGTCTCTGTGGA
18961	Yes	AATCCAGCCATCTGAGTTCGAGTCTCTGTGGAA
18962	Yes	AATCCAGCCATCTGAGTTCGAGTCTCTGTGGAAC
18963	Yes	AATCCAGCCATCTGAGTTCGAGTCTCTGTGGAACC
18964	Yes	AATCCAGCCATCTGAGTTCGAGTCTCTGTGGAACCT
18965	Yes	AATCCAGCCATCTGAGTTCGAGTCTCTGTGGAACCTC
18966	Yes	AATCCAGCCATCTGAGTTCGAGTCTCTGTGGAACCTCC
18967	Yes	AATCCAGCCATCTGAGTTCGAGTCTCTGTGGAACCTCCA
18968	Yes	AATCCAGCGATCCGAG
18969	Yes	AATCCAGCGATCCGAGT
18970	Yes	AATCCAGCGATCCGAGTT
18971	Yes	AATCCAGCGATCCGAGTTC
18972	Yes	AATCCAGCGATCCGAGTTCA
18973	Yes	AATCCAGCGATCCGAGTTCAA
18974	Yes	AATCCAGCGATCCGAGTTCAAA
18975	Yes	AATCCAGCGATCCGAGTTCAAAT
18976	Yes	AATCCAGCGATCCGAGTTCAAATC
18977	Yes	AATCCAGCGATCCGAGTTCAAATCT

18978	Yes	AATCCAGCGATCCGAGTTCAAATCTC
18979	Yes	AATCCAGCGATCCGAGTTCAAATCTCG
18980	Yes	AATCCAGCGATCCGAGTTCAAATCTCGG
18981	Yes	AATCCAGCGATCCGAGTTCAAATCTCGGT
18982	Yes	AATCCAGCGATCCGAGTTCAAATCTCGGTG
18983	Yes	AATCCAGCGATCCGAGTTCAAATCTCGGTGG
18984	Yes	AATCCAGCGATCCGAGTTCAAATCTCGGTGGA
18985	Yes	AATCCAGCGATCCGAGTTCAAATCTCGGTGGAA
18986	Yes	AATCCAGCGATCCGAGTTCAAATCTCGGTGGAAC
18987	Yes	AATCCAGCGATCCGAGTTCAAATCTCGGTGGAACC
18988	Yes	AATCCAGCGATCCGAGTTCAAATCTCGGTGGAACCT
18989	Yes	AATCCAGCGATCCGAGTTCAAATCTCGGTGGAACCTC
18990	Yes	AATCCAGCGATCCGAGTTCAAATCTCGGTGGAACCTCC
18991	Yes	AATCCAGCGATCCGAGTTCAAATCTCGGTGGAACCTCCA
18992	Yes	AATCCAGCGATCCGAGTTCAAATCTCGGTGGG
18993	Yes	AATCCAGCGATCCGAGTTCAAATCTCGGTGGGA
18994	Yes	AATCCAGCGATCCGAGTTCAAATCTCGGTGGGAC
18995	Yes	AATCCAGCGATCCGAGTTCAAATCTCGGTGGGACC
18996	Yes	AATCCAGCGATCCGAGTTCAAATCTCGGTGGGACCC
18997	Yes	AATCCAGCGATCCGAGTTCAAATCTCGGTGGGACCCC
18998	Yes	AATCCAGCGATCCGAGTTCAAATCTCGGTGGGACCCCC
18999	Yes	AATCCAGCGATCCGAGTTCAAATCTCGGTGGGACCCCCA
19000	Yes	AATCCAGCGATCCGAGTTCAAATCTCGGTGGGACCT
19001	Yes	AATCCAGCGATCCGAGTTCAAATCTCGGTGGGACCTC
19002	Yes	AATCCAGCGATCCGAGTTCAAATCTCGGTGGGACCTCC
19003	Yes	AATCCAGCGATCCGAGTTCAAATCTCGGTGGGACCTCCA
19004	Yes	AATCCAGCGATCCGAGTTCAAG
19005	Yes	AATCCAGCGATCCGAGTTCAAGT
19006	Yes	AATCCAGCGATCCGAGTTCAAGTC
19007	Yes	AATCCAGCGATCCGAGTTCAAGTCT
19008	Yes	AATCCAGCGATCCGAGTTCAAGTCTC
19009	Yes	AATCCAGCGATCCGAGTTCAAGTCTCG
19010	Yes	AATCCAGCGATCCGAGTTCAAGTCTCGG
19011	Yes	AATCCAGCGATCCGAGTTCAAGTCTCGGT
19012	Yes	AATCCAGCGATCCGAGTTCAAGTCTCGGTG
19013	Yes	AATCCAGCGATCCGAGTTCAAGTCTCGGTGG
19014	Yes	AATCCAGCGATCCGAGTTCAAGTCTCGGTGGA
19015	Yes	AATCCAGCGATCCGAGTTCAAGTCTCGGTGGAA
19016	Yes	AATCCAGCGATCCGAGTTCAAGTCTCGGTGGAAC
19017	Yes	AATCCAGCGATCCGAGTTCAAGTCTCGGTGGAACC
19018	Yes	AATCCAGCGATCCGAGTTCAAGTCTCGGTGGAACCT
19019	Yes	AATCCAGCGATCCGAGTTCAAGTCTCGGTGGAACCTC

19020	Yes	AATCCAGCGATCCGAGTTCAAGTCTCGGTGGAACCTCC
19021	Yes	AATCCAGCGATCCGAGTTCAAGTCTCGGTGGAACCTCCA
19022	Yes	AATCCAGCGATCCGAGTTCG
19023	Yes	AATCCAGCGATCCGAGTTCGA
19024	Yes	AATCCAGCGATCCGAGTTCGAG
19025	Yes	AATCCAGCGATCCGAGTTCGAGT
19026	Yes	AATCCAGCGATCCGAGTTCGAGTC
19027	Yes	AATCCAGCGATCCGAGTTCGAGTCT
19028	Yes	AATCCAGCGATCCGAGTTCGAGTCTC
19029	Yes	AATCCAGCGATCCGAGTTCGAGTCTCG
19030	Yes	AATCCAGCGATCCGAGTTCGAGTCTCGG
19031	Yes	AATCCAGCGATCCGAGTTCGAGTCTCGGT
19032	Yes	AATCCAGCGATCCGAGTTCGAGTCTCGGTG
19033	Yes	AATCCAGCGATCCGAGTTCGAGTCTCGGTGG
19034	Yes	AATCCAGCGATCCGAGTTCGAGTCTCGGTGGA
19035	Yes	AATCCAGCGATCCGAGTTCGAGTCTCGGTGGAA
19036	Yes	AATCCAGCGATCCGAGTTCGAGTCTCGGTGGAAC
19037	Yes	AATCCAGCGATCCGAGTTCGAGTCTCGGTGGAACC
19038	Yes	AATCCAGCGATCCGAGTTCGAGTCTCGGTGGAACCT
19039	Yes	AATCCAGCGATCCGAGTTCGAGTCTCGGTGGAACCTC
19040	Yes	AATCCAGCGATCCGAGTTCGAGTCTCGGTGGAACCTCC
19041	Yes	AATCCAGCGATCCGAGTTCGAGTCTCGGTGGAACCTCCA
19042	Yes	AATCCAGGTGCCCCCC
19043	Yes	AATCCAGGTGCCCCCCC
19044	Yes	AATCCAGGTGCCCCCCCC
19045	Yes	AATCCAGGTGCCCCCCCCA
19046	No	AATCCAGGTGCCCCCT
19047	Yes	AATCCAGGTGCCCCCTC
19048	Yes	AATCCAGGTGCCCCCTCC
19049	Yes	AATCCAGGTGCCCCCTCCA
19050	Yes	AATCCAGGTGCCCCTT
19051	Yes	AATCCAGGTGCCCCTTC
19052	Yes	AATCCAGGTGCCCCTTCC
19053	Yes	AATCCAGGTGCCCCTTCCA
19054	No	AATCCAGGTTCTCCCT
19055	No	AATCCAGGTTCTCCCTC
19056	Yes	AATCCAGGTTCTCCCTCC
19057	Yes	AATCCAGGTTCTCCCTCCA
19058	Yes	AATCCATTGGGGTCTC
19059	Yes	AATCCATTGGGGTCTCC
19060	Yes	AATCCATTGGGGTCTCCC
19061	Yes	AATCCATTGGGGTCTCCCC

19062	Yes	AATCCATTGGGGTCTCCCCG
19063	Yes	AATCCATTGGGGTCTCCCCGC
19064	Yes	AATCCATTGGGGTCTCCCCGCG
19065	Yes	AATCCATTGGGGTCTCCCCGCGC
19066	Yes	AATCCATTGGGGTCTCCCCGCGCA
19067	Yes	AATCCATTGGGGTCTCCCCGCGCAG
19068	Yes	AATCCATTGGGGTCTCCCCGCGCAGG
19069	Yes	AATCCATTGGGGTCTCCCCGCGCAGGT
19070	Yes	AATCCATTGGGGTCTCCCCGCGCAGGTT
19071	Yes	AATCCATTGGGGTCTCCCCGCGCAGGTTC
19072	Yes	AATCCATTGGGGTCTCCCCGCGCAGGTTCG
19073	Yes	AATCCATTGGGGTCTCCCCGCGCAGGTTCGA
19074	Yes	AATCCATTGGGGTCTCCCCGCGCAGGTTCGAA
19075	Yes	AATCCATTGGGGTCTCCCCGCGCAGGTTCGAAT
19076	Yes	AATCCATTGGGGTCTCCCCGCGCAGGTTCGAATC
19077	Yes	AATCCATTGGGGTCTCCCCGCGCAGGTTCGAATCC
19078	Yes	AATCCATTGGGGTCTCCCCGCGCAGGTTCGAATCCT
19079	Yes	AATCCATTGGGGTCTCCCCGCGCAGGTTCGAATCCTG
19080	Yes	AATCCATTGGGGTCTCCCCGCGCAGGTTCGAATCCTGC
19081	Yes	AATCCATTGGGGTCTCCCCGCGCAGGTTCGAATCCTGCC
19082	Yes	AATCCATTGGGGTCTCCCCGCGCAGGTTCGAATCCTGCCG
19083	Yes	AATCCATTGGGGTCTCCCCGCGCAGGTTCGAATCCTGCCGA
19084	Yes	AATCCATTGGGGTCTCCCCGCGCAGGTTCGAATCCTGCCGAC
19085	Yes	AATCCATTGGGGTCTCCCCGCGCAGGTTCGAATCCTGCCGACT
19086	Yes	AATCCATTGGGGTCTCCCCGCGCAGGTTCGAATCCTGCCGACTA
19087	Yes	AATCCATTGGGGTCTCCCCGCGCAGGTTCGAATCCTGCCGACTAC
19088	Yes	AATCCATTGGGGTCTCCCCGCGCAGGTTCGAATCCTGCCGACTACG
19089	Yes	AATCCATTGGGGTCTCCCCGCGCAGGTTCGAATCCTGCCGACTACGC
19090	Yes	AATCCATTGGGGTCTCCCCGCGCAGGTTCGAATCCTGCCGACTACGCC
19091	Yes	AATCCATTGGGGTCTCCCCGCGCAGGTTCGAATCCTGCCGACTACGCCA
19092	No	AATCCATTGGGGTTTC
19093	No	AATCCATTGGGGTTTCC
19094	No	AATCCATTGGGGTTTCCC
19095	Yes	AATCCATTGGGGTTTCCCC
19096	Yes	AATCCATTGGGGTTTCCCCA
19097	Yes	AATCCATTGGGGTTTCCCCAC
19098	Yes	AATCCATTGGGGTTTCCCCACG
19099	Yes	AATCCATTGGGGTTTCCCCACGC
19100	Yes	AATCCATTGGGGTTTCCCCACGCA
19101	Yes	AATCCATTGGGGTTTCCCCACGCAG
19102	Yes	AATCCATTGGGGTTTCCCCACGCAGG
19103	Yes	AATCCATTGGGGTTTCCCCACGCAGGT

19104	Yes	AATCCATTGGGGTTTCCCCACGCAGGTT
19105	Yes	AATCCATTGGGGTTTCCCCACGCAGGTTC
19106	Yes	AATCCATTGGGGTTTCCCCACGCAGGTTCG
19107	Yes	AATCCATTGGGGTTTCCCCACGCAGGTTCGA
19108	Yes	AATCCATTGGGGTTTCCCCACGCAGGTTCGAA
19109	Yes	AATCCATTGGGGTTTCCCCACGCAGGTTCGAAT
19110	Yes	AATCCATTGGGGTTTCCCCACGCAGGTTCGAATC
19111	Yes	AATCCATTGGGGTTTCCCCACGCAGGTTCGAATCC
19112	Yes	AATCCATTGGGGTTTCCCCACGCAGGTTCGAATCCT
19113	Yes	AATCCATTGGGGTTTCCCCACGCAGGTTCGAATCCTG
19114	Yes	AATCCATTGGGGTTTCCCCACGCAGGTTCGAATCCTGC
19115	Yes	AATCCATTGGGGTTTCCCCACGCAGGTTCGAATCCTGCC
19116	Yes	AATCCATTGGGGTTTCCCCACGCAGGTTCGAATCCTGCCG
19117	Yes	AATCCATTGGGGTTTCCCCACGCAGGTTCGAATCCTGCCGA
19118	Yes	AATCCATTGGGGTTTCCCCACGCAGGTTCGAATCCTGCCGAC
19119	Yes	AATCCATTGGGGTTTCCCCACGCAGGTTCGAATCCTGCCGACT
19120	Yes	AATCCATTGGGGTTTCCCCACGCAGGTTCGAATCCTGCCGACTA
19121	Yes	AATCCATTGGGGTTTCCCCACGCAGGTTCGAATCCTGCCGACTAC
19122	Yes	AATCCATTGGGGTTTCCCCACGCAGGTTCGAATCCTGCCGACTACG
19123	Yes	AATCCATTGGGGTTTCCCCACGCAGGTTCGAATCCTGCCGACTACGC
19124	Yes	AATCCATTGGGGTTTCCCCACGCAGGTTCGAATCCTGCCGACTACGCC
19125	Yes	AATCCATTGGGGTTTCCCCACGCAGGTTCGAATCCTGCCGACTACGCCA
19126	Yes	AATCCATTGGGGTTTCCCCG
19127	Yes	AATCCATTGGGGTTTCCCCGC
19128	Yes	AATCCATTGGGGTTTCCCCGCG
19129	Yes	AATCCATTGGGGTTTCCCCGCGC
19130	Yes	AATCCATTGGGGTTTCCCCGCGCA
19131	Yes	AATCCATTGGGGTTTCCCCGCGCAG
19132	Yes	AATCCATTGGGGTTTCCCCGCGCAGG
19133	Yes	AATCCATTGGGGTTTCCCCGCGCAGGT
19134	Yes	AATCCATTGGGGTTTCCCCGCGCAGGTT
19135	Yes	AATCCATTGGGGTTTCCCCGCGCAGGTTC
19136	Yes	AATCCATTGGGGTTTCCCCGCGCAGGTTCG
19137	Yes	AATCCATTGGGGTTTCCCCGCGCAGGTTCGA
19138	Yes	AATCCATTGGGGTTTCCCCGCGCAGGTTCGAA
19139	Yes	AATCCATTGGGGTTTCCCCGCGCAGGTTCGAAT
19140	Yes	AATCCATTGGGGTTTCCCCGCGCAGGTTCGAATC
19141	Yes	AATCCATTGGGGTTTCCCCGCGCAGGTTCGAATCC
19142	Yes	AATCCATTGGGGTTTCCCCGCGCAGGTTCGAATCCT
19143	Yes	AATCCATTGGGGTTTCCCCGCGCAGGTTCGAATCCTG
19144	Yes	AATCCATTGGGGTTTCCCCGCGCAGGTTCGAATCCTGC
19145	Yes	AATCCATTGGGGTTTCCCCGCGCAGGTTCGAATCCTGCC

19146	Yes	AATCCATTGGGGTTTCCCCGCGCAGGTTCGAATCCTGCCG
19147	Yes	AATCCATTGGGGTTTCCCCGCGCAGGTTCGAATCCTGCCGA
19148	Yes	AATCCATTGGGGTTTCCCCGCGCAGGTTCGAATCCTGCCGAC
19149	Yes	AATCCATTGGGGTTTCCCCGCGCAGGTTCGAATCCTGCCGACT
19150	Yes	AATCCATTGGGGTTTCCCCGCGCAGGTTCGAATCCTGCCGACTA
19151	Yes	AATCCATTGGGGTTTCCCCGCGCAGGTTCGAATCCTGCCGACTAC
19152	Yes	AATCCATTGGGGTTTCCCCGCGCAGGTTCGAATCCTGCCGACTACG
19153	Yes	AATCCATTGGGGTTTCCCCGCGCAGGTTCGAATCCTGCCGACTACGC
19154	Yes	AATCCATTGGGGTTTCCCCGCGCAGGTTCGAATCCTGCCGACTACGCC
19155	Yes	AATCCATTGGGGTTTCCCCGCGCAGGTTCGAATCCTGCCGACTACGCCA
19156	Yes	AATCCATTGGGGTTTCCCCGCGCAGGTTCGAATCCTGT
19157	Yes	AATCCATTGGGGTTTCCCCGCGCAGGTTCGAATCCTGTC
19158	Yes	AATCCATTGGGGTTTCCCCGCGCAGGTTCGAATCCTGTCG
19159	Yes	AATCCATTGGGGTTTCCCCGCGCAGGTTCGAATCCTGTCGG
19160	Yes	AATCCATTGGGGTTTCCCCGCGCAGGTTCGAATCCTGTCGGC
19161	Yes	AATCCATTGGGGTTTCCCCGCGCAGGTTCGAATCCTGTCGGCT
19162	Yes	AATCCATTGGGGTTTCCCCGCGCAGGTTCGAATCCTGTCGGCTA
19163	Yes	AATCCATTGGGGTTTCCCCGCGCAGGTTCGAATCCTGTCGGCTAC
19164	Yes	AATCCATTGGGGTTTCCCCGCGCAGGTTCGAATCCTGTCGGCTACG
19165	Yes	AATCCATTGGGGTTTCCCCGCGCAGGTTCGAATCCTGTCGGCTACGC
19166	Yes	AATCCATTGGGGTTTCCCCGCGCAGGTTCGAATCCTGTCGGCTACGCC
19167	Yes	AATCCATTGGGGTTTCCCCGCGCAGGTTCGAATCCTGTCGGCTACGCCA
19168	No	AATCCATTGTGCTCTG
19169	Yes	AATCCATTGTGCTCTGC
19170	Yes	AATCCATTGTGCTCTGCA
19171	Yes	AATCCATTGTGCTCTGCAC
19172	Yes	AATCCATTGTGCTCTGCACA
19173	Yes	AATCCATTGTGCTCTGCACAC
19174	Yes	AATCCATTGTGCTCTGCACACG
19175	Yes	AATCCATTGTGCTCTGCACACGT
19176	Yes	AATCCATTGTGCTCTGCACACGTG
19177	Yes	AATCCATTGTGCTCTGCACACGTGG
19178	Yes	AATCCATTGTGCTCTGCACACGTGGG
19179	Yes	AATCCATTGTGCTCTGCACACGTGGGT
19180	Yes	AATCCATTGTGCTCTGCACACGTGGGTT
19181	Yes	AATCCATTGTGCTCTGCACACGTGGGTTC
19182	Yes	AATCCATTGTGCTCTGCACACGTGGGTTCG
19183	Yes	AATCCATTGTGCTCTGCACACGTGGGTTCGA
19184	Yes	AATCCATTGTGCTCTGCACACGTGGGTTCGAA
19185	Yes	AATCCATTGTGCTCTGCACACGTGGGTTCGAAT
19186	Yes	AATCCATTGTGCTCTGCACACGTGGGTTCGAATC
19187	Yes	AATCCATTGTGCTCTGCACACGTGGGTTCGAATCC

19188	Yes	AATCCATTGTGCTCTGCACACGTGGGTTCGAATCCC
19189	Yes	AATCCATTGTGCTCTGCACACGTGGGTTCGAATCCCA
19190	Yes	AATCCATTGTGCTCTGCACACGTGGGTTCGAATCCCAT
19191	Yes	AATCCATTGTGCTCTGCACACGTGGGTTCGAATCCCATC
19192	Yes	AATCCATTGTGCTCTGCACACGTGGGTTCGAATCCCATCC
19193	Yes	AATCCATTGTGCTCTGCACACGTGGGTTCGAATCCCATCCT
19194	Yes	AATCCATTGTGCTCTGCACACGTGGGTTCGAATCCCATCCTC
19195	Yes	AATCCATTGTGCTCTGCACACGTGGGTTCGAATCCCATCCTCG
19196	Yes	AATCCATTGTGCTCTGCACACGTGGGTTCGAATCCCATCCTCGT
19197	Yes	AATCCATTGTGCTCTGCACACGTGGGTTCGAATCCCATCCTCGTC
19198	Yes	AATCCATTGTGCTCTGCACACGTGGGTTCGAATCCCATCCTCGTCG
19199	Yes	AATCCATTGTGCTCTGCACACGTGGGTTCGAATCCCATCCTCGTCGC
19200	Yes	AATCCATTGTGCTCTGCACACGTGGGTTCGAATCCCATCCTCGTCGCC
19201	Yes	AATCCATTGTGCTCTGCACACGTGGGTTCGAATCCCATCCTCGTCGCCA
19202	Yes	AATCCATTGTGCTCTGCACG
19203	Yes	AATCCATTGTGCTCTGCACGC
19204	Yes	AATCCATTGTGCTCTGCACGCG
19205	Yes	AATCCATTGTGCTCTGCACGCGT
19206	Yes	AATCCATTGTGCTCTGCACGCGTG
19207	Yes	AATCCATTGTGCTCTGCACGCGTGG
19208	Yes	AATCCATTGTGCTCTGCACGCGTGGG
19209	Yes	AATCCATTGTGCTCTGCACGCGTGGGT
19210	Yes	AATCCATTGTGCTCTGCACGCGTGGGTT
19211	Yes	AATCCATTGTGCTCTGCACGCGTGGGTTC
19212	Yes	AATCCATTGTGCTCTGCACGCGTGGGTTCG
19213	Yes	AATCCATTGTGCTCTGCACGCGTGGGTTCGA
19214	Yes	AATCCATTGTGCTCTGCACGCGTGGGTTCGAA
19215	Yes	AATCCATTGTGCTCTGCACGCGTGGGTTCGAAT
19216	Yes	AATCCATTGTGCTCTGCACGCGTGGGTTCGAATC
19217	Yes	AATCCATTGTGCTCTGCACGCGTGGGTTCGAATCC
19218	Yes	AATCCATTGTGCTCTGCACGCGTGGGTTCGAATCCC
19219	Yes	AATCCATTGTGCTCTGCACGCGTGGGTTCGAATCCCA
19220	Yes	AATCCATTGTGCTCTGCACGCGTGGGTTCGAATCCCAC
19221	Yes	AATCCATTGTGCTCTGCACGCGTGGGTTCGAATCCCACC
19222	Yes	AATCCATTGTGCTCTGCACGCGTGGGTTCGAATCCCACCC
19223	Yes	AATCCATTGTGCTCTGCACGCGTGGGTTCGAATCCCACCCT
19224	Yes	AATCCATTGTGCTCTGCACGCGTGGGTTCGAATCCCACCCTC
19225	Yes	AATCCATTGTGCTCTGCACGCGTGGGTTCGAATCCCACCCTCG
19226	Yes	AATCCATTGTGCTCTGCACGCGTGGGTTCGAATCCCACCCTCGT
19227	Yes	AATCCATTGTGCTCTGCACGCGTGGGTTCGAATCCCACCCTCGTC
19228	Yes	AATCCATTGTGCTCTGCACGCGTGGGTTCGAATCCCACCCTCGTCG
19229	Yes	AATCCATTGTGCTCTGCACGCGTGGGTTCGAATCCCACCCTCGTCGC

19230	Yes	AATCCATTGTGCTCTGCACGCGTGGGTTCGAATCCCACCCTCGTCGCC
19231	Yes	AATCCATTGTGCTCTGCACGCGTGGGTTCGAATCCCACCCTCGTCGCCA
19232	Yes	AATCCATTGTGCTCTGCACGCGTGGGTTCGAATCCCACCT
19233	Yes	AATCCATTGTGCTCTGCACGCGTGGGTTCGAATCCCACCTT
19234	Yes	AATCCATTGTGCTCTGCACGCGTGGGTTCGAATCCCACCTTC
19235	Yes	AATCCATTGTGCTCTGCACGCGTGGGTTCGAATCCCACCTTCG
19236	Yes	AATCCATTGTGCTCTGCACGCGTGGGTTCGAATCCCACCTTCGT
19237	Yes	AATCCATTGTGCTCTGCACGCGTGGGTTCGAATCCCACCTTCGTC
19238	Yes	AATCCATTGTGCTCTGCACGCGTGGGTTCGAATCCCACCTTCGTCG
19239	Yes	AATCCATTGTGCTCTGCACGCGTGGGTTCGAATCCCACCTTCGTCGC
19240	Yes	AATCCATTGTGCTCTGCACGCGTGGGTTCGAATCCCACCTTCGTCGCC
19241	Yes	AATCCATTGTGCTCTGCACGCGTGGGTTCGAATCCCACCTTCGTCGCCA
19242	Yes	AATCCATTGTGCTCTGCACGCGTGGGTTCGAATCCCAT
19243	Yes	AATCCATTGTGCTCTGCACGCGTGGGTTCGAATCCCATC
19244	Yes	AATCCATTGTGCTCTGCACGCGTGGGTTCGAATCCCATCC
19245	Yes	AATCCATTGTGCTCTGCACGCGTGGGTTCGAATCCCATCCT
19246	Yes	AATCCATTGTGCTCTGCACGCGTGGGTTCGAATCCCATCCTC
19247	Yes	AATCCATTGTGCTCTGCACGCGTGGGTTCGAATCCCATCCTCG
19248	Yes	AATCCATTGTGCTCTGCACGCGTGGGTTCGAATCCCATCCTCGT
19249	Yes	AATCCATTGTGCTCTGCACGCGTGGGTTCGAATCCCATCCTCGTC
19250	Yes	AATCCATTGTGCTCTGCACGCGTGGGTTCGAATCCCATCCTCGTCG
19251	Yes	AATCCATTGTGCTCTGCACGCGTGGGTTCGAATCCCATCCTCGTCGC
19252	Yes	AATCCATTGTGCTCTGCACGCGTGGGTTCGAATCCCATCCTCGTCGCC
19253	Yes	AATCCATTGTGCTCTGCACGCGTGGGTTCGAATCCCATCCTCGTCGCCA
19254	No	AATCCATTGTGCTTTG
19255	No	AATCCATTGTGCTTTGC
19256	Yes	AATCCATTGTGCTTTGCA
19257	Yes	AATCCATTGTGCTTTGCAC
19258	Yes	AATCCATTGTGCTTTGCACG
19259	Yes	AATCCATTGTGCTTTGCACGC
19260	Yes	AATCCATTGTGCTTTGCACGCG
19261	Yes	AATCCATTGTGCTTTGCACGCGT
19262	Yes	AATCCATTGTGCTTTGCACGCGTG
19263	Yes	AATCCATTGTGCTTTGCACGCGTGG
19264	Yes	AATCCATTGTGCTTTGCACGCGTGGG
19265	Yes	AATCCATTGTGCTTTGCACGCGTGGGT
19266	Yes	AATCCATTGTGCTTTGCACGCGTGGGTT
19267	Yes	AATCCATTGTGCTTTGCACGCGTGGGTTC
19268	Yes	AATCCATTGTGCTTTGCACGCGTGGGTTCG
19269	Yes	AATCCATTGTGCTTTGCACGCGTGGGTTCGA
19270	Yes	AATCCATTGTGCTTTGCACGCGTGGGTTCGAA
19271	Yes	AATCCATTGTGCTTTGCACGCGTGGGTTCGAAT

19272	Yes	AATCCATTGTGCTTTGCACGCGTGGGTTCGAATC
19273	Yes	AATCCATTGTGCTTTGCACGCGTGGGTTCGAATCC
19274	Yes	AATCCATTGTGCTTTGCACGCGTGGGTTCGAATCCC
19275	Yes	AATCCATTGTGCTTTGCACGCGTGGGTTCGAATCCCA
19276	Yes	AATCCATTGTGCTTTGCACGCGTGGGTTCGAATCCCAT
19277	Yes	AATCCATTGTGCTTTGCACGCGTGGGTTCGAATCCCATC
19278	Yes	AATCCATTGTGCTTTGCACGCGTGGGTTCGAATCCCATCC
19279	Yes	AATCCATTGTGCTTTGCACGCGTGGGTTCGAATCCCATCCT
19280	Yes	AATCCATTGTGCTTTGCACGCGTGGGTTCGAATCCCATCCTC
19281	Yes	AATCCATTGTGCTTTGCACGCGTGGGTTCGAATCCCATCCTCG
19282	Yes	AATCCATTGTGCTTTGCACGCGTGGGTTCGAATCCCATCCTCGT
19283	Yes	AATCCATTGTGCTTTGCACGCGTGGGTTCGAATCCCATCCTCGTC
19284	Yes	AATCCATTGTGCTTTGCACGCGTGGGTTCGAATCCCATCCTCGTCG
19285	Yes	AATCCATTGTGCTTTGCACGCGTGGGTTCGAATCCCATCCTCGTCGC
19286	Yes	AATCCATTGTGCTTTGCACGCGTGGGTTCGAATCCCATCCTCGTCGCC
19287	Yes	AATCCATTGTGCTTTGCACGCGTGGGTTCGAATCCCATCCTCGTCGCCA
19288	No	AATCCCACCACTGCCA
19289	No	AATCCCACCACTGCCAC
19290	No	AATCCCACCACTGCCACC
19291	No	AATCCCACCACTGCCACCA
19292	Yes	AATCCCACCAGAGTCG
19293	Yes	AATCCCACCAGAGTCGC
19294	Yes	AATCCCACCAGAGTCGCC
19295	Yes	AATCCCACCAGAGTCGCCA
19296	Yes	AATCCCACCCTCGTCG
19297	Yes	AATCCCACCCTCGTCGC
19298	Yes	AATCCCACCCTCGTCGCC
19299	Yes	AATCCCACCCTCGTCGCCA
19300	Yes	AATCCCACCGCTGCCA
19301	Yes	AATCCCACCGCTGCCAC
19302	Yes	AATCCCACCGCTGCCACC
19303	Yes	AATCCCACCGCTGCCACCA
19304	Yes	AATCCCACCGCTGCTA
19305	Yes	AATCCCACCGCTGCTAC
19306	Yes	AATCCCACCGCTGCTACC
19307	Yes	AATCCCACCGCTGCTACCA
19308	Yes	AATCCCACCTTCGTCG
19309	Yes	AATCCCACCTTCGTCGC
19310	Yes	AATCCCACCTTCGTCGCC
19311	Yes	AATCCCACCTTCGTCGCCA
19312	No	AATCCCACTCCTGACA
19313	No	AATCCCACTCCTGACAC

19314	No	AATCCCACTCCTGACACC
19315	No	AATCCCACTCCTGACACCA
19316	No	AATCCCACTGCTGCAA
19317	No	AATCCCACTGCTGCAAC
19318	No	AATCCCACTGCTGCAACC
19319	No	AATCCCACTGCTGCAACCA
19320	No	AATCCCACTGCTGCCA
19321	No	AATCCCACTGCTGCCAC
19322	Yes	AATCCCACTGCTGCCACC
19323	Yes	AATCCCACTGCTGCCACCA
19324	No	AATCCCACTTCTGACA
19325	No	AATCCCACTTCTGACAC
19326	No	AATCCCACTTCTGACACC
19327	No	AATCCCACTTCTGACACCA
19328	No	AATCCCACTTTTGACA
19329	No	AATCCCACTTTTGACAC
19330	No	AATCCCACTTTTGACACC
19331	No	AATCCCACTTTTGACACCA
19332	No	AATCCCAGAGGAGCCC
19333	No	AATCCCAGAGGAGCCCC
19334	No	AATCCCAGAGGAGCCCCC
19335	No	AATCCCAGAGGAGCCCCCA
19336	No	AATCCCAGAGTCAGTG
19337	Yes	AATCCCAGAGTCAGTGG
19338	Yes	AATCCCAGAGTCAGTGGG
19339	Yes	AATCCCAGAGTCAGTGGGT
19340	Yes	AATCCCAGAGTCAGTGGGTT
19341	Yes	AATCCCAGAGTCAGTGGGTTC
19342	Yes	AATCCCAGAGTCAGTGGGTTCA
19343	Yes	AATCCCAGAGTCAGTGGGTTCAA
19344	Yes	AATCCCAGAGTCAGTGGGTTCAAG
19345	Yes	AATCCCAGAGTCAGTGGGTTCAAGC
19346	Yes	AATCCCAGAGTCAGTGGGTTCAAGCC
19347	Yes	AATCCCAGAGTCAGTGGGTTCAAGCCT
19348	Yes	AATCCCAGAGTCAGTGGGTTCAAGCCTC
19349	Yes	AATCCCAGAGTCAGTGGGTTCAAGCCTCA
19350	Yes	AATCCCAGAGTCAGTGGGTTCAAGCCTCAC
19351	Yes	AATCCCAGAGTCAGTGGGTTCAAGCCTCACA
19352	Yes	AATCCCAGAGTCAGTGGGTTCAAGCCTCACAT
19353	Yes	AATCCCAGAGTCAGTGGGTTCAAGCCTCACATT
19354	Yes	AATCCCAGAGTCAGTGGGTTCAAGCCTCACATTG
19355	Yes	AATCCCAGAGTCAGTGGGTTCAAGCCTCACATTGA

19356	Yes	AATCCCAGAGTCAGTGGGTTCAAGCCTCACATTGAG
19357	Yes	AATCCCAGAGTCAGTGGGTTCAAGCCTCACATTGAGT
19358	Yes	AATCCCAGAGTCAGTGGGTTCAAGCCTCACATTGAGTG
19359	Yes	AATCCCAGAGTCAGTGGGTTCAAGCCTCACATTGAGTGC
19360	Yes	AATCCCAGAGTCAGTGGGTTCAAGCCTCACATTGAGTGCC
19361	Yes	AATCCCAGAGTCAGTGGGTTCAAGCCTCACATTGAGTGCCA
19362	No	AATCCCAGCACTGTTG
19363	No	AATCCCAGCACTGTTGC
19364	No	AATCCCAGCACTGTTGCC
19365	Yes	AATCCCAGCACTGTTGCCA
19366	No	AATCCCAGCCCCGCCA
19367	No	AATCCCAGCCCCGCCAC
19368	No	AATCCCAGCCCCGCCACC
19369	Yes	AATCCCAGCCCCGCCACCA
19370	Yes	AATCCCAGCGAGGCCT
19371	Yes	AATCCCAGCGAGGCCTC
19372	Yes	AATCCCAGCGAGGCCTCC
19373	Yes	AATCCCAGCGAGGCCTCCA
19374	No	AATCCCAGCGGGGCCT
19375	No	AATCCCAGCGGGGCCTC
19376	Yes	AATCCCAGCGGGGCCTCC
19377	Yes	AATCCCAGCGGGGCCTCCA
19378	Yes	AATCCCAGCGGTGCCT
19379	Yes	AATCCCAGCGGTGCCTC
19380	Yes	AATCCCAGCGGTGCCTCC
19381	Yes	AATCCCAGCGGTGCCTCCA
19382	No	AATCCCAGCTGTATGC
19383	Yes	AATCCCAGCTGTATGCC
19384	Yes	AATCCCAGCTGTATGCCC
19385	Yes	AATCCCAGCTGTATGCCCA
19386	No	AATCCCAGCTTATTCA
19387	No	AATCCCAGCTTATTCAC
19388	No	AATCCCAGCTTATTCACC
19389	Yes	AATCCCAGCTTATTCACCA
19390	No	AATCCCAGCTTCTACA
19391	Yes	AATCCCAGCTTCTACAC
19392	Yes	AATCCCAGCTTCTACACC
19393	Yes	AATCCCAGCTTCTACACCA
19394	No	AATCCCAGCTTGGCCT
19395	No	AATCCCAGCTTGGCCTC
19396	No	AATCCCAGCTTGGCCTCC
19397	No	AATCCCAGCTTGGCCTCCA

19398	No	AATCCCAGCTTTGTCA
19399	No	AATCCCAGCTTTGTCAC
19400	No	AATCCCAGCTTTGTCACC
19401	Yes	AATCCCAGCTTTGTCACCA
19402	No	AATCCCAGGGTCGTGG
19403	No	AATCCCAGGGTCGTGGG
19404	No	AATCCCAGGGTCGTGGGT
19405	No	AATCCCAGGGTCGTGGGTT
19406	No	AATCCCAGGGTCGTGGGTTC
19407	Yes	AATCCCAGGGTCGTGGGTTCG
19408	Yes	AATCCCAGGGTCGTGGGTTCGA
19409	Yes	AATCCCAGGGTCGTGGGTTCGAG
19410	Yes	AATCCCAGGGTCGTGGGTTCGAGC
19411	Yes	AATCCCAGGGTCGTGGGTTCGAGCC
19412	Yes	AATCCCAGGGTCGTGGGTTCGAGCCC
19413	Yes	AATCCCAGGGTCGTGGGTTCGAGCCCC
19414	Yes	AATCCCAGGGTCGTGGGTTCGAGCCCCA
19415	Yes	AATCCCAGGGTCGTGGGTTCGAGCCCCAC
19416	Yes	AATCCCAGGGTCGTGGGTTCGAGCCCCACG
19417	Yes	AATCCCAGGGTCGTGGGTTCGAGCCCCACGT
19418	Yes	AATCCCAGGGTCGTGGGTTCGAGCCCCACGTT
19419	Yes	AATCCCAGGGTCGTGGGTTCGAGCCCCACGTTG
19420	Yes	AATCCCAGGGTCGTGGGTTCGAGCCCCACGTTGG
19421	Yes	AATCCCAGGGTCGTGGGTTCGAGCCCCACGTTGGG
19422	Yes	AATCCCAGGGTCGTGGGTTCGAGCCCCACGTTGGGC
19423	Yes	AATCCCAGGGTCGTGGGTTCGAGCCCCACGTTGGGCG
19424	Yes	AATCCCAGGGTCGTGGGTTCGAGCCCCACGTTGGGCGC
19425	Yes	AATCCCAGGGTCGTGGGTTCGAGCCCCACGTTGGGCGCC
19426	Yes	AATCCCAGGGTCGTGGGTTCGAGCCCCACGTTGGGCGCCA
19427	Yes	AATCCCAGGGTCGTGGGTTT
19428	Yes	AATCCCAGGGTCGTGGGTTTG
19429	Yes	AATCCCAGGGTCGTGGGTTTGA
19430	Yes	AATCCCAGGGTCGTGGGTTTGAG
19431	Yes	AATCCCAGGGTCGTGGGTTTGAGC
19432	Yes	AATCCCAGGGTCGTGGGTTTGAGCC
19433	Yes	AATCCCAGGGTCGTGGGTTTGAGCCC
19434	Yes	AATCCCAGGGTCGTGGGTTTGAGCCCC
19435	Yes	AATCCCAGGGTCGTGGGTTTGAGCCCCA
19436	Yes	AATCCCAGGGTCGTGGGTTTGAGCCCCAT
19437	Yes	AATCCCAGGGTCGTGGGTTTGAGCCCCATG
19438	Yes	AATCCCAGGGTCGTGGGTTTGAGCCCCATGT
19439	Yes	AATCCCAGGGTCGTGGGTTTGAGCCCCATGTT

19440	Yes	AATCCCAGGGTCGTGGGTTTGAGCCCCATGTTG
19441	Yes	AATCCCAGGGTCGTGGGTTTGAGCCCCATGTTGG
19442	Yes	AATCCCAGGGTCGTGGGTTTGAGCCCCATGTTGGG
19443	Yes	AATCCCAGGGTCGTGGGTTTGAGCCCCATGTTGGGC
19444	Yes	AATCCCAGGGTCGTGGGTTTGAGCCCCATGTTGGGCA
19445	Yes	AATCCCAGGGTCGTGGGTTTGAGCCCCATGTTGGGCAC
19446	Yes	AATCCCAGGGTCGTGGGTTTGAGCCCCATGTTGGGCACC
19447	Yes	AATCCCAGGGTCGTGGGTTTGAGCCCCATGTTGGGCACCA
19448	No	AATCCCAGGTCTAGCG
19449	Yes	AATCCCAGGTCTAGCGC
19450	Yes	AATCCCAGGTCTAGCGCC
19451	Yes	AATCCCAGGTCTAGCGCCA
19452	Yes	AATCCCAGTAGAGCCT
19453	Yes	AATCCCAGTAGAGCCTC
19454	Yes	AATCCCAGTAGAGCCTCC
19455	Yes	AATCCCAGTAGAGCCTCCA
19456	No	AATCCCAGTGCTGTCA
19457	No	AATCCCAGTGCTGTCAC
19458	No	AATCCCAGTGCTGTCACC
19459	Yes	AATCCCAGTGCTGTCACCA
19460	Yes	AATCCCATCCTCGTCG
19461	Yes	AATCCCATCCTCGTCGC
19462	Yes	AATCCCATCCTCGTCGCC
19463	Yes	AATCCCATCCTCGTCGCCA
19464	Yes	AATCCCCAATACCTCC
19465	Yes	AATCCCCAATACCTCCA
19466	Yes	AATCCCCAATACCTCCAC
19467	Yes	AATCCCCAATACCTCCACC
19468	Yes	AATCCCCAATACCTCCACCA
19469	Yes	AATCCCCACCGGGCTA
19470	Yes	AATCCCCACCGGGCTAT
19471	Yes	AATCCCCACCGGGCTATC
19472	Yes	AATCCCCACCGGGCTATCC
19473	Yes	AATCCCCACCGGGCTATCCA
19474	No	AATCCCCAGCACCTCC
19475	No	AATCCCCAGCACCTCCA
19476	Yes	AATCCCCAGCACCTCCAC
19477	Yes	AATCCCCAGCACCTCCACC
19478	Yes	AATCCCCAGCACCTCCACCA
19479	Yes	AATCCCCGGCACCTCC
19480	Yes	AATCCCCGGCACCTCCA
19481	Yes	AATCCCCGGCACCTCCAC

19482	Yes	AATCCCCGGCACCTCCACC
19483	Yes	AATCCCCGGCACCTCCACCA
19484	Yes	AATCCCCGGCATCTCC
19485	Yes	AATCCCCGGCATCTCCA
19486	Yes	AATCCCCGGCATCTCCAC
19487	Yes	AATCCCCGGCATCTCCACC
19488	Yes	AATCCCCGGCATCTCCACCA
19489	No	AATCCCGGACGAGCCC
19490	No	AATCCCGGACGAGCCCC
19491	No	AATCCCGGACGAGCCCCC
19492	No	AATCCCGGACGAGCCCCCA
19493	No	AATCCCGGCTCTGCCG
19494	No	AATCCCGGCTCTGCCGC
19495	No	AATCCCGGCTCTGCCGCC
19496	No	AATCCCGGCTCTGCCGCCA
19497	No	AATCCCGGGTTTCGGC
19498	Yes	AATCCCGGGTTTCGGCA
19499	Yes	AATCCCGGGTTTCGGCAC
19500	Yes	AATCCCGGGTTTCGGCACC
19501	Yes	AATCCCGGGTTTCGGCACCA
19502	No	AATCCCTCCGTGGTTA
19503	No	AATCCCTCCGTGGTTAC
19504	No	AATCCCTCCGTGGTTACC
19505	No	AATCCCTCCGTGGTTACCA
19506	No	AATCCCTGGCACCTCC
19507	Yes	AATCCCTGGCACCTCCA
19508	Yes	AATCCCTGGCACCTCCAC
19509	Yes	AATCCCTGGCACCTCCACC
19510	Yes	AATCCCTGGCACCTCCACCA
19511	No	AATCCCTGGTACCTCC
19512	No	AATCCCTGGTACCTCCA
19513	Yes	AATCCCTGGTACCTCCAC
19514	Yes	AATCCCTGGTACCTCCACC
19515	Yes	AATCCCTGGTACCTCCACCA
19516	No	AATCCCTTCGTGGTTA
19517	No	AATCCCTTCGTGGTTAC
19518	No	AATCCCTTCGTGGTTACC
19519	No	AATCCCTTCGTGGTTACCA
19520	No	AATCCCTTCGTGGTTG
19521	No	AATCCCTTCGTGGTTGC
19522	No	AATCCCTTCGTGGTTGCC
19523	No	AATCCCTTCGTGGTTGCCA

19524	Yes	AATCCCTTGCTGGGGC
19525	Yes	AATCCCTTGCTGGGGCG
19526	Yes	AATCCCTTGCTGGGGCGC
19527	Yes	AATCCCTTGCTGGGGCGCC
19528	Yes	AATCCCTTGCTGGGGCGCCA
19529	No	AATCCCTTGTGCACTG
19530	No	AATCCCTTGTGCACTGC
19531	Yes	AATCCCTTGTGCACTGCT
19532	Yes	AATCCCTTGTGCACTGCTT
19533	Yes	AATCCCTTGTGCACTGCTTC
19534	Yes	AATCCCTTGTGCACTGCTTCC
19535	Yes	AATCCCTTGTGCACTGCTTCCA
19536	No	AATCCGAGTCACGGCA
19537	No	AATCCGAGTCACGGCAC
19538	No	AATCCGAGTCACGGCACC
19539	No	AATCCGAGTCACGGCACCA
19540	No	AATCCGAGTTCAAATC
19541	Yes	AATCCGAGTTCAAATCT
19542	Yes	AATCCGAGTTCAAATCTC
19543	Yes	AATCCGAGTTCAAATCTCG
19544	Yes	AATCCGAGTTCAAATCTCGG
19545	Yes	AATCCGAGTTCAAATCTCGGT
19546	Yes	AATCCGAGTTCAAATCTCGGTG
19547	Yes	AATCCGAGTTCAAATCTCGGTGG
19548	Yes	AATCCGAGTTCAAATCTCGGTGGA
19549	Yes	AATCCGAGTTCAAATCTCGGTGGAA
19550	Yes	AATCCGAGTTCAAATCTCGGTGGAAC
19551	Yes	AATCCGAGTTCAAATCTCGGTGGAACC
19552	Yes	AATCCGAGTTCAAATCTCGGTGGAACCT
19553	Yes	AATCCGAGTTCAAATCTCGGTGGAACCTC
19554	Yes	AATCCGAGTTCAAATCTCGGTGGAACCTCC
19555	Yes	AATCCGAGTTCAAATCTCGGTGGAACCTCCA
19556	Yes	AATCCGAGTTCGAATC
19557	Yes	AATCCGAGTTCGAATCT
19558	Yes	AATCCGAGTTCGAATCTC
19559	Yes	AATCCGAGTTCGAATCTCG
19560	Yes	AATCCGAGTTCGAATCTCGG
19561	Yes	AATCCGAGTTCGAATCTCGGT
19562	Yes	AATCCGAGTTCGAATCTCGGTG
19563	Yes	AATCCGAGTTCGAATCTCGGTGG
19564	Yes	AATCCGAGTTCGAATCTCGGTGGG
19565	Yes	AATCCGAGTTCGAATCTCGGTGGGA

19566	Yes	AATCCGAGTTCGAATCTCGGTGGGAC
19567	Yes	AATCCGAGTTCGAATCTCGGTGGGACC
19568	Yes	AATCCGAGTTCGAATCTCGGTGGGACCT
19569	Yes	AATCCGAGTTCGAATCTCGGTGGGACCTC
19570	Yes	AATCCGAGTTCGAATCTCGGTGGGACCTCC
19571	Yes	AATCCGAGTTCGAATCTCGGTGGGACCTCCA
19572	Yes	AATCCGAGTTCGAATCTT
19573	Yes	AATCCGAGTTCGAATCTTG
19574	Yes	AATCCGAGTTCGAATCTTGG
19575	Yes	AATCCGAGTTCGAATCTTGGT
19576	Yes	AATCCGAGTTCGAATCTTGGTG
19577	Yes	AATCCGAGTTCGAATCTTGGTGG
19578	Yes	AATCCGAGTTCGAATCTTGGTGGG
19579	Yes	AATCCGAGTTCGAATCTTGGTGGGA
19580	Yes	AATCCGAGTTCGAATCTTGGTGGGAC
19581	Yes	AATCCGAGTTCGAATCTTGGTGGGACC
19582	Yes	AATCCGAGTTCGAATCTTGGTGGGACCT
19583	Yes	AATCCGAGTTCGAATCTTGGTGGGACCTC
19584	Yes	AATCCGAGTTCGAATCTTGGTGGGACCTCC
19585	Yes	AATCCGAGTTCGAATCTTGGTGGGACCTCCA
19586	Yes	AATCCGGCTCGAAGGA
19587	Yes	AATCCGGCTCGAAGGAC
19588	Yes	AATCCGGCTCGAAGGACC
19589	Yes	AATCCGGCTCGAAGGACCA
19590	Yes	AATCCGGCTCGGAGGA
19591	Yes	AATCCGGCTCGGAGGAC
19592	Yes	AATCCGGCTCGGAGGACC
19593	Yes	AATCCGGCTCGGAGGACCA
19594	No	AATCCGGGTGCCCCCC
19595	No	AATCCGGGTGCCCCCCC
19596	No	AATCCGGGTGCCCCCCCC
19597	Yes	AATCCGGGTGCCCCCCCCA
19598	Yes	AATCCGGGTGCCCCCT
19599	Yes	AATCCGGGTGCCCCCTC
19600	Yes	AATCCGGGTGCCCCCTCC
19601	Yes	AATCCGGGTGCCCCCTCCA
19602	Yes	AATCCGGGTGCTGGAT
19603	Yes	AATCCGGGTGCTGGATC
19604	Yes	AATCCGGGTGCTGGATCC
19605	Yes	AATCCGGGTGCTGGATCCA
19606	Yes	AATCCGGTAATCCGAG
19607	Yes	AATCCGGTAATCCGAGT

19608	Yes	AATCCGGTAATCCGAGTT
19609	Yes	AATCCGGTAATCCGAGTTC
19610	Yes	AATCCGGTAATCCGAGTTCA
19611	Yes	AATCCGGTAATCCGAGTTCAA
19612	Yes	AATCCGGTAATCCGAGTTCAAA
19613	Yes	AATCCGGTAATCCGAGTTCAAAT
19614	Yes	AATCCGGTAATCCGAGTTCAAATC
19615	Yes	AATCCGGTAATCCGAGTTCAAATCT
19616	Yes	AATCCGGTAATCCGAGTTCAAATCTC
19617	Yes	AATCCGGTAATCCGAGTTCAAATCTCG
19618	Yes	AATCCGGTAATCCGAGTTCAAATCTCGG
19619	Yes	AATCCGGTAATCCGAGTTCAAATCTCGGT
19620	Yes	AATCCGGTAATCCGAGTTCAAATCTCGGTG
19621	Yes	AATCCGGTAATCCGAGTTCAAATCTCGGTGG
19622	Yes	AATCCGGTAATCCGAGTTCAAATCTCGGTGGA
19623	Yes	AATCCGGTAATCCGAGTTCAAATCTCGGTGGAA
19624	Yes	AATCCGGTAATCCGAGTTCAAATCTCGGTGGAAC
19625	Yes	AATCCGGTAATCCGAGTTCAAATCTCGGTGGAACC
19626	Yes	AATCCGGTAATCCGAGTTCAAATCTCGGTGGAACCT
19627	Yes	AATCCGGTAATCCGAGTTCAAATCTCGGTGGAACCTC
19628	Yes	AATCCGGTAATCCGAGTTCAAATCTCGGTGGAACCTCC
19629	Yes	AATCCGGTAATCCGAGTTCAAATCTCGGTGGAACCTCCA
19630	No	AATCCGGTTACTCCCT
19631	Yes	AATCCGGTTACTCCCTC
19632	Yes	AATCCGGTTACTCCCTCC
19633	Yes	AATCCGGTTACTCCCTCCA
19634	No	AATCCTATATATCTTA
19635	Yes	AATCCTATATATCTTAC
19636	Yes	AATCCTATATATCTTACC
19637	Yes	AATCCTATATATCTTACCA
19638	No	AATCCTGACTCTGTCA
19639	No	AATCCTGACTCTGTCAC
19640	No	AATCCTGACTCTGTCACC
19641	No	AATCCTGACTCTGTCACCA
19642	Yes	AATCCTGCCGACTACG
19643	Yes	AATCCTGCCGACTACGC
19644	Yes	AATCCTGCCGACTACGCC
19645	Yes	AATCCTGCCGACTACGCCA
19646	No	AATCCTGCTAATTATG
19647	No	AATCCTGCTAATTATGC
19648	Yes	AATCCTGCTAATTATGCC
19649	Yes	AATCCTGCTAATTATGCCA

19650	Yes	AATCCTGCTCACAGCG
19651	Yes	AATCCTGCTCACAGCGC
19652	Yes	AATCCTGCTCACAGCGCC
19653	Yes	AATCCTGCTCACAGCGCCA
19654	No	AATCCTGGCTTAGGGT
19655	No	AATCCTGGCTTAGGGTA
19656	Yes	AATCCTGGCTTAGGGTAC
19657	Yes	AATCCTGGCTTAGGGTACC
19658	Yes	AATCCTGGCTTAGGGTACCA
19659	Yes	AATCCTGTCGGCTACG
19660	Yes	AATCCTGTCGGCTACGC
19661	Yes	AATCCTGTCGGCTACGCC
19662	Yes	AATCCTGTCGGCTACGCCA
19663	No	AATCCTGTTCGTGACG
19664	No	AATCCTGTTCGTGACGC
19665	No	AATCCTGTTCGTGACGCC
19666	No	AATCCTGTTCGTGACGCCA
19667	No	AATCCTGTTTCTGCCA
19668	No	AATCCTGTTTCTGCCAC
19669	No	AATCCTGTTTCTGCCACC
19670	No	AATCCTGTTTCTGCCACCA
19671	Yes	AATCGCACCCTCCGCT
19672	Yes	AATCGCACCCTCCGCTG
19673	Yes	AATCGCACCCTCCGCTGG
19674	Yes	AATCGCACCCTCCGCTGGA
19675	Yes	AATCGCACCCTCCGCTGGAG
19676	Yes	AATCGCACCCTCCGCTGGAGG
19677	Yes	AATCGCACCCTCCGCTGGAGGC
19678	Yes	AATCGCACCCTCCGCTGGAGGCG
19679	Yes	AATCGCACCCTCCGCTGGAGGCGT
19680	Yes	AATCGCACCCTCCGCTGGAGGCGTG
19681	Yes	AATCGCACCCTCCGCTGGAGGCGTGG
19682	Yes	AATCGCACCCTCCGCTGGAGGCGTGGG
19683	Yes	AATCGCACCCTCCGCTGGAGGCGTGGGT
19684	Yes	AATCGCACCCTCCGCTGGAGGCGTGGGTT
19685	Yes	AATCGCACCCTCCGCTGGAGGCGTGGGTTC
19686	Yes	AATCGCACCCTCCGCTGGAGGCGTGGGTTCG
19687	Yes	AATCGCACCCTCCGCTGGAGGCGTGGGTTCGA
19688	Yes	AATCGCACCCTCCGCTGGAGGCGTGGGTTCGAA
19689	Yes	AATCGCACCCTCCGCTGGAGGCGTGGGTTCGAAT
19690	Yes	AATCGCACCCTCCGCTGGAGGCGTGGGTTCGAATC
19691	Yes	AATCGCACCCTCCGCTGGAGGCGTGGGTTCGAATCC

19692	Yes	AATCGCACCCTCCGCTGGAGGCGTGGGTTCGAATCCC
19693	Yes	AATCGCACCCTCCGCTGGAGGCGTGGGTTCGAATCCCA
19694	Yes	AATCGCACCCTCCGCTGGAGGCGTGGGTTCGAATCCCAC
19695	Yes	AATCGCACCCTCCGCTGGAGGCGTGGGTTCGAATCCCACT
19696	Yes	AATCGCACCCTCCGCTGGAGGCGTGGGTTCGAATCCCACTT
19697	Yes	AATCGCACCCTCCGCTGGAGGCGTGGGTTCGAATCCCACTTT
19698	Yes	AATCGCACCCTCCGCTGGAGGCGTGGGTTCGAATCCCACTTTT
19699	Yes	AATCGCACCCTCCGCTGGAGGCGTGGGTTCGAATCCCACTTTTG
19700	Yes	AATCGCACCCTCCGCTGGAGGCGTGGGTTCGAATCCCACTTTTGA
19701	Yes	AATCGCACCCTCCGCTGGAGGCGTGGGTTCGAATCCCACTTTTGAC
19702	Yes	AATCGCACCCTCCGCTGGAGGCGTGGGTTCGAATCCCACTTTTGACA
19703	Yes	AATCGCACCCTCCGCTGGAGGCGTGGGTTCGAATCCCACTTTTGACAC
19704	Yes	AATCGCACCCTCCGCTGGAGGCGTGGGTTCGAATCCCACTTTTGACACC
19705	Yes	AATCGCACCCTCCGCTGGAGGCGTGGGTTCGAATCCCACTTTTGACACCA
19706	Yes	AATCGCATAAAACTTA
19707	Yes	AATCGCATAAAACTTAA
19708	Yes	AATCGCATAAAACTTAAA
19709	Yes	AATCGCATAAAACTTAAAA
19710	Yes	AATCGCATAAAACTTAAAAC
19711	Yes	AATCGCATAAAACTTAAAACT
19712	Yes	AATCGCATAAAACTTAAAACTT
19713	Yes	AATCGCATAAAACTTAAAACTTT
19714	Yes	AATCGCATAAAACTTAAAACTTTA
19715	Yes	AATCGCATAAAACTTAAAACTTTAC
19716	Yes	AATCGCATAAAACTTAAAACTTTACA
19717	Yes	AATCGCATAAAACTTAAAACTTTACAG
19718	Yes	AATCGCATAAAACTTAAAACTTTACAGT
19719	Yes	AATCGCATAAAACTTAAAACTTTACAGTC
19720	Yes	AATCGCATAAAACTTAAAACTTTACAGTCA
19721	Yes	AATCGCATAAAACTTAAAACTTTACAGTCAG
19722	Yes	AATCGCATAAAACTTAAAACTTTACAGTCAGA
19723	Yes	AATCGCATAAAACTTAAAACTTTACAGTCAGAG
19724	Yes	AATCGCATAAAACTTAAAACTTTACAGTCAGAGG
19725	Yes	AATCGCATAAAACTTAAAACTTTACAGTCAGAGGT
19726	Yes	AATCGCATAAAACTTAAAACTTTACAGTCAGAGGTT
19727	Yes	AATCGCATAAAACTTAAAACTTTACAGTCAGAGGTTC
19728	Yes	AATCGCATAAAACTTAAAACTTTACAGTCAGAGGTTCA
19729	Yes	AATCGCATAAAACTTAAAACTTTACAGTCAGAGGTTCAA
19730	Yes	AATCGCATAAAACTTAAAACTTTACAGTCAGAGGTTCAAT
19731	Yes	AATCGCATAAAACTTAAAACTTTACAGTCAGAGGTTCAATT
19732	Yes	AATCGCATAAAACTTAAAACTTTACAGTCAGAGGTTCAATTC
19733	Yes	AATCGCATAAAACTTAAAACTTTACAGTCAGAGGTTCAATTCC

19734	Yes	AATCGCATAAAACTTAAAACTTTACAGTCAGAGGTTCAATTCCT
19735	Yes	AATCGCATAAAACTTAAAACTTTACAGTCAGAGGTTCAATTCCTC
19736	Yes	AATCGCATAAAACTTAAAACTTTACAGTCAGAGGTTCAATTCCTCT
19737	Yes	AATCGCATAAAACTTAAAACTTTACAGTCAGAGGTTCAATTCCTCTT
19738	Yes	AATCGCATAAAACTTAAAACTTTACAGTCAGAGGTTCAATTCCTCTTC
19739	Yes	AATCGCATAAAACTTAAAACTTTACAGTCAGAGGTTCAATTCCTCTTCT
19740	Yes	AATCGCATAAAACTTAAAACTTTACAGTCAGAGGTTCAATTCCTCTTCTT
19741	Yes	AATCGGCTAGCGCGTT
19742	Yes	AATCGGCTAGCGCGTTT
19743	Yes	AATCGGCTAGCGCGTTTG
19744	Yes	AATCGGCTAGCGCGTTTGG
19745	Yes	AATCGGCTAGCGCGTTTGGC
19746	Yes	AATCGGCTAGCGCGTTTGGCT
19747	Yes	AATCGGCTAGCGCGTTTGGCTG
19748	Yes	AATCGGCTAGCGCGTTTGGCTGT
19749	Yes	AATCGGCTAGCGCGTTTGGCTGTT
19750	Yes	AATCGGCTAGCGCGTTTGGCTGTTA
19751	Yes	AATCGGCTAGCGCGTTTGGCTGTTAA
19752	Yes	AATCGGCTAGCGCGTTTGGCTGTTAAC
19753	Yes	AATCGGCTAGCGCGTTTGGCTGTTAACT
19754	Yes	AATCGGCTAGCGCGTTTGGCTGTTAACTA
19755	Yes	AATCGGCTAGCGCGTTTGGCTGTTAACTAA
19756	Yes	AATCGGCTAGCGCGTTTGGCTGTTAACTAAA
19757	Yes	AATCGGCTAGCGCGTTTGGCTGTTAACTAAAA
19758	Yes	AATCGGCTAGCGCGTTTGGCTGTTAACTAAAAA
19759	Yes	AATCGGCTAGCGCGTTTGGCTGTTAACTAAAAAG
19760	Yes	AATCGGCTAGCGCGTTTGGCTGTTAACTAAAAAGT
19761	Yes	AATCGGCTAGCGCGTTTGGCTGTTAACTAAAAAGTT
19762	Yes	AATCGGCTAGCGCGTTTGGCTGTTAACTAAAAAGTTG
19763	Yes	AATCGGCTAGCGCGTTTGGCTGTTAACTAAAAAGTTGG
19764	Yes	AATCGGCTAGCGCGTTTGGCTGTTAACTAAAAAGTTGGT
19765	Yes	AATCGGCTAGCGCGTTTGGCTGTTAACTAAAAAGTTGGTG
19766	Yes	AATCGGCTAGCGCGTTTGGCTGTTAACTAAAAAGTTGGTGG
19767	Yes	AATCGGCTAGCGCGTTTGGCTGTTAACTAAAAAGTTGGTGGT
19768	Yes	AATCGGCTAGCGCGTTTGGCTGTTAACTAAAAAGTTGGTGGTT
19769	Yes	AATCGGCTAGCGCGTTTGGCTGTTAACTAAAAAGTTGGTGGTTC
19770	Yes	AATCGGCTAGCGCGTTTGGCTGTTAACTAAAAAGTTGGTGGTTCG
19771	Yes	AATCGGCTAGCGCGTTTGGCTGTTAACTAAAAAGTTGGTGGTTCGA
19772	Yes	AATCGGCTAGCGCGTTTGGCTGTTAACTAAAAAGTTGGTGGTTCGAA
19773	Yes	AATCGGCTAGCGCGTTTGGCTGTTAACTAAAAAGTTGGTGGTTCGAAC
19774	Yes	AATCGGCTAGCGCGTTTGGCTGTTAACTAAAAAGTTGGTGGTTCGAACA
19775	Yes	AATCGGCTAGCGCGTTTGGCTGTTAACTAAAAAGTTGGTGGTTCGAACAC

19776	Yes	AATCGGCTAGCGCGTTTGGCTGTTAACTAAAAG
19777	Yes	AATCGGCTAGCGCGTTTGGCTGTTAACTAAAAGG
19778	Yes	AATCGGCTAGCGCGTTTGGCTGTTAACTAAAAGGT
19779	Yes	AATCGGCTAGCGCGTTTGGCTGTTAACTAAAAGGTT
19780	Yes	AATCGGCTAGCGCGTTTGGCTGTTAACTAAAAGGTTG
19781	Yes	AATCGGCTAGCGCGTTTGGCTGTTAACTAAAAGGTTGG
19782	Yes	AATCGGCTAGCGCGTTTGGCTGTTAACTAAAAGGTTGGC
19783	Yes	AATCGGCTAGCGCGTTTGGCTGTTAACTAAAAGGTTGGCG
19784	Yes	AATCGGCTAGCGCGTTTGGCTGTTAACTAAAAGGTTGGCGG
19785	Yes	AATCGGCTAGCGCGTTTGGCTGTTAACTAAAAGGTTGGCGGT
19786	Yes	AATCGGCTAGCGCGTTTGGCTGTTAACTAAAAGGTTGGCGGTT
19787	Yes	AATCGGCTAGCGCGTTTGGCTGTTAACTAAAAGGTTGGCGGTTC
19788	Yes	AATCGGCTAGCGCGTTTGGCTGTTAACTAAAAGGTTGGCGGTTCG
19789	Yes	AATCGGCTAGCGCGTTTGGCTGTTAACTAAAAGGTTGGCGGTTCGA
19790	Yes	AATCGGCTAGCGCGTTTGGCTGTTAACTAAAAGGTTGGCGGTTCGAA
19791	Yes	AATCGGCTAGCGCGTTTGGCTGTTAACTAAAAGGTTGGCGGTTCGAAC
19792	Yes	AATCGGCTAGCGCGTTTGGCTGTTAACTAAAAGGTTGGCGGTTCGAACC
19793	Yes	AATCGGCTAGCGCGTTTGGCTGTTAACTAAAAGGTTGGCGGTTCGAACCC
19794	Yes	AATCGGCTAGCGCGTTTGGCTGTTAACTAAAAGGTTGGT
19795	Yes	AATCGGCTAGCGCGTTTGGCTGTTAACTAAAAGGTTGGTG
19796	Yes	AATCGGCTAGCGCGTTTGGCTGTTAACTAAAAGGTTGGTGG
19797	Yes	AATCGGCTAGCGCGTTTGGCTGTTAACTAAAAGGTTGGTGGT
19798	Yes	AATCGGCTAGCGCGTTTGGCTGTTAACTAAAAGGTTGGTGGTT
19799	Yes	AATCGGCTAGCGCGTTTGGCTGTTAACTAAAAGGTTGGTGGTTC
19800	Yes	AATCGGCTAGCGCGTTTGGCTGTTAACTAAAAGGTTGGTGGTTCG
19801	Yes	AATCGGCTAGCGCGTTTGGCTGTTAACTAAAAGGTTGGTGGTTCGA
19802	Yes	AATCGGCTAGCGCGTTTGGCTGTTAACTAAAAGGTTGGTGGTTCGAA
19803	Yes	AATCGGCTAGCGCGTTTGGCTGTTAACTAAAAGGTTGGTGGTTCGAAC
19804	Yes	AATCGGCTAGCGCGTTTGGCTGTTAACTAAAAGGTTGGTGGTTCGAACC
19805	Yes	AATCGGCTAGCGCGTTTGGCTGTTAACTAAAAGGTTGGTGGTTCGAACCC
19806	Yes	AATCGGTCAGAGCGTT
19807	Yes	AATCGGTCAGAGCGTTC
19808	Yes	AATCGGTCAGAGCGTTCG
19809	Yes	AATCGGTCAGAGCGTTCGG
19810	Yes	AATCGGTCAGAGCGTTCGGC
19811	Yes	AATCGGTCAGAGCGTTCGGCT
19812	Yes	AATCGGTCAGAGCGTTCGGCTA
19813	Yes	AATCGGTCAGAGCGTTCGGCTAT
19814	Yes	AATCGGTCAGAGCGTTCGGCTATT
19815	Yes	AATCGGTCAGAGCGTTCGGCTATTA
19816	Yes	AATCGGTCAGAGCGTTCGGCTATTAA
19817	Yes	AATCGGTCAGAGCGTTCGGCTATTAAC

19818	Yes	AATCGGTCAGAGCGTTCGGCTATTAACC
19819	Yes	AATCGGTCAGAGCGTTCGGCTATTAACCG
19820	Yes	AATCGGTCAGAGCGTTCGGCTATTAACCGA
19821	Yes	AATCGGTCAGAGCGTTCGGCTATTAACCGAA
19822	Yes	AATCGGTCAGAGCGTTCGGCTATTAACCGAAC
19823	Yes	AATCGGTCAGAGCGTTCGGCTATTAACCGAACG
19824	Yes	AATCGGTCAGAGCGTTCGGCTATTAACCGAACGG
19825	Yes	AATCGGTCAGAGCGTTCGGCTATTAACCGAACGGT
19826	Yes	AATCGGTCAGAGCGTTCGGCTATTAACCGAACGGTG
19827	Yes	AATCGGTCAGAGCGTTCGGCTATTAACCGAACGGTGA
19828	Yes	AATCGGTCAGAGCGTTCGGCTATTAACCGAACGGTGAG
19829	Yes	AATCGGTCAGAGCGTTCGGCTATTAACCGAACGGTGAGT
19830	Yes	AATCGGTCAGAGCGTTCGGCTATTAACCGAACGGTGAGTA
19831	Yes	AATCGGTCAGAGCGTTCGGCTATTAACCGAACGGTGAGTAG
19832	Yes	AATCGGTCAGAGCGTTCGGCTATTAACCGAACGGTGAGTAGT
19833	Yes	AATCGGTCAGAGCGTTCGGCTATTAACCGAACGGTGAGTAGTT
19834	Yes	AATCGGTCAGAGCGTTCGGCTATTAACCGAACGGTGAGTAGTTC
19835	Yes	AATCGGTCAGAGCGTTCGGCTATTAACCGAACGGTGAGTAGTTCA
19836	Yes	AATCGGTCAGAGCGTTCGGCTATTAACCGAACGGTGAGTAGTTCAA
19837	Yes	AATCGGTCAGAGCGTTCGGCTATTAACCGAACGGTGAGTAGTTCAAG
19838	Yes	AATCGGTCAGAGCGTTCGGCTATTAACCGAACGGTGAGTAGTTCAAGA
19839	Yes	AATCGGTCAGAGCGTTCGGCTATTAACCGAACGGTGAGTAGTTCAAGAC
19840	Yes	AATCGGTCAGAGCGTTCGGCTATTAACCGAACGGTGAGTAGTTCAAGACC
19841	Yes	AATCGGTTAGAGCGTT
19842	Yes	AATCGGTTAGAGCGTTC
19843	Yes	AATCGGTTAGAGCGTTCG
19844	Yes	AATCGGTTAGAGCGTTCGG
19845	Yes	AATCGGTTAGAGCGTTCGGC
19846	Yes	AATCGGTTAGAGCGTTCGGCT
19847	Yes	AATCGGTTAGAGCGTTCGGCTG
19848	Yes	AATCGGTTAGAGCGTTCGGCTGT
19849	Yes	AATCGGTTAGAGCGTTCGGCTGTT
19850	Yes	AATCGGTTAGAGCGTTCGGCTGTTA
19851	Yes	AATCGGTTAGAGCGTTCGGCTGTTAA
19852	Yes	AATCGGTTAGAGCGTTCGGCTGTTAAT
19853	Yes	AATCGGTTAGAGCGTTCGGCTGTTAATC
19854	Yes	AATCGGTTAGAGCGTTCGGCTGTTAATCT
19855	Yes	AATCGGTTAGAGCGTTCGGCTGTTAATCTA
19856	Yes	AATCGGTTAGAGCGTTCGGCTGTTAATCTAA
19857	Yes	AATCGGTTAGAGCGTTCGGCTGTTAATCTAAA
19858	Yes	AATCGGTTAGAGCGTTCGGCTGTTAATCTAAAG
19859	Yes	AATCGGTTAGAGCGTTCGGCTGTTAATCTAAAGG

19860	Yes	AATCGGTTAGAGCGTTCGGCTGTTAATCTAAAGGT
19861	Yes	AATCGGTTAGAGCGTTCGGCTGTTAATCTAAAGGTT
19862	Yes	AATCGGTTAGAGCGTTCGGCTGTTAATCTAAAGGTTG
19863	Yes	AATCGGTTAGAGCGTTCGGCTGTTAATCTAAAGGTTGG
19864	Yes	AATCGGTTAGAGCGTTCGGCTGTTAATCTAAAGGTTGGT
19865	Yes	AATCGGTTAGAGCGTTCGGCTGTTAATCTAAAGGTTGGTG
19866	Yes	AATCGGTTAGAGCGTTCGGCTGTTAATCTAAAGGTTGGTGG
19867	Yes	AATCGGTTAGAGCGTTCGGCTGTTAATCTAAAGGTTGGTGGC
19868	Yes	AATCGGTTAGAGCGTTCGGCTGTTAATCTAAAGGTTGGTGGCT
19869	Yes	AATCGGTTAGAGCGTTCGGCTGTTAATCTAAAGGTTGGTGGCTA
19870	Yes	AATCGGTTAGAGCGTTCGGCTGTTAATCTAAAGGTTGGTGGCTAG
19871	Yes	AATCGGTTAGAGCGTTCGGCTGTTAATCTAAAGGTTGGTGGCTAGA
19872	Yes	AATCGGTTAGAGCGTTCGGCTGTTAATCTAAAGGTTGGTGGCTAGAA
19873	Yes	AATCGGTTAGAGCGTTCGGCTGTTAATCTAAAGGTTGGTGGCTAGAAC
19874	Yes	AATCGGTTAGAGCGTTCGGCTGTTAATCTAAAGGTTGGTGGCTAGAACC
19875	Yes	AATCGGTTAGAGCGTTCGGCTGTTAATCTAAAGGTTGGTGGCTAGAACCC
19876	Yes	AATCGGTTAGCGCATT
19877	Yes	AATCGGTTAGCGCATTC
19878	Yes	AATCGGTTAGCGCATTCG
19879	Yes	AATCGGTTAGCGCATTCGG
19880	Yes	AATCGGTTAGCGCATTCGGC
19881	Yes	AATCGGTTAGCGCATTCGGCT
19882	Yes	AATCGGTTAGCGCATTCGGCTG
19883	Yes	AATCGGTTAGCGCATTCGGCTGT
19884	Yes	AATCGGTTAGCGCATTCGGCTGTT
19885	Yes	AATCGGTTAGCGCATTCGGCTGTTA
19886	Yes	AATCGGTTAGCGCATTCGGCTGTTAA
19887	Yes	AATCGGTTAGCGCATTCGGCTGTTAAC
19888	Yes	AATCGGTTAGCGCATTCGGCTGTTAACC
19889	Yes	AATCGGTTAGCGCATTCGGCTGTTAACCG
19890	Yes	AATCGGTTAGCGCATTCGGCTGTTAACCGA
19891	Yes	AATCGGTTAGCGCATTCGGCTGTTAACCGAA
19892	Yes	AATCGGTTAGCGCATTCGGCTGTTAACCGAAA
19893	Yes	AATCGGTTAGCGCATTCGGCTGTTAACCGAAAG
19894	Yes	AATCGGTTAGCGCATTCGGCTGTTAACCGAAAGG
19895	Yes	AATCGGTTAGCGCATTCGGCTGTTAACCGAAAGGT
19896	Yes	AATCGGTTAGCGCATTCGGCTGTTAACCGAAAGGTT
19897	Yes	AATCGGTTAGCGCATTCGGCTGTTAACCGAAAGGTTG
19898	Yes	AATCGGTTAGCGCATTCGGCTGTTAACCGAAAGGTTGG
19899	Yes	AATCGGTTAGCGCATTCGGCTGTTAACCGAAAGGTTGGT
19900	Yes	AATCGGTTAGCGCATTCGGCTGTTAACCGAAAGGTTGGTG
19901	Yes	AATCGGTTAGCGCATTCGGCTGTTAACCGAAAGGTTGGTGG

19902	Yes	AATCGGTTAGCGCATTCGGCTGTTAACCGAAAGGTTGGTGGT
19903	Yes	AATCGGTTAGCGCATTCGGCTGTTAACCGAAAGGTTGGTGGTT
19904	Yes	AATCGGTTAGCGCATTCGGCTGTTAACCGAAAGGTTGGTGGTTC
19905	Yes	AATCGGTTAGCGCATTCGGCTGTTAACCGAAAGGTTGGTGGTTCG
19906	Yes	AATCGGTTAGCGCATTCGGCTGTTAACCGAAAGGTTGGTGGTTCGA
19907	Yes	AATCGGTTAGCGCATTCGGCTGTTAACCGAAAGGTTGGTGGTTCGAG
19908	Yes	AATCGGTTAGCGCATTCGGCTGTTAACCGAAAGGTTGGTGGTTCGAGC
19909	Yes	AATCGGTTAGCGCATTCGGCTGTTAACCGAAAGGTTGGTGGTTCGAGCC
19910	Yes	AATCGGTTAGCGCATTCGGCTGTTAACCGAAAGGTTGGTGGTTCGAGCCC
19911	No	AATCGGTTAGCGCGCG
19912	No	AATCGGTTAGCGCGCGG
19913	No	AATCGGTTAGCGCGCGGT
19914	No	AATCGGTTAGCGCGCGGTA
19915	No	AATCGGTTAGCGCGCGGTAC
19916	No	AATCGGTTAGCGCGCGGTACT
19917	No	AATCGGTTAGCGCGCGGTACTT
19918	No	AATCGGTTAGCGCGCGGTACTTA
19919	No	AATCGGTTAGCGCGCGGTACTTAT
19920	No	AATCGGTTAGCGCGCGGTACTTATA
19921	No	AATCGGTTAGCGCGCGGTACTTATAA
19922	Yes	AATCGGTTAGCGCGCGGTACTTATAAT
19923	Yes	AATCGGTTAGCGCGCGGTACTTATAATG
19924	Yes	AATCGGTTAGCGCGCGGTACTTATAATGC
19925	Yes	AATCGGTTAGCGCGCGGTACTTATAATGCC
19926	Yes	AATCGGTTAGCGCGCGGTACTTATAATGCCG
19927	Yes	AATCGGTTAGCGCGCGGTACTTATAATGCCGA
19928	Yes	AATCGGTTAGCGCGCGGTACTTATAATGCCGAG
19929	Yes	AATCGGTTAGCGCGCGGTACTTATAATGCCGAGG
19930	Yes	AATCGGTTAGCGCGCGGTACTTATAATGCCGAGGT
19931	Yes	AATCGGTTAGCGCGCGGTACTTATAATGCCGAGGTT
19932	Yes	AATCGGTTAGCGCGCGGTACTTATAATGCCGAGGTTG
19933	Yes	AATCGGTTAGCGCGCGGTACTTATAATGCCGAGGTTGT
19934	Yes	AATCGGTTAGCGCGCGGTACTTATAATGCCGAGGTTGTG
19935	Yes	AATCGGTTAGCGCGCGGTACTTATAATGCCGAGGTTGTGA
19936	Yes	AATCGGTTAGCGCGCGGTACTTATAATGCCGAGGTTGTGAG
19937	Yes	AATCGGTTAGCGCGCGGTACTTATAATGCCGAGGTTGTGAGT
19938	Yes	AATCGGTTAGCGCGCGGTACTTATAATGCCGAGGTTGTGAGTT
19939	Yes	AATCGGTTAGCGCGCGGTACTTATAATGCCGAGGTTGTGAGTTC
19940	Yes	AATCGGTTAGCGCGCGGTACTTATAATGCCGAGGTTGTGAGTTCA
19941	Yes	AATCGGTTAGCGCGCGGTACTTATAATGCCGAGGTTGTGAGTTCAA
19942	Yes	AATCGGTTAGCGCGCGGTACTTATAATGCCGAGGTTGTGAGTTCAAG
19943	Yes	AATCGGTTAGCGCGCGGTACTTATAATGCCGAGGTTGTGAGTTCAAGC

19944	Yes	AATCGGTTAGCGCGCGGTACTTATAATGCCGAGGTTGTGAGTTCAAGCC
19945	Yes	AATCGGTTAGCGCGCGGTACTTATAATGCCGAGGTTGTGAGTTCAAGCCT
19946	Yes	AATCGGTTAGCGCGCGGTACTTATAATGCCGAGGTTGTGAGTTCG
19947	Yes	AATCGGTTAGCGCGCGGTACTTATAATGCCGAGGTTGTGAGTTCGA
19948	Yes	AATCGGTTAGCGCGCGGTACTTATAATGCCGAGGTTGTGAGTTCGAG
19949	Yes	AATCGGTTAGCGCGCGGTACTTATAATGCCGAGGTTGTGAGTTCGAGC
19950	Yes	AATCGGTTAGCGCGCGGTACTTATAATGCCGAGGTTGTGAGTTCGAGCC
19951	Yes	AATCGGTTAGCGCGCGGTACTTATAATGCCGAGGTTGTGAGTTCGAGCCT
19952	Yes	AATCGGTTAGCGCGCGGTACTTATAATGCCGAGGTTGTGAGTTCGAT
19953	Yes	AATCGGTTAGCGCGCGGTACTTATAATGCCGAGGTTGTGAGTTCGATC
19954	Yes	AATCGGTTAGCGCGCGGTACTTATAATGCCGAGGTTGTGAGTTCGATCC
19955	Yes	AATCGGTTAGCGCGCGGTACTTATAATGCCGAGGTTGTGAGTTCGATCCT
19956	Yes	AATCGGTTAGCGCGTT
19957	Yes	AATCGGTTAGCGCGTTC
19958	Yes	AATCGGTTAGCGCGTTCC
19959	Yes	AATCGGTTAGCGCGTTCCG
19960	Yes	AATCGGTTAGCGCGTTCCGC
19961	Yes	AATCGGTTAGCGCGTTCCGCT
19962	Yes	AATCGGTTAGCGCGTTCCGCTG
19963	Yes	AATCGGTTAGCGCGTTCCGCTGT
19964	Yes	AATCGGTTAGCGCGTTCCGCTGTT
19965	Yes	AATCGGTTAGCGCGTTCCGCTGTTA
19966	Yes	AATCGGTTAGCGCGTTCCGCTGTTAA
19967	Yes	AATCGGTTAGCGCGTTCCGCTGTTAAC
19968	Yes	AATCGGTTAGCGCGTTCCGCTGTTAACC
19969	Yes	AATCGGTTAGCGCGTTCCGCTGTTAACCG
19970	Yes	AATCGGTTAGCGCGTTCCGCTGTTAACCGA
19971	Yes	AATCGGTTAGCGCGTTCCGCTGTTAACCGAA
19972	Yes	AATCGGTTAGCGCGTTCCGCTGTTAACCGAAA
19973	Yes	AATCGGTTAGCGCGTTCCGCTGTTAACCGAAAG
19974	Yes	AATCGGTTAGCGCGTTCCGCTGTTAACCGAAAGC
19975	Yes	AATCGGTTAGCGCGTTCCGCTGTTAACCGAAAGCT
19976	Yes	AATCGGTTAGCGCGTTCCGCTGTTAACCGAAAGCTT
19977	Yes	AATCGGTTAGCGCGTTCCGCTGTTAACCGAAAGCTTG
19978	Yes	AATCGGTTAGCGCGTTCCGCTGTTAACCGAAAGCTTGG
19979	Yes	AATCGGTTAGCGCGTTCCGCTGTTAACCGAAAGCTTGGT
19980	Yes	AATCGGTTAGCGCGTTCCGCTGTTAACCGAAAGCTTGGTG
19981	Yes	AATCGGTTAGCGCGTTCCGCTGTTAACCGAAAGCTTGGTGG
19982	Yes	AATCGGTTAGCGCGTTCCGCTGTTAACCGAAAGCTTGGTGGT
19983	Yes	AATCGGTTAGCGCGTTCCGCTGTTAACCGAAAGCTTGGTGGTT
19984	Yes	AATCGGTTAGCGCGTTCCGCTGTTAACCGAAAGCTTGGTGGTTC
19985	Yes	AATCGGTTAGCGCGTTCCGCTGTTAACCGAAAGCTTGGTGGTTCG

19986	Yes	AATCGGTTAGCGCGTTCCGCTGTTAACCGAAAGCTTGGTGGTTCGA
19987	Yes	AATCGGTTAGCGCGTTCCGCTGTTAACCGAAAGCTTGGTGGTTCGAG
19988	Yes	AATCGGTTAGCGCGTTCCGCTGTTAACCGAAAGCTTGGTGGTTCGAGC
19989	Yes	AATCGGTTAGCGCGTTCCGCTGTTAACCGAAAGCTTGGTGGTTCGAGCC
19990	Yes	AATCGGTTAGCGCGTTCCGCTGTTAACCGAAAGCTTGGTGGTTCGAGCCC
19991	Yes	AATCGGTTAGCGCGTTCCGCTGTTAACCGAAAGCTTGGTGGTTT
19992	Yes	AATCGGTTAGCGCGTTCCGCTGTTAACCGAAAGCTTGGTGGTTTG
19993	Yes	AATCGGTTAGCGCGTTCCGCTGTTAACCGAAAGCTTGGTGGTTTGA
19994	Yes	AATCGGTTAGCGCGTTCCGCTGTTAACCGAAAGCTTGGTGGTTTGAG
19995	Yes	AATCGGTTAGCGCGTTCCGCTGTTAACCGAAAGCTTGGTGGTTTGAGC
19996	Yes	AATCGGTTAGCGCGTTCCGCTGTTAACCGAAAGCTTGGTGGTTTGAGCC
19997	Yes	AATCGGTTAGCGCGTTCCGCTGTTAACCGAAAGCTTGGTGGTTTGAGCCC
19998	Yes	AATCGGTTAGCGCGTTCG
19999	Yes	AATCGGTTAGCGCGTTCGG
20000	Yes	AATCGGTTAGCGCGTTCGGC
20001	Yes	AATCGGTTAGCGCGTTCGGCT
20002	Yes	AATCGGTTAGCGCGTTCGGCTG
20003	Yes	AATCGGTTAGCGCGTTCGGCTGT
20004	Yes	AATCGGTTAGCGCGTTCGGCTGTT
20005	Yes	AATCGGTTAGCGCGTTCGGCTGTTA
20006	Yes	AATCGGTTAGCGCGTTCGGCTGTTAA
20007	Yes	AATCGGTTAGCGCGTTCGGCTGTTAAC
20008	Yes	AATCGGTTAGCGCGTTCGGCTGTTAACC
20009	Yes	AATCGGTTAGCGCGTTCGGCTGTTAACCA
20010	Yes	AATCGGTTAGCGCGTTCGGCTGTTAACCAT
20011	Yes	AATCGGTTAGCGCGTTCGGCTGTTAACCATA
20012	Yes	AATCGGTTAGCGCGTTCGGCTGTTAACCATAA
20013	Yes	AATCGGTTAGCGCGTTCGGCTGTTAACCATAAG
20014	Yes	AATCGGTTAGCGCGTTCGGCTGTTAACCATAAGG
20015	Yes	AATCGGTTAGCGCGTTCGGCTGTTAACCATAAGGT
20016	Yes	AATCGGTTAGCGCGTTCGGCTGTTAACCATAAGGTT
20017	Yes	AATCGGTTAGCGCGTTCGGCTGTTAACCATAAGGTTG
20018	Yes	AATCGGTTAGCGCGTTCGGCTGTTAACCATAAGGTTGG
20019	Yes	AATCGGTTAGCGCGTTCGGCTGTTAACCATAAGGTTGGT
20020	Yes	AATCGGTTAGCGCGTTCGGCTGTTAACCATAAGGTTGGTG
20021	Yes	AATCGGTTAGCGCGTTCGGCTGTTAACCATAAGGTTGGTGG
20022	Yes	AATCGGTTAGCGCGTTCGGCTGTTAACCATAAGGTTGGTGGT
20023	Yes	AATCGGTTAGCGCGTTCGGCTGTTAACCATAAGGTTGGTGGTT
20024	Yes	AATCGGTTAGCGCGTTCGGCTGTTAACCATAAGGTTGGTGGTTA
20025	Yes	AATCGGTTAGCGCGTTCGGCTGTTAACCATAAGGTTGGTGGTTAC
20026	Yes	AATCGGTTAGCGCGTTCGGCTGTTAACCATAAGGTTGGTGGTTACA
20027	Yes	AATCGGTTAGCGCGTTCGGCTGTTAACCATAAGGTTGGTGGTTACAG

20028	Yes	AATCGGTTAGCGCGTTCGGCTGTTAACCATAAGGTTGGTGGTTACAGA
20029	Yes	AATCGGTTAGCGCGTTCGGCTGTTAACCATAAGGTTGGTGGTTACAGAC
20030	Yes	AATCGGTTAGCGCGTTCGGCTGTTAACCATAAGGTTGGTGGTTACAGACC
20031	Yes	AATCGGTTAGCGCGTTCGGCTGTTAACCATAAGGTTGGTGGTTAG
20032	Yes	AATCGGTTAGCGCGTTCGGCTGTTAACCATAAGGTTGGTGGTTAGA
20033	Yes	AATCGGTTAGCGCGTTCGGCTGTTAACCATAAGGTTGGTGGTTAGAG
20034	Yes	AATCGGTTAGCGCGTTCGGCTGTTAACCATAAGGTTGGTGGTTAGAGA
20035	Yes	AATCGGTTAGCGCGTTCGGCTGTTAACCATAAGGTTGGTGGTTAGAGAC
20036	Yes	AATCGGTTAGCGCGTTCGGCTGTTAACCATAAGGTTGGTGGTTAGAGACC
20037	Yes	AATCGGTTAGCGCGTTCGGCTGTTAACCG
20038	Yes	AATCGGTTAGCGCGTTCGGCTGTTAACCGA
20039	Yes	AATCGGTTAGCGCGTTCGGCTGTTAACCGAA
20040	Yes	AATCGGTTAGCGCGTTCGGCTGTTAACCGAAA
20041	Yes	AATCGGTTAGCGCGTTCGGCTGTTAACCGAAAG
20042	Yes	AATCGGTTAGCGCGTTCGGCTGTTAACCGAAAGA
20043	Yes	AATCGGTTAGCGCGTTCGGCTGTTAACCGAAAGAT
20044	Yes	AATCGGTTAGCGCGTTCGGCTGTTAACCGAAAGATT
20045	Yes	AATCGGTTAGCGCGTTCGGCTGTTAACCGAAAGATTG
20046	Yes	AATCGGTTAGCGCGTTCGGCTGTTAACCGAAAGATTGG
20047	Yes	AATCGGTTAGCGCGTTCGGCTGTTAACCGAAAGATTGGT
20048	Yes	AATCGGTTAGCGCGTTCGGCTGTTAACCGAAAGATTGGTG
20049	Yes	AATCGGTTAGCGCGTTCGGCTGTTAACCGAAAGATTGGTGG
20050	Yes	AATCGGTTAGCGCGTTCGGCTGTTAACCGAAAGATTGGTGGT
20051	Yes	AATCGGTTAGCGCGTTCGGCTGTTAACCGAAAGATTGGTGGTT
20052	Yes	AATCGGTTAGCGCGTTCGGCTGTTAACCGAAAGATTGGTGGTTC
20053	Yes	AATCGGTTAGCGCGTTCGGCTGTTAACCGAAAGATTGGTGGTTCG
20054	Yes	AATCGGTTAGCGCGTTCGGCTGTTAACCGAAAGATTGGTGGTTCGA
20055	Yes	AATCGGTTAGCGCGTTCGGCTGTTAACCGAAAGATTGGTGGTTCGAG
20056	Yes	AATCGGTTAGCGCGTTCGGCTGTTAACCGAAAGATTGGTGGTTCGAGC
20057	Yes	AATCGGTTAGCGCGTTCGGCTGTTAACCGAAAGATTGGTGGTTCGAGCC
20058	Yes	AATCGGTTAGCGCGTTCGGCTGTTAACCGAAAGATTGGTGGTTCGAGCCC
20059	Yes	AATCGGTTAGCGCGTTCGGCTGTTAACCGAAAGG
20060	Yes	AATCGGTTAGCGCGTTCGGCTGTTAACCGAAAGGT
20061	Yes	AATCGGTTAGCGCGTTCGGCTGTTAACCGAAAGGTT
20062	Yes	AATCGGTTAGCGCGTTCGGCTGTTAACCGAAAGGTTG
20063	Yes	AATCGGTTAGCGCGTTCGGCTGTTAACCGAAAGGTTGG
20064	Yes	AATCGGTTAGCGCGTTCGGCTGTTAACCGAAAGGTTGGT
20065	Yes	AATCGGTTAGCGCGTTCGGCTGTTAACCGAAAGGTTGGTG
20066	Yes	AATCGGTTAGCGCGTTCGGCTGTTAACCGAAAGGTTGGTGG
20067	Yes	AATCGGTTAGCGCGTTCGGCTGTTAACCGAAAGGTTGGTGGT
20068	Yes	AATCGGTTAGCGCGTTCGGCTGTTAACCGAAAGGTTGGTGGTT
20069	Yes	AATCGGTTAGCGCGTTCGGCTGTTAACCGAAAGGTTGGTGGTTC

20070	Yes	AATCGGTTAGCGCGTTCGGCTGTTAACCGAAAGGTTGGTGGTTCG
20071	Yes	AATCGGTTAGCGCGTTCGGCTGTTAACCGAAAGGTTGGTGGTTCGA
20072	Yes	AATCGGTTAGCGCGTTCGGCTGTTAACCGAAAGGTTGGTGGTTCGAG
20073	Yes	AATCGGTTAGCGCGTTCGGCTGTTAACCGAAAGGTTGGTGGTTCGAGC
20074	Yes	AATCGGTTAGCGCGTTCGGCTGTTAACCGAAAGGTTGGTGGTTCGAGCC
20075	Yes	AATCGGTTAGCGCGTTCGGCTGTTAACCGAAAGGTTGGTGGTTCGAGCCC
20076	Yes	AATCGGTTAGCGCGTTCGGCTGTTAACCGAAAGGTTGGTGGTTCGAT
20077	Yes	AATCGGTTAGCGCGTTCGGCTGTTAACCGAAAGGTTGGTGGTTCGATC
20078	Yes	AATCGGTTAGCGCGTTCGGCTGTTAACCGAAAGGTTGGTGGTTCGATCC
20079	Yes	AATCGGTTAGCGCGTTCGGCTGTTAACCGAAAGGTTGGTGGTTCGATCCC
20080	Yes	AATCGGTTAGCGCGTTCGGCTGTTAACT
20081	Yes	AATCGGTTAGCGCGTTCGGCTGTTAACTG
20082	Yes	AATCGGTTAGCGCGTTCGGCTGTTAACTGA
20083	Yes	AATCGGTTAGCGCGTTCGGCTGTTAACTGAA
20084	Yes	AATCGGTTAGCGCGTTCGGCTGTTAACTGAAA
20085	Yes	AATCGGTTAGCGCGTTCGGCTGTTAACTGAAAG
20086	Yes	AATCGGTTAGCGCGTTCGGCTGTTAACTGAAAGG
20087	Yes	AATCGGTTAGCGCGTTCGGCTGTTAACTGAAAGGT
20088	Yes	AATCGGTTAGCGCGTTCGGCTGTTAACTGAAAGGTT
20089	Yes	AATCGGTTAGCGCGTTCGGCTGTTAACTGAAAGGTTA
20090	Yes	AATCGGTTAGCGCGTTCGGCTGTTAACTGAAAGGTTAG
20091	Yes	AATCGGTTAGCGCGTTCGGCTGTTAACTGAAAGGTTAGT
20092	Yes	AATCGGTTAGCGCGTTCGGCTGTTAACTGAAAGGTTAGTG
20093	Yes	AATCGGTTAGCGCGTTCGGCTGTTAACTGAAAGGTTAGTGG
20094	Yes	AATCGGTTAGCGCGTTCGGCTGTTAACTGAAAGGTTAGTGGT
20095	Yes	AATCGGTTAGCGCGTTCGGCTGTTAACTGAAAGGTTAGTGGTT
20096	Yes	AATCGGTTAGCGCGTTCGGCTGTTAACTGAAAGGTTAGTGGTTC
20097	Yes	AATCGGTTAGCGCGTTCGGCTGTTAACTGAAAGGTTAGTGGTTCG
20098	Yes	AATCGGTTAGCGCGTTCGGCTGTTAACTGAAAGGTTAGTGGTTCGA
20099	Yes	AATCGGTTAGCGCGTTCGGCTGTTAACTGAAAGGTTAGTGGTTCGAG
20100	Yes	AATCGGTTAGCGCGTTCGGCTGTTAACTGAAAGGTTAGTGGTTCGAGC
20101	Yes	AATCGGTTAGCGCGTTCGGCTGTTAACTGAAAGGTTAGTGGTTCGAGCC
20102	Yes	AATCGGTTAGCGCGTTCGGCTGTTAACTGAAAGGTTAGTGGTTCGAGCCC
20103	Yes	AATCGGTTAGCGCGTTCGGCTGTTAACTGAAAGGTTG
20104	Yes	AATCGGTTAGCGCGTTCGGCTGTTAACTGAAAGGTTGG
20105	Yes	AATCGGTTAGCGCGTTCGGCTGTTAACTGAAAGGTTGGT
20106	Yes	AATCGGTTAGCGCGTTCGGCTGTTAACTGAAAGGTTGGTG
20107	Yes	AATCGGTTAGCGCGTTCGGCTGTTAACTGAAAGGTTGGTGG
20108	Yes	AATCGGTTAGCGCGTTCGGCTGTTAACTGAAAGGTTGGTGGT
20109	Yes	AATCGGTTAGCGCGTTCGGCTGTTAACTGAAAGGTTGGTGGTT
20110	Yes	AATCGGTTAGCGCGTTCGGCTGTTAACTGAAAGGTTGGTGGTTC
20111	Yes	AATCGGTTAGCGCGTTCGGCTGTTAACTGAAAGGTTGGTGGTTCG

20112	Yes	AATCGGTTAGCGCGTTCGGCTGTTAACTGAAAGGTTGGTGGTTCGA
20113	Yes	AATCGGTTAGCGCGTTCGGCTGTTAACTGAAAGGTTGGTGGTTCGAG
20114	Yes	AATCGGTTAGCGCGTTCGGCTGTTAACTGAAAGGTTGGTGGTTCGAGC
20115	Yes	AATCGGTTAGCGCGTTCGGCTGTTAACTGAAAGGTTGGTGGTTCGAGCC
20116	Yes	AATCGGTTAGCGCGTTCGGCTGTTAACTGAAAGGTTGGTGGTTCGAGCCC
20117	Yes	AATCGGTTAGCGCGTTCGGCTGTTAAT
20118	Yes	AATCGGTTAGCGCGTTCGGCTGTTAATC
20119	Yes	AATCGGTTAGCGCGTTCGGCTGTTAATCT
20120	Yes	AATCGGTTAGCGCGTTCGGCTGTTAATCTA
20121	Yes	AATCGGTTAGCGCGTTCGGCTGTTAATCTAG
20122	Yes	AATCGGTTAGCGCGTTCGGCTGTTAATCTAGA
20123	Yes	AATCGGTTAGCGCGTTCGGCTGTTAATCTAGAG
20124	Yes	AATCGGTTAGCGCGTTCGGCTGTTAATCTAGAGG
20125	Yes	AATCGGTTAGCGCGTTCGGCTGTTAATCTAGAGGT
20126	Yes	AATCGGTTAGCGCGTTCGGCTGTTAATCTAGAGGTT
20127	Yes	AATCGGTTAGCGCGTTCGGCTGTTAATCTAGAGGTTG
20128	Yes	AATCGGTTAGCGCGTTCGGCTGTTAATCTAGAGGTTGG
20129	Yes	AATCGGTTAGCGCGTTCGGCTGTTAATCTAGAGGTTGGT
20130	Yes	AATCGGTTAGCGCGTTCGGCTGTTAATCTAGAGGTTGGTG
20131	Yes	AATCGGTTAGCGCGTTCGGCTGTTAATCTAGAGGTTGGTGG
20132	Yes	AATCGGTTAGCGCGTTCGGCTGTTAATCTAGAGGTTGGTGGT
20133	Yes	AATCGGTTAGCGCGTTCGGCTGTTAATCTAGAGGTTGGTGGTT
20134	Yes	AATCGGTTAGCGCGTTCGGCTGTTAATCTAGAGGTTGGTGGTTA
20135	Yes	AATCGGTTAGCGCGTTCGGCTGTTAATCTAGAGGTTGGTGGTTAG
20136	Yes	AATCGGTTAGCGCGTTCGGCTGTTAATCTAGAGGTTGGTGGTTAGA
20137	Yes	AATCGGTTAGCGCGTTCGGCTGTTAATCTAGAGGTTGGTGGTTAGAG
20138	Yes	AATCGGTTAGCGCGTTCGGCTGTTAATCTAGAGGTTGGTGGTTAGAGC
20139	Yes	AATCGGTTAGCGCGTTCGGCTGTTAATCTAGAGGTTGGTGGTTAGAGCC
20140	Yes	AATCGGTTAGCGCGTTCGGCTGTTAATCTAGAGGTTGGTGGTTAGAGCCC
20141	Yes	AATCGGTTAGCGCGTTT
20142	Yes	AATCGGTTAGCGCGTTTG
20143	Yes	AATCGGTTAGCGCGTTTGA
20144	Yes	AATCGGTTAGCGCGTTTGAC
20145	Yes	AATCGGTTAGCGCGTTTGACT
20146	Yes	AATCGGTTAGCGCGTTTGACTG
20147	Yes	AATCGGTTAGCGCGTTTGACTGT
20148	Yes	AATCGGTTAGCGCGTTTGACTGTT
20149	Yes	AATCGGTTAGCGCGTTTGACTGTTA
20150	Yes	AATCGGTTAGCGCGTTTGACTGTTAA
20151	Yes	AATCGGTTAGCGCGTTTGACTGTTAAC
20152	Yes	AATCGGTTAGCGCGTTTGACTGTTAACT
20153	Yes	AATCGGTTAGCGCGTTTGACTGTTAACTG

20154	Yes	AATCGGTTAGCGCGTTTGACTGTTAACTGA
20155	Yes	AATCGGTTAGCGCGTTTGACTGTTAACTGAA
20156	Yes	AATCGGTTAGCGCGTTTGACTGTTAACTGAAA
20157	Yes	AATCGGTTAGCGCGTTTGACTGTTAACTGAAAG
20158	Yes	AATCGGTTAGCGCGTTTGACTGTTAACTGAAAGG
20159	Yes	AATCGGTTAGCGCGTTTGACTGTTAACTGAAAGGT
20160	Yes	AATCGGTTAGCGCGTTTGACTGTTAACTGAAAGGTT
20161	Yes	AATCGGTTAGCGCGTTTGACTGTTAACTGAAAGGTTG
20162	Yes	AATCGGTTAGCGCGTTTGACTGTTAACTGAAAGGTTGG
20163	Yes	AATCGGTTAGCGCGTTTGACTGTTAACTGAAAGGTTGGT
20164	Yes	AATCGGTTAGCGCGTTTGACTGTTAACTGAAAGGTTGGTG
20165	Yes	AATCGGTTAGCGCGTTTGACTGTTAACTGAAAGGTTGGTGG
20166	Yes	AATCGGTTAGCGCGTTTGACTGTTAACTGAAAGGTTGGTGGT
20167	Yes	AATCGGTTAGCGCGTTTGACTGTTAACTGAAAGGTTGGTGGTG
20168	Yes	AATCGGTTAGCGCGTTTGACTGTTAACTGAAAGGTTGGTGGTGC
20169	Yes	AATCGGTTAGCGCGTTTGACTGTTAACTGAAAGGTTGGTGGTGCA
20170	Yes	AATCGGTTAGCGCGTTTGACTGTTAACTGAAAGGTTGGTGGTGCAA
20171	Yes	AATCGGTTAGCGCGTTTGACTGTTAACTGAAAGGTTGGTGGTGCAAG
20172	Yes	AATCGGTTAGCGCGTTTGACTGTTAACTGAAAGGTTGGTGGTGCAAGC
20173	Yes	AATCGGTTAGCGCGTTTGACTGTTAACTGAAAGGTTGGTGGTGCAAGCC
20174	Yes	AATCGGTTAGCGCGTTTGACTGTTAACTGAAAGGTTGGTGGTGCAAGCCC
20175	Yes	AATCGGTTAGCTTGTT
20176	Yes	AATCGGTTAGCTTGTTC
20177	Yes	AATCGGTTAGCTTGTTCG
20178	Yes	AATCGGTTAGCTTGTTCGG
20179	Yes	AATCGGTTAGCTTGTTCGGC
20180	Yes	AATCGGTTAGCTTGTTCGGCT
20181	Yes	AATCGGTTAGCTTGTTCGGCTG
20182	Yes	AATCGGTTAGCTTGTTCGGCTGT
20183	Yes	AATCGGTTAGCTTGTTCGGCTGTT
20184	Yes	AATCGGTTAGCTTGTTCGGCTGTTA
20185	Yes	AATCGGTTAGCTTGTTCGGCTGTTAA
20186	Yes	AATCGGTTAGCTTGTTCGGCTGTTAAT
20187	Yes	AATCGGTTAGCTTGTTCGGCTGTTAATC
20188	Yes	AATCGGTTAGCTTGTTCGGCTGTTAATCT
20189	Yes	AATCGGTTAGCTTGTTCGGCTGTTAATCTA
20190	Yes	AATCGGTTAGCTTGTTCGGCTGTTAATCTAG
20191	Yes	AATCGGTTAGCTTGTTCGGCTGTTAATCTAGA
20192	Yes	AATCGGTTAGCTTGTTCGGCTGTTAATCTAGAG
20193	Yes	AATCGGTTAGCTTGTTCGGCTGTTAATCTAGAGG
20194	Yes	AATCGGTTAGCTTGTTCGGCTGTTAATCTAGAGGT
20195	Yes	AATCGGTTAGCTTGTTCGGCTGTTAATCTAGAGGTT

20196	Yes	AATCGGTTAGCTTGTTCGGCTGTTAATCTAGAGGTTG
20197	Yes	AATCGGTTAGCTTGTTCGGCTGTTAATCTAGAGGTTGG
20198	Yes	AATCGGTTAGCTTGTTCGGCTGTTAATCTAGAGGTTGGT
20199	Yes	AATCGGTTAGCTTGTTCGGCTGTTAATCTAGAGGTTGGTG
20200	Yes	AATCGGTTAGCTTGTTCGGCTGTTAATCTAGAGGTTGGTGG
20201	Yes	AATCGGTTAGCTTGTTCGGCTGTTAATCTAGAGGTTGGTGGT
20202	Yes	AATCGGTTAGCTTGTTCGGCTGTTAATCTAGAGGTTGGTGGTT
20203	Yes	AATCGGTTAGCTTGTTCGGCTGTTAATCTAGAGGTTGGTGGTTA
20204	Yes	AATCGGTTAGCTTGTTCGGCTGTTAATCTAGAGGTTGGTGGTTAG
20205	Yes	AATCGGTTAGCTTGTTCGGCTGTTAATCTAGAGGTTGGTGGTTAGA
20206	Yes	AATCGGTTAGCTTGTTCGGCTGTTAATCTAGAGGTTGGTGGTTAGAG
20207	Yes	AATCGGTTAGCTTGTTCGGCTGTTAATCTAGAGGTTGGTGGTTAGAGC
20208	Yes	AATCGGTTAGCTTGTTCGGCTGTTAATCTAGAGGTTGGTGGTTAGAGCC
20209	Yes	AATCGGTTAGCTTGTTCGGCTGTTAATCTAGAGGTTGGTGGTTAGAGCCC
20210	No	AATCTAAAGACAGGGG
20211	No	AATCTAAAGACAGGGGT
20212	No	AATCTAAAGACAGGGGTT
20213	No	AATCTAAAGACAGGGGTTA
20214	Yes	AATCTAAAGACAGGGGTTAG
20215	Yes	AATCTAAAGACAGGGGTTAGG
20216	Yes	AATCTAAAGACAGGGGTTAGGC
20217	Yes	AATCTAAAGACAGGGGTTAGGCC
20218	Yes	AATCTAAAGACAGGGGTTAGGCCT
20219	Yes	AATCTAAAGACAGGGGTTAGGCCTC
20220	Yes	AATCTAAAGACAGGGGTTAGGCCTCT
20221	Yes	AATCTAAAGACAGGGGTTAGGCCTCTT
20222	Yes	AATCTAAAGACAGGGGTTAGGCCTCTTT
20223	Yes	AATCTAAAGACAGGGGTTAGGCCTCTTTT
20224	Yes	AATCTAAAGACAGGGGTTAGGCCTCTTTTT
20225	Yes	AATCTAAAGACAGGGGTTAGGCCTCTTTTTA
20226	Yes	AATCTAAAGACAGGGGTTAGGCCTCTTTTTAC
20227	Yes	AATCTAAAGACAGGGGTTAGGCCTCTTTTTACC
20228	Yes	AATCTAAAGACAGGGGTTAGGCCTCTTTTTACCA
20229	Yes	AATCTAAAGACAGGGGTTAGGCCTCTTTTTACCAC
20230	Yes	AATCTAAAGACAGGGGTTAGGCCTCTTTTTACCACC
20231	Yes	AATCTAAAGACAGGGGTTAGGCCTCTTTTTACCACCA
20232	No	AATCTAAAGGTTGGTG
20233	No	AATCTAAAGGTTGGTGG
20234	Yes	AATCTAAAGGTTGGTGGC
20235	Yes	AATCTAAAGGTTGGTGGCT
20236	Yes	AATCTAAAGGTTGGTGGCTA
20237	Yes	AATCTAAAGGTTGGTGGCTAG

20238	Yes	AATCTAAAGGTTGGTGGCTAGA
20239	Yes	AATCTAAAGGTTGGTGGCTAGAA
20240	Yes	AATCTAAAGGTTGGTGGCTAGAAC
20241	Yes	AATCTAAAGGTTGGTGGCTAGAACC
20242	Yes	AATCTAAAGGTTGGTGGCTAGAACCC
20243	Yes	AATCTAAAGGTTGGTGGCTAGAACCCA
20244	Yes	AATCTAAAGGTTGGTGGCTAGAACCCAC
20245	Yes	AATCTAAAGGTTGGTGGCTAGAACCCACT
20246	Yes	AATCTAAAGGTTGGTGGCTAGAACCCACTG
20247	Yes	AATCTAAAGGTTGGTGGCTAGAACCCACTGA
20248	Yes	AATCTAAAGGTTGGTGGCTAGAACCCACTGAG
20249	Yes	AATCTAAAGGTTGGTGGCTAGAACCCACTGAGG
20250	Yes	AATCTAAAGGTTGGTGGCTAGAACCCACTGAGGG
20251	Yes	AATCTAAAGGTTGGTGGCTAGAACCCACTGAGGGA
20252	Yes	AATCTAAAGGTTGGTGGCTAGAACCCACTGAGGGAC
20253	Yes	AATCTAAAGGTTGGTGGCTAGAACCCACTGAGGGACG
20254	Yes	AATCTAAAGGTTGGTGGCTAGAACCCACTGAGGGACGC
20255	Yes	AATCTAAAGGTTGGTGGCTAGAACCCACTGAGGGACGCC
20256	Yes	AATCTAAAGGTTGGTGGCTAGAACCCACTGAGGGACGCCA
20257	No	AATCTAGAGGTTGGTG
20258	Yes	AATCTAGAGGTTGGTGG
20259	Yes	AATCTAGAGGTTGGTGGT
20260	Yes	AATCTAGAGGTTGGTGGTT
20261	Yes	AATCTAGAGGTTGGTGGTTA
20262	Yes	AATCTAGAGGTTGGTGGTTAG
20263	Yes	AATCTAGAGGTTGGTGGTTAGA
20264	Yes	AATCTAGAGGTTGGTGGTTAGAG
20265	Yes	AATCTAGAGGTTGGTGGTTAGAGC
20266	Yes	AATCTAGAGGTTGGTGGTTAGAGCC
20267	Yes	AATCTAGAGGTTGGTGGTTAGAGCCC
20268	Yes	AATCTAGAGGTTGGTGGTTAGAGCCCA
20269	Yes	AATCTAGAGGTTGGTGGTTAGAGCCCAC
20270	Yes	AATCTAGAGGTTGGTGGTTAGAGCCCACT
20271	Yes	AATCTAGAGGTTGGTGGTTAGAGCCCACTG
20272	Yes	AATCTAGAGGTTGGTGGTTAGAGCCCACTGA
20273	Yes	AATCTAGAGGTTGGTGGTTAGAGCCCACTGAG
20274	Yes	AATCTAGAGGTTGGTGGTTAGAGCCCACTGAGG
20275	Yes	AATCTAGAGGTTGGTGGTTAGAGCCCACTGAGGG
20276	Yes	AATCTAGAGGTTGGTGGTTAGAGCCCACTGAGGGA
20277	Yes	AATCTAGAGGTTGGTGGTTAGAGCCCACTGAGGGAT
20278	Yes	AATCTAGAGGTTGGTGGTTAGAGCCCACTGAGGGATG
20279	Yes	AATCTAGAGGTTGGTGGTTAGAGCCCACTGAGGGATGC

20280	Yes	AATCTAGAGGTTGGTGGTTAGAGCCCACTGAGGGATGCC
20281	Yes	AATCTAGAGGTTGGTGGTTAGAGCCCACTGAGGGATGCCA
20282	Yes	AATCTAGGTGCCCCCT
20283	Yes	AATCTAGGTGCCCCCTC
20284	Yes	AATCTAGGTGCCCCCTCC
20285	Yes	AATCTAGGTGCCCCCTCCA
20286	No	AATCTCACCAGGGCCT
20287	Yes	AATCTCACCAGGGCCTC
20288	Yes	AATCTCACCAGGGCCTCC
20289	Yes	AATCTCACCAGGGCCTCCA
20290	No	AATCTCAGACCTGCTA
20291	Yes	AATCTCAGACCTGCTAC
20292	Yes	AATCTCAGACCTGCTACC
20293	Yes	AATCTCAGACCTGCTACCA
20294	No	AATCTCAGGGTAGTGG
20295	No	AATCTCAGGGTAGTGGG
20296	Yes	AATCTCAGGGTAGTGGGT
20297	Yes	AATCTCAGGGTAGTGGGTT
20298	Yes	AATCTCAGGGTAGTGGGTTC
20299	Yes	AATCTCAGGGTAGTGGGTTCA
20300	Yes	AATCTCAGGGTAGTGGGTTCAA
20301	Yes	AATCTCAGGGTAGTGGGTTCAAG
20302	Yes	AATCTCAGGGTAGTGGGTTCAAGC
20303	Yes	AATCTCAGGGTAGTGGGTTCAAGCC
20304	Yes	AATCTCAGGGTAGTGGGTTCAAGCCC
20305	Yes	AATCTCAGGGTAGTGGGTTCAAGCCCC
20306	Yes	AATCTCAGGGTAGTGGGTTCAAGCCCCA
20307	Yes	AATCTCAGGGTAGTGGGTTCAAGCCCCAC
20308	Yes	AATCTCAGGGTAGTGGGTTCAAGCCCCACA
20309	Yes	AATCTCAGGGTAGTGGGTTCAAGCCCCACAT
20310	Yes	AATCTCAGGGTAGTGGGTTCAAGCCCCACATT
20311	Yes	AATCTCAGGGTAGTGGGTTCAAGCCCCACATTG
20312	Yes	AATCTCAGGGTAGTGGGTTCAAGCCCCACATTGG
20313	Yes	AATCTCAGGGTAGTGGGTTCAAGCCCCACATTGGA
20314	Yes	AATCTCAGGGTAGTGGGTTCAAGCCCCACATTGGAC
20315	Yes	AATCTCAGGGTAGTGGGTTCAAGCCCCACATTGGACA
20316	Yes	AATCTCAGGGTAGTGGGTTCAAGCCCCACATTGGACAC
20317	Yes	AATCTCAGGGTAGTGGGTTCAAGCCCCACATTGGACACC
20318	Yes	AATCTCAGGGTAGTGGGTTCAAGCCCCACATTGGACACCA
20319	No	AATCTCAGGGTCATGG
20320	No	AATCTCAGGGTCATGGG
20321	No	AATCTCAGGGTCATGGGT

20322	Yes	AATCTCAGGGTCATGGGTT
20323	Yes	AATCTCAGGGTCATGGGTTC
20324	Yes	AATCTCAGGGTCATGGGTTCG
20325	Yes	AATCTCAGGGTCATGGGTTCGT
20326	Yes	AATCTCAGGGTCATGGGTTCGTG
20327	Yes	AATCTCAGGGTCATGGGTTCGTGC
20328	Yes	AATCTCAGGGTCATGGGTTCGTGCC
20329	Yes	AATCTCAGGGTCATGGGTTCGTGCCC
20330	Yes	AATCTCAGGGTCATGGGTTCGTGCCCC
20331	Yes	AATCTCAGGGTCATGGGTTCGTGCCCCA
20332	Yes	AATCTCAGGGTCATGGGTTCGTGCCCCAT
20333	Yes	AATCTCAGGGTCATGGGTTCGTGCCCCATG
20334	Yes	AATCTCAGGGTCATGGGTTCGTGCCCCATGT
20335	Yes	AATCTCAGGGTCATGGGTTCGTGCCCCATGTT
20336	Yes	AATCTCAGGGTCATGGGTTCGTGCCCCATGTTG
20337	Yes	AATCTCAGGGTCATGGGTTCGTGCCCCATGTTGG
20338	Yes	AATCTCAGGGTCATGGGTTCGTGCCCCATGTTGGG
20339	Yes	AATCTCAGGGTCATGGGTTCGTGCCCCATGTTGGGT
20340	Yes	AATCTCAGGGTCATGGGTTCGTGCCCCATGTTGGGTG
20341	Yes	AATCTCAGGGTCATGGGTTCGTGCCCCATGTTGGGTGC
20342	Yes	AATCTCAGGGTCATGGGTTCGTGCCCCATGTTGGGTGCC
20343	Yes	AATCTCAGGGTCATGGGTTCGTGCCCCATGTTGGGTGCCA
20344	Yes	AATCTCAGGGTCATGGGTTT
20345	Yes	AATCTCAGGGTCATGGGTTTG
20346	Yes	AATCTCAGGGTCATGGGTTTGA
20347	Yes	AATCTCAGGGTCATGGGTTTGAG
20348	Yes	AATCTCAGGGTCATGGGTTTGAGC
20349	Yes	AATCTCAGGGTCATGGGTTTGAGCC
20350	Yes	AATCTCAGGGTCATGGGTTTGAGCCC
20351	Yes	AATCTCAGGGTCATGGGTTTGAGCCCC
20352	Yes	AATCTCAGGGTCATGGGTTTGAGCCCCA
20353	Yes	AATCTCAGGGTCATGGGTTTGAGCCCCAC
20354	Yes	AATCTCAGGGTCATGGGTTTGAGCCCCACG
20355	Yes	AATCTCAGGGTCATGGGTTTGAGCCCCACGT
20356	Yes	AATCTCAGGGTCATGGGTTTGAGCCCCACGTT
20357	Yes	AATCTCAGGGTCATGGGTTTGAGCCCCACGTTT
20358	Yes	AATCTCAGGGTCATGGGTTTGAGCCCCACGTTTG
20359	Yes	AATCTCAGGGTCATGGGTTTGAGCCCCACGTTTGG
20360	Yes	AATCTCAGGGTCATGGGTTTGAGCCCCACGTTTGGT
20361	Yes	AATCTCAGGGTCATGGGTTTGAGCCCCACGTTTGGTG
20362	Yes	AATCTCAGGGTCATGGGTTTGAGCCCCACGTTTGGTGC
20363	Yes	AATCTCAGGGTCATGGGTTTGAGCCCCACGTTTGGTGCC

20364	Yes	AATCTCAGGGTCATGGGTTTGAGCCCCACGTTTGGTGCCA
20365	Yes	AATCTCAGGGTCATGGGTTTGAGCCCT
20366	Yes	AATCTCAGGGTCATGGGTTTGAGCCCTA
20367	Yes	AATCTCAGGGTCATGGGTTTGAGCCCTAC
20368	Yes	AATCTCAGGGTCATGGGTTTGAGCCCTACG
20369	Yes	AATCTCAGGGTCATGGGTTTGAGCCCTACGT
20370	Yes	AATCTCAGGGTCATGGGTTTGAGCCCTACGTT
20371	Yes	AATCTCAGGGTCATGGGTTTGAGCCCTACGTTG
20372	Yes	AATCTCAGGGTCATGGGTTTGAGCCCTACGTTGG
20373	Yes	AATCTCAGGGTCATGGGTTTGAGCCCTACGTTGGG
20374	Yes	AATCTCAGGGTCATGGGTTTGAGCCCTACGTTGGGC
20375	Yes	AATCTCAGGGTCATGGGTTTGAGCCCTACGTTGGGCG
20376	Yes	AATCTCAGGGTCATGGGTTTGAGCCCTACGTTGGGCGC
20377	Yes	AATCTCAGGGTCATGGGTTTGAGCCCTACGTTGGGCGCC
20378	Yes	AATCTCAGGGTCATGGGTTTGAGCCCTACGTTGGGCGCCA
20379	No	AATCTCAGGGTCGTGG
20380	No	AATCTCAGGGTCGTGGG
20381	Yes	AATCTCAGGGTCGTGGGC
20382	Yes	AATCTCAGGGTCGTGGGCT
20383	Yes	AATCTCAGGGTCGTGGGCTC
20384	Yes	AATCTCAGGGTCGTGGGCTCG
20385	Yes	AATCTCAGGGTCGTGGGCTCGA
20386	Yes	AATCTCAGGGTCGTGGGCTCGAG
20387	Yes	AATCTCAGGGTCGTGGGCTCGAGC
20388	Yes	AATCTCAGGGTCGTGGGCTCGAGCT
20389	Yes	AATCTCAGGGTCGTGGGCTCGAGCTC
20390	Yes	AATCTCAGGGTCGTGGGCTCGAGCTCC
20391	Yes	AATCTCAGGGTCGTGGGCTCGAGCTCCA
20392	Yes	AATCTCAGGGTCGTGGGCTCGAGCTCCAT
20393	Yes	AATCTCAGGGTCGTGGGCTCGAGCTCCATG
20394	Yes	AATCTCAGGGTCGTGGGCTCGAGCTCCATGT
20395	Yes	AATCTCAGGGTCGTGGGCTCGAGCTCCATGTT
20396	Yes	AATCTCAGGGTCGTGGGCTCGAGCTCCATGTTG
20397	Yes	AATCTCAGGGTCGTGGGCTCGAGCTCCATGTTGG
20398	Yes	AATCTCAGGGTCGTGGGCTCGAGCTCCATGTTGGG
20399	Yes	AATCTCAGGGTCGTGGGCTCGAGCTCCATGTTGGGC
20400	Yes	AATCTCAGGGTCGTGGGCTCGAGCTCCATGTTGGGCG
20401	Yes	AATCTCAGGGTCGTGGGCTCGAGCTCCATGTTGGGCGC
20402	Yes	AATCTCAGGGTCGTGGGCTCGAGCTCCATGTTGGGCGCC
20403	Yes	AATCTCAGGGTCGTGGGCTCGAGCTCCATGTTGGGCGCCA
20404	No	AATCTCAGGGTCGTGGGT
20405	No	AATCTCAGGGTCGTGGGTT

20406	No	AATCTCAGGGTCGTGGGTTC
20407	No	AATCTCAGGGTCGTGGGTTCA
20408	Yes	AATCTCAGGGTCGTGGGTTCAA
20409	Yes	AATCTCAGGGTCGTGGGTTCAAG
20410	Yes	AATCTCAGGGTCGTGGGTTCAAGC
20411	Yes	AATCTCAGGGTCGTGGGTTCAAGCC
20412	Yes	AATCTCAGGGTCGTGGGTTCAAGCCC
20413	Yes	AATCTCAGGGTCGTGGGTTCAAGCCCC
20414	Yes	AATCTCAGGGTCGTGGGTTCAAGCCCCA
20415	Yes	AATCTCAGGGTCGTGGGTTCAAGCCCCAC
20416	Yes	AATCTCAGGGTCGTGGGTTCAAGCCCCACA
20417	Yes	AATCTCAGGGTCGTGGGTTCAAGCCCCACAT
20418	Yes	AATCTCAGGGTCGTGGGTTCAAGCCCCACATT
20419	Yes	AATCTCAGGGTCGTGGGTTCAAGCCCCACATTG
20420	Yes	AATCTCAGGGTCGTGGGTTCAAGCCCCACATTGG
20421	Yes	AATCTCAGGGTCGTGGGTTCAAGCCCCACATTGGG
20422	Yes	AATCTCAGGGTCGTGGGTTCAAGCCCCACATTGGGC
20423	Yes	AATCTCAGGGTCGTGGGTTCAAGCCCCACATTGGGCG
20424	Yes	AATCTCAGGGTCGTGGGTTCAAGCCCCACATTGGGCGC
20425	Yes	AATCTCAGGGTCGTGGGTTCAAGCCCCACATTGGGCGCC
20426	Yes	AATCTCAGGGTCGTGGGTTCAAGCCCCACATTGGGCGCCA
20427	No	AATCTCAGGGTCGTGGGTTCG
20428	No	AATCTCAGGGTCGTGGGTTCGA
20429	No	AATCTCAGGGTCGTGGGTTCGAG
20430	No	AATCTCAGGGTCGTGGGTTCGAGC
20431	No	AATCTCAGGGTCGTGGGTTCGAGCC
20432	No	AATCTCAGGGTCGTGGGTTCGAGCCC
20433	No	AATCTCAGGGTCGTGGGTTCGAGCCCC
20434	No	AATCTCAGGGTCGTGGGTTCGAGCCCCA
20435	No	AATCTCAGGGTCGTGGGTTCGAGCCCCAC
20436	Yes	AATCTCAGGGTCGTGGGTTCGAGCCCCACG
20437	Yes	AATCTCAGGGTCGTGGGTTCGAGCCCCACGT
20438	Yes	AATCTCAGGGTCGTGGGTTCGAGCCCCACGTT
20439	Yes	AATCTCAGGGTCGTGGGTTCGAGCCCCACGTTG
20440	Yes	AATCTCAGGGTCGTGGGTTCGAGCCCCACGTTGG
20441	Yes	AATCTCAGGGTCGTGGGTTCGAGCCCCACGTTGGG
20442	Yes	AATCTCAGGGTCGTGGGTTCGAGCCCCACGTTGGGC
20443	Yes	AATCTCAGGGTCGTGGGTTCGAGCCCCACGTTGGGCG
20444	Yes	AATCTCAGGGTCGTGGGTTCGAGCCCCACGTTGGGCGC
20445	Yes	AATCTCAGGGTCGTGGGTTCGAGCCCCACGTTGGGCGCC
20446	Yes	AATCTCAGGGTCGTGGGTTCGAGCCCCACGTTGGGCGCCA
20447	Yes	AATCTCAGGGTCGTGGGTTCGAGCCG

20448	Yes	AATCTCAGGGTCGTGGGTTCGAGCCGC
20449	Yes	AATCTCAGGGTCGTGGGTTCGAGCCGCA
20450	Yes	AATCTCAGGGTCGTGGGTTCGAGCCGCAC
20451	Yes	AATCTCAGGGTCGTGGGTTCGAGCCGCACG
20452	Yes	AATCTCAGGGTCGTGGGTTCGAGCCGCACGT
20453	Yes	AATCTCAGGGTCGTGGGTTCGAGCCGCACGTT
20454	Yes	AATCTCAGGGTCGTGGGTTCGAGCCGCACGTTG
20455	Yes	AATCTCAGGGTCGTGGGTTCGAGCCGCACGTTGG
20456	Yes	AATCTCAGGGTCGTGGGTTCGAGCCGCACGTTGGG
20457	Yes	AATCTCAGGGTCGTGGGTTCGAGCCGCACGTTGGGC
20458	Yes	AATCTCAGGGTCGTGGGTTCGAGCCGCACGTTGGGCG
20459	Yes	AATCTCAGGGTCGTGGGTTCGAGCCGCACGTTGGGCGC
20460	Yes	AATCTCAGGGTCGTGGGTTCGAGCCGCACGTTGGGCGCC
20461	Yes	AATCTCAGGGTCGTGGGTTCGAGCCGCACGTTGGGCGCCA
20462	Yes	AATCTCAGGGTCGTGGGTTT
20463	Yes	AATCTCAGGGTCGTGGGTTTG
20464	Yes	AATCTCAGGGTCGTGGGTTTGA
20465	Yes	AATCTCAGGGTCGTGGGTTTGAG
20466	Yes	AATCTCAGGGTCGTGGGTTTGAGC
20467	Yes	AATCTCAGGGTCGTGGGTTTGAGCC
20468	Yes	AATCTCAGGGTCGTGGGTTTGAGCCC
20469	Yes	AATCTCAGGGTCGTGGGTTTGAGCCCT
20470	Yes	AATCTCAGGGTCGTGGGTTTGAGCCCTG
20471	Yes	AATCTCAGGGTCGTGGGTTTGAGCCCTGT
20472	Yes	AATCTCAGGGTCGTGGGTTTGAGCCCTGTG
20473	Yes	AATCTCAGGGTCGTGGGTTTGAGCCCTGTGT
20474	Yes	AATCTCAGGGTCGTGGGTTTGAGCCCTGTGTT
20475	Yes	AATCTCAGGGTCGTGGGTTTGAGCCCTGTGTTG
20476	Yes	AATCTCAGGGTCGTGGGTTTGAGCCCTGTGTTGA
20477	Yes	AATCTCAGGGTCGTGGGTTTGAGCCCTGTGTTGAG
20478	Yes	AATCTCAGGGTCGTGGGTTTGAGCCCTGTGTTGAGC
20479	Yes	AATCTCAGGGTCGTGGGTTTGAGCCCTGTGTTGAGCA
20480	Yes	AATCTCAGGGTCGTGGGTTTGAGCCCTGTGTTGAGCAC
20481	Yes	AATCTCAGGGTCGTGGGTTTGAGCCCTGTGTTGAGCACC
20482	Yes	AATCTCAGGGTCGTGGGTTTGAGCCCTGTGTTGAGCACCA
20483	No	AATCTCAGGGTTGTGG
20484	Yes	AATCTCAGGGTTGTGGA
20485	Yes	AATCTCAGGGTTGTGGAT
20486	Yes	AATCTCAGGGTTGTGGATT
20487	Yes	AATCTCAGGGTTGTGGATTC
20488	Yes	AATCTCAGGGTTGTGGATTCG
20489	Yes	AATCTCAGGGTTGTGGATTCGT

20490	Yes	AATCTCAGGGTTGTGGATTCGTG
20491	Yes	AATCTCAGGGTTGTGGATTCGTGC
20492	Yes	AATCTCAGGGTTGTGGATTCGTGCC
20493	Yes	AATCTCAGGGTTGTGGATTCGTGCCC
20494	Yes	AATCTCAGGGTTGTGGATTCGTGCCCC
20495	Yes	AATCTCAGGGTTGTGGATTCGTGCCCCA
20496	Yes	AATCTCAGGGTTGTGGATTCGTGCCCCAT
20497	Yes	AATCTCAGGGTTGTGGATTCGTGCCCCATG
20498	Yes	AATCTCAGGGTTGTGGATTCGTGCCCCATGC
20499	Yes	AATCTCAGGGTTGTGGATTCGTGCCCCATGCT
20500	Yes	AATCTCAGGGTTGTGGATTCGTGCCCCATGCTG
20501	Yes	AATCTCAGGGTTGTGGATTCGTGCCCCATGCTGG
20502	Yes	AATCTCAGGGTTGTGGATTCGTGCCCCATGCTGGG
20503	Yes	AATCTCAGGGTTGTGGATTCGTGCCCCATGCTGGGT
20504	Yes	AATCTCAGGGTTGTGGATTCGTGCCCCATGCTGGGTG
20505	Yes	AATCTCAGGGTTGTGGATTCGTGCCCCATGCTGGGTGC
20506	Yes	AATCTCAGGGTTGTGGATTCGTGCCCCATGCTGGGTGCC
20507	Yes	AATCTCAGGGTTGTGGATTCGTGCCCCATGCTGGGTGCCA
20508	Yes	AATCTCCGTGGAGCAA
20509	Yes	AATCTCCGTGGAGCAAT
20510	Yes	AATCTCCGTGGAGCAATT
20511	Yes	AATCTCCGTGGAGCAATTG
20512	Yes	AATCTCCGTGGAGCAATTGG
20513	Yes	AATCTCCGTGGAGCAATTGGT
20514	Yes	AATCTCCGTGGAGCAATTGGTT
20515	Yes	AATCTCCGTGGAGCAATTGGTTA
20516	Yes	AATCTCCGTGGAGCAATTGGTTAG
20517	Yes	AATCTCCGTGGAGCAATTGGTTAGC
20518	Yes	AATCTCCGTGGAGCAATTGGTTAGCG
20519	Yes	AATCTCCGTGGAGCAATTGGTTAGCGC
20520	Yes	AATCTCCGTGGAGCAATTGGTTAGCGCG
20521	Yes	AATCTCCGTGGAGCAATTGGTTAGCGCGT
20522	Yes	AATCTCCGTGGAGCAATTGGTTAGCGCGTT
20523	Yes	AATCTCCGTGGAGCAATTGGTTAGCGCGTTC
20524	Yes	AATCTCCGTGGAGCAATTGGTTAGCGCGTTCG
20525	Yes	AATCTCCGTGGAGCAATTGGTTAGCGCGTTCGG
20526	Yes	AATCTCCGTGGAGCAATTGGTTAGCGCGTTCGGC
20527	Yes	AATCTCCGTGGAGCAATTGGTTAGCGCGTTCGGCC
20528	Yes	AATCTCCGTGGAGCAATTGGTTAGCGCGTTCGGCCG
20529	Yes	AATCTCCGTGGAGCAATTGGTTAGCGCGTTCGGCCGT
20530	Yes	AATCTCCGTGGAGCAATTGGTTAGCGCGTTCGGCCGTT
20531	Yes	AATCTCCGTGGAGCAATTGGTTAGCGCGTTCGGCCGTTA

20532	Yes	AATCTCCGTGGAGCAATTGGTTAGCGCGTTCGGCCGTTAA
20533	Yes	AATCTCCGTGGAGCAATTGGTTAGCGCGTTCGGCCGTTAAC
20534	Yes	AATCTCCGTGGAGCAATTGGTTAGCGCGTTCGGCCGTTAACC
20535	Yes	AATCTCCGTGGAGCAATTGGTTAGCGCGTTCGGCCGTTAACCG
20536	Yes	AATCTCCGTGGAGCAATTGGTTAGCGCGTTCGGCCGTTAACCGG
20537	Yes	AATCTCCGTGGAGCAATTGGTTAGCGCGTTCGGCCGTTAACCGGA
20538	Yes	AATCTCCGTGGAGCAATTGGTTAGCGCGTTCGGCCGTTAACCGGAA
20539	Yes	AATCTCCGTGGAGCAATTGGTTAGCGCGTTCGGCCGTTAACCGGAAA
20540	Yes	AATCTCCGTGGAGCAATTGGTTAGCGCGTTCGGCCGTTAACCGGAAAG
20541	Yes	AATCTCCGTGGAGCAATTGGTTAGCGCGTTCGGCCGTTAACCGGAAAGT
20542	Yes	AATCTCCGTGGAGCAATTGGTTAGCGCGTTCGGCCGTTAACCGGAAAGTT
20543	Yes	AATCTCCGTGGAGCAATTGGTTAGCGCGTTCGGCT
20544	Yes	AATCTCCGTGGAGCAATTGGTTAGCGCGTTCGGCTG
20545	Yes	AATCTCCGTGGAGCAATTGGTTAGCGCGTTCGGCTGT
20546	Yes	AATCTCCGTGGAGCAATTGGTTAGCGCGTTCGGCTGTT
20547	Yes	AATCTCCGTGGAGCAATTGGTTAGCGCGTTCGGCTGTTA
20548	Yes	AATCTCCGTGGAGCAATTGGTTAGCGCGTTCGGCTGTTAA
20549	Yes	AATCTCCGTGGAGCAATTGGTTAGCGCGTTCGGCTGTTAAC
20550	Yes	AATCTCCGTGGAGCAATTGGTTAGCGCGTTCGGCTGTTAACC
20551	Yes	AATCTCCGTGGAGCAATTGGTTAGCGCGTTCGGCTGTTAACCG
20552	Yes	AATCTCCGTGGAGCAATTGGTTAGCGCGTTCGGCTGTTAACCGG
20553	Yes	AATCTCCGTGGAGCAATTGGTTAGCGCGTTCGGCTGTTAACCGGA
20554	Yes	AATCTCCGTGGAGCAATTGGTTAGCGCGTTCGGCTGTTAACCGGAA
20555	Yes	AATCTCCGTGGAGCAATTGGTTAGCGCGTTCGGCTGTTAACCGGAAA
20556	Yes	AATCTCCGTGGAGCAATTGGTTAGCGCGTTCGGCTGTTAACCGGAAAG
20557	Yes	AATCTCCGTGGAGCAATTGGTTAGCGCGTTCGGCTGTTAACCGGAAAGT
20558	Yes	AATCTCCGTGGAGCAATTGGTTAGCGCGTTCGGCTGTTAACCGGAAAGTT
20559	No	AATCTCGCTGGGGCCT
20560	Yes	AATCTCGCTGGGGCCTC
20561	Yes	AATCTCGCTGGGGCCTCC
20562	Yes	AATCTCGCTGGGGCCTCCA
20563	Yes	AATCTCGGTGGAACCT
20564	Yes	AATCTCGGTGGAACCTC
20565	Yes	AATCTCGGTGGAACCTCC
20566	Yes	AATCTCGGTGGAACCTCCA
20567	Yes	AATCTCGGTGGGACCC
20568	Yes	AATCTCGGTGGGACCCC
20569	Yes	AATCTCGGTGGGACCCCC
20570	Yes	AATCTCGGTGGGACCCCCA
20571	Yes	AATCTCGGTGGGACCT
20572	Yes	AATCTCGGTGGGACCTC
20573	Yes	AATCTCGGTGGGACCTCC

20574	Yes	AATCTCGGTGGGACCTCCA
20575	Yes	AATCTCGGTGGTACCT
20576	Yes	AATCTCGGTGGTACCTC
20577	Yes	AATCTCGGTGGTACCTCC
20578	Yes	AATCTCGGTGGTACCTCCA
20579	No	AATCTGAAAGTCCTGA
20580	No	AATCTGAAAGTCCTGAG
20581	No	AATCTGAAAGTCCTGAGT
20582	Yes	AATCTGAAAGTCCTGAGTT
20583	Yes	AATCTGAAAGTCCTGAGTTC
20584	Yes	AATCTGAAAGTCCTGAGTTCA
20585	Yes	AATCTGAAAGTCCTGAGTTCAA
20586	Yes	AATCTGAAAGTCCTGAGTTCAAG
20587	Yes	AATCTGAAAGTCCTGAGTTCAAGC
20588	Yes	AATCTGAAAGTCCTGAGTTCAAGCC
20589	Yes	AATCTGAAAGTCCTGAGTTCAAGCCT
20590	Yes	AATCTGAAAGTCCTGAGTTCAAGCCTC
20591	Yes	AATCTGAAAGTCCTGAGTTCAAGCCTCA
20592	Yes	AATCTGAAAGTCCTGAGTTCAAGCCTCAG
20593	Yes	AATCTGAAAGTCCTGAGTTCAAGCCTCAGA
20594	Yes	AATCTGAAAGTCCTGAGTTCAAGCCTCAGAG
20595	Yes	AATCTGAAAGTCCTGAGTTCAAGCCTCAGAGA
20596	Yes	AATCTGAAAGTCCTGAGTTCAAGCCTCAGAGAG
20597	Yes	AATCTGAAAGTCCTGAGTTCAAGCCTCAGAGAGG
20598	Yes	AATCTGAAAGTCCTGAGTTCAAGCCTCAGAGAGGG
20599	Yes	AATCTGAAAGTCCTGAGTTCAAGCCTCAGAGAGGGC
20600	Yes	AATCTGAAAGTCCTGAGTTCAAGCCTCAGAGAGGGCA
20601	Yes	AATCTGAAAGTCCTGAGTTCAAGCCTCAGAGAGGGCAC
20602	Yes	AATCTGAAAGTCCTGAGTTCAAGCCTCAGAGAGGGCACC
20603	Yes	AATCTGAAAGTCCTGAGTTCAAGCCTCAGAGAGGGCACCA
20604	No	AATCTGAAGGTCCTGA
20605	No	AATCTGAAGGTCCTGAG
20606	No	AATCTGAAGGTCCTGAGT
20607	Yes	AATCTGAAGGTCCTGAGTT
20608	Yes	AATCTGAAGGTCCTGAGTTC
20609	Yes	AATCTGAAGGTCCTGAGTTCA
20610	Yes	AATCTGAAGGTCCTGAGTTCAA
20611	Yes	AATCTGAAGGTCCTGAGTTCAAG
20612	Yes	AATCTGAAGGTCCTGAGTTCAAGC
20613	Yes	AATCTGAAGGTCCTGAGTTCAAGCC
20614	Yes	AATCTGAAGGTCCTGAGTTCAAGCCT
20615	Yes	AATCTGAAGGTCCTGAGTTCAAGCCTC

20616	Yes	AATCTGAAGGTCCTGAGTTCAAGCCTCA
20617	Yes	AATCTGAAGGTCCTGAGTTCAAGCCTCAG
20618	Yes	AATCTGAAGGTCCTGAGTTCAAGCCTCAGA
20619	Yes	AATCTGAAGGTCCTGAGTTCAAGCCTCAGAG
20620	Yes	AATCTGAAGGTCCTGAGTTCAAGCCTCAGAGA
20621	Yes	AATCTGAAGGTCCTGAGTTCAAGCCTCAGAGAG
20622	Yes	AATCTGAAGGTCCTGAGTTCAAGCCTCAGAGAGG
20623	Yes	AATCTGAAGGTCCTGAGTTCAAGCCTCAGAGAGGG
20624	Yes	AATCTGAAGGTCCTGAGTTCAAGCCTCAGAGAGGGC
20625	Yes	AATCTGAAGGTCCTGAGTTCAAGCCTCAGAGAGGGCA
20626	Yes	AATCTGAAGGTCCTGAGTTCAAGCCTCAGAGAGGGCAC
20627	Yes	AATCTGAAGGTCCTGAGTTCAAGCCTCAGAGAGGGCACC
20628	Yes	AATCTGAAGGTCCTGAGTTCAAGCCTCAGAGAGGGCACCA
20629	Yes	AATCTGAAGGTCCTGAGTTCG
20630	Yes	AATCTGAAGGTCCTGAGTTCGA
20631	Yes	AATCTGAAGGTCCTGAGTTCGAA
20632	Yes	AATCTGAAGGTCCTGAGTTCGAAC
20633	Yes	AATCTGAAGGTCCTGAGTTCGAACC
20634	Yes	AATCTGAAGGTCCTGAGTTCGAACCT
20635	Yes	AATCTGAAGGTCCTGAGTTCGAACCTC
20636	Yes	AATCTGAAGGTCCTGAGTTCGAACCTCA
20637	Yes	AATCTGAAGGTCCTGAGTTCGAACCTCAG
20638	Yes	AATCTGAAGGTCCTGAGTTCGAACCTCAGA
20639	Yes	AATCTGAAGGTCCTGAGTTCGAACCTCAGAG
20640	Yes	AATCTGAAGGTCCTGAGTTCGAACCTCAGAGG
20641	Yes	AATCTGAAGGTCCTGAGTTCGAACCTCAGAGGG
20642	Yes	AATCTGAAGGTCCTGAGTTCGAACCTCAGAGGGG
20643	Yes	AATCTGAAGGTCCTGAGTTCGAACCTCAGAGGGGG
20644	Yes	AATCTGAAGGTCCTGAGTTCGAACCTCAGAGGGGGC
20645	Yes	AATCTGAAGGTCCTGAGTTCGAACCTCAGAGGGGGCA
20646	Yes	AATCTGAAGGTCCTGAGTTCGAACCTCAGAGGGGGCAC
20647	Yes	AATCTGAAGGTCCTGAGTTCGAACCTCAGAGGGGGCACC
20648	Yes	AATCTGAAGGTCCTGAGTTCGAACCTCAGAGGGGGCACCA
20649	Yes	AATCTGAAGGTCCTGAGTTCGAG
20650	Yes	AATCTGAAGGTCCTGAGTTCGAGC
20651	Yes	AATCTGAAGGTCCTGAGTTCGAGCC
20652	Yes	AATCTGAAGGTCCTGAGTTCGAGCCT
20653	Yes	AATCTGAAGGTCCTGAGTTCGAGCCTC
20654	Yes	AATCTGAAGGTCCTGAGTTCGAGCCTCA
20655	Yes	AATCTGAAGGTCCTGAGTTCGAGCCTCAG
20656	Yes	AATCTGAAGGTCCTGAGTTCGAGCCTCAGA
20657	Yes	AATCTGAAGGTCCTGAGTTCGAGCCTCAGAG

20658	Yes	AATCTGAAGGTCCTGAGTTCGAGCCTCAGAGA
20659	Yes	AATCTGAAGGTCCTGAGTTCGAGCCTCAGAGAG
20660	Yes	AATCTGAAGGTCCTGAGTTCGAGCCTCAGAGAGG
20661	Yes	AATCTGAAGGTCCTGAGTTCGAGCCTCAGAGAGGG
20662	Yes	AATCTGAAGGTCCTGAGTTCGAGCCTCAGAGAGGGC
20663	Yes	AATCTGAAGGTCCTGAGTTCGAGCCTCAGAGAGGGCA
20664	Yes	AATCTGAAGGTCCTGAGTTCGAGCCTCAGAGAGGGCAC
20665	Yes	AATCTGAAGGTCCTGAGTTCGAGCCTCAGAGAGGGCACC
20666	Yes	AATCTGAAGGTCCTGAGTTCGAGCCTCAGAGAGGGCACCA
20667	Yes	AATCTGAAGGTCGTGA
20668	Yes	AATCTGAAGGTCGTGAG
20669	Yes	AATCTGAAGGTCGTGAGT
20670	Yes	AATCTGAAGGTCGTGAGTT
20671	Yes	AATCTGAAGGTCGTGAGTTC
20672	Yes	AATCTGAAGGTCGTGAGTTCG
20673	Yes	AATCTGAAGGTCGTGAGTTCGA
20674	Yes	AATCTGAAGGTCGTGAGTTCGAG
20675	Yes	AATCTGAAGGTCGTGAGTTCGAGC
20676	Yes	AATCTGAAGGTCGTGAGTTCGAGCC
20677	Yes	AATCTGAAGGTCGTGAGTTCGAGCCT
20678	Yes	AATCTGAAGGTCGTGAGTTCGAGCCTC
20679	Yes	AATCTGAAGGTCGTGAGTTCGAGCCTCA
20680	Yes	AATCTGAAGGTCGTGAGTTCGAGCCTCAC
20681	Yes	AATCTGAAGGTCGTGAGTTCGAGCCTCACA
20682	Yes	AATCTGAAGGTCGTGAGTTCGAGCCTCACAC
20683	Yes	AATCTGAAGGTCGTGAGTTCGAGCCTCACACG
20684	Yes	AATCTGAAGGTCGTGAGTTCGAGCCTCACACGG
20685	Yes	AATCTGAAGGTCGTGAGTTCGAGCCTCACACGGG
20686	Yes	AATCTGAAGGTCGTGAGTTCGAGCCTCACACGGGG
20687	Yes	AATCTGAAGGTCGTGAGTTCGAGCCTCACACGGGGC
20688	Yes	AATCTGAAGGTCGTGAGTTCGAGCCTCACACGGGGCA
20689	Yes	AATCTGAAGGTCGTGAGTTCGAGCCTCACACGGGGCAC
20690	Yes	AATCTGAAGGTCGTGAGTTCGAGCCTCACACGGGGCACC
20691	Yes	AATCTGAAGGTCGTGAGTTCGAGCCTCACACGGGGCACCA
20692	Yes	AATCTGAAGGTCGTGAGTTCGAT
20693	Yes	AATCTGAAGGTCGTGAGTTCGATC
20694	Yes	AATCTGAAGGTCGTGAGTTCGATCC
20695	Yes	AATCTGAAGGTCGTGAGTTCGATCCT
20696	Yes	AATCTGAAGGTCGTGAGTTCGATCCTC
20697	Yes	AATCTGAAGGTCGTGAGTTCGATCCTCA
20698	Yes	AATCTGAAGGTCGTGAGTTCGATCCTCAC
20699	Yes	AATCTGAAGGTCGTGAGTTCGATCCTCACA

20700	Yes	AATCTGAAGGTCGTGAGTTCGATCCTCACAC
20701	Yes	AATCTGAAGGTCGTGAGTTCGATCCTCACACG
20702	Yes	AATCTGAAGGTCGTGAGTTCGATCCTCACACGG
20703	Yes	AATCTGAAGGTCGTGAGTTCGATCCTCACACGGG
20704	Yes	AATCTGAAGGTCGTGAGTTCGATCCTCACACGGGG
20705	Yes	AATCTGAAGGTCGTGAGTTCGATCCTCACACGGGGC
20706	Yes	AATCTGAAGGTCGTGAGTTCGATCCTCACACGGGGCA
20707	Yes	AATCTGAAGGTCGTGAGTTCGATCCTCACACGGGGCAC
20708	Yes	AATCTGAAGGTCGTGAGTTCGATCCTCACACGGGGCACC
20709	Yes	AATCTGAAGGTCGTGAGTTCGATCCTCACACGGGGCACCA
20710	No	AATCTGAATGGTCCTG
20711	Yes	AATCTGAATGGTCCTGA
20712	Yes	AATCTGAATGGTCCTGAG
20713	Yes	AATCTGAATGGTCCTGAGT
20714	Yes	AATCTGAATGGTCCTGAGTT
20715	Yes	AATCTGAATGGTCCTGAGTTC
20716	Yes	AATCTGAATGGTCCTGAGTTCA
20717	Yes	AATCTGAATGGTCCTGAGTTCAA
20718	Yes	AATCTGAATGGTCCTGAGTTCAAG
20719	Yes	AATCTGAATGGTCCTGAGTTCAAGC
20720	Yes	AATCTGAATGGTCCTGAGTTCAAGCC
20721	Yes	AATCTGAATGGTCCTGAGTTCAAGCCT
20722	Yes	AATCTGAATGGTCCTGAGTTCAAGCCTC
20723	Yes	AATCTGAATGGTCCTGAGTTCAAGCCTCA
20724	Yes	AATCTGAATGGTCCTGAGTTCAAGCCTCAG
20725	Yes	AATCTGAATGGTCCTGAGTTCAAGCCTCAGA
20726	Yes	AATCTGAATGGTCCTGAGTTCAAGCCTCAGAG
20727	Yes	AATCTGAATGGTCCTGAGTTCAAGCCTCAGAGG
20728	Yes	AATCTGAATGGTCCTGAGTTCAAGCCTCAGAGGG
20729	Yes	AATCTGAATGGTCCTGAGTTCAAGCCTCAGAGGGG
20730	Yes	AATCTGAATGGTCCTGAGTTCAAGCCTCAGAGGGGG
20731	Yes	AATCTGAATGGTCCTGAGTTCAAGCCTCAGAGGGGGC
20732	Yes	AATCTGAATGGTCCTGAGTTCAAGCCTCAGAGGGGGCA
20733	Yes	AATCTGAATGGTCCTGAGTTCAAGCCTCAGAGGGGGCAC
20734	Yes	AATCTGAATGGTCCTGAGTTCAAGCCTCAGAGGGGGCACC
20735	Yes	AATCTGAATGGTCCTGAGTTCAAGCCTCAGAGGGGGCACCA
20736	No	AATCTGACAACAGAGG
20737	No	AATCTGACAACAGAGGC
20738	No	AATCTGACAACAGAGGCT
20739	Yes	AATCTGACAACAGAGGCTT
20740	Yes	AATCTGACAACAGAGGCTTA
20741	Yes	AATCTGACAACAGAGGCTTAC

20742	Yes	AATCTGACAACAGAGGCTTACG
20743	Yes	AATCTGACAACAGAGGCTTACGA
20744	Yes	AATCTGACAACAGAGGCTTACGAC
20745	Yes	AATCTGACAACAGAGGCTTACGACC
20746	Yes	AATCTGACAACAGAGGCTTACGACCC
20747	Yes	AATCTGACAACAGAGGCTTACGACCCC
20748	Yes	AATCTGACAACAGAGGCTTACGACCCCT
20749	Yes	AATCTGACAACAGAGGCTTACGACCCCTT
20750	Yes	AATCTGACAACAGAGGCTTACGACCCCTTA
20751	Yes	AATCTGACAACAGAGGCTTACGACCCCTTAT
20752	Yes	AATCTGACAACAGAGGCTTACGACCCCTTATT
20753	Yes	AATCTGACAACAGAGGCTTACGACCCCTTATTT
20754	Yes	AATCTGACAACAGAGGCTTACGACCCCTTATTTA
20755	Yes	AATCTGACAACAGAGGCTTACGACCCCTTATTTAC
20756	Yes	AATCTGACAACAGAGGCTTACGACCCCTTATTTACC
20757	Yes	AATCTGACAACAGAGGCTTACGACCCCTTATTTACCC
20758	Yes	AATCTGACAACAGAGGCTTACGACCCCTTATTTACCCC
20759	Yes	AATCTGACAACAGAGGCTTACGACCCCTTATTTACCCCA
20760	No	AATCTGACGGTGCAGG
20761	Yes	AATCTGACGGTGCAGGG
20762	Yes	AATCTGACGGTGCAGGGT
20763	Yes	AATCTGACGGTGCAGGGTT
20764	Yes	AATCTGACGGTGCAGGGTTC
20765	Yes	AATCTGACGGTGCAGGGTTCA
20766	Yes	AATCTGACGGTGCAGGGTTCAA
20767	Yes	AATCTGACGGTGCAGGGTTCAAG
20768	Yes	AATCTGACGGTGCAGGGTTCAAGT
20769	Yes	AATCTGACGGTGCAGGGTTCAAGTC
20770	Yes	AATCTGACGGTGCAGGGTTCAAGTCC
20771	Yes	AATCTGACGGTGCAGGGTTCAAGTCCC
20772	Yes	AATCTGACGGTGCAGGGTTCAAGTCCCT
20773	Yes	AATCTGACGGTGCAGGGTTCAAGTCCCTG
20774	Yes	AATCTGACGGTGCAGGGTTCAAGTCCCTGT
20775	Yes	AATCTGACGGTGCAGGGTTCAAGTCCCTGTT
20776	Yes	AATCTGACGGTGCAGGGTTCAAGTCCCTGTTC
20777	Yes	AATCTGACGGTGCAGGGTTCAAGTCCCTGTTCA
20778	Yes	AATCTGACGGTGCAGGGTTCAAGTCCCTGTTCAG
20779	Yes	AATCTGACGGTGCAGGGTTCAAGTCCCTGTTCAGG
20780	Yes	AATCTGACGGTGCAGGGTTCAAGTCCCTGTTCAGGC
20781	Yes	AATCTGACGGTGCAGGGTTCAAGTCCCTGTTCAGGCG
20782	Yes	AATCTGACGGTGCAGGGTTCAAGTCCCTGTTCAGGCGC
20783	Yes	AATCTGACGGTGCAGGGTTCAAGTCCCTGTTCAGGCGCC

20784	Yes	AATCTGACGGTGCAGGGTTCAAGTCCCTGTTCAGGCGCCA
20785	Yes	AATCTGAGGGTCCAAG
20786	Yes	AATCTGAGGGTCCAAGG
20787	Yes	AATCTGAGGGTCCAAGGT
20788	Yes	AATCTGAGGGTCCAAGGTT
20789	Yes	AATCTGAGGGTCCAAGGTTC
20790	Yes	AATCTGAGGGTCCAAGGTTCA
20791	Yes	AATCTGAGGGTCCAAGGTTCAT
20792	Yes	AATCTGAGGGTCCAAGGTTCATG
20793	Yes	AATCTGAGGGTCCAAGGTTCATGT
20794	Yes	AATCTGAGGGTCCAAGGTTCATGTC
20795	Yes	AATCTGAGGGTCCAAGGTTCATGTCC
20796	Yes	AATCTGAGGGTCCAAGGTTCATGTCCC
20797	Yes	AATCTGAGGGTCCAAGGTTCATGTCCCT
20798	Yes	AATCTGAGGGTCCAAGGTTCATGTCCCTT
20799	Yes	AATCTGAGGGTCCAAGGTTCATGTCCCTTT
20800	Yes	AATCTGAGGGTCCAAGGTTCATGTCCCTTTT
20801	Yes	AATCTGAGGGTCCAAGGTTCATGTCCCTTTTT
20802	Yes	AATCTGAGGGTCCAAGGTTCATGTCCCTTTTTG
20803	Yes	AATCTGAGGGTCCAAGGTTCATGTCCCTTTTTGG
20804	Yes	AATCTGAGGGTCCAAGGTTCATGTCCCTTTTTGGG
20805	Yes	AATCTGAGGGTCCAAGGTTCATGTCCCTTTTTGGGT
20806	Yes	AATCTGAGGGTCCAAGGTTCATGTCCCTTTTTGGGTG
20807	Yes	AATCTGAGGGTCCAAGGTTCATGTCCCTTTTTGGGTGC
20808	Yes	AATCTGAGGGTCCAAGGTTCATGTCCCTTTTTGGGTGCC
20809	Yes	AATCTGAGGGTCCAAGGTTCATGTCCCTTTTTGGGTGCCA
20810	No	AATCTGAGGGTCCAGG
20811	No	AATCTGAGGGTCCAGGG
20812	No	AATCTGAGGGTCCAGGGT
20813	No	AATCTGAGGGTCCAGGGTT
20814	No	AATCTGAGGGTCCAGGGTTC
20815	No	AATCTGAGGGTCCAGGGTTCA
20816	Yes	AATCTGAGGGTCCAGGGTTCAA
20817	Yes	AATCTGAGGGTCCAGGGTTCAAG
20818	Yes	AATCTGAGGGTCCAGGGTTCAAGT
20819	Yes	AATCTGAGGGTCCAGGGTTCAAGTC
20820	Yes	AATCTGAGGGTCCAGGGTTCAAGTCC
20821	Yes	AATCTGAGGGTCCAGGGTTCAAGTCCC
20822	Yes	AATCTGAGGGTCCAGGGTTCAAGTCCCT
20823	Yes	AATCTGAGGGTCCAGGGTTCAAGTCCCTG
20824	Yes	AATCTGAGGGTCCAGGGTTCAAGTCCCTGT
20825	Yes	AATCTGAGGGTCCAGGGTTCAAGTCCCTGTC

20826	Yes	AATCTGAGGGTCCAGGGTTCAAGTCCCTGTCC
20827	Yes	AATCTGAGGGTCCAGGGTTCAAGTCCCTGTCCA
20828	Yes	AATCTGAGGGTCCAGGGTTCAAGTCCCTGTCCAG
20829	Yes	AATCTGAGGGTCCAGGGTTCAAGTCCCTGTCCAGG
20830	Yes	AATCTGAGGGTCCAGGGTTCAAGTCCCTGTCCAGGC
20831	Yes	AATCTGAGGGTCCAGGGTTCAAGTCCCTGTCCAGGCG
20832	Yes	AATCTGAGGGTCCAGGGTTCAAGTCCCTGTCCAGGCGC
20833	Yes	AATCTGAGGGTCCAGGGTTCAAGTCCCTGTCCAGGCGCC
20834	Yes	AATCTGAGGGTCCAGGGTTCAAGTCCCTGTCCAGGCGCCA
20835	Yes	AATCTGAGGGTCCAGGGTTCAAGTCCCTGTT
20836	Yes	AATCTGAGGGTCCAGGGTTCAAGTCCCTGTTC
20837	Yes	AATCTGAGGGTCCAGGGTTCAAGTCCCTGTTCA
20838	Yes	AATCTGAGGGTCCAGGGTTCAAGTCCCTGTTCAG
20839	Yes	AATCTGAGGGTCCAGGGTTCAAGTCCCTGTTCAGG
20840	Yes	AATCTGAGGGTCCAGGGTTCAAGTCCCTGTTCAGGC
20841	Yes	AATCTGAGGGTCCAGGGTTCAAGTCCCTGTTCAGGCA
20842	Yes	AATCTGAGGGTCCAGGGTTCAAGTCCCTGTTCAGGCAC
20843	Yes	AATCTGAGGGTCCAGGGTTCAAGTCCCTGTTCAGGCACC
20844	Yes	AATCTGAGGGTCCAGGGTTCAAGTCCCTGTTCAGGCACCA
20845	Yes	AATCTGAGGGTCCAGGGTTCAAGTCCCTGTTCAGGCG
20846	Yes	AATCTGAGGGTCCAGGGTTCAAGTCCCTGTTCAGGCGC
20847	Yes	AATCTGAGGGTCCAGGGTTCAAGTCCCTGTTCAGGCGCC
20848	Yes	AATCTGAGGGTCCAGGGTTCAAGTCCCTGTTCAGGCGCCA
20849	Yes	AATCTGAGGGTCCAGGGTTCAAGTCCCTGTTCG
20850	Yes	AATCTGAGGGTCCAGGGTTCAAGTCCCTGTTCGG
20851	Yes	AATCTGAGGGTCCAGGGTTCAAGTCCCTGTTCGGG
20852	Yes	AATCTGAGGGTCCAGGGTTCAAGTCCCTGTTCGGGC
20853	Yes	AATCTGAGGGTCCAGGGTTCAAGTCCCTGTTCGGGCG
20854	Yes	AATCTGAGGGTCCAGGGTTCAAGTCCCTGTTCGGGCGC
20855	Yes	AATCTGAGGGTCCAGGGTTCAAGTCCCTGTTCGGGCGCC
20856	Yes	AATCTGAGGGTCCAGGGTTCAAGTCCCTGTTCGGGCGCCA
20857	Yes	AATCTGAGGGTCCAGGGTTCAAGTCCT
20858	Yes	AATCTGAGGGTCCAGGGTTCAAGTCCTC
20859	Yes	AATCTGAGGGTCCAGGGTTCAAGTCCTCG
20860	Yes	AATCTGAGGGTCCAGGGTTCAAGTCCTCGT
20861	Yes	AATCTGAGGGTCCAGGGTTCAAGTCCTCGTT
20862	Yes	AATCTGAGGGTCCAGGGTTCAAGTCCTCGTTC
20863	Yes	AATCTGAGGGTCCAGGGTTCAAGTCCTCGTTCG
20864	Yes	AATCTGAGGGTCCAGGGTTCAAGTCCTCGTTCGG
20865	Yes	AATCTGAGGGTCCAGGGTTCAAGTCCTCGTTCGGG
20866	Yes	AATCTGAGGGTCCAGGGTTCAAGTCCTCGTTCGGGC
20867	Yes	AATCTGAGGGTCCAGGGTTCAAGTCCTCGTTCGGGCA

20868	Yes	AATCTGAGGGTCCAGGGTTCAAGTCCTCGTTCGGGCAC
20869	Yes	AATCTGAGGGTCCAGGGTTCAAGTCCTCGTTCGGGCACC
20870	Yes	AATCTGAGGGTCCAGGGTTCAAGTCCTCGTTCGGGCACCA
20871	No	AATCTGAGGGTCCAGGGTTCAT
20872	Yes	AATCTGAGGGTCCAGGGTTCATG
20873	Yes	AATCTGAGGGTCCAGGGTTCATGT
20874	Yes	AATCTGAGGGTCCAGGGTTCATGTC
20875	Yes	AATCTGAGGGTCCAGGGTTCATGTCC
20876	Yes	AATCTGAGGGTCCAGGGTTCATGTCCC
20877	Yes	AATCTGAGGGTCCAGGGTTCATGTCCCT
20878	Yes	AATCTGAGGGTCCAGGGTTCATGTCCCTG
20879	Yes	AATCTGAGGGTCCAGGGTTCATGTCCCTGT
20880	Yes	AATCTGAGGGTCCAGGGTTCATGTCCCTGTT
20881	Yes	AATCTGAGGGTCCAGGGTTCATGTCCCTGTTC
20882	Yes	AATCTGAGGGTCCAGGGTTCATGTCCCTGTTCC
20883	Yes	AATCTGAGGGTCCAGGGTTCATGTCCCTGTTCCT
20884	Yes	AATCTGAGGGTCCAGGGTTCATGTCCCTGTTCCTT
20885	Yes	AATCTGAGGGTCCAGGGTTCATGTCCCTGTTCCTTA
20886	Yes	AATCTGAGGGTCCAGGGTTCATGTCCCTGTTCCTTAA
20887	Yes	AATCTGAGGGTCCAGGGTTCATGTCCCTGTTCCTTAAC
20888	Yes	AATCTGAGGGTCCAGGGTTCATGTCCCTGTTCCTTAACC
20889	Yes	AATCTGAGGGTCCAGGGTTCATGTCCCTGTTCCTTAACCA
20890	Yes	AATCTGAGGGTCCGGG
20891	Yes	AATCTGAGGGTCCGGGG
20892	Yes	AATCTGAGGGTCCGGGGT
20893	Yes	AATCTGAGGGTCCGGGGTT
20894	Yes	AATCTGAGGGTCCGGGGTTC
20895	Yes	AATCTGAGGGTCCGGGGTTCA
20896	Yes	AATCTGAGGGTCCGGGGTTCAA
20897	Yes	AATCTGAGGGTCCGGGGTTCAAG
20898	Yes	AATCTGAGGGTCCGGGGTTCAAGT
20899	Yes	AATCTGAGGGTCCGGGGTTCAAGTC
20900	Yes	AATCTGAGGGTCCGGGGTTCAAGTCC
20901	Yes	AATCTGAGGGTCCGGGGTTCAAGTCCC
20902	Yes	AATCTGAGGGTCCGGGGTTCAAGTCCCT
20903	Yes	AATCTGAGGGTCCGGGGTTCAAGTCCCTG
20904	Yes	AATCTGAGGGTCCGGGGTTCAAGTCCCTGT
20905	Yes	AATCTGAGGGTCCGGGGTTCAAGTCCCTGTT
20906	Yes	AATCTGAGGGTCCGGGGTTCAAGTCCCTGTTC
20907	Yes	AATCTGAGGGTCCGGGGTTCAAGTCCCTGTTCG
20908	Yes	AATCTGAGGGTCCGGGGTTCAAGTCCCTGTTCGG
20909	Yes	AATCTGAGGGTCCGGGGTTCAAGTCCCTGTTCGGG

20910	Yes	AATCTGAGGGTCCGGGGTTCAAGTCCCTGTTCGGGC
20911	Yes	AATCTGAGGGTCCGGGGTTCAAGTCCCTGTTCGGGCG
20912	Yes	AATCTGAGGGTCCGGGGTTCAAGTCCCTGTTCGGGCGC
20913	Yes	AATCTGAGGGTCCGGGGTTCAAGTCCCTGTTCGGGCGCC
20914	Yes	AATCTGAGGGTCCGGGGTTCAAGTCCCTGTTCGGGCGCCA
20915	No	AATCTGAGGGTCTAGG
20916	Yes	AATCTGAGGGTCTAGGG
20917	Yes	AATCTGAGGGTCTAGGGT
20918	Yes	AATCTGAGGGTCTAGGGTT
20919	Yes	AATCTGAGGGTCTAGGGTTC
20920	Yes	AATCTGAGGGTCTAGGGTTCA
20921	Yes	AATCTGAGGGTCTAGGGTTCAA
20922	Yes	AATCTGAGGGTCTAGGGTTCAAG
20923	Yes	AATCTGAGGGTCTAGGGTTCAAGT
20924	Yes	AATCTGAGGGTCTAGGGTTCAAGTC
20925	Yes	AATCTGAGGGTCTAGGGTTCAAGTCC
20926	Yes	AATCTGAGGGTCTAGGGTTCAAGTCCC
20927	Yes	AATCTGAGGGTCTAGGGTTCAAGTCCCT
20928	Yes	AATCTGAGGGTCTAGGGTTCAAGTCCCTG
20929	Yes	AATCTGAGGGTCTAGGGTTCAAGTCCCTGT
20930	Yes	AATCTGAGGGTCTAGGGTTCAAGTCCCTGTC
20931	Yes	AATCTGAGGGTCTAGGGTTCAAGTCCCTGTCC
20932	Yes	AATCTGAGGGTCTAGGGTTCAAGTCCCTGTCCA
20933	Yes	AATCTGAGGGTCTAGGGTTCAAGTCCCTGTCCAG
20934	Yes	AATCTGAGGGTCTAGGGTTCAAGTCCCTGTCCAGG
20935	Yes	AATCTGAGGGTCTAGGGTTCAAGTCCCTGTCCAGGC
20936	Yes	AATCTGAGGGTCTAGGGTTCAAGTCCCTGTCCAGGCG
20937	Yes	AATCTGAGGGTCTAGGGTTCAAGTCCCTGTCCAGGCGC
20938	Yes	AATCTGAGGGTCTAGGGTTCAAGTCCCTGTCCAGGCGCC
20939	Yes	AATCTGAGGGTCTAGGGTTCAAGTCCCTGTCCAGGCGCCA
20940	No	AATCTGAGTCATGACA
20941	Yes	AATCTGAGTCATGACAC
20942	Yes	AATCTGAGTCATGACACC
20943	Yes	AATCTGAGTCATGACACCA
20944	No	AATCTGCCTGGGTTCT
20945	Yes	AATCTGCCTGGGTTCTA
20946	Yes	AATCTGCCTGGGTTCTAG
20947	Yes	AATCTGCCTGGGTTCTAGT
20948	Yes	AATCTGCCTGGGTTCTAGTC
20949	Yes	AATCTGCCTGGGTTCTAGTCC
20950	Yes	AATCTGCCTGGGTTCTAGTCCC
20951	Yes	AATCTGCCTGGGTTCTAGTCCCA

20952	Yes	AATCTGCCTGGGTTCTAGTCCCAG
20953	Yes	AATCTGCCTGGGTTCTAGTCCCAGC
20954	Yes	AATCTGCCTGGGTTCTAGTCCCAGCT
20955	Yes	AATCTGCCTGGGTTCTAGTCCCAGCTG
20956	Yes	AATCTGCCTGGGTTCTAGTCCCAGCTGT
20957	Yes	AATCTGCCTGGGTTCTAGTCCCAGCTGTC
20958	Yes	AATCTGCCTGGGTTCTAGTCCCAGCTGTCT
20959	Yes	AATCTGCCTGGGTTCTAGTCCCAGCTGTCTC
20960	Yes	AATCTGCCTGGGTTCTAGTCCCAGCTGTCTCA
20961	Yes	AATCTGCCTGGGTTCTAGTCCCAGCTGTCTCAC
20962	Yes	AATCTGCCTGGGTTCTAGTCCCAGCTGTCTCACC
20963	Yes	AATCTGCCTGGGTTCTAGTCCCAGCTGTCTCACCA
20964	Yes	AATCTGGGTGCCCACT
20965	Yes	AATCTGGGTGCCCACTC
20966	Yes	AATCTGGGTGCCCACTCC
20967	Yes	AATCTGGGTGCCCACTCCA
20968	No	AATCTGGGTGCCCCCT
20969	No	AATCTGGGTGCCCCCTC
20970	Yes	AATCTGGGTGCCCCCTCC
20971	Yes	AATCTGGGTGCCCCCTCCA
20972	No	AATCTTAAAACTTTAT
20973	No	AATCTTAAAACTTTATA
20974	No	AATCTTAAAACTTTATAA
20975	No	AATCTTAAAACTTTATAAT
20976	Yes	AATCTTAAAACTTTATAATC
20977	Yes	AATCTTAAAACTTTATAATCA
20978	Yes	AATCTTAAAACTTTATAATCAG
20979	Yes	AATCTTAAAACTTTATAATCAGA
20980	Yes	AATCTTAAAACTTTATAATCAGAG
20981	Yes	AATCTTAAAACTTTATAATCAGAGG
20982	Yes	AATCTTAAAACTTTATAATCAGAGGT
20983	Yes	AATCTTAAAACTTTATAATCAGAGGTT
20984	Yes	AATCTTAAAACTTTATAATCAGAGGTTC
20985	Yes	AATCTTAAAACTTTATAATCAGAGGTTCG
20986	Yes	AATCTTAAAACTTTATAATCAGAGGTTCGA
20987	Yes	AATCTTAAAACTTTATAATCAGAGGTTCGAC
20988	Yes	AATCTTAAAACTTTATAATCAGAGGTTCGACT
20989	Yes	AATCTTAAAACTTTATAATCAGAGGTTCGACTC
20990	Yes	AATCTTAAAACTTTATAATCAGAGGTTCGACTCC
20991	Yes	AATCTTAAAACTTTATAATCAGAGGTTCGACTCCT
20992	Yes	AATCTTAAAACTTTATAATCAGAGGTTCGACTCCTC
20993	Yes	AATCTTAAAACTTTATAATCAGAGGTTCGACTCCTCT

20994	Yes	AATCTTAAAACTTTATAATCAGAGGTTCGACTCCTCTT
20995	Yes	AATCTTAAAACTTTATAATCAGAGGTTCGACTCCTCTTC
20996	Yes	AATCTTAAAACTTTATAATCAGAGGTTCGACTCCTCTTCT
20997	Yes	AATCTTAAAACTTTATAATCAGAGGTTCGACTCCTCTTCTT
20998	Yes	AATCTTAAAACTTTATAATCAGAGGTTCGACTCCTCTTCTTA
20999	Yes	AATCTTAAAACTTTATAATCAGAGGTTCGACTCCTCTTCTTAA
21000	Yes	AATCTTAAAACTTTATAATCAGAGGTTCGACTCCTCTTCTTAAC
21001	Yes	AATCTTAAAACTTTATAATCAGAGGTTCGACTCCTCTTCTTAACA
21002	Yes	AATCTTAAAACTTTATAATCAGAGGTTCGACTCCTCTTCTTAACAC
21003	Yes	AATCTTAAAACTTTATAATCAGAGGTTCGACTCCTCTTCTTAACACC
21004	Yes	AATCTTAAAACTTTATAATCAGAGGTTCGACTCCTCTTCTTAACACCA
21005	No	AATCTTAGCTTTGGGT
21006	No	AATCTTAGCTTTGGGTG
21007	No	AATCTTAGCTTTGGGTGC
21008	No	AATCTTAGCTTTGGGTGCT
21009	Yes	AATCTTAGCTTTGGGTGCTA
21010	Yes	AATCTTAGCTTTGGGTGCTAA
21011	Yes	AATCTTAGCTTTGGGTGCTAAT
21012	Yes	AATCTTAGCTTTGGGTGCTAATG
21013	Yes	AATCTTAGCTTTGGGTGCTAATGG
21014	Yes	AATCTTAGCTTTGGGTGCTAATGGT
21015	Yes	AATCTTAGCTTTGGGTGCTAATGGTG
21016	Yes	AATCTTAGCTTTGGGTGCTAATGGTGG
21017	Yes	AATCTTAGCTTTGGGTGCTAATGGTGGA
21018	Yes	AATCTTAGCTTTGGGTGCTAATGGTGGAG
21019	Yes	AATCTTAGCTTTGGGTGCTAATGGTGGAGT
21020	Yes	AATCTTAGCTTTGGGTGCTAATGGTGGAGTT
21021	Yes	AATCTTAGCTTTGGGTGCTAATGGTGGAGTTA
21022	Yes	AATCTTAGCTTTGGGTGCTAATGGTGGAGTTAA
21023	Yes	AATCTTAGCTTTGGGTGCTAATGGTGGAGTTAAA
21024	Yes	AATCTTAGCTTTGGGTGCTAATGGTGGAGTTAAAG
21025	Yes	AATCTTAGCTTTGGGTGCTAATGGTGGAGTTAAAGA
21026	Yes	AATCTTAGCTTTGGGTGCTAATGGTGGAGTTAAAGAC
21027	Yes	AATCTTAGCTTTGGGTGCTAATGGTGGAGTTAAAGACT
21028	Yes	AATCTTAGCTTTGGGTGCTAATGGTGGAGTTAAAGACTT
21029	Yes	AATCTTAGCTTTGGGTGCTAATGGTGGAGTTAAAGACTTT
21030	Yes	AATCTTAGCTTTGGGTGCTAATGGTGGAGTTAAAGACTTTT
21031	Yes	AATCTTAGCTTTGGGTGCTAATGGTGGAGTTAAAGACTTTTT
21032	Yes	AATCTTAGCTTTGGGTGCTAATGGTGGAGTTAAAGACTTTTTC
21033	Yes	AATCTTAGCTTTGGGTGCTAATGGTGGAGTTAAAGACTTTTTCT
21034	Yes	AATCTTAGCTTTGGGTGCTAATGGTGGAGTTAAAGACTTTTTCTC
21035	Yes	AATCTTAGCTTTGGGTGCTAATGGTGGAGTTAAAGACTTTTTCTCT

21036	Yes	AATCTTAGCTTTGGGTGCTAATGGTGGAGTTAAAGACTTTTTCTCTG
21037	Yes	AATCTTAGCTTTGGGTGCTAATGGTGGAGTTAAAGACTTTTTCTCTGA
21038	Yes	AATCTTAGCTTTGGGTGCTAATGGTGGAGTTAAAGACTTTTTCTCTGAC
21039	Yes	AATCTTAGCTTTGGGTGCTAATGGTGGAGTTAAAGACTTTTTCTCTGACC
21040	No	AATCTTGGTGGGACCT
21041	No	AATCTTGGTGGGACCTC
21042	No	AATCTTGGTGGGACCTCC
21043	No	AATCTTGGTGGGACCTCCA
21044	Yes	AATGAGGCTGAGGTCG
21045	Yes	AATGAGGCTGAGGTCGT
21046	Yes	AATGAGGCTGAGGTCGTG
21047	Yes	AATGAGGCTGAGGTCGTGG
21048	Yes	AATGAGGCTGAGGTCGTGGG
21049	Yes	AATGAGGCTGAGGTCGTGGGT
21050	Yes	AATGAGGCTGAGGTCGTGGGTT
21051	Yes	AATGAGGCTGAGGTCGTGGGTTC
21052	Yes	AATGAGGCTGAGGTCGTGGGTTCA
21053	Yes	AATGAGGCTGAGGTCGTGGGTTCAA
21054	Yes	AATGAGGCTGAGGTCGTGGGTTCAAT
21055	Yes	AATGAGGCTGAGGTCGTGGGTTCAATC
21056	Yes	AATGAGGCTGAGGTCGTGGGTTCAATCC
21057	Yes	AATGAGGCTGAGGTCGTGGGTTCAATCCC
21058	Yes	AATGAGGCTGAGGTCGTGGGTTCAATCCCC
21059	Yes	AATGAGGCTGAGGTCGTGGGTTCAATCCCCA
21060	Yes	AATGAGGCTGAGGTCGTGGGTTCAATCCCCAC
21061	Yes	AATGAGGCTGAGGTCGTGGGTTCAATCCCCACC
21062	Yes	AATGAGGCTGAGGTCGTGGGTTCAATCCCCACCG
21063	Yes	AATGAGGCTGAGGTCGTGGGTTCAATCCCCACCGG
21064	Yes	AATGAGGCTGAGGTCGTGGGTTCAATCCCCACCGGG
21065	Yes	AATGAGGCTGAGGTCGTGGGTTCAATCCCCACCGGGC
21066	Yes	AATGAGGCTGAGGTCGTGGGTTCAATCCCCACCGGGCT
21067	Yes	AATGAGGCTGAGGTCGTGGGTTCAATCCCCACCGGGCTA
21068	Yes	AATGAGGCTGAGGTCGTGGGTTCAATCCCCACCGGGCTAT
21069	Yes	AATGAGGCTGAGGTCGTGGGTTCAATCCCCACCGGGCTATC
21070	Yes	AATGAGGCTGAGGTCGTGGGTTCAATCCCCACCGGGCTATCC
21071	Yes	AATGAGGCTGAGGTCGTGGGTTCAATCCCCACCGGGCTATCCA
21072	No	AATGATTTCGACTCAT
21073	No	AATGATTTCGACTCATT
21074	No	AATGATTTCGACTCATTA
21075	No	AATGATTTCGACTCATTAA
21076	No	AATGATTTCGACTCATTAAA
21077	No	AATGATTTCGACTCATTAAAT

21078	No	AATGATTTCGACTCATTAAATT
21079	No	AATGATTTCGACTCATTAAATTA
21080	No	AATGATTTCGACTCATTAAATTAT
21081	No	AATGATTTCGACTCATTAAATTATG
21082	No	AATGATTTCGACTCATTAAATTATGA
21083	No	AATGATTTCGACTCATTAAATTATGAT
21084	No	AATGATTTCGACTCATTAAATTATGATA
21085	No	AATGATTTCGACTCATTAAATTATGATAA
21086	No	AATGATTTCGACTCATTAAATTATGATAAT
21087	No	AATGATTTCGACTCATTAAATTATGATAATC
21088	No	AATGATTTCGACTCATTAAATTATGATAATCA
21089	No	AATGATTTCGACTCATTAAATTATGATAATCAT
21090	No	AATGATTTCGACTCATTAAATTATGATAATCATA
21091	No	AATGATTTCGACTCATTAAATTATGATAATCATAT
21092	No	AATGATTTCGACTCATTAAATTATGATAATCATATT
21093	No	AATGATTTCGACTCATTAAATTATGATAATCATATTT
21094	No	AATGATTTCGACTCATTAAATTATGATAATCATATTTA
21095	No	AATGATTTCGACTCATTAAATTATGATAATCATATTTAC
21096	No	AATGATTTCGACTCATTAAATTATGATAATCATATTTACC
21097	No	AATGATTTCGACTCATTAAATTATGATAATCATATTTACCA
21098	No	AATGATTTCGACTCATTAAATTATGATAATCATATTTACCAA
21099	Yes	AATGATTTCGACTCATTAAATTATGATAATCATATTTACCAAC
21100	Yes	AATGATTTCGACTCATTAAATTATGATAATCATATTTACCAACC
21101	Yes	AATGATTTCGACTCATTAAATTATGATAATCATATTTACCAACCA
21102	Yes	AATGCCAGGGTCGAGG
21103	Yes	AATGCCAGGGTCGAGGT
21104	Yes	AATGCCAGGGTCGAGGTT
21105	Yes	AATGCCAGGGTCGAGGTTT
21106	Yes	AATGCCAGGGTCGAGGTTTC
21107	Yes	AATGCCAGGGTCGAGGTTTCG
21108	Yes	AATGCCAGGGTCGAGGTTTCGA
21109	Yes	AATGCCAGGGTCGAGGTTTCGAT
21110	Yes	AATGCCAGGGTCGAGGTTTCGATC
21111	Yes	AATGCCAGGGTCGAGGTTTCGATCC
21112	Yes	AATGCCAGGGTCGAGGTTTCGATCCC
21113	Yes	AATGCCAGGGTCGAGGTTTCGATCCCC
21114	Yes	AATGCCAGGGTCGAGGTTTCGATCCCCG
21115	Yes	AATGCCAGGGTCGAGGTTTCGATCCCCGT
21116	Yes	AATGCCAGGGTCGAGGTTTCGATCCCCGTA
21117	Yes	AATGCCAGGGTCGAGGTTTCGATCCCCGTAC
21118	Yes	AATGCCAGGGTCGAGGTTTCGATCCCCGTACG
21119	Yes	AATGCCAGGGTCGAGGTTTCGATCCCCGTACGG

21120	Yes	AATGCCAGGGTCGAGGTTTCGATCCCCGTACGGG
21121	Yes	AATGCCAGGGTCGAGGTTTCGATCCCCGTACGGGC
21122	Yes	AATGCCAGGGTCGAGGTTTCGATCCCCGTACGGGCC
21123	Yes	AATGCCAGGGTCGAGGTTTCGATCCCCGTACGGGCCT
21124	Yes	AATGCCAGGGTCGAGGTTTCGATCCCCGTACGGGCCTC
21125	Yes	AATGCCAGGGTCGAGGTTTCGATCCCCGTACGGGCCTCC
21126	Yes	AATGCCAGGGTCGAGGTTTCGATCCCCGTACGGGCCTCCA
21127	No	AATGCCCACATTCTCC
21128	No	AATGCCCACATTCTCCA
21129	No	AATGCCCACATTCTCCAC
21130	Yes	AATGCCCACATTCTCCACC
21131	Yes	AATGCCCACATTCTCCACCA
21132	No	AATGCCGAGGTTGTGA
21133	No	AATGCCGAGGTTGTGAG
21134	No	AATGCCGAGGTTGTGAGT
21135	No	AATGCCGAGGTTGTGAGTT
21136	No	AATGCCGAGGTTGTGAGTTC
21137	No	AATGCCGAGGTTGTGAGTTCA
21138	No	AATGCCGAGGTTGTGAGTTCAA
21139	No	AATGCCGAGGTTGTGAGTTCAAG
21140	No	AATGCCGAGGTTGTGAGTTCAAGC
21141	No	AATGCCGAGGTTGTGAGTTCAAGCC
21142	No	AATGCCGAGGTTGTGAGTTCAAGCCT
21143	No	AATGCCGAGGTTGTGAGTTCAAGCCTC
21144	No	AATGCCGAGGTTGTGAGTTCAAGCCTCA
21145	No	AATGCCGAGGTTGTGAGTTCAAGCCTCAC
21146	No	AATGCCGAGGTTGTGAGTTCAAGCCTCACC
21147	No	AATGCCGAGGTTGTGAGTTCAAGCCTCACCT
21148	No	AATGCCGAGGTTGTGAGTTCAAGCCTCACCTG
21149	No	AATGCCGAGGTTGTGAGTTCAAGCCTCACCTGG
21150	No	AATGCCGAGGTTGTGAGTTCAAGCCTCACCTGGA
21151	No	AATGCCGAGGTTGTGAGTTCAAGCCTCACCTGGAG
21152	No	AATGCCGAGGTTGTGAGTTCAAGCCTCACCTGGAGC
21153	No	AATGCCGAGGTTGTGAGTTCAAGCCTCACCTGGAGCA
21154	Yes	AATGCCGAGGTTGTGAGTTCAAGCCTCACCTGGAGCAC
21155	Yes	AATGCCGAGGTTGTGAGTTCAAGCCTCACCTGGAGCACC
21156	Yes	AATGCCGAGGTTGTGAGTTCAAGCCTCACCTGGAGCACCA
21157	No	AATGCCGAGGTTGTGAGTTCG
21158	No	AATGCCGAGGTTGTGAGTTCGA
21159	No	AATGCCGAGGTTGTGAGTTCGAG
21160	No	AATGCCGAGGTTGTGAGTTCGAGC
21161	No	AATGCCGAGGTTGTGAGTTCGAGCC

21162	No	AATGCCGAGGTTGTGAGTTCGAGCCT
21163	No	AATGCCGAGGTTGTGAGTTCGAGCCTC
21164	No	AATGCCGAGGTTGTGAGTTCGAGCCTCA
21165	No	AATGCCGAGGTTGTGAGTTCGAGCCTCAC
21166	No	AATGCCGAGGTTGTGAGTTCGAGCCTCACC
21167	No	AATGCCGAGGTTGTGAGTTCGAGCCTCACCT
21168	No	AATGCCGAGGTTGTGAGTTCGAGCCTCACCTG
21169	No	AATGCCGAGGTTGTGAGTTCGAGCCTCACCTGG
21170	No	AATGCCGAGGTTGTGAGTTCGAGCCTCACCTGGA
21171	No	AATGCCGAGGTTGTGAGTTCGAGCCTCACCTGGAG
21172	No	AATGCCGAGGTTGTGAGTTCGAGCCTCACCTGGAGC
21173	No	AATGCCGAGGTTGTGAGTTCGAGCCTCACCTGGAGCA
21174	Yes	AATGCCGAGGTTGTGAGTTCGAGCCTCACCTGGAGCAC
21175	Yes	AATGCCGAGGTTGTGAGTTCGAGCCTCACCTGGAGCACC
21176	Yes	AATGCCGAGGTTGTGAGTTCGAGCCTCACCTGGAGCACCA
21177	No	AATGCCGAGGTTGTGAGTTCGAT
21178	No	AATGCCGAGGTTGTGAGTTCGATC
21179	No	AATGCCGAGGTTGTGAGTTCGATCC
21180	No	AATGCCGAGGTTGTGAGTTCGATCCT
21181	No	AATGCCGAGGTTGTGAGTTCGATCCTC
21182	No	AATGCCGAGGTTGTGAGTTCGATCCTCA
21183	No	AATGCCGAGGTTGTGAGTTCGATCCTCAC
21184	No	AATGCCGAGGTTGTGAGTTCGATCCTCACC
21185	No	AATGCCGAGGTTGTGAGTTCGATCCTCACCT
21186	No	AATGCCGAGGTTGTGAGTTCGATCCTCACCTG
21187	No	AATGCCGAGGTTGTGAGTTCGATCCTCACCTGG
21188	No	AATGCCGAGGTTGTGAGTTCGATCCTCACCTGGA
21189	No	AATGCCGAGGTTGTGAGTTCGATCCTCACCTGGAG
21190	No	AATGCCGAGGTTGTGAGTTCGATCCTCACCTGGAGC
21191	No	AATGCCGAGGTTGTGAGTTCGATCCTCACCTGGAGCA
21192	No	AATGCCGAGGTTGTGAGTTCGATCCTCACCTGGAGCAC
21193	Yes	AATGCCGAGGTTGTGAGTTCGATCCTCACCTGGAGCACC
21194	Yes	AATGCCGAGGTTGTGAGTTCGATCCTCACCTGGAGCACCA
21195	Yes	AATGCGTCTGACTAAG
21196	Yes	AATGCGTCTGACTAAGA
21197	Yes	AATGCGTCTGACTAAGAA
21198	Yes	AATGCGTCTGACTAAGAAT
21199	Yes	AATGCGTCTGACTAAGAATC
21200	Yes	AATGCGTCTGACTAAGAATCA
21201	Yes	AATGCGTCTGACTAAGAATCAG
21202	Yes	AATGCGTCTGACTAAGAATCAGA
21203	Yes	AATGCGTCTGACTAAGAATCAGAA

21204	Yes	AATGCGTCTGACTAAGAATCAGAAG
21205	Yes	AATGCGTCTGACTAAGAATCAGAAGA
21206	Yes	AATGCGTCTGACTAAGAATCAGAAGAT
21207	Yes	AATGCGTCTGACTAAGAATCAGAAGATT
21208	Yes	AATGCGTCTGACTAAGAATCAGAAGATTC
21209	Yes	AATGCGTCTGACTAAGAATCAGAAGATTCC
21210	Yes	AATGCGTCTGACTAAGAATCAGAAGATTCCA
21211	Yes	AATGCGTCTGACTAAGAATCAGAAGATTCCAG
21212	Yes	AATGCGTCTGACTAAGAATCAGAAGATTCCAGC
21213	Yes	AATGCGTCTGACTAAGAATCAGAAGATTCCAGCC
21214	Yes	AATGCGTCTGACTAAGAATCAGAAGATTCCAGCCT
21215	Yes	AATGCGTCTGACTAAGAATCAGAAGATTCCAGCCTT
21216	Yes	AATGCGTCTGACTAAGAATCAGAAGATTCCAGCCTTG
21217	Yes	AATGCGTCTGACTAAGAATCAGAAGATTCCAGCCTTGA
21218	Yes	AATGCGTCTGACTAAGAATCAGAAGATTCCAGCCTTGAC
21219	Yes	AATGCGTCTGACTAAGAATCAGAAGATTCCAGCCTTGACT
21220	Yes	AATGCGTCTGACTAAGAATCAGAAGATTCCAGCCTTGACTC
21221	Yes	AATGCGTCTGACTAAGAATCAGAAGATTCCAGCCTTGACTCC
21222	Yes	AATGCGTCTGACTAAGAATCAGAAGATTCCAGCCTTGACTCCT
21223	Yes	AATGCGTCTGACTAAGAATCAGAAGATTCCAGCCTTGACTCCTG
21224	Yes	AATGCGTCTGACTAAGAATCAGAAGATTCCAGCCTTGACTCCTGG
21225	Yes	AATGCGTCTGACTAAGAATCAGAAGATTCCAGCCTTGACTCCTGGC
21226	Yes	AATGCGTCTGACTAAGAATCAGAAGATTCCAGCCTTGACTCCTGGCT
21227	Yes	AATGCGTCTGACTAAGAATCAGAAGATTCCAGCCTTGACTCCTGGCTG
21228	Yes	AATGCGTCTGACTAAGAATCAGAAGATTCCAGCCTTGACTCCTGGCTGG
21229	Yes	AATGCGTCTGACTAAGAATCAGAAGATTCCAGCCTTGACTCCTGGCTGGC
21230	No	AATGCTGGTTCTGTCA
21231	No	AATGCTGGTTCTGTCAC
21232	Yes	AATGCTGGTTCTGTCACC
21233	Yes	AATGCTGGTTCTGTCACCA
21234	Yes	AATGGACAGGTGTCCG
21235	Yes	AATGGACAGGTGTCCGC
21236	Yes	AATGGACAGGTGTCCGCG
21237	Yes	AATGGACAGGTGTCCGCGT
21238	Yes	AATGGACAGGTGTCCGCGTG
21239	Yes	AATGGACAGGTGTCCGCGTGG
21240	Yes	AATGGACAGGTGTCCGCGTGGG
21241	Yes	AATGGACAGGTGTCCGCGTGGGT
21242	Yes	AATGGACAGGTGTCCGCGTGGGTT
21243	Yes	AATGGACAGGTGTCCGCGTGGGTTC
21244	Yes	AATGGACAGGTGTCCGCGTGGGTTCG
21245	Yes	AATGGACAGGTGTCCGCGTGGGTTCGA

21246	Yes	AATGGACAGGTGTCCGCGTGGGTTCGAG
21247	Yes	AATGGACAGGTGTCCGCGTGGGTTCGAGC
21248	Yes	AATGGACAGGTGTCCGCGTGGGTTCGAGCC
21249	Yes	AATGGACAGGTGTCCGCGTGGGTTCGAGCCC
21250	Yes	AATGGACAGGTGTCCGCGTGGGTTCGAGCCCC
21251	Yes	AATGGACAGGTGTCCGCGTGGGTTCGAGCCCCA
21252	Yes	AATGGACAGGTGTCCGCGTGGGTTCGAGCCCCAC
21253	Yes	AATGGACAGGTGTCCGCGTGGGTTCGAGCCCCACT
21254	Yes	AATGGACAGGTGTCCGCGTGGGTTCGAGCCCCACTC
21255	Yes	AATGGACAGGTGTCCGCGTGGGTTCGAGCCCCACTCC
21256	Yes	AATGGACAGGTGTCCGCGTGGGTTCGAGCCCCACTCCC
21257	Yes	AATGGACAGGTGTCCGCGTGGGTTCGAGCCCCACTCCCG
21258	Yes	AATGGACAGGTGTCCGCGTGGGTTCGAGCCCCACTCCCGG
21259	Yes	AATGGACAGGTGTCCGCGTGGGTTCGAGCCCCACTCCCGGT
21260	Yes	AATGGACAGGTGTCCGCGTGGGTTCGAGCCCCACTCCCGGTA
21261	Yes	AATGGACAGGTGTCCGCGTGGGTTCGAGCCCCACTCCCGGTAC
21262	Yes	AATGGACAGGTGTCCGCGTGGGTTCGAGCCCCACTCCCGGTACC
21263	Yes	AATGGACAGGTGTCCGCGTGGGTTCGAGCCCCACTCCCGGTACCA
21264	No	AATGGACATATGTCCG
21265	Yes	AATGGACATATGTCCGC
21266	Yes	AATGGACATATGTCCGCG
21267	Yes	AATGGACATATGTCCGCGT
21268	Yes	AATGGACATATGTCCGCGTG
21269	Yes	AATGGACATATGTCCGCGTGG
21270	Yes	AATGGACATATGTCCGCGTGGG
21271	Yes	AATGGACATATGTCCGCGTGGGT
21272	Yes	AATGGACATATGTCCGCGTGGGTT
21273	Yes	AATGGACATATGTCCGCGTGGGTTC
21274	Yes	AATGGACATATGTCCGCGTGGGTTCG
21275	Yes	AATGGACATATGTCCGCGTGGGTTCGA
21276	Yes	AATGGACATATGTCCGCGTGGGTTCGAA
21277	Yes	AATGGACATATGTCCGCGTGGGTTCGAAC
21278	Yes	AATGGACATATGTCCGCGTGGGTTCGAACC
21279	Yes	AATGGACATATGTCCGCGTGGGTTCGAACCC
21280	Yes	AATGGACATATGTCCGCGTGGGTTCGAACCCC
21281	Yes	AATGGACATATGTCCGCGTGGGTTCGAACCCCA
21282	Yes	AATGGACATATGTCCGCGTGGGTTCGAACCCCAC
21283	Yes	AATGGACATATGTCCGCGTGGGTTCGAACCCCACT
21284	Yes	AATGGACATATGTCCGCGTGGGTTCGAACCCCACTC
21285	Yes	AATGGACATATGTCCGCGTGGGTTCGAACCCCACTCC
21286	Yes	AATGGACATATGTCCGCGTGGGTTCGAACCCCACTCCT
21287	Yes	AATGGACATATGTCCGCGTGGGTTCGAACCCCACTCCTG

21288	Yes	AATGGACATATGTCCGCGTGGGTTCGAACCCCACTCCTGG
21289	Yes	AATGGACATATGTCCGCGTGGGTTCGAACCCCACTCCTGGT
21290	Yes	AATGGACATATGTCCGCGTGGGTTCGAACCCCACTCCTGGTA
21291	Yes	AATGGACATATGTCCGCGTGGGTTCGAACCCCACTCCTGGTAC
21292	Yes	AATGGACATATGTCCGCGTGGGTTCGAACCCCACTCCTGGTACC
21293	Yes	AATGGACATATGTCCGCGTGGGTTCGAACCCCACTCCTGGTACCA
21294	Yes	AATGGACGAGCGCGCT
21295	Yes	AATGGACGAGCGCGCTG
21296	Yes	AATGGACGAGCGCGCTGG
21297	Yes	AATGGACGAGCGCGCTGGA
21298	Yes	AATGGACGAGCGCGCTGGAC
21299	Yes	AATGGACGAGCGCGCTGGACT
21300	Yes	AATGGACGAGCGCGCTGGACTT
21301	Yes	AATGGACGAGCGCGCTGGACTTC
21302	Yes	AATGGACGAGCGCGCTGGACTTCT
21303	Yes	AATGGACGAGCGCGCTGGACTTCTA
21304	Yes	AATGGACGAGCGCGCTGGACTTCTAA
21305	Yes	AATGGACGAGCGCGCTGGACTTCTAAT
21306	Yes	AATGGACGAGCGCGCTGGACTTCTAATC
21307	Yes	AATGGACGAGCGCGCTGGACTTCTAATCC
21308	Yes	AATGGACGAGCGCGCTGGACTTCTAATCCA
21309	Yes	AATGGACGAGCGCGCTGGACTTCTAATCCAG
21310	Yes	AATGGACGAGCGCGCTGGACTTCTAATCCAGA
21311	Yes	AATGGACGAGCGCGCTGGACTTCTAATCCAGAG
21312	Yes	AATGGACGAGCGCGCTGGACTTCTAATCCAGAGG
21313	Yes	AATGGACGAGCGCGCTGGACTTCTAATCCAGAGGT
21314	Yes	AATGGACGAGCGCGCTGGACTTCTAATCCAGAGGTT
21315	Yes	AATGGACGAGCGCGCTGGACTTCTAATCCAGAGGTTC
21316	Yes	AATGGACGAGCGCGCTGGACTTCTAATCCAGAGGTTCC
21317	Yes	AATGGACGAGCGCGCTGGACTTCTAATCCAGAGGTTCCG
21318	Yes	AATGGACGAGCGCGCTGGACTTCTAATCCAGAGGTTCCGG
21319	Yes	AATGGACGAGCGCGCTGGACTTCTAATCCAGAGGTTCCGGG
21320	Yes	AATGGACGAGCGCGCTGGACTTCTAATCCAGAGGTTCCGGGT
21321	Yes	AATGGACGAGCGCGCTGGACTTCTAATCCAGAGGTTCCGGGTT
21322	Yes	AATGGACGAGCGCGCTGGACTTCTAATCCAGAGGTTCCGGGTTC
21323	Yes	AATGGACGAGCGCGCTGGACTTCTAATCCAGAGGTTCCGGGTTCG
21324	Yes	AATGGACGAGCGCGCTGGACTTCTAATCCAGAGGTTCCGGGTTCGA
21325	Yes	AATGGACGAGCGCGCTGGACTTCTAATCCAGAGGTTCCGGGTTCGAG
21326	Yes	AATGGACGAGCGCGCTGGACTTCTAATCCAGAGGTTCCGGGTTCGAGT
21327	Yes	AATGGACGAGCGCGCTGGACTTCTAATCCAGAGGTTCCGGGTTCGAGTC
21328	Yes	AATGGACGAGCGCGCTGGACTTCTAATCCAGAGGTTCCGGGTTCGAGTCC
21329	No	AATGGAGAATTTTGAA

21330	No	AATGGAGAATTTTGAAT
21331	No	AATGGAGAATTTTGAATT
21332	Yes	AATGGAGAATTTTGAATTC
21333	Yes	AATGGAGAATTTTGAATTCT
21334	Yes	AATGGAGAATTTTGAATTCTC
21335	Yes	AATGGAGAATTTTGAATTCTCA
21336	Yes	AATGGAGAATTTTGAATTCTCAG
21337	Yes	AATGGAGAATTTTGAATTCTCAGG
21338	Yes	AATGGAGAATTTTGAATTCTCAGGG
21339	Yes	AATGGAGAATTTTGAATTCTCAGGGG
21340	Yes	AATGGAGAATTTTGAATTCTCAGGGGT
21341	Yes	AATGGAGAATTTTGAATTCTCAGGGGTA
21342	Yes	AATGGAGAATTTTGAATTCTCAGGGGTAG
21343	Yes	AATGGAGAATTTTGAATTCTCAGGGGTAGG
21344	Yes	AATGGAGAATTTTGAATTCTCAGGGGTAGGT
21345	Yes	AATGGAGAATTTTGAATTCTCAGGGGTAGGTT
21346	Yes	AATGGAGAATTTTGAATTCTCAGGGGTAGGTTC
21347	Yes	AATGGAGAATTTTGAATTCTCAGGGGTAGGTTCA
21348	Yes	AATGGAGAATTTTGAATTCTCAGGGGTAGGTTCAA
21349	Yes	AATGGAGAATTTTGAATTCTCAGGGGTAGGTTCAAT
21350	Yes	AATGGAGAATTTTGAATTCTCAGGGGTAGGTTCAATT
21351	Yes	AATGGAGAATTTTGAATTCTCAGGGGTAGGTTCAATTC
21352	Yes	AATGGAGAATTTTGAATTCTCAGGGGTAGGTTCAATTCC
21353	Yes	AATGGAGAATTTTGAATTCTCAGGGGTAGGTTCAATTCCT
21354	Yes	AATGGAGAATTTTGAATTCTCAGGGGTAGGTTCAATTCCTA
21355	Yes	AATGGAGAATTTTGAATTCTCAGGGGTAGGTTCAATTCCTAT
21356	Yes	AATGGAGAATTTTGAATTCTCAGGGGTAGGTTCAATTCCTATA
21357	Yes	AATGGAGAATTTTGAATTCTCAGGGGTAGGTTCAATTCCTATAG
21358	Yes	AATGGAGAATTTTGAATTCTCAGGGGTAGGTTCAATTCCTATAGT
21359	Yes	AATGGAGAATTTTGAATTCTCAGGGGTAGGTTCAATTCCTATAGTT
21360	Yes	AATGGAGAATTTTGAATTCTCAGGGGTAGGTTCAATTCCTATAGTTC
21361	Yes	AATGGAGAATTTTGAATTCTCAGGGGTAGGTTCAATTCCTATAGTTCT
21362	Yes	AATGGAGAATTTTGAATTCTCAGGGGTAGGTTCAATTCCTATAGTTCTA
21363	Yes	AATGGAGAATTTTGAATTCTCAGGGGTAGGTTCAATTCCTATAGTTCTAG
21364	Yes	AATGGAGGCGTGGGTT
21365	Yes	AATGGAGGCGTGGGTTC
21366	Yes	AATGGAGGCGTGGGTTCG
21367	Yes	AATGGAGGCGTGGGTTCGA
21368	Yes	AATGGAGGCGTGGGTTCGAA
21369	Yes	AATGGAGGCGTGGGTTCGAAT
21370	Yes	AATGGAGGCGTGGGTTCGAATC
21371	Yes	AATGGAGGCGTGGGTTCGAATCC

21372	Yes	AATGGAGGCGTGGGTTCGAATCCC
21373	Yes	AATGGAGGCGTGGGTTCGAATCCCA
21374	Yes	AATGGAGGCGTGGGTTCGAATCCCAC
21375	Yes	AATGGAGGCGTGGGTTCGAATCCCACT
21376	Yes	AATGGAGGCGTGGGTTCGAATCCCACTT
21377	Yes	AATGGAGGCGTGGGTTCGAATCCCACTTC
21378	Yes	AATGGAGGCGTGGGTTCGAATCCCACTTCT
21379	Yes	AATGGAGGCGTGGGTTCGAATCCCACTTCTG
21380	Yes	AATGGAGGCGTGGGTTCGAATCCCACTTCTGA
21381	Yes	AATGGAGGCGTGGGTTCGAATCCCACTTCTGAC
21382	Yes	AATGGAGGCGTGGGTTCGAATCCCACTTCTGACA
21383	Yes	AATGGAGGCGTGGGTTCGAATCCCACTTCTGACAC
21384	Yes	AATGGAGGCGTGGGTTCGAATCCCACTTCTGACACC
21385	Yes	AATGGAGGCGTGGGTTCGAATCCCACTTCTGACACCA
21386	No	AATGGAGTAGTGGCTG
21387	Yes	AATGGAGTAGTGGCTGT
21388	Yes	AATGGAGTAGTGGCTGTG
21389	Yes	AATGGAGTAGTGGCTGTGT
21390	Yes	AATGGAGTAGTGGCTGTGTG
21391	Yes	AATGGAGTAGTGGCTGTGTGG
21392	Yes	AATGGAGTAGTGGCTGTGTGGG
21393	Yes	AATGGAGTAGTGGCTGTGTGGGT
21394	Yes	AATGGAGTAGTGGCTGTGTGGGTT
21395	Yes	AATGGAGTAGTGGCTGTGTGGGTTT
21396	Yes	AATGGAGTAGTGGCTGTGTGGGTTTA
21397	Yes	AATGGAGTAGTGGCTGTGTGGGTTTAA
21398	Yes	AATGGAGTAGTGGCTGTGTGGGTTTAAA
21399	Yes	AATGGAGTAGTGGCTGTGTGGGTTTAAAC
21400	Yes	AATGGAGTAGTGGCTGTGTGGGTTTAAACC
21401	Yes	AATGGAGTAGTGGCTGTGTGGGTTTAAACCC
21402	Yes	AATGGAGTAGTGGCTGTGTGGGTTTAAACCCC
21403	Yes	AATGGAGTAGTGGCTGTGTGGGTTTAAACCCCA
21404	Yes	AATGGAGTAGTGGCTGTGTGGGTTTAAACCCCAC
21405	Yes	AATGGAGTAGTGGCTGTGTGGGTTTAAACCCCACT
21406	Yes	AATGGAGTAGTGGCTGTGTGGGTTTAAACCCCACTA
21407	Yes	AATGGAGTAGTGGCTGTGTGGGTTTAAACCCCACTAC
21408	Yes	AATGGAGTAGTGGCTGTGTGGGTTTAAACCCCACTACT
21409	Yes	AATGGAGTAGTGGCTGTGTGGGTTTAAACCCCACTACTG
21410	Yes	AATGGAGTAGTGGCTGTGTGGGTTTAAACCCCACTACTGG
21411	Yes	AATGGAGTAGTGGCTGTGTGGGTTTAAACCCCACTACTGGT
21412	Yes	AATGGAGTAGTGGCTGTGTGGGTTTAAACCCCACTACTGGTA
21413	Yes	AATGGAGTAGTGGCTGTGTGGGTTTAAACCCCACTACTGGTAC

21414	Yes	AATGGAGTAGTGGCTGTGTGGGTTTAAACCCCACTACTGGTACC
21415	Yes	AATGGAGTAGTGGCTGTGTGGGTTTAAACCCCACTACTGGTACCA
21416	No	AATGGATAACGCGTCT
21417	No	AATGGATAACGCGTCTG
21418	No	AATGGATAACGCGTCTGA
21419	No	AATGGATAACGCGTCTGAC
21420	No	AATGGATAACGCGTCTGACT
21421	No	AATGGATAACGCGTCTGACTA
21422	No	AATGGATAACGCGTCTGACTAC
21423	No	AATGGATAACGCGTCTGACTACG
21424	No	AATGGATAACGCGTCTGACTACGG
21425	No	AATGGATAACGCGTCTGACTACGGA
21426	No	AATGGATAACGCGTCTGACTACGGAT
21427	No	AATGGATAACGCGTCTGACTACGGATC
21428	No	AATGGATAACGCGTCTGACTACGGATCA
21429	No	AATGGATAACGCGTCTGACTACGGATCAG
21430	No	AATGGATAACGCGTCTGACTACGGATCAGA
21431	No	AATGGATAACGCGTCTGACTACGGATCAGAA
21432	No	AATGGATAACGCGTCTGACTACGGATCAGAAG
21433	No	AATGGATAACGCGTCTGACTACGGATCAGAAGA
21434	No	AATGGATAACGCGTCTGACTACGGATCAGAAGAT
21435	No	AATGGATAACGCGTCTGACTACGGATCAGAAGATT
21436	No	AATGGATAACGCGTCTGACTACGGATCAGAAGATTC
21437	Yes	AATGGATAACGCGTCTGACTACGGATCAGAAGATTCC
21438	Yes	AATGGATAACGCGTCTGACTACGGATCAGAAGATTCCA
21439	Yes	AATGGATAACGCGTCTGACTACGGATCAGAAGATTCCAG
21440	Yes	AATGGATAACGCGTCTGACTACGGATCAGAAGATTCCAGG
21441	Yes	AATGGATAACGCGTCTGACTACGGATCAGAAGATTCCAGGT
21442	Yes	AATGGATAACGCGTCTGACTACGGATCAGAAGATTCCAGGTT
21443	Yes	AATGGATAACGCGTCTGACTACGGATCAGAAGATTCCAGGTTC
21444	Yes	AATGGATAACGCGTCTGACTACGGATCAGAAGATTCCAGGTTCG
21445	Yes	AATGGATAACGCGTCTGACTACGGATCAGAAGATTCCAGGTTCGA
21446	Yes	AATGGATAACGCGTCTGACTACGGATCAGAAGATTCCAGGTTCGAC
21447	Yes	AATGGATAACGCGTCTGACTACGGATCAGAAGATTCCAGGTTCGACT
21448	Yes	AATGGATAACGCGTCTGACTACGGATCAGAAGATTCCAGGTTCGACTC
21449	Yes	AATGGATAACGCGTCTGACTACGGATCAGAAGATTCCAGGTTCGACTCC
21450	Yes	AATGGATAACGCGTCTGACTACGGATCAGAAGATTCCAGGTTCGACTCCT
21451	No	AATGGATAACGCGTCTGACTACGGATCAGAAGATTCT
21452	No	AATGGATAACGCGTCTGACTACGGATCAGAAGATTCTA
21453	No	AATGGATAACGCGTCTGACTACGGATCAGAAGATTCTAG
21454	Yes	AATGGATAACGCGTCTGACTACGGATCAGAAGATTCTAGG
21455	Yes	AATGGATAACGCGTCTGACTACGGATCAGAAGATTCTAGGT

21456	Yes	AATGGATAACGCGTCTGACTACGGATCAGAAGATTCTAGGTT
21457	Yes	AATGGATAACGCGTCTGACTACGGATCAGAAGATTCTAGGTTC
21458	Yes	AATGGATAACGCGTCTGACTACGGATCAGAAGATTCTAGGTTCG
21459	Yes	AATGGATAACGCGTCTGACTACGGATCAGAAGATTCTAGGTTCGA
21460	Yes	AATGGATAACGCGTCTGACTACGGATCAGAAGATTCTAGGTTCGAC
21461	Yes	AATGGATAACGCGTCTGACTACGGATCAGAAGATTCTAGGTTCGACT
21462	Yes	AATGGATAACGCGTCTGACTACGGATCAGAAGATTCTAGGTTCGACTC
21463	Yes	AATGGATAACGCGTCTGACTACGGATCAGAAGATTCTAGGTTCGACTCC
21464	Yes	AATGGATAACGCGTCTGACTACGGATCAGAAGATTCTAGGTTCGACTCCT
21465	No	AATGGATAAGGCACTG
21466	No	AATGGATAAGGCACTGG
21467	No	AATGGATAAGGCACTGGC
21468	No	AATGGATAAGGCACTGGCC
21469	No	AATGGATAAGGCACTGGCCT
21470	No	AATGGATAAGGCACTGGCCTC
21471	Yes	AATGGATAAGGCACTGGCCTCC
21472	Yes	AATGGATAAGGCACTGGCCTCCT
21473	Yes	AATGGATAAGGCACTGGCCTCCTA
21474	Yes	AATGGATAAGGCACTGGCCTCCTAA
21475	Yes	AATGGATAAGGCACTGGCCTCCTAAG
21476	Yes	AATGGATAAGGCACTGGCCTCCTAAGC
21477	Yes	AATGGATAAGGCACTGGCCTCCTAAGCC
21478	Yes	AATGGATAAGGCACTGGCCTCCTAAGCCA
21479	Yes	AATGGATAAGGCACTGGCCTCCTAAGCCAG
21480	Yes	AATGGATAAGGCACTGGCCTCCTAAGCCAGG
21481	Yes	AATGGATAAGGCACTGGCCTCCTAAGCCAGGG
21482	Yes	AATGGATAAGGCACTGGCCTCCTAAGCCAGGGA
21483	Yes	AATGGATAAGGCACTGGCCTCCTAAGCCAGGGAT
21484	Yes	AATGGATAAGGCACTGGCCTCCTAAGCCAGGGATT
21485	Yes	AATGGATAAGGCACTGGCCTCCTAAGCCAGGGATTG
21486	Yes	AATGGATAAGGCACTGGCCTCCTAAGCCAGGGATTGT
21487	Yes	AATGGATAAGGCACTGGCCTCCTAAGCCAGGGATTGTG
21488	Yes	AATGGATAAGGCACTGGCCTCCTAAGCCAGGGATTGTGG
21489	Yes	AATGGATAAGGCACTGGCCTCCTAAGCCAGGGATTGTGGG
21490	Yes	AATGGATAAGGCACTGGCCTCCTAAGCCAGGGATTGTGGGT
21491	Yes	AATGGATAAGGCACTGGCCTCCTAAGCCAGGGATTGTGGGTT
21492	Yes	AATGGATAAGGCACTGGCCTCCTAAGCCAGGGATTGTGGGTTC
21493	Yes	AATGGATAAGGCACTGGCCTCCTAAGCCAGGGATTGTGGGTTCG
21494	Yes	AATGGATAAGGCACTGGCCTCCTAAGCCAGGGATTGTGGGTTCGA
21495	Yes	AATGGATAAGGCACTGGCCTCCTAAGCCAGGGATTGTGGGTTCGAG
21496	Yes	AATGGATAAGGCACTGGCCTCCTAAGCCAGGGATTGTGGGTTCGAGT
21497	Yes	AATGGATAAGGCACTGGCCTCCTAAGCCAGGGATTGTGGGTTCGAGTC

21498	Yes	AATGGATAAGGCACTGGCCTCCTAAGCCAGGGATTGTGGGTTCGAGTCC
21499	Yes	AATGGATAAGGCACTGGCCTCCTAAGCCAGGGATTGTGGGTTCGAGTCCC
21500	No	AATGGATAAGGCATCA
21501	Yes	AATGGATAAGGCATCAG
21502	Yes	AATGGATAAGGCATCAGC
21503	Yes	AATGGATAAGGCATCAGCC
21504	Yes	AATGGATAAGGCATCAGCCT
21505	Yes	AATGGATAAGGCATCAGCCTC
21506	Yes	AATGGATAAGGCATCAGCCTCC
21507	Yes	AATGGATAAGGCATCAGCCTCCG
21508	Yes	AATGGATAAGGCATCAGCCTCCGG
21509	Yes	AATGGATAAGGCATCAGCCTCCGGA
21510	Yes	AATGGATAAGGCATCAGCCTCCGGAG
21511	Yes	AATGGATAAGGCATCAGCCTCCGGAGC
21512	Yes	AATGGATAAGGCATCAGCCTCCGGAGCT
21513	Yes	AATGGATAAGGCATCAGCCTCCGGAGCTG
21514	Yes	AATGGATAAGGCATCAGCCTCCGGAGCTGG
21515	Yes	AATGGATAAGGCATCAGCCTCCGGAGCTGGG
21516	Yes	AATGGATAAGGCATCAGCCTCCGGAGCTGGGG
21517	Yes	AATGGATAAGGCATCAGCCTCCGGAGCTGGGGA
21518	Yes	AATGGATAAGGCATCAGCCTCCGGAGCTGGGGAT
21519	Yes	AATGGATAAGGCATCAGCCTCCGGAGCTGGGGATT
21520	Yes	AATGGATAAGGCATCAGCCTCCGGAGCTGGGGATTG
21521	Yes	AATGGATAAGGCATCAGCCTCCGGAGCTGGGGATTGT
21522	Yes	AATGGATAAGGCATCAGCCTCCGGAGCTGGGGATTGTG
21523	Yes	AATGGATAAGGCATCAGCCTCCGGAGCTGGGGATTGTGG
21524	Yes	AATGGATAAGGCATCAGCCTCCGGAGCTGGGGATTGTGGG
21525	Yes	AATGGATAAGGCATCAGCCTCCGGAGCTGGGGATTGTGGGT
21526	Yes	AATGGATAAGGCATCAGCCTCCGGAGCTGGGGATTGTGGGTT
21527	Yes	AATGGATAAGGCATCAGCCTCCGGAGCTGGGGATTGTGGGTTC
21528	Yes	AATGGATAAGGCATCAGCCTCCGGAGCTGGGGATTGTGGGTTCG
21529	Yes	AATGGATAAGGCATCAGCCTCCGGAGCTGGGGATTGTGGGTTCGA
21530	Yes	AATGGATAAGGCATCAGCCTCCGGAGCTGGGGATTGTGGGTTCGAG
21531	Yes	AATGGATAAGGCATCAGCCTCCGGAGCTGGGGATTGTGGGTTCGAGT
21532	Yes	AATGGATAAGGCATCAGCCTCCGGAGCTGGGGATTGTGGGTTCGAGTC
21533	Yes	AATGGATAAGGCATCAGCCTCCGGAGCTGGGGATTGTGGGTTCGAGTCC
21534	Yes	AATGGATAAGGCATCAGCCTCCGGAGCTGGGGATTGTGGGTTCGAGTCCC
21535	No	AATGGATAAGGCATTG
21536	No	AATGGATAAGGCATTGG
21537	No	AATGGATAAGGCATTGGC
21538	No	AATGGATAAGGCATTGGCC
21539	No	AATGGATAAGGCATTGGCCT

21540	No	AATGGATAAGGCATTGGCCTC
21541	No	AATGGATAAGGCATTGGCCTCC
21542	No	AATGGATAAGGCATTGGCCTCCT
21543	Yes	AATGGATAAGGCATTGGCCTCCTA
21544	Yes	AATGGATAAGGCATTGGCCTCCTAA
21545	Yes	AATGGATAAGGCATTGGCCTCCTAAG
21546	Yes	AATGGATAAGGCATTGGCCTCCTAAGC
21547	Yes	AATGGATAAGGCATTGGCCTCCTAAGCC
21548	Yes	AATGGATAAGGCATTGGCCTCCTAAGCCA
21549	Yes	AATGGATAAGGCATTGGCCTCCTAAGCCAG
21550	Yes	AATGGATAAGGCATTGGCCTCCTAAGCCAGG
21551	Yes	AATGGATAAGGCATTGGCCTCCTAAGCCAGGG
21552	Yes	AATGGATAAGGCATTGGCCTCCTAAGCCAGGGA
21553	Yes	AATGGATAAGGCATTGGCCTCCTAAGCCAGGGAT
21554	Yes	AATGGATAAGGCATTGGCCTCCTAAGCCAGGGATT
21555	Yes	AATGGATAAGGCATTGGCCTCCTAAGCCAGGGATTG
21556	Yes	AATGGATAAGGCATTGGCCTCCTAAGCCAGGGATTGT
21557	Yes	AATGGATAAGGCATTGGCCTCCTAAGCCAGGGATTGTG
21558	Yes	AATGGATAAGGCATTGGCCTCCTAAGCCAGGGATTGTGG
21559	Yes	AATGGATAAGGCATTGGCCTCCTAAGCCAGGGATTGTGGG
21560	Yes	AATGGATAAGGCATTGGCCTCCTAAGCCAGGGATTGTGGGT
21561	Yes	AATGGATAAGGCATTGGCCTCCTAAGCCAGGGATTGTGGGTT
21562	Yes	AATGGATAAGGCATTGGCCTCCTAAGCCAGGGATTGTGGGTTC
21563	Yes	AATGGATAAGGCATTGGCCTCCTAAGCCAGGGATTGTGGGTTCG
21564	Yes	AATGGATAAGGCATTGGCCTCCTAAGCCAGGGATTGTGGGTTCGA
21565	Yes	AATGGATAAGGCATTGGCCTCCTAAGCCAGGGATTGTGGGTTCGAG
21566	Yes	AATGGATAAGGCATTGGCCTCCTAAGCCAGGGATTGTGGGTTCGAGT
21567	Yes	AATGGATAAGGCATTGGCCTCCTAAGCCAGGGATTGTGGGTTCGAGTC
21568	Yes	AATGGATAAGGCATTGGCCTCCTAAGCCAGGGATTGTGGGTTCGAGTCC
21569	Yes	AATGGATAAGGCATTGGCCTCCTAAGCCAGGGATTGTGGGTTCGAGTCCC
21570	Yes	AATGGATAAGGCGTCT
21571	Yes	AATGGATAAGGCGTCTG
21572	Yes	AATGGATAAGGCGTCTGA
21573	Yes	AATGGATAAGGCGTCTGAC
21574	Yes	AATGGATAAGGCGTCTGACT
21575	Yes	AATGGATAAGGCGTCTGACTT
21576	Yes	AATGGATAAGGCGTCTGACTTC
21577	Yes	AATGGATAAGGCGTCTGACTTCG
21578	Yes	AATGGATAAGGCGTCTGACTTCGG
21579	Yes	AATGGATAAGGCGTCTGACTTCGGA
21580	Yes	AATGGATAAGGCGTCTGACTTCGGAT
21581	Yes	AATGGATAAGGCGTCTGACTTCGGATC

21582	Yes	AATGGATAAGGCGTCTGACTTCGGATCA
21583	Yes	AATGGATAAGGCGTCTGACTTCGGATCAA
21584	Yes	AATGGATAAGGCGTCTGACTTCGGATCAAA
21585	Yes	AATGGATAAGGCGTCTGACTTCGGATCAAAA
21586	Yes	AATGGATAAGGCGTCTGACTTCGGATCAAAAG
21587	Yes	AATGGATAAGGCGTCTGACTTCGGATCAAAAGA
21588	Yes	AATGGATAAGGCGTCTGACTTCGGATCAAAAGAT
21589	Yes	AATGGATAAGGCGTCTGACTTCGGATCAAAAGATT
21590	Yes	AATGGATAAGGCGTCTGACTTCGGATCAAAAGATTG
21591	Yes	AATGGATAAGGCGTCTGACTTCGGATCAAAAGATTGC
21592	Yes	AATGGATAAGGCGTCTGACTTCGGATCAAAAGATTGCA
21593	Yes	AATGGATAAGGCGTCTGACTTCGGATCAAAAGATTGCAG
21594	Yes	AATGGATAAGGCGTCTGACTTCGGATCAAAAGATTGCAGG
21595	Yes	AATGGATAAGGCGTCTGACTTCGGATCAAAAGATTGCAGGT
21596	Yes	AATGGATAAGGCGTCTGACTTCGGATCAAAAGATTGCAGGTT
21597	Yes	AATGGATAAGGCGTCTGACTTCGGATCAAAAGATTGCAGGTTT
21598	Yes	AATGGATAAGGCGTCTGACTTCGGATCAAAAGATTGCAGGTTTG
21599	Yes	AATGGATAAGGCGTCTGACTTCGGATCAAAAGATTGCAGGTTTGA
21600	Yes	AATGGATAAGGCGTCTGACTTCGGATCAAAAGATTGCAGGTTTGAG
21601	Yes	AATGGATAAGGCGTCTGACTTCGGATCAAAAGATTGCAGGTTTGAGT
21602	Yes	AATGGATAAGGCGTCTGACTTCGGATCAAAAGATTGCAGGTTTGAGTT
21603	Yes	AATGGATAAGGCGTCTGACTTCGGATCAAAAGATTGCAGGTTTGAGTTC
21604	Yes	AATGGATAAGGCGTCTGACTTCGGATCAAAAGATTGCAGGTTTGAGTTCT
21605	Yes	AATGGATAAGGCGTCTGACTTCGGATCAG
21606	Yes	AATGGATAAGGCGTCTGACTTCGGATCAGA
21607	Yes	AATGGATAAGGCGTCTGACTTCGGATCAGAA
21608	Yes	AATGGATAAGGCGTCTGACTTCGGATCAGAAG
21609	Yes	AATGGATAAGGCGTCTGACTTCGGATCAGAAGA
21610	Yes	AATGGATAAGGCGTCTGACTTCGGATCAGAAGAT
21611	Yes	AATGGATAAGGCGTCTGACTTCGGATCAGAAGATT
21612	Yes	AATGGATAAGGCGTCTGACTTCGGATCAGAAGATTG
21613	Yes	AATGGATAAGGCGTCTGACTTCGGATCAGAAGATTGA
21614	Yes	AATGGATAAGGCGTCTGACTTCGGATCAGAAGATTGAG
21615	Yes	AATGGATAAGGCGTCTGACTTCGGATCAGAAGATTGAGG
21616	Yes	AATGGATAAGGCGTCTGACTTCGGATCAGAAGATTGAGGG
21617	Yes	AATGGATAAGGCGTCTGACTTCGGATCAGAAGATTGAGGGT
21618	Yes	AATGGATAAGGCGTCTGACTTCGGATCAGAAGATTGAGGGTT
21619	Yes	AATGGATAAGGCGTCTGACTTCGGATCAGAAGATTGAGGGTTC
21620	Yes	AATGGATAAGGCGTCTGACTTCGGATCAGAAGATTGAGGGTTCG
21621	Yes	AATGGATAAGGCGTCTGACTTCGGATCAGAAGATTGAGGGTTCGA
21622	Yes	AATGGATAAGGCGTCTGACTTCGGATCAGAAGATTGAGGGTTCGAA
21623	Yes	AATGGATAAGGCGTCTGACTTCGGATCAGAAGATTGAGGGTTCGAAT

21624	Yes	AATGGATAAGGCGTCTGACTTCGGATCAGAAGATTGAGGGTTCGAATC
21625	Yes	AATGGATAAGGCGTCTGACTTCGGATCAGAAGATTGAGGGTTCGAATCC
21626	Yes	AATGGATAAGGCGTCTGACTTCGGATCAGAAGATTGAGGGTTCGAATCCC
21627	Yes	AATGGATAAGGCGTCTGACTTCGGATCAGAAGATTGAGGGTTCGAG
21628	Yes	AATGGATAAGGCGTCTGACTTCGGATCAGAAGATTGAGGGTTCGAGT
21629	Yes	AATGGATAAGGCGTCTGACTTCGGATCAGAAGATTGAGGGTTCGAGTC
21630	Yes	AATGGATAAGGCGTCTGACTTCGGATCAGAAGATTGAGGGTTCGAGTCC
21631	Yes	AATGGATAAGGCGTCTGACTTCGGATCAGAAGATTGAGGGTTCGAGTCCC
21632	Yes	AATGGATAAGGCGTCTGACTTCGGATCAGAAGATTGC
21633	Yes	AATGGATAAGGCGTCTGACTTCGGATCAGAAGATTGCA
21634	Yes	AATGGATAAGGCGTCTGACTTCGGATCAGAAGATTGCAG
21635	Yes	AATGGATAAGGCGTCTGACTTCGGATCAGAAGATTGCAGG
21636	Yes	AATGGATAAGGCGTCTGACTTCGGATCAGAAGATTGCAGGT
21637	Yes	AATGGATAAGGCGTCTGACTTCGGATCAGAAGATTGCAGGTT
21638	Yes	AATGGATAAGGCGTCTGACTTCGGATCAGAAGATTGCAGGTTC
21639	Yes	AATGGATAAGGCGTCTGACTTCGGATCAGAAGATTGCAGGTTCG
21640	Yes	AATGGATAAGGCGTCTGACTTCGGATCAGAAGATTGCAGGTTCGA
21641	Yes	AATGGATAAGGCGTCTGACTTCGGATCAGAAGATTGCAGGTTCGAG
21642	Yes	AATGGATAAGGCGTCTGACTTCGGATCAGAAGATTGCAGGTTCGAGT
21643	Yes	AATGGATAAGGCGTCTGACTTCGGATCAGAAGATTGCAGGTTCGAGTC
21644	Yes	AATGGATAAGGCGTCTGACTTCGGATCAGAAGATTGCAGGTTCGAGTCC
21645	Yes	AATGGATAAGGCGTCTGACTTCGGATCAGAAGATTGCAGGTTCGAGTCCT
21646	Yes	AATGGATAAGGCGTCTGAT
21647	Yes	AATGGATAAGGCGTCTGATT
21648	Yes	AATGGATAAGGCGTCTGATTC
21649	Yes	AATGGATAAGGCGTCTGATTCC
21650	Yes	AATGGATAAGGCGTCTGATTCCG
21651	Yes	AATGGATAAGGCGTCTGATTCCGG
21652	Yes	AATGGATAAGGCGTCTGATTCCGGA
21653	Yes	AATGGATAAGGCGTCTGATTCCGGAT
21654	Yes	AATGGATAAGGCGTCTGATTCCGGATC
21655	Yes	AATGGATAAGGCGTCTGATTCCGGATCA
21656	Yes	AATGGATAAGGCGTCTGATTCCGGATCAG
21657	Yes	AATGGATAAGGCGTCTGATTCCGGATCAGA
21658	Yes	AATGGATAAGGCGTCTGATTCCGGATCAGAA
21659	Yes	AATGGATAAGGCGTCTGATTCCGGATCAGAAG
21660	Yes	AATGGATAAGGCGTCTGATTCCGGATCAGAAGA
21661	Yes	AATGGATAAGGCGTCTGATTCCGGATCAGAAGAT
21662	Yes	AATGGATAAGGCGTCTGATTCCGGATCAGAAGATT
21663	Yes	AATGGATAAGGCGTCTGATTCCGGATCAGAAGATTG
21664	Yes	AATGGATAAGGCGTCTGATTCCGGATCAGAAGATTGA
21665	Yes	AATGGATAAGGCGTCTGATTCCGGATCAGAAGATTGAG

21666	Yes	AATGGATAAGGCGTCTGATTCCGGATCAGAAGATTGAGG
21667	Yes	AATGGATAAGGCGTCTGATTCCGGATCAGAAGATTGAGGG
21668	Yes	AATGGATAAGGCGTCTGATTCCGGATCAGAAGATTGAGGGT
21669	Yes	AATGGATAAGGCGTCTGATTCCGGATCAGAAGATTGAGGGTT
21670	Yes	AATGGATAAGGCGTCTGATTCCGGATCAGAAGATTGAGGGTTC
21671	Yes	AATGGATAAGGCGTCTGATTCCGGATCAGAAGATTGAGGGTTCG
21672	Yes	AATGGATAAGGCGTCTGATTCCGGATCAGAAGATTGAGGGTTCGA
21673	Yes	AATGGATAAGGCGTCTGATTCCGGATCAGAAGATTGAGGGTTCGAG
21674	Yes	AATGGATAAGGCGTCTGATTCCGGATCAGAAGATTGAGGGTTCGAGT
21675	Yes	AATGGATAAGGCGTCTGATTCCGGATCAGAAGATTGAGGGTTCGAGTC
21676	Yes	AATGGATAAGGCGTCTGATTCCGGATCAGAAGATTGAGGGTTCGAGTCC
21677	Yes	AATGGATAAGGCGTCTGATTCCGGATCAGAAGATTGAGGGTTCGAGTCCC
21678	No	AATGGATAATGCGTCT
21679	No	AATGGATAATGCGTCTG
21680	No	AATGGATAATGCGTCTGA
21681	No	AATGGATAATGCGTCTGAC
21682	No	AATGGATAATGCGTCTGACT
21683	No	AATGGATAATGCGTCTGACTA
21684	Yes	AATGGATAATGCGTCTGACTAA
21685	Yes	AATGGATAATGCGTCTGACTAAG
21686	Yes	AATGGATAATGCGTCTGACTAAGA
21687	Yes	AATGGATAATGCGTCTGACTAAGAA
21688	Yes	AATGGATAATGCGTCTGACTAAGAAT
21689	Yes	AATGGATAATGCGTCTGACTAAGAATC
21690	Yes	AATGGATAATGCGTCTGACTAAGAATCA
21691	Yes	AATGGATAATGCGTCTGACTAAGAATCAG
21692	Yes	AATGGATAATGCGTCTGACTAAGAATCAGA
21693	Yes	AATGGATAATGCGTCTGACTAAGAATCAGAA
21694	Yes	AATGGATAATGCGTCTGACTAAGAATCAGAAG
21695	Yes	AATGGATAATGCGTCTGACTAAGAATCAGAAGA
21696	Yes	AATGGATAATGCGTCTGACTAAGAATCAGAAGAT
21697	Yes	AATGGATAATGCGTCTGACTAAGAATCAGAAGATT
21698	Yes	AATGGATAATGCGTCTGACTAAGAATCAGAAGATTC
21699	Yes	AATGGATAATGCGTCTGACTAAGAATCAGAAGATTCC
21700	Yes	AATGGATAATGCGTCTGACTAAGAATCAGAAGATTCCA
21701	Yes	AATGGATAATGCGTCTGACTAAGAATCAGAAGATTCCAG
21702	Yes	AATGGATAATGCGTCTGACTAAGAATCAGAAGATTCCAGC
21703	Yes	AATGGATAATGCGTCTGACTAAGAATCAGAAGATTCCAGCC
21704	Yes	AATGGATAATGCGTCTGACTAAGAATCAGAAGATTCCAGCCT
21705	Yes	AATGGATAATGCGTCTGACTAAGAATCAGAAGATTCCAGCCTT
21706	Yes	AATGGATAATGCGTCTGACTAAGAATCAGAAGATTCCAGCCTTG
21707	Yes	AATGGATAATGCGTCTGACTAAGAATCAGAAGATTCCAGCCTTGA

21708	Yes	AATGGATAATGCGTCTGACTAAGAATCAGAAGATTCCAGCCTTGAC
21709	Yes	AATGGATAATGCGTCTGACTAAGAATCAGAAGATTCCAGCCTTGACT
21710	Yes	AATGGATAATGCGTCTGACTAAGAATCAGAAGATTCCAGCCTTGACTC
21711	Yes	AATGGATAATGCGTCTGACTAAGAATCAGAAGATTCCAGCCTTGACTCC
21712	Yes	AATGGATAATGCGTCTGACTAAGAATCAGAAGATTCCAGCCTTGACTCCT
21713	No	AATGGATAGCGCATTG
21714	No	AATGGATAGCGCATTGG
21715	No	AATGGATAGCGCATTGGA
21716	No	AATGGATAGCGCATTGGAC
21717	No	AATGGATAGCGCATTGGACT
21718	No	AATGGATAGCGCATTGGACTT
21719	No	AATGGATAGCGCATTGGACTTC
21720	No	AATGGATAGCGCATTGGACTTCT
21721	No	AATGGATAGCGCATTGGACTTCTA
21722	No	AATGGATAGCGCATTGGACTTCTAA
21723	Yes	AATGGATAGCGCATTGGACTTCTAAT
21724	Yes	AATGGATAGCGCATTGGACTTCTAATT
21725	Yes	AATGGATAGCGCATTGGACTTCTAATTC
21726	Yes	AATGGATAGCGCATTGGACTTCTAATTCA
21727	Yes	AATGGATAGCGCATTGGACTTCTAATTCAA
21728	Yes	AATGGATAGCGCATTGGACTTCTAATTCAAA
21729	Yes	AATGGATAGCGCATTGGACTTCTAATTCAAAG
21730	Yes	AATGGATAGCGCATTGGACTTCTAATTCAAAGG
21731	Yes	AATGGATAGCGCATTGGACTTCTAATTCAAAGGT
21732	Yes	AATGGATAGCGCATTGGACTTCTAATTCAAAGGTT
21733	Yes	AATGGATAGCGCATTGGACTTCTAATTCAAAGGTTC
21734	Yes	AATGGATAGCGCATTGGACTTCTAATTCAAAGGTTCC
21735	Yes	AATGGATAGCGCATTGGACTTCTAATTCAAAGGTTCCG
21736	Yes	AATGGATAGCGCATTGGACTTCTAATTCAAAGGTTCCGG
21737	Yes	AATGGATAGCGCATTGGACTTCTAATTCAAAGGTTCCGGG
21738	Yes	AATGGATAGCGCATTGGACTTCTAATTCAAAGGTTCCGGGT
21739	Yes	AATGGATAGCGCATTGGACTTCTAATTCAAAGGTTCCGGGTT
21740	Yes	AATGGATAGCGCATTGGACTTCTAATTCAAAGGTTCCGGGTTC
21741	Yes	AATGGATAGCGCATTGGACTTCTAATTCAAAGGTTCCGGGTTCG
21742	Yes	AATGGATAGCGCATTGGACTTCTAATTCAAAGGTTCCGGGTTCGA
21743	Yes	AATGGATAGCGCATTGGACTTCTAATTCAAAGGTTCCGGGTTCGAG
21744	Yes	AATGGATAGCGCATTGGACTTCTAATTCAAAGGTTCCGGGTTCGAGT
21745	Yes	AATGGATAGCGCATTGGACTTCTAATTCAAAGGTTCCGGGTTCGAGTC
21746	Yes	AATGGATAGCGCATTGGACTTCTAATTCAAAGGTTCCGGGTTCGAGTCC
21747	Yes	AATGGATAGCGCATTGGACTTCTAATTCAAAGGTTCCGGGTTCGAGTCCC
21748	Yes	AATGGATAGCGCATTGGACTTCTAATTCAAAGGTTG
21749	Yes	AATGGATAGCGCATTGGACTTCTAATTCAAAGGTTGC

21750	Yes	AATGGATAGCGCATTGGACTTCTAATTCAAAGGTTGCG
21751	Yes	AATGGATAGCGCATTGGACTTCTAATTCAAAGGTTGCGG
21752	Yes	AATGGATAGCGCATTGGACTTCTAATTCAAAGGTTGCGGG
21753	Yes	AATGGATAGCGCATTGGACTTCTAATTCAAAGGTTGCGGGT
21754	Yes	AATGGATAGCGCATTGGACTTCTAATTCAAAGGTTGCGGGTT
21755	Yes	AATGGATAGCGCATTGGACTTCTAATTCAAAGGTTGCGGGTTC
21756	Yes	AATGGATAGCGCATTGGACTTCTAATTCAAAGGTTGCGGGTTCG
21757	Yes	AATGGATAGCGCATTGGACTTCTAATTCAAAGGTTGCGGGTTCGA
21758	Yes	AATGGATAGCGCATTGGACTTCTAATTCAAAGGTTGCGGGTTCGAG
21759	Yes	AATGGATAGCGCATTGGACTTCTAATTCAAAGGTTGCGGGTTCGAGT
21760	Yes	AATGGATAGCGCATTGGACTTCTAATTCAAAGGTTGCGGGTTCGAGTC
21761	Yes	AATGGATAGCGCATTGGACTTCTAATTCAAAGGTTGCGGGTTCGAGTCC
21762	Yes	AATGGATAGCGCATTGGACTTCTAATTCAAAGGTTGCGGGTTCGAGTCCC
21763	Yes	AATGGATAGCGCATTGGACTTCTAATTCAAAGGTTGT
21764	Yes	AATGGATAGCGCATTGGACTTCTAATTCAAAGGTTGTG
21765	Yes	AATGGATAGCGCATTGGACTTCTAATTCAAAGGTTGTGG
21766	Yes	AATGGATAGCGCATTGGACTTCTAATTCAAAGGTTGTGGG
21767	Yes	AATGGATAGCGCATTGGACTTCTAATTCAAAGGTTGTGGGT
21768	Yes	AATGGATAGCGCATTGGACTTCTAATTCAAAGGTTGTGGGTT
21769	Yes	AATGGATAGCGCATTGGACTTCTAATTCAAAGGTTGTGGGTTC
21770	Yes	AATGGATAGCGCATTGGACTTCTAATTCAAAGGTTGTGGGTTCG
21771	Yes	AATGGATAGCGCATTGGACTTCTAATTCAAAGGTTGTGGGTTCGA
21772	Yes	AATGGATAGCGCATTGGACTTCTAATTCAAAGGTTGTGGGTTCGAA
21773	Yes	AATGGATAGCGCATTGGACTTCTAATTCAAAGGTTGTGGGTTCGAAT
21774	Yes	AATGGATAGCGCATTGGACTTCTAATTCAAAGGTTGTGGGTTCGAATC
21775	Yes	AATGGATAGCGCATTGGACTTCTAATTCAAAGGTTGTGGGTTCGAATCC
21776	Yes	AATGGATAGCGCATTGGACTTCTAATTCAAAGGTTGTGGGTTCGAATCCC
21777	Yes	AATGGATAGCGCATTGGACTTCTAATTCAAAGGTTGTGGGTTCGAG
21778	Yes	AATGGATAGCGCATTGGACTTCTAATTCAAAGGTTGTGGGTTCGAGT
21779	Yes	AATGGATAGCGCATTGGACTTCTAATTCAAAGGTTGTGGGTTCGAGTC
21780	Yes	AATGGATAGCGCATTGGACTTCTAATTCAAAGGTTGTGGGTTCGAGTCC
21781	Yes	AATGGATAGCGCATTGGACTTCTAATTCAAAGGTTGTGGGTTCGAGTCCC
21782	Yes	AATGGATCGAAGCCAT
21783	Yes	AATGGATCGAAGCCATC
21784	Yes	AATGGATCGAAGCCATCC
21785	Yes	AATGGATCGAAGCCATCCT
21786	Yes	AATGGATCGAAGCCATCCTT
21787	Yes	AATGGATCGAAGCCATCCTTG
21788	Yes	AATGGATCGAAGCCATCCTTGG
21789	Yes	AATGGATCGAAGCCATCCTTGGC
21790	Yes	AATGGATCGAAGCCATCCTTGGCT
21791	Yes	AATGGATCGAAGCCATCCTTGGCTA

21792	Yes	AATGGATCGAAGCCATCCTTGGCTAC
21793	Yes	AATGGATCGAAGCCATCCTTGGCTACC
21794	Yes	AATGGATCGAAGCCATCCTTGGCTACCA
21795	Yes	AATGGATTCATATCCG
21796	Yes	AATGGATTCATATCCGC
21797	Yes	AATGGATTCATATCCGCG
21798	Yes	AATGGATTCATATCCGCGT
21799	Yes	AATGGATTCATATCCGCGTG
21800	Yes	AATGGATTCATATCCGCGTGG
21801	Yes	AATGGATTCATATCCGCGTGGG
21802	Yes	AATGGATTCATATCCGCGTGGGT
21803	Yes	AATGGATTCATATCCGCGTGGGTT
21804	Yes	AATGGATTCATATCCGCGTGGGTTC
21805	Yes	AATGGATTCATATCCGCGTGGGTTCG
21806	Yes	AATGGATTCATATCCGCGTGGGTTCGA
21807	Yes	AATGGATTCATATCCGCGTGGGTTCGAA
21808	Yes	AATGGATTCATATCCGCGTGGGTTCGAAC
21809	Yes	AATGGATTCATATCCGCGTGGGTTCGAACC
21810	Yes	AATGGATTCATATCCGCGTGGGTTCGAACCC
21811	Yes	AATGGATTCATATCCGCGTGGGTTCGAACCCC
21812	Yes	AATGGATTCATATCCGCGTGGGTTCGAACCCCA
21813	Yes	AATGGATTCATATCCGCGTGGGTTCGAACCCCAC
21814	Yes	AATGGATTCATATCCGCGTGGGTTCGAACCCCACT
21815	Yes	AATGGATTCATATCCGCGTGGGTTCGAACCCCACTT
21816	Yes	AATGGATTCATATCCGCGTGGGTTCGAACCCCACTTC
21817	Yes	AATGGATTCATATCCGCGTGGGTTCGAACCCCACTTCT
21818	Yes	AATGGATTCATATCCGCGTGGGTTCGAACCCCACTTCTG
21819	Yes	AATGGATTCATATCCGCGTGGGTTCGAACCCCACTTCTGG
21820	Yes	AATGGATTCATATCCGCGTGGGTTCGAACCCCACTTCTGGT
21821	Yes	AATGGATTCATATCCGCGTGGGTTCGAACCCCACTTCTGGTA
21822	Yes	AATGGATTCATATCCGCGTGGGTTCGAACCCCACTTCTGGTAC
21823	Yes	AATGGATTCATATCCGCGTGGGTTCGAACCCCACTTCTGGTACC
21824	Yes	AATGGATTCATATCCGCGTGGGTTCGAACCCCACTTCTGGTACCA
21825	Yes	AATGGCCGAGTGGTTA
21826	Yes	AATGGCCGAGTGGTTAA
21827	Yes	AATGGCCGAGTGGTTAAG
21828	Yes	AATGGCCGAGTGGTTAAGG
21829	Yes	AATGGCCGAGTGGTTAAGGC
21830	Yes	AATGGCCGAGTGGTTAAGGCG
21831	Yes	AATGGCCGAGTGGTTAAGGCGT
21832	Yes	AATGGCCGAGTGGTTAAGGCGTT
21833	Yes	AATGGCCGAGTGGTTAAGGCGTTG

21834	Yes	AATGGCCGAGTGGTTAAGGCGTTGG
21835	Yes	AATGGCCGAGTGGTTAAGGCGTTGGA
21836	Yes	AATGGCCGAGTGGTTAAGGCGTTGGAC
21837	Yes	AATGGCCGAGTGGTTAAGGCGTTGGACT
21838	Yes	AATGGCCGAGTGGTTAAGGCGTTGGACTT
21839	Yes	AATGGCCGAGTGGTTAAGGCGTTGGACTTA
21840	Yes	AATGGCCGAGTGGTTAAGGCGTTGGACTTAA
21841	Yes	AATGGCCGAGTGGTTAAGGCGTTGGACTTAAG
21842	Yes	AATGGCCGAGTGGTTAAGGCGTTGGACTTAAGA
21843	Yes	AATGGCCGAGTGGTTAAGGCGTTGGACTTAAGAT
21844	Yes	AATGGCCGAGTGGTTAAGGCGTTGGACTTAAGATC
21845	Yes	AATGGCCGAGTGGTTAAGGCGTTGGACTTAAGATCC
21846	Yes	AATGGCCGAGTGGTTAAGGCGTTGGACTTAAGATCCA
21847	Yes	AATGGCCGAGTGGTTAAGGCGTTGGACTTAAGATCCAA
21848	Yes	AATGGCCGAGTGGTTAAGGCGTTGGACTTAAGATCCAAT
21849	Yes	AATGGCCGAGTGGTTAAGGCGTTGGACTTAAGATCCAATG
21850	Yes	AATGGCCGAGTGGTTAAGGCGTTGGACTTAAGATCCAATGG
21851	Yes	AATGGCCGAGTGGTTAAGGCGTTGGACTTAAGATCCAATGGA
21852	Yes	AATGGCCGAGTGGTTAAGGCGTTGGACTTAAGATCCAATGGAT
21853	Yes	AATGGCCGAGTGGTTAAGGCGTTGGACTTAAGATCCAATGGATT
21854	Yes	AATGGCCGAGTGGTTAAGGCGTTGGACTTAAGATCCAATGGATTC
21855	Yes	AATGGCCGAGTGGTTAAGGCGTTGGACTTAAGATCCAATGGATTCA
21856	Yes	AATGGCCGAGTGGTTAAGGCGTTGGACTTAAGATCCAATGGATTCAT
21857	Yes	AATGGCCGAGTGGTTAAGGCGTTGGACTTAAGATCCAATGGATTCATA
21858	Yes	AATGGCCGAGTGGTTAAGGCGTTGGACTTAAGATCCAATGGATTCATAT
21859	Yes	AATGGCCGAGTGGTTAAGGCGTTGGACTTAAGATCCAATGGATTCATATC
21860	No	AATGGCTGAGTGAAGC
21861	No	AATGGCTGAGTGAAGCA
21862	No	AATGGCTGAGTGAAGCAT
21863	No	AATGGCTGAGTGAAGCATT
21864	No	AATGGCTGAGTGAAGCATTG
21865	No	AATGGCTGAGTGAAGCATTGG
21866	No	AATGGCTGAGTGAAGCATTGGA
21867	No	AATGGCTGAGTGAAGCATTGGAC
21868	No	AATGGCTGAGTGAAGCATTGGACT
21869	No	AATGGCTGAGTGAAGCATTGGACTG
21870	No	AATGGCTGAGTGAAGCATTGGACTGT
21871	No	AATGGCTGAGTGAAGCATTGGACTGTA
21872	No	AATGGCTGAGTGAAGCATTGGACTGTAA
21873	No	AATGGCTGAGTGAAGCATTGGACTGTAAA
21874	No	AATGGCTGAGTGAAGCATTGGACTGTAAAT
21875	No	AATGGCTGAGTGAAGCATTGGACTGTAAATC

21876	No	AATGGCTGAGTGAAGCATTGGACTGTAAATCT
21877	No	AATGGCTGAGTGAAGCATTGGACTGTAAATCTA
21878	No	AATGGCTGAGTGAAGCATTGGACTGTAAATCTAA
21879	No	AATGGCTGAGTGAAGCATTGGACTGTAAATCTAAA
21880	No	AATGGCTGAGTGAAGCATTGGACTGTAAATCTAAAG
21881	No	AATGGCTGAGTGAAGCATTGGACTGTAAATCTAAAGA
21882	No	AATGGCTGAGTGAAGCATTGGACTGTAAATCTAAAGAC
21883	No	AATGGCTGAGTGAAGCATTGGACTGTAAATCTAAAGACA
21884	No	AATGGCTGAGTGAAGCATTGGACTGTAAATCTAAAGACAG
21885	No	AATGGCTGAGTGAAGCATTGGACTGTAAATCTAAAGACAGG
21886	No	AATGGCTGAGTGAAGCATTGGACTGTAAATCTAAAGACAGGG
21887	No	AATGGCTGAGTGAAGCATTGGACTGTAAATCTAAAGACAGGGG
21888	No	AATGGCTGAGTGAAGCATTGGACTGTAAATCTAAAGACAGGGGT
21889	No	AATGGCTGAGTGAAGCATTGGACTGTAAATCTAAAGACAGGGGTT
21890	No	AATGGCTGAGTGAAGCATTGGACTGTAAATCTAAAGACAGGGGTTA
21891	Yes	AATGGCTGAGTGAAGCATTGGACTGTAAATCTAAAGACAGGGGTTAG
21892	Yes	AATGGCTGAGTGAAGCATTGGACTGTAAATCTAAAGACAGGGGTTAGG
21893	Yes	AATGGCTGAGTGAAGCATTGGACTGTAAATCTAAAGACAGGGGTTAGGC
21894	Yes	AATGGCTGAGTGAAGCATTGGACTGTAAATCTAAAGACAGGGGTTAGGCC
21895	Yes	AATGGGCTGGTGCCCG
21896	Yes	AATGGGCTGGTGCCCGC
21897	Yes	AATGGGCTGGTGCCCGCG
21898	Yes	AATGGGCTGGTGCCCGCGT
21899	Yes	AATGGGCTGGTGCCCGCGTG
21900	Yes	AATGGGCTGGTGCCCGCGTGG
21901	Yes	AATGGGCTGGTGCCCGCGTGGG
21902	Yes	AATGGGCTGGTGCCCGCGTGGGT
21903	Yes	AATGGGCTGGTGCCCGCGTGGGTT
21904	Yes	AATGGGCTGGTGCCCGCGTGGGTTC
21905	Yes	AATGGGCTGGTGCCCGCGTGGGTTCG
21906	Yes	AATGGGCTGGTGCCCGCGTGGGTTCGA
21907	Yes	AATGGGCTGGTGCCCGCGTGGGTTCGAA
21908	Yes	AATGGGCTGGTGCCCGCGTGGGTTCGAAC
21909	Yes	AATGGGCTGGTGCCCGCGTGGGTTCGAACC
21910	Yes	AATGGGCTGGTGCCCGCGTGGGTTCGAACCC
21911	Yes	AATGGGCTGGTGCCCGCGTGGGTTCGAACCCC
21912	Yes	AATGGGCTGGTGCCCGCGTGGGTTCGAACCCCA
21913	Yes	AATGGGCTGGTGCCCGCGTGGGTTCGAACCCCAC
21914	Yes	AATGGGCTGGTGCCCGCGTGGGTTCGAACCCCACT
21915	Yes	AATGGGCTGGTGCCCGCGTGGGTTCGAACCCCACTC
21916	Yes	AATGGGCTGGTGCCCGCGTGGGTTCGAACCCCACTCT
21917	Yes	AATGGGCTGGTGCCCGCGTGGGTTCGAACCCCACTCTC

21918	Yes	AATGGGCTGGTGCCCGCGTGGGTTCGAACCCCACTCTCG
21919	Yes	AATGGGCTGGTGCCCGCGTGGGTTCGAACCCCACTCTCGG
21920	Yes	AATGGGCTGGTGCCCGCGTGGGTTCGAACCCCACTCTCGGT
21921	Yes	AATGGGCTGGTGCCCGCGTGGGTTCGAACCCCACTCTCGGTA
21922	Yes	AATGGGCTGGTGCCCGCGTGGGTTCGAACCCCACTCTCGGTAC
21923	Yes	AATGGGCTGGTGCCCGCGTGGGTTCGAACCCCACTCTCGGTACC
21924	Yes	AATGGGCTGGTGCCCGCGTGGGTTCGAACCCCACTCTCGGTACCA
21925	No	AATGGGGGTTCCCCGC
21926	Yes	AATGGGGGTTCCCCGCG
21927	Yes	AATGGGGGTTCCCCGCGC
21928	Yes	AATGGGGGTTCCCCGCGCA
21929	Yes	AATGGGGGTTCCCCGCGCAG
21930	Yes	AATGGGGGTTCCCCGCGCAGG
21931	Yes	AATGGGGGTTCCCCGCGCAGGT
21932	Yes	AATGGGGGTTCCCCGCGCAGGTT
21933	Yes	AATGGGGGTTCCCCGCGCAGGTTC
21934	Yes	AATGGGGGTTCCCCGCGCAGGTTCA
21935	Yes	AATGGGGGTTCCCCGCGCAGGTTCAA
21936	Yes	AATGGGGGTTCCCCGCGCAGGTTCAAA
21937	Yes	AATGGGGGTTCCCCGCGCAGGTTCAAAT
21938	Yes	AATGGGGGTTCCCCGCGCAGGTTCAAATC
21939	Yes	AATGGGGGTTCCCCGCGCAGGTTCAAATCC
21940	Yes	AATGGGGGTTCCCCGCGCAGGTTCAAATCCT
21941	Yes	AATGGGGGTTCCCCGCGCAGGTTCAAATCCTG
21942	Yes	AATGGGGGTTCCCCGCGCAGGTTCAAATCCTGC
21943	Yes	AATGGGGGTTCCCCGCGCAGGTTCAAATCCTGCT
21944	Yes	AATGGGGGTTCCCCGCGCAGGTTCAAATCCTGCTC
21945	Yes	AATGGGGGTTCCCCGCGCAGGTTCAAATCCTGCTCA
21946	Yes	AATGGGGGTTCCCCGCGCAGGTTCAAATCCTGCTCAC
21947	Yes	AATGGGGGTTCCCCGCGCAGGTTCAAATCCTGCTCACA
21948	Yes	AATGGGGGTTCCCCGCGCAGGTTCAAATCCTGCTCACAG
21949	Yes	AATGGGGGTTCCCCGCGCAGGTTCAAATCCTGCTCACAGC
21950	Yes	AATGGGGGTTCCCCGCGCAGGTTCAAATCCTGCTCACAGCG
21951	Yes	AATGGGGGTTCCCCGCGCAGGTTCAAATCCTGCTCACAGCGC
21952	Yes	AATGGGGGTTCCCCGCGCAGGTTCAAATCCTGCTCACAGCGCC
21953	Yes	AATGGGGGTTCCCCGCGCAGGTTCAAATCCTGCTCACAGCGCCA
21954	Yes	AATGGGGTCTCCCCGC
21955	Yes	AATGGGGTCTCCCCGCG
21956	Yes	AATGGGGTCTCCCCGCGC
21957	Yes	AATGGGGTCTCCCCGCGCA
21958	Yes	AATGGGGTCTCCCCGCGCAG
21959	Yes	AATGGGGTCTCCCCGCGCAGG

21960	Yes	AATGGGGTCTCCCCGCGCAGGT
21961	Yes	AATGGGGTCTCCCCGCGCAGGTT
21962	Yes	AATGGGGTCTCCCCGCGCAGGTTC
21963	Yes	AATGGGGTCTCCCCGCGCAGGTTCA
21964	Yes	AATGGGGTCTCCCCGCGCAGGTTCAA
21965	Yes	AATGGGGTCTCCCCGCGCAGGTTCAAA
21966	Yes	AATGGGGTCTCCCCGCGCAGGTTCAAAT
21967	Yes	AATGGGGTCTCCCCGCGCAGGTTCAAATC
21968	Yes	AATGGGGTCTCCCCGCGCAGGTTCAAATCC
21969	Yes	AATGGGGTCTCCCCGCGCAGGTTCAAATCCT
21970	Yes	AATGGGGTCTCCCCGCGCAGGTTCAAATCCTG
21971	Yes	AATGGGGTCTCCCCGCGCAGGTTCAAATCCTGC
21972	Yes	AATGGGGTCTCCCCGCGCAGGTTCAAATCCTGCT
21973	Yes	AATGGGGTCTCCCCGCGCAGGTTCAAATCCTGCTC
21974	Yes	AATGGGGTCTCCCCGCGCAGGTTCAAATCCTGCTCA
21975	Yes	AATGGGGTCTCCCCGCGCAGGTTCAAATCCTGCTCAC
21976	Yes	AATGGGGTCTCCCCGCGCAGGTTCAAATCCTGCTCACA
21977	Yes	AATGGGGTCTCCCCGCGCAGGTTCAAATCCTGCTCACAG
21978	Yes	AATGGGGTCTCCCCGCGCAGGTTCAAATCCTGCTCACAGC
21979	Yes	AATGGGGTCTCCCCGCGCAGGTTCAAATCCTGCTCACAGCG
21980	Yes	AATGGGGTCTCCCCGCGCAGGTTCAAATCCTGCTCACAGCGC
21981	Yes	AATGGGGTCTCCCCGCGCAGGTTCAAATCCTGCTCACAGCGCC
21982	Yes	AATGGGGTCTCCCCGCGCAGGTTCAAATCCTGCTCACAGCGCCA
21983	Yes	AATGGGGTCTCCCCGCGCAGGTTCG
21984	Yes	AATGGGGTCTCCCCGCGCAGGTTCGA
21985	Yes	AATGGGGTCTCCCCGCGCAGGTTCGAA
21986	Yes	AATGGGGTCTCCCCGCGCAGGTTCGAAC
21987	Yes	AATGGGGTCTCCCCGCGCAGGTTCGAACC
21988	Yes	AATGGGGTCTCCCCGCGCAGGTTCGAACCC
21989	Yes	AATGGGGTCTCCCCGCGCAGGTTCGAACCCT
21990	Yes	AATGGGGTCTCCCCGCGCAGGTTCGAACCCTG
21991	Yes	AATGGGGTCTCCCCGCGCAGGTTCGAACCCTGC
21992	Yes	AATGGGGTCTCCCCGCGCAGGTTCGAACCCTGCT
21993	Yes	AATGGGGTCTCCCCGCGCAGGTTCGAACCCTGCTC
21994	Yes	AATGGGGTCTCCCCGCGCAGGTTCGAACCCTGCTCG
21995	Yes	AATGGGGTCTCCCCGCGCAGGTTCGAACCCTGCTCGC
21996	Yes	AATGGGGTCTCCCCGCGCAGGTTCGAACCCTGCTCGCT
21997	Yes	AATGGGGTCTCCCCGCGCAGGTTCGAACCCTGCTCGCTG
21998	Yes	AATGGGGTCTCCCCGCGCAGGTTCGAACCCTGCTCGCTGC
21999	Yes	AATGGGGTCTCCCCGCGCAGGTTCGAACCCTGCTCGCTGCG
22000	Yes	AATGGGGTCTCCCCGCGCAGGTTCGAACCCTGCTCGCTGCGC
22001	Yes	AATGGGGTCTCCCCGCGCAGGTTCGAACCCTGCTCGCTGCGCC

22002	Yes	AATGGGGTCTCCCCGCGCAGGTTCGAACCCTGCTCGCTGCGCCA
22003	Yes	AATGGGGTCTCCCCGCGCAGGTTCGAAT
22004	Yes	AATGGGGTCTCCCCGCGCAGGTTCGAATC
22005	Yes	AATGGGGTCTCCCCGCGCAGGTTCGAATCC
22006	Yes	AATGGGGTCTCCCCGCGCAGGTTCGAATCCT
22007	Yes	AATGGGGTCTCCCCGCGCAGGTTCGAATCCTG
22008	Yes	AATGGGGTCTCCCCGCGCAGGTTCGAATCCTGC
22009	Yes	AATGGGGTCTCCCCGCGCAGGTTCGAATCCTGCT
22010	Yes	AATGGGGTCTCCCCGCGCAGGTTCGAATCCTGCTC
22011	Yes	AATGGGGTCTCCCCGCGCAGGTTCGAATCCTGCTCA
22012	Yes	AATGGGGTCTCCCCGCGCAGGTTCGAATCCTGCTCAC
22013	Yes	AATGGGGTCTCCCCGCGCAGGTTCGAATCCTGCTCACA
22014	Yes	AATGGGGTCTCCCCGCGCAGGTTCGAATCCTGCTCACAG
22015	Yes	AATGGGGTCTCCCCGCGCAGGTTCGAATCCTGCTCACAGC
22016	Yes	AATGGGGTCTCCCCGCGCAGGTTCGAATCCTGCTCACAGCG
22017	Yes	AATGGGGTCTCCCCGCGCAGGTTCGAATCCTGCTCACAGCGC
22018	Yes	AATGGGGTCTCCCCGCGCAGGTTCGAATCCTGCTCACAGCGCC
22019	Yes	AATGGGGTCTCCCCGCGCAGGTTCGAATCCTGCTCACAGCGCCA
22020	Yes	AATGGGGTTTCCCCGC
22021	Yes	AATGGGGTTTCCCCGCA
22022	Yes	AATGGGGTTTCCCCGCAC
22023	Yes	AATGGGGTTTCCCCGCACA
22024	Yes	AATGGGGTTTCCCCGCACAG
22025	Yes	AATGGGGTTTCCCCGCACAGG
22026	Yes	AATGGGGTTTCCCCGCACAGGT
22027	Yes	AATGGGGTTTCCCCGCACAGGTT
22028	Yes	AATGGGGTTTCCCCGCACAGGTTC
22029	Yes	AATGGGGTTTCCCCGCACAGGTTCG
22030	Yes	AATGGGGTTTCCCCGCACAGGTTCGA
22031	Yes	AATGGGGTTTCCCCGCACAGGTTCGAA
22032	Yes	AATGGGGTTTCCCCGCACAGGTTCGAAT
22033	Yes	AATGGGGTTTCCCCGCACAGGTTCGAATC
22034	Yes	AATGGGGTTTCCCCGCACAGGTTCGAATCC
22035	Yes	AATGGGGTTTCCCCGCACAGGTTCGAATCCT
22036	Yes	AATGGGGTTTCCCCGCACAGGTTCGAATCCTG
22037	Yes	AATGGGGTTTCCCCGCACAGGTTCGAATCCTGT
22038	Yes	AATGGGGTTTCCCCGCACAGGTTCGAATCCTGTT
22039	Yes	AATGGGGTTTCCCCGCACAGGTTCGAATCCTGTTC
22040	Yes	AATGGGGTTTCCCCGCACAGGTTCGAATCCTGTTCG
22041	Yes	AATGGGGTTTCCCCGCACAGGTTCGAATCCTGTTCGT
22042	Yes	AATGGGGTTTCCCCGCACAGGTTCGAATCCTGTTCGTG
22043	Yes	AATGGGGTTTCCCCGCACAGGTTCGAATCCTGTTCGTGA

22044	Yes	AATGGGGTTTCCCCGCACAGGTTCGAATCCTGTTCGTGAC
22045	Yes	AATGGGGTTTCCCCGCACAGGTTCGAATCCTGTTCGTGACG
22046	Yes	AATGGGGTTTCCCCGCACAGGTTCGAATCCTGTTCGTGACGC
22047	Yes	AATGGGGTTTCCCCGCACAGGTTCGAATCCTGTTCGTGACGCC
22048	Yes	AATGGGGTTTCCCCGCACAGGTTCGAATCCTGTTCGTGACGCCA
22049	No	AATGGGTAGCACAGAG
22050	Yes	AATGGGTAGCACAGAGA
22051	Yes	AATGGGTAGCACAGAGAA
22052	Yes	AATGGGTAGCACAGAGAAT
22053	Yes	AATGGGTAGCACAGAGAATT
22054	Yes	AATGGGTAGCACAGAGAATTT
22055	Yes	AATGGGTAGCACAGAGAATTTT
22056	Yes	AATGGGTAGCACAGAGAATTTTG
22057	Yes	AATGGGTAGCACAGAGAATTTTGG
22058	Yes	AATGGGTAGCACAGAGAATTTTGGA
22059	Yes	AATGGGTAGCACAGAGAATTTTGGAT
22060	Yes	AATGGGTAGCACAGAGAATTTTGGATT
22061	Yes	AATGGGTAGCACAGAGAATTTTGGATTC
22062	Yes	AATGGGTAGCACAGAGAATTTTGGATTCT
22063	Yes	AATGGGTAGCACAGAGAATTTTGGATTCTC
22064	Yes	AATGGGTAGCACAGAGAATTTTGGATTCTCA
22065	Yes	AATGGGTAGCACAGAGAATTTTGGATTCTCAG
22066	Yes	AATGGGTAGCACAGAGAATTTTGGATTCTCAGG
22067	Yes	AATGGGTAGCACAGAGAATTTTGGATTCTCAGGG
22068	Yes	AATGGGTAGCACAGAGAATTTTGGATTCTCAGGGG
22069	Yes	AATGGGTAGCACAGAGAATTTTGGATTCTCAGGGGT
22070	Yes	AATGGGTAGCACAGAGAATTTTGGATTCTCAGGGGTG
22071	Yes	AATGGGTAGCACAGAGAATTTTGGATTCTCAGGGGTGG
22072	Yes	AATGGGTAGCACAGAGAATTTTGGATTCTCAGGGGTGGG
22073	Yes	AATGGGTAGCACAGAGAATTTTGGATTCTCAGGGGTGGGT
22074	Yes	AATGGGTAGCACAGAGAATTTTGGATTCTCAGGGGTGGGTT
22075	Yes	AATGGGTAGCACAGAGAATTTTGGATTCTCAGGGGTGGGTTC
22076	Yes	AATGGGTAGCACAGAGAATTTTGGATTCTCAGGGGTGGGTTCA
22077	Yes	AATGGGTAGCACAGAGAATTTTGGATTCTCAGGGGTGGGTTCAA
22078	Yes	AATGGGTAGCACAGAGAATTTTGGATTCTCAGGGGTGGGTTCAAT
22079	Yes	AATGGGTAGCACAGAGAATTTTGGATTCTCAGGGGTGGGTTCAATT
22080	Yes	AATGGGTAGCACAGAGAATTTTGGATTCTCAGGGGTGGGTTCAATTC
22081	Yes	AATGGGTAGCACAGAGAATTTTGGATTCTCAGGGGTGGGTTCAATTCC
22082	Yes	AATGGGTAGCACAGAGAATTTTGGATTCTCAGGGGTGGGTTCAATTCCT
22083	Yes	AATGGGTAGCACAGAGAATTTTGGATTCTCAGGGGTGGGTTCAATTCCTT
22084	Yes	AATGGGTTAGCGCGTT
22085	Yes	AATGGGTTAGCGCGTTC

22086	Yes	AATGGGTTAGCGCGTTCG
22087	Yes	AATGGGTTAGCGCGTTCGG
22088	Yes	AATGGGTTAGCGCGTTCGGC
22089	Yes	AATGGGTTAGCGCGTTCGGCT
22090	Yes	AATGGGTTAGCGCGTTCGGCTG
22091	Yes	AATGGGTTAGCGCGTTCGGCTGT
22092	Yes	AATGGGTTAGCGCGTTCGGCTGTT
22093	Yes	AATGGGTTAGCGCGTTCGGCTGTTA
22094	Yes	AATGGGTTAGCGCGTTCGGCTGTTAA
22095	Yes	AATGGGTTAGCGCGTTCGGCTGTTAAC
22096	Yes	AATGGGTTAGCGCGTTCGGCTGTTAACC
22097	Yes	AATGGGTTAGCGCGTTCGGCTGTTAACCG
22098	Yes	AATGGGTTAGCGCGTTCGGCTGTTAACCGA
22099	Yes	AATGGGTTAGCGCGTTCGGCTGTTAACCGAA
22100	Yes	AATGGGTTAGCGCGTTCGGCTGTTAACCGAAA
22101	Yes	AATGGGTTAGCGCGTTCGGCTGTTAACCGAAAG
22102	Yes	AATGGGTTAGCGCGTTCGGCTGTTAACCGAAAGG
22103	Yes	AATGGGTTAGCGCGTTCGGCTGTTAACCGAAAGGT
22104	Yes	AATGGGTTAGCGCGTTCGGCTGTTAACCGAAAGGTT
22105	Yes	AATGGGTTAGCGCGTTCGGCTGTTAACCGAAAGGTTG
22106	Yes	AATGGGTTAGCGCGTTCGGCTGTTAACCGAAAGGTTGG
22107	Yes	AATGGGTTAGCGCGTTCGGCTGTTAACCGAAAGGTTGGT
22108	Yes	AATGGGTTAGCGCGTTCGGCTGTTAACCGAAAGGTTGGTG
22109	Yes	AATGGGTTAGCGCGTTCGGCTGTTAACCGAAAGGTTGGTGG
22110	Yes	AATGGGTTAGCGCGTTCGGCTGTTAACCGAAAGGTTGGTGGT
22111	Yes	AATGGGTTAGCGCGTTCGGCTGTTAACCGAAAGGTTGGTGGTT
22112	Yes	AATGGGTTAGCGCGTTCGGCTGTTAACCGAAAGGTTGGTGGTTC
22113	Yes	AATGGGTTAGCGCGTTCGGCTGTTAACCGAAAGGTTGGTGGTTCG
22114	Yes	AATGGGTTAGCGCGTTCGGCTGTTAACCGAAAGGTTGGTGGTTCGA
22115	Yes	AATGGGTTAGCGCGTTCGGCTGTTAACCGAAAGGTTGGTGGTTCGAG
22116	Yes	AATGGGTTAGCGCGTTCGGCTGTTAACCGAAAGGTTGGTGGTTCGAGC
22117	Yes	AATGGGTTAGCGCGTTCGGCTGTTAACCGAAAGGTTGGTGGTTCGAGCC
22118	Yes	AATGGGTTAGCGCGTTCGGCTGTTAACCGAAAGGTTGGTGGTTCGAGCCC
22119	Yes	AATGGTAAGCACCCTG
22120	Yes	AATGGTAAGCACCCTGG
22121	Yes	AATGGTAAGCACCCTGGA
22122	Yes	AATGGTAAGCACCCTGGAC
22123	Yes	AATGGTAAGCACCCTGGACT
22124	Yes	AATGGTAAGCACCCTGGACTC
22125	Yes	AATGGTAAGCACCCTGGACTCT
22126	Yes	AATGGTAAGCACCCTGGACTCTG
22127	Yes	AATGGTAAGCACCCTGGACTCTGA

22128	Yes	AATGGTAAGCACCCTGGACTCTGAA
22129	Yes	AATGGTAAGCACCCTGGACTCTGAAT
22130	Yes	AATGGTAAGCACCCTGGACTCTGAATC
22131	Yes	AATGGTAAGCACCCTGGACTCTGAATCC
22132	Yes	AATGGTAAGCACCCTGGACTCTGAATCCA
22133	Yes	AATGGTAAGCACCCTGGACTCTGAATCCAG
22134	Yes	AATGGTAAGCACCCTGGACTCTGAATCCAGC
22135	Yes	AATGGTAAGCACCCTGGACTCTGAATCCAGCA
22136	Yes	AATGGTAAGCACCCTGGACTCTGAATCCAGCAA
22137	Yes	AATGGTAAGCACCCTGGACTCTGAATCCAGCAAC
22138	Yes	AATGGTAAGCACCCTGGACTCTGAATCCAGCAACC
22139	Yes	AATGGTAAGCACCCTGGACTCTGAATCCAGCAACCA
22140	Yes	AATGGTAAGCACCCTGGACTCTGAATCCAGCAACCAG
22141	Yes	AATGGTAAGCACCCTGGACTCTGAATCCAGCAACCAGA
22142	Yes	AATGGTAAGCACCCTGGACTCTGAATCCAGCAACCAGAG
22143	Yes	AATGGTAAGCACCCTGGACTCTGAATCCAGCAACCAGAGT
22144	Yes	AATGGTAAGCACCCTGGACTCTGAATCCAGCAACCAGAGTT
22145	Yes	AATGGTAAGCACCCTGGACTCTGAATCCAGCAACCAGAGTTC
22146	Yes	AATGGTAAGCACCCTGGACTCTGAATCCAGCAACCAGAGTTCC
22147	Yes	AATGGTAAGCACCCTGGACTCTGAATCCAGCAACCAGAGTTCCA
22148	Yes	AATGGTAAGCACCCTGGACTCTGAATCCAGCAACCAGAGTTCCAG
22149	Yes	AATGGTAAGCACCCTGGACTCTGAATCCAGCAACCAGAGTTCCAGT
22150	Yes	AATGGTAAGCACCCTGGACTCTGAATCCAGCAACCAGAGTTCCAGTC
22151	Yes	AATGGTAAGCACCCTGGACTCTGAATCCAGCAACCAGAGTTCCAGTCT
22152	Yes	AATGGTAAGCACCCTGGACTCTGAATCCAGCAACCAGAGTTCCAGTCTC
22153	Yes	AATGGTAAGCACCCTGGACTCTGAATCCAGCAACCAGAGTTCCAGTCTCA
22154	No	AATGGTAAGCACTCTG
22155	No	AATGGTAAGCACTCTGG
22156	No	AATGGTAAGCACTCTGGA
22157	No	AATGGTAAGCACTCTGGAC
22158	No	AATGGTAAGCACTCTGGACT
22159	No	AATGGTAAGCACTCTGGACTC
22160	No	AATGGTAAGCACTCTGGACTCT
22161	No	AATGGTAAGCACTCTGGACTCTG
22162	No	AATGGTAAGCACTCTGGACTCTGA
22163	No	AATGGTAAGCACTCTGGACTCTGAA
22164	No	AATGGTAAGCACTCTGGACTCTGAAT
22165	Yes	AATGGTAAGCACTCTGGACTCTGAATC
22166	Yes	AATGGTAAGCACTCTGGACTCTGAATCC
22167	Yes	AATGGTAAGCACTCTGGACTCTGAATCCA
22168	Yes	AATGGTAAGCACTCTGGACTCTGAATCCAG
22169	Yes	AATGGTAAGCACTCTGGACTCTGAATCCAGC

22170	Yes	AATGGTAAGCACTCTGGACTCTGAATCCAGCC
22171	Yes	AATGGTAAGCACTCTGGACTCTGAATCCAGCCA
22172	Yes	AATGGTAAGCACTCTGGACTCTGAATCCAGCCAT
22173	Yes	AATGGTAAGCACTCTGGACTCTGAATCCAGCCATC
22174	Yes	AATGGTAAGCACTCTGGACTCTGAATCCAGCCATCT
22175	Yes	AATGGTAAGCACTCTGGACTCTGAATCCAGCCATCTG
22176	Yes	AATGGTAAGCACTCTGGACTCTGAATCCAGCCATCTGA
22177	Yes	AATGGTAAGCACTCTGGACTCTGAATCCAGCCATCTGAG
22178	Yes	AATGGTAAGCACTCTGGACTCTGAATCCAGCCATCTGAGT
22179	Yes	AATGGTAAGCACTCTGGACTCTGAATCCAGCCATCTGAGTT
22180	Yes	AATGGTAAGCACTCTGGACTCTGAATCCAGCCATCTGAGTTC
22181	Yes	AATGGTAAGCACTCTGGACTCTGAATCCAGCCATCTGAGTTCG
22182	Yes	AATGGTAAGCACTCTGGACTCTGAATCCAGCCATCTGAGTTCGA
22183	Yes	AATGGTAAGCACTCTGGACTCTGAATCCAGCCATCTGAGTTCGAG
22184	Yes	AATGGTAAGCACTCTGGACTCTGAATCCAGCCATCTGAGTTCGAGT
22185	Yes	AATGGTAAGCACTCTGGACTCTGAATCCAGCCATCTGAGTTCGAGTC
22186	Yes	AATGGTAAGCACTCTGGACTCTGAATCCAGCCATCTGAGTTCGAGTCT
22187	Yes	AATGGTAAGCACTCTGGACTCTGAATCCAGCCATCTGAGTTCGAGTCTC
22188	Yes	AATGGTAAGCACTCTGGACTCTGAATCCAGCCATCTGAGTTCGAGTCTCT
22189	Yes	AATGGTAAGCACTCTGGACTCTGAATCCAGCG
22190	Yes	AATGGTAAGCACTCTGGACTCTGAATCCAGCGA
22191	Yes	AATGGTAAGCACTCTGGACTCTGAATCCAGCGAT
22192	Yes	AATGGTAAGCACTCTGGACTCTGAATCCAGCGATC
22193	Yes	AATGGTAAGCACTCTGGACTCTGAATCCAGCGATCC
22194	Yes	AATGGTAAGCACTCTGGACTCTGAATCCAGCGATCCG
22195	Yes	AATGGTAAGCACTCTGGACTCTGAATCCAGCGATCCGA
22196	Yes	AATGGTAAGCACTCTGGACTCTGAATCCAGCGATCCGAG
22197	Yes	AATGGTAAGCACTCTGGACTCTGAATCCAGCGATCCGAGT
22198	Yes	AATGGTAAGCACTCTGGACTCTGAATCCAGCGATCCGAGTT
22199	Yes	AATGGTAAGCACTCTGGACTCTGAATCCAGCGATCCGAGTTC
22200	Yes	AATGGTAAGCACTCTGGACTCTGAATCCAGCGATCCGAGTTCG
22201	Yes	AATGGTAAGCACTCTGGACTCTGAATCCAGCGATCCGAGTTCGA
22202	Yes	AATGGTAAGCACTCTGGACTCTGAATCCAGCGATCCGAGTTCGAG
22203	Yes	AATGGTAAGCACTCTGGACTCTGAATCCAGCGATCCGAGTTCGAGT
22204	Yes	AATGGTAAGCACTCTGGACTCTGAATCCAGCGATCCGAGTTCGAGTC
22205	Yes	AATGGTAAGCACTCTGGACTCTGAATCCAGCGATCCGAGTTCGAGTCT
22206	Yes	AATGGTAAGCACTCTGGACTCTGAATCCAGCGATCCGAGTTCGAGTCTC
22207	Yes	AATGGTAAGCACTCTGGACTCTGAATCCAGCGATCCGAGTTCGAGTCTCG
22208	No	AATGGTAGAATTCTCG
22209	No	AATGGTAGAATTCTCGC
22210	No	AATGGTAGAATTCTCGCC
22211	No	AATGGTAGAATTCTCGCCT

22212	No	AATGGTAGAATTCTCGCCTC
22213	No	AATGGTAGAATTCTCGCCTCC
22214	No	AATGGTAGAATTCTCGCCTCCC
22215	No	AATGGTAGAATTCTCGCCTCCCA
22216	Yes	AATGGTAGAATTCTCGCCTCCCAC
22217	Yes	AATGGTAGAATTCTCGCCTCCCACG
22218	Yes	AATGGTAGAATTCTCGCCTCCCACGC
22219	Yes	AATGGTAGAATTCTCGCCTCCCACGCA
22220	Yes	AATGGTAGAATTCTCGCCTCCCACGCAG
22221	Yes	AATGGTAGAATTCTCGCCTCCCACGCAGG
22222	Yes	AATGGTAGAATTCTCGCCTCCCACGCAGGA
22223	Yes	AATGGTAGAATTCTCGCCTCCCACGCAGGAG
22224	Yes	AATGGTAGAATTCTCGCCTCCCACGCAGGAGA
22225	Yes	AATGGTAGAATTCTCGCCTCCCACGCAGGAGAC
22226	Yes	AATGGTAGAATTCTCGCCTCCCACGCAGGAGACC
22227	Yes	AATGGTAGAATTCTCGCCTCCCACGCAGGAGACCC
22228	Yes	AATGGTAGAATTCTCGCCTCCCACGCAGGAGACCCA
22229	Yes	AATGGTAGAATTCTCGCCTCCCACGCAGGAGACCCAG
22230	Yes	AATGGTAGAATTCTCGCCTCCCACGCAGGAGACCCAGG
22231	Yes	AATGGTAGAATTCTCGCCTCCCACGCAGGAGACCCAGGT
22232	Yes	AATGGTAGAATTCTCGCCTCCCACGCAGGAGACCCAGGTT
22233	Yes	AATGGTAGAATTCTCGCCTCCCACGCAGGAGACCCAGGTTC
22234	Yes	AATGGTAGAATTCTCGCCTCCCACGCAGGAGACCCAGGTTCG
22235	Yes	AATGGTAGAATTCTCGCCTCCCACGCAGGAGACCCAGGTTCGA
22236	Yes	AATGGTAGAATTCTCGCCTCCCACGCAGGAGACCCAGGTTCGAT
22237	Yes	AATGGTAGAATTCTCGCCTCCCACGCAGGAGACCCAGGTTCGATT
22238	Yes	AATGGTAGAATTCTCGCCTCCCACGCAGGAGACCCAGGTTCGATTC
22239	Yes	AATGGTAGAATTCTCGCCTCCCACGCAGGAGACCCAGGTTCGATTCC
22240	Yes	AATGGTAGAATTCTCGCCTCCCACGCAGGAGACCCAGGTTCGATTCCT
22241	Yes	AATGGTAGAATTCTCGCCTCCCACGCAGGAGACCCAGGTTCGATTCCTG
22242	Yes	AATGGTAGAATTCTCGCCTCCCACGCAGGAGACCCAGGTTCGATTCCTGG
22243	Yes	AATGGTAGAGCGCTCG
22244	Yes	AATGGTAGAGCGCTCGC
22245	Yes	AATGGTAGAGCGCTCGCT
22246	Yes	AATGGTAGAGCGCTCGCTT
22247	Yes	AATGGTAGAGCGCTCGCTTA
22248	Yes	AATGGTAGAGCGCTCGCTTAG
22249	Yes	AATGGTAGAGCGCTCGCTTAGC
22250	Yes	AATGGTAGAGCGCTCGCTTAGCA
22251	Yes	AATGGTAGAGCGCTCGCTTAGCAT
22252	Yes	AATGGTAGAGCGCTCGCTTAGCATG
22253	Yes	AATGGTAGAGCGCTCGCTTAGCATGC

22254	Yes	AATGGTAGAGCGCTCGCTTAGCATGCG
22255	Yes	AATGGTAGAGCGCTCGCTTAGCATGCGA
22256	Yes	AATGGTAGAGCGCTCGCTTAGCATGCGAG
22257	Yes	AATGGTAGAGCGCTCGCTTAGCATGCGAGA
22258	Yes	AATGGTAGAGCGCTCGCTTAGCATGCGAGAG
22259	Yes	AATGGTAGAGCGCTCGCTTAGCATGCGAGAGG
22260	Yes	AATGGTAGAGCGCTCGCTTAGCATGCGAGAGGT
22261	Yes	AATGGTAGAGCGCTCGCTTAGCATGCGAGAGGTA
22262	Yes	AATGGTAGAGCGCTCGCTTAGCATGCGAGAGGTAG
22263	Yes	AATGGTAGAGCGCTCGCTTAGCATGCGAGAGGTAGC
22264	Yes	AATGGTAGAGCGCTCGCTTAGCATGCGAGAGGTAGCG
22265	Yes	AATGGTAGAGCGCTCGCTTAGCATGCGAGAGGTAGCGG
22266	Yes	AATGGTAGAGCGCTCGCTTAGCATGCGAGAGGTAGCGGG
22267	Yes	AATGGTAGAGCGCTCGCTTAGCATGCGAGAGGTAGCGGGA
22268	Yes	AATGGTAGAGCGCTCGCTTAGCATGCGAGAGGTAGCGGGAT
22269	Yes	AATGGTAGAGCGCTCGCTTAGCATGCGAGAGGTAGCGGGATC
22270	Yes	AATGGTAGAGCGCTCGCTTAGCATGCGAGAGGTAGCGGGATCG
22271	Yes	AATGGTAGAGCGCTCGCTTAGCATGCGAGAGGTAGCGGGATCGA
22272	Yes	AATGGTAGAGCGCTCGCTTAGCATGCGAGAGGTAGCGGGATCGAT
22273	Yes	AATGGTAGAGCGCTCGCTTAGCATGCGAGAGGTAGCGGGATCGATG
22274	Yes	AATGGTAGAGCGCTCGCTTAGCATGCGAGAGGTAGCGGGATCGATGC
22275	Yes	AATGGTAGAGCGCTCGCTTAGCATGCGAGAGGTAGCGGGATCGATGCC
22276	Yes	AATGGTAGAGCGCTCGCTTAGCATGCGAGAGGTAGCGGGATCGATGCCC
22277	Yes	AATGGTAGAGCGCTCGCTTAGCATGCGAGAGGTAGCGGGATCGATGCCCG
22278	Yes	AATGGTAGCGCGTCTG
22279	Yes	AATGGTAGCGCGTCTGA
22280	Yes	AATGGTAGCGCGTCTGAC
22281	Yes	AATGGTAGCGCGTCTGACT
22282	Yes	AATGGTAGCGCGTCTGACTC
22283	Yes	AATGGTAGCGCGTCTGACTCC
22284	Yes	AATGGTAGCGCGTCTGACTCCA
22285	Yes	AATGGTAGCGCGTCTGACTCCAG
22286	Yes	AATGGTAGCGCGTCTGACTCCAGA
22287	Yes	AATGGTAGCGCGTCTGACTCCAGAT
22288	Yes	AATGGTAGCGCGTCTGACTCCAGATC
22289	Yes	AATGGTAGCGCGTCTGACTCCAGATCA
22290	Yes	AATGGTAGCGCGTCTGACTCCAGATCAG
22291	Yes	AATGGTAGCGCGTCTGACTCCAGATCAGA
22292	Yes	AATGGTAGCGCGTCTGACTCCAGATCAGAA
22293	Yes	AATGGTAGCGCGTCTGACTCCAGATCAGAAG
22294	Yes	AATGGTAGCGCGTCTGACTCCAGATCAGAAGG
22295	Yes	AATGGTAGCGCGTCTGACTCCAGATCAGAAGGT

22296	Yes	AATGGTAGCGCGTCTGACTCCAGATCAGAAGGTT
22297	Yes	AATGGTAGCGCGTCTGACTCCAGATCAGAAGGTTG
22298	Yes	AATGGTAGCGCGTCTGACTCCAGATCAGAAGGTTGC
22299	Yes	AATGGTAGCGCGTCTGACTCCAGATCAGAAGGTTGCG
22300	Yes	AATGGTAGCGCGTCTGACTCCAGATCAGAAGGTTGCGT
22301	Yes	AATGGTAGCGCGTCTGACTCCAGATCAGAAGGTTGCGTG
22302	Yes	AATGGTAGCGCGTCTGACTCCAGATCAGAAGGTTGCGTGT
22303	Yes	AATGGTAGCGCGTCTGACTCCAGATCAGAAGGTTGCGTGTT
22304	Yes	AATGGTAGCGCGTCTGACTCCAGATCAGAAGGTTGCGTGTTC
22305	Yes	AATGGTAGCGCGTCTGACTCCAGATCAGAAGGTTGCGTGTTCA
22306	Yes	AATGGTAGCGCGTCTGACTCCAGATCAGAAGGTTGCGTGTTCAA
22307	Yes	AATGGTAGCGCGTCTGACTCCAGATCAGAAGGTTGCGTGTTCAAG
22308	Yes	AATGGTAGCGCGTCTGACTCCAGATCAGAAGGTTGCGTGTTCAAGT
22309	Yes	AATGGTAGCGCGTCTGACTCCAGATCAGAAGGTTGCGTGTTCAAGTC
22310	Yes	AATGGTAGCGCGTCTGACTCCAGATCAGAAGGTTGCGTGTTCAAGTCA
22311	Yes	AATGGTAGCGCGTCTGACTCCAGATCAGAAGGTTGCGTGTTCAAGTCAC
22312	Yes	AATGGTAGCGCGTCTGACTCCAGATCAGAAGGTTGCGTGTTCAAGTCACG
22313	Yes	AATGGTCAGCACTCTG
22314	Yes	AATGGTCAGCACTCTGG
22315	Yes	AATGGTCAGCACTCTGGA
22316	Yes	AATGGTCAGCACTCTGGAC
22317	Yes	AATGGTCAGCACTCTGGACT
22318	Yes	AATGGTCAGCACTCTGGACTC
22319	Yes	AATGGTCAGCACTCTGGACTCT
22320	Yes	AATGGTCAGCACTCTGGACTCTG
22321	Yes	AATGGTCAGCACTCTGGACTCTGA
22322	Yes	AATGGTCAGCACTCTGGACTCTGAA
22323	Yes	AATGGTCAGCACTCTGGACTCTGAAT
22324	Yes	AATGGTCAGCACTCTGGACTCTGAATC
22325	Yes	AATGGTCAGCACTCTGGACTCTGAATCC
22326	Yes	AATGGTCAGCACTCTGGACTCTGAATCCA
22327	Yes	AATGGTCAGCACTCTGGACTCTGAATCCAG
22328	Yes	AATGGTCAGCACTCTGGACTCTGAATCCAGC
22329	Yes	AATGGTCAGCACTCTGGACTCTGAATCCAGCG
22330	Yes	AATGGTCAGCACTCTGGACTCTGAATCCAGCGA
22331	Yes	AATGGTCAGCACTCTGGACTCTGAATCCAGCGAT
22332	Yes	AATGGTCAGCACTCTGGACTCTGAATCCAGCGATC
22333	Yes	AATGGTCAGCACTCTGGACTCTGAATCCAGCGATCC
22334	Yes	AATGGTCAGCACTCTGGACTCTGAATCCAGCGATCCG
22335	Yes	AATGGTCAGCACTCTGGACTCTGAATCCAGCGATCCGA
22336	Yes	AATGGTCAGCACTCTGGACTCTGAATCCAGCGATCCGAG
22337	Yes	AATGGTCAGCACTCTGGACTCTGAATCCAGCGATCCGAGT

22338	Yes	AATGGTCAGCACTCTGGACTCTGAATCCAGCGATCCGAGTT
22339	Yes	AATGGTCAGCACTCTGGACTCTGAATCCAGCGATCCGAGTTC
22340	Yes	AATGGTCAGCACTCTGGACTCTGAATCCAGCGATCCGAGTTCA
22341	Yes	AATGGTCAGCACTCTGGACTCTGAATCCAGCGATCCGAGTTCAA
22342	Yes	AATGGTCAGCACTCTGGACTCTGAATCCAGCGATCCGAGTTCAAA
22343	Yes	AATGGTCAGCACTCTGGACTCTGAATCCAGCGATCCGAGTTCAAAT
22344	Yes	AATGGTCAGCACTCTGGACTCTGAATCCAGCGATCCGAGTTCAAATC
22345	Yes	AATGGTCAGCACTCTGGACTCTGAATCCAGCGATCCGAGTTCAAATCT
22346	Yes	AATGGTCAGCACTCTGGACTCTGAATCCAGCGATCCGAGTTCAAATCTC
22347	Yes	AATGGTCAGCACTCTGGACTCTGAATCCAGCGATCCGAGTTCAAATCTCG
22348	No	AATGGTCCTGAGTTCA
22349	Yes	AATGGTCCTGAGTTCAA
22350	Yes	AATGGTCCTGAGTTCAAG
22351	Yes	AATGGTCCTGAGTTCAAGC
22352	Yes	AATGGTCCTGAGTTCAAGCC
22353	Yes	AATGGTCCTGAGTTCAAGCCT
22354	Yes	AATGGTCCTGAGTTCAAGCCTC
22355	Yes	AATGGTCCTGAGTTCAAGCCTCA
22356	Yes	AATGGTCCTGAGTTCAAGCCTCAG
22357	Yes	AATGGTCCTGAGTTCAAGCCTCAGA
22358	Yes	AATGGTCCTGAGTTCAAGCCTCAGAG
22359	Yes	AATGGTCCTGAGTTCAAGCCTCAGAGG
22360	Yes	AATGGTCCTGAGTTCAAGCCTCAGAGGG
22361	Yes	AATGGTCCTGAGTTCAAGCCTCAGAGGGG
22362	Yes	AATGGTCCTGAGTTCAAGCCTCAGAGGGGG
22363	Yes	AATGGTCCTGAGTTCAAGCCTCAGAGGGGGC
22364	Yes	AATGGTCCTGAGTTCAAGCCTCAGAGGGGGCA
22365	Yes	AATGGTCCTGAGTTCAAGCCTCAGAGGGGGCAC
22366	Yes	AATGGTCCTGAGTTCAAGCCTCAGAGGGGGCACC
22367	Yes	AATGGTCCTGAGTTCAAGCCTCAGAGGGGGCACCA
22368	No	AATGGTCTAAGGCACT
22369	Yes	AATGGTCTAAGGCACTG
22370	Yes	AATGGTCTAAGGCACTGA
22371	Yes	AATGGTCTAAGGCACTGAA
22372	Yes	AATGGTCTAAGGCACTGAAT
22373	Yes	AATGGTCTAAGGCACTGAATT
22374	Yes	AATGGTCTAAGGCACTGAATTT
22375	Yes	AATGGTCTAAGGCACTGAATTTA
22376	Yes	AATGGTCTAAGGCACTGAATTTAG
22377	Yes	AATGGTCTAAGGCACTGAATTTAGG
22378	Yes	AATGGTCTAAGGCACTGAATTTAGGC
22379	Yes	AATGGTCTAAGGCACTGAATTTAGGCT

22380	Yes	AATGGTCTAAGGCACTGAATTTAGGCTC
22381	Yes	AATGGTCTAAGGCACTGAATTTAGGCTCC
22382	Yes	AATGGTCTAAGGCACTGAATTTAGGCTCCA
22383	Yes	AATGGTCTAAGGCACTGAATTTAGGCTCCAG
22384	Yes	AATGGTCTAAGGCACTGAATTTAGGCTCCAGT
22385	Yes	AATGGTCTAAGGCACTGAATTTAGGCTCCAGTC
22386	Yes	AATGGTCTAAGGCACTGAATTTAGGCTCCAGTCT
22387	Yes	AATGGTCTAAGGCACTGAATTTAGGCTCCAGTCTC
22388	Yes	AATGGTCTAAGGCACTGAATTTAGGCTCCAGTCTCT
22389	Yes	AATGGTCTAAGGCACTGAATTTAGGCTCCAGTCTCTT
22390	Yes	AATGGTCTAAGGCACTGAATTTAGGCTCCAGTCTCTTT
22391	Yes	AATGGTCTAAGGCACTGAATTTAGGCTCCAGTCTCTTTG
22392	Yes	AATGGTCTAAGGCACTGAATTTAGGCTCCAGTCTCTTTGG
22393	Yes	AATGGTCTAAGGCACTGAATTTAGGCTCCAGTCTCTTTGGG
22394	Yes	AATGGTCTAAGGCACTGAATTTAGGCTCCAGTCTCTTTGGGG
22395	Yes	AATGGTCTAAGGCACTGAATTTAGGCTCCAGTCTCTTTGGGGA
22396	Yes	AATGGTCTAAGGCACTGAATTTAGGCTCCAGTCTCTTTGGGGAC
22397	Yes	AATGGTCTAAGGCACTGAATTTAGGCTCCAGTCTCTTTGGGGACG
22398	Yes	AATGGTCTAAGGCACTGAATTTAGGCTCCAGTCTCTTTGGGGACGT
22399	Yes	AATGGTCTAAGGCACTGAATTTAGGCTCCAGTCTCTTTGGGGACGTG
22400	Yes	AATGGTCTAAGGCACTGAATTTAGGCTCCAGTCTCTTTGGGGACGTGG
22401	Yes	AATGGTCTAAGGCACTGAATTTAGGCTCCAGTCTCTTTGGGGACGTGGG
22402	Yes	AATGGTCTAAGGCACTGAATTTAGGCTCCAGTCTCTTTGGGGACGTGGGT
22403	No	AATGGTGACCACTTTG
22404	No	AATGGTGACCACTTTGG
22405	No	AATGGTGACCACTTTGGA
22406	Yes	AATGGTGACCACTTTGGAC
22407	Yes	AATGGTGACCACTTTGGACT
22408	Yes	AATGGTGACCACTTTGGACTC
22409	Yes	AATGGTGACCACTTTGGACTCT
22410	Yes	AATGGTGACCACTTTGGACTCTG
22411	Yes	AATGGTGACCACTTTGGACTCTGA
22412	Yes	AATGGTGACCACTTTGGACTCTGAA
22413	Yes	AATGGTGACCACTTTGGACTCTGAAT
22414	Yes	AATGGTGACCACTTTGGACTCTGAATA
22415	Yes	AATGGTGACCACTTTGGACTCTGAATAC
22416	Yes	AATGGTGACCACTTTGGACTCTGAATACA
22417	Yes	AATGGTGACCACTTTGGACTCTGAATACAG
22418	Yes	AATGGTGACCACTTTGGACTCTGAATACAGT
22419	Yes	AATGGTGACCACTTTGGACTCTGAATACAGTG
22420	Yes	AATGGTGACCACTTTGGACTCTGAATACAGTGA
22421	Yes	AATGGTGACCACTTTGGACTCTGAATACAGTGAT

22422	Yes	AATGGTGACCACTTTGGACTCTGAATACAGTGATC
22423	Yes	AATGGTGACCACTTTGGACTCTGAATACAGTGATCA
22424	Yes	AATGGTGACCACTTTGGACTCTGAATACAGTGATCAG
22425	Yes	AATGGTGACCACTTTGGACTCTGAATACAGTGATCAGA
22426	Yes	AATGGTGACCACTTTGGACTCTGAATACAGTGATCAGAG
22427	Yes	AATGGTGACCACTTTGGACTCTGAATACAGTGATCAGAGT
22428	Yes	AATGGTGACCACTTTGGACTCTGAATACAGTGATCAGAGTT
22429	Yes	AATGGTGACCACTTTGGACTCTGAATACAGTGATCAGAGTTC
22430	Yes	AATGGTGACCACTTTGGACTCTGAATACAGTGATCAGAGTTCA
22431	Yes	AATGGTGACCACTTTGGACTCTGAATACAGTGATCAGAGTTCAA
22432	Yes	AATGGTGACCACTTTGGACTCTGAATACAGTGATCAGAGTTCAAG
22433	Yes	AATGGTGACCACTTTGGACTCTGAATACAGTGATCAGAGTTCAAGT
22434	Yes	AATGGTGACCACTTTGGACTCTGAATACAGTGATCAGAGTTCAAGTC
22435	Yes	AATGGTGACCACTTTGGACTCTGAATACAGTGATCAGAGTTCAAGTCT
22436	Yes	AATGGTGACCACTTTGGACTCTGAATACAGTGATCAGAGTTCAAGTCTC
22437	Yes	AATGGTGACCACTTTGGACTCTGAATACAGTGATCAGAGTTCAAGTCTCA
22438	No	AATGGTGAGCACCCTG
22439	No	AATGGTGAGCACCCTGG
22440	No	AATGGTGAGCACCCTGGA
22441	No	AATGGTGAGCACCCTGGAC
22442	No	AATGGTGAGCACCCTGGACT
22443	No	AATGGTGAGCACCCTGGACTC
22444	No	AATGGTGAGCACCCTGGACTCT
22445	No	AATGGTGAGCACCCTGGACTCTG
22446	No	AATGGTGAGCACCCTGGACTCTGA
22447	Yes	AATGGTGAGCACCCTGGACTCTGAA
22448	Yes	AATGGTGAGCACCCTGGACTCTGAAT
22449	Yes	AATGGTGAGCACCCTGGACTCTGAATC
22450	Yes	AATGGTGAGCACCCTGGACTCTGAATCA
22451	Yes	AATGGTGAGCACCCTGGACTCTGAATCAA
22452	Yes	AATGGTGAGCACCCTGGACTCTGAATCAAG
22453	Yes	AATGGTGAGCACCCTGGACTCTGAATCAAGC
22454	Yes	AATGGTGAGCACCCTGGACTCTGAATCAAGCG
22455	Yes	AATGGTGAGCACCCTGGACTCTGAATCAAGCGA
22456	Yes	AATGGTGAGCACCCTGGACTCTGAATCAAGCGAT
22457	Yes	AATGGTGAGCACCCTGGACTCTGAATCAAGCGATC
22458	Yes	AATGGTGAGCACCCTGGACTCTGAATCAAGCGATCC
22459	Yes	AATGGTGAGCACCCTGGACTCTGAATCAAGCGATCCG
22460	Yes	AATGGTGAGCACCCTGGACTCTGAATCAAGCGATCCGA
22461	Yes	AATGGTGAGCACCCTGGACTCTGAATCAAGCGATCCGAG
22462	Yes	AATGGTGAGCACCCTGGACTCTGAATCAAGCGATCCGAGT
22463	Yes	AATGGTGAGCACCCTGGACTCTGAATCAAGCGATCCGAGTT

22464	Yes	AATGGTGAGCACCCTGGACTCTGAATCAAGCGATCCGAGTTC
22465	Yes	AATGGTGAGCACCCTGGACTCTGAATCAAGCGATCCGAGTTCA
22466	Yes	AATGGTGAGCACCCTGGACTCTGAATCAAGCGATCCGAGTTCAA
22467	Yes	AATGGTGAGCACCCTGGACTCTGAATCAAGCGATCCGAGTTCAAA
22468	Yes	AATGGTGAGCACCCTGGACTCTGAATCAAGCGATCCGAGTTCAAAT
22469	Yes	AATGGTGAGCACCCTGGACTCTGAATCAAGCGATCCGAGTTCAAATC
22470	Yes	AATGGTGAGCACCCTGGACTCTGAATCAAGCGATCCGAGTTCAAATCT
22471	Yes	AATGGTGAGCACCCTGGACTCTGAATCAAGCGATCCGAGTTCAAATCTC
22472	Yes	AATGGTGAGCACCCTGGACTCTGAATCAAGCGATCCGAGTTCAAATCTCG
22473	No	AATGGTGAGCACTCTG
22474	No	AATGGTGAGCACTCTGG
22475	No	AATGGTGAGCACTCTGGA
22476	No	AATGGTGAGCACTCTGGAC
22477	No	AATGGTGAGCACTCTGGACT
22478	No	AATGGTGAGCACTCTGGACTC
22479	No	AATGGTGAGCACTCTGGACTCT
22480	No	AATGGTGAGCACTCTGGACTCTG
22481	No	AATGGTGAGCACTCTGGACTCTGA
22482	No	AATGGTGAGCACTCTGGACTCTGAA
22483	No	AATGGTGAGCACTCTGGACTCTGAAT
22484	No	AATGGTGAGCACTCTGGACTCTGAATC
22485	No	AATGGTGAGCACTCTGGACTCTGAATCC
22486	No	AATGGTGAGCACTCTGGACTCTGAATCCA
22487	No	AATGGTGAGCACTCTGGACTCTGAATCCAG
22488	No	AATGGTGAGCACTCTGGACTCTGAATCCAGC
22489	No	AATGGTGAGCACTCTGGACTCTGAATCCAGCG
22490	No	AATGGTGAGCACTCTGGACTCTGAATCCAGCGA
22491	No	AATGGTGAGCACTCTGGACTCTGAATCCAGCGAT
22492	No	AATGGTGAGCACTCTGGACTCTGAATCCAGCGATC
22493	Yes	AATGGTGAGCACTCTGGACTCTGAATCCAGCGATCC
22494	Yes	AATGGTGAGCACTCTGGACTCTGAATCCAGCGATCCG
22495	Yes	AATGGTGAGCACTCTGGACTCTGAATCCAGCGATCCGA
22496	Yes	AATGGTGAGCACTCTGGACTCTGAATCCAGCGATCCGAG
22497	Yes	AATGGTGAGCACTCTGGACTCTGAATCCAGCGATCCGAGT
22498	Yes	AATGGTGAGCACTCTGGACTCTGAATCCAGCGATCCGAGTT
22499	Yes	AATGGTGAGCACTCTGGACTCTGAATCCAGCGATCCGAGTTC
22500	Yes	AATGGTGAGCACTCTGGACTCTGAATCCAGCGATCCGAGTTCG
22501	Yes	AATGGTGAGCACTCTGGACTCTGAATCCAGCGATCCGAGTTCGA
22502	Yes	AATGGTGAGCACTCTGGACTCTGAATCCAGCGATCCGAGTTCGAG
22503	Yes	AATGGTGAGCACTCTGGACTCTGAATCCAGCGATCCGAGTTCGAGT
22504	Yes	AATGGTGAGCACTCTGGACTCTGAATCCAGCGATCCGAGTTCGAGTC
22505	Yes	AATGGTGAGCACTCTGGACTCTGAATCCAGCGATCCGAGTTCGAGTCT

22506	Yes	AATGGTGAGCACTCTGGACTCTGAATCCAGCGATCCGAGTTCGAGTCTC
22507	Yes	AATGGTGAGCACTCTGGACTCTGAATCCAGCGATCCGAGTTCGAGTCTCG
22508	No	AATGGTGAGCACTTTG
22509	No	AATGGTGAGCACTTTGG
22510	No	AATGGTGAGCACTTTGGA
22511	Yes	AATGGTGAGCACTTTGGAC
22512	Yes	AATGGTGAGCACTTTGGACT
22513	Yes	AATGGTGAGCACTTTGGACTC
22514	Yes	AATGGTGAGCACTTTGGACTCT
22515	Yes	AATGGTGAGCACTTTGGACTCTG
22516	Yes	AATGGTGAGCACTTTGGACTCTGA
22517	Yes	AATGGTGAGCACTTTGGACTCTGAA
22518	Yes	AATGGTGAGCACTTTGGACTCTGAAT
22519	Yes	AATGGTGAGCACTTTGGACTCTGAATA
22520	Yes	AATGGTGAGCACTTTGGACTCTGAATAC
22521	Yes	AATGGTGAGCACTTTGGACTCTGAATACA
22522	Yes	AATGGTGAGCACTTTGGACTCTGAATACAG
22523	Yes	AATGGTGAGCACTTTGGACTCTGAATACAGT
22524	Yes	AATGGTGAGCACTTTGGACTCTGAATACAGTG
22525	Yes	AATGGTGAGCACTTTGGACTCTGAATACAGTGA
22526	Yes	AATGGTGAGCACTTTGGACTCTGAATACAGTGAT
22527	Yes	AATGGTGAGCACTTTGGACTCTGAATACAGTGATC
22528	Yes	AATGGTGAGCACTTTGGACTCTGAATACAGTGATCA
22529	Yes	AATGGTGAGCACTTTGGACTCTGAATACAGTGATCAG
22530	Yes	AATGGTGAGCACTTTGGACTCTGAATACAGTGATCAGA
22531	Yes	AATGGTGAGCACTTTGGACTCTGAATACAGTGATCAGAG
22532	Yes	AATGGTGAGCACTTTGGACTCTGAATACAGTGATCAGAGT
22533	Yes	AATGGTGAGCACTTTGGACTCTGAATACAGTGATCAGAGTT
22534	Yes	AATGGTGAGCACTTTGGACTCTGAATACAGTGATCAGAGTTC
22535	Yes	AATGGTGAGCACTTTGGACTCTGAATACAGTGATCAGAGTTCA
22536	Yes	AATGGTGAGCACTTTGGACTCTGAATACAGTGATCAGAGTTCAA
22537	Yes	AATGGTGAGCACTTTGGACTCTGAATACAGTGATCAGAGTTCAAG
22538	Yes	AATGGTGAGCACTTTGGACTCTGAATACAGTGATCAGAGTTCAAGT
22539	Yes	AATGGTGAGCACTTTGGACTCTGAATACAGTGATCAGAGTTCAAGTC
22540	Yes	AATGGTGAGCACTTTGGACTCTGAATACAGTGATCAGAGTTCAAGTCT
22541	Yes	AATGGTGAGCACTTTGGACTCTGAATACAGTGATCAGAGTTCAAGTCTC
22542	Yes	AATGGTGAGCACTTTGGACTCTGAATACAGTGATCAGAGTTCAAGTCTCA
22543	Yes	AATGGTGAGCGCTTTG
22544	Yes	AATGGTGAGCGCTTTGG
22545	Yes	AATGGTGAGCGCTTTGGA
22546	Yes	AATGGTGAGCGCTTTGGAC
22547	Yes	AATGGTGAGCGCTTTGGACT

22548	Yes	AATGGTGAGCGCTTTGGACTC
22549	Yes	AATGGTGAGCGCTTTGGACTCT
22550	Yes	AATGGTGAGCGCTTTGGACTCTG
22551	Yes	AATGGTGAGCGCTTTGGACTCTGA
22552	Yes	AATGGTGAGCGCTTTGGACTCTGAG
22553	Yes	AATGGTGAGCGCTTTGGACTCTGAGT
22554	Yes	AATGGTGAGCGCTTTGGACTCTGAGTA
22555	Yes	AATGGTGAGCGCTTTGGACTCTGAGTAC
22556	Yes	AATGGTGAGCGCTTTGGACTCTGAGTACG
22557	Yes	AATGGTGAGCGCTTTGGACTCTGAGTACGG
22558	Yes	AATGGTGAGCGCTTTGGACTCTGAGTACGGT
22559	Yes	AATGGTGAGCGCTTTGGACTCTGAGTACGGTG
22560	Yes	AATGGTGAGCGCTTTGGACTCTGAGTACGGTGA
22561	Yes	AATGGTGAGCGCTTTGGACTCTGAGTACGGTGAT
22562	Yes	AATGGTGAGCGCTTTGGACTCTGAGTACGGTGATC
22563	Yes	AATGGTGAGCGCTTTGGACTCTGAGTACGGTGATCA
22564	Yes	AATGGTGAGCGCTTTGGACTCTGAGTACGGTGATCAG
22565	Yes	AATGGTGAGCGCTTTGGACTCTGAGTACGGTGATCAGC
22566	Yes	AATGGTGAGCGCTTTGGACTCTGAGTACGGTGATCAGCG
22567	Yes	AATGGTGAGCGCTTTGGACTCTGAGTACGGTGATCAGCGT
22568	Yes	AATGGTGAGCGCTTTGGACTCTGAGTACGGTGATCAGCGTT
22569	Yes	AATGGTGAGCGCTTTGGACTCTGAGTACGGTGATCAGCGTTC
22570	Yes	AATGGTGAGCGCTTTGGACTCTGAGTACGGTGATCAGCGTTCA
22571	Yes	AATGGTGAGCGCTTTGGACTCTGAGTACGGTGATCAGCGTTCAA
22572	Yes	AATGGTGAGCGCTTTGGACTCTGAGTACGGTGATCAGCGTTCAAG
22573	Yes	AATGGTGAGCGCTTTGGACTCTGAGTACGGTGATCAGCGTTCAAGT
22574	Yes	AATGGTGAGCGCTTTGGACTCTGAGTACGGTGATCAGCGTTCAAGTC
22575	Yes	AATGGTGAGCGCTTTGGACTCTGAGTACGGTGATCAGCGTTCAAGTCT
22576	Yes	AATGGTGAGCGCTTTGGACTCTGAGTACGGTGATCAGCGTTCAAGTCTC
22577	Yes	AATGGTGAGCGCTTTGGACTCTGAGTACGGTGATCAGCGTTCAAGTCTCA
22578	No	AATGGTGAGGGCTTTG
22579	No	AATGGTGAGGGCTTTGG
22580	Yes	AATGGTGAGGGCTTTGGA
22581	Yes	AATGGTGAGGGCTTTGGAC
22582	Yes	AATGGTGAGGGCTTTGGACT
22583	Yes	AATGGTGAGGGCTTTGGACTC
22584	Yes	AATGGTGAGGGCTTTGGACTCT
22585	Yes	AATGGTGAGGGCTTTGGACTCTG
22586	Yes	AATGGTGAGGGCTTTGGACTCTGA
22587	Yes	AATGGTGAGGGCTTTGGACTCTGAC
22588	Yes	AATGGTGAGGGCTTTGGACTCTGACT
22589	Yes	AATGGTGAGGGCTTTGGACTCTGACTA

22590	Yes	AATGGTGAGGGCTTTGGACTCTGACTAC
22591	Yes	AATGGTGAGGGCTTTGGACTCTGACTACA
22592	Yes	AATGGTGAGGGCTTTGGACTCTGACTACAG
22593	Yes	AATGGTGAGGGCTTTGGACTCTGACTACAGT
22594	Yes	AATGGTGAGGGCTTTGGACTCTGACTACAGTG
22595	Yes	AATGGTGAGGGCTTTGGACTCTGACTACAGTGA
22596	Yes	AATGGTGAGGGCTTTGGACTCTGACTACAGTGAT
22597	Yes	AATGGTGAGGGCTTTGGACTCTGACTACAGTGATC
22598	Yes	AATGGTGAGGGCTTTGGACTCTGACTACAGTGATCA
22599	Yes	AATGGTGAGGGCTTTGGACTCTGACTACAGTGATCAG
22600	Yes	AATGGTGAGGGCTTTGGACTCTGACTACAGTGATCAGA
22601	Yes	AATGGTGAGGGCTTTGGACTCTGACTACAGTGATCAGAG
22602	Yes	AATGGTGAGGGCTTTGGACTCTGACTACAGTGATCAGAGT
22603	Yes	AATGGTGAGGGCTTTGGACTCTGACTACAGTGATCAGAGTT
22604	Yes	AATGGTGAGGGCTTTGGACTCTGACTACAGTGATCAGAGTTC
22605	Yes	AATGGTGAGGGCTTTGGACTCTGACTACAGTGATCAGAGTTCA
22606	Yes	AATGGTGAGGGCTTTGGACTCTGACTACAGTGATCAGAGTTCAA
22607	Yes	AATGGTGAGGGCTTTGGACTCTGACTACAGTGATCAGAGTTCAAG
22608	Yes	AATGGTGAGGGCTTTGGACTCTGACTACAGTGATCAGAGTTCAAGT
22609	Yes	AATGGTGAGGGCTTTGGACTCTGACTACAGTGATCAGAGTTCAAGTC
22610	Yes	AATGGTGAGGGCTTTGGACTCTGACTACAGTGATCAGAGTTCAAGTCT
22611	Yes	AATGGTGAGGGCTTTGGACTCTGACTACAGTGATCAGAGTTCAAGTCTC
22612	Yes	AATGGTGAGGGCTTTGGACTCTGACTACAGTGATCAGAGTTCAAGTCTCA
22613	No	AATGGTGGAGTTAAAG
22614	Yes	AATGGTGGAGTTAAAGA
22615	Yes	AATGGTGGAGTTAAAGAC
22616	Yes	AATGGTGGAGTTAAAGACT
22617	Yes	AATGGTGGAGTTAAAGACTT
22618	Yes	AATGGTGGAGTTAAAGACTTT
22619	Yes	AATGGTGGAGTTAAAGACTTTT
22620	Yes	AATGGTGGAGTTAAAGACTTTTT
22621	Yes	AATGGTGGAGTTAAAGACTTTTTC
22622	Yes	AATGGTGGAGTTAAAGACTTTTTCT
22623	Yes	AATGGTGGAGTTAAAGACTTTTTCTC
22624	Yes	AATGGTGGAGTTAAAGACTTTTTCTCT
22625	Yes	AATGGTGGAGTTAAAGACTTTTTCTCTG
22626	Yes	AATGGTGGAGTTAAAGACTTTTTCTCTGA
22627	Yes	AATGGTGGAGTTAAAGACTTTTTCTCTGAC
22628	Yes	AATGGTGGAGTTAAAGACTTTTTCTCTGACC
22629	Yes	AATGGTGGAGTTAAAGACTTTTTCTCTGACCA
22630	No	AATGGTGGTTCAGTGG
22631	No	AATGGTGGTTCAGTGGT

22632	No	AATGGTGGTTCAGTGGTA
22633	No	AATGGTGGTTCAGTGGTAG
22634	Yes	AATGGTGGTTCAGTGGTAGA
22635	Yes	AATGGTGGTTCAGTGGTAGAA
22636	Yes	AATGGTGGTTCAGTGGTAGAAT
22637	Yes	AATGGTGGTTCAGTGGTAGAATT
22638	Yes	AATGGTGGTTCAGTGGTAGAATTC
22639	Yes	AATGGTGGTTCAGTGGTAGAATTCT
22640	Yes	AATGGTGGTTCAGTGGTAGAATTCTC
22641	Yes	AATGGTGGTTCAGTGGTAGAATTCTCG
22642	Yes	AATGGTGGTTCAGTGGTAGAATTCTCGC
22643	Yes	AATGGTGGTTCAGTGGTAGAATTCTCGCC
22644	Yes	AATGGTGGTTCAGTGGTAGAATTCTCGCCT
22645	Yes	AATGGTGGTTCAGTGGTAGAATTCTCGCCTT
22646	Yes	AATGGTGGTTCAGTGGTAGAATTCTCGCCTTT
22647	Yes	AATGGTGGTTCAGTGGTAGAATTCTCGCCTTTC
22648	Yes	AATGGTGGTTCAGTGGTAGAATTCTCGCCTTTCA
22649	Yes	AATGGTGGTTCAGTGGTAGAATTCTCGCCTTTCAC
22650	Yes	AATGGTGGTTCAGTGGTAGAATTCTCGCCTTTCACA
22651	Yes	AATGGTGGTTCAGTGGTAGAATTCTCGCCTTTCACAC
22652	Yes	AATGGTGGTTCAGTGGTAGAATTCTCGCCTTTCACACA
22653	Yes	AATGGTGGTTCAGTGGTAGAATTCTCGCCTTTCACACAG
22654	Yes	AATGGTGGTTCAGTGGTAGAATTCTCGCCTTTCACACAGG
22655	Yes	AATGGTGGTTCAGTGGTAGAATTCTCGCCTTTCACACAGGA
22656	Yes	AATGGTGGTTCAGTGGTAGAATTCTCGCCTTTCACACAGGAG
22657	Yes	AATGGTGGTTCAGTGGTAGAATTCTCGCCTTTCACACAGGAGA
22658	Yes	AATGGTGGTTCAGTGGTAGAATTCTCGCCTTTCACACAGGAGAC
22659	Yes	AATGGTGGTTCAGTGGTAGAATTCTCGCCTTTCACACAGGAGACC
22660	Yes	AATGGTGGTTCAGTGGTAGAATTCTCGCCTTTCACACAGGAGACCC
22661	Yes	AATGGTGGTTCAGTGGTAGAATTCTCGCCTTTCACACAGGAGACCCG
22662	Yes	AATGGTGGTTCAGTGGTAGAATTCTCGCCTTTCACACAGGAGACCCGG
22663	Yes	AATGGTGGTTCAGTGGTAGAATTCTCGCCTTTCACACAGGAGACCCGGG
22664	Yes	AATGGTGGTTCAGTGGTAGAATTCTCGCCTTTCACACAGGAGACCCGGGT
22665	No	AATGGTTAGCACTCTG
22666	No	AATGGTTAGCACTCTGG
22667	No	AATGGTTAGCACTCTGGA
22668	No	AATGGTTAGCACTCTGGAC
22669	No	AATGGTTAGCACTCTGGACT
22670	No	AATGGTTAGCACTCTGGACTC
22671	No	AATGGTTAGCACTCTGGACTCT
22672	No	AATGGTTAGCACTCTGGACTCTG
22673	No	AATGGTTAGCACTCTGGACTCTGA

22674	No	AATGGTTAGCACTCTGGACTCTGAA
22675	Yes	AATGGTTAGCACTCTGGACTCTGAAT
22676	Yes	AATGGTTAGCACTCTGGACTCTGAATC
22677	Yes	AATGGTTAGCACTCTGGACTCTGAATCC
22678	Yes	AATGGTTAGCACTCTGGACTCTGAATCCA
22679	Yes	AATGGTTAGCACTCTGGACTCTGAATCCAG
22680	Yes	AATGGTTAGCACTCTGGACTCTGAATCCAGC
22681	Yes	AATGGTTAGCACTCTGGACTCTGAATCCAGCG
22682	Yes	AATGGTTAGCACTCTGGACTCTGAATCCAGCGA
22683	Yes	AATGGTTAGCACTCTGGACTCTGAATCCAGCGAT
22684	Yes	AATGGTTAGCACTCTGGACTCTGAATCCAGCGATC
22685	Yes	AATGGTTAGCACTCTGGACTCTGAATCCAGCGATCC
22686	Yes	AATGGTTAGCACTCTGGACTCTGAATCCAGCGATCCG
22687	Yes	AATGGTTAGCACTCTGGACTCTGAATCCAGCGATCCGA
22688	Yes	AATGGTTAGCACTCTGGACTCTGAATCCAGCGATCCGAG
22689	Yes	AATGGTTAGCACTCTGGACTCTGAATCCAGCGATCCGAGT
22690	Yes	AATGGTTAGCACTCTGGACTCTGAATCCAGCGATCCGAGTT
22691	Yes	AATGGTTAGCACTCTGGACTCTGAATCCAGCGATCCGAGTTC
22692	Yes	AATGGTTAGCACTCTGGACTCTGAATCCAGCGATCCGAGTTCA
22693	Yes	AATGGTTAGCACTCTGGACTCTGAATCCAGCGATCCGAGTTCAA
22694	Yes	AATGGTTAGCACTCTGGACTCTGAATCCAGCGATCCGAGTTCAAA
22695	Yes	AATGGTTAGCACTCTGGACTCTGAATCCAGCGATCCGAGTTCAAAT
22696	Yes	AATGGTTAGCACTCTGGACTCTGAATCCAGCGATCCGAGTTCAAATC
22697	Yes	AATGGTTAGCACTCTGGACTCTGAATCCAGCGATCCGAGTTCAAATCT
22698	Yes	AATGGTTAGCACTCTGGACTCTGAATCCAGCGATCCGAGTTCAAATCTC
22699	Yes	AATGGTTAGCACTCTGGACTCTGAATCCAGCGATCCGAGTTCAAATCTCG
22700	Yes	AATGGTTAGCACTCTGGACTCTGAATCCAGCGATCCGAGTTCAAG
22701	Yes	AATGGTTAGCACTCTGGACTCTGAATCCAGCGATCCGAGTTCAAGT
22702	Yes	AATGGTTAGCACTCTGGACTCTGAATCCAGCGATCCGAGTTCAAGTC
22703	Yes	AATGGTTAGCACTCTGGACTCTGAATCCAGCGATCCGAGTTCAAGTCT
22704	Yes	AATGGTTAGCACTCTGGACTCTGAATCCAGCGATCCGAGTTCAAGTCTC
22705	Yes	AATGGTTAGCACTCTGGACTCTGAATCCAGCGATCCGAGTTCAAGTCTCG
22706	Yes	AATGGTTAGCACTCTGGACTCTGAATCCG
22707	Yes	AATGGTTAGCACTCTGGACTCTGAATCCGG
22708	Yes	AATGGTTAGCACTCTGGACTCTGAATCCGGT
22709	Yes	AATGGTTAGCACTCTGGACTCTGAATCCGGTA
22710	Yes	AATGGTTAGCACTCTGGACTCTGAATCCGGTAA
22711	Yes	AATGGTTAGCACTCTGGACTCTGAATCCGGTAAT
22712	Yes	AATGGTTAGCACTCTGGACTCTGAATCCGGTAATC
22713	Yes	AATGGTTAGCACTCTGGACTCTGAATCCGGTAATCC
22714	Yes	AATGGTTAGCACTCTGGACTCTGAATCCGGTAATCCG
22715	Yes	AATGGTTAGCACTCTGGACTCTGAATCCGGTAATCCGA

22716	Yes	AATGGTTAGCACTCTGGACTCTGAATCCGGTAATCCGAG
22717	Yes	AATGGTTAGCACTCTGGACTCTGAATCCGGTAATCCGAGT
22718	Yes	AATGGTTAGCACTCTGGACTCTGAATCCGGTAATCCGAGTT
22719	Yes	AATGGTTAGCACTCTGGACTCTGAATCCGGTAATCCGAGTTC
22720	Yes	AATGGTTAGCACTCTGGACTCTGAATCCGGTAATCCGAGTTCA
22721	Yes	AATGGTTAGCACTCTGGACTCTGAATCCGGTAATCCGAGTTCAA
22722	Yes	AATGGTTAGCACTCTGGACTCTGAATCCGGTAATCCGAGTTCAAA
22723	Yes	AATGGTTAGCACTCTGGACTCTGAATCCGGTAATCCGAGTTCAAAT
22724	Yes	AATGGTTAGCACTCTGGACTCTGAATCCGGTAATCCGAGTTCAAATC
22725	Yes	AATGGTTAGCACTCTGGACTCTGAATCCGGTAATCCGAGTTCAAATCT
22726	Yes	AATGGTTAGCACTCTGGACTCTGAATCCGGTAATCCGAGTTCAAATCTC
22727	Yes	AATGGTTAGCACTCTGGACTCTGAATCCGGTAATCCGAGTTCAAATCTCG
22728	Yes	AATGGTTAGCACTCTGGACTT
22729	Yes	AATGGTTAGCACTCTGGACTTT
22730	Yes	AATGGTTAGCACTCTGGACTTTG
22731	Yes	AATGGTTAGCACTCTGGACTTTGA
22732	Yes	AATGGTTAGCACTCTGGACTTTGAA
22733	Yes	AATGGTTAGCACTCTGGACTTTGAAT
22734	Yes	AATGGTTAGCACTCTGGACTTTGAATC
22735	Yes	AATGGTTAGCACTCTGGACTTTGAATCC
22736	Yes	AATGGTTAGCACTCTGGACTTTGAATCCA
22737	Yes	AATGGTTAGCACTCTGGACTTTGAATCCAG
22738	Yes	AATGGTTAGCACTCTGGACTTTGAATCCAGC
22739	Yes	AATGGTTAGCACTCTGGACTTTGAATCCAGCA
22740	Yes	AATGGTTAGCACTCTGGACTTTGAATCCAGCAA
22741	Yes	AATGGTTAGCACTCTGGACTTTGAATCCAGCAAT
22742	Yes	AATGGTTAGCACTCTGGACTTTGAATCCAGCAATC
22743	Yes	AATGGTTAGCACTCTGGACTTTGAATCCAGCAATCC
22744	Yes	AATGGTTAGCACTCTGGACTTTGAATCCAGCAATCCG
22745	Yes	AATGGTTAGCACTCTGGACTTTGAATCCAGCAATCCGA
22746	Yes	AATGGTTAGCACTCTGGACTTTGAATCCAGCAATCCGAG
22747	Yes	AATGGTTAGCACTCTGGACTTTGAATCCAGCAATCCGAGT
22748	Yes	AATGGTTAGCACTCTGGACTTTGAATCCAGCAATCCGAGTT
22749	Yes	AATGGTTAGCACTCTGGACTTTGAATCCAGCAATCCGAGTTC
22750	Yes	AATGGTTAGCACTCTGGACTTTGAATCCAGCAATCCGAGTTCG
22751	Yes	AATGGTTAGCACTCTGGACTTTGAATCCAGCAATCCGAGTTCGA
22752	Yes	AATGGTTAGCACTCTGGACTTTGAATCCAGCAATCCGAGTTCGAA
22753	Yes	AATGGTTAGCACTCTGGACTTTGAATCCAGCAATCCGAGTTCGAAT
22754	Yes	AATGGTTAGCACTCTGGACTTTGAATCCAGCAATCCGAGTTCGAATC
22755	Yes	AATGGTTAGCACTCTGGACTTTGAATCCAGCAATCCGAGTTCGAATCT
22756	Yes	AATGGTTAGCACTCTGGACTTTGAATCCAGCAATCCGAGTTCGAATCTC
22757	Yes	AATGGTTAGCACTCTGGACTTTGAATCCAGCAATCCGAGTTCGAATCTCG

22758	Yes	AATGGTTAGCACTCTGGACTTTGAATCCAGCG
22759	Yes	AATGGTTAGCACTCTGGACTTTGAATCCAGCGA
22760	Yes	AATGGTTAGCACTCTGGACTTTGAATCCAGCGAT
22761	Yes	AATGGTTAGCACTCTGGACTTTGAATCCAGCGATC
22762	Yes	AATGGTTAGCACTCTGGACTTTGAATCCAGCGATCC
22763	Yes	AATGGTTAGCACTCTGGACTTTGAATCCAGCGATCCG
22764	Yes	AATGGTTAGCACTCTGGACTTTGAATCCAGCGATCCGA
22765	Yes	AATGGTTAGCACTCTGGACTTTGAATCCAGCGATCCGAG
22766	Yes	AATGGTTAGCACTCTGGACTTTGAATCCAGCGATCCGAGT
22767	Yes	AATGGTTAGCACTCTGGACTTTGAATCCAGCGATCCGAGTT
22768	Yes	AATGGTTAGCACTCTGGACTTTGAATCCAGCGATCCGAGTTC
22769	Yes	AATGGTTAGCACTCTGGACTTTGAATCCAGCGATCCGAGTTCA
22770	Yes	AATGGTTAGCACTCTGGACTTTGAATCCAGCGATCCGAGTTCAA
22771	Yes	AATGGTTAGCACTCTGGACTTTGAATCCAGCGATCCGAGTTCAAA
22772	Yes	AATGGTTAGCACTCTGGACTTTGAATCCAGCGATCCGAGTTCAAAT
22773	Yes	AATGGTTAGCACTCTGGACTTTGAATCCAGCGATCCGAGTTCAAATC
22774	Yes	AATGGTTAGCACTCTGGACTTTGAATCCAGCGATCCGAGTTCAAATCT
22775	Yes	AATGGTTAGCACTCTGGACTTTGAATCCAGCGATCCGAGTTCAAATCTC
22776	Yes	AATGGTTAGCACTCTGGACTTTGAATCCAGCGATCCGAGTTCAAATCTCG
22777	Yes	AATGGTTAGCACTCTGGG
22778	Yes	AATGGTTAGCACTCTGGGC
22779	Yes	AATGGTTAGCACTCTGGGCT
22780	Yes	AATGGTTAGCACTCTGGGCTT
22781	Yes	AATGGTTAGCACTCTGGGCTTT
22782	Yes	AATGGTTAGCACTCTGGGCTTTG
22783	Yes	AATGGTTAGCACTCTGGGCTTTGA
22784	Yes	AATGGTTAGCACTCTGGGCTTTGAA
22785	Yes	AATGGTTAGCACTCTGGGCTTTGAAT
22786	Yes	AATGGTTAGCACTCTGGGCTTTGAATC
22787	Yes	AATGGTTAGCACTCTGGGCTTTGAATCC
22788	Yes	AATGGTTAGCACTCTGGGCTTTGAATCCA
22789	Yes	AATGGTTAGCACTCTGGGCTTTGAATCCAG
22790	Yes	AATGGTTAGCACTCTGGGCTTTGAATCCAGC
22791	Yes	AATGGTTAGCACTCTGGGCTTTGAATCCAGCA
22792	Yes	AATGGTTAGCACTCTGGGCTTTGAATCCAGCAA
22793	Yes	AATGGTTAGCACTCTGGGCTTTGAATCCAGCAAT
22794	Yes	AATGGTTAGCACTCTGGGCTTTGAATCCAGCAATC
22795	Yes	AATGGTTAGCACTCTGGGCTTTGAATCCAGCAATCC
22796	Yes	AATGGTTAGCACTCTGGGCTTTGAATCCAGCAATCCG
22797	Yes	AATGGTTAGCACTCTGGGCTTTGAATCCAGCAATCCGA
22798	Yes	AATGGTTAGCACTCTGGGCTTTGAATCCAGCAATCCGAG
22799	Yes	AATGGTTAGCACTCTGGGCTTTGAATCCAGCAATCCGAGT

22800	Yes	AATGGTTAGCACTCTGGGCTTTGAATCCAGCAATCCGAGTT
22801	Yes	AATGGTTAGCACTCTGGGCTTTGAATCCAGCAATCCGAGTTC
22802	Yes	AATGGTTAGCACTCTGGGCTTTGAATCCAGCAATCCGAGTTCG
22803	Yes	AATGGTTAGCACTCTGGGCTTTGAATCCAGCAATCCGAGTTCGA
22804	Yes	AATGGTTAGCACTCTGGGCTTTGAATCCAGCAATCCGAGTTCGAA
22805	Yes	AATGGTTAGCACTCTGGGCTTTGAATCCAGCAATCCGAGTTCGAAT
22806	Yes	AATGGTTAGCACTCTGGGCTTTGAATCCAGCAATCCGAGTTCGAATC
22807	Yes	AATGGTTAGCACTCTGGGCTTTGAATCCAGCAATCCGAGTTCGAATCT
22808	Yes	AATGGTTAGCACTCTGGGCTTTGAATCCAGCAATCCGAGTTCGAATCTT
22809	Yes	AATGGTTAGCACTCTGGGCTTTGAATCCAGCAATCCGAGTTCGAATCTTG
22810	No	AATGGTTAGGAGTCGG
22811	No	AATGGTTAGGAGTCGGC
22812	Yes	AATGGTTAGGAGTCGGCA
22813	Yes	AATGGTTAGGAGTCGGCAC
22814	Yes	AATGGTTAGGAGTCGGCACT
22815	Yes	AATGGTTAGGAGTCGGCACTC
22816	Yes	AATGGTTAGGAGTCGGCACTCT
22817	Yes	AATGGTTAGGAGTCGGCACTCTC
22818	Yes	AATGGTTAGGAGTCGGCACTCTCA
22819	Yes	AATGGTTAGGAGTCGGCACTCTCAC
22820	Yes	AATGGTTAGGAGTCGGCACTCTCACC
22821	Yes	AATGGTTAGGAGTCGGCACTCTCACCG
22822	Yes	AATGGTTAGGAGTCGGCACTCTCACCGC
22823	Yes	AATGGTTAGGAGTCGGCACTCTCACCGCC
22824	Yes	AATGGTTAGGAGTCGGCACTCTCACCGCCG
22825	Yes	AATGGTTAGGAGTCGGCACTCTCACCGCCGC
22826	Yes	AATGGTTAGGAGTCGGCACTCTCACCGCCGCG
22827	Yes	AATGGTTAGGAGTCGGCACTCTCACCGCCGCGG
22828	Yes	AATGGTTAGGAGTCGGCACTCTCACCGCCGCGGC
22829	Yes	AATGGTTAGGAGTCGGCACTCTCACCGCCGCGGCT
22830	Yes	AATGGTTAGGAGTCGGCACTCTCACCGCCGCGGCTG
22831	Yes	AATGGTTAGGAGTCGGCACTCTCACCGCCGCGGCTGG
22832	Yes	AATGGTTAGGAGTCGGCACTCTCACCGCCGCGGCTGGG
22833	Yes	AATGGTTAGGAGTCGGCACTCTCACCGCCGCGGCTGGGG
22834	Yes	AATGGTTAGGAGTCGGCACTCTCACCGCCGCGGCTGGGGT
22835	Yes	AATGGTTAGGAGTCGGCACTCTCACCGCCGCGGCTGGGGTT
22836	Yes	AATGGTTAGGAGTCGGCACTCTCACCGCCGCGGCTGGGGTTT
22837	Yes	AATGGTTAGGAGTCGGCACTCTCACCGCCGCGGCTGGGGTTTG
22838	Yes	AATGGTTAGGAGTCGGCACTCTCACCGCCGCGGCTGGGGTTTGA
22839	Yes	AATGGTTAGGAGTCGGCACTCTCACCGCCGCGGCTGGGGTTTGAT
22840	Yes	AATGGTTAGGAGTCGGCACTCTCACCGCCGCGGCTGGGGTTTGATT
22841	Yes	AATGGTTAGGAGTCGGCACTCTCACCGCCGCGGCTGGGGTTTGATTC

22842	Yes	AATGGTTAGGAGTCGGCACTCTCACCGCCGCGGCTGGGGTTTGATTCC
22843	Yes	AATGGTTAGGAGTCGGCACTCTCACCGCCGCGGCTGGGGTTTGATTCCC
22844	Yes	AATGGTTAGGAGTCGGCACTCTCACCGCCGCGGCTGGGGTTTGATTCCCA
22845	No	AATGGTTATGAGGGTG
22846	No	AATGGTTATGAGGGTGG
22847	Yes	AATGGTTATGAGGGTGGA
22848	Yes	AATGGTTATGAGGGTGGAC
22849	Yes	AATGGTTATGAGGGTGGACT
22850	Yes	AATGGTTATGAGGGTGGACTT
22851	Yes	AATGGTTATGAGGGTGGACTTT
22852	Yes	AATGGTTATGAGGGTGGACTTTA
22853	Yes	AATGGTTATGAGGGTGGACTTTAA
22854	Yes	AATGGTTATGAGGGTGGACTTTAAC
22855	Yes	AATGGTTATGAGGGTGGACTTTAACC
22856	Yes	AATGGTTATGAGGGTGGACTTTAACCA
22857	Yes	AATGGTTATGAGGGTGGACTTTAACCAC
22858	Yes	AATGGTTATGAGGGTGGACTTTAACCACA
22859	Yes	AATGGTTATGAGGGTGGACTTTAACCACAC
22860	Yes	AATGGTTATGAGGGTGGACTTTAACCACACT
22861	Yes	AATGGTTATGAGGGTGGACTTTAACCACACTG
22862	Yes	AATGGTTATGAGGGTGGACTTTAACCACACTGC
22863	Yes	AATGGTTATGAGGGTGGACTTTAACCACACTGCC
22864	Yes	AATGGTTATGAGGGTGGACTTTAACCACACTGCCT
22865	Yes	AATGGTTATGAGGGTGGACTTTAACCACACTGCCTA
22866	Yes	AATGGTTATGAGGGTGGACTTTAACCACACTGCCTAG
22867	Yes	AATGGTTATGAGGGTGGACTTTAACCACACTGCCTAGG
22868	Yes	AATGGTTATGAGGGTGGACTTTAACCACACTGCCTAGGT
22869	Yes	AATGGTTATGAGGGTGGACTTTAACCACACTGCCTAGGTT
22870	Yes	AATGGTTATGAGGGTGGACTTTAACCACACTGCCTAGGTTC
22871	Yes	AATGGTTATGAGGGTGGACTTTAACCACACTGCCTAGGTTCA
22872	Yes	AATGGTTATGAGGGTGGACTTTAACCACACTGCCTAGGTTCAA
22873	Yes	AATGGTTATGAGGGTGGACTTTAACCACACTGCCTAGGTTCAAA
22874	Yes	AATGGTTATGAGGGTGGACTTTAACCACACTGCCTAGGTTCAAAT
22875	Yes	AATGGTTATGAGGGTGGACTTTAACCACACTGCCTAGGTTCAAATC
22876	Yes	AATGGTTATGAGGGTGGACTTTAACCACACTGCCTAGGTTCAAATCC
22877	Yes	AATGGTTATGAGGGTGGACTTTAACCACACTGCCTAGGTTCAAATCCT
22878	Yes	AATGGTTATGAGGGTGGACTTTAACCACACTGCCTAGGTTCAAATCCTG
22879	Yes	AATGGTTATGAGGGTGGACTTTAACCACACTGCCTAGGTTCAAATCCTGA
22880	No	AATGTAGCATTGTGGC
22881	Yes	AATGTAGCATTGTGGCT
22882	Yes	AATGTAGCATTGTGGCTA
22883	Yes	AATGTAGCATTGTGGCTAA

22884	Yes	AATGTAGCATTGTGGCTAAG
22885	Yes	AATGTAGCATTGTGGCTAAGT
22886	Yes	AATGTAGCATTGTGGCTAAGTG
22887	Yes	AATGTAGCATTGTGGCTAAGTGC
22888	Yes	AATGTAGCATTGTGGCTAAGTGCA
22889	Yes	AATGTAGCATTGTGGCTAAGTGCAC
22890	Yes	AATGTAGCATTGTGGCTAAGTGCACA
22891	Yes	AATGTAGCATTGTGGCTAAGTGCACAG
22892	Yes	AATGTAGCATTGTGGCTAAGTGCACAGG
22893	Yes	AATGTAGCATTGTGGCTAAGTGCACAGGC
22894	Yes	AATGTAGCATTGTGGCTAAGTGCACAGGCT
22895	Yes	AATGTAGCATTGTGGCTAAGTGCACAGGCTT
22896	Yes	AATGTAGCATTGTGGCTAAGTGCACAGGCTTT
22897	Yes	AATGTAGCATTGTGGCTAAGTGCACAGGCTTTG
22898	Yes	AATGTAGCATTGTGGCTAAGTGCACAGGCTTTGG
22899	Yes	AATGTAGCATTGTGGCTAAGTGCACAGGCTTTGGA
22900	Yes	AATGTAGCATTGTGGCTAAGTGCACAGGCTTTGGAA
22901	Yes	AATGTAGCATTGTGGCTAAGTGCACAGGCTTTGGAAA
22902	Yes	AATGTAGCATTGTGGCTAAGTGCACAGGCTTTGGAAAC
22903	Yes	AATGTAGCATTGTGGCTAAGTGCACAGGCTTTGGAAACT
22904	Yes	AATGTAGCATTGTGGCTAAGTGCACAGGCTTTGGAAACTG
22905	Yes	AATGTAGCATTGTGGCTAAGTGCACAGGCTTTGGAAACTGG
22906	Yes	AATGTAGCATTGTGGCTAAGTGCACAGGCTTTGGAAACTGGC
22907	Yes	AATGTAGCATTGTGGCTAAGTGCACAGGCTTTGGAAACTGGCA
22908	Yes	AATGTAGCATTGTGGCTAAGTGCACAGGCTTTGGAAACTGGCAG
22909	Yes	AATGTAGCATTGTGGCTAAGTGCACAGGCTTTGGAAACTGGCAGG
22910	Yes	AATGTAGCATTGTGGCTAAGTGCACAGGCTTTGGAAACTGGCAGGC
22911	Yes	AATGTAGCATTGTGGCTAAGTGCACAGGCTTTGGAAACTGGCAGGCC
22912	Yes	AATGTAGCATTGTGGCTAAGTGCACAGGCTTTGGAAACTGGCAGGCCT
22913	Yes	AATGTAGCATTGTGGCTAAGTGCACAGGCTTTGGAAACTGGCAGGCCTG
22914	Yes	AATGTAGCATTGTGGCTAAGTGCACAGGCTTTGGAAACTGGCAGGCCTGG
22915	No	AATGTAGCCTCGTGGT
22916	Yes	AATGTAGCCTCGTGGTT
22917	Yes	AATGTAGCCTCGTGGTTA
22918	Yes	AATGTAGCCTCGTGGTTAG
22919	Yes	AATGTAGCCTCGTGGTTAGG
22920	Yes	AATGTAGCCTCGTGGTTAGGG
22921	Yes	AATGTAGCCTCGTGGTTAGGGG
22922	Yes	AATGTAGCCTCGTGGTTAGGGGC
22923	Yes	AATGTAGCCTCGTGGTTAGGGGCT
22924	Yes	AATGTAGCCTCGTGGTTAGGGGCTG
22925	Yes	AATGTAGCCTCGTGGTTAGGGGCTGC

22926	Yes	AATGTAGCCTCGTGGTTAGGGGCTGCA
22927	Yes	AATGTAGCCTCGTGGTTAGGGGCTGCAT
22928	Yes	AATGTAGCCTCGTGGTTAGGGGCTGCATT
22929	Yes	AATGTAGCCTCGTGGTTAGGGGCTGCATTC
22930	Yes	AATGTAGCCTCGTGGTTAGGGGCTGCATTCT
22931	Yes	AATGTAGCCTCGTGGTTAGGGGCTGCATTCTA
22932	Yes	AATGTAGCCTCGTGGTTAGGGGCTGCATTCTAG
22933	Yes	AATGTAGCCTCGTGGTTAGGGGCTGCATTCTAGA
22934	Yes	AATGTAGCCTCGTGGTTAGGGGCTGCATTCTAGAG
22935	Yes	AATGTAGCCTCGTGGTTAGGGGCTGCATTCTAGAGC
22936	Yes	AATGTAGCCTCGTGGTTAGGGGCTGCATTCTAGAGCT
22937	Yes	AATGTAGCCTCGTGGTTAGGGGCTGCATTCTAGAGCTA
22938	Yes	AATGTAGCCTCGTGGTTAGGGGCTGCATTCTAGAGCTAT
22939	Yes	AATGTAGCCTCGTGGTTAGGGGCTGCATTCTAGAGCTATG
22940	Yes	AATGTAGCCTCGTGGTTAGGGGCTGCATTCTAGAGCTATGC
22941	Yes	AATGTAGCCTCGTGGTTAGGGGCTGCATTCTAGAGCTATGCT
22942	Yes	AATGTAGCCTCGTGGTTAGGGGCTGCATTCTAGAGCTATGCTG
22943	Yes	AATGTAGCCTCGTGGTTAGGGGCTGCATTCTAGAGCTATGCTGC
22944	Yes	AATGTAGCCTCGTGGTTAGGGGCTGCATTCTAGAGCTATGCTGCC
22945	Yes	AATGTAGCCTCGTGGTTAGGGGCTGCATTCTAGAGCTATGCTGCCC
22946	Yes	AATGTAGCCTCGTGGTTAGGGGCTGCATTCTAGAGCTATGCTGCCCA
22947	Yes	AATGTAGCCTCGTGGTTAGGGGCTGCATTCTAGAGCTATGCTGCCCAG
22948	Yes	AATGTAGCCTCGTGGTTAGGGGCTGCATTCTAGAGCTATGCTGCCCAGG
22949	Yes	AATGTAGCCTCGTGGTTAGGGGCTGCATTCTAGAGCTATGCTGCCCAGGT
22950	No	AATGTAGTGGTTAAAG
22951	No	AATGTAGTGGTTAAAGG
22952	Yes	AATGTAGTGGTTAAAGGC
22953	Yes	AATGTAGTGGTTAAAGGCA
22954	Yes	AATGTAGTGGTTAAAGGCAT
22955	Yes	AATGTAGTGGTTAAAGGCATG
22956	Yes	AATGTAGTGGTTAAAGGCATGG
22957	Yes	AATGTAGTGGTTAAAGGCATGGG
22958	Yes	AATGTAGTGGTTAAAGGCATGGGC
22959	Yes	AATGTAGTGGTTAAAGGCATGGGCT
22960	Yes	AATGTAGTGGTTAAAGGCATGGGCTC
22961	Yes	AATGTAGTGGTTAAAGGCATGGGCTCT
22962	Yes	AATGTAGTGGTTAAAGGCATGGGCTCTA
22963	Yes	AATGTAGTGGTTAAAGGCATGGGCTCTAG
22964	Yes	AATGTAGTGGTTAAAGGCATGGGCTCTAGA
22965	Yes	AATGTAGTGGTTAAAGGCATGGGCTCTAGAG
22966	Yes	AATGTAGTGGTTAAAGGCATGGGCTCTAGAGC
22967	Yes	AATGTAGTGGTTAAAGGCATGGGCTCTAGAGCC

22968	Yes	AATGTAGTGGTTAAAGGCATGGGCTCTAGAGCCA
22969	Yes	AATGTAGTGGTTAAAGGCATGGGCTCTAGAGCCAG
22970	Yes	AATGTAGTGGTTAAAGGCATGGGCTCTAGAGCCAGA
22971	Yes	AATGTAGTGGTTAAAGGCATGGGCTCTAGAGCCAGAC
22972	Yes	AATGTAGTGGTTAAAGGCATGGGCTCTAGAGCCAGACT
22973	Yes	AATGTAGTGGTTAAAGGCATGGGCTCTAGAGCCAGACTT
22974	Yes	AATGTAGTGGTTAAAGGCATGGGCTCTAGAGCCAGACTTC
22975	Yes	AATGTAGTGGTTAAAGGCATGGGCTCTAGAGCCAGACTTCC
22976	Yes	AATGTAGTGGTTAAAGGCATGGGCTCTAGAGCCAGACTTCCT
22977	Yes	AATGTAGTGGTTAAAGGCATGGGCTCTAGAGCCAGACTTCCTG
22978	Yes	AATGTAGTGGTTAAAGGCATGGGCTCTAGAGCCAGACTTCCTGG
22979	Yes	AATGTAGTGGTTAAAGGCATGGGCTCTAGAGCCAGACTTCCTGGG
22980	Yes	AATGTAGTGGTTAAAGGCATGGGCTCTAGAGCCAGACTTCCTGGGT
22981	Yes	AATGTAGTGGTTAAAGGCATGGGCTCTAGAGCCAGACTTCCTGGGTT
22982	Yes	AATGTAGTGGTTAAAGGCATGGGCTCTAGAGCCAGACTTCCTGGGTTC
22983	Yes	AATGTAGTGGTTAAAGGCATGGGCTCTAGAGCCAGACTTCCTGGGTTCA
22984	Yes	AATGTAGTGGTTAAAGGCATGGGCTCTAGAGCCAGACTTCCTGGGTTCAA
22985	No	AATGTAGTGGTTATCA
22986	No	AATGTAGTGGTTATCAC
22987	Yes	AATGTAGTGGTTATCACA
22988	Yes	AATGTAGTGGTTATCACAT
22989	Yes	AATGTAGTGGTTATCACATT
22990	Yes	AATGTAGTGGTTATCACATTC
22991	Yes	AATGTAGTGGTTATCACATTCG
22992	Yes	AATGTAGTGGTTATCACATTCGC
22993	Yes	AATGTAGTGGTTATCACATTCGCC
22994	Yes	AATGTAGTGGTTATCACATTCGCCT
22995	Yes	AATGTAGTGGTTATCACATTCGCCTC
22996	Yes	AATGTAGTGGTTATCACATTCGCCTCA
22997	Yes	AATGTAGTGGTTATCACATTCGCCTCAC
22998	Yes	AATGTAGTGGTTATCACATTCGCCTCACA
22999	Yes	AATGTAGTGGTTATCACATTCGCCTCACAC
23000	Yes	AATGTAGTGGTTATCACATTCGCCTCACACA
23001	Yes	AATGTAGTGGTTATCACATTCGCCTCACACAT
23002	Yes	AATGTAGTGGTTATCACATTCGCCTCACACATG
23003	Yes	AATGTAGTGGTTATCACATTCGCCTCACACATGA
23004	Yes	AATGTAGTGGTTATCACATTCGCCTCACACATGAA
23005	Yes	AATGTAGTGGTTATCACATTCGCCTCACACATGAAA
23006	Yes	AATGTAGTGGTTATCACATTCGCCTCACACATGAAAG
23007	Yes	AATGTAGTGGTTATCACATTCGCCTCACACATGAAAGG
23008	Yes	AATGTAGTGGTTATCACATTCGCCTCACACATGAAAGGT
23009	Yes	AATGTAGTGGTTATCACATTCGCCTCACACATGAAAGGTC

23010	Yes	AATGTAGTGGTTATCACATTCGCCTCACACATGAAAGGTCA
23011	Yes	AATGTAGTGGTTATCACATTCGCCTCACACATGAAAGGTCAC
23012	Yes	AATGTAGTGGTTATCACATTCGCCTCACACATGAAAGGTCACC
23013	Yes	AATGTAGTGGTTATCACATTCGCCTCACACATGAAAGGTCACCA
23014	Yes	AATGTAGTGGTTATCACATTCGCCTCACACATGAAAGGTCACCAG
23015	Yes	AATGTAGTGGTTATCACATTCGCCTCACACATGAAAGGTCACCAGT
23016	Yes	AATGTAGTGGTTATCACATTCGCCTCACACATGAAAGGTCACCAGTT
23017	Yes	AATGTAGTGGTTATCACATTCGCCTCACACATGAAAGGTCACCAGTTT
23018	Yes	AATGTAGTGGTTATCACATTCGCCTCACACATGAAAGGTCACCAGTTTG
23019	Yes	AATGTAGTGGTTATCACATTCGCCTCACACATGAAAGGTCACCAGTTTGA
23020	Yes	AATGTCAAGGTTGCGG
23021	Yes	AATGTCAAGGTTGCGGG
23022	Yes	AATGTCAAGGTTGCGGGT
23023	Yes	AATGTCAAGGTTGCGGGTT
23024	Yes	AATGTCAAGGTTGCGGGTTG
23025	Yes	AATGTCAAGGTTGCGGGTTGG
23026	Yes	AATGTCAAGGTTGCGGGTTGGA
23027	Yes	AATGTCAAGGTTGCGGGTTGGAT
23028	Yes	AATGTCAAGGTTGCGGGTTGGATC
23029	Yes	AATGTCAAGGTTGCGGGTTGGATCC
23030	Yes	AATGTCAAGGTTGCGGGTTGGATCCC
23031	Yes	AATGTCAAGGTTGCGGGTTGGATCCCC
23032	Yes	AATGTCAAGGTTGCGGGTTGGATCCCCG
23033	Yes	AATGTCAAGGTTGCGGGTTGGATCCCCGA
23034	Yes	AATGTCAAGGTTGCGGGTTGGATCCCCGAA
23035	Yes	AATGTCAAGGTTGCGGGTTGGATCCCCGAAC
23036	Yes	AATGTCAAGGTTGCGGGTTGGATCCCCGAACG
23037	Yes	AATGTCAAGGTTGCGGGTTGGATCCCCGAACGG
23038	Yes	AATGTCAAGGTTGCGGGTTGGATCCCCGAACGGG
23039	Yes	AATGTCAAGGTTGCGGGTTGGATCCCCGAACGGGC
23040	Yes	AATGTCAAGGTTGCGGGTTGGATCCCCGAACGGGCC
23041	Yes	AATGTCAAGGTTGCGGGTTGGATCCCCGAACGGGCCA
23042	Yes	AATGTCAAGGTTGCGGGTTGGATCCCCGAACGGGCCAC
23043	Yes	AATGTCAAGGTTGCGGGTTGGATCCCCGAACGGGCCACC
23044	Yes	AATGTCAAGGTTGCGGGTTGGATCCCCGAACGGGCCACCA
23045	No	AATGTGCGCTCTGGGG
23046	No	AATGTGCGCTCTGGGGC
23047	No	AATGTGCGCTCTGGGGCT
23048	Yes	AATGTGCGCTCTGGGGCTG
23049	Yes	AATGTGCGCTCTGGGGCTGC
23050	Yes	AATGTGCGCTCTGGGGCTGCT
23051	Yes	AATGTGCGCTCTGGGGCTGCTG

23052	Yes	AATGTGCGCTCTGGGGCTGCTGA
23053	Yes	AATGTGCGCTCTGGGGCTGCTGAT
23054	Yes	AATGTGCGCTCTGGGGCTGCTGATC
23055	Yes	AATGTGCGCTCTGGGGCTGCTGATCC
23056	Yes	AATGTGCGCTCTGGGGCTGCTGATCCC
23057	Yes	AATGTGCGCTCTGGGGCTGCTGATCCCA
23058	Yes	AATGTGCGCTCTGGGGCTGCTGATCCCAG
23059	Yes	AATGTGCGCTCTGGGGCTGCTGATCCCAGG
23060	Yes	AATGTGCGCTCTGGGGCTGCTGATCCCAGGC
23061	Yes	AATGTGCGCTCTGGGGCTGCTGATCCCAGGCT
23062	Yes	AATGTGCGCTCTGGGGCTGCTGATCCCAGGCTC
23063	Yes	AATGTGCGCTCTGGGGCTGCTGATCCCAGGCTCA
23064	Yes	AATGTGCGCTCTGGGGCTGCTGATCCCAGGCTCAA
23065	Yes	AATGTGCGCTCTGGGGCTGCTGATCCCAGGCTCAAA
23066	Yes	AATGTGCGCTCTGGGGCTGCTGATCCCAGGCTCAAAC
23067	Yes	AATGTGCGCTCTGGGGCTGCTGATCCCAGGCTCAAACC
23068	Yes	AATGTGCGCTCTGGGGCTGCTGATCCCAGGCTCAAACCC
23069	Yes	AATGTGCGCTCTGGGGCTGCTGATCCCAGGCTCAAACCCT
23070	Yes	AATGTGCGCTCTGGGGCTGCTGATCCCAGGCTCAAACCCTG
23071	Yes	AATGTGCGCTCTGGGGCTGCTGATCCCAGGCTCAAACCCTGG
23072	Yes	AATGTGCGCTCTGGGGCTGCTGATCCCAGGCTCAAACCCTGGC
23073	Yes	AATGTGCGCTCTGGGGCTGCTGATCCCAGGCTCAAACCCTGGCG
23074	Yes	AATGTGCGCTCTGGGGCTGCTGATCCCAGGCTCAAACCCTGGCGC
23075	Yes	AATGTGCGCTCTGGGGCTGCTGATCCCAGGCTCAAACCCTGGCGCT
23076	Yes	AATGTGCGCTCTGGGGCTGCTGATCCCAGGCTCAAACCCTGGCGCTG
23077	Yes	AATGTGCGCTCTGGGGCTGCTGATCCCAGGCTCAAACCCTGGCGCTGT
23078	Yes	AATGTGCGCTCTGGGGCTGCTGATCCCAGGCTCAAACCCTGGCGCTGTC
23079	Yes	AATGTGCGCTCTGGGGCTGCTGATCCCAGGCTCAAACCCTGGCGCTGTCA
23080	No	AATGTGGACTTTGAAG
23081	No	AATGTGGACTTTGAAGT
23082	Yes	AATGTGGACTTTGAAGTT
23083	Yes	AATGTGGACTTTGAAGTTA
23084	Yes	AATGTGGACTTTGAAGTTAG
23085	Yes	AATGTGGACTTTGAAGTTAGA
23086	Yes	AATGTGGACTTTGAAGTTAGAT
23087	Yes	AATGTGGACTTTGAAGTTAGATA
23088	Yes	AATGTGGACTTTGAAGTTAGATAC
23089	Yes	AATGTGGACTTTGAAGTTAGATACA
23090	Yes	AATGTGGACTTTGAAGTTAGATACAC
23091	Yes	AATGTGGACTTTGAAGTTAGATACACC
23092	Yes	AATGTGGACTTTGAAGTTAGATACACCT
23093	Yes	AATGTGGACTTTGAAGTTAGATACACCTG

23094	Yes	AATGTGGACTTTGAAGTTAGATACACCTGG
23095	Yes	AATGTGGACTTTGAAGTTAGATACACCTGGG
23096	Yes	AATGTGGACTTTGAAGTTAGATACACCTGGGT
23097	Yes	AATGTGGACTTTGAAGTTAGATACACCTGGGTT
23098	Yes	AATGTGGACTTTGAAGTTAGATACACCTGGGTTC
23099	Yes	AATGTGGACTTTGAAGTTAGATACACCTGGGTTCA
23100	Yes	AATGTGGACTTTGAAGTTAGATACACCTGGGTTCAA
23101	Yes	AATGTGGACTTTGAAGTTAGATACACCTGGGTTCAAA
23102	Yes	AATGTGGACTTTGAAGTTAGATACACCTGGGTTCAAAT
23103	Yes	AATGTGGACTTTGAAGTTAGATACACCTGGGTTCAAATC
23104	Yes	AATGTGGACTTTGAAGTTAGATACACCTGGGTTCAAATCC
23105	Yes	AATGTGGACTTTGAAGTTAGATACACCTGGGTTCAAATCCC
23106	Yes	AATGTGGACTTTGAAGTTAGATACACCTGGGTTCAAATCCCA
23107	Yes	AATGTGGACTTTGAAGTTAGATACACCTGGGTTCAAATCCCAG
23108	Yes	AATGTGGACTTTGAAGTTAGATACACCTGGGTTCAAATCCCAGT
23109	Yes	AATGTGGACTTTGAAGTTAGATACACCTGGGTTCAAATCCCAGTG
23110	Yes	AATGTGGACTTTGAAGTTAGATACACCTGGGTTCAAATCCCAGTGC
23111	Yes	AATGTGGACTTTGAAGTTAGATACACCTGGGTTCAAATCCCAGTGCT
23112	Yes	AATGTGGACTTTGAAGTTAGATACACCTGGGTTCAAATCCCAGTGCTG
23113	Yes	AATGTGGACTTTGAAGTTAGATACACCTGGGTTCAAATCCCAGTGCTGT
23114	Yes	AATGTGGACTTTGAAGTTAGATACACCTGGGTTCAAATCCCAGTGCTGTC
23115	No	AATGTGGCCTCTGCAA
23116	No	AATGTGGCCTCTGCAAG
23117	Yes	AATGTGGCCTCTGCAAGC
23118	Yes	AATGTGGCCTCTGCAAGCA
23119	Yes	AATGTGGCCTCTGCAAGCAG
23120	Yes	AATGTGGCCTCTGCAAGCAGA
23121	Yes	AATGTGGCCTCTGCAAGCAGAC
23122	Yes	AATGTGGCCTCTGCAAGCAGACA
23123	Yes	AATGTGGCCTCTGCAAGCAGACAG
23124	Yes	AATGTGGCCTCTGCAAGCAGACAGC
23125	Yes	AATGTGGCCTCTGCAAGCAGACAGCC
23126	Yes	AATGTGGCCTCTGCAAGCAGACAGCCT
23127	Yes	AATGTGGCCTCTGCAAGCAGACAGCCTG
23128	Yes	AATGTGGCCTCTGCAAGCAGACAGCCTGG
23129	Yes	AATGTGGCCTCTGCAAGCAGACAGCCTGGG
23130	Yes	AATGTGGCCTCTGCAAGCAGACAGCCTGGGT
23131	Yes	AATGTGGCCTCTGCAAGCAGACAGCCTGGGTT
23132	Yes	AATGTGGCCTCTGCAAGCAGACAGCCTGGGTTC
23133	Yes	AATGTGGCCTCTGCAAGCAGACAGCCTGGGTTCA
23134	Yes	AATGTGGCCTCTGCAAGCAGACAGCCTGGGTTCAA
23135	Yes	AATGTGGCCTCTGCAAGCAGACAGCCTGGGTTCAAT

23136	Yes	AATGTGGCCTCTGCAAGCAGACAGCCTGGGTTCAATT
23137	Yes	AATGTGGCCTCTGCAAGCAGACAGCCTGGGTTCAATTC
23138	Yes	AATGTGGCCTCTGCAAGCAGACAGCCTGGGTTCAATTCC
23139	Yes	AATGTGGCCTCTGCAAGCAGACAGCCTGGGTTCAATTCCC
23140	Yes	AATGTGGCCTCTGCAAGCAGACAGCCTGGGTTCAATTCCCA
23141	Yes	AATGTGGCCTCTGCAAGCAGACAGCCTGGGTTCAATTCCCAG
23142	Yes	AATGTGGCCTCTGCAAGCAGACAGCCTGGGTTCAATTCCCAGC
23143	Yes	AATGTGGCCTCTGCAAGCAGACAGCCTGGGTTCAATTCCCAGCT
23144	Yes	AATGTGGCCTCTGCAAGCAGACAGCCTGGGTTCAATTCCCAGCTT
23145	Yes	AATGTGGCCTCTGCAAGCAGACAGCCTGGGTTCAATTCCCAGCTTG
23146	Yes	AATGTGGCCTCTGCAAGCAGACAGCCTGGGTTCAATTCCCAGCTTGG
23147	Yes	AATGTGGCCTCTGCAAGCAGACAGCCTGGGTTCAATTCCCAGCTTGGC
23148	Yes	AATGTGGCCTCTGCAAGCAGACAGCCTGGGTTCAATTCCCAGCTTGGCC
23149	Yes	AATGTGGCCTCTGCAAGCAGACAGCCTGGGTTCAATTCCCAGCTTGGCCA
23150	No	AATGTTGAATTCTCAG
23151	No	AATGTTGAATTCTCAGG
23152	No	AATGTTGAATTCTCAGGG
23153	Yes	AATGTTGAATTCTCAGGGG
23154	Yes	AATGTTGAATTCTCAGGGGT
23155	Yes	AATGTTGAATTCTCAGGGGTA
23156	Yes	AATGTTGAATTCTCAGGGGTAG
23157	Yes	AATGTTGAATTCTCAGGGGTAGG
23158	Yes	AATGTTGAATTCTCAGGGGTAGGT
23159	Yes	AATGTTGAATTCTCAGGGGTAGGTT
23160	Yes	AATGTTGAATTCTCAGGGGTAGGTTC
23161	Yes	AATGTTGAATTCTCAGGGGTAGGTTCA
23162	Yes	AATGTTGAATTCTCAGGGGTAGGTTCAA
23163	Yes	AATGTTGAATTCTCAGGGGTAGGTTCAAT
23164	Yes	AATGTTGAATTCTCAGGGGTAGGTTCAATT
23165	Yes	AATGTTGAATTCTCAGGGGTAGGTTCAATTC
23166	Yes	AATGTTGAATTCTCAGGGGTAGGTTCAATTCC
23167	Yes	AATGTTGAATTCTCAGGGGTAGGTTCAATTCCT
23168	Yes	AATGTTGAATTCTCAGGGGTAGGTTCAATTCCTA
23169	Yes	AATGTTGAATTCTCAGGGGTAGGTTCAATTCCTAT
23170	Yes	AATGTTGAATTCTCAGGGGTAGGTTCAATTCCTATA
23171	Yes	AATGTTGAATTCTCAGGGGTAGGTTCAATTCCTATAG
23172	Yes	AATGTTGAATTCTCAGGGGTAGGTTCAATTCCTATAGT
23173	Yes	AATGTTGAATTCTCAGGGGTAGGTTCAATTCCTATAGTT
23174	Yes	AATGTTGAATTCTCAGGGGTAGGTTCAATTCCTATAGTTC
23175	Yes	AATGTTGAATTCTCAGGGGTAGGTTCAATTCCTATAGTTCT
23176	Yes	AATGTTGAATTCTCAGGGGTAGGTTCAATTCCTATAGTTCTA
23177	Yes	AATGTTGAATTCTCAGGGGTAGGTTCAATTCCTATAGTTCTAG

23178	Yes	AATGTTGAATTCTCAGGGGTAGGTTCAATTCCTATAGTTCTAGC
23179	Yes	AATGTTGAATTCTCAGGGGTAGGTTCAATTCCTATAGTTCTAGCC
23180	Yes	AATGTTGAATTCTCAGGGGTAGGTTCAATTCCTATAGTTCTAGCCA
23181	No	AATGTTGGTTATACCC
23182	No	AATGTTGGTTATACCCT
23183	No	AATGTTGGTTATACCCTT
23184	No	AATGTTGGTTATACCCTTC
23185	No	AATGTTGGTTATACCCTTCC
23186	No	AATGTTGGTTATACCCTTCCC
23187	No	AATGTTGGTTATACCCTTCCCG
23188	No	AATGTTGGTTATACCCTTCCCGT
23189	Yes	AATGTTGGTTATACCCTTCCCGTA
23190	Yes	AATGTTGGTTATACCCTTCCCGTAC
23191	Yes	AATGTTGGTTATACCCTTCCCGTACT
23192	Yes	AATGTTGGTTATACCCTTCCCGTACTA
23193	Yes	AATGTTGGTTATACCCTTCCCGTACTAC
23194	Yes	AATGTTGGTTATACCCTTCCCGTACTACC
23195	Yes	AATGTTGGTTATACCCTTCCCGTACTACCA
23196	Yes	AATGTTTAGACGGGCT
23197	Yes	AATGTTTAGACGGGCTC
23198	Yes	AATGTTTAGACGGGCTCA
23199	Yes	AATGTTTAGACGGGCTCAC
23200	Yes	AATGTTTAGACGGGCTCACA
23201	Yes	AATGTTTAGACGGGCTCACAT
23202	Yes	AATGTTTAGACGGGCTCACATC
23203	Yes	AATGTTTAGACGGGCTCACATCA
23204	Yes	AATGTTTAGACGGGCTCACATCAC
23205	Yes	AATGTTTAGACGGGCTCACATCACC
23206	Yes	AATGTTTAGACGGGCTCACATCACCC
23207	Yes	AATGTTTAGACGGGCTCACATCACCCC
23208	Yes	AATGTTTAGACGGGCTCACATCACCCCA
23209	Yes	AATGTTTAGACGGGCTCACATCACCCCAT
23210	Yes	AATGTTTAGACGGGCTCACATCACCCCATA
23211	Yes	AATGTTTAGACGGGCTCACATCACCCCATAA
23212	Yes	AATGTTTAGACGGGCTCACATCACCCCATAAA
23213	Yes	AATGTTTAGACGGGCTCACATCACCCCATAAAC
23214	Yes	AATGTTTAGACGGGCTCACATCACCCCATAAACA
23215	Yes	AATGTTTAGACGGGCTCACATCACCCCATAAACAC
23216	Yes	AATGTTTAGACGGGCTCACATCACCCCATAAACACC
23217	Yes	AATGTTTAGACGGGCTCACATCACCCCATAAACACCA
23218	No	AATTAAAGTGGCTGAT
23219	No	AATTAAAGTGGCTGATT

23220	No	AATTAAAGTGGCTGATTT
23221	No	AATTAAAGTGGCTGATTTG
23222	No	AATTAAAGTGGCTGATTTGC
23223	No	AATTAAAGTGGCTGATTTGCG
23224	No	AATTAAAGTGGCTGATTTGCGT
23225	No	AATTAAAGTGGCTGATTTGCGTT
23226	No	AATTAAAGTGGCTGATTTGCGTTC
23227	No	AATTAAAGTGGCTGATTTGCGTTCA
23228	No	AATTAAAGTGGCTGATTTGCGTTCAG
23229	No	AATTAAAGTGGCTGATTTGCGTTCAGT
23230	No	AATTAAAGTGGCTGATTTGCGTTCAGTT
23231	No	AATTAAAGTGGCTGATTTGCGTTCAGTTG
23232	No	AATTAAAGTGGCTGATTTGCGTTCAGTTGA
23233	No	AATTAAAGTGGCTGATTTGCGTTCAGTTGAT
23234	No	AATTAAAGTGGCTGATTTGCGTTCAGTTGATG
23235	No	AATTAAAGTGGCTGATTTGCGTTCAGTTGATGC
23236	No	AATTAAAGTGGCTGATTTGCGTTCAGTTGATGCA
23237	No	AATTAAAGTGGCTGATTTGCGTTCAGTTGATGCAG
23238	No	AATTAAAGTGGCTGATTTGCGTTCAGTTGATGCAGA
23239	No	AATTAAAGTGGCTGATTTGCGTTCAGTTGATGCAGAG
23240	No	AATTAAAGTGGCTGATTTGCGTTCAGTTGATGCAGAGT
23241	Yes	AATTAAAGTGGCTGATTTGCGTTCAGTTGATGCAGAGTG
23242	Yes	AATTAAAGTGGCTGATTTGCGTTCAGTTGATGCAGAGTGG
23243	Yes	AATTAAAGTGGCTGATTTGCGTTCAGTTGATGCAGAGTGGG
23244	Yes	AATTAAAGTGGCTGATTTGCGTTCAGTTGATGCAGAGTGGGG
23245	Yes	AATTAAAGTGGCTGATTTGCGTTCAGTTGATGCAGAGTGGGGT
23246	Yes	AATTAAAGTGGCTGATTTGCGTTCAGTTGATGCAGAGTGGGGTT
23247	Yes	AATTAAAGTGGCTGATTTGCGTTCAGTTGATGCAGAGTGGGGTTT
23248	Yes	AATTAAAGTGGCTGATTTGCGTTCAGTTGATGCAGAGTGGGGTTTT
23249	Yes	AATTAAAGTGGCTGATTTGCGTTCAGTTGATGCAGAGTGGGGTTTTG
23250	Yes	AATTAAAGTGGCTGATTTGCGTTCAGTTGATGCAGAGTGGGGTTTTGC
23251	Yes	AATTAAAGTGGCTGATTTGCGTTCAGTTGATGCAGAGTGGGGTTTTGCA
23252	Yes	AATTAAAGTGGCTGATTTGCGTTCAGTTGATGCAGAGTGGGGTTTTGCAG
23253	No	AATTAACTAGTTTTGA
23254	No	AATTAACTAGTTTTGAC
23255	No	AATTAACTAGTTTTGACA
23256	No	AATTAACTAGTTTTGACAA
23257	Yes	AATTAACTAGTTTTGACAAC
23258	Yes	AATTAACTAGTTTTGACAACA
23259	Yes	AATTAACTAGTTTTGACAACAT
23260	Yes	AATTAACTAGTTTTGACAACATT
23261	Yes	AATTAACTAGTTTTGACAACATTC

23262	Yes	AATTAACTAGTTTTGACAACATTCA
23263	Yes	AATTAACTAGTTTTGACAACATTCAA
23264	Yes	AATTAACTAGTTTTGACAACATTCAAA
23265	Yes	AATTAACTAGTTTTGACAACATTCAAAA
23266	Yes	AATTAACTAGTTTTGACAACATTCAAAAA
23267	Yes	AATTAACTAGTTTTGACAACATTCAAAAAA
23268	Yes	AATTAACTAGTTTTGACAACATTCAAAAAAG
23269	Yes	AATTAACTAGTTTTGACAACATTCAAAAAAGA
23270	Yes	AATTAACTAGTTTTGACAACATTCAAAAAAGAG
23271	Yes	AATTAACTAGTTTTGACAACATTCAAAAAAGAGT
23272	Yes	AATTAACTAGTTTTGACAACATTCAAAAAAGAGTA
23273	Yes	AATTAACTAGTTTTGACAACATTCAAAAAAGAGTAC
23274	Yes	AATTAACTAGTTTTGACAACATTCAAAAAAGAGTACC
23275	Yes	AATTAACTAGTTTTGACAACATTCAAAAAAGAGTACCA
23276	No	AATTAAGTCTCCAGTC
23277	No	AATTAAGTCTCCAGTCT
23278	No	AATTAAGTCTCCAGTCTC
23279	Yes	AATTAAGTCTCCAGTCTCT
23280	Yes	AATTAAGTCTCCAGTCTCTT
23281	Yes	AATTAAGTCTCCAGTCTCTTT
23282	Yes	AATTAAGTCTCCAGTCTCTTTG
23283	Yes	AATTAAGTCTCCAGTCTCTTTGG
23284	Yes	AATTAAGTCTCCAGTCTCTTTGGG
23285	Yes	AATTAAGTCTCCAGTCTCTTTGGGG
23286	Yes	AATTAAGTCTCCAGTCTCTTTGGGGG
23287	Yes	AATTAAGTCTCCAGTCTCTTTGGGGGC
23288	Yes	AATTAAGTCTCCAGTCTCTTTGGGGGCG
23289	Yes	AATTAAGTCTCCAGTCTCTTTGGGGGCGT
23290	Yes	AATTAAGTCTCCAGTCTCTTTGGGGGCGTG
23291	Yes	AATTAAGTCTCCAGTCTCTTTGGGGGCGTGG
23292	Yes	AATTAAGTCTCCAGTCTCTTTGGGGGCGTGGT
23293	Yes	AATTAAGTCTCCAGTCTCTTTGGGGGCGTGGTT
23294	Yes	AATTAAGTCTCCAGTCTCTTTGGGGGCGTGGTTT
23295	Yes	AATTAAGTCTCCAGTCTCTTTGGGGGCGTGGTTTT
23296	Yes	AATTAAGTCTCCAGTCTCTTTGGGGGCGTGGTTTTC
23297	Yes	AATTAAGTCTCCAGTCTCTTTGGGGGCGTGGTTTTCA
23298	Yes	AATTAAGTCTCCAGTCTCTTTGGGGGCGTGGTTTTCAA
23299	Yes	AATTAAGTCTCCAGTCTCTTTGGGGGCGTGGTTTTCAAT
23300	Yes	AATTAAGTCTCCAGTCTCTTTGGGGGCGTGGTTTTCAATC
23301	Yes	AATTAAGTCTCCAGTCTCTTTGGGGGCGTGGTTTTCAATCC
23302	Yes	AATTAAGTCTCCAGTCTCTTTGGGGGCGTGGTTTTCAATCCC
23303	Yes	AATTAAGTCTCCAGTCTCTTTGGGGGCGTGGTTTTCAATCCCA

23304	Yes	AATTAAGTCTCCAGTCTCTTTGGGGGCGTGGTTTTCAATCCCAC
23305	Yes	AATTAAGTCTCCAGTCTCTTTGGGGGCGTGGTTTTCAATCCCACC
23306	Yes	AATTAAGTCTCCAGTCTCTTTGGGGGCGTGGTTTTCAATCCCACCG
23307	Yes	AATTAAGTCTCCAGTCTCTTTGGGGGCGTGGTTTTCAATCCCACCGC
23308	Yes	AATTAAGTCTCCAGTCTCTTTGGGGGCGTGGTTTTCAATCCCACCGCT
23309	Yes	AATTAAGTCTCCAGTCTCTTTGGGGGCGTGGTTTTCAATCCCACCGCTG
23310	Yes	AATTAAGTCTCCAGTCTCTTTGGGGGCGTGGTTTTCAATCCCACCGCTGC
23311	No	AATTAGAAGTTCAACA
23312	No	AATTAGAAGTTCAACAC
23313	Yes	AATTAGAAGTTCAACACC
23314	Yes	AATTAGAAGTTCAACACCT
23315	Yes	AATTAGAAGTTCAACACCTC
23316	Yes	AATTAGAAGTTCAACACCTCT
23317	Yes	AATTAGAAGTTCAACACCTCTT
23318	Yes	AATTAGAAGTTCAACACCTCTTC
23319	Yes	AATTAGAAGTTCAACACCTCTTCT
23320	Yes	AATTAGAAGTTCAACACCTCTTCTT
23321	Yes	AATTAGAAGTTCAACACCTCTTCTTA
23322	Yes	AATTAGAAGTTCAACACCTCTTCTTAA
23323	Yes	AATTAGAAGTTCAACACCTCTTCTTAAC
23324	Yes	AATTAGAAGTTCAACACCTCTTCTTAACA
23325	Yes	AATTAGAAGTTCAACACCTCTTCTTAACAC
23326	Yes	AATTAGAAGTTCAACACCTCTTCTTAACACC
23327	Yes	AATTAGAAGTTCAACACCTCTTCTTAACACCA
23328	No	AATTAGAATCTTAGCT
23329	No	AATTAGAATCTTAGCTT
23330	No	AATTAGAATCTTAGCTTT
23331	No	AATTAGAATCTTAGCTTTG
23332	No	AATTAGAATCTTAGCTTTGG
23333	No	AATTAGAATCTTAGCTTTGGG
23334	No	AATTAGAATCTTAGCTTTGGGT
23335	No	AATTAGAATCTTAGCTTTGGGTG
23336	No	AATTAGAATCTTAGCTTTGGGTGC
23337	No	AATTAGAATCTTAGCTTTGGGTGCT
23338	Yes	AATTAGAATCTTAGCTTTGGGTGCTA
23339	Yes	AATTAGAATCTTAGCTTTGGGTGCTAA
23340	Yes	AATTAGAATCTTAGCTTTGGGTGCTAAT
23341	Yes	AATTAGAATCTTAGCTTTGGGTGCTAATG
23342	Yes	AATTAGAATCTTAGCTTTGGGTGCTAATGG
23343	Yes	AATTAGAATCTTAGCTTTGGGTGCTAATGGT
23344	Yes	AATTAGAATCTTAGCTTTGGGTGCTAATGGTG
23345	Yes	AATTAGAATCTTAGCTTTGGGTGCTAATGGTGG

23346	Yes	AATTAGAATCTTAGCTTTGGGTGCTAATGGTGGA
23347	Yes	AATTAGAATCTTAGCTTTGGGTGCTAATGGTGGAG
23348	Yes	AATTAGAATCTTAGCTTTGGGTGCTAATGGTGGAGT
23349	Yes	AATTAGAATCTTAGCTTTGGGTGCTAATGGTGGAGTT
23350	Yes	AATTAGAATCTTAGCTTTGGGTGCTAATGGTGGAGTTA
23351	Yes	AATTAGAATCTTAGCTTTGGGTGCTAATGGTGGAGTTAA
23352	Yes	AATTAGAATCTTAGCTTTGGGTGCTAATGGTGGAGTTAAA
23353	Yes	AATTAGAATCTTAGCTTTGGGTGCTAATGGTGGAGTTAAAG
23354	Yes	AATTAGAATCTTAGCTTTGGGTGCTAATGGTGGAGTTAAAGA
23355	Yes	AATTAGAATCTTAGCTTTGGGTGCTAATGGTGGAGTTAAAGAC
23356	Yes	AATTAGAATCTTAGCTTTGGGTGCTAATGGTGGAGTTAAAGACT
23357	Yes	AATTAGAATCTTAGCTTTGGGTGCTAATGGTGGAGTTAAAGACTT
23358	Yes	AATTAGAATCTTAGCTTTGGGTGCTAATGGTGGAGTTAAAGACTTT
23359	Yes	AATTAGAATCTTAGCTTTGGGTGCTAATGGTGGAGTTAAAGACTTTT
23360	Yes	AATTAGAATCTTAGCTTTGGGTGCTAATGGTGGAGTTAAAGACTTTTT
23361	Yes	AATTAGAATCTTAGCTTTGGGTGCTAATGGTGGAGTTAAAGACTTTTTC
23362	Yes	AATTAGAATCTTAGCTTTGGGTGCTAATGGTGGAGTTAAAGACTTTTTCT
23363	No	AATTAGCAAGCACAGT
23364	No	AATTAGCAAGCACAGTT
23365	No	AATTAGCAAGCACAGTTG
23366	No	AATTAGCAAGCACAGTTGG
23367	No	AATTAGCAAGCACAGTTGGC
23368	Yes	AATTAGCAAGCACAGTTGGCT
23369	Yes	AATTAGCAAGCACAGTTGGCTA
23370	Yes	AATTAGCAAGCACAGTTGGCTAG
23371	Yes	AATTAGCAAGCACAGTTGGCTAGA
23372	Yes	AATTAGCAAGCACAGTTGGCTAGAA
23373	Yes	AATTAGCAAGCACAGTTGGCTAGAAC
23374	Yes	AATTAGCAAGCACAGTTGGCTAGAACA
23375	Yes	AATTAGCAAGCACAGTTGGCTAGAACAT
23376	Yes	AATTAGCAAGCACAGTTGGCTAGAACATG
23377	Yes	AATTAGCAAGCACAGTTGGCTAGAACATGG
23378	Yes	AATTAGCAAGCACAGTTGGCTAGAACATGGT
23379	Yes	AATTAGCAAGCACAGTTGGCTAGAACATGGTG
23380	Yes	AATTAGCAAGCACAGTTGGCTAGAACATGGTGC
23381	Yes	AATTAGCAAGCACAGTTGGCTAGAACATGGTGCT
23382	Yes	AATTAGCAAGCACAGTTGGCTAGAACATGGTGCTA
23383	Yes	AATTAGCAAGCACAGTTGGCTAGAACATGGTGCTAA
23384	Yes	AATTAGCAAGCACAGTTGGCTAGAACATGGTGCTAAT
23385	Yes	AATTAGCAAGCACAGTTGGCTAGAACATGGTGCTAATA
23386	Yes	AATTAGCAAGCACAGTTGGCTAGAACATGGTGCTAATAA
23387	Yes	AATTAGCAAGCACAGTTGGCTAGAACATGGTGCTAATAAG

23388	Yes	AATTAGCAAGCACAGTTGGCTAGAACATGGTGCTAATAAGG
23389	Yes	AATTAGCAAGCACAGTTGGCTAGAACATGGTGCTAATAAGGC
23390	Yes	AATTAGCAAGCACAGTTGGCTAGAACATGGTGCTAATAAGGCC
23391	Yes	AATTAGCAAGCACAGTTGGCTAGAACATGGTGCTAATAAGGCCA
23392	Yes	AATTAGCAAGCACAGTTGGCTAGAACATGGTGCTAATAAGGCCAC
23393	Yes	AATTAGCAAGCACAGTTGGCTAGAACATGGTGCTAATAAGGCCACG
23394	Yes	AATTAGCAAGCACAGTTGGCTAGAACATGGTGCTAATAAGGCCACGG
23395	Yes	AATTAGCAAGCACAGTTGGCTAGAACATGGTGCTAATAAGGCCACGGT
23396	Yes	AATTAGCAAGCACAGTTGGCTAGAACATGGTGCTAATAAGGCCACGGTC
23397	Yes	AATTAGCAAGCACAGTTGGCTAGAACATGGTGCTAATAAGGCCACGGTCA
23398	No	AATTAGCCCAAGTGGT
23399	Yes	AATTAGCCCAAGTGGTA
23400	Yes	AATTAGCCCAAGTGGTAG
23401	Yes	AATTAGCCCAAGTGGTAGA
23402	Yes	AATTAGCCCAAGTGGTAGAG
23403	Yes	AATTAGCCCAAGTGGTAGAGC
23404	Yes	AATTAGCCCAAGTGGTAGAGCG
23405	Yes	AATTAGCCCAAGTGGTAGAGCGC
23406	Yes	AATTAGCCCAAGTGGTAGAGCGCT
23407	Yes	AATTAGCCCAAGTGGTAGAGCGCTT
23408	Yes	AATTAGCCCAAGTGGTAGAGCGCTTG
23409	Yes	AATTAGCCCAAGTGGTAGAGCGCTTGC
23410	Yes	AATTAGCCCAAGTGGTAGAGCGCTTGCT
23411	Yes	AATTAGCCCAAGTGGTAGAGCGCTTGCTT
23412	Yes	AATTAGCCCAAGTGGTAGAGCGCTTGCTTA
23413	Yes	AATTAGCCCAAGTGGTAGAGCGCTTGCTTAG
23414	Yes	AATTAGCCCAAGTGGTAGAGCGCTTGCTTAGC
23415	Yes	AATTAGCCCAAGTGGTAGAGCGCTTGCTTAGCA
23416	Yes	AATTAGCCCAAGTGGTAGAGCGCTTGCTTAGCAT
23417	Yes	AATTAGCCCAAGTGGTAGAGCGCTTGCTTAGCATG
23418	Yes	AATTAGCCCAAGTGGTAGAGCGCTTGCTTAGCATGC
23419	Yes	AATTAGCCCAAGTGGTAGAGCGCTTGCTTAGCATGCA
23420	Yes	AATTAGCCCAAGTGGTAGAGCGCTTGCTTAGCATGCAA
23421	Yes	AATTAGCCCAAGTGGTAGAGCGCTTGCTTAGCATGCAAG
23422	Yes	AATTAGCCCAAGTGGTAGAGCGCTTGCTTAGCATGCAAGA
23423	Yes	AATTAGCCCAAGTGGTAGAGCGCTTGCTTAGCATGCAAGAG
23424	Yes	AATTAGCCCAAGTGGTAGAGCGCTTGCTTAGCATGCAAGAGG
23425	Yes	AATTAGCCCAAGTGGTAGAGCGCTTGCTTAGCATGCAAGAGGT
23426	Yes	AATTAGCCCAAGTGGTAGAGCGCTTGCTTAGCATGCAAGAGGTA
23427	Yes	AATTAGCCCAAGTGGTAGAGCGCTTGCTTAGCATGCAAGAGGTAG
23428	Yes	AATTAGCCCAAGTGGTAGAGCGCTTGCTTAGCATGCAAGAGGTAGT
23429	Yes	AATTAGCCCAAGTGGTAGAGCGCTTGCTTAGCATGCAAGAGGTAGTG

23430	Yes	AATTAGCCCAAGTGGTAGAGCGCTTGCTTAGCATGCAAGAGGTAGTGG
23431	Yes	AATTAGCCCAAGTGGTAGAGCGCTTGCTTAGCATGCAAGAGGTAGTGGG
23432	Yes	AATTAGCCCAAGTGGTAGAGCGCTTGCTTAGCATGCAAGAGGTAGTGGGA
23433	Yes	AATTAGCGCAAGTGGT
23434	Yes	AATTAGCGCAAGTGGTA
23435	Yes	AATTAGCGCAAGTGGTAG
23436	Yes	AATTAGCGCAAGTGGTAGA
23437	Yes	AATTAGCGCAAGTGGTAGAG
23438	Yes	AATTAGCGCAAGTGGTAGAGT
23439	Yes	AATTAGCGCAAGTGGTAGAGTG
23440	Yes	AATTAGCGCAAGTGGTAGAGTGC
23441	Yes	AATTAGCGCAAGTGGTAGAGTGCT
23442	Yes	AATTAGCGCAAGTGGTAGAGTGCTT
23443	Yes	AATTAGCGCAAGTGGTAGAGTGCTTG
23444	Yes	AATTAGCGCAAGTGGTAGAGTGCTTGC
23445	Yes	AATTAGCGCAAGTGGTAGAGTGCTTGCT
23446	Yes	AATTAGCGCAAGTGGTAGAGTGCTTGCTT
23447	Yes	AATTAGCGCAAGTGGTAGAGTGCTTGCTTA
23448	Yes	AATTAGCGCAAGTGGTAGAGTGCTTGCTTAG
23449	Yes	AATTAGCGCAAGTGGTAGAGTGCTTGCTTAGC
23450	Yes	AATTAGCGCAAGTGGTAGAGTGCTTGCTTAGCA
23451	Yes	AATTAGCGCAAGTGGTAGAGTGCTTGCTTAGCAT
23452	Yes	AATTAGCGCAAGTGGTAGAGTGCTTGCTTAGCATG
23453	Yes	AATTAGCGCAAGTGGTAGAGTGCTTGCTTAGCATGC
23454	Yes	AATTAGCGCAAGTGGTAGAGTGCTTGCTTAGCATGCA
23455	Yes	AATTAGCGCAAGTGGTAGAGTGCTTGCTTAGCATGCAA
23456	Yes	AATTAGCGCAAGTGGTAGAGTGCTTGCTTAGCATGCAAG
23457	Yes	AATTAGCGCAAGTGGTAGAGTGCTTGCTTAGCATGCAAGA
23458	Yes	AATTAGCGCAAGTGGTAGAGTGCTTGCTTAGCATGCAAGAG
23459	Yes	AATTAGCGCAAGTGGTAGAGTGCTTGCTTAGCATGCAAGAGG
23460	Yes	AATTAGCGCAAGTGGTAGAGTGCTTGCTTAGCATGCAAGAGGT
23461	Yes	AATTAGCGCAAGTGGTAGAGTGCTTGCTTAGCATGCAAGAGGTA
23462	Yes	AATTAGCGCAAGTGGTAGAGTGCTTGCTTAGCATGCAAGAGGTAG
23463	Yes	AATTAGCGCAAGTGGTAGAGTGCTTGCTTAGCATGCAAGAGGTAGT
23464	Yes	AATTAGCGCAAGTGGTAGAGTGCTTGCTTAGCATGCAAGAGGTAGTG
23465	Yes	AATTAGCGCAAGTGGTAGAGTGCTTGCTTAGCATGCAAGAGGTAGTGG
23466	Yes	AATTAGCGCAAGTGGTAGAGTGCTTGCTTAGCATGCAAGAGGTAGTGGG
23467	Yes	AATTAGCGCAAGTGGTAGAGTGCTTGCTTAGCATGCAAGAGGTAGTGGGA
23468	No	AATTAGCTCAAATGGT
23469	Yes	AATTAGCTCAAATGGTA
23470	Yes	AATTAGCTCAAATGGTAG
23471	Yes	AATTAGCTCAAATGGTAGA

23472	Yes	AATTAGCTCAAATGGTAGAG
23473	Yes	AATTAGCTCAAATGGTAGAGC
23474	Yes	AATTAGCTCAAATGGTAGAGCG
23475	Yes	AATTAGCTCAAATGGTAGAGCGC
23476	Yes	AATTAGCTCAAATGGTAGAGCGCT
23477	Yes	AATTAGCTCAAATGGTAGAGCGCTC
23478	Yes	AATTAGCTCAAATGGTAGAGCGCTCG
23479	Yes	AATTAGCTCAAATGGTAGAGCGCTCGC
23480	Yes	AATTAGCTCAAATGGTAGAGCGCTCGCT
23481	Yes	AATTAGCTCAAATGGTAGAGCGCTCGCTT
23482	Yes	AATTAGCTCAAATGGTAGAGCGCTCGCTTA
23483	Yes	AATTAGCTCAAATGGTAGAGCGCTCGCTTAG
23484	Yes	AATTAGCTCAAATGGTAGAGCGCTCGCTTAGC
23485	Yes	AATTAGCTCAAATGGTAGAGCGCTCGCTTAGCA
23486	Yes	AATTAGCTCAAATGGTAGAGCGCTCGCTTAGCAT
23487	Yes	AATTAGCTCAAATGGTAGAGCGCTCGCTTAGCATG
23488	Yes	AATTAGCTCAAATGGTAGAGCGCTCGCTTAGCATGC
23489	Yes	AATTAGCTCAAATGGTAGAGCGCTCGCTTAGCATGCG
23490	Yes	AATTAGCTCAAATGGTAGAGCGCTCGCTTAGCATGCGA
23491	Yes	AATTAGCTCAAATGGTAGAGCGCTCGCTTAGCATGCGAG
23492	Yes	AATTAGCTCAAATGGTAGAGCGCTCGCTTAGCATGCGAGA
23493	Yes	AATTAGCTCAAATGGTAGAGCGCTCGCTTAGCATGCGAGAG
23494	Yes	AATTAGCTCAAATGGTAGAGCGCTCGCTTAGCATGCGAGAGG
23495	Yes	AATTAGCTCAAATGGTAGAGCGCTCGCTTAGCATGCGAGAGGT
23496	Yes	AATTAGCTCAAATGGTAGAGCGCTCGCTTAGCATGCGAGAGGTA
23497	Yes	AATTAGCTCAAATGGTAGAGCGCTCGCTTAGCATGCGAGAGGTAG
23498	Yes	AATTAGCTCAAATGGTAGAGCGCTCGCTTAGCATGCGAGAGGTAGC
23499	Yes	AATTAGCTCAAATGGTAGAGCGCTCGCTTAGCATGCGAGAGGTAGCG
23500	Yes	AATTAGCTCAAATGGTAGAGCGCTCGCTTAGCATGCGAGAGGTAGCGG
23501	Yes	AATTAGCTCAAATGGTAGAGCGCTCGCTTAGCATGCGAGAGGTAGCGGG
23502	Yes	AATTAGCTCAAATGGTAGAGCGCTCGCTTAGCATGCGAGAGGTAGCGGGA
23503	Yes	AATTAGCTCAAGCGGT
23504	Yes	AATTAGCTCAAGCGGTA
23505	Yes	AATTAGCTCAAGCGGTAG
23506	Yes	AATTAGCTCAAGCGGTAGA
23507	Yes	AATTAGCTCAAGCGGTAGAG
23508	Yes	AATTAGCTCAAGCGGTAGAGC
23509	Yes	AATTAGCTCAAGCGGTAGAGCG
23510	Yes	AATTAGCTCAAGCGGTAGAGCGC
23511	Yes	AATTAGCTCAAGCGGTAGAGCGCT
23512	Yes	AATTAGCTCAAGCGGTAGAGCGCTC
23513	Yes	AATTAGCTCAAGCGGTAGAGCGCTCC

23514	Yes	AATTAGCTCAAGCGGTAGAGCGCTCCC
23515	Yes	AATTAGCTCAAGCGGTAGAGCGCTCCCT
23516	Yes	AATTAGCTCAAGCGGTAGAGCGCTCCCTT
23517	Yes	AATTAGCTCAAGCGGTAGAGCGCTCCCTTA
23518	Yes	AATTAGCTCAAGCGGTAGAGCGCTCCCTTAG
23519	Yes	AATTAGCTCAAGCGGTAGAGCGCTCCCTTAGC
23520	Yes	AATTAGCTCAAGCGGTAGAGCGCTCCCTTAGCA
23521	Yes	AATTAGCTCAAGCGGTAGAGCGCTCCCTTAGCAT
23522	Yes	AATTAGCTCAAGCGGTAGAGCGCTCCCTTAGCATG
23523	Yes	AATTAGCTCAAGCGGTAGAGCGCTCCCTTAGCATGC
23524	Yes	AATTAGCTCAAGCGGTAGAGCGCTCCCTTAGCATGCG
23525	Yes	AATTAGCTCAAGCGGTAGAGCGCTCCCTTAGCATGCGA
23526	Yes	AATTAGCTCAAGCGGTAGAGCGCTCCCTTAGCATGCGAG
23527	Yes	AATTAGCTCAAGCGGTAGAGCGCTCCCTTAGCATGCGAGA
23528	Yes	AATTAGCTCAAGCGGTAGAGCGCTCCCTTAGCATGCGAGAG
23529	Yes	AATTAGCTCAAGCGGTAGAGCGCTCCCTTAGCATGCGAGAGG
23530	Yes	AATTAGCTCAAGCGGTAGAGCGCTCCCTTAGCATGCGAGAGGT
23531	Yes	AATTAGCTCAAGCGGTAGAGCGCTCCCTTAGCATGCGAGAGGTA
23532	Yes	AATTAGCTCAAGCGGTAGAGCGCTCCCTTAGCATGCGAGAGGTAG
23533	Yes	AATTAGCTCAAGCGGTAGAGCGCTCCCTTAGCATGCGAGAGGTAGC
23534	Yes	AATTAGCTCAAGCGGTAGAGCGCTCCCTTAGCATGCGAGAGGTAGCG
23535	Yes	AATTAGCTCAAGCGGTAGAGCGCTCCCTTAGCATGCGAGAGGTAGCGG
23536	Yes	AATTAGCTCAAGCGGTAGAGCGCTCCCTTAGCATGCGAGAGGTAGCGGG
23537	Yes	AATTAGCTCAAGCGGTAGAGCGCTCCCTTAGCATGCGAGAGGTAGCGGGA
23538	Yes	AATTAGCTCAAGCGGTAGAGCGCTT
23539	Yes	AATTAGCTCAAGCGGTAGAGCGCTTG
23540	Yes	AATTAGCTCAAGCGGTAGAGCGCTTGC
23541	Yes	AATTAGCTCAAGCGGTAGAGCGCTTGCT
23542	Yes	AATTAGCTCAAGCGGTAGAGCGCTTGCTT
23543	Yes	AATTAGCTCAAGCGGTAGAGCGCTTGCTTA
23544	Yes	AATTAGCTCAAGCGGTAGAGCGCTTGCTTAG
23545	Yes	AATTAGCTCAAGCGGTAGAGCGCTTGCTTAGC
23546	Yes	AATTAGCTCAAGCGGTAGAGCGCTTGCTTAGCA
23547	Yes	AATTAGCTCAAGCGGTAGAGCGCTTGCTTAGCAT
23548	Yes	AATTAGCTCAAGCGGTAGAGCGCTTGCTTAGCATG
23549	Yes	AATTAGCTCAAGCGGTAGAGCGCTTGCTTAGCATGC
23550	Yes	AATTAGCTCAAGCGGTAGAGCGCTTGCTTAGCATGCA
23551	Yes	AATTAGCTCAAGCGGTAGAGCGCTTGCTTAGCATGCAA
23552	Yes	AATTAGCTCAAGCGGTAGAGCGCTTGCTTAGCATGCAAG
23553	Yes	AATTAGCTCAAGCGGTAGAGCGCTTGCTTAGCATGCAAGA
23554	Yes	AATTAGCTCAAGCGGTAGAGCGCTTGCTTAGCATGCAAGAG
23555	Yes	AATTAGCTCAAGCGGTAGAGCGCTTGCTTAGCATGCAAGAGG

23556	Yes	AATTAGCTCAAGCGGTAGAGCGCTTGCTTAGCATGCAAGAGGT
23557	Yes	AATTAGCTCAAGCGGTAGAGCGCTTGCTTAGCATGCAAGAGGTA
23558	Yes	AATTAGCTCAAGCGGTAGAGCGCTTGCTTAGCATGCAAGAGGTAG
23559	Yes	AATTAGCTCAAGCGGTAGAGCGCTTGCTTAGCATGCAAGAGGTAGT
23560	Yes	AATTAGCTCAAGCGGTAGAGCGCTTGCTTAGCATGCAAGAGGTAGTG
23561	Yes	AATTAGCTCAAGCGGTAGAGCGCTTGCTTAGCATGCAAGAGGTAGTGG
23562	Yes	AATTAGCTCAAGCGGTAGAGCGCTTGCTTAGCATGCAAGAGGTAGTGGG
23563	Yes	AATTAGCTCAAGCGGTAGAGCGCTTGCTTAGCATGCAAGAGGTAGTGGGA
23564	Yes	AATTAGCTCAAGTGGT
23565	Yes	AATTAGCTCAAGTGGTA
23566	Yes	AATTAGCTCAAGTGGTAG
23567	Yes	AATTAGCTCAAGTGGTAGA
23568	Yes	AATTAGCTCAAGTGGTAGAG
23569	Yes	AATTAGCTCAAGTGGTAGAGC
23570	Yes	AATTAGCTCAAGTGGTAGAGCG
23571	Yes	AATTAGCTCAAGTGGTAGAGCGC
23572	Yes	AATTAGCTCAAGTGGTAGAGCGCT
23573	Yes	AATTAGCTCAAGTGGTAGAGCGCTC
23574	Yes	AATTAGCTCAAGTGGTAGAGCGCTCG
23575	Yes	AATTAGCTCAAGTGGTAGAGCGCTCGC
23576	Yes	AATTAGCTCAAGTGGTAGAGCGCTCGCT
23577	Yes	AATTAGCTCAAGTGGTAGAGCGCTCGCTT
23578	Yes	AATTAGCTCAAGTGGTAGAGCGCTCGCTTA
23579	Yes	AATTAGCTCAAGTGGTAGAGCGCTCGCTTAG
23580	Yes	AATTAGCTCAAGTGGTAGAGCGCTCGCTTAGC
23581	Yes	AATTAGCTCAAGTGGTAGAGCGCTCGCTTAGCA
23582	Yes	AATTAGCTCAAGTGGTAGAGCGCTCGCTTAGCAT
23583	Yes	AATTAGCTCAAGTGGTAGAGCGCTCGCTTAGCATG
23584	Yes	AATTAGCTCAAGTGGTAGAGCGCTCGCTTAGCATGC
23585	Yes	AATTAGCTCAAGTGGTAGAGCGCTCGCTTAGCATGCG
23586	Yes	AATTAGCTCAAGTGGTAGAGCGCTCGCTTAGCATGCGA
23587	Yes	AATTAGCTCAAGTGGTAGAGCGCTCGCTTAGCATGCGAG
23588	Yes	AATTAGCTCAAGTGGTAGAGCGCTCGCTTAGCATGCGAGA
23589	Yes	AATTAGCTCAAGTGGTAGAGCGCTCGCTTAGCATGCGAGAG
23590	Yes	AATTAGCTCAAGTGGTAGAGCGCTCGCTTAGCATGCGAGAGG
23591	Yes	AATTAGCTCAAGTGGTAGAGCGCTCGCTTAGCATGCGAGAGGT
23592	Yes	AATTAGCTCAAGTGGTAGAGCGCTCGCTTAGCATGCGAGAGGTA
23593	Yes	AATTAGCTCAAGTGGTAGAGCGCTCGCTTAGCATGCGAGAGGTAG
23594	Yes	AATTAGCTCAAGTGGTAGAGCGCTCGCTTAGCATGCGAGAGGTAGT
23595	Yes	AATTAGCTCAAGTGGTAGAGCGCTCGCTTAGCATGCGAGAGGTAGTG
23596	Yes	AATTAGCTCAAGTGGTAGAGCGCTCGCTTAGCATGCGAGAGGTAGTGG
23597	Yes	AATTAGCTCAAGTGGTAGAGCGCTCGCTTAGCATGCGAGAGGTAGTGGG

23598	Yes	AATTAGCTCAAGTGGTAGAGCGCTCGCTTAGCATGCGAGAGGTAGTGGGA
23599	Yes	AATTAGCTCAAGTGGTAGAGCGCTT
23600	Yes	AATTAGCTCAAGTGGTAGAGCGCTTG
23601	Yes	AATTAGCTCAAGTGGTAGAGCGCTTGC
23602	Yes	AATTAGCTCAAGTGGTAGAGCGCTTGCT
23603	Yes	AATTAGCTCAAGTGGTAGAGCGCTTGCTT
23604	Yes	AATTAGCTCAAGTGGTAGAGCGCTTGCTTA
23605	Yes	AATTAGCTCAAGTGGTAGAGCGCTTGCTTAG
23606	Yes	AATTAGCTCAAGTGGTAGAGCGCTTGCTTAGC
23607	Yes	AATTAGCTCAAGTGGTAGAGCGCTTGCTTAGCA
23608	Yes	AATTAGCTCAAGTGGTAGAGCGCTTGCTTAGCAC
23609	Yes	AATTAGCTCAAGTGGTAGAGCGCTTGCTTAGCACG
23610	Yes	AATTAGCTCAAGTGGTAGAGCGCTTGCTTAGCACGC
23611	Yes	AATTAGCTCAAGTGGTAGAGCGCTTGCTTAGCACGCA
23612	Yes	AATTAGCTCAAGTGGTAGAGCGCTTGCTTAGCACGCAA
23613	Yes	AATTAGCTCAAGTGGTAGAGCGCTTGCTTAGCACGCAAG
23614	Yes	AATTAGCTCAAGTGGTAGAGCGCTTGCTTAGCACGCAAGA
23615	Yes	AATTAGCTCAAGTGGTAGAGCGCTTGCTTAGCACGCAAGAG
23616	Yes	AATTAGCTCAAGTGGTAGAGCGCTTGCTTAGCACGCAAGAGG
23617	Yes	AATTAGCTCAAGTGGTAGAGCGCTTGCTTAGCACGCAAGAGGT
23618	Yes	AATTAGCTCAAGTGGTAGAGCGCTTGCTTAGCACGCAAGAGGTA
23619	Yes	AATTAGCTCAAGTGGTAGAGCGCTTGCTTAGCACGCAAGAGGTAG
23620	Yes	AATTAGCTCAAGTGGTAGAGCGCTTGCTTAGCACGCAAGAGGTAGT
23621	Yes	AATTAGCTCAAGTGGTAGAGCGCTTGCTTAGCACGCAAGAGGTAGTG
23622	Yes	AATTAGCTCAAGTGGTAGAGCGCTTGCTTAGCACGCAAGAGGTAGTGG
23623	Yes	AATTAGCTCAAGTGGTAGAGCGCTTGCTTAGCACGCAAGAGGTAGTGGG
23624	Yes	AATTAGCTCAAGTGGTAGAGCGCTTGCTTAGCACGCAAGAGGTAGTGGGA
23625	Yes	AATTAGCTCAAGTGGTAGAGCGCTTGCTTAGCAT
23626	Yes	AATTAGCTCAAGTGGTAGAGCGCTTGCTTAGCATG
23627	Yes	AATTAGCTCAAGTGGTAGAGCGCTTGCTTAGCATGC
23628	Yes	AATTAGCTCAAGTGGTAGAGCGCTTGCTTAGCATGCA
23629	Yes	AATTAGCTCAAGTGGTAGAGCGCTTGCTTAGCATGCAA
23630	Yes	AATTAGCTCAAGTGGTAGAGCGCTTGCTTAGCATGCAAG
23631	Yes	AATTAGCTCAAGTGGTAGAGCGCTTGCTTAGCATGCAAGA
23632	Yes	AATTAGCTCAAGTGGTAGAGCGCTTGCTTAGCATGCAAGAG
23633	Yes	AATTAGCTCAAGTGGTAGAGCGCTTGCTTAGCATGCAAGAGG
23634	Yes	AATTAGCTCAAGTGGTAGAGCGCTTGCTTAGCATGCAAGAGGT
23635	Yes	AATTAGCTCAAGTGGTAGAGCGCTTGCTTAGCATGCAAGAGGTA
23636	Yes	AATTAGCTCAAGTGGTAGAGCGCTTGCTTAGCATGCAAGAGGTAG
23637	Yes	AATTAGCTCAAGTGGTAGAGCGCTTGCTTAGCATGCAAGAGGTAGT
23638	Yes	AATTAGCTCAAGTGGTAGAGCGCTTGCTTAGCATGCAAGAGGTAGTG
23639	Yes	AATTAGCTCAAGTGGTAGAGCGCTTGCTTAGCATGCAAGAGGTAGTGG

23640	Yes	AATTAGCTCAAGTGGTAGAGCGCTTGCTTAGCATGCAAGAGGTAGTGGG
23641	Yes	AATTAGCTCAAGTGGTAGAGCGCTTGCTTAGCATGCAAGAGGTAGTGGGA
23642	No	AATTAGCTCAGGCGGT
23643	Yes	AATTAGCTCAGGCGGTA
23644	Yes	AATTAGCTCAGGCGGTAG
23645	Yes	AATTAGCTCAGGCGGTAGA
23646	Yes	AATTAGCTCAGGCGGTAGAG
23647	Yes	AATTAGCTCAGGCGGTAGAGC
23648	Yes	AATTAGCTCAGGCGGTAGAGCG
23649	Yes	AATTAGCTCAGGCGGTAGAGCGC
23650	Yes	AATTAGCTCAGGCGGTAGAGCGCT
23651	Yes	AATTAGCTCAGGCGGTAGAGCGCTC
23652	Yes	AATTAGCTCAGGCGGTAGAGCGCTCG
23653	Yes	AATTAGCTCAGGCGGTAGAGCGCTCGC
23654	Yes	AATTAGCTCAGGCGGTAGAGCGCTCGCT
23655	Yes	AATTAGCTCAGGCGGTAGAGCGCTCGCTT
23656	Yes	AATTAGCTCAGGCGGTAGAGCGCTCGCTTA
23657	Yes	AATTAGCTCAGGCGGTAGAGCGCTCGCTTAG
23658	Yes	AATTAGCTCAGGCGGTAGAGCGCTCGCTTAGC
23659	Yes	AATTAGCTCAGGCGGTAGAGCGCTCGCTTAGCA
23660	Yes	AATTAGCTCAGGCGGTAGAGCGCTCGCTTAGCAT
23661	Yes	AATTAGCTCAGGCGGTAGAGCGCTCGCTTAGCATG
23662	Yes	AATTAGCTCAGGCGGTAGAGCGCTCGCTTAGCATGC
23663	Yes	AATTAGCTCAGGCGGTAGAGCGCTCGCTTAGCATGCG
23664	Yes	AATTAGCTCAGGCGGTAGAGCGCTCGCTTAGCATGCGA
23665	Yes	AATTAGCTCAGGCGGTAGAGCGCTCGCTTAGCATGCGAG
23666	Yes	AATTAGCTCAGGCGGTAGAGCGCTCGCTTAGCATGCGAGA
23667	Yes	AATTAGCTCAGGCGGTAGAGCGCTCGCTTAGCATGCGAGAG
23668	Yes	AATTAGCTCAGGCGGTAGAGCGCTCGCTTAGCATGCGAGAGG
23669	Yes	AATTAGCTCAGGCGGTAGAGCGCTCGCTTAGCATGCGAGAGGT
23670	Yes	AATTAGCTCAGGCGGTAGAGCGCTCGCTTAGCATGCGAGAGGTA
23671	Yes	AATTAGCTCAGGCGGTAGAGCGCTCGCTTAGCATGCGAGAGGTAG
23672	Yes	AATTAGCTCAGGCGGTAGAGCGCTCGCTTAGCATGCGAGAGGTAGC
23673	Yes	AATTAGCTCAGGCGGTAGAGCGCTCGCTTAGCATGCGAGAGGTAGCG
23674	Yes	AATTAGCTCAGGCGGTAGAGCGCTCGCTTAGCATGCGAGAGGTAGCGG
23675	Yes	AATTAGCTCAGGCGGTAGAGCGCTCGCTTAGCATGCGAGAGGTAGCGGG
23676	Yes	AATTAGCTCAGGCGGTAGAGCGCTCGCTTAGCATGCGAGAGGTAGCGGGA
23677	No	AATTATAGCTCAGTGG
23678	Yes	AATTATAGCTCAGTGGT
23679	Yes	AATTATAGCTCAGTGGTA
23680	Yes	AATTATAGCTCAGTGGTAG
23681	Yes	AATTATAGCTCAGTGGTAGA

23682	Yes	AATTATAGCTCAGTGGTAGAG
23683	Yes	AATTATAGCTCAGTGGTAGAGC
23684	Yes	AATTATAGCTCAGTGGTAGAGCA
23685	Yes	AATTATAGCTCAGTGGTAGAGCAT
23686	Yes	AATTATAGCTCAGTGGTAGAGCATT
23687	Yes	AATTATAGCTCAGTGGTAGAGCATTT
23688	Yes	AATTATAGCTCAGTGGTAGAGCATTTA
23689	Yes	AATTATAGCTCAGTGGTAGAGCATTTAA
23690	Yes	AATTATAGCTCAGTGGTAGAGCATTTAAC
23691	Yes	AATTATAGCTCAGTGGTAGAGCATTTAACT
23692	Yes	AATTATAGCTCAGTGGTAGAGCATTTAACTG
23693	Yes	AATTATAGCTCAGTGGTAGAGCATTTAACTGT
23694	Yes	AATTATAGCTCAGTGGTAGAGCATTTAACTGTA
23695	Yes	AATTATAGCTCAGTGGTAGAGCATTTAACTGTAG
23696	Yes	AATTATAGCTCAGTGGTAGAGCATTTAACTGTAGA
23697	Yes	AATTATAGCTCAGTGGTAGAGCATTTAACTGTAGAT
23698	Yes	AATTATAGCTCAGTGGTAGAGCATTTAACTGTAGATC
23699	Yes	AATTATAGCTCAGTGGTAGAGCATTTAACTGTAGATCA
23700	Yes	AATTATAGCTCAGTGGTAGAGCATTTAACTGTAGATCAA
23701	Yes	AATTATAGCTCAGTGGTAGAGCATTTAACTGTAGATCAAG
23702	Yes	AATTATAGCTCAGTGGTAGAGCATTTAACTGTAGATCAAGA
23703	Yes	AATTATAGCTCAGTGGTAGAGCATTTAACTGTAGATCAAGAG
23704	Yes	AATTATAGCTCAGTGGTAGAGCATTTAACTGTAGATCAAGAGG
23705	Yes	AATTATAGCTCAGTGGTAGAGCATTTAACTGTAGATCAAGAGGT
23706	Yes	AATTATAGCTCAGTGGTAGAGCATTTAACTGTAGATCAAGAGGTC
23707	Yes	AATTATAGCTCAGTGGTAGAGCATTTAACTGTAGATCAAGAGGTCC
23708	Yes	AATTATAGCTCAGTGGTAGAGCATTTAACTGTAGATCAAGAGGTCCC
23709	Yes	AATTATAGCTCAGTGGTAGAGCATTTAACTGTAGATCAAGAGGTCCCT
23710	Yes	AATTATAGCTCAGTGGTAGAGCATTTAACTGTAGATCAAGAGGTCCCTG
23711	Yes	AATTATAGCTCAGTGGTAGAGCATTTAACTGTAGATCAAGAGGTCCCTGG
23712	No	AATTATAGGCTAAATC
23713	No	AATTATAGGCTAAATCC
23714	No	AATTATAGGCTAAATCCT
23715	No	AATTATAGGCTAAATCCTA
23716	No	AATTATAGGCTAAATCCTAT
23717	No	AATTATAGGCTAAATCCTATA
23718	No	AATTATAGGCTAAATCCTATAT
23719	No	AATTATAGGCTAAATCCTATATA
23720	No	AATTATAGGCTAAATCCTATATAT
23721	No	AATTATAGGCTAAATCCTATATATC
23722	No	AATTATAGGCTAAATCCTATATATCT
23723	No	AATTATAGGCTAAATCCTATATATCTT

23724	No	AATTATAGGCTAAATCCTATATATCTTA
23725	Yes	AATTATAGGCTAAATCCTATATATCTTAC
23726	Yes	AATTATAGGCTAAATCCTATATATCTTACC
23727	Yes	AATTATAGGCTAAATCCTATATATCTTACCA
23728	No	AATTATGATAATCATA
23729	No	AATTATGATAATCATAT
23730	No	AATTATGATAATCATATT
23731	No	AATTATGATAATCATATTT
23732	No	AATTATGATAATCATATTTA
23733	No	AATTATGATAATCATATTTAC
23734	No	AATTATGATAATCATATTTACC
23735	No	AATTATGATAATCATATTTACCA
23736	No	AATTATGATAATCATATTTACCAA
23737	Yes	AATTATGATAATCATATTTACCAAC
23738	Yes	AATTATGATAATCATATTTACCAACC
23739	Yes	AATTATGATAATCATATTTACCAACCA
23740	Yes	AATTCAAAGGTTCCGG
23741	Yes	AATTCAAAGGTTCCGGG
23742	Yes	AATTCAAAGGTTCCGGGT
23743	Yes	AATTCAAAGGTTCCGGGTT
23744	Yes	AATTCAAAGGTTCCGGGTTC
23745	Yes	AATTCAAAGGTTCCGGGTTCG
23746	Yes	AATTCAAAGGTTCCGGGTTCGA
23747	Yes	AATTCAAAGGTTCCGGGTTCGAG
23748	Yes	AATTCAAAGGTTCCGGGTTCGAGT
23749	Yes	AATTCAAAGGTTCCGGGTTCGAGTC
23750	Yes	AATTCAAAGGTTCCGGGTTCGAGTCC
23751	Yes	AATTCAAAGGTTCCGGGTTCGAGTCCC
23752	Yes	AATTCAAAGGTTCCGGGTTCGAGTCCCG
23753	Yes	AATTCAAAGGTTCCGGGTTCGAGTCCCGG
23754	Yes	AATTCAAAGGTTCCGGGTTCGAGTCCCGGC
23755	Yes	AATTCAAAGGTTCCGGGTTCGAGTCCCGGCG
23756	Yes	AATTCAAAGGTTCCGGGTTCGAGTCCCGGCGG
23757	Yes	AATTCAAAGGTTCCGGGTTCGAGTCCCGGCGGA
23758	Yes	AATTCAAAGGTTCCGGGTTCGAGTCCCGGCGGAG
23759	Yes	AATTCAAAGGTTCCGGGTTCGAGTCCCGGCGGAGT
23760	Yes	AATTCAAAGGTTCCGGGTTCGAGTCCCGGCGGAGTC
23761	Yes	AATTCAAAGGTTCCGGGTTCGAGTCCCGGCGGAGTCG
23762	Yes	AATTCAAAGGTTCCGGGTTCGAGTCCCGGCGGAGTCGC
23763	Yes	AATTCAAAGGTTCCGGGTTCGAGTCCCGGCGGAGTCGCC
23764	Yes	AATTCAAAGGTTCCGGGTTCGAGTCCCGGCGGAGTCGCCA
23765	Yes	AATTCAAAGGTTGCGG

23766	Yes	AATTCAAAGGTTGCGGG
23767	Yes	AATTCAAAGGTTGCGGGT
23768	Yes	AATTCAAAGGTTGCGGGTT
23769	Yes	AATTCAAAGGTTGCGGGTTC
23770	Yes	AATTCAAAGGTTGCGGGTTCG
23771	Yes	AATTCAAAGGTTGCGGGTTCGA
23772	Yes	AATTCAAAGGTTGCGGGTTCGAG
23773	Yes	AATTCAAAGGTTGCGGGTTCGAGT
23774	Yes	AATTCAAAGGTTGCGGGTTCGAGTC
23775	Yes	AATTCAAAGGTTGCGGGTTCGAGTCC
23776	Yes	AATTCAAAGGTTGCGGGTTCGAGTCCC
23777	Yes	AATTCAAAGGTTGCGGGTTCGAGTCCCT
23778	Yes	AATTCAAAGGTTGCGGGTTCGAGTCCCTC
23779	Yes	AATTCAAAGGTTGCGGGTTCGAGTCCCTCC
23780	Yes	AATTCAAAGGTTGCGGGTTCGAGTCCCTCCA
23781	Yes	AATTCAAAGGTTGCGGGTTCGAGTCCCTCCAG
23782	Yes	AATTCAAAGGTTGCGGGTTCGAGTCCCTCCAGA
23783	Yes	AATTCAAAGGTTGCGGGTTCGAGTCCCTCCAGAG
23784	Yes	AATTCAAAGGTTGCGGGTTCGAGTCCCTCCAGAGT
23785	Yes	AATTCAAAGGTTGCGGGTTCGAGTCCCTCCAGAGTC
23786	Yes	AATTCAAAGGTTGCGGGTTCGAGTCCCTCCAGAGTCG
23787	Yes	AATTCAAAGGTTGCGGGTTCGAGTCCCTCCAGAGTCGC
23788	Yes	AATTCAAAGGTTGCGGGTTCGAGTCCCTCCAGAGTCGCC
23789	Yes	AATTCAAAGGTTGCGGGTTCGAGTCCCTCCAGAGTCGCCA
23790	No	AATTCAAAGGTTGTGG
23791	No	AATTCAAAGGTTGTGGG
23792	No	AATTCAAAGGTTGTGGGT
23793	No	AATTCAAAGGTTGTGGGTT
23794	No	AATTCAAAGGTTGTGGGTTC
23795	No	AATTCAAAGGTTGTGGGTTCG
23796	No	AATTCAAAGGTTGTGGGTTCGA
23797	No	AATTCAAAGGTTGTGGGTTCGAA
23798	No	AATTCAAAGGTTGTGGGTTCGAAT
23799	No	AATTCAAAGGTTGTGGGTTCGAATC
23800	No	AATTCAAAGGTTGTGGGTTCGAATCC
23801	No	AATTCAAAGGTTGTGGGTTCGAATCCC
23802	No	AATTCAAAGGTTGTGGGTTCGAATCCCA
23803	No	AATTCAAAGGTTGTGGGTTCGAATCCCAC
23804	No	AATTCAAAGGTTGTGGGTTCGAATCCCACC
23805	No	AATTCAAAGGTTGTGGGTTCGAATCCCACCA
23806	No	AATTCAAAGGTTGTGGGTTCGAATCCCACCAG
23807	No	AATTCAAAGGTTGTGGGTTCGAATCCCACCAGA

23808	No	AATTCAAAGGTTGTGGGTTCGAATCCCACCAGAG
23809	No	AATTCAAAGGTTGTGGGTTCGAATCCCACCAGAGT
23810	No	AATTCAAAGGTTGTGGGTTCGAATCCCACCAGAGTC
23811	No	AATTCAAAGGTTGTGGGTTCGAATCCCACCAGAGTCG
23812	Yes	AATTCAAAGGTTGTGGGTTCGAATCCCACCAGAGTCGC
23813	Yes	AATTCAAAGGTTGTGGGTTCGAATCCCACCAGAGTCGCC
23814	Yes	AATTCAAAGGTTGTGGGTTCGAATCCCACCAGAGTCGCCA
23815	No	AATTCAAAGGTTGTGGGTTCGAG
23816	No	AATTCAAAGGTTGTGGGTTCGAGT
23817	No	AATTCAAAGGTTGTGGGTTCGAGTC
23818	No	AATTCAAAGGTTGTGGGTTCGAGTCC
23819	No	AATTCAAAGGTTGTGGGTTCGAGTCCC
23820	No	AATTCAAAGGTTGTGGGTTCGAGTCCCA
23821	No	AATTCAAAGGTTGTGGGTTCGAGTCCCAC
23822	No	AATTCAAAGGTTGTGGGTTCGAGTCCCACC
23823	No	AATTCAAAGGTTGTGGGTTCGAGTCCCACCA
23824	No	AATTCAAAGGTTGTGGGTTCGAGTCCCACCAG
23825	No	AATTCAAAGGTTGTGGGTTCGAGTCCCACCAGA
23826	No	AATTCAAAGGTTGTGGGTTCGAGTCCCACCAGAG
23827	No	AATTCAAAGGTTGTGGGTTCGAGTCCCACCAGAGT
23828	No	AATTCAAAGGTTGTGGGTTCGAGTCCCACCAGAGTC
23829	No	AATTCAAAGGTTGTGGGTTCGAGTCCCACCAGAGTCG
23830	No	AATTCAAAGGTTGTGGGTTCGAGTCCCACCAGAGTCGC
23831	Yes	AATTCAAAGGTTGTGGGTTCGAGTCCCACCAGAGTCGCC
23832	Yes	AATTCAAAGGTTGTGGGTTCGAGTCCCACCAGAGTCGCCA
23833	No	AATTCAGCACTCTCAC
23834	No	AATTCAGCACTCTCACT
23835	Yes	AATTCAGCACTCTCACTG
23836	Yes	AATTCAGCACTCTCACTGC
23837	Yes	AATTCAGCACTCTCACTGCC
23838	Yes	AATTCAGCACTCTCACTGCCA
23839	Yes	AATTCAGCACTCTCACTGCCAC
23840	Yes	AATTCAGCACTCTCACTGCCACA
23841	Yes	AATTCAGCACTCTCACTGCCACAG
23842	Yes	AATTCAGCACTCTCACTGCCACAGC
23843	Yes	AATTCAGCACTCTCACTGCCACAGCC
23844	Yes	AATTCAGCACTCTCACTGCCACAGCCC
23845	Yes	AATTCAGCACTCTCACTGCCACAGCCCA
23846	Yes	AATTCAGCACTCTCACTGCCACAGCCCAG
23847	Yes	AATTCAGCACTCTCACTGCCACAGCCCAGG
23848	Yes	AATTCAGCACTCTCACTGCCACAGCCCAGGT
23849	Yes	AATTCAGCACTCTCACTGCCACAGCCCAGGTT

23850	Yes	AATTCAGCACTCTCACTGCCACAGCCCAGGTTC
23851	Yes	AATTCAGCACTCTCACTGCCACAGCCCAGGTTCA
23852	Yes	AATTCAGCACTCTCACTGCCACAGCCCAGGTTCAA
23853	Yes	AATTCAGCACTCTCACTGCCACAGCCCAGGTTCAAT
23854	Yes	AATTCAGCACTCTCACTGCCACAGCCCAGGTTCAATT
23855	Yes	AATTCAGCACTCTCACTGCCACAGCCCAGGTTCAATTC
23856	Yes	AATTCAGCACTCTCACTGCCACAGCCCAGGTTCAATTCC
23857	Yes	AATTCAGCACTCTCACTGCCACAGCCCAGGTTCAATTCCC
23858	Yes	AATTCAGCACTCTCACTGCCACAGCCCAGGTTCAATTCCCT
23859	Yes	AATTCAGCACTCTCACTGCCACAGCCCAGGTTCAATTCCCTG
23860	Yes	AATTCAGCACTCTCACTGCCACAGCCCAGGTTCAATTCCCTGT
23861	Yes	AATTCAGCACTCTCACTGCCACAGCCCAGGTTCAATTCCCTGTC
23862	Yes	AATTCAGCACTCTCACTGCCACAGCCCAGGTTCAATTCCCTGTCA
23863	Yes	AATTCAGCACTCTCACTGCCACAGCCCAGGTTCAATTCCCTGTCAG
23864	Yes	AATTCAGCACTCTCACTGCCACAGCCCAGGTTCAATTCCCTGTCAGA
23865	Yes	AATTCAGCACTCTCACTGCCACAGCCCAGGTTCAATTCCCTGTCAGAG
23866	Yes	AATTCAGCACTCTCACTGCCACAGCCCAGGTTCAATTCCCTGTCAGAGA
23867	Yes	AATTCAGCACTCTCACTGCCACAGCCCAGGTTCAATTCCCTGTCAGAGAA
23868	No	AATTCAGTGTTTTCAG
23869	No	AATTCAGTGTTTTCAGT
23870	No	AATTCAGTGTTTTCAGTG
23871	Yes	AATTCAGTGTTTTCAGTGC
23872	Yes	AATTCAGTGTTTTCAGTGCT
23873	Yes	AATTCAGTGTTTTCAGTGCTC
23874	Yes	AATTCAGTGTTTTCAGTGCTCT
23875	Yes	AATTCAGTGTTTTCAGTGCTCTA
23876	Yes	AATTCAGTGTTTTCAGTGCTCTAG
23877	Yes	AATTCAGTGTTTTCAGTGCTCTAGT
23878	Yes	AATTCAGTGTTTTCAGTGCTCTAGTC
23879	Yes	AATTCAGTGTTTTCAGTGCTCTAGTCC
23880	Yes	AATTCAGTGTTTTCAGTGCTCTAGTCCA
23881	Yes	AATTCAGTGTTTTCAGTGCTCTAGTCCAG
23882	Yes	AATTCAGTGTTTTCAGTGCTCTAGTCCAGG
23883	Yes	AATTCAGTGTTTTCAGTGCTCTAGTCCAGGT
23884	Yes	AATTCAGTGTTTTCAGTGCTCTAGTCCAGGTT
23885	Yes	AATTCAGTGTTTTCAGTGCTCTAGTCCAGGTTC
23886	Yes	AATTCAGTGTTTTCAGTGCTCTAGTCCAGGTTCA
23887	Yes	AATTCAGTGTTTTCAGTGCTCTAGTCCAGGTTCAA
23888	Yes	AATTCAGTGTTTTCAGTGCTCTAGTCCAGGTTCAAT
23889	Yes	AATTCAGTGTTTTCAGTGCTCTAGTCCAGGTTCAATT
23890	Yes	AATTCAGTGTTTTCAGTGCTCTAGTCCAGGTTCAATTC
23891	Yes	AATTCAGTGTTTTCAGTGCTCTAGTCCAGGTTCAATTCC

23892	Yes	AATTCAGTGTTTTCAGTGCTCTAGTCCAGGTTCAATTCCT
23893	Yes	AATTCAGTGTTTTCAGTGCTCTAGTCCAGGTTCAATTCCTG
23894	Yes	AATTCAGTGTTTTCAGTGCTCTAGTCCAGGTTCAATTCCTGG
23895	Yes	AATTCAGTGTTTTCAGTGCTCTAGTCCAGGTTCAATTCCTGGT
23896	Yes	AATTCAGTGTTTTCAGTGCTCTAGTCCAGGTTCAATTCCTGGTC
23897	Yes	AATTCAGTGTTTTCAGTGCTCTAGTCCAGGTTCAATTCCTGGTCA
23898	Yes	AATTCAGTGTTTTCAGTGCTCTAGTCCAGGTTCAATTCCTGGTCAG
23899	Yes	AATTCAGTGTTTTCAGTGCTCTAGTCCAGGTTCAATTCCTGGTCAGG
23900	Yes	AATTCAGTGTTTTCAGTGCTCTAGTCCAGGTTCAATTCCTGGTCAGGG
23901	Yes	AATTCAGTGTTTTCAGTGCTCTAGTCCAGGTTCAATTCCTGGTCAGGGA
23902	Yes	AATTCAGTGTTTTCAGTGCTCTAGTCCAGGTTCAATTCCTGGTCAGGGAC
23903	No	AATTCCAATGGTGCCT
23904	Yes	AATTCCAATGGTGCCTC
23905	Yes	AATTCCAATGGTGCCTCC
23906	Yes	AATTCCAATGGTGCCTCCA
23907	No	AATTCCACCTCTGCCA
23908	No	AATTCCACCTCTGCCAC
23909	No	AATTCCACCTCTGCCACC
23910	No	AATTCCACCTCTGCCACCA
23911	No	AATTCCCAGCCAACGC
23912	Yes	AATTCCCAGCCAACGCA
23913	Yes	AATTCCCAGCCAACGCAC
23914	Yes	AATTCCCAGCCAACGCACC
23915	Yes	AATTCCCAGCCAACGCACCA
23916	No	AATTCCCAGCTTGGCC
23917	No	AATTCCCAGCTTGGCCA
23918	Yes	AATTCCCAGCTTGGCCAC
23919	Yes	AATTCCCAGCTTGGCCACC
23920	Yes	AATTCCCAGCTTGGCCACCA
23921	No	AATTCCCAGTCAGGGA
23922	No	AATTCCCAGTCAGGGAA
23923	No	AATTCCCAGTCAGGGAAC
23924	No	AATTCCCAGTCAGGGAACC
23925	No	AATTCCCAGTCAGGGAACCA
23926	No	AATTCCCAGTTCTGCA
23927	No	AATTCCCAGTTCTGCAT
23928	No	AATTCCCAGTTCTGCATC
23929	Yes	AATTCCCAGTTCTGCATCC
23930	Yes	AATTCCCAGTTCTGCATCCA
23931	No	AATTCCCATCTCTGCC
23932	Yes	AATTCCCATCTCTGCCG
23933	Yes	AATTCCCATCTCTGCCGC

23934	Yes	AATTCCCATCTCTGCCGCC
23935	Yes	AATTCCCATCTCTGCCGCCA
23936	No	AATTCCCCGACGGGGA
23937	Yes	AATTCCCCGACGGGGAG
23938	Yes	AATTCCCCGACGGGGAGC
23939	Yes	AATTCCCCGACGGGGAGCC
23940	Yes	AATTCCCCGACGGGGAGCCA
23941	Yes	AATTCCCGATGAAGGA
23942	Yes	AATTCCCGATGAAGGAA
23943	Yes	AATTCCCGATGAAGGAAC
23944	Yes	AATTCCCGATGAAGGAACC
23945	Yes	AATTCCCGATGAAGGAACCA
23946	No	AATTCCCGGCCAATGC
23947	No	AATTCCCGGCCAATGCA
23948	No	AATTCCCGGCCAATGCAC
23949	No	AATTCCCGGCCAATGCACC
23950	No	AATTCCCGGCCAATGCACCA
23951	No	AATTCCCGGCCACTGC
23952	Yes	AATTCCCGGCCACTGCA
23953	Yes	AATTCCCGGCCACTGCAC
23954	Yes	AATTCCCGGCCACTGCACC
23955	Yes	AATTCCCGGCCACTGCACCA
23956	Yes	AATTCCCGGTCAAGAT
23957	Yes	AATTCCCGGTCAAGATA
23958	Yes	AATTCCCGGTCAAGATAC
23959	Yes	AATTCCCGGTCAAGATACC
23960	Yes	AATTCCCGGTCAAGATACCA
23961	Yes	AATTCCCGGTCAAGGC
23962	Yes	AATTCCCGGTCAAGGCA
23963	Yes	AATTCCCGGTCAAGGCAC
23964	Yes	AATTCCCGGTCAAGGCACC
23965	Yes	AATTCCCGGTCAAGGCACCA
23966	Yes	AATTCCCGGTCAGGGA
23967	Yes	AATTCCCGGTCAGGGAA
23968	Yes	AATTCCCGGTCAGGGAAC
23969	Yes	AATTCCCGGTCAGGGAACC
23970	Yes	AATTCCCGGTCAGGGAACCA
23971	No	AATTCCCTGATGGGGA
23972	Yes	AATTCCCTGATGGGGAG
23973	Yes	AATTCCCTGATGGGGAGC
23974	Yes	AATTCCCTGATGGGGAGCC
23975	Yes	AATTCCCTGATGGGGAGCCA

23976	No	AATTCCCTGTCAGAGA
23977	Yes	AATTCCCTGTCAGAGAA
23978	Yes	AATTCCCTGTCAGAGAAC
23979	Yes	AATTCCCTGTCAGAGAACC
23980	Yes	AATTCCCTGTCAGAGAACCA
23981	No	AATTCCCTTATGGGCT
23982	No	AATTCCCTTATGGGCTG
23983	Yes	AATTCCCTTATGGGCTGC
23984	Yes	AATTCCCTTATGGGCTGCC
23985	Yes	AATTCCCTTATGGGCTGCCA
23986	Yes	AATTCCGGCTCGAAGG
23987	Yes	AATTCCGGCTCGAAGGA
23988	Yes	AATTCCGGCTCGAAGGAC
23989	Yes	AATTCCGGCTCGAAGGACC
23990	Yes	AATTCCGGCTCGAAGGACCA
23991	Yes	AATTCCGGGTTTCGAC
23992	Yes	AATTCCGGGTTTCGACA
23993	Yes	AATTCCGGGTTTCGACAC
23994	Yes	AATTCCGGGTTTCGACACC
23995	Yes	AATTCCGGGTTTCGACACCA
23996	No	AATTCCTACAGTTCTA
23997	Yes	AATTCCTACAGTTCTAG
23998	Yes	AATTCCTACAGTTCTAGC
23999	Yes	AATTCCTACAGTTCTAGCC
24000	Yes	AATTCCTACAGTTCTAGCCA
24001	Yes	AATTCCTATAGAACCT
24002	Yes	AATTCCTATAGAACCTA
24003	Yes	AATTCCTATAGAACCTAG
24004	Yes	AATTCCTATAGAACCTAGG
24005	Yes	AATTCCTATAGAACCTAGGC
24006	Yes	AATTCCTATAGAACCTAGGCC
24007	Yes	AATTCCTATAGAACCTAGGCCA
24008	Yes	AATTCCTATAGTACTA
24009	Yes	AATTCCTATAGTACTAG
24010	Yes	AATTCCTATAGTACTAGC
24011	Yes	AATTCCTATAGTACTAGCC
24012	Yes	AATTCCTATAGTACTAGCCA
24013	No	AATTCCTATAGTCCTA
24014	No	AATTCCTATAGTCCTAG
24015	Yes	AATTCCTATAGTCCTAGC
24016	Yes	AATTCCTATAGTCCTAGCC
24017	Yes	AATTCCTATAGTCCTAGCCA

24018	No	AATTCCTATAGTTCCA
24019	Yes	AATTCCTATAGTTCCAG
24020	Yes	AATTCCTATAGTTCCAGC
24021	Yes	AATTCCTATAGTTCCAGCC
24022	Yes	AATTCCTATAGTTCCAGCCA
24023	No	AATTCCTATAGTTCTA
24024	No	AATTCCTATAGTTCTAG
24025	Yes	AATTCCTATAGTTCTAGC
24026	Yes	AATTCCTATAGTTCTAGCC
24027	Yes	AATTCCTATAGTTCTAGCCA
24028	No	AATTCCTATAGTTCTG
24029	No	AATTCCTATAGTTCTGG
24030	Yes	AATTCCTATAGTTCTGGC
24031	Yes	AATTCCTATAGTTCTGGCC
24032	Yes	AATTCCTATAGTTCTGGCCA
24033	No	AATTCCTATATTCTAG
24034	No	AATTCCTATATTCTAGA
24035	Yes	AATTCCTATATTCTAGAA
24036	Yes	AATTCCTATATTCTAGAAC
24037	Yes	AATTCCTATATTCTAGAACC
24038	Yes	AATTCCTATATTCTAGAACCA
24039	Yes	AATTCCTATATTCTAGAG
24040	Yes	AATTCCTATATTCTAGAGC
24041	Yes	AATTCCTATATTCTAGAGCC
24042	Yes	AATTCCTATATTCTAGAGCCA
24043	No	AATTCCTCTTCTTAAC
24044	No	AATTCCTCTTCTTAACA
24045	No	AATTCCTCTTCTTAACAC
24046	Yes	AATTCCTCTTCTTAACACC
24047	Yes	AATTCCTCTTCTTAACACCA
24048	No	AATTCCTGACCCATGT
24049	No	AATTCCTGACCCATGTA
24050	Yes	AATTCCTGACCCATGTAC
24051	Yes	AATTCCTGACCCATGTACC
24052	Yes	AATTCCTGACCCATGTACCA
24053	No	AATTCCTGGCCAATGC
24054	No	AATTCCTGGCCAATGCA
24055	No	AATTCCTGGCCAATGCAC
24056	No	AATTCCTGGCCAATGCACC
24057	No	AATTCCTGGCCAATGCACCA
24058	No	AATTCCTGGTCAGGGA
24059	Yes	AATTCCTGGTCAGGGAC

24060	Yes	AATTCCTGGTCAGGGACC
24061	Yes	AATTCCTGGTCAGGGACCA
24062	Yes	AATTCCTGGTTAGAGA
24063	Yes	AATTCCTGGTTAGAGAC
24064	Yes	AATTCCTGGTTAGAGACC
24065	Yes	AATTCCTGGTTAGAGACCA
24066	No	AATTCCTGTAGTTCTA
24067	Yes	AATTCCTGTAGTTCTAG
24068	Yes	AATTCCTGTAGTTCTAGC
24069	Yes	AATTCCTGTAGTTCTAGCC
24070	Yes	AATTCCTGTAGTTCTAGCCA
24071	No	AATTCCTTCCTTTTTT
24072	No	AATTCCTTCCTTTTTTG
24073	No	AATTCCTTCCTTTTTTGC
24074	Yes	AATTCCTTCCTTTTTTGCC
24075	Yes	AATTCCTTCCTTTTTTGCCA
24076	Yes	AATTCCTTTCGTCCTA
24077	Yes	AATTCCTTTCGTCCTAG
24078	Yes	AATTCCTTTCGTCCTAGC
24079	Yes	AATTCCTTTCGTCCTAGCC
24080	Yes	AATTCCTTTCGTCCTAGCCA
24081	No	AATTCGAAGAAGCAGC
24082	No	AATTCGAAGAAGCAGCT
24083	No	AATTCGAAGAAGCAGCTT
24084	No	AATTCGAAGAAGCAGCTTC
24085	No	AATTCGAAGAAGCAGCTTCA
24086	No	AATTCGAAGAAGCAGCTTCAA
24087	No	AATTCGAAGAAGCAGCTTCAAA
24088	No	AATTCGAAGAAGCAGCTTCAAAC
24089	No	AATTCGAAGAAGCAGCTTCAAACC
24090	No	AATTCGAAGAAGCAGCTTCAAACCT
24091	No	AATTCGAAGAAGCAGCTTCAAACCTG
24092	No	AATTCGAAGAAGCAGCTTCAAACCTGC
24093	No	AATTCGAAGAAGCAGCTTCAAACCTGCC
24094	No	AATTCGAAGAAGCAGCTTCAAACCTGCCG
24095	No	AATTCGAAGAAGCAGCTTCAAACCTGCCGG
24096	No	AATTCGAAGAAGCAGCTTCAAACCTGCCGGG
24097	No	AATTCGAAGAAGCAGCTTCAAACCTGCCGGGG
24098	No	AATTCGAAGAAGCAGCTTCAAACCTGCCGGGGC
24099	No	AATTCGAAGAAGCAGCTTCAAACCTGCCGGGGCT
24100	No	AATTCGAAGAAGCAGCTTCAAACCTGCCGGGGCTT
24101	No	AATTCGAAGAAGCAGCTTCAAACCTGCCGGGGCTTC

24102	Yes	AATTCGAAGAAGCAGCTTCAAACCTGCCGGGGCTTCC
24103	Yes	AATTCGAAGAAGCAGCTTCAAACCTGCCGGGGCTTCCA
24104	No	AATTCGTTGGTTTCTC
24105	Yes	AATTCGTTGGTTTCTCT
24106	Yes	AATTCGTTGGTTTCTCTC
24107	Yes	AATTCGTTGGTTTCTCTCT
24108	Yes	AATTCGTTGGTTTCTCTCTG
24109	Yes	AATTCGTTGGTTTCTCTCTGT
24110	Yes	AATTCGTTGGTTTCTCTCTGTG
24111	Yes	AATTCGTTGGTTTCTCTCTGTGC
24112	Yes	AATTCGTTGGTTTCTCTCTGTGCA
24113	Yes	AATTCGTTGGTTTCTCTCTGTGCAG
24114	Yes	AATTCGTTGGTTTCTCTCTGTGCAGG
24115	Yes	AATTCGTTGGTTTCTCTCTGTGCAGGT
24116	Yes	AATTCGTTGGTTTCTCTCTGTGCAGGTT
24117	Yes	AATTCGTTGGTTTCTCTCTGTGCAGGTTT
24118	Yes	AATTCGTTGGTTTCTCTCTGTGCAGGTTTG
24119	Yes	AATTCGTTGGTTTCTCTCTGTGCAGGTTTGA
24120	Yes	AATTCGTTGGTTTCTCTCTGTGCAGGTTTGAA
24121	Yes	AATTCGTTGGTTTCTCTCTGTGCAGGTTTGAAT
24122	Yes	AATTCGTTGGTTTCTCTCTGTGCAGGTTTGAATC
24123	Yes	AATTCGTTGGTTTCTCTCTGTGCAGGTTTGAATCC
24124	Yes	AATTCGTTGGTTTCTCTCTGTGCAGGTTTGAATCCT
24125	Yes	AATTCGTTGGTTTCTCTCTGTGCAGGTTTGAATCCTG
24126	Yes	AATTCGTTGGTTTCTCTCTGTGCAGGTTTGAATCCTGC
24127	Yes	AATTCGTTGGTTTCTCTCTGTGCAGGTTTGAATCCTGCT
24128	Yes	AATTCGTTGGTTTCTCTCTGTGCAGGTTTGAATCCTGCTA
24129	Yes	AATTCGTTGGTTTCTCTCTGTGCAGGTTTGAATCCTGCTAA
24130	Yes	AATTCGTTGGTTTCTCTCTGTGCAGGTTTGAATCCTGCTAAT
24131	Yes	AATTCGTTGGTTTCTCTCTGTGCAGGTTTGAATCCTGCTAATT
24132	Yes	AATTCGTTGGTTTCTCTCTGTGCAGGTTTGAATCCTGCTAATTA
24133	Yes	AATTCGTTGGTTTCTCTCTGTGCAGGTTTGAATCCTGCTAATTAT
24134	Yes	AATTCGTTGGTTTCTCTCTGTGCAGGTTTGAATCCTGCTAATTATG
24135	Yes	AATTCGTTGGTTTCTCTCTGTGCAGGTTTGAATCCTGCTAATTATGC
24136	Yes	AATTCGTTGGTTTCTCTCTGTGCAGGTTTGAATCCTGCTAATTATGCC
24137	Yes	AATTCGTTGGTTTCTCTCTGTGCAGGTTTGAATCCTGCTAATTATGCCA
24138	Yes	AATTCTCACCGCCTGC
24139	Yes	AATTCTCACCGCCTGCA
24140	Yes	AATTCTCACCGCCTGCAT
24141	Yes	AATTCTCACCGCCTGCATA
24142	Yes	AATTCTCACCGCCTGCATAG
24143	Yes	AATTCTCACCGCCTGCATAGG

24144	Yes	AATTCTCACCGCCTGCATAGGA
24145	Yes	AATTCTCACCGCCTGCATAGGAG
24146	Yes	AATTCTCACCGCCTGCATAGGAGA
24147	Yes	AATTCTCACCGCCTGCATAGGAGAC
24148	Yes	AATTCTCACCGCCTGCATAGGAGACC
24149	Yes	AATTCTCACCGCCTGCATAGGAGACCC
24150	Yes	AATTCTCACCGCCTGCATAGGAGACCCT
24151	Yes	AATTCTCACCGCCTGCATAGGAGACCCTG
24152	Yes	AATTCTCACCGCCTGCATAGGAGACCCTGG
24153	Yes	AATTCTCACCGCCTGCATAGGAGACCCTGGT
24154	Yes	AATTCTCACCGCCTGCATAGGAGACCCTGGTT
24155	Yes	AATTCTCACCGCCTGCATAGGAGACCCTGGTTT
24156	Yes	AATTCTCACCGCCTGCATAGGAGACCCTGGTTTA
24157	Yes	AATTCTCACCGCCTGCATAGGAGACCCTGGTTTAA
24158	Yes	AATTCTCACCGCCTGCATAGGAGACCCTGGTTTAAT
24159	Yes	AATTCTCACCGCCTGCATAGGAGACCCTGGTTTAATT
24160	Yes	AATTCTCACCGCCTGCATAGGAGACCCTGGTTTAATTC
24161	Yes	AATTCTCACCGCCTGCATAGGAGACCCTGGTTTAATTCC
24162	Yes	AATTCTCACCGCCTGCATAGGAGACCCTGGTTTAATTCCT
24163	Yes	AATTCTCACCGCCTGCATAGGAGACCCTGGTTTAATTCCTG
24164	Yes	AATTCTCACCGCCTGCATAGGAGACCCTGGTTTAATTCCTGG
24165	Yes	AATTCTCACCGCCTGCATAGGAGACCCTGGTTTAATTCCTGGC
24166	Yes	AATTCTCACCGCCTGCATAGGAGACCCTGGTTTAATTCCTGGCC
24167	Yes	AATTCTCACCGCCTGCATAGGAGACCCTGGTTTAATTCCTGGCCA
24168	Yes	AATTCTCACCGCCTGCATAGGAGACCCTGGTTTAATTCCTGGCCAA
24169	Yes	AATTCTCACCGCCTGCATAGGAGACCCTGGTTTAATTCCTGGCCAAT
24170	Yes	AATTCTCACCGCCTGCATAGGAGACCCTGGTTTAATTCCTGGCCAATG
24171	Yes	AATTCTCACCGCCTGCATAGGAGACCCTGGTTTAATTCCTGGCCAATGC
24172	Yes	AATTCTCACCGCCTGCATAGGAGACCCTGGTTTAATTCCTGGCCAATGCA
24173	No	AATTCTCAGGGGTAGG
24174	Yes	AATTCTCAGGGGTAGGT
24175	Yes	AATTCTCAGGGGTAGGTT
24176	Yes	AATTCTCAGGGGTAGGTTC
24177	Yes	AATTCTCAGGGGTAGGTTCA
24178	Yes	AATTCTCAGGGGTAGGTTCAA
24179	Yes	AATTCTCAGGGGTAGGTTCAAT
24180	Yes	AATTCTCAGGGGTAGGTTCAATT
24181	Yes	AATTCTCAGGGGTAGGTTCAATTC
24182	Yes	AATTCTCAGGGGTAGGTTCAATTCC
24183	Yes	AATTCTCAGGGGTAGGTTCAATTCCT
24184	Yes	AATTCTCAGGGGTAGGTTCAATTCCTA
24185	Yes	AATTCTCAGGGGTAGGTTCAATTCCTAT

24186	Yes	AATTCTCAGGGGTAGGTTCAATTCCTATA
24187	Yes	AATTCTCAGGGGTAGGTTCAATTCCTATAG
24188	Yes	AATTCTCAGGGGTAGGTTCAATTCCTATAGT
24189	Yes	AATTCTCAGGGGTAGGTTCAATTCCTATAGTT
24190	Yes	AATTCTCAGGGGTAGGTTCAATTCCTATAGTTC
24191	Yes	AATTCTCAGGGGTAGGTTCAATTCCTATAGTTCT
24192	Yes	AATTCTCAGGGGTAGGTTCAATTCCTATAGTTCTA
24193	Yes	AATTCTCAGGGGTAGGTTCAATTCCTATAGTTCTAG
24194	Yes	AATTCTCAGGGGTAGGTTCAATTCCTATAGTTCTAGC
24195	Yes	AATTCTCAGGGGTAGGTTCAATTCCTATAGTTCTAGCC
24196	Yes	AATTCTCAGGGGTAGGTTCAATTCCTATAGTTCTAGCCA
24197	Yes	AATTCTCGCCTCACAC
24198	Yes	AATTCTCGCCTCACACG
24199	Yes	AATTCTCGCCTCACACGC
24200	Yes	AATTCTCGCCTCACACGCG
24201	Yes	AATTCTCGCCTCACACGCGG
24202	Yes	AATTCTCGCCTCACACGCGGG
24203	Yes	AATTCTCGCCTCACACGCGGGA
24204	Yes	AATTCTCGCCTCACACGCGGGAC
24205	Yes	AATTCTCGCCTCACACGCGGGACA
24206	Yes	AATTCTCGCCTCACACGCGGGACAC
24207	Yes	AATTCTCGCCTCACACGCGGGACACC
24208	Yes	AATTCTCGCCTCACACGCGGGACACCC
24209	Yes	AATTCTCGCCTCACACGCGGGACACCCG
24210	Yes	AATTCTCGCCTCACACGCGGGACACCCGG
24211	Yes	AATTCTCGCCTCACACGCGGGACACCCGGG
24212	Yes	AATTCTCGCCTCACACGCGGGACACCCGGGT
24213	Yes	AATTCTCGCCTCACACGCGGGACACCCGGGTT
24214	Yes	AATTCTCGCCTCACACGCGGGACACCCGGGTTC
24215	Yes	AATTCTCGCCTCACACGCGGGACACCCGGGTTCA
24216	Yes	AATTCTCGCCTCACACGCGGGACACCCGGGTTCAA
24217	Yes	AATTCTCGCCTCACACGCGGGACACCCGGGTTCAAT
24218	Yes	AATTCTCGCCTCACACGCGGGACACCCGGGTTCAATT
24219	Yes	AATTCTCGCCTCACACGCGGGACACCCGGGTTCAATTC
24220	Yes	AATTCTCGCCTCACACGCGGGACACCCGGGTTCAATTCC
24221	Yes	AATTCTCGCCTCACACGCGGGACACCCGGGTTCAATTCCC
24222	Yes	AATTCTCGCCTCACACGCGGGACACCCGGGTTCAATTCCCG
24223	Yes	AATTCTCGCCTCACACGCGGGACACCCGGGTTCAATTCCCGG
24224	Yes	AATTCTCGCCTCACACGCGGGACACCCGGGTTCAATTCCCGGT
24225	Yes	AATTCTCGCCTCACACGCGGGACACCCGGGTTCAATTCCCGGTC
24226	Yes	AATTCTCGCCTCACACGCGGGACACCCGGGTTCAATTCCCGGTCA
24227	Yes	AATTCTCGCCTCACACGCGGGACACCCGGGTTCAATTCCCGGTCAA

24228	Yes	AATTCTCGCCTCACACGCGGGACACCCGGGTTCAATTCCCGGTCAAG
24229	Yes	AATTCTCGCCTCACACGCGGGACACCCGGGTTCAATTCCCGGTCAAGG
24230	Yes	AATTCTCGCCTCACACGCGGGACACCCGGGTTCAATTCCCGGTCAAGGC
24231	Yes	AATTCTCGCCTCACACGCGGGACACCCGGGTTCAATTCCCGGTCAAGGCA
24232	No	AATTCTCGCCTCCCAC
24233	No	AATTCTCGCCTCCCACG
24234	No	AATTCTCGCCTCCCACGC
24235	No	AATTCTCGCCTCCCACGCA
24236	Yes	AATTCTCGCCTCCCACGCAG
24237	Yes	AATTCTCGCCTCCCACGCAGG
24238	Yes	AATTCTCGCCTCCCACGCAGGA
24239	Yes	AATTCTCGCCTCCCACGCAGGAG
24240	Yes	AATTCTCGCCTCCCACGCAGGAGA
24241	Yes	AATTCTCGCCTCCCACGCAGGAGAC
24242	Yes	AATTCTCGCCTCCCACGCAGGAGACC
24243	Yes	AATTCTCGCCTCCCACGCAGGAGACCC
24244	Yes	AATTCTCGCCTCCCACGCAGGAGACCCA
24245	Yes	AATTCTCGCCTCCCACGCAGGAGACCCAG
24246	Yes	AATTCTCGCCTCCCACGCAGGAGACCCAGG
24247	Yes	AATTCTCGCCTCCCACGCAGGAGACCCAGGT
24248	Yes	AATTCTCGCCTCCCACGCAGGAGACCCAGGTT
24249	Yes	AATTCTCGCCTCCCACGCAGGAGACCCAGGTTC
24250	Yes	AATTCTCGCCTCCCACGCAGGAGACCCAGGTTCG
24251	Yes	AATTCTCGCCTCCCACGCAGGAGACCCAGGTTCGA
24252	Yes	AATTCTCGCCTCCCACGCAGGAGACCCAGGTTCGAT
24253	Yes	AATTCTCGCCTCCCACGCAGGAGACCCAGGTTCGATT
24254	Yes	AATTCTCGCCTCCCACGCAGGAGACCCAGGTTCGATTC
24255	Yes	AATTCTCGCCTCCCACGCAGGAGACCCAGGTTCGATTCC
24256	Yes	AATTCTCGCCTCCCACGCAGGAGACCCAGGTTCGATTCCT
24257	Yes	AATTCTCGCCTCCCACGCAGGAGACCCAGGTTCGATTCCTG
24258	Yes	AATTCTCGCCTCCCACGCAGGAGACCCAGGTTCGATTCCTGG
24259	Yes	AATTCTCGCCTCCCACGCAGGAGACCCAGGTTCGATTCCTGGC
24260	Yes	AATTCTCGCCTCCCACGCAGGAGACCCAGGTTCGATTCCTGGCC
24261	Yes	AATTCTCGCCTCCCACGCAGGAGACCCAGGTTCGATTCCTGGCCA
24262	Yes	AATTCTCGCCTCCCACGCAGGAGACCCAGGTTCGATTCCTGGCCAA
24263	Yes	AATTCTCGCCTCCCACGCAGGAGACCCAGGTTCGATTCCTGGCCAAT
24264	Yes	AATTCTCGCCTCCCACGCAGGAGACCCAGGTTCGATTCCTGGCCAATG
24265	Yes	AATTCTCGCCTCCCACGCAGGAGACCCAGGTTCGATTCCTGGCCAATGC
24266	Yes	AATTCTCGCCTCCCACGCAGGAGACCCAGGTTCGATTCCTGGCCAATGCA
24267	Yes	AATTCTCGCCTCCCACGCG
24268	Yes	AATTCTCGCCTCCCACGCGG
24269	Yes	AATTCTCGCCTCCCACGCGGG

24270	Yes	AATTCTCGCCTCCCACGCGGGA
24271	Yes	AATTCTCGCCTCCCACGCGGGAG
24272	Yes	AATTCTCGCCTCCCACGCGGGAGA
24273	Yes	AATTCTCGCCTCCCACGCGGGAGAC
24274	Yes	AATTCTCGCCTCCCACGCGGGAGACC
24275	Yes	AATTCTCGCCTCCCACGCGGGAGACCC
24276	Yes	AATTCTCGCCTCCCACGCGGGAGACCCG
24277	Yes	AATTCTCGCCTCCCACGCGGGAGACCCGG
24278	Yes	AATTCTCGCCTCCCACGCGGGAGACCCGGG
24279	Yes	AATTCTCGCCTCCCACGCGGGAGACCCGGGT
24280	Yes	AATTCTCGCCTCCCACGCGGGAGACCCGGGTT
24281	Yes	AATTCTCGCCTCCCACGCGGGAGACCCGGGTTC
24282	Yes	AATTCTCGCCTCCCACGCGGGAGACCCGGGTTCA
24283	Yes	AATTCTCGCCTCCCACGCGGGAGACCCGGGTTCAA
24284	Yes	AATTCTCGCCTCCCACGCGGGAGACCCGGGTTCAAT
24285	Yes	AATTCTCGCCTCCCACGCGGGAGACCCGGGTTCAATT
24286	Yes	AATTCTCGCCTCCCACGCGGGAGACCCGGGTTCAATTC
24287	Yes	AATTCTCGCCTCCCACGCGGGAGACCCGGGTTCAATTCC
24288	Yes	AATTCTCGCCTCCCACGCGGGAGACCCGGGTTCAATTCCC
24289	Yes	AATTCTCGCCTCCCACGCGGGAGACCCGGGTTCAATTCCCG
24290	Yes	AATTCTCGCCTCCCACGCGGGAGACCCGGGTTCAATTCCCGG
24291	Yes	AATTCTCGCCTCCCACGCGGGAGACCCGGGTTCAATTCCCGGC
24292	Yes	AATTCTCGCCTCCCACGCGGGAGACCCGGGTTCAATTCCCGGCC
24293	Yes	AATTCTCGCCTCCCACGCGGGAGACCCGGGTTCAATTCCCGGCCA
24294	Yes	AATTCTCGCCTCCCACGCGGGAGACCCGGGTTCAATTCCCGGCCAA
24295	Yes	AATTCTCGCCTCCCACGCGGGAGACCCGGGTTCAATTCCCGGCCAAT
24296	Yes	AATTCTCGCCTCCCACGCGGGAGACCCGGGTTCAATTCCCGGCCAATG
24297	Yes	AATTCTCGCCTCCCACGCGGGAGACCCGGGTTCAATTCCCGGCCAATGC
24298	Yes	AATTCTCGCCTCCCACGCGGGAGACCCGGGTTCAATTCCCGGCCAATGCA
24299	Yes	AATTCTCGCCTCCCACGCGGGAGACCCGGGTTT
24300	Yes	AATTCTCGCCTCCCACGCGGGAGACCCGGGTTTA
24301	Yes	AATTCTCGCCTCCCACGCGGGAGACCCGGGTTTAA
24302	Yes	AATTCTCGCCTCCCACGCGGGAGACCCGGGTTTAAT
24303	Yes	AATTCTCGCCTCCCACGCGGGAGACCCGGGTTTAATT
24304	Yes	AATTCTCGCCTCCCACGCGGGAGACCCGGGTTTAATTC
24305	Yes	AATTCTCGCCTCCCACGCGGGAGACCCGGGTTTAATTCC
24306	Yes	AATTCTCGCCTCCCACGCGGGAGACCCGGGTTTAATTCCC
24307	Yes	AATTCTCGCCTCCCACGCGGGAGACCCGGGTTTAATTCCCG
24308	Yes	AATTCTCGCCTCCCACGCGGGAGACCCGGGTTTAATTCCCGG
24309	Yes	AATTCTCGCCTCCCACGCGGGAGACCCGGGTTTAATTCCCGGT
24310	Yes	AATTCTCGCCTCCCACGCGGGAGACCCGGGTTTAATTCCCGGTC
24311	Yes	AATTCTCGCCTCCCACGCGGGAGACCCGGGTTTAATTCCCGGTCA

24312	Yes	AATTCTCGCCTCCCACGCGGGAGACCCGGGTTTAATTCCCGGTCAA
24313	Yes	AATTCTCGCCTCCCACGCGGGAGACCCGGGTTTAATTCCCGGTCAAG
24314	Yes	AATTCTCGCCTCCCACGCGGGAGACCCGGGTTTAATTCCCGGTCAAGA
24315	Yes	AATTCTCGCCTCCCACGCGGGAGACCCGGGTTTAATTCCCGGTCAAGAT
24316	Yes	AATTCTCGCCTCCCACGCGGGAGACCCGGGTTTAATTCCCGGTCAAGATA
24317	Yes	AATTCTCGCCTCCCACGT
24318	Yes	AATTCTCGCCTCCCACGTG
24319	Yes	AATTCTCGCCTCCCACGTGG
24320	Yes	AATTCTCGCCTCCCACGTGGG
24321	Yes	AATTCTCGCCTCCCACGTGGGA
24322	Yes	AATTCTCGCCTCCCACGTGGGAG
24323	Yes	AATTCTCGCCTCCCACGTGGGAGA
24324	Yes	AATTCTCGCCTCCCACGTGGGAGAC
24325	Yes	AATTCTCGCCTCCCACGTGGGAGACC
24326	Yes	AATTCTCGCCTCCCACGTGGGAGACCC
24327	Yes	AATTCTCGCCTCCCACGTGGGAGACCCG
24328	Yes	AATTCTCGCCTCCCACGTGGGAGACCCGG
24329	Yes	AATTCTCGCCTCCCACGTGGGAGACCCGGG
24330	Yes	AATTCTCGCCTCCCACGTGGGAGACCCGGGT
24331	Yes	AATTCTCGCCTCCCACGTGGGAGACCCGGGTT
24332	Yes	AATTCTCGCCTCCCACGTGGGAGACCCGGGTTC
24333	Yes	AATTCTCGCCTCCCACGTGGGAGACCCGGGTTCA
24334	Yes	AATTCTCGCCTCCCACGTGGGAGACCCGGGTTCAA
24335	Yes	AATTCTCGCCTCCCACGTGGGAGACCCGGGTTCAAT
24336	Yes	AATTCTCGCCTCCCACGTGGGAGACCCGGGTTCAATT
24337	Yes	AATTCTCGCCTCCCACGTGGGAGACCCGGGTTCAATTC
24338	Yes	AATTCTCGCCTCCCACGTGGGAGACCCGGGTTCAATTCC
24339	Yes	AATTCTCGCCTCCCACGTGGGAGACCCGGGTTCAATTCCC
24340	Yes	AATTCTCGCCTCCCACGTGGGAGACCCGGGTTCAATTCCCG
24341	Yes	AATTCTCGCCTCCCACGTGGGAGACCCGGGTTCAATTCCCGG
24342	Yes	AATTCTCGCCTCCCACGTGGGAGACCCGGGTTCAATTCCCGGC
24343	Yes	AATTCTCGCCTCCCACGTGGGAGACCCGGGTTCAATTCCCGGCC
24344	Yes	AATTCTCGCCTCCCACGTGGGAGACCCGGGTTCAATTCCCGGCCA
24345	Yes	AATTCTCGCCTCCCACGTGGGAGACCCGGGTTCAATTCCCGGCCAA
24346	Yes	AATTCTCGCCTCCCACGTGGGAGACCCGGGTTCAATTCCCGGCCAAT
24347	Yes	AATTCTCGCCTCCCACGTGGGAGACCCGGGTTCAATTCCCGGCCAATG
24348	Yes	AATTCTCGCCTCCCACGTGGGAGACCCGGGTTCAATTCCCGGCCAATGC
24349	Yes	AATTCTCGCCTCCCACGTGGGAGACCCGGGTTCAATTCCCGGCCAATGCA
24350	Yes	AATTCTCGCCTCCCAT
24351	Yes	AATTCTCGCCTCCCATG
24352	Yes	AATTCTCGCCTCCCATGC
24353	Yes	AATTCTCGCCTCCCATGCG

24354	Yes	AATTCTCGCCTCCCATGCGG
24355	Yes	AATTCTCGCCTCCCATGCGGG
24356	Yes	AATTCTCGCCTCCCATGCGGGA
24357	Yes	AATTCTCGCCTCCCATGCGGGAG
24358	Yes	AATTCTCGCCTCCCATGCGGGAGA
24359	Yes	AATTCTCGCCTCCCATGCGGGAGAC
24360	Yes	AATTCTCGCCTCCCATGCGGGAGACC
24361	Yes	AATTCTCGCCTCCCATGCGGGAGACCC
24362	Yes	AATTCTCGCCTCCCATGCGGGAGACCCG
24363	Yes	AATTCTCGCCTCCCATGCGGGAGACCCGG
24364	Yes	AATTCTCGCCTCCCATGCGGGAGACCCGGG
24365	Yes	AATTCTCGCCTCCCATGCGGGAGACCCGGGT
24366	Yes	AATTCTCGCCTCCCATGCGGGAGACCCGGGTT
24367	Yes	AATTCTCGCCTCCCATGCGGGAGACCCGGGTTC
24368	Yes	AATTCTCGCCTCCCATGCGGGAGACCCGGGTTCA
24369	Yes	AATTCTCGCCTCCCATGCGGGAGACCCGGGTTCAA
24370	Yes	AATTCTCGCCTCCCATGCGGGAGACCCGGGTTCAAT
24371	Yes	AATTCTCGCCTCCCATGCGGGAGACCCGGGTTCAATT
24372	Yes	AATTCTCGCCTCCCATGCGGGAGACCCGGGTTCAATTC
24373	Yes	AATTCTCGCCTCCCATGCGGGAGACCCGGGTTCAATTCC
24374	Yes	AATTCTCGCCTCCCATGCGGGAGACCCGGGTTCAATTCCC
24375	Yes	AATTCTCGCCTCCCATGCGGGAGACCCGGGTTCAATTCCCG
24376	Yes	AATTCTCGCCTCCCATGCGGGAGACCCGGGTTCAATTCCCGG
24377	Yes	AATTCTCGCCTCCCATGCGGGAGACCCGGGTTCAATTCCCGGC
24378	Yes	AATTCTCGCCTCCCATGCGGGAGACCCGGGTTCAATTCCCGGCC
24379	Yes	AATTCTCGCCTCCCATGCGGGAGACCCGGGTTCAATTCCCGGCCA
24380	Yes	AATTCTCGCCTCCCATGCGGGAGACCCGGGTTCAATTCCCGGCCAC
24381	Yes	AATTCTCGCCTCCCATGCGGGAGACCCGGGTTCAATTCCCGGCCACT
24382	Yes	AATTCTCGCCTCCCATGCGGGAGACCCGGGTTCAATTCCCGGCCACTG
24383	Yes	AATTCTCGCCTCCCATGCGGGAGACCCGGGTTCAATTCCCGGCCACTGC
24384	Yes	AATTCTCGCCTCCCATGCGGGAGACCCGGGTTCAATTCCCGGCCACTGCA
24385	No	AATTCTCGCCTGCCAC
24386	Yes	AATTCTCGCCTGCCACG
24387	Yes	AATTCTCGCCTGCCACGC
24388	Yes	AATTCTCGCCTGCCACGCG
24389	Yes	AATTCTCGCCTGCCACGCGG
24390	Yes	AATTCTCGCCTGCCACGCGGG
24391	Yes	AATTCTCGCCTGCCACGCGGGA
24392	Yes	AATTCTCGCCTGCCACGCGGGAG
24393	Yes	AATTCTCGCCTGCCACGCGGGAGG
24394	Yes	AATTCTCGCCTGCCACGCGGGAGGC
24395	Yes	AATTCTCGCCTGCCACGCGGGAGGCC

24396	Yes	AATTCTCGCCTGCCACGCGGGAGGCCC
24397	Yes	AATTCTCGCCTGCCACGCGGGAGGCCCG
24398	Yes	AATTCTCGCCTGCCACGCGGGAGGCCCGG
24399	Yes	AATTCTCGCCTGCCACGCGGGAGGCCCGGG
24400	Yes	AATTCTCGCCTGCCACGCGGGAGGCCCGGGT
24401	Yes	AATTCTCGCCTGCCACGCGGGAGGCCCGGGTT
24402	Yes	AATTCTCGCCTGCCACGCGGGAGGCCCGGGTTC
24403	Yes	AATTCTCGCCTGCCACGCGGGAGGCCCGGGTTCG
24404	Yes	AATTCTCGCCTGCCACGCGGGAGGCCCGGGTTCGA
24405	Yes	AATTCTCGCCTGCCACGCGGGAGGCCCGGGTTCGAT
24406	Yes	AATTCTCGCCTGCCACGCGGGAGGCCCGGGTTCGATT
24407	Yes	AATTCTCGCCTGCCACGCGGGAGGCCCGGGTTCGATTC
24408	Yes	AATTCTCGCCTGCCACGCGGGAGGCCCGGGTTCGATTCC
24409	Yes	AATTCTCGCCTGCCACGCGGGAGGCCCGGGTTCGATTCCC
24410	Yes	AATTCTCGCCTGCCACGCGGGAGGCCCGGGTTCGATTCCCG
24411	Yes	AATTCTCGCCTGCCACGCGGGAGGCCCGGGTTCGATTCCCGG
24412	Yes	AATTCTCGCCTGCCACGCGGGAGGCCCGGGTTCGATTCCCGGC
24413	Yes	AATTCTCGCCTGCCACGCGGGAGGCCCGGGTTCGATTCCCGGCC
24414	Yes	AATTCTCGCCTGCCACGCGGGAGGCCCGGGTTCGATTCCCGGCCA
24415	Yes	AATTCTCGCCTGCCACGCGGGAGGCCCGGGTTCGATTCCCGGCCAA
24416	Yes	AATTCTCGCCTGCCACGCGGGAGGCCCGGGTTCGATTCCCGGCCAAT
24417	Yes	AATTCTCGCCTGCCACGCGGGAGGCCCGGGTTCGATTCCCGGCCAATG
24418	Yes	AATTCTCGCCTGCCACGCGGGAGGCCCGGGTTCGATTCCCGGCCAATGC
24419	Yes	AATTCTCGCCTGCCACGCGGGAGGCCCGGGTTCGATTCCCGGCCAATGCA
24420	Yes	AATTCTCGCCTGCCACGCGGGAGGCCCGGGTTCGATTCCCGGCCC
24421	Yes	AATTCTCGCCTGCCACGCGGGAGGCCCGGGTTCGATTCCCGGCCCA
24422	Yes	AATTCTCGCCTGCCACGCGGGAGGCCCGGGTTCGATTCCCGGCCCAT
24423	Yes	AATTCTCGCCTGCCACGCGGGAGGCCCGGGTTCGATTCCCGGCCCATG
24424	Yes	AATTCTCGCCTGCCACGCGGGAGGCCCGGGTTCGATTCCCGGCCCATGC
24425	Yes	AATTCTCGCCTGCCACGCGGGAGGCCCGGGTTCGATTCCCGGCCCATGCA
24426	Yes	AATTCTCGCCTGCCACGCGGGAGGCCCGGGTTT
24427	Yes	AATTCTCGCCTGCCACGCGGGAGGCCCGGGTTTG
24428	Yes	AATTCTCGCCTGCCACGCGGGAGGCCCGGGTTTGA
24429	Yes	AATTCTCGCCTGCCACGCGGGAGGCCCGGGTTTGAT
24430	Yes	AATTCTCGCCTGCCACGCGGGAGGCCCGGGTTTGATT
24431	Yes	AATTCTCGCCTGCCACGCGGGAGGCCCGGGTTTGATTC
24432	Yes	AATTCTCGCCTGCCACGCGGGAGGCCCGGGTTTGATTCC
24433	Yes	AATTCTCGCCTGCCACGCGGGAGGCCCGGGTTTGATTCCC
24434	Yes	AATTCTCGCCTGCCACGCGGGAGGCCCGGGTTTGATTCCCG
24435	Yes	AATTCTCGCCTGCCACGCGGGAGGCCCGGGTTTGATTCCCGG
24436	Yes	AATTCTCGCCTGCCACGCGGGAGGCCCGGGTTTGATTCCCGGC
24437	Yes	AATTCTCGCCTGCCACGCGGGAGGCCCGGGTTTGATTCCCGGCC

24438	Yes	AATTCTCGCCTGCCACGCGGGAGGCCCGGGTTTGATTCCCGGCCA
24439	Yes	AATTCTCGCCTGCCACGCGGGAGGCCCGGGTTTGATTCCCGGCCAG
24440	Yes	AATTCTCGCCTGCCACGCGGGAGGCCCGGGTTTGATTCCCGGCCAGT
24441	Yes	AATTCTCGCCTGCCACGCGGGAGGCCCGGGTTTGATTCCCGGCCAGTG
24442	Yes	AATTCTCGCCTGCCACGCGGGAGGCCCGGGTTTGATTCCCGGCCAGTGC
24443	Yes	AATTCTCGCCTGCCACGCGGGAGGCCCGGGTTTGATTCCCGGCCAGTGCA
24444	No	AATTCTCGCCTGCCAT
24445	Yes	AATTCTCGCCTGCCATG
24446	Yes	AATTCTCGCCTGCCATGC
24447	Yes	AATTCTCGCCTGCCATGCG
24448	Yes	AATTCTCGCCTGCCATGCGG
24449	Yes	AATTCTCGCCTGCCATGCGGG
24450	Yes	AATTCTCGCCTGCCATGCGGGC
24451	Yes	AATTCTCGCCTGCCATGCGGGCG
24452	Yes	AATTCTCGCCTGCCATGCGGGCGG
24453	Yes	AATTCTCGCCTGCCATGCGGGCGGC
24454	Yes	AATTCTCGCCTGCCATGCGGGCGGCC
24455	Yes	AATTCTCGCCTGCCATGCGGGCGGCCG
24456	Yes	AATTCTCGCCTGCCATGCGGGCGGCCGG
24457	Yes	AATTCTCGCCTGCCATGCGGGCGGCCGGG
24458	Yes	AATTCTCGCCTGCCATGCGGGCGGCCGGGC
24459	Yes	AATTCTCGCCTGCCATGCGGGCGGCCGGGCT
24460	Yes	AATTCTCGCCTGCCATGCGGGCGGCCGGGCTT
24461	Yes	AATTCTCGCCTGCCATGCGGGCGGCCGGGCTTC
24462	Yes	AATTCTCGCCTGCCATGCGGGCGGCCGGGCTTCG
24463	Yes	AATTCTCGCCTGCCATGCGGGCGGCCGGGCTTCGA
24464	Yes	AATTCTCGCCTGCCATGCGGGCGGCCGGGCTTCGAT
24465	Yes	AATTCTCGCCTGCCATGCGGGCGGCCGGGCTTCGATT
24466	Yes	AATTCTCGCCTGCCATGCGGGCGGCCGGGCTTCGATTC
24467	Yes	AATTCTCGCCTGCCATGCGGGCGGCCGGGCTTCGATTCC
24468	Yes	AATTCTCGCCTGCCATGCGGGCGGCCGGGCTTCGATTCCT
24469	Yes	AATTCTCGCCTGCCATGCGGGCGGCCGGGCTTCGATTCCTG
24470	Yes	AATTCTCGCCTGCCATGCGGGCGGCCGGGCTTCGATTCCTGG
24471	Yes	AATTCTCGCCTGCCATGCGGGCGGCCGGGCTTCGATTCCTGGC
24472	Yes	AATTCTCGCCTGCCATGCGGGCGGCCGGGCTTCGATTCCTGGCC
24473	Yes	AATTCTCGCCTGCCATGCGGGCGGCCGGGCTTCGATTCCTGGCCA
24474	Yes	AATTCTCGCCTGCCATGCGGGCGGCCGGGCTTCGATTCCTGGCCAA
24475	Yes	AATTCTCGCCTGCCATGCGGGCGGCCGGGCTTCGATTCCTGGCCAAT
24476	Yes	AATTCTCGCCTGCCATGCGGGCGGCCGGGCTTCGATTCCTGGCCAATG
24477	Yes	AATTCTCGCCTGCCATGCGGGCGGCCGGGCTTCGATTCCTGGCCAATGC
24478	Yes	AATTCTCGCCTGCCATGCGGGCGGCCGGGCTTCGATTCCTGGCCAATGCA
24479	Yes	AATTCTCGCCTTTCAC

24480	Yes	AATTCTCGCCTTTCACA
24481	Yes	AATTCTCGCCTTTCACAC
24482	Yes	AATTCTCGCCTTTCACACA
24483	Yes	AATTCTCGCCTTTCACACAG
24484	Yes	AATTCTCGCCTTTCACACAGG
24485	Yes	AATTCTCGCCTTTCACACAGGA
24486	Yes	AATTCTCGCCTTTCACACAGGAG
24487	Yes	AATTCTCGCCTTTCACACAGGAGA
24488	Yes	AATTCTCGCCTTTCACACAGGAGAC
24489	Yes	AATTCTCGCCTTTCACACAGGAGACC
24490	Yes	AATTCTCGCCTTTCACACAGGAGACCC
24491	Yes	AATTCTCGCCTTTCACACAGGAGACCCG
24492	Yes	AATTCTCGCCTTTCACACAGGAGACCCGG
24493	Yes	AATTCTCGCCTTTCACACAGGAGACCCGGG
24494	Yes	AATTCTCGCCTTTCACACAGGAGACCCGGGT
24495	Yes	AATTCTCGCCTTTCACACAGGAGACCCGGGTT
24496	Yes	AATTCTCGCCTTTCACACAGGAGACCCGGGTTC
24497	Yes	AATTCTCGCCTTTCACACAGGAGACCCGGGTTCA
24498	Yes	AATTCTCGCCTTTCACACAGGAGACCCGGGTTCAA
24499	Yes	AATTCTCGCCTTTCACACAGGAGACCCGGGTTCAAT
24500	Yes	AATTCTCGCCTTTCACACAGGAGACCCGGGTTCAATT
24501	Yes	AATTCTCGCCTTTCACACAGGAGACCCGGGTTCAATTC
24502	Yes	AATTCTCGCCTTTCACACAGGAGACCCGGGTTCAATTCC
24503	Yes	AATTCTCGCCTTTCACACAGGAGACCCGGGTTCAATTCCT
24504	Yes	AATTCTCGCCTTTCACACAGGAGACCCGGGTTCAATTCCTG
24505	Yes	AATTCTCGCCTTTCACACAGGAGACCCGGGTTCAATTCCTGA
24506	Yes	AATTCTCGCCTTTCACACAGGAGACCCGGGTTCAATTCCTGAC
24507	Yes	AATTCTCGCCTTTCACACAGGAGACCCGGGTTCAATTCCTGACC
24508	Yes	AATTCTCGCCTTTCACACAGGAGACCCGGGTTCAATTCCTGACCC
24509	Yes	AATTCTCGCCTTTCACACAGGAGACCCGGGTTCAATTCCTGACCCA
24510	Yes	AATTCTCGCCTTTCACACAGGAGACCCGGGTTCAATTCCTGACCCAT
24511	Yes	AATTCTCGCCTTTCACACAGGAGACCCGGGTTCAATTCCTGACCCATG
24512	Yes	AATTCTCGCCTTTCACACAGGAGACCCGGGTTCAATTCCTGACCCATGT
24513	Yes	AATTCTCGCCTTTCACACAGGAGACCCGGGTTCAATTCCTGACCCATGTA
24514	No	AATTCTCGCTGGGGCC
24515	Yes	AATTCTCGCTGGGGCCT
24516	Yes	AATTCTCGCTGGGGCCTC
24517	Yes	AATTCTCGCTGGGGCCTCC
24518	Yes	AATTCTCGCTGGGGCCTCCA
24519	Yes	AATTCTGCGGTCTCAC
24520	Yes	AATTCTGCGGTCTCACA
24521	Yes	AATTCTGCGGTCTCACAG

24522	Yes	AATTCTGCGGTCTCACAGC
24523	Yes	AATTCTGCGGTCTCACAGCC
24524	Yes	AATTCTGCGGTCTCACAGCCG
24525	Yes	AATTCTGCGGTCTCACAGCCGC
24526	Yes	AATTCTGCGGTCTCACAGCCGCG
24527	Yes	AATTCTGCGGTCTCACAGCCGCGG
24528	Yes	AATTCTGCGGTCTCACAGCCGCGGC
24529	Yes	AATTCTGCGGTCTCACAGCCGCGGCC
24530	Yes	AATTCTGCGGTCTCACAGCCGCGGCCC
24531	Yes	AATTCTGCGGTCTCACAGCCGCGGCCCG
24532	Yes	AATTCTGCGGTCTCACAGCCGCGGCCCGG
24533	Yes	AATTCTGCGGTCTCACAGCCGCGGCCCGGG
24534	Yes	AATTCTGCGGTCTCACAGCCGCGGCCCGGGT
24535	Yes	AATTCTGCGGTCTCACAGCCGCGGCCCGGGTT
24536	Yes	AATTCTGCGGTCTCACAGCCGCGGCCCGGGTTC
24537	Yes	AATTCTGCGGTCTCACAGCCGCGGCCCGGGTTCG
24538	Yes	AATTCTGCGGTCTCACAGCCGCGGCCCGGGTTCGA
24539	Yes	AATTCTGCGGTCTCACAGCCGCGGCCCGGGTTCGAT
24540	Yes	AATTCTGCGGTCTCACAGCCGCGGCCCGGGTTCGATT
24541	Yes	AATTCTGCGGTCTCACAGCCGCGGCCCGGGTTCGATTC
24542	Yes	AATTCTGCGGTCTCACAGCCGCGGCCCGGGTTCGATTCC
24543	Yes	AATTCTGCGGTCTCACAGCCGCGGCCCGGGTTCGATTCCC
24544	Yes	AATTCTGCGGTCTCACAGCCGCGGCCCGGGTTCGATTCCCA
24545	Yes	AATTCTGCGGTCTCACAGCCGCGGCCCGGGTTCGATTCCCAT
24546	Yes	AATTCTGCGGTCTCACAGCCGCGGCCCGGGTTCGATTCCCATT
24547	Yes	AATTCTGCGGTCTCACAGCCGCGGCCCGGGTTCGATTCCCATTC
24548	Yes	AATTCTGCGGTCTCACAGCCGCGGCCCGGGTTCGATTCCCATTCC
24549	Yes	AATTCTGCGGTCTCACAGCCGCGGCCCGGGTTCGATTCCCATTCCG
24550	Yes	AATTCTGCGGTCTCACAGCCGCGGCCCGGGTTCGATTCCCATTCCGG
24551	Yes	AATTCTGCGGTCTCACAGCCGCGGCCCGGGTTCGATTCCCATTCCGGG
24552	Yes	AATTCTGCGGTCTCACAGCCGCGGCCCGGGTTCGATTCCCATTCCGGGA
24553	Yes	AATTCTGCGGTCTCACAGCCGCGGCCCGGGTTCGATTCCCATTCCGGGAA
24554	No	AATTCTGGATTCTCAG
24555	No	AATTCTGGATTCTCAGG
24556	No	AATTCTGGATTCTCAGGG
24557	Yes	AATTCTGGATTCTCAGGGG
24558	Yes	AATTCTGGATTCTCAGGGGT
24559	Yes	AATTCTGGATTCTCAGGGGTA
24560	Yes	AATTCTGGATTCTCAGGGGTAG
24561	Yes	AATTCTGGATTCTCAGGGGTAGG
24562	Yes	AATTCTGGATTCTCAGGGGTAGGT
24563	Yes	AATTCTGGATTCTCAGGGGTAGGTT

24564	Yes	AATTCTGGATTCTCAGGGGTAGGTTC
24565	Yes	AATTCTGGATTCTCAGGGGTAGGTTCA
24566	Yes	AATTCTGGATTCTCAGGGGTAGGTTCAA
24567	Yes	AATTCTGGATTCTCAGGGGTAGGTTCAAT
24568	Yes	AATTCTGGATTCTCAGGGGTAGGTTCAATT
24569	Yes	AATTCTGGATTCTCAGGGGTAGGTTCAATTC
24570	Yes	AATTCTGGATTCTCAGGGGTAGGTTCAATTCC
24571	Yes	AATTCTGGATTCTCAGGGGTAGGTTCAATTCCT
24572	Yes	AATTCTGGATTCTCAGGGGTAGGTTCAATTCCTA
24573	Yes	AATTCTGGATTCTCAGGGGTAGGTTCAATTCCTAT
24574	Yes	AATTCTGGATTCTCAGGGGTAGGTTCAATTCCTATA
24575	Yes	AATTCTGGATTCTCAGGGGTAGGTTCAATTCCTATAG
24576	Yes	AATTCTGGATTCTCAGGGGTAGGTTCAATTCCTATAGA
24577	Yes	AATTCTGGATTCTCAGGGGTAGGTTCAATTCCTATAGAA
24578	Yes	AATTCTGGATTCTCAGGGGTAGGTTCAATTCCTATAGAAC
24579	Yes	AATTCTGGATTCTCAGGGGTAGGTTCAATTCCTATAGAACC
24580	Yes	AATTCTGGATTCTCAGGGGTAGGTTCAATTCCTATAGAACCT
24581	Yes	AATTCTGGATTCTCAGGGGTAGGTTCAATTCCTATAGAACCTA
24582	Yes	AATTCTGGATTCTCAGGGGTAGGTTCAATTCCTATAGAACCTAG
24583	Yes	AATTCTGGATTCTCAGGGGTAGGTTCAATTCCTATAGAACCTAGG
24584	Yes	AATTCTGGATTCTCAGGGGTAGGTTCAATTCCTATAGAACCTAGGC
24585	Yes	AATTCTGGATTCTCAGGGGTAGGTTCAATTCCTATAGAACCTAGGCC
24586	Yes	AATTCTGGATTCTCAGGGGTAGGTTCAATTCCTATAGAACCTAGGCCA
24587	No	AATTCTGGGTTTTGGC
24588	No	AATTCTGGGTTTTGGCA
24589	Yes	AATTCTGGGTTTTGGCAC
24590	Yes	AATTCTGGGTTTTGGCACC
24591	Yes	AATTCTGGGTTTTGGCACCA
24592	No	AATTCTGGTGCCACTC
24593	No	AATTCTGGTGCCACTCC
24594	No	AATTCTGGTGCCACTCCC
24595	Yes	AATTCTGGTGCCACTCCCA
24596	No	AATTCTTATAGTTCTA
24597	No	AATTCTTATAGTTCTAG
24598	Yes	AATTCTTATAGTTCTAGC
24599	Yes	AATTCTTATAGTTCTAGCC
24600	Yes	AATTCTTATAGTTCTAGCCA
24601	Yes	AATTCTTGCCTGCCAC
24602	Yes	AATTCTTGCCTGCCACG
24603	Yes	AATTCTTGCCTGCCACGC
24604	Yes	AATTCTTGCCTGCCACGCA
24605	Yes	AATTCTTGCCTGCCACGCAG

24606	Yes	AATTCTTGCCTGCCACGCAGG
24607	Yes	AATTCTTGCCTGCCACGCAGGA
24608	Yes	AATTCTTGCCTGCCACGCAGGAG
24609	Yes	AATTCTTGCCTGCCACGCAGGAGG
24610	Yes	AATTCTTGCCTGCCACGCAGGAGGC
24611	Yes	AATTCTTGCCTGCCACGCAGGAGGCC
24612	Yes	AATTCTTGCCTGCCACGCAGGAGGCCC
24613	Yes	AATTCTTGCCTGCCACGCAGGAGGCCCA
24614	Yes	AATTCTTGCCTGCCACGCAGGAGGCCCAG
24615	Yes	AATTCTTGCCTGCCACGCAGGAGGCCCAGG
24616	Yes	AATTCTTGCCTGCCACGCAGGAGGCCCAGGT
24617	Yes	AATTCTTGCCTGCCACGCAGGAGGCCCAGGTT
24618	Yes	AATTCTTGCCTGCCACGCAGGAGGCCCAGGTTT
24619	Yes	AATTCTTGCCTGCCACGCAGGAGGCCCAGGTTTG
24620	Yes	AATTCTTGCCTGCCACGCAGGAGGCCCAGGTTTGA
24621	Yes	AATTCTTGCCTGCCACGCAGGAGGCCCAGGTTTGAT
24622	Yes	AATTCTTGCCTGCCACGCAGGAGGCCCAGGTTTGATT
24623	Yes	AATTCTTGCCTGCCACGCAGGAGGCCCAGGTTTGATTC
24624	Yes	AATTCTTGCCTGCCACGCAGGAGGCCCAGGTTTGATTCC
24625	Yes	AATTCTTGCCTGCCACGCAGGAGGCCCAGGTTTGATTCCT
24626	Yes	AATTCTTGCCTGCCACGCAGGAGGCCCAGGTTTGATTCCTG
24627	Yes	AATTCTTGCCTGCCACGCAGGAGGCCCAGGTTTGATTCCTGG
24628	Yes	AATTCTTGCCTGCCACGCAGGAGGCCCAGGTTTGATTCCTGGC
24629	Yes	AATTCTTGCCTGCCACGCAGGAGGCCCAGGTTTGATTCCTGGCC
24630	Yes	AATTCTTGCCTGCCACGCAGGAGGCCCAGGTTTGATTCCTGGCCC
24631	Yes	AATTCTTGCCTGCCACGCAGGAGGCCCAGGTTTGATTCCTGGCCCA
24632	Yes	AATTCTTGCCTGCCACGCAGGAGGCCCAGGTTTGATTCCTGGCCCAT
24633	Yes	AATTCTTGCCTGCCACGCAGGAGGCCCAGGTTTGATTCCTGGCCCATG
24634	Yes	AATTCTTGCCTGCCACGCAGGAGGCCCAGGTTTGATTCCTGGCCCATGC
24635	Yes	AATTCTTGCCTGCCACGCAGGAGGCCCAGGTTTGATTCCTGGCCCATGCA
24636	Yes	AATTGACCCGACTTCA
24637	Yes	AATTGACCCGACTTCAA
24638	Yes	AATTGACCCGACTTCAAT
24639	Yes	AATTGACCCGACTTCAATT
24640	Yes	AATTGACCCGACTTCAATTC
24641	Yes	AATTGACCCGACTTCAATTCC
24642	Yes	AATTGACCCGACTTCAATTCCC
24643	Yes	AATTGACCCGACTTCAATTCCCA
24644	Yes	AATTGACCCGACTTCAATTCCCAG
24645	Yes	AATTGACCCGACTTCAATTCCCAGC
24646	Yes	AATTGACCCGACTTCAATTCCCAGCC
24647	Yes	AATTGACCCGACTTCAATTCCCAGCCA

24648	Yes	AATTGACCCGACTTCAATTCCCAGCCAA
24649	Yes	AATTGACCCGACTTCAATTCCCAGCCAAC
24650	Yes	AATTGACCCGACTTCAATTCCCAGCCAACG
24651	Yes	AATTGACCCGACTTCAATTCCCAGCCAACGC
24652	Yes	AATTGACCCGACTTCAATTCCCAGCCAACGCA
24653	Yes	AATTGACCCGACTTCAATTCCCAGCCAACGCAC
24654	Yes	AATTGACCCGACTTCAATTCCCAGCCAACGCACC
24655	Yes	AATTGACCCGACTTCAATTCCCAGCCAACGCACCA
24656	Yes	AATTGACCCGACTTCG
24657	Yes	AATTGACCCGACTTCGA
24658	Yes	AATTGACCCGACTTCGAT
24659	Yes	AATTGACCCGACTTCGATT
24660	Yes	AATTGACCCGACTTCGATTC
24661	Yes	AATTGACCCGACTTCGATTCC
24662	Yes	AATTGACCCGACTTCGATTCCC
24663	Yes	AATTGACCCGACTTCGATTCCCA
24664	Yes	AATTGACCCGACTTCGATTCCCAG
24665	Yes	AATTGACCCGACTTCGATTCCCAGC
24666	Yes	AATTGACCCGACTTCGATTCCCAGCC
24667	Yes	AATTGACCCGACTTCGATTCCCAGCCA
24668	Yes	AATTGACCCGACTTCGATTCCCAGCCAA
24669	Yes	AATTGACCCGACTTCGATTCCCAGCCAAT
24670	Yes	AATTGACCCGACTTCGATTCCCAGCCAATG
24671	Yes	AATTGACCCGACTTCGATTCCCAGCCAATGC
24672	Yes	AATTGACCCGACTTCGATTCCCAGCCAATGCA
24673	Yes	AATTGACCCGACTTCGATTCCCAGCCAATGCAC
24674	Yes	AATTGACCCGACTTCGATTCCCAGCCAATGCACC
24675	Yes	AATTGACCCGACTTCGATTCCCAGCCAATGCACCA
24676	No	AATTGCAAATTCGAAG
24677	No	AATTGCAAATTCGAAGA
24678	No	AATTGCAAATTCGAAGAA
24679	No	AATTGCAAATTCGAAGAAG
24680	No	AATTGCAAATTCGAAGAAGC
24681	No	AATTGCAAATTCGAAGAAGCA
24682	No	AATTGCAAATTCGAAGAAGCAG
24683	No	AATTGCAAATTCGAAGAAGCAGC
24684	No	AATTGCAAATTCGAAGAAGCAGCT
24685	No	AATTGCAAATTCGAAGAAGCAGCTT
24686	No	AATTGCAAATTCGAAGAAGCAGCTTC
24687	No	AATTGCAAATTCGAAGAAGCAGCTTCA
24688	No	AATTGCAAATTCGAAGAAGCAGCTTCAA
24689	No	AATTGCAAATTCGAAGAAGCAGCTTCAAA

24690	No	AATTGCAAATTCGAAGAAGCAGCTTCAAAC
24691	No	AATTGCAAATTCGAAGAAGCAGCTTCAAACC
24692	No	AATTGCAAATTCGAAGAAGCAGCTTCAAACCT
24693	No	AATTGCAAATTCGAAGAAGCAGCTTCAAACCTG
24694	No	AATTGCAAATTCGAAGAAGCAGCTTCAAACCTGC
24695	No	AATTGCAAATTCGAAGAAGCAGCTTCAAACCTGCC
24696	No	AATTGCAAATTCGAAGAAGCAGCTTCAAACCTGCCG
24697	No	AATTGCAAATTCGAAGAAGCAGCTTCAAACCTGCCGG
24698	No	AATTGCAAATTCGAAGAAGCAGCTTCAAACCTGCCGGG
24699	No	AATTGCAAATTCGAAGAAGCAGCTTCAAACCTGCCGGGG
24700	No	AATTGCAAATTCGAAGAAGCAGCTTCAAACCTGCCGGGGC
24701	No	AATTGCAAATTCGAAGAAGCAGCTTCAAACCTGCCGGGGCT
24702	No	AATTGCAAATTCGAAGAAGCAGCTTCAAACCTGCCGGGGCTT
24703	No	AATTGCAAATTCGAAGAAGCAGCTTCAAACCTGCCGGGGCTTC
24704	Yes	AATTGCAAATTCGAAGAAGCAGCTTCAAACCTGCCGGGGCTTCC
24705	Yes	AATTGCAAATTCGAAGAAGCAGCTTCAAACCTGCCGGGGCTTCCA
24706	No	AATTGCATAAAACTTA
24707	No	AATTGCATAAAACTTAA
24708	No	AATTGCATAAAACTTAAA
24709	No	AATTGCATAAAACTTAAAA
24710	No	AATTGCATAAAACTTAAAAC
24711	No	AATTGCATAAAACTTAAAACT
24712	No	AATTGCATAAAACTTAAAACTT
24713	No	AATTGCATAAAACTTAAAACTTT
24714	No	AATTGCATAAAACTTAAAACTTTA
24715	Yes	AATTGCATAAAACTTAAAACTTTAC
24716	Yes	AATTGCATAAAACTTAAAACTTTACA
24717	Yes	AATTGCATAAAACTTAAAACTTTACAA
24718	Yes	AATTGCATAAAACTTAAAACTTTACAAT
24719	Yes	AATTGCATAAAACTTAAAACTTTACAATC
24720	Yes	AATTGCATAAAACTTAAAACTTTACAATCA
24721	Yes	AATTGCATAAAACTTAAAACTTTACAATCAG
24722	Yes	AATTGCATAAAACTTAAAACTTTACAATCAGA
24723	Yes	AATTGCATAAAACTTAAAACTTTACAATCAGAG
24724	Yes	AATTGCATAAAACTTAAAACTTTACAATCAGAGG
24725	Yes	AATTGCATAAAACTTAAAACTTTACAATCAGAGGT
24726	Yes	AATTGCATAAAACTTAAAACTTTACAATCAGAGGTT
24727	Yes	AATTGCATAAAACTTAAAACTTTACAATCAGAGGTTC
24728	Yes	AATTGCATAAAACTTAAAACTTTACAATCAGAGGTTCA
24729	Yes	AATTGCATAAAACTTAAAACTTTACAATCAGAGGTTCAA
24730	Yes	AATTGCATAAAACTTAAAACTTTACAATCAGAGGTTCAAC
24731	Yes	AATTGCATAAAACTTAAAACTTTACAATCAGAGGTTCAACT

24732	Yes	AATTGCATAAAACTTAAAACTTTACAATCAGAGGTTCAACTC
24733	Yes	AATTGCATAAAACTTAAAACTTTACAATCAGAGGTTCAACTCC
24734	Yes	AATTGCATAAAACTTAAAACTTTACAATCAGAGGTTCAACTCCT
24735	Yes	AATTGCATAAAACTTAAAACTTTACAATCAGAGGTTCAACTCCTC
24736	Yes	AATTGCATAAAACTTAAAACTTTACAATCAGAGGTTCAACTCCTCT
24737	Yes	AATTGCATAAAACTTAAAACTTTACAATCAGAGGTTCAACTCCTCTT
24738	Yes	AATTGCATAAAACTTAAAACTTTACAATCAGAGGTTCAACTCCTCTTC
24739	Yes	AATTGCATAAAACTTAAAACTTTACAATCAGAGGTTCAACTCCTCTTCT
24740	Yes	AATTGCATAAAACTTAAAACTTTACAATCAGAGGTTCAACTCCTCTTCTT
24741	Yes	AATTGCATAAAACTTAAAAT
24742	Yes	AATTGCATAAAACTTAAAATT
24743	Yes	AATTGCATAAAACTTAAAATTT
24744	Yes	AATTGCATAAAACTTAAAATTTT
24745	Yes	AATTGCATAAAACTTAAAATTTTA
24746	Yes	AATTGCATAAAACTTAAAATTTTAT
24747	Yes	AATTGCATAAAACTTAAAATTTTATA
24748	Yes	AATTGCATAAAACTTAAAATTTTATAA
24749	Yes	AATTGCATAAAACTTAAAATTTTATAAT
24750	Yes	AATTGCATAAAACTTAAAATTTTATAATC
24751	Yes	AATTGCATAAAACTTAAAATTTTATAATCA
24752	Yes	AATTGCATAAAACTTAAAATTTTATAATCAG
24753	Yes	AATTGCATAAAACTTAAAATTTTATAATCAGA
24754	Yes	AATTGCATAAAACTTAAAATTTTATAATCAGAG
24755	Yes	AATTGCATAAAACTTAAAATTTTATAATCAGAGG
24756	Yes	AATTGCATAAAACTTAAAATTTTATAATCAGAGGT
24757	Yes	AATTGCATAAAACTTAAAATTTTATAATCAGAGGTT
24758	Yes	AATTGCATAAAACTTAAAATTTTATAATCAGAGGTTC
24759	Yes	AATTGCATAAAACTTAAAATTTTATAATCAGAGGTTCA
24760	Yes	AATTGCATAAAACTTAAAATTTTATAATCAGAGGTTCAA
24761	Yes	AATTGCATAAAACTTAAAATTTTATAATCAGAGGTTCAAC
24762	Yes	AATTGCATAAAACTTAAAATTTTATAATCAGAGGTTCAACT
24763	Yes	AATTGCATAAAACTTAAAATTTTATAATCAGAGGTTCAACTC
24764	Yes	AATTGCATAAAACTTAAAATTTTATAATCAGAGGTTCAACTCC
24765	Yes	AATTGCATAAAACTTAAAATTTTATAATCAGAGGTTCAACTCCT
24766	Yes	AATTGCATAAAACTTAAAATTTTATAATCAGAGGTTCAACTCCTC
24767	Yes	AATTGCATAAAACTTAAAATTTTATAATCAGAGGTTCAACTCCTCT
24768	Yes	AATTGCATAAAACTTAAAATTTTATAATCAGAGGTTCAACTCCTCTT
24769	Yes	AATTGCATAAAACTTAAAATTTTATAATCAGAGGTTCAACTCCTCTTC
24770	Yes	AATTGCATAAAACTTAAAATTTTATAATCAGAGGTTCAACTCCTCTTCT
24771	Yes	AATTGCATAAAACTTAAAATTTTATAATCAGAGGTTCAACTCCTCTTCTT
24772	Yes	AATTGCATAAAACTTAAG
24773	Yes	AATTGCATAAAACTTAAGA

24774	Yes	AATTGCATAAAACTTAAGAC
24775	Yes	AATTGCATAAAACTTAAGACT
24776	Yes	AATTGCATAAAACTTAAGACTT
24777	Yes	AATTGCATAAAACTTAAGACTTT
24778	Yes	AATTGCATAAAACTTAAGACTTTA
24779	Yes	AATTGCATAAAACTTAAGACTTTAT
24780	Yes	AATTGCATAAAACTTAAGACTTTATA
24781	Yes	AATTGCATAAAACTTAAGACTTTATAA
24782	Yes	AATTGCATAAAACTTAAGACTTTATAAT
24783	Yes	AATTGCATAAAACTTAAGACTTTATAATT
24784	Yes	AATTGCATAAAACTTAAGACTTTATAATTA
24785	Yes	AATTGCATAAAACTTAAGACTTTATAATTAG
24786	Yes	AATTGCATAAAACTTAAGACTTTATAATTAGA
24787	Yes	AATTGCATAAAACTTAAGACTTTATAATTAGAA
24788	Yes	AATTGCATAAAACTTAAGACTTTATAATTAGAAG
24789	Yes	AATTGCATAAAACTTAAGACTTTATAATTAGAAGT
24790	Yes	AATTGCATAAAACTTAAGACTTTATAATTAGAAGTT
24791	Yes	AATTGCATAAAACTTAAGACTTTATAATTAGAAGTTC
24792	Yes	AATTGCATAAAACTTAAGACTTTATAATTAGAAGTTCA
24793	Yes	AATTGCATAAAACTTAAGACTTTATAATTAGAAGTTCAA
24794	Yes	AATTGCATAAAACTTAAGACTTTATAATTAGAAGTTCAAC
24795	Yes	AATTGCATAAAACTTAAGACTTTATAATTAGAAGTTCAACA
24796	Yes	AATTGCATAAAACTTAAGACTTTATAATTAGAAGTTCAACAC
24797	Yes	AATTGCATAAAACTTAAGACTTTATAATTAGAAGTTCAACACC
24798	Yes	AATTGCATAAAACTTAAGACTTTATAATTAGAAGTTCAACACCT
24799	Yes	AATTGCATAAAACTTAAGACTTTATAATTAGAAGTTCAACACCTC
24800	Yes	AATTGCATAAAACTTAAGACTTTATAATTAGAAGTTCAACACCTCT
24801	Yes	AATTGCATAAAACTTAAGACTTTATAATTAGAAGTTCAACACCTCTT
24802	Yes	AATTGCATAAAACTTAAGACTTTATAATTAGAAGTTCAACACCTCTTC
24803	Yes	AATTGCATAAAACTTAAGACTTTATAATTAGAAGTTCAACACCTCTTCT
24804	Yes	AATTGCATAAAACTTAAGACTTTATAATTAGAAGTTCAACACCTCTTCTT
24805	No	AATTGCATAAATCTTA
24806	No	AATTGCATAAATCTTAA
24807	No	AATTGCATAAATCTTAAA
24808	No	AATTGCATAAATCTTAAAA
24809	No	AATTGCATAAATCTTAAAAC
24810	No	AATTGCATAAATCTTAAAACT
24811	No	AATTGCATAAATCTTAAAACTT
24812	No	AATTGCATAAATCTTAAAACTTT
24813	Yes	AATTGCATAAATCTTAAAACTTTA
24814	Yes	AATTGCATAAATCTTAAAACTTTAT
24815	Yes	AATTGCATAAATCTTAAAACTTTATA

24816	Yes	AATTGCATAAATCTTAAAACTTTATAA
24817	Yes	AATTGCATAAATCTTAAAACTTTATAAT
24818	Yes	AATTGCATAAATCTTAAAACTTTATAATC
24819	Yes	AATTGCATAAATCTTAAAACTTTATAATCA
24820	Yes	AATTGCATAAATCTTAAAACTTTATAATCAG
24821	Yes	AATTGCATAAATCTTAAAACTTTATAATCAGA
24822	Yes	AATTGCATAAATCTTAAAACTTTATAATCAGAG
24823	Yes	AATTGCATAAATCTTAAAACTTTATAATCAGAGG
24824	Yes	AATTGCATAAATCTTAAAACTTTATAATCAGAGGT
24825	Yes	AATTGCATAAATCTTAAAACTTTATAATCAGAGGTT
24826	Yes	AATTGCATAAATCTTAAAACTTTATAATCAGAGGTTC
24827	Yes	AATTGCATAAATCTTAAAACTTTATAATCAGAGGTTCG
24828	Yes	AATTGCATAAATCTTAAAACTTTATAATCAGAGGTTCGA
24829	Yes	AATTGCATAAATCTTAAAACTTTATAATCAGAGGTTCGAC
24830	Yes	AATTGCATAAATCTTAAAACTTTATAATCAGAGGTTCGACT
24831	Yes	AATTGCATAAATCTTAAAACTTTATAATCAGAGGTTCGACTC
24832	Yes	AATTGCATAAATCTTAAAACTTTATAATCAGAGGTTCGACTCC
24833	Yes	AATTGCATAAATCTTAAAACTTTATAATCAGAGGTTCGACTCCT
24834	Yes	AATTGCATAAATCTTAAAACTTTATAATCAGAGGTTCGACTCCTC
24835	Yes	AATTGCATAAATCTTAAAACTTTATAATCAGAGGTTCGACTCCTCT
24836	Yes	AATTGCATAAATCTTAAAACTTTATAATCAGAGGTTCGACTCCTCTT
24837	Yes	AATTGCATAAATCTTAAAACTTTATAATCAGAGGTTCGACTCCTCTTC
24838	Yes	AATTGCATAAATCTTAAAACTTTATAATCAGAGGTTCGACTCCTCTTCT
24839	Yes	AATTGCATAAATCTTAAAACTTTATAATCAGAGGTTCGACTCCTCTTCTT
24840	No	AATTGCATAAGACTTA
24841	Yes	AATTGCATAAGACTTAA
24842	Yes	AATTGCATAAGACTTAAA
24843	Yes	AATTGCATAAGACTTAAAA
24844	Yes	AATTGCATAAGACTTAAAAC
24845	Yes	AATTGCATAAGACTTAAAACT
24846	Yes	AATTGCATAAGACTTAAAACTT
24847	Yes	AATTGCATAAGACTTAAAACTTT
24848	Yes	AATTGCATAAGACTTAAAACTTTA
24849	Yes	AATTGCATAAGACTTAAAACTTTAT
24850	Yes	AATTGCATAAGACTTAAAACTTTATA
24851	Yes	AATTGCATAAGACTTAAAACTTTATAA
24852	Yes	AATTGCATAAGACTTAAAACTTTATAAT
24853	Yes	AATTGCATAAGACTTAAAACTTTATAATC
24854	Yes	AATTGCATAAGACTTAAAACTTTATAATCA
24855	Yes	AATTGCATAAGACTTAAAACTTTATAATCAG
24856	Yes	AATTGCATAAGACTTAAAACTTTATAATCAGA
24857	Yes	AATTGCATAAGACTTAAAACTTTATAATCAGAG

24858	Yes	AATTGCATAAGACTTAAAACTTTATAATCAGAGG
24859	Yes	AATTGCATAAGACTTAAAACTTTATAATCAGAGGT
24860	Yes	AATTGCATAAGACTTAAAACTTTATAATCAGAGGTT
24861	Yes	AATTGCATAAGACTTAAAACTTTATAATCAGAGGTTC
24862	Yes	AATTGCATAAGACTTAAAACTTTATAATCAGAGGTTCA
24863	Yes	AATTGCATAAGACTTAAAACTTTATAATCAGAGGTTCAA
24864	Yes	AATTGCATAAGACTTAAAACTTTATAATCAGAGGTTCAAC
24865	Yes	AATTGCATAAGACTTAAAACTTTATAATCAGAGGTTCAACT
24866	Yes	AATTGCATAAGACTTAAAACTTTATAATCAGAGGTTCAACTC
24867	Yes	AATTGCATAAGACTTAAAACTTTATAATCAGAGGTTCAACTCC
24868	Yes	AATTGCATAAGACTTAAAACTTTATAATCAGAGGTTCAACTCCT
24869	Yes	AATTGCATAAGACTTAAAACTTTATAATCAGAGGTTCAACTCCTC
24870	Yes	AATTGCATAAGACTTAAAACTTTATAATCAGAGGTTCAACTCCTCT
24871	Yes	AATTGCATAAGACTTAAAACTTTATAATCAGAGGTTCAACTCCTCTC
24872	Yes	AATTGCATAAGACTTAAAACTTTATAATCAGAGGTTCAACTCCTCTCA
24873	Yes	AATTGCATAAGACTTAAAACTTTATAATCAGAGGTTCAACTCCTCTCAT
24874	Yes	AATTGCATAAGACTTAAAACTTTATAATCAGAGGTTCAACTCCTCTCATT
24875	Yes	AATTGCGTAAAATTTA
24876	Yes	AATTGCGTAAAATTTAC
24877	Yes	AATTGCGTAAAATTTACA
24878	Yes	AATTGCGTAAAATTTACAA
24879	Yes	AATTGCGTAAAATTTACAAC
24880	Yes	AATTGCGTAAAATTTACAACT
24881	Yes	AATTGCGTAAAATTTACAACTT
24882	Yes	AATTGCGTAAAATTTACAACTTT
24883	Yes	AATTGCGTAAAATTTACAACTTTA
24884	Yes	AATTGCGTAAAATTTACAACTTTAT
24885	Yes	AATTGCGTAAAATTTACAACTTTATG
24886	Yes	AATTGCGTAAAATTTACAACTTTATGG
24887	Yes	AATTGCGTAAAATTTACAACTTTATGGG
24888	Yes	AATTGCGTAAAATTTACAACTTTATGGGC
24889	Yes	AATTGCGTAAAATTTACAACTTTATGGGCA
24890	Yes	AATTGCGTAAAATTTACAACTTTATGGGCAG
24891	Yes	AATTGCGTAAAATTTACAACTTTATGGGCAGA
24892	Yes	AATTGCGTAAAATTTACAACTTTATGGGCAGAG
24893	Yes	AATTGCGTAAAATTTACAACTTTATGGGCAGAGG
24894	Yes	AATTGCGTAAAATTTACAACTTTATGGGCAGAGGT
24895	Yes	AATTGCGTAAAATTTACAACTTTATGGGCAGAGGTT
24896	Yes	AATTGCGTAAAATTTACAACTTTATGGGCAGAGGTTC
24897	Yes	AATTGCGTAAAATTTACAACTTTATGGGCAGAGGTTCA
24898	Yes	AATTGCGTAAAATTTACAACTTTATGGGCAGAGGTTCAA
24899	Yes	AATTGCGTAAAATTTACAACTTTATGGGCAGAGGTTCAAT

24900	Yes	AATTGCGTAAAATTTACAACTTTATGGGCAGAGGTTCAATT
24901	Yes	AATTGCGTAAAATTTACAACTTTATGGGCAGAGGTTCAATTC
24902	Yes	AATTGCGTAAAATTTACAACTTTATGGGCAGAGGTTCAATTCC
24903	Yes	AATTGCGTAAAATTTACAACTTTATGGGCAGAGGTTCAATTCCT
24904	Yes	AATTGCGTAAAATTTACAACTTTATGGGCAGAGGTTCAATTCCTC
24905	Yes	AATTGCGTAAAATTTACAACTTTATGGGCAGAGGTTCAATTCCTCT
24906	Yes	AATTGCGTAAAATTTACAACTTTATGGGCAGAGGTTCAATTCCTCTT
24907	Yes	AATTGCGTAAAATTTACAACTTTATGGGCAGAGGTTCAATTCCTCTTC
24908	Yes	AATTGCGTAAAATTTACAACTTTATGGGCAGAGGTTCAATTCCTCTTCT
24909	Yes	AATTGCGTAAAATTTACAACTTTATGGGCAGAGGTTCAATTCCTCTTCTT
24910	No	AATTGCTCAGTTGGGA
24911	No	AATTGCTCAGTTGGGAG
24912	No	AATTGCTCAGTTGGGAGA
24913	No	AATTGCTCAGTTGGGAGAG
24914	Yes	AATTGCTCAGTTGGGAGAGC
24915	Yes	AATTGCTCAGTTGGGAGAGCG
24916	Yes	AATTGCTCAGTTGGGAGAGCGT
24917	Yes	AATTGCTCAGTTGGGAGAGCGTT
24918	Yes	AATTGCTCAGTTGGGAGAGCGTTA
24919	Yes	AATTGCTCAGTTGGGAGAGCGTTAG
24920	Yes	AATTGCTCAGTTGGGAGAGCGTTAGA
24921	Yes	AATTGCTCAGTTGGGAGAGCGTTAGAC
24922	Yes	AATTGCTCAGTTGGGAGAGCGTTAGACT
24923	Yes	AATTGCTCAGTTGGGAGAGCGTTAGACTG
24924	Yes	AATTGCTCAGTTGGGAGAGCGTTAGACTGA
24925	Yes	AATTGCTCAGTTGGGAGAGCGTTAGACTGAA
24926	Yes	AATTGCTCAGTTGGGAGAGCGTTAGACTGAAG
24927	Yes	AATTGCTCAGTTGGGAGAGCGTTAGACTGAAGA
24928	Yes	AATTGCTCAGTTGGGAGAGCGTTAGACTGAAGAT
24929	Yes	AATTGCTCAGTTGGGAGAGCGTTAGACTGAAGATC
24930	Yes	AATTGCTCAGTTGGGAGAGCGTTAGACTGAAGATCT
24931	Yes	AATTGCTCAGTTGGGAGAGCGTTAGACTGAAGATCTA
24932	Yes	AATTGCTCAGTTGGGAGAGCGTTAGACTGAAGATCTAA
24933	Yes	AATTGCTCAGTTGGGAGAGCGTTAGACTGAAGATCTAAA
24934	Yes	AATTGCTCAGTTGGGAGAGCGTTAGACTGAAGATCTAAAG
24935	Yes	AATTGCTCAGTTGGGAGAGCGTTAGACTGAAGATCTAAAGG
24936	Yes	AATTGCTCAGTTGGGAGAGCGTTAGACTGAAGATCTAAAGGT
24937	Yes	AATTGCTCAGTTGGGAGAGCGTTAGACTGAAGATCTAAAGGTC
24938	Yes	AATTGCTCAGTTGGGAGAGCGTTAGACTGAAGATCTAAAGGTCC
24939	Yes	AATTGCTCAGTTGGGAGAGCGTTAGACTGAAGATCTAAAGGTCCC
24940	Yes	AATTGCTCAGTTGGGAGAGCGTTAGACTGAAGATCTAAAGGTCCCT
24941	Yes	AATTGCTCAGTTGGGAGAGCGTTAGACTGAAGATCTAAAGGTCCCTG

24942	Yes	AATTGCTCAGTTGGGAGAGCGTTAGACTGAAGATCTAAAGGTCCCTGG
24943	Yes	AATTGCTCAGTTGGGAGAGCGTTAGACTGAAGATCTAAAGGTCCCTGGT
24944	Yes	AATTGCTCAGTTGGGAGAGCGTTAGACTGAAGATCTAAAGGTCCCTGGTT
24945	Yes	AATTGGCTCAAGCGGT
24946	Yes	AATTGGCTCAAGCGGTA
24947	Yes	AATTGGCTCAAGCGGTAG
24948	Yes	AATTGGCTCAAGCGGTAGA
24949	Yes	AATTGGCTCAAGCGGTAGAG
24950	Yes	AATTGGCTCAAGCGGTAGAGC
24951	Yes	AATTGGCTCAAGCGGTAGAGCG
24952	Yes	AATTGGCTCAAGCGGTAGAGCGC
24953	Yes	AATTGGCTCAAGCGGTAGAGCGCT
24954	Yes	AATTGGCTCAAGCGGTAGAGCGCTT
24955	Yes	AATTGGCTCAAGCGGTAGAGCGCTTG
24956	Yes	AATTGGCTCAAGCGGTAGAGCGCTTGC
24957	Yes	AATTGGCTCAAGCGGTAGAGCGCTTGCT
24958	Yes	AATTGGCTCAAGCGGTAGAGCGCTTGCTT
24959	Yes	AATTGGCTCAAGCGGTAGAGCGCTTGCTTA
24960	Yes	AATTGGCTCAAGCGGTAGAGCGCTTGCTTAG
24961	Yes	AATTGGCTCAAGCGGTAGAGCGCTTGCTTAGC
24962	Yes	AATTGGCTCAAGCGGTAGAGCGCTTGCTTAGCA
24963	Yes	AATTGGCTCAAGCGGTAGAGCGCTTGCTTAGCAT
24964	Yes	AATTGGCTCAAGCGGTAGAGCGCTTGCTTAGCATG
24965	Yes	AATTGGCTCAAGCGGTAGAGCGCTTGCTTAGCATGC
24966	Yes	AATTGGCTCAAGCGGTAGAGCGCTTGCTTAGCATGCA
24967	Yes	AATTGGCTCAAGCGGTAGAGCGCTTGCTTAGCATGCAA
24968	Yes	AATTGGCTCAAGCGGTAGAGCGCTTGCTTAGCATGCAAG
24969	Yes	AATTGGCTCAAGCGGTAGAGCGCTTGCTTAGCATGCAAGA
24970	Yes	AATTGGCTCAAGCGGTAGAGCGCTTGCTTAGCATGCAAGAG
24971	Yes	AATTGGCTCAAGCGGTAGAGCGCTTGCTTAGCATGCAAGAGG
24972	Yes	AATTGGCTCAAGCGGTAGAGCGCTTGCTTAGCATGCAAGAGGT
24973	Yes	AATTGGCTCAAGCGGTAGAGCGCTTGCTTAGCATGCAAGAGGTA
24974	Yes	AATTGGCTCAAGCGGTAGAGCGCTTGCTTAGCATGCAAGAGGTAG
24975	Yes	AATTGGCTCAAGCGGTAGAGCGCTTGCTTAGCATGCAAGAGGTAGC
24976	Yes	AATTGGCTCAAGCGGTAGAGCGCTTGCTTAGCATGCAAGAGGTAGCA
24977	Yes	AATTGGCTCAAGCGGTAGAGCGCTTGCTTAGCATGCAAGAGGTAGCAG
24978	Yes	AATTGGCTCAAGCGGTAGAGCGCTTGCTTAGCATGCAAGAGGTAGCAGG
24979	Yes	AATTGGCTCAAGCGGTAGAGCGCTTGCTTAGCATGCAAGAGGTAGCAGGA
24980	No	AATTGGGAGAGTGTTA
24981	Yes	AATTGGGAGAGTGTTAG
24982	Yes	AATTGGGAGAGTGTTAGA
24983	Yes	AATTGGGAGAGTGTTAGAC

24984	Yes	AATTGGGAGAGTGTTAGACT
24985	Yes	AATTGGGAGAGTGTTAGACTG
24986	Yes	AATTGGGAGAGTGTTAGACTGA
24987	Yes	AATTGGGAGAGTGTTAGACTGAA
24988	Yes	AATTGGGAGAGTGTTAGACTGAAG
24989	Yes	AATTGGGAGAGTGTTAGACTGAAGA
24990	Yes	AATTGGGAGAGTGTTAGACTGAAGAT
24991	Yes	AATTGGGAGAGTGTTAGACTGAAGATC
24992	Yes	AATTGGGAGAGTGTTAGACTGAAGATCT
24993	Yes	AATTGGGAGAGTGTTAGACTGAAGATCTT
24994	Yes	AATTGGGAGAGTGTTAGACTGAAGATCTTC
24995	Yes	AATTGGGAGAGTGTTAGACTGAAGATCTTCT
24996	Yes	AATTGGGAGAGTGTTAGACTGAAGATCTTCTG
24997	Yes	AATTGGGAGAGTGTTAGACTGAAGATCTTCTGC
24998	Yes	AATTGGGAGAGTGTTAGACTGAAGATCTTCTGCA
24999	Yes	AATTGGGAGAGTGTTAGACTGAAGATCTTCTGCAG
25000	Yes	AATTGGGAGAGTGTTAGACTGAAGATCTTCTGCAGG
25001	Yes	AATTGGGAGAGTGTTAGACTGAAGATCTTCTGCAGGT
25002	Yes	AATTGGGAGAGTGTTAGACTGAAGATCTTCTGCAGGTC
25003	Yes	AATTGGGAGAGTGTTAGACTGAAGATCTTCTGCAGGTCT
25004	Yes	AATTGGGAGAGTGTTAGACTGAAGATCTTCTGCAGGTCTC
25005	Yes	AATTGGGAGAGTGTTAGACTGAAGATCTTCTGCAGGTCTCT
25006	Yes	AATTGGGAGAGTGTTAGACTGAAGATCTTCTGCAGGTCTCTG
25007	Yes	AATTGGGAGAGTGTTAGACTGAAGATCTTCTGCAGGTCTCTGG
25008	Yes	AATTGGGAGAGTGTTAGACTGAAGATCTTCTGCAGGTCTCTGGT
25009	Yes	AATTGGGAGAGTGTTAGACTGAAGATCTTCTGCAGGTCTCTGGTT
25010	Yes	AATTGGGAGAGTGTTAGACTGAAGATCTTCTGCAGGTCTCTGGTTC
25011	Yes	AATTGGGAGAGTGTTAGACTGAAGATCTTCTGCAGGTCTCTGGTTCA
25012	Yes	AATTGGGAGAGTGTTAGACTGAAGATCTTCTGCAGGTCTCTGGTTCAA
25013	Yes	AATTGGGAGAGTGTTAGACTGAAGATCTTCTGCAGGTCTCTGGTTCAAT
25014	Yes	AATTGGGAGAGTGTTAGACTGAAGATCTTCTGCAGGTCTCTGGTTCAATT
25015	Yes	AATTGGTTAGAGCGTG
25016	Yes	AATTGGTTAGAGCGTGG
25017	Yes	AATTGGTTAGAGCGTGGT
25018	Yes	AATTGGTTAGAGCGTGGTG
25019	Yes	AATTGGTTAGAGCGTGGTGC
25020	Yes	AATTGGTTAGAGCGTGGTGCT
25021	Yes	AATTGGTTAGAGCGTGGTGCTA
25022	Yes	AATTGGTTAGAGCGTGGTGCTAA
25023	Yes	AATTGGTTAGAGCGTGGTGCTAAT
25024	Yes	AATTGGTTAGAGCGTGGTGCTAATA
25025	Yes	AATTGGTTAGAGCGTGGTGCTAATAA

25026	Yes	AATTGGTTAGAGCGTGGTGCTAATAAT
25027	Yes	AATTGGTTAGAGCGTGGTGCTAATAATG
25028	Yes	AATTGGTTAGAGCGTGGTGCTAATAATGT
25029	Yes	AATTGGTTAGAGCGTGGTGCTAATAATGTC
25030	Yes	AATTGGTTAGAGCGTGGTGCTAATAATGTCA
25031	Yes	AATTGGTTAGAGCGTGGTGCTAATAATGTCAA
25032	Yes	AATTGGTTAGAGCGTGGTGCTAATAATGTCAAG
25033	Yes	AATTGGTTAGAGCGTGGTGCTAATAATGTCAAGG
25034	Yes	AATTGGTTAGAGCGTGGTGCTAATAATGTCAAGGT
25035	Yes	AATTGGTTAGAGCGTGGTGCTAATAATGTCAAGGTT
25036	Yes	AATTGGTTAGAGCGTGGTGCTAATAATGTCAAGGTTG
25037	Yes	AATTGGTTAGAGCGTGGTGCTAATAATGTCAAGGTTGC
25038	Yes	AATTGGTTAGAGCGTGGTGCTAATAATGTCAAGGTTGCG
25039	Yes	AATTGGTTAGAGCGTGGTGCTAATAATGTCAAGGTTGCGG
25040	Yes	AATTGGTTAGAGCGTGGTGCTAATAATGTCAAGGTTGCGGG
25041	Yes	AATTGGTTAGAGCGTGGTGCTAATAATGTCAAGGTTGCGGGT
25042	Yes	AATTGGTTAGAGCGTGGTGCTAATAATGTCAAGGTTGCGGGTT
25043	Yes	AATTGGTTAGAGCGTGGTGCTAATAATGTCAAGGTTGCGGGTTG
25044	Yes	AATTGGTTAGAGCGTGGTGCTAATAATGTCAAGGTTGCGGGTTGG
25045	Yes	AATTGGTTAGAGCGTGGTGCTAATAATGTCAAGGTTGCGGGTTGGA
25046	Yes	AATTGGTTAGAGCGTGGTGCTAATAATGTCAAGGTTGCGGGTTGGAT
25047	Yes	AATTGGTTAGAGCGTGGTGCTAATAATGTCAAGGTTGCGGGTTGGATC
25048	Yes	AATTGGTTAGAGCGTGGTGCTAATAATGTCAAGGTTGCGGGTTGGATCC
25049	Yes	AATTGGTTAGAGCGTGGTGCTAATAATGTCAAGGTTGCGGGTTGGATCCC
25050	No	AATTGGTTAGCGCGTT
25051	No	AATTGGTTAGCGCGTTC
25052	No	AATTGGTTAGCGCGTTCG
25053	No	AATTGGTTAGCGCGTTCGG
25054	No	AATTGGTTAGCGCGTTCGGC
25055	Yes	AATTGGTTAGCGCGTTCGGCC
25056	Yes	AATTGGTTAGCGCGTTCGGCCG
25057	Yes	AATTGGTTAGCGCGTTCGGCCGT
25058	Yes	AATTGGTTAGCGCGTTCGGCCGTT
25059	Yes	AATTGGTTAGCGCGTTCGGCCGTTA
25060	Yes	AATTGGTTAGCGCGTTCGGCCGTTAA
25061	Yes	AATTGGTTAGCGCGTTCGGCCGTTAAC
25062	Yes	AATTGGTTAGCGCGTTCGGCCGTTAACC
25063	Yes	AATTGGTTAGCGCGTTCGGCCGTTAACCG
25064	Yes	AATTGGTTAGCGCGTTCGGCCGTTAACCGG
25065	Yes	AATTGGTTAGCGCGTTCGGCCGTTAACCGGA
25066	Yes	AATTGGTTAGCGCGTTCGGCCGTTAACCGGAA
25067	Yes	AATTGGTTAGCGCGTTCGGCCGTTAACCGGAAA

25068	Yes	AATTGGTTAGCGCGTTCGGCCGTTAACCGGAAAG
25069	Yes	AATTGGTTAGCGCGTTCGGCCGTTAACCGGAAAGT
25070	Yes	AATTGGTTAGCGCGTTCGGCCGTTAACCGGAAAGTT
25071	Yes	AATTGGTTAGCGCGTTCGGCCGTTAACCGGAAAGTTG
25072	Yes	AATTGGTTAGCGCGTTCGGCCGTTAACCGGAAAGTTGG
25073	Yes	AATTGGTTAGCGCGTTCGGCCGTTAACCGGAAAGTTGGT
25074	Yes	AATTGGTTAGCGCGTTCGGCCGTTAACCGGAAAGTTGGTG
25075	Yes	AATTGGTTAGCGCGTTCGGCCGTTAACCGGAAAGTTGGTGG
25076	Yes	AATTGGTTAGCGCGTTCGGCCGTTAACCGGAAAGTTGGTGGT
25077	Yes	AATTGGTTAGCGCGTTCGGCCGTTAACCGGAAAGTTGGTGGTT
25078	Yes	AATTGGTTAGCGCGTTCGGCCGTTAACCGGAAAGTTGGTGGTTC
25079	Yes	AATTGGTTAGCGCGTTCGGCCGTTAACCGGAAAGTTGGTGGTTCG
25080	Yes	AATTGGTTAGCGCGTTCGGCCGTTAACCGGAAAGTTGGTGGTTCGA
25081	Yes	AATTGGTTAGCGCGTTCGGCCGTTAACCGGAAAGTTGGTGGTTCGAG
25082	Yes	AATTGGTTAGCGCGTTCGGCCGTTAACCGGAAAGTTGGTGGTTCGAGC
25083	Yes	AATTGGTTAGCGCGTTCGGCCGTTAACCGGAAAGTTGGTGGTTCGAGCC
25084	Yes	AATTGGTTAGCGCGTTCGGCCGTTAACCGGAAAGTTGGTGGTTCGAGCCT
25085	No	AATTGGTTAGCGCGTTCGGCT
25086	No	AATTGGTTAGCGCGTTCGGCTG
25087	No	AATTGGTTAGCGCGTTCGGCTGT
25088	No	AATTGGTTAGCGCGTTCGGCTGTT
25089	No	AATTGGTTAGCGCGTTCGGCTGTTA
25090	No	AATTGGTTAGCGCGTTCGGCTGTTAA
25091	No	AATTGGTTAGCGCGTTCGGCTGTTAAC
25092	No	AATTGGTTAGCGCGTTCGGCTGTTAACC
25093	No	AATTGGTTAGCGCGTTCGGCTGTTAACCG
25094	Yes	AATTGGTTAGCGCGTTCGGCTGTTAACCGG
25095	Yes	AATTGGTTAGCGCGTTCGGCTGTTAACCGGA
25096	Yes	AATTGGTTAGCGCGTTCGGCTGTTAACCGGAA
25097	Yes	AATTGGTTAGCGCGTTCGGCTGTTAACCGGAAA
25098	Yes	AATTGGTTAGCGCGTTCGGCTGTTAACCGGAAAG
25099	Yes	AATTGGTTAGCGCGTTCGGCTGTTAACCGGAAAGT
25100	Yes	AATTGGTTAGCGCGTTCGGCTGTTAACCGGAAAGTT
25101	Yes	AATTGGTTAGCGCGTTCGGCTGTTAACCGGAAAGTTG
25102	Yes	AATTGGTTAGCGCGTTCGGCTGTTAACCGGAAAGTTGG
25103	Yes	AATTGGTTAGCGCGTTCGGCTGTTAACCGGAAAGTTGGT
25104	Yes	AATTGGTTAGCGCGTTCGGCTGTTAACCGGAAAGTTGGTG
25105	Yes	AATTGGTTAGCGCGTTCGGCTGTTAACCGGAAAGTTGGTGG
25106	Yes	AATTGGTTAGCGCGTTCGGCTGTTAACCGGAAAGTTGGTGGT
25107	Yes	AATTGGTTAGCGCGTTCGGCTGTTAACCGGAAAGTTGGTGGTT
25108	Yes	AATTGGTTAGCGCGTTCGGCTGTTAACCGGAAAGTTGGTGGTTC
25109	Yes	AATTGGTTAGCGCGTTCGGCTGTTAACCGGAAAGTTGGTGGTTCG

25110	Yes	AATTGGTTAGCGCGTTCGGCTGTTAACCGGAAAGTTGGTGGTTCGA
25111	Yes	AATTGGTTAGCGCGTTCGGCTGTTAACCGGAAAGTTGGTGGTTCGAG
25112	Yes	AATTGGTTAGCGCGTTCGGCTGTTAACCGGAAAGTTGGTGGTTCGAGC
25113	Yes	AATTGGTTAGCGCGTTCGGCTGTTAACCGGAAAGTTGGTGGTTCGAGCC
25114	Yes	AATTGGTTAGCGCGTTCGGCTGTTAACCGGAAAGTTGGTGGTTCGAGCCT
25115	No	AATTTACAACTTTATG
25116	No	AATTTACAACTTTATGG
25117	No	AATTTACAACTTTATGGG
25118	Yes	AATTTACAACTTTATGGGC
25119	Yes	AATTTACAACTTTATGGGCA
25120	Yes	AATTTACAACTTTATGGGCAG
25121	Yes	AATTTACAACTTTATGGGCAGA
25122	Yes	AATTTACAACTTTATGGGCAGAG
25123	Yes	AATTTACAACTTTATGGGCAGAGG
25124	Yes	AATTTACAACTTTATGGGCAGAGGT
25125	Yes	AATTTACAACTTTATGGGCAGAGGTT
25126	Yes	AATTTACAACTTTATGGGCAGAGGTTC
25127	Yes	AATTTACAACTTTATGGGCAGAGGTTCA
25128	Yes	AATTTACAACTTTATGGGCAGAGGTTCAA
25129	Yes	AATTTACAACTTTATGGGCAGAGGTTCAAT
25130	Yes	AATTTACAACTTTATGGGCAGAGGTTCAATT
25131	Yes	AATTTACAACTTTATGGGCAGAGGTTCAATTC
25132	Yes	AATTTACAACTTTATGGGCAGAGGTTCAATTCC
25133	Yes	AATTTACAACTTTATGGGCAGAGGTTCAATTCCT
25134	Yes	AATTTACAACTTTATGGGCAGAGGTTCAATTCCTC
25135	Yes	AATTTACAACTTTATGGGCAGAGGTTCAATTCCTCT
25136	Yes	AATTTACAACTTTATGGGCAGAGGTTCAATTCCTCTT
25137	Yes	AATTTACAACTTTATGGGCAGAGGTTCAATTCCTCTTC
25138	Yes	AATTTACAACTTTATGGGCAGAGGTTCAATTCCTCTTCT
25139	Yes	AATTTACAACTTTATGGGCAGAGGTTCAATTCCTCTTCTT
25140	Yes	AATTTACAACTTTATGGGCAGAGGTTCAATTCCTCTTCTTA
25141	Yes	AATTTACAACTTTATGGGCAGAGGTTCAATTCCTCTTCTTAA
25142	Yes	AATTTACAACTTTATGGGCAGAGGTTCAATTCCTCTTCTTAAC
25143	Yes	AATTTACAACTTTATGGGCAGAGGTTCAATTCCTCTTCTTAACA
25144	Yes	AATTTACAACTTTATGGGCAGAGGTTCAATTCCTCTTCTTAACAC
25145	Yes	AATTTACAACTTTATGGGCAGAGGTTCAATTCCTCTTCTTAACACC
25146	Yes	AATTTACAACTTTATGGGCAGAGGTTCAATTCCTCTTCTTAACACCA
25147	No	AATTTAGATGTCCTGA
25148	Yes	AATTTAGATGTCCTGAG
25149	Yes	AATTTAGATGTCCTGAGT
25150	Yes	AATTTAGATGTCCTGAGTT
25151	Yes	AATTTAGATGTCCTGAGTTC

25152	Yes	AATTTAGATGTCCTGAGTTCA
25153	Yes	AATTTAGATGTCCTGAGTTCAA
25154	Yes	AATTTAGATGTCCTGAGTTCAAA
25155	Yes	AATTTAGATGTCCTGAGTTCAAAT
25156	Yes	AATTTAGATGTCCTGAGTTCAAATC
25157	Yes	AATTTAGATGTCCTGAGTTCAAATCC
25158	Yes	AATTTAGATGTCCTGAGTTCAAATCCC
25159	Yes	AATTTAGATGTCCTGAGTTCAAATCCCA
25160	Yes	AATTTAGATGTCCTGAGTTCAAATCCCAG
25161	Yes	AATTTAGATGTCCTGAGTTCAAATCCCAGC
25162	Yes	AATTTAGATGTCCTGAGTTCAAATCCCAGCT
25163	Yes	AATTTAGATGTCCTGAGTTCAAATCCCAGCTG
25164	Yes	AATTTAGATGTCCTGAGTTCAAATCCCAGCTGT
25165	Yes	AATTTAGATGTCCTGAGTTCAAATCCCAGCTGTA
25166	Yes	AATTTAGATGTCCTGAGTTCAAATCCCAGCTGTAT
25167	Yes	AATTTAGATGTCCTGAGTTCAAATCCCAGCTGTATG
25168	Yes	AATTTAGATGTCCTGAGTTCAAATCCCAGCTGTATGC
25169	Yes	AATTTAGATGTCCTGAGTTCAAATCCCAGCTGTATGCC
25170	Yes	AATTTAGATGTCCTGAGTTCAAATCCCAGCTGTATGCCC
25171	Yes	AATTTAGATGTCCTGAGTTCAAATCCCAGCTGTATGCCCA
25172	Yes	AATTTAGCGCTTTCAC
25173	Yes	AATTTAGCGCTTTCACC
25174	Yes	AATTTAGCGCTTTCACCG
25175	Yes	AATTTAGCGCTTTCACCGC
25176	Yes	AATTTAGCGCTTTCACCGCC
25177	Yes	AATTTAGCGCTTTCACCGCCG
25178	Yes	AATTTAGCGCTTTCACCGCCGC
25179	Yes	AATTTAGCGCTTTCACCGCCGCA
25180	Yes	AATTTAGCGCTTTCACCGCCGCAG
25181	Yes	AATTTAGCGCTTTCACCGCCGCAGC
25182	Yes	AATTTAGCGCTTTCACCGCCGCAGCT
25183	Yes	AATTTAGCGCTTTCACCGCCGCAGCTC
25184	Yes	AATTTAGCGCTTTCACCGCCGCAGCTCG
25185	Yes	AATTTAGCGCTTTCACCGCCGCAGCTCGG
25186	Yes	AATTTAGCGCTTTCACCGCCGCAGCTCGGG
25187	Yes	AATTTAGCGCTTTCACCGCCGCAGCTCGGGT
25188	Yes	AATTTAGCGCTTTCACCGCCGCAGCTCGGGTT
25189	Yes	AATTTAGCGCTTTCACCGCCGCAGCTCGGGTTG
25190	Yes	AATTTAGCGCTTTCACCGCCGCAGCTCGGGTTGG
25191	Yes	AATTTAGCGCTTTCACCGCCGCAGCTCGGGTTGGA
25192	Yes	AATTTAGCGCTTTCACCGCCGCAGCTCGGGTTGGAT
25193	Yes	AATTTAGCGCTTTCACCGCCGCAGCTCGGGTTGGATT

25194	Yes	AATTTAGCGCTTTCACCGCCGCAGCTCGGGTTGGATTA
25195	Yes	AATTTAGCGCTTTCACCGCCGCAGCTCGGGTTGGATTAC
25196	Yes	AATTTAGCGCTTTCACCGCCGCAGCTCGGGTTGGATTACC
25197	Yes	AATTTAGCGCTTTCACCGCCGCAGCTCGGGTTGGATTACCA
25198	Yes	AATTTAGCGCTTTCACCGCCGCAGCTCGGGTTGGATTACCAG
25199	Yes	AATTTAGCGCTTTCACCGCCGCAGCTCGGGTTGGATTACCAGT
25200	Yes	AATTTAGCGCTTTCACCGCCGCAGCTCGGGTTGGATTACCAGTC
25201	Yes	AATTTAGCGCTTTCACCGCCGCAGCTCGGGTTGGATTACCAGTCA
25202	Yes	AATTTAGCGCTTTCACCGCCGCAGCTCGGGTTGGATTACCAGTCAG
25203	Yes	AATTTAGCGCTTTCACCGCCGCAGCTCGGGTTGGATTACCAGTCAGG
25204	Yes	AATTTAGCGCTTTCACCGCCGCAGCTCGGGTTGGATTACCAGTCAGGG
25205	Yes	AATTTAGCGCTTTCACCGCCGCAGCTCGGGTTGGATTACCAGTCAGGGA
25206	Yes	AATTTAGCGCTTTCACCGCCGCAGCTCGGGTTGGATTACCAGTCAGGGAA
25207	No	AATTTAGGCTCCAGTC
25208	No	AATTTAGGCTCCAGTCT
25209	Yes	AATTTAGGCTCCAGTCTC
25210	Yes	AATTTAGGCTCCAGTCTCT
25211	Yes	AATTTAGGCTCCAGTCTCTT
25212	Yes	AATTTAGGCTCCAGTCTCTTT
25213	Yes	AATTTAGGCTCCAGTCTCTTTG
25214	Yes	AATTTAGGCTCCAGTCTCTTTGG
25215	Yes	AATTTAGGCTCCAGTCTCTTTGGG
25216	Yes	AATTTAGGCTCCAGTCTCTTTGGGG
25217	Yes	AATTTAGGCTCCAGTCTCTTTGGGGA
25218	Yes	AATTTAGGCTCCAGTCTCTTTGGGGAC
25219	Yes	AATTTAGGCTCCAGTCTCTTTGGGGACG
25220	Yes	AATTTAGGCTCCAGTCTCTTTGGGGACGT
25221	Yes	AATTTAGGCTCCAGTCTCTTTGGGGACGTG
25222	Yes	AATTTAGGCTCCAGTCTCTTTGGGGACGTGG
25223	Yes	AATTTAGGCTCCAGTCTCTTTGGGGACGTGGG
25224	Yes	AATTTAGGCTCCAGTCTCTTTGGGGACGTGGGT
25225	Yes	AATTTAGGCTCCAGTCTCTTTGGGGACGTGGGTT
25226	Yes	AATTTAGGCTCCAGTCTCTTTGGGGACGTGGGTTT
25227	Yes	AATTTAGGCTCCAGTCTCTTTGGGGACGTGGGTTTA
25228	Yes	AATTTAGGCTCCAGTCTCTTTGGGGACGTGGGTTTAA
25229	Yes	AATTTAGGCTCCAGTCTCTTTGGGGACGTGGGTTTAAA
25230	Yes	AATTTAGGCTCCAGTCTCTTTGGGGACGTGGGTTTAAAT
25231	Yes	AATTTAGGCTCCAGTCTCTTTGGGGACGTGGGTTTAAATC
25232	Yes	AATTTAGGCTCCAGTCTCTTTGGGGACGTGGGTTTAAATCC
25233	Yes	AATTTAGGCTCCAGTCTCTTTGGGGACGTGGGTTTAAATCCC
25234	Yes	AATTTAGGCTCCAGTCTCTTTGGGGACGTGGGTTTAAATCCCA
25235	Yes	AATTTAGGCTCCAGTCTCTTTGGGGACGTGGGTTTAAATCCCAC

25236	Yes	AATTTAGGCTCCAGTCTCTTTGGGGACGTGGGTTTAAATCCCACT
25237	Yes	AATTTAGGCTCCAGTCTCTTTGGGGACGTGGGTTTAAATCCCACTG
25238	Yes	AATTTAGGCTCCAGTCTCTTTGGGGACGTGGGTTTAAATCCCACTGC
25239	Yes	AATTTAGGCTCCAGTCTCTTTGGGGACGTGGGTTTAAATCCCACTGCT
25240	Yes	AATTTAGGCTCCAGTCTCTTTGGGGACGTGGGTTTAAATCCCACTGCTG
25241	Yes	AATTTAGGCTCCAGTCTCTTTGGGGACGTGGGTTTAAATCCCACTGCTGC
25242	No	AATTTAGGTTAAATAC
25243	Yes	AATTTAGGTTAAATACA
25244	Yes	AATTTAGGTTAAATACAG
25245	Yes	AATTTAGGTTAAATACAGA
25246	Yes	AATTTAGGTTAAATACAGAC
25247	Yes	AATTTAGGTTAAATACAGACC
25248	Yes	AATTTAGGTTAAATACAGACCA
25249	Yes	AATTTAGGTTAAATACAGACCAA
25250	Yes	AATTTAGGTTAAATACAGACCAAG
25251	Yes	AATTTAGGTTAAATACAGACCAAGA
25252	Yes	AATTTAGGTTAAATACAGACCAAGAG
25253	Yes	AATTTAGGTTAAATACAGACCAAGAGC
25254	Yes	AATTTAGGTTAAATACAGACCAAGAGCC
25255	Yes	AATTTAGGTTAAATACAGACCAAGAGCCT
25256	Yes	AATTTAGGTTAAATACAGACCAAGAGCCTT
25257	Yes	AATTTAGGTTAAATACAGACCAAGAGCCTTC
25258	Yes	AATTTAGGTTAAATACAGACCAAGAGCCTTCA
25259	Yes	AATTTAGGTTAAATACAGACCAAGAGCCTTCAA
25260	Yes	AATTTAGGTTAAATACAGACCAAGAGCCTTCAAA
25261	Yes	AATTTAGGTTAAATACAGACCAAGAGCCTTCAAAG
25262	Yes	AATTTAGGTTAAATACAGACCAAGAGCCTTCAAAGC
25263	Yes	AATTTAGGTTAAATACAGACCAAGAGCCTTCAAAGCC
25264	Yes	AATTTAGGTTAAATACAGACCAAGAGCCTTCAAAGCCC
25265	Yes	AATTTAGGTTAAATACAGACCAAGAGCCTTCAAAGCCCT
25266	Yes	AATTTAGGTTAAATACAGACCAAGAGCCTTCAAAGCCCTC
25267	Yes	AATTTAGGTTAAATACAGACCAAGAGCCTTCAAAGCCCTCA
25268	Yes	AATTTAGGTTAAATACAGACCAAGAGCCTTCAAAGCCCTCAG
25269	Yes	AATTTAGGTTAAATACAGACCAAGAGCCTTCAAAGCCCTCAGT
25270	Yes	AATTTAGGTTAAATACAGACCAAGAGCCTTCAAAGCCCTCAGTA
25271	Yes	AATTTAGGTTAAATACAGACCAAGAGCCTTCAAAGCCCTCAGTAA
25272	Yes	AATTTAGGTTAAATACAGACCAAGAGCCTTCAAAGCCCTCAGTAAG
25273	Yes	AATTTAGGTTAAATACAGACCAAGAGCCTTCAAAGCCCTCAGTAAGT
25274	Yes	AATTTAGGTTAAATACAGACCAAGAGCCTTCAAAGCCCTCAGTAAGTT
25275	Yes	AATTTAGGTTAAATACAGACCAAGAGCCTTCAAAGCCCTCAGTAAGTTG
25276	Yes	AATTTAGGTTAAATACAGACCAAGAGCCTTCAAAGCCCTCAGTAAGTTGC
25277	No	AATTTCCAATCAGGGA

25278	No	AATTTCCAATCAGGGAA
25279	Yes	AATTTCCAATCAGGGAAC
25280	Yes	AATTTCCAATCAGGGAACC
25281	Yes	AATTTCCAATCAGGGAACCA
25282	Yes	AATTTCGTCTCCTACG
25283	Yes	AATTTCGTCTCCTACGC
25284	Yes	AATTTCGTCTCCTACGCG
25285	Yes	AATTTCGTCTCCTACGCGG
25286	Yes	AATTTCGTCTCCTACGCGGG
25287	Yes	AATTTCGTCTCCTACGCGGGA
25288	Yes	AATTTCGTCTCCTACGCGGGAG
25289	Yes	AATTTCGTCTCCTACGCGGGAGA
25290	Yes	AATTTCGTCTCCTACGCGGGAGAC
25291	Yes	AATTTCGTCTCCTACGCGGGAGACT
25292	Yes	AATTTCGTCTCCTACGCGGGAGACTC
25293	Yes	AATTTCGTCTCCTACGCGGGAGACTCG
25294	Yes	AATTTCGTCTCCTACGCGGGAGACTCGG
25295	Yes	AATTTCGTCTCCTACGCGGGAGACTCGGG
25296	Yes	AATTTCGTCTCCTACGCGGGAGACTCGGGT
25297	Yes	AATTTCGTCTCCTACGCGGGAGACTCGGGTT
25298	Yes	AATTTCGTCTCCTACGCGGGAGACTCGGGTTC
25299	Yes	AATTTCGTCTCCTACGCGGGAGACTCGGGTTCG
25300	Yes	AATTTCGTCTCCTACGCGGGAGACTCGGGTTCGA
25301	Yes	AATTTCGTCTCCTACGCGGGAGACTCGGGTTCGAC
25302	Yes	AATTTCGTCTCCTACGCGGGAGACTCGGGTTCGACT
25303	Yes	AATTTCGTCTCCTACGCGGGAGACTCGGGTTCGACTT
25304	Yes	AATTTCGTCTCCTACGCGGGAGACTCGGGTTCGACTTC
25305	Yes	AATTTCGTCTCCTACGCGGGAGACTCGGGTTCGACTTCG
25306	Yes	AATTTCGTCTCCTACGCGGGAGACTCGGGTTCGACTTCGG
25307	Yes	AATTTCGTCTCCTACGCGGGAGACTCGGGTTCGACTTCGGC
25308	Yes	AATTTCGTCTCCTACGCGGGAGACTCGGGTTCGACTTCGGCC
25309	Yes	AATTTCGTCTCCTACGCGGGAGACTCGGGTTCGACTTCGGCCA
25310	Yes	AATTTCGTCTCCTACGCGGGAGACTCGGGTTCGACTTCGGCCAT
25311	Yes	AATTTCGTCTCCTACGCGGGAGACTCGGGTTCGACTTCGGCCATG
25312	Yes	AATTTCGTCTCCTACGCGGGAGACTCGGGTTCGACTTCGGCCATGC
25313	Yes	AATTTCGTCTCCTACGCGGGAGACTCGGGTTCGACTTCGGCCATGCA
25314	Yes	AATTTCGTCTCCTACGCGGGAGACTCGGGTTCGACTTCGGCCATGCAC
25315	Yes	AATTTCGTCTCCTACGCGGGAGACTCGGGTTCGACTTCGGCCATGCACC
25316	Yes	AATTTCGTCTCCTACGCGGGAGACTCGGGTTCGACTTCGGCCATGCACCA
25317	No	AATTTGAATGCTGGTT
25318	No	AATTTGAATGCTGGTTC
25319	No	AATTTGAATGCTGGTTCT

25320	Yes	AATTTGAATGCTGGTTCTG
25321	Yes	AATTTGAATGCTGGTTCTGT
25322	Yes	AATTTGAATGCTGGTTCTGTC
25323	Yes	AATTTGAATGCTGGTTCTGTCA
25324	Yes	AATTTGAATGCTGGTTCTGTCAC
25325	Yes	AATTTGAATGCTGGTTCTGTCACC
25326	Yes	AATTTGAATGCTGGTTCTGTCACCA
25327	Yes	AATTTGACTAGAGATC
25328	Yes	AATTTGACTAGAGATCA
25329	Yes	AATTTGACTAGAGATCAA
25330	Yes	AATTTGACTAGAGATCAAG
25331	Yes	AATTTGACTAGAGATCAAGA
25332	Yes	AATTTGACTAGAGATCAAGAG
25333	Yes	AATTTGACTAGAGATCAAGAGG
25334	Yes	AATTTGACTAGAGATCAAGAGGT
25335	Yes	AATTTGACTAGAGATCAAGAGGTC
25336	Yes	AATTTGACTAGAGATCAAGAGGTCC
25337	Yes	AATTTGACTAGAGATCAAGAGGTCCC
25338	Yes	AATTTGACTAGAGATCAAGAGGTCCCT
25339	Yes	AATTTGACTAGAGATCAAGAGGTCCCTG
25340	Yes	AATTTGACTAGAGATCAAGAGGTCCCTGG
25341	Yes	AATTTGACTAGAGATCAAGAGGTCCCTGGT
25342	Yes	AATTTGACTAGAGATCAAGAGGTCCCTGGTT
25343	Yes	AATTTGACTAGAGATCAAGAGGTCCCTGGTTC
25344	Yes	AATTTGACTAGAGATCAAGAGGTCCCTGGTTCA
25345	Yes	AATTTGACTAGAGATCAAGAGGTCCCTGGTTCAA
25346	Yes	AATTTGACTAGAGATCAAGAGGTCCCTGGTTCAAA
25347	Yes	AATTTGACTAGAGATCAAGAGGTCCCTGGTTCAAAT
25348	Yes	AATTTGACTAGAGATCAAGAGGTCCCTGGTTCAAATC
25349	Yes	AATTTGACTAGAGATCAAGAGGTCCCTGGTTCAAATCC
25350	Yes	AATTTGACTAGAGATCAAGAGGTCCCTGGTTCAAATCCA
25351	Yes	AATTTGACTAGAGATCAAGAGGTCCCTGGTTCAAATCCAG
25352	Yes	AATTTGACTAGAGATCAAGAGGTCCCTGGTTCAAATCCAGG
25353	Yes	AATTTGACTAGAGATCAAGAGGTCCCTGGTTCAAATCCAGGT
25354	Yes	AATTTGACTAGAGATCAAGAGGTCCCTGGTTCAAATCCAGGTG
25355	Yes	AATTTGACTAGAGATCAAGAGGTCCCTGGTTCAAATCCAGGTGC
25356	Yes	AATTTGACTAGAGATCAAGAGGTCCCTGGTTCAAATCCAGGTGCC
25357	Yes	AATTTGACTAGAGATCAAGAGGTCCCTGGTTCAAATCCAGGTGCCC
25358	Yes	AATTTGACTAGAGATCAAGAGGTCCCTGGTTCAAATCCAGGTGCCCC
25359	Yes	AATTTGACTAGAGATCAAGAGGTCCCTGGTTCAAATCCAGGTGCCCCC
25360	Yes	AATTTGACTAGAGATCAAGAGGTCCCTGGTTCAAATCCAGGTGCCCCCT
25361	Yes	AATTTGACTAGAGATCAAGAGGTCCCTGGTTCAAATCCAGGTGCCCCCTC

25362	No	AATTTGAGGGCCCAGG
25363	Yes	AATTTGAGGGCCCAGGT
25364	Yes	AATTTGAGGGCCCAGGTT
25365	Yes	AATTTGAGGGCCCAGGTTT
25366	Yes	AATTTGAGGGCCCAGGTTTC
25367	Yes	AATTTGAGGGCCCAGGTTTCA
25368	Yes	AATTTGAGGGCCCAGGTTTCAA
25369	Yes	AATTTGAGGGCCCAGGTTTCAAG
25370	Yes	AATTTGAGGGCCCAGGTTTCAAGT
25371	Yes	AATTTGAGGGCCCAGGTTTCAAGTC
25372	Yes	AATTTGAGGGCCCAGGTTTCAAGTCC
25373	Yes	AATTTGAGGGCCCAGGTTTCAAGTCCC
25374	Yes	AATTTGAGGGCCCAGGTTTCAAGTCCCT
25375	Yes	AATTTGAGGGCCCAGGTTTCAAGTCCCTG
25376	Yes	AATTTGAGGGCCCAGGTTTCAAGTCCCTGT
25377	Yes	AATTTGAGGGCCCAGGTTTCAAGTCCCTGTT
25378	Yes	AATTTGAGGGCCCAGGTTTCAAGTCCCTGTTT
25379	Yes	AATTTGAGGGCCCAGGTTTCAAGTCCCTGTTTG
25380	Yes	AATTTGAGGGCCCAGGTTTCAAGTCCCTGTTTGG
25381	Yes	AATTTGAGGGCCCAGGTTTCAAGTCCCTGTTTGGG
25382	Yes	AATTTGAGGGCCCAGGTTTCAAGTCCCTGTTTGGGT
25383	Yes	AATTTGAGGGCCCAGGTTTCAAGTCCCTGTTTGGGTG
25384	Yes	AATTTGAGGGCCCAGGTTTCAAGTCCCTGTTTGGGTGC
25385	Yes	AATTTGAGGGCCCAGGTTTCAAGTCCCTGTTTGGGTGCC
25386	Yes	AATTTGAGGGCCCAGGTTTCAAGTCCCTGTTTGGGTGCCA
25387	No	AATTTTATAATCAGAG
25388	No	AATTTTATAATCAGAGG
25389	No	AATTTTATAATCAGAGGT
25390	No	AATTTTATAATCAGAGGTT
25391	No	AATTTTATAATCAGAGGTTC
25392	No	AATTTTATAATCAGAGGTTCA
25393	No	AATTTTATAATCAGAGGTTCAA
25394	Yes	AATTTTATAATCAGAGGTTCAAC
25395	Yes	AATTTTATAATCAGAGGTTCAACT
25396	Yes	AATTTTATAATCAGAGGTTCAACTC
25397	Yes	AATTTTATAATCAGAGGTTCAACTCC
25398	Yes	AATTTTATAATCAGAGGTTCAACTCCT
25399	Yes	AATTTTATAATCAGAGGTTCAACTCCTC
25400	Yes	AATTTTATAATCAGAGGTTCAACTCCTCT
25401	Yes	AATTTTATAATCAGAGGTTCAACTCCTCTT
25402	Yes	AATTTTATAATCAGAGGTTCAACTCCTCTTC
25403	Yes	AATTTTATAATCAGAGGTTCAACTCCTCTTCT

25404	Yes	AATTTTATAATCAGAGGTTCAACTCCTCTTCTT
25405	Yes	AATTTTATAATCAGAGGTTCAACTCCTCTTCTTA
25406	Yes	AATTTTATAATCAGAGGTTCAACTCCTCTTCTTAA
25407	Yes	AATTTTATAATCAGAGGTTCAACTCCTCTTCTTAAC
25408	Yes	AATTTTATAATCAGAGGTTCAACTCCTCTTCTTAACA
25409	Yes	AATTTTATAATCAGAGGTTCAACTCCTCTTCTTAACAC
25410	Yes	AATTTTATAATCAGAGGTTCAACTCCTCTTCTTAACACC
25411	Yes	AATTTTATAATCAGAGGTTCAACTCCTCTTCTTAACACCA
25412	No	AATTTTCACCTGCCAT
25413	No	AATTTTCACCTGCCATG
25414	Yes	AATTTTCACCTGCCATGC
25415	Yes	AATTTTCACCTGCCATGCA
25416	Yes	AATTTTCACCTGCCATGCAG
25417	Yes	AATTTTCACCTGCCATGCAGG
25418	Yes	AATTTTCACCTGCCATGCAGGA
25419	Yes	AATTTTCACCTGCCATGCAGGAG
25420	Yes	AATTTTCACCTGCCATGCAGGAGG
25421	Yes	AATTTTCACCTGCCATGCAGGAGGT
25422	Yes	AATTTTCACCTGCCATGCAGGAGGTC
25423	Yes	AATTTTCACCTGCCATGCAGGAGGTCC
25424	Yes	AATTTTCACCTGCCATGCAGGAGGTCCA
25425	Yes	AATTTTCACCTGCCATGCAGGAGGTCCAG
25426	Yes	AATTTTCACCTGCCATGCAGGAGGTCCAGG
25427	Yes	AATTTTCACCTGCCATGCAGGAGGTCCAGGT
25428	Yes	AATTTTCACCTGCCATGCAGGAGGTCCAGGTT
25429	Yes	AATTTTCACCTGCCATGCAGGAGGTCCAGGTTC
25430	Yes	AATTTTCACCTGCCATGCAGGAGGTCCAGGTTCA
25431	Yes	AATTTTCACCTGCCATGCAGGAGGTCCAGGTTCAT
25432	Yes	AATTTTCACCTGCCATGCAGGAGGTCCAGGTTCATT
25433	Yes	AATTTTCACCTGCCATGCAGGAGGTCCAGGTTCATTT
25434	Yes	AATTTTCACCTGCCATGCAGGAGGTCCAGGTTCATTTC
25435	Yes	AATTTTCACCTGCCATGCAGGAGGTCCAGGTTCATTTCC
25436	Yes	AATTTTCACCTGCCATGCAGGAGGTCCAGGTTCATTTCCT
25437	Yes	AATTTTCACCTGCCATGCAGGAGGTCCAGGTTCATTTCCTG
25438	Yes	AATTTTCACCTGCCATGCAGGAGGTCCAGGTTCATTTCCTGG
25439	Yes	AATTTTCACCTGCCATGCAGGAGGTCCAGGTTCATTTCCTGGC
25440	Yes	AATTTTCACCTGCCATGCAGGAGGTCCAGGTTCATTTCCTGGCC
25441	Yes	AATTTTCACCTGCCATGCAGGAGGTCCAGGTTCATTTCCTGGCCT
25442	Yes	AATTTTCACCTGCCATGCAGGAGGTCCAGGTTCATTTCCTGGCCTA
25443	Yes	AATTTTCACCTGCCATGCAGGAGGTCCAGGTTCATTTCCTGGCCTAT
25444	Yes	AATTTTCACCTGCCATGCAGGAGGTCCAGGTTCATTTCCTGGCCTATG
25445	Yes	AATTTTCACCTGCCATGCAGGAGGTCCAGGTTCATTTCCTGGCCTATGC

25446	Yes	AATTTTCACCTGCCATGCAGGAGGTCCAGGTTCATTTCCTGGCCTATGCA
25447	No	AATTTTGAATTCTCAG
25448	No	AATTTTGAATTCTCAGG
25449	No	AATTTTGAATTCTCAGGG
25450	No	AATTTTGAATTCTCAGGGG
25451	No	AATTTTGAATTCTCAGGGGT
25452	No	AATTTTGAATTCTCAGGGGTA
25453	No	AATTTTGAATTCTCAGGGGTAG
25454	Yes	AATTTTGAATTCTCAGGGGTAGG
25455	Yes	AATTTTGAATTCTCAGGGGTAGGT
25456	Yes	AATTTTGAATTCTCAGGGGTAGGTT
25457	Yes	AATTTTGAATTCTCAGGGGTAGGTTC
25458	Yes	AATTTTGAATTCTCAGGGGTAGGTTCA
25459	Yes	AATTTTGAATTCTCAGGGGTAGGTTCAA
25460	Yes	AATTTTGAATTCTCAGGGGTAGGTTCAAT
25461	Yes	AATTTTGAATTCTCAGGGGTAGGTTCAATT
25462	Yes	AATTTTGAATTCTCAGGGGTAGGTTCAATTC
25463	Yes	AATTTTGAATTCTCAGGGGTAGGTTCAATTCC
25464	Yes	AATTTTGAATTCTCAGGGGTAGGTTCAATTCCT
25465	Yes	AATTTTGAATTCTCAGGGGTAGGTTCAATTCCTA
25466	Yes	AATTTTGAATTCTCAGGGGTAGGTTCAATTCCTAT
25467	Yes	AATTTTGAATTCTCAGGGGTAGGTTCAATTCCTATA
25468	Yes	AATTTTGAATTCTCAGGGGTAGGTTCAATTCCTATAG
25469	Yes	AATTTTGAATTCTCAGGGGTAGGTTCAATTCCTATAGT
25470	Yes	AATTTTGAATTCTCAGGGGTAGGTTCAATTCCTATAGTT
25471	Yes	AATTTTGAATTCTCAGGGGTAGGTTCAATTCCTATAGTTC
25472	Yes	AATTTTGAATTCTCAGGGGTAGGTTCAATTCCTATAGTTCT
25473	Yes	AATTTTGAATTCTCAGGGGTAGGTTCAATTCCTATAGTTCTA
25474	Yes	AATTTTGAATTCTCAGGGGTAGGTTCAATTCCTATAGTTCTAG
25475	Yes	AATTTTGAATTCTCAGGGGTAGGTTCAATTCCTATAGTTCTAGC
25476	Yes	AATTTTGAATTCTCAGGGGTAGGTTCAATTCCTATAGTTCTAGCC
25477	Yes	AATTTTGAATTCTCAGGGGTAGGTTCAATTCCTATAGTTCTAGCCA
25478	No	AATTTTGGAGTCTCAG
25479	Yes	AATTTTGGAGTCTCAGG
25480	Yes	AATTTTGGAGTCTCAGGG
25481	Yes	AATTTTGGAGTCTCAGGGA
25482	Yes	AATTTTGGAGTCTCAGGGAT
25483	Yes	AATTTTGGAGTCTCAGGGATA
25484	Yes	AATTTTGGAGTCTCAGGGATAG
25485	Yes	AATTTTGGAGTCTCAGGGATAGG
25486	Yes	AATTTTGGAGTCTCAGGGATAGGT
25487	Yes	AATTTTGGAGTCTCAGGGATAGGTT

25488	Yes	AATTTTGGAGTCTCAGGGATAGGTTC
25489	Yes	AATTTTGGAGTCTCAGGGATAGGTTCA
25490	Yes	AATTTTGGAGTCTCAGGGATAGGTTCAA
25491	Yes	AATTTTGGAGTCTCAGGGATAGGTTCAAT
25492	Yes	AATTTTGGAGTCTCAGGGATAGGTTCAATT
25493	Yes	AATTTTGGAGTCTCAGGGATAGGTTCAATTC
25494	Yes	AATTTTGGAGTCTCAGGGATAGGTTCAATTCC
25495	Yes	AATTTTGGAGTCTCAGGGATAGGTTCAATTCCT
25496	Yes	AATTTTGGAGTCTCAGGGATAGGTTCAATTCCTA
25497	Yes	AATTTTGGAGTCTCAGGGATAGGTTCAATTCCTAT
25498	Yes	AATTTTGGAGTCTCAGGGATAGGTTCAATTCCTATA
25499	Yes	AATTTTGGAGTCTCAGGGATAGGTTCAATTCCTATAG
25500	Yes	AATTTTGGAGTCTCAGGGATAGGTTCAATTCCTATAGT
25501	Yes	AATTTTGGAGTCTCAGGGATAGGTTCAATTCCTATAGTT
25502	Yes	AATTTTGGAGTCTCAGGGATAGGTTCAATTCCTATAGTTC
25503	Yes	AATTTTGGAGTCTCAGGGATAGGTTCAATTCCTATAGTTCC
25504	Yes	AATTTTGGAGTCTCAGGGATAGGTTCAATTCCTATAGTTCCA
25505	Yes	AATTTTGGAGTCTCAGGGATAGGTTCAATTCCTATAGTTCCAG
25506	Yes	AATTTTGGAGTCTCAGGGATAGGTTCAATTCCTATAGTTCCAGC
25507	Yes	AATTTTGGAGTCTCAGGGATAGGTTCAATTCCTATAGTTCCAGCC
25508	Yes	AATTTTGGAGTCTCAGGGATAGGTTCAATTCCTATAGTTCCAGCCA
25509	No	AATTTTGGATTATCAG
25510	No	AATTTTGGATTATCAGG
25511	Yes	AATTTTGGATTATCAGGG
25512	Yes	AATTTTGGATTATCAGGGG
25513	Yes	AATTTTGGATTATCAGGGGT
25514	Yes	AATTTTGGATTATCAGGGGTA
25515	Yes	AATTTTGGATTATCAGGGGTAG
25516	Yes	AATTTTGGATTATCAGGGGTAGG
25517	Yes	AATTTTGGATTATCAGGGGTAGGT
25518	Yes	AATTTTGGATTATCAGGGGTAGGTT
25519	Yes	AATTTTGGATTATCAGGGGTAGGTTC
25520	Yes	AATTTTGGATTATCAGGGGTAGGTTCA
25521	Yes	AATTTTGGATTATCAGGGGTAGGTTCAA
25522	Yes	AATTTTGGATTATCAGGGGTAGGTTCAAT
25523	Yes	AATTTTGGATTATCAGGGGTAGGTTCAATT
25524	Yes	AATTTTGGATTATCAGGGGTAGGTTCAATTC
25525	Yes	AATTTTGGATTATCAGGGGTAGGTTCAATTCC
25526	Yes	AATTTTGGATTATCAGGGGTAGGTTCAATTCCT
25527	Yes	AATTTTGGATTATCAGGGGTAGGTTCAATTCCTA
25528	Yes	AATTTTGGATTATCAGGGGTAGGTTCAATTCCTAT
25529	Yes	AATTTTGGATTATCAGGGGTAGGTTCAATTCCTATA

25530	Yes	AATTTTGGATTATCAGGGGTAGGTTCAATTCCTATAG
25531	Yes	AATTTTGGATTATCAGGGGTAGGTTCAATTCCTATAGT
25532	Yes	AATTTTGGATTATCAGGGGTAGGTTCAATTCCTATAGTT
25533	Yes	AATTTTGGATTATCAGGGGTAGGTTCAATTCCTATAGTTC
25534	Yes	AATTTTGGATTATCAGGGGTAGGTTCAATTCCTATAGTTCT
25535	Yes	AATTTTGGATTATCAGGGGTAGGTTCAATTCCTATAGTTCTA
25536	Yes	AATTTTGGATTATCAGGGGTAGGTTCAATTCCTATAGTTCTAG
25537	Yes	AATTTTGGATTATCAGGGGTAGGTTCAATTCCTATAGTTCTAGC
25538	Yes	AATTTTGGATTATCAGGGGTAGGTTCAATTCCTATAGTTCTAGCC
25539	Yes	AATTTTGGATTATCAGGGGTAGGTTCAATTCCTATAGTTCTAGCCA
25540	No	AATTTTGGATTCTCAG
25541	No	AATTTTGGATTCTCAGG
25542	No	AATTTTGGATTCTCAGGG
25543	No	AATTTTGGATTCTCAGGGA
25544	No	AATTTTGGATTCTCAGGGAT
25545	Yes	AATTTTGGATTCTCAGGGATG
25546	Yes	AATTTTGGATTCTCAGGGATGG
25547	Yes	AATTTTGGATTCTCAGGGATGGG
25548	Yes	AATTTTGGATTCTCAGGGATGGGT
25549	Yes	AATTTTGGATTCTCAGGGATGGGTT
25550	Yes	AATTTTGGATTCTCAGGGATGGGTTC
25551	Yes	AATTTTGGATTCTCAGGGATGGGTTCA
25552	Yes	AATTTTGGATTCTCAGGGATGGGTTCAA
25553	Yes	AATTTTGGATTCTCAGGGATGGGTTCAAT
25554	Yes	AATTTTGGATTCTCAGGGATGGGTTCAATT
25555	Yes	AATTTTGGATTCTCAGGGATGGGTTCAATTC
25556	Yes	AATTTTGGATTCTCAGGGATGGGTTCAATTCC
25557	Yes	AATTTTGGATTCTCAGGGATGGGTTCAATTCCT
25558	Yes	AATTTTGGATTCTCAGGGATGGGTTCAATTCCTA
25559	Yes	AATTTTGGATTCTCAGGGATGGGTTCAATTCCTAT
25560	Yes	AATTTTGGATTCTCAGGGATGGGTTCAATTCCTATA
25561	Yes	AATTTTGGATTCTCAGGGATGGGTTCAATTCCTATAG
25562	Yes	AATTTTGGATTCTCAGGGATGGGTTCAATTCCTATAGT
25563	Yes	AATTTTGGATTCTCAGGGATGGGTTCAATTCCTATAGTC
25564	Yes	AATTTTGGATTCTCAGGGATGGGTTCAATTCCTATAGTCC
25565	Yes	AATTTTGGATTCTCAGGGATGGGTTCAATTCCTATAGTCCT
25566	Yes	AATTTTGGATTCTCAGGGATGGGTTCAATTCCTATAGTCCTA
25567	Yes	AATTTTGGATTCTCAGGGATGGGTTCAATTCCTATAGTCCTAG
25568	Yes	AATTTTGGATTCTCAGGGATGGGTTCAATTCCTATAGTCCTAGC
25569	Yes	AATTTTGGATTCTCAGGGATGGGTTCAATTCCTATAGTCCTAGCC
25570	Yes	AATTTTGGATTCTCAGGGATGGGTTCAATTCCTATAGTCCTAGCCA
25571	Yes	AATTTTGGATTCTCAGGGATGGGTTCAATTCCTG

25572	Yes	AATTTTGGATTCTCAGGGATGGGTTCAATTCCTGT
25573	Yes	AATTTTGGATTCTCAGGGATGGGTTCAATTCCTGTA
25574	Yes	AATTTTGGATTCTCAGGGATGGGTTCAATTCCTGTAG
25575	Yes	AATTTTGGATTCTCAGGGATGGGTTCAATTCCTGTAGT
25576	Yes	AATTTTGGATTCTCAGGGATGGGTTCAATTCCTGTAGTT
25577	Yes	AATTTTGGATTCTCAGGGATGGGTTCAATTCCTGTAGTTC
25578	Yes	AATTTTGGATTCTCAGGGATGGGTTCAATTCCTGTAGTTCT
25579	Yes	AATTTTGGATTCTCAGGGATGGGTTCAATTCCTGTAGTTCTA
25580	Yes	AATTTTGGATTCTCAGGGATGGGTTCAATTCCTGTAGTTCTAG
25581	Yes	AATTTTGGATTCTCAGGGATGGGTTCAATTCCTGTAGTTCTAGC
25582	Yes	AATTTTGGATTCTCAGGGATGGGTTCAATTCCTGTAGTTCTAGCC
25583	Yes	AATTTTGGATTCTCAGGGATGGGTTCAATTCCTGTAGTTCTAGCCA
25584	Yes	AATTTTGGATTCTCAGGGATGGGTTCG
25585	Yes	AATTTTGGATTCTCAGGGATGGGTTCGA
25586	Yes	AATTTTGGATTCTCAGGGATGGGTTCGAT
25587	Yes	AATTTTGGATTCTCAGGGATGGGTTCGATT
25588	Yes	AATTTTGGATTCTCAGGGATGGGTTCGATTC
25589	Yes	AATTTTGGATTCTCAGGGATGGGTTCGATTCT
25590	Yes	AATTTTGGATTCTCAGGGATGGGTTCGATTCTC
25591	Yes	AATTTTGGATTCTCAGGGATGGGTTCGATTCTCA
25592	Yes	AATTTTGGATTCTCAGGGATGGGTTCGATTCTCAT
25593	Yes	AATTTTGGATTCTCAGGGATGGGTTCGATTCTCATA
25594	Yes	AATTTTGGATTCTCAGGGATGGGTTCGATTCTCATAG
25595	Yes	AATTTTGGATTCTCAGGGATGGGTTCGATTCTCATAGT
25596	Yes	AATTTTGGATTCTCAGGGATGGGTTCGATTCTCATAGTC
25597	Yes	AATTTTGGATTCTCAGGGATGGGTTCGATTCTCATAGTCC
25598	Yes	AATTTTGGATTCTCAGGGATGGGTTCGATTCTCATAGTCCT
25599	Yes	AATTTTGGATTCTCAGGGATGGGTTCGATTCTCATAGTCCTA
25600	Yes	AATTTTGGATTCTCAGGGATGGGTTCGATTCTCATAGTCCTAG
25601	Yes	AATTTTGGATTCTCAGGGATGGGTTCGATTCTCATAGTCCTAGC
25602	Yes	AATTTTGGATTCTCAGGGATGGGTTCGATTCTCATAGTCCTAGCC
25603	Yes	AATTTTGGATTCTCAGGGATGGGTTCGATTCTCATAGTCCTAGCCA
25604	No	AATTTTGGATTCTCAGGGG
25605	No	AATTTTGGATTCTCAGGGGT
25606	No	AATTTTGGATTCTCAGGGGTA
25607	No	AATTTTGGATTCTCAGGGGTAG
25608	No	AATTTTGGATTCTCAGGGGTAGG
25609	Yes	AATTTTGGATTCTCAGGGGTAGGT
25610	Yes	AATTTTGGATTCTCAGGGGTAGGTT
25611	Yes	AATTTTGGATTCTCAGGGGTAGGTTA
25612	Yes	AATTTTGGATTCTCAGGGGTAGGTTAA
25613	Yes	AATTTTGGATTCTCAGGGGTAGGTTAAA

25614	Yes	AATTTTGGATTCTCAGGGGTAGGTTAAAT
25615	Yes	AATTTTGGATTCTCAGGGGTAGGTTAAATT
25616	Yes	AATTTTGGATTCTCAGGGGTAGGTTAAATTC
25617	Yes	AATTTTGGATTCTCAGGGGTAGGTTAAATTCC
25618	Yes	AATTTTGGATTCTCAGGGGTAGGTTAAATTCCT
25619	Yes	AATTTTGGATTCTCAGGGGTAGGTTAAATTCCTA
25620	Yes	AATTTTGGATTCTCAGGGGTAGGTTAAATTCCTAT
25621	Yes	AATTTTGGATTCTCAGGGGTAGGTTAAATTCCTATA
25622	Yes	AATTTTGGATTCTCAGGGGTAGGTTAAATTCCTATAG
25623	Yes	AATTTTGGATTCTCAGGGGTAGGTTAAATTCCTATAGT
25624	Yes	AATTTTGGATTCTCAGGGGTAGGTTAAATTCCTATAGTA
25625	Yes	AATTTTGGATTCTCAGGGGTAGGTTAAATTCCTATAGTAC
25626	Yes	AATTTTGGATTCTCAGGGGTAGGTTAAATTCCTATAGTACT
25627	Yes	AATTTTGGATTCTCAGGGGTAGGTTAAATTCCTATAGTACTA
25628	Yes	AATTTTGGATTCTCAGGGGTAGGTTAAATTCCTATAGTACTAG
25629	Yes	AATTTTGGATTCTCAGGGGTAGGTTAAATTCCTATAGTACTAGC
25630	Yes	AATTTTGGATTCTCAGGGGTAGGTTAAATTCCTATAGTACTAGCC
25631	Yes	AATTTTGGATTCTCAGGGGTAGGTTAAATTCCTATAGTACTAGCCA
25632	Yes	AATTTTGGATTCTCAGGGGTAGGTTC
25633	Yes	AATTTTGGATTCTCAGGGGTAGGTTCA
25634	Yes	AATTTTGGATTCTCAGGGGTAGGTTCAA
25635	Yes	AATTTTGGATTCTCAGGGGTAGGTTCAAT
25636	Yes	AATTTTGGATTCTCAGGGGTAGGTTCAATT
25637	Yes	AATTTTGGATTCTCAGGGGTAGGTTCAATTC
25638	Yes	AATTTTGGATTCTCAGGGGTAGGTTCAATTCC
25639	Yes	AATTTTGGATTCTCAGGGGTAGGTTCAATTCCT
25640	Yes	AATTTTGGATTCTCAGGGGTAGGTTCAATTCCTA
25641	Yes	AATTTTGGATTCTCAGGGGTAGGTTCAATTCCTAC
25642	Yes	AATTTTGGATTCTCAGGGGTAGGTTCAATTCCTACA
25643	Yes	AATTTTGGATTCTCAGGGGTAGGTTCAATTCCTACAG
25644	Yes	AATTTTGGATTCTCAGGGGTAGGTTCAATTCCTACAGT
25645	Yes	AATTTTGGATTCTCAGGGGTAGGTTCAATTCCTACAGTT
25646	Yes	AATTTTGGATTCTCAGGGGTAGGTTCAATTCCTACAGTTC
25647	Yes	AATTTTGGATTCTCAGGGGTAGGTTCAATTCCTACAGTTCT
25648	Yes	AATTTTGGATTCTCAGGGGTAGGTTCAATTCCTACAGTTCTA
25649	Yes	AATTTTGGATTCTCAGGGGTAGGTTCAATTCCTACAGTTCTAG
25650	Yes	AATTTTGGATTCTCAGGGGTAGGTTCAATTCCTACAGTTCTAGC
25651	Yes	AATTTTGGATTCTCAGGGGTAGGTTCAATTCCTACAGTTCTAGCC
25652	Yes	AATTTTGGATTCTCAGGGGTAGGTTCAATTCCTACAGTTCTAGCCA
25653	Yes	AATTTTGGATTCTCAGGGGTAGGTTCAATTCCTAT
25654	Yes	AATTTTGGATTCTCAGGGGTAGGTTCAATTCCTATA
25655	Yes	AATTTTGGATTCTCAGGGGTAGGTTCAATTCCTATAT

25656	Yes	AATTTTGGATTCTCAGGGGTAGGTTCAATTCCTATATT
25657	Yes	AATTTTGGATTCTCAGGGGTAGGTTCAATTCCTATATTC
25658	Yes	AATTTTGGATTCTCAGGGGTAGGTTCAATTCCTATATTCT
25659	Yes	AATTTTGGATTCTCAGGGGTAGGTTCAATTCCTATATTCTA
25660	Yes	AATTTTGGATTCTCAGGGGTAGGTTCAATTCCTATATTCTAG
25661	Yes	AATTTTGGATTCTCAGGGGTAGGTTCAATTCCTATATTCTAGA
25662	Yes	AATTTTGGATTCTCAGGGGTAGGTTCAATTCCTATATTCTAGAA
25663	Yes	AATTTTGGATTCTCAGGGGTAGGTTCAATTCCTATATTCTAGAAC
25664	Yes	AATTTTGGATTCTCAGGGGTAGGTTCAATTCCTATATTCTAGAACC
25665	Yes	AATTTTGGATTCTCAGGGGTAGGTTCAATTCCTATATTCTAGAACCA
25666	Yes	AATTTTGGATTCTCAGGGGTAGGTTCAATTCT
25667	Yes	AATTTTGGATTCTCAGGGGTAGGTTCAATTCTT
25668	Yes	AATTTTGGATTCTCAGGGGTAGGTTCAATTCTTA
25669	Yes	AATTTTGGATTCTCAGGGGTAGGTTCAATTCTTAT
25670	Yes	AATTTTGGATTCTCAGGGGTAGGTTCAATTCTTATA
25671	Yes	AATTTTGGATTCTCAGGGGTAGGTTCAATTCTTATAG
25672	Yes	AATTTTGGATTCTCAGGGGTAGGTTCAATTCTTATAGT
25673	Yes	AATTTTGGATTCTCAGGGGTAGGTTCAATTCTTATAGTT
25674	Yes	AATTTTGGATTCTCAGGGGTAGGTTCAATTCTTATAGTTC
25675	Yes	AATTTTGGATTCTCAGGGGTAGGTTCAATTCTTATAGTTCT
25676	Yes	AATTTTGGATTCTCAGGGGTAGGTTCAATTCTTATAGTTCTA
25677	Yes	AATTTTGGATTCTCAGGGGTAGGTTCAATTCTTATAGTTCTAG
25678	Yes	AATTTTGGATTCTCAGGGGTAGGTTCAATTCTTATAGTTCTAGC
25679	Yes	AATTTTGGATTCTCAGGGGTAGGTTCAATTCTTATAGTTCTAGCC
25680	Yes	AATTTTGGATTCTCAGGGGTAGGTTCAATTCTTATAGTTCTAGCCA
25681	Yes	AATTTTGGATTCTCAGGGGTAGGTTCG
25682	Yes	AATTTTGGATTCTCAGGGGTAGGTTCGA
25683	Yes	AATTTTGGATTCTCAGGGGTAGGTTCGAT
25684	Yes	AATTTTGGATTCTCAGGGGTAGGTTCGATT
25685	Yes	AATTTTGGATTCTCAGGGGTAGGTTCGATTC
25686	Yes	AATTTTGGATTCTCAGGGGTAGGTTCGATTCC
25687	Yes	AATTTTGGATTCTCAGGGGTAGGTTCGATTCCT
25688	Yes	AATTTTGGATTCTCAGGGGTAGGTTCGATTCCTA
25689	Yes	AATTTTGGATTCTCAGGGGTAGGTTCGATTCCTAT
25690	Yes	AATTTTGGATTCTCAGGGGTAGGTTCGATTCCTATA
25691	Yes	AATTTTGGATTCTCAGGGGTAGGTTCGATTCCTATAA
25692	Yes	AATTTTGGATTCTCAGGGGTAGGTTCGATTCCTATAAT
25693	Yes	AATTTTGGATTCTCAGGGGTAGGTTCGATTCCTATAATT
25694	Yes	AATTTTGGATTCTCAGGGGTAGGTTCGATTCCTATAATTC
25695	Yes	AATTTTGGATTCTCAGGGGTAGGTTCGATTCCTATAATTCT
25696	Yes	AATTTTGGATTCTCAGGGGTAGGTTCGATTCCTATAATTCTA
25697	Yes	AATTTTGGATTCTCAGGGGTAGGTTCGATTCCTATAATTCTAC

25698	Yes	AATTTTGGATTCTCAGGGGTAGGTTCGATTCCTATAATTCTACC
25699	Yes	AATTTTGGATTCTCAGGGGTAGGTTCGATTCCTATAATTCTACCC
25700	Yes	AATTTTGGATTCTCAGGGGTAGGTTCGATTCCTATAATTCTACCCA
25701	Yes	AATTTTGGATTCTCAGGGGTG
25702	Yes	AATTTTGGATTCTCAGGGGTGG
25703	Yes	AATTTTGGATTCTCAGGGGTGGG
25704	Yes	AATTTTGGATTCTCAGGGGTGGGT
25705	Yes	AATTTTGGATTCTCAGGGGTGGGTT
25706	Yes	AATTTTGGATTCTCAGGGGTGGGTTC
25707	Yes	AATTTTGGATTCTCAGGGGTGGGTTCA
25708	Yes	AATTTTGGATTCTCAGGGGTGGGTTCAA
25709	Yes	AATTTTGGATTCTCAGGGGTGGGTTCAAT
25710	Yes	AATTTTGGATTCTCAGGGGTGGGTTCAATT
25711	Yes	AATTTTGGATTCTCAGGGGTGGGTTCAATTC
25712	Yes	AATTTTGGATTCTCAGGGGTGGGTTCAATTCC
25713	Yes	AATTTTGGATTCTCAGGGGTGGGTTCAATTCCT
25714	Yes	AATTTTGGATTCTCAGGGGTGGGTTCAATTCCTA
25715	Yes	AATTTTGGATTCTCAGGGGTGGGTTCAATTCCTAT
25716	Yes	AATTTTGGATTCTCAGGGGTGGGTTCAATTCCTATA
25717	Yes	AATTTTGGATTCTCAGGGGTGGGTTCAATTCCTATAG
25718	Yes	AATTTTGGATTCTCAGGGGTGGGTTCAATTCCTATAGT
25719	Yes	AATTTTGGATTCTCAGGGGTGGGTTCAATTCCTATAGTT
25720	Yes	AATTTTGGATTCTCAGGGGTGGGTTCAATTCCTATAGTTC
25721	Yes	AATTTTGGATTCTCAGGGGTGGGTTCAATTCCTATAGTTCT
25722	Yes	AATTTTGGATTCTCAGGGGTGGGTTCAATTCCTATAGTTCTA
25723	Yes	AATTTTGGATTCTCAGGGGTGGGTTCAATTCCTATAGTTCTAG
25724	Yes	AATTTTGGATTCTCAGGGGTGGGTTCAATTCCTATAGTTCTAGC
25725	Yes	AATTTTGGATTCTCAGGGGTGGGTTCAATTCCTATAGTTCTAGCC
25726	Yes	AATTTTGGATTCTCAGGGGTGGGTTCAATTCCTATAGTTCTAGCCA
25727	Yes	AATTTTGGATTCTCAGGGGTGGGTTCAATTCCTT
25728	Yes	AATTTTGGATTCTCAGGGGTGGGTTCAATTCCTTT
25729	Yes	AATTTTGGATTCTCAGGGGTGGGTTCAATTCCTTTC
25730	Yes	AATTTTGGATTCTCAGGGGTGGGTTCAATTCCTTTCG
25731	Yes	AATTTTGGATTCTCAGGGGTGGGTTCAATTCCTTTCGT
25732	Yes	AATTTTGGATTCTCAGGGGTGGGTTCAATTCCTTTCGTC
25733	Yes	AATTTTGGATTCTCAGGGGTGGGTTCAATTCCTTTCGTCC
25734	Yes	AATTTTGGATTCTCAGGGGTGGGTTCAATTCCTTTCGTCCT
25735	Yes	AATTTTGGATTCTCAGGGGTGGGTTCAATTCCTTTCGTCCTA
25736	Yes	AATTTTGGATTCTCAGGGGTGGGTTCAATTCCTTTCGTCCTAG
25737	Yes	AATTTTGGATTCTCAGGGGTGGGTTCAATTCCTTTCGTCCTAGC
25738	Yes	AATTTTGGATTCTCAGGGGTGGGTTCAATTCCTTTCGTCCTAGCC
25739	Yes	AATTTTGGATTCTCAGGGGTGGGTTCAATTCCTTTCGTCCTAGCCA

25740	Yes	AATTTTGGATTCTCAGGGT
25741	Yes	AATTTTGGATTCTCAGGGTT
25742	Yes	AATTTTGGATTCTCAGGGTTA
25743	Yes	AATTTTGGATTCTCAGGGTTAG
25744	Yes	AATTTTGGATTCTCAGGGTTAGG
25745	Yes	AATTTTGGATTCTCAGGGTTAGGT
25746	Yes	AATTTTGGATTCTCAGGGTTAGGTT
25747	Yes	AATTTTGGATTCTCAGGGTTAGGTTC
25748	Yes	AATTTTGGATTCTCAGGGTTAGGTTCA
25749	Yes	AATTTTGGATTCTCAGGGTTAGGTTCAA
25750	Yes	AATTTTGGATTCTCAGGGTTAGGTTCAAT
25751	Yes	AATTTTGGATTCTCAGGGTTAGGTTCAATT
25752	Yes	AATTTTGGATTCTCAGGGTTAGGTTCAATTC
25753	Yes	AATTTTGGATTCTCAGGGTTAGGTTCAATTCC
25754	Yes	AATTTTGGATTCTCAGGGTTAGGTTCAATTCCT
25755	Yes	AATTTTGGATTCTCAGGGTTAGGTTCAATTCCTA
25756	Yes	AATTTTGGATTCTCAGGGTTAGGTTCAATTCCTAT
25757	Yes	AATTTTGGATTCTCAGGGTTAGGTTCAATTCCTATA
25758	Yes	AATTTTGGATTCTCAGGGTTAGGTTCAATTCCTATAG
25759	Yes	AATTTTGGATTCTCAGGGTTAGGTTCAATTCCTATAGT
25760	Yes	AATTTTGGATTCTCAGGGTTAGGTTCAATTCCTATAGTT
25761	Yes	AATTTTGGATTCTCAGGGTTAGGTTCAATTCCTATAGTTC
25762	Yes	AATTTTGGATTCTCAGGGTTAGGTTCAATTCCTATAGTTCT
25763	Yes	AATTTTGGATTCTCAGGGTTAGGTTCAATTCCTATAGTTCTA
25764	Yes	AATTTTGGATTCTCAGGGTTAGGTTCAATTCCTATAGTTCTAG
25765	Yes	AATTTTGGATTCTCAGGGTTAGGTTCAATTCCTATAGTTCTAGC
25766	Yes	AATTTTGGATTCTCAGGGTTAGGTTCAATTCCTATAGTTCTAGCC
25767	Yes	AATTTTGGATTCTCAGGGTTAGGTTCAATTCCTATAGTTCTAGCCA
25768	Yes	AATTTTGGATTCTCAGGT
25769	Yes	AATTTTGGATTCTCAGGTG
25770	Yes	AATTTTGGATTCTCAGGTGC
25771	Yes	AATTTTGGATTCTCAGGTGCA
25772	Yes	AATTTTGGATTCTCAGGTGCAG
25773	Yes	AATTTTGGATTCTCAGGTGCAGG
25774	Yes	AATTTTGGATTCTCAGGTGCAGGT
25775	Yes	AATTTTGGATTCTCAGGTGCAGGTT
25776	Yes	AATTTTGGATTCTCAGGTGCAGGTTC
25777	Yes	AATTTTGGATTCTCAGGTGCAGGTTCA
25778	Yes	AATTTTGGATTCTCAGGTGCAGGTTCAA
25779	Yes	AATTTTGGATTCTCAGGTGCAGGTTCAAT
25780	Yes	AATTTTGGATTCTCAGGTGCAGGTTCAATT
25781	Yes	AATTTTGGATTCTCAGGTGCAGGTTCAATTC

25782	Yes	AATTTTGGATTCTCAGGTGCAGGTTCAATTCC
25783	Yes	AATTTTGGATTCTCAGGTGCAGGTTCAATTCCT
25784	Yes	AATTTTGGATTCTCAGGTGCAGGTTCAATTCCTA
25785	Yes	AATTTTGGATTCTCAGGTGCAGGTTCAATTCCTAT
25786	Yes	AATTTTGGATTCTCAGGTGCAGGTTCAATTCCTATA
25787	Yes	AATTTTGGATTCTCAGGTGCAGGTTCAATTCCTATAT
25788	Yes	AATTTTGGATTCTCAGGTGCAGGTTCAATTCCTATATT
25789	Yes	AATTTTGGATTCTCAGGTGCAGGTTCAATTCCTATATTC
25790	Yes	AATTTTGGATTCTCAGGTGCAGGTTCAATTCCTATATTCT
25791	Yes	AATTTTGGATTCTCAGGTGCAGGTTCAATTCCTATATTCTA
25792	Yes	AATTTTGGATTCTCAGGTGCAGGTTCAATTCCTATATTCTAG
25793	Yes	AATTTTGGATTCTCAGGTGCAGGTTCAATTCCTATATTCTAGA
25794	Yes	AATTTTGGATTCTCAGGTGCAGGTTCAATTCCTATATTCTAGAG
25795	Yes	AATTTTGGATTCTCAGGTGCAGGTTCAATTCCTATATTCTAGAGC
25796	Yes	AATTTTGGATTCTCAGGTGCAGGTTCAATTCCTATATTCTAGAGCC
25797	Yes	AATTTTGGATTCTCAGGTGCAGGTTCAATTCCTATATTCTAGAGCCA
25798	No	AATTTTGGTGCAACTC
25799	No	AATTTTGGTGCAACTCC
25800	No	AATTTTGGTGCAACTCCA
25801	No	AATTTTGGTGCAACTCCAA
25802	No	AATTTTGGTGCAACTCCAAA
25803	No	AATTTTGGTGCAACTCCAAAT
25804	No	AATTTTGGTGCAACTCCAAATA
25805	No	AATTTTGGTGCAACTCCAAATAA
25806	No	AATTTTGGTGCAACTCCAAATAAA
25807	No	AATTTTGGTGCAACTCCAAATAAAA
25808	No	AATTTTGGTGCAACTCCAAATAAAAG
25809	No	AATTTTGGTGCAACTCCAAATAAAAGT
25810	No	AATTTTGGTGCAACTCCAAATAAAAGTA
25811	Yes	AATTTTGGTGCAACTCCAAATAAAAGTAC
25812	Yes	AATTTTGGTGCAACTCCAAATAAAAGTACC
25813	Yes	AATTTTGGTGCAACTCCAAATAAAAGTACCA
25814	No	ACAAAACGAATGATTT
25815	No	ACAAAACGAATGATTTC
25816	No	ACAAAACGAATGATTTCG
25817	No	ACAAAACGAATGATTTCGA
25818	No	ACAAAACGAATGATTTCGAC
25819	No	ACAAAACGAATGATTTCGACT
25820	No	ACAAAACGAATGATTTCGACTC
25821	No	ACAAAACGAATGATTTCGACTCA
25822	No	ACAAAACGAATGATTTCGACTCAT
25823	No	ACAAAACGAATGATTTCGACTCATT

25824	No	ACAAAACGAATGATTTCGACTCATTA
25825	No	ACAAAACGAATGATTTCGACTCATTAA
25826	No	ACAAAACGAATGATTTCGACTCATTAAA
25827	No	ACAAAACGAATGATTTCGACTCATTAAAT
25828	No	ACAAAACGAATGATTTCGACTCATTAAATT
25829	No	ACAAAACGAATGATTTCGACTCATTAAATTA
25830	No	ACAAAACGAATGATTTCGACTCATTAAATTAT
25831	No	ACAAAACGAATGATTTCGACTCATTAAATTATG
25832	No	ACAAAACGAATGATTTCGACTCATTAAATTATGA
25833	No	ACAAAACGAATGATTTCGACTCATTAAATTATGAT
25834	No	ACAAAACGAATGATTTCGACTCATTAAATTATGATA
25835	No	ACAAAACGAATGATTTCGACTCATTAAATTATGATAA
25836	No	ACAAAACGAATGATTTCGACTCATTAAATTATGATAAT
25837	No	ACAAAACGAATGATTTCGACTCATTAAATTATGATAATC
25838	No	ACAAAACGAATGATTTCGACTCATTAAATTATGATAATCA
25839	No	ACAAAACGAATGATTTCGACTCATTAAATTATGATAATCAT
25840	No	ACAAAACGAATGATTTCGACTCATTAAATTATGATAATCATA
25841	No	ACAAAACGAATGATTTCGACTCATTAAATTATGATAATCATAT
25842	No	ACAAAACGAATGATTTCGACTCATTAAATTATGATAATCATATT
25843	No	ACAAAACGAATGATTTCGACTCATTAAATTATGATAATCATATTT
25844	No	ACAAAACGAATGATTTCGACTCATTAAATTATGATAATCATATTTA
25845	No	ACAAAACGAATGATTTCGACTCATTAAATTATGATAATCATATTTAC
25846	No	ACAAAACGAATGATTTCGACTCATTAAATTATGATAATCATATTTACC
25847	No	ACAAAACGAATGATTTCGACTCATTAAATTATGATAATCATATTTACCA
25848	No	ACAAAACGAATGATTTCGACTCATTAAATTATGATAATCATATTTACCAA
25849	No	ACAAAGCACCCAACTT
25850	No	ACAAAGCACCCAACTTA
25851	No	ACAAAGCACCCAACTTAC
25852	No	ACAAAGCACCCAACTTACA
25853	No	ACAAAGCACCCAACTTACAC
25854	No	ACAAAGCACCCAACTTACACT
25855	No	ACAAAGCACCCAACTTACACTT
25856	No	ACAAAGCACCCAACTTACACTTA
25857	No	ACAAAGCACCCAACTTACACTTAG
25858	No	ACAAAGCACCCAACTTACACTTAGG
25859	No	ACAAAGCACCCAACTTACACTTAGGA
25860	No	ACAAAGCACCCAACTTACACTTAGGAG
25861	No	ACAAAGCACCCAACTTACACTTAGGAGA
25862	No	ACAAAGCACCCAACTTACACTTAGGAGAT
25863	No	ACAAAGCACCCAACTTACACTTAGGAGATT
25864	No	ACAAAGCACCCAACTTACACTTAGGAGATTT
25865	No	ACAAAGCACCCAACTTACACTTAGGAGATTTC

25866	No	ACAAAGCACCCAACTTACACTTAGGAGATTTCA
25867	No	ACAAAGCACCCAACTTACACTTAGGAGATTTCAA
25868	No	ACAAAGCACCCAACTTACACTTAGGAGATTTCAAC
25869	No	ACAAAGCACCCAACTTACACTTAGGAGATTTCAACT
25870	Yes	ACAAAGCACCCAACTTACACTTAGGAGATTTCAACTT
25871	Yes	ACAAAGCACCCAACTTACACTTAGGAGATTTCAACTTA
25872	Yes	ACAAAGCACCCAACTTACACTTAGGAGATTTCAACTTAA
25873	Yes	ACAAAGCACCCAACTTACACTTAGGAGATTTCAACTTAAC
25874	Yes	ACAAAGCACCCAACTTACACTTAGGAGATTTCAACTTAACT
25875	Yes	ACAAAGCACCCAACTTACACTTAGGAGATTTCAACTTAACTT
25876	Yes	ACAAAGCACCCAACTTACACTTAGGAGATTTCAACTTAACTTG
25877	Yes	ACAAAGCACCCAACTTACACTTAGGAGATTTCAACTTAACTTGA
25878	Yes	ACAAAGCACCCAACTTACACTTAGGAGATTTCAACTTAACTTGAC
25879	Yes	ACAAAGCACCCAACTTACACTTAGGAGATTTCAACTTAACTTGACC
25880	Yes	ACAAAGCACCCAACTTACACTTAGGAGATTTCAACTTAACTTGACCG
25881	Yes	ACAAAGCACCCAACTTACACTTAGGAGATTTCAACTTAACTTGACCGC
25882	Yes	ACAAAGCACCCAACTTACACTTAGGAGATTTCAACTTAACTTGACCGCT
25883	Yes	ACAAAGCACCCAACTTACACTTAGGAGATTTCAACTTAACTTGACCGCTC
25884	No	ACAAAGTTCCTTGGCT
25885	No	ACAAAGTTCCTTGGCTC
25886	No	ACAAAGTTCCTTGGCTCA
25887	No	ACAAAGTTCCTTGGCTCAA
25888	No	ACAAAGTTCCTTGGCTCAAT
25889	Yes	ACAAAGTTCCTTGGCTCAATC
25890	Yes	ACAAAGTTCCTTGGCTCAATCC
25891	Yes	ACAAAGTTCCTTGGCTCAATCCA
25892	Yes	ACAAAGTTCCTTGGCTCAATCCAG
25893	Yes	ACAAAGTTCCTTGGCTCAATCCAGA
25894	Yes	ACAAAGTTCCTTGGCTCAATCCAGAG
25895	Yes	ACAAAGTTCCTTGGCTCAATCCAGAGT
25896	Yes	ACAAAGTTCCTTGGCTCAATCCAGAGTT
25897	Yes	ACAAAGTTCCTTGGCTCAATCCAGAGTTT
25898	Yes	ACAAAGTTCCTTGGCTCAATCCAGAGTTTG
25899	Yes	ACAAAGTTCCTTGGCTCAATCCAGAGTTTGG
25900	Yes	ACAAAGTTCCTTGGCTCAATCCAGAGTTTGGG
25901	Yes	ACAAAGTTCCTTGGCTCAATCCAGAGTTTGGGG
25902	Yes	ACAAAGTTCCTTGGCTCAATCCAGAGTTTGGGGG
25903	Yes	ACAAAGTTCCTTGGCTCAATCCAGAGTTTGGGGGC
25904	Yes	ACAAAGTTCCTTGGCTCAATCCAGAGTTTGGGGGCC
25905	Yes	ACAAAGTTCCTTGGCTCAATCCAGAGTTTGGGGGCCA
25906	Yes	ACAACAGAGGCTTACG
25907	Yes	ACAACAGAGGCTTACGA

25908	Yes	ACAACAGAGGCTTACGAC
25909	Yes	ACAACAGAGGCTTACGACC
25910	Yes	ACAACAGAGGCTTACGACCC
25911	Yes	ACAACAGAGGCTTACGACCCC
25912	Yes	ACAACAGAGGCTTACGACCCCT
25913	Yes	ACAACAGAGGCTTACGACCCCTT
25914	Yes	ACAACAGAGGCTTACGACCCCTTA
25915	Yes	ACAACAGAGGCTTACGACCCCTTAT
25916	Yes	ACAACAGAGGCTTACGACCCCTTATT
25917	Yes	ACAACAGAGGCTTACGACCCCTTATTT
25918	Yes	ACAACAGAGGCTTACGACCCCTTATTTA
25919	Yes	ACAACAGAGGCTTACGACCCCTTATTTAC
25920	Yes	ACAACAGAGGCTTACGACCCCTTATTTACC
25921	Yes	ACAACAGAGGCTTACGACCCCTTATTTACCC
25922	Yes	ACAACAGAGGCTTACGACCCCTTATTTACCCC
25923	Yes	ACAACAGAGGCTTACGACCCCTTATTTACCCCA
25924	No	ACAACATGGCTTTCTC
25925	No	ACAACATGGCTTTCTCA
25926	Yes	ACAACATGGCTTTCTCAC
25927	Yes	ACAACATGGCTTTCTCACC
25928	Yes	ACAACATGGCTTTCTCACCA
25929	No	ACAACATTCAAAAAAG
25930	No	ACAACATTCAAAAAAGA
25931	No	ACAACATTCAAAAAAGAG
25932	No	ACAACATTCAAAAAAGAGT
25933	Yes	ACAACATTCAAAAAAGAGTA
25934	Yes	ACAACATTCAAAAAAGAGTAC
25935	Yes	ACAACATTCAAAAAAGAGTACC
25936	Yes	ACAACATTCAAAAAAGAGTACCA
25937	No	ACAACGATGGTTTTTC
25938	No	ACAACGATGGTTTTTCA
25939	No	ACAACGATGGTTTTTCAT
25940	No	ACAACGATGGTTTTTCATA
25941	No	ACAACGATGGTTTTTCATAT
25942	No	ACAACGATGGTTTTTCATATC
25943	No	ACAACGATGGTTTTTCATATCA
25944	No	ACAACGATGGTTTTTCATATCAT
25945	No	ACAACGATGGTTTTTCATATCATT
25946	No	ACAACGATGGTTTTTCATATCATTG
25947	No	ACAACGATGGTTTTTCATATCATTGG
25948	No	ACAACGATGGTTTTTCATATCATTGGT
25949	No	ACAACGATGGTTTTTCATATCATTGGTC

25950	No	ACAACGATGGTTTTTCATATCATTGGTCG
25951	No	ACAACGATGGTTTTTCATATCATTGGTCGT
25952	No	ACAACGATGGTTTTTCATATCATTGGTCGTG
25953	No	ACAACGATGGTTTTTCATATCATTGGTCGTGG
25954	No	ACAACGATGGTTTTTCATATCATTGGTCGTGGT
25955	No	ACAACGATGGTTTTTCATATCATTGGTCGTGGTT
25956	No	ACAACGATGGTTTTTCATATCATTGGTCGTGGTTG
25957	No	ACAACGATGGTTTTTCATATCATTGGTCGTGGTTGT
25958	No	ACAACGATGGTTTTTCATATCATTGGTCGTGGTTGTA
25959	No	ACAACGATGGTTTTTCATATCATTGGTCGTGGTTGTAG
25960	Yes	ACAACGATGGTTTTTCATATCATTGGTCGTGGTTGTAGT
25961	Yes	ACAACGATGGTTTTTCATATCATTGGTCGTGGTTGTAGTC
25962	Yes	ACAACGATGGTTTTTCATATCATTGGTCGTGGTTGTAGTCC
25963	Yes	ACAACGATGGTTTTTCATATCATTGGTCGTGGTTGTAGTCCG
25964	Yes	ACAACGATGGTTTTTCATATCATTGGTCGTGGTTGTAGTCCGT
25965	Yes	ACAACGATGGTTTTTCATATCATTGGTCGTGGTTGTAGTCCGTG
25966	Yes	ACAACGATGGTTTTTCATATCATTGGTCGTGGTTGTAGTCCGTGC
25967	Yes	ACAACGATGGTTTTTCATATCATTGGTCGTGGTTGTAGTCCGTGCG
25968	Yes	ACAACGATGGTTTTTCATATCATTGGTCGTGGTTGTAGTCCGTGCGA
25969	Yes	ACAACGATGGTTTTTCATATCATTGGTCGTGGTTGTAGTCCGTGCGAG
25970	Yes	ACAACGATGGTTTTTCATATCATTGGTCGTGGTTGTAGTCCGTGCGAGA
25971	Yes	ACAACGATGGTTTTTCATATCATTGGTCGTGGTTGTAGTCCGTGCGAGAA
25972	No	ACAACGGTTAAGTGAA
25973	Yes	ACAACGGTTAAGTGAAG
25974	Yes	ACAACGGTTAAGTGAAGA
25975	Yes	ACAACGGTTAAGTGAAGAG
25976	Yes	ACAACGGTTAAGTGAAGAGG
25977	Yes	ACAACGGTTAAGTGAAGAGGC
25978	Yes	ACAACGGTTAAGTGAAGAGGCT
25979	Yes	ACAACGGTTAAGTGAAGAGGCTG
25980	Yes	ACAACGGTTAAGTGAAGAGGCTGT
25981	Yes	ACAACGGTTAAGTGAAGAGGCTGTA
25982	Yes	ACAACGGTTAAGTGAAGAGGCTGTAA
25983	Yes	ACAACGGTTAAGTGAAGAGGCTGTAAA
25984	Yes	ACAACGGTTAAGTGAAGAGGCTGTAAAC
25985	Yes	ACAACGGTTAAGTGAAGAGGCTGTAAACC
25986	Yes	ACAACGGTTAAGTGAAGAGGCTGTAAACCC
25987	Yes	ACAACGGTTAAGTGAAGAGGCTGTAAACCCA
25988	Yes	ACAACGGTTAAGTGAAGAGGCTGTAAACCCAG
25989	Yes	ACAACGGTTAAGTGAAGAGGCTGTAAACCCAGA
25990	Yes	ACAACGGTTAAGTGAAGAGGCTGTAAACCCAGAC
25991	Yes	ACAACGGTTAAGTGAAGAGGCTGTAAACCCAGACT

25992	Yes	ACAACGGTTAAGTGAAGAGGCTGTAAACCCAGACTG
25993	Yes	ACAACGGTTAAGTGAAGAGGCTGTAAACCCAGACTGG
25994	Yes	ACAACGGTTAAGTGAAGAGGCTGTAAACCCAGACTGGA
25995	Yes	ACAACGGTTAAGTGAAGAGGCTGTAAACCCAGACTGGAT
25996	Yes	ACAACGGTTAAGTGAAGAGGCTGTAAACCCAGACTGGATG
25997	Yes	ACAACGGTTAAGTGAAGAGGCTGTAAACCCAGACTGGATGG
25998	Yes	ACAACGGTTAAGTGAAGAGGCTGTAAACCCAGACTGGATGGG
25999	Yes	ACAACGGTTAAGTGAAGAGGCTGTAAACCCAGACTGGATGGGT
26000	Yes	ACAACGGTTAAGTGAAGAGGCTGTAAACCCAGACTGGATGGGTT
26001	Yes	ACAACGGTTAAGTGAAGAGGCTGTAAACCCAGACTGGATGGGTTC
26002	Yes	ACAACGGTTAAGTGAAGAGGCTGTAAACCCAGACTGGATGGGTTCA
26003	Yes	ACAACGGTTAAGTGAAGAGGCTGTAAACCCAGACTGGATGGGTTCAA
26004	Yes	ACAACGGTTAAGTGAAGAGGCTGTAAACCCAGACTGGATGGGTTCAAT
26005	Yes	ACAACGGTTAAGTGAAGAGGCTGTAAACCCAGACTGGATGGGTTCAATT
26006	Yes	ACAACGGTTAAGTGAAGAGGCTGTAAACCCAGACTGGATGGGTTCAATTC
26007	Yes	ACAACTTTATGGGCAG
26008	Yes	ACAACTTTATGGGCAGA
26009	Yes	ACAACTTTATGGGCAGAG
26010	Yes	ACAACTTTATGGGCAGAGG
26011	Yes	ACAACTTTATGGGCAGAGGT
26012	Yes	ACAACTTTATGGGCAGAGGTT
26013	Yes	ACAACTTTATGGGCAGAGGTTC
26014	Yes	ACAACTTTATGGGCAGAGGTTCA
26015	Yes	ACAACTTTATGGGCAGAGGTTCAA
26016	Yes	ACAACTTTATGGGCAGAGGTTCAAT
26017	Yes	ACAACTTTATGGGCAGAGGTTCAATT
26018	Yes	ACAACTTTATGGGCAGAGGTTCAATTC
26019	Yes	ACAACTTTATGGGCAGAGGTTCAATTCC
26020	Yes	ACAACTTTATGGGCAGAGGTTCAATTCCT
26021	Yes	ACAACTTTATGGGCAGAGGTTCAATTCCTC
26022	Yes	ACAACTTTATGGGCAGAGGTTCAATTCCTCT
26023	Yes	ACAACTTTATGGGCAGAGGTTCAATTCCTCTT
26024	Yes	ACAACTTTATGGGCAGAGGTTCAATTCCTCTTC
26025	Yes	ACAACTTTATGGGCAGAGGTTCAATTCCTCTTCT
26026	Yes	ACAACTTTATGGGCAGAGGTTCAATTCCTCTTCTT
26027	Yes	ACAACTTTATGGGCAGAGGTTCAATTCCTCTTCTTA
26028	Yes	ACAACTTTATGGGCAGAGGTTCAATTCCTCTTCTTAA
26029	Yes	ACAACTTTATGGGCAGAGGTTCAATTCCTCTTCTTAAC
26030	Yes	ACAACTTTATGGGCAGAGGTTCAATTCCTCTTCTTAACA
26031	Yes	ACAACTTTATGGGCAGAGGTTCAATTCCTCTTCTTAACAC
26032	Yes	ACAACTTTATGGGCAGAGGTTCAATTCCTCTTCTTAACACC
26033	Yes	ACAACTTTATGGGCAGAGGTTCAATTCCTCTTCTTAACACCA

26034	No	ACAAGAACTGCTAACT
26035	No	ACAAGAACTGCTAACTC
26036	No	ACAAGAACTGCTAACTCA
26037	No	ACAAGAACTGCTAACTCAT
26038	No	ACAAGAACTGCTAACTCATG
26039	No	ACAAGAACTGCTAACTCATGC
26040	No	ACAAGAACTGCTAACTCATGCC
26041	No	ACAAGAACTGCTAACTCATGCCC
26042	No	ACAAGAACTGCTAACTCATGCCCC
26043	No	ACAAGAACTGCTAACTCATGCCCCC
26044	No	ACAAGAACTGCTAACTCATGCCCCCA
26045	No	ACAAGAACTGCTAACTCATGCCCCCAT
26046	No	ACAAGAACTGCTAACTCATGCCCCCATG
26047	Yes	ACAAGAACTGCTAACTCATGCCCCCATGT
26048	Yes	ACAAGAACTGCTAACTCATGCCCCCATGTC
26049	Yes	ACAAGAACTGCTAACTCATGCCCCCATGTCT
26050	Yes	ACAAGAACTGCTAACTCATGCCCCCATGTCTA
26051	Yes	ACAAGAACTGCTAACTCATGCCCCCATGTCTAA
26052	Yes	ACAAGAACTGCTAACTCATGCCCCCATGTCTAAC
26053	Yes	ACAAGAACTGCTAACTCATGCCCCCATGTCTAACA
26054	Yes	ACAAGAACTGCTAACTCATGCCCCCATGTCTAACAA
26055	Yes	ACAAGAACTGCTAACTCATGCCCCCATGTCTAACAAC
26056	Yes	ACAAGAACTGCTAACTCATGCCCCCATGTCTAACAACA
26057	Yes	ACAAGAACTGCTAACTCATGCCCCCATGTCTAACAACAT
26058	Yes	ACAAGAACTGCTAACTCATGCCCCCATGTCTAACAACATG
26059	Yes	ACAAGAACTGCTAACTCATGCCCCCATGTCTAACAACATGG
26060	Yes	ACAAGAACTGCTAACTCATGCCCCCATGTCTAACAACATGGC
26061	Yes	ACAAGAACTGCTAACTCATGCCCCCATGTCTAACAACATGGCT
26062	Yes	ACAAGAACTGCTAACTCATGCCCCCATGTCTAACAACATGGCTT
26063	Yes	ACAAGAACTGCTAACTCATGCCCCCATGTCTAACAACATGGCTTT
26064	Yes	ACAAGAACTGCTAACTCATGCCCCCATGTCTAACAACATGGCTTTC
26065	Yes	ACAAGAACTGCTAACTCATGCCCCCATGTCTAACAACATGGCTTTCT
26066	Yes	ACAAGAACTGCTAACTCATGCCCCCATGTCTAACAACATGGCTTTCTC
26067	Yes	ACAAGAACTGCTAACTCATGCCCCCATGTCTAACAACATGGCTTTCTCA
26068	Yes	ACAAGAACTGCTAACTCATGCCCCCATGTCTAACAACATGGCTTTCTCAC
26069	Yes	ACAAGACTCTTAATCT
26070	Yes	ACAAGACTCTTAATCTC
26071	Yes	ACAAGACTCTTAATCTCA
26072	Yes	ACAAGACTCTTAATCTCAG
26073	Yes	ACAAGACTCTTAATCTCAGG
26074	Yes	ACAAGACTCTTAATCTCAGGG
26075	Yes	ACAAGACTCTTAATCTCAGGGT

26076	Yes	ACAAGACTCTTAATCTCAGGGTC
26077	Yes	ACAAGACTCTTAATCTCAGGGTCG
26078	Yes	ACAAGACTCTTAATCTCAGGGTCGT
26079	Yes	ACAAGACTCTTAATCTCAGGGTCGTG
26080	Yes	ACAAGACTCTTAATCTCAGGGTCGTGG
26081	Yes	ACAAGACTCTTAATCTCAGGGTCGTGGG
26082	Yes	ACAAGACTCTTAATCTCAGGGTCGTGGGT
26083	Yes	ACAAGACTCTTAATCTCAGGGTCGTGGGTT
26084	Yes	ACAAGACTCTTAATCTCAGGGTCGTGGGTTT
26085	Yes	ACAAGACTCTTAATCTCAGGGTCGTGGGTTTG
26086	Yes	ACAAGACTCTTAATCTCAGGGTCGTGGGTTTGA
26087	Yes	ACAAGACTCTTAATCTCAGGGTCGTGGGTTTGAG
26088	Yes	ACAAGACTCTTAATCTCAGGGTCGTGGGTTTGAGC
26089	Yes	ACAAGACTCTTAATCTCAGGGTCGTGGGTTTGAGCC
26090	Yes	ACAAGACTCTTAATCTCAGGGTCGTGGGTTTGAGCCC
26091	Yes	ACAAGACTCTTAATCTCAGGGTCGTGGGTTTGAGCCCT
26092	Yes	ACAAGACTCTTAATCTCAGGGTCGTGGGTTTGAGCCCTG
26093	Yes	ACAAGACTCTTAATCTCAGGGTCGTGGGTTTGAGCCCTGT
26094	Yes	ACAAGACTCTTAATCTCAGGGTCGTGGGTTTGAGCCCTGTG
26095	Yes	ACAAGACTCTTAATCTCAGGGTCGTGGGTTTGAGCCCTGTGT
26096	Yes	ACAAGACTCTTAATCTCAGGGTCGTGGGTTTGAGCCCTGTGTT
26097	Yes	ACAAGACTCTTAATCTCAGGGTCGTGGGTTTGAGCCCTGTGTTG
26098	Yes	ACAAGACTCTTAATCTCAGGGTCGTGGGTTTGAGCCCTGTGTTGA
26099	Yes	ACAAGACTCTTAATCTCAGGGTCGTGGGTTTGAGCCCTGTGTTGAG
26100	Yes	ACAAGACTCTTAATCTCAGGGTCGTGGGTTTGAGCCCTGTGTTGAGC
26101	Yes	ACAAGACTCTTAATCTCAGGGTCGTGGGTTTGAGCCCTGTGTTGAGCA
26102	Yes	ACAAGACTCTTAATCTCAGGGTCGTGGGTTTGAGCCCTGTGTTGAGCAC
26103	Yes	ACAAGACTCTTAATCTCAGGGTCGTGGGTTTGAGCCCTGTGTTGAGCACC
26104	Yes	ACAATCAGAGGTTCAA
26105	Yes	ACAATCAGAGGTTCAAC
26106	Yes	ACAATCAGAGGTTCAACT
26107	Yes	ACAATCAGAGGTTCAACTC
26108	Yes	ACAATCAGAGGTTCAACTCC
26109	Yes	ACAATCAGAGGTTCAACTCCT
26110	Yes	ACAATCAGAGGTTCAACTCCTC
26111	Yes	ACAATCAGAGGTTCAACTCCTCT
26112	Yes	ACAATCAGAGGTTCAACTCCTCTT
26113	Yes	ACAATCAGAGGTTCAACTCCTCTTC
26114	Yes	ACAATCAGAGGTTCAACTCCTCTTCT
26115	Yes	ACAATCAGAGGTTCAACTCCTCTTCTT
26116	Yes	ACAATCAGAGGTTCAACTCCTCTTCTTA
26117	Yes	ACAATCAGAGGTTCAACTCCTCTTCTTAA

26118	Yes	ACAATCAGAGGTTCAACTCCTCTTCTTAAC
26119	Yes	ACAATCAGAGGTTCAACTCCTCTTCTTAACA
26120	Yes	ACAATCAGAGGTTCAACTCCTCTTCTTAACAC
26121	Yes	ACAATCAGAGGTTCAACTCCTCTTCTTAACACC
26122	Yes	ACAATCAGAGGTTCAACTCCTCTTCTTAACACCA
26123	Yes	ACAATCGGTTAGAGCG
26124	Yes	ACAATCGGTTAGAGCGT
26125	Yes	ACAATCGGTTAGAGCGTT
26126	Yes	ACAATCGGTTAGAGCGTTC
26127	Yes	ACAATCGGTTAGAGCGTTCG
26128	Yes	ACAATCGGTTAGAGCGTTCGG
26129	Yes	ACAATCGGTTAGAGCGTTCGGC
26130	Yes	ACAATCGGTTAGAGCGTTCGGCT
26131	Yes	ACAATCGGTTAGAGCGTTCGGCTG
26132	Yes	ACAATCGGTTAGAGCGTTCGGCTGT
26133	Yes	ACAATCGGTTAGAGCGTTCGGCTGTT
26134	Yes	ACAATCGGTTAGAGCGTTCGGCTGTTA
26135	Yes	ACAATCGGTTAGAGCGTTCGGCTGTTAA
26136	Yes	ACAATCGGTTAGAGCGTTCGGCTGTTAAT
26137	Yes	ACAATCGGTTAGAGCGTTCGGCTGTTAATC
26138	Yes	ACAATCGGTTAGAGCGTTCGGCTGTTAATCT
26139	Yes	ACAATCGGTTAGAGCGTTCGGCTGTTAATCTA
26140	Yes	ACAATCGGTTAGAGCGTTCGGCTGTTAATCTAA
26141	Yes	ACAATCGGTTAGAGCGTTCGGCTGTTAATCTAAA
26142	Yes	ACAATCGGTTAGAGCGTTCGGCTGTTAATCTAAAG
26143	Yes	ACAATCGGTTAGAGCGTTCGGCTGTTAATCTAAAGG
26144	Yes	ACAATCGGTTAGAGCGTTCGGCTGTTAATCTAAAGGT
26145	Yes	ACAATCGGTTAGAGCGTTCGGCTGTTAATCTAAAGGTT
26146	Yes	ACAATCGGTTAGAGCGTTCGGCTGTTAATCTAAAGGTTG
26147	Yes	ACAATCGGTTAGAGCGTTCGGCTGTTAATCTAAAGGTTGG
26148	Yes	ACAATCGGTTAGAGCGTTCGGCTGTTAATCTAAAGGTTGGT
26149	Yes	ACAATCGGTTAGAGCGTTCGGCTGTTAATCTAAAGGTTGGTG
26150	Yes	ACAATCGGTTAGAGCGTTCGGCTGTTAATCTAAAGGTTGGTGG
26151	Yes	ACAATCGGTTAGAGCGTTCGGCTGTTAATCTAAAGGTTGGTGGC
26152	Yes	ACAATCGGTTAGAGCGTTCGGCTGTTAATCTAAAGGTTGGTGGCT
26153	Yes	ACAATCGGTTAGAGCGTTCGGCTGTTAATCTAAAGGTTGGTGGCTA
26154	Yes	ACAATCGGTTAGAGCGTTCGGCTGTTAATCTAAAGGTTGGTGGCTAG
26155	Yes	ACAATCGGTTAGAGCGTTCGGCTGTTAATCTAAAGGTTGGTGGCTAGA
26156	Yes	ACAATCGGTTAGAGCGTTCGGCTGTTAATCTAAAGGTTGGTGGCTAGAA
26157	Yes	ACAATCGGTTAGAGCGTTCGGCTGTTAATCTAAAGGTTGGTGGCTAGAAC
26158	Yes	ACAATCGGTTAGCGCG
26159	Yes	ACAATCGGTTAGCGCGT

26160	Yes	ACAATCGGTTAGCGCGTT
26161	Yes	ACAATCGGTTAGCGCGTTC
26162	Yes	ACAATCGGTTAGCGCGTTCG
26163	Yes	ACAATCGGTTAGCGCGTTCGG
26164	Yes	ACAATCGGTTAGCGCGTTCGGC
26165	Yes	ACAATCGGTTAGCGCGTTCGGCT
26166	Yes	ACAATCGGTTAGCGCGTTCGGCTG
26167	Yes	ACAATCGGTTAGCGCGTTCGGCTGT
26168	Yes	ACAATCGGTTAGCGCGTTCGGCTGTT
26169	Yes	ACAATCGGTTAGCGCGTTCGGCTGTTA
26170	Yes	ACAATCGGTTAGCGCGTTCGGCTGTTAA
26171	Yes	ACAATCGGTTAGCGCGTTCGGCTGTTAAT
26172	Yes	ACAATCGGTTAGCGCGTTCGGCTGTTAATC
26173	Yes	ACAATCGGTTAGCGCGTTCGGCTGTTAATCT
26174	Yes	ACAATCGGTTAGCGCGTTCGGCTGTTAATCTA
26175	Yes	ACAATCGGTTAGCGCGTTCGGCTGTTAATCTAG
26176	Yes	ACAATCGGTTAGCGCGTTCGGCTGTTAATCTAGA
26177	Yes	ACAATCGGTTAGCGCGTTCGGCTGTTAATCTAGAG
26178	Yes	ACAATCGGTTAGCGCGTTCGGCTGTTAATCTAGAGG
26179	Yes	ACAATCGGTTAGCGCGTTCGGCTGTTAATCTAGAGGT
26180	Yes	ACAATCGGTTAGCGCGTTCGGCTGTTAATCTAGAGGTT
26181	Yes	ACAATCGGTTAGCGCGTTCGGCTGTTAATCTAGAGGTTG
26182	Yes	ACAATCGGTTAGCGCGTTCGGCTGTTAATCTAGAGGTTGG
26183	Yes	ACAATCGGTTAGCGCGTTCGGCTGTTAATCTAGAGGTTGGT
26184	Yes	ACAATCGGTTAGCGCGTTCGGCTGTTAATCTAGAGGTTGGTG
26185	Yes	ACAATCGGTTAGCGCGTTCGGCTGTTAATCTAGAGGTTGGTGG
26186	Yes	ACAATCGGTTAGCGCGTTCGGCTGTTAATCTAGAGGTTGGTGGT
26187	Yes	ACAATCGGTTAGCGCGTTCGGCTGTTAATCTAGAGGTTGGTGGTT
26188	Yes	ACAATCGGTTAGCGCGTTCGGCTGTTAATCTAGAGGTTGGTGGTTA
26189	Yes	ACAATCGGTTAGCGCGTTCGGCTGTTAATCTAGAGGTTGGTGGTTAG
26190	Yes	ACAATCGGTTAGCGCGTTCGGCTGTTAATCTAGAGGTTGGTGGTTAGA
26191	Yes	ACAATCGGTTAGCGCGTTCGGCTGTTAATCTAGAGGTTGGTGGTTAGAG
26192	Yes	ACAATCGGTTAGCGCGTTCGGCTGTTAATCTAGAGGTTGGTGGTTAGAGC
26193	Yes	ACAATCGGTTAGCTTG
26194	Yes	ACAATCGGTTAGCTTGT
26195	Yes	ACAATCGGTTAGCTTGTT
26196	Yes	ACAATCGGTTAGCTTGTTC
26197	Yes	ACAATCGGTTAGCTTGTTCG
26198	Yes	ACAATCGGTTAGCTTGTTCGG
26199	Yes	ACAATCGGTTAGCTTGTTCGGC
26200	Yes	ACAATCGGTTAGCTTGTTCGGCT
26201	Yes	ACAATCGGTTAGCTTGTTCGGCTG

26202	Yes	ACAATCGGTTAGCTTGTTCGGCTGT
26203	Yes	ACAATCGGTTAGCTTGTTCGGCTGTT
26204	Yes	ACAATCGGTTAGCTTGTTCGGCTGTTA
26205	Yes	ACAATCGGTTAGCTTGTTCGGCTGTTAA
26206	Yes	ACAATCGGTTAGCTTGTTCGGCTGTTAAT
26207	Yes	ACAATCGGTTAGCTTGTTCGGCTGTTAATC
26208	Yes	ACAATCGGTTAGCTTGTTCGGCTGTTAATCT
26209	Yes	ACAATCGGTTAGCTTGTTCGGCTGTTAATCTA
26210	Yes	ACAATCGGTTAGCTTGTTCGGCTGTTAATCTAG
26211	Yes	ACAATCGGTTAGCTTGTTCGGCTGTTAATCTAGA
26212	Yes	ACAATCGGTTAGCTTGTTCGGCTGTTAATCTAGAG
26213	Yes	ACAATCGGTTAGCTTGTTCGGCTGTTAATCTAGAGG
26214	Yes	ACAATCGGTTAGCTTGTTCGGCTGTTAATCTAGAGGT
26215	Yes	ACAATCGGTTAGCTTGTTCGGCTGTTAATCTAGAGGTT
26216	Yes	ACAATCGGTTAGCTTGTTCGGCTGTTAATCTAGAGGTTG
26217	Yes	ACAATCGGTTAGCTTGTTCGGCTGTTAATCTAGAGGTTGG
26218	Yes	ACAATCGGTTAGCTTGTTCGGCTGTTAATCTAGAGGTTGGT
26219	Yes	ACAATCGGTTAGCTTGTTCGGCTGTTAATCTAGAGGTTGGTG
26220	Yes	ACAATCGGTTAGCTTGTTCGGCTGTTAATCTAGAGGTTGGTGG
26221	Yes	ACAATCGGTTAGCTTGTTCGGCTGTTAATCTAGAGGTTGGTGGT
26222	Yes	ACAATCGGTTAGCTTGTTCGGCTGTTAATCTAGAGGTTGGTGGTT
26223	Yes	ACAATCGGTTAGCTTGTTCGGCTGTTAATCTAGAGGTTGGTGGTTA
26224	Yes	ACAATCGGTTAGCTTGTTCGGCTGTTAATCTAGAGGTTGGTGGTTAG
26225	Yes	ACAATCGGTTAGCTTGTTCGGCTGTTAATCTAGAGGTTGGTGGTTAGA
26226	Yes	ACAATCGGTTAGCTTGTTCGGCTGTTAATCTAGAGGTTGGTGGTTAGAG
26227	Yes	ACAATCGGTTAGCTTGTTCGGCTGTTAATCTAGAGGTTGGTGGTTAGAGC
26228	No	ACACAAAGCACCCAAC
26229	No	ACACAAAGCACCCAACT
26230	No	ACACAAAGCACCCAACTT
26231	No	ACACAAAGCACCCAACTTA
26232	No	ACACAAAGCACCCAACTTAC
26233	No	ACACAAAGCACCCAACTTACA
26234	No	ACACAAAGCACCCAACTTACAC
26235	No	ACACAAAGCACCCAACTTACACT
26236	No	ACACAAAGCACCCAACTTACACTT
26237	No	ACACAAAGCACCCAACTTACACTTA
26238	No	ACACAAAGCACCCAACTTACACTTAG
26239	No	ACACAAAGCACCCAACTTACACTTAGG
26240	No	ACACAAAGCACCCAACTTACACTTAGGA
26241	No	ACACAAAGCACCCAACTTACACTTAGGAG
26242	No	ACACAAAGCACCCAACTTACACTTAGGAGA
26243	No	ACACAAAGCACCCAACTTACACTTAGGAGAT

26244	No	ACACAAAGCACCCAACTTACACTTAGGAGATT
26245	No	ACACAAAGCACCCAACTTACACTTAGGAGATTT
26246	No	ACACAAAGCACCCAACTTACACTTAGGAGATTTC
26247	No	ACACAAAGCACCCAACTTACACTTAGGAGATTTCA
26248	No	ACACAAAGCACCCAACTTACACTTAGGAGATTTCAA
26249	No	ACACAAAGCACCCAACTTACACTTAGGAGATTTCAAC
26250	No	ACACAAAGCACCCAACTTACACTTAGGAGATTTCAACT
26251	Yes	ACACAAAGCACCCAACTTACACTTAGGAGATTTCAACTT
26252	Yes	ACACAAAGCACCCAACTTACACTTAGGAGATTTCAACTTA
26253	Yes	ACACAAAGCACCCAACTTACACTTAGGAGATTTCAACTTAA
26254	Yes	ACACAAAGCACCCAACTTACACTTAGGAGATTTCAACTTAAC
26255	Yes	ACACAAAGCACCCAACTTACACTTAGGAGATTTCAACTTAACT
26256	Yes	ACACAAAGCACCCAACTTACACTTAGGAGATTTCAACTTAACTT
26257	Yes	ACACAAAGCACCCAACTTACACTTAGGAGATTTCAACTTAACTTG
26258	Yes	ACACAAAGCACCCAACTTACACTTAGGAGATTTCAACTTAACTTGA
26259	Yes	ACACAAAGCACCCAACTTACACTTAGGAGATTTCAACTTAACTTGAC
26260	Yes	ACACAAAGCACCCAACTTACACTTAGGAGATTTCAACTTAACTTGACC
26261	Yes	ACACAAAGCACCCAACTTACACTTAGGAGATTTCAACTTAACTTGACCG
26262	Yes	ACACAAAGCACCCAACTTACACTTAGGAGATTTCAACTTAACTTGACCGC
26263	Yes	ACACACCCAGAGGCGC
26264	Yes	ACACACCCAGAGGCGCC
26265	Yes	ACACACCCAGAGGCGCCA
26266	No	ACACAGGAGACCCGGG
26267	Yes	ACACAGGAGACCCGGGT
26268	Yes	ACACAGGAGACCCGGGTT
26269	Yes	ACACAGGAGACCCGGGTTC
26270	Yes	ACACAGGAGACCCGGGTTCA
26271	Yes	ACACAGGAGACCCGGGTTCAA
26272	Yes	ACACAGGAGACCCGGGTTCAAT
26273	Yes	ACACAGGAGACCCGGGTTCAATT
26274	Yes	ACACAGGAGACCCGGGTTCAATTC
26275	Yes	ACACAGGAGACCCGGGTTCAATTCC
26276	Yes	ACACAGGAGACCCGGGTTCAATTCCT
26277	Yes	ACACAGGAGACCCGGGTTCAATTCCTG
26278	Yes	ACACAGGAGACCCGGGTTCAATTCCTGA
26279	Yes	ACACAGGAGACCCGGGTTCAATTCCTGAC
26280	Yes	ACACAGGAGACCCGGGTTCAATTCCTGACC
26281	Yes	ACACAGGAGACCCGGGTTCAATTCCTGACCC
26282	Yes	ACACAGGAGACCCGGGTTCAATTCCTGACCCA
26283	Yes	ACACAGGAGACCCGGGTTCAATTCCTGACCCAT
26284	Yes	ACACAGGAGACCCGGGTTCAATTCCTGACCCATG
26285	Yes	ACACAGGAGACCCGGGTTCAATTCCTGACCCATGT

26286	Yes	ACACAGGAGACCCGGGTTCAATTCCTGACCCATGTA
26287	Yes	ACACAGGAGACCCGGGTTCAATTCCTGACCCATGTAC
26288	Yes	ACACAGGAGACCCGGGTTCAATTCCTGACCCATGTACC
26289	Yes	ACACAGGAGACCCGGGTTCAATTCCTGACCCATGTACCA
26290	No	ACACATGAAAGGTCAC
26291	Yes	ACACATGAAAGGTCACC
26292	Yes	ACACATGAAAGGTCACCA
26293	Yes	ACACATGAAAGGTCACCAG
26294	Yes	ACACATGAAAGGTCACCAGT
26295	Yes	ACACATGAAAGGTCACCAGTT
26296	Yes	ACACATGAAAGGTCACCAGTTT
26297	Yes	ACACATGAAAGGTCACCAGTTTG
26298	Yes	ACACATGAAAGGTCACCAGTTTGA
26299	Yes	ACACATGAAAGGTCACCAGTTTGAG
26300	Yes	ACACATGAAAGGTCACCAGTTTGAGA
26301	Yes	ACACATGAAAGGTCACCAGTTTGAGAC
26302	Yes	ACACATGAAAGGTCACCAGTTTGAGACC
26303	Yes	ACACATGAAAGGTCACCAGTTTGAGACCG
26304	Yes	ACACATGAAAGGTCACCAGTTTGAGACCGG
26305	Yes	ACACATGAAAGGTCACCAGTTTGAGACCGGG
26306	Yes	ACACATGAAAGGTCACCAGTTTGAGACCGGGC
26307	Yes	ACACATGAAAGGTCACCAGTTTGAGACCGGGCC
26308	Yes	ACACATGAAAGGTCACCAGTTTGAGACCGGGCCA
26309	Yes	ACACATGAAAGGTCACCAGTTTGAGACCGGGCCAA
26310	Yes	ACACATGAAAGGTCACCAGTTTGAGACCGGGCCAAA
26311	Yes	ACACATGAAAGGTCACCAGTTTGAGACCGGGCCAAAA
26312	Yes	ACACATGAAAGGTCACCAGTTTGAGACCGGGCCAAAAC
26313	Yes	ACACATGAAAGGTCACCAGTTTGAGACCGGGCCAAAACA
26314	Yes	ACACATGAAAGGTCACCAGTTTGAGACCGGGCCAAAACAC
26315	Yes	ACACATGAAAGGTCACCAGTTTGAGACCGGGCCAAAACACC
26316	Yes	ACACATGAAAGGTCACCAGTTTGAGACCGGGCCAAAACACCA
26317	Yes	ACACCAAGGTCGCGGG
26318	Yes	ACACCAAGGTCGCGGGC
26319	Yes	ACACCAAGGTCGCGGGCT
26320	Yes	ACACCAAGGTCGCGGGCTC
26321	Yes	ACACCAAGGTCGCGGGCTCG
26322	Yes	ACACCAAGGTCGCGGGCTCGA
26323	Yes	ACACCAAGGTCGCGGGCTCGAC
26324	Yes	ACACCAAGGTCGCGGGCTCGACT
26325	Yes	ACACCAAGGTCGCGGGCTCGACTC
26326	Yes	ACACCAAGGTCGCGGGCTCGACTCC
26327	Yes	ACACCAAGGTCGCGGGCTCGACTCCC

26328	Yes	ACACCAAGGTCGCGGGCTCGACTCCCG
26329	Yes	ACACCAAGGTCGCGGGCTCGACTCCCGC
26330	Yes	ACACCAAGGTCGCGGGCTCGACTCCCGCA
26331	Yes	ACACCAAGGTCGCGGGCTCGACTCCCGCAC
26332	Yes	ACACCAAGGTCGCGGGCTCGACTCCCGCACC
26333	Yes	ACACCAAGGTCGCGGGCTCGACTCCCGCACCG
26334	Yes	ACACCAAGGTCGCGGGCTCGACTCCCGCACCGG
26335	Yes	ACACCAAGGTCGCGGGCTCGACTCCCGCACCGGC
26336	Yes	ACACCAAGGTCGCGGGCTCGACTCCCGCACCGGCC
26337	Yes	ACACCAAGGTCGCGGGCTCGACTCCCGCACCGGCCA
26338	Yes	ACACCAAGGTCGCGGGCTCGACTCCCGCACCGGCCAC
26339	Yes	ACACCAAGGTCGCGGGCTCGACTCCCGCACCGGCCACC
26340	Yes	ACACCAAGGTCGCGGGCTCGACTCCCGCACCGGCCACCA
26341	No	ACACCAGTCTTGTAAA
26342	Yes	ACACCAGTCTTGTAAAC
26343	Yes	ACACCAGTCTTGTAAACC
26344	Yes	ACACCAGTCTTGTAAACCG
26345	Yes	ACACCAGTCTTGTAAACCGG
26346	Yes	ACACCAGTCTTGTAAACCGGA
26347	Yes	ACACCAGTCTTGTAAACCGGAG
26348	Yes	ACACCAGTCTTGTAAACCGGAGA
26349	Yes	ACACCAGTCTTGTAAACCGGAGAT
26350	Yes	ACACCAGTCTTGTAAACCGGAGATG
26351	Yes	ACACCAGTCTTGTAAACCGGAGATGA
26352	Yes	ACACCAGTCTTGTAAACCGGAGATGAA
26353	Yes	ACACCAGTCTTGTAAACCGGAGATGAAA
26354	Yes	ACACCAGTCTTGTAAACCGGAGATGAAAA
26355	Yes	ACACCAGTCTTGTAAACCGGAGATGAAAAC
26356	Yes	ACACCAGTCTTGTAAACCGGAGATGAAAACC
26357	Yes	ACACCAGTCTTGTAAACCGGAGATGAAAACCT
26358	Yes	ACACCAGTCTTGTAAACCGGAGATGAAAACCTT
26359	Yes	ACACCAGTCTTGTAAACCGGAGATGAAAACCTTT
26360	Yes	ACACCAGTCTTGTAAACCGGAGATGAAAACCTTTT
26361	Yes	ACACCAGTCTTGTAAACCGGAGATGAAAACCTTTTT
26362	Yes	ACACCAGTCTTGTAAACCGGAGATGAAAACCTTTTTC
26363	Yes	ACACCAGTCTTGTAAACCGGAGATGAAAACCTTTTTCC
26364	Yes	ACACCAGTCTTGTAAACCGGAGATGAAAACCTTTTTCCA
26365	Yes	ACACCAGTCTTGTAAACCGGAGATGAAAACCTTTTTCCAA
26366	Yes	ACACCAGTCTTGTAAACCGGAGATGAAAACCTTTTTCCAAG
26367	Yes	ACACCAGTCTTGTAAACCGGAGATGAAAACCTTTTTCCAAGG
26368	Yes	ACACCAGTCTTGTAAACCGGAGATGAAAACCTTTTTCCAAGGA
26369	Yes	ACACCAGTCTTGTAAACCGGAGATGAAAACCTTTTTCCAAGGAC

26370	Yes	ACACCAGTCTTGTAAACCGGAGATGAAAACCTTTTTCCAAGGACA
26371	Yes	ACACCAGTCTTGTAAACCGGAGATGAAAACCTTTTTCCAAGGACAC
26372	Yes	ACACCAGTCTTGTAAACCGGAGATGAAAACCTTTTTCCAAGGACACC
26373	Yes	ACACCAGTCTTGTAAACCGGAGATGAAAACCTTTTTCCAAGGACACCA
26374	Yes	ACACCCAGAGGCGCCA
26375	Yes	ACACCCGGGTTCAATT
26376	Yes	ACACCCGGGTTCAATTC
26377	Yes	ACACCCGGGTTCAATTCC
26378	Yes	ACACCCGGGTTCAATTCCC
26379	Yes	ACACCCGGGTTCAATTCCCG
26380	Yes	ACACCCGGGTTCAATTCCCGG
26381	Yes	ACACCCGGGTTCAATTCCCGGT
26382	Yes	ACACCCGGGTTCAATTCCCGGTC
26383	Yes	ACACCCGGGTTCAATTCCCGGTCA
26384	Yes	ACACCCGGGTTCAATTCCCGGTCAA
26385	Yes	ACACCCGGGTTCAATTCCCGGTCAAG
26386	Yes	ACACCCGGGTTCAATTCCCGGTCAAGG
26387	Yes	ACACCCGGGTTCAATTCCCGGTCAAGGC
26388	Yes	ACACCCGGGTTCAATTCCCGGTCAAGGCA
26389	Yes	ACACCCGGGTTCAATTCCCGGTCAAGGCAC
26390	Yes	ACACCCGGGTTCAATTCCCGGTCAAGGCACC
26391	Yes	ACACCCGGGTTCAATTCCCGGTCAAGGCACCA
26392	No	ACACCTCTAGGCCTGG
26393	No	ACACCTCTAGGCCTGGG
26394	Yes	ACACCTCTAGGCCTGGGT
26395	Yes	ACACCTCTAGGCCTGGGTT
26396	Yes	ACACCTCTAGGCCTGGGTTC
26397	Yes	ACACCTCTAGGCCTGGGTTCA
26398	Yes	ACACCTCTAGGCCTGGGTTCAA
26399	Yes	ACACCTCTAGGCCTGGGTTCAAA
26400	Yes	ACACCTCTAGGCCTGGGTTCAAAT
26401	Yes	ACACCTCTAGGCCTGGGTTCAAATC
26402	Yes	ACACCTCTAGGCCTGGGTTCAAATCC
26403	Yes	ACACCTCTAGGCCTGGGTTCAAATCCC
26404	Yes	ACACCTCTAGGCCTGGGTTCAAATCCCA
26405	Yes	ACACCTCTAGGCCTGGGTTCAAATCCCAG
26406	Yes	ACACCTCTAGGCCTGGGTTCAAATCCCAGG
26407	Yes	ACACCTCTAGGCCTGGGTTCAAATCCCAGGT
26408	Yes	ACACCTCTAGGCCTGGGTTCAAATCCCAGGTC
26409	Yes	ACACCTCTAGGCCTGGGTTCAAATCCCAGGTCT
26410	Yes	ACACCTCTAGGCCTGGGTTCAAATCCCAGGTCTA
26411	Yes	ACACCTCTAGGCCTGGGTTCAAATCCCAGGTCTAG

26412	Yes	ACACCTCTAGGCCTGGGTTCAAATCCCAGGTCTAGC
26413	Yes	ACACCTCTAGGCCTGGGTTCAAATCCCAGGTCTAGCG
26414	Yes	ACACCTCTAGGCCTGGGTTCAAATCCCAGGTCTAGCGC
26415	Yes	ACACCTCTAGGCCTGGGTTCAAATCCCAGGTCTAGCGCC
26416	Yes	ACACCTCTAGGCCTGGGTTCAAATCCCAGGTCTAGCGCCA
26417	No	ACACCTCTTCTTAACA
26418	Yes	ACACCTCTTCTTAACAC
26419	Yes	ACACCTCTTCTTAACACC
26420	Yes	ACACCTCTTCTTAACACCA
26421	No	ACACCTCTTTACAGTG
26422	No	ACACCTCTTTACAGTGA
26423	Yes	ACACCTCTTTACAGTGAC
26424	Yes	ACACCTCTTTACAGTGACC
26425	Yes	ACACCTCTTTACAGTGACCA
26426	Yes	ACACCTGGCGGCCTGG
26427	Yes	ACACCTGGCGGCCTGGG
26428	Yes	ACACCTGGCGGCCTGGGT
26429	Yes	ACACCTGGCGGCCTGGGTT
26430	Yes	ACACCTGGCGGCCTGGGTTC
26431	Yes	ACACCTGGCGGCCTGGGTTCA
26432	Yes	ACACCTGGCGGCCTGGGTTCAA
26433	Yes	ACACCTGGCGGCCTGGGTTCAAA
26434	Yes	ACACCTGGCGGCCTGGGTTCAAAT
26435	Yes	ACACCTGGCGGCCTGGGTTCAAATC
26436	Yes	ACACCTGGCGGCCTGGGTTCAAATCC
26437	Yes	ACACCTGGCGGCCTGGGTTCAAATCCC
26438	Yes	ACACCTGGCGGCCTGGGTTCAAATCCCA
26439	Yes	ACACCTGGCGGCCTGGGTTCAAATCCCAG
26440	Yes	ACACCTGGCGGCCTGGGTTCAAATCCCAGC
26441	Yes	ACACCTGGCGGCCTGGGTTCAAATCCCAGCT
26442	Yes	ACACCTGGCGGCCTGGGTTCAAATCCCAGCTT
26443	Yes	ACACCTGGCGGCCTGGGTTCAAATCCCAGCTTC
26444	Yes	ACACCTGGCGGCCTGGGTTCAAATCCCAGCTTCT
26445	Yes	ACACCTGGCGGCCTGGGTTCAAATCCCAGCTTCTA
26446	Yes	ACACCTGGCGGCCTGGGTTCAAATCCCAGCTTCTAC
26447	Yes	ACACCTGGCGGCCTGGGTTCAAATCCCAGCTTCTACA
26448	Yes	ACACCTGGCGGCCTGGGTTCAAATCCCAGCTTCTACAC
26449	Yes	ACACCTGGCGGCCTGGGTTCAAATCCCAGCTTCTACACC
26450	Yes	ACACCTGGCGGCCTGGGTTCAAATCCCAGCTTCTACACCA
26451	No	ACACCTGGGTTCAAAT
26452	No	ACACCTGGGTTCAAATC
26453	No	ACACCTGGGTTCAAATCC

26454	No	ACACCTGGGTTCAAATCCC
26455	No	ACACCTGGGTTCAAATCCCA
26456	No	ACACCTGGGTTCAAATCCCAG
26457	Yes	ACACCTGGGTTCAAATCCCAGT
26458	Yes	ACACCTGGGTTCAAATCCCAGTG
26459	Yes	ACACCTGGGTTCAAATCCCAGTGC
26460	Yes	ACACCTGGGTTCAAATCCCAGTGCT
26461	Yes	ACACCTGGGTTCAAATCCCAGTGCTG
26462	Yes	ACACCTGGGTTCAAATCCCAGTGCTGT
26463	Yes	ACACCTGGGTTCAAATCCCAGTGCTGTC
26464	Yes	ACACCTGGGTTCAAATCCCAGTGCTGTCA
26465	Yes	ACACCTGGGTTCAAATCCCAGTGCTGTCAC
26466	Yes	ACACCTGGGTTCAAATCCCAGTGCTGTCACC
26467	Yes	ACACCTGGGTTCAAATCCCAGTGCTGTCACCA
26468	Yes	ACACGAGAAAAGTCCC
26469	Yes	ACACGAGAAAAGTCCCT
26470	Yes	ACACGAGAAAAGTCCCTG
26471	Yes	ACACGAGAAAAGTCCCTGA
26472	Yes	ACACGAGAAAAGTCCCTGAT
26473	Yes	ACACGAGAAAAGTCCCTGATT
26474	Yes	ACACGAGAAAAGTCCCTGATTC
26475	Yes	ACACGAGAAAAGTCCCTGATTCG
26476	Yes	ACACGAGAAAAGTCCCTGATTCGA
26477	Yes	ACACGAGAAAAGTCCCTGATTCGAG
26478	Yes	ACACGAGAAAAGTCCCTGATTCGAGA
26479	Yes	ACACGAGAAAAGTCCCTGATTCGAGAC
26480	Yes	ACACGAGAAAAGTCCCTGATTCGAGACT
26481	Yes	ACACGAGAAAAGTCCCTGATTCGAGACTG
26482	Yes	ACACGAGAAAAGTCCCTGATTCGAGACTGG
26483	Yes	ACACGAGAAAAGTCCCTGATTCGAGACTGGG
26484	Yes	ACACGAGAAAAGTCCCTGATTCGAGACTGGGT
26485	Yes	ACACGAGAAAAGTCCCTGATTCGAGACTGGGTG
26486	Yes	ACACGAGAAAAGTCCCTGATTCGAGACTGGGTGG
26487	Yes	ACACGAGAAAAGTCCCTGATTCGAGACTGGGTGGG
26488	Yes	ACACGAGAAAAGTCCCTGATTCGAGACTGGGTGGGA
26489	Yes	ACACGAGAAAAGTCCCTGATTCGAGACTGGGTGGGAA
26490	Yes	ACACGAGAAAAGTCCCTGATTCGAGACTGGGTGGGAAC
26491	Yes	ACACGAGAAAAGTCCCTGATTCGAGACTGGGTGGGAACG
26492	Yes	ACACGAGAAAAGTCCCTGATTCGAGACTGGGTGGGAACGC
26493	Yes	ACACGAGAAAAGTCCCTGATTCGAGACTGGGTGGGAACGCC
26494	Yes	ACACGAGAAAAGTCCCTGATTCGAGACTGGGTGGGAACGCCA
26495	Yes	ACACGCAGAAGGTCCT

26496	Yes	ACACGCAGAAGGTCCTG
26497	Yes	ACACGCAGAAGGTCCTGG
26498	Yes	ACACGCAGAAGGTCCTGGG
26499	Yes	ACACGCAGAAGGTCCTGGGT
26500	Yes	ACACGCAGAAGGTCCTGGGTT
26501	Yes	ACACGCAGAAGGTCCTGGGTTC
26502	Yes	ACACGCAGAAGGTCCTGGGTTCA
26503	Yes	ACACGCAGAAGGTCCTGGGTTCAA
26504	Yes	ACACGCAGAAGGTCCTGGGTTCAAG
26505	Yes	ACACGCAGAAGGTCCTGGGTTCAAGC
26506	Yes	ACACGCAGAAGGTCCTGGGTTCAAGCC
26507	Yes	ACACGCAGAAGGTCCTGGGTTCAAGCCC
26508	Yes	ACACGCAGAAGGTCCTGGGTTCAAGCCCC
26509	Yes	ACACGCAGAAGGTCCTGGGTTCAAGCCCCA
26510	Yes	ACACGCAGAAGGTCCTGGGTTCAAGCCCCAG
26511	Yes	ACACGCAGAAGGTCCTGGGTTCAAGCCCCAGT
26512	Yes	ACACGCAGAAGGTCCTGGGTTCAAGCCCCAGTG
26513	Yes	ACACGCAGAAGGTCCTGGGTTCAAGCCCCAGTGG
26514	Yes	ACACGCAGAAGGTCCTGGGTTCAAGCCCCAGTGGA
26515	Yes	ACACGCAGAAGGTCCTGGGTTCAAGCCCCAGTGGAA
26516	Yes	ACACGCAGAAGGTCCTGGGTTCAAGCCCCAGTGGAAC
26517	Yes	ACACGCAGAAGGTCCTGGGTTCAAGCCCCAGTGGAACC
26518	Yes	ACACGCAGAAGGTCCTGGGTTCAAGCCCCAGTGGAACCA
26519	Yes	ACACGCAGAAGGTCCTGGGTTCAAGCCCCAGTGGAACCAC
26520	Yes	ACACGCAGAAGGTCCTGGGTTCAAGCCCCAGTGGAACCACC
26521	Yes	ACACGCAGAAGGTCCTGGGTTCAAGCCCCAGTGGAACCACCA
26522	Yes	ACACGCAGAAGGTCCTGGGTTCG
26523	Yes	ACACGCAGAAGGTCCTGGGTTCGA
26524	Yes	ACACGCAGAAGGTCCTGGGTTCGAG
26525	Yes	ACACGCAGAAGGTCCTGGGTTCGAGC
26526	Yes	ACACGCAGAAGGTCCTGGGTTCGAGCC
26527	Yes	ACACGCAGAAGGTCCTGGGTTCGAGCCC
26528	Yes	ACACGCAGAAGGTCCTGGGTTCGAGCCCC
26529	Yes	ACACGCAGAAGGTCCTGGGTTCGAGCCCCA
26530	Yes	ACACGCAGAAGGTCCTGGGTTCGAGCCCCAG
26531	Yes	ACACGCAGAAGGTCCTGGGTTCGAGCCCCAGT
26532	Yes	ACACGCAGAAGGTCCTGGGTTCGAGCCCCAGTG
26533	Yes	ACACGCAGAAGGTCCTGGGTTCGAGCCCCAGTGG
26534	Yes	ACACGCAGAAGGTCCTGGGTTCGAGCCCCAGTGGA
26535	Yes	ACACGCAGAAGGTCCTGGGTTCGAGCCCCAGTGGAA
26536	Yes	ACACGCAGAAGGTCCTGGGTTCGAGCCCCAGTGGAAC
26537	Yes	ACACGCAGAAGGTCCTGGGTTCGAGCCCCAGTGGAACC

26538	Yes	ACACGCAGAAGGTCCTGGGTTCGAGCCCCAGTGGAACCA
26539	Yes	ACACGCAGAAGGTCCTGGGTTCGAGCCCCAGTGGAACCAC
26540	Yes	ACACGCAGAAGGTCCTGGGTTCGAGCCCCAGTGGAACCACC
26541	Yes	ACACGCAGAAGGTCCTGGGTTCGAGCCCCAGTGGAACCACCA
26542	Yes	ACACGCGAAAAGTCCC
26543	Yes	ACACGCGAAAAGTCCCC
26544	Yes	ACACGCGAAAAGTCCCCG
26545	Yes	ACACGCGAAAAGTCCCCGG
26546	Yes	ACACGCGAAAAGTCCCCGGT
26547	Yes	ACACGCGAAAAGTCCCCGGTT
26548	Yes	ACACGCGAAAAGTCCCCGGTTC
26549	Yes	ACACGCGAAAAGTCCCCGGTTCG
26550	Yes	ACACGCGAAAAGTCCCCGGTTCGA
26551	Yes	ACACGCGAAAAGTCCCCGGTTCGAA
26552	Yes	ACACGCGAAAAGTCCCCGGTTCGAAA
26553	Yes	ACACGCGAAAAGTCCCCGGTTCGAAAT
26554	Yes	ACACGCGAAAAGTCCCCGGTTCGAAATC
26555	Yes	ACACGCGAAAAGTCCCCGGTTCGAAATCA
26556	Yes	ACACGCGAAAAGTCCCCGGTTCGAAATCAG
26557	Yes	ACACGCGAAAAGTCCCCGGTTCGAAATCAGG
26558	Yes	ACACGCGAAAAGTCCCCGGTTCGAAATCAGGC
26559	Yes	ACACGCGAAAAGTCCCCGGTTCGAAATCAGGCG
26560	Yes	ACACGCGAAAAGTCCCCGGTTCGAAATCAGGCGG
26561	Yes	ACACGCGAAAAGTCCCCGGTTCGAAATCAGGCGGG
26562	Yes	ACACGCGAAAAGTCCCCGGTTCGAAATCAGGCGGGA
26563	Yes	ACACGCGAAAAGTCCCCGGTTCGAAATCAGGCGGGAA
26564	Yes	ACACGCGAAAAGTCCCCGGTTCGAAATCAGGCGGGAAC
26565	Yes	ACACGCGAAAAGTCCCCGGTTCGAAATCAGGCGGGAACA
26566	Yes	ACACGCGAAAAGTCCCCGGTTCGAAATCAGGCGGGAACAC
26567	Yes	ACACGCGAAAAGTCCCCGGTTCGAAATCAGGCGGGAACACC
26568	Yes	ACACGCGAAAAGTCCCCGGTTCGAAATCAGGCGGGAACACCA
26569	Yes	ACACGCGAAAGGTCCC
26570	Yes	ACACGCGAAAGGTCCCC
26571	Yes	ACACGCGAAAGGTCCCCG
26572	Yes	ACACGCGAAAGGTCCCCGG
26573	Yes	ACACGCGAAAGGTCCCCGGT
26574	Yes	ACACGCGAAAGGTCCCCGGTT
26575	Yes	ACACGCGAAAGGTCCCCGGTTC
26576	Yes	ACACGCGAAAGGTCCCCGGTTCG
26577	Yes	ACACGCGAAAGGTCCCCGGTTCGA
26578	Yes	ACACGCGAAAGGTCCCCGGTTCGAA
26579	Yes	ACACGCGAAAGGTCCCCGGTTCGAAA

26580	Yes	ACACGCGAAAGGTCCCCGGTTCGAAAC
26581	Yes	ACACGCGAAAGGTCCCCGGTTCGAAACC
26582	Yes	ACACGCGAAAGGTCCCCGGTTCGAAACCG
26583	Yes	ACACGCGAAAGGTCCCCGGTTCGAAACCGG
26584	Yes	ACACGCGAAAGGTCCCCGGTTCGAAACCGGG
26585	Yes	ACACGCGAAAGGTCCCCGGTTCGAAACCGGGC
26586	Yes	ACACGCGAAAGGTCCCCGGTTCGAAACCGGGCA
26587	Yes	ACACGCGAAAGGTCCCCGGTTCGAAACCGGGCAG
26588	Yes	ACACGCGAAAGGTCCCCGGTTCGAAACCGGGCAGA
26589	Yes	ACACGCGAAAGGTCCCCGGTTCGAAACCGGGCAGAA
26590	Yes	ACACGCGAAAGGTCCCCGGTTCGAAACCGGGCAGAAA
26591	Yes	ACACGCGAAAGGTCCCCGGTTCGAAACCGGGCAGAAAC
26592	Yes	ACACGCGAAAGGTCCCCGGTTCGAAACCGGGCAGAAACA
26593	Yes	ACACGCGAAAGGTCCCCGGTTCGAAACCGGGCAGAAACAC
26594	Yes	ACACGCGAAAGGTCCCCGGTTCGAAACCGGGCAGAAACACC
26595	Yes	ACACGCGAAAGGTCCCCGGTTCGAAACCGGGCAGAAACACCA
26596	Yes	ACACGCGAAAGGTCCCCGGTTCGAAACCGGGCAGAAG
26597	Yes	ACACGCGAAAGGTCCCCGGTTCGAAACCGGGCAGAAGC
26598	Yes	ACACGCGAAAGGTCCCCGGTTCGAAACCGGGCAGAAGCA
26599	Yes	ACACGCGAAAGGTCCCCGGTTCGAAACCGGGCAGAAGCAC
26600	Yes	ACACGCGAAAGGTCCCCGGTTCGAAACCGGGCAGAAGCACC
26601	Yes	ACACGCGAAAGGTCCCCGGTTCGAAACCGGGCAGAAGCACCA
26602	Yes	ACACGCGAAAGGTCCCCGGTTCGAAACCGGGCG
26603	Yes	ACACGCGAAAGGTCCCCGGTTCGAAACCGGGCGG
26604	Yes	ACACGCGAAAGGTCCCCGGTTCGAAACCGGGCGGA
26605	Yes	ACACGCGAAAGGTCCCCGGTTCGAAACCGGGCGGAA
26606	Yes	ACACGCGAAAGGTCCCCGGTTCGAAACCGGGCGGAAA
26607	Yes	ACACGCGAAAGGTCCCCGGTTCGAAACCGGGCGGAAAC
26608	Yes	ACACGCGAAAGGTCCCCGGTTCGAAACCGGGCGGAAACA
26609	Yes	ACACGCGAAAGGTCCCCGGTTCGAAACCGGGCGGAAACAC
26610	Yes	ACACGCGAAAGGTCCCCGGTTCGAAACCGGGCGGAAACACC
26611	Yes	ACACGCGAAAGGTCCCCGGTTCGAAACCGGGCGGAAACACCA
26612	Yes	ACACGCGAAAGGTCCCCGGTTCGAAACT
26613	Yes	ACACGCGAAAGGTCCCCGGTTCGAAACTG
26614	Yes	ACACGCGAAAGGTCCCCGGTTCGAAACTGG
26615	Yes	ACACGCGAAAGGTCCCCGGTTCGAAACTGGG
26616	Yes	ACACGCGAAAGGTCCCCGGTTCGAAACTGGGC
26617	Yes	ACACGCGAAAGGTCCCCGGTTCGAAACTGGGCG
26618	Yes	ACACGCGAAAGGTCCCCGGTTCGAAACTGGGCGG
26619	Yes	ACACGCGAAAGGTCCCCGGTTCGAAACTGGGCGGA
26620	Yes	ACACGCGAAAGGTCCCCGGTTCGAAACTGGGCGGAA
26621	Yes	ACACGCGAAAGGTCCCCGGTTCGAAACTGGGCGGAAA

26622	Yes	ACACGCGAAAGGTCCCCGGTTCGAAACTGGGCGGAAAC
26623	Yes	ACACGCGAAAGGTCCCCGGTTCGAAACTGGGCGGAAACA
26624	Yes	ACACGCGAAAGGTCCCCGGTTCGAAACTGGGCGGAAACAC
26625	Yes	ACACGCGAAAGGTCCCCGGTTCGAAACTGGGCGGAAACACC
26626	Yes	ACACGCGAAAGGTCCCCGGTTCGAAACTGGGCGGAAACACCA
26627	Yes	ACACGCGAAAGGTCCCCGGTTCGAT
26628	Yes	ACACGCGAAAGGTCCCCGGTTCGATC
26629	Yes	ACACGCGAAAGGTCCCCGGTTCGATCC
26630	Yes	ACACGCGAAAGGTCCCCGGTTCGATCCC
26631	Yes	ACACGCGAAAGGTCCCCGGTTCGATCCCG
26632	Yes	ACACGCGAAAGGTCCCCGGTTCGATCCCGG
26633	Yes	ACACGCGAAAGGTCCCCGGTTCGATCCCGGG
26634	Yes	ACACGCGAAAGGTCCCCGGTTCGATCCCGGGC
26635	Yes	ACACGCGAAAGGTCCCCGGTTCGATCCCGGGCG
26636	Yes	ACACGCGAAAGGTCCCCGGTTCGATCCCGGGCGG
26637	Yes	ACACGCGAAAGGTCCCCGGTTCGATCCCGGGCGGA
26638	Yes	ACACGCGAAAGGTCCCCGGTTCGATCCCGGGCGGAA
26639	Yes	ACACGCGAAAGGTCCCCGGTTCGATCCCGGGCGGAAA
26640	Yes	ACACGCGAAAGGTCCCCGGTTCGATCCCGGGCGGAAAC
26641	Yes	ACACGCGAAAGGTCCCCGGTTCGATCCCGGGCGGAAACA
26642	Yes	ACACGCGAAAGGTCCCCGGTTCGATCCCGGGCGGAAACAC
26643	Yes	ACACGCGAAAGGTCCCCGGTTCGATCCCGGGCGGAAACACC
26644	Yes	ACACGCGAAAGGTCCCCGGTTCGATCCCGGGCGGAAACACCA
26645	Yes	ACACGCGAAAGGTCCCCGGTTT
26646	Yes	ACACGCGAAAGGTCCCCGGTTTG
26647	Yes	ACACGCGAAAGGTCCCCGGTTTGA
26648	Yes	ACACGCGAAAGGTCCCCGGTTTGAA
26649	Yes	ACACGCGAAAGGTCCCCGGTTTGAAA
26650	Yes	ACACGCGAAAGGTCCCCGGTTTGAAAC
26651	Yes	ACACGCGAAAGGTCCCCGGTTTGAAACC
26652	Yes	ACACGCGAAAGGTCCCCGGTTTGAAACCA
26653	Yes	ACACGCGAAAGGTCCCCGGTTTGAAACCAG
26654	Yes	ACACGCGAAAGGTCCCCGGTTTGAAACCAGG
26655	Yes	ACACGCGAAAGGTCCCCGGTTTGAAACCAGGC
26656	Yes	ACACGCGAAAGGTCCCCGGTTTGAAACCAGGCG
26657	Yes	ACACGCGAAAGGTCCCCGGTTTGAAACCAGGCGG
26658	Yes	ACACGCGAAAGGTCCCCGGTTTGAAACCAGGCGGA
26659	Yes	ACACGCGAAAGGTCCCCGGTTTGAAACCAGGCGGAA
26660	Yes	ACACGCGAAAGGTCCCCGGTTTGAAACCAGGCGGAAA
26661	Yes	ACACGCGAAAGGTCCCCGGTTTGAAACCAGGCGGAAAC
26662	Yes	ACACGCGAAAGGTCCCCGGTTTGAAACCAGGCGGAAACA
26663	Yes	ACACGCGAAAGGTCCCCGGTTTGAAACCAGGCGGAAACAC

26664	Yes	ACACGCGAAAGGTCCCCGGTTTGAAACCAGGCGGAAACACC
26665	Yes	ACACGCGAAAGGTCCCCGGTTTGAAACCAGGCGGAAACACCA
26666	Yes	ACACGCGAAAGGTCCCT
26667	Yes	ACACGCGAAAGGTCCCTG
26668	Yes	ACACGCGAAAGGTCCCTGG
26669	Yes	ACACGCGAAAGGTCCCTGGA
26670	Yes	ACACGCGAAAGGTCCCTGGAT
26671	Yes	ACACGCGAAAGGTCCCTGGATC
26672	Yes	ACACGCGAAAGGTCCCTGGATCA
26673	Yes	ACACGCGAAAGGTCCCTGGATCAA
26674	Yes	ACACGCGAAAGGTCCCTGGATCAAA
26675	Yes	ACACGCGAAAGGTCCCTGGATCAAAA
26676	Yes	ACACGCGAAAGGTCCCTGGATCAAAAC
26677	Yes	ACACGCGAAAGGTCCCTGGATCAAAACC
26678	Yes	ACACGCGAAAGGTCCCTGGATCAAAACCA
26679	Yes	ACACGCGAAAGGTCCCTGGATCAAAACCAG
26680	Yes	ACACGCGAAAGGTCCCTGGATCAAAACCAGG
26681	Yes	ACACGCGAAAGGTCCCTGGATCAAAACCAGGC
26682	Yes	ACACGCGAAAGGTCCCTGGATCAAAACCAGGCG
26683	Yes	ACACGCGAAAGGTCCCTGGATCAAAACCAGGCGG
26684	Yes	ACACGCGAAAGGTCCCTGGATCAAAACCAGGCGGA
26685	Yes	ACACGCGAAAGGTCCCTGGATCAAAACCAGGCGGAA
26686	Yes	ACACGCGAAAGGTCCCTGGATCAAAACCAGGCGGAAA
26687	Yes	ACACGCGAAAGGTCCCTGGATCAAAACCAGGCGGAAAC
26688	Yes	ACACGCGAAAGGTCCCTGGATCAAAACCAGGCGGAAACA
26689	Yes	ACACGCGAAAGGTCCCTGGATCAAAACCAGGCGGAAACAC
26690	Yes	ACACGCGAAAGGTCCCTGGATCAAAACCAGGCGGAAACACC
26691	Yes	ACACGCGAAAGGTCCCTGGATCAAAACCAGGCGGAAACACCA
26692	Yes	ACACGCGAAAGGTCCG
26693	Yes	ACACGCGAAAGGTCCGC
26694	Yes	ACACGCGAAAGGTCCGCG
26695	Yes	ACACGCGAAAGGTCCGCGG
26696	Yes	ACACGCGAAAGGTCCGCGGT
26697	Yes	ACACGCGAAAGGTCCGCGGTT
26698	Yes	ACACGCGAAAGGTCCGCGGTTC
26699	Yes	ACACGCGAAAGGTCCGCGGTTCG
26700	Yes	ACACGCGAAAGGTCCGCGGTTCGA
26701	Yes	ACACGCGAAAGGTCCGCGGTTCGAA
26702	Yes	ACACGCGAAAGGTCCGCGGTTCGAAA
26703	Yes	ACACGCGAAAGGTCCGCGGTTCGAAAC
26704	Yes	ACACGCGAAAGGTCCGCGGTTCGAAACC
26705	Yes	ACACGCGAAAGGTCCGCGGTTCGAAACCG

26706	Yes	ACACGCGAAAGGTCCGCGGTTCGAAACCGG
26707	Yes	ACACGCGAAAGGTCCGCGGTTCGAAACCGGG
26708	Yes	ACACGCGAAAGGTCCGCGGTTCGAAACCGGGC
26709	Yes	ACACGCGAAAGGTCCGCGGTTCGAAACCGGGCG
26710	Yes	ACACGCGAAAGGTCCGCGGTTCGAAACCGGGCGG
26711	Yes	ACACGCGAAAGGTCCGCGGTTCGAAACCGGGCGGA
26712	Yes	ACACGCGAAAGGTCCGCGGTTCGAAACCGGGCGGAA
26713	Yes	ACACGCGAAAGGTCCGCGGTTCGAAACCGGGCGGAAA
26714	Yes	ACACGCGAAAGGTCCGCGGTTCGAAACCGGGCGGAAAC
26715	Yes	ACACGCGAAAGGTCCGCGGTTCGAAACCGGGCGGAAACA
26716	Yes	ACACGCGAAAGGTCCGCGGTTCGAAACCGGGCGGAAACAC
26717	Yes	ACACGCGAAAGGTCCGCGGTTCGAAACCGGGCGGAAACACC
26718	Yes	ACACGCGAAAGGTCCGCGGTTCGAAACCGGGCGGAAACACCA
26719	Yes	ACACGCGAAAGGTCCT
26720	Yes	ACACGCGAAAGGTCCTC
26721	Yes	ACACGCGAAAGGTCCTCG
26722	Yes	ACACGCGAAAGGTCCTCGG
26723	Yes	ACACGCGAAAGGTCCTCGGG
26724	Yes	ACACGCGAAAGGTCCTCGGGT
26725	Yes	ACACGCGAAAGGTCCTCGGGTC
26726	Yes	ACACGCGAAAGGTCCTCGGGTCG
26727	Yes	ACACGCGAAAGGTCCTCGGGTCGA
26728	Yes	ACACGCGAAAGGTCCTCGGGTCGAA
26729	Yes	ACACGCGAAAGGTCCTCGGGTCGAAA
26730	Yes	ACACGCGAAAGGTCCTCGGGTCGAAAC
26731	Yes	ACACGCGAAAGGTCCTCGGGTCGAAACC
26732	Yes	ACACGCGAAAGGTCCTCGGGTCGAAACCG
26733	Yes	ACACGCGAAAGGTCCTCGGGTCGAAACCGA
26734	Yes	ACACGCGAAAGGTCCTCGGGTCGAAACCGAG
26735	Yes	ACACGCGAAAGGTCCTCGGGTCGAAACCGAGC
26736	Yes	ACACGCGAAAGGTCCTCGGGTCGAAACCGAGCG
26737	Yes	ACACGCGAAAGGTCCTCGGGTCGAAACCGAGCGG
26738	Yes	ACACGCGAAAGGTCCTCGGGTCGAAACCGAGCGGA
26739	Yes	ACACGCGAAAGGTCCTCGGGTCGAAACCGAGCGGAA
26740	Yes	ACACGCGAAAGGTCCTCGGGTCGAAACCGAGCGGAAA
26741	Yes	ACACGCGAAAGGTCCTCGGGTCGAAACCGAGCGGAAAC
26742	Yes	ACACGCGAAAGGTCCTCGGGTCGAAACCGAGCGGAAACA
26743	Yes	ACACGCGAAAGGTCCTCGGGTCGAAACCGAGCGGAAACAC
26744	Yes	ACACGCGAAAGGTCCTCGGGTCGAAACCGAGCGGAAACACC
26745	Yes	ACACGCGAAAGGTCCTCGGGTCGAAACCGAGCGGAAACACCA
26746	Yes	ACACGCGAAAGGTCCTT
26747	Yes	ACACGCGAAAGGTCCTTG

26748	Yes	ACACGCGAAAGGTCCTTGG
26749	Yes	ACACGCGAAAGGTCCTTGGT
26750	Yes	ACACGCGAAAGGTCCTTGGTT
26751	Yes	ACACGCGAAAGGTCCTTGGTTT
26752	Yes	ACACGCGAAAGGTCCTTGGTTTG
26753	Yes	ACACGCGAAAGGTCCTTGGTTTGA
26754	Yes	ACACGCGAAAGGTCCTTGGTTTGAA
26755	Yes	ACACGCGAAAGGTCCTTGGTTTGAAA
26756	Yes	ACACGCGAAAGGTCCTTGGTTTGAAAC
26757	Yes	ACACGCGAAAGGTCCTTGGTTTGAAACC
26758	Yes	ACACGCGAAAGGTCCTTGGTTTGAAACCA
26759	Yes	ACACGCGAAAGGTCCTTGGTTTGAAACCAG
26760	Yes	ACACGCGAAAGGTCCTTGGTTTGAAACCAGG
26761	Yes	ACACGCGAAAGGTCCTTGGTTTGAAACCAGGC
26762	Yes	ACACGCGAAAGGTCCTTGGTTTGAAACCAGGCA
26763	Yes	ACACGCGAAAGGTCCTTGGTTTGAAACCAGGCAG
26764	Yes	ACACGCGAAAGGTCCTTGGTTTGAAACCAGGCAGA
26765	Yes	ACACGCGAAAGGTCCTTGGTTTGAAACCAGGCAGAA
26766	Yes	ACACGCGAAAGGTCCTTGGTTTGAAACCAGGCAGAAA
26767	Yes	ACACGCGAAAGGTCCTTGGTTTGAAACCAGGCAGAAAC
26768	Yes	ACACGCGAAAGGTCCTTGGTTTGAAACCAGGCAGAAACA
26769	Yes	ACACGCGAAAGGTCCTTGGTTTGAAACCAGGCAGAAACAC
26770	Yes	ACACGCGAAAGGTCCTTGGTTTGAAACCAGGCAGAAACACC
26771	Yes	ACACGCGAAAGGTCCTTGGTTTGAAACCAGGCAGAAACACCA
26772	Yes	ACACGCGGGACACCCG
26773	Yes	ACACGCGGGACACCCGG
26774	Yes	ACACGCGGGACACCCGGG
26775	Yes	ACACGCGGGACACCCGGGT
26776	Yes	ACACGCGGGACACCCGGGTT
26777	Yes	ACACGCGGGACACCCGGGTTC
26778	Yes	ACACGCGGGACACCCGGGTTCA
26779	Yes	ACACGCGGGACACCCGGGTTCAA
26780	Yes	ACACGCGGGACACCCGGGTTCAAT
26781	Yes	ACACGCGGGACACCCGGGTTCAATT
26782	Yes	ACACGCGGGACACCCGGGTTCAATTC
26783	Yes	ACACGCGGGACACCCGGGTTCAATTCC
26784	Yes	ACACGCGGGACACCCGGGTTCAATTCCC
26785	Yes	ACACGCGGGACACCCGGGTTCAATTCCCG
26786	Yes	ACACGCGGGACACCCGGGTTCAATTCCCGG
26787	Yes	ACACGCGGGACACCCGGGTTCAATTCCCGGT
26788	Yes	ACACGCGGGACACCCGGGTTCAATTCCCGGTC
26789	Yes	ACACGCGGGACACCCGGGTTCAATTCCCGGTCA

26790	Yes	ACACGCGGGACACCCGGGTTCAATTCCCGGTCAA
26791	Yes	ACACGCGGGACACCCGGGTTCAATTCCCGGTCAAG
26792	Yes	ACACGCGGGACACCCGGGTTCAATTCCCGGTCAAGG
26793	Yes	ACACGCGGGACACCCGGGTTCAATTCCCGGTCAAGGC
26794	Yes	ACACGCGGGACACCCGGGTTCAATTCCCGGTCAAGGCA
26795	Yes	ACACGCGGGACACCCGGGTTCAATTCCCGGTCAAGGCAC
26796	Yes	ACACGCGGGACACCCGGGTTCAATTCCCGGTCAAGGCACC
26797	Yes	ACACGCGGGACACCCGGGTTCAATTCCCGGTCAAGGCACCA
26798	Yes	ACACGCGTAAAGGTCC
26799	Yes	ACACGCGTAAAGGTCCC
26800	Yes	ACACGCGTAAAGGTCCCC
26801	Yes	ACACGCGTAAAGGTCCCCG
26802	Yes	ACACGCGTAAAGGTCCCCGG
26803	Yes	ACACGCGTAAAGGTCCCCGGT
26804	Yes	ACACGCGTAAAGGTCCCCGGTT
26805	Yes	ACACGCGTAAAGGTCCCCGGTTC
26806	Yes	ACACGCGTAAAGGTCCCCGGTTCG
26807	Yes	ACACGCGTAAAGGTCCCCGGTTCGA
26808	Yes	ACACGCGTAAAGGTCCCCGGTTCGAA
26809	Yes	ACACGCGTAAAGGTCCCCGGTTCGAAA
26810	Yes	ACACGCGTAAAGGTCCCCGGTTCGAAAC
26811	Yes	ACACGCGTAAAGGTCCCCGGTTCGAAACC
26812	Yes	ACACGCGTAAAGGTCCCCGGTTCGAAACCG
26813	Yes	ACACGCGTAAAGGTCCCCGGTTCGAAACCGG
26814	Yes	ACACGCGTAAAGGTCCCCGGTTCGAAACCGGG
26815	Yes	ACACGCGTAAAGGTCCCCGGTTCGAAACCGGGC
26816	Yes	ACACGCGTAAAGGTCCCCGGTTCGAAACCGGGCG
26817	Yes	ACACGCGTAAAGGTCCCCGGTTCGAAACCGGGCGG
26818	Yes	ACACGCGTAAAGGTCCCCGGTTCGAAACCGGGCGGA
26819	Yes	ACACGCGTAAAGGTCCCCGGTTCGAAACCGGGCGGAA
26820	Yes	ACACGCGTAAAGGTCCCCGGTTCGAAACCGGGCGGAAA
26821	Yes	ACACGCGTAAAGGTCCCCGGTTCGAAACCGGGCGGAAAC
26822	Yes	ACACGCGTAAAGGTCCCCGGTTCGAAACCGGGCGGAAACA
26823	Yes	ACACGCGTAAAGGTCCCCGGTTCGAAACCGGGCGGAAACAC
26824	Yes	ACACGCGTAAAGGTCCCCGGTTCGAAACCGGGCGGAAACACC
26825	Yes	ACACGCGTAAAGGTCCCCGGTTCGAAACCGGGCGGAAACACCA
26826	Yes	ACACGCTTAAAGTTCT
26827	Yes	ACACGCTTAAAGTTCTC
26828	Yes	ACACGCTTAAAGTTCTCT
26829	Yes	ACACGCTTAAAGTTCTCTG
26830	Yes	ACACGCTTAAAGTTCTCTGG
26831	Yes	ACACGCTTAAAGTTCTCTGGT

26832	Yes	ACACGCTTAAAGTTCTCTGGTT
26833	Yes	ACACGCTTAAAGTTCTCTGGTTG
26834	Yes	ACACGCTTAAAGTTCTCTGGTTGG
26835	Yes	ACACGCTTAAAGTTCTCTGGTTGGA
26836	Yes	ACACGCTTAAAGTTCTCTGGTTGGAT
26837	Yes	ACACGCTTAAAGTTCTCTGGTTGGATA
26838	Yes	ACACGCTTAAAGTTCTCTGGTTGGATAC
26839	Yes	ACACGCTTAAAGTTCTCTGGTTGGATACC
26840	Yes	ACACGCTTAAAGTTCTCTGGTTGGATACCA
26841	Yes	ACACGCTTAAAGTTCTCTGGTTGGATACCAG
26842	Yes	ACACGCTTAAAGTTCTCTGGTTGGATACCAGA
26843	Yes	ACACGCTTAAAGTTCTCTGGTTGGATACCAGAT
26844	Yes	ACACGCTTAAAGTTCTCTGGTTGGATACCAGATG
26845	Yes	ACACGCTTAAAGTTCTCTGGTTGGATACCAGATGG
26846	Yes	ACACGCTTAAAGTTCTCTGGTTGGATACCAGATGGA
26847	Yes	ACACGCTTAAAGTTCTCTGGTTGGATACCAGATGGAA
26848	Yes	ACACGCTTAAAGTTCTCTGGTTGGATACCAGATGGAAA
26849	Yes	ACACGCTTAAAGTTCTCTGGTTGGATACCAGATGGAAAT
26850	Yes	ACACGCTTAAAGTTCTCTGGTTGGATACCAGATGGAAATG
26851	Yes	ACACGCTTAAAGTTCTCTGGTTGGATACCAGATGGAAATGC
26852	Yes	ACACGCTTAAAGTTCTCTGGTTGGATACCAGATGGAAATGCC
26853	Yes	ACACGCTTAAAGTTCTCTGGTTGGATACCAGATGGAAATGCCA
26854	Yes	ACACGTGAAAGGTCCC
26855	Yes	ACACGTGAAAGGTCCCT
26856	Yes	ACACGTGAAAGGTCCCTG
26857	Yes	ACACGTGAAAGGTCCCTGG
26858	Yes	ACACGTGAAAGGTCCCTGGT
26859	Yes	ACACGTGAAAGGTCCCTGGTT
26860	Yes	ACACGTGAAAGGTCCCTGGTTC
26861	Yes	ACACGTGAAAGGTCCCTGGTTCG
26862	Yes	ACACGTGAAAGGTCCCTGGTTCGA
26863	Yes	ACACGTGAAAGGTCCCTGGTTCGAA
26864	Yes	ACACGTGAAAGGTCCCTGGTTCGAAA
26865	Yes	ACACGTGAAAGGTCCCTGGTTCGAAAC
26866	Yes	ACACGTGAAAGGTCCCTGGTTCGAAACC
26867	Yes	ACACGTGAAAGGTCCCTGGTTCGAAACCA
26868	Yes	ACACGTGAAAGGTCCCTGGTTCGAAACCAG
26869	Yes	ACACGTGAAAGGTCCCTGGTTCGAAACCAGG
26870	Yes	ACACGTGAAAGGTCCCTGGTTCGAAACCAGGT
26871	Yes	ACACGTGAAAGGTCCCTGGTTCGAAACCAGGTG
26872	Yes	ACACGTGAAAGGTCCCTGGTTCGAAACCAGGTGG
26873	Yes	ACACGTGAAAGGTCCCTGGTTCGAAACCAGGTGGA

26874	Yes	ACACGTGAAAGGTCCCTGGTTCGAAACCAGGTGGAA
26875	Yes	ACACGTGAAAGGTCCCTGGTTCGAAACCAGGTGGAAA
26876	Yes	ACACGTGAAAGGTCCCTGGTTCGAAACCAGGTGGAAAC
26877	Yes	ACACGTGAAAGGTCCCTGGTTCGAAACCAGGTGGAAACA
26878	Yes	ACACGTGAAAGGTCCCTGGTTCGAAACCAGGTGGAAACAC
26879	Yes	ACACGTGAAAGGTCCCTGGTTCGAAACCAGGTGGAAACACC
26880	Yes	ACACGTGAAAGGTCCCTGGTTCGAAACCAGGTGGAAACACCA
26881	Yes	ACACGTGGGTTCGAAT
26882	Yes	ACACGTGGGTTCGAATC
26883	Yes	ACACGTGGGTTCGAATCC
26884	Yes	ACACGTGGGTTCGAATCCC
26885	Yes	ACACGTGGGTTCGAATCCCA
26886	Yes	ACACGTGGGTTCGAATCCCAT
26887	Yes	ACACGTGGGTTCGAATCCCATC
26888	Yes	ACACGTGGGTTCGAATCCCATCC
26889	Yes	ACACGTGGGTTCGAATCCCATCCT
26890	Yes	ACACGTGGGTTCGAATCCCATCCTC
26891	Yes	ACACGTGGGTTCGAATCCCATCCTCG
26892	Yes	ACACGTGGGTTCGAATCCCATCCTCGT
26893	Yes	ACACGTGGGTTCGAATCCCATCCTCGTC
26894	Yes	ACACGTGGGTTCGAATCCCATCCTCGTCG
26895	Yes	ACACGTGGGTTCGAATCCCATCCTCGTCGC
26896	Yes	ACACGTGGGTTCGAATCCCATCCTCGTCGCC
26897	Yes	ACACGTGGGTTCGAATCCCATCCTCGTCGCCA
26898	Yes	ACACGTTCGCCTCACA
26899	Yes	ACACGTTCGCCTCACAC
26900	Yes	ACACGTTCGCCTCACACG
26901	Yes	ACACGTTCGCCTCACACGC
26902	Yes	ACACGTTCGCCTCACACGCT
26903	Yes	ACACGTTCGCCTCACACGCTT
26904	Yes	ACACGTTCGCCTCACACGCTTA
26905	Yes	ACACGTTCGCCTCACACGCTTAA
26906	Yes	ACACGTTCGCCTCACACGCTTAAA
26907	Yes	ACACGTTCGCCTCACACGCTTAAAG
26908	Yes	ACACGTTCGCCTCACACGCTTAAAGT
26909	Yes	ACACGTTCGCCTCACACGCTTAAAGTT
26910	Yes	ACACGTTCGCCTCACACGCTTAAAGTTC
26911	Yes	ACACGTTCGCCTCACACGCTTAAAGTTCT
26912	Yes	ACACGTTCGCCTCACACGCTTAAAGTTCTC
26913	Yes	ACACGTTCGCCTCACACGCTTAAAGTTCTCT
26914	Yes	ACACGTTCGCCTCACACGCTTAAAGTTCTCTG
26915	Yes	ACACGTTCGCCTCACACGCTTAAAGTTCTCTGG

26916	Yes	ACACGTTCGCCTCACACGCTTAAAGTTCTCTGGT
26917	Yes	ACACGTTCGCCTCACACGCTTAAAGTTCTCTGGTT
26918	Yes	ACACGTTCGCCTCACACGCTTAAAGTTCTCTGGTTG
26919	Yes	ACACGTTCGCCTCACACGCTTAAAGTTCTCTGGTTGG
26920	Yes	ACACGTTCGCCTCACACGCTTAAAGTTCTCTGGTTGGA
26921	Yes	ACACGTTCGCCTCACACGCTTAAAGTTCTCTGGTTGGAT
26922	Yes	ACACGTTCGCCTCACACGCTTAAAGTTCTCTGGTTGGATA
26923	Yes	ACACGTTCGCCTCACACGCTTAAAGTTCTCTGGTTGGATAC
26924	Yes	ACACGTTCGCCTCACACGCTTAAAGTTCTCTGGTTGGATACC
26925	Yes	ACACGTTCGCCTCACACGCTTAAAGTTCTCTGGTTGGATACCA
26926	Yes	ACACGTTCGCCTCACACGCTTAAAGTTCTCTGGTTGGATACCAG
26927	Yes	ACACGTTCGCCTCACACGCTTAAAGTTCTCTGGTTGGATACCAGA
26928	Yes	ACACGTTCGCCTCACACGCTTAAAGTTCTCTGGTTGGATACCAGAT
26929	Yes	ACACGTTCGCCTCACACGCTTAAAGTTCTCTGGTTGGATACCAGATG
26930	Yes	ACACGTTCGCCTCACACGCTTAAAGTTCTCTGGTTGGATACCAGATGG
26931	Yes	ACACGTTCGCCTCACACGCTTAAAGTTCTCTGGTTGGATACCAGATGGA
26932	Yes	ACACGTTCGCCTCACACGCTTAAAGTTCTCTGGTTGGATACCAGATGGAA
26933	No	ACACTCTCACCGCCGC
26934	No	ACACTCTCACCGCCGCA
26935	Yes	ACACTCTCACCGCCGCAG
26936	Yes	ACACTCTCACCGCCGCAGC
26937	Yes	ACACTCTCACCGCCGCAGCC
26938	Yes	ACACTCTCACCGCCGCAGCCC
26939	Yes	ACACTCTCACCGCCGCAGCCCG
26940	Yes	ACACTCTCACCGCCGCAGCCCGG
26941	Yes	ACACTCTCACCGCCGCAGCCCGGG
26942	Yes	ACACTCTCACCGCCGCAGCCCGGGT
26943	Yes	ACACTCTCACCGCCGCAGCCCGGGTT
26944	Yes	ACACTCTCACCGCCGCAGCCCGGGTTT
26945	Yes	ACACTCTCACCGCCGCAGCCCGGGTTTG
26946	Yes	ACACTCTCACCGCCGCAGCCCGGGTTTGA
26947	Yes	ACACTCTCACCGCCGCAGCCCGGGTTTGAT
26948	Yes	ACACTCTCACCGCCGCAGCCCGGGTTTGATT
26949	Yes	ACACTCTCACCGCCGCAGCCCGGGTTTGATTC
26950	Yes	ACACTCTCACCGCCGCAGCCCGGGTTTGATTCC
26951	Yes	ACACTCTCACCGCCGCAGCCCGGGTTTGATTCCC
26952	Yes	ACACTCTCACCGCCGCAGCCCGGGTTTGATTCCCA
26953	Yes	ACACTCTCACCGCCGCAGCCCGGGTTTGATTCCCAG
26954	Yes	ACACTCTCACCGCCGCAGCCCGGGTTTGATTCCCAGG
26955	Yes	ACACTCTCACCGCCGCAGCCCGGGTTTGATTCCCAGGC
26956	Yes	ACACTCTCACCGCCGCAGCCCGGGTTTGATTCCCAGGCA
26957	Yes	ACACTCTCACCGCCGCAGCCCGGGTTTGATTCCCAGGCAG

26958	Yes	ACACTCTCACCGCCGCAGCCCGGGTTTGATTCCCAGGCAGG
26959	Yes	ACACTCTCACCGCCGCAGCCCGGGTTTGATTCCCAGGCAGGG
26960	Yes	ACACTCTCACCGCCGCAGCCCGGGTTTGATTCCCAGGCAGGGA
26961	Yes	ACACTCTCACCGCCGCAGCCCGGGTTTGATTCCCAGGCAGGGAA
26962	Yes	ACACTCTCACCGCCGCAGCCCGGGTTTGATTCCCAGGCAGGGAAG
26963	Yes	ACACTCTCACCGCCGCAGCCCGGGTTTGATTCCCAGGCAGGGAAGC
26964	Yes	ACACTCTCACCGCCGCAGCCCGGGTTTGATTCCCAGGCAGGGAAGCC
26965	Yes	ACACTCTCACCGCCGCAGCCCGGGTTTGATTCCCAGGCAGGGAAGCCA
26966	No	ACACTGAAAATGTTTA
26967	No	ACACTGAAAATGTTTAG
26968	No	ACACTGAAAATGTTTAGA
26969	Yes	ACACTGAAAATGTTTAGAC
26970	Yes	ACACTGAAAATGTTTAGACG
26971	Yes	ACACTGAAAATGTTTAGACGG
26972	Yes	ACACTGAAAATGTTTAGACGGG
26973	Yes	ACACTGAAAATGTTTAGACGGGC
26974	Yes	ACACTGAAAATGTTTAGACGGGCT
26975	Yes	ACACTGAAAATGTTTAGACGGGCTC
26976	Yes	ACACTGAAAATGTTTAGACGGGCTCA
26977	Yes	ACACTGAAAATGTTTAGACGGGCTCAC
26978	Yes	ACACTGAAAATGTTTAGACGGGCTCACA
26979	Yes	ACACTGAAAATGTTTAGACGGGCTCACAT
26980	Yes	ACACTGAAAATGTTTAGACGGGCTCACATC
26981	Yes	ACACTGAAAATGTTTAGACGGGCTCACATCA
26982	Yes	ACACTGAAAATGTTTAGACGGGCTCACATCAC
26983	Yes	ACACTGAAAATGTTTAGACGGGCTCACATCACC
26984	Yes	ACACTGAAAATGTTTAGACGGGCTCACATCACCC
26985	Yes	ACACTGAAAATGTTTAGACGGGCTCACATCACCCC
26986	Yes	ACACTGAAAATGTTTAGACGGGCTCACATCACCCCA
26987	Yes	ACACTGAAAATGTTTAGACGGGCTCACATCACCCCAT
26988	Yes	ACACTGAAAATGTTTAGACGGGCTCACATCACCCCATA
26989	Yes	ACACTGAAAATGTTTAGACGGGCTCACATCACCCCATAA
26990	Yes	ACACTGAAAATGTTTAGACGGGCTCACATCACCCCATAAA
26991	Yes	ACACTGAAAATGTTTAGACGGGCTCACATCACCCCATAAAC
26992	Yes	ACACTGAAAATGTTTAGACGGGCTCACATCACCCCATAAACA
26993	Yes	ACACTGAAAATGTTTAGACGGGCTCACATCACCCCATAAACAC
26994	Yes	ACACTGAAAATGTTTAGACGGGCTCACATCACCCCATAAACACC
26995	Yes	ACACTGAAAATGTTTAGACGGGCTCACATCACCCCATAAACACCA
26996	No	ACACTGCCTAGGTTCA
26997	No	ACACTGCCTAGGTTCAA
26998	No	ACACTGCCTAGGTTCAAA
26999	No	ACACTGCCTAGGTTCAAAT

27000	No	ACACTGCCTAGGTTCAAATC
27001	No	ACACTGCCTAGGTTCAAATCC
27002	Yes	ACACTGCCTAGGTTCAAATCCT
27003	Yes	ACACTGCCTAGGTTCAAATCCTG
27004	Yes	ACACTGCCTAGGTTCAAATCCTGA
27005	Yes	ACACTGCCTAGGTTCAAATCCTGAC
27006	Yes	ACACTGCCTAGGTTCAAATCCTGACT
27007	Yes	ACACTGCCTAGGTTCAAATCCTGACTC
27008	Yes	ACACTGCCTAGGTTCAAATCCTGACTCT
27009	Yes	ACACTGCCTAGGTTCAAATCCTGACTCTG
27010	Yes	ACACTGCCTAGGTTCAAATCCTGACTCTGT
27011	Yes	ACACTGCCTAGGTTCAAATCCTGACTCTGTC
27012	Yes	ACACTGCCTAGGTTCAAATCCTGACTCTGTCA
27013	Yes	ACACTGCCTAGGTTCAAATCCTGACTCTGTCAC
27014	Yes	ACACTGCCTAGGTTCAAATCCTGACTCTGTCACC
27015	Yes	ACACTGCCTAGGTTCAAATCCTGACTCTGTCACCA
27016	No	ACACTGTAAAGCTAAC
27017	No	ACACTGTAAAGCTAACT
27018	Yes	ACACTGTAAAGCTAACTT
27019	Yes	ACACTGTAAAGCTAACTTA
27020	Yes	ACACTGTAAAGCTAACTTAG
27021	Yes	ACACTGTAAAGCTAACTTAGC
27022	Yes	ACACTGTAAAGCTAACTTAGCA
27023	Yes	ACACTGTAAAGCTAACTTAGCAT
27024	Yes	ACACTGTAAAGCTAACTTAGCATT
27025	Yes	ACACTGTAAAGCTAACTTAGCATTA
27026	Yes	ACACTGTAAAGCTAACTTAGCATTAA
27027	Yes	ACACTGTAAAGCTAACTTAGCATTAAC
27028	Yes	ACACTGTAAAGCTAACTTAGCATTAACC
27029	Yes	ACACTGTAAAGCTAACTTAGCATTAACCT
27030	Yes	ACACTGTAAAGCTAACTTAGCATTAACCTT
27031	Yes	ACACTGTAAAGCTAACTTAGCATTAACCTTT
27032	Yes	ACACTGTAAAGCTAACTTAGCATTAACCTTTT
27033	Yes	ACACTGTAAAGCTAACTTAGCATTAACCTTTTA
27034	Yes	ACACTGTAAAGCTAACTTAGCATTAACCTTTTAA
27035	Yes	ACACTGTAAAGCTAACTTAGCATTAACCTTTTAAG
27036	Yes	ACACTGTAAAGCTAACTTAGCATTAACCTTTTAAGT
27037	Yes	ACACTGTAAAGCTAACTTAGCATTAACCTTTTAAGTT
27038	Yes	ACACTGTAAAGCTAACTTAGCATTAACCTTTTAAGTTA
27039	Yes	ACACTGTAAAGCTAACTTAGCATTAACCTTTTAAGTTAA
27040	Yes	ACACTGTAAAGCTAACTTAGCATTAACCTTTTAAGTTAAA
27041	Yes	ACACTGTAAAGCTAACTTAGCATTAACCTTTTAAGTTAAAG

27042	Yes	ACACTGTAAAGCTAACTTAGCATTAACCTTTTAAGTTAAAGA
27043	Yes	ACACTGTAAAGCTAACTTAGCATTAACCTTTTAAGTTAAAGAT
27044	Yes	ACACTGTAAAGCTAACTTAGCATTAACCTTTTAAGTTAAAGATT
27045	Yes	ACACTGTAAAGCTAACTTAGCATTAACCTTTTAAGTTAAAGATTA
27046	Yes	ACACTGTAAAGCTAACTTAGCATTAACCTTTTAAGTTAAAGATTAA
27047	Yes	ACACTGTAAAGCTAACTTAGCATTAACCTTTTAAGTTAAAGATTAAG
27048	Yes	ACACTGTAAAGCTAACTTAGCATTAACCTTTTAAGTTAAAGATTAAGA
27049	Yes	ACACTGTAAAGCTAACTTAGCATTAACCTTTTAAGTTAAAGATTAAGAG
27050	Yes	ACACTGTAAAGCTAACTTAGCATTAACCTTTTAAGTTAAAGATTAAGAGA
27051	No	ACACTTAGGAGATTTC
27052	No	ACACTTAGGAGATTTCA
27053	No	ACACTTAGGAGATTTCAA
27054	No	ACACTTAGGAGATTTCAAC
27055	No	ACACTTAGGAGATTTCAACT
27056	No	ACACTTAGGAGATTTCAACTT
27057	No	ACACTTAGGAGATTTCAACTTA
27058	No	ACACTTAGGAGATTTCAACTTAA
27059	No	ACACTTAGGAGATTTCAACTTAAC
27060	No	ACACTTAGGAGATTTCAACTTAACT
27061	No	ACACTTAGGAGATTTCAACTTAACTT
27062	No	ACACTTAGGAGATTTCAACTTAACTTG
27063	No	ACACTTAGGAGATTTCAACTTAACTTGA
27064	No	ACACTTAGGAGATTTCAACTTAACTTGAC
27065	No	ACACTTAGGAGATTTCAACTTAACTTGACC
27066	No	ACACTTAGGAGATTTCAACTTAACTTGACCG
27067	No	ACACTTAGGAGATTTCAACTTAACTTGACCGC
27068	No	ACACTTAGGAGATTTCAACTTAACTTGACCGCT
27069	No	ACACTTAGGAGATTTCAACTTAACTTGACCGCTC
27070	No	ACACTTAGGAGATTTCAACTTAACTTGACCGCTCT
27071	No	ACACTTAGGAGATTTCAACTTAACTTGACCGCTCTG
27072	No	ACACTTAGGAGATTTCAACTTAACTTGACCGCTCTGA
27073	Yes	ACACTTAGGAGATTTCAACTTAACTTGACCGCTCTGAC
27074	Yes	ACACTTAGGAGATTTCAACTTAACTTGACCGCTCTGACC
27075	Yes	ACACTTAGGAGATTTCAACTTAACTTGACCGCTCTGACCA
27076	No	ACACTTTATAATCAGA
27077	Yes	ACACTTTATAATCAGAG
27078	Yes	ACACTTTATAATCAGAGG
27079	Yes	ACACTTTATAATCAGAGGT
27080	Yes	ACACTTTATAATCAGAGGTT
27081	Yes	ACACTTTATAATCAGAGGTTC
27082	Yes	ACACTTTATAATCAGAGGTTCA
27083	Yes	ACACTTTATAATCAGAGGTTCAA

27084	Yes	ACACTTTATAATCAGAGGTTCAAC
27085	Yes	ACACTTTATAATCAGAGGTTCAACT
27086	Yes	ACACTTTATAATCAGAGGTTCAACTC
27087	Yes	ACACTTTATAATCAGAGGTTCAACTCC
27088	Yes	ACACTTTATAATCAGAGGTTCAACTCCT
27089	Yes	ACACTTTATAATCAGAGGTTCAACTCCTC
27090	Yes	ACACTTTATAATCAGAGGTTCAACTCCTCT
27091	Yes	ACACTTTATAATCAGAGGTTCAACTCCTCTT
27092	Yes	ACACTTTATAATCAGAGGTTCAACTCCTCTTC
27093	Yes	ACACTTTATAATCAGAGGTTCAACTCCTCTTCT
27094	Yes	ACACTTTATAATCAGAGGTTCAACTCCTCTTCTT
27095	Yes	ACACTTTATAATCAGAGGTTCAACTCCTCTTCTTA
27096	Yes	ACACTTTATAATCAGAGGTTCAACTCCTCTTCTTAA
27097	Yes	ACACTTTATAATCAGAGGTTCAACTCCTCTTCTTAAC
27098	Yes	ACACTTTATAATCAGAGGTTCAACTCCTCTTCTTAACA
27099	Yes	ACACTTTATAATCAGAGGTTCAACTCCTCTTCTTAACAC
27100	Yes	ACACTTTATAATCAGAGGTTCAACTCCTCTTCTTAACACC
27101	Yes	ACACTTTATAATCAGAGGTTCAACTCCTCTTCTTAACACCA
27102	No	ACAGACCAAGAGCCTT
27103	No	ACAGACCAAGAGCCTTC
27104	Yes	ACAGACCAAGAGCCTTCA
27105	Yes	ACAGACCAAGAGCCTTCAA
27106	Yes	ACAGACCAAGAGCCTTCAAA
27107	Yes	ACAGACCAAGAGCCTTCAAAG
27108	Yes	ACAGACCAAGAGCCTTCAAAGC
27109	Yes	ACAGACCAAGAGCCTTCAAAGCC
27110	Yes	ACAGACCAAGAGCCTTCAAAGCCC
27111	Yes	ACAGACCAAGAGCCTTCAAAGCCCT
27112	Yes	ACAGACCAAGAGCCTTCAAAGCCCTC
27113	Yes	ACAGACCAAGAGCCTTCAAAGCCCTCA
27114	Yes	ACAGACCAAGAGCCTTCAAAGCCCTCAG
27115	Yes	ACAGACCAAGAGCCTTCAAAGCCCTCAGT
27116	Yes	ACAGACCAAGAGCCTTCAAAGCCCTCAGTA
27117	Yes	ACAGACCAAGAGCCTTCAAAGCCCTCAGTAA
27118	Yes	ACAGACCAAGAGCCTTCAAAGCCCTCAGTAAG
27119	Yes	ACAGACCAAGAGCCTTCAAAGCCCTCAGTAAGT
27120	Yes	ACAGACCAAGAGCCTTCAAAGCCCTCAGTAAGTT
27121	Yes	ACAGACCAAGAGCCTTCAAAGCCCTCAGTAAGTTG
27122	Yes	ACAGACCAAGAGCCTTCAAAGCCCTCAGTAAGTTGC
27123	Yes	ACAGACCAAGAGCCTTCAAAGCCCTCAGTAAGTTGCA
27124	Yes	ACAGACCAAGAGCCTTCAAAGCCCTCAGTAAGTTGCAA
27125	Yes	ACAGACCAAGAGCCTTCAAAGCCCTCAGTAAGTTGCAAT

27126	Yes	ACAGACCAAGAGCCTTCAAAGCCCTCAGTAAGTTGCAATA
27127	Yes	ACAGACCAAGAGCCTTCAAAGCCCTCAGTAAGTTGCAATAC
27128	Yes	ACAGACCAAGAGCCTTCAAAGCCCTCAGTAAGTTGCAATACT
27129	Yes	ACAGACCAAGAGCCTTCAAAGCCCTCAGTAAGTTGCAATACTT
27130	Yes	ACAGACCAAGAGCCTTCAAAGCCCTCAGTAAGTTGCAATACTTA
27131	Yes	ACAGACCAAGAGCCTTCAAAGCCCTCAGTAAGTTGCAATACTTAA
27132	Yes	ACAGACCAAGAGCCTTCAAAGCCCTCAGTAAGTTGCAATACTTAAT
27133	Yes	ACAGACCAAGAGCCTTCAAAGCCCTCAGTAAGTTGCAATACTTAATT
27134	Yes	ACAGACCAAGAGCCTTCAAAGCCCTCAGTAAGTTGCAATACTTAATTT
27135	Yes	ACAGACCAAGAGCCTTCAAAGCCCTCAGTAAGTTGCAATACTTAATTTC
27136	Yes	ACAGACCAAGAGCCTTCAAAGCCCTCAGTAAGTTGCAATACTTAATTTCT
27137	No	ACAGACCACCCAGGGA
27138	No	ACAGACCACCCAGGGAC
27139	Yes	ACAGACCACCCAGGGACG
27140	Yes	ACAGACCACCCAGGGACGC
27141	Yes	ACAGACCACCCAGGGACGCC
27142	Yes	ACAGACCACCCAGGGACGCCA
27143	No	ACAGAGAATAGTTTAA
27144	No	ACAGAGAATAGTTTAAA
27145	Yes	ACAGAGAATAGTTTAAAT
27146	Yes	ACAGAGAATAGTTTAAATT
27147	Yes	ACAGAGAATAGTTTAAATTA
27148	Yes	ACAGAGAATAGTTTAAATTAG
27149	Yes	ACAGAGAATAGTTTAAATTAGA
27150	Yes	ACAGAGAATAGTTTAAATTAGAA
27151	Yes	ACAGAGAATAGTTTAAATTAGAAT
27152	Yes	ACAGAGAATAGTTTAAATTAGAATC
27153	Yes	ACAGAGAATAGTTTAAATTAGAATCT
27154	Yes	ACAGAGAATAGTTTAAATTAGAATCTT
27155	Yes	ACAGAGAATAGTTTAAATTAGAATCTTA
27156	Yes	ACAGAGAATAGTTTAAATTAGAATCTTAG
27157	Yes	ACAGAGAATAGTTTAAATTAGAATCTTAGC
27158	Yes	ACAGAGAATAGTTTAAATTAGAATCTTAGCT
27159	Yes	ACAGAGAATAGTTTAAATTAGAATCTTAGCTT
27160	Yes	ACAGAGAATAGTTTAAATTAGAATCTTAGCTTT
27161	Yes	ACAGAGAATAGTTTAAATTAGAATCTTAGCTTTG
27162	Yes	ACAGAGAATAGTTTAAATTAGAATCTTAGCTTTGG
27163	Yes	ACAGAGAATAGTTTAAATTAGAATCTTAGCTTTGGG
27164	Yes	ACAGAGAATAGTTTAAATTAGAATCTTAGCTTTGGGT
27165	Yes	ACAGAGAATAGTTTAAATTAGAATCTTAGCTTTGGGTG
27166	Yes	ACAGAGAATAGTTTAAATTAGAATCTTAGCTTTGGGTGC
27167	Yes	ACAGAGAATAGTTTAAATTAGAATCTTAGCTTTGGGTGCT

27168	Yes	ACAGAGAATAGTTTAAATTAGAATCTTAGCTTTGGGTGCTA
27169	Yes	ACAGAGAATAGTTTAAATTAGAATCTTAGCTTTGGGTGCTAA
27170	Yes	ACAGAGAATAGTTTAAATTAGAATCTTAGCTTTGGGTGCTAAT
27171	Yes	ACAGAGAATAGTTTAAATTAGAATCTTAGCTTTGGGTGCTAATG
27172	Yes	ACAGAGAATAGTTTAAATTAGAATCTTAGCTTTGGGTGCTAATGG
27173	Yes	ACAGAGAATAGTTTAAATTAGAATCTTAGCTTTGGGTGCTAATGGT
27174	Yes	ACAGAGAATAGTTTAAATTAGAATCTTAGCTTTGGGTGCTAATGGTG
27175	Yes	ACAGAGAATAGTTTAAATTAGAATCTTAGCTTTGGGTGCTAATGGTGG
27176	Yes	ACAGAGAATAGTTTAAATTAGAATCTTAGCTTTGGGTGCTAATGGTGGA
27177	Yes	ACAGAGAATAGTTTAAATTAGAATCTTAGCTTTGGGTGCTAATGGTGGAG
27178	No	ACAGAGAATTCTGGAT
27179	Yes	ACAGAGAATTCTGGATT
27180	Yes	ACAGAGAATTCTGGATTC
27181	Yes	ACAGAGAATTCTGGATTCT
27182	Yes	ACAGAGAATTCTGGATTCTC
27183	Yes	ACAGAGAATTCTGGATTCTCA
27184	Yes	ACAGAGAATTCTGGATTCTCAG
27185	Yes	ACAGAGAATTCTGGATTCTCAGG
27186	Yes	ACAGAGAATTCTGGATTCTCAGGG
27187	Yes	ACAGAGAATTCTGGATTCTCAGGGG
27188	Yes	ACAGAGAATTCTGGATTCTCAGGGGT
27189	Yes	ACAGAGAATTCTGGATTCTCAGGGGTA
27190	Yes	ACAGAGAATTCTGGATTCTCAGGGGTAG
27191	Yes	ACAGAGAATTCTGGATTCTCAGGGGTAGG
27192	Yes	ACAGAGAATTCTGGATTCTCAGGGGTAGGT
27193	Yes	ACAGAGAATTCTGGATTCTCAGGGGTAGGTT
27194	Yes	ACAGAGAATTCTGGATTCTCAGGGGTAGGTTC
27195	Yes	ACAGAGAATTCTGGATTCTCAGGGGTAGGTTCA
27196	Yes	ACAGAGAATTCTGGATTCTCAGGGGTAGGTTCAA
27197	Yes	ACAGAGAATTCTGGATTCTCAGGGGTAGGTTCAAT
27198	Yes	ACAGAGAATTCTGGATTCTCAGGGGTAGGTTCAATT
27199	Yes	ACAGAGAATTCTGGATTCTCAGGGGTAGGTTCAATTC
27200	Yes	ACAGAGAATTCTGGATTCTCAGGGGTAGGTTCAATTCC
27201	Yes	ACAGAGAATTCTGGATTCTCAGGGGTAGGTTCAATTCCT
27202	Yes	ACAGAGAATTCTGGATTCTCAGGGGTAGGTTCAATTCCTA
27203	Yes	ACAGAGAATTCTGGATTCTCAGGGGTAGGTTCAATTCCTAT
27204	Yes	ACAGAGAATTCTGGATTCTCAGGGGTAGGTTCAATTCCTATA
27205	Yes	ACAGAGAATTCTGGATTCTCAGGGGTAGGTTCAATTCCTATAG
27206	Yes	ACAGAGAATTCTGGATTCTCAGGGGTAGGTTCAATTCCTATAGA
27207	Yes	ACAGAGAATTCTGGATTCTCAGGGGTAGGTTCAATTCCTATAGAA
27208	Yes	ACAGAGAATTCTGGATTCTCAGGGGTAGGTTCAATTCCTATAGAAC
27209	Yes	ACAGAGAATTCTGGATTCTCAGGGGTAGGTTCAATTCCTATAGAACC

27210	Yes	ACAGAGAATTCTGGATTCTCAGGGGTAGGTTCAATTCCTATAGAACCT
27211	Yes	ACAGAGAATTCTGGATTCTCAGGGGTAGGTTCAATTCCTATAGAACCTA
27212	Yes	ACAGAGAATTCTGGATTCTCAGGGGTAGGTTCAATTCCTATAGAACCTAG
27213	No	ACAGAGAATTTTGGAT
27214	Yes	ACAGAGAATTTTGGATT
27215	Yes	ACAGAGAATTTTGGATTC
27216	Yes	ACAGAGAATTTTGGATTCT
27217	Yes	ACAGAGAATTTTGGATTCTC
27218	Yes	ACAGAGAATTTTGGATTCTCA
27219	Yes	ACAGAGAATTTTGGATTCTCAG
27220	Yes	ACAGAGAATTTTGGATTCTCAGG
27221	Yes	ACAGAGAATTTTGGATTCTCAGGG
27222	Yes	ACAGAGAATTTTGGATTCTCAGGGG
27223	Yes	ACAGAGAATTTTGGATTCTCAGGGGT
27224	Yes	ACAGAGAATTTTGGATTCTCAGGGGTA
27225	Yes	ACAGAGAATTTTGGATTCTCAGGGGTAG
27226	Yes	ACAGAGAATTTTGGATTCTCAGGGGTAGG
27227	Yes	ACAGAGAATTTTGGATTCTCAGGGGTAGGT
27228	Yes	ACAGAGAATTTTGGATTCTCAGGGGTAGGTT
27229	Yes	ACAGAGAATTTTGGATTCTCAGGGGTAGGTTA
27230	Yes	ACAGAGAATTTTGGATTCTCAGGGGTAGGTTAA
27231	Yes	ACAGAGAATTTTGGATTCTCAGGGGTAGGTTAAA
27232	Yes	ACAGAGAATTTTGGATTCTCAGGGGTAGGTTAAAT
27233	Yes	ACAGAGAATTTTGGATTCTCAGGGGTAGGTTAAATT
27234	Yes	ACAGAGAATTTTGGATTCTCAGGGGTAGGTTAAATTC
27235	Yes	ACAGAGAATTTTGGATTCTCAGGGGTAGGTTAAATTCC
27236	Yes	ACAGAGAATTTTGGATTCTCAGGGGTAGGTTAAATTCCT
27237	Yes	ACAGAGAATTTTGGATTCTCAGGGGTAGGTTAAATTCCTA
27238	Yes	ACAGAGAATTTTGGATTCTCAGGGGTAGGTTAAATTCCTAT
27239	Yes	ACAGAGAATTTTGGATTCTCAGGGGTAGGTTAAATTCCTATA
27240	Yes	ACAGAGAATTTTGGATTCTCAGGGGTAGGTTAAATTCCTATAG
27241	Yes	ACAGAGAATTTTGGATTCTCAGGGGTAGGTTAAATTCCTATAGT
27242	Yes	ACAGAGAATTTTGGATTCTCAGGGGTAGGTTAAATTCCTATAGTA
27243	Yes	ACAGAGAATTTTGGATTCTCAGGGGTAGGTTAAATTCCTATAGTAC
27244	Yes	ACAGAGAATTTTGGATTCTCAGGGGTAGGTTAAATTCCTATAGTACT
27245	Yes	ACAGAGAATTTTGGATTCTCAGGGGTAGGTTAAATTCCTATAGTACTA
27246	Yes	ACAGAGAATTTTGGATTCTCAGGGGTAGGTTAAATTCCTATAGTACTAG
27247	Yes	ACAGAGAATTTTGGATTCTCAGGGGTAGGTTAAATTCCTATAGTACTAGC
27248	Yes	ACAGAGAATTTTGGATTCTCAGGGGTAGGTTC
27249	Yes	ACAGAGAATTTTGGATTCTCAGGGGTAGGTTCG
27250	Yes	ACAGAGAATTTTGGATTCTCAGGGGTAGGTTCGA
27251	Yes	ACAGAGAATTTTGGATTCTCAGGGGTAGGTTCGAT

27252	Yes	ACAGAGAATTTTGGATTCTCAGGGGTAGGTTCGATT
27253	Yes	ACAGAGAATTTTGGATTCTCAGGGGTAGGTTCGATTC
27254	Yes	ACAGAGAATTTTGGATTCTCAGGGGTAGGTTCGATTCC
27255	Yes	ACAGAGAATTTTGGATTCTCAGGGGTAGGTTCGATTCCT
27256	Yes	ACAGAGAATTTTGGATTCTCAGGGGTAGGTTCGATTCCTA
27257	Yes	ACAGAGAATTTTGGATTCTCAGGGGTAGGTTCGATTCCTAT
27258	Yes	ACAGAGAATTTTGGATTCTCAGGGGTAGGTTCGATTCCTATA
27259	Yes	ACAGAGAATTTTGGATTCTCAGGGGTAGGTTCGATTCCTATAA
27260	Yes	ACAGAGAATTTTGGATTCTCAGGGGTAGGTTCGATTCCTATAAT
27261	Yes	ACAGAGAATTTTGGATTCTCAGGGGTAGGTTCGATTCCTATAATT
27262	Yes	ACAGAGAATTTTGGATTCTCAGGGGTAGGTTCGATTCCTATAATTC
27263	Yes	ACAGAGAATTTTGGATTCTCAGGGGTAGGTTCGATTCCTATAATTCT
27264	Yes	ACAGAGAATTTTGGATTCTCAGGGGTAGGTTCGATTCCTATAATTCTA
27265	Yes	ACAGAGAATTTTGGATTCTCAGGGGTAGGTTCGATTCCTATAATTCTAC
27266	Yes	ACAGAGAATTTTGGATTCTCAGGGGTAGGTTCGATTCCTATAATTCTACC
27267	Yes	ACAGAGAATTTTGGATTCTCAGGGGTG
27268	Yes	ACAGAGAATTTTGGATTCTCAGGGGTGG
27269	Yes	ACAGAGAATTTTGGATTCTCAGGGGTGGG
27270	Yes	ACAGAGAATTTTGGATTCTCAGGGGTGGGT
27271	Yes	ACAGAGAATTTTGGATTCTCAGGGGTGGGTT
27272	Yes	ACAGAGAATTTTGGATTCTCAGGGGTGGGTTC
27273	Yes	ACAGAGAATTTTGGATTCTCAGGGGTGGGTTCA
27274	Yes	ACAGAGAATTTTGGATTCTCAGGGGTGGGTTCAA
27275	Yes	ACAGAGAATTTTGGATTCTCAGGGGTGGGTTCAAT
27276	Yes	ACAGAGAATTTTGGATTCTCAGGGGTGGGTTCAATT
27277	Yes	ACAGAGAATTTTGGATTCTCAGGGGTGGGTTCAATTC
27278	Yes	ACAGAGAATTTTGGATTCTCAGGGGTGGGTTCAATTCC
27279	Yes	ACAGAGAATTTTGGATTCTCAGGGGTGGGTTCAATTCCT
27280	Yes	ACAGAGAATTTTGGATTCTCAGGGGTGGGTTCAATTCCTT
27281	Yes	ACAGAGAATTTTGGATTCTCAGGGGTGGGTTCAATTCCTTT
27282	Yes	ACAGAGAATTTTGGATTCTCAGGGGTGGGTTCAATTCCTTTC
27283	Yes	ACAGAGAATTTTGGATTCTCAGGGGTGGGTTCAATTCCTTTCG
27284	Yes	ACAGAGAATTTTGGATTCTCAGGGGTGGGTTCAATTCCTTTCGT
27285	Yes	ACAGAGAATTTTGGATTCTCAGGGGTGGGTTCAATTCCTTTCGTC
27286	Yes	ACAGAGAATTTTGGATTCTCAGGGGTGGGTTCAATTCCTTTCGTCC
27287	Yes	ACAGAGAATTTTGGATTCTCAGGGGTGGGTTCAATTCCTTTCGTCCT
27288	Yes	ACAGAGAATTTTGGATTCTCAGGGGTGGGTTCAATTCCTTTCGTCCTA
27289	Yes	ACAGAGAATTTTGGATTCTCAGGGGTGGGTTCAATTCCTTTCGTCCTAG
27290	Yes	ACAGAGAATTTTGGATTCTCAGGGGTGGGTTCAATTCCTTTCGTCCTAGC
27291	Yes	ACAGAGAATTTTGGATTCTCAGGT
27292	Yes	ACAGAGAATTTTGGATTCTCAGGTG
27293	Yes	ACAGAGAATTTTGGATTCTCAGGTGC

27294	Yes	ACAGAGAATTTTGGATTCTCAGGTGCA
27295	Yes	ACAGAGAATTTTGGATTCTCAGGTGCAG
27296	Yes	ACAGAGAATTTTGGATTCTCAGGTGCAGG
27297	Yes	ACAGAGAATTTTGGATTCTCAGGTGCAGGT
27298	Yes	ACAGAGAATTTTGGATTCTCAGGTGCAGGTT
27299	Yes	ACAGAGAATTTTGGATTCTCAGGTGCAGGTTC
27300	Yes	ACAGAGAATTTTGGATTCTCAGGTGCAGGTTCA
27301	Yes	ACAGAGAATTTTGGATTCTCAGGTGCAGGTTCAA
27302	Yes	ACAGAGAATTTTGGATTCTCAGGTGCAGGTTCAAT
27303	Yes	ACAGAGAATTTTGGATTCTCAGGTGCAGGTTCAATT
27304	Yes	ACAGAGAATTTTGGATTCTCAGGTGCAGGTTCAATTC
27305	Yes	ACAGAGAATTTTGGATTCTCAGGTGCAGGTTCAATTCC
27306	Yes	ACAGAGAATTTTGGATTCTCAGGTGCAGGTTCAATTCCT
27307	Yes	ACAGAGAATTTTGGATTCTCAGGTGCAGGTTCAATTCCTA
27308	Yes	ACAGAGAATTTTGGATTCTCAGGTGCAGGTTCAATTCCTAT
27309	Yes	ACAGAGAATTTTGGATTCTCAGGTGCAGGTTCAATTCCTATA
27310	Yes	ACAGAGAATTTTGGATTCTCAGGTGCAGGTTCAATTCCTATAT
27311	Yes	ACAGAGAATTTTGGATTCTCAGGTGCAGGTTCAATTCCTATATT
27312	Yes	ACAGAGAATTTTGGATTCTCAGGTGCAGGTTCAATTCCTATATTC
27313	Yes	ACAGAGAATTTTGGATTCTCAGGTGCAGGTTCAATTCCTATATTCT
27314	Yes	ACAGAGAATTTTGGATTCTCAGGTGCAGGTTCAATTCCTATATTCTA
27315	Yes	ACAGAGAATTTTGGATTCTCAGGTGCAGGTTCAATTCCTATATTCTAG
27316	Yes	ACAGAGAATTTTGGATTCTCAGGTGCAGGTTCAATTCCTATATTCTAGA
27317	Yes	ACAGAGAATTTTGGATTCTCAGGTGCAGGTTCAATTCCTATATTCTAGAG
27318	Yes	ACAGAGCACGTGGTTA
27319	Yes	ACAGAGCACGTGGTTAG
27320	Yes	ACAGAGCACGTGGTTAGC
27321	Yes	ACAGAGCACGTGGTTAGCA
27322	Yes	ACAGAGCACGTGGTTAGCAT
27323	Yes	ACAGAGCACGTGGTTAGCATG
27324	Yes	ACAGAGCACGTGGTTAGCATGC
27325	Yes	ACAGAGCACGTGGTTAGCATGCG
27326	Yes	ACAGAGCACGTGGTTAGCATGCGT
27327	Yes	ACAGAGCACGTGGTTAGCATGCGTG
27328	Yes	ACAGAGCACGTGGTTAGCATGCGTGA
27329	Yes	ACAGAGCACGTGGTTAGCATGCGTGAG
27330	Yes	ACAGAGCACGTGGTTAGCATGCGTGAGG
27331	Yes	ACAGAGCACGTGGTTAGCATGCGTGAGGT
27332	Yes	ACAGAGCACGTGGTTAGCATGCGTGAGGTC
27333	Yes	ACAGAGCACGTGGTTAGCATGCGTGAGGTCC
27334	Yes	ACAGAGCACGTGGTTAGCATGCGTGAGGTCCT
27335	Yes	ACAGAGCACGTGGTTAGCATGCGTGAGGTCCTG

27336	Yes	ACAGAGCACGTGGTTAGCATGCGTGAGGTCCTGC
27337	Yes	ACAGAGCACGTGGTTAGCATGCGTGAGGTCCTGCG
27338	Yes	ACAGAGCACGTGGTTAGCATGCGTGAGGTCCTGCGT
27339	Yes	ACAGAGCACGTGGTTAGCATGCGTGAGGTCCTGCGTT
27340	Yes	ACAGAGCACGTGGTTAGCATGCGTGAGGTCCTGCGTTC
27341	Yes	ACAGAGCACGTGGTTAGCATGCGTGAGGTCCTGCGTTCA
27342	Yes	ACAGAGCACGTGGTTAGCATGCGTGAGGTCCTGCGTTCAA
27343	Yes	ACAGAGCACGTGGTTAGCATGCGTGAGGTCCTGCGTTCAAC
27344	Yes	ACAGAGCACGTGGTTAGCATGCGTGAGGTCCTGCGTTCAACT
27345	Yes	ACAGAGCACGTGGTTAGCATGCGTGAGGTCCTGCGTTCAACTT
27346	Yes	ACAGAGCACGTGGTTAGCATGCGTGAGGTCCTGCGTTCAACTTC
27347	Yes	ACAGAGCACGTGGTTAGCATGCGTGAGGTCCTGCGTTCAACTTCC
27348	Yes	ACAGAGCACGTGGTTAGCATGCGTGAGGTCCTGCGTTCAACTTCCA
27349	Yes	ACAGAGCACGTGGTTAGCATGCGTGAGGTCCTGCGTTCAACTTCCAG
27350	Yes	ACAGAGCACGTGGTTAGCATGCGTGAGGTCCTGCGTTCAACTTCCAGT
27351	Yes	ACAGAGCACGTGGTTAGCATGCGTGAGGTCCTGCGTTCAACTTCCAGTA
27352	Yes	ACAGAGCACGTGGTTAGCATGCGTGAGGTCCTGCGTTCAACTTCCAGTAT
27353	Yes	ACAGAGGCTTACGACC
27354	Yes	ACAGAGGCTTACGACCC
27355	Yes	ACAGAGGCTTACGACCCC
27356	Yes	ACAGAGGCTTACGACCCCT
27357	Yes	ACAGAGGCTTACGACCCCTT
27358	Yes	ACAGAGGCTTACGACCCCTTA
27359	Yes	ACAGAGGCTTACGACCCCTTAT
27360	Yes	ACAGAGGCTTACGACCCCTTATT
27361	Yes	ACAGAGGCTTACGACCCCTTATTT
27362	Yes	ACAGAGGCTTACGACCCCTTATTTA
27363	Yes	ACAGAGGCTTACGACCCCTTATTTAC
27364	Yes	ACAGAGGCTTACGACCCCTTATTTACC
27365	Yes	ACAGAGGCTTACGACCCCTTATTTACCC
27366	Yes	ACAGAGGCTTACGACCCCTTATTTACCCC
27367	Yes	ACAGAGGCTTACGACCCCTTATTTACCCCA
27368	No	ACAGAGGTTATGGGAT
27369	No	ACAGAGGTTATGGGATT
27370	Yes	ACAGAGGTTATGGGATTG
27371	Yes	ACAGAGGTTATGGGATTGG
27372	Yes	ACAGAGGTTATGGGATTGGC
27373	Yes	ACAGAGGTTATGGGATTGGCT
27374	Yes	ACAGAGGTTATGGGATTGGCTT
27375	Yes	ACAGAGGTTATGGGATTGGCTTG
27376	Yes	ACAGAGGTTATGGGATTGGCTTGA
27377	Yes	ACAGAGGTTATGGGATTGGCTTGAA

27378	Yes	ACAGAGGTTATGGGATTGGCTTGAAA
27379	Yes	ACAGAGGTTATGGGATTGGCTTGAAAC
27380	Yes	ACAGAGGTTATGGGATTGGCTTGAAACC
27381	Yes	ACAGAGGTTATGGGATTGGCTTGAAACCA
27382	Yes	ACAGAGGTTATGGGATTGGCTTGAAACCAG
27383	Yes	ACAGAGGTTATGGGATTGGCTTGAAACCAGT
27384	Yes	ACAGAGGTTATGGGATTGGCTTGAAACCAGTC
27385	Yes	ACAGAGGTTATGGGATTGGCTTGAAACCAGTCT
27386	Yes	ACAGAGGTTATGGGATTGGCTTGAAACCAGTCTG
27387	Yes	ACAGAGGTTATGGGATTGGCTTGAAACCAGTCTGT
27388	Yes	ACAGAGGTTATGGGATTGGCTTGAAACCAGTCTGTG
27389	Yes	ACAGAGGTTATGGGATTGGCTTGAAACCAGTCTGTGG
27390	Yes	ACAGAGGTTATGGGATTGGCTTGAAACCAGTCTGTGGG
27391	Yes	ACAGAGGTTATGGGATTGGCTTGAAACCAGTCTGTGGGG
27392	Yes	ACAGAGGTTATGGGATTGGCTTGAAACCAGTCTGTGGGGG
27393	Yes	ACAGAGGTTATGGGATTGGCTTGAAACCAGTCTGTGGGGGG
27394	Yes	ACAGAGGTTATGGGATTGGCTTGAAACCAGTCTGTGGGGGGT
27395	Yes	ACAGAGGTTATGGGATTGGCTTGAAACCAGTCTGTGGGGGGTT
27396	Yes	ACAGAGGTTATGGGATTGGCTTGAAACCAGTCTGTGGGGGGTTC
27397	Yes	ACAGAGGTTATGGGATTGGCTTGAAACCAGTCTGTGGGGGGTTCG
27398	Yes	ACAGAGGTTATGGGATTGGCTTGAAACCAGTCTGTGGGGGGTTCGA
27399	Yes	ACAGAGGTTATGGGATTGGCTTGAAACCAGTCTGTGGGGGGTTCGAT
27400	Yes	ACAGAGGTTATGGGATTGGCTTGAAACCAGTCTGTGGGGGGTTCGATT
27401	Yes	ACAGAGGTTATGGGATTGGCTTGAAACCAGTCTGTGGGGGGTTCGATTC
27402	Yes	ACAGAGGTTATGGGATTGGCTTGAAACCAGTCTGTGGGGGGTTCGATTCC
27403	No	ACAGAGTGTAGCTTAA
27404	Yes	ACAGAGTGTAGCTTAAC
27405	Yes	ACAGAGTGTAGCTTAACA
27406	Yes	ACAGAGTGTAGCTTAACAC
27407	Yes	ACAGAGTGTAGCTTAACACA
27408	Yes	ACAGAGTGTAGCTTAACACAA
27409	Yes	ACAGAGTGTAGCTTAACACAAA
27410	Yes	ACAGAGTGTAGCTTAACACAAAG
27411	Yes	ACAGAGTGTAGCTTAACACAAAGC
27412	Yes	ACAGAGTGTAGCTTAACACAAAGCA
27413	Yes	ACAGAGTGTAGCTTAACACAAAGCAC
27414	Yes	ACAGAGTGTAGCTTAACACAAAGCACC
27415	Yes	ACAGAGTGTAGCTTAACACAAAGCACCC
27416	Yes	ACAGAGTGTAGCTTAACACAAAGCACCCA
27417	Yes	ACAGAGTGTAGCTTAACACAAAGCACCCAA
27418	Yes	ACAGAGTGTAGCTTAACACAAAGCACCCAAC
27419	Yes	ACAGAGTGTAGCTTAACACAAAGCACCCAACT

27420	Yes	ACAGAGTGTAGCTTAACACAAAGCACCCAACTT
27421	Yes	ACAGAGTGTAGCTTAACACAAAGCACCCAACTTA
27422	Yes	ACAGAGTGTAGCTTAACACAAAGCACCCAACTTAC
27423	Yes	ACAGAGTGTAGCTTAACACAAAGCACCCAACTTACA
27424	Yes	ACAGAGTGTAGCTTAACACAAAGCACCCAACTTACAC
27425	Yes	ACAGAGTGTAGCTTAACACAAAGCACCCAACTTACACT
27426	Yes	ACAGAGTGTAGCTTAACACAAAGCACCCAACTTACACTT
27427	Yes	ACAGAGTGTAGCTTAACACAAAGCACCCAACTTACACTTA
27428	Yes	ACAGAGTGTAGCTTAACACAAAGCACCCAACTTACACTTAG
27429	Yes	ACAGAGTGTAGCTTAACACAAAGCACCCAACTTACACTTAGG
27430	Yes	ACAGAGTGTAGCTTAACACAAAGCACCCAACTTACACTTAGGA
27431	Yes	ACAGAGTGTAGCTTAACACAAAGCACCCAACTTACACTTAGGAG
27432	Yes	ACAGAGTGTAGCTTAACACAAAGCACCCAACTTACACTTAGGAGA
27433	Yes	ACAGAGTGTAGCTTAACACAAAGCACCCAACTTACACTTAGGAGAT
27434	Yes	ACAGAGTGTAGCTTAACACAAAGCACCCAACTTACACTTAGGAGATT
27435	Yes	ACAGAGTGTAGCTTAACACAAAGCACCCAACTTACACTTAGGAGATTT
27436	Yes	ACAGAGTGTAGCTTAACACAAAGCACCCAACTTACACTTAGGAGATTTC
27437	Yes	ACAGAGTGTAGCTTAACACAAAGCACCCAACTTACACTTAGGAGATTTCA
27438	No	ACAGATAATTTTGGAT
27439	No	ACAGATAATTTTGGATT
27440	No	ACAGATAATTTTGGATTC
27441	Yes	ACAGATAATTTTGGATTCT
27442	Yes	ACAGATAATTTTGGATTCTC
27443	Yes	ACAGATAATTTTGGATTCTCA
27444	Yes	ACAGATAATTTTGGATTCTCAG
27445	Yes	ACAGATAATTTTGGATTCTCAGG
27446	Yes	ACAGATAATTTTGGATTCTCAGGG
27447	Yes	ACAGATAATTTTGGATTCTCAGGGG
27448	Yes	ACAGATAATTTTGGATTCTCAGGGGT
27449	Yes	ACAGATAATTTTGGATTCTCAGGGGTA
27450	Yes	ACAGATAATTTTGGATTCTCAGGGGTAG
27451	Yes	ACAGATAATTTTGGATTCTCAGGGGTAGG
27452	Yes	ACAGATAATTTTGGATTCTCAGGGGTAGGT
27453	Yes	ACAGATAATTTTGGATTCTCAGGGGTAGGTT
27454	Yes	ACAGATAATTTTGGATTCTCAGGGGTAGGTTC
27455	Yes	ACAGATAATTTTGGATTCTCAGGGGTAGGTTCA
27456	Yes	ACAGATAATTTTGGATTCTCAGGGGTAGGTTCAA
27457	Yes	ACAGATAATTTTGGATTCTCAGGGGTAGGTTCAAT
27458	Yes	ACAGATAATTTTGGATTCTCAGGGGTAGGTTCAATT
27459	Yes	ACAGATAATTTTGGATTCTCAGGGGTAGGTTCAATTC
27460	Yes	ACAGATAATTTTGGATTCTCAGGGGTAGGTTCAATTCT
27461	Yes	ACAGATAATTTTGGATTCTCAGGGGTAGGTTCAATTCTT

27462	Yes	ACAGATAATTTTGGATTCTCAGGGGTAGGTTCAATTCTTA
27463	Yes	ACAGATAATTTTGGATTCTCAGGGGTAGGTTCAATTCTTAT
27464	Yes	ACAGATAATTTTGGATTCTCAGGGGTAGGTTCAATTCTTATA
27465	Yes	ACAGATAATTTTGGATTCTCAGGGGTAGGTTCAATTCTTATAG
27466	Yes	ACAGATAATTTTGGATTCTCAGGGGTAGGTTCAATTCTTATAGT
27467	Yes	ACAGATAATTTTGGATTCTCAGGGGTAGGTTCAATTCTTATAGTT
27468	Yes	ACAGATAATTTTGGATTCTCAGGGGTAGGTTCAATTCTTATAGTTC
27469	Yes	ACAGATAATTTTGGATTCTCAGGGGTAGGTTCAATTCTTATAGTTCT
27470	Yes	ACAGATAATTTTGGATTCTCAGGGGTAGGTTCAATTCTTATAGTTCTA
27471	Yes	ACAGATAATTTTGGATTCTCAGGGGTAGGTTCAATTCTTATAGTTCTAG
27472	Yes	ACAGATAATTTTGGATTCTCAGGGGTAGGTTCAATTCTTATAGTTCTAGC
27473	Yes	ACAGATTCTAGATCGA
27474	Yes	ACAGATTCTAGATCGAG
27475	Yes	ACAGATTCTAGATCGAGA
27476	Yes	ACAGATTCTAGATCGAGAC
27477	Yes	ACAGATTCTAGATCGAGACA
27478	Yes	ACAGATTCTAGATCGAGACAT
27479	Yes	ACAGATTCTAGATCGAGACATT
27480	Yes	ACAGATTCTAGATCGAGACATTC
27481	Yes	ACAGATTCTAGATCGAGACATTCC
27482	Yes	ACAGATTCTAGATCGAGACATTCCT
27483	Yes	ACAGATTCTAGATCGAGACATTCCTG
27484	Yes	ACAGATTCTAGATCGAGACATTCCTGG
27485	Yes	ACAGATTCTAGATCGAGACATTCCTGGG
27486	Yes	ACAGATTCTAGATCGAGACATTCCTGGGT
27487	Yes	ACAGATTCTAGATCGAGACATTCCTGGGTT
27488	Yes	ACAGATTCTAGATCGAGACATTCCTGGGTTC
27489	Yes	ACAGATTCTAGATCGAGACATTCCTGGGTTCA
27490	Yes	ACAGATTCTAGATCGAGACATTCCTGGGTTCAA
27491	Yes	ACAGATTCTAGATCGAGACATTCCTGGGTTCAAA
27492	Yes	ACAGATTCTAGATCGAGACATTCCTGGGTTCAAAT
27493	Yes	ACAGATTCTAGATCGAGACATTCCTGGGTTCAAATC
27494	Yes	ACAGATTCTAGATCGAGACATTCCTGGGTTCAAATCC
27495	Yes	ACAGATTCTAGATCGAGACATTCCTGGGTTCAAATCCC
27496	Yes	ACAGATTCTAGATCGAGACATTCCTGGGTTCAAATCCCA
27497	Yes	ACAGATTCTAGATCGAGACATTCCTGGGTTCAAATCCCAG
27498	Yes	ACAGATTCTAGATCGAGACATTCCTGGGTTCAAATCCCAGC
27499	Yes	ACAGATTCTAGATCGAGACATTCCTGGGTTCAAATCCCAGCA
27500	Yes	ACAGATTCTAGATCGAGACATTCCTGGGTTCAAATCCCAGCAC
27501	Yes	ACAGATTCTAGATCGAGACATTCCTGGGTTCAAATCCCAGCACT
27502	Yes	ACAGATTCTAGATCGAGACATTCCTGGGTTCAAATCCCAGCACTG
27503	Yes	ACAGATTCTAGATCGAGACATTCCTGGGTTCAAATCCCAGCACTGT

27504	Yes	ACAGATTCTAGATCGAGACATTCCTGGGTTCAAATCCCAGCACTGTT
27505	Yes	ACAGATTCTAGATCGAGACATTCCTGGGTTCAAATCCCAGCACTGTTG
27506	Yes	ACAGATTCTAGATCGAGACATTCCTGGGTTCAAATCCCAGCACTGTTGC
27507	Yes	ACAGATTCTAGATCGAGACATTCCTGGGTTCAAATCCCAGCACTGTTGCC
27508	No	ACAGCAACCATGGTTC
27509	Yes	ACAGCAACCATGGTTCA
27510	Yes	ACAGCAACCATGGTTCAA
27511	Yes	ACAGCAACCATGGTTCAAA
27512	Yes	ACAGCAACCATGGTTCAAAT
27513	Yes	ACAGCAACCATGGTTCAAATC
27514	Yes	ACAGCAACCATGGTTCAAATCT
27515	Yes	ACAGCAACCATGGTTCAAATCTG
27516	Yes	ACAGCAACCATGGTTCAAATCTGA
27517	Yes	ACAGCAACCATGGTTCAAATCTGAG
27518	Yes	ACAGCAACCATGGTTCAAATCTGAGT
27519	Yes	ACAGCAACCATGGTTCAAATCTGAGTC
27520	Yes	ACAGCAACCATGGTTCAAATCTGAGTCA
27521	Yes	ACAGCAACCATGGTTCAAATCTGAGTCAT
27522	Yes	ACAGCAACCATGGTTCAAATCTGAGTCATG
27523	Yes	ACAGCAACCATGGTTCAAATCTGAGTCATGA
27524	Yes	ACAGCAACCATGGTTCAAATCTGAGTCATGAC
27525	Yes	ACAGCAACCATGGTTCAAATCTGAGTCATGACA
27526	Yes	ACAGCAACCATGGTTCAAATCTGAGTCATGACAC
27527	Yes	ACAGCAACCATGGTTCAAATCTGAGTCATGACACC
27528	Yes	ACAGCAACCATGGTTCAAATCTGAGTCATGACACCA
27529	No	ACAGCCCAGGTTCAAT
27530	No	ACAGCCCAGGTTCAATT
27531	No	ACAGCCCAGGTTCAATTC
27532	No	ACAGCCCAGGTTCAATTCC
27533	Yes	ACAGCCCAGGTTCAATTCCC
27534	Yes	ACAGCCCAGGTTCAATTCCCT
27535	Yes	ACAGCCCAGGTTCAATTCCCTG
27536	Yes	ACAGCCCAGGTTCAATTCCCTGT
27537	Yes	ACAGCCCAGGTTCAATTCCCTGTC
27538	Yes	ACAGCCCAGGTTCAATTCCCTGTCA
27539	Yes	ACAGCCCAGGTTCAATTCCCTGTCAG
27540	Yes	ACAGCCCAGGTTCAATTCCCTGTCAGA
27541	Yes	ACAGCCCAGGTTCAATTCCCTGTCAGAG
27542	Yes	ACAGCCCAGGTTCAATTCCCTGTCAGAGA
27543	Yes	ACAGCCCAGGTTCAATTCCCTGTCAGAGAA
27544	Yes	ACAGCCCAGGTTCAATTCCCTGTCAGAGAAC
27545	Yes	ACAGCCCAGGTTCAATTCCCTGTCAGAGAACC

27546	Yes	ACAGCCCAGGTTCAATTCCCTGTCAGAGAACCA
27547	No	ACAGCCCAGGTTCAATTCCT
27548	No	ACAGCCCAGGTTCAATTCCTG
27549	Yes	ACAGCCCAGGTTCAATTCCTGG
27550	Yes	ACAGCCCAGGTTCAATTCCTGGT
27551	Yes	ACAGCCCAGGTTCAATTCCTGGTT
27552	Yes	ACAGCCCAGGTTCAATTCCTGGTTA
27553	Yes	ACAGCCCAGGTTCAATTCCTGGTTAG
27554	Yes	ACAGCCCAGGTTCAATTCCTGGTTAGA
27555	Yes	ACAGCCCAGGTTCAATTCCTGGTTAGAG
27556	Yes	ACAGCCCAGGTTCAATTCCTGGTTAGAGA
27557	Yes	ACAGCCCAGGTTCAATTCCTGGTTAGAGAC
27558	Yes	ACAGCCCAGGTTCAATTCCTGGTTAGAGACC
27559	Yes	ACAGCCCAGGTTCAATTCCTGGTTAGAGACCA
27560	No	ACAGCCCAGTGGTGCA
27561	Yes	ACAGCCCAGTGGTGCAG
27562	Yes	ACAGCCCAGTGGTGCAGG
27563	Yes	ACAGCCCAGTGGTGCAGGG
27564	Yes	ACAGCCCAGTGGTGCAGGGC
27565	Yes	ACAGCCCAGTGGTGCAGGGCA
27566	Yes	ACAGCCCAGTGGTGCAGGGCAT
27567	Yes	ACAGCCCAGTGGTGCAGGGCATG
27568	Yes	ACAGCCCAGTGGTGCAGGGCATGG
27569	Yes	ACAGCCCAGTGGTGCAGGGCATGGC
27570	Yes	ACAGCCCAGTGGTGCAGGGCATGGCT
27571	Yes	ACAGCCCAGTGGTGCAGGGCATGGCTC
27572	Yes	ACAGCCCAGTGGTGCAGGGCATGGCTCT
27573	Yes	ACAGCCCAGTGGTGCAGGGCATGGCTCTG
27574	Yes	ACAGCCCAGTGGTGCAGGGCATGGCTCTGA
27575	Yes	ACAGCCCAGTGGTGCAGGGCATGGCTCTGAC
27576	Yes	ACAGCCCAGTGGTGCAGGGCATGGCTCTGACA
27577	Yes	ACAGCCCAGTGGTGCAGGGCATGGCTCTGACAC
27578	Yes	ACAGCCCAGTGGTGCAGGGCATGGCTCTGACACC
27579	Yes	ACAGCCCAGTGGTGCAGGGCATGGCTCTGACACCT
27580	Yes	ACAGCCCAGTGGTGCAGGGCATGGCTCTGACACCTG
27581	Yes	ACAGCCCAGTGGTGCAGGGCATGGCTCTGACACCTGG
27582	Yes	ACAGCCCAGTGGTGCAGGGCATGGCTCTGACACCTGGC
27583	Yes	ACAGCCCAGTGGTGCAGGGCATGGCTCTGACACCTGGCG
27584	Yes	ACAGCCCAGTGGTGCAGGGCATGGCTCTGACACCTGGCGG
27585	Yes	ACAGCCCAGTGGTGCAGGGCATGGCTCTGACACCTGGCGGC
27586	Yes	ACAGCCCAGTGGTGCAGGGCATGGCTCTGACACCTGGCGGCC
27587	Yes	ACAGCCCAGTGGTGCAGGGCATGGCTCTGACACCTGGCGGCCT

27588	Yes	ACAGCCCAGTGGTGCAGGGCATGGCTCTGACACCTGGCGGCCTG
27589	Yes	ACAGCCCAGTGGTGCAGGGCATGGCTCTGACACCTGGCGGCCTGG
27590	Yes	ACAGCCCAGTGGTGCAGGGCATGGCTCTGACACCTGGCGGCCTGGG
27591	Yes	ACAGCCCAGTGGTGCAGGGCATGGCTCTGACACCTGGCGGCCTGGGT
27592	Yes	ACAGCCCAGTGGTGCAGGGCATGGCTCTGACACCTGGCGGCCTGGGTT
27593	Yes	ACAGCCCAGTGGTGCAGGGCATGGCTCTGACACCTGGCGGCCTGGGTTC
27594	Yes	ACAGCCCAGTGGTGCAGGGCATGGCTCTGACACCTGGCGGCCTGGGTTCA
27595	Yes	ACAGCCGCGGCCCGGG
27596	Yes	ACAGCCGCGGCCCGGGT
27597	Yes	ACAGCCGCGGCCCGGGTT
27598	Yes	ACAGCCGCGGCCCGGGTTC
27599	Yes	ACAGCCGCGGCCCGGGTTCG
27600	Yes	ACAGCCGCGGCCCGGGTTCGA
27601	Yes	ACAGCCGCGGCCCGGGTTCGAT
27602	Yes	ACAGCCGCGGCCCGGGTTCGATT
27603	Yes	ACAGCCGCGGCCCGGGTTCGATTC
27604	Yes	ACAGCCGCGGCCCGGGTTCGATTCC
27605	Yes	ACAGCCGCGGCCCGGGTTCGATTCCC
27606	Yes	ACAGCCGCGGCCCGGGTTCGATTCCCA
27607	Yes	ACAGCCGCGGCCCGGGTTCGATTCCCAT
27608	Yes	ACAGCCGCGGCCCGGGTTCGATTCCCATT
27609	Yes	ACAGCCGCGGCCCGGGTTCGATTCCCATTC
27610	Yes	ACAGCCGCGGCCCGGGTTCGATTCCCATTCC
27611	Yes	ACAGCCGCGGCCCGGGTTCGATTCCCATTCCG
27612	Yes	ACAGCCGCGGCCCGGGTTCGATTCCCATTCCGG
27613	Yes	ACAGCCGCGGCCCGGGTTCGATTCCCATTCCGGG
27614	Yes	ACAGCCGCGGCCCGGGTTCGATTCCCATTCCGGGA
27615	Yes	ACAGCCGCGGCCCGGGTTCGATTCCCATTCCGGGAA
27616	Yes	ACAGCCGCGGCCCGGGTTCGATTCCCATTCCGGGAAC
27617	Yes	ACAGCCGCGGCCCGGGTTCGATTCCCATTCCGGGAACC
27618	Yes	ACAGCCGCGGCCCGGGTTCGATTCCCATTCCGGGAACCA
27619	No	ACAGCCTGGGTTCAAT
27620	No	ACAGCCTGGGTTCAATT
27621	No	ACAGCCTGGGTTCAATTC
27622	No	ACAGCCTGGGTTCAATTCC
27623	Yes	ACAGCCTGGGTTCAATTCCC
27624	Yes	ACAGCCTGGGTTCAATTCCCA
27625	Yes	ACAGCCTGGGTTCAATTCCCAG
27626	Yes	ACAGCCTGGGTTCAATTCCCAGC
27627	Yes	ACAGCCTGGGTTCAATTCCCAGCT
27628	Yes	ACAGCCTGGGTTCAATTCCCAGCTT
27629	Yes	ACAGCCTGGGTTCAATTCCCAGCTTG

27630	Yes	ACAGCCTGGGTTCAATTCCCAGCTTGG
27631	Yes	ACAGCCTGGGTTCAATTCCCAGCTTGGC
27632	Yes	ACAGCCTGGGTTCAATTCCCAGCTTGGCC
27633	Yes	ACAGCCTGGGTTCAATTCCCAGCTTGGCCA
27634	Yes	ACAGCCTGGGTTCAATTCCCAGCTTGGCCAC
27635	Yes	ACAGCCTGGGTTCAATTCCCAGCTTGGCCACC
27636	Yes	ACAGCCTGGGTTCAATTCCCAGCTTGGCCACCA
27637	Yes	ACAGCTATCCATTGGT
27638	Yes	ACAGCTATCCATTGGTC
27639	Yes	ACAGCTATCCATTGGTCT
27640	Yes	ACAGCTATCCATTGGTCTT
27641	Yes	ACAGCTATCCATTGGTCTTA
27642	Yes	ACAGCTATCCATTGGTCTTAG
27643	Yes	ACAGCTATCCATTGGTCTTAGG
27644	Yes	ACAGCTATCCATTGGTCTTAGGC
27645	Yes	ACAGCTATCCATTGGTCTTAGGCC
27646	Yes	ACAGCTATCCATTGGTCTTAGGCCC
27647	Yes	ACAGCTATCCATTGGTCTTAGGCCCC
27648	Yes	ACAGCTATCCATTGGTCTTAGGCCCCA
27649	Yes	ACAGCTATCCATTGGTCTTAGGCCCCAA
27650	Yes	ACAGCTATCCATTGGTCTTAGGCCCCAAA
27651	Yes	ACAGCTATCCATTGGTCTTAGGCCCCAAAA
27652	Yes	ACAGCTATCCATTGGTCTTAGGCCCCAAAAA
27653	Yes	ACAGCTATCCATTGGTCTTAGGCCCCAAAAAT
27654	Yes	ACAGCTATCCATTGGTCTTAGGCCCCAAAAATT
27655	Yes	ACAGCTATCCATTGGTCTTAGGCCCCAAAAATTT
27656	Yes	ACAGCTATCCATTGGTCTTAGGCCCCAAAAATTTT
27657	Yes	ACAGCTATCCATTGGTCTTAGGCCCCAAAAATTTTG
27658	Yes	ACAGCTATCCATTGGTCTTAGGCCCCAAAAATTTTGG
27659	Yes	ACAGCTATCCATTGGTCTTAGGCCCCAAAAATTTTGGT
27660	Yes	ACAGCTATCCATTGGTCTTAGGCCCCAAAAATTTTGGTG
27661	Yes	ACAGCTATCCATTGGTCTTAGGCCCCAAAAATTTTGGTGC
27662	Yes	ACAGCTATCCATTGGTCTTAGGCCCCAAAAATTTTGGTGCA
27663	Yes	ACAGCTATCCATTGGTCTTAGGCCCCAAAAATTTTGGTGCAA
27664	Yes	ACAGCTATCCATTGGTCTTAGGCCCCAAAAATTTTGGTGCAAC
27665	Yes	ACAGCTATCCATTGGTCTTAGGCCCCAAAAATTTTGGTGCAACT
27666	Yes	ACAGCTATCCATTGGTCTTAGGCCCCAAAAATTTTGGTGCAACTC
27667	Yes	ACAGCTATCCATTGGTCTTAGGCCCCAAAAATTTTGGTGCAACTCC
27668	Yes	ACAGCTATCCATTGGTCTTAGGCCCCAAAAATTTTGGTGCAACTCCA
27669	Yes	ACAGCTATCCATTGGTCTTAGGCCCCAAAAATTTTGGTGCAACTCCAA
27670	Yes	ACAGCTATCCATTGGTCTTAGGCCCCAAAAATTTTGGTGCAACTCCAAA
27671	Yes	ACAGCTATCCATTGGTCTTAGGCCCCAAAAATTTTGGTGCAACTCCAAAT

27672	No	ACAGCTGCCTTTCAAG
27673	No	ACAGCTGCCTTTCAAGC
27674	No	ACAGCTGCCTTTCAAGCA
27675	No	ACAGCTGCCTTTCAAGCAG
27676	Yes	ACAGCTGCCTTTCAAGCAGT
27677	Yes	ACAGCTGCCTTTCAAGCAGTT
27678	Yes	ACAGCTGCCTTTCAAGCAGTTA
27679	Yes	ACAGCTGCCTTTCAAGCAGTTAA
27680	Yes	ACAGCTGCCTTTCAAGCAGTTAAC
27681	Yes	ACAGCTGCCTTTCAAGCAGTTAACG
27682	Yes	ACAGCTGCCTTTCAAGCAGTTAACGC
27683	Yes	ACAGCTGCCTTTCAAGCAGTTAACGCG
27684	Yes	ACAGCTGCCTTTCAAGCAGTTAACGCGG
27685	Yes	ACAGCTGCCTTTCAAGCAGTTAACGCGGG
27686	Yes	ACAGCTGCCTTTCAAGCAGTTAACGCGGGT
27687	Yes	ACAGCTGCCTTTCAAGCAGTTAACGCGGGTT
27688	Yes	ACAGCTGCCTTTCAAGCAGTTAACGCGGGTTC
27689	Yes	ACAGCTGCCTTTCAAGCAGTTAACGCGGGTTCG
27690	Yes	ACAGCTGCCTTTCAAGCAGTTAACGCGGGTTCGA
27691	Yes	ACAGCTGCCTTTCAAGCAGTTAACGCGGGTTCGAT
27692	Yes	ACAGCTGCCTTTCAAGCAGTTAACGCGGGTTCGATT
27693	Yes	ACAGCTGCCTTTCAAGCAGTTAACGCGGGTTCGATTC
27694	Yes	ACAGCTGCCTTTCAAGCAGTTAACGCGGGTTCGATTCC
27695	Yes	ACAGCTGCCTTTCAAGCAGTTAACGCGGGTTCGATTCCC
27696	Yes	ACAGCTGCCTTTCAAGCAGTTAACGCGGGTTCGATTCCCG
27697	Yes	ACAGCTGCCTTTCAAGCAGTTAACGCGGGTTCGATTCCCGG
27698	Yes	ACAGCTGCCTTTCAAGCAGTTAACGCGGGTTCGATTCCCGGG
27699	Yes	ACAGCTGCCTTTCAAGCAGTTAACGCGGGTTCGATTCCCGGGT
27700	Yes	ACAGCTGCCTTTCAAGCAGTTAACGCGGGTTCGATTCCCGGGTA
27701	Yes	ACAGCTGCCTTTCAAGCAGTTAACGCGGGTTCGATTCCCGGGTAA
27702	Yes	ACAGCTGCCTTTCAAGCAGTTAACGCGGGTTCGATTCCCGGGTAAC
27703	Yes	ACAGCTGCCTTTCAAGCAGTTAACGCGGGTTCGATTCCCGGGTAACG
27704	Yes	ACAGCTGCCTTTCAAGCAGTTAACGCGGGTTCGATTCCCGGGTAACGA
27705	Yes	ACAGCTGCCTTTCAAGCAGTTAACGCGGGTTCGATTCCCGGGTAACGAA
27706	Yes	ACAGCTGCCTTTCAAGCAGTTAACGCGGGTTCGATTCCCGGGTAACGAAC
27707	Yes	ACAGGAGACCCGGGTT
27708	Yes	ACAGGAGACCCGGGTTC
27709	Yes	ACAGGAGACCCGGGTTCA
27710	Yes	ACAGGAGACCCGGGTTCAA
27711	Yes	ACAGGAGACCCGGGTTCAAT
27712	Yes	ACAGGAGACCCGGGTTCAATT
27713	Yes	ACAGGAGACCCGGGTTCAATTC

27714	Yes	ACAGGAGACCCGGGTTCAATTCC
27715	Yes	ACAGGAGACCCGGGTTCAATTCCT
27716	Yes	ACAGGAGACCCGGGTTCAATTCCTG
27717	Yes	ACAGGAGACCCGGGTTCAATTCCTGA
27718	Yes	ACAGGAGACCCGGGTTCAATTCCTGAC
27719	Yes	ACAGGAGACCCGGGTTCAATTCCTGACC
27720	Yes	ACAGGAGACCCGGGTTCAATTCCTGACCC
27721	Yes	ACAGGAGACCCGGGTTCAATTCCTGACCCA
27722	Yes	ACAGGAGACCCGGGTTCAATTCCTGACCCAT
27723	Yes	ACAGGAGACCCGGGTTCAATTCCTGACCCATG
27724	Yes	ACAGGAGACCCGGGTTCAATTCCTGACCCATGT
27725	Yes	ACAGGAGACCCGGGTTCAATTCCTGACCCATGTA
27726	Yes	ACAGGAGACCCGGGTTCAATTCCTGACCCATGTAC
27727	Yes	ACAGGAGACCCGGGTTCAATTCCTGACCCATGTACC
27728	Yes	ACAGGAGACCCGGGTTCAATTCCTGACCCATGTACCA
27729	No	ACAGGAGATCCTGAGT
27730	No	ACAGGAGATCCTGAGTT
27731	No	ACAGGAGATCCTGAGTTT
27732	Yes	ACAGGAGATCCTGAGTTTC
27733	Yes	ACAGGAGATCCTGAGTTTCA
27734	Yes	ACAGGAGATCCTGAGTTTCAA
27735	Yes	ACAGGAGATCCTGAGTTTCAAT
27736	Yes	ACAGGAGATCCTGAGTTTCAATT
27737	Yes	ACAGGAGATCCTGAGTTTCAATTC
27738	Yes	ACAGGAGATCCTGAGTTTCAATTCC
27739	Yes	ACAGGAGATCCTGAGTTTCAATTCCA
27740	Yes	ACAGGAGATCCTGAGTTTCAATTCCAA
27741	Yes	ACAGGAGATCCTGAGTTTCAATTCCAAT
27742	Yes	ACAGGAGATCCTGAGTTTCAATTCCAATG
27743	Yes	ACAGGAGATCCTGAGTTTCAATTCCAATGG
27744	Yes	ACAGGAGATCCTGAGTTTCAATTCCAATGGT
27745	Yes	ACAGGAGATCCTGAGTTTCAATTCCAATGGTG
27746	Yes	ACAGGAGATCCTGAGTTTCAATTCCAATGGTGC
27747	Yes	ACAGGAGATCCTGAGTTTCAATTCCAATGGTGCC
27748	Yes	ACAGGAGATCCTGAGTTTCAATTCCAATGGTGCCT
27749	Yes	ACAGGAGATCCTGAGTTTCAATTCCAATGGTGCCTC
27750	Yes	ACAGGAGATCCTGAGTTTCAATTCCAATGGTGCCTCC
27751	Yes	ACAGGAGATCCTGAGTTTCAATTCCAATGGTGCCTCCA
27752	No	ACAGGAGATCCTGGGT
27753	No	ACAGGAGATCCTGGGTT
27754	Yes	ACAGGAGATCCTGGGTTC
27755	Yes	ACAGGAGATCCTGGGTTCG

27756	Yes	ACAGGAGATCCTGGGTTCGA
27757	Yes	ACAGGAGATCCTGGGTTCGAA
27758	Yes	ACAGGAGATCCTGGGTTCGAAT
27759	Yes	ACAGGAGATCCTGGGTTCGAATC
27760	Yes	ACAGGAGATCCTGGGTTCGAATCC
27761	Yes	ACAGGAGATCCTGGGTTCGAATCCC
27762	Yes	ACAGGAGATCCTGGGTTCGAATCCCA
27763	Yes	ACAGGAGATCCTGGGTTCGAATCCCAG
27764	Yes	ACAGGAGATCCTGGGTTCGAATCCCAGC
27765	Yes	ACAGGAGATCCTGGGTTCGAATCCCAGCG
27766	Yes	ACAGGAGATCCTGGGTTCGAATCCCAGCGA
27767	Yes	ACAGGAGATCCTGGGTTCGAATCCCAGCGAG
27768	Yes	ACAGGAGATCCTGGGTTCGAATCCCAGCGAGG
27769	Yes	ACAGGAGATCCTGGGTTCGAATCCCAGCGAGGC
27770	Yes	ACAGGAGATCCTGGGTTCGAATCCCAGCGAGGCC
27771	Yes	ACAGGAGATCCTGGGTTCGAATCCCAGCGAGGCCT
27772	Yes	ACAGGAGATCCTGGGTTCGAATCCCAGCGAGGCCTC
27773	Yes	ACAGGAGATCCTGGGTTCGAATCCCAGCGAGGCCTCC
27774	Yes	ACAGGAGATCCTGGGTTCGAATCCCAGCGAGGCCTCCA
27775	Yes	ACAGGAGATCCTGGGTTCGAATCCCAGCGG
27776	Yes	ACAGGAGATCCTGGGTTCGAATCCCAGCGGG
27777	Yes	ACAGGAGATCCTGGGTTCGAATCCCAGCGGGG
27778	Yes	ACAGGAGATCCTGGGTTCGAATCCCAGCGGGGC
27779	Yes	ACAGGAGATCCTGGGTTCGAATCCCAGCGGGGCC
27780	Yes	ACAGGAGATCCTGGGTTCGAATCCCAGCGGGGCCT
27781	Yes	ACAGGAGATCCTGGGTTCGAATCCCAGCGGGGCCTC
27782	Yes	ACAGGAGATCCTGGGTTCGAATCCCAGCGGGGCCTCC
27783	Yes	ACAGGAGATCCTGGGTTCGAATCCCAGCGGGGCCTCCA
27784	Yes	ACAGGAGATCCTGGGTTCGAATCCCAGCGGT
27785	Yes	ACAGGAGATCCTGGGTTCGAATCCCAGCGGTG
27786	Yes	ACAGGAGATCCTGGGTTCGAATCCCAGCGGTGC
27787	Yes	ACAGGAGATCCTGGGTTCGAATCCCAGCGGTGCC
27788	Yes	ACAGGAGATCCTGGGTTCGAATCCCAGCGGTGCCT
27789	Yes	ACAGGAGATCCTGGGTTCGAATCCCAGCGGTGCCTC
27790	Yes	ACAGGAGATCCTGGGTTCGAATCCCAGCGGTGCCTCC
27791	Yes	ACAGGAGATCCTGGGTTCGAATCCCAGCGGTGCCTCCA
27792	Yes	ACAGGAGATCCTGGGTTCGAATCCCAGT
27793	Yes	ACAGGAGATCCTGGGTTCGAATCCCAGTA
27794	Yes	ACAGGAGATCCTGGGTTCGAATCCCAGTAG
27795	Yes	ACAGGAGATCCTGGGTTCGAATCCCAGTAGA
27796	Yes	ACAGGAGATCCTGGGTTCGAATCCCAGTAGAG
27797	Yes	ACAGGAGATCCTGGGTTCGAATCCCAGTAGAGC

27798	Yes	ACAGGAGATCCTGGGTTCGAATCCCAGTAGAGCC
27799	Yes	ACAGGAGATCCTGGGTTCGAATCCCAGTAGAGCCT
27800	Yes	ACAGGAGATCCTGGGTTCGAATCCCAGTAGAGCCTC
27801	Yes	ACAGGAGATCCTGGGTTCGAATCCCAGTAGAGCCTCC
27802	Yes	ACAGGAGATCCTGGGTTCGAATCCCAGTAGAGCCTCCA
27803	Yes	ACAGGAGATCCTGGGTTCGAC
27804	Yes	ACAGGAGATCCTGGGTTCGACT
27805	Yes	ACAGGAGATCCTGGGTTCGACTC
27806	Yes	ACAGGAGATCCTGGGTTCGACTCC
27807	Yes	ACAGGAGATCCTGGGTTCGACTCCC
27808	Yes	ACAGGAGATCCTGGGTTCGACTCCCA
27809	Yes	ACAGGAGATCCTGGGTTCGACTCCCAG
27810	Yes	ACAGGAGATCCTGGGTTCGACTCCCAGC
27811	Yes	ACAGGAGATCCTGGGTTCGACTCCCAGCG
27812	Yes	ACAGGAGATCCTGGGTTCGACTCCCAGCGG
27813	Yes	ACAGGAGATCCTGGGTTCGACTCCCAGCGGG
27814	Yes	ACAGGAGATCCTGGGTTCGACTCCCAGCGGGG
27815	Yes	ACAGGAGATCCTGGGTTCGACTCCCAGCGGGGC
27816	Yes	ACAGGAGATCCTGGGTTCGACTCCCAGCGGGGCC
27817	Yes	ACAGGAGATCCTGGGTTCGACTCCCAGCGGGGCCT
27818	Yes	ACAGGAGATCCTGGGTTCGACTCCCAGCGGGGCCTC
27819	Yes	ACAGGAGATCCTGGGTTCGACTCCCAGCGGGGCCTCC
27820	Yes	ACAGGAGATCCTGGGTTCGACTCCCAGCGGGGCCTCCA
27821	Yes	ACAGGAGATCCTGGGTTCGACTCCCAGT
27822	Yes	ACAGGAGATCCTGGGTTCGACTCCCAGTG
27823	Yes	ACAGGAGATCCTGGGTTCGACTCCCAGTGG
27824	Yes	ACAGGAGATCCTGGGTTCGACTCCCAGTGGG
27825	Yes	ACAGGAGATCCTGGGTTCGACTCCCAGTGGGG
27826	Yes	ACAGGAGATCCTGGGTTCGACTCCCAGTGGGGC
27827	Yes	ACAGGAGATCCTGGGTTCGACTCCCAGTGGGGCC
27828	Yes	ACAGGAGATCCTGGGTTCGACTCCCAGTGGGGCCT
27829	Yes	ACAGGAGATCCTGGGTTCGACTCCCAGTGGGGCCTC
27830	Yes	ACAGGAGATCCTGGGTTCGACTCCCAGTGGGGCCTCC
27831	Yes	ACAGGAGATCCTGGGTTCGACTCCCAGTGGGGCCTCCA
27832	No	ACAGGCTTTGGAAACT
27833	No	ACAGGCTTTGGAAACTG
27834	Yes	ACAGGCTTTGGAAACTGG
27835	Yes	ACAGGCTTTGGAAACTGGC
27836	Yes	ACAGGCTTTGGAAACTGGCA
27837	Yes	ACAGGCTTTGGAAACTGGCAG
27838	Yes	ACAGGCTTTGGAAACTGGCAGG
27839	Yes	ACAGGCTTTGGAAACTGGCAGGC

27840	Yes	ACAGGCTTTGGAAACTGGCAGGCC
27841	Yes	ACAGGCTTTGGAAACTGGCAGGCCT
27842	Yes	ACAGGCTTTGGAAACTGGCAGGCCTG
27843	Yes	ACAGGCTTTGGAAACTGGCAGGCCTGG
27844	Yes	ACAGGCTTTGGAAACTGGCAGGCCTGGG
27845	Yes	ACAGGCTTTGGAAACTGGCAGGCCTGGGT
27846	Yes	ACAGGCTTTGGAAACTGGCAGGCCTGGGTT
27847	Yes	ACAGGCTTTGGAAACTGGCAGGCCTGGGTTC
27848	Yes	ACAGGCTTTGGAAACTGGCAGGCCTGGGTTCA
27849	Yes	ACAGGCTTTGGAAACTGGCAGGCCTGGGTTCAA
27850	Yes	ACAGGCTTTGGAAACTGGCAGGCCTGGGTTCAAA
27851	Yes	ACAGGCTTTGGAAACTGGCAGGCCTGGGTTCAAAT
27852	Yes	ACAGGCTTTGGAAACTGGCAGGCCTGGGTTCAAATC
27853	Yes	ACAGGCTTTGGAAACTGGCAGGCCTGGGTTCAAATCC
27854	Yes	ACAGGCTTTGGAAACTGGCAGGCCTGGGTTCAAATCCC
27855	Yes	ACAGGCTTTGGAAACTGGCAGGCCTGGGTTCAAATCCCA
27856	Yes	ACAGGCTTTGGAAACTGGCAGGCCTGGGTTCAAATCCCAG
27857	Yes	ACAGGCTTTGGAAACTGGCAGGCCTGGGTTCAAATCCCAGC
27858	Yes	ACAGGCTTTGGAAACTGGCAGGCCTGGGTTCAAATCCCAGCT
27859	Yes	ACAGGCTTTGGAAACTGGCAGGCCTGGGTTCAAATCCCAGCTT
27860	Yes	ACAGGCTTTGGAAACTGGCAGGCCTGGGTTCAAATCCCAGCTTA
27861	Yes	ACAGGCTTTGGAAACTGGCAGGCCTGGGTTCAAATCCCAGCTTAT
27862	Yes	ACAGGCTTTGGAAACTGGCAGGCCTGGGTTCAAATCCCAGCTTATT
27863	Yes	ACAGGCTTTGGAAACTGGCAGGCCTGGGTTCAAATCCCAGCTTATTC
27864	Yes	ACAGGCTTTGGAAACTGGCAGGCCTGGGTTCAAATCCCAGCTTATTCA
27865	Yes	ACAGGCTTTGGAAACTGGCAGGCCTGGGTTCAAATCCCAGCTTATTCAC
27866	Yes	ACAGGCTTTGGAAACTGGCAGGCCTGGGTTCAAATCCCAGCTTATTCACC
27867	No	ACAGGGGCTAATATGC
27868	No	ACAGGGGCTAATATGCT
27869	Yes	ACAGGGGCTAATATGCTG
27870	Yes	ACAGGGGCTAATATGCTGG
27871	Yes	ACAGGGGCTAATATGCTGGG
27872	Yes	ACAGGGGCTAATATGCTGGGC
27873	Yes	ACAGGGGCTAATATGCTGGGCC
27874	Yes	ACAGGGGCTAATATGCTGGGCCT
27875	Yes	ACAGGGGCTAATATGCTGGGCCTT
27876	Yes	ACAGGGGCTAATATGCTGGGCCTTT
27877	Yes	ACAGGGGCTAATATGCTGGGCCTTTA
27878	Yes	ACAGGGGCTAATATGCTGGGCCTTTAC
27879	Yes	ACAGGGGCTAATATGCTGGGCCTTTACC
27880	Yes	ACAGGGGCTAATATGCTGGGCCTTTACCA
27881	Yes	ACAGGGGCTAATATGCTGGGCCTTTACCAC

27882	Yes	ACAGGGGCTAATATGCTGGGCCTTTACCACT
27883	Yes	ACAGGGGCTAATATGCTGGGCCTTTACCACTT
27884	Yes	ACAGGGGCTAATATGCTGGGCCTTTACCACTTC
27885	Yes	ACAGGGGCTAATATGCTGGGCCTTTACCACTTCA
27886	Yes	ACAGGGGCTAATATGCTGGGCCTTTACCACTTCAG
27887	Yes	ACAGGGGCTAATATGCTGGGCCTTTACCACTTCAGC
27888	Yes	ACAGGGGCTAATATGCTGGGCCTTTACCACTTCAGCC
27889	Yes	ACAGGGGCTAATATGCTGGGCCTTTACCACTTCAGCCC
27890	Yes	ACAGGGGCTAATATGCTGGGCCTTTACCACTTCAGCCCA
27891	Yes	ACAGGGGCTAATATGCTGGGCCTTTACCACTTCAGCCCAG
27892	Yes	ACAGGGGCTAATATGCTGGGCCTTTACCACTTCAGCCCAGG
27893	Yes	ACAGGGGCTAATATGCTGGGCCTTTACCACTTCAGCCCAGGT
27894	Yes	ACAGGGGCTAATATGCTGGGCCTTTACCACTTCAGCCCAGGTT
27895	Yes	ACAGGGGCTAATATGCTGGGCCTTTACCACTTCAGCCCAGGTTC
27896	Yes	ACAGGGGCTAATATGCTGGGCCTTTACCACTTCAGCCCAGGTTCG
27897	Yes	ACAGGGGCTAATATGCTGGGCCTTTACCACTTCAGCCCAGGTTCGA
27898	Yes	ACAGGGGCTAATATGCTGGGCCTTTACCACTTCAGCCCAGGTTCGAT
27899	Yes	ACAGGGGCTAATATGCTGGGCCTTTACCACTTCAGCCCAGGTTCGATT
27900	Yes	ACAGGGGCTAATATGCTGGGCCTTTACCACTTCAGCCCAGGTTCGATTC
27901	Yes	ACAGGGGCTAATATGCTGGGCCTTTACCACTTCAGCCCAGGTTCGATTCC
27902	No	ACAGGGGTTAGGCCTC
27903	Yes	ACAGGGGTTAGGCCTCT
27904	Yes	ACAGGGGTTAGGCCTCTT
27905	Yes	ACAGGGGTTAGGCCTCTTT
27906	Yes	ACAGGGGTTAGGCCTCTTTT
27907	Yes	ACAGGGGTTAGGCCTCTTTTT
27908	Yes	ACAGGGGTTAGGCCTCTTTTTA
27909	Yes	ACAGGGGTTAGGCCTCTTTTTAC
27910	Yes	ACAGGGGTTAGGCCTCTTTTTACC
27911	Yes	ACAGGGGTTAGGCCTCTTTTTACCA
27912	Yes	ACAGGGGTTAGGCCTCTTTTTACCAC
27913	Yes	ACAGGGGTTAGGCCTCTTTTTACCACC
27914	Yes	ACAGGGGTTAGGCCTCTTTTTACCACCA
27915	Yes	ACAGGTAGAGCATTTG
27916	Yes	ACAGGTAGAGCATTTGA
27917	Yes	ACAGGTAGAGCATTTGAC
27918	Yes	ACAGGTAGAGCATTTGACT
27919	Yes	ACAGGTAGAGCATTTGACTG
27920	Yes	ACAGGTAGAGCATTTGACTGC
27921	Yes	ACAGGTAGAGCATTTGACTGCA
27922	Yes	ACAGGTAGAGCATTTGACTGCAG
27923	Yes	ACAGGTAGAGCATTTGACTGCAGA

27924	Yes	ACAGGTAGAGCATTTGACTGCAGAT
27925	Yes	ACAGGTAGAGCATTTGACTGCAGATC
27926	Yes	ACAGGTAGAGCATTTGACTGCAGATCA
27927	Yes	ACAGGTAGAGCATTTGACTGCAGATCAA
27928	Yes	ACAGGTAGAGCATTTGACTGCAGATCAAG
27929	Yes	ACAGGTAGAGCATTTGACTGCAGATCAAGA
27930	Yes	ACAGGTAGAGCATTTGACTGCAGATCAAGAG
27931	Yes	ACAGGTAGAGCATTTGACTGCAGATCAAGAGG
27932	Yes	ACAGGTAGAGCATTTGACTGCAGATCAAGAGGT
27933	Yes	ACAGGTAGAGCATTTGACTGCAGATCAAGAGGTC
27934	Yes	ACAGGTAGAGCATTTGACTGCAGATCAAGAGGTCC
27935	Yes	ACAGGTAGAGCATTTGACTGCAGATCAAGAGGTCCC
27936	Yes	ACAGGTAGAGCATTTGACTGCAGATCAAGAGGTCCCC
27937	Yes	ACAGGTAGAGCATTTGACTGCAGATCAAGAGGTCCCCG
27938	Yes	ACAGGTAGAGCATTTGACTGCAGATCAAGAGGTCCCCGG
27939	Yes	ACAGGTAGAGCATTTGACTGCAGATCAAGAGGTCCCCGGT
27940	Yes	ACAGGTAGAGCATTTGACTGCAGATCAAGAGGTCCCCGGTT
27941	Yes	ACAGGTAGAGCATTTGACTGCAGATCAAGAGGTCCCCGGTTC
27942	Yes	ACAGGTAGAGCATTTGACTGCAGATCAAGAGGTCCCCGGTTCA
27943	Yes	ACAGGTAGAGCATTTGACTGCAGATCAAGAGGTCCCCGGTTCAA
27944	Yes	ACAGGTAGAGCATTTGACTGCAGATCAAGAGGTCCCCGGTTCAAA
27945	Yes	ACAGGTAGAGCATTTGACTGCAGATCAAGAGGTCCCCGGTTCAAAT
27946	Yes	ACAGGTAGAGCATTTGACTGCAGATCAAGAGGTCCCCGGTTCAAATC
27947	Yes	ACAGGTAGAGCATTTGACTGCAGATCAAGAGGTCCCCGGTTCAAATCC
27948	Yes	ACAGGTAGAGCATTTGACTGCAGATCAAGAGGTCCCCGGTTCAAATCCG
27949	Yes	ACAGGTAGAGCATTTGACTGCAGATCAAGAGGTCCCCGGTTCAAATCCGG
27950	Yes	ACAGGTAGAGCATTTGACTGCAGATCAAGAGGTCCCCGGTTCAAATCT
27951	Yes	ACAGGTAGAGCATTTGACTGCAGATCAAGAGGTCCCCGGTTCAAATCTG
27952	Yes	ACAGGTAGAGCATTTGACTGCAGATCAAGAGGTCCCCGGTTCAAATCTGG
27953	No	ACAGGTAGCATGGAGA
27954	Yes	ACAGGTAGCATGGAGAA
27955	Yes	ACAGGTAGCATGGAGAAT
27956	Yes	ACAGGTAGCATGGAGAATT
27957	Yes	ACAGGTAGCATGGAGAATTT
27958	Yes	ACAGGTAGCATGGAGAATTTT
27959	Yes	ACAGGTAGCATGGAGAATTTTG
27960	Yes	ACAGGTAGCATGGAGAATTTTGG
27961	Yes	ACAGGTAGCATGGAGAATTTTGGA
27962	Yes	ACAGGTAGCATGGAGAATTTTGGAT
27963	Yes	ACAGGTAGCATGGAGAATTTTGGATT
27964	Yes	ACAGGTAGCATGGAGAATTTTGGATTC
27965	Yes	ACAGGTAGCATGGAGAATTTTGGATTCT

27966	Yes	ACAGGTAGCATGGAGAATTTTGGATTCTC
27967	Yes	ACAGGTAGCATGGAGAATTTTGGATTCTCA
27968	Yes	ACAGGTAGCATGGAGAATTTTGGATTCTCAG
27969	Yes	ACAGGTAGCATGGAGAATTTTGGATTCTCAGG
27970	Yes	ACAGGTAGCATGGAGAATTTTGGATTCTCAGGG
27971	Yes	ACAGGTAGCATGGAGAATTTTGGATTCTCAGGGT
27972	Yes	ACAGGTAGCATGGAGAATTTTGGATTCTCAGGGTT
27973	Yes	ACAGGTAGCATGGAGAATTTTGGATTCTCAGGGTTA
27974	Yes	ACAGGTAGCATGGAGAATTTTGGATTCTCAGGGTTAG
27975	Yes	ACAGGTAGCATGGAGAATTTTGGATTCTCAGGGTTAGG
27976	Yes	ACAGGTAGCATGGAGAATTTTGGATTCTCAGGGTTAGGT
27977	Yes	ACAGGTAGCATGGAGAATTTTGGATTCTCAGGGTTAGGTT
27978	Yes	ACAGGTAGCATGGAGAATTTTGGATTCTCAGGGTTAGGTTC
27979	Yes	ACAGGTAGCATGGAGAATTTTGGATTCTCAGGGTTAGGTTCA
27980	Yes	ACAGGTAGCATGGAGAATTTTGGATTCTCAGGGTTAGGTTCAA
27981	Yes	ACAGGTAGCATGGAGAATTTTGGATTCTCAGGGTTAGGTTCAAT
27982	Yes	ACAGGTAGCATGGAGAATTTTGGATTCTCAGGGTTAGGTTCAATT
27983	Yes	ACAGGTAGCATGGAGAATTTTGGATTCTCAGGGTTAGGTTCAATTC
27984	Yes	ACAGGTAGCATGGAGAATTTTGGATTCTCAGGGTTAGGTTCAATTCC
27985	Yes	ACAGGTAGCATGGAGAATTTTGGATTCTCAGGGTTAGGTTCAATTCCT
27986	Yes	ACAGGTAGCATGGAGAATTTTGGATTCTCAGGGTTAGGTTCAATTCCTA
27987	Yes	ACAGGTAGCATGGAGAATTTTGGATTCTCAGGGTTAGGTTCAATTCCTAT
27988	No	ACAGGTAGGTAGATCT
27989	Yes	ACAGGTAGGTAGATCTG
27990	Yes	ACAGGTAGGTAGATCTGG
27991	Yes	ACAGGTAGGTAGATCTGGG
27992	Yes	ACAGGTAGGTAGATCTGGGT
27993	Yes	ACAGGTAGGTAGATCTGGGTT
27994	Yes	ACAGGTAGGTAGATCTGGGTTC
27995	Yes	ACAGGTAGGTAGATCTGGGTTCA
27996	Yes	ACAGGTAGGTAGATCTGGGTTCAA
27997	Yes	ACAGGTAGGTAGATCTGGGTTCAAA
27998	Yes	ACAGGTAGGTAGATCTGGGTTCAAAC
27999	Yes	ACAGGTAGGTAGATCTGGGTTCAAACC
28000	Yes	ACAGGTAGGTAGATCTGGGTTCAAACCC
28001	Yes	ACAGGTAGGTAGATCTGGGTTCAAACCCT
28002	Yes	ACAGGTAGGTAGATCTGGGTTCAAACCCTA
28003	Yes	ACAGGTAGGTAGATCTGGGTTCAAACCCTAG
28004	Yes	ACAGGTAGGTAGATCTGGGTTCAAACCCTAGC
28005	Yes	ACAGGTAGGTAGATCTGGGTTCAAACCCTAGCC
28006	Yes	ACAGGTAGGTAGATCTGGGTTCAAACCCTAGCCC
28007	Yes	ACAGGTAGGTAGATCTGGGTTCAAACCCTAGCCCT

28008	Yes	ACAGGTAGGTAGATCTGGGTTCAAACCCTAGCCCTG
28009	Yes	ACAGGTAGGTAGATCTGGGTTCAAACCCTAGCCCTGG
28010	Yes	ACAGGTAGGTAGATCTGGGTTCAAACCCTAGCCCTGGC
28011	Yes	ACAGGTAGGTAGATCTGGGTTCAAACCCTAGCCCTGGCA
28012	Yes	ACAGGTAGGTAGATCTGGGTTCAAACCCTAGCCCTGGCAC
28013	Yes	ACAGGTAGGTAGATCTGGGTTCAAACCCTAGCCCTGGCACC
28014	Yes	ACAGGTAGGTAGATCTGGGTTCAAACCCTAGCCCTGGCACCA
28015	No	ACAGGTCATATGTAGC
28016	No	ACAGGTCATATGTAGCT
28017	Yes	ACAGGTCATATGTAGCTG
28018	Yes	ACAGGTCATATGTAGCTGG
28019	Yes	ACAGGTCATATGTAGCTGGG
28020	Yes	ACAGGTCATATGTAGCTGGGT
28021	Yes	ACAGGTCATATGTAGCTGGGTT
28022	Yes	ACAGGTCATATGTAGCTGGGTTC
28023	Yes	ACAGGTCATATGTAGCTGGGTTCA
28024	Yes	ACAGGTCATATGTAGCTGGGTTCAA
28025	Yes	ACAGGTCATATGTAGCTGGGTTCAAA
28026	Yes	ACAGGTCATATGTAGCTGGGTTCAAAT
28027	Yes	ACAGGTCATATGTAGCTGGGTTCAAATC
28028	Yes	ACAGGTCATATGTAGCTGGGTTCAAATCC
28029	Yes	ACAGGTCATATGTAGCTGGGTTCAAATCCT
28030	Yes	ACAGGTCATATGTAGCTGGGTTCAAATCCTG
28031	Yes	ACAGGTCATATGTAGCTGGGTTCAAATCCTGT
28032	Yes	ACAGGTCATATGTAGCTGGGTTCAAATCCTGTT
28033	Yes	ACAGGTCATATGTAGCTGGGTTCAAATCCTGTTT
28034	Yes	ACAGGTCATATGTAGCTGGGTTCAAATCCTGTTTC
28035	Yes	ACAGGTCATATGTAGCTGGGTTCAAATCCTGTTTCT
28036	Yes	ACAGGTCATATGTAGCTGGGTTCAAATCCTGTTTCTG
28037	Yes	ACAGGTCATATGTAGCTGGGTTCAAATCCTGTTTCTGC
28038	Yes	ACAGGTCATATGTAGCTGGGTTCAAATCCTGTTTCTGCC
28039	Yes	ACAGGTCATATGTAGCTGGGTTCAAATCCTGTTTCTGCCA
28040	Yes	ACAGGTCATATGTAGCTGGGTTCAAATCCTGTTTCTGCCAC
28041	Yes	ACAGGTCATATGTAGCTGGGTTCAAATCCTGTTTCTGCCACC
28042	Yes	ACAGGTCATATGTAGCTGGGTTCAAATCCTGTTTCTGCCACCA
28043	Yes	ACAGGTGTCCGCGTGG
28044	Yes	ACAGGTGTCCGCGTGGG
28045	Yes	ACAGGTGTCCGCGTGGGT
28046	Yes	ACAGGTGTCCGCGTGGGTT
28047	Yes	ACAGGTGTCCGCGTGGGTTC
28048	Yes	ACAGGTGTCCGCGTGGGTTCG
28049	Yes	ACAGGTGTCCGCGTGGGTTCGA

28050	Yes	ACAGGTGTCCGCGTGGGTTCGAG
28051	Yes	ACAGGTGTCCGCGTGGGTTCGAGC
28052	Yes	ACAGGTGTCCGCGTGGGTTCGAGCC
28053	Yes	ACAGGTGTCCGCGTGGGTTCGAGCCC
28054	Yes	ACAGGTGTCCGCGTGGGTTCGAGCCCC
28055	Yes	ACAGGTGTCCGCGTGGGTTCGAGCCCCA
28056	Yes	ACAGGTGTCCGCGTGGGTTCGAGCCCCAC
28057	Yes	ACAGGTGTCCGCGTGGGTTCGAGCCCCACT
28058	Yes	ACAGGTGTCCGCGTGGGTTCGAGCCCCACTC
28059	Yes	ACAGGTGTCCGCGTGGGTTCGAGCCCCACTCC
28060	Yes	ACAGGTGTCCGCGTGGGTTCGAGCCCCACTCCC
28061	Yes	ACAGGTGTCCGCGTGGGTTCGAGCCCCACTCCCG
28062	Yes	ACAGGTGTCCGCGTGGGTTCGAGCCCCACTCCCGG
28063	Yes	ACAGGTGTCCGCGTGGGTTCGAGCCCCACTCCCGGT
28064	Yes	ACAGGTGTCCGCGTGGGTTCGAGCCCCACTCCCGGTA
28065	Yes	ACAGGTGTCCGCGTGGGTTCGAGCCCCACTCCCGGTAC
28066	Yes	ACAGGTGTCCGCGTGGGTTCGAGCCCCACTCCCGGTACC
28067	Yes	ACAGGTGTCCGCGTGGGTTCGAGCCCCACTCCCGGTACCA
28068	No	ACAGGTTCGAATCCTG
28069	Yes	ACAGGTTCGAATCCTGT
28070	Yes	ACAGGTTCGAATCCTGTT
28071	Yes	ACAGGTTCGAATCCTGTTC
28072	Yes	ACAGGTTCGAATCCTGTTCG
28073	Yes	ACAGGTTCGAATCCTGTTCGT
28074	Yes	ACAGGTTCGAATCCTGTTCGTG
28075	Yes	ACAGGTTCGAATCCTGTTCGTGA
28076	Yes	ACAGGTTCGAATCCTGTTCGTGAC
28077	Yes	ACAGGTTCGAATCCTGTTCGTGACG
28078	Yes	ACAGGTTCGAATCCTGTTCGTGACGC
28079	Yes	ACAGGTTCGAATCCTGTTCGTGACGCC
28080	Yes	ACAGGTTCGAATCCTGTTCGTGACGCCA
28081	No	ACAGTCAGAGGTTCAA
28082	No	ACAGTCAGAGGTTCAAT
28083	Yes	ACAGTCAGAGGTTCAATT
28084	Yes	ACAGTCAGAGGTTCAATTC
28085	Yes	ACAGTCAGAGGTTCAATTCC
28086	Yes	ACAGTCAGAGGTTCAATTCCT
28087	Yes	ACAGTCAGAGGTTCAATTCCTC
28088	Yes	ACAGTCAGAGGTTCAATTCCTCT
28089	Yes	ACAGTCAGAGGTTCAATTCCTCTT
28090	Yes	ACAGTCAGAGGTTCAATTCCTCTTC
28091	Yes	ACAGTCAGAGGTTCAATTCCTCTTCT

28092	Yes	ACAGTCAGAGGTTCAATTCCTCTTCTT
28093	Yes	ACAGTCAGAGGTTCAATTCCTCTTCTTA
28094	Yes	ACAGTCAGAGGTTCAATTCCTCTTCTTAA
28095	Yes	ACAGTCAGAGGTTCAATTCCTCTTCTTAAC
28096	Yes	ACAGTCAGAGGTTCAATTCCTCTTCTTAACA
28097	Yes	ACAGTCAGAGGTTCAATTCCTCTTCTTAACAC
28098	Yes	ACAGTCAGAGGTTCAATTCCTCTTCTTAACACC
28099	Yes	ACAGTCAGAGGTTCAATTCCTCTTCTTAACACCA
28100	No	ACAGTCATGTGCCTGG
28101	No	ACAGTCATGTGCCTGGG
28102	No	ACAGTCATGTGCCTGGGT
28103	Yes	ACAGTCATGTGCCTGGGTT
28104	Yes	ACAGTCATGTGCCTGGGTTC
28105	Yes	ACAGTCATGTGCCTGGGTTCA
28106	Yes	ACAGTCATGTGCCTGGGTTCAA
28107	Yes	ACAGTCATGTGCCTGGGTTCAAT
28108	Yes	ACAGTCATGTGCCTGGGTTCAATT
28109	Yes	ACAGTCATGTGCCTGGGTTCAATTC
28110	Yes	ACAGTCATGTGCCTGGGTTCAATTCC
28111	Yes	ACAGTCATGTGCCTGGGTTCAATTCCC
28112	Yes	ACAGTCATGTGCCTGGGTTCAATTCCCA
28113	Yes	ACAGTCATGTGCCTGGGTTCAATTCCCAG
28114	Yes	ACAGTCATGTGCCTGGGTTCAATTCCCAGT
28115	Yes	ACAGTCATGTGCCTGGGTTCAATTCCCAGTT
28116	Yes	ACAGTCATGTGCCTGGGTTCAATTCCCAGTTC
28117	Yes	ACAGTCATGTGCCTGGGTTCAATTCCCAGTTCT
28118	Yes	ACAGTCATGTGCCTGGGTTCAATTCCCAGTTCTG
28119	Yes	ACAGTCATGTGCCTGGGTTCAATTCCCAGTTCTGC
28120	Yes	ACAGTCATGTGCCTGGGTTCAATTCCCAGTTCTGCA
28121	Yes	ACAGTCATGTGCCTGGGTTCAATTCCCAGTTCTGCAT
28122	Yes	ACAGTCATGTGCCTGGGTTCAATTCCCAGTTCTGCATC
28123	Yes	ACAGTCATGTGCCTGGGTTCAATTCCCAGTTCTGCATCC
28124	Yes	ACAGTCATGTGCCTGGGTTCAATTCCCAGTTCTGCATCCA
28125	No	ACAGTGATCAGAGTTC
28126	No	ACAGTGATCAGAGTTCA
28127	Yes	ACAGTGATCAGAGTTCAA
28128	Yes	ACAGTGATCAGAGTTCAAG
28129	Yes	ACAGTGATCAGAGTTCAAGT
28130	Yes	ACAGTGATCAGAGTTCAAGTC
28131	Yes	ACAGTGATCAGAGTTCAAGTCT
28132	Yes	ACAGTGATCAGAGTTCAAGTCTC
28133	Yes	ACAGTGATCAGAGTTCAAGTCTCA

28134	Yes	ACAGTGATCAGAGTTCAAGTCTCAC
28135	Yes	ACAGTGATCAGAGTTCAAGTCTCACT
28136	Yes	ACAGTGATCAGAGTTCAAGTCTCACTG
28137	Yes	ACAGTGATCAGAGTTCAAGTCTCACTGG
28138	Yes	ACAGTGATCAGAGTTCAAGTCTCACTGGA
28139	Yes	ACAGTGATCAGAGTTCAAGTCTCACTGGAA
28140	Yes	ACAGTGATCAGAGTTCAAGTCTCACTGGAAC
28141	Yes	ACAGTGATCAGAGTTCAAGTCTCACTGGAACC
28142	Yes	ACAGTGATCAGAGTTCAAGTCTCACTGGAACCT
28143	Yes	ACAGTGATCAGAGTTCAAGTCTCACTGGAACCTC
28144	Yes	ACAGTGATCAGAGTTCAAGTCTCACTGGAACCTCC
28145	Yes	ACAGTGATCAGAGTTCAAGTCTCACTGGAACCTCCA
28146	Yes	ACAGTGATCAGAGTTCAAGTCTCACTGGG
28147	Yes	ACAGTGATCAGAGTTCAAGTCTCACTGGGA
28148	Yes	ACAGTGATCAGAGTTCAAGTCTCACTGGGAC
28149	Yes	ACAGTGATCAGAGTTCAAGTCTCACTGGGACC
28150	Yes	ACAGTGATCAGAGTTCAAGTCTCACTGGGACCT
28151	Yes	ACAGTGATCAGAGTTCAAGTCTCACTGGGACCTC
28152	Yes	ACAGTGATCAGAGTTCAAGTCTCACTGGGACCTCC
28153	Yes	ACAGTGATCAGAGTTCAAGTCTCACTGGGACCTCCA
28154	Yes	ACAGTGATCAGAGTTCAAGTCTCAG
28155	Yes	ACAGTGATCAGAGTTCAAGTCTCAGT
28156	Yes	ACAGTGATCAGAGTTCAAGTCTCAGTG
28157	Yes	ACAGTGATCAGAGTTCAAGTCTCAGTGG
28158	Yes	ACAGTGATCAGAGTTCAAGTCTCAGTGGG
28159	Yes	ACAGTGATCAGAGTTCAAGTCTCAGTGGGA
28160	Yes	ACAGTGATCAGAGTTCAAGTCTCAGTGGGAC
28161	Yes	ACAGTGATCAGAGTTCAAGTCTCAGTGGGACC
28162	Yes	ACAGTGATCAGAGTTCAAGTCTCAGTGGGACCT
28163	Yes	ACAGTGATCAGAGTTCAAGTCTCAGTGGGACCTC
28164	Yes	ACAGTGATCAGAGTTCAAGTCTCAGTGGGACCTCC
28165	Yes	ACAGTGATCAGAGTTCAAGTCTCAGTGGGACCTCCA
28166	No	ACAGTGGTTAAACCAT
28167	Yes	ACAGTGGTTAAACCATG
28168	Yes	ACAGTGGTTAAACCATGG
28169	Yes	ACAGTGGTTAAACCATGGT
28170	Yes	ACAGTGGTTAAACCATGGTC
28171	Yes	ACAGTGGTTAAACCATGGTCT
28172	Yes	ACAGTGGTTAAACCATGGTCTT
28173	Yes	ACAGTGGTTAAACCATGGTCTTT
28174	Yes	ACAGTGGTTAAACCATGGTCTTTG
28175	Yes	ACAGTGGTTAAACCATGGTCTTTGG

28176	Yes	ACAGTGGTTAAACCATGGTCTTTGGA
28177	Yes	ACAGTGGTTAAACCATGGTCTTTGGAG
28178	Yes	ACAGTGGTTAAACCATGGTCTTTGGAGC
28179	Yes	ACAGTGGTTAAACCATGGTCTTTGGAGCC
28180	Yes	ACAGTGGTTAAACCATGGTCTTTGGAGCCA
28181	Yes	ACAGTGGTTAAACCATGGTCTTTGGAGCCAG
28182	Yes	ACAGTGGTTAAACCATGGTCTTTGGAGCCAGA
28183	Yes	ACAGTGGTTAAACCATGGTCTTTGGAGCCAGAC
28184	Yes	ACAGTGGTTAAACCATGGTCTTTGGAGCCAGACT
28185	Yes	ACAGTGGTTAAACCATGGTCTTTGGAGCCAGACTG
28186	Yes	ACAGTGGTTAAACCATGGTCTTTGGAGCCAGACTGC
28187	Yes	ACAGTGGTTAAACCATGGTCTTTGGAGCCAGACTGCC
28188	Yes	ACAGTGGTTAAACCATGGTCTTTGGAGCCAGACTGCCT
28189	Yes	ACAGTGGTTAAACCATGGTCTTTGGAGCCAGACTGCCTG
28190	Yes	ACAGTGGTTAAACCATGGTCTTTGGAGCCAGACTGCCTGG
28191	Yes	ACAGTGGTTAAACCATGGTCTTTGGAGCCAGACTGCCTGGG
28192	Yes	ACAGTGGTTAAACCATGGTCTTTGGAGCCAGACTGCCTGGGG
28193	Yes	ACAGTGGTTAAACCATGGTCTTTGGAGCCAGACTGCCTGGGGT
28194	Yes	ACAGTGGTTAAACCATGGTCTTTGGAGCCAGACTGCCTGGGGTC
28195	Yes	ACAGTGGTTAAACCATGGTCTTTGGAGCCAGACTGCCTGGGGTCG
28196	Yes	ACAGTGGTTAAACCATGGTCTTTGGAGCCAGACTGCCTGGGGTCGG
28197	Yes	ACAGTGGTTAAACCATGGTCTTTGGAGCCAGACTGCCTGGGGTCGGA
28198	Yes	ACAGTGGTTAAACCATGGTCTTTGGAGCCAGACTGCCTGGGGTCGGAT
28199	Yes	ACAGTGGTTAAACCATGGTCTTTGGAGCCAGACTGCCTGGGGTCGGATC
28200	Yes	ACAGTGGTTAAACCATGGTCTTTGGAGCCAGACTGCCTGGGGTCGGATCC
28201	No	ACAGTTGGCTAGAACA
28202	No	ACAGTTGGCTAGAACAT
28203	Yes	ACAGTTGGCTAGAACATG
28204	Yes	ACAGTTGGCTAGAACATGG
28205	Yes	ACAGTTGGCTAGAACATGGT
28206	Yes	ACAGTTGGCTAGAACATGGTG
28207	Yes	ACAGTTGGCTAGAACATGGTGC
28208	Yes	ACAGTTGGCTAGAACATGGTGCT
28209	Yes	ACAGTTGGCTAGAACATGGTGCTA
28210	Yes	ACAGTTGGCTAGAACATGGTGCTAA
28211	Yes	ACAGTTGGCTAGAACATGGTGCTAAT
28212	Yes	ACAGTTGGCTAGAACATGGTGCTAATA
28213	Yes	ACAGTTGGCTAGAACATGGTGCTAATAA
28214	Yes	ACAGTTGGCTAGAACATGGTGCTAATAAG
28215	Yes	ACAGTTGGCTAGAACATGGTGCTAATAAGG
28216	Yes	ACAGTTGGCTAGAACATGGTGCTAATAAGGC
28217	Yes	ACAGTTGGCTAGAACATGGTGCTAATAAGGCC

28218	Yes	ACAGTTGGCTAGAACATGGTGCTAATAAGGCCA
28219	Yes	ACAGTTGGCTAGAACATGGTGCTAATAAGGCCAC
28220	Yes	ACAGTTGGCTAGAACATGGTGCTAATAAGGCCACG
28221	Yes	ACAGTTGGCTAGAACATGGTGCTAATAAGGCCACGG
28222	Yes	ACAGTTGGCTAGAACATGGTGCTAATAAGGCCACGGT
28223	Yes	ACAGTTGGCTAGAACATGGTGCTAATAAGGCCACGGTC
28224	Yes	ACAGTTGGCTAGAACATGGTGCTAATAAGGCCACGGTCA
28225	Yes	ACAGTTGGCTAGAACATGGTGCTAATAAGGCCACGGTCAG
28226	Yes	ACAGTTGGCTAGAACATGGTGCTAATAAGGCCACGGTCAGG
28227	Yes	ACAGTTGGCTAGAACATGGTGCTAATAAGGCCACGGTCAGGG
28228	Yes	ACAGTTGGCTAGAACATGGTGCTAATAAGGCCACGGTCAGGGG
28229	Yes	ACAGTTGGCTAGAACATGGTGCTAATAAGGCCACGGTCAGGGGT
28230	Yes	ACAGTTGGCTAGAACATGGTGCTAATAAGGCCACGGTCAGGGGTT
28231	Yes	ACAGTTGGCTAGAACATGGTGCTAATAAGGCCACGGTCAGGGGTTC
28232	Yes	ACAGTTGGCTAGAACATGGTGCTAATAAGGCCACGGTCAGGGGTTCA
28233	Yes	ACAGTTGGCTAGAACATGGTGCTAATAAGGCCACGGTCAGGGGTTCAA
28234	Yes	ACAGTTGGCTAGAACATGGTGCTAATAAGGCCACGGTCAGGGGTTCAAT
28235	Yes	ACAGTTGGCTAGAACATGGTGCTAATAAGGCCACGGTCAGGGGTTCAATT
28236	Yes	ACATAGGGTAGTGGCT
28237	Yes	ACATAGGGTAGTGGCTA
28238	Yes	ACATAGGGTAGTGGCTAA
28239	Yes	ACATAGGGTAGTGGCTAAG
28240	Yes	ACATAGGGTAGTGGCTAAGA
28241	Yes	ACATAGGGTAGTGGCTAAGAA
28242	Yes	ACATAGGGTAGTGGCTAAGAAC
28243	Yes	ACATAGGGTAGTGGCTAAGAACC
28244	Yes	ACATAGGGTAGTGGCTAAGAACCT
28245	Yes	ACATAGGGTAGTGGCTAAGAACCTA
28246	Yes	ACATAGGGTAGTGGCTAAGAACCTAA
28247	Yes	ACATAGGGTAGTGGCTAAGAACCTAAA
28248	Yes	ACATAGGGTAGTGGCTAAGAACCTAAAC
28249	Yes	ACATAGGGTAGTGGCTAAGAACCTAAACT
28250	Yes	ACATAGGGTAGTGGCTAAGAACCTAAACTC
28251	Yes	ACATAGGGTAGTGGCTAAGAACCTAAACTCT
28252	Yes	ACATAGGGTAGTGGCTAAGAACCTAAACTCTA
28253	Yes	ACATAGGGTAGTGGCTAAGAACCTAAACTCTAA
28254	Yes	ACATAGGGTAGTGGCTAAGAACCTAAACTCTAAA
28255	Yes	ACATAGGGTAGTGGCTAAGAACCTAAACTCTAAAT
28256	Yes	ACATAGGGTAGTGGCTAAGAACCTAAACTCTAAATT
28257	Yes	ACATAGGGTAGTGGCTAAGAACCTAAACTCTAAATTT
28258	Yes	ACATAGGGTAGTGGCTAAGAACCTAAACTCTAAATTTA
28259	Yes	ACATAGGGTAGTGGCTAAGAACCTAAACTCTAAATTTAG

28260	Yes	ACATAGGGTAGTGGCTAAGAACCTAAACTCTAAATTTAGA
28261	Yes	ACATAGGGTAGTGGCTAAGAACCTAAACTCTAAATTTAGAT
28262	Yes	ACATAGGGTAGTGGCTAAGAACCTAAACTCTAAATTTAGATG
28263	Yes	ACATAGGGTAGTGGCTAAGAACCTAAACTCTAAATTTAGATGT
28264	Yes	ACATAGGGTAGTGGCTAAGAACCTAAACTCTAAATTTAGATGTC
28265	Yes	ACATAGGGTAGTGGCTAAGAACCTAAACTCTAAATTTAGATGTCC
28266	Yes	ACATAGGGTAGTGGCTAAGAACCTAAACTCTAAATTTAGATGTCCT
28267	Yes	ACATAGGGTAGTGGCTAAGAACCTAAACTCTAAATTTAGATGTCCTG
28268	Yes	ACATAGGGTAGTGGCTAAGAACCTAAACTCTAAATTTAGATGTCCTGA
28269	Yes	ACATAGGGTAGTGGCTAAGAACCTAAACTCTAAATTTAGATGTCCTGAG
28270	Yes	ACATAGGGTAGTGGCTAAGAACCTAAACTCTAAATTTAGATGTCCTGAGT
28271	Yes	ACATAGTGTAATAGGT
28272	Yes	ACATAGTGTAATAGGTA
28273	Yes	ACATAGTGTAATAGGTAG
28274	Yes	ACATAGTGTAATAGGTAGC
28275	Yes	ACATAGTGTAATAGGTAGCA
28276	Yes	ACATAGTGTAATAGGTAGCAC
28277	Yes	ACATAGTGTAATAGGTAGCACA
28278	Yes	ACATAGTGTAATAGGTAGCACAG
28279	Yes	ACATAGTGTAATAGGTAGCACAGA
28280	Yes	ACATAGTGTAATAGGTAGCACAGAT
28281	Yes	ACATAGTGTAATAGGTAGCACAGATA
28282	Yes	ACATAGTGTAATAGGTAGCACAGATAA
28283	Yes	ACATAGTGTAATAGGTAGCACAGATAAT
28284	Yes	ACATAGTGTAATAGGTAGCACAGATAATT
28285	Yes	ACATAGTGTAATAGGTAGCACAGATAATTT
28286	Yes	ACATAGTGTAATAGGTAGCACAGATAATTTT
28287	Yes	ACATAGTGTAATAGGTAGCACAGATAATTTTG
28288	Yes	ACATAGTGTAATAGGTAGCACAGATAATTTTGG
28289	Yes	ACATAGTGTAATAGGTAGCACAGATAATTTTGGA
28290	Yes	ACATAGTGTAATAGGTAGCACAGATAATTTTGGAT
28291	Yes	ACATAGTGTAATAGGTAGCACAGATAATTTTGGATT
28292	Yes	ACATAGTGTAATAGGTAGCACAGATAATTTTGGATTC
28293	Yes	ACATAGTGTAATAGGTAGCACAGATAATTTTGGATTCT
28294	Yes	ACATAGTGTAATAGGTAGCACAGATAATTTTGGATTCTC
28295	Yes	ACATAGTGTAATAGGTAGCACAGATAATTTTGGATTCTCA
28296	Yes	ACATAGTGTAATAGGTAGCACAGATAATTTTGGATTCTCAG
28297	Yes	ACATAGTGTAATAGGTAGCACAGATAATTTTGGATTCTCAGG
28298	Yes	ACATAGTGTAATAGGTAGCACAGATAATTTTGGATTCTCAGGG
28299	Yes	ACATAGTGTAATAGGTAGCACAGATAATTTTGGATTCTCAGGGG
28300	Yes	ACATAGTGTAATAGGTAGCACAGATAATTTTGGATTCTCAGGGGT
28301	Yes	ACATAGTGTAATAGGTAGCACAGATAATTTTGGATTCTCAGGGGTA

28302	Yes	ACATAGTGTAATAGGTAGCACAGATAATTTTGGATTCTCAGGGGTAG
28303	Yes	ACATAGTGTAATAGGTAGCACAGATAATTTTGGATTCTCAGGGGTAGG
28304	Yes	ACATAGTGTAATAGGTAGCACAGATAATTTTGGATTCTCAGGGGTAGGT
28305	Yes	ACATAGTGTAATAGGTAGCACAGATAATTTTGGATTCTCAGGGGTAGGTT
28306	No	ACATATGAAAGGTCTC
28307	No	ACATATGAAAGGTCTCT
28308	No	ACATATGAAAGGTCTCTG
28309	Yes	ACATATGAAAGGTCTCTGG
28310	Yes	ACATATGAAAGGTCTCTGGT
28311	Yes	ACATATGAAAGGTCTCTGGTT
28312	Yes	ACATATGAAAGGTCTCTGGTTC
28313	Yes	ACATATGAAAGGTCTCTGGTTCG
28314	Yes	ACATATGAAAGGTCTCTGGTTCGA
28315	Yes	ACATATGAAAGGTCTCTGGTTCGAG
28316	Yes	ACATATGAAAGGTCTCTGGTTCGAGA
28317	Yes	ACATATGAAAGGTCTCTGGTTCGAGAC
28318	Yes	ACATATGAAAGGTCTCTGGTTCGAGACT
28319	Yes	ACATATGAAAGGTCTCTGGTTCGAGACTG
28320	Yes	ACATATGAAAGGTCTCTGGTTCGAGACTGC
28321	Yes	ACATATGAAAGGTCTCTGGTTCGAGACTGCG
28322	Yes	ACATATGAAAGGTCTCTGGTTCGAGACTGCGT
28323	Yes	ACATATGAAAGGTCTCTGGTTCGAGACTGCGTG
28324	Yes	ACATATGAAAGGTCTCTGGTTCGAGACTGCGTGG
28325	Yes	ACATATGAAAGGTCTCTGGTTCGAGACTGCGTGGG
28326	Yes	ACATATGAAAGGTCTCTGGTTCGAGACTGCGTGGGA
28327	Yes	ACATATGAAAGGTCTCTGGTTCGAGACTGCGTGGGAA
28328	Yes	ACATATGAAAGGTCTCTGGTTCGAGACTGCGTGGGAAC
28329	Yes	ACATATGAAAGGTCTCTGGTTCGAGACTGCGTGGGAACA
28330	Yes	ACATATGAAAGGTCTCTGGTTCGAGACTGCGTGGGAACAC
28331	Yes	ACATATGAAAGGTCTCTGGTTCGAGACTGCGTGGGAACACC
28332	Yes	ACATATGAAAGGTCTCTGGTTCGAGACTGCGTGGGAACACCA
28333	Yes	ACATATGTCCGCGTGG
28334	Yes	ACATATGTCCGCGTGGG
28335	Yes	ACATATGTCCGCGTGGGT
28336	Yes	ACATATGTCCGCGTGGGTT
28337	Yes	ACATATGTCCGCGTGGGTTC
28338	Yes	ACATATGTCCGCGTGGGTTCG
28339	Yes	ACATATGTCCGCGTGGGTTCGA
28340	Yes	ACATATGTCCGCGTGGGTTCGAA
28341	Yes	ACATATGTCCGCGTGGGTTCGAAC
28342	Yes	ACATATGTCCGCGTGGGTTCGAACC
28343	Yes	ACATATGTCCGCGTGGGTTCGAACCC

28344	Yes	ACATATGTCCGCGTGGGTTCGAACCCC
28345	Yes	ACATATGTCCGCGTGGGTTCGAACCCCA
28346	Yes	ACATATGTCCGCGTGGGTTCGAACCCCAC
28347	Yes	ACATATGTCCGCGTGGGTTCGAACCCCACT
28348	Yes	ACATATGTCCGCGTGGGTTCGAACCCCACTC
28349	Yes	ACATATGTCCGCGTGGGTTCGAACCCCACTCC
28350	Yes	ACATATGTCCGCGTGGGTTCGAACCCCACTCCT
28351	Yes	ACATATGTCCGCGTGGGTTCGAACCCCACTCCTG
28352	Yes	ACATATGTCCGCGTGGGTTCGAACCCCACTCCTGG
28353	Yes	ACATATGTCCGCGTGGGTTCGAACCCCACTCCTGGT
28354	Yes	ACATATGTCCGCGTGGGTTCGAACCCCACTCCTGGTA
28355	Yes	ACATATGTCCGCGTGGGTTCGAACCCCACTCCTGGTAC
28356	Yes	ACATATGTCCGCGTGGGTTCGAACCCCACTCCTGGTACC
28357	Yes	ACATATGTCCGCGTGGGTTCGAACCCCACTCCTGGTACCA
28358	No	ACATCACAGGTAGGTA
28359	No	ACATCACAGGTAGGTAG
28360	Yes	ACATCACAGGTAGGTAGA
28361	Yes	ACATCACAGGTAGGTAGAT
28362	Yes	ACATCACAGGTAGGTAGATC
28363	Yes	ACATCACAGGTAGGTAGATCT
28364	Yes	ACATCACAGGTAGGTAGATCTG
28365	Yes	ACATCACAGGTAGGTAGATCTGG
28366	Yes	ACATCACAGGTAGGTAGATCTGGG
28367	Yes	ACATCACAGGTAGGTAGATCTGGGT
28368	Yes	ACATCACAGGTAGGTAGATCTGGGTT
28369	Yes	ACATCACAGGTAGGTAGATCTGGGTTC
28370	Yes	ACATCACAGGTAGGTAGATCTGGGTTCA
28371	Yes	ACATCACAGGTAGGTAGATCTGGGTTCAA
28372	Yes	ACATCACAGGTAGGTAGATCTGGGTTCAAA
28373	Yes	ACATCACAGGTAGGTAGATCTGGGTTCAAAC
28374	Yes	ACATCACAGGTAGGTAGATCTGGGTTCAAACC
28375	Yes	ACATCACAGGTAGGTAGATCTGGGTTCAAACCC
28376	Yes	ACATCACAGGTAGGTAGATCTGGGTTCAAACCCT
28377	Yes	ACATCACAGGTAGGTAGATCTGGGTTCAAACCCTA
28378	Yes	ACATCACAGGTAGGTAGATCTGGGTTCAAACCCTAG
28379	Yes	ACATCACAGGTAGGTAGATCTGGGTTCAAACCCTAGC
28380	Yes	ACATCACAGGTAGGTAGATCTGGGTTCAAACCCTAGCC
28381	Yes	ACATCACAGGTAGGTAGATCTGGGTTCAAACCCTAGCCC
28382	Yes	ACATCACAGGTAGGTAGATCTGGGTTCAAACCCTAGCCCT
28383	Yes	ACATCACAGGTAGGTAGATCTGGGTTCAAACCCTAGCCCTG
28384	Yes	ACATCACAGGTAGGTAGATCTGGGTTCAAACCCTAGCCCTGG
28385	Yes	ACATCACAGGTAGGTAGATCTGGGTTCAAACCCTAGCCCTGGC

28386	Yes	ACATCACAGGTAGGTAGATCTGGGTTCAAACCCTAGCCCTGGCA
28387	Yes	ACATCACAGGTAGGTAGATCTGGGTTCAAACCCTAGCCCTGGCAC
28388	Yes	ACATCACAGGTAGGTAGATCTGGGTTCAAACCCTAGCCCTGGCACC
28389	Yes	ACATCACAGGTAGGTAGATCTGGGTTCAAACCCTAGCCCTGGCACCA
28390	No	ACATCACCCCATAAAC
28391	No	ACATCACCCCATAAACA
28392	Yes	ACATCACCCCATAAACAC
28393	Yes	ACATCACCCCATAAACACC
28394	Yes	ACATCACCCCATAAACACCA
28395	No	ACATCAGACTTTTAAT
28396	No	ACATCAGACTTTTAATC
28397	No	ACATCAGACTTTTAATCT
28398	No	ACATCAGACTTTTAATCTG
28399	No	ACATCAGACTTTTAATCTGA
28400	Yes	ACATCAGACTTTTAATCTGAC
28401	Yes	ACATCAGACTTTTAATCTGACG
28402	Yes	ACATCAGACTTTTAATCTGACGG
28403	Yes	ACATCAGACTTTTAATCTGACGGT
28404	Yes	ACATCAGACTTTTAATCTGACGGTG
28405	Yes	ACATCAGACTTTTAATCTGACGGTGC
28406	Yes	ACATCAGACTTTTAATCTGACGGTGCA
28407	Yes	ACATCAGACTTTTAATCTGACGGTGCAG
28408	Yes	ACATCAGACTTTTAATCTGACGGTGCAGG
28409	Yes	ACATCAGACTTTTAATCTGACGGTGCAGGG
28410	Yes	ACATCAGACTTTTAATCTGACGGTGCAGGGT
28411	Yes	ACATCAGACTTTTAATCTGACGGTGCAGGGTT
28412	Yes	ACATCAGACTTTTAATCTGACGGTGCAGGGTTC
28413	Yes	ACATCAGACTTTTAATCTGACGGTGCAGGGTTCA
28414	Yes	ACATCAGACTTTTAATCTGACGGTGCAGGGTTCAA
28415	Yes	ACATCAGACTTTTAATCTGACGGTGCAGGGTTCAAG
28416	Yes	ACATCAGACTTTTAATCTGACGGTGCAGGGTTCAAGT
28417	Yes	ACATCAGACTTTTAATCTGACGGTGCAGGGTTCAAGTC
28418	Yes	ACATCAGACTTTTAATCTGACGGTGCAGGGTTCAAGTCC
28419	Yes	ACATCAGACTTTTAATCTGACGGTGCAGGGTTCAAGTCCC
28420	Yes	ACATCAGACTTTTAATCTGACGGTGCAGGGTTCAAGTCCCT
28421	Yes	ACATCAGACTTTTAATCTGACGGTGCAGGGTTCAAGTCCCTG
28422	Yes	ACATCAGACTTTTAATCTGACGGTGCAGGGTTCAAGTCCCTGT
28423	Yes	ACATCAGACTTTTAATCTGACGGTGCAGGGTTCAAGTCCCTGTT
28424	Yes	ACATCAGACTTTTAATCTGACGGTGCAGGGTTCAAGTCCCTGTTC
28425	Yes	ACATCAGACTTTTAATCTGACGGTGCAGGGTTCAAGTCCCTGTTCA
28426	Yes	ACATCAGACTTTTAATCTGACGGTGCAGGGTTCAAGTCCCTGTTCAG
28427	Yes	ACATCAGACTTTTAATCTGACGGTGCAGGGTTCAAGTCCCTGTTCAGG

28428	Yes	ACATCAGACTTTTAATCTGACGGTGCAGGGTTCAAGTCCCTGTTCAGGC
28429	Yes	ACATCAGACTTTTAATCTGACGGTGCAGGGTTCAAGTCCCTGTTCAGGCG
28430	Yes	ACATCAGACTTTTAATCTGAG
28431	Yes	ACATCAGACTTTTAATCTGAGG
28432	Yes	ACATCAGACTTTTAATCTGAGGG
28433	Yes	ACATCAGACTTTTAATCTGAGGGT
28434	Yes	ACATCAGACTTTTAATCTGAGGGTC
28435	Yes	ACATCAGACTTTTAATCTGAGGGTCT
28436	Yes	ACATCAGACTTTTAATCTGAGGGTCTA
28437	Yes	ACATCAGACTTTTAATCTGAGGGTCTAG
28438	Yes	ACATCAGACTTTTAATCTGAGGGTCTAGG
28439	Yes	ACATCAGACTTTTAATCTGAGGGTCTAGGG
28440	Yes	ACATCAGACTTTTAATCTGAGGGTCTAGGGT
28441	Yes	ACATCAGACTTTTAATCTGAGGGTCTAGGGTT
28442	Yes	ACATCAGACTTTTAATCTGAGGGTCTAGGGTTC
28443	Yes	ACATCAGACTTTTAATCTGAGGGTCTAGGGTTCA
28444	Yes	ACATCAGACTTTTAATCTGAGGGTCTAGGGTTCAA
28445	Yes	ACATCAGACTTTTAATCTGAGGGTCTAGGGTTCAAG
28446	Yes	ACATCAGACTTTTAATCTGAGGGTCTAGGGTTCAAGT
28447	Yes	ACATCAGACTTTTAATCTGAGGGTCTAGGGTTCAAGTC
28448	Yes	ACATCAGACTTTTAATCTGAGGGTCTAGGGTTCAAGTCC
28449	Yes	ACATCAGACTTTTAATCTGAGGGTCTAGGGTTCAAGTCCC
28450	Yes	ACATCAGACTTTTAATCTGAGGGTCTAGGGTTCAAGTCCCT
28451	Yes	ACATCAGACTTTTAATCTGAGGGTCTAGGGTTCAAGTCCCTG
28452	Yes	ACATCAGACTTTTAATCTGAGGGTCTAGGGTTCAAGTCCCTGT
28453	Yes	ACATCAGACTTTTAATCTGAGGGTCTAGGGTTCAAGTCCCTGTC
28454	Yes	ACATCAGACTTTTAATCTGAGGGTCTAGGGTTCAAGTCCCTGTCC
28455	Yes	ACATCAGACTTTTAATCTGAGGGTCTAGGGTTCAAGTCCCTGTCCA
28456	Yes	ACATCAGACTTTTAATCTGAGGGTCTAGGGTTCAAGTCCCTGTCCAG
28457	Yes	ACATCAGACTTTTAATCTGAGGGTCTAGGGTTCAAGTCCCTGTCCAGG
28458	Yes	ACATCAGACTTTTAATCTGAGGGTCTAGGGTTCAAGTCCCTGTCCAGGC
28459	Yes	ACATCAGACTTTTAATCTGAGGGTCTAGGGTTCAAGTCCCTGTCCAGGCG
28460	No	ACATCAGATTGTGAAT
28461	No	ACATCAGATTGTGAATC
28462	No	ACATCAGATTGTGAATCT
28463	No	ACATCAGATTGTGAATCTG
28464	No	ACATCAGATTGTGAATCTGA
28465	No	ACATCAGATTGTGAATCTGAC
28466	No	ACATCAGATTGTGAATCTGACA
28467	No	ACATCAGATTGTGAATCTGACAA
28468	No	ACATCAGATTGTGAATCTGACAAC
28469	No	ACATCAGATTGTGAATCTGACAACA

28470	No	ACATCAGATTGTGAATCTGACAACAG
28471	No	ACATCAGATTGTGAATCTGACAACAGA
28472	No	ACATCAGATTGTGAATCTGACAACAGAG
28473	No	ACATCAGATTGTGAATCTGACAACAGAGG
28474	No	ACATCAGATTGTGAATCTGACAACAGAGGC
28475	No	ACATCAGATTGTGAATCTGACAACAGAGGCT
28476	Yes	ACATCAGATTGTGAATCTGACAACAGAGGCTT
28477	Yes	ACATCAGATTGTGAATCTGACAACAGAGGCTTA
28478	Yes	ACATCAGATTGTGAATCTGACAACAGAGGCTTAC
28479	Yes	ACATCAGATTGTGAATCTGACAACAGAGGCTTACG
28480	Yes	ACATCAGATTGTGAATCTGACAACAGAGGCTTACGA
28481	Yes	ACATCAGATTGTGAATCTGACAACAGAGGCTTACGAC
28482	Yes	ACATCAGATTGTGAATCTGACAACAGAGGCTTACGACC
28483	Yes	ACATCAGATTGTGAATCTGACAACAGAGGCTTACGACCC
28484	Yes	ACATCAGATTGTGAATCTGACAACAGAGGCTTACGACCCC
28485	Yes	ACATCAGATTGTGAATCTGACAACAGAGGCTTACGACCCCT
28486	Yes	ACATCAGATTGTGAATCTGACAACAGAGGCTTACGACCCCTT
28487	Yes	ACATCAGATTGTGAATCTGACAACAGAGGCTTACGACCCCTTA
28488	Yes	ACATCAGATTGTGAATCTGACAACAGAGGCTTACGACCCCTTAT
28489	Yes	ACATCAGATTGTGAATCTGACAACAGAGGCTTACGACCCCTTATT
28490	Yes	ACATCAGATTGTGAATCTGACAACAGAGGCTTACGACCCCTTATTT
28491	Yes	ACATCAGATTGTGAATCTGACAACAGAGGCTTACGACCCCTTATTTA
28492	Yes	ACATCAGATTGTGAATCTGACAACAGAGGCTTACGACCCCTTATTTAC
28493	Yes	ACATCAGATTGTGAATCTGACAACAGAGGCTTACGACCCCTTATTTACC
28494	Yes	ACATCAGATTGTGAATCTGACAACAGAGGCTTACGACCCCTTATTTACCC
28495	No	ACATCTCACTTTGGGT
28496	Yes	ACATCTCACTTTGGGTC
28497	Yes	ACATCTCACTTTGGGTCA
28498	Yes	ACATCTCACTTTGGGTCAG
28499	Yes	ACATCTCACTTTGGGTCAGA
28500	Yes	ACATCTCACTTTGGGTCAGAA
28501	Yes	ACATCTCACTTTGGGTCAGAAG
28502	Yes	ACATCTCACTTTGGGTCAGAAGA
28503	Yes	ACATCTCACTTTGGGTCAGAAGAT
28504	Yes	ACATCTCACTTTGGGTCAGAAGATT
28505	Yes	ACATCTCACTTTGGGTCAGAAGATTG
28506	Yes	ACATCTCACTTTGGGTCAGAAGATTGA
28507	Yes	ACATCTCACTTTGGGTCAGAAGATTGAG
28508	Yes	ACATCTCACTTTGGGTCAGAAGATTGAGG
28509	Yes	ACATCTCACTTTGGGTCAGAAGATTGAGGG
28510	Yes	ACATCTCACTTTGGGTCAGAAGATTGAGGGT
28511	Yes	ACATCTCACTTTGGGTCAGAAGATTGAGGGTT

28512	Yes	ACATCTCACTTTGGGTCAGAAGATTGAGGGTTC
28513	Yes	ACATCTCACTTTGGGTCAGAAGATTGAGGGTTCA
28514	Yes	ACATCTCACTTTGGGTCAGAAGATTGAGGGTTCAA
28515	Yes	ACATCTCACTTTGGGTCAGAAGATTGAGGGTTCAAG
28516	Yes	ACATCTCACTTTGGGTCAGAAGATTGAGGGTTCAAGT
28517	Yes	ACATCTCACTTTGGGTCAGAAGATTGAGGGTTCAAGTC
28518	Yes	ACATCTCACTTTGGGTCAGAAGATTGAGGGTTCAAGTCC
28519	Yes	ACATCTCACTTTGGGTCAGAAGATTGAGGGTTCAAGTCCT
28520	Yes	ACATCTCACTTTGGGTCAGAAGATTGAGGGTTCAAGTCCTT
28521	Yes	ACATCTCACTTTGGGTCAGAAGATTGAGGGTTCAAGTCCTTT
28522	Yes	ACATCTCACTTTGGGTCAGAAGATTGAGGGTTCAAGTCCTTTC
28523	Yes	ACATCTCACTTTGGGTCAGAAGATTGAGGGTTCAAGTCCTTTCA
28524	Yes	ACATCTCACTTTGGGTCAGAAGATTGAGGGTTCAAGTCCTTTCAT
28525	Yes	ACATCTCACTTTGGGTCAGAAGATTGAGGGTTCAAGTCCTTTCATG
28526	Yes	ACATCTCACTTTGGGTCAGAAGATTGAGGGTTCAAGTCCTTTCATGG
28527	Yes	ACATCTCACTTTGGGTCAGAAGATTGAGGGTTCAAGTCCTTTCATGGT
28528	Yes	ACATCTCACTTTGGGTCAGAAGATTGAGGGTTCAAGTCCTTTCATGGTC
28529	Yes	ACATCTCACTTTGGGTCAGAAGATTGAGGGTTCAAGTCCTTTCATGGTCA
28530	Yes	ACATCTGCTTTACACG
28531	Yes	ACATCTGCTTTACACGC
28532	Yes	ACATCTGCTTTACACGCA
28533	Yes	ACATCTGCTTTACACGCAG
28534	Yes	ACATCTGCTTTACACGCAGA
28535	Yes	ACATCTGCTTTACACGCAGAA
28536	Yes	ACATCTGCTTTACACGCAGAAG
28537	Yes	ACATCTGCTTTACACGCAGAAGG
28538	Yes	ACATCTGCTTTACACGCAGAAGGT
28539	Yes	ACATCTGCTTTACACGCAGAAGGTC
28540	Yes	ACATCTGCTTTACACGCAGAAGGTCC
28541	Yes	ACATCTGCTTTACACGCAGAAGGTCCT
28542	Yes	ACATCTGCTTTACACGCAGAAGGTCCTG
28543	Yes	ACATCTGCTTTACACGCAGAAGGTCCTGG
28544	Yes	ACATCTGCTTTACACGCAGAAGGTCCTGGG
28545	Yes	ACATCTGCTTTACACGCAGAAGGTCCTGGGT
28546	Yes	ACATCTGCTTTACACGCAGAAGGTCCTGGGTT
28547	Yes	ACATCTGCTTTACACGCAGAAGGTCCTGGGTTC
28548	Yes	ACATCTGCTTTACACGCAGAAGGTCCTGGGTTCA
28549	Yes	ACATCTGCTTTACACGCAGAAGGTCCTGGGTTCAA
28550	Yes	ACATCTGCTTTACACGCAGAAGGTCCTGGGTTCAAG
28551	Yes	ACATCTGCTTTACACGCAGAAGGTCCTGGGTTCAAGC
28552	Yes	ACATCTGCTTTACACGCAGAAGGTCCTGGGTTCAAGCC
28553	Yes	ACATCTGCTTTACACGCAGAAGGTCCTGGGTTCAAGCCC

28554	Yes	ACATCTGCTTTACACGCAGAAGGTCCTGGGTTCAAGCCCC
28555	Yes	ACATCTGCTTTACACGCAGAAGGTCCTGGGTTCAAGCCCCA
28556	Yes	ACATCTGCTTTACACGCAGAAGGTCCTGGGTTCAAGCCCCAG
28557	Yes	ACATCTGCTTTACACGCAGAAGGTCCTGGGTTCAAGCCCCAGT
28558	Yes	ACATCTGCTTTACACGCAGAAGGTCCTGGGTTCAAGCCCCAGTG
28559	Yes	ACATCTGCTTTACACGCAGAAGGTCCTGGGTTCAAGCCCCAGTGG
28560	Yes	ACATCTGCTTTACACGCAGAAGGTCCTGGGTTCAAGCCCCAGTGGA
28561	Yes	ACATCTGCTTTACACGCAGAAGGTCCTGGGTTCAAGCCCCAGTGGAA
28562	Yes	ACATCTGCTTTACACGCAGAAGGTCCTGGGTTCAAGCCCCAGTGGAAC
28563	Yes	ACATCTGCTTTACACGCAGAAGGTCCTGGGTTCAAGCCCCAGTGGAACC
28564	Yes	ACATCTGCTTTACACGCAGAAGGTCCTGGGTTCAAGCCCCAGTGGAACCA
28565	No	ACATGAAAGGTCACCA
28566	Yes	ACATGAAAGGTCACCAG
28567	Yes	ACATGAAAGGTCACCAGT
28568	Yes	ACATGAAAGGTCACCAGTT
28569	Yes	ACATGAAAGGTCACCAGTTT
28570	Yes	ACATGAAAGGTCACCAGTTTG
28571	Yes	ACATGAAAGGTCACCAGTTTGA
28572	Yes	ACATGAAAGGTCACCAGTTTGAG
28573	Yes	ACATGAAAGGTCACCAGTTTGAGA
28574	Yes	ACATGAAAGGTCACCAGTTTGAGAC
28575	Yes	ACATGAAAGGTCACCAGTTTGAGACC
28576	Yes	ACATGAAAGGTCACCAGTTTGAGACCG
28577	Yes	ACATGAAAGGTCACCAGTTTGAGACCGG
28578	Yes	ACATGAAAGGTCACCAGTTTGAGACCGGG
28579	Yes	ACATGAAAGGTCACCAGTTTGAGACCGGGC
28580	Yes	ACATGAAAGGTCACCAGTTTGAGACCGGGCC
28581	Yes	ACATGAAAGGTCACCAGTTTGAGACCGGGCCA
28582	Yes	ACATGAAAGGTCACCAGTTTGAGACCGGGCCAA
28583	Yes	ACATGAAAGGTCACCAGTTTGAGACCGGGCCAAA
28584	Yes	ACATGAAAGGTCACCAGTTTGAGACCGGGCCAAAA
28585	Yes	ACATGAAAGGTCACCAGTTTGAGACCGGGCCAAAAC
28586	Yes	ACATGAAAGGTCACCAGTTTGAGACCGGGCCAAAACA
28587	Yes	ACATGAAAGGTCACCAGTTTGAGACCGGGCCAAAACAC
28588	Yes	ACATGAAAGGTCACCAGTTTGAGACCGGGCCAAAACACC
28589	Yes	ACATGAAAGGTCACCAGTTTGAGACCGGGCCAAAACACCA
28590	No	ACATGCTTTGCATGTG
28591	No	ACATGCTTTGCATGTGT
28592	Yes	ACATGCTTTGCATGTGTG
28593	Yes	ACATGCTTTGCATGTGTGA
28594	Yes	ACATGCTTTGCATGTGTGAG
28595	Yes	ACATGCTTTGCATGTGTGAGG

28596	Yes	ACATGCTTTGCATGTGTGAGGC
28597	Yes	ACATGCTTTGCATGTGTGAGGCC
28598	Yes	ACATGCTTTGCATGTGTGAGGCCC
28599	Yes	ACATGCTTTGCATGTGTGAGGCCCC
28600	Yes	ACATGCTTTGCATGTGTGAGGCCCCG
28601	Yes	ACATGCTTTGCATGTGTGAGGCCCCGG
28602	Yes	ACATGCTTTGCATGTGTGAGGCCCCGGG
28603	Yes	ACATGCTTTGCATGTGTGAGGCCCCGGGT
28604	Yes	ACATGCTTTGCATGTGTGAGGCCCCGGGTT
28605	Yes	ACATGCTTTGCATGTGTGAGGCCCCGGGTTC
28606	Yes	ACATGCTTTGCATGTGTGAGGCCCCGGGTTCG
28607	Yes	ACATGCTTTGCATGTGTGAGGCCCCGGGTTCGA
28608	Yes	ACATGCTTTGCATGTGTGAGGCCCCGGGTTCGAT
28609	Yes	ACATGCTTTGCATGTGTGAGGCCCCGGGTTCGATC
28610	Yes	ACATGCTTTGCATGTGTGAGGCCCCGGGTTCGATCC
28611	Yes	ACATGCTTTGCATGTGTGAGGCCCCGGGTTCGATCCC
28612	Yes	ACATGCTTTGCATGTGTGAGGCCCCGGGTTCGATCCCC
28613	Yes	ACATGCTTTGCATGTGTGAGGCCCCGGGTTCGATCCCCG
28614	Yes	ACATGCTTTGCATGTGTGAGGCCCCGGGTTCGATCCCCGG
28615	Yes	ACATGCTTTGCATGTGTGAGGCCCCGGGTTCGATCCCCGGC
28616	Yes	ACATGCTTTGCATGTGTGAGGCCCCGGGTTCGATCCCCGGCA
28617	Yes	ACATGCTTTGCATGTGTGAGGCCCCGGGTTCGATCCCCGGCAC
28618	Yes	ACATGCTTTGCATGTGTGAGGCCCCGGGTTCGATCCCCGGCACC
28619	Yes	ACATGCTTTGCATGTGTGAGGCCCCGGGTTCGATCCCCGGCACCT
28620	Yes	ACATGCTTTGCATGTGTGAGGCCCCGGGTTCGATCCCCGGCACCTC
28621	Yes	ACATGCTTTGCATGTGTGAGGCCCCGGGTTCGATCCCCGGCACCTCC
28622	Yes	ACATGCTTTGCATGTGTGAGGCCCCGGGTTCGATCCCCGGCACCTCCA
28623	Yes	ACATGCTTTGCATGTGTGAGGCCCCGGGTTCGATCCCCGGCACCTCCAC
28624	Yes	ACATGCTTTGCATGTGTGAGGCCCCGGGTTCGATCCCCGGCACCTCCACC
28625	No	ACATGGACTCTGGAGC
28626	No	ACATGGACTCTGGAGCC
28627	No	ACATGGACTCTGGAGCCA
28628	No	ACATGGACTCTGGAGCCAA
28629	No	ACATGGACTCTGGAGCCAAA
28630	No	ACATGGACTCTGGAGCCAAAT
28631	Yes	ACATGGACTCTGGAGCCAAATC
28632	Yes	ACATGGACTCTGGAGCCAAATCT
28633	Yes	ACATGGACTCTGGAGCCAAATCTG
28634	Yes	ACATGGACTCTGGAGCCAAATCTGC
28635	Yes	ACATGGACTCTGGAGCCAAATCTGCC
28636	Yes	ACATGGACTCTGGAGCCAAATCTGCCT
28637	Yes	ACATGGACTCTGGAGCCAAATCTGCCTG

28638	Yes	ACATGGACTCTGGAGCCAAATCTGCCTGG
28639	Yes	ACATGGACTCTGGAGCCAAATCTGCCTGGG
28640	Yes	ACATGGACTCTGGAGCCAAATCTGCCTGGGT
28641	Yes	ACATGGACTCTGGAGCCAAATCTGCCTGGGTT
28642	Yes	ACATGGACTCTGGAGCCAAATCTGCCTGGGTTC
28643	Yes	ACATGGACTCTGGAGCCAAATCTGCCTGGGTTCT
28644	Yes	ACATGGACTCTGGAGCCAAATCTGCCTGGGTTCTA
28645	Yes	ACATGGACTCTGGAGCCAAATCTGCCTGGGTTCTAG
28646	Yes	ACATGGACTCTGGAGCCAAATCTGCCTGGGTTCTAGT
28647	Yes	ACATGGACTCTGGAGCCAAATCTGCCTGGGTTCTAGTC
28648	Yes	ACATGGACTCTGGAGCCAAATCTGCCTGGGTTCTAGTCC
28649	Yes	ACATGGACTCTGGAGCCAAATCTGCCTGGGTTCTAGTCCC
28650	Yes	ACATGGACTCTGGAGCCAAATCTGCCTGGGTTCTAGTCCCA
28651	Yes	ACATGGACTCTGGAGCCAAATCTGCCTGGGTTCTAGTCCCAG
28652	Yes	ACATGGACTCTGGAGCCAAATCTGCCTGGGTTCTAGTCCCAGC
28653	Yes	ACATGGACTCTGGAGCCAAATCTGCCTGGGTTCTAGTCCCAGCT
28654	Yes	ACATGGACTCTGGAGCCAAATCTGCCTGGGTTCTAGTCCCAGCTG
28655	Yes	ACATGGACTCTGGAGCCAAATCTGCCTGGGTTCTAGTCCCAGCTGT
28656	Yes	ACATGGACTCTGGAGCCAAATCTGCCTGGGTTCTAGTCCCAGCTGTC
28657	Yes	ACATGGACTCTGGAGCCAAATCTGCCTGGGTTCTAGTCCCAGCTGTCT
28658	Yes	ACATGGACTCTGGAGCCAAATCTGCCTGGGTTCTAGTCCCAGCTGTCTC
28659	Yes	ACATGGACTCTGGAGCCAAATCTGCCTGGGTTCTAGTCCCAGCTGTCTCA
28660	No	ACATGGCTTTCTCACC
28661	No	ACATGGCTTTCTCACCA
28662	Yes	ACATGGTCTAGCGGTT
28663	Yes	ACATGGTCTAGCGGTTA
28664	Yes	ACATGGTCTAGCGGTTAG
28665	Yes	ACATGGTCTAGCGGTTAGG
28666	Yes	ACATGGTCTAGCGGTTAGGA
28667	Yes	ACATGGTCTAGCGGTTAGGAT
28668	Yes	ACATGGTCTAGCGGTTAGGATT
28669	Yes	ACATGGTCTAGCGGTTAGGATTC
28670	Yes	ACATGGTCTAGCGGTTAGGATTCC
28671	Yes	ACATGGTCTAGCGGTTAGGATTCCT
28672	Yes	ACATGGTCTAGCGGTTAGGATTCCTG
28673	Yes	ACATGGTCTAGCGGTTAGGATTCCTGG
28674	Yes	ACATGGTCTAGCGGTTAGGATTCCTGGT
28675	Yes	ACATGGTCTAGCGGTTAGGATTCCTGGTT
28676	Yes	ACATGGTCTAGCGGTTAGGATTCCTGGTTT
28677	Yes	ACATGGTCTAGCGGTTAGGATTCCTGGTTTT
28678	Yes	ACATGGTCTAGCGGTTAGGATTCCTGGTTTTC
28679	Yes	ACATGGTCTAGCGGTTAGGATTCCTGGTTTTCA

28680	Yes	ACATGGTCTAGCGGTTAGGATTCCTGGTTTTCAC
28681	Yes	ACATGGTCTAGCGGTTAGGATTCCTGGTTTTCACC
28682	Yes	ACATGGTCTAGCGGTTAGGATTCCTGGTTTTCACCC
28683	Yes	ACATGGTCTAGCGGTTAGGATTCCTGGTTTTCACCCA
28684	Yes	ACATGGTCTAGCGGTTAGGATTCCTGGTTTTCACCCAG
28685	Yes	ACATGGTCTAGCGGTTAGGATTCCTGGTTTTCACCCAGG
28686	Yes	ACATGGTCTAGCGGTTAGGATTCCTGGTTTTCACCCAGGC
28687	Yes	ACATGGTCTAGCGGTTAGGATTCCTGGTTTTCACCCAGGCG
28688	Yes	ACATGGTCTAGCGGTTAGGATTCCTGGTTTTCACCCAGGCGG
28689	Yes	ACATGGTCTAGCGGTTAGGATTCCTGGTTTTCACCCAGGCGGC
28690	Yes	ACATGGTCTAGCGGTTAGGATTCCTGGTTTTCACCCAGGCGGCC
28691	Yes	ACATGGTCTAGCGGTTAGGATTCCTGGTTTTCACCCAGGCGGCCC
28692	Yes	ACATGGTCTAGCGGTTAGGATTCCTGGTTTTCACCCAGGCGGCCCG
28693	Yes	ACATGGTCTAGCGGTTAGGATTCCTGGTTTTCACCCAGGCGGCCCGG
28694	Yes	ACATGGTCTAGCGGTTAGGATTCCTGGTTTTCACCCAGGCGGCCCGGG
28695	Yes	ACATGGTCTAGCGGTTAGGATTCCTGGTTTTCACCCAGGCGGCCCGGGT
28696	Yes	ACATGGTCTAGCGGTTAGGATTCCTGGTTTTCACCCAGGCGGCCCGGGTT
28697	No	ACATGGTGCTAATAAG
28698	Yes	ACATGGTGCTAATAAGG
28699	Yes	ACATGGTGCTAATAAGGC
28700	Yes	ACATGGTGCTAATAAGGCC
28701	Yes	ACATGGTGCTAATAAGGCCA
28702	Yes	ACATGGTGCTAATAAGGCCAC
28703	Yes	ACATGGTGCTAATAAGGCCACG
28704	Yes	ACATGGTGCTAATAAGGCCACGG
28705	Yes	ACATGGTGCTAATAAGGCCACGGT
28706	Yes	ACATGGTGCTAATAAGGCCACGGTC
28707	Yes	ACATGGTGCTAATAAGGCCACGGTCA
28708	Yes	ACATGGTGCTAATAAGGCCACGGTCAG
28709	Yes	ACATGGTGCTAATAAGGCCACGGTCAGG
28710	Yes	ACATGGTGCTAATAAGGCCACGGTCAGGG
28711	Yes	ACATGGTGCTAATAAGGCCACGGTCAGGGG
28712	Yes	ACATGGTGCTAATAAGGCCACGGTCAGGGGT
28713	Yes	ACATGGTGCTAATAAGGCCACGGTCAGGGGTT
28714	Yes	ACATGGTGCTAATAAGGCCACGGTCAGGGGTTC
28715	Yes	ACATGGTGCTAATAAGGCCACGGTCAGGGGTTCA
28716	Yes	ACATGGTGCTAATAAGGCCACGGTCAGGGGTTCAA
28717	Yes	ACATGGTGCTAATAAGGCCACGGTCAGGGGTTCAAT
28718	Yes	ACATGGTGCTAATAAGGCCACGGTCAGGGGTTCAATT
28719	Yes	ACATGGTGCTAATAAGGCCACGGTCAGGGGTTCAATTC
28720	Yes	ACATGGTGCTAATAAGGCCACGGTCAGGGGTTCAATTCC
28721	Yes	ACATGGTGCTAATAAGGCCACGGTCAGGGGTTCAATTCCC

28722	Yes	ACATGGTGCTAATAAGGCCACGGTCAGGGGTTCAATTCCCT
28723	Yes	ACATGGTGCTAATAAGGCCACGGTCAGGGGTTCAATTCCCTT
28724	Yes	ACATGGTGCTAATAAGGCCACGGTCAGGGGTTCAATTCCCTTA
28725	Yes	ACATGGTGCTAATAAGGCCACGGTCAGGGGTTCAATTCCCTTAT
28726	Yes	ACATGGTGCTAATAAGGCCACGGTCAGGGGTTCAATTCCCTTATG
28727	Yes	ACATGGTGCTAATAAGGCCACGGTCAGGGGTTCAATTCCCTTATGG
28728	Yes	ACATGGTGCTAATAAGGCCACGGTCAGGGGTTCAATTCCCTTATGGG
28729	Yes	ACATGGTGCTAATAAGGCCACGGTCAGGGGTTCAATTCCCTTATGGGC
28730	Yes	ACATGGTGCTAATAAGGCCACGGTCAGGGGTTCAATTCCCTTATGGGCT
28731	Yes	ACATGGTGCTAATAAGGCCACGGTCAGGGGTTCAATTCCCTTATGGGCTG
28732	Yes	ACATGGTGTAATAGGT
28733	Yes	ACATGGTGTAATAGGTA
28734	Yes	ACATGGTGTAATAGGTAG
28735	Yes	ACATGGTGTAATAGGTAGA
28736	Yes	ACATGGTGTAATAGGTAGAA
28737	Yes	ACATGGTGTAATAGGTAGAAT
28738	Yes	ACATGGTGTAATAGGTAGAATG
28739	Yes	ACATGGTGTAATAGGTAGAATGG
28740	Yes	ACATGGTGTAATAGGTAGAATGGA
28741	Yes	ACATGGTGTAATAGGTAGAATGGAG
28742	Yes	ACATGGTGTAATAGGTAGAATGGAGA
28743	Yes	ACATGGTGTAATAGGTAGAATGGAGAA
28744	Yes	ACATGGTGTAATAGGTAGAATGGAGAAT
28745	Yes	ACATGGTGTAATAGGTAGAATGGAGAATT
28746	Yes	ACATGGTGTAATAGGTAGAATGGAGAATTT
28747	Yes	ACATGGTGTAATAGGTAGAATGGAGAATTTT
28748	Yes	ACATGGTGTAATAGGTAGAATGGAGAATTTTG
28749	Yes	ACATGGTGTAATAGGTAGAATGGAGAATTTTGA
28750	Yes	ACATGGTGTAATAGGTAGAATGGAGAATTTTGAA
28751	Yes	ACATGGTGTAATAGGTAGAATGGAGAATTTTGAAT
28752	Yes	ACATGGTGTAATAGGTAGAATGGAGAATTTTGAATT
28753	Yes	ACATGGTGTAATAGGTAGAATGGAGAATTTTGAATTC
28754	Yes	ACATGGTGTAATAGGTAGAATGGAGAATTTTGAATTCT
28755	Yes	ACATGGTGTAATAGGTAGAATGGAGAATTTTGAATTCTC
28756	Yes	ACATGGTGTAATAGGTAGAATGGAGAATTTTGAATTCTCA
28757	Yes	ACATGGTGTAATAGGTAGAATGGAGAATTTTGAATTCTCAG
28758	Yes	ACATGGTGTAATAGGTAGAATGGAGAATTTTGAATTCTCAGG
28759	Yes	ACATGGTGTAATAGGTAGAATGGAGAATTTTGAATTCTCAGGG
28760	Yes	ACATGGTGTAATAGGTAGAATGGAGAATTTTGAATTCTCAGGGG
28761	Yes	ACATGGTGTAATAGGTAGAATGGAGAATTTTGAATTCTCAGGGGT
28762	Yes	ACATGGTGTAATAGGTAGAATGGAGAATTTTGAATTCTCAGGGGTA
28763	Yes	ACATGGTGTAATAGGTAGAATGGAGAATTTTGAATTCTCAGGGGTAG

28764	Yes	ACATGGTGTAATAGGTAGAATGGAGAATTTTGAATTCTCAGGGGTAGG
28765	Yes	ACATGGTGTAATAGGTAGAATGGAGAATTTTGAATTCTCAGGGGTAGGT
28766	Yes	ACATGGTGTAATAGGTAGAATGGAGAATTTTGAATTCTCAGGGGTAGGTT
28767	No	ACATGGTGTGATAGGT
28768	No	ACATGGTGTGATAGGTA
28769	No	ACATGGTGTGATAGGTAG
28770	No	ACATGGTGTGATAGGTAGC
28771	No	ACATGGTGTGATAGGTAGCA
28772	No	ACATGGTGTGATAGGTAGCAT
28773	No	ACATGGTGTGATAGGTAGCATG
28774	No	ACATGGTGTGATAGGTAGCATGG
28775	Yes	ACATGGTGTGATAGGTAGCATGGA
28776	Yes	ACATGGTGTGATAGGTAGCATGGAG
28777	Yes	ACATGGTGTGATAGGTAGCATGGAGA
28778	Yes	ACATGGTGTGATAGGTAGCATGGAGAA
28779	Yes	ACATGGTGTGATAGGTAGCATGGAGAAT
28780	Yes	ACATGGTGTGATAGGTAGCATGGAGAATT
28781	Yes	ACATGGTGTGATAGGTAGCATGGAGAATTT
28782	Yes	ACATGGTGTGATAGGTAGCATGGAGAATTTT
28783	Yes	ACATGGTGTGATAGGTAGCATGGAGAATTTTG
28784	Yes	ACATGGTGTGATAGGTAGCATGGAGAATTTTGG
28785	Yes	ACATGGTGTGATAGGTAGCATGGAGAATTTTGGA
28786	Yes	ACATGGTGTGATAGGTAGCATGGAGAATTTTGGAT
28787	Yes	ACATGGTGTGATAGGTAGCATGGAGAATTTTGGATT
28788	Yes	ACATGGTGTGATAGGTAGCATGGAGAATTTTGGATTC
28789	Yes	ACATGGTGTGATAGGTAGCATGGAGAATTTTGGATTCT
28790	Yes	ACATGGTGTGATAGGTAGCATGGAGAATTTTGGATTCTC
28791	Yes	ACATGGTGTGATAGGTAGCATGGAGAATTTTGGATTCTCA
28792	Yes	ACATGGTGTGATAGGTAGCATGGAGAATTTTGGATTCTCAG
28793	Yes	ACATGGTGTGATAGGTAGCATGGAGAATTTTGGATTCTCAGG
28794	Yes	ACATGGTGTGATAGGTAGCATGGAGAATTTTGGATTCTCAGGG
28795	Yes	ACATGGTGTGATAGGTAGCATGGAGAATTTTGGATTCTCAGGGG
28796	Yes	ACATGGTGTGATAGGTAGCATGGAGAATTTTGGATTCTCAGGGGT
28797	Yes	ACATGGTGTGATAGGTAGCATGGAGAATTTTGGATTCTCAGGGGTA
28798	Yes	ACATGGTGTGATAGGTAGCATGGAGAATTTTGGATTCTCAGGGGTAG
28799	Yes	ACATGGTGTGATAGGTAGCATGGAGAATTTTGGATTCTCAGGGGTAGG
28800	Yes	ACATGGTGTGATAGGTAGCATGGAGAATTTTGGATTCTCAGGGGTAGGT
28801	Yes	ACATGGTGTGATAGGTAGCATGGAGAATTTTGGATTCTCAGGGGTAGGTT
28802	No	ACATGTAATGGTTATG
28803	No	ACATGTAATGGTTATGA
28804	Yes	ACATGTAATGGTTATGAG
28805	Yes	ACATGTAATGGTTATGAGG

28806	Yes	ACATGTAATGGTTATGAGGG
28807	Yes	ACATGTAATGGTTATGAGGGT
28808	Yes	ACATGTAATGGTTATGAGGGTG
28809	Yes	ACATGTAATGGTTATGAGGGTGG
28810	Yes	ACATGTAATGGTTATGAGGGTGGA
28811	Yes	ACATGTAATGGTTATGAGGGTGGAC
28812	Yes	ACATGTAATGGTTATGAGGGTGGACT
28813	Yes	ACATGTAATGGTTATGAGGGTGGACTT
28814	Yes	ACATGTAATGGTTATGAGGGTGGACTTT
28815	Yes	ACATGTAATGGTTATGAGGGTGGACTTTA
28816	Yes	ACATGTAATGGTTATGAGGGTGGACTTTAA
28817	Yes	ACATGTAATGGTTATGAGGGTGGACTTTAAC
28818	Yes	ACATGTAATGGTTATGAGGGTGGACTTTAACC
28819	Yes	ACATGTAATGGTTATGAGGGTGGACTTTAACCA
28820	Yes	ACATGTAATGGTTATGAGGGTGGACTTTAACCAC
28821	Yes	ACATGTAATGGTTATGAGGGTGGACTTTAACCACA
28822	Yes	ACATGTAATGGTTATGAGGGTGGACTTTAACCACAC
28823	Yes	ACATGTAATGGTTATGAGGGTGGACTTTAACCACACT
28824	Yes	ACATGTAATGGTTATGAGGGTGGACTTTAACCACACTG
28825	Yes	ACATGTAATGGTTATGAGGGTGGACTTTAACCACACTGC
28826	Yes	ACATGTAATGGTTATGAGGGTGGACTTTAACCACACTGCC
28827	Yes	ACATGTAATGGTTATGAGGGTGGACTTTAACCACACTGCCT
28828	Yes	ACATGTAATGGTTATGAGGGTGGACTTTAACCACACTGCCTA
28829	Yes	ACATGTAATGGTTATGAGGGTGGACTTTAACCACACTGCCTAG
28830	Yes	ACATGTAATGGTTATGAGGGTGGACTTTAACCACACTGCCTAGG
28831	Yes	ACATGTAATGGTTATGAGGGTGGACTTTAACCACACTGCCTAGGT
28832	Yes	ACATGTAATGGTTATGAGGGTGGACTTTAACCACACTGCCTAGGTT
28833	Yes	ACATGTAATGGTTATGAGGGTGGACTTTAACCACACTGCCTAGGTTC
28834	Yes	ACATGTAATGGTTATGAGGGTGGACTTTAACCACACTGCCTAGGTTCA
28835	Yes	ACATGTAATGGTTATGAGGGTGGACTTTAACCACACTGCCTAGGTTCAA
28836	Yes	ACATGTAATGGTTATGAGGGTGGACTTTAACCACACTGCCTAGGTTCAAA
28837	No	ACATGTGAAAGACCCT
28838	No	ACATGTGAAAGACCCTT
28839	Yes	ACATGTGAAAGACCCTTG
28840	Yes	ACATGTGAAAGACCCTTGG
28841	Yes	ACATGTGAAAGACCCTTGGC
28842	Yes	ACATGTGAAAGACCCTTGGCT
28843	Yes	ACATGTGAAAGACCCTTGGCTC
28844	Yes	ACATGTGAAAGACCCTTGGCTCG
28845	Yes	ACATGTGAAAGACCCTTGGCTCGA
28846	Yes	ACATGTGAAAGACCCTTGGCTCGAG
28847	Yes	ACATGTGAAAGACCCTTGGCTCGAGA

28848	Yes	ACATGTGAAAGACCCTTGGCTCGAGAC
28849	Yes	ACATGTGAAAGACCCTTGGCTCGAGACT
28850	Yes	ACATGTGAAAGACCCTTGGCTCGAGACTG
28851	Yes	ACATGTGAAAGACCCTTGGCTCGAGACTGG
28852	Yes	ACATGTGAAAGACCCTTGGCTCGAGACTGGA
28853	Yes	ACATGTGAAAGACCCTTGGCTCGAGACTGGAG
28854	Yes	ACATGTGAAAGACCCTTGGCTCGAGACTGGAGG
28855	Yes	ACATGTGAAAGACCCTTGGCTCGAGACTGGAGGG
28856	Yes	ACATGTGAAAGACCCTTGGCTCGAGACTGGAGGGA
28857	Yes	ACATGTGAAAGACCCTTGGCTCGAGACTGGAGGGAA
28858	Yes	ACATGTGAAAGACCCTTGGCTCGAGACTGGAGGGAAA
28859	Yes	ACATGTGAAAGACCCTTGGCTCGAGACTGGAGGGAAAC
28860	Yes	ACATGTGAAAGACCCTTGGCTCGAGACTGGAGGGAAACA
28861	Yes	ACATGTGAAAGACCCTTGGCTCGAGACTGGAGGGAAACAC
28862	Yes	ACATGTGAAAGACCCTTGGCTCGAGACTGGAGGGAAACACC
28863	Yes	ACATGTGAAAGACCCTTGGCTCGAGACTGGAGGGAAACACCA
28864	Yes	ACATTAACGTCCTGGG
28865	Yes	ACATTAACGTCCTGGGT
28866	Yes	ACATTAACGTCCTGGGTT
28867	Yes	ACATTAACGTCCTGGGTTC
28868	Yes	ACATTAACGTCCTGGGTTCA
28869	Yes	ACATTAACGTCCTGGGTTCAA
28870	Yes	ACATTAACGTCCTGGGTTCAAA
28871	Yes	ACATTAACGTCCTGGGTTCAAAT
28872	Yes	ACATTAACGTCCTGGGTTCAAATC
28873	Yes	ACATTAACGTCCTGGGTTCAAATCC
28874	Yes	ACATTAACGTCCTGGGTTCAAATCCC
28875	Yes	ACATTAACGTCCTGGGTTCAAATCCCA
28876	Yes	ACATTAACGTCCTGGGTTCAAATCCCAG
28877	Yes	ACATTAACGTCCTGGGTTCAAATCCCAGC
28878	Yes	ACATTAACGTCCTGGGTTCAAATCCCAGCT
28879	Yes	ACATTAACGTCCTGGGTTCAAATCCCAGCTT
28880	Yes	ACATTAACGTCCTGGGTTCAAATCCCAGCTTT
28881	Yes	ACATTAACGTCCTGGGTTCAAATCCCAGCTTTG
28882	Yes	ACATTAACGTCCTGGGTTCAAATCCCAGCTTTGT
28883	Yes	ACATTAACGTCCTGGGTTCAAATCCCAGCTTTGTC
28884	Yes	ACATTAACGTCCTGGGTTCAAATCCCAGCTTTGTCA
28885	Yes	ACATTAACGTCCTGGGTTCAAATCCCAGCTTTGTCAC
28886	Yes	ACATTAACGTCCTGGGTTCAAATCCCAGCTTTGTCACC
28887	Yes	ACATTAACGTCCTGGGTTCAAATCCCAGCTTTGTCACCA
28888	No	ACATTCAAAAAAGAGT
28889	No	ACATTCAAAAAAGAGTA

28890	No	ACATTCAAAAAAGAGTAC
28891	No	ACATTCAAAAAAGAGTACC
28892	Yes	ACATTCAAAAAAGAGTACCA
28893	Yes	ACATTCACCTAACACG
28894	Yes	ACATTCACCTAACACGC
28895	Yes	ACATTCACCTAACACGCG
28896	Yes	ACATTCACCTAACACGCGA
28897	Yes	ACATTCACCTAACACGCGAA
28898	Yes	ACATTCACCTAACACGCGAAA
28899	Yes	ACATTCACCTAACACGCGAAAG
28900	Yes	ACATTCACCTAACACGCGAAAGG
28901	Yes	ACATTCACCTAACACGCGAAAGGT
28902	Yes	ACATTCACCTAACACGCGAAAGGTC
28903	Yes	ACATTCACCTAACACGCGAAAGGTCC
28904	Yes	ACATTCACCTAACACGCGAAAGGTCCT
28905	Yes	ACATTCACCTAACACGCGAAAGGTCCTT
28906	Yes	ACATTCACCTAACACGCGAAAGGTCCTTG
28907	Yes	ACATTCACCTAACACGCGAAAGGTCCTTGG
28908	Yes	ACATTCACCTAACACGCGAAAGGTCCTTGGT
28909	Yes	ACATTCACCTAACACGCGAAAGGTCCTTGGTT
28910	Yes	ACATTCACCTAACACGCGAAAGGTCCTTGGTTT
28911	Yes	ACATTCACCTAACACGCGAAAGGTCCTTGGTTTG
28912	Yes	ACATTCACCTAACACGCGAAAGGTCCTTGGTTTGA
28913	Yes	ACATTCACCTAACACGCGAAAGGTCCTTGGTTTGAA
28914	Yes	ACATTCACCTAACACGCGAAAGGTCCTTGGTTTGAAA
28915	Yes	ACATTCACCTAACACGCGAAAGGTCCTTGGTTTGAAAC
28916	Yes	ACATTCACCTAACACGCGAAAGGTCCTTGGTTTGAAACC
28917	Yes	ACATTCACCTAACACGCGAAAGGTCCTTGGTTTGAAACCA
28918	Yes	ACATTCACCTAACACGCGAAAGGTCCTTGGTTTGAAACCAG
28919	Yes	ACATTCACCTAACACGCGAAAGGTCCTTGGTTTGAAACCAGG
28920	Yes	ACATTCACCTAACACGCGAAAGGTCCTTGGTTTGAAACCAGGC
28921	Yes	ACATTCACCTAACACGCGAAAGGTCCTTGGTTTGAAACCAGGCA
28922	Yes	ACATTCACCTAACACGCGAAAGGTCCTTGGTTTGAAACCAGGCAG
28923	Yes	ACATTCACCTAACACGCGAAAGGTCCTTGGTTTGAAACCAGGCAGA
28924	Yes	ACATTCACCTAACACGCGAAAGGTCCTTGGTTTGAAACCAGGCAGAA
28925	Yes	ACATTCACCTAACACGCGAAAGGTCCTTGGTTTGAAACCAGGCAGAAA
28926	Yes	ACATTCACCTAACACGCGAAAGGTCCTTGGTTTGAAACCAGGCAGAAAC
28927	Yes	ACATTCACCTAACACGCGAAAGGTCCTTGGTTTGAAACCAGGCAGAAACA
28928	No	ACATTCATGAGGCTCT
28929	Yes	ACATTCATGAGGCTCTG
28930	Yes	ACATTCATGAGGCTCTGG
28931	Yes	ACATTCATGAGGCTCTGGG

28932	Yes	ACATTCATGAGGCTCTGGGT
28933	Yes	ACATTCATGAGGCTCTGGGTT
28934	Yes	ACATTCATGAGGCTCTGGGTTC
28935	Yes	ACATTCATGAGGCTCTGGGTTCG
28936	Yes	ACATTCATGAGGCTCTGGGTTCGA
28937	Yes	ACATTCATGAGGCTCTGGGTTCGAT
28938	Yes	ACATTCATGAGGCTCTGGGTTCGATC
28939	Yes	ACATTCATGAGGCTCTGGGTTCGATCC
28940	Yes	ACATTCATGAGGCTCTGGGTTCGATCCC
28941	Yes	ACATTCATGAGGCTCTGGGTTCGATCCCC
28942	Yes	ACATTCATGAGGCTCTGGGTTCGATCCCCA
28943	Yes	ACATTCATGAGGCTCTGGGTTCGATCCCCAG
28944	Yes	ACATTCATGAGGCTCTGGGTTCGATCCCCAGC
28945	Yes	ACATTCATGAGGCTCTGGGTTCGATCCCCAGCA
28946	Yes	ACATTCATGAGGCTCTGGGTTCGATCCCCAGCAC
28947	Yes	ACATTCATGAGGCTCTGGGTTCGATCCCCAGCACT
28948	Yes	ACATTCATGAGGCTCTGGGTTCGATCCCCAGCACTT
28949	Yes	ACATTCATGAGGCTCTGGGTTCGATCCCCAGCACTTC
28950	Yes	ACATTCATGAGGCTCTGGGTTCGATCCCCAGCACTTCC
28951	Yes	ACATTCATGAGGCTCTGGGTTCGATCCCCAGCACTTCCA
28952	Yes	ACATTCATGAGGCTCTGGGTTCGATCCCCAGCACTTCCAC
28953	Yes	ACATTCATGAGGCTCTGGGTTCGATCCCCAGCACTTCCACC
28954	Yes	ACATTCATGAGGCTCTGGGTTCGATCCCCAGCACTTCCACCA
28955	No	ACATTCCTGGGTTCAA
28956	No	ACATTCCTGGGTTCAAA
28957	No	ACATTCCTGGGTTCAAAT
28958	Yes	ACATTCCTGGGTTCAAATC
28959	Yes	ACATTCCTGGGTTCAAATCC
28960	Yes	ACATTCCTGGGTTCAAATCCC
28961	Yes	ACATTCCTGGGTTCAAATCCCA
28962	Yes	ACATTCCTGGGTTCAAATCCCAG
28963	Yes	ACATTCCTGGGTTCAAATCCCAGC
28964	Yes	ACATTCCTGGGTTCAAATCCCAGCA
28965	Yes	ACATTCCTGGGTTCAAATCCCAGCAC
28966	Yes	ACATTCCTGGGTTCAAATCCCAGCACT
28967	Yes	ACATTCCTGGGTTCAAATCCCAGCACTG
28968	Yes	ACATTCCTGGGTTCAAATCCCAGCACTGT
28969	Yes	ACATTCCTGGGTTCAAATCCCAGCACTGTT
28970	Yes	ACATTCCTGGGTTCAAATCCCAGCACTGTTG
28971	Yes	ACATTCCTGGGTTCAAATCCCAGCACTGTTGC
28972	Yes	ACATTCCTGGGTTCAAATCCCAGCACTGTTGCC
28973	Yes	ACATTCCTGGGTTCAAATCCCAGCACTGTTGCCA

28974	Yes	ACATTCGCCTCACACA
28975	Yes	ACATTCGCCTCACACAT
28976	Yes	ACATTCGCCTCACACATG
28977	Yes	ACATTCGCCTCACACATGA
28978	Yes	ACATTCGCCTCACACATGAA
28979	Yes	ACATTCGCCTCACACATGAAA
28980	Yes	ACATTCGCCTCACACATGAAAG
28981	Yes	ACATTCGCCTCACACATGAAAGG
28982	Yes	ACATTCGCCTCACACATGAAAGGT
28983	Yes	ACATTCGCCTCACACATGAAAGGTC
28984	Yes	ACATTCGCCTCACACATGAAAGGTCA
28985	Yes	ACATTCGCCTCACACATGAAAGGTCAC
28986	Yes	ACATTCGCCTCACACATGAAAGGTCACC
28987	Yes	ACATTCGCCTCACACATGAAAGGTCACCA
28988	Yes	ACATTCGCCTCACACATGAAAGGTCACCAG
28989	Yes	ACATTCGCCTCACACATGAAAGGTCACCAGT
28990	Yes	ACATTCGCCTCACACATGAAAGGTCACCAGTT
28991	Yes	ACATTCGCCTCACACATGAAAGGTCACCAGTTT
28992	Yes	ACATTCGCCTCACACATGAAAGGTCACCAGTTTG
28993	Yes	ACATTCGCCTCACACATGAAAGGTCACCAGTTTGA
28994	Yes	ACATTCGCCTCACACATGAAAGGTCACCAGTTTGAG
28995	Yes	ACATTCGCCTCACACATGAAAGGTCACCAGTTTGAGA
28996	Yes	ACATTCGCCTCACACATGAAAGGTCACCAGTTTGAGAC
28997	Yes	ACATTCGCCTCACACATGAAAGGTCACCAGTTTGAGACC
28998	Yes	ACATTCGCCTCACACATGAAAGGTCACCAGTTTGAGACCG
28999	Yes	ACATTCGCCTCACACATGAAAGGTCACCAGTTTGAGACCGG
29000	Yes	ACATTCGCCTCACACATGAAAGGTCACCAGTTTGAGACCGGG
29001	Yes	ACATTCGCCTCACACATGAAAGGTCACCAGTTTGAGACCGGGC
29002	Yes	ACATTCGCCTCACACATGAAAGGTCACCAGTTTGAGACCGGGCC
29003	Yes	ACATTCGCCTCACACATGAAAGGTCACCAGTTTGAGACCGGGCCA
29004	Yes	ACATTCGCCTCACACATGAAAGGTCACCAGTTTGAGACCGGGCCAA
29005	Yes	ACATTCGCCTCACACATGAAAGGTCACCAGTTTGAGACCGGGCCAAA
29006	Yes	ACATTCGCCTCACACATGAAAGGTCACCAGTTTGAGACCGGGCCAAAA
29007	Yes	ACATTCGCCTCACACATGAAAGGTCACCAGTTTGAGACCGGGCCAAAAC
29008	Yes	ACATTCGCCTCACACATGAAAGGTCACCAGTTTGAGACCGGGCCAAAACA
29009	Yes	ACATTCGCCTCACACG
29010	Yes	ACATTCGCCTCACACGC
29011	Yes	ACATTCGCCTCACACGCG
29012	Yes	ACATTCGCCTCACACGCGA
29013	Yes	ACATTCGCCTCACACGCGAA
29014	Yes	ACATTCGCCTCACACGCGAAA
29015	Yes	ACATTCGCCTCACACGCGAAAG

29016	Yes	ACATTCGCCTCACACGCGAAAGG
29017	Yes	ACATTCGCCTCACACGCGAAAGGT
29018	Yes	ACATTCGCCTCACACGCGAAAGGTC
29019	Yes	ACATTCGCCTCACACGCGAAAGGTCC
29020	Yes	ACATTCGCCTCACACGCGAAAGGTCCC
29021	Yes	ACATTCGCCTCACACGCGAAAGGTCCCC
29022	Yes	ACATTCGCCTCACACGCGAAAGGTCCCCG
29023	Yes	ACATTCGCCTCACACGCGAAAGGTCCCCGG
29024	Yes	ACATTCGCCTCACACGCGAAAGGTCCCCGGT
29025	Yes	ACATTCGCCTCACACGCGAAAGGTCCCCGGTT
29026	Yes	ACATTCGCCTCACACGCGAAAGGTCCCCGGTTC
29027	Yes	ACATTCGCCTCACACGCGAAAGGTCCCCGGTTCG
29028	Yes	ACATTCGCCTCACACGCGAAAGGTCCCCGGTTCGA
29029	Yes	ACATTCGCCTCACACGCGAAAGGTCCCCGGTTCGAT
29030	Yes	ACATTCGCCTCACACGCGAAAGGTCCCCGGTTCGATC
29031	Yes	ACATTCGCCTCACACGCGAAAGGTCCCCGGTTCGATCC
29032	Yes	ACATTCGCCTCACACGCGAAAGGTCCCCGGTTCGATCCC
29033	Yes	ACATTCGCCTCACACGCGAAAGGTCCCCGGTTCGATCCCG
29034	Yes	ACATTCGCCTCACACGCGAAAGGTCCCCGGTTCGATCCCGG
29035	Yes	ACATTCGCCTCACACGCGAAAGGTCCCCGGTTCGATCCCGGG
29036	Yes	ACATTCGCCTCACACGCGAAAGGTCCCCGGTTCGATCCCGGGC
29037	Yes	ACATTCGCCTCACACGCGAAAGGTCCCCGGTTCGATCCCGGGCG
29038	Yes	ACATTCGCCTCACACGCGAAAGGTCCCCGGTTCGATCCCGGGCGG
29039	Yes	ACATTCGCCTCACACGCGAAAGGTCCCCGGTTCGATCCCGGGCGGA
29040	Yes	ACATTCGCCTCACACGCGAAAGGTCCCCGGTTCGATCCCGGGCGGAA
29041	Yes	ACATTCGCCTCACACGCGAAAGGTCCCCGGTTCGATCCCGGGCGGAAA
29042	Yes	ACATTCGCCTCACACGCGAAAGGTCCCCGGTTCGATCCCGGGCGGAAAC
29043	Yes	ACATTCGCCTCACACGCGAAAGGTCCCCGGTTCGATCCCGGGCGGAAACA
29044	Yes	ACATTCGCCTTACACG
29045	Yes	ACATTCGCCTTACACGC
29046	Yes	ACATTCGCCTTACACGCG
29047	Yes	ACATTCGCCTTACACGCGA
29048	Yes	ACATTCGCCTTACACGCGAA
29049	Yes	ACATTCGCCTTACACGCGAAA
29050	Yes	ACATTCGCCTTACACGCGAAAG
29051	Yes	ACATTCGCCTTACACGCGAAAGG
29052	Yes	ACATTCGCCTTACACGCGAAAGGT
29053	Yes	ACATTCGCCTTACACGCGAAAGGTC
29054	Yes	ACATTCGCCTTACACGCGAAAGGTCC
29055	Yes	ACATTCGCCTTACACGCGAAAGGTCCT
29056	Yes	ACATTCGCCTTACACGCGAAAGGTCCTC
29057	Yes	ACATTCGCCTTACACGCGAAAGGTCCTCG

29058	Yes	ACATTCGCCTTACACGCGAAAGGTCCTCGG
29059	Yes	ACATTCGCCTTACACGCGAAAGGTCCTCGGG
29060	Yes	ACATTCGCCTTACACGCGAAAGGTCCTCGGGT
29061	Yes	ACATTCGCCTTACACGCGAAAGGTCCTCGGGTC
29062	Yes	ACATTCGCCTTACACGCGAAAGGTCCTCGGGTCG
29063	Yes	ACATTCGCCTTACACGCGAAAGGTCCTCGGGTCGA
29064	Yes	ACATTCGCCTTACACGCGAAAGGTCCTCGGGTCGAA
29065	Yes	ACATTCGCCTTACACGCGAAAGGTCCTCGGGTCGAAA
29066	Yes	ACATTCGCCTTACACGCGAAAGGTCCTCGGGTCGAAAC
29067	Yes	ACATTCGCCTTACACGCGAAAGGTCCTCGGGTCGAAACC
29068	Yes	ACATTCGCCTTACACGCGAAAGGTCCTCGGGTCGAAACCG
29069	Yes	ACATTCGCCTTACACGCGAAAGGTCCTCGGGTCGAAACCGA
29070	Yes	ACATTCGCCTTACACGCGAAAGGTCCTCGGGTCGAAACCGAG
29071	Yes	ACATTCGCCTTACACGCGAAAGGTCCTCGGGTCGAAACCGAGC
29072	Yes	ACATTCGCCTTACACGCGAAAGGTCCTCGGGTCGAAACCGAGCG
29073	Yes	ACATTCGCCTTACACGCGAAAGGTCCTCGGGTCGAAACCGAGCGG
29074	Yes	ACATTCGCCTTACACGCGAAAGGTCCTCGGGTCGAAACCGAGCGGA
29075	Yes	ACATTCGCCTTACACGCGAAAGGTCCTCGGGTCGAAACCGAGCGGAA
29076	Yes	ACATTCGCCTTACACGCGAAAGGTCCTCGGGTCGAAACCGAGCGGAAA
29077	Yes	ACATTCGCCTTACACGCGAAAGGTCCTCGGGTCGAAACCGAGCGGAAAC
29078	Yes	ACATTCGCCTTACACGCGAAAGGTCCTCGGGTCGAAACCGAGCGGAAACA
29079	No	ACCAAAACATCAGATT
29080	No	ACCAAAACATCAGATTG
29081	No	ACCAAAACATCAGATTGT
29082	No	ACCAAAACATCAGATTGTG
29083	No	ACCAAAACATCAGATTGTGA
29084	No	ACCAAAACATCAGATTGTGAA
29085	No	ACCAAAACATCAGATTGTGAAT
29086	No	ACCAAAACATCAGATTGTGAATC
29087	No	ACCAAAACATCAGATTGTGAATCT
29088	No	ACCAAAACATCAGATTGTGAATCTG
29089	No	ACCAAAACATCAGATTGTGAATCTGA
29090	No	ACCAAAACATCAGATTGTGAATCTGAC
29091	No	ACCAAAACATCAGATTGTGAATCTGACA
29092	No	ACCAAAACATCAGATTGTGAATCTGACAA
29093	No	ACCAAAACATCAGATTGTGAATCTGACAAC
29094	No	ACCAAAACATCAGATTGTGAATCTGACAACA
29095	No	ACCAAAACATCAGATTGTGAATCTGACAACAG
29096	No	ACCAAAACATCAGATTGTGAATCTGACAACAGA
29097	No	ACCAAAACATCAGATTGTGAATCTGACAACAGAG
29098	No	ACCAAAACATCAGATTGTGAATCTGACAACAGAGG
29099	No	ACCAAAACATCAGATTGTGAATCTGACAACAGAGGC

29100	No	ACCAAAACATCAGATTGTGAATCTGACAACAGAGGCT
29101	Yes	ACCAAAACATCAGATTGTGAATCTGACAACAGAGGCTT
29102	Yes	ACCAAAACATCAGATTGTGAATCTGACAACAGAGGCTTA
29103	Yes	ACCAAAACATCAGATTGTGAATCTGACAACAGAGGCTTAC
29104	Yes	ACCAAAACATCAGATTGTGAATCTGACAACAGAGGCTTACG
29105	Yes	ACCAAAACATCAGATTGTGAATCTGACAACAGAGGCTTACGA
29106	Yes	ACCAAAACATCAGATTGTGAATCTGACAACAGAGGCTTACGAC
29107	Yes	ACCAAAACATCAGATTGTGAATCTGACAACAGAGGCTTACGACC
29108	Yes	ACCAAAACATCAGATTGTGAATCTGACAACAGAGGCTTACGACCC
29109	Yes	ACCAAAACATCAGATTGTGAATCTGACAACAGAGGCTTACGACCCC
29110	Yes	ACCAAAACATCAGATTGTGAATCTGACAACAGAGGCTTACGACCCCT
29111	Yes	ACCAAAACATCAGATTGTGAATCTGACAACAGAGGCTTACGACCCCTT
29112	Yes	ACCAAAACATCAGATTGTGAATCTGACAACAGAGGCTTACGACCCCTTA
29113	Yes	ACCAAAACATCAGATTGTGAATCTGACAACAGAGGCTTACGACCCCTTAT
29114	No	ACCAACACCTCTTTAC
29115	No	ACCAACACCTCTTTACA
29116	No	ACCAACACCTCTTTACAG
29117	No	ACCAACACCTCTTTACAGT
29118	No	ACCAACACCTCTTTACAGTG
29119	No	ACCAACACCTCTTTACAGTGA
29120	Yes	ACCAACACCTCTTTACAGTGAC
29121	Yes	ACCAACACCTCTTTACAGTGACC
29122	Yes	ACCAACACCTCTTTACAGTGACCA
29123	No	ACCAAGAGCCTTCAAA
29124	No	ACCAAGAGCCTTCAAAG
29125	No	ACCAAGAGCCTTCAAAGC
29126	No	ACCAAGAGCCTTCAAAGCC
29127	No	ACCAAGAGCCTTCAAAGCCC
29128	No	ACCAAGAGCCTTCAAAGCCCT
29129	Yes	ACCAAGAGCCTTCAAAGCCCTC
29130	Yes	ACCAAGAGCCTTCAAAGCCCTCA
29131	Yes	ACCAAGAGCCTTCAAAGCCCTCAG
29132	Yes	ACCAAGAGCCTTCAAAGCCCTCAGT
29133	Yes	ACCAAGAGCCTTCAAAGCCCTCAGTA
29134	Yes	ACCAAGAGCCTTCAAAGCCCTCAGTAA
29135	Yes	ACCAAGAGCCTTCAAAGCCCTCAGTAAG
29136	Yes	ACCAAGAGCCTTCAAAGCCCTCAGTAAGT
29137	Yes	ACCAAGAGCCTTCAAAGCCCTCAGTAAGTT
29138	Yes	ACCAAGAGCCTTCAAAGCCCTCAGTAAGTTG
29139	Yes	ACCAAGAGCCTTCAAAGCCCTCAGTAAGTTGC
29140	Yes	ACCAAGAGCCTTCAAAGCCCTCAGTAAGTTGCA
29141	Yes	ACCAAGAGCCTTCAAAGCCCTCAGTAAGTTGCAA

29142	Yes	ACCAAGAGCCTTCAAAGCCCTCAGTAAGTTGCAAT
29143	Yes	ACCAAGAGCCTTCAAAGCCCTCAGTAAGTTGCAATA
29144	Yes	ACCAAGAGCCTTCAAAGCCCTCAGTAAGTTGCAATAC
29145	Yes	ACCAAGAGCCTTCAAAGCCCTCAGTAAGTTGCAATACT
29146	Yes	ACCAAGAGCCTTCAAAGCCCTCAGTAAGTTGCAATACTT
29147	Yes	ACCAAGAGCCTTCAAAGCCCTCAGTAAGTTGCAATACTTA
29148	Yes	ACCAAGAGCCTTCAAAGCCCTCAGTAAGTTGCAATACTTAA
29149	Yes	ACCAAGAGCCTTCAAAGCCCTCAGTAAGTTGCAATACTTAAT
29150	Yes	ACCAAGAGCCTTCAAAGCCCTCAGTAAGTTGCAATACTTAATT
29151	Yes	ACCAAGAGCCTTCAAAGCCCTCAGTAAGTTGCAATACTTAATTT
29152	Yes	ACCAAGAGCCTTCAAAGCCCTCAGTAAGTTGCAATACTTAATTTC
29153	Yes	ACCAAGAGCCTTCAAAGCCCTCAGTAAGTTGCAATACTTAATTTCT
29154	Yes	ACCAAGAGCCTTCAAAGCCCTCAGTAAGTTGCAATACTTAATTTCTG
29155	Yes	ACCAAGAGCCTTCAAAGCCCTCAGTAAGTTGCAATACTTAATTTCTGC
29156	Yes	ACCAAGAGCCTTCAAAGCCCTCAGTAAGTTGCAATACTTAATTTCTGCC
29157	Yes	ACCAAGAGCCTTCAAAGCCCTCAGTAAGTTGCAATACTTAATTTCTGCCA
29158	Yes	ACCAAGGTCGCGGGCT
29159	Yes	ACCAAGGTCGCGGGCTC
29160	Yes	ACCAAGGTCGCGGGCTCG
29161	Yes	ACCAAGGTCGCGGGCTCGA
29162	Yes	ACCAAGGTCGCGGGCTCGAC
29163	Yes	ACCAAGGTCGCGGGCTCGACT
29164	Yes	ACCAAGGTCGCGGGCTCGACTC
29165	Yes	ACCAAGGTCGCGGGCTCGACTCC
29166	Yes	ACCAAGGTCGCGGGCTCGACTCCC
29167	Yes	ACCAAGGTCGCGGGCTCGACTCCCG
29168	Yes	ACCAAGGTCGCGGGCTCGACTCCCGC
29169	Yes	ACCAAGGTCGCGGGCTCGACTCCCGCA
29170	Yes	ACCAAGGTCGCGGGCTCGACTCCCGCAC
29171	Yes	ACCAAGGTCGCGGGCTCGACTCCCGCACC
29172	Yes	ACCAAGGTCGCGGGCTCGACTCCCGCACCG
29173	Yes	ACCAAGGTCGCGGGCTCGACTCCCGCACCGG
29174	Yes	ACCAAGGTCGCGGGCTCGACTCCCGCACCGGC
29175	Yes	ACCAAGGTCGCGGGCTCGACTCCCGCACCGGCC
29176	Yes	ACCAAGGTCGCGGGCTCGACTCCCGCACCGGCCA
29177	Yes	ACCAAGGTCGCGGGCTCGACTCCCGCACCGGCCAC
29178	Yes	ACCAAGGTCGCGGGCTCGACTCCCGCACCGGCCACC
29179	Yes	ACCAAGGTCGCGGGCTCGACTCCCGCACCGGCCACCA
29180	No	ACCAATTAGCAAGCAC
29181	No	ACCAATTAGCAAGCACA
29182	No	ACCAATTAGCAAGCACAG
29183	Yes	ACCAATTAGCAAGCACAGT

29184	Yes	ACCAATTAGCAAGCACAGTT
29185	Yes	ACCAATTAGCAAGCACAGTTG
29186	Yes	ACCAATTAGCAAGCACAGTTGG
29187	Yes	ACCAATTAGCAAGCACAGTTGGC
29188	Yes	ACCAATTAGCAAGCACAGTTGGCT
29189	Yes	ACCAATTAGCAAGCACAGTTGGCTA
29190	Yes	ACCAATTAGCAAGCACAGTTGGCTAG
29191	Yes	ACCAATTAGCAAGCACAGTTGGCTAGA
29192	Yes	ACCAATTAGCAAGCACAGTTGGCTAGAA
29193	Yes	ACCAATTAGCAAGCACAGTTGGCTAGAAC
29194	Yes	ACCAATTAGCAAGCACAGTTGGCTAGAACA
29195	Yes	ACCAATTAGCAAGCACAGTTGGCTAGAACAT
29196	Yes	ACCAATTAGCAAGCACAGTTGGCTAGAACATG
29197	Yes	ACCAATTAGCAAGCACAGTTGGCTAGAACATGG
29198	Yes	ACCAATTAGCAAGCACAGTTGGCTAGAACATGGT
29199	Yes	ACCAATTAGCAAGCACAGTTGGCTAGAACATGGTG
29200	Yes	ACCAATTAGCAAGCACAGTTGGCTAGAACATGGTGC
29201	Yes	ACCAATTAGCAAGCACAGTTGGCTAGAACATGGTGCT
29202	Yes	ACCAATTAGCAAGCACAGTTGGCTAGAACATGGTGCTA
29203	Yes	ACCAATTAGCAAGCACAGTTGGCTAGAACATGGTGCTAA
29204	Yes	ACCAATTAGCAAGCACAGTTGGCTAGAACATGGTGCTAAT
29205	Yes	ACCAATTAGCAAGCACAGTTGGCTAGAACATGGTGCTAATA
29206	Yes	ACCAATTAGCAAGCACAGTTGGCTAGAACATGGTGCTAATAA
29207	Yes	ACCAATTAGCAAGCACAGTTGGCTAGAACATGGTGCTAATAAG
29208	Yes	ACCAATTAGCAAGCACAGTTGGCTAGAACATGGTGCTAATAAGG
29209	Yes	ACCAATTAGCAAGCACAGTTGGCTAGAACATGGTGCTAATAAGGC
29210	Yes	ACCAATTAGCAAGCACAGTTGGCTAGAACATGGTGCTAATAAGGCC
29211	Yes	ACCAATTAGCAAGCACAGTTGGCTAGAACATGGTGCTAATAAGGCCA
29212	Yes	ACCAATTAGCAAGCACAGTTGGCTAGAACATGGTGCTAATAAGGCCAC
29213	Yes	ACCAATTAGCAAGCACAGTTGGCTAGAACATGGTGCTAATAAGGCCACG
29214	Yes	ACCAATTAGCAAGCACAGTTGGCTAGAACATGGTGCTAATAAGGCCACGG
29215	Yes	ACCACACTGCCTAGGT
29216	Yes	ACCACACTGCCTAGGTT
29217	Yes	ACCACACTGCCTAGGTTC
29218	Yes	ACCACACTGCCTAGGTTCA
29219	Yes	ACCACACTGCCTAGGTTCAA
29220	Yes	ACCACACTGCCTAGGTTCAAA
29221	Yes	ACCACACTGCCTAGGTTCAAAT
29222	Yes	ACCACACTGCCTAGGTTCAAATC
29223	Yes	ACCACACTGCCTAGGTTCAAATCC
29224	Yes	ACCACACTGCCTAGGTTCAAATCCT
29225	Yes	ACCACACTGCCTAGGTTCAAATCCTG

29226	Yes	ACCACACTGCCTAGGTTCAAATCCTGA
29227	Yes	ACCACACTGCCTAGGTTCAAATCCTGAC
29228	Yes	ACCACACTGCCTAGGTTCAAATCCTGACT
29229	Yes	ACCACACTGCCTAGGTTCAAATCCTGACTC
29230	Yes	ACCACACTGCCTAGGTTCAAATCCTGACTCT
29231	Yes	ACCACACTGCCTAGGTTCAAATCCTGACTCTG
29232	Yes	ACCACACTGCCTAGGTTCAAATCCTGACTCTGT
29233	Yes	ACCACACTGCCTAGGTTCAAATCCTGACTCTGTC
29234	Yes	ACCACACTGCCTAGGTTCAAATCCTGACTCTGTCA
29235	Yes	ACCACACTGCCTAGGTTCAAATCCTGACTCTGTCAC
29236	Yes	ACCACACTGCCTAGGTTCAAATCCTGACTCTGTCACC
29237	Yes	ACCACACTGCCTAGGTTCAAATCCTGACTCTGTCACCA
29238	Yes	ACCACCATGGCCCGGA
29239	Yes	ACCACCATGGCCCGGAT
29240	Yes	ACCACCATGGCCCGGATT
29241	Yes	ACCACCATGGCCCGGATTT
29242	Yes	ACCACCATGGCCCGGATTTG
29243	Yes	ACCACCATGGCCCGGATTTGA
29244	Yes	ACCACCATGGCCCGGATTTGAT
29245	Yes	ACCACCATGGCCCGGATTTGATT
29246	Yes	ACCACCATGGCCCGGATTTGATTC
29247	Yes	ACCACCATGGCCCGGATTTGATTCC
29248	Yes	ACCACCATGGCCCGGATTTGATTCCC
29249	Yes	ACCACCATGGCCCGGATTTGATTCCCG
29250	Yes	ACCACCATGGCCCGGATTTGATTCCCGG
29251	Yes	ACCACCATGGCCCGGATTTGATTCCCGGT
29252	Yes	ACCACCATGGCCCGGATTTGATTCCCGGTC
29253	Yes	ACCACCATGGCCCGGATTTGATTCCCGGTCA
29254	Yes	ACCACCATGGCCCGGATTTGATTCCCGGTCAG
29255	Yes	ACCACCATGGCCCGGATTTGATTCCCGGTCAGG
29256	Yes	ACCACCATGGCCCGGATTTGATTCCCGGTCAGGG
29257	Yes	ACCACCATGGCCCGGATTTGATTCCCGGTCAGGGA
29258	Yes	ACCACCATGGCCCGGATTTGATTCCCGGTCAGGGAA
29259	Yes	ACCACCATGGCCCGGATTTGATTCCCGGTCAGGGAAC
29260	Yes	ACCACCATGGCCCGGATTTGATTCCCGGTCAGGGAACC
29261	Yes	ACCACCATGGCCCGGATTTGATTCCCGGTCAGGGAACCA
29262	No	ACCACCCAGGGACGCC
29263	No	ACCACCCAGGGACGCCA
29264	No	ACCACGTGGCCTAATG
29265	Yes	ACCACGTGGCCTAATGG
29266	Yes	ACCACGTGGCCTAATGGA
29267	Yes	ACCACGTGGCCTAATGGAT

29268	Yes	ACCACGTGGCCTAATGGATA
29269	Yes	ACCACGTGGCCTAATGGATAA
29270	Yes	ACCACGTGGCCTAATGGATAAG
29271	Yes	ACCACGTGGCCTAATGGATAAGG
29272	Yes	ACCACGTGGCCTAATGGATAAGGC
29273	Yes	ACCACGTGGCCTAATGGATAAGGCG
29274	Yes	ACCACGTGGCCTAATGGATAAGGCGT
29275	Yes	ACCACGTGGCCTAATGGATAAGGCGTC
29276	Yes	ACCACGTGGCCTAATGGATAAGGCGTCT
29277	Yes	ACCACGTGGCCTAATGGATAAGGCGTCTG
29278	Yes	ACCACGTGGCCTAATGGATAAGGCGTCTGA
29279	Yes	ACCACGTGGCCTAATGGATAAGGCGTCTGAC
29280	Yes	ACCACGTGGCCTAATGGATAAGGCGTCTGACT
29281	Yes	ACCACGTGGCCTAATGGATAAGGCGTCTGACTT
29282	Yes	ACCACGTGGCCTAATGGATAAGGCGTCTGACTTC
29283	Yes	ACCACGTGGCCTAATGGATAAGGCGTCTGACTTCG
29284	Yes	ACCACGTGGCCTAATGGATAAGGCGTCTGACTTCGG
29285	Yes	ACCACGTGGCCTAATGGATAAGGCGTCTGACTTCGGA
29286	Yes	ACCACGTGGCCTAATGGATAAGGCGTCTGACTTCGGAT
29287	Yes	ACCACGTGGCCTAATGGATAAGGCGTCTGACTTCGGATC
29288	Yes	ACCACGTGGCCTAATGGATAAGGCGTCTGACTTCGGATCA
29289	Yes	ACCACGTGGCCTAATGGATAAGGCGTCTGACTTCGGATCAG
29290	Yes	ACCACGTGGCCTAATGGATAAGGCGTCTGACTTCGGATCAGA
29291	Yes	ACCACGTGGCCTAATGGATAAGGCGTCTGACTTCGGATCAGAA
29292	Yes	ACCACGTGGCCTAATGGATAAGGCGTCTGACTTCGGATCAGAAG
29293	Yes	ACCACGTGGCCTAATGGATAAGGCGTCTGACTTCGGATCAGAAGA
29294	Yes	ACCACGTGGCCTAATGGATAAGGCGTCTGACTTCGGATCAGAAGAT
29295	Yes	ACCACGTGGCCTAATGGATAAGGCGTCTGACTTCGGATCAGAAGATT
29296	Yes	ACCACGTGGCCTAATGGATAAGGCGTCTGACTTCGGATCAGAAGATTG
29297	Yes	ACCACGTGGCCTAATGGATAAGGCGTCTGACTTCGGATCAGAAGATTGA
29298	Yes	ACCACGTGGCCTAATGGATAAGGCGTCTGACTTCGGATCAGAAGATTGAG
29299	Yes	ACCACGTGGTCTGGGT
29300	Yes	ACCACGTGGTCTGGGTT
29301	Yes	ACCACGTGGTCTGGGTTT
29302	Yes	ACCACGTGGTCTGGGTTTA
29303	Yes	ACCACGTGGTCTGGGTTTAA
29304	Yes	ACCACGTGGTCTGGGTTTAAG
29305	Yes	ACCACGTGGTCTGGGTTTAAGT
29306	Yes	ACCACGTGGTCTGGGTTTAAGTC
29307	Yes	ACCACGTGGTCTGGGTTTAAGTCT
29308	Yes	ACCACGTGGTCTGGGTTTAAGTCTC
29309	Yes	ACCACGTGGTCTGGGTTTAAGTCTCA

29310	Yes	ACCACGTGGTCTGGGTTTAAGTCTCAG
29311	Yes	ACCACGTGGTCTGGGTTTAAGTCTCAGC
29312	Yes	ACCACGTGGTCTGGGTTTAAGTCTCAGCT
29313	Yes	ACCACGTGGTCTGGGTTTAAGTCTCAGCTG
29314	Yes	ACCACGTGGTCTGGGTTTAAGTCTCAGCTGT
29315	Yes	ACCACGTGGTCTGGGTTTAAGTCTCAGCTGTG
29316	Yes	ACCACGTGGTCTGGGTTTAAGTCTCAGCTGTGC
29317	Yes	ACCACGTGGTCTGGGTTTAAGTCTCAGCTGTGCT
29318	Yes	ACCACGTGGTCTGGGTTTAAGTCTCAGCTGTGCTA
29319	Yes	ACCACGTGGTCTGGGTTTAAGTCTCAGCTGTGCTAC
29320	Yes	ACCACGTGGTCTGGGTTTAAGTCTCAGCTGTGCTACC
29321	Yes	ACCACGTGGTCTGGGTTTAAGTCTCAGCTGTGCTACCA
29322	No	ACCACTGCTACCCATG
29323	Yes	ACCACTGCTACCCATGC
29324	Yes	ACCACTGCTACCCATGCT
29325	Yes	ACCACTGCTACCCATGCTC
29326	Yes	ACCACTGCTACCCATGCTCG
29327	Yes	ACCACTGCTACCCATGCTCGA
29328	Yes	ACCACTGCTACCCATGCTCGAT
29329	Yes	ACCACTGCTACCCATGCTCGATT
29330	Yes	ACCACTGCTACCCATGCTCGATTC
29331	Yes	ACCACTGCTACCCATGCTCGATTCC
29332	Yes	ACCACTGCTACCCATGCTCGATTCCT
29333	Yes	ACCACTGCTACCCATGCTCGATTCCTG
29334	Yes	ACCACTGCTACCCATGCTCGATTCCTGG
29335	Yes	ACCACTGCTACCCATGCTCGATTCCTGGT
29336	Yes	ACCACTGCTACCCATGCTCGATTCCTGGTC
29337	Yes	ACCACTGCTACCCATGCTCGATTCCTGGTCA
29338	Yes	ACCACTGCTACCCATGCTCGATTCCTGGTCAG
29339	Yes	ACCACTGCTACCCATGCTCGATTCCTGGTCAGG
29340	Yes	ACCACTGCTACCCATGCTCGATTCCTGGTCAGGG
29341	Yes	ACCACTGCTACCCATGCTCGATTCCTGGTCAGGGA
29342	Yes	ACCACTGCTACCCATGCTCGATTCCTGGTCAGGGAA
29343	Yes	ACCACTGCTACCCATGCTCGATTCCTGGTCAGGGAAC
29344	Yes	ACCACTGCTACCCATGCTCGATTCCTGGTCAGGGAACC
29345	Yes	ACCACTGCTACCCATGCTCGATTCCTGGTCAGGGAACCA
29346	No	ACCACTTCAGCCCAGG
29347	No	ACCACTTCAGCCCAGGT
29348	Yes	ACCACTTCAGCCCAGGTT
29349	Yes	ACCACTTCAGCCCAGGTTC
29350	Yes	ACCACTTCAGCCCAGGTTCG
29351	Yes	ACCACTTCAGCCCAGGTTCGA

29352	Yes	ACCACTTCAGCCCAGGTTCGAT
29353	Yes	ACCACTTCAGCCCAGGTTCGATT
29354	Yes	ACCACTTCAGCCCAGGTTCGATTC
29355	Yes	ACCACTTCAGCCCAGGTTCGATTCC
29356	Yes	ACCACTTCAGCCCAGGTTCGATTCCT
29357	Yes	ACCACTTCAGCCCAGGTTCGATTCCTG
29358	Yes	ACCACTTCAGCCCAGGTTCGATTCCTGG
29359	Yes	ACCACTTCAGCCCAGGTTCGATTCCTGGT
29360	Yes	ACCACTTCAGCCCAGGTTCGATTCCTGGTC
29361	Yes	ACCACTTCAGCCCAGGTTCGATTCCTGGTCA
29362	Yes	ACCACTTCAGCCCAGGTTCGATTCCTGGTCAG
29363	Yes	ACCACTTCAGCCCAGGTTCGATTCCTGGTCAGG
29364	Yes	ACCACTTCAGCCCAGGTTCGATTCCTGGTCAGGG
29365	Yes	ACCACTTCAGCCCAGGTTCGATTCCTGGTCAGGGA
29366	Yes	ACCACTTCAGCCCAGGTTCGATTCCTGGTCAGGGAA
29367	Yes	ACCACTTCAGCCCAGGTTCGATTCCTGGTCAGGGAAC
29368	Yes	ACCACTTCAGCCCAGGTTCGATTCCTGGTCAGGGAACC
29369	Yes	ACCACTTCAGCCCAGGTTCGATTCCTGGTCAGGGAACCA
29370	No	ACCACTTTGGACTCTG
29371	Yes	ACCACTTTGGACTCTGA
29372	Yes	ACCACTTTGGACTCTGAA
29373	Yes	ACCACTTTGGACTCTGAAT
29374	Yes	ACCACTTTGGACTCTGAATA
29375	Yes	ACCACTTTGGACTCTGAATAC
29376	Yes	ACCACTTTGGACTCTGAATACA
29377	Yes	ACCACTTTGGACTCTGAATACAG
29378	Yes	ACCACTTTGGACTCTGAATACAGT
29379	Yes	ACCACTTTGGACTCTGAATACAGTG
29380	Yes	ACCACTTTGGACTCTGAATACAGTGA
29381	Yes	ACCACTTTGGACTCTGAATACAGTGAT
29382	Yes	ACCACTTTGGACTCTGAATACAGTGATC
29383	Yes	ACCACTTTGGACTCTGAATACAGTGATCA
29384	Yes	ACCACTTTGGACTCTGAATACAGTGATCAG
29385	Yes	ACCACTTTGGACTCTGAATACAGTGATCAGA
29386	Yes	ACCACTTTGGACTCTGAATACAGTGATCAGAG
29387	Yes	ACCACTTTGGACTCTGAATACAGTGATCAGAGT
29388	Yes	ACCACTTTGGACTCTGAATACAGTGATCAGAGTT
29389	Yes	ACCACTTTGGACTCTGAATACAGTGATCAGAGTTC
29390	Yes	ACCACTTTGGACTCTGAATACAGTGATCAGAGTTCA
29391	Yes	ACCACTTTGGACTCTGAATACAGTGATCAGAGTTCAA
29392	Yes	ACCACTTTGGACTCTGAATACAGTGATCAGAGTTCAAG
29393	Yes	ACCACTTTGGACTCTGAATACAGTGATCAGAGTTCAAGT

29394	Yes	ACCACTTTGGACTCTGAATACAGTGATCAGAGTTCAAGTC
29395	Yes	ACCACTTTGGACTCTGAATACAGTGATCAGAGTTCAAGTCT
29396	Yes	ACCACTTTGGACTCTGAATACAGTGATCAGAGTTCAAGTCTC
29397	Yes	ACCACTTTGGACTCTGAATACAGTGATCAGAGTTCAAGTCTCA
29398	Yes	ACCACTTTGGACTCTGAATACAGTGATCAGAGTTCAAGTCTCAC
29399	Yes	ACCACTTTGGACTCTGAATACAGTGATCAGAGTTCAAGTCTCACT
29400	Yes	ACCACTTTGGACTCTGAATACAGTGATCAGAGTTCAAGTCTCACTG
29401	Yes	ACCACTTTGGACTCTGAATACAGTGATCAGAGTTCAAGTCTCACTGG
29402	Yes	ACCACTTTGGACTCTGAATACAGTGATCAGAGTTCAAGTCTCACTGGA
29403	Yes	ACCACTTTGGACTCTGAATACAGTGATCAGAGTTCAAGTCTCACTGGAA
29404	Yes	ACCACTTTGGACTCTGAATACAGTGATCAGAGTTCAAGTCTCACTGGAAC
29405	No	ACCAGAATGGCCGAGT
29406	Yes	ACCAGAATGGCCGAGTG
29407	Yes	ACCAGAATGGCCGAGTGG
29408	Yes	ACCAGAATGGCCGAGTGGT
29409	Yes	ACCAGAATGGCCGAGTGGTT
29410	Yes	ACCAGAATGGCCGAGTGGTTA
29411	Yes	ACCAGAATGGCCGAGTGGTTAA
29412	Yes	ACCAGAATGGCCGAGTGGTTAAG
29413	Yes	ACCAGAATGGCCGAGTGGTTAAGG
29414	Yes	ACCAGAATGGCCGAGTGGTTAAGGC
29415	Yes	ACCAGAATGGCCGAGTGGTTAAGGCG
29416	Yes	ACCAGAATGGCCGAGTGGTTAAGGCGT
29417	Yes	ACCAGAATGGCCGAGTGGTTAAGGCGTT
29418	Yes	ACCAGAATGGCCGAGTGGTTAAGGCGTTG
29419	Yes	ACCAGAATGGCCGAGTGGTTAAGGCGTTGG
29420	Yes	ACCAGAATGGCCGAGTGGTTAAGGCGTTGGA
29421	Yes	ACCAGAATGGCCGAGTGGTTAAGGCGTTGGAC
29422	Yes	ACCAGAATGGCCGAGTGGTTAAGGCGTTGGACT
29423	Yes	ACCAGAATGGCCGAGTGGTTAAGGCGTTGGACTT
29424	Yes	ACCAGAATGGCCGAGTGGTTAAGGCGTTGGACTTA
29425	Yes	ACCAGAATGGCCGAGTGGTTAAGGCGTTGGACTTAA
29426	Yes	ACCAGAATGGCCGAGTGGTTAAGGCGTTGGACTTAAG
29427	Yes	ACCAGAATGGCCGAGTGGTTAAGGCGTTGGACTTAAGA
29428	Yes	ACCAGAATGGCCGAGTGGTTAAGGCGTTGGACTTAAGAT
29429	Yes	ACCAGAATGGCCGAGTGGTTAAGGCGTTGGACTTAAGATC
29430	Yes	ACCAGAATGGCCGAGTGGTTAAGGCGTTGGACTTAAGATCC
29431	Yes	ACCAGAATGGCCGAGTGGTTAAGGCGTTGGACTTAAGATCCA
29432	Yes	ACCAGAATGGCCGAGTGGTTAAGGCGTTGGACTTAAGATCCAA
29433	Yes	ACCAGAATGGCCGAGTGGTTAAGGCGTTGGACTTAAGATCCAAT
29434	Yes	ACCAGAATGGCCGAGTGGTTAAGGCGTTGGACTTAAGATCCAATG
29435	Yes	ACCAGAATGGCCGAGTGGTTAAGGCGTTGGACTTAAGATCCAATGG

29436	Yes	ACCAGAATGGCCGAGTGGTTAAGGCGTTGGACTTAAGATCCAATGGA
29437	Yes	ACCAGAATGGCCGAGTGGTTAAGGCGTTGGACTTAAGATCCAATGGAT
29438	Yes	ACCAGAATGGCCGAGTGGTTAAGGCGTTGGACTTAAGATCCAATGGATT
29439	Yes	ACCAGAATGGCCGAGTGGTTAAGGCGTTGGACTTAAGATCCAATGGATTC
29440	No	ACCAGAGTTCCAGTCT
29441	No	ACCAGAGTTCCAGTCTC
29442	Yes	ACCAGAGTTCCAGTCTCA
29443	Yes	ACCAGAGTTCCAGTCTCAG
29444	Yes	ACCAGAGTTCCAGTCTCAGC
29445	Yes	ACCAGAGTTCCAGTCTCAGCG
29446	Yes	ACCAGAGTTCCAGTCTCAGCGT
29447	Yes	ACCAGAGTTCCAGTCTCAGCGTG
29448	Yes	ACCAGAGTTCCAGTCTCAGCGTGG
29449	Yes	ACCAGAGTTCCAGTCTCAGCGTGGA
29450	Yes	ACCAGAGTTCCAGTCTCAGCGTGGAC
29451	Yes	ACCAGAGTTCCAGTCTCAGCGTGGACC
29452	Yes	ACCAGAGTTCCAGTCTCAGCGTGGACCT
29453	Yes	ACCAGAGTTCCAGTCTCAGCGTGGACCTC
29454	Yes	ACCAGAGTTCCAGTCTCAGCGTGGACCTCC
29455	Yes	ACCAGAGTTCCAGTCTCAGCGTGGACCTCCA
29456	No	ACCAGATGGAAATGCC
29457	No	ACCAGATGGAAATGCCA
29458	No	ACCAGCATGTCTCAGT
29459	Yes	ACCAGCATGTCTCAGTC
29460	Yes	ACCAGCATGTCTCAGTCG
29461	Yes	ACCAGCATGTCTCAGTCGG
29462	Yes	ACCAGCATGTCTCAGTCGGT
29463	Yes	ACCAGCATGTCTCAGTCGGTA
29464	Yes	ACCAGCATGTCTCAGTCGGTAT
29465	Yes	ACCAGCATGTCTCAGTCGGTATA
29466	Yes	ACCAGCATGTCTCAGTCGGTATAG
29467	Yes	ACCAGCATGTCTCAGTCGGTATAGT
29468	Yes	ACCAGCATGTCTCAGTCGGTATAGTG
29469	Yes	ACCAGCATGTCTCAGTCGGTATAGTGT
29470	Yes	ACCAGCATGTCTCAGTCGGTATAGTGTG
29471	Yes	ACCAGCATGTCTCAGTCGGTATAGTGTGA
29472	Yes	ACCAGCATGTCTCAGTCGGTATAGTGTGAG
29473	Yes	ACCAGCATGTCTCAGTCGGTATAGTGTGAGA
29474	Yes	ACCAGCATGTCTCAGTCGGTATAGTGTGAGAC
29475	Yes	ACCAGCATGTCTCAGTCGGTATAGTGTGAGACT
29476	Yes	ACCAGCATGTCTCAGTCGGTATAGTGTGAGACTC
29477	Yes	ACCAGCATGTCTCAGTCGGTATAGTGTGAGACTCT

29478	Yes	ACCAGCATGTCTCAGTCGGTATAGTGTGAGACTCTT
29479	Yes	ACCAGCATGTCTCAGTCGGTATAGTGTGAGACTCTTA
29480	Yes	ACCAGCATGTCTCAGTCGGTATAGTGTGAGACTCTTAA
29481	Yes	ACCAGCATGTCTCAGTCGGTATAGTGTGAGACTCTTAAT
29482	Yes	ACCAGCATGTCTCAGTCGGTATAGTGTGAGACTCTTAATC
29483	Yes	ACCAGCATGTCTCAGTCGGTATAGTGTGAGACTCTTAATCT
29484	Yes	ACCAGCATGTCTCAGTCGGTATAGTGTGAGACTCTTAATCTC
29485	Yes	ACCAGCATGTCTCAGTCGGTATAGTGTGAGACTCTTAATCTCA
29486	Yes	ACCAGCATGTCTCAGTCGGTATAGTGTGAGACTCTTAATCTCAG
29487	Yes	ACCAGCATGTCTCAGTCGGTATAGTGTGAGACTCTTAATCTCAGG
29488	Yes	ACCAGCATGTCTCAGTCGGTATAGTGTGAGACTCTTAATCTCAGGG
29489	Yes	ACCAGCATGTCTCAGTCGGTATAGTGTGAGACTCTTAATCTCAGGGT
29490	Yes	ACCAGCATGTCTCAGTCGGTATAGTGTGAGACTCTTAATCTCAGGGTC
29491	Yes	ACCAGCATGTCTCAGTCGGTATAGTGTGAGACTCTTAATCTCAGGGTCG
29492	Yes	ACCAGCATGTCTCAGTCGGTATAGTGTGAGACTCTTAATCTCAGGGTCGT
29493	No	ACCAGCCTTAGGAGGT
29494	No	ACCAGCCTTAGGAGGTT
29495	Yes	ACCAGCCTTAGGAGGTTC
29496	Yes	ACCAGCCTTAGGAGGTTCA
29497	Yes	ACCAGCCTTAGGAGGTTCAA
29498	Yes	ACCAGCCTTAGGAGGTTCAAT
29499	Yes	ACCAGCCTTAGGAGGTTCAATT
29500	Yes	ACCAGCCTTAGGAGGTTCAATTC
29501	Yes	ACCAGCCTTAGGAGGTTCAATTCC
29502	Yes	ACCAGCCTTAGGAGGTTCAATTCCT
29503	Yes	ACCAGCCTTAGGAGGTTCAATTCCTT
29504	Yes	ACCAGCCTTAGGAGGTTCAATTCCTTC
29505	Yes	ACCAGCCTTAGGAGGTTCAATTCCTTCC
29506	Yes	ACCAGCCTTAGGAGGTTCAATTCCTTCCT
29507	Yes	ACCAGCCTTAGGAGGTTCAATTCCTTCCTT
29508	Yes	ACCAGCCTTAGGAGGTTCAATTCCTTCCTTT
29509	Yes	ACCAGCCTTAGGAGGTTCAATTCCTTCCTTTT
29510	Yes	ACCAGCCTTAGGAGGTTCAATTCCTTCCTTTTT
29511	Yes	ACCAGCCTTAGGAGGTTCAATTCCTTCCTTTTTT
29512	Yes	ACCAGCCTTAGGAGGTTCAATTCCTTCCTTTTTTG
29513	Yes	ACCAGCCTTAGGAGGTTCAATTCCTTCCTTTTTTGC
29514	Yes	ACCAGCCTTAGGAGGTTCAATTCCTTCCTTTTTTGCC
29515	Yes	ACCAGCCTTAGGAGGTTCAATTCCTTCCTTTTTTGCCA
29516	No	ACCAGCTTTGGGGGGT
29517	No	ACCAGCTTTGGGGGGTT
29518	No	ACCAGCTTTGGGGGGTTC
29519	Yes	ACCAGCTTTGGGGGGTTCG

29520	Yes	ACCAGCTTTGGGGGGTTCGA
29521	Yes	ACCAGCTTTGGGGGGTTCGAT
29522	Yes	ACCAGCTTTGGGGGGTTCGATT
29523	Yes	ACCAGCTTTGGGGGGTTCGATTC
29524	Yes	ACCAGCTTTGGGGGGTTCGATTCC
29525	Yes	ACCAGCTTTGGGGGGTTCGATTCCT
29526	Yes	ACCAGCTTTGGGGGGTTCGATTCCTT
29527	Yes	ACCAGCTTTGGGGGGTTCGATTCCTTC
29528	Yes	ACCAGCTTTGGGGGGTTCGATTCCTTCC
29529	Yes	ACCAGCTTTGGGGGGTTCGATTCCTTCCT
29530	Yes	ACCAGCTTTGGGGGGTTCGATTCCTTCCTT
29531	Yes	ACCAGCTTTGGGGGGTTCGATTCCTTCCTTT
29532	Yes	ACCAGCTTTGGGGGGTTCGATTCCTTCCTTTT
29533	Yes	ACCAGCTTTGGGGGGTTCGATTCCTTCCTTTTT
29534	Yes	ACCAGCTTTGGGGGGTTCGATTCCTTCCTTTTTT
29535	Yes	ACCAGCTTTGGGGGGTTCGATTCCTTCCTTTTTTG
29536	Yes	ACCAGCTTTGGGGGGTTCGATTCCTTCCTTTTTTGC
29537	Yes	ACCAGCTTTGGGGGGTTCGATTCCTTCCTTTTTTGCC
29538	Yes	ACCAGCTTTGGGGGGTTCGATTCCTTCCTTTTTTGCCA
29539	Yes	ACCAGGATGGCCGAGT
29540	Yes	ACCAGGATGGCCGAGTG
29541	Yes	ACCAGGATGGCCGAGTGG
29542	Yes	ACCAGGATGGCCGAGTGGT
29543	Yes	ACCAGGATGGCCGAGTGGTT
29544	Yes	ACCAGGATGGCCGAGTGGTTA
29545	Yes	ACCAGGATGGCCGAGTGGTTAA
29546	Yes	ACCAGGATGGCCGAGTGGTTAAG
29547	Yes	ACCAGGATGGCCGAGTGGTTAAGG
29548	Yes	ACCAGGATGGCCGAGTGGTTAAGGC
29549	Yes	ACCAGGATGGCCGAGTGGTTAAGGCG
29550	Yes	ACCAGGATGGCCGAGTGGTTAAGGCGT
29551	Yes	ACCAGGATGGCCGAGTGGTTAAGGCGTT
29552	Yes	ACCAGGATGGCCGAGTGGTTAAGGCGTTG
29553	Yes	ACCAGGATGGCCGAGTGGTTAAGGCGTTGG
29554	Yes	ACCAGGATGGCCGAGTGGTTAAGGCGTTGGA
29555	Yes	ACCAGGATGGCCGAGTGGTTAAGGCGTTGGAC
29556	Yes	ACCAGGATGGCCGAGTGGTTAAGGCGTTGGACT
29557	Yes	ACCAGGATGGCCGAGTGGTTAAGGCGTTGGACTT
29558	Yes	ACCAGGATGGCCGAGTGGTTAAGGCGTTGGACTTA
29559	Yes	ACCAGGATGGCCGAGTGGTTAAGGCGTTGGACTTAA
29560	Yes	ACCAGGATGGCCGAGTGGTTAAGGCGTTGGACTTAAG
29561	Yes	ACCAGGATGGCCGAGTGGTTAAGGCGTTGGACTTAAGA

29562	Yes	ACCAGGATGGCCGAGTGGTTAAGGCGTTGGACTTAAGAT
29563	Yes	ACCAGGATGGCCGAGTGGTTAAGGCGTTGGACTTAAGATC
29564	Yes	ACCAGGATGGCCGAGTGGTTAAGGCGTTGGACTTAAGATCC
29565	Yes	ACCAGGATGGCCGAGTGGTTAAGGCGTTGGACTTAAGATCCA
29566	Yes	ACCAGGATGGCCGAGTGGTTAAGGCGTTGGACTTAAGATCCAA
29567	Yes	ACCAGGATGGCCGAGTGGTTAAGGCGTTGGACTTAAGATCCAAT
29568	Yes	ACCAGGATGGCCGAGTGGTTAAGGCGTTGGACTTAAGATCCAATG
29569	Yes	ACCAGGATGGCCGAGTGGTTAAGGCGTTGGACTTAAGATCCAATGG
29570	Yes	ACCAGGATGGCCGAGTGGTTAAGGCGTTGGACTTAAGATCCAATGGA
29571	Yes	ACCAGGATGGCCGAGTGGTTAAGGCGTTGGACTTAAGATCCAATGGAC
29572	Yes	ACCAGGATGGCCGAGTGGTTAAGGCGTTGGACTTAAGATCCAATGGACA
29573	Yes	ACCAGGATGGCCGAGTGGTTAAGGCGTTGGACTTAAGATCCAATGGACAT
29574	No	ACCAGGCAGAAACACC
29575	Yes	ACCAGGCAGAAACACCA
29576	No	ACCAGGCGGAAACACC
29577	No	ACCAGGCGGAAACACCA
29578	Yes	ACCAGGGGTCGCGAGT
29579	Yes	ACCAGGGGTCGCGAGTT
29580	Yes	ACCAGGGGTCGCGAGTTC
29581	Yes	ACCAGGGGTCGCGAGTTCA
29582	Yes	ACCAGGGGTCGCGAGTTCAA
29583	Yes	ACCAGGGGTCGCGAGTTCAAA
29584	Yes	ACCAGGGGTCGCGAGTTCAAAT
29585	Yes	ACCAGGGGTCGCGAGTTCAAATC
29586	Yes	ACCAGGGGTCGCGAGTTCAAATCT
29587	Yes	ACCAGGGGTCGCGAGTTCAAATCTC
29588	Yes	ACCAGGGGTCGCGAGTTCAAATCTCG
29589	Yes	ACCAGGGGTCGCGAGTTCAAATCTCGC
29590	Yes	ACCAGGGGTCGCGAGTTCAAATCTCGCT
29591	Yes	ACCAGGGGTCGCGAGTTCAAATCTCGCTG
29592	Yes	ACCAGGGGTCGCGAGTTCAAATCTCGCTGG
29593	Yes	ACCAGGGGTCGCGAGTTCAAATCTCGCTGGG
29594	Yes	ACCAGGGGTCGCGAGTTCAAATCTCGCTGGGG
29595	Yes	ACCAGGGGTCGCGAGTTCAAATCTCGCTGGGGC
29596	Yes	ACCAGGGGTCGCGAGTTCAAATCTCGCTGGGGCC
29597	Yes	ACCAGGGGTCGCGAGTTCAAATCTCGCTGGGGCCT
29598	Yes	ACCAGGGGTCGCGAGTTCAAATCTCGCTGGGGCCTC
29599	Yes	ACCAGGGGTCGCGAGTTCAAATCTCGCTGGGGCCTCC
29600	Yes	ACCAGGGGTCGCGAGTTCAAATCTCGCTGGGGCCTCCA
29601	Yes	ACCAGGGGTCGCGAGTTCAAT
29602	Yes	ACCAGGGGTCGCGAGTTCAATT
29603	Yes	ACCAGGGGTCGCGAGTTCAATTC

29604	Yes	ACCAGGGGTCGCGAGTTCAATTCT
29605	Yes	ACCAGGGGTCGCGAGTTCAATTCTC
29606	Yes	ACCAGGGGTCGCGAGTTCAATTCTCG
29607	Yes	ACCAGGGGTCGCGAGTTCAATTCTCGC
29608	Yes	ACCAGGGGTCGCGAGTTCAATTCTCGCT
29609	Yes	ACCAGGGGTCGCGAGTTCAATTCTCGCTG
29610	Yes	ACCAGGGGTCGCGAGTTCAATTCTCGCTGG
29611	Yes	ACCAGGGGTCGCGAGTTCAATTCTCGCTGGG
29612	Yes	ACCAGGGGTCGCGAGTTCAATTCTCGCTGGGG
29613	Yes	ACCAGGGGTCGCGAGTTCAATTCTCGCTGGGGC
29614	Yes	ACCAGGGGTCGCGAGTTCAATTCTCGCTGGGGCC
29615	Yes	ACCAGGGGTCGCGAGTTCAATTCTCGCTGGGGCCT
29616	Yes	ACCAGGGGTCGCGAGTTCAATTCTCGCTGGGGCCTC
29617	Yes	ACCAGGGGTCGCGAGTTCAATTCTCGCTGGGGCCTCC
29618	Yes	ACCAGGGGTCGCGAGTTCAATTCTCGCTGGGGCCTCCA
29619	Yes	ACCAGGGGTCGCGAGTTCG
29620	Yes	ACCAGGGGTCGCGAGTTCGA
29621	Yes	ACCAGGGGTCGCGAGTTCGAT
29622	Yes	ACCAGGGGTCGCGAGTTCGATC
29623	Yes	ACCAGGGGTCGCGAGTTCGATCC
29624	Yes	ACCAGGGGTCGCGAGTTCGATCCT
29625	Yes	ACCAGGGGTCGCGAGTTCGATCCTC
29626	Yes	ACCAGGGGTCGCGAGTTCGATCCTCG
29627	Yes	ACCAGGGGTCGCGAGTTCGATCCTCGC
29628	Yes	ACCAGGGGTCGCGAGTTCGATCCTCGCT
29629	Yes	ACCAGGGGTCGCGAGTTCGATCCTCGCTG
29630	Yes	ACCAGGGGTCGCGAGTTCGATCCTCGCTGG
29631	Yes	ACCAGGGGTCGCGAGTTCGATCCTCGCTGGG
29632	Yes	ACCAGGGGTCGCGAGTTCGATCCTCGCTGGGG
29633	Yes	ACCAGGGGTCGCGAGTTCGATCCTCGCTGGGGC
29634	Yes	ACCAGGGGTCGCGAGTTCGATCCTCGCTGGGGCC
29635	Yes	ACCAGGGGTCGCGAGTTCGATCCTCGCTGGGGCCT
29636	Yes	ACCAGGGGTCGCGAGTTCGATCCTCGCTGGGGCCTC
29637	Yes	ACCAGGGGTCGCGAGTTCGATCCTCGCTGGGGCCTCC
29638	Yes	ACCAGGGGTCGCGAGTTCGATCCTCGCTGGGGCCTCCA
29639	Yes	ACCAGGGTCGCGAGTT
29640	Yes	ACCAGGGTCGCGAGTTC
29641	Yes	ACCAGGGTCGCGAGTTCA
29642	Yes	ACCAGGGTCGCGAGTTCAA
29643	Yes	ACCAGGGTCGCGAGTTCAAA
29644	Yes	ACCAGGGTCGCGAGTTCAAAT
29645	Yes	ACCAGGGTCGCGAGTTCAAATC

29646	Yes	ACCAGGGTCGCGAGTTCAAATCT
29647	Yes	ACCAGGGTCGCGAGTTCAAATCTC
29648	Yes	ACCAGGGTCGCGAGTTCAAATCTCG
29649	Yes	ACCAGGGTCGCGAGTTCAAATCTCGC
29650	Yes	ACCAGGGTCGCGAGTTCAAATCTCGCT
29651	Yes	ACCAGGGTCGCGAGTTCAAATCTCGCTG
29652	Yes	ACCAGGGTCGCGAGTTCAAATCTCGCTGG
29653	Yes	ACCAGGGTCGCGAGTTCAAATCTCGCTGGG
29654	Yes	ACCAGGGTCGCGAGTTCAAATCTCGCTGGGG
29655	Yes	ACCAGGGTCGCGAGTTCAAATCTCGCTGGGGC
29656	Yes	ACCAGGGTCGCGAGTTCAAATCTCGCTGGGGCC
29657	Yes	ACCAGGGTCGCGAGTTCAAATCTCGCTGGGGCCT
29658	Yes	ACCAGGGTCGCGAGTTCAAATCTCGCTGGGGCCTC
29659	Yes	ACCAGGGTCGCGAGTTCAAATCTCGCTGGGGCCTCC
29660	Yes	ACCAGGGTCGCGAGTTCAAATCTCGCTGGGGCCTCCA
29661	No	ACCAGGTGGAAACACC
29662	Yes	ACCAGGTGGAAACACCA
29663	No	ACCAGTCAGGGAACCA
29664	Yes	ACCAGTCTCTGGGGGG
29665	Yes	ACCAGTCTCTGGGGGGT
29666	Yes	ACCAGTCTCTGGGGGGTT
29667	Yes	ACCAGTCTCTGGGGGGTTC
29668	Yes	ACCAGTCTCTGGGGGGTTCG
29669	Yes	ACCAGTCTCTGGGGGGTTCGA
29670	Yes	ACCAGTCTCTGGGGGGTTCGAT
29671	Yes	ACCAGTCTCTGGGGGGTTCGATT
29672	Yes	ACCAGTCTCTGGGGGGTTCGATTC
29673	Yes	ACCAGTCTCTGGGGGGTTCGATTCC
29674	Yes	ACCAGTCTCTGGGGGGTTCGATTCCC
29675	Yes	ACCAGTCTCTGGGGGGTTCGATTCCCT
29676	Yes	ACCAGTCTCTGGGGGGTTCGATTCCCTC
29677	Yes	ACCAGTCTCTGGGGGGTTCGATTCCCTCC
29678	Yes	ACCAGTCTCTGGGGGGTTCGATTCCCTCCT
29679	Yes	ACCAGTCTCTGGGGGGTTCGATTCCCTCCTT
29680	Yes	ACCAGTCTCTGGGGGGTTCGATTCCCTCCTTT
29681	Yes	ACCAGTCTCTGGGGGGTTCGATTCCCTCCTTTT
29682	Yes	ACCAGTCTCTGGGGGGTTCGATTCCCTCCTTTTT
29683	Yes	ACCAGTCTCTGGGGGGTTCGATTCCCTCCTTTTTC
29684	Yes	ACCAGTCTCTGGGGGGTTCGATTCCCTCCTTTTTCA
29685	Yes	ACCAGTCTCTGGGGGGTTCGATTCCCTCCTTTTTCAC
29686	Yes	ACCAGTCTCTGGGGGGTTCGATTCCCTCCTTTTTCACC
29687	Yes	ACCAGTCTCTGGGGGGTTCGATTCCCTCCTTTTTCACCA

29688	No	ACCAGTCTGTGGGGGG
29689	Yes	ACCAGTCTGTGGGGGGT
29690	Yes	ACCAGTCTGTGGGGGGTT
29691	Yes	ACCAGTCTGTGGGGGGTTC
29692	Yes	ACCAGTCTGTGGGGGGTTCG
29693	Yes	ACCAGTCTGTGGGGGGTTCGA
29694	Yes	ACCAGTCTGTGGGGGGTTCGAT
29695	Yes	ACCAGTCTGTGGGGGGTTCGATT
29696	Yes	ACCAGTCTGTGGGGGGTTCGATTC
29697	Yes	ACCAGTCTGTGGGGGGTTCGATTCC
29698	Yes	ACCAGTCTGTGGGGGGTTCGATTCCC
29699	Yes	ACCAGTCTGTGGGGGGTTCGATTCCCT
29700	Yes	ACCAGTCTGTGGGGGGTTCGATTCCCTC
29701	Yes	ACCAGTCTGTGGGGGGTTCGATTCCCTCC
29702	Yes	ACCAGTCTGTGGGGGGTTCGATTCCCTCCT
29703	Yes	ACCAGTCTGTGGGGGGTTCGATTCCCTCCTT
29704	Yes	ACCAGTCTGTGGGGGGTTCGATTCCCTCCTTT
29705	Yes	ACCAGTCTGTGGGGGGTTCGATTCCCTCCTTTT
29706	Yes	ACCAGTCTGTGGGGGGTTCGATTCCCTCCTTTTT
29707	Yes	ACCAGTCTGTGGGGGGTTCGATTCCCTCCTTTTTC
29708	Yes	ACCAGTCTGTGGGGGGTTCGATTCCCTCCTTTTTCA
29709	Yes	ACCAGTCTGTGGGGGGTTCGATTCCCTCCTTTTTCAC
29710	Yes	ACCAGTCTGTGGGGGGTTCGATTCCCTCCTTTTTCACC
29711	Yes	ACCAGTCTGTGGGGGGTTCGATTCCCTCCTTTTTCACCA
29712	No	ACCAGTCTTGTAAACC
29713	Yes	ACCAGTCTTGTAAACCG
29714	Yes	ACCAGTCTTGTAAACCGG
29715	Yes	ACCAGTCTTGTAAACCGGA
29716	Yes	ACCAGTCTTGTAAACCGGAG
29717	Yes	ACCAGTCTTGTAAACCGGAGA
29718	Yes	ACCAGTCTTGTAAACCGGAGAT
29719	Yes	ACCAGTCTTGTAAACCGGAGATG
29720	Yes	ACCAGTCTTGTAAACCGGAGATGA
29721	Yes	ACCAGTCTTGTAAACCGGAGATGAA
29722	Yes	ACCAGTCTTGTAAACCGGAGATGAAA
29723	Yes	ACCAGTCTTGTAAACCGGAGATGAAAA
29724	Yes	ACCAGTCTTGTAAACCGGAGATGAAAAC
29725	Yes	ACCAGTCTTGTAAACCGGAGATGAAAACC
29726	Yes	ACCAGTCTTGTAAACCGGAGATGAAAACCT
29727	Yes	ACCAGTCTTGTAAACCGGAGATGAAAACCTT
29728	Yes	ACCAGTCTTGTAAACCGGAGATGAAAACCTTT
29729	Yes	ACCAGTCTTGTAAACCGGAGATGAAAACCTTTT

29730	Yes	ACCAGTCTTGTAAACCGGAGATGAAAACCTTTTT
29731	Yes	ACCAGTCTTGTAAACCGGAGATGAAAACCTTTTTC
29732	Yes	ACCAGTCTTGTAAACCGGAGATGAAAACCTTTTTCC
29733	Yes	ACCAGTCTTGTAAACCGGAGATGAAAACCTTTTTCCA
29734	Yes	ACCAGTCTTGTAAACCGGAGATGAAAACCTTTTTCCAA
29735	Yes	ACCAGTCTTGTAAACCGGAGATGAAAACCTTTTTCCAAG
29736	Yes	ACCAGTCTTGTAAACCGGAGATGAAAACCTTTTTCCAAGG
29737	Yes	ACCAGTCTTGTAAACCGGAGATGAAAACCTTTTTCCAAGGA
29738	Yes	ACCAGTCTTGTAAACCGGAGATGAAAACCTTTTTCCAAGGAC
29739	Yes	ACCAGTCTTGTAAACCGGAGATGAAAACCTTTTTCCAAGGACA
29740	Yes	ACCAGTCTTGTAAACCGGAGATGAAAACCTTTTTCCAAGGACAC
29741	Yes	ACCAGTCTTGTAAACCGGAGATGAAAACCTTTTTCCAAGGACACC
29742	Yes	ACCAGTCTTGTAAACCGGAGATGAAAACCTTTTTCCAAGGACACCA
29743	Yes	ACCAGTTTGAGACCGG
29744	Yes	ACCAGTTTGAGACCGGG
29745	Yes	ACCAGTTTGAGACCGGGC
29746	Yes	ACCAGTTTGAGACCGGGCC
29747	Yes	ACCAGTTTGAGACCGGGCCA
29748	Yes	ACCAGTTTGAGACCGGGCCAA
29749	Yes	ACCAGTTTGAGACCGGGCCAAA
29750	Yes	ACCAGTTTGAGACCGGGCCAAAA
29751	Yes	ACCAGTTTGAGACCGGGCCAAAAC
29752	Yes	ACCAGTTTGAGACCGGGCCAAAACA
29753	Yes	ACCAGTTTGAGACCGGGCCAAAACAC
29754	Yes	ACCAGTTTGAGACCGGGCCAAAACACC
29755	Yes	ACCAGTTTGAGACCGGGCCAAAACACCA
29756	Yes	ACCATAAGGTTGGTGG
29757	Yes	ACCATAAGGTTGGTGGT
29758	Yes	ACCATAAGGTTGGTGGTT
29759	Yes	ACCATAAGGTTGGTGGTTA
29760	Yes	ACCATAAGGTTGGTGGTTAC
29761	Yes	ACCATAAGGTTGGTGGTTACA
29762	Yes	ACCATAAGGTTGGTGGTTACAG
29763	Yes	ACCATAAGGTTGGTGGTTACAGA
29764	Yes	ACCATAAGGTTGGTGGTTACAGAC
29765	Yes	ACCATAAGGTTGGTGGTTACAGACC
29766	Yes	ACCATAAGGTTGGTGGTTACAGACCA
29767	Yes	ACCATAAGGTTGGTGGTTACAGACCAC
29768	Yes	ACCATAAGGTTGGTGGTTACAGACCACC
29769	Yes	ACCATAAGGTTGGTGGTTACAGACCACCC
29770	Yes	ACCATAAGGTTGGTGGTTACAGACCACCCA
29771	Yes	ACCATAAGGTTGGTGGTTACAGACCACCCAG

29772	Yes	ACCATAAGGTTGGTGGTTACAGACCACCCAGG
29773	Yes	ACCATAAGGTTGGTGGTTACAGACCACCCAGGG
29774	Yes	ACCATAAGGTTGGTGGTTACAGACCACCCAGGGA
29775	Yes	ACCATAAGGTTGGTGGTTACAGACCACCCAGGGAC
29776	Yes	ACCATAAGGTTGGTGGTTACAGACCACCCAGGGACG
29777	Yes	ACCATAAGGTTGGTGGTTACAGACCACCCAGGGACGC
29778	Yes	ACCATAAGGTTGGTGGTTACAGACCACCCAGGGACGCC
29779	Yes	ACCATAAGGTTGGTGGTTACAGACCACCCAGGGACGCCA
29780	Yes	ACCATAAGGTTGGTGGTTAG
29781	Yes	ACCATAAGGTTGGTGGTTAGA
29782	Yes	ACCATAAGGTTGGTGGTTAGAG
29783	Yes	ACCATAAGGTTGGTGGTTAGAGA
29784	Yes	ACCATAAGGTTGGTGGTTAGAGAC
29785	Yes	ACCATAAGGTTGGTGGTTAGAGACC
29786	Yes	ACCATAAGGTTGGTGGTTAGAGACCA
29787	Yes	ACCATAAGGTTGGTGGTTAGAGACCAC
29788	Yes	ACCATAAGGTTGGTGGTTAGAGACCACC
29789	Yes	ACCATAAGGTTGGTGGTTAGAGACCACCC
29790	Yes	ACCATAAGGTTGGTGGTTAGAGACCACCCA
29791	Yes	ACCATAAGGTTGGTGGTTAGAGACCACCCAG
29792	Yes	ACCATAAGGTTGGTGGTTAGAGACCACCCAGG
29793	Yes	ACCATAAGGTTGGTGGTTAGAGACCACCCAGGG
29794	Yes	ACCATAAGGTTGGTGGTTAGAGACCACCCAGGGA
29795	Yes	ACCATAAGGTTGGTGGTTAGAGACCACCCAGGGAC
29796	Yes	ACCATAAGGTTGGTGGTTAGAGACCACCCAGGGACG
29797	Yes	ACCATAAGGTTGGTGGTTAGAGACCACCCAGGGACGC
29798	Yes	ACCATAAGGTTGGTGGTTAGAGACCACCCAGGGACGCC
29799	Yes	ACCATAAGGTTGGTGGTTAGAGACCACCCAGGGACGCCA
29800	No	ACCATCCTCTGCTACC
29801	No	ACCATCCTCTGCTACCA
29802	No	ACCATGACCTGGGTTC
29803	No	ACCATGACCTGGGTTCA
29804	Yes	ACCATGACCTGGGTTCAA
29805	Yes	ACCATGACCTGGGTTCAAT
29806	Yes	ACCATGACCTGGGTTCAATA
29807	Yes	ACCATGACCTGGGTTCAATAC
29808	Yes	ACCATGACCTGGGTTCAATACC
29809	Yes	ACCATGACCTGGGTTCAATACCC
29810	Yes	ACCATGACCTGGGTTCAATACCCA
29811	Yes	ACCATGACCTGGGTTCAATACCCAG
29812	Yes	ACCATGACCTGGGTTCAATACCCAGT
29813	Yes	ACCATGACCTGGGTTCAATACCCAGTC

29814	Yes	ACCATGACCTGGGTTCAATACCCAGTCA
29815	Yes	ACCATGACCTGGGTTCAATACCCAGTCAG
29816	Yes	ACCATGACCTGGGTTCAATACCCAGTCAGG
29817	Yes	ACCATGACCTGGGTTCAATACCCAGTCAGGG
29818	Yes	ACCATGACCTGGGTTCAATACCCAGTCAGGGA
29819	Yes	ACCATGACCTGGGTTCAATACCCAGTCAGGGAA
29820	Yes	ACCATGACCTGGGTTCAATACCCAGTCAGGGAAC
29821	Yes	ACCATGACCTGGGTTCAATACCCAGTCAGGGAACC
29822	Yes	ACCATGACCTGGGTTCAATACCCAGTCAGGGAACCA
29823	No	ACCATGGATTCTAGAG
29824	No	ACCATGGATTCTAGAGC
29825	No	ACCATGGATTCTAGAGCC
29826	Yes	ACCATGGATTCTAGAGCCA
29827	Yes	ACCATGGATTCTAGAGCCAG
29828	Yes	ACCATGGATTCTAGAGCCAGA
29829	Yes	ACCATGGATTCTAGAGCCAGAT
29830	Yes	ACCATGGATTCTAGAGCCAGATG
29831	Yes	ACCATGGATTCTAGAGCCAGATGG
29832	Yes	ACCATGGATTCTAGAGCCAGATGGG
29833	Yes	ACCATGGATTCTAGAGCCAGATGGGT
29834	Yes	ACCATGGATTCTAGAGCCAGATGGGTT
29835	Yes	ACCATGGATTCTAGAGCCAGATGGGTTC
29836	Yes	ACCATGGATTCTAGAGCCAGATGGGTTCA
29837	Yes	ACCATGGATTCTAGAGCCAGATGGGTTCAA
29838	Yes	ACCATGGATTCTAGAGCCAGATGGGTTCAAA
29839	Yes	ACCATGGATTCTAGAGCCAGATGGGTTCAAAT
29840	Yes	ACCATGGATTCTAGAGCCAGATGGGTTCAAATC
29841	Yes	ACCATGGATTCTAGAGCCAGATGGGTTCAAATCC
29842	Yes	ACCATGGATTCTAGAGCCAGATGGGTTCAAATCCC
29843	Yes	ACCATGGATTCTAGAGCCAGATGGGTTCAAATCCCG
29844	Yes	ACCATGGATTCTAGAGCCAGATGGGTTCAAATCCCGG
29845	Yes	ACCATGGATTCTAGAGCCAGATGGGTTCAAATCCCGGC
29846	Yes	ACCATGGATTCTAGAGCCAGATGGGTTCAAATCCCGGCT
29847	Yes	ACCATGGATTCTAGAGCCAGATGGGTTCAAATCCCGGCTC
29848	Yes	ACCATGGATTCTAGAGCCAGATGGGTTCAAATCCCGGCTCT
29849	Yes	ACCATGGATTCTAGAGCCAGATGGGTTCAAATCCCGGCTCTG
29850	Yes	ACCATGGATTCTAGAGCCAGATGGGTTCAAATCCCGGCTCTGC
29851	Yes	ACCATGGATTCTAGAGCCAGATGGGTTCAAATCCCGGCTCTGCC
29852	Yes	ACCATGGATTCTAGAGCCAGATGGGTTCAAATCCCGGCTCTGCCG
29853	Yes	ACCATGGATTCTAGAGCCAGATGGGTTCAAATCCCGGCTCTGCCGC
29854	Yes	ACCATGGATTCTAGAGCCAGATGGGTTCAAATCCCGGCTCTGCCGCC
29855	Yes	ACCATGGATTCTAGAGCCAGATGGGTTCAAATCCCGGCTCTGCCGCCA

29856	Yes	ACCATGGCCCGGATTT
29857	Yes	ACCATGGCCCGGATTTG
29858	Yes	ACCATGGCCCGGATTTGA
29859	Yes	ACCATGGCCCGGATTTGAT
29860	Yes	ACCATGGCCCGGATTTGATT
29861	Yes	ACCATGGCCCGGATTTGATTC
29862	Yes	ACCATGGCCCGGATTTGATTCC
29863	Yes	ACCATGGCCCGGATTTGATTCCC
29864	Yes	ACCATGGCCCGGATTTGATTCCCG
29865	Yes	ACCATGGCCCGGATTTGATTCCCGG
29866	Yes	ACCATGGCCCGGATTTGATTCCCGGT
29867	Yes	ACCATGGCCCGGATTTGATTCCCGGTC
29868	Yes	ACCATGGCCCGGATTTGATTCCCGGTCA
29869	Yes	ACCATGGCCCGGATTTGATTCCCGGTCAG
29870	Yes	ACCATGGCCCGGATTTGATTCCCGGTCAGG
29871	Yes	ACCATGGCCCGGATTTGATTCCCGGTCAGGG
29872	Yes	ACCATGGCCCGGATTTGATTCCCGGTCAGGGA
29873	Yes	ACCATGGCCCGGATTTGATTCCCGGTCAGGGAA
29874	Yes	ACCATGGCCCGGATTTGATTCCCGGTCAGGGAAC
29875	Yes	ACCATGGCCCGGATTTGATTCCCGGTCAGGGAACC
29876	Yes	ACCATGGCCCGGATTTGATTCCCGGTCAGGGAACCA
29877	No	ACCATGGCTTAGCTGG
29878	No	ACCATGGCTTAGCTGGT
29879	No	ACCATGGCTTAGCTGGTT
29880	Yes	ACCATGGCTTAGCTGGTTA
29881	Yes	ACCATGGCTTAGCTGGTTAA
29882	Yes	ACCATGGCTTAGCTGGTTAAA
29883	Yes	ACCATGGCTTAGCTGGTTAAAG
29884	Yes	ACCATGGCTTAGCTGGTTAAAGC
29885	Yes	ACCATGGCTTAGCTGGTTAAAGCA
29886	Yes	ACCATGGCTTAGCTGGTTAAAGCAC
29887	Yes	ACCATGGCTTAGCTGGTTAAAGCACC
29888	Yes	ACCATGGCTTAGCTGGTTAAAGCACCT
29889	Yes	ACCATGGCTTAGCTGGTTAAAGCACCTG
29890	Yes	ACCATGGCTTAGCTGGTTAAAGCACCTGT
29891	Yes	ACCATGGCTTAGCTGGTTAAAGCACCTGTC
29892	Yes	ACCATGGCTTAGCTGGTTAAAGCACCTGTCT
29893	Yes	ACCATGGCTTAGCTGGTTAAAGCACCTGTCTA
29894	Yes	ACCATGGCTTAGCTGGTTAAAGCACCTGTCTAG
29895	Yes	ACCATGGCTTAGCTGGTTAAAGCACCTGTCTAGT
29896	Yes	ACCATGGCTTAGCTGGTTAAAGCACCTGTCTAGTA
29897	Yes	ACCATGGCTTAGCTGGTTAAAGCACCTGTCTAGTAA

29898	Yes	ACCATGGCTTAGCTGGTTAAAGCACCTGTCTAGTAAA
29899	Yes	ACCATGGCTTAGCTGGTTAAAGCACCTGTCTAGTAAAC
29900	Yes	ACCATGGCTTAGCTGGTTAAAGCACCTGTCTAGTAAACA
29901	Yes	ACCATGGCTTAGCTGGTTAAAGCACCTGTCTAGTAAACAG
29902	Yes	ACCATGGCTTAGCTGGTTAAAGCACCTGTCTAGTAAACAGG
29903	Yes	ACCATGGCTTAGCTGGTTAAAGCACCTGTCTAGTAAACAGGA
29904	Yes	ACCATGGCTTAGCTGGTTAAAGCACCTGTCTAGTAAACAGGAG
29905	Yes	ACCATGGCTTAGCTGGTTAAAGCACCTGTCTAGTAAACAGGAGA
29906	Yes	ACCATGGCTTAGCTGGTTAAAGCACCTGTCTAGTAAACAGGAGAT
29907	Yes	ACCATGGCTTAGCTGGTTAAAGCACCTGTCTAGTAAACAGGAGATC
29908	Yes	ACCATGGCTTAGCTGGTTAAAGCACCTGTCTAGTAAACAGGAGATCC
29909	Yes	ACCATGGCTTAGCTGGTTAAAGCACCTGTCTAGTAAACAGGAGATCCT
29910	Yes	ACCATGGCTTAGCTGGTTAAAGCACCTGTCTAGTAAACAGGAGATCCTG
29911	Yes	ACCATGGCTTAGCTGGTTAAAGCACCTGTCTAGTAAACAGGAGATCCTGA
29912	No	ACCATGGTCTTTGGAG
29913	Yes	ACCATGGTCTTTGGAGC
29914	Yes	ACCATGGTCTTTGGAGCC
29915	Yes	ACCATGGTCTTTGGAGCCA
29916	Yes	ACCATGGTCTTTGGAGCCAG
29917	Yes	ACCATGGTCTTTGGAGCCAGA
29918	Yes	ACCATGGTCTTTGGAGCCAGAC
29919	Yes	ACCATGGTCTTTGGAGCCAGACT
29920	Yes	ACCATGGTCTTTGGAGCCAGACTG
29921	Yes	ACCATGGTCTTTGGAGCCAGACTGC
29922	Yes	ACCATGGTCTTTGGAGCCAGACTGCC
29923	Yes	ACCATGGTCTTTGGAGCCAGACTGCCT
29924	Yes	ACCATGGTCTTTGGAGCCAGACTGCCTG
29925	Yes	ACCATGGTCTTTGGAGCCAGACTGCCTGG
29926	Yes	ACCATGGTCTTTGGAGCCAGACTGCCTGGG
29927	Yes	ACCATGGTCTTTGGAGCCAGACTGCCTGGGG
29928	Yes	ACCATGGTCTTTGGAGCCAGACTGCCTGGGGT
29929	Yes	ACCATGGTCTTTGGAGCCAGACTGCCTGGGGTC
29930	Yes	ACCATGGTCTTTGGAGCCAGACTGCCTGGGGTCG
29931	Yes	ACCATGGTCTTTGGAGCCAGACTGCCTGGGGTCGG
29932	Yes	ACCATGGTCTTTGGAGCCAGACTGCCTGGGGTCGGA
29933	Yes	ACCATGGTCTTTGGAGCCAGACTGCCTGGGGTCGGAT
29934	Yes	ACCATGGTCTTTGGAGCCAGACTGCCTGGGGTCGGATC
29935	Yes	ACCATGGTCTTTGGAGCCAGACTGCCTGGGGTCGGATCC
29936	Yes	ACCATGGTCTTTGGAGCCAGACTGCCTGGGGTCGGATCCC
29937	Yes	ACCATGGTCTTTGGAGCCAGACTGCCTGGGGTCGGATCCCA
29938	Yes	ACCATGGTCTTTGGAGCCAGACTGCCTGGGGTCGGATCCCAG
29939	Yes	ACCATGGTCTTTGGAGCCAGACTGCCTGGGGTCGGATCCCAGC

29940	Yes	ACCATGGTCTTTGGAGCCAGACTGCCTGGGGTCGGATCCCAGCT
29941	Yes	ACCATGGTCTTTGGAGCCAGACTGCCTGGGGTCGGATCCCAGCTC
29942	Yes	ACCATGGTCTTTGGAGCCAGACTGCCTGGGGTCGGATCCCAGCTCT
29943	Yes	ACCATGGTCTTTGGAGCCAGACTGCCTGGGGTCGGATCCCAGCTCTC
29944	Yes	ACCATGGTCTTTGGAGCCAGACTGCCTGGGGTCGGATCCCAGCTCTCA
29945	Yes	ACCATGGTCTTTGGAGCCAGACTGCCTGGGGTCGGATCCCAGCTCTCAC
29946	Yes	ACCATGGTCTTTGGAGCCAGACTGCCTGGGGTCGGATCCCAGCTCTCACA
29947	No	ACCATGGTTCAAATCT
29948	Yes	ACCATGGTTCAAATCTG
29949	Yes	ACCATGGTTCAAATCTGA
29950	Yes	ACCATGGTTCAAATCTGAG
29951	Yes	ACCATGGTTCAAATCTGAGT
29952	Yes	ACCATGGTTCAAATCTGAGTC
29953	Yes	ACCATGGTTCAAATCTGAGTCA
29954	Yes	ACCATGGTTCAAATCTGAGTCAT
29955	Yes	ACCATGGTTCAAATCTGAGTCATG
29956	Yes	ACCATGGTTCAAATCTGAGTCATGA
29957	Yes	ACCATGGTTCAAATCTGAGTCATGAC
29958	Yes	ACCATGGTTCAAATCTGAGTCATGACA
29959	Yes	ACCATGGTTCAAATCTGAGTCATGACAC
29960	Yes	ACCATGGTTCAAATCTGAGTCATGACACC
29961	Yes	ACCATGGTTCAAATCTGAGTCATGACACCA
29962	No	ACCATGTGGCCTAAGG
29963	No	ACCATGTGGCCTAAGGG
29964	No	ACCATGTGGCCTAAGGGA
29965	Yes	ACCATGTGGCCTAAGGGAA
29966	Yes	ACCATGTGGCCTAAGGGAAA
29967	Yes	ACCATGTGGCCTAAGGGAAAA
29968	Yes	ACCATGTGGCCTAAGGGAAAAG
29969	Yes	ACCATGTGGCCTAAGGGAAAAGA
29970	Yes	ACCATGTGGCCTAAGGGAAAAGAC
29971	Yes	ACCATGTGGCCTAAGGGAAAAGACA
29972	Yes	ACCATGTGGCCTAAGGGAAAAGACAT
29973	Yes	ACCATGTGGCCTAAGGGAAAAGACATC
29974	Yes	ACCATGTGGCCTAAGGGAAAAGACATCT
29975	Yes	ACCATGTGGCCTAAGGGAAAAGACATCTC
29976	Yes	ACCATGTGGCCTAAGGGAAAAGACATCTCA
29977	Yes	ACCATGTGGCCTAAGGGAAAAGACATCTCAC
29978	Yes	ACCATGTGGCCTAAGGGAAAAGACATCTCACT
29979	Yes	ACCATGTGGCCTAAGGGAAAAGACATCTCACTT
29980	Yes	ACCATGTGGCCTAAGGGAAAAGACATCTCACTTT
29981	Yes	ACCATGTGGCCTAAGGGAAAAGACATCTCACTTTG

29982	Yes	ACCATGTGGCCTAAGGGAAAAGACATCTCACTTTGG
29983	Yes	ACCATGTGGCCTAAGGGAAAAGACATCTCACTTTGGG
29984	Yes	ACCATGTGGCCTAAGGGAAAAGACATCTCACTTTGGGT
29985	Yes	ACCATGTGGCCTAAGGGAAAAGACATCTCACTTTGGGTC
29986	Yes	ACCATGTGGCCTAAGGGAAAAGACATCTCACTTTGGGTCA
29987	Yes	ACCATGTGGCCTAAGGGAAAAGACATCTCACTTTGGGTCAG
29988	Yes	ACCATGTGGCCTAAGGGAAAAGACATCTCACTTTGGGTCAGA
29989	Yes	ACCATGTGGCCTAAGGGAAAAGACATCTCACTTTGGGTCAGAA
29990	Yes	ACCATGTGGCCTAAGGGAAAAGACATCTCACTTTGGGTCAGAAG
29991	Yes	ACCATGTGGCCTAAGGGAAAAGACATCTCACTTTGGGTCAGAAGA
29992	Yes	ACCATGTGGCCTAAGGGAAAAGACATCTCACTTTGGGTCAGAAGAT
29993	Yes	ACCATGTGGCCTAAGGGAAAAGACATCTCACTTTGGGTCAGAAGATT
29994	Yes	ACCATGTGGCCTAAGGGAAAAGACATCTCACTTTGGGTCAGAAGATTG
29995	Yes	ACCATGTGGCCTAAGGGAAAAGACATCTCACTTTGGGTCAGAAGATTGA
29996	Yes	ACCATGTGGCCTAAGGGAAAAGACATCTCACTTTGGGTCAGAAGATTGAG
29997	No	ACCATTTCATAACTTT
29998	No	ACCATTTCATAACTTTG
29999	No	ACCATTTCATAACTTTGT
30000	No	ACCATTTCATAACTTTGTC
30001	No	ACCATTTCATAACTTTGTCA
30002	No	ACCATTTCATAACTTTGTCAA
30003	No	ACCATTTCATAACTTTGTCAAA
30004	No	ACCATTTCATAACTTTGTCAAAG
30005	No	ACCATTTCATAACTTTGTCAAAGT
30006	No	ACCATTTCATAACTTTGTCAAAGTT
30007	No	ACCATTTCATAACTTTGTCAAAGTTA
30008	No	ACCATTTCATAACTTTGTCAAAGTTAA
30009	No	ACCATTTCATAACTTTGTCAAAGTTAAA
30010	No	ACCATTTCATAACTTTGTCAAAGTTAAAT
30011	No	ACCATTTCATAACTTTGTCAAAGTTAAATT
30012	No	ACCATTTCATAACTTTGTCAAAGTTAAATTA
30013	No	ACCATTTCATAACTTTGTCAAAGTTAAATTAT
30014	No	ACCATTTCATAACTTTGTCAAAGTTAAATTATA
30015	No	ACCATTTCATAACTTTGTCAAAGTTAAATTATAG
30016	No	ACCATTTCATAACTTTGTCAAAGTTAAATTATAGG
30017	No	ACCATTTCATAACTTTGTCAAAGTTAAATTATAGGC
30018	No	ACCATTTCATAACTTTGTCAAAGTTAAATTATAGGCT
30019	No	ACCATTTCATAACTTTGTCAAAGTTAAATTATAGGCTA
30020	No	ACCATTTCATAACTTTGTCAAAGTTAAATTATAGGCTAA
30021	No	ACCATTTCATAACTTTGTCAAAGTTAAATTATAGGCTAAA
30022	No	ACCATTTCATAACTTTGTCAAAGTTAAATTATAGGCTAAAT
30023	No	ACCATTTCATAACTTTGTCAAAGTTAAATTATAGGCTAAATC

30024	No	ACCATTTCATAACTTTGTCAAAGTTAAATTATAGGCTAAATCC
30025	No	ACCATTTCATAACTTTGTCAAAGTTAAATTATAGGCTAAATCCT
30026	No	ACCATTTCATAACTTTGTCAAAGTTAAATTATAGGCTAAATCCTA
30027	No	ACCATTTCATAACTTTGTCAAAGTTAAATTATAGGCTAAATCCTAT
30028	No	ACCATTTCATAACTTTGTCAAAGTTAAATTATAGGCTAAATCCTATA
30029	No	ACCATTTCATAACTTTGTCAAAGTTAAATTATAGGCTAAATCCTATAT
30030	No	ACCATTTCATAACTTTGTCAAAGTTAAATTATAGGCTAAATCCTATATA
30031	No	ACCATTTCATAACTTTGTCAAAGTTAAATTATAGGCTAAATCCTATATAT
30032	No	ACCCAACTTACACTTA
30033	No	ACCCAACTTACACTTAG
30034	No	ACCCAACTTACACTTAGG
30035	No	ACCCAACTTACACTTAGGA
30036	No	ACCCAACTTACACTTAGGAG
30037	No	ACCCAACTTACACTTAGGAGA
30038	No	ACCCAACTTACACTTAGGAGAT
30039	No	ACCCAACTTACACTTAGGAGATT
30040	No	ACCCAACTTACACTTAGGAGATTT
30041	No	ACCCAACTTACACTTAGGAGATTTC
30042	No	ACCCAACTTACACTTAGGAGATTTCA
30043	No	ACCCAACTTACACTTAGGAGATTTCAA
30044	No	ACCCAACTTACACTTAGGAGATTTCAAC
30045	No	ACCCAACTTACACTTAGGAGATTTCAACT
30046	No	ACCCAACTTACACTTAGGAGATTTCAACTT
30047	No	ACCCAACTTACACTTAGGAGATTTCAACTTA
30048	No	ACCCAACTTACACTTAGGAGATTTCAACTTAA
30049	No	ACCCAACTTACACTTAGGAGATTTCAACTTAAC
30050	No	ACCCAACTTACACTTAGGAGATTTCAACTTAACT
30051	No	ACCCAACTTACACTTAGGAGATTTCAACTTAACTT
30052	No	ACCCAACTTACACTTAGGAGATTTCAACTTAACTTG
30053	No	ACCCAACTTACACTTAGGAGATTTCAACTTAACTTGA
30054	No	ACCCAACTTACACTTAGGAGATTTCAACTTAACTTGAC
30055	No	ACCCAACTTACACTTAGGAGATTTCAACTTAACTTGACC
30056	No	ACCCAACTTACACTTAGGAGATTTCAACTTAACTTGACCG
30057	No	ACCCAACTTACACTTAGGAGATTTCAACTTAACTTGACCGC
30058	No	ACCCAACTTACACTTAGGAGATTTCAACTTAACTTGACCGCT
30059	No	ACCCAACTTACACTTAGGAGATTTCAACTTAACTTGACCGCTC
30060	No	ACCCAACTTACACTTAGGAGATTTCAACTTAACTTGACCGCTCT
30061	No	ACCCAACTTACACTTAGGAGATTTCAACTTAACTTGACCGCTCTG
30062	No	ACCCAACTTACACTTAGGAGATTTCAACTTAACTTGACCGCTCTGA
30063	Yes	ACCCAACTTACACTTAGGAGATTTCAACTTAACTTGACCGCTCTGAC
30064	Yes	ACCCAACTTACACTTAGGAGATTTCAACTTAACTTGACCGCTCTGACC
30065	Yes	ACCCAACTTACACTTAGGAGATTTCAACTTAACTTGACCGCTCTGACCA

30066	Yes	ACCCAAGAGGTCCCGG
30067	Yes	ACCCAAGAGGTCCCGGG
30068	Yes	ACCCAAGAGGTCCCGGGT
30069	Yes	ACCCAAGAGGTCCCGGGTT
30070	Yes	ACCCAAGAGGTCCCGGGTTC
30071	Yes	ACCCAAGAGGTCCCGGGTTCA
30072	Yes	ACCCAAGAGGTCCCGGGTTCAA
30073	Yes	ACCCAAGAGGTCCCGGGTTCAAA
30074	Yes	ACCCAAGAGGTCCCGGGTTCAAAT
30075	Yes	ACCCAAGAGGTCCCGGGTTCAAATC
30076	Yes	ACCCAAGAGGTCCCGGGTTCAAATCC
30077	Yes	ACCCAAGAGGTCCCGGGTTCAAATCCC
30078	Yes	ACCCAAGAGGTCCCGGGTTCAAATCCCG
30079	Yes	ACCCAAGAGGTCCCGGGTTCAAATCCCGG
30080	Yes	ACCCAAGAGGTCCCGGGTTCAAATCCCGGA
30081	Yes	ACCCAAGAGGTCCCGGGTTCAAATCCCGGAC
30082	Yes	ACCCAAGAGGTCCCGGGTTCAAATCCCGGACG
30083	Yes	ACCCAAGAGGTCCCGGGTTCAAATCCCGGACGA
30084	Yes	ACCCAAGAGGTCCCGGGTTCAAATCCCGGACGAG
30085	Yes	ACCCAAGAGGTCCCGGGTTCAAATCCCGGACGAGC
30086	Yes	ACCCAAGAGGTCCCGGGTTCAAATCCCGGACGAGCC
30087	Yes	ACCCAAGAGGTCCCGGGTTCAAATCCCGGACGAGCCC
30088	Yes	ACCCAAGAGGTCCCGGGTTCAAATCCCGGACGAGCCCC
30089	Yes	ACCCAAGAGGTCCCGGGTTCAAATCCCGGACGAGCCCCC
30090	Yes	ACCCAAGAGGTCCCGGGTTCAAATCCCGGACGAGCCCCCA
30091	Yes	ACCCACCCAGAGGCGC
30092	Yes	ACCCACCCAGAGGCGCC
30093	Yes	ACCCACCCAGAGGCGCCA
30094	Yes	ACCCACTGAGGGACGC
30095	Yes	ACCCACTGAGGGACGCC
30096	Yes	ACCCACTGAGGGACGCCA
30097	No	ACCCAGACTGGATGGG
30098	Yes	ACCCAGACTGGATGGGT
30099	Yes	ACCCAGACTGGATGGGTT
30100	Yes	ACCCAGACTGGATGGGTTC
30101	Yes	ACCCAGACTGGATGGGTTCA
30102	Yes	ACCCAGACTGGATGGGTTCAA
30103	Yes	ACCCAGACTGGATGGGTTCAAT
30104	Yes	ACCCAGACTGGATGGGTTCAATT
30105	Yes	ACCCAGACTGGATGGGTTCAATTC
30106	Yes	ACCCAGACTGGATGGGTTCAATTCC
30107	Yes	ACCCAGACTGGATGGGTTCAATTCCC

30108	Yes	ACCCAGACTGGATGGGTTCAATTCCCA
30109	Yes	ACCCAGACTGGATGGGTTCAATTCCCAT
30110	Yes	ACCCAGACTGGATGGGTTCAATTCCCATC
30111	Yes	ACCCAGACTGGATGGGTTCAATTCCCATCT
30112	Yes	ACCCAGACTGGATGGGTTCAATTCCCATCTC
30113	Yes	ACCCAGACTGGATGGGTTCAATTCCCATCTCT
30114	Yes	ACCCAGACTGGATGGGTTCAATTCCCATCTCTG
30115	Yes	ACCCAGACTGGATGGGTTCAATTCCCATCTCTGC
30116	Yes	ACCCAGACTGGATGGGTTCAATTCCCATCTCTGCC
30117	Yes	ACCCAGACTGGATGGGTTCAATTCCCATCTCTGCCG
30118	Yes	ACCCAGACTGGATGGGTTCAATTCCCATCTCTGCCGC
30119	Yes	ACCCAGACTGGATGGGTTCAATTCCCATCTCTGCCGCC
30120	Yes	ACCCAGACTGGATGGGTTCAATTCCCATCTCTGCCGCCA
30121	No	ACCCAGAGGTCAATGG
30122	Yes	ACCCAGAGGTCAATGGA
30123	Yes	ACCCAGAGGTCAATGGAT
30124	Yes	ACCCAGAGGTCAATGGATC
30125	Yes	ACCCAGAGGTCAATGGATCG
30126	Yes	ACCCAGAGGTCAATGGATCGA
30127	Yes	ACCCAGAGGTCAATGGATCGAA
30128	Yes	ACCCAGAGGTCAATGGATCGAAG
30129	Yes	ACCCAGAGGTCAATGGATCGAAGC
30130	Yes	ACCCAGAGGTCAATGGATCGAAGCC
30131	Yes	ACCCAGAGGTCAATGGATCGAAGCCA
30132	Yes	ACCCAGAGGTCAATGGATCGAAGCCAT
30133	Yes	ACCCAGAGGTCAATGGATCGAAGCCATC
30134	Yes	ACCCAGAGGTCAATGGATCGAAGCCATCC
30135	Yes	ACCCAGAGGTCAATGGATCGAAGCCATCCT
30136	Yes	ACCCAGAGGTCAATGGATCGAAGCCATCCTT
30137	Yes	ACCCAGAGGTCAATGGATCGAAGCCATCCTTG
30138	Yes	ACCCAGAGGTCAATGGATCGAAGCCATCCTTGG
30139	Yes	ACCCAGAGGTCAATGGATCGAAGCCATCCTTGGC
30140	Yes	ACCCAGAGGTCAATGGATCGAAGCCATCCTTGGCT
30141	Yes	ACCCAGAGGTCAATGGATCGAAGCCATCCTTGGCTA
30142	Yes	ACCCAGAGGTCAATGGATCGAAGCCATCCTTGGCTAC
30143	Yes	ACCCAGAGGTCAATGGATCGAAGCCATCCTTGGCTACC
30144	Yes	ACCCAGAGGTCAATGGATCGAAGCCATCCTTGGCTACCA
30145	No	ACCCAGAGGTCGATGG
30146	No	ACCCAGAGGTCGATGGA
30147	No	ACCCAGAGGTCGATGGAT
30148	No	ACCCAGAGGTCGATGGATC
30149	Yes	ACCCAGAGGTCGATGGATCG

30150	Yes	ACCCAGAGGTCGATGGATCGA
30151	Yes	ACCCAGAGGTCGATGGATCGAA
30152	Yes	ACCCAGAGGTCGATGGATCGAAA
30153	Yes	ACCCAGAGGTCGATGGATCGAAAC
30154	Yes	ACCCAGAGGTCGATGGATCGAAACC
30155	Yes	ACCCAGAGGTCGATGGATCGAAACCA
30156	Yes	ACCCAGAGGTCGATGGATCGAAACCAT
30157	Yes	ACCCAGAGGTCGATGGATCGAAACCATC
30158	Yes	ACCCAGAGGTCGATGGATCGAAACCATCC
30159	Yes	ACCCAGAGGTCGATGGATCGAAACCATCCT
30160	Yes	ACCCAGAGGTCGATGGATCGAAACCATCCTC
30161	Yes	ACCCAGAGGTCGATGGATCGAAACCATCCTCT
30162	Yes	ACCCAGAGGTCGATGGATCGAAACCATCCTCTG
30163	Yes	ACCCAGAGGTCGATGGATCGAAACCATCCTCTGC
30164	Yes	ACCCAGAGGTCGATGGATCGAAACCATCCTCTGCT
30165	Yes	ACCCAGAGGTCGATGGATCGAAACCATCCTCTGCTA
30166	Yes	ACCCAGAGGTCGATGGATCGAAACCATCCTCTGCTAC
30167	Yes	ACCCAGAGGTCGATGGATCGAAACCATCCTCTGCTACC
30168	Yes	ACCCAGAGGTCGATGGATCGAAACCATCCTCTGCTACCA
30169	Yes	ACCCAGAGGTCGATGGATCT
30170	Yes	ACCCAGAGGTCGATGGATCTA
30171	Yes	ACCCAGAGGTCGATGGATCTAA
30172	Yes	ACCCAGAGGTCGATGGATCTAAA
30173	Yes	ACCCAGAGGTCGATGGATCTAAAC
30174	Yes	ACCCAGAGGTCGATGGATCTAAACC
30175	Yes	ACCCAGAGGTCGATGGATCTAAACCA
30176	Yes	ACCCAGAGGTCGATGGATCTAAACCAT
30177	Yes	ACCCAGAGGTCGATGGATCTAAACCATC
30178	Yes	ACCCAGAGGTCGATGGATCTAAACCATCC
30179	Yes	ACCCAGAGGTCGATGGATCTAAACCATCCT
30180	Yes	ACCCAGAGGTCGATGGATCTAAACCATCCTC
30181	Yes	ACCCAGAGGTCGATGGATCTAAACCATCCTCT
30182	Yes	ACCCAGAGGTCGATGGATCTAAACCATCCTCTG
30183	Yes	ACCCAGAGGTCGATGGATCTAAACCATCCTCTGC
30184	Yes	ACCCAGAGGTCGATGGATCTAAACCATCCTCTGCT
30185	Yes	ACCCAGAGGTCGATGGATCTAAACCATCCTCTGCTA
30186	Yes	ACCCAGAGGTCGATGGATCTAAACCATCCTCTGCTAC
30187	Yes	ACCCAGAGGTCGATGGATCTAAACCATCCTCTGCTACC
30188	Yes	ACCCAGAGGTCGATGGATCTAAACCATCCTCTGCTACCA
30189	No	ACCCAGAGGTTGATGG
30190	Yes	ACCCAGAGGTTGATGGA
30191	Yes	ACCCAGAGGTTGATGGAT

30192	Yes	ACCCAGAGGTTGATGGATG
30193	Yes	ACCCAGAGGTTGATGGATGG
30194	Yes	ACCCAGAGGTTGATGGATGGA
30195	Yes	ACCCAGAGGTTGATGGATGGAA
30196	Yes	ACCCAGAGGTTGATGGATGGAAA
30197	Yes	ACCCAGAGGTTGATGGATGGAAAC
30198	Yes	ACCCAGAGGTTGATGGATGGAAACC
30199	Yes	ACCCAGAGGTTGATGGATGGAAACCA
30200	Yes	ACCCAGAGGTTGATGGATGGAAACCAT
30201	Yes	ACCCAGAGGTTGATGGATGGAAACCATC
30202	Yes	ACCCAGAGGTTGATGGATGGAAACCATCC
30203	Yes	ACCCAGAGGTTGATGGATGGAAACCATCCT
30204	Yes	ACCCAGAGGTTGATGGATGGAAACCATCCTC
30205	Yes	ACCCAGAGGTTGATGGATGGAAACCATCCTCT
30206	Yes	ACCCAGAGGTTGATGGATGGAAACCATCCTCTG
30207	Yes	ACCCAGAGGTTGATGGATGGAAACCATCCTCTGC
30208	Yes	ACCCAGAGGTTGATGGATGGAAACCATCCTCTGCT
30209	Yes	ACCCAGAGGTTGATGGATGGAAACCATCCTCTGCTA
30210	Yes	ACCCAGAGGTTGATGGATGGAAACCATCCTCTGCTAC
30211	Yes	ACCCAGAGGTTGATGGATGGAAACCATCCTCTGCTACC
30212	Yes	ACCCAGAGGTTGATGGATGGAAACCATCCTCTGCTACCA
30213	No	ACCCAGATAGCTCAGT
30214	No	ACCCAGATAGCTCAGTC
30215	No	ACCCAGATAGCTCAGTCA
30216	No	ACCCAGATAGCTCAGTCAG
30217	No	ACCCAGATAGCTCAGTCAGT
30218	No	ACCCAGATAGCTCAGTCAGTA
30219	No	ACCCAGATAGCTCAGTCAGTAG
30220	No	ACCCAGATAGCTCAGTCAGTAGA
30221	No	ACCCAGATAGCTCAGTCAGTAGAG
30222	No	ACCCAGATAGCTCAGTCAGTAGAGC
30223	No	ACCCAGATAGCTCAGTCAGTAGAGCA
30224	No	ACCCAGATAGCTCAGTCAGTAGAGCAT
30225	No	ACCCAGATAGCTCAGTCAGTAGAGCATC
30226	No	ACCCAGATAGCTCAGTCAGTAGAGCATCA
30227	Yes	ACCCAGATAGCTCAGTCAGTAGAGCATCAG
30228	Yes	ACCCAGATAGCTCAGTCAGTAGAGCATCAGA
30229	Yes	ACCCAGATAGCTCAGTCAGTAGAGCATCAGAC
30230	Yes	ACCCAGATAGCTCAGTCAGTAGAGCATCAGACT
30231	Yes	ACCCAGATAGCTCAGTCAGTAGAGCATCAGACTT
30232	Yes	ACCCAGATAGCTCAGTCAGTAGAGCATCAGACTTT
30233	Yes	ACCCAGATAGCTCAGTCAGTAGAGCATCAGACTTTT

30234	Yes	ACCCAGATAGCTCAGTCAGTAGAGCATCAGACTTTTA
30235	Yes	ACCCAGATAGCTCAGTCAGTAGAGCATCAGACTTTTAA
30236	Yes	ACCCAGATAGCTCAGTCAGTAGAGCATCAGACTTTTAAT
30237	Yes	ACCCAGATAGCTCAGTCAGTAGAGCATCAGACTTTTAATC
30238	Yes	ACCCAGATAGCTCAGTCAGTAGAGCATCAGACTTTTAATCT
30239	Yes	ACCCAGATAGCTCAGTCAGTAGAGCATCAGACTTTTAATCTG
30240	Yes	ACCCAGATAGCTCAGTCAGTAGAGCATCAGACTTTTAATCTGA
30241	Yes	ACCCAGATAGCTCAGTCAGTAGAGCATCAGACTTTTAATCTGAG
30242	Yes	ACCCAGATAGCTCAGTCAGTAGAGCATCAGACTTTTAATCTGAGG
30243	Yes	ACCCAGATAGCTCAGTCAGTAGAGCATCAGACTTTTAATCTGAGGG
30244	Yes	ACCCAGATAGCTCAGTCAGTAGAGCATCAGACTTTTAATCTGAGGGT
30245	Yes	ACCCAGATAGCTCAGTCAGTAGAGCATCAGACTTTTAATCTGAGGGTC
30246	Yes	ACCCAGATAGCTCAGTCAGTAGAGCATCAGACTTTTAATCTGAGGGTCC
30247	Yes	ACCCAGATAGCTCAGTCAGTAGAGCATCAGACTTTTAATCTGAGGGTCCA
30248	Yes	ACCCAGATAGCTCAGTT
30249	Yes	ACCCAGATAGCTCAGTTG
30250	Yes	ACCCAGATAGCTCAGTTGA
30251	Yes	ACCCAGATAGCTCAGTTGAT
30252	Yes	ACCCAGATAGCTCAGTTGATA
30253	Yes	ACCCAGATAGCTCAGTTGATAG
30254	Yes	ACCCAGATAGCTCAGTTGATAGA
30255	Yes	ACCCAGATAGCTCAGTTGATAGAG
30256	Yes	ACCCAGATAGCTCAGTTGATAGAGC
30257	Yes	ACCCAGATAGCTCAGTTGATAGAGCA
30258	Yes	ACCCAGATAGCTCAGTTGATAGAGCAT
30259	Yes	ACCCAGATAGCTCAGTTGATAGAGCATC
30260	Yes	ACCCAGATAGCTCAGTTGATAGAGCATCA
30261	Yes	ACCCAGATAGCTCAGTTGATAGAGCATCAG
30262	Yes	ACCCAGATAGCTCAGTTGATAGAGCATCAGA
30263	Yes	ACCCAGATAGCTCAGTTGATAGAGCATCAGAC
30264	Yes	ACCCAGATAGCTCAGTTGATAGAGCATCAGACT
30265	Yes	ACCCAGATAGCTCAGTTGATAGAGCATCAGACTT
30266	Yes	ACCCAGATAGCTCAGTTGATAGAGCATCAGACTTT
30267	Yes	ACCCAGATAGCTCAGTTGATAGAGCATCAGACTTTT
30268	Yes	ACCCAGATAGCTCAGTTGATAGAGCATCAGACTTTTA
30269	Yes	ACCCAGATAGCTCAGTTGATAGAGCATCAGACTTTTAA
30270	Yes	ACCCAGATAGCTCAGTTGATAGAGCATCAGACTTTTAAT
30271	Yes	ACCCAGATAGCTCAGTTGATAGAGCATCAGACTTTTAATC
30272	Yes	ACCCAGATAGCTCAGTTGATAGAGCATCAGACTTTTAATCT
30273	Yes	ACCCAGATAGCTCAGTTGATAGAGCATCAGACTTTTAATCTG
30274	Yes	ACCCAGATAGCTCAGTTGATAGAGCATCAGACTTTTAATCTGA
30275	Yes	ACCCAGATAGCTCAGTTGATAGAGCATCAGACTTTTAATCTGAG

30276	Yes	ACCCAGATAGCTCAGTTGATAGAGCATCAGACTTTTAATCTGAGG
30277	Yes	ACCCAGATAGCTCAGTTGATAGAGCATCAGACTTTTAATCTGAGGG
30278	Yes	ACCCAGATAGCTCAGTTGATAGAGCATCAGACTTTTAATCTGAGGGT
30279	Yes	ACCCAGATAGCTCAGTTGATAGAGCATCAGACTTTTAATCTGAGGGTC
30280	Yes	ACCCAGATAGCTCAGTTGATAGAGCATCAGACTTTTAATCTGAGGGTCC
30281	Yes	ACCCAGATAGCTCAGTTGATAGAGCATCAGACTTTTAATCTGAGGGTCCA
30282	No	ACCCAGGCGGCCCGGG
30283	Yes	ACCCAGGCGGCCCGGGT
30284	Yes	ACCCAGGCGGCCCGGGTT
30285	Yes	ACCCAGGCGGCCCGGGTTC
30286	Yes	ACCCAGGCGGCCCGGGTTCG
30287	Yes	ACCCAGGCGGCCCGGGTTCGA
30288	Yes	ACCCAGGCGGCCCGGGTTCGAC
30289	Yes	ACCCAGGCGGCCCGGGTTCGACT
30290	Yes	ACCCAGGCGGCCCGGGTTCGACTC
30291	Yes	ACCCAGGCGGCCCGGGTTCGACTCC
30292	Yes	ACCCAGGCGGCCCGGGTTCGACTCCC
30293	Yes	ACCCAGGCGGCCCGGGTTCGACTCCCG
30294	Yes	ACCCAGGCGGCCCGGGTTCGACTCCCGG
30295	Yes	ACCCAGGCGGCCCGGGTTCGACTCCCGGT
30296	Yes	ACCCAGGCGGCCCGGGTTCGACTCCCGGTG
30297	Yes	ACCCAGGCGGCCCGGGTTCGACTCCCGGTGT
30298	Yes	ACCCAGGCGGCCCGGGTTCGACTCCCGGTGTG
30299	Yes	ACCCAGGCGGCCCGGGTTCGACTCCCGGTGTGG
30300	Yes	ACCCAGGCGGCCCGGGTTCGACTCCCGGTGTGGG
30301	Yes	ACCCAGGCGGCCCGGGTTCGACTCCCGGTGTGGGA
30302	Yes	ACCCAGGCGGCCCGGGTTCGACTCCCGGTGTGGGAA
30303	Yes	ACCCAGGCGGCCCGGGTTCGACTCCCGGTGTGGGAAC
30304	Yes	ACCCAGGCGGCCCGGGTTCGACTCCCGGTGTGGGAACC
30305	Yes	ACCCAGGCGGCCCGGGTTCGACTCCCGGTGTGGGAACCA
30306	No	ACCCAGGTGGCCCGGG
30307	Yes	ACCCAGGTGGCCCGGGT
30308	Yes	ACCCAGGTGGCCCGGGTT
30309	Yes	ACCCAGGTGGCCCGGGTTC
30310	Yes	ACCCAGGTGGCCCGGGTTCG
30311	Yes	ACCCAGGTGGCCCGGGTTCGA
30312	Yes	ACCCAGGTGGCCCGGGTTCGAC
30313	Yes	ACCCAGGTGGCCCGGGTTCGACT
30314	Yes	ACCCAGGTGGCCCGGGTTCGACTC
30315	Yes	ACCCAGGTGGCCCGGGTTCGACTCC
30316	Yes	ACCCAGGTGGCCCGGGTTCGACTCCC
30317	Yes	ACCCAGGTGGCCCGGGTTCGACTCCCG

30318	Yes	ACCCAGGTGGCCCGGGTTCGACTCCCGG
30319	Yes	ACCCAGGTGGCCCGGGTTCGACTCCCGGT
30320	Yes	ACCCAGGTGGCCCGGGTTCGACTCCCGGTA
30321	Yes	ACCCAGGTGGCCCGGGTTCGACTCCCGGTAT
30322	Yes	ACCCAGGTGGCCCGGGTTCGACTCCCGGTATG
30323	Yes	ACCCAGGTGGCCCGGGTTCGACTCCCGGTATGG
30324	Yes	ACCCAGGTGGCCCGGGTTCGACTCCCGGTATGGG
30325	Yes	ACCCAGGTGGCCCGGGTTCGACTCCCGGTATGGGA
30326	Yes	ACCCAGGTGGCCCGGGTTCGACTCCCGGTATGGGAA
30327	Yes	ACCCAGGTGGCCCGGGTTCGACTCCCGGTATGGGAAC
30328	Yes	ACCCAGGTGGCCCGGGTTCGACTCCCGGTATGGGAACC
30329	Yes	ACCCAGGTGGCCCGGGTTCGACTCCCGGTATGGGAACCA
30330	Yes	ACCCAGGTTCGATTCC
30331	Yes	ACCCAGGTTCGATTCCT
30332	Yes	ACCCAGGTTCGATTCCTG
30333	Yes	ACCCAGGTTCGATTCCTGG
30334	Yes	ACCCAGGTTCGATTCCTGGC
30335	Yes	ACCCAGGTTCGATTCCTGGCC
30336	Yes	ACCCAGGTTCGATTCCTGGCCA
30337	Yes	ACCCAGGTTCGATTCCTGGCCAA
30338	Yes	ACCCAGGTTCGATTCCTGGCCAAT
30339	Yes	ACCCAGGTTCGATTCCTGGCCAATG
30340	Yes	ACCCAGGTTCGATTCCTGGCCAATGC
30341	Yes	ACCCAGGTTCGATTCCTGGCCAATGCA
30342	Yes	ACCCAGGTTCGATTCCTGGCCAATGCAC
30343	Yes	ACCCAGGTTCGATTCCTGGCCAATGCACC
30344	Yes	ACCCAGGTTCGATTCCTGGCCAATGCACCA
30345	Yes	ACCCAGTCAGGGAACC
30346	Yes	ACCCAGTCAGGGAACCA
30347	No	ACCCAGTGGCCTAATG
30348	No	ACCCAGTGGCCTAATGG
30349	No	ACCCAGTGGCCTAATGGA
30350	No	ACCCAGTGGCCTAATGGAT
30351	No	ACCCAGTGGCCTAATGGATA
30352	Yes	ACCCAGTGGCCTAATGGATAA
30353	Yes	ACCCAGTGGCCTAATGGATAAG
30354	Yes	ACCCAGTGGCCTAATGGATAAGG
30355	Yes	ACCCAGTGGCCTAATGGATAAGGC
30356	Yes	ACCCAGTGGCCTAATGGATAAGGCA
30357	Yes	ACCCAGTGGCCTAATGGATAAGGCAT
30358	Yes	ACCCAGTGGCCTAATGGATAAGGCATC
30359	Yes	ACCCAGTGGCCTAATGGATAAGGCATCA

30360	Yes	ACCCAGTGGCCTAATGGATAAGGCATCAG
30361	Yes	ACCCAGTGGCCTAATGGATAAGGCATCAGC
30362	Yes	ACCCAGTGGCCTAATGGATAAGGCATCAGCC
30363	Yes	ACCCAGTGGCCTAATGGATAAGGCATCAGCCT
30364	Yes	ACCCAGTGGCCTAATGGATAAGGCATCAGCCTC
30365	Yes	ACCCAGTGGCCTAATGGATAAGGCATCAGCCTCC
30366	Yes	ACCCAGTGGCCTAATGGATAAGGCATCAGCCTCCG
30367	Yes	ACCCAGTGGCCTAATGGATAAGGCATCAGCCTCCGG
30368	Yes	ACCCAGTGGCCTAATGGATAAGGCATCAGCCTCCGGA
30369	Yes	ACCCAGTGGCCTAATGGATAAGGCATCAGCCTCCGGAG
30370	Yes	ACCCAGTGGCCTAATGGATAAGGCATCAGCCTCCGGAGC
30371	Yes	ACCCAGTGGCCTAATGGATAAGGCATCAGCCTCCGGAGCT
30372	Yes	ACCCAGTGGCCTAATGGATAAGGCATCAGCCTCCGGAGCTG
30373	Yes	ACCCAGTGGCCTAATGGATAAGGCATCAGCCTCCGGAGCTGG
30374	Yes	ACCCAGTGGCCTAATGGATAAGGCATCAGCCTCCGGAGCTGGG
30375	Yes	ACCCAGTGGCCTAATGGATAAGGCATCAGCCTCCGGAGCTGGGG
30376	Yes	ACCCAGTGGCCTAATGGATAAGGCATCAGCCTCCGGAGCTGGGGA
30377	Yes	ACCCAGTGGCCTAATGGATAAGGCATCAGCCTCCGGAGCTGGGGAT
30378	Yes	ACCCAGTGGCCTAATGGATAAGGCATCAGCCTCCGGAGCTGGGGATT
30379	Yes	ACCCAGTGGCCTAATGGATAAGGCATCAGCCTCCGGAGCTGGGGATTG
30380	Yes	ACCCAGTGGCCTAATGGATAAGGCATCAGCCTCCGGAGCTGGGGATTGT
30381	Yes	ACCCAGTGGCCTAATGGATAAGGCATCAGCCTCCGGAGCTGGGGATTGTG
30382	Yes	ACCCATGCTCGATTCC
30383	Yes	ACCCATGCTCGATTCCT
30384	Yes	ACCCATGCTCGATTCCTG
30385	Yes	ACCCATGCTCGATTCCTGG
30386	Yes	ACCCATGCTCGATTCCTGGT
30387	Yes	ACCCATGCTCGATTCCTGGTC
30388	Yes	ACCCATGCTCGATTCCTGGTCA
30389	Yes	ACCCATGCTCGATTCCTGGTCAG
30390	Yes	ACCCATGCTCGATTCCTGGTCAGG
30391	Yes	ACCCATGCTCGATTCCTGGTCAGGG
30392	Yes	ACCCATGCTCGATTCCTGGTCAGGGA
30393	Yes	ACCCATGCTCGATTCCTGGTCAGGGAA
30394	Yes	ACCCATGCTCGATTCCTGGTCAGGGAAC
30395	Yes	ACCCATGCTCGATTCCTGGTCAGGGAACC
30396	Yes	ACCCATGCTCGATTCCTGGTCAGGGAACCA
30397	Yes	ACCCATTGGGGTCTCC
30398	Yes	ACCCATTGGGGTCTCCC
30399	Yes	ACCCATTGGGGTCTCCCC
30400	Yes	ACCCATTGGGGTCTCCCCG
30401	Yes	ACCCATTGGGGTCTCCCCGC

30402	Yes	ACCCATTGGGGTCTCCCCGCG
30403	Yes	ACCCATTGGGGTCTCCCCGCGC
30404	Yes	ACCCATTGGGGTCTCCCCGCGCA
30405	Yes	ACCCATTGGGGTCTCCCCGCGCAG
30406	Yes	ACCCATTGGGGTCTCCCCGCGCAGG
30407	Yes	ACCCATTGGGGTCTCCCCGCGCAGGT
30408	Yes	ACCCATTGGGGTCTCCCCGCGCAGGTT
30409	Yes	ACCCATTGGGGTCTCCCCGCGCAGGTTC
30410	Yes	ACCCATTGGGGTCTCCCCGCGCAGGTTCG
30411	Yes	ACCCATTGGGGTCTCCCCGCGCAGGTTCGA
30412	Yes	ACCCATTGGGGTCTCCCCGCGCAGGTTCGAA
30413	Yes	ACCCATTGGGGTCTCCCCGCGCAGGTTCGAAT
30414	Yes	ACCCATTGGGGTCTCCCCGCGCAGGTTCGAATC
30415	Yes	ACCCATTGGGGTCTCCCCGCGCAGGTTCGAATCC
30416	Yes	ACCCATTGGGGTCTCCCCGCGCAGGTTCGAATCCT
30417	Yes	ACCCATTGGGGTCTCCCCGCGCAGGTTCGAATCCTG
30418	Yes	ACCCATTGGGGTCTCCCCGCGCAGGTTCGAATCCTGC
30419	Yes	ACCCATTGGGGTCTCCCCGCGCAGGTTCGAATCCTGCC
30420	Yes	ACCCATTGGGGTCTCCCCGCGCAGGTTCGAATCCTGCCG
30421	Yes	ACCCATTGGGGTCTCCCCGCGCAGGTTCGAATCCTGCCGA
30422	Yes	ACCCATTGGGGTCTCCCCGCGCAGGTTCGAATCCTGCCGAC
30423	Yes	ACCCATTGGGGTCTCCCCGCGCAGGTTCGAATCCTGCCGACT
30424	Yes	ACCCATTGGGGTCTCCCCGCGCAGGTTCGAATCCTGCCGACTA
30425	Yes	ACCCATTGGGGTCTCCCCGCGCAGGTTCGAATCCTGCCGACTAC
30426	Yes	ACCCATTGGGGTCTCCCCGCGCAGGTTCGAATCCTGCCGACTACG
30427	Yes	ACCCATTGGGGTCTCCCCGCGCAGGTTCGAATCCTGCCGACTACGC
30428	Yes	ACCCATTGGGGTCTCCCCGCGCAGGTTCGAATCCTGCCGACTACGCC
30429	Yes	ACCCATTGGGGTCTCCCCGCGCAGGTTCGAATCCTGCCGACTACGCCA
30430	No	ACCCCACTACTGGTAC
30431	No	ACCCCACTACTGGTACC
30432	No	ACCCCACTACTGGTACCA
30433	No	ACCCCACTCCTGGTAC
30434	No	ACCCCACTCCTGGTACC
30435	No	ACCCCACTCCTGGTACCA
30436	No	ACCCCACTCTCGGTAC
30437	No	ACCCCACTCTCGGTACC
30438	No	ACCCCACTCTCGGTACCA
30439	No	ACCCCACTTCTGGTAC
30440	No	ACCCCACTTCTGGTACC
30441	No	ACCCCACTTCTGGTACCA
30442	No	ACCCCAGCTGCGCCCC
30443	Yes	ACCCCAGCTGCGCCCCC

30444	Yes	ACCCCAGCTGCGCCCCCA
30445	No	ACCCCCGGCTCCTCCA
30446	No	ACCCCCGGCTCCTCCAC
30447	Yes	ACCCCCGGCTCCTCCACC
30448	Yes	ACCCCCGGCTCCTCCACCA
30449	No	ACCCCCTTATTTCTAC
30450	Yes	ACCCCCTTATTTCTACC
30451	Yes	ACCCCCTTATTTCTACCA
30452	No	ACCCCGAAAATGTTGG
30453	No	ACCCCGAAAATGTTGGT
30454	No	ACCCCGAAAATGTTGGTT
30455	No	ACCCCGAAAATGTTGGTTA
30456	No	ACCCCGAAAATGTTGGTTAT
30457	No	ACCCCGAAAATGTTGGTTATA
30458	No	ACCCCGAAAATGTTGGTTATAC
30459	No	ACCCCGAAAATGTTGGTTATACC
30460	No	ACCCCGAAAATGTTGGTTATACCC
30461	No	ACCCCGAAAATGTTGGTTATACCCT
30462	No	ACCCCGAAAATGTTGGTTATACCCTT
30463	No	ACCCCGAAAATGTTGGTTATACCCTTC
30464	No	ACCCCGAAAATGTTGGTTATACCCTTCC
30465	No	ACCCCGAAAATGTTGGTTATACCCTTCCC
30466	No	ACCCCGAAAATGTTGGTTATACCCTTCCCG
30467	No	ACCCCGAAAATGTTGGTTATACCCTTCCCGT
30468	Yes	ACCCCGAAAATGTTGGTTATACCCTTCCCGTA
30469	Yes	ACCCCGAAAATGTTGGTTATACCCTTCCCGTAC
30470	Yes	ACCCCGAAAATGTTGGTTATACCCTTCCCGTACT
30471	Yes	ACCCCGAAAATGTTGGTTATACCCTTCCCGTACTA
30472	Yes	ACCCCGAAAATGTTGGTTATACCCTTCCCGTACTAC
30473	Yes	ACCCCGAAAATGTTGGTTATACCCTTCCCGTACTACC
30474	Yes	ACCCCGAAAATGTTGGTTATACCCTTCCCGTACTACCA
30475	Yes	ACCCCGGGTGGTGTAG
30476	Yes	ACCCCGGGTGGTGTAGT
30477	Yes	ACCCCGGGTGGTGTAGTG
30478	Yes	ACCCCGGGTGGTGTAGTGG
30479	Yes	ACCCCGGGTGGTGTAGTGGA
30480	Yes	ACCCCGGGTGGTGTAGTGGAT
30481	Yes	ACCCCGGGTGGTGTAGTGGATG
30482	Yes	ACCCCGGGTGGTGTAGTGGATGG
30483	Yes	ACCCCGGGTGGTGTAGTGGATGGG
30484	Yes	ACCCCGGGTGGTGTAGTGGATGGGA
30485	Yes	ACCCCGGGTGGTGTAGTGGATGGGAT

30486	Yes	ACCCCGGGTGGTGTAGTGGATGGGATT
30487	Yes	ACCCCGGGTGGTGTAGTGGATGGGATTT
30488	Yes	ACCCCGGGTGGTGTAGTGGATGGGATTTG
30489	Yes	ACCCCGGGTGGTGTAGTGGATGGGATTTGG
30490	Yes	ACCCCGGGTGGTGTAGTGGATGGGATTTGGC
30491	Yes	ACCCCGGGTGGTGTAGTGGATGGGATTTGGCG
30492	Yes	ACCCCGGGTGGTGTAGTGGATGGGATTTGGCGC
30493	Yes	ACCCCGGGTGGTGTAGTGGATGGGATTTGGCGCT
30494	Yes	ACCCCGGGTGGTGTAGTGGATGGGATTTGGCGCTC
30495	Yes	ACCCCGGGTGGTGTAGTGGATGGGATTTGGCGCTCT
30496	Yes	ACCCCGGGTGGTGTAGTGGATGGGATTTGGCGCTCTC
30497	Yes	ACCCCGGGTGGTGTAGTGGATGGGATTTGGCGCTCTCA
30498	Yes	ACCCCGGGTGGTGTAGTGGATGGGATTTGGCGCTCTCAC
30499	Yes	ACCCCGGGTGGTGTAGTGGATGGGATTTGGCGCTCTCACC
30500	Yes	ACCCCGGGTGGTGTAGTGGATGGGATTTGGCGCTCTCACCA
30501	Yes	ACCCCGGGTGGTGTAGTGGATGGGATTTGGCGCTCTCACCAC
30502	Yes	ACCCCGGGTGGTGTAGTGGATGGGATTTGGCGCTCTCACCACC
30503	Yes	ACCCCGGGTGGTGTAGTGGATGGGATTTGGCGCTCTCACCACCA
30504	Yes	ACCCCGGGTGGTGTAGTGGATGGGATTTGGCGCTCTCACCACCAT
30505	Yes	ACCCCGGGTGGTGTAGTGGATGGGATTTGGCGCTCTCACCACCATG
30506	Yes	ACCCCGGGTGGTGTAGTGGATGGGATTTGGCGCTCTCACCACCATGG
30507	Yes	ACCCCGGGTGGTGTAGTGGATGGGATTTGGCGCTCTCACCACCATGGC
30508	Yes	ACCCCGGGTGGTGTAGTGGATGGGATTTGGCGCTCTCACCACCATGGCC
30509	Yes	ACCCCGGGTGGTGTAGTGGATGGGATTTGGCGCTCTCACCACCATGGCCC
30510	Yes	ACCCCGTACGGGCCAC
30511	Yes	ACCCCGTACGGGCCACC
30512	Yes	ACCCCGTACGGGCCACCA
30513	Yes	ACCCCGTCCGTGCCTC
30514	Yes	ACCCCGTCCGTGCCTCC
30515	Yes	ACCCCGTCCGTGCCTCCA
30516	Yes	ACCCCTGGCGGTCTAG
30517	Yes	ACCCCTGGCGGTCTAGT
30518	Yes	ACCCCTGGCGGTCTAGTG
30519	Yes	ACCCCTGGCGGTCTAGTGG
30520	Yes	ACCCCTGGCGGTCTAGTGGT
30521	Yes	ACCCCTGGCGGTCTAGTGGTT
30522	Yes	ACCCCTGGCGGTCTAGTGGTTA
30523	Yes	ACCCCTGGCGGTCTAGTGGTTAG
30524	Yes	ACCCCTGGCGGTCTAGTGGTTAGG
30525	Yes	ACCCCTGGCGGTCTAGTGGTTAGGA
30526	Yes	ACCCCTGGCGGTCTAGTGGTTAGGAT
30527	Yes	ACCCCTGGCGGTCTAGTGGTTAGGATT

30528	Yes	ACCCCTGGCGGTCTAGTGGTTAGGATTC
30529	Yes	ACCCCTGGCGGTCTAGTGGTTAGGATTCG
30530	Yes	ACCCCTGGCGGTCTAGTGGTTAGGATTCGG
30531	Yes	ACCCCTGGCGGTCTAGTGGTTAGGATTCGGC
30532	Yes	ACCCCTGGCGGTCTAGTGGTTAGGATTCGGCG
30533	Yes	ACCCCTGGCGGTCTAGTGGTTAGGATTCGGCGC
30534	Yes	ACCCCTGGCGGTCTAGTGGTTAGGATTCGGCGCT
30535	Yes	ACCCCTGGCGGTCTAGTGGTTAGGATTCGGCGCTC
30536	Yes	ACCCCTGGCGGTCTAGTGGTTAGGATTCGGCGCTCT
30537	Yes	ACCCCTGGCGGTCTAGTGGTTAGGATTCGGCGCTCTC
30538	Yes	ACCCCTGGCGGTCTAGTGGTTAGGATTCGGCGCTCTCA
30539	Yes	ACCCCTGGCGGTCTAGTGGTTAGGATTCGGCGCTCTCAT
30540	Yes	ACCCCTGGCGGTCTAGTGGTTAGGATTCGGCGCTCTCATC
30541	Yes	ACCCCTGGCGGTCTAGTGGTTAGGATTCGGCGCTCTCATCC
30542	Yes	ACCCCTGGCGGTCTAGTGGTTAGGATTCGGCGCTCTCATCCA
30543	Yes	ACCCCTGGCGGTCTAGTGGTTAGGATTCGGCGCTCTCATCCAC
30544	Yes	ACCCCTGGCGGTCTAGTGGTTAGGATTCGGCGCTCTCATCCACC
30545	Yes	ACCCCTGGCGGTCTAGTGGTTAGGATTCGGCGCTCTCATCCACCG
30546	Yes	ACCCCTGGCGGTCTAGTGGTTAGGATTCGGCGCTCTCATCCACCGC
30547	Yes	ACCCCTGGCGGTCTAGTGGTTAGGATTCGGCGCTCTCATCCACCGCG
30548	Yes	ACCCCTGGCGGTCTAGTGGTTAGGATTCGGCGCTCTCATCCACCGCGG
30549	Yes	ACCCCTGGCGGTCTAGTGGTTAGGATTCGGCGCTCTCATCCACCGCGGC
30550	Yes	ACCCCTGGCGGTCTAGTGGTTAGGATTCGGCGCTCTCATCCACCGCGGCC
30551	No	ACCCCTGGTAGTCTAG
30552	Yes	ACCCCTGGTAGTCTAGT
30553	Yes	ACCCCTGGTAGTCTAGTG
30554	Yes	ACCCCTGGTAGTCTAGTGG
30555	Yes	ACCCCTGGTAGTCTAGTGGT
30556	Yes	ACCCCTGGTAGTCTAGTGGTT
30557	Yes	ACCCCTGGTAGTCTAGTGGTTA
30558	Yes	ACCCCTGGTAGTCTAGTGGTTAG
30559	Yes	ACCCCTGGTAGTCTAGTGGTTAGG
30560	Yes	ACCCCTGGTAGTCTAGTGGTTAGGC
30561	Yes	ACCCCTGGTAGTCTAGTGGTTAGGCT
30562	Yes	ACCCCTGGTAGTCTAGTGGTTAGGCTT
30563	Yes	ACCCCTGGTAGTCTAGTGGTTAGGCTTT
30564	Yes	ACCCCTGGTAGTCTAGTGGTTAGGCTTTG
30565	Yes	ACCCCTGGTAGTCTAGTGGTTAGGCTTTGC
30566	Yes	ACCCCTGGTAGTCTAGTGGTTAGGCTTTGCC
30567	Yes	ACCCCTGGTAGTCTAGTGGTTAGGCTTTGCCG
30568	Yes	ACCCCTGGTAGTCTAGTGGTTAGGCTTTGCCGC
30569	Yes	ACCCCTGGTAGTCTAGTGGTTAGGCTTTGCCGCT

30570	Yes	ACCCCTGGTAGTCTAGTGGTTAGGCTTTGCCGCTC
30571	Yes	ACCCCTGGTAGTCTAGTGGTTAGGCTTTGCCGCTCT
30572	Yes	ACCCCTGGTAGTCTAGTGGTTAGGCTTTGCCGCTCTC
30573	Yes	ACCCCTGGTAGTCTAGTGGTTAGGCTTTGCCGCTCTCA
30574	Yes	ACCCCTGGTAGTCTAGTGGTTAGGCTTTGCCGCTCTCAG
30575	Yes	ACCCCTGGTAGTCTAGTGGTTAGGCTTTGCCGCTCTCAGT
30576	Yes	ACCCCTGGTAGTCTAGTGGTTAGGCTTTGCCGCTCTCAGTG
30577	Yes	ACCCCTGGTAGTCTAGTGGTTAGGCTTTGCCGCTCTCAGTGC
30578	Yes	ACCCCTGGTAGTCTAGTGGTTAGGCTTTGCCGCTCTCAGTGCC
30579	Yes	ACCCCTGGTAGTCTAGTGGTTAGGCTTTGCCGCTCTCAGTGCCG
30580	Yes	ACCCCTGGTAGTCTAGTGGTTAGGCTTTGCCGCTCTCAGTGCCGC
30581	Yes	ACCCCTGGTAGTCTAGTGGTTAGGCTTTGCCGCTCTCAGTGCCGCT
30582	Yes	ACCCCTGGTAGTCTAGTGGTTAGGCTTTGCCGCTCTCAGTGCCGCTG
30583	Yes	ACCCCTGGTAGTCTAGTGGTTAGGCTTTGCCGCTCTCAGTGCCGCTGC
30584	Yes	ACCCCTGGTAGTCTAGTGGTTAGGCTTTGCCGCTCTCAGTGCCGCTGCC
30585	Yes	ACCCCTGGTAGTCTAGTGGTTAGGCTTTGCCGCTCTCAGTGCCGCTGCCT
30586	Yes	ACCCCTGGTGGTCTAG
30587	Yes	ACCCCTGGTGGTCTAGT
30588	Yes	ACCCCTGGTGGTCTAGTG
30589	Yes	ACCCCTGGTGGTCTAGTGC
30590	Yes	ACCCCTGGTGGTCTAGTGCT
30591	Yes	ACCCCTGGTGGTCTAGTGCTT
30592	Yes	ACCCCTGGTGGTCTAGTGCTTA
30593	Yes	ACCCCTGGTGGTCTAGTGCTTAG
30594	Yes	ACCCCTGGTGGTCTAGTGCTTAGG
30595	Yes	ACCCCTGGTGGTCTAGTGCTTAGGA
30596	Yes	ACCCCTGGTGGTCTAGTGCTTAGGAT
30597	Yes	ACCCCTGGTGGTCTAGTGCTTAGGATT
30598	Yes	ACCCCTGGTGGTCTAGTGCTTAGGATTC
30599	Yes	ACCCCTGGTGGTCTAGTGCTTAGGATTCG
30600	Yes	ACCCCTGGTGGTCTAGTGCTTAGGATTCGG
30601	Yes	ACCCCTGGTGGTCTAGTGCTTAGGATTCGGT
30602	Yes	ACCCCTGGTGGTCTAGTGCTTAGGATTCGGTG
30603	Yes	ACCCCTGGTGGTCTAGTGCTTAGGATTCGGTGC
30604	Yes	ACCCCTGGTGGTCTAGTGCTTAGGATTCGGTGCT
30605	Yes	ACCCCTGGTGGTCTAGTGCTTAGGATTCGGTGCTC
30606	Yes	ACCCCTGGTGGTCTAGTGCTTAGGATTCGGTGCTCT
30607	Yes	ACCCCTGGTGGTCTAGTGCTTAGGATTCGGTGCTCTC
30608	Yes	ACCCCTGGTGGTCTAGTGCTTAGGATTCGGTGCTCTCA
30609	Yes	ACCCCTGGTGGTCTAGTGCTTAGGATTCGGTGCTCTCAC
30610	Yes	ACCCCTGGTGGTCTAGTGCTTAGGATTCGGTGCTCTCACC
30611	Yes	ACCCCTGGTGGTCTAGTGCTTAGGATTCGGTGCTCTCACCG

30612	Yes	ACCCCTGGTGGTCTAGTGCTTAGGATTCGGTGCTCTCACCGC
30613	Yes	ACCCCTGGTGGTCTAGTGCTTAGGATTCGGTGCTCTCACCGCT
30614	Yes	ACCCCTGGTGGTCTAGTGCTTAGGATTCGGTGCTCTCACCGCTG
30615	Yes	ACCCCTGGTGGTCTAGTGCTTAGGATTCGGTGCTCTCACCGCTGC
30616	Yes	ACCCCTGGTGGTCTAGTGCTTAGGATTCGGTGCTCTCACCGCTGCT
30617	Yes	ACCCCTGGTGGTCTAGTGCTTAGGATTCGGTGCTCTCACCGCTGCTG
30618	Yes	ACCCCTGGTGGTCTAGTGCTTAGGATTCGGTGCTCTCACCGCTGCTGC
30619	Yes	ACCCCTGGTGGTCTAGTGCTTAGGATTCGGTGCTCTCACCGCTGCTGCC
30620	Yes	ACCCCTGGTGGTCTAGTGCTTAGGATTCGGTGCTCTCACCGCTGCTGCCT
30621	Yes	ACCCCTGGTGGTCTAGTGCTTAGGATTT
30622	Yes	ACCCCTGGTGGTCTAGTGCTTAGGATTTG
30623	Yes	ACCCCTGGTGGTCTAGTGCTTAGGATTTGG
30624	Yes	ACCCCTGGTGGTCTAGTGCTTAGGATTTGGC
30625	Yes	ACCCCTGGTGGTCTAGTGCTTAGGATTTGGCA
30626	Yes	ACCCCTGGTGGTCTAGTGCTTAGGATTTGGCAC
30627	Yes	ACCCCTGGTGGTCTAGTGCTTAGGATTTGGCACT
30628	Yes	ACCCCTGGTGGTCTAGTGCTTAGGATTTGGCACTC
30629	Yes	ACCCCTGGTGGTCTAGTGCTTAGGATTTGGCACTCT
30630	Yes	ACCCCTGGTGGTCTAGTGCTTAGGATTTGGCACTCTC
30631	Yes	ACCCCTGGTGGTCTAGTGCTTAGGATTTGGCACTCTCG
30632	Yes	ACCCCTGGTGGTCTAGTGCTTAGGATTTGGCACTCTCGC
30633	Yes	ACCCCTGGTGGTCTAGTGCTTAGGATTTGGCACTCTCGCC
30634	Yes	ACCCCTGGTGGTCTAGTGCTTAGGATTTGGCACTCTCGCCA
30635	Yes	ACCCCTGGTGGTCTAGTGCTTAGGATTTGGCACTCTCGCCAC
30636	Yes	ACCCCTGGTGGTCTAGTGCTTAGGATTTGGCACTCTCGCCACC
30637	Yes	ACCCCTGGTGGTCTAGTGCTTAGGATTTGGCACTCTCGCCACCG
30638	Yes	ACCCCTGGTGGTCTAGTGCTTAGGATTTGGCACTCTCGCCACCGC
30639	Yes	ACCCCTGGTGGTCTAGTGCTTAGGATTTGGCACTCTCGCCACCGCA
30640	Yes	ACCCCTGGTGGTCTAGTGCTTAGGATTTGGCACTCTCGCCACCGCAG
30641	Yes	ACCCCTGGTGGTCTAGTGCTTAGGATTTGGCACTCTCGCCACCGCAGC
30642	Yes	ACCCCTGGTGGTCTAGTGCTTAGGATTTGGCACTCTCGCCACCGCAGCC
30643	Yes	ACCCCTGGTGGTCTAGTGCTTAGGATTTGGCACTCTCGCCACCGCAGCCT
30644	Yes	ACCCCTGGTGGTCTAT
30645	Yes	ACCCCTGGTGGTCTATC
30646	Yes	ACCCCTGGTGGTCTATCG
30647	Yes	ACCCCTGGTGGTCTATCGG
30648	Yes	ACCCCTGGTGGTCTATCGGT
30649	Yes	ACCCCTGGTGGTCTATCGGTT
30650	Yes	ACCCCTGGTGGTCTATCGGTTA
30651	Yes	ACCCCTGGTGGTCTATCGGTTAG
30652	Yes	ACCCCTGGTGGTCTATCGGTTAGG
30653	Yes	ACCCCTGGTGGTCTATCGGTTAGGA

30654	Yes	ACCCCTGGTGGTCTATCGGTTAGGAT
30655	Yes	ACCCCTGGTGGTCTATCGGTTAGGATT
30656	Yes	ACCCCTGGTGGTCTATCGGTTAGGATTC
30657	Yes	ACCCCTGGTGGTCTATCGGTTAGGATTCA
30658	Yes	ACCCCTGGTGGTCTATCGGTTAGGATTCAG
30659	Yes	ACCCCTGGTGGTCTATCGGTTAGGATTCAGA
30660	Yes	ACCCCTGGTGGTCTATCGGTTAGGATTCAGAC
30661	Yes	ACCCCTGGTGGTCTATCGGTTAGGATTCAGACC
30662	Yes	ACCCCTGGTGGTCTATCGGTTAGGATTCAGACCT
30663	Yes	ACCCCTGGTGGTCTATCGGTTAGGATTCAGACCTC
30664	Yes	ACCCCTGGTGGTCTATCGGTTAGGATTCAGACCTCT
30665	Yes	ACCCCTGGTGGTCTATCGGTTAGGATTCAGACCTCTC
30666	Yes	ACCCCTGGTGGTCTATCGGTTAGGATTCAGACCTCTCA
30667	Yes	ACCCCTGGTGGTCTATCGGTTAGGATTCAGACCTCTCAC
30668	Yes	ACCCCTGGTGGTCTATCGGTTAGGATTCAGACCTCTCACC
30669	Yes	ACCCCTGGTGGTCTATCGGTTAGGATTCAGACCTCTCACCA
30670	Yes	ACCCCTGGTGGTCTATCGGTTAGGATTCAGACCTCTCACCAC
30671	Yes	ACCCCTGGTGGTCTATCGGTTAGGATTCAGACCTCTCACCACT
30672	Yes	ACCCCTGGTGGTCTATCGGTTAGGATTCAGACCTCTCACCACTG
30673	Yes	ACCCCTGGTGGTCTATCGGTTAGGATTCAGACCTCTCACCACTGC
30674	Yes	ACCCCTGGTGGTCTATCGGTTAGGATTCAGACCTCTCACCACTGCT
30675	Yes	ACCCCTGGTGGTCTATCGGTTAGGATTCAGACCTCTCACCACTGCTA
30676	Yes	ACCCCTGGTGGTCTATCGGTTAGGATTCAGACCTCTCACCACTGCTAC
30677	Yes	ACCCCTGGTGGTCTATCGGTTAGGATTCAGACCTCTCACCACTGCTACC
30678	Yes	ACCCCTGGTGGTCTATCGGTTAGGATTCAGACCTCTCACCACTGCTACCC
30679	Yes	ACCCCTTATTTACCCC
30680	Yes	ACCCCTTATTTACCCCA
30681	Yes	ACCCCTTGTAGTCTAG
30682	Yes	ACCCCTTGTAGTCTAGT
30683	Yes	ACCCCTTGTAGTCTAGTG
30684	Yes	ACCCCTTGTAGTCTAGTGG
30685	Yes	ACCCCTTGTAGTCTAGTGGT
30686	Yes	ACCCCTTGTAGTCTAGTGGTT
30687	Yes	ACCCCTTGTAGTCTAGTGGTTA
30688	Yes	ACCCCTTGTAGTCTAGTGGTTAG
30689	Yes	ACCCCTTGTAGTCTAGTGGTTAGA
30690	Yes	ACCCCTTGTAGTCTAGTGGTTAGAA
30691	Yes	ACCCCTTGTAGTCTAGTGGTTAGAAT
30692	Yes	ACCCCTTGTAGTCTAGTGGTTAGAATT
30693	Yes	ACCCCTTGTAGTCTAGTGGTTAGAATTC
30694	Yes	ACCCCTTGTAGTCTAGTGGTTAGAATTCT
30695	Yes	ACCCCTTGTAGTCTAGTGGTTAGAATTCTG

30696	Yes	ACCCCTTGTAGTCTAGTGGTTAGAATTCTGC
30697	Yes	ACCCCTTGTAGTCTAGTGGTTAGAATTCTGCG
30698	Yes	ACCCCTTGTAGTCTAGTGGTTAGAATTCTGCGG
30699	Yes	ACCCCTTGTAGTCTAGTGGTTAGAATTCTGCGGT
30700	Yes	ACCCCTTGTAGTCTAGTGGTTAGAATTCTGCGGTC
30701	Yes	ACCCCTTGTAGTCTAGTGGTTAGAATTCTGCGGTCT
30702	Yes	ACCCCTTGTAGTCTAGTGGTTAGAATTCTGCGGTCTC
30703	Yes	ACCCCTTGTAGTCTAGTGGTTAGAATTCTGCGGTCTCA
30704	Yes	ACCCCTTGTAGTCTAGTGGTTAGAATTCTGCGGTCTCAC
30705	Yes	ACCCCTTGTAGTCTAGTGGTTAGAATTCTGCGGTCTCACA
30706	Yes	ACCCCTTGTAGTCTAGTGGTTAGAATTCTGCGGTCTCACAG
30707	Yes	ACCCCTTGTAGTCTAGTGGTTAGAATTCTGCGGTCTCACAGC
30708	Yes	ACCCCTTGTAGTCTAGTGGTTAGAATTCTGCGGTCTCACAGCC
30709	Yes	ACCCCTTGTAGTCTAGTGGTTAGAATTCTGCGGTCTCACAGCCG
30710	Yes	ACCCCTTGTAGTCTAGTGGTTAGAATTCTGCGGTCTCACAGCCGC
30711	Yes	ACCCCTTGTAGTCTAGTGGTTAGAATTCTGCGGTCTCACAGCCGCG
30712	Yes	ACCCCTTGTAGTCTAGTGGTTAGAATTCTGCGGTCTCACAGCCGCGG
30713	Yes	ACCCCTTGTAGTCTAGTGGTTAGAATTCTGCGGTCTCACAGCCGCGGC
30714	Yes	ACCCCTTGTAGTCTAGTGGTTAGAATTCTGCGGTCTCACAGCCGCGGCC
30715	Yes	ACCCCTTGTAGTCTAGTGGTTAGAATTCTGCGGTCTCACAGCCGCGGCCC
30716	Yes	ACCCGACTTCAATTCC
30717	Yes	ACCCGACTTCAATTCCC
30718	Yes	ACCCGACTTCAATTCCCA
30719	Yes	ACCCGACTTCAATTCCCAG
30720	Yes	ACCCGACTTCAATTCCCAGC
30721	Yes	ACCCGACTTCAATTCCCAGCC
30722	Yes	ACCCGACTTCAATTCCCAGCCA
30723	Yes	ACCCGACTTCAATTCCCAGCCAA
30724	Yes	ACCCGACTTCAATTCCCAGCCAAC
30725	Yes	ACCCGACTTCAATTCCCAGCCAACG
30726	Yes	ACCCGACTTCAATTCCCAGCCAACGC
30727	Yes	ACCCGACTTCAATTCCCAGCCAACGCA
30728	Yes	ACCCGACTTCAATTCCCAGCCAACGCAC
30729	Yes	ACCCGACTTCAATTCCCAGCCAACGCACC
30730	Yes	ACCCGACTTCAATTCCCAGCCAACGCACCA
30731	Yes	ACCCGACTTCGATTCC
30732	Yes	ACCCGACTTCGATTCCC
30733	Yes	ACCCGACTTCGATTCCCA
30734	Yes	ACCCGACTTCGATTCCCAG
30735	Yes	ACCCGACTTCGATTCCCAGC
30736	Yes	ACCCGACTTCGATTCCCAGCC
30737	Yes	ACCCGACTTCGATTCCCAGCCA

30738	Yes	ACCCGACTTCGATTCCCAGCCAA
30739	Yes	ACCCGACTTCGATTCCCAGCCAAT
30740	Yes	ACCCGACTTCGATTCCCAGCCAATG
30741	Yes	ACCCGACTTCGATTCCCAGCCAATGC
30742	Yes	ACCCGACTTCGATTCCCAGCCAATGCA
30743	Yes	ACCCGACTTCGATTCCCAGCCAATGCAC
30744	Yes	ACCCGACTTCGATTCCCAGCCAATGCACC
30745	Yes	ACCCGACTTCGATTCCCAGCCAATGCACCA
30746	Yes	ACCCGGGCTCGATTCC
30747	Yes	ACCCGGGCTCGATTCCC
30748	Yes	ACCCGGGCTCGATTCCCG
30749	Yes	ACCCGGGCTCGATTCCCGC
30750	Yes	ACCCGGGCTCGATTCCCGCC
30751	Yes	ACCCGGGCTCGATTCCCGCCC
30752	Yes	ACCCGGGCTCGATTCCCGCCCA
30753	Yes	ACCCGGGCTCGATTCCCGCCCAA
30754	Yes	ACCCGGGCTCGATTCCCGCCCAAC
30755	Yes	ACCCGGGCTCGATTCCCGCCCAACG
30756	Yes	ACCCGGGCTCGATTCCCGCCCAACGC
30757	Yes	ACCCGGGCTCGATTCCCGCCCAACGCA
30758	Yes	ACCCGGGCTCGATTCCCGCCCAACGCAC
30759	Yes	ACCCGGGCTCGATTCCCGCCCAACGCACC
30760	Yes	ACCCGGGCTCGATTCCCGCCCAACGCACCA
30761	No	ACCCGGGTTCAATTCC
30762	Yes	ACCCGGGTTCAATTCCC
30763	Yes	ACCCGGGTTCAATTCCCG
30764	Yes	ACCCGGGTTCAATTCCCGG
30765	Yes	ACCCGGGTTCAATTCCCGGC
30766	Yes	ACCCGGGTTCAATTCCCGGCC
30767	Yes	ACCCGGGTTCAATTCCCGGCCA
30768	Yes	ACCCGGGTTCAATTCCCGGCCAA
30769	Yes	ACCCGGGTTCAATTCCCGGCCAAT
30770	Yes	ACCCGGGTTCAATTCCCGGCCAATG
30771	Yes	ACCCGGGTTCAATTCCCGGCCAATGC
30772	Yes	ACCCGGGTTCAATTCCCGGCCAATGCA
30773	Yes	ACCCGGGTTCAATTCCCGGCCAATGCAC
30774	Yes	ACCCGGGTTCAATTCCCGGCCAATGCACC
30775	Yes	ACCCGGGTTCAATTCCCGGCCAATGCACCA
30776	Yes	ACCCGGGTTCAATTCCCGGCCAC
30777	Yes	ACCCGGGTTCAATTCCCGGCCACT
30778	Yes	ACCCGGGTTCAATTCCCGGCCACTG
30779	Yes	ACCCGGGTTCAATTCCCGGCCACTGC

30780	Yes	ACCCGGGTTCAATTCCCGGCCACTGCA
30781	Yes	ACCCGGGTTCAATTCCCGGCCACTGCAC
30782	Yes	ACCCGGGTTCAATTCCCGGCCACTGCACC
30783	Yes	ACCCGGGTTCAATTCCCGGCCACTGCACCA
30784	Yes	ACCCGGGTTCAATTCCCGGT
30785	Yes	ACCCGGGTTCAATTCCCGGTC
30786	Yes	ACCCGGGTTCAATTCCCGGTCA
30787	Yes	ACCCGGGTTCAATTCCCGGTCAA
30788	Yes	ACCCGGGTTCAATTCCCGGTCAAG
30789	Yes	ACCCGGGTTCAATTCCCGGTCAAGG
30790	Yes	ACCCGGGTTCAATTCCCGGTCAAGGC
30791	Yes	ACCCGGGTTCAATTCCCGGTCAAGGCA
30792	Yes	ACCCGGGTTCAATTCCCGGTCAAGGCAC
30793	Yes	ACCCGGGTTCAATTCCCGGTCAAGGCACC
30794	Yes	ACCCGGGTTCAATTCCCGGTCAAGGCACCA
30795	Yes	ACCCGGGTTCAATTCCCGGTCAG
30796	Yes	ACCCGGGTTCAATTCCCGGTCAGG
30797	Yes	ACCCGGGTTCAATTCCCGGTCAGGG
30798	Yes	ACCCGGGTTCAATTCCCGGTCAGGGA
30799	Yes	ACCCGGGTTCAATTCCCGGTCAGGGAA
30800	Yes	ACCCGGGTTCAATTCCCGGTCAGGGAAC
30801	Yes	ACCCGGGTTCAATTCCCGGTCAGGGAACC
30802	Yes	ACCCGGGTTCAATTCCCGGTCAGGGAACCA
30803	Yes	ACCCGGGTTCAATTCCT
30804	Yes	ACCCGGGTTCAATTCCTG
30805	Yes	ACCCGGGTTCAATTCCTGA
30806	Yes	ACCCGGGTTCAATTCCTGAC
30807	Yes	ACCCGGGTTCAATTCCTGACC
30808	Yes	ACCCGGGTTCAATTCCTGACCC
30809	Yes	ACCCGGGTTCAATTCCTGACCCA
30810	Yes	ACCCGGGTTCAATTCCTGACCCAT
30811	Yes	ACCCGGGTTCAATTCCTGACCCATG
30812	Yes	ACCCGGGTTCAATTCCTGACCCATGT
30813	Yes	ACCCGGGTTCAATTCCTGACCCATGTA
30814	Yes	ACCCGGGTTCAATTCCTGACCCATGTAC
30815	Yes	ACCCGGGTTCAATTCCTGACCCATGTACC
30816	Yes	ACCCGGGTTCAATTCCTGACCCATGTACCA
30817	No	ACCCGGGTTCGATTCC
30818	Yes	ACCCGGGTTCGATTCCC
30819	Yes	ACCCGGGTTCGATTCCCG
30820	Yes	ACCCGGGTTCGATTCCCGG
30821	Yes	ACCCGGGTTCGATTCCCGGC

30822	Yes	ACCCGGGTTCGATTCCCGGCC
30823	Yes	ACCCGGGTTCGATTCCCGGCCA
30824	Yes	ACCCGGGTTCGATTCCCGGCCAA
30825	Yes	ACCCGGGTTCGATTCCCGGCCAAC
30826	Yes	ACCCGGGTTCGATTCCCGGCCAACG
30827	Yes	ACCCGGGTTCGATTCCCGGCCAACGC
30828	Yes	ACCCGGGTTCGATTCCCGGCCAACGCA
30829	Yes	ACCCGGGTTCGATTCCCGGCCAACGCAC
30830	Yes	ACCCGGGTTCGATTCCCGGCCAACGCACC
30831	Yes	ACCCGGGTTCGATTCCCGGCCAACGCACCA
30832	Yes	ACCCGGGTTCGATTCCCGGG
30833	Yes	ACCCGGGTTCGATTCCCGGGC
30834	Yes	ACCCGGGTTCGATTCCCGGGCG
30835	Yes	ACCCGGGTTCGATTCCCGGGCGG
30836	Yes	ACCCGGGTTCGATTCCCGGGCGGC
30837	Yes	ACCCGGGTTCGATTCCCGGGCGGCG
30838	Yes	ACCCGGGTTCGATTCCCGGGCGGCGC
30839	Yes	ACCCGGGTTCGATTCCCGGGCGGCGCA
30840	Yes	ACCCGGGTTCGATTCCCGGGCGGCGCAC
30841	Yes	ACCCGGGTTCGATTCCCGGGCGGCGCACC
30842	Yes	ACCCGGGTTCGATTCCCGGGCGGCGCACCA
30843	Yes	ACCCGGGTTTAATTCC
30844	Yes	ACCCGGGTTTAATTCCC
30845	Yes	ACCCGGGTTTAATTCCCG
30846	Yes	ACCCGGGTTTAATTCCCGG
30847	Yes	ACCCGGGTTTAATTCCCGGT
30848	Yes	ACCCGGGTTTAATTCCCGGTC
30849	Yes	ACCCGGGTTTAATTCCCGGTCA
30850	Yes	ACCCGGGTTTAATTCCCGGTCAA
30851	Yes	ACCCGGGTTTAATTCCCGGTCAAG
30852	Yes	ACCCGGGTTTAATTCCCGGTCAAGA
30853	Yes	ACCCGGGTTTAATTCCCGGTCAAGAT
30854	Yes	ACCCGGGTTTAATTCCCGGTCAAGATA
30855	Yes	ACCCGGGTTTAATTCCCGGTCAAGATAC
30856	Yes	ACCCGGGTTTAATTCCCGGTCAAGATACC
30857	Yes	ACCCGGGTTTAATTCCCGGTCAAGATACCA
30858	Yes	ACCCTAGCCCTGGCAC
30859	Yes	ACCCTAGCCCTGGCACC
30860	Yes	ACCCTAGCCCTGGCACCA
30861	Yes	ACCCTCCGCTGGAGGC
30862	Yes	ACCCTCCGCTGGAGGCG
30863	Yes	ACCCTCCGCTGGAGGCGT

30864	Yes	ACCCTCCGCTGGAGGCGTG
30865	Yes	ACCCTCCGCTGGAGGCGTGG
30866	Yes	ACCCTCCGCTGGAGGCGTGGG
30867	Yes	ACCCTCCGCTGGAGGCGTGGGT
30868	Yes	ACCCTCCGCTGGAGGCGTGGGTT
30869	Yes	ACCCTCCGCTGGAGGCGTGGGTTC
30870	Yes	ACCCTCCGCTGGAGGCGTGGGTTCG
30871	Yes	ACCCTCCGCTGGAGGCGTGGGTTCGA
30872	Yes	ACCCTCCGCTGGAGGCGTGGGTTCGAA
30873	Yes	ACCCTCCGCTGGAGGCGTGGGTTCGAAT
30874	Yes	ACCCTCCGCTGGAGGCGTGGGTTCGAATC
30875	Yes	ACCCTCCGCTGGAGGCGTGGGTTCGAATCC
30876	Yes	ACCCTCCGCTGGAGGCGTGGGTTCGAATCCC
30877	Yes	ACCCTCCGCTGGAGGCGTGGGTTCGAATCCCA
30878	Yes	ACCCTCCGCTGGAGGCGTGGGTTCGAATCCCAC
30879	Yes	ACCCTCCGCTGGAGGCGTGGGTTCGAATCCCACT
30880	Yes	ACCCTCCGCTGGAGGCGTGGGTTCGAATCCCACTT
30881	Yes	ACCCTCCGCTGGAGGCGTGGGTTCGAATCCCACTTT
30882	Yes	ACCCTCCGCTGGAGGCGTGGGTTCGAATCCCACTTTT
30883	Yes	ACCCTCCGCTGGAGGCGTGGGTTCGAATCCCACTTTTG
30884	Yes	ACCCTCCGCTGGAGGCGTGGGTTCGAATCCCACTTTTGA
30885	Yes	ACCCTCCGCTGGAGGCGTGGGTTCGAATCCCACTTTTGAC
30886	Yes	ACCCTCCGCTGGAGGCGTGGGTTCGAATCCCACTTTTGACA
30887	Yes	ACCCTCCGCTGGAGGCGTGGGTTCGAATCCCACTTTTGACAC
30888	Yes	ACCCTCCGCTGGAGGCGTGGGTTCGAATCCCACTTTTGACACC
30889	Yes	ACCCTCCGCTGGAGGCGTGGGTTCGAATCCCACTTTTGACACCA
30890	Yes	ACCCTGCTCGCTGCGC
30891	Yes	ACCCTGCTCGCTGCGCC
30892	Yes	ACCCTGCTCGCTGCGCCA
30893	No	ACCCTGGACTCTGAAT
30894	No	ACCCTGGACTCTGAATC
30895	Yes	ACCCTGGACTCTGAATCA
30896	Yes	ACCCTGGACTCTGAATCAA
30897	Yes	ACCCTGGACTCTGAATCAAG
30898	Yes	ACCCTGGACTCTGAATCAAGC
30899	Yes	ACCCTGGACTCTGAATCAAGCG
30900	Yes	ACCCTGGACTCTGAATCAAGCGA
30901	Yes	ACCCTGGACTCTGAATCAAGCGAT
30902	Yes	ACCCTGGACTCTGAATCAAGCGATC
30903	Yes	ACCCTGGACTCTGAATCAAGCGATCC
30904	Yes	ACCCTGGACTCTGAATCAAGCGATCCG
30905	Yes	ACCCTGGACTCTGAATCAAGCGATCCGA

30906	Yes	ACCCTGGACTCTGAATCAAGCGATCCGAG
30907	Yes	ACCCTGGACTCTGAATCAAGCGATCCGAGT
30908	Yes	ACCCTGGACTCTGAATCAAGCGATCCGAGTT
30909	Yes	ACCCTGGACTCTGAATCAAGCGATCCGAGTTC
30910	Yes	ACCCTGGACTCTGAATCAAGCGATCCGAGTTCA
30911	Yes	ACCCTGGACTCTGAATCAAGCGATCCGAGTTCAA
30912	Yes	ACCCTGGACTCTGAATCAAGCGATCCGAGTTCAAA
30913	Yes	ACCCTGGACTCTGAATCAAGCGATCCGAGTTCAAAT
30914	Yes	ACCCTGGACTCTGAATCAAGCGATCCGAGTTCAAATC
30915	Yes	ACCCTGGACTCTGAATCAAGCGATCCGAGTTCAAATCT
30916	Yes	ACCCTGGACTCTGAATCAAGCGATCCGAGTTCAAATCTC
30917	Yes	ACCCTGGACTCTGAATCAAGCGATCCGAGTTCAAATCTCG
30918	Yes	ACCCTGGACTCTGAATCAAGCGATCCGAGTTCAAATCTCGG
30919	Yes	ACCCTGGACTCTGAATCAAGCGATCCGAGTTCAAATCTCGGT
30920	Yes	ACCCTGGACTCTGAATCAAGCGATCCGAGTTCAAATCTCGGTG
30921	Yes	ACCCTGGACTCTGAATCAAGCGATCCGAGTTCAAATCTCGGTGG
30922	Yes	ACCCTGGACTCTGAATCAAGCGATCCGAGTTCAAATCTCGGTGGT
30923	Yes	ACCCTGGACTCTGAATCAAGCGATCCGAGTTCAAATCTCGGTGGTA
30924	Yes	ACCCTGGACTCTGAATCAAGCGATCCGAGTTCAAATCTCGGTGGTAC
30925	Yes	ACCCTGGACTCTGAATCAAGCGATCCGAGTTCAAATCTCGGTGGTACC
30926	Yes	ACCCTGGACTCTGAATCAAGCGATCCGAGTTCAAATCTCGGTGGTACCT
30927	Yes	ACCCTGGACTCTGAATCAAGCGATCCGAGTTCAAATCTCGGTGGTACCTC
30928	Yes	ACCCTGGACTCTGAATCC
30929	Yes	ACCCTGGACTCTGAATCCA
30930	Yes	ACCCTGGACTCTGAATCCAG
30931	Yes	ACCCTGGACTCTGAATCCAGC
30932	Yes	ACCCTGGACTCTGAATCCAGCA
30933	Yes	ACCCTGGACTCTGAATCCAGCAA
30934	Yes	ACCCTGGACTCTGAATCCAGCAAC
30935	Yes	ACCCTGGACTCTGAATCCAGCAACC
30936	Yes	ACCCTGGACTCTGAATCCAGCAACCA
30937	Yes	ACCCTGGACTCTGAATCCAGCAACCAG
30938	Yes	ACCCTGGACTCTGAATCCAGCAACCAGA
30939	Yes	ACCCTGGACTCTGAATCCAGCAACCAGAG
30940	Yes	ACCCTGGACTCTGAATCCAGCAACCAGAGT
30941	Yes	ACCCTGGACTCTGAATCCAGCAACCAGAGTT
30942	Yes	ACCCTGGACTCTGAATCCAGCAACCAGAGTTC
30943	Yes	ACCCTGGACTCTGAATCCAGCAACCAGAGTTCC
30944	Yes	ACCCTGGACTCTGAATCCAGCAACCAGAGTTCCA
30945	Yes	ACCCTGGACTCTGAATCCAGCAACCAGAGTTCCAG
30946	Yes	ACCCTGGACTCTGAATCCAGCAACCAGAGTTCCAGT
30947	Yes	ACCCTGGACTCTGAATCCAGCAACCAGAGTTCCAGTC

30948	Yes	ACCCTGGACTCTGAATCCAGCAACCAGAGTTCCAGTCT
30949	Yes	ACCCTGGACTCTGAATCCAGCAACCAGAGTTCCAGTCTC
30950	Yes	ACCCTGGACTCTGAATCCAGCAACCAGAGTTCCAGTCTCA
30951	Yes	ACCCTGGACTCTGAATCCAGCAACCAGAGTTCCAGTCTCAG
30952	Yes	ACCCTGGACTCTGAATCCAGCAACCAGAGTTCCAGTCTCAGC
30953	Yes	ACCCTGGACTCTGAATCCAGCAACCAGAGTTCCAGTCTCAGCG
30954	Yes	ACCCTGGACTCTGAATCCAGCAACCAGAGTTCCAGTCTCAGCGT
30955	Yes	ACCCTGGACTCTGAATCCAGCAACCAGAGTTCCAGTCTCAGCGTG
30956	Yes	ACCCTGGACTCTGAATCCAGCAACCAGAGTTCCAGTCTCAGCGTGG
30957	Yes	ACCCTGGACTCTGAATCCAGCAACCAGAGTTCCAGTCTCAGCGTGGA
30958	Yes	ACCCTGGACTCTGAATCCAGCAACCAGAGTTCCAGTCTCAGCGTGGAC
30959	Yes	ACCCTGGACTCTGAATCCAGCAACCAGAGTTCCAGTCTCAGCGTGGACC
30960	Yes	ACCCTGGACTCTGAATCCAGCAACCAGAGTTCCAGTCTCAGCGTGGACCT
30961	No	ACCCTGGCGCTGTCAC
30962	Yes	ACCCTGGCGCTGTCACC
30963	Yes	ACCCTGGCGCTGTCACCA
30964	No	ACCCTGGGTTTCAGCC
30965	Yes	ACCCTGGGTTTCAGCCC
30966	Yes	ACCCTGGGTTTCAGCCCC
30967	Yes	ACCCTGGGTTTCAGCCCCA
30968	Yes	ACCCTGGTTTAATTCC
30969	Yes	ACCCTGGTTTAATTCCT
30970	Yes	ACCCTGGTTTAATTCCTG
30971	Yes	ACCCTGGTTTAATTCCTGG
30972	Yes	ACCCTGGTTTAATTCCTGGC
30973	Yes	ACCCTGGTTTAATTCCTGGCC
30974	Yes	ACCCTGGTTTAATTCCTGGCCA
30975	Yes	ACCCTGGTTTAATTCCTGGCCAA
30976	Yes	ACCCTGGTTTAATTCCTGGCCAAT
30977	Yes	ACCCTGGTTTAATTCCTGGCCAATG
30978	Yes	ACCCTGGTTTAATTCCTGGCCAATGC
30979	Yes	ACCCTGGTTTAATTCCTGGCCAATGCA
30980	Yes	ACCCTGGTTTAATTCCTGGCCAATGCAC
30981	Yes	ACCCTGGTTTAATTCCTGGCCAATGCACC
30982	Yes	ACCCTGGTTTAATTCCTGGCCAATGCACCA
30983	Yes	ACCCTGTGGTACAGGG
30984	Yes	ACCCTGTGGTACAGGGG
30985	Yes	ACCCTGTGGTACAGGGGC
30986	Yes	ACCCTGTGGTACAGGGGCT
30987	Yes	ACCCTGTGGTACAGGGGCTA
30988	Yes	ACCCTGTGGTACAGGGGCTAA
30989	Yes	ACCCTGTGGTACAGGGGCTAAT

30990	Yes	ACCCTGTGGTACAGGGGCTAATA
30991	Yes	ACCCTGTGGTACAGGGGCTAATAT
30992	Yes	ACCCTGTGGTACAGGGGCTAATATG
30993	Yes	ACCCTGTGGTACAGGGGCTAATATGC
30994	Yes	ACCCTGTGGTACAGGGGCTAATATGCT
30995	Yes	ACCCTGTGGTACAGGGGCTAATATGCTG
30996	Yes	ACCCTGTGGTACAGGGGCTAATATGCTGG
30997	Yes	ACCCTGTGGTACAGGGGCTAATATGCTGGG
30998	Yes	ACCCTGTGGTACAGGGGCTAATATGCTGGGC
30999	Yes	ACCCTGTGGTACAGGGGCTAATATGCTGGGCC
31000	Yes	ACCCTGTGGTACAGGGGCTAATATGCTGGGCCT
31001	Yes	ACCCTGTGGTACAGGGGCTAATATGCTGGGCCTT
31002	Yes	ACCCTGTGGTACAGGGGCTAATATGCTGGGCCTTT
31003	Yes	ACCCTGTGGTACAGGGGCTAATATGCTGGGCCTTTA
31004	Yes	ACCCTGTGGTACAGGGGCTAATATGCTGGGCCTTTAC
31005	Yes	ACCCTGTGGTACAGGGGCTAATATGCTGGGCCTTTACC
31006	Yes	ACCCTGTGGTACAGGGGCTAATATGCTGGGCCTTTACCA
31007	Yes	ACCCTGTGGTACAGGGGCTAATATGCTGGGCCTTTACCAC
31008	Yes	ACCCTGTGGTACAGGGGCTAATATGCTGGGCCTTTACCACT
31009	Yes	ACCCTGTGGTACAGGGGCTAATATGCTGGGCCTTTACCACTT
31010	Yes	ACCCTGTGGTACAGGGGCTAATATGCTGGGCCTTTACCACTTC
31011	Yes	ACCCTGTGGTACAGGGGCTAATATGCTGGGCCTTTACCACTTCA
31012	Yes	ACCCTGTGGTACAGGGGCTAATATGCTGGGCCTTTACCACTTCAG
31013	Yes	ACCCTGTGGTACAGGGGCTAATATGCTGGGCCTTTACCACTTCAGC
31014	Yes	ACCCTGTGGTACAGGGGCTAATATGCTGGGCCTTTACCACTTCAGCC
31015	Yes	ACCCTGTGGTACAGGGGCTAATATGCTGGGCCTTTACCACTTCAGCCC
31016	Yes	ACCCTGTGGTACAGGGGCTAATATGCTGGGCCTTTACCACTTCAGCCCA
31017	Yes	ACCCTGTGGTACAGGGGCTAATATGCTGGGCCTTTACCACTTCAGCCCAG
31018	No	ACCCTGTGGTCTAGTG
31019	No	ACCCTGTGGTCTAGTGG
31020	No	ACCCTGTGGTCTAGTGGC
31021	No	ACCCTGTGGTCTAGTGGCT
31022	Yes	ACCCTGTGGTCTAGTGGCTA
31023	Yes	ACCCTGTGGTCTAGTGGCTAA
31024	Yes	ACCCTGTGGTCTAGTGGCTAAG
31025	Yes	ACCCTGTGGTCTAGTGGCTAAGA
31026	Yes	ACCCTGTGGTCTAGTGGCTAAGAC
31027	Yes	ACCCTGTGGTCTAGTGGCTAAGACT
31028	Yes	ACCCTGTGGTCTAGTGGCTAAGACTT
31029	Yes	ACCCTGTGGTCTAGTGGCTAAGACTTT
31030	Yes	ACCCTGTGGTCTAGTGGCTAAGACTTTG
31031	Yes	ACCCTGTGGTCTAGTGGCTAAGACTTTGT

31032	Yes	ACCCTGTGGTCTAGTGGCTAAGACTTTGTG
31033	Yes	ACCCTGTGGTCTAGTGGCTAAGACTTTGTGC
31034	Yes	ACCCTGTGGTCTAGTGGCTAAGACTTTGTGCT
31035	Yes	ACCCTGTGGTCTAGTGGCTAAGACTTTGTGCTT
31036	Yes	ACCCTGTGGTCTAGTGGCTAAGACTTTGTGCTTT
31037	Yes	ACCCTGTGGTCTAGTGGCTAAGACTTTGTGCTTTC
31038	Yes	ACCCTGTGGTCTAGTGGCTAAGACTTTGTGCTTTCA
31039	Yes	ACCCTGTGGTCTAGTGGCTAAGACTTTGTGCTTTCAT
31040	Yes	ACCCTGTGGTCTAGTGGCTAAGACTTTGTGCTTTCATT
31041	Yes	ACCCTGTGGTCTAGTGGCTAAGACTTTGTGCTTTCATTG
31042	Yes	ACCCTGTGGTCTAGTGGCTAAGACTTTGTGCTTTCATTGC
31043	Yes	ACCCTGTGGTCTAGTGGCTAAGACTTTGTGCTTTCATTGCT
31044	Yes	ACCCTGTGGTCTAGTGGCTAAGACTTTGTGCTTTCATTGCTG
31045	Yes	ACCCTGTGGTCTAGTGGCTAAGACTTTGTGCTTTCATTGCTGC
31046	Yes	ACCCTGTGGTCTAGTGGCTAAGACTTTGTGCTTTCATTGCTGCA
31047	Yes	ACCCTGTGGTCTAGTGGCTAAGACTTTGTGCTTTCATTGCTGCAT
31048	Yes	ACCCTGTGGTCTAGTGGCTAAGACTTTGTGCTTTCATTGCTGCATC
31049	Yes	ACCCTGTGGTCTAGTGGCTAAGACTTTGTGCTTTCATTGCTGCATCC
31050	Yes	ACCCTGTGGTCTAGTGGCTAAGACTTTGTGCTTTCATTGCTGCATCCT
31051	Yes	ACCCTGTGGTCTAGTGGCTAAGACTTTGTGCTTTCATTGCTGCATCCTA
31052	Yes	ACCCTGTGGTCTAGTGGCTAAGACTTTGTGCTTTCATTGCTGCATCCTAG
31053	Yes	ACCCTGTGGTCTAGTGGT
31054	Yes	ACCCTGTGGTCTAGTGGTT
31055	Yes	ACCCTGTGGTCTAGTGGTTA
31056	Yes	ACCCTGTGGTCTAGTGGTTAG
31057	Yes	ACCCTGTGGTCTAGTGGTTAGG
31058	Yes	ACCCTGTGGTCTAGTGGTTAGGA
31059	Yes	ACCCTGTGGTCTAGTGGTTAGGAG
31060	Yes	ACCCTGTGGTCTAGTGGTTAGGAGT
31061	Yes	ACCCTGTGGTCTAGTGGTTAGGAGTT
31062	Yes	ACCCTGTGGTCTAGTGGTTAGGAGTTG
31063	Yes	ACCCTGTGGTCTAGTGGTTAGGAGTTGG
31064	Yes	ACCCTGTGGTCTAGTGGTTAGGAGTTGGT
31065	Yes	ACCCTGTGGTCTAGTGGTTAGGAGTTGGTG
31066	Yes	ACCCTGTGGTCTAGTGGTTAGGAGTTGGTGC
31067	Yes	ACCCTGTGGTCTAGTGGTTAGGAGTTGGTGCT
31068	Yes	ACCCTGTGGTCTAGTGGTTAGGAGTTGGTGCTT
31069	Yes	ACCCTGTGGTCTAGTGGTTAGGAGTTGGTGCTTT
31070	Yes	ACCCTGTGGTCTAGTGGTTAGGAGTTGGTGCTTTC
31071	Yes	ACCCTGTGGTCTAGTGGTTAGGAGTTGGTGCTTTCG
31072	Yes	ACCCTGTGGTCTAGTGGTTAGGAGTTGGTGCTTTCGT
31073	Yes	ACCCTGTGGTCTAGTGGTTAGGAGTTGGTGCTTTCGTC

31074	Yes	ACCCTGTGGTCTAGTGGTTAGGAGTTGGTGCTTTCGTCA
31075	Yes	ACCCTGTGGTCTAGTGGTTAGGAGTTGGTGCTTTCGTCAT
31076	Yes	ACCCTGTGGTCTAGTGGTTAGGAGTTGGTGCTTTCGTCATG
31077	Yes	ACCCTGTGGTCTAGTGGTTAGGAGTTGGTGCTTTCGTCATGA
31078	Yes	ACCCTGTGGTCTAGTGGTTAGGAGTTGGTGCTTTCGTCATGAC
31079	Yes	ACCCTGTGGTCTAGTGGTTAGGAGTTGGTGCTTTCGTCATGACA
31080	Yes	ACCCTGTGGTCTAGTGGTTAGGAGTTGGTGCTTTCGTCATGACAG
31081	Yes	ACCCTGTGGTCTAGTGGTTAGGAGTTGGTGCTTTCGTCATGACAGC
31082	Yes	ACCCTGTGGTCTAGTGGTTAGGAGTTGGTGCTTTCGTCATGACAGCC
31083	Yes	ACCCTGTGGTCTAGTGGTTAGGAGTTGGTGCTTTCGTCATGACAGCCC
31084	Yes	ACCCTGTGGTCTAGTGGTTAGGAGTTGGTGCTTTCGTCATGACAGCCCA
31085	Yes	ACCCTGTGGTCTAGTGGTTAGGAGTTGGTGCTTTCGTCATGACAGCCCAG
31086	No	ACCCTTAATCTCAGGG
31087	Yes	ACCCTTAATCTCAGGGT
31088	Yes	ACCCTTAATCTCAGGGTC
31089	Yes	ACCCTTAATCTCAGGGTCG
31090	Yes	ACCCTTAATCTCAGGGTCGT
31091	Yes	ACCCTTAATCTCAGGGTCGTG
31092	Yes	ACCCTTAATCTCAGGGTCGTGG
31093	Yes	ACCCTTAATCTCAGGGTCGTGGG
31094	Yes	ACCCTTAATCTCAGGGTCGTGGGT
31095	Yes	ACCCTTAATCTCAGGGTCGTGGGTT
31096	Yes	ACCCTTAATCTCAGGGTCGTGGGTTC
31097	Yes	ACCCTTAATCTCAGGGTCGTGGGTTCG
31098	Yes	ACCCTTAATCTCAGGGTCGTGGGTTCGA
31099	Yes	ACCCTTAATCTCAGGGTCGTGGGTTCGAG
31100	Yes	ACCCTTAATCTCAGGGTCGTGGGTTCGAGC
31101	Yes	ACCCTTAATCTCAGGGTCGTGGGTTCGAGCC
31102	Yes	ACCCTTAATCTCAGGGTCGTGGGTTCGAGCCC
31103	Yes	ACCCTTAATCTCAGGGTCGTGGGTTCGAGCCCC
31104	Yes	ACCCTTAATCTCAGGGTCGTGGGTTCGAGCCCCA
31105	Yes	ACCCTTAATCTCAGGGTCGTGGGTTCGAGCCCCAC
31106	Yes	ACCCTTAATCTCAGGGTCGTGGGTTCGAGCCCCACG
31107	Yes	ACCCTTAATCTCAGGGTCGTGGGTTCGAGCCCCACGT
31108	Yes	ACCCTTAATCTCAGGGTCGTGGGTTCGAGCCCCACGTT
31109	Yes	ACCCTTAATCTCAGGGTCGTGGGTTCGAGCCCCACGTTG
31110	Yes	ACCCTTAATCTCAGGGTCGTGGGTTCGAGCCCCACGTTGG
31111	Yes	ACCCTTAATCTCAGGGTCGTGGGTTCGAGCCCCACGTTGGG
31112	Yes	ACCCTTAATCTCAGGGTCGTGGGTTCGAGCCCCACGTTGGGC
31113	Yes	ACCCTTAATCTCAGGGTCGTGGGTTCGAGCCCCACGTTGGGCG
31114	Yes	ACCCTTAATCTCAGGGTCGTGGGTTCGAGCCCCACGTTGGGCGC
31115	Yes	ACCCTTAATCTCAGGGTCGTGGGTTCGAGCCCCACGTTGGGCGCC

31116	Yes	ACCCTTAATCTCAGGGTCGTGGGTTCGAGCCCCACGTTGGGCGCCA
31117	Yes	ACCCTTCCCGTACTAC
31118	Yes	ACCCTTCCCGTACTACC
31119	Yes	ACCCTTCCCGTACTACCA
31120	Yes	ACCCTTGGCTCGAGAC
31121	Yes	ACCCTTGGCTCGAGACT
31122	Yes	ACCCTTGGCTCGAGACTG
31123	Yes	ACCCTTGGCTCGAGACTGG
31124	Yes	ACCCTTGGCTCGAGACTGGA
31125	Yes	ACCCTTGGCTCGAGACTGGAG
31126	Yes	ACCCTTGGCTCGAGACTGGAGG
31127	Yes	ACCCTTGGCTCGAGACTGGAGGG
31128	Yes	ACCCTTGGCTCGAGACTGGAGGGA
31129	Yes	ACCCTTGGCTCGAGACTGGAGGGAA
31130	Yes	ACCCTTGGCTCGAGACTGGAGGGAAA
31131	Yes	ACCCTTGGCTCGAGACTGGAGGGAAAC
31132	Yes	ACCCTTGGCTCGAGACTGGAGGGAAACA
31133	Yes	ACCCTTGGCTCGAGACTGGAGGGAAACAC
31134	Yes	ACCCTTGGCTCGAGACTGGAGGGAAACACC
31135	Yes	ACCCTTGGCTCGAGACTGGAGGGAAACACCA
31136	Yes	ACCGAAAAGTTGGTGG
31137	Yes	ACCGAAAAGTTGGTGGT
31138	Yes	ACCGAAAAGTTGGTGGTT
31139	Yes	ACCGAAAAGTTGGTGGTTC
31140	Yes	ACCGAAAAGTTGGTGGTTCG
31141	Yes	ACCGAAAAGTTGGTGGTTCGA
31142	Yes	ACCGAAAAGTTGGTGGTTCGAG
31143	Yes	ACCGAAAAGTTGGTGGTTCGAGC
31144	Yes	ACCGAAAAGTTGGTGGTTCGAGCC
31145	Yes	ACCGAAAAGTTGGTGGTTCGAGCCC
31146	Yes	ACCGAAAAGTTGGTGGTTCGAGCCCA
31147	Yes	ACCGAAAAGTTGGTGGTTCGAGCCCAC
31148	Yes	ACCGAAAAGTTGGTGGTTCGAGCCCACC
31149	Yes	ACCGAAAAGTTGGTGGTTCGAGCCCACCC
31150	Yes	ACCGAAAAGTTGGTGGTTCGAGCCCACCCA
31151	Yes	ACCGAAAAGTTGGTGGTTCGAGCCCACCCAG
31152	Yes	ACCGAAAAGTTGGTGGTTCGAGCCCACCCAGG
31153	Yes	ACCGAAAAGTTGGTGGTTCGAGCCCACCCAGGA
31154	Yes	ACCGAAAAGTTGGTGGTTCGAGCCCACCCAGGAA
31155	Yes	ACCGAAAAGTTGGTGGTTCGAGCCCACCCAGGAAC
31156	Yes	ACCGAAAAGTTGGTGGTTCGAGCCCACCCAGGAACG
31157	Yes	ACCGAAAAGTTGGTGGTTCGAGCCCACCCAGGAACGC

31158	Yes	ACCGAAAAGTTGGTGGTTCGAGCCCACCCAGGAACGCC
31159	Yes	ACCGAAAAGTTGGTGGTTCGAGCCCACCCAGGAACGCCA
31160	Yes	ACCGAAAGATTGGTGG
31161	Yes	ACCGAAAGATTGGTGGT
31162	Yes	ACCGAAAGATTGGTGGTT
31163	Yes	ACCGAAAGATTGGTGGTTC
31164	Yes	ACCGAAAGATTGGTGGTTCG
31165	Yes	ACCGAAAGATTGGTGGTTCGA
31166	Yes	ACCGAAAGATTGGTGGTTCGAG
31167	Yes	ACCGAAAGATTGGTGGTTCGAGC
31168	Yes	ACCGAAAGATTGGTGGTTCGAGCC
31169	Yes	ACCGAAAGATTGGTGGTTCGAGCCC
31170	Yes	ACCGAAAGATTGGTGGTTCGAGCCCA
31171	Yes	ACCGAAAGATTGGTGGTTCGAGCCCAC
31172	Yes	ACCGAAAGATTGGTGGTTCGAGCCCACC
31173	Yes	ACCGAAAGATTGGTGGTTCGAGCCCACCC
31174	Yes	ACCGAAAGATTGGTGGTTCGAGCCCACCCA
31175	Yes	ACCGAAAGATTGGTGGTTCGAGCCCACCCAG
31176	Yes	ACCGAAAGATTGGTGGTTCGAGCCCACCCAGG
31177	Yes	ACCGAAAGATTGGTGGTTCGAGCCCACCCAGGG
31178	Yes	ACCGAAAGATTGGTGGTTCGAGCCCACCCAGGGA
31179	Yes	ACCGAAAGATTGGTGGTTCGAGCCCACCCAGGGAC
31180	Yes	ACCGAAAGATTGGTGGTTCGAGCCCACCCAGGGACG
31181	Yes	ACCGAAAGATTGGTGGTTCGAGCCCACCCAGGGACGC
31182	Yes	ACCGAAAGATTGGTGGTTCGAGCCCACCCAGGGACGCC
31183	Yes	ACCGAAAGATTGGTGGTTCGAGCCCACCCAGGGACGCCA
31184	Yes	ACCGAAAGCTTGGTGG
31185	Yes	ACCGAAAGCTTGGTGGT
31186	Yes	ACCGAAAGCTTGGTGGTT
31187	Yes	ACCGAAAGCTTGGTGGTTC
31188	Yes	ACCGAAAGCTTGGTGGTTCG
31189	Yes	ACCGAAAGCTTGGTGGTTCGA
31190	Yes	ACCGAAAGCTTGGTGGTTCGAG
31191	Yes	ACCGAAAGCTTGGTGGTTCGAGC
31192	Yes	ACCGAAAGCTTGGTGGTTCGAGCC
31193	Yes	ACCGAAAGCTTGGTGGTTCGAGCCC
31194	Yes	ACCGAAAGCTTGGTGGTTCGAGCCCA
31195	Yes	ACCGAAAGCTTGGTGGTTCGAGCCCAC
31196	Yes	ACCGAAAGCTTGGTGGTTCGAGCCCACC
[truncated: 358,446 more chars]
